# DNA Satellites Are Transcribed as Part of the Non-Coding Genome in Eukaryotes and Bacteria

**DOI:** 10.3390/genes12111651

**Published:** 2021-10-20

**Authors:** Juan A. Subirana, Xavier Messeguer

**Affiliations:** Department of Computer Science, Universitat Politècnica de Catalunya, 08034 Barcelona, Spain; peypoch@lsi.upc.edu

**Keywords:** tandem repeats, satellites, *Caenorhabditis elegans*, *Bacillus coagulans*, non-coding DNA, small RNA, RNA interference, RNA-seq, non-coding genome

## Abstract

It has been shown in recent years that many repeated sequences in the genome are expressed as RNA transcripts, although the role of such RNAs is poorly understood. Some isolated and tandem repeats (satellites) have been found to be transcribed, such as mammalian Alu sequences and telomeric/centromeric satellites in different species. However, there is no detailed study on the eventual transcription of the interspersed satellites found in many species. Therefore, we decided to study for the first time the transcription of the abundant DNA satellites in the bacterium *Bacillus coagulans* and in the nematode *Caenorhabditis elegans*. We have updated the data for *C. elegans* satellites using the latest version of the genome. We analyzed the transcription of satellites in both species in available RNA-seq results and found that they are widely transcribed. Our demonstration that satellite RNAs are transcribed adds a new family of non-coding RNAs. This is a field that requires further investigation and will provide a deeper understanding of gene expression and control.

## 1. Introduction

DNA tandem repeats (satellites) are present in most eukaryotic species, but their amount and composition vary significantly, even in closely related species. Centromere and telomere repeats have been studied in great detail [1]. These repeats are frequently expressed as RNA transcripts [2], although the role of such RNAs is poorly understood. A thorough study of repeat transcription in the pericentric heterochromatin of *Drosophila* has been recently published [3]; previous studies in *Drosophila* have been reviewed by different authors [4,5]. In the case of human centromeric satellites, it appears that α-satellite RNA transcripts are involved in centromere–nucleolus interactions [6]. Transcription of telomeric satellites has also been described [7]. A few other repetitive sequences have also been found to be transcribed, such as mammalian Alu sequences [8]. However, there is no detailed study on the transcription of the interspersed satellites found in many species. Therefore, we decided to study the abundant satellites in two species for which RNA-seq data are available: the free-living model nematode *C. elegans* and the bacterium *B. coagulans*. We have analyzed these satellites in available RNA-seq results [9,10,11] and found that they are widely transcribed. Our results add a new group of RNA molecules that might play a role in RNA interference.

## 2. Materials and Methods

We first determined the distribution of satellites and their families in an updated genome sequence of *C. elegans* [12]. We used the methodology described in detail elsewhere [13]. A complete list of satellites and their families is given in the Supplementary Materials (Tables S1–S4). Each family is formed by satellites with the same repeat length and a similar sequence; characterized by three values: Fam_a_b_c. The order in the list of families is given by a, starting with those families with the largest number of members. The second value, b, gives the size of the repeat; c gives the number of members in the family.

We have next aligned the consensus repeat of the main *C. elegans* satellite families with the RNA-seq data [9,10], using the Blastn facility in the SRA-NCBI website [14]. Sequence Read Archive (SRA) is the largest publicly available repository of high throughput sequencing data. As a query, we used two repeats for repeat lengths over 30 nucleotides (nt), and three repeats for shorter lengths; six repeats were used for the telomere repeat Fam_1_12_169. Five hundred hits with the highest identity score were collected and filtered by the percentage of sequence similarity. Each hit provides a read sequence (called spot) which contains a few repeats of the satellites. The number of repeats is limited by the short length of the RNA-seq spots, a maximum of 140 nt in this case. The RNA-seq data published by Kaletsky et al. [9] have several libraries from different replicate experiments carried out with four tissues of *C. elegans*. For our study, we have chosen two replicates for each tissue, three for neurons, as described in the results section.

For *B. coagulans* we used the same procedure, with the satellite data previously reported [15] and the RNA-seq data of Qin et al. [11]. We enclose the list of *B. coagulans* satellite families in Supplementary Table S5.

## 3. Results

### 3.1. Caenorhabditis Elegans

We performed our search for the expression of satellites as described in the previous section. The results obtained are presented in Table 1. In the upper half of Table 1, we compare the results available in different tissues, using the second-largest satellite family found in the *C*. *elegans* genome: Fam_2_35_166. This family has 166 satellites distributed throughout the genome, although it is absent in the X chromosome. This absence suggests a specific function for this family of satellites. Its consensus repeat length of 35 nt is: AAtTgAAAATTTCCGGCAAATCGGCAAaTTGCCGg. The satellites in this family have a highly variable length (4–214 repeats), with an average length of 15.4 repeats. From the results shown in Table 1, it is clear that these satellites are expressed in all tissues, but their expression appears to be more extensive in neurons.

We studied in detail the actual sequence of individual spots in the RNA-seq results. A few examples are given in Supplementary Table S6. We find that most individual spots cover a continuous fragment of satellite repeats, which clearly shows that either multiple repeats or whole satellites are simultaneously expressed; however, each spot covers only a few repeats of a satellite, a maximum of four in this case, since the RNA-seq data have a maximum length of 140 nt. It is equally possible that tandem repeats are expressed as a log RNA transcript including neighboring regions of the genome.

In Table 1 we present the results of a search for the presence of the consensus repeat of *C. elegans* satellite families in a selection of RNA-seq experiments. The table has two parts: in the upper half we compare the expression of a single satellite family in different tissues; in the lower half we compare the expression of different satellites in a single neurons_3 library. The sequence of the consensus repeat of all families is given in Supplementary Table S4. The search was carried out with BLASTN in the SRA-NCBI site, as described in the methods section. In each case we only retrieved the five hundred hits with the highest similarity score; the number of hits column represents the number of cases above the indicated percentage of sequence identity. Most searches were carried out with the RNA-seq files obtained by Kaletski et al. [9]. Two additional searches were carried out with the data of Miki et al. [10]; practically identical values were obtained. For comparison, we also carried out a search for a transfer RNA gene (Wormbase: ZK970.t1). This gene has a length of 72 nt, practically identical to two repeats of the consensus sequence of the 2_35_166 family.

We also compared different satellite families, as shown in the lower half of Table 1; we find that most satellites are clearly expressed. These results should be analyzed with care since they are strongly influenced by the number of satellites in each family and by the variability of individual repeats in a satellite. For example, the consensus repeat of Fam_14_43_26 has five variable bases in its consensus repeat (Supplementary Table S4), so that it is statistically unlikely that a spot sequence coincides over 95% with the consensus sequence.

Once we demonstrated that satellites are transcribed as non-coding RNA molecules, we searched the Rfam database [16] to determine if these RNA molecules had been previously described. The Rfam database is a collection of all non-coding RNAs previously described, grouped in families and including miRNA and other small RNA families. We searched the database with the consensus sequence of satellite Fam_2_35_166. We found a partial sequence correspondence in 65 RNAs, described as unclassified non-coding RNAs. These RNAs had a small size of 50–200 nt, none of them contained a long string of repeats. In summary, we conclude that tandem repeat RNAs have not yet been described and introduced in the Rfam database.

Non-coding RNA linc-95 is the only related case that has been thoroughly described for *C. elegans* in the Rfam database: it has a length of 784 nt, transcribed from chromosome III: 3,633,005–3,635,788. This RNA contains a sequence of four imperfect satellite repeats with a length of 35–43 nt each. This observation shows that the satellite repeat sequence is also found in a modified form in other locations of the genome. It is not clear which is the relation of these imperfect repeats with the satellite RNAs we have described.

### 3.2. Bacillus coagulans

In this case, we used the satellite families previously described [15]. An intriguing feature of satellites in bacteria is their absence in most species. Only a few species do contain satellites, usually with a variable sequence and a constant repeat length of 52 nt [15]. The sequence of the consensus repeat of all satellite families in *B. coagulans* is given in Supplementary Table S5. We determined their expression with the RNA-seq data of Qin et al. [11]. These authors studied lactate fermentation in bacterial cultures in the presence of either Na or Ca lactate. The results obtained are presented in Table 2. It is clear that under all conditions a substantial expression of satellite DNA is observed, although expression varies in different conditions; in the presence of Ca lactate a lower expression is observed. Expression is observed for all satellites, even in those cases in which there is a single satellite in the strain 2–6 used in these experiments. Further work is required to determine if the differences in satellite expression are correlated with the differences in gene expression observed [11].

In Table 2 we present the results of a search for the presence of the consensus repeat of *B.coagulans* satellite families in published RNA-seq results [11]. The search was carried out with BLASTN in the SRA-NCBI site, as described in the methods section. Five hundred hits were retrieved in each case; the number of hits columns gives the number of cases above 80% sequence identity. The maximum length of the RNA-seq data is 110 nt in this case, so that a maximum of two satellite repeats can be present in each spot. The number of satellites row gives the number of satellites present in the 2–6 strain used by Qin et al. [11].

## 4. Discussion

Our results are limited by the short length of the RNA-seq spots (140 nt in *C. elegans*). Most of the spots we have analyzed coincide in sequence with several repeats of a satellite, which demonstrate that satellite DNAs are transcribed as long fragments; they may cover a whole satellite or at least several repeats. Some examples are given in Table S6. We have recently discussed the eventual function of these transcribed DNA satellites in *B. coagulans* [17], so that here we will concentrate on *C. elegans* which has many similarities, with the expected differences between bacteria and eukaryotes. We should first note that satellite repeats possess extreme diversity in their length, monomer size, nucleotide sequence, complexity, genomic distribution, and abundance even in closely related species [5]. The different *Caenorhabditis* species are a good example; each of them has a unique distribution of abundant satellites [13].

**Figure 1:**
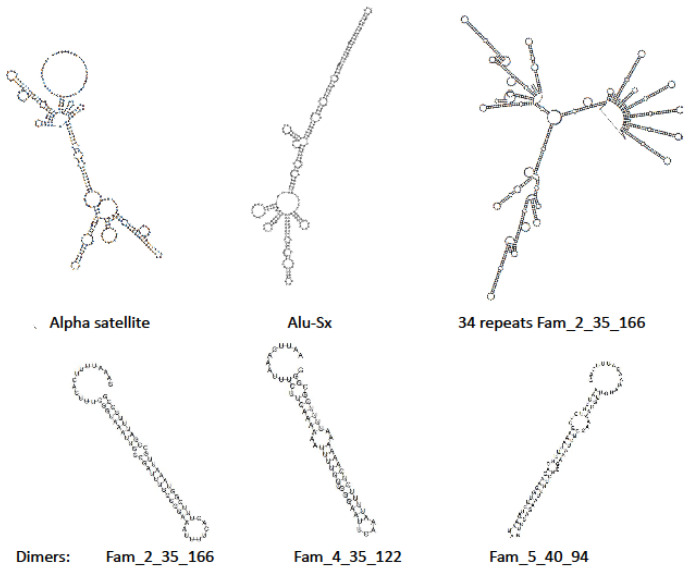
Predicted 2D structure of satellite RNAs. In the upper row, we present the structure of a single repeat of human α satellite (NCBI code: DAAF01000002.1), one Alu sequence, and 34 repeats of one *C. elegans* satellite. In that case, the 34 repeats are not identical, they present minor variations. In the lower row we present the structure of two repeats of three different *C. elegans* satellites; all of them have an approximate duplex conformation, similar to the structures found in micro and short RNAs^,^ as discussed in the text.

In order to find a role for transcribed DNA satellites, we show in Figure 1 the conformation of different satellite RNAs, predicted with RNA-fold [18], which may provide a clue of their eventual function. The different types of satellites give rise to similar structures, with many double-stranded RNA branches. Once transcribed, satellite RNA may remain as such in the cell or be degraded into small duplexes by specific ribonucleases [19]; they may have a function as either micro or short RNAs. Small non-coding RNAs exert their regulatory function by directly base pairing with mRNA targets to alter their stability and/or affect their translation [20]. Different classes of these RNAs have been described in *C. elegans* [21,22,23]. The size of the duplex branches apparent in Figure 1 is indeed similar to that found in many short RNAs [21,24]. Short RNAs act in a complex with Argonaute proteins and regulate gene expression by recognizing complementary RNA targets. Three classes of small non-coding RNAs involved in RNA interference include short interfering RNAs (siRNAs), microRNAs (miRNAs), and PIWI-interacting RNAs (piRNAs). These RNAs differ in the mechanism of their biogenesis and function [25]. These processes are collectively called RNA interference.

Alternatively, whole satellite RNAs may act as a sponge, as described in circular RNAs [26,27], trapping either microRNA or Argonaute and other proteins with an affinity for RNA, and thus play a role in the control of transcription. A long satellite RNA, similar to the one represented in 2D in Figure 1, will have a complex 3D structure; it will have many exposed sites suitable for a specific interaction with proteins and different kinds of RNA.

It has also been suggested that RNA, along with RNA-binding proteins, might be mediating chromatin organization [28]. Long satellite RNAs will form complex secondary structures that provide unique domains for interaction with specific proteins and other RNA molecules. A single satellite RNA may act as an RNA scaffold either by interacting with multiple copies of the same protein or several different proteins at once. Satellite RNA associated with chromatin modifier proteins may contribute to stabilize and control chromosome structure.

## 5. Conclusions

Our results demonstrate for the first time that interspersed DNA satellites are transcribed in different tissues. DNA satellites can no longer be considered a useless feature of the genome. They may be transcribed as small RNAs and play a role in RNA interference. Alternatively, they may have a structural role or act as a sponge to trap other RNAs and proteins. To find out the exact mode of action of these non-coding RNAs, further experimental studies are required; new bioinformatics tools have to be developed, given the repetitive nature of satellite RNAs.

As noted many years ago by Mattick and collaborators [29], the genomes of all studied eukaryotes are almost entirely transcribed, generating an enormous number of non-coding RNAs. Our demonstration that satellite DNAs are transcribed adds a new family of non-coding RNAs. The eukaryotic genome may indeed be considered an RNA machine.

## Figures and Tables

**Table 1:** Transcription of satellites in *Caenorhabditis elegans*.

Experiment	Average Spot Length	Bases (Gb)	Library Name	Satellite Family	Number of Hits	
					**85%**	**95%**
SRX4314529	139	34.44	hypodermis_1	2_35_166	494	44
SRX4314521	85	33.14	hypodermis_7		500	157
SRX4314518	115	22.00	intestine_2		500	107
SRX4314515	117	24.28	intestine_3		500	134
SRX4314514	103	31.28	neurons_1		500	233
SRX4314512	113	28.26	neurons_3		500	402
SRX4314519	115	37.93	neurons_4		500	315
SRX4314505	117	22.97	muscle_6		495	130
SRX4314522	112	25.57	muscle_1		494	85
		**Average values**				
		24.3	muscle		494	107
		33.2	neurons		499	317
		23.1	intestine		500	120
		33.7	hypodermis		497	101
**Comparison of satellite families**						
SRX4314512	113	28.26	neurons_3	1_12_169	500	364
SRX4314512	113	28.26	neurons_3	2_35_166	500	402
SRX4314512	113	28.26	neurons_3	4_35_122	500	73
SRX4314512	113	28.26	neurons_3	5_40_94	317	10
SRX4314512	113	28.26	neurons_3	9_20_48	500	441
SRX4314512	113	28.26	neurons_3	10_25_41	500	143
SRX4314512	113	28.26	neurons_3	11_45_30	289	8
SRX4314512	113	28.26	neurons_3	12_20_29	74	3
SRX4314512	113	28.26	neurons_3	13_31_27	324	49
SRX4314512	113	28.26	neurons_3	14_43_26	500	0
SRX4314512	113	28.26	neurons_3	15_26_22	500	174
SRX4314512	113	28.26	neurons_3	22_59_13	194	3
SRX4314512	113	28.26	neurons_3	24_32_11	500	330
SRX4314512	113	28.26	neurons_3	Transfer RNA	500	330
**Results obtained by Miki et al.** [10]						
SRX3104615	51	4.5	Whole worms	2_35_166	500	138
SRX2737099	100	3.8	Whole body	2_35_166	496	119

**Table 2:** Transcription of satellites in *B. coagulans*.

Conditions	SRX Code	Number of Hits in Each Repeat Family			
		1_52_139	2_52_35	8_52_18	360_52_1
No stress	700697	500	482	399	341
Ca lactate	700698	500	142	203	290
Na lactate	700710	500	500	498	499
Number of satellites		9	4	1	1

## Data Availability

The sequences of all tandem repeats and a list of all tandem repeat families and their members are available in the Supplementary Materials.

## Supplementary Materials

The following are available online at https://www.mdpi.com/article/10.3390/genes12111651/s1, Table S1: A list of all satellites in *C. elegans*, Table S2: Sequence of all satellites in *C. elegans*, Table S3: Alignment of satellites in families, Table S4: Satellite families in *C. elegans*, Table S5: Sequence of main satellite families in *B. coagulans*, Table S6: Example of perfect RNA-seq hits.
