# Supplementary material for: DNA Satellites Are Transcribed as Part of the Non-Coding Genome in Eukaryotes and Bacteria
Source: Genes (Basel). 2021 Oct 20;12(11):1651. doi: 10.3390/genes12111651 (PMC8625621; doi:10.3390/genes12111651)
Supplement: Supplementary file 1 [file Table S2. Sequence of all satellites in C.elegans.pdf › Table S2. Sequence of all satellites in C.elegans.pdf]

Table S2. Sequence of all satellites in *C.elegans*

>Cele-UNSB01_1:1-2668 Satlength=2668 Nr of Repeats=204 RepeatLength=12
seed=CTAAGCCTAA
CTAAGCCTAAAGC
CTAAGCCTAAGC
CTAAGCCTAAGC
CTAAGCCTAAGC
CTAAGCCTAAGC
CTAAGCCTAAGC
CTAAGCCTAAGC
CTAAGCCTAAGC
CTAAGCCTAAGC
CTAAGCCTAAGC
CTAAGCCTAAGC
CTAAGCCTAAGC
CTAAGCCTAAGC
CTAAGCCTAAGC
CTAAGCCTAAGC
CTAAGCCTAAGC
CTAAGCCTAAGGC
CTAAGCCTAAGC
CTAAGCCTAAGC
CTAAGCCTAAGC
CTAAGCCTAAGC
CTAAGCCTAAGCGC
CTAAGCCTAAGCTAG
CTAAGCCTAAG
CTAAGCTAAGC
CTAAGCCTAAGC
CTAAGCTAAGC
CTAAGCCTAAGC
CTAAGCCTAAGC
CTAAGCCTAAGC
CTAAGCCTAAGC
CTAAGCCTAAGC
CTAAGCCTAAGC
CTAAGCCTAAGC
CTAAGCCTAAGC
CTAAGGCCTAAGC
CTAAGCCTAAGC
CTAAGCCTAAGC
CTAAGCCTAAGC
CTAAGCCTAAGC
CTAAGCCTAAGC
CTAAGCCTAAGC
CTAAGCCTAAGC
CTAAGCCTAAGC
CTAAGCCTAAGC
CTAAGCCTAAGC
CTAAGCCTAAGCC
CTAAGCCTAAGC
CTAAGCCTAAGC

CTAAGCCTAAGC
CTAAGCCTAAGC
CTAAGCCTAAGC
CTAAGCCTAAGC
CTAAGCCTAAGC
CTAAGCCTAAGC
CTAAGCCTAAGCC
CTAAGCCTAAGC
CTAAGCCTAAGC
CTAAGCCTAAGC
CTAAGCCTAAGC
CTAAGCCTAAGC
CTAAGCCTAAGC
CTAAGCCTAAGCGC
CTAAGCCTAAGCCTG
TAAGCCTAAGCCTAAGCTGC
CTAAGCCTAAGCCTAAG
CTAGCCTAAGC
CTAAGCCTAAG
CTAAGCCTAAGC
CTAAGCCTAAGCCTAG
CTAAGCCTAAGC
CTAAGCCTAAGC
CTAAGCCTAAGC
CTAAGCCTAAGC
CTAAGCCTAAGC
CTAAGCCTAAGC
CTAAGCCTAAGC
CTAAGCCTAAGC
CTAAGCCTAAGC
CTAAGCCTAAGC
CTAAGCCTAAGC
CTAAGCCTAAGC
CTAAGCCTAAGC
CTAAGCCTAAGC
CTAAGCCTAAGC
CTAAGCCTAAGC
CTAAAGCCTAAGC
CTAAGCCTAAGC
CTAAGCCTAAGC
CTAAGCCTAAGC
CTAAGCCTAAGC
CTAAGCCTAAGC
CTAAGCCTAAGC
CTAAGCCCTAAGC
CTAAGCCTAAGC
CTAAGCCTAAGC
CTAAGCCTAAGC
CTAAGCCTAAGC
CTAAGCCTAAGC
CTAAGCCTAAGC
CTAAGCCTAAGC
CTAAGCCTAAGC
CTAAGCCTAAGC

CTAAGCCTAAGC
CTAAGCCCTAAGCCTAAGCTAG
CTAGCCTAAGC
CTAAGCCTAAGC
CTAAGCCTAAGC
CTAAGCCTAAGC
CTAAGCCTAAGC
CTAAGCCTAAGC
CTAAGCCTAAGC
CTAAGCCTAAGC
CTAAGCCTAAGC
CTAAGCCTAAGC
CTAAGCCTAAGC
CTAAGCCTAAGC
CTAAGCCTAAGC
CTAAGCCTAAGC
CTAAGCCTAAGC
CTAAGCCTAAGC
CTAAGCCTAAGC
CTAAGCCTAAGC
CTAAGCCTAAGC
CTAAGCCTAAGC
CTAAGCCTAAGC
CTAAGCCTAAGC
CTAAGCCTAAGC
CTAAGCCTAAGC
CTAAGCCTAAGCC
CTAAGCCTAAGC
CTAAGCCTAAGC
CTAAGCCTAAGC
CTAAGCCTAAGC
CTAAGCCTAAGC
CTAAGCCTAAGC
CTAAGCCTAAGC
CTAAGCCTAAGC
CTAAGCCTAAGC
CTAAGCCTAAGCC
CTAAGCCTAAGC
CTAAGCCTAAGC
CTAAGCCTAAGC
CTAAGCCTAAGC
CTAAGCCTAAGC
CTAAGCCTAAGC
CTAAGCCTAAGC
CTAAGCCTAAGC
CTAAGCCTAAGC
CTAAGCCTAAGC
CTAAGCCTAAGC
CTAAGCCTAAGC
CTAAGCCTAAGC
CTAAGCCTAAGC
CTAAGCCTAAGC
CTAAGCCTAAGC
CTAAGCCTAAGC

CTAAGCCTAAGC
CTAAGCCTAAGC
CTAAGCCTAAGC
CTAAGCCTAAGC
CTAAGCCTAAGC
CTAAGCCTAAGC
CTAAGCCTAAGC
CTAAGCCTAAGC
CTAAGCCTAAGC
CTAAAGCCTAAGC
CTAAGCCTAAGC
CTAAGCCTAAGC
CTAAGCCTAAGC
CTAAGCCTAAGC
CTAAGCCTAAGC
CTAAGCCTAAGC
CTAAGCCTAAG
CTAAGCCTAAGC
CTAAGCCTAAGC
CTAAGCCTAAGC
CTAAGCCTAAG
CTAAGCCTAAGC
CTAAGCCTAAGC
CTAAGCCTAAGC
CTAAGCCTAAGC
CTAAGCCTAAGC
CTAAGCCTAAGC
CTAAGCCTAAGCCTAAAAAATTGAGATAAGAAAACATTTTACTTTTTCAAAATTGTTTTCATGCTAAATTCAAAACGTT
TTTTTTTTAGTGAAGCTTCTAGATATTTGGCGGGTACCTCTAATTTTGCCTGCCTGCCAACCTATATGCTCCTGTGTTT
AGGCCTAATA
CTAAGCCTAAGC
CTAAGCCTAATA
CTAAGCCTAAGC
CTAAGACTAAGCCTAATA
CTAAGCCTAAGC
CTAAGACTAAGC
CTAAGACTAAGC
CTAAGACTAAGCCTAATA
CTAAGCCTAAGC
CTAAGACTAAGC
CTAAGCCTAATA
CTAAGCCTAAGC
CTAAGACTAAGCCTAATA
CTAAGCCTAAGC
CTAAGACTAAGC
CTAAGACTAAGC
CTAAGACTAAGCCTAATA
CTAAGCCTAAGC
CTAAGACTAAGC
Nr of selected repeats 173 Similarity 0.987409
Consensus:
CTAAGCCTAAGC
>Cele-UNSB01_1:224582-224687 Satlength=106 Nr of Repeats=5 RepeatLength=15
seed=AGACCCATCG

AGACCCATCGTGGCGAGACATATCGTGGTGATATCCTTCGTGGCG
AGACCCATCGTGGTG
AGACCCATCGTGGCG
AGACCCATCGTGGTG
AGACCCATCGTGGTA
Nr of selected repeats 4 Similarity 0.911111
Consensus:
AGACCCATCGTGGtg
>Cele-UNSB01_1:282371-283239 Satlength=869 Nr of Repeats=23 RepeatLength=38
seed=AAATTTTAAT
AAATTTTAATTTTTTGAAAATATTTTGGCGGGAATTTA
AAATTTTAATTTTTTGAAAATATTTTGGCGGGAATTTA
AAATTTTAATTTTTTGAAAATATTTTGGCGGGAATTTA
AAATTTTAATTTTTTGAAAATATTTTGGCGGGAATTC
AAATTTTAATTTTTTGAAAATATTTTGGCGGGAATTTA
AAATTTTAATTTTTTGAAAATATTTTGACGGGAATTTA
AAATTTTAATTTTCTGAAAATATTTTGGCGGGAATTC
AAATTTTAATTTTTTGAAAATATTTTGGCGGGAATTC
AAATTTTAATTTTTTGAAAATATTTTGGCGGGAATTTA
AAATTTTAATTTTTTGAAAATATTTTGGCGGGAATTTA
AAATTTTAATTTTTTGAAAATATTTTGGCGGGAATTTA
AAATTTTAATTTTTTGAAAATATTTTGGCGGGAATTTA
AAATTTTAATTTTTTGAAAATATTTTGGCGGGAATTTA
AAATTTTAATTTTTTGAAAATATTTTGGCGGGAATTTA
AAATTTTAATTTTTTGAAAATATTTTGGCGGGAATTC
AAATTTTAATTTTTTGAAAATATTTTGGCGGGAATTTA
AAATTTTAATTTTTTGAAAATATTTTGGCGGGAATTTA
AAATTTTAATTTTTTGAAAATATTTTGGCGGGAATTTA
AAATTTTAATTTTTTGAAAATATTTTGGCGGGAATTTA
AAATTTTAATTTTTTGAAAATATTTTGGCGGGAATTC
AAATTTTAATTTTTTGAAAATATTTTGGCGGGAATTTA
AAATTTTAATTTTCTGAAAATATTTTGGCGGGAATTC
AAATTTTAATTTTTTGAAAATATTTTGGCGGGAATTTA
Nr of selected repeats 17 Similarity 0.995872
Consensus:
AAATTTTAATTTTTTGAAAATATTTTGGCGGGAATTTA
>Cele-UNSB01_1:283314-284124 Satlength=811 Nr of Repeats=52 RepeatLength=15
seed=TCGTGGTGAG
TCGTGGTGAGACCCT
TCGTGGTGAGACCCA
TCGTGGTGACACCCA
TCGTGGTGAGACCCT
TCGTAGTGAGACCCT
TCGTGGTGAGACCTT
TCGTGGTGAGACCCA
TCGTGGTGAGACCCA
TCGTGGTGAGACTCA
TCGTGGTGAGACCCT
TCGTGGTGATACCCA
TCGTGGTGAGACCTT
TCGTGGTGAGACCCAC
CGTGGTGAGACCCA
TCGTGGTGAGACTCA
TCGTGGTGAGACCCT

TCGTGGTGAGACCCA
TCGTGGTGAGACCTT
TCGTGGTGAGACCTT
TCGTGGTGAGACCCA
TCGTGGTGAGACCTT
TCGTGGTGAGACCTT
TCGTGGTGAGACCTT
TCGTGGTGAGACCCA
TCGTGGTGAGACCCT
TCGTGGTGAGACCCA
TCGTGGTGAGACCTT
TCGTGGTGAGACCCA
TCGTGGTGAGACCCA
TCGTGGTGAGACCCA
TCGTGGTGAGACCCA
TCGTGGTGAGACCCTTCATGGTTAGACCCA
TCGTGGTGAGACCCT
TCGTGCTGAGACCATTCATGGAGAGACCCA
TCGTGGTGAGACCCT
TCGTGGTTAGACCCA
TCGTGGTGAGATCTT
TCGTGGTGAGACCCA
TCGTGGTGAGACTCA
TCGTGGTGAGACCCT
TCGTGGGGAGACTCT
TCGTGGTGAGACCCT
TCGTGGTTAGACCCA
TCGTGGTTAGACCCA
TCGTGGTGAGATCTT
TCGTGGTGAGCCCCA
TCGTGGTGAGACCCA
TCGTGGTGAGACTTT
TCGTGGTGAGACCCA
TCGTGGTGAGACTTT
TCGTGGTGAGACCCA
TCGTGGTGAGACCTT
Nr of selected repeats 48 Similarity 0.862569
Consensus:
TCGTGGTGAGACCca
>Cele-UNSB01_1:309874-309994 Satlength=121 Nr of Repeats=7 RepeatLength=15
seed=GAGACCCATC
GAGACCCATCGTGGC
GAGACCCATCGTGGC
GAGACCCATCGTGAC
GAGACCCATCGTGACGAGGCCTATCGTGGT
GAGACCCATCGTGGC
GAGACCCTCCGTTGA
GAGACCCATCGTGAT
Nr of selected repeats 6 Similarity 0.764583
Consensus:
GAGACCCATCGTGac
>Cele-UNSB01_1:336527-337247 Satlength=721 Nr of Repeats=18 RepeatLength=40
seed=AATTTTCTCG
AATTTTCTCGAAATTTTCAGAAGGTTCTAGAATATTTCAG

AATTTTCTCGAAATTTCCAAAAGGTTCTAGAACATTTCAG
AATTTTCTCGAAATTTTCAGAAGGTTCTAGAACATTCCAG
AATTTTCTCGAAATTTTCAGAAGGTTCTAGAATAGTTCAG
AATTTTCTCGAAATTTCCAAAAGGTTCTAGAACATTACAT
AATTTTCTCGAAATTTCCAGAAGGTTCTAGAACATTCCAG
AATTTTCTCGAAATTTTCAGAAGGTTCTAGAATATTTCAG
AATTTTCTCGAAATTTCCAAAAGGTTCTAGAACATTACAG
AATTTTCTCGAAATTTCCAAAAGGTTCTAGAACATTACAG
AATTTTCTCGAAATTTTCAGAAGGTTCTAGAATATTTCAG
AATTTTCTCGAAATTTCCAAAAGGTTCTAGAACATTACAG
AATTTTCTCGAAATTTCCAAAAGGTTCTAGAACATTACAG
AATTTTCTCGAAATTTTCAGAAGGTTCTAGAATATTTCAG
AATTTTCTCGAAATTTCCAAAAGGTTCTAGAACATTTCAG
AATTTTCTCGAAATTTTCAGAAGGTTCTAGAATATTTCAG
AATTTTCTCGAAATTTCCAAAAGGTTCTAGAACATTACAG
AATTTTCTCGAAATTTCCAAAAGGTTCTAGAACATTACAG
AATTTTCTCGAAATTTTCAGAAGGTTCTAGAATATTTCAG
Nr of selected repeats 18 Similarity 0.920044
Consensus:
AATTTTCTCGAAATTTcCAaAAGGTTCTAGAAcATTtCAG
>Cele-UNSB01_1:340649-342857 Satlength=2209 Nr of Repeats=12 RepeatLength=184
seed=GAATACAGAA
GAATACAGAACACCAATTATGCCCGAGAAAAAGATCCTACTCAGAATATAAACATAGTCAAATTTATCGGATGTATAAA
GATTCCCGAAGACACTTTCCAATTACCCAAATTGTTCATATTCTAAATCAAATTCTCTTACTAGAACGCTCTTGGCCAA
TGTACGCAGCCGAACGTATCATAAGT
GAATACAGAACACCAATTATGCCCGAGAAAAAGATCCTACTCAGAATATAAACATAGTCAAATTTATCGGATGTATAAA
GATTCCCGAAGACACTTTCCAATTACCCAAATTGTTCATATTCTAAATGAAATTCTTTTACTAGAACACTCTTGGCCAA
TGTACGCAGCCGAACGTATCATAAGT
GAATACAGAACACCAATTATGCCCGAGAAAAAGATCCTACTCAGAATATAAACATAGTTAAATTTATTGGATGTATAAA
GATTCCCGAAGACACTTTCCAATTACCCAAATTGTTCATATTCTAAATGAAATTCTCTTACTAGAACACTCTTGGCCAA
TGTACGCAGCCGAACGTATCATAAGT
GAATACAGAACACCAATTATGCCCGAGAAAAAGATCCTACTCAGAATATAAACATAGTCGAATTTATCGGATGTATAAA
GATTCCCGAAGACACTTTCCAATTACCCAAATTGTTCATATTCTAAATGAAATTCTCTTACTAGAACACTCTTGGCCAA
TGTACGCAGCCGAACGTATCATAAGT
GAATACAGAACACCAATTATGCCCGAGAAAAAGATCCTACTCAGAATATAAACATATTCGAATTTAACGGATGTATAAA
GATTCCCGAAGACACTTTCCAATTACCCAAATTGTTCATATTCTAAATGAAATTCTCTTACTAGAACACTCTTGGCCAA
TGTACGCAGCCGAACGTATCATAAGT
GAATACAGAACACCAATTATGCCCGGGAAAAAGATCCTACTCAGAATATAAACATATTCGAATTTATCGGATGTATAAA
GATTCCCGAAGACACTTTCCAATTACCCAAATTGTTCATATTCTAAATGAAATTCTCTTACTAGAACACTCTTGGCCAA
TGTACGCAGCCGAACGTATCATAAGT
GAATACAGAACACCAATTATGCCCGAGAAAAAGATCCTACTCAGAATATAAACATAGTCAAATTTATCGGATGTATAAA
GATTCCCGAAGACACTTTCCAATTACCCAAATTGTTCATATTCTAAATGAAATTCTCTTACTAGAACACTCTTGGCCAA
TGTACGCAGCCTAACGTATCATAAGT
GAATACAGAACACCAATTATGCCCGAGAAAAAGATCCAACTCAGAATAAAAACATATTCGAATTTACCGGATGTATAAA
GATTCCCGAAGACACTTTCCAATTACCCAAATTGTTCATATTCTGAATGAAATTCTCTTACTAGAACACTCTTGGCCAA
TGTACGCAGCCGAACGTATCATAAGT
GAATACAGAACACCAATTATGCCCGAGAAAAAGATCCTACTCAGAATATAAACATAGTCGAATTTATCGAATGTATAAA
GATTCCCGAAGACACTTTCCAATTACCCAAATTGTTCATATTCTAAATGAAATTCTCTTACTAGAACACTCTTGGCCAA
TGTACGCAGCCGAACGTATCATAAGT
GAATACAGAACACCAATTATGCCCGAGAAAAAGATCCTACTCAGAATATAAACATATTCGAATTTAACGGATGTATAAA
GATTCCCGAAGACACTTTCCAATTACCCAAATTGTTCATATTCTAAATGAAATTCTCTTACTAGAACACTCTTGGCCAA
TGTACGCAGCCGAACGTATCATAAGT

GAATACAGAACACCAATTATGCCCGGGAAAAAGATCCTACTCAGAATATAAACATATTCGAATTTATCGGATGTATAAA
GATTCCCGAAGACACTTTCCAATTACCCAAATTGTTCATATTCTAAATGAAATTCTCTTACTAGAACACTCTTGGCCAA
TGTACGCAGCCGAACGTATCATAAGT
GAATACAGAACACCAATTATGCCCGAGAAAAAGATCCAACTCAGAATATAAACATATTCGAATTTATCGGATGTATAAA
GATTCCCGAAGACACTTTCCAATTACCCAAATTGTTCATATTCTAAATGAAATTCTCTTACTAGAACACTCTTGGCCAA
TGTACGCAGCCGAACGTATCATAAGT
Nr of selected repeats 12 Similarity 0.974089
Consensus:
GAATACAGAACACCAATTATGCCCGAGAAAAAGATCCTACTCAGAATATAAACATAgTCgAATTTAtCGGATGTATAAA
GATTCCCGAAGACACTTTCCAATTACCCAAATTGTTCATATTCTAAATGAAATTCTCTTACTAGAACACTCTTGGCCAA
TGTACGCAGCCGAACGTATCATAAGT
>Cele-UNSB01_1:356425-357011 Satlength=587 Nr of Repeats=29 RepeatLength=19
seed=TATCGATTTT
TATCGATTTTTATG
TATCGAATTTACTGGAAAACTATCAAAAGTTTAGGAAAAA
TATCGATTTTCCTGAAATT
TATCGATTTTCCTGAAATT
TATCGATTTTCCTGAAATT
TATCGATTTTCCTGAAATT
TATCGATTTTTCTGGAATT
TATCGATTTTTCTGGAATT
TATCGATTTTCCTGAAATT
TATCAATTTTTCTGGAATT
TATCGATTTTTCTGGAATT
TATCGATTTTTCTGGAATT
TATCGATTTTTCTGGAATT
TATCGATTTTTCTGGAATT
TATCGATTTTTCTGGAATA
TATCGATTTTTCTGGAATT
TATCGATTTTTCTGGAATT
TATCGATTTTTCTGGAATT
TATCGATTTTTCTGGAATA
TATCGATTTTCCTGAAATT
TATCGATTTTTCTGGAATT
TATCGATTTTCCTGAAATT
TATCAATTTTTCTGGAATT
TATCGATTTTTCTGGAATT
TATCGATTTTTCTGGAATT
TATCGATTTTTCTGGAATT
TATCGATTTTTCTGGAATA
TATCGATTTTTCTGGAATTC
ATCGATTTTTCTGGAATTCATCGAGTTTTCCGAGATT
Nr of selected repeats 25 Similarity 0.914854
Consensus:
TATCGATTTTtCTGgAATT
>Cele-UNSB01_1:360798-360950 Satlength=153 Nr of Repeats=8 RepeatLength=19
seed=GTACTACCTT
GTACTACCTTTTAATGTAT
GTACTACCTTTTAATGTAT
GTACTACCTTTTAATGTAT
GTACTACCTTTTAATGTAA
GTACTACCTTTTAATGTAT
GTACTACCTTTTAATGTAT
GTACTACCTTTTAATGTAT

GTACTACCTTTTAATGTAT
Nr of selected repeats 8 Similarity 0.982456
Consensus:
GTACTACCTTTTAATGTAT
>Cele-UNSB01_1:479600-479769 Satlength=170 Nr of Repeats=13 RepeatLength=12
seed=TAGGCTTAGG
TAGGCTTAGGTT
TAGGCCTAGGCT
TAGGCTTAGGCTA
AGGCTTAGGCT
TAGGCGTAGGCT
TAGGCATAGGCG
TAGGCTTTAGGTTTAGGCTAAAGCG
TAGGCTTAGGTT
TAGGCCTAGGCT
TAGGCTTAGGCTA
AGGCTTAGGCT
TAGGCGTAGGCT
TAGGCGTAGGCG
Nr of selected repeats 8 Similarity 0.813492
Consensus:
TAGGCnTAGGct
>Cele-UNSB01_1:558852-559000 Satlength=149 Nr of Repeats=8 RepeatLength=12
seed=CTAAGCCTAA
CTAAGCCTAAGC
CTAAGCCTAACCTAAATCGCGTCAGAGATAACGTTCGCCACTGACGCCAAGCCTCAAC
CTAAGCCTAAGC
CTAAGCCTAACA
CTAAGCCTAAGTA
TAAGCCTAAATCTAGGC
CTCAGCCTAATA
CTAAGCCTAAGC
Nr of selected repeats 5 Similarity 0.811111
Consensus:
CTaAGCCTAAgc
>Cele-UNSB01_1:564264-564319 Satlength=56 Nr of Repeats=5 RepeatLength=11
seed=GTACTCCTAC
GTACTCCTACC
GTACTCCTAAA
GTACTCCTACC
GTACTCCTACA
GTACTCCGACC
Nr of selected repeats 5 Similarity 0.830303
Consensus:
GTACTCCtAcc
>Cele-UNSB01_1:567454-567526 Satlength=73 Nr of Repeats=6 RepeatLength=12
seed=GCGTTGCGTG
GCGTTGCGTGCT
GCGTTGCGTGCC
GCGTTGCGTGCC
GCGTTGCGTGCT
GCGTTGCGGGCT
GCGTTGCGTGCC
Nr of selected repeats 6 Similarity 0.896296

Consensus:
GCGTTGCGTGCc
>Cele-UNSB01_1:568805-568943 Satlength=139 Nr of Repeats=8 RepeatLength=12
seed=AGGCTTAGGC
AGGCTTAGGCTTAGGTCTAGACTTAAGGCTACACTTAAATTT
AGGCCTAGGCTT
AGGCTTAGGCTT
AGGCTTAGGCTG
AGGCTTAGGCTG
AGGCTTAGGCTTATGCTC
AGACTTAGGCTT
AGGCTTACGCTTAGGCTT
Nr of selected repeats 5 Similarity 0.844444
Consensus:
AGgCtTAGGCTt
>Cele-UNSB01_1:625093-626033 Satlength=941 Nr of Repeats=22 RepeatLength=23
seed=AAAAAAAATT
AAAAAAAATTAGAGTTTGCGCGTCAAATATGATGTATGACCGGCAGTGTTTGCACACACATAGCCTAGACACGATAAAA
TACATCAGATTTTACGCGCAAACTGGC
AAAAAAATTGATTTTTTGGCTTTTTCGGACTAAACATTAGAAAAAAAGACTATACTCCAACAATTTTTTTCAGTTTTTT
TTTGAAAAATTCCATATTTTCTGAAGTTCAAATAAATATATATTTTTTTAAAGTTCAAAAAATTCAAATTAGCGCGTTA
AAAACGACGTATTCAGGCATCACATTTGACGCGCAATTTTTTTTTTAAAGAAGGATTTGCGCGTCAAATCTGGCGAATT
AGGTTTGTCGGCGGAATACACCGAATTTCACGCGCAAAATTTCAAACCTCAATGATAAAATCGGAGAGCGTATTCGAGG
AAATTACGTCTTTCAACGTCTCCGCCGGAACCACGACAGCGAACAAGTTTTGTGTTGACTGA
AAAAAAAATTAAATTTTTCGAC
AAAAAAAATTAAATTTTTCAACG
AAAAAAAATTAAATTTTTCAACG
AAAAAAATTAAATTTTTCAACA
AAAAAAAATTAAATTTTTCAACA
AAAAAAAATTAAATTTTTCAACA
AAAAAAAATTTAATTTTTCAACA
AAAAAAAATTAAATTTTTCAACG
AAAAAAAATTAAATTTTTCAACG
AAAAAAATTAAATTTTTCAACA
AAAAAAAATTAAATTTTTCAACA
AAAAAAAATTAAATTTTTCAACA
AAAAAAAATTTAATTTTTCAACA
AAAAAAAATTAAATTTTCCAACA
AAAAAAAATTAAATTTTTCAACA
AAAAAAAATTAAATTTTTCAACA
AAAAAAAATTAAATTTTTCAAC
AAAAAAAATTAAATTTTTCAACA
AAAAAAAATTAAAATTTTCAACGA
AAAAAAAATAAATTTTTCATCA
Nr of selected repeats 14 Similarity 0.950948
Consensus:
AAAAAAAATTAAATTTTTCAACa
>Cele-UNSB01_1:642767-642875 Satlength=109 Nr of Repeats=6 RepeatLength=12
seed=TAAGCCTAAG
TAAGCCTAAGCC
TAAGCCTAAGACTAGGTC
TAAGCCTAAGCCTGAGCCTATGCCTCAGCCTGAGAATAAGCG
TGAGCCTAAGCG
TGAGCCTAAGTC

TAAACCTAAGCC
Nr of selected repeats 4 Similarity 0.759259
Consensus:
TaAgCCTAAGcc
>Cele-UNSB01_1:671513-680928 Satlength=9416 Nr of Repeats=294 RepeatLength=32
seed=GAATTCAAAT
GAATTCAAATTTTCTGAGAAAAATTTAGGCGA
GAATTCAAATTTTCTGAGAAAAATTTAGGCGA
GAATTCAAATTTTCAGAGAAAAAATTTTGGCGG
GAATTCAAATATTCTGAAAAAAAATTTGCCGG
GAATTCAAAATTTCTGAAAATAAATTTAGGCGA
GAATTCAAATTTTCAGAGAAAAATTTTGGCGG
GAATTCAAATTTTCTGAAAAAAATTTAGGCGA
GAATTCAAATTTTCTGAGAAAAATTTTGGCGA
GAATTCAAATTTTCAGAGAAAAAATTTTGGCGG
GAATTCAATTATTCTGAGTAAAACTTTGGCGG
GAATTCAAATTTTCTGAGAAAAATTTAGGCGA
GAATTCAAATTTTCAGAGAAAAATTTTGGCGG
GAATTCAAATTTTCTAAGAAAAATTTTGGCGG
GAATTCAAATTTTCTGAGTAAAACTTTGGCGG
GAATTCAAATTTTCTGAGAAAAATTTAGGCGA
GAATTCAAATTTTCAGAGAAAAAATTTTGGCGG
GAATTCAAATATTCTGAAAAAAAATTTGCCGG
GAATTCAAATTTTCTGAAAATAAATTTAGGCGA
GAATTCAAATTTTCAGAGAAAAATTTTGGCGG
GAATTCAAATTTTCTAAGAAAAATTTTGGCGG
GAATTCAAATTTTCTGAGAAAAATTTGGCGG
GAATTCAAATTTTCTGAGAAAAATTTAGGCGA
GAATTCAAATTTTCAGAGAAAAATTTTGGCGG
GAATTCAAATTTTCTGAAAAAAATTTGGCG
GAATTCAAATTTCGAAAAAATTTGCG
GAATTCAAATTTCTGAAAAAAATTTAGGCGA
GAATTCAAATTTTCAGAGAAAAATTTTGGCGG
GAATTCAAATTTTCTGAAAAAAATTTAGGCGA
GAATTCAAATTTTCAGAGAAAAATTTTGGCGG
GAATTCAAATTTTCTGAGAAAAATTTAGGCGA
GAATTCAAATTTTCAGAGAAAAATTTTGGCGG
GAATTCAAATTTTCTGAGGAAAAATTTTGGCGG
GAATTCAAATTTTCTGAAAAAAATTTAGGCGA
GAATTCAAATTTTCAGAGAAAAATTTTGGCGG
GAATTCAAATATTCTGAAAAAAAATTTGGCGA
GAATTCAAATTTTCAGAGAAAAATTTTGGCGG
GAATTCAAATTTCTGAAAAAAATTTGGCG
GAATTCAAATTTTCAGAGAAAAATTTGGCGG
GAATTCAAATTTTCTAAGAAAAATTTTGGCGG
GAATTCAAATTTTCTGAGAAAAATTTGGCGG
GAATTCAAATTTTCAGAGAAAAATTTGGCGG
GAATTCAAATTTTCAGAGAAAAATTTTGGCGG
GAATTCAAATTTTCTAAGAAAAATTTTGGCGG
GAATTCAAATTTTCTGAGTAAAACTTTGGCGG
GAATTCAAATTTTCTGAGAAAAATTTAGGCGA
GAATTCAAATTTTCAGAGAAAAATTTTGGCGG
GAATTCAAATATTCTGAAAAAAAATTTGCGG
GAATTCAAAATTTCTGAAAATAAATTTAGGCGA

GAATTCAAATTTTCAGAGAAAAATTTTGGCGG
GAATTCAAATTTTCTAAGAAAAATTTTGGCGG
GAATTCAATTTTCTGAGAAAAATTTTGGCGG
GAATTCAAATTTTCTGAGAAAAATTTAGGCG
GAATTCAAATTTTCTGAGAAAAATTTTGGCGG
GAATTCAAATTTTCTGAAAAAAATTTTGGCGG
GAATTCAAATTTCTGAAAAAATTTGGCGG
GAATTCAAATTTCTGAAAAAAATTTGGCG
GAATTCAAATTTTCAGAGAAAAAATTTTGGCGG
GAATTCAAATTTTCTGAAAAAATTTTGGCGG
GAATTCAAATTTTCTAAGAAAAATTTTGGCGG
GAATTCAAATTTTCTGAGAAAAATTTTGGCGG
GAATTCAAATTTTCTGAGAAAAATTTTGGCGG
GAATTCAAATTTTCTGAGAAAAATTTAGGCGA
GAATTCAAATTTTCAGAGAAAAAATTTTGGCGG
GAATTCAAATATTCTGAAAAAAAATTTAGGCGA
GAATTCAAATTTTCTGAGAAAAATTTAGGCGA
GAATTCAAATTTTCAGAGAAAAATTTTGGCGG
GAATTCAAATATTCTGAAAAAAAATTTTGGCGA
GAATTCAAATTTTCAGAGAAAAATTCTGGCGG
GAATTCAAATTTTCTAAGAAAAATTTTGGCGG
GAATTCAAATTTTCTGAGTAAAATTTGGCGG
GAATTCAAATTTTCAGAGAAAAATTTAGGCGA
GAATTCAAATTTTCAGAGAAAAAATTTTGGCGG
GAATTCAAATTTTCTAAGAAAAATTTTGGCGG
GAATTCAAATTTTCTGAGAAAAATTTGGCGG
GAATTCAAATTTTCTGAGAAAAATTTGGCGA
GAATTCAAATTTTCAGAGAAAAATTTGGCGG
GAATTCAAATTTTCAGAGAAAAAATTTTGGCGG
GAATTCAAATTTCTGAAAAAAATTTAGGCGA
GAATTCAAATTTTCAGAGAAAAATTTGGCG
GAATTCAAATTTTCAGAGAAAAATTTTGGCGG
GAATTCAAATTTTCTAAGAAAAATTTTGGCGG
GAATTCAAATTTTCTGAGAAAAATTTTGGCGG
GAATTCAAATTTTCTGAGAAAAATTTTGGCGG
GAATTCAAATTTTCTGAGAAAAATTTTGGCGG
GAATTCAAATTTTCTGAGAAAAATTTTGGCGG
GAATTCAAATTTTCTGAGAAAAATTTTGGCGG
GAATTCAAATTTTCTGAAAAAATTTTGGCGG
GAATTCAAAATTTCTGAAAAAAAATTTGCCGG
GAATTCAAAATTTCTGAAAATAAATTTTGGCGG
GAATTCAAATTTTCTGAAAAAATTTTGGCGG
GAATTCAAATTTTCTGAAAAAATTTTGGCGG
GAATTCAAATTTTCTGAGAAAAATTTTGGCGG
GAATTCAAATTTTCTGAGAAAAATTTTGGCGG
GAATTCAAATTTTCTGAGAAAAATTTAGGCGA
GAATTCAAATTTTCAGAGAAAAATTTTGGCGG
GAATTCAAATATTCTGAAAAAAAATTTAGGCGA
GAATTCAAATTTTCTGAGAAAAATTTAGGCGA
GAATTCAAATTTTCAGAGAAAAATTTTGGCGG
GAATTCAAATATTCTGAAAAAAAATTTTGGCGA
GAATTCAAATTTTCAGAGAAAAATTTGGCGG
GAATTCAAATTTTCTAAGAAAAATTTTGGCGG
GAATTCAAATTTTCTGAGTAAAACTTTGGCGG

GAATTCAAATTTTCTGAGAAAAAATTTAGGCGA
GAATTCAAATTTTCAGAGAAAAAAATTTTGGCGG
GAATTCAAATATTCTGAAAAAAAAATTTGCCGG
GAATTCAAAATTTCTGAAAAAAAATTTAGGCGA
GAATTCAAATTTTCAGAGAAAAAATTTTGGCGG
GAATTCAAATTTTCTAAGAAAAAATTTTGGCGG
GAATTCAATTTTCTGAGAAAAAATTTTGGCGG
GAATTCAAATTTTCTGAGAAAAAATTTAGGCGA
GAATTCAAATTTTCAGAGAAAAAATTTTGGCGG
GAATTCAAATTTCTGAAAAAAAATTTTGGCGG
GAATTCAAATTTTCTGAAAAAAATTTTGGCGG
GAATTCAAATTTCTGAAAAAAAATTTGCGG
GAATTCAAATTTCTGAAAAAAAATTTTGGCGG
GAATTCAAATTTTCTGAAAAAAATTTTGGCGG
GAATTCAAATTTTCTGAAAAAAATTTTGGCGG
GAATTCAAATTTTCTGAGAAAAAATTTTGGCGG
GAATTCAAATTTTCTGAGAAAAAATTTTGGCGG
GAATTCAATTTTCTGAGAAATTTTGGCGGA
AATTCAAATTTTCTGAGAAAATTTTGGCGG
GAATTCAAATTTTCTGAGAAAAAATTTTGGCGG
GAATTCAAATTTTCTGAGAAAAAATTTAGGCGA
GAATTCAAATTTTCAGAGAAAAAAATTTTGGCGG
GAATTCAAATATTCTGAAAAAAAAATTTGCCGG
GAATTCAAAATTTCTGAAAATAAATTTAGGCGA
GAATTCAAATTTTCAGAGAAAAAATTTTGGCGG
GAATTCAAATTTTCAGAGAAAAAAATTTTGGCGG
GAATTCAAATTTTCTAAGAAAAAATTTTGGCGG
GAATTCAAATTTTCTAAGAAAAAATTTTGGCGG
GAATTCAAATTTTCAGAGAAAAAATTTTGGCGG
GAATTCAAATTTTCTAAGAAAAAATTTTGGCGG
GAATTCAAATTTTCTAAGAAAAAATTTTGGCGG
GAATTCACATTTTCTGAGTAAAACTTTGGCGG
GAATTCAAATTTTCTGAGAAAAAATTTAGGCGA
GAATTTAAATTTTCAGAGAAAAAAATTTTGGCGG
GAATTCAAATATTCTGAAAAAAAAATTTTGGCGA
GAATTCAAATTTTCAGAGAAAAAATTTGGCGG
GAATTCAAATTTTCTAAGAAAAAATTTTGGCGG
GAATTCACATTTTCTGAGAAAAAATTTTGGCGG
GAATTCAAATTTTCTAAGAAAAAATTTTGGCGG
GAATTCACATTTTCTGAGAAAAAATTTTGGCGG
GAATTCAAATTTTCTGAGAAAAAATTTAGGCGA
GAATTCAAATTTTCAGAGAAAAAAATTTTGGCGG
GAATTCAAATATTCTGAAAAAAAAATTTAGGCGA
GAATTCAAATTTTCAGAGAAAAAATTTAGGCGA
GAATTCAAATTTTCAGAGAAAAAAATTTTGGCGG
GAATTCAAATATTCTGAAAAAAAAATTTGCCGG
GAATTCAAAATTTCTGAAAATAAATTTAGGCGA
GAATTCAAATTTTCAGAGAAAAAATTTTGGCGG
GAATTCAAATTTTCAGAGAAAAAAATTTAGGCGA
GAATTCAAATTTTCTGAGAAAAAATTTAGGCGA
GAATTCAAATTTTCAGAGAAAAAATTTTTGGCGG
GAATTCAAATATTCTGAAAAAAAAATTTTGGCGA
GAATTCAAATTTTCAGAGAAAAAATTCTGGCGG
GAATTCAAATTTTCTAAGAAAAAATTTTGGCGG

GAATTCAAATTTTCTGAGTAAAACTTTGGCGG
GAATTCAAATTTTCTGAGAAAAATTTAGGCGA
GAATTCAAATTTTCAGAGAAAAAATTTTGGCGG
GAATTCAAATTTTCTAAGAAAAATTTTGGCGG
GAATTCACATTTTCTGAGTAAAACTTTGGCGG
GAATTCAAATTTTCTGAGAAAAATTTAGGCGA
GAATTCAAATTTTCAGAGAAAAAATTTTGGCGG
GAATTCAAATATTCTGAAAAAAAATTTGCCGG
GAATTCAAAATTTCTGAAAATAAATTTAGGCGA
GAATTCAAATTTTCAGAGAAAAATTTTGGCGG
GAATTCAAATTTTCAGAGAAAAAATTTTGGCGG
GAATTCAAATTTTCTAAGAAAAATTTTGGCGG
GAATTCAAATTTTCTAAGAAAAATTTTGGCGG
GAATTCAAATTTTCAGAGAAAAATTTTGGCGG
GAATTCAAATTTTCTAAGAAAAATTTTGGCGG
GAATTCAAATTTTCTAAGAAAAATTTTGGCGG
GAATTCACATTTTCTGAGTAAAACTTTGGCGG
GAATTCAAATTTTCTGAGAAAAATTTAGGCGA
GAATTTAAATTTTCAGAGAAAAAATTTTGGCGG
GAATTCAAATATTCTGAAAAAAAATTTTGGCGA
GAATTCAAATTTTCAGAGAAAAATTCTGGCGG
GAATTCAAATTTTCTAAGAAAAATTTTGGCGG
GAATTCAAATTTTCTGAGTAAAACTTTGGCGG
GAATTCAAATTTTCTGAGAAAAATTTAGGCGA
GAATTCAAATTTTCAGAGAAAAAATTTTGGCGG
GAATTCAAATTTTCTAAGAAAAATTTTGGCGG
GAATTCACATTTTCTGAGAAAATTTGGCGG
GAATTCAAATTTTCTGAGAAAAATTTAGGCGA
GAATTCAAATTTTCAGAGAAAAAATTTTGGCGG
GAATTCAAATATTCTGAAAAAAAATTTGCCGG
GAATTCAAAATTTCTGAAAATAAATTTAGGCGA
GAATTCAAATTTTCAGAGAAAAATTTTGGCGG
GAATTCAAATTTTCAGAGAAAAAATTTTGGCGG
GAATTCAAATTTTCTAAGAAAAATTTTGGCGG
GAATTCAAATTTTCTAAGAAAAATTTTGGCGG
GAATTCAAATTTTCAGAGAAAAATTTTGGCGG
GAATTCAAATTTTCTAAGAAAAATTTTGGCGG
GAATTCAAATTTTCTAAGAAAAATTTTGGCGG
GAATTCAAATTTTCTGAGTAAAACTTTGGCGG
GAATTCAAATTTTCTGAGAAAAATTTAGGCGA
GAATTCAAATTTTCAGAGAAAAAATTTTGGCGG
GAATTCAAATATTCTGAAAAAAAATTTGCCGG
GAATTCAAAATTTCTGAAAATAAATTTAGGCGA
GAATTCAAATTTTCAGAGAAAAATTTTGGCGG
GAATTCAAATTTTCTAAGAAAAATTTTGGCGG
GAATTCACATTTTCTGAGAAAAATTTTGGCGG
GAATTCAAATTTTCTGAGAAAAATTTAGGCGA
GAATTCAAATTTTCTGAGAAAAATTTTGGCGG
GAATTCAAAATTTCTGAAAATAAATTTTGGCGG
GAATTCAAATTTTCTGCAAAAAATTTTGGCGG
GAATTCAAATATTCTGAAAAAAAATTTGCCGG
GAATTCAAAATTTCTGAAAATAAATTTAGGCGA
GAATTCAAATTTTCAGAGAAAAATTTTGGCGG
GAATTCAAATTTTCAGAGAAAAAATTTTGGCGG

GAATTCAAATTTTCTAAGAAAAATTTTGGCGG
GAATTCAAATTTTCTAAGAAAAATTTTGGCGG
GAATTCAAATTTTCAGAGAAAAATTTTGGCGG
GAATTCAAATTTTCTAAGAAAAATTTTGGCGG
GAATTCAAATTTTCTAAGAAAAATTTTGGCGG
GAATTCAAATTTTCTGAGTAAAACTTTGGCGG
GAATTCAAATTTTCTGAGAAAAATTTAGGCGA
GAATTCAAATTTTCAGAGAAAAAATTTTGGCGG
GAATTCAAATATTCTGAAAAAAAATTTGCCGG
GAATTCAAAATTTCTGAAAATAAATTTAGGCGA
GAATTCAAATTTTCAGAGAAAAATTTTGGCGG
GAATTCAAATTTTCTAAGAAAAATTTTGGCGG
GAATTCAAATTTTCTAAGAAAAATTTTGGCGG
GAATTCAAATTTTCTGAGTAAAACTTTGGCGG
GAATTCAAATTTTCTGAGAAAAATTTAGGCGA
GAATTCAAATTTTCAGAGAAAAAATTTTGGCGG
GAATTCAAATATTCTGAAAAAAAATTTGCCGG
GAATTCAAAATTTCTGAAAATAAATTTAGGCGA
GAATTCAAATTTTCAGAGAAAAATTTTGGCGG
GAATTCAAATTTTCTAAGAAAAATTTTGGCGG
GAATTCACATTTTCTGAGAAAAATTTTGGCGG
GAATTCAAATTTTCTGAGAAAAATTTAGGCGA
GAATTCAAATTTTCTGAGAAAAATTTTGGCGG
GAATTCAAAATTTCTGAAAATAAATTTTGGCGG
GAATTCAAATTTTCTGCAAAAAATTTTGGCGG
GAATTCAAATATTCTGAAAAAAAATTTGCCGG
GAATTCAAAATTTCTGAAAATAAATTTAGGCGA
GAATTCAAATTTTCAGAGAAAAATTTTGGCGG
GAATTCAAATTTTCAGAGAAAAAATTTTGGCGG
GAATTCAAATTTTCTAAGAAAAATTTTGGCGG
GAATTCAAATTTTCTAAGAAAAATTTTGGCGG
GAATTCAAATTTTCAGAGAAAAATTTTGGCGG
GAATTCAAATTTTCTAAGAAAAATTTTGGCGG
GAATTCAAATTTTCTAAGAAAAATTTTGGCGG
GAATTCAAATTTTCTGAGTAAAACTTTGGCGG
GAATTCAAATTTTCTGAGAAAAATTTAGGCGA
GAATTCAAATTTTCAGAGAAAAAATTTTGGCGG
GAATTCAAATATTCTGAAAAAAAATTTGCCGG
GAATTCAAAATTTCTGAAAATAAATTTAGGCGA
GAATTCAAATTTTCAGAGAAAAATTTTGGCGG
GAATTCAAATTTTCTAAGAAAAATTTTGGCGG
GAATTCACATTTTCTGAGAAAAATTTTGGCGG
GAATTCAAATTTTCTGAGAAAAATTTAGGCGA
GAATTCAAATTTTCTGAGAAAAATTTTGGCGG
GAATTCAAAATTTCTGAAAATAAATTTTGGCGG
GAATTCAAATTTTCTGCAAAAAATTTTGGCGG
GAATTCAAATTTTCTGCAAAAAATTTTGGCGG
GAATTCAAAATTTCTGAAAAAAAATTTGCCGG
GAATTCAAAATTTCTGAAAATAAATTTTGGCGG
GAATTCAAATTTTCTGCAAAAAATTTTGGCGG
GAATTCAAATTTTCTGCAAAAAATTTTGGCGG
GAATTCAAATTTTCTGAGAAAAATTTTGGCGG
GAATTCAAATTTTCTGAGAAAAATTTTGGCGG
GAATTCAAATTTTCTGAGAAAAATTTAGGCGA

GAATTCAAATTTTCAGAGAAAAAATTTTGGCGG
GAATTCAAATATTCTGAAAAAAAATTTAGGCGA
GAATTCAAATTTTCTGAGAAAAAATTTAGGCGA
GAATTCAAATTTTCAGAGAAAAATTTTTGGCGG
GAATTCAAATATTCTGAAAAAAAATTTTGGCGA
GAATTCAAATTTTCAGAGAAAAAATTCTGGCGG
GAATTCAAATTTTCTAAGAAAAAATTTTGGCGG
GAATTCAAATTTTCTGAGTAAAACTTTGGCGG
GAATTCAAATTTTCTGAGAAAAAATTTAGGCGA
GAATTCAAATTTTCAGAGAAAAAATTTTGGCGG
GAATTCAAATATTCTGAAAAAAAATTTGCCGG
GAATTCAAAATTTCTGAAAATAAATTTAGGCGA
GAATTCAAATTTTCAGAGAAAAAATTTTGGCGG
GAATTCAAATTTTCTAAGAAAAAATTTTGGCGG
GAATTCACATTTTCTGAGAAAAAATTTTGGCGG
GAATTCAAATTTTCTGAGAAAAAATTTAGGCGA
GAATTCAAATTTTCTGAGAAAAAATTTTGGCGG
GAATTCAAAATTTCTGAAAATAAATTTTGGCGG
GAATTCAAATTTTCTGCAAAAAAATTTTGGCGG
GAATTCAAAATTTCTGAAAAAAAATTTGCCGG
GAATTCAAAATTTCTGAAAATAAATTTTGGCGG
GAATTCAAATTTTCTGCAAAAAAATTTTGGCGG
GAATTCAAATTTTCTGCAAAAAAATTTTGGCGG
GAATTCAAATTTTCTGAGAAAAAATTTTGGCGG
GAATTCAAATTTTCTGAGAAAAAATTTTGGCGG
GAATTCTAATTTTCTGAGAACATTTTTGGCGGA
AATTCAAATTTTCTGAGAACATTTTTGGCGG
GAATTCAAATTTTCTGAGAAAAAATTGTGGCGG
GAATTCAAATTTTCTGAGAAAAAATTTAGGCGA
GAATTCAAATTTTCAGAGAAAAAATTTTGGCGG
Nr of selected repeats 192 Similarity 0.889807
Consensus:
GAATTCAAATTTTCtgAGAAAAAATTTtGGCGg
>Cele-UNSB01_1:685710-685978 Satlength=269 Nr of Repeats=6 RepeatLength=45
seed=TCTGATCCTT
TCTGATCCTTCAGCTCCGAAGGATCACATGGGTACCTTCTGATGT
TCTGATCCTTCAGATAAGAAGGATCGTAAGGGTACCTCCTGATGG
TCTGATCCTTCAGATCCAAAGGATCACGAGGCTCTTCCTGATGT
TCTGATCCTTCAGCTCCGAAGGATCACATGGGCACCTTCTGATGA
TCTGATCCTTCAGATAAGAAGGATCGTAAGGGTACCACTTGATGG
TCTGATCCTTCAGCTCCAAAGGATCACGAGGCTCTTCCTGATGT
Nr of selected repeats 4 Similarity 0.792593
Consensus:
TCTGATCCTTCAGaTaaGAAGGATCacAaGGGtACCtccTGATGg
>Cele-UNSB01_1:696260-697073 Satlength=814 Nr of Repeats=14 RepeatLength=44
seed=TTTGCCGGTT
TTTGCCGGTTTGCCGATTTGCCCGACATTTTTCAATTTCGGCAA
TTTGCCGGTTTGGCGATTTGCCCGAAATTTTTCAATTTCGGCAA
TTTGCCGGTTTGCCGATTTGCCCGACATTTTTCATTTTCGGCAA
TTTGCCGGTTTGCCGATTTGCCCGAAATTTTTCAATTTCGGCAA
TTTGCCGGTGTGCCGATTTGCCAAAAATTTTCATTTTCGGCAA
TTTGCCGGTTTGCCGATTTGCCGGAAATTTTCATTTTCGGCAA
TTCGCCGGTTTGCCGATTTGCCCGAAATTTTTCAATTTCGGCAA
TTTGCCGGTTTGCCGATTTGCCGGAATATTTCATTTTCGGCAA

TTTGCCGGTTTGCCGATTTGCCCGAAGTTTTTCAATTTCGGCAA
TTTGCCGGTTTGCCGATTTGTCGAAATTTTTCATTTTCGGCAG
TTTGCCGGTTTGCCGATTTGCCCGAAATTTTTCAATTTCGGCAG
TTTGCCGGTTTGCCGATTAGCCCGAAATTTTTAAATTTCGGTAA
TTTGCCGGTTTGCTGATTTGCCAGAAATTTTTCAATTTCAGCAATTTGTCGATTTGCCATTTGCCGGAAGTGTTCAGAA
GGATTTTTTTATAATAAGGAACCACTTAAAACTGTGCCGTTTTGAAACTTTTCCCCGTTTTTTTAAGATGATTTCATAG
AATTTGCTTACTTGGTTTGCCGAATTGCCAGAAGTTTTTCAATTCCTACAAGTTGCCAATTTGCCGGAAACTTTTTAAT
TATGGCAA
TTTGCCGGTTTGCCGATTTGCCAGAAATTTTTCAAATTCGGCAA
Nr of selected repeats 9 Similarity 0.920875
Consensus:
TTTGCCGGTTTGCCGATTTGCCCGAaATTTTTCAATTTCGGCAA
>Cele-UNSB01_1:698928-699108 Satlength=181 Nr of Repeats=12 RepeatLength=15
seed=GGTGAGACCC
GGTGAGACCCATCGT
GGTGAGACACATCGT
GGTGAGACCCATCGT
GGTGAGACCCTTCGT
GGTGAGACCCTTCGT
GGTGAGACCCATAGT
GGTGAGACCCATCGA
GGTGAGACCCTTCGT
GGTGAGACCCATCGT
GGTGAGACCCATCGA
GGTGAGACCCTTCGT
GGTGAGACCCATCGT
Nr of selected repeats 12 Similarity 0.900337
Consensus:
GGTGAGACCCaTCGT
>Cele-UNSB01_1:733992-734160 Satlength=169 Nr of Repeats=4 RepeatLength=42
seed=TTTGATCTAC
TTTGATCTACAAAAAAATGCGGGAGTTTTCCCAGCATTGCTCA
TTTGATCTACAAAAAAATGCGGGAATTTTCCCAGCATTGCTCA
TTTGATCTACAAAAAAATGCGGGAATTTTCCCAGCATTGCTCA
TTTGATCTACAAAAAAATGCGGGAATTCTCCCAGCATTGCTCA
Nr of selected repeats 4 Similarity 0.968254
Consensus:
TTTGATCTACAAAAAAATGCGGGAaTTtTCCCAGCATTGCTCA
>Cele-UNSB01_1:736250-736486 Satlength=237 Nr of Repeats=5 RepeatLength=12
seed=GCTTAGGCTT
GCTTAGGCTTAA
GCTTAGGCCTGG
GCTTAGTCTTAGGTTTATGCTTAAATTTGAAAAAAAAAAATTTCTAATTTTTTCCAGATTTTTCCGTTTTTTTTTTCAGA
GAAAATCTAAAATTTTCGATTTCTACCTGTTTTTCAAAAAAAGTTAAAAAAAACATTTTCGCAAATTTTGTAGAAATTT
TTCTTTTTTTTTTCTTAAAATTAG
GCTTAGGCTTAG
GCTTATGCTTAGGCTTAG
Nr of selected repeats 3 Similarity 0.777778
Consensus:
GCTTAGGCtTag
>Cele-UNSB01_1:742856-743285 Satlength=430 Nr of Repeats=11 RepeatLength=12
seed=GCAGGTAGGT
GCAGGTAGGTAG
GTAGGTAGGTAG

GTAGGTAGGTAG
GTAGGTAGGTAG
GTAGGTAGGTAGGTAAGTAGGTAAAGCGAGACACGGTGCATCGATCTGACTTTAAAAATCTCAATCTCGTATCTTAAGC
GTCTTTGGTTCTGTCAGGGAA
GCAGGTAGGGGG
GAGGTAGGTGTGATCAGGCAGAGCACCGGAGTTAATATCTGGTGGATTGTGGGCTGCTAATAGGTAGGTAGGCAGGTAA
GCAAGCAAGCAA
GCAGGCAGGTAG
GTAGGTAGGTAGGTAG
GCAGGCAGGTATCAGTTGACAACTTTTTGATGAAACTAAACGGAGCCGAGTTATAAGGTGCCAAAGTTGCACTAGACAT
GGTGCATCGACCTAACTTGAACAACTCGTATCTCAAGTGTCTTTGGTTCTATTTGGGAG
GCAGGTAGGTAG
Nr of selected repeats 7 Similarity 0.841270
Consensus:
GcAGGTAGGTAG
>Cele-UNSB01_1:758474-759042 Satlength=569 Nr of Repeats=35 RepeatLength=16
seed=TTTCAGGCTT
TTTCAGGCTTTCAGGC
TTTCAGGCTTTCAGGC
TTTCAGGCTTTCAGGC
TTTCAGGCTTTCAGGC
TTTCAGGCTTTCAGGC
TTTCAGGCTTTCAGGC
TTTCAGGCTTTCAGGC
TTTCAGGCTTTCAGGC
TTTCAGGCTTTCAGGC
TTTCAGGCTTTCAGGC
TTTCAGGCTTTCAGGC
TTTCAGGCTTTCAGGC
TTTCAGGCTTTCAGGC
TTTCAGGCTTTCAAGC
TTTCAGGGTTTCAGGG
TTTCAGGCTTTTAGGC
TTTCAGGCTTTCAGGC
TTTCAGGCTTTCAGGC
TTTCAGGCTTTCAGGC
TTTCAGGCTTTCAGGC
TTTCAGGCTTTCAGGCTTTCAAAC
TTTCAGGCTTTCAGGC
TTTCAGACTTTCAGGC
TTTCAGGCTTTCAGGC
TTTCAGGCTTTCAGGC
TTTCAGGCTTTCAGGC
TTTCAGGCTTTCAGGC
TTTCAAGCTTTCAGGC
TTTCAGGCTTTCAGGC
TTTCAGGCTTTCAGGC
TTTCAGGCTTTCAAAC
TTTCAGGCTTTCAAAC
TTTCAGGCTTTCAGGC
TTTCAGACTTTCAGGC
TTTCAGGCTTTCAGGC
Nr of selected repeats 34 Similarity 0.947564
Consensus:

TTTCAGGCTTTCAGGC
>Cele-UNSB01_1:768290-768741 Satlength=452 Nr of Repeats=13 RepeatLength=34
seed=TTTCTCAGAA
TTTCTCAGAAAATTTGAATTTCCCGCCAAAATTT
TTTATCAGAAAATTTGAATTTCCCACCAAAATTT
TTTATCAGAAGATTTGAATTTCCCACCAAAATTT
TTTCTCAGAAAATTTGAATTTCCCACCAAAATTT
TTTCTCAGAAAACTTGAAATTCCCGCCAAAAACTT
TTTCTCAGAATTTTGAAATTCCCGCTAAAAAATG
TTTCTCAGAAAATTTGAAATTCCCGCTAAAAAATT
TTTCTCAGAAAATTAAAAATCCCCGCCAAAACTT
TTTCTCAGAGAATTTGAATTTGCCGCCAAAAAATTGT
TTTCTCATAAAATCTGAATTTCCCACCAAAATTT
TTTCTCAGAAAATTTGAAATTCCCGCTAAAAAATT
TTTCTCAGAAAATTTGAATTTCCCGCCAAAAAATTGT
TTTCTCATAAAATCTGAATTTCCCACCAAAATTT
Nr of selected repeats 8 Similarity 0.787245
Consensus:
TTTcTCAgAAaATtTGAAtTTCCCaCCAAAAtTT
>Cele-UNSB01_1:774871-775189 Satlength=319 Nr of Repeats=26 RepeatLength=12
seed=TCTAGGTCTA
TCTAGGTCTAAG
TCTAGGTTTAGG
TCTAGGTCTAAG
TCTAGGTCTAGG
TCTAGGTCTAGG
TCTAGGTCTAGG
TCTAGGTCTAGG
TCTAGGTCTAGG
TCTAGGTCTAGG
TCTAGGTCTAGG
TCTAGGTCTAGG
TCTAGGTCTAGG
TCTAGGTCTAGG
TCTAGGTCTAGG
TCTAGGTCTAGG
TCTAGGTCTAGG
TCTAGGTCTATG
TCTAGGTCTAGG
TCTAGGTCTAGG
TCTAGGTCTAGG
TCTAGGTCTAGA
TCTAGGTCTAGG
TCTAGGTCTAGG
TCTAGGTCTAGG
TCTATGTCTAGG
TCTAGGTCTAGGTCTAGG
Nr of selected repeats 25 Similarity 0.948148
Consensus:
TCTAGGTCTAGG
>Cele-UNSB01_1:787252-787531 Satlength=280 Nr of Repeats=6 RepeatLength=40
seed=GAACCTTCTG
GAACCTTCTGGAATATTTGAGAAAACTCTAGAATGTTCTA

GAACCTTCTGAAAAATTCGAAAAAAGTCTAGAATGTTCTAGAGCCTTTTGGAAAATTCGAAAAAAATCTGGAATATTCT
A
GAACCTTTTGGAAATTTTGAGAAAATTCTGGAATGTTTTG
GAACCTTCTGGAAAATTCGAGAAAATTCTGGAATGTTCTA
GAACCTTCTGAAAAATTTGAGAAAATTCTGGAATGTTCTA
GACCCTTCTGGAAATCCGAGAAAATTCTGGAATGTTCTG
Nr of selected repeats 4 Similarity 0.850000
Consensus:
GAACCTTcTGgAAaaTTtGAGAAAAtTCTgGAATGTTcTa
>Cele-UNSB01_1:788353-788527 Satlength=175 Nr of Repeats=12 RepeatLength=12
seed=GCCTAAGCCT
GCCTAAGCCTAA
GCCTAAGCCTAA
GCCTAAGCCTAA
GCCTAAGCCTAA
GCCTAAGCCTAA
GCTTAAGCCTAA
GCCTAAGCCTAA
GCCTAAGCCTAG
GCCTAAGCCTAA
GTCTAAGCCTAAGCCTGAGCTTAAGCTTAA
GCCAAAGCCTAA
GCCTAAGCCTACTCCTATGCCTGA
Nr of selected repeats 10 Similarity 0.933333
Consensus:
GCCTAAGCCTAA
>Cele-UNSB01_1:789928-790208 Satlength=281 Nr of Repeats=7 RepeatLength=40
seed=TCTAGAACAT
TCTAGAACATTCCAGAATTTTCTCGAATTTTCCAAAAAGT
TCTAGAACATTCCAAACTTTTCTCAAAGTTTCCAAAAGAC
TCTAGAACATTCCAGAATATTCTCGAATTTTTCAGAAGGT
TCTAGAACATTCCTGAATTTTCTCGAATTTTCCAAAAGGT
TCTAGAACATTTCAGAATTTTCTCAAATTTTCCAAAAGGT
TCTAGAACATTCCAGAATTTTCTCGAATTTCCCAAAAGAT
TCTAGAACATTCCAGAATATTCTCGAATTTTTCAGAAGGC
Nr of selected repeats 7 Similarity 0.838095
Consensus:
TCTAGAACATTCCAGAATtTTCTCgAATTTTcCAaAAGgt
>Cele-UNSB01_1:815618-815840 Satlength=223 Nr of Repeats=16 RepeatLength=12
seed=GCCTAAGCCT
GCCTAAGCCTGAACTTAAGTTTAA
GCCAAAGCCTGA
GCCTAAGCCTAA
GCCTAACCCTAA
GCCTCAGCCTCA
GCCTAAGCCTAA
GCCTAAGCATAAG
GCTAAGCCTAA
GCCTAAGCCTGA
GCCTAAGCTTTAGTCTAA
GCGTAAGCCTAG
GCCTAACCCTAACCCTAA
GCTTAAGCCTAA
GCCTAACCCTAA

GCCTAACCCTAA
GCCTGAGCCTAAGCCTAA
Nr of selected repeats 10 Similarity 0.782716
Consensus:
GCcTAAgCCTaA
>Cele-UNSB01_1:817628-817952 Satlength=325 Nr of Repeats=8 RepeatLength=22
seed=TTAAAATTTT
TTAAAATTTTGTTAGTTTGACC
TTAAAATTTTGTCAGTTTGACA
TTAAAAATTTGTCAGTTTGACC
TTAAAATTTTGTCAGACATATAATTTTTTTGTGTTTTTTGGTTAAAAAATTGAAGGTTTTTCGCGATATTTAACACATT
ACATGGAAAAATTCTTTTGATCGAGTTTTCTAATTTGTTCGTGTAGCAAGTCGATGCACCATGTCCAATTTT
TTAAAATTTTACTTTTTTTAAATTATTTGCCCAGTTTGACC
TTAAAATTTTGTCAATTTTACC
TTAAAATTTTGTCAGTTTTACC
TTAAAATTTTGTCAGTTTGACC
Nr of selected repeats 6 Similarity 0.886869
Consensus:
TTAAAATTTTGTCAGTTTgACC
>Cele-UNSB01_1:823660-823840 Satlength=181 Nr of Repeats=13 RepeatLength=12
seed=GCTTAGGCTT
GCTTAGGCTTAG
GTTTAGGCTTAGACTTAA
GCTTAGGCTTAG
GCTTAGGCTTAG
GCTTAGGCTTAGA
CTTAGGCTTAAGCTTATTCTTGA
GCTTAGGCTTAA
GCTTAGGCTTAA
GCTTAGGCTTCG
GCTTGGGCTTAG
GCTTAGGCTTAG
GCTTAGGCTTAA
GCTTAGGATTAGGCTTAG
Nr of selected repeats 9 Similarity 0.895062
Consensus:
GCTTAGGCTTAg
>Cele-UNSB01_1:833000-833653 Satlength=654 Nr of Repeats=29 RepeatLength=21
seed=TGGCCTAGAT
TGGCCTAGATTCTCTATAGGG
TGGCCTACATTTTTACACTGGTTGTCTAGATTCTTAAACAGGG
TGGCCTCGATTCTGTACAGGG
TGGCCTAGATTTTCTACACGG
TGGTCTAGATTTTCACTGG
TGGCCTAGATTCTCACACTAGG
TGGCCTAGATTTTTCCACTAA
TAGCCTAGATTCTTTACAGGG
TGTCCTAGATTTTCACACTGG
TGTCCTAGATTCTACACAGGG
TGACCTAGATTTTTTACACTGG
TGGCCTAGATTCTCTACAGGG
TGGCCTAAATTCCTTATAGGA
TGACCTAGATTCTTAAACAGGA
TGACCTAGATCTTGACACTAG

TGGCCTTGATTTTTTACAGGG
TGGCCTAGATTTTCTACAA
TGGTCTAGATTTTTTGCAGGGTGGCTTGGATTCTTAAACAAGG
TGGCCTAGATTTTTCACAGGG
TGGCCTAGATTTTATACAGGG
TGGTCTAGATTTTTACACTGG
TGGCCTAGATTCTTTACAGGT
TGGCCTAGATTTTCTATAGGA
TGGCCTAGATTCTTAAACAGGG
TGGCCTAGATTCTTTACAGGG
TAGCCTAGATTCTTCACAGGG
TAGCCTAGATTTTTACACTGG
TGGCCTAGATTTTCTAAAGGG
TGGTCTAGATTTTTGCACTGG
Nr of selected repeats 21 Similarity 0.658586
Consensus:
TGGCCTAGATTnTttACAgGG
>Cele-UNSB01_1:842713-843650 Satlength=938 Nr of Repeats=48 RepeatLength=12
seed=CTTAGGCTTA
CTTAGGCTTAAGCTTAGGCCTTTT
CTCAGGCTTAGG
CTTAGGCTTATG
CTCAGGCTTAGGCCTTTT
CTTAGGCTTATG
CTTGGGCTTAGG
CTTAGGCGTAGG
CTTAGGCTTAGG
CTTAGGCTTATG
CTTAGACTTAGTCTCACTATCAGT
CTTAGGCTTAGG
CTTAGACTTAGG
CTTAAGCTTAGG
CTTAAGCTTAGA
CTTAGGCTTAGG
CTTAGGCTTAGG
CTTAGGCTTAGGTTTGGG
CTTAGGCTTAGGCTTAAC
CTCAGGCTTAGG
CTTAGGTTTAGG
CTCAGGCTTAAA
CTTAGGCTTAGGCCCAGG
CTTAGGCTTAGGCTCACACGTTCTCACGTTCTTAACCATACAAAATCAGCTAAGAACTCTGCGTCTTTTCTCCCGCATT
TTTTGTAGATCTACGTAGATCAAACCGAAATGAGGCACTTTCTGAATCCACGAG
CTAGGCTTAAG
CTTAGGCTTAAGCTTAGGCCTTTT
CTCAGGCTTAGG
CTTAGGCTTATGCCCAGGCTTAGGCCTTTT
CTTAGGCTTATG
CTTGGGCTTAGG
CTTAGGCGTAGG
CTTAGGCTTAGG
CTTAGGCTTATG
CTTAGACTTAGTCTCACTATCAGT
CTTAGGCTTAGG

CTTAGACTTAGG
CTTAAGCTTAGG
CTTAAGCTTAGA
CTTAGGCTTAGG
CTTAGGCTTAGG
CTTAGGCTTAGGTTTGGG
CTTAGGCTTAGGCTTAAC
CTCAGGCTTAGG
CTTAGGTTTAGG
CTCAGGCTTAAA
CTTAGGCTTAGGCCCAGG
CTTAGGCTTAGGCTCACACGTTCTCACGTTCTTAACCATACAAAATCAGCTAAGAACTCTGCGTCTTTTCTCTCGCATT
TTTTGTAGATCAACGAAGATCGAGCCGAAATGAGGCACTCTGACATCACGTGGGCTAAGA
CTTAGGCTTAGG
CTTAGGCTTAGGCTTAGG
Nr of selected repeats 32 Similarity 0.819668
Consensus:
CTTAGGCTTAgG
>Cele-UNSB01_1:855372-855570 Satlength=199 Nr of Repeats=12 RepeatLength=12
seed=GCTTAGGCTT
GCTTAGGCTTAG
GCTTAGACTTAG
GCTTAGGCTTAGTCTTCG
GCTTAGGCTTAG
GCTTAGGCTTAG
GCTTAGGCTTAG
GCTTAGGTTTAG
GCTTAGGCTTAG
GCTTAGGCTTGGGACTGG
GCTTAGGCTTGAGCTTTGGCCTAA
GCTTAGGCTTAGGCTTACGCTCAGGCGTAGGATTAGGCTCTG
GCTTTGGCTTTG
Nr of selected repeats 8 Similarity 0.888889
Consensus:
GCTTAGGCTTAG
>Cele-UNSB01_1:907957-908415 Satlength=459 Nr of Repeats=21 RepeatLength=22
seed=TTTTAGCCAA
TTTTAGCCAAAAAATTCGCTA
TTTTAACCAAAAAATCCGTTGA
TTTTAGTCAAAAAATTCGCTAT
TTTTAGCCAAAAAATCGCTAT
TTTTAGCCAAAAAATCCGTTAA
TTTTAGCCAAAAATTTCGCTAT
TTTTAGCCAAAAAATCCGTTAA
TTTTAGGCAAAAAATCCGTTAA
TTTTAGTCAAAAAATCCGCTA
TTTTAACCAAAAAATCCGCTA
TTTTAACCAAAAAATCCGTTAA
TTTTAGCCAAAAAATTCGCTAT
TTTTAGCCAAAAAATCCGTTAA
TTTTAGCCAAAAAATTCGCTAT
TTTTAGCCAAAAAATCCGTTAA
TTTTAGCCAAAAAATTCGCTAT
TTTTAGCCAAAAAATCCGTTAA

TTTTAGCCAAAAAATTCGCTAT
TTTTAGCCAAAAAATTCGCTAT
TTTTAGCCAAAAAATCCGTTAA
TTTTAGCCAAAAAATTCGCTAT
Nr of selected repeats 17 Similarity 0.862299
Consensus:
TTTTAGCCAAAAAATcCGtTAa
>Cele-UNSB01_1:911764-911915 Satlength=152 Nr of Repeats=8 RepeatLength=15
seed=ATCTTCTTTT
ATCTTCTTTTCGTAGATTTTCAGAATGTAG
ATCTTCTTTTGGTAA
ATCTTCTTTTCGTAG
ATCTTCTTTTCGTCGATCTTCAAAATGTAG
ATCTTCTTTTGGTAG
ATCTTCTTTTCGTAG
ATCTTCTTTTCGTAG
ATCTTCTTTTTCGTAG
Nr of selected repeats 5 Similarity 0.911111
Consensus:
ATCTTCTTTTcGTAg
>Cele-UNSB01_1:927465-927693 Satlength=229 Nr of Repeats=6 RepeatLength=32
seed=GAAAATTTGA
GAAAATTTGAATTCCCGCCAAAATGTTTCTTT
GAAAGTTTGAATTCCCGCCAAAAACATTCTCAGAAAATTCAAGTTCCCGCCAAAAATGTTTCTTT
GAAAATTTGAATTCCCGCCAAGATTTTTTACA
GAAAATTTGAATTTCCGCCTAAAATATTCTCG
GAAAATTTGAATTCCCGCCAAAATTTTTCTTT
GAAAATTTGAACTCTCGCCAAAATGTTTTTTTTT
Nr of selected repeats 4 Similarity 0.727941
Consensus:
GAAAATTTGAATTcCCGCCAAaATttTTcTtt
>Cele-UNSB01_1:932742-934750 Satlength=2009 Nr of Repeats=89 RepeatLength=21
seed=AATTTCTAGG
AATTTCTAGGCCATCAAG
AATTTCTAGGCCACATCAGACTACTTTGAA
AATTTCTAGGCCACAATGGGAACTAATTTCTGAACCTAATTTCTGA
AATTTCTTGGCCACGATCTGAAATTTCGAAGCCATAATTTGA
AATTTCTAGGCCACGATCTGA
AATTTCTAGACCATCAAC
AATTTCTAGGCCACGATTTGA
AATTTCTAGGCCACGATTTGA
AATTTGTAGGTCAAGGTTTGA
AATTTCTAAGCCACGATTTGA
AATTTCTAGGCCACGATCTGA
AATTTTTAGGCCACGATTTGA
AATTTCTAGGCCACGATTTGA
AATTTTTAGGCCACGATCTGAAGTTTTTAGGTCATCAAA
AATTTCTAGGCCACGATTTGA
AATTTCTAGGCCACGATCTGA
AATTTCTAGGCCACGATTTGA
AATTTCTAGGCCACGATTTGA
AATTTTTAGGCCACGATCTGA
AATTTCTAGGCCATCAAA
AATTTATAGGCCACGATTTGA

AATTTCTAGGCCACGATTTGA
AATTTCTGGGCCACGATCTGA
AATTTCTAGGTCATCAAA
AATTTCTAGGCCACGATTTGA
AATTTCTAGGCCACGATTTGA
AATTTCTAGGCCGCGATCTGA
AATTTCTAAGCCATCAAA
AATTTCTAGGCCACGATTTGA
AATTTCTAGGCCGCGATCTGA
AATTTCTAAGCCATCAAA
AATTTCTAGGCCACGTTTTAAAATTTTTAAAATGTTTAAAATTATAGGTTTTCAAAAA
AATTCTAGGCCATCAAA
AATTTCTAGGCCACGATTTGA
AATTTCTAGGCCACGATCTGA
AATTTCTAGGCCACGATTTGA
AATTTCTAGGCCACGATTTGAAATTTTTAGACCACGATCTGA
AATTTCTAGGCCATCAAA
AATTTATAGGCCACGATTTGA
AATTTCTAGGCCACGATTTGA
AATTTCTGGGCCACGATCTGA
AATTTCTAGGTCATCAAA
AATTTCTAGGCCACGATTTGA
AATTTCTAGGCCACGATTTGA
AATTTCTAGGCCGCGATCTGA
AATTTCTAAGCCATCAAA
AATTTCTAGGCCACGTTTTAAAATTTTTAAAATGTTTAAAATTATAGGTTTTCAAAAA
AATTCTAGGCCATCAAA
AATTTCTAGGCCACGATTTGA
AATTTCTAGGCCACGATCTGA
ACTTTCTAGGCCACGATTTGA
AATTTCTAGGCCACGATTTGA
AATTTTTAGGCCACGATCTGA
AATTTCTAGGCCATCAAA
AATTTATAGGCCACGATTTGA
AATTTCTAGGCCACGATTTGA
AATTTTTAGGCCACGATCTGA
AATTTCTAGGTCATCAAA
AATTTATAGGCCACGATTTGA
AATTTCTAGGCCACGATTTGA
AATTTCTAGGCCACGATTTGA
AATTTCTGGGCCACGATCTGA
AATTTCTAGGTCATCAAA
AATTTCTAGGCCACGATTTGA
AATTTCTAGGCCGCGATTTGA
AATTTCTAGGCCGCGATCTGA
AATTTCTAAGCCATCAAA
AATTTCTAGGCCACGTTTTAAAATTTTTAAAATGTTCAAAATTATAGGTTTTCAAAAA
AATTCTAGGCCATCAAA
AATTTCTAGGCCACGATTTGA
AATTTCTAGGCCACGATTTGA
AATTTTTAGGCCACGATCTGA
AATTTCTAGGCCATCAAA
AATTTATAGGCCACGATTTGA
AATTTCTAGGCCACGATTTGA

AATTTCTGGGCCACGATCTGA
AATTTATAGGTCATCAAA
AATTTCTAGGCCACGATTTGA
AATTTCTAGGCCACGATTTGA
AATTTCTAGGCCGCGATCTGA
AATTTCTAAGCCATCAAA
AATTTCTAGGCCACGATTTGA
AATTTCTAGGCCACGATTTGA
AATTTCTAGGTCACGATTTGA
AATTTCTAGGCCACGATTTGA
AATTTATAGGCCGTGATCTGA
AAGTTCTAGGTCATCAAA
AATTTCTAGGCCACGATTTAA
AATTTCTAGTCCATCAAT
Nr of selected repeats 60 Similarity 0.911757
Consensus:
AATTTcTAGGCCACGATtTGA
>Cele-UNSB01_1:943054-953346 Satlength=10293 Nr of Repeats=369 RepeatLength=27
seed=TTTTACTCTC
TTTTACTCTCTATGGCTTCACCAATTATCTTTCTCTCTGTGGCTTCCCACTATA
TTTTACTCTCTGTGGCTTCACCAATTA
TTTTACTCTCTGTGGCTTCCCACTATA
TTTTACTCTCTGTGGCTTCCCACTATA
TTTTACTCTCTGTGGCTTCACCAATTA
TTTTACTCTCTGTGGCTTCCCACTATA
TTTTACTCTCTGTGGCTTCACCAATTA
TTTTACTCTCTGTGGCTTCCCACTATA
TTTTACTCTCTGTGGCTTCCCACTATA
TTTTACTCTCTGTGGCTTCACCAATTA
TTTTACTCTCTGTGGCTTCCCACTATA
TTTTACTCTCTGTGGCTTCACCAATTA
TTTTACTCTCTGTGGCTTCCCACTATA
TTTTACTCTCTGTGGCTTCCCACTATA
TTTTACTCTCTGTGGCTTCCCACTATA
TTTTACTCTCTGTGGCTTCACCAATTA
TTTTACTCTCTGTGGCTTCCCACTATA
TTTTACTCTCTGTGGCTTCACCAATTA
TTTTACTCTCTGTGGCTTCCCACTATA
TTTTACTCTCTGTGGCTTCACCAATTATCTTTCTCTCTGTGGCTTCCCACTATA
TTTTACTCTCTGTGGCTTCCCACTATA
TTTTACTCTCTGTGGCTTCCCACTATA
TTTTACTCTCTGTGGCTTCACCAATTA
TTTTACTCTCTGTGGCTTCCCACTATA
TTTTACTCTCTGTGGCTTCACCAATTA
TTTTACTCTCTGTGGCTTTCCACTATA
TTTTACTCTCTGTGGCTTCACCAATTATCTTTCTCTCTGTGGCTTCACCAATTA
TTTTACTCTCTGTGGCTTCCCACTATA
TTTTACTCTCTGTGGCTTCCCACTATA
TTTTACTCTCTGTGGCTTCACCAATTA
TTTTACTCTCTGTGGCTTCCCACTATA
TTTTACTCTCTGTGGCTTCCCACTATA
TTTTACTCTCTGTGGCTTCCCACTATA
TTTTACTCTCTGTGGCTTCCCACTATA
TTTTGCTCTCTGTGGCTTCACCAACTA

TTTTACTCTCTGTGGCTTCCCACTATA
TTTTACTCTCTGTGGCTTCCCACTATA
TTTTACTCTCTGTGGCTTCCCACTATA
TTTTACTCTCTGTGGCTTCACCAACTA
TTTTACTCTCTGTGGCTTCCCACTATA
TTTTACTCTCTGTGGCTTCCCACTATA
TTTTACTCTCTGTGGCTTCCCACTATA
TTTTACTCTCTGTGGCTTCACCAATTA
TTTTACTCTCTGTGGCTTCCCACTATA
TTTTACTCTCTGTGGCTTCCCACTATA
TTTTACTCTCTGTGGCTTCCCACTATA
TTTTACTCTCTGTGGCTTCCCACTATA
TTTTGCTCTCTGTGGCTTCACCAACTA
TTTTACTCTCTGTGGCTTCCCACTATA
TTTTACTCTCTGTGGCTTCCCACTATA
TTTTACTCTCTGTGGCTTCACCAACTA
TTTTACTCTCTGTGGCTTCACCAATTA
TTTTACTCTCTGTGGCTTCCCACTATA
TTTTACTCTCTGTGGCTTCACCAATTA
TTTTACTCTCTGTGGCTTCCCACTATA
TTTTACTCTCTGTGGCTTCCCACTATA
TTTTACTCTCTGTGGCTTCCCACTATA
TTTTACTCTCTGTGGCTTCCCACTATA
TTTTACTCTCTGTGGCTTCACCAATTA
TTTTACTCTCTGTGGCTTCCCACTATA
TTTTACTCTCTGTGGCTTCCCACTATA
TTTTACTCTCTGTGGCTTCCCACTATA
TTTTACTCTCTGTGGCTTCACCAATTA
TTTTACTCTCTGTGGCTTCCCACTATA
TTTTACTCTCTGTGGCTTCCCACTATA
TTTTACTCTCTGTGGCTTCCCACTATA
TTTTACTCTCTGTGGCTTCACCAATTA
TTTTACTCTCTGTGGCTTCCCACTATA
TTTTACTCTCTGTGGCTTCACCAACTA
TTTTACTCTCTGTGGCTTCCCACTATA
TTTTACTCTCTGTGGCTTCCCACTATA
TTTTACTCTCTGTGGCTTCACCAACTA
TTTTACTCTCTGTGGCTTCCCACTATA
TTTTACTCTCTGTGGCTTCACCAACTA
TTTTACTCTCTGTGGCTTCCCACTATA
TTTTACTCTCTGTGGCTTCACCAATTA
TTTTACTCTCTGTGGCTTCCCACTATA
TTTTACTCTCTGTGGCTTCCCACTATA
TTTTACTCTCTGTGGCTTCACCAATTA
TTTTACTCTCTGTGGCTTCCCACTATA
TTTTACTCTCTGTGGCTTCACCAATTATCTTTCTCTCTGTGGCTTCCCACTATA
TTTTACTCTCTGTGGCTTCCCACTATA
TTTTACTCTCTGTGGCTTCACCAATTA
TTTTACTCTCTGTGGCTTCCCACTATA
TTTTACTCTCTGTGGCTTCACCAACTA
TTTTACTCTCTGTGGCTTCACCAACTA
TTTTACTCTCTGTGGCTTCCCACTATA
TTTTACTCTCTGTGGCTTCCCACTATA
TTTTACTCTCTGTGGCTTCACCAATTATCTTTCTCTCTGTGGCTTCCCACTATA

TTTTACTCTCTGTGGCTTCACCAACTA
TTTTACTCTCTGTGGCTTCACCAATTATCTTTCTCTCTGTGGCTTCCCACTATA
TTTTACTCTCTGTGGCTTCCCACTATA
TTTTACTCTCTGTGGCTTCACCAATTATCTTTCTCTCTGTGGCTTCCCACTATA
TTTTACTCTCTGTGGCTTCACCAACTA
TTTTACTCTCTGTGGCTTCACCAATTA
TTTTACTCTCTGTGGCTTCCCACTATA
TTTTACTCTCTGTGGCTTCACCAATTA
TTTTACTCTCTGTGGCTTCCCACTATA
TTTTACTCTCTGTGGCTTCCCACTATA
TTTTACTCTCTGTGGCTTCACCAACTA
TTTTACTCTCTGTGGCTTCCCACTATA
TTTTACTCTCTGTGGCTTCACCAACTA
TTTTACTCTCTGTGGCTTCCCACTATA
TTTTACTCTCTGTGGCTTCACCAACTA
TTTTACTCTCTGTGGCTTCCCACTATA
TTTTACTCTCTGTGGCTTCCCACTATA
TTTTACTCTCTGTGGCTTCACCAACTA
TTTTACTCTCTGTGGCTTCCCACTATA
TTTTACTCTCTGTGGCTTCCCACTATA
TTTTACTCTCTGTGGCTTCCCACTATA
TTTTACTCTCTGTGGCTTCACCAATTA
TTTTACTCTCTGTGGCTTCCCACTATA
TTTTACTCTCTGTGGCTTCCCACTATA
TTTTACTCTCTGTGGCTTCACCAACTATA
TTTTACTCTCTGTGGCTTCACCAATTA
TTTTACTCTCTGTGGCTTCCCACTATA
TTTTACTCTCTGTGGCTTCACCAATTA
TTTTACTCTCTGTGGCTTCCCACTATA
TTTTACTCTCTGTGGCTTCCCACTATA
TTTTACTCTCTGTGGCTTCACCAACTATA
TTTTACTCTCTGTGGCTTCCCACTATA
TTTTACTCTCTGTGGCTTCACCAATTA
TTTTACTCTCTGTGGCTTCCCACTATA
TTTTACTCTCTGTGGCTTCCCACTATA
TTTTACTCTCTGTGGCTTCACCGATTA
TTTTACTCTCTGTGGCTTCCCACTATA
TTTTACTCTCTGTGGCTTCACCAATTA
TTTTACTCTCTGTGGCTTCCCACTATA
TTTTACTCTCTGTGGCTTCACCACTTA
TTTTACTCTCTGTGGCTTCACCAATTA
TTTTACTCTCTGTGGCTTCCCACTATA
TTTTACTCTCTGTGGCTTCACCAATA
TTTTACTCTCTGTGGCTTCCCACTATA
TTTTACTCTCTGTGGCTTCACCAATTA
TTTTACTCTCTGTGGCTTCCCACTATA
TTTTACTCTCTGTGGCTTCACCGATTA
TTTTACTCTCTGTGGCTTCCCACTATA
TTTTACTCTCTGTGGCTTCACCAATA
TTTTACTCTCTGTGGCTTCCCACTATA
TTTTACTCTCTGTGGCTTCACCAATTA
TTTTACTCTCTGTGGCTTCCCACTATA
TTTTACTCTCTGTGGCTTCACCAATA
TTTTACTCTCTGTGGCTTCCCACTATA

TTTTACTCTCTGTGGCTTCACCAATTA
TTTTACTCTCTGTGGCTTCCCACTATA
TTTTACTCTCTGTGGCTTCCCACTATA
TTTTACTCTCTGTGGCTTCCCACTATA
TTTTACTCTCTGTGGCTTCACCAACTATA
TTTTACTCTCTGTGGCTTCCCACTATA
TTTTACTCTCTGTGGCTTCACCAATTA
TTTTACTCTCTGTGGCTTCCCACTATA
TTTTACTCTCTGTGGCTTCCCACTATA
TTTTACTCTCTGTGGCTTCCCACTATA
TTTTACTCTCTGTGGCTTCCCACTATA
TTTTACTCTCTGTGGCTTCACCAATTA
TTTTACTCTCTGTGGCTTCCCACTATA
TTTTACTCTCTGTGGCTTCACCAATTA
TTTTACTCTCTGTGGCTTCCCACTATA
TTTTACTCTCTGTGGCTTCCCACTATA
TTTTACTCTCTGTGGCTTCCCACTATA
TTTTACTCTCTGTGGCTTCACCAATTA
TTTTACTCTCTGTGGCTTCCCACTATA
TTTTACTCTCTGTGGCTTCACCAATTA
TTTTACTCTCTGTGGCTTCCCACTATA
TTTTACTCTCTGTGGCTTCCCACTATA
TTTTACTCTCTGTGGCTTCACCAATTA
TTTTACTCTCTGTGGCTTCCCACTATA
TTTTACTCTCTGTGGCTTCACCAATTA
TTTTACTCTCTGTGGCTTCCCACTATA
TTTTACTCTCTGTGGCTTCCCACTATA
TTTTACTCTCTGTGGCTTCCCACTATA
TTTTACTCTCTGTGGCTTCACCGATTA
TTTTACTCTCTGTGGCTTCCCACTATA
TTTTACTCTCTGTGGCTTCACCAATA
TTTTACTCTCTGTGGCTTCCCACTATA
TTTTACTCTCTGTGGCTTCACCAATA
TTTTACTCTCTGTGGCTTCCCACTATA
TTTTACTCTCTGTGGCTTCACCAATTA
TTTTACTCTCTGTGGCTTCCCACTATA
TTTTACTCTCTGTGGCTTCCCACTATA
TTTTACTCTCTGTGGCTTCACCAATA
TTTTACTCTCTGTGGCTTCCCACTATA
TTTTACTCTCTGTGGCTTCACCAATA
TTTTACTCTCTGTGGCTTCCCACTATA
TTTTACTCTCTGTGGCTTCCCACTATA
TTTTACTCTCTGTGGCTTCCCACTATA
TTTTACTCTCTGTGGCTTCACCAATA
TTTTACTCTCTGTGGCTTCCCACTATA
TTTTACTCTCTGTGGCTTCCCACTATA
TTTTACTCTCTGTGGCTTCCCACTATA
TTTTACTCTCTGTGGCTTCCCACTATA
TTTTACTCTCTGTGGCTTCCCATTA
TTTTACTCTCTGTGGCTTCCCACTATA
TTTTACTCTCTGTGGCTTCACCAATTA
TTTTACTCTCTGTGGCTTCCCACTATA
TTTTACTCTCTGTGGCTTCCCACTATA
TTTTACTCTCTGTGGCTTCACCAATA

TTTTACTCTCTGTGGCTTCCCACTATA
TTTTACTCTCTGTGGCTTCACCAATA
TTTTACTCTCTGTGGCTTCCCACTATA
TTTTACTCTCTGTGGCTTCCCACTATA
TTTTACTCTCTGTGGCTTCCCAATATA
TTTTACTCTCTGTGGCTTCCCACTATA
TTTTACTCTCTGTGGCTTCCCAATA
TTTTACTCTCTGTGGCTTCACCAATA
TTTTACTCTCTGTGGCTTCCCACTATA
TTTTACTCTCTGTGGCTTCCCACTATA
TTTTACTCTCTGTGGCTTCACCAATTA
TTTTACTCTCTGTGGCTTCCCACTATA
TTTTACTCTCTGTGGCTTCCCACTATA
TTTTACTCTCTGTGGCTTCACCAATTA
TTTTACTCTCTGTGGCTTCCCACTATA
TTTTACTCTCTGTGGCTTCCCACTA
TTTTACTCTCTGTGGCTTCACCAATT
TTTTACTCTCTGTGGCTTCCCACTATA
TTTTACTCTCTGTGGCTTCACCAATA
TTTTACTCTCTGTGGCTTCCCACTATA
TTTTACTCTCTGTGGCTTCCCACTATA
TTTTACTCTCTGTGGCTTCCCAATA
TTTTACTCTCTGTGGCTTCCCACTATA
TTTTACTCTCTGTGGCTTCACCAATA
TTTTACTCTCTGTGGCTTCCCACTATA
TTTTACTCTCTGTGGCTTCACCACTA
TTTTACTCTCTGTGGCTTCCCACTATA
TTTTACTCTCTGTGGCTTCCCACTATA
TTTTACTCTCTGTGGCTTCCCACTATA
TTTTACTCTCTGTGGCTTCCCACTATA
TTTTACTCTCTGTGGCTTCCCACTATA
TTTTACTCTCTGTGGCTTCCCACTATA
TTTTACTCTCTGTGGCTTCCCACTATA
TTTTACTCTCTGTGGCTTCCCATA
TTTTACTCTCTGTCTTAATA
TTTTACTCTCTGTGGCTTCCCACTATA
TTTTACTCTCTGTGGCTTCCCACTATA
TTTTACTCTCTGTGGCTTCCCACTATA
TTTTACTCTCTGTGGCTTCACCGATTA
TTTTACTCTCTGTGGCTTCCCACTATA
TTTTACTCTCTGTGGCTTCCCACTATA
TTTTACTCTCTGTGGCTTCACCGATTA
TTTTACTCTCTGTGGCTTCCCACTATA
TTTTACTCTCTGTGGCTTCCCACTATA
TTTTACTCTCTGTGGCTTCCCACTATA
TTTTACTCTCTGTGGCTTCCCACTATA
TTTTACTCTCTGTGGCTTCACCAATA
TTTTACTCTCTGTGGCTTCCCACTATA
TTTTACTCTCTGTGGCTTCCCAATA
TTTTACTCTCTGTGGCTTCCCACTATA
TTTTACTCTCTGTGGCTTCCCACTATA
TTTTACTCTCTGTGGCTTCCCACTATA
TTTTACTCTCTGTGGCTTCCCACTATA
TTTTACTCTCTGTGGCTTCCCACTATA

TTTTACTCTCTGTGGCTTCCCACTATA
TTTTACTCTCTGTGGCTTCACATA
TTTTACTCTCTGTGGCTTCCCACTATA
TTTTACTCTCTGTGGCTTCCCACTATA
TTTTACTCTCTGTATA
TTTTACTCTCTGTGGCTTCCCACTATA
TTTTACTCTCTGTGGCTTCACCGATTA
TTTTACTCTCTGTGGCTTCCCACTATA
TTTTACTCTCTGTGGCTTCACCGATTA
TTTTACTCTCTGTGGCTTCCCACTATA
TTTTACTCTCTGTGGCTTCCCATTTTTTTTTTTCACTATA
TTTTACTCTCTGTGGCTTCCCATA
TTTTACTCTCTGTATA
TTTTACTCTCTGTGGCTTCCCACTATA
TTTTACTCTCTGTGGCTTCCCACTATA
TTTTACTCTCTGTGGCTTCCCACTATA
TTTTACTCTCTGTGGCTTCCCACTATA
TTTTACTCTCTGTGGCTTCCCACTATA
TTTTACTCTCTGTGGCTTCCCACTATA
TTTTACTCTCTGTGGCTTCACCAATA
TTTTACTCTCTGTGGCTTCCCACTATA
TTTTACTCTCTGTGGCTTCACCAATA
TTTTACTCTCTGTGGCTTCCCACTATA
TTTTACTCTCTGTGGCTTCCCACTATA
TTTTACTCTCTGTGGCTTCCCACTATA
TTTTACTCTCTGTGGCTTCCCACTATA
TTTTATTCTCTGTGCTTACCATTA
TTTTACTCTCTGTGGCTTCCCACTATA
TTTTACTCTCTGTGGCTTCACCAATTA
TTTTACTCTCTGTGGCTTCCCACTATA
TTTTACTCTCTGTGGCTTCACCGATTA
TTTTACTCTCTGTGGCTTCCCACTATA
TTTTACTCTCTGTATA
TTTTACTCTCTGTGGCTTCCCACTATA
TTTTACTCTCTGTGGCTTCCCACTATA
TTTTACTCTCTGTGGCTTCCCATTA
TTTTACTCTCTGTGGCTTCCCACTATA
TTTTACTCTCTGTGGCTTCACCAATTA
TTTTACTCTCTGTGGCTTCCCACTATA
TTTTACTCTCTGTGGCTTCACCAATA
TTTTACTCTCTGTGGCTTCCCACTATA
TTTTACTCTCTATGGCTTCACCTATTA
TTTTACTCTCTGTGGCTTCCCACTATA
TTTTACTCTCTGTGGCTTCCCACTATA
TTTTACTCTCTGTATA
TTTTACTCTCTGTGGCTTCCCACTATA
TTTTACTCTCTGTGGCTTCCCACTATA
TTTTACTCTCTGTGGCTTCCCACTATA
TTTTACTCTCTATGGCTTCACCAATTA
TTTTACTCTCTGTGGCTTCCCACTATA
TTTTACTCTCTGTGGCTTCACTTATTTTTTTTCTTACTATTTTTTTTTTTTT
TTTTACTCTCTGTGGCTTCCCACTATA
TTTTACTCTCTGTGGCTTCACCAATTA
TTTTACTCTCTGTGGCTTCCCACTATA

TTTTACTCTCTGTGGCTTCCCACTATA
TTTTACTCTCTGTGGCTTCCCACTATA
TTTTACTCTCTATGGCTTCACCTATTA
TTTTACTCTCTGTGGCTTCCCACTATA
TTTTACTCTCTATGGCTTCACCTATTA
TTTTACTCTCTGTGGCTTCCCACTATA
TTTTACTCTCTGTGGCTTCACCAATTA
TTTTACTCTCTGTGGCTTCCCACTATA
TTTTACTCTCTGTGGCTTCCCATTA
TTTTACTCTCTGTGGCTTCACCAATTA
TTTTACTCTCTGTGGCTTCCCACTATA
TTTTACTCTCTGTGGCTTCACCAATTA
TTTTACTCTCTTGGCTTTTTTTTTTTTTTTTTTTTTTTTTTTTTTTTTTTTTTCTCTA
TTTTACTCTCTGTGGCTTCCCACTATA
TTTTACTCTCTGTGGCTTCACCAATTA
TTTTACTCTCTGTGGCTTCACCAATTA
TTTTACTCTCTGTGGCTTCACCAATTA
TTTTACTCTCTGTGGCTTCCCACTATA
TTTTACTCTCTATGGCTTCACCTATTA
TTTTACTCTCTGTGGCTTCCCACTATA
TTTTACTCTCTATGGCTTCACCAATTATCTTTCTCTCTGTGGCTTCCCACTATA
TTTTACTCTCTATGGCTTCACCAATTA
TTTTACTCTCTGTGGCTTCCCACTATATTTTTTTTTTTTTTTTTTTTTTTTTTTTTTTTTCTTTA
TTTTACTCTCTGTGGCTTCACCAATTA
TTTTACTCTCTGTGGCTTCCCACTATA
TTTTACTCTCTGTGGCTTCACCAATTA
TTTTACTCTCTGTGGCTTCCCATTA
TTTTACTCTCTGTGGCTTCACCAATTA
TTTTACTCTCTGTGGCTTCACCAATTA
TTTTACTCTCTGTGGCTTCACCAATTA
TTTTACTCTCTGTGGCTTCCCTCTATA
TTTTACTCTCTGTGGCTTCCCACTATA
TTTTACTCTCTATGGCTTCACCAATTATCTTTCTCTCTGTGGCTTCCCACTATA
TTTTACTCTCTGTGGCTTCACCAAGTA
TTTTACTCTCTGTGGCTTCACCAATTA
TTTTACTCTCTGTGGCTTCACCAATTA
TTTTACTCTCTGTGGCTTCCCTCTATA
TTTTACTCTCTGTGGCTTCCCACTATA
TTTTACTCTCTATGGCTTCACCAATTATCTTTCTCTCTGTGGCTTCCCACTATA
TTTTACTCTCTGTGGCTTCACCAATTA
TTTTACTCTCTGTGGCTTCACCTATTA
TTTTACTCTCTGTGGCTTCCCACTATA
TTTTACTCTCTATGGCTTCACCAATTATCTTTCTCTCTGTGGCTTCCCACTATA
TTTTACTCTCTGTGGCTTCACCAATTA
TTTTACTCTCTGTGGCTTCCCACTATA
TTTTACTCTCTGTGGCTTCCCACTATA
TTTTACTCTCTATGGCTTCACCAATTATCTTTCTCTCTGTGGCTTCCCACTATA
TTTTACTCTCTGTGGCTTCACCAATTA
TTTTACTCTCTGTGGCTTCACCAATTA
TTTTACTCTCTGTGGCTTCCCACTATA
TTTTACTCTCTGTGGCTTCACCAATTA
TTTTACTCTCTGTGGCTTCACCAATTA
TTTTACTCTCTGTGGCTTCACCAATTA
TTTTACTCTCTGTGGCTTCACCAATTA

TTTTACTCTCTGTGGCTTCACCAATTA
TTTTACTCTCTGTGGCTTCACCAATTA
TTTTACTCTCTGTGGCTTCCCACTATA
TTTTACTCTCTGTGGCTTCCCACTATA
TTTTGCTCTCTGTGGCTTCCCTCTATA
TTTTACTCTCTGGCTTCACAGTATA
TTTTATTCTCTGGCATCACAATATA
TTTTACTCTTTGGCTTCGCAGAATA
TTTTACACTCTGGCTTCACAGAATA
TTTTACTCTCTGGCTTCGCAGAATA
Nr of selected repeats 309 Similarity 0.884164
Consensus:
TTTTACTCTCTGTGGCTTCCCAcTaTA
>Cele-UNSB01_1:961004-961082 Satlength=79 Nr of Repeats=6 RepeatLength=12
seed=AGCCTAAGCC
AGCCTAAGCCTA
AGCCTAAGCCTA
AGCCTCAGCCTA
AGCTTAAGCCTA
AGCCTAAGCCTC
AGCCTAAGCCTAAGCCTA
Nr of selected repeats 5 Similarity 0.866667
Consensus:
AGCcTaAGCCTa
>Cele-UNSB01_1:961195-961471 Satlength=277 Nr of Repeats=23 RepeatLength=12
seed=CTAAGTCTAA
CTAAGTCTAAGT
CTAAGTCTAAGT
CTAAGTCTAAGT
CTAAGTCTAAGT
CTAAGTCTAAGT
CTAAGTCTAAGT
CTAAGTCTAAGT
CTAAGTCTAAGT
CTAAGTCTAAGT
CTAAGTCTAAGT
CTAAGTCTAAGT
CTAAGTCTAAGT
CTAAGTCTAAGT
CTAAGTCTAAGT
CTAAGTCTAAGT
CTAAGTCTAAGT
CTAAGTCTAAGT
CTAAGTCTAAGT
CTAAGTCTAAGT
CTAAGTCTAAGT
CTAAGTCTAAGT
CTAAGTCTAAGT
CTAAGTCTAAGT
Nr of selected repeats 23 Similarity 1.000000
Consensus:
CTAAGTCTAAGT
>Cele-UNSB01_1:961002-961808 Satlength=807 Nr of Repeats=44 RepeatLength=12
seed=TAAGCCTAAG

TAAGCCTAAGCC
TAAGCCTAAGCC
TAAGCCTCAGCC
TAAGCTTAAGCC
TAAGCCTAAGCC
TCAGCCTAAGCC
TAAGCCTAAGCC
TAAGCCTAAACTTAAGTC
TAATCCTAAGTCTATTCACTAAAATTTTGACGTAAAACTTGGGACTAAAAATTTTGGACACAAATTTTTTACCTAATTT
TTTGGTC
TAAGCCTAAGTC
TAAGTCTAAGTC
TAAGTCTAAGTC
TAAGTCTAAGTC
TAAGTCTAAGTC
TAAGTCTAAGTC
TAAGTCTAAGTC
TAAGTCTAAGTC
TAAGTCTAAGTC
TAAGTCTAAGTC
TAAGTCTAAGTC
TAAGTCTAAGTC
TAAGTCTAAGTC
TAAGTCTAAGTC
TAAGTCTAAGTC
TAAGTCTAAGTC
TAAGTCTAAGTC
TAAGTCTAAGTC
TAAGTCTAAGTC
TAAGTCTAAGTC
TAAGTCTAAGTC
TAAGTCTAAGTC
TAAGTCTAAGTC
TAAGTCTAAGTC
TAAGCCTAATAAGCCTAAATAGGGTCTAACGCTTGCCACTGACGCCGCTAACGCTCGCCACTGAAGCCACGCC
TAAGCCTAAGCATAAATCAATTTGAAACTTCAGACTTAAATTTCTTGGTGGCCTATACGACTCTCTGTTAAATTTCGCT
TTGATCAGCAAACGGGAACCCTTTGAAAAATTAAAAAACTAACGCTCGCCACTGACGCCAAGTC
TAAACCTAAGGGTAATAC
TAAGCCTAAGCC
TAAGCCTAAGCC
TAAGCGTAAGCT
TAAGCGTAAGCG
TAAGCGTAAGCG
TAAGCCTAAGCC
TAAGCCTAAGTG
TAAGCGTAAGCG
Nr of selected repeats 39 Similarity 0.765770
Consensus:
TAAGtCTAAGtC
>Cele-UNSB01_1:980318-980990 Satlength=673 Nr of Repeats=16 RepeatLength=42
seed=CCGGAAATTT
CCGGAAATTTCGAAAACCGGCAATTGCCGAAATTGCCCTTTG
CCGGAAATTTCGAAAACCGGCAATTGCCGAAATTGCCCATTG
CCGGAAATTTCGAAAACCGGCAATTGCCGAAGTTGCCGACTC

CCGGAAATTTTGAAAACCGGCAATTGCCGAAATTGCCGACTC
CCGGAAATTTTGAAAACCGGCAATTGCCGAAGTTGCCGACTC
CCGGAAATTTTGAAAACCGGCAATTGCCGAAATTGCCGATTG
CCGGAAATTTCGAAAACCGGCAATTGCCGAAATTGCCGCTTG
CCGGAAATTTCGAAAACCGGCAATTGCCGAAATTGCCGATTG
CCGGAAATTTTGAAAACCGGCAATTGCCGAAATTGCCCATTG
CCGGAAATTTCGAAAACCGGCAATTGCCGAAATTGCCGCTTG
CCGGAAATTTTGAAAACCGGCAATTGCCGAAACTGCCCTTTG
CCGGAAATTTCGAAAACCGGCAATTGCCGAAGTTGCCGACTC
CCGGAAATTTCGAAAACCGGCAATTGCCAAAATTGCCGATTG
CCGGAAATTTTGAAAACCGGCCACTGCCGAAATTGCCGACTC
CCGGAAATTTCGAAAACCGGCCACTGCCGAAATTGCCGACTC
CCGGAAATTTCGAAAACCGGCAATTGCCGAAATTGTCGATTG
Nr of selected repeats 16 Similarity 0.888889
Consensus:
CCGGAAATTTcGAAAACCGGCAATTGCCGAAATTGCCgatTg
>Cele-UNSB01_1:995161-995964 Satlength=804 Nr of Repeats=5 RepeatLength=162
seed=ATCATATGAA
ATCATATGAAGCGATTATTTTTTCTTTTATAAACCTGTTCAGGATTTGCATTATTATGAGAAAATCCTAGGCCAATTAG
GCTAGCATGTACGGAAGTATTTTTTAAAAATTTATAAAAGTAATGAAAAGCTGATATTTTCAAAATTCCAGAATGACGG
AAAA
ATCATATGAAATGCTTTTTTTTTCTTTTTTAAACCTATTTAGAATTGTCAAAACTACCAGAAAATACTAAACAAAATAG
CTTAGCTTGACCGGAAATATTTTTAAAAAATTGATAAAAATAATGAAAAGCTGATATTTTCAAAATTCCAGAATGACGG
AAAA
ATCATATGAAGCGATTCTTTTTTCTTTTATAAACCTCTTTAGAATTGTCAAAACTACCAGAAAATACTAAACAAAGTAG
GTTAGCTTAACCGGAAGTAATTTTTACAAATTGATAAAAATAATAAAAAGATGATTTTTTCAAAATTCCAAAATGACTG
AAAA
ATCATATGAAGCGATTCGTTTTTCTTTCATAAAATAGTTTAGAATTGGAATAATGATCAGAAAATACTAAACAAAGTAG
GTTAGCTTGACCGGAAGTATTTTTAAAAAATTGATTAAAAAAACTGATTTTTAAAAAATTCCAGAATGACGGAAAA
ATCATATGAAGCGATTCTTTTTTCTTTTATAAAGTAGTTCAGAATTAGCATTATTATGAGAAAATCCTAGGCGAATAAG
GCTAGCATGTACGAAAGTATTATTTAAAAATTTATAAAAGTAATGAAAAGCTGATTTTTTAAAAATTCCAGAATGACGG
AAAA
Nr of selected repeats 4 Similarity 0.781893
Consensus:
ATCATATGAAgcGaTTcTTTTTTCTTTTaTAAAcctgTTcAGaATTggCAaaAcTAccAGAAAATaCTAaaCaAAttAG
gcTAGCaTgaaCGgAAgTAtTtTTtAaAAATTgATAAAAaTAATgAAAAGcTGATaTTTTcAAAATTCCAgAATGACgG
AAAA
>Cele-UNSB01_1:1009660-1009762 Satlength=103 Nr of Repeats=8 RepeatLength=12
seed=GGTCTAGGTC
GGTCTAGGTCTA
GGTCTAGGTCTA
GGTCTAGGTCTA
GGTCTAGGTCTA
GGTCTTGGTCTA
GGTCTAGGTCTA
GGTCTAGGTCTT
GGTCTAGGTCTAGTTCTA
Nr of selected repeats 7 Similarity 0.936508
Consensus:
GGTCTAGGTCTA
>Cele-UNSB01_1:1012680-1013098 Satlength=419 Nr of Repeats=4 RepeatLength=32
seed=CAAAATTTTT
CAAAATTTTTGACGTGGAAGAATTTTAAGATTTTCGAGGCCAAGAGGTGGCCTAGAATTCAACATTTATTGGCAATTCT
TATCTAGATCCCGAACCCCGACCAGTCAGTTTTTAATTTCCGGTGTCTGAATTTTTAATTAAAAATTATTTTTGGGAGG

AGCTCCTGTTTTTTTTTCGTCAAAAAATGAAGGTGGCCTAGCTTTCTCATTTTAAGGCCACGAATTCTAGTTAATTTTT
TGTTGGCTCATTTTTTGTTTCAAAAAACGAAAGTTAGTCAAAAACTTTTCTGCATATCAAATGCTCTTTTTGAGGTTTT
TTCCTG
CAAAATTTTTCTAAGAAAATTTGAATTCCCGC
CAAAATTTTTCTCAGAAAATTTGAATCGCCGC
CAAAATTTTTCTCTGAAAATTTGAATCGCCGC
Nr of selected repeats 3 Similarity 0.888889
Consensus:
CAAAATTTTTCTcaGAAAATTTGAATcgCCGC
>Cele-UNSB01_1:1018800-1018890 Satlength=91 Nr of Repeats=5 RepeatLength=12
seed=AGCCTAAGCC
AGCCTAAGCCTAAGCCTAAGTTTGAGCATA
AGCATAAGCCTA
AGCCTATGCCTA
AGCCTACGCCTC
AGCCTAAGCCAAATGCGGAGCCTA
Nr of selected repeats 3 Similarity 0.740741
Consensus:
AGCcTAnGCCTa
>Cele-UNSB01_1:1018801-1019027 Satlength=227 Nr of Repeats=14 RepeatLength=12
seed=GCCTAAGCCT
GCCTAAGCCTAAGCCTAAGTTTGAGCATAA
GCATAAGCCTAA
GCCTATGCCTAA
GCCTACGCCTCA
GCCTAAGCCAAATGCGGA
GCCTAAGCCTAA
GCCTAAGTCTAAGTCTAAGTCTAA
GTCTAAGCCTAA
GCCTAAGCCTAA
GCCTAAGCCTGCCGGAGCCTAAGTATAA
GCCTAAGCCTAA
GCCTAAGCCTAA
GCCTAAGCCTAT
GCCTAAGCCTAAGCCGAT
Nr of selected repeats 9 Similarity 0.854938
Consensus:
GCCTAaGCCTAA
>Cele-UNSB01_1:1031726-1032601 Satlength=876 Nr of Repeats=25 RepeatLength=35
seed=ATTTTCAATT
ATTTTCAATTCCGGCAATTTTCCGATTTGCCGGAA
ATTTTCAATTCCGGCAATTTGCCGATTTGCCGGAA
ATTTTCAATTCCTGCAGTTTGCCGATTTGCCGGAA
ATTTTCAATTCCGGCAGTTTGCCGATTTGCCGGAA
ATTTTCAATTCCGGCAGTTTGCCGATTTGCCGGAA
ATTTTCAATTCCGGCAGTTTGCCGATTTGCCGAAA
ATTTTCAATTCCGGCAATTTTCCGATTTGCCGGAT
ATTTTCAATTCCGGCAGTTTGCCGATTTGCCGGAA
ATTTTCAATTCCGGCAATTTGCTGATTCGCCGGAA
ATTTTCAATTCCTGCAGTTTGCCGATTTGCCGGAA
ATTTTCAATTCCGGCAGTTTGCCGATTTGCCGGAA
ATTTTCAATTCCGGCAGTTTGCCGATTTGCCGGAA
ATTTTCAATTCCGGCAGTTTGCCGATTTGCCGGAA
ATTTTCAATTCCGGCAGTTTGCCGATTTGCCGAAA

ATTTTCAATTCCGGCAATTTTCCGATTTGCCGGAT
ATTTTCAATTCCGGCAGTTTGCCGATTTGCCGGAA
ATTTTCAATTCCGGATATTTGCCGATTTGCCGGAA
ATTTTCAATTCCGGCAATTTGCTGATTCGCCGGAA
ATTTTCAATTCCAGCAGTTTGCCGATTTGCCGGAA
ATTTTCAATTCCGGATATTTGCCGATTTGCCGGAA
ATTTTCAATTCCGGCAATTTTCCGATTTGCCGGAA
ATTTTCAATTCCGGCAGTTTGCCGATTTGCCGGGA
ATTTTCAATTCCGGCAATTTGCCGATTTGCCGGAA
ATTTTCAATTCCGGCATTTTGCCGATTTGCCGGAA
ATTTTCAATTCCGGCAATTTGCCGATTTGCCGGAA
Nr of selected repeats 25 Similarity 0.921397
Consensus:
ATTTTCAATTCCGGCAgTTTGCCGATTTGCCGGAA
>Cele-UNSB01_1:1103105-1103421 Satlength=317 Nr of Repeats=8 RepeatLength=35
seed=CGGCAAATCG
CGGCAAATCGGCATTTTGCCGGAATTGAAAATATC
CGGCAAATCGGTATTTTGCCGGAATTGAAAATTTT
CGGCAAATCGGCAATTTGTCGGAATCGAAAATTTC
CGGCAAATCGGCATTTTGCCGGAATTGAAAATATC
CGGCAAATCGGTATTTTGCCGGAATTGAAAATTTTCGGCAAATTTAGCAGTTTGCCGGAATGGAAAATATC
CGGCAAATCGGTATTTGGCCGGAATTGAAAATTTT
CGGCAAATCGGCAATTTGTCGGATTTGAAAATTTC
CGGCAAATCGGCATTTTGCCGGAATTGAAAATATC
Nr of selected repeats 7 Similarity 0.873016
Consensus:
CGGCAAATCGGcAtTTTGcCGGAATTGAAAATtTc
>Cele-UNSB01_1:1117625-1118154 Satlength=530 Nr of Repeats=23 RepeatLength=23
seed=GGCCTAGAAA
GGCCTAGAAATACTAAACGTGAT
GGCCTAGAAATACTGAAAGTGGT
GGCCTAGAAATACTGAGCGTGGT
GGCCTAGAAATTCTAAAAGTGGT
GGCCTAGAAATACTGAACGTGGT
GGCCTAGAAATACTGAACGTGGT
GACCTAGAAAAACTAAACGTGGT
GGCCTAGAAATACTGAGCGTGGT
GGCCTAGAAATACCGAGCGTGGT
GGCCTAGAAATTCTAAAAGTGGT
GGCCTAGAAATACTGAACGTGGT
GGCCTAGAAATTCTGAAAATGGT
GGCCTAGAAATACTGAATGTGGG
GGCCTAGAAATGCTGAACGTGAT
GGCCTAGAAATACTGAAAATGGT
GACCTAGAAATACTGAATGTGGG
GGCCTAGAAATGCTGAACGTGAT
GGCCTAGAAATACTGAGCGTGGT
GGCCTAGAAATTCTAAAAGTGAT
GGCCTAGAAATACTGAAAATGGT
GGCCTAGAAACACTGAATGTGGG
GGCCTAGAAATGCTGAACGTGAT
GGCCTAGAAATACTGAACGGGAT
Nr of selected repeats 23 Similarity 0.818525
Consensus:

GGCCTAGAAATaCTgAAcGTGgT
>Cele-UNSB01_1:1127917-1128577 Satlength=661 Nr of Repeats=21 RepeatLength=20
seed=ATTTCTAGGC
ATTTCTAGGCCATCAATATAAATTCTAGGTTATATTTCAAATATCTAGGGGACCAATGATGCTTCTATGTTATATATTA
A
ATTTCTAGGCTATCGATGAG
ATTTCTAGGCCATGTTCAGA
ATTTCTAGGCTATCAATAAAT
TTTCTAGGCTATCGATGAG
ATTTCTAGGCCATATTCAGA
ATTTCTAGGTAATCCATAAA
ATTTCTAGGCCATCGATAAG
ATTTCTAGGCCAGATTTAGA
ATTTTTAGGCCATCGATGAA
ATTTCTAGGCCATCAATATG
ATTTCTAGGCCATGTTCAGA
ATTTCTAGACTATCAATAAATTTTCTAAGCCATCGATAAGATTTCTGGACCATATTTAGA
ATTTCTAGGCCACCAATGAA
ATTTCTGGGCCAAATTTTCAATTTCTTTTTTTTTTA
ATTTCTAGGTCATATTCAAA
ACTTCTAGGCCGCCAATAAA
ATTTCTAGGCCAAATCTTTAAATTCTAGGTCATATTTTGA
ATTTCTAGGCCACCAATGAGATTTCTAGAACAAAAAAGCTTAAGTTCTAGGTCACATTTTGA
ATTTCTAGGCCACCAATAAAATTTCTAGATAATATTTTAAATGTCTAAAACACATTTAAAAATTTTCTAAGCCACTAAT
AAA
AGTTCTAGGCCACGGATAAG
Nr of selected repeats 13 Similarity 0.625153
Consensus:
ATTTCTAGGCCAtcnaTnAg
>Cele-UNSB01_1:1149569-1149724 Satlength=156 Nr of Repeats=10 RepeatLength=12
seed=CCTAAGCCTA
CCTAAGCCTAAG
CCTAAGCCTATGCCTAGCACAAG
CCTAAGCCTAAG
CCTCAGCCTAAACCTAAGCAAAAG
CCTAAACCTAAG
CCTAAACCTAAGCAAAAG
CCTAAGTCTAGG
CCTAAGCCTAAG
CCTAAACCTAAG
CCTAAGCCTAAGCCTAAG
Nr of selected repeats 6 Similarity 0.866667
Consensus:
CCTAAgCCTAAG
>Cele-UNSB01_1:1158888-1159076 Satlength=189 Nr of Repeats=5 RepeatLength=31
seed=ATTCCCGCCA
ATTCCCGCCAATTTTTTTCTCAGGAAATGTGA
ATTCCCGCCAATTTTTTCTCAGAAATTTTGA
ATCCCCGCCATTTTTTCCTAGCGAAATTTGA
ATTCCCGCCAAACTTTTTCTCAGAAAGTTGAATTCGCGTCAAATTTGTTCACAGGAAATTTGA
ATTCCCACCAAAATTTTCTCAGGAAATCTGC
Nr of selected repeats 3 Similarity 0.628472
Consensus:
ATtCCCgCCaAttTTTTCtcAGgAAaTtTGa

>Cele-UNSB01_1:1232170-1233535 Satlength=1366 Nr of Repeats=32 RepeatLength=43
seed=CCAGAAATTA
CCAGAAATTAGAATTTTCGGCAAATCGGCAAGCCGGCAAATTG
CCAGAAATTAGAATTTTCGGCAAATCAACAAACTGACAAATTG
CCAGAAATTAAAATCTCCGGCAAATCGGCAAATTG
CCAGAAATTAAAATTTTCGGCAAATCAACAAACCGGCAAATTG
CCAGAAATTAGAATTTCCGGCAAATCAGCAAACTGACAAATTG
CCAGAAATTAAAATCTCCGGCAAATCGGCAAATTG
CCAGAAATTAGAATTTTCGGCAAATCAGCAAACCGGCAAATTG
CCAGAAATTAGAATTTTCGGCAAATCGGCAAGCCGGCAAATTT
CCAGAAATTAGAATTTTCGGCAAATCGGCAAGCCGGCAAATTT
CCAGAAATTAGAATTTTCGGCAAATCAACAAACTGACAAATTG
CCAGAAATTAAAATCTCCGGCAAATCAGCAAATTG
CCAGAAATTAAAATCTCCGGCAAATCGGCAAGTTG
CCAGAAATTAAAATTTCCGGCAAACCGAAAAATTG
CCAGAAATTAAAATTTCCGGCAAATCAGCAAATTGCCAGATAGTAGAATTTCCGGCAAATCAGCAAACCGGCAAATTG
CCAGAAATTAGAATTTCCGGCAAATCGGCAAGCCGGCAAATTG
CCAGAAATTAGAATTTCCGGCAAATCAGCAAACCGGCAAATTG
CCAGAAATTAGAATTTCCGGCAAATCAGCAAACCGGCAAATTT
CCAGAAATTAGAATTTTCGGCAAATCGGCAAGCCGGCAAATTG
CCAGAAATTAGAATTTTCGGCAAATCAACAAACTGACAAATTG
CCAGAAATTAAAATCTCCGGCAAATCAGCAAATTG
CCAGAAATTAGAATTTTCGGCAAATCAGCAAACCGGCAAATTG
CCAGAAATTAGAATTTCCGGCAAATCAGCAAACTGACAAATTG
CCAGAAATTAAAATCTCCGGCAAATCGGCAAATTG
CCAGAAATTAGAATTTTCGGCAAATCAGCAAACCGGCAAATTG
CCAGAAATTAGAATTTTCGGCAAATCGGCAAGCCGGCAAATTT
CCAGAAATTAGAATTTTCGGCAAATCGGCAAGCCGGCAAATTT
CCAGAAATTAGAATTTTCGGCAAATCGGCAAACTGACAAATTG
CCAGAAATTAAAATCTCCGGCAAATTGGCAAATTG
CCAGAAATTAAAATTTCCGGCAAATCGGCAAACCGCCAGATTG
CCAGAAATCAAATTTTCTGCAAATTGCCAGAATTTTAATTTTCCGGCAAATCGGCAAATTG
CGAGAAATTAGAATTTCCGGCAAATCAGCAAACTGGCAAATTG
CCAGAAATTAGAATTTTCGGCAAATCAGCAAACCGGCGAATTG
Nr of selected repeats 22 Similarity 0.892211
Consensus:
CCAGAAATTAGAATTTtCGGCAAATCaGCAAaCcGgCAAATTg
>Cele-UNSB01_1:1234705-1235262 Satlength=558 Nr of Repeats=15 RepeatLength=35
seed=AAATCGGCAA
AAATCGGCAATATGCCTAAAATGAAAATTTCCGGC
AAATCGGCAATTTGCCTAAAATGAAAGGTTCCGG
AAATCGGCAATTTTCCTAAAATGAAAGGTTCCGGC
AAATCGGCAATTTTCCTAAAATGAAAGATTCCGGC
AAATCGGCAAGTTGTCAAAAATGATAGATTCCGGC
AAATCGGCAAAAAGCCAGAAATTAAAATTTTCTGCAAATCCACAATTTGCCTAAAATTAAAATTTCCGG
AAATCGGCAATTTGCCGAAAATGAGAGATTCCGGC
AAATCGGCAATTTGTCAAAAATGATAGATTCCGGC
AAATCGGCAAAAAGCCAGAAATTAAAATTTCCGGC
AAATCGGCAATTTGCCGAAAATGATAGATTCCGGC
AAACCGGCAAAAAGCCAGAACTTAAAATTTCCGG
AAATCGGCAATTTGCCGAAAATGAGAGATTCCGGC
AAATCGGCAATTTTTCAAAAATGATAGATTCCGGC
AAATCGGCAAAAAGCCAGAAATTAAAATTTCCGGC
AAATCGGCAATTTGCCTAAAATGAAAATTTCCAGC

Nr of selected repeats 12 Similarity 0.802020
Consensus:
AAATCGGCAAttTgcCnAAAATGAaAgaTTCCGGC
>Cele-UNSB01_1:1236946-1237103 Satlength=158 Nr of Repeats=7 RepeatLength=11
seed=GTACTGTAGG
GTACTGTAGGG
GTACTGTAGGA
GTACGGTAGGATTACTGTAGTTTAGGGAAATTTGACTTTTTCTCTTTTGAATGGATATTGGTTTGGGGTTAGTGGGGGG
GATATGGTCGGG
GTACTGTAGTA
GTACTGTAGGG
GTACTGTAGGA
GTACTGTAGTA
Nr of selected repeats 6 Similarity 0.870707
Consensus:
GTACTGTAGga
>Cele-UNSB01_1:1302534-1303556 Satlength=1023 Nr of Repeats=33 RepeatLength=19
seed=TTCTAGGCCA
TTCTAGGCCACGCAGCAAAAAAAAAAACAAAATCCAATTTTGGAATACTATTTTTATGTCCGCAAAATAGATTAATTTC
CTTGAAAATAGTAA
TCTAGGCCACCACTAGATTTGTAAACTATGTGGGAAGAAAAGATGAACCTCGGCCACCTTGGGAAAACCTAGGCCGCGG
GTCAAAGCAAGGCCTCACTCCTAGACCACATTAAGCTTTAATTTATCGGATTGTCAAGGCCTGCTGATAAT
TTCTAGGCCATCTGATAAT
TTCTAGGCCATCTGAGGAT
TTCTAGGCCATCTGAGGAT
TTCTAGGCCATCTGAGGATTTCTAGGATATCTGAGGAT
TTCTAGGCCATCTGAGGAT
TTCTAGGCCACCTGGGGAA
TTCTAGGCCATCTGAGGAT
TTCAAGGCCATCTGAAAATTTCTGGGTTATCTGAGGAT
TTCTAGGCCATTTCATGAT
TTCTAGGCCATTTGAGGAT
TTCTAGGCCATCTGAGGAT
TTCTAGGCCACCTGGGGAA
TTCTAGGCCATCTGAGGAT
TTCTAGGCCATCTGAGGAT
TTCTAGGCCATCTGGGGAAT
TTTAGGCCATCTGTGGAT
TTCTAGGCCATCTGAGGATTTCTAGGATATCTGAGGATTTCTAGTCCCACTGAAGAT
TTCTAGACCACCTGGGGAA
TTCTAGGCCAGCAATGGATT
TTTAGGCCATCTGTGGAT
TTCTAGGCCATCTGAGGATTTCTAGGATATCTGAGGAT
TTCTAGGCCCACTGAAGAT
TTCTAGACCACCTGGGGAA
TTCTAGGCCAGCAATGGATTTTTAGGCCTTCAGAGGATTTGTAGGCCCTTTGAGGATTTCTAGGATATCTGAGGAT
TTCTAGGCCATTTGAGGATTTTTTGGCCATCTGAAAATTTCTGTGCTATCTGAGGAT
TTCTAGACCACCTGGGGAT
TTCTTGGCCACCTGGGTAA
TTCTAGGCCATTTGAGGAT
TTCTAGGCCAGCAATGGAT
TTCTAGGCCTTCCGAGGAT
TTCTATGCCAGCAATGGGT
Nr of selected repeats 21 Similarity 0.753383

Consensus:
TTCTAGGCCAtCTGaGGAt
>Cele-UNSB01_1:1341786-1343571 Satlength=1786 Nr of Repeats=47 RepeatLength=38
seed=GGGAATTCAA
GGGAATTCAAATTTTAATTGTTTTGAAAATATTTTGGC
GGGAATTCAAACTTTAATTTTTTTGAAAACATTTTGGC
GGGAATTCAAACTTTAATTTTTTTGAAAACATTTTAGC
GGGAATTCAAATTTTAATTTTTTTGAAAACATTTTGAC
GGGAATTCAAATTTTAATTTTTTTGAAAACATTTTGGC
GGGAATTCAAATTTTAATTGTTTTGAAAATATTTTGGC
GGGAATTCAAACTTTAATTTTTTTGAAAACATTTTGGC
GGGAATTCAAACTTTAATTTTTTTGAAAACATTTTAGC
GGGAATTCAAATTTTAATTTTTTTGAAAACATTTTGAC
GGGAATTCAAATTTTAATTTTTTTGAAAACATTTTGGC
GGGAATTCAAATTTTAATTGTTTTGAAAATATTTTGGC
GGGAATTCAAACTTTAATTTTTTTGAAAACATTTTAGC
GGGAATTCAAATTTTAATTTTTTTGAAAATATTTTAGC
GGGAATTCAAATTTTAATTGTTTTGAAAATATTTTGGC
GGGAATTCAAACTTTAATTTTTTTGAAAACATTTTGGC
GGGAATTCAAACTTTAATTTTTTTGAAAACATTTTAGC
GGGAATTCAAATTTTAATTTTTTTGAAAACATTTTGAC
GGGAATTCAAATTTTAATTTTTTTGAAAACATTTTGGC
GGGAATTCAAATTTTAATTGTTTTGAAAATATTTTGGC
GGGAATTCAAACTTTAATTTTTTTGAAAACATTTTAGC
GGGAATTCAAATTTTAATTTTTTTGAAAATATTTTAGC
GGGAATTCAAATTTTAATTTTTTTGAAAACATTTTGGC
GGGAATTCAAATTTTAATTTTTTTGAAAACATTTTGGC
GGGAATTCAAATTTTAATTTTTTTGAAAACATTTTAGC
GGGAATTCAAATTTTAATTTTTTTGAAAGCATTTTGGC
GGGAATTCAAATTTTAATTTTTTTGAAAACATTTTGGC
GGGAATTCAAATTTTAATTTTTTTGAAAACATTTTGGC
GGGAATTCAAATTTTAATTTTTTTGAAAATATTTTAGC
GGGAATTCAAATTTTAATTTTTTTGAAAACATTTTAGC
GGGAATTCAAATTTTAATTTTTTTGAAAGCATTTTGGC
GGGAATTCAAATTTTAATTTTTTTGAAAATATTTTGGC
GGGAATTCAAATTTTAATTTTTTTGAAAACATTTTAGC
GGGAATTCAAATTTTAATTTTTTTGAAAACATTTTAGC
GGGAATTCAAATTTTAATTTTTTTGAAAGCATTTTGGC
GGGAATTCAAATTTTAATTTTTTTGAAAATATTTTGGC
GGGAATTCAAATTTTAATTGTTTTGAAAATATTTTGGC
GGGAATTCAAATTTTAATTTTTTTGAAATTATTTTAGC
GGGAATTCAAATTTTAATTTTTTTGAAAGCATTTTGGT
GGGAATTCAAATTTTAATTTTTTTGAAAGCATTTTGGC
GGGAATTCAAATTTTAATTTTTTTGAAAGCATTTTGGC
GGGAATTCAAATTTTAATTTTTTTGAAAACATTTTGGC
GGGAATTCAAATTTTAATTTTTTGAAAACATTTTGGC
GGGAATTCAAATTTTAATTTTTTTGAAAACATTTTGGC
GGGAATTCAAATTTTAATTTTTTTGAAAACATTTTGGC
GGGAATTCAAATTTTAATTTTTTTGAAAACATTTTGGC
GGGAATTCAAATTTTAATTTTTTTGAAAACATTTTGGC
GGGAATTCAAATTTTAATTTTTTTGAAAACATTTTGGC
Nr of selected repeats 46 Similarity 0.937825
Consensus:
GGGAATTCAAATTTTAATTTTTTTGAAAAcATTTTgGC

>Cele-UNSB01_1:1346901-1347222 Satlength=322 Nr of Repeats=5 RepeatLength=42
seed=TTTGCCGGAA
TTTGCCGGAAATTTTCATTTCAGGCAAATTGCCGGTTTGACG
TTTCCCGGAAGTTTTCATTTTCGACAAATTACCGATTTGTCG
TTTGCCGAATATCAGA
TTTGCCGGAAATGTATAGATGGATTTTTTATAAGACAGAAACATTTAAAACTGTGCCTTTTTGAATTTTTTCCCGTTTT
TTCTACATATTTTCATGGAATTTGCTTACTTTCGAAAATTTTCGGCAAATTGCCGTTTTTCTGACAAATTCGGCAAATC
GACAGTTTGCCAGTTTGCCGG
TTTGCCGGAAATTTTAATTCCGGAAAATTGCCGATTTGCCAA
Nr of selected repeats 3 Similarity 0.705426
Consensus:
TTTgCCGGAAaTTTTcAtTTccGgcAAATTgCCGaTTTGnCg
>Cele-UNSB01_1:1386048-1386208 Satlength=161 Nr of Repeats=5 RepeatLength=32
seed=AAAATTTTGG
AAAATTTTGGCGGGAATTCAAATTTTCTGAGA
AAAATTTTGGCGGGAATTCAAATTTTCTGAGA
AAAATTTTGGCGGGAATTCAAATTTTCTGAGA
AAAATTTTGGCGGGAATTCAAATTTTCTGAGA
AAAATTTTGGCGGGAATTTAAATTTTCTGAGA
Nr of selected repeats 5 Similarity 0.983333
Consensus:
AAAATTTTGGCGGGAATTcAAATTTTCTGAGA
>Cele-UNSB01_1:1435639-1436319 Satlength=681 Nr of Repeats=8 RepeatLength=71
seed=ATTTATGTGA
ATTTATGTGAAAATTCAGGTTTTTAGGGCATTTTTTGATGGAAATCTGAATATTTCCGCTTTTTCTGATGG
ATTTATGTGAAAATTCAGGTTTTTAGGGCATTTTTTGAGGGAAATCTGATTTTTTTTCGATTTTCTGGCGA
ATTTATGTGAAAATTCAGGTTTTTAGGGCATTTTTTGAGGGAAATCTGAATTTTTTCGGTTTTTCGGATGG
ATTTATGTGAAAATTTTAAGGTTTAGGGTATTTTTACGGGAAAAATGGATTTTTAGCCGTTTTTTGTGGAAAATTCCGA
TTTTTTGCCGAATTTTTAAGAAAATTTCGGATCAAAAAAGACTTTTCAGGCTTTTAGGAACATTTTTTAAAGGAAATCT
GAATATTTCCGCTTTTTCTGATGG
ATTTATGTGAAAATTCAGGTTTTTAGGGCATTTTTTGATGGAAATCTGAATATTTCCGCTTTTTCTGATGG
ATTTATGTGAAAATTCAGGTTTTTAGGGCATTTTTTGAGGGAAATCTGATTTTTTTTCGATTTTCTGGCGA
ATTTATGTGAAAATTCAGGTTTTTAGGGCATTTTTTGAGGGAAATCTGAACTTTTCCGATTTTTCCAACCA
ATTTCTGTGAAAATGCAGGTTTTCGAGACACATTTTTGAAGGAAATCGTAAAATGTTCGGTTTTTCTGAAGG
Nr of selected repeats 6 Similarity 0.847840
Consensus:
ATTTATGTGAAAATTCAGGTTTTTAGGGCATTTTTTGAgGGAAATCTGAaTtTTTcCGatTTTTCtGacGa
>Cele-UNSB01_1:1456315-1457241 Satlength=927 Nr of Repeats=24 RepeatLength=35
seed=AAATTTGAAT
AAATTTGAATTTCCCGCCAAAATCTTTTTTCTCAGA
AAATCTGAATTTCCCGCAAAATTTTTTTTCTCAGA
AAATTTGAATTCCCGCCAAAATTTTTCAAAGA
AAATTTGAATTCCCGCCAAAATTTCTTTTCTCATAAATTTTGAAGTTCCCGCCCATATTTTTAAGGGTCTCACCACGAT
GGGTCTCGCCAGTTCCAGGTGGTACTTAAGCTAACAAAAAGTTTCTTTGA
AAATTTGAATTTCCCGGCAAAATGTTTTTCACTGA
AAATTTGAAATTCCCGCCAAAATGTTTTTCACTGA
AAATTTGAATTTCCCGCCAAAATTTTTTTCACTGA
AAATTTGAACTTCCCCCCAAAATTTTTCTCACAGA
AAATTTGAATTTCCCGCCAAAATTTTTTTCACTGA
AAATTTGAATTTCCCGCCAGAAATTTTTTTACTGA
AAATTTGAATTTCCCGCTAAAATTTTTTTTCACTG
AAATTTGAATTTCCGCTAAAAATTTTTTTCACTG
AAATTTGAATTTCCCGCCAAAATTTTTTCACTGA
AAATTTGAATTTCCCGCCAAAATTTTTTTCACTGA

AAATTTGAATTTCCCGCCAGAAATTTTTTTACTGA
AAATTTGAATTTCCCGCTAAAATTTTTTTTCACTG
AAATTTGAATTTCCGCTAAAAATTTTTTTCACTG
AAATTTGAATTTCCCGCCAAAATTTTTTCACTGA
AAATTTGAATTTCCCGCCAAAATTTTTTTCACTGA
AAATTTGAATTTCCCGCCAAAAATTTTTTCACTGA
AAATTTGAATTTCCGCTAAAAATTTTTTTCACTG
AAATTTGAATTTCCCACCAAAAATTTTTTTCACTG
AAATTTGAATTTCCCGCCAAAATTTTTTCACTGA
AAATTTGAATTTCCCGCCAAAATTTTTTTCACTGA
Nr of selected repeats 15 Similarity 0.827628
Consensus:
AAATTTGAATTTCCCGCcAAAATTTTTTTCACTGa
>Cele-UNSB01_1:1457508-1457876 Satlength=369 Nr of Repeats=14 RepeatLength=11
seed=CTACAGTACT
CTACAGTACTA
CTACAGTACCCCGACCACATCCCATCACTAACCTCAAACCAATATCCCTTCAAAGGCGAAAAGTCAATTTTTCTCAAA
CTACAGTAATT
CTACAGTACTC
CTACAGTACCT
CTACAGTACTA
CTACAGTACCCCGACCATATCCAACCACTAACCTCAAACCAATATCCCATCAAAAGGCGAAAAGTCACATTTTCCCAAA
CTACAGTAATC
CTACAGTACTC
CTACAGTACCT
CTACAGTACTA
CTACAGTACCCCGACCATATCCCACCACTAACCTAAACCAATATCCCATCAAAAGACGAAAAGTCAATTTTTCCCAAA
CTACAGTAATC
CTACAGTACTCCTACAGTACCT
Nr of selected repeats 10 Similarity 0.753704
Consensus:
CTACAGTAcTn
>Cele-UNSB01_1:1530606-1531290 Satlength=685 Nr of Repeats=18 RepeatLength=31
seed=GGTTACTGTA
GGTTACTGTAGATCGATCCTGAAATTCTAAAAAAGTACTGTAGATCAATTTGGGGATACTTCA
GGTTACTGTAACTCAATTATAGGATTCTTCG
GGTTACTGTAGATAGATCCTAGGATTCTGAAA
GTTACTGTAGATCGGTTCTAGGATACTTTG
GGTTACTGTAGATCGGGTCTAGGATACTTTG
GGTTATTGTAACTCAATTATAGGATTCATCG
GGTTACTGTAGATCGGTTCTAGGATACTTTG
GGTTACTGTAGATCAGGTCTAGGATACTTTG
GGTTACTGTAACTCAATTATAGGATTCATCG
GGTTACTGTAGATCGATCCTAGGATTCTAAAATTTACTGTAGATCGGTTCTAGGATACTTTT
GGTTACTGTAACTCAATTATAAAATACTTCG
GGTTACTGTAACTCAATTATAAGATTCATCG
GGTTACTGTAGATCGATCCTAGAATTCTGAAAGTAACTGTAGATCGGTTCTAGGATTCTTCG
GGTTACTGTAGATCGGGTCTAGGATACTTTG
GGTTACTGTAACTCAATTATAAAATACTTTG
GGTTACTGTAACTCAATTATAAAATACTTTGGGTTCCTGTGGCCCGATTCTAGGATTATTCG
GGTTACTGTAGATCGGGTCTAGGATACTAAA
GGTTACTCTAGGATCAATCCTAGGATTCTAGG
Nr of selected repeats 11 Similarity 0.738025
Consensus:

GGTTACTGTAacTCaatTaTAgGATaCtTnG
>Cele-UNSB01_1:1591838-1592129 Satlength=292 Nr of Repeats=7 RepeatLength=35
seed=CGGCAAATCG
CGGCAAATCGGCAAATTGTCGGAATTGAAAATTTC
CGGCAAATCGGCAAATTGCCGGAATTGAAAATTTC
CGGCAAATCGGCAAATTGCCGGAATTGAAAATTTC
CGGCAAATCGACAAATTGCCGAAATTGGAAATTTC
CGGCAAATCGGCAAATTGCCGGAATTGAAAATTTC
CGGAAAATCGACAAATTGCCGAAATTGAAATCTTC
CGGCAAATCGGCAAATTGCCGGAAGACAATCCCTTCAGAAAAATCCGGTAAACTGCCGGAATTGAAAATTTCCGGCAAT
TT
Nr of selected repeats 6 Similarity 0.895873
Consensus:
CGGCAAATCGgCAAATTGCCGgAATTGAAAATTTC
>Cele-UNSB01_1:1620106-1621181 Satlength=1076 Nr of Repeats=5 RepeatLength=189
seed=TTTTTTTTGA
TTTTTTTTGAGAAGTTTTTAAAAAGTTTCATTATGAAATTCGATGTTTTCAGACAATTTTGAGTCTAATTAAGCAATAA
AAAAAATTCCACTACACCACCTTTAAATTTTGCCACGTCGCTAAATAAGCACTAGTTTTATCAATTTTAAAACTTGGAA
AAATCCAGAAAGAAAAATATGTTTTCTGGAAATTAAAATCAAATCCTTAATGATGTTTTGATTTTCCAGGTATCACCAT
CTCCAAATGAACGAATGCAGATGGGCCTGAACCCACGTGGCTCAATGAGCCCATGTCCTGTACACGGTGGAAGTCAGCA
ACTATCCCACTTATAGTTTTTTGACTATTCCTTTCGAATTTCCTTTCAATTTGTTACGTGTTACCAAGATTCACCTCTT
TCCGTTC
TTTTTTTTAAATATCAATTGGTTTGCATTGTTTCAAGTGCCCCCTGATTTCTGCTCCTTAATTCACTTATCTGTTTTCT
CACGGGTTTAGGATATTTTTTCTGTTA
TTTTTTTTGACTATGGAATTATAAAGAAAATCGAATTTCAAAAAAGCATGTATTAGCCAATGTAAACGCGCTCCAATGA
TGATGTAGCCCACGTGGTATCAGAGTGTCTCTTTACGGCTTGATTTAAATACGTAGATCTACAAATTCACTTATCTGTT
TTCTCACGGGTTTAGGATATTTTTTCTGTTA
TTTTTTTTGACTATGGAATTATAAAGAAAATCGAATTTCAAAAAAGCATGTATTAGCCAATGTAAACGCGCTCCAATGA
TGATGTAGCCCACGTGGTATCAGAGTGTCTCTTTACGGCTTGATTTAAATACGTAGATCTACAAATTCACTTATCTGTT
TTCTCACGGGTTTAGGATATTTTTTCTGTTA
TTTTTTTTGACTATGGAATTATAAAGAAAATCGAATTTCAAAAAAGCATGTATTAGCCAATGTAAACGCGCTCCAATGA
TGATGTAGCCCACGTGGTATCAGAGTGTCTCTTTACGGCTTGATTTAAATACGTAGATCTACAAATTCACTTATCTGTT
TTCTCACGGGTTTAGGATATTTTTTCTGTTA
Nr of selected repeats 3 Similarity 1.000000
Consensus:
TTTTTTTTGACTATGGAATTATAAAGAAAATCGAATTTCAAAAAAGCATGTATTAGCCAATGTAAACGCGCTCCAATGA
TGATGTAGCCCACGTGGTATCAGAGTGTCTCTTTACGGCTTGATTTAAATACGTAGATCTACAAATTCACTTATCTGTT
TTCTCACGGGTTTAGGATATTTTTTCTGTTA
>Cele-UNSB01_1:1632570-1633118 Satlength=549 Nr of Repeats=6 RepeatLength=35
seed=AAATTTTCAA
AAATTTTCAAACATTTCCATATCGTGCGCCTTTAAAATAACGGAAATCGCGGCGAGACCCATTCCATGCACCTTTAAAG
TAGCACCACCCAGCGTTGGATTTCTGCTTTAAAATCGGTTTTTTACCATAATATCTAGTTTTTTGGCTGAAAAATCGAT
TTTTTCGCAAATTTCACGAAAAAAATCGCACAAAAATGTTTTTTTTGCTCATTTTTTCCTCAAAAATCCGAATTTCCAG
GCAATTTCCATTAAAAACCCCGTTTTTTGCCGCAAAATTCTTAATTTTTCACTAAAAATCGTAAGCTTAAAATTTTTTA
AATTGAAATTCCAAAATTTCCCGCAAAAATCGCTCCGGCAATTTGCTGATTTGCCGG
AAATTTTCAATTCCGGCATTTTGCCGATTTGCCGG
AAATTTTTAATTCCGCCAATTTGCCGATTTGCCGG
AAATTTTCAATTCCGGCATTTTGCCGATTTGCCGG
AAATTTTTAATTCCGCCAATTTGCCGATGTGCCGA
AAATTTTCAATTCCGGCAAATTGCCGATTTGCCGA
Nr of selected repeats 5 Similarity 0.878095
Consensus:
AAATTTTcAATTCCGgCAatTTGCCGATtTGCCGg

>Cele-UNSB01_1:1647038-1647577 Satlength=540 Nr of Repeats=9 RepeatLength=49
seed=CAATAATTTT
CAATAATTTTAAATTAGATTATCAATTTTTCTAAATTTTTCCGAAAAAT
CAATAATTTTAAATTAAATTATCAATTTTTCTAAATTATGCCGAAAAATCGATAATTTCAAATTTAATTATCGATTTTT
TTAAATTTTTTCGAAAAAT
CAATAATTTTAAATGTAATTATCGATTTTTCTAAATTTTGCCGAAAAAT
CAATAATTTTAAATTAAATTATCGATTTTTCTAAATTTTTCCGAAAAAT
CAATAATTTTAAATTAAATGATCGATTTTTCTAAATTTTTCCGAAAAAT
CAATAATTTTAAATTTACTTATCGATTTTTCTAAATTATGCCGAAAAATCGATAATTTCAAATTTAATTATCGATTTTT
TAAAATTTTTTCGAAAAAT
CAATAATTTTAAATTTAATTATCGATTTTTCTAAATTTTGCCGAAAAAC
CAATAATTTTAAATTAAATTATCGATTTTTCTGAATTTTTCCGAAAAAT
CAATAATTTTAAATTAAATTATTGACTTTTCTAAATTTTGCCGAAAAAT
Nr of selected repeats 7 Similarity 0.909297
Consensus:
CAATAATTTTAAATTaAATTATCGATTTTTCTAAATTTTtCCGAAAAAT
>Cele-UNSB01_1:1679297-1679633 Satlength=337 Nr of Repeats=16 RepeatLength=21
seed=TCTCCTGGTA
TCTCCTGGTAAATTTAAAAAT
TCTCCTGGTAAATTTAAAAAT
TCTCCTGGTAAATTTAAAAAT
TCTCCTGGTAAATTTAAAAAT
TCTCCTGGTAAATTTAAAAAG
TCTCCTGGTAAATTTAAAAAT
TCTCCTGGTAAATTTAAAAAT
TCTCCTGGTAAATTAAAAAAT
TCTCCTGGTAAATTTTAAAAT
TCTCCTGGTAAATTTAAAAAT
TCTCCTGGTAAATTTTAAAAT
TCTCCTGGTAAATTTAAAAAT
TCTCCTGGTAAATTTAAAAAT
TCTCCTGGTAAATTTAAAAAT
TCTCCTGGTAAATTTAAAAAT
TCTCCTGGTAAATTTTAAAAT
Nr of selected repeats 16 Similarity 0.963492
Consensus:
TCTCCTGGTAAATTTAAAAAT
>Cele-UNSB01_1:1680231-1680351 Satlength=121 Nr of Repeats=7 RepeatLength=12
seed=AGCCTAAACC
AGCCTAAACCTA
AGCCTAAACCTG
AGCCTGAACCAT
AGCCTAAACCTGAAACTGAACCATAGCCTAAGCAAT
AGCCTAAGCCTG
AGCTTAAACCTG
AGCCTGAACCATAGCCTAAGAAAT
Nr of selected repeats 5 Similarity 0.702564
Consensus:
AGCcTaAaCCTg
>Cele-UNSB01_1:1685357-1686631 Satlength=1275 Nr of Repeats=49 RepeatLength=26
seed=CAAAACCGGC
CAAAACCGGCAATTGCCGAAAACTTC
CAAAACCGGCAATTGCCGAAAATTCC
CAAAACCGGCAATCGCCGAAAATTCC

CAAAACCGGCAATTGCCGAAAATTCC
CAAAACCGGCAATTGCCGAAAATTCC
CAAAACCGGCAATTGCCGAAAACTTC
CAAAACCGGCAATTGCCGAAAATTCC
CAAAACCGGCAATTGCCGAAAACTTC
CAAAACCGGCAATTGCCGAAAATTCC
CAAAACCGGCAATTGCCAAAAATTCC
CAAAACCGGCAATTGCCAAAAATTCC
CAAAACCAGCAATTGCCAAAAATTCC
CAAAACCGGCAATTGCCGAAAATTTC
CAAAACCGGCAATTGCCGAAAATTCC
CAAAACCGGCAATTGCCAAAAATTCC
CAAAACCGGCAATTGCCGAAAATTCC
CAAAACCGGCAATTGCCAAAAATTCC
CAAAACCGGCTATTGCCGAAAATTTC
CAAAACCGGCAATTGCCGAAAATTCC
CAAAACCGGCAATTGCCAAAAATTCC
CAAAACCGGCAATTGCCGAAAATTCC
CAAAACCGGCAATTGCCAAAAATTCC
CAAAACCGGCAATTGCCGAAAATTCC
CAAAACCGGCAATTGCCAAAATTTCC
CAAAGCCGGCAATTGCCGAAAATTTC
CAAAACCGGCAATTGCCAAAAATTCC
CAAAACCCGCAATTGCCGAAAATTCC
CAAAACCGGCAATTGCCAAAAATTCC
CAAAACCGGCAATTGCCGAAAATTCC
CAAAACCGGCAATTGCCAAAATTTCC
CAAAGCCGGCAATTGCCGAAAATTTC
CAAAACCGGCAATTGCCAAAAATTCC
CAAAACCCGCAATTGCCGAAAATTCC
CAAAACCGGCAATTGCCAAAAATTCC
CAAAACCGGCTATTGCCGAAAATTTC
CAAAACCGGCAATTGCCGAAAATTCC
CAAAACCGGCAATTGCCAAAAATTCC
CAAAACCGGCAATTGCCAAAAATTCC
CAAAACCGGCTATTGCCGAAAATTTT
CAAAACCGGCAATTGCCGAAAATTCC
CAAAACCGGCAATTGCCGAAAATTCC
CAAAACCGGCAATTGCCAAAAATTCC
CAAAACCGGCAATTGCCGAAAATTCC
CAAAACCGGCAATTGCCAAAAATTCC
CAAAACCGGCTATTGCCAAAAATTCC
CAAAACCGGCAATTGCCGAAAATTCC
CAAAACCGGCAATTGCCAAAAATTCC
CAAAACCGGCTATTGCCGAAAATTTC
CAAAACCGGCAATTGCCGAAAATTCC
Nr of selected repeats 49 Similarity 0.924036
Consensus:
CAAAACCGGCAATTGCCgAAAATTcC
>Cele-UNSB01_1:1691009-1691188 Satlength=180 Nr of Repeats=4 RepeatLength=45
seed=TCTGATCCTT
TCTGATCCTTCAGCTCCGAAGGATCACATGGGTACCTTCTGATGT
TCTGATCCTTCAGATAAGAAGGATCGTAAGGGTGCCACTTGATGG
TCTGATCCTTCAGATCCAAAGGATCACGAGGCTCTTCCTGATGT

TCTGATCCTTTAGCTCCAAAGGATCACATGGGTACCTTCTGATGT
Nr of selected repeats 3 Similarity 0.743210
Consensus:
TCTGATCCTTcAGcTccgAAGGATCacAtGGGTaCCttcTGATGt
>Cele-UNSB01_1:1730783-1731662 Satlength=880 Nr of Repeats=24 RepeatLength=30
seed=AATTTGAATT
AATTTGAATTTTCCTGGATTTTTAAACGGA
AATTTGATTATTTCCGAATTTTTTAGCGAA
AATTTGAATTTTCCCGACTTTTTAGTTGAAATTTAAACTTTCCCAAAGTTTTAAACGGA
AATTTGAATTTTCCCGAACTTTTTAACGAA
AATTTGAATTTTCCCGAATTTTTAAATCTA
AATTTGATTTTTCTCGAATTTTTTAAAAGGA
AATCTGAATTTTCCCGAATTTTTAAGCGAAA
AATTGAATTTTTCCGATTTTTAAGCGAA
AATTTAAATTTTCCGAAATTTTTAATCGAT
AATTTGAATTTTCCCGAATTTTTTAGTGAA
AACTTGAATTTTCCTGGATTTTTCATCGAT
AATTTGAATTTTCTCGAAGTTTTCAACGGA
AATCTGAATTTTCCCGAACTTTTTAACGAA
AATTTGAATTTACCCGAGTTTTTAAGCGAAAAGTTGAGTTTTTTTCGAATTTTTAAGCGGA
AATTTGAATTTTCTCGAAGTTTTCAACGGA
AATTTGAATTTTCTCGAAATGTTTACCAAAAGACTGAATTTTCCCGAATTTTTTAGTGAA
AATTTGAATTTTCCTGGATTTTTAGTTGAA
AATTCGAATTTTCTCGAAGTTTTCAACGGAAATCTGCGTTTTCCCGATTTTTTAGCAAA
AATTTGACTTTTCCCGAACTTTTTAAAGAA
AATTTGAATTTTCCCGAATTTTTTAGTGAA
AATTTGAGTTTTTCCGAGTTTTTTTAGCAAA
AATTTGAAATTTCCCGAATTTTTTAGTGAAAATTTCAGTTTTGCCGAATTTTGGAATGAA
AATTTGAATTTCTTCAAAGTTTCCCGTATTTTTGAGCGAA
AATTTGAATTTTCCCGATTTTTTTACTGGA
Nr of selected repeats 15 Similarity 0.732487
Consensus:
AATTTGAaTTTTCccGaAtTTTTnAacGaA
>Cele-UNSB01_1:1781850-1784996 Satlength=3147 Nr of Repeats=154 RepeatLength=20
seed=CCACCAAAAA
CCACCAAAAAGGTTTCTAGG
CCACCAAAAAGATTTCTAGG
CCACCAAAAATGTTTCTAGG
CCACCAAAAATGTTTCTAGG
CCACCAAAAATGTTTCTAGG
CCACCAAAAATGTTTCTAGG
CCACCAAAAATGTTTCTAGG
CCACCAAACAGGTTTCAATG
CCACCAAAAATGTTTCTAGG
CCACCAAAAATGTTTCTAGGC
CCCCAAAAAATTTTTCTAGG
CCACCAAAAAGGTTTCTAGG
CCACCAAAAATGTTTCTAGG
CCACCAAAAAGGTTTCTAGG
CCACCAAACAGGTTTCAATG
CCACCAAAAAGGTTTCTAGGCCACCAACCAGGTTTCAATG
CCACCAAAAATGTTTCTAGG
CCACCAAAAAGGTTTCTAGG
CCACCAAAAATGTTTCTAGG

CCACCAAAAAATGTTTCTAGG
CCACCAAAAAAGGTTTCTAGG
CCACCAAACAGGTTTCAATG
CCACCAAAAAATGTTTCTAGG
CCACCAAACAGGTTTCAATG
CCACCAAAAAAGGTTTCTAGG
CCACCAAAAAAGGTTTCTAGG
CCACCAAAAAATGTTTCTAGG
CCACCAAAAAAGGTTTCTAGG
CCACCAAACAGGTTTCAATG
CCACCAAAAAATGTTTCTAGG
CCACCAAACAGGTTTCAATG
CCACCAAAAAATGTTTCTAGG
CCACCAAACAGGTTTCAATG
CCACCAAAAAATGTTTCTAGG
CCACCAAAAAAGGTTTCTAGG
CCACCAAAAAATGTTTCTAGG
CCACCAAAAAATGTTTCTAGG
CCACCAAAAAAGGTTTCTAGG
CCACCAAACAGGTTTCAATG
CCACCAAAAAATGTTTCTAGG
CCACCAAACAGGTTTCAATG
CCACCAAAAAATGTTTCTAGG
CCACCAAAAAATGTTTCTAGGC
CCCCAAAAAAATTTTTCTAGG
CCACCAAAAAAGGTTTCTAGG
CCACCAAAAAATGTTTCTAGG
CCACCAAAAAAGGTTTCTAGG
CCACCAAACAGGTTTCAATG
CCACCAAAAAAGGTTTCTAGGCCACCAACCAGGTTTCAATG
CCACCAAAAAATGTTTCTAGG
CCACCAAAAAAGGTTTCTAGG
CCACCAAAAAATGTTTCTAGG
CCACCAAAAAATGTTTCTAGG
CCACCAAAAAAGGTTTCTAGG
CCACCAAAAAAGGTTTCAAGG
CCACCAAAAAAGGTTTCAATG
CCACCAAAAAATGTTTCTAGG
CCACCAAACAGGTTTCAATG
CCACCAAAAAAGGTTTCTAGG
CCACCAAAAAATGTTTCTAGA
CCACCAAAAAAGGTTTCTAGG
CCACCAAACAGGTTTCAATG
CCACCAAAAAAGGTTTCTAGG
CCACCAAACAGGTTTCAATG
CCACCAAAAAATGTTTCTAGG
CCACCAAAAAAGGTTTCTAGG
CCACCAAAAAATGTTTCTAGG
CCACCAAAAAATGTTTCTAGG
CCACCAAAAAAGGTTTCTAGG
CCACCAAACAGGTTTCAATG
CCACCAAAAAATGTTTCTAGG
CCACCAAACAGGTTTCAATGC
CCCCAAAAAAATTTTTCTAGG

CCACCAAAAAGGTTTCTAGG
CCATCAAAAATGTTTCTAGA
CCACCAAAAAGGTTTCTAGG
CCACCAAAAATGTTTCTAGA
CCACCAAAAAGGTTTCTAGG
CCACCAAAAATGTTTCTAGG
CCACCAAAAAGGTTTCTAGG
CCACCAAAAATGTTTCTAGG
CCACCAAAAAGGTTTCTAGG
CCACCAAACAGGTTTCAATG
CCACCAAAAAGGTTTCTAGGCCACCAACCAGGTTTCAATG
CCACCAAAAATGTTTCTAGG
CCACCAAAAAGGTTTCTAGG
CCACCAAAAATGTTTCTAGG
CCACCAAAAATGTTTCTAGG
CCACCAAAAAGGTTTCTAGG
CCACCAAAAAGGTTTCAAGG
CCACCAAAAAGGTTTCAATG
CCACCAAAAATGTTTCTAGG
CCACCAAACAGGTTTCAATG
CCACCAAAAAGGTTTCTAGG
CCACCAAACAGGTTTCAATG
CCACCAAAAAGGTTTCTAGA
CCACCAAAAAGGTTTCTAGG
CCACCAAACAGGTTTCAATG
CCACCAAAAAGGTTTCTAGG
CCACCAAACAGGTTTCAATG
CCACCAAAAATGTTTCTAGG
CCACCAAAAAGGTTTCTAGG
CCACCAAAAATGTTTCTAGG
CCACCAAAAATGTTTCTAGG
CCACCAAAAAGGTTTCTAGG
CCACCAAACAGGTTTCAATG
CCACCAAAAATGTTTCTAGG
CCACCAAACAGGTTTCAATGC
CCCCAAAAAATTTTTCTAGG
CCACCAAAAAGGTTTCTAGG
CCACCAAAAATGTTTCTAGA
CCACCAAAAAGGTTTCTAGG
CCACCAAAAATGTTTCTAGA
CCACCAAAAAGGTTTCTAGG
CCACCAAAAATGTTTCTAGG
CCACCAAAAAGGTTTCTAGG
CCACCAAACAGGTTTCAATG
CCACCAAAAATGTTTCTAGG
CCACCAAAAATGTTTCTAGGC
CCCCAAAAAATTTTTCTAGG
CCACCAAAAAGGTTTCAATG
CCACCAAAAATGTTTCTAGG
CCACCAAAAAGGTTTCTAGG
CCACCAAAAATGTTTCTAGG
CCACCAAAAATGTTTCTAGG
CCACCAAAAAGGTTTCTAGG
CCACCAAACAGGTTTCAATG

CCACCAAAAATGTTTCTAGG
CCACCAAACAGGTTTCAATG
CCACCAAAAAGGTTTCTAGG
CCACCAAAAATGTTTCTAGA
CCACCAAAAAGGTTTCTAGG
CCACCAAACAGGTTTCAATG
CCACCAAAAAGGTTTCTAGG
CCACCAAACAGGTTTCAATG
CCACCAAAAATGTTTCTAGG
CCACCAAAAAGGTTTCTAGG
CCACCAAAAATGTTTCTAGG
CCACCAAAAATGTTTCTAGG
CCACCAAAAAGGTTTCTAGG
CCACCAAACAGGTTTCAATG
CCACCAAAAATGTTTCTAGG
CCACCAAACAGGTTTCAATG
CCACCAAAAATGTTTCTAGG
CCACCAAAAATGTTTCTAGGC
CCCCAAAAAATTTTTCTAGG
CCACCAAAAAGGTTTCTAGG
CCACCAAAAATGTTTCTAGA
CCACCAAAAAGGTTTCTAGG
CCACCAAAAATGTTTCTAGA
CCACCAAAAAGGTTTCTAGG
CCACCAAAAATGTTTCTAGG
CCACCAAAAAGGTTTCTAGG
CCACCAAAAATGCTTCTAGG
Nr of selected repeats 145 Similarity 0.874815
Consensus:
CCACCAAAAAgGTTTCtAgG
>Cele-UNSB01_1:1787086-1788479 Satlength=1394 Nr of Repeats=64 RepeatLength=20
seed=TTCTAGGCCA
TTCTAGGCCACCAAAAAGGT
TTCTAGGCCACCAAGAAAGT
TTCTAGGCCACCAAAGTAT
TTATAGGCCACCTAAGATGT
TTCTAGGCCACCTGAGATGT
TTCTAGGTCACCAAAAATGTTTCTCGGTCACCAAAAATGT
TTCAAGGCCACCGAAAAGGT
TTCTAGGCCACCTAAGTAT
TTCTAGGCCACCTAAGATGT
TTCTAGGCCACCTGAGATGT
TTCTAGGTCACCAAAAATGTTTCTAGGTTACCAAAAATGT
TTCAAGGCCATCGAAAAGGT
TTCTAGGCCACCAAAGTAT
TTCTAGGCCACCTAAGATGT
TTCTAGGCCACCTGAGATGT
TTCTAGGTCACCAAAAATGT
TTCAAGGCCACCGAAAAGGT
TTCTAGGCCACCAAAAAGGT
TTCTAGGCCACCAAAAATAT
TTCTAGGCCACCTAAGATGT
TTCTAGGCCACCTGAGATGT
TTCTAGGCCACCTGAGATGT

TTCTAGGCCACCTGAGATGT
TTCTAGGTCACCAAAAATGTTTCTCGGTCACCAAAAATGT
TTCAAGGCCACCGAAAAGGT
TTCTAGGCCACCTAAGTAT
TTCTAGGCCACCTAAGATGT
TTCTAGGCCACCTGAGATGT
TTCTAGGTCACCAAAAATGTTTCTAGGTTACCAAAAATGT
TTCAAGGCCATCGAAAAGGT
TTCTAGGCCACCAAAGTAT
TTCTAGGCCACCTAAGATGT
TTCTAGGCCACCTGAGATGT
TTCTAGGTCACCAAAAATGT
TTCAAGGCCACCGAAAAGGT
TTCTAGGCCACCAAAAAGGT
TTCTAGGCCACCAAAAATAT
TTCTAGGCCACCAAAAATGT
TTCTAGGTCACCAAAAATGT
TTCTAGGTCACCAAAAATGTATCAAGGCCACCAAAAAGGT
TTCTAGGTCACCAAAAATGT
TTCTAGGCCACCAAAAATGT
TTCTAGGTCACCAAAAATGT
TTCTAGGCCACCAAAAAGGT
TTCTAGGCCACCAAAAAGGT
TTCTAGGCCACCAAAAAGGT
TTCTAGGCCACCAAAAAGGT
TTCAAGGCCACCAAAAAGGT
TTCTAGGCCACCAAAAATGT
TTCTAGGTCACCAAAAATGT
TTCTAGGCCACCAAAGTAT
TTCTAGGCCACCTAAAAGGT
TTCTAGGCCATCAAAAAGGT
TTCTAGGCCATCAAAAAGGA
TTCTAGGCCACCAAAAATAT
TTCTAGGCCACCTAAGATGT
TTCTAGGCCACCAGAGTAT
TTCTAGGCCACCTAAGAGGT
TTCTGGGCCATCAAAAAGGTTTCAAGTCCATCAAAAAGGT
TTCTAGGCCACCAAAAAGGT
TTCTAGGCCACCGAAAAGGT
TTCTAGGCCACCAAAAAGGT
TTCTAGACCACCTAAGACAT
TTCTAGGCCAACAAAAAGGT
Nr of selected repeats 51 Similarity 0.812235
Consensus:
TTCTAGGCCACCaAAaAtGT
>Cele-UNSB01_1:1822719-1823169 Satlength=451 Nr of Repeats=9 RepeatLength=43
seed=AAATCGGCAA
AAATCGGCAAACCGGCAATTTGCCGAAATTGGAAATTTCTGGC
AAATCGGCAACCCCGCAATTTGCCAGAATTGAAAATTTCCGGA
AAATCGGCAAATCGGCAAACCAGCAATTTGCCGGAATTTAAAATTTTCAGC
AAATCGGCAAACCGGCAATTTGCCGGAATTGAAAATTTCCGGA
AAATCGACAAACCGGCAGATTGCCGGAATTTAAAATTTCCGGC
AAATCGGCAAACCGGTGAATAGCCGCAATTTGAAATTTCCGGC

AAATCGGCAAAACTGGCAAATTGCCGTAATTTAAAATTTCTGGCAAATTACCAGAATTGAAAATTTACGGCAAATTAAC
GGAATTGAAAATTTCTGCC
AAATCGGCAAACCGGCAATTTACCGGAAATGAAAATTTCCGGC
AAATCGACAACCCGGCAAATTGCCGAAAATGACAATTTCCGGC
Nr of selected repeats 7 Similarity 0.793282
Consensus:
AAATCGgCAAaCCGGCAAtTTGCCGgAAtTgaAAATTTCCGGc
>Cele-UNSB01_1:1824052-1824465 Satlength=414 Nr of Repeats=9 RepeatLength=43
seed=GCCGGAATTG
GCCGGAATTGAAAATTTCGGGCAAATCGGTAAGCCGGCCAATT
GCCGGAATTGAAAATTTCTGGCAAATCGGCAAACCGACACATA
GCCGGAATTGAAAATTTCTGGCAAATCAAAAAACCGGCAAATTGCCGAAGTTTAAAATTTCTGGAAATCGGCAAATCGG
CAAACT
GCCGGAATTGAAAATTTCTGGCAAATT
GCCGGAATTGAAAATTTCTGGCAAGTCGGCTAACCGGCAAATT
GCCGGAATTGAAAGTTCCTGGCAAATCGGCAAATCGGCACATA
GCCGGAATTGAAAATGTCTGGCAAATCGGCAAACCGGCACATA
GCCGGAATTGAAAATGTCTGGCAAATCGGCAAACCGGCACATA
GCCGGAATTGAAAATGTCTGGCAAATCGGCAAACCGGCACATA
Nr of selected repeats 7 Similarity 0.864157
Consensus:
GCCGGAATTGAAAATtTCTGGCAAATCGGCAAACCGGCAcATa
>Cele-UNSB01_1:1833668-1834030 Satlength=363 Nr of Repeats=11 RepeatLength=26
seed=TTGCCGGTTT
TTGCCGGTTTTACGAAATTTCGGCAATTGTCTGTTTTGGGAATTATCGGCAA
TTGCTGGTTTTGGGAATTATCGGCAC
TTGCCGGTTTTCAAAATTTCCGGCAATTGTCAGTTTTGGAAATTTCGGCAATTGTCTGTTTTGGGAATTATCGGCAA
TTGCTGGTTTTGGGAATTATCGGCAC
TTGCCGGTTTTCAAAATTTCCGGCAA
TTGCCGGTTTTGGGAATTTTTGGCAA
TTGCCGGTTTTGGAAATTTTCGGCTA
TTGCCGATTTTGGGAAATTTCGGCAA
TTGCCGGTTTTGGGAATTTTTGGCTA
TTGCCGGTTTTGGGAATTTTCGGCTA
TTGCCGGTTTTGGGAATTTTTGAAA
Nr of selected repeats 8 Similarity 0.798535
Consensus:
TTGCcGGTTTTGGgAATTtTcGGCaa
>Cele-UNSB01_1:1840655-1841028 Satlength=374 Nr of Repeats=9 RepeatLength=35
seed=TTTGCCGGAA
TTTGCCGGAAATGTTTAAAGGAGGATTTTTTTATACTTTGGAAGCACTTCAAACTGTGCCTTTTTGAAATTTTTTCCCG
TTTTTTGTTGCCAA
TTTGCCGGAAATTTTCAAATCCGGCAATTTGCCGA
TTTGCCGGAAAATTTAAATTCTGGCAAATTACCGA
TTTGCCGGAAATTTTCAATACCGGCAAGTTGCCAA
TTTGCCGGAAATTTTCAATTCCGGCAAGTTGCCGA
TTTACCGGAAATGTTCAATACCGGCAAGTTGCCGA
TTTACCGGAAATTTTCAAATCCGGCAGTTTGCCGA
TTTGCCGGAAAATTTAAATTCTGGCAAATTACCGA
TTTGCCGGAAATTTTAAATACCGGTAAGTTGACAT
Nr of selected repeats 8 Similarity 0.766700
Consensus:
TTTgCCGGAAAtTTTcAAttCcGGCAAgTTgCCgA

>Cele-UNSB01_1:1841619-1842039 Satlength=421 Nr of Repeats=18 RepeatLength=19
seed=AAAATCAATA
AAAATCAATAAATCCTAAG
AAAATCAATAAATCCTAAG
AAAATCAATAAATCCTAAG
AAAATCAATAAATCCTAAG
AAAATCAATAAATTCACAG
AAAATCAATAAACTCCAAT
AAAATCAATAAATTCTAAG
AAAATCAATAAATTCGGATACAATCGATAATTTCCGAG
AAAATCAATAAATCATGAG
AAAATCAATAAATTCCGAGAAAATTATTAAGTTCTGAGAAAATCGGTAAATTCCCAG
AAAATCAAAAAATTTAAAATA
AAAATCAATAAATCATGAG
AAAATCAATAAATTCCTAG
AAAATCAATAAATTCCTAG
AAAATCAATAAATTCCAAG
AAAATCGATAAATTCTGAG
AAAATCAATAAATTCCGAGAAAGTCGATAAATTCTGGG
AAAATCGATATGTTCCGAG
Nr of selected repeats 14 Similarity 0.796414
Consensus:
AAAATCAATAAATtCtaAG
>Cele-UNSB01_1:1841619-1843394 Satlength=1776 Nr of Repeats=44 RepeatLength=19
seed=AAAATCAATA
AAAATCAATAAATCCTAAG
AAAATCAATAAATCCTAAG
AAAATCAATAAATCCTAAG
AAAATCAATAAATCCTAAG
AAAATCAATAAATTCACAG
AAAATCAATAAACTCCAAT
AAAATCAATAAATTCTAAG
AAAATCAATAAATTCGGATACAATCGATAATTTCCGAG
AAAATCAATAAATCATGAG
AAAATCAATAAATTCCGAGAAAATTATTAAGTTCTGAGAAAATCGGTAAATTCCCAG
AAAATCAAAAAATTTAAAATA
AAAATCAATAAATCATGAG
AAAATCAATAAATTCCTAG
AAAATCAATAAATTCCTAG
AAAATCAATAAATTCCAAG
AAAATCGATAAATTCTGAG
AAAATCAATAAATTCCGAGAAAGTCGATAAATTCTGGG
AAAATCGATATGTTCCGAG
AAAATCAATAAATTCCAAGCTTACTTTCCCACGAGGACTACACAAAATTACCTTTGAATGGTTACCGGACCACCTGCCG
TCGATGTCAAATAAATAATATCTTCATCGAAAACTATTGAAATATTGGCCTAAATATTGATGGACTAGTTTTTTTTCTA
GGCCACCGAAAAACTCACCACGTCATCGTGAATACTCTCATAAACAGCAAACCGCTTAGCTTTTGGCATTTTCCTTCTT
TTTTTTTGGCAAAAAAAGTGACGAAAGTAAGCAAATACGGAAAGTAAATACTACAAAAAAAGTGATAAGAAACTCGATG
ATTTTCTTTTTGTTATGAAATTTTAAAAATTCGACTAGAAATCATGTCATGTTGGCAAAATATTCCGATTCTGGTCAAG
CGGTATTTGGTGGGATACCTGGATTACGAGGCGCGGTGGGTTTTTTCTTCAAAAAAAATTTGTAAAAAATGTGCAAAAA
ATGTGCAAAAAATGTGCAAAAAATGTGCAAAAAATGTGCAAAAATGTCAAT
AAAATCAATAAATTCGTAG
AAAATAAATAAATTCCCAGAATTCCGAG
AAAATCAATAATTCTCAG
AAAATCGATAAATTATAAG

AACATCAATAATTTACTAG
AAAATCAATAAAATCTCACAATCAGAAATCCAATAAATTCGTAGAAAATCGATTTATTCCGAG
AAAATCAATAAATTCTCAG
AAAATCAATAAAATCTCAG
AAAATCAATAAAACCTCAA
AAAATCAATAAAATCTCAG
AAAATTAATAAATTTCCAG
AAAATCAATAAATTTTTAG
AAAATCAATATTTTTCCAGAAAAGCGATAAATTTTCAG
AAAATCAATAAATTTCAAG
AAAATCGATAAATTCCTAG
AAAATCGATATTTTTCCAGAAAAGCGATAAATTTTCAG
AAAATCAATAAATTTCAAG
AAAATCGATAAATTCCTAG
AAAATCGATATTTTTCCAGAAAAGCGATAAATTTTCAG
AAAATCAATAAATTTCAAG
AAAATCGATAAATTCCTAG
AAAATCGATAAATTCTCAG
AAAATCAATAAATTTCCAG
AAAATCAATAATTTACTAG
AAAATCAATAATTCTCAGAAAATCGTTAAACTGTAAAAAACTCGATACTATGGTCTCATTTTCCAGCTGCAACCTAAAA
CTATGCTCGAAATCCGACCAATTTCTTGTGACCTCCACCCGACACGTGGCACAAATCATATCGATTGCTGAACAATCGA
ACTCTTCGTGTAACGAATTATCAACAATTATTCTACGATACGAATTCCGGGTACTACAATTAGAGGAGATTGACGGAGT
TACTACAATGACTAGCGGAGAGTATCAA
Nr of selected repeats 32 Similarity 0.769949
Consensus:
AAAATCaATAAATtcnnAG
>Cele-UNSB01_1:1878726-1878810 Satlength=85 Nr of Repeats=4 RepeatLength=21
seed=CCTACTCTAG
CCTACTCTAGGCAGCCCCTAG
CCTACTCTAGGCAGCCCCTAG
CCTACTCTAGGCAGCCCCTAG
CCTACTCTAGGCAGCCCCTAG
Nr of selected repeats 4 Similarity 1.000000
Consensus:
CCTACTCTAGGCAGCCCCTAG
>Cele-UNSB01_1:1878816-1878916 Satlength=101 Nr of Repeats=5 RepeatLength=20
seed=CTAGGCAGAC
CTAGGCAGACTTAGCCTACC
CTAGGCAGACTTAGCCTACC
CTAGGCAGACTTAGCCTACC
CTAGGCAGACTTAGCCTACC
CTAGGCAGACTTAGCCTACC
Nr of selected repeats 5 Similarity 1.000000
Consensus:
CTAGGCAGACTTAGCCTACC
>Cele-UNSB01_1:1910732-1911187 Satlength=456 Nr of Repeats=11 RepeatLength=35
seed=AAATCGGCAA
AAATCGGCAAATTGGCGGAATTGAAAATCTCCGGC
AAATCGGCAAATTGCCGAAACTAAAAATTTCTGGT
AAATCGGCAATTTGCCGAAACTGAAAATTTCTGGCAACACGGCAAATTGCCGGAATTGAAAATTTCCGGC
ATATCGGCAATAAGCCAAAATTGAAAATTTCCGGC
AAATCGGCAAAATGCCGGAATTGAAAATTTCCGGC
AAATCGGCAAATTGTCGGAATCGAAAATTTCCGTC

AAATTGGCAAATGGACAAAATTTAAAATTTCCGGT
AAATCGGCAATACGCCGAAATTGAAAATTTCCAGC
AAATCGGCAATTTGCCGGAATTGAAAATTTCCGAC
AAATCGGCAATTTGGCGAAAATGAAAATTTCCGGCAAACTGGC
AAATTGGCAAAAATGAAAAATTTCCGGCAAGTTGGCAATTTGCCGAAACTGGAAATTCCGGT
Nr of selected repeats 8 Similarity 0.753742
Consensus:
AAATCGGCAAattGcCgaAATTgAAAATTTCCGgc
>Cele-UNSB01_1:1946077-1948348 Satlength=2272 Nr of Repeats=60 RepeatLength=35
seed=TTGCCGATTT
TTGCCGATTTGCCGATTTGCCGGAAATTTTCTAATCCGGCAAT
TTGCCGATTTGCCGGAAATTTTCATTTTTCGCAAA
TTGCCGATTTGCTGATTTGCCGGAATTTTTCATTTTTCGCAATTTGCCCGT
TTGCCGATTTACCTGAAATTTTCATTTTTGGCAAAA
TGCCGATTTGCCGGAAATTTTCTATTCCGGCAAT
TTGCCGATTTGCCGGAAATTTTCATTTTTGGCAAT
TTACCGATTTTCCGGAATTTTTCATTTTTGGCAATTTGCCCGT
TTGCCGATTTTCCGGAAATTTTCATTTTTCGCAAA
TTGCCGATTTGCCGGAATTTTTCATTTTTGGCAAA
TTGCCGATTTGCCGGAAATTTTCATTTTTGGCAAT
TTGTCGATTTGCCGGAATTTTTCATTTTTGGCAATTTGCCCGT
TTGCCGATTTTCCGGAAATTTTCATTTTTCGCAAA
TTGCCGATTTGCCGGAAATTTTCATTTTTCGCAAA
TTGCCGATTTGCCGGAATTTTTAATTTTTGGCAAA
TTGCCGATTTGCCGGAATTTTTAATTTTTGGCAAA
TTGCCGATTTGCCGGAAATTTTCATTTTTCACAAA
TTGCCGATTTGCCGGAAATTTTCATTTTTCACAAA
TTGCCGATTTGCCGGAAATTTTCATTTTTTGCAAA
TTGCCGATTTTCCGAAAATTTTCATTTTTCACAAA
TTGCCGATTTGCCGATTTGCTGGAAATTTTCATTTTTGGCAAA
TTGGCGATTTGCCGATTTTCCGGAAATTTTCATTTTTGGCAATTTGGCGGTTTGCCGGAATTTTTAATTTTTGGCAAA
TTGCCGATTTGCCGGAAATTTTCATTTTTGGCAAT
TTGTCGATTTGCCGGAATTTTTCATTTTTGGCAATTTGCCCGT
TTGCCGATTTTCCGGAAATTTTCATTTTTCGCAAA
TTGCCGATTTGCCGGAAATTTTCATTTTTCGCAAA
TTGCCGATTTGCCGAAAATTTTCATTTTTCGCAAA
TTGCCGATTTGCCGGAATTTTTAATTTTTGGCAAA
TTGCCGATTTGCCGGAATTTTTAATTTTTGGCAAA
TTGCCGATTTGCCGGAAATTTTCATTTTTCACAAA
TTGCCGATTTGCCGGAAATTTTCATTTTTTGCAAA
TTGCCGATTTTCCGAAAATTTTCATTTTTCACAAA
TTGCCGATTTGCCGATTTGCTGGAAATTTTCATTTTTGGCAAA
TTGGCGATTTGCCGATTTTCCGGAAATTTTCATTTTTGGCAAT
TTGGCGATTTGCCGATTTTCCGGAAATTTTCATTTTTGGCAAT
TTGCCCATTTGCCGATTTTCCGGAAATTTTCATTTTTCGCAAA
TTGCCGATTTGCCGGAATTTTTAATTTTTGGCAAA
TTGCCGATTTGCCGGAAATTTTCATTTTTCGCAAA
TTGCCGATTTGCCGGAATTTTTAATTTTTGGCAAA
TTGCCGATTTGCCGGAATTTTTCACTTTTGCCCGT
TTGCCGATTTTCCGGAAATTTTCATTTTTCGCAAA
TTGCCGATTTGCCGGAAATTTTCATTTTTGGCAAA
TTGCCGATTTGCCGGAATTTTTAATTTTTGGCAAA
TTGCCGATTTGCCGGAAATTTTCATTTTTCACAAA
TTGCCGATTTGCCGGAAATTTTCATTTTTTGCAAA

TTGCCGATTTTCCGAAAATTTTCATTTTTCACAAA
TTGCCGATTTGCCGATTTGCTGCAAATTTTCATTTTTGGCAAT
TTGGCGATTTGCCGATTTTCCGGAAATTTTCATTTTTGGCAATTTGCCCGT
TTGCCGATTTTCCGGAAATTTTCATTTTTCGCAAA
TTGCCGATTTGCCGGAATTTTTAATTTTTGGCAAA
TTGCCGATTTGCCGGAAATTTTCATTTTTCGCAAA
TTGCCGATTTGCCGGAATTTTTAATTTTTGGCAAA
TTGCCGATTTGCCGGAATTTTTCACTTTTGCCCGT
TTGCCGATTTTCCGGAAATTTTCATTTTTCGCAAA
TTGCCGATTTGCCGGAAATTTTCATTTTTGGCAAA
TTGCCGATTTGCCGGAATTTTTAATTTTTGGCAAA
TTGCCGATTTGCCGGAATTTTTAATTTTTGGCAAA
TTGCCGATTTGCCGGAAATTTTCATTTTTCACAAA
TTGCCGATTTGCCGGAAATTTTCATTTTTTGCAAA
TTGCCGATTTTCCGAAAATTTTCATTTTTCACAAA
TTGCCGATTTGCCGATTTGCTGGAAATTTTCATTTTTGGCAAATTGGCGAT
Nr of selected repeats 44 Similarity 0.891070
Consensus:
TTGCCGATTTgCCGGAAaTTTTcATTTTTngCAAA
>Cele-UNSB01_1:1961569-1963172 Satlength=1604 Nr of Repeats=40 RepeatLength=35
seed=GAAATTTTCA
GAAATTTTCAGTTTCGGCAAATTGCCGATTTGCCC
GAAATTTTCAGTTTCGGCAAATTGCCGATTTGCCC
GAAATTTTCAGTTTCGGCAAATTGCCGATTTGCCG
GAAATTTTCAGTTTTGTCACTTTGCCAATTTACCG
GAAATTTTCAATTCCGGCAATTTGCCTGTTTGCCGGAATTTTCGTTTTCGGCAAATTGCCGATTTGCCC
GAAATTTTCAGTTTCGGCAAATTGCCGATTTGTCC
GAAATTTTCAGTTTCGGCAAATTGCCGATTTGCCG
GAAATTTTCAGTTTCGGCAAATTGCCGATTTGTCC
GAAATTTTCAGTTTCGGCAAATTGCCGATTTGCCC
GAAATTTTCAGTTTCGGCAAATTGCTGATTTGCCC
GAAATTTTCAGTTTCGGCAAATTGCCTGTTTGCCGGAATTTTCGTTTTCGGCAAATTGCCGATTTGCCC
GAAATTTTCAGTTTCGGCAAATTGCCGATTTGTCC
GAAATTTTCAGTTTCGGCAAATTGCCGATTTGCCG
GAAATTTTCAGTTTTGTCACTTTGCCAATTTACCG
GAAATTTTCAATTCCGGCAATTTGCCTGTTTGCCGGAATTTTCGTTTTCGGCAAATTGCCGATTTGCCC
GAAATTTTCAGTTTCGGCAAATTGCCGATTTGTCC
GAAATTTTCAGTTTCGGCAAATTGCCGATTTGCCC
GAAATTTTCAGTTTCGGCAAATTGCCGATTTGTCC
GAAATTTTCAGTTTCGGCAAATTGCCGATTTGCCC
GAAATTTTCAGTTTCGGCAAATTGCTGATTTGCCC
GAAATTTTCAGTTTCGGCAAATTGCCGATTTGCCG
GAAATTTTCAGTTTTGTCACTTTGCCAATTTACCG
GAAATTTTCAATTCCGGCAATTTGCCTGTTTGCCGGAATTTTCGTTTTCGGCAAATTGCCGATTTGCCC
GAAATTTTCAGTTTCGGCAAATTGCCGATTTGTCC
GAAATTTTCAGTTTCGGCAAATTGCCGATTTGCCG
GAAATTTTCAGTTTTGTCACTTTGCCAATTTACCG
GAAATTTTCAATTCCGGCAATTTGCCTGTTTGCCGGAATTTTCGTTTTCGGCAAACTGCCGATTTGCCC
GAAATTTTCAGTTTCGGCAAATTGCCGATTTGCCC
GAAATTTTCAGTTTCGGCGAATTGTCGATTTGTCC
GAAATTTTCAGTTTCGGCAAATTGCTGATTTGTCC
GAAATTTTCAGTTTCGGCAAATTGCCGATTTGCCG
GAAATTTTCAGTTTTGTCACTTTGCCAATTTACCG
GAAATTTTCAATTCCGGCAATTTGCCTGTTTGCCGGAATTTTCGTTTTCGGCAAATTGCCGATTTGCCC

GAAATTTTCAGTTTCGGCAAATTGCCGATTTGTCC
GAAATTTTCAGTTTCGGCAAATTGCCGATTTGCCC
GAAATTTTCAGTTTCGGCAAATTGCCGATTTGCCC
GAAATTTTCAGTTTCGGCAAATTGCCGATTTGCCC
GAAATTTTCAGTTTCGGCAAATTGCCGATTTACTG
GAAATTTTCAATCCGGCAATTTGCCGATTTGCCC
GAAATTTTCAGTTTCGGCAAATTGCCGATTTGCCC
Nr of selected repeats 33 Similarity 0.890620
Consensus:
GAAATTTTCAGTTTCGGCAAATTGCCGATTTGcCc
>Cele-UNSB01_1:1961569-1964216 Satlength=2648 Nr of Repeats=64 RepeatLength=35
seed=GAAATTTTCA
GAAATTTTCAGTTTCGGCAAATTGCCGATTTGCCC
GAAATTTTCAGTTTCGGCAAATTGCCGATTTGCCC
GAAATTTTCAGTTTCGGCAAATTGCCGATTTGCCG
GAAATTTTCAGTTTTGTCACTTTGCCAATTTACCG
GAAATTTTCAATTCCGGCAATTTGCCTGTTTGCCGGAATTTTCGTTTTCGGCAAATTGCCGATTTGCCC
GAAATTTTCAGTTTCGGCAAATTGCCGATTTGTCC
GAAATTTTCAGTTTCGGCAAATTGCCGATTTGCCG
GAAATTTTCAGTTTCGGCAAATTGCCGATTTGTCC
GAAATTTTCAGTTTCGGCAAATTGCCGATTTGCCC
GAAATTTTCAGTTTCGGCAAATTGCTGATTTGCCC
GAAATTTTCAGTTTCGGCAAATTGCCTGTTTGCCGGAATTTTCGTTTTCGGCAAATTGCCGATTTGCCC
GAAATTTTCAGTTTCGGCAAATTGCCGATTTGTCC
GAAATTTTCAGTTTCGGCAAATTGCCGATTTGCCG
GAAATTTTCAGTTTTGTCACTTTGCCAATTTACCG
GAAATTTTCAATTCCGGCAATTTGCCTGTTTGCCGGAATTTTCGTTTTCGGCAAATTGCCGATTTGCCC
GAAATTTTCAGTTTCGGCAAATTGCCGATTTGTCC
GAAATTTTCAGTTTCGGCAAATTGCCGATTTGCCC
GAAATTTTCAGTTTCGGCAAATTGCCGATTTGTCC
GAAATTTTCAGTTTCGGCAAATTGCCGATTTGCCC
GAAATTTTCAGTTTCGGCAAATTGCTGATTTGCCC
GAAATTTTCAGTTTCGGCAAATTGCCGATTTGCCG
GAAATTTTCAGTTTTGTCACTTTGCCAATTTACCG
GAAATTTTCAATTCCGGCAATTTGCCTGTTTGCCGGAATTTTCGTTTTCGGCAAATTGCCGATTTGCCC
GAAATTTTCAGTTTCGGCAAATTGCCGATTTGTCC
GAAATTTTCAGTTTCGGCAAATTGCCGATTTGCCG
GAAATTTTCAGTTTTGTCACTTTGCCAATTTACCG
GAAATTTTCAATTCCGGCAATTTGCCTGTTTGCCGGAATTTTCGTTTTCGGCAAACTGCCGATTTGCCC
GAAATTTTCAGTTTCGGCAAATTGCCGATTTGCCC
GAAATTTTCAGTTTCGGCGAATTGTCGATTTGTCC
GAAATTTTCAGTTTCGGCAAATTGCTGATTTGTCC
GAAATTTTCAGTTTCGGCAAATTGCCGATTTGCCG
GAAATTTTCAGTTTTGTCACTTTGCCAATTTACCG
GAAATTTTCAATTCCGGCAATTTGCCTGTTTGCCGGAATTTTCGTTTTCGGCAAATTGCCGATTTGCCC
GAAATTTTCAGTTTCGGCAAATTGCCGATTTGTCC
GAAATTTTCAGTTTCGGCAAATTGCCGATTTGCCC
GAAATTTTCAGTTTCGGCAAATTGCCGATTTGCCC
GAAATTTTCAGTTTCGGCAAATTGCCGATTTGCCC
GAAATTTTCAGTTTCGGCAAATTGCCGATTTACTG
GAAATTTTCAATCCGGCAATTTGCCGATTTGCCC
GAAATTTTCAGTTTCGGCAAATTGCCGATTTGCCC
GAAATTTTCAGTTTCGGCAAATTGCCGATTTGCCGT
AAATTTTCAATTCCGGCAGTCTGCCGATTTGCCGTAAATTTTCGTTTTTGGCAAATTACCGATTTGCCG

GAAATGTTCAATCCCGGCAATTTGCCAATTTGCCGTAAATTTTCGTTTTTGGAAAATTACCGATTTGCCGTAAATTTTC
GTTTTTGGCAAATTACCGATTTGCCG
GAAATGTTCAATCCCGGCAATTTGCCAATTTGCCGTAAATTTTCGTTTTTGGAAAATTACCGATTTGCCG
GAAATTTTCAGTTTCGGCAATTTGCCAATTCGCCG
GAAATTTTCAATTCCGGCAATTTGGTGATTTGCCG
GAAATGTTCAATCCGGCAATTTGCCAATTTGCCG
GAAATTTTCAGTTTCTGCAATTTGCAGATTTGACG
GAAATTTCAATTCCGGCAATTTCCCTGTTTGCCG
GAAATTTTCAGTTTCGTCACTTTGCCAATTTACCG
GAAATTTTCAATTCCGGCAATTTGCCTGTTTGCCGGAATTTTCGTTTTCGGCAAATTGCCGATTTGCCC
GAAATTTTCAGTTTCGGCAAATTGCCGATTTACTG
GAAATTTTCAATCCGGAAATTTGCCGATTTGCCC
GAAATTTTCAGTTTCGGCAAATTGCCGATTTGCCC
GAAATTTTCAGTTTCGGCAAATTGCCGATTTGTCC
GAAATTTTCAGTTTCGGCAAATTGCCGATTTGTCC
GAAATTTTCAATTCCGGCAATTTGCCTGTTTGCCGGAATTTTCGTTTTCGGCAAATTGCCGATTTGCCC
GAAATTTTCAGTTTCGGCAAATTGCCGATTTGTCC
GAAATTTTCAGTTTCGGCAAATTGCCGATTTGCCC
GAAATTTTCAGTTTCGGCAAATTGCCGATTTGCCC
GAAATTTTCAGTTTCGGCAAATTGCCGATTTGCCC
GAAATTTTCAGTTTCGGCAAATTGCCGATTTACTG
GAAATTTTCAATCCGGCAATTTGCCGATTTGCCC
GAAATTTTCAGTTTCGGCAAATTGCCGATTTGCCC
Nr of selected repeats 47 Similarity 0.883388
Consensus:
GAAATTTTCAGTTTCGGCAAATTGCCGATTTGcCc
>Cele-UNSB01_1:1961569-1966139 Satlength=4571 Nr of Repeats=108 RepeatLength=35
seed=GAAATTTTCA
GAAATTTTCAGTTTCGGCAAATTGCCGATTTGCCC
GAAATTTTCAGTTTCGGCAAATTGCCGATTTGCCC
GAAATTTTCAGTTTCGGCAAATTGCCGATTTGCCG
GAAATTTTCAGTTTTGTCACTTTGCCAATTTACCG
GAAATTTTCAATTCCGGCAATTTGCCTGTTTGCCGGAATTTTCGTTTTCGGCAAATTGCCGATTTGCCC
GAAATTTTCAGTTTCGGCAAATTGCCGATTTGTCC
GAAATTTTCAGTTTCGGCAAATTGCCGATTTGCCG
GAAATTTTCAGTTTCGGCAAATTGCCGATTTGTCC
GAAATTTTCAGTTTCGGCAAATTGCCGATTTGCCC
GAAATTTTCAGTTTCGGCAAATTGCTGATTTGCCC
GAAATTTTCAGTTTCGGCAAATTGCCTGTTTGCCGGAATTTTCGTTTTCGGCAAATTGCCGATTTGCCC
GAAATTTTCAGTTTCGGCAAATTGCCGATTTGTCC
GAAATTTTCAGTTTCGGCAAATTGCCGATTTGCCG
GAAATTTTCAGTTTTGTCACTTTGCCAATTTACCG
GAAATTTTCAATTCCGGCAATTTGCCTGTTTGCCGGAATTTTCGTTTTCGGCAAATTGCCGATTTGCCC
GAAATTTTCAGTTTCGGCAAATTGCCGATTTGTCC
GAAATTTTCAGTTTCGGCAAATTGCCGATTTGCCC
GAAATTTTCAGTTTCGGCAAATTGCCGATTTGTCC
GAAATTTTCAGTTTCGGCAAATTGCCGATTTGCCC
GAAATTTTCAGTTTCGGCAAATTGCTGATTTGCCC
GAAATTTTCAGTTTCGGCAAATTGCCGATTTGCCG
GAAATTTTCAGTTTTGTCACTTTGCCAATTTACCG
GAAATTTTCAATTCCGGCAATTTGCCTGTTTGCCGGAATTTTCGTTTTCGGCAAATTGCCGATTTGCCC
GAAATTTTCAGTTTCGGCAAATTGCCGATTTGTCC
GAAATTTTCAGTTTCGGCAAATTGCCGATTTGCCG
GAAATTTTCAGTTTTGTCACTTTGCCAATTTACCG

GAAATTTTCAATTCCGGCAATTTGCCTGTTTGCCGGAATTTTCGTTTTCGGCAAACTGCCGATTTGCCC
GAAATTTTCAGTTTCGGCAAATTGCCGATTTGCCC
GAAATTTTCAGTTTCGGCGAATTGTCGATTTGTCC
GAAATTTTCAGTTTCGGCAAATTGCTGATTTGTCC
GAAATTTTCAGTTTCGGCAAATTGCCGATTTGCCG
GAAATTTTCAGTTTTGTCACTTTGCCAATTTACCG
GAAATTTTCAATTCCGGCAATTTGCCTGTTTGCCGGAATTTTCGTTTTCGGCAAATTGCCGATTTGCCC
GAAATTTTCAGTTTCGGCAAATTGCCGATTTGTCC
GAAATTTTCAGTTTCGGCAAATTGCCGATTTGCCC
GAAATTTTCAGTTTCGGCAAATTGCCGATTTGCCC
GAAATTTTCAGTTTCGGCAAATTGCCGATTTGCCC
GAAATTTTCAGTTTCGGCAAATTGCCGATTTACTG
GAAATTTTCAATCCGGCAATTTGCCGATTTGCCC
GAAATTTTCAGTTTCGGCAAATTGCCGATTTGCCC
GAAATTTTCAGTTTCGGCAAATTGCCGATTTGCCGT
AAATTTTCAATTCCGGCAGTCTGCCGATTTGCCGTAAATTTTCGTTTTTGGCAAATTACCGATTTGCCG
GAAATGTTCAATCCCGGCAATTTGCCAATTTGCCGTAAATTTTCGTTTTTGGAAAATTACCGATTTGCCGTAAATTTTC
GTTTTTGGCAAATTACCGATTTGCCG
GAAATGTTCAATCCCGGCAATTTGCCAATTTGCCGTAAATTTTCGTTTTTGGAAAATTACCGATTTGCCG
GAAATTTTCAGTTTCGGCAATTTGCCAATTCGCCG
GAAATTTTCAATTCCGGCAATTTGGTGATTTGCCG
GAAATGTTCAATCCGGCAATTTGCCAATTTGCCG
GAAATTTTCAGTTTCTGCAATTTGCAGATTTGACG
GAAATTTCAATTCCGGCAATTTCCCTGTTTGCCG
GAAATTTTCAGTTTCGTCACTTTGCCAATTTACCG
GAAATTTTCAATTCCGGCAATTTGCCTGTTTGCCGGAATTTTCGTTTTCGGCAAATTGCCGATTTGCCC
GAAATTTTCAGTTTCGGCAAATTGCCGATTTACTG
GAAATTTTCAATCCGGAAATTTGCCGATTTGCCC
GAAATTTTCAGTTTCGGCAAATTGCCGATTTGCCC
GAAATTTTCAGTTTCGGCAAATTGCCGATTTGTCC
GAAATTTTCAGTTTCGGCAAATTGCCGATTTGTCC
GAAATTTTCAATTCCGGCAATTTGCCTGTTTGCCGGAATTTTCGTTTTCGGCAAATTGCCGATTTGCCC
GAAATTTTCAGTTTCGGCAAATTGCCGATTTGTCC
GAAATTTTCAGTTTCGGCAAATTGCCGATTTGCCC
GAAATTTTCAGTTTCGGCAAATTGCCGATTTGCCC
GAAATTTTCAGTTTCGGCAAATTGCCGATTTGCCC
GAAATTTTCAGTTTCGGCAAATTGCCGATTTACTG
GAAATTTTCAATCCGGCAATTTGCCGATTTGCCC
GAAATTTTCAGTTTCGGCAAATTGCCGATTTGCCC
GAAATTTTCAGTTTCGGCAAATTGCCGATTTGCCGT
AAATTTTCAATTCCGGCAGTCTGCCGATTTGCCGTAAATTTTCGTTTTTGGCAAATTACCGATTTGCCG
GAAATGTTCAATCCCGGCAATTTGCCAATTTGCCGTAAATTTTCGTTTTTGGAAAATTACCGATTTGCCGTAAATTTTC
GTTTTTGGCAAATTACCGATTTGCCG
GAAATGTTCAATCCCGGCAATTTGCCAATTTGCCGTAAATTTTCGTTTTTGGAAAATTACCGATTTGCCG
GAAATTTTCAGTTTCGGCAATTTGCCAATTCGCCG
GAAATTTTCAATTCCGGCAATTTGGTGATTTGCCG
GAAATGTTCAATCCGGCAATTTGCCAATTTGCCG
GAAATTTTCAGTTTCTGCAATTTGCAGATTTGACG
GAAATTTCAATTCCGGCAATTTCCCTGTTTGCCG
GAAATTTTCAGTTTCGTCACTTTGCCAATTTACCG
GAAATTTTCAATTCCGGCAATTTGCCTGTTTGCCGGAATTTTCGTTTTCGGCAAATTGCCGATTTGCCC
GAAATTTTCAGTTTCGGCAAATTGCCGATTTACTG
GAAATTTTCAATCCGGAAATTTGCCGATTTGCCC
GAAATTTTCAGTTTCGGCAAATTGCCGATTTGCCC

GAAATTTTCAGTTTCGGCAAATTGCCGATTTGCCG
GAAATTTTCAATTCCGGCAGTCTGCCGATTTGCCGTAAATTTTCGTTTTTGGCAAATTACCGATTTGCCG
GAAATGTTCAATCCCGGCAATTTGCCAATTTGCCGTAAATTTTCGTTTTTGGAAAATTACCGATTTGCCG
GAAATTTTCAGTTTCGGCAATTTGCCAATTCGCCG
GAAATTTTCAATTCCGGCAATTTGGTGATTTGCCG
GAAATGTTCAATCCGGCAATTTGCCAATTTGCCG
GAAATTTTCAGTTTCTGCAATTTGCAGATTTGACG
GAAATTTCAATTCCGGCAATTTCCCTGTTTGCCG
GAAATTTTCAGTTTCGTCACTTTGCCAATTTACCGAA
AAATTTTCAATTCCGGCAATTTGCCTGTTTGCCGGAATTTTCGTTTTCGGCAAATTGCCGATTTGCCC
GAAATTTTCAGTTTCGGCGAATTGTCGATTTACCG
GAAATTTTCAATCCGGCAATTTGCCAATTTGCCGTAAATTTTCGTTTTTGGAAAATTGCCGGTTTGCCGATTCGCCG
GAAATTTTCAGTTTCGGCAATTTGCCAATTTGCCG
GAAATTTTCAGTTTCGGCGAATTGTCGATTTGCCG
GAAATTTTCAGTTTCGGCAATTTGCCAATTTGCCG
GAAATTTTCAGTTTCGGCGAATTGTCGATTTGCCG
GAAATTTTCAGTTTTGTCACTTTGCCAATTTACCG
GAAATTTTCAATTCCGGCAATTTGCCTGTTTGCCGGAATTTTCGTTTTCGGCAAACTGCCGATTTGCCC
GAAATTTTCAGTTTCGGCAAATTGCCGATTTGCCC
GAAATTTTCAGTTTCGGCGAATTGTCGATTTGTCC
GAAATTTTCAGTTTCGGCAAATTGCCGATTTGCCG
GAAATTTTCAGTTTTGTCACTTTGCCAATTTACCG
GAAATTTTCAATTCCGGCAATTTGCCTGTTTGCCGGAATTTTCGTTTTCGGCAAATTGCCGATTTGCCC
GAAATTTTCAGTTTCGGCAAATTGCCGATTTGTCC
GAAATTTTCAGTTTCGGCAAATTGCCGATTTGCCC
GAAATTTTCAGTTTCGGCAAATTGCCGATTTGCCC
GAAATTTTCAGTTTCGGCAAATTGCCGATTTGCCC
GAAATTTTCAGTTTCGGCAAATTGCCGATTTACTG
GAAATTTTCAATCCGGCAATTTGCCGATTTGCCC
GAAATTTTCAGTTTCGGCAAATTGCCGATTTGCCC
Nr of selected repeats 73 Similarity 0.839524
Consensus:
GAAATTTTCAGTTTCGGCAAaTTGCCGATTTgcCn
>Cele-UNSB01_1:1961569-1968269 Satlength=6701 Nr of Repeats=154 RepeatLength=35
seed=GAAATTTTCA
GAAATTTTCAGTTTCGGCAAATTGCCGATTTGCCC
GAAATTTTCAGTTTCGGCAAATTGCCGATTTGCCC
GAAATTTTCAGTTTCGGCAAATTGCCGATTTGCCG
GAAATTTTCAGTTTTGTCACTTTGCCAATTTACCG
GAAATTTTCAATTCCGGCAATTTGCCTGTTTGCCGGAATTTTCGTTTTCGGCAAATTGCCGATTTGCCC
GAAATTTTCAGTTTCGGCAAATTGCCGATTTGTCC
GAAATTTTCAGTTTCGGCAAATTGCCGATTTGCCG
GAAATTTTCAGTTTCGGCAAATTGCCGATTTGTCC
GAAATTTTCAGTTTCGGCAAATTGCCGATTTGCCC
GAAATTTTCAGTTTCGGCAAATTGCTGATTTGCCC
GAAATTTTCAGTTTCGGCAAATTGCCTGTTTGCCGGAATTTTCGTTTTCGGCAAATTGCCGATTTGCCC
GAAATTTTCAGTTTCGGCAAATTGCCGATTTGTCC
GAAATTTTCAGTTTCGGCAAATTGCCGATTTGCCG
GAAATTTTCAGTTTTGTCACTTTGCCAATTTACCG
GAAATTTTCAATTCCGGCAATTTGCCTGTTTGCCGGAATTTTCGTTTTCGGCAAATTGCCGATTTGCCC
GAAATTTTCAGTTTCGGCAAATTGCCGATTTGTCC
GAAATTTTCAGTTTCGGCAAATTGCCGATTTGCCC
GAAATTTTCAGTTTCGGCAAATTGCCGATTTGTCC
GAAATTTTCAGTTTCGGCAAATTGCCGATTTGCCC

GAAATTTTCAGTTTCGGCAAATTGCTGATTTGCCC
GAAATTTTCAGTTTCGGCAAATTGCCGATTTGCCG
GAAATTTTCAGTTTTGTCACTTTGCCAATTTACCG
GAAATTTTCAATTCCGGCAATTTGCCTGTTTGCCGGAATTTTCGTTTTCGGCAAATTGCCGATTTGCCC
GAAATTTTCAGTTTCGGCAAATTGCCGATTTGTCC
GAAATTTTCAGTTTCGGCAAATTGCCGATTTGCCG
GAAATTTTCAGTTTTGTCACTTTGCCAATTTACCG
GAAATTTTCAATTCCGGCAATTTGCCTGTTTGCCGGAATTTTCGTTTTCGGCAAACTGCCGATTTGCCC
GAAATTTTCAGTTTCGGCAAATTGCCGATTTGCCC
GAAATTTTCAGTTTCGGCGAATTGTCGATTTGTCC
GAAATTTTCAGTTTCGGCAAATTGCTGATTTGTCC
GAAATTTTCAGTTTCGGCAAATTGCCGATTTGCCG
GAAATTTTCAGTTTTGTCACTTTGCCAATTTACCG
GAAATTTTCAATTCCGGCAATTTGCCTGTTTGCCGGAATTTTCGTTTTCGGCAAATTGCCGATTTGCCC
GAAATTTTCAGTTTCGGCAAATTGCCGATTTGTCC
GAAATTTTCAGTTTCGGCAAATTGCCGATTTGCCC
GAAATTTTCAGTTTCGGCAAATTGCCGATTTGCCC
GAAATTTTCAGTTTCGGCAAATTGCCGATTTGCCC
GAAATTTTCAGTTTCGGCAAATTGCCGATTTACTG
GAAATTTTCAATCCGGCAATTTGCCGATTTGCCC
GAAATTTTCAGTTTCGGCAAATTGCCGATTTGCCC
GAAATTTTCAGTTTCGGCAAATTGCCGATTTGCCGT
AAATTTTCAATTCCGGCAGTCTGCCGATTTGCCGTAAATTTTCGTTTTTGGCAAATTACCGATTTGCCG
GAAATGTTCAATCCCGGCAATTTGCCAATTTGCCGTAAATTTTCGTTTTTGGAAAATTACCGATTTGCCGTAAATTTTC
GTTTTTGGCAAATTACCGATTTGCCG
GAAATGTTCAATCCCGGCAATTTGCCAATTTGCCGTAAATTTTCGTTTTTGGAAAATTACCGATTTGCCG
GAAATTTTCAGTTTCGGCAATTTGCCAATTCGCCG
GAAATTTTCAATTCCGGCAATTTGGTGATTTGCCG
GAAATGTTCAATCCGGCAATTTGCCAATTTGCCG
GAAATTTTCAGTTTCTGCAATTTGCAGATTTGACG
GAAATTTCAATTCCGGCAATTTCCCTGTTTGCCG
GAAATTTTCAGTTTCGTCACTTTGCCAATTTACCG
GAAATTTTCAATTCCGGCAATTTGCCTGTTTGCCGGAATTTTCGTTTTCGGCAAATTGCCGATTTGCCC
GAAATTTTCAGTTTCGGCAAATTGCCGATTTACTG
GAAATTTTCAATCCGGAAATTTGCCGATTTGCCC
GAAATTTTCAGTTTCGGCAAATTGCCGATTTGCCC
GAAATTTTCAGTTTCGGCAAATTGCCGATTTGTCC
GAAATTTTCAGTTTCGGCAAATTGCCGATTTGTCC
GAAATTTTCAATTCCGGCAATTTGCCTGTTTGCCGGAATTTTCGTTTTCGGCAAATTGCCGATTTGCCC
GAAATTTTCAGTTTCGGCAAATTGCCGATTTGTCC
GAAATTTTCAGTTTCGGCAAATTGCCGATTTGCCC
GAAATTTTCAGTTTCGGCAAATTGCCGATTTGCCC
GAAATTTTCAGTTTCGGCAAATTGCCGATTTGCCC
GAAATTTTCAGTTTCGGCAAATTGCCGATTTACTG
GAAATTTTCAATCCGGCAATTTGCCGATTTGCCC
GAAATTTTCAGTTTCGGCAAATTGCCGATTTGCCC
GAAATTTTCAGTTTCGGCAAATTGCCGATTTGCCGT
AAATTTTCAATTCCGGCAGTCTGCCGATTTGCCGTAAATTTTCGTTTTTGGCAAATTACCGATTTGCCG
GAAATGTTCAATCCCGGCAATTTGCCAATTTGCCGTAAATTTTCGTTTTTGGAAAATTACCGATTTGCCGTAAATTTTC
GTTTTTGGCAAATTACCGATTTGCCG
GAAATGTTCAATCCCGGCAATTTGCCAATTTGCCGTAAATTTTCGTTTTTGGAAAATTACCGATTTGCCG
GAAATTTTCAGTTTCGGCAATTTGCCAATTCGCCG
GAAATTTTCAATTCCGGCAATTTGGTGATTTGCCG
GAAATGTTCAATCCGGCAATTTGCCAATTTGCCG

GAAATTTTCAGTTTCTGCAATTTGCAGATTTGACG
GAAATTTCAATTCCGGCAATTTCCCTGTTTGCCG
GAAATTTTCAGTTTCGTCACTTTGCCAATTTACCG
GAAATTTTCAATTCCGGCAATTTGCCTGTTTGCCGGAATTTTCGTTTTCGGCAAATTGCCGATTTGCCC
GAAATTTTCAGTTTCGGCAAATTGCCGATTTACTG
GAAATTTTCAATCCGGAAATTTGCCGATTTGCCC
GAAATTTTCAGTTTCGGCAAATTGCCGATTTGCCC
GAAATTTTCAGTTTCGGCAAATTGCCGATTTGCCG
GAAATTTTCAATTCCGGCAGTCTGCCGATTTGCCGTAAATTTTCGTTTTTGGCAAATTACCGATTTGCCG
GAAATGTTCAATCCCGGCAATTTGCCAATTTGCCGTAAATTTTCGTTTTTGGAAAATTACCGATTTGCCG
GAAATTTTCAGTTTCGGCAATTTGCCAATTCGCCG
GAAATTTTCAATTCCGGCAATTTGGTGATTTGCCG
GAAATGTTCAATCCGGCAATTTGCCAATTTGCCG
GAAATTTTCAGTTTCTGCAATTTGCAGATTTGACG
GAAATTTCAATTCCGGCAATTTCCCTGTTTGCCG
GAAATTTTCAGTTTCGTCACTTTGCCAATTTACCGAA
AAATTTTCAATTCCGGCAATTTGCCTGTTTGCCGGAATTTTCGTTTTCGGCAAATTGCCGATTTGCCC
GAAATTTTCAGTTTCGGCGAATTGTCGATTTACCG
GAAATTTTCAATCCGGCAATTTGCCAATTTGCCGTAAATTTTCGTTTTTGGAAAATTGCCGGTTTGCCGATTCGCCG
GAAATTTTCAGTTTCGGCAATTTGCCAATTTGCCG
GAAATTTTCAGTTTCGGCGAATTGTCGATTTGCCG
GAAATTTTCAGTTTCGGCAATTTGCCAATTTGCCG
GAAATTTTCAGTTTCGGCGAATTGTCGATTTGCCG
GAAATTTTCAGTTTTGTCACTTTGCCAATTTACCG
GAAATTTTCAATTCCGGCAATTTGCCTGTTTGCCGGAATTTTCGTTTTCGGCAAACTGCCGATTTGCCC
GAAATTTTCAGTTTCGGCAAATTGCCGATTTGCCC
GAAATTTTCAGTTTCGGCGAATTGTCGATTTGTCC
GAAATTTTCAGTTTCGGCAAATTGCCGATTTGCCG
GAAATTTTCAGTTTTGTCACTTTGCCAATTTACCG
GAAATTTTCAATTCCGGCAATTTGCCTGTTTGCCGGAATTTTCGTTTTCGGCAAATTGCCGATTTGCCC
GAAATTTTCAGTTTCGGCAAATTGCCGATTTGTCC
GAAATTTTCAGTTTCGGCAAATTGCCGATTTGCCC
GAAATTTTCAGTTTCGGCAAATTGCCGATTTGCCC
GAAATTTTCAGTTTCGGCAAATTGCCGATTTGCCC
GAAATTTTCAGTTTCGGCAAATTGCCGATTTACTG
GAAATTTTCAATCCGGCAATTTGCCGATTTGCCC
GAAATTTTCAGTTTCGGCAAATTGCCGATTTGCCC
GAAATTTTCAGTTTCGGCAAATTGCCGATTTGCCGT
AAATTTTCAATTCCGGCAGTCTGCCGATTTGCCGTAAATTTTCGTTTTTGGCAAATTACCGATTTGCCG
GAAATGTTCAATCCCGGCAATTTGCCAATTTGCCGTAAATTTTCGTTTTTGGAAAATTACCGATTTGCCGTAAATTTTC
GTTTTTGGCAAATTACCGATTTGCCG
GAAATGTTCAATCCCGGCAATTTGCCAATTTGCCGTAAATTTTCGTTTTTGGAAAATTACCGATTTGCCG
GAAATTTTCAGTTTCGGCAATTTGCCAATTCGCCG
GAAATTTTCAATTCCGGCAATTTGGTGATTTGCCG
GAAATGTTCAATCCGGCAATTTGCCAATTTGCCG
GAAATTTTCAGTTTCTGCAATTTGCAGATTTGACG
GAAATTTCAATTCCGGCAATTTCCCTGTTTGCCG
GAAATTTTCAGTTTCGTCACTTTGCCAATTTACCG
GAAATTTTCAATTCCGGCAATTTGCCTGTTTGCCGGAATTTTCGTTTTCGGCAAATTGCCGATTTGCCC
GAAATTTTCAGTTTCGGCAAATTGCCGATTTACTG
GAAATTTTCAATCCGGAAATTTGCCGATTTGCCC
GAAATTTTCAGTTTCGGCAAATTGCCGATTTGCCC
GAAATTTTCAGTTTCGGCAAATTGCCGATTTACTG
GAAATTTTCAATCCGGCAATTTGCCGATTTGCCC

GAAATTTTCAGTTTCGGCAAATTGCCGATTTGCCC
GAAATTTTCAGTTTCGGCAAATTGCCGATTTGCCGT
AAATTTTCAATTCCGGCAGTCTGCCGATTTGCCGTAAATTTTCGTTTTTGGCAAATTACCGATTTGCCG
GAAATGTTCAATCCCGGCAATTTGCCAATTTGCCGTAAATTTTCGTTTTTGGAAAATTACCGATTTGCCGTAAATTTTC
GTTTTTGGCAAATTACCGATTTGCCG
GAAATGTTCAATCCCGGCAATTTGCCAATTTGCCGTAAATTTTCGTTTTTGGAAAATTACCGATTTGCCG
GAAATTTTCAGTTTCGGCAATTTGCCAATTCGCCG
GAAATTTTCAATTCCGGCAATTTGGTGATTTGCCG
GAAATGTTCAATCCGGCAATTTGCCAATTTGCCG
GAAATTTTCAGTTTCTGCAATTTGCAGATTTGACG
GAAATTTCAATTCCGGCAATTTCCCTGTTTGCCG
GAAATTTTCAGTTTCGTCACTTTGCCAATTTACCG
GAAATTTTCAATTCCGGCAATTTGCCTGTTTGCCGGAATTTTCGTTTTCGGCAAATTGCCGATTTGCCC
GAAATTTTCAGTTTCGGCAAATTGCCGATTTACTG
GAAATTTTCAATCCGGAAATTTGCCGATTTGCCC
GAAATTTTCAGTTTCGGCAAATTGCCGATTTGCCC
GAAATTTTCAGTTTCGGCAAATTGCCGATTTGCCG
GAAATTTTCAATTCCGGCAGTCTGCCGATTTGCCGTAAATTTTCGTTTTTGGCAAATTACCGATTTGCCG
GAAATGTTCAATCCCGGCAATTTGCCAATTTGCCGTAAATTTTCGTTTTTGGAAAATTACCGATTTGCCG
GAAATTTTCAGTTTCGGCAATTTGCCAATTCGCCG
GAAATTTTCAATTCCGGCAATTTGGTGATTTGCCG
GAAATGTTCAATCCGGCAATTTGCCAATTTGCCG
GAAATTTTCAGTTTCTGCAATTTGCAGATTTGACG
GAAATTTCAATTCCGGCAATTTCCCTGTTTGCCG
GAAATTTTCAGTTTCGTCACTTTGCCAATTTACCGAA
AAATTTTCAATTCCGGCAATTTGCCTGTTTGCCGGAATTTTCGTTTTCGGCAAATTGCCGATTTGCCC
GAAATTTTCAGTTTCGGCGAATTGTCGATTTACCG
GAAATTTTCAATCCGGCAATTTGCCAATTTGCCGTAAATTTTCGTTTTTGGAAAATTGCCGGTTTGCCGATTCGCCG
GAAATTTTCAGTTTCGGCAATTTGCCAATTTGCCG
GAAATTTTCAGTTTCGGCGAATTGTCGATTTGCCG
GAAATTTTCAGTTTCGGCAATTTGCCAATTTGCCGGAATTTTCGTTTTCGGCAAATTGTCGATTTGCCG
Nr of selected repeats 94 Similarity 0.828463
Consensus:
GAAATTTTCAGTTTCGGCAAaTTGCCgATTTgCCg
>Cele-UNSB01_1:1976543-1977056 Satlength=514 Nr of Repeats=5 RepeatLength=35
seed=TTTGCCGGAA
TTTGCCGGAATTTTTTTTTCGATTTTTCGGTAGAAAAAAAAAATCGAATTCAGTTTTTCACATACGGAATCTGAATAA
TATTTATGTCAGGGGTGCGCGGCGATTGCCGTTCGGCAAATTGATTTGTCGACAAATTCGGCAAATCGGCAAATTGCCG
GTTTGCCGA
TTTGCCGGAAATTTTCATTTTTGGCAAAGTGCCGA
TTTGCCGGAAATGTTAGAGGGATTTTTTGAAAGACGGAAACACTTAAAACTGTGCCTTTTTGTATTTTTTCCCGTTTTT
TTCTACATATTTTCATAGAATTTGCTTACTTTTCAAAATAGATGTAGGAACATTTATAGAATGCGTTTAATTTTGCCAT
TTGAAATTGAAATTCTGAAATTTCCAAAAAATGTGCAAAACCACAATTTGGCGAAAATTTTCGGCAAATCGGCAATTTG
CCGG
TTTGCCGGAAAATTTCAATTCCGGCAATTTACCTG
TTTGCCGGAAATTTTCAATTCCGGCAATTTGCCGA
Nr of selected repeats 3 Similarity 0.759259
Consensus:
TTTGCCGGAAAtTTTCAaTTccGGCAAttTgCCGa
>Cele-UNSB01_1:1980049-1980327 Satlength=279 Nr of Repeats=8 RepeatLength=35
seed=TTGCCGGAAA
TTGCCGGAAATTTTTAATTCTGACAAATTGCCGAT
TTGCCGGAAATTCCAATTCCGGCAATTTGCCGAT
TTGCCGGAAAGTATCAATTTCGGCTGATTGCCGAT

TTGCCGGAAACGTTCAATTTCCGCACTTTGCCGAT
TTGCCGGAAATGTTCAATTTCGGCGATTTGCCGCT
TTGCAGGAAATTTTCTTTTTCGGCAGTTTGGCGAT
TTGCCGGAAATTCCAATTCCGGCAATTTGTCGAT
TTGCCGGAAATGTTCAATTCTGATAAATTGCCGAA
Nr of selected repeats 6 Similarity 0.654655
Consensus:
TTGCCGGAAAtgTTCAAtTTCgGCaaaTTGCCGAT
>Cele-UNSB01_1:2017348-2017684 Satlength=337 Nr of Repeats=5 RepeatLength=38
seed=AAAATCGATA
AAAATCGATATTTTAGTATTGCTCTCAAAATTTTTTTGATTTTCCAGGAAATTATCAAAATTTTTAGTTTTCAAAAAAA
TTGTCGAAATTTTCAATTTATTTTTTGAATAGAATTTGTTTTTTTAAAAATTATTTCTAACTCTTGGCATTTCCTTTTT
TAAAAATAGAGG
AAAATCGATAATTCCAAGTGATTTTTTGCAATCCAAAAAAAATTCTTCGAAA
AAAATCGATAATTTTCAGTATGCAGTGAAATTCTTGGA
AAAATCGATAATTTTCAGTATGCAGTGAAATTCTTGGA
AAAATCGATAATTTTCAGTATGCAGTGAAATTCTTGGA
Nr of selected repeats 3 Similarity 1.000000
Consensus:
AAAATCGATAATTTTCAGTATGCAGTGAAATTCTTGGA
>Cele-UNSB01_1:2044939-2045157 Satlength=219 Nr of Repeats=13 RepeatLength=11
seed=TTTTGCCGAA
TTTTGCCGAAG
TTTTGCCGATCTTTTGTTGAAC
TTTCGCCGAAC
TTTTGCCGAAC
TCTTGCCGAAG
TTTTGCCGAAG
TTTTGCCGAAG
TTTTGCCGAACTTTCACCAACCTTTCGTCAAAATTCGCCAAACTTTCGTCAAAATTCGCCAAAC
TTTTGCCAAAT
TTTCGCCGAAC
TTTTGCCGAAC
TTTTGCCAAAC
TTTTGCCCAATTTTCGCCAAAC
Nr of selected repeats 10 Similarity 0.811448
Consensus:
TTTtGCCgAAc
>Cele-UNSB01_1:2141918-2142329 Satlength=412 Nr of Repeats=7 RepeatLength=35
seed=CAAACCGGCA
CAAACCGGCAACCTGCCGAAAATCAATATTTTGAG
CAAACCGGCATTTTGCCGGAAATTAACATTTAGAG
CAAATCGGCAAACCGGCAATTTGCCGAAAAGCAACATTTCCGGCAAATCAG
CAAACCGGCAAACTACCGAAATTAAGAATTTTCGG
CAAACCGGGAATTTTTGTCGAAAACTTTACATTCCGACAATTTGTCAGTTTGCCGATTTTCTCTAAATGTTGATTTTCG
GCAAATTGCCAGTTTGCCGGAAATTTTGAATTCCAGAAATTTGCCGGTTTGCCGATTTGCTGGAAATTTTGATTTTGGG
CAAACTGCCGGAAGTGAAAATTTCCCA
CAAACCGGCAATTTGCAGAAAAATAACATTTCCAG
CAAACCGGCAAATTGCCGAAAAGCATCATTTAGAG
Nr of selected repeats 5 Similarity 0.675926
Consensus:
CAAACCGGCAantTgCcGaAAAttAacATTTngaG
>Cele-UNSB01_1:2169410-2169690 Satlength=281 Nr of Repeats=8 RepeatLength=35
seed=CGGCAAATTG

CGGCAAATTGATTTGTCGGAAAATT
CGGCAAATTTTGCTGATTTGACGGAAATTTTCAATTC
CGGCAAATTGCCGATTTGCCGGAAACTTTCAATTC
CGGCAAATTGCCGGTTTGCCGATTTGCCGGAAATCTTCGATTC
CGGCAAATTGTCGATTTGCCGGATATTCTCAATTC
CAGCAAATTGCCGATTTGCCGGAAATTTTCAATTC
CGGCAAATTGCCCGTTTGCCGGAAATTTTCAATTC
CGGCAAATTGCCCGTTTGCCGGAAATTTTCAATTC
Nr of selected repeats 5 Similarity 0.878095
Consensus:
CgGCAAATTGcCgaTTTGCCGGAaAtTtTCAATTC
>Cele-UNSB01_1:2174082-2174248 Satlength=167 Nr of Repeats=10 RepeatLength=15
seed=AGATCTTGTT
AGATCTTGTTTTCGT
AGATCATGTTTTCGG
AGATCTTGTTGTCGT
AGATCTTGTTTCCGT
AGATCTCGTTTTTGTAGTTCTCCAAAATGT
AGATCTTGTTCTGGT
AGATCTTGTTTTCGT
AGGTCTTGTTTTCGT
AGGTCTTGTTTTCGT
AGATCTTTTTTTTCGT
Nr of selected repeats 8 Similarity 0.831746
Consensus:
AGaTCTTGTTtTCGT
>Cele-UNSB01_1:2195476-2195914 Satlength=439 Nr of Repeats=10 RepeatLength=35
seed=TTTGCCGGAA
TTTGCCGGAAATATCCAATTCAGGCAATTTGGCCGTTTGCTGAA
TTTGCCGGAATTTCGGAATTCTGACAGTTTGCCGG
TTTGCCGAATCTGTCAACAATTTTCAATTCCGGCAAATTACCGATTTGTCGGGAATTCGGAATTCCGACAA
TTTGCCGAATTCGCCGGGAATTTTCATTCCGGCAATTTACCGA
TTTGCTGGAAGTTGTCATTTTCGGCAAATTGCCGATGTGCTAGAAATTTTTATTTTTGGCAAATGGCCAA
TTTGCAGGAAATTTTTATTTTTGGCAAATTGTTGA
TTTGCCTGAAATTTTGAATTCCGACAATTTGCTAA
TTTGCCGGAAATTTTGAATTCCAGCAATTTGCCTA
TTTGCCGGAAATTTTGAATTCCGACAATTTGCCGG
TTTGCCGGAAATTTTGAATTCCGACAATTTGCTAA
Nr of selected repeats 6 Similarity 0.735873
Consensus:
TTTGCCGGAAATTTTGAATTCcGaCAATTTGCcga
>Cele-UNSB01_1:2263475-2263726 Satlength=252 Nr of Repeats=6 RepeatLength=43
seed=CAAATCGGCA
CAAATCGGCAACTCGACAAATTTCCGAAATTAAAAATTTCCAG
CAAATCGGCAAACCGGCTGGAATTGAACATTTCCGG
CAAATCGGCAACTCGGCAAATTTCCGAAATTAAAAAATTTCAG
CAAATTGGCAAACCGGAAAATTGCCGGAATTGAACATTTCCGG
CAAATCGGCAACTCGGCAAATTTCCGAAATTAAAAAATTTCAG
CAAATTGGCAAACCGGAAAATTGCCGGAATTGAACATTTCCGG
Nr of selected repeats 5 Similarity 0.782946
Consensus:
CAAATcGGCAActCGgcAAATTtCCGaAATTaAAaAtTTcCaG
>Cele-UNSB01_1:2281114-2281247 Satlength=134 Nr of Repeats=4 RepeatLength=33
seed=TAAGAAAAGT

TAAGAAAAGTTAGGCCACCAAACTTTGGTGAAC
TAAGAAAAGTTAGGCCACCAAACATTGGTGGAC
TAAGAAAAGTTAGGCCACCAAACTTTGGCGAAT
TAAGAAAAGTTAAGTCACAAAATTTTTGGTGGAC
Nr of selected repeats 3 Similarity 0.892256
Consensus:
TAAGAAAAGTTAGGCCACCAAACtTTGGtGaAc
>Cele-UNSB01_1:2284293-2285881 Satlength=1589 Nr of Repeats=82 RepeatLength=12
seed=CACGCAACGC
CACGCAACGCAG
CACGCAACGCTG
CACGCAGCGCAG
CACGCAACGCAG
CACGCAACGTGACACGCAATGAGG
CACGCACCGCAGCGCGAAACGCAG
CACGCAACGCGA
CACGCAACGCAG
CACGCAACGCAG
CACGCAACGCAG
CACGCAACGCGAT
ACGCAACGCAG
CACGCGACGCAGT
ACGCAACGCGA
CACGCAACGCAACACAGTTCCCTTTTTCAGGAAGAACCGAGCCTTGGGGAGAGACACCGTCAAATGCCACGTCAGCAAG
ACTCTCCACTTCGGTAACAATGTGTGAGGATGGAATGTGCAAGCTGACCCTTTTGGAGAAACATAATCACGAATTCCGA
ACAGAATCAAAAATCTCATCCGGGTCAGGGAATTTGAAAAAGAAGGTACGTTGTCCACGTGGAGTACACGATTTTGCCA
AATTTATTTTCACAAAAATTTGAATTTTCCGCGGAGCCAACAAGCGGAGTGTCGTTGCAACAAATTTCTAAATTCCAGG
TGTCACGAGTATCGTATGCCGGTGAGGAAATTGTGATACCCGGTCGGATAGAGGATCTCGAGAATGACGTCATTTATAG
TATAGAAGAAGAACGTGAAGATTAATTGTCAATTTTGTATCCAAAAATTATTATTTGTGATCTCTTTCTCTAATTGTAA
TCTACTAAATTAATTTTTTTGAACTATGAATAAATTCGGTAATTGTTAGTAAAATCATTAAAAAAGGTTCGAATTTAGC
GCCAAAAATATTCAATTTAAAATTGGAGTTTTGTCGGCAG
CACGCAACGCAG
CACGCAACGCAG
CACGCAACGCAG
CACGCAACGCAG
CACGCAACGCAG
CACGCAACGCAG
CACGCAACGCAG
CACGCAACGCAG
CACGCAACGCAG
CACGCAACGCAG
CACGCAACGCAG
CACGCAACGCAG
CACGCAACGCAG
CACGCAACGCAG
CACGCAACGCAG
CACGCAACGCAG
CACGCAACGCAG
CACGCAACGCAG
CACGCAACGCAG
CACGCAACGCAG
CACGCAACGCAG
CACGCAACGCAA
CACGCAACGCAA

CACGCAACGCAA
CACGCAACGCAA
CACGCAACGCAA
CACGCAACGCAA
CACGCAACGCAA
CACGCAACGCAA
CACGCAACGCAA
CACGCAACGCAA
CACGCAACGCAA
CACGCAACGCAA
CACGCAACGCAA
CACGCAACGCAA
CACGCAACGCAG
CACGCAACGCAG
CACGCAACGCAG
CACGCAACGCAG
CACGCAACGCAG
CACGCAACGCAG
CACGCAACGCAG
CACGCAACGCA
CACGCAACGCAA
CACGCAACGCAA
CACGCAACGCAA
CACGCAACGCAA
CACGCAACGCAA
CACGCAACGCAA
CACGCAACGCAA
CACGCAACGCAA
CACGCAACGCAA
CACGCAACGCAA
CACGCAACGCAA
CACGCAACGCAA
CACGCAACGCAA
CACGCAACGCAA
CACGCAACGCAA
CACGCAGCGCAG
CACGCAACGCAG
CACGCAACGCAG
CACGCAACGCAG
CACGCAACGCAG
CACGCAACGCAG
CACGCAACGCAG
CACGCAACGCAG
CACGCAACGCAG
Nr of selected repeats 74 Similarity 0.933811
Consensus:
CACGCAACGCAg
>Cele-UNSB01_1:2293586-2294094 Satlength=509 Nr of Repeats=12 RepeatLength=43
seed=AATTTCCGGC
AATTTCCGGCAAATGGGCAAGTCGGCAAATTGCCGAAATTAAA
AATTTCCGGCAAACCGGCAAATTGCCGAAATTAAA
AATTTCCGGCAAACCGGCAAACCGACATGTTGCCGAAAAAGAA
AATTTCCGGCAAATCGGTAAACCGGCAATTTTCCGAAAATAAA
AATTTCCGGCAAATCGGCAAACCGGCAATTTTCCGAAAATGAG

AATTTCCGGCAAATGGGCAAGTCGGCAAATTGCCGAAATTAAA
AATTTCCAGCAAACCGGCAAACCGACATGTTGCCGAAAAAGAA
AATTTCCGGCAAATCGGTAAACCGGCAATTTTCCGAAAATAAA
AATTTCCGGCAAATCGGCAAACCGGCAATTTGCCGAAAATAAG
AATTTCCGGCAAATCGGCAAGTCGGCAAACTGCCGGAATTAAA
AATTTCCGACAAATCGGCATGTCGGCAAATTGCCGGAATTGAA
AATTTCCTGCAAATCGGCAAACCGGCAATTTTCCGAAAATAAA
Nr of selected repeats 11 Similarity 0.790839
Consensus:
AATTTCCGGCAAATCGGCAAacCGGCAAnTTgCCGAAAaTaAA
>Cele-UNSB01_1:2301010-2301088 Satlength=79 Nr of Repeats=6 RepeatLength=12
seed=TAGGCTTAGG
TAGGCTTAGGCT
TAGGCTTAGGCT
TAGGCTTAGGCA
TAGGTTTAGGCTTAAGCT
TAGACTTAGGCT
TACGCTTAGGCA
Nr of selected repeats 5 Similarity 0.844444
Consensus:
TAggCTTAGGCt
>Cele-UNSB01_1:2308046-2308235 Satlength=190 Nr of Repeats=5 RepeatLength=12
seed=AGGCTTAGGC
AGGCTTAGGCTCCTTAAGTTTGAGTTTATGAAAAAAAAGTTGTGAAAAAAAACATAGAATCTAAAAATAATTTTCTCTT
CGAAATTTTAAAAATTTCAGAATGTTCAAAAGTTTTCCAAAGTCTTTAAT
AGGCTCAGGCTC
AGGTTTAGGCTT
AGGCTTAGGCCT
AGGCTCAGGCTTAGGCTTTGGTTT
Nr of selected repeats 3 Similarity 0.703704
Consensus:
AGGcTtAGGCtt
>Cele-UNSB01_1:2365340-2366051 Satlength=712 Nr of Repeats=8 RepeatLength=20
seed=GGACTAGTTT
GGACTAGTTTTCTACCAAATTTCAAATTTTCAGACATTTCTCGTCTCCGTTGCTGACGTCACCAGGAGGCACGAAATCG
GTGTGAAGCTCACCAAAAATGTGATAATTGTGGATGTAAATTTACAGTTTTTCTTAAAATGTTTTTCCTAATTTTTGAA
TTTTCAGAAACCAAATTTCAGCCAAGATGACGTGGAAATTGATATGGACGAGGTTTTAAGACCATTATGGACCATTCAA
AATAGGCCATCTGACTTTGCAACGGTGAGTTCTGGTGGTGGCCTAGAAATCCCATAGGTGGCCTAGAAGTTTCAAATGA
TGAATTTTCACATTTTAAATTCGATTATTCTTTGAAGTGCGCAAAAACTCTTCCACGGCCATATTCAAATTTCTAGGCC
ACCAACCTCAAAACTTAGACCATGTATTTCAGTGCTTCGAATTTTTCATTCAAATGATGCAAACAACTTGTGCGGAATT
TACATCAAACCGAGAAATTTTCAAAAAACTTTCAAAAACGGAATTCCACGTGGCAATTGCGGAACCTGTTTCAATTTGC
GGCCTCGGTATGGCTGGAG
GGACTAGTTTTTGAGTAGAG
GGACTAGGTTTTGAGTAGAG
GGACTAGTTTTTGAGTAGAA
GGACTAGTTTCCAGTTGGG
GGACTAGTTTTCAAGTAGAG
GGACTAGTTTTCGAGTAGAG
GGACTAGGTTTCGAGTAGAG
Nr of selected repeats 6 Similarity 0.880000
Consensus:
GGACTAGtTTTcGAGTAGAG
>Cele-UNSB01_1:2365340-2366212 Satlength=873 Nr of Repeats=11 RepeatLength=20
seed=GGACTAGTTT

GGACTAGTTTTCTACCAAATTTCAAATTTTCAGACATTTCTCGTCTCCGTTGCTGACGTCACCAGGAGGCACGAAATCG
GTGTGAAGCTCACCAAAAATGTGATAATTGTGGATGTAAATTTACAGTTTTTCTTAAAATGTTTTTCCTAATTTTTGAA
TTTTCAGAAACCAAATTTCAGCCAAGATGACGTGGAAATTGATATGGACGAGGTTTTAAGACCATTATGGACCATTCAA
AATAGGCCATCTGACTTTGCAACGGTGAGTTCTGGTGGTGGCCTAGAAATCCCATAGGTGGCCTAGAAGTTTCAAATGA
TGAATTTTCACATTTTAAATTCGATTATTCTTTGAAGTGCGCAAAAACTCTTCCACGGCCATATTCAAATTTCTAGGCC
ACCAACCTCAAAACTTAGACCATGTATTTCAGTGCTTCGAATTTTTCATTCAAATGATGCAAACAACTTGTGCGGAATT
TACATCAAACCGAGAAATTTTCAAAAAACTTTCAAAAACGGAATTCCACGTGGCAATTGCGGAACCTGTTTCAATTTGC
GGCCTCGGTATGGCTGGAG
GGACTAGTTTTTGAGTAGAG
GGACTAGGTTTTGAGTAGAG
GGACTAGTTTTTGAGTAGAA
GGACTAGTTTCCAGTTGGG
GGACTAGTTTTCAAGTAGAG
GGACTAGTTTTCGAGTAGAG
GGACTAGGTTTCGAGTAGAG
GGACTAGTTTTCGAGTAGAGGGACTAACTTTCCAATTGGTGGACTAACTTTCCAATTGGTGGACTAACTTTCCAATTGG
CGGACTAACTTTCTAATTAGG
GGACTAATTTTGCGCTTGGG
GGACTAGTTTTCCAACTGATGGCCTAACTTTTTCAAAACAG
Nr of selected repeats 7 Similarity 0.745276
Consensus:
GGACTAGtTTTnGAGTAGAG
>Cele-UNSB01_1:2396315-2396849 Satlength=535 Nr of Repeats=20 RepeatLength=20
seed=TTATTGATTT
TTATTGATTTTTCTAAAAAA
TTATTAATTTTTCTAGGACA
TTATTGATTTGTCTAGGTCA
TTATTGATTTTTCTAGAACA
TTATTGATTTTTCTAGGACA
TTATTGATTTTTAGGACA
TTATTGATTTTCCTATAACAC
TATTGATTTTTCTAAAAAA
TTATTAATTTTTCTAGGACA
TTATTGATTTGTCTAGGTCA
TTATTGATTTTTCTACGACA
TTATTAATTTTTTTTGGCCA
TTATTGATTTTTCTAGAACAA
TATTGATTTTTCTAGGTCA
TTATTGATTTGTCTAGGTCA
TTATTGATTTTTCTACGACA
TTATTGATTTTTCTAGAACAA
TATTGATTTTTCTAGAACA
TTATTGATTTTTCTAGGACT
TTATTGATTTTTCAGTGTAATCTGCTGTGATCGGGGGACCTTATTGACATTTGGTACTGCAAAGAACCTCGGTTTGGGG
AAAGATGCAAAAGATTCAGCGAAGCCGATGCTCCTCGAGCAACTTTTGAGGTATTTATTTGGCTTCCACTACACGAA
Nr of selected repeats 12 Similarity 0.816162
Consensus:
TTATTgATTTtTCTAgGaCA
>Cele-UNSB01_1:2402282-2402378 Satlength=97 Nr of Repeats=6 RepeatLength=12
seed=AAGCCTAAGC
AAGCCTAAGCAC
AAGCCTAAGCCT
AAGCCTAAGCCT
AAGCCTAAGCCT

AAGCCTAAGCCCTAGCATAAACCAAAGCCCTAGCCTA
AACCTAAGCCT
Nr of selected repeats 4 Similarity 0.839744
Consensus:
AAGCCTAAGCCt
>Cele-UNSB01_1:2461739-2462720 Satlength=982 Nr of Repeats=5 RepeatLength=175
seed=GATGCACCAT
GATGCACCATGAGGCTAAATTTAGTTTGACAGCCCATATCTCGGTGAATTCTGGTGCTATCAAAAACTTTTTGTATTTG
TATTTGTATTTATTACGATTGAAATCGTGATTAAAAATAAATAGTGAATAGACATGAATAAGTTTTTAACTACAGAGCT
ATAGAAAATTATGCGCGCAATATTTTACCAGTTGACGGGTTTTTGACAACTGCAAAGAAAATCGAGATATGAGCTTCCA
AAGTTAGCTGGGCTACTAGTGATC
GATGCACCATGTCTTTGAAAGCCTATACTTCGGTTCATTTCGTAGCTGTCAAAAACTTTTTAACAACACATCTATAGAA
ATCAACATGCTTAATATTTCGTCAGTTGACAGTTTTTTGATAACTGCAATGAAATCCGAGATATGAGCTTTCAAAGTTG
AGAGACCTCACTCGGTT
GATGCACCATGTCTTTGACAGTCAATATCTCGGTTTATTTTGTAGCTATCAAAAAATTTTCAACTACAAATCTATAGAA
AATTAAATGTTCAACATTTTCTCAGTTGACAGTTTTTTGATAACTGCAATGACATCCGAGATATGAGCTTTCAAAGTTG
AGAGACCTCACTCGGTT
GATGCACCATGTCTTTGACAGTCAATATCTCGGTTTATTTTGTAGCTATCAAAAAATTTTCAACTACAAATCTATAGAA
AATTAAATGTTCAACATTTTCTCAGTTGACAGTTTTTTGATAACTGCAATGACATCCGAGATATAAGCTTTCAAAGTTG
AGTAAACCCATTTTGTA
GATGCACCATGAGGCTAAATCACTTTTGACCGCCCATATCTCGGTGAATTTTATAGCTATCAAAAACTTATAAATTACA
AAACTATAGAAAATCATATGCGCAATATTTTAATATTTTGCTAGTTGACGGGTTTTTGATAACTGCAAAGAAAATTGAG
AAATGAGCTTTCAAAGTTGAGTGTGCTACTTGTTATC
Nr of selected repeats 3 Similarity 0.840552
Consensus:
GATGCACCATGTCTTTGAcAGtCaATAtcTCGGTTtATTTtGTAGCTaTCAAAAAaTTTTcAACtACAaATCTATAGaA
AATtAAATGtTcAAcATTTtcTCAGTTGACAGTTTTTTGATAACTGCAATGAcATCCGAGATATgAGCTTTCAAAGTTG
AGAgACCtCAcTcgGTt
>Cele-UNSB01_1:2556234-2556523 Satlength=290 Nr of Repeats=10 RepeatLength=18
seed=GCCTAGGAAT
GCCTAGGAATACGAAAACTTCCACCCATGCAAACTGAGGGAGGTACCTCGTGACCTCCATTTTGGCATATATCCTAGCA
GCACGGAAACATCTTCAATCGCCTAGTACG
GACTAGGAATCCAATATG
GCCTAGGAATCCTATGTG
GCCTAGAAATCCAATATG
GCCTAGGAATGCTATGTGGCCTTGGAACCCCATGTG
GCCTAGAAATCCAATATG
GCCTAGGAATCCCATGTG
GCCTAAGAATCCTATGTG
GCCTAGGAATCCCATGTG
GCCTAGAAATTCAATACA
Nr of selected repeats 8 Similarity 0.772487
Consensus:
GCCTAGgAATCCaATaTG
>Cele-UNSB01_1:2574462-2585290 Satlength=10829 Nr of Repeats=565
RepeatLength=19 seed=TACACGAAAA
TACACGAAAAATAGAATGT
TACACGAAAAATAGATTGT
TACACGAAAAATAGATTGT
TACACGAAAAATAGAATGT
TACACGAAAAATAGATTGT
TACACGAAAAAAAATAAGAAAAATAGATTGT
TACACGAAAAATAGAATGT
TACACGAAAAATAGATTGT

TACACGAAAAATAGATTGT
TACACGAAAAATAGAATGT
TACACGAAAAATAGATTGT
TACACGAAAAAAAATGTTAAGAAAAATAGATTGT
TACACGAAAAATAGATTGT
TACACGAAAAATAGA
TACACGAAAAATAGATTGT
TACACGAAAAAATAGAATGAACGAAAAATAGATTGT
TACACGAAAAATAGATTGT
TACACGAAAAATAGATTGT
TACACGAAAAATAGATTGT
TACACGAAAAATAGATTGT
TACACGAAAAATAGAATGT
TACACGAAAAATAGAATGT
TACACGAAAAATAGATTGT
TACACGAAAAATAGAATGT
TACACGAAAAAAAGTAAGAAAAATAGA
TACACGAAAAATAGATTGT
TACACGAAAAATAGAATGT
TACACGAAAAATAGATTGT
TACACGAAAAATAGATTGT
TACACGAAAAATAGATTGT
TACACGAAAAATAGATTGT
TACACGAAAAATAGATTGT
TACACGAAAAATAGAATGT
TACACGAAAAATAGATTGT
TACACGAAAAATAGAATGT
TACACGAAAAATAGATTGT
TACACGAAAAATAGATTGT
TACACGAAAAATAGATTGT
TACACGAAAAATAGATTGT
TACACGAAAAATAGATTGT
TACACGAAAAATAGATTGT
TACACGAAAAATAGATTGT
TACACGAAAAATAGATTGT
TACACGAAAAATAGATTGT
TACACGAAAAATAGATTGT
TACACGAAAAATAGATTGT
TACACGAAAAATAGATTGT
TACACGAAAAATAGATTGT
TACACGAAAAATAGATTGT
TACACGAAAAATAGATTGT
TACACGAAAAATAGATTGT
TACACGAAAAATAGATGT
TACACGAAAAATAGATTGT
TACACGAAAAATAGATGT
TACACGAAAAATAGAATGT
TACACGAAAAATAGATTGT
TACACGAAAAATAGATTGT
TACACGAAAAATAGATTGT
TACACGAAAAATAGAATGT
TACACGAAAAATAGATTGT
TACACGAAAAATAGATTGT
TACACGAAAAATAGAATGT

TACACGAAAAATAGATTGT
TACACGAAAAATAGATTGT
TACACGAAAAATAGATTGT
TACACGAAAAATAGAATGT
TACACGAAAAATAGATTGT
TACACGAAAAATAGATTGT
TACACGAAAAATAGAATGT
TACACGAAAAATAGATTGT
TACACGAAAAATAGATTGT
TACACGAAAAATAGAATGT
TACACGAAAAATAGATTGT
TACACGAAAAATAGATTGT
TACACGAAAAAATAGACTGT
TACACGAAAAATAGATTGT
TACACGAAAAATAGATTGT
TACACGAAAAATAGAATGT
TACACGAAAAATAGATTGT
TACACGAAAAATAGATTGT
TACACGAAAAATAGATTGT
TACACGAAAAATAGATTGT
TACACGAAAAATAGATTGT
TACACGAAAAATAGATTGT
TACACGAAAAAATAGATTGT
TACACGAAAAATAGATTGT
TACACGAAAAATAGATTGT
TACACGAAAAATAGATTGT
TACACGAAAAATAGATTGT
TACACGAAAAATAGAATGT
TACACGAAAAATAGATTGT
TACACGAAAAATAGATTGT
TACACGAAAAATAGATTGT
TACACGAAAAATAGATTGT
TACACGAAAAATAGATTGT
TACACGAAAAATAGAATGT
TACACGAAAAATAGATTGT
TACACGAAAAATAGATTGT
TACACGAAAAATAGATTGT
TACACGAAAAATAGAATTGT
TACACGAAAAATAGATTGT
TACACGAAAAATAGATTGT
TACACGAAAAATAGATTGT
TACACGAAAAATAGATTGT
TACACGAAAAATAGATTGT
TACACGAAAAATAGATTGT
TACACGAAAAATAGAATGT
TACACGAAAAATAGATTGT
TACACGAAAAATAGATTGT
TACACGAAAAATAGATTGT
TACACGAAAAATAGATTGT
TACACGAAAAATAGATTGT
TACACGAAAAATAGATTGT
TACACGAAAAATAGATTGT
TACACGAAAAATAGATTGT
TACACGAAAAATAGATTGT

TACACGAAAAATAGATTGT
TACACGAAAAATAGATTGT
TACACGAAAAATAGATTGT
TACACGAAAAATAGATTGT
TACACGAAAAATAGAATGT
TACACGAAAAATAGATTGT
TACACGAAAAATAGATTGT
TACACGAAAAATAGATTGT
TACACGAAAAATAGATTGT
TACACGAAAAATAGAATGT
TACACGAAAAATAGATTGT
TACACGAAAAATAGATTGT
TACACGAAAAATAGATTGT
TACACGAAAAATAGATTGT
TACACGAAAAATAGATTGT
TACACGAAAAATAGATTGT
TACACGAAAAATAGATTGT
TACACGAAAAATAGATTGT
TACACGAAAAATAGATTGT
TACACGAAAAATAGATTGT
TACACGAAAAATAGATTGT
TACACGAAAAATAGATTGT
TACACGAAAAATAGAATGT
TACACGAAAAATAGATTGT
TACACGAAAAATAGATTGT
TACACGAAAAATAAGAAAAAAATAGATTGT
TACACGAAAAATAGATTGT
TACACGAAAAATAGATTGT
TACACGAAAAATAGATTGT
TACACGAAAAATAGATTGT
TACACGAAAAATAGAATGT
TACACGAAAAATAGATTGT
TACACGAAAAATAGATTGT
TACACGAAAAATAGATTGT
TACACGAAAAATAGATTGT
TACACGAAAAAATAGATTGT
TACACGAAAAATAGATTGT
TACACGAAAAATAGAATGT
TACACGAAAAATAGATTGT
TACACGAAAAATAGATTGT
TACACGAAAAATAGATTGT
TACACGAAAAATAGATTGT
TACACGAAAAATAGAATGT
TACACGAAAAATAGATTGT
TACACGAAAAATAGATTGT
TACACGAAAAATAGAATGT
TACACGAAAAATAGATTGT
TACACGAAAAATAGATTGT
TACACGAAAAATAGATTGT
TACACGAAAAATAGATTGT
TACACGAAAAATAGATTGT
TACACGAAAAATAGATTGT
TACACGAAAAATAGATTGT
TACACGAACACGAAAATAGATTGT

TACACGAAAAATAGATTGT
TACACGAAAAATAGATTGT
TACACGAAAAATAGATTGT
TACACGAAAAATAGATTGT
TACACGAAAAATAGATTGT
TACACGAAAAATAGAATGT
TACACGAAAAATAGATTGT
TACACGAAAAATAGATTGT
TACACGAAAAATAGATTGT
TACACGAAAAATAGATTGT
TACACGAAAAATAGATTGT
TACACGAAAAATAGATTGT
TACACGAAAAATAGATTGT
TACACGAAAAATAGAATGT
TACACGAAAAATAGATTGT
TACACGAAAAATAGAATGT
TACACGAAAAATAGATTGT
TACACGAAAAATAGATTGT
TACACGAAAAATAGATTGT
TACACGAAAAATAGATTGT
TACACGAAAAATAGATTGT
TACACGAAAAATAGAATGT
TACACGAAAAATAGATTGT
TACACGAAAAATAGATTGT
TACACGAAAAATAGATTGT
TACACGAAAAATAGATTGT
TACACGAAAAATAGATTGT
TACACGAAAAATAGATTGT
TACACGAAAAATAGATTGT
TACACGAAAAATAGATTGT
TACACGAAAAATAGATTGT
TACACGAAAAATAGATTGT
TACACGAAAAATAGATTGT
TACACGAAAAATAGAATGT
TACACGAAAAATAGATTGT
TACACGAAAAATAGATGT
TACACGAAAAATAGATTGT
TACACGAAAAATAGATTGT
TACACGAAAAATAGATTGT
TACACGAAAAATAGAATGT
TACACGAAAAATAGATTGT
TACACGAAAAATAGATTGT
TACACGAAAAATAGATTGT
TACACGAAAAATAGAATGT
TACACGAAAAATAGATTGT
TACACGAAAAATAGATTGT
TACACGAAAAATAGATTGT
TACACGAAAAATAGATTGT
TACACGAAAAATAGATTGT
TACACGAAAAATAGATTGT
TACACGAAAAATAGATTGT
TACACGAAAAATAGATTGT
TACACGAAAAATAGATTGT
TACACGAAAAATAGATTGT

TACACGAAAAATAGATTGT
TACACGAAAAATAGATTGT
TACACGAAAAATAGATTGT
TACACGAAAAATAGATTGT
TACACGAAAAATAGATTGT
TACACGAAAAATAGATTGT
TACACGAAAAATAGATTGT
TACACGAAAAATAGATTGT
TACACGAAAAATAGATTGT
TACACGAAAAATAGATTGT
TACACGAAAAATAGATTGT
TACACGAAAAATAGATTGT
TACACGAAAAATAGATTGT
TACACGAAAAATAGATTGT
TACACGAAAAATAGATTGT
TACACGAAAAATAGAATGT
TACACGAAAAATAGATTGT
TACACGAAAAATAGATTGT
TACACGAAAAATAGATTGT
TACACGAAAAATAGATTGT
TACACGAAAAATAGATTGT
TACACGAAAAATAGAATGT
TACACGAAAAATAGATTGT
TACACGAAAAATAGAATGT
TACACGAAAAATAGATTGT
TACACGAAAAATAGAATGT
TACACGAAAAATAGATTGT
TACACGAAAAATAGATTGT
TACACGAAAAATAGATTGT
TACACGAAAAATAGATTGT
TACACGAAAAATAGATTGT
TACACGAAAAATAGATTGT
TACACGAAAAATAGATTGT
TACACGAAAAATAGATTGT
TACACGAAAAATAGATTGT
TACACGAAAAATAGATTGT
TACACGAAAAATAGATTGT
TACACGAAAAATAGATTGT
TACACGAAAAATAGATTGT
TACACGAAAAATAGATTGT
TACACGAAAAATAGATTGT
TACACGAAAAATAGATTGT
TACACGAAAAATAGAATGT
TACACGAAAAATAGATTGT
TACACGAAAAATAGAATGT
TACACGAAAAATAGATTGT
TACACGAAAAATAGAATGT
TACACGAAAAATAGATTGT
TACACGAAAAATAGAATGT
TACACGAAAAATAGATTGT
TACACGAAAAATAGATTGT
TACACGAAAAATAGATTGT
TACACGAAAAATAGATTGT
TACACGAAAAATAGATTGT

TACACGAAAAATAGATTGT
TACACGAAAAATAGATTGT
TACACGAAAAATAGAATGT
TACACGAAAAATAGATTGT
TACACGAAAAATAGATTGT
TACACGAAAAATAGATTGT
TACACGAAAAATAGATTGT
TACACGAAAAATAGATTGT
TACACGAAAAATAGATTGT
TACACGAAAAATAGATTGT
TACACGAAAAATAGATTGT
TACACGAAAAATAGATTGT
TACACGAAAAATAGATTGT
TACACGAAAAATAGATTGT
TACACGAAAAATAGATTGT
TACACGAAAAATAGATTGT
TACACGAAAAATAGATTGT
TACACGAAAAATAGATTGT
TACACGAAAAATAGATTGT
TACACGAAAAATAGATTGT
TACACGAAAAATAGAATGT
TACACGAAAAATAGATTGT
TACACGAAAAATAGATTGT
TACACGAAAAATAGAAAAAAAAATAGATTGT
TACACGAAAAATAGATTGT
TACACGAAAAATAGATTGT
TACACGAAAAATAGATTGT
TACACGAAAAATAGATTGT
TACACGAAAAATAGAATGT
TACACGAAAAATAGATTGT
TACACGAAAAATAGATTGT
TACACGAAAAATAGATTGT
TACACGAAAAATAGATTGT
TACACGAAAAAATAGATTGT
TACACGAAAAATAGATTGT
TACACGAAAAATAGATTGT
TACACGAAAAATAGATTGT
TACACGAAAAATAGATTGT
TACACGAAAAATAGATTGT
TACACGAAAAATAGATTGT
TACACGAAAAATAGATTGT
TACACGAAAAATAGATTGT
TACACGAAAAATAGATGT
TACACGAAAAAAAAAAAAATAGATTGT
TACACGAAAAATAGATTGT
TACACGAAAAATAGAATGT
TACACGAAAAATAGATTGT
TACACGAAAAATAGATTGT
TACACGAAAAATAGATGT
TACACGAAAAATAGATTGT
TACACGAAAAATAGATTGT
TACACGAAAAATAGATGT
TACACGAAAAATAGATTGT
TACACGAAAAATAGATTGT

TACACGAAAAAAAAAAAAATAGATTGT
TACACGAAAAATAGATTGT
TACACGAAAAATAGATTGT
TACACGAAAAATAGATTGT
TACACGAAAAATAGATTGT
TACACGAAAAATAGATTGT
TACACGAAAAATAGATTGT
TACACGAAAAATAGAATGT
TACACGAAAAATAGATTGT
TACACGAAAAATAGATTGT
TACACGAAAAATAGATTGT
TACACGAAAAATAGATTGT
TACACGAAAAATAGATTGT
TACACGAAAAATAGATTGT
TACACGAAAAATAGATTGT
TACACGAAAAATAGATTGT
TACACGAAAAATAGATTGT
TACACGAAAAATAGATTGT
TACACGAAAAATAGATTGT
TACACGAAAAATAGATTGT
TACACGAAAAATAGATTGT
TACACGAAAAATAGATTGT
TACACGAAAAATAGATTGT
TACACGAAAAATAGATTGT
TACACGAAAAATAGAATGT
TACACGAAAAATAGATTGT
TACACGAAAAATAGATTGT
TACACGAAAAATAGAATGT
TACACGAAAAATAGATTGT
TACACGAAAAATAGATTGT
TACACGAAAAATAGATTGT
TACACGAAAAATAGAATGT
TACACGAAAAATAGATTGT
TACACGAAAAATAGATTGT
TACACGAAAAATAGATTGT
TACACGAAAAATAGAATGT
TACACGAAAAATAGATTGT
TACACGAAAAATAGATTGT
TACACGAAAAATAGATTGT
TACACGAAAAATAGAATGT
TACACGAAAAATAGATTGT
TACACGAAAAATAGATTGT
TACACGAAAAATAGATTGT
TACACGAAAAATAGATTGT
TACACGAAAAATAGATTGT
TACACGAAAAATAGATTGT
TACACGAAAAATAGATTGT
TACACGAAAAATAGAATGT
TACACGAAAAATAGATTGT
TACACGAAAAATAGAATGT
TACACGAAAAATAGATTGT
TACACGAAAAATAGATTGT
TACACGAAAAATAGAATGT
TACACGAAAAATAGATTGT

TACACGAAAAATAGATTGT
TACACGAAAAATAGAATGT
TACACGAAAAATAGATTGT
TACACGAAAAAAAAAAATAGATTGT
TACACGAAAAATAGATTGT
TACACGAAAAATAGAATGT
TACACGAAAAATAGATTGT
TACACGAAAAATAGATTGT
TACACGAAAAATAGATTGT
TACACGAAAAATAGATGT
TACACGAAAAATAGATTGT
TACACGAAAAATAGATTGT
TACACGAAAAATAGAATGT
TACACGAAAAATAGATTGT
TACACGAAAAATAGATTGT
TACACGAAAAATAGATTGT
TACACGAAAAATAGATTGT
TACACGAAAAATAGATTGT
TACACGAAAAATAGATTGT
TACACGAAAAATAGATTGT
TACACGAAAAATAGATTGT
TACACGAAAAATAGATTGT
TACACGAAAAATAGATTGT
TACACGAAAAATAGATTGT
TACACGAAAAATAGATGT
TACACGAAAAATAGATTGT
TACACGAAAAATAGATTGT
TACACGAAAAATAGATTGT
TACACGAAAAATAGATTGT
TACACGAAAAATAGATTGT
TACACGAAAAATAGAATGT
TACACGAAAAATAGATTGT
TACACGAAAAATAGATTGT
TACACGAAAAATAGAATGT
TACACGAAAAATAGATTGT
TACACGAAAAATAGATTGT
TACACGAAAAATAGAATGT
TACACGAAAAATAGATTGT
TACACGAAAAATAGATTGT
TACACGAAAAATAGAATGT
TACACGAAAAATAGATTGT
TACACGAAAAATAGATTGT
TACACGAAAAATAGAATGT
TACACGAAAAATAGATTGT
TACACGAAAAATAGATTGT
TACACGAAAAATAGATTGT
TACACGAAAAATAGATTGT
TACACGAAAAATAGATTGT
TACACGAAAAATAGATTGT
TACACGAAAAATAGATTGT
TACACGAAAAATAGATTGT
TACACGAAAAATAGATTGT
TACACGAAAAATAGATTGT
TACACGAAAAATAGATTGT

TACACGAAAAATAGATTGT
TACACGAAAAATAGATTGT
TACACGAAAAATAGATTGT
TACACGAAAAATAGATTGT
TACACGAAAAATAGATTGT
TACACGAAAAATAGATTGT
TACACGAAAAATAGATTGT
TACACGAAAAATAGATTGT
TACACGAAAAATAGATTGT
TACACGAAAAATAGATTGT
TACACGAAAAATAGATTGT
TACACGAAAAATAGATTGT
TACACGAAAAATAGAATGT
TACACGAAAAATAGATTGT
TACACGAAAAATAGATTGT
TACACGAAAAATAGATTGT
TACACGAAAAATAGATTGT
TACACGAAAAATAGATTGT
TACACGAAAAATAGAATGT
TACACGAAAAATAGATTGT
TACACGAAAAATAGATTGT
TACACGAAAAATAGATTGT
TACACGAAAAATAGATTGT
TACACGAAAAATAGATTGT
TACACGAAAAATAGATTGT
TACACGAAAAATAGATTGT
TACACGAAAAATAGAATGT
TACACGAAAAATAGATTGT
TACACGAAAAATAGATTGT
TACACGAAAAATAGATTGT
TACACGAAAAATAGATTGT
TACACGAAAAATAGATTGT
TACACGAAAAATAGATTGT
TACACAAAAAAATAGAATGT
TACACGAAAAATAGATTGT
TACACGAAAAATAGAATGT
TACACGAAAAATAGATTGT
TACACGAAAAATAGATTGT
TACACGAAAAATAGATTGT
TACACGAAAAATAGATTGT
TACACGAAAAATAGATTGT
TACACGAAAAATAGAATGT
TACACGAAAAATAGATTGT
TACACGAAAAATAGATTGT
TACACGAAAAATAGATTGT
TACACGAAAAATAGAATGT
TACACGAAAAATAGATTGT
TACACGAAAAATAGAATGT
TACACGAAAAATAGATTGT
TACACGAAAAATAGATTGT
TACACGAAAAATAGATTGT
TACACGAAAAATAGAATGT
TACACGAAAAATAGATTGT
TACACGAAAAATAGATTGT

TACACGAAAAATAGAATGT
TACACGAAAAATAGATTGT
TACACGAAAAATAGAATGT
TACACGAAAAATAGAATGT
TACACGAAAAATAGATTGT
TACACGAAAAATAGAATGT
TACACGAAAAATAGATTGT
TACACGAAAAATAGATTGT
TACACGAAAAATAGAATGT
TACACGAAAAATAGATTGT
TACACGAAAAATAGATTGT
TACACGAAAAATAGAATGT
TACACGAAAAATAGAATGT
TACACGAAAAATAGATTGT
TACACGAAAAATAGATTGT
TACACGAAAAATAGATTGT
TACACGAAAAATAGATTGT
TACACGAAAAATAGATTGT
TACACGAAAAATAGATTGT
TACACGAAAAATAGATTGT
TACACGAAAAATAGAATGT
TACACGAAAAATAGATTGT
TACACGAAAAATAGAATGT
TACACGAAAAATAGATTGT
TACACGAAAAATAGAATGT
TACACGAAAAATAGATTGT
TACACGAAAAATAGATTGT
TACACGAAAAATAGATTGT
TACACGAAAAATAGATTGT
TACACGAAAAATAGATTGT
TACACGAAAAATAGATTGT
TACACGAAAAATAGAATGT
TACACGAAAAATAGATTGT
TACACGAAAAATAGATTGT
TACACGAAAAATAGAATGT
TACACGAAAAATAGATTGT
TACACGAAAAATAGATTGT
TACACGAAAAATAGAATGT
TACACGAAAAATAGATTGT
TACACGAAAAATAGAATGT
TACACGAAAAATAGATTGT
TACACGAAAAATAGATTGT
TACACGAAAAATAGATTGT
TACACGAAAAATAGATTGT
TACACGAAAAATAGATTGT
TACACGAAAAATAGATTGT
TACACGAAAAATAGAATGT
TACACGAAAAATAGATTGT
TACACGAAAAATAGATTGT
TACACGAAAAATAGATTGT
TACACGAAAAATAGATTGT
TACACGAAAAATAGATTGT
TACACGAAAAATAGATGT
TACACGAAAAATAGAATGT

TACACGAAAAATAGATTGT
TACACGAAAAATAGAATGT
TACACGAAAAATAGAATGT
TACACGAAAAATAGATTGT
TACACGAAAAATAGATTGT
TACACGAAAAATAGAATGT
TACACGAAAAATAGAATGT
TACACGAAAAATAGATTGT
TACACGAAAAATAGAATGT
TACACGAAAAATAGAATGT
TACACGAAAAATAGATTGT
TACACGAAAAATAGATTGT
TACACGAAAAATAGATTGT
TACACGAAAAATAGATTGT
TACACGAAAAATAGAATGT
TACACGAAAAATAGATTGT
TACACGAAAAATAGATTGT
Nr of selected repeats 539 Similarity 0.979754
Consensus:
TACACGAAAAATAGATTGT
>Cele-UNSB01_1:2599082-2599291 Satlength=210 Nr of Repeats=6 RepeatLength=35
seed=TTGCCGGAAT
TTGCCGGAATTAAAAATTTCTGGAAAATTGGCAAT
TTGCCGGAATGGAAATTTTTTGGTAAATCGGCAAT
TTGCCGGAATTGAAAATTTTTGGAAAATTGGCAAA
TTGCGGAATTAAAAATTTCTGGAAAATCGGCAAT
TTGCCGGAATTGAAATTTTCTGGCAAATCGGTAAA
TTGCCGGAATTAAACATTTTTGGTAAATCGGCCAT
Nr of selected repeats 5 Similarity 0.794286
Consensus:
TTGCCGGAATtgAAaaTTTtTGGnAAATcGGcaAt
>Cele-UNSB01_1:2691842-2691886 Satlength=45 Nr of Repeats=4 RepeatLength=11
seed=AAACTTGGGC
AAACTTGGGCC
AAACTTGGGTA
AAACTTGGGCT
AAACTTGGGCT
Nr of selected repeats 4 Similarity 0.770833
Consensus:
AAACTTGGGct
>Cele-UNSB01_1:2695367-2695539 Satlength=173 Nr of Repeats=4 RepeatLength=43
seed=TTGCCGATTT
TTGCCGATTTTCCTGAATTGTTCATTTCCGACAATATGACGAT
TTGCCGATTTGCCGGAAATGTTTATTTCCGACAGTTGGCAGAT
TTGCCGATTTGCCGGAAATATTTATTTCCGACAGTTGGCAGAT
TTGCCGATTTGCCGGAAATGTTTGTTTCCGACAATTTGCCCAT
Nr of selected repeats 4 Similarity 0.798450
Consensus:
TTGCCGATTTgCCgGAAaTgTTtaTTTCCGACAaTtgGcagAT
>Cele-UNSB01_1:2780250-2780460 Satlength=211 Nr of Repeats=6 RepeatLength=35
seed=AATTTCAATT
AATTTCAATTCCGGCAATTTGCCGATTTGCCGGAA
AATTTCAATTTCGGCAATTTGCCGATTTGCCGGAA
AATTTCAATTCTGGCAACTTTCCGATTTGCCGGAA

AATTTCAATTCCGGCAATTTGCGGATTTGCTGGAT
AATTTCAATTCCGGCAACTTTCCGATTTGCCGGAG
AATTTCAATTCTGGCAACTTTCCGATTTGCCGAAA
Nr of selected repeats 6 Similarity 0.860317
Consensus:
AATTTCAATTCcGGCAAcTTgCCGATTTGCCGGAa
>Cele-UNSB01_1:2850958-2851167 Satlength=210 Nr of Repeats=5 RepeatLength=35
seed=GGAATTGAAA
GGAATTGAAAATTTCCGGCAAATTGGCAAATTGCCGTAATTGAACACTTCCGGCAAATTGGCAACTTGTA
GGAATTGAAAATTCCCGGCAAATCGGCAAATTGCC
GGAATTGAAATTTCCAGCGAATCGGCAACTTGTA
GGAATTGAAAATTTCCGGCAAATCGGCAAATTGTC
GGAATTGAAATTTTCCGGCGAATCGGCAAATTGCC
Nr of selected repeats 3 Similarity 0.898413
Consensus:
GGAATTGAAAaTTtCCGGCaAATCGGCAAATTGcC
>Cele-UNSB01_1:2859395-2859671 Satlength=277 Nr of Repeats=5 RepeatLength=43
seed=TTGCCGATTT
TTGCCGATTTGCCAATTTGCCGGAAATTTACAATTCCACTATT
TTGCCGGTTTCTCGATTTGCCGGATATTTACAATTTCACTATT
TTGCCGGTTTGCCGATTTACCAGAAATTTACAATTCCACTATT
TTGCCGGTTTGCCGAT
TTGCCGATTTGTCGGAAGTTTTTAGACGAATTTTTACGACTGAAACTATTTCGAAAAAAAAAGTGCAGAACCACAACTT
GCCGAAAATTTTCGGCAAGTTCGCCAACTCGGCGATTTGCCATCTTGCCGAT
Nr of selected repeats 3 Similarity 0.834625
Consensus:
TTGCCGgTTTgcCgATTTgCCgGAaATTTACAATTcCACTATT
>Cele-UNSB01_1:2881736-2881825 Satlength=90 Nr of Repeats=5 RepeatLength=12
seed=TAGGCTTAGG
TAGGCTTAGGCT
TAGGCTGAGGCG
TAGGCTTAGGCT
TAGGCCTAGGCT
TAGGCTTAGGCGTAAAGTCAGGCTTACGGTAGGCGCAGGGT
Nr of selected repeats 4 Similarity 0.833333
Consensus:
TAGGCttAGGCt
>Cele-UNSB01_1:2886442-2886617 Satlength=176 Nr of Repeats=4 RepeatLength=35
seed=TTTGCCGATT
TTTGCCGATTTGCCAAAAAATTTTAATTCCGGCAA
TTTGCCGACTTGTCAAAAATTGTTAATTTTAGCAC
TTTGCCGATTTGCCAAAAAATTTTAATTTCGGCAATTTTCCGACTTGTCAAAAATTGTTAATTTCAGCAA
TTTGCCGATTTGACGGAAATTTTTAATTCCGGCAA
Nr of selected repeats 3 Similarity 0.733333
Consensus:
TTTGCCGAtTTGnCaaAAAtTtTTAATTccgGCAa
>Cele-UNSB01_1:2899706-2899878 Satlength=173 Nr of Repeats=4 RepeatLength=43
seed=AAATCGGCAA
AAATCGGCAATCCGGCAAATTGCGAAAAATGAAAATTTCCGGC
AAATCGGCAATCCGGCCAATTGCAAAAAATGAAAATTCCCGGC
AAATCGGCAAACCGGCAAATTGCGAAAAATGAAAATTTCCGGC
AAATCGGCAATTCGGCCAATTGCAAAAAATGAAAATTCCCGGC
Nr of selected repeats 4 Similarity 0.906977
Consensus:

AAATCGGCAAtcCGGCaAATTGCaAAAAATGAAAATTcCCGGC
>Cele-UNSB01_1:3070958-3071149 Satlength=192 Nr of Repeats=5 RepeatLength=35
seed=TTGCCGGAAA
TTGCCGGAAATTTTCAATTCCGGCAATTTGCCATT
TTGCCGGAAATTTTCAATTCCGGCAAATTGTTGTTTTTCCAAT
TTGCCGGAAATTTGTAATTCCGACATATTGCCGAT
TTGCCGGAAATTTGTAATTCCGACAAATTGCCGAT
TTGCCGGAAATTGTTTATTCCGGCAATTTGTCGTTTTGCCAGT
Nr of selected repeats 3 Similarity 0.808642
Consensus:
TTGCCGGAAATTTgtAATTCCGaCAaaTTGCCgAT
>Cele-UNSB01_1:3156606-3157000 Satlength=395 Nr of Repeats=8 RepeatLength=35
seed=CGATTTGCCG
CGATTTGCCGGAAGTTTCTGAAATTATTTTAATAAGACGGAAACATTTAAAACTGTGCCTTTTTGATTTTTTTTTTCA
TTTTCTTCAGATATTTTCATAAAACTTACTTACTTTCAAGTCGGTTTTTTTTGTCGGTTTTCCGGAAATTTTCATTTCC
GGCAAACTGT
CGATTTGCCGGAAATATTCATTTTCTGCAAATTGC
CGATTTTCCGGAAATTTTCATTTCCGGCAAACTGT
CGATTTGCCGGAAATATTCATTTTCTGCAAATTGC
CGATTTTCCGGAAATTTTCATTTCCGGCAAACTGT
CGATTTGCCGGAAATATTCATTTTCTGCAAATTGC
CGATTTTCCGGAAATTTTCATTTCCGGCAAACTGT
CGATTTGCCGATTTGC
Nr of selected repeats 6 Similarity 0.862857
Consensus:
CGATTTgCCGGAAATaTTCATTTcCgGCAAAcTGc
>Cele-UNSB01_1:3158079-3158367 Satlength=289 Nr of Repeats=21 RepeatLength=12
seed=CTAAGCCTAA
CTAAGCCTAATTCTAAGG
CTATGCCTAAAC
CTAAGCCTAGAC
CTAAGCCTAACA
CTAAGCTTAAAC
CTAAGCCTAAGC
CTAAGCCTAAGC
CTAAACCTAAACCTGAGT
CTAAGCCTAAGA
CTAAGCTTAAGCCTGAGC
CTAGGCCTAAGT
CTAAGCCTAAGC
CTCAGCCTAAGCCAAAGACTACGCG
TAAGCCTAAGC
CTAAGTCTAATC
CTAAGCCTAAGC
CTAAGCATAAGC
CTAAACCTAAGC
CTAAGCCTAAGT
CTGAGCCTAAGCCAAAGC
CAAAGCCTAAAC
Nr of selected repeats 15 Similarity 0.766138
Consensus:
CTAAGCCTAAgc
>Cele-UNSB01_1:3159205-3159335 Satlength=131 Nr of Repeats=4 RepeatLength=26
seed=GGCAGGCGTC

GGCAGGCGTCAAGCAGGCAGGTTTCA
GGCAGGCGTCAAGCAGGCAGGTTTTA
GGCAGGCGTCAAGCAGGCAGGTTTCT
GGCAGGCGTCCGGCAAGCAAAATTCAAGCAGGCGTAAGGCGGGTAGGTTTCA
Nr of selected repeats 3 Similarity 0.931624
Consensus:
GGCAGGCGTCAAGCAGGCAGGTTTca
>Cele-UNSB01_1:3213789-3217715 Satlength=3927 Nr of Repeats=151 RepeatLength=26
seed=CAGAGTCACT
CAGAGTCACTAAATTTGGTGAACGGT
CAGAGTCACTAAATTTGGTGAACGGC
CAGAGTCACTATTTTTGGGGAACGGC
CAGAGTCACTATTTTTGGGGAACGGC
CAGAGTCACTATTTTTGGTGAACGGC
CAGAGTCACTATTTTTGGGGAACGGC
CAGAGTCACTATTTTTGGGGAACGGC
CAGAGTCACTATTTTTGGTGAACGGT
CAGAGTCACTAAATTTGGTGAACGGC
CAGAGTCACTATTTTTGGTGAACGGC
CAGAGTCACTATTTTTGGGGAACGGC
CAGAGTCACTATTTTTGGTGAACGGC
CAGAGTCACTATTTTTGGTGAACGGC
CAGAGTCACTATTTTTGGGGAACGGC
CAGAGTCACTATTTTTGGGGAACGGC
CAGAGTCACTATTTTTGGTGAACGGC
CAGAGTCACTATTTTTGGTGAACGGC
CAGAGTCACTATTTTTGGGGAACGGC
CAGAGTCACTATTTTTGGGGAACGGC
CAGAGTCACTATTTTTGGGGAACGGC
CAGAGTCACTAAATTTGGTGAACGGC
CAGAGTCACTATTTTTGGGGAACGGC
CAGAGTCACTATTTTTGGGGAACGGC
CAGAGTCACTATTTTTGGGGAACGGC
CAGAGTCACTATTTTTGGGGAACGGC
CAGAGTCACTATTTTTGGTGAACGGC
CAGAGTCACTATTTTTGGGGAACGGC
CAGAGTCACTATTTTTGGGGAACGGC
CAGAGTCACTAAATTTGGTGAACGGC
CAGAGTCACTATTTTTGGGGAACGGC
CAGAGTCACTATTTTTGGGGAACGGC
CAGAGTCACTATTTTTGGTGAACGGC
CAGAGTCACTATTTTTGGTGAACGGC
CAGAGTCACTATTTTTGGTGAACGGC
CAGAGTCACTATTTTTGGGGAACGGC
CAGAGTCACTATTTTTGGTGAACGGC
CAGAGTCACTATTTTTGGGGAACGGC
CAGAGTCACTAAATTTGGTGAACGGC
CAGAGTCACTATTTTTGGGGAACGGC
CAGAGTCACTATTTTTGGGGAACGAC
CAGAGTCACTATTTTTGGGCAACGAC
CAGAGTCACTATTTTTGGTGAACGGC
CAGAGTCACTATTTTTGGGGAACGGC
CAGAGTCACTATTTTTGGGGAACGGC
CAGAGTCACTATTTTTGGGGAACGGC

CAGAGTCACTATTTTTGGTGAACGGC
CAGAGTCACTAGTTTTGGTGAACGGC
CAGAGTCACTATTTTTGGGGAACGGC
CAGAGTCACTAAATTTGGTGAACGGC
CAGAGTCACTATTTTTGGGGAACGGC
CAGAGTCACTATTTTTGGGGAACGGC
CAGAGTCACTATTTTTGGGGAACGGC
CAGAGTCACTATTTTTGGTGAACGGC
CAGAGTCACTATTTTTGGGGAACGGC
CAGAGTCACTAAATTTGGTGAACGGC
CAGAGTCACTATTTTTGGGGAACGGC
CAGAGTCACTATTTTTGGGGAACGAC
CAGAGTCACTATTTTTGGGCAACGAC
CAGAGTCACTATTTTTGGTGAACGGC
CAGAGTCACTATTTTTGGGGAACGGC
CAGAGTCACTAAATTTGGTGAACGGC
CAGAGTCACTATTTTTGGGGAACGAC
CAGAGTCACTATTTTTGGTGAACGGC
CAGAGTCACTATTTTTGGGGAACGGC
CAGAGTCACTAAATTTGGTGAACGGC
CAGAGTCACTATTTTTGGTGAACGGC
CAGAGTCACTATTTTTGGGGAACGGC
CAGAGTCACTATTTTTGGTGAACGGC
CAGAGTCACTATTTTTGGGGAACGGC
CAGAGTCACTATTTTTGGGGAACGGC
CAGAGTCACTAAATTTGGTGAACAGC
CAGAGTCACTATTTTTGGGGAACGGC
CAGAGTCACTATTTTTGGTGAACGGC
CAGAGTCACTATTTTTGGGGAACGAC
CAGAGTCACTATTTTTGGGGAACGAC
CAGAGTCACTATTTTTGGTGAACGGC
CAGAGTCACTATTTTTGGTGAACGGC
CAGAGTCACTATTTTTGGGGAACGAC
CAGAGTCACTATTTTTGGGGAACGAC
CAGAGTCACTATTTTTGGGGAACGAC
CAGAGTCACTATTTTTGGTGAACGGC
CAGAGTCACTATTTTTGGTGAACGGC
CAGAGTCACTATTTTTGGTGAACGGC
CAGAGTCACTATTTTTGGGGAACGGC
CAGAGTCACTATTTTTGGTGAACGGC
CAGAGTCACTAGTTTTGGTGAACGGC
CAGAGTCACTATTTTTGGTGAACGGC
CAGAGTCACTATTTTTGGGGAACGAC
CAGAGTCACTATTTTTGGTGAACGGC
CAGAGTCACTATTTTTGGTGAACGGC
CAGAGTCACTATTTTTGGGGAACGAC
CAGAGTCACTATTTTTGGTGAACGGC
CAGAGTCACTATTTTTGGGGAACGGC
CAGAGTCACTAAATTTGGTGAACGGC
CAGAGTCACTAAATTTGGTGAACGGC
CAGAGTCACTATTTTTGGGGAACGGC
CAGAGTCACTATTTTTGGGGAACGGC
CAGAGTCACTATTTTTGGGGAACGAC
CAGAGTCACTATTTTTGGTGAACGGC

CAGAGTCACTATTTTTGGTGAACGGC
CAGAGTCACTATTTTTGGGGAACGAC
CAGAGTCACTATTTTTGGTGAACGGC
CAGAGTCACTATTTTTGGGGAACGAC
CAGAGTCACTATTTTTGGTGAACGGC
CAGAGTCACTATTTTTGGGGAACGGC
CAGAGTCACTAAATTTGGTGAACGGC
CAGAGTCACTATTTTTGGGGAACGGC
CAGAGTCACTATTTTTGGTGAACGGC
CAGAGTCACTATTTTTGGGGAACGGC
CAGAGTCACTAAATTTGGTGAACGGC
CAGAGTCACTATTTTTGGGGAACGGC
CAGAGTCACTATTTTTGGGGAACGAC
CAGAGTCACTATTTTTGGGCAACGAC
CAGAGTCACTATTTTTGGTGAACGGC
CAGAGTCACTATTTTTGGGGAACGAC
CAGAGTCACTATTTTTGGGGAACGAC
CAGAGTCACTATTTTTGGTGAACGGC
CAGAGTCACTATTTTTGGGGAACGGC
CAGAGTCACTAAATTTGGTGAACGGC
CAGAGTCACTATTTTTGGGGAACGGC
CAGAGTCACTATTTTTGGGGAACGGC
CAGAGTCACTATTTTTGGGGAACGGC
CAGAGTCACTATTTTTGGTGAACGGC
CAGAGTCACTAGTTTTGGTGAACGGC
CAGAGTCACTATTTTTGGTGAACGGC
CAGAGTCACTATTTTTGGGGAACGAC
CAGAGTCACTATTTTTGGTGAACGGC
CAGAGTCACTATTTTTGGTGAACGGC
CAGAGTCACTATTTTTGGGGAACGAC
CAGAGTCACTATTTTTGGTGAACGGC
CAGAGTCACTATTTTTGGGGAACGAC
CAGAGTCACTATTTTTGGTGAACGGC
CAGAGTCACTATTTTTGGGGAACGGC
CAGAGTCACTAAATTTGGTGAACGGC
CAGAGTCACTAAATTTGGTGAACGGC
CAGAGTCACTATTTTTGGGGAACGGC
CAGAGTCACTATTTTTGGTGAACGGC
CAGAGTCACTATTTTTGGGGAACGGC
CAGAGTCACTATTTTTGGGGAACGGC
CAGAGTCACTATTTTTGGTGAACGGC
CAGAGTCACTAAATTTGGTGAACGGC
CAGAGTCACTAAATTTGGTGAACGGC
CAGAGTCACTATTTTTGGGGAACGGC
CAGAGTCACTATTTTTGGGGAACGGC
CAGAGTCACTATTTTTGGGGAACGAC
CAGAGTCACTATTTTTGGTGAACGGC
CAGAGTCACTATTTTTGGTGAACGGC
CAGAGTCACTATTTTTGGGGAACGAC
CAGAGTCACTATTTTTGGTGAACGGC
CAGAGTCACTATTTTTGGGGAACGAC
CAGAGTCACTATTTTTGGTGAACGGC
Nr of selected repeats 151 Similarity 0.930474
Consensus:

CAGAGTCACTATTTTTGGgGAACGGC
>Cele-UNSB01_1:3303901-3304753 Satlength=853 Nr of Repeats=71 RepeatLength=12
seed=CTAAGTCTAA
CTAAGTCTAAGC
CTAAGTCTAAGC
CTAAGTCTAAGC
CTAAGTCTAAGC
CTAAGCCTAAGT
CTAAGCCTAAGT
CTAAGCCTAAGT
CTAAGCCTAAGT
CTAAGCCTAAGT
CTAAGCCTAAGC
CTAAGTCTAAGC
CTAAGTCTAAGC
CTAAGCCTAAGT
CTAAGTCTAAGT
CTAAGTCTAAGT
CTAAGCCTAAGT
CTAAGCCTAAGT
CTAAGTCTAAGC
CTAAGTCTAAGC
CTAAGTCTAAGC
CTAAGTCTAAGC
CTAAGCCTAAGT
CTAAGCCTAAGC
CTAAGTCTAAGC
CTAAGCCTAAGT
CTAAGCCTAAGC
CTAAGTCTAAGC
CTAAGTCTAAGC
CTAAGTCTAAGC
CTAAGCCTAAGT
CTAAGCCTAAGT
CTAAGCCTAAGT
CTAAGCCTAAGC
CTAAGTCTAAGC
CTAAGTCTAAGC
CTAAGTCTAAGT
CTAAGTCTAAG
CTAAGTCTAAGT
CTAAGTCTAAGT
CTAAGTCTAAGT
CTAAGTCTAAGT
CTAAGTCTAAGT
CTAAGTCTAAGT
CTAAGTCTAAGT
CTAAGTCTAAGT
CTAAGTCTAAGT
CTAAGTCTAAGT
CTAAGTCTAAGT
CTAAGTCTAAGT
CTAAGTCTAAGT
CTAAGTCTAAGT

CTAAGTCTAAGT
CTAAGTCTAAGT
CTAAGTCTAAGT
CTAAGTCTAAGT
CTAAGTCTAAGT
CTAAGTCTAAGT
CTAAGTCTAAGT
CTAAGTCTAAGT
CTAAGTCTAAGT
CTAAGTCTAAGT
CTAAGTCTAAGT
CTAAGGTCTAAGT
CTAAGTCTAAGT
CTAAGTCTAAGT
CTAAGTCTAAGT
CTAAGTCTAAGT
CTAAGTCTAAGT
CTAAGTCTAAGT
CTAAGTCTAAGT
CTAAGTCTAATT
Nr of selected repeats 69 Similarity 0.908497
Consensus:
CTAAGtCTAAGt
>Cele-UNSB01_1:3325616-3325694 Satlength=79 Nr of Repeats=6 RepeatLength=12
seed=AGGCTTAGGC
AGGCTTAGGCTT
AGGCTTAGTCTTCAACCT
AGGCTTAGGCTT
AGGCTCAGGCTT
AGGCTCAGGCTT
AGGCTTAGGCTC
Nr of selected repeats 5 Similarity 0.888889
Consensus:
AGGCTtAGGCTt
>Cele-UNSB01_1:3430435-3430513 Satlength=79 Nr of Repeats=6 RepeatLength=12
seed=GGCTTAGGCT
GGCTTAGGCTCA
GGCTTAGGCTTC
GGCTTAGGCTTA
GGCTTAGGCTTAAGCTCA
GGCTTTGGCTTA
GGCTCAGGCTTT
Nr of selected repeats 5 Similarity 0.788889
Consensus:
GGCTtaGGCTta
>Cele-UNSB01_1:3435421-3440265 Satlength=4845 Nr of Repeats=323 RepeatLength=15
seed=CTCACCACGA
CTCACCACGATGGGT
CTCACCACGAAGGGTT
TCACCACGAAAGGT
CTCGCCACGATGGGT
CTCACCACGAAGTGT
CTCACCACGAAAGGT
CTCGCCACGAAAGGT

CTCGCCACGATGGGT
CTCACCACGAAAGGT
CTCGCCACGATGGGT
CTCACCACGAAGTGT
CTCACCACGAAAGGT
CTCGCCACGATGGGT
CTCACCACGAAAGGT
CTCACCACGATGGGA
CTCACCACGATGGGT
CTCACCACGAAAGGT
CTCACCACGAAAGGT
CTCGCCACGATGGGT
CTCACCACGAAAGGT
CTCACCACGATGGGT
CTCACCACGATGGGT
CTCACCACGATGGGT
CTCACCACGAAAGGTT
TCACCACGACGGGT
CTCACCACGATGGGT
CTCACCACGAAAGGT
CTCGCCACGATGGGT
CTCACCACGAAGGGT
CTCACCACGATGGGT
CTCACCACGATGGGT
CTCACCACGAAAGGT
CTCACCACGATGAGT
CTCACCACGATGGGT
CTCACCACGATGGGT
CTCACCACGAAAGGT
CTCGCCACGATGGGC
CTCACCACGAAGGGT
CTCACCACGATGGGT
CTCACCACGAAGGGT
CTCACCACGAAGGGT
CTCACCACGAAGGGT
CTCACCACGAAAGGT
CTCGCCACGATGGGC
CTCACCACGAAGGGT
CTCACCACGATGGGT
CTCACCACGAAGGGT
CTCACCACGATGGGC
CTCACCACGAAGGGT
CTCACCACGATGGGT
CTCACCACGAAAGGT
CTCACCACGATGGGT
CTCACCACGATGGGT
CTCACCACGAAAGGT
CTCACCACGAAAGGT
CTCGCCACGAAGTGT
CTCACCACGAAAGGT
CTCGCCACGATGGGT
CTCACCACGAAGTGT
CTCACCACGAAAGGT
CTCGCCACGATGGGT

CTCACCACGAAGTGT
CTCACCACGAAAGGT
CTCGCCACGATGGGT
CTCACCACGATGGGT
CTCACCACGATGGGT
CTCACCACGAAAGGTT
TCACCACGACGGGT
CTCACCACGATGGGT
CTCACCACGAAAGGT
CTCGCCACGATGGGT
CTCACCACGAAGGGT
CTCACCACGATGGGT
CTCACCACGATGGGT
CTCACCACGAAAGGT
CTCACCACGATGAGT
CTCACCACGATGGGT
CTCACCACGATGGGT
CTCACCACGAAAGGC
CTCGCCACGATGGGC
CTCACCACGAAGGGT
CTCACCACGATGGGT
CTCACCACGAAAGGT
CTCACCACGAAGGGT
CTCACCACGAAGGGT
CTCACCACGAAAGGT
CTCGCCACGATGGGC
CTCACCACGAAGGGT
CTCACCACGAAGGGT
CTCACCACGAAGGGT
CTCACCACGATGGGT
CTCACCACGATGGGT
CTCACCACGAAGGGT
CTCACCACGAAGGGT
CTCACCACGTTGGT
CTCACCACGAAGGGT
CTCACCACGAAGGGT
CTCACCACGATGGGT
CTCACCACGATGGGT
CTCACCACGAAAGGTT
TCACCACGACGGGT
CTCACCACGATGGGT
CTCACCACGAAAGGT
CTCGCCACGATGGGT
CTCACCACGAAGGGT
CTCACCACGATGGGT
CTCACCACGATGGGT
CTCACCACGAAAGGT
CTCACCACGAAGTGT
CTCACCACGAAAGGT
CTCGCCACGATGGGT
CTCACCACGAAGTGT
CTCACCACGAAAGGT
CTCGCCACGATGGGT
CTCACCACGAAAGGT

CTCACCACGATGGGT
CTCACCACGATGGGT
CTCACCACGAAAGGT
CTCACCACGAAAGGT
CTCGCCACGAAGTGT
CTCACCACGAAAGGT
CTCGCCACGATGGGT
CTCACCACGAAGTGT
CTCACCACGAAAGGT
CTCGCCACGATGGGT
CTCACCACGAAGTGT
CTCACCACGAAAGGT
CTCGCCACGATGGGT
CTCACCACGAAAGGT
CTCACCACGATGGGT
CTCACCACGAAAGGT
CTCACCACGAAAGGT
CTCGCCACGATGGGT
CTCACCACGAAAGGT
CTCACCACGATGGGT
CTCACCACGATGGGT
CTCACCACGATGGGT
CTCACCACGAAAGGTT
TCACCACGACGGGT
CTCACCACGATGGGT
CTCACCACGATGGGT
CTCACCACGAAGGGT
CTCACCACGAAGGGT
CTCACCACGATGGGT
CTCACCACGATGGGT
CTCACCACGAAAGGT
CTCACAACGATGGGT
CTCACCACGATGGGT
CTCACCACGAAGGGT
CTCACCACGAAGGGT
CTCACCACGATGGGT
CTCACCACGATGGGT
CTCACCACGAAGGGT
CTCACCACGAAGGGT
CTCACCACGATGGGT
CTCACCACGAAGGGT
CTCACCACGAAGGGT
CTCACCACGATGGGT
CTCACCACGATGGGT
CTCACCACGAAAGGT
CTCACCACGACGGGT
CTCACCACGAAAGGT
CTCACCACGAAAGGT
CTCACCACGACGGGT
CTCACCACGAAAGGT
CTCACCACGATGGGT
CTCACCACGATGGGT
CTCACCACGATGGGT
CTCACCACGAAAGGT

CTCACCACGACGGGT
CTCACCACGAAAGGT
CTCGCCACGATGGGC
CTCACCACGAAGGGT
CTCACCACGATGGGT
CTCACCACGAAGGGT
CTCACCACGAAGGGT
CTCACCACGAAGGGT
CTCACCACGAAAGGT
CTCGCCACGATGGGC
CTCACCACGAAGGGT
CTCACCACGATGGGT
CTCACCACGAAGGGT
CTCACCACGAAGGGT
CTCACCACGATGGGT
CTCACCACGATGGGT
CTCACCACGAAGGGT
CTCACCACGAAGGGT
CTCACCACGATGGGT
CTCACCACGAAGGGT
CTCACCACGATGGGT
CTCACCACGATGGGT
CTCACCACGAAAGGTT
TCACCACGACGGGT
CTCACCACGATGGGT
CTCACCACGAAAGGT
CTCACCACGATGGGT
CTCACCACGATGGGT
CTCACCACGAAAGGT
CTCACCACGAAGTGT
CTCACCACGAAAGGT
CTCGCCACGATGGGT
CTCACCACGAAGTGT
CTCACCACGAAAGGT
CTCGCCACGATGGGT
CTCACCACGATGGGT
CTCACCACGATGGGT
CTCACCACGAAAGGTT
TCACCACGACGGGT
CTCACCACGATGGGT
CTCACCACGAAAGGT
CTCACCACGATGGGT
CTCACCACGAAAGGT
CTCACCACGATGGGT
CTCACCACGATGGGT
CTCACCACGATGGGT
CTCACCACGAAAGGTT
TCACCACGACGGGT
CTCACCACGATGGGT
CTCACCACGAAAGGT
CTCACCACGATGGGT
CTCACCACGAAAGGT
CTCACCACGATGGGT
CTCACCACGAAGGGT

CTCACCACGAAGGGT
CTCACCACGATGGGT
CTCACCACGAAGGGT
CTCACCACGAAGGGT
CTCACCACGATGGGT
CTCACCACGAAGGGT
CTCACCACGAAGGGT
CTCACCACGATGGGT
CTCACCACGATGGGT
CTCACCACGAAAGGTT
TCACCACGACGGGT
CTCACCACGATGGGT
CTCACCACGAAAGGT
CTCGCCACGATGGGT
CTCACCACGAAGGGT
CTCACCACGATGGGT
CTCACCACGATGGGT
CTCACCACGAAAGGT
CTCACCACGAAGTGT
CTCACCACGAAGTGT
CTCACCACGAAAGGT
CTCGCCACGATGGGT
CTCACCACGAAGTGT
CTCTCCACGAAAGGT
CTCGCCACGATGGGT
CTCACCACGAAAGGT
CTCACCACGAAAGGT
CTCGCCACGATGGGT
CTCACCACGAAAGGT
CTCACCACGATGGGT
CTCACCACGATGGGT
CTCACCACGATGGGT
CTCACCACGAAAGGTT
TCACCACGACGGGT
CTCACCACGATGGGT
CTCACCACGATGGGT
CTCACCACGATGGGT
CTCACCACGAAAGGT
CTCACCACGACGGGT
CTCACCACGAAAGGT
CTCACCACGATGGGT
CTCACCACGAAGGGT
CTCACCACGAAGGGT
CTCACCACGATGGGT
CTCACCACGATGGGT
CTCACCACGAAGGGT
CTCACCACGATGGGT
CTCACCACGAAGGGT
CTCACCACGAAGGGT
CTCACCACGATGGGT
CTCACCACGATGGGT
CTCACCACGAAAGGTT
TCACCACGACGGGT
CTCACCACGATGGGT

CTCACCACGAAAGGT
CTCGCCACGATGGGT
CTCACCACGAAGGGT
CTCACCACGATGGGTA
TCACCACGATGGGT
CTCACCACGAAAGGT
CTCACCACGAAGTGT
CTCACCACGAAAGGT
CTCGCCACGATGGGT
CTCACCACGAAGTGT
CTCACCACGAAAGGT
CTCGCCACGATGGGT
CTCACCACGAAAGGT
CTCACCACGAAAGGT
CTCGCCACGATGGGT
CTCACCACGAAAGGT
CTCACCACGATGGGT
CTCACCACGATGGGT
CTCACCACGATGGGT
CTCACCACGAAAGGTT
TCACCACGACGGGT
CTCACCACGATGGGT
CTCACCACGATGGGT
CTCACCACGATGGGT
CTCACCACGAAAGGT
CTCACCACGACGGGT
CTCACCACGAAAGGT
CTCACCACGATGGGT
CTCACCACGAAAGGT
CTCACCACGACGGGT
CTCACCACGAAAGGT
CTCGCCACGATGGGTG
TCACCACGAAGGGT
CTCACCACGATGGGT
CTCACCACGAAGGGT
CTCACCACGATGGGT
CTCACCACGAAGGGT
CTCACCACGATGGGT
CTCACCACGATGGGT
CTCACCACGAAAGGT
CTCACCACGACGGGT
CTCACCACGAAAGGT
CTCACCACGACGGGT
CTCACCACGATGGGT
CTCACCACGAAAGGC
CTCACCACGATCGGT
Nr of selected repeats 294 Similarity 0.881418
Consensus:
CTCACCACGAngGGT
>Cele-UNSB01_1:3453387-3453600 Satlength=214 Nr of Repeats=5 RepeatLength=32
seed=AATTCAAATT
AATTCAAATTTTCAAAAATAATATATGCGGGA
AATTCAAATTGTCAAGAATAATTTAGGTGGAA

AATTTAAATTTCCTCAGAAAAAAAGCTTTGGCAGGAAATTAGATTTTTTTCAGAACTTTTTTCAGAAAAAATATATGGC
GTGG
AATTCAAATTTTCTCTGTAAAATTTTGGTTGA
AATTCAAATTATCTTTAACAATGTTTTGGCGGGA
Nr of selected repeats 3 Similarity 0.602694
Consensus:
AATTCAAATTtTCaagaAtAATtTagGtgGaa
>Cele-UNSB01_1:3471234-3471484 Satlength=251 Nr of Repeats=6 RepeatLength=43
seed=TTGAAAATTT
TTGAAAATTTCCGACAAACCGGCAACTTGCCGGAA
TTGAAAATTTTCTAAAAATCGGCAAACCGGCAACTTGCCGGAA
TTGAAAATTTTCGGCAAATCGACAATCCGGCAAATTGTCGGAA
TTGAAAATTTTCTAAAAATTGGCAAACTGGCAAATTGGCGGAA
TTGAAAATTTCCGGCAAATCAGCAAATCGGCAAATTGGCGGAA
TTGAAAATTTCCGGCAAATCGGCAAATCGGCAAATTGGCGGAA
Nr of selected repeats 5 Similarity 0.810853
Consensus:
TTGAAAATTTtCggcAAATcggCAAaccGGCAAaTTGgCGGAA
>Cele-UNSB01_1:3474380-3475480 Satlength=1101 Nr of Repeats=54 RepeatLength=20
seed=CTAGGCAGAC
CTAGGCAGACCTAGCCTACT
CTAGGCAGACCTAGCCTACT
CTAGGCAGACCTAGCCTACT
CTAGGCAGACCCAGTCCACC
CTAGGCAGACCCAGTCCACCCTAGGCAGGGCTAGCCTTCC
CTAGGCAGACCTAGCCTACC
CTAGGCAGACCTGGCCCACC
CTAGGCAGACCTAGCCTACT
CTAGGCAGACCTAGCCTGCT
CTAGGCAGACCTAGCCTGCC
CTAGGCAGACCCAGTCTACC
CTAGGCAGACCTAGCCTTCC
CTAGGCAGACCTAGCCTTCC
CTAGGCTGACCTGGCCTTTC
CTAGGCAGACCTAGCCTACC
CTAGGCAGACCTAGCCTACT
CTAGGCAGACCTAGTCTACC
CTAGGCAGACCTAGCCTACT
CTAGGCAGACCTAGCCTACT
CTAGGCAGACCTAGCCTACT
CTAGGCAGACCCAGTCCACC
CTAGGCAGGCCTAGCCTTCC
CTAGGCAGACCTAGCCTACC
CTAGGCAGACCTGGCCCACC
CTAGACAGACCTAGCCTACT
CTAGGCAAACCTAGCCTACT
CTAGGAAGACCTAGTCCACC
CTAGGCAGACCTAGCATTCC
CTAGGCAGACCTAGCCTTCC
CTAGGCAGACCTGGCCTTTC
CTAGGCAGACCTAGCCTACC
CTAGGCAGACCTAGCCTACC
CTAGGCAGACCTAGCCTACC
CTAGGCAGACCTAGCCTACT

CTAGGCAGACCTAGTCTACC
CTAGGCAGACCTAGCCTACT
CTAGGCAGACCTAGCCTACT
CTAGGCAGACCTAGCCTACT
CTAGGCAGACCCAGTCCACC
CTAGGCAGACCCAGTCCACC
CTAGGCAGGCCTAGCCTTCC
CTAGGCAGGCCTAGCCTACC
CTAGGCAGACCTGGCCCACC
CTAGGCAGACCTAGCCTACT
CTAGGCAAACCTAGCCTACT
CTAGGCAGACCTAGTCCACC
CTAGGCAGACCTAGCATTCC
CTAGGCAGACCTAGCCTTCC
CTAGGCAGACCTGGCCTTTC
CTAGGCAGACCTAGCCTACC
CTAGGCAGACCTAGCCTACC
CTAGGCAGACCTAGCCTACC
CTAGGCAGACCTAGCCTACC
CTAGGCAGACCTGGCCTACC
Nr of selected repeats 53 Similarity 0.846734
Consensus:
CTAGGCAGACCTAGCCTaCc
>Cele-UNSB01_1:3474383-3476103 Satlength=1721 Nr of Repeats=83 RepeatLength=20
seed=GGCAGACCTA
GGCAGACCTAGCCTACTCTA
GGCAGACCTAGCCTACTCTA
GGCAGACCTAGCCTACTCTA
GGCAGACCCAGTCCACCCTA
GGCAGACCCAGTCCACCCTAGGCAGGGCTAGCCTTCCCTA
GGCAGACCTAGCCTACCCTA
GGCAGACCTGGCCCACCCTA
GGCAGACCTAGCCTACTCTA
GGCAGACCTAGCCTGCTCTA
GGCAGACCTAGCCTGCCCTA
GGCAGACCCAGTCTACCCTA
GGCAGACCTAGCCTTCCCTA
GGCAGACCTAGCCTTCCCTAGGCTGACCTGGCCTTTCCTA
GGCAGACCTAGCCTACCCTA
GGCAGACCTAGCCTACTCTA
GGCAGACCTAGTCTACCCTA
GGCAGACCTAGCCTACTCTA
GGCAGACCTAGCCTACTCTA
GGCAGACCTAGCCTACTCTA
GGCAGACCCAGTCCACCCTA
GGCAGGCCTAGCCTTCCCTA
GGCAGACCTAGCCTACCCTA
GGCAGACCTGGCCCACCCTA
GACAGACCTAGCCTACTCTA
GGCAAACCTAGCCTACTCTA
GGAAGACCTAGTCCACCCTA
GGCAGACCTAGCATTCCCTA
GGCAGACCTAGCCTTCCCTA
GGCAGACCTGGCCTTTCCTA

GGCAGACCTAGCCTACCCTA
GGCAGACCTAGCCTACCCTA
GGCAGACCTAGCCTACCCTA
GGCAGACCTAGCCTACTCTA
GGCAGACCTAGTCTACCCTA
GGCAGACCTAGCCTACTCTA
GGCAGACCTAGCCTACTCTA
GGCAGACCTAGCCTACTCTA
GGCAGACCCAGTCCACCCTA
GGCAGACCCAGTCCACCCTA
GGCAGGCCTAGCCTTCCCTA
GGCAGGCCTAGCCTACCCTA
GGCAGACCTGGCCCACCCTA
GGCAGACCTAGCCTACTCTA
GGCAAACCTAGCCTACTCTA
GGCAGACCTAGTCCACCCTA
GGCAGACCTAGCATTCCCTA
GGCAGACCTAGCCTTCCCTA
GGCAGACCTGGCCTTTCCTA
GGCAGACCTAGCCTACCCTA
GGCAGACCTAGCCTACCCTA
GGCAGACCTAGCCTACCCTA
GGCAGACCTAGCCTACCCTA
GGCAGACCTGGCCTACCCTA
GGCAGACCTAGCCCACCCTG
GGCAGACCTAGCCCACCCTG
GGCAGACCTAGCCCACCCTG
GGCAGACCTAGCCCACCCTA
GGCAAACCTAGCCCACCCTA
GGCAGACCTAGCCCACCCTG
GGCAGACCTAGCCCACCCTG
GGCAGACCTAGCCCACCCTG
GGCAGACCTAGCCCACCCTG
GGCAGACCTAGCCCACCCTG
GGCAGACCTAGCCCACCCTGGGCAGCACTAGCCCACCCTA
GGCAGACCTAGCCTACCCTA
GGCAGGCCTAGCCCACCCTG
GGCAGACCTAGCCCACCCTG
GGCAGACCTAGCCCACCCTG
GGCAGACCTAGCCCACCCTA
GGCAGACCTGGCCTACCCTG
GGCAGCCCTAGCCCACCCTA
GGCAGACCTAGCCTACCCTG
GGCAGACCTAGCCCACCCTA
GGCAAACCTAGCCCACCCTA
GGCAGACCTAGCCCACCCTG
GGCAGACCTAGCCCACCCTA
GGCAAACCTAGCCCACCCTA
GGCAGACCTAGCCCACCCTG
GGCAGACCTAGCCCACCCTA
GGCAAACCTAGCCCACCCTA
GGCAGACCTAGCCCACCCTG
GGCAGACCTAGCCCACCCTG
GGCAGACCTAGCCCACCCTG

Nr of selected repeats 80 Similarity 0.844789
Consensus:
GGCAGACCTAGCCtACcCTa
>Cele-UNSB01_1:3489580-3490945 Satlength=1366 Nr of Repeats=56 RepeatLength=35
seed=TTTCGGCAAT
TTTCGGCAATTGCCGGTATCGTGAAT
TTTCGGCAATTTCGGCAACTGCCGGTATCGTGAAT
TTTCGGCAATTTCGGCAATTGCCGGTTTTGTGAAT
TTTCGGCAATTTCGGCAACTGCCGGTATCGTGAAT
TTTCGGCAATTTCGATAATTGCCAGTTCTGTAAAA
TTCCGGCAATTTCAGCAATTGCCGGTTTCATGATT
TTTCGGCAATTTCGGCAATTGCCGGTTTCAGGATT
TTTCGGCAATTTCGGCAATTGCCGGTTTCTTGAGT
TTCCGGCAATTTCGGCAATTGCCGGTTTTGTGAAT
TTTCGGCAATTTCGGCAACTGCCGGTATCGTGAAT
TTTCGGCAATTTCGATAATTGCCAGTTCTGTAAAA
TTGCGGCAATTTCAGCAATTGCCGGTTTCATGATT
TTTCGGCAATTTCGGCAATTGCCGGTTTCTTGAAT
TTCCGGCAATTTCGGCAATTGCCGGTTTTGTGAATT
TTTGGCAATTTCGATAATTGCCAGTTCTGTAAAA
TTCCGGCAATTTCAGCAATTGCCGGTTTCATGATT
TTTCGGCAATTTCGGCAATTGCCGGTTTCATGATT
TTTCGGCAATTTCGGCAATTGCCGGTTTCGTGAAT
TTTCGGCAATTTCAGCAATTGCCGGTTTCATGATT
TTTCGGCAATTTCGGCAATTGCCGGTTTCTTGAGT
TTCCGGCAATTTCGGCAATTGCCGGTTTCGTGAAT
TTTCGGCAATTTCGATAATTGCCAGTTCTGTAAAA
TTGCGGCAATTTCAGCAATTGCCGGTTTCATGATT
TTTCGGCAATTTTGGCAATTGCCGGTTTCATGATT
TTTCGGCAATTTCGGCAATTGCCGGTTTCTTGAGT
TTCCGGCAATTTCGACAATTGCCGGTTTCGTGAAT
TTTCGGCAATTTCGATAATTGCCAGTTCTGTAAAA
TTGCGGCAATTTCAGCAATTGCCGGTTTCATGATT
TTTCGGCAATTTTGGCAATTGCCGGTTTCATGATT
TTTCGGCAATTTTGGCAATTGCCGGTTTCATGCTT
TTTCGGCAATTTCGGCAATTGCCGGTTTCGTGAAT
TTTCGGCAATTTCACCAATTGCCGGTATCGTGAAT
TTTCGGCAATTTCGATAATTGCCAGTTCTGTAAAA
TTGCGGCAATTTCAGCAATTGCCGGTTTCATGATT
TTTCGACAATTTCGGCAATTGCCGGTTTCTTGAAT
TTCCGGCAATTTCGGCAATTGCCGGTTTTGTGAAT
TTTCGGCAATTTTGGCAATTGCCGGTTTTTTGAAT
TTTCGGCAATTTCGATAATTGCCAGTTCTGTAAAA
TTCCGGCAATTTCCGCAATTGCCGGTTTCTTAAATTTTCTGCAG
Nr of selected repeats 35 Similarity 0.808371
Consensus:
TTtCGGCAATTTCggCAATTGCCGGTTTcnTGAaT
>Cele-UNSB01_1:3506179-3506592 Satlength=414 Nr of Repeats=8 RepeatLength=35
seed=TCCGGCAAAT
TCCGGCAAATCGACAAATCGCCGGAATTGAAATT
TCCGGCAAATCGACAGA
CCGGCAAATTGCCGAATTTGCCTGAAAAACGGCAAAAATGTTCAGCAAATTATGGATTTGCACTTTTTTTTTTTTGGAA
ATTTCGGAATATCAATTTTAAACGAAAAAAATTTCAAAAAGGCACAGTTTTAAGTATTTCCGTCTTATAAAAAATCCCT
CTAAAAACTTCTGTTTTTTTTTTTCAAAT

TCCGGCAAATCGGAAATCTGCCGGAAGTCAAAAAT
TCCGGCAAATCGGAAATCTGCCGGAAGTCAAAAAT
TCCGGCAAATCGGCAATCTGCCGGAAATCAAAAAT
TCCGGAAAATCGGAAATCTGCCGGAAGTCAAAAAT
TCCGGCAAATCGGAAATCTGCCGGAAGTCAAAAAC
Nr of selected repeats 5 Similarity 0.939048
Consensus:
TCCGGcAAATCGGaAATCTGCCGGAAgTCAAAAAt
>Cele-UNSB01_1:3532132-3534432 Satlength=2301 Nr of Repeats=115 RepeatLength=20
seed=AAAAAATAGA
AAAAAATAGATTGACATACC
AAAAAATAGATTGATATACC
AAAAAATAGATTGACATACC
AAAAAATAGATTGACATACC
AAAAAATAGATTGACATACC
AAAAAATAGATTGACATACC
AAAAAATAGATTGACATACC
AAAAAATAGATTGACATACC
AAAAAATAGATTGACATACC
AAAAAATAGATTGACATACC
AAAAAATAGATTGACATACC
AAAAAATAGATTGACATACC
AAAAAATAGATTGGCATACC
AAAAAATAGATTGACATACC
AAAAAATAGATTGATATACC
AAAAAATAGATTGATATACC
AAAAAATAGATTGACATACC
AAAAAATAGATTGACATACC
AAAAAATAGATTGATATACC
AAAAAATAGATTGACATACC
AAAAAATAGATTGACATACC
AAAAAATAGATTGACATACC
AAAAAATAGATTGACATACC
AAAAAATAGATTGACATACC
AAAAAATAGATTGACATACC
AAAAAATAGATTGACATACC
AAAAAATAGATTGACATACC
AAAAAATAGATTGACATACC
AAAAAATAGATTGACATACC
AAAAAATAGATTGATATACC
AAAAAATAGATTGACATACC
AAAAAATAGATTGACATACC
AAAAAATAGATTGACATACC
AAAAAATAGATTGACATACC
AAAAAATAGATTGACATACC
AAAAAATAGATTGGTATACC
AAAAAATAGATTGATATACC
AAAAAATAGATTGACATACC
AAAAAATAGATTGACATACC
AAAAAATAGATTGACATACC
AAAAAATAGATTGACATACC
AAAAAATAGATTGACATACC
AAAAAATAGATTGACATACC
AAAAAATAGATTGACATACC

AAAAAATAGATTGACATACC
AAAAAATAGATTGGTATACC
AAAAAATAGATTGGCATACC
AAAAAATAGATTGACATACC
AAAAAATAGATTGACATACC
AAAAAATAGATTGACATACC
AAAAAATAGATTGACATACC
AAAAAATAGATTGACATACC
AAAAAATAGATTGACATACC
AAAAAATAGATTGACATACC
AAAAAATAGATTGACATACC
AAAAAATAGATTGACATACC
AAAAAATAGATTGACATACC
AAAAAATAGATTGACATACC
AAAAAATAGATTGATATACC
AAAAAATAGATTGACATACC
AAAAAATAGATTGACATACC
AAAAAATAGATTGACATACC
AAAAAATAGATTGACATACC
AAAAAATAGATTGACATACC
AAAAAATAGATTGGTATACC
AAAAAATAGATTGGCATACC
AAAAAATAGATTGACATACC
AAAAAATAGATTGACATACC
AAAAAATAGATTGATATACC
AAAAAATAGATTGACATACC
AAAAAATAGATTGACATACC
AAAAAATAGATTGACATACC
AAAAAATAGATTGACATACC
AAAAAATAGATTGACATACC
AAAAAATAGATTGACATACC
AAAAAATAGATTGACATACC
AAAAAATAGATTGACATACC
AAAAAATAGATTGACATACC
AAAAAATAGATTGATATACC
AAAAAATAGATTGACATACC
AAAAAATAGATTGACATACC
AAAAAATAGATTGACATACC
AAAAAATAGATTGACATACC
AAAAAATAGATTGACATACC
AAAAAATAGATTGACATACC
AAAAAATAGATTGACATACC
AAAAAATAGATTGACATACC
AAAAAATAGATTGACATACC
AAAAAATAGATTGACATACC
AAAAAATAGATTGACATACC
AAAAAATAGATTGACATACC
AAAAAATAGATTGACATACC
AAAAAATAGATTGACATACC
AAAAAATAGATTGGTATACC
AAAAAATAGATTGGCATACC
AAAAAATAGATTGACATACC
AAAAAATAGATTGGTATACC
AAAAAATAGATTGGCATACC

AAAAAATAGATTGACATACC
AAAAAATAGATTGACATACC
AAAAAATAGATTGACATACC
AAAAAATAGATTGACATACC
AAAAAATAGATTGACATACC
AAAAAATAGATTGACATACC
AAAAAATAGATTGACATACC
AAAAAATAGATTGACATACC
AAAAAATAGATTGACATACC
AAAAAATAGATTGACATACC
AAAAAATAGATTGGTATACC
AAAAAATAGATTGGCATACC
AAAAAATAGATTGACATACC
AAAAAATAGATTGATATACC
AAAAAATAGATTGACATACC
AAAAAATAGATTGACATACC
AAAAAATAGATTGACATACC
Nr of selected repeats 115 Similarity 0.971320
Consensus:
AAAAAATAGATTGACATACC
>Cele-UNSB01_1:3600639-3600909 Satlength=271 Nr of Repeats=6 RepeatLength=45
seed=CTGAATCTTC
CTGAATCTTCATCTCCAAAGGATCACAAGGATCCTCCTTTATGAT
CTGAATCTTCAGCTCCAAAGGATCACAAGGATCCTCCTTTATGAT
CTGAATCTTCAGCTCCAAAGGATCACAAGGATCCTCCTTTATGAT
CTGAATCTTCAGCTCCAAAGGATCACAAGGATCCTCCTTTATGAT
CTGAATCTTCAGCTCCAAAGGATCACAAGGATCCTCCTTTATGAT
CTGAATCTTCAGCTCCAAAGGATCACAAGGATCCTCCTTTATGAT
Nr of selected repeats 6 Similarity 0.990123
Consensus:
CTGAATCTTCAGCTCCAAAGGATCACAAGGATCCTCCTTTATGAT
>Cele-UNSB01_1:3650776-3650949 Satlength=174 Nr of Repeats=5 RepeatLength=35
seed=TTTGCCGGAA
TTTGCCGGAAATGTTCAATTCCGACAATTTGCCGG
TTTGCCGGAAAATTTTAATTCCAGCAATTTGCCGA
TTTGCCGGAAATTTCAAATCCGGCAATTTGCCGA
TTTGCCGGAAATGTTCAATTCCGACAATTTTCCGG
TTTGCCGGAAATTTCAAATCCGGCAATTTACCGA
Nr of selected repeats 3 Similarity 0.822222
Consensus:
TTTGCCGGAAAtgTTcAATTCCgaCAATTTgCCGg
>Cele-UNSB01_1:3711978-3718275 Satlength=6298 Nr of Repeats=242 RepeatLength=26
seed=ATGGTTAGAG
ATGGTTAGAGTCATAATTTTAGGTGA
ATGGTTAGAGTCATATTTTTAGGTGA
ATGGTTAGAGTCATATTTTTAGGTGT
ATGGTTAGAGTCATATTTTTAGGTGA
ATGGTTAGAGTCATATTTTTAGGTGA
ATGGTTAGAGTCATATTTTTAGGTGA
ATGGTTAGAGTCATATTTTTAGGTGA
ATGGTTAGAGTCATATTTTTAGGTGA
ATGGTTAGAGTCATATTTTTAGGTGT
ATGGTTAGAGTCATATTTTTAGGTGT
ATGGTTAGAGTCATATTTTTAGGTGT

ATGGTTAGAGTCATATTTTTAGGTGT
ATGGTTAGAGTCATATTTTTAGGTGA
ATGGTTAGAGTCATATTTTTAGGTGA
ATGGTTAGAGTCATATTTTTAGGTGA
ATGGTTAGAGTCATATTTTTAGGTGA
ATGGTTAGAGTCATATTTTTAGGTGA
ATGGTTAGAGTCATATTTTTAGGTGA
ATGGTTAGAGTCATATTTTTAGGTGA
ATGGTTAGAGTCATATTTTTAGGTGT
ATGGTTAGAGTCATATTTTTAGGTGA
ATGGTTAGAGTCATATTTTTAGGTGA
ATGGTTAGAGTCATATTTTTAGGTGA
ATGGTTAGAGTCATATTTTTAGGTGT
ATGGTTAGAGTCATATTTTTAGGTGA
ATGGTTAGAGTCATATTTTTAGGTGA
ATGGTTAGAGTCATTATTTTAGGTGA
ATGGTTAGAGTCATATTTTTAGGTGA
ATGGTTAGAGTCATATTTTTAGGTGA
ATGGTTAGAGTCATTATTTTAGGTGA
ATGGTTAGAGTCATATTTTTAGGTGT
ATGGTTAGAGTCATATTTTTAGGTGA
ATGGTTAGAGTCATATTTTTAGGTGA
ATGGTTAGAGTCATATTTTTAGGTGA
ATGGTTAGAGTCATTATTTTAGGTGA
ATGGTTAGAGTCATATTTTTAGGTGA
ATGGTTAGAGTCATTATTTTAGGTGA
ATGGTTAGAGTCATATTTTTAGGTGT
ATGGTTAGAGTCATATTTTTAGGTGA
ATGGTTAGAGTCATTATTTTAGGTGA
ATGGTTAGAGTCATATTTTTAGGTGT
ATGGTTAGAGTCATATTTTTAGGTGA
ATGGTTAGAGTCATATTTTTAGGTGA
ATGGTTAGAGTCATTATTTTAGGTGA
ATGGTTAGAGTCATATTTTTAGGTGA
ATGGTTAGAGTCATATTTTTAGGTGA
ATGGTTAGAGTCATATTTTTAGGTGA
ATGGTTAGAGTCATATTTTTAGGTGA
ATGGTTAGAGTCATATTTTTAGGTGA
ATGGTTAGAGTCATATTTTTAGGTGA
ATGGTTAGAGTCATATTTTTAGGTGA
ATGGTTAGAGTCATATTTTTAGGTGA
ATGGTTAGAGTCATATTTTTAGGTGA
ATGGTTAGAGTCATATTTTTAGGTGA
ATGGTTAGAGTCATATTTTTAGGTGA
ATGGTTAGAGTCATATTTTTAGGTGA
ATGGTTAGAGTCATATTTTTAGGTGA
ATGGTTAGAGTCATATTTTTAGGTGA
ATGGTTAGAGTCATATTTTTAGGTGA
ATGGTTAGAGTCATATTTTTAGGTGA
ATGGTTAGAGTCATATTTTTAGGTGA
ATGGTTAGAGTCATATTTTTAGGTGA
ATGGTTAGAGTCATATTTTTAGGTGA
ATGGTTAGAGTCATATTTTTAGGTGA
ATGGTTAGAGTCATATTTTTAGGTGA

ATGGTTAGAGTCATATTTTTAGGTGA
ATGGTTAGAGTCATATTTTTAGGTGA
ATGGTTAGAGTCATATTTTTAGGTGA
ATGGTTAGAGTCATATTTTTAGGTGA
ATGGTTAGAGTCATATTTTTAGGTGA
ATGGTTAGAGTCATATTTTTAGGTGA
ATGGTTAGAGTCATTATTTTAGGTGA
ATGGTTAGAGTCATATTTTTAGGTGA
ATGGTTAGAGTCATATTTTTAGGTGA
ATGGTTAGAGTCATATTTTTT
ATGGTTAGAGTCATATTTTTAGGTGA
ATGGTTAGAGTCATATTTTTAGGTGT
ATGGTTAGAGTCATATTTTTAGGTGA
ATGGTTAGAGTCATATTTTTAGGTGT
ATGGTTAGAGTCATATTTTTAGGTGA
ATGGTTAGAGTCATATTTTTAGGTG
ATGGTTAGAGTCATATTTTTAGGTGA
ATGGTTAGAGTCATATTTTTAGGTGT
ATGGTTAGAGTCATATTTTTAGGTGA
ATGGTTAGAGTCATATTTTTAGGTGA
ATGGTTAGAGTCATATTTTTAGGTGA
ATGGTTAGAGTCATATTTTTAGGTGA
ATGGTTAGAGTCATATTTTTAGGTGA
ATGGTTAGAGTCATATTTTTAGGTGA
ATGGTTAGAGTCATATTTTTAGGTGA
ATGGTTAGAGTCATATTTTTAGGTGA
ATGGTTAGAGTCATATTTTTAGGTGA
ATGGTTAGAGTCATATTTTTAGGTGA
ATGGTTAGAGTCATATTTTTAGGTGA
ATGGTTAGAGTCATATTTTTAGGTGA
ATGGTTAGAGTCATATTTTTAGGTGA
ATGGTTAGAGTCATATTTTTAGGTGA
ATGGTTAGAGTCATATTTTTAGGTGA
ATGGTTAGAGTCATATTTTTAGGTGA
ATGGTTAGAGTCATATTTTTAGGTGA
ATGGTTAGAGTCATATTTTTAGGTGA
ATGGTTAGAGTCATATTTTTAGGTGA
ATGGTTAGAGTCATATTTTTAGGTGA
ATGGTTAGAGTCATATTTTTAGGTGA
ATGGTTAGAGTCATATTTTTAGGTGA
ATGGTTAGAGTCATATTTTTAGGTGA
ATGGTTAGAGTCATATTTTTAGGTGA
ATGGTTAGAGTCATATTTTTAGGTGA
ATGGTTAGAGTCATATTTTTAGGTGA
ATGGTTAGAGTCATTATTTTAGGTGA
ATGGTTAGAGTCATATTTTTAGGTGA
ATGGTTAGAGTCATATTTTTAGGTGT
ATGGTTAGAGTCATATTTTTAGGTGT
ATGGTTAGAGTCATATTTTTAGGTGA
ATGGTTAGAGTCATATTTTTAGGTGA
ATGGTTAGAGTCATATTTTTAGGTGA
ATGGTTAGAGTCATTATTTTTAGGTGA
ATGGTTAGAGTCATATTTTTAGGTGA

ATGGTTAGAGTCATATTTTTAGGTGA
ATGGTTAGAGTCATATTTTTAGGTGA
ATGGTTAGAGTCATATTTTTAGGTGA
ATGGTTAGAGTCATTATTTTAGGTGA
ATGGTTAGAGTCATATTTTTAGGTGA
ATGGTTAGAGTCATATTTTTAGGTGA
ATGGTTAGAGTCATATTTTTAGGTGA
ATGGTTAGAGTCATATTTTTAGGTGA
ATGGTTAGAGTCATATTTTTAGGTGA
ATGGTTAGAGTCATATTTTTAGGTGA
ATGGTTAGAGTCATATTTTTAGGTGT
ATGGTTAGAGTCATATTTTTAGGTGA
ATGGTTAGAGTCATATTTTTAGGTGA
ATGGTTAGAGTCATATTTTTAGGTGA
ATGGTTAGAGTCATATTTTTAGGTGA
ATGGTTAGAGTCATATTTTTAGGTGA
ATGGTTAGAGTCATATTTTTAGGTGA
ATGGTTAGAGTCATTATTTTAGGTGA
ATGGTTAGAGTCATATTTTTAGGTGA
ATGGTTAGAGTCATTATTTTAGGTGA
ATGGTTAGAGTCATATTTTTAGGTGA
ATGGTTAGAGTCATATTTTTAGGTGA
ATGGTTAGAGTCATATTTTTAGGTGA
ATGGTTAGAGTCATATTTTTAGGTGA
ATGGTTAGAGTCATATTTTTAGGTGA
ATGGTTAGAGTCATATTTTTAGGTGA
ATGGTTAGAGTCATATTTTTAGGTGA
ATGGTTAGAGTCATATTTTTAGGTGA
ATGGTTAGAGTCATATTTTTAGGTGA
ATGGTTAGAGTCATATTTTTAGGTGA
ATGGTTAGAGTCATATTTTTAGGTGA
ATGGTTAGAGTCATATTTTTAGGTGA
ATGGTTAGAGTCATATTTTTAGGTGA
ATGGTTAGAGTCATATTTTTAGGTGA
ATGGTTAGAGTCATATTTTTAGGTGA
ATGGTTAGAGTCATATTTTTAGGTGA
ATGGTTAGAGTCATATTTTTAGGTGA
ATGGTTAGAGTCATATTTTTAGGTGA
ATGGTTAGAGTCATATTTTTAGGTGA
ATGGTTAGAGTCATATTTTTAGGTGA
ATGGTTAGAGTCATATTTTTAGGTGA
ATGGTTAGAGTCATATTTTTAGGTGA
ATGGTTAGAGTCATATTTTTAGGTGA
ATGGTTAGAGTCATATTTTTAGGTGA
ATGGTTAGAGTCATATTTTTAGGTGA
ATGGTTAGAGTCATATTTTTAGGTGA
ATGGTTAGAGTCATATTTTTAGGTGA
ATGGTTAGAGTCATATTTTTAGGTGA
ATGGTTAGAGTCATATTTTTAGGTGA
ATGGTTAGAGTCATATTTTTAGGTGA
ATGGTTAGAGTCATATTTTTAGGTGA
ATGGTTAGAGTCATATTTTTAGGTGA
ATGGTTAGAGTCATATTTTTAGGTGA
ATGGTTAGAGTCATATTTTTAGGTGA

ATGGTTAGAGTCATATTTTTAGGTGA
ATGGTTAGAGTCATATTTTTAGGTGA
ATGGTTAGAGTCATTATTTTAGGTGA
ATGGTTAGAGTCATATTTTTAGGTGA
ATGGTTAGAGTCATTATTTTAGGTGA
ATGGTTAGAGTCATTATTTTAGGTGAATGGTTAGTA
ATGGTTAGAGTCATATTTTTAGGTGT
ATGGTTAGAGTCATATTTTTAGGTGA
ATGGTTAGAGTCATATTTTTAGGTGA
ATGGTTAGAGTCATATTTTTAGGTGA
ATGGTTAGAGTCATATTTTTAGGTGA
ATGGTTAGAGTCATATTTTTAGGTGA
ATGGTTAGAGTCATATTTTTAGGTGA
ATGGTTAGAGTCATATTTTTAGGTGT
ATGGTTAGAGTCATATTTTTAGGTGT
ATGGTTAGAGTCATATTTTTAGGTGA
ATGGTTAGAGTCATATTTTTAGGTGA
ATGGTTAGAGTCATTATTTTAGGTGA
ATGGTTAGAGTCATATTTTTAGGTGA
ATGGTTAGAGTCATTATTTTAGGTGA
ATGGTTAGAGTCATATTTTTAGGTGA
ATGGTTAGAGTCATATTTTTAGGTGA
ATGGTTAGAGTCATATTTTTAGGTGA
ATGGTTAGAGTCATATTTTTAGGTGA
ATGGTTAGAGTCATTATTTTAGGTGA
ATGGTTAGAGTCATATTTTTAGGTGA
ATGGTTAGAGTCATTATTTTAGGTGA
ATGGTTAGAGTCATATTTTTAGGTGA
ATGGTTAGAGTCATATTTTTAGGTGT
ATGGTTAGAGTCATATTTTTAGGTGT
ATGGTTAGAGTCATATTTTTAGGTGA
ATGGTTAGAGTCATATTTTTAGGTGT
ATGGTTAGAGTCATATTTTTAGGTGT
ATGGTTAGAGTCATATTTTTAGGTGA
ATGGTTAGAGTCATATTTTTAGGTGA
ATGGTTAGAGTCATATTTTTAGGTGT
ATGGTTAGAGTCATATTTTTAGGTGT
ATGGTTAGAGTCATATTTTTAGGTGA
ATGGTTAGAGTCATATTTTTAGGTGA
ATGGTTAGAGTCATTATTTTAGGTGA
ATGGTTAGAGTCATATTTTTAGGTGA
ATGGTTAGAGTCATTATTTTAGGTGA
ATGGTTAGAGTCATATTTTTAGGTGA
ATGGTTAGAGTCATATTTTTAGGTGA
ATGGTTAGAGTCATATTTTTAGGTGA
ATGGTTAGAGTCATATTTTTAGGTGA
ATGGTTAGAGTCATTATTTTAGGTGA
ATGGTTAGAGTCATATTTTTAGGTGA
ATGGTTAGAGTCATTATTTTAGGTGA
ATGGTTAGAGTCATATTTTTAGGTGA
ATGGTTAGAGTCATATTTTTAGGTGA
ATGGTTAGAGTCATTATTTTAGGTGA
ATGGTTAGAGTCATATTTTTAGGTGA
ATGGTTAGAGTCATATTTTTAGGTGT

ATGGTTAGAGTCATATTTTTAGGTGT
ATGGTTAGAGTCATATTTTTAGGTGA
ATGGTTAGAGTCATATTTTTAGGTGT
ATGGTTAGAGTCATATTTTTAGGTGT
ATGGTTAGAGTCATATTTTTAGGTGA
ATGGTTAGAGTCATATTTTTAGGTGT
ATGGTTAGAGTCATATTTTTAGGTGA
ATGGTTAGAGTCATATTTTTAGGTGA
ATGGTTAGAGTCATATTTTTAGGTGA
ATGGTTAGAGTCATATTTTTAGGTGT
ATGGTTAGAGTCATATTTTTAGGTGT
ATGGTTAGAGTCATATTTTTAGGTGT
ATGGTTAGAGTCATATTTTTAGGTGA
ATGGTTAGAGTCATATTTTTAGGTGA
ATGGTTAGAGTCATATTTTTAGGTGA
Nr of selected repeats 238 Similarity 0.970067
Consensus:
ATGGTTAGAGTCATATTTTTAGGTGA
>Cele-UNSB01_1:3764869-3765030 Satlength=162 Nr of Repeats=5 RepeatLength=14
seed=TTTTTGTAGA
TTTTTGTAGATCCCATTTTCATAGATTCCCCA
TTTTGTAGAACGTC
TTTTCGTAGATCTACTTTTCGTGGAACCTCTTTTCATTTAACCTCTTTCGTAGAACCTTTTTCGTAAGTCCCACTTCAT
AGATCATC
TTTTTGTAGATACT
TTTTTGTAGATCAC
Nr of selected repeats 3 Similarity 0.588235
Consensus:
tTTTTGTAGAtACn
>Cele-UNSB01_1:3798271-3798674 Satlength=404 Nr of Repeats=9 RepeatLength=45
seed=TCTGATCCTT
TCTGATCCTTCAGCTCCGAAGGATCACATGGGTACCTTCTGATGT
TCTGATCCTTCAGATAAGAAGGATCGTAAGGGTACCTCCTGATGG
TCTGATACTTCAGCTCCAAAGGATCACGAGGCTCTTCCTGATGT
TCTGATCCTTCAGCTCCGAAGGATCACATGGGCATCTTCTGATGA
TCTGATCCTTCAGATAAGAAGGATCGTAAGGGTACCACCTGATGG
TCTGATACTTCAGCTCCAAAGGATCACGAGGCTCTTCCTGATGT
TCTGATCCTTCAGCTCCGAAGGATCACATGGGCACCTTCTGATGT
TCTGATCCTTCAGATAAGAAGGATCGTAAGGGTACCTCCTGATGT
TCTGATCCTTCAGCTCCGAAGGATCACATGGGCACCTTCTGATGA
Nr of selected repeats 7 Similarity 0.825044
Consensus:
TCTGATCCTTCAGcTccGAAGGATCacAtGGGtACCTtCTGATGn
>Cele-UNSB01_1:3799094-3799407 Satlength=314 Nr of Repeats=7 RepeatLength=45
seed=CTGAAGGATC
CTGAAGGATCAGAACATCAGAAGGTGCCCATGTGATCCTTCGGAG
CTGAAGGATCAGAACATCAGGAAGAGCCTCGTGATCCTTTGGAG
CTGAAGTATCAGACCATCAGGTGGTACCCTTACGATCCTTCTTAT
CTGAAGGATCAGATCATCAGAAGATGCCCATGTGATCCTTCGGAG
CTGAAGGATCAGAACATCAGGAAGAGCCTCGTGATCCTTTGGAG
CTGAAGTATCAGACCATCAGGAGGTACCCTTACGATCCTTCTTAT
CTGAAGGATCAGAACATCAGAAGGTACCCATGTGATCCTTCGGAG
Nr of selected repeats 5 Similarity 0.792593
Consensus:

CTGAAGgATCAGAnCATCAGaaGgTaCCCaTgtGATCCTTCggAg
>Cele-UNSB01_1:3815334-3818659 Satlength=3326 Nr of Repeats=133 RepeatLength=25
seed=TTTACTCTCT
TTTACTCTCTGGCTTCACAGAATAT
TTTACTCTCTGGCTTCACCAAATAT
TTTACTCTCTGGCTTCACAGAATAT
TTTACTCTCTGGCTTCACCAAATAA
TTTACTCTCTGGCTTCACAGAATAT
TTTACTCTCTGGCTTCACCAAATAT
TTTACTCTCTGGCTTCACAGTATAT
TTTACTCTCTGGCTTCACCAAATAA
TTTACTCTCTGGCTTCACCAAATAA
TTTACTCTCTCGCTTCACAGTATAT
TTTACTCTCTGGCTTCACCAAATAT
TTTACTCTCTCGCTTCACAGTATAT
TTTACTCTCTGGCTTCACCAAATAT
TTTACTCTCTCGCTTCACAGTATAT
TTTACTCTCTGGCTTCACCAAATAT
TTTACTCTCTCGCTTCACAGTATAT
TTTACTCTCTGGCTTCACCAAATAA
TTTACTCTCTGGCTTCACCAAATAT
TTTACTCTCTGGCTTCACCAAATAT
TTTACTCTCTCGCTTCACAGTATAT
TTTACTCTCTGGCTTCACCAAATAA
TTTACTCTCTGGCTTCACAGAATAT
TTTACTCTCTGGCTTCACCAAATAT
TTTACTCTCTGGCTTCACCAAATAT
TTTACTCTCTGGCTTCACCAAATAA
TTTACTCTCTCGCTTCACAGTATAT
TTTACTCTCTGGCTTCACCAAATAA
TTTACTCACTGGCTTCACCAAATAT
TTTACTCTCTGGCTTCACCAAATAT
TTTACTCTCTCGCTTCACAGAATAT
TTTACTCTCTGGCTTCACCAAATAT
TTTACTCTCTGGCTTCACCAAATAT
TTTACTCTCTGGCTTCACCATATAT
TTTACTCTCTGGCTTCACCAAATAT
TTTACTCTCTGGCTTCACCAAATAT
TTTACTCTCTGGCTTCACCAAATAT
TTTACTCTCTGGCTTCACCAAATAT
TTTACTCTCTGGCTTCACCAAATAA
TTTACTCTCTGGCTTCACAGAATAT
TTTACTCTCTGGCTTCACCAAATAT
TTTACTCTCTGGCTTCACCAAATAA
TTTACTCTCTCGCTTCACAGTATAT
TTTACTCTCTGGCTTCACCAAATAA
TTTACTCTCTGGCTTCACCAAATAT
TTTACTCTCTGGCTTCACCAAATAT
TTTACTCTCTGGCTTCACAGTATAT
TTTACTCTCTGGCTTCACCAAATAT
TTTACTCTCTGGCTTCACAGTATAT
TTTACTCTCTGGCTTCACCAAATAT
TTTACTCTCTGGCTTCACAGAATAT
TTTACTCTCTGGCTTCACCAAATAT

TTTACTCTCTGGCTTCACAGAATAT
TTTACTCTCTGGCTTCACCAAATAT
TTTACTCTCTGGCTTCACAGAATAT
TTTACTCTCTGGCTTCACCAAATAA
TTTACTCTCTGGCTTCACAGAATAT
TTTACTCTCTGGCTTCACCAAATAT
TTTACTCTCTGGCTTCACAGTATAT
TTTACTCTCTGGCTTCACCAAATAA
TTTACTCTCTGGCTTCACCAAATAA
TTTACTCTCTCGCTTCACAGTATAT
TTTACTCTCTGGCTTCACCAAATAT
TTTACTCTCTCGCTTCACAGTATAT
TTTACTCTCTGGCTTCACCAAATAA
TTTACTCTCTGGCTTCACCAAATAT
TTTACTCTCTGGCTTCACCAAATAT
TTTACTCTCTCGCTTCACAGTATAT
TTTACTCTCTGGCTTCACCAAATAA
TTTACTCTCTGGCTTCACAGAATAT
TTTACTCTCTGGCTTCACCAAATAT
TTTACTCTCTGGCTTCACCAAATAT
TTTACTCTCTGGCTTCACCAAATAA
TTTACTCTCTCGCTTCACAGTATAT
TTTACTCTCTGGCTTCACCAAATAA
TTTACTCACTGGCTTCACCAAATAT
TTTACTCTCTGGCTTCACCAAATAT
TTTACTCTCTCGCTTCACAGTATAT
TTTACTCTCTGGCTTCACCAAATAA
TTTACTCTCTGGCTTCACAGAATAT
TTTACTCTCTGGCTTCACCAAATAT
TTTACTCTCTGGCTTCACCAAATAT
TTTACTCTCTGGCTTCACCATATAT
TTTACTCTCTGGCTTCACCAAATAT
TTTACTCTCTGGCTTCACCAAATAT
TTTACTCTCTGGCTTCACCAAATAT
TTTACTCTCTGGCTTCACCAAATAT
TTTACTCTCTGGCTTCACCAAATAA
TTTACTCTCTGGCTTCACAGAATAT
TTTACTCTCTGGCTTCACCAAATAT
TTTACTCTCTGGCTTCACCAAATAA
TTTACTCTCTGGCTTCACAGTATAT
TTTACTCTCTGGCTTCACCAAATAT
TTTACTCTCTGGCTTCACCAAATAT
TTTACTCTCTGGCTTCACCAAATAA
TTTACTCTCTGGCTTCACAGTATAT
TTTACTCTCTGGCTTCACCAAATAT
TTTACTCTCTGGCTTCACCAAATAA
TTTACTCTCTCGCTTCACAGTATAT
TTTACTCTCTGGCTTCACCAAATAT
TTTACTCTCTGGCTTCACCAAATAT
TTTACTCTCTGGCTTCACAGTATAT
TTTACTCTCTGGCTTCACCAAATAT
TTTACTCTCTGGCTTCACAGAATAT
TTTACTCTCTGGCTTCACCAAATAT
TTTACTCTCTGGCTTCACAGAATAT

TTTACTCTCTCGCTTCACAGTATAT
TTTACTCTCTGGCTTCACCAAATAA
TTTACTCTCTGGCTTCACAGAATAT
TTTACTCTCTGGCTTCACCAAATAT
TTTACTCTCTGGCTTCACCAAATAA
TTTACTCTCTCGCTTCACAGTATAT
TTTACTCTCTGGCTTCACCAAATAT
TTTACTCTCTGGCTTCACCAAATAT
TTTACTCTCTGGCTTCACAGTATAT
TTTACTCTCTGGCTTCACAGTATAT
TTTACTCTCTGGCTTCACCAAATAT
TTTACTCTCTCGCTTCACAGTATAT
TTTACTCTCTGGCTTCACCAAATAA
TTTACTCTCTGGCTTCACCAAATAT
TTTACTCTCTGGCTTCACCAAATAT
TTTACTCTCTCGCTTCACAGTATAT
TTTACTCTCTGGCTTCACCAAATAA
TTTACTCTCTGGCTTCACAGAATAT
TTTACTCTCTGGCTTCACCAAATAT
TTTACTCTCTGGCTTCACCAAATAT
TTTACTCTCTGGCTTCACCAAATAA
TTTACTCTCTCGCTTCACAGTATAT
TTTACTCTCTGGCTTCACCAAATAA
TTTACTCACTGGCTTCACCAAATAT
TTTACTCTCTGGCTTCACCAAATAT
TTTACTCTCTCGCTTCACAGAATAT
TTTACTCTCTGGCTTCACCAAATAA
TTTACTCTCTGGCTTCACCAAATAA
Nr of selected repeats 133 Similarity 0.898680
Consensus:
TTTACTCTCTGGCTTCACcaaATAt
>Cele-UNSB01_1:3888641-3889727 Satlength=1087 Nr of Repeats=26 RepeatLength=34
seed=TGCCGGAATT
TGCCGGAATTGAAATTTCCGGCAAATCGGCAAATTACCGTAATTGAAATTTCCAGCCAATCGGCAAAT
TGTCGGAATTGAAATTTCCAGCAAATCGGCAAAT
TGTCGGAATTGAAATTTCCGGCAAATCGACAAAT
TGCCGGAATTGGAGTTTCCGGCAAATCGGTAAAT
TGCCGGAATTGAAATTTCCGGCAAATCGGCAATCCGGCAAAT
TGCCGGAATTGAAATTTCCAGAAAATCGGCAAAT
TGCCGGAATTGAAATTTCATGCAAATCGGCAAATTGTGGGAATTGAAATTTCCGGCAAATCGACAAAT
TGCCGGAATTGGAGTTTCCGGCAAATCGGTAAAT
TGCCGGAATTGAAATTTCCGGCAAATCGGCAATCCGGCAAAT
TGCCGGAATTGAAATTTCCAGAAAATCGGCAAAT
TGCCGGAATTGAAATTTCATGCAAATCGGCAAATTGTGGGAATTTAAATTTCCAGCAATTGGGCAAAG
TGCCGGAATTTAAATTTCCAGCAAATCGGCAAAT
TGCCGGAATTGAAATTTCATGCAAATCGGCAAATTGTGGGAATTTAAATTTCCAGCAATTGGGCAAAT
TGCCGGAATTGAAATTTCCAGAAAATCGGCAAAT
TGCCGGAATTGAAATTTCATGCAAATCGGCAAATTGTGGGAATTTAAATTTCCAGCAATTGGGCAAAG
TGCCGGAATTTAAATTTCCAGCAAATCGGCAAAT
TGCCGGAATTGAAATTTCCAGCAAATCGGCAAAT
TGCCGGAATTGAAATTTCCAGAAAATCGGCAAAT
TGCCGGAATTTAAATTTCCAGCAAATCGGCAAAT
TGCCGGAATTGAAATTTCCAGCAAATCGGCAAAT
TGCCGGAATTTAAATTTCCAGAAAATCGGCAAAT

TGCCGGAATTGAAATTTCCGGCAAATCGACAAATT
TCCGGAATTGGAGTTTCCGGCAAATCGGCCATCCGGCACGT
TGCCGGAATTGAAATTTCCAGCAAATCGGCAAACCGGCAAAT
TGCCGGAATTGAAATTTCTGACAAATCGGCAAAC
TGCCGGAATTGAGATTTTTGGCAAATCGGCAAAT
Nr of selected repeats 16 Similarity 0.878105
Consensus:
TGCCGGAATTgAAATTTCCaGcAAATCGGCAAAT
>Cele-UNSB01_1:4033028-4033133 Satlength=106 Nr of Repeats=7 RepeatLength=15
seed=TCGTGGTGAG
TCGTGGTGAGACCCA
TCGTGGTGAGACCCT
TCGTGGTGAGACCCT
TCGTGGTGAGACCCA
TCGTGGTGAGACCCT
TCGTGGTGAGACCCA
TCGTGGTGAGACCCT
Nr of selected repeats 7 Similarity 0.949206
Consensus:
TCGTGGTGAGACCCt
>Cele-UNSB01_1:4033525-4033725 Satlength=201 Nr of Repeats=6 RepeatLength=40
seed=TTTCTAGAAA
TTTCTAGAAAATTCGAGAAAATTCCGGAATGGTCCAGAAT
TTTCTAGAAAA
TTCTAGAAAGTTCTGGAATGGTCCAGAAT
TTTCTAGAAAATTCGGGAAAACTCTGGAATATTCCAGAAC
TTTCTAGAAAAAATCGAGAAATGTCTGCAATGTTCCAGAAG
TTTCTAGAAAATTCGAGAAAATTCCGGAATGGTCCAGAAT
Nr of selected repeats 4 Similarity 0.816667
Consensus:
TTTCTAGAAAAtTCGaGAAAatTCcGgAATggTCCAGAAt
>Cele-UNSB01_1:4042754-4055380 Satlength=12627 Nr of Repeats=214
RepeatLength=59 seed=AAAACCAGTG
AAAACCAGTGCACATTTGAAATTCCCAATTCTCCATAAATTTCAGTTTAAAAAAATTTG
AAAACCAGTGCGCATTTGAAATTCCAAATTCTCCATTAATTTCAGTTTAAAAAATTTTG
AAAACCAGTGCACATTTGAAATTTTATATTCTCCATAATTCTCGGTTTAAAATATTTTA
AAAACCAGTGCACATTTGAAATTCCCAATTCTCCACAGTTTTCAGTTTAAAAAATTTTG
AAAACCAGTGCACATTTGAAATTTTATATTCTCCATAATTCTCGGTTTAAAATATTTTA
AAAACCAGTGCACATTTGAAATTCCTAATTCTCCACAGTTTTCAGTTAAAAAAATTTTG
AAAACCAGTGCACATTTGAAATTTTATATTCTCCATAATTCTCGGTTTTAAATATTTTG
AAAACCAGTGCACATTTGAAATTCCCAATTCTCCACAGTTTTCAGTTAAAAAAATTTTG
AAAACCAGTGCACATTTGAAATTTTATATTCTCCATAATTCTCGGTTTTAAATATTTTG
AAAACCAGTGCACATTTGAAATTCCCAATTCTCCACAGTTTTCAGTTAAAAAAATTTTG
AAAACCAGTGCACATTTGAAATTTTATATTCTCCATAATTCTCGGTTTTAAATATTTTG
AAAACCAGTGCACATTTGAAATTCCAAATTCTCCATTAATTTCAGTTTAAAAAATTTTG
AAAACCAGTGCACATTTCAAATTTTATATTCTCCATAATTCTCGGTTTTAAATATTTTG
AAAACCAGTGCACATTTGAAATTCCCAATTCTCCACAGTTTTCAGTTAAAAAAATTTTG
AAAACCAGTGCACATTTGAAATTTTATATTCTCCATAATTCTCGGTTTTAAATATTTTG
AAAACCAGTGCACATTTGAAATTCCAAATTCTCCATTAATTTCAGTTTAAAAAATTTTG
AAAACCAGTGCACATTTGAAATTCCAAATTCTCCATAAATTTCAGTTTAAAAAAATTTG
AAAACCAGTGCGCATTTGAAATTTTATATTCTCCATAATTCTCAGTTTGAAAAATTTTG
AAAACCAGTGCACATTTCAAATTTTATATTCTCCATAATTCTCGGTTTTAAATATTTTG
AAAACCAGTGCACATTTCAAATGTTATATTCTCCATAATTCTCGGTTTTAAATATTTTG
AAAACCAGTGCACATTTGAAATTCCTAATTCTCCACAGTTTTCAGTTAAAAAAATTTTG

AAAACCAGTGCACATTTGAAATTTTATATTCTCCATAATTCTCGGTTTTAAATATTTTG
AAAACCAGTGCACATTTGAAATTCCCAATTCTCCACAGTTTTCAGTTAAAAAAATTTTG
AAAACCAGTGCACATTTGAAATTTTATATTCTCCATAATTCTCGGTTTTAAATATTTTG
AAAACCAGTGCACATTTGAAATTCCCAATTCTCCACAGTTTTCAGTTAAAAAAATTTTG
AAAACCAGTGCACATTTGAAATTTTATATTCTCCATAATTCTCGGTTTTAAATATTTTG
AAAACCAGTGCACATTTGAAATTCCAAATTCTCCACAGTTTTCAGTTTGAAAAATTTTG
AAAACCAGTGCACATTTCAAATGTTATATTCTCCATAATTCTCGGTTTTAAATATTTTG
AAAACCAGTGCACATTTGAAATTCCCAATTCTCCACAGTTTTCAGTTAAAAAAATTTTG
AAAACCAGTGCACATTTGAAATTTTATATTCTCCATAATTCTCGGTTTTAAATATTTTG
AAAACCAGTGCACATTTGAAATTCCAAATTCTCCACAGTTTTCAGTTTGAAAAATTTTG
AAAACCAGTGCACATTTCAAATTTTATATTCTCCATAATTCTCGGTTTTAAATATTTTG
AAAACCAGTGCACATTTGAAATTCCCAATTCTCCACAGTTTTCAGTTAAAAAAATTTTG
AAAACCAGTGCACATTTGAAATTTTATATTCTCCATAATTCTCGGTTTTAAATATTTTG
AAAACCAGTGCACATTTGAAATTCCCAATTCTCCACAGTTTTCAGTTTAAAAAATTTTG
AAAACCAGTGCACATTTCAAATTTTATATTCTCCATAATTCTCGCTTTTAAATATTTTG
AAAACCAGTGCACATTTGAAATTCCAAATTCTCCACAGTTTTCAGTTTGAAAAATTTTG
AAAACCAGTGCACATTTCAAATTTTATATTCTCCATAATTCTCGGTTTTAAATATTTTG
AAAACCAGTGCACATTTCAAATTTTATATTCTCCATAATTCTCGGTTTAAAATATTTTA
AAAACCAGTGCACATTTGAAATTTTATATTCTCCATAATTCTCGGTTTAAAATATTTTA
AAAACCAGTGCACATTTGAAATTTTATATTCTCCATAATTCTCGGTTTTAAATATTTTG
AAAACCAGTGCACATTTGAAATTCCCAATTCTCCATTAATTTCAGTTTAAAAAAATTTG
AAAACCAGTGCACATTTCAAATTTTATATTCTCTATAATTCACGGTTTAAAATATTTTA
AAAACCAGTGCACATTTGAAATTTTATATTCTCTATAATTCACGGTTTAAAATATTTTA
AAAACCAGTGCACATTTCAAATTTTATATTCTCTATAATTCACGGTTTTAAATATTTTG
AAAACCAGTGCACATTTCAAATTTTATATTCTCCATAATTCTCGGTTTTAAATATTTTG
AAAACCAGTGCACATTTCAAATTTTATATTCTCCATAATTCTCGGTTTTAAATATTTTG
AAAACCAGTGCACATTTGAAATTTTATATTCTCCATAATTCTCGGTTTTAAATATTTTG
AAAACCAGTGCACATTTCAAATTTTATATTCTCCATAATTCTCGGTTTTAAATATTTTG
AAAACCAGTGCACATTTGAAATTTTATATTCTCCATAATTGTCGGTTTTAAATATTTTG
AAAACCAGTGCACATTTCAAATTTTATATTCTCCATAATTCTCGGTTTTAAATATTTTG
AAAACCAGTGCACATTTGAAATTTTATATTCTCCATAATTCTCGGTTTTAAATATTTTG
AAAACCAGTGCACATTTCAAATTTTATATTCTCCATAATTCTCGGTTTTAAATATTTTG
AAAACCAGTGCACATTTCAAATTTTATATTCTCCATAATTCTCGGTTTTAAATATTTTG
AAAACCAGTGCACATTTGAAATTCCCAATTCTCCATTAATTTCAGTTTAAAAAAATTTG
AAAACCAGTGCACATTTGAAATTTTATATTCCCCATAATTCTCGGTTTTAAATATTTTA
AAAACCAGTGCACATTTGAAATTTTATATTCTCTATAATTCTCGGTTTTAAATATTTTG
AAAACCAGTGCACATTTCAAATTTTATATTCTCCATAATTCTCGGTTTTAAATATTTTG
AAAACCTGTGCACATTTCAAATTTTATATTCTCCATAATTCTCGGTTTTAAATATTTTA
AAAACCAGTGCACATTTGAAATTTTATATTCTCTATAATTCTCGGTTTTAAATATTTTG
AAAACCAGTGCACATTTCAAATTTTATATTCTCCATAATTCTCGCTTTTAAATATTTTG
AAAACCAGTGCACATTTGAAATTCCCAATTCTCCATTAATTTCAGTTTAAAAAATTTTA
AAAACCAGTGCACATTTGAAATTTTATATTCTCCATAATTCTCGGTTTTAAATATTTTA
AAAACCAGTGCACATTTCAAATTTTATATTCTCTATAATTCACGGTTTTAAATATTTTG
AAAACCAGTGCACATTTCAAATTTTATATTCTCCATAATTCTCGGTTTTAAATATTTTG
AAAACCAGTGCACATTTCAAATTTTATATTCTCCATAATTCTCGGTTTTAAATATTTTG
AAAACCAGTGCACATTTGAAATTTTATATTCTCCATAATTCTCGGTTTTAAATATTTTG
AAAACCAGTGCACATTTCAAATTTTATATTCTCCATAATTCTCGGTTTTAAATATTTTG
AAAACCAGTGCACATTTGAAATTTTATATTCTCCATAATTCTCGGTTTTAAATATTTTG
AAAACCAGTGCACATTTCAAATTTTATATTCTCTATAATTCACGGTTTTAAATATTTTG
AAAACCAGTGCACATTTCAAATTTTATATTCTCCATAATTCTCGGTTTTAAATATTTTG
AAAACCAGTGCACATTTCAAATTTTATATTCTCTATAATTCACGGTTTTAAATATTTTG
AAAACCAGTGCACATTTCAAATTTTATATTCTCCATAATTCTCGGTTTTAAATATTTTG
AAAACCAGTGCACATTTCAAATTTTATATTCTCTATAATTCACGGTTTTAAATATTTTG
AAAACCAGTGCACATTTCAAATTTTATATTCTCCATAATTCTCGGTTTTAAATATTTTG

AAAACCAGTGCACATTTCAAATTTTATATTCTCCATAATTCTCGGTTTTAAATATTTTG
AAAACCAGTGCACATTTGAAATTTTATATTCTCCATAATTCTCGGTTTTAAATATTTTG
AAAACCAGTGCACATTTCAAATTTTATATTCTCCATAATTCTCGGTTTTAAATATTTTG
AAAACCAGTGCACATTTGAAATTTTATATTCTCCATAATTGTCGGTTTTAAATATTTTG
AAAACCAGTGCACATTTCAAATTTTATATTCTCCATAATTCTCGGTTTTAAATATTTTG
AAAACCAGTGCACATTTGAAATTTTATATTCTCCATAATTCTCGGTTTTAAATATTTTG
AAAACCAGTGCACATTTCAAATTTTATATTCTCCATAATTCTCGGTTTTAAATATTTTG
AAAACCAGTGCACATTTCAAATTTTATATTCTCCATAATTCTCGGTTTTAAATATTTTG
AAAACCAGTGCACATTTGAAATTCCCAATTCTCCATTAATTTCAGTTTAAAAAATTTG
AAAACCAGTGCACATTTGAAATTTTATATTCTCCATAATTCTCGGTTTTAAATATTTTA
AAAACCAGTGCACATTTGAAATTTTATATTCTCTATAATTCTCGGTTTTAAATATTTTG
AAAACCAGTGCACATTTCAAATTTTATATTCTCCATAATTCTCGCTTTTAAATATTTTG
AAAACCAGTGCACATTTGAAATTCCCAATTCTCCACAGTTTTCAGTTAAAAAAATTTTG
AAAACCAGTGCACATTTGAAATTTTATATTCTCCATAATTCTCGGTTTTAAATATTTTG
AAAACCAGTGCACATTTGAAATTCCCAATTCTCCACAGTTTTCAGTTAAAAAAATTTTG
AAAACCAGTGCACATTTGAAATTTTATATTCTCCATAATTCTCGGTTTTAAATATTTTG
AAAACCAGTGCACATTTGAAATTCCCAATTCTCCACAGTTTTCAGTTTAAAAAATTTTG
AAAACCAGTGCACATTTGAAATTTTATATTCTCCATAATTCTCGGTTTAAAATATTTTA
AAAACCAGTGCACATTTGAAATTCCTAATTCTCCACAGTTTTCAGTTAAAAAAATTTTG
AAAACCAGTGCACATTTGAAATTTTATATTCTCTATAATTCTCGGTTTTAAATATTTTG
AAAACCAGTGCACATTTGAAATTCCCAATTCTCCACAGTTTTCAGTTAAAAAAATTTTG
AAAACCAGTGCACATTTGAAATTCCAAATTCTCCATAAATTTCAGTTTAAAAAAATTTG
AAAACCAGTGCGCATTTGAAATTCCAAATTCTCCATTAATTTCAGTTTAAAAAATTTTG
AAAACCAGTGCACATTTGAAATTTTATATTCTCCATAATTCTCGGTTTAAAATATTTTA
AAAACCAGTGCACATTTGAAATTCCCAATTCTCCACAGTTTTCAGTTTAAAAAATTTTG
AAAACCAGTGCACATTTGAAATTTTATATTCTCCATAATTCTCGGTTTAAAATATTTTA
AAAACCAGTGCACATTTGAAATTCCTAATTCTCCACAGTTTTCAGTTAAAAAAATTTTG
AAAACCAGTGCACATTTGAAATTTTATATTCTCCATAATTCTCGGTTTTAAATATTTTG
AAAACCAGTGCACATTTGAAATTCCCAATTCTCCACAGTTTTCAGTTAAAAAAATTTTG
AAAACCAGTGCACATTTGAAATTTTATATTCTCCATAATTCTCGGTTTTAAATATTTTG
AAAACCAGTGCACATTTGAAATTCCCAATTCTCCACAGTTTTCAGTTAAAAAAATTTTG
AAAACCAGTGCACATTTGAAATTTTATATTCTCCATAATTCTCGGTTTTAAATATTTTG
AAAACCAGTGCACATTTGAAATTCCAAATTCTCCATTAATTTCAGTTTAAAAAATTTTG
AAAACCAGTGCACATTTCAAATTTTATATTCTCCATAATTCTCGCTTTTAAATATTTTG
AAAACCAGTGCACATTTGAAATTCCAAATTCTCCATAAATTTCAGTTTAAAAAAATTTG
AAAACCAGTGCGCATTTGAAATTTTATATTCTCCATAATTCTCAGTTTGAAAAATTTTG
AAAACCAGTGCACATTTCAAATTTTATATTCTCCATAATTCTCGGTTTTAAATATTTTG
AAAACCAGTGCACATTTGAAATTTTATATTCTCCATAATTCTCGGTTTTAAATATTTTG
AAAACCAGTGCACATTTGAAATTCCCAATTCTCCACAGTTTTCAGTTAAAAAAATTTTG
AAAACCAGTGCACATTTGAAATTTTATATTCTCCATAATTCTCGGTTTTAAATATTTTG
AAAACCAGTGCACATTTGAAATTCCCAATTCTCCACAGTTTTCAGTTAAAAAAATTTTG
AAAACCAGTGCACATTTGAAATTTTATATTCTCCATAATTCTCGGTTTTAAATATTTTG
AAAACCAGTGCACATTTGAAATTCCAAATTCTCCACAGTTTTCAATTTGAAAAATTTTG
AAAACCAGTGCACATTTCAAATGTTATATTCTCCATAATTCTCGGTTTTAAATATTTTG
AAAACCAGTGCACATTTGAAATTCCCAATTCTCCACAGTTTTCAGTTAAAAAAATTTTG
AAAACCAGTGCACATTTGAAATTTTATATTCTCCATAATTCTCGGTTTTAAATATTTTG
AAAACCAGTGCACATTTGAAATTCCAAATTCTCCACAGTTTTCAGTTTGAAAAATTTTG
AAAACCAGTGCACATTTCAAATTTTATATTCTCCATAATTCTCGGTTTTAAATATTTTG
AAAACCAGTGCACATTTCAAATGTTATATTCTCCATAATTCTCGGTTTTAAATATTTTG
AAAACCAGTGCACATTTGAAATTTTATATTCTCCATAATTCTCGGTTTTAAATATTTTG
AAAACCAGTGCACATTTCAAATGTTATATTCTCCATAATTCTCGGTTTTAAATATTTTG
AAAACCAGTGCACATTTGAAATTCCCAATTCTCCACAGTTTTCAGTTAAAAAAATTTTG
AAAACCAGTGCACATTTCAAATTTTATATTCTCCATAATTCTCGCTTTTAAATATTTTG
AAAACCAGTGCACATTTGAAATTCCAAATTCTCCACAGTTTTCAGTTTGAAAAATTTTG

AAAACCAGTGCACATTTCAAATTTTATATTCTCCATAATTCTCGGTTTTAAATATTTTG
AAAACCAGTGCACATTTCAAATTTTATATTCTCCATAATTCTCGGTTTAAAATATTTTA
AAAACCAGTGCACATTTGAAATTTTATATTCTCCATAATTCTCGGTTTTAAATATTTTG
AAAACCAGTGCACATTTGAAATTCCCAATTCTCCATTAATTTCAGTTTAAAAAAATTTG
AAAACCAGTGCACATTTCAAATTTTATATTCTCTATAATTCACGGTTTAAAATATTTTA
AAAACCAGTGCACATTTGAAATTTTATATTCTCTATAATTCACGGTTTAAAATATTTTA
AAAACCAGTGCACATTTCAAATTTTATATTCTCTATAATTCACGGTTTTAAATATTTTG
AAAACCAGTGCACATTTCAAATTTTATATTCTCCATAATTCTCGGTTTTAAATATTTTG
AAAACCAGTGCACATTTCAAATTTTATATTCTCCATAATTGTCGGTTTTAAATATTTTG
AAAACCAGTGCACATTTCAAATTTTATATTCTCCATAATTCTCGGTTTTAAATATTTTG
AAAACCAGTGCACATTTGAAATTTTATATTCTCCATAATTCTCGGTTTTAAATATTTTG
AAAACCAGTGCACATTTCAAATTTTATATTCTCCATAATTCTCGGTTTTAAATATTTTG
AAAACCAGTGCACATTTGAAATTTTATATTCTCCATAATTCTCGGTTTTAAATATTTTG
AAAACCAGTGCACATTTCAAATTTTATATTCTCCATAATTCTCGGTTTTAAATATTTTG
AAAACCAGTGCACATTTCAAATTTTATATTCTCCATAATTCTCGGTTTTAAATATTTTG
AAAACCAGTGCACATTTGAAATTCCCAATTCTCCATTAATTTCAGTTTAAAAAAATTTG
AAAACCAGTGCACATTTGAAATTTTATATTCTCCATAATTCTCGGTTTTAAATATTTTG
AAAACCAGTGCACATTTCAAATTTTATATTCTCCATAATTCTCGGTTTTAAATATTTTG
AAAACCAGTGCACATTTGAAATTTTATATTCTCCATAATTCTCGGTTTTAAATATTTTG
AAAACCAGTGCACATTTCAAATTTTATATTCTCCATAATTCTCGGTTTAAAATATTTTA
AAAACCAGTGCACATTTGAAATTTTATATTCTCCATAATTCTCGGTTTTAAATATTTTG
AAAACCAGTGCACATTTGAAATTCCCAGTTCTCCATTAATTTCAGTTTAAAATATTTTA
AAAACCAGTGCACATTTGAAATTTTATATTCTCCATAATTCTCGGTTTAAAATATTTTA
AAAACCAGTGCACATTTGAAATTTTATATTCTCCATAATTCTCGGTTTTAAATATTTTG
AAAACCAGTGCACATTTGAAATTCCCAATTCTCCATTAATTTCAGTTTAAAATATTTTA
AAAACCAGTGCACATTTGAAATTTTATATTCTCCATAATTCTCGGTTTAAAATATTTTA
AAAACCAGTGCACATTTCAAATTTTATATTCTCCATAATTCTCGGTTTTAAATATTTTG
AAAACCAGTGCACATTTGAAATTCTCAATTCTCCACAGTTTTCAGTTTAAAATATTTTA
AAAACCAGTGCACATTTGAAATTTTATATTCTCCATAAATTTCAGTTTAAAAAAATTTG
AAAACCAGTGCACATTTGAAATTTTATATTCTCCATAATTCTCGGTTTTAAATATTTTG
AAAACCAGTGCACATTTGAAATTCTCAATTCTCCACAGTTTTCAGTTTAAAATATTTTA
AAAACCAGTGCACATTTGAAATTTTATATTCTCCATAAATTTCAGTTTAAAAAAATTTG
AAAACCAGTGCACATTTGAAATTTTATATTCTCTATAATTCTCGGTTTAAAATATTTTA
AAAACCAGTGCACATTTGAAATTCCCAATTCTCCACAGTTTTCAGTTTGAAAAATTTTG
AAAACCAGTGCACATTTGAAATTTTATATTCTCTATAATTCTCGGTTTTAAATATTTTG
AAAACCAGTGCACATTTGAAATTCCCAATTCTCCACAGTTTTCAGTTTGAAAAATTTTG
AAAACCAGTGCACATTTGAAATTTTATATTCTCTATAATTCTCGGTTTAAAATATTTTA
AAAACCAGTGCACATTTGAAATTCCCAATTCTCCACAGTTTTCAGTTTAAAAAATTTTG
AAAACCAGTGCACATTTGAAATTTTATATTCTCTATAATTCTCGGTTTAAAATATTTTA
AAAACCAGTGCACATTTGAAATTCCCAATTCTCCACAGTTTTCAGTTTAAAATATTTTA
AAAACCAGTGCACATTTGAAATTCCCAATTCTCCACAGTTTTCAGTTTAAAATATTTTA
AAAACCAGTGCACATTTGAAATTCTCAATTCTCCACAGTTTTCAGTTTAAAATATTTTA
AAAACCAGTGCACATTTGAAATTTTATATTCTCTATAATTCTCGGTTTAAAATATTTTA
AAAACCAGTGCACATTTGAAATTCCCAATTCTCCACAGTGTTCAGTTTAAAAAATTTTA
AAAACCAGTACACATTTGAAATTTTATATTCTCCATAATTCTCGGTTTTAAATATTTTG
AAAACCAGTGCACATTTGAAATTCTCAATTCTCCACAGTTTTCAGTTTAAAATATTTTA
AAAACCAGTGCACATTTGAAATTTTATATTCTCCATAAATTTCAGTTTAAAAAAATTTG
AAAACCAGTGCACATTTGAAATTTTATATTCTCTATAATTCTCGGTTTAAAATATTTTA
AAAACCAGTGCACATTTGAAATTCCCAATTCTCCACAGTTTTCAGTTTAAAAAATTTTG
AAAACCAGTGCACATTTGAAATTTTATATTCTCTATAATTCTCGGTTTAAAATATTTTA
AAAACCAGTGCACATTTGAAATTCTCAATTCTCCACAGTTTTCAGTTTAAAATATTTTA
AAAACCAGTGCACATTTGAAATTCCCAATTCTCCATAAATTTCAGTTTAAAAAAATTTG
AAAACCAGTGCACATTTGAAATTTTATATTCTCCATAATTCTCGGTTTTAAATATTTTG
AAAACCAGTGCACATTTGAAATTCTCAATTCTCCACAGTTTTCAGTTTAAAATATTTTA

AAAACCAGTGCACATTTGAAATTCTCAATTCTCCACAGTTTTCAGTTTAAAATATTTTA
AAAACCAGTGCACATTTGAAATTTTATATTCTCTATAATTCTCGGTTTTAAATATTTTA
AAAACCAGTGCACATTTGAAATTCCCAATTCTCCACAGTTTTCAGTTTAAAAAAATTTTA
AAAACCAGTACACATTTGAAATTTTATATTCTCCATAATTCTCGGTTTTAAATATTTTG
AAAACCAGTGCACATTTGAAATTCTCAATTCTCCACAGTTTTCAGTTTAAAATATTTTA
AAAACCAGTGCACATTTGAAATTTTATATTCTCCATAAATTTCAGTTTAAAAAAAATTTG
AAAACCAGTGCACATTTGAAATTTTATATTCTCTATAATTCTCGGTTTTAAATATTTTA
AAAACCAGTGCACATTTGAAATTCCCAATTCTCCACAGTTTTCAGTTTAAAAAAAATTTG
AAAACCAGTGCACATTTGAAATTTTATATTCTCTATAATTCTCGGTTTTAAATATTTTA
AAAACCAGTGCACATTTGAAATTCTCAATTCTCCACAGTTTTCAGTTTAAAATATTTTA
AAAACCAGTGCACATTTGAAATTCCCAATTCTCCATAAATTTCAGTTTAAAAAAAATTTG
AAAACCAGTGCACATTTGAAATTTTATATTCTCTATAATTCTCGGTTTTAAATATTTTA
AAAACCAGTGCACATTTGAAATTCCCAATTCTCCACAGTTTTCAGTTTAAAAAAATTTTA
AAAACCAGTGCACATTTGAAATTTTATATTCTCCATAATTCTCGGTTTTAAATATTTTG
AAAACCAGTGCACATTTGAAATTCTCAATTCTCCACAGTTTTCAGTTTAAAATATTTTA
AAAACCAGTGCACATTTGAAATTTTATATTCTCCATAATTCTCGGTTTTAAATATTTTG
AAAACCAGTGCACATTTGAAATTCTCAATTCTCCACAGTTTTCAGTTTAAAATATTTTA
AAAACCAGTGCACATTTGAAATTTTATATTCTCCATAAATTTCAGTTTAAAAAAAATTTG
AAAACCAGTGCACATTTGAAATTTTATATTCTCCATAATTCTCGGTTTTAAATATTTTG
AAAACCAGTGCACATTTGAAATTCCCAATTCTCCACAGTTTTCAGTTTAAAAAAATTTTG
AAAACCAGTGCACATTTGAAATTTTATATTCTCTATAATGCTCGGTTTTAAATATTTTA
AAAACCAGTGCACATTTGAAATTCCCAATTCTCCACAGTTTTCAGTTTAAAAAAATTTTG
AAAACCAGTGCACATTTGAAATTTTATATTCTCTATAATTCTCGGTTTTAAATATTTTA
AAAACCAGTGCACATTTGAAATTCCCAATTCTCCACAGTTTTCAGTTTAAAAAAATTTTG
AAAACCAGTGCACATTTGAAATTTTATATTCTCTATAATTCTCGGTTTTAAATATTTTG
AAAACCAGTGCACATTTGAAATTCCCAATTCTCCACAGTTTTCAGTTTAAAAAAATTTTG
AAAACCAGTGCACATTTGAAATTTTATATTCTCTATAATGCTCGGTTTTAAATATTTTA
AAAACCAGTGCACATTTGAAATTCCCAATTCTCCACAGTTTTCAGTTTAAAAAAATTTTG
AAAACCAGTGCACATTTGAAATTTTATATTCTCTATAATTCTCGGTTTTAAATATTTTG
AAAACCAGTGAAGTCTTGAATTTCCATATTCCCCATAATTCTCGGTTTTAAATATTTTG
AAAACCAGTGAAGTCTTGAATTTCCATATTCTCCATAATTTTCAGTTTAAAAAAAATTTG
Nr of selected repeats 214 Similarity 0.852508
Consensus:
AAAACCAGTGCACATTTgAAATTttatATTCTCCAtAaTTcTCgGTTTtAAAtATTTTg
>Cele-UNSB01_1:4183555-4184714 Satlength=1160 Nr of Repeats=5 RepeatLength=223
seed=TTTCAGAATT
TTTCAGAATTTCAATTTTAATCGGCAAAATTGTACGCATCCTATGAATGTTCCTACATCTATTTTGAAAATAAGCAAGT
TTTATGAAAATATCTAAAGAATACGGGAAGCGGCAAACCGGCATCTTGGCAAAAATGAAAATTTCCGGCAAACCGGCAA
ACGGGAAATTGCCGTTTTGCTAAAATAATTTCAGAACGGCAATTGCCGCCCACCACTGATAAACATTATGCTGAGCGGA
CTAAACAACCTCTTAAAATCGCTAAACTTC
TTTCAGAATTTAAAAACGGCATTCCAAAAACTTTCACAACCGCCAAGCTCAAGCAAAAAAAGTAAACTTCAGTCATACC
TTTCAGAGCAAACTTTGAAATTGAATGAAATAGACCATTTATTTGCAAAATTAGTAGATTTTCCAATTTTTAAAAATTA
ATATCTGGCTTCTAAATAACTTTTTTGAAAATCCCCGTAGCCATTTTGTTGCAAATTTTGTGCTC
TTTCAGAATTTAAAAACGACATTCCAAAAACTTTCACAACCGCCAAGTTCAAGCAAAAAAAGTAAACTTCAGTCATACC
TTTCAGACCAAACTTTGAAATTGAATGAAATAAATCATTTATTTGCAAAATTAGTAGATTTTCCAATTTTTAAAAATTA
ATATCTGGCTTCAAACTAACTTTTTTGAAAATCCCCGTAGCCATTTTGTTGCAAATTTTGTGCTC
TTTCAGAATTTAAAAACGGCATTCCAAAAACTATCATAACCGCCAAGTTCAAGCAAAAAAAGTAAACTTCAGTCATACC
TTTCAGAGCAAACTTTGAAATTGAATGAAATAGACCATTTATTTGCAAAATTAGTAGATTTTCCAATTTTTAAAAATTA
ATATCTGGCTTCAAACTAACTTTTTTGAAAATCCCCGTAGCCATTTTGTTGCAAATTTTGTGCTC
TTTCAGAATTTAAAAACGGCATTCCAAAAACTTTCACAACCGCCAAGTTCAAGCAAAAAAAGTAAACTTCAGTCATACC
TTTCAGAGCAAACTTTGAAATTGAATGAAATAGACCATTTATTTGCAAAATTAGTAGATTTTCCAATTTTTAAAAATTA
ATATCTGGCTTCTAAATAACTTTTTTGAAAATCCCCGTAGCCATTTTGTTGCAAATTTTGTGCTC
Nr of selected repeats 4 Similarity 0.971101
Consensus:

TTTCAGAATTTAAAAACGgCATTCCAAAAACTtTCAcAACCGCCAAGtTCAAGCAAAAAAAGTAAACTTCAGTCATACC
TTTCAGAgCAAACTTTGAAATTGAATGAAATAgAcCATTTATTTGCAAAATTAGTAGATTTTCCAATTTTTAAAAATTA
ATATCTGGCTTCaAAaTAACTTTTTTGAAAATCCCCGTAGCCATTTTGTTGCAAATTTTGTGCTC
>Cele-UNSB01_1:4232491-4233003 Satlength=513 Nr of Repeats=13 RepeatLength=39
seed=TATTTTTAAT
TATTTTTAATTTGGCCTACTTAAAAAGTAGGTCATGACC
TATTTTTAATTTGGCCTACTTAAAAAGTAGGTCATGACT
TATTTTTAATTTGACCTACTTAAAAAGTAGGTCATGACC
TATTTTTAATTTGGCCTACTTAAAAAGTAGGTCATGACC
TATTTTTAATTTAACCTACTTAAAAAAGTAGGTCACGACC
TATTTTTAATTTGGCCTACTTAAAAAGTAGGTCATGACT
TATTTTTAATTTGACCTACTTAAAAAGTAGGTCATGACC
TATTTTTAATTTGGCCTACTTAAAAAGTAGGTCATGACC
TATTTTTAATTTAACCTACTTAAAAAAGTAGGTCACGACC
TATTTTTAATTTGACCTACTTAAAAAACTAGGTCATGGCC
TATTTTTAATTTGACCTACTTAAAAAAGTAGGTCATGACC
TATTTTTAATTTGACCTACTTAAAAAACTAGGTCATGACC
TATTTTTAATTTGACCTACCTAAAAAGTAGGTCATGACT
Nr of selected repeats 8 Similarity 0.954823
Consensus:
TATTTTTAATTTGgCCTACTTAAAAAGTAGGTCATGACc
>Cele-UNSB01_1:4250268-4250328 Satlength=61 Nr of Repeats=4 RepeatLength=15
seed=TTTCCGGATC
TTTCCGGATCTACCA
TTTCCGGATCTACGT
TTTCCGGATCTACGT
TTTCCGGATCTACGT
Nr of selected repeats 4 Similarity 0.911111
Consensus:
TTTCCGGATCTACgt
>Cele-UNSB01_1:4250510-4250645 Satlength=136 Nr of Repeats=9 RepeatLength=15
seed=AGATCCGGAA
AGATCCGGAAAACGT
AGATCCGGAAAACGT
AGATCCGGAAAACGT
AGATCCGGAAAACGT
AGATCCGGAAAACGT
AGATCCGGAAAACGT
AGATCCGGAAAACGT
AGATCCGGAAAACGT
AGATCCGGAAAACGT
Nr of selected repeats 9 Similarity 1.000000
Consensus:
AGATCCGGAAAACGT
>Cele-UNSB01_1:4344690-4345849 Satlength=1160 Nr of Repeats=6 RepeatLength=226
seed=ACAAGAAGCT
ACAAGAAGCTTTACAATATTCCAGTAGGT
ACAAGAAGCTTCACGTAGTTACAGAAATAGTAGATTTTTAGCCCTACCTTTTAGAGCGTATTTTATTATTTAATGAAAA
CTACCACTTATAGGCAAAATAGAGGGATTTTCGTAATTTTGAAAATTCATAAATCTCTTCAAAGTAATTTTTTTGCGAA
ATGTCTTTCAGAAACTTTGTAGTAAATTTTAAGCTCTTTTAGAATATATTAACAATATTCCAGTAGGT
ACAAGAAGCTTCACGTAGTTACAGAAATAGTACATTTTTAGCCCTACCTTTTAGCGCGTATTTTATTATTTAATGAAAA
CTACCATTTATAGGCAAAAATAAATGGATTTTCCAAACTTTGAAAATTCATAAATCTCTTCAAAGTAACTTTTTTTGAA
ATGTCTTTTAGAAACTTTGTAGTAAATTTTATGCTCTTTTAGAATATATTAACATTATTTCAGTAGGT

ACAAGAAGCTTCACGTAGTTACAGAAATTGTACATTTTCAGCCCTACCTTTTAGTGCGTATTTTATTAATTAATGAAAA
CTACCACTTATAGGCAAAATAGAGGGATTTTCGTAATTTTGAAAATTCATAAATCTCTTCAAAGTAATTTTTTTGCGAA
ATGTCTTTCAGAAACTTTGTAGTAAATTTTAAGCTCTTTTAGAATATATTAACAATATTCCAGTAGGT
ACAAGAAGCTTCACGTAGTTACAGAAATAGTACATTTTCAGCCCTACCTTTTAGTGCGTATTTTATTAATTAATGAAAA
CTACCACTTATAGGCAAAATAGAGGGATTTTCGTAATTTTGAAAATTCATAAATCTCTTCAAAGTAATTTTTTTGCGAA
ATGTCTTTCAGAAACTTTGTAGTAAATTTTAAGCTCTTTTAGAATATATTAACAATATTCCAGTAGGT
ACAAGAAGCTTCACGTAGTTACAGAAATAGTACATTTTCAGCCCTACCTTTTAGTGCATATTTTATTATTTAATGAAAA
CTACCATTTATAGGCAAAAATAGATGGATTTTCCAAAATTTGAAAATTCATAAATCTCTTCACAGTAACTTTTTTTGAA
ATGTCTTTCAGAAACCATGGAGTAAATTTTAAGCTCTTTCAGAATATATTAACAATATTACAGTAGGT
Nr of selected repeats 5 Similarity 0.915789
Consensus:
ACAAGAAGCTTCACGTAGTTACAGAAATaGTAcATTTTcAGCCCTACCTTTTAGtGCgTATTTTATTAtTTAATGAAAA
CTACCAcTTATAGGCAAAATAgAgGGATTTTCgtAAtTTTGAAAATTCATAAATCTCTTCAaAGTAATTTTTTTGcgAA
ATGTCTTTcAGAAACttTGtAGTAAATTTTAaGCTCTTTtAGAATATATTAACAaTATTcCAGTAGGT
>Cele-UNSB01_1:4346155-4359862 Satlength=13708 Nr of Repeats=146
RepeatLength=94 seed=TCAAAATCTT
TCAAAATCTTCTGTTCTCTAAATATTGGGTGCTTTTCAATGCTCTTTGTAGACAAATCAATGGGAAAATGATCAATTTC
TGGAGGCAGTAAATC
TCAAAATCTTCTGTTCTCTAAATATTGGGTGCTTTTCGATGCTCTTTGTAGACAAATCAATGGGAAAATTGTCAATTTC
TGAAGGCAGTAATTC
TCAAAATCTTCTGTTCTCTAAATATTGGGTGCTTTTCGATGCCCTATGTAGAAAAATCAATGGGAAAATCATCAATTTC
TGAAGGCAGTAATTC
TCAAAATCTTCTGTTCTCTAAATATTGGGTGCTTTTCGATGCCCTATGTAGACAAATCAATGGGAAAATCATCAATTTC
TGAAGGCAGTAATTC
TCAAAATCTTCTGTTCTCTAAATATTGGGTGCTTTTCGATGCTCTTTGTAGACAAATCAATGGGAAAATCATAAATTTC
TGAAGGCAGTAATTC
TCAAAATCTTCTGTTCTCTAAATATTGGGTGCTTTTCGATGCTCTTTGTAGACAAATCAATGGGAAAATCATAAATTTC
TGAAGGCAGTAATTC
TCAAAATCTTCTGTTCTCTAAATATTGGGTGCTTTTCGATGCTCTTTGTAGACAAATCAATGGGAAAATCATAAATTTC
TGAAGGCAGTAATTC
TCAAAATCTTCTGTTCTCTAAATATTGGGTGCTTTTCGATGCCCTATGTAGACAAATCAATGGGAAAATCATCAATTTC
TGAAGGAAGTAATTC
TCAAAATCTTCTGTTCTCTAAATATTGGGTGCTTTTCGATGCTCTTTGTAGACAAATCAATGGGAAAATCATAAATTTC
TGAAGGCAGTAATTC
TCAAAATCTTCTGTTCTCTAAATATTGGGTGCTTTTCGATGCCCTATGTAGACAAATCAATGGGAAAATCATCAATTTC
TGAAGGCAGTAATTC
TCAAAATCTTCTGTTCTCTAAATATTGGGTGCTTTTCGATGCTCTTTGTAGACAAATCAATGGGAAAATCATAAATTTC
TGAAGGAAGTAATTC
TCAAAATCTTCTGTTCTCTAAATATTGGGTGCTTTTCGATGCCCTATGTAGACAAATCAATGGGAAAATCATCAATTTC
TGAAGGCAGTAATTC
TCAAAATCTTCTGTTCTCTAAATATTGGGTGCTTTTCGATGCCCTATGTAGACAAATCAATGGGAAAATCATCAATTTC
TGAAGGAAGTAATTC
TCAAAATCTTCTGTTCTCTAAATATTGGGTGCTTTTCGATGCTCTTTGTAGACAAATCAATGGGAAAATCATAAATTTC
TGAAGGCAGTAATTC
TCAAAATCTTCTGTTCTCTAAATATTGGGTGCTTTTCGATGCCCTATGTAGACAAATCAATGGGAAAATCATCAATTTC
TGAAGGAAGTAATTC
TCAAAATCTTCTGTTCTCTAAATATTGGGTGCTTTTCGATGCTCTTTGTAGACAAATCAATGGGAAAATCATAAATTTC
TGAAGGCAGTAATTC
TCAAAATCTTCTGTTCTCTAAATATTGGGTGCTTTTCGATGCCCTATGTAGACAAATCAATGGGAAAATCATCAATTTC
TGAAGGCAGTAATTC
TCAAAATCTTCTGTTCTCTAAATATTGGGTGCTTTTCGATGCTCTTTGTAGACAAATCAATGGGAAAATCATAAATTTC
TGAAGGAAGTAATTC
TCAAAATCTTCTGTTCTCTAAATATTGGGTGCTTTTCGATGCCCTATGTAGACAAATCAATGGGAAAATCATCAATTTC
TGAAGGCAGTAATTC

TCAAAATCTTCTGTTCTCTAAATATTGGGTGCTTTTCGATGCCCTATGTAGACAAATCAATGGGAAAATCATCAATTTC
TGAAGGCAGTAATTC
TCAAAATCTTCTGTTCTCTAAATATTGGGTGCTTTTCGATGCCCTATGTAGACAAATCAATGGGAAAATCATCAATTTC
TGAAGGCAGTAATTC
TCAAAATCTTCTGTTCTCTAAATATTGGGTGCTTTTCGATGCTCTTTGTAGACAAATCAATGGGAAAATCATAAATTTC
TGAAGGAAGTAATTC
TCAAAATCTTCTGTTCTCTAAATATTGGGTGCTTTTCGATGCCCTATGTAGACAAATCAATGGGAAAATCATCAATTTC
TGAAGGAAGTAATTC
TCAAAATCTTCTGTTCTCTAAATATTGGGTGCTTTTCGATGCCCTATGTAGACAAATCAATGGGAAAATCATCAATTTC
TGAAGGAAGTAATTC
TCAAAATCTTCTGTTCTCTAAATATTGGGTGCTTTTCGATGCCCTATGTAGACAAATCAATGGGAAAATTGTCAATTTC
TGAAGGCAGTAATTC
TCAAAATCTTCTGTTCTCTAAATATTGGGTGCTTTTCGATGCCCTATGTAGACAAATCAATGGGAAAATTGTCAATTTC
TGAAGGCAGTAATTC
TCAAAATCTTCTGTTCTCTAAATATTGGGTGCTTTTCGATGATCTTTGTAGACAAATCAATGGGAAAATTGTCAATTTC
TGAAGGCAGTAATTC
TCAAAATCTTCTGTTCTCTAAATATTGGGTACTTTTCGATGCTCTTTGTAGACAAATCAATGGGAAAATTGTCAATTTC
TGAAGGCAGTAATTC
TCAAAATCTTCTGTTCTCTAAATATTGGGTGCTTTTCGATGCCCTATGTAGACAAATCAATGGGAAAATTGTCAATTTC
TGAAGGCAGTAATTC
TCAAAATCTTCTGTTCTCTAAATATTGGGTGCTTTTCGATGCCCTATGTAGACAAATCAATGGGAAAATTGTCAATTTC
TGAAGGCAGTAATTC
TCAAAATCTTCTGTTCTCTAAATATTGGGTGCTTTTCGTTGCCCTATGTAGACAAATCAATGGGAAAATCATCAATTTC
TGAAGGCAGTAATTC
TCAAAATCTTCTGTTCTCTAAATATTGGGTGCTTTTCGATGCCCTATGTAGACAAATCAATGGGAAAATTGTCAATTTC
TGAAGGCAGTAATTC
TCAAAATCTTCTGTTCTCTAAATATTGGGTGCTTTTCGATGCCCTATGTAGACAAATCAATGGGAAAATCATCAATTTC
TGAAGGCAGTATTTC
TCAAAATCTTCTGTTCTCTAAATATTGGGTGCTTTTCGATGCCCTATGTAGACAAATCAATGGGAAAATTGTCAATTTC
TGAAGGCAGTAATTC
TCAAAATCTTCTGTTCTCTAAATATTGGGTGCTTTTCGATGATCTTTGTAGACAAATCAATGGGAAAATTGTCAATTTC
TGAAGGCAGTAATTC
TCAAAATCTTCTGTTCTCTAAATATTGGGTGCTTTTCGATGCTCTTTGTAGACAAATCAATGGGAAAATCATCAATTTC
TGAAGGCAGTATTTC
TCAAAATCTTCTGTTCTCTAAATATTGGGTACTTTTCGATGCCCTTTGTAGACAAATCAATGGGAAAATCATCAATTTC
TGAAGGCAGTAATTC
TCAAAATCTTCTGTTCTCTAAATATTGGGTGCTTTTCGATGCTCTTTGTAGACAAATCAATGGGAAAATCATCAATTTC
TGAAGGCAGTAATTC
TCAAAATCTTCTGTTCTCTAAATATTGGGTGCTTTTCGATGCTCTTTGTAGACAAATCAATGGGAAAATCATAAATTTC
TGAAGGAAGTAATTC
TCAAAATCTTCTGTTCTCTAAATATTGGGTGCTTTTCGATGCTCTTTGTAGACAAATCAATGGGAAAATCATCAATTTC
TGAAGGCAGTATTTC
TCAAAATCTTCTGTTCTCTAAATATTGGGTGCTTTTCGATGCCCTATGTAGACAAATCAATGGGAAAATCATCAATTTC
TGAAGGCAGTAATTC
TCAAAATCTTCTGTTCTCTAAATATTGGGTGCTTTTCGATGCTCTTTGTAGACAAATCAATGGGAAAATCATCAATTTC
TGAAGGCAGTAATTC
TCAAAATCTTCTGTTCTCTAAATATTGGGTGCTTTTCGATGCTCTTTGTAGACAAATCAATGGGAAAATCATCAATTTC
TGAAGGCAGTAATTC
TCAAAATCTTCTGTTCTCTAAATATTGGGTGCTTTTCGATGCTCTTTGTAGACAAATCAATGGGAAAATCATCAATTTC
TGAAGGCAGTAATTC
TCAAAATCTTCTGTTCTCTAAATATTGGGTGCTTTTCGATGCTCTTTGTAGACAAATCAATGGGAAAATCATAAATTTC
TGAAGGAAGTAATTC
TCAAAATCTTCTGTTCTCTAAATATTGGGTGCTTTTCGATGCTCTTTGTAGACAAATCAATGGGAAAATCATCAATTTC
TGAAGGCAGTAATTC

TCAAAATCTTCTGTTCTCTAAATATTGGGTGCTTTTCGATGCTCTTTGAAGACAAATCAATGGGAAAATCATCAATTTC
TGGAGGCAGTAATTC
TCAAAATCTTCTGTTCTCTAAATATTGGGTGCTTTCGATGCTCTTTGTAGACAAATCAATAGGAAAATCATCAATTTCT
GAAGGCAGTAATTC
TCAAAATCTTCTGTTCTCTAAATATTGGGTGCTTTTCGATGCTCTTTGTAGACAAATCAATGGGAAAATCATCAATTTC
TGAAGGAAGTAATTC
TCAAAATCTTCTGTTCTCTAAATATTGGGTGCTTTTCGATGCCCTATGTAGACAAATCAATGGGAAAATTGTCAATTTC
TGAAGGCAGTAATTC
TCAAAATCTTCTGTTCTCTAAATATTGGGTGCTTTTCGATGCCCTATGTAGACAAATCAATGGGAAAATTGTCAATTTC
TGAAGGCAGTAGTTC
TCAAAATCTTCCGTTCTCTAAATATTGGGTGCTTTTCGATGCCCTATGTAGACAAATCAATGGGAAAATTGTCAATTTC
TGAAGGCAGTAGTTC
TCAAAATCTTCTGTTCTCTAAATATTGGGTGCTTTTCGATGCCCTATGTAGACAAATCAATGGGAAAATTGTCAATTTC
TGAAGGCAGTAATTC
TCAAAATCTTCTGTTCTCTAAATATTGGGTGCTTTTCGATGCTCTTTGTAGACAAATCAATGGGAAAATCATCAATTTC
TGAAGGCAGTAATTC
TCAAAATCTTCTGTTCTCTAAATATTGGGTGCTTTTCGATGCTCTTTGTAGACAAATCAATGGGAAAATCATCAATTTC
TGAAGGCAGTAATTC
TCAAAATCTTCTGTTCTCTAAATATTGGGTGCTTTTCGATGCCCTATGTAGACAAATCAATGGGAAAATCATCAATTTC
TGAAGGCAGTAATTT
TCAAAATCTTCTGTTCTCTAAATATTGGGTGCTTTTCGATGCTCTTTGTAGACAAATCAATGGGAAAATCATCAATTTC
TGAAGGCAGTAATTC
TCAAAATCTTCTGTTCTCTAAATATTGGGTGCTTTTCGATGCTCTTTGTAGACAAATCAATGGGAAAATCATCAATTTC
TGAAGGCAGTAATTC
TCAAAATCTTCTGTTCTCTAAATATTGGGTGCTTTTCGATGCTCTTTGTAGACAAATCAATGGGAAAATCATCAATTTC
TGAAGGCAGTAATTC
TCAAAATCTTCTGTTCTCTAAATATTGGGTGCTTTTCGATGCCCTATGTAGACAAATCAATGGGAAAATCATCAATTTC
TGAAGGCAGTAATTT
TCAAAATCTTCTGTTCTCTAAATATTGGGTGCTTTTCGATGCTCTTTGTAGACAAATCAATGGGAAAATCATCAATTTC
TGAAGGCAGTAATTC
TCAAAATCTTCTGTTCTCTAAATATTGGGTGCTTTCGATGCTCTTTGTAGACAAATCAATGGGAAAATTGTCAATTTCT
GAAGGCAGTAATTC
TCAAAATCTTCTGTTCTCTAAATATTGGGTGCTTTTCGATGCTCTTTGTAGACAAATCAATGGGAAAATCATCAATTTC
TGAAGGCAGTAATTC
TCAAAATCTTCTGTTCTCTAAATATTGGGTGCTTTTCGATGCTCTTTGTAGACAAATCAATGGGAAAATCATCAATTTC
TGAAGGCAGTAATTC
TCAAAATCTTCTGTTCTCTAAATATTGGGTGCTTTTCGATGCTCTTTGTAGACAAATCAATGGGAAAATCATCAATTTC
TGAAGGCAGTAATTC
TCAAAATCTTCTGTTCTCTAAATATTGGGTGCTTTTCGATGCTCTTTGTAGACAAATCAATGGGAAAATCATCAATTTC
TGAAGGCAGTAATTC
TCAAAATCTTCTGTTCTCTAAATATTGGGTGCTTTTCGATGCCCTATGTAGACAAATCAATGGGAAAATCATCAATTTC
TGAAGGCAGTAATTC
TCAAAATCTTCTGTTCTTTAAATATTGGGTGCTTTCGATGCTCTTTGTAGACAAATCAATGGGAAAATCATCAATTTCT
GAAGGCAGTAATTC
TCAAAATCTTCTGTTCTCTAAATATTGGGTGCTTTTCGATGCCCTATGTAGACAAATCAATGGGAAAATCATCAATTTC
TGAAGGCAGTAATTC
TCAAAATCTTCTGTTCTTTAAATATTGGGTGCTTTCGATGCTCTTTGTAGACAAATCAATGGGAAAATCATCAATTTCT
GAAGGCAGTAATTC
TCAAAATCTTCTGTTCTCTAAATATTGGGTGCTTTTCGATGCCCTATGTAGACAAATCAATGGGAAAATCATCAATTTC
TGAAGGCAGTAATTC
TCAAAATCTTCTGTTCTCTAAATATTGGGTGCTTTTCGATGCTCTTTGTAGACAAATCAATGGGAAAATTGTCAATTTC
TGAAGGCAGTAATTT
TCAAAATCTTCTGTTCTCTAAATATTGGGTGCTTTTCGATGCCCTATGTAGACAAATCAATGGGAAAATCATCAATTTC
TGAAGGCAGTAATTC

TCAAAATCTTCTGTTCTCTAAATATTGGGTGCTTTTCGATGCCCTATGTAGACAAATCAATGGGAAAATCATCAATTTC
TGGAGGCAGTAATTC
TCAAAATCTTCTGTTCTCTAAATATTGGGTGCTTTTCGATGCCCTATGTAGACAAATCAATGGGAAAATCATCAATTTC
TGAAGGCAGTAATTT
TCAAAATCTTCTGTTCTCTAAATATTGGGTGCTTTTCGATGCCCTATGTAGACAAATCAATGGGAAAATCATCAATTTC
TGGAGGCAGTAATTC
TCAAAATCTTCTGTTCTCTAAATATTGGGTGCTTTTCGATGCCCTATGTAGACAAATCAATGGGAAAATCATCAATTTC
TGAAGGCAGTAATTC
TCAAAATCTTCTGTTCTCTAAATATTGGGTGCTTTCGATGCTCTTTGTAGACAAATCAATGGGAAAATGTCAATTTCTG
AAGGCAGTAATTC
TCAAAATCTTCTGTTCTCTAAATATTGGGTGCTTTTCGATGCTCTTTGTAGACAAATCAATGGGAAAATCATCAATTTC
TGAAGGCAGTAATTC
TCAAAATCTTCTGTTCTCTAAATATTGGGTGCTTTTCGATGCTCTTTGTAGACAAATCAATGGGAAAATCATCAATTTC
TGAAGGCAGTAATTC
TCAAAATCTTCTGTTCTCTAAATATTGGGTGCTTTTCGATGCTCTTTGTAGACAAATCAATGGGAAAATCATCAATTTC
TGAAGGCAGTAATTC
TCAAAATCTTCTGTTCTCTAAATATTGGGTGCTTTTCGATGCTCTTTGTAGACAAATCAATGGGAAAATCATCAATTTC
TGAAGGCAGTAATTC
TCAAAATCTTCTGTTCTCTAAATATTGGGTGCTTTTCGATGCCCTATGTAGACAAATCAATGGGAAAATCATCAATTTC
TGAAGGCAGTAATTC
TCAAAATCTTCTGTTCTTTAAATATTGGGTGCTTTCGATGCTCTTTGTAGACAAATCAATGGGAAAATCATCAATTTCT
GAAGGCAGTAATTC
TCAAAATCTTCTGTTCTCTAAATATTGGGTGCTTTTCGATGCCCTATGTAGACAAATCAATGGGAAAATCATCAATTTC
TGAAGGCAGTAATTC
TCAAAATCTTCTGTTCTTTAAATATTGGGTGCTTTCGATGCTCTTTGTAGACAAATCAATGGGAAAATCATCAATTTCT
GAAGGCAGTAATTC
TCAAAATCTTCTGTTCTCTAAATATTGGGTGCTTTTCGATGCTCTTTGTAGACAAATCAATGGGAAAATCATCAATTTC
TGAAGGCAGTAATTC
TCAAAATCTTCTGTTCTCTAAATATTGGGTGCTTTTCGATGCCCTATGTAGACAAATCAATGGGAAAATCATCAATTTC
TGAAGGCAGTAATTC
TCAAAATCTTCTGTTCTTTAAATATTGGGTGCTTTCGATGCTCTTTGTAGACAAATCAATGGGAAAATCATCAATTTCT
GAAGGCAGTAATTC
TCAAAATCTTCTGTTCTCTAAATATTGGGTGCTTTTCGATGCCCTATGTAGACAAATCAATGGGAAAATCATCAATTTC
TGAAGGCAGTAATTC
TCAAAATCTTCTGTTCTTTAAATATTGGGTGCTTTCGATGCTCTTTGTAGACAAATCAATGGGAAAATCATCAATTTCT
GAAGGCAGTAATTC
TCAAAATCTTCTGTTCTCTAAATATTGGGTGCTTTTCGATGCCCTATGTAGACAAATCAATGGGAAAATCATCAATTTC
TGAAGGCAGTAATTC
TCAAAATCTTCTGTTCTCTAAATATTGGGTGCTTTTCGATGCTCTTTGTAGACAAATCAATGGGAAAATGTCAATTTCT
GAAGGCAGTAATTT
TCAAAATCTTCTGTTCTCTAAATATTGGGTGCTTTTCGATGCCCTATGTAGACAAATCAATGGGAAAATCATCAATTTC
TGAAGGCAGTAATTC
TCAAAATCTTCTGTTCTCTAAATATTGGGTGCTTTTCGATGCCCTATGTAGACAAATCAATGGGAAAATCATCAATTTC
TGGAGGCAGTAATTC
TCAAAATCTTCTGTTCTCTAAATATTGGGTGCTTTTCGATGCCCTATGTAGACAAATCAATGGGAAAATCATCAATTTC
TGAAGGCAGTAATTT
TCAAAATCTTCTGTTCTCTAAATATTGGGTGCTTTTCGATGCCCTATGTAGACAAATCAATGGGAAAATCATCAATTTC
TGGAGGCAGTAATTC
TCAAAATCTTCTGTTCTCTAAATATTGGGTGCTTTTCGATGCCCTATGTAGACAAATCAATGGGAAAATCATCAATTTC
TGAAGGCAGTAATTC
TCAAAATCTTCTGTTCTCTAAATATTGGGTGCTTTTCGATGCCCTATGTAGACAAATCAATGGGAAAATCATCAATTTC
TGAAGGCAGTAATTT
TCAAAATCTTCTGTTCTCTAAATATTGGGTGCTTTTCGATGCTCTTTGTAGACAAATCAATGGGAAAATCATCAATTTC
TGAAGGCAGTAATTC

TCAAAATCTTCTGTTCTCTAAATATTGGGTGCTTTTCGATGCCCTATGTAGACAAATCAATGGGAAAATCATCAATTTC
TGAAGGCAGTAATTC
TCAAAATCTTCTGTTCTCTAAATATTGGGTGCTTTTCGATGCTCTTTGTAGACAAATCAATGGGAAAATTGTCAATTTC
TGAAGGCAGTAATTT
TCAAAATCTTCTGTTCTCTAAATATTGGGTGCTTTTCGATGCCCTATGTAGACAAATCAATGGGAAAATCATCAATTTC
TGAAGGCAGTAATTC
TCAAAATCTTCTGTTCTCTAAATATTGGGTGCTTTTCGATGCTCTTTGTAGACAAATCAATGGGAAAATCATCAATTTC
TGAAGGCAGTAATTC
TCAAAATCTTCTGTTCTCTAAATATTGGGTGCTTTTCGATGCTCTTTGAAGACAAATCAATGGGAAAATCATCAATTTC
TGGAGGCAGTAATTC
TCAAAATCTTCTGTTCTCTAAATATTGGGTGCTTTTCGATGCCCTATGTAGACAAATCAATGGGAAAATCATCAATTTC
TGAAGGCAGTAATTC
TCAAAATCTTCTGTTCTCTAAATATTGGGTGCTTTTCGATGCCCTATGTAGACAAATCAATGGGAAAATCATCAATTTC
TGGAGGCAGTAATTC
TCAAAATCTTCTGTTCTCTAAATATTGGGTGCTTTTCGATGCCCTATGTAGACAAATCAATGGGAAAATCATCAATTTC
TGAAGGCAGTAATTT
TCAAAATCTTCTGTTCTCTAAATATTGGGTGCTTTTCGATGCCCTATGTAGACAAATCAATGGGAAAATCATCAATTTC
TGAAGGCAGTAATTC
TCAAAATCTTCTGTTCTCTAAATATTGGGTGCTTTTCGATGCCCTATGTAGACAAATCAATGGGAAAATCGTCAATTTC
TGAAGGCAGTAATTC
TCAAAATCTTCTGTTCTCTAAATATTGGGTGCTTTTCGATGCCCTATGTAGACAAATCAATGGGAAAATCATCAATTTC
TGGAGGCAGTAATTC
TCAAAATCTTCTGTTCTCTAAATATTGGGTGCTTTTCGATGCCCTATGTAGACAAATCAATGGGAAAATCATCAATTTC
TGAAGGCAGTAATTT
TCAAAATCTTCTGTTCTCTAAATATTGGGTGCTTTTCGATGCCCTATGTAGACAAATCAATGGGAAAATCATCAATTTC
TGGAGGCAGTAATTC
TCAAAATCTTCTGTTCTCTAAATATTGGGTGCTTTTCGATGCCCTATGTAGACAAATCAATGGGAAAATCATCAATTTC
TGAAGGCAGTAATTC
TCAAAATCTTCTGTTCTCTAAATATTGGGTGCTTTTCGATGCCCTATGTAGACAAATCAATGGGAAAATCATCAATTTC
TGAAGGCAGTAATTT
TCAAAATCTTCTGTTCTCTAAATATTGGGTGCTTTTCGATGCTCTTTGTAGACAAATCAATGGGAAAATTGTCAATTTC
TGAAGGCAGTAATTC
TCAAAATCTTCTGTTCTCTAAATATTGGGTGCTTTTCGATGCTCTTTGTAGACAAATCAATGGGAAAATCATCAATTTC
TGAAGGCAGTAATTC
TCAAAATCTTCTGTTCTCTAAATATTGGGTGCTTTTCGATGCTCTTTGAAGACAAATCAATGGGAAAATTGTCAATTTC
TGAAGGCAGTAATTC
TCAAAATCTTCTGTTCTCTAAATATTGGGTGCTTTTCGATGCTCTTTGAAGACAAATCAATGGGAAAATCATCAATTTC
TGGAGGCAGTAATTC
TCAAAATCTTCTGTTCTCTAAATATTGGGTGCTTTTCGATGCCCTATGTAGACAAATCAATGGGAAAATCATCAATTTC
TGAAGGCAGTAATTC
TCAAAATCTTCTGTTCTCTAAATATTGGGTGCTTTTCGATGCCCTATGTAGACAAATCAATGGGAAAATCATCAATTTC
TGAAGGCAGTAATTC
TCAAAATCTTCTGTTCTCTAAATATTGGGTGCTTTTCGATGCCCTATGTAGACAAATCAATGGGAAAATCATCAATTTC
TGGAGGCAGTAATTC
TCAAAATCTTCTGTTCTCTAAATATTGGGTGCTTTTCGATGCCCTATGTAGACAAATCAATGGGAAAATCATCAATTTC
TGAAGGCAGTAATTT
TCAAAATCTTCTGTTCTCTAAATATTGGGTGCTTTTCGATGCCCTATGTAGACAAATCAATGGGAAAATCATCAATTTC
TGAAGGCAGTAATTC
TCAAAATCTTCTGTTCTCTAAATATTGGGTGCTTTTCGATGCCCTATGTAGACAAATCAATGGGAAAATCATCAATTTC
TGAAGGCAGTAATTC
TCAAAATCTTCTGTTCTCTAAATATTGGGTGCTTTTCGATGCCCTATGTAGACAAATCAATGGGAAAATCATCAATTTC
TGGAGGCAGTAATTC
TCAAAATCTTCTGTTCTCTAAATATTGGGTGCTTTTCGATGCCCTATGTAGACAAATCAATGGGAAAATCATCAATTTC
TGAAGGCAGTAATTT

TCAAAATCTTCTGTTCTCTAAATATTGGGTGCTTTTCGATGCCCTATGTAGACAAATCAATGGGAAAATCATCAATTTC
TGGAGGCAGTAATTC
TCAAAATCTTCTGTTCTCTAAATATTGGGTGCTTTTCGATGCCCTATGTAGACAAATCAATGGGAAAATCATCAATTTC
TGAAGGCAGTAATTT
TCAAAATCTTCTGTTCTCTAAATATTGGGTGCTTTTCGATGCCCTATGTAGACAAATCAATGGGAAAATCATCAATTTC
TGGAGGCAGTAATTC
TCAAAATCTTCTGTTCTCTAAATATTGGGTGCTTTTCGATGCCCTATGTAGACAAATCAATGGGAAAATCATCAATTTC
TGAAGGCAGTAATTT
TCAAAATCTTCTGTTCTCTAAATATTGGGTGCTTTTCGATGCTCTTTGTAGACAAATCAATGGGAAAATTGTCAATTTC
TGAAGGCAGTAATTC
TCAAAATCTTCTGTTCTCTAAATATTGGGTGCTTTTCGATGCTCTTTGTAGACAAATCAATGGGAAAATTGTCAATTTC
TGAAGGCAGTAATTC
TCAAAATCTTCTGTTCTCTAAATATTGGGTGCTTTTCGATGCTCTTTGTAGACAAATCAATGGGAAAATCATCAATTTC
TGGAGGCAGTAATTC
TCAAAATCTTCGGTTTTCTAAATATTGGGTGCTTTCGATGCTCTTTGTAGACAAATCAATAGGAAAATCATCAATTTCT
GAAGGCAGTAATTC
TCAAAATCTTCTGTTCTCTAAATATTGGGTGCTTTTCGATGCCCTTTGTAGACAAATCAATGGGAAAATCATCAATTTC
TGGAGGCAGTAATTC
TCAAAATCTTCTGTTCTCTAAATATTGGGTGCTTTCGATGCTCTTTGTAGACAAATCAATAGGAAAATCATCAATTTCT
GAAGGCAGTAATTC
TCAAAATCTTCTGTTCTCTAAATATTGGGTGCTTTTCGATGCCCTTTGTAGACAAATCAATGGGAAAATCATCAATTTC
TGGAGGCAGTAATTC
TCAAAATCTTCTGTTCTCTAAATATTGGGTGCTTTCGATGCTCTTTGTAGACAAATCAATGGGAAAATCATCAATTTCT
GAAGGCAGTAATTC
TCAAAATCTTCTGTTCTCTAAATATTGGGTGCTTTCGATGCTCTTTGTAGACAAATCAATGGGAAAATTGTCAATTTCT
GAAGGCAGTAATTC
TCAAAATCTTCTGTTCTCTAAATATTGGGTGCTTTCGATGCTCTTTGTAGACAAATCAATGGGAAAATTGTCAATTTCT
GAAGGCAGTAATTC
TCAAAATCTTCTGTTCTCTAAATATTGGGTGCTTTCGATGCTCTTTGTAGACAAATCAATGGGAAAATTGTCAATTTCT
GAAGGCAGTAATTC
TCAAAATCTTCTGTTCTCTAAATATTGGGTGCTTTTCGATGCCCTATGTAGACAAATCAATGGGAAAATCATCAATTTC
TGAAGGCAGTAATTC
TCAAAATCTTCTGTTCTCTAAATATTGGGTGCTTTTCGATGCTCTTTGTAGACAAATCAATGGGAAAATTGTCAATTTC
TGGAGGGAGTAATTC
TCAAAATCTTCTGTTCTCTAAATATTGGGTGCTTTTCGATGCCCTATGTAGACAAATCAATGGGAAAATCATCAATTTC
TGAAGGCAGTAATTC
TCAAAATCTTCTGTTCTCTAAATATTGGGTGCTTTTCGATGCTCTTTGTAGACAAATCAATGGGAAAATTGTCAATTTC
TGGAGGGAGTAATTC
Nr of selected repeats 130 Similarity 0.962485
Consensus:
TCAAAATCTTCTGTTCTCTAAATATTGGGTGCTTTTCGATGCcCTaTGTAGACAAATCAATGGGAAAATCATCAATTTC
TGAAGGCAGTAATTC
>Cele-UNSB01_1:4404167-4404863 Satlength=697 Nr of Repeats=4 RepeatLength=174
seed=CTTCAAGAGC
CTTCAAGAGCTCAGTGTTATTTCTGACATGCCAATCTCGACTCTCGACTTGAACATGTTACAAGGAATCGAAGACAACT
TAAACAGCCTCCCAGCTGAAGAATCTGATGAGATCCGAGAGAAGTTAAATGAACTGAGAAGAAGAAAGCAAGAAAGTGA
CCAAGCTGAAGCTCTA
CTTCAAGAGCTCAGTGTTATTTCTGACATGCCAATCTCGACTCTAGACCTGAACATGTTGCAAGGAGTTGAAGACAACT
TGAACAGTCTTCCAACTGAAGAAGCTGATAAGATCCGCGAGAAGATTAACGATCTGAGAAGAAGGAAACAAGAAAGCGA
TCTGGCTGAAGCTCTT
CTTCAAGAGCTCAGTGTTATTTATGACATGCCAACTTCTACAATTGACCTGAACATGCTCCAAGGAATCGAAGACAACT
TGAACAGCCTTCCAGCTGAAGAATCTGATAAGATCCGAGAGAAGATTAACGATCTGAGAAGAAGGAAACAAGAAAGCGA
TCTGGCTGAAGCTCTT

CTTCAAGAGCTCAGTGTTATTTATGACATGCCAACTTCTACAATTGACCTGAACATGCTCCAAGGAATCGAAGACAACT
TGAACAGCCTTCCAGCTGAAGAATCTGATAAGATCCGAGAGAAGATTAACGATCTGAGAAGAAGGAAACAAGAAAGCGA
TCAAGCTGAAGCTCTT
Nr of selected repeats 4 Similarity 0.868455
Consensus:
CTTCAAGAGCTCAGTGTTATTTaTGACATGCCAAccTCgACaaTtGACcTGAACATGcTcCAAGGAaTcGAAGACAACT
TgAACAGcCTtCCAgCTGAAGAAtCTGATaAGATCCGaGAGAAGaTtAAcGAtCTGAGAAGAAGgAAaCAAGAAAGcGA
tCaaGCTGAAGCTCTt
>Cele-UNSB01_1:4530676-4530829 Satlength=154 Nr of Repeats=8 RepeatLength=19
seed=TTTTTCGTGT
TTTTTCGTGTAACAATCTA
TTTTTCGTGTAACAATCTA
TTTTTCGTGTAACAATCTA
TTTTTCGTGTAACAATTAA
TTTTTCGTGTAACAATCAA
TTTTTCGTGTAACAATCTA
TTTTTCGTGTAACAATCTA
TTTTTCGTGTAACAATCTAT
Nr of selected repeats 7 Similarity 0.900000
Consensus:
TTTTTCGTGTAACAATCTA
>Cele-UNSB01_1:4605678-4635032 Satlength=29355 Nr of Repeats=1129
RepeatLength=26 seed=TTTTCTAAAA
TTTTCTAAAATCCGCCGCAGTGCCAT
TTTTCTAAAATCCGCCGCAATGCCAT
TTTTCTAAAATCCGCCGCAATGCCAT
TTTTCTAAAATCCGCCGCAATGCCAT
TTTTCTAAAATCCGCCGCAATGCCAT
TTTTCTAAAATCCGCCGCAATGCCAT
TTTTCTAAAATCCGCCGCAATGCCAT
TTTTCTAAAATCCGCCGCAATGCCAT
TTTTCTAAAATCCGCCGCAATGCCAT
TTTTCTAAAATCCGCCGCAATGCCAT
TTTTCTAAAATCCGCCGCAATGCCAT
TTTTCTAAAATCCGCCGCAATGCCAT
TTTTCTAAAATCCGCCGCAATGCCAT
TTTTCTAAAATCCGCCGCAATGCCAT
TTTTCTAAAATCCGCCGCAATGCCAT
TTTTCTAAAATCCGCCGCAATGCCAT
TTTTCTAAAATCCGCCGCAATGCCAT
TTTTCTAAAATCCGCCGCAATGCCAT
TTTTCTAAAATCCGCCGCAATGCCAT
TTTTCTAAAATCCGCCGCAATGCCAT
TTTTCTAAAATCCGCCGCAATGCCAT
TTTTCTAAAATCCGCCGCAATGCCAT
TTTTCTAAAATCCGCCGCAATGCCAT
TTTTCTAAAATCCGCCGCAATGCCAT
TTTTCTAAAATCCGCCGCAATGCCAT
TTTTCTAAAATCCGCCGCAATGCCAT
TTTTCTAAAATCCGCCGCAATGCCAT
TTTTCTAAAATCCGCCGCAATGCCAT
TTTTCTAAAATCCGCCGCAATGCCAT
TTTTCTAAAATCCGCCGCAATGCCAT
TTTTCTAAAATCCGCCGCAATGCCAT

TTTTCTAAAATCCGCCGCAATGCCAT
TTTTCTAAAATCCGCCGCAATGCCAT
TTTTCTAAAATCCGCCGCAATGCCAT
TTTTCTAAAATCCGCCGCAATGCCAT
TTTTCTAAAATCCGCCGCAATGCCAT
TTTTCTAAAATCCGCCGCAATGCCAT
TTTTCTAAAATCCGCCGCAATGCCAT
TTTTCTAAAATCCGCCGCAATGCCAT
TTTTCTAAAATCCGCCGCAATGCCAT
TTTTCTAAAATCCGCCGCAATGCCAT
TTTTCTAAAATCCGCCGCAATGCCAT
TTTTCTAAAATCCGCCGCAATGCCAT
TTTTCTAAAATCCGCCGCAATGCCAT
TTTTCTAAAATCCGCCGCAATGCCAT
TTTTCTAAAATCCGCCGCAATGCCAT
TTTTCTAAAATCCGCCGCAATGCCAT
TTTTCTAAAATCCGCCGCAATGCCAT
TTTTCTAAAATCCGCCGCAATGCCAT
TTTTCTAAAATCCGCCGCAATGCCAT
TTTTCTAAAATCCGCCGCAATGCCAT
TTTTCTAAAATCCGCCGCAATGCCAT
TTTTCTAAAATCCGCCGCAATGCCAT
TTTTCTAAAATCCGCCGCAATGCCAT
TTTTCTAAAATCCGCCGCAATGCCAT
TTTTCTAAAATCCGCCGCAATGCCAT
TTTTCTAAAATCCGCCGCAATGCCAT
TTTTCTAAAATCCGCCGCAATGCCAT
TTTTCTAAAATCCGCCGCAATGCCAT
TTTTCTAAAATCCGCCGCAATGCCAT
TTTTCTAAAATCCGCCGCAATGCCAT
TTTTCTAAAATCCGCCGCAATGCCAT
TTTTCTAAAATCCGCCGCAATGCCAT
TTTTCTAAAATCCGCCGCAATGCCAT
TTTTCTAAAATCCGCCGCAATGCCAT
TTTTCTAAAATCCGCCGCAATGCCAT
TTTTCTAAAATCCGCCGCAATGCCAT
TTTTCTAAAATCCGCCGCAATGCCAT
TTTTCTAAAATCCGCCGCAATGCCAT
TTTTCTAAAATCCGCCGCAATGCCAT
TTTTCTAAAATCCGCCGCAATGCCAT
TTTTCTAAAATCCGCCGCAATGCCAT
TTTTCTAAAATCCGCCGCAATGCCAT
TTTTCTAAAATCCGCCGCAATGCCAT
TTTTCTAAAATCCGCCGCAATGCCAT
TTTTCTAAAATCCGCCGCAATGCCAT
TTTTCTAAAATCCGCCGCAATGCCAT
TTTTCTAAAATCCGCCGCAATGCCAT
TTTTCTAAAATCCGCCGCAATGCCAT
TTTTCTAAAATCCGCCGCAATGCCAT
TTTTCTAAAATCCGCCGCAATGCCAT
TTTTCTAAAATCCGCCGCAATGCCAT
TTTTCTAAAATCCGCCGCAATGCCAT
TTTTCTAAAATCCGCCGCAATGCCAT
TTTTCTAAAATCCGCCGCAATGCCAT

TTTTCTAAAATCCGCCGCAATGCCAT
TTTTCTAAAATCCGCCGCAATGCCAT
TTTTCTAAAATCCGCCGCAATGCCAT
TTTTCTAAAATCCGCCGCAATGCCAT
TTTTCTAAAATCCGCCGCAATGCCAT
TTTTCTAAAATCCGCCGCAATGCCAT
TTTTCTAAAATCCGCCGCAATGCCAT
TTTTCTAAAATCCGCCGCAATGCCAT
TTTTCTAAAATCCGCCGCAATGCCAT
TTTTCTAAAATCCGCCGCAATGCCAT
TTTTCTAAAATCCGCCGCAATGCCAT
TTTTCTAAAATCCGCCGCAATGCCAT
TTTTCTAAAATCCGCCGCAATGCCAT
TTTTCTAAAATCCGCCGCAATGCCAT
TTTTCTAAAATCCGCCGCAATGCCAT
TTTTCTAAAATCCGCCGCAATGCCAT
TTTTCTAAAATCCGCCGCAATGCCAT
TTTTCTAAAATCCGCCGCAATGCCAT
TTTTCTAAAATCCGCCGCAATGCCAT
TTTTCTAAAATCCGCCGCAATGCCAT
TTTTCTAAAATCCGCCGCAATGCCAT
TTTTCTAAAATCCGCCGCAATGCCAT
TTTTCTAAAATCCGCCGCAATGCCAT
TTTTCTAAAATCCGCCGCAATGCCAT
TTTTCTAAAATCCGCCGCAATGCCAT
TTTTCTAAAATCCGCCGCAATGCCAT
TTTTCTAAAATCCGCCGCAATGCCAT
TTTTCTAAAATCCGCCGCAATGCCAT
TTTTCTAAAATCCGCCGCAATGCCAT
TTTTCTAAAATCCGCCGCAATGCCAT
TTTTCTAAAATCCGCCGCAATGCCAT
TTTTCTAAAATCCGCCGCAATGCCAT
TTTTCTAAAATCCGCCGCAATGCCAT
TTTTCTAAAATCCGCCGCAATGCCAT
TTTTCTAAAATCCGCCGCAATGCCAT
TTTTCTAAAATCCGCCGCAATGCCAT
TTTTCTAAAATCCGCCGCAATGCCAT
TTTTCTAAAATCCGCCGCAATGCCAT
TTTTCTAAAATCCGCCGCAATGCCAT
TTTTCTAAAATCCGCCGCAATGCCAT
TTTTCTAAAATCCGCCGCAATGCCAT
TTTTCTAAAATCCGCCGCAATGCCAT
TTTTCTAAAATCCGCCGCAATGCCAT
TTTTCTAAAATCCGCCGCAATGCCAT
TTTTCTAAAATCCGCCGCAATGCCAT
TTTTCTAAAATCCGCCGCAATGCCAT
TTTTCTAAAATCCGCCGCAATGCCAT
TTTTCTAAAATCCGCCGCAATGCCAT
TTTTCTAAAATCCGCCGCAATGCCAT
TTTTCTAAAATCCGCCGCAATGCCAT
TTTTCTAAAATCCGCCGCAATGCCAT
TTTTCTAAAATCCGCCGCAATGCCAT
TTTTCTAAAATCCGCCGCAATGCCAT
TTTTCTAAAATCCGCCGCAATGCCAT

TTTTCTAAAATCCGCCGCAATGCCAT
TTTTCTAAAATCCGCCGCAATGCCAT
TTTTCTAAAATCCGCCGCAATGCCAT
TTTTCTAAAATCCGCCGCAATGCCAT
TTTTCTAAAATCCGCCGCAATGCCAT
TTTTCTAAAATCCGCCGCAATGCCAT
TTTTCTAAAATCCGCCGCAATGCCAT
TTTTCTAAAATCCGCCGCAATGCCAT
TTTTCTAAAATCCGCCGCAATGCCAT
TTTTCTAAAATCCGCCGCAATGCCAT
TTTTCTAAAATCCGCCGCAATGCCAT
TTTTCTAAAATCCGCCGCAATGCCAT
TTTTCTAAAATCCGCCGCAATGCCAT
TTTTCTAAAATCCGCCGCAATGCCAT
TTTTCTAAAATCCGCCGCAATGCCAT
TTTTCTAAAATCCGCCGCAATGCCAT
TTTTCTAAAATCCGCCGCAATGCCAT
TTTTCTAAAATCCGCCGCAATGCCAT
TTTTCTAAAATCCGCCGCAATGCCAT
TTTTCTAAAATCCGCCGCAATGCCAT
TTTTCTAAAATCCGCCGCAATGCCAT
TTTTCTAAAATCCGCCGCAATGCCAT
TTTTCTAAAATCCGCCGCAATGCCAT
TTTTCTAAAATCCGCCGCAATGCCAT
TTTTCTAAAATCCGCCGCAATGCCAT
TTTTCTAAAATCCGCCGCAATGCCAT
TTTTCTAAAATCCGCCGCAATGCCAT
TTTTCTAAAATCCGCCGCAATGCCAT
TTTTCTAAAATCCGCCGCAATGCCAT
TTTTCTAAAATCCGCCGCAATGCCAT
TTTTCTAAAATCCGCCGCAATGCCAT
TTTTCTAAAATCCGCCGCAATGCCAT
TTTTCTAAAATCCGCCGCAATGCCAT
TTTTCTAAAATCCGCCGCAATGCCAT
TTTTCTAAAATCCGCCGCAATGCCAT
TTTTCTAAAATCCGCCGCAATGCCAT
TTTTCTAAAATCCGCCGCAATGCCAT
TTTTCTAAAATCCGCCGCAATGCCAT
TTTTCTAAAATCCGCCGCAATGCCAT
TTTTCTAAAATCCGCCGCAATGCCAT
TTTTCTAAAATCCGCCGCAATGCCAT
TTTTCTAAAATCCGCCGCAATGCCAT
TTTTCTAAAATCCGCCGCAATGCCAT
TTTTCTAAAATCCGCCGCAATGCCAT
TTTTCTAAAATCCGCCGCAATGCCAT
TTTTCTAAAATCCGCCGCAATGCCAT
TTTTCTAAAATCCGCCGCAATGCCAT
TTTTCTAAAATCCGCCGCAATGCCAT
TTTTCTAAAATCCGCCGCAATGCCAT
TTTTCTAAAATCCGCCGCAATGCCAT
TTTTCTAAAATCCGCCGCAATGCCAT
TTTTCTAAAATCCGCCGCAATGCCAT
TTTTCTAAAATCCGCCGCAATGCCAT
TTTTCTAAAATCCGCCGCAATGCCAT

TTTTCTAAAATCCGCCGCAATGCCAT
TTTTCTAAAATCCGCCGCAATGCCAT
TTTTCTAAAATCCGCCGCAATGCCAT
TTTTCTAAAATCCGCCGCAATGCCAT
TTTTCTAAAATCCGCCGCAATGCCAT
TTTTCTAAAATCCGCCGCAATGCCAT
TTTTCTAAAATCCGCCGCAATGCCAT
TTTTCTAAAATCCGCCGCAATGCCAT
TTTTCTAAAATCCGCCGCAATGCCAT
TTTTCTAAAATCCGCCGCAATGCCAT
TTTTCTAAAATCCGCCGCAATGCCAT
TTTTCTAAAATCCGCCGCAATGCCAT
TTTTCTAAAATCCGCCGCAATGCCAT
TTTTCTAAAATCCGCCGCAATGCCAT
TTTTCTAAAATCCGCCGCAATGCCAT
TTTTCTAAAATCCGCCGCAATGCCAT
TTTTCTAAAATCCGCCGCAATGCCAT
TTTTCTAAAATCCGCCGCAATGCCAT
TTTTCTAAAATCCGCCGCAATGCCAT
TTTTCTAAAATCCGCCGCAATGCCAT
TTTTCTAAAATCCGCCGCAATGCCAT
TTTTCTAAAATCCGCCGCAATGCCAT
TTTTCTAAAATCCGCCGCAATGCCAT
TTTTCTAAAATCCGCCGCAATGCCAT
TTTTCTAAAATCCGCCGCAATGCCAT
TTTTCTAAAATCCGCCGCAATGCCAT
TTTTCTAAAATCCGCCGCAATGCCAT
TTTTCTAAAATCCGCCGCAATGCCAT
TTTTCTAAAATCCGCCGCAATGCCAT
TTTTCTAAAATCCGCCGCAATGCCAT
TTTTCTAAAATCCGCCGCAATGCCAT
TTTTCTAAAATCCGCCGCAATGCCAT
TTTTCTAAAATCCGCCGCAATGCCAT
TTTTCTAAAATCCGCCGCAATGCCAT
TTTTCTAAAATCCGCCGCAATGCCAT
TTTTCTAAAATCCGCCGCAATGCCAT
TTTTCTAAAATCCGCCGCAATGCCAT
TTTTCTAAAATCCGCCGCAATGCCAT
TTTTCTAAAATCCGCCGCAATGCCAT
TTTTCTAAAATCCGCCGCAATGCCAT
TTTTCTAAAATCCGCCGCAATGCCAT
TTTTCTAAAATCCGCCGCAATGCCAT
TTTTCTAAAATCCGCCGCAATGCCAT
TTTTCTAAAATCCGCCGCAATGCCAT
TTTTCTAAAATCCGCCGCAATGCCAT
TTTTCTAAAATCCGCCGCAATGCCAT
TTTTCTAAAATCCGCCGCAATGCCAT
TTTTCTAAAATCCGCCGCAATGCCAT
TTTTCTAAAATCCGCCGCAATGCCAT
TTTTCTAAAATCCGCCGCAATGCCAT
TTTTCTAAAATCCGCCGCAATGCCAT
TTTTCTAAAATCCGCCGCAATGCCAT
TTTTCTAAAATCCGCCGCAATGCCAT
TTTTCTAAAATCCGCCGCAATGCCAT

TTTTCTAAAATCCGCCGCAATGCCAT
TTTTCTAAAATCCGCCGCAATGCCAT
TTTTCTAAAATCCGCCGCAATGCCAT
TTTTCTAAAATCCGCCGCAATGCCAT
TTTTCTAAAATCCGCCGCAATGCCAT
TTTTCTAAAATCCGCCGCAATGCCAT
TTTTCTAAAATCCGCCGCAATGCCAT
TTTTCTAAAATCCGCCGCAATGCCAT
TTTTCTAAAATCCGCCGCAATGCCAT
TTTTCTAAAATCCGCCGCAATGCCAT
TTTTCTAAAATCCGCCGCAATGCCAT
TTTTCTAAAATCCGCCGCAATGCCAT
TTTTCTAAAATCCGCCGCAATGCCAT
TTTTCTAAAATCCGCCGCAATGCCAT
TTTTCTAAAATCCGCCGCAATGCCAT
TTTTCTAAAATCCGCCGCAATGCCAT
TTTTCTAAAATCCGCCGCAATGCCAT
TTTTCTAAAATCCGCCGCAATGCCAT
TTTTCTAAAATCCGCCGCAATGCCAT
TTTTCTAAAATCCGCCGCAATGCCAT
TTTTCTAAAATCCGCCGCAATGCCAT
TTTTCTAAAATCCGCCGCAATGCCAT
TTTTCTAAAATCCGCCGCAATGCCAT
TTTTCTAAAATCCGCCGCAATGCCAT
TTTTCTAAAATCCGCCGCAATGCCAT
TTTTCTAAAATCCGCCGCAATGCCAT
TTTTCTAAAATCCGCCGCAATGCCAT
TTTTCTAAAATCCGCCGCAATGCCAT
TTTTCTAAAATCCGCCGCAATGCCAT
TTTTCTAAAATCCGCCGCAATGCCAT
TTTTCTAAAATCCGCCGCAATGCCAT
TTTTCTAAAATCCGCCGCAATGCCAT
TTTTCTAAAATCCGCCGCAATGCCAT
TTTTCTAAAATCCGCCGCAATGCCAT
TTTTCTAAAATCCGCCGCAATGCCAT
TTTTCTAAAATCCGCCGCAATGCCAT
TTTTCTAAAATCCGCCGCAATGCCAT
TTTTCTAAAATCCGCCGCAATGCCAT
TTTTCTAAAATCCGCCGCAATGCCAT
TTTTCTAAAATCCGCCGCAATGCCAT
TTTTCTAAAATCCGCCGCAATGCCAT
TTTTCTAAAATCCGCCGCAATGCCAT
TTTTCTAAAATCCGCCGCAATGCCAT
TTTTCTAAAATCCGCCGCAATGCCAT
TTTTCTAAAATCCGCCGCAATGCCAT
TTTTCTAAAATCCGCCGCAATGCCAT
TTTTCTAAAATCCGCCGCAATGCCAT
TTTTCTAAAATCCGCCGCAATGCCAT
TTTTCTAAAATCCGCCGCAATGCCAT
TTTTCTAAAATCCGCCGCAATGCCAT
TTTTCTAAAATCCGCCGCAATGCCAT
TTTTCTAAAATCCGCCGCAATGCCAT
TTTTCTAAAATCCGCCGCAATGCCAT
TTTTCTAAAATCCGCCGCAATGCCAT

TTTTCTAAAATCCGCCGCAATGCCAT
TTTTCTAAAATCCGCCGCAATGCCAT
TTTTCTAAAATCCGCCGCAATGCCAT
TTTTCTAAAATCCGCCGCAATGCCAT
TTTTCTAAAATCCGCCGCAATGCCAT
TTTTCTAAAATCCGCCGCAATGCCAT
TTTTCTAAAATCCGCCGCAATGCCAT
TTTTCTAAAATCCGCCGCAATGCCAT
TTTTCTAAAATCCGCCGCAATGCCAT
TTTTCTAAAATCCGCCGCAATGCCAT
TTTTCTAAAATCCGCCGCAATGCCAT
TTTTCTAAAATCCGCCGCAATGCCAT
TTTTCTAAAATCCGCCGCAATGCCAT
TTTTCTAAAATCCGCCGCAATGCCAT
TTTTCTAAAATCCGCCGCAATGCCAT
TTTTCTAAAATCCGCCGCAATGCCAT
TTTTCTAAAATCCGCCGCAATGCCAT
TTTTCTAAAATCCGCCGCAATGCCAT
TTTTCTAAAATCCGCCGCAATGCCAT
TTTTCTAAAATCCGCCGCAATGCCAT
TTTTCTAAAATCCGCCGCAATGCCAT
TTTTCTAAAATCCGCCGCAATGCCAT
TTTTCTAAAATCCGCCGCAATGCCAT
TTTTCTAAAATCCGCCGCAATGCCAT
TTTTCTAAAATCCGCCGCAATGCCAT
TTTTCTAAAATCCGCCGCAATGCCAT
TTTTCTAAAATCCGCCGCAATGCCAT
TTTTCTAAAATCCGCCGCAATGCCAT
TTTTCTAAAATCCGCCGCAATGCCAT
TTTTCTAAAATCCGCCGCAATGCCAT
TTTTCTAAAATCCGCCGCAATGCCAT
TTTTCTAAAATCCGCCGCAATGCCAT
TTTTCTAAAATCCGCCGCAATGCCAT
TTTTCTAAAATCCGCCGCAATGCCAT
TTTTCTAAAATCCGCCGCAATGCCAT
TTTTCTAAAATCCGCCGCAATGCCAT
TTTTCTAAAATCCGCCGCAATGCCAT
TTTTCTAAAATCCGCCGCAATGCCAT
TTTTCTAAAATCCGCCGCAATGCCAT
TTTTCTAAAATCCGCCGCAATGCCAT
TTTTCTAAAATCCGCCGCAATGCCAT
TTTTCTAAAATCCGCCGCAATGCCAT
TTTTCTAAAATCCGCCGCAATGCCAT
TTTTCTAAAATCCGCCGCAATGCCAT
TTTTCTAAAATCCGCCGCAATGCCAT
TTTTCTAAAATCCGCCGCAATGCCAT
TTTTCTAAAATCCGCCGCAATGCCAT
TTTTCTAAAATCCGCCGCAATGCCAT
TTTTCTAAAATCCGCCGCAATGCCAT
TTTTCTAAAATCCGCCGCAATGCCAT
TTTTCTAAAATCCGCCGCAATGCCAT
TTTTCTAAAATCCGCCGCAATGCCAT
TTTTCTAAAATCCGCCGCAATGCCAT
TTTTCTAAAATCCGCCGCAATGCCAT

TTTTCTAAAATCCGCCGCAATGCCAT
TTTTCTAAAATCCGCCGCAATGCCAT
TTTTCTAAAATCCGCCGCAATGCCAT
TTTTCTAAAATCCGCCGCAATGCCAT
TTTTCTAAAATCCGCCGCAATGCCAT
TTTTCTAAAATCCGCCGCAATGCCAT
TTTTCTAAAATCCGCCGCAATGCCAT
TTTTCTAAAATCCGCCGCAATGCCAT
TTTTCTAAAATCCGCCGCAATGCCAT
TTTTCTAAAATCCGCCGCAATGCCAT
TTTTCTAAAATCCGCCGCAATGCCAT
TTTTCTAAAATCCGCCGCAATGCCAT
TTTTCTAAAATCCGCCGCAATGCCAT
TTTTCTAAAATCCGCCGCAATGCCAT
TTTTCTAAAATCCGCCGCAATGCCAT
TTTTCTAAAATCCGCCGCAATGCCAT
TTTTCTAAAATCCGCCGCAATGCCAT
TTTTCTAAAATCCGCCGCAATGCCAT
TTTTCTAAAATCCGCCGCAATGCCAT
TTTTCTAAAATCCGCCGCAATGCCAT
TTTTCTAAAATCCGCCGCAATGCCAT
TTTTCTAAAATCCGCCGCAATGCCAT
TTTTCTAAAATCCGCCGCAATGCCAT
TTTTCTAAAATCCGCCGCAATGCCAT
TTTTCTAAAATCCGCCGCAATGCCAT
TTTTCTAAAATCCGCCGCAATGCCAT
TTTTCTAAAATCCGCCGCAATGCCAT
TTTTCTAAAATCCGCCGCAATGCCAT
TTTTCTAAAATCCGCCGCAATGCCAT
TTTTCTAAAATCCGCCGCAATGCCAT
TTTTCTAAAATCCGCCGCAATGCCAT
TTTTCTAAAATCCGCCGCAATGCCAT
TTTTCTAAAATCCGCCGCAATGCCAT
TTTTCTAAAATCCGCCGCAATGCCAT
TTTTCTAAAATCCGCCGCAATGCCAT
TTTTCTAAAATCCGCCGCAATGCCAT
TTTTCTAAAATCCGCCGCAATGCCAT
TTTTCTAAAATCCGCCGCAATGCCAT
TTTTCTAAAATCCGCCGCAATGCCAT
TTTTCTAAAATCCGCCGCAATGCCAT
TTTTCTAAAATCCGCCGCAATGCCAT
TTTTCTAAAATCCGCCGCAATGCCAT
TTTTCTAAAATCCGCCGCAATGCCAT
TTTTCTAAAATCCGCCGCAATGCCAT
TTTTCTAAAATCCGCCGCAATGCCAT
TTTTCTAAAATCCGCCGCAATGCCAT
TTTTCTAAAATCCGCCGCAATGCCAT
TTTTCTAAAATCCGCCGCAATGCCAT
TTTTCTAAAATCCGCCGCAATGCCAT
TTTTCTAAAATCCGCCGCAATGCCAT
TTTTCTAAAATCCGCCGCAATGCCAT
TTTTCTAAAATCCGCCGCAATGCCAT
TTTTCTAAAATCCGCCGCAATGCCAT
TTTTCTAAAATCCGCCGCAATGCCAT

TTTTCTAAAATCCGCCGCAATGCCAT
TTTTCTAAAATCCGCCGCAATGCCAT
TTTTCTAAAATCCGCCGCAATGCCAT
TTTTCTAAAATCCGCCGCAATGCCAT
TTTTCTAAAATCCGCCGCAATGCCAT
TTTTCTAAAATCCGCCGCAATGCCAT
TTTTCTAAAATCCGCCGCAATGCCAT
TTTTCTAAAATCCGCCGCAATGCCAT
TTTTCTAAAATCCGCCGCAATGCCAT
TTTTCTAAAATCCGCCGCAATGCCAT
TTTTCTAAAATCCGCCGCAATGCCAT
TTTTCTAAAATCCGCCGCAATGCCAT
TTTTCTAAAATCCGCCGCAATGCCAT
TTTTCTAAAATCCGCCGCAATGCCAT
TTTTCTAAAATCCGCCGCAATGCCAT
TTTTCTAAAATCCGCCGCAATGCCAT
TTTTCTAAAATCCGCCGCAATGCCAT
TTTTCTAAAATCCGCCGCAATGCCAT
TTTTCTAAAATCCGCCGCAATGCCAT
TTTTCTAAAATCCGCCGCAATGCCAT
TTTTCTAAAATCCGCCGCAATGCCAT
TTTTCTAAAATCCGCCGCAATGCCAT
TTTTCTAAAATCCGCCGCAATGCCAT
TTTTCTAAAATCCGCCGCAATGCCAT
TTTTCTAAAATCCGCCGCAATGCCAT
TTTTCTAAAATCCGCCGCAATGCCAT
TTTTCTAAAATCCGCCGCAATGCCAT
TTTTCTAAAATCCGCCGCAATGCCAT
TTTTCTAAAATCCGCCGCAATGCCAT
TTTTCTAAAATCCGCCGCAATGCCAT
TTTTCTAAAATCCGCCGCAATGCCAT
TTTTCTAAAATCCGCCGCAATGCCAT
TTTTCTAAAATCCGCCGCAATGCCAT
TTTTCTAAAATCCGCCGCAATGCCAT
TTTTCTAAAATCCGCCGCAATGCCAT
TTTTCTAAAATCCGCCGCAATGCCAT
TTTTCTAAAATCCGCCGCAATGCCAT
TTTTCTAAAATCCGCCGCAATGCCAT
TTTTCTAAAATCCGCCGCAATGCCAT
TTTTCTAAAATCCGCCGCAATGCCAT
TTTTCTAAAATCCGCCGCAATGCCAT
TTTTCTAAAATCCGCCGCAATGCCAT
TTTTCTAAAATCCGCCGCAATGCCAT
TTTTCTAAAATCCGCCGCAATGCCAT
TTTTCTAAAATCCGCCGCAATGCCAT
TTTTCTAAAATCCGCCGCAATGCCAT
TTTTCTAAAATCCGCCGCAATGCCAT
TTTTCTAAAATCCGCCGCAATGCCAT
TTTTCTAAAATCCGCCGCAATGCCAT
TTTTCTAAAATCCGCCGCAATGCCAT
TTTTCTAAAATCCGCCGCAATGCCAT
TTTTCTAAAATCCGCCGCAATGCCAT
TTTTCTAAAATCCGCCGCAATGCCAT
TTTTCTAAAATCCGCCGCAATGCCAT

TTTTCTAAAATCCGCCGCAATGCCAT
TTTTCTAAAATCCGCCGCAATGCCAT
TTTTCTAAAATCCGCCGCAATGCCAT
TTTTCTAAAATCCGCCGCAATGCCAT
TTTTCTAAAATCCGCCGCAATGCCAT
TTTTCTAAAATCCGCCGCAATGCCAT
TTTTCTAAAATCCGCCGCAATGCCAT
TTTTCTAAAATCCGCCGCAATGCCAT
TTTTCTAAAATCCGCCGCAATGCCAT
TTTTCTAAAATCCGCCGCAATGCCAT
TTTTCTAAAATCCGCCGCAATGCCAT
TTTTCTAAAATCCGCCGCAATGCCAT
TTTTCTAAAATCCGCCGCAATGCCAT
TTTTCTAAAATCCGCCGCAATGCCAT
TTTTCTAAAATCCGCCGCAATGCCAT
TTTTCTAAAATCCGCCGCAATGCCAT
TTTTCTAAAATCCGCCGCAATGCCAT
TTTTCTAAAATCCGCCGCAATGCCAT
TTTTCTAAAATCCGCCGCAATGCCAT
TTTTCTAAAATCCGCCGCAATGCCAT
TTTTCTAAAATCCGCCGCAATGCCAT
TTTTCTAAAATCCGCCGCAATGCCAT
TTTTCTAAAATCCGCCGCAATGCCAT
TTTTCTAAAATCCGCCGCAATGCCAT
TTTTCTAAAATCCGCCGCAATGCCAT
TTTTCTAAAATCCGCCGCAATGCCAT
TTTTCTAAAATCCGCCGCAATGCCAT
TTTTCTAAAATCCGCCGCAATGCCAT
TTTTCTAAAATCCGCCGCAATGCCAT
TTTTCTAAAATCCGCCGCAATGCCAT
TTTTCTAAAATCCGCCGCAATGCCAT
TTTTCTAAAATCCGCCGCAATGCCAT
TTTTCTAAAATCCGCCGCAATGCCAT
TTTTCTAAAATCCGCCGCAATGCCAT
TTTTCTAAAATCCGCCGCAATGCCAT
TTTTCTAAAATCCGCCGCAATGCCAT
TTTTCTAAAATCCGCCGCAATGCCAT
TTTTCTAAAATCCGCCGCAATGCCAT
TTTTCTAAAATCCGCCGCAATGCCAT
TTTTCTAAAATCCGCCGCAATGCCAT
TTTTCTAAAATCCGCCGCAATGCCAT
TTTTCTAAAATCCGCCGCAATGCCAT
TTTTCTAAAATCCGCCGCAATGCCAT
TTTTCTAAAATCCGCCGCAATGCCAT
TTTTCTAAAATCCGCCGCAATGCCAT
TTTTCTAAAATCCGCCGCAATGCCAT
TTTTCTAAAATCCGCCGCAATGCCAT
TTTTCTAAAATCCGCCGCAATGCCAT
TTTTCTAAAATCCGCCGCAATGCCAT
TTTTCTAAAATCCGCCGCAATGCCAT
TTTTCTAAAATCCGCCGCAATGCCAT
TTTTCTAAAATCCGCCGCAATGCCAT
TTTTCTAAAATCCGCCGCAATGCCAT
TTTTCTAAAATCCGCCGCAATGCCAT

TTTTCTAAAATCCGCCGCAATGCCAT
TTTTCTAAAATCCGCCGCAATGCCAT
TTTTCTAAAATCCGCCGCAATGCCAT
TTTTCTAAAATCCGCCGCAATGCCAT
TTTTCTAAAATCCGCCGCAATGCCAT
TTTTCTAAAATCCGCCGCAATGCCAT
TTTTCTAAAATCCGCCGCAATGCCAT
TTTTCTAAAATCCGCCGCAATGCCAT
TTTTCTAAAATCCGCCGCAATGCCAT
TTTTCTAAAATCCGCCGCAATGCCAT
TTTTCTAAAATCCGCCGCAATGCCAT
TTTTCTAAAATCCGCCGCAATGCCAT
TTTTCTAAAATCCGCCGCAATGCCAT
TTTTCTAAAATCCGCCGCAATGCCAT
TTTTCTAAAATCCGCCGCAATGCCAT
TTTTCTAAAATCCGCCGCAATGCCAT
TTTTCTAAAATCCGCCGCAATGCCAT
TTTTCTAAAATCCGCCGCAATGCCAT
TTTTCTAAAATCCGCCGCAATGCCAT
TTTTCTAAAATCCGCCGCAATGCCAT
TTTTCTAAAATCCGCCGCAATGCCAT
TTTTCTAAAATCCGCCGCAATGCCAT
TTTTCTAAAATCCGCCGCAATGCCAT
TTTTCTAAAATCCGCCGCAATGCCAT
TTTTCTAAAATCCGCCGCAATGCCAT
TTTTCTAAAATCCGCCGCAATGCCAT
TTTTCTAAAATCCGCCGCAATGCCAT
TTTTCTAAAATCCGCCGCAATGCCAT
TTTTCTAAAATCCGCCGCAATGCCAT
TTTTCTAAAATCCGCCGCAATGCCAT
TTTTCTAAAATCCGCCGCAATGCCAT
TTTTCTAAAATCCGCCGCAATGCCAT
TTTTCTAAAATCCGCCGCAATGCCAT
TTTTCTAAAATCCGCCGCAATGCCAT
TTTTCTAAAATCCGCCGCAATGCCAT
TTTTCTAAAATCCGCCGCAATGCCAT
TTTTCTAAAATCCGCCGCAATGCCAT
TTTTCTAAAATCCGCCGCAATGCCAT
TTTTCTAAAATCCGCCGCAATGCCAT
TTTTCTAAAATCCGCCGCAATGCCAT
TTTTCTAAAATCCGCCGCAATGCCAT
TTTTCTAAAATCCGCCGCAATGCCAT
TTTTCTAAAATCCGCCGCAATGCCAT
TTTTCTAAAATCCGCCGCAATGCCAT
TTTTCTAAAATCCGCCGCAATGCCAT
TTTTCTAAAATCCGCCGCAATGCCAT
TTTTCTAAAATCCGCCGCAATGCCAT
TTTTCTAAAATCCGCCGCAATGCCAT
TTTTCTAAAATCCGCCGCAATGCCAT
TTTTCTAAAATCCGCCGCAATGCCAT
TTTTCTAAAATCCGCCGCAATGCCAT
TTTTCTAAAATCCGCCGCAATGCCAT
TTTTCTAAAATCCGCCGCAATGCCAT
TTTTCTAAAATCCGCCGCAATGCCAT

TTTTCTAAAATCCGCCGCAATGCCAT
TTTTCTAAAATCCGCCGCAATGCCAT
TTTTCTAAAATCCGCCGCAATGCCAT
TTTTCTAAAATCCGCCGCAATGCCAT
TTTTCTAAAATCCGCCGCAATGCCAT
TTTTCTAAAATCCGCCGCAATGCCAT
TTTTCTAAAATCCGCCGCAATGCCAT
TTTTCTAAAATCCGCCGCAATGCCAT
TTTTCTAAAATCCGCCGCAATGCCAT
TTTTCTAAAATCCGCCGCAATGCCAT
TTTTCTAAAATCCGCCGCAATGCCAT
TTTTCTAAAATCCGCCGCAATGCCAT
TTTTCTAAAATCCGCCGCAATGCCAT
TTTTCTAAAATCCGCCGCAATGCCAT
TTTTCTAAAATCCGCCGCAATGCCAT
TTTTCTAAAATCCGCCGCAATGCCAT
TTTTCTAAAATCCGCCGCAATGCCAT
TTTTCTAAAATCCGCCGCAATGCCAT
TTTTCTAAAATCCGCCGCAATGCCAT
TTTTCTAAAATCCGCCGCAATGCCAT
TTTTCTAAAATCCGCCGCAATGCCAT
TTTTCTAAAATCCGCCGCAATGCCAT
TTTTCTAAAATCCGCCGCAATGCCAT
TTTTCTAAAATCCGCCGCAATGCCAT
TTTTCTAAAATCCGCCGCAATGCCAT
TTTTCTAAAATCCGCCGCAATGCCAT
TTTTCTAAAATCCGCCGCAATGCCAT
TTTTCTAAAATCCGCCGCAATGCCAT
TTTTCTAAAATCCGCCGCAATGCCAT
TTTTCTAAAATCCGCCGCAATGCCAT
TTTTCTAAAATCCGCCGCAATGCCAT
TTTTCTAAAATCCGCCGCAATGCCAT
TTTTCTAAAATCCGCCGCAATGCCAT
TTTTCTAAAATCCGCCGCAATGCCAT
TTTTCTAAAATCCGCCGCAATGCCAT
TTTTCTAAAATCCGCCGCAATGCCAT
TTTTCTAAAATCCGCCGCAATGCCAT
TTTTCTAAAATCCGCCGCAATGCCAT
TTTTCTAAAATCCGCCGCAATGCCAT
TTTTCTAAAATCCGCCGCAATGCCAT
TTTTCTAAAATCCGCCGCAATGCCAT
TTTTCTAAAATCCGCCGCAATGCCAT
TTTTCTAAAATCCGCCGCAATGCCAT
TTTTCTAAAATCCGCCGCAATGCCAT
TTTTCTAAAATCCGCCGCAATGCCAT
TTTTCTAAAATCCGCCGCAATGCCAT
TTTTCTAAAATCCGCCGCAATGCCAT
TTTTCTAAAATCCGCCGCAATGCCAT
TTTTCTAAAATCCGCCGCAATGCCAT
TTTTCTAAAATCCGCCGCAATGCCAT
TTTTCTAAAATCCGCCGCAATGCCAT
TTTTCTAAAATCCGCCGCAATGCCAT
TTTTCTAAAATCCGCCGCAATGCCAT
TTTTCTAAAATCCGCCGCAATGCCAT

TTTTCTAAAATCCGCCGCAATGCCAT
TTTTCTAAAATCCGCCGCAATGCCAT
TTTTCTAAAATCCGCCGCAATGCCAT
TTTTCTAAAATCCGCCGCAATGCCAT
TTTTCTAAAATCCGCCGCAATGCCAT
TTTTCTAAAATCCGCCGCAATGCCAT
TTTTCTAAAATCCGCCGCAATGCCAT
TTTTCTAAAATCCGCCGCAATGCCAT
TTTTCTAAAATCCGCCGCAATGCCAT
TTTTCTAAAATCCGCCGCAATGCCAT
TTTTCTAAAATCCGCCGCAATGCCAT
TTTTCTAAAATCCGCCGCAATGCCAT
TTTTCTAAAATCCGCCGCAATGCCAT
TTTTCTAAAATCCGCCGCAATGCCAT
TTTTCTAAAATCCGCCGCAATGCCAT
TTTTCTAAAATCCGCCGCAATGCCAT
TTTTCTAAAATCCGCCGCAATGCCAT
TTTTCTAAAATCCGCCGCAATGCCAT
TTTTCTAAAATCCGCCGCAATGCCAT
TTTTCTAAAATCCGCCGCAATGCCAT
TTTTCTAAAATCCGCCGCAATGCCAT
TTTTCTAAAATCCGCCGCAATGCCAT
TTTTCTAAAATCCGCCGCAATGCCAT
TTTTCTAAAATCCGCCGCAATGCCAT
TTTTCTAAAATCCGCCGCAATGCCAT
TTTTCTAAAATCCGCCGCAATGCCAT
TTTTCTAAAATCCGCCGCAATGCCAT
TTTTCTAAAATCCGCCGCAATGCCAT
TTTTCTAAAATCCGCCGCAATGCCAT
TTTTCTAAAATCCGCCGCAATGCCAT
TTTTCTAAAATCCGCCGCAATGCCAT
TTTTCTAAAATCCGCCGCAATGCCAT
TTTTCTAAAATCCGCCGCAATGCCAT
TTTTCTAAAATCCGCCGCAATGCCAT
TTTTCTAAAATCCGCCGCAATGCCAT
TTTTCTAAAATCCGCCGCAATGCCAT
TTTTCTAAAATCCGCCGCAATGCCAT
TTTTCTAAAATCCGCCGCAATGCCAT
TTTTCTAAAATCCGCCGCAATGCCAT
TTTTCTAAAATCCGCCGCAATGCCAT
TTTTCTAAAATCCGCCGCAATGCCAT
TTTTCTAAAATCCGCCGCAATGCCAT
TTTTCTAAAATCCGCCGCAATGCCAT
TTTTCTAAAATCCGCCGCAATGCCAT
TTTTCTAAAATCCGCCGCAATGCCAT
TTTTCTAAAATCCGCCGCAATGCCAT
TTTTCTAAAATCCGCCGCAATGCCAT
TTTTCTAAAATCCGCCGCAATGCCAT
TTTTCTAAAATCCGCCGCAATGCCAT
TTTTCTAAAATCCGCCGCAATGCCAT
TTTTCTAAAATCCGCCGCAATGCCAT
TTTTCTAAAATCCGCCGCAATGCCAT
TTTTCTAAAATCCGCCGCAATGCCAT
TTTTCTAAAATCCGCCGCAATGCCAT

TTTTCTAAAATCCGCCGCAATGCCAT
TTTTCTAAAATCCGCCGCAATGCCAT
TTTTCTAAAATCCGCCGCAATGCCAT
TTTTCTAAAATCCGCCGCAATGCCAT
TTTTCTAAAATCCGCCGCAATGCCAT
TTTTCTAAAATCCGCCGCAATGCCAT
TTTTCTAAAATCCGCCGCAATGCCAT
TTTTCTAAAATCCGCCGCAATGCCAT
TTTTCTAAAATCCGCCGCAATGCCAT
TTTTCTAAAATCCGCCGCAATGCCAT
TTTTCTAAAATCCGCCGCAATGCCAT
TTTTCTAAAATCCGCCGCAATGCCAT
TTTTCTAAAATCCGCCGCAATGCCAT
TTTTCTAAAATCCGCCGCAATGCCAT
TTTTCTAAAATCCGCCGCAATGCCAT
TTTTCTAAAATCCGCCGCAATGCCAT
TTTTCTAAAATCCGCCGCAATGCCAT
TTTTCTAAAATCCGCCGCAATGCCAT
TTTTCTAAAATCCGCCGCAATGCCAT
TTTTCTAAAATCCGCCGCAATGCCAT
TTTTCTAAAATCCGCCGCAATGCCAT
TTTTCTAAAATCCGCCGCAATGCCAT
TTTTCTAAAATCCGCCGCAATGCCAT
TTTTCTAAAATCCGCCGCAATGCCAT
TTTTCTAAAATCCGCCGCAATGCCAT
TTTTCTAAAATCCGCCGCAATGCCAT
TTTTCTAAAATCCGCCGCAATGCCAT
TTTTCTAAAATCCGCCGCAATGCCAT
TTTTCTAAAATCCGCCGCAATGCCAT
TTTTCTAAAATCCGCCGCAATGCCAT
TTTTCTAAAATCCGCCGCAATGCCAT
TTTTCTAAAATCCGCCGCAATGCCAT
TTTTCTAAAATCCGCCGCAATGCCAT
TTTTCTAAAATCCGCCGCAATGCCAT
TTTTCTAAAATCCGCCGCAATGCCAT
TTTTCTAAAATCCGCCGCAATGCCAT
TTTTCTAAAATCCGCCGCAATGCCAT
TTTTCTAAAATCCGCCGCAATGCCAT
TTTTCTAAAATCCGCCGCAATGCCAT
TTTTCTAAAATCCGCCGCAATGCCAT
TTTTCTAAAATCCGCCGCAATGCCAT
TTTTCTAAAATCCGCCGCAATGCCAT
TTTTCTAAAATCCGCCGCAATGCCAT
TTTTCTAAAATCCGCCGCAATGCCAT
TTTTCTAAAATCCGCCGCAATGCCAT
TTTTCTAAAATCCGCCGCAATGCCAT
TTTTCTAAAATCCGCCGCAATGCCAT
TTTTCTAAAATCCGCCGCAATGCCAT
TTTTCTAAAATCCGCCGCAATGCCAT
TTTTCTAAAATCCGCCGCAATGCCAT
TTTTCTAAAATCCGCCGCAATGCCAT
TTTTCTAAAATCCGCCGCAATGCCAT
TTTTCTAAAATCCGCCGCAATGCCAT
TTTTCTAAAATCCGCCGCAATGCCAT

TTTTCTAAAATCCGCCGCAATGCCAT
TTTTCTAAAATCCGCCGCAATGCCAT
TTTTCTAAAATCCGCCGCAATGCCAT
TTTTCTAAAATCCGCCGCAATGCCAT
TTTTCTAAAATCCGCCGCAATGCCAT
TTTTCTAAAATCCGCCGCAATGCCAT
TTTTCTAAAATCCGCCGCAATGCCAT
TTTTCTAAAATCCGCCGCAATGCCAT
TTTTCTAAAATCCGCCGCAATGCCAT
TTTTCTAAAATCCGCCGCAATGCCAT
TTTTCTAAAATCCGCCGCAATGCCAT
TTTTCTAAAATCCGCCGCAATGCCAT
TTTTCTAAAATCCGCCGCAATGCCAT
TTTTCTAAAATCCGCCGCAATGCCAT
TTTTCTAAAATCCGCCGCAATGCCAT
TTTTCTAAAATCCGCCGCAATGCCAT
TTTTCTAAAATCCGCCGCAATGCCAT
TTTTCTAAAATCCGCCGCAATGCCAT
TTTTCTAAAATCCGCCGCAATGCCAT
TTTTCTAAAATCCGCCGCAATGCCAT
TTTTCTAAAATCCGCCGCAATGCCAT
TTTTCTAAAATCCGCCGCAATGCCAT
TTTTCTAAAATCCGCCGCAATGCCAT
TTTTCTAAAATCCGCCGCAATGCCAT
TTTTCTAAAATCCGCCGCAATGCCAT
TTTTCTAAAATCCGCCGCAATGCCAT
TTTTCTAAAATCCGCCGCAATGCCAT
TTTTCTAAAATCCGCCGCAATGCCAT
TTTTCTAAAATCCGCCGCAATGCCAT
TTTTCTAAAATCCGCCGCAATGCCAT
TTTTCTAAAATCCGCCGCAATGCCAT
TTTTCTAAAATCCGCCGCAATGCCAT
TTTTCTAAAATCCGCCGCAATGCCAT
TTTTCTAAAATCCGCCGCAATGCCAT
TTTTCTAAAATCCGCCGCAATGCCAT
TTTTCTAAAATCCGCCGCAATGCCAT
TTTTCTAAAATCCGCCGCAATGCCAT
TTTTCTAAAATCCGCCGCAATGCCAT
TTTTCTAAAATCCGCCGCAATGCCAT
TTTTCTAAAATCCGCCGCAATGCCAT
TTTTCTAAAATCCGCCGCAATGCCAT
TTTTCTAAAATCCGCCGCAATGCCAT
TTTTCTAAAATCCGCCGCAATGCCAT
TTTTCTAAAATCCGCCGCAATGCCAT
TTTTCTAAAATCCGCCGCAATGCCAT
TTTTCTAAAATCCGCCGCAATGCCAT
TTTTCTAAAATCCGCCGCAATGCCAT
TTTTCTAAAATCCGCCGCAATGCCAT
TTTTCTAAAATCCGCCGCAATGCCAT
TTTTCTAAAATCCGCCGCAATGCCAT
TTTTCTAAAATCCGCCGCAATGCCAT
TTTTCTAAAATCCGCCGCAATGCCAT
TTTTCTAAAATCCGCCGCAATGCCAT
TTTTCTAAAATCCGCCGCAATGCCAT

TTTTCTAAAATCCGCCGCAATGCCAT
TTTTCTAAAATCCGCCGCAATGCCAT
TTTTCTAAAATCCGCCGCAATGCCAT
TTTTCTAAAATCCGCCGCAATGCCAT
TTTTCTAAAATCCGCCGCAATGCCAT
TTTTCTAAAATCCGCCGCAATGCCAT
TTTTCTAAAATCCGCCGCAATGCCAT
TTTTCTAAAATCCGCCGCAATGCCAT
TTTTCTAAAATCCGCCGCAATGCCAT
TTTTCTAAAATCCGCCGCAATGCCAT
TTTTCTAAAATCCGCCGCAATGCCAT
TTTTCTAAAATCCGCCGCAATGCCAT
TTTTCTAAAATCCGCCGCAATGCCAT
TTTTCTAAAATCCGCCGCAATGCCAT
TTTTCTAAAATCCGCCGCAATGCCAT
TTTTCTAAAATCCGCCGCAATGCCAT
TTTTCTAAAATCCGCCGCAATGCCAT
TTTTCTAAAATCCGCCGCAATGCCAT
TTTTCTAAAATCCGCCGCAATGCCAT
TTTTCTAAAATCCGCCGCAATGCCAT
TTTTCTAAAATCCGCCGCAATGCCAT
TTTTCTAAAATCCGCCGCAATGCCAT
TTTTCTAAAATCCGCCGCAATGCCAT
TTTTCTAAAATCCGCCGCAATGCCAT
TTTTCTAAAATCCGCCGCAATGCCAT
TTTTCTAAAATCCGCCGCAATGCCAT
TTTTCTAAAATCCGCCGCAATGCCAT
TTTTCTAAAATCCGCCGCAATGCCAT
TTTTCTAAAATCCGCCGCAATGCCAT
TTTTCTAAAATCCGCCGCAATGCCAT
TTTTCTAAAATCCGCCGCAATGCCAT
TTTTCTAAAATCCGCCGCAATGCCAT
TTTTCTAAAATCCGCCGCAATGCCAT
TTTTCTAAAATCCGCCGCAATGCCAT
TTTTCTAAAATCCGCCGCAATGCCAT
TTTTCTAAAATCCGCCGCAATGCCAT
TTTTCTAAAATCCGCCGCAATGCCAT
TTTTCTAAAATCCGCCGCAATGCCAT
TTTTCTAAAATCCGCCGCAATGCCAT
TTTTCTAAAATCCGCCGCAATGCCAT
TTTTCTAAAATCCGCCGCAATGCCAT
TTTTCTAAAATCCGCCGCAATGCCAT
TTTTCTAAAATCCGCCGCAATGCCAT
TTTTCTAAAATCCGCCGCAATGCCAT
TTTTCTAAAATCCGCCGCAATGCCAT
TTTTCTAAAATCCGCCGCAATGCCAT
TTTTCTAAAATCCGCCGCAATGCCAT
TTTTCTAAAATCCGCCGCAATGCCAT
TTTTCTAAAATCCGCCGCAATGCCAT
TTTTCTAAAATCCGCCGCAATGCCAT
TTTTCTAAAATCCGCCGCAATGCCAT
TTTTCTAAAATCCGCCGCAATGCCAT
TTTTCTAAAATCCGCCGCAATGCCAT
TTTTCTAAAATCCGCCGCAATGCCAT

TTTTCTAAAATCCGCCGCAATGCCAT
TTTTCTAAAATCCGCCGCAATGCCAT
TTTTCTAAAATCCGCCGCAATGCCAT
TTTTCTAAAATCCGCCGCAATGCCAT
TTTTCTAAAATCCGCCGCAATGCCAT
TTTTCTAAAATCCGCCGCAATGCCAT
TTTTCTAAAATCCGCCGCAATGCCAT
TTTTCTAAAATCCGCCGCAATGCCAT
TTTTCTAAAATCCGCCGCAATGCCAT
TTTTCTAAAATCCGCCGCAATGCCAT
TTTTCTAAAATCCGCCGCAATGCCAT
TTTTCTAAAATCCGCCGCAATGCCAT
TTTTCTAAAATCCGCCGCAATGCCAT
TTTTCTAAAATCCGCCGCAATGCCAT
TTTTCTAAAATCCGCCGCAATGCCAT
TTTTCTAAAATCCGCCGCAATGCCAT
TTTTCTAAAATCCGCCGCAATGCCAT
TTTTCTAAAATCCGCCGCAATGCCAT
TTTTCTAAAATCCGCCGCAATGCCAT
TTTTCTAAAATCCGCCGCAATGCCAT
TTTTCTAAAATCCGCCGCAATGCCAT
TTTTCTAAAATCCGCCGCAATGCCAT
TTTTCTAAAATCCGCCGCAATGCCAT
TTTTCTAAAATCCGCCGCAATGCCAT
TTTTCTAAAATCCGCCGCAATGCCAT
TTTTCTAAAATCCGCCGCAATGCCAT
TTTTCTAAAATCCGCCGCAATGCCAT
TTTTCTAAAATCCGCCGCAATGCCAT
TTTTCTAAAATCCGCCGCAATGCCAT
TTTTCTAAAATCCGCCGCAATGCCAT
TTTTCTAAAATCCGCCGCAATGCCAT
TTTTCTAAAATCCGCCGCAATGCCAT
TTTTCTAAAATCCGCCGCAATGCCAT
TTTTCTAAAATCCGCCGCAATGCCAT
TTTTCTAAAATCCGCCGCAATGCCAT
TTTTCTAAAATCCGCCGCAATGCCAT
TTTTCTAAAATCCGCCGCAATGCCAT
TTTTCTAAAATCCGCCGCAATGCCAT
TTTTCTAAAATCCGCCGCAATGCCAT
TTTTCTAAAATCCGCCGCAATGCCAT
TTTTCTAAAATCCGCCGCAATGCCAT
TTTTCTAAAATCCGCCGCAATGCCAT
TTTTCTAAAATCCGCCGCAATGCCAT
TTTTCTAAAATCCGCCGCAATGCCAT
TTTTCTAAAATCCGCCGCAATGCCAT
TTTTCTAAAATCCGCCGCAATGCCAT
TTTTCTAAAATCCGCCGCAATGCCAT
TTTTCTAAAATCCGCCGCAATGCCAT
TTTTCTAAAATCCGCCGCAATGCCAT
TTTTCTAAAATCCGCCGCAATGCCAT
TTTTCTAAAATCCGCCGCAATGCCAT
TTTTCTAAAATCCGCCGCAATGCCAT
TTTTCTAAAATCCGCCGCAATGCCAT
TTTTCTAAAATCCGCCGCAATGCCAT

TTTTCTAAAATCCGCCGCAATGCCAT
TTTTCTAAAATCCGCCGCAATGCCAT
TTTTCTAAAATCCGCCGCAATGCCAT
TTTTCTAAAATCCGCCGCAATGCCAT
TTTTCTAAAATCCGCCGCAATGCCAT
TTTTCTAAAATCCGCCGCAATGCCAT
TTTTCTAAAATCCGCCGCAATGCCAT
TTTTCTAAAATCCGCCGCAATGCCAT
TTTTCTAAAATCCGCCGCAATGCCAT
TTTTCTAAAATCCGCCGCAATGCCAT
TTTTCTAAAATCCGCCGCAATGCCAT
TTTTCTAAAATCCGCCGCAATGCCAT
TTTTCTAAAATCCGCCGCAATGCCAT
TTTTCTAAAATCCGCCGCAATGCCAT
TTTTCTAAAATCCGCCGCAATGCCAT
TTTTCTAAAATCCGCCGCAATGCCAT
TTTTCTAAAATCCGCCGCAATGCCAT
TTTTCTAAAATCCGCCGCAATGCCAT
TTTTCTAAAATCCGCCGCAATGCCAT
TTTTCTAAAATCCGCCGCAATGCCAT
TTTTCTAAAATCCGCCGCAATGCCAT
TTTTCTAAAATCCGCCGCAATGCCAT
TTTTCTAAAATCCGCCGCAATGCCAT
TTTTCTAAAATCCGCCGCAATGCCAT
TTTTCTAAAATCCGCCGCAATGCCAT
TTTTCTAAAATCCGCCGCAATGCCAT
TTTTCTAAAATCCGCCGCAATGCCAT
TTTTCTAAAATCCGCCGCAATGCCAT
TTTTCTAAAATCCGCCGCAATGCCAT
TTTTCTAAAATCCGCCGCAATGCCAT
TTTTCTAAAATCCGCCGCAATGCCAT
TTTTCTAAAATCCGCCGCAATGCCAT
TTTTCTAAAATCCGCCGCAATGCCAT
TTTTCTAAAATCCGCCGCAATGCCAT
TTTTCTAAAATCCGCCGCAATGCCAT
TTTTCTAAAATCCGCCGCAATGCCAT
TTTTCTAAAATCCGCCGCAATGCCAT
TTTTCTAAAATCCGCCGCAATGCCAT
TTTTCTAAAATCCGCCGCAATGCCAT
TTTTCTAAAATCCGCCGCAATGCCAT
TTTTCTAAAATCCGCCGCAATGCCAT
TTTTCTAAAATCCGCCGCAATGCCAT
TTTTCTAAAATCCGCCGCAATGCCAT
TTTTCTAAAATCCGCCGCAATGCCAT
TTTTCTAAAATCCGCCGCAATGCCAT
TTTTCTAAAATCCGCCGCAATGCCAT
TTTTCTAAAATCCGCCGCAATGCCAT
TTTTCTAAAATCCGCCGCAATGCCAT
TTTTCTAAAATCCGCCGCAATGCCAT
TTTTCTAAAATCCGCCGCAATGCCAT
TTTTCTAAAATCCGCCGCAATGCCAT
TTTTCTAAAATCCGCCGCAATGCCAT
TTTTCTAAAATCCGCCGCAATGCCAT
TTTTCTAAAATCCGCCGCAATGCCAT

TTTTCTAAAATCCGCCGCAATGCCAT
TTTTCTAAAATCCGCCGCAATGCCAT
TTTTCTAAAATCCGCCGCAATGCCAT
TTTTCTAAAATCCGCCGCAATGCCAT
TTTTCTAAAATCCGCCGCAATGCCAT
TTTTCTAAAATCCGCCGCAATGCCAT
TTTTCTAAAATCCGCCGCAATGCCAT
TTTTCTAAAATCCGCCGCAATGCCAT
TTTTCTAAAATCCGCCGCAATGCCAT
TTTTCTAAAATCCGCCGCAATGCCAT
TTTTCTAAAATCCGCCGCAATGCCAT
TTTTCTAAAATCCGCCGCAATGCCAT
TTTTCTAAAATCCGCCGCAATGCCAT
TTTTCTAAAATCCGCCGCAATGCCAT
TTTTCTAAAATCCGCCGCAATGCCAT
TTTTCTAAAATCCGCCGCAATGCCAT
TTTTCTAAAATCCGCCGCAATGCCAT
TTTTCTAAAATCCGCCGCAATGCCAT
TTTTCTAAAATCCGCCGCAATGCCAT
TTTTCTAAAATCCGCCGCAATGCCAT
TTTTCTAAAATCCGCCGCAATGCCAT
TTTTCTAAAATCCGCCGCAATGCCAT
TTTTCTAAAATCCGCCGCAATGCCAT
TTTTCTAAAATCCGCCGCAATGCCAT
TTTTCTAAAATCCGCCGCAATGCCAT
TTTTCTAAAATCCGCCGCAATGCCAT
TTTTCTAAAATCCGCCGCAATGCCAT
TTTTCTAAAATCCGCCGCAATGCCAT
TTTTCTAAAATCCGCCGCAATGCCAT
TTTTCTAAAATCCGCCGCAATGCCAT
TTTTCTAAAATCCGCCGCAATGCCAT
TTTTCTAAAATCCGCCGCAATGCCAT
TTTTCTAAAATCCGCCGCAATGCCAT
TTTTCTAAAATCCGCCGCAATGCCAT
TTTTCTAAAATCCGCCGCAATGCCAT
TTTTCTAAAATCCGCCGCAATGCCAT
TTTTCTAAAATCCGCCGCAATGCCAT
TTTTCTAAAATCCGCCGCAATGCCAT
TTTTCTAAAATCCGCCGCAATGCCAT
TTTTCTAAAATCCGCCGCAATGCCAT
TTTTCTAAAATCCGCCGCAATGCCAT
TTTTCTAAAATCCGCCGCAATGCCAT
TTTTCTAAAATCCGCCGCAATGCCAT
TTTTCTAAAATCCGCCGCAATGCCAT
TTTTCTAAAATCCGCCGCAATGCCAT
TTTTCTAAAATCCGCCGCAATGCCAT
TTTTCTAAAATCCGCCGCAATGCCAT
TTTTCTAAAATCCGCCGCAATGCCAT
TTTTCTAAAATCCGCCGCAATGCCAT
TTTTCTAAAATCCGCCGCAATGCCAT
TTTTCTAAAATCCGCCGCAATGCCAT
TTTTCTAAAATCCGCCGCAATGCCAT
TTTTCTAAAATCCGCCGCAATGCCAT
TTTTCTAAAATCCGCCGCAATGCCAT

TTTTCTAAAATCCGCCGCAATGCCAT
TTTTCTAAAATCCGCCGCAATGCCAT
TTTTCTAAAATCCGCCGCAATGCCAT
TTTTCTAAAATCCGCCGCAATGCCAT
TTTTCTAAAATCCGCCGCAATGCCAT
TTTTCTAAAATCCGCCGCAATGCCAT
TTTTCTAAAATCCGCCGCAATGCCAT
TTTTCTAAAATCCGCCGCAATGCCAT
TTTTCTAAAATCCGCCGCAATGCCAT
TTTTCTAAAATCCGCCGCAATGCCAT
TTTTCTAAAATCCGCCGCAATGCCAT
TTTTCTAAAATCCGCCGCAATGCCAT
TTTTCTAAAATCCGCCGCAATGCCAT
TTTTCTAAAATCCGCCGCAATGCCAT
TTTTCTAAAATCCGCCGCAATGCCAT
TTTTCTAAAATCCGCCGCAATGCCAT
TTTTCTAAAATCCGCCGCAATGCCAT
TTTTCTAAAATCCGCCGCAATGCCAT
TTTTCTAAAATCCGCCGCAATGCCAT
TTTTCTAAAATCCGCCGCAATGCCAT
TTTTCTAAAATCCGCCGCAATGCCAT
TTTTCTAAAATCCGCCGCAATGCCAT
TTTTCTAAAATCCGCCGCAATGCCAT
TTTTCTAAAATCCGCCGCAATGCCAT
TTTTCTAAAATCCGCCGCAATGCCAT
TTTTCTAAAATCCGCCGCAATGCCAT
TTTTCTAAAATCCGCCGCAATGCCAT
TTTTCTAAAATCCGCCGCAATGCCAT
TTTTCTAAAATCCGCCGCAATGCCAT
TTTTCTAAAATCCGCCGCAATGCCAT
TTTTCTAAAATCCGCCGCAATGCCAT
TTTTCTAAAATCCGCCGCAATGCCAT
TTTTCTAAAATCCGCCGCAATGCCAT
TTTTCTAAAATCCGCCGCAATGCCAT
TTTTCTAAAATCCGCCGCAATGCCAT
TTTTCTAAAATCCGCCGCAATGCCAT
TTTTCTAAAATCCGCCGCAATGCCAT
TTTTCTAAAATCCGCCGCAATGCCAT
TTTTCTAAAATCCGCCGCAATGCCAT
TTTTCTAAAATCCGCCGCAATGCCAT
TTTTCTAAAATCCGCCGCAATGCCAT
TTTTCTAAAATCCGCCGCAATGCCAT
TTTTCTAAAATCCGCCGCAATGCCAT
TTTTCTAAAATCCGCCGCAATGCCAT
TTTTCTAAAATCCGCCGCAATGCCAT
TTTTCTAAAATCCGCCGCAATGCCAT
TTTTCTAAAATCCGCCGCAATGCCAT
TTTTCTAAAATCCGCCGCAATGCCAT
TTTTCTAAAATCCGCCGCAATGCCAT
TTTTCTAAAATCCGCCGCAATGCCAT
TTTTCTAAAATCCGCCGCAATGCCAT
TTTTCTAAAATCCGCCGCAATGCCAT
TTTTCTAAAATCCGCCGCAATGCCAT
TTTTCTAAAATCCGCCGCAATGCCAT

TTTTCTAAAATCCGCCGCAATGCCAT
TTTTCTAAAATCCGCCGCAATGCCAT
TTTTCTAAAATCCGCCGCAATGCCAT
TTTTCTAAAATCCGCCGCAATGCCAT
TTTTCTAAAATCCGCCGCAATGCCAT
TTTTCTAAAATCCGCCGCAATGCCAT
TTTTCTAAAATCCGCCGCAATGCCAT
TTTTCTAAAATCCGCCGCAATGCCAT
TTTTCTAAAATCCGCCGCAATGCCAT
TTTTCTAAAATCCGCCGCAATGCCAT
TTTTCTAAAATCCGCCGCAATGCCAT
TTTTCTAAAATCCGCCGCAATGCCAT
TTTTCTAAAATCCGCCGCAATGCCAT
TTTTCTAAAATCCGCCGCAATGCCAT
TTTTCTAAAATCCGCCGCAATGCCAT
TTTTCTAAAATCCGCCGCAATGCCAT
TTTTCTAAAATCCGCCGCAATGCCAT
TTTTCTAAAATCCGCCGCAATGCCAT
TTTTCTAAAATCCGCCGCAATGCCAT
TTTTCTAAAATCCGCCGCAATGCCAT
TTTTCTAAAATCCGCCGCAATGCCAT
TTTTCTAAAATCCGCCGCAATGCCAT
TTTTCTAAAATCCGCCGCAATGCCAT
TTTTCTAAAATCCGCCGCAATGCCAT
TTTTCTAAAATCCGCCGCAATGCCAT
TTTTCTAAAATCCGCCGCAATGCCAT
TTTTCTAAAATCCGCCGCAATGCCAT
TTTTCTAAAATCCGCCGCAATGCCAT
TTTTCTAAAATCCGCCGCAATGCCAT
TTTTCTAAAATCCGCCGCAATGCCAT
TTTTCTAAAATCCGCCGCAATGCCAT
TTTTCTAAAATCCGCCGCAATGCCAT
TTTTCTAAAATCCGCCGCAATGCCAT
TTTTCTAAAATCCGCCGCAATGCCAT
TTTTCTAAAATCCGCCGCAATGCCAT
TTTTCTAAAATCCGCCGCAATGCCAT
TTTTCTAAAATCCGCCGCAATGCCAT
TTTTCTAAAATCCGCCGCAATGCCAT
TTTTCTAAAATCCGCCGCAATGCCAT
TTTTCTAAAATCCGCCGCAATGCCAT
TTTTCTAAAATCCGCCGCAATGCCAT
TTTTCTAAAATCCGCCGCAATGCCAT
TTTTCTAAAATCCGCCGCAATGCCAT
TTTTCTAAAATCCGCCGCAATGCCAT
TTTTCTAAAATCCGCCGCAATGCCAT
TTTTCTAAAATCCGCCGCAATGCCAT
TTTTCTAAAATCCGCCGCAATGCCAT
TTTTCTAAAATCCGCCGCAATGCCAT
TTTTCTAAAATCCGCCGCAATGCCAT
TTTTCTAAAATCCGCCGCAATGCCAT
TTTTCTAAAATCCGCCGCAATGCCAT
TTTTCTAAAATCCGCCGCAATGCCAT
TTTTCTAAAATCCGCCGCAATGCCAT
TTTTCTAAAATCCGCCGCAATGCCAT

TTTTCTAAAATCCGCCGCAATGCCAT
TTTTCTAAAATCCGCCGCAATGCCAT
TTTTCTAAAATCCGCCGCAATGCCAT
TTTTCTAAAATCCGCCGCAATGCCAT
TTTTCTAAAATCCGCCGCAATGCCAT
TTTTCTAAAATCCGCCGCAATGCCAT
TTTTCTAAAATCCGCCGCAATGCCAT
TTTTCTAAAATCCGCCGCAATGCCAT
TTTTCTAAAATCCGCCGCAATGCCAT
TTTTCTAAAATCCGCCGCAATGCCAT
TTTTCTAAAATCCGCCGCAATGCCAT
TTTTCTAAAATCCGCCGCAATGCCAT
TTTTCTAAAATCCGCCGCAATGCCAT
TTTTCTAAAATCCGCCGCAATGCCAT
TTTTCTAAAATCCGCCGCAATGCCAT
TTTTCTAAAATCCGCCGCAATGCCAT
TTTTCTAAAATCCGCCGCAATGCCAT
TTTTCTAAAATCCGCCGCAATGCCAT
Nr of selected repeats 1129 Similarity 0.999909
Consensus:
TTTTCTAAAATCCGCCGCAATGCCAT
>Cele-UNSB01_1:4635066-4668893 Satlength=33828 Nr of Repeats=1253
RepeatLength=27 seed=TGGCTTCCCA
TGGCTTCCCACTATATTTTACTCTCTG
TGGCTTCCCACTATATTTTACTCTCTG
TGGCTTCCCACTATATTTTACTCTCTG
TGGCTTCCCACTATATTTTACTCTCTG
TGGCTTCCCACTATATTTTACTCTCTG
TGGCTTCCCACTATATTTTACTCTCTG
TGGCTTCCCACTATATTTTACTCTCTG
TGGCTTCCCACTATATTTTACTCTCTG
TGGCTTCCCACTATATTTTACTCTCTG
TGGCTTCCCACTATATTTTACTCTCTG
TGGCTTCCCACTATATTTTACTCTCTG
TGGCTTCCCACTATATTTTACTCTCTG
TGGCTTCCCACTATATTTTACTCTCTG
TGGCTTCCCACTATATTTTACTCTCTG
TGGCTTCCCACTATATTTTACTCTCTG
TGGCTTCCCACTATATTTTACTCTCTG
TGGCTTCCCACTATATTTTACTCTCTG
TGGCTTCCCACTATATTTTACTCTCTG
TGGCTTCCCACTATATTTTACTCTCTG
TGGCTTCCCACTATATTTTACTCTCTG
TGGCTTCCCACTATATTTTACTCTCTG
TGGCTTCCCACTATATTTTACTCTCTG
TGGCTTCCCACTATATTTTACTCTCTG
TGGCTTCCCACTATATTTTACTCTCTG
TGGCTTCCCACTATATTTTACTCTCTG
TGGCTTCCCACTATATTTTACTCTCTG
TGGCTTCCCACTATATTTTACTCTCTG
TGGCTTCCCACTATATTTTACTCTCTG
TGGCTTCCCACTATATTTTACTCTCTG
TGGCTTCCCACTATATTTTACTCTCTG
TGGCTTCCCACTATATTTTACTCTCTG

TGGCTTCCCACTATATTTTACTCTCTG
TGGCTTCCCACTATATTTTACTCTCTG
TGGCTTCCCACTATATTTTACTCTCTG
TGGCTTCCCACTATATTTTACTCTCTG
TGGCTTCCCACTATATTTTACTCTCTG
TGGCTTCCCACTATATTTTACTCTCTG
TGGCTTCCCACTATATTTTACTCTCTG
TGGCTTCCCACTATATTTTACTCTCTG
TGGCTTCCCACTATATTTTACTCTCTG
TGGCTTCCCACTATATTTTACTCTCTG
TGGCTTCCCACTATATTTTACTCTCTG
TGGCTTCCCACTATATTTTACTCTCTG
TGGCTTCCCACTATATTTTACTCTCTG
TGGCTTCCCACTATATTTTACTCTCTG
TGGCTTCCCACTATATTTTACTCTCTG
TGGCTTCCCACTATATTTTACTCTCTG
TGGCTTCCCACTATATTTTACTCTCTG
TGGCTTCCCACTATATTTTACTCTCTG
TGGCTTCCCACTATATTTTACTCTCTG
TGGCTTCCCACTATATTTTACTCTCTG
TGGCTTCCCACTATATTTTACTCTCTG
TGGCTTCCCACTATATTTTACTCTCTG
TGGCTTCCCACTATATTTTACTCTCTG
TGGCTTCCCACTATATTTTACTCTCTG
TGGCTTCCCACTATATTTTACTCTCTG
TGGCTTCCCACTATATTTTACTCTCTG
TGGCTTCCCACTATATTTTACTCTCTG
TGGCTTCCCACTATATTTTACTCTCTG
TGGCTTCCCACTATATTTTACTCTCTG
TGGCTTCCCACTATATTTTACTCTCTG
TGGCTTCCCACTATATTTTACTCTCTG
TGGCTTCCCACTATATTTTACTCTCTG
TGGCTTCCCACTATATTTTACTCTCTG
TGGCTTCCCACTATATTTTACTCTCTG
TGGCTTCCCACTATATTTTACTCTCTG
TGGCTTCCCACTATATTTTACTCTCTG
TGGCTTCCCACTATATTTTACTCTCTG
TGGCTTCCCACTATATTTTACTCTCTG
TGGCTTCCCACTATATTTTACTCTCTG
TGGCTTCCCACTATATTTTACTCTCTG
TGGCTTCCCACTATATTTTACTCTCTG
TGGCTTCCCACTATATTTTACTCTCTG
TGGCTTCCCACTATATTTTACTCTCTG
TGGCTTCCCACTATATTTTACTCTCTG
TGGCTTCCCACTATATTTTACTCTCTG
TGGCTTCCCACTATATTTTACTCTCTG
TGGCTTCCCACTATATTTTACTCTCTG
TGGCTTCCCACTATATTTTACTCTCTG
TGGCTTCCCACTATATTTTACTCTCTG
TGGCTTCCCACTATATTTTACTCTCTG
TGGCTTCCCACTATATTTTACTCTCTG
TGGCTTCCCACTATATTTTACTCTCTG
TGGCTTCCCACTATATTTTACTCTCTG
TGGCTTCCCACTATATTTTACTCTCTG

TGGCTTCCCACTATATTTTACTCTCTG
TGGCTTCCCACTATATTTTACTCTCTG
TGGCTTCCCACTATATTTTACTCTCTG
TGGCTTCCCACTATATTTTACTCTCTG
TGGCTTCCCACTATATTTTACTCTCTG
TGGCTTCCCACTATATTTTACTCTCTG
TGGCTTCCCACTATATTTTACTCTCTG
TGGCTTCCCACTATATTTTACTCTCTG
TGGCTTCCCACTATATTTTACTCTCTG
TGGCTTCCCACTATATTTTACTCTCTG
TGGCTTCCCACTATATTTTACTCTCTG
TGGCTTCCCACTATATTTTACTCTCTG
TGGCTTCCCACTATATTTTACTCTCTG
TGGCTTCCCACTATATTTTACTCTCTG
TGGCTTCCCACTATATTTTACTCTCTG
TGGCTTCCCACTATATTTTACTCTCTG
TGGCTTCCCACTATATTTTACTCTCTG
TGGCTTCCCACTATATTTTACTCTCTG
TGGCTTCCCACTATATTTTACTCTCTG
TGGCTTCCCACTATATTTTACTCTCTG
TGGCTTCCCACTATATTTTACTCTCTG
TGGCTTCCCACTATATTTTACTCTCTG
TGGCTTCCCACTATATTTTACTCTCTG
TGGCTTCCCACTATATTTTACTCTCTG
TGGCTTCCCACTATATTTTACTCTCTG
TGGCTTCCCACTATATTTTACTCTCTG
TGGCTTCCCACTATATTTTACTCTCTG
TGGCTTCCCACTATATTTTACTCTCTG
TGGCTTCCCACTATATTTTACTCTCTG
TGGCTTCCCACTATATTTTACTCTCTG
TGGCTTCCCACTATATTTTACTCTCTG
TGGCTTCCCACTATATTTTACTCTCTG
TGGCTTCCCACTATATTTTACTCTCTG
TGGCTTCCCACTATATTTTACTCTCTG
TGGCTTCCCACTATATTTTACTCTCTG
TGGCTTCCCACTATATTTTACTCTCTG
TGGCTTCCCACTATATTTTACTCTCTG
TGGCTTCCCACTATATTTTACTCTCTG
TGGCTTCCCACTATATTTTACTCTCTG
TGGCTTCCCACTATATTTTACTCTCTG
TGGCTTCCCACTATATTTTACTCTCTG
TGGCTTCCCACTATATTTTACTCTCTG
TGGCTTCCCACTATATTTTACTCTCTG
TGGCTTCCCACTATATTTTACTCTCTG
TGGCTTCCCACTATATTTTACTCTCTG
TGGCTTCCCACTATATTTTACTCTCTG
TGGCTTCCCACTATATTTTACTCTCTG
TGGCTTCCCACTATATTTTACTCTCTG
TGGCTTCCCACTATATTTTACTCTCTG
TGGCTTCCCACTATATTTTACTCTCTG
TGGCTTCCCACTATATTTTACTCTCTG
TGGCTTCCCACTATATTTTACTCTCTG
TGGCTTCCCACTATATTTTACTCTCTG
TGGCTTCCCACTATATTTTACTCTCTG
TGGCTTCCCACTATATTTTACTCTCTG

TGGCTTCCCACTATATTTTACTCTCTG
TGGCTTCCCACTATATTTTACTCTCTG
TGGCTTCCCACTATATTTTACTCTCTG
TGGCTTCCCACTATATTTTACTCTCTG
TGGCTTCCCACTATATTTTACTCTCTG
TGGCTTCCCACTATATTTTACTCTCTG
TGGCTTCCCACTATATTTTACTCTCTG
TGGCTTCCCACTATATTTTACTCTCTG
TGGCTTCCCACTATATTTTACTCTCTG
TGGCTTCCCACTATATTTTACTCTCTG
TGGCTTCCCACTATATTTTACTCTCTG
TGGCTTCCCACTATATTTTACTCTCTG
TGGCTTCCCACTATATTTTACTCTCTG
TGGCTTCCCACTATATTTTACTCTCTG
TGGCTTCCCACTATATTTTACTCTCTG
TGGCTTCCCACTATATTTTACTCTCTG
TGGCTTCCCACTATATTTTACTCTCTG
TGGCTTCCCACTATATTTTACTCTCTG
TGGCTTCCCACTATATTTTACTCTCTG
TGGCTTCCCACTATATTTTACTCTCTG
TGGCTTCCCACTATATTTTACTCTCTG
TGGCTTCCCACTATATTTTACTCTCTG
TGGCTTCCCACTATATTTTACTCTCTG
TGGCTTCCCACTATATTTTACTCTCTG
TGGCTTCCCACTATATTTTACTCTCTG
TGGCTTCCCACTATATTTTACTCTCTG
TGGCTTCCCACTATATTTTACTCTCTG
TGGCTTCCCACTATATTTTACTCTCTG
TGGCTTCCCACTATATTTTACTCTCTG
TGGCTTCCCACTATATTTTACTCTCTG
TGGCTTCCCACTATATTTTACTCTCTG
TGGCTTCCCACTATATTTTACTCTCTG
TGGCTTCCCACTATATTTTACTCTCTG
TGGCTTCCCACTATATTTTACTCTCTG
TGGCTTCCCACTATATTTTACTCTCTG
TGGCTTCCCACTATATTTTACTCTCTG
TGGCTTCCCACTATATTTTACTCTCTG
TGGCTTCCCACTATATTTTACTCTCTG
TGGCTTCCCACTATATTTTACTCTCTG
TGGCTTCCCACTATATTTTACTCTCTG
TGGCTTCCCACTATATTTTACTCTCTG
TGGCTTCCCACTATATTTTACTCTCTG
TGGCTTCCCACTATATTTTACTCTCTG
TGGCTTCCCACTATATTTTACTCTCTG
TGGCTTCCCACTATATTTTACTCTCTG
TGGCTTCCCACTATATTTTACTCTCTG
TGGCTTCCCACTATATTTTACTCTCTG
TGGCTTCCCACTATATTTTACTCTCTG
TGGCTTCCCACTATATTTTACTCTCTG
TGGCTTCCCACTATATTTTACTCTCTG
TGGCTTCCCACTATATTTTACTCTCTG
TGGCTTCCCACTATATTTTACTCTCTG
TGGCTTCCCACTATATTTTACTCTCTG
TGGCTTCCCACTATATTTTACTCTCTG

TGGCTTCCCACTATATTTTACTCTCTG
TGGCTTCCCACTATATTTTACTCTCTG
TGGCTTCCCACTATATTTTACTCTCTG
TGGCTTCCCACTATATTTTACTCTCTG
TGGCTTCCCACTATATTTTACTCTCTG
TGGCTTCCCACTATATTTTACTCTCTG
TGGCTTCCCACTATATTTTACTCTCTG
TGGCTTCCCACTATATTTTACTCTCTG
TGGCTTCCCACTATATTTTACTCTCTG
TGGCTTCCCACTATATTTTACTCTCTG
TGGCTTCCCACTATATTTTACTCTCTG
TGGCTTCCCACTATATTTTACTCTCTG
TGGCTTCCCACTATATTTTACTCTCTG
TGGCTTCCCACTATATTTTACTCTCTG
TGGCTTCCCACTATATTTTACTCTCTG
TGGCTTCCCACTATATTTTACTCTCTG
TGGCTTCCCACTATATTTTACTCTCTG
TGGCTTCCCACTATATTTTACTCTCTG
TGGCTTCCCACTATATTTTACTCTCTG
TGGCTTCCCACTATATTTTACTCTCTG
TGGCTTCCCACTATATTTTACTCTCTG
TGGCTTCCCACTATATTTTACTCTCTG
TGGCTTCCCACTATATTTTACTCTCTG
TGGCTTCCCACTATATTTTACTCTCTG
TGGCTTCCCACTATATTTTACTCTCTG
TGGCTTCCCACTATATTTTACTCTCTG
TGGCTTCCCACTATATTTTACTCTCTG
TGGCTTCCCACTATATTTTACTCTCTG
TGGCTTCCCACTATATTTTACTCTCTG
TGGCTTCCCACTATATTTTACTCTCTG
TGGCTTCCCACTATATTTTACTCTCTG
TGGCTTCCCACTATATTTTACTCTCTG
TGGCTTCCCACTATATTTTACTCTCTG
TGGCTTCCCACTATATTTTACTCTCTG
TGGCTTCCCACTATATTTTACTCTCTG
TGGCTTCCCACTATATTTTACTCTCTG
TGGCTTCCCACTATATTTTACTCTCTG
TGGCTTCCCACTATATTTTACTCTCTG
TGGCTTCCCACTATATTTTACTCTCTG
TGGCTTCCCACTATATTTTACTCTCTG
TGGCTTCCCACTATATTTTACTCTCTG
TGGCTTCCCACTATATTTTACTCTCTG
TGGCTTCCCACTATATTTTACTCTCTG
TGGCTTCCCACTATATTTTACTCTCTG
TGGCTTCCCACTATATTTTACTCTCTG
TGGCTTCCCACTATATTTTACTCTCTG
TGGCTTCCCACTATATTTTACTCTCTG
TGGCTTCCCACTATATTTTACTCTCTG
TGGCTTCCCACTATATTTTACTCTCTG
TGGCTTCCCACTATATTTTACTCTCTG
TGGCTTCCCACTATATTTTACTCTCTG
TGGCTTCCCACTATATTTTACTCTCTG
TGGCTTCCCACTATATTTTACTCTCTG
TGGCTTCCCACTATATTTTACTCTCTG

TGGCTTCCCACTATATTTTACTCTCTG
TGGCTTCCCACTATATTTTACTCTCTG
TGGCTTCCCACTATATTTTACTCTCTG
TGGCTTCCCACTATATTTTACTCTCTG
TGGCTTCCCACTATATTTTACTCTCTG
TGGCTTCCCACTATATTTTACTCTCTG
TGGCTTCCCACTATATTTTACTCTCTG
TGGCTTCCCACTATATTTTACTCTCTG
TGGCTTCCCACTATATTTTACTCTCTG
TGGCTTCCCACTATATTTTACTCTCTG
TGGCTTCCCACTATATTTTACTCTCTG
TGGCTTCCCACTATATTTTACTCTCTG
TGGCTTCCCACTATATTTTACTCTCTG
TGGCTTCCCACTATATTTTACTCTCTG
TGGCTTCCCACTATATTTTACTCTCTG
TGGCTTCCCACTATATTTTACTCTCTG
TGGCTTCCCACTATATTTTACTCTCTG
TGGCTTCCCACTATATTTTACTCTCTG
TGGCTTCCCACTATATTTTACTCTCTG
TGGCTTCCCACTATATTTTACTCTCTG
TGGCTTCCCACTATATTTTACTCTCTG
TGGCTTCCCACTATATTTTACTCTCTG
TGGCTTCCCACTATATTTTACTCTCTG
TGGCTTCCCACTATATTTTACTCTCTG
TGGCTTCCCACTATATTTTACTCTCTG
TGGCTTCCCACTATATTTTACTCTCTG
TGGCTTCCCACTATATTTTACTCTCTG
TGGCTTCCCACTATATTTTACTCTCTG
TGGCTTCCCACTATATTTTACTCTCTG
TGGCTTCCCACTATATTTTACTCTCTG
TGGCTTCCCACTATATTTTACTCTCTG
TGGCTTCCCACTATATTTTACTCTCTG
TGGCTTCCCACTATATTTTACTCTCTG
TGGCTTCCCACTATATTTTACTCTCTG
TGGCTTCCCACTATATTTTACTCTCTG
TGGCTTCCCACTATATTTTACTCTCTG
TGGCTTCCCACTATATTTTACTCTCTG
TGGCTTCCCACTATATTTTACTCTCTG
TGGCTTCCCACTATATTTTACTCTCTG
TGGCTTCCCACTATATTTTACTCTCTG
TGGCTTCCCACTATATTTTACTCTCTG
TGGCTTCCCACTATATTTTACTCTCTG
TGGCTTCCCACTATATTTTACTCTCTG
TGGCTTCCCACTATATTTTACTCTCTG
TGGCTTCCCACTATATTTTACTCTCTG
TGGCTTCCCACTATATTTTACTCTCTG
TGGCTTCCCACTATATTTTACTCTCTG
TGGCTTCCCACTATATTTTACTCTCTG
TGGCTTCCCACTATATTTTACTCTCTG
TGGCTTCCCACTATATTTTACTCTCTG
TGGCTTCCCACTATATTTTACTCTCTG
TGGCTTCCCACTATATTTTACTCTCTG
TGGCTTCCCACTATATTTTACTCTCTG
TGGCTTCCCACTATATTTTACTCTCTG

TGGCTTCCCACTATATTTTACTCTCTG
TGGCTTCCCACTATATTTTACTCTCTG
TGGCTTCCCACTATATTTTACTCTCTG
TGGCTTCCCACTATATTTTACTCTCTG
TGGCTTCCCACTATATTTTACTCTCTG
TGGCTTCCCACTATATTTTACTCTCTG
TGGCTTCCCACTATATTTTACTCTCTG
TGGCTTCCCACTATATTTTACTCTCTG
TGGCTTCCCACTATATTTTACTCTCTG
TGGCTTCCCACTATATTTTACTCTCTG
TGGCTTCCCACTATATTTTACTCTCTG
TGGCTTCCCACTATATTTTACTCTCTG
TGGCTTCCCACTATATTTTACTCTCTG
TGGCTTCCCACTATATTTTACTCTCTG
TGGCTTCCCACTATATTTTACTCTCTG
TGGCTTCCCACTATATTTTACTCTCTG
TGGCTTCCCACTATATTTTACTCTCTG
TGGCTTCCCACTATATTTTACTCTCTG
TGGCTTCCCACTATATTTTACTCTCTG
TGGCTTCCCACTATATTTTACTCTCTG
TGGCTTCCCACTATATTTTACTCTCTG
TGGCTTCCCACTATATTTTACTCTCTG
TGGCTTCCCACTATATTTTACTCTCTG
TGGCTTCCCACTATATTTTACTCTCTG
TGGCTTCCCACTATATTTTACTCTCTG
TGGCTTCCCACTATATTTTACTCTCTG
TGGCTTCCCACTATATTTTACTCTCTG
TGGCTTCCCACTATATTTTACTCTCTG
TGGCTTCCCACTATATTTTACTCTCTG
TGGCTTCCCACTATATTTTACTCTCTG
TGGCTTCCCACTATATTTTACTCTCTG
TGGCTTCCCACTATATTTTACTCTCTG
TGGCTTCCCACTATATTTTACTCTCTG
TGGCTTCCCACTATATTTTACTCTCTG
TGGCTTCCCACTATATTTTACTCTCTG
TGGCTTCCCACTATATTTTACTCTCTG
TGGCTTCCCACTATATTTTACTCTCTG
TGGCTTCCCACTATATTTTACTCTCTG
TGGCTTCCCACTATATTTTACTCTCTG
TGGCTTCCCACTATATTTTACTCTCTG
TGGCTTCCCACTATATTTTACTCTCTG
TGGCTTCCCACTATATTTTACTCTCTG
TGGCTTCCCACTATATTTTACTCTCTG
TGGCTTCCCACTATATTTTACTCTCTG
TGGCTTCCCACTATATTTTACTCTCTG
TGGCTTCCCACTATATTTTACTCTCTG
TGGCTTCCCACTATATTTTACTCTCTG
TGGCTTCCCACTATATTTTACTCTCTG
TGGCTTCCCACTATATTTTACTCTCTG
TGGCTTCCCACTATATTTTACTCTCTG
TGGCTTCCCACTATATTTTACTCTCTG
TGGCTTCCCACTATATTTTACTCTCTG
TGGCTTCCCACTATATTTTACTCTCTG
TGGCTTCCCACTATATTTTACTCTCTG

TGGCTTCCCACTATATTTTACTCTCTG
TGGCTTCCCACTATATTTTACTCTCTG
TGGCTTCCCACTATATTTTACTCTCTG
TGGCTTCCCACTATATTTTACTCTCTG
TGGCTTCCCACTATATTTTACTCTCTG
TGGCTTCCCACTATATTTTACTCTCTG
TGGCTTCCCACTATATTTTACTCTCTG
TGGCTTCCCACTATATTTTACTCTCTG
TGGCTTCCCACTATATTTTACTCTCTG
TGGCTTCCCACTATATTTTACTCTCTG
TGGCTTCCCACTATATTTTACTCTCTG
TGGCTTCCCACTATATTTTACTCTCTG
TGGCTTCCCACTATATTTTACTCTCTG
TGGCTTCCCACTATATTTTACTCTCTG
TGGCTTCCCACTATATTTTACTCTCTG
TGGCTTCCCACTATATTTTACTCTCTG
TGGCTTCCCACTATATTTTACTCTCTG
TGGCTTCCCACTATATTTTACTCTCTG
TGGCTTCCCACTATATTTTACTCTCTG
TGGCTTCCCACTATATTTTACTCTCTG
TGGCTTCCCACTATATTTTACTCTCTG
TGGCTTCCCACTATATTTTACTCTCTG
TGGCTTCCCACTATATTTTACTCTCTG
TGGCTTCCCACTATATTTTACTCTCTG
TGGCTTCCCACTATATTTTACTCTCTG
TGGCTTCCCACTATATTTTACTCTCTG
TGGCTTCCCACTATATTTTACTCTCTG
TGGCTTCCCACTATATTTTACTCTCTG
TGGCTTCCCACTATATTTTACTCTCTG
TGGCTTCCCACTATATTTTACTCTCTG
TGGCTTCCCACTATATTTTACTCTCTG
TGGCTTCCCACTATATTTTACTCTCTG
TGGCTTCCCACTATATTTTACTCTCTG
TGGCTTCCCACTATATTTTACTCTCTG
TGGCTTCCCACTATATTTTACTCTCTG
TGGCTTCCCACTATATTTTACTCTCTG
TGGCTTCCCACTATATTTTACTCTCTG
TGGCTTCCCACTATATTTTACTCTCTG
TGGCTTCCCACTATATTTTACTCTCTG
TGGCTTCCCACTATATTTTACTCTCTG
TGGCTTCCCACTATATTTTACTCTCTG
TGGCTTCCCACTATATTTTACTCTCTG
TGGCTTCCCACTATATTTTACTCTCTG
TGGCTTCCCACTATATTTTACTCTCTG
TGGCTTCCCACTATATTTTACTCTCTG
TGGCTTCCCACTATATTTTACTCTCTG
TGGCTTCCCACTATATTTTACTCTCTG
TGGCTTCCCACTATATTTTACTCTCTG
TGGCTTCCCACTATATTTTACTCTCTG
TGGCTTCCCACTATATTTTACTCTCTG
TGGCTTCCCACTATATTTTACTCTCTG
TGGCTTCCCACTATATTTTACTCTCTG
TGGCTTCCCACTATATTTTACTCTCTG
TGGCTTCCCACTATATTTTACTCTCTG

TGGCTTCCCACTATATTTTACTCTCTG
TGGCTTCCCACTATATTTTACTCTCTG
TGGCTTCCCACTATATTTTACTCTCTG
TGGCTTCCCACTATATTTTACTCTCTG
TGGCTTCCCACTATATTTTACTCTCTG
TGGCTTCCCACTATATTTTACTCTCTG
TGGCTTCCCACTATATTTTACTCTCTG
TGGCTTCCCACTATATTTTACTCTCTG
TGGCTTCCCACTATATTTTACTCTCTG
TGGCTTCCCACTATATTTTACTCTCTG
TGGCTTCCCACTATATTTTACTCTCTG
TGGCTTCCCACTATATTTTACTCTCTG
TGGCTTCCCACTATATTTTACTCTCTG
TGGCTTCCCACTATATTTTACTCTCTG
TGGCTTCCCACTATATTTTACTCTCTG
TGGCTTCCCACTATATTTTACTCTCTG
TGGCTTCCCACTATATTTTACTCTCTG
TGGCTTCCCACTATATTTTACTCTCTG
TGGCTTCCCACTATATTTTACTCTCTG
TGGCTTCCCACTATATTTTACTCTCTG
TGGCTTCCCACTATATTTTACTCTCTG
TGGCTTCCCACTATATTTTACTCTCTG
TGGCTTCCCACTATATTTTACTCTCTG
TGGCTTCCCACTATATTTTACTCTCTG
TGGCTTCCCACTATATTTTACTCTCTG
TGGCTTCCCACTATATTTTACTCTCTG
TGGCTTCCCACTATATTTTACTCTCTG
TGGCTTCCCACTATATTTTACTCTCTG
TGGCTTCCCACTATATTTTACTCTCTG
TGGCTTCCCACTATATTTTACTCTCTG
TGGCTTCCCACTATATTTTACTCTCTG
TGGCTTCCCACTATATTTTACTCTCTG
TGGCTTCCCACTATATTTTACTCTCTG
TGGCTTCCCACTATATTTTACTCTCTG
TGGCTTCCCACTATATTTTACTCTCTG
TGGCTTCCCACTATATTTTACTCTCTG
TGGCTTCCCACTATATTTTACTCTCTG
TGGCTTCCCACTATATTTTACTCTCTG
TGGCTTCCCACTATATTTTACTCTCTG
TGGCTTCCCACTATATTTTACTCTCTG
TGGCTTCCCACTATATTTTACTCTCTG
TGGCTTCCCACTATATTTTACTCTCTG
TGGCTTCCCACTATATTTTACTCTCTG
TGGCTTCCCACTATATTTTACTCTCTG
TGGCTTCCCACTATATTTTACTCTCTG
TGGCTTCCCACTATATTTTACTCTCTG
TGGCTTCCCACTATATTTTACTCTCTG
TGGCTTCCCACTATATTTTACTCTCTG
TGGCTTCCCACTATATTTTACTCTCTG
TGGCTTCCCACTATATTTTACTCTCTG
TGGCTTCCCACTATATTTTACTCTCTG
TGGCTTCCCACTATATTTTACTCTCTG
TGGCTTCCCACTATATTTTACTCTCTG
TGGCTTCCCACTATATTTTACTCTCTG

TGGCTTCCCACTATATTTTACTCTCTG
TGGCTTCCCACTATATTTTACTCTCTG
TGGCTTCCCACTATATTTTACTCTCTG
TGGCTTCCCACTATATTTTACTCTCTG
TGGCTTCCCACTATATTTTACTCTCTG
TGGCTTCCCACTATATTTTACTCTCTG
TGGCTTCCCACTATATTTTACTCTCTG
TGGCTTCCCACTATATTTTACTCTCTG
TGGCTTCCCACTATATTTTACTCTCTG
TGGCTTCCCACTATATTTTACTCTCTG
TGGCTTCCCACTATATTTTACTCTCTG
TGGCTTCCCACTATATTTTACTCTCTG
TGGCTTCCCACTATATTTTACTCTCTG
TGGCTTCCCACTATATTTTACTCTCTG
TGGCTTCCCACTATATTTTACTCTCTG
TGGCTTCCCACTATATTTTACTCTCTG
TGGCTTCCCACTATATTTTACTCTCTG
TGGCTTCCCACTATATTTTACTCTCTG
TGGCTTCCCACTATATTTTACTCTCTG
TGGCTTCCCACTATATTTTACTCTCTG
TGGCTTCCCACTATATTTTACTCTCTG
TGGCTTCCCACTATATTTTACTCTCTG
TGGCTTCCCACTATATTTTACTCTCTG
TGGCTTCCCACTATATTTTACTCTCTG
TGGCTTCCCACTATATTTTACTCTCTG
TGGCTTCCCACTATATTTTACTCTCTG
TGGCTTCCCACTATATTTTACTCTCTG
TGGCTTCCCACTATATTTTACTCTCTG
TGGCTTCCCACTATATTTTACTCTCTG
TGGCTTCCCACTATATTTTACTCTCTG
TGGCTTCCCACTATATTTTACTCTCTG
TGGCTTCCCACTATATTTTACTCTCTG
TGGCTTCCCACTATATTTTACTCTCTG
TGGCTTCCCACTATATTTTACTCTCTG
TGGCTTCCCACTATATTTTACTCTCTG
TGGCTTCCCACTATATTTTACTCTCTG
TGGCTTCCCACTATATTTTACTCTCTG
TGGCTTCCCACTATATTTTACTCTCTG
TGGCTTCCCACTATATTTTACTCTCTG
TGGCTTCCCACTATATTTTACTCTCTG
TGGCTTCCCACTATATTTTACTCTCTG
TGGCTTCCCACTATATTTTACTCTCTG
TGGCTTCCCACTATATTTTACTCTCTG
TGGCTTCCCACTATATTTTACTCTCTG
TGGCTTCCCACTATATTTTACTCTCTG
TGGCTTCCCACTATATTTTACTCTCTG
TGGCTTCCCACTATATTTTACTCTCTG
TGGCTTCCCACTATATTTTACTCTCTG
TGGCTTCCCACTATATTTTACTCTCTG
TGGCTTCCCACTATATTTTACTCTCTG
TGGCTTCCCACTATATTTTACTCTCTG
TGGCTTCCCACTATATTTTACTCTCTG
TGGCTTCCCACTATATTTTACTCTCTG
TGGCTTCCCACTATATTTTACTCTCTG

TGGCTTCCCACTATATTTTACTCTCTG
TGGCTTCCCACTATATTTTACTCTCTG
TGGCTTCCCACTATATTTTACTCTCTG
TGGCTTCCCACTATATTTTACTCTCTG
TGGCTTCCCACTATATTTTACTCTCTG
TGGCTTCCCACTATATTTTACTCTCTG
TGGCTTCCCACTATATTTTACTCTCTG
TGGCTTCCCACTATATTTTACTCTCTG
TGGCTTCCCACTATATTTTACTCTCTG
TGGCTTCCCACTATATTTTACTCTCTG
TGGCTTCCCACTATATTTTACTCTCTG
TGGCTTCCCACTATATTTTACTCTCTG
TGGCTTCCCACTATATTTTACTCTCTG
TGGCTTCCCACTATATTTTACTCTCTG
TGGCTTCCCACTATATTTTACTCTCTG
TGGCTTCCCACTATATTTTACTCTCTG
TGGCTTCCCACTATATTTTACTCTCTG
TGGCTTCCCACTATATTTTACTCTCTG
TGGCTTCCCACTATATTTTACTCTCTG
TGGCTTCCCACTATATTTTACTCTCTG
TGGCTTCCCACTATATTTTACTCTCTG
TGGCTTCCCACTATATTTTACTCTCTG
TGGCTTCCCACTATATTTTACTCTCTG
TGGCTTCCCACTATATTTTACTCTCTG
TGGCTTCCCACTATATTTTACTCTCTG
TGGCTTCCCACTATATTTTACTCTCTG
TGGCTTCCCACTATATTTTACTCTCTG
TGGCTTCCCACTATATTTTACTCTCTG
TGGCTTCCCACTATATTTTACTCTCTG
TGGCTTCCCACTATATTTTACTCTCTG
TGGCTTCCCACTATATTTTACTCTCTG
TGGCTTCCCACTATATTTTACTCTCTG
TGGCTTCCCACTATATTTTACTCTCTG
TGGCTTCCCACTATATTTTACTCTCTG
TGGCTTCCCACTATATTTTACTCTCTG
TGGCTTCCCACTATATTTTACTCTCTG
TGGCTTCCCACTATATTTTACTCTCTG
TGGCTTCCCACTATATTTTACTCTCTG
TGGCTTCCCACTATATTTTACTCTCTG
TGGCTTCCCACTATATTTTACTCTCTG
TGGCTTCCCACTATATTTTACTCTCTG
TGGCTTCCCACTATATTTTACTCTCTG
TGGCTTCCCACTATATTTTACTCTCTG
TGGCTTCCCACTATATTTTACTCTCTG
TGGCTTCCCACTATATTTTACTCTCTG
TGGCTTCCCACTATATTTTACTCTCTG
TGGCTTCCCACTATATTTTACTCTCTG
TGGCTTCCCACTATATTTTACTCTCTG
TGGCTTCCCACTATATTTTACTCTCTG
TGGCTTCCCACTATATTTTACTCTCTG
TGGCTTCCCACTATATTTTACTCTCTG
TGGCTTCCCACTATATTTTACTCTCTG
TGGCTTCCCACTATATTTTACTCTCTG
TGGCTTCCCACTATATTTTACTCTCTG

TGGCTTCCCACTATATTTTACTCTCTG
TGGCTTCCCACTATATTTTACTCTCTG
TGGCTTCCCACTATATTTTACTCTCTG
TGGCTTCCCACTATATTTTACTCTCTG
TGGCTTCCCACTATATTTTACTCTCTG
TGGCTTCCCACTATATTTTACTCTCTG
TGGCTTCCCACTATATTTTACTCTCTG
TGGCTTCCCACTATATTTTACTCTCTG
TGGCTTCCCACTATATTTTACTCTCTG
TGGCTTCCCACTATATTTTACTCTCTG
TGGCTTCCCACTATATTTTACTCTCTG
TGGCTTCCCACTATATTTTACTCTCTG
TGGCTTCCCACTATATTTTACTCTCTG
TGGCTTCCCACTATATTTTACTCTCTG
TGGCTTCCCACTATATTTTACTCTCTG
TGGCTTCCCACTATATTTTACTCTCTG
TGGCTTCCCACTATATTTTACTCTCTG
TGGCTTCCCACTATATTTTACTCTCTG
TGGCTTCCCACTATATTTTACTCTCTG
TGGCTTCCCACTATATTTTACTCTCTG
TGGCTTCCCACTATATTTTACTCTCTG
TGGCTTCCCACTATATTTTACTCTCTG
TGGCTTCCCACTATATTTTACTCTCTG
TGGCTTCCCACTATATTTTACTCTCTG
TGGCTTCCCACTATATTTTACTCTCTG
TGGCTTCCCACTATATTTTACTCTCTG
TGGCTTCCCACTATATTTTACTCTCTG
TGGCTTCCCACTATATTTTACTCTCTG
TGGCTTCCCACTATATTTTACTCTCTG
TGGCTTCCCACTATATTTTACTCTCTG
TGGCTTCCCACTATATTTTACTCTCTG
TGGCTTCCCACTATATTTTACTCTCTG
TGGCTTCCCACTATATTTTACTCTCTG
TGGCTTCCCACTATATTTTACTCTCTG
TGGCTTCCCACTATATTTTACTCTCTG
TGGCTTCCCACTATATTTTACTCTCTG
TGGCTTCCCACTATATTTTACTCTCTG
TGGCTTCCCACTATATTTTACTCTCTG
TGGCTTCCCACTATATTTTACTCTCTG
TGGCTTCCCACTATATTTTACTCTCTG
TGGCTTCCCACTATATTTTACTCTCTG
TGGCTTCCCACTATATTTTACTCTCTG
TGGCTTCCCACTATATTTTACTCTCTG
TGGCTTCCCACTATATTTTACTCTCTG
TGGCTTCCCACTATATTTTACTCTCTG
TGGCTTCCCACTATATTTTACTCTCTG
TGGCTTCCCACTATATTTTACTCTCTG
TGGCTTCCCACTATATTTTACTCTCTG
TGGCTTCCCACTATATTTTACTCTCTG
TGGCTTCCCACTATATTTTACTCTCTG
TGGCTTCCCACTATATTTTACTCTCTG
TGGCTTCCCACTATATTTTACTCTCTG
TGGCTTCCCACTATATTTTACTCTCTG
TGGCTTCCCACTATATTTTACTCTCTG

TGGCTTCCCACTATATTTTACTCTCTG
TGGCTTCCCACTATATTTTACTCTCTG
TGGCTTCCCACTATATTTTACTCTCTG
TGGCTTCCCACTATATTTTACTCTCTG
TGGCTTCCCACTATATTTTACTCTCTG
TGGCTTCCCACTATATTTTACTCTCTG
TGGCTTCCCACTATATTTTACTCTCTG
TGGCTTCCCACTATATTTTACTCTCTG
TGGCTTCCCACTATATTTTACTCTCTG
TGGCTTCCCACTATATTTTACTCTCTG
TGGCTTCCCACTATATTTTACTCTCTG
TGGCTTCCCACTATATTTTACTCTCTG
TGGCTTCCCACTATATTTTACTCTCTG
TGGCTTCCCACTATATTTTACTCTCTG
TGGCTTCCCACTATATTTTACTCTCTG
TGGCTTCCCACTATATTTTACTCTCTG
TGGCTTCCCACTATATTTTACTCTCTG
TGGCTTCCCACTATATTTTACTCTCTG
TGGCTTCCCACTATATTTTACTCTCTG
TGGCTTCCCACTATATTTTACTCTCTG
TGGCTTCCCACTATATTTTACTCTCTG
TGGCTTCCCACTATATTTTACTCTCTG
TGGCTTCCCACTATATTTTACTCTCTG
TGGCTTCCCACTATATTTTACTCTCTG
TGGCTTCCCACTATATTTTACTCTCTG
TGGCTTCCCACTATATTTTACTCTCTG
TGGCTTCCCACTATATTTTACTCTCTG
TGGCTTCCCACTATATTTTACTCTCTG
TGGCTTCCCACTATATTTTACTCTCTG
TGGCTTCCCACTATATTTTACTCTCTG
TGGCTTCCCACTATATTTTACTCTCTG
TGGCTTCCCACTATATTTTACTCTCTG
TGGCTTCCCACTATATTTTACTCTCTG
TGGCTTCCCACTATATTTTACTCTCTG
TGGCTTCCCACTATATTTTACTCTCTG
TGGCTTCCCACTATATTTTACTCTCTG
TGGCTTCCCACTATATTTTACTCTCTG
TGGCTTCCCACTATATTTTACTCTCTG
TGGCTTCCCACTATATTTTACTCTCTG
TGGCTTCCCACTATATTTTACTCTCTG
TGGCTTCCCACTATATTTTACTCTCTG
TGGCTTCCCACTATATTTTACTCTCTG
TGGCTTCCCACTATATTTTACTCTCTG
TGGCTTCCCACTATATTTTACTCTCTG
TGGCTTCCCACTATATTTTACTCTCTG
TGGCTTCCCACTATATTTTACTCTCTG
TGGCTTCCCACTATATTTTACTCTCTG
TGGCTTCCCACTATATTTTACTCTCTG
TGGCTTCCCACTATATTTTACTCTCTG
TGGCTTCCCACTATATTTTACTCTCTG
TGGCTTCCCACTATATTTTACTCTCTG
TGGCTTCCCACTATATTTTACTCTCTG
TGGCTTCCCACTATATTTTACTCTCTG
TGGCTTCCCACTATATTTTACTCTCTG

TGGCTTCCCACTATATTTTACTCTCTG
TGGCTTCCCACTATATTTTACTCTCTG
TGGCTTCCCACTATATTTTACTCTCTG
TGGCTTCCCACTATATTTTACTCTCTG
TGGCTTCCCACTATATTTTACTCTCTG
TGGCTTCCCACTATATTTTACTCTCTG
TGGCTTCCCACTATATTTTACTCTCTG
TGGCTTCCCACTATATTTTACTCTCTG
TGGCTTCCCACTATATTTTACTCTCTG
TGGCTTCCCACTATATTTTACTCTCTG
TGGCTTCCCACTATATTTTACTCTCTG
TGGCTTCCCACTATATTTTACTCTCTG
TGGCTTCCCACTATATTTTACTCTCTG
TGGCTTCCCACTATATTTTACTCTCTG
TGGCTTCCCACTATATTTTACTCTCTG
TGGCTTCCCACTATATTTTACTCTCTG
TGGCTTCCCACTATATTTTACTCTCTG
TGGCTTCCCACTATATTTTACTCTCTG
TGGCTTCCCACTATATTTTACTCTCTG
TGGCTTCCCACTATATTTTACTCTCTG
TGGCTTCCCACTATATTTTACTCTCTG
TGGCTTCCCACTATATTTTACTCTCTG
TGGCTTCCCACTATATTTTACTCTCTG
TGGCTTCCCACTATATTTTACTCTCTG
TGGCTTCCCACTATATTTTACTCTCTG
TGGCTTCCCACTATATTTTACTCTCTG
TGGCTTCCCACTATATTTTACTCTCTG
TGGCTTCCCACTATATTTTACTCTCTG
TGGCTTCCCACTATATTTTACTCTCTG
TGGCTTCCCACTATATTTTACTCTCTG
TGGCTTCCCACTATATTTTACTCTCTG
TGGCTTCCCACTATATTTTACTCTCTG
TGGCTTCCCACTATATTTTACTCTCTG
TGGCTTCCCACTATATTTTACTCTCTG
TGGCTTCCCACTATATTTTACTCTCTG
TGGCTTCCCACTATATTTTACTCTCTG
TGGCTTCCCACTATATTTTACTCTCTG
TGGCTTCCCACTATATTTTACTCTCTG
TGGCTTCCCACTATATTTTACTCTCTG
TGGCTTCCCACTATATTTTACTCTCTG
TGGCTTCCCACTATATTTTACTCTCTG
TGGCTTCCCACTATATTTTACTCTCTG
TGGCTTCCCACTATATTTTACTCTCTG
TGGCTTCCCACTATATTTTACTCTCTG
TGGCTTCCCACTATATTTTACTCTCTG
TGGCTTCCCACTATATTTTACTCTCTG
TGGCTTCCCACTATATTTTACTCTCTG
TGGCTTCCCACTATATTTTACTCTCTG
TGGCTTCCCACTATATTTTACTCTCTG
TGGCTTCCCACTATATTTTACTCTCTG
TGGCTTCCCACTATATTTTACTCTCTG
TGGCTTCCCACTATATTTTACTCTCTG
TGGCTTCCCACTATATTTTACTCTCTG
TGGCTTCCCACTATATTTTACTCTCTG

TGGCTTCCCACTATATTTTACTCTCTG
TGGCTTCCCACTATATTTTACTCTCTG
TGGCTTCCCACTATATTTTACTCTCTG
TGGCTTCCCACTATATTTTACTCTCTG
TGGCTTCCCACTATATTTTACTCTCTG
TGGCTTCCCACTATATTTTACTCTCTG
TGGCTTCCCACTATATTTTACTCTCTG
TGGCTTCCCACTATATTTTACTCTCTG
TGGCTTCCCACTATATTTTACTCTCTG
TGGCTTCCCACTATATTTTACTCTCTG
TGGCTTCCCACTATATTTTACTCTCTG
TGGCTTCCCACTATATTTTACTCTCTG
TGGCTTCCCACTATATTTTACTCTCTG
TGGCTTCCCACTATATTTTACTCTCTG
TGGCTTCCCACTATATTTTACTCTCTG
TGGCTTCCCACTATATTTTACTCTCTG
TGGCTTCCCACTATATTTTACTCTCTG
TGGCTTCCCACTATATTTTACTCTCTG
TGGCTTCCCACTATATTTTACTCTCTG
TGGCTTCCCACTATATTTTACTCTCTG
TGGCTTCCCACTATATTTTACTCTCTG
TGGCTTCCCACTATATTTTACTCTCTG
TGGCTTCCCACTATATTTTACTCTCTG
TGGCTTCCCACTATATTTTACTCTCTG
TGGCTTCCCACTATATTTTACTCTCTG
TGGCTTCCCACTATATTTTACTCTCTG
TGGCTTCCCACTATATTTTACTCTCTG
TGGCTTCCCACTATATTTTACTCTCTG
TGGCTTCCCACTATATTTTACTCTCTG
TGGCTTCCCACTATATTTTACTCTCTG
TGGCTTCCCACTATATTTTACTCTCTG
TGGCTTCCCACTATATTTTACTCTCTG
TGGCTTCCCACTATATTTTACTCTCTG
TGGCTTCCCACTATATTTTACTCTCTG
TGGCTTCCCACTATATTTTACTCTCTG
TGGCTTCCCACTATATTTTACTCTCTG
TGGCTTCCCACTATATTTTACTCTCTG
TGGCTTCCCACTATATTTTACTCTCTG
TGGCTTCCCACTATATTTTACTCTCTG
TGGCTTCCCACTATATTTTACTCTCTG
TGGCTTCCCACTATATTTTACTCTCTG
TGGCTTCCCACTATATTTTACTCTCTG
TGGCTTCCCACTATATTTTACTCTCTG
TGGCTTCCCACTATATTTTACTCTCTG
TGGCTTCCCACTATATTTTACTCTCTG
TGGCTTCCCACTATATTTTACTCTCTG
TGGCTTCCCACTATATTTTACTCTCTG
TGGCTTCCCACTATATTTTACTCTCTG
TGGCTTCCCACTATATTTTACTCTCTG
TGGCTTCCCACTATATTTTACTCTCTG
TGGCTTCCCACTATATTTTACTCTCTG
TGGCTTCCCACTATATTTTACTCTCTG
TGGCTTCCCACTATATTTTACTCTCTG
TGGCTTCCCACTATATTTTACTCTCTG

TGGCTTCCCACTATATTTTACTCTCTG
TGGCTTCCCACTATATTTTACTCTCTG
TGGCTTCCCACTATATTTTACTCTCTG
TGGCTTCCCACTATATTTTACTCTCTG
TGGCTTCCCACTATATTTTACTCTCTG
TGGCTTCCCACTATATTTTACTCTCTG
TGGCTTCCCACTATATTTTACTCTCTG
TGGCTTCCCACTATATTTTACTCTCTG
TGGCTTCCCACTATATTTTACTCTCTG
TGGCTTCCCACTATATTTTACTCTCTG
TGGCTTCCCACTATATTTTACTCTCTG
TGGCTTCCCACTATATTTTACTCTCTG
TGGCTTCCCACTATATTTTACTCTCTG
TGGCTTCCCACTATATTTTACTCTCTG
TGGCTTCCCACTATATTTTACTCTCTG
TGGCTTCCCACTATATTTTACTCTCTG
TGGCTTCCCACTATATTTTACTCTCTG
TGGCTTCCCACTATATTTTACTCTCTG
TGGCTTCCCACTATATTTTACTCTCTG
TGGCTTCCCACTATATTTTACTCTCTG
TGGCTTCCCACTATATTTTACTCTCTG
TGGCTTCCCACTATATTTTACTCTCTG
TGGCTTCCCACTATATTTTACTCTCTG
TGGCTTCCCACTATATTTTACTCTCTG
TGGCTTCCCACTATATTTTACTCTCTG
TGGCTTCCCACTATATTTTACTCTCTG
TGGCTTCCCACTATATTTTACTCTCTG
TGGCTTCCCACTATATTTTACTCTCTG
TGGCTTCCCACTATATTTTACTCTCTG
TGGCTTCCCACTATATTTTACTCTCTG
TGGCTTCCCACTATATTTTACTCTCTG
TGGCTTCCCACTATATTTTACTCTCTG
TGGCTTCCCACTATATTTTACTCTCTG
TGGCTTCCCACTATATTTTACTCTCTG
TGGCTTCCCACTATATTTTACTCTCTG
TGGCTTCCCACTATATTTTACTCTCTG
TGGCTTCCCACTATATTTTACTCTCTG
TGGCTTCCCACTATATTTTACTCTCTG
TGGCTTCCCACTATATTTTACTCTCTG
TGGCTTCCCACTATATTTTACTCTCTG
TGGCTTCCCACTATATTTTACTCTCTG
TGGCTTCCCACTATATTTTACTCTCTG
TGGCTTCCCACTATATTTTACTCTCTG
TGGCTTCCCACTATATTTTACTCTCTG
TGGCTTCCCACTATATTTTACTCTCTG
TGGCTTCCCACTATATTTTACTCTCTG
TGGCTTCCCACTATATTTTACTCTCTG
TGGCTTCCCACTATATTTTACTCTCTG
TGGCTTCCCACTATATTTTACTCTCTG
TGGCTTCCCACTATATTTTACTCTCTG
TGGCTTCCCACTATATTTTACTCTCTG
TGGCTTCCCACTATATTTTACTCTCTG
TGGCTTCCCACTATATTTTACTCTCTG
TGGCTTCCCACTATATTTTACTCTCTG

TGGCTTCCCACTATATTTTACTCTCTG
TGGCTTCCCACTATATTTTACTCTCTG
TGGCTTCCCACTATATTTTACTCTCTG
TGGCTTCCCACTATATTTTACTCTCTG
TGGCTTCCCACTATATTTTACTCTCTG
TGGCTTCCCACTATATTTTACTCTCTG
TGGCTTCCCACTATATTTTACTCTCTG
TGGCTTCCCACTATATTTTACTCTCTG
TGGCTTCCCACTATATTTTACTCTCTG
TGGCTTCCCACTATATTTTACTCTCTG
TGGCTTCCCACTATATTTTACTCTCTG
TGGCTTCCCACTATATTTTACTCTCTG
TGGCTTCCCACTATATTTTACTCTCTG
TGGCTTCCCACTATATTTTACTCTCTG
TGGCTTCCCACTATATTTTACTCTCTG
TGGCTTCCCACTATATTTTACTCTCTG
TGGCTTCCCACTATATTTTACTCTCTG
TGGCTTCCCACTATATTTTACTCTCTG
TGGCTTCCCACTATATTTTACTCTCTG
TGGCTTCCCACTATATTTTACTCTCTG
TGGCTTCCCACTATATTTTACTCTCTG
TGGCTTCCCACTATATTTTACTCTCTG
TGGCTTCCCACTATATTTTACTCTCTG
TGGCTTCCCACTATATTTTACTCTCTG
TGGCTTCCCACTATATTTTACTCTCTG
TGGCTTCCCACTATATTTTACTCTCTG
TGGCTTCCCACTATATTTTACTCTCTG
TGGCTTCCCACTATATTTTACTCTCTG
TGGCTTCCCACTATATTTTACTCTCTG
TGGCTTCCCACTATATTTTACTCTCTG
TGGCTTCCCACTATATTTTACTCTCTG
TGGCTTCCCACTATATTTTACTCTCTG
TGGCTTCCCACTATATTTTACTCTCTG
TGGCTTCCCACTATATTTTACTCTCTG
TGGCTTCCCACTATATTTTACTCTCTG
TGGCTTCCCACTATATTTTACTCTCTG
TGGCTTCCCACTATATTTTACTCTCTG
TGGCTTCCCACTATATTTTACTCTCTG
TGGCTTCCCACTATATTTTACTCTCTG
TGGCTTCCCACTATATTTTACTCTCTG
TGGCTTCCCACTATATTTTACTCTCTG
TGGCTTCCCACTATATTTTACTCTCTG
TGGCTTCCCACTATATTTTACTCTCTG
TGGCTTCCCACTATATTTTACTCTCTG
TGGCTTCCCACTATATTTTACTCTCTG
TGGCTTCCCACTATATTTTACTCTCTG
TGGCTTCCCACTATATTTTACTCTCTG
TGGCTTCCCACTATATTTTACTCTCTG
TGGCTTCCCACTATATTTTACTCTCTG
TGGCTTCCCACTATATTTTACTCTCTG
TGGCTTCCCACTATATTTTACTCTCTG
TGGCTTCCCACTATATTTTACTCTCTG
TGGCTTCCCACTATATTTTACTCTCTG
TGGCTTCCCACTATATTTTACTCTCTG

TGGCTTCCCACTATATTTTACTCTCTG
TGGCTTCCCACTATATTTTACTCTCTG
TGGCTTCCCACTATATTTTACTCTCTG
TGGCTTCCCACTATATTTTACTCTCTG
TGGCTTCCCACTATATTTTACTCTCTG
TGGCTTCCCACTATATTTTACTCTCTG
TGGCTTCCCACTATATTTTACTCTCTG
TGGCTTCCCACTATATTTTACTCTCTG
TGGCTTCCCACTATATTTTACTCTCTG
TGGCTTCCCACTATATTTTACTCTCTG
TGGCTTCCCACTATATTTTACTCTCTG
TGGCTTCCCACTATATTTTACTCTCTG
TGGCTTCCCACTATATTTTACTCTCTG
TGGCTTCCCACTATATTTTACTCTCTG
TGGCTTCCCACTATATTTTACTCTCTG
TGGCTTCCCACTATATTTTACTCTCTG
TGGCTTCCCACTATATTTTACTCTCTG
TGGCTTCCCACTATATTTTACTCTCTG
TGGCTTCCCACTATATTTTACTCTCTG
TGGCTTCCCACTATATTTTACTCTCTG
TGGCTTCCCACTATATTTTACTCTCTG
TGGCTTCCCACTATATTTTACTCTCTG
TGGCTTCCCACTATATTTTACTCTCTG
TGGCTTCCCACTATATTTTACTCTCTG
TGGCTTCCCACTATATTTTACTCTCTG
TGGCTTCCCACTATATTTTACTCTCTG
TGGCTTCCCACTATATTTTACTCTCTG
TGGCTTCCCACTATATTTTACTCTCTG
TGGCTTCCCACTATATTTTACTCTCTG
TGGCTTCCCACTATATTTTACTCTCTG
TGGCTTCCCACTATATTTTACTCTCTG
TGGCTTCCCACTATATTTTACTCTCTG
TGGCTTCCCACTATATTTTACTCTCTG
TGGCTTCCCACTATATTTTACTCTCTG
TGGCTTCCCACTATATTTTACTCTCTG
TGGCTTCCCACTATATTTTACTCTCTG
TGGCTTCCCACTATATTTTACTCTCTG
TGGCTTCCCACTATATTTTACTCTCTG
TGGCTTCCCACTATATTTTACTCTCTG
TGGCTTCCCACTATATTTTACTCTCTG
TGGCTTCCCACTATATTTTACTCTCTG
TGGCTTCCCACTATATTTTACTCTCTG
TGGCTTCCCACTATATTTTACTCTCTG
TGGCTTCCCACTATATTTTACTCTCTG
TGGCTTCCCACTATATTTTACTCTCTG
TGGCTTCCCACTATATTTTACTCTCTG
TGGCTTCCCACTATATTTTACTCTCTG
TGGCTTCCCACTATATTTTACTCTCTG
TGGCTTCCCACTATATTTTACTCTCTG
TGGCTTCCCACTATATTTTACTCTCTG
TGGCTTCCCACTATATTTTACTCTCTG
TGGCTTCCCACTATATTTTACTCTCTG
TGGCTTCCCACTATATTTTACTCTCTG
TGGCTTCCCACTATATTTTACTCTCTG

TGGCTTCCCACTATATTTTACTCTCTG
TGGCTTCCCACTATATTTTACTCTCTG
TGGCTTCCCACTATATTTTACTCTCTG
TGGCTTCCCACTATATTTTACTCTCTG
TGGCTTCCCACTATATTTTACTCTCTG
TGGCTTCCCACTATATTTTACTCTCTG
TGGCTTCCCACTATATTTTACTCTCTG
TGGCTTCCCACTATATTTTACTCTCTG
TGGCTTCCCACTATATTTTACTCTCTG
TGGCTTCCCACTATATTTTACTCTCTG
TGGCTTCCCACTATATTTTACTCTCTG
TGGCTTCCCACTATATTTTACTCTCTG
TGGCTTCCCACTATATTTTACTCTCTG
TGGCTTCCCACTATATTTTACTCTCTG
TGGCTTCCCACTATATTTTACTCTCTG
TGGCTTCCCACTATATTTTACTCTCTG
TGGCTTCCCACTATATTTTACTCTCTG
TGGCTTCCCACTATATTTTACTCTCTG
TGGCTTCCCACTATATTTTACTCTCTG
TGGCTTCCCACTATATTTTACTCTCTG
TGGCTTCCCACTATATTTTACTCTCTG
TGGCTTCCCACTATATTTTACTCTCTG
TGGCTTCCCACTATATTTTACTCTCTG
TGGCTTCCCACTATATTTTACTCTCTG
TGGCTTCCCACTATATTTTACTCTCTG
TGGCTTCCCACTATATTTTACTCTCTG
TGGCTTCCCACTATATTTTACTCTCTG
TGGCTTCCCACTATATTTTACTCTCTG
TGGCTTCCCACTATATTTTACTCTCTG
TGGCTTCCCACTATATTTTACTCTCTG
TGGCTTCCCACTATATTTTACTCTCTG
TGGCTTCCCACTATATTTTACTCTCTG
TGGCTTCCCACTATATTTTACTCTCTG
TGGCTTCCCACTATATTTTACTCTCTG
TGGCTTCCCACTATATTTTACTCTCTG
TGGCTTCCCACTATATTTTACTCTCTG
TGGCTTCCCACTATATTTTACTCTCTG
TGGCTTCCCACTATATTTTACTCTCTG
TGGCTTCCCACTATATTTTACTCTCTG
TGGCTTCCCACTATATTTTACTCTCTG
TGGCTTCCCACTATATTTTACTCTCTG
TGGCTTCCCACTATATTTTACTCTCTG
TGGCTTCCCACTATATTTTACTCTCTG
TGGCTTCCCACTATATTTTACTCTCTG
TGGCTTCCCACTATATTTTACTCTCTG
TGGCTTCCCACTATATTTTACTCTCTG
TGGCTTCCCACTATATTTTACTCTCTG
TGGCTTCCCACTATATTTTACTCTCTG
TGGCTTCCCACTATATTTTACTCTCTG
TGGCTTCCCACTATATTTTACTCTCTG
TGGCTTCCCACTATATTTTACTCTCTG
TGGCTTCCCACTATATTTTACTCTCTG
TGGCTTCCCACTATATTTTACTCTCTG
TGGCTTCCCACTATATTTTACTCTCTG

TGGCTTCCCACTATATTTTACTCTCTG
TGGCTTCCCACTATATTTTACTCTCTG
TGGCTTCCCACTATATTTTACTCTCTG
TGGCTTCCCACTATATTTTACTCTCTG
TGGCTTCCCACTATATTTTACTCTCTG
TGGCTTCCCACTATATTTTACTCTCTG
TGGCTTCCCACTATATTTTACTCTCTG
TGGCTTCCCACTATATTTTACTCTCTG
TGGCTTCCCACTATATTTTACTCTCTG
TGGCTTCCCACTATATTTTACTCTCTG
TGGCTTCCCACTATATTTTACTCTCTG
TGGCTTCCCACTATATTTTACTCTCTG
TGGCTTCCCACTATATTTTACTCTCTG
TGGCTTCCCACTATATTTTACTCTCTG
TGGCTTCCCACTATATTTTACTCTCTG
TGGCTTCCCACTATATTTTACTCTCTG
TGGCTTCCCACTATATTTTACTCTCTG
TGGCTTCCCACTATATTTTACTCTCTG
TGGCTTCCCACTATATTTTACTCTCTG
TGGCTTCCCACTATATTTTACTCTCTG
TGGCTTCCCACTATATTTTACTCTCTG
TGGCTTCCCACTATATTTTACTCTCTG
TGGCTTCCCACTATATTTTACTCTCTG
TGGCTTCCCACTATATTTTACTCTCTG
TGGCTTCCCACTATATTTTACTCTCTG
TGGCTTCCCACTATATTTTACTCTCTG
TGGCTTCCCACTATATTTTACTCTCTG
TGGCTTCCCACTATATTTTACTCTCTG
TGGCTTCCCACTATATTTTACTCTCTG
TGGCTTCCCACTATATTTTACTCTCTG
TGGCTTCCCACTATATTTTACTCTCTG
TGGCTTCCCACTATATTTTACTCTCTG
TGGCTTCCCACTATATTTTACTCTCTG
TGGCTTCCCACTATATTTTACTCTCTG
TGGCTTCCCACTATATTTTACTCTCTG
TGGCTTCCCACTATATTTTACTCTCTG
TGGCTTCCCACTATATTTTACTCTCTG
TGGCTTCCCACTATATTTTACTCTCTG
TGGCTTCCCACTATATTTTACTCTCTG
TGGCTTCCCACTATATTTTACTCTCTG
TGGCTTCCCACTATATTTTACTCTCTG
TGGCTTCCCACTATATTTTACTCTCTG
TGGCTTCCCACTATATTTTACTCTCTG
TGGCTTCCCACTATATTTTACTCTCTG
TGGCTTCCCACTATATTTTACTCTCTG
TGGCTTCCCACTATATTTTACTCTCTG
TGGCTTCCCACTATATTTTACTCTCTG
TGGCTTCCCACTATATTTTACTCTCTG
TGGCTTCCCACTATATTTTACTCTCTG
TGGCTTCCCACTATATTTTACTCTCTG
TGGCTTCCCACTATATTTTACTCTCTG
TGGCTTCCCACTATATTTTACTCTCTG
TGGCTTCCCACTATATTTTACTCTCTG
TGGCTTCCCACTATATTTTACTCTCTG

TGGCTTCCCACTATATTTTACTCTCTG
TGGCTTCCCACTATATTTTACTCTCTG
TGGCTTCCCACTATATTTTACTCTCTG
TGGCTTCCCACTATATTTTACTCTCTG
TGGCTTCCCACTATATTTTACTCTCTG
TGGCTTCCCACTATATTTTACTCTCTG
TGGCTTCCCACTATATTTTACTCTCTG
TGGCTTCCCACTATATTTTACTCTCTG
TGGCTTCCCACTATATTTTACTCTCTG
TGGCTTCCCACTATATTTTACTCTCTG
TGGCTTCCCACTATATTTTACTCTCTG
TGGCTTCCCACTATATTTTACTCTCTG
TGGCTTCCCACTATATTTTACTCTCTG
TGGCTTCCCACTATATTTTACTCTCTG
TGGCTTCCCACTATATTTTACTCTCTG
TGGCTTCCCACTATATTTTACTCTCTG
TGGCTTCCCACTATATTTTACTCTCTG
TGGCTTCCCACTATATTTTACTCTCTG
TGGCTTCCCACTATATTTTACTCTCTG
TGGCTTCCCACTATATTTTACTCTCTG
TGGCTTCCCACTATATTTTACTCTCTG
TGGCTTCCCACTATATTTTACTCTCTG
TGGCTTCCCACTATATTTTACTCTCTG
TGGCTTCCCACTATATTTTACTCTCTG
TGGCTTCCCACTATATTTTACTCTCTG
TGGCTTCCCACTATATTTTACTCTCTG
TGGCTTCCCACTATATTTTACTCTCTG
TGGCTTCCCACTATATTTTACTCTCTG
TGGCTTCCCACTATATTTTACTCTCTG
TGGCTTCCCACTATATTTTACTCTCTG
TGGCTTCCCACTATATTTTACTCTCTG
TGGCTTCCCACTATATTTTACTCTCTG
TGGCTTCCCACTATATTTTACTCTCTG
TGGCTTCCCACTATATTTTACTCTCTG
TGGCTTCCCACTATATTTTACTCTCTG
TGGCTTCCCACTATATTTTACTCTCTG
TGGCTTCCCACTATATTTTACTCTCTG
TGGCTTCCCACTATATTTTACTCTCTG
TGGCTTCCCACTATATTTTACTCTCTG
TGGCTTCCCACTATATTTTACTCTCTG
TGGCTTCCCACTATATTTTACTCTCTG
TGGCTTCCCACTATATTTTACTCTCTG
TGGCTTCCCACTATATTTTACTCTCTG
TGGCTTCCCACTATATTTTACTCTCTG
TGGCTTCCCACTATATTTTACTCTCTG
TGGCTTCCCACTATATTTTACTCTCTG
TGGCTTCCCACTATATTTTACTCTCTG
TGGCTTCCCACTATATTTTACTCTCTG
TGGCTTCCCACTATATTTTACTCTCTG
TGGCTTCCCACTATATTTTACTCTCTG
TGGCTTCCCACTATATTTTACTCTCTG
TGGCTTCCCACTATATTTTACTCTCTG
TGGCTTCCCACTATATTTTACTCTCTG
TGGCTTCCCACTATATTTTACTCTCTG

TGGCTTCCCACTATATTTTACTCTCTG
TGGCTTCCCACTATATTTTACTCTCTG
TGGCTTCCCACTATATTTTACTCTCTG
TGGCTTCCCACTATATTTTACTCTCTG
TGGCTTCCCACTATATTTTACTCTCTG
TGGCTTCCCACTATATTTTACTCTCTG
TGGCTTCCCACTATATTTTACTCTCTG
TGGCTTCCCACTATATTTTACTCTCTG
TGGCTTCCCACTATATTTTACTCTCTG
TGGCTTCCCACTATATTTTACTCTCTG
TGGCTTCCCACTATATTTTACTCTCTG
TGGCTTCCCACTATATTTTACTCTCTG
TGGCTTCCCACTATATTTTACTCTCTG
TGGCTTCCCACTATATTTTACTCTCTG
TGGCTTCCCACTATATTTTACTCTCTG
TGGCTTCCCACTATATTTTACTCTCTG
TGGCTTCCCACTATATTTTACTCTCTG
TGGCTTCCCACTATATTTTACTCTCTG
TGGCTTCCCACTATATTTTACTCTCTG
TGGCTTCCCACTATATTTTACTCTCTG
TGGCTTCCCACTATATTTTACTCTCTG
TGGCTTCCCACTATATTTTACTCTCTG
TGGCTTCCCACTATATTTTACTCTCTG
TGGCTTCCCACTATATTTTACTCTCTG
TGGCTTCCCACTATATTTTACTCTCTG
TGGCTTCCCACTATATTTTACTCTCTG
TGGCTTCCCACTATATTTTACTCTCTG
TGGCTTCCCACTATATTTTACTCTCTG
TGGCTTCCCACTATATTTTACTCTCTG
TGGCTTCCCACTATATTTTACTCTCTG
TGGCTTCCCACTATATTTTACTCTCTG
TGGCTTCCCACTATATTTTACTCTCTG
TGGCTTCCCACTATATTTTACTCTCTG
TGGCTTCCCACTATATTTTACTCTCTG
TGGCTTCCCACTATATTTTACTCTCTG
TGGCTTCCCACTATATTTTACTCTCTG
TGGCTTCCCACTATATTTTACTCTCTG
TGGCTTCCCACTATATTTTACTCTCTG
TGGCTTCCCACTATATTTTACTCTCTG
TGGCTTCCCACTATATTTTACTCTCTG
TGGCTTCCCACTATATTTTACTCTCTG
TGGCTTCCCACTATATTTTACTCTCTG
TGGCTTCCCACTATATTTTACTCTCTG
TGGCTTCCCACTATATTTTACTCTCTG
TGGCTTCCCACTATATTTTACTCTCTG
TGGCTTCCCACTATATTTTACTCTCTG
TGGCTTCCCACTATATTTTACTCTCTG
TGGCTTCCCACTATATTTTACTCTCTG
TGGCTTCCCACTATATTTTACTCTCTG
TGGCTTCCCACTATATTTTACTCTCTG
TGGCTTCCCACTATATTTTACTCTCTG
TGGCTTCCCACTATATTTTACTCTCTG
TGGCTTCCCACTATATTTTACTCTCTG
TGGCTTCCCACTATATTTTACTCTCTG

TGGCTTCCCACTATATTTTACTCTCTG
TGGCTTCCCACTATATTTTACTCTCTG
TGGCTTCCCACTATATTTTACTCTCTG
TGGCTTCCCACTATATTTTACTCTCTG
TGGCTTCCCACTATATTTTACTCTCTG
TGGCTTCCCACTATATTTTACTCTCTG
TGGCTTCCCACTATATTTTACTCTCTG
TGGCTTCCCACTATATTTTACTCTCTG
TGGCTTCCCACTATATTTTACTCTCTG
TGGCTTCCCACTATATTTTACTCTCTG
TGGCTTCCCACTATATTTTACTCTCTG
TGGCTTCCCACTATATTTTACTCTCTG
TGGCTTCCCACTATATTTTACTCTCTG
TGGCTTCCCACTATATTTTACTCTCTG
TGGCTTCCCACTATATTTTACTCTCTG
TGGCTTCCCACTATATTTTACTCTCTG
TGGCTTCCCACTATATTTTACTCTCTG
TGGCTTCCCACTATATTTTACTCTCTG
TGGCTTCCCACTATATTTTACTCTCTG
TGGCTTCCCACTATATTTTACTCTCTG
TGGCTTCCCACTATATTTTACTCTCTG
TGGCTTCCCACTATATTTTACTCTCTG
TGGCTTCCCACTATATTTTACTCTCTG
TGGCTTCCCACTATATTTTACTCTCTG
TGGCTTCCCACTATATTTTACTCTCTG
TGGCTTCCCACTATATTTTACTCTCTG
TGGCTTCCCACTATATTTTACTCTCTG
TGGCTTCCCACTATATTTTACTCTCTG
TGGCTTCCCACTATATTTTACTCTCTG
TGGCTTCCCACTATATTTTACTCTCTG
TGGCTTCCCACTATATTTTACTCTCTG
TGGCTTCCCACTATATTTTACTCTCTG
TGGCTTCCCACTATATTTTACTCTCTG
TGGCTTCCCACTATATTTTACTCTCTG
TGGCTTCCCACTATATTTTACTCTCTG
TGGCTTCCCACTATATTTTACTCTCTG
TGGCTTCCCACTATATTTTACTCTCTG
TGGCTTCCCACTATATTTTACTCTCTG
TGGCTTCCCACTATATTTTACTCTCTG
TGGCTTCCCACTATATTTTACTCTCTG
TGGCTTCCCACTATATTTTACTCTCTG
TGGCTTCCCACTATATTTTACTCTCTG
TGGCTTCCCACTATATTTTACTCTCTG
TGGCTTCCCACTATATTTTACTCTCTG
TGGCTTCCCACTATATTTTACTCTCTG
TGGCTTCCCACTATATTTTACTCTCTG
TGGCTTCCCACTATATTTTACTCTCTG
TGGCTTCCCACTATATTTTACTCTCTG
TGGCTTCCCACTATATTTTACTCTCTG
TGGCTTCCCACTATATTTTACTCTCTG
TGGCTTCCCACTATATTTTACTCTCTG
TGGCTTCCCACTATATTTTACTCTCTG
TGGCTTCCCACTATATTTTACTCTCTG
TGGCTTCCCACTATATTTTACTCTCTG
TGGCTTCCCACTATATTTTACTCTCTG

TGGCTTCCCACTATATTTTACTCTCTG
TGGCTTCCCACTATATTTTACTCTCTG
TGGCTTCCCACTATATTTTACTCTCTG
TGGCTTCCCACTATATTTTACTCTCTG
TGGCTTCCCACTATATTTTACTCTCTG
TGGCTTCCCACTATATTTTACTCTCTG
TGGCTTCCCACTATATTTTACTCTCTG
TGGCTTCCCACTATATTTTACTCTCTG
TGGCTTCCCACTATATTTTACTCTCTG
TGGCTTCCCACTATATTTTACTCTCTG
TGGCTTCCCACTATATTTTACTCTCTG
TGGCTTCCCACTATATTTTACTCTCTG
TGGCTTCCCACTATATTTTACTCTCTG
TGGCTTCCCACTATATTTTACTCTCTG
TGGCTTCCCACTATATTTTACTCTCTG
TGGCTTCCCACTATATTTTACTCTCTG
TGGCTTCCCACTATATTTTACTCTCTG
TGGCTTCCCACTATATTTTACTCTCTG
TGGCTTCCCACTATATTTTACTCTCTG
TGGCTTCCCACTATATTTTACTCTCTG
TGGCTTCCCACTATATTTTACTCTCTG
TGGCTTCCCACTATATTTTACTCTCTG
TGGCTTCCCACTATATTTTACTCTCTG
TGGCTTCCCACTATATTTTACTCTCTG
TGGCTTCCCACTATATTTTACTCTCTG
TGGCTTCCCACTATATTTTACTCTCTG
TGGCTTCCCACTATATTTTACTCTCTG
TGGCTTCCCACTATATTTTACTCTCTG
TGGCTTCCCACTATATTTTACTCTCTG
TGGCTTCCCACTATATTTTACTCTCTG
TGGCTTCCCACTATATTTTACTCTCTG
TGGCTTCCCACTATATTTTACTCTC
TGGCTTCCCAGTATTTTTTACTCAC
Nr of selected repeats 1251 Similarity 1.000000
Consensus:
TGGCTTCCCACTATATTTTACTCTCTG
>Cele-UNSB01_1:4669638-4670054 Satlength=417 Nr of Repeats=33 RepeatLength=12
seed=TTTTTATTTT
TTTTTATTTTTA
TTTTTATTTTTA
TTTTTATTTTTA
TTTTTATTTTTA
TTTTTATTTTTA
TTTTTATTTTTA
TTTTTATTTTTA
TTTTTATTTTTA
TTTTTATTTTTA
TTTTTATTTTTT
TTTTTATTTTTATT
TTTTTTTTTA
TTTTTATTTTTA
TTTTTATTTTTTTT
TTTTTTTTTATTTTTTTTTT

TTTTTTTTTA
TTTTTATTTTTA
TTTTTATTTTTA
TTTTTATTTTTA
TTTTTATTTTTA
TTTTTATTTTTA
TTTTTATTTTTA
TTTTTATTTTTA
TTTTTATTTTTA
TTTTTATTTTTA
TTTTTATTTTTA
TTTTTATTTTTA
TTTTTATTTTTG
TTTTTATTTTTA
TTTTTATTTGTG
TTTTTATTTTTA
TTTTTATTTTTATTCTCA
TTTTTATTTTTATTTTTA
Nr of selected repeats 26 Similarity 0.967179
Consensus:
TTTTTATTTTTA
>Cele-UNSB01_1:4669638-4670196 Satlength=559 Nr of Repeats=41 RepeatLength=12
seed=TTTTTATTTT
TTTTTATTTTTA
TTTTTATTTTTA
TTTTTATTTTTA
TTTTTATTTTTA
TTTTTATTTTTA
TTTTTATTTTTA
TTTTTATTTTTA
TTTTTATTTTTA
TTTTTATTTTTA
TTTTTATTTTTT
TTTTTATTTTTATT
TTTTTTTTTA
TTTTTATTTTTA
TTTTTATTTTTTTT
TTTTTTTTTATTTTTTTTTT
TTTTTTTTTA
TTTTTATTTTTA
TTTTTATTTTTA
TTTTTATTTTTA
TTTTTATTTTTA
TTTTTATTTTTA
TTTTTATTTTTA
TTTTTATTTTTA
TTTTTATTTTTA
TTTTTATTTTTA
TTTTTATTTTTA
TTTTTATTTTTA
TTTTTATTTTTG
TTTTTATTTTTA
TTTTTATTTGTG
TTTTTATTTTTA

TTTTTATTTTTATTCTCA
TTTTTATTTTTA
TTTTTATTTTTATTTTATTATTTATTTATTTATT
TATTTATTTTTAA
TTTTAATTTTAATTTCAA
TTTTAATTTTAATTTTAATTTCAA
TTTTAATTTTAATTTCAA
TTTTATTTTTA
TTTTTATTTTTAT
TTTTTATTTATTTTTTA
Nr of selected repeats 27 Similarity 0.968344
Consensus:
TTTTTATTTTTA
>Cele-UNSB01_1:4669638-4670946 Satlength=1309 Nr of Repeats=43 RepeatLength=12
seed=TTTTTATTTT
TTTTTATTTTTA
TTTTTATTTTTA
TTTTTATTTTTA
TTTTTATTTTTA
TTTTTATTTTTA
TTTTTATTTTTA
TTTTTATTTTTA
TTTTTATTTTTA
TTTTTATTTTTA
TTTTTATTTTTT
TTTTTATTTTTATT
TTTTTTTTTA
TTTTTATTTTTA
TTTTTATTTTTTTT
TTTTTTTTTATTTTTTTTTT
TTTTTTTTTA
TTTTTATTTTTA
TTTTTATTTTTA
TTTTTATTTTTA
TTTTTATTTTTA
TTTTTATTTTTA
TTTTTATTTTTA
TTTTTATTTTTA
TTTTTATTTTTA
TTTTTATTTTTA
TTTTTATTTTTA
TTTTTATTTTTA
TTTTTATTTTTG
TTTTTATTTTTA
TTTTTATTTGTG
TTTTTATTTTTA
TTTTTATTTTTATTCTCA
TTTTTATTTTTA
TTTTTATTTTTATTTTATTATTTATTTATTTATT
TATTTATTTTTAA
TTTTAATTTTAATTTCAA
TTTTAATTTTAATTTTAATTTCAA
TTTTAATTTTAATTTCAA
TTTTATTTTTA

TTTTTATTTTTAT
TTTTTATTTAT
TTTTTATTTTTAT
TTTTTATTCTCATTTTTACCAGAATATATTCCAGAATATATTCCAAAATATATTCCAGAATATATTCCAGAATATACTC
CAAAATATATTCCAGAGTATATTCCAGAATATATTCCAGAATTTATTCCAGAATATATTCCAGAATATATTCCAAAATA
TATTTCAGAATATATTCCAAAATATATTCCAGAATTTATTTCAGAATATATTCCAGAATATATTCCAGAATATATTCCA
GAATATATTCCAAAATATATTCCAAAATATATTCCAGAATATATTCCAGAATATATTCCAAAATATATTCCAGAATATA
TTCCTTATTGATCTTACGAAAACCTGAAACATGAAACTAGATTAGAAATGACCTATCTAACTGTTATACGGCTACAGTC
CATTTTTAGCAGGCTTGGTTTCAGGGACCTGTCGTCCTCACACGAAGCTGGTTTTTGGCGCCTATATGGAAGCCCTGTC
TGCCTCATGCCTTTACCAATTTTCTATGAAATCTCCGGAGTTCGGTGTTTGCACTGTTAATTATAAGTTTATCATTTCA
ATTAATTTTTTGAAAATTCAATTCTAAAAACTTCAAGGTTCACGGTCATTCTGGCAATAGGCACACCATTTTTTTTATT
GGATAAAATAGAAAATAGTGTCATTAGAGGTAATTTGAAATTTTAGAATCTGAAATTTTCAGACTCGATTCTTAAAATC
AATGAAATGACGGATCTTTGTCAAGTTCGTCC
Nr of selected repeats 27 Similarity 0.968344
Consensus:
TTTTTATTTTTA
>Cele-UNSB01_1:4694849-4695647 Satlength=799 Nr of Repeats=20 RepeatLength=40
seed=CATTCCAGAA
CATTCCAGAATTTTCTCGATTTTTCTAGAAAGTTCTGGAA
CATTCCAGAATTTTCTCGATTTTTCTAGAAAGTTCTGGAG
CATTCCAGAATTTTCTCGATTTTTCTAGAAAGTTCTGGAA
CATTCCAGAATTTTCCCGATTTTTCTAGAAAGTTCTGGAA
CATTCCAGAATTTTCTCGATTTTTCTAGAAAGTTCTGGAA
CATTCCAGAATTTTCCCGATTTTTCTAGAAAGTTCTGGAA
CATTCCAGAATTTTCTCGATTTTTCTAGAAAGTTCTGGAA
CATTCCAGAATTTTCCCGATTTTTCTAGAAAGTTCTGGAA
CATTCCAGAAATTTCCCGATTTTTCTAGAAAGTTCTGGAA
CATTCCAGAATTTTCCCGATTTTTCTAGAAAGTTCTGGAA
CATTCCAGAATTTTCTCGATTTTCTAGAAAGTTCTGGAA
CATTCCAGAAATTTCCCGATTTTTCTAGAAAGTTCTGGAA
CATTCCAGAATTTTCTCGATTTTTCTAGAAAGTTCTGGAG
CATTCCAGAATTTTCTCGATTTTTCTAGAAAGTTCTGGAA
CATTCCAGAATTTTCCCGATTTTTCTAGAAAGTTCTGGAA
CATTCCAGAAATTTCCCGATTTTTCTAGAAAGTTCTGGAA
CATTCCAGAATTTTCCCGATTTTTCTAGAAAGTTCTGGAA
CATTCCAGAATTTTCTCGATTTTCTAGAAAGTTCTGGAA
CATTCCAGAAATTTCCCGATTTTTCTAGAAAGTTCTGGAA
CATTCCAGAATTTTCCCGATTTTTCTAGAAAGTTCTGGAA
Nr of selected repeats 18 Similarity 0.964052
Consensus:
CATTCCAGAAtTTTCcCGATTTTTCTAGAAAGTTCTGGAA
>Cele-UNSB01_1:4695896-4696451 Satlength=556 Nr of Repeats=34 RepeatLength=15
seed=GGGTCTCACC
GGGTCTCACCACGAC
GGGTCTCACCACGGT
GGGTCTCACCACGAT
GGGTCTCACCACGAT
GGGTCTCACCACGATGGGTTTCGCCACGAT
GGGTCTCGCCACGACGGGTCTTATCACGGT
GGGTCTCGCCACGAT
GGGTCTCACCACGAT
GGGTCTTACCACGAT
GGGTCTCACCACGAC
GGGTCTCACCACGGT

GGGTCTCACCACGAT
GGGTCTCACCACGAT
GGGTCTCACCACGAT
GGGTCTCGCCACGACGGGTCTTATCACGGT
GGGTCTCACCACGGT
GGGTCTCACCACGGT
GGGTCTCGCCACGAT
GGGTCTCACCACGAT
GGGTCTCACCACGGT
GGGTGTCACCACGAT
GGGTCTCACCACGGT
GGGTCTCACCACGAT
GGGTATCACCACGAC
GGGTCTCACCACGAT
GGGTCTCACCACGAC
GGGTCTCACCACGGT
GGGTCTCGCCACGAC
GGGTCTCACCACGAT
GGGTCTCACCACGGT
GGGTCTCGCCACGAT
GGGTCTCACCACGAT
GGGCCTCACCACGAT
GGGTCTCACCACGAT
Nr of selected repeats 31 Similarity 0.896583
Consensus:
GGGTCTCACCACGaT
>Cele-UNSB01_1:5080040-5080117 Satlength=78 Nr of Repeats=5 RepeatLength=15
seed=CGAAAAGGGG
CGAAAAGGGGAGCAGAA
CGAAAAGGGGATCTG
CGAAAAGGGGAGATA
CGAAAAGGGGAGATA
CGAAAAGGGGAGCAA
Nr of selected repeats 4 Similarity 0.807407
Consensus:
CGAAAAGGGGAgata
>Cele-UNSB01_1:5080289-5080381 Satlength=93 Nr of Repeats=5 RepeatLength=15
seed=CTCCCCTTTT
CTCCCCTTTTCGTTG
CTCCCCTTTTCGTAT
CTCCCCTTTTCGTAT
CTCCCCTTTTCGCAGA
TCCCCTTTTTGCAGATCGCCTTTTCGTTCTG
Nr of selected repeats 3 Similarity 0.847222
Consensus:
CTCCCCTTTTCGTaT
>Cele-UNSB01_1:5082880-5082987 Satlength=108 Nr of Repeats=6 RepeatLength=15
seed=CTCCCCTTTT
CTCCCCTTTTCGTTG
CTCCCCTTTTCGTAT
CTCCCCTTTTCGTAT
CTCCCCTTTTCGTAT
CTCCCCTTTTCGCAGA
TCCCCTTTTTGCAGATCGCCTTTTCGTTCTG

Nr of selected repeats 4 Similarity 0.869792
Consensus:
CTCCCCTTTTCGTaT
>Cele-UNSB01_1:5266039-5266863 Satlength=825 Nr of Repeats=51 RepeatLength=15
seed=CGTGGTGAGA
CGTGGTGAGACCCAT
CGTGGTGAGACCTTT
CGTGGTGAGACCCTT
CGTGGTGAGACCCAT
CGTGGTGAGACCTTT
CGTGTTGAGACCCAT
CGGGGTGAGACCTTT
CGTGGTGAGACCTTTCGTGTTGAACCTTT
CGTGGTGAGACCTTTCGTGTTGAGGCCTTT
CGTGGTGAGACCCAT
CGTGGTGAGACCCTC
CGTGGTCAGAACTTT
CGTGTTGAGATCCAT
CGTGGTGAGACCCTC
CGTTGTGAGACCTTT
CGTGGTGAGACCTTT
CGTGTTGAGACCCAT
CGGGGTGAGACCTTT
CGTGGTGAGACCCTT
CGTGGTGAGACCCAT
CGTGGTGAGACCCAT
CGTGGTGAGACCTTT
CGTGGTGAGACCTTT
CGTGGTGAGACCTTT
CGTGGTGAGACCTTT
CGTGGTGAGACCTTTCGTGTTGAGGCCTTT
CGTGGTGAGACCCAT
CGTGGTGAGACCCTC
CGTGGTGAGAACTTT
CGTGTTGAGACCCAT
CGTGGTCAGACCCTCCGTTGTGAAACCTTT
CGTGGTGAGACCTTT
CGTGTTGAGACCCAT
CGGGGTGAGACCTTT
CGTGGTGAGACCTTT
CGTGGTGAGACCCTC
CGTGGTGAGACCTTT
CGTGTTGAGACCCAT
CGTGGTGAGACCTTT
CGTGGTGAGACCTTT
CGTGGTGAGACCCAC
CGTGGTGAGACCCTC
CGTGGTGAGACCTTT
CGTGTTGAGACCCAT
CGTGGTGAGACCCTC
CGTTGTGAGACCTTT
CGTGGTGAGACCTTT
CGTGGTGAGACCCTC
CGTGGTGAGACCTTT

CGTGTTGAGACCCAT
CGGGGTGAGACCTTT
Nr of selected repeats 47 Similarity 0.827485
Consensus:
CGTGGTGAGACCctT
>Cele-UNSB01_1:5273525-5290948 Satlength=17424 Nr of Repeats=599
RepeatLength=29 seed=TTGTAAGGAT
TTGTAAGGATTTCCCATTATGTCAATACA
TTGTAAGGATTTCCCATTATGTCAATACA
TTGTAAGGATTTCCCACTATGTCAATACA
TTGTAAGGATTTCCCATTATGTCAATACA
TTGTAAGGATTTCCCACTATGTCAATACA
TTGTAAGGATTTCCCACTATGTCAATACA
TTGTAAGGATTTCCCATTATGTCAATACA
TTGTAAGGATTTCCCATTATGTCAATACA
TTGTAAGGATTTCCCATTATGTCAATACA
TTGTAAGGATTTCCCATTATGTCAATACA
TTGTAAGGATTTCCCATTATGTCAATACA
TTGTAAGGATTTCCCATTATGTCAATACA
TTGTAAGGATTTCCCATTATGTCAATACA
TTGTAAGGATTTCCCATTATGTCAATACA
TTGTAAGGATTTCCCATTATGTCAATACA
TTGTAAGGATTTCCCATTATGTCAATACA
TTGTAAGGATTTCCCACTATGTCAATACA
TTGTAAGGATTTCCCATTATGTCAATACA
TTGTAAGGATATCCCATTATGTCAATACA
TTGTAAGGATTTCCCACTATGTCAATACA
TTGTAAGGATTTCCCATTATGTCAATACA
TTGTAAGGATTTCCCATTATGTCAATACA
TTGTAAGGATTTCCCACTATGTCAATACA
TTGTAAGGATTTCCCACTATGTCAATACA
TTGTAAGGATTTCCCATTATGTCAATACA
TTGTAAGGATTTCCCATTATGTCAATACA
TTGTAAGGATTTCCCATTATGTCAATACA
TTGTAAGGATTTCCCACTATGTCAATACA
TTGTAAGGATTTCCCACTTTGTCAATACA
TTGTAAGGATTTCCCATTATGTCAATACA
TTGTAAGGATTTCCCACTATGTCAATACA
TTGTAAGGATTTCCCATTATGTCAATACA
TTGTAAGGATTTCCCATTATGTCAATACA
TTGTAAGGATTTCCCACTATGTCAATACA
TTGTAAGGATTTCCCACTTTGTCAATACA
TTGTAAGGATTTCCCATTATGTCAATACA
TTGTAAGGATTTCCCATTATGTCAATACA
TTGTAAGGATTTCCCATTATGTCAATACA
TTGTAAGGATTTCCCACTATGTCAATACA
TTGTAAGATACAATACA
TTGTAAGGATTTCCCATTATGTCAATACA
TTGTAAGGATTTCCCACTATGTCAATACA
TTGTAAGGATTTCCCATTATGTCAATACA
TTGTAAGGATTTCCATTATGTCATACA
TTGTAAGGATTTCCCACTATGTCAATACA
TTGTAAGGATTTCCCATTATGTCAATACA
TTGTAAGGATTTCCCATTATGTCAATACA

TTGTAAGGATTTCCCACTATGTCAATACA
TTGTAAGGATTTCCCACTATGTCAATACA
TTGTAAGGATTTCCCATTATGTCAATACA
TTGTAAGGATTTCCCACTATGTCAATACA
TTGTAAGGATTTCCCATTATGTCAATACA
TTGTAAGGATTTCCCATTATGTCAATACA
TTGTAAGGATTTCCATTTGTCAATACA
TTGTAAGGATTTCCCATTATGTCAATACA
TTGTAAGGATTTCCCATATGTCAATACA
TTGTAAGGATTTCAATACA
TTGTAAGGATTTCCCATTATGTCAATACA
TTGTAAGGATTTCCCATTATGTCAATACA
TTGTAAGGATTTCCCATTATGTCAATACA
TTGTAAGGATTTCCCATTATGTCAATACA
TTGTAAGGATTTCCCATTATGTCAATACA
TTGTAAGGATTTCCCATTATGTCAATACA
TTGTAAGGATTTCCCATTATGTCAATACA
TTGTAAGGATTTCCCATTATGTCAATACA
TTGTAAGGATTTCCCATTATGTCAATACA
TTGTAAGGATTTCCCACTATGTCAATACA
TTGTAAGGATTTCCCATTATGTCAATACA
TTGTAAGGATTTCCCATTATGTCAATACA
TTGTAAGGATTTCCCACTATGTCAATACA
TTGTAAGGATTTCCCACTATGTCAATACA
TTGTAAGGATTTCCCATTATGTCAATACA
TTGTAAGGATTTCCCACTATGTCAATACA
TTGTAAGGATTTCCCACTATGTCAATACA
TTGTAAGGATTTCCCATTATGTCAATACA
TTGTAAGGATTTCCCATTATGTCAATACA
TTGTAAGGATTTCCCATTATGTCAATACA
TTGTAAGGATTTCCCACTATGTCAATACA
TTGTAAGGATTTCCCACTTTGTCAATACA
TTGTAAGGATTTCCCACTTTGTCAATACA
TTGTAAGGATTTCCCATTATGTCAATACA
TTGTAAGGATTTCCCACTATGTCAATACA
TTGTAAGGATTTCCCATTATGTCAATACA
TTGTAAGGATTTCCCACTATGTCAATACA
TTGTAAGGATTTCCCATTATGTCAATACA
TTGTAAGGATTTCCCATTATGTCAATACA
TTGTAAGGATTTCCCATTATGTCAATACA
TTGTAAGGATTTCCCATTATGTCAATACA
TTGTAAGGATTTCCCATTATGTCAATACA
TTGTAAGGATTTCCCATTATGTCAATACA
TTGTAAGGATTTCCCATTATGTCAATACA
TTGTAAGGATTTCCCATTATGTCAATACA
TTGTAAGGATTTCCCATTATGTCAATACA
TTGTAAGGATTTCCCATTATGTCAATACA
TTGTAAGGATTTCCCATTATGTCAATACA
TTGTAAGGATTTCCCACTATGTCAATACA
TTGTAAGGATTTCCCATTATGTCAATACA
TTGTAAGGATATCCCATTATGTCAATACA
TTGTAAGGATTTCCCACTATGTCAATACA
TTGTAAGGATTTCCCATTATGTCAATACA
TTGTAAGGATTTCCCACTTTGTCAATACA

TTGTAAGGATTTCCCACTATGTCAATACA
TTGTAAGGATTTCCCATTATGTCAATACA
TTGTAAGGATTTCCCACTATGTCAATACA
TTGTAAGGATTTCCCACTTTGTCAATACA
TTGTAAGGATTTCCCATTATGTCAATACA
TTGTAAGGATTTCCCACTATGTCAATACA
TTGTAAGGATTTCCCATTATGTCAATACA
TTGTAAGGATTTCCCATTATGTCAATACA
TTGTAAGGATTTCCCACTATGTCAATACA
TTGTAAGGATTTCCCACTTTGTCAATACA
TTGTAAGGATTTCCCATTATGTCAATACA
TTGTAAGGATTTCCCATTATGTCAATACA
TTGTAAGGATTTCCCATTATGTCAATACA
TTGTAAGGATTTCCCACTATGTCAATACA
TTGTAAGGATTTCCCATTATGTCAATACA
TTGTAAGGATTTCCCATTATGTCAATACA
TTGTAAGGATTTCCCATTATGTCAATACA
TTGTAAGGATTTCCCATTATGTCAATACA
TTGTAAGGATTTCCCACTATGTCAATACA
TTGTAAGGATTTCCCACTATGTCAATACA
TTGTAAGGATTTCCCACTATGTCAATACA
TTGTAAGGATTTCCCACTTTGTCAATACA
TTGTAAGGATTTCCCATTATGTCAATACA
TTGTAAGGATTTCCCATTATGTCAATACA
TTGTAAGGATTTCCCATTATGTCAATACA
TTGTAAGGATTTCCCACTATGTCAATACA
TTGTAAGGATTTCCCACTATGTCAATACA
TTGTAAGGATTTCCCATTATGTCAATACA
TTGTAAGGATTTCCCACTATGTCAATACA
TTGTAAGGATTTCCCACTATGTCAATACA
TTGTAAGGATTTCCCATTATGTCAATACA
TTGTAAGGATTTCCCATTATGTCAATACA
TTGTAAGGATTTCCCATTATGTCAATACA
TTGTAAGGATTTCCCACTATGTCAATACA
TTGTAAGGATTTCCCATTATGTCAATACA
TTGTAAGGATTTCCCATTATGTCAATACA
TTGTAAGGATTTCCCATTATGTCAATACA
TTGTAAGGATTTCCCATTATGTCAATACA
TTGTAAGGATTTCCCACTATGTCAATACA
TTGTAAGGATTTCCCACTATGTCAATACA
TTGTAAGGATTTCCCATTATGTCAATACA
TTGTAAGGATTTCCCATTATGTCAATACA
TTGTAAGGATTTCCCATTATGTCAATACA
TTGTAAGGATTTCCCACTATGTCAATACA
TTGTAAGGATTTCCCACTATGTCAATACA
TTGTAAGGATTTCCCACTTTGTCAATACA
TTGTAAGGATTTCCCATTATGTCAATACA
TTGTAAGGATTTCCCATTATGTCAATACA
TTGTAAGGATTTCCCATTATGTCAATACA
TTGTAAGGATTTCCCACTATGTCAATACA
TTGTAAGGATTTCCCACTATGTCAATACA
TTGTAAGGATTTCCCATTATGTCAATACA
TTGTAAGGATTTCCCACTATGTCAATACA
TTGTAAGGATTTCCCACTATGTCAATACA

TTGTAAGGATTTCCCATTATGTCAATACA
TTGTAAGGATTTCCCATTATGTCAATACA
TTGTAAGGATTTCCCATTATGTCAATACA
TTGTAAGGATTTCCCACTATGTCAATACA
TTGTAAGGATTTCCCATTATGTCAATACA
TTGTAAGGATTTCCCATTATGTCAATACA
TTGTAAGGATTTCCCATTATGTCAATACA
TTGTAAGGATTTCCCATTATGTCAATACA
TTGTAAGGATTTCCCACTATGTCAATACA
TTGTAAGGATTTCCCACTATGTCAATACA
TTGTAAGGATTTCCCATTATGTCAATACA
TTGTAAGGATTTCCCATTATGTCAATACA
TTGTAAGGATTTCCCATTATGTCAATACA
TTGTAAGGATTTCCCATTATGTCAATACA
TTGTAAGGATTTCCCATTATGTCAATACA
TTGTAAGGATTTCCCACTATGTCAATACA
TTGTAAGGATTTCCCACTATGTCAATACA
TTGTAAGGATTTCCCATTATGTCAATACA
TTGTAAGGATTTCCCATTATGTCAATACA
TTGTAAGGATTTCCCATTATGTCAATACA
TTGTAAGGATTTCCCATTATGTCAATACA
TTGTAAGGATTTCCCATTATGTCAATACA
TTGTAAGGATTTCCCATTATGTCAATACA
TTGTAAGGATTTCCCATTATGTCAATACA
TTGTAAGGATTTCCCATTATGTCAATACA
TTGTAAGGATTTCCCACTATGTCAATACA
TTGTAAGGATTTCCCATTATGTCAATACA
TTGTAAGGATTTCCCATTATGTCAATACA
TTGTAAGGATTTCCCATTATGTCAATACA
TTGTAAGGATTTCCCATTATGTCAATACA
TTGTAAGAATTTCCCACTATGTCAATACA
TTGTAAGGATTTCCCATTATGTCAATACA
TTGTAAGGATTTCCCACTATGTCAATACA
TTGTAAGGATTTCCCACTTTGTCAATACA
TTGTAAGGATTTCCCACTATGTCAATACA
TTGTAAGGATTTCCCACTATGTCAATACA
TTGTAAGGATTTCCCACTTTGTCAATACA
TTGTAAGGATTTCCCACTATGTCAATACA
TTGTAAGGATTTCCCACTTTGTCAATACA
TTGTAAGGATTTCCCACTTTGTCAATACA
TTGTAAGGATTTCCCATTATGTCAATACA
TTGTAAGGATTTCCCACTTTGTCAATACA
TTGTAAGGATTTCCCACTATGTCAATACA
TTGTAAGGATTTCCCACTATGTCAATACA
TTGTAAGGATTTCCCACTATGTCAATACA
TTGTAAGGATTTCCCATTATGTCAATACA
TTGTAAGGATTTCCCACTTTATCAATACA
TTGTAAGGATTTCCCACTTTATCAATACA
TTGTAAGGATTTCCCACTATGTCAATACA
TTGTAAGGATTTCCCACTATGTCAATACA
TTGTAAGGATTTCCCACTATGTCAATACA
TTGTAAGGATTTCCCACTTTATCAATACA
TTGTAAGGATTTCCCACTATGTCAATACA
TTGTAAGGATTTCCCACTATGTCAATACA

TTGTAAGGATTTCCCACTATGTCAATACA
TTGTAAGGATTTCCCACTATGTCAATACA
TTGTAAGGATTTCCCACTATGTCAATACA
TTGTAAGGATTTCCCACTATGTCAATACA
TTGTAAGGATTTCCCACTATGTCAATACA
TTGTAAGGATTTCCCATTATGTCAATACA
TTGTAAGGATTTCCCATTATGTCAATACA
TTGTAAGGATTTCCCACTATGTCAATACA
TTGTAAGGATTTCCCACTATGTCAATACA
TTGTAAGGATTTCCCACTATGTCAATACA
TTGTAAGGATTTCCCATTATGTCAATACA
TTGTAAGGATTTCCCATTATGTCAATACA
TTGTAAGGATTTCCCACTATGTCAATACA
TTGTAAGGATTTCCCACTATGTCAATACA
TTGTAAGGATTTCCCACTATGTCAATACA
TTGTAAGGATTTCCCATTATGTCAATACA
TTGTAAGGATTTCCCACTATGTCAATACA
TTGTAAGGATTTCCCACTTTGTCAATACA
TTGTAAGGATTTCCCACTTTGTCAATACA
TTGTAAGGATTTCCCACTTTGTCAATACA
TTGTAAGGATTTCCCACTTTGTCAATACA
TTGTAAGGATTTCCCACTTTGTCAATACA
TTGTAAGGATTTCCCACTTTGTCAATACA
TTGTAAGGATTTCCCACTTTGTCAATACA
TTGTAAGGATTTCCCACTTTGTCAATACA
TTGTAAGGATTTCCCACTTTGTCAATACA
TTGTAAGGATTTCCCACTTTGTCAATACA
TTGTAAGGATTTCCCACTATGTCAATACA
TTGTAAGGATTTCCCACTTTGTCAATACA
TTGTAAGGATTTCCCACTTTATCAATACA
TTGTAAGGATTTCCCACTATGTCAATACT
TTGTAAGGATTTCCCATTATGTCAATACA
TTGTAAGGATTTCCCACTTTGTCAATACA
TTGTAAGGATTTCCCACTTTGTCAATACA
TTGTAAGGATTTCCCACTTTGTCAATACA
TTGTAAGGATTTCCCACTTTGTCAATACA
TTGTAAGGATTTCCCACTTTGTCAATACA
TTGTAAGGATTTCCCACTTTGTCAATACA
TTGTAAGGATTTCCCACTTTGTCAATACA
TTGTAAGGATTTCCCACTTTGTCAATACA
TTGTAAGGATTTCCCACTTTGTCAATACA
TTGTAAGGATTTCCCACTTTGTCAATACA
TTGTAAGGATTTCCCACTTTGTCAATACA
TTGTAAGGATTTCCCACTTTGTCAATACA
TTGTAAGGATTTCCCACTTTGTCAATACA
TTGTAAGGATTTCCCACTATGTCAATACA
TTGTAAGGATTTCCCACTTTGTCAATACA
TTGTAAGGATTTCCCACTTTGTCAATACA
TTGTAAGGATTTCCCACTTTGTCAATACA
TTGTAAGGATTTCCCACTATGTCAATACT
TTGTAAGGATTTCCCATTATGTCAATACA
TTGTAAGGATTTCCCACTTTGTCAATACA
TTGTAAGGATTTCCCATTATGTCAATACA
TTGTAAGGATTTCCCACTTTGTCAATACA

TTGTAAGGATTTCCCACTTTGTCAATACA
TTGTAAGGATTTCCCACTTTGTCAATACA
TTGTAAGGATTTCCCACTTTGTCAATACA
TTGTAAGGATTTCCCACTTTGTCAATACA
TTGTAAGGATTTCCCACTTTGTCAATACA
TTGTAAGGATTTCCCACTTTGTCAATACA
TTGTAAGGATTTCCCACTTTGTCAATACA
TTGTAAGGATTTCCCACTTTGTCAATACA
TTGTAAGGATTTCCCACTTTGTCAATACA
TTGTAAGGATTTCCCACTTTGTCAATACA
TTGTAAGGATTTCCCACTTTGTCAATACA
TTGTAAGGATTTCCCACTATGTCAATACA
TTGTAAGGATTTCCCACTTTGTCAATACA
TTGTAAGGATTTCCCACTTTATCAATACA
TTGTAAGGATTTCCCACTATGTCAATACT
TTGTAAGGATTTCCCATTATGTCAATACA
TTGTAAGGATTTCCCACTTTGTCAATACA
TTGTAAGGATTTCCCACTTTGTCAATACA
TTGTAAGGATTTCCCACTTTGTCAATACA
TTGTAAGGATTTCCCACTTTGTCAATACA
TTGTAAGGATTTCCCACTTTGTCAATACA
TTGTAAGGATTTCCCACTTTGTCAATACA
TTGTAAGGATTTCCCACTTTGTCAATACA
TTGTAAGGATTTCCCACTTTGTCAATACA
TTGTAAGGATTTCCCACTTTGTCAATACA
TTGTAAGGATTTCCCACTTTGTCAATACA
TTGTAAGGATTTCCCACTTTGTCAATACA
TTGTAAGGATTTCCCACTTTGTCAATACA
TTGTAAGGATTTCCCACTATGTCAATACA
TTGTAAGGATTTCCCACTTTGTCAATACA
TTGTAAGGATTTCCCACTTTATCAATACA
TTGTAAGGATTTCCCACTATGTCAATACT
TTGTAAGGATTTCCCATTATGTCAATACA
TTGTAAGGATTTCCCACTTTGTCAATACA
TTGTAAGGATTTCCCACTTTGTCAATACA
TTGTAAGGATTTCCCACTTTGTCAATACA
TTGTAAGGATTTCCCACTTTGTCAATACA
TTGTAAGGATTTCCCACTTTGTCAATACA
TTGTAAGGATTTCCCACTTTGTCAATACA
TTGTAAGGATTTCCCACTATGTCAATACA
TTGTAAGGATTTCCCACTATGTCAATACA
TTGTAAGGATTTCCCACTTTGTCAATACA
TTGTAAGGATTTCCCACTATGTCAATACA
TTGTAAGGATTTCCCACTATGTCAATACA
TTGTAAGGATTTCCCACTTTGTCAATACA
TTGTAAGGATTTCCCACTATGTCAATACA
TTGTAAGGATTTCCCACTATGTCAATACA
TTGTAAGGATTTCCCACTTTGTCAATACA
TTGTAAGGATTTCCCACTATGTCAATACA
TTGTAAGGATTTCCCACTATGTCAATACA
TTGTAAGGATTTCCCACTTTGTCAATACA
TTGTAAGGATTTCCCACTTTGTCAATACA
TTGTAAGGATTTCCCACTATGTCAATACA
TTGTAAGGATTTCCCACTTTGTCAATACA

TTGTAAGGATTTCCCACTTTATCAATACA
TTGTAAGGATTTCCCACTATGTCAATACA
TTGTAAGGATTTCCCACTATGTCAATACA
TTGTAAGGATTTCCCACTTTGTCAATACA
TTGTAAGGATTTCCCATTATGTCAATACA
TTGTAAGGATTTCCCACTATGTCAATACA
TTGTAAGGATTTCCCATTATGTCAATACA
TTGTAAGGATTTCCCACTTTGTCAATACA
TTGTAAGGATTTCCCACTTTGTCAATACA
TTGTAAGGATTTCCCATTATGTCAATACA
TTGTAAGGATTTCCCATTATGTCAATACA
TTGTAAGGATTTCCCACTATGTCAATACA
TTGTAAGGATTTCCCATTATGTCAATACA
TTGTAAGGATTTCCCATTATGTCAATACA
TTGTAAGGATTTCCCACTATGTCAATACA
TTGTAAGGATTTCCCATTATGTCAATACA
TTGTAAGGATTTCCCATTATGTCAATACA
TTGTAAGGATTTCCCATTATGTCAATACA
TTGTAAGGATTTCCCACTATGTCAATACA
TTGTAAGGATTTCCCACTTTGTCAATACA
TTGTAAGGATTTCCATTATGTCAATACA
TTGTAAGGATTTCCCACTATGTCAATACA
TTGTAAGGATTTCCCACTATGTCAATACA
TTGTAAGGATTTCCCACTATGTCAATACA
TTGTAAGGATTTCCCATATGTCAATACA
TTGTAAGGATTTCCCATTATGTCAATACA
TTGTAAGGATTTCCTTATGTCAATACA
TTGTAAGGATTTCCCACTATGTCAATACA
TTGTAAGGATTTCCCATTATGTCAATACA
TTGTAAGGATTTCCCATTATGTCAATACA
TTGTAAGGATTTCCCATTATGTCAATACA
TTGTAAGGATTTCCCACTATGTCAATACA
TTGTAAGGATTTCCCATTATGTCAATACA
TTGTAAGGATTTCCCATTATGTCAATACA
TTGTAAGGATTTCCCATTATGTCAATACA
TTGTAAGGATTTCCCACTATGTCAATACA
TTGTAAGGATTTCCCATTATGTCAATACA
TTGTAAGGATTTCCCATTATGTCAATACA
TTGTAAGGATTTCCCACTATGTCAATACA
TTGTAAGGATTTCCCATTATGTCAATACA
TTGTAAGGATTTCCCATTATGTCAATACA
TTGTAAGGATTTCCCATTATGTCAATACA
TTGTAAGGATTTCCCATTATGTCAATACA
TTGTAAGGATTTCCCATTATGTCAATACA
TTGTAAGGATTTCCCATTATGTCAATACA
TTGTAAGGATTTCCCATTATGTCAATACA
TTGTAAGGATTTCCCATTATGTCAATACA
TTGTAAGGATTTCCCATTATGTCAATACA
TTGTAAGGATTTCCCACTTTGTCAATACA
TTGTAAGGATTTCCCACTTTGTCAATACA
TTGTAAGGATTTCCCACTTTTTCCCATTATGTCAATACA
TTGTAAGGATTTCCCACTTATGTCAATACA
TTGTAAGGATTTCCCACTTTGTCAATACA
TTGTAAGGATTTCCCACTTATGTCAATACA

TTGTAAGGATTTCCCACTTATGTCAATACA
TTGTAAGGATTTCCCATTATGTCAATACA
TTGTAAGGATTTCCCATTTGTCAATACA
TTGTAAGGATTTCCCACTTTGTCAATACA
TTGTAAGGATTTCCCATTATGTCAATACA
TTGTAAGGATTTCCCACTTATGTCAATACA
TTGTAAGGATTTCCCATTATGTCAATACA
TTGTAAGGATTTCCCACTTTGTCAATACA
TTGTAAGGATTTCCCATTATGTCAATACA
TTGTAAGGATTTCCCACTTTGTCAATACA
TTGTAAGGATTTCCCACTTTGTCAATACA
TTGTAAGGATTTCCCACTTTGTCAATACA
TTGTAAGGATTTCCCACTTTGTCAATACA
TTGTAAGGATTTCCCACTATGTCAATACA
TTGTAAGGATTTCCCATTATGTCAATACA
TTGTAAGGATTTCCCACTTTGTCAATACA
TTGTAGGATTCCCATTATGTCAATACA
TTGTAAGGATTTCCCACTTATGTCAATACA
TTGTAAGGATTTCCCACTTATGTCAATACA
TTGTAAGGATTTCCCATTATGTCAATACA
TTGTAAGGATTTCCCATTATGTCAATACA
TTGTAAGGATTTCCCATTATGTCAATACA
TTGTAAGGATTTCCCACTTTGTCAATACA
TTGTAAGGATTTCCCACTTTGTCAATACA
TTGTAAGGATTTCCCACTTTGTCAATACA
TTGTAAGGATTTCCCACTTTGTCAATACA
TTGTAAGGATTTCCCACTTTGTCAATACA
TTGTAAGGATTTCCCACTTTGTCAATACA
TTGTAAGGATTTCCCACTTTGTCAATACA
TTGTAAGGATTTCCCACTTTGTCAATACA
TTGTAAGGATTTCCCACTTATGTCAATACA
TTGTAAGGATTTCCCACTATGTCAATACA
TTGTAAGGATTTCCCACTTATGTCAATACA
TTGTAAGGATTTCCCACTATGTCAATACA
TTGTAAGGATTTCCCACTTTGTCAATACA
TTGTAAGGATTTCCCATTATGTCAATACA
TTGTAAGGATTTCCCACTTATGTCAATACA
TTGTAAGGATTTCCCACTATGTCAATACA
TTGTAAGGATTTCCCACTTTGTCAATACA
TTGTAAGGATTTCCCACTTTGTCAATACA
TTGTAAGGATTTCCCACTATGTCAATACA
TTGTAAGGATTTCCCATTCATACATTGTAAATTCCATTGTCAATACA
TTGTAAGGATTTCCCATTATGTCAATACA
TTGTAAGGATTTCCCATTATGTCAATACA
TTGTAAGGATTTCCCACTATGTCAATACA
TTGTAAGGATTTCCCACTATGTCAATACA
TTGTAAGGATTTCCCACTTTGTCAATACA
TTGTAAGGATTTCCCACTTTGTCAATACA
TTGTAAGGATTTCCCACTTTGTCAATACA
TTGTAAGGATTTCCCACTTTGTCAATACA
TTGTAAGGATTTCCCACTATGTCAATACA
TTGTAAGGATTTCCCACTTTGTCAATACA
TTGTAAGGATTTCCCACTTTGTCAATACA
TTGTAAGGATTTCCCACTATGTCAATACA

TTGTAAGGATTTCCCACTTTGTCAATACA
TTGTAAGGATTTCCCACTTTGTCAATACA
TTGTAAGGATTTCCCATTATGTCAATACA
TTGTAAGGATTTCCCATTATGTCAATACA
TTGTAAGGATTTCCCATTATGTCAATACA
TTGTAAGGATTTCCCACTTTGTCAATACA
TTGTAAGGATTTCCCACTTTGTCAATACA
TTGTAAGGATTTCCCATTATGTCAATACA
TTGTAAGGATTTCCCATTATGTCAATACA
TTGTAAGGATTTCCCACTTATGTCAATACA
TTGTAAGGATTTCCCATTATGTCAATACA
TTGTAAGGATTTCCCACTTTGTCAATACA
TTGTAAGGATTTCCCATTATGTCAATACA
TTGTAAGGATTTCCCATTATGTCAATACA
TTGTAAGGATTTCCCACTATGTCAATACA
TTGTAAGGATTTCCCATTATGTCAATACA
TTGTAAGGATTTCCCATTATGTCAATACA
TTGTAAGGATTTCCCATTATGTCAATACA
TTGTAAGGATTTCCCACTATGTCAATACA
TTGTAAGGATTTCCCATTATGTCAATACA
TTGTAAGGATTTCCCATTATGTCAATACA
TTGTAAGGATTTCCCATTATGTCAATACA
TTGTAAGGATTTCCCATTATGTCAATACA
TTGTAAGGATTTCCCACTTATGTCAATACA
TTGTAAGGATTTCCCATTATGTCAATACA
TTGTAAGGATTTCCCATTATGTCAATACA
TTGTAAGGATTTCCCACTATGTCAATACA
TTGTAAGGATTTCCCATTATGTCAATACA
TTGTAAGGATTTCCCACTATGTCAATACA
TTGTAAGGATTTCCCATTATGTCAATACA
TTGTAAGGATTTCCCACTATGTCAATACA
TTGTAAGGATTTCCCATTATGTCAATACA
TTGTAAGGATTTCCCACTATGTCAATACA
TTGTAAGGATTTCCCATTATGTCAATACA
TTGTAAGGATTTCCCATTATGTCAATACA
TTGTAAGGATTTCCCATTATGTCAATACA
TTGTAAGGATTTCCCATTATGTCAATACA
TTGTAAGGATTTCCCACTATGTCAATACA
TTGTAAGGATTTCCCACTATGTCAATACA
TTGTAAGGATTTCCCACTTTGTCAATACA
TTGTAAGGATTTCCCATTATGTCAATACA
TTGTAAGGATTTCCCACTTTGTCAATACA
TTGTAAGGATTTCCCATTATGTCAATACA
TTGTAAGGATTTCCCACTATGTCAATACA
TTGTAAGGATTTCCCACTATGTCAATACA
TTGTAAGGATTTCCCATTATGTCAATACA
TTGTAAGGATTTCCCACTTTGTCAATACA
TTGTAAGGATTTCCCACTTTGTCAATACA
TTGTAAGGATTTCCCATTATGTCAATACA
TTGTAAGGATTTCCCACTATGTCAATACA
TTGTAAGGATTTCCCACTTTGTCAATACA
TTGTAAGGATTTCCCACTTTGTCAATACA
TTGTAAGGATTTCCCACTATGTCAATACA
TTGTAAGGATTTCCCACTTTGTCAATACA

TTGTAAGGATTTCCCACTTTGTCAATACA
TTGTAAGGATTTCCCATTATGTCAATACA
TTGTAAGGATTTCCCATTATGTCAATACA
TTGTAAGGATTTCCCATTATGTCAATACA
TTGTAAGGATTTCCCACTTTGTCAATACA
TTGTAAGGATTTCCCACTATGTCAATACA
TTGTAAGGATTTCCCACTTTGTCAATACA
TTGTAAGGATTTCCCACTTGGTCAATACA
TTGTAAGGATTTCCCATTATGTCAATACA
TTGTAAGGATTTCCCACTTTGTCAATACA
TTGTAAGGATTTCCCACTTTGTCAATACA
TTGTAAGGATTTCCCATTTGTCAATACA
TTGTAAGGATTTCCCACTTTGTCAATACA
TTGTAAGGATTTCCCATTATGTCAATACA
TTGTAAGGATTTCCCACTATGTCAATACA
TTGTAAGGATTTCCCACTATGTCAATACA
TTGTAAGGATTTCCCATTATGTCAATACA
TTGTAAGGATTTCCCACTTTGTCAATACA
TTGTAAGGATTTCCCACTATGTCAATACA
TTGTAAGGATTTCCCACTATGTCAATACA
TTGTAAGGATTTCCCATTATGTCAATACA
TTGTAAGGATTTCCCACTTTGTCAATACA
TTGTAAGGATTTCCCACTTTGTCAATACA
TTGTAAGGATTTCCCATTATGTCAATACA
TTGTAAGGATTTCCCATTTGTCAATACA
TTGTAAGGATTTCCCACTTTGTCAATACA
TTGTAAGGATTTCCCATTATGTCAATACA
TTGTAAGGATTTCCCATTATGTCAATACA
TTGTAAGGATTTCCCACTTGGTCAATACA
TTGTAAGGATTTCCCACTTTGTCAATACA
TTGTAAGGATTTCCCATTATGTCAATACATAAAATAACA
TTGTAAGGATTTCCCACTTTGTCAATACA
TTGTAAGGATTTCCCACTTTGTCAATACA
TTGTAAGGATTTCCCACTATGTCAATACA
TTGTAAGGATTTCCCACTTGTCAATACATTTAAATTTATTGTCAATACA
TTGTAAGGATTTCCCACTTTGTCAATACA
TTGTAAGGATTTCCCATTTGTCAATACA
TTGTAAGGATTTCCCACTATGTCAATACA
TTGTAAGGATTTCCCACTATGTCAATACA
TTGTAAGGATTTCCCATTATGTCAATACA
TTGTAAGGATTTCCCACTATGTCAATACA
TTGTAAGGATTTCCCATTTGTCAATACATTGTAATTTTTTTACA
TTGTAAGGATTTCCCACTATGTCAATACA
TTGTAAGGATTTCCCACTATGTCAATACA
TTGTAAGGATTTCCCATTTGTCAATACA
TTGTAAGGATTTCCCACTTTGTCAATACA
TTGTAAGGATTTCCCACTATGTCAATACA
TTGTAAGGATTTCCCACTTTGTCAATACA
TTGTAAGGATTTCCCACTATGTCAATACA
TTGTAAGGATTTCCCACTTTGTCAATACA
TTGTAAGGATTTCCCATTATGTCAATACA
TTGTAAGGATTTCCCACTTTGTCAATACA
TTGTAAGGATTTCCCACTATGTCAATACA
TTGTAAGGATTTCCCACTTGGTCAATACA

TTGTAAGGATTTCCCACTATGTCAATACA
TTGTAAGGATTTCCCACTTTGTCAATACA
TTGTAAGGATTTCCCACTATGTCAATACA
TTGTAAGGATTTCCCACTATGTCAATACA
TTGTAAGGATTTCCCACTTTGTCAATACA
TTGTAAGGATTTCCCACTATGTCAATACA
TTGTAAGGATTTCCCACTTTGTCAATACA
TTGTAAGGATTTCCCACTATGTCAATACA
TTGTAAGGATTTCCCACTATGTCAATACA
TTGTAAGGATTTCCCACTTTGTCAATACA
TTGTAAGGATTTCCCACTTTGTCAATACA
TTGTAAGGATTTCCCATTATGTCAATACA
TTGTAAGGATTTCCCACTATGTCAATACA
TTGTAAGGATTTCCCACTATGTCAATACA
TTGTAAGGATTTCCCACTTTGTCAATACA
TTGTAAGGATTTCCCACTTTGTCAATACA
TTGTAAGGATTTCCCACTATGTCAATACA
TTGTAAGGATTTCCCACTTTGTCAATACA
TTGTAAGGATTTCCCATTATGTCAATACA
TTGTAAGGATTTCCCACTATGTCAATACA
TTGTAAGGATTTCCCACTTTGTCAATACA
TTGTAAGGATTTCCCACTTTGTCAATGTCAATACA
TTGTAAGGATTTCCCACTATGTCAATACA
TTGTAAGGATTTCCCACTATGTCAATACA
TTGTAAGGATTTCCCATTATGTCAATACA
TTGTAAGGATTTCCCACTTTGTCAATACA
TTGTAAGGATTTCCCATTATGTCAATACA
TTGTAAGGATTTCCCATTATGTCAATACA
TTGTAAGGATTTCCCACTTTGTCAATACA
TTGTAAGGATTTCCCATTTGTCAATACA
TTGTAAGGATTTCCCATTATGTCAATACA
TTGTAAGGATTTCCCACTTATGTCAATACA
TTGTAAGGATTTCCCATTATGTCAATACA
TTGTAAGGATTTCCCATTATGTCAATACA
TTGTAAGGATTTCCCACTTTGTCAATACA
TTGTAAGGATTTCCCATTATGTCAATACA
TTGTAAGGATTTCCCATTATGTCAATACA
TTGTAAGGATTTCCCATTATGTCAATACA
TTGTAAGGATTTCCCATTATGTCAATACA
TTGTAAGGATTTCCCACTATGTCAATACA
TTGTAAGGATTTCCCATTATGTCAATACA
TTGTAAGGATTTCCCATTATGTCAATACA
TTGTAAGGATTTCCCATTATGTCAATACA
TTGTAAGGATTTCCCATTATGTCAATACA
TTGTAAGGATTTCCCATTATGTCAATACA
TTGTAAGGATTTCCCATTATGTCAATACA
TTGTAAGGATTTCCCATTATGTCAATACA
TTGTAAGGATTTCCCATTATGTCAATACA
TTGTAAGGATTTCCCATTATGTCAATACA
TTGTAAGGATTTCCCATTATGTCAATACA
TTGTAAGGATTTCCCATTATGTCAATACA
TTGTAAGGATTTCCCATTATGTCAATACA
TTGTAAGGATTTCCCATTATGTCAATACA
TTGTAAGGATTTCCCATTATGTCAATACA

TTGTAAGGATTTCCCATTATGTCAATACA
TTGTAAGGATTTCCCATTATGTCAATACA
TTGTAAGGATTTCCCATTATGTCAATACA
TTGTAAGGATTTCCCATTTGTCAATACA
TTGTAAGGATTTCCCATTATGTCAATACA
TTGTAAGGATTTCCCATTATGTCAATACA
TTGTAAGGATTTCCCACTTTGTCAATACA
TTGTAAGGATTTCCCATTATGTCAATACA
TTGTAAGGATTTCCCACTTTGTCAATACA
TTGTAAGGATTTCCCACTTTGTCAATACA
TTGTAAGGATTTCCCATTATGTCAATACA
TTGTAAGGATTTCCCACTTTGTCAATACA
Nr of selected repeats 565 Similarity 0.955515
Consensus:
TTGTAAGGATTTCCCAcTaTGTCAATACA
>Cele-UNSB01_1:5313372-5313447 Satlength=76 Nr of Repeats=5 RepeatLength=15
seed=TCCCCTTTTC
TCCCCTTTTCGTTGC
TCCCCTTTTCGTATC
TCCCCTTTTCGTATC
TCCCCTTTTCGCAGA
TCCCCTTTTTGCAGA
Nr of selected repeats 5 Similarity 0.768889
Consensus:
TCCCCTTTTcGtagc
>Cele-UNSB01_1:5700313-5700505 Satlength=193 Nr of Repeats=16 RepeatLength=12
seed=GAAATTTTTT
GAAATTTTTTCC
GAAATTTTTTCT
GAAATTTTTTCC
GAAATTTTTTCC
GAAATTTTTTCT
GAAATTTTTTCC
GAAATTTTTTCC
GAAATTTTTTCT
GAAATTTTTTCC
GAAATTTTTTCC
GAAATTTTTTCT
GAAATTTTTTCC
GAAATTTTTTCC
GAAATTTTTTCT
GAAATTTTTTCC
GAAATTTTTTCC
Nr of selected repeats 16 Similarity 0.949074
Consensus:
GAAATTTTTTCc
>Cele-UNSB01_1:5758991-5759083 Satlength=93 Nr of Repeats=6 RepeatLength=15
seed=TCCCCTTTTC
TCCCCTTTTCGTTGC
TCCCCTTTTCGTATC
TCCCCTTTTCGCAGA
TCCCCTTTTTGCAGA
TCCCCTTTTTGCAGA
TCCCCTTTTCGTTCTGC

Nr of selected repeats 5 Similarity 0.768889
Consensus:
TCCCCTTTTcGcaga
>Cele-UNSB01_1:5777645-5778359 Satlength=715 Nr of Repeats=6 RepeatLength=119
seed=GCCACCTTTA
GCCACCTTTAACCCTAATCCAAGCAGAGCAGGCCGTAGGCCTGCTAGGCGGTAGCGACACGCTCAAGCCCAACAGTCCG
GGATGAGGGTCGCAGGCCCTCTTACTCTTAGAAACTTTAG
GCCACCTTTAACCCTAATCCAAGCAGAGCAGGCTGTAGGCCTGCTATGCGGTAGCGACACGCTCAGGCCCAATAGTCCG
GGATGAGGGCCGCAGGCCCTCTTACTCTTAGAAACTTTAG
GCCACCTTTAACCCTAATCCAAGCAGAGCAGGCTGTAGGCCTGCTATGCGGTAGCGACACGCTCAGGCCCAATAGTCCG
GGATGAGGGCCGCAGGCCCTCTTACTCTTAGAAACTTTAG
GCCACCTTTAACCCTAATCCAAGCAGAGCAGGCTGTAGGCCTGCTATGCTGTAGCGACACGCTCAGGCCCAATAGTCCG
GGATGAGGGCCGCAGGCCCTCTTACTCTTAGAAACTTTAG
GCCACCTTTAACCCTAATCCAAGCAGAGCAGGCCGTAGGCCTGCTATGCCGTAGCGACACGCTCAGGCCCAATAGTCCG
GGATGAGGGCCGCAGGCCCTCTTACTCTTAGAAACTTTAG
GCCACCTTTAACCCTAATCCAAGCAGAGCAGGCCGTAGGCCTGCTATGCGGTAGCGACACGCTTAGGCCCAATAGTCCG
GGATGAGGGCCGCAGGCCCTCTTACTCTTAGAAACTTTAG
Nr of selected repeats 6 Similarity 0.967880
Consensus:
GCCACCTTTAACCCTAATCCAAGCAGAGCAGGCcGTAGGCCTGCTATGCgGTAGCGACACGCTCAGGCCCAATAGTCCG
GGATGAGGGCCGCAGGCCCTCTTACTCTTAGAAACTTTAG
>Cele-UNSB01_1:6112428-6113232 Satlength=805 Nr of Repeats=18 RepeatLength=45
seed=TCTGATCCTT
TCTGATCCTTCAGCTCCGAAGGATCACATGGGTACCTTCTGATGT
TCTGATCCTTCAGATAAGAAGGATCGTAAGGGTACCTTCTGATGG
TCTGATCCTTTAGCTCCAAAGGATCACGAGGCTCTTCCTGATGT
TCTGTTCCTTCAGCTCCGAAGGATCACAAGGGTACCTTCTGATGT
TCTGATCCTTCAGCTCCAAAGGATCACGACGCTCTTCCTGATGT
TCTGAACCTTCAGCTCCGAAGGATCACATGGGTACCTTCTGATGT
TCTGATCCTTCAGATAAGAAGGATCGTAAGGGTACCTCCTGATGG
TCTGATACTTCAGCTCCAAAGGATCACGAGGCTCTTCCTGATGT
TCTGAACCTTCAGCTCCGAAGGATCACATGGGTACCTTCTGATGT
TCTGATCCTTCAGATAAGAAGGATCGTAAGGGTACCTTCTGATGG
TCTGATCCTTTAGCTCCAAAGGATCACGACGCTCTTCCTGATGT
TCTGAACCTTCAGCTCCGAAGGATCACATGGGTACCTTCTGATGT
TCTGATCCTTCAGATAAGAAGGATCGTAAGGGTACCTCCTGATGG
TCTGATCCTTCAGCTCTAAAAGAACATGAGGCTCTGCCTGATGA
TCTGATCCTTCCGATCCGAAGGATCACATGGGTACCTTCTGATGA
TCTGATCCTTCAGATAAGAAGGATCGTAAGGGTACCTCCTGATGG
TCTGATCCTTCAGCTCCAAAAGAACATGAGGCTCTGCCTGATGA
TCTGATCTTTCAGAGCCGAATGATCACATGGGTACCTCCTGATGT
Nr of selected repeats 12 Similarity 0.835690
Consensus:
TCTGAtCCTTCAGaTccGAAGGATCacAaGGGTACCTtCTGATGt
>Cele-UNSB01_1:6114632-6114855 Satlength=224 Nr of Repeats=5 RepeatLength=45
seed=AAGGATCAGA
AAGGATCAGACCATCAGGAGGTACCCTTACGATCCTTCTTATCTG
AAGGATCAGATCATCAGAAGGTACCCATGTGATCCTTCGGAGCTG
AAGGTTCAGAACATCAGGCAGAGCCTCATGTTCTTTTAGAGCTG
AAGGATCAGAACATCAGGAAGAGCCTCGTGATCCTTTGGAGCTA
AAGGATCAGACCATCAGAAGGTACCCTTACGATCCTTCTTATCTG
Nr of selected repeats 3 Similarity 0.841975
Consensus:
AAGGATCAGAcCATCAGaAGGTACCCtTacGATCCTTCttAtCTG

>Cele-UNSB01_1:6226496-6226583 Satlength=88 Nr of Repeats=5 RepeatLength=17
seed=AGGTAGTCAA
AGGTAGTCAAAGAGCATTTGA
AGGTAGTCAATGAGCAA
AGGTAGTCAATGAGCAA
AGGTAGTCAATGAGCAA
AGGTAGTCAATGAGA
Nr of selected repeats 3 Similarity 1.000000
Consensus:
AGGTAGTCAATGAGCAA
>Cele-UNSB01_1:6651710-6652545 Satlength=836 Nr of Repeats=13 RepeatLength=30
seed=TGGTGGAACT
TGGTGGAACTGGATTCGGCAGTGGAAATGC
TGGTGGAACTGGATTCGGCAGTGGAAATGC
TGGTGGAACTGGATTCGGCAGTGGAAATGC
TGGTGGAACTGGGTTCGGCAGCGGAAATGC
TGGTGGAACTGGATTCGGCGGTGGAAATGC
TGGTGGAACTGGATTCGGCAGTGGAAATGC
TGGTGGAACTGGATTGGGCAGTGGAAATGC
TGGTGGAACTGGATTCGGCAGTGGAAATGC
TGGTGGAACTGGATTCGGCAGTGGAAATGC
TGGTGGAACTGGATTCGGCAGTGGAAAAGC
TGGTGGAACTGGATTCGGCAGTGGAAAAGC
TGGTGGAACTGGATTTGGCGGTAATAGCAACAGTGGATCTGGTTTTGGTAGCGGATTGACCAATGGATTTGGTAGTGGC
AACAATCATGAATCTGGGTTTGGTGGAGGAAAATCAGGAGGTTTCGGTGGAGGCAACTC
TGGTGGAAGTGGCTTCGGTTCAGGAGGTAACTCGAATGGATTCGGAAGTGGTGGTGGGGGACAAGATCGAGGCGAAAGG
AATAATAGTATGTTTGCTACTTGAAATCTATATCTTCACGTTGCTTTTAGATTGTTTCAATTGCCAGCAGCCAGGACAT
CGATCGAATGACTGTCCAGAGCCGAAAAAGGAAAGGGAACCCAGAGGTTTTAGCTTAAAATGTTTTTAGACGTAATCTG
TTTTATTTTTAGTGTGTTACAATTGCCAGCAGCCTGGTCACAACTCTCGCGATTGTCCAGAAGAGCGGAAACCGCGTGA
AGGCCGAAATGGATTCACCGGTGGAAGCTCTGGTTTCGGAGGAGGAAATGG
Nr of selected repeats 11 Similarity 0.953131
Consensus:
TGGTGGAACTGGATTCGGCAGTGGAAATGC
>Cele-UNSB01_1:6785229-6785394 Satlength=166 Nr of Repeats=11 RepeatLength=15
seed=GAGACCCATC
GAGACCCATCGTGGC
GAGACCCACCGTGGT
GAGACCCATCGTGGC
GAGACCCATCGTGGC
GAGACCCATCGTGGC
GAGACCCGTCGTGGC
GAGACCCATCGTGGT
GAGACCCATCGTGGC
GAGACCCACCGTGGT
GAGACCCGTCGTGGT
GAGACCCGTCGTGGT
Nr of selected repeats 11 Similarity 0.883636
Consensus:
GAGACCCaTCGTGGc
>Cele-UNSB01_1:6785543-6785983 Satlength=441 Nr of Repeats=11 RepeatLength=40
seed=TTCTGGAATG
TTCTGGAATGTTCCAGAACTTTCTAGAAAAAATCGAGAAAA
TTCTGGAATGTTCCAGAACTTTCTAGAAAAAATCGAGAAAA
TTCTGGAATGTTCCAGAACTTTCTAAAAAAAATCGAGAAAA

TTCTGGAATGTTCCAGAACTTTCTAGAAAAAATCGGGAAAT
TTCTGGAATGTTCCAGAACTTTCTAGAAAAAATCGGGAAAA
TTCTGGAATGTTCCAGAACTTTCTAGAAAAAATCGAGAAAA
TTCTGGAATGTTCCAGAACTTTCTAAAAAAAATCGAGAAAA
TTCTGGAATGTTCCAGAACTTTCTAAAAAAAATCGAGAAAA
TTCTGGAATGTTCCAGAACTTTCTAGAAAAAATCGAGAAAA
TTCTGGAATGTTCCAGAACTTTCTAAAAAAAATCGAGAAAA
TTCTGGAATGTTCCAGAACTTTCTAGAAAAAATTGAGAAAA
Nr of selected repeats 11 Similarity 0.960000
Consensus:
TTCTGGAATGTTCCAGAACTTTCTAgAAAAAATCGAGAAAA
>Cele-UNSB01_1:6909627-6919602 Satlength=9976 Nr of Repeats=399 RepeatLength=25
seed=GAAGCCAGAG
GAAGCCAGAGAGTAAAATATACTGT
GAAGCCAGAGAGTAAATTATTATGT
GAAGCCAGAGAGTAAAATATACTGT
GAAGCCAGAGAGTAAATTATTATGT
GAAGCCAGAGAGTAAAATATACTGT
GAAGCCAGAGAGTAAATTATTATGT
GAAGCCAGAGAGTAAAATATACTGT
GAAGCCAGAGAGTAAATTATTATGT
GAAGCCAGAGAGTAAAATATACTGT
GAAGCCAGAGAGTAAATTATTATGT
GAAGCCAGAGAGTAAAATATACTGT
GAAGCCAGAGAGTAAATTATTATGT
GAAGCCAGAGAGTAAAATATACTGT
GAAGCCAGAGAGTAAATTATTCTGT
GAAGCCAGAGAGTAAATTATTCTGT
GAAGCCAGAGAGCAAAATATACTGT
GAAGCCAGAGAGTAAATTATTATGT
GAAGCCAGAGAGTAAAATATACTGT
GAAGCCAGAGAGTAAATTATTATGT
GAAGCCAGAGAGTAAAATATACTGT
GAAGCCAGAGAGTAAATTATTCTGT
GAAGCCAGAGAGTAAAATATACTGT
GAAGCCAGAGAGTAAATTATTATGT
GAAGCCAGAGAGTAAAATATACTGT
GAAGCCAGAGAGTAAATTATTATGT
GAAGCCAGAGAGTAAAATATACTGT
GAAGCCAGAGAGTAAATTATTATGT
GAAGCCAGAGAGTAAAATATACTGT
GAAGCCAGAGAGTAAATTATTATGT
GAAGCCAGAGAGTAAAATATACTGT
GAAGCCAGAGAGTAAATTATTATGT
GAAGCCAGAGAGTAAAATATAGTGG
GAAGCCAGAGAGTAAATTATTCTGT
GAAGCCAGAGAGTAAATTATTATGT
GAAGCCAGAGAGTAAAATATACTGT
GAAGCCAGAGAGTAAATTATTATGT
GAAGCCAGAGAGTAAAATATACTGT
GAAGCCAGAGAGTAAATTATTATGT
GAAGCCAGAGAGTAAAATATACTGT
GAAGCCAGAGAGTAAATTATTCTGT
GAAGCCAGAGAGTAAAATATACTGT

GAAGCCAGAGAGTAAATTATTATGT
GAAGCCAGAGAGTAAAATATACTGT
GAAGCCAGAGAGTAAATTATTATGT
GAAGCCAGAGAGTAAAATATACTGT
GAAGCCAGAGAGTAAAATATACTGT
GAAGCCAGAGAGTAAAATAATCGGT
GAAGCCAGAGAGTAAAATATAGTGG
GAAGCCAGAGAGTAAATTATTCTGT
GAAGCCAGAGAGTAAATTATTCTGT
GAAGCCAGAGAGTAAAATATACTGT
GAAGCCAGAGAGTAAATTATTATGT
GAAGCCAGAGAGTAAAATATACTGT
GAAGCCAGAGAGTAAATTATTATGT
GAAGCCAGAGAGTAAAATATACTGT
GAAGCCAGAGAGTAAAATATACTGT
GAAGCCAGAGAGTAAATTATTTTGT
GAAGCCAGAGAGTAAAATATACTGT
GAAGCCAGAGAGTAAATTATTATGT
GAAGCCAGAGAGTAAAATATACTGT
GAAGCCAGAGAGTAAATTATTATGT
GAAGCCAGAGAGTAAAATATACTGT
GAAGCCAGAGAGTAAATTATTATGT
GAAGCCAGAGAGTAAAATATACTGT
GAAGCCAGAGAGTAAATTATTATGT
GAAGCCAGAGAGTAAAATATACTGT
GAAGCCAGAGAGTAAATTATTATGT
GAAGCCAGAGAGTAAAATATACTGT
GAAGCCAGAGAGTAAAATATACTGT
GAAGCCAGAGAGTAAAATAATCGGT
GAAGCCAGAGAGTAAAATATAGTGG
GAAGCCAGAGAGTAAAATATACTGT
GAAGCCAGAGAGTAAATTATTATGT
GAAGCCAGAGAGTAAAATATACTGT
GAAGCCAGAGAGTAAATTATTATGT
GAAGCCAGAGAGTAAAATATACTGT
GAAGCCAGAGAGTAAAATATACTGT
GAAGCCAGAGAGTAAAATATACTGT
GAAGCCAGAGAGTAAAATATACTGT
GAAGCCAGAGAGTAAATTATTCTGT
GAAGCCAGAGAGTAAAATATACTGT
GAAGCCAGAGAGTAAATTATTATGT
GAAGCCAGAGAGTAAAATATACTGT
GAAGCCAGAGAGTAAATTATTATGT
GAAGCCAGAGAGTAAAATATACTGT
GAAGCCAGAGAGTAAATTATTATGT
GAAGCCAGAGAGTAAATTATTATGT
GAAGCCAGAGAGTAAAATATACTGT
GAAGCCAGAGAGTAAAATATACTGT
GAAGCCAGAGAGTAAATTATTCTGT
GAAGCCAGAGAGTAAAATATACTGT
GAAGCCAGAGAGTAAATTATTATGT
GAAGCCAGAGAGTAAAATATACTGT
GAAGCCAGAGAGTAAATTATTATGT
GAAGCCAGAGAGTAAAATATACTGT

GAAGCCAGAGAGTAAATTATTATGT
GAAGCCAGAGAGTAAAATATACTGT
GAAGCCAGAGAGTAAATTATTCTGT
GAAGCCAGAGAGTAAATTATACTGT
GAAGCCAGAGAGTAAAATAATCGGT
GAAGCCAGAGAGTAAAATATAGTGG
GAAGCCAGAGAGTAAATTATTCTGT
GAAGCCAGAGAGTAAATTATTCTGT
GAAGCCAGAGAGTAAATTATTCTGT
GAAGCCAGAGAGTAAAATATACTGT
GAAGCCAGAGAGTAAAATAATCGGT
GAAGCCAGAGAGTAAAATATAGTGG
GAAGCCAGAGAGTAAATTATTCTGT
GAAGCCAGAGAGTAAATTATTCTGT
GAAGCCAGAGAGTAAAATATACTGT
GAAGCCAGAGAGTAAAATATACTGT
GAAGCCAGAGAGTAAATTATTATGT
GAAGCCAGAGAGTAAAATATACTGT
GAAGCCAGAGAGTAAATTTTTCTGT
GAAGCCAGAGAGTAAAATATACTGT
GAAGCCAGAGAGTAAATTATTATGT
GAAGCCAGAGAGTAAAATATACTGT
GAAGCCAGAGAGTAAAATATACTGT
GAAGCCAGAGAGTAAAATATACTGT
GAAGCCAGAGAGTAAATTATTATGT
GAAGCCAGAGAGTAAAATATACTGT
GAAGCCAGAGAGTAAATTATTATGT
GAAGCCAGAGAGTAAAATATACTGT
GAAGCCAGAGAGTAAAATATACTGT
GAAGCCAGAGAGTAAAATATACTGT
GAAGCCAGAGAGTAAAATATACTGT
GAAGCCAGAGAGTAAATTATTCTGT
GAAGCCAGAGAGTAAAATATACTGT
GAAGCCAGAGAGTAAATTATTATGT
GAAGCCAGAGAGTAAAATATACTGT
GAAGCCAGAGAGTAAATTATTATGT
GAAGCCAGAGAGTAAAATATACTGT
GAAGCCAGAGAGTAAAATATACTGT
GAAGCCAGAGAGTAAAATAATCGGT
GAAGCCAGAGAGTAAAATATAGTGG
GAAGCCAGAGAGTAAATTATTCTGT
GAAGCCAGAGAGTAAATTATTCTGT
GAAGCCAGAGAGTAAAATATACTGT
GAAGCCAGAGAGTAAATTATTATGT
GAAGCCAGAGAGTAAAATATACTGT
GAAGCCAGAGAGTAAAATATACTGT
GAAGCCAGAGAGTAAAATATACTGT
GAAGCCAGAGAGTAAAATATACTGT
GAAGCCAGAGAGTAAATTATTCTGT
GAAGCCAGAGAGTAAAATATACTGT
GAAGCCAGAGAGTAAATTATTATGT
GAAGCCAGAGAGTAAAATATACTGT
GAAGCCAGAGAGTAAATTATTATGT
GAAGCCAGAGAGTAAAATATACTGT

GAAGCCAGAGAGTAAAATATACTGT
GAAGCCAGAGAGTAAAATAATCGGT
GAAGCCAGAGAGTAAAATATAGTGG
GAAGCCAGAGAGTAAATTATTCTGT
GAAGCCAGAGAGTAAATTATTCTGT
GAAGCCAGAGAGTAAATTATTCTGT
GAAGCCAGAGAGTAAAATATACTGT
GAAGCCAGAGAGTAAATTATTCTGT
GAAGCCAGAGAGTAAAATATACTGT
GAAGCCAGAGAGTAAATTATTATGT
GAAGCCAGAGAGTAAAATATACTGT
GAAGCCAGAGAGTAAATTATTATGT
GAAGCCAGAGAGTAAAATATACTGT
GAAGCCAGAGAGTAAAATATACTGT
GAAGCCAGAGAGTAAAATAATCGGT
GAAGCCAGAGAGTAAAATATAGTGG
GAAGCCAGAGAGTAAATTATTCTGT
GAAGCCAGAGAGTAAATTATTCTGT
GAAGCCAGAGAGTAAAATATACTGT
GAAGCCAGAGAGTAAATTATTATGT
GAAGCCAGAGAGTAAAATATACTGT
GAAGCCAGAGAGTAAATTATTATGT
GAAGCCAGAGAGTAAAATATACTGT
GAAGCCAGAGAGTAAAATATACTGT
GAAGCCAGAGAGTAAATTATTATGT
GAAGCCAGAGAGTAAAATATACTGT
GAAGCCAGAGAGTAAATTATTATGT
GAAGCCAGAGAGTAAAATATACTGT
GAAGCCAGAGAGTAAATTATTATGT
GAAGCCAGAGAGTAAAATATACTGT
GAAGTCAGAGAGTAAATTATTATGT
GAAGCCAGAGAGTAAAATATACTGT
GAAGCCAGAGAGTAAATTATTATGT
GAAGCCAGAGAGTAAAATATACTGT
GAAGCCAGAGAGTAAAATATACTGT
GAAGCCAGAGAGTAAAATATACTGT
GAAGCCAGAGAGTAAATTATTATGT
GAAGCCAGAGAGTAAAATATACTGT
GAAGCCAGAGAGTAAAATATACTGT
GAAGCCAGAGAGTAAAATATACTGT
GAAGCCAGAGAGTAAATTATTATGT
GAAGCCAGAGAGTAAAATATACTGT
GAAGCCAGAGAGTAAATTATTATGT
GAAGCCAGAGAGTAAAATATACTGT
GAAGCCAGAGAGTAAAATATACTGT
GAAGCCAGAGAGTAAAATAATCGGT
GAAGCCAGAGAGTAAAATATAGTGG
GAAGCCAGAGAGTAAATTATTCTGT
GAAGCCAGAGAGTAAATTATTCTGT
GAAGCCAGAGAGTAAAATATACTGT
GAAGCCAGAGAGTAAATTATTATGT
GAAGCCAGAGAGTAAAATATACTGT
GAAGCCAGAGAGTAAATTATTATGT
GAAGCCAGAGAGTAAAATATACTGT

GAAGCCAGAGAGTAAATTATTATGT
GAAGCCAGAGAGTAAAATATACTGT
GAAGCCAGAGAGTAAATTATTATGT
GAAGCCAGAGAGTAAAATATACTGT
GAAGCCAGAGAGTAAATTATTATGT
GAAGCCAGAGAGTAAAATATACTGT
GAAGCCAGAGAGTAAAATATACTGT
GAAGCCAGAGAGTAAAATAATCGGT
GAAGCCAGAGAGTAAAATATAGTGG
GAAGCCAGAGAGTAAAATATACTGT
GAAGCCAGAGAGTAAATTATTATGT
GAAGCCAGAGAGTAAAATATACTGT
GAAGCCAGAGAGTAAATTATTATGT
GAAGCCAGAGAGTAAAATATACTGT
GAAGCCAGAGAGTAAAATATTATGT
GAAGCCAGAGAGTAAAATATACTGT
GAAGCCAGAGAGTAAAATATTATGT
GAAGCCAGAGAGTAAAATATACTGT
GAAGCCAGAGAGTAAATTATTATGT
GAAGCCAGAGAGTAAAATATACTGT
GAAGCCAGAGAGTAAATTATTATGT
GAAGCCAGAGAGTAAAATATACTGT
GAAGCCAGAGAGTAAAATATACTGT
GAAGCCAGAGAGTAAAATATACTGT
GAAGCCAGAGAGTAAAATATACTGT
GAAGCCAGAGAGTAAATTATTCTGT
GAAGCCAGAGAGTAAAATATACTGT
GAAGCCAGAGAGTAAATTATTATGT
GAAGCCAGAGAGTAAAATATACTGT
GAAGCCAGAGAGTAAATTATTATGT
GAAGCCAGAGAGTAAAATATACTGT
GAAGCCAGAGAGTAAATTATTATGT
GAAGCCAGAGAGTAAAATATACTGT
GAAGCCAGAGAGTAAAATATACTGT
GAAGCCAGAGAGTAAATTATTCTGT
GAAGCCAGAGAGTAAATTATACTGT
GAAGCCAGAGAGTAAAATAATCGGT
GAAGCCAGAGAGTAAAATATAGTGG
GAAGCCAGAGAGTAAATTATTATGT
GAAGCCAGAGAGTAAAATATACTGT
GAAGCCAGAGAGTAAAATATACTGT
GAAGCCAGAGAGTAAAATAATCGGT
GAAGCCAGAGAGTAAAATATAGTGG
GAAGCCAGAGAGTAAATTATTCTGT
GAAGCCAGAGAGTAAATTATTCTGT
GAAGCCAGAGAGTAAAATATACTGT
GAAGCCAGAGAGTAAATTATTATGT
GAAGCCAGAGAGTAAAATATACTGT
GAAGCCAGAGAGTAAATTATTATGT
GAAGCCAGAGAGTAAAATATACTGT
GAAGCCAGAGAGTAAATTATTATGT
GAAGCCAGAGAGTAAAATATACTGT
GAAGCCAGAGAGTAAATTATTATGT
GAAGCCAGAGAGTAAAATATACTGT

GAAGCCAGAGAGTAAATTATTATGT
GAAGCCAGAGAGTAAAATATACTGT
GAAGCCAGAGAGTAAATTATTATGT
GAAGCCAGAGAGTAAAATATACTGT
GAAGCCAGAGAGTAAATTATTATGT
GAAGCCAGAGAGTAAAATATACTGT
GAAGCCAGAGAGTAAAATATACTGT
GAAGCCAGAGAGTAAAATAATCGGT
GAAGCCAGAGAGTAAAATATAGTGG
GAAGCCAGAGAGTAAAATATACTGT
GAAGCCAGAGAGTAAATTATTATGT
GAAGCCAGAGAGTAAAATATACTGT
GAAGCCAGAGAGTAAATTATTATGT
GAAGCCAGAGAGTAAAATATACTGT
GAAGCCAGAGAGTAAAATATTATGT
GAAGCCAGAGAGTAAAATATACTGT
GAAGCCAGAGAGTAAAATATTATGT
GAAGCCAGAGAGTAAAATATACTGT
GAAGCCAGAGAGTAAATTATTATGT
GAAGCCAGAGAGTAAAATATACTGT
GAAGCCAGAGAGTAAATTATTATGT
GAAGCCAGAGAGTAAAATATAGTGG
GAAGCCAGAGAGTAAAATATACTGT
GAAGCCAGAGAGTAAAATATTATGT
GAAGCCAGAGAGTAAAATATACTGT
GAAGCCAGAGAGTAAAATATTATGT
GAAGCCAGAGAGTAAAATATACTGT
GAAGCCAGAGAGTAAATTATTATGT
GAAGCCAGAGAGTAAAATATACTGT
GAAGCCAGAGAGTAAATTATTATGT
GAAGCCAGAGAGTAAAATATACTGT
GAAGCCAGAGAGTAAAATATACTGT
GAAGCCAGAGAGTAAAATATACTGT
GAAGCCAGAGAGTAAATTATTATGT
GAAGCCAGAGAGTAAAATATACTGT
GAAGCCAGAGAGTAAATTATTATGT
GAAGCCAGAGAGTAAAATATACTGT
GAAGCCAGAGAGTAAAATATACTGT
GAAGCCAGAGAGTAAAATATACTGT
GAAGCCAGAGAGTAAAATATACTGT
GAAGCCAGAGAGTAAATTATTCTGT
GAAGCCAGAGAGTAAAATATACTGT
GAAGCCAGAGAGTAAATTATTATGT
GAAGCCAGAGAGTAAAATATACTGT
GAAGCCAGAGAGTAAATTATTATGT
GAAGCCAGAGAGTAAAATATACTGT
GAAGCCAGAGAGTAAATTATTATGT
GAAGCCAGAGAGTAAAATATACTGT
GAAGCCAGAGAGTAAAATATACTGT
GAAGCCAGAGAGTAAATTATTCTGT
GAAGCCAGAGAGTAAATTATACTGT
GAAGCCAGAGAGTAAAATAATCGGT
GAAGCCAGAGAGTAAAATATAGTGG
GAAGCCAGAGAGTAAATTATTCTGT

GAAGCCAGAGAGTAAATTATTCTGT
GAAGCCAGAGAGTAAATTATTCTGT
GAAGCCAGAGAGTAAAATATACTGT
GAAGCCAGAGAGTAAAATAATCGGT
GAAGCCAGAGAGTAAAATATAGTGG
GAAGCCAGAGAGTAAATTATTCTGT
GAAGCCAGAGAGTAAATTATTCTGT
GAAGCCAGAGAGTAAAATATACTGT
GAAGCCAGAGAGTAAAATATACTGT
GAAGCCAGAGAGTAAATTATTATGT
GAAGCCAGAGAGTAAAATATACTGT
GAAGCCAGAGAGTAAATTTTTCTGT
GAAGCCAGAGAGTAAAATATACTGT
GAAGCCAGAGAGTAAATTATTATGT
GAAGCCAGAGAGTAAAATATACTGT
GAAGCCAGAGAGTAAAATATACTGT
GAAGCCAGAGAGTAAAATAATCGGT
GAAGCCAGAGAGTAAAATATAGTGG
GAAGCCAGAGAGTAAATTATTATGT
GAAGCCAGAGAGTAAATTATTATGT
GAAGCCAGAGAGTAAATTATTATGT
GAAGCCAGAGAGTAAAATATACTGT
GAAGCCAGAGAGTAAATTATTATGT
GAAGCCAGAGAGTAAAATATACTGT
GAAGCCAGAGAGTAAAATATACTGT
GAAGCCAGAGAGTAAAATATACTGT
GAAGCCAGAGAGTAAAATATACTGT
GAAGCCAGAGAGTAAATTATTCTGT
GAAGCCAGAGAGTAAAATATACTGT
GAAGCCAGAGAGTAAATTATTATGT
GAAGCCAGAGAGTAAAATATACTGT
GAAGCCAGAGAGTAAATTATTATGT
GAAGCCAGAGAGTAAAATATACTGT
GAAGCCAGAGAGTAAATTATTATGT
GAAGCCAGAGAGTAAAATATACTGT
GAAGCCAGAGAGTAAAATATACTGT
GAAGCCAGAGAGTAAAATAATCGGT
GAAGCCAGAGAGTAAAATATAGTGG
GAAGCCAGAGAGTAAATTATTCTGT
GAAGCCAGAGAGTAAATTATTCTGT
GAAGCCAGAGAGTAAAATATACTGT
GAAGCCAGAGAGTAAAATATACTGT
GAAGCCAGAGAGTAAAATATACTGT
GAAGCCAGAGAGTAAAATAATCGGT
GAAGCCAGAGAGTAAATTATTCTGT
GAAGCCAGAGAGTAAATTATTCTGT
GAAGCCAGAAAGTAAAATATACTGT
GAAGCCAGAGAGTAAATTATTATGT
GAAGCCAGAGAGTAAAATATACTGT
GAAGCCAGAGAGTAAATTATTATGT
GAAGCCAGAGAGTAAAATATACTGT
GAAGCCAGAGAGTAAATTATTATGT
GAAGCCAGAGAGTAAAATATACTGT
GAAGCCAGAGAGTAAAATATACTGT

GAAGCCAGAGAGTAAAATAATCGGT
GAAGCCAGAGAGTAAAATATAGTGG
GAAGCCAGAGAGTAAATTATTCTGT
GAAGCCAGAGAGTAAATTATTCTGT
GAAGCCAGAGAGTAAATTATTCTGT
GAAGCCAGAGAGTAAATTATTCTGT
GAAGCCAGAAAGTAAAATATACTGT
GAAGCCAGAGAGTAAAATATACTGT
GAAGCCAGAGAGTAAAATAATCGGT
GAAGCCAGAGAGTAAATTATTCTGT
GAAGCCAGAGAGTAAATTATTCTGT
GAAGCCAGAAAGTAAAATATACTGT
GAAGCCAGAGAGTAAATTATTATGT
GAAGCCAGAGAGTAAAATATACTGT
GAAGCCAGAGAGTAAAATAATCGGT
GAAGCCAGAGAGTAAATTATTCTGT
GAAGCCAGAGAGTAAATTATTCTGT
GAAGCCAGAAAGTAAAATATAGTGG
GAAGCCAGAGAGTAAATTATTCTGT
GAAGCCAGAAAGTAAAATATACTGT
GAAGCCAGAGAGTAAAATATACTGT
GAAGCCAGAGAGTAAAATAATCGGT
GAAGCCAGAGAGTAAATTATTCTGT
GAAGCCAGAGAGTAAATTATTCTGT
GAAGCCAGAAAGTAAAATATAGTGG
GAAGCCAGAGAGTAAATTATTCTGT
GAAGCCAGAAAGTAAAATATAGTGG
GAAGCCAGAGAGTAAATTATTCTGT
GAAGCCAGAGAGTAAATTATTCTGT
GAAGCCAGAGAGTAAATTATTCTGT
GAAGCCAGAGAGTAAAATATACTGT
GAAGCCAGAGAGTAAATTATTCTGT
GAAGCCAGAGAGTAAATTATTCTGT
GAAGCCAGAGAGTAAATTATTCTGT
Nr of selected repeats 399 Similarity 0.903264
Consensus:
GAAGCCAGAGAGTAAAaTATacTGT
>Cele-UNSB01_1:7000180-7000255 Satlength=76 Nr of Repeats=5 RepeatLength=15
seed=AGATCCGGAA
AGATCCGGAAAACGT
AGATCCGGAAAACGT
AGATCCGGAAAACGT
AGATCCGGAAATGGT
AGATCCGGAAAACGT
Nr of selected repeats 5 Similarity 0.928889
Consensus:
AGATCCGGAAAacGT
>Cele-UNSB01_1:7149236-7149296 Satlength=61 Nr of Repeats=4 RepeatLength=15
seed=CGAAAAGGGG
CGAAAAGGGGATCAG
CAAAAAGGGGATCTG
CGAAAAGGGGATCTG
CGAAAAGGGGAGCAA
Nr of selected repeats 4 Similarity 0.807407

Consensus:
CgAAAAGGGGAtCag
>Cele-UNSB01_1:7886332-7886407 Satlength=76 Nr of Repeats=5 RepeatLength=15
seed=TCGTGGTGAG
TCGTGGTGAGACCCA
TCGTGGTGAGACCCT
TCGTGGTGAGACCCT
TCGTGGTAAGACCCA
TCGTGGTGAGACCCT
Nr of selected repeats 5 Similarity 0.911111
Consensus:
TCGTGGTgAGACCCt
>Cele-UNSB01_1:7886430-7887346 Satlength=917 Nr of Repeats=26 RepeatLength=35
seed=GCGGGAAATT
GCGGGAAATTCAAATTTTCAGTAAAAAAAATTTTTG
GCGGGAAATTCAAATTTTAAGGGAAAAAATTTTTG
GCGGGAAATTCAAATTTTCAGTGAAAAAAATTTTG
GCGGGAAATTCAAATTTTAAGTGAAAAAAATTTTTGA
CGGGAAATTCAAATTTTAAGTGAAAAAAAATTTTG
GCGGGAAATTCAAATTTTCAGTAAAAAAAATTTTTG
GCGGGAAATTCAAATTTTCAGTGAAAAAAATTTTG
GCGGGAAATTCAAATTTTAAGTGAAAAAAAATTTTTG
GCGGGAAATTCAAATTTTCAGTGAAAAAAATTTTG
GCGGGAAATTCAAATTTTAAGGGAAAAAATTTTTG
GCGGGAAATTCAAATTTTAAGTGAAAAAAATTTTG
GCGGGAAATTCAAATTTTAAGTGAAAAAAAATTTTG
GCGGGAAATTCAAATTTTAAGTGAAAAAAAATTTTTG
GCGGGAAATTCAAATTTTCAGTGAAAAAAATTTTG
GCGGGAAATTCAAATTTTAAGGGAAAAAATTTTTG
GCGGGAAATTCAAATTTTCAATAAAAAAAATTTTG
GCGGGAAATTCAAATTTTCAGTGAAAAATATTTTG
GCGGAAATTTAAATTTTCTGAGAAAAATATTTTG
GCGGAAATTTAAATTTTCTGAGAAAAATATTTTG
GAGGGAAATTTAAATTTTCTGTGAAAAATATTTTG
GCGGGAAATTTAAATTTTCTGAGAAAAATATTTTG
GCGGGAAATTCAAATTTTCAGTGAAAAAAATTTTG
GCGGGAAATTCAAATTTTAAGGGAAAAAATTTTTG
GCGGGAAATTCAAATTTTCAGTGAAAAAAATTTTG
GCGGGAAATTCAAATTTTCAGTGAAAACAATTTTG
GCGGGAAATTCAAATTTTCAGTGAAAAAAATTTA
Nr of selected repeats 17 Similarity 0.870370
Consensus:
GCGGGAAATTCAAATTTTcAGtGAAAAAAaTTTTG
>Cele-UNSB01_1:7887436-7887916 Satlength=481 Nr of Repeats=13 RepeatLength=40
seed=TTTCTAGAAA
TTTCTAGAAAATTCGGGAAAACTCTGGAATATTCCAGAAC
TTTCTAGAAAAATCGAGAAATGTCTGCAATGTTCCAGAAG
TTTCTAGAAAATTCGAGAAAATTCCGGAATGGACCAGAAT
TTTCTAGAAAA
TTCTAGAAAGTTCTGGAATGGTCCAGAAT
TTTCTAGAAAATTCGGGAAAACTCTGGAATATTCCAGAAC
TTTCTAGAAAATTCGAGAAAATTCCGGAATGGTCCAGAAT
TTTCTAGAAAATTCGGGAAAACTCTGGAATATTCCAGAAC
TTTCTAGAAAAATCGGGAAAAGTCGGCAATGTTCTTGAAC

TTTCTAGAAAATTCGAGAAAATTCCGGAATGGTCCAGAAT
TTTCTAGAAAATTCGGGAAAACTCTGGAATATTCCAGAAG
TTTCTAGAAAATCCGAGAAAGTTCTGGAATGGTCCAGAAT
TTTCTAGAAAATTCGGAAAAAATTCTGGAATATTCCAGAAC
Nr of selected repeats 11 Similarity 0.818182
Consensus:
TTTCTAGAAAATTCGgGAAAAnTCtGGAATgtTCCAGAAn
>Cele-UNSB01_1:7925712-7926382 Satlength=671 Nr of Repeats=39 RepeatLength=16
seed=AATTTTTCCG
AATTTTTCCGCCACGA
AATTTTGCCGCGTCGA
AATTTTTCCGCGGCGA
AATTTTTCAGCAGCAAC
ATTTTTCCGCGTCGA
AATTTTTCCGCAGCAA
AATTTTTCCGCCACG
AATTTTTGCCGCGTCGA
AATTTTTCCGCGGCGA
AATTTTTCAGCAGCAACATTTTTTCGCGGCGA
AATTTTTCCGCAGCAA
AATTTTTCCGCGTCGA
AATTTTTCCGCAGCAA
AATTTTTCCGCGTCGA
AATTTTTCCGCAGCAA
AATTTTTCCGCCACGA
AATTTTTCAGCAGCAA
AATTTTTCGGCGTCGA
AATTTTTCAGCAGCAA
AATTTTTCCGCGTCGAAATTTTCCAGCAGCAA
AATTTTTCCGCCACGA
AATTTTTCCGCAGCAA
AATTTTTCCGCGTCGA
AATTTTTCAGCAGCAA
AATTTTTCCGCGACGA
AATTTTTCAGCAGCAA
AATTTTTCCGCGGCGA
AATTTTTCCGCGACG
AATTTTTCCGCAGCAATTTTTTCAGCAGCAA
AATTTTTCCGCGTCGA
AATTTTTCCGCGGCGA
AATTTTTCAGCAGCAA
AATTTTTCCGCGGCGA
AATTTTTCCGCAGCAA
AATTTTTCCGCGTCGA
AATTTTTCAGCAGCAA
AATTTTTCCGCGACGA
AATTTTTCAGCAGCAA
AATTTTTCCGTGTCGA
Nr of selected repeats 31 Similarity 0.813978
Consensus:
AATTTTTCcGCngCgA
>Cele-UNSB01_1:8014213-8014468 Satlength=256 Nr of Repeats=17 RepeatLength=15
seed=ACCCATCGTG
ACCCATCGTGGCGAG

ACCCATCGTGGCGAA
ACCCATCGTGGTGAG
ACCCATCGTGGTGAG
ACCCATCGTGGCGAT
ACCCATCGTGGCCAG
ACCCATCGTGGTGAG
ACCCGTCGTGGCGAG
ACCCATCGTGGTGAG
ACCCATCGTGGTGAG
ACCCACCGTGGTGAG
ACCCGTCGTGGTGAG
ACCCATCGTGGTAAG
ACCCATCGTGGTGAA
ACCCATCGTGGCGAG
ACCCACCGTGATGAG
ACCCGTCGTGGCGAG
Nr of selected repeats 17 Similarity 0.847712
Consensus:
ACCCATCGTGGtGAG
>Cele-UNSB01_1:8014573-8014958 Satlength=386 Nr of Repeats=11 RepeatLength=35
seed=GGGAAATTTA
GGGAAATTTAAATTTTCTGTGAAAAAATATTTTGGC
GGGAAATTTAAATTTTCTGTGAAAAAATATTTTGGC
GGGAAATTTAAATTTTCTGAGAAAAAATATTTTGGA
GGGAAATTTAAATTTTCTGTGAAAAAATATTTTGGC
GGGAAATTTAAATTTTCTGAGAAAAAATTTTTTGGC
GGGAAATTTAAATATTCTGTGAAAAAATATTTTGGA
GGGAAATTTAAATTTTCTGTGAAAAAATATTTTGGC
GGGAAATTTAAATTTTCTGTGAAAAAATATTTTGGC
GGGAAATTTAAATTTTCTGAGAAAAAATATTTTGGA
GGGAAATTTAAATTTTCTGTGAAAAAATATTTTGGC
GGGAAATTTAAATTTTCTGAGAAAAAATTTTTTGGC
Nr of selected repeats 11 Similarity 0.944589
Consensus:
GGGAAATTTAAATTTTCTGtGAAAAAATATTTTGGc
>Cele-UNSB01_1:8015067-8016984 Satlength=1918 Nr of Repeats=48 RepeatLength=40
seed=GAATGTTCCA
GAATGTTCCAGAACTTTCTAGAAAAAATCGAGAAAATTCTG
GAATGTTCCAGAACTTTCTAGAAAAAATCGAGAAAATTCTG
GAATGTTCCAGAACTCCTAGAAAAAATCGAGAAAATTCTG
GAATGTTCCAGAACTTTCTAGAAAAAATTGGGAAAGTTCTG
GAATGTTCCAGAACTTTCTAGAAAAAATCGAGAAAATTCTG
GAATGTTCCAGAACTTTCTAGAAAAAATTGGGAAAAGTCTG
GAATGTTCCAGAACTTTCTAGAAAAAATCGAGAAAATTCTG
GAATGTTCCAGAACTTTCTAGAAAAAATCGAGAAAATTCTG
GAATGTTCCAGAACTTTCTAGAAAAAATTGGGAAAGTTCTG
GAATGTTCCAGAACTTTCTAGAAAAAATCGAGAAAATTCTG
GAATGTTCCAGAACTTTCTAGAAAAAATTGGGAAAGTTCTG
GAATGTTCCAGAACTTTCTAGAAAAAATCGAGAAAATTCTG
GAATGTTCCAGAACTTTCTAGAAAAAATTGGGAAAAGTCTG
GAATGTTCCAGAACTTTCTAGAAAAAATCGAGAAAATTCTG
GAATGTTCCAGAACTTTCTAGAAAAAATCGAGAAAATTCTG
GAATGTTCCAGAACTCCTAGAAAAAATCGAGAAAATTCTG
GAATGTTCCAGAACTTTCTAGAAAAAATTGGGAAAGTTCTG

GAATGTTCCAGAACTTTCTAGAAAAAATCGAGAAAATTCTG
GAATGTTCCAGAACTTTCTAGAAAAAATTGGGAAAAGTCTG
GAATGTTCCAGAACTTTCTAGAAAAAATCGAGAAAATTCTG
GAATGTTCCAGAACTTTCTAGAAAAAATCGAGAAAATTCTG
GAATGTTCCAGAACTTTCTAGAAAAAATTGGGAAAGTTCTG
GAATGTTCCAGAACTTTCTAGAAAAAATCGAGAAAATTCTG
GAATGTTCCAGAACTTTCTAGAAAAAATTGGGAAAGTTCTG
GAATGTTCCAGAACTTTCTAGAAAAAATCGAGAAAATTCTG
GAATGTTCCAGAACTTTCTAGAAAAAATTGGGAAAAGTCTG
GAATGTTCCAGAACTTTCTAGAAAAAATCGAGAAAATTCTG
GAATGTTCCAGAACTTTCTAGAAAAAATTGGGAAAATTCTG
GAATGTTCCAGAACTTTCTAGAAAAAATCGAGAAAATTCTG
GAATGTTCCAGAACTTTCTAGAAAAAATTGGGAAAGTTCTG
GAATGTTCCAGAACTTTCTAGAAAAAATCGAGAAAATTCTG
GAATGTTCCAGAACTTTCTAGAAAAAATCGAGAAAATTCTG
GAATGTTCCAGAACTCCTAGAAAAAATCGAGAAAATTCTG
GAATGTTCCAGAACTTTCTAGAAAAAATTGGGAAAGTTCTG
GAATGTTCCAGAACTTTCTAGAAAAAATCGAGAAAATTCTG
GAATGTTCCAGAACTTTCTAGAAAAAATTGGGAAAAGTCTG
GAATGTTCCAGAACTTTCTAGAAAAAATCGAGAAAATTCTG
GAATGTTCCAGAACTTTCTAGAAAAAATCGAGAAAATTCTG
GAATGTTCCAGAACTTTCTAGAAAAAATTGGGAAAGTTCTG
GAATGTTCCAGAACTTTCTAGAAAAAATCGAGAAAATTCTG
GAATGTTCCAGAACTTTCTAGAACAATTGGGAAAGTTCTG
GAATGTTCCAGAACTTTCTAGAAAAAATCGAGAAAATTCTG
GAATGTTCCAGAACTTTCTAGAAAAAATTGGGAAAAGTCTG
GAATGTTCCAGAACTTTCTAGAAAAAATCGAGAAAATTCTG
GAATGTTCCAGAACTTTCTAGAAAAAATTGGGAAAATTCTG
GAATGTTCCAGAACTTTCTAGAAAAAATCGAGAAAATTCTG
GAATGTTCCAGAACTTTCTAGAAAAAATTGGGAAAAGTCTG
GAATGTTCCAGAACTTTCTAGAAAAAATCGAGAAAATTCTG
Nr of selected repeats 45 Similarity 0.944512
Consensus:
GAATGTTCCAGAACTTTCTAGAAAAAATcGaGAAAaTTCTG
>Cele-UNSB01_1:8640900-8641029 Satlength=130 Nr of Repeats=4 RepeatLength=32
seed=AAAATCGAAA
AAAATCGAAAAATTCTCGTTTTTCAATTTTTA
AAAATCGAAAAATTCTCGTTTTTCAATTTTTA
AAAATCGAAAAATTCTCGTTTTTCAATTTTTA
AAAATCGAAAAATTCTCGTTTTTCAATTTTTAA
Nr of selected repeats 3 Similarity 1.000000
Consensus:
AAAATCGAAAAATTCTCGTTTTTCAATTTTTA
>Cele-UNSB01_1:9793063-9794825 Satlength=1763 Nr of Repeats=41 RepeatLength=44
seed=CTGTTTTCTG
CTGTTTTCTGAGCATTGTAGATTTTCGGATTGATGGCCGAAAGG
CTGTTTTCTGAGCATTGTAGATTTTCGGATTGATGGCCGAAAGG
CTGTTTTCTGAGAATTTTAGATTTTCGGATTGATGGCCGAAAGG
CTGTTTTCTGAGAATTTTAGATTTTCGGATTGATGGCCGAAAGG
CTGTTTTCTGAGCATTTTAGATTTTCGGATTGATGGCCGAAAGG
CTGTTTTCTGAGCATTGTAGATTTTCGGATTGATGGCCGAAAGG
CTGTTTTCTGAGCATTTTAGATTTTCGGATTGATGGCCGAAAGG
CTGTTTTCTGAGCATTTTAGATTTTCGGATTGATGGCCGAAAGG
CTGTTTTCTGAGCATTTTAGATTTTCGGATTGATGGCCGAAAGG

CTGTTTTCTGAGCATTTTAGATTTTCGGATTGATGGCCGAAAGG
CTGTTTTCTGAGCATTTTAGATTTTCGGATTGATGGCCGAAAGG
CTGTTTTCTGAGCATTTTAGATTTTCGGATTGATGGCCGAAAGG
CTGTTTTCTGAGCATTTTAGATTTTCGGATTGATGGCCGAAAGG
CTGTTTTCTGAGCATTTTAGATTTTCGGATTGATGGCCGAAAGG
CTGTTTTCTGAGCATTTTAGATTTTCGGATTGATGGCCGAAAGG
CTGTTTTCTGAGCATTTTAGATTTTCGGATTGATGGCCGAAAGG
CTGTTTTCTGAGCATTGTAGATTTTCGGATTGATAGG
CTGTTTTCTGAGCATTGTAGATTTTCGGATTGATAGG
CTGTTTTCTGAGCATTTTAGATTTTCGGATTGATGGCCGAAAGG
CTGTTTTCTGAGCATTGTAGATTTTCGGATTGATGGCCGAAAGG
CTGTTTTCTGAGCATTGTAGATTTTCGGATTGATGGCCGAAAGG
CTGTTTTCTGAGCATTTTAGATTTTCGGATTGATGGCCGAAAGG
CTGTTTTCTGAGCATTTTAGATTTTCGGATTGATGGCCGAAAGG
CTGTTTTCTGAGCATTTTAGATTTTCGGATTGATGGCCGAAAGG
CTGTTTTCTGAGCATTTTAGATTTTCGGATTGATGGCCGAAAGG
CTGTTTTCTGAGCATTTTAGATTTTCGGATTGATGGCCGAAAGG
CTGTTTTCTGAGCATTTTAGATTTTCGGATTGATGGCCGAAAGG
CTGTTTTCTGAGCATTTTAGATTTTCGGATTGATGGCCGAAAGG
CTGTTTTCTGAGCATTTTAGATTTTCGGATTGATGGCCGAAAGG
CTGTTTTCTGAGCATTGTAGATTTTCGGATTGATAGG
CTGTTTTCTGAGCATTGTAGATTTTCGGATTGATAGG
CTGTTTTCTGAGCATTTTAGATTTTCGGATTGATGGCCGAAAGG
CTGTTTTCTGAGCATTTTAGATTTTCGGATTGATGGCCGAAAGG
CTGTTTTCTGAGCATTTTAGATTTTCGGATTGATGGCCGAAAGG
CTGTTTTCTGAGCATTTTAGATTTTCGGATTGATGGCCGAAAGG
CTGTTTTCTGAGCATTTTAGATTTTCGGATTGATGGCCGAAAGG
CTGTTTTCTGAGCATTTTAGATTTTCGGATTGATGGCCGAAAGG
CTGTTTTCTGAGCATTGTAGATTTTCGGATTGATAGG
CTGTTTTCTGAGCATTTTAGATTTTCGGATTGATGGCCGAAAGG
CTGTTTTCTGAGCATTGTAGATTTTCGGATTGATAGG
CTGTTTTCTGAGCATTTTAGATTTTCGGATTGATGGCCGAAAGG
Nr of selected repeats 35 Similarity 0.988999
Consensus:
CTGTTTTCTGAGCATTTTAGATTTTCGGATTGATGGCCGAAAGG
>Cele-UNSB01_1:9974009-9976809 Satlength=2801 Nr of Repeats=15 RepeatLength=200 seed=AACAGAATTT
AACAGAATTTTGAAAAATTTCGAAACCGCCAAGTTCAAGCAGAAAAAGTGAATTTTCAGTCATACCTTGTAGACCACAA
TTTATATACAAAATTAATAGATTTTCCAAACTTTGAAAATTAATTTCTGGCTTCAAACTAACTTTTTTAGAAATTCCCG
TTGCCATTTTGTAGCAAATTTTATGTTCTTTCAGAATTTGAA
AACAGAATTTTGAAAAATTTCGAAACCGCCAAGTTCAAGCAGAAAAAGTGAATTTTCAGTCATACCTTGTAGACCACAA
TTTATATACAAAATTAATAGATTTTCCAAACTTTGAAAATTAATTTCTGGCTTCAAACTAACTTTTTTAGAAATTCCCG
TTGCCATTTTGTAGCACATTTTATGTTCTTTCAGAATTTGAA
AACAGAATTTTAAAAAATTTCGAAACCGCCAAGTTCAAGCATTAAAAGTGAATTTTCAGTCATACCTTGTAGACCACAA
TTTATATGCAAAATTAATAGATTTTCCAAACTTTGAAAATTAATTTCTGGCTTCAAACTAACTTTTTTAGAAATTCCCG
TTGCCATTTTGTAGCAAATTTTATGTTCTTTCAGAATTTGAA
AACAGAATTTTAAAAAATTTCGAAACCGCCAAGTTCAAGCATTAAAAGTGAATTTTCAGTCATACCTTGTAGACCACAA
TTTATATACAAAATTAATAGATTTTCCAAACTTTGAAAATTAATTTCTGGCTTCAAACTAACTTTTTTAGAAATTCCCG
TTGCCATTTTGTAGCAAATTTTATGTTCTTTCAGAATTTGAA
AACAGAATTTCAAAAAATTTCGAAACCGCCAAGTTCAAGCATTAAAAGTGAATTTTCAGTCATACCTTGTAGACCACAA
TTTATATGCAAAATTAATAGATTTTCCAAACTTTGAAAATTAATTTCTGGCTTCAAACTAACTTTTTTAGAAATTCCCG
TAGCCATTTTGTAGCAAATTTTATGTTCT
AACAGAATTTGAA

AACAGAATTTTAAAAAATTTCGAAAATGCCAAGTTCAAGCATTAAAAGTGAATTTTCAGTCATACCTTGTAGACCACAA
TTTATATGCAAAATTAATAGATTTTCCAAACTTTGAAAATTAATTTCTGGCTTCAAACTAACTTTTTTAGAAATTCCCG
TTGCCATTTTGTAGCAAATTTTATGTTCTTTCAGAATTTGAA
AACAGAATTTTAAAAAATTTCGAAACCGCCAAGTTCAAGCATTAAAAGTGAATTTTCAGTCATACCTTGTAGACCACAA
TTTATATGCAAAATTAATAGATTTTCCAAACTTTGAAAATTAATTTCTGGCTTCAAACTAACTTTTTTAGAAATTCCCG
TTGCCATTTTGTAGCAAATTTTATGTTCTTTCAGAATTTGAA
AACAGAATTTTGAAAAAATTTCGAAACCGCCAAGTTCAAGCAGAAAAAGTGAATTTTCAGTCATACCTTGTAGACCACAA
TTTATATACAAAATTAATAGATTTTCCAAACTTTGAAAATTAATTTCTGGCTTCAAACTAACTTTTTTAGAAATTCCCG
TTGCCATTTTGTAGCACATTTTATGTTCTTTCAGAATTTGAA
AACAGAATTTTAAAAAATTTCGAAACCGCCAAGTTCAAGCATTAAAAGTGAATTTTCAGTCATACCTTGTAGACCACAA
TTTATATGCAAAATTAATAGATTTTCCAAACTTTGAAAATTAATTTCTGGCTTCAAACTAACTTTTTTAGAAATTCCCG
TTGCCATTTTGTAGCAAATTTTATGTTCTTTCAGAATTTGAA
AACAGAATTTTAAAAAATTTCGAAACCGCCAAGTTCAAGCATTAAAAGTGAATTTTCAGTCATACCTTGTAGACCACAA
TTTATATACAAAATTAATAGATTTTCCAAACTTTGAAAATTAATTTCTGGCTTCAAACTAACTTTTTTAGAAATTCCCG
TTGCCATTTTGTAGCAAATTTTATGTTCTTTCAGAATTTGAA
AACAGAATTTTGAAAAAATTTCGAAACCGCCAAGTTCAAGCAGAAAAAGTGAATTTTCAGTCATACCTTGTAGACCACAA
TTTATATACAAAATTAATAGATTTTCCAAACTTTGAAATTTAATTTCTGGCTTCAAACTAACTTTTTTAGAAATTCCCG
TAGCCATTTTGTAGCAAATTTTATGTTCTTTCAGAATTTGAA
AACAGAATTTCAAAAAATTTCGAAACCGCCAAGTTCAAGCAGAAAAAGTGAATTTTCAGTCATACCTTGTAGACCACAA
TTTATATGCAAAATTAATAGATTTTCCAAACTTTGAAAATTAATTTCTGGCTTCAAACTAACTTTTTTAGAAATTCCCG
TAGCCATTTTGTAGCAAATTTTATGTTCTTTCAGAATTTGAA
AACAGAATTTTAAAAAATTTCGAAACCGCCAAGTTCAAGCAGAAAAAGTGAATTTTCAGTCATACCTTGTAGACCACAA
TTTATATACAAAATTAATAGATTTTCCAAACTTTGAAATTTAATTTCTGGCTTCAAACTAACTTTTTTAGAAATTCCCG
TAGCCATTTTGTAGCAAATTTTATGTTCTTTCAGAATTTGAA
AACAGAATTTCAAAAAATTTCGAAACCGCCAAGTTCAAGCAGAAAAAGTGAATTTTCAGTCATACCTTGTAGACCACAA
TTTATATGCAAAATTAATAGATTTTCCAAACTTTGAAAATTAATTTCTGGCTTCAAACTAACTTTTTTAGAAATTCCCG
TAGCCATTTTGTAGCAAATTTTATGTTCTTTCAGAATTTGAA
Nr of selected repeats 13 Similarity 0.975385
Consensus:
AACAGAATTTTaAAAAAATTTCGAAACCGCCAAGTTCAAGCAgaAAAAGTGAATTTTCAGTCATACCTTGTAGACCACAA
TTTATATaCAAAATTAATAGATTTTCCAAACTTTGAAAATTAATTTCTGGCTTCAAACTAACTTTTTTAGAAATTCCCG
TtGCCATTTTGTAGCAAATTTTATGTTCTTTCAGAATTTGAA
>Cele-UNSB01_1:9977112-9977772 Satlength=661 Nr of Repeats=41 RepeatLength=15
seed=CACGATGGGT
CACGATGGGTCTCAC
CACGAAGGGTATCAC
CACGATGGGTCTCAC
CACGAAGGGTATCAC
CACGATGGGTCTCAC
CACGATGGGTCTCAC
CACGATGGGTCTCGT
CACGATGGGTCTCAC
CACGATGGGTCTCAC
CACGATGGGTCTCAG
CACGACGGGTCTCAC
CACGATGGGTCTCAC
CACAATGGGTCTCGT
CACGATGGGTCTCAC
CACGATGGGTCTCGT
CACGATAGGTCTCAC
CACGATGGGTCTAAC
CACGATGGGTCTCAC
CACGATGGGTCTCACCACGATGAGACTCAC
CACGACGGGTCTTAC

CACGATGGGTCTCAGCACAACGGGTCTCAC
CACGATGGGTCTCAC
CACAATGGGTCTCGT
CACGATGGGTCTCAC
CACGATGGGTCTCGT
CACGATAGGTCTCAC
CACGATGGGTCTCAC
CACGATGGGTCTCAC
CACAATGGGTCTCGT
CACGATGGGTCTCAC
CACGATGGGTCTCGT
CACGATAGGTCTCAC
CACGATGGGTCTAAC
CACGATGGGTCTCAC
CACGATGGGTCTCAC
CACGATGGGTCTCGT
CACGATAGGTCTCAC
CACGATGGGTCTAAC
CACGATGGGTCTCAC
CACGATGGGTCTCGTCACAATGGCTCTCAC
CACGACGGGTCTTAC
Nr of selected repeats 38 Similarity 0.852821
Consensus:
CACGATGGGTCTCac
>Cele-UNSB01_1:10089590-10089728 Satlength=139 Nr of Repeats=5 RepeatLength=37
seed=ATAATTTTTC
ATAATTTTTCAC
ATACTTTTTCATAAT
ATAATTTTTCTTGAGTAATGAGAGACTTTTTCATAAT
ATAATTTTTCTTGAGTAATGAGAGACTTTTTCATAAT
ATAATTTTTCTTGAGTAATGAGAGACTTTTTCATAAT
Nr of selected repeats 3 Similarity 1.000000
Consensus:
ATAATTTTTCTTGAGTAATGAGAGACTTTTTCATAAT
>Cele-UNSB01_1:10295164-10303189 Satlength=8026 Nr of Repeats=319
RepeatLength=25 seed=TTACTCTAAT
TTACTCTAATGTTCTGCCAATTTAT
TTACTCTAATTTTCTGCCAATTTAT
TTACTCTAATGTTCTGCCAATTTAT
TTACTCTAATGTTCTGCCAATTTAT
TTACTCTAATTTTCTGCCAATATAT
TTACTCTAATTTTCTGCCAATTTAT
TTACTCTAATGTTCTGCCAATATAT
TTACTCTAATGTTCTGCCAATATAT
TTACTCTAATGTTCTGCCAATTTTTATTTTTTCTATAT
TTACTCTAATTTTCTGCCAATTTAT
TTACTCTAATGTTCTGCCAATTTAT
TTACTCTAATGTTCTGCCAATATAT
TTACTCTAATGTTCTGCCAATATAT
TTACTCTAATTTTCTGCCAATTTAT
TTACTCTAATGTTCTGCCAATTTAT
TTACTCTAATGTTCTGCCAATTTAT
TTACTCTAATTTTCTGCCAATTTAT
TTACTCTAATTTTCTGCCAATTTAT

TTACTCTAATGTTCTGCCAATTTAT
TTACTCTAATGTTCTGCCAATATAT
TTACTCTAATGTTCTGCCAATTTAT
TTACTCTAATTTTCTGCCAATTTAT
TTACTCTAATGTTCTGCCAATTTAT
TTACTCTAATGTTCTGCCAATTTAT
TTACTCTAATTTTCTGCCAATTTAT
TTACTCTAATGTTCTGCCAATTTAT
TTACTCTAATGTTCTGCCAATATAT
TTACTCTAATGTTCTGCCAATTTAT
TTACTCTAATTTTCTGCCAATTTAT
TTACTCTAATGTTCTGCCAATTTAT
TTACTCTAATGTTCTGCCAATTTAT
TTACTCTAATGTTCTGCCAATTTAT
TTACTCTAATGTTCTGCCAATTTAT
TTACTCTAATGTTCTGCCAATGCCAATATAT
TTACTCTAATGTTCTGCCAATTTAT
TTACTCTAATTTTCTGCCAATATAT
TTACTCTAATGTTCTGCCAATTTAT
TTACTCTAATTTTCTGCCAATATAT
TTACTCTAATGTTCTGCCAATTTAT
TTACTCTAATTTTCTGCCAATATAT
TTACTCTAATGTTCTGCCAATTTAT
TTACTCTAATGTTCTGCCAATATAT
TTACTCTAATGTTCTGCCAATTTAT
TTACTCTAATGTTCTGCCAATTTAT
TTACTCTAATGTTCTGCCAATATAT
TTACTCTAATGTTCTGCCAATTTAT
TTACTCTAATGTTCTGCCAATATAT
TTACTCTAATGTTCTGCCAATATAT
TTACTCTAATTTTCTGCCAATATAT
TTACTCTAATGTTCTGCCAATTTAT
TTACTCTAATGTTCTGCCAATATAT
TTACTCTAATTTTCTGCCAATTTAT
TTACTCTAATGTTCTGCCAATTTAT
TTACTCTAATGTTCTGCCAATTTAT
TTACTCTAATGTTCTGCCAATATAT
TTACTCTAATGTTCTGCCAATAT
TTACTCTAATTTAT
TTACTCTAATGTTCTGCCAATATAT
TTACTCTAATGTTCTGCCAATATAT
TTACTCTAATGTTCTGCCAATTTAT
TTACTCTAATGTTCTGCCAATATAT
TTACTCTAATGTTCTGCCAATAT
TTACTCTAATGTTCTGCCAATATAT
TTACTCTAATGTTCTGCCAATATAT
TTACTCTAATGTTCTGCCAATTTAT
TTACTCTAATGTTCTGCCAATTTAT
TTACTCTAATTTCTGCCAATATAT
TTACTCTAATGTTCTGCCAATTTAT
TTACTCTAATGTTCTGCCAATA
TTACTCTAATGTTCTGCCAATATAT
TTACTCTAATGTTCTGCCAATATAT
TTACTCTAATGTTCTGCCAATATAT

TTACTCTAATGTTCTGCCAATAT
TTACTCTAATGTTCTGCCAATAT
TTACTCTAATGTTCTGCCAATATAT
TTACTCTAATGTTCTGCCAATATAT
TTACTCTAATGTTCTGCCAATATAT
TTACTCTAATGTTCTGCCAATATAT
TTACTCTAATGTTCTGCCAATATAT
TTACTCTAATGTTCTGCCAATATAT
TTACTCTAATGTTCTGCCAATATAT
TTACTCTAATTTTCTGCCAATTTAT
TTACTCTAATGTTCTGCCAATTTAT
TTACTCTAATGTTCTGCCAATTTAT
TTACTCTAATTTTCTGCCAATATAT
TTACTCTAATGTTCTGCCAATTTAT
TTACTCTAATGTTCTGCCAATATAT
TTACTCTAATGTTCTGCCAATTTAT
TTACTCTAATTTTCTGCCAATATAT
TTACTCTAATGTTCTGCCAATTATTTTTATTTCTCTATTTATTTTTCTGCCAATTTAT
TTACTCTAATTTCTGCCAATATAT
TTACTCTAATGTTCTGCCAATTTAT
TTACTCTAATTTTCTGCCAATATAT
TTACTCTAATGTTCTGCCAATTTAT
TTACTCTAATTTTCTGCCAATATAT
TTACTCTAATGTTCTGCCAATTTAT
TTACTCTAATGTTCTGCCAATATAT
TTACTCTAATGTTCTGCCAATTTAT
TTACTCTAATTTCTGCCAATATAT
TTACTCTAATGTTCTGCCAATTTAT
TTACTCTAATTTTCTGCCAATATAT
TTACTCTAATGTTCTGCCAATTTAT
TTACTCTAATTTTCTGCCAATATAT
TTACTCTAATGTTCTGCCAATTTAT
TTACTCTAATTTTCTGCCAATATAT
TTACTCTAATGTTCTGCCAATTTAT
TTACTCTAATTTTCTGCCAATATAT
TTACTCTAATGTTCTGCCAATTTAT
TTACTCTAATTTTCTGCCAATATAT
TTACTCTAATGTTCTGCCAATTTAT
TTACTCTAATTTTCTGCCAATATAT
TTACTCTAATGTTCTGCCAATTTAT
TTACTCTAATTTTCTGCCAATATAT
TTACTCTAATGTTCTGCCAATTTAT
TTACTCTAATTTTCTGCCAATATAT
TTACTCTAATGTTCTGCCAATTTAT
TTACTCTAATTTTCTGCCAATATAT
TTACTCTAATGTTCTGCCAATTTAT
TTACTCTAATTTTTTTAT
TTACTCTAATTTTCTGCCAATATAT
TTACTCTAATGTTCTGCCAATTTAT
TTACTCTAATTTTCTGCCAATATAT
TTACTCTAATGTTCTGCCAATTAT
TTACTCTAATTTCTGCCAATTAT
TTACTCTAATTTCTGCCAATATAT
TTACTCTAATGTTCTGCCAATTTAT

TTACTCTAATTTTCTGCCAATATAT
TTACTCTAATGTTCTGCCAATTTAT
TTACTCTAATTTTCTGCCAATATAT
TTACTCTAATGTTCTGCCAATTTAT
TTACTCTAATTTCTGCCAATTTAT
TTACTCTAATTTTCTGCCAATATAT
TTACTCTAATGTTCTGCCAATTTAT
TTACTCTAATTTTCTGCCAATATAT
TTACTCTAATGTTCTGCCAATTTAT
TTACTCTAATTTTCTGCCAATATAT
TTACTCTAATGTTCTGCCAATTTAT
TTACTCTAATTTTCTGCCAATATAT
TTACTCTAATGTTCTGCCAATTTAT
TTACTCTAATTTTCTGCCAATATAT
TTACTCTAATGTTCTGCCAATTTAT
TTACTCTAATTTTCTGCCAATATAT
TTACTCTAATGTTCTGCCAATTTAT
TTACTCTAATTTTCTGCCAATATAT
TTACTCTAATGTTCTGCCAATTTAT
TTACTCTAATTTTCTGCCAATATAT
TTACTCTAATGTTCTGCCAATTTAT
TTACTCTAATGTTCTGCCAATTTAT
TTACTCTAATGTTCTGCCAATATAT
TTACTCTAATGTTCTGCCAATATAT
TTACTCTAATGTTCTGCCAATTTAT
TTACTCTAATGTTCTGCCAATTTAT
TTACTCTAATGTTCTGCCAATATAT
TTACTCTAATGTTCTGCCAATATAT
TTACTCTAATGTTCTGCCAATATAT
TTACTCTAATGTTCTGCCAATTTAT
TTACTCTAATGTTCTGCCAATATAT
TTACTCTAATGTTCTGCCAATATAT
TTACTCTAATGTTCTGCCAATTTAT
TTACTCTAATGTTCTGCCAATATAT
TTACTCTAATGTTCTGCCAATATAT
TTACTCTAATTTCTGCCAATATAT
TTACTCTAATGTTCTGCCAATATAT
TTACTCTAATGTTCTGCCAATTTAT
TTACTCTAATGTTCTGCCAATATAT
TTACTCTAATGTTCTGCCAATTTAT
TTACTCTAATTTCTGCCAATTAT
TTACTCTAATTTCTGCCAATATAT
TTACTCTAATGTTCTGCCAATTTAT
TTACTCTAATTTTCTGCCAATATAT
TTACTCTAATGTTCTGCCAATTTAT
TTACTCTAATGTTCTGCCAATTTAT
TTACTCTAATTTTCTGCCAATATAT
TTACTCTAATGTTCTGCCAATTTAT
TTACTCTAATTTTCTGCCAATTTAT
TTACTCTAATTTTCTGCCAATATAT
TTACTCTAATGTTCTGCCAATTTAT
TTACTCTAATTTTCTGCCAATATAT
TTACTCTAATGTTCTGCCAATTTAT
TTACTCTAATTTTCTGCCAATATAT

TTACTCTAATGTTCTGCCAATTTAT
TTACTCTAATGTTCTGCCAATTTAT
TTACTCTAATTTTCTGCCAATATAT
TTACTCTAATGTTCTGCCAATTTAT
TTACTCTAATTTTCTGCCAATATAT
TTACTCTAATGTTCTGCCAATTTAT
TTACTCTAATTTTCTGCCAATATAT
TTACTCTAATGTTCTGCCAATATAT
TTACTCTAATGTTCTGCCAATATAT
TTACTCTAATTTCTGCCAATATAT
TTACTCTAATGTTCTGCCAATTTAT
TTACTCTAATGTTCTGCCAATTTAT
TTACTCTAATGTTCTGCCAATATAT
TTACTCTAATGTTCTGCCAATATAT
TTACTCTAATGTTCTGCCAATTTAT
TTACTCTAATGTTCTGCCAATTTAT
TTACTCTAATTTTCTGCCAATATAT
TTACTCTAATGTTCTGCCAATTTAT
TTACTCTAATTTCTGCCAATATAT
TTACTCTAATGTTCTGCCAATTTAT
TTACTCTAATTTTCTGCCAATATAT
TTACTCTAATGTTCTGCCAATTTAT
TTACTCTAATTTTCTGCCAATATAT
TTACTCTAATGTTCTGCCAATTTAT
TTACTCTAATTTTCTGCCAATATAT
TTACTCTAATGTTCTGCCAATTTAT
TTACTCTAATTTTCTGCCAATTTAT
TTACTCTAATGTTCTGCCAATTTAT
TTACTCTAATTTTCTGCCAATATAT
TTACTCTAATGTTCTGCCAATTTAT
TTACTCTAATTTTCTGCCAATATAT
TTACTCTAATGTTCTGCCAATTTAT
TTACTCTAATTTTCTGCCAATATAT
TTACTCTAATGTTCTGCCAATTTAT
TTACTCTAATTTTCTGCCAATATAT
TTACTCTAATGTTCTGCCAATTTAT
TTACTCTAATTTTCTGCCAATATAT
TTACTCTAATGTTCTGCCAATTTAT
TTACTCTAATTTCTGCCAATATAT
TTACTCTAATGTTCTGCCAATTTAT
TTACTCTAATTTTCTGCCAATATAT
TTACTCTAATGTTCTGCCAAATAGTTTGATCTAATGTTCTGCCAATATAT
TTACTCTAATGTTCTGCCAATTTAT
TTACTCTAATTTTCTGCCAATATAT
TTACTCTAATGTTCTGCCAATATAT
TTACTCTAATTTTCTGCCAATATAT
TTACTCTAATGTTCTGCCAATTTAT
TTACTCTAATGTTCTGCCAATATAT
TTACTCTAATGTTCTGCCAATATAT
TTACTCTAATTTTCTGCCAATATAT
TTACTCTAATGTTCTGCCAATTTAT
TTACTCTAATGTTCTGCCAATATAT
TTACTCTAATGTTCTGCCAATATAT
TTACTCTAATTTTCTGCCAATATAT

TTACTCTAATGTTCTGCCAATATAT
TTACTCTAATTTTCTGCCAATATAT
TTACTCTAATGTTCTGCCAATTTAT
TTACTCTAATGTTCTGCCAATTTAT
TTACTCTAATGTTCTGCCAATATAT
TTACTCTAATGTTCTGCCAATTTAT
TTACTCTAATGTTCTGCCAATATAT
TTACTCTAATGTTCTGCCAATATAT
TTACTCTAATGTTCTGCCAATATAT
TTACTCTAATTTTCTGCCAATATAT
TTACTCTAATGTTCTGCCAATTTATTTACTTTTTCTGCAATTAT
TTACTCTAATGTTCTGCCAATTTAT
TTACTCTAATGTTCTGCCAATATAT
TTACTCTAATGTTCTGCCAATTTAT
TTACTCTAATTTTCTGCCAATATAT
TTACTCTAATGTTCTGCCAATATAT
TTACTCTAATGTTCTGCCAATATAT
TTACTCTAATGTTCTGCCAATATAT
TTACTCTAATGTTCTGCCAATATAT
TTACTCTAATGTTCTGCCAATATAT
TTACTCTAATGTTCTGCCAATATAT
TTACTCTAATGTTCTGCCAATATAT
TTACTCTAATGTTCTGCCAATTTAT
TTACTCTAATGTTCTGCCAATTTAT
TTACTCTAATGTTCTGCCAATATAT
TTACTCTAATGTTCTGCCAATATAT
TTACTCTAATGTTCTGCCAATATAT
TTACTCTAATGTTCTGCCAATATAT
TTACTCTAATGTTCTGCCAATATAT
TTACTCTAATGTTCTGCCAATTTAT
TTACTCTAATGTTCTGCCAATATAT
TTACTCTAATGTTCTGCCAATATAT
TTACTCTAATGTTCTGCCAATTTAT
TTACTCTAATGTTCTGCCAATTTAT
TTACTCTAATGTTCTGCCAATATAT
TTACTCTAATGTTCTGCCAATTTAT
TTACTCTAATGTTCTGCCAATATAT
TTACTCTAATGTTCTGCCAATATAT
TTACTCTAATGTTCTGCCAATATAT
TTACTCTAATGTTCTGCCAATATAT
TTACTCTAATTTTCTGCCAATATAT
TTACTCTAATGTTCTGCCAATTTAT
TTACTCTAATGTTCTGCCAATTTAT
TTACTCTAATGTTCTGCCAATATAT
TTACTCTAATGTTCTGCCAATTTAT
TTACTCTAATTTTCTGCCAATATAT
TTACTCTAATGTTCTGCCAATATAT
TTACTCTAATGTTCTGCCAATATAT
TTACTCTAATGTTCTGCCAATATAT
TTACTCTAATGTTCTGCCAATATAT
TTACTCTAATGTTCTGCCAATATAT
TTACTCTAATGTTCTGCCAATATAT
TTACTCTAATGTTCTGCCAATATAT
TTACTCTAATGTTCTGCCAATATAT

TTACTCTAATGTTCTGCCAATTTAT
TTACTCTAATGTTCTGCCAATTTAT
TTACTCTAATGTTCTGCCAATTTAT
TTACTCTAATGTTCTGCCAATATAT
TTACTCTAATGTTCTGCCAATATAT
TTACTCTAATGTTCTGCCAATATAT
TTACTCTAATGTTCTGCCAATATAT
TTACTCTAATGTTCTGCCAATTTAT
TTACTCTAATGTTCTGCCAATATAT
TTACTCTAATGTTCTGCCAATATAT
TTACTCTAATGTTCTGCCAATTTAT
TTACTCTAATGTTCTGCCAATATAT
TTACTCTAATGTTCTGCCAATATAT
TTACTCTAATGTTCTGCCAATATAT
TTACTCTAATGTTCTGCCAATATAT
TTACTCTAATGTTCTGCCAATTTAT
TTACTCTAATGTTCTGCCAATTTAT
TTACTCTAATGTTCTGCCAATTTAT
TTACTCTAATGTTCTGCCAATTTAT
TTACTCTAATGTTCTGCCAATTTAT
TTACTCTAATTTTCTGCCAATATAT
TTACTCTAATTTTCTGCCAATTTAT
TTACTCTAATGTTCTGCCAATAT
TTACTCTAATGTTCTGCCAATATAT
TTACTCTAATGTTCTGCCAATATAT
TTACTCTAATGTTCTGCCAATTTAT
TTACTCTAATGTTCTGCCAATTTAT
TTACTCTAATTTTCTGCCAATTTAT
TTACTCTAATGTTCTGCCAATATAT
TTACTCTAATTTTCTGCCAATATAT
TTACTCTAATGTTCTGCCAATTTAT
Nr of selected repeats 293 Similarity 0.953127
Consensus:
TTACTCTAATgTTCTGCCAATaTAT
>Cele-UNSB01_1:10374816-10387323 Satlength=12508 Nr of Repeats=186
RepeatLength=68 seed=AAAACCATTG
AAAACCATTGCCAACTGAATAGGTTGATTTGTGTTTTCTTTCTGAAATTCTAAGAATTTTGGTAAAAG
AAAACCATTGTCAACTGAATAGGTTGATTTGTGTTTTCTTTCTGAAATTCTAAGAATTTTGTTAAAAG
AAAACCATTGTCAACTGAATAGGTTATTTGTGTTTTCTTTCTGAAATTCTAAGAATTTTGGTAAAAG
AAAACCATTGTCAACTGAATAGGTTGATTTGTGTTTTCTTTCTGAAATTCTAAGAATTTTGTTAAAAG
AAAACCATTGTCAACTGAATAGGTTGATTTGTGTTTTCTTTCTGAAATTCTAAGAATTTTGGTAAAAG
AAAACCATTGTCAACTGAATAGGTTGATTTGTGTTTTCTTTCTGAAATTCTAAGAATTTTGTTAAAAG
AAAACCATTGTCAACTGAATAGGTTGATTTGTGTTTTCTTTAAAATTTTAAAAAATTTGGTAAAAG
AAAACCATTGTCAACTGAATAGGTTGATTTGTGTTTTCTTTAAAATTTTAAAAAATTTGGTAAAAG
AAAACCATTGTCAACTGAATAGGTTGATTTGTGTTTTCTTTCTGAAATTCTAAGAATTTTGGTAAAAG
AAAACCATTGTCAACTGAATAGGTTGATTTGTGTTTTCTTTCTGAAATTCTAAGAATTTTGTTAAAAG
AAAACCATTGTCAACTGAATAGGTTGATTTGTGTTTTCTTTAAAATTTTAAAAAATTTGTTAAAAG
AAAACCATTGTCAACTGAATAGGTTATTTGTGTTTTCTTTCTGAAATTCTAAGAATTTTGGTAAAAG
AAAACCATTGTCAACTGAATAGGTTGATTTGTGTTTTCTTTCTGAAATTCTAAGAATTTTGTTAAAAG
AAAACCATTGTCAACTGAATAGGTTGATTTGTGTTTTCTTTCTGAAATTCTAAGAATTTTGGTAAAAG
AAAACCATTGTCAACTGAATAGGTTGATTTGTGTTTTCTTTCTGAAATTCTAAGAATTTTGTTAAAAG
AAAACCATTGTCAACTGAATAGGTTGATTTGTGTTTTCTTTCTGAAATTCTAAGAATTTTGGTAAAAG
AAAACCATTGTCAACTGAATAGGTTGATTTGTGTTTTCTTTCTGAAATTCTAAGAATTTTGTTAAAAG
AAAACCATTGTCAACTGAATAGGTTGATTTGTGTTTTCTTTAAAATTTTAAAAAATTTGGTAAAAG

AAAACCATTGTCAACTGAATAGGTTGATTTGTGTTTTCTTTAAAATTTTAAAAAATTTGGTAAAAG
AAAACCATTGTCAACTGAATAGGTTGATTTGTGTTTTCTTTAAAATTTTAAAAAATTTGGTAAAAG
AAAACCATTGTCAACTGAATAGGTTGATTTGTGTTTTCTTTAAAATTTTAAAAAATTTGGTAAAAG
AAAACCATTGTCAACTGAATAGGTTGATTTGTGTTTTCTTTCTGAAATTCTAAGAATTTTGGTAAAAG
AAAACCATTGTCAACTGAATAGGTTGATTTGTGTTTTCTTTCTGAAATTCTAAGAACTTTGTTAAAAG
AAAACCATTGTCAACTGAATAGGTTGATTTGTGTTTTCTTTCTGAAATTCTAAGAATTTTGTTAAAAG
AAAACCATTGTCAACTGAATAGGTTGATTTGTGTTTTCTTTAAAATTTTAAAAAATTTGGTAAAAG
AAAACCATTGTCAACTGAATAGGTTGATTTGTGTTTTCTTTCTGAAATTCTAAGAATTTTGTTAAAAG
AAAACCATTGTCAACTGAATAGGTTGATTTGTGTTTTCTTTCTGAAATTCTAAGAATTTTGGTAAAAG
AAAACCATTGTCAACTGAATAGGTTGATTTGTGTTTTCTTTCTGAAATTCTAAGAACTTTGTTAAAAG
AAAACCATTGTCAACTGAATAGGTTGATTTGTGTTTTCTTTCTGAAATTCTAAGAATTTTGTTAAAAG
AAAACCATTGTCAACTGAATAGGTTGATTTGTGTTTTCTTTAAAATTTTAAAAAATTTGGTAAAAG
AAAACCATTGTCAACTGAATAGGTTGATTTGTGTTTTCTTTCTGAAATTCTAAGAATTTTGTTAAAAG
AAAACCATTGTCAACTGAATAGGTTGATTTGTGTTTTCTTTCTGAAATTCTAAGAATTTTGGTAAAAG
AAAACCATTGTCAACTGAATAGGTTGATTTGTGTTTTCTTTCTGAAATTCTAAGAACTTTGTTAAAAG
AAAACCATTGTCAACTGAATAGGTTGATTTGTGTTTTCTTTAAAATTTTACAAAATTTGGTAAAAG
AAAACCATTGTCAACTGAATAGGTTGATTTGTGTTTTCTTTCTGAAATTCTAAGAATTTTGTTAAAAG
AAAACCATTGTCAACTGAATAGGTTGATTTGTGTTTTCTTTAAAATTTTAAAAAATTTGGTAAAAG
AAAACCATTGTCAACTGAATAGGTTGATTTGTGTTTTCTTTCTGAAATTCTAAGAATTTTGTTAAAAG
AAAACCATTGTCAACTGAATAGGTTGATTTGTGTTTTCTTTAAAATTTTAAAAAATTTGGTAAAAG
AAAACCATTGTCAACTGAATAGGTTGATTTGTGTTTTCTTTAAAATTTTAAAAAATTTGGTAAAAG
AAAACCATTGTCAACTGAATAGGTTGATTTGTGTTTTCTTTCTGAAATTCTAAGAATTTTGTTAAAAG
AAAACCATTGTCAACTGAATAGGTTGATTTGTGTTTTCTTTAAAATTTTAAAAAATTTGGTAAAAG
AAAACCATTGTCAACTGAATAGGTTGATTTGTGTTTTCTTTCTGAAATTCTAAGAATTTTGTTAAAAG
AAAACCATTGTCAACTGAATAGGTTGATTTGTGTTTTCTTTCTGAAATTCTAAGAATTTTGGTAAAAG
AAAACCATTGTCAACTGAATAGGTTGATTTGTGTTTTCTTTAAAATTTTACAAAATTTGGTAAAAG
AAAACCATTGTCAACTGAATAGGTTGATTTGTGTTTTCTTTCTGAAATTCTAAGAATTTTGTTAAAAG
AAAACCATTGTCAACTGAATAGGTTGATTTGTGTTTTCTTTAAAATTTTAAAAAATTTGGTAAAAG
AAAACCATTGTCAACTGAATAGGTTGATTTGTGTTTTCTTTAAAATTTTAAAAAATTTGGTAAAAG
AAAACCATTGTCAACTGAATAGGTTGATTTGTGTTTTCTTTAAAATTTTAAAAAATTTGGTAAAAG
AAAACCATTGTCAACTGAATAGGTTGATTTGTGTTTTCTTTAAAATTTTAAAAAATTTGGTAAAAG
AAAACCATTGTCAACTGAATAGGTTGATTTGTGTTTTCTTTCTGAAATTCTAAGAATTTTGGTAAAAG
AAAACCATTGTCAATGAATTTTTTTTTTTTTTTTTTTGGTAAAAG
AAAACCATTGTCAACTGAATAGGTTGATTTGTGTTTTCTTTCTGAAATTCTAAGAATTTGGTAAAAG
AAAACCATTGTCAACTGAATAGGTTGATTTGTGTTTTCTTTCTGAAATTCTAAGAATTTGGTTAAAAG
AAAACCATTGTCAACTGAATAGGTTGATTTGTGTTTTCTTTCTGAAATTCTAAGAATTTGGTAAAAG
AAAACCATTGTCAACTGAATAGGTTGATTTGTGTTTTCTTTAAAATTTCAAAAAATTTGATAAAAG
AAAACCATTGTCAACTGAATAGGTTGATTTGTGTTTTCTTTCTGAAATTCTAAGAATTTTGGTAAAAG
AAAACCATTGTCAACTGAATAGGTTGATTTGTGTTTTCTTTCTGAAATTCTAAGAATTTGGTAAAAG
AAAACCATTGTCAACTGAATAGGTTGATTTGTGTTTTCTTTCTGAAATTCTAAGAATTTTGGTAAAAG
AAAACCATTGTCAACTGAATAGGTTGATTTGTGTTTTCTTTCTGAAATTCTAAGAATTTTGGTAAAAG
AAAACCATTGTCAACTGAATAGGTTGATTTGTGTTTTCTTTCTGAAATTCTAAGAATTTTGGTAAAAG
AAAACCATTGTCAACTGAATAGGTTGATTTGTGTTTTCTTTCTGAAATTCTAAGAATTTTGTTAAAAG
AAAACCATTGTCAACTGAATAGGTTGATTTGTGTTTTCTTTCTGAAATTCTAAGAATTTTGTTAAAAG
AAAACCATTGTCAACTGAATAGGTTGATTTGTGTTTTCTTTAAATTTAAAAATTTGGTAAAAG
AAAACCATTGTCAACTGAATAGGTTGATTTGTGTTTTCTTTCTGAAATTCTAAGAATTTTGGTAAAAA
AAAACCATTGTCAACTGAATAGGTTGATTTATGTTTTCTTTCTGAAATTCTAAGAATTTTGGTAAAAG
AAAACCATTGTCAACTGAATAGGTTGATTTGTGTTTTCTTTCTGAAATTCTAAGAATTTTGGTAAAAG
AAAACCATTGTCAACTGAATAGGTTGATTTGTGTTTTCTTTCTGAAATTCTAAGAATTTTTAAAAG
AAAACCATTGTCAACTGAATAGGTTGATTTGTGTTTTCTTTCTGAAATTCTAAGAATTTTGGTAAAAG
AAAACCATTGTCAACTGAATAGGTTGATTTGTGTTTTCTTTCTGAAATTCTAAGAATTTTGTAAAAG
AAAACCATTGTCAACTGAATAGGTTGATTTGTTTTTCTTTCTGAAATTCTAAGAATTTTGGTAAAA
AAAACCATTGTCAACTGAATAGGTTGATTTGTGTTTTCTTTCTGAAATTCTAAGAATTTTGGTAAAAG
AAAACCATTGTCAACTGAATAGGTTGATTTGTGTTTTCTTTCTGAAATTCTAAGAATTTTGGTAAAAG

AAAACCATTGTCAACTGAATAGGTTGATTTGTGTTTTCTTTCTGAAATTCTAAGAATTTTGGTAAAAG
AAAACCATTGTCAACTGAATAGGTTGATTTGTGTTTTCTTTCTGAAATTCTAAGAATTTTGGTAAAAG
AAAACCATTGTCAACTGAATAGGTTGATTTGTGTTTTCTTTCTGAAATTCTAAGAATTTTGGTAAAAG
AAAACCATTGTCAACTGAATAGGTTGATTTGTGTTTTCTTTCTGAAATTCTAAGAATTTTGGTAAAAG
AAAACCATTGTCAACTGAATAGGTTGATTTGTGTTTTCTTTCTGAAATTCTAAGAATTTTGGTAAAAG
AAAACCATTGTCAACTGAATAGGTTGATTTGTGTTTTCTTTCTGAAATTCTAAGAATTTTGGTAAAAG
AAAACCATTGTCAACTGAATAGGTTGATTTGTGTTTTCTTTCTGAAATTCTAAGAATTTTGGTAAAAG
AAAACCATTGTCAACTGAATAGGTTGATTTGTGTTTTCTTTCTGAAATTCTAAGAATTTTGGTTAAAAG
AAAACCATTGTCAACTGAATAGGTTGATTTATGTTTTCTTTCTGAAATTCTAAGAATTTTGGTAAAAG
AAAACCATTGTCAACTGAATAGGTTGATTTATGTTTTCTTTCTGAAATTCTAAGAATTTTGGTAAAAG
AAAACCATTGTCAACTGAATAGGTTGATTTGTGTTTTCTTTCTGAAATTCTAAGAATTTTGTTAAAAG
AAAACCATTGTCAACTGAATAGGTTGATTTGTGTTTTCTTTAAAATTTTAAAAATTTGTTAAAAG
AAAACCATTGTCAACTGAATAGGTTGATTTGTGTTTTCTTTCTGAAATTCTAAGAATTTTGGTAAAAG
AAAACCATTGTCAACTGAATAGGTTGATTTGTGTTTTCTTTCTGAAATTCTAAGAATTTTGGTAAAAG
AAAACCATTGTCAACTGAATAGGTTGATTTGTGTTTTCTTTCTGAAATTCTAAGAATTTTGTAAAAG
AAAACCATTGTCAACTGAATAGGTTGATTTATGTTTTCTTTCTGAAATTCTAAGAATTTTGTTAAAAG
AAAACCATTGTCAACTGAATAGGTTGATTTGTGTTTTCTTTAAAATTTTAAAAATTTGGTAAAAG
AAAACCATTGTCAACTGAATAGGTTGATTTGTGTTTTCTTTAAAATTTTACAAAATTTGGTAAAAG
AAAACCATTGTCAACTGAATAGGTTGATTTGTGTTTTCTTTCTGAAATTCTAAGAATTTTGGTAAAAG
AAAACCATTGTCAACTGAATAGGTTGATTTATGTTTTCTTTCTGAAATTCTAAGAATTTTGTTAAAAG
AAAACCATTGTCAACTGAATAGGTTGATTTATGTTTTCTTTCTGAAATTCTAAGAATTTTGGTAAAAG
AAAACCATTGTCAACTGAATAGGTTGATTTATGTTTTCTTTCTGAAATTCTAAGAATTTTGTTAAAAG
AAAACCATTGTCAACTGAATAGGTTGATTTATGTTTTCTTTCTGAAATTCTAAGAATTTTGTTAAAAG
AAAACCATTGTCAACTGAATAGGTTGATTTGTGTTTTCTTTAAAATTTTACAAAATTTGTTAAAAG
AAAACCATTGTCAACTGAATAGGTTGATTTGTGTTTTCTTTCTGAATTCTAAGAATTTTGGTAAAAG
AAAACCATTGTCAACTGAATAGGTTGATTTGTGTTTTCTTTAAAATTTTAAAAATTTGGTAAAAG
AAAACCATTGTCAACTGAATAGGTTGATTTGTGTTTTCTTTAAAATTTTAAAAAATTTGGTAAAAG
AAAACCATTGTCAACTGAATAGGTTGATTTGTGTTTTCTTTAAAATTTTACAAAATTTGGTAAAAG
AAAACCATTGTCAACTGAATAGGTTGATTTGTGTTTTCTTTCTGAAATTCTAAGAATTTTGGTAAAAG
AAAACCATTGTCAACTGAATAGGTTGATTTATGTTTTCTTTCTGAAATTCTAAGAATTTTGTTAAAAG
AAAACCATTGTCAACTGAATAGGTTGATTTATGTTTTCTTTCTGAAATTCTAAGAATTTTGTTAAAAG
AAAACCATTGTCAACTGAATAGGTTGATTTATGTTTTCTTTCTGAAATTCTAAGAATTTTGTTAAAAG
AAAACCATTGTCAACTGAATAGGTTGATTTGTGTTTTCTTTCTGAAATTCTAAGAATTTTGTTAAAAG
AAAACCATTGTCAACTGAATAGGTTGATTTGTGTTTTCTTTCTGAAATTCTAAGAATTTTGGTAAAAG
AAAACCATTGTCAACTGAATAGGTTGATTTATGTTTTCTTTCTGAAATTCTAAGAATTTTGTTAAAAG
AAAACCATTGTCAACTGAATAGGTTGATTTATGTTTTCTTTCTGAAATTCTAAGAATTTTGGTAAAAG
AAAACCATTGTCAACTGAATAGGTTGATTTATGTTTTCTTTCTGAAATTCTAAGAATTTTGTTAAAAG
AAAACCATTGTCAACTGAATAGGTTGATTTATGTTTTCTTTCTGAAATTCTAAGAATTTTGGTAAAAG
AAAACCATTGTCAACTGAATAGGTTGATTTATGTTTTCTTTCTGAAATTCTAAGAATTTTGTTAAAAG
AAAACCATTGTCAACTGAATAGGTTGATTTATGTTTTCTTTCTGAAATTCTAAGAATTTTGTTAAAAG
AAAACCATTGTCAACTGAATAGGTTGATTTATGTTTTCTTTCTGAAATTCTAAGAATTTTGGTAAAAG
AAAACCATTGTCAACTGAATAGGTTGATTTGTGTTTTCTTTCTGAAATTCTAAGAATTTTGTTAAAAG
AAAACCATTGTCAACTGAATAGGTTGATTTGTGTTTTCTTTCTGAAATTCTAAGAATTTTGGTAAAAG
AAAACCATTGTCAACTGAATAGGTTGATTTGTGTTTTCTTTCTGAAATTCTAAGAATTTTGTTAAAAG
AAAACCATTGTCAACTGAATAGGTTGATTTGTGTTTTCTTTCTGAAATTCTAAGAATTTTGTTAAAAG
AAAACCATTGTCAACTGAATAGGTTGATTTATGTTTTCTTTCTGAAATTCTAAGAATTTTGGTAAAAG
AAAACCATTGTCAACTGAATAGGTTGATTTGTGTTTTCTTTCTGAAATTCTAAGAATTTTGTTAAAAG
AAAACCATTGTCAACTGAATAGGTTGATTTGTGTTTTCTTTCTGAAATTCTAAGAATTTTGTTAAAAG
AAAACCATTGTCAACTGAATAGGTTGATTTGTGTTTTCTTTAAAATTTTAAAAAATTTGGTAAAAG
AAAACCATTGTCAACTGAATAGGTTGATTTGTGTTTTCTTTCTGAAATTCTAAGAATTTTGGTAAAAG
AAAACCATTGTCAACTGAATAGGTTGATTTGTGTTTTCTTTAAAATTTTAAAAAATTTGGTAAAAG
AAAACCATTGTCAACTGAATAGGTTGATTTGTGTTTTCTTTCTGAAATTCTAAGAATTTTGGTAAAAG
AAAACCATTGTCAACTGAATAGGTTGATTTGTGTTTTCTTTAAAATTTTACAAAATTTGGTAAAAG
AAAACCATTGTCAACTGAATAGGTTGATTTGTGTTTTCTTTCTGAAATTCTAAGAATTTTGTTAAAAG

AAAACCATTGTCAACTGAATAGGTTGATTTGTGTTTTCTTTCTGAAATTCTAAGAATTTTGGTAAAAG
AAAACCATTGTCAACTGAATAGGTTGATTTGTGTTTTCTTTCTGAAATTCTAAGAATTTTGGTAAAAG
AAAACCATTGTCAACTGAATAGGTTGATTTGTGTTTTCTTTCTGAAATTCTAAGAATTTTGGTAAAAG
AAAACCATTGTCAACTGAATAGGTTGATTTGTGTTTTCTTTAAAATTTTAAAAAATTTGGTAAAAG
AAAACCATTGTCAACTGAATAGGTTGATTTGTGTTTTCTTTAAAATTTTAAAAAATTTGGTAAAAG
AAAACCATTGTCAACTGAATAGGTTGATTTGTGTTTTCTTTCTGAAATTCTAAGAATTTTGGTAAAAG
AAAACCATTGTCAACTGAATAGGTTGATTTGTGTTTTCTTTAAAATTTTACAAAATTTGGTAAAAG
AAAACCATTGTCAACTGAATAGGTTGATTTGTGTTTTCTTTCTGAAATTCTAAGAATTTTGTTAAAAG
AAAACCATTGTCAACTGAATAGGTTGATTTGTGTTTTCTTTCTGAAATTCTAAGAATTTTGGTAAAAG
AAAACCATTGTCAACTGAATAGGTTGATTTGTGTTTTCTTTCTGAAATTCTAAGAATTTTGGTAAAAG
AAAACCATTGTCAACTGAATAGGTTGATTTGTGTTTTCTTTCTGAAATTCTAAGAATTTTGGTAAAAG
AAAACCATTGTCAACTGAATAGGTTGATTTGTGTTTTCTTTAAAATTTTTAAAAATTTGGTAAAAG
AAAACCATTGTCAACTGAATAGGTTGATTTGTGTTTTCTTTCTGAAATTCTAAGAATTTTGTTAAAAG
AAAACCATTGTCAACTGAATAGGTTGATTTATGTTTTCTTTCTGAAATTCTAAGAATTTTGGTAAAAG
AAAACCATTGTCAACTGAATAGGTTGATTTATGTTTTCTTTCTGAAATTCTAAGAATTTTGTTAAAAG
AAAACCATTGTCAACTGAATAGGTTGATTTATGTTTTCTTTCTGAAATTCTAAGAATTTTGTTAAAAG
AAAACCATTGTCAACTGAATAGGTTGATTTGTGTTTTCTTTAAAATTTTACAAAATTTGTTAAAAG
AAAACCATTGTCAACTGAATAGGTTGATTTGTGTTTTCTTTCTGAAATTCTAAGAATTTTGGTAAAAG
AAAACCATTGTCAACTGAATAGGTTGATTTGTGTTTTCTTTAAAATTTTAAAAAATTTGGTAAAAG
AAAACCATTGTCAACTGAATAGGTTGATTTGTGTTTTCTTTAAAATTTTACAAAATTTGGTAAAAG
AAAACCATTGTCAACTGAATAGGTTGATTTGTGTTTTCTTTAAAATTTTAAAAAATTTGGTAAAAG
AAAACCATTGTCAACTGAATAGGTTGATTTGTGTTTTCTTTAAAATTTTACAAAATTTGGTAAAAG
AAAACCATTGTCAACTGAATAGGTTGATTTGTGTTTTCTTTCTGAAATTCTAAGAATTTTGGTAAAAG
AAAACCATTGTCAACTGAATAGGTTGATTTATGTTTTCTTTCTGAAATTCTAAGAATTTTGTTAAAAG
AAAACCATTGTCAACTGAATAGGTTGATTTATGTTTTCTTTCTGAAATTCTAAGAATTTTGTTAAAAG
AAAACCATTGTCAACTGAATAGGTTGATTTATGTTTTCTTTCTGAAATTCTAAGAATTTTGTTAAAAG
AAAACCATTGTCAACTGAATAGGTTGATTTATGTTTTCTTTCTGAAATTCTAAGAATTTTGTTAAAAG
AAAACCATTGTCAACTGAATAGGTTGATTTATGTTTTCTTTCTGAAATTCTAAGAATTTTGTTAAAAG
AAAACCATTGTCAACTGAATAGGTTGATTTGTGTTTTCTTTAAAATTTTACAAAATTTGTTAAAAG
AAAACCATTGTCAACTGAATAGGTTGATTTGTGTTTTCTTTCTGAAATTCTAAGAATTTTGTTAAAAG
AAAACCATTGTCAACTGAATAGGTTGATTTGTGTTTTCTTTAAAATTTTAAAAAATTTGGTAAAAG
AAAACCATTGTCAACTGAATAGGTTGATTTGTGTTTTCTTTAAAATTTTACAAAATTTGGTAAAAG
AAAACCATTGTCAACTGAATAGGTTGATTTGTGTTTTCTTTCTGAAATTCTAAGAATTTTGGTAAAAG
AAAACCATTGTCAACTGAATAGGTTGATTTATGTTTTCTTTCTGAAATTCTAAGAATTTTGTTAAAAG
AAAACCATTGTCAACTGAATAGGTTGATTTATGTTTTCTTTCTGAAATTCTAAGAATTTTGTTAAAAG
AAAACCATTGTCAACTGAATAGGTTGATTTGTGTTTTCTTTCTGAAATTCTAAGAATTTTGTTAAAAG
AAAACCATTGTCAACTGAATAGGTTGATTTGTGTTTTCTTTCTGAAATTCTAAGAATTTTGGTAAAAG
AAAACCATTGTCAACTGAATAGGTTGATTTATGTTTTCTTTCTGAAATTCTAAGAATTTTGTTAAAAG
AAAACCATTGTCAACTGAATAGGTTGATTTATGTTTTCTTTCTGAAATTCTAAGAATTTTGGTAAAAG
AAAACCATTGTCAACTGAATAGGTTGATTTATGTTTTCTTTCTGAAATTCTAAGAATTTTGTTAAAAG
AAAACCATTGTCAACTGAATAGGTTGATTTATGTTTTCTTTCTGAAATTCTAAGAATTTTGGTAAAAG
AAAACCATTGTCAACTGAATAGGTTGATTTATGTTTTCTTTCTGAAATTCTAAGAATTTTGTTAAAAG
AAAACCATTGTCAACTGAATAGGTTGATTTATGTTTTCTTTCTGAAATTCTAAGAATTTTGTTAAAAG
AAAACCATTGTCAACTGAATAGGTTGATTTATGTTTTCTTTCTGAAATTCTAAGAATTTTGGTAAAAG
AAAACCATTGTCAACTGAATAGGTTGATTTATGTTTTCTTTCTGAAATTCTAAGAATTTTGGTAAAAG
AAAACCATTGTCAACTGAATAGGTTGATTTGTGTTTTCTTTCTGAAATTCTAAGAATTTTGTTAAAAG
AAAACCATTGTCAACTGAATAGGTTGATTTGTGTTTTCTTTAAAATTTTAAAAAATTTGGTAAAAG
AAAACCATTGTCAACTGAATAGGTTGATTTGTGTTTTCTTTAAAATTTTAAAAAATTTGGTAAAAG
AAAACCATTGTCAACTGAATAGGTTGATTTGTGTTTTCTTTCTGAAATTCTAAGAATTTTGGTAAAAG
AAAACCATTGTCAACTGAATAGGTTGATTTGTGTTTTCTTTAAAATTTTACAAAATTTGGTAAAAG
AAAACCATTGTCAACTGAATAGGTTGATTTGTGTTTTCTTTCTGAAATTCTAAGAATTTTGTTAAAAG
AAAACCATTGTCAACTGAATAGGTTGATTTGTGTTTTCTTTAAAATTTTACAAAATTTGGTAAAAG
AAAACCATTGTCAACTGAATAGGTTGATTTGTGTTTTCTTTCTGAAATTCTAAGAATTTTGGTAAAAG
AAAACCATTGTCAACTGAATAGGTTGATTTGTGTTTTCTTTAAAATTTTACAAAATTTGGTAAAAG

AAAACCATTGTCAACTGAATAGGTTGATTTGTGTTTTCTTTCTGAAATTCTAAGAATTTTGGTAAAAG
AAAACCATTGTCAACTGAATAGGTTGATTTGTGTTTTCTTTCTGAAATTCTAAGAATTTTGGTAAAAG
AAAACCATTGTCAACTGAATAGGTTGATTTGTGTTTTCTTTCTGAAATTCTAAGAATTTTGGTAAAAG
AAAACCATTGTCAACTGAATAGGTTGATTTGTGTTTTCTTTAAAATTTTAAAAAATTTGGTAAAAG
AAAACCATTGTCAACTGAATAGGTTGATTTGTGTTTTCTTTAAAATTTTTAAAAATTTGGTAAAAG
AAAACCATTGTCAACTGAATAGGTTGATTTGTGTTTTCTTTAAAATTTTTAAAAATTTGGTAAAAG
Nr of selected repeats 123 Similarity 0.979909
Consensus:
AAAACCATTGTCAACTGAATAGGTTGATTTgTGTTTTCTTTCTGAAATTCTAAGAATTTTGgTAAAAG
>Cele-UNSB01_1:10434433-10461186 Satlength=26754 Nr of Repeats=116
RepeatLength=231 seed=GCTGAATTCA
GCTGAATTCAGTTGGGTAGGATGTGACTTCATGGCTGAGGGGAACATCTTGAACATCATCAGCACGATGTGTAGTGTCG
AGTGGTCCATCGTATTGATCAGAAATCGGGTAATCAGAATGAGCAGAAGACTTCTTGAACAATCCAGTAATCTTCAATT
TCAATGATGGCTCGTCCTTCTTCTCCGATTCTTCAATCACAACTGGGATGACATACTCTGGCAGCTTCTCATA
GCTGAATTCAGTTGGGTAAGATGTGACTTCATGGCTGAGTGGAACATCTTGAACATCATCAGCACGATGTGTAGTGTCG
AGTGGTCCATCGTATTGATCAGAAATCGGGTAATCAGAATGAGCAGAAGACTTCTTGAACAATCCAGTAATCTTCAATT
TCAATGATGGCTCGTCCTTCTTCTCCGATTCTTCAATCACAACTGGGATGACATACTCTGGCAGCTTCTCATA
GCTGAATTCAGTTGGGTAGGATGTGACTTCATGGCTGAGTGGGATGTCGTTCAGGTTATCAGAACGATGTGTAGAGTCG
AGTGGTCCATCGTATTGCTCAGAAATTGGATAATCAAGATGTGATGGTGATTTCTTGAACAATCCAGTGATCTTCGATT
TCAATGATGGCTCGTCCTTCTTTTCTGTCTCTTCAATCACAACTGGGATGACATGCTCTGGCAGCTTCTCATA
GCTGAATTCAGTTGGGTAAGATGTGACTTCATGGCTGAATGGGATGTCGTTCAGGTTATCAGCACGATGTGTAGAGTCG
AGTGGTCCATCGTATTGTTCAGAAATCGGGTAATCAGAATGAGCAGAAGATTTCTTGAAGAGTCCAGTGATCTTCGATT
TCAATGATGGCTCGTCCTTCTTCTCCGATTCTTCAATCACAACTGGGATGACATGCTCTGGCAGCTTCTCATA
GCTGAATTCAGTTGGGTAAGATGTGACTTCATGGCTGAGTGGGATGTCGTTCAGGTTATCAGCACGATGTGTAGAGTCG
AGTGGTCCATCGTATTGTTCAGAAATCGGGTAATCAGAATGAGCAGAAGATTTCTTGAACAATCCAGTGATCTTCGATT
TCAATAATGGCTCGTCCTTCTTCTCCGATTCTTCAATCACAACTGGGATGAAATGCTCTGGCAGCTTCTCATA
GCTGAATTCAGTTGGGTAGGATGTGACTTCATGGCTGAGTGGGATGTCGTTCAAGTTATCAGCACGATGTGTAGAGTCG
AGTGGTCCATCGTATTGTTCAGAAATCGGGTAATCAGAATGAGCAGAAGATTTCTTGAACAATCCAGTGATCTTCGATT
TCAATGATGGCTCGTCCTTCTTTTCTGTCTCTTCAATCACAACTGGGATGACATGCTCTGGCAGCTTCTCATA
GCTGAATTCAGTTGGGTAAGATGTGACTTCATGGCTGAGTGGAACATCTTGGACATCATCAGCACGATGTGTAGAGTCG
AGTGGTCCATCGTATTGTTCAGAAATCGGGTAATCAGAATGAGCAGAAGATTTCTTGAACCATCCAGTGATCTTCGATT
TCAATGATGGCTCGTCCTTCTTTTCTGTCTCTTCAATCACAACTGGGATGACATGCTCTGGCAGCTTCTCATA
GCTGAATTCAGTTGGGTAGGATGTGACTTCATGGCTGAGTGGGATGTCGTTCAAGTTATCAGCACGATGTGTAGAGTCG
AGTGGTCCATCGTATTGTTCAGAAATCGGGTAATCAGAATGAGCAGAAGATTTCTTGAACAATCCAGTGATCTTCGATT
TCAATGATGGCTCGTCCTTCTTTTCTGTCTCTTCAATCACAACTGGGATGACATGCTCTGGCAGCTTCTCATA
GCTGAATTCAGTTGGGTAGGATGTGACTTCATGGCTGAGTGGGATGTCGTTCAAGTTATCAGCACGATGTGTAGAGTCG
AGTGGTCCATCGTATTGTTCAGAAATCGGGTAATCAGAATGAGCAGAAGATTTCTTGAACAATCCAGTGATCTTCGATT
TCAATGATGGCTCGTCCTTCTTTTCTGTCTCTTCAATCACAACTGGGATGACATGCTCTGGCAGCTTCTCATA
GCTGAATTCAGTTGGGTAGGATGTGACTTCATGGCTGAGTGGGATGTCGTTCAAGTTATCAGCACGATGTGTAGAGTCG
AGCGGTCCATCGTATTGTTCAGAAATCGGGTAATCAAGATGTGATGGTGATTTCTTGAACAATCCAGTGATCTTCGATT
TCAATGATGGCTCGTCCTTCTTTTCTGTCTCTTCAATCACAACTGGGATGACATGCTCTGGCAGCTTCTCATA
GCTGAATTCAGTTGGGTAGGATGTGACTTCATGGCTGAGTGGGATGTCGTTCAAGTTATCAGCACGATGTGTAGAGTCG
AGCGGTCCATCGTATTGTTCAGAAATCGGGTAATCAAGATGTGATGGTGATTTCTTGAACAATCCAGTGATCTTCGATT
TCAATGATGGCTCGTTCTTCTTCTCCGTTCTTCAATCACAACTGGGATGACATGCTCTGGCAGCTTCTCATA
GCTGAATTCAGTTGGGTAGGATGTGACTTCATGGCTGAGTGGGATGTCGTTCAAGTTATCAGCACGATGTGTAGAGTCG
AGTGGTCCATCGTATTGTTCAGAAATCGGGTAATCAGAATGAGCAGAAGATTTCTTGAACAATCCAGTGATCTTCGATT
TCAATGATGGCTCGTCCTTCTTTTCTGTCTCTTCAATCACAACTGGGATGACATGCTCTGGCAGCTTCTCATA
GCTGAATTCAGTTGGGTAAGATGTGACTTCATGGCTGAGTGGAACATCTTGGACATCATCAGCACGATGTGTAGAGTCG
AGTGGTCCATCGTATTGTTCAGAAATCGGGTAATCAGAATGAGCAGAAGATTTCTTGAACAATCCAGTGATCTTCGATT
TCAATGATGGCTCGTCCTTCTTTTCTGTCTCTTCAATCACAACTGGGATGACATGCTCTGGCAGCTTCTCATA
GCTGAATTCAGTTGGGTAGGATGTGACTTCATGGCTGAGTGGGATGTCGTTCAAGTTATCAGCACGATGTGTAGAGTCG
AGTGGTCCATCGTATTGTTCAGAAATCGGGTAATCAGAATGAGCAGAAGATTTCTTGAACAATCCAGTGATCTTCGATT
TCAATGATGGCTCGTCCTTCTTTTCCGTTCTTCAATCACAACTGGGATGACATGCTCTGGCAGCTTCTCATA

GCTGAATTCAGTTGGGTAGGATGTGACTTCATGGCTGAGTGGGATGTCGTTCAAGTTATCAGCACGATGTGTAGAGTCG
AGTGGTCCATCGTATTGTTCAGAAATCGGGTAATCAGAATGAGCAGAAGATTTCTTGAACAATCCAGTGATCTTCGATT
TCAATGATGGCTCGTCCTTCTTTTCTGTCTCTTCAATCACAACTGGGATGACATGCTCTGGCAGCTTCTCATA
GCTGAATTCAGTTGGGTAGGATGTGACTTCATGGCTGAGTGGGATGTCGTTCAGTTATCAGCACGATGTGTAGAGTCGA
GTGGTCCATCGTATTGTTCAGAAATCGGGTAATCAGAATGAGCAGAAGATTTCTTGAACAATCCAGTGATCTTCGATTT
CAATGATGGCTCGTCCTTCTTCTCCGATTCTTCAATCACAACTGGGATGACATGCTCTGGCAGCTTCTCATA
GCTGAATTCAGTTGGGTAGGATGTGACTTCATGGCTGAGTGGGATGTCGTTCAAGTTATCAGCACGATGTGTAGAGTCG
AGTGGTCCATCGTATTGTTCAGAAATCGGGTAATCAGAATGAGCAGAAGATTTCTTGAACAATCCAGTGATCTTCGATT
TCAATGATGGCTCGTCCTTCTTTTCTGTCTCTTCAATCACAACTGGGATGACATGCTCTGGCAGCTTCTCATA
GCTGAATTCAGTTGGGTAGGATGTGACTTCATGGCTGAGTGGGATGTCGTTCAAGTTATCAGCACGATGTGTAGAGTCG
AGTGGTCCATCGTATTGTTCAGAAATCGGGTAATCAGAATGAGCAGAAGATTTCTTGAACAATCCAGTGATCTTCGATT
TCAATGATGGCTCGTCCTTCTTTTCTGTCTCTTCAATCACAACTGGGATGACATGCTCTGGCAGCTTCTCATA
GCTGAATTCAGTTGGGTAGGATGTGACTTCATGGCTGAGTGGGATGTCGTTCAAGTTATCAGCACGATGTGTAGAGTCG
AGTGGTCCATCGTATTGTTCAGAAATCGGGTAATCAGAATGAGCAGAAGATTTCTTGAACAATCCAGTGATCTTCGATT
TCAATGATGGCTCGTCCTTCTTTTCTGTCTCTTCAATCACAACTGGGATGACATGCTCTGGCAGCTTCTCATA
GCTGAATTCAGTTGGGTAGGATGTGACTTCATGGCTGAGTGGGATGTCGTTCAAGTTATCAGCACGATGTGTAGAGTCG
AGTGGTCCATCGTATTGTTCAGAAATCGGGTAATCAGAATGAGCAGAAGATTTCTTGAACAATCCAGTGATCTTCGATT
TCAATGATGGCTCGTCCTTCTTTTCTGTCTCTTCAATCACAACTGGGATGACATGCTCTGGCAGCTTCTCATA
GCTGAATTCAGTTGGGTAAGATGTGACTTCATGGCTGAGTGGAATCTTGACATCATCAGCACGATGTGTAGAGTCGAGT
GGTCCATCGTATTGTTCAGAAATCGGGTAATCAGAATGAGCAGAAGATTTCTTGAACAATCCAGTGATCTTCGATTTCA
ATGATGGCTCGTCCTTCTTCTCCGTTCTTCAATCACAACTGGGATGACATGCTCTGGCAGCTTCTCATA
GCTGAATTCAGTTGGGTAAGATGTGACTTCATGGCTGAGTGGAACATCTTGACATCATCAGCACGATGTGTAGAGTCGA
GTGGTCCATCGTATTGTTCAGAAATCGGGTAATCAGAATGAGCAGAAGATTTCTTGAACAATCCAGTGATCTTCGATTT
CAATGATGGCTCGTCCTTCTTCTCCGATTCTTCAATCACAACTGGGATGACATGCTCTGGCAGCTTCTCATA
GCTGAATTCAGTTGGGTAAGATGTGACTTCATGGCTGAGTGGAAATCTTGACATCATCAGCACGATGTGTAGAGTCGAG
TGGTCCATCGTATTGTTCAGAAATCGGGTAATCAGAATGAGCAGAAGATTTCTTGAACAATCCAGTGATCTTCGATTTC
AATGATGGCTCGTCCTTCTTTTCTGTCTCTTCAATCACAACTGGGATGACATGCTCTGGCAGCTTCTCATA
GCTGAATTCAGTTGGGTAGGATGTGACTTCATGGCTGAGTGGGATGTCGTTCAAGTTATCAGCACGATGTGTAGAGTCG
AGTGGTCCATCGTATTGTTCAGAAATCGGGTAATCAGAATGAGCAGAAGATTTCTTGAACAATCCAGTGATCTTCGATT
TCAATGATGGCTCGTCCTTCTTTTCTGTCTCTTCAATCACAACTGGGATGACATGCTCTGGCAGCTTCTCATA
GCTGAATTCAGTTGGGTAGGATGTGACTTCATGGCTGAGTGGGATGTCGTTCAAGTTATCAGCACGATGTGTAGAGTCG
AGTGGTCCATCGTATTGTTCAGAAATCGGGTAATCAGAATGAGCAGAAGATTTCTTGAACAATCCAGTGATCTTCGATT
TCAATGATGGCTCGTCCTTCTTTTCTGTCTCTTCAATCACAACTGGGATGACATGCTCTGGCAGCTTCTCATA
GCTGAATTCAGTTGGGTAGGATGTGACTTCATGGCTGAGTGGGATGTCGTTCAAGTTATCAGCACGATGTGTAGAGTCG
AGTGGTCCATCGTATTGTTCAGAAATCGGGTAATCAGAATGAGCAGAAGATTTCTTGAACAATCCAGTGATCTTCGATT
TCAATGATGGCTCGTCCTTCTTTTCTGTCTCTTCAATCACAACTGGGATGACATGCTCTGGCAGCTTCTCATA
GCTGAATTCAGTTGGGTAGGATGTGACTTCATGGCTGAGTGGGATGTCGTTCAAGTTATCAGCACGATGTGTAGAGTCG
AGCGGTCCATCGTATTGTTCAGAAATCGGGTAATCAAGATGTGATGGTGATTTCTTGAACAATCCAGTGATCTTCGATT
TCAATGATGGCTCGTCCTTCTTTTCTGTCTCTTCAATCACAACTGGGATGACATGCTCTGGCAGCTTCTCATA
GCTGAATTCAGTTGGGTAGGATGTGACTTCATGGCTGAGTGGGATGTCGTTCAAGTTATCAGCACGATGTGTAGAGTCG
AGCGGTCCATCGTATTGTTCAGAAATCGGGTAATCAAGATGTGATGGTGATTTCTTGAACAATCCAGTGATCTTCGATT
TCAATGATGGCTCGTCCTTCTTTCTGTTCTTCAATCACAACTGGGATGACATGCTCTGGCAGCTTCTCATA
GCTGAATTCAGTTGGGTAGGATGTGACTTCATGGCTGAGTGGGATGTCGTTCAAGTTATCAGCACGATGTGTAGAGTCG
AGTGGTCCATCGTATTGTTCAGAAATCGGGTAATCAGAATGAGCAGAAGATTTCTTGAACAATCCAGTGATCTTCGATT
TCAATGATGGCTCGTCCTTCTTTTCTGTCTCTTCAATCACAACTGGGATGACATGCTCTGGCAGCTTCTCATA
GCTGAATTCAGTTGGGTAAGATGTGACTTCATGGCTGAGTGGAACATCTTGGACATCATCAGCACGATGTGTAGAGTCG
AGTGGTCCATCGTATTGTTCAGAAATCGGGTAATCAGAATGAGCAGAAGATTTCTTGAACAATCCAGTGATCTTCGATT
TCAATGATGGCTCGTCCTTCTTCTCCGATTCTTCAATCACAACTGGGATGACATGCTCTGGCAGCTTCTCATA
GCTGAATTCAGTTGGGTAGGATGTGACTTCATGGCTGAGTGGGATGTCGTTCAAGTTATCAGCACGATGTGTAGAGTCG
AGTGGTCCATCGTATTGTTCAGAAATCGGGTAATCAGAATGAGCAGAAGATTTCTTGAACAATCCAGTGATCTTCGATT
TCAATGATGGCTCGTCCTTCTTTTCTGTCTCTTCAATCACAACTGGGATGACATGCTCTGGCAGCTTCTCATA
GCTGAATTCAGTTGGGTAAGATGTGACTTCATGGCTGAGTGGAACATCTTGACATTATCAGCACGATGTGTAGAGTCGA
GTGGTCCATCGTATTGTTCAGAAATCGGGTAATCAGAATGAGCAGAAGATTTCTTGAACAATCCAGTGATCTTCGATTT
CAATGATGGCTCGTCCTTCTTTTCTGTCTCTTCAATCACAACTGGGATGACATGCTCTGGCAGCTTCTCATA

GCTGAATTCAGTTGGGTAAGATGTGACTTCATGGCTGAGTGGAACATCTTGGACATCATCAGCACGATGTGTAGAGTCG
AGTGGTCCATCGTATTGTTCAGAAATCGGGTAATCAGAATGAGCAGAAGATTTCTTGAACAATCCAGTGATCTTCGATT
TCAATGATGGCTCGTCCTTCTTCTCCGATTCTTCAATCACAACTGGGATGACATGCTCTGGCAGCTTCTCATA
GCTGAATTCAGTTGGGTAAGATGTGACTTCATGGCTGAGTGGAACATCTTGGACATCATCAGCACGATGTGTAGAGTCG
AGTGGTCCATCGTATTGTTCAGAAATCGGGTAATCAGAATGAGCAGAAGATTTCTTGAACAATCCAGTGATCTTCGATT
TCAATGATGGCTCGTCCTTCTTTTCTGTCTCTTCAATCACAACTGGGATGACATGCTCTGGCAGCTTCTCATA
GCTGAATTCAGTTGGGTAGGATGTGACTTCATGGCTGAGTGGGATGTCGTTCAAGTTATCAGCACGATGTGTAGAGTCG
AGTGGTCCATCGTATTGTTCAGAAATCGGGTAATCAGAATGAGCAGAAGATTTCTTGAACAATCCAGTGATCTTCGATT
TCAATGATGGCTCGTCCTTCTTTTCTGTCTCTTCAATCACAACTGGGATGACATGCTCTGGCAGCTTCTCATA
GCTGAATTCAGTTGGGTAAGATGTGACTTCATGGCTGAGTGGGATGTCGTTCAAGTTATCAGCACGATGTGTAGAGTCG
AGTGGTCCATCGTATTGTTCAGAAATCGGGTAATCAGAATGAGCAGAAGATTTCTTGAACAATCCAGTGATCTTCGATT
TCAATGATGGCTCGTCCTTCTTTTCTGTCTCTTCAATCACAACTGGGATGACATGCTCTGGCAGCTTCTCATA
GCTGAATTCAGTTGGGTAGGATGTGACTTCATGGCTGAGTGGAACATCTTGGACATCATCAGCACGATGTGTAGAGTCG
AGTGGTCCATCGTATTGATCAGAAATCGGGTAATCAGAATGAGCAGAAGATTTCTTGAACAATCCAGTGATCTTCGATT
TCAATGATGGCTCGTCCTTCTTTTCTGTCTCTTCAATCACAACTGGGATGACATGCTCTGGCAGCTTCTCATA
GCTGAATTCAGTTGGGTAAGATGTGACTTCATGGCTGAGTGGACATCTTGACATCATCAGCACGATGTGTAGAGTCGAG
TGGTCCATCGTATTGTTCAGAAATCGGGTAATCAGAATGAGCAGAAGATTTCTTGAACAATCCAGTGATCTTCGATTTC
AATGATGGCTCGTCCTTCTTCTCCGATTCTTCAATCACAACTGGGATGACATGCTCTGGCAGCTTCTCATA
GCTGAATTCAGTTGGGTAGGATGTGACTTCATGGCTGAGTGGGATGTCGTTCAAGTTATCAGCACGATGTGTAGAGTCG
AGTGGTCCATCGTATTGTTCAGAAATCGGGTAATCAGAATGAGCAGAAGATTTCTTGAACAATCCAGTGATCTTCGATT
TCAATGATGGCTCGTCCTTCTTTTCTGTCTCTTCAATCACAACTGGGATGACATGCTCTGGCAGCTTCTCATA
GCTGAATTCAGTTGGGTAAGATGTGACTTCATGGCTGAGTGGGATGTCGTTCAAGTTATCAGCACGATGTGTAGAGTCG
AGCGGTCCATCGTATTGTTCAGAAATCGGGTAATCAAGATGTGATGGTGATTTCTTGAACAATCCAGTGATCTTCGATT
TCAATGATGGCTCGTCCTTCTTCTCCGATTCTTCAATCACAACTGGGATGACATGCTCTGGCAGCTTCTCATA
GCTGAATTCAGTTGGGTAGGATGTGACTTCATGGCTGAGTGGGATGTCGTTCAAGTTATCAGCACGATGTGTAGAGTCG
AGTGGTCCATCGTATTGTTCAGAAATCGGGTAATCAGAATGAGCAGAAGATTTCTTGAACAATCCAGTGATCTTCGATT
TCAATGATGGCTCGTCCTTCTTCTCCGTTCTTCAATCACAACTGGGATGACATGCTCTGGCAGCTTCTCATA
GCTGAATTCAGTTGGGTAAGATGTGACTTCATGGCTGAGTGGAAATCTTGAATTATCAGCACGATGTGTAGAGTCGAGT
GGTCCATCGTATTGTCAGAAATCGGGTAATCAGAATGAGCAGAAGATTTCTTGAACAATCCAGTGATCTTCGATTTCAA
TGATGGCTCGTCCTTCTTTTCTGTCTCTTCAATCACAACTGGGATGACATGCTCTGGCAGCTTCTCATA
GCTGAATTCAGTTGGGTAGGATGTGACTTCATGGCTGAGTGGGATGTCGTTCAAGTTATCAGCACGATGTGTAGAGTCG
AGTGGTCCATCGTATTGTTCAGAAATCGGGTAATCAGAATGAGCAGAAGATTTCTTGAACAATCCAGTGATCTTCGATT
TCAATGATGGCTCGTCCTTCTTTTCTGTCTCTTCAATCACAACTGGGATGACATGCTCTGGCAGCTTCTCATA
GCTGAATTCAGTTGGGTAAGATGTGACTTCATGGCTGAGTGGAATGTCTTCAAGTTATCAGCACGATGTGTAGAGTCGA
GTGGTCCATCGTATTGTTCAGAAATCGGGTAATCAGAATGAGCAGAAGATTTCTTGAACAATCCAGTGATCTTCGATTT
CAATGATGGCTCGTCCTTCTTCTCCGATTCTTCAATCACAACTGGGATGACATGCTCTGGCAGCTTCTCATA
GCTGAATTCAGTTGGGTAGGATGTGACTTCATGGCTGAGTGGGATGTCGTTCAAGTTATCAGCACGATGTGTAGAGTCG
AGTGGTCCATCGTATTGTTCAGAAATCGGGTAATCAGAATGAGCAGAAGATTTCTTGAACAATCCAGTGATCTTCGATT
TCAATGATGGCTCGTCCTTCTTTTCTGTCTCTTCAATCACAACTGGGATGACATGCTCTGGCAGCTTCTCATA
GCTGAATTCAGTTGGGTAAGATGTGACTTCATGGCTGAGTGGGATGTCGTTCAAGTTATCAGCACGATGTGTAGAGTCG
AGCGGTCCATCGTATTGTTCAGAAATCGGGTAATCAAGATGTGATGGTGATTTCTTGAACAATCCAGTGATCTTCGATT
TCAATGATGGCTCGTCCTTCTTCTCCGTTCTTCAATCACAACTGGGATGACATGCTCTGGCAGCTTCTCATA
GCTGAATTCAGTTGGGTAGGATGTGACTTCATGGCTGAGTGGGATGTCGTTCAAGTTATCAGCACGATGTGTAGAGTCG
AGTGGTCCATCGTATTGTTCAGAAATCGGGTAATCAGAATGAGCAGAGATTTCTTGAACAATCCAGTGATCTTCGATTT
CAATGATGGCTCGTCCTTCTTTTCTGTCTCTTCAATCACAACTGGGATGACATGCTCTGGCAGCTTCTCATA
GCTGAATTCAGTTGGGTAAGATGTGACTTCATGGCTGAGTGGGATGTCGTTCAATTATCAGCACGATGTGTAGAGTCGA
GTGGTCCATCGTATTGTCAGAAATCGGGTAATCAGAATGAGCAGAAGATTTCTTGAACAATCCAGTGATCTTCGATTTC
AATGATGGCTCGTCCTTCTTTTCTGTCTCTTCAATCACAACTGGGATGACATGCTCTGGCAGCTTCTCATA
GCTGAATTCAGTTGGGTAGGATGTGACTTCATGGCTGAGTGGGATGTCGTTCAAGTTATCAGCACGATGTGTAGAGTCG
AGTGGTCCATCGTATTGTTCAGAAATCGGGTAATCAGAATGAGCAGAAGATTTCTTGAACAATCCAGTGATCTTCGATT
TCAATGATGGCTCGTCCTTCTTTTCTGTCTCTTCAATCACAACTGGGATGACATGCTCTGGCAGCTTCTCATA
GCTGAATTCAGTTGGGTAGATGTGACTTCATGGCTGAGTGGGATGTCGTTCAAGTTATCAGCACGATGTGTAGAGTCGA
GTGGTCCATCGTATTGTTCAGAAATCGGGTAATCAGAATGAGCAGAAGATTTCTTGAACAATCCAGTGATCTTCGATTT
CAATGATGGCTCGTCCTTCTTCTCCGATTCTTCAATCACAACTGGGATGACATGCTCTGGCAGCTTCTCATA

GCTGAATTCAGTTGGGTAAGATGTGACTTCATGGCTGAGTGGGATGTCGTTCAAGTTATCAGCACGATGTGTAGAGTCG
AGTGGTCCATCGTATTGATTCAGAAATCGGGTAATCAAGATGAGATGGTGATTTCTTGAACAATCCAGTGATCTTAGAT
TTCAATGATGGCTCGTCCTTCTTCTCTGTCTCTTCAATCACAACTGGGATGACATGCTCTGGCAGCTTCTCATA
GCTGAATTCAGTTGGATAGATGTGACTTCATGGTGAGTGGAATATCTTGAAGTTATAGCTCGTGAAGTAGATCCAATGG
TCCATCGTAAGATTCAGACACTGGATAATCAAGATGAGATGGTGATTTCTTGAACAATCCAGTGATCTTAGATTTCAGT
GATGGCTCGTCCTTCTTCTCTGTTCCTCGATCACAACTGGGATGACATGCTCTGGCAGCTTCTCATA
GCTGAATTCAGTTGGATATGAATGCACTTCGTTGTGAGTGGAATATCATGAATGTTGGATACTCGTGAGTAGATTCGAT
GGTCCATCATATGATTCAGACACTGGATAATCAAGATGAGATGGTGATTTCTTGAACAATCCAGTGATCTTAGATTTCA
GTGATGGCTCGTCCTTCTTCTCTGTCTCCTCGATCACAACTGGAATGACATGCTCTGGCAGTTTCTCATA
GCTGAATTCAGTTGGATATGAATGCACTTCGTTGTGAGTGGAATATCATGAATGTTGGATACTCGTGAGTAGAGTCCAA
TGGTCCATCGTATGATTCAGAACTGGATAATCAAGATGAGATGGTGATTTCTTGAACAATCCAGTGATCTTAGATTTCA
ATGATGGCTCGTCCTTCTTCTCTGTCTCCTCGATCACAACTGGAATGACATGCTCTGGCAGTTTCTCATA
GCTGAATTCAGTTGGATATGAATGCACTTCCGTTGTGAGTGGAATATCATGAATGTTGGATACTCGTGAAGTAGATTCC
AATGGTCCATCATATGATTCAGACACTGGATAATCAAGATGAGATGGTGATTTCTTGAACAATCCAGTGATCTTAGATT
TCAGTGATGGCTCGTCCTTCTTCTCCGATTCCTCGATCACAACTGGAATGACATGCTCTGGCAGTTTCTCATA
GCTGAATTCAGTTGGATATGAATGCACTTCCGTTGTGAGTGGAATATCATGAATGTTGATACTCGTGAAGTAGATCAAG
TGGTCCATCATATGATTCAGACACTGGATAATCAAGATGAGATGGTGATTTCTTGAACAATCCAGTGATCTTAGATTTC
AGTGATGGCTCGTCCTTCTTCTCCGATTCCTCGATCACAACTGGAATGACATGCTCTGGCAGTTTCTCATA
GCTGAATTCAGTTGGATATGAATGCACTTCCGTTGTGAGTGGAATATCATGAATGTTGGATACTCGTGAAGTAGATTCC
AATGGTCCATCATAAGATTCAGACACTGGATAATCAAGATGAGATGGTGATTTCTTGAACAATCCAGTGATCTTAGATT
TCAGTGATGGCTCGTCCTTCTTCTCTGTCTCCTCGATCACAACTGGAATGACATGCTCTGGCAGTTTCTCATA
GCTGAATTCAGTTGGATATGAATGCACTTCCGTTGTGAGTGGAATATCATGAATGTTGATGCTCGTGAAGTAGATTCCA
ATGGTCCATCATAAGATTCAGACACTGGATAATCAAGATGTGATGGTGATTTCTTGAACAATCCAGTGATCTTAGATTT
CAGTGATGGCTCGTCCTTCTTCTTCGATTCCTCGATCACAACTGGGATGACATGCTCTGGCAGCTTCTCATA
GCTGAATTCATTTGGATATGAATGCACTTCCGTTGTGAGTGGAATATCATGAATGTTCGATGCTCGTGAAGTAGATTCC
AATGGTCCATCATAAGATTCAGACACTGGATAATCAAGATGTGATGGTGATTTCTTGAACAATCCAGTGATCTTAGATT
TCAGTGATGGCTCGTCCTTCTTCTTCGATTCCTCGATCACAACTGGGATGACATGCTCTGGCAGCTTCTCATA
GCTGAATTCATTTGGATATGAATGCACTTCCGTTGTGAGTGGAATATCATGAATGTTCGATGCTCGTGAAGTAGATTCC
AATGGTCCATCATAAGATTCAGACACTGGATAATCAAGATGTGATGGTGATTTCTTGAACAATCCAGTGATCTTAGATT
TCAGTGATGGCTCGTCCTTCTTCTCTGTCTCCTCGATCACAACTGGAATGACATGCTCTGGCAGTTTCTCATA
GCTGAATTCAGTTGGATATGAATGCACTTCCGTTGTGAGTGGAATATCATGAATGTTGATGCTCGTGAAGTAGATCAAG
TGGTCCATCATAAGATTCAGACACTGGATAATCAAGATGTGATGGTGATTTTTTGAACAATCCAGTGATCTTAGATTTC
AGTGATGGCTCGTCCTTCTTCTCCGATTCCTCGATCACAACTGGAATGACATGCTCTGGCAGTTTCTCATA
GCTGAATTCAGTTGGATATGAATGCACTTCCGTTGTGAGTGGAATATCATGAATGTTGGATGCTCGTGAAGTTGAATCA
AGTGGTCCATCATAAGATTCAGACACTGGATAATCAAGATGTGATGGTGATTTTTTGAACAATCCAGTGATCTTAGATT
TCAGTGATGGCTCGTCCTTCTTCTCCGATTCCTCGATCACAACTGGAATGACATGCTCTGGCAGTTTCTCATA
GCTGAATTCAGTTGGATATGAATGCACTTCCGTTGTGAGTGGAATATCATGAATGTTCGATGCTCGTGAAGTAGATTCC
AATGGTCCATCATAAGATTCAGACACTGGATAATCAAGATGTGATGGTGATTTCTTGAACAATCCAGTGATCTTAGATT
TCAGTGATGGCTCGTCCTTCTTCTCTGTCTCCTCGATCACAACTGGAATGACATGCTCTGGCAGTTTCTCATA
GCTGAATTCATTTGGATATGAATGCACTTCCGTTGTGAGTGGAATATCATGAATGTTGATGCTCGTGAAGTTGAATCAA
GTGGTCCATCATAAGATTCAGACACTGGATAATCAAGATGTGATGGTGATTTCTTGAACAATCCAGTGATCTTAGATTT
CAGTGATGGCTCGTCCTTCTTCTCCGATTCCTCGATCACAACTGGAATGACATGCTCTGGCAGTTTCTCATA
GCTGAATTCATTTGGATATGAATGCACTTCCGTTGTGAGTGGAATATCATGAATGTTCGATGCTCGTGAAGTAGATTCC
AATGGTCCATCATAAGATTCAGACACTGGATAATCAAGATGAGATGGTGATTTCTTGAACAATCCAGTGATCTTAGATT
TCAGTGATGGCTCGTCCTTCTTCTCCGATTCCTCGATCACAACTGGAATGACATGCTCTGGCAGTTTCTCATA
GCTGAATTCATTTGGATATGAATGCACTTCCGTTGTGAGTGGAATATCATGAATGTTGGATGCTCGTGAAGTTGAATCA
AGTGGTCCATCATAAGATTCAGACACTCGATAATCAAGATGAGATGGAGATTTCTTGAACAATCCAGTGATCTTAGATT
TCAGTGATGGCTCGTCCTTCTTCTCCGATTCCTCGATCACAACTGGAATGACATGCTCTGGCAGTTTCTCATA
GCTGAATTCATTTGGATATGAATGCACTTCCGTTGTGAGTGGAATATCATGAATGTTCGATGCTCGTGAAGTAGATTCC
AATGGTCCATCATAAGATTCAGACACTGGATAATCAAGATGAGATGGAGATTTCTTGAACAATCCAGTGATCTTAGATT
TCAGTGATGGCTCGTCCTTCTTCTCTGTCTCCTCGATCACAACTGGAATGACATGCTCTGGCAGTTTCTCATA
GCTGAATTCATTTGGATATGAATGCACTTCCGTTGTGAGTGGAATATCATGAATGTTCGATGCTCGTGAAGTAGATTCC
AATGGTCCATCATAAGATTCAGACACTGGATAATCAAGATGTGATGGTGATTTCTTGAACAATCCAGTGATCTTAGATT
TCAGTGATGGCTCGTCCTTCTTCTCCGATTCCTCGATCACAACTGGAATGACATGCTCTGGCAGTTTCTCATA

GCTGAATTCAGTTGGATATGAATGCACTTCCGTTGTGAGTGGAATATCATGAATGTTCGATGCTCGTGAAGTAGATTCC
AATGGTCCATCATAAGATTCAGACACTGGATAATCAAGATGTGATGGAGATTTCTTGAACAATCCAGTGATCTTAGATT
TCAGTGATGGCTCGTCCTTCTTCTCCGATTCCTCGATCACAACTGGAATGACATGCTCTGGCAGTTTCTCATA
GCTGAATTCATTTGGATATGAATGCACTTCCGTTGTGAGTGGAATATCATGAATGTTCGATGCTCGTGAAGTTGAATCA
AGTGGTCCATCATAAGATTCAGACACTGGATAATCAAGATGTGATGGTGATTTCTTGAACAATCCAGTGATCTTAGATT
TCAGTGATGGCTCGTCCTTCTTCTCTGTCTCCTCGATCACAACTGGAATGACATGCTCTGGCAGTTTCTCATA
GCTGAATTCATTTGGATATGAATGCACTTCCGTTGTGAGTGGAATATCATGAATGTTCGATGCTCGTGAAGTTGAATCA
AGTGGTCCATCATAAGATTCAGACACTGGATAATCAAGATGTGATGGTGATTTCTTGAACAATCCAGTGATCTTAGATT
TCAGTGATGGCTCGTCCTTCTTCTCTGTCTCCTCGATCACAACTGGAATGACATGCTCTGGCAGTTTCTCATA
GCTGAATTCAGTTGGATATGAATGCACTTCCGTTGTGAGTGGAATATCATGAATGTTCGATGCTCGTGAAGTAGATTCC
AATGGTCCATCATAAGATTCAGACACTGGATAATCAAGATGTGATGGTGATTTCTTGAACAATCCAGTGATCTTAGATT
TCAGTGATGGCTCGTCCTTCTTCTCCGATTCCTCGATCACAACTGGAATGACATGCTCTGGCAGTTTCTCATA
GCTGAATTCAGTTGGATATGAATGCACTTCCGTTGTGAGTGGAATATCATGAATGTTCGATGCTCGTGAAGTAGATTCC
AATGGTCCATCATAAGATTCAGACACTGGATAATCAAGATGTGATGGTGATTTCTTGAACAATCCAGTGATCTTAGATT
TCAGTGATGGCTCGTCCTTCTTCTCCGATTCCTCGATCACAACTGGAATGACATGCTCTGGCAGTTTCTCATA
GCTGAATTCAGTTGGATATGAATGCACTTCCGTTGTGAGTGGAATATCATGAATGTTCGATGCTCGTGAAGTAGATTCC
AATGGTCCATCATAAGATTCAGACACTGGATAATCAAGATGAGATGGAGATTTCTTGAACAATCCAGTGATCTTAGATT
TCAGTGATGGCTCGTCCTTCTTCTCCGATTCCTCGATCACAACTGGAATGACATGCTCTGGCAGCTTCTCATA
GCTGAATTCAGTTGGATATGAATGCACTTCCGTTGTGAGTGGAATATCATGAATGTTCGATGCTCGTGAAGTAGATTCC
AATGGTCCATCATAAGATTCAGACACTGGATAATCAAGATGAGATGGTGATTTCTTGAACAATCCAGTGATCTTAGATT
TCAGTGATGGCTCGTCCTTCTTCTCTGTCTCCTCGATCACAACTGGAATGACATGCTCTGGCAGCTTCTCATA
GCTGAATTCAGTTGGATATGAATGCACTAGCGTTGTGAGTGGAATATCATGAATGTTCGATGCTCGTGAAGTAGATTCC
AATGGTCCATCATAAGATTCAGACACTGGATAATCAAGATGAGATGGTGATTTCTTGAACAATCCAGTGATCTTAGATT
TCAGTGATGGCTCGTCCTTCTTCTCTGTCTCCTCGATCACAACTGGAATGACATGCTCTGGCTGCTTCTCATA
GCTGAATTCAGTTGGATATGAATGCACTAGCGTTGTGAGTGGAATATCATGAATGTTCGATGCTCGTGAAGTAGATTCC
AATGGTCCATCATAAGATTCAGACACTGGATAATCAAGATGAGATGGAGATTTCTTGAACAATCCAGTGATCTTAGATT
TCAGTGATGGCTCGTCCTTCTTCTCTGTCTCCTCGATCACAACTGGAATGACATGCTCAGGCTGCTTCTCATA
GCTGAATTCAGTTGGATATGAATGCACTTCCGTTGTGAGTGGAATATCATGAATGTTCGATGCTCGTGAAGTAGATTCC
AATGGTCCATCATAAGATTCAGACACTGGATAATCAAGATGAGATGGTGATTTCTTGAACAATCCAGTGATCTTAGATT
TCAGTGATGGCTCGTCCTTGTTCTCCGATTCCTCGATCACAACTGGAATGACATGCTCTGGCAGTTTCTCATA
GCTGAATTCAGTTGGATATGAATGCACTTCCGTTGTGAGTGGAATATCATGAATGTTCGATGCTCGTGAAGTAGATTCC
AATGGTCCATCATAAGATTCAGACACTGGATAATCAAGATGAGATGGTGATTTCTTGAACAATCCAGTGATCTTAGATT
TCAGTGATGGCTCGTCCTTCTTCTCCGATTCCTCGATCACAACTGGAATGACATGCTCTGGCAGTTTCTCATA
GCTGAATTCAGTTGGATATGAATGCACTTCCGTTGTGAGTGGAATATCATGAATGTTCGATGCTCGTGAAGTAGATTCC
AATGGTCCATCATAAGATTCAGACACTGGATAATCAAGATGAGATGGTGATTTCTTGAACAATCCAGTGATCTTAGATT
TCAGTGATGGCTCGTCCTTCTTCTCTGTCTCCTCGATCACAACTGGAATGACATGCTCTGGCAGCTTCTCATA
GCTGAATTCAGTTGGATATGAATGCACTTCCGTTGTGAGTGGAATATCATGAATGTTCGATGCTCGTGAAGTAGATTCC
AATGGTCCATCATAAGATTCAGACACTGGATAATCAAGATGAGATGGTGATTTCTTGAACAATCCAGTGATCTTAGATT
TCAGTGATGGCTCGTCCTTCTTCTCCGATTCCTCGATCACAACTGGAATGACATGCTCTGGCAGTTTCTCATA
GCTGAATTCAGTTGGATATGAATGCACTTCCGTTGTGAGTGGAATATCATGAATGTTGGATGCTCGTGAAGTAGATTCC
AATGGTCCATCATAAGATTCAGACACTGGATAATCAAGATGAGATGGAGATTTCTTGAACAATCCAGTGATCTTAGATT
TCAGTGATGGCTCGTCCTTCTTCTCCGATTCCTCGATCACAACTGGAATGACATGCTCTGGCAGTTTCTCATA
GCTGAATTCAGTTGGATATGAATGCACTTCCGTTGTGAGTGGAATATCATGAATGTTGGATGCTCGTGAAGTAGATTCC
AATGGTCCATCATAAGATTCAGACACTGGATAATCAAGATGAGATGGAGATTTCTTGAACAATCCAGTGATCTTAGATT
TCAGTGATGGCTCGTCCTTCTTCTCCGATTCCTCGATCACAACTGGAATGACATGCTCTGGCAGCTTCTCATA
GCTGAATTCAGTTGGATATGAATGCACTTCCGTTGTGAGTGGAATATCATGAATGTTGGATGCTCGTGAAGTTGAATCA
AGTGGTCCATCATAAGATTCAGACACTGGATAATCAAGATGAGATGGTGATTTCTTGAACAATCCAGTGATCTTAGATT
TCAGTGATGGCTCGTCCTTCTTCTCCGATTCCTCGATCACAACTGGAATGACATGCTCTGGCAGTTTCTCATA
GCTGAATTCAGTTGGATATGAATGCACTTCCGTTGTGAGTGGAATATCATGAATGTTGGATGCTCGTGAAGTAGATTCC
AATGGTCCATCGTAAGATTCAGACACTGGATAATCAAGATGAGATGGAGATTTCTTGAACAATCCAGTGATCTTAGATT
TCAGTGATGGCTCGTCCTTCTTCTCCGATTCCTCGATCACAACTGGAATGACATGCTCTGGCAGTTTCTCATA
GCTGAATTCAGTTGGATATGAATGCACTTCCGTTGTGAGTGGAATATCATGAATGTTGGATGCTCGTGAAGTAGATTCC
AATGGTCCATCATAAGATTCAGACACTGGATAATCAAGATGAGATGGTGATTTCTTGAACAATCCAGTGATCTTAGATT
TCAGTGATGGCTCGTCCTTCTTCTCCGATTCCTCGATCACAACTGGAATGACATGCTCTGGCAGTTTCTCATA

GCTGAATTCAGTTGGATATGAATGCACTTCCGTTGTGAGTGGAATATCATGAATGTTCGATGCTCGTGAAGTAGATTCC
AATGGTCCATCATAAGATTCAGACACTGGATAATCAAGATGAGATGGTGATTTCTTGAACAATCCAGTGATCTTAGATT
TCAGTGATGGCTCGTCCTTCTTCTCTGTCTCCTCGATCACAACTGGAATGACATGCTCTGGCAGCTTCTCATA
GCTGAATTCAGTTGGATATGAATGCACTTCCGTTGTGAGTGGAATATCATGAATGTTCGATGCTCGTGAAGTAGATTCC
AATGGTCCATCATAAGATTCAGACACTGGATAATCAAGATGAGATGGTGATTTCTTGAACAATCCAGTGATCTTAGATT
TCAGTGATGGCTCGTCCTTCTTCTCTGTCTCCTCCATCACAACTGGAATGACATGCTCAGGCTGCTTCTCATA
GCTGAATTCAGTTGGATATGAATGCACTTCCGTTGTGAGTGGAATATCATGAATGTTCGATGCTCGTGAAGTAGATTCC
AATGGTCCATCATAAGATTCAGACACTGGATAATCAAGATGAGATGGTGATTTCTTGAACAATCCAGTGATCTTAGATT
TCAGTGATGGCTCGTCCTTCTTCTCTGTCTCCTCGATCACAACTGGAATGACATGCTCTGGCAGCTTCTCATA
GCTGAATTCAGTTGGATATGAATGCACTTCCGTTGTGAGTGGAATATCATGAATGTTGGATGCTCGTGAAGTTGAATCA
AGTGGTCCATCATAAGATTCAGACACTGGATAATCAAGATGAGATGGTGATTTCTTGAACAATCCAGTGATCTTAGATT
TCAGTGATGGCTCGTCCTTCTTCTCCGATTCCTCGATCACAACTGGAATGACATGCTCTGGCAGTTTCTCATA
GCTGAATTCAGTTGGATATGAATGCACTTCCGTTGTGAGTGGAATATCATGAATGTTGGATGCTCGTGAAGTTGAATCA
AGTGGTCCATCATAAGATTCAGACACTGGATAATCAAGATGAGATGGTGATTTCTTGAACAATCCAGTGATCTTAGATT
TCAGTGATGGCTCGTCCTTCTTCTCCGATTCCTCGATCACAACTGGAATGACATGCTCTGGCAGTTTCTCATA
GCTGAATTCAGTTGGATATGAATGCACTTCCGTTGTGAGTGGAATATCATGAATGTTCGATGCTCGTGAAGTAGATTCC
AATGGTCCATCATAAGATTCAGACACTGGATAATCAAGATGAGATGGTGATTTCTTGAACAATCCAGTGATCTTAGATT
TCAGTGATGGCTCGTCCTTCTTCTCCGATTCCTCGATCACAACTGGAATGACATGCTCTGGCAGCTTCTCATA
GCTGAATTCAGTTGGATATGAATGCACTTCCGTTGTGAGTGGAATATCATGAATGTTCGATGCTCGTGAAGTAGATTCC
AATGGTCCATCATAAGATTCAGACACTGGATAATCAAGATGAGATGGTGATTTCTTGAACAATCCAGTGATCTTAGATT
TCAGTGATGGCTCGTCCTTCTTCTCTGTCTCCTCGATCACAACTGGAATGACATGCTCTGGCAGCTTCTCATA
GCTGAATTCAGTTGGATATGAATGCACTTCCGTTGTGAGTGGAATATCATGAATGTTCGATGCTCGTGAAGTAGATTCC
AATGGTCCATCATAAGATTCAGACACTGGATAATCAAGATGAGATGGTGATTTCTTGAACAATCCAGTGATCTTAGATT
TCAGTGATGGCTCGTCCTTCTTCTCCGATTCCTCGATCACAACTGGAATGACATGCTCTGGCAGTTTCTCATA
GCTGAATTCAGTTGGATATGAATGCACTTCCGTTGTGAGTGGAATATCATGAATGTTGGATGCTCGTGAAGTAGATTCC
AATGGTCCATCGTAAGATTCAGACACTGGATAATCAAGATGAGATGGTGATTTCTTGAACAATCCAGTGATCTTAGATT
TCAGTGATGGCTCGTCCTTCTTCTCCGATTCCTCGATCACAACTGGAATGACATGCTCTGGCAGCTTCTCATA
GCTGAATTCAGTTGGATATGAATGCACTTCCGTTGTGAATGGAATATCATGAATGTTGGATGCTCGTGAAGTTGAATCA
AGTGGTCCATCATAAGATTCAGACACTGGATAATCAAGATGAGATGGTGATTTCTTGAACAATCCAGTGATCTTAGATT
TCAGTGATGGCTCGTCCTTCTTCTCCGATTCCTCGATCACAACTGGAATGACATGCTCTGGCAGCTTCTCATA
GCTGAATTCAGTTGGATATGAATGCACTTCCGTTGTGAGTGGAATATCATGAATGTTGGATGCTCGTGAAGTTGAATCA
AGTGGTCCATCATAAGATTCAGACACTGGATAATCAAGATGAGATGGTGATTTCTTGAACAATCCAGTGATCTTAGATT
TCAGTGATGGCTCGTCCTTCTTCTCCGATTCCTCGATCACAACTGGAATGACATGCTCTGGCAGTTTCTCATA
GCTGAATTCAGTTGGATATGAATGCACTTCCGTTGTGAGTGGAATATCATGAATGTTCGATGCTCGTGAAGTAGATTCC
AATGGTCCATCATAAGATTCAGACACTGGATAATCAAGATGAGATGGAGATTTCTTGAACAATCCAGTGATCTTAGATT
TCAGTGATGGCTCGTCCTTCTTCTCCGATTCCTCGATCACAACTGGAATGACATGCTCTGGCAGCTTCTCATA
GCTGAATTCAGTTGGATATGAATGCACTTCCGTTGTGAGTGGAATATCATGAATGTTCGATGCTCGTGAAGTAGATTCC
AATGGTCCATCATAAGATTCAGACACTGGATAATCAAGATGAGATGGTGATTTCTTGAACAATCCAGTGATCTTAGATT
TCAGTGATGGCTCGTCCTTCTTCTCTGTCTCCTCGATCACAACTGGAATGACATGCTCAGGCTGCTTCTCATA
GCTGAATTCAGTTGGATATGAATGCACTTCCGTTGTGAGTGGAATATCATGAATGTTCGATGCTCGTGAAGTAGATTCC
AATGGTCCATCATAAGATTCAGACACTGGATAATCAAGATGAGATGGTGATTTCTTGAACAATCCAGTGATCTTAGATT
TCAGTGATGGCTCGTCCTTCTTCTCCGATTCCTCGATCACAACTGGAATGACATGCTCTGGCAGTTTCTCATA
GCTGAATTCAGTTGGATATGAATGCACTTCCGTTGTGAGTGGAATATCATGAATGTTCGATGCTCGTGAAGTAGATTCC
AATGGTCCATCATAAGATTCAGACACTGGATAATCAAGATGAGATGGTGATTTCTTGAACAATCCAGTGATCTTAGATT
TCAGTGATGGCTCGTCCTTCTTCTCTGTCTCCTCGATCACAACTGGAATGACATGCTCAGGCTGCTTCTCATA
GCTGAATTCAGTTGGATATGAATGCACTTCCGTTGTGAGTGGAATATCATGAATGTTCGATGCTCGTGAAGTAGTTTCC
AATGGTCCATCATAAGATTCAGACACTGGATAATCAAGATGAGATGGTGATTTCTTGAACAATCCAGTGATCTTAGATT
TCAGTGATGGCTCGTCCTTCTTCTCCGATTCCTCGATCACAACTGGAATGACATGCTCTGGCAGTTTCTCATA
GCTGAATTCAGTTGGATATGAATGCACTTCCGTTGTGAGTGGAATATCATGAATGTTCGATGCTCGTGAAGTAGTTTCC
AATGGTCCATCATAAGATTCAGACACTGGATAATCAAGATGAGATGGTGATTTCTTGAACAATCCAGTGATCTTAGATT
TCAGTGATGGCTCGTCCTTCTTCTCCGATTCCTCGATCACAACTGGAATGACATGCTCTGGCAGCTTCTCATA
GCTGAATTCAGTTGGATATGAATGCACTTCCGTTGTGAGTGGAATATCATGAATGTTGGATGCTCGTGAAGTTGAATCA
AGTGGTCCATCATAAGATTCAGACACTGGATAATCAAGATGAGATGGTGATTTCTTGAACAATCCAGTGATCTTAGATT
TCAGTGATGGCTCGTCCTTCTTCTCCGATTCCTCGATCACAACTGGAATGACATGCTCTGGCAGTTTCTCATA

GCTGAATTCAGTTGGATATGAATGCACTTCCGTTGTGAGTGGAATATCATGAATGTTGGATGCTCGTGAAGTAGATTCC
AATGGTCCATCGTAAGATTCAGACACTGGATAATCAAGATGAGATGGAGATTTCTTGAACAATCCAGTGATCTTAGATT
TCAGTGATGGCTCGTCCTTCTTCTCCGATTCCTCGATCACAACTGGAATGACATGCTCTGGCAGCTTCTCATA
GCTGAATTCAGTTGGATATGAATGCACTTCCGTTGTGAGTGGAATATCATGAATGTTGGATGCTCGTGAAGTTGAATCA
AGTGGTCCATCATAAGATTCAGACACTGGATAATCAAGATGAGATGGTGATTTCTTGAACAATCCAGTGATCTTAGATT
TCAGTGATGGCTCGTCCTTCTTCTCCGATTCCTCGATCACAACTGGAATGACATGCTCTGGCAGCTTCTCATA
GCTGAATTCAGTTGGATATGAATGCACTTCCGTTGTGAGTGGAATATCATGAATGTTCGATGCTCGTGAAGTAGATTCC
AATGGTCCATCATAAGATTCAGACACTGGATAATCAAGATGAGATGGTGATTTCTTGAACAATCCAGTGATCTTAGATT
TCAGTGATGGCTCGTCCTTCTTCTCTGTCTCCTCGATCACAACTGGAATGACATGCTCAGGCTGCTTCTCATA
GCTGAATTCAGTTGGATATGAATGCACTTCCGTTGTGAGTGGAATATCATGAATGTTCGATGCTCGTGAAGTAGATTCC
AATGGTCCATCATAAGATTCAGACACTGGATAATCAAGATGAGATGGAGATTTCTTGAACAATCCAGTGATCTTAGATT
TCAGTGATGGCTCGTCCTTCTTCTCCGATTCCTCGATCACAACTGGAATGACATGCTCTGGCAGCTTCTCATA
GCTGAATTCAGTTGGATATGAATGCACTTCCGTTGTGAGTGGAATATCATGAATGTTCGATGCTCGTGAAGTAGATTCC
AATGGTCCATCATAAGATTCAGACACTGGATAATCAAGATGAGATGGTGATTTCTTGAACAATCCAGTGATCTTAGATT
TCAGTGATGGCTCGTCCTTCTTCTCTGTCTCCTCGATCACAACTGGAATGACATGCTCTGGCAGCTTCTCATA
GCTGAATTCAGTTGGATATGAATGCACTTCCGTTGTGAGTGGAATATCATGAATGTTCGATGCTCGTGAAGTAGATTCC
AATGGTCCATCATAAGATTCAGACACTGGATAATCAAGATGAGATGGTGATTTCTTGAACAATCCAGTGATCTTAGATT
TCAGTGATGGCTCGTCCTTCTTCTCTGTCTCCTCGATCACAACTGGAATGACATGCTCAGGCTGCTTCTCATA
GCTGAATTCAGTTGGATATGAATGCACTTCCGTTGTGAGTGGAATATCATGAATGTTCGATGCTCGTGAAGTTGAATCA
AGTGGTCCATCATAAGATTCAGACACTGGATAATCAAGATGAGATGGAGATTTCTTGAACAATCCAGTGATCTTAAATT
TCAGTGATGGCTCGTCCTTCTTCTCTGACTCCTCGATCACAACTGGAATGACATGCTCTGGCAGCTTCTCATA
GCTGAATTCAGTTGGATATGAATGCACTTCCGTTGTGAGTGGAATATCATGAATGTTCGATGCTCGTGAAGTAGATTCC
AATGGTCCATCATAAGATTCAGACACTGGATAATCAAGATGAGATGGAGATTTCTTGAACAATCCAGTGATCTTAAATT
TCAGTGATGGCTCGTCCTTCTTCTCTGACTCCTCGATCACAACTGGAATGACATGCTCTGGCAGCTTCTCATA
GCTGAATTCAGTTGGATATGAATGCACTTCCGTTGTGAGTGGAATATCATGAATGTTCGATGCTCGTGAAGTAGATTCC
AATGGTCCATCGTAAGATTCAGACACTGGATAATCAAGATGAGATGGAGATTTCTTGAACAATCCAGTGATCTTAAATT
TCAGTGATGGCTCGTCCTTCTTCTCTGACTCCTCGATCACAACTGGAATGACATGCTCTGGCAGCTTCTCATA
GCTGAATTCAGTTGGATATGAATGCACTTCCGTTGTGAGTGGAATATCATGAATGTTCGATGCTCGTGAAGTAGATTCC
AATGGTCCATCATAAGATTCAGACACTGGATAATCAAGATGAGATGGTGATTTCTTGAACAATCCAGTGATCTTAGATT
TCAGTGATGGCTCGTCCTTCTTCTCTGTCTCCTCGATCACAACTGGAATGACATGCTCAGGCTGCTTCTCATA
GCTGAATTCAGTTGGATATGAATGCACTTCCGTTGTGAGTGGAATATCATGAATGTTCGATGCTCGTGAAGTAGATTCC
AATGGTCCATCATAAGATTCAGACACTGGATAATCAAGATGAGATGGTGATTTCTTGAACAATCCAGTGATCTTAGATT
TCAGTGATGGCTCGTCCTTCTTCTCTGACTCCTCGATCACAACTGGAATGACATGCTCTGGCAGTTTCTCATA
GCTGAATTCAGTTGGATATGAATGCACTTCCGTTGTGAGTGGAATATCATGAATGTTGGATGCTCGTGAAGTTGAATCA
AGTGGTCCATCATAAGATTCAGACACTGGATAATCAAGATGAGATGGTGATTTCTTGAACAATCCAGTGATCTTAGATT
TCAGTGATGGCTCGTCCTTCTTCTCTGACTCCTCGATCACAACTGGAATGACATGCTCTGGCAGTTTCTCATA
Nr of selected repeats 92 Similarity 0.829532
Consensus:
GCTGAATTCAGTTGGaTAtGaATGcACTTcCgTtGTGAGTGGaATaTCaTgaAtGTTngATGCtCGTGaaGTAGAnTCn
AgTGGTCCATCaTAagaTTCAGAcActGGaTAATCAagATGAGatGgtGATTTCTTGAACAATCCAGTGATCTTaGATT
TCAgTGATGGCTCGTCCTTCTTcTCtGtcTCcTCgATCACAACTGGaATGACATGCTCTGGCAGcTTCTCATA
>Cele-UNSB01_1:10475895-10475986 Satlength=92 Nr of Repeats=6 RepeatLength=14
seed=GCTATACGCT
GCTATACGCTATAC
GCTATACGCTATAC
GCTATACGCTATAC
GCTATACGCTATAC
GCTATACGCTATAC
GCTATACGCTATACGCTATAC
Nr of selected repeats 5 Similarity 1.000000
Consensus:
GCTATACGCTATAC
>Cele-UNSB01_1:10588945-10589028 Satlength=84 Nr of Repeats=6 RepeatLength=12
seed=TAGGCTTAGG

TAGGCTTAGGCTGAGGC
TAGGCTTAGGATTGGGCG
TAGGCTTAGTCT
TAGGCTTAGGCA
TAGGCTTAAGCT
TAGGCTTAGGTT
Nr of selected repeats 4 Similarity 0.777778
Consensus:
TAGGCTTAggct
>Cele-UNSB01_1:10648848-10654019 Satlength=5172 Nr of Repeats=115
RepeatLength=45 seed=AAGGATCACA
AAGGATCACAAGGGTAGCTCCTGATGATCTGATCCTTCAGCTTCG
AAGGATCACAAGGGTAGCTCCTGATGATCTGATCCTTCAGCTTCG
AAGGATCACAAGGGTAGTTTCTGATGATATGATCCTTCAGCTTCG
AAGGATCACAAGGGTAGTTTCTGATGATCTGATCCTTCAGCTCTG
AAGGATCACAAGAGTAGTTTCTGATGATCTGATCCTTCAGCTTCG
AAGGATCACAAGGGTAGCTCCTGATGATCTGATCCTTCAGCTTCG
AAGGATCACAAGGGTAGCTCCTGATGATCTGATCCTTCAGCTTCG
AAGGATCACAAGGGTAGCTCCTGATGATCTGATCCTTCAGCTTCG
AAGGATCACAAGGGTAGTTTCTGATGATCTGATCCTTCAGCTCTG
AAGGATCACAAGGGTAGTTTCTGATGATCTGATCCTTCAGCTCTG
AAGGATCACAAGGGTATTTTCTGATGATCTGATCCTTCAGCTCTG
AAGGATCACAAGGGTAGTTTCTGATGATCTGATCCTTCAGCTTCG
AAGGATCACAAGGGTAGCTCCTGATGATCTGATCCTTCAGCTTCG
AAGGATCACAAGGGTAGCTCCTGATGATCTGATCATTCAGCTTCC
AAGGATCACAAGGGTAGCTCCTGATGATCTGATCCTTCAGCTTCG
AAGGATCACAAGGGTAGTTTCTGATGATCTGATCCTTCAGCTTCG
AAGGATCACAAGGGTAGCTCCTGATGATCTGATCCTTCAGCTTCG
AAGGATCACAAGGGTAGTTTCTGATGATCTGATCCTTCAGCTCTG
AAGGATCACAAGGGTAGTTTCTGATGATCTGATCCTTCAGCTCTG
AAGGATCACAAGGGTATTTTCTGATGATCTGATCCTTCAGCTCTG
AAGGATCACAAGGGTAGTTTCTGATGATCTGATCCTTCAGCTTCG
AAGGATCACAAGGGTAGTTTCTGATGATCTGATCCTTCAGCTTCG
AAGGATCACAAGGGTAGCTCCTGATGATCTGATCCTTCAGCTTCG
AAGGATCACAAGGGTAACTCCTGATGATCTGATCCTTCAGCTTCG
AAGGATCACAAGGGTAGTTTCTGATGATCTGATCCTTCAGCTCTG
AAGGATCACAAGGGTAGTTTCTGATGATCTGATCCTTCAGCTCTG
AAGGATCACAAGGGTAGTTTCTGATGATCTGATCCTTCAGCTCTG
AAGGATCACAAGGGTAGTTTCTGATGATCTGATCCTTCAGCTTCG
AAGGATCACAAGGGTAGCTCCTGATGATCTGATCCTTCAGCTTCG
AAGGATCACAAGGGTAGTTTCTGATGATCTGATCCTTCAGCTCTG
AAGGATCACAAGGGTAGTTTCTGATGATCTGATCCTTCAGCTCTG
AAGGATCACAAGGGTAGTTTCTGATGATCTGATCCTTCAGCTCTG
AAGGATCACAAGGGTAGTTTCTGATGATCTGATCCTTCAGCTTCG
AAGGATCACAAGGGTAGTTTCTGATGATCTGATCCTTCAGCTTCG
AAGGATCACAAGGGTAGCTCCTGATGATCTGATCCTTCAGCTTCG
AAGGATCACAAGGGTAGTTTCTGATGATCTGATCCTTCAGCTCTG
AAGGATCACAAGGGTAGTTTCTGATGATCTGATCCTTCAGCTCTG
AAGGATCACAAGGGTAGTTTCTGATGATCTGATCCTTCAGCTCTG
AAGGATCACAAGGGTAGTTTCTGATGATCTGATCCTTCAGCTTCG
AAGGATCACAAGGGTAGTTTCTGATGATCTGATCCTTCAGCTTCG
AAGGATCACAAGGGTAGCTCCTGATGATCTGATCCTTCAGCTTCG
AAGGATCACAAGGGTAGTTTCTGATGATCTGATCCTTCAGCTTCG
AAGGATCACAAGGGTAGTTTCTGATGATCTGATCAGCTCTG

AAGGATCACAAGGGTAGTTTCTGATGATCTGATCCTTCAGCTTCG
AAGGATCACAAGGGTAGCTCCTGATGATCTGATCCTTCAGCTTCG
AAGGATCACAAGGGTAGTTTCTGATGATCTGATCCTTCAGCTCTG
AAGGATCACAAGGGTAGTTTCTGATGATCTGATCCTTCAGCTCTG
AAGGATCACAAGGGTAGTTTCTGATGATCTGATCCTTCAGCTCTG
AAGGATCACAAGGGTAGTTTCTGATGATCTGATCCTTCAGCTTCG
AAGGATCACAAGGGTAGTTTCTGATGATCTGATCCTTCAGCTTCG
AAGGATCACAAGGGTAGCTCCTGATGATCTGATCCTTCAGCTTCG
AAGGATCACAAGGGTAGTTTCTGATGATCTGATCCTTCAGCTTCG
AAGGATCACAAGGGTAGCTCCTGATGATCTGATCCTTCAGCTTCG
AAGGATCACAAGGGTAGTTTCTGATGATCTGATCCTTCAGCTTCG
AAGGATCACAAGGGTAGTTTCTGATGATCTGATCCTTCAGCTCTG
AAGGATCACAAGGGTAGTTTCTGATGATCTGATCCTTCAGCTTCG
AAGGATCACAAGGGTAGCTCCTGATGATCTGATCCTTCAGCTTCG
AAGGATCACAAGGGTAGTTTCTGATGATCTGATCCTTCAGCTTCG
AAGGATCACAAGGGTAGTTTCTGATGATCTGATCCTTCAGCTCTG
AAGGATCACAAGGGTAGCTCCTGATGATCTGATCCTTCAGCTTCG
AAGGATCACAAGGGTAGCTCCTGATGATCTGATCCTTCAGCTTCG
AAGGATCACAAGGGTAGCTCCTGATGATCTGATCCTTCAGCTTCG
AAGGATCACAAGGGTAGTTTCTGATGATCTGATCCTTCAGCTTCG
AAGGATCACAAGGGTAGTTTCTGATGATCTGATCCTTCAGCTTCG
AAGGATCACAAGGGTAGCTCCTGATGATCTGATCCTTCAGCTTCG
AAGGATCACAAGGGTAGTTTCTGATGATCTGATCCTTCAGCTTCG
AAGGATCACAAGGGTAGTTTCTGATGATCTGATCCTTCAGCTCTG
AAGGATCACAAGGGTAGTTTCTGATGATCTGATCCTTCAGCTTCG
AAGGATCACAAGGGTAGCTCCTGATGATCTGATCCTTCAGCTTCG
AAGGATCACAAGGGTAGTTTCTGATGATTTGATCCTTCAGCTTCG
AAGGATCACAAGGGTAGTTTCTGATGATCTGATCCTTCAGCTCTG
AAGGATCACAAGGGTAGCTCCTGATGATCTGATCCTTCAGCTTCG
AAGGATCACAAGGGTAGCTCCTGATGATCTGATCCTTCAGCTTCG
AAGGATCACAAGGGTAGCTCCTGATGATCTGATCCTTCAGCTTCG
AAGGATCACAAGGGTAGTTTCTGATGATCTGATCCTTCAGCTTCG
AAGGATCACAAGGGTAGTTTCTGATGATTTGATCCTTCAGCTTCG
AAGGATCACAAGGGTAGTTTCTGATGATCTGATCCTTCAGCTCTG
AAGGATCACAAGGGTAGCTCCTGATGATCTGATCCTTCAGCTTCG
AAGGATCACAAGGGTAGCTCCTGATGATCTGATCCTTCAGCTTCG
AAGGATCACAAGGGTAGCTCCTGATGATCTGATCCTTCAGCTTCG
AAGGATCACAAGGGTAGTTTCTGATGATCTGATCCTTCAGCTTCG
AAGGATCACAAGGGTAGTTTCTGATGATCTGATCCTTCAGCTCTG
AAGGATCACAAGGGTAGCTCCTGATGATCTGATCCTTCAGCTTCG
AAGGATCACAAGGGTAGCTCCTGATGATCTGATCCTTCAGCTTCG
AAGGATCACAAGGGTAGCTCCTGATGATCTGATCCTTCAGCTTCG
AAGGATCACAAGGGTAGCTCCTGATGATCTGATCCTTCAGCTTCG
AAGGATCACAAGGGTAGTTTCTGATGATCTGATCCTTCAGCTTCG
AAGGATCACAAGGGTAGCTCCTGATGATCTGATCCTTCAGCTTCG
AAGGATCACAAGGGTAGTTTCTGATGATCTGATCCTTCAGCTTCG
AAGGATCACAAGGGTAGTTTCTGATGATCTGATCCTTCAGCTCTG
AAGGATCACAAGGGTAGTTTCTGATGATCTGATCCTTCAGCTTCG
AAGGATCACAAGGGTAGCTCCTGATGATCTGATCCTTCAGCTTCG
AAGGATCACAAGGGTAGTTTCTGATGATCTGATCCTTCAGCTTCG
AAGGATCACAAGGGTAGTTTCTGATGATCTGATCCTTCAGCTCTG
AAGGATCACAAGGGTAGCTCCTGATGATCTGATCCTTCAGCTTCG
AAGGATCACAAGGGTAGCTCCTGATGATCTGATCCTTCAGCTTCG
AAGGATCACAAGGGTAGCTCCTGATGATCTGATCCTTCAGCTTCG

AAGGATCACAAGGGTAGTTTCTGATGATCTGATCCTTCAGCTTCG
AAGGATCACAAGGGTAGTTTCTGATGATCTGATCCTTCAGCTCTG
AAGGATCACAAGGGTAGCTCCTGATGATCTGATCCTTCAGCTTCG
AAGGATCACAAGGGTAGCTCCTGATGATCTGATCCTTCAGCTTCG
AAGGATCACAAGGGTAGCTCCTGATGATCTGATCCTTCAGCTTCG
AAGGATCACAAGGGTAGCTCCTGATGATCTGATCCTTCAGCTTCG
AAGGATCACAAGGGTAGTTTCTGATGATCTGATCCTTCAGCTTCG
AAGGATCACAAGGGTAGCTCCTGATGATCTGATCCTTCAGCTTCG
AAGGATCACAAGGGTAGCTCCTGATGATCTGATCCTTCAGCTTCG
AAGGATCACAAGGGTAGCTCCTGATGATCTGATCCTTCAGCTTCG
AAGGATCACAAGGGTAGCTCCTGATGATCTGATCCTTCAGCTTCG
AAGGATCACAAGAGTAGTTTCTGATGATCTGATCCTTCAGCTTCG
AAGGATCACAAGGGTAGCTCCTGATGATCTGATCCTTCAGCTTCG
AAGGATCACAAGGGTAGCTCCTGATGATCTGATCCTTCAGCTTCG
AAGGATCACAAGAGTAGTTTCTGATGATCTGATCCTTCAGCTTCG
AAGGATCACAAGGGTAGCTCCTGATGATCTGATCCTTCAGCTTCG
AAGGATCACAAGGGTAGCTCCTGATGATCTGATCCTTCAGCTTCG
AAGGATCACAAGAGTAGTTTCTGATGATCTGATCCTTCAGCTCTG
Nr of selected repeats 114 Similarity 0.941734
Consensus:
AAGGATCACAAGGGTAGtTtCTGATGATCTGATCCTTCAGCTtcG
>Cele-UNSB01_1:10660173-10660265 Satlength=93 Nr of Repeats=6 RepeatLength=15
seed=CGAAAAGGGG
CGAAAAGGGGAGCAGAA
CGAAAAGGGGATCTG
CAAAAAGGGGATCTG
CGAAAAGGGGAGATA
CGAAAAGGGGAGATA
CGAAAAGGGGAGCAA
Nr of selected repeats 5 Similarity 0.768889
Consensus:
CgAAAAGGGGAgcta
>Cele-UNSB01_1:10660440-10660532 Satlength=93 Nr of Repeats=6 RepeatLength=15
seed=TCCCCTTTTC
TCCCCTTTTCGTTGC
TCCCCTTTTCGTATC
TCCCCTTTTCGTATC
TCCCCTTTTCGCAGA
TCCCCTTTTTGCAGA
TCCCCTTTTCGTTCTGC
Nr of selected repeats 5 Similarity 0.768889
Consensus:
TCCCCTTTTcGtagc
>Cele-UNSB01_1:10842122-10842194 Satlength=73 Nr of Repeats=4 RepeatLength=18
seed=AAAAGTTACA
AAAAGTTACAGTGTCCTT
AAAAGTTACAGTGTCCTT
AAAAGTTACAGTGTCCTT
AAAAGTTACAGTGTCCTT
Nr of selected repeats 4 Similarity 1.000000
Consensus:
AAAAGTTACAGTGTCCTT
>Cele-UNSB01_1:11042845-11046757 Satlength=3913 Nr of Repeats=24
RepeatLength=163 seed=GCATAGTCAA

GCATAGTCAAAAATACAAGAATATGCCTATCAAATTATAATAGTTTGTACGGAAGTATTTTTTAAAAACTTGATAAAAA
TATATAAAAGCTGATTTTTTAAAAAATTCAAAAGTATGGGAAAATCATATGGAATCATTCTTTTTTATCTCAAAAAACT
GTTCA
GCATAGTCAAAAACACCAGCAAATACCAAACAGAGTATATTAGCTTTTACGGAAGTATTTTTTAGAAAATTGATAAAAA
TATATAAAAGCTGATTTTTTCAAAAATTCAAAAGTATGGGAAAATCATATGGAGTCATTCTTTTTTATCTTAAAAAACT
GTTCG
GCATAGTCAAAAATACCAGCAAATACCAAACAGAGTATATTAGCTTTTACGGAAGTATTTTTTAGAAAATTGATAAAAA
TATATAAAAGCTGATTTTTTCAAAAATTCAAAAGTATGGGAAAATCATATGGAGTCATTCTTTTTTATCTTAAAAAACT
GTTCG
GCATAGTCAAAAATACCAGCAAATACCAAACAGAGTATATTAGCTTTTACGGAAGTATTTTTTAGAAAATTGATAAAAA
TATATAAAAGCTGATTTTTTCAAAAATTCAAAAGTATGGGAAAATCATATGGAGTCATTCTTTTTTATCTTAAAAAACT
GTTCG
GCATAGTCAAAAATACCAGCAAATACCAAACAGAGTATATTAGCTTTTACGGAAGTATTTTTTAGAAAATTGATAAAAA
TATATAAAAGCTGATTTTTTCAAAAATTCAAAAGTATGGGAAAATCATATGGAGTCATTCTTTTTTATCTTAAAAAACT
GTTCG
GCATAGTCAAAAATACCAGCAAATACCAAACAGAGTATATTAGCTTTTACGGAAGTATTTTTTAGAAAATTGATAAAAA
TATATAAAAGCTGATTTTTTCAAAAATTCAAAAGTATGGGAAAATCATATGGAGTCATTCTTTTTTATCTCAAAAAACT
GTTCA
GCATAGTCAAGAATACCAGCAAATACCAAACAGAGTATATTAGCTTGTACGGAAGTATTTTTTAGAAAATTGATAAAAA
TATATAAAAGCTGATTTTTTCAAAAATTCAAAAGTATGGGAAAATCATATGGAATCATTCTTTTTTATCTTAAAAAACT
GTTCG
GCATAGTCAAAAACACCAGCAAATACCAAACAGAGTATATTAGCTTTTACGGAAGTATTTTTTAGAAAATTGATAAAAA
TATATAAAAGCTGATTTTTTCAAAAATTCAAAAGTATGGGAAAATCATATGGAGTCATTCTTTTTTATCTTAAAAAACT
GTTCG
GCATAGTCAAAAATACCAGCAAATACCAAACAGAGTATATTAGCTTTTACGGAAGTATTTTTTAGAAAATTGATAAAAA
TATATAAAAGCTGATTTTTTCAAAAATTCAAAAGTATGGGAAAATCATATGGAGTCATTCTTTTTTATCTCAAAAAACT
GTTCA
GCATAGTCAAGAATACCAGAATATGCCTATCAAATTATAATAGTTTGTACGGAAGTATTTTTTAAAAAATTGATAAAAA
TCTATAAAAGCTGATTTTTTCAAAAATTCAAAAGTATGGGAAAATCATATGGAGTCATTCTTTTTTATCTCAAAAAACT
GTTCA
GCATAGTCAAGAATACCAGAATATGCCTATCAAATTATAATAGTTTGTACGGAAGTATTTTTTAAAAAATTGATAAAAA
TATATAAAAGCTGATTTTTTCAAAAATTCAAAAGTATGGGAAAATCATATGGAGTCATTCTTTTTTATCTCAAAAAACT
GTTCA
GCATAGTCAAAAACACCAGCAAATACCAAACAGAGTATATTAGCTTGTACGGAAGTATTTTTTAGAAAATTGATAAAAA
TATATAAAAGCTGATTTTTTCAAAAATTCAAAAGTATGGGAAAACCATATGGAGTCATTCTTTTTTATCTCAAAAAACT
GTTCA
GCATAGTCAAGAATACCAGAATATGCCTATCAAATTATAATAGTTTGTACGGAAGTATTTTTTAAAAAATTGATAAAAA
TATATAAAAGCTGATTTTTTCAAAAATTCAAAAGTATGGGAAAATCATATGGAGTCATTCTTTTTTATCTCAAAAAACT
GTTCA
GCATAGTCAAGAATACCAGCAAATACCAAACAGAGTATATTAGCTTGTACGGAAGTATTTTTTAGAAAATTGATAAAAA
TATATAAAAGCTGATTTTTTCAAAAATTCAAAAGTATGGGAAAATCATATGGAGTCATTCTTTTTTATCTCAAAAAACT
GTTCA
GCATAGTCAAGAATACCAGCAAATACCAAACAGAGTATATTAGCTTGTACGGAAGTATTTTTTAGAAAATTGATAAAAA
TATATAAAAGCTGATTTTTTCAAAAATTCAAAAGTATGGGAAAATCATATGGAGTCATTCTTTTTTATCTCAAAAAACT
GTTCA
GCATAGTCAAGAATACCAGCAAATACCAAACAGAGTATATTAGCTTGTACGGAAGTATTTTTTAGAAAATTGATAAAAA
TATATAAAAGCTGATTTTTTCAAAAATTCAAAAGTATGGGAAAATCATATGGAGTCATTCTTTTTTATCTCAAAAAACT
GTTCA
GCATAGTCAAGAATACCAGAATATGCCTATCAAATTATAATAGTTTGTACGGAAGTATTTTTTAAAAAATTGATAAAAA
TATATAAAAGCTGATTTTTTAAAAAATTCAAAAGTATGGGAAAATCATATGGAATCATTCTTTTTTATCTCAAAAAACT
GTTCA
GCATAGTCAAAAACACCAGCAAATACCAAACAGAGTATATTAGCTTGTACGGAAGTATTTTTTAGAAAATTGATAAAAA
TATATAAAAGCTGATTTTTTCAAAAATTCAAAAGTATGGGAAAACCATATGGAGTCATTCTTTTTTATCTCAAAAAACT
GTTCA

GCATAGTCAAGAATACCAGAATATGCCTATCAAATTAAAATAGTTTGTACGGAAGTATTTTTTAAAAAATTGATAAAAA
TATATAAAAGCTGATTTTTTAAAAAATTCAAAAGTATGGGAAAATCATATGGAATCATTCTTTTTTATCTCAAAAAACT
GTTCA
GCATAGTCAAAAACACCAGCAAATACCAAACAGAGTATATTAGCTTGTACGGAAGTATTTTTTAGAAAATTGATAAAAA
TATATAAAAGCTGATTTTTTCAAAAATTCAAAAGTATGGGAAAATCATATGGAATCATTCTTTTTTATCTTAAAAAACT
GTTCG
GCATAGTCAAAAACACCAGCAAATACCAAACAGAGTATATTAGCTTGTACGGAAGTATTTTTTAGAAAATTGATAAAAA
TATATAAAAGCTGATTTTTTCAAAAATTCAAAAGTATGGGAAAATCATATGGAATCATTCTTTTTTATCTTAAAAAACT
GTTCG
GCATAGTCAAAAATACCAGCAAATACCAAACAGAGTATATTAGCTTGTACGGAAGTATTTTTTAGAAAATTGATAAAAA
TATATAAAAGCTGATTTTTTCAAAAATTCAAAAGTATGGGAAAATCATATGGAGTCATTCTTTTTTATCTCAAAAAACT
GTTCA
GCATAGTCAAGAATACCAGCAAATACCAAACAGAGTATATTAGCTTGTACGGAAGTATTTTTTAGAAAATTGATAAAAA
TATATAAAAGCTGATTTTTTCAAAAATTCAAAAGTATGGGAAAACCATATGGAGTCATTCTTTTTTATCTCAAAAAACT
GTTCA
GCATAGTCAAGAATACCAGAATATGCCTATCAAATTATAATAGTTTGTACGGAAGTATTTTTTAAAAAATTGATAAAAA
TATATAAAAGCTGATTTTTTCAAAAATTCAAAAGTATGGGAAAATCATATGGAGTCATTCTTTTTTATCTCAAAAAACT
GTTCA
Nr of selected repeats 24 Similarity 0.936516
Consensus:
GCATAGTCAAaAAtACCAGcAaATaCCaAaCAgAgTATAtTAGcTTgTACGGAAGTATTTTTTAgAAAATTGATAAAAA
TATATAAAAGCTGATTTTTTCAAAAATTCAAAAGTATGGGAAAATCATATGGAgTCATTCTTTTTTATCTcAAAAAACT
GTTCa
>Cele-UNSB01_1:11104747-11105884 Satlength=1138 Nr of Repeats=5 RepeatLength=94
seed=TGTAGACAAA
TGTAGACAAATCTATGTTAGCACTAACAATTGCGGCGGATGAAATTGAGGTTCAAAGAGAGGTCGATAAGTGTCTAAAC
AAAATAGACAAAGTCACAGATTTAATGGAAGAAAGCCAAGAAATCCGTGACACGCTTCATGCATCCGTCAAAAGGTCAC
AAGTAAGAATACAAAATCTTACAAGACCAACAACAGTAGCACCGATTCTGAATGAAAATAAATCATCGGATTCAGCGCT
ACCAAAGATAAAGATTCCCACTTTCAATGGAGAAAGATGGGAATTTGACAGTTTCTGGACAATCTTCGAAGAGCTCGTG
CACAAAAAAGAGTTACCAGATATGGTTAAATTCATGCACTTAATCAACGCTCTTCAAGGAGAAGCCAAAACACTGGTGA
GTAAAATTCAGGTTACTCCTGAACACTACACCACAGCCATTGAGGCACTAAAGAAAAAGTATACTGTAGGCGGGGTGCT
CCGCTCCTGCCTATTAGTGTGGATCTCTTTATTCCACTCTCGTATGGTTAGGGACCGAGCCCAGGTTCTAGGTCCAATA
GTAGAATATATAAGAATTTATATAAGAAAGATATAATAAGAAACAGTAGAGTAAGTTAGATACAATAGAAGTAGGATCA
TATGCGAGTCGGGCTCAACCATTACCTCTCGAGGTTGCCGACAAATCAATGGGAAAATCATCAATTTCTGAAGGCAGTA
ATTCTCAAAATCTTCTGTTCTCTAAATATTGGGTGCGTTTCGATGTCCTT
TGTAGACAAATCAATGGGAGAATCATCAATTTCTGAAGGCAGTAATTCTCAAAATCTTCTGTTCTCTAAATATTGGGTG
CTTTTCGATGTCCTT
TGTAGACAAATCAATGGGAGAATCATCAATTTCTGAAGGCAGTAATTCTCAAAATCTTCTGTTCTCTAAATATTGGGTG
CTTTTCGATGTCCTT
TGTAGACAAATCAATGGGAGAATCATCAATTTCTGAAGGCAGTAATTCTCAAAATCTTCTGTTCTCTAAATATTGGGTG
CTTTTCGATGTCCTT
TGTAGACAAATCAATGGAAAAATCATCAATTTCTGAAGGCAGTAATTCTCAAAATCTTCTGTTCTCTAAATATTGGGTG
CTTTTCGATGCCCTA
Nr of selected repeats 4 Similarity 0.971631
Consensus:
TGTAGACAAATCAATGGgAgAATCATCAATTTCTGAAGGCAGTAATTCTCAAAATCTTCTGTTCTCTAAATATTGGGTG
CTTTTCGATGtCCTt
>Cele-UNSB01_1:11137320-11159590 Satlength=22271 Nr of Repeats=783
RepeatLength=32 seed=GCACCAAATA
GCACCAAATAGCACCGATGTA
GCACCAAATATCACCAATTTAGCACCGATAGA
GCACCAAATAGCACCAATTTAGCACCAATGGA
GCACCAAATAGCACCAATTTAGCACCAATGGA
GCACCAAATAGCACCGATGGA

GCACCAAATAGCACCAATTTAACACCGATAGA
GCACCAAATAGCACCAATTTAGCACCAATGGA
GCACCAAATAGCACCGATGGA
GCACCAAATAGCACCAATTTAGCACCAATGGA
GCACCAAATAGCACCGATGGA
GCACCAAATAGCACCAATTTAACACCGATAGA
GCACCAAATAGCCCCAATTTAGCACCGATGGA
GCACCAAATAGCACCAATTTAGCACCAATGGA
GCACCAAATAGCACCGATGTA
GCACCAAATAGCACCAATTTAGCACCGACAGA
GCACCAAATAGCACCAATTTAGCACCAATGAA
GCACCAAATA
GCACCAAATAGCACCAATTTAGCACCAATGGA
GCACCAAATAGCACCGATGGA
GCACCAAATAGCACCGATGGA
GCACCAAATAGCACCAATTTAGCACCAATGGA
GCACCAAATAGCACCAATTTAGCACCGACAGA
GCACCAAATAGCACCAATTTAGCACCGACAGA
GCACCAAATA
GCACCAAATATCACCAATTTAGCACCAATGGA
GCACCAAATAGCACCGATGGA
GCACCAAATAGCACCGATGGA
GCACCAAATAGCACCAATTTAGCACCAATGGA
GCACCAAATAGCACCGATGGA
GCACCAAATAGCCCCAATTTAGCACCAATGGA
GCACCAAATAGCACCAATTTAGCACCAATGGA
GCACCAAATAGCACCAATTTAGCACCGACAGA
GCACCAAATAGCACCAATTTAGCACCAATGGA
GCACCAAATAGCACCAATTTAGCACCGACAGA
GCACCAAATAGCACCAATTTAGCACCGACAGA
GCACCAAATA
GCACCAAATAGCACCAATTTAGCACCAATGGA
GCACCAAATAGCACCAATTTAGCACCGACAGA
GCACCAAATAGCACCAATTTAGCACCGACAGA
GCACCAAATA
GCACCAAATATCACCAATTTAGCACCAATGGA
GCACCAAATAGCACCGATGGA
GCACCAAATAGCACCGATGGA
GCACCAAATAGCACCAATTTAGCACCAATGGA
GCACCAAATAGCACCAATTTAGCACCGACAGA
GCACCAAATAGCACCAATTTAGCACCGACAGA
GCACCAAATAGCACCAATTTAGCACCGACAGA
GCACCAAATA
GCACCAAATAGCACCAATTTAGCACCAATGGA
GCACCAAATAGCACCGATGGA
GCACCAAATAGCACCGATGGA
GCACCAAATAGCACCAATTTAGCACCAATGGA
GCACCAAATAGCACCAATTTAGCACCAATGGA
GCACCAAATAGCACCAATTTAGCACCGATGGA
GCACCAAATAGCACCGATGGA
GCACCAAATAGCACCAATTTAACACCGATAGA
GCACCAAATAGCACCAATTTAGCACCAATGGA
GCACCAAATAGCACCAATTTAGCACCGATGGA
GCACCAAATAGCACCAATTTAGCACCGACAGA

GCACCAAATAGCACCAATTTAGCACCAATGGA
GCACCAAATAGCACCGATGGA
GCACCAAATAGCCCCAATTTAGCACCAATGGA
GCACCAAATAGCACCGATGTA
GCACCAAATAGCACCAATTTAGCACCAATGGA
GCACCAAATAGCACCAATTTAGCACCAATGGA
GCACCAAATAGCACCAATTTAGCACCAATGGA
GCACCAAATAGCACCAATTTAGCACCGATGGA
GCACCAAATAGCACCAATTTAGCACCAATGGA
GCACCAAATAGCACCGATGTA
GCACCTAATAGCACCAATTTAGCACCGACAGA
GCACCAAATAGCACCAATTTAGCACCGACAGA
GCACCAAATAGCACCAATTTAGCACCAATGGA
GCACCAAATAGCACCGATGTA
GCACCAAATAGCACCGATGTA
GCACCAAATAGCACCAATTTAGCACCGACAGA
GCACCAAATAGCACCAATTTAGCACCGACAGA
GCACCAAATAGCACCAATTTAGCACCAATGGA
GCACCAAATAGCACCGATGTA
GCACCAAATAGCACCAATTTAGCACCGACAGA
GCACCAAATAGCACCAATTTAGCACCAATGGA
GCACCAAATAGCACCGATGTA
GCACCAAATAGCACCAATTTAGCACCGACAGA
GCACCAAATAGCACCAATTTAGCACCAATGGA
GCACCAAATAGCACCGATGTA
GCACCAAATAGCACCAATTTAGCACCGACAGA
GCACCAACTAGCACCGATGTA
GCACCAAATAGCACCAATTTAGCACCGACAGA
GCACCAACTAGCACCGATGTA
GCACCAAATAGCACCAATTTAGCACCAATGGA
GCACCAAATAGCACCAATTTAGCACCGACAGA
GCACCAAATAGCACCAATTTAGCACCAATGGA
GCACCAAATAGCACCGATGTA
GCACCAAATAGCACCAATTTAGCACCGACAGA
GCACCAAATAGCACCAATTTAGCACCAATGGA
GCACCAAATAGCACCGATGTA
GCACCAAATAGCACCAATTTAGCACCGACAGA
GCACCAAATAGCACCAATTTAGCACCAATGGA
GCACCAAATAGCACCGATGTA
GCACCAAATAGCACCAATTTAGCACCGACAGA
GCACCAACTAGCACCGATGTA
GCACCAAATAGCACCAATTTAGCACCAATGGA
GCACCAAATAGCACCAATTTAGCACCGACAGA
GCACCAAATAGCACCAATTTAGCACCAATGGA
GCACCAAATAGCACCGATGTA
GCACCAAATAGCACCAATTTAGCACCGACAGA
GCACCAAATAGCACCAATTTAGCACCAATGGA
GCACCAAATAGCACCGATGTA
GCACCAAATAGCACCAATTTAGCACCGACAGA
GCACCAACTAGCACCGATGTA
GCACCAAATAGCACCAATTTAGCACCGACAGA
GCACCAAATAGCACCGATGTA
GCACCAAATAGCACCAATTTAGCACCGACAGA
GCACCAACTAGCACCGATGTA

GCACCAAATAGCACCAATTTAGCACCAATGGA
GCACCAAATAGCACCAATTTAGCACCGACAGA
GCACCAAATAGCACCAATTTAGCACCAATGGA
GCACCAAATAGCACCGATGTA
GCACCAAATAGCACCAATTTAGCACCGACAGA
GCACCAACTAGCACCGATGTA
GCACCAAATAGCACCAATTTAGCACCAATGGA
GCACCAAATAGCACCAATTTAGCACCGACAGA
GCACCAACTAGCACCGATGTA
GCACCAAATAGCACCAATTTAGCACCGACAGA
GCACCAAATAGCACCAATTTAGCACCAATGGA
GCACCAAATAGCACCGATGTA
GCACCAAATAGCACCAATTTAGCACCGACAGA
GCACCAAATAGCACCAATTTAGCACCAATGGA
GCACCAAATAGCACCGATGTA
GCACCAAATAGCACCAATTTAGCACCGACAGA
GCACCAACTAGCACCAATTTAGCACCGACAGA
GCACCAAATAGCACCAATTTAGCACCAATGGA
GCACCAAATAGCACCGATGTA
GCACCAAATAGCACCAATTTAGCACCAATGGA
GCACCAAATAGCACCGATGTA
GCACCAAATAGCACCAATTTAGCACCGACAGA
GCACCAAATAGCACCAATTTAGCACCAATGGA
GCACCAAATAGCACCGATGTA
GCACCAAATAGCACCAATTTAGCACCAATGGA
GCACCAAATAGCACCGATGGA
GCACCAAATAGCACCAATTTAGCACCAATGGA
GCACCAAATAGCACCGATGTA
GCACCAAATAGCACCAATTTAGCACCGACAGA
GCACCAAATAGCACCAATTTAGCACCAATGGA
GCACCAAATAGCACCGATGTA
GCACCAAATAGCACCAATTTAGCACCGACAGA
GCACCAACTAGCACCGATGTA
GCACCAAATAGCACCAATTTAGCACCGACAGA
GCACCAACTAGCACCGATGTA
GCACCAAATAGCACCAATTTAGCACCAATGGA
GCACCAAATAGCACCAATTTAGCACCGACAGA
GCACCAAATAGCACCAATTTAGCACCAATGGA
GCACCAAATAGCACCGATGTA
GCACCAAATAGCACCAATTTAGCACCGACAGA
GCACCAAATAGCACCAATTTAGCACCAATGGA
GCACCAAATAGCACCGATGTA
GCACCAAATAGCACCAATTTAGCACCAATGGA
GCACCAAATAGCACCGATGGA
GCACCAAATAGCACCAATTTAGCACCAATGGA
GCACCAAATAGCACCGATGTA
GCACCAAATAGCACCAATTTAGCACCGACAGA
GCACCAAATAGCACCAATTTAGCACCAATGGA
GCACCAAATAGCACCGATGTA
GCACCAAATAGCACCAATTTAGCACCGACAGA
GCACCAAATAGCACCAATTTAGCACCAATGGA
GCACCAAATAGCACCGATGTA
GCACCAAATAGCACCAATTTAGCACCAATGGA
GCACCAAATAGCACCGATGGA

GCACCAAATAGCACCAATTTAGCACCAATGGA
GCACCAAATAGCACCGATGTA
GCACCAAATAGCACCAATTTAGCACCGACAGA
GCACCAAATAGCACCAATTTAGCACCAATGGA
GCACCAAATAGCACCGATGTA
GCACCAAATAGCACCAATTTAGCACCGACAGA
GCACCAACTAGCACCGATGTA
GCACCAAATAGCACCAATTTAGCACCGACAGA
GCACCAACTAGCACCGATGTA
GCACCAAATAGCACCAATTTAGCACCAATGGA
GCACCAAATAGCACCAATTTAGCACCGACAGA
GCACCAAATAGCACCAATTTAGCACCAATGGA
GCACCAAATAGCACCGATGTA
GCACCAAATAGCACCAATTTAGCACCGACAGA
GCACCAAATAGCACCAATTTAGCACCAATGGA
GCACCAAATAGCACCGATGTA
GCACCAAATAGCACCAATTTAGCACCAATGGA
GCACCAAATAGCACCGATGGA
GCACCAAATAGCACCAATTTAGCACCAATGGA
GCACCAAATAGCACCGATGTA
GCACCAAATAGCACCAATTTAGCACCGACAGA
GCACCAAATAGCACCAATTTAGCACCAATGGA
GCACCAAATAGCACCGATGTA
GCACCAAATAGCACCAATTTAGCACCGACAGA
GCACCAAATAGCACCAATTTAGCACCAATGGA
GCACCAAATAGCACCGATGTA
GCACCAAATAGCACCAATTTAGCACCAATGGA
GCACCAAATAGCACCGATGGA
GCACCAAATAGCACCAATTTAGCACCAATGGA
GCACCAAATAGCACCGATGTA
GCACCAAATAGCACCAATTTAGCACCGACAGA
GCACCAAATAGCACCAATTTAGCACCAATGGA
GCACCAAATAGCACCGATGTA
GCACCAAATAGCACCAATTTAGCACCGACAGA
GCACCAACTAGCACCGATGTA
GCACCAAATAGCACCAATTTAGCACCGACAGA
GCACCAACTAGCACCGATGTA
GCACCAAATAGCACCAATTTAGCACCGACAGA
GCACCAAATAGCACCAATTTAGCACCAATGGA
GCACCAAATAGCACCGATGTA
GCACCAAATAGCACCAATTTAGCACCGACAGA
GCACCAACTAGCACCGATGTA
GCACCAAATAGCACCAATTTAGCACCGACAGA
GCACCAACTAGCACCGATGTA
GCACCAAATAGCACCAATTTAGCACCAATGGA
GCACCAAATAGCACCAATTTAGCACCGACAGA
GCACCAAATAGCACCAATTTAGCACCAATGGA
GCACCAAATAGCACCGATGTA
GCACCAAATAGCACCAATTTAGCACCGACAGA
GCACCAAATAGCACCAATTTAGCACCAATGGA
GCACCAAATAGCACCGATGTA
GCACCAAATAGCACCAATTTAGCACCGACAGA
GCACCAAATAGCACCAATTTAGCACCAATGGA
GCACCAAATAGCACCGATGTA

GCACCAAATAGCACCAATTTAGCACCGACAGA
GCACCAACTAGCACCGATGTA
GCACCAAATAGCACCAATTTAGCACCGACAGA
GCACCAACTAGCACCGATGTA
GCACCAAATAGCACCAATTTAGCACCGACAGA
GCACCAAATAGCACCAATTTAGCACCAATGGA
GCACCAAATAGCACCGATGTA
GCACCAAATAGCACCAATTTAGCACCGACAGA
GCACCAACTAGCACCGATGTA
GCACCAAATAGCACCAATTTAGCACCGACAGA
GCACCAACTAGCACCGATGTA
GCACCAAATAGCATCAATTTAGCACCGACAGA
GCACCAAATAGCACCAATTTAGCACCAATGGA
GCACCAAATAGCACCGATGTA
GCACCAAATAGCACCAATTTAGCACCGACAGA
GCACCAACTAGCACCGATGTA
GCACCAAATAGCACCAATTTAGCACCGACAGA
GCACCAACTAGCACCGATGTA
GCACCAAATAGCACCAATTTAGCACCAATGGA
GCACCAAATAGCACCAATTTAGCACCGACAGA
GCACCAAATAGCACCAATTTAGCACCAATGGA
GCACCAAATAGCACCGATGTA
GCACCAAATAGCACCAATTTAGCACCGACAGA
GCACCAAATAGCACCAATTTAGCACCAATGGA
GCACCAAATAGCACCGATGTA
GCACCAAATAGCACCAATTTAGCACCGACAGA
GCACCAAATAGCACCAATTTAGCACCAATGGA
GCACCAAATAGCACCGATGTA
GCACCAAATAGCACCAATTTAGCACCGACAGA
GCACCAACTAGCACCGATGTA
GCACCAAATAGCACCAATTTAGCACCAATGGA
GCACCAAATAGCACCAATTTAGCACCGACAGA
GCACCAAATAGCACCAATTTAGCACCAATGGA
GCACCAAATAGCACCGATGTA
GCACCAAATAGCACCAATTTAGCACCGACAGA
GCACCAAATAGCACCAATTTAGCACCAATGGA
GCACCAAATAGCACCGATGTA
GCACCAAATAGCACCAATTTAGCACCGACAGA
GCACCAACTAGCACCGATGTA
GCACCAAATAGCACCAATTTAGCACCGACAGA
GCACCAAATAGCACCGATGTA
GCACCAAATAGCACCAATTTAGCACCGACAGA
GCACCAACTAGCACCGATGTA
GCACCAAATAGCACCAATTTAGCACCGACAGA
GCACCAAATAGCACCAATGGA
GCACCAAATAGCACCAATTTAGCACCGACAGA
GCACCAAATAGCACCAATTTAGCACCAATGGA
GCACCAAATAGCACCGATGTA
GCACCAAATAGCACCAATTTAGCACCGACAGA
GCACCAACTAGCACCGATGTA
GCACCAAATAGCACCAATTTAGCACCAATGGA
GCACCAAATAGCACCAATTTAGCACCGACAGA
GCACCAACTAGCACCGATGTA
GCACCAAATAGCACCAATTTAGCACCGACAGA

GCACCAAATAGCACCAATTTAGCACCAATGGA
GCACCAAATAGCACCGATGTA
GCACCAAATAGCACCAATTTAGCACCGACAGA
GCACCAAATAGCACCAATTTAGCACCAATGGA
GCACCAAATAGCACCGATGTA
GCACCAAATAGCACCAATTTAGCACCGACAGA
GCACCAACTAGCACCAATTTAGCACCGACAGA
GCACCAAATAGCACCAATTTAGCACCAATGGA
GCACCAAATAGCACCGATGTA
GCACCAAATAGCACCAATTTAGCACCAATGGA
GCACCAAATAGCACCGATGTA
GCACCAAATAGCACCAATTTAGCACCGACAGA
GCACCAAATAGCACCAATTTAGCACCAATGGA
GCACCAAATAGCACCGATGTA
GCACCAAATAGCACCAATTTAGCACCAATGGA
GCACCAAATAGCACCGATGGA
GCACCAAATAGCACCAATTTAGCACCAATGGA
GCACCAAATAGCACCGATGTA
GCACCAAATAGCACCAATTTAGCACCGACAGA
GCACCAAATAGCACCAATTTAGCACCAATGGA
GCACCAAATAGCACCGATGTA
GCACCAAATAGCACCAATTTAGCACCGACAGA
GCACCAACTAGCACCGATGTA
GCACCAAATAGCACCAATTTAGCACCGACAGA
GCACCAACTAGCACCGATGTA
GCACCAAATAGCACCAATTTAGCACCAATGGA
GCACCAAATAGCACCAATTTAGCACCGACAGA
GCACCAACTAGCACCGATGTA
GCACCAAATAGCACCAATTTAGCACCGACAGA
GCACCAACTAGCACCAATTTAGCACCGACAGA
GCACCAAATAGCACCAATTTAGCACCAATGGA
GCACCAAATAGCACCGATGTA
GCACCAAATAGCACCAATTTAGCACCAATGGA
GCACCAAATAGCACCGATGTA
GCACCAAATAGCACCAATTTAGCACCGACAGA
GCACCAAATAGCACCAATTTAGCACCAATGGA
GCACCAAATAGCACCGATGTA
GCACCAAATAGCACCAATTTAGCACCAATGGA
GCACCAAATAGCACCGATGGA
GCACCAAATAGCACCAATTTAGCACCAATGGA
GCACCAAATAGCACCGATGTA
GCACCAAATAGCACCAATTTAGCACCGACAGA
GCACCAAATAGCACCAATTTAGCACCAATGGA
GCACCAAATAGCACCGATGTA
GCACCAAATAGCACCAATTTAGCACCGACAGA
GCACCAACTAGCACCGATGTA
GCACCAAATAGCACCAATTTAGCACCGACAGA
GCACCAACTAGCACCGATGTA
GCACCAAATAGCACCAATTTAGCACCAATGGA
GCACCAAATAGCACCAATTTAGCACCGACAGA
GCACCAACTAGCACCGATGTA
GCACCAAATAGCACCAATTTAGCACCGACAGA
GCACCAACTAGCACCAATTTAGCACCGACAGA
GCACCAAATAGCACCAATTTAGCACCAATGGA

GCACCAAATAGCACCGATGTA
GCACCAAATAGCACCAATTTAGCACCAATGGA
GCACCAAATAGCACCGATGTA
GCACCAAATAGCACCAATTTAGCACCGACAGA
GCACCAAATAGCACCAATTTAGCACCAATGGA
GCACCAAATAGCACCGATGTA
GCACCAAATAGCACCAATTTAGCACCAATGGA
GCACCAAATAGCACCGATGGA
GCACCAAATAGCACCAATTTAGCACCAATGGA
GCACCAAATAGCACCGATGTA
GCACCAAATAGCACCAATTTAGCACCGACAGA
GCACCAAATAGCACCAATTTAGCACCAATGGA
GCACCAAATAGCACCGATGTA
GCACCAAATAGCACCAATTTAGCACCGACAGA
GCACCAACTAGCACCGATGTA
GCACCAAATAGCACCAATTTAGCACCGACAGA
GCACCAACTAGCACCGATGTA
GCACCAAATAGCACCAATTTAGCACCAATGGA
GCACCAAATAGCACCAATTTAGCACCGACAGA
GCACCAACTAGCACCGATGTA
GCACCAAATAGCACCAATTTAGCACCGACAGA
GCACCAAATAGCACCAATTTAGCACCAATGGA
GCACCAAATAGCACCGATGTA
GCACCAAATAGCACCAATTTAGCACCGACAGA
GCACCAACTAGCACCGATGTA
GCACCAAATAGCACCAATTTAGCACCAATGGA
GCACCAAATAGCACCAATTTAGCACCGACAGA
GCACCAAATAGCACCGATGTA
GCACCAAATAGCACCGATGTA
GCACCAAATAGCACCAATTTAGCACCGACAGA
GCACCAAATAGCACCAATTTAGCACCAATGGA
GCACCAAATAGCACCGATGTA
GCACCAAATAGCACCAATTTAGCACCGACAGA
GCACCAACTAGCACCAATTTAGCACCGACAGA
GCACCAAATAGCACCAATTTAGCACCGACAGA
GCACCAACTAGCACCAATTTAGCACCGACAGA
GCACCAAATAGCACCAATTTAGCACCAATGGA
GCACCAAATAGCACCGATGTA
GCACCAAATAGCACCAATTTAGCACCAATGGA
GCACCAAATAGCACCGATGTA
GCACCAAATAGCACCAATTTAGCACCAATGGA
GCACCAAATAGCACCGATGTA
GCACCAAATAGCACCAATTTAGCACCGACAGA
GCACCAAATAGCACCAATTTAGCACCAATGGA
GCACCAAATAGCACCGATGTA
GCACCAAATAGCACCAATTTAGCACCAATGGA
GCACCAAATAGCACCGATGTA
GCACCAAATAGCACCAATTTAGCACCAATGGA
GCACCAAATAGCACCGATGGA
GCACCAAATAGCACCAATTTAGCACCAATGGA
GCACCAAATAGCACCGATGTA
GCACCAAATAGCACCAATTTAGCACCGACAGA
GCACCAAATAGCACCAATTTAGCACCAATGGA
GCACCAAATAGCACCGATGTA

GCACCAAATAGCACCAATTTAGCACCGACAGA
GCACCAACTAGCACCGATGTA
GCACCAAATAGCACCAATTTAGCACCGACAGA
GCACCAACTAGCACCGATGTA
GCACCAAATAGCACCAATTTAGCACCAATGGA
GCACCAAATAGCACCAATTTAGCACCGACAGA
GCACCAACTAGCACCGATGTA
GCACCAAATAGCACCAATTTAGCACCGACAGA
GCACCAAATAGCACCAATTTAGCACCAATGGA
GCACCAAATAGCACCGATGTA
GCACCAAATAGCACCAATTTAGCACCGACAGA
GCACCAAATAGCACCAATTGAGCACCAATGGA
GCACCAAATAGCACCGATGTA
GCACCAAATAGCACCAATTTAGCACCGACAGA
GCACCAACTAGCACCGATGTA
GCACCAAATAGCACCAATTTAGCACCAATGGA
GCACCAAATAGCACCAATTTAGCACCGACAGA
GCACCAACTAGCACCGATGTA
GCACCAAATAGCACCAATTTAGCACCGACAGA
GCACCAAATAGCACCAATTTAGCACCAATGGA
GCACCAAATAGCACCGATGTA
GCACCAAATAGCACCAATTTAGCACCGACAGA
GCACCAAATAGCACCAATTTAGCACCAATGGA
GCACCAAATAGCACCGATGTA
GCACCAAATAGCACCAATTTAGCACCGACAGA
GCACCAACTAGCACCAATTTAGCACCGACAGA
GCACCAAATAGCACCAATTTAGCACCGACAGA
GCACCAACTAGCACCAATTTAGCACCGACAGA
GCACCAAATAGCACCAATTTAGCACCAATGGA
GCACCAAATAGCACCGATGTA
GCACCAAATAGCACCAATTTAGCACCAATGGA
GCACCAAATAGCACCGATGGA
GCACCAAATAGCACCAATTTAGCACCAATGGA
GCACCAAATAGCACCGATGTA
GCACCAAATAGCACCAATTTAGCACCGACAGA
GCACCAAATAGCACCAATTTAGCACCAATGGA
GCACCAAATAGCACCGATGTA
GCACCAAATAGCACCAATTTAGCACCGACAGA
GCACCAAATAGCACCAATTTAGCACCAATGGA
GCACCAAATAGCACCGATGTA
GCACCAAATAGCACCAATTTAGCACCGACAGA
GCACCAACTAGCACCGATGTA
GCACCAAATAGCACCAATTTAGCACCGACAGA
GCACCAACTAGCACCGATGA
GCACCAAATAGCACCAATTTAGCACCAATGGA
GCACCAAATAGCACCGATGTA
GCACCAAATAGCACCAATTTAGCACCGACAGA
GCACCAAATAGCACCAATTTAGCACCAATGGA
GCACCAAATAGCACCGATGTA
GCACCAAATAGCACCAATTTAGCACCGACAGA
GCACCAAATAGCACCGATGTA
GCACCAAATAGCACCAATTTAGCACCGACAGA
GCACCAACTAGCACCGATGTA
GCACCAAATAGCACCAATTTAGCACCGACAGA

GCACCAAATAGCACCAATTTAGCACCAATGGA
GCACCAAATAGCACCGATGTA
GCACCAAATAGCACCAATTTAGCACCGACAGA
GCACCAAATAGCACCAATTTAGCACCAATGGA
GCACCAAATAGCACCGATGTA
GCACCAAATAGCACCAATTTAGCACCAATGGA
GCACCAAATAGCACCGATGTA
GCACCAAATAGCACCAATTTAGCACCGACAGA
GCACCAACTAGCACCGATGTA
GCACCAAATAGCACCAATTTAGCACCGACAGA
GCACCAAATAGCACCAATTTAGCACCAATGGA
GCACCAAATAGCACCAATTTAGCACCGACAGA
GCACCAACTAGCACCGATGTA
GCACCAAATAGCACCAATTTAGCACCGACAGA
GCACCAAATAGCACCAATTTAGCACCAATGGA
GCACCAAATAGCACCGATGTA
GCACCAAATAGCACCAATTTAGCACCGACAGA
GCACCAAATAGCACCAATTTAGCACCAATGGA
GCACCAAATAGCACCGATGTA
GCACCAAATAGCACCAATTTAGCACCGACAGA
GCACCAAATAGCACCAATGGA
GCACCAAATAGCACCAATTTAGCACCAATGGA
GCACCAAATAGCACCAATTTAGCACCGACAGA
GCACCAACTAGCACCGATGTA
GCACCAAATAGCACCAATTTAGCACCGACAGA
GCACCAAATAGCACCAATTTAGCACCAATGGA
GCACCAAATAGCACCGATGTA
GCACCAAATAGCACCAATTTAGCACCGACAGA
GCACCAAATAGCACCAATTTAGCACCAATGGA
GCACCAAATAGCACCGATGA
GCACCAAATAGCACCAATTTAGCACCGACAGA
GCACCAATAGCACCAATTTAGCACCGACAGA
GCACCAAATAGCACCAATTTAGCACCAATGGA
GCACCAAATAGCACCGATGTA
GCACCAAATAGCACCAATTTAGCACCAATGGA
GCACCAAATAGCACCGATGGA
GCACCAAATAGCACCAATTTAGCACCAATGGA
GCACCAAATAGCACCGATGTA
GCACCAAATAGCACCAATTTAGCACCGACAGA
GCACCAAATAGCACCAATTTAGCACCAATGGA
GCACCAAATAGCACCGATGTA
GCACCAAATAGCACCAATTTAGCACCGACAGA
GCACCAAATAGCACCAATTTAGCACCGACAGA
GCACCAAATAGCACCAATTTAGCACCGACAGA
GCACCAAATAGCACCAATGGA
GCACCAAATAGCACCAATTTAGCACCAATGGA
GCACCAAATAGCACCAATTTAGCACCGACAGA
GCACCAAATAGCACCAATTTAGCACCAATGGA
GCACCAAATAGCACCAATTTAGCACCAATGGA
GCACCAAATAGCACCGATGA
GCACCAAATAGCACCAATTTAGCACCGACAGA
GCACCAAATAGCACCAATTTA
GCACCAATAGCACCAATAGCACATGA
GCACCAAATAGCACCAATTTAGCACCATGGA

GCACCAAATAGCACCAATTTAGCACCGACAGA
GCACCAAATAGCACCAATTTAGCACCAATGGA
GCACCAAATAGCACCGATGGA
GCACCAAATAGCACCAATTTAGCACCGACAGA
GCACCAAATAGCACCAATTTAGCACCAATGAGCACAATAGACCAATAGCCCAAAA
GCACCAAATAGCACCAATTTAGCACCGATGGA
GCACCAAATAGCACCAATTTAGCACCGACAGA
GCACCAAATA
GCACCAATTAGCACCAATGGA
GCACCAAATAGCACCGATGTA
GCACCCAAATAGCACCAATTTAGCACCGACAGA
GCACCAAATAGCACCAATTTAGCTCCAATGAA
GCACCAAATAGCACCAATTTAGCACCGATGGA
GCACCAAATAGCACCAATTTAGCACCGACAGA
GCACCAAATAGCACCAATTTAGCACCGATGGA
GCACCAAATAGCACCAATTTAGCTCCAATGAA
GCACCAAATAGCACCAATTTAGCTCCAATGAA
GCACCAAATAGCACCAATTTAGCTCCAATGAA
GCACCAAATAGCACCAATTTAGCACCGATGGA
GCACCAAATAGCACCGATGGA
GCACCAAATAGCACCGATGGA
GCACCAAATAGCACCAATTTAACACCGATAGA
GCACCAAATAGCCCCAAATTAGCACCAATGGA
GCACCAAATAGCACCGATGTA
GCACCAAATAGCACCAATTTAGCACCGACAGA
GCACCAAATAGCACCAATTTAGCTCCAATGAA
GCACCAAATAGCACCAATTTAGCACCGATGGA
GCACCAAATAGCACCAATTTAGCACCGACAGA
GCACCAAATAGCACCAATTTAGCACCGACAGA
GCACCAAATAGCACCAATTTAGCTCCAATGAA
GCACCAAATAGCACCAATTTAGCTCCAATGAA
GCACCAAATAGCACCAATTTAGCTCCAATGAA
GCACCAAATAGCCCCAATTTAGCACCAATGGA
GCACCAAATAGCACCGATGTA
GCACCAAATAGCACCAATTTAGCACCAATGGA
GCACCAAATAGCACCGATGGA
GCACCAAATAGCCCCAATTTAGCTCCAATGAA
GCACCTAATAGCACCAATTTAGCTCCAATGAA
GCACCAAATAGCACCAATTTAGCACCGATGGA
GCACCAAATAGCACCGATGGA
GCACCAAATAGCACCAATTTAACACCGATAGA
GCACCAAATAGCACCGATGTA
GCACCAAATAGCACCAATTTAGCACCGACAGA
GCACCAAATAGCACCAATTTAACACCGATAGA
GCACCAAATAGCACCAATTTAGCTCCAATGAA
GCACCAAATAGCACCAATTTAGCACCGATGGA
GCACCAAATAGCACCGATGGA
GCACCAAATAGCACCAATTTAGCACCGATGGA
GCACCAAATAGCACCAATTTAGCACCGATGGA
GCACCAAATAGCACCAATTTAGCACCGATGGA
GCACCAAATAGCACCAATTTAGCACCGATGGA
GCACCAAATAGCACCGATGGA
GCACCAAATAGCACCAATTTAACACCGATAGA
GCACCAAATAGCACCGATGTA

GCACCAAATAGCACCAATTTAGCACCGACAGA
GCACCAAATAGCACCAATTTAGCACCGACAGA
GCACCAAATAGCACCAATTTAGCTCCAATGAA
GCACCAAATAGCACCAATTTAGCACCGATGGA
GCACCAAATAGCACCGATGGA
GCACCAAATAGCACCAATTTAACACCGATAGA
GCACCAAATAGCACCAATTTAGCTCCAATGAA
GCACCAAATAGCACCAATTTAGCACCGATGGA
GCACCAAATAGCACCGATGTAGCACCGACAGA
GCACCAAATAGCACCAATTTAGCTCCAATGAA
GCACCAAATAGCACCAATTTAGCACCGATGGA
GCACCAAATAGCACCGATGGA
GCACCAAATAGCACCAATTTAACACCGATAGA
GCACCAAATAGCACCAATTTAGCTCCAATGAA
GCACCAAATAGCACCAATTTAGCACCGATGGA
GCACCAAATAGCACCAATTTAGCACCGACAGA
GCACCAAATAGCACCAATTTAGCACCGATGGA
GCACCAAATAGCACCAATTTAGCACCGACAGA
GCACCAAATAGCACCAATTTAGCACCGACAGA
GCACCAAATAGCACCAATTTAGCTTCAATGAAACACCTAATAGCACCAATTTAGCTCCAATGAA
GCACCAAATAGCACCAATTTAGCACCGATGGA
GCACCAAATAGCACCGATGGA
GCACCAAATAGCACCAATTTAACACCGATAGA
GCACCAAATAGCCCCAATTTAGCACCAATGGA
GCACCAAATAGCACAAATTTAGCACCGACAGA
GCACCAAATAGCACCAATTTAGCTCCAATGAA
GCACCAAATAGCACCAATTTAGCTCCAATGAA
GCACCAAATAGCACCAATTTAGCACCGATGGA
GCACCAAATAGCACCAATTTAACACCGATAGA
GCACCAAATAGCACCGATGTA
GCACCAAATAGCACCAATTTAGCACCGACAGA
GCACCAAATAGCACCAATTTAGCTCCAATGAA
GCACCAAATAGCACCAATTTAGCACCGATGGA
GCACCAAATAGCACCGATGGA
GCACCAAATAGCACCGATGGA
GCACCAAATAGCACCAATTTAACACCGATAGA
GCACCAAATAGCACCGATGTA
GCACCAAATAGCACCAATTTAGCTCCAATGAA
GCACCAAATAGTACCAATTTAGCACCGATGGA
GCACCAAATAGCACCAATTTAGCACCGATGGA
GCACCAAATAGCACCGATGTA
GCACCAAATAGCACCAATTTAGCACCGACAGA
GCACCAAATAGCACCAATTTAGCTCCAATGAA
GCACCAAATAGCACCAATTTAGCACCGATGGA
GCACCAAATAGCACCGATGGA
GCACCAAATAGCACCGATGGA
GCACCAAATAGCACCAATTTAACACCGATAGA
GCACCAAATAGCACCGATGTA
GCACCAAATAGCACCAATTTAGCTCCAATGAA
GCACCAAATAGTACCAATTTAGCACCGATGGA
GCACCAAATAGCACCAATTTAGCACCGATGGA
GCACCAAATAGCACCGATGTA
GCACCAAATAGCACCAATTTAGCACCGACAGA
GCACCAAATAGCACCAATTTAGCTCCAATGAA

GCACCAAATAGCACCAATTTAGCACCGATGGA
GCACCAAATAGCACCGATGGA
GCACCAAATAGCACCGATGGA
GCACCAAATAGCACCAATTTAACACCGATAGA
GCACCAAATAGCACCGATGTA
GCACCAAATAGCACCAATTTAGCTCCAATGAA
GCACCAAATAGTACCAATTTAGCACCGATGGA
GCACCAAATAGCACCAATTTAGCACCGATGGA
GCACCAAATAGCACCAATTTAGCACCGATGGA
GCACCAAATAGCACCAATTTAGCTCCAATGAA
GCACCAAATAGCACCAATTTAGCACCGATGGA
GCACCAAATAGCACCGATGGA
GCACCAAATAGCACCGATGGA
GCACCAAATAGCACCAATTTAACACCGATAGA
GCACCAAATAGCACCGATGTA
GCACCAAATAGCACCAATTTAGCTCCAATGAA
GCACCAAATAGTACCAATTTAGCACCGATGGA
GCACCAAATAGCACCAATTTAGCACCGATGGA
GCACCAAATAGCACCGATGTA
GCACCAAATAGCACCAATTTAGCACCGACAGA
GCACCAAATAGCACCAATTTAGCTCCAATGAA
GCACCAAATAGCACCAATTTAGCACCGATGGA
GCACCAAATAGCACCGATGGA
GCACCAAATAGCACCGATGGA
GCACCAAATAGCACCAATTTAACACCGATAGA
GCACCAAATAGCACCGATGTA
GCACCAAATAGCACCAATTTAGCTCCAATGAA
GCACCAAATAGCACCAATTTAGCACCGATGGA
GCACCAAATAGCACCAATTTAACACCGATAGA
GCACCAAATAGCACCGATGTA
GCACCAAATAGCACCAATTTAGCACCGACAGA
GCACCAAATAGCACCAATTTAGCTCCAATGAA
GCACCAAATAGCACCAATTTAGCACCGATGGA
GCACCAAATAGCACCGATGGA
GCACCAAATAGCACCGATGGA
GCACCAAATAGCACCAATTTAACACCGATAGA
GCACCAAATAGCACCGATGTA
GCACCAAATAGCACCAATTTAGCTCCAATGAA
GCACCAAATAGTACCAATTTAGCACCGATGGA
GCACCAAATAGCACCAATTTAGCACCGATGGA
GCACCAAATAGCACCAATTTAGCACCGATGGA
GCACCAAATAGCACCAATTTAGCACCGATGGA
GCACCAAATAGCACCGATGTA
GCACCAAATAGCACCAATTTAACACCGATAGA
GCACCAAATAGCCCCAATTTAGCACCAATGGA
GCACCAAATAGCACCGATGTA
GCACCAAATAGCACCAATTTAGCTCCAATGAA
GCACCAAATAGTACCAATTTAGCACCGATGGA
GCACCAAATAGCACCAATTTAGCACCGATGGA
GCACCAAATAGCACCAATTTAGCTCCAATGAA
GCACCAAATAGCACCAATTTAGCACCGATGGA
GCACCAAATAGCACCAATTTAGCACCGATGGA
GCACCAAATAGCACCGATGTA
GCACCAAATAGCACCAATTTAACACCGATAGA

GCACCAAATAGCCCCAATTTAGCACCAATGGA
GCACCAAATAGCACCGATGTA
GCACCAAATAGCACCAATTTAGCTCCAATGAA
GCACCAAATACCACCAATTTAGCACCGACAGA
GCACCAAATAGCACCAATTTAGCTCCAATGAA
GCACCAAATAGCACCAATTTAGCACCAATGGA
GCACCAAATAGCACCAATTTAGCTCCAATGAA
GCACCAAATACCACCAATTTAGCACCGACAGA
GCACCAAATAGCACCAATTTAGCTCCAATGAA
GCACCAAATAGCACCAATTTAACACCGATAGT
GCACCAAATAGCCCCAAGTTAGCACCAATGGA
GCACCAAATAGCACCAATTTAGCTCCAATGAA
GCACCAAATACCACCAATTTAGCACCGACAGA
GCACCAAATAGCACCAATTTAGCTCCAATGAA
GCACCAAATACCACCAATTTAGCACCGACAGA
GCACCAAATAGCACCAATTTAGCTCCAATGAA
GCACCAAATAGCACCAATTTAACACCGATAGT
GCACCAAATAGCCCCAAGTTAGCACCAATGGA
GCACCAAATAGCACCAATTTAGCTCCAATGAA
GCACCAAATAGCACCAATTTAGCTCCAATGAA
GCACCAAATAGCACCAATTTAGCACCAATGGA
GCACCAAATAGCACCGATGGA
GCACCAAATAGCACCAATTTAACACCGATAGA
GCACCAAATAGCCCCAAGTTAGCACCAATGGA
GCACCAAATAGCACCGATGTAGCACCAATTTAGCTCCAATGAA
GCACCAAATAGCACCAATTTAGCACCGATGGA
GCACCAAATAGCACCGATGGA
GCACCAAATAGCACCGATGGA
GCACCAAATAGCACCAATTTAACACCGATAGA
GCACCAAATAGCACCAATTTAGCTCCAATGAA
GCACCAAATA
GCACCAAATAGCACCGATGGA
GCACCAAATAGCACCGATGGA
GCACCAAATAGCACCAATTTAACACCGATAGA
GCACCAAATAGCACCAATTTAGCTCCAATGAA
GCACCAAATAGCACCAATTTAGCACCGATGGA
GCACCAAATAGCACCGATGGA
GCACCAAATAGCACCAATTTAACACCGATAGA
GCACCAAATAGCACCAATTTAGCTCCAATGAA
GCACCAAATAGCACCAATTTAGCACCGATGGA
GCACCAAATA
GCACCAAATAGCACCAATTTAACACCGATAGA
GCACCTAATAGCACCAATTTAGCTCCAATGAA
GCACCAAATAGCACCAATTTAGCACCGATGGA
GCACCAAATAGCACCGATGGA
GCACCAAATAGCACCAATTTAGCACCGACAGA
GCACCAAATAGCACCAATTTAGCTCCAATGAA
GCACCAAATAGCACCAATTTAGCTCCAATGAA
GCACCAAATAGCACCAATTTAGCACCGATGGA
GCACCAAATAGCACCGATGGA
GCACCAAATAGCACCAATTTAACACCGATAGA
GCACCAAATAGCCCCAATTTAGCACCAATGGA
GCACCAAATAGCACCGATGTAGCACCGACAGA
GCACCAAATAGCACCAATTTAGCTCCAATGAA

GCACCAAATA
GCACCAAATAGCACCAATTTAGCACCGATGGA
GCACCAAATAGCACCAATTTAGCACCGACAGA
GCACCAAATAGCACCAATTTA
GCACCAAATAGCACCAATTTAGCACCGACAGA
GCACCAAATAGCACCAATTTAGCTCCAATGAAACACCTAATAGCACCAATTTAGCTCCAATGAA
GCACCAAATAGCACCAATTTAGCACCGATGGA
GCACCAAATAGCACCGATGGA
GCACCAAATAGCACCGATGGA
GCACCAAATA
GCACCAAATAGCACCGATGGA
GCACCAAATAGCCCCAATTTAGCACCAATGGA
GCACCAAATAGCACAAATTTAGCACCGACAGA
GCACCAAATAGCACCAATTTAGCTCCAATGAA
GCACCTAATAGCACCAATTTAGCTCCAATGAA
GCACCAAATA
GCACCAAATAGCACCGATGTA
GCACCAAATAGCACCAATTTAACACCAATGGA
GCACCAAATAGCACCAATTTAGCTCCAATGAA
GCACCAAATAGCACCAATTTAGCTCCAATGAA
GCACCAAATAGCCCCAATTTAGCACCAATGGA
GCACCAAATAGCACCGATGTA
GCACCAAATAGCACCAATTTAGCACCGACAGA
GCACCAAATAGCACCAATTTAGCTCCAATGAA
GCACCAAATAGCACCAATTTAACACCGATAGA
GCACCAAATAGCCCCAATTTAGCACCGATGGA
GCACCAAATAGCACCAATTTAGCACCGACAGA
GCACCGAATAGCACCAATTTAGCACCGACAGA
GCACCAAATAGCACCAATTTAGCTCCAATGAA
GCACCAAATAGCACCAATTTAGCACCGATGGA
GCACCAAATAGCACCAATTTAGCTCCAATGAA
GCACCAAATA
GCACCAAATAGCACCAATTTAGCTCCAATGAAACACCTAATAGCACCAATTTAGCTCCAATGAA
GCACCAAATA
GCACCAAATAGCACCAATTTAGCTCCAATGAAACACCTAATAGCACCAATTTAGCTCCAATGAA
GCACCAAATAGCACCAATTTAGCACCGATGGA
GCACCAAATAGCACCGATGGA
GCACCAAATAGCACCGATGGA
GCACCAAATAGCACCGATAGA
GCACCAAATAGCCCCAATTTAGCACCAATGGA
GCACCAAATAGCACAAATTTAGCACCGACAGA
GCACCAAATAGCACCAATTTAGCTCCAATGAAGCACCTATTAGCACCAATTTAGCTCCAATGAA
GCACCAAATA
GCACCAAATAGCACCGATGGA
GCACCAAATAGCACCAATTTAGCACCGACAGA
GCACCGAATAGCACCAATTTAGCACCGACAGA
GCACCAAATAGCACCAATTTAGCACCGACAGA
GCACCAAATAGCACCGATGTA
GCACCAAATAGCACCAATTTAACACCGATAGT
GCACCAAATAGCCCCAATTTAGCACCAATGGA
GCACCAAATAGCACCAATTTAGCTCCAATGAA
GCACCAAATAGCCCCAATTTAGCACCAATGGA
GCACCAAATAGCACCGATGTA
GCACCAAATAGCACCAATTTAGCACCGACAGA

GCACCAAATAGCACCAATTTAGCTCCAATGAA
GCACCAAATAGCACCAATTTAACACCGATAGA
GCACCAAATAGCCCCAATTTAGCACCGATGGA
GCACCAAATAGCACCAATTTAGCACCGACAGA
GCACCGAATAGCACCAATTTAGCACCGACAGA
GCACCAAATAGCACCAATTTAGCTCCAATGAA
GCACCAAATAGCACCAATTTAGCTCCAATGAA
GCACCAAATAGCCCCAATTTAGCACCAATGGA
GCACCAAATAGCACCGATGTA
GCACCAAATAGCACCAATTTAGCACCGACAGA
GCACCAAATAGCACCAATTTAGCTCCAATGAA
GCACCAAATAGCACCAATTTAACACCGATAGA
GCACCAAATAGCCCCAATTTAGCACCGATGGA
GCACCAAATAGCACCAATTTAGCACCGACAGA
GCACCGAATAGCACCAATTTAGCACCGACAGA
GCACCAAATAGCACCAATTTAGCTCCAATGAA
GCACCAAATAGCACCAATTTAGCTCCAATGAA
GCACCAAATAGCACCAATTTAGCACCGATGGA
GCACCAAATAGCACCGATGGA
GCACCAAATAGCACCAATTTAACACCGATAGA
GCACCAAATAGCCCCAATTTAGCACCAATGGA
GCACCAAATAGCACCGATGTA
Nr of selected repeats 516 Similarity 0.905773
Consensus:
GCACCAAATAGCACCAATTTAGCACCgAtgGA
>Cele-UNSB01_1:11159617-11160082 Satlength=466 Nr of Repeats=4 RepeatLength=26
seed=TTTTTGAAAA
TTTTTGAAAAATGGCATTGCGGCGGA
TTTTTGAAAAATGGCATTGCGGCGGA
TTTTTGAAAAATGGCATTGCGGCACA
TTTTTCAAAAATCCGCCGCAATGCCATTTTTCTAAAATCCACCTGTGCCATACGTGTTTACATATCGAAAGAACACCCT
GAAATTCCACAGTTTTCCATATTACTAACCATCTAGTTGTCTTACATATACTGTTACGTCGGGACAAGTCATTCCGACT
GGAGGTCCACAAAACAAGTTTTGCTCTATGCAATCTTCTCCTGTTGGTGTTGTTGGTCTGAAATGATCAAAACAATTTT
GGAGCATTAAGATGTATTTAGAGTATTTAGTATAGTTTGTTTAAAGGTGGACTACGCTCGGTGGGGAAATTTCTTTGAA
CGCGCATATGGGGTCACAATGACCGAATATCATAATAAAAAAATTCAAAAAAATTTCTAAATTTTATGAT
Nr of selected repeats 3 Similarity 0.931624
Consensus:
TTTTTGAAAAATGGCATTGCGGCggA
>Cele-UNSB01_1:11183622-11184094 Satlength=473 Nr of Repeats=10 RepeatLength=35
seed=TTGCCGATTT
TTGCCGATTTGATGGAATTTTTCAGTACCGGCAAT
TTACCGATTTGCCGGAATTTTTCAATTCCGGCAAT
TTGCCGACTTGCCGGAAGTTTTCGTTCCGGCAAATTTATCGGT
TTGCCGATTTGCCGGCAATTTTCAATTTCGGCAAA
TTGCCGATTTATCGAAAAATTATTAATCCGGCAAT
TTGCCGATTTGCCGAAAATTTTCAATTTCGGAAAGCTGTCGGTTTTCCAAAAATTTTCAATTCTGGCAAA
TTGCCGGTTTGCCGGAAAGTTTCAATTCCTGCAAA
TTGCCGGTTTGCCGATTTGCCGAAATTTTTCAATTCCTGCAAA
TTGCCGGTTTGTCGATTTGCCAAAAATTTTCTATTCCGCCAATTTACCGTTTTGCCGAAAATTTTTAAATTTTGGCAAT
TTGCCGGAAAGTTTCAATTCCTGCAAA
TTGCCGATTTGCCGGAAATTTTCAATTCTGCCAAT
Nr of selected repeats 6 Similarity 0.702469
Consensus:
TTGCCGATTTGcCGGAAatTTTCAATtCCGGCAAt

>Cele-UNSB01_1:11199905-11200227 Satlength=323 Nr of Repeats=8 RepeatLength=35
seed=GCCGGAATTG
GCCGGAATTGAAATTTTCCGGCAAATTGGTAAATT
GCCGGAATCGAAATTTTCCGGCAAATCTACAAACT
GTCGGAATTGAAATTTTCCGACAAATCGGCAGATT
GCCGGAATTGAGATTTTTCGGCAAATCGGCAAATT
GCCGGAATTGAAATTTTCCGACAAATCGGCAGAGT
GCCGGAATTGAACATTTCCGGAAAATCAGCAAACCGGCAAATTGCCAGAATTCAAAATTCCGGCAAATCTGTAAATT
GCCGGAATTGAAAATTTCCGGCAAATCGGCAAATT
GCCGGAATTGAAAATTTCCGGCAAATCAGCAAATT
Nr of selected repeats 7 Similarity 0.829478
Consensus:
GCCGGAATTGAAAtTTTCCGgCAAATCgGCAaAtT
>Cele-UNSB01_1:11239237-11239369 Satlength=133 Nr of Repeats=12 RepeatLength=11
seed=TATGCCTAGC
TATGCCTAGCC
TATGCCTAGCC
TATGCCTAGCC
TATGCCTAGCC
TATGCCTAGCC
TATGCCTAGCC
TATGCCTAGCC
TATGCCTAGCC
TATGCCTAGCC
TATGCCTAGCC
TATGCCTAGCC
TATGCCTAGCC
Nr of selected repeats 12 Similarity 1.000000
Consensus:
TATGCCTAGCC
>Cele-UNSB01_1:11267568-11267793 Satlength=226 Nr of Repeats=15 RepeatLength=15
seed=CGATGCTCCA
CGATGCTCCACTCGA
CGATGCTCCACCCGG
CGATGCTCCACCCGA
CGATGCTCCACCCGG
CGATGCTCCACCCGA
CGATGCTCCACCCGA
CGATGCTCTACCCGG
CGATGCTCCACCCTG
CGATGCTCCACCCGA
CGATGCTCCACTCGA
CGATGCTCCACCCGA
CGATGCTCCACTCGA
CGATGCTCCACCCGG
CGATGCTCCACCCGG
CGATGCTCCACCCGA
Nr of selected repeats 15 Similarity 0.900106
Consensus:
CGATGCTCCACcCGa
>Cele-UNSB01_1:11268535-11268966 Satlength=432 Nr of Repeats=11 RepeatLength=34
seed=TTCCGGCAAA
TTCCGGCAAATCGGAAAATTGCCGGATATTAAAT
TTCCGGCAAATCGGAAAATTGCTGGAAACTAAAA

TTCCTGCAAATCGGCAAATTGCCGGAATTTAAAT
TTCCGGCAAATCGGCAAATCGGCAAATAGCCGGAATTTAAATTTCCAGCGAATCGGTAGATTGCCGGAATTTAAAT
TTCCGGCAAATCGGAAAATTGCCGGAATTTAAAT
TTCCGGCAAATCGGCAAAACGCTGGAAATTAAAC
TTCCGGCAAATCGGAAAATTGTCGGAATTTAAAT
TTCCGGCAAATCGGAAAAATGCTGGAAATTAAATTGCCGGAATTTAAAT
TTCCGGCAAATCAGAAAATTGCCGGAATTTAAAT
TTCCGGCAAATCGGCAAATAGCCGGAATTTAAAT
TTCCGGCAAATCGGCAAATTGCTGGAAAATAAAT
Nr of selected repeats 9 Similarity 0.844227
Consensus:
TTCCGGCAAATCGGaAAATtGCcGGAAttTAAAt
>Cele-UNSB01_1:11276130-11276202 Satlength=73 Nr of Repeats=6 RepeatLength=12
seed=CGATCTCCAC
CGATCTCCACCT
CGATCTCCACCA
CGATCTCCACCT
CGATCTCCACCA
CGATCTCCACCT
CGATCTCCACCA
Nr of selected repeats 6 Similarity 0.933333
Consensus:
CGATCTCCACCa
>Cele-UNSB01_1:11291527-11291639 Satlength=113 Nr of Repeats=7 RepeatLength=14
seed=AGCTTGAAGC
AGCTTGAAGCTTGG
AGCTTGAAGCTGGA
AGCTTGGAGCTTGA
AGCTGGAAGCTTGG
AGCTTGAAGCTAGG
AGCTTGGAGCTTAAAGCGTGG
AGCTTGAAGCTTGGAGTTTTC
Nr of selected repeats 5 Similarity 0.800000
Consensus:
AGCTtGaAGCTtGg
>Cele-UNSB01_1:11320046-11320214 Satlength=169 Nr of Repeats=4 RepeatLength=42
seed=TGCCGGAAAT
TGCCGGAAATTTCAAACCCGGTAGTTTGCCCATTTGCCGAGC
TGCCGGAAATTTCAAACCCGTCAATTTGTTGATTTGCCGATT
TGCCGGAAATTTGAAAGCCGGTAGTTTGCCCATTTGCCGAGC
TGCCGGAAATTTCAAACCCGGCAATTTGTTGATTTGTCGATT
Nr of selected repeats 4 Similarity 0.788360
Consensus:
TGCCGGAAATTTcAAAcCCGgcAaTTTGcccATTTGcCGAgc
>Cele-UNSB01_1:11351419-11351614 Satlength=196 Nr of Repeats=13 RepeatLength=15
seed=TCGTGGTGAG
TCGTGGTGAGACCCA
TCGTAGTGAGACCCA
TCGTAGTGAGACCCA
TCGTAGTGAGACCCT
TCGTGGTGAGGCCAA
TCGTGGTGAGACCCT
TCGTGGTGAGATCCA
TCGTGGTGAGACCCA

TCGTGGTGAGACCCA
TCGTAGTGAGACCCT
TCGTGGTGAGGCCAA
TCGTGGTGAGACCCT
TCGTGGTGAGATCCA
Nr of selected repeats 13 Similarity 0.842735
Consensus:
TCGTgGTGAGACCCa
>Cele-UNSB01_1:11352081-11352141 Satlength=61 Nr of Repeats=5 RepeatLength=12 seed=TATACATATA
TATACATATACA
TATACATATACA
TATACATATACA
TATACATATACA
TATACATATACA
Nr of selected repeats 5 Similarity 1.000000
Consensus:
TATACATATACA
>Cele-UNSB01_1:11361560-11361658 Satlength=99 Nr of Repeats=7 RepeatLength=14 seed=AACTACTAAC
AACTACTAACTACT
AACTACAAACTACA
AACTACAAACTATC
AACTACTAACTACT
AACTACTAACGACT
AACTACTAACTACT
AACTACTAACTACT
Nr of selected repeats 7 Similarity 0.803175
Consensus:
AACTACtAACTACt
>Cele-UNSB01_1:11393763-11394894 Satlength=1132 Nr of Repeats=15 RepeatLength=35 seed=TTCCGGCAAA
TTCCGGCAAAATCGGCAAATTGTCGGAACTGAAAGCTTCTGACAAACTGGCGAATTGCCGGAATTGAAAAG
TTCCGGCAAATCGGCAAATTGCCGGGACTGAAAAT
TTCCGACAAATGGGCAAATTGCCGGAATTGAAAAT
TTCCGGCAAACCGGCAAACGGCCAGAACTGAACAT
TTCCGGCAAATCGGCCAATTGTCTGAACTGAAAATATCTGGCAAATTGACAAATTGCCGGAATTGAAAAT
TTCGGCAAATTAACCAGTTGTCTGAACTGAAAATATCTGGCAAATAGGCAAATTGCCGAAACTAAGTAT
TTCCGGCAAATCAGCAAATTGCATGAACTTAAAAT
TTCCGGCAAATCGGCAAATTGCTGGAATTGTGAAT
TTCCGGCAAATCAGCAACTTGCTAGAACTGAAAGT
TTACGGCAAATCGGCAAATTGCCAGAACTGGGAAT
TTCCGGCAAATTGACAAAACGCAAGAACTGAAAAT
TTCCGGCAAACCGACAAATTGCCGAAACTGAAAAT
TTCCGGCAAATCGGCAAAATGCCGAAAATAAAAATATCTGACAAGTCGGCAAGCGTCGCTCTACATGACGAGTATTTAA
CTTTTTAACTCAAAATCAAAGCAAATTCACAGTTTAATTGAACTATAACCAAAAGTCCTCCATTTCCCATCAAATTCAC
CCAAAAAAAGTTCCAAAAAGCACTCTAAATCCCCCGATCCAGCCTGGAAAAACCCTCTAAAATTTCGGAAAAAAGATGC
TAAAATTTCAAGATGGGTGAGCGGAGATAGTGAGAGGCGGCTGGATGAGGCTGGGGAAGGGACGAGTGAATTTCAAGTC
ATCTTTCTTTTTTTTTTGCTGCCTTTTCTTCTTTTTTCCCCAATTTTCTTCGACTTTTCGGACATTCAAAGGGTCGGTG
AGACGCAGAGAACTGCAGTGTAATCGGACGTTAAATGTTGTCTTGAATTGTGTATGTTGAAATTTCGTAAGACGAAGAC
TACAAACTACAAATTGCGGTTTGTCTGAATTATTTCTAAAACAAACAAACAAAGACAAACTGT
TTCGGCAAATTGGCAAATTGCCGGAATTGTAAATTTACGGCAA
TTCGGCAAATTGCCGGAATTGTAAGT
Nr of selected repeats 9 Similarity 0.743915

Consensus:
TTCCGGCAAAtcggCAAAttGCcnGAAcTGaaAAT
>Cele-UNSB01_1:11398081-11398840 Satlength=760 Nr of Repeats=4 RepeatLength=35
seed=TCGGCAAATT
TCGGCAAATTACGGAAAATGAAAATTTCCGGCAAA
TCGGCAAATTACGGAAAATGAAAATTTCCGGCAAA
TCGGCAAATTACGGAAAATGAAAATTTCCGGCAAA
TCGGCAAATTGTCGGAATTGAAGGCGGAAGTCGGCGCGGCTGCCGTTTTGGCGCCTGCATGGAAGCCCCACAGCCTCAA
TCAAAAATCGCTACTCCCTTTTCTTTAACCTCCGGCTCTTTTTTTGTGCTAAAACTTGCTCTCTGAATAGAAAATCAAA
TCTTTTCAGAAGTTTCTTGAAGGGGGCCCTGACCTCTTGAAACTTTTCCACAACCCTCCGCCGCTTTCTTCTTCTTATT
CCCTTTTGCTCTCTCCGCATTTTTATGAATTTCTGAATAGAGGGAAACTGCGGCTCCGTGAGCACAAAGGTACTTTTTT
GATGGTGCCTGAGAGCCTGCTGAAACGGGAAGGGAATCGTAGGAGTTTTCGTCCAAATTCCACAATTTTCTCTTACTTT
ACACTCTGGGGGCTCCATCACCAAGTGACACTTACCCACAATGCCCTGGAAATGCACTTTACTCTGTCAGGATTTTTTT
GTTGTTGAAAAATCTGGATTTTCTAGGTGAAACTCTCTTGAAAATGTGGAATTTTTGACGGTTCTTTTTTTTTGCTCTT
TTCTGAAATTTTTCAGCTGTCAAAATTGACAATTTTCAGTTTTCACTTAAGTTTGACTGGAAATTTCGCCTGAAAATTA
AAGATTGCGGTGTGAAGCAGGT
Nr of selected repeats 3 Similarity 1.000000
Consensus:
TCGGCAAATTACGGAAAATGAAAATTTCCGGCAAA
>Cele-UNSB01_1:11408393-11409029 Satlength=637 Nr of Repeats=9 RepeatLength=35
seed=AATTGCCGAA
AATTGCCGAATTTCTCGTTTCTGCCAATTTACCAA
AATTGCCAAATTTCTCGTTTTTGGCAATTTTCAAA
AATTGCCAAGTTTCCGATTTCTGCCAATTTACCAA
AATTGCCGAATTTCTCGTTTTTGACAATTTTCAAA
AATTGCCGAATTTTTTCGGTTTTGGCAATTTATCAA
AATTGCCGAATTTCTCGGTTTTGGCAATTTACCAT
AATTGCCGAATTTCTCGTTTTTGGCAATTTACCAA
AATTGCCGAATTGCTCTTTTTTGGTCATTTCTAAAAATAGCCAAAGTCCTTATTTTTGGCAATTTACTAAAATTGAGTA
GTTGCAAATTTTCGGCAACTTTACAAAAATTGCACACATCGAAAGCCTGTGATTTTTTTTAAATCTCCAACTTTTAGAG
ACTTTCCAAAATGCCAAAATGTAAAAATTTCGGCAATTTTCAAAAATAGCCAAAACTCTCCTTTTTGGCAATTTACGAA
AATTTAATAGTTCCCATACTTCGGCAATTTTCAGAAAATTGCACAAATTTTTAAAAAATGTTCAGAATTTTGGGCAACT
TGGCGTTTTTTAAA
AATTGCCCAATTTCCCACTGTCCGGCAAGTTGCAAGTTCTCGATTTCCGGCAATTTTTGAG
Nr of selected repeats 7 Similarity 0.793210
Consensus:
AATTGCCgAATTTCTCGtTTtTGgCAATTTaCcAA
>Cele-UNSB01_1:11460977-11461721 Satlength=745 Nr of Repeats=60 RepeatLength=12
seed=CGTTGCGTGT
CGTTGCGTGTCG
CGTTGCATGTCG
CGTTGCGTGTCG
CGTTGCGTGTCG
CGTTGCGTGTGG
CGTTGCGTGTCG
CGTTGCGTGTGG
CGTTGCGTGTGG
CGTTGCGTGTTG
CGTTGTGTGTCGCTTTGTGTGTCG
CGTTGCGTGTCG
CGTTGCGTGTCG
CGTTGCATGTCG
CGTTGCGTGTCG
CGTTGCGTGTCG

CGTTGCATGTCG
CGTTGCGTGTCG
CGTTGTGTGTCG
CGTTGCGTGTCG
CGTTGCGTGTCG
CGTTGCGTGTGG
CGTTGCGTGTCG
CGTTGCGTGCCG
CGTTGCGTGTGG
CGTTGCGTGTCG
CGTTGCATGTCG
CGTTGCGTGTAG
CGTTGCGTGTGG
CGTTGCGTGTCG
CGTTGCGTGTCG
CGTTGCGTGCCG
CGTTGCGTGTCG
CGTTGCGTGTGG
CGTTGCGTGTCG
CGTTGCGTGTCGCGATGCATGTCG
CGTTGCGTGTGG
CGTTGCGTGTCG
CGTTGCGTGTGG
CGTTGCGTGTCG
CGTTGCATGTCG
CGTTGCGTGTCG
CGTTGCGTGTGG
CGTTGCGTGTCG
CGTTGCATGTCG
CGTTGCGTGTCA
CGTTGCGTGTGG
CGTTGCGTGTCG
CGTTGCGTGTGG
CGTTGCGTGTCG
CGTTGCATGTCG
CGTTGCGTGTCG
CGTTGCGTGTGG
CGTTGCGTGTCG
CGTTGCATGTCG
CGTTGCGTGTCA
CGTTGCGTGTGG
CGTTGCGTGTCG
CGTTGCGTGTCG
CGTTGCGTGTCA
CGTTGCGTGTGG
Nr of selected repeats 58 Similarity 0.901727
Consensus:
CGTTGCGTGTcG
>Cele-UNSB01_1:11481110-11481222 Satlength=113 Nr of Repeats=7 RepeatLength=16
seed=CTACATGCCT
CTACATGCCTAAATTT
CTACATGCCTATATGC
CTACATGCCTACATGC
CTACATTCCTACATGC

CTACATGGCTACATTT
CTACATGCCTACATGC
CTACACGCCTACATGC
Nr of selected repeats 7 Similarity 0.805556
Consensus:
CTACATGCCTAcATgc
>Cele-UNSB01_1:11481110-11482230 Satlength=1121 Nr of Repeats=68
RepeatLength=16 seed=CTACATGCCT
CTACATGCCTAAATTT
CTACATGCCTATATGC
CTACATGCCTACATGC
CTACATTCCTACATGC
CTACATGGCTACATTT
CTACATGCCTACATGC
CTACACGCCTACATGC
CTACATGCCTACATTC
CTACATGCCGACATGG
CTACATGGCTACATGG
CTACATGGCTACATGC
CTACATGTCTACATGCCTACATTT
CTACATGCCTAAATGC
CTACATGCCTACATGC
CTACATGCCTACATGC
CTACATGCCTACATGC
CTACATGCCTACATGC
CTACATGTCTACATGC
CTACATGCCTACATGC
CTACATTCCTACATGC
CTATATGCCTACATGC
CTACATGCCTACATTC
CTACAGGCCTACATGGCTACATTT
CTACATGCCTACATGC
CTACATTCCTACATGCCGACATGG
CTACATGGCTACATGG
CTACATGCCTACATGT
CTACATGCCTACATTT
CTACATGCCTAAATGC
CTACATGCCTACATGC
CTACATGCCTACATGC
CTACATGCCTACATGC
CTACATGCCTACATTC
CTACATGCCTATATGC
CTACATGCCTACATGC
CTACATTCCTACATGC
CTACATGGCTACATTT
CTACATGCCTACATGC
CTACACGCCTACATGC
CTACATGCCTACATTC
CTACATGCCGACATGG
CTACATGGCTACATGG
CTACATGCCTACATGT
CTACATGCCTACATTT
CTACATGCCTAAATGC
CTACATGCCTACATGC

CTACATGCCTACATGC
CTACATGCCTACATGC
CTACATGCCTACATGC
CTACATGCCTACATGC
CTACATGCCTACATGC
CTACACGCCTACATGC
CTACATTCCTACATGCCGACATGG
CTACATGGCTACATGG
CTACATGCCTACATGT
CTACATGCCTACATTT
CTACATGCCTACATTT
CTACATGCCTACATGC
CTACATGCCTATATGC
CTACATTCCTACATGC
CTACATGCCTACATGC
CTACATTCCTACATGC
CTATATGCCTACATGC
CTACATGCCTACATTC
CTACATGCCTACATGG
CTACATGGCTACATGC
CTACATGTCTACATGC
CTACATGCCTACATGC
Nr of selected repeats 64 Similarity 0.868304
Consensus:
CTACATGCCTACATGc
>Cele-UNSB01_1:11519985-11520529 Satlength=545 Nr of Repeats=18 RepeatLength=26 seed=ACCTACAGTA
ACCTACAGTAACCCAGGCATATAGTAGGATAAAGTACTTATAAGCTATAGTA
ATCTACAGTACCCCTATTCTAGAGTG
ACCTACAGTAACCTACAGAGAATCGAAGGATTCCAATCGAGCTATAGTAACCCACCGTAACCCGTAGCCGCTAGCTACA
GAACCCATATGCTACAGTA
ACCTACAGTATCCTCGACATTCCGCTAA
AGCTACAGTACCCCTGCTCTAGAATG
ACCTACAGTACCCTTGTCCTAGAGTG
ACCTACAGTACCCCTGTCCTAGAATG
ACCTACAGTATGCCTGTG
ACCTACAGTACCCCTGCCCTAGAATG
ACCTACAGTACCCTTGTCCTAGAGTG
ACCTACAGTACCCCTGTCCTAGAATG
ACCTACAGTATGCCTGTG
ACCTACAGTACCCCTGCCCTAGAATG
ACCTACAGTACCCTTGTCCTAGAGTG
ACCTACAGTACCCCTGTCCTAGAATG
ACCTACAGTATGCCTGTG
ACCTACAGTACCCCTGCCCTAGAGTG
ACCTACAGTACCCTTGTCCTAGAGTG
Nr of selected repeats 12 Similarity 0.881896
Consensus:
ACCTACAGTACCCcTGtCCTAGAaTG
>Cele-UNSB01_1:11521003-11522550 Satlength=1548 Nr of Repeats=48 RepeatLength=32 seed=GCAATTTTTA
GCAATTTTTAAGCTAAAAATCACGAAAAAAACC
GCAATTTTTAGGCTAAAAATCACTAAAAAACT
GCAATTTTTAGGATAAAAATCACTAAAAAACT

GCAATTTTTAGGCTAAAAATCACTTAAAAAACT
GCAATTTTTAGTCTAAAAATCACTAAACAACT
GCAATTTTTAGGCTAAAAATCACTAAACAACT
GCAATTTTTAGGCTAAAAATCACTAAAAAACT
GCAATTTTTAGGATAAAAATCACTAAAAAACT
GCAATCTTTAGGCTAAAAATCACTAAAAAACT
GCAATTTTTAGGATAAAAATCACTAAACAACC
GCAATTTTTAGGATAAAAATCACTAAACAACC
GCAATTTTTAGGATAAAAATCACTAAACAACC
GCAATTTTTAGGCTAAAAATCACTAAAAAACT
GCAATTTTTAGGCTAAAAATCACTTAAAAAACT
GCAATTTTTAGTCTAAAAATCACTAAACAACT
GCAATTTTTAGGCTAAAAATCACTAAACAACT
GCAATTTTTAGGCTAAAAATCACTAAAAAACT
GCAATTTTTAGGATAAAAATCACTAAAAAACT
GCAATTTTTAGGATAAAAATCACTAAACAACC
GCAATTTTTAGGATAAAAATCACTAAAAAACT
GCAATTTTTAGGATAAAAATCACTAAAAAACT
GCAATTTTTAGTCTAAAAATCACTAAAAAACT
GCAATTTTTAGTCTAAAAATCACTAAACAACT
GCAATTTTTAGGCTAAAAATCACTAAAAAACT
GCAATTTTTAGGATAAAAATCACTAAAAAACT
GCAATTTTTAGGCTAAAAATCACTTAAAAAACT
GCAATTTTTAGGCTAAAAATCACGAAAAAATT
GTAATTTTTAAGCAAAAAATCACAAAAAAACC
GCAATTTTTAAGCTAAAAATCACGAAAAAACT
GCAATTTTTAGGCTAAAAATCACTTAAAAAACC
GCAATTTTTAAGCTGAAAATCACAAAAAAAACT
GCAATTTTTAGGATAAAAATCAGTAAAAAACT
GCAATTTTTAGGCTAAAAATCACTTAAAAAACT
GCAATTTTTAGGCTAAAAATCACGAAAAAATT
GTAATTTTTAAGCAAAAAATCACGAAAAAACT
GCAATTTTTAGGATAAAAATCACTAAACAACC
GCAATTTTTAGGCTAAAAATCACTAAAAAACT
GCAATTTTTAGGATAAAAATCACTAAAAAACT
GCAATTTTTAGGCTAAAAATCACTTAAAAAACT
GCAATTTTTAGTCTAAAAATCACGAAAAAACC
GCAATTTTTAAGCTAAAAATCACGAAAAAACA
GCAATTTTTAAGCTGAAAATCACAAAAAAAAACT
GCAATTTTTAAGCTAAAAATCACTAAAAAACT
GCATTTTTTAAGCTAAAATCACTAAAAAACT
GCAATTTTTAGGATAAAAATCACTAAAAAACC
GCAATTTTTAAGCTGAAAATCACAAAAAAAAACT
GCAATTTTTAAGCTAAAAATCACTAAAAAACT
GCATTTTTTAAGCTAAAAATCACTAAAAAACT
Nr of selected repeats 37 Similarity 0.890140
Consensus:
GCAATTTTTAGGcTAAAAATCACTAAAaAACt
>Cele-UNSB01_1:11550058-11550130 Satlength=73 Nr of Repeats=6 RepeatLength=12
seed=TATATGTATA
TATATGTATATG
TATATGTATATG
TATATGTATATG
TATATGTATATG

TATATGTATATG
TATATGTATATG
Nr of selected repeats 6 Similarity 1.000000
Consensus:
TATATGTATATG
>Cele-UNSB01_1:11556393-11558176 Satlength=1784 Nr of Repeats=47
RepeatLength=35 seed=GGCAAATCAG
GGCAAATCAGCAAATTGCGGGAATTGAAAATTTCC
GGCAAATCAGCAAGTTACCGGAATTGAAAATTTCC
GGCAAATCAGCAAATTGCCGGAATTGAAAATTTCC
GGCAAATCAGCAAGTTGCCGGAATTGAAAATTTCG
GGCAAATCAGCAAATTGCGGGAATTGAAAATTTCC
GGCAAATCAGCAAGTTGCCGGAATTGAAAATTTCC
GGCAAATCAGCAAATTGCCGGAATTGAAAATTTTC
GGCAAATCAGCAAGATGCCGGAATTGAAAATTTCC
GGCAAATCAGCAAATTGCCGGAATTGAAAATTTCC
GGCAAATCAGCAAATTGTCGGAATTGAAAATTTCC
GGCAAATCAGCAAATTCCCGGAATTGAAAATTTCC
GGCAAATCAACAAATTGCCGGAATTGAAAATTTCC
GGCAAATCAGCAAATTGCCGGAATTGAAATATTCC
GGCAAATCAGCAAATTGCCGGAGTTGAAAATTTTT
GGCAAATCAGCAAATTGCCGGAGTTGAAAATTTTT
GGCAAATCAGCAAATTGCCGGAATTGAAAATTTCG
GGCAAATCAGCAAATTGCCGGAATTGAAAATTTCTTACTAATAAGCAAATTGCCGGAATTGAAAATTTCC
GGCAAATCAGCTAATTACCGGAATTGAAAATTTCC
GGCAAATCAGCAAATTGCCGGAATTAAAAATTTCC
GGCAAATCAGCAAATTGCCGGAATTGAAAATTTCG
GGCAAATCAGCAAATTGCCGGAATTGAAAATTTCC
GGCAAATCAGCAAATTGCCGGAATTGAAAGTTTCG
GGCAAATCAGCAAATTGTCGGAATTGAAAATTTCG
GGCAAATCAGCAAATTGCCGGAATTGAAAATTTCG
GGCAAATCAGCAAATTGACGGAATTGAAAATTTCG
GGCAAATCAGCAAATTGCCGGAATTGAAAATTTCG
GGCAAATCAGCAAATTGCCGGAGTTGAAAATTTTT
GGCAAATCAGCAAATTGCCGGAGTTGAAAATTTTT
GGCAAATCAGCAAATTGCCGGAATTGAAAATTTCG
GGCAAATCAGCAAATTGCCGGAATTGAAAATTTCTTACTAATAAGCAAATTGCCGGAATTGAAAATTTCC
GGCAAATCAGCTAATTACCGGAATTGAAAATTTCC
GGCAAATCAGCAAATTGCCGGAATTAAAAATTTCC
GGCAAATCAGCAAATTGCCGGAATTGAAAATTTCG
GGCAAATCAGCAAATTGCCGGAATTGAAAATTTCC
GGCAAATCAGCAAATTGCCGGAATTGAAAGTTTCG
GGCAAATCAGCAAATTGTCGGAATTGAAAATTTCG
GGCAAATCAGCAAATTGCCGGAATTGAAAATTTCG
GGCAAATCAGCAAATTGACGGAATTGAAAATTTCG
GGCAAATCAGCAAATTGCCGGAATTGAAAATTTCG
GGCAAATCAGCAAATTGCCGGAATTGAAAATTTCTTAGTAATAAGCAAATTGCCGGAATTGAAAATTTCC
GGCAAATCAGCTAATTACCGGAATTGAAAATTTCC
GGCAAATCAGCAAATTGCCGGAATTAAAAATTTCC
GGCAAATCAGCAAATTGCCGGAATTGAAAATTTCC
GGCAAATCAGCAAATTGCCGGAATTGAAAATTTCG
GGCAAATCAGCAAATTGCCGGAATTGAAAATTCCGTGCAAATAGGCAAATTACCGGTTTTGAAAATTTC
GGCAAATCGGCAAATTCGGAAATTGAACATTTCT
GGCAAATCGGCAAATTGCCGGAATTGATAATTTCC

Nr of selected repeats 42 Similarity 0.911023
Consensus:
GGCAAATCAGCAAATTGCCGGAATTGAAAATTTCc
>Cele-UNSB01_1:11564527-11564628 Satlength=102 Nr of Repeats=5 RepeatLength=20 seed=TGTAACAATC
TGTAACAATCTATTTTTTTG
TGTAACAATCTATTTTTTCG
TGTAACAATCTATTTTTTCG
TGTAACAATCTATTTTTTCAT
TGTAACAATCTATTCTTTCG
Nr of selected repeats 4 Similarity 0.933333
Consensus:
TGTAACAATCTATTtTTTcG
>Cele-UNSB01_1:11591612-11591946 Satlength=335 Nr of Repeats=14 RepeatLength=20 seed=AAAAATCAAT
AAAAATCAATAATTTCATCG
AAAAATCAATAATTTCATCG
AAAAATCAATAATTTCATCG
AAAAATCAATAATTTCATCG
AAAAATCAATAATTTCATCG
AAAAATCAATTATTTTAAC
AAAAATCAATAATTTCCACA
AAAAATCGATAACTTCATCA
AAAAATCGATAATTTCATCA
AAAAATCAATAATTTCATCA
AAAAATCAATAATTTCATCAAAAAGTCGATAATTTCAACA
AAAAATCAATATTTTCATCG
AAAAATCAATAATTTCAACA
AAAAATCAATAATTTCATCAAAAAGTCGATAATTTTACCGAATTTTCCACAACAA
Nr of selected repeats 11 Similarity 0.883636
Consensus:
AAAAATCAATAATTTCATCg
>Cele-UNSB01_1:11592271-11597890 Satlength=5620 Nr of Repeats=277 RepeatLength=20 seed=ATTGATTTTT
ATTGATTTTTTGAGAAAATT
ATTGATTTTTTGAGAAAATT
ATTGATTTTTTGAGAAAATT
ATTGATTTTTTGAGAAAATT
ATTGATTTTTTGAGAAAATT
ATTGATTTTTTGAGAAAATT
ATTGATTTTTTGAGAAAATT
ATTGATTTTTTGAGAAAATT
ATTGATTTTTTGAGAAAATT
ATTGATTTTTTGAGAAAATT
ATTGATTTTTTGAGAAAATT
ATTGATTTTTTGAGAAAATT
ATTGATTTTTTGAGAAAATT
ATTGATTTTTTGAGAAAATT
ATTGATTTTTTGAGAAAATT
ATTGATTTTTTGAGAAAATT
ATTGATTTTTTGAGAAAATT
ATTGATTTTTTGAGAAAATT
ATTGATTTTTTGAGAAAATT
ATTGATTTTTTGAGAAAATT

ATTGATTTTTTGAGAAAATT
ATTGATTTTTTGAGAAAATT
ATTGATTTTTTGAGAAAATT
ATTGATTTTTTGAGAAAATT
ATTGATTTTTTGAGAAAATT
ATTGATTTTTTGAGAAAATT
ATTGATTTTTCGATGAAATT
ATTGATTTTTCGATGAAATT
ATTGATTTTTCGATGAAATT
ATTGATTTTTCGATGAAATT
ATTGATTTTTCGATGAAGTT
ATTGATTTTTCGATGAAATTATAGACTTTTCGATGAAATT
ATCGATTTTTCGATGAAATTATAGACTTTTCGATGAAATTATAGACTTTTCGATGAAATT
ATCGATTTTTTGATGAAGTT
ATTGATTTTTTGAGGAAATT
ATTGATTTTTTGAGGAAATT
ATTGATTTTTTGAGGAAATT
ATCGATTTTTTGAGGAAATT
ATTGATTTTTTGAGGAAATT
ATCGATTTTTTGGTGAAATT
ATTGATTTTTTGGTGAAATT
ATTGATTTTTTGATGAAATT
ATCGATTTTTTGAGGAAATT
ATTGATTTTTTGGTGAAATT
ATTGATTTTTTGATGAAATT
ATCGATTTTTTGATGAAGTT
ATTGATTTTTTGAGGAAATT
ATTGATTTTTTGGTGAAATT
ATTGATTTTTTGAGGAAATT
ATTGATTTTTTGGTGAAATT
ATTGATTTTTTGAGGAAGTT
ATTGATTTTTTGGTGAAATT
ATTGATTTTTTGAGGAAATT
ATTGATTTTTTGGTGAAATT
ATTGATTTTTTGATGAAATT
ATCGATTTTTTGATGAAATT
ATCGATTTTTTGATGAAGTT
ATTGATTTTTTGAGGAAATT
ATTGATTTTTTGGTGAAATT
ATTGATTTTTTGAGGAAGTT
ATTGATTTTTTGGTGAAATT
ATTGATTTTTTGAGGAAATT
ATTGATTTTTTGAGGAAATT
ATTGATTTTTTGAGGCAATT
ATTGATTTTTTGAGGAAATT
ATTGATTTTTTGAGGAAATT
ATTGATTTTTTGATGAAATT
ATCGATTTTTTGATGAAGTT
ATTGATTTTTTGAGGAAATT
ATTGATTTTTTGATGAAATT
ATCGATTTTTTGATGAAGCT
ATTGATTTTTTGAGGAAATT
ATTGATTTTTTGAGAAAATT
ATTGATTTTTTGAGAAAATT

ATTGATTTTTTGAGAAAATT
ATTGATTTTTTGAGAAAATT
ATTGATTTTTTGAGAAAATT
ATTGATTTTTTGAGAAAATT
ATTGATTTTTTGAGAAAATT
ATTGATTTTTTGAGAAAATT
ATTGATTTTTTGAGAAAATT
ATTGATTTTTTGAGAAAATT
ATTGATTTTTTGAGAAAATT
ATTGATTTTTTGAGAAAATT
ATTGATTTTTTGAGAAAATT
ATTGATTTTTTGAGAAAATT
ATTGATTTTTTGATGAAGTT
ATTGATTTTTTGAGGAAATT
ATTGATTTTTTGAGAAAATT
ATTGATTTTTTGAGAAAATT
ATTGATTTTTTGAGAAAATT
ATTGATTTTTTGAGAAAATT
ATTGATTTTTTGAGAAAATT
ATTGATTTTTTGAGAAAATT
ATTGATTTTTTGAGAAAATT
ATTGATTTTTTGAGAAAATT
ATTGATTTTTTGAGAAAATT
ATTGATTTTTTGAGAAAATT
ATTGATTTTTTGAGAAAATT
ATTGATTTTTTGATGAAGTT
ATTGATTTTTTGAGGAAATT
ATTGATTTTTTGAGAAAATT
ATTGATTTTTTGAGAAAATT
ATTGATTTTTTGAGAAAATT
ATTGATTTTTTGAGAAAATT
ATTGATTTTTTGAAAAAATT
ATTGATTTTTTGAGAAAATT
ATTGATTTTTTGATGAAGTT
ATTGATTTTTTGAGGAAATT
ATTGATTTTTTGAGAAAATT
ATTGATTTTTTGATGAAGTT
ATTGATTTTTTGATGAAGTT
ATTGATTTTTTGAGAAAATT
ATTGATTTTTTGAGAAAATT
ATTGATTTTTTGAGAAAATT
ATTGATTTTTTGAGAAAATT
ATTGATTTTTTGATGAAGTT
ATTGATTTTTTGAGGAAATT
ATTGATTTTTTGAGAAAATT
ATTGATTTTTTGAGAAAATT
ATTGATTTTTTGATGAAATT
ATCGATTTTTTGATGAAGTT
ATTGATTTTTTGATGAAGTT
ATTGATTTTTTGAGGAAATT
ATTGATTTTTTGAGGAAATT
ATTGATTTTTTGAGGAAATT
ATTGATTTTTTGGTGAAATT
ATTGATTTTTTGATGAAATT

ATCGATTTTTTGAGGAAATT
ATTGATTTTTTGGTGAAATT
ATTGATTTTTTGATGAAATT
ATCGATTTTTTGATGAAGTT
ATTGATTTTTTGGTGAAATT
ATTGATTTTTTGAGGAAGTT
ATTGATTTTTTGGTGAAATT
ATTGATTTTTTGAGGAAATT
ATTGATTTTTTGAGGAAATT
ATTGATTTTTTGAGGCAATT
ATTGATTTTTTGAGGAAATT
ATTGATTTTTTGAGGAAATT
ATTGATTTTTTGAGGAAATT
ATTGATTTTTTGAGGAAGTT
ATTGATTTTTTGAGGAAATT
ATTGATTTTTTGGTGAAATT
ATTGATTTTTTGATGAAATT
ATTGATTTTTTGAGGAAGTT
ATTGATTTTTTGAGGAAATT
ATTGATTTTTTGAGGAAATT
ATTGATTTTTTGATGAAATT
ATCGATTTTTTGATGAAGCT
ATTGATTTTTTGAGGAAATT
ATTGATTTTTTGATGAAATT
ATCGATTTTTTGATGAAGCT
ATTGACTTTTTGAGGAAATT
ATTGATTTTTTGATGAAATT
ATTGATTTTTTGAGAAAATT
ATTGATTTTTTGAGAAAATT
ATTGATTTTTTGAGAAAATT
ATTGATTTTTTGAGAAAATT
ATTGATTTTTTGAGAAAATT
ATTGATTTTTTGATGAAGTT
ATTGATTTTTTGAGGAAATT
ATTGATTTTTTGGTGAAATT
ATTGATTTTTTGATGAAATT
ATCGATTTTTTGATGAAATT
ATCGATTTTTTGATGAAATT
ATCGATTTTTTGATGAAGCT
ATTGACTTTTTGAGGAAATT
ATTGATTTTTTGATGAAGTT
ATTGATTTTTTGAGGAAATT
ATTGATTTTTTGGTGAAATT
ATTGATTTTTTGATGAAATT
ATCGATTTTTTGATGAAATT
ATCGATTTTTTGATGAAGTT
ATTGATTTTTTGATGAAGTT
ATTGATTTTTTGAGGAAATT
ATTGATTTTTTGAGGAAATT
ATTGATTTTTTGAGGAAATT
ATTGATTTTTTGGTGAAATT
ATTGATTTTTTGATGAAATT
ATCGATTTTTTGAGGAAATT
ATTGATTTTTTGGTGAAATT

ATTGATTTTTTGATGAAATT
ATCGATTTTTTGATGAAGTT
ATTGATTTTTTGGTGAAATT
ATTGATTTTTTGAGGAAGTT
ATTGATTTTTTGGTGAAATT
ATTGATTTTTTGAGGAAATT
ATTGATTTTTTGAGGAAATT
ATTGATTTTTTGAGGCAATT
ATTGATTTTTTGAGGAAATT
ATTGATTTTTTGAGGAAATT
ATTGATTTTTTGAGGAAATT
ATTGATTTTTTGAGGAAGTT
ATTGATTTTTTGAGGAAATT
ATTGATTTTTTGGTGAAATT
ATTGATTTTTTGGTGAAATT
ATTGATTTTTTGATGAAATT
ATTGATTTTTTGAGGAAGTT
ATTGATTTTTTGAGGAAATT
ATTGATTTTTTGAGGAAATT
ATTGATTTTTTGATGAAATT
ATCGATTTTTTGATGAAGCT
ATTGATTTTTTGAGGAAATT
ATTGATTTTTTGATGAAATT
ATCGATTTTTTGATGAAGCT
ATTGACTTTTTGAGGAAATT
ATTGATTTTTTGATGAAATT
ATTGATTTTTTGAGAAAATT
ATTGATTTTTTGAGAAAATT
ATTGATTTTTTGAGAAAATT
ATTGATTTTTTGAGAAAATT
ATTGATTTTTTGAGAAAATT
ATTGATTTTTTGATGAAGTT
ATTGATTTTTTGAGGAAATT
ATTGATTTTTTGGTGAAATT
ATTGATTTTTTGAGGAAGTT
ATTGATTTTTTGGTGAAATT
ATTGATTTTTTGAGGAAATT
ATTGATTTTTTGAGGAAATT
ATTGATTTTTTGAGGAAATT
ATTGATTTTTTGAGGAAATT
ATTGATTTTTTGAGGAAATT
ATTGATTTTTTGAGGAAGTT
ATTGATTTTTTGAGGAAATT
ATTGATTTTTTGGTGAAATT
ATTGATTTTTTGATGAAATT
ATTGATTTTTTGAGGAAATT
ATTGATTTTTTGAGGAAATT
ATTGATTTTTTGAGGAAGTT
ATTGATTTTTTGAGGAAATT
ATTGATTTTTTGAGGAAATT
ATTGATTTTTTGATGAAATT
ATCGATTTTTTGATGAAGCT
ATTGATTTTTTGAGGAAATT
ATTGATTTTTTGATGAAATT

ATCGATTTTTTGATGAAGCT
ATTGATTTTTTGAGGAAATT
ATTGATTTTTTGATGAAATT
ATTGATTTTTTGATGAAGTT
ATTGATTTTTTGAGGAAATT
ATTGATTTTTTGAGGAAATT
ATTGATTTTTTGAGGAAATT
ATTGATTTTTTGATGAAGTT
ATTGATTTTTTGAGGAAATT
ATTGATTTTTTGAGGAAATT
ATTGATTTTTTGGTGAAATT
ATTGATTTTTTGAGGAAATT
ATTGATTTTTTGGTGAAATT
ATTGATTTTTTGGTGAAATT
ATTGATTTTTTGATGAAATT
ATAGATTTTTTGTGGAAATT
ATTGATTTTTTGATGAAGTT
ATCGATTTTTGTTGAAATA
ATTGATTTTTCGATGAAATT
ATTGATTTTTTGAGGAAATT
ATTGATTTTTCGATGAAATTATCGGTTTTTTGATGAAGTT
ATTGATTTTTTGATGAAGTT
ATTGATTTTTTGAGGAAATT
ATTGATTTTTTGAGGAAATT
ATTGATTTTTTGGTGAAATT
ATTGATTTTTTGAGGAAATT
ATTGATTTTTTGAGGAAATT
ATTGATTTTTTGGTGAAATT
ATTGATTTTTTGGTGAAATT
ATTGATTTTTTGATGAAATT
ATAGATTTTTTGATGAAGTT
ATTGATTTTTTGAGGAAGTT
ATTGATTTTTTGAGGAAATT
ATTGATTTTTTGGTGAAATT
ATTGATTTTTTGATGAAATT
ATCGATTTTTTTATGAAGTT
ATTGATTTTTTGAGGAAATT
ATTGATTTTTTGGTGAAATT
ATTGATTTTTTGATGAAATT
ATCGATTTTTTGATGAAGCT
ATTGATTTTTTGAGGAAATT
Nr of selected repeats 273 Similarity 0.884518
Consensus:
ATTGATTTTTTGAggAAATT
>Cele-UNSB01_1:11615647-11616001 Satlength=355 Nr of Repeats=10 RepeatLength=30
seed=CCTTGGGCTC
CCTTGGGCTCCGACTTCAGCTCCAGGGGGTC
CCTGGGCTCCGTTTTGATATCCATGTGCTCCTGGAGCAGGTCCATAGGAAGCTTGAGGTGGTGTGGGGGCTCCATAGCT
TGCCGGTGGTGGA
CCTTGGGCTCCCTGAGCTCCAAATTGTGTT
CCTTGGGCTCCTTGTGCTCCAAAATGTGCT
CCTTGGGCTCCTTGTGCTCCAAAATGTGCT
CCTTGGGCTCCCTGAGCTCCAAATTGTTCT
CCTTGGGCTCCCTGAGCTCCGAATTGTGCT

CCTTGGGCTCCTTGTGCTCCAAAATGTGCT
CCTTGGGCTCCCTGAGCTCCAAATTGTACT
CCTTGTGCTCCAAAATGTGCT
Nr of selected repeats 7 Similarity 0.875132
Consensus:
CCTTGGGCTCCcTGaGCTCCAAAtTGTgCT
>Cele-UNSB01_1:11631541-11632264 Satlength=724 Nr of Repeats=15 RepeatLength=11
seed=GTACTGTAGG
GTACTGTAGGG
GTACTGTAGGTATACGGTAGGATTACTGTAGTTTGAGAAAATTTGAATTTTCAGCTTTTGAACAGGTATTGGGTTGGGA
GTTGGTGGGGGATAATGTCAAG
GTACTGTAGTG
GTACTGTAGGG
GTACTGTAAGTATACGGTAGGGTTACTGTAGTTTGGGAAAATTTGAATTTTAGCTTTTGAACAGATATTGGGTCGGGAG
TTGATGTGGGATAATATCAAGGTATTGTGGTG
GTACTGTAGGC
GTACTGTAGGTATACGGTAGGATTACTGTAGTTTAGGAAAATTTGAATCTTTAGCTTTTGAAGAGATAGTGGGTTGGGA
GTTGGTGGGGGATAATGTCAAG
GTACTGTAGGT
GCACTGTAGGG
GTACTGTAGGTATACGGTAGGATTACTGTAGTTGGAGAAAATTTGAATTTTAGCTTTTGAACAGGTATTGGGTTGGGAG
TTGGTGGGGATGATGTCAAG
GTACTGTAGTA
GTACTGTAGGG
GTACTGTAGGTATACGGTAGGATTACTGTAGTTTGAGAAAATTTGAATTTTAGCTTTTGAACAGATATTGGGTCGGGAG
TTGATGTGGGATAATATCAAGGTATTGTGGTG
GTACTGTAGGC
GTACTGTAGGTATACGGTAGGATTACTGTAGTTTAGGAAAGTTTGAATCTTTAGCTTTTGAAGAGATAGTGGGTTGGGA
GTTGGTGGGGGATAATGTCAAG
Nr of selected repeats 9 Similarity 0.841751
Consensus:
GTACTGTAGgg
>Cele-UNSB01_1:11636321-11636670 Satlength=350 Nr of Repeats=9 RepeatLength=35
seed=ATTCCGGCAA
ATTCCGGCAATTTGCCGGTCTGCCGATTTGCCGGAAATTTTTA
ATTCCGGCAATTTGCTAAATGTTTTCA
ATTCCGGCAATTTGTCGATTTAACGAAAATTTACATTTCCGGAAATTTGCCATTTTCCAGAAATTTTCA
ATTCCGGCAATTTGCCGATTTTCCAGAAATTTTCA
ATTCCGGCAATTTGCTGATTTTCCAAAAATTTACA
ATTCCGGCAATTTGCCGATTTTCCAGAAATTTTCA
ATTCCGGCAATTTGCTGATTTTCCAAAAATTTACA
ATTCCGGCAATTTGCCGATTTTCCAGAAATTTTCA
ATTCCGGCCATTTGCCGATTTGCCGGAAATTTTCC
Nr of selected repeats 6 Similarity 0.888254
Consensus:
ATTCCGGCAATTTGCcGATTTTCCAgAAATTTtCA
>Cele-UNSB01_1:11643779-11644036 Satlength=258 Nr of Repeats=6 RepeatLength=34
seed=ATGCACCATG
ATGCACCATGTTTGGCTCACTCTCAATAGTTTTAAAATGGTTTGATAAATTTAGGAGGAACCAATGTATAAGATGTTAA
AATTATTCGGGGTCATATCA
ATGCACCATGCCAAAAAATATATCCAAAACCCCG
ATGCACCGTGTCCAAAAGTATATATAAAAGTGCG
ATGCACCATGTTCAAAAATATATTCAAAAATATG
ATGCTCCATGTTCAAAAATACG

ATGCACCATGTCAAAAAGTATATCTTGAAGTATG
Nr of selected repeats 4 Similarity 0.679739
Consensus:
ATGCACCaTGtcaAAAAaTATATccaaAAgtacG
>Cele-UNSB01_1:11661653-11661731 Satlength=79 Nr of Repeats=6 RepeatLength=12
seed=AAGCCTAAGC
AAGCCTAAGCCT
AAGCCTAAGCCT
AAGCCTAAGCCT
AAGCCTAAGCCT
AAGCCTAAGCCT
AAGCCTAAGCCTAAGCCA
Nr of selected repeats 5 Similarity 1.000000
Consensus:
AAGCCTAAGCCT
>Cele-UNSB01_1:11662428-11662542 Satlength=115 Nr of Repeats=9 RepeatLength=12
seed=GCTTAGGCTT
GCTTAGGCTTTG
GCTTGGGCTTAG
GCTTACGCTTAG
GCTTAGGCTTAG
GTTTAGGCTTAGGCACAT
GCTTAGGTTTAG
GCTTAGGCTTAG
GCTTTGGCTTGG
GTTTAGGCTTAG
Nr of selected repeats 8 Similarity 0.813492
Consensus:
GCTTaGGCTTaG
>Cele-UNSB01_1:11667166-11667423 Satlength=258 Nr of Repeats=6 RepeatLength=34
seed=ATGCACCATG
ATGCACCATGTTTGGCTCACTCTCAATAGTTTTAAAATGGTTTGATAAATTTAGGAGGAACCAATGTATAAGATGTTAA
AATTATTCGGGGTCATATCA
ATGCACCATGCCAAAAAATATATCCAAAACCCCG
ATGCACCGTGTCCAAAAGTATATATAAAAGTGCG
ATGCACCATGTTCAAAAATATATTCAAAAATATG
ATGCTCCATGTTCAAAAATACG
ATGCACCATGTCAAAAAGTATATCTTGAAGTATG
Nr of selected repeats 4 Similarity 0.679739
Consensus:
ATGCACCaTGtcaAAAAaTATATccaaAAgtacG
>Cele-UNSB01_1:11682141-11682225 Satlength=85 Nr of Repeats=7 RepeatLength=12
seed=GGCTTAGGCT
GGCTTAGGCTTT
GGCTTAGGCTTA
GGCTTAGGCTTA
GGCTTAGGCTTA
GGCTTAGGCTTA
GGCTTAGGCTTA
GGCTTAGGCTTA
Nr of selected repeats 7 Similarity 0.968254
Consensus:
GGCTTAGGCTTA

>Cele-UNSB01_1:11685636-11685851 Satlength=216 Nr of Repeats=5 RepeatLength=43
seed=CGGCAAATCG
CGGCAAATCGGCAAATCGGCAACTTGCCGGAATTGAAAATTTC
CGGCAAATCGGCAAACCAGCAAGTTGCCAAAAATCAAAATTTC
CGGCAAATCGGCAAAGTAGCAAATTTCTGGAATTGAAAATTTC
CGGCAAATCGGCAAACCGCCAATTTGCTGAAAATGAAACTTTC
CGGCAAATCGGCAAACCGGCAATTTGCCGAAAATGAAAATTTC
Nr of selected repeats 5 Similarity 0.801550
Consensus:
CGGCAAATCGGCAAAccggCAAnTTgCcgaAAaTgAAAaTTTC
>Cele-UNSB01_1:11691383-11691503 Satlength=121 Nr of Repeats=8 RepeatLength=15
seed=TCGTGGTGAG
TCGTGGTGAGACCCA
TCGTGGTGAGACCCT
TCGTGGTGAGACCCA
TCGTGGTGAGACCCT
TCGTGGTGAGACCCA
TCGTGGTGAGATCCA
TCGTGGTGAGACCCT
TCGTGGTGAGACCCA
Nr of selected repeats 8 Similarity 0.930159
Consensus:
TCGTGGTGAGACCCa
>Cele-UNSB01_1:11691539-11692754 Satlength=1216 Nr of Repeats=24
RepeatLength=35 seed=TGGCGGGAAA
TGGCGGGAAATTCAAATTTTCAGTGAAAAAATTT
TGGCGGGAAATTCAAATTTTCAGTGAAAAAAATTT
TGGCGGGAAATTCAAATTTTCAGTGAAAAAATTTG
GGCGGGAAATTCAAATTTTCAGTGAAAAAAATTT
TAGCGGGAAAATCAAGTTTTCAGTAAAAAAATTTT
TGGCGGGAAATTCAAATTTTCAGTGAAAAAAATTT
TGGCGGGAAATTCAAATTTTCAGTGAAAAAATTTG
GGCGGGAAATTCAAATTTTCAGTGAAAAAAATTT
TAGCGGGAAAATCAAGTTTTCAGTAAAAAAATTTT
TGGCGGGAAATTCAAATTTTCAGTGAAAAAAATTT
TGGCAGGAAATTCAAATTTTCAGTGAAAAAAAATTT
TGGCGGGAAATTAAAATTTTCAGTGAAAAAAATTT
TAGCGGGAAAATCAAGTTTTCAGTAAAAAAATTTT
TGGCGGGAAATTCAAATTTTCAGTGAAAAAAATTT
TGGCAGGAAATTCAAATTTTCAGTGAAAAAAAATTT
TGGCGGGAAATTAAAATTTTCAGTGAAAAAAATTT
TAGCGGGAAATTCAAATTTGCAGTGTAAAAAATTT
TGGCAGGAAATTCAAATTTTCAGTGAAAAAAAATT
TGGCGGGAAATTCAAATTTTCAGTGAAAAAAAATT
TAGCGGGAAATTCAAATTTTCAGTGAAAAAAATTT
TAGCGGGAAAATCAAGTTTTCAGTAAAAAAATTTT
TGGCGGGAAATTCAAATTTTCAGTGAAAAAAATTT
TGGCAGGAAATTCAAATTTTCAGTGAAAAAAAATTT
TGGCGGAAAATTCCAATTTCTGAGAAAAATCGAGAAATGTCTGCAATGTTCCAGAAGTTTCTAGAAAATTCGAGAAAAC
TCCGGAATGGTCCAGAATTTTCTAGAAAATTCTAGAAAGTTCTGGAATGGTCCAGAAGTTTCTAGAAAATTCGAGAAAA
TTCCGGAATGGTCCAGAATTTTCTAGAAAATTCTAGAAAGTTCTGGAATGGTCCAGAATTTTCTAGAAAATTCGGGAAA
ATTCTGGAATATTCCAGAACTTTCTAGAAAAATCGGGAAAAGTCTGCAATGTTCCAGAACTAAAAAATTCGATAAAACT
CTGAAATGTTCAATTTCGTGGAAAAAATTCAAGAAACTTCTGCAAAGTTCTACACGGGGTTCTGGCTCGATCCCGCGCTC
CATTGGACACTGAAA

Nr of selected repeats 17 Similarity 0.860771
Consensus:
TgGCGGGAAAtTCAAaTTTTCAGTgAAAAAAATTT
>Cele-UNSB01_1:11696177-11696255 Satlength=79 Nr of Repeats=6 RepeatLength=12
seed=TAGGTCTAGG
TAGGTCTAGGTC
TAGGTCTAGGTC
TAGGTCTAGGTC
TAGGTCTAGGTC
TAGGTCTAGGTC
TAGGTCTAGGTCTAGGTC
Nr of selected repeats 5 Similarity 1.000000
Consensus:
TAGGTCTAGGTC
>Cele-UNSB01_1:11696998-11697105 Satlength=108 Nr of Repeats=5 RepeatLength=19
seed=TCAAAAATCA
TCAAAAATCAAAAATTTCATCAAAAATTTCA
TCAAAAATCAATAATTTCA
TCAAAAATCAATAATTTCA
TCAAAAATCAATAATTTGC
TCAAAAATCAATAATTTCT
Nr of selected repeats 4 Similarity 0.873611
Consensus:
TCAAAAATCAATAATTTCa
>Cele-UNSB01_1:11697315-11697448 Satlength=134 Nr of Repeats=7 RepeatLength=19
seed=TTATCGATTT
TTATCGATTTTCCGAGAAAT
TTTCGATTTTTTATGAAA
TTATCGATTTTTCCGGAAA
TTATCGATTTTTCAAGAAT
TTATCGATTTTTCAAGAAA
TTATCGATTTTTCCAGAAA
TTATCGATTTTTCTGGAAA
Nr of selected repeats 5 Similarity 0.873684
Consensus:
TTATCGATTTTTCnaGAAa
>Cele-UNSB01_1:11696961-11698223 Satlength=1263 Nr of Repeats=20
RepeatLength=19 seed=AAAAATCGAT
AAAAATCGATAATTTCATCA
AAAAATCGATAATTTGCTCAAAAATCAAAAATTTCATCAAAAATTTCATC
AAAAATCAATAATTTCATC
AAAAATCAATAATTTCATC
AAAAATCAATAATTTGCTC
AAAAATCAATAATTTCTTC
AAAAATCAATAAAATACTC
AAAAATCGATATATCCGCTA
AAAAATCAATATTTTCCCACTTACATAACGCCAATCATTGCGTATTTATAACTATCCCTGTTGCCTGTACGCAAATTGT
CCACCAAAATCATAGTGTTGTGGAAATCACCTTCGGCCGCCGGAACAACCATCTG
AAAAATGGATTTTTATGAGAAATTTCTCAATTAAATTATCGATTTTCCGAGAAATTTTCGATTTTTTATGAAATTATCG
ATTTTTCCGGAAATTATCGATTTTTCAAGAATTTATCGATTTTTCAAGAAATTATCGATTTTTCCAGAAATTATCGATT
TTTCTGGAAATTATCGATTTTCCGAGAAATTATCAATATTTTCTCAATGGAGCGCACTTACTCACCGGTTTTTTCAATT
CGAAGCCCACTTGGTACATCGGGTTGCTGTGCTTTGGCTTCTGGGTCGCCGAGTCTGCTGCCAACGAGAATTGCACAAG
TTGGAGCATCAAAAAGACTGCGAGAAATGATGACGTCATCGTGTTGAGGCGGCGAGTTTCGGTTGGTTGGGTTATCCAA

TTCACACCGATGAAAATCCGATGAATGAAAATCGAATACTGATATCGATTATTGATTGATTCGATCGGATTGAACTGAT
ATCGATGATTTGGAAGAAATGACGTGGCTTAATTGTACCGGGGGTTTGGCCGAAAATTGGAATTTTTCCAG
AAAAATCGATAATTTCTTC
AAAAATCGATAATTTCATC
AAAAATCGATAATTTCCAG
AAAAATCGATAATTTCCAG
AAAAATCAATAAATTTCTTC
AAAAGTCGATAATTTCATC
AAAAATCAATAATTTCATCAGAATTCGATAATTTCCAG
AAAAATCGATAATTCCTTC
AAAAATCGATAATTTCATC
AAAAGTCGATAATTTCTTCCAAAGTCGATAATTTTTGAAAACTGTAGATAATATTAGAAAATTAAATTTTCCTTCTTTT
TTAAATCAATAAATTGAGCTCCCGTCAATCAATCAATACTTTATTCAGACCCAAGTCAACCCAAAAATTGCAATAAATA
GTTCTATTTATTCGTGTAGAATGGCTTCTTGCAGAACACTTCAGCCTGA
Nr of selected repeats 12 Similarity 0.725830
Consensus:
AAAAATCgATAATTTCnTC
>Cele-UNSB01_1:11744458-11744933 Satlength=476 Nr of Repeats=17 RepeatLength=19
seed=TTTCACAAAA
TTTCACAAAAATCGATAATTTTGATGAAAACCAATAAT
TTTCACAAAAATCGATAAT
TTTCACAAAAATCAATAAT
TTTCACAAAAATCAATAAT
TTTCACAAAAATCGATAAT
TTTCACAAAAATCAATAAT
TTTCACAAAAATCCATCATTTTGACGAAAATCAATAAT
TTTCACAAAAATCAATAAT
TTTCACAAAAATCCATCATTTTGACGAAAATCAATAATTTTGACGAAAATCAATAAT
TTTCACAAAACTCGATAAT
TTTCACAAAAATCAATAAT
TTTCACAAAAATCCATCATTTTGACGAAAATCAATAATTTTGACGAAAATCCATCATTTTGACGCAAATCAATAAT
TTTCACAAAAATCGATAAT
TTTCACAAAAATCAATAAT
TTTCACAAAAATCAATAAT
TTTCACAAAAGTCAATAAT
TTTCACAAAAATCGATAATATTGATGAAATCCAATAAT
Nr of selected repeats 12 Similarity 0.943647
Consensus:
TTTCACAAAAATCaATAAT
>Cele-UNSB01_1:11743735-11745857 Satlength=2123 Nr of Repeats=45
RepeatLength=19 seed=AAAATCGATA
AAAATCGATATTTCTCAGCGATAAGGCTTACCGCATGCTTTGTGCTTTCGGAGATGACTAATCGATGCATGACAGCTAG
CCAGACTATTGATTGAGGTTCCTGAAAAACAAAAATTTGAAAGGAAAAACTTTTGAAAAAATCACCTTCTTGACCCAAT
CGATAAAAGCGCCTTCCGATATTGTTGGCGCCAATCGTACAGTTTCGAAGCACGATCGGACCGATGGCTCCACATTGGT
ACCTCCAAATGCGGCGGATTCACCGACCAAACGAGGAATCTGAGAGTCAAA
AAAATCGATAATTCACCA
AAAATCGATTTTTTTTAAAAACCAAAATGAATTGTTACCTAAAAATAAATCAATAAAACACTTACGTGGATAAGATCAT
ATAAAAGCAATGCAATTTGTTTTTGGGTCGCATGACCGTCCTGGGAAACTAATTTGAAAAGATCTGGAAATTAAAAACC
GATTTCAAGGTACAGGGAGTCAGAAAAAAAGCGTACATCTATATTTTTCCTCTAATGGAATGTTCGAGAATACGATCAT
GGCAATTTTAAAGGAGAGAACACGCATTATTCCGTCGCGGGATCTGCAAAAATATGTTAGTTAGTTTTAAAAA
AAAATCGATAATTTTGTCGAAAGGCGGAATTTTTATCGGAAATCCATAAATTTGCCGGAAATATATGATTTTCTGG
AAAATCGATAATTTCTCAT
AAAGTCGATAAATTTCACA
AAAATCGATAATTTTGATGAAAACCAATAATTTTCACA

AAAATCGATAATTTTCACA
AAAATCAATAATTTTCACA
AAAATCAATAATTTTCACA
AAAATCGATAATTTTCACA
AAAATCAATAATTTTCACAAAAATCCATCATTTTGACG
AAAATCAATAATTTTCACA
AAAATCAATAATTTTCACAAAAATCCATCATTTTGACG
AAAATCAATAATTTTGACG
AAAATCAATAATTTTCACA
AAACTCGATAATTTTCACA
AAAATCAATAATTTTCACAAAAATCCATCATTTTGACG
AAAATCAATAATTTTGACGAAAATCCATCATTTTGACGCAAATCAATAATTTTCACA
AAAATCGATAATTTTCACA
AAAATCAATAATTTTCACA
AAAATCAATAATTTTCACAAAAGTCAATAATTTTCACA
AAAATCGATAATATTGATGAAATCCAATAATTTTCACA
AAAATCGATAATTTTGACG
AAAATCGATAATTTTCCGAATTTTTTTTTAATCTATAAATTTCAAGAAAATCTATCATAATTTTTTTAAAATTTTTTCC
TTCACGTTCAAAAATTATCAAATTGTA
AAAATCGATAACTCACTGATCAAAAAGGTTCAACAAGAAATTAATGCAAATATCGACAGCCAACGAGACACTTTGCACT
TGATTTGGATATTTTGCATGAAGAGCTTCATACATCGGCAGCAGAGCACAAACCATACCCTCCAGTCCCGGACATTCTT
CAGCTGAAAGTCCTTTCAGTCGAACAAACGCCTTTTCCAAAAGTGTTAGGTCGACTAGATCGAGACAGAGCCGTTTTTG
AAGGGCTCGGAGCTTCATGGCGGTACGGTAGGCGAGGAATTTGACGCGGTTGAATTGTGAGAGCTCTTTTACGATTTCC
ACCCATACTGGATGCTCCCATTGTGTTTTTTCACTGGTTTGTCTGGAATTTGTTCACATTCGGTTATTTTTCCAAAAAA
TAAATTTCTGG
AAAATCGATAATTTCCCGA
AAAACCGATAATTTTCCGA
AAAATCGATAATTTCCGGAAAA
AAAGTCGATAATTTCCCGA
AAAATCGATAATTTCCAAA
AAAATCGATACTTCTCCGA
AAAATCGATAATTTCCCGA
AAAATCGATAATTTCTCGA
AAAATCGATAATTTCCAAA
AAAATCGATACTTCTCCGA
AAAATCGATAATTTCCCGA
AAAATCGATAATTTCCCGA
AAAATCGATAATTTTCCGAAAAATCATTAATTTTCAGA
AAAATCAATAATTTTCCGA
AAAATCGAAACTTTTCCGA
AAAATCGATATTTTCCCGA
AAAATCGATAATTTCCGGAAAA
AAAGTCGATAATTTCCCGA
AAAATCGATAATTTCCAGA
Nr of selected repeats 29 Similarity 0.730706
Consensus:
AAAATCgATAATTTtCanA
>Cele-UNSB01_1:11752079-11752337 Satlength=259 Nr of Repeats=16 RepeatLength=12
seed=AAGCCAAAGC
AAGCCAAAGCCA
AAGCCAAAGAAT
AAGCCGAAGCCTCAGCCTAAGAAC
AAGCCTAAGCCTCAGCCTAAGCCTAAGAGTGAGCCTAAGTCT
AAGCCTAAGCCA

AAGCCACAGCCA
AAGCCAAAGCCA
AAGCCAAAGCCA
AAGCCAAAGCCA
AAGCCAAAGCCA
AAGCCAAAGCCA
AAGCCAAAGCCA
AAGCCCAAGCCA
AGGCCAAAGCATAGGCCTAAGCCTCAGCCTAAGAAC
AAGCCAAAGTCT
AAGCCAAAGCAA
Nr of selected repeats 13 Similarity 0.854701
Consensus:
AAGCCAAAGCCA
>Cele-UNSB01_1:11868010-11868741 Satlength=732 Nr of Repeats=60 RepeatLength=12
seed=AGGCTCAGGC
AGGCTCAGGCTC
AGGGTCAGGCTC
AGGCTCAGGCTT
AGGCTCAGGTTC
AGGCTCAGGCTT
AGGCTCAGGCTC
AGGCTCAGGCTT
AGGCTCAGGCTC
AGGCTCAGGCTC
AGGCTCAGGCTC
AGGCTCAGGCTC
AGGCTCAGGCTC
AGGCTCAGGCTC
AGGCTCAGGCTC
AGGCTCAGGCTC
AGGCTCAGGCTC
AGGCTCAGGCTC
AGGCTCAGGCTC
AGGCTCAGGCTC
AGGCTCAGGCTC
AGGCTCAGGCTC
AGGCTCAGGCTC
AGGCTCAGGCTC
AGGCTCAGGCTC
AGGCTCAGGCTC
AGGCTCAGGCTC
AGGCTCAGGCTC
AGGCTCAGGCTC
AGGCTCAGGCTC
AGGCTCAGGTC
AGGCTCAGGCTC
AGGCTCAGGCTC
AGGCTCAGGCTC
AGGCTCAGGCTC
AGGCTCAGGCTC
AGGCTCAGGCTC
AGGCTCAGGCTC
AGGCTCAGGCTC

AGGCTCAGGCTC
AGGCTCAGGCTC
AGGCTCAGGCTC
AGGCTCAGGCTC
AGGCTCAGGCTC
AGGCTCAGGCTC
AGGCTCAGGCTC
AGGCTCAGGCTC
AGGCTCAGGCTC
AGGCTCAGGCTC
AGGCTCAGGCTT
AGGCTTAGGCTC
AGGCTCAGACTCAGGCCT
AGGCTCAGGCTC
AGGCTCAGGCTC
AGGCTCAGGCTC
AGGCTCAGGCTC
AGGCTCAGGCTC
AGGCTCAGGCTT
AGGCTTAGGCTC
AGGCTCAGACTC
AGGCTCAGGCTTAGGCTT
Nr of selected repeats 57 Similarity 0.962545
Consensus:
AGGCTCAGGCTC
>Cele-UNSB01_1:11869240-11870103 Satlength=864 Nr of Repeats=53 RepeatLength=16
seed=GCATGTAGGC
GCATGTAGGCATGTAG
GCATGTAGGCATGTG
GCATGTAGGCATGTAG
GCATGTAGGCATGTAG
GCATGTAGGCATGTAG
GCTTGTAGGCATGTAG
GCATGTAGGCATGTAG
GCATGTAGGCATGTAG
GCATGTAAGCATGTAG
GCATGTAGGCATGTAG
GCATGTAGGCATGTAG
GCATGTAGGCATGTAA
GCATGTAGGCATGTAG
GCATGTAGGCATGTAG
GCATGTAGGCATGTAG
GCTTGTAGGCATGTAG
GCATGTAGGCATGTAG
GCATGTAGGCATGTAG
GCATGTAAGCATGTAG
GCATGTAGGCATGTAG
GCATGTAGGCATGTAG
GCATGTAGGCATGTAG
GCATGTAGGCATGTAG
GCATGTAGGCATGTAG
GCATGTAGGCATGTAG
GCATGTAGGCATGTAG
GCATCTAGGCATGTGG

GCATGTGGGCATGTAG
GCATGTAGGCATGTAG
GCATGTAGGCATGTAG
GCATGTAGGCATGTAG
GCATGTAGGCATGTAG
GCATGTAGGCATGTAG
GCTTGTAGGCATGTAG
GCATGTAGGCATGTAG
GCATGTAGGCATGTAG
GCATGTAGGCATGTAG
GCATGTAGGCATGTAG
GCATGTAGGCATGTAA
GCATGTAGGCATGTAG
GCATGTAGGCATGTAG
GCATGTAGGCATGTAG
GCATGTAGGCATGTAG
GCATGTAGGCATGTAG
GCATGTAGGCATGTAG
GCATCTAGGCATGTGG
GCATGTAGGCATGTGG
GCATGTAGGTATGTAGGCATGCCG
GCATGTAGGCATGTAG
GCATGTAGGCATGTAG
GCATGTAGGCATGTAG
GCATGTAGGCATGTAG
GTATGTAGGCATGTAGGCATGTAG
Nr of selected repeats 50 Similarity 0.957891
Consensus:
GCATGTAGGCATGTAG
>Cele-UNSB01_1:11907174-11907313 Satlength=140 Nr of Repeats=13 RepeatLength=11
seed=TTTTGCACAT
TTTTGCACATT
TTTTGCACAT
TTTTCCACATT
TTTTGCACTTT
TTTTGGACATT
TTTTGCACATT
TTTTGCACAC
TTTTGCACATA
TTTTGCACAT
TTTTGCACATT
TCTTGCACATT
TTTTGCAAAT
TTATGCACATT
Nr of selected repeats 9 Similarity 0.838384
Consensus:
TTTTGCACATT
>Cele-UNSB01_1:11925845-11930260 Satlength=4416 Nr of Repeats=27
RepeatLength=164 seed=ATACCAGAAA
ATACCAGAAAATGCCAATCAAAGTATATTAGCTTGTACGGAAGTAATTTTTTTTTAATTGATAAAAATATATAAAAGCT
GATTTTTTCAAAAATTCAAAAGTATGGGAAAATCATATGGAGTCATTCTTTTTTATTCCATAAAACTGTTCAGCATAGT
CAAAA

ATACCAGAAAATGCCAATCAAAGTATATTAGCTTGTACGGAAGTAATTTTTTTTTTAATTGATAAAAATATATAAAAGC
TGATTTTTTCAAAAATTCAAAAGTATGGGAAAGTCAAATGGAGTCATTCTTTTTTTATTCTATAAAACTGTTTACCATAG
TCAAAA
ATACCAGAAAATGCCGATCAAAGTATATTAGCTTGTACGGAAGTAATTTTTTTAAAAATTGATAAAAATATATAAAAGC
TGATTTTTTCAAAAATTCAAAAGTATGGGAAAGTCAAATGGAGTCATTCTTTTTTTATTCCATAAAACTGTTCAGCATAG
TCAAAA
ATACCAGAAAATGCCAATCAAAGTATATTAGCTTGTACGGAAGTAATTTTTTTTTTAATTGATAAAAATATATAAAAGC
TGATTTTTTCAAAAATTCAAAAGTATGGGAAAGTCAAATGGAGTCATTCTTTTTTTATTCTATAAAACTGTTTACCATAG
TCAAAA
ATACCAGAAAATGCCGATCAAAGTATATTAGCTTGTACGGAAGTAATTTTTTTTTTAATTGATAAAAATATATAAAAGC
TGATTTTTTCAAAAATTCAAAAGTATGGGAAAGTCAAATGGAGTCATTCTTTTTTTATTCTATAAAACTGTTTACCATAG
TCAAAA
ATACCAGAAAATGCCGATCAAAGTATATTAGCTTGTACGGAAGTAATTTTTTTAAAAATTGATAAAAATATATAAAAGC
TGATTTTTTCAAAAATTCAAAAGTATGGGAAAGTCAAATGGAGTCATTCTTTTTTTATTCCATAAAACTGTTTACCATAG
TCAAAA
ATACCAGAAAATGCCGATCAAAGTATATTAGCTTGTACGGAAGTAATTTTTTTAAAAATTGATAAAAATATATAAAAGC
TGATTTTTTCAAAAATTCAAAAGTATGGGAAAGTCAAATGGAGTCATTCTTTTTTTATTCCATAAAACTGTTCAGCATAG
TCAAAA
ATACCAGAAAATGCCAATCAAAGTATATTAGCTTGTACGGAAGTAATTTTTTTTTTAATTGATAAAAATATATAAAAGC
TGATTTTTTCAAAAATTCAAAAGTATGGGAAAGTCAAATGGAGTCATTCTTTTTTTATTCTATAAAACTGTTTACCATAG
TCAAAA
ATACCAGAAAATGCCGATCAAAGTATATTAGCTTGTACGGAAGTAATTTTTTTAAAAATTGATAAAAATATATAAAAGC
TGATTTTTTCAAAAATTCAAAAGTATGGGAAAGTCAAATGGAGTCATTCTTTTTTTATTCCATAAAACTGTTTACCATAG
TCAAAA
ATACCAGAAAATGCCGATCAAAGTATATTAGCTTGTACGGAAGTAATTTTTTTAAAAATTGATAAAAATATATAAAAGC
TGATTTTTTCAAAAATTCAAAAGTATGGGAAAATCATATGGAGTCATTCTTTTTTTATTTCATAAAATTCTTCAGCATAG
TCAAAA
ATACCAGAAAATGCCAATCAAAGTATATTAGCTTGTACGGAAGTAATTTTTTTTTAAATTGATAAAAATATATAAAAGC
TGATTTTTTCAAAAATTCAAAAGTATGGGAAAATCATATGGAGTCATTCTTTTTTTATTTCATAAAATTCTTCAGCATAG
TCAAAA
ATACCAGAAAATGCCAATCAAAGTATATTAGCTTGTACGGAAGTAATTTTTTTTTTAATTGATAAAAATATATAAAAGCT
GATTTTTTCAAAAATTCAAAAGTATGGGAAAATCATATGGAGTCATTCTTTTTTTATTCCATAAAACTGTTCAGCATAGT
CAAAA
ATACCAGAAAATGCCAATCAAAGTATATTAGCTTGTACGGAAGTAATTTTTTTAAAAATTGATAAAAATATATAAAAGC
TGATTTTTTCAAAAATTCAAAAGTATGGGAAAATCATATGGAGTCATTCTTTTTTTATTCCATAAAACTGTTTACCATAG
TCAAAA
ATACCAGAAAATGCCGATCAAAGTATATTAGCTTGTACGGAAGTAATTTTTTTTTAAATTGATAAAAATATATAAAAGC
TGATTTTTTCAAAAATTCAAAAGTATGGGAAAGTCAAATGGAGTCATTCTTTTTTTATTCTATAAAACTGTTTACCATAG
TCAAAA
ATACCAGAAAATGCCGATCAAAGTATATTAGCTTGTACGGAAGTAATTTTTTTAAAAATTGATAAAAATATATAAAAGC
TGATTTTTTCAAAAATTCAAAAGTATGGGAAAATCATATGGAGTCATTCTTTTTTTATTTCATAAAATTCTTCAGCATAG
TCAAAA
ATACCAGAAAATGCCAATCAAAGTATAATAGCTTGTACGGAAGTAATTTTTTTTTAAATTGATAAAAATATATAAAAGC
TGATTTTTTCAAAAATTCAAAAGTATGGGAAAATCATATGGAGTCATTCTTTTTTTATTTCATAAAATTCTTCAGCATAG
TCAAAA
ATACCAGAAAATGCCAATCAAAGTATATTAGCTTGTACGGAAGTAATTTTTTTTTAAATTGATAAAAATATATAAAAGC
TGATTTTTTCAAAAATTCAAAAGTATGGGAAAATCATATGGAGTCATTCTTTTTTTATTTCATAAAACTGTTCAGCATAG
TCAAAA
ATACCAGAAAATGCCAATAAAAGAATATCAGCTTGTACGGAAGTATTTTTTAAAAATTGATAAAAATATATAAAAGCTG
ATTTTTTCAAAAATTCAAAAGTGTGGGAAAATCATATGGAGTCATTCTTTTTTTATTTCATAAAACTGTTCAGCATAGTC
AAAA
ATACCAGAAAATGCCAATAAAAGTATATCAGCTTGTACGGAAGTATTTTTTTTTAATTGATAAAAATATATAAAAGCTG
ATTTTTTGAAACTTCGAAAAGTACGATAAAATCATATGAAGTGCTTTTTTTTTCTTTCATAAAACTGTTAAGTATTGCC
GAAT

ATACCAGAAAATACCAAACTAAGTAGGCTAGCTTGTACGGAAGTAATTTTTTTTTAAATTGATAAAAATATATAAAAGC
TGATTTTTTCAAAAATTCAAAAGTATGGGAAAGTCAAATGGAGTCATTCTTTTTTATTCTATAAAACTGTTTACCATAG
TCAAAA
ATACCAGAAAATGCCGATCAAAGTATATTAGCTTGTACGGAAGTAATTTTTTTAAAAATTGATAAAAATATATAAAAGC
TGATTTTTTCAAAAATTCAAAAGTATGGGAAAATCATATGGAGTCATTCTTTTTTATTTCATAAAATTCTTCAGCATAG
TCAAAA
ATACCAGAAAATGCCAATCAAAGTATATTAGCTTGTACGGAAGTAATTTTTTTTTAAATTGATAAAAATATATAAAAGC
TGATTTTTTCAAAAATTCAAAAGTATGGGAAAATCATATGGAGTCATTCTTTTTTATTTCATAAAATTCTTCAGCATAG
TCAAAA
ATACCAGAAAATGCCAATCAAAGTATATTAGCTTGTACGGAAGTAATTTTTTTTTAAATTGATAAAAATATATAAAAGC
TGATTTTTTCAAAAATTCAAAAGTATGGGAAAATCATATGGAGTCATTCTTTTTTATTTCATAAAACTGTTCAGCATAG
TCAAAA
ATACCAGAAAATGCCAATAAAAGAATATCAGCTTGTACGGAAGTATTTTTTTAAAAATTGATAAAAATATATAAAAGCTG
ATTTTTTCAAAAATTCAAAAGTGTGGGAAAATCATATGGAGTCATTCTTTTTTATTTCATAAAACTGTTCAGCATAGTC
AAAA
ATACCAGAAAATGCCAATAAAAGTATATCAGCTTGTACGGAAGTATTTTTTTTTAATTGATAAAAATATATAAAAGCTG
ATTTTTTGAAACTTCGAAAAGTACGATAAAATCATATGAAGTGCTTTTTTTTTCTTTCATAAAACTGTTAAGTATTGCC
GAAT
ATACCAGAAAATACCAAACTAAGTAGGCTAGCTTGTACGGAAGTAATTTTTTTTTAAATTGATAAAAATATATAAAAGC
TGATTTTTTCAAAAATTCAAAAGTATGGGAAAATCATATGGAGTCATTCTTTTTTATTTCATAAAATTCTTCAGCATAG
TCAAAA
ATACCAGAAAATGCCGATCAAAGTATATTAGCTTGTACGGAAGTAATTTTTTTAAAATTGATAAAAATATATAAAAGCTG
ATTTTTTGAAACTTCGAAAAGTACGATAAAATCATATGAAGTGCTTTTTTTTTCTTTCATAAAACTGTTAAGTATTGCCG
AAT
Nr of selected repeats 20 Similarity 0.942191
Consensus:
ATACCAGAAAATGCCaATCAAAGTATATTAGCTTGTACGGAAGTAATTTTTTTttaAATTGATAAAAATATATAAAAGC
TGATTTTTTCAAAAATTCAAAAGTATGGGAAAaTCAaATGGAGTCATTCTTTTTTATTccATAAAAcTgTTcAgCATAG
TCAAAA
>Cele-UNSB01_1:11979429-11979575 Satlength=147 Nr of Repeats=6 RepeatLength=11
seed=AGTACTGTAG
AGTACTGTAGT
AGTACTGTAGG
AGTACTGTAGGATTACTGTATTTTTGAAAAAAATTGGCTTTTCGTCTTTTGAAGTGATATTGGTTTGAGGTTAGTGGTG
GGATATGGTTGGG
GTACTGTAGTT
GTACTGTAGAG
GTACTGTAGG
Nr of selected repeats 4 Similarity 0.731481
Consensus:
aGTACTGTAGtn
>Cele-UNSB01_1:12045808-12046235 Satlength=428 Nr of Repeats=5 RepeatLength=42
seed=TCGGCAAATC
TCGGCAAATCGGCTATTCCTGGTTTTTCAAAATTTGCCGAGC
TCGGCAAATTCGGCAAATCTCTTTTTTCAATATTTGCCGAGCACGGCAAAT
TCGGCAAATTTGCCGTGCTTAACAAACTCGGAAAATTTTGATTTTTTTAAATGTTTTTTGGAGCACCAAAACTACTGAA
TTCTTAACATCTGGTTTCTGAATAAGTTCCGCGTAGTATGTCTGTTTAAGCATCAAAATAGCTCAATTTTGTGTCATTT
TACTAAATTTTTGGCTAAAAAATCAATAGTTTTAGTCAAAATTGTACTGTCAAATTTTTGACGTGTGCGGCAAATTTCG
AAATTTGCCGAGC
TCGGCAAATTCGGCAAATCTACTTTTTTGAAAATTGCCGAGC
TCGGCAAATCGGCAATTGCTGGTTTTTTTTAAATTTGCCGAAT
Nr of selected repeats 3 Similarity 0.723514
Consensus:
TCGGCAAATCGGCaAtTnCTggTTTTTtnAAAtTTGCCGAgc

>Cele-UNSB01_1:12056400-12056477 Satlength=78 Nr of Repeats=7 RepeatLength=11 seed=CAAAAAATGT
CAAAAAATGTC
CAAAAAATGTC
CAAAAAATGTC
CAAAAAATGTC
CAAAAAATGTC
CAAAAAATGTG
CAAAAAATGTG
Nr of selected repeats 7 Similarity 0.942280
Consensus:
CAAAAAATGTc
>Cele-UNSB01_1:12149612-12149804 Satlength=193 Nr of Repeats=15 RepeatLength=12 seed=TTAGGCTTAG
TTAGGCTTAGACTTAGGA
TTACGCTTAGGC
TTAGGCTTAGGC
TTAGGCTTAGACTATGGC
TTAGGCTTAGGC
TTAGGCTAAGGC
TAAGGCTTAGGCA
TAGGCTTAGTC
TTAAGCTTAGCT
TTAGGCTTAGGC
TTAGGCTTAGGC
TTAGGCTTAGGC
TTAGGCTTAGGC
TTAGGCTTAGGC
TTAGGCTTAGGC
Nr of selected repeats 11 Similarity 0.838695
Consensus:
TTAGGCTTAGGC
>Cele-UNSB01_1:12200484-12200598 Satlength=115 Nr of Repeats=7 RepeatLength=12 seed=CTAAGCCTAA
CTAAGCCTAAGCCTTTGTCTAAGT
CGAAGCCTAAGC
CTAAGCCTAAAC
CTAAGACTAAGC
CTAAACCTAAAC
CAAAGCCTAAAC
CTAAGCCTAAGACAAAGCCTAGACTTAAGC
Nr of selected repeats 5 Similarity 0.766667
Consensus:
CtAAgcCTAAaC
>Cele-UNSB01_1:12310642-12315079 Satlength=4438 Nr of Repeats=373 RepeatLength=11 seed=CACATTTTTT
CACATTTTTTA
CACATTTTTTG
CACAATTTTTA
CACAATTTTTA
CACATTTTTTG
CACATTTTTTG
CACATTTTTTA
CACATTTTTTGAACATTTTTCG

CACATTTTTA
CACATTTTTG
CACATTTTCG
CACATTTTTTG
CACATTTTTG
CACATTTTTG
CACAATTTTG
CACATTTTTA
CACATTTTCG
CACATTTTTTG
CACATTTTTG
CACCTTTTTG
CACATTTTTG
CACATTTTTA
CACAATTTTA
CACATTTTTA
CACAATTTTA
CACATTTTTG
CACATTTTCG
CACATTTTTTG
CACATTTTCG
CACATTTTTTG
CACATTTTCG
CACATTTTTTG
CACATTTTCG
CACATTTTTTG
CACATTTTTA
CACAATTTTA
CACATTTTTA
CACAATTTTA
CACATTTTTA
CACAATTTTA
CACATTTTTG
CACATTTTCG
CACATTTTTTG
CACATTTTTG
CACCTTTTTG
CACAATTTTG
CACATTTTA
CACAATTTTA
CAAATTTTTA
CACAATTTTA
CACATTTTTA
CACAATTTTA
CACATTTTTG
CACATTTTTG
CACATTTTTA
CACATTTTTGAACATTTTCG
CACATTTTTTG
CACATTTTTG
CACAATTTTG
CACATTTTTA
CACATTTTTG
CACATTTTCG

CACATTTTTTTG
CACATTTTTTG
CACATGTTTTG
CACAATTTTTG
CACATTTTTTA
CACAATTTTTG
CACATTTTTTA
CACAATTTTTA
CACTTTTTTTG
CACATTTTTTACAAATTTTTCG
CACATTTTTTG
CACATTTTTTA
CACAATTTTTA
CACATTTTTTG
CACATTTTTTG
CACATTTTTTG
CACATTTTTTG
CACATTTTTTACAAATTTTTCG
CACATTTTTTG
CACATTTTTAA
CACTTTTTTTGA
ACATTTTTTG
CACATTTTTTACAAATTTTTCG
CACATTTTTTG
CACATTTTTTA
CACTTTTTTTGAACATTTTTCG
CACTTTTTTTG
CACAATTTTTG
CACTTTTTTTA
CACATTTTTTGAACATTTTTCG
CACATTTTTTTG
CACATTTTTTG
CACAATTTTTG
CACATTTTTTA
CACATTTTTTG
CACATTTTTCG
CACATTTTTTTG
CACATTTTTCG
CACATTTTTTTG
CACATTTTTTA
CACAATTTTTA
CACTTTTTTTG
CACATTTTTTACAAATTTTTCG
CACATTTTTTG
CACATTTTTTA
CACAATTTTTA
CACATTTTTTG
CACATTTTTTG
CACATTTTTTG
CACATTTTTTG
CACATTTTTTACAAATTTTTCG
CACATTTTTTG
CACATTTTTAA
CACTTTTTTTGA

ACATTTTTTG
CACATTTTTTACAAATTTTTCG
CACATTTTTTG
CACATTTTTA
CACTTTTTTTGAACATTTTTCG
CACTTTTTTG
CACAATTTTTG
CACTTTTTTA
CACATTTTTTGAACATTTTTCG
CACATTTTTTTG
CACATTTTTTG
CACAATTTTTG
CACATTTTTTA
CACATTTTTTG
CACATTTTTCG
CACATTTTTTTG
CACATTTTTCG
CACATTTTTTTG
CACATTTTTTA
CACAATTTTTA
CACATTTTTTA
CACAATTTTTA
CACATTTTTTA
CACAATTTTTA
CACAATTTTTA
CACATTTTTTG
CACATTTTTTG
CACATTTTTTA
CACATTTTTTGAACATTTTTCG
CACATTTTTTTG
CACATTTTTTG
CACAATTTTTG
CACATTTTTTA
CACATTTTTTG
CACATTTTTCG
CACATTTTTTTG
CACATTTTTTG
CACCTTTTTTG
CACATTTTTTG
CACATTTTTTA
CACAATTTTTA
CACATTTTTTA
CACAATTTTTA
CACATTTTTTG
CACATTTTTCG
CACATTTTTTTG
CACATTTTTCG
CACATTTTTTTG
CACATTTTTCG
CACATTTTTTTG
CACATTTTTCG
CACATTTTTTTG
CACATTTTTTA
CACAATTTTTA

CACATTTTTTA
CACAATTTTTA
CACATTTTTTA
CACAATTTTTA
CACATTTTTTG
CACATTTTTCG
CACATTTTTTTG
CACATTTTTTG
CACCTTTTTTG
CACAATTTTTG
CACATTTTTA
CACAATTTTTA
CAAATTTTTTA
CACAATTTTTA
CACATTTTTTA
CACAATTTTTA
CACATTTTTTG
CACATTTTTTG
CACATTTTTTA
CACATTTTTTGAACATTTTTCG
CACATTTTTTTG
CACATTTTTTG
CACAATTTTTG
CACATTTTTTA
CACATTTTTTG
CACATTTTTCG
CACATTTTTTTG
CACATTTTTTG
CACATGTTTTG
CACAATTTTTG
CACATTTTTTA
CACAATTTTTG
CACATTTTTTA
CACAATTTTTA
CACTTTTTTTG
CACATTTTTTACAAATTTTTCG
CACATTTTTTG
CACATTTTTTA
CACAATTTTTG
CACATTTTTTA
CACAATTTTTG
CACTTTTTTTGA
ACATTTTTTG
CACATTTTTTACAAATTTTTCG
CACATTTTTTG
CACATTTTTTA
CACTTTTTTTGAACATTTTTCG
CACTTTTTTTG
CACAATTTTTG
CACTTTTTTTA
CACATTTTTCG
CACATTTTTTTG
CACATTTTTTG
CACAATTTTTG

CACATTTTTA
CACATTTTTG
CACATTTTCG
CACATTTTTTG
CACATTTTCG
CACATTTTTTG
CACATTTTTA
CACAATTTTA
CACATTTTTA
CACAATTTTA
CACATTTTTA
CACAATTTTA
CACATTTTTA
CACAATTTTA
CACATTTTTG
CACATTTTCG
CACATTTTTG
CACATTTTTA
CACATTTTTGAACATTTTTCG
CACATTTTTTG
CACATTTTTG
CACAATTTTG
CACATTTTTA
CACATTTTTG
CACATTTTCG
CACATTTTTTG
CACATTTTTG
CACCTTTTTG
CACATTTTTG
CACATTTTTA
CACAATTTTA
CACATTTTTA
CACAATTTTA
CACATTTTTG
CACATTTTCG
CACATTTTTTG
CACATTTTCG
CACATTTTTTG
CACATTTTCG
CACATTTTTTG
CACATTTTCG
CACATTTTTTG
CACATTTTTA
CACAATTTTA
CACATTTTTA
CACAATTTTA
CACATTTTTA
CACAATTTTA
CACATTTTTG
CACATTTTCG
CACATTTTTTG
CACATTTTTG
CACCTTTTTG
CACAATTTTG

CACATTTTTA
CACAATTTTTA
CAAATTTTTTA
CACAATTTTTA
CACATTTTTTA
CACAATTTTTA
CACATTTTTTG
CACATTTTTTG
CACATTTTTTA
CACATTTTTTGAACATTTTTCG
CACATTTTTTTG
CACATTTTTTG
CACAATTTTTG
CACATTTTTTA
CACATTTTTTG
CACATTTTTCG
CACATTTTTTTG
CACATTTTTTG
CACATGTTTTG
CACAATTTTTG
CACATTTTTTA
CACAATTTTTG
CACATTTTTTA
CACAATTTTTA
CACTTTTTTTG
CACATTTTTTACAAATTTTTCG
CACATTTTTTG
CACATTTTTTA
CACAATTTTTA
CACAATTTTTA
CACATTTTTTA
CACAATTTTTA
CACATTTTTTA
CACAATTTTTA
CACATTTTTTG
CACATTTTTCG
CACATTTTTTTG
CACATTTTTTG
CACCTTTTTTG
CACAATTTTTG
CACATTTTTA
CACAATTTTTA
CAAATTTTTTA
CACAATTTTTA
CACATTTTTTA
CACAATTTTTA
CACATTTTTTG
CACATTTTTTG
CACATTTTTTA
CACATTTTTTGAACATTTTTCG
CACATTTTTTTG
CACATTTTTTG
CACAATTTTTG
CACATTTTTTA

CACATTTTTTG
CACATTTTTCG
CACATTTTTTTG
CACATTTTTTG
CACATGTTTTG
CACAATTTTTG
CACATTTTTTA
CACAATTTTTG
CACATTTTTTA
CACAATTTTTA
CACTTTTTTTG
CACATTTTTTACAAATTTTTCG
CACATTTTTTG
CACATTTTTTA
CACAATTTTTG
CACATTTTTTA
CACAATTTTTG
CACATTTTTTG
CACATTTTTTG
CACATTTTTTG
CACATTTTTTG
CACATTTTTTACAAATTTTTCG
CACATTTTTTG
CACATTTTTAA
CACTTTTTTTGA
ACATTTTTTG
CACATTTTTTACAAATTTTTCG
CACATTTTTTG
CACATTTTTTA
CACTTTTTTTGAACATTTTTCG
CACTTTTTTTG
CACAATTTTTG
CACTTTTTTTA
CACATTTTTT
CACATTTTTCG
CACATTTTTTTG
CACAATTTTTCGCA
CACTTTTTTG
CACATTTTTTG
CACATTTTTTG
CACAATTTTTGCAGAATTTTAGCACAATCTTAG
Nr of selected repeats 292 Similarity 0.843776
Consensus:
CACAtTTTTTg
>Cele-UNSB01_1:12330775-12330985 Satlength=211 Nr of Repeats=7 RepeatLength=30
seed=GTCATTATCA
GTCATTATCAGCTTCTAGTGTCTTAGAAGA
GTCATTATCAGCTTCTGGTGTCTTAGAAGA
GTCATTATCAGCTTCTGGTGTCTTAGAAGA
GTCATTATCAGCTTCTGGTGTCTTAGAAGA
GTCATTATCAGCTTCTGGTGTCTTAGAAGA
GTCATTATCAGCTTCTGGTGTCTTAGAAGA
GTCATTATCAGCTTCTGGTGTCTTCGAAGA
Nr of selected repeats 7 Similarity 0.974603

Consensus:
GTCATTATCAGCTTCTGGTGTCTTAGAAGA
>Cele-UNSB01_1:12340678-12340816 Satlength=139 Nr of Repeats=10 RepeatLength=12
seed=TAAGCCTAAG
TAAGCCTAAGCG
TAAGACTAAGCCTGTGCC
TAAGCCTAAGCT
TAAGCCTAAGCCTAAGCCA
TAGCCTAAGCC
TAAGCCTAAGCC
TAAGACTAAGCC
TAAGCCTAAGCC
TAAGCCTAAGAC
TAAGCCTAAGCCTAAGCC
Nr of selected repeats 6 Similarity 0.859259
Consensus:
TAAGCCTAAGCc
>Cele-UNSB01_1:12350087-12350549 Satlength=463 Nr of Repeats=41 RepeatLength=11
seed=TTGCACATTT
TTGCACATTTT
TTGCACATTTC
TTGCACATTTTT
TTCACATTTTTGCACATTTC
TTGCACATTTT
TTGCACATTTT
TTGCACATTTT
TTGCACATTTT
TTGCACATTTT
TTGCACATTTT
TTGCACATTTTT
TTCACATTTTTGCACATTTC
TTGCACATTTT
TTGCACATTTT
TTGCCCATTTTTAGCCCATTTTT
TTGCACATTTT
TTGCACATTTT
TCGCACATTTG
TTGCACATTTT
TTGCACATTTT
TTGCACATTTT
TTGCACATTTT
TTGCACATTTT
TTGCCCATTTT
TTGCCCATTTTT
TTGCACATTTT
TTGCACATTTT
TTGCACATTTT
TTGCACATTTT
TTGCACATTT
TTGCACATTTT
TTGCACATTTT
TTGCACATTTT
TTGCCCATTTT
TTGCCCATTTTT

TTGCACATTTT
TTGCACATTTT
TTGCACATTTT
TTGCACATTTT
Nr of selected repeats 31 Similarity 0.961681
Consensus:
TTGCACATTTT
>Cele-UNSB01_1:12396670-12396802 Satlength=133 Nr of Repeats=9 RepeatLength=12
seed=GCCTAAGCCT
GCCTAAGCCTAA
GCCTAAGCCTAT
GCCTATGCCTTAGACCAATCCCAA
GCCTAAGCCTAT
GCCTAAGCCTGG
GCCTAAGACTGG
GTCTAAGCCTAA
GCATAAGCCTAA
GCCTAATCCTAAGCTTAAGCATAA
Nr of selected repeats 7 Similarity 0.767196
Consensus:
GCCTAAGCCTan
>Cele-UNSB01_1:12413207-12413533 Satlength=327 Nr of Repeats=4 RepeatLength=73
seed=AATAAAATAT
AATAAAATATATGAAACCCTCTTAAAAAGTTACAAGAAGGTTTTTCCTTGCGCTTTGAGCGCAAAAGAAAAGAAAAAGA
GCTATTTAGACTTAGGGTGCCCAACTGG
AATAAAATATAGAAAATCCTTATGACACACTTAAGCCTAAAGGCCCGAAAAACATACTAGGATGCCCAACTGG
AATAAAATATTGGAAATCCCTATGACACACTTAAGCCTAAAGGCCCGAAAAACATACTAGGATGCCCAACTGG
AATAAAATATTGGAAATCCCTATGACACACTTAAGCCTAAAGGCCCGAAAAACATACTAGGATGCCCAACTGG
Nr of selected repeats 3 Similarity 0.963470
Consensus:
AATAAAATATtGgAAATCCcTATGACACACTTAAGCCTAAAGGCCCGAAAAACATACTAGGATGCCCAACTGG
>Cele-UNSB01_1:12444291-12444339 Satlength=49 Nr of Repeats=4 RepeatLength=12
seed=GCCTAAGCCT
GCCTAAGCCTAA
GCCTAAGCCTAG
GCCTAAGCCTGA
GCCTAGGCCTAT
Nr of selected repeats 4 Similarity 0.796296
Consensus:
GCCTAaGCCTaa
>Cele-UNSB01_1:12478910-12479068 Satlength=159 Nr of Repeats=5 RepeatLength=34
seed=ATGCACCATG
ATGCACCATGCCAAAAAATATATCCAAAACTCCG
ATGCACCGTGTCCAAAAGTATATATAAAATTGCG
ATGCACCATGTTAAAAAATATATCCAAAAATATG
ATGCTCCATGTTTAAAAATACG
ATGCACCATGTCAAAAAGTATATCTTGAAGTATG
Nr of selected repeats 4 Similarity 0.712418
Consensus:
ATGCACCaTGtcaAAAAaTATATccaaAAnTacG
>Cele-UNSB01_1:12487096-12487168 Satlength=73 Nr of Repeats=5 RepeatLength=12
seed=GGCTTAGGCT
GGCTTAGGCTTA
GGCTTCGGCTTA

GGCTTCGGCTTC
GGTTTAGGCTTA
GGCTTAGGTTTTGGTTGTGGCTTT
Nr of selected repeats 4 Similarity 0.814815
Consensus:
GGcTTaGGCTTa
>Cele-UNSB01_1:12700590-12700802 Satlength=213 Nr of Repeats=16 RepeatLength=12
seed=GAAATTTTTT
GAAATTTTTTCC
GAAATTTTTTCC
GAACTTTTTTCC
GAAATTTTTTCA
GAAATTTTTTCT
GAAATTTTTTTAAATTTTTCC
GAAATTTTTTCC
GAAATTTTTTCT
GAAATTTTTTCC
GAAATTTTTTCT
GAAATTTTTTCC
GAAATTTTTTCT
GAAATTTTTTTAAATTTTTCCGA
AAATTTTTTCC
GAAATTTTTTCC
GAAATTTTTTTCA
Nr of selected repeats 12 Similarity 0.915825
Consensus:
GAAATTTTTTCc
>Cele-UNSB01_1:12700867-12701490 Satlength=624 Nr of Repeats=50 RepeatLength=12
seed=AAAAAATTTC
AAAAAATTTCGGAA
AAAAAATTTCGGAAAAAATTAAAA
AAAAAATTTCGG
AAAAAATTTCAG
AAGAAATTTCCGAA
AAAAATTTTCGGAAAATTTTC
AAAAATTTCCG
AAAAAATTTCGGT
AAAAATTTCAG
AAAAAATTTCGG
AAAAAATTTCAG
AAAAAATTTCGGT
AAAAATTTCAG
AAAAAATTTCGA
AAAAAATTTCAG
AAAAAATTTCGG
AAAAAATTACAG
AAAAAATTTCGG
AAAAAATTTCGG
AAAAAATTTCAG
AAAAAATTTCGG
AAAAAATTTCGG
AAAAAATTTCAG
AAAAAATTTCGA
AAAAAATTTCAG

AAAAAATTTCGG
AAAAAATTTCAG
AAAAAATTTCGG
AAAAAATTACAG
AAAAAATTTCGG
AAAAAATTTCGG
AAAAAATTTCAG
AAAAAATTTCGG
AAAAAATTTCGG
AAAAAATTTCAG
AAAAAATTTCGA
AAAAAATTTCGGA
AAAAAATTTCAG
AAAAAATTTCGG
AAAAAATTTCAG
AAAAAATTTCAG
AAAAAATTTCAG
AAAAAATTTCGGT
AAAAATTTCAG
AAAAAATTTCGG
AAAAAATTTCAG
AAAAAATTTCGG
AAAAATTTCAG
AAAAAATTTCGA
AAAAAATTTCGA
Nr of selected repeats 37 Similarity 0.905572
Consensus:
AAAAAATTTCgG
>Cele-UNSB01_1:12702448-12702698 Satlength=251 Nr of Repeats=5 RepeatLength=50
seed=ACACATGCTA
ACACATGCTATAACATTGTCTCCTTCCCCTAGTGCACGAGAGCGGCCGTT
ACACATGCTATAACATTGTCTCCTTCCTCTAGTGCACGAGAGCGGCCGCC
ACACATGCTATAACATTGTCTCCTTCCCCTAGTGCACGAGAGCGGCCGTT
ACACATGCTATAACATTGTCTCCTTCCTCTAGTGCACGAGAGCGGCCGCC
ACACATGCTATAACATTGTCTCCTTCCCCTAGTGCACGAGAGCGGCCGTT
Nr of selected repeats 5 Similarity 0.952000
Consensus:
ACACATGCTATAACATTGTCTCCTTCCcCTAGTGCACGAGAGCGGCCGtt
>Cele-UNSB01_1:12783664-12784000 Satlength=337 Nr of Repeats=20 RepeatLength=16
seed=TGATCTGATG
TGATCTGATGATCTGA
TGATCTGATGATCTCA
TGATCTGATGGTCTGA
TGATCTGTTGATCTGA
TGGTCTGATGGTCTGA
TGATCTCATGATCTGA
TGATCTGTTGATCTGT
TGATCTGATGATCTGA
TGATCTGATGATCTGATGACCTGA
TCATCTGATGGTCTGA
TGATCTGTTGATCTGA
TGGTCTGATGGTCTGA
TAATCTGATGATCTGA
TGATCTGTTGATCTGT

TGATCTGATGATCTGA
TGATCTGTTGATCTGT
TGATCTGATGATTTGA
TGATCTGTTGATCTGT
TGATCTGATGATCTGT
TGATCTGATGATCTGATGACCTGA
Nr of selected repeats 18 Similarity 0.831699
Consensus:
TGATCTGaTGaTCTGa
>Cele-UNSB01_1:12807224-12807329 Satlength=106 Nr of Repeats=5 RepeatLength=21
seed=ACCACCCCTA
ACCACCCCTACAACTACCACC
ACCACTCCTACAACAACCACT
ACCACCCCAACAACCACCACG
ACCACCCCTACAACCACGACC
ACCACCCCTACAACTACGACC
Nr of selected repeats 5 Similarity 0.815873
Consensus:
ACCACcCCtACAACnACcACc
>Cele-UNSB01_1:12834373-12834766 Satlength=394 Nr of Repeats=5 RepeatLength=32
seed=GCTGATTGGT
GCTGATTGGTTCAAAAGTGGGCGTGGCCAATC
GCTGATTGGTTTCAAGTTTGTGAAGGGTTTTTTGTGTTTCATGTTGAATTTTTGAAATACTGGAATGACACAAAAACAA
GAAACTTTCCAATAAACAGCGTATCTTTTGGGCTTAACCGTTTTGCAGATAGTACGCC
GCTGATTGGTTCAAAAGTGGGCTTGGCTTATG
GCTGATTGGTCTACGTTTTATGGACGTTTTCATGAAGCTAATATCGATTTTTTTTTTTTTTTAAAGAACTTCAAATTATT
ACCCGAATCGCTAGATCACCAGAAAAAGTGAGATTTTTCAACAGTAGCTCATCTTTTGAATCTCCTGGCAGATGGGACG
CC
GCTGATTGGTTCAAAAGTGGGCGTGGCTATTC
Nr of selected repeats 3 Similarity 0.861111
Consensus:
GCTGATTGGTTCAAAAGTGGGCgTGGCtaaTc
>Cele-UNSB01_1:12842011-12842077 Satlength=67 Nr of Repeats=5 RepeatLength=12
seed=TACCTATACC
TACCTATACCTA
TACCTATACCTT
TACCTATACCTA
TACCTATACCTA
TACCTATACCTATACCTA
Nr of selected repeats 4 Similarity 0.944444
Consensus:
TACCTATACCTa
>Cele-UNSB01_1:12905958-12906024 Satlength=67 Nr of Repeats=5 RepeatLength=11
seed=TTTGGAGCGG
TTTGGAGCGGA
TTTGGAGCGGTTTCAGAGCGAT
TTCGGAGCGGT
TTTGGAGCGCA
TTTGGAGCGGG
Nr of selected repeats 4 Similarity 0.777778
Consensus:
TTtGGAGCGga
>Cele-UNSB01_1:13114799-13114994 Satlength=196 Nr of Repeats=5 RepeatLength=39
seed=TATTTTTAAT

TATTTTTAATTTAACCTACTTAAAAAGTAGGTCATGACC
TATTTTTAATTTAACCTACTTAAAAAGTAGGTCATGACC
TATTTTTAATTTGACCTACTTAAAAAGTAGGTCATGACC
TATTTTTAATTTGACCTACTTAAAAAGTAGGTCATGACC
TATTTTTAATTTAACCTACCTAAAAAGTAGGTCATGACC
Nr of selected repeats 5 Similarity 0.965812
Consensus:
TATTTTTAATTTaACCTACtTAAAAAGTAGGTCATGACC
>Cele-UNSB01_1:13115312-13116088 Satlength=777 Nr of Repeats=11 RepeatLength=23
seed=ACTACAAACT
ACTACAAACTACAAACTATGAAA
ACTACAAACTACAAACTATGAAA
ACTACAAACTACAAACTATGAAA
ACTACAAACTACAAACTATGAAA
ACTACAAACTACAAACTATGAAA
ACTACAAACTACAAACTATGAAA
ACTACAAACTACAAACTATGAAA
ACTACAAACTACAAACTATGAAA
ACTACAAACTACAAACTATGAAA
ACTACAAACTACAAACTATGAAA
ACTACAAACTTGCAGGTAATCCACGAGAACGCTACTCCAACACCATCATCAACATCAATGGTTGGTCATGCAATGTCAG
TTATTCCCGGATCAAAAGTAGCGACAGAACTATTCTCCCAATTGGGAAAAGTATTCTCAATGAGTGGAGACTCTGGCGG
TGTCATATCAAAACTCGACAGTTCCCGACAACATGGAAATGGATGGCGTCAAGCACAATCAGACATGCTCTCATCACTT
CCACACTCTCTTGCCACTATTTGTAATGTATGGACTGTTGTCCGCAGGGCTCAGCCACCGATTGTACCAATTGGAACGA
ATAGTCAGTTGAGACGGCTTGTGCTCCATCTCCTATCACCTATTGCTCAATATCATAAGCACGCATTCCTAACGTCTCT
TGCGTTGGTTTGGCTCACGCGGAGCACTGCGAAACCTACAGTAACCCTTCGGAAACAGGATCCAGATCGAGCGACGTTT
GAGTATTCATCTGCACAATTGGATATTACTAATCTTTTGCTATCACTTCAAGTTATTCCATTTGAGGTAGAC
Nr of selected repeats 10 Similarity 1.000000
Consensus:
ACTACAAACTACAAACTATGAAA
>Cele-UNSB01_1:13121380-13121635 Satlength=256 Nr of Repeats=17 RepeatLength=15
seed=AAAAGGGGAT
AAAAGGGGATCTGCA
AAAAGGGGATCTGCG
AAAAGGGGATCGGCA
AAAAGGGGATCTGCG
AAAAGGGGATCGGCA
AAAAGGGGATCTGCA
AAAAGGGGATCTGCA
AAAAGGGGATCTGCG
AAAAGGGATCTGCA
AAAAGGGGATCTGCA
AAAAGGGGATCTGCA
AAAAGGGGATCTGCG
AAAAGGGGATCTGCA
AAAAGGGGATCTGCGA
AAAAGGGGATCTGCA
AAAAGGGGATCTGCG
AAAAGGGGATCTGCA
Nr of selected repeats 15 Similarity 0.935661
Consensus:
AAAAGGGGATCTGCa
>Cele-UNSB01_1:13132164-13132288 Satlength=125 Nr of Repeats=6 RepeatLength=21
seed=AATCCAATAT

AATCCAATATGGATTTTCT
AATCAAATATGGTTGAAATCT
AATCCAATATGGCCGAAATCT
AATCCAATATGGAACAATTCA
AATCCAATATGGACGAAATCT
AATCCAATATGGAACAAATCT
Nr of selected repeats 5 Similarity 0.790476
Consensus:
AATCcAATATGGangAAaTCt
>Cele-UNSB01_1:13177981-13178228 Satlength=248 Nr of Repeats=13 RepeatLength=19
seed=CAAGGCACTA
CAAGGCACTAGAAAAGCGC
CAAGGTACTAGAAATGCGC
CAATGCACTAGAAATGCGT
CAAGGCACTAGGAATGCGC
CAAGGCAATAAAAATGCGT
CAAGGCACTAGGAATGCGC
CAAGGCAATAAAAATGCGT
CAAGGCACTAGGAATGCGC
CAAGGCAATAAAAATGCGT
CAAGGCACTAGGAATGCGC
CAAGGCAATAAAGATGCGC
CAAGGCACTAGAAATGCGT
CAAGGCAATAAAAATGCGC
Nr of selected repeats 13 Similarity 0.816464
Consensus:
CAAGGCAcTAgaAATGCGc
>Cele-UNSB01_1:13199796-13200258 Satlength=463 Nr of Repeats=10 RepeatLength=35
seed=GCCGGAAATT
GCCGGAAATTTTAATTTTCGGAAAATTAACGAAATGCCGATTTGCCGAAAATGTTTGATTCCGGCAAATT
GCCGGAAATTTTCAATTCTGGCAGTTTACCAATTT
GCCGGAAATTTTCAATTCTGGCAGTTTACCAATTT
GCCGGAAATTTTCATTTTCGGAAAATTAACGGTATGCCGATTT
GCTGGAAATTGTTAATTCCGGCAATTTGCCGATTT
GCAGGAAATTATCAATTCCGGCAAATTACCGGTTT
GCCGGAACTTTTGAATTCCAGCAATTTGCCGATTC
GCCGGAAATTCTAAATTCCAGCAATTTGCCGATTT
GCCGGAAATTCTGAATTCCGGCAATTTGCCAATTT
GCCGGAAATTTTCAATTCCGCAATTTGCCGGTTCTCTGGAAATTTTCATTTTCGGCAAATCGGCAAAGTGCCGGAAAAA
TCAAATTCCGGCGAATTACCGAGTT
Nr of selected repeats 7 Similarity 0.776871
Consensus:
GCcGGAAATTnTnAATTCcgGCAaTTTgCCgATTT
>Cele-UNSB01_1:13236201-13236854 Satlength=654 Nr of Repeats=16 RepeatLength=35
seed=ATTGCCGGAA
ATTGCCGGAATTAAAAATTTCGGAAAATAGGCAT
ATTGCCGGAATTGAAAAATGCAGGCAAATCGGCAT
ATTGCCGGAATTGAAAAATGCAGGCAAATCGGCAA
ATTGCCGGAATTGAAAATTTTCGGCAAATCGGCAAACCGGTAA
ATTGCCGGAATTGAAAATTAGCTGCAAATTGCCAA
ATTGCCGGAATTGAAAATTTTCGGCAAATCGGCAAACCGGTAA
ATTGCCGGAATTAAAATTTTCCGGCAAATAGGTAA
ATTGCCAGAATTGAAAATTTCGGCAATTCGGCAAATTGACAATTTACTGAAAACAAAAAATTCCGGCAAATCGGCAAAC
CGGCAATTATTAAAAAATTTCGGAAAATAGGCAA

ATTGCCGGAATTAAAAATTTTGGGCAAATCGGAAA
ATTGCCGGACATGAGAATTTCCGGCAAATCGGCAT
ATTGCCGGAATTGAAAATTTTCGGCAAATCGGCAAACCGGTAA
ATTGCCGGAATTGAAAATTAGCTGCAA
ATTGCCGGAATTGAACATTTCCGACAAATCGGTAA
ATTGCCGGAATTGAAAATTTTCGGCAAACCGGTAA
ATTGCCGGAATTGAAAATTTCCGGCAAATCGGAAA
ATTGCCGGAATTAAAATTTTCCGGCAAATCGGTAA
Nr of selected repeats 10 Similarity 0.780741
Consensus:
ATTGCCGGAATTgAAAatTtccGGCAAATcGGnAa
>Cele-UNSB01_1:13241763-13242154 Satlength=392 Nr of Repeats=8 RepeatLength=14
seed=AGTTTGTAGT
AGTTTGTAGTCTGT
AGTCTGTAGTTTGT
AGTTTGTAGTTTGTAGTTTGATTCTCCAAAACTACACGATTTTCAAAAATGCAATTTTTGAGTAATATCCGAAAAATCT
AAATTTTCACCCAAATTTTGATGTATTTAAAAAGTTTGATCTCCACCTAAATGACAATGAAACTTCAA
AGTTTGTAGTTTGT
AGTTTGTAGTTTGT
AGTCTGTAGTCTGT
AGTCTGTAGTCTGTAGTTTATAGCTGAATTTCCAAAACTACACGATTTCCAAAAAAGAAAATTCTTATGTCAAATCTCA
AAAATCTGAATATTCACTTAAATTTTACAATCACTGAAAGGCTTTTTGCACCTAAATAAAGACTGAAACTTCAA
AGTTTGTAGTTTGTAGTTTGT
Nr of selected repeats 5 Similarity 0.885714
Consensus:
AGTtTGTAGTtTGT
>Cele-UNSB01_1:13307625-13307715 Satlength=91 Nr of Repeats=4 RepeatLength=22
seed=TCGAGAAAAA
TCGAGAAAAAATCTGAAAAAAT
TCGAGAAAAAAAAATGGAAAAAATTC
TCGAGAAAAAATCTGAAAAAAT
TCGAGAAAAAATCTGAAAAAAT
Nr of selected repeats 3 Similarity 1.000000
Consensus:
TCGAGAAAAAATCTGAAAAAAT
>Cele-UNSB01_1:13315136-13315216 Satlength=81 Nr of Repeats=5 RepeatLength=16
seed=AATACGGGAA
AATACGGGAATACGGG
AATACGGGAATACGGG
AATACGGGAATACGGG
AATACGGGAATACGGG
AATACGGGAATACGGG
Nr of selected repeats 5 Similarity 1.000000
Consensus:
AATACGGGAATACGGG
>Cele-UNSB01_1:13324034-13324147 Satlength=114 Nr of Repeats=5 RepeatLength=22
seed=TTTTTTCTCG
TTTTTTCTCGAATTTTTTCAGA
TTTTTTCTCGAATTTTTTCAGA
TTTTTTCTCGAATTTTTTCAGA
TTTTTTCTCGAGAATTTTTCCATTT
TTTTTTCTCGAATTTTTTCAGA
Nr of selected repeats 4 Similarity 1.000000
Consensus:

TTTTTTCTCGAATTTTTTCAGA
>Cele-UNSB01_1:13379937-13380822 Satlength=886 Nr of Repeats=18 RepeatLength=39 seed=TTAAAAAGTA
TTAAAAAGTAGGTCATGACCTAATTTTTAATTTGACCTAC
TTAAAAAGTAGGTCATGACCTAATTTTAATATGACCTAC
TTAAAAAGTAGGTCATGACCTACTTTTTTAAACGTGACCCACT
TTAAAAGTAGGCCATGACCTACTTTTTTTAACTTGACCTAC
TTAAAAAGTAGGTCATGACCTAATTTTAATTTAGGTCAC
TTAAAAAGTAGGTCATGACCTAGATTTAATTTGACCTAC
TTAAAAAGTAGGTCATGACCTAAATTTAATTGGGCATAC
TTAAAAAGTAGGTCATCACCTAATTTTAATTTAGGTCAA
TTAAAAAGTAGGTCATGACCTAAATTTAATTTGGCCTAC
TTAAAAAGTAGGTCATGACCTAATTTTAATTTAGGTCAC
TTAAAAAGTAGGTCATGACCTAAATTTAATTTGGCCTAC
TTAAAAAGTAGGTCGTGATCTACTTTTAATTTAGGTCAC
TTAAAAAGTAGGTCATGACCTAAATTTAATTTGGCCTAC
TTAAAAAGTAGGTCGTGATCTACTTTTAATTTAGGTCAC
TTAAAAAGTAGGTCATGACCTAAATTTAATTTGACCTAC
TTAAAAAGTAGGTCATGACTTAATTTTAATTTGACCTAC
TTAAAAAATAGGTCATGACCTACAGTGCATTTTTTTTCCACTTCTACGACTTTAAAGGCGCGCGCATTTATACAAAATG
ATCCCGGCATTGGTCTCGCCACGCGCTCAACAAATCAATGGGATGTGCGTGGCGAGACCATTGCGCGAAAATGCGCGCG
CCTTTAAAGTCGTAGAAGTTGGAAAAAAAATGCACTGTAATTTTAATTTAGGTCAC
TTAAAAAGTAGGTCATGACCTACTTTTCAACTTGACCTAC
Nr of selected repeats 13 Similarity 0.822863
Consensus:
TTAAAAAGTAGGTCATGACCTAatTTTAATTTGgcCtAC
>Cele-UNSB01_1:13456302-13456675 Satlength=374 Nr of Repeats=12 RepeatLength=11 seed=GTACTGTAGG
GTACTGTAGGA
GTACTGTAGGA
GTACGGTAGGATTACTGTAGTTTAGGAAAAATTGAGTTTTGTCTTTTGAAGAGATATAGGTTTGGGGTTATTAGTGGGA
TATGGTCGGG
GTACTGTAGTA
GTACTGTAGGA
GTACTGTAGGA
GTACGGTAGGATTACTGTAGTTAATGAATAATTGTGTTTTTGTCTTTTGAAGAGATATAGGTTTGGGGTTATTAGTGGG
ATATGGTCGGG
GTACTGTAGTA
GTACTGTAGGA
GTACTGTAGGA
GTACGGTAGGATTACTGTAGTTAATGAATAATTGTGTTTTTGTCTTTTGAAGAGATATAGGTTTGGGGTTAGTAGTGGG
ATATGGTCGGGGTACT
GTACTGTAGGA
Nr of selected repeats 9 Similarity 0.952862
Consensus:
GTACTGTAGGgA
>Cele-UNSB01_1:13456767-13461871 Satlength=5105 Nr of Repeats=124 RepeatLength=40 seed=AATGTTCCAG
AATGTTCCAGAACTTTCTAGAAAAATCGAGAAAATTCTGA
AATGTTCCAGAACTTTCTAGAAAAATCGAGAAAATTCTGA
AATGTTCCAGAACTTTCTAGAAAAATCGAGAAAATTCTGA
AATGTTCCAGAACTTTCTAGAAAAATTGAGAAAATTCTGA
AATGTTCCAGAACTTTCTAGAAAAATCGAGAAAATTCTGG
AATGTTCCAGAACTTTCTAGAAAAATAGAGAAAATTCTGGAAGTGTATGTGTGCCAAATTCTGA

AATGTTCCAGAACTTTCTAGAAAAATTGAGAAAATTCTGA
AATGTTCCAGAACTTTCTAGAAAAATCGAGAAAATTCTGG
AATGTTCCAGAACTTTCTAGAAAAATCGAGAAAATTCTGGAAGTGTATGTGTGCCAAATTCTGA
AATGTTCCAGAACTTTCTAGAAAAATTGAGAAAATTCTGA
AATGTTCCAGAACTTTCTAGAAAAATTGAGAAAATTCTGA
AATGTTCCAGAACTTTCTAGAAAAATCGAGAAAATTCTGG
AATGTTCCAGAACTTTCTAGAAAAATCGAGAAAATTCTGGAAGTGTATGTGTGCCAAATTCTGA
AATGTTCCAGAACTTTCTAGAAAAATTGAGAAAATTCTGA
AATGTTCCAGAACTTTCTAGAAAAATCGAGAAAATTCTGG
AATGTTCCAGAACTTTCTAGAAAAATCGAGAAAATTCTGGAAGTGTATGTGTGCCAAATTCTGA
AATGTTCCAGAACTTTCTAGAAAAATTGAGAAAATTCTGA
AATGTTCCAGAACTTTCTAGAAAAATTGAGAAAATTCTGA
AATGTTCCAGAACTTTCTAGAAAAATCGAGAAAATTCTGG
AATGTTCCAGAACTTTCTAGAAAAATCGAGAAAATTCTGGAAGTGTATGTGTGCCAAATTCTGA
AATGTTCCAGAACTTTCTAGAAAAATTGAGAAAATTCTGA
AATGTTCCAGAACTTTCTAGAAAAATCGAGAAAATTCTGG
AATGTTCCAGAACTTTCTAGAAAAATCGAGAAAATTCTGGAAGTGTATGTGTGCCAAATTCTGA
AATGTTCCAGAACTTTCTAGAAAAATCGAGAAAATTCTGA
AATGTTCCAGAACTTTCTAGAAAAATCGAGAAAATTCTGA
AATGTTCCAGAACTTTCTAGAAAAATCGAGAAAATTCTGA
AATGTTCCAGAACTTTCTAGAAAAATTGAGAAAATTCTGA
AATGTTCCAGAACTTTCTAGAAAAATCGAGAAAATTCTGA
AATGTTCCAGAACTTTCTAGAAAAATCGAGAAAATTCTGA
AATGTTCCAGAACTTTCTAGAAAAATCGAGAAAATTCTGA
AATGTTCCAGAACTTTCTAGAAAAATCGAGAAAATTCTGA
AATGTTCCAGAACTTTCTAGAAAAATTGAGAAAATTCTGA
AATGTTCCAGAACTTTCTAGAAAAATCGAGAAAATTCTGA
AATGTTCCAGAACTTTCTAGAAAAATCGAGAAAATTCTGA
AATGTTCCAGAACTTTCTAGAAAAATCGAGAAAATTCTGA
AATGTTCCAGAACTTTCTAGAAAAATCGAGAAAATTCTGA
AATGTTCCAGAACTTTCTAGAAAAATCGAGAAAATTCTGA
AATGTTCCAGAACTTTCTAGAAAAATCGAGAAAATTCTGA
AATGTTCCAGAACTTTCTAGAAAAATTGAGAAAATTCTGA
AATGTTCCAGAACTTTCTAGAAAAATCGAGAAAATTCTGA
AATGTTCCAGAACTTTCTAGAAAAATCGAGAAAATTCTGA
AATGTTCCAGAACTTTCTAGAAAAATCGAGAAAATTCTGA
AATGTTCCAGAACTTTCTAGAAAAATCGAGAAAATTCTGA
AATGTTCCAGAACTTTCTAGAAAAATTGAGAAAATTCTGA
AATGTTCCAGAACTTTCTAGAAAAATCGAGAAAATTCTGG
AATGTTCCAGAACTTTCTAGAAAAATCGAGAAAATTCTGG
AATGTTCCAGAACCTTCTAGAAAAATCGAGAAAATTCTGA
AATGTTCCAGAACTTTCTAGAAAAATCGAGAAAATTCTGA
AATGTTCCAGAACTTTCTAGAAAAATCGAGAAAATTCTGA
AATGTTCCAGAACTTTCTAGAAAAATTGAGAAAATTCTTA
AATGTTCCAGAACTTTCTAGAAAAATCGAGAAAATTCTGA
AATGTTCCAGAACTTTCTAGAAAAATCGAGAAAATTCTGA
AATGTTCCAGAACTTTCTAGAAAAATCGAGAAAATTCTGA
AATGTTCCAGAACTTTCTAGAAAAATCGAGAAAATTCTGA
AATGTTCCAGAACTTTCTAGAAAAATCGAGAAAATTCTGA
AATGTTCCAGAACTTTCTAGAAAAATCGAGAAAATTCTGA
AATGTTCCAGAACTTTCTAGAAAAATCGAGAAAATTCTGA
AATGTTCCAGAACTTTCTAGAAAAATCGAGAAAATTCTGA
AATGTTCCAGAACTTTCTAGAAAAATCGAGAAAATTCTGA
AATGTTCCAGAACTTTCTAGAAAAATCGAGAAAATTCTGA

AATGTTCCAGAACTTTCTAGAAAAATTGAGAAAATTCTGA
AATGTTCCAGAACTTTCTAGAAAAATCGAGAAAATTCTGA
AATGTTCCAGAACTTTCTAGAAAAATCGAGAAAATTCTGA
AATGTTCCAGAACTTTCTAGAAAAATCGAGAAAATTCTGA
AATGTTCCAGAACTTTCTAGAAAAATTGAGAAAATTCTGA
AATGTTCCAGAACTTTCTAGAAAAATCGAGAAAATTCTGA
AATGTTCCAGAACTTTCTAGAAAAATTGAGAAAATTCTGA
AATGTTCCAGAACTTTCTAGAAAAATCGAGAAAATTCTGG
AATGTTCCAGAACTTTCTAGAAAAATCGAGAAAATTCTGA
AATGTTCCAGAACTTTCTAGAAAAATCGAGAAAATTCTGA
AATGTTCCAGAACTTTCTAGAAAAATTGAGAAAATTCTGA
AATGTTCCAGAACTTTCTAGAAAAATCGAGAAAATTCTGA
AATGTTCCAGAACTTTCTAGAAAAATTGAGAAAATTCTGA
AATGTTCCAGAACTTTCTAGAAAAATCGAGAAAATTCTGG
AATGTTCCAGAACTTTCTAGAAAAATCGAGAAAATTCTGA
AATGTTCCAGAACTTTCTAGAAAAATCGAGAAAATTCTGA
AATGTTCCAGAACTTTCTAGAAAAATCGAGAAAATTCTGA
AATGTTCCAGAACTTTCTAGAAAAATTGAGAAAATTCTTA
AATGTTCCAGAACTTTCTAGAAAAATCGAGAAAATTCTGA
AATGTTCCAGAACTTTCTAGAAAAATCGAGAAAATTCTGA
AATGTTCCAGAACTTTCTAGAAAAATCGAGAAAATTCTGA
AATGTTCCAGAACTTTCTAGAAAAATCGAGAAAATTCTGA
AATGTTCCAGAACTTTCTAGAAAAATCGAGAAAATTCTGA
AATGTTCCAGAACTTTCTAGAAAAATCGAGAAAATTCTGA
AATGTTCCAGAACTTTCTAGAAAAATCGAGAAAATTCTGA
AATGTTCCAGAACTTTCTAGAAAAATCGAGAAAATTCTGA
AATGTTCCAGAACTTTCTAGAAAAATCGAGAAAATTCTGA
AATGTTCCAGAACTTTCTAGAAAAATTGAGAAAATTCTGA
AATGTTCCAGAACTTTCTAGAAAAATCGAGAAAATTCTGA
AATGTTCCAGAACTTTCTAGAAAAATCGAGAAAATTCTGA
AATGTTCCAGAACTTTCTAGAAAAATTGAGAAAATTCTGA
AATGTTCCAGAACTTTCTAGAAAAATCGAGAAAATTCTGA
AATGTTCCAGAACTTTCTAGAAAAATCGAGAAAATTCTGA
AATGTTCCAGAACTTTCTAGAAAAATCGAGAAAATTCTGA
AATGTTCCAGAACTTTCTAGAAAAATCGAGAAAATTCTGA
AATGTTCCAGAACTTTCTAGAAAAATCGAGAAAATTCTGA
AATGTTCCAGAACTTTCTAGAAAAATTGAGAAAATTCTGA
AATGTTCCAGAACTTTCTAGAAAAATCGAGAAAATTCTGA
AATGTTCCAGAACTTTCTAGAAAAATCGAGAAAATTCTGA
AATGTTCCAGAACTTTCTAGAAAAATTGAGAAAATTCTGA
AATGTTCCAGAACTTTCTAGAAAAATCGAGAAAATTCTGG
AATGTTCCAGAACTTTCTAGAAAAATCGAGAAAATTCTGG
AATGTTCCAGAACCTTCTAGAAAAATCGAGAAAATTCTGA
AATGTTCCAGAACTTTCTAGAAAAATCGAGAAAATTCTGG
AATGTTCCAGAACTTTCTAGAAAAATCGAGAAAATTCTGG
AATGTTCCAGAACTTTCTAGAAAAATTGAGAAAATTCTGA
AATGTTCCAGAACTTTCTAGAAAAATCGAGAAAATTCTGA
AATGTTCCAGAACTTTCTAGAAAAATCGAGAAAATTCTGG
AATGTTCCAGAACTTTCTAGAAAAATCGAGAAAATTCTGG
AATGTTCCAGAACTTTCTAGAAAAATTGAGAAAATTCTGA
AATGTTCCAGAACTTTCTAGAAAAATCGAGAAAATTCTGA
AATGTTCCAGAACTTTCTAGAAAAATCGAGAAAATTCTGA
AATGTTCCAGAACTTTCTAGAAAAATTGAGAAAATTCTGG
AATGTTCCAGAACCTTCTAGAAAAATCGAGAAAATTCTGA

AATGTTCCAGAACTTTCTAGAAAAATCGAGAAAATTCTGG
AATGTTCCAGAACTTTCTAGAAAAATCGAGAAAATTCTGG
AATGTTCCAGAACTTTCTAGAAAAATTGAGAAAATTCTGA
AATGTTCCAGAACTTTCTAGAAAAATCGAGAAAATTCTGA
AATGTTCCAGAACTTTCTAGAAAAATCGAGAAAATTCTGA
AATGTTCCAGAACTTTCTAGAAAAATTGAGAAAATTCTGA
AATGTTCCAGAACTTTCTAGAAAAATCGAGAAAATTCTGG
AATGTTCCAGAACTTTCTAGAAAAATCGAGAAAATTCTGG
AATGTTCCAGAACCTTCTAGAAAAATCGAGAAAATTCTGA
AATGTTCCAGAACTTTCTAGAAAAATCGAGAAAATTCTGG
Nr of selected repeats 118 Similarity 0.974311
Consensus:
AATGTTCCAGAACTTTCTAGAAAAATcGAGAAAATTCTGA
>Cele-UNSB01_1:13507191-13507550 Satlength=360 Nr of Repeats=18 RepeatLength=20
seed=AAAATAGATT
AAAATAGATTGTTACACGAA
AAAATAGATTGTTACACGAA
AAAATAGATTGTTACACGAA
AAAATAGATTGTTACACGAA
AAAATAGATTGTTACACGAA
AAAATAGATTGTTACACGAA
AAAATAGATTGTTACACGAA
AAAATAGATTGTTACACGAA
AAAATAGATTGTTACACGAA
AAAATAGATTGTTACACGAT
AAAATAGATTGTTACACGAA
AAAATAGATTGTTACACGAA
AAAATAGATTGTTACACGAA
AAAATAGATTGTTACACGAA
AAAATAGATTGTTACACGAA
AAAATAGATTGTTACACGAA
AAAATAGATTGTTACACGAA
AAAATAGATTGTTTCACGA
Nr of selected repeats 17 Similarity 0.992157
Consensus:
AAAATAGATTGTTACACGAA
>Cele-UNSB01_1:13507576-13507758 Satlength=183 Nr of Repeats=7 RepeatLength=26
seed=CTCTGGCAGT
CTCTGGCAGTTCACTAAAATAAGTGG
CTCTGGCAGTTCACTAAAATAAGTGA
CTCTGGCAGTTCACTAAAATAAGTGG
CTCTGGCAGTTCACTAAAATAAGTGG
CTCTGGCAGTTCACTAAAATAAGTGG
CTCTGGCAGTTCACTAAAATAAGTGA
CTCTGGCAGTTCACTAAAAATAGTGA
Nr of selected repeats 7 Similarity 0.941392
Consensus:
CTCTGGCAGTTCACTAAAATAAGTGg
>Cele-UNSB01_1:13634561-13638668 Satlength=4108 Nr of Repeats=158
RepeatLength=26 seed=TTTTAGGTGA
TTTTAGGTGAATGGTTAGAGTCATAA
TTTTAGGTGAATGGTTAGAGTCATAA
TTTTAGGTGAATGGTTAGAGTCATAA
TTTTAGGTGAATGGTTAAAGTCATAT

TTTTAGGTGAATGGTTAGAGTCATAT
TTTTAGGTGAATGGTTAGAGTCATAT
TTTTAGGTGAATGGTTAGAGTCATAA
TTTTAGGTGAATGGTTAGAGTCATAA
TTTTAGGTGAATGGTTAGAGTCATAA
TTTTAGGTGAATGGTTAGAGTCATAT
TTTTAGGTGAATGGTTAGAGTCATAA
TTTTAGGTGAATGGTTAGAGTCATAC
TTTTAGGTGAATGGTTAGAGTCATAA
TTTTAGGTGAATGGTTAGAGTCATAA
TTTTAGGTGAATGGTTAGAGTCATAT
TTTTAGGTGAATGGTTAGAGTCATAT
TTTTAGGTGAATGGTTAGAGTCATAT
TTTTAGGTGAATGGTTAGAGTCATAA
TTTTAGGTGAATGGTTAGAGTCATAT
TTTTAGGTGAATGGTTAGAGTCATAA
TTTTAGGTGAATGGTTAGAGTCATAT
TTTTAGGTGAATGGTTAGAGTCATAA
TTTTAGGTGAATGGTTAGAGTCATAA
TTTTAGGTGAATGGTTAGAGTCATAA
TTTTAGGTGAATGGTTAGAGTCATAA
TTTTAGGTGAATGGTTAGAGTCATAT
TTTTAGGTGAATGGTTAGAGTCATAA
TTTTAGGTGAATGGTTAGAGTCATAA
TTTTAGGTGAATGGTTAGAGTCATAT
TTTTAGGTGAATGGTTAGAGTCATAA
TTTTAGGTGAATGGTTAGAGTCATAT
TTTTAGGTGAATGGTTAGAGTCATAA
TTTTAGGTGAATGGTTAGAGTCATAT
TTTTAGGTGAATGGTTAGAGTCATAA
TTTTAGGTGAATGGTTAGAGTCATAT
TTTTAGGTGAATGGTTAGAGTCATAA
TTTTAGGTGAATGGTTAGAGTCATAT
TTTTAGGTGAATGGTTAGAGTCATA
TTTTAGGTGAATGGTTAGAGTCATAA
TTTTAGGTGAATGGTTAGAGTCATAT
TTTTAGGTGAATGGTTAGAGTCATAA
TTTTAGGTGAATGGTTAGAGTCATAT
TTTTAGGTGAATGGTTAGAGTCATAA
TTTTAGGTGAATGGTTAGAGTCATAT
TTTTAGGTGAATGGTTAGAGTCATAA
TTTTAGGTGAATGGTTAGAGTCATAT
TTTTAGGTGAATGGTTAGAGTCATAA
TTTTAGGTGAATGGTTAGAGTCATAA
TTTTAGGTGAATGGTTAGAGTCATAA
TTTTAGGTGAATGGTTAGAGTCATAA
TTTTAGGTGAATGGTTAGAGTCATAT
TTTTAGGTGAATGGTTAGAGTCATAA
TTTTAGGTGAATGGTTAGAGTCATAT
TTTTAGGTGAATGGTTAGAGTCATAA
TTTTAGGTGAATGGTTAGAGTCATAA
TTTTAGGTGAATGGTTAGAGTCATAA
TTTTAGGTGAATGGTTAGAGTCATAA
TTTTAGGTGAATGGTTAGAGTCATAA

TTTTAGGTGAATGGTTAGAGTCATAA
TTTTAGGTGAATGGTTAGAGTCATAT
TTTTAGGTGAATGGTTAGAGTCATAA
TTTTAGGTGAATGGTTAGAGTCATAT
TTTTAGGTGAATGGTTAGAGTCATAA
TTTTAGGTGAATGGTTAGAGTCATAT
TTTTAGGTGAATGGTTAGAGTCATAT
TTTTAGGTGAATGGTTAGAGTCATAA
TTTTAGGTGAATGGTTAGAGTCATAT
TTTTAGGTGAATGGTTAGAGTCATAT
TTTTAGGTGAATGGTTAGAGTCATAA
TTTTAGGTGAATGGTTAGAGTCATAT
TTTTAGGTGAATGGTTAGAGTCATAA
TTTTAGGTGAATGGTTAGAGTCATAT
TTTTAGGTGAATGGTTAGAGTCATAA
TTTTAGGTGAATGGTTAGAGTCATAA
TTTTAGGTGAATGGTTAGAGTCATAT
TTTTAGGTGAATGGTTAGAGTCATAT
TTTTAGGTGAATGGTTAGAGTCATAT
TTTTAGGTGAATGGTTAGAGTCATAA
TTTTAGGTGAATGGTTAGAGTCATAT
TTTTAGGTGAATGGTTAGAGTCATAA
TTTTAGGTGAATGGTTAGAGTCATAA
TTTTAGGTGAATGGTTAGAGTCATAA
TTTTAGGTGAATGGTTAGAGTCATAT
TTTTAGGTGAATGGTTAGAGTCATAA
TTTTAGGTGAATGGTTAGAGTCATAA
TTTTAGGTGAATGGTTAGAGTCATAT
TTTTAGGTGAATGGTTAGAGTCATAT
TTTTAGGTGAATGGTTAGAGTCATAT
TTTTAGGTGAATGGTTAGAGTCATAT
TTTTAGGTGAATGGTTAGAGTCATAT
TTTTAGGTGAATGGTTAGAGTCATAA
TTTTAGGTGAATGGTTAGAGTCATAT
TTTTAGGTGAATGGTTAGAGTCATAA
TTTTAGGTGAATGGTTAGAGTCATAT
TTTTAGGTGAATGGTTAGAGTCATAA
TTTTAGGTGAATGGTTAGAGTCATAT
TTTTAGGTGAATGGTTAGAGTCATAA
TTTTAGGTGAATGGTTAGAGTCATAT
TTTTAGGTGAATGGTTAGAGTCATAA
TTTTAGGTGAATGGTTAGAGTCATAT
TTTTAGGTGAATGGTTAGAGTCATAA
TTTTAGGTGAATGGTTAGAGTCATAT
TTTTAGGTGAATGGTTAGAGTCATAA
TTTTAGGTGAATGGTTAGAGTCATAA
TTTTAGGTGAATGGTTAGAGTCATAT
TTTTAGGTGAATGGTTAGAGTCATAA
TTTTAGGTGAATGGTTAGAGTCATAA
TTTTAGGTGAATGGTTAGAGTCATAT
TTTTAGGTGAATGGTTAGAGTCATAA
TTTTAGGTGAATGGTTAGAGTCATAA
TTTTAGGTGAATGGTTAGAGTCATAT
TTTTAGGTGAATGGTTAGAGTCATAT

TTTTAGGTGAATGGTTAGAGTCATAT
TTTTAGGTGAATGGTTAGAGTCATAT
TTTTAGGTGAATGGTTAGAGTCATAT
TTTTAGGTGAATGGTTAGAGTCATAT
TTTTAGGTGAATGGTTAGAGTCATAT
TTTTAGGTGAATGGTTAGAGTCATAA
TTTTAGGTGAATGGTTAGAGTCATAA
TTTTAGGTGAATGGTTAGAGTCATAT
TTTTAGGTGAATGGTTAGAGTCATAT
TTTTAGGTGAATGGTTAGAGTCATAT
TTTTAGGTGAATGGTTAGAGTCATAA
TTTTAGGTGAATGGTTAGAGTCATAT
TTTTAGGTGAATGGTTAGAGTCATAA
TTTTAGGTGAATGGTTAGAGTCATAA
TTTTAGGTGAATGGTTAGAGTCATAT
TTTTAGGTGAATGGTTAGAGTCATAA
TTTTAGGTGAATGGTTAGAGTCATAT
TTTTAGGTGAATGGTTAGAGTCATAT
TTTTAGGTGAATGGTTAGAGTCATAA
TTTTAGGTGAATGGTTAGAGTCATAA
TTTTAGGTGAATGGTTAGAGTCATAA
TTTTAGGTGAATGGTTAGAGTCATAT
TTTTAGGTGAATGGTTAGAGTCATAT
TTTTAGGTGAATGGTTAGAGTCATAA
TTTTAGGTGAATGGTTAGAGTCATAA
TTTTAGGTGAATGGTTAGAGTCATAA
TTTTAGGTGAATGGTTAGAGTCATAT
TTTTAGGTGAATGGTTAGAGTCATAA
TTTTAGGTGAATGGTTAGAGTCATAT
TTTTAGGTGAATGGTTAGAGTCATAA
TTTTAGGTGAATGGTTAGAGTCATAT
TTTTAGGTGAATGGTTAGAGTCATAT
TTTTAGGTGAATGGTTAGAGTCATAT
TTTTAGGTGAATGGTTAGAGTCATAT
TTTTAGGTGAATGGTTAGAGTCATAA
TTTTAGGTGAATGGTTAGAGTCATAT
TTTTAGGTGAATGGTTAGAGTCATAA
TTTTAGGTGAATGGTTAGAGTCATAT
TTTTAGGTGAATGGTTAGAGTCATAT
TTTTAGGTGAATGGTTAGAGTCATAT
TTTTAGGTGAATGGTTAGAGTCATAA
TTTTAGGTGAATGGTTAGAGTCATAT
TTTTAGGTGAATGGTTAGAGTCATAA
TTTTAGGTGAATGGTTAGAGTCATAA
TTTTAGGTGAATGGTTAGAGTCATAA
TTTTAGGTGAATGGTTAGAGTCATAC
Nr of selected repeats 157 Similarity 0.972948
Consensus:
TTTTAGGTGAATGGTTAGAGTCATAa
>Cele-UNSB01_1:13683275-13683450 Satlength=176 Nr of Repeats=5 RepeatLength=35
seed=TGAAAATTTC
TGAAAATTTCCGGCAAATCGGCAAATTGTCGGAAT
TGAAAATTTCCGGCAAAACGGCAAATTTTCGAAAA
TGAAAATTTCCGGCAAATCGGCAAATTGTCGGAAT

TGAAAATTTCCGGCAAAACGGCAAATTTTCGAAAA
TGAAAATTTCCGGCAAATCGGCAAACTGCCGGAAT
Nr of selected repeats 5 Similarity 0.878095
Consensus:
TGAAAATTTCCGGCAAAtCGGCAAAtTgtCGgAAt
>Cele-UNSB01_1:13729567-13730578 Satlength=1012 Nr of Repeats=11
RepeatLength=14 seed=TAGTTTGTAG
TAGTTTGTAGTCACTGAGCCTCAATTAATATGCTATTGACAACCTTATGAATTAGGTGTTGGTATTAATTTTAATTTAA
GAAAAGTTTCAAAATTTGGTTGAGGCTAAAAGGTTTTG
TAGTTTGTAGGAGGTTTAGAGACTACAAACTACACAATAATTCCCCACCACCCATAATTCATAAGAAACAAAGGAGATA
GGTGTAATTTATGACAGGGGGGGGGGGGGGGGGGTCACATGGACATCTGGTGGAAGGGAGGTATCCGTGTAATAAGATAC
CTCCCAGTTGAAGACCAAAAAAAAACTTTTTGATGGTTGAGGCTATCGGAGACTACAAACTACAAAGCTGCAAAGCCTT
AACCAATTGTCCTTTTTAAGGCTATTTACACTATTTATATACAACCTTACATTTGACAAACGGTTAGTCACCACAAAAA
TGACTAAGATATTAACTTGATTTGTGAGAAACTGGTTGAGGCTAACACTACAAACTACAATATCGGCTTAGCCTCACCC
AATTTTTTTTTTCAAAATTTTATCAAGTTCATTGCTCCGTAGAATATTTGAGCATACAGTATTGTGTTTCAAAAATTAT
TGAAAAAGTGTTGCCACGTCACAAAGTTTTATAGCTAA
TAGTTTGTAGTTTG
TAGTTTGTAGTTTG
TAGTTTGAAGTTGG
TAGTTTGTAGTTTG
TAGTTTGTAGTCTTTAGCCAAATGCTCTGCTCGTATTAAAATCAAGTTGCTCCGTGCAACAATTTTCCACAAAAAAACT
TGCCAAAAAATGTTAAAATGCAACGTGTCTAATGCAATTAGAGTCTTACTATT
TAATTTGTAGTCTA
TAGTTTGTAGTTTG
TAGTTTGTAGTTTA
TAGTTTGTAATACTTAATACAAAAATCAACTTGGTCAATGTAAAAATTTTCAAAAACAACAACTTTTTTTTTGTTGGAA
AAACTCGTTGTGTTGCACATTATTAAAACTATTGAATAACTATTAAACGTATTACAATACCTTAAAAATTATG
Nr of selected repeats 7 Similarity 0.845805
Consensus:
TAGTTTGTAGTTTg
>Cele-UNSB01_1:13730896-13731271 Satlength=376 Nr of Repeats=11 RepeatLength=14
seed=TACAAACTAC
TACAAACTACAAACTATAAAC
TATAAACTACAAAC
TACAACTACAAAC
TACAAGCTACAAAC
TACAAACTACAAACTACAAGTAATTATTTCACATAGTTAAGTCTATCTCATATGGTTTTAAAATTAAATGTATGTTGAG
TTAACACAATGTGGCAATTGTTTATCGTTCTATGAAATGATGGAC
TACAAACTACAAAT
TACAAACTACAAAC
TACAAACTACAAAC
TACAAACTACAAAC
TACAAATCTACAATTTTACATATTAAGTTTATAGAAGTTTTAAAACAATCTCGAATGTTTTTAAAATTCAATGTATGTT
GAGTTAACACAATTATTTATCGTTTTACAAGATGATAGAC
TACAAACTACAAAC
Nr of selected repeats 7 Similarity 0.918367
Consensus:
TACAAACTACAAAC
>Cele-UNSB01_1:13831060-13832042 Satlength=983 Nr of Repeats=52 RepeatLength=14
seed=TGTAGTATGT
TGTAGTATGTAGTT
TGTAGTATGTAGTT
TGTAGTATGTAGTT
TGTAGTATGTAGTA

TGTAGTATGTAGTA
TGTAGTATGTAGTA
TGTAGTATGTAGTA
TGTAGTATGTAGTA
TGTAGTATGTAGTA
TGTAGTATGTAGTA
TGTAGTATGTAGTA
TGTAGTATGTAGTA
TGTAGTATGTAGTA
TGTAGTATGTAGTA
TGTAGTATGTAGTA
TGTAGTATGTAGTA
TGTAGTATGTAGTA
TGTAGTATGTAGTA
TGTAGTATGTAGTA
TGTAGTATGTAGTA
TGTAGTATGTAGTA
TGTAGTATGTAGTA
TGTAGTATGTAGTA
TGTAGTATGTAGTA
TGTAGTATGTAGTA
TGTAGTATGTAGTA
TGTAGTATGTAGTA
TGTAGTATGTAGTA
TGTAGTATGTAGTA
TGTAGTATGTAGTA
TGTAGTATGTAGTA
TGTTGTATGTTGTATGATGTA
TGTAGTATGATGTA
TGTAGTATGTAGCA
TGTAGTATGTAGTA
TGTAGTATGTAGTA
TGTAGTATGTAGTA
TGTAGTATGTTGTATGATGTATGTTGTATGATGTATGTTGTATGATGTA
TGTAGTATGTAGTA
TGTAGTATGTAGTA
TGTAGTATGTAGTATGTTGTATGATGTATGTTGTATGATGTATGTTGTATGATGTA
TGTAGTATGTAGCATGTGCATATGTTAAACGTTGCA
TGTAGTATGTTATATGAAGAATGTTATATGTAGCATTCAAAATGGAATGTTTAAAATTCAATATGTATTT
TGTAGTATGTAGTA
TGTAGTATGTAGTA
TGTAGTATGTAGTA
TGTAGTATGTAGTA
TGTAGTATGTAGTA
TGTAGTATGTAGTATGTTGTATGATGTA
TGTAGTATGATGTA
TGTAGTATGTAGCATGTGCATATGTTAAACGTTGCA
TGTAGTATGTTATATGAAGAATGTTATATGTAGCATGCAAAATGGAATGTTTAAAATTCAATATGTATTT
Nr of selected repeats 44 Similarity 0.966375
Consensus:
TGTAGTATGTAGTA
>Cele-UNSB01_1:13840435-13852243 Satlength=11809 Nr of Repeats=369
RepeatLength=32 seed=TGAATTCCCG
TGAATTCCCGCCAAAATTTTTCACAGAAATTT

TGAATTCCCGCCAAAACTTTTCACAGAAAATT
TGAATTCCCGCCAAAATTTTTCACAGAAATTT
TGAATTCCCGCCAAAACTTTTCACAGAAATTT
TGAATTCCCGCCAAAATTTTTCATAGAAATTT
TGAATTCCCGCCAAAATTTTTCATAGAAATTT
TGAATTCCCGCCAAAATTTTTCACAGAAATTT
TGAATTCCCGCCAAAATTTTTCATAGAAATTT
TGAATTCCCGCCAAAATTTTTCACAGAAATTT
TGAATTCCCGCCAAAATTTTTCATAGAAATTT
TGAATTCCCGCCAAAATTTTTCACAGAAATTT
TGAATTCCCGCCAAAATTTTTCATAGAAATTT
TGAATTCCCGCCAAAATTTTTCACAGAAATTT
TGAATTCCCGCCAAAATTTTTCACAGAAATTT
TGAATTCCCGCCAAAATTTTTCACAGAAATTT
TGAATTCCCGTTATAATTTTTCACAGAAATTT
TGAATTCCCGTTATAATTTTTCACAGAAATTC
TGAATTCCCGCCAAAATTTTTCACAGAAAATT
TGAATTCCCGCCAAAATTTTTCACAGAAATTT
TGAATTCCCGCCAAAATTTTTCATAGAAATTT
TGAATTCCCGCCAAAATTTTTCACAGAAATTT
TGAATTCCCGCCAAAATTTTTCATAGAAATTT
TGAATTCCCGCCAAAATTTTTCATAGAAATTT
TGAATTCCCGCCAAAATTTTTCACAGAAATTT
TGAATTCCCGCCAAAATTTTTCATAGAAATTT
TGAATTCCCGTTATAATTTTTCACAGAAATTT
TGAATTCCCGCCAAAATTTTTCACAGAAATTT
TGAATTCCCGTTATAATTTTTCACAGAAATTT
TGAATTCCCGCCAAAATTTTTCACAGAAATTT
TGAATTCCCGCCAAAATTTTTCACAGAAAACT
TGAATTCCCGTTATAATTTTTCACAGAAATTC
TGAATTCCCGTTATAATTTTTCACAGAAATTC
TGAATTCCCGCCAAAATTTTTCACAGAAATTT
TGAATTCCCGCCAAAATTTTTCATAGAAATTT
TGAATTCCCGCCAAAATTTTTCATAGAAATTT
TGAATTCCCGCCAAAATTTTTCACAGAAATTT
TGAATTCCCGCCAAAATTTTTCATAGAAATTT
TGAATTCCCGCCAAAATTTTTCACAGAAATTT
TGAATTCCCGCCAAAATTTTTCATAGAAATTT
TGAATTCCCGCCAAAATTTTTCACAGAAATTT
TGAATTCCCGCCAAAATTTTTCATAGAAATTT
TGAATTCCCGCCAAAATTTTTCACAGAAATTT
TGAATTCCCGCCAAAATTTTTCATAGAAATTT
TGAATTCCCGCCAAAATTTTTCACAGAAATTT
TGAATTCCCGTTATAATTTTTCACAGAAATTT
TGAATTCCCGCCAAAATTTTTCACAGAAATTT
TGAATTCCCGCCAAAATTTTTCACAGAAATTT
TGAATTCCCGTTATAATTTTTCACAGAAATTC
TGAATTCCCGCCAAAATTTTTCATAGAAATTT
TGAATTCCCGCCAAAATTTTTCATAGAAATTT
TGAATTCCCGCCAAAATTTTTCACAGAAATTT
TGAATTCCCGTTATAATTTTTCACAGAAATTT
TGAATTCCCGCCAAAATTTTTCATAGAAATTT
TGAATTCCCGCCAAAATTTTTCATAGAAATTT
TGAATTCCCGCCAAAATTTTTCATAGAAATTT

TGAATTCCCGCCAAAATTTTTCATAGAAATTT
TGAATTCCCGCCAAAATTTTTCACAGAAAACT
TGAATTCCCGTTATAATTTTTCACAGAAATTC
TGAATTCCCGCCAAAATTTTTCACAGAAATTT
TGAATTCCCGCCAAAATTTTTCACAGAAAACT
TGAATTCCCGTTATAATTTTTCACAGAAATTC
TGAATTCCCGCCAAAATTTTTCACAGAAAACT
TGAATTCCCGTTATAATTTTTCACAGAAATTC
TGAATTCCCGCCAAAATTTTTCACAGAAATTT
TGAATTCCCGCCAAAATTTTTCATAGAAATTT
TGAATTCCCGCCAAAATTTTTCATAGAAATTT
TGAATTCCCGCCAAAATTTTTCACAGAAATTT
TGAATTCCCGCCAAAATTTTTCACAGAAATTT
TGAATTCCCGTTATAATTTTTCACAGAAATTC
TGAATTCCCGCCAAAATTTTTCATAGAAATTT
TGAATTCCCGCCAAAATTTTTCATAGAAATTT
TGAATTCCCGCCAAAATTTTTCACAGAAATTT
TGAATTCCCGTTATAATTTTTCACAGAAATTT
TGAATTCCCGCCAAAATTTTTCACAGAAAACT
TGAATTCCCGCCAAAATTTTTCACAGAAATTT
TGAATTCCCGCCAAAATTTTTCATAGAAAACT
TGAATTCCCGTTATAATTTTTCACAGAAATTT
TGAATTCCCGCCAAAACTTTTCACAGAAATTT
TGAATTCCCGCCAAAATTTTTCATAGAAATTT
TGAATTCCCGCCAAAATTTTTCATAGAAATTT
TGAATTCCCGCCAAAATTTTTCACAGAAAACT
TGAATTCCCGTTATAATTTTTCACAGAAATTC
TGAATTCCCGCCAAAATTTTTCACAGAAATTT
TGAATTCCCGCCAAAATTTTTCACAGAAAACT
TGAATTCCCGTTATAATTTTTCACAGAAATTC
TGAATTCCCGCCAAAATTTTTCACAGAAATTT
TGAATTCCCGCCAAAATTTTTCATAGAAATTT
TGAATTCCCGCCAAAATTTTTCATAGAAATTT
TGAATTCCCGCCAAAATTTTTCACAGAAAACT
TGAATTCCCGTTATAATTTTTCACAGAAATTC
TGAATTCCCGCCAAAATTTTTCACAGAAATTT
TGAATTCCCGCCAAAATTTTTCATAGAAATTT
TGAATTCCCGCCAAAATTTTTCATAGAAATTT
TGAATTCCCGCCAAAATTTTTCACAGAAAACT
TGAATTCCCGTTATAATTTTTCACAGAAATTC
TGAATTCCCGCCAAAATTTTTCACAGAAATTT
TGAATTCCCGCCAAAATTTTTCATAGAAATTT
TGAATTCCCGCCAAAATTTTTCATAGAAATTT
TGAATTCCCGCCAAAATTTTTCACAGAAATTT
TGAATTCCCGCCAAAATTTTTCACAGAAATTT
TGAATTCCCGTTATAATTTTTCACAGAAATTC
TGAATTCCCGCCAAAATTTTTCATAGAAATTT
TGAATTCCCGCCAAAATTTTTCATAGAAATTT
TGAATTCCCGCCAAAATTTTTCACAGAAATTT
TGAATTCCCGTTATAATTTTTCACAGAAATTT
TGAATTCCCGCCAAAATTTTTCACAGAAAACT
TGAATTCCCGCCAAAATTTTTCACAGAAATTT
TGAATTCCCGCCAAAATTTTTCATAGAAAACT
TGAATTCCCGTTATAATTTTTCACAGAAATTT

TGAATTCCCGCCAAAACTTTTCACAGAAATTT
TGAATTCCCGCCAAAATTTTTCATAGAAATTT
TGAATTCCCGCCAAAATTTTTCATAGAAATTT
TGAATTCCCGCCAAAATTTTTCACAGAAAACT
TGAATTCCCGTTATAATTTTTCACAGAAATTC
TGAATTCCCGCCAAAATTTTTCACAGAAATTT
TGAATTCCCGCCAAAATTTTTCACAGAAAACT
TGAATTCCCGTTATAATTTTTCACAGAAATTC
TGAATTCCCGCCAAAATTTTTCACAGAAATTT
TGAATTCCCGCCAAAATTTTTCATAGAAATTT
TGAATTCCCGCCAAAATTTTTCATAGAAATTT
TGAATTCCCGCCAAAATTTTTCACAGAAAACT
TGAATTCCCGTTATAATTTTTCACAGAAATTC
TGAATTCCCGCCAAAATTTTTCACAGAAATTT
TGAATTCCCGCCAAAATTTTTCATAGAAATTT
TGAATTCCCGCCAAAATTTTTCATAGAAATTT
TGAATTCCCGCCAAAATTTTTCACAGAAAACT
TGAATTCCCGTTATAATTTTTCACAGAAATTC
TGAATTCCCGCCAAAATTTTTCACAGAAATTT
TGAATTCCCGCCAAAATTTTTCATAGAAATTT
TGAATTCCCGCCAAAATTTTTCACAGAAATTT
TGAATTCCCGCCAAAATTTTTCATAGAAATTT
TGAATTCCCGCCAAAATTTTTCACAGAAATTT
TGAATTCCCGCCAAAATTTTTCATAGAAATTT
TGAATTCCCGCCAAAATTTTTCATAGAAATTT
TGAATTCCCGCCAAAATTTTTCACAGAAATTC
TGAATTCCCGCCAAAATTTTTCATAGAAATTT
TGAATTCCCGCCAAAATTTTTCATAGAAATTT
TGAATTCCCGCCAAAATTTTTCACAGAAATTT
TGAATTCCCGTTATAATTTTTCACAGAAATTT
TGAATTCCCGCCAAAATTTTTCACAGAAAACT
TGAATTCCCGCCAAAATTTTTCACAGAAATTT
TGAATTCCCGCCAAAATTTTTCATAGAAATTT
TGAATTCCCGCCAAAATTTTTCATAGAAATTT
TGAATTCCCGCCAAAATTTTTCATAGAAATTT
TGAATTCCCGCCAAAATTTTTCATAGAAATTT
TGAATTCCCGCCAAAATTTTTCACAGAAAACT
TGAATTCCCGTTATAATTTTTCACAGAAATTC
TGAATTCCCGCCAAAATTTTTCACAGAAATTT
TGAATTCCCGCCAAAATTTTTCACAGAAATTC
TGAATTCCCGCCAAAATTTTTCATAGAAATTT
TGAATTCCCGCCAAAATTTTTCATAGAAATTT
TGAATTCCCGCCAAAATTTTTCACAGAAAACT
TGAATTCCCGTTATAATTTTTCACAGAAATTC
TGAATTCCCGCCAAAATTTTTCACAGAAAACT
TGAATTCCCGTTATAATTTTTCACAGAAATTC
TGAATTCCCGCCAAAATTTTTCACAGAAAACT
TGAATTCCCGTTATAATTTTTCACAGAAATTC
TGAATTCCCGCCAAAATTTTTCACAGAAAACT
TGAATTCCCGTTATAATTTTTCACAGAAATTC
TGAATTCCCGCCAAAATTTTTCACAGAAATTT
TGAATTCCCGCCAAAATTTTTCATAGAAATTT
TGAATTCCCGCCAAAATTTTTCATAGAAATTT
TGAATTCCCGCCAAAATTTTTCACAGAAAACT

TGAATTCCCGTTATAATTTTTCACAGAAATTC
TGAATTCCCGCCAAAATTTTTCACAGAAATTT
TGAATTCCCGCCAAAATTTTTCATAGAAATTT
TGAATTCCCGCCAAAATTTTTCATAGAAATTT
TGAATTCCCGCCAAAATTTTTCACAGAAATTT
TGAATTCCCGCCAAAATTTTTCATAGAAATTT
TGAATTCCCGCCAAAATTTTTCACAGAAATTT
TGAATTCCCGCCAAAATTTTTCATAGAAATTT
TGAATTCCCGCCAAAATTTTTCACAGAAATTT
TGAATTCCCGTTATAATTTTTCACAGAAATTC
TGAATTCCCGCCAAAATTTTTCACAGAAATTT
TGAATTCCCGCCAAAATTTTTCATAGAAATTT
TGAATTCCCGCCAAAATTTTTCACAGAAATTT
TGAATTCCCGCCAAAATTTTTCATAGAAATTT
TGAATTCCCGCCAAAATTTTTCACAGAAATTT
TGAATTCCCGCCAAAATTTTTCACAGAAATTT
TGAATTCCCGCCAAAATTTTTCATAGAAATTT
TGAATTCCCGCCAAAATTTTTCACAGAAATTT
TGAATTCCCGCCAAAATTTTTCATAGAAATTT
TGAATTCCCGCCAAAATTTTTCATAGAAATTT
TGAATTCCCGCCAAAATTTTTCACAGAAATTT
TGAATTCCCGCCAAAATTTTTCATAGAAATTT
TGAATTCCCGCCAAAATTTTTCATAGAAATTT
TGAATTCCCGCCAAAATTTTTCACAGAAATTC
TGAATTCCCGCCAAAATTTTTCACAGAAATTT
TGAATTCCCGCCAAAATTTTTCATAGAAATTT
TGAATTCCCGCCAAAATTTTTCATAGAAATTT
TGAATTCCCGCCAAAATTTTTCACAGAAATTC
TGAATTCCCGCCAAAATTTTTCACAGAAATTT
TGAATTCCCGCCAAAATTTTTCATAGAAATTT
TGAATTCCCGCCAAAATTTTTCATAGAAATTT
TGAATTCCCGCCAAAATTTTTCACAGAAATTC
TGAATTCCCGCCAAAATTTTTCACAGAAATTT
TGAATTCCCGCCAAAATTTTTCATAGAAATTT
TGAATTCCCGCCAAAATTTTTCATAGAAATTT
TGAATTCCCGCCAAAATTTTTCATAGAAATTT
TGAATTCCCGCCAAAATTTTTCACAGAAATTC
TGAATTCCCGCCAAAATTTTTCATAGAAATTT
TGAATTCCCGCCAAAATTTTTCACAGAAATTT
TGAATTCCCGCCAAAATTTTTCATAGAAATTT
TGAATTCCCGCCAAAATTTTTCACAGAAATTT
TGAATTCCCGTTATAATTTTTCACAGAAATTT
TGAATTCCCGCCAAAATTTTTCACAGAAATTT
TGAATTCCCGCCAAAATTTTTCACAGAAATTT
TGAATTCCCGTTATAATTTTTCACAGAAATTC
TGAATTCCCGCCAAAATTTTTCATAGAAATTT
TGAATTCCCGCCAAAATTTTTCATAGAAATTT
TGAATTCCCGCCAAAATTTTTCACAGAAATTT
TGAATTCCCGCCAAAATTTTTCATAGAAATTT
TGAATTCCCGCCAAAATTTTTCATAGAAATTT
TGAATTCCCGCCAAAATTTTTCATAGAAATTT
TGAATTCCCGCCAAAATTTTTCACAGAAATTC
TGAATTCCCGCCAAAATTTTTCATAGAAATTT
TGAATTCCCGCCAAAATTTTTCACAGAAATTT

TGAATTCCCGCCAAAATTTTTCATAGAAATTT
TGAATTCCCGCCAAAATTTTTCACAGAAATTT
TGAATTCCCGTTATAATTTTTCACAGAAATTT
TGAATTCCCGCCAAAATTTTTCACAGAAATTT
TGAATTCCCGCCAAAATTTTTCACAGAAATTT
TGAATTCCCGTTATAATTTTTCACAGAAATTC
TGAATTCCCGCCAAAATTTTTCATAGAAATTT
TGAATTCCCGCCAAAATTTTTCATAGAAATTT
TGAATTCCCGCCAAAATTTTTCACAGAAATTT
TGAATTCCCGCCAAAATTTTTCATAGAAATTT
TGAATTCCCGCCAAAATTTTTCACAGAAATTT
TGAATTCCCGTTATAATTTTTCACAGAAATTT
TGAATTCCCGCCAAAATTTTTCACAGAAATTT
TGAATTCCCGCCAAAATTTTTCACAGAAATTT
TGAATTCCCGCCAAAATTTTTCATAGAAATTT
TGAATTCCCGCCAAAATTTTTCACAGAAATTT
TGAATTCCCGCCAAAATTTTTCATAGAAATTT
TGAATTCCCGCCAAAATTTTTCACAGAAATTT
TGAATTCCCGCCAAAATTTTTCATAGAAATTT
TGAATTCCCGCCAAAATTTTTCACAGAAATTT
TGAATTCCCGTTATAATTTTTCACAGAAATTC
TGAATTCCCGCCAAAATTTTTCACAGAAATTT
TGAATTCCCGCCAAAATTTTTCATAGAAATTT
TGAATTCCCGCCAAAATTTTTCACAGAAATTT
TGAATTCCCGCCAAAATTTTTCACAGAAATTT
TGAATTCCCGCCAAAATTTTTCATAGAAATTT
TGAATTCCCGCCAAAATTTTTCACAGAAATTT
TGAATTCCCGTTATAATTTTTCACAGAAATTC
TGAATTCCCGCCAAAATTTTTCACAGAAATTT
TGAATTCCCGCCAAAATTTTTCACAGAAAATT
TGAATTCCCGCCAAAATTTTTCACAGAAATTT
TGAATTCCCGCCAAAATTTTTCACAGAAATTT
TGAATTCCCGCCAAAATTTTTCATAGAAATTT
TGAATTCCCGCCAAAATTTTTCACAGAAATTT
TGAATTCCCGTTATAATTTTTCACAGAAATTC
TGAATTCCCGCCAAAATTTTTCACAGAAATTT
TGAATTCCCGCCAAAATTTTTCACAGAAAATT
TGAATTCCCGCCAAAATTTTTCACAGAAATTT
TGAATTCCCGCCAAAATTTTTCACAGAAATTT
TGAATTCCCGCCAAAATTTTTCACAGAAATTT
TGAATTCCCGCCAAAATTTTTCACAGAAATTT
TGAATTCCCGCCAAAATTTTTCACAGAAATTT
TGAATTCCCGCCAAAATTTTTCACAGAAATTT
TGAATTCCCGCCAAAATTTTTCACAGAAAATT
TGAATTCCCGCTAAAAATTTTCACAGAAATTT
TGAATTCCCGCCAAAATTTTTCACAGAAAATT
TGAATTCCCGCCAAAACTTTTCACAGAAATTT
TGAATTCCCGCCAAAATTTTTCACAGAAATTT
TGAATTCCCGCCAAAATTTTTCACAGAAATTT
TGAATTCCCGCCAAAACTTTTCACAGAAAATT
TGAATTCCCGCCAAAATTTTTCACTGAAATTT
TGAATTCCCGCCAAAATTTTTCACAGAAATTT
TGAATTCCCGTTATAATTTTTCACAGAAATTT
TGAATTCCCGCCAAAATTTTTCACAGAAAATT

TGAATTCCCGCCAAAATTTTTCACTGAAATTT
TGAATTCCCGCCAAAATTTTTCACAGAAATTT
TGAATTCCCGCCAAAATTTTTCACAGAAATTT
TGAATTCCCGCCAAAATTTTTCACAGAAATTT
TGAATTCCCGCCAAAATTTTTCATAGAAATTT
TGAATTCCCGTTATAATTTTTCACAGAAATTC
TGAATTCCCGCCAAAATTTTTCATAGAAATTT
TGAATTCCCGCCAAAATTTTTCACAGAAATTT
TGAATTCCCGTTATAATTTTTCACAAAAATTC
TGAATTCCCGCCAAAATTTTTCACAGAAAATTA
GAATTCCCGCTAAAATTTTTCACAGAAATTT
TGAATTCCCGCCAAAATTTTTAACAGAAATTT
TGAATTCCCGCCAAAATTTTTCATAGAAATTT
TGAATTCCCGCCAAAACTTTTCACAGAAATTT
TGAATTCCCGCCAAAATTTTTCACAGAAATTT
TGAATTCCCGCCAAAATTTTTCACAGAAATTT
TGAATTCCCGCCAAAATTTTTCACAGAAATTT
TGAATTCCCGCCAAAATTTTTCACAGAAATTT
TGAATTCCCGCCAAAATTTTTAACAGAAATTT
TGAATTCCCGCCAAAATTTTTCATAGAAATTT
TGAATTCCCGCCAAAACTTTTCACAGAAATTT
TGAATTCCCGCCAAAATTTTTCACAGAAATTT
TGAATTCCCGCCAAAATTTTTCATAGAAATTT
TGAATTCCCGCCAAAATTTTTCACAGAAATTT
TGAATTCCCGCCAAAATTTTTCACAGAAATTT
TGAATTCCCGCCAAAATTTTTCACAGAAAATT
TGAATTCCCGCCAAAATTTTTCACAGAAATTT
TGAATTCCCGCCAAAATTTTTCACAGAAATTT
TGAATTCCCGCCAAAATTTTTCACAGAAATTT
TGAATTCCCGCCAAAATTTTTCACAGAAATTT
TGAATTCCCGCCAAAATTTTTCATAGAAATTT
TGAATTCCCGCCAAAATTTTTCACAGAAATTT
TGAATTCCCGCCAAAATTTTTCACAGAAATTT
TGAATTCCCGCCAAAATTTTTCACAGAAATTT
TGAATTCCCGCCAAAATTTTTCACAGAAATTT
TGAATTCCCGCCAAAATTTTTAACAGAAATTT
TGAATTCCCGCCAAAATTTTTCATAGAAATTT
TGAATTCCCGCCAAAACTTTTCACAGAAATTT
TGAATTCCCGCCAAAATTTTTCACAGAAATTT
TGAATTCCCGCCAAAATTTTTCATAGAAATTT
TGAATTCCCGCCAAAATTTTTCACAGAAATTT
TGAATTCCCGCCAAAATTTTTCACAGAAATTT
TGAATTCCCGCCAAAATTTTTCACAGAAATTT
TGAATTCCCGCCAAAATTTTTCACAGAAATTT
TGAATTCCCGCCAAAATTTTTAACAGAAATTT
TGAATTCCCGCCAAAATTTTTCATAGAAATTT
TGAATTCCCGCCAAAACTTTTCACAGAAATTT
TGAATTCCCGCCAAAATTTTTCACAGAAATTT
TGAATTCCCGCCAAAATTTTTCATAGAAATTT
TGAATTCCCGCCAAAATTTTTCACAGAAATTT
TGAATTCCCGTTATAATTTTTCACAGAAATTC
TGAATTCCCGCCAAAATTTTTCACAGAAAATTA
GAATTCCCGCTAAAAATTTTCACAGAAATTT
TGAATTCCCGCCAAAATTTTTCACAGAAATTT

TGAATTCCCGCCAAAATTTTTCACAGAAATTT
TGAATTCCCGCCAAAATTTTTCATAGAAATTT
TGAATTCCCGCCAAAATTTTTCACAGAAATTT
TGAATTCCCGCCAAAATTTTTCACAGAAATTT
TGAATTCCCGCCAAAACTTTTCACAGAAATTT
TGAATTCCCGCCAAAATTTTTCATAGAAATTT
TGAATTCCCGCCAAAATTTTTCACAGAAATTT
TGAATTCCAGTTATAATTTTTCACAGAAATTC
TGAATTCCCGCCAAAATTTTTCACAGAAATTT
TGAATTCCCGCCAAAATTTTTCACAGAAAATT
TGAATTCCCGCCAAAATTTTTCACAGAAATTT
TGAATTCCCGCCAAAATTTTTCACAGAAATTT
TGAATTCCCGCCAAAATTTTTCACAGAAATTT
TGAATTCCCGCCAAAATTTTTCACAGAAATTT
TGAATTCCCGCCAAAATTTTTCACAGAAATTT
TGAATTCCCGCCAAAATTTTTCACAGAAATTT
TGAATTCCCGCCAAAATTTTTCACAGAAAATT
TGAATTCCCGCTAAAAATTTTCACAGAAATTT
TGAATTCCCGCCAAAATTTTTCACAGAAAATT
TGAATTCCCGCCAAAACTTTTCACAGAAATTT
TGAATTCCCGCCAAAATTTTTCACAGAAATTT
TGAATTCCCGCCAAAATTTTTCACAGAAATTT
TGAATTCCCGCCAAAACTTTTCACAGAAAATT
TGAATTCCCGCCAAAATTTTTCACTGAAATTT
TGAATTCCCGCCAAAATTTTTCACAGAAATTT
TGAATTCCCGTTATAATTTTTCACAGAAATTT
TGAATTCCCGCCAAAATTTTTCACAGAAAATT
TGAATTCCCGCCAAAATTTTTCACTGAAATTT
TGAATTCCCGCCAAAATTTTTCACAGAAATTT
TGAATTCCCGCCAAAACTTTTCACAGAAAATT
TGAATTCCCGCCAAAATTTTTCACTGAAATTT
TGAATTCCCGCCAAAATTTTTCACAGAAAATT
TGAATTCCCGCCAAAATTTTTCACAGAAATTT
TGAATTCCCGCCAAAATTTTTCACAGAAAATT
TGAATTCCCGCCAAAATTTTTCACTGAAATTT
TGAATTCCCGTTATAATTTTTCACAGAAATTT
TGAATTCCCGCCAAAATTTTTCACAGAAATTT
TGAATTCCCGCCAAAATTTTTCACAGAAATTT
TGAATTCCCGCCAAAATTTTTCACAGAAAATT
TGAATTCCCGCCAAAATTTTTCACTGAAATTT
TGAATTCCCGCCAAAATTTTTCACAGAAATTT
TGAATTCCCGCCAAAATTTTTCACAGAAAATT
TGAATTCCCGCTAAAAATTTTCACAGAAATTC
TGAATTCCCGCCAAAATTTTTCACAGAAAATT
Nr of selected repeats 365 Similarity 0.924622
Consensus:
TGAATTCCCGCCAAAATTTTTCAcAGAAATTT
>Cele-UNSB01_1:13852339-13853459 Satlength=1121 Nr of Repeats=28
RepeatLength=40 seed=GAATTTTCTC
GAATTTTCTCGAATTTTCCAGAAGGTTCTGGAACATTCCA
GAATTTTCTCGAATTTTCCAGAAGGTTCTGGAGCATTCCA
GAATTTTCTCGGATTTTCCAGAAGGTTCTGGAACATTCCA
GAATTTTCTCGAATTTTCCAGAAGGTTCTGGAGCATTCCA
GAATTTTCTCGAATTTTCCAGAAGGTTCTGGAGCATTCCA

GAATTTTCTCGAATTTTCCAGAAGGTTCTGGAGCATTCCA
GAATTTTCTCGGATTTTCCAGAAGGTTCTGGAGCATTCCA
GAATTTTCTCGGATTTTCCAGAAGGTTCTGGAACATTCCA
GAATTTTCTCGAATTTTCCAGAAGGTTCTGGAGCATTCCA
GAATTTTCTCGAATTTTCCAGAAGGTTCTGGAGCATTCCA
GAATTTTCTCGAATTTTCCAGAAGGTTCTGGAGCATTCCA
GAATTTTCTCGGATTTTCCAGAAGGTTCTGGAACATTCCA
GAATTTTCTCGAATTTTCCAGTAGGTTCTGGAGCATTCCA
GAATTTTCTCGGATTTTCCAGAAGGTTCTGGAGCATTCCA
GAATTTTCTCGGATTTTCCAGAAGGTTCTGGAACATTCCA
GAATTTTCTCGAATTTTCCAGAAGGTTCTGGAGCATTCCA
GAATTTTCTCGAATTTTCCAGAAGGTTCTGGAGCATTCCA
GAATTTTCTCGAATTTTCCAGAAGGTTCTGGAGCATTCCA
GAATTTTCTCGGATTTTCCAGAAGGTTCTGGAACATTCCA
GAATTTTCTCGAATTTTCCAGTAGGTTCTGGAGCATTCCA
GAATTTTCTCGGATTTTCCAGAAGGTTCTGGAACATTCCA
GAATTTTCTCAAATTTTCCAGAAGGTTCTGGAGCATTCCA
GAATTTTCTCGGATTTTCCAGAAGGTTCTGGAACATTCCA
GAATTTTCTCGAATTTTCCAGAAGGTTCTGGAGCATTCCA
GAATTTTCTCGGATTTTCCAGAAGGTTCTGGAACATTCCA
GAATTTTCTCAAATTTTCCAGAAGGTTCTGGAGCATTCCA
GAATTTTCTCGAATTTTCCAGAAGGTTCTGGAACATTCCA
GAATTTTCTCGAATTTTCCAGAAGGTTCTGGAGCATTCCG
Nr of selected repeats 28 Similarity 0.956702
Consensus:
GAATTTTCTCGaATTTTCCAGAAGGTTCTGGAgCATTCCA
>Cele-UNSB01_1:13927551-13927713 Satlength=163 Nr of Repeats=13 RepeatLength=12
seed=TATACCTATA
TATACCTATACC
TATACCTATACC
TATACTTATACC
TATACCTATACC
TATACCTATACC
TATACCTATACC
TATACCTATACC
TATACCTATATC
TATACCTATACCT
TTACCTATACC
TATACCTATACC
TATACCTATACC
TATACCTATACCTATACC
Nr of selected repeats 10 Similarity 0.955556
Consensus:
TATACCTATACC
>Cele-UNSB01_1:13927986-13928124 Satlength=139 Nr of Repeats=11 RepeatLength=12
seed=ATAGGTATAG
ATAGGTATAGGT
ATAGGTATAGGC
ATAGGTATAGGT
ATAGTTATAGGT
ATAGGTATAGGT
ATAGGTATAGGT
ATAGGTATAGGT
ATAGGTATAGGT

ATAGGTATAGGT
ATAGGTATAGGT
ATAGGTATAGGTATAGGT
Nr of selected repeats 10 Similarity 0.955556
Consensus:
ATAGGTATAGGT
>Cele-UNSB01_1:14006347-14006515 Satlength=169 Nr of Repeats=7 RepeatLength=21 seed=CAAAATTCGT
CAAAATTCGTCCGAAAATGCT
CAAAATTCGTCCGAAAATGGCCAAAATACGACCAAAAAAGGC
CAAAATTCGCCCAAAAATGCT
CAAAATTCGTCCGAAAATGCT
CAAAATTCGTCCAAAAATGGC
CAAAATTCGTCCGAAAATGAC
CAAAATTCGTCCGAAAATAGC
Nr of selected repeats 6 Similarity 0.814646
Consensus:
CAAAATTCGTCCgAAAATnGCt
>Cele-UNSB01_1:14038349-14038662 Satlength=314 Nr of Repeats=9 RepeatLength=35 seed=AATTCCGGCA
AATTCCGGCAATTTGCCAATTTACCGGACAATTTA
AATTCCGCCAATTTGCCGATTTGCCGGAAAATTTA
AATTCCGGCAATTTGCCCATTTGCCGGAAAATTTA
AATTCCGGCAATTTGCCCATTTGCCGGAAAATTTA
AATTCCGGCAATATTCCAATTTGCCGGAAACTTTC
AATTCCGGCAATTTGCCGGTTTGCCGGAAATTTC
AATTCCGGCAATTTGCAAGCTAGACGGAAATGTTC
AATTCCGACAATTTGCCGATCTGGCGGAAAATTTT
AATTCCGGCAATTTGCCGGTTTGCCGGAAATTTC
Nr of selected repeats 7 Similarity 0.756173
Consensus:
AATTCCGgCAATTTGCCnATtTGcCGGAAAaTTTa
>Cele-UNSB01_1:14122632-14123925 Satlength=1294 Nr of Repeats=19 RepeatLength=68 seed=TTGGTTGAGG
TTGGTTGAGGCTAGAAAATGTTGTAGTTTGTAGTGTGTTAGCCTCAACCAAAATTAATTTTTTTTTAA
TTGGTTGAGGCTAGAAAATGTTGTAGTTTGTAGTGTGTTAGCCTCAACCAAAATTAATTTTTTTTTAA
TTGGTTGAGGCTAGAAAATGTTGTAGTTTGTAGTGTGTTAGCCTCAACCAAAATTAATTTTTTTTTTAA
TTGGTTGAGGCTAGAAAATGTTGTAGTTTGTAGTGTGCTAGCCTCAACCAAAATTAATTTTTTTTTAA
TTGGTTGAGGCTAGAAAATGTTGTAGTTTGTAGTGTGTTAGCCTCAACCAAAATTAATTTTTTTTTAA
TTGGTTGAGGCTAGAAAATGTTGTAGTTTGTAGTGTGTTAGCCTCAACCAAAATTAATTTTTTTTTAA
TTGGTTGAGGCTAGAAAATGTTGTAGTTTGTAGTGTGTTAGCCTCAACCAAAATTAATTTTTTTTTTAA
TTGGTTGAGGCTAGAAAATGTTGTAGTTTGTAGTGTGCTAGCCTCAACCAAAATTAATTTTTTTTTAA
TTGGTTGAGGCTAGAAAATGTTGTAGTTTGTAGTGTGTTAGCCTCAACCAAAATTAATTTTTTTTTAA
TTGGTTGAGGCTAGAAAATGTTGTAGTTTGTAGTGTGTTAGCCTCAACCAAAATTAATTTTTTTTTAA
TTGGTTGAGGCTAGAAAATGTTGTAGTTTGTAGTGTGTTAGCCTCAACCAAAATTAATTTTTTTTTTAA
TTGGTTGAGGCTAGAAAATGTTGTAGTTTGTAGTGTGCTAGCCTCAACCAAAATTAATTTTTTTTTAA
TTGGTTGAGGCTAGAAAATGTTGTAGTTTGTAGTGTGTTAGCCTCAACCAAAATTAATTTTTTTTTAA
TTGGTTGAGGCTAGAAAATGTTGTAGTTTGTAGTGTGCTAGCCTCAACCAAAATTAATTTTTTTTTAA
TTGGTTGAGGCTAGAAAATGTTGTAGTTTGTAGTGTGCTAGCCTCAACCAAAATTAATTTTTTTTTAA
TTGGTTGAGGCTAGAAAATGTTGTAGTTTGTAGTGTGTTAGCCTCAACCAAAATTAATTTTTTTTTAA
TTGGTTGAGGCTAGAAAATGTTGTGGTTTGTAGTGTGTTAGCCTCAACCAAAATTAATTTTTTTTTAA
TTGGTTGAGGCTAGAAAATGTTGTAGTTTGTAGTGTGTTAGCCTCAACCAAAATTAATTTTTTTTAA
TTGGTTGAGGCTAGAAAATGTTGTAGTTTGTAGTGTGAGCCTCAACCAAAATTAATTTTTTTTTTAA
Nr of selected repeats 14 Similarity 0.987503

Consensus:
TTGGTTGAGGCTAGAAAATGTTGTAGTTTGTAGTGTGtTAGCCTCAACCAAAATTAATTTTTTTTTAA
>Cele-UNSB01_1:14164675-14164835 Satlength=161 Nr of Repeats=8 RepeatLength=20
seed=AAAAAATAGA
AAAAAATAGATTGACATACG
AAAAAATAGATTAGCATACC
AAAAAATAGATGGACATACC
AAAAAATAGATTGACATACC
AAAAAATAGATTGATATACC
AAAAAATAGATTGACATACC
AAAAAATAGATTGACATACC
AAAAAATAGATTGACATGCC
Nr of selected repeats 8 Similarity 0.900000
Consensus:
AAAAAATAGATTGACATACC
>Cele-UNSB01_1:14166580-14166640 Satlength=61 Nr of Repeats=5 RepeatLength=12
seed=GCCTAAGCCT
GCCTAAGCCTGA
GCCTAAGCCTAC
GCCTAAGCCTAA
GCCTGAGCCTAA
GCCTAAGCCTAA
Nr of selected repeats 5 Similarity 0.866667
Consensus:
GCCTaAGCCTaa
>Cele-UNSB01_1:14168111-14168331 Satlength=221 Nr of Repeats=5 RepeatLength=63
seed=TTTTTGACTA
TTTTTGACTACTGGA
TTTTTGACTACATTGC
TTTTTGACTACGAGTGTCTTTTTCGCTACTGAGCGAAAAAATAGCTACATTCGCACTTTTGTC
TTTTTGACTACGGAATGTCTTTTTCGCTACTGACTGAAAAATAGCTACATTCGCACTTTTGCC
TTTTTGACTACGGAATGTCTTTTTCGCTACTGACTGAAAAATAGCTACATTCGCACTTTTGAA
Nr of selected repeats 3 Similarity 0.876736
Consensus:
TTTTTGACTACgGAaTGTCTTTTTCGCTACTGACtgAAAAATAGCTACATTCGCACTTTTGnc
>Cele-UNSB01_1:14174364-14175224 Satlength=861 Nr of Repeats=15 RepeatLength=64
seed=GTAGTCAAAA
GTAGTCAAAAAGTCAAAAGTGCGAATGTAGCTATTTTTCAGTCAGTAGCGAAAAAGACATTCA
GTAGTCAAAAAGTCAAAAGTGCGAATGTAGCTATTTTTTCAGTCAGTAGCGAAAAAGACATTCA
GTAGTCAAAAAGACAAAAGTGCGAATGTAGCTATTTTTTCAGTCAGTAGCGAAAAAGACATTCA
GTAGTCAAAAAGTCAAAAGTGCGAATGTAGCTATTTTTTCAGTCAGTAGCGAAAAAGACATTCA
GTAGTCAAAAAGACAAAAGTGCGAATGTAGCTATTTTTTCAGTCAGTAGCGAAAAAGACATTCA
GTAGTCAAAAAGACAAAAGTGCGAATGTAGCTATTTTTTCAGTCAGTAGCGAAAAAGACATTCA
GTAGTCAAAAAGTCAAAAGTGCGAATGTAGCTATTTTTCAGTCAGTAGCGAAAAAGACATTCA
GTAGTCAAAAAGTCAAAAGTGCGAATGTAGCTATTTTTTCAGTCAGTAGCGAAAAAGACATTCA
GTAGTCAAAAAGACAAAAGTGCGAATGTAGCTATTTTTTCAGTCAGTAGCGAAAAAGACATTCA
GTAGTCAAAAAGACAAAAGTGCGAATGTAGCTATTTTTTCGGTCAGTAGCGAAAAAGACATTCA
GTAGTCAAAAAGACAAAAGTGCGAATGTAGCTATTTTTTCAGTCAGTAGCGAAAAAGACATTCA
GTAGTCAAAAAGACAAAAGTGCGAATGTAGCTATTTTTTCGGTCAGTAGCGAAAAAGACATTCA
GTAGTCAAAAAGACAAAAGTGCGAATGTAGCTATTTTTTCGGTCAGTAGCGAAAAAGACACTC
GTAGTCAAAAAGCAAT
GTAGTCAAAAATCCA
Nr of selected repeats 10 Similarity 0.982870
Consensus:

GTAGTCAAAAAGaCAAAAGTGCGAATGTAGCTATTTTTTCaGTCAGTAGCGAAAAAGACATTCA
>Cele-UNSB01_1:14199204-14199288 Satlength=85 Nr of Repeats=4 RepeatLength=21 seed=ATATTTTGTG
ATATTTTGTGACATTTTTATT
ATATTTTGTGACATTTTTATT
ATATTTTGTGACATTTTGATT
ATATTTTGTGACATTTTTATG
Nr of selected repeats 4 Similarity 0.936508
Consensus:
ATATTTTGTGACATTTTtATt
>Cele-UNSB01_1:14207225-14207295 Satlength=71 Nr of Repeats=6 RepeatLength=10 seed=GATTTTCATA
GATTTTCATA
GATTTTCATA
GATTTTCATA
GATTTTCATA
GATTTTCATA
GATTTTCATAGATTTTCATA
Nr of selected repeats 5 Similarity 1.000000
Consensus:
GATTTTCATA
>Cele-UNSB01_1:14240945-14242222 Satlength=1278 Nr of Repeats=80 RepeatLength=16 seed=ATCTAAGGAT
ATCTAAGGATCTAAGG
ATCTAAGGATCTAAGG
ATCTAAGGATCTAAGT
ATCTAAGTATCTAAAG
ATCTAAGGATCTAAGG
ATCTAAGGATCTAAGG
ATCTAAGGATCTAAGG
ATCTAAGGATCTAAGG
ATCTAAGGATCTAAGG
ATCTAAGGATCTAAGG
ATCTAAGGATCTAAGG
ATCTAAGGATCTAAGG
ATCTAAGGATCTAAGG
ATCTAAGTATCTAAGT
ATCTAAAGATCTAAAG
ATCTAAGGATCTAAGG
ATCTAAGGATCTAAGG
ATCTAAGGATCTAAGT
ATCTAAGTATCTAAAG
ATCTAAGGATCTAAGG
ATCTAAGGATCTAAGG
ATCTAAGGATCTAAGG
ATCTAAGGATCTAAGG
ATCTAAGGATCTAAGG
ATCTAAGGATCTAAGG
ATCTAAGGATCTAAGG
ATCTAAGGATCTAAGG
ATCTAAGGATCTAAGG
ATCTAAGGATCTAAGG
ATCTAAGGATCTAAGG
ATCTAAGGATCTAAGG

ATCTAAGGATCTAAGG
ATCTAAGGATCTAAGG
ATCTAAGGATCTAAGG
ATCTAAGGATCTAAGG
ATCTAAGGATCTAAGG
ATCTAAAGATCTAAGT
ATCTAAGTATCTAAAG
ATCTAAAGATCTAAGG
ATCTAAGGATCTAAGG
ATCTAAGGATCTAAGG
ATCTAAGGATCTAAGG
ATCTAAGGATCTAAGG
ATCTAAGGATCTAAGG
ATCTAAGGATCTAAGG
ATCTAAGGATCTAAGG
ATCTAAGGATCTAAGG
ATCTAAGGTAAAG
ATCTAAGTATCTAAAG
ATCTAAAGATCTAAGG
ATCTAAGGATCTAAGG
ATCTAAGGATCTAAGG
ATCTAAGTATCTAAGT
ATCTAAAGATCTAAGG
ATCTAAGGATCTAAGG
ATCTAAGGATCTAAGG
ATCTAAGGATCTAAGG
ATCTAAGGATCTAAGG
ATCTAAGGATCTAAGG
ATCTAAGGATCTAAGG
ATCTAAGGATCTAAGG
ATCTAAGGATCTAAGG
ATCTAAGGATCTAAGG
ATCTAAGGATCTAAGG
ATCTAAGGATCTAAGG
ATCTAAGGATCTAAGG
ATCTAAGGATCTAAGG
ATCTAAGGATCTAAGG
ATCTAAGGATCTAAGG
ATCTAAGGATCTAAGG
ATCTAAGGATCTAAGG
ATCTAAGGATCTAAGG
ATCTAAGGATCTAAGG
ATCTAAGGATCTAAGG
ATCTAAGGATCTAAGG
ATCTAAGGATCTAAGG
ATCTAAGGATCTAAGG
ATCTAAGGATCTAAGG
ATCTAAGGATCTAAGG
ATCTAAGAATCTAAGG
Nr of selected repeats 79 Similarity 0.956183
Consensus:
ATCTAAGGATCTAAGG
>Cele-UNSB01_1:14240945-14243302 Satlength=2358 Nr of Repeats=147
RepeatLength=16 seed=ATCTAAGGAT

ATCTAAGGATCTAAGG
ATCTAAGGATCTAAGG
ATCTAAGGATCTAAGT
ATCTAAGTATCTAAAG
ATCTAAGGATCTAAGG
ATCTAAGGATCTAAGG
ATCTAAGGATCTAAGG
ATCTAAGGATCTAAGG
ATCTAAGGATCTAAGG
ATCTAAGGATCTAAGG
ATCTAAGGATCTAAGG
ATCTAAGGATCTAAGG
ATCTAAGGATCTAAGG
ATCTAAGTATCTAAGT
ATCTAAAGATCTAAAG
ATCTAAGGATCTAAGG
ATCTAAGGATCTAAGG
ATCTAAGGATCTAAGT
ATCTAAGTATCTAAAG
ATCTAAGGATCTAAGG
ATCTAAGGATCTAAGG
ATCTAAGGATCTAAGG
ATCTAAGGATCTAAGG
ATCTAAGGATCTAAGG
ATCTAAGGATCTAAGG
ATCTAAGGATCTAAGG
ATCTAAGGATCTAAGG
ATCTAAGGATCTAAGG
ATCTAAGGATCTAAGG
ATCTAAGGATCTAAGG
ATCTAAGGATCTAAGG
ATCTAAGGATCTAAGG
ATCTAAGGATCTAAGG
ATCTAAGGATCTAAGG
ATCTAAGGATCTAAGG
ATCTAAGGATCTAAGG
ATCTAAAGATCTAAGT
ATCTAAGTATCTAAAG
ATCTAAAGATCTAAGG
ATCTAAGGATCTAAGG
ATCTAAGGATCTAAGG
ATCTAAGGATCTAAGG
ATCTAAGGATCTAAGG
ATCTAAGGATCTAAGG
ATCTAAGGATCTAAGG
ATCTAAGGATCTAAGG
ATCTAAGGATCTAAGG
ATCTAAGGTAAAG
ATCTAAGTATCTAAAG
ATCTAAAGATCTAAGG
ATCTAAGGATCTAAGG
ATCTAAGGATCTAAGG
ATCTAAGTATCTAAGT
ATCTAAAGATCTAAGG

ATCTAAGGATCTAAGG
ATCTAAGGATCTAAGG
ATCTAAGGATCTAAGG
ATCTAAGGATCTAAGG
ATCTAAGGATCTAAGG
ATCTAAGGATCTAAGG
ATCTAAGGATCTAAGG
ATCTAAGGATCTAAGG
ATCTAAGGATCTAAGG
ATCTAAGGATCTAAGG
ATCTAAGGATCTAAGG
ATCTAAGGATCTAAGG
ATCTAAGGATCTAAGG
ATCTAAGGATCTAAGG
ATCTAAGGATCTAAGG
ATCTAAGGATCTAAGG
ATCTAAGGATCTAAGG
ATCTAAGGATCTAAGG
ATCTAAGGATCTAAGG
ATCTAAGGATCTAAGG
ATCTAAGGATCTAAGG
ATCTAAGGATCTAAGG
ATCTAAGGATCTAAGG
ATCTAAGGATCTAAGG
ATCTAAGGATCTAAGG
ATCTAAGAATCTAAGG
ATCTAAGGATCAAAGG
ATCAAAGGATCAAAGG
ATCAAAGGATCAAAGA
ATCTAAGGATCTAAGG
ATCTAAGGATCTAAGG
ATCTAAGGATCTAAGT
ATCTAAGTATCTAAAG
ATCTAAAGATCTAAGG
ATCTAAGGATCTAAGG
ATCTAAGGATCTAAGG
ATCTAAGTATCTAAGT
ATCTAAAGATCTAAGG
ATCTAAGTATCTAAGT
ATCTAAAGATCTAAGG
ATCTAAGGATCTAAGG
ATCTAAGTATCTAAGT
ATCTAAAGATCTAAGG
ATCTAAGGATCTAAGG
ATCTAAGGATCTAAGG
ATCTAAGGATCTAAGG
ATCTAAGGATCTAAGG
ATCTAAGGATCTAAGG
ATCTAAGGATCTAAGG
ATCTAAGGATCTAAGG
ATCTAAGGATCTAAGG
ATCTAAGGATCTAAGG
ATCTAAGAATCTAAGG
ATCTAAGGATCAAAGG

ATCAAAGGATCAAAGG
ATCAAAGGATCAAAGA
ATCTAAGGATCTAAGG
ATCTAAGGATCTAAGG
ATCTAAGTATCTAAGT
ATCTAAAGATCTAAAG
ATCTAAGGATCTAAGG
ATCTAAGGATCTAAGG
ATCTAAGGATCTAAGT
ATCTAAGTATCTAAAG
ATCTAAGGATCTAAGT
ATCTAAGTATCTAAAG
ATCTAAGGATCTAAGG
ATCTAAGGATCTAAGT
ATCTAAGTATCTAAAG
ATCTAAAGATCTAAGG
ATCTAAGGATCTAAGG
ATCTAAGGATCTAAGG
ATCTAAAGATCTAAGG
ATCTAAGTATCTAAGT
ATCTAAAGATCTAAGG
ATCTAAGGATCTAAGG
ATCTAAGTATCTAAGT
ATCTAAAGATCTAAGG
ATCTAAGGATCTAAGG
ATCTAAGGATCTAAGG
ATCTAAGGATCTAAGG
ATCTAAGGATCTAAGG
ATCTAAGGATCTAAGG
ATCTAAGGATCTAAGG
ATCTAAGGATCTAAGG
ATCTAAGGATCTAAGG
ATCTAAGGATCTAAGG
ATCTAAGGATCTAAGA
ATCTAAGGATCTAAGG
ATCAAAGGATCAAAGG
ATCAAAGGATCAAAGGATCAAAGA
ATCTAAGGATCTAAGG
ATCTAAGGATCTAAGG
Nr of selected repeats 145 Similarity 0.924178
Consensus:
ATCTAAGGATCTAAGG
>Cele-UNSB01_1:14245984-14247232 Satlength=1249 Nr of Repeats=59
RepeatLength=16 seed=AGATCCTTAG
AGATCCTTAGGCTTAGGTTGCAAAGCTAAATTTCTTTGGGCTTATTTTGTCAAGCGAGTTCATGCAAATTCTCTTACAA
CTAGGGGAATTATGTTTACTATCAGTCTTTTAGGCTTCAGACTCTTACTATTAGGCTCAGACTCTTTGACGCAAATTAT
CAAGCTAGGGCCCTGAAAATTTCAATTTTGATAGTTATACATTTTGGATCAGCCCCCTTCGTTTTGGACCTAAGATTAA
ATGCTAGGGTTT
GATCCTTAGATCCTTAGATTATTT
GATCCTTAGTTCCTT
AGATCCTTAGATCCTT
AGATCCTTAGATCCAT
AGATCCATAGATACCT
AGATCCATAGATCCTT

AGATCCTTAGATCCTT
AGATCCATAGATCCATAGATACCT
AGATCCATAGATCCTT
AGATCCTTAGATCCTT
AGATCCATAGATCCTT
AGATCCTTAGATCCTT
AGATCCTTAGATCCTT
AGATCCTTAGATCCAT
AGATCCATAGATACCT
AGATCCATAGATCCTT
AGATCCTTAGATCCTT
AGATCCATAGATCCAT
AGATCCTTCGACCCAT
AGATCCTTAGATCCTT
AGATCCTTAGTTCCTT
AGATCCTTAGATCCTT
AGATCCTTAGATCCTT
AGATCCATAGATCCATAGATACCT
AGATCCATAGATCCTT
AGATCCTTAGATCCTT
AGATCCTTAGATCCTT
AGATCCATAGATCCATAGATACCT
AGATCCATAGATCCTT
AGATCCTTAGATCCTT
AGATCCTTAGATCCTT
AGATCCATAGATCCATAGATACCT
AGATCCATAGATCCTT
AGATCCTTAGATCCTT
AGATCCTTAGATCCTT
AGATCCTTAGATCCTT
AGATCCTTAGATCCTT
AGATCCATAGATCCATAGATACCT
AGATCCATAGATCCTT
AGATCCTTAGATCCTT
AGATCCTTAGATCCTT
AGATCCATAGATCCATAGATACCT
AGATCCATAGATCCTT
AGATCCTTAGATCCTT
AGATCCTTAGATCCTT
AGATCCATAGATCCATAGATACCT
AGATCCATAGATCCTT
AGATCCTTAGATCCTT
AGATCCTTAGATCCTT
AGATCCTTAGATCCTT
AGATCCTTAGATCCTT
AGATCCTTAGATCCTT
AGATCCATAGATCCATAGATACCT
AGATCCATAGATCCTT
AGATCCTTAGATCCTT
AGATCCATAGATCCAT
AGATCCTTCGACCCAT
AGATCCTTAGATCCTT
Nr of selected repeats 48 Similarity 0.862780
Consensus:

AGATCCtTAGATCCTT
>Cele-UNSB01_1:14248002-14248098 Satlength=97 Nr of Repeats=6 RepeatLength=16
seed=AGGATCTAAG
AGGATCTAAGGATCTA
AGGATCTAAGGATCTA
AGGATCTAAGGATCTA
AGGATCTAAGGATCTA
AGGATCTAAGGATCTA
AGAATCTAAGGATCTA
Nr of selected repeats 6 Similarity 0.972222
Consensus:
AGGATCTAAGGATCTA
>Cele-UNSB01_1:14266018-14266935 Satlength=918 Nr of Repeats=5 RepeatLength=179
seed=CTACCCAAAT
CTACCCAAATTTTTGAAATTTGGAAATTTTTCAAAAATTCTCAAATTTTAAAAATCCCATAATTCAGCCAATTTTTGAG
ATTTTCAGATATTTTTTAATTGAAAATTCTGTTTGTCTTATCCATTAAACTATATCGCTGCATCTACATTTTTCACAGT
ACCCCATGACTTTTCCCGATT
CTACCCAAATTTTTGAAATTTGGAAATTTTTCAAAAATTCTCAAATTTTCAAAATCCCATAATTCAGCCAATTTTTGAG
ATTTTCAGATATTTTTTAATTGAAAATTCTGTTTGTCTTATCCATTAAACTATATCGCTGCATCTACATTTTTCACAGT
ACCCCATGACTTTTCCCGATT
CTACCCAAATTTTTGAAATTTGGAAATTTTTCAAAAATTCTCAAATTTTCAAAATCCCATAATTCAGCCAATTTTTGAG
ATTTTCAGATATTTTTTAATTGAAAATTCTGTTTGTCTTATCCATTAAACTATATCGCTGCATCTACATTTTTCACAGT
ACCCCATGACTTTTCCCGATT
CTACCCAAATTTTTGAAATTTTGAAATTTTTTCAAAAATTCAAAACTGCAAAACCCCATAACTCTGCCAAATTTGAAGA
TTTTCAGATATTTTCTAATTCAATTTACACATGAATTCTCAGTTTTTTTCCAAAAAAAAATTGCCGCATCTACATCTTC
CACAGTACCCCATGACTTTTCGCATAAATAG
CTACCCAAATTTTTGAAACTTGGAGATTTTTCAAAAATTCCAAAATTTTAAAATCCCATAATTCAGCCAATTTTTGAGA
TTTTCCAATACTTTTTACTTCAAATTACACATGAATTCTTAATTTTTTTTTACTAAAAAAATATATTGCCGCATCTAC
ATTTTCCACACTACCCCAGAACTTTTCGCAATG
Nr of selected repeats 3 Similarity 0.995034
Consensus:
CTACCCAAATTTTTGAAATTTGGAAATTTTTCAAAAATTCTCAAATTTTcAAAATCCCATAATTCAGCCAATTTTTGAG
ATTTTCAGATATTTTTTAATTGAAAATTCTGTTTGTCTTATCCATTAAACTATATCGCTGCATCTACATTTTTCACAGT
ACCCCATGACTTTTCCCGATT
>Cele-UNSB01_1:14275073-14275913 Satlength=841 Nr of Repeats=24 RepeatLength=40
seed=TTCTGGAAAA
TTCTGGAAAAGTCGGTAAAATTTTGGAGTGTTCCCGAACT
TTCTGGAAAATTTTAGAAAATTCGGGAATGTTCTGGAACC
TTCTTGAAAATTCGAGGAAATTCTGGAATGTTCTAGAACC
TTCTGGAAAATTCGATAAGAATCTGGAGTGTTCCAGAACT
TTCTGGAAAATTCGATAAAATTCTGGAATGCTCTGGAACT
TTCTGGAAAATTCCAGAAAATTCCGGAATGCTCTAGAACC
TACTGGAAAATTTGATAAAA
TTCTGGAATATTCTACAACA
TTCTGCAAAAGTCGGTAAAATTCTGGAATGTTCTAGAACT
TTCTGGAAAATTCCAGAAAATTCCGGAATGCTCTAGAACC
TTCTGGAAAATTCGCTAAAA
TTCTGGAATATTCTACAACA
TTCTGGAAAAGTCGGTAAAATTCTGGAATGCTCTGGAACT
TTCAGGAAAATTTTAGAAAATCCTGGAATGTTCTGGAACC
TTCTGGAAAATTCGCAAAAATTCTGGAGCGTTCTAGAACC
TACTGGAAAATTTGATAAAATTCTGGCATATTCTACAACA
TTCTGCAAAAGTCGGTAAAATTCTGGAATGTTCTAGAACT
TTCTTGAAAATTCCAGAAAATTCCGGAATGCTCTAGAACC

TTCTGGAAAATTCGGTAAAA
TTCTGGAATATTGTACAACA
TTCTGGAAAAGTCGGAAAAAATTTTGGAGTGTTCCCGAACT
TTCTGGAAAATTTTAGAAAATTCTGGAATGTTATGGAACC
TTCTGGAAAATTCAATAAATTTCTGGAATGTTCCAGAACC
TTCTGGAAAATTCGGTGAAATTCTGGAATGTTCTGGAACT
Nr of selected repeats 18 Similarity 0.755338
Consensus:
TTCTGGAAAAtTcgatAAAATTCtGGAaTGtTCtaGAACc
>Cele-UNSB01_1:14343732-14344621 Satlength=890 Nr of Repeats=4 RepeatLength=62
seed=GTAGTTTGTA
GTAGTTTGTAGCCTGAGCCTCACCAAAATTCGGATTCTGTGTTTAATTCTTGATTTCTAGTTGATTTTCGCCGGTTTTT
TTTTGCTTTTTTCTTTCTTTTTCTCCCGCAAAACCCAATTTTCCCTTTAAAAAACCCCCGTTTTTTCCAGATGGTGCAT
TCAAACGGAGCCATCACGAGCACAATTCTGAAGGCTCGTCACGCCGACGTCGTCCGCAAGACGTCACTTCACCACGCCA
ACGACTTGACACTCATCGATCTCGTGCTCAATGTGCAGCGGCTTTTGGCTCTCCCATCCGACGCGAATTTCGTGTTGAA
GTACAAGGATGAAGGTAGGAATTGCACTGAAAATTGCGATTTTAATTCAATTTTTGTCAATTTTAGAAGGCGATTTGGT
GACACTCGCCGAGGATAGCGACCTCCTGCTGGCTCTTCACACGTCTGGAGCCACGCTCGACGTGACCGTCGTTGTCGAC
TCGAGAGCTCGTGAGGCTGTGCACGATGTGCAGAAGCAAGTCGAGCAGATTAAGGTAAGGAGGATTTTGAGCGAAAAAA
AACGATTTTTGAGCTGAAAAGCGCAATTTTGAGCGGGAAAATGCAAAATGTTGCAGAATTTTCATCGAAAATGCTGCAT
TTTTGAGCTTTTTAGACAAAAAAAAATCCGGAAAAACACTGAAAAATGTTGGTTGAGGCTAAGAAACAAGT
GTAGTTTGTAGTGTGAGCCTCAACCAAACTAGCCAAAGCTTGAGTGAGGCTATGGTGATTTT
GTAGTTTGTAGCTTTAGCCTCAACCAAATTAGCTAAAGCTTGAGTGAGGCTATGGTGATTTT
GTAGTTTGTAGCTTTAGCCTCAACCAAATTAGCTAAAGCTTGAGTGAGGCTATGGTGATTTT
Nr of selected repeats 3 Similarity 0.928315
Consensus:
GTAGTTTGTAGctTtAGCCTCAACCAAAtTAGCtAAAGCTTGAGTGAGGCTATGGTGATTTT
>Cele-UNSB01_1:14381938-14382071 Satlength=134 Nr of Repeats=7 RepeatLength=19
seed=CCCTGATACC
CCCTGATACCCCCTGGTCC
CCCTGATACCCCCGGTCCC
CCCTGATACCCCCTAGCCT
CCCTGATGCCCCCTAGTCT
CCCTGATACCCCCTAGTCT
CCCTGATACCCCCTAGTCC
CCCTGATACCCCCGGGTCC
Nr of selected repeats 7 Similarity 0.788889
Consensus:
CCCTGATACCCCCtaGTCc
>Cele-UNSB01_1:14392151-14393037 Satlength=887 Nr of Repeats=5 RepeatLength=177
seed=CTAACAAAAT
CTAACAAAATAATTAGCGTAAAATTTCCTACATTTTACTAGTTAACAATATTTTGATAGCATCAGTGGTTTTTGAGATA
CAGAAGTTATAAGATGTACGTCCAAAAATGATTGTGAGCTGCTCTTATCAAAAATTACCATAACTTTTTACTCACTAAC
GGTATCAAAAATGTTTTAA
CTAACAAAATAATCAGCGTAAAATTTCCTACATTTCACTAGTTATTAATATTTTGATAACATCAGTGGTTTTTAAGTTA
CGCAAGTTTTAAGGTGAGCGCCCAAAAATAGTTGTGAGAAGGTTTAATCAAAAACCGGTATAATTTTTTACACACTAAC
GATATCAAAAATGTTTTAA
CTAACAAAATAATCAGCGTAAAATTTCCTACATTTTACTAGTTAACAATGTTTTGATAGCATTAGTGGTTTTTGAGATA
CGCAAGTTTTAAGGTGGGCGCCCAAAAATAGTTGTGAGCAGGTTTAAATAAAAACCGACATAACTTTTTACTCACTAAC
GGTATTAAAAATGTTTTAA
CTAACAAAATAATCAACGTAAAATTTTCTATATTTTATTAGTTAGCAACTTTCAAATAACATTACTGTTTTTTGAGATA
AGCATGGTTAAAGGTAGACGCTCAAAAATGTCTGCGAGCGGCTTTTCCTTTAGAGTGATATAACTCCTTGCTCATGAAC
GCTAGCAAAAATCTTTCAA

CTAACAAAATAATTAACGTAAAATTTTTCTATATTTTCTTTCTGAACAACTTTCTTAAAGCGTTAGTAGTTTTTAAGTT
ACGCAAGTTATAAGATATAGTGCCGAAAATGTCTGCGTGCTGCTTTAATCAAAAACCTACATAACTTTTTACAGAATAA
CGACATCAAAAATATTTCAG
Nr of selected repeats 4 Similarity 0.721286
Consensus:
CTAACAAAATAATcAgCGTAAAATTTcCTAcATTTtAcTAGTTAacAAtaTTttgATAaCATcAgTGgTTTTTgAGaTA
cgcAaGtTttAAGgTggaCGccCAAAAATagTTGtGAGcaGcTtTaatcaaAaAccgacATAAcTttTTaCtCActAAC
GgTAtcAAAAATgTTTtAA
>Cele-UNSB01_1:14511380-14511819 Satlength=440 Nr of Repeats=17 RepeatLength=22
seed=GCACGGCCTG
GCACGGCCTGGTAGTCCTGAGGGCGCGACCTTTCGGTCTGAGG
GCATGGCCTGACGGTCCTGAGG
GCATGGCCTGGCAGTCCAGAGG
GCATGGCCTGGCAGTCCTGAGA
GCACGGCCTGGTGGTCCTGAAG
GCACGGCATGGCAGTCCTGAGA
GCACGGCCTGTCATTACTGACG
GCACGGCCTGGCGGTCCGGAGG
GCACGGCCTGGCGGTCCTGAGG
GCACGGCCTCACGGTCTTGAGG
GCACGACCTGGGGGGCCCTGAAAACACTCTTTGGGGGCCCAGAAAA
CACGGCCTGACGGTCCTGAGG
GCACGGCCTGAGAGTCCTGAAGGCCCGGAGTGTCGGTCTGAGG
GCATGGCCTGGTAGTCCTGAGA
GCACGGCCTGGTGGTCCTGATG
GCACGGCCTGTCATTACTGAGA
GCACGGCCTGGTGGTCCTCAGG
Nr of selected repeats 13 Similarity 0.701226
Consensus:
GCAcGGCCTGgcgGTCCTGAGg
>Cele-UNSB01_1:14535288-14535435 Satlength=148 Nr of Repeats=8 RepeatLength=15
seed=GGGGGCAACA
GGGGGCAACAAAAAA
GGGGGCAACATTTGAACTCTCCCT
GGGGGCAACAAATTA
GGGGGCAACATTTTCATAAATGTA
GGGGGCAGCAAAAAA
GGGGGCAACATAATA
GGGGTCAACATTTGAACTCTCCCC
GGGGGCAACAAATTA
Nr of selected repeats 5 Similarity 0.822222
Consensus:
GGGGGCAaCAaAatA
>Cele-UNSB01_1:14537614-14537761 Satlength=148 Nr of Repeats=8 RepeatLength=15
seed=TGTTGCCCCC
TGTTGCCCCCTAATT
TGTTGCCCCCGGGGAGAGTTCAAA
TGTTGACCCCTATTA
TGTTGCCCCCTTTTT
TGCTGCCCCCTACATTTATGAAAA
TGTTGCCCCCTAATT
TGTTGCCCCCAGGGAGAGTTCAAA
TGTTGCCCCCTTTTT
Nr of selected repeats 5 Similarity 0.822222

Consensus:
TGTTGcCCCCTatTt
>Cele-UNSB01_1:14566637-14566717 Satlength=81 Nr of Repeats=4 RepeatLength=20
seed=ATATCAAAAA
ATATCAAAAAATAGATTGAC
ATACCAAAAAATAGATTGGC
ATATCAAAAAATAAATTGGC
ATATCAAAAAACAAATTGGC
Nr of selected repeats 4 Similarity 0.855556
Consensus:
ATAtCAAAAAAtAaATTGgC
>Cele-UNSB01_1:14568940-14569168 Satlength=229 Nr of Repeats=4 RepeatLength=14
seed=TGTAGTTTGT
TGTAGTTTGTAGTT
TGTAGTTTGTAGTG
TGTAGTTTGTAGTT
TGTAGTTTGTAGTTTGTAGAAAACCTTTCAAAGCTCTTTTGAGAGTTACCAAAAAACGTTTTTTTGGGAATTTTGAATT
TTTCAAATTTATAATATTTCAGTATTTTTTTTTCCTTTGTAACATTCCAAAAACTGTCAAAAAAAAAACAAACTCATCA
AATTCCAAGTTTTCTTAAATTTGAAGAT
Nr of selected repeats 3 Similarity 0.936508
Consensus:
TGTAGTTTGTAGTt
>Cele-UNSB01_1:14574460-14574865 Satlength=406 Nr of Repeats=4 RepeatLength=101
seed=GCATTTGCAT
GCATTTGCATATAGAGATCAGATAAAAGTTAGAATTTTCCAGCCGTTGCAAGTGCGCTCTATTGAGAACATTTTGAAAA
TTGAATGTTTTCAAATTAGAGC
GCATTTGCATATCGAGATCAGATAAAAGTTAGAATTTTCCAGCCGTTGCAAGTGCGCTCTATTGAGAACATTTTGAAAA
TTGAATGTTTTCACATTAGAGC
GCATTTGCATATGAAGGTCAGATAAAAATTTGAATATTCGAGTAGTTGCAAGTGCGCTTTAATGAGAAAATCTTAAAAT
CTGCATGTTTTCACATTAGAGA
GCATTTGCATATCGAGGTTAGATAAAAAGTTTGAATTTTCCAGTTGTTGCAAGTCCGCTCTATTGAGAAAATCTTGAAA
ATTGAATGTTTTCACATTAGAGC
Nr of selected repeats 3 Similarity 0.826797
Consensus:
GCATTTGCATATngAGaTCAGATAAAAgTTaGAATtTTCcAGccGTTGCAAGTGCGCTcTAtTGAGAAcATtTTgAAAA
TTGaATGTTTTCAcATTAGAGc
>Cele-UNSB01_1:14577219-14577388 Satlength=170 Nr of Repeats=5 RepeatLength=23
seed=GCACCATAAG
GCACCATAAGGATCACATAATCATACGAACCTTAGAGCATCACAGTAGAT
GCATCATAAGGACTCTAGTTGAT
GCATCATAAGGATCATAGTTGAT
GCACCATAAGGATTATAGTAGAA
GCACCATAAGAACCACAGAACCATACGGATCTCAGAGCACCACTGTAGAT
Nr of selected repeats 3 Similarity 0.726852
Consensus:
GCAtCATAAGGATcaTAGTtGAt
>Cele-UNSB01_1:14582954-14583490 Satlength=537 Nr of Repeats=26 RepeatLength=18
seed=TTGTAGATCA
TTGTAGATCAGCGGGGTC
TTGTAGATCAACGGGGTC
TTGTAGATTAACGGGGTC
TTGTAGATCAACGGGGTA
TTGTAGATCAACGGGGTC
TTATAGATCAACGGGGTA

TCGTAGATCAACGGGGTC
TTATAGATCAACGGGGGA
TTGTAGATCAACGGAGTC
TTGTAGATCATTGTTCAGACTAGCTTAACGGGGTC
TTGTAGATCAACGGGGTC
TTGTAGATCATTGTTCAGTGTAGCTTAACGGGGTC
TTGTAGATTAACGGGGTC
TTGTAAATCAACGGGGTC
TTGTAAATCAACGGGGTA
TTGTAGATCAACGGGGTG
TTGTAGATCAACGGGGTG
TTGTAGATCAACGGAGTC
TTGTAGATCATTGTTCAGACTAGCTTAACGGGGTC
TTGTAGATCAACGGGGTC
TTGTAGATCATTGTTCAGTGTAGCTTAACGGGGTC
TTGTAGATTAACGGGGTC
TTGTAAATCAACGGGGTC
TTGTAAATCAACGGGGTA
TTGTAGATCAACGGGGTC
TTGTAGATCAACGGGGCC
Nr of selected repeats 22 Similarity 0.869489
Consensus:
TTGTAGATCAACGGGGTc
>Cele-UNSB01_1:14647518-14647641 Satlength=124 Nr of Repeats=4 RepeatLength=11
seed=GTACTGTAGG
GTACTGTAGGA
GTACTGTAGGA
GTACTGTAGGATTACTGTAGTTTAGGAAGAATTGAGTTTTTGTCTTTTGAAGAGATATAGGTTTGCGGTTAGTGGTGGG
ATATGGTCGGG
GTACTGTAGTA
Nr of selected repeats 3 Similarity 0.919192
Consensus:
GTACTGTAGgA
>Cele-UNSB01_1:14648049-14648258 Satlength=210 Nr of Repeats=6 RepeatLength=35
seed=AATTTAAATT
AATTTAAATTTTCTGCGAAAAATGTTTTAGCGGGA
AATTTAAATTTTCTGAAAAAAAATATTTTGGAGGGA
AATTTAAATTTTCTGAAAAAAATATTTTGGAGGGA
AATTTAAATTTTCTGTGAAAAATATTTTGGCGGGT
AATTTAAATTTTCGGAGAAAAAAAATTTTGGAGGGA
AATTTAAATTTTCTGAGAAAAATATTTTGGCGGGA
Nr of selected repeats 5 Similarity 0.859048
Consensus:
AATTTAAATTTTCtGagAAAAAtaTTTTgGcGGGa
>Cele-UNSB01_1:14674978-14675094 Satlength=117 Nr of Repeats=10 RepeatLength=12
seed=ATATGTATAT
ATATGTATAT
ATATATATAT
ATATGTATTTGT
ATATGTATATGT
ATATGTATATGT
ATATGCATATGC
ATATGTATATGT
ATATGTATATG

TATGTATATGTAT
ATATGTATATGT
Nr of selected repeats 6 Similarity 0.888889
Consensus:
ATATGTATATGT
>Cele-UNSB01_1:14687699-14688279 Satlength=581 Nr of Repeats=8 RepeatLength=73
seed=TGTCGGCCGC
TGTCGGCCGCTACCAAATAAGCACCTTTTAGCACTACGTTGCGCACACACACCAAACTGCGGAACCCCTAACA
TGTCGGCCGCTCTAAACTATTTGCATACACACCAAGCTACTTGTTTCACGCCAAGCTGCGGAACCTCGAAAG
TGTCGGCCGCTTCCAAATAACTACCTTTTCACATAATGTTGCGCACACCTCAAGCTGCGGGACCCTGAACG
TGTCGGCCGCTACCAAATAAGCACCTTTTAGCACTACGTTGCGCACACACACCAAACTGCGGAACCCCTAACA
TGTCGGCCGCTCTAAACTATTTGCATACACACCAAGCTACTTGTTTCACGCCAAGCTGCGGAACCTCGAAAG
TGTCGGCCGCTTCCAAATAACTACCTTTTCACATAATGTTGCGCACACCTCAAGCTGCGGGACCCTGAACG
TGTCGGCCGCTACCAAATAAGCACCTTCTAGCACTACGTTGCGCACACACACCAAACTGCGGAACCCCTAACA
TGTCGGCCGCTCTAAACTATTTGCACACACACCAAGCTACTTGTTTCACGCCAAGCTGCGGAACCTCGAAAG
Nr of selected repeats 6 Similarity 0.624829
Consensus:
TGTCGGCCGCTaCcAAAcTAtttaGCanACaCaccaAGcaCTACgTTgcGcacaCACaCCAAaCTGCGGAACCcCgAAc
Ag
>Cele-UNSB01_1:14694045-14694325 Satlength=281 Nr of Repeats=8 RepeatLength=35
seed=AAAATTTCCG
AAAATTTCCGGCAAATCGGCAATTTACCGAAAGTG
AAAATTTCCGGCAAATCGGCAATTTACCGAAAGTG
AAAATTTCCGGCAAATCGGCAATTTACCGAAAGTG
AAAATTTCCGGCAAATCGGCAATTTACCGAAAGTG
AAAATTTCCGGCAAATCGGCAATTTACCGAAAGTG
AAAATTTCCGGCAAATCGGCAATTTGTCGAAAACG
AAAATTTCCGGCAAATCGGCAGTTTACCGAAAGTG
AAAATTTCCGGCAAATCGGCAATTTACCGAAAGTG
Nr of selected repeats 8 Similarity 0.952381
Consensus:
AAAATTTCCGGCAAATCGGCAATTTACCGAAAGTG
>Cele-UNSB01_1:14697056-14704541 Satlength=7486 Nr of Repeats=214
RepeatLength=35 seed=GAAATTTTCA
GAAATTTTCATTTTCGGCGAAGTGCCGATTTGCCG
GAAATTTTCACTTTCGGTAAACTGCCGATTTACCG
GAAATTTTCACTTTCGGTAAATTATTGCCG
GAAATTTTCATTTTCGGCGAAGTGCCGATTTGCCG
GAAATTTTCACTCTCGGTAAATTGCCGATTAGCCG
GAAATTTTCACTTTCGGTAAATATCCGATTTGCCA
GAAATTTTCACTTTCGGTAAATTGCCGATTTGGCA
GAAATTTTCACTTTCGGTAAATTGCCGATTTGCCG
GAAATTTTCACTTTCGGTAAATTGCCGATTTGCCG
GAAATTTTCACTTTCGGTAAATATCCGATTTGCCA
GAAATTTTCACTTTCGGTAAATTGCCGATTTGCCG
GAAATTTTCACTTTCGGTAAATTGCCGATTTGGCA
GAAATTTTCACTTTCGGTAAATTGCCGATTTGCCG
GAAATTTTCACTTTCGGTAAATTGCCGATTTGGCA
GAAATTTTCACTTTCGGTAAATTGCCGATTTGCCG
GAAATTTTCACTCTCGGTAAATTGCCGATTAGCCG
GAAATTTTCACTTTCGGTAAATATCCGATTTGCCA
GAAATTTTCACTTTCGGTAAATTGCCGATTTGGCA
GAAATTTTCACTTTCGGTAAATTGCCGATTTGCCG
GAAATTTTCACTTTCGGTAAATTGCCGATTTGCCG

GAAATTTTCACTTTCGGTAAATATCCGATTTGCCA
GAAATTTTCACTTTCGGTGAATTGCCGATTTGCCG
GAAATTTTCACTTTCGGTAAATTGCCGATTTGGCA
GAAATTTTCACTTTCGGTAAATTGCCGATTTGCCG
GAAATTTTCACTTTCGGTAAATTGCCGATTTGGCA
GAAATTTTCACTTTCGGTAAATTGCCGATTTGCCG
GAAATTTTCACTTTCGGTAAATTGCCGATTTGCCG
GAAATTTTCACTTTCGGTAAATTGCCGATTTGCCG
GAAATTTTCACTTTCGGTAAATTGCCGATTTGCCG
GAAATTTTCACTTTCGGTAAATTGCCGATTTGCCG
GAAATTTTCACTTTCGGTAAATTGCCGATTTGCCG
GAAATTTTCACTTTCGGTAAATTGCCGATTTGCCG
GAAATTTTCACTTTCGGTAAATTGCCGATTTGCCG
GAAATTTTCACTTTCGGTAAATATCCGATTTGCCA
GAAATTTTCACTTTCGGTAAATTGCCGATTTGCCG
GAAATTTTCACTTTCGGTAAATTGCCGATTTGCCG
GAAATTTTCACTTTCGGTAAATATCCGATTTGCCA
GAAATTTTCACTTTCGGTAAATTGCCGATTTGGCA
GAAATTTTCACTTTCGGTAAATTGCCGATTTGCCG
GAAATTTTCACTTTCGGTAAATTGCCGATTTGCCG
GAAATTTTCACTTTCGGTAAATTGCCGATTTGCCG
GAAATTTTCACTTTCGGTAAATATCCGATTTGCCG
GAAAATTTCACTTTCGGTAAATTGCCGATTTGCCA
GAAATTTTCACTTTCGGTAAATTGCCGATTTGCCG
GAAATTTTCACTTTCGGTAAATTGCCGATTTGCCG
GAAATTTTCACTTTCGGTAAATTGCCGATTTGCCG
GAAATTTTCACTTTCGGTAAATATCCGATTTGCCA
GAAATTTTCACTTTCGGTAAATTGCCGATTTGCCG
GAAATTTTCACTTTCGGTAAATTGCCGATTTGCCG
GAAATTTTCACTTTCGGTAAATTGCCGATTTGCCG
GAAATTTTCACTTTCGGTAAATTGCCGATTTGCCG
GAAATTTTCACTTTCGGTAAATTGCCGATTTGCCG
GAAATTTTCACTTTCGGTAAATTGCCGATTTGCCG
GAAATTTTCACTTTCGGTAAATTGCCGATTTGGCA
GAAATTTTCACTTTCGGTAAATTGCCGATTTGCCG
GAAATTTTCACTTTCGGTAAATTGCCGATTTGCCG
GAAATTTTCACTTTCGGTAAATTGCCGATTTGCCG
GAAATTTTCACTTTCGGTAAATATCCGATTTGCCA
GAAATTTTCACTTTCGGTAAATTGCCGATTTGCCG
GAAATTTTCACTTTCGGTAAATTGCCGATTTGCCG
GAAATTTTCACTTTCGGTAAATTGCCGATTTGCCG
GAAATTTTCACTTTCGGTAAATTGCCGATTTGGCA
GAAATTTTCACTTTCGGTAAATTGCCGATTTGCCG
GAAATTTTCACTTTCGGTAAATTGCCGATTTGGCA
GAAATTTTCACTTTCGGTAAATTGCCGATTTGCCG
GAAATTTTCACTTTCGGTAAATTGCCGATTTGCCG
GAAATTTTCACTTTCGGTAAATTGCCGATTTGCCG
GAAATTTTCACTTTCGGTAAATTGCCGATTTGCCG
GAAATTTTCACTTTCGGTAAATTGCCGATTTGCCG
GAAATTTTCACTTTCGGTAAATTGCCGATTTGCCG
GAAATTTTCACTTTCGGTAAATATCCGATTTGCCA
GAAATTTTCACTTTCGGTAAATTGCCGATTTGCCG
GAAATTTTCACTTTCGGTAAATTGCCGATTTGCCG
GAAATTTTCACTTTCGGTAAATATCCGATTTGCCA

GAAATTTTCACTTTCGGTAAATTGCCGATTTGGCA
GAAATTTTCACTTTCGGTAAATTGCCGATTTGCCG
GAAATTTTCACTTTCGGTAAATTGCCAATTTGCCG
GAAATTTTCACTTTCGGTAAATTGCCGATTTGCCG
GAAATTTTCACTTTCGGTAAATATCCGATTTGCCG
GAAAATTTCACTTTCGGTAAATTGCCGATTTGCCG
GAAATTTTCACTTTCGGTAAATATCCGATTTGCCA
GAAATTTTCACTTTCGGTAAATTGCCGATTTGCCG
GAAATTTTCACTTTCGGTAAATTGCCGATTTGCCG
GAAATTTTCACTTTCGGTAAATATCCGATTTGGCA
GAAATTTTCACTTTCGGTAAATATCCGATTTGGCA
GAAATTTTCACTTTCGGTAAATTGCCGATTTGGCA
GAAATTTTCACTTTCGGTAAATTGCCGATTTGGCA
GAAATTTTCACTTTCGGTAAATTGCCGATTTGCCG
GAAATTTTCACTTTCGGTAAATTGCCGATTTGCCG
GAAATTTTCACTTTCGGTAAATTGCCGATTTGCCG
GAAATTTTCACTTTCGGTAAATTGCCGATTTGCCA
GAAATTTTCACTTTCGGTAAATATCCGATTTGCCA
GAAATTTTCACTTTCGGTAATTATCCGATTTGCCA
GAAATTTTCACTTTCGGTAAATTGCCGATTTGGCA
GAAATTTTCACTTTCGGTAAATATCCGATTTGGCA
GAAATTTTCACTTTCGGTAAATTGCCGATTTGCCG
GAAATTTTCACTTTCGGTAAATTGCCGATTTGCCG
GAAATTTTCACTTTCGGTAATTATCCGATTTGGCA
GAAATTTTCACTTTCGGTAAATTGCCGATTTGCCA
GAAATTTTCACTTTCGGTAAATATCCGATTTGCCG
GAAATTTTCACTTTCGGTAAATTGCCGATTTGCCG
GAAATTTTCACTTTCGGTAAATTGCCGATTTGCCG
GAAATTTTCACTTTCGGTAAATATCCGATTTGCCG
GAAATTTTCACTTTCGGTAAATTGCCGATTTGCCG
GAAATTTTCACTTTCGGTAAATTGCCGATTTGCCG
GAAATTTTCACTTTCGGTAAATATCCGATTTGCCA
GAAATTTTCACTTTCGGTAAATTGCCGATTTGCCG
GAAATTTTCACTTTCGGTAAATTGCCGATTTGCCG
GAAATTTTCACTTTCGGTAAATATCCGATTTGCCA
GAAATTTTCACTTTCGGTAAATTGCCGATTTGGCA
GAAATTTTCACTTTCGGTAAATTGCCGATTTGCCG
GAAATTTTCACTTTCGGTAAATTGCCAATTTGCCG
GAAATTTTCACTTTCGGTAAATTGCCGATTTGCCG
GAAATTTTCACTTTCGGTAAATATCCGATTTGCCG
GAAAATTTCACTTTCGGTAAATTGCCGATTTGCCG
GAAATTTTCACTTTCGGTAAATATCCGATTTGCCA
GAAATTTTCACTTTCGGTAAATTGCCGATTTGCCG
GAAATTTTCACTTTCGGTAAATTGCCGATTTGCCG
GAAATTTTCACTTTCGGTAAATATCCGATTTGGCA
GAAATTTTCACTTTCGGTAAATATCCGATTTGGCA
GAAATTTTCACTTTCGGTAAATTGCCGATTTGGCA
GAAATTTTCACTTTCGGTAAATTGCCGATTTGGCA
GAAATTTTCACTTTCGGTAAATTGCCGATTTGCCG
GAAATTTTCACTTTCGGTAAATTGCCGATTTGCCG
GAAATTTTCACTTTCGGTAAATTGCCGATTTGCCG
GAAATTTTCACTTTCGGTAAATTGCCGATTTGCCA
GAAATTTTCACTTTCGGTAAATATCCGATTTGCCA
GAAATTTTCACTTTCGGTAATTATCCGATTTGCCA

GAAATTTTCACTTTCGGTAAATTGCCGATTTGGCA
GAAATTTTCACTTTCGGTAAATATCCGATTTGGCA
GAAATTTTCACTTTCGGTAAATTGCCGATTTGCCG
GAAATTTTCACTTTCGGTAAATTGCCGATTTGCCG
GAAATTTTCACTTTCGGTAATTATCCGATTTGGCA
GAAATTTTCACTTTCGGTAAATTGCCGATTTGCCA
GAAATTTTCACTTTCGGTAAATATCCGATTTGCCA
GAAATTTTCACTTTCGGTAATTATCCGATTTGCCA
GAAATTTTCACTTTCGGTAAATTGCCGATTTGGCA
GAAATTTTCACTTTCGGTAATTATCCGATTTGCCA
GAAATTTTCACTTTCGGTAAATATCCGATTTGGCA
GAAATTTTCACTTTCGGTAAATTGCCGATTTGCCA
GAAATTTTCACTTTCGGTAAATATCCGATTTGCCA
GAAATTTTCACTTTCGGTAATTATCCGATTTGCCA
GAAATTTTCACTTTCGGTAAATATCCGATTTGCCA
GAAATTTTCACTTTCGGTAATTATCCGATTTGCCA
GAAATTTTCACTTTCGGTAAATATCCGATTTGCCA
GAAATTTTCACTTTCGGTAAATTGCCGATTTGGCA
GAAATTTTCACTTTCGGTAAATATCCGATTTGGCA
GAAATTTTCACTTTCGGTAAATTGCCGATTTGCCG
GAAATTTTCACTTTCGGTAAATATCCGATTTGCCA
GAAATTTTCACTTTCGGTAAATTGCCGATTTGGCA
GAAATTTTCACTTTCGGTAAATTGCCGATTTGGCA
GAAATTTTCACTTTCGGTAAATTGCCGATTTGCCG
GAAATTTTCACTTTCGGTAAATTGCCGATTTGCCG
GAAATTTTCACTTTCGGTAAATTGCCGATTTGCCA
GAAATTTTCACTTTCGGTAAATTGCCGATTTGCCG
GAAATTTTCACTTTCGGTAAATTGCCGATTTGCCG
GAAATTTTCACTTTCGGTAAATTGCCGATTTGCCG
GAAATTTTCACTTTCGGTAAATTGCCGATTTGGCA
GAAATTTTCACTTTCGGTAAATTGCCGATTTGCCG
GAAATTTTCACTTTCGGTAAATTGCCGATTTGGCA
GAAATTTTCACTTTCGGTAAATTGCCGATTTGCCG
GAAATTTTCACTTTCGGTAAATTGCCGATTTGCCG
GAAATTTTCACTTTCGGTAAATTGCCGATTTGCCG
GAAATTTTCACTTTCGGTAAATATCCGATTTGCCA
GAAATTTTCACTTTCGGTAAATTGCCGATTTGCCG
GAAATTTTCACTTTCGGTAAATTGCCGATTTGCCG
GAAATTTTCACTTTCGGTAAATTGCCGATTTGCCG
GAAATTTTCACTTTCGGTAAATATCCGATTTGGCA
GAAATTTTCACTTTCGGTAAATTGCCGATTTGCCG
GAAATTTTCACTTTCGGTAAATATCCGATTTGCCA
GAAATTTTCACTTTCGGTAAATTGCCGATTTGCCA
GAAATTTTCACTTTCGGTAAATTGCCGATTTGCCG
GAAATTTTCACTTTCGGTAAATTGCCGATTTGCCG
GAAATTTTCACTTTCGGTAAATTGCCGATTTGCCG
GAAATTTTCACTTTCGGTAAATATCCGATTTGGCA
GAAATTTTCACTTTCGGTAAATTGCCGATTTGCCG
GAAATTTTCACTTTCGGTAAATATCCGATTTGCCA
GAAATTTTCACTTTCGGTAAATTGCCGATTTGCCA
GAAATTTTCACTTTCGGTAAATTGCCGATTTGCCG
GAAATTTTCACTTTCGGTAAATTGCCGATTTGCCG
GAAATTTTCACTTTCGGTAAATTGCCGATTTGCCG
GAAATTTTCACTTTCGGTAAATTGCCGATTTGCCG

GAAATTTTCACTTTCGGTAAATTGCCGATTTGCCG
GAAATTTTCACTTTCGGTAAATATCCGATTTGGCA
GAAATTTTCACTTTCGGTAAATTGCCGATTTGCCG
GAAATTTTCACTTTCGGTAAATATCCGATTTGCCA
GAAATTTTCACTTTCGGTAAATTGCCGATTTGCCG
GAAATTTTCACTTTCGGTAAATTGCCGATTTGCCG
GAAATTTTCACTTTCGGTAAATATCCGATTTGGCA
GAAATTTTCACTTTCGGTAAATTGCCGATTTGCCG
GAAATTTTCACTTTCGGTAAATATCCGATTTGCCA
GAAATTTTCACTTTCGGTAAATTGCCGATTTGCCA
GAAATTTTCACTTTCGGTAAATTGCCGATTTGCCG
GAAATTTTCACTTTCGGTAAATTGCCGATTTGCCG
GAAATTTTCACTTTCGGTAAATTGCCGATTTGCCG
GAAATTTTCACTTTCGGTAAATATCCGATTTGGCA
GAAATTTTCACTTTCGGTAAATTGCCGATTTGCCG
GAAATTTTCACTTTCGGTAAATATCCGATTTGGCA
GAAATTTTCACTTTCGGTAAATTGCCGATTTGCCA
GAAATTTTCACTTTCGGTAAATTGCCGATTTTCCG
GAAATTTTCACTTTCGGTAAATTGCCGATTTGCCA
GAAATTTTCACTTTCGGTAAATTGCCGATTTGCCG
GAAATTTTCACTTTCGGTAAATTGCCGATTTGCCA
GAAATTTTCACTTTCGGTAAATTGCCGATTTTCCG
GAAATTTTCACTTTCGGTAAATTGCCGATTTGCCA
GAAATTTTCACTTTCGGTAAATTGCCGATTTGCCA
GAAATTTTCACTTTCGGTAAATTGCCGATTTTCCA
GAAATTTTCACTTTCGGTAAATTGCCGATTTGCCG
GAAATTTTCACTTTCGGTAAATTGCCGATTTGCCA
GAAATTTTCACTTTCGGTAAATTGCCGATTTGCCG
GAAATTTTCACTTTCGGTAAATTGCCGATTTGCCG
GAAATTTTCACTTTCGGTAAATTGCCGATTTGCCG
GAAATTTTCACTTTCGGTAAATATCCGATTTGCCG
GAAAATTTCACTTTCGGTAAATTGCCGATTTGCCG
Nr of selected repeats 213 Similarity 0.929447
Consensus:
GAAATTTTCACTTTCGGTAAATtgCCGATTTGCCg
>Cele-UNSB01_1:14726982-14727102 Satlength=121 Nr of Repeats=6 RepeatLength=20
seed=GTGAAATTTT
GTGAAATTTTGAAGTTTTAG
GTGAAATTTTAAATTTTTGA
GTGAAAATTTTGATTTCCGA
GTGAAATTTTGCATTTTTAA
GTGAAATTTTAAATTTTTAA
GTGAAATTTTAAAGTTTTAG
Nr of selected repeats 6 Similarity 0.705820
Consensus:
GTGAAATTTTaaAtTTTTaa
>Cele-UNSB01_1:14731269-14731934 Satlength=666 Nr of Repeats=19 RepeatLength=35
seed=GAAAATTTCC
GAAAATTTCCGGCAAACCGGCAAATTGCCGGAATT
GAAAATTTCCGGCAACCCGGCAAATTGCCGGAATC
GAAAATTTCCGGCAAACCGGCAAATTGTCGGAATT
GAAAATTTCCGGCAAACCGGCAAATTGCCGGAATC
GAAAATTTCCGTCAAACCGGCAAATTGCCGGAATT
GAAAATTTCCGGCAAACCGGCAAATTGCCGGAATT

GAAAATTTCCGGCAAATCGGCAAATTGCCGGAATC
GAAAATTTCCGGCAAACCGGCAAATTGCCGGAATT
GAAAATTTCCGGCAAATCGGCAAATTGCCGGAATT
GAAAATTTCCGGCAAACCAGCAAATTGCCGGAATT
GAAAATTTCCGGCAAATCGGCAAATTGCCGGAATC
GAAAATTTCCGGCAAACCGGCAAATTGCCGGAATC
GAAAATTTCCGGCAAACCGGCAAATTGCCGGAATT
GAAAATTTCCGGCAAATCGGCAAATTGCCGGAATC
GAAAATTTCCGGCAAACCGGCAAATTGCCGGAATT
GAAAATTTCCGTCAAACCGGCAAATTGCCGGAAGC
GAAAATTTCCGGCAAACCGGCAAATTGCCGGAATC
GAAAATTTCCGGCAAACCGGCAAATTGCCGGAATT
GAAAATTTCCGTCAAACCGGCAAATTGCCGGAATC
Nr of selected repeats 19 Similarity 0.939850
Consensus:
GAAAATTTCCGGCAAAcCGGCAAATTGCCGGAATt
>Cele-UNSB01_1:14804070-14804449 Satlength=380 Nr of Repeats=9 RepeatLength=43
seed=TTTGCCGGTT
TTTGCCGGTTTGCCGATTTGCCGGAAATTTTCATTTTCGGCAA
TTTGCCGGTTCGACGGAAATTTTTATTTTCGGCAC
TTTGCCGGTTTGCCGATTTGCCGGAAATTTTTATTTTCGGCAC
TTTGCCGGTTTGCCGGTTCGACGGAAATTTTTATTTTCGGCAC
TTTGCCGGTTTGCCGGTTCGACGGAAATTTTTATTTTCGGCAC
TTTGCCGGTTTGCCGGTTCGACGGAAATTTTTATTTTCGGCAC
TTTGCCGGTTTGCCGCTTCGATGGAAATTTTTATTTTCGGCAC
TTTGCCGGTTTGCCGGTTCGACGGAAATTTTTATTTTCGGCAC
TTTGCCGGTTTGCCGGTTCGACGGAAATTTTTATTTTCGGCAC
Nr of selected repeats 8 Similarity 0.931340
Consensus:
TTTGCCGGTTTGCCGgTTcGaCGGAAATTTTTATTTTCGGCAC
>Cele-UNSB01_1:14869199-14869444 Satlength=246 Nr of Repeats=6 RepeatLength=35
seed=CAAATCGGCA
CAAATCGGCAAATTTTCGGTGTTGAAAAATTCCGG
CAAATCGGCAAATTTTCGGAATTGAAAATTTTCGG
CAAATCGACAAGTTGGCGGAATTGAAAATTTCCGT
CAAATCGGCAAATTGCCGGAATTGAAAATTTCCCGG
CAAATCGGCAGCTTGCTGGAATTGAACATTTTCGG
CAAATCGGCAAATTTTCGGAATTAAAAATTTCCGGCAAATTGCCAAGTTGTCAGAATTGAAATTTCTGG
Nr of selected repeats 4 Similarity 0.733333
Consensus:
CAAATCGgCAaaTTgtcGGaaTTGAAaAtTTcCGg
>Cele-UNSB01_1:14918515-14918575 Satlength=61 Nr of Repeats=4 RepeatLength=15
seed=CCACGATGGG
CCACGATGGGTCTCG
CCACGATGGGTCTCT
CCACGATGGGTCTTT
CCACGATGGGTCTCA
Nr of selected repeats 4 Similarity 0.881481
Consensus:
CCACGATGGGTCTct
>Cele-UNSB01_1:14918627-14918817 Satlength=191 Nr of Repeats=5 RepeatLength=40
seed=CCAGAAGGTT
CCAGAAGGTTCTAGAACAATCCAGAATTTTTTCGAATTTT
CCAGAAGGTTCTAGAACAATCCAGAATTTTTTCGAATTTT

CCAGAAGGTTTTGGAACATTCCAGAATTTTCTCGATTGTT
CCAGAAGGTTCTAAAGCTTTTCAGCATTTT
CCAGAAGGTTCTGGAACATTCTAGAATTTTCCAGAAATTC
Nr of selected repeats 4 Similarity 0.800000
Consensus:
CCAGAAGGTTcTaGAACAaTCcAGAATTTTctcGAattTt
>Cele-UNSB01_1:14936456-14937856 Satlength=1401 Nr of Repeats=35
RepeatLength=40 seed=TTTCTAGAAA
TTTCTAGAAAGTTCTGGAACATTCCAGAACTTTTTCGAAA
TTTCTAGAAAGTTCTGGAGCATTCCAGAATTTTCTCGATT
TTTCTAGAAAGTTCTGGAACGTTCTAGAATTTTCTCGATT
TTTCTAGAAAGTTCTGGAACATTCCAGAACTTTTTCGAAA
TTTCTAGAAAGTTCTGGAGCATTCCAGAATTTTCTCGATT
TTTCTAGAAAGTTCTGGAACGTTCTAGAATTTTCTCGATT
TTTCTAGAAAGTTCTGGAACATTCCAGAATTTTCCCGATT
TTTCTAGAAAGTTCTGGAACGTTCTAGAATTTTCTCGATT
TTTCTAGAAAGTTCTGGAACGTTCTAGAATTTTCTCGATT
TTTCTAGAAAGTTCTGGAACATTCCAGAACTTTTTCGAAA
TTTCTAGAAAGTTCTGGAGCATTCCAGAATTTTCTCGATT
TTTCTAGAAAGTTCTGGAACGTTCTAGAATTTTCTCGATT
TTTCTAGAAAGTTCTGGAACATTCCAGAATTTTCCCGATT
TTTCTAGAAAGTTCTGGAACGTTCTAGAATTTTCTCGATT
TTTCTAGAAAGTTCTGGAACGTTCTAGAATTTTCTCGATT
TTTCTAGAAAGTTCTGGAACATTCCAGAACTTTTTCGAAA
TTTCTAGAAAGTTCTGGAGCATTCCAGAATTTTCTCGATT
TTTCTAGAAAGTTCTGGAACGTTCTAGAATTTTCTCGATT
TTTCTAGAAAGTTCTGGAACATTCCAGAATTTTTTTCGAAA
TTTCTAGAAAGTTCTGGAGCATTCCAGAATTTTCTCGATT
TTTCTAGAAAGTTCTGGAACATTCCAGAATTTTCTCGATT
TTTCTAGAAAGTTCTGGAACGTTCTAGAATTTTCTCGATT
TTTCTAGAAAGTTCTGGAACGTTCTAGAATTTTCTCGATT
TTTCTAGAAAGTTCTGGAACATTCCAGAATTTTTTTCGAAA
TTTCTAGAAAGTTCTGGAACATTCCAGAATTTTCCCGATT
TTTCTAGAAAGTTCTGGAACGTTCTAGAATTTTCTCGATT
TTTCTAGAAAGTTCTGGAACATTCCAGAATTTTCCCGATT
TTTCTAGAAAGTTCTGGAACGTTCTAGAATTTTCTCGATT
TTTCTAGAAAGTTCTGGAACGTTCTAGAATTTTCTCGATT
TTTCTAGAAAGTTCTGGAACATTCCAGAATTTTCCCGATT
TTTCTAGAAAGTTCTGGAACGTTCTAGAATTTTCTCGATT
TTTCTAGAAAGTTCTGGAACATTCCAGAATTTTTTTCGAAA
TTTCTAGAAAGTTCTGGAGCATTCCAGAATTTTCTCGATT
TTTCTAGAAAGTTCTGGAACATTCCAGAATTTTCTCGATT
TTTCTAGAAAGTTCTGGAACATTCCAGAATTTTCCCGATT
Nr of selected repeats 35 Similarity 0.907675
Consensus:
TTTCTAGAAAGTTCTGGAACaTTCcAGAATTTTcTCGAtt
>Cele-UNSB01_1:15014974-15015203 Satlength=230 Nr of Repeats=5 RepeatLength=34
seed=GAAATTTCAA
GAAATTTCAAAAAAAAAAAATATGCAAAACCTCAATTTGCCGAAAATTTCCAAATTTCCGGCAAATCGATAGTTTGCCGG
TTTTCTGATTTGCG
GAAACTTCAACTCCTGCAAATTGCCGATTTGCCG
GAAATTTCAACTCCGGCAATTAGCCAATTTGCCG
GAAATTTCAACTCCGGCAATTAGCCAATTTGCCG
GAAATTTCAACTCCGGCAATTAGCCAATTTGCCG

Nr of selected repeats 4 Similarity 0.901961
Consensus:
GAAAtTTCAACTCCgGCAAtTaGCCaATTTGCCG
>Cele-UNSB01_1:15023397-15024044 Satlength=648 Nr of Repeats=19 RepeatLength=34
seed=CGGAAATTTC
CGGAAATTTCCAATTCCGGCAAATTGTCGATTTAT
CGGTAATTTCAATTCCGGCAAGTTGCCGATTTCC
CGGAAATTTCCATTCCAGAAATTTGTCGATTTGC
CGGAAATTTCAATTCCAGCAATTTGCCGATTTGC
CGGAAATTTCAATTCCAGCAATTTGCCGATTTGC
CGGAAATTTCAATTCCAGCAATTTGCCGATTTGC
CGGAAATTTCAATTCCAGCAATTTGCCGATTTGC
CGGAAATTTCAATTCCAGCAATTTGCCGATTTGC
CGGAAATTTCAATTCCAGCAATTTGCCGATTTGC
CGGAAATTTCAATTCCAGCAATTTGCCGATTTGC
CGGAAATTTCAATTCCAGCAATTTGCCGATTTGC
CGGAAATTTCAATTCCAGCAATTTGCCGATTTGT
CGGAAATTTCAATTCCAGCAATTTGCCGATTTGC
CGGAAATTTCAATTCCAGCAATTTGCCGATTTGC
CGGAAATTTCAATTCCAGCAATTTGCCGATTTGC
CGGAAATTTCAATTCCAGCAATTTGCCGATTTGC
CGGAAATTTCAATTCCAGCAATTTGCCGATTTGC
CGGAAATTTCAATTCCAGCAATTTGCCGATTTGC
CGGAAATTTCAATTCCAGCAATTTGCCGATTTGC
Nr of selected repeats 18 Similarity 0.965142
Consensus:
CGGAAATTTCAATTCCAGCAATTTGCCGATTTGC
>Cele-UNSB01_1:15062491-15062841 Satlength=351 Nr of Repeats=10 RepeatLength=35
seed=AATTCCGGCA
AATTCCGGCAAGTTGTCGTTTTGTCGGAAATTTTT
AATTACGGCACTTTGTCGATTTTCCTGAAATTTTC
AATTCCGGCAATGTGCCGATTTTCCTGAAATTTTC
AATTCCGGCAAGTTGCCGTTTTGTCGGAAATTTTT
AATTCCGGCAATGTGCCGATTTTCCTGAAATTTTC
AATTCCGGCAATTTGACGATTTTCCTGAAATTTTA
AATTCCGGCAAGTTGCCGATTTTCCTGAAATTTTC
AATTCCGGCAATTTGCCGATTTTCCTGAAATTTTC
AATTCCGGCAATTGCCCGAGTTGCCTGAAATTTTC
AATTCCGGCACTTTGCCGATTTTCCTGAAATTTTC
Nr of selected repeats 10 Similarity 0.804115
Consensus:
AATTCCGGCAattTGcCGaTTTtcCtGAAATTTTc
>Cele-UNSB01_1:15073460-15073709 Satlength=250 Nr of Repeats=7 RepeatLength=35
seed=CGGTTTTGGA
CGGTTTTGGAAAATTTCGGCAATTTCGATAATTGC
CGGTTTTGGAAATTTTCGGCAATTTCGACAATTTTGT
CGGTTTGGGAAATTTTCGGTAATTTTGACAATTGC
CGGTTTTGGAAAATTTCGGCAATTTTGACAATTGC
CGGTTTTGGAAATTTTCGGCAATTTCGATAATTGC
CGGTTTTGGAAAATTTCGGCAATTTCGATAATTTC
CGGTTTTGGAAATTTTCGGCAAATTTCACAATTTTGT
Nr of selected repeats 5 Similarity 0.885714
Consensus:
CGGTTTtGGAAAaTTTCGGcAATTTcGAtAATTgC

>Cele-UNSB01_1:15094323-15094532 Satlength=210 Nr of Repeats=6 RepeatLength=35
seed=TTTCAATTCC
TTTCAATTCCGACATTTTGCCGATTTGCCGAAAAC
TTTCAATTCCGGCAATTCGCCGATTTACCAAAAAC
TTTCAATTCCGGCAATTTGCCGATTTGCCGAAAAC
TTTCAATTCCGGCAATTTGCCGATTTGCCGAAAAC
TTTCAATTCCGGCAATTTGCCGACTTGCCGGAAAT
TTTCAATTCCGGCAATTTGACGATTTACCGAAAA
Nr of selected repeats 5 Similarity 0.878095
Consensus:
TTTCAATTCCGgCAaTTtGCCGAtTTgCCgaAAAc
>Cele-UNSB01_1:15278589-15278861 Satlength=273 Nr of Repeats=10 RepeatLength=21
seed=AACTTTTTGG
AACTTTTTGGCAGGTTTCCAAATTTTGTTGGCAGAGTTTCAA
AACTTTTCGGAAATTTTCAAAAATTTTTCTGCAATTTTCCAA
ACCTTTTTGGCAGATTTTCAA
AACTTTTTGGCTATTTTCCGA
AACTTTTCGGCAATTTTCCAG
AACTTTTTGGCAATTTTCCAA
AACGTTTTGGTAGATTTTCA
AACTTTTTGGCAATTTTCCAA
AACTTTTTGGCAATTTTTCAA
AACTTTTCGGCAATTATCCAAAATTTTATGGCAATTTTCCAA
Nr of selected repeats 6 Similarity 0.791919
Consensus:
AACTTTTTGGCAATTTTcCAA
>Cele-UNSB01_2:2-3054 Satlength=3053 Nr of Repeats=243 RepeatLength=12
seed=AAGCCTAAGC
AAGCCTAAGCCT
AAGCCTAGCCT
AAGCCTAAGGAGCCTAACT
AAGCCTAAGAGCCCCT
AAGCCTAAGCGAGCCTAA
AAGCCTAAGGCCTAGT
AAGCCTAAGCCTAGCT
AAGCCTAAGCCT
AAGCTAAGCCT
AAGCCCTAAGCTAGTGCC
AAGCTAAGCCT
AAGCCTAAGCCT
AAGCTAAGCCT
AAGCCTAAGCCT
AAGCCTAAAGCCT
AAGCCTAAGCCGCCT
AAGCCTAAGCCT
AAGCCTTAAGCCTAAGCCCCT
AAGCCTAAGCTCCTAAGCCTAG
AAGCCTAAGCCT
AAGCTAAGCCT
AAGCCTAAGCCT
AAGCCTAAGCCT
AAGCCCAAGCCT
AAGCCTAAGCCT
AAGCCTAAGCCT

AAGCCTAAGCCT
AAGCCTAAGCCT
AAGCCTAAGCCT
AAGCTAAGCCT
AAGCCTAAAGCCT
AAGCCTAAGCCT
AAGCCTAAGCCT
AAGCCTAAGCCT
AAGCCTAAGCCT
AAGCCTAAGCCT
AAGCCTAAGCCCT
AAGCCTAAGCCT
AAGCCTAAGCCT
AAGCCTAAGCCT
AAGCCTAAGCCT
AAGCCTAAGCCT
AAGCCTAAGCCT
AAGCCTAAGCCT
AAGCCTAAGCCT
AAGCCTAAGCCT
AAGCCTAAGCCTAA
AACCTAAGCCT
AAGCCTAAGCCT
AAGCCTAAGCCT
AAGCCTAAGCCT
AAGCCTAAGCCT
AAGCCTAAGCCT
AAGCCTAAGCCT
AAGCCTAAGCCT
AAGCCTAAGCCT
AAGCCTAAGCCT
AAGCCTAAGCCCT
AAGCCTAAGCCT
AAGCCTAAGCCT
AAGCCTAAGCCT
AAGCCTAAGCCT
AAGCCTAAGCCT
AAGCCTAAGCCT
AAGCCTAAGCCT
AAGCCTAAGCCT
AAGCCTAAGCCT
AAGCCTAAGCCT
AAGCCTAAGCCT
AAGCCTAAGCCT
AAGCCTAAAGCCT
AAGCCTAAGCCT
AAGCCTAAGCCT
AAGCCTAAGCCT
AAGCCTAAGCCT
AAGCCTAAGCCCT
AAGCCTAAGCCT
AAGCCTAAGCCT
AAGCCTAAGCCT
AAGCCTAAGCCT

AAGCCTAAGCCT
AAGCCTAAGCCT
AAGCCTAAGCCT
AAGCCTAAGCCT
AAGCCCTAAGCCT
AAGCCTAAGCCT
AAGCCTAAGCCT
AAGCCTAAGCCT
AAGCCTAAGCCT
AAGCCTAAGCCT
AAGCCTAAGCCT
AAGCCTAAGCCT
AAGCCTAAGCCT
AAGCCTAAGCCT
AAGCCTAAGCCT
AAGCCCTAAGCCT
AAGCCTAAGCCT
AAGCCTAAGCCT
AAGCCTAAGCCT
AAGCCTAAGCCT
AAGCCTAAGCCT
AAGCCTAAGCCT
AAGCCTAAGCCT
AAGCCTAAGCCT
AAGCCTAAGCCT
AAGCCTAAGCCT
AAGCCTAAGCCT
AAGCCTAAGCCT
AAGCCTAAGCCT
AAGCCTAAGCCT
AAGCCTAAGCCT
AAGCCTAAGCCT
AAGCCTAAGCCT
AAGCTAAGCCT
AAGCCTAAGCCT
AAGCTAAGCCT
AAGCCTAAGCCT
AAGCCTAAGCCT
AAGCCTAAGCCT
AAGCCTAAGCCT
AAGCCTAAGCCT
AAGCCTAAGCCT
AAGCCTAAGCT
AAGCCTAAGCCT
AAGCCTAAGCCT
AAGCCTAAGCCT
AAGCCTAAGCCT
AAGCCTAAGCCT
AAGCCTAAGCT
AAGCCTAAGCCT
AAGCCTAAGCCT
AAGCCTAAGCCT
AAGCCTAAGCCT
AAGCCTAAGCCT

AAGCTAAGCCT
AAGCCTAAGCCT
AAGCCTAAGCCT
AAGCCTAAGCCT
AAGCCTAAGCCT
AAGCCTAAGCCT
AAGCCTAAGCCT
AAGCCTAAGCCT
AAGCCTAAGCCT
AAGCCTAAGCCT
AAGCCTAAGCCT
AAGCCTAAGCCT
AAGCCTAAGCCT
AAGCCTAAGCCT
AAGCCTAAGCCT
AAGCCTAAGCCT
AAGCCTAAGCCT
AAGCCTAAGCCT
AAGCCTAAGCCT
AAGCCTAAGCCT
AAGCCTAAGCCT
AAGCCTAAGCCT
AAGCCTAAGCCT
AAGCCTAAGCCT
AAGCCTAAGCCT
AAGCCTAAGCCT
AAGCCTAAGCCT
AAGCCTAAGCCT
AAGCCTAAGCCT
AAGCCTAAGCCT
AAGCCTAAGCCT
AAGCCTAAGCCT
AAGCCTAAGCCT
AAGCCTAAGCCT
AAGCCTAAGCCTAAGCCCCT
AAGCCTAAGCCT
AAGCCTAAGCCT
AAGCCTAAGCCT
AAGCCTAAGCCT
AAGCCTAAGCCT
AAGCCTAAGCCT
AAGCCTAAGCCT
AAGCCTAAGCCT
AAGCCCTAAGCCT
AAGCCTAAGCCT
AAGCCTAAGCCT
AAGCCTAAGCCT
AAGCCTAAGCCT
AAGCCTAAGCCTAAGCCTAA
AAGCCTAAGCCT
AAGCCTAAGCCT
AAGCCTAAGCCTAAGCCTAA
AAGCCTAAGCCT
AAGCCTAAGCCT

AAGCCTAAGCCT
AAGCCTAAGCCT
AAGCCTAAGCCT
AAGCCTAAGCCT
AAGCCTAAGC
AAGCCTAAGCCT
AAGCCTAAGCCT
AAGCTAAGCCT
AAGCCTAAGCCT
AAGCCTAAGCCT
AAGCCTAAGCCT
AAGCCTAAGCCT
AAGCCTAAGCCT
AAGCCTAAGCCT
AAGCCTAAGCCTAAGCT
AGCCTAAGCCT
AAGCCTAAGCCT
AAGCCTAAGCCT
AAGCCTAAGCCT
AAGCCTAAGCCT
AAGCCTAAGCCT
AAGCCTAAGCCT
AAGCCTAAGCCT
AAGCCTAAGCT
AAGCCTAAGCCT
AAGCCTAAGCCTA
AAGCCTAAGCCT
AAGCCTAAGCCT
AAGCCTAAGCCT
AAGCCTAAGCCT
AAGCCTAAGCCCT
AAGCCTAAGCCT
AAGCCTAAGCCT
AAGCCTAAGCCCT
AAGCCTAAGCCT
AAGCCTAAGCCT
AAGCCTAAGCCTA
AAGCCTAAGCCT
AAGCCTAAGCCT
AAGCCTAAGCCT
AAGCCTAAGCCTAGT
AAGCCTAAGCCT
AAGCCTAAGCCT
AAGCCTAAGCCT
AAGCCTAAGCCT
AAGCCTAAGCCT
AAGCCTAAGCCT
AAGCCTAAGCCT
AAGCCTAAGCCT
AAGCCTAAGCCT
AAGCCTAAGCCTAAAAAAAAAAAAAAAAAAAAAAAAAAAAAAAAAAAAAAAAAAAAAAAAAAAAAA
AAGCTAAGCT
AAGCCTAAGCCT
AAGCCTAAGCCT

AAGCCTAAGCCT
Nr of selected repeats 197 Similarity 0.998872
Consensus:
AAGCCTAAGCCT
>Cele-UNSB01_2:3110-3266 Satlength=157 Nr of Repeats=6 RepeatLength=26
seed=GTTCACCAAA
GTTCACCAAAAATTGTGACTCTGACC
GTTCACCAAAAATAGTGACTCTGACC
GTTCACCAAAAATAGTGACTCTGACC
GTTCACCAAAAATAGTGACTCTGACC
GTTCACCAAATATAGTGACTCTGACC
GTTCACCAAAAATTGTGACAATGACC
Nr of selected repeats 6 Similarity 0.921368
Consensus:
GTTCACCAAAAATaGTGACTCTGACC
>Cele-UNSB01_2:146899-146982 Satlength=84 Nr of Repeats=6 RepeatLength=12
seed=ATTTCTTGCA
ATTTCTTGCAAC
ATTTCTTGCAAC
ATTTTTTGCAAA
ATTTCTTGAAAATTTCCAGCAAC
ATTTCTTCCAAA
ATTTCTTGCAAA
Nr of selected repeats 5 Similarity 0.844444
Consensus:
ATTTcTTgCAAa
>Cele-UNSB01_2:236549-238078 Satlength=1530 Nr of Repeats=40 RepeatLength=35
seed=TTTCCGGCAA
TTTCCGGCAAATCGGCCACTTGCCGGATTTGAAAA
TTTCCGGCAAAACGCCGGAATTGAATG
TTTCCGGCAAATTGGCAAGTTGTCGGAATTGAAAATGACAAATCGGCAAATTGCTGGAATTGAAAA
TTTCCGGCAAATCGGCAGCCTGTCGGCATTGAAAA
TTTCCGGAAAATCAGTATATTACCAGAATTGAAAA
TTTCCGGCAATCGACAAATTGCCGAATTTAAAAAA
TTCCGGCAAAACGGCAAATTGCCGGATTTGAAAA
TTTCCGGCATATCGGCAGCCTGTCGGAATTGAAAA
TTTCCGGCAAATCGGCAGCCTGTCGGCATTGAAAA
TTTCCGGCAAATCAGTATATTACCAGAATTGAAAA
TTTCCGGCAATCGGCAAATTGCCGAATTTAAAAAA
TTCCGGCAAAACGGCAAATTGCCGGATTTGAAAA
TTTCCGGCAAATCGGCAGCCTGTCGGAATTGAAAA
TTTCCGGCAAATCAGTAAACTGCCAGAATTGAAAT
TTTCCGGCAAATCGACAAATTGCCGAAATTGAAAAATTCCGACAAATCGGAAAATTGCCGGATTTGAAAT
TTTCCGGCAAATCGGTAGATTGCCGGATTTGAAAT
TTTCCGGCAAATCGGTAAATTGTCGTAATTGAAAA
TTTCCGGCAAACCGGTAAATTGTCGTAATTGAAAA
TTTCCGGCAAATCGGCATACCGGTAAATTGTCGGAATTGAAAA
TTTCCGGCAAATCGGCATACCGGAATATTTTCGGAATTGAAAA
TTTCCGGCAAATCGGCATACCGGAATATTTTCGGAATTGAAAA
TTTCCGGCAAATCGGCAGCCTGTCGGCATTGAAAA
TTTCCGGAAAATCAGTATATTACCAGAATTGAAAA
TTTCCGGCAATCGACAAATTGCCGAATTTAAAAAA
TTCCGGCAAAACGGCAAATTGCCGGATTTGAAAA
TTTCCGGCATATCGGCAGCCTGTCGGAATTGAAAA

TTTCCGGCAAATCGGCAGCCTGTCGACATTGAAAA
TTTCCGGCAAATCAGTATATTACCAGAATTGAAAA
TTTCCGGCAATCGGCAAATTGCCGAATTTAAAAAA
TTCCGGCAAAACGGCAAATTGCCGGATTTGAAAA
TTTCCGGCAAATCGGCAGCCTGTCGGAATTGAAAA
TTTCCGGCAAATCAGTAAACTGCCAGAATTGAAAT
TTTCCGGCAAATCGACAAATTGCCGAAATTGAAAAATTCCGACAAATCGGAAAATTGCCGGATTTGAAAT
TTTCCGGCAAATCGGTAGATTGCCGGATTTGAAAT
TTTCCGGCAAATCGGTAAATTGTCATAATTGAAAA
TTTCCGGCAAACCGGTAAATTGTCGTAATTGAAAA
TTTCCGGCAAATCGGCATACCGGTAAATTGTCGGAATTGAAAA
TTTCCGGCAAATCGGCAAGTTACCGGAATTGAAAA
TTTCCGGCAAATTGGCAAGTTACCGGAATTGAAAA
TTTCCGGCAAATCGGCATACCGGAATATTTTCGGAATTGAAAA
Nr of selected repeats 27 Similarity 0.708272
Consensus:
TTTCCGGCAaATCgGcAnatTgcCgGAATTGAAAA
>Cele-UNSB01_2:371286-371494 Satlength=209 Nr of Repeats=10 RepeatLength=18
seed=TAAGCCTAAG
TAAGCCTAAGAC
TAAGCCTAAGTT
TAAGCCTATGTCTAAGAA
TAAGCCTAAGCATCGCTC
TAAGTCTAAGTCCAAGTCTAATAAACCCAAGCCTAAATAGCGTCACTGACGCCAAGCCCACGCA
TAAGCCTAAGTC
TGAGCCTAAGCTTAAGTC
TACGCCTAAGCCTAAACC
TAAACCTAAGCCTAAGTC
TAAACCTAAGCCGAAGCC
Nr of selected repeats 6 Similarity 0.619753
Consensus:
TAAgCCTAAGCcTAAgtC
>Cele-UNSB01_2:454595-455440 Satlength=846 Nr of Repeats=32 RepeatLength=15
seed=TCTCACCACG
TCTCACCACGATGGG
TCTCGCCACGAAGGG
TCTCACCACGATGTG
TCTCACCACGATGGG
TCTCACCACGAAAGG
TCTCGCCACGAAGGG
TCTCACCACGATGTG
TCTCACCACGAAGGG
TCTCACCACGAAGGG
TCTCACCACGATAATTTGATTTTCCCGCCAAAATATTTTTCTGAGAAAATTTGAATTTCCCGCCAAAATATTTTTCTGA
GAAAATTTGAATTTCCCACCAAAATATTTTTCTCAGAAAATTTAAATTTCCCGCCAAAATATTTTTCTCAGAAAATTTG
AATTTCCCGCCAAAATATTTTTCTCAGAAAATTTGAATTTCCCGCCGGAATATTTTTCTGAGAAAATTTGAATTTCCCG
CCAAAATGTTTTTCTCAGAAAATTTAAATTTCCCGCTAAAGTTTTGGA
TCTCACCACGATGGG
TCTCACCACGATGGG
TCTCGCCACGAAGGG
TCTCACCACGATGTG
TCTCACCACGATGGG
TCTCACCACGAAAGG
TCTCGCCACGATGGG

TCTCACCACGAAGGG
TCTCGCCACGAAGGG
TCTCACCACGAAGGG
TCTCACCACGATGGG
TCTCGCCACGATGGG
TCTCGCCACGATGGG
TCTCACCACGATGGG
TCTCACCACGATGGG
TCTCGCCACGATGGG
TCTCGCCACGATGGG
TCTCACCACGAAGGG
TCTCACCACGAAAGG
TCTCACCACGATGGG
TCTCACCACGATGGG
TCTCGCCACGAAAATTTGAATTTCCCGCCAAAATGTTTTTTTCAGAAAATTTGAATTTCCCGCCCAAATTTTTATCTGA
GAAAATTTGAATTTCCCGCCAAAGTTTTGGG
Nr of selected repeats 30 Similarity 0.884138
Consensus:
TCTCaCCACGAtGGG
>Cele-UNSB01_2:466682-467257 Satlength=576 Nr of Repeats=9 RepeatLength=20
seed=CAATTTTTGA
CAATTTTTGAGCTGGAAATCAAGAAAATTTCAATTTTCTGTTTTTTTTGTGCTGTAAAAATCTAATTTTTCTAATTTTT
AAGGAAAAAAAATGAGAAATTTTCTGGAAACGCCACAAAATCGGCGAAACTTCGATTTTTCCGGAATTTTCACAAAAAAA
AATCGATTTTTCTCAATTTTTCCAATTTTCACGCTGGAAAATTCAGAATTTCATAAAAAAATTCCGTACTTTCTTTCGCT
GCAAAATGTTGTTTCGTCTTTGGATCCACATAAATCTGCATTTGGCCTTGTTGCTCCAAGGCGATGGCTCCAAGTGAAT
CTTGCTCAATAATTTCCTCGTGTTCTTCAATTTCTACTTCTTCCATCGGCATCGGCTCCATTGGCTCCTGAAAAATCGA
ATTTTTTGAGTTAAAAAAT
CAATTTTTGAGTTAAAAAAT
CAATTTTTGAGTTAAAAATT
CAATTTTTGAGTTAAAAATT
CAATTTTTGAATTAAAAATT
CAATTTTTGAGTTAAAAATT
CAATTTTTGAATTAAAAATT
CAATTTTTGAATTAAAAATT
CAATTTTTGAGTTAAAAAAAT
Nr of selected repeats 7 Similarity 0.942857
Consensus:
CAATTTTTGAgTTAAAAATT
>Cele-UNSB01_2:577469-577719 Satlength=251 Nr of Repeats=20 RepeatLength=12
seed=TAGGCTTAGG
TAGGCTTAGGCA
TAGGCTTAGGCT
TAGGTTTAGGCT
TAGGCCTAGGCT
TAGGCCTAGGAT
TAGGCCTAGGCT
TAGGCCTAGGAT
TAGGCCTAGGCT
TAGGCCTAGGAT
TAGGCCTAGGCT
TAGGCTTAGGCC
TAGGATTAGGCTTAGGTTTAGCA
TAGGTTTAGGCA
TAGGTTAGGTT

TAGGCTTAGGCT
TAGGCTTAGGTT
TAGGCTTAGGCC
TAGGCTTAGGCC
TAGGCTTAGGCT
TAGGCCTAGGAT
Nr of selected repeats 18 Similarity 0.816993
Consensus:
TAGGCtTAGGct
>Cele-UNSB01_2:590141-590909 Satlength=769 Nr of Repeats=12 RepeatLength=64
seed=AAATTTTGTA
AAATTTTGTAGTTTGTAGTCTAGCAGACCCATATTGAATACCAGCAAGTTGGGTCTGCCAAATC
AAATTTTGTAGTTTGTAGTCTAGCAGACCCATTTAGAATACCAACAAGTTGGGTCTGCCAGATC
AAATTTTGTAGTTTGTAGTCTAGCAGACCCATTTAGAATACCAATAAATTGGGTATGCCAAATC
AAATTTTGTAGTTTGTAGTCTAGCAGACCCATTTAGAATACCAATAAATTAGGTCTGCCAGATC
AAATTTTGTAGTTTGTAGTCTAGAAGACCCATTTAGAATACCAACAAGTTGGGTCTGCCAGATC
AAATTTTGTAGTTTGTAGTCTAGCAGACCCATATTGAATACCAATAAATTGGGTCTGCCAGATC
AAATTTTGTAGTTTGTAGTCTAGAAGACCCATTTAGAATACCAACAAGTTGGGTCTGCCAGATC
AAATTTTGTAGTTTGTAGTCTAGCAGACCCATTTAGAATACCAATAAATTGGGTCTGCCAAATC
AAATTTTGTAGTTTGTAGTCTAGCAGACCCATTTAGAATACCAACAAGTTGGGTCTGCCAGATC
AAATTTTGTAGTTTGTAGTCTAGCAGACCCATATTGAATACCAGCAAGTTGGGTCTGCCAGATC
AAATTTTGTAGTTTATAGTCTAGAAGACCCATATTGAATACCAATAAATTGGGTCTGCCAGATC
AAATTTTGTAGTTTGTAGTCTAGCAGACCCATATTGAATACCAATAAATTGGGTCTGCCAGATC
Nr of selected repeats 12 Similarity 0.921402
Consensus:
AAATTTTGTAGTTTGTAGTCTAGcAGACCCATtTaGAATACCAAcAAaTTGGGTCTGCCAgATC
>Cele-UNSB01_2:636344-636664 Satlength=321 Nr of Repeats=8 RepeatLength=40
seed=TTTCTAGAAA
TTTCTAGAAAGTTCTGGAACATTCGAGAATTTTCTCGATT
TTTCTAGAAAATTCTGGAACATTCCAGAATTTTCCCGATT
TTTCTAGAAAATTCTGGAACATTCCAGAATTTTCCCGATT
TTTCTAGAAAGTTCTGGAACATTCGAGAATTTTTTCGAGA
TTTCTAGAAAGTTCTGGAACATTCGAGAATTTTCTCGATT
TTTCTAGAAAATTCTGGAACATTCCAGAATTTTCCCGATT
TTTCTAGAAAGTTCTGGAACATTCGAGAATTTTCTCGATT
TTTCTAGAAAATTCTGGAACATTCCAGAATTTTCCCGATT
Nr of selected repeats 8 Similarity 0.917857
Consensus:
TTTCTAGAAAaTTCTGGAACATTCcAGAATTTTCcCGATT
>Cele-UNSB01_2:635957-636850 Satlength=894 Nr of Repeats=4 RepeatLength=35
seed=GAAAATTTAA
GAAAATTTAAATATCTTTTAAAAAACCAGAAATCAGCTTTTGCCAGTGCAAAACTAATAGAAGGCGTAATATTAATAGG
AAAACTCTCTTTCGAGCAAATAATATTTTTGCTTGGAAAATGTTTTCCCAACAAAAAAACAACTTTATTGGGATTTTAA
AGATTTAAAAAATTTTTTATGACGAATCAGTGTATGACTAACTAAAAACATTGTTCATTGTCATGGGTCCAAGCACAAC
AATATTTATCAACTCAGACAAAAAAGGAAAACAATTTTCAGCGTGAAATTAGTTATTTTGGACCAGCCGCTGACCGCGC
CTACGGCGCGGGCAACGACTGGCACCATTAAAAGTATTTGACTCACATACACTTCCAGAATTTTCTCGATTTTTCTAGA
AAGTTCTGGAACATTCGAGAATTTTCTCGATTTTTCTAGAAAATTCTGGAACATTCCAGAATTTTCCCGATTTTTCTAG
AAAATTCTGGAACATTCCAGAATTTTCCCGATTTTTCTAGAAAGTTCTGGAACATTCGAGAATTTTTTCGAGATTTCTA
GAAAGTTCTGGAACATTCGAGAATTTTCTCGATTTTTCTAGAAAATTCTGGAACATTCCAGAATTTTCCCGATTTTTCT
AGAAAGTTCTGGAACATTCGAGAATTTTCTCGATTTTTCTAGAAAATTCTGGAACATTCCAGAATTTTCCCGATTTTTC
TAGAAAGTTCTGGAACATTCGAGAATTTTTTCGAAATTTCCAAAAGATTCTAGATTTCCAGAATTTTAGAACCTTCA
GAAAATTTAAATTTCCCTCCAAAATTTTTTTCTCA
GAAAATTTAAATTTCCCGCCAAAATATTTTTCACA
GAAAATTTAAATTTCCCTCCAAAATATTTTTCACA

Nr of selected repeats 3 Similarity 0.923810
Consensus:
GAAAATTTAAATTTCCCtCCAAAATaTTTTTCaCA
>Cele-UNSB01_2:636908-637328 Satlength=421 Nr of Repeats=27 RepeatLength=15
seed=GGGTCTCACC
GGGTCTCACCACGAT
GGGTCTCACCACGAT
GGGTCTCACCACGAT
GGGTCTCACCACGAT
GGGTCTCACCTCGAT
GGGTCTCACCACGAT
GGGTCTCACCACGAC
GGGTCTCACCTCGAT
GGGTCTCACCACGAT
GGGTCACACCGCGGT
GGGTCTCACCGCGAT
GGGTCTCACCACGAT
GGGTCTCATCACGAT
GGGTCTCACCACGAT
GGGTCTCACCACGAT
GGGTCTCACCACGAT
GGGTCTCACCACGAT
GGGTCTCACCTCGAT
GGGTCTCACCACGAT
GGGTCACACCGCGGT
GGGTCTCACCGCGAT
GGGTCTCACCACGAT
GGGTCTCATCACGAT
GGGTCTCACCACGAT
GGGTCTCACCACGAC
GGGTCTCACCGCGGT
GGGTCTCGCCACGATGGGTCTTTCCACGAT
Nr of selected repeats 26 Similarity 0.898256
Consensus:
GGGTCTCACCaCGAT
>Cele-UNSB01_2:637418-637568 Satlength=151 Nr of Repeats=4 RepeatLength=40
seed=CAGAAGGTTC
CAGAAGGTTCTAGAACAATCCAGAATTTTTTCGAATTTTC
CAGAAGGTTCTGCAACATTCCAGAATTTTCTAGAATTTTC
CAGAAGGTTCTAAAGCTTTTCAGAATTTTC
CAGAAGGTTCTGGAACATTCTAGAATTTTCCAGAAATTCC
Nr of selected repeats 3 Similarity 0.800000
Consensus:
CAGAAGGTTCTggAACAtTCcAGAATTTTctaGAAtTTtC
>Cele-UNSB01_2:637664-637798 Satlength=135 Nr of Repeats=4 RepeatLength=11
seed=GTACTCCTAC
GTACTCCTACA
GTACTCCTACAGTACTACAACAGTACCCCGACCATATCCCACTACTAACCCCAAATCTATATCTCTTCAAAAGACTAAA
ACACAATTTTTCCTAAACTACA
GTAATCCTACC
GTACTCCTACA
Nr of selected repeats 3 Similarity 0.838384
Consensus:
GTAcTCCTACa

>Cele-UNSB01_2:646191-646835 Satlength=645 Nr of Repeats=25 RepeatLength=23
seed=GGCCACCAAT
GGCCACCAATTTCTTAAAAATTC
GGCCACCGATCTATAAAAATCTA
GGCCACCAATTTCTTAAAAGTTC
GGCCACCGATCTATAAAAAGCTA
GGCCACCAATCTGTTAAAAATTC
GGCCACCGATCTATAAAAAGCTA
GGCCACGAATCTGTTAAAAACTC
GGCCACCGATCTATAAAAAGCTA
GGCCACCAATCTGTTAAAAATTC
GGCCACCGATCTATAAAAATCTAA
GCCACCAATTTCTTAAAAATTC
GGCCACCGATCTATAAAAATCTA
GGCCACCAATTTCTTAAAAACTCGGCCACCGGTCAATAAAAAGCTA
GGCCACCAATCTGTTAAAAGCTCGTCCACCGATCTATAAAAATCTA
GGCCACCAATTTGTTAAAAATTC
GGCCACCGATCTATAAAAAACTA
GGCCACCAATTTGTTAAAAATTC
GGCCACCGATCTATAAAAATCTA
GGCCACCAATTTCTTAAAAATTC
GGCCACCGATCTATAAAGAGTTA
GGCCACCAATCTTTTAAAAATTCGGCCAAGAATCTATAAAAATCAA
GGCCACCAATTTCTTAAAAACTC
GGCCACCGATCAATAAAAAGCTG
GGCCACCAATCTGTTAAAAATTC
GGCCACCAATCTATAAAAACCTT
Nr of selected repeats 20 Similarity 0.753776
Consensus:
GGCCACCaATcTaTaAAAAacTc
>Cele-UNSB01_2:649028-649082 Satlength=55 Nr of Repeats=4 RepeatLength=12
seed=GGCTCAGGCT
GGCTCAGGCTCA
GGCTCAGGCTCA
GGCTCAGGCTCA
GGCTCAGGCTCAGGCTCC
Nr of selected repeats 3 Similarity 1.000000
Consensus:
GGCTCAGGCTCA
>Cele-UNSB01_2:677942-678110 Satlength=169 Nr of Repeats=9 RepeatLength=16
seed=CCTACGTGCC
CCTACGTGCCTTCGTG
CCTAGGTGCCTTCGTG
CCTACGTGCCTACGTG
CCTACGTTCCTACGTGCCTGCGTA
CCTACGTTCCTACGTGCCTGCGTA
CCTAAGTGCCTTCGTA
CCTACGTGCCTACGTG
CCTACGTACCTACGTGCCTTCGTA
CCTACGTGCCTAAGTG
Nr of selected repeats 6 Similarity 0.844444
Consensus:
CCTAcGTGCCTaCGTG

>Cele-UNSB01_2:694600-694660 Satlength=61 Nr of Repeats=4 RepeatLength=15
seed=GTAGATCTTG
GTAGATCTTGTTTTG
GTAGATCTTGAAAAT
GTAGATCTTGTTTTG
GTGGATCTTGTTTTC
Nr of selected repeats 4 Similarity 0.675347
Consensus:
GTaGATCTTGtttTg
>Cele-UNSB01_2:698255-706115 Satlength=7861 Nr of Repeats=432 RepeatLength=15
seed=GGTGAGACCC
GGTGAGACCCATCGT
GGTGAGACCCATCGT
GGTGAGACCCATCGTGACGAGACCCATCGT
GGTGAGACCCATCGTGACGAGACCCATCGT
GGTGAGACCCATCGT
GGTGAGACCCATCGT
GGTGAGACCCACCGT
GGTGAGACCCATCGTGACGAGACCCACCGT
GGTGAGACCCATCGTGACGAGACCCATCGT
GGTGAGACCCACCGT
GGTGAGACCCATCGT
GGTGAGACCCACCGT
GGTGAGACCCATCGTGACGAGACCCATCGT
GGTGAGACCCACCGT
GGTGAGACCCATCGT
GGTGAGACCCATCGT
GGTGAGACCCATCGT
GGTGAGACCCATCGT
GCTGAGACCCATCGT
GGTGAGACCCATCGT
GCTGAGACCCATCGT
GGTGAGACCAATCGT
GGTGAGACCCATCGT
GGTGAGACCCATCGT
GGTGAGACCCATCGTGGTGATACCAATCGT
GGTGAGACCCATCGT
GGTGAGACCCATCGT
GGTGAGACCCATCGT
GGTGAGACCCATCGTGCTGATACCCATCGTGACGAGACCCATCGT
GGTGAGACCCATCGTGACGAGACCCATCGT
GGTGAGACCCATCGT
GGTGAGACCCATCGTGACGAGACCCATCGT
GGTGAGACCCACCGT
GGTGAGACCCATCGT
GGTGAGACCCATCGT
GGTGAGACCCATCGTGACGAGACCCATCGT
GGTGAGACCCACCGT
GGTGAGACCCATCGTGACGATACCCATCGT
GGTGAGACCCATCGT
GGTGAGACCCATCGT
GGTGAGACCCACCGT
GGTGAGACCCATCGT
GGTGAGACCCATCGT

GGTGAGACCCATCGT
GGTGAGACCCATCGT
GGTGAGACCCATCGT
GGTGAGACCCATCGT
GGTGAGACCCATCGC
GGTGAGACCCATTGTGACGATACCCATCGT
GGTGAGACCCGTCGT
GCTGAGACCCATCGT
GGTGAGACCCATCGC
GGTGAGACCCATCGTGACGAGACCCATCGTGACGATACCCATCGT
GGTGAGACCCATCGT
GGTGAGACCCATCGT
GGTGAGACCCACCGT
GGTGAGACCCATCGT
GGTGAGACCCATCGTGACGAGACCCATCGT
GGTGAGACCCATCGTGACGAGACCCATCGT
GGTGAGACCCATCGT
GATGAGACCCATCGT
GGTGAGACCCATCGT
GATGAGACCCATCGT
GGTGAGACCCATCGT
GGTGAGACCCATCGT
GGTGAGACCCATCGTGACGATACCTTTCGT
GGTGAGACCTTTCGT
GGTGAGACCCATCGTGACGAGACCCATCGT
GGTGAGACCCATCGT
GATGAGACCCATCGT
GGTGAGACCCATCGT
GGTGAGACCCATCGT
GGTGAGACCCATTGTGACGATACCCATCGT
GGTGAGACCCGTCGT
GCTGAGACCCATCGT
GGTGAGACCCATCGC
GGTGAGACCCATCGTGACGAGACCCATCGTGACGATACCCATCGT
GGTGAGACCCATCGT
GGTGAGACCCATCGT
GGTGAGACCCACCGT
GGTGAGACCCATCGT
GGTGAGACCCATCGTGACGAGACCCATCGT
GGTGAGACCCATCGT
GGTGATACCCATCGT
GGTGAGACCCATCGC
GGTGAGACCCATCGTGACGATACCTTTCGT
GGTGAGACCCTTCGT
GGTGAGACCCATCTTGACGAGACCCATCGT
GGTGAGACCCATCGT
GATGAGACCCATCGT
GGTGAGACCCATCGT
GGTGAGACCCATCGC
GGTGAGACCCATCGT
GGTGAGACCCATCGT
GGTGAGACCCACCGT
GGTGAGACCCATCGT
GGTGAGACCCACCGT

GGTGAGACCCATCGC
GGTGAGACCCATCGTGACGATACCTTTCGT
GGTGAGACCCTTCGT
GGTGAGACCCATCTTGACGAGACCCATCAT
GGTGAGACCCATCGT
GATGAGACCCATCGT
GGTGAGACCAATCGT
GGTGAGACCCATCGC
GGTGAGACCCATCGT
GGTGAGACCCATCGT
GGTGAGACCCACCGT
GGTGAGACCCATCGT
GGTGAGACCCACCGT
GGTGAGACCCATCGTGACGATACCCATCGT
GGTGAGACCCATCGT
GGTGAGACCCATCGT
GGTGAGACCCACCAT
GGTGAGACCCATCGT
GGTGAGACCCATCGT
GGTGAGACCCATCGT
GGTGAGACCCATCGT
GGTGAGACCCATCGT
GGTGAGACCCATCGT
GGTGAGACCCATCGC
GGTGAGACCCATTGTGACGATACCCATCGT
GGTGAGACCCGTCGT
GCTGAGACCCATCGT
GGTGAGACCCATCGC
GGTGAGACCCATCGTGACGAGACCCATCGT
GGTGAGACCCATCGT
GGTGAGACCCATCGT
GGTGAGACCCACCGT
GGTGAGACCCATCGT
GGTGAGACCCATCGTGACGAGACCCATCGTGACGATACCTTTCGT
GGTGAGACCCTTCGT
GGTGAGACCCATCGTGACGAGACCCATCGT
GGTGAGACCCATCGTGACGATACCTTTCGT
GGTGAGACCCTTCGT
GGTGAGACCCATCGTGACGAGACCCATCGT
GGTGAGACCCATCGT
GATGAGACCCATCGT
GGTGAGACCCATCGT
GGTGAGACCCATCGT
GGTGAGACCCATCGT
GGTGAGACCCATCGT
GGTGAGACCCATCGT
GGTGAGACCCATCGT
GGTGAGACCCATCGC
GGTGAGACCCATTGTGACGATACCCATCGT
GGTGAGACCCGTCGT
GCTGAGACCCATCGT
GGTGAGACCCATCGC
GGTGAGACCCATCGTGACGAGACCCATCGT
GGTGAGACCCATCGT

GGTGAGACCCATCGT
GGTGAGACCCACCGT
GGTGAGACCCATCGT
GGTGAGACCCATCGTGACGAGACCCATCGTGACGATACCTTTCGT
GGTGAGACCCTTCGT
GGTGAGACCCATCGTGACGAGACCCATCGT
GGTGAGACCCATCGTGACGATACCTTTCGT
GGTGAGACCCTTCGT
GGTGAGACCCATCTTGACGAGACCCATCGT
GGTGAGACCCATCGT
GATGAGACCCATCGT
GGTGAGACCCATCGT
GGTGAGACCCATCGC
GGTGAGACCCATCGT
GATGAGACCCATCGT
GGTGAGACCCACCGT
GGTGAGACCCATCGT
GGTGAGACCCATCGC
GGTGAGACCCATCGTGACGATACCTTTCGT
GGTGAGACCCATCGT
GATGAGACCCATCGT
GGTGAGACCCATCGT
GGTGAGACCCATCGC
GGTGAGACCCATCGT
GGTGAGACCCATCGT
GGTGAGACCCACCGT
GGTGAGACCCATCGT
GGTGAGACCCATCGT
GGTGAGACCCATCGT
GCTGAGACCCATCGT
GGTGAGACCCATCGT
GCTGAGACCCATCGT
GGTGAGACCAATCGT
GGTGAGACCCATCGT
GGTGAGACCCATCGT
GGTGAGACCCATCGTGGTGACACCAATCGT
GGTGAGACCCATCGT
GGTGAGACCCATCGT
GGTGAGACCCATCGT
GGTGAGACCCATCGTGCTGATACCCATCGTGACGAGACCCATCGT
GGTGAGACCCATCGTGACGAGACCCATCGT
GGTGAGACCCATCGT
GGTGAGACCCATCGTGACGAGACCCATCGT
GGTGAGACCCACCGT
GGTGAGACCCATCGT
GGTGAGACCCATCGT
GGTGAGACCCATCGTGACGAGACCCATCGT
GGTGAGACCCACCGT
GGTGAGACCCATCGTGACGATACCCATCGT
GGTGAGACCCATCGT
GGTGAGACCCATCGT
GGTGAGACCCACCGT
GGTGAGACCCATCGT
GGTGAGACCCATCGT

GGTGAGACCCATCGT
GGTGAGACCCATCGT
GGTGAGACCCATCGT
GGTGAGACCCATCGT
GGTGAGACCCATCGC
GGTGAGACCCATTGTGACGATACCCATCGT
GGTGAGACCCGTCGT
GCTGAGACCCATCGT
GGTGAGACCCATCGC
GGTGAGACCCATCGTGACGAGACCCATCGT
GGTGAGACCCATCGT
GGTGATACCCATCGT
GGTGAGACCCATCGC
GGTGAGACCCACCGT
GGTGAGACCCATCGC
GGTGAGACCCATCGTGACGATACCTTTCGT
GGTGAGACCCTTCGT
GGTGAGACCCATCTTGACGAGACCCATCGT
GGTGAGACCCATCGT
GATGAGACCCATCGT
GGTGAGACCCATCGT
GGTGAGACCCATCGC
GGTGAGACCCATCGT
GGTGAGACCCATCGT
GGTGAGACCCACCGT
GGTGAGACCCATCGT
GGTGAGACCCATCGC
GGTGAGACCCATCGTGACGATACCTTTCGT
GGTGAGACCCATCGT
GGTGAGACCCATCGT
GGTGAGACCCATCGC
GGTGAGACCCATCGTGACGATACCTTTCGT
GGTGAGACCCTTCGT
GGTGAGACCCATCGTGACGAGACCCATCGT
GGTGAGACCCATCGT
GATGAGACCCATCGT
GGTGAGACCCTTCGT
GGTGAGACCCACCGT
GGTGAGACCCATCGT
GGTGAGACCCATCGC
GGTGATACCCATCGTGACGATACCTTTCGT
GGTGAGACCCTTCGT
GGTGAGACCCATCGTGACGAGACCCATCGT
GGTGAGACCCATCGT
GATGAGACCCATCGT
GGTGAGACCCATCGT
GGTGAGACCCATCGT
GGTGAGACCCATCGT
GGTGAGACCCACCGT
GGTGAGACCCATCGT
GGTGAGACCCATCGC
GGTGAGACCCATCGTGACGATACCTTTCGT
GGTGAGACCCTTCGT
GGTGAGACCCATCGTGACGAGACCCATCGT

GGTGAGACCCATCGT
GATGAGACCCATCGC
GGTGAGACCTATCGT
GGTGAGACCCATCGC
GGTGAGACCCATCGT
GGTGAGACCCATCGT
GGTGAGACCCACCGT
GGTGAGACCCATCGT
GGTGAGACCCATCGC
GGTGAGACCCATCGTGACGATACCTTTCGT
GGTGAGACCCATCGT
GGTGAGACCCATCGT
GGTGAGACCCATCGC
GGTGAGACCCATCGTGACGATACCTTTCGT
GGTGAGACCCTTCGT
GGTGAGACCCATCGTGACGAGACCCATCGT
GGTGAGACCCATCGT
GATGAGACCCATCGT
GGTGAGACCCTTCGT
GGTGAGACCCACCGT
GGTGAGACCCATCGT
GGTGAGACCCATCGC
GGTGATACCCATCGTGACGATACCTTTCGT
GGTGAGACCCTTCGT
GGTGAGACCCATCGTGACGAGACCCATCGT
GGTGAGACCCATCGT
GATGAGACCCATCGT
GGTGAGACCCATCGC
GGTGAGACCCATCGC
GGTGAGATCCATCGT
GGTGAGACCCATCGT
GGTGAGACCCACCGT
GGTGAGACCCATCGT
GGTGAGACCCATCGC
GGTGAGACCCATCGTGACGAAACCTTTCGT
GGTGAGACCCATCGT
GGTGAGACCCATCGC
GGTGAGACCCATCGT
GGTGAGACCCATCGT
GGTGAGACCCATCGTGACGAGACCCATCGT
GGTGAGACCCATCGT
GGTGAGACCCATCGTGACGAGACCCATCGT
GGTGAGACCCACCGT
GGTGAGACCCATCGT
GGTGAGACCCATCGT
GGTGAGACCCATCGTGACGAGACCCATCGT
GGTGAGACCCACCGT
GGTGAGACCCATCGTGACGATACCCATCGT
GGTGAGACCCATCGT
GGTGAGACCCATCGT
GGTGAGACCCACCGT
GGTGAGACCCATCGT
GGTGAGACCCATCGT
GGTGAGACCCATCGT

GGTGAGACCCATCGT
GGTGAGACCCATCGT
GGTGAGACCCATCGT
GGTGAGACCCATCGC
GGTGAGACCCATTGTGACGATACCCATCGT
GGTGAGACCCGTCGT
GCTGAGACCCATCGT
GGTGAGACCCATCGC
GGTGAGACCCATCGTGACGAGACCCATCGT
GGTGAGACCCATCGT
GGTGATACCCATCGT
GGTGAGACCCATCGC
GGTGAGACCCACCGT
GGTGAGACCCATCGC
GGTGAGACCCATCGTGACGATACCTTTCGT
GGTGAGACCCTTCGT
GGTGAGACCCATCTTGACGAGACCCATCGT
GGTGAGACCCATCGT
GATGAGACCCATCGT
GGTGAGACCCATCGT
GGTGAGACCCATCGC
GGTGAGACCCATCGT
GGTGAGACCCATCGT
GGTGAGACCCACCGT
GGTGAGACCCATCGT
GGTGAGACCCATCGC
GGTGAGACCCATCGTGACGATACCTTTCGT
GGTGAGACCCATCGT
GGTGAGACCCATCGT
GGTGAGACCCATCGC
GGTGAGACCCATCGTGACGATACCTTTCGT
GGTGAGACCCTTCGT
GGTGAGACCCATCGTGACGAGACCCATCGT
GGTGAGACCCATCGT
GATGAGACCCATCGT
GGTGAGACCCTTCGT
GGTGAGACCCACCGT
GGTGAGACCCATCGT
GGTGAGACCCATCGC
GGTGATACCCATCGTGACGATACCTTTCGT
GGTGAGACCCTTCGT
GGTGAGACCCATCGTGACGAGACCCATCGT
GGTGAGACCCATCGT
GATGAGACCCATCGT
GGTGAGACCCATCGT
GGTGAGACCCATCGT
GGTGAGACCCATCGT
GGTGAGACCCACCGT
GGTGAGACCCATCGT
GGTGAGACCCATCGC
GGTGAGACCCATCGTGACGATACCTTTCGT
GGTGAGACCCTTCGT
GGTGAGACCCATCGTGACGAGACCCATCGT
GGTGAGACCCATCGT

GATGAGACCCATCGC
GGTGAGACCCATCGT
GGTGAGACCCATCGC
GGTGAGACCCATCGT
GGTGAGACCCATCGT
GGTGAGACCCACCGT
GGTGAGACCCATCGT
GGTGAGACCCATCGC
GGTGAGACCCATCGTGACGATACCTTTCGT
GGTGAGACCCATCGT
GGTGAGACCCATCGT
GGTGAGACCCATCGC
GGTGAGACCCATCGTGACGATACCTTTCGT
GGTGAGACCCTTCGT
GGTGAGACCCATCGTGACGAGACCCATCGT
GGTGAGACCCATCGT
GATGAGACCCATCGT
GGTGAGACCCTTCGT
GGTGAGACCCACCGT
GGTGAGACCCATCGT
GGTGAGACCCATCGC
GGTGATACCCATCGTGACGATACCTTTCGT
GGTGAGACCCTTCGT
GGTGAGACCCATCGTGACGAGACCCATCGT
GGTGAGACCCATCGT
GATGAGACCCATCGT
GGTGAGACCCATCGC
GGTGAGACCCATCGC
GGTGAGATCCATCGT
GGTGAGACCCATCGT
GGTGAGACCCACCGT
GGTGAGACCCATCGT
GGTGAGACCCATCGC
GGTGAGACCCATCGTGACGAAACCTTTCGT
GGTGAGACCCATCGT
GGTGAGACCCATCGC
GGTGAGACCCATCGT
GGTGAGACCCATCGT
GGTGAGACCCACCGT
GGTGAGACCCATCGT
GGTGAGACCCATCGC
GGTGAGACCCATCGTGACGATACCTTTCGT
GGTGAGACCCATCGT
GGTGAGACCCATTGT
GGTGAGACCCATCGT
GGTGAGACCCATCGT
GGTGAGACCCTTCGT
GGTGAGACCCTTCGT
GGTGAGACCCATCGTGACGAGACCCATCGT
GGTGAGACCCATCGT
GATGAGACCCATCGT
GGTGAGACCCTTCGT
GGTGAGACCCATCGT
GGTGAGACCCATCGC

GGTGAGACCCATCGTGACGATACCTTTCGT
GGTGAGACCCATCGT
GGTGAGACCCATTGT
GGTGAGACCCATCGT
GGTGAGACCCATCGT
GGTGAGACCCTTCGT
GGTGAGACCCTTCGT
GGTGAGACCCATCGTGACGAGACCCATCGT
GGTGAGACCCATCGT
GATGAGACCCATCGT
GGTGAGACCCTTCGT
Nr of selected repeats 346 Similarity 0.920561
Consensus:
GGTGAGACCCATCGT
>Cele-UNSB01_2:698255-706481 Satlength=8227 Nr of Repeats=433 RepeatLength=15
seed=GGTGAGACCC
GGTGAGACCCATCGT
GGTGAGACCCATCGT
GGTGAGACCCATCGTGACGAGACCCATCGT
GGTGAGACCCATCGTGACGAGACCCATCGT
GGTGAGACCCATCGT
GGTGAGACCCATCGT
GGTGAGACCCACCGT
GGTGAGACCCATCGTGACGAGACCCACCGT
GGTGAGACCCATCGTGACGAGACCCATCGT
GGTGAGACCCACCGT
GGTGAGACCCATCGT
GGTGAGACCCACCGT
GGTGAGACCCATCGTGACGAGACCCATCGT
GGTGAGACCCACCGT
GGTGAGACCCATCGT
GGTGAGACCCATCGT
GGTGAGACCCATCGT
GGTGAGACCCATCGT
GCTGAGACCCATCGT
GGTGAGACCCATCGT
GCTGAGACCCATCGT
GGTGAGACCAATCGT
GGTGAGACCCATCGT
GGTGAGACCCATCGT
GGTGAGACCCATCGTGGTGATACCAATCGT
GGTGAGACCCATCGT
GGTGAGACCCATCGT
GGTGAGACCCATCGT
GGTGAGACCCATCGTGCTGATACCCATCGTGACGAGACCCATCGT
GGTGAGACCCATCGTGACGAGACCCATCGT
GGTGAGACCCATCGT
GGTGAGACCCATCGTGACGAGACCCATCGT
GGTGAGACCCACCGT
GGTGAGACCCATCGT
GGTGAGACCCATCGT
GGTGAGACCCATCGTGACGAGACCCATCGT
GGTGAGACCCACCGT
GGTGAGACCCATCGTGACGATACCCATCGT

GGTGAGACCCATCGT
GGTGAGACCCATCGT
GGTGAGACCCACCGT
GGTGAGACCCATCGT
GGTGAGACCCATCGT
GGTGAGACCCATCGT
GGTGAGACCCATCGT
GGTGAGACCCATCGT
GGTGAGACCCATCGT
GGTGAGACCCATCGC
GGTGAGACCCATTGTGACGATACCCATCGT
GGTGAGACCCGTCGT
GCTGAGACCCATCGT
GGTGAGACCCATCGC
GGTGAGACCCATCGTGACGAGACCCATCGTGACGATACCCATCGT
GGTGAGACCCATCGT
GGTGAGACCCATCGT
GGTGAGACCCACCGT
GGTGAGACCCATCGT
GGTGAGACCCATCGTGACGAGACCCATCGT
GGTGAGACCCATCGTGACGAGACCCATCGT
GGTGAGACCCATCGT
GATGAGACCCATCGT
GGTGAGACCCATCGT
GATGAGACCCATCGT
GGTGAGACCCATCGT
GGTGAGACCCATCGT
GGTGAGACCCATCGTGACGATACCTTTCGT
GGTGAGACCTTTCGT
GGTGAGACCCATCGTGACGAGACCCATCGT
GGTGAGACCCATCGT
GATGAGACCCATCGT
GGTGAGACCCATCGT
GGTGAGACCCATCGT
GGTGAGACCCATTGTGACGATACCCATCGT
GGTGAGACCCGTCGT
GCTGAGACCCATCGT
GGTGAGACCCATCGC
GGTGAGACCCATCGTGACGAGACCCATCGTGACGATACCCATCGT
GGTGAGACCCATCGT
GGTGAGACCCATCGT
GGTGAGACCCACCGT
GGTGAGACCCATCGT
GGTGAGACCCATCGTGACGAGACCCATCGT
GGTGAGACCCATCGT
GGTGATACCCATCGT
GGTGAGACCCATCGC
GGTGAGACCCATCGTGACGATACCTTTCGT
GGTGAGACCCTTCGT
GGTGAGACCCATCTTGACGAGACCCATCGT
GGTGAGACCCATCGT
GATGAGACCCATCGT
GGTGAGACCCATCGT
GGTGAGACCCATCGC

GGTGAGACCCATCGT
GGTGAGACCCATCGT
GGTGAGACCCACCGT
GGTGAGACCCATCGT
GGTGAGACCCACCGT
GGTGAGACCCATCGC
GGTGAGACCCATCGTGACGATACCTTTCGT
GGTGAGACCCTTCGT
GGTGAGACCCATCTTGACGAGACCCATCAT
GGTGAGACCCATCGT
GATGAGACCCATCGT
GGTGAGACCAATCGT
GGTGAGACCCATCGC
GGTGAGACCCATCGT
GGTGAGACCCATCGT
GGTGAGACCCACCGT
GGTGAGACCCATCGT
GGTGAGACCCACCGT
GGTGAGACCCATCGTGACGATACCCATCGT
GGTGAGACCCATCGT
GGTGAGACCCATCGT
GGTGAGACCCACCAT
GGTGAGACCCATCGT
GGTGAGACCCATCGT
GGTGAGACCCATCGT
GGTGAGACCCATCGT
GGTGAGACCCATCGT
GGTGAGACCCATCGT
GGTGAGACCCATCGC
GGTGAGACCCATTGTGACGATACCCATCGT
GGTGAGACCCGTCGT
GCTGAGACCCATCGT
GGTGAGACCCATCGC
GGTGAGACCCATCGTGACGAGACCCATCGT
GGTGAGACCCATCGT
GGTGAGACCCATCGT
GGTGAGACCCACCGT
GGTGAGACCCATCGT
GGTGAGACCCATCGTGACGAGACCCATCGTGACGATACCTTTCGT
GGTGAGACCCTTCGT
GGTGAGACCCATCGTGACGAGACCCATCGT
GGTGAGACCCATCGTGACGATACCTTTCGT
GGTGAGACCCTTCGT
GGTGAGACCCATCGTGACGAGACCCATCGT
GGTGAGACCCATCGT
GATGAGACCCATCGT
GGTGAGACCCATCGT
GGTGAGACCCATCGT
GGTGAGACCCATCGT
GGTGAGACCCATCGT
GGTGAGACCCATCGT
GGTGAGACCCATCGT
GGTGAGACCCATCGC
GGTGAGACCCATTGTGACGATACCCATCGT

GGTGAGACCCGTCGT
GCTGAGACCCATCGT
GGTGAGACCCATCGC
GGTGAGACCCATCGTGACGAGACCCATCGT
GGTGAGACCCATCGT
GGTGAGACCCATCGT
GGTGAGACCCACCGT
GGTGAGACCCATCGT
GGTGAGACCCATCGTGACGAGACCCATCGTGACGATACCTTTCGT
GGTGAGACCCTTCGT
GGTGAGACCCATCGTGACGAGACCCATCGT
GGTGAGACCCATCGTGACGATACCTTTCGT
GGTGAGACCCTTCGT
GGTGAGACCCATCTTGACGAGACCCATCGT
GGTGAGACCCATCGT
GATGAGACCCATCGT
GGTGAGACCCATCGT
GGTGAGACCCATCGC
GGTGAGACCCATCGT
GATGAGACCCATCGT
GGTGAGACCCACCGT
GGTGAGACCCATCGT
GGTGAGACCCATCGC
GGTGAGACCCATCGTGACGATACCTTTCGT
GGTGAGACCCATCGT
GATGAGACCCATCGT
GGTGAGACCCATCGT
GGTGAGACCCATCGC
GGTGAGACCCATCGT
GGTGAGACCCATCGT
GGTGAGACCCACCGT
GGTGAGACCCATCGT
GGTGAGACCCATCGT
GGTGAGACCCATCGT
GCTGAGACCCATCGT
GGTGAGACCCATCGT
GCTGAGACCCATCGT
GGTGAGACCAATCGT
GGTGAGACCCATCGT
GGTGAGACCCATCGT
GGTGAGACCCATCGTGGTGACACCAATCGT
GGTGAGACCCATCGT
GGTGAGACCCATCGT
GGTGAGACCCATCGT
GGTGAGACCCATCGTGCTGATACCCATCGTGACGAGACCCATCGT
GGTGAGACCCATCGTGACGAGACCCATCGT
GGTGAGACCCATCGT
GGTGAGACCCATCGTGACGAGACCCATCGT
GGTGAGACCCACCGT
GGTGAGACCCATCGT
GGTGAGACCCATCGT
GGTGAGACCCATCGTGACGAGACCCATCGT
GGTGAGACCCACCGT
GGTGAGACCCATCGTGACGATACCCATCGT

GGTGAGACCCATCGT
GGTGAGACCCATCGT
GGTGAGACCCACCGT
GGTGAGACCCATCGT
GGTGAGACCCATCGT
GGTGAGACCCATCGT
GGTGAGACCCATCGT
GGTGAGACCCATCGT
GGTGAGACCCATCGT
GGTGAGACCCATCGC
GGTGAGACCCATTGTGACGATACCCATCGT
GGTGAGACCCGTCGT
GCTGAGACCCATCGT
GGTGAGACCCATCGC
GGTGAGACCCATCGTGACGAGACCCATCGT
GGTGAGACCCATCGT
GGTGATACCCATCGT
GGTGAGACCCATCGC
GGTGAGACCCACCGT
GGTGAGACCCATCGC
GGTGAGACCCATCGTGACGATACCTTTCGT
GGTGAGACCCTTCGT
GGTGAGACCCATCTTGACGAGACCCATCGT
GGTGAGACCCATCGT
GATGAGACCCATCGT
GGTGAGACCCATCGT
GGTGAGACCCATCGC
GGTGAGACCCATCGT
GGTGAGACCCATCGT
GGTGAGACCCACCGT
GGTGAGACCCATCGT
GGTGAGACCCATCGC
GGTGAGACCCATCGTGACGATACCTTTCGT
GGTGAGACCCATCGT
GGTGAGACCCATCGT
GGTGAGACCCATCGC
GGTGAGACCCATCGTGACGATACCTTTCGT
GGTGAGACCCTTCGT
GGTGAGACCCATCGTGACGAGACCCATCGT
GGTGAGACCCATCGT
GATGAGACCCATCGT
GGTGAGACCCTTCGT
GGTGAGACCCACCGT
GGTGAGACCCATCGT
GGTGAGACCCATCGC
GGTGATACCCATCGTGACGATACCTTTCGT
GGTGAGACCCTTCGT
GGTGAGACCCATCGTGACGAGACCCATCGT
GGTGAGACCCATCGT
GATGAGACCCATCGT
GGTGAGACCCATCGT
GGTGAGACCCATCGT
GGTGAGACCCATCGT
GGTGAGACCCACCGT

GGTGAGACCCATCGT
GGTGAGACCCATCGC
GGTGAGACCCATCGTGACGATACCTTTCGT
GGTGAGACCCTTCGT
GGTGAGACCCATCGTGACGAGACCCATCGT
GGTGAGACCCATCGT
GATGAGACCCATCGC
GGTGAGACCTATCGT
GGTGAGACCCATCGC
GGTGAGACCCATCGT
GGTGAGACCCATCGT
GGTGAGACCCACCGT
GGTGAGACCCATCGT
GGTGAGACCCATCGC
GGTGAGACCCATCGTGACGATACCTTTCGT
GGTGAGACCCATCGT
GGTGAGACCCATCGT
GGTGAGACCCATCGC
GGTGAGACCCATCGTGACGATACCTTTCGT
GGTGAGACCCTTCGT
GGTGAGACCCATCGTGACGAGACCCATCGT
GGTGAGACCCATCGT
GATGAGACCCATCGT
GGTGAGACCCTTCGT
GGTGAGACCCACCGT
GGTGAGACCCATCGT
GGTGAGACCCATCGC
GGTGATACCCATCGTGACGATACCTTTCGT
GGTGAGACCCTTCGT
GGTGAGACCCATCGTGACGAGACCCATCGT
GGTGAGACCCATCGT
GATGAGACCCATCGT
GGTGAGACCCATCGC
GGTGAGACCCATCGC
GGTGAGATCCATCGT
GGTGAGACCCATCGT
GGTGAGACCCACCGT
GGTGAGACCCATCGT
GGTGAGACCCATCGC
GGTGAGACCCATCGTGACGAAACCTTTCGT
GGTGAGACCCATCGT
GGTGAGACCCATCGC
GGTGAGACCCATCGT
GGTGAGACCCATCGT
GGTGAGACCCATCGTGACGAGACCCATCGT
GGTGAGACCCATCGT
GGTGAGACCCATCGTGACGAGACCCATCGT
GGTGAGACCCACCGT
GGTGAGACCCATCGT
GGTGAGACCCATCGT
GGTGAGACCCATCGTGACGAGACCCATCGT
GGTGAGACCCACCGT
GGTGAGACCCATCGTGACGATACCCATCGT
GGTGAGACCCATCGT

GGTGAGACCCATCGT
GGTGAGACCCACCGT
GGTGAGACCCATCGT
GGTGAGACCCATCGT
GGTGAGACCCATCGT
GGTGAGACCCATCGT
GGTGAGACCCATCGT
GGTGAGACCCATCGT
GGTGAGACCCATCGC
GGTGAGACCCATTGTGACGATACCCATCGT
GGTGAGACCCGTCGT
GCTGAGACCCATCGT
GGTGAGACCCATCGC
GGTGAGACCCATCGTGACGAGACCCATCGT
GGTGAGACCCATCGT
GGTGATACCCATCGT
GGTGAGACCCATCGC
GGTGAGACCCACCGT
GGTGAGACCCATCGC
GGTGAGACCCATCGTGACGATACCTTTCGT
GGTGAGACCCTTCGT
GGTGAGACCCATCTTGACGAGACCCATCGT
GGTGAGACCCATCGT
GATGAGACCCATCGT
GGTGAGACCCATCGT
GGTGAGACCCATCGC
GGTGAGACCCATCGT
GGTGAGACCCATCGT
GGTGAGACCCACCGT
GGTGAGACCCATCGT
GGTGAGACCCATCGC
GGTGAGACCCATCGTGACGATACCTTTCGT
GGTGAGACCCATCGT
GGTGAGACCCATCGT
GGTGAGACCCATCGC
GGTGAGACCCATCGTGACGATACCTTTCGT
GGTGAGACCCTTCGT
GGTGAGACCCATCGTGACGAGACCCATCGT
GGTGAGACCCATCGT
GATGAGACCCATCGT
GGTGAGACCCTTCGT
GGTGAGACCCACCGT
GGTGAGACCCATCGT
GGTGAGACCCATCGC
GGTGATACCCATCGTGACGATACCTTTCGT
GGTGAGACCCTTCGT
GGTGAGACCCATCGTGACGAGACCCATCGT
GGTGAGACCCATCGT
GATGAGACCCATCGT
GGTGAGACCCATCGT
GGTGAGACCCATCGT
GGTGAGACCCATCGT
GGTGAGACCCACCGT
GGTGAGACCCATCGT

GGTGAGACCCATCGC
GGTGAGACCCATCGTGACGATACCTTTCGT
GGTGAGACCCTTCGT
GGTGAGACCCATCGTGACGAGACCCATCGT
GGTGAGACCCATCGT
GATGAGACCCATCGC
GGTGAGACCCATCGT
GGTGAGACCCATCGC
GGTGAGACCCATCGT
GGTGAGACCCATCGT
GGTGAGACCCACCGT
GGTGAGACCCATCGT
GGTGAGACCCATCGC
GGTGAGACCCATCGTGACGATACCTTTCGT
GGTGAGACCCATCGT
GGTGAGACCCATCGT
GGTGAGACCCATCGC
GGTGAGACCCATCGTGACGATACCTTTCGT
GGTGAGACCCTTCGT
GGTGAGACCCATCGTGACGAGACCCATCGT
GGTGAGACCCATCGT
GATGAGACCCATCGT
GGTGAGACCCTTCGT
GGTGAGACCCACCGT
GGTGAGACCCATCGT
GGTGAGACCCATCGC
GGTGATACCCATCGTGACGATACCTTTCGT
GGTGAGACCCTTCGT
GGTGAGACCCATCGTGACGAGACCCATCGT
GGTGAGACCCATCGT
GATGAGACCCATCGT
GGTGAGACCCATCGC
GGTGAGACCCATCGC
GGTGAGATCCATCGT
GGTGAGACCCATCGT
GGTGAGACCCACCGT
GGTGAGACCCATCGT
GGTGAGACCCATCGC
GGTGAGACCCATCGTGACGAAACCTTTCGT
GGTGAGACCCATCGT
GGTGAGACCCATCGC
GGTGAGACCCATCGT
GGTGAGACCCATCGT
GGTGAGACCCACCGT
GGTGAGACCCATCGT
GGTGAGACCCATCGC
GGTGAGACCCATCGTGACGATACCTTTCGT
GGTGAGACCCATCGT
GGTGAGACCCATTGT
GGTGAGACCCATCGT
GGTGAGACCCATCGT
GGTGAGACCCTTCGT
GGTGAGACCCTTCGT
GGTGAGACCCATCGTGACGAGACCCATCGT

GGTGAGACCCATCGT
GATGAGACCCATCGT
GGTGAGACCCTTCGT
GGTGAGACCCATCGT
GGTGAGACCCATCGC
GGTGAGACCCATCGTGACGATACCTTTCGT
GGTGAGACCCATCGT
GGTGAGACCCATTGT
GGTGAGACCCATCGT
GGTGAGACCCATCGT
GGTGAGACCCTTCGT
GGTGAGACCCTTCGT
GGTGAGACCCATCGTGACGAGACCCATCGT
GGTGAGACCCATCGT
GATGAGACCCATCGT
GGTGAGACCCTTCGT
GGTGAGACCCAAATTTTTGGCGGGAAATTCAAATTTTCAGTGAAAAAAATTTTGGCGGGAAATTCAAATCAGTGAAAAA
AAATTTTGGCGGGAAATTCAAATTTTCAGTGAAAAAGTTTTGGCGGGAAATTCAAATTTTCTGTGAGAAAAATTTTGGG
GGAAATTCAAATTTTCAGTGAAAAAAATTTTGGCGGGAAATTCAAATTTTCAGTGAAAAACATTTTGGCGGGAATTTCA
AATTTTCAGTGAAAAACATTTTGCCGGGAAATTCAAATTTCTGAGAAAAAAATTTTTGGCGGGAAATTCAAATTTTCTGA
GAAACTTTTTGTTAGCTTAAGTACCACCTGGAACTGGCGAGACCTATCGT
Nr of selected repeats 346 Similarity 0.920561
Consensus:
GGTGAGACCCATCGT
>Cele-UNSB01_2:858289-858649 Satlength=361 Nr of Repeats=5 RepeatLength=31
seed=TGTCTAGCGA
TGTCTAGCGAGACCCAGATAAGAGCAGAGGTGCTTCTGAGAGGGTTGGTGATTCAGGGGTAGTGCGAGCACTATCTCCT
GTCTCTTATGTGGCACATTGTTGTGTTTCTGTGGTTATAATAATACTCTCTCCCCCCATACTCCTACATATGGGGGCGA
GTGAGAAGGCTGGAGT
TGTCGAGCGAGACCTCAGAAGAGAGCTGAGT
TGTCTAGCGAGATAGCAGAGAAGAGATGAGT
TGTCGAGCGAGACCTCAGAAGAGAGCTGAGT
TGTCTAGCGAGATAGCAGAGAAGAGATGAGTTGTCGAGTGAGATAGCAGAGAAGAGATGAGTTGTGGAGTGAAACCTCA
GAAGAGAGCTGAGT
Nr of selected repeats 3 Similarity 0.799283
Consensus:
TGTCgAGCGAGAcctCAGAagAGAGcTGAGT
>Cele-UNSB01_2:863874-864986 Satlength=1113 Nr of Repeats=34 RepeatLength=32
seed=AATTCCCGCC
AATTCCCGCCAAAAATTTTTCAGAAAATTTG
AATTCCCGCCAAAATTTTTCACAGAAAATTTG
AATTCCCGCCAAAATTTTTCAGAGAAAATTTG
AATTCCCGCCAAAAATGTTTCAGAAAATTTG
AATTCACGCCAAAAATTTTCAAAGAAAATTTG
AATTCCCGCCAAAAAATTTTGAAGAAAATTTG
AATTCCCGCCAAAAATTTTCAAAGAAAATTTG
AATTCCCGCCAAAAAATTTTGAAGGAAATTTA
AATTCCCGCCAAAATTTTTCAGAGAAAATTTG
AATTCCCGCCAAAAATTTTTCAGAAAATTTG
AATTCCCGCCAAAATTTTTCACAGAAAATTTG
AATTCCCGCCAAAAAATTTTAAAGAAAATTAA
AATTCCCGCCAAAAATTTTCAGAGAAATTTTG
AATTCCCGCCAAAAAATTTTAAAGAAAATTTA
AATTCCCGCCAAAAATTTTCAGAGAAATTTTG

AATTCCCGCCAAAAAATTTTAAAGAAAATTTA
AATTCCCGCCAAAAATTTTCAGAGAAAATTTG
AATTCCCGCCAAAAATGTTTCAGAAAATTTG
AATTCACGCCAAAAATGTTTCAGAAAATTTG
AATTCACGCCAAAAATTTTCAAAGAAAATTTG
AATTCCCGCCAAAAAATTTTTCAGAAAATTTG
AATTCCCGCCAAAAATTTTCAAAGAAAATTTA
AATTCCCGCCAAAAATTTTCAGAGAAATTTTG
AATTCCCGCCAAAAAATTTTAAAGAAAATTTA
AATTCCCGCCAAAAATTTTCAGAGAAAATTTG
AATTCCCGCCAAAAATGTTTCAGAAAATTTG
AATTCACGCCAAAAATGTTTCAGAAAATTTG
AATTCACGCCAAAAATTTTCAAAGAAAATTTG
AATTCCCGCCAAAAAATTTTTCAGAAAATTTG
AATTCCCGCCAAAAATTTTCAAAGAAAATTTG
AATTCCCGCCAAAAAATTTTGAAGGAAATTTA
AATTCCCGCCAAATTTTTTCAGAGAAAATTTG
AATTCCCGCCAAAAATTTTTCAGAAAATTTGAACAACCGCCAAAAATTTTCACAGAAAATTTG
AATTCCCGCCGAAAAATTTCAAAGAAAATTTA
Nr of selected repeats 26 Similarity 0.854615
Consensus:
AATTCCCGCCAAAAAtTTTcAaAGAAAATTTg
>Cele-UNSB01_2:865003-865078 Satlength=76 Nr of Repeats=5 RepeatLength=15
seed=ATGGGTCTCG
ATGGGTCTCGCCACG
ATGGGTCTCGCCACG
ATGGGTCTCGCCACG
ATGGGTCTCGCCACGA
AGGGTCTCGCAACG
Nr of selected repeats 3 Similarity 1.000000
Consensus:
ATGGGTCTCGCCACG
>Cele-UNSB01_2:870904-873513 Satlength=2610 Nr of Repeats=35 RepeatLength=72
seed=TCCATCCAGC
TCCATCCAGCTGTGGTTGGTCTCTGGGTGAATCCTCGAGAAGTATCACGAGTAGTACCATTGGCTCCGTTTG
TCCATCCAGCTGTGGTTGGTCGATGGGTGGGTCCTCGAGTAGTTCCAGGGACTCTCGTTTGTCCACGAGTAATGCCATT
GGCTCTAATTG
TCCATCCAGCTGCGGTTGGTCGCTGGGTGAATCCTCGAGAAGTACCACTAGTAGTACCATTGGCTCCGTTCG
TCCATCCAGCTGCGGTTGGTCGCTGGGTGAATCCTCGAGAAGTACCACTAGTAGTACCATTGGCTCCGTTCG
TCCATCCAGCTGCGGTTGGTCGTGGGCGAATCCTCGAGAAGTACCACTAGTAGTACCATTGGCTCCGTTCG
TCCATCCAGCTGCGGTTGGTCGCTGGGCGAATCCTCGAGAAGTACCACTAGTAGTACCATTGGCTCCGTTCG
TCCATCCAGCTGCGGTTGGTCGCTGGGTGAATCCTCGAGAAGTACCACTAGTAGTACCATTGGCTCCGTTCG
TCCATCCAGCTGTGGTTGGTCTCCGGGTGAATCCTCGAGAAGTATCACGAGTAGTACCATTGGCTCCGTTTG
TCCATCCAGCTGTGGTTGGTCGATGGGTGGGTCCTCGAGTAGTTCCAGGGACTCTCGTTTGTCCACGAGTAATGCCATT
GGCTCTAATTG
TCCATCCAGCTGCGGTTGGTCGCTGGGTGAATCCTCGAGAAGTACCACTAGTAGTACCATTGGCTCCGTTCG
TCCATCCAGCTATGGTTGGTCTCTGGGTGAATCCTCGAGAAGTACCACTAGTAGTACCATTGGCTCCGTTCG
TCCATCCAGCTGTGGTTGGTCTCTGGGTGAATCCTCGAGAAGTATCACGAGTAGTACCATTGGCTCCGTTTG
TCCATCCAGCTGTGGTTGGTCGATGGGTGGGTCCTCGAGTAGTTCCAGGGACTCTCGTTTGTCCGCGAGTAATGCCATT
GGCTCCGTTCG
TCCATCCAGCTATGGTTGGTCTCTGGGTGAATCCTCGAGAAGTACCACTAGTAGTACCATTGGCTCCGTTCG
TCCATCCAGCTGTGGTTGGTCTCTGGGTGAATCCTCGAGAAGTACCACTAGTAGTACCATTGGCTCCGTTTG
TCCATCCAGCTGTGGTTGGTCTCTGGGTGAATCCTCGAGAAGTACCACTAGTAGTACCATTGGCTCCGTTCG
TCCATCCAGCTATGGTTGGTCTCTGGGTGAATCCTCGAGAAGTACCACTAGTAGTACCATTGGCTCCGTTCG

TCCATCCAGCTGTGGTTGGTCTCTGGGTGAATCCTCGAGAAGTATCAATAGTAGTACCATTGGCTCCGTTCG
TCCATCCAGCTATGGTTGGTCTCTGGGTGAATCCTCGAGAAGTACCACTAGTAGTACCATTGGCTCCGTTCG
TCCATCCAGCTGTGGTTGGTCTCTGGGTGAATCCTCGAGAAGTATCACGAGTAGTACCATTGGCTCCGTTTG
TCCATCCAGCTGTGGTTGGTCGATGGGTGGGTCCTCGAGTAGTTCCAGGGACTCTCGTTTGTCCGCGAGTAATGCCATT
GGCTCCGTTCG
TCCATCCAGCTATGGTTGGTCTCTGGGTGAATCCTCGAGAAGTACCACTAGTAGTACCATTGGCTCCGTTCG
TCCATCCAGCTGTGGTTGGTCTCTGGGTGAATCCTCGAGAAGTATCAATAGTAGTACCATTGGCTCCGTTCG
TCCATCCAGCTATGGTTGGTCTCTGGGTGAATCCTCGAGAAGTACCACTAGTAGTACCATTGGCTCCGTTCG
TCCATCCAGCTGTGGTTGGTCTCTGGGTGAATCCTCGAGAAGTATCAATAGTAGTACCATTGGCTCCGTTTG
TCCATCCAGCTGTGGTTGGTCTCTGGGTGAATTCTCGAGAAGTATCAATAGTAGTACCATTGGCTCCGTTTC
TCCATCCAGCTGTGGTTGGTCTCTGGGTGAATCCTCGAGAAGTATCACGAGTAGTACCATTGGCTCCGTTTC
TCCATCCAGCTGTGGTTGGTCGATGGGTGGGTCCTCGAGAAGTATCAATAGTAGTACCATTGGCTCCGTTTG
TCCATCCAGCTGTGGTTGGTCTCTGGGTGAATTCTCGAGAAGTATCAATAGTAGTACCATTGGCTCCGTTTG
TCCATCCAGCTGTGGTTGGTCGATGGGTGGGTCCTCGAGAAGTATCAATAGTAGTACCATTGGCTCCGTTCG
TCCATCCAGCTGTGGTTGGTCTCTGGGTGAATCCTCGAGAAGTATCAATAGTAGTACCATTGGCTCCGTTTG
TCCATCCAGCTGTGGTTGGTCTCTGGGTGAATTCTCGAGAAGTATCAATAGTAGTACCATTGGCTCCGTTTC
TCCATCCAGCTGTGGTTGGTCTCTGGGTGAATCCTCGAGAAGTATCACGAGTAGTACCATTGGCTCCGTTTC
TCCATCCAGCTGTGGTTGGTCTCTGGGTGAATCCTCGAGAAGTATCACGAGTAGTACCATTGGCTCCGTTTG
TCCATCCAGCTGTGGTTGGTCGATGGGTGGGTTCTCGAGTAGTTCCAGGGACTCTCGTTTGTCCGCGAGTAATGCCATT
GGCTCTAATTG
Nr of selected repeats 29 Similarity 0.928845
Consensus:
TCCATCCAGCTgTGGTTGGTCtCTGGGTGAATCCTCGAGAAGTAtCActAGTAGTACCATTGGCTCCGTTcG
>Cele-UNSB01_2:882806-882988 Satlength=183 Nr of Repeats=12 RepeatLength=14
seed=TAGGATCTAG
TAGGATCTAGAGTA
TAGGATCTACGATC
TAGGGTCTAGGATC
TAGGATCTAGGATC
TAGGATCTAGGATC
TAGGATCTAGGGTC
TAGGGTCTAGGATC
TGGGATCTAGGATCTAGGGTG
TAGGATCTAGGATC
TAGGATCCAGAATC
TAGAATCTAGGATCTAGGCTC
TGGGATCTAGGCTC
Nr of selected repeats 10 Similarity 0.807407
Consensus:
TAGGaTCTAGgaTC
>Cele-UNSB01_2:885470-885668 Satlength=199 Nr of Repeats=15 RepeatLength=12
seed=ACCTAGACCT
ACCTAGACCTAGACCTATACCGAGA
ACTAGACCTAG
ACCTAGACCTAG
ACCTGGACCTAG
ACCTAGACCTAG
ACCTAGACCTAG
ACCTAGACCTAG
ACCTAGACCTAG
ACCTAAACCTAG
ACCTAGACCTAG
ACCTAGACCTAA
ACCTAGAACTAG

ACCTAAACCTTG
ATCTAGACCTTG
ATCTAGACCTAGACCTAG
Nr of selected repeats 12 Similarity 0.858586
Consensus:
ACCTAGACCTAG
>Cele-UNSB01_2:889690-889842 Satlength=153 Nr of Repeats=5 RepeatLength=11
seed=TGTAGGGGTA
TGTAGGGGTAC
TGTAGGGGTAC
TGTAGGGGTAC
TGTAGGGGTACTCTAGGAGTAATTTGGAAATGAACAGAAAGGATGGTAT
TGTAGGGGTACTGTAGGCCTATCTTGGGAGCAGAGAAGGAGTATTGTAAGGGTTCATACTATAAAGGTTT
Nr of selected repeats 3 Similarity 1.000000
Consensus:
TGTAGGGGTAC
>Cele-UNSB01_2:891851-892689 Satlength=839 Nr of Repeats=4 RepeatLength=184
seed=GTTCAAAAGA
GTTCAAAAGACCCCATACGGTGGTACGTAAGCTTCTGGTCAACTGGCATAATGTTAATGTAAGTTTCCCATAAAATTAA
CCTGCGCGGCCGACAATTTACGAGCCTAACCCATACATTGTCCGCTGCTAATTTCTAACAGTTTTTTTAGTATTTTTC
GGCGCAGAAGGTCCATATTCCTCTTTCTTCTCATTCTAGTTCTAATTTTATAAATTTGCTCCCGCATACATTTTTGCAT
ACATTTTTATCTCATGACGCGGACATGCCACAAATGTCTAGTTAGGCTT
GTTCAAAAGATACAAAATTTATCTACACATCTACAAGTTAAACTTGCAACTTAGTTAACCAAATTCAAAAGTTATGAGC
CGGGGAACTATAATAACCAACGTTTCACACTAAAAAAGCCCAAAAAAAATTCGAAAATCATTTTTATCTCATGACGCGGA
CAAGCTACAAATGTCTAGTTAGGCTT
GTTCAAAAGATACAAAATTTATCTACACATCTACAAGTTAAACTTGCAACTTAGTTAACCAAATTCAAAAGTTATGAGC
CGGGGAACTATAATAACCAACGTTTCACACTAAAAAAGCCCAAAAAAAATTCGAAAATCATTTTTATCTCATGACGCGGA
CAAGCTACAAATGTCTAGTTAGGCTT
GTTCAAAAGATACAAAATTTATCTACACATCTACAAGTTAAACTTGCAACTTAGTTAACCAAATTCAAAAGTTATGAGC
CGGGGAACTATAATAACCAACGTTTCACACTAAAAAAGCCCAAAAAAAATTCGAAAATCATTTTTATCTCATGACGCGGA
CAAGCTACAAATGTCTAGTTAGGCTT
Nr of selected repeats 3 Similarity 1.000000
Consensus:
GTTCAAAAGATACAAAATTTATCTACACATCTACAAGTTAAACTTGCAACTTAGTTAACCAAATTCAAAAGTTATGAGC
CGGGGAACTATAATAACCAACGTTTCACACTAAAAAAGCCCAAAAAAAATTCGAAAATCATTTTTATCTCATGACGCGGA
CAAGCTACAAATGTCTAGTTAGGCTT
>Cele-UNSB01_2:894739-894896 Satlength=158 Nr of Repeats=8 RepeatLength=20
seed=CAATCTATTT
CAATCTATTTTTGTCTGT
CAATCTATTTTCTGGTATGC
CAATCTATTTTTCGTGTAA
CAATCTATTTTTCGGTATGC
CAATCTATTTTTTGGTATGA
CAATCTATTTTTTGGTATGA
CAATCTATTTTTTGGTATGT
CAATCTATTTTTTGGTATGT
Nr of selected repeats 6 Similarity 0.902222
Consensus:
CAATCTATTTTTTGGTATGn
>Cele-UNSB01_2:898652-902061 Satlength=3410 Nr of Repeats=58 RepeatLength=59
seed=TATGGAAAAT
TATGGAAAATATGGTGTTCCAAATGAGCAATGGTTTTCAAATTTTTTTAAACTGAGAAT
TATGGAAAATATGGATTTTTAAATGTGCACTGGTTTTCAAATTTTTTTAAACTGAGAAT
TATGGAAAATATGGATTTTTAAATGTGAACTGGTTTTCAAATTTTTTTAAACTGAGAAT

TATGGAAAATATGGATTTTTAAATGTGAACTGGTTTTCAAATTTTTTTAAACTGAGAAT
TATGGAAAATATGGATTTTTAAATGTGCACTGGTTTTCAAATTTTTTTAAACTGAGAAT
TATGGAAAATATGGATTTTTAAATGTGAACTGGTTTTCAAATTTTTTTAAACTGAGAAT
TATGGAAAATATGGATTTTTAAATGTGCACTGGTTTTCAAATTTTTTTAAACTGAGAAT
TATGGAAAATATGGATTTTTAAATGTGCACTGGTTTTCAAATTTTTTTAAACTGAGAAT
TATGGAAAATATGGATTTTTAAATGTGAACTGGTTTTCAAATTTTTTTAAACTGAGAAT
TATGGAAAATATGGATTTTTAAATGTGAACTGGTTTTCAAATTTTTTTAAACTGAGAAT
TATGGAAAATATGGATTTTTAAATGTGAACTGGTTTTCAAATTTTTTTAAACTGAGAAT
TATGGAAAATATGGATTTTTAAATGTGCACTGGTTTTCAAATTTTTTTAAACTGAGAAT
TATGGAAAATATGGATTTTTAAATGTGAACTGGTTTTCAAATTTTTTTAAACTGAGAAT
TATGGAAAATATGGATTTTTAAATGTGCACTGGTTTTCAAATTTTTTTAAACTGAGAAT
TATGGAAAATATGGATTTTTAAATGTGCACTGGTTTTCAAATTTTTTTAAACTGAGAAT
TATGGAAAATATGGATTTTTAAATGTGAACTGGTTTTCAAATTTTTTTAAACTGAGAAT
TATGGAAAATATGGATTTTTAAATGTGCACTGGTTTTCAAATTTTTTTAAACTGAGAAT
TATGGAAAATATGGATTTTTAAATGTGCACTGGTTTTCAAATTTTTTTAAACTGAGAAT
TATGGAAAATATGGATTTTTAAATGTGAACTGGTTTTCAAATTTTTTTAAACTGAGAAT
TATGGAAAATATGGATTTTTAAATGTGCACTGGTTTTCAAATTTTTTTAAACTGAGAAT
TATGGAAAATATGGATTTTTAAATGTGAACTGGTTTTCAAATTTTTTTAAACTGAGAAT
TATGGAAAATATGGATTTTTAAATGTGCACTGGTTTTCAAATTTTTTTAAACTGAGAAT
TATGGAAAATATGGATTTTTAAATGTGCACTGGTTTTCAAATTTTTTTAAACTGAGAAT
TATGGAAAATATGGATTTTTAAATGTGAACTGGTTTTCAAATTTTTTTAAACTGAGAAT
TATGGAAAATATGGATTTTTAATGTTTTTTTTTTTTTAACTGAGAAT
TATGGAAAATATGGATTTTTAAATGTGAACTGGTTTTCAAATTTTTTTAAACTGAGAAT
TATGGAAAATATGGATTTTTAAATGTGCACTGGTTTTCAAATTTTTTTAAACTGAGAAT
TATGGAAAATATGGATTTTTAAATGTGCACTGGTTTTCAAATTTTTTTAAACTGAGAAT
TATGGAAAATATGGATTTTTAAATGTGAACTGGTTTTCAAATTTTTTTAAACTGAGAAT
TATGGAAAATATGGATTTTTAAATGTGCACTGGTTTTCAAATTTTTTTAAACTGAGAAT
TATGGAAAATATGGATTTTTAAATGTGCACTGGTTTTCAAATTTTTTTAAACTGAGAAT
TATGGAAAATATGGATTTTTAATGTGAACTGGTTTTCAAATTTTTTTAAACTGAGAAT
TATGGAAAATATGGATTTTTAAATGTGCACTGGTTTTCAAATTTTTTTAAACTGAGAAT
TATGGAAAATATGGATTTTTAAATGTGCACTGGTTTTCAAATTTTTTTAAACTGAGAAT
TATGGAAAATATGGATTTTTAAATGTGCACTGGTTTTCAAATTTTTTTAAACTGAGAAT
TATGGAAAATATGGATTTTTAAATGTGAACTGGTTTTCAAATTTTTTTAAACTGAGAAT
TATGGAAAATATGGATTTTTAAATGTGCACTGGTTTTCAAATTTTTTTAAACTGAGAAT
TATGGAAAATATGGATTTTTAAATGTGCACTGGTTTTCAAATTTTTTTAAACTGAGAAT
TATGGAAAATATGGATTTTTTAATGTGAACTGGTTTTCAAATTTTTTTAAACTGAGAAT
TATGGAAAATATGGATTTTTAAATGTGCACTGGTTTTCAAATTTTTTTAAACTGAGAAT
TATGGAAAATATGGATTTTTTAATGTGAACTGGTTTTCAAATTTTTTTAAACTGAGAAT
TATGGAAAATATGGATTTTTAAATGTGCACTGGTTTTCAAATTTTTTTAAACTGAGAAT
TATGGAAAATATGGATTTTTAAATGTGCACTGGTTTTCAAATTTTTTTAAACTGAGAAT
TATGGAAAATATGGATTTTTAAATGTGCACTGGTTTTCAAATTTTTTTAAACTGAGAAT
TATGGAAAATATGGATTTTTAAATGTGAACTGGTTTTCAAATTTTTTTAAACTGAGAAT
TATGGAAAATATGGATTTTTAAATGTGCACTGGTTTTCAAATTTTTTTAAACTGAGAAT
TATGGAAAATATGGATTTTTAAATGTGCACTGGTTTTCAAATTTTTTTAAACTGAGAAT
TATGGAAAATATGGATTTTTTAATGTGAACTGGTTTTCAAATTTTTTTAAACTGAGAAT
TATGGAAAATATGGATTTTTAAATGTGCACTGGTTTTCAAATTTTTTTAAACTGAGAAT
TATGGAAAATATGGATTTTTAAATGTGCACTGGTTTTCAAATTTTTTTAAACTGAGAAT
TATGGAAAATATGGATTTTTAAATGTGCACTGGTTTTCAAATTTTTTTAAACTGAGAAT
TATGGAAAATATGGATTTTTAAATGTGAACTGGTTTTCAAATTTTTTTAAACTGAGAAT
TATGGAAAATATGGATTTTTAAATGTGCACTGGTTTTCAAATTTTTTTAAACTGAGAAT
TATGGAAAATATGGATTTTTAAATGTGCACTGGTTTTCAAATTTTTTTAAACTGAGAAT
TATGGAAAATATGGATTTTTAAATGTGAACTGGTTTTCAAATTTTTTTAAACTGAGAAT
TATGGAAAATATGGATTTTTAAATGTGAACTGGTTTTCAAATTTTTTTAAACTGAGAAT
TATGGAAAATATGGATTTTTAAATGTGAACTGGTTTTCAAATTTTTTTAAACTGAGAAT

TATGGAAAATATGGATTTTTAAATGTGCACTGGTTTTCAAATTTTTTTAAACTGAGAAT
Nr of selected repeats 56 Similarity 0.981848
Consensus:
TATGGAAAATATGGATTTTTAAATGTGcACTGGTTTTCAAATTTTTTTAAACTGAGAAT
>Cele-UNSB01_2:1043954-1044457 Satlength=504 Nr of Repeats=17 RepeatLength=11
seed=GTACTGTAGG
GTACTGTAGGG
GTACTGTAGGGT
TACTGTAGGATTACTGTAGTTTGGGAAAAATGGACTTTTTGTCTTTTGAAGTGATATTGCTTTGGGGTTAGTGGCGGGA
TATGGTCAGG
GTACTGTAGTA
GTACTGTAGGG
GTACTGTAGGGT
TACTGTAGGATTACTGTAGTTTGGGAAAAATGGACTTTTTGTCTTTTGAAGTGATATTGCTTTGGGGTTAGTGGCGGGA
TATGGTCAGG
GTACTGTAGTA
GTACTGTAGAG
GTACTGTAGGA
GTACTGTAGGA
GTACTGTTGGATTACTGTAGTTTGGGAAAAATGGACTTTTTGTCTTTTGAAGTGATATTGCTTTGGGGTTAGTGGCGGG
ATATGGTCAGG
GTACTGTGGTA
GTACTGTAGAG
GTACTGTAGGA
GTACTGTAGGATTACTGTAGTTTGGGAAAAATTGGCTTTTCGTCTTTTGAAGTGATATTGCTTTGGGGTTAGTGGTGGG
ATATGGTTGGG
GTACTGTAGTT
Nr of selected repeats 11 Similarity 0.819284
Consensus:
GTACTGTAGGna
>Cele-UNSB01_2:1044642-1047492 Satlength=2851 Nr of Repeats=181 RepeatLength=15
seed=TCGTGGTGAG
TCGTGGTGAGACCCA
TCGTGGTGAGATCCA
TCGTGGTGAGACCCT
TCGTGGTGAGACCCA
TCGTGGTGAGTCCCA
TCGTGGTGAGACCCA
TCGTAGTGAGACCCA
TCGTTGTGAGACCCA
TCGTTGTGAGACCCA
TCGTGGTGAGACCCA
TCGTGGTGAGACCCA
TCGTGGTGAGACCCT
TCGTGGTGAGACCCT
TCGTGGTGAGACCCT
TCGTGGTGAGACCCT
TCGTGGTGAGACCCA
TCGTGGTGAGACCCA
TCGTGGCGAGACCCATCATGTTGAGACCCTTCGTGATGAAACCCT
TCGTGGTGAGACCCA
TCGTGGTGAGACCCT
TCGTGGTGAGACCCA
TCGTGGCGAGACCCA

TCGTGGTGAGACCCT
TCGTGGTGAAACCCT
TCGTGGTGAGACCCA
TCGTGGTGAGACCCT
TCGTGGTGAGACCCT
TCGTGGTGAGACCCT
TCGTGGTGAGACCCA
TCGTGGTGAGACCCA
TCGTGGCGAGACCCA
TCGTGGTGAGACCCTTCGTGATGAAACCCT
TCGTGGTGAGACCCA
TCGTGGTGAGACCCT
TCGTGGTGAGACCCA
TCGTGGCGAGACCCA
TCGTGGTGAGACCCT
TCGTGGTGAAACCCT
TCGTGGTGAGACCCA
TCGTGGTGAGACCCT
TCGTGGTGAGACCTT
TCGTGGTGAGACCCT
TCGTGGTGAGACCCT
TCGTGGTGAGACCCA
TCGTGGTGAGACCCA
TCGTGGTGAGACCCATCGTGACGAGACCCA
TCGTGGTGAGACCCT
TCGTGGTGAGACCTT
TCGTGGTGAGACCCA
TCGTGGTGAGACCCT
TCGTGGTGAGACCTT
TCGTGGTGAGACCCA
TCGTGGTGAGACCCA
TCGTGGTGAGACCCT
TCGTGGTGAGACCCT
TCGTGGTGAGACCCT
TCGTGGTGAGACCCA
TCGTGGTGAGACCCA
TCGTGGCGAGACCCA
TCGTGTTGAGACCCTTCGTGATGAAACCCT
TCGTGGTGAGACCCA
TCGTGGTGAGACCCT
TCGTGGTGAGACCCA
TCGTGGCGAGACCCA
TCGTGGTGAGACCCT
TCGTGGTGAAACCCT
TCGTGGTGAGACCCA
TCGTGGTGAGACCCT
TCGTGGTGAGACCCT
TCGTGGTGAGACCCT
TCGTGGTGAGACCCA
TCGTGGTGAGACCCA
TCGTGGCGAGACCCA
TCGTGGTGAGACCCTTCGTGATGAAACCCT
TCGTGGTGAGACCCA
TCGTGGTGAGACCCT

TCGTGGTGAGACCCA
TCGTGGCGAGACCCA
TCGTGGTGAGACCCT
TCGTGGTGAAACCCT
TCGTGGTGAGACCCA
TCGTGGTGAGACCCT
TCGTGGTGAGACCTT
TCGTGGTGAGACCCA
TCGTGGTGAGACCCT
TCGTGGTGAGACCTT
TCGTGGTGAGACCCA
TCGTGGTGAGTCCCA
TCGTGGTGAGACCCA
TCGTAGTGAGACCCA
TCGTTGTGAGACCCA
TCGTTGTGAGACCCA
TCGTGGTGAGACCCA
TCGTGGTGAGACCCT
TCGTGGTGAGACCCT
TCGTGGTGAGACCCT
TCGTGGTGAGACCCA
TCGTGGTGAGACCCA
TCGTGGCGAGACCCA
TCGTGGTGAGACCCTTCGTGATGAAACCCT
TCGTGGTGAGACCCA
TCGTGGTGAGACCCT
TCGTGGTGAGACCCA
TCGTGGCGAGACCCA
TCGTGGTGAGACCCT
TCGTGGTGAAACCCT
TCGTGGTGAGACCCA
TCGTGGTGAGACCCT
TCGTGGTGAGACCTT
TCGTGGTGAGACCCT
TCGTGGTGAGACCCT
TCGTGGTGAGACCCA
TCGTGGTGAGACCCA
TCGTGGTGAGACCCT
TCGTGGTGAAACCCT
TCGTGGTGAGACCCA
TCGTGGTGAGACCCT
TCGTGGTGAGACCTT
TCGTGGTGAGACCCT
TCGTGGTGAGACCCT
TCGTGGTGAGACCCA
TCGTGGTGAGACCCA
TCGTGGCGAGACCCA
TCGTGGCGAGACCCA
TCGTGGTGAGACCCT
TCGTGGTGAAACCCT
TCGTGGTGAGACCCA
TCGTGGTGAGACCCT
TCGTGGTGAGACCTT
TCGTGGTGAGACCCT

TCGTGGTGAGACCCT
TCGTGGTGAGACCCT
TCGTGGTGAGACCCA
TCGTGGTGAGACCCA
TCGTGGTGAGACCCT
TCGTGGTGAGACCCA
TCGTGGTGAGACCCA
TCGTGGCGAGACCCA
TCGTGGTGAGACCCTTCGTGATGAAACCCT
TCGTGGTGAGACCCA
TCGTGGTGAGACCCT
TCGTGGTGAGACCCT
TCGTGGTGAGACCCT
TCGTGGTGAGACCCA
TCGTGGTGAGACCCA
TCGTGGCGAGACCCA
TCGTGGTGAGACCCTTCGTGATGAAACCCT
TCGTGGTGAGACCCA
TCGTGGTGAGACCCT
TCGTGGTGAGACCCA
TCGTGGCGAGACCCA
TCGTGGTGAGACCCA
TCGTGGTGAGACCCT
TCGTGGTGAAACCCT
TCGTGGTGAGACCCA
TCGTGGTGAGACCCT
TCGTGGTGAGACCTT
TCGTGGTGAGACCCT
TCGTGGTGAGACCCT
TCGTGGTGAGACCCT
TCGTGGTGAGACCCA
TCGTGGTGAGACCCA
TCGTGGTGAGACCCT
TCGTGGTGAGACCCA
TCGTGGCGAGACCCA
TCGTGGTGAGACCCT
TCGTGGTGAAACCCT
TCGTGGTGAGACCCT
TCGTGGTGAGACCCT
TCGTGGTGAGACCCA
TCGTGGTGAGACCCT
TCGTGGTGACACCCA
TCGTGGTGAGACCCT
TCGTGGTGAAACCCT
TCGTGGTGAGACCCA
TCGTGGTGAGACCCT
TCGTGGTGAGACCCT
TCGTGGTGAGACCCA
TCGTGGTGAGACCCT
TCGTGGTGAGACCCA
TCGTAGTGAGACCCA
Nr of selected repeats 173 Similarity 0.911565
Consensus:
TCGTGGTGAGACCCa

```
>Cele-UNSB01_2:1097712-1098091 Satlength=380 Nr of Repeats=5 RepeatLength=26
seed=GTCGGCTGCT
GTCGGCTGCTCACAAACCCTCGAGGT
GTCGGCTGCTTTTTTTTTGCAAAAGT
GCCGGCTGCTTCCAAACCTCTGATTT
GTCGTCTGCTACTAATTATAGCACACTTTATGTTCCTTGTCTACTCCCCGCAATTCCCATTCCAATATCAAAATAAGAA
AGCAAATAACAATTCTAAGTGGGTTAGTAGTAATAGTAGCAGGAGACCTGTTTTACTATTTTTACCGAAAATTTTCAAA
ATGCCTGCTAATCGATTAGAAGAATTAGGGAATTACAAGGCTTTTTAACTGTAATTCTATTAAATTTGAGAACACTGTA
GCATTGCCTCAGAACCCATCAGGT
GTCGGCTGCTTAATCATGTATAAAATGTTGCCTGGGAGGT
Nr of selected repeats 3 Similarity 0.339869
Consensus:
GtCGGCTGCTtnCAAAccctgAgtt
>Cele-UNSB01_2:1142725-1142802 Satlength=78 Nr of Repeats=5 RepeatLength=15
seed=CGAAAAGGGG
CGAAAAGGGGAGCAGAA
CGAAAAGGGGATCTG
CAAAAAGGGGATCTG
CGAAAAGGGGAGATA
CGAAAAGGGGAGATA
Nr of selected repeats 4 Similarity 0.777778
Consensus:
CgAAAAGGGGAgaTa
>Cele-UNSB01_2:1142992-1143084 Satlength=93 Nr of Repeats=6 RepeatLength=15
seed=TCCCCTTTTC
TCCCCTTTTCGTTGC
TCCCCTTTTCGTATC
TCCCCTTTTCGTATC
TCCCCTTTTCGCAGA
TCCCCTTTTTGCAGA
TCCCCTTTTCGTTCTGC
Nr of selected repeats 5 Similarity 0.768889
Consensus:
TCCCCTTTTcGtagc
>Cele-UNSB01_2:1186731-1187015 Satlength=285 Nr of Repeats=8 RepeatLength=26
seed=TACTGTAGTT
TACTGTAGTTTGACCTTCTAAACGAT
TACTGTAGTTTGACTAACTTAGGACT
TACTGTAGTTTGACCTACTTGTGGGTTACTGTAACTATACTTAAGTATACCTGCGGT
TACTGTAGGTTGACCTACTTAGGGGA
TACTGTAGTTTGATATTCATAAGGGT
TACTGTAGCTAGATCTAAGTAAAGCTACGGT
TACAGTAGTTTGACCTACTTAGGGGA
TACTGTAGATTGACCTAATTTCCATAAACCTTACTGCACTTTGACCTACTATCTAAAAATATAAAT
Nr of selected repeats 5 Similarity 0.621429
Consensus:
TACtGTAGtTTGAcctaCttAngGat
>Cele-UNSB01_2:1212949-1213169 Satlength=221 Nr of Repeats=4 RepeatLength=55
seed=GGGATTATTG
GGGATTATTGGAGTTTCTTTTTCTAGAATTTTTTGAAAATTTTGGACAGTTTTTC
GGGATTATTGGAGTTTCTTTTTCTAGAATTTTTTGAAAATTTTGGACAGTTTTTT
GGGATTATTGGAGTTTCTTTTTCTAGAATTTTTTGAAAATTTTAGACCCTTTTCT
GGGATTATTGGAGTTTCTTTTTCTAGAATTTTCTGAAAATTTGGAACAGTTTTTT
Nr of selected repeats 4 Similarity 0.903030
```

Consensus:
GGGATTATTGGAGTTTCTTTTTCTAGAATTTTtTGAAAATTTtggACagTTTTtt
>Cele-UNSB01_2:1233155-1233235 Satlength=81 Nr of Repeats=5 RepeatLength=12
seed=TTAGGCTTAG
TTAGGCTTAGGC
TTAGGCTTAGGT
TTAGGCTTATGCATAGGCTTATGCGC
TTAGGCTTAGGT
TTAGGCTTAGGATTATGT
Nr of selected repeats 3 Similarity 0.925926
Consensus:
TTAGGCTTAGGt
>Cele-UNSB01_2:1258661-1259567 Satlength=907 Nr of Repeats=73 RepeatLength=12
seed=CTATGCCTAT
CTATGCCTATAC
CTATGCCTATGC
CTATACCTATGCCCATAC
CTATGCCTATGCCTAAGCCAAAGC
CTATGCCTATGC
CTATGCCTATGC
CTATTCCTATGC
CTATGCCTATAC
CTATGCCCATAC
CTATGCCTATGCCTAAGCCAAAGC
CTATGCCTATGC
CTATGCCTATGC
CTATGCCTATGC
CTATGCCTATGC
CTATGCCTATGC
CTATGCCTATGC
CTATGCCTATGT
CTATGCCTATGC
CTATGCCTATGC
CTATGCCTCTGC
CTATGCCTATGC
CTATGCCTATGC
CTATGCCTATGC
CTATGCCTATGC
CTATGCCTATGT
CTATGCCTATGC
CTATGCCTATGC
CTATGTCTATTC
CTATGCCTATGC
CTATGCCTATGT
CTATGCCTATGC
CTATGCCTATGC
CTATGCCTATGC
CTATGCCTATGC
CTATGCCTATGC
CTATGCCTATGC
CTATGCCTATGC
CTATGCCTATGC
CTATGCCTATGC
CTATGCCTATGC

CTATGCCTATGC
CTATGCCTATGC
CTATGCCTATGC
CTATGTCTATGC
CTATGCCTATGC
CTATGCCTATGC
CTCTGCCTATGC
CTATGCCTATGC
CTATGCCTATGC
CTATGCCTATGC
CTATGCCTATGC
CTATGCCTATGC
CTATGCCTATGC
CTATGCCTATGT
CTATGCCTATGC
CTATGCCTATGC
CTATGCCTCTGC
CTATGCCTATGC
CTATGCCTATGC
CTATGCCTATGC
CTATGCCTATGC
CTATGCCTATGC
CTATGTCTATGC
CTATGCCTATGC
CTATGCCTATGT
CTATTCCTATGC
CTATGCCTATGC
CTATGTCTATGC
CTATGCCTATGT
CTATGCCTATGC
CTATGCCTATGT
CTATGCCTATGC
CTATGCCTATGC
Nr of selected repeats 70 Similarity 0.936416
Consensus:
CTATGCCTATGC
>Cele-UNSB01_2:1261170-1261684 Satlength=515 Nr of Repeats=42 RepeatLength=12
seed=CTATGCCTAT
CTATGCCTATGCC
CTATGCCTATGC
CTATGCCTGTGC
CTATGCCTATGC
CTATTCCTATTC
CTATGCCTATGC
CTATGCCTATTC
CTATGCCTATGC
CTATGCCTTTGC
CTTTGCCTATGC
CAATGCCTATGC
CTATGCCTATGC
CTGTGCCTATGC
CTATGCCTATGC
CTATGCCTATGC
CTATGCCTATGC

CTATGCCTATGC
CTATGCCTATGC
CTATGCCTATGC
CTATGCCTATGT
CTATGTCTATGC
CTATGCCTATGC
CTATGTCTATGC
CTATGCCTATGC
CTATGCCTATGC
CTATGCCTATGC
CTATGCCTATGC
CTATGCCTATGC
CTATGCCTATGCCTAG
CTATGCCTATGC
CTATGCCTATGC
CTATGCCTATGC
CTATGCCTATGC
CTATGCCTATGC
CTATGCCTATGC
CTATGCCTATC
CTATGCCTATGC
CTATGCCTATGC
CTATGCCTATGC
CTATGCCTATGC
CTATGCCTATGC
CTATGCCTATGCCTATGC
Nr of selected repeats 38 Similarity 0.936621
Consensus:
CTATGCCTATGC
>Cele-UNSB01_2:1316328-1316758 Satlength=431 Nr of Repeats=7 RepeatLength=63
seed=GGTCTGCTAA
GGTCTGCTAAATCGAAACTTGTAGTTTGTAGCCTAGCAGACCCAATTTCTTCAAAACTACTTG
GGTCTGCTAAATCGAAACTTGTAGTTTGTAGTCTAGCAGACCAAATTTCTTCAAAACTACTTG
GGTCTGCTAAATCGAAACTTGTAGTTTGTAGCCTAGCAGACCCAATTTCTTCAAAACTACTTG
GGTCTGCTAAATCGAAACTTGTAGTTTGTAGTCTAGCAGACCAAATTTATTTAAAACTACTTG
GGTCTGCTAAATTGAAAATTGTAGTTTGTAGTCTAGCAGACCCATAGAATTG
GGTCTGCTAAATCAAAACTTGTAGTTTGTAGCCTAGCAGACCAAATCTCTTCAAAACCACTTT
GGTCTGCTAAATCAAATTTTGTAGTTTGTAGTCCAGCAGACCCAATCTTATCAAAATCACATT
Nr of selected repeats 6 Similarity 0.855035
Consensus:
GGTCTGCTAAATCgAAACTTGTAGTTTGTAGcCTAGCAGACCaAATtTcTTCAAAACtACTTg
>Cele-UNSB01_2:1327419-1327790 Satlength=372 Nr of Repeats=5 RepeatLength=73
seed=AGTTTGTAGT
AGTTTGTAGTTTGTAGTCTAGCAGACCAAAATTTCCTAAAAAGCGCAGTAATTGATTGGTCTGCTAAATAAG
ACTTTGTAGTTTGTAGTCTAGCAGACCAAAATTTATTAAAAACTGCAGAAAATTGATGGGTCTGCTAATTAAG
AGTTTGTAGTTTGTAGTCTAGCAGACCAAAATTTATTAAAAACTGCAGAAAATTGATAGGTCTGCTAAATTTG
ATTTTGTAGTTTGTAGTGTAGCAGACCAAAATTTATTAAAAACTGCAGAAAATTGATGGGTCTGCTAATTAAG
AGTTTGTAGTTTGTAGTCTAGCAGACCAAAATTTATTAAAAACTGCAGAAAATTGATAGGTCTGCTAAATTTGATTTTG
T
Nr of selected repeats 3 Similarity 0.920852
Consensus:
AnTTTGTAGTTTGTAGTcTAGCAGACCAAAATTTATTAAAAACTGCAGAAAATTGATgGGTCTGCTAAtTaaG
>Cele-UNSB01_2:1412228-1413706 Satlength=1479 Nr of Repeats=12 RepeatLength=63
seed=TAGTTTGTAG

TAGTTTGTAGTTTGTAGTCTGGCAGACCACCCATAAAACTTGGGTTTTCAGGAGTTTGCGCGGAAGGAATTGACGACGC
AATTCGCTCTGAAGACTTTGAAACAGCTTCCCAACATATTCATCGCTTCTTAACACTCGATCAGGCTGTATTTCAGATT
CGAGAGTTCAAGCAGAAAGGTTTTTTCGCCTTTAATAGCTAATTTTACGTCAAATCCTGCCCATTTTCCAGACGCCACT
GACTCAATTCGCCACAGCTACGAAGTGCTCTCATCGGCGAAAGAACGCTTGTCGAAAATTCTGAAATCCCGTCTAACAG
AGTCTGTTCAAAAGGGAGACGTCGCAGAAATGCAGCGATTTATTAAAATGTTCCCGTTGATTCATGAACCCGACGAGGG
ATTACAGAGATATTCGGTTTTCTTGAATCAGAAAATCGATAAACTGGCCGAGGATAATTTGGCGGTAAGAAATCGGCTA
TTTTTGTAGGAAATATTCTTTAAAAAAGAGGAAAAAATACCAAAATTGATCAAAAAATTGGGTCTGCTAATTATTTTTT
G
TAGTTTGTAGTCTAGCAGACCATATTTCTTCAAAACCCCATAGGTCTGCTAAATCGAAACTTT
TAGTTTGTAGTCTAGCAGACCGAATTTCGACAAAACCCCATTGGTCTGCTAAATCGAAATTTG
TAGTTTGTAGTCTAGCAGACCAAAGTTCGACAAAACCCCATGGGTCTGCTAAATCGAAACTTG
TAGTTTGTAGTCTAGCAGACCATATTTCTTCAAAACCCCATTGGTCTGCTAAATCGAAACTTG
TAGTTTGTAGTCTAGCAGACCAAATTTCTTCAAAACCCCATAGGTCTGCTAAATCGAAGCTTG
TAGTTTGTAGTCTAGCAGACCAAATTTCTTCAAAATCTTATTGGTTTGCTAAATCGAAAAGTG
TAGTTTGTAGTCTAGCAGACCGAATTTCTTCAAAACCCAATGGGTCTGCTAAATCGAAACTTG
TAGTTTGTAGTCTAGCAGACCAACTTTCGCCAAAACCTCATGGGTCTGCTAAATATTTTATG
TAGTTTGTAGTCTAGCAGACCGAATTTCTTCGAAATCCTATTGGTCTGCTAAATCGAAACCTG
TAGTTTGTAGTCTAGCAGACCAATACATTTTTTTCTTCAATTATCCATCAAACAACGGCGAGAAAAATTTCAAAATTT
ACCCAAAATTAGTGTTTAGCGCACTAATTTTTCAGGAAAATTACGAAAATTGCCACTTCTGCTAGATTTTTGCTGCAAA
TTTGCAGCCATTGGTCTGCGAAATGCGCTTTG
TAGTTTGTAGCCTAGCAGACCAAACTCATTTTTCTTCGATTTTCAGCAAGAAATCTACGAAAAAATATCAAAAACTATT
GATTTTGCTCTTAAATTTGCTATTAAAACCTGTTTTTAATTAAAATTGTGGAAAAAATCCCCGATATTGGTCTGCTAAA
TCGAAATTCG
Nr of selected repeats 8 Similarity 0.873016
Consensus:
TAGTTTGTAGTCTAGCAGACCaaATTTCttCAAAAcCCcATtGGTCTGCTAAATCGAAActTG
>Cele-UNSB01_2:1416594-1416934 Satlength=341 Nr of Repeats=5 RepeatLength=59
seed=CTACAAACTA
CTACAAACTACACGAAATATTTAGCAGACCCGCTCTAGAAATTCCAAAAAAGTTGAGAAAAATTGGAAAAAAATGGCAA
TTTTCTCTTGGGTCTGCTAGA
CTACAAACTACAAAAGCCTATTTAGCAGACCCACGCCAACTTCTGATGGGTCTGCTAGA
CTACAAACTACAAAAGCCTATTTAGCAGACCCACGCCAACTTCTGATGGGTCTGCTAGG
CTACAAACTACAAAAAATATTTAGCAGACCCAACTAATGTCTTATAAATTTGGGCCTGCTAGA
CTACAAACTACAAAAGCCTATTTAGCAGACCCACGCCAATTTCTGATGGGTCTGCTAGA
Nr of selected repeats 3 Similarity 0.969868
Consensus:
CTACAAACTACAAAAGCCTATTTAGCAGACCCACGCCAAcTTCTGATGGGTCTGCTAGa
>Cele-UNSB01_2:1422798-1423926 Satlength=1129 Nr of Repeats=9 RepeatLength=63
seed=TTGGTCTGCT
TTGGTCTGCTCTGGACAACAAACTACAACTTTTGATTTAGCAGACCCATGGGGTTTTGAAGAAAA
TTGGTCTGCTAGACTACAAACTACAACTTTTGATTTAGCAGACCCATGGGGTTTTGAAGAAAA
TTGGTCTGCTAGACTACATACTACAACTTTTGATTTAGCAGACCCATGGTGTTTTGAAGAAAA
TTGGTCTGCTAGACTACAAACTACAACTTTTGATTTAGCAGACCCATGGGGTTTTGAAGAAAA
TTGGTCTGCTAGACTACATACTACAACTTTTGATTTAGCAGACCCATGGTGTTTTGAAGAAAA
TTGGTCTGCTAGACTACAAACTACAACTTTTGATTTAGCAGACCCATGGTGTTTTGAAGAAAA
TTGGTCTGCTAGACTACAAACTACAACTTTTGATTTAGCAGACCCATGGGGTTTTGAAGAAAA
TTGGTCTGCTAGACTACAAACTACAACTTTTTTTCCAAAAATCTGAAGAAAAGTCAATTTTTCTATGTTTTTTTTTTTG
AAGAAAAAAGCTAAAAAATATTTTTAAAAACAAGATTTTTTTCAGAAAATCACAAAAAAAAAACCCCCCGAAAATTGTT
GTTTTCTAGTGTTTTTTTAACAAAAAATTTCCAATTTTCAGAAAAATAAAGTACAAATATGAAAATTTTCCAAAAAATC
GGAATTTCAATGTTTTTTTAGAAAAAAGTTGGAAAAATCAAAAAAAAAATTGAATTGTAGATTTTTTGTGTTTTTTTAG
AGAAAAAAATGGAAAAAAAACTTATCAAATTTATTAATTTTTTTTGTCAATTTAAATTGTTTTTATTAATTTAAACAAA
T
TGGGTCTGCTAGTAGACTACAAACTACACATTTTCTATTAATAACTGGAACAATTTTCGATTTTCAAAAAATCCCAGGA
AAAACGATAATTATCGATTTTTGTCTATCTTTGAAAAAAAAATCGGAAACATTTTTTTCTGAAAAATTGAAATTTAAAA

AAATAGAAAAAATGGCTGAAAAACTCAATTTTCAAAGCAATAAATCAAAAAAAATTGTAGATTTTTAGTTTTTTTTAAA
GAAAAAAATCCATTTTAAACGATTTTTATATTTTTTCAACAACAAAAAAATT
Nr of selected repeats 6 Similarity 0.976014
Consensus:
TTGGTCTGCTAGACTACAaACTACAACTTTTGATTTAGCAGACCCATGGgGTTTTGAAGAAAA
>Cele-UNSB01_2:1429641-1430207 Satlength=567 Nr of Repeats=41 RepeatLength=12
seed=CCTATGCCTA
CCTATGCCTATG
CCTATGCCTATG
CCTATGCCTATG
CCTATGCCTATG
CCTATGCCTATG
CCTATGCCTAAA
CCTAAGCCTATGCTTGAGCCTATGAT
CTATGCCTATGCCTAAA
CCTAAGCCTATG
CCTATGCCTATG
CCTATGCCTATG
CCTATGACCTATG
CCTAAGCCTATG
CCTATGCCTAAG
CCTATGCCTAAG
CCTATACCTATG
CCTATGCCTAAG
CATATGCCTATGCCAAAGCATATT
CCTATGCCTAGGCCCATA
CCTATGCCTATC
CCTATGCCTAAG
CCTATGCCTATG
CCTATGCCTAAG
CCTAAGCCTATG
CCTATGCCTATG
CCTATGCCTATG
CCTAAGCCTATG
CCTAAGCCTAAG
CCTATGCCTATG
CCTATGCCTCAGCCTAAGCCTGTGT
CTATGCCTATG
CCTATGCCTATG
CCTAAGCCTATG
CCTAAGCCTAAG
CCTATGCCTATG
CCTATGCCTCAGCCTAAG
CCTGTGCCTATG
CCTATGCCTATGCCCAAGA
CTATGCCTATGCCCATA
CCTATGCCTAAA
CCTAAGCCTAAGCCTATG
Nr of selected repeats 30 Similarity 0.874585
Consensus:
CCTAtGCCTAtG
>Cele-UNSB01_2:1488365-1488455 Satlength=91 Nr of Repeats=5 RepeatLength=18
seed=GGAGGTCCAG
GGAGGTCCAGGTACCCCG

GGAGGTCCAGGAACCCCT
GGAGGTCCAGGTACTCCA
GGAGGTCTAGGTACCCCG
GGAGGTCCAGGTACCCCG
Nr of selected repeats 5 Similarity 0.859259
Consensus:
GGAGGTCcAGGtACcCCg
>Cele-UNSB01_2:1507976-1508324 Satlength=349 Nr of Repeats=19 RepeatLength=15
seed=GGGGGCAACA
GGGGGCAACAAAATA
GGGGGCAACATTTTCATAAATGTA
GGGGACAACAAAAAA
GGGGGCAACAAAAAT
GGGGGCAACATTTTCATAAATGTA
GGGGGCAGCAAAAAA
GGGGGCAACATAATA
GGGGTCAACATTTGAACTCTCCCC
GGGGGCAACAAATTA
GGGGGCAACATTTGAACTCTCCCT
GGGGGCAACAAATTA
GGGGGCAACATTTTCATAAATGTA
GGGGACAACAAAAAA
GGGGGCAACAAAAAT
GGGGGCAACATTTTCATAAATGTA
GGGGGCAGCAAAAAA
GGGGGCAACATAATA
GGGGTCAACATTTGAACTCTCCCC
GGGGGCAACAAATTA
Nr of selected repeats 12 Similarity 0.807407
Consensus:
GGGGGCAACAAAaaA
>Cele-UNSB01_2:1517382-1518287 Satlength=906 Nr of Repeats=6 RepeatLength=150
seed=TTGCAACATG
TTGCAACATGCGGTCGGCGGGAAATTCAAATTTTTAGATCAAAAACAGGTTTTTCGACCAATTTTTCATATTTTCTAGT
GAAATTATGCTAGAAAAATCAATTTTTCACTCAAAATCTTCAGTTTTCGACATTTTCTCATTGGAGCGCAC
TTGCAACATGCAGTTGGCGGGAAATTTGAATTTTTGGGTCCAAAACACGTTTTTCGACTATTTTTTCTATATTTTCAAG
TAAAATTAGGCTAGAAAATTCCAATTTTCACGAAAAATCACCAATTTTCGACATTTTCTCATTGGGGCGCGC
TTGCAACATGCGGCCGGCGGGAAATTTGAATTTTTGGGTCCAAAACACGTTTTTCGACTAATTTTTCATATTTTCAAGC
TAAATTATGATAGAAAATTCCAATTTTCACTAAAAATCGGCAATTTTCAGGATTTTCTCACTGGGGCGCAT
TTGCAACATGCGGTCGGCGGAAAATTCGAATTTTTCAGTCCAAAACAGGTTTTTCGACCAATTTTTCATATTTTCTAGT
GAAATTATGCTCGAAAAGTCAATTTTTCACTAAAAATCGCCAATTTTCCACATTTTCTCATTAGAGCGCAC
TTGCAACATGCGATCCGCGGGAAATTCGAAATTTTGGGTCAAAAACACGGTTTTCGACTATTTTTTTTTCTATACTTTC
AAGTGAAATTATGCTCGAAAAGTCAATTTTTCACTAAAAATCGATATTTTTCTACTTTTTCTCATTGGGGCGCGC
TTGCAACATGCGGTCGGCGGAAAATTCGAATTTTTCAGTCCAAAACATGCTTTTCGACCAATTTTTCATATTTTCAAGC
TAAATTATGATAGAAAATTCAAATTTTCATAAAAAACCGCCAATTTTCCACATTTTCTCATTGGGGCGCGC
Nr of selected repeats 4 Similarity 0.817145
Consensus:
TTGCAACATGCGGtCGGCGGaAAATTcgAATTTTTcaGTCcAAAACAgGtTTTTCGACcAATTTTTCATATTTTCaAGc
gAAATTATGaTaGAAAAtTCaAaTTTTCActaAAAAtCgcCAaTTTTCcacATTTTCTCAtTgGaGCGCac
>Cele-UNSB01_2:1585165-1585860 Satlength=696 Nr of Repeats=7 RepeatLength=96
seed=GGCCTGCGGC
GGCCTGCGGCCCTCAAACCTGGTTATTAGGCTTGAGTGAATAACATATCTGCTTAGAATAGGATCAAAGCCAGCCCATA
GTTACCAAGAGTGTTCG

GGCCTGCGGCCCTCGAACCTGGTTGTTAGGTTTGAGTAAATAACAACCGCGTCGCAGGCCTACAGCCTGCTCTGCTTAG
AATAGGATTAAAGCCAGCCCACAGTTTTCGAGAGTATTCT
GGCCTGCGGCCCTCAAACCTGGTTATTAGGCTTGAGTGAATAACATATCTGCTTAGAATAGGATCAAAGCCAGCCCATA
GTTTCCAAGAGTGTTCG
GGCCTGCGGCCCTCGAACCTGGTTGTTAGGCTTGAGTGAATAACATATCTGCTTAGAATAGGATCAAAGCCAGCCCATA
GTTTTCAAGAGTGTTCG
GGCCTGCGGCCCTCGAACCTGGTTGTTAGGCTTGAGTGAATAACATATCTGCTTAGAATAGGATCAAAACCAGCCCATA
GTTTCCAAGAGTGTTCG
GGCCTGCGGCCCTCGAACCTGGTTGTTAGGCTTGAGTGAATAACATATCTGCTTAGAATAGGATCAAAGCCAGCCCATA
GTTTCCAAGAGTGTTCG
GGCCTGCGGCCCTCGAACCTGGTTGTTAGGCTTGAGTGAATAACATATCTGCTTAGAATAGGATCAAAGCCAGCCCATA
GTTTTCAAGAGTGTTCG
Nr of selected repeats 6 Similarity 0.968518
Consensus:
GGCCTGCGGCCCTCgAACCTGGTTgTTAGGCTTGAGTGAATAACATATCTGCTTAGAATAGGATCAAAGCCAGCCCATA
GTTTcCAAGAGTGTTCG
>Cele-UNSB01_2:1599041-1599824 Satlength=784 Nr of Repeats=24 RepeatLength=23
seed=CGCTGTAAGA
CGCTGTAAGACCCTTCGCTGTATCGCTGCGAGACCCTCCGCTGTAC
CGCTGTAAGACCATTTGCTGTAT
CGCTGTAACACCCTTCTCTGTAT
CGCTGTAAGACCCTTCGCTGTATCGCTGCGAGCCCCTTCGCTATATCGTTGCGAGACCCTTCACTATTT
CGCTGTAAGACCCTTCGCTGTAC
CGCTGTAAGATCCTTCGCTGTTC
CGCAGTAAGATCCTTCGCTGTTC
CGCTGTAAGACACTTCACTATTC
CGCTGTAAGACCCTTCGCTGTAC
CGCTGTAAGATCCTTCGCTTTTC
CGTTGTAAGACCGTTTGCTGTATCGCTGCGAGCCCCTTTGCTGTAC
CGCTGTAAGATCTTTCGCTGTTC
CGTTGTAAGACCCTTCGCTGTAC
CGCTGTAAGAGCCTTTGCTGTAC
CGCTGTAAGATCTTTCGCTGTTC
CGTTGTAAGACCTTTCGCTGTAC
CGCTGTAAGACCCTTTGCTGTAC
CGCTGTAAGATCTTTCGCTGTTC
CGTTGTAAGACCCTTCGCTGTAC
CGCTGTAAGACCCTTTGCTGTATCGCTGCGAGCCCCTTCGCTGTATCGTTGTGAGACCCTTCACTATTTCGCTGCGAGA
CCCTTCGCTGTACA
GCTGTAAGATCCTTCGCTGTTC
CGCTGTAAGACCCTTCGCTGTATCGTTGTGAGACCCTTCACTATTTCGCTGCGAGACCCTTCACTATAT
CGCTGTAAGACTCTTTGCTGTATCGTCGCGAGACCTTTCGATGTAT
CGCTGCAAGACCTCTTCGCTCTTT
Nr of selected repeats 16 Similarity 0.806763
Consensus:
CGCTGTAAGAcCcTTCGCTGTaC
>Cele-UNSB01_2:1617453-1617521 Satlength=69 Nr of Repeats=4 RepeatLength=17
seed=TCATTCCGTG
TCATTCCGTGATTATTT
TCATTCCGTGATTATTT
TCATTCCGTGATTATTT
TCATTCCGTGATTATTT
Nr of selected repeats 4 Similarity 1.000000
Consensus:

TCATTCCGTGATTATTT
>Cele-UNSB01_2:1625642-1627656 Satlength=2015 Nr of Repeats=131 RepeatLength=14
seed=GATCCTAGAT
GATCCTAGATCCTG
GATCCTAGATCCTA
GATCCGAGATCCTA
GAGCCTAGATCCTA
GATCCTAGATCCTA
GATCCCAGATCCTA
GATCCCAGATCCTA
GATCCCAGATCCTA
GATCCTAGATCCTA
GATCCTAGATCCTA
GATCCTAGATCCTA
GATCCTAGATCCTA
GATCCTAGATCCTA
GATCCTAGTTCCTA
GATCCTAGATCCTA
GATCCTAGATCCTA
GATCCTAGATCCTA
GATCCTAGATCCTA
GATCCTAGATCCTA
GATCCTAGATCCTA
GATCCCAGATCCTA
GATCCTAGATCCTA
GATCCTAGATCCTA
GATCCTAGATCCTA
GATCCTAGATCCCA
GATCCTAGATCCCA
GATCCTAGATCCTA
GATCCTAGATCCTA
GATCCTAGATCCTA
GATCCTAGATCCTA
GATCCTAGATCCTA
GATCCTAGATCCTA
GATCCTAGATCCTA
GATCCTAGATCCTA
GATCCTAGATCCTA
GATCCTAGATCCTAGATCC
GATCCTAGATCCTA
GATCCTAGATCCTA
GATCCTAGATCCTA
GATCCTAGATCCTA
GATCCTAGATCCTA
GATCCTAGATCCTA
GATACTAGATCATA
GAGCCTAGATCCTA
GATCCTAGATCCTA
GATCCTAGATACTAGATCATA
GAGCCTAGATCCTGGTGTCTA
GATCCTAGATCCTA
GATCCTAGATCCTA
GAGCCTAGATCCTGGTGTCTA
GATCCTAGATCCTA

GATCCTAGATCCTA
GATCCTAGTCTA
GATCCTAGATCCTA
GATCCTAGATCCTA
GATCCTAGATCCTA
GATCCTAGATCCTA
GATCCTAGATCCTA
GATCCTAGATCCTA
GATCCTAGATCCTA
GATCCTAGATCCTA
GATCCTAGATCCTA
GATCCTAGATCCTA
GATCCTAGATCCTA
GATCCTAGATCCTA
GATCCTAGATCCTA
GATCCTAGATCCTA
GATCCTAGATCCTA
GATCCTAGATCCTA
GATCCTAGATCCTA
GATCCTAGATCCTA
GATCCTAGATCCTA
GATCCTAGATCCTA
GATCCTAGATCCTA
GATCCTAGATCCTA
GATCCTAGATCCTA
GATCCTAGATCCTA
GATCCTAGATCCTA
GATCCTAGATCCTA
GATCCTAGATCCTA
GATCCTAGATCCTA
GATCCTAGATCTA
GATCCTAGATCCTA
GATCCTAGATCCTA
GATCCTAGATCCTA
GATCCTAGATCCTA
GATCCTAGATCCTA
GATCCTAGATCCTAGATCCTGGTATCTA
GATCCTAGATCCTA
GATCCTAGATCCTA
GATCCTAGATCCTA
GATCCTAGATCCTA
GATCCTAGATCCTA
GATCCTAGATCCTA
GATCCTAGATCCTA
GATCCTAGATCCTA
GATCCTAGATCCTA
GATCCTAGATCCTA
GATCCTAGATCCTA
GATCCTAGATCCTA
GATCCTAGATCCTA
GATCCTAGATCCTA
GATCCTAGATCCTA
GATCCTAGATCCTA
GATCCTAGATCCTA

GATCCTAGATCCTA
GATCCTAGATCCTAGATTA
ATCCTAGATCCTA
GATCCTAGATCCTA
GATCTAGATCCTA
GATCCTAGATCCTA
GATCCTAGATCCTA
GATACTAGATCATA
GAGCCTAGATCCTGGTGTCTA
GATCCTAGAACCTA
GATCCTAGATACTAAATCATA
GAGCCTAGATCCTGGTGTCTA
GATCCTAGATCCTA
GATCCTAGATCCTA
GATCCTAGTTCCTA
GATCCTAGATCCTA
GATCCTAGATCCTA
GATCCTAGTTCCTA
GATCCTAGATCCTAGAGCCTAGAGCCTAGAGCCTAAAGCCTAAAGCCTA
GATCCTAGATCCTAGAGCCTAGAGCTTA
GAACCTAGATCCTAGATCATAGAACCTAAATCTTG
GATCCTAGATCCTAGATAATC
GATCCTAGATAATAGATCCTGAAGCCTAGAGCGTAGATCCAAGACACTT
GATCCTAGATTTTA
GATCCTAGATCCTAGGTCTTA
GATCCTAGATACTT
Nr of selected repeats 112 Similarity 0.963106
Consensus:
GATCCTAGATCCTA
>Cele-UNSB01_2:1652802-1653206 Satlength=405 Nr of Repeats=5 RepeatLength=42
seed=TTGCCGGTTG
TTGCCGGTTGCCGAAATTTTTTGGAATCAGCAACTTCGGCAA
TTGCCGGTTGCCGAACTCCAAAAATTTTTGGAGTTCGGCAACTTCGGCAATTGCCGAACTTTTTTTCAGCAACTTCGGC
AATTGCTGATATCAATGTCAAACATTCTGTAGCTCAGAAAAAGCATTCAGAGAGCATGAAAAATGCTAATATTTTGCTG
TAAACGAGAAAACAACCATTGCTTTTTGCCAAAACTCAAAAATATATTTTCAACAGAAACAAAAATTCGAAACCTGG
TTGCCGGTTGTCGAAAAGTTGCCGAAGTTTCTGCAACCGAAAG
TTGCCGGTTGCCGAAGTTTTCAGCGTTCGACAACTTCGGCAA
TTGCCGGTTGCCGAAATGTTTTGGGTTCGGCAACTTCGGCAA
Nr of selected repeats 3 Similarity 0.809524
Consensus:
TTGCCGGTTGCCGAAaTtTTttGggtTCggCAACTTCGGCAA
>Cele-UNSB01_2:1657514-1657909 Satlength=396 Nr of Repeats=5 RepeatLength=42
seed=CAACCGGCAA
CAACCGGCAATTGCCGAAGTTGCCGAACCCAAAACATTTCGG
CAACCGGCAATTGCCGAAGTTGTCGAACGCTGAAAACTTCGG
CAACCGGCAACTTTCGGTTGCAGAAACTTCGGCAACTTTTCGA
CAACCGGCAACCAGGTTTCGAATTTTTGTTTCTGTTGAAAATATATTTTTGAGTTTTGGCAAAAAGCAATGGTTGTTTT
CTCGTTTACAGCAAAATATTAGCATTTTTCATGCTCTCTGAATGCTTTTTCTGAGCTACAGAATGTTTGACATTGATAT
CAGCAATTGCCGAAGTTGCTGAAAAAAAGTTCGGCAATTGCCGAAGTTGCCGAACTCCAAAAATTCGG
CAACCGGCAATTGCCGAAGTTGCTGATTCCAAAAAATTTCGG
Nr of selected repeats 3 Similarity 0.809524
Consensus:
CAACCGGCAATTGCCGAAGTTGccGAaccCaaAAaAtTTCGG

>Cele-UNSB01_2:1681965-1682083 Satlength=119 Nr of Repeats=7 RepeatLength=17
seed=GATGCCCCCT
GATGCCCCCTCTGAACG
GATGCCCCCTCTGAACG
GATGCCCCATTTGAACG
GATGCCCCCTACGAACAA
ATGCCCCCTTTGACCTA
ATGCCCCCTTAGAACGC
GATGCCCCCTCTCTC
Nr of selected repeats 5 Similarity 0.729630
Consensus:
gATGCCCCcTttGAaCg
>Cele-UNSB01_2:1815608-1815814 Satlength=207 Nr of Repeats=6 RepeatLength=34
seed=ATTTGAATTT
ATTTGAATTTCCCGCCAAAATTTTTTCACTGAAA
ATTTGAATTTCCCGCCAAAATTTTTTTCACTGAAT
ATTTGAATTTCCCGCCAAAATTTTTTCACTGAAA
ATTTGAATTTCCCGCCAAAATTTTTTCACTGAAA
ATTTGAATTTCCCGGCAAAATTTTTTCACTGAAA
ATTTGAATTTCCCGCCAAAATGCTTTTCACAGAAC
Nr of selected repeats 4 Similarity 0.980392
Consensus:
ATTTGAATTTCCCGcCAAAATTTTTTCACTGAAA
>Cele-UNSB01_2:1818966-1819342 Satlength=377 Nr of Repeats=11 RepeatLength=34
seed=CGGGAAATTC
CGGGAAATTCAAATGTTCTGTGAAAAGCATTTTGG
CGGGAAATTCAAATTTTCAGTGAAAAAATTTTGC
CGGGAAATTCAAATTTTCAGTGAAAAAATTTTGG
CGGGAAATTCAAATTTTCAGTGAAAAAATTTTGG
CGGGAAATTCAAATATTCAGTGAAAAAAATTTTGG
CGGGAAATTCAAATTTTCAGCGAAAAAATTTTGG
CGGGAAATTCAAATTTTCAGTGAAAAAATTTTGG
CGGGAAATTCAAATTTTCAGTGAAAAAAAATTTGG
CGGGAAATTCAAATTTTCAGTGAAAAAATTTTGG
CGGGAAATTCAAATTTTCAGTGAAACAATTTTGG
CGGGAAATTCAAATTTTCAGTAAAAAAAATTTTGG
Nr of selected repeats 9 Similarity 0.956427
Consensus:
CGGGAAATTCAAATTTTCAGTGAAAAAATTTTGG
>Cele-UNSB01_2:1881035-1881350 Satlength=316 Nr of Repeats=5 RepeatLength=65
seed=TATCGAGAGA
TATCGAGAGAATTTCCAATGTTTCCAACAAAATCGATTTCCAGTTCCAATAAGACTCTCAGTAAG
TATCGAGAGAATGCTCTACATTCACTTTGAATTTCTTTTCCAATTTCAATAAGACTCTCAGTAAG
TATCGAGAGAATTCCACATTCACTTTGAATGCCCGTTCCAATTTCAGTAAAACTCTCTATAAG
TATCGAGAGAATGTTCCAAAGAATTCAATTCCAATTTTCAATAAGACTCCCAGTAGG
TATCGAGAGAATATTACACATTCATTTTGAATGCCCATTCCAATTTCAATAAGACTCTCAGTAAG
Nr of selected repeats 3 Similarity 0.658497
Consensus:
TATCGAGAGAATntTccAcATTCactttgAAttccntTTCCAaTTtCAATAAGACTCTCAGTAAG
>Cele-UNSB01_2:1915179-1915855 Satlength=677 Nr of Repeats=26 RepeatLength=26
seed=ATTTACGGAG
ATTTACGGAGGTTCGATCCTGTGTCG
ATTTACGGAGCTTCAAGTTAATGTTG
ATTCACGGAGCTTCAAGCTTGTGCCAA

ATTACGGAGCTTCACGTTTGTGTCG
ATTTATGGAGCTTCAAGCTTGTGTCG
ATTTACGGAGTTTCAAGCTTGTGTCGG
TTTACGGAGCTTCAAGCTTGTGCCA
ATTTACGGAACTTCAAGGTTGTGTCG
ATTTACGGAGCTTCAATCTTGTGTCG
ATTTACGGATCTCCAAGCTTGTGTAG
ATTTACGGAGCTTTAGGATTATGTCG
ATTTACGGAGCTTCAAGGTTGTATCA
ATTCACGGAGCTTTGGGATTGTGTTG
ATTTACGGAGCTTCAAGCTTGTGTCG
ATTTACGGAGCTTCAAGCTTGTGTCG
ATTTACGGAGCTACAAGCTTGTGTCG
ATTTACGGAGCTTCAAGCTTGTGTCG
ATTTACGGAGCTTCGAGCTTGTGTCG
ATTTACGGAGCTACAAGCTTGTGTCG
ATTTATGGAGCTACAAGCTTGTGTCA
ATTTACGGAGCTTCGAGCTTGTGTCG
ATTTACGGAGCTTCAAGCTTGTGTCG
ATTTACGGAGCTTCGAGCTTGTGTCG
ATTTACGGAGCTACAAGCTTGTGTCG
ATTTATGGAGCTACAAGCTTGTGTCA
ATTTACGGAGCTTCGAGCTTGTGTCG
Nr of selected repeats 22 Similarity 0.829282
Consensus:
ATTTACGGAGCTtCaAGcTTGTGTCG
>Cele-UNSB01_2:1984417-1985378 Satlength=962 Nr of Repeats=31 RepeatLength=31
seed=AGAGCTGAGT
AGAGCTGAGTTGTCTAGCGAGATAGCAGAGA
AGAGATGAGTTGTCGAGCGAGACCTCAGAAG
AGAGCTGAGTTGTCTAGCGAGATAGCAGAGA
AGAGATGAGTTGTCGAGTGAGACCTCAGAAG
AGAGCTGAGTTGTCTAGCGAGATAGCAGAAA
AGAGCTGAGTTGTCTAGCGAGATAGCAGAGA
AGAGATGAGTTGTCGAGTGAGACCTCAGAAG
AGAGCTGAGTTGTCTAGCGAGATAGCAGAGA
AGAGATGAGTTGTCGAGCGAGACCTCAGAAG
AGAGCTGAGTTGTCTAGCGAGATAGCAGAGA
AGAGATGAGTTGTCGAGTGAGACCTCAGAAG
AGAGCTGAGTTGTCTAGCGAGATAGCAGAGA
AGAGATGAGTTGTCGAGCGAGACCTCAGAAG
AGAGCTGAGTTGTCTAGCGAGATAGCAGAGA
AGAGATGAGTTGTCGAGCGAGACCTCAGAAG
AGAGCTGAGTTGTCTAGCGAGATAGCAGAGA
AGAGCTGAGTTGTCGAGCGAGACCTCAGAAG
AGAGCTGAGTTGTCTAGCGAGATAGCAGAGA
AGAGATGAGTTGTCGAGCGAGACCTCAGAAG
AGAGCTGAGTTGTCTAGCGAGATAGCAGAGA
AGAGATGAGTTGTCGGGCGAGACCTCAGAAG
AGAGCTGAGTTGTCTAGCGAGATAGCAGAGA
AGAGATGAGTTGTCGAGTGAGACCTCAGAAG
AGAGCTGAGTTGTCTAGCGAGATAGCAGAGA
AGAGCTGAGTTGTCGAGCGAGACCTCAGAAG
AGAGCTGAGTTGTCTAGCGAGATAGCAGAGA

AGAGATGAGTTGTCGAGCGAGACCTCAGAAG
AGAGCTGAGTTGTCTAGCGAGATAGCAGAGA
AGAGATGAGTTGTCGAGTGAGACCTCAGAAG
AGAGCTGAGTTGTCTAGCGAGATAGCAGAGA
AGAGATGAGTTGTCGAGTGAGACCTCAGAAG
Nr of selected repeats 31 Similarity 0.809341
Consensus:
AGAGcTGAGTTGTCtAGCGAGAtagCAGAnA
>Cele-UNSB01_2:1991206-1991967 Satlength=762 Nr of Repeats=23 RepeatLength=32
seed=AATTCCCGCC
AATTCCCGCCAAAAATTTTTCAGAAAATTTG
AATTCCCGCCAAAAAAATTGTAAAGAAAATTTA
AATTCCCGCCAAAAATTTTCAGAGAAAATTTG
AATTCCCGCCAAAAATTTTTCAGAAAATTTG
AATTCCCGCCAAAATTTTTCACAGAAAATTTG
AATTCCCGCCAAAAAAATTTTAAAGAAAATTTA
AATTCCCGCCAAAAATTTTCAGAGAAAATTTG
AATTCCCGCCAAAAATTTTTCAGAAAATTTG
AATTCCCGCCAAAATTTTTCACAGAAAATTTG
AATTCCCGCCAAAAAAATTTTAAAGAAAATTTA
AATTCCCGCCAAAAATTTTCAGAGAAAATTTG
AATTCCCGCCAAAAATGTTTCAGAAAATTTG
AATTCACGCCAAAAATTTTCAAAGAAAATTTG
AATTCCCGCCAAAAAAATTTTGAAGGAAATTTA
AATTCCCGCCAAAATTTTTCAGAGAAAATTTG
AATTCCCGCCAAAAATTTTTCAGAAAATTTG
AATTCCCGCCAAAAAAATTTTGAAGGAAATTTA
AATTCCCGCCAAAATTTTTCAGAGAAAATTTG
AATTCCCGCCAAAAATTTTTCAGAAAATTTG
AATTCCCGCCAAAATTTTTCACAGAAAATTTG
AATTCCCGCCAAAAAAATTTTAAAGAAAATTTA
AATTCCCGCCAAAAATTTTCAGAGAAATTTTG
AATTCCCGCCAAAAATGTTTCAGAAAATTTGAGCTACCGCCAAAAATTTTCACAGAAAATTTG
Nr of selected repeats 16 Similarity 0.855208
Consensus:
AATTCCCGCCAAAAatTTTcAnAGAAAATTTg
>Cele-UNSB01_2:1992839-1994265 Satlength=1427 Nr of Repeats=46 RepeatLength=31
seed=AGAGCTGAGT
AGAGCTGAGTTGTCTAGCGAGATAGCAGAGA
AGAGATGAGTTGTCGAGCGAGACCTCAGAAG
AGAGCTGAGTTGTCTAGCGAGATAGCAGAGA
AGAGATGAGTTGTCGAGTGAGACCTCAGAAG
AGAGCTGAGTTGTCTAGCGAGATAGCAGAAA
AGAGCTGAGTTGTCTAGCGAGATAGCAGAGA
AGAGATGAGTTGTCGAGTGAGACCTCAGAAG
AGAGCTGAGTTGTCTAGCGAGATAGCAGAGA
AGAGATGAGTTGTCGAGCGAGACCTCAGAAG
AGAGCTGAGTTGTCTAGCGAGATAGCAGAAA
AGAGCTGAGTTGTCTAGCGAGATAGCAGAGA
AGAGATGAGTTGTCGAGTGAGACCTCAGAAG
AGAGCTGAGTTGTCTAGCGAGATAGCAGAGA
AGAGATGAGTTGTCGAGCGAGACCTCAGAAG
AGAGCTGAGTTGTCTAGCGAGATAGCAGAGA
AGAGATGAGTTGTCGAGCGAGACCTCAGAAG

AGAGCTGAGTTGTCTAGCGAGATAGCAGAGA
AGAGATGAGTTGTCGAGTGAGACCTCAGAAG
AGAGCTGAGTTGTCTAGCGAGATAGCAGAGA
AGAGATGAGTTGTCGAGTGAGACCTCAGAAG
AGAGCTGAGTTGTCTAGCGAGATAGCAGAGA
AGAGATGAGTTGTCGAGCGAGACCTCAGAAG
AGAGCTGAGTTGTCTAGCGAGATAGCAGAGA
AGAGATGAGTTGTCGAGTGAGACCTCAGAAG
AGAGCTGAGTTGTCTAGCGAGATAGCAGAGA
AGAGATGAGTTGTCGAGTGAGACCTCAGAAG
AGAGCTGAGTTGTCTAGCGAGATAGCAGAGA
AGAGATGAGTTGTCGAGCGAGACCTCAGAAG
AGAGCTGAGTTGTCTAGCGAGATAGCAGAGA
AGAGATGAGTTGTCGAGCGAGACCTCAGAAG
AGAGCTGAGTTGTCTAGCGAGATAGCAGAGA
AGAGCTGAGTTGTCGAGCGAGACCTCAGAAG
AGAGCTGAGTTGTCTAGCGAGATAGCAGAGA
AGAGATGAGTTGTCGAGCGAGACCTCAGAAG
AGAGCTGAGTTGTCTAGCGAGATAGCAGAGA
AGAGATGAGTTGTCGGGCGAGACCTCAGAAG
AGAGCTGAGTTGTCTAGCGAGATAGCAGAGA
AGAGATGAGTTGTCGAGTGAGACCTCAGAAG
AGAGCTGAGTTGTCTAGCGAGATAGCAGAGA
AGAGCTGAGTTGTCGAGCGAGACCTCAGAAG
AGAGCTGAGTTGTCTAGCGAGATAGCAGAGA
AGAGATGAGTTGTCGAGCGAGACCTCAGAAG
AGAGCTGAGTTGTCTAGCGAGATAGCAGAGA
AGAGATGAGTTGTCGAGCGAGACCTCAGAAG
AGAGCTGAGTTGTCTAGCGAGATAGCAGAGA
AGAGATGAGTTGTCGAGTGAGACCTCAGAAG
Nr of selected repeats 46 Similarity 0.811262
Consensus:
AGAGcTGAGTTGTCtAGCGAGAtagCAGAnA
>Cele-UNSB01_2:2000093-2000918 Satlength=826 Nr of Repeats=25 RepeatLength=32
seed=AATTCCCGCC
AATTCCCGCCAAAAATTTTTCAGAAAATTTG
AATTCCCGCCAAAAAAATTTTAAAGAAAATTTA
AATTCCCGCCAAAAATTTTCAGAGAAAATTTG
AATTCCCGCCAAAAATTTTTCAGAAAATTTG
AATTCCCGCCAAAATTTTTCACAGAAAATTTG
AATTCCCGCCAAAAAAATTTTAAAGAAAATTTA
AATTCCCGCCAAAAATTTTCAGAGAAAATTTG
AATTCCCGCCAAAAATTTTTCAGAAAATTTG
AATTCCCGCCAAAATTTTTCACAGAAAATTTG
AATTCCCGCCAAAAAAATTTTAAAGAAAATTTA
AATTCCCGCCAAAAATTTTCAGAGAAAATTTG
AATTCCCGCCAAAAATGTTTCAGAAAATTTG
AATTCACGCCAAAAATTTTCAAAGAAAATTTG
AATTCCCGCCAAAAAAATTTTGAAGGAAATTTA
AATTCCCGCCAAAATTTTTCAGAGAAAATTTG
AATTCCCGCCAAAAATTTTTCAGAAAATTTG
AATTCCCGCCAAAAAAATTTTGAAGGAAATTTA
AATTCCCGCCAAAATTTTTCAGAGAAAATTTG
AATTCCCGCCAAAAATTTTTCAGAAAATTTG

AATTCCCGCCAAAATTTTTCACAGAAAATTTG
AATTCCCGCCAAAAAAATTTTAAAGAAAATTTA
AATTCCCGCCAAAAAATTTTCAGAGAAATTTTG
AATTCCCGCCAAAAAAATTTTAAAGAAAATTTA
AATTCCCGCCAAAAAATTTTCAGAGAAAATTTG
AATTCCCGCCAAAAAATGTTTCAGAAAATTTGAGCTACCGCCAAAAATTTTCACAGAAAATTTG
Nr of selected repeats 18 Similarity 0.865196
Consensus:
AATTCCCGCCAAAAatTTTcAnAGAAAATTTg
>Cele-UNSB01_2:2015956-2016256 Satlength=301 Nr of Repeats=25 RepeatLength=12
seed=ATGTATATGT
ATGTATATGTAT
ATGTATATGTAT
ATGTATATGTAT
ATGTTTATGTAT
ATGTATATGTAT
ATGTATATGTAT
ATGTATATGTAA
ATGTAAATGTAA
ATGTATATGTAT
ATGTATATGTAT
ATGTATATGTAT
ATGTATATGTAT
ATGTATATGTAT
ATGTATATGTAT
ATGTATATGTAT
ATGTATATGTAT
ATGTATATGTAT
ATGTATATGTTT
ATGTATATGTAT
ATGTATATGTAA
ATGTATATGTAT
ATGTATATGTAT
ATGTATATGTAT
ATGTATATGTAT
ATGTATATGTAT
Nr of selected repeats 25 Similarity 0.948889
Consensus:
ATGTATATGTAT
>Cele-UNSB01_2:2017071-2017146 Satlength=76 Nr of Repeats=5 RepeatLength=15
seed=TCGTGGCGAG
TCGTGGCGAGACCCA
TCGTTGCGAGACCCT
TCGTGGCGAGACCCA
TCGTGGCGAGACCCA
TCGTGGCGAGACCCA
Nr of selected repeats 5 Similarity 0.928889
Consensus:
TCGTgGCGAGACCCa
>Cele-UNSB01_2:2017169-2017519 Satlength=351 Nr of Repeats=10 RepeatLength=32
seed=GGCGGGAATT
GGCGGGAATTTAAATTTTCTTTGAAATTTTTC
GGCGGGAATTCAAATTTTCTGTGAAAATTTTTGGCGGTAGTTCAAATTTTCTTCAAAATTTTTT
GGCGGGAATTCAAATTTTCTTTGAAAATTTTT

GGCGTGAATTCAAATTTTCTGAAACATTTTT
GGCGGGAATTCAAATTTTCTCTGAAAATTTTT
GGCGGGAATTTAAATTTTCTTTGAAATTTTTC
GGCGGGAATTCAAAATTTCTCTGAAAATTTTT
GGCGGGAATTTAAATTTTCTTTAAAATTTTTT
GGCGGGAATTCAAATTTTCTGTGAAAAAATTTT
GGCGGGAATTCAAATTTTCTGAAAAAATTTTT
Nr of selected repeats 7 Similarity 0.843675
Consensus:
GGCGGGAATTcAAATTTTCTtTGaAAATTTTT
>Cele-UNSB01_2:2023342-2024644 Satlength=1303 Nr of Repeats=42 RepeatLength=31
seed=GACAACTCAG
GACAACTCAGCTCTCTTCTGAGGTCTCACTC
GACAACTCATCTCTTCTCTGCTATCTCGCTA
GACAACTCAGCTCTCTTCTGAGGTCTCACTC
GACAACTCATCTCTTCTCTGCTATCTCGCTA
GACAACTCAGCTCTCTTCTGAGGTCTCACTC
GACAACTCATCTCTTCTCTGCTATCTCGCTA
GACAACTCAGCTCTCTTCTGAGGTCTCACTC
GACAACTCATCTCTTCTCTGCTATCTCGCTA
GACAACTCAGCTCTCTTCTGAGGTCTCACTC
GACAACTCATCTCTTCTCTGCTATCTCGCTA
GACAACTCAGCTCTCTTCTGAGGTCTCACTC
GACAACTCATCTCTTCTCTGCTATCTCGCTA
GACAACTCAGCTCTCTTCTGAGGTCTCACTC
GACAACTCATCTCTTCTCTGCTATCTCGCTA
GACAACTCAGCTCTCTTCTGAGGTCTCACTC
GACAACTCAGCTCTTCTCTGCTATCTCGCTA
GACAACTCAGCTCTCTTCTGAGGTCTCACTC
GACAACTCAGCTCTTCTCTGCTATCTCGCTA
GACAACTCAGCTCTCTTCTGAGGTCTCACTC
GACAACTCAGCTCTTCTCTGCTATCTCGCTA
GACAACTCAGCTCTCTTCTGAGGTCTCACTC
GACAACTCATCTCTTCTCTGCTATCTCGCTA
GACAACTCAGCTCTCTTCTGAGGTCTCGCTC
GACAACTCATCTCTTCTCTGCTATCTCGCTA
GACAACTCAGCTCTCTTCTGAGGTCTCACTC
GACAACTCATCTCTTCTCTGCTATCTCGCTA
GACAACTCAGCTCTTTTCTGCTATCTCGCTA
GACAACTCAGCTCTCTTCTGAGGTCTCACTC
GACAACTCATCTCTTCTCTGCTATCTCGCTA
GACAACTCAGCTCTCTTCTGAGGTCTCGCTC
GACAACTCATCTCTTCTCTGCTATCTCGCTA
GACAACTCAGCTCTCTTCTGAGGTCTCGCTC
GACAACTCAGCTCTTCTCTGCTATCTCGCTA
GACAACTCAGCTCTCTTCTGAGGTCTCACTC
GACAACTCATCTCTTCTCTGCTATCTCGCTA
GACAACTCAGCTCTCTTCTGAGGTCTCACTC
GACAACTCATCTCTTCTCTGCTATCTCGCTA
GACAACTCAGCTCTTTTCTGCTATCTCGCTA
GACAACTCAGCTCTCTTCTGAGGTCTCACTC
GACAACTCATCTCTTCTCTGCTATCTCGCTA
GACAACTCAGCTCTCTTCTGAGGTCTCGCTC
GACAACTCATCTCTTCTCTGCTATCTCGCTA

Nr of selected repeats 42 Similarity 0.826558
Consensus:
GACAACTCAgCTCTttTCTGctaTCTCgCTa
>Cele-UNSB01_2:2065528-2066342 Satlength=815 Nr of Repeats=5 RepeatLength=162
seed=AGTTTTATGA
AGTTTTATGAAATAAAAAAGAATGACTCCATATGATTTTCCAGGACTTTTGCATTTTTGAAAAAATCAGCTTTTATATA
TTTTTATCATTAAAAAAAAAAAAATACTTCCGTACAAGCTATTATAATTTGATTAGCATATTCTGGTATTTTTGACTATG
CTGAAG
AGTTTTATGAAATAAAAAAGAATGACTCTATATGATTTTCCCATACTTTTGAATTTTTGAAAAAATCAGCTTTTATATA
TTTTTATCCATTTTTAAAAAATACTTCCGTACAAACTATTATACTTTGTTTAGCATTTTCTGGTATTTTTGACTATGCT
GAAG
AGTTTTATGAAATAAAAAAGAATGACTCTATATGATTTTCCCATACTTTTGAATTTTTGAAAAAATCAGCTTTTATATA
TTTTTATCCATTTTTAAAAAATACTTCCGTACAAACTATTATACTTTGTTTAGCATTTTCTGGTATTTTTGACTATGCT
GAAG
AGTTTTATGAAATAAAAAAGAATGACTCTATATGATTTTCCCATACTTTTGAATTTTTGAAAAAATCAGCTTTTATATA
TTTTTATCATTAAAAAAAAAAAAATACTTCCGTACAAGCTATTATAATTTGATTAGCATATTCTGGTATTTTTGACTATG
CTGAAG
AGTTTTATGAAACAAAAAAGAATGACTCCATATGATTTTCCCATACTTTTGAATTTTTGAAAAAATCAGCTTTTATATA
TTTTTATCCATTTTTAAAAAATACTTCCGTACAAACTATTATACTTTGTTTAGCATTTTCTGGTATTTTTGACTATGCT
AAAG
Nr of selected repeats 3 Similarity 0.983539
Consensus:
AGTTTTATGAAAtAAAAAAGAATGACTCtATATGATTTTCCCATACTTTTGAATTTTTGAAAAAATCAGCTTTTATATA
TTTTTATCCATTTTTAAAAAATACTTCCGTACAAACTATTATACTTTGTTTAGCATTTTCTGGTATTTTTGACTATGCT
gAAG
>Cele-UNSB01_2:2125481-2125594 Satlength=114 Nr of Repeats=5 RepeatLength=22
seed=TGGTCAGTGC
TGGTCAGTGCAAAAAAAAAACTAA
TGGTCAGTGCAAAAAATACTGAT
TGGTCAGTGCAAACAATTTTAT
TGGTCAGTGCAAACATTTTTAT
TGGTCAGTGCAAACATTTTTAT
Nr of selected repeats 3 Similarity 0.959596
Consensus:
TGGTCAGTGCAAACAtTTTTAT
>Cele-UNSB01_2:2168167-2168227 Satlength=61 Nr of Repeats=4 RepeatLength=11
seed=ACCGCAACGC
ACCGCAACGCA
ACCGCAACGCA
ACCGCAACGCA
ACCGCAACGCAACCGCACCTTAACGCG
Nr of selected repeats 3 Similarity 1.000000
Consensus:
ACCGCAACGCA
>Cele-UNSB01_2:2174137-2174267 Satlength=131 Nr of Repeats=5 RepeatLength=26
seed=TCAAGGCGAA
TCAAGGCGAAACGGGAGGTGAATCGC
TCAAGGCGAAACGGGAGGTGAATCGC
TCAAGGCGAAACGGGAGGTGAATCGA
TCAAGGCGAAACGGGAGGTGAATCGC
TCAAGGCGAAACGGGAGGTGAATCGC
Nr of selected repeats 5 Similarity 0.979487
Consensus:
TCAAGGCGAAACGGGAGGTGAATCGc

>Cele-UNSB01_2:2187430-2189107 Satlength=1678 Nr of Repeats=38 RepeatLength=43
seed=CGAGTTTGGC
CGAGTTTGGCCTACAGCAAATTTTGCAAATCCGCCAAATTTGGCGAGGTCGGCAAACAAAAAATTTGGCAAATTCGGCA
TATTTGC
CGAGTTTGGCCTACAGCAAATTTGGCAAAATCGGCAAATTTGC
CGAGTTTGGCCTACATCAAATTTTGCAAATTTAGCAAATTTGC
CGAGTTTGGCCTACATCAAATTTGGCCAATTCGGCAATTTTGC
CGAGTTTGGCAAACAAAAAATTTGGCAAATTCGGCAAATTTGC
CGAGTTTGGCAAACAAAAAATTTGACAAATTCGGCAAATTTGT
CGAGTTTGGCCTACAGCAAATTTTGCAAATCCGTCAAATTTGG
CGAGGTTGGCAAACAAAAAATTTGGCAAATTCGGCAAATTTGC
CGACTTTGGCCTACAGCAAATTTGGCAAAATCGGCAAATTTGC
CGAGTTTGGCCTACATCAAATTTTGCAAATTTAGCAAATTTGC
CGAGTTTGGCCTACATCAAATTTGGCCAATTCGGCAATTTTGC
CGAGTTTGGCAAACAAAAAATTTGGCAAATTCGGCAAATTTGC
CGAGTTTGGCAAACAAAAAATTTGACAAATTCGGCAAATTTGT
CGAGTTTGGCCTACAGCAAATTTTGCAAATCCGTCAAATTTGG
CGAGGTTGGCAAACAAAAAATTTGGCAAATTCGGCAAATTTGC
CGACTTTGGCCTACAGCAAATTTGGCAAAATCGGCAAATTTGC
CGAGTTTGGCCTACATCAAATTTTGCAAATTTAGCAAATTTGC
CGAGTTTGGCCTACATCAAATTTGGCCAATTCGGCAATTTTGC
CGAGTTTGGCAAACAAAAAATTTGGCAAATTCGGCAAATTTGC
CGAGTTTGGCAAACAAAAAATTTGACAAATTCGGCAAATTTGT
CGAGTTTGGCCTACAGCAAATTTTGCAAATCCGTCAAATTTGG
CGAGGTTGGCAAACAAAAAATTTGGCAAATTCGGCAAATTTGC
CGACTTTGGCCTACAGCAAATTTGGCAAAATCGGCAAATTTGC
CGAGTTTGGCCTACATCAAATTTTGCAAATTTAGCAAATTTGC
CGAGTTTGGCCTACATCAAATTTGGCCAATTCGGCAATTTTGC
CGAGTTTGGCAAACAAAAAATTTGGCAAATTCGGCAAATTTGC
CGAGTTTGGCAAACAAAAAATTTGACAAATTCGGCAAATTTGT
CGAGTTTGGCCTACAGCAAATTTTGCAAATCCGTCAAATTTGG
CGAGGTTGGCAAACAAAAAATTTGGCAAATTCGGCAAATTTGC
CGACTTTGGCCTACAGCAAATTTGGCAAAATCGGCAAATTTGC
CGAGTTTGGCCTACATCAAATTTTGCAAATTTAGCAAATTTGC
CGAGTTTGGCAAACAAAAAATTTGGCAAATTCGGCAAATTTGC
CGACTTTGGCCTACAGCAAATTTGGCAAAATCGGCAAATTTGC
CGAGTTTGGCCTACATCAAATTTTGCAAATTTAGCAAATTTGC
CGAGTTTGGCCTACATCAAATTTGGCCAATTCGGCAATTTTGC
CGAGTTTGGCAAACAAAAAATTTGGCAAATTCGGCAAATGTGC
CGAGTCTGGCAAACAGCAAATTTGGCCAATTCAGCAAATTTGC
CGATTTTGGCAAACAGGAAATTTGGCAAATCCGCCAAATTTGA
Nr of selected repeats 37 Similarity 0.823312
Consensus:
CGAGTTTGGCctACAncAAATTTgGCAAATTCGGCAAATTTGc
>Cele-UNSB01_2:2291158-2291371 Satlength=214 Nr of Repeats=8 RepeatLength=21
seed=TGTGCCCTGG
TGTGCCCTGGAGAGCTGTGGGA
TGTGCCCTGGAAAGCTGTGGAACGCAGATATATTTTTTCTGCGGTGTGCCGCGGAAAGCTGGCGG
TGTGCCCTGGAAAGCTGGTGG
TGCGCCCTGGAAAGCTGGCAG
TGTGCCCTGGAAAGCTGGCAG
TGTGCCCTGGAAAGCTGGCAG
TGTGCCCTGGAAAGCTGGCAG
TGTGCCCTGGAAAGCTGGCAG

Nr of selected repeats 6 Similarity 0.936508
Consensus:
TGTGCCCTGGAAAGCTGGCAG
>Cele-UNSB01_2:2296694-2296754 Satlength=61 Nr of Repeats=4 RepeatLength=10
seed=CAGCAGAGTG
CAGCAGAGTG
CAGCAGAGTG
CAGCAGAGTG
CAGCAGAGTGCAGCGAAGTACAGCAGTGTG
Nr of selected repeats 3 Similarity 1.000000
Consensus:
CAGCAGAGTG
>Cele-UNSB01_2:2366031-2366422 Satlength=392 Nr of Repeats=13 RepeatLength=11
seed=CTACAGTACC
CTACAGTACCTTTACAGCACTA
CTACAGTACCCCGACCATACCCCATCACTAACCTCAAACCAATATCCCTTCAAAAGGCGAAAAGTCAATTTTTCCCAAA
CTACAGTAATC
CTACAGTACTC
CTACAGTACCT
CTACAGTACTA
CTACAGTACCCCGACCATATCCCACCACTAACCTCAAACCAATATCCCTTCAAAAGGCGAAAAGTCAATTTTTCCCAAA
CTACAGTAATC
CTACAGTAACC
CTACAGTACCT
CTACAGTACTA
CTACAGTACCCCGACCATATCCCACCACTAACCTCAAACCAATATCCCTTCAAAAGGCGAAAAGTCAGTTTTTCCCAAA
CTACAGTAATC
CTACAGTACTC
CTACAGTACCT
CTACAGTACTA
Nr of selected repeats 9 Similarity 0.763889
Consensus:
CTACAGTACTn
>Cele-UNSB01_2:2379059-2380853 Satlength=1795 Nr of Repeats=9 RepeatLength=163
seed=TGAATTTTTG
TGAATTTTTGAAAATAATCTTCCGTACGAGCTACTATCAAAATTTATGAATCGATCCTTCGATTTTGCAAAAATCTTTG
CCGCACAGAAAGTTGGGCGGAGCGTCCTTTCGCAACACTGCCGCGCGGGGTTTGCCCCCGGCCCTTTTCCGCTGCTTTT
CTTGTTGATTTTTTCAATAAAGTTTCGATTTTTCTTTTTTTTTTTCAAATTGGATAACATTTTCATTCGAAAAAAAAAG
AAAAATCGAAAATTTATTGAAAAAATCAACAAGAAAAGCAGCGGAAAATGGCCGGGGGCAAACCCCGCGCGGCAGTGTT
GCGAAAGGACGCTCCTCCCAACTTTCTGTGCGGCAAAGATTTTTGCAAAATCGAAGGATCGATTCATAAATTTTGATAG
TAGCACAATTTGTTTGGTATTTTCTGGTACATTTGACTATGCTGAACAGTTTTATAAAATAAAAAAGAATGACTTCATA
TAATTTTGAGGTACTTT
TGAATTTTTGAATATATCAGCTTTTGGTTAAATTCTGCATTTTTTAAAAATAAACTTCCGTACGAGCTAGCACCTTTTT
TTAAGTATTTTCTGGTACATTTGACTATGCTGAACAGTTTTATAAAATAAAAAAGAATGACTTCATATAATTTTGAGGT
ACTTT
TGAATTTTTGAAAATAACAGCTTTTGGTTAAATTCTGCATTTTTTAAAAATAAACTTCCGTACGAGCTAGCACCTTTTT
TTAAGTATTTTCTGGTACATTTGACTATGTTGAACAGTTTTATAAAATAAAAAAAAATGACTTCATATGATTTTGAGGT
ACTTT
TGAATTTTTGAAAATAAGAGCTTGTGGTTAAATTCTGCATTTTTTAAAAATAAACTTCCGTACGAGCTATTGTATATTT
TTTGGTATTTTCTGGTACATTTGACTATGATGAACAGTTTTATGAAATAAAAAAGAATGACTTCATATGATTTTGAGGT
ACTTT
TGAATTTTTGAAAATAAGAGCTTTTGGTTAAATTCTGCATTTTTTAAAAATAAACTTCCGTACGAGCTAGCACCTTTTT
TTAAGTATTTTCTGGTACATTTGACTATGCTGAACAGTTTTATAAAATAAAAAAGAATGACTTCATATAATTTTGAGGT
ACTTT

TGAATTTTTGAAAAATACAGCTTTTGGTTAAATTCTGCATTTTTTAAAAATAAACTTCCGTACGAGCTAGCACCTTTTT
TTAAGTATTTTCTGGTACATTTGACTATGCTGAACAGTTTTATAAAATAAAAAAAAATGACTTCATATGATTTTGACGT
ACTTT
TGAATTTTTGAAAATAAGAGCTTTTGGTTAAATTCTGCATTTTTTAAAAATAAACTTCCGTACGAGCTAGCACCTTTTT
TTAAGTATTTTCTGGTACATTTGACTATGCTGAACAGTTTTATAAAATAAAAAAGAATGACTTCATATGATTTTGACGT
ACTTT
TGAATTTTTGAAAATAACAGCTTTTGGTTAAATTCAGCATTTTTTTTAATAAACTTCCGTACGAGCTATTGTATATTTT
TTGGTATTTTCTGGTACATTTGACTATGATGAACAGTTTTATGAAATAAAAAAGAATCGCTTCATATGATTTTTCAGTA
CTTT
TGAATTTTTGAATATATCAGCTTTTGGTTAAATTCTGCATTTTTTTTAAATAAACTTTGGTACGAGCTAGCATTCTTTG
TTTAGTATTTTCTGGTATATTTGACTATGTTGAACAGTTTTATAAAATAAAAAAGAATGACTTCATATAATTTTGAGGT
ACTTT
Nr of selected repeats 7 Similarity 0.906387
Consensus:
TGAATTTTTGAAaATAacAGCTTTTGGTTAAATTCTGCATTTTTTAAAAATAAACTTCCGTACGAGCTAGCAcCTTTTT
TTaAGTATTTTCTGGTACATTTGACTATGcTGAACAGTTTTATAAAATAAAAAAgAATGACTTCATATgATTTTGAgGT
ACTTT
>Cele-UNSB01_2:2403604-2403679 Satlength=76 Nr of Repeats=5 RepeatLength=15
seed=TCGTGGCGAG
TCGTGGCGAGACCCA
TCGTTGCGAGACCCT
TCGTGGCGAGACCCA
TCGTGGCGAGACCCA
TCGTGGCGAGACCCA
Nr of selected repeats 5 Similarity 0.928889
Consensus:
TCGTgGCGAGACCCa
>Cele-UNSB01_2:2547654-2548528 Satlength=875 Nr of Repeats=24 RepeatLength=35
seed=CCGGAAATTT
CCGGAAATTTTCATTTTCGGCAATTTGCCGATTTG
CCGGAAGTTTTAAATTCCGGCACTTTGTCGGTTTGCCGAAAACTTTTGTTTTCGGAAATGTGCCGCTTTG
CCGGAAATTTTCATTTTCAACGATTTGCCGATTTG
CCGGAAATTTTAAACT
CCGGCAATTTGCCGGTTTG
CCGGAAATTTTCATTTTTGGTGATTTGCTGATTTT
CCGGAAATTTTCATTTTTCGTGATTTGCCGATTGT
CCGGAAATTTTCATTTTTGGTGATTTTCCGATTGT
CCGGAAATTTTCATTTTCGACAATTTGCCGATTTG
CCGGAAATTTTAAATTCCGGCACTTTGCCGGTTTGCCGAAAACTTTTATTTTCGGAAATGTGCCGCTTTG
CCGGAAATTTTCATTTTCAACGATTTGCCGATTTG
CCGGAAATTTTAAACT
CCGGCAATTTGCCGGTTTG
CCGGAAATTTTCATTTTTGGTGATTTGCTGATTTT
CCGGAAATTTTCATTTTTCGTGATTTGCCGAGTGT
CCGGAAATTTTCATTTTTGGTGATTTTCCGATTGT
CCGGAAATTTTCATTTTCGACAATTTGCCGATTTG
CCGGAAATTTTAAATTCCGGCACTTTGCCGGTTTGCCGAAAACTTTTATTTTCGGAAATGTGCCGATTTG
CCGGAAATTTCATCATCGGCACATTATCGATTTG
CCGGAAATTTTCATCATCGGCACATTGCCGATTTG
CCGGAAATTTTCATTTTTGGTGATTTGCTGATTTG
CCGGAAATTTTCATTTTCGGCGAATTGCCGATTTG
CCGGAAATTTTCATCATCGGCACATTGCCGGTTTG
CCGGAAATTTTCATTTTTGGTGATGTGCTGATTTG
Nr of selected repeats 16 Similarity 0.798413

Consensus:
CCGGAAATTTTCATTTTcggcgATTTGCcGATTtg
>Cele-UNSB01_2:2568294-2568366 Satlength=73 Nr of Repeats=6 RepeatLength=12
seed=TTAGGCTTAG
TTAGGCTTAGGA
TTAGGCTTAGGC
TTGGGCTTAGGT
TTAGGTTTAGTC
TTAGTCTTAGGC
TTAGGCTTAGGC
Nr of selected repeats 6 Similarity 0.785185
Consensus:
TTAGGCTTAGGc
>Cele-UNSB01_2:2602717-2603767 Satlength=1051 Nr of Repeats=24 RepeatLength=31
seed=CTGAAATTCC
CTGAAATTCCAATTTTCCAGTGTAAAAATGT
CTGAAATTCCAATTTTCCACAGTAAAAATGC
CTGAAATTCCAATTTTCCAGTGTAAAAATGT
CTGAAATTCCGATTTTCCAGTATAGAAATGT
CTGAAATTTCGATTTTCCAGTGTAAAAATGTCTGGAATTTCGATTTTCCAGTATAGAAATGT
CTGAAATTCCGATTTTCCAGTGTAAAAATGTCTGGAATTTCAAATTTCCACTGAAAAATGT
CTGAAATTCCGAATTTCCAGTGCAAAAATGT
CTGAAATTTCGATTTTCCAATGTAAAAATCT
CTGAAAATCCGATTTTCCAGCGTAAAACGTCTGAAATTTTGATTTTCCACTGTAAAAATGTCTAAAATCCCGAATTTCC
ACTGAAAAAATGT
CTGAAATTCCGATTTTCCAGTGTAAAAATGC
CTGAAATTCCGAATTTCCAGTGCAAAAATGTCAGAAATTTTGATTTTCCAATGTAAAAATCT
CTGAAATTCCAATTTTCCAGTGTAAAAATGT
CTGAAATTCCAATTTTCCACAGTAAAAATGC
CTGAAATTCCAATTTTCCAGTGTAAAAATGT
CTGAAATTCCGATTTTCCAGTATAGAAATGT
CTGAAATTTCGATTTTCCAGTGTAAAAATGTCTGGAATTTCGATTTTCCAGTATAGAAATGT
CTGAAATTCCGATTTTCCAGTGTAAAAATGTCTGGAATTTCAAATTTCCACTGAAAAATGT
CTGAAATTCCGAATTTCCAGTGCAAAAATGT
CTGAAATTTCGATTTTCCAATGTAAAAATCT
CTGAAAATCCGATTTTCCAGCGTAAAACGTCTGAAATTTTGATTTTCCACTGTAAAAATGTCTAAAATCCCGAATTTCC
ACTGAAAAAATGT
CTGAAATTCCGATTTTCCAGTGTAAAAATGC
CTGAAATTCCGAATTTCCAGTGCAAAAATGTCAGAAATTTTGATTTTCCAATGTAAAAATCT
CTGAAATTCCAATTTTCCACTGTTAAAATGT
CTGAAATTCCAATTTTCCAATGTAAAAATCT
Nr of selected repeats 16 Similarity 0.855556
Consensus:
CTGAAATTCCaATTTTCCAgTGTAAAAATGt
>Cele-UNSB01_2:2619434-2619682 Satlength=249 Nr of Repeats=8 RepeatLength=31
seed=AGAGCTGAGT
AGAGCTGAGTTGTCTAGCGAGATAGCAGAGA
AGAGATGAGTTGTCGAGCGAGACCTCAGAAG
AGAGCTGAGTTGTCTAGCGAGATAGCAGAGA
AGAGATGAGTTGTCGAGTGAGACCTCAGAAG
AGAGCTGAGTTGTCTAGCGAGATAGCAGAGA
AGAGCTGAGTTGTCGAGCGAGACCTCAGAAG
AGAGCTGAGTTGTCTAGCGAGATAGCAGAGA
AGAGCTGAGTTGTCGAGCGAGACCTCAGAAG

Nr of selected repeats 8 Similarity 0.806548
Consensus:
AGAGcTGAGTTGTCgAGCGAGAcagCAGaAGa
>Cele-UNSB01_2:2625510-2625733 Satlength=224 Nr of Repeats=7 RepeatLength=32
seed=AATTCCCGCC
AATTCCCGCCAAAATTTTTCACAGAAAATTTG
AATTCCCGCCAAAAAATTTTAAAGAAAATTTA
AATTCCCGCCAAAAATTTTCAGAGAAAATTTG
AATTCCCGCCAAAAATGTTTCAGAAATTTTG
AATTCCCGCCAAAAAATTTTAAAGAAAATTTA
AATTCCCGCCAAAAATTTTCAGAGAAATTTTG
AATTCCCGCCGAAAAATTTCAAAGAAAATTTA
Nr of selected repeats 6 Similarity 0.855556
Consensus:
AATTCCCGCCAAAAAaTTTcAaAGAAAATTTa
>Cele-UNSB01_2:2625547-2626102 Satlength=556 Nr of Repeats=7 RepeatLength=32
seed=CCGCCAAAAA
CCGCCAAAAAATTTTAAAGAAAATTTAAATTC
CCGCCAAAAATTTTCAGAGAAAATTTGAATTC
CCGCCAAAAATGTTTCAGAAATTTTGAATTC
CCGCCAAAAAATTTTAAAGAAAATTTAAATTC
CCGCCAAAAATTTTCAGAGAAATTTTGAATTC
CCGCCGAAAAATTTCAAAGAAAATTTAAATTC
CCGCCAAAATGTAGGGGTCTCGCCACGATGGGTCTCGCCACGATGGGTCTCGCCACGATGGGTCTCGCCACGAAGGGTC
TCGCATCGATGGGTCTCGCCACGAAGGGTGACTGGCCCCGCCCACAACTCTCAGTGACTTTAGGGGTGACGTCACAGAC
TACAAAGACTACATAGACTACAAACTATGGACAGACCCACACTATTTTTTTATATATAAGTAATGATAATGATATGATC
ACATGTTTGTCCTCTTAAATCCTTTGATATTCTTTTTTGCCTTGTCTTCAAAATGTGAACGAATAAATACATATAATAT
AAATTATTTTCTTCAATTCTGATTTTCTGATTCCAAAAGATTGCAAAA
Nr of selected repeats 5 Similarity 0.866667
Consensus:
CCGCCaAAAAaTTTcAaAGAAAaTTTaAATTC
>Cele-UNSB01_2:2648748-2648862 Satlength=115 Nr of Repeats=6 RepeatLength=19
seed=AAAATCGATA
AAAATCGATAATTTTGAGA
AAAATCAATAGTTTCATCG
AAAATAGATAATTTCGTCG
AAAATAGATAATTTCATGG
AAAATCGATAATATCTTCA
AAAATCGATAACTTAATTG
Nr of selected repeats 6 Similarity 0.601058
Consensus:
AAAATcGATAATTTcaTcg
>Cele-UNSB01_2:2655599-2656120 Satlength=522 Nr of Repeats=10 RepeatLength=14
seed=CTACAAACTA
CTACAAACTATACTCTACAGAATACAATTTGAGCACAAAAAACTACAAAAAAAATTTTGGCAATAGCTGAAATAGTTTC
CTTGTTTTTTCTCGCGATTTTATTGGAGAAATTCGATTTATTTTGAAACTCGAATTTTGAATAACTTGTTTTGTTCTTG
AGTTTAAATTCAGTGACAG
CTACAACCTATAAA
CTACAAACTACAAG
CTACAAACTACAAAATACATA
CTACAAACTAGAAACTACAACACAATAGAAAATGACTCTCATTTTGAAAAAAAAATGTTAGAATAGGTAGCCACTGTAG
TTAGAAAAGGTCACAACAATGAACAAGAATGAGCCCTAAA
CTACAGACTACAGA
CTACAAACTACAAA

CTACAGACTACAAACTACAACAACATAGAAAATAATTCTCATTTTGAAAAAAGAAATGTGAGAAGCGGTGGCCACTGTT
GTTAGAAAAGATCACAACAATGTTCAATAATGAACACTAAG
CTACAGACTACAAC
CTACAAACTACAAA
Nr of selected repeats 6 Similarity 0.796825
Consensus:
CTACAaACTACAAa
>Cele-UNSB01_2:2657840-2658242 Satlength=403 Nr of Repeats=31 RepeatLength=13
seed=TAAGCCTAAG
TAAGCCTAAGCTC
TAAGCCTAAGCTC
TAAGCCTAAGCTC
TAAGCCTAAGCTC
TAAGCCTAAGCTC
TAAGCCTAAGCTC
TAAGCCTAAGCTC
TAAGCCTAAGCTC
TAAGCCTAAGCTA
TAAGCCTAAGCTC
TAAGCCTAAGCTC
TAAGCCTAAGCTC
TGAGCCTAAGCTC
TAAGCCTAAGCTC
TAAGCCTAAGCTC
TAAGCCTAAGCTC
TAAGCCTAAGCTC
TAAGCCTAAGCC
TAAGCCTAAGCC
TAAGCTCTAAGCC
TAAGCTCTAAGCC
TAAGCTCTAAGCC
TAAGCTCTAAGCTC
TAAGCCTAAGCTC
TAAGCCTAAGCTC
TAAGCCTAAGCTC
TAAGCCTAAGCTC
TAAGCCTAAGCTC
TAAGCCTAAGCTC
TAAGCCTAAGCTC
TAAGCCTAAGCTC
Nr of selected repeats 28 Similarity 0.872449
Consensus:
TAAGCCTAAGCTC
>Cele-UNSB01_2:2659185-2659251 Satlength=67 Nr of Repeats=5 RepeatLength=12
seed=CCTAAGCCTA
CCTAAGCCTAAG
CTTAAGCCTATG
CATAAGCCTAAGCCTAGT
CCTAAGCCTAAG
CCTAAGCCTAAC
Nr of selected repeats 4 Similarity 0.833333
Consensus:
CcTAAGCCTAag

>Cele-UNSB01_2:2738496-2739194 Satlength=699 Nr of Repeats=7 RepeatLength=95
seed=TTCGGGCCTG
TTCGGGCCTGCGGCCCTCAACCTGACTTATTGGGCTCAGAATAGGTTTCACGGTTGCCCAACACTTCTTCGTGTATTGC
CTGTTTTGCTGAGAATGTGGTTAGAAGTTCCCTACAGTTTTAATGAGTA
TTCGGGCCTGCGGCCCTCAAACCTGGTTTGTAGGCTTGAGCGAATAACAATCTGCTTGGAATAGGATTAAAGTTGGCCC
ACAGTTTCCAAGAGTG
TTCGGGCCTGCGGCCCTCAAACCTGGTTTGTAGGCTTGAGCGAATAACAGTCTGCTTGGAATAGGATTAAAGCTGGCCC
ACAGTTTCCATGAGTA
TTCGGGCCTGCGGCCCTCAAACCTGGTTTGTAGGCTTGAGCGAATAACAATCTGCTTGGAATAGGATTAAAGCTGGCCC
ACAGTTTCCATGAGTA
TTCGGGCCTGCGGCCCTCAAACCTGGTTTGTAGGCTTGAGCGAATAACAATCTGCTTGGAATAGGATTAAAGCTGGCCC
ACAGTTTCCATGAGTA
TTCGGGCCTGCGGCCCTCAAACCTGGTTTGTAGGCTTGAGCGAATAACAATCTGCTTGGAATAGGATTAAAGCTGGCCC
ACAGTTTCCATGAGTA
TTCGGGCCTGCGGCCCTCAAACCTGGTTTGTAGGCTTGAGCGAATAACAATCTGCTTGGAATAGGATTAAAGCTGGCCC
ACAGTTTCCATGAGTA
Nr of selected repeats 6 Similarity 0.981287
Consensus:
TTCGGGCCTGCGGCCCTCAAACCTGGTTTGTAGGCTTGAGCGAATAACAATCTGCTTGGAATAGGATTAAAGCTGGCCC
ACAGTTTCCATGAGTA
>Cele-UNSB01_2:2756689-2756749 Satlength=61 Nr of Repeats=4 RepeatLength=12
seed=GCCTAAGCCT
GCCTAAGCCTAAGCCCAAACCTAG
GCCTAAGCCTAA
GCCTATGCCTAA
GCCTAATCCTGA
Nr of selected repeats 3 Similarity 0.777778
Consensus:
GCCTAagCCTaA
>Cele-UNSB01_2:2758878-2759540 Satlength=663 Nr of Repeats=24 RepeatLength=22
seed=ACTACAGTAA
ACTACAGTAATGCTACAGTACC
ACTACAGTAATTCTACGTCGCT
ACTACAGTAACCTAACAATGACCCTACAGTAGCTCTATGGTACC
ACTACAGTAACCCTACATCACC
ACTACAGTAATCCTACAGTACCATAACAGTAGACCTCCATTGCCAATACATTAATCCAGTATC
ACTACAGTAAGCCTAGAGTATC
ATTACAGTAACTATACAGTACT
ACTACAGTAACCCTACATCAGC
ACTACAGTAATTCTACAATACC
ACTACAGTAACCCTACATCACC
ACTACAGTAATCCTACAGTACCATAACAGTAGACCTCCATTGCCAATACATCAATCCAGTATC
ACTACAGTAAGCCTAGAGTATC
ATTACAGTAACTATACAGTACT
ACTACAGTAACCCTACATCAAC
ACTACAGTAATTCTACAATACC
ACTACAGTAACCCTACATCACC
ACTACAGTACCATAACAGTAGACCTCCATTGCCAATACATTAATCCAGTATC
ACTACAGTAAGCCTAGAGTACC
ATTACAGTAACTATACAGTACT
ACTACAGTAACCCTACATCAGC
ACTACAGTAATTCTACAATACC
ACTACAGTAACCTAACAGTCATC
CTACAGTAACTCTATAGAGCC

ACTACCGTAATCCTACATCACC
Nr of selected repeats 18 Similarity 0.729847
Consensus:
ACTACAGTAAccCTACAntAcc
>Cele-UNSB01_2:2787652-2787836 Satlength=185 Nr of Repeats=11 RepeatLength=16
seed=CACGTAGGCA
CACGTAGGCACGTAGG
CACGTAGGCAAGTAGG
CACGTAGGCACGTAGG
CACGTAGGCACATAGACACATAGA
CACGTAGGCACGTAGG
CACGTAGGCACGTAGG
CAAGTAGGCACGTAGG
CACGTAGGCACGTAGG
CACGTAGGCACGTAGG
CACGTAGGCACGTAGG
CACGTAGGCACGTAGG
Nr of selected repeats 10 Similarity 0.966667
Consensus:
CACGTAGGCACGTAGG
>Cele-UNSB01_2:2836927-2837967 Satlength=1041 Nr of Repeats=6 RepeatLength=144
seed=TGGCCTAGAA
TGGCCTAGAAATCCAAGTTTGCAAAAGTTAGTCCACCAACTTCAAATGCCTATAACTTTGCGGAAAATCAAGATTTTTC
AGTGATTTTAACGTAACTATGATGCTACTGGCTTTGCGCATCTTTTTGCCCTACTTAACGTTTGG
TGGCCTAGAAATCCAAGTTTGCAAAAGTTAGGCCATCAACTTCAAATGCCTATAACTTTGCGGAAAATCTATATTTCAA
ATTAATTTTTGAACTTCTGTGATGCCATTAACATTGCGCATTTTTTTGGCCTAGTTAACGTTTGA
TGACCTAGAAATTTAAAATTTGCAAAAGTTAGGTTATCAACTCAAAATATTTCTATCTTTGTGAAAAATCAAGATTTTT
CTGTGATTTTTACATTAACTTCATGGCATTTGTTTAGCGCATTTTTTTTTGAGTACAGACCGATTGG
TGGCCTAGAAATCCACAGTTCGGCCACCAAATTCAATCAACCATAACTTGGCTTAAAGTTCATATTTTTCCGTTCTGAA
AAAAACATTTTGGTTCAAAAACTCGTGCACTTTCCTTCTAATTTCCTAAAATTGCAGACAATCACCAACTTGAAAACCT
ACCACTGTTACGAATGTCGTCAAGATGTTCAGTGTGTTCTGGGCGCTGAAAATCTCACGGCAGGACCACTAATCAGTCA
TTTGAGTACCAAACACTCCTTCCAGTGTGTTCCATGGAAATGTGATGAGTGTGGATACAAAAATGCGGTCCAGGTAAGC
GG
TGGCCTAGAAATCTCGATTTGCAAAAGTTAGGCCACCAACTTTAAATTTCTGTAATTCAGCTGAAAATCGATATTTTCC
TATGTTTTTAGTGCCAAGATGTAGCGAATTATGTAACGCTACTTTTTGGGGTACTTAACAATTGG
TGGCCTAGAAATCCCAACTTGCAAAAGTTAGGCCACCAACTTCAAATCGCCGTTACTCAGCTGAAAATCAATATTTTCC
ATTGTTCTTGATGCTAAAATGTAGCCAATTTTGTAGCGCAACTTTTTGGGGTACTTAACAATTGG
Nr of selected repeats 4 Similarity 0.634815
Consensus:
TGGCCTAGAAATCnCaAgtTTGCAAAAGTTAGgCCAcCAACTTcAAATgcCtaTaAcTcaGCgGAAAATCaAtATTTtc
catTgaTtTTaacTgCtaagATGtagCcAaTtacgTagCGCaacTTTTTGgccTAcTTAACaaTTGg
>Cele-UNSB01_2:2842834-2842918 Satlength=85 Nr of Repeats=6 RepeatLength=12
seed=CCTAAGCCTA
CCTAAGCCTAGT
CCTAAGTCTAAG
CCTAAACCTAAGCCTAATTCTAAGG
CCAAGCCTAAG
CCTAAGCCTACG
CCTAAGCCTACG
Nr of selected repeats 4 Similarity 0.754274
Consensus:
CCTAAGcCTAcG
>Cele-UNSB01_2:2923591-2929183 Satlength=5593 Nr of Repeats=231 RepeatLength=21
seed=CCAAAAATTG

CCAAAAATTGTGATTTTTAGT
CCAAAAATTGAGATTTTTAGT
CCTAAAATTGAGGCTTTTAGT
CCAAAAATTGAGATTTTTAGT
CCAAAAATTGTGATTTTTAGT
CCAAAAATTGAGATTTGTAGT
CCTAAAATTGTGATTTTTAGT
CCATAAATTGAGGTTTTTAGT
CCAAAAATTGTGATTTTTAGT
CCAAAAATTGAGATTTTTAGT
CCAAAAATTGTGATTTTTAGT
CCTAAAATTGAGGCTTTTAGT
CCAAAAATTGAGATTTATAGTCCTAAAATCGAGGTTTTTAGT
CCAAAAATTGAGATTTTTAGT
CCAAAAATTGTGATTTTTAGT
CCAAAAATTGAGATTTTTAGT
CCTAAAATTGAGGCTTTTAGT
CCAAAAATTGAGATTTTTAGT
CCAAAAATTGAGATTTGTAGT
CCTAAAATTGTGATTTTTAGT
CCAAAAATTGAGATTTGTAGTCC
CCAAAAATTGAGGTTTATAGT
CCTAAAATTGAGATTTATAGT
CCTAAAATTGAGGCTTTTAGT
CCAAAAATTGAGATTTTTAGT
CCAAAAATTGAGATTTGTAGTCC
CCAAAAATTGAGGTTTATAGT
CCTAAAATTGAGATTTATAGT
CCTAAAATTGAGGCTTTTAGT
CCAAAAATTGAGGTTTATAGT
CCAAAAATTGAGATTTTTAGT
CCATAAATTGAGGCTTTTAGG
CCTAAAATTGAGGTTTATAGT
CCTAAAATTGAGATTTTTAGT
CCTAAAATTGAGGCTTTTAGT
CCAAAAATTGAGATTTTTAGT
CCAAAAATTGTGATTTTTAGT
CCAAAAATTGAGATTTGTAGT
CCTAAAATTGAGGCTTTTAGT
CCAAAAATTGAGGTTTTTAGT
CCAAAAATTGTGATTTTTAGT
CCAAAAATTGAGATTTTTAGT
CCAAAAATTGTGATTTTTAGT
CCTAAAATTGAGGCTTTTAGT
CCAAAAATTGAGATTTATAGTCCTAAAATCGAGGTTTTTAGT
CCAAAAATTGAGATTTTTAGT
CCAAAAATTGTGATTTTTAGT
CCAAAAATTGAGATTTTTAGT
CCTAAAATTGAGGCTTTTAGT
CCAAAAATTGAGATTTTTAGT
CCAAAAATTGAGATTTGTAGT
CCTAAAATTGTGATTTTTAGT
CCAAAAATTGAGATTTGTAGTCC
CCAAAAATTGAGGTTTATAGT

CCTAAAATTGAGATTTATAGT
CCTAAAATTGAGGCTTTTAGT
CCAAAAATTGAGATTTTTAGT
CCAAAAATTGAGATTTGTAGTCC
CCAAAAATTGAGGTTTATAGT
CCTAAAATTGAGATTTATAGT
CCTAAAATTGAGGCTTTTAGT
CCAAAAATTGAGGTTTATAGT
CCAAAAATTGAGGCTTTTAGT
CCAAAAATTGAGATTTTTAGT
CCAAAAATTGAGATTTATAGTCCTAAAATCGAGGTTTTTAGT
CCAAAAATTGAGGTTTGTAGT
CCTAAAATTGTGATTTTTAGT
CCATAAATTGAGGTTTTTAGT
CCAAAAATTGAGGTTTGTAGT
CCTAAAATTGTGATTTTTAGT
CCAAAAATTGAGATTTATAGT
CCTAAAATTGAGGCTTTTAGT
CCAAAAATTGAGGTTTATAGT
CCAAAAATTGAGATTTTTAGT
CCATAAATTGAGGCTTTTAGG
CCTAAAATTGAGGCTTTTAGT
CCAAAAATTGAGGCTTTTAGT
CCAAAAATTGAGATTTATAGTCCTAAAATCGAGGTTTTTAGT
CCAAAAATTGAGGTTTGTAGT
CCTAAAATTGTGATTTTTAGT
CCAAAAATTGAGGTTTATAGT
CCAAAAATTGAGATTTTTAGT
CCATAAATTGAGGCTTTTAGG
CCTAAAATTGAGGTTTATAGT
CCTAAAATTGAGTTTTTTAGG
CCTAAAATTGAGGCTTTTAGT
CCAAAAATTGAGATTTTTAGT
CCAAAAATTGAGATTTATAGTCCTAAAATCGAGGTTTTTAGT
CCAAAAATTGAGGTTTGTAGT
CCTAAAATTGTGATTTTTAGT
CCATAAATTGAGGTTTTTAGT
CCAAAAATTGAGGTTTGTAGT
CCTAAAATTGTGATTTTTAGT
CCAAAAATTGAGATTTATAGT
CCTAAAATTGAGGCTTTTAGT
CCAAAAATTGAGGTTTATAGT
CCAAAAATTGAGATTTTTAGT
CCATAAATTGAGGCTTTTAGG
CCTAAAATTGAGGCTTTTAGT
CCAAAAATTGAGGCTTTTAGT
CCAAAAATTGAGATTTATAGTCCTAAAATCGAGGTTTTTAGT
CCAAAAATTGAGGTTTGTAGT
CCTAAAATTGTGATTTTTAGT
CCAAAAATTGAGGTTTATAGT
CCAAAAATTGAGATTTTTAGT
CCATAAATTGAGGCTTTTAGG
CCTAAAATTGAGGTTTATAGT
CCTAAAATTGAGTTTTTTAGG

CCTAAAATTGAGGCTTTTAGT
CCAAAAATTGAGATTTTTAGT
CCAAAAATTGAGTTTTGAAGACCACAAAAATTTAGGTTTTTAGT
CCATAAATTGAGGCTTTTAGT
CCTAAAATTGAGGTTTATAGT
CCTAAAATTGTGATTTTTAGT
CCAAAAATTGAGATTTGTAGTCC
CCAAAAATTGAGGTTTATAGT
CCTAAAATTGAGGTTTATAGT
CCTAAAATTGAGGCTTTTAGT
CCAAAAATTGAGATTTGTAGTCC
CCAAAAATTGAGGTTTATAGT
CCAAAAATTGAGATTTATAGT
CCTAAAATTGAGGCTTTTAGT
CCAAAAATTGAGGTTTATAGT
CCAAAAATTGAGATTTTTAGT
CCATAAATTGAGGCTTTTAGG
CCTAAAATTGAGGCTTTTAGT
CCAAAAATTGAGGCTTTTAGT
CCAAAAATTGAGATTTATAGTCCTAAAATCGAGGTTTTTAGT
CCAAAAATTGAGGTTTGTAGT
CCTAAAATTGTGATTTTTAGT
CCAAAAATTGAGGTTTATAGT
CCAAAAATTGAGATTTTTAGT
CCATAAATTGAGGCTTTTAGG
CCTAAAATTGAGGTTTATAGT
CCTAAAATTGAGTTTTTTAGG
CCTAAAATTGAGGCTTTTAGT
CCAAAAATTGAGATTTTTAGT
CCAAAAATTGAGATTTATAGTCCTAAAATCGAGGTTTTTAGT
CCAAAAATTGAGGTTTGTAGT
CCTAAAATTGTGATTTTTAGT
CCAAAAATTGAGATTTTTAGT
CCAAAAATTGAGTTTTGAAGACCACAAAAATTTAGGTTTTTAGT
CCATAAATTGAGGCTTTTAGT
CCTAAAATTGAGGTTTATAGT
CCTAAAATTGTGATTTTTAGT
CCAAAAATTGAGATTTGTAGTCC
CCAAAAATTGAGGTTTATAGT
CCTAAAATTGAGGTTTATAGT
CCTAAAATTGAGGCTTTTAGT
CCAAAAATTGAGATTTGTAGTCC
CCAAAAATTGAGGTTTATAGT
CCAAAAATTGAGATTTATAGT
CCTAAAATTGAGGCTTTTAGT
CCAAAAATTGAGGTTTATAGT
CCAAAAATTGAGATTTTTAGT
CCATAAATTGAGGCTTTTAGG
CCTAAAATTGAGGCTTTTAGT
CCAAAAATTGAGGCTTTTAGT
CCAAAAATTGAGATTTATAGTCCTAAAATCGAGGTTTTTAGT
CCAAAAATTGAGGTTTGTAGT
CCTAAAATTGTGATTTTTAGT
CCAAAAATTGAGGTTTATAGT

CCAAAAATTGAGATTTTTAGT
CCATAAATTGAGGCTTTTAGG
CCTAAAATTGAGGTTTATAGT
CCTAAAATTGAGTTTTTTAGG
CCTAAAATTGAGGCTTTTAGT
CCAAAAATTGAGATTTTTAGT
CCAAAAATTGAGATTTATAGTCCTAAAATCGAGGTTTTTAGT
CCAAAAATTGAGGTTTGTAGT
CCTAAAATTGTGATTTTTAGT
CCATAAATTGAGGTTTTTAGT
CCAAAAATTGAGGTTTGTAGT
CCTAAAATTGTGATTTTTAGT
CCAAAAATTGAGATTTATAGT
CCTAAAATTGAGGCTTTTAGT
CCAAAAATTGAGGTTTATAGT
CCAAAAATTGAGATTTTTAGT
CCATAAATTGAGGCTTTTAGG
CCTAAAATTGAGGCTTTTAGT
CCAAAAATTGAGGCTTTTAGT
CCAAAAATTGAGATTTATAGTCCTAAAATCGAGGTTTTTAGT
CCAAAAATTGAGGTTTGTAGT
CCTAAAATTGTGATTTTTAGT
CCAAAAATTGAGGTTTATAGT
CCAAAAATTGAGATTTTTAGT
CCATAAATTGAGGCTTTTAGG
CCTAAAATTGAGGTTTATAGT
CCTAAAATTGAGTTTTTTAGG
CCTAAAATTGAGGCTTTTAGT
CCAAAAATTGAGATTTTTAGT
CCAAAAATTGAGTTTTGAAGACCACAAAAATTTAGGTTTTTAGT
CCATAAATTGAGGCTTTTAGT
CCTAAAATTGAGGTTTATAGT
CCTAAAATTGTGATTTTTAGT
CCAAAAATTGAGATTTGTAGTCC
CCAAAAATTGAGGTTTATAGT
CCTAAAATTGAGGTTTATAGT
CCTAAAATTGAGGCTTTTAGT
CCAAAAATTGAGATTTGTAGTCC
CCAAAAATTGAGGTTTATAGT
CCAAAAATTGAGATTTATAGT
CCTAAAATTGAGGCTTTTAGT
CCAAAAATTGAGGCTTTTAGT
CCAAAAATTGAGATTTTTAGT
CCTAAAATTGAGATTTATAGT
CCTAAAATTGAGATTTATAGT
CCTAAAATTGAGGCTTTTAGT
CCAAAAATTGAGGCTTTTAGT
CCAAAAATTGAGATTTTTAGT
CCAAAAATTGAGTTTTGTAGACCACAAAAATTTAGGTTTTTAGT
CCATAAATTGAGGCTTTTAGT
CCTAAAATTGAGGTTTATAGT
CCTAAAATTGTGATTTTTAGG
CCTAAAATTGTGATTTTTAGT
CCGAAAATTGAGATTTGTAGTCC

CCAAAATTTGAGGTTTATAGT
CCTAAAATTGAGGTTTATAGT
CCTAAAATTGAGGCTTTTAGT
CCAAAAATTGAGGTTTATAGT
CCTAAAATTGAGATTTTTTAGT
CCAAAAATTGAGATTTGTAGT
CCTAAAATTGAGGTTTTTAGT
CCAAAAATTGAGATTTGTAGT
CCTAAAATTGAGATTTTCTTTTGGATCCAAAAAACCAAAATTTCGAGCTTTTTGTGTTTGTCAAGCTCAATTTTGCTCC
AATTTGCCGGAAAATAGCTCAATTTTTTGGCCAAAATTCACCTGAAATCTGCCATTCGGTGTTAGCATAAGAATTGCGG
GCGGTTTGAATGGAAAATCTTTGGGAAAAAGCAATTTTCCCATATAAATTCCACCTTCGTACGGTGTTTTTGGCGCTCC
AATGATAATATATCGCCATTCGAGAATATTCGTCTCCAGTGGTGCAGCTTTCATGAATGGAACGGGTTCTCTGCAAAAA
ATTAGGCTTTTTAGG
CCAAAAATTGAAATTTTTAGT
CCAAAATTTGGGCTTTTTAGGCTAGAAATTGAGTGTTTTAGTATAAAAGTTCAGGTTTTTAGCCTGAAAAATAGGGGTT
TTAGT
CCAAAAATTAAGGGTTTTAGG
CCACAAATTGAGGGTTTAAAACCTGAAAATTGAGGTTTTCAGG
CCAAAAATTGACATTTATAAG
CCAAAAAATGAGATTTCAATA
Nr of selected repeats 201 Similarity 0.731595
Consensus:
CCaAAAATTGAGgtTTtTAGT
>Cele-UNSB01_2:2979318-2980713 Satlength=1396 Nr of Repeats=9 RepeatLength=155
seed=TCCCGCTGTG
TCCCGCTGTGGTCAAAAGTTTTTTGTGTGCTCAGTTCTGTACGACAAATGTGTTAAAATCTTCTGAAAACTCAAAATTC
CAAGTTTTCCCAAAAAATGGGTCAAATGGTACAGTGGGATTTTCCACGAATTCTAAGTAGATGGTCGTGAGTTCAAC
TCCCGCTGTGGTCAAAATCTTTTTGCATGTTCAGTTCTGTGTGCGGTAAATATGTTTAAAATTTCTGGAAACTTGAAAT
TCCAAGTTATTCAGAAATTTGTCCAAATGGCGCAGTGGGAAATTCGATTTCTCTGACCCAAGAGGCCACGGGTTCAAT
TCCCGCTGTGGTTAAAAGTTTTTTGCGTGCTCAGTTCTGTACGGTAAATGTGTTAAAAACTTCTGGAAACTTGAAATTC
CAAGTTATTCAGAAATTTGTCCAAATGGCGCAGTGGGAAATTCGATTTCTCTGACCCAAGAGGCCACGGGTTCAAT
TCCCGCTGTGGTTAAAAGTTTTTTGCGTGCTCAGTTCTGTACGGTAAATGTGTTAAAAACTTCTGGAAACTTGAAATTC
CAAGTTATTCAGAAATTTGTTCAAATGGCGCAGTGGGAAATTCGATTTCTCTGACCCAAGAGGCCACGGGTTCAAT
TCCCGCTGTGGTTAAAAGTTTTTTGCGTGCTCAGTTCTGTACGGTAAATGTGTTAAAAACTTCTGGAAACTTGAAATTC
CAAGTTATTCAGAAATTTGTTCAAATGGCGCAGTGGGAAATTCGATTTCTCTGACCCAAGAGGCCACGGGTTCAAT
TCCCGCTGTGGTTAAAAGTTTTTTGCGTGCTCAGTTCTGTACGGTAAATGTGTTAAAAACTTCTGAAAACTTCAAATTC
CAAATTTTTCAAAAAGTGTTCAAATAGCGCAGTGGGGTTTTCGAGTTCTCTGTCGCAAAAGGTCACGGGTTCAAT
TCCCGCTGTGGTTAAAAGTTTTTTGCGTGCTCAGTTCTGTACGGTAAATGTGTTAAAAACTTCTGAAAACTTCAAATTC
CAAATTTTTCAAAAAGTGTTCAAATAGCGCAGTGGGGTTTTCGAGTTCTCTGTCGCAAAAGAACACGGGTTCAAT
TCCCGCTGTGGATAAAGGGTTTTTACATGTTCAGTTCTATATGGTAAATATGTTTAAAATTTCTGGAAACTTGAAATTC
CAAGATTTTCGGAAAATCGTTCAAATGGCGCAGTGGGGGTTTCGATTTCTTAGGCGCAAAAGGTCACGGGTTCAAT
TCCCGCTGTGGATAAAGGGTTTTTACATGTTCAGTTCTATATGGTAAATATGTTTAAAATTTCTGGAAACTTGAAATTC
CAAGATTTTCGGAAAATCGTTCAAATGGCGCAGTGGGGGTTTCGATTTCTTAGGCGCAAAAGGTCACGGGTTCAAT
Nr of selected repeats 6 Similarity 0.781691
Consensus:
TCCCGCTGTGGtTAAAaGtTTTTTgCgTGcTCAGTTCTgTAcGGTAAATgTGTTaAAAAcTTCTGGAAACTTGAAATTC
CAAGtTaTTCaGAAAaTtGTTCAAATGGCGCAGTGGGaaaTTCGATTTCTcaGaCcCAAgAGGcCACGGGTTCAAT
>Cele-UNSB01_2:2986853-2986937 Satlength=85 Nr of Repeats=7 RepeatLength=12
seed=ATAGGCATAG
ATAGGCATAGGC
ATAGGCATAGGC
ATAGGCATAGGC
ATAGGCATAGGC
ATAGGCATAGGC

ATAGGCATAGGC
ATAGGCATAGGC
Nr of selected repeats 7 Similarity 1.000000
Consensus:
ATAGGCATAGGC
>Cele-UNSB01_2:2995760-2997536 Satlength=1777 Nr of Repeats=53 RepeatLength=14
seed=CTACCTGCCT
CTACCTGCCTATGC
CTACCTGCCTATGGCTGCATGCATTTGC
CTACCTGCTTGTAC
CTAACTGCCTATGACCATCTGCCGTCGT
CTACCTGCCTATGC
CTACCTGTCTGTGC
CTACCTGCCTATGC
CTACCTGCCTATGCA
TACCTGCCTATGC
CTACCTGCCTATGC
CTACCTGCCTATGTCTTTCTGCTATGCACACCTGTTATAC
CTACCTGCCTATGC
CTACCTGCTTATGC
CTACCTGCCTATGCCTAATTACCTATGCCTAACTGCCCATGTCTAAATACTTATGT
CTATCTGCCTAGGG
CTACCTGCCTATGC
CTATCTGCCTATTC
CTACCTGCCTATGAATACCTTCCTATGC
CTACCTGCCTATGCACACCTGTTATAC
CTACCTGCCTATGC
CTGCCTGCCTATGCCTACCTGATATAC
CTACCTTCCTATGCCTTTCTGCTATGCA
CACCTGCCTATGC
CTACCTGCCTATGC
CTACCTGCTTATGC
CTACCTACCTATGCCTAATTGCCTATGCCTAACTGCCCATGTCTAAATACTTATGT
CTATCTGCCTAGGG
CTACCTGTCTATTTCAATCTGCCACTGC
CTACCTGCCTTTGA
CTACCCGCCTATTC
CTGCCTGCCTATGA
CTACCTGCCTATGCCCACCTGCCCATGC
CTACCTGCCCATGC
CTACCCGCCTATGC
CTACCTGCTTATGC
CTACCTGCCTATGC
CTACCTGCCTTACATGCCCCTGAAGTCCTTAACATGTTCTATCTAATAAATACACATATGGTCAAGTCCCCCCGGCCGG
CCGGGAAAAGTCGAAAATGCGCTCCACTTGAAATGTTGTTCCCGTCCGTCCTATATATGTTTCTATTCTCGCCCCCCCC
CTCTCCTGCCCCCCGTCCAAATCCTCTTTGCAGACGGGGGTTGTATTTGTTCGTTTTTTGTCAAAAAAATTTTCCTCTC
TTTCTCTAGCCCACTCTCTCGATTTCCTATACTATTTTTTTCGCAAGAGTTTCGTTTTCTGTTTGTATAAACAGACAGT
GTCTGTTCCCACATAACCTCTTAAATTTTTGTGTTATTTTTTTCTATTTGAAACACCAAAACCACCACCCGAAATTTTA
TGTATCAAATGGTTTTTTCTGCGCCCCCCCCCCCCCACCGCTTATAGGCTGCCTATAAACGTGTCTAGTAGTAAGTGTA
TGTAGGTTGATCGAGGATTACTGTAGGGAATTATGCGGACGCTGGAGACGGGCAAATCTTTGCAAATGACTTGAGCATA
TAAGTCTTCTCTAGAAGGCTTCCGTGGAAGGCACGCCCTGCCTTCTTTGAAATGTCATGAATTTTCCAATTTTCGAACG
GTTTTTTTTTCGAAAATTTTTCGAATTTCTAAGATTCTGAGATTTCTGAATTTCTGAGACTTTCATAGTTTCCAGAACT
TTTTTTAATATTCTTTTTTTCAAAATCTTGAATTTTTAATGCCTATGC
CTACCTGCCTATGC

CTACCTGCCTATTC
CTACCTGCCTATTC
CTACCTGCCTATGC
CTACCTGCCTATTC
CTACCTGCCTATGCCGGCCTTCCTATGCCTACCTTCTTATGC
CTACTTGCCTCTGC
CTACCTGTCTATAC
CTACCTGTCTATAC
CTACCTGCCTATGC
CTACCTGCCTAGAG
CTATCTGCCTAGCG
CTACCTGCTTATGCCTAACTGCTTAAGCCTTTCTGCCTATGC
CTACCTGCCAATGCCTACCTTCTTATGCT
TACCTGCCTATGC
CTACCTGCCTATGC
Nr of selected repeats 34 Similarity 0.798659
Consensus:
CTACCTGCCTATgC
>Cele-UNSB01_2:3009682-3009822 Satlength=141 Nr of Repeats=5 RepeatLength=28
seed=AATGTCGGCT
AATGTCGGCTGCTTCTAGAAGCCCGGCA
AATGTCGGCTGCTTCTAGAAACTAAGCA
AATGTCGGCTGCTTCTAGAAACTAAGCA
AATGTCGGCTGCTTCTAGAAGCCCGGCG
AATGTCGGCTGTTTCTAGAAACCAGGCA
Nr of selected repeats 5 Similarity 0.847619
Consensus:
AATGTCGGCTGcTTCTAGAAaCcagGCa
>Cele-UNSB01_2:3063296-3063566 Satlength=271 Nr of Repeats=18 RepeatLength=14
seed=TTAGGGCCTT
TTAGGGCCTTGGGT
TTAGGGCCTTGGGC
TTAGGGCCTTGGGT
TTAGGGCCTTGGGC
TTAGGGCCTTGGGC
TTAGGGCCTTGGGC
TTAGGGCCTTGGGC
TTAGGGCCTTGGGC
TTAGGGCCTTGGGCTTAGGCAAGGCCTTGGGC
TTAGGGCCTTGGGC
TTAGGGCCTTGGGC
TTAGGGCCTTGGGC
TTAGGGCCTTGGGC
TTAGGGCCTTGGGC
TTACGGCCTTGGGC
TTAGGGCCTTGGGC
TTAGGGCCTTGGGA
TTAGGGCCTTGGGA
Nr of selected repeats 17 Similarity 0.949580
Consensus:
TTAGGGCCTTGGGc
>Cele-UNSB01_2:3065991-3066701 Satlength=711 Nr of Repeats=18 RepeatLength=19
seed=GCCAAGGCAC
GCCAAGGCACTTGATAAGG

GCCAAGGCACTGGAAATGC
GCCAAGGCACTGGAAATGC
GCCAAGGCACGAGAAACGC
GCCAAGGCACTAGGAATGG
GCCAAGTCACTAGAAACGC
GCCAAGGCACTGGAAATGT
GCCAAGGCACTGGAAATGCGCCAAATCACGAGAAACTCGCCAATGCCTTAGAAATGCGTCAAGTCACTAGGAATGTGTC
AAGTCACAAAAGGAACAAAAAACTC
GTCAAGGCACTACAAAAACGTCAAGTTACCAGAAACGTGCCAAGACATAAAGAATGC
GCCAAGGCACGCAAAAGGC
GCCAAGTCACAATAAATGCGCCAAGTCAGTAGAAACGAGTCAAAACACTAAGAGTGC
GCCAAGTCACGAGAATCGC
GCCAAGGTACGATATATGTGTCAAGTCATTAAAAATGTGCAGTCACTAGAAACTCGGCAAGTCACTAGGAATGC
GTCAAGGCACGCAAAAAAC
GCCAAGGCACGAGAAACGC
GCCAAAGCACAAGAAATGCGTCAAGTCACGATAAATGC
GCCAAGGCACTCAAAACGAGCCAAGTCTTGATAAATGC
GCCAGGGCACCGAAAAAGCGTCAAGACACTACAAAAGCGTGAAGTTACTAAAAAGGCGCCAGATGACTAAAAACGCGCC
AAGTCTTGATAATTGCGCCAGGTCACCAAAAAAGCGTTAAGTCACGAAAAAGGC
Nr of selected repeats 11 Similarity 0.680606
Consensus:
GCCAAGGCACtnGAAAnGc
>Cele-UNSB01_2:3077809-3078033 Satlength=225 Nr of Repeats=15 RepeatLength=14
seed=CTTATACCTT
CTTATACCTTATAC
CTTATACCTTATAC
CTTATACATTATAA
CTTATGCCTTATAC
CTTATACCTTATAC
CTTATACCTTATACCTTATGCCTAATAT
CTTATACCTTATAC
CTTATACCTTATAC
CTTATACCTTATAC
CTTATACCTTATAC
CTTATACCTTATAC
CTTATACCTTATAC
CTTATACCTTATAC
CTTATACCTTATAC
CTTATGCCTTATAC
Nr of selected repeats 14 Similarity 0.947671
Consensus:
CTTATACCTTATAC
>Cele-UNSB01_2:3099938-3100072 Satlength=135 Nr of Repeats=4 RepeatLength=31
seed=ATCGATTTTT
ATCGATTTTTTCGGTAAAAAATCAATAAAAT
ATCGATTTTTTAGTGAAATTTAACAAAAAAAAAATTCAAATT
ATCGATTTTTTGGTTAAAAAATCAATAAAAT
ATCGATTTTTTCAGTGAAAATAGTCCAAAAA
Nr of selected repeats 3 Similarity 0.700337
Consensus:
ATCGATTTTTTcGttAAAAAaTCaAtAAAAt
>Cele-UNSB01_2:3132033-3132195 Satlength=163 Nr of Repeats=6 RepeatLength=27
seed=GCTGCTGGAG
GCTGCTGGAGGATCCACTGCATCAACT

GCTGCTGGAGGATCAACTTCAACAACT
GCTGCTGGAGGATCTACTGCATCAACT
GCTGCTGGAGGACCCACTGGATCAACT
GCTGCTGGAGGATCTACTGCATCAACT
GCTGCTGGAGGATCCACTGCATCAACC
Nr of selected repeats 6 Similarity 0.881481
Consensus:
GCTGCTGGAGGATCcACTGCATCAACT
>Cele-UNSB01_2:3135702-3137322 Satlength=1621 Nr of Repeats=60 RepeatLength=27
seed=TGCTGGAGGA
TGCTGGAGGATCCACTGCATCAACTGC
TGCTGGAGGATCAACTTCAACAACTGC
TGCTGGAGGATCTACTGTATCAACCGC
TGCTGGAGGATCCACTGCATCAACTGC
TGCTGGAGGATCCACTGCATCAACCGC
TGCTGGAGGATCAACTGCAACAACCGC
TGCTGGAGGATCAACTGCAACAACCGC
TGCTGGAGGATCCACTGCATCAACTGC
TGCTGGAGGATCAACTGCAACAACCGC
TGCTGGAGGATCCACTGCATCAACTGC
TGCTGGAGGATCCACTGCATCAACTGC
TGCTGGAGGATCAACTGCATCAACCGC
TGCTGGAGGATCAACTGCAACAACCGG
TGCTGGAGGATCAACTGCAACAACCGC
TGCTGGAGGATCCACTGCATCAACTGC
TGCTGGAGGATCAACTGCAACAACCGC
TGCTGGAGGATCCACTGCATCAACTGC
TGCTGGAGGATCCACTGCATCAACCGC
TGCTGGAGGATCAACTGCAACAACCGC
TGCTGGAGGATCAACTGCAACAACCGC
TGCTGGAGGATCCACTGCATCAACTGC
TGCTGGAGGATCAACTGCAACAACCGC
TGCTGGAGGATCAACTGCAACAACCGC
TGCTGGAGGATCAACTGCAACAACCGC
TGCTGGAGGATCAACTGCATCAACCGC
TGCTGGAGGATCAACTGCAACAACCGC
TGCTGGAGGATCAACTGCAACAACCGC
TGCTGGAGGATCCACTGCATCAACTGC
TGCTGGAGGATCAACTGCAACAACCGC
TGCTGGAGGATCCACTGCATCAACTGC
TGCTGGAGGATCAACTGCATCAACCGC
TGCTGGAGGATCAACTGCAACAACCGC
TGCTGGAGGATCAACTGCATCAACCGC
TGCTGGAGGATCAACTGCAACAACCGC
TGCTGGAGGATCCACTGCATCAACTGC
TGCTGGAGGATCAACTGCAACAACCGC
TGCTGGAGGATCCACTGCATCAACTGC
TGCTGGAGGATCAACTGCATCAACCGC
TGCTGGAGGATCAACTGCAACAACCGC
TGCTGGAGGATCAACTGCAACAACCGC
TGCTGGAGGATCCACTGCATCAACTGC
TGCTGGAGGATCCACTGCATCAACCGC
TGCTGGAGGATCAACTGCAACAACCGC
TGCTGGAGGATCCACTGCATCAACTGC

TGCTGGAGGATCCACTGCATCAACCGC
TGCTGGAGGATCAACTGCAACAACCGC
TGCTGGAGGATCAACTGCAACAACCGC
TGCTGGAGGATCCACTGCATCAACCGC
TGCTGGAGGATCAACTGCATCAACCGC
TGCTGGAGGATCAACTGCAACAACCGC
TGCTGGAGGATCAACTGCAACAACCGC
TGCTGGAGGATCCACTGCATCAACTGC
TGCTGGAGGATCAACTGCAACAACCGC
TGCTGGAGGATCCACTGCATCAACTGC
TGCTGGAGGATCCACTGCATCAACCGC
TGCTGGAGGATCAACTGCAACAACCGC
TGCTGGAGGATCAACTGCAACAACCGC
TGCTGGAGGATCCACTGCATCAACTGC
TGCTGGAGGATCCACTGCATCAACCGC
TGCTGGAGGATCAACTGCAACAACCGC
Nr of selected repeats 60 Similarity 0.923806
Consensus:
TGCTGGAGGATCaACTGCAtCAACcGC
>Cele-UNSB01_2:3141695-3142468 Satlength=774 Nr of Repeats=16 RepeatLength=43
seed=TTGCCGGAAT
TTGCCGGAATTGACAATTCTCGGCAAACTGCAAACCAGCAAC
TTGCCGGAGTTGAAAATTTCCTGTAAATCGGCAAACCGGCAAC
TTGCCGGAATTGACAATTTCCTGTAAATCGGCAAACCGGGAAA
TTGTCGGAATTGAAAATTTCCTGTAAATCGGCAAACCGGGAAA
TTGCCGGAATTGAAAATTTCCTGTAAATCGGCAAAACGGCAAC
TTGCCGGAATTAAAAATTTCCTTTAAATCGGCAAACCGGCAAG
TTGCCGGAATTGACAATTCCGGGCACATCTGCAAACCGGCAAC
TTGCCGGAATTGACAATTTCCTGTAAATCGGCAAACCGGGAAA
TTGCCGGAATTGACAATTCTCGGCAAATCTGCAAACCGGCAACTTCCTGGAATGGAAAATTTCCTGTAAATCGGCAAAC
CGGGAAA
TTGCCGGAATTTAAAATTTTCTGTCAATCTGCAAGCCGGCAAC
TTGCCGGAATTAAAAATTTCCTTTAAAGCGGCAAACCGGGAAATTACCGAAATTGAAAATTTCCGGCAAGTCTGCAAAC
TGTCAAC
TTGCCAGAATTGAAAATTTCCTGTAAATCGGCAAACCGGCAAC
TTGTCGGAATTGAAAATTTCCGGCAAATCTGCAAACCGGCAAC
TTGCCGGAATTGAAAACTTCCTGTAAATCGGCAAACCGGGAGT
TTGCTGGAATTGAAAACTTCCTGTAAATCTGCAAACCGGCAAC
TTGTCGGAATTGAAAATTTCCGGCAAATCTGCAAACCGGGAAA
Nr of selected repeats 13 Similarity 0.825084
Consensus:
TTGcCGGAATTGAaAATTTCCtGtAAATCgGCAAACCGGcAAc
>Cele-UNSB01_2:3154398-3154558 Satlength=161 Nr of Repeats=9 RepeatLength=16
seed=TAGGCACGTA
TAGGCACGTAGGCATG
TAGGCACGTAAGCATG
TAGGCATGTAGGCACG
TAGGCACATAGGCACA
TAGGCACGTAGGCACG
TAGGCACATAGGCACG
TAGGCACGTAGGCACG
TAGGCACGTAGGCACGTAGACATG
TAGGCACCTAGGCACCTATGCACC
Nr of selected repeats 7 Similarity 0.849206

Consensus:
TAGGCACgTAGGCAcG
>Cele-UNSB01_2:3155808-3156160 Satlength=353 Nr of Repeats=18 RepeatLength=16
seed=TACGTGCCTA
TACGTGCCTACGTGCCTATGTACCTACCTGTT
TACGTGCCTACGTGCC
TACGTGCATACGTGCA
TACGTGCATACGTGCC
TACGTGCCTATGTGGCAATGTGCC
TACGTGCCTACGTGCC
TACGTGCCTACGTGCC
TACGTGCCTACGTGCC
TACGTGCCTACGTGCC
TATGTGCCTATGTGCC
TACGTGCCTATGTGCCAACGTGCC
TGCGTGCCTACGTACC
TACGTGCCTACGTGCCTATGTGCCAATGTGCC
TACGTGCCTACGTGCC
TACGTGCCTAGGTACC
TACGTGCCTAGGTTAC
TACGTGCCTACGCGACTACGCGAC
TACGTGCCTACGTGGCTACGTACC
Nr of selected repeats 12 Similarity 0.845960
Consensus:
TACGTGCCTAcGTgCC
>Cele-UNSB01_2:3166478-3166668 Satlength=191 Nr of Repeats=8 RepeatLength=12
seed=GGCTTAGGCT
GGCTTAGGCTTAGGTTTAGACTTAGGCCTAGACTTAAATTTA
GGCTTAGGCTTA
GGCTGAGGCTGAGGCTGAGCTTCGAAATTTTGTTTTTTTTTGTTTTAAATTTTTTTTTAAATACGATTTTGGTATG
GGCTTAGGCTTA
GGCTTAAGCTTA
GGCTTAGACTTA
GGCTTAGGCTAA
GGCTTTGGCTCG
Nr of selected repeats 6 Similarity 0.785185
Consensus:
GGCTTAGGCTtA
>Cele-UNSB01_2:3175289-3175339 Satlength=51 Nr of Repeats=4 RepeatLength=10
seed=AACTTTCGAA
AACTTTCGAA
AACTTTCGAA
AACTTTCGAA
AACTTTCGAAAACTTTCGAA
Nr of selected repeats 3 Similarity 1.000000
Consensus:
AACTTTCGAA
>Cele-UNSB01_2:3182927-3183063 Satlength=137 Nr of Repeats=8 RepeatLength=16
seed=AGGCACGTAG
AGGCACGTAGGCAAGT
AGGCACGTAGGCATAT
AGGCACGTAGGCACGTAGGCATAT
AGGCACGTAGGCACGT
AGGCACGTAGGCAAGT

AGGCAAGTAGGCGCGT
AGGCACGTAGGCAAGT
AGGCAAGTAGGCACGT
Nr of selected repeats 7 Similarity 0.853175
Consensus:
AGGCAcGTAGGCAnGT
>Cele-UNSB01_2:3184073-3184138 Satlength=66 Nr of Repeats=4 RepeatLength=14
seed=CCAAGGCCCA
CCAAGGCCCAAGGCCCAAAACCC
CCAAAGCCCAAGGC
CCAAGGCCGAAGCC
CCAAGGCCCAATGC
Nr of selected repeats 3 Similarity 0.746032
Consensus:
CCAAgGCCcAAggC
>Cele-UNSB01_2:3220556-3221123 Satlength=568 Nr of Repeats=21 RepeatLength=16
seed=GTAGGCACGT
GTAGGCACGTAGGCGC
GTAGGCACGTAGGCAC
GTAGGCACGTAGGCAC
GTAGACACGTAGGCAC
GTAGGCAAGTAGGCAC
GTGGGCACGTAGGCAC
GTAGGCACGTAGGCAC
GTAGGCACGTAGGCAC
GTAGGCACGTAGGCAC
GTAGGCACGTTTTAATTTATACATTTATATTTTTAGACCTATGTATTTTTATATTAGAGCAAGTTTGGACACCCGTCGG
CTTCCTGAGGCAAA
GTAGGCACGTAGACAC
GTAGGCACGTAGGCAC
GTAGACACGTAGGCAC
GTAGGCAGGTAGGCAC
GTAGGCACGTAGGCAC
GTAGGCACGTAGGCAC
GTAGGCACGTTTTAATTTATAAATTTATATTTTTAGACCTATTTTTATAGTAAAGCAAGTTTGGACACTCGTCGGCTTC
CTGAGGCAAA
GTAGGCACGTAGGCAC
GTAGGCACGTAGGCAC
GTAGGCACGTAGGCAC
GTAGGCACGTTTTAATTTATAAATTTATATTTTTAGACCTATTTTTATAGTAAAGCAAGTTTGGACACTCGTCGGCTTC
CTGAGGCAAAGTAGGCAC
Nr of selected repeats 18 Similarity 0.936819
Consensus:
GTAGGCACGTAGGCAC
>Cele-UNSB01_2:3287465-3288008 Satlength=544 Nr of Repeats=19 RepeatLength=20
seed=GTAAATCTAC
GTAAATCTACAAGAAAACATTTACAATTTGGCAGAAATTTAA
GTAAATCTACATGAAAATCCGCAAGTCTACACAAAGATCCGTAAATCTTTACGTAGGTTC
GTAAATCTACACTTGCTTCC
GTAAATCTACACGTCCTTCC
GTAAATCTACACTGGCGTCC
GTAAATCTACACCTACCGTAAGTTCACACATTCCTCTT
GTAAATCCACATAGTCTTCC
GTAAATCTACACTATCTACTTCCCGTTTATCTACGCCACCTACCGCAAGTTCACTCATTCCTTCC

GTAAATCTATGTGTAGATCC
GTAAATCTACACAGTCCCC
GTAAATCCACACTAACCCCC
GTAAATCTACACTAGCCCCC
GTAAATCTTCACGGGCTCCT
GTAAATCTACACTAGCTTCC
GTAAACCTACACAATCTCCC
GTAAAACTACATGTGCTTTC
GTAAATCTACTCAACTTTTC
GTGAATCTACACAGACCACCTATATCCTCACATTCCTCC
GTAAATCTACACCTATTCCCGTAAACCCACACTAAAAACC
Nr of selected repeats 12 Similarity 0.565197
Consensus:
GTAAATCTACActagCttCC
>Cele-UNSB01_2:3312389-3313069 Satlength=681 Nr of Repeats=42 RepeatLength=16
seed=CGTAGGCACG
CGTAGGCACGAAGGCA
CGTAGGCACGTGGGCG
CGTAGGCACGAAGGCA
CGTAGGCACGTGGGCG
CGTAGGCACGTAGGCA
CGTAGGCACGTAGGCA
CGAAGGCACGTAGGCACGTGGGCG
CGTAGGCACGTAGGCA
CGTAGGCACGAAGGCA
CGTAGGCACGTAGGCA
CGTAGGCACGAAGGCA
CGTAGGCACGTAGGCAT
GTAGGCACGTAGGCA
CGTAGGCACGTAGGCA
CGTAGGCACGTAGGCA
CGTAGGCACGTAGGCA
CGTAGGCACGTAGGCA
CGTAGGCACGTAGGCA
CGTAGGCACGTAGGCA
CGTAGGCACGTAGGCA
CGTAGGCACGTAGGCA
CGTAGGCACGTAGGCA
CGTAGGCACGTAGGCA
CGTAGGCACGTAGGCA
CGTAGGCACGTAGGCA
CGTAGGCACGTAGGCA
CGTAGGCACGTAGGCA
CGTAGGCACGTAGGCA
CGTAGGCACGTAGGCA
CGTAGGCACGTAGGCA
CGTAGGCACGTAGGCA
CGTAGGCACGTAGGCA
CGTAGGCACGTAGGCA
CGTAGGCACGTAGGCA
CGTAGGCACGTAGGCA
CGTAGGCACGTAGGCA
CGTAGGCACGTAGGCA

CGTAGGCACGTAGGCA
CGTAGGCACGTAGGCA
CGTAGGCACGTAGGCA
CGTAGGCACGTAGGCA
Nr of selected repeats 39 Similarity 0.967611
Consensus:
CGTAGGCACGTAGGCA
>Cele-UNSB01_2:3331473-3331608 Satlength=136 Nr of Repeats=5 RepeatLength=27
seed=TTTACTCTCT
TTTACTCTCTGTGGCTTCCCACTATAT
TTTACTCTCTGTGGCTTCCCACTATAT
TTTACTCTCTGTGGCTTCCCACTATAT
TTTACTCTCTGTGGCTTCCCACTATAT
TTTACTCTCTGTGGCTTCCCACTATAT
Nr of selected repeats 5 Similarity 1.000000
Consensus:
TTTACTCTCTGTGGCTTCCCACTATAT
>Cele-UNSB01_2:3341963-3342088 Satlength=126 Nr of Repeats=4 RepeatLength=22
seed=ATTCTGCGCA
ATTCTGCGCACCCCCTGCGCCG
ATTCTGCGCACATTGATGTTGTTGTGCCCTTTTTCTTGGTGGCATAGCCTCGTGCGCAC
ATTCTGCGCACCCCCTGAGCGG
ATTCTGCGCACGTTCAGCTCAC
Nr of selected repeats 3 Similarity 0.656566
Consensus:
ATTCTGCGCACccccCtGcgCng
>Cele-UNSB01_2:3406743-3406803 Satlength=61 Nr of Repeats=4 RepeatLength=15
seed=GGTGAGACCC
GGTGAGACCCATCGT
GGTGAGACCCATCGC
GGTGAGACCCATCGT
GGTGAGACCCGTCGT
Nr of selected repeats 4 Similarity 0.911111
Consensus:
GGTGAGACCCaTCGt
>Cele-UNSB01_2:3456136-3456276 Satlength=141 Nr of Repeats=4 RepeatLength=28
seed=TGCCTCGCCT
TGCCTCGCCTACGTGCCTATTTCAGGAATTCCTTGCCTACGTGGCTATTTCAGGCT
TGCCTCGCCTACGTGCCTATTTCAGGCA
TGCCTCGCCTACGTGCTTATTTCAGGCA
TGCCTCGCCTACGTGCTTATTTCAGGCA
Nr of selected repeats 3 Similarity 0.968254
Consensus:
TGCCTCGCCTACGTGCtTATTTCAGGCA
>Cele-UNSB01_2:3460910-3461075 Satlength=166 Nr of Repeats=5 RepeatLength=16
seed=GCACGTAGGC
GCACGTAGGCACGCAGGTACATAAGAATCTAGGCGGATACCTAGGCACCTTAACATCCTGGAACCTAGGCATCTAAACA
TCCAGGACACCCAG
GCACGTAAGCACGTAG
GCACGTAGGCACGTAG
GCACGTAGGCACGTAG
GCACGTAGGCATGTAGGCACGTAG
Nr of selected repeats 3 Similarity 0.944444
Consensus:

GCACGTAgGCACGTAG
>Cele-UNSB01_2:3500187-3500295 Satlength=109 Nr of Repeats=5 RepeatLength=18
seed=GGCTTAGGCT
GGCTTAGGCTTA
GGCTTAGGCTTAGGCTCA
GGATTAGGCTTAAGCTCA
GGCTTAGGCTTAAGCTCA
GGCTTAGGCTTGTGCTTGTGCTTAGGCCTAGGTCTTGGCTTA
Nr of selected repeats 3 Similarity 0.901235
Consensus:
GGcTTAGGCTTAaGCTCA
>Cele-UNSB01_2:3515774-3515844 Satlength=71 Nr of Repeats=4 RepeatLength=12
seed=ATACATATAC
ATACATATACAT
ATACATATACAT
ATACATATACAT
ATACATATACATATACAGTCCATATACAGTATAC
Nr of selected repeats 3 Similarity 1.000000
Consensus:
ATACATATACAT
>Cele-UNSB01_2:3535061-3535729 Satlength=669 Nr of Repeats=54 RepeatLength=12
seed=TAGGTATAGG
TAGGTATAGGTA
TAGGTAAAGGCA
TAGGCATAGGCA
TAGGTATAGGTTT
TGGTATAGGTA
TAGGTATAGGTA
TAGGTATAGGTATAGGAC
TAGGTATAGGTA
TAGGTATAGGTA
TAGGTATAGGTA
TCGGTATAGGTA
TCGGTATAGGTA
TAGGTATAGGTA
TAGGGATAGGTA
TAGGTATAGGTA
TAGGTATAGGAA
TAGGTATAGGTA
TAGGTATAGGTA
TAGGTATAGGTA
TAGGTATCGGTA
TAGGTATAGGTA
TCGGTATAGGTA
TAGGTAAAGGGA
TAGGTATAGGTAAAGGGA
TAGGTATAGGTA
TAGGTAAAGGGA
TAGGTATAGGTA
TAGGTAAAGGCA
TAGGCATAGGCA
TAGGTATAGGTTT
TGGTATAGGTA
TAGGTATAGGTA

TAGGTTTAGGTA
TAGGTATAGGAA
TAGGTATAGGTGTA
TAGGAATAGGTA
TAGGTATAGGTA
TAGGTATAGGTA
TAGGTATAGGTA
TCGGTATAGGTA
TCGGTATAGGTA
TAGGTAAAGGAA
TAGATATAGGTA
TAGGTATAGGTA
TAGGTATAGGTA
TAGGTATAGGTA
TAGGTATAGGTA
TAGGTATAGGTA
TAGGTAAAGGGA
TAGGTATAGGTA
TAGGTATAGGTA
TAGGTATAGGTA
TAGGTATAGGTA
TAGGTATAGGTATAGGTA
Nr of selected repeats 46 Similarity 0.876973
Consensus:
TAGGTATAGGtA
>Cele-UNSB01_2:3539245-3539476 Satlength=232 Nr of Repeats=5 RepeatLength=39
seed=AATTTCCCGC
AATTTCCCGCCAAAAATTTTACGTTTTTCAGAAAATTCA
AATTTCCCGCCAAAATTGAATTTTTTAAAAGAAAATTTA
AATTTCCCGCCAAAATTGAATTTTTTTAAAGAAAATTTA
AATTTCCCGCCAGAACTGTTTTTTTTGGGAAAATTTGAATTAGCCGCCTAAATTAAATTTTTTTTTAGAAAATCTG
AATTTCCCGCCAAAATTAATTTTTTTAAAGAAAATTCA
Nr of selected repeats 3 Similarity 0.769444
Consensus:
AATTTCCCGCCAAAATTgaAttTTTTtaaAGAAAATTtA
>Cele-UNSB01_2:3540761-3541492 Satlength=732 Nr of Repeats=18 RepeatLength=31
seed=GGTTACTGTA
GGTTACTGTAGGACTACTGTAGCTTCCAA
GGTACTGTACTGTACCTTTAAGACAAAGCCAACAGAATTCTGCTCCGCACAGTGTACAGTGCATATGATTACATGAACC
ATCGTTTTCCTTCACAATTAATGTCTTGCAACGTGGACACGGGTGCGCTGAGACGTCTGAAAAAAACATTTGTAGG
GGTACTGTAGGAGTACTGTAGGGTTTTAAAAAAACTCAGTGCAGTGCTGTAG
GATTACTGTATATCGTCCTAGGACACTTTG
GGTTACTGTAACTCAAGCCTGAGATTCTTCG
GGTTACTGTAACTCAATCCTAGCCATCTAAAGGGTACTGTGGATCGGTTCTAGGATATGTTG
GGTTACTGTAGATCGGTTCTAGGATACACTA
GGTTACTGTATATCGATCCTAGAATACGTTG
GGTTACTGTAGCTTAATCCTAAGGTTCTAGT
GGTTACTGTAGATCGATCCTAGGATACTTTG
GGTTACTGTAGATCGATCCTAGGATACTTTG
GGTTACTGTAGATCGGTTCTAGGAAATTTTG
GGTTACTGTAGCTTAATCCTAAGATTCTAAG
GGTTACTGTAGATCGGTTCTAGGATACGTTG
GGTTACTGTAGATCGGTTCTAGGATACGTCG
GGTTACTGTAGATCGATCCTAGAATTCTGAAG

GGTACTGTAGATCGATTTGGGGATGCTTTG
GGTTACTGTAGCCCAATCCTGGGATTCTAAG
Nr of selected repeats 11 Similarity 0.669318
Consensus:
GGTTACTGTAGaTCgaTcCTAgGATaCtttG
>Cele-UNSB01_2:3557247-3557399 Satlength=153 Nr of Repeats=9 RepeatLength=16
seed=GTAGGCACGT
GTAGGCACGTAGGCAC
GTAGGTACGTAGGCAC
GTAGGCACGTAGGCAC
GTAGGCACGTAGGCAC
GTAGGCTCGTAGGCAC
GTAGGCACGTAGGCAC
GTAGGCACGTAGGCAC
GTAGGCACGTAGGCAC
GTAGGCACGTAAGCACGTAGGCAC
Nr of selected repeats 8 Similarity 0.958333
Consensus:
GTAGGCACGTAGGCAC
>Cele-UNSB01_2:3617397-3617684 Satlength=288 Nr of Repeats=6 RepeatLength=42
seed=TTTGAAAACC
TTTGAAAACCTGCAATATCCGGAATTGCCGATTGCCGGAAAT
TTTGAAAACCTACATTTGCCGATTTGCCGATTTGCCAAAAACATTCAATTTGCTCAATTTGCCGATTTGCCGATTTGCC
GAAAAT
TTTGAAAACCTACGTTTGCTGATTTGCCGAAAAA
TTTGAAAACCGGCAATTGCCGAAATTGTCGAGTGCCGGAAAT
TTTGAAAACCTACAATTGCCGAAATTGCCGAGTGCCGGAAAT
TTTGAAAACCGGCGATAGCCGGAATTGCCGATTGCCGGAAAT
Nr of selected repeats 4 Similarity 0.851852
Consensus:
TTTGAAAACCggCaATagCCGaAATTGcCGAgTGCCGGAAAT
>Cele-UNSB01_2:3687013-3687103 Satlength=91 Nr of Repeats=5 RepeatLength=20
seed=CTATCGCCTG
CTATCGCCTGCTAGCGCCTA
CTATCGCCTGCTATTGCCTA
CTATTGCCTG
CTATCGCCTGCTGTCGCCTA
CTATCGCCTACTATCGCCTG
Nr of selected repeats 4 Similarity 0.833333
Consensus:
CTATCGCCTgCTatcGCCTa
>Cele-UNSB01_2:3736361-3736609 Satlength=249 Nr of Repeats=8 RepeatLength=31
seed=GCTGATTGGT
GCTGATTGGTCGGCATGGTCTGCTCATCATC
GCTGATTGGTCGGAATAACTCTCCCAGAATC
GCTGATTGGTTGGTGAGGTTTTCACATCTTT
GCTGATTGGTCAACGGGTTGCACCTACTGTT
GCTGATTGGTCGGCGCGGTTCGCCTATCACC
GCTGATTGGTCGGAGGGACTCGCCCAACGTT
GCTGATTGGTCGGTGAGCTTTGCCCATCTTT
GCTGATTGGTCGGCGGATTGCACCCACTGTT
Nr of selected repeats 8 Similarity 0.553571
Consensus:
GCTGATTGGTCGGcgnGgtTcgCccAtcnTt

>Cele-UNSB01_2:3799242-3799338 Satlength=97 Nr of Repeats=4 RepeatLength=24
seed=TGCATCTGCC
TGCATCTGCCACAGGAGCTGGCGT
TGCATCTGCCGCTGGAGTTGTTGC
TGCATCTGCCGCTGGAACTGGCGC
TGCATCTACCGCTGGAGTTGTTGC
Nr of selected repeats 4 Similarity 0.750000
Consensus:
TGCATCTgCCgCtGGAgcTGgcGc
>Cele-UNSB01_2:3989433-3989722 Satlength=290 Nr of Repeats=6 RepeatLength=45
seed=AAGGATCAGA
AAGGATCAGAAGGGTACCTCCTGATGATCTGATCCTTCAGCTTCA
AAGGATCATAAGGGGTATAAAAGCAATAAATCCCCTTCTGTACCCTTCTGATCCTTTGAAGCTG
AAGGATCAGATCATTAGGAGGTACCCTTCTGATCCTTCGGAGCTG
AAGGATCAGATCACCAGGAGGTACCCTTGTGATCCTTCGGAGCTG
AAGGATCAGATCACCAGGAGGTACCCTTGTGATCCTTCGGAGCTT
AAGGATCAGATCATCAGGAGGTACCCTTCTGATCCTTCGGAGCTG
Nr of selected repeats 5 Similarity 0.643210
Consensus:
AAGGATCAGatcAncAGGagGTACCCTTcTGATCCTTCggAGCTg
>Cele-UNSB01_2:3993365-3994945 Satlength=1581 Nr of Repeats=20 RepeatLength=79
seed=GTGAATCTCC
GTGAATCTCCTCTCTACCTAATGAGTACTGACAGAATACCGGCAGTATTCTGGGGTCTCATTAGGTTTTAGTTTCTTCT
GTGAATCTCCTCTCCACCTAATGATTCCTGACAGAATACCGGCAGTATTCTGGGGTCTCATTAGCTTTTAGTTTCTTCT
GTGAATCTCCTCTCAACCTAATGATTCCTGACAGAATACCGGCAGTATTCTGGGGTCTCATTAGGTTTTAGTTTCTTCT
GTGAATCTCCTCTCAACCTAATGATTCCTGACAGAATACCGGCAGTATTCTGGGGTCTCATTAGGTTTTAGTTTCTTCT
GTGAATCTCCTCTCAACCTAATGATTCCTGACAGAATACCGGCAGTATTCTGGGGTCTCATTAGGTTTTAGTTTCTTCT
GTGAATCTCCTCTCCACCTAATGATTCCTGACAGAATACCGGCAGTATTCTGGGGTCTCATTAGGTTTTAGTTTCTTCT
GTGAATCTCCTCTCCACCTAATGATTCCTGACAGAATACCGGCAGTATTCTGGGGTCTCATTAGGTTTTAGTTTCTTCT
GTGAATCTCCTCTCCACCTAATGATTCCTGACAGAATACCGGCAGTATTCTGGGGTCTCATTAGGTTTTAGTTTCTTCT
GTGAATCTCCTCTCCACCTAATGATTCCTGACAGAATACCGGCAGTATTCTGGGGTCTCATTAGGTTTTAGTTTCTTCT
GTGAATCTCCTCTCCACCTAATGATTCCTGACAGAATACCGGCAGTATTCTGGGGTCTCATTAGGTTTTAGTTTCTTCT
GTGAATCTCCTCTCCACCTAATGATTCCTGACAGAATACCGGCAGTATTCTGGGGTCTCATTAGGTTTTAGTTTCTTCT
GTGAATCTCCTCTCCACCTAATGATTCCTGACAGAATACCGGCAGTATTCTGGGGTCTCATTAGGTTTTAGTTTCTTCT
GTGAATCTCCTCTCCACCTAATGATTCCTGACAGAATACCGGCAGTATTCTGGGGTCTCATTAGGTTTTAGTTTCTTCT
GTGAATCTCCTCTCCACCTAATGATTCCTGACAGAATACCGGCAGTATTCTGGGGTCTCATTAGGTTTTAGTTTCTTCT
GTGAATCTCCTCTCCACCTAATGATTCCTGACAGAATACCGGCAGTATTCTGGGGTCTCATTAGGTTTTAGTTTCTTCT
GTGAATCTCCTCTCCACCTAATGATTCCTGACAGAATACCGGCAGTATTCTGGGGTCTCATTAGGTTTTAGTTTCTTCT
GTGAATCTCCTCTCCACCTAATGATTCCTGACAGAATACCGGCAGTATTCTGGGGTCTCATTAGGTTTTAGTTTCTTCT
GTGAATCTCCTCTCCACCTAATGATTCCTGACAGAATACCGGCAGTATTCTGGGGTCTCATTAGGTTTTAGTTTCTTCT
GTGAATCTCCTCTCTACCTAGTGAGTCCTGACAGAATACCGGCAGTATTCTGGGGTCTCATTAGGTTTTAGTTTCTTCT
GTGAATCTCCTCTCCACCTAATGATTCCTGACAGAATACCGGCAGTATTCTGGGGTCTCATTAGGTTTTAGTTTCTTCT
Nr of selected repeats 20 Similarity 0.984544
Consensus:
GTGAATCTCCTCTCcACCTAATGATTCCTGACAGAATACCGGCAGTATTCTGGGGTCTCATTAGGTTTTAGTTTCTTCT
>Cele-UNSB01_2:4043072-4059727 Satlength=16656 Nr of Repeats=833
RepeatLength=20 seed=ATTGTTACAC
ATTGTTACACGAAAAATAG
ATTGTTACACGAAAAAATAG
ATTGTTACACTAAAAAATAG
ATTGTTACACGAAAAAATAG
ATTGTTACACTAAAAAATAG
ATTGTTACACTAAAAAATAG
ATTGTTACACGAAAAAATAG

ATTGTTACACTAAAAAAATAG
ATTGTTACACTAAAAAAATAG
ATTGTTACACGAAAAAAATAG
ATTGTTACACTAAAAAAATAG
ATTGTTACACTAAAAAAATAG
ATTGTTACACGAAAAAAATAG
ATTGTTACACTAAAAAAATAG
ATTGTTACACTAAAAAAATAG
ATTGTTACACTAAAAAAATAG
ATTGTTACACGAAAAAAATAG
ATTGTTACACTAAAAAAATAG
ATTGTTACACTAAAAAAATAG
ATTGTTACACGAAAAAAATAG
ATTGTTACACTAAAAAAATAG
ATTGTTACACTAAAAAAATAG
ATTGTTACACGAAAAAAATAG
ATTGTTACACGAAAAAAATAG
ATTGTTACACGAAAAAAATAG
ATTGTTACACGAAAAAAATAG
ATTGTTACACTAAAAAAATAG
ATTGTTACACGAAAAAAATAG
ATTGTTACACTAAAAAAATAG
ATTGTTACACTAAAAAAATAG
ATTGTTACACGAAAAAAATAG
ATTGTTACACGAAAAAAATAG
ATTGTTACACGAAAAAAATAG
ATTGTTACACGAAAAAAATAG
ATTGTTACACTAAAAAAATAG
ATTGTTACACGAAAAAAATAG
ATTGTTACACGAAAAAAATAG
ATTGTTACACTAAAAAAATAG
ATTGTTACACTAAAAAAATAG
ATTGTTACACTAAAAAAATAG
ATTGTTACACTAAAAAAATAG
ATTGTTACACGAAAAAAATAG
ATTGTTACACGAAAAAAATAG
ATTGTTACACGAAAAAAATAG
ATTGTTACACTAAAAAAATAG
ATTGTTACACGAAAAAAATAG
ATTGTTACACTAAAAAAATAG
ATTGTTACACGAAAAAAATAG
ATTGTTACACGAAAAAAATAG
ATTGTTACACTAAAAAAATAG
ATTGTTACACTAAAAAAATAG
ATTGTTACACGAAAAAAATAG
ATTGTTACACTAAAAAAATAG
ATTGTTACACGAAAAAAATAG
ATTGTTACACTAAAAAAATAG
ATTGTTACACGAAAAAAATAG
ATTGTTACACTAAAAAAATAG
ATTGTTACACGAAAAAAATAG
ATTGTTACACTAAAAAAATAG
ATTGTTACACGAAAAAAATAG
ATTGTTACACTAAAAAAATAG

ATTGTTACACGAAAAAAATAG
ATTGTTACACTAAAAAAATAG
ATTGTTACACGAAAAAAATAG
ATTGTTACACTAAAAAAATAG
ATTGTTACACGAAAAAAATAG
ATTGTTACACTAAAAAAATAG
ATTGTTACACGAAAAAAATAG
ATTGTTACACTAAAAAAATAG
ATTGTTACACGAAAAAAATAG
ATTGTTACACTAAAAAAATAG
ATTGTTACACGAAAAAAATAG
ATTGTTACACTAAAAAAATAG
ATTGTTACACGAAAAAAATAG
ATTGTTACACTAAAAAAATAG
ATTGTTACACGAAAAAAATAG
ATTGTTACACTAAAAAAATAG
ATTGTTACACGAAAAAAATAG
ATTGTTACACTAAAAAAATAG
ATTGTTACACGAAAAAAATAG
ATTGTTACACTAAAAAAATAG
ATTGTTACACGAAAAAAATAG
ATTGTTACACTAAAAAAATAG
ATTGTTACACTAAAAAAATAG
ATTGTTACACGAAAAAAATAG
ATTGTTACACTAAAAAAATAG
ATTGTTACACTAAAAAAATAG
ATTGTTACACTAAAAAAATAG
ATTGTTACACGAAAAAAATAG
ATTGTTACACTAAAAAAATAG
ATTGTTACACTAAAAAAATAG
ATTGTTACACTAAAAAAATAG
ATTGTTACACTAAAAAAATAG
ATTGTTACACGAAAAAAATAG
ATTGTTACACGAAAAAAATAG
ATTGTTACACTAAAAAAATAG
ATTGTTACACGAAAAAAATAG
ATTGTTACACTAAAAAAATAG
ATTGTTACACGAAAAAAATAG
ATTGTTACACTAAAAAAATAG
ATTGTTACACGAAAAAAATAG
ATTGTTACACTAAAAAAATAG
ATTGTTACACGAAAAAAATAG
ATTGTTACACTAAAAAAATAG
ATTGTTACACGAAAAAAATAG
ATTGTTACACTAAAAAAATAG
ATTGTTACACGAAAAAAATAG
ATTGTTACACGAAAAAAATAG
ATTGTTACACGAAAAAAATAG
ATTGTTACACTAAAAAAATAG
ATTGTTACACTAAAAAAATAG
ATTGTTACACTAAAAAAATAG
ATTGTTACACTAAAAAAATAG
ATTGTTACACTAAAAAAATAG
ATTGTTACACAAAAAAATAG
ATTGTTACACTAAAAAAATAG
ATTGTTACACGAAAAAAATAG

ATTGTTACACTAAAAAAATAG
ATTGTTACACGAAAAAAATAG
ATTGTTACACTAAAAAAATAG
ATTGTTACACGAAAAAAATAG
ATTGTTACACTAAAAAAATAG
ATTGTTACACGAAAAAAATAG
ATTGTTACACTAAAAAAATAG
ATTGTTACACTAAAAAAATAG
ATTGTTACACTAAAAAAATAG
ATTGTTACACGAAAAAAATAG
ATTGTTACACGAAAAAAATAG
ATTGTTACACGAAAAAAATAG
ATTGTTACACGAAAAAAATAG
ATTGTTACACTAAAAAAATAG
ATTGTTACACGAAAAAAATAG
ATTGTTACACGAAAAAAATAG
ATTGTTACACGAAAAAAATAG
ATTGTTACACGAAAAAAATAG
ATTGTTACACTAAAAAAATAG
ATTGTTACACGAAAAAAATAG
ATTGTTACACTAAAAAAATAG
ATTGTTACACGAAAAAAATAG
ATTGTTACACGAAAAAAATAG
ATTGTTACACTAAAAAAATAG
ATTGTTACACTAAAAAAATAG
ATTGTTACACTAAAAAAATAG
ATTGTTACACGAAAAAAATAG
ATTGTTACACTAAAAAAATAG
ATTGTTACACTAAAAAAATAG
ATTGTTACACGAAAAAAATAG
ATTGTTACACTAAAAAAATAG
ATTGTTACACGAAAAAAATAG
ATTGTTACACTAAAAAAATAG
ATTGTTACACGAAAAAAATAG
ATTGTTACACTAAAAAAATAG
ATTGTTACACGAAAAAAATAG
ATTGTTACACTAAAAAAATAG
ATTGTTACACGAAAAAAATAG
ATTGTTACACTAAAAAAATAG
ATTGTTACACGAAAAAAATAG
ATTGTTACACTAAAAAAATAG
ATTGTTACACGAAAAAAATAG
ATTGTTACACTAAAAAAATAG
ATTGTTACACGAAAAAAATAG
ATTGTTACACTAAAAAAATAG
ATTGTTACACGAAAAAAATAG
ATTGTTACACTAAAAAAATAG
ATTGTTACACGAAAAAAATAG
ATTGTTACACTAAAAAAATAG
ATTGTTACACGAAAAAAATAG
ATTGTTACACTAAAAAAATAG
ATTGTTACACGAAAAAAATAG
ATTGTTACACTAAAAAAATAG
ATTGTTACACGAAAAAAATAG

ATTGTTACACTAAAAAAATAG
ATTGTTACACAAAAAAATAG
ATTGTTACACTAAAAAAATAG
ATTGTTACACTAAAAAAATAG
ATTGTTACACTAAAAAAATAG
ATTGTTACACTAAAAAAATAG
ATTGTTACACTAAAAAAATAG
ATTGTTACACTAAAAAAATAG
ATTGTTACACTAAAAAAATAG
ATTGTTACACGAAAAAAATAG
ATTGTTACACTAAAAAAATAG
ATTGTTACACGAAAAAAATAG
ATTGTTACACTAAAAAAATAG
ATTGTTACACTAAAAAAATAG
ATTGTTACACTAAAAAAATAG
ATTGTTACACGAAAAAAATAG
ATTGTTACACGAAAAAAATAG
ATTGTTACACTAAAAAAATAG
ATTGTTACACGAAAAAAATAG
ATTGTTACACTAAAAAAATAG
ATTGTTACACGAAAAAAATAG
ATTGTTACACTAAAAAAATAG
ATTGTTACACGAAAAAAATAG
ATTGTTACACTAAAAAAATAG
ATTGTTACACGAAAAAAATAG
ATTGTTACACTAAAAAAATAG
ATTGTTACACGAAAAAAATAG
ATTGTTACACTAAAAAAATAG
ATTGTTACACGAAAAAAATAG
ATTGTTACACTAAAAAAATAG
ATTGTTACACTAAAAAAATAG
ATTGTTACACTAAAAAAATAG
ATTGTTACACTAAAAAAATAG
ATTGTTACACGAAAAAAATAG
ATTGTTACACAAAAAAATAG
ATTGTTACACTAAAAAAATAG
ATTGTTACACTAAAAAAATAG
ATTGTTACACGAAAAAAATAG
ATTGTTACACGAAAAAAATAG
ATTGTTACACGAAAAAAATAG
ATTGTTACACGAAAAAAATAG
ATTGTTACACGAAAAAAATAG
ATTGTTACACGAAAAAAATAG
ATTGTTACACGAAAAAAATAG
ATTGTTACACGAAAAAAATAG
ATTGTTACACTAAAAAAATAG
ATTGTTACACGAAAAAAATAG
ATTGTTACACTAAAAAAATAG
ATTGTTACACGAAAAAAATAG
ATTGTTACACTAAAAAAATAG
ATTGTTACACGAAAAAAATAG
ATTGTTACACTAAAAAAATAG
ATTGTTACACGAAAAAATAG
ATTGTTACACTAAAAAAATAG

ATTGTTACACGAAAAAAATAG
ATTGTTACACTAAAAAAATAG
ATTGTTACACTAAAAAAATAG
ATTGTTACACTAAAAAAATAG
ATTGTTACACTAAAAAAATAG
ATTGTTACACGAAAAAAATAG
ATTGTTACACTAAAAAAATAG
ATTGTTACACTAAAAAAATAG
ATTGTTACACTAAAAAAATAG
ATTGTTACACGAAAAAAATAG
ATTGTTACACGAAAAAAATAG
ATTGTTACACGAAAAAAATAG
ATTGTTACACGAAAAAAATAG
ATTGTTACACTAAAAAAATAG
ATTGTTACACTAAAAAAATAG
ATTGTTACACTAAAAAAATAG
ATTGTTACACGAAAAAAATAG
ATTGTTACACTAAAAAAATAG
ATTGTTACACTAAAAAAATAG
ATTGTTACACTAAAAAAATAG
ATTGTTACACTAAAAAAATAG
ATTGTTACACGAAAAAAATAC
ATTGTTACACGAAAAAAATAG
ATTGTTACACTAAAAAAATAG
ATTGTTACACGAAAAAAATAG
ATTGTTACACTAAAAAAATAG
ATTGTTACACGAAAAAAATAG
ATTGTTAAACTAAAAAAATAG
ATTGTTACACGAAAAAAATAG
ATTGTTACACTAAAAAAATAG
ATTGTTACACGAAAAAAATAG
ATTGTTACACGAAAAAAATAG
ATTGTTACACGAAAAAAATAG
ATTGTTACACGAAAAAAATAG
ATTGTTACACGAAAAAAATAG
ATTGTTACACTAAAAAAATAG
ATTGTTACACTAAAAAAATAG
ATTGTTACACGAAAAAAATAG
ATTGTTACACTAAAAAAATAG
ATTGTTACACTAAAAAAATAG
ATTGTTACACTAAAAAAATAG
ATTGTTACACTAAAAAAATAG
ATTGTTACACGAAAAAAATAG
ATTGTTACACTAAAAAAATAG
ATTGTTACACGAAAAAAATAG
ATTGTTACACTAAAAAAATAG
ATTGTTACACGAAAAAAATAG
ATTGTTACACTAAAAAAATAG
ATTGTTACACGAAAAAAATAG
ATTGTTACACTAAAAAAATAG
ATTGTTACACGAAAAAAATAG
ATTGTTACACGAAAAAAATAG
ATTGTTACACGAAAAAAATAG
ATTGTTACACGAAAAGAAAAAAATAG

ATTGTTACACAAAAAATAG
ATTGTTACACGAAAAAATAG
ATTGTTACACGAAAAAATAG
ATTGTTACACGAAAAAATAG
ATTGTTACACGAAAAAATAG
ATTGTTACACGAAAAAATAG
ATTGTTACACGAAAAAATAG
ATTGTTACACGAAAAAATAG
ATTGTTACACGAAAAAAATAAAAAAAATAG
ATTGTTACACGAAAAAATAG
ATTGTTACACTAAAAAATAG
ATTGTTACACGAAAAAATAG
ATTGTTACACGAAAAAATAG
ATTGTTACACGAAAAAATAG
ATTGTTACACGAAAAAATAG
ATTGTTACACTAAAAAATAG
ATTGTTACACGAAAAAATAG
ATTGTTACACGAAAAAATAG
ATTGTTACACTAAAAAATAG
ATTGTTACACTAAAAAATAG
ATTGTTACACTAAAAAATAG
ATTGTTACACGAAAAAATAG
ATTGTTACACTAAAAAATAG
ATTGTTACACGAAAAAATAG
ATTGTTACACTAAAAAATAG
ATTGTTACACGAAAAAATAG
ATTGTTACACTAAAAAATAG
ATTGTTACACGAAAAAATAG
ATTGTACACAAAAAATAG
ATTGTTACACTAAAAAATAG
ATTGTTACACGAAAAAATAG
ATTGTTACACTAAAAAATAG
ATTGTTACACGAAAAAATAG
ATTGTTACACTAAAAAATAG
ATTGTTACACGAAAAAATAG
ATTGTTACACTAAAAAATAG
ATTGTTACACTAAAAAATAG
ATTGTTACACTAAAAAATAG
ATTGTTACACTAAAAAATAG
ATTGTTACACGAAAAAATAG
ATTGTTACACTAAAAAATAG
ATTGTTACACTAAAAAATAG
ATTGTTACACGAAAAAATAG
ATTGTTACACGAAAAAATAG
ATTGTTACACGAAAAAATAG
ATTGTTACACGAAAAAATAG
ATTGTTACACGAAAAAATAG
ATTGTTACACGAAAAAATAG
ATTGTTACACGAAAAAATAG
ATTGTTACACGAAAAAATAG
ATTGTTACACGAAAAAATAG
ATTGTTACACTAAAAAATAG
ATTGTTACACGAAAAAATAG
ATTGTTACACTAAAAAATAG

ATTGTTACACGAAAAAAATAG
ATTGTTACACTAAAAAAATAG
ATTGTTACACGAAAAAAATAG
ATTGTTACACTAAAAAAATAG
ATTGTTACACGAAAAAAATAG
ATTGTTACACTAAAAAAATAG
ATTGTTACACTAAAAAAATAG
ATTGTTACACGAAAAAAATAG
ATTGTTACACTAAAAAAATAG
ATTGTTACACTAAAAAAATAG
ATTGTTACACTAAAAAAATAG
ATTGTTACACTAAAAAAATAG
ATTGTTACACGAAAAAAATAG
ATTGTTACACTAAAAAAATAG
ATTGTTACACTAAAAAAATAG
ATTGTTACACGAAAAAAATAG
ATTGTTACACGAAAAAAATAG
ATTGTTACACGAAAAAAATAG
ATTGTTACACGAAAAAAATAG
ATTGTTACACGAAAAAAATAG
ATTGTTACACGAAAAAAATAG
ATTGTTACACGAAAAAAATAG
ATTGTTACACGAAAAAAATAG
ATTGTTACACTAAAAAAATAG
ATTGTTACACGAAAAAAATAG
ATTGTTACACTAAAAAAATAG
ATTGTTACACGAAAAAAATAG
ATTGTTACACTAAAAAAATAG
ATTGTTACACGAAAAAAATAG
ATTGTTACACTAAAAAAATAG
ATTGTTACACTAAAAAAATAG
ATTGTTACACTAAAAAAATAG
ATTGTTACACTAAAAAAATAG
ATTGTTACACGAAAAAAATAG
ATTGTTACACGAAAAAAATAG
ATTGTTACACTAAAAAAATAG
ATTGTTACACTAAAAAAATAG
ATTGTTACACGAAAAAAATAG
ATTGTTACACTAAAAAAATAG
ATTGTTACACTAAAAAAATAG
ATTGTTACACTAAAAAAATAG
ATTGTTACACTAAAAAAATAG
ATTGTTACACTAAAAAAATAG
ATTGTTACACGAAAAAAATAG
ATTGTTACACTAAAAAAATAG
ATTGTTACACGAAAAAAATAG
ATTGTTACACGAAAAAAATAG
ATTGTTACACTAAAAAAATAG
ATTGTTACACGAAAAAAATAG
ATTGTTACACTAAAAAAATAG
ATTGTTACACGAAAAAAATAG
ATTGTTACACTAAAAAAATAG
ATTGTTACACGAAAAAAATAG
ATTGTTACACTAAAAAAATAG

ATTGTTACACTAAAAAAATAG
ATTGTTACACTAAAAAAATAG
ATTGTTACACTAAAAAAATAG
ATTGTTACACGAAAAAAATAG
ATTGTTACACGAAAAAAATAG
ATTGTTACACTAAAAAAATAG
ATTGTTACACTAAAAAAATAG
ATTGTTACACGAAAAAAATAG
ATTGTTACACTAAAAAAATAG
ATTGTTACACTAAAAAAATAG
ATTGTTACACTAAAAAAATAG
ATTGTTACACTAAAAAAATAG
ATTGTTACACTAAAAAAATAG
ATTGTTACACGAAAAAAATAG
ATTGTTACACTAAAAAAATAG
ATTGTTACACGAAAAAAATAG
ATTGTTACACTAAAAAAATAG
ATTGTTACACTAAAAAAATAG
ATTGTTACACTAAAAAAATAG
ATTGTTACACTAAAAAAATAG
ATTGTTACACTAAAAAAATAG
ATTGTTACACTAAAAAAATAG
ATTGTTACACGAAAAAAATAG
ATTGTTACACTAAAAAAATAG
ATTGTTACACGAAAAAAATAG
ATTGTTACACTAAAAAAATAG
ATTGTTACACGAAAAAAATAG
ATTGTTACACTAAAAAAATAG
ATTGTTACACTAAAAAAATAG
ATTGTTACACTAAAAAAATAG
ATTGTTACACTAAAAAAATAG
ATTGTTACACGAAAAAAATAG
ATTGTTACACTAAAAAAATAG
ATTGTTACACTAAAAAAATAG
ATTGTTACACTAAAAAAATAG
ATTGTTACACGAAAAAAATAG
ATTGTTACACGAAAAAAATAG
ATTGTTACACAAAAAAAATAG
ATTGTTACACGAAAAAATAG
ATTGTTACACGAAAAAAATAG
ATTGTTACACTAAAAAAATAG
ATTGTTACACTAAAAAAATAG
ATTGTTACACTAAAAAAATAG
ATTGTTACACTAAAAAAATAG
ATTGTTACACTAAAAAAATAG
ATTGTTACACTAAAAAAATAG
ATTGTTACACTAAAAAAATAG
ATTGTTACACTAAAAAAATAG
ATTGTTACACTAAAAAAATAG
ATTGTTACACGAAAAAAATAG
ATTGTTAAACTAAAAAAATAG
ATTGTTACACGAAAAAAATAG
ATTGTTACACTAAAAAAATAG
ATTGTTACACGAAAAAAATAG

ATTGTTACACTAAAAAATAG
ATTGTTACACGAAAAAATAG
ATTGTTACACGAAAAAATAG
ATTGTTACACGAAAAAATAG
ATTGTTACACTAAAAAATAG
ATTGTTACACGAAAAAATAC
ATTGTTACACTAAAAAATAG
ATTGTTACACGAAAAAATAC
ATTGTTACACTAAAAAATAG
ATTGTTACACTAAAAAATAG
ATTGTTACACGAAAAAATAC
ATTGTTACACTAAAAAATAG
ATTGTTACACTAAAAAATAG
ATTGTTACACGAAAAAATAG
ATTGTTACACTAAAAAATAG
ATTGTTACACGAAAAAATAC
ATTGTTACACTAAAAAATAG
ATTGTTACACGAAAAAATAC
ATTGTTACACTAAAAAATAG
ATTGTTACACTAAAAAATAG
ATTGTTACACGAAAAAATAC
ATTGTTACACTAAAAAATAG
ATTGTTACACGAAAAAATAC
ATTGTTACACTAAAAAATAG
ATTGTTACACGAAAAAATAG
ATTGTTACACTAAAAAATAG
ATTGTTACACGAAAAAATAC
ATTGTTACACTAAAAAATAG
ATTGTTACACGAAAAAATAC
ATTGTTACACGAAAAAATAG
ATTGTTACACTAAAAAATAG
ATTGTTACACTAAAAAATAG
ATTGTTACACTAAAAAATAG
ATTGTTACACTAAAAAATAG
ATTGTTACACTAAAAAATAG
ATTGTTACACGAAAAAATAG
ATTGTTAAACTAAAAAATAG
ATTGTTACACGAAAAAATAG
ATTGTTACACTAAAAAATAG
ATTGTTACACGAAAAAATAG
ATTGTTACACTAAAAAATAG
ATTGTTACACGAAAAAATAG
ATTGTTACACGAAAAAATAG
ATTGTTACACGAAAAAATAG
ATTGTTACACTAAAAAATAG
ATTGTTACACTAAAAAATAG
ATTGTTACACGAAAAAATAG
ATTGTTACACTAAAAAATAG
ATTGTTACACTAAAAAATAG
ATTGTTACACTAAAAAATAG
ATTGTTACACTAAAAAATAG
ATTGTTACACGAAAAAATAG
ATTGTTACACTAAAAAATAG
ATTGTTACACGAAAAAATAG

ATTGTTACACGAAAAAAATAG
ATTGTTACACGAAAAAAATAG
ATTGTTACACTAAAAAAATAG
ATTGTTACACGAAAAAAATAC
ATTGTTACACTAAAAAAATAG
ATTGTTACACTAAAAAAATAG
ATTGTTACACGAAAAAAATAC
ATTGTTACACTAAAAAAATAG
ATTGTTACACGAAAAAAATAC
ATTGTTACACTAAAAAAATAG
ATTGTTACACGAAAAAAATAG
ATTGTTACACTAAAAAAATAG
ATTGTTACACGAAAAAAATAC
ATTGTTACACTAAAAAAATAG
ATTGTTACACGAAAAAAATAC
ATTGTTACACGAAAAAAATAG
ATTGTTACACTAAAAAAATAG
ATTGTTACACTAAAAAAATAG
ATTGTTACACTAAAAAAATAG
ATTGTTACACTAAAAAAATAG
ATTGTTACACTAAAAAAATAG
ATTGTTACACGAAAAAAATAG
ATTGTTAAACTAAAAAAATAG
ATTGTTACACGAAAAAAATAG
ATTGTTACACTAAAAAAATAG
ATTGTTACACGAAAAAAATAG
ATTGTTACACTAAAAAAATAG
ATTGTTACACGAAAAAAATAG
ATTGTTACACGAAAAAAATAG
ATTGTTACACGAAAAAAATAG
ATTGTTACACTAAAAAAATAG
ATTGTTACACTAAAAAAATAG
ATTGTTACACGAAAAAAATAG
ATTGTTACACTAAAAAAATAG
ATTGTTACACTAAAAAAATAG
ATTGTTACACTAAAAAAATAG
ATTGTTACACGAAAAAAATAG
ATTGTTACACTAAAAAAATAG
ATTGTTACACTAAAAAAATAG
ATTGTTACACTAAAAAAATAG
ATTGTTACACGAAAAAAATAG
ATTGTTACACGAAAAAAATAG
ATTGTTACACTAAAAAAATAG
ATTGTTACACTAAAAAAATAG
ATTGTTACACGAAAAAAATAG
ATTGTTACACTAAAAAAATAG
ATTGTTACACTAAAAAAATAG
ATTGTTACACTAAAAAAATAG
ATTGTTACACTAAAAAAATAG
ATTGTTACACGAAAAAAATAG
ATTGTTACACGAAAAAAATAG
ATTGTTACACTAAAAAAATAG
ATTGTTACACTAAAAAAATAG
ATTGTTACACTAAAAAAATAG

ATTGTTACACTAAAAAAATAG
ATTGTTACACGAAAAAAATAG
ATTGTTACACTAAAAAAATAG
ATTGTTACACGAAAAAAATAG
ATTGTTACACTAAAAAAATAG
ATTGTTACACTAAAAAAATAG
ATTGTTACACGAAAAAAATAG
ATTGTTACACTAAAAAAATAG
ATTGTTACACGAAAAAAATAG
ATTGTTAAACTAAAAAAATAG
ATTGTTACACGAAAAAAATAG
ATTGTTACACTAAAAAAATAG
ATTGTTACACGAAAAAAATAG
ATTGTTACACTAAAAAAATAG
ATTGTTACACGAAAAAAATAG
ATTGTTACACGAAAAAAATAG
ATTGTTACACGAAAAAAATAG
ATTGTTACACGAAAAAAATAG
ATTGTTACACTAAAAAAATAG
ATTGTTACACTAAAAAAATAG
ATTTTTACACGAAAAAAATAG
ATTGTTACACTAAAAAAATAG
ATTGTTACACGAAAAAAATAG
ATTGTTACACTAAAAAAATAG
ATTGTTACACGAAAAAAATAG
ATTGTTACACTAAAAAAATAG
ATTGTTACACGAAAAAAATAG
ATTGTTACACTAAAAAAATAG
ATTGTTACACGAAAAAAATAG
ATTGTTACACTAAAAAAATAG
ATTGTTACACGAAAAAAATAG
ATTGTTACACTAAAAAAATAG
ATTGTTACACGAAAAAAATAG
ATTGTTACACTAAAAAAATAG
ATTGTTACACGAAAAAAATAG
ATTGTTACACTAAAAAAATAG
ATTGTTACACTAAAAAAATAG
ATTGTTACACTAAAAAAATAG
ATTGTTACACGAAAAAAATAG
ATTGTTACACTAAAAAAATAG
ATTGTTACACTAAAAAAATAG
ATTGTTACACGAAAAAAATAC
ATTGTTACACTAAAAAAATAG
ATTGTTACACGAAAAAAATAC
ATTGTTACACGAAAAAAATAG
ATTGTTACACTAAAAAAATAG
ATTGTTACACTAAAAAAATAG
ATTGTTACACTAAAAAAATAG
ATTGTTACACTAAAAAAATAG
ATTGTTACACTAAAAAAATAG
ATTGTTACACTAAAAAAATAG
ATTGTTACACGAAAAAAATAG
ATTGTTAAACTAAAAAAATAG
ATTGTTACACGAAAAAAATAG

ATTGTTACACTAAAAATAG
ATTGTTACACGAAAAAATAG
ATTGTTACACTAAAAAATAG
ATTGTTACACGAAAAAATAG
ATTGTTACACGAAAAAATAG
ATTGTTACACGAAAAAATAG
ATTGTTACACTAAAAAATAG
ATTGTTACACTAAAAAATAG
ATTGTTACACGAAAAAATAC
ATTGTTACACTAAAAAATAG
ATTGTTACACGAAAAAATAC
ATTGTTACACGAAAAAATAG
ATTGTTACACTAAAAAATAG
ATTGTTACACTAAAAAATAG
ATTGTTACACTAAAAAATAG
ATTGTTACACTAAAAAATAG
ATTGTTACACTAAAAAATAG
ATTGTTACACTAAAAAATAG
ATTGTTACACTAAAAAATAG
ATTGTTACACGAAAAAATAG
ATTGTTAAACTAAAAAATAG
ATTGTTACACGAAAAAATAG
ATTGTTACACTAAAAAATAG
ATTGTTACACGAAAAAATAG
ATTGTTACACTAAAAAATAG
ATTGTTACACGAAAAAATAG
ATTGTTACACGAAAAAATAG
ATTGTTACACGAAAAAATAG
ATTGTTACACTAAAAAATAG
ATTGTTACACTAAAAAATAG
ATTGTTACACGAAAAAATAG
ATTGTTACACTAAAAAATAG
ATTGTTACACTAAAAAATAG
ATTGTTACACTAAAAAATAG
ATTGTTACACGAAAAAATAG
ATTGTTACACGAAAAAATAG
ATTGTTACACTAAAAAATAG
ATTGTTACACGAAAAAATAG
ATTGTTACACTAAAAAATAG
ATTGTTACACGAAAAAATAG
ATTGTTACACTAAAAAATAG
ATTGTTACACTAAAAAATAG
ATTGTTACACGAAAAAATAC
ATTGTTACACTAAAAAATAG
ATTGTTACACGAAAAAATAC
ATTGTTACACGAAAAAATAG
ATTGTTACACTAAAAAATAG
ATTGTTACACTAAAAAATAG
ATTGTTACACTAAAAAATAG
ATTGTTACACTAAAAAATAG
ATTGTTACACTAAAAAATAG
ATTGTTACACTAAAAAATAG
ATTGTTACACGAAAAAATAG
ATTGTTAAACTAAAAAATAG

ATTGTTACACGAAAAAATAG
ATTGTTACACTAAAAAATAG
ATTGTTACACGAAAAAATAG
ATTGTTACACTAAAAAATAG
ATTGTTACACGAAAAAATAG
ATTGTTACACGAAAAAATAG
ATTGTTACACGAAAAAATAG
ATTGTTACACTAAAAAATAG
ATTGTTACACTAAAAAATAG
ATTGTTACACGAAAAAATAC
ATTGTTACACTAAAAAATAG
ATTGTTACACGAAAAAATAC
ATTGTTACACGAAAAAATAG
ATTGTTACACTAAAAAATAG
ATTGTTACACTAAAAAATAG
ATTGTTACACTAAAAAATAG
ATTGTTACACTAAAAAATAG
ATTGTTACACTAAAAAATAG
ATTGTTACACTAAAAAATAG
ATTGTTACACTAAAAAATAG
ATTGTTACACGAAAAAATAG
ATTGTTAAACTAAAAAATAG
ATTGTTACACGAAAAAATAG
ATTGTTACACTAAAAAATAG
ATTGTTACACGAAAAAATAG
ATTGTTACACTAAAAAATAG
ATTGTTACACGAAAAAATAG
ATTGTTACACGAAAAAATAG
ATTGTTACACGAAAAAATAG
ATTGTTACACTAAAAAATAG
ATTGTTACACTAAAAAATAG
ATTGTTACACGAAAAAATAG
ATTGTTACACTAAAAAATAG
ATTGTTACACTAAAAAATAG
ATTGTTACACTAAAAAATAG
ATTGTTACACGAAAAAATAG
ATTGTTACACGAAAAAATAG
ATTGTTACACTAAAAAATAG
ATTGTTACACGAAAAAATAG
ATTGTTACACTAAAAAATAG
ATTGTTACACGAAAAAATAG
ATTGTTACACTAAAAAATAG
ATTGTTACACGAAAAAATAG
ATTGTTACACGAAAAAATAG
ATTGTTACACGAAAAAATAG
ATTGTTACACGAAAAAATAG
ATTGTTACACGAAAAAATAG
ATTGTTACACTAAAAAATAG
ATTGTTACACGAAAAAATAG
ATTGTTACACGAAAAAATAG
ATTGTTACACGAAAAAATAG
ATTGTTACACGAAAAAATAG
ATTGTTACACGAAAAAATAG
ATTGTTACACGAAAAAATAG

ATTGTTACACGAAAAAAATAG
ATTGTTACACTAAAAAAATAG
ATTGTTACACGAAAAAAATAG
ATTGTTACACGAAAAAAATAG
ATTGTTACACGAAAAAAATAG
ATTGTTACACGAAAAAAATAG
ATTGTTACACTAAAAAAATAG
ATTGTTACACGAAAAAAATAG
ATTGTTACACGAAAAAAATAG
ATTGTTACACTAAAAAAATAG
ATTGTTACACGAAAAAAATAG
ATTGTTACACTAAAAAAATAG
ATTGTTACACGAAAAAAATAG
ATTGTTACACTAAAAAAATAG
ATTGTTACACGAAAAAAATAG
ATTGTTACACTAAAAAAATAG
ATTGTTACACTAAAAAAATAG
ATTGTTACACTAAAAAAATAG
ATTGTTACACTAAAAAAATAG
ATTGTTACACGAAAAAAATAG
ATTGTTACACTAAAAAAATAG
ATTGTTACACGAAAAAAATAG
ATTGTTACACGAAACATAG
ATTGTTACACGAAAAAAATAG
ATTGTTACACTAAAAAAATAG
ATTGTTACACTAAAAAAATAA
ATTGTTACACTAAAAAAATAG
ATTGTTACACTAAAAAAATAG
ATTGTTACACTAAAAAAATAG
ATTGTTACACTAAAAAAATAG
ATTGTTACACTAAAAAAATAG
ATTGTTACACTAAAAAAATAG
ATTGTTACACTAAAAAAATAA
ATTGTTAAACTAAAAAAATAG
ATTGTTACACTAAAAAAATAG
ATTGTTACACTAAAAAAATAA
ATTGTTACACTAAAAAAATAG
ATTGTTACACTAAAAAAATAG
ATTGTTACACTAAAAAAATAG
ATTGTTACACTAAAAAAATAG
ATTGTTACACGAAACATAG
ATTGTTACACGAAAAAAATAG
ATTGTTACACGAAACATAG
ATTGTTACACGAAAAAAATAG
ATTGTTACACTAAAAAAATAG
ATTGTTACACTAAAAAAATAG
ATTGTTACACGAAAAAAATAG
ATTGTTACACGAAACATAG
ATTGTTACACGAAAAAAATAG
ATTGTTACACTAAAAAAATAG
ATTGTTAAACTAAAAAAATAG
ATTGTTACACTAAAAAAATAG
ATTGTTACACTAAAAAAATAG
ATTGTTACACTAAAAAAATAG

ATTGTTACACTAAAAAAATAG
ATTGTTACACTAAAAAAATAG
ATTGTTACACTAAAAAAATAG
ATTGTTACACTAAAAAAATAG
ATTGTTACACTAAAAAAATAG
ATTGTTACACTAAAAAAATAG
ATTGTTACACGAAAAAAATAG
ATTGTTACACTAAAAAAATAG
ATTGTTACACGAAAAAAATAG
ATTGTTACACGAAACATAG
ATTGTTACACGAAAAAAATAG
ATTGTTACACTAAAAAAATAG
ATTGTTACACTAAAAAAATAG
ATTGTTACACTAAAAAAATAG
ATTGTTACACGAAAAAAATAG
ATTGTTACACTAAAAAAATAG
ATTGTTACACGAAAAAAATAG
ATTGTTACACTAAAAAAATAG
ATTGTTACACTAAAAAAATAA
ATTGTTAAACTAAAAAAATAG
ATTGTTACACTAAAAAAATAG
ATTGTTACACTAAAAAAATAA
ATTGTTACACTAAAAAAATAG
ATTGTTACACTAAAAAAATAG
ATTGTTACACGAAACATAG
ATTGTTACACGAAAAAAATAG
ATTGTTACACGAAACATAG
ATTGTTACACGAAAAAAATAG
ATTGTTACACTAAAAAAATAG
ATTGTTACACTAAAAAAATAG
ATTGTTACACGAAAAAAATAG
ATTGTTACACGAAACATAG
ATTGTTACACGAAAAAAATAG
ATTGTTACACTAAAAAAATAG
ATTGTTAAACTAAAAAAATAG
ATTGTTACACTAAAAAAATAG
ATTGTTACACTAAAAAAATAG
ATTGTTACACTAAAAAAATAG
ATTGTTACACTAAAAAAATAG
ATTGTTACACTAAAAAAATAG
ATTGTTACACTAAAAAAATAG
ATTGTTACACTAAAAAAATAG
ATTGTTACACTAAAAAAATAG
ATTGTTACACTAAAAAAATAG
ATTGTTACACGAAAAAAATAG
ATTGTTACACTAAAAAAATAG
ATTGTTACACGAAAAAAATAG
ATTGTTACACGAAACATAG
ATTGTTACACGAAAAAAATAG
ATTGTTACACTAAAAAAATAG
ATTGTTACACTAAAAAAATAG
ATTGTTACACTAAAAAAATAG
ATTGTTACACGAAAAAAATAG
ATTGTTACACTAAAAAAATAG

ATTGTTACACTAAAAAAATAG
ATTGTTACACTAAAAAAATAG
ATTGTTACACTAAAAAAATAG
ATTGTTACACGAAAAAAATAG
ATTGTTACACTAAAAAAATAG
ATTGTTACACGAAAAAAATAG
ATTGTTACACTAAAAAAATAG
ATTGTTACACGAAAAAAATAG
ATTGTTACACTAAAAAAATAG
ATTGTTACACGAAACATAG
ATTGTTACACGAAAAAAATAG
ATTGTTACACTAAAAAAATAG
ATTGTTACACTAAAAAAATAG
ATTGTTACACTAAAAAAATAG
ATTGTTACACTAAAAAAATAG
ATTGTTACACTAAAAAAATAG
Nr of selected repeats 812 Similarity 0.960325
Consensus:
ATTGTTACACtAAAAAAATAG
>Cele-UNSB01_2:4095823-4096643 Satlength=821 Nr of Repeats=4 RepeatLength=16
seed=TGCCTACGTG
TGCCTACGTGCCTACG
TGCCTACGTGCCTACG
TGCCTACGTGCCTACG
TGCCTACCTGAAGTATGCGCAAGACCAGCAGGCAGGAACGTAAAAGGCATACTTTTTCTAGATTGTCCCCTGAAATTTT
TTAATATCTTCATATTTCTGTAGTTCAAAAAACTTGTTTATTACATCTTCAAATTATTATTATTATTAGGATTATCGTG
TGTCACAAAATAAAGAAATAAATTATTCAATTATTTGTCTGTTTTAATTTTCACCAGCTCACGGACGTTGGGAATAAGG
ATAGAGAAATGAGAAATACAACAAAAAATAATAAATATTTCAAATGAAAATGAAACTTGAGCAAAACAAAAAAAAAACA
TATTTATTTGATTTTGCCGGCAGCGGAGGTGGCAAAAAGGCATTGTCTGGGGTGGGGGGAATTCTAAATTTAGAATTTC
ATAAATTCTTCGGGAAAAAAAAATTTAAAATTCCATCATCTATAGTCAGATACGATAGCGTCCAAAGTTGAGAAGAAGT
CTTCTGGAGCGTAAATTCGGGAGTTTTGGAGCCGGAACTTCTTCGATTTCTCGACGTGTTGGACCCCGTCGCTTCTCGG
TTTGCCTTGAGTCGAAGACGGTTTTGAGCACGTTTACCATCTTCCAGAATGTGTAATCAGGTCGGAGAGCTACGATGTG
GGACGAGAAGCTGGAAATAAAAATTTTGGTTTTTGAAGGAAATAAAATATTTGCCCACAAGGTTTCAGAATTTTGTAAT
ATTCAAAAATTATAATTTTAGATGTTAGGATTTTCATGATAGAAACGCCTGCCTGCCTGCG
Nr of selected repeats 3 Similarity 1.000000
Consensus:
TGCCTACGTGCCTACG
>Cele-UNSB01_2:4119390-4119751 Satlength=362 Nr of Repeats=4 RepeatLength=21
seed=CTACGTAAGA
CTACGTAAGAATGACACAGGA
CTACGTAAGAGTAGCGCAGGA
CTACGTAAGAGTAGCGCAGGA
CTACGTAGGAGCGGTTCATGGCCATACTAGAAGTCCGATTTCGAATCGGCTCCCCGGAAACCATTGTACAGGTAATATA
GTCATACGATCTGGAATAGTTGCACGATCGAGTTCCATGCCGAACCGTCGGCAGATAATCCAGACTTAACATTGCAAGT
GTGATCTTCTCCAGGTTTACCGAGTGTTTCGACATGGCGTTCCTGTATATTCCTTCTAGAGTCTATCTCTTGCACTCTC
TCCCGCGCGACTTTCACGTTTTCAGTTTTCAAATGCCCCCGTTCCCGCTACAGTCGTCAGG
Nr of selected repeats 3 Similarity 0.830688
Consensus:
CTACGTAAGAgTagCgCAGGA
>Cele-UNSB01_2:4146856-4147072 Satlength=217 Nr of Repeats=14 RepeatLength=12
seed=CTTAGGCTTA
CTTAGGCTTAGA
CTTAGGTTTAGA
CTGAGGCTTAAA

CTTAGGCTGAGACTGAGGT
TTAGGCTTAGG
CTTAGGCTGAGGCTTGGATTTAAGTTTGGG
CTTAGGCTTATG
CTTAGGCTCAGG
CTCAGGCTTAGG
CTTAGACTTAGA
CTTAGTCTTAGG
CTTAGGCTTAGG
CTTAAGCTTAGG
CATAGGCTTAGGCTTAGACATAGGCGTAGACTTAAG
Nr of selected repeats 10 Similarity 0.748148
Consensus:
CTtAGgCTTAgg
>Cele-UNSB01_2:4159337-4159593 Satlength=257 Nr of Repeats=13 RepeatLength=11
seed=GTACTGTAGG
GTACTGTAGGACAGCTGTAGGG
GTACTGTAGGA
GTACTGTAGGAAAACTGTAGGAGCACTGTAAGAGTACTGTAAAG
GTACTGTAGGA
GTACTGTAGGT
GTACTGTAGGG
GTACTTTAGGAGTTCTGTGAGATACTGTAACTCTTGAAAATTGAGTTTTTGGTTTTTGAAGAAAGAGTG
GTACTGTAGGA
GTACTGTAGAG
GTACTGTTGGAGTACTCTACGA
GTACTGTAGGT
GTACTGTAAGG
GTACTGTAGGA
Nr of selected repeats 9 Similarity 0.735043
Consensus:
GTACTGTAGGn
>Cele-UNSB01_2:4203805-4204480 Satlength=676 Nr of Repeats=4 RepeatLength=11
seed=TGCCTGCCTA
TGCCTGCCTAT
TGCCTGCCTAC
TGCCCGCCTAACCATGAAATAATTTTATAAACTGAAATAAATTTTGTTAATTTCAGTATAAAATACAGGAATCTTGAAA
AAACTCCAAACATCTAAAAATACTCTCAAAATATAGAAAACAAAGCGAAAGAAAAAAGAAAAGAAAAAAGAAGCGATAA
AGATTGGAACGGAACTTGAATATAATTAACTAGAAATTTGGAGAAAGTGGGTGAGAGGGGCAAGGAAAATGAATGTGTT
GAAGAGGCAGAATGGTTTGTTGGACATCAAATCCACGATTACTGTTCACCACATTTTTTATTTATTTTGTGGCTTTAGT
GTTAATATTTTATTCCAGAAAGAAACTATATGTTCCGGAAATAATGTTGCTCAAATTTTAATTATACGACCTAGAACTT
ATATTTTTCGCTGAAAATTCAAATTTTTCATATGGAATTTCTAATGTTTCAAGCTAAAATTTCACATTTTGACAGAAGT
TTCAGATTTAAGATTCTTGATTATGATTTTAAAAAAGGATTTAAAAATTTAACTAAACAGGTTTTGCAAAAGTTGAACT
TAAGGTTCTCAACTTTTCAACTATTTTTTTGGATATAATATTAACTCAGTCTCTTTTATATTACTCACATTGATAGCAG
TTTGCCTTTC
TGCCTGCCTAC
Nr of selected repeats 3 Similarity 0.919192
Consensus:
TGCCTGCCTAc
>Cele-UNSB01_2:4205915-4205959 Satlength=45 Nr of Repeats=4 RepeatLength=11
seed=GGCAGGCAGT
GGCAGGCAGTG
GGCAGGCAGTA
GGCAGGCAGTA

GGCAGGCAGTC
Nr of selected repeats 4 Similarity 0.898990
Consensus:
GGCAGGCAGTa
>Cele-UNSB01_2:4270872-4271040 Satlength=169 Nr of Repeats=5 RepeatLength=24
seed=GTGAGGTACG
GTGAGGTACGCA
GTGAGGTACGAGATGAGGTACAAG
GTGAGATACGATGTGAGGTCTGGG
GTGAGGTGCGAGGCGAGGCAGGAG
GTGAGGTACGAGGCCAGGTACTATGCGAGGTATGAGGTGAGGGGCGAGATGAGGTACTAGGCGAGGTGGGAGGCATGAT
ATGAG
Nr of selected repeats 3 Similarity 0.611111
Consensus:
GTGAGgTaCGAggtGAGGtangaG
>Cele-UNSB01_2:4272676-4272936 Satlength=261 Nr of Repeats=25 RepeatLength=10
seed=GCGTTAGCAG
GCGTTAGCAG
GCGTTAGCAG
GCATTAGCAG
GCGTTAGCAG
GCGTTAGCAG
GCGTAAGCAG
GCGTTAGCAG
GCGTTAGCAG
GCGTTAGCAG
GCGTTAGCAG
GCGTTAGCAG
GCGTTAGCAG
GCGTTAGCAG
GCGTTAGCAG
GCGTTAGCAG
GCGTTAGCAG
GCGTTAGCAG
GCGTTAGCAG
GCGTTAGCAG
GCGTTAGCAG
GCGTTAGCAG
GCGTTAGCAG
GCGTTAGCAG
GCGTTAGCAG
GCGTTAGCAGGCGTTAGCAG
Nr of selected repeats 24 Similarity 0.977778
Consensus:
GCGTTAGCAG
>Cele-UNSB01_2:4275898-4276001 Satlength=104 Nr of Repeats=5 RepeatLength=18
seed=TGCAAGACTA
TGCAAGACTAATAGAGGC
TGCAAGACTAATTTTCGATTGATCCGTAGGGG
TGCAAGACTAATAGGGG
TGCAAGACTAATAGAGAC
TGCAAGACTAATAGAGAC
Nr of selected repeats 3 Similarity 0.950617
Consensus:

TGCAAGACTAATAGAGaC
>Cele-UNSB01_2:4325046-4325721 Satlength=676 Nr of Repeats=27 RepeatLength=25
seed=GAGAGTAAAA
GAGAGTAAAATATACGGTGAAGCCA
GAGAGTAAAATATACGGTGAAGCCA
GAGAGTAAAACATACGGTGAAGCCA
GAGAGTAAAATATTCTGTGAAGCCA
GAGAGTAAAATATTCTGTGAAGCCA
GAGAGTAAAATATTCTGTGAAGCCA
GAGAGTAAAATATACGATGAAGCCA
GAGAGTAAAATATACGGTGAAGCCA
GAGAGTAAAATATACGGTGAAGCCA
GAGAGTAAAATATACGGTGAAGCCA
GAGAGTAAAATATACGGTGAAGCCA
GAGAGTAAAATATACGGTGAAGCCA
GAGAGTAAAATATTCTGTGAAGCCA
GAGAGTAAAATATTCTGTGAAGCCA
GAGAGTAAAATATTCTGTGAAGCCA
GAGAGTAAAATATTCTGTGAAGCCA
GAGAGTAAAATATTCTGTGAAGCCA
GAGAGTAAAATATACGGTGAAGCCA
GAGAGTAAAATATACGGTGAAGCCA
GAGAGTAAAATATACGGTGAAGCCA
GAGAGTAAAATATACGGTGAAGCCA
GAGAGTAAAATATACGGTGAAGCCA
GAGAGTAAAATATACGGTGAAGCCA
GAGAGTAAAATATACGGTGAAGCCA
GAGAGTAAAATATACGGTGAAGCCA
GAGAGTAAAATATTCTGTGAAGCCA
GAGAGTAAAATATTCTGTGAAGGCA
Nr of selected repeats 27 Similarity 0.936486
Consensus:
GAGAGTAAAATATaCgGTGAAGCCA
>Cele-UNSB01_2:4325748-4326453 Satlength=706 Nr of Repeats=45 RepeatLength=15
seed=CTCACCACGA
CTCACCACGACGGGT
CTCACCACGAAGGGT
CTCACCACGAAAGGA
CTTACCACGATGGGT
CTCACCACGATAAGT
CTCACCACGAAGGGTA
TCACCACGAAGGGTCTTACCATGAAGGGT
CTCACCACGAAGGGT
CTCACCACGATGGGTCTGACTACGATGCGT
CTCACCACGAAGGGC
CTCACCACGAAAGGT
CTCACCACGAAGGGC
CTTACCACGAAAGGT
CTCACCACGAAGGGT
CTCACCACGAAAGGT
CTCACCACGAATGGT
CTCACCACGAAGGGT
CTCATCACGAAAGGT
CTCACCACGAAGGGC

CTTACCACGAAAGGT
CTCACCACGAAGGGT
CTCACCACGAAAGGT
CTCACCACGAATGGT
CTCACCACGAAGGGT
CTCATCACGAAAGGT
CTCACCACGAAGGGC
CTCACCACGAATGGT
CTCACCACGAAGGGT
CTCACCACGAAAGGT
CTCACCACGATGGGT
CTCACCACGAAGGGT
CTCACCACGGAAGGT
CTCACCACGAAGGGT
CTCATCACGAAAGGT
CTCACCACGAAGGGCT
TCACCACGAATGGT
CTCACCACGAAGGGT
CTCACCACGGAAGGT
CTCACCACGAAGGGT
CTCATCACGAAAGGT
CTCACCACGAAGGGC
CTCACCACGAATGGT
CTCACCACGAAGGGT
CTCACCACGGAAGGT
CTCACCACGAAGTTC
Nr of selected repeats 40 Similarity 0.798251
Consensus:
CTCACCACGAAgGGT
>Cele-UNSB01_2:4361834-4362159 Satlength=326 Nr of Repeats=8 RepeatLength=35
seed=TTCCGGCAAA
TTCCGGCAAATCGGCAAAACTGAACAT
TTCCGGCAAAACGGCAAATTGTCAAAATTGAAAAT
TTCCGGCAAATCCGCAATATGCCGAAATTGGAAAACGCCGGCAAAACGGCAAAAGCCAAAATTGAAAAC
TTCCGGCAAAATGGCAAGTTGCCAAATTTGAAAAT
TTCCGGCAAGACGGCAATATGCCAAAATTAAAAAA
TTCCGGCAAAACGGCAAATTGCCAAAATTTAAAAAATCCGGCAGAACGGCAAAC
TTCCGGGAAAACGGCAAATTGCCAAATATGGAAAT
TTCCGGGAAAACGGCAAATTGCCAAAATTAAAAAA
Nr of selected repeats 5 Similarity 0.790476
Consensus:
TTCCGGcAAaAcGGCAAatTGcCAAAatTgaAAAt
>Cele-UNSB01_2:4377229-4377482 Satlength=254 Nr of Repeats=23 RepeatLength=11
seed=CAGTTGTCAC
CAGTTGTCACT
CAGTTATCACC
CAGTTGTCACT
CAGTTGTCACT
CAGTTGTCACC
CAGTTGTCACC
CAGTAGTCACT
CAGTTGTCACC
CAGTTGTCACT
CAGTTGTCACC

CAGTTGTCACT
CAGTTGTCACC
CAGTTGTCACT
CAGTTGTCACT
CAGTTGTCACC
CAGTTGTCACC
CAGTTGTCACTC
CGTTGTCACC
CGGTTGTCACT
CAGTTGTCACT
CAGTTGTCACC
CAGTTGTCACTC
CGTTGTCACC
Nr of selected repeats 19 Similarity 0.897927
Consensus:
CAGTTGTCACt
>Cele-UNSB01_2:4380400-4380472 Satlength=73 Nr of Repeats=4 RepeatLength=12
seed=AGCCTAAGCC
AGCCTAAGCCTA
AGCCTTAGCCTA
AGCCTAAGCCTG
AGCCTAGGCCTGAGCCTGATCCTAGGCCTGAGCCTG
Nr of selected repeats 3 Similarity 0.851852
Consensus:
AGCCTaAGCCTa
>Cele-UNSB01_2:4433247-4433329 Satlength=83 Nr of Repeats=4 RepeatLength=12
seed=TAAGCCTAAG
TAAGCCTAAGTCTATGCTTATGTCTAAGACTGAGCCTGAGCCTAGT
TAAGCCTAAGAC
TAAGCCTAAGAC
TAAGCCTAAGCC
Nr of selected repeats 3 Similarity 0.925926
Consensus:
TAAGCCTAAGaC
>Cele-UNSB01_2:4510577-4510967 Satlength=391 Nr of Repeats=36 RepeatLength=11
seed=TGCACATTTT
TGCACATTTTT
TGCACATTTTT
TGCACATTTTT
TGCACATTTTT
TGCACATTTTT
TGCACATTTTT
TGCACATTTTT
TGCACATTTTT
TGCACATTTT
TGCACATTTTT
TGCACATTTT
TGCACATTTTGCACATTTTT
TGCACATTTTT
TGCACATTTTT
TGCACATTTTT
TGCACATTTTT
TGCACATTTTT
TGCACATTTTT

TGCACATTTTT
TGCACATTTTT
TGCACATTTTT
TGCACATTTTT
TGCACATTTTT
TGCACATTTTT
TGCACATTTT
TGCACATTTTT
TGCACATTTTT
TGCACATTTTT
TGCACATTTTT
TGCACACTTT
TGCACATTTTT
TGCACATTTTT
TGCACATTTTT
TGCACATTTTC
TGCACATTTTT
Nr of selected repeats 30 Similarity 0.991919
Consensus:
TGCACATTTTT
>Cele-UNSB01_2:4622617-4623337 Satlength=721 Nr of Repeats=18 RepeatLength=40
seed=TCCAGAACCT
TCCAGAACCTTCTGGAAAATTCGAGAAAATTCTGGAATGT
TCCAGAACCTTCTGGAAAATCCGAGAAAATTCTGGAATGT
TCCAGAACCTTCTGGAAAATTTGAGAAAATTCTGGAATGT
TCCAGAACCTTCTGGAAAATCCGAGAAAATTCTGGAATGT
TCCAGAACCTTCTGGAAAATTTGAGAAAATTCTGGAATGT
TCCAGAACCTTCTGGAAAATTTGAGAAAATGCTGGAATGT
TCCAGAACCTTCTGGAAAATCCGAGAAAATTCTGGAATGT
TCCAGAACCTTCTGGAAAATTTGAGAAAATTCTGGAATGT
TCCAGAACCTTCTGGAAAATCCGAGAAAATTCTGGAATGT
TCCAGAACCTTCTGGAAAATTTGAGAAAATTCTGGAATGT
TCCAGAACCTTCTGGAAAATTTGAGAAAATGCTGGAATGT
TCCAGAACCTTCTGGAAAATCCGAGAAAATTCTGGAATGT
TCCAGAACCTTCTGGAAAATTTGAGAAAATTCTGGAATGT
TCCAGAACCTTCTGGAAAATCCGAGAAAATTCTGGAATGT
TCCAGAACCTTCTGGAAAATTTGAGAAAATTCTGGAATGT
TCCAGAACCTTCTGGAAAATTTGAGAAAATGCTGGAATGT
TCCAGAACCTTCTGGAAAATTTGAGAAAATTCTGGAATGT
TCCAGAACCTTCTGGAAAATTTGAGAAAATTCTGGAATGT
Nr of selected repeats 18 Similarity 0.957734
Consensus:
TCCAGAACCTTCTGGAAAATttGAGAAAATTCTGGAATGT
>Cele-UNSB01_2:4623470-4624110 Satlength=641 Nr of Repeats=20 RepeatLength=32
seed=CGGGAATTCA
CGGGAATTCAAAATTTCTGTGAAAAATTTTGG
CGGGAATTCAAAATTTCTGTGAAAAATTTTGG
CGGGAATTCAAAATTTCTGTGAAAATTTTTAG
CGGGAATTCAAATTTTCTGTGAAAAATTTTGG
CGGGAATTCAAAATTTCTGTGAAAAATTTTGG
CGGGAATTCAGAATTTCTGTGAAAATTTTTGG
CGGGAATTCAAATTTTCTGTGAAAAATTTTGG
CGGGAATTCAAAATTTCTGTGAAAAAAATTTGG
CGGGAATTCAAAATTTCTGTGAAAAATTTTGG

CGAGAATTCAAAATTTCTGTGAAAAAAATTTGG
CGGGAATTCAAAATTTCTGTGAAAAAAATTTGG
CGGGAATTCAAAATTTCTGTGAAAAAATTTTGG
CGGGAATTCAAAATTTCTGTGAAAAAATTTTGG
CGGGAATTCAAATTTTCTGTGAAAAAATTTTGG
CGGGAATTCAAAATTTCTGTGAAAAAATTTTGG
CGGGAATTCAAAATTTTTATGAAAAAATTTTGG
CGGGAATTCAAATTTTCTGTGAAAAAATTATAA
CGGGAATTCAAGTTTTCTGTGAAAAAATTTTGG
CGGGAATTCAAATTTTCTGTGAAAAAATTTTGG
CGGGAATTCAAAATTTCTTTGAAAAAATTTTGG
Nr of selected repeats 20 Similarity 0.921491
Consensus:
CGGGAATTCAAAaTTTCTGTGAAAAAATTTTGG
>Cele-UNSB01_2:4637434-4637644 Satlength=211 Nr of Repeats=5 RepeatLength=35
seed=TTTGCCGGAA
TTTGCCGGAATTTTCAATTTTCGGAAAATTGCCGG
TTTGCCGGAAAATTTAATTTTCGGAAAATCACGGATTTTCCTGAAATATTCGATTCCGGCAAATTTCTGA
TTTGCCAGAAAATTTCATTTTCGGCAAATTACCGA
TTTGCCGGAAAATTTCAATTCCGGCAATTTACCGG
TTTCCCGGAAAATTTCATTTTCGGCAAATTACCGA
Nr of selected repeats 4 Similarity 0.757716
Consensus:
TTTgCCgGaAAaTTTCAtTTtCGGcAAaTTaCCGa
>Cele-UNSB01_2:4751417-4751507 Satlength=91 Nr of Repeats=6 RepeatLength=15
seed=GAGACCCATC
GAGACCCATCGTGGC
GAGACCCAGCGTGGT
GAGACCCATCGTGGC
GAGACCCAGCGTGGT
GAGACCCATCGTGGC
GAGACCCATCGTGGT
Nr of selected repeats 6 Similarity 0.899259
Consensus:
GAGACCCAtCGTGGc
>Cele-UNSB01_2:4751413-4751956 Satlength=544 Nr of Repeats=21 RepeatLength=15
seed=TGGTGAGACC
TGGTGAGACCCATCG
TGGCGAGACCCAGCG
TGGTGAGACCCATCG
TGGCGAGACCCAGCG
TGGTGAGACCCATCG
TGGCGAGACCCATCG
TGGTGAGACCCATCG
TGGTGAGACCCATAAGTTTTGGCGGGAATTCAAATTTTCTGTGAAAATTTTTTGGTGGGAATTCAAATTTTATGTGAAA
AATTTTGGCGGCAATTCAAATTTTATGTGAAAAATTTTGGCGGGAATTCAAATTTTCTGTGAAAAATTTCGGCGGGAAA
TTCAAATTTTCTGAGAAAAATTTTTGGCGGGAAATTAAAATTTTCTTAGAAATAATTTTTGCCGGGAAAT
TGGTGAGACCCTTCG
TGGTGAGACCCTTCG
TGGTGAGACCCATCGT
TGTGAGACCCTTCG
TGGTGAGACCCTTAG
TGGTGAGACCCATCGT
TGTGAGACCTTTCG

TGGTGAGACCCTTCG
TGGTGAGACCCATCGTTGCGAGACCCAGTG
TGGTGAGACCCTTCG
TGGTGAGACCCTTCG
TGGTGAGACCCATCGT
TGTGAGACCCTTCG
Nr of selected repeats 13 Similarity 0.879202
Consensus:
TGGtGAGACCCaTCG
>Cele-UNSB01_2:5140446-5146375 Satlength=5930 Nr of Repeats=77 RepeatLength=76
seed=CAATAGAAAA
CAATAGAAAAAAGGAAGACTACAGAAAAACTAATGAGAGACCCCGAAATACTGGCTGTATTTTGAAAGGTCATCT
CAATAGAAAAAATGGAAGAAGTGGAAGACTACAGAAAAACTATTGAGAGACCCCGAAATACTGGCTGTATTTTGAAAGG
TCATCT
CAATAGAAAAGATGGAAGACTACAGAAAAACTATTGAGAGACCCCGAAATACTGGCTGTATTTTGAAAGGTCATCT
CAATAGAAAAGATGGAAGACTACAGAAAAACTATTGAGAGACCCCGAAATACTGGCTGTATTTTGAAAGGTCATCT
CAATAGAAAAAATGGAAGAAGTGGACTACAGAAAAACTATTGAGAGACCCCGAAATACTGGCTGTATTTTGAAAGGTCAT
CT
CAATAGAAAAGATGGAAGACTACAGAAAAACTATTGAGAGACCCCGAAATACTGGCTGTATTTTGAAAGGTCATCT
CAATAGAAAAGATGGAAGACTACAGAAAACTATTGAGAGACCCCGAAATACTGGCTGTATTTTGAAAGGTCATCT
CAATAGAAAAAATGGAAGACTACAGAAAAAAAAAAAAAAAAAAAAAAAAAAAAAAAAAAAAAAAAAAAAAAAAAAAA
AAAAAAAAAAAAAAAAAAAAAAAT
CAATAGAAAAGAGGAAGACTACAGAAAAACTATTGAGAGACCCCGAAATACTGGCTGTATTTTGAAAGGTCATCT
CAATAGAAAAAAGAAGTGGAAGACTACAGAAAAACTATTGAGAGCCCCGAAATACTGGCTGTATTTTGAAAGGTATCT
CAATAGAAAAGATGGAAGACTACAGAAAAACTATTGTGAGACCCCGAAATACTGGCTGTATTTTGAAAGGTCATCT
CAATAGAAAAGATGGAAGACTACAGAAAAACTATTGAGAGACCCCGAAATACTGGCTGTATTTTGAAAGGTCATCT
CAATAGAAAAGATGGAAGACTACAGAAAAACTATTGAGAGACCCCGAAATACTGGCTGTATTTTGAAAGGTCATCT
CAATAGAAAAGATGGAAGACTACAGAAAAACTATTGAGAGACCCCGAAATACTGGATGTATTTTGAAAGGTATCT
CAATAGAAAAGATGGAAGACTACAGAAAAACTATTGAGAGACCCCGAAATACTGGCTGTATTTTGAAAGGTCATCT
CAATAGAAAAGATGGAAGACTACAGAAAAACTATTGAGAGACCCCGAAATACTGGCTGTATTTTGAAAGGTCATCT
CAATAGAAAAGATGGAAGACTACAGAAAAACTATTGTGAGACCCCGAAATACTGGCTGTATTTTGAAAGGTCATCT
CAATAGAAAAGATGGAAGACTACAGAAAAACTATTGAGAGACCCCGAAATACTGGCTGTATTTTGAAAGGTCATCT
CAATAGAAAAGATGGAAGACTACAGAAAAACATTGAGAGACCCCGAAATACTGGCTGTATTTTGAAAGGTCATCT
CAATAGAAAAAGGAGGAAGACTACAGAAAAACATTGGAGACCGAAATACTGCTGTATTTGAAAGGTCATCT
CAATAGAAAAGAGGAAGACTACAGAAAAACTATTGAGAGACCCCGAAATACTGGCTGTATTTTGAAAGGTCATCT
CAATAGAAAAAATGGAAGAAGTGGAAGACTACAGAAAAACTATTGGAGACCCCGAAATACTGCTGTATTTTGAAAGGTTA
TCT
CAATAGAAAAGATGGAAGACTACAGAAAAACTATTGAGAGACCCCGAAATACTGGCTGTATTTTGAAAGGTCATCT
CAATAGAAAAGATGGAAGACTACAGAAAAACTATTGTGAGACCCCGAAATACTGGCTGTATTTTGAAAGGTCATCT
CAATAGAAAAGATGGAAGACTACAGAAAAACTATTGAGAGACCCCGAAATACTGGCTGTATTTTGAAAGGTCATCT
CAATAGAAAAGATGGAAGACTACAGAAAAACTATTGAGAGACCCCGAAATACTGGCTGTATTTTGAAAGGTCATCT
CAATAGAAAAGATGGAAGACTACAGAAAAACTATTGAGAGACCCCGAAATACTGGCTGTATTTTGAAAGGTCATCT
CAATAGAAAAGATGGAAGACTACAGAAAAACTATTGAGAGACCCCGAAATACTGGCTGTATTTTGAAAGGTCATCT
CAATAGAAAAGATGGAAGACTACAGAAAAACTATTGAGAGACCCCGAAATACTGGCTGTATTTTGAAAGGTCATCT
CAATAGAAAAGATGGAAGACTACAGAAAAACTATTGAGAGACCCCGAAATACTGGCTGTATTTTGAAAGGTCATCT
CAATAGAAAAGATGGAAGACTACAGAAAAACTATTGAGAGACCCCGAAATACTGGCTGTATTTTGAAAGGTCATCT
CAATAGAAAAGATGGAAGACTACAGAAAAACTATTGAGAGACCCCGAAATACTGGCTGTATTTTGAAAGGTCATCT
CAATAGAAAAGATGGAAGACTACAGAAAAACTATTGAGAGACCCCGAAATACTGGCTGTATTTTGAAAGGTCATCT
CAATAGAAAAGATGGAAGACTACAGAAAAACTATTGGAGACCCCGAAATACTGCTGTATTTTGAAAGGTATCT
CAATAGAAAAGATGGAAGACTACAGAAAAACTATTGAGAGACCCCGAAATACTGGCTGTATTTTGAAAGGTCATCT
CAATAGAAAAGATGGAAGACTACAGAAAAACTATTGGAGACCCCGAAATACTGGCTGTATTTTGAAAGGTCATCT
CAATAGAAAAGATGGAAGACTACAGAAAAACTATTGAGAGACCCCGAAATACTGGCTGTATTTTGAAAGGTCATCT
CAATAGAAAAGAGGAAGACTACAGAGAAACTGTTGAGAGACCCCGAAATACTGGCTGTATTTTGAAAGGTCATCT
CAATAGAAAAGATGGAAACTACAGAGAAACTGTTGAGAGACCCCGAAATACTGGCTGTATTTTGAAAGGTCATCT

CAATAGAAAAGATGGAAGACTACAGAAAAACTATTGAGAGACCCCGAAATACTGGCTGTATTTTGAAAGGTCATCT
CAATAGAAAAAAATGGAACAAGTGGAAGACTACAGAAAAACTATTGAGAGACCCCGAAATACTGGCTGTATTTTGAAAGG
TTATCT
CAATAGAAAAGATGGAAGACTACAGAAAAACTATTGAGAGACCCCGAAATACTGGCTGTATTTTGAAAGGTCATCT
CAATAGAAAAGATGGAAGACTACAGAAAAACTATTGAGAGACCCCGAAATACTGGCTGTATTTTGAAAGGTCATCT
CAATAGAAAAGATGGAAGACTACAGAAAAACTATTGAGAGACCCCGAAATACTGGCTGTATTTTGAAAGGTCATCT
CAATAGAAAAGATGGAAGACTACAGAAAAACTATTGAGAGACCCCGAAATACTGGCTGTATTTTGAAAGGTCATCT
CAATAGAAAAGATGGAAGACTACAGAAAAACTATTGAGAGACCCCGAAATACTGGCTGTATTTTGAAAGGTCATCT
CAATAGAAAAAAATGGAAGAAGTGGAAGACTACAGAAAAACTATTGTGAGACCCCGAAATACTGACTGTATTTTGAAAGG
TTATCT
CAATAGAAAAGATGGAAGACTACAGAAAAACTATTGAGAGACCCCGAAATACTGGCTGTATTTTGAAAGGTCATCT
CAATAGAAAAGATGGAAGAAGTGGAAGACTACAGAAAAACTATTGAGAGACCCCGAAATACTGGCTGTATTTTGAAAGG
TCATCT
CAATAGAAAAGATGGAAGACTACAGAAAAACTATTGAGAGACCCCGAAATACTGGCTGTATTTTGAAAGGTCATCT
CAATAGAAAAGATGGAAGACTACAGAATAACTATTAAGAGACCCCGAAATACTGGCTGTATTTTGAAAGGTCATCT
CAATAGAAAAGATGGAAGACTACAGAAAAACTATTGAGAGACCCCGAAATACTGGCTGTATTTTGAAAGGTCATCT
CAATAGAAAAGATGGAAGACTACAGAAAAACTATTGAGAGACCCCGAAATACTGGCTGTATTTTGAAAGGTTATCT
CAATAGAAAAGATGGAAGACTACAGAAAAACTATTGAGAGACCCCGAAATACTGGCTGTATTTTGAAAGGTCATCT
CAATAGAAAAGATGGAAGACTACAGAAAAACTATTAAGAGACCCCGAAATACTGGCTGTATTTTGAAAGGTTATCT
CAATAGAAAAGATGGAAGACTACAGAAAAACTATTGAGAGACCCCGAAATACTGGCTGTATTTTGAAAGGTCATCT
CAATAGAAAAAAATGGAAGAAGTGGAAGACTACAGAAAAACTATTGAGAGGCCCCGAAATACTGGCTGTATTTTGAAAGG
TTATCT
CAATAGAAAAGATGGAAGACTACAGAAAAACTATTGTGAGACCCCGAAATACTGGCTGTATTTTGAAAGGTCATCT
CAATAGAAAAGATGGAAGACTACAGAAAAACTATTGAGAGACCCCGAAATACTGGCTGTATTTTGAAAGGTCATCT
CAATAGAAAAGATGGAAGACTACAGAAAAACTATTGAGAGACCCCGAAATACTGGCTGTATTTTGAAAGGTCATCT
CAATAGAAAAGATGGAAGACTACAGAAAAACTATTGAGAGACCCCGAAATACTGGCTGTATTTTGAAAGGTAATCT
CAATAGAAAAGATGGAAGACTACAGAAAAACTATTGAGAGACCCCGAAATACTGGATGTATTTTGAAAGGTTATCT
CAATAGAAAAGATGGAAGACTACAGAAAAACTATTGAGAGACCCCGAAATACTGGCTGTATTTTGAAAGGTCATCT
CAATAGAAAAGATGGAAGACTACAGAAAAACTATTGTGAGACCCCGAAATACTGGCTGTATTTTGAAAGGTCATCT
CAATAGAAAAGATGGAAGACTACAGAAAAACTATTAAGAGACCCCGAAATACTGGCTGTATTTTGAAAGGTCATCT
CAATAGAAAAGATGGAAGACTACAGAAAAACTATTGAGAGACCCCGAAATACTGGCTGTATTTTGAAAGGTCATCT
CAATAGAAAAAAATGGAAGAAGTGGAAGACTACAGAAAAACTATTGAGAGGCCCCGAAATACTGGCTGTATTTTGAAAGG
TTATCT
CAATAGAAAAGATGGAAGACTACAGAAAAACTATTGTGAGACCCCGAAATACTGGCTGTATTTTGAAAGGTCATCT
CAATAGAAAAGATGGAAGACTACAGAAAAACTATTGAGAGACCCCGAAATACTGGCTGTATTTTGAAAGGTCATCT
CAATAGAAAAGATGGAAGACTACAGAAAAACTATTGAGAGACCCCGAAATACTGGCTGTATTTTGAAAGGTCATCT
CAATAGAAAAGATGGAAGACTACAGAAAAACTATTGAGAGACCCCGAAATACTGGCTGTATTTTGAAAGGTCATCT
CAATAGAAAAGATGGAAGACTACAGAAAAACTATTGAGAGACCCCGAAATACTGGATGTATTTTGAAAGGTTATCT
CAATAGAAAAGATGGAAGACTACAGAAAAACTATTGAGAGACCCCGAAATACTGGCTGTATTTTGAAAGGTCATCT
CAATAGAAAAGATGGAAGACTACAGAAAAACTATTGTGAGACCCCGAAATACTGGCTGTATTTTGAAAGGTCATCT
CAATAGAAAAGATGGAAGACTACAGAAAAACTATTGAGAGACCCCGAAATACTGGCTGTATTTTGAAAGGTCATCT
CAATAGAAAAGATGGAAGACTACAGAAAAACTATTGAGAGACCCCGAAATACTGGCTGTATTTTGAAAGGTCATCT
CAATAGAAAAGATGGAAGACTACAGAAAAACTATTGTGAGACCCCGAAATACTGACTGTATTTTGAAAGGTCATCT
Nr of selected repeats 56 Similarity 0.988380
Consensus:
CAATAGAAAAGATGGAAGACTACAGAAAAACTATTGAGAGACCCCGAAATACTGGCTGTATTTTGAAAGGTCATCT
>Cele-UNSB01_2:5166221-5171016 Satlength=4796 Nr of Repeats=184 RepeatLength=26
seed=TAGACCATTA
TAGACCATTAGATGAATTCCCAAAGT
TAGACCATTAGATGAATTCCCAAAGT
TAGACCATTAGATGAATTTCCAAAGT
TAGACCATTAGATGAATTCCCAAAGT
TAGACCATTAGATGAATTTCCAAAGT
TAGACCATTAGATGAATTTCCAAAGT

TAGACCATTAGATAAATTTCCAAAGT
TAGACCATTAGATGAATTTCCAAAGT
TAGACCATTAGATGAATTCCCAAAGT
TAGACCATTAGATGAATTCCCAAAGT
TAGACCATTAGATGAATTTCCAAAGT
TAGACCATTAGATGAATTTCCAAAGT
TAGACCATTAGATGAATTCCCAAAGT
TAGACCATTAGATGAATTTCCAAAGT
TAGACCATTAGATGAATTCCAAAGT
TAGACCATTAGATAAATAAATTAAAA
TAGACCATTAGATGAATTCCAAAGT
TAGACCATTAGATGAATTCCAAAGT
TAGACCATTAGATGAATTCCAAAGTTAGACAAAGT
TAGACCATTAGATGAATTCCCAAAGT
TAGACCATTAGATGAATTCCCAAAGT
TAGACCATTAGATGAATTTCCAAAGT
TAGACCATTAGATGAATTTCCAAAGT
TAGACCATTAGATGAATTCCAAAGT
TAGACCATTAGATGAATTTCCAAAGT
TAGACCATTAGATGAATTCCAAAGT
TAGACCATTAGATGAATTTCCAAAGT
TAGACCATTAGATGAATTTCCAAAGT
TAGACCATTAGATGAATTTCCAAAGT
TAGACCATTAGATGAATTTCCAAAGT
TAGACCATTAGATGAATTTCCAAAGT
TAGACCATTAGATGAATTTCCAAAGT
TAGACCATTAGATGAATTCCAAAGT
TAGACCATTAGATGAATTTCCAAAGT
TAGACCATTAGATAATTTCAAAGT
TAGACCATTAGATGAATTTCCAAAGT
TAGACCATTAGATGAATTTCCAAAGT
TAGACCATTAGATGAATTTCCAAAGT
TAGACCATTAGATGAATTTCCAAAGT
TAGACCATTAGATGAATTTCCAAAGT
TAGACCATTAGATGAATTTCCAAAGT
TAGACCATTAGATGAATTTCCAAAGT
TAGACCATTAGATGAATTTCCAAAGT
TAGACCATTAGATGAATTTCCAAAGT
TAGACCATTAGATAATTTCAAAGT
TAGACCATTAGATGAATTTCCAAAGT
TAGACCATTAGATGAATTTCCAAAGT
TAGACCATTAGATGAATTTCCAAAGT
TAGACCATTAGATGAATTTCCAAAGT
TAGACCATTAGATGAATTTCCAAAGT
TAGACCATTAGATGAATTTCCAAAGT
TAGACCATTAGATGAATTTCCAAAGT
TAGACCATTAGATGAATTTCCAAAGT
TAGACCATTAGATGAATTTCCAAAGT
TAGACCATTAGATGAATTTCCAAAGT
TAGACCATTAGATGAATTTCCAAAGT
TAGACCATTAGATAAATTTCCAAAGT
TAGACCATTAGATAAATTTTCAAAGT
TAGACCATTAGATGAATTTCCAAAGT
TAGACCATTAGATGAATTTCAAAGT

TAGACCATTAGATGAATTTCAAAGT
TAGACCATTAGATGAATTTCCAAAGT
TAGACCATTAGATGAATTTCCAAAGT
TAGACCATTAGATGAATTTCCAAAGT
TAGACCATTAGATGAATTTCCAAAGT
TAGACCATTAGATGAATTTCCAAAGT
TAGACCATTAGATGAATTTCCAAAGT
TAGACCATTAGATAAATTTCCAAAGT
TAGACCATTAGATGAATTCCAAAGT
TAGACCATTAGATGAATTTCCAAAGT
TAGACCATTAGATGAATTTCCAAAGT
TAGACCATTAGATGAATTTCCAAAGT
TAGACCATTAGATGAATTTCCAAAGT
TAGACCATTAGATGAATTTCCAAAGT
TAGACCATTAGATAAATTTCAAAGT
TAGACCATTAGATGAATTTCCAAAGT
TAGACCATTAGATGAATTTCCAAAGT
TAGACCATTAGATGAATTTTCAAAGT
TAGACCATTAGATGAATTTCCAAAGT
TAGACCATTAGATGAATTTCAAAGT
TAGACCATTAGATGAATTTCCAAAGT
TAGACCATTAGATGAATTTCTAACTTTGATTCAAAGT
TAGACCATTAGATGAATTTCCAAAGT
TAGACCATTAGATGAATTTCCAAAGT
TAGACCATTAGAAATTTCCAAAGT
TAGACCATTAGATTTTCAAAGT
TAGACCATTAGATGAATTTCCAAAGT
TAGACCATTAGATGAATTTCCAAAGT
TAGACCATTAGATAATTAAAAACATTAGATGAATTTCCAAAGT
TAGACCATTAGATGAATTTCCAAAGT
TAGACCATTAGATGAATTTCCAAAGT
TAGACCATTAGATGAATTTCCAAAGT
TAGACCATTAGATGAATTTCCAAAGT
TAGACCATTAGATGAATTCTCAAAGT
TAGACCATTAGATGAATTTCCAAAGT
TAGACCATTAGATGAATTTCCAAAGT
TAGACCATTAGATGAATTTCCAAAGT
TAGACCATTAGATGAATTTCCAAAGT
TAGACCATTAGATGAATTTCCAAAGT
TAGACCATTAGATGAATTTCCAAAGT
TAGACCATTAGATGAATTTCAAAGT
TAGACCATTAGATGAATTTCCAAAGT
TAGACCATTAGATGAATTTCCAAAGT
TAGACCATTAGATGAATTTCCAAAGT
TAGACCATTAGATAATTTCCAAAGT
TAGACCATTAGATGAATTTCCAAAGT
TAGACCATTAGATGAATTTCCAAAGT
TAGACCATTAGATGAATTCTCAAAGT
TAGACCATTAGATGAATTTCCAAAGT
TAGACCATTAGATGAATTTCCAAAGT
TAGACCATTAGATGAATTTCCAAAGT
TAGACCATTAGATGAATTTCCAAAGT
TAGACCATTAGATGAATTTCCAAAGT
TAGACCATTAGATGAATTTCCAAAGT

TAGACCATTAGATGAATTTCCAAAGT
TAGACCATTAGATGAATTTCCAAAGT
TAGACCATTAGATGAATTTCCAAAGT
TAGACCATTAGATGAATTTCCAAAGT
TAGACCATTAGATGAATTTCCAAAGT
TAGACCATTAGATGAATTTCAAAGT
TAGACCATTAGATGAATTTCCAAAGT
TAGACCATTAGATGAATTTCCAAAGT
TAGACCATTAGATGAATTTCCAAAGT
TAGACCATTAGATGAATTTCCAAAGT
TAGACCATTAGATGAATTTCCAAAGT
TAGACCATTAGATGAATTTCCAAAGT
TAGACCATTAGATGAATTCCAAAGT
TAGACCATTAGATGAATTTCCAAAGT
TAGACCATTAGATGAATTTCCAAAGT
TAGACCATTAGATGAATTTCCAAAGT
TAGACCATTAGATGAATTTCCAAAGT
TAGACCATTAGATGAATTTCCAAAGT
TAGACCATTAGATGAATTCCAAAGT
TAGACCATTAGATGAATTTCCAAAGT
TAGACCATTAGATGAATTTCCAAAGT
TAGACCATTAGATGAATTTCCAAAGT
TAGACCATTAGATGAATTTCCAAAGT
TAGACCATTAGATGAATTTCCAAAGT
TAGACCATTAGATGAATTTCCAAAGT
TAGACCATTAGATGAATTTCCAAAGT
TAGACCATTAGATGAATTTCCAAAGT
TAGACCATTAGATGAATTTCCAAAGT
TAGACCATTAGATAATTTCAAAGT
TAGACCATTAGATGAATTTCCAAAGT
TAGACCATTAGATGAATTTCCAAAGT
TAGACCATTAGATGAATTTCCAAAGT
TAGACCATTAGATGAATTTCCAAAGT
TAGACCATTAGATGAATTTCCAAAGT
TAGACCATTAGATGAATTTCCAAAGT
TAGACCATTAGATAAAAAAAAAAATTTCAAAGT
TAGACCATTAGATAATTTCAAAGT
TAGACCATTAGATGAATTTCCAAAGT
TAGACCATTAGATGAATTTCCAAAGT
TAGACCATTAGATGAATTTCCAAAGT
TAGACCATTAGATGAATTTCCAAAGT
TAGACCATTAGATGAATTTCAAAGT
TAGACCATTAGATGAATTCCAAAGT
TAGACCATTAGATGAATTTCCAAAGT
TAGACCATTAGATGAATTTCCAAAGT
TAGACCATTAGATGAATTTCCAAAGT
TAGACCATTAGATGAATTTCCAAAGT
TAGACCATTAGATGAATTCCAAAGT
TAGACCATTAGATGAATTTCCAAAGT
TAGACCATTAGATGAATTTCCAAAGT
TAGACCATTAGATGAATTTCCAAAGT
TAGACCATTAGATGAATTCTCAAAGT
TAGACCATTAGATAAATTTTCAAAGT
TAGACCATTAGATGAATTTCCAAAGT

TAGACCATTAGATGAATTCTCAAAGT
TAGACCATTAGATGAATTTCCAAAAT
TAGACCATTAGATGAATTTCCAAAGT
TAGACCATTAGATGAATTCTCAAAGT
TAGACCATTAGATGAATTCTCAAAGT
TAGACCATTAGATGAATTTCCAAAGT
TAGACCATTAGATGAATTTCCAAAGT
TAGACCATTAGATGAATTTCCAAAGT
TAGACCATTAGATAAATTTCCAAAGT
TAGACCATTAGATAAATTTTCAAAGT
TAGACCATTAGATGAATTTCCAAAGT
TAGACCATTAGATGAATTCTCAAAGT
TAGACCATTAGATAAATTTTCAAAGT
TAGACCATTAGATGAATTTCCAAAGT
TAGACCATTAGATGAATTCTCAAAGT
TAGACCATTAGATGAATTCTCAAAGT
Nr of selected repeats 155 Similarity 0.871050
Consensus:
TAGACCATTAGATGAATTTCCAAAGT
>Cele-UNSB01_2:5217429-5217681 Satlength=253 Nr of Repeats=9 RepeatLength=25
seed=AGAGTAAAAT
AGAGTAAAATATAGTGGGAAGTCAG
AGAGTAAAATAATTGGTGAAGCCAG
AGAATAAAATATGGTGGGAAGCCACAG
AGAGTAAAATATAGTGGGAAAGCCAG
AGAGTAAAATATTTGGGAAAGCCAC
AGAGTAAAATAATTGGGAAAGCCAG
AGAGTAAAATAATTGGGAAAGCCAG
AGAGTAAAATAATTGGGAAAGCCAG
AGAGTAAAATATTTGGGAAAGCCACAGAGCAAATAATTGGTGAAGCCAG
Nr of selected repeats 6 Similarity 0.823932
Consensus:
AGAGTAAAATAATTGGGaAAGCCAG
>Cele-UNSB01_2:5217526-5241162 Satlength=23637 Nr of Repeats=927
RepeatLength=25 seed=AGCCAGAGAG
AGCCAGAGAGTAAAATATTTGGGAA
AGCCACAGAGTAAAATAATTGGGAA
AGCCAGAGAGTAAAATAATTGGGAA
AGCCAGAGAGTAAAATAATTGGGAA
AGCCAGAGAGTAAAATATTTGGGAA
AGCCACAGAGCAAATAATTGGTGA
AGCCAGAGAGTAAAATATCCTGTGA
AGCCAGAGAGTTACACGAAAAATAGATTGTTACACGAAAAATAGATTGTTACACGAAAAATAGATTGTTACACGAAAAA
TAGATTGTTACACGAGAAATGGATTGTTACAAGAAAAATAGATTGTTACACGAAAAATAGATTGTTACACGAAAAATAG
ATTGTTACACGAAAAATAGATTGTTACACGAAAAATAGATTGTTACACGAAAAATAGATTGTTACACGAAAAATAGATT
GTTACACGAAAAATAGATTGTTACACGAAAAATAGATTGTTACACGAGAAATGGATTGTTACACGAAAAATAGATTGTT
ACACGAAAAATAGATTGTTACACGAAAAATAGATTGTTACACGAAAAATAGTAAAATATTTGGAAA
AGCCACAGAGTAAAATAATTGGTGAA
ACCAGAGAGTAAAATAATTGGTGA
AGCCAGAGAGTAAAATAATTGGTGA
AGCCAGAGAGTAAAATAATTGGTGA
AGCCAGAGAGTAAAATAATTGGTGA
AGCCAGAGAGTAAAATAATTGGTGA
AGCCAGAGAGTGAAATATTGGGTGA

AGCCAGAGAGTAAAATATCCTGTGA
AGCCAGAGAGTAAAATAATTGGTGA
AGCCAGAGAGTGAAATATTTGGTGA
AGCCAGAGAGTAAAATATCCTGTGA
AGCCAGAGAGTAAAATAATTGATGAA
ACCAGAGAGTAAAATATCCTGTGA
AGCCAGAGAGTAAAATAATTGGTGA
AGCCAGAGAGTGAAATATTTGGTGA
AGCCAGAGAGTAAAATATCCTGTGA
AGCCAGAGAGTAAAATAATTGGTGA
AGCCAGAGAGTAAAATAATTGGTGA
AGCCAGAGAGTAAAATAATTGGTGA
AGCCAGAGAGTAAAATAATTGGTGA
AGCCAGAGAGTAAAATAATTGGTGA
AGCCAGAGAGTAAAATATTTGGTGA
AGCCAGAGAGTAAAATAATTGGTGA
AGCCAGAGAGTAAAATAATTGGTGA
AGCCAGAGAGTGAAATATTTGGTGA
AGCCAGAGAGTAAAATATTTGGTGA
AGCCAGAGAGTAAAATATTTGGTGA
AGCCAGAGAGTAAAACAATTGGTGA
AGCCAGAGAGTAAAACAATTGGTGA
AGCCAGAGAGTAAAATATTTGGTGA
AGCCAGAGAGTAAAATATTTGGTGA
AGCCAGAGAGTAAAACAATTGGTGA
AGCCAGAGAGTAAAACAATTGGTGA
AGCCAGAGAGTAAAACAATTGGTGA
AGCCAGAGAGTAAAATATTTGGTGA
AGCCAGAGAGTAAAACAATTGGTGA
AGCCAGAGAGTAAAACAATTGGTGA
AGCCAGAGAGTAAAATATTTGGTGA
AGCCAGAGAGTAAAACAATTGGTGT
AGCCAGAGAGTAAAATAATTGGTGA
AGCCAGAGAGTAAAATAATTGGTGA
AGCCAGAGAGTAAAATATTTGGTGA
AGCCAGAGAGTAAAATAATTGGTGA
AGCCAGAGAGTAAAATAATTGGTGA
AGCCAGAGAGTGAAATATTTGGTGA
AGCCAGAGAGTAAAATAATTGGTGA
AGCCAGAGAGTAAAATATTTGGTGA
AGCCAGAGAGTAAAATATTTGGTGA
AGCCAGAGAGTAAAATATTTGGTGA
AGCCAGAGAGTAAAATATTTGGTGA
AGCCAGAGAGTAAAATATTTGGTGA
AGCCAGAGAGTAAAATATTTGGTGA
AGCCAGAGAGTAAAATATTTGGTGA
AGCCAGAGAGTAAAATATTTGGTGA
AGCCAGAGAGTAAAATATTTGGTGA
AGCCAGAGAGTAAAATATTTGGTGA
AGCCAGAGAGTAAAATATTTGGTGA
AGCCAGAGAGTAAAATATTTGGTGA
AGCCAGAGAGTAAAACAATTGGTGA
AGCCAGAGAGTAAAATATTTGGTGA
AGCCAGAGAGTAAAATAATTGGTGA

AGCCAGAGAGTAAAATATTTGGTGA
AGCCAGAGAGTAAAATAATTGGTGA
AGCCAGAGAGTAAAATAATTGGTGA
AGCCAGAGAGTAAAATATTTGGTGA
AGCCAGAGAGTAAAATAATTTGGTGA
AGCCAGAGAGTAAAATATTTGGTGA
AGCCAGAGAGTAAAACAATTGGTGA
AGCCAGAGAGTAAAATATTTGGTGA
AGCCAGAGAGTAAAACAATTGGTGA
AGCCAGAGAGTAAAATATTTGGTGA
AGCCAGAGAGTAAAATAATTGGTGA
AGCCAGAGAGTAAAATAATTGGTGA
AGCCAGAGAGTAAAATATTTGGTGA
AGCCAGAGAGTAAAATAATTGGTGA
AGCCAGAGAGTAAAAATTGGTGA
AGCCAGAGAGTAAAATAATTGGTGA
AGCCAGAGAGTAAAATAATTGGTGA
AGCCAGAGAGTAAAATAATTGGTGA
AGCCAGAGAGTAAAATAATTGGTGA
AGCCAGAGAGTAAAATAATTGGTGA
AGCCAGAGAGTAAAATAATTGGTGA
AGCCAGAGAGTAAAATAATTGGTGA
AGCCAGAGAGTAAAATAAAAGAAAAATAATTGGTGA
AGCCAGAGAGTAAAATAATTGGTGA
AGCCAGAGAGTGAAATATTTGGTGA
AGCCAGAGAGTAAAATATCCTGTGA
AGCCAGAGAGTAAAATAATTGGTGA
AGCCAGAGAGTGAAATATTTGGTGA
AGCCAGAGAGTAAAATATCCTGTGA
AGCCAGAGAGTAAAATAATTGATGAA
ACCAGAGAGTAAAATATCCTGTGA
AGCCAGAGAGTAAAATAATTGGTGA
AGCCAGAGAGTGAAATATTTGGTGA
AGCCAGAGAGTAAAATATCCTGTGA
AGCCAGAGAGTAAAATAATTGGTGA
AGCCAGAGAGTAAAATAATTGGTGA
AGCCAGAGAGTAAAATATTTGGTGA
AGCCAGAGAGTAAAATAATTGGTGA
AGCCAGAGAGTAAAATAATTGGTGA
AGCCAGAGAGTAAAACAATTGGTGA
AGCCAGAGAGTACAATAATTGGTGA
AGCCAGAGAGTAAAATAATTGGTGA
AGCCAGAGAGTAAAATAATTGGTGA
AGCCAGAGAGTAAAATATTTGGTGA
AGCCAGAGAGTAAAACAATTGGTGA
AGCCAGAGAGTAAAATAATTGGTGA
AGCCAGAGAGTAAAATATTTGGTGA
AGCCAGAGAGTAAAATATTTGGTGA
AGCCAGAGAGTAAAATATTTGGTGA
AGCCAGAGAGTAAAACAATTGGTGA
AGCCAGAGAGTAAAATATTTGGTGA
AGCCAGAGAGTAAAACAATTGGTGA
AGCCAGAGAGTAAAATATTTGGTGA
AGCCAGAGAGTAAAATAATTGGTGA

AGCCAGAGAGTAAAATAATTGGTGA
AGCCAGAGAGTAAAATATTTTGTGA
AGCCAGAGAGTAAAATAATTGGTGA
AGCCAGAGAGTAAAATAATTGGTGA
AGCCAGAGAGTAAAATATTTGGTGA
AGCCAGAGAGTAAAATAATTAGTGA
AGCCAGAGAGTGAAATATTTGGTGA
AGCCAGAGAGTAAAATATCCTGTGA
AGCCAGAGAGTAAAATAATTGGTGA
AGCCAGAGAGTGAAATATTTGGTGA
AGCCAGAGAGTAAAATATCCTGTGA
AGCCAGAGAGTAAAATAATTGATGAA
ACCAGAGAGTAAAATATCCTGTGA
AGCCAGAGAGTAAAATAATTGGTGA
AGCCAGAGAGTGAAATATTTGGTGA
AGCCAGAGAGTAAAATATCCTGTGA
AGCCAGAGAGTAAAATAATTGGTGA
AGCCAGAGAGTAAAATAATTGGTGA
AGCCAGAGAGTAAAATATTTGGTGA
AGCCAGAGAGTAAAATAATTGGTGA
AGCCAGAGAGTAAAATAATTGGTGA
AGCCAGAGAGTAAAACAATTGGTGA
AGCCAGAGAGTACAATAATTGGTGA
AGCCAGAGAGTAAAATAATTGGTGA
AGCCAGAGAGTAAAATAATTGGTGA
AGCCAGAGAGTAAAATATTTGGTGA
AGCCAGAGAGTAAAACAATTGGTGA
AGCCAGAGAGTAAAATAATTGGTGA
AGCCAGAGAGTAAAATATTTGGTGA
AGCCAGAGAGTAAAATATTTGGTGA
AGCCAGAGAGTAAAATATTTGGTGA
AGCCAGAGAGTAAAACAATTGGTGA
AGCCAGAGAGTAAAATATTTGGTGA
AGCCAGAGAGTAAAACAATTGGTGA
AGCCAGAGAGTAAAATATTTGGTGA
AGCCAGAGAGTAAAATAATTGGTGA
AGCCAGAGAGTAAAATAATTGGTGA
AGCCAGAGAGTAAAATATTTTGTGA
AGCCAGAGAGTAAAATAATTGGTGA
AGCCAGAGAGTAAAATAATTGGTGA
AGCCAGAGAGTAAAATATTTGGTGA
AGCCAGAGAGTAAAATAATTAGTGA
AGCCAGAGAGTAAAATATTTTGTGA
AGCCAGAGAGTAAAATATTTGGTGA
AGCCAGAGAGTAAAATAATTGGTGA
AGCCAGAGAGTAAAATATTTGGTGA
AGCCAGAGAGTAAAACAATTGGTGA
AGCCAGAGAGTAAAATATTTGGTGA
AGCCAGAGAGTAAAACAATTGGTGA
AGCCAGAGAGTAAAACAATTAGTGA
AGCCAGAGAGTAAAATATTTGGTGA
AGCCAGAGAGTAAAACAATTGGTGA
AGCCAGAGAGTAAAATATTTGGTGA
AGCCAGAGAGTAAAACAATTGGTGA

AGCCAGAGAGTAAAACAATTGGTGA
AGCCAGAGAGTAAAACAATGGTGA
AGCCAGAGAGTAAAACAATTGGTGA
AGCCAGAGAGTAAAATATTTGGTGA
AGCCAGAGAGTAAAATATTTGGTGA
AGCCAGAGAGTAAAATAATTGGTGA
AGCCAGAGAGTAAAATAATTGGTGA
AGCCAGAGAGTAAAATAATTGGTGA
AGCCAGAGAGTAAAATAATTGGTGA
AGCCAGAGAGTAAAATAATTGGTGA
AGCCAGAGAGTGAAATATTTGGTGA
AGCCAGAGAGTAAAATAATTGGTGA
AGCCAGAGAGTAAAATATTTGGTGA
AGCCAGAGAGTAAAATATTTGGTGA
AGCCAGAGAGTAAAATATTTGGTGA
AGCCAGAGAGTAAAATATTTGGTGA
AGCCAGAGAGTAAAATATTTGGTGA
AGCCAGAGAGTAAAATATTTGGTGA
AGCCAGAGAGTAAAATATTTGGTGA
AGCCAGAGAGTAAAACAATTGGTGA
AGCCAGAGAGTAAAACAATTGGTGA
AGCCAGAGAGTAAAACAATTGGTGA
AGCCAGAGAGTAAAATATTTGGTGA
AGCCAGAGAGTAAAACAATTGGTGA
AGCCAGAGAGTAAAATATTGGTGA
AGCCAGAGAGTAAAACAATTGGTGAATTGGTGA
AGCCAGAGAGTAAAATAATTGGTGA
AGCCAGAGAGTAAAATATTTGGTGA
AGCCAGAGAGTAAAATAATTGGTGA
AGCCAGAGAGTAAAATAATTGGTGA
AGCCAGAGAGTAAAATATTTGGTGA
AGCCAGAGAGTAAAATAATTGGTGA
AGCCAGAGAGTAAAATATTTGGTGA
AGCCAGAGAGTAAAATATTTGGTGA
AGCCAGAGAGTAAAATATTTGGTGA
AGCCAGAGAGTAAAATATTTGGTGA
AGCCAGAGAGTAAAATATTTGGTGA
AGCCAGAGAGTAAAATATTTGGTGA
AGCCAGAGAGTAAAATATTTGGTGA
AGCCAGAGAGTAAAATATTTGGTGA
AGCCAGAGAGTAAAATATTTGGTGA
AGCCAGAGAGTAAAATATTTGGTGA
AGCCAGAGAGTAAAATATTTGGTGA
AGCCAGAGAGTAAAATATTGAGTGA
AGCCAGAGAGTAAAATATTTGGTGA
AGCCAGAGAGTAAAAAATATGGTGA
AGCCAGAGAGTAAAATATTTGGTGA
AGCCAGAGAGTAAAACAATTGGTGA
AGCCAGAGAGTAAAATTGGTGA
AGCCAGAGAGTAAAAAATTGGTGA
AGCCAGAGAGTAAAATATTTGGTGA
AGCCAGAGAGTAAAATATTTGGTGA
AGCCAGAGAGTAAAACAATTGGTGA
AGCCAGAGAGTAAAATATTTGGTGA

AGCCAGAGAGTAAAATAATTGGTGA
AGCCAGAGAGTAAAATATTTGGTGA
AGCCAGAGAGTAAAATAATTGGTGA
AGCCAGAGAGTAAAATAATTGGTGA
AGCCAGAGAGTAAAATAATTGGTGA
AGCCAGAGAGTAAAATATTGGTGA
AGCCAGAGAGTAAAATATTTGGTGA
AGCCAGAGAGTAAAATATTTGGTGA
AGCCAGAGAGTAAAAAATTGGTGA
AGCCAGAGAGTAAAATATTTGGTGA
AGCCAGAGAGTAAAACAATTGGTGA
AGCCAGAGAGTAAAATATTTGGTGA
AGCCAGAGAGTAAAACAATTGGTGA
AGCCAGAGAGTAAAATATTTGGTGA
AGCCAGAGAGTAAAAATTGGTGA
AGCCAGAGAGTAAAATATTTGGTGA
AGCCAGAGAGTAAAATATTTGGTGA
AGCCAGAGAGTAAAACAATTGGTGA
AGCCAGAGAGTAAAATATTTGGTGA
AGCCAGAGAGTAAAATATTGGTGA
AGCCAGAGAGTAAAATATTGGTGA
AGCCAGAGAGTAAAATATTGGTGA
AGCCAGAGAGTAAAATATTGGTGA
AGCCAGAGAGTAAAAATTTGGTGA
AGCCAGAGAGTAAAATATTGGTGA
AGCCAGAGAGTAAAATATTTGGTGA
AGCCAGAGAGTAAAAATATGGTGA
AGCCAGAGAGTAAAATATTGGTGA
AGCCAGAGAGTAAAATATTGGTGA
AGCCAGAGAGTAAAATATTGGTGA
AGCCAGAGAGTAAAATATTGGTGA
AGCCAGAGAGTAAAATATTGGTGA
AGCCAGAGAGTAAAATATTGGTGA
AGCCAGAGAGTAAAATATTGGTGA
AGCCAGAGAGTAAAATATTGGTGA
AGCCAGAGAGTAAAAATTTGGTGA
AGCCAGAGAGTAAAATATTGGTGA
AGCCAGAGAGTAAAATATTGGTGA
AGCCAGAGAGTAAAATTTGGTGA
AGCCAGAGAGTAAAATATTTGGTGA
AGCCAGAGAGTAAAATAATTGGTGA
AGCCAGAGAGTAAAATATTGGTGA
AGCCAGAGAGTAAAATAATTGGTGA
AGCCAGAGAGTAAAATATTGGTGA
AGCCAGAGAGTAAAATATTGGTGA
AGCCAGAGAGTAAAATATTTGGTGA
AGCCAGAGAGTAAAAATTGGTGA
AGCCAGAGAGTAAAATATTTGGTGA
AGCCAGAGAGTAAAATATTTGGTGA
AGCAGAGAGTAAATAATTGGTGA
AGCCAGAGAGTAAAATATTGGTGA
AGCCAGAGAGTAAAATATTGGTGA
AGCCAGAGAGTAAAATAATTGGTGA
AGCCAGAGAGTAAAATATTTGGTGA

AGCCAGAGAGTAAAATAATTGGTGA
AGCCAGAGAGTAAAATAATTGGTGA
AGCCAGAGAGTAAAATATTTGGTGA
AGCCAGAGAGTAAAATTGTGA
AGCAGAGAGTAAAATATTTGGTGA
AGCCAGAGAGTAAAATAATTGGTGA
AGCCAGAGAGTAAAAATTGGTGA
AGCCAGAGAGTAAAACAATTGGTGA
AGCCAGAGAGTAAAATATTTGGTGA
AGCCAGAGAGTAAAACAATTGGTGA
AGCCAGAGAGTAAAATATTTGGTGA
AGCCAGAGAGTAAAATATTGGTGA
AGCCAGAGAGTAAAATAATTGGTGA
AGCCAGAGAGTAAAATATTGGTGA
AGCCAGAGAGTAAAACAATTGGTGA
AGCCAGAGAGTAAAATAATTGGTGA
AGCCAGAGAGTAAAATAATTGGTGA
AGCCAGAGAGTAAAATATTTGGTGA
AGCCAGAGAGTAAAATATTGGTGA
AGCCAGAGAGTAAAATATTTGGTGA
AGCCAGAGAGTAAAATAATTTGGTGA
AGCCAGAGAGTAAAATATTTGGTGA
AGCCAGAGAGTAAAATAATTGGTGA
AGCCAGAGAGTAAAATATTTGGTGA
AGCCAGAGAGTAAAATATTTGGTGA
AGCCAGAGAGTAAAATATTGGTGA
AGCCAGAGAGTAAAATAATTTGGTGA
AGCCAGAGAGTAAAAATTGTGA
AGCCAGAGAGTAAAATATTTGGTGA
AGCCAGAGAGTAAAATAATTGGTGA
AGCCAGAGAGTAAAATATTTGGTGA
AGCCAGAGAGTAAAATATTTGGTGA
AGCCAGAGAGTAAAACAATATTTGGTGA
AGCCAGAGAGTAAAATATTGGTGA
AGCCAGAGAGTAAAATATTTGGTGA
AGCCAGAGAGTAAAATAATTGGTGA
AGCCAGAGAGTAAAATATTTGGTGA
AGCCAGAGAGTAAAAATTGGTGA
AGCCAGAGAGTAAAAATTGGTGA
AGCCAGAGAGTAAATATTTGGTGA
AGCCAGAGAGTAAAATAATTGGTGA
AGCCAGAGAGTAAAATATTTGGTGA
AGCCAGAGAGTAAAATATTGGTGAA
AGCAGAGAGTAAAAAATTGGTGA
AGCCAGAGAGTAAAATATTTGTGA
AGCCAGAGAGTAAAATTGTAAGCGGGTGGA
AGCCAGAGAGTAAAACAATTGGTGA
AGCCAAGAGGAGGTAAAAATATTTGGTAAA
AGCCCAGAGAGTAAATATTTGGTGA
AGCCAGAGAGTAAAACAATTTGGTGA
AGCCAGAGAGTTAAAATATTGGTGA
AGCCAGAGAGTAAAATAATTGGGTGA
AGCCAGAGAGTAAAATATTTGGTGA
AGCCAGAGAGTAAAATATTTGGTGA

AGCCAGAGAGTAAAATAATTGTGGCGAATGGTGA
AGCCAGAGAGTAAAATCAATTTGGTGA
AGCCAGAGAGTAAAATAATTGGTGA
AGCCAGAGAGTAAAATATTTGGTGA
AGCCAGAGAGTAAAAATATTTGGTGA
AGCCAGAGAGTAAAATATTTGGTGA
AGCCAGAGAGTAAAATAATTGGTGA
AGCCAGAGAGTAAAATATTTGGTGA
AGCCAGAGAGTAAAACAATTGGTGA
AGCCAGAGAGTAAAATATTTGGTGA
AGCCAGAGAGTAAAATATTGGGTG
AGCCAGAGAGTAAAATATTTGGTGA
AGCCAGAGAGTAAAATATTTGGTGA
AGCCAGAGAGTAAAATATTGGTGA
AGCCAGAGAGTAAAATAATTGGTGA
AGCCAGAGAGTTAAAACAATTGGTGA
AGCCAGAGAGTAAAATATTTGGTGGA
AGCCAGAGAGTAAAACAATTGGTGA
AGCCAGAGAGTAAAATATTTGGTGA
AGCCAAGAGAGTAAAATAAAATATTGGTGA
AGCCAGAGAGTAAAATATTGGTGA
AGCCAGAGAGTAAAATATTTGGTGA
AGCCAGAGAGTAAAATATATGGTGA
AGCCAGAGAGTAAAATATATGGTGA
AGCCAGAGAGTAAAATATTGGTGA
AGCCAGAGAGTAAAATATTGGTGA
AGCCAGAGAGTAAAATATTTGTGA
AGCCAGAGAGTAAAATATTGGTGA
AGCCAGAGAGTAAAATATTGGTGA
AGCCAGAGAGTAAAATATTTGGTGA
AGCCAGAGAGTAAAATATTTGGTG
AGCCAGAGAGTAAAAATTGGTGA
AGCCAGAGAGAAAACAATTGGGA
AGCCAGAGAGTAAAATATATGGTGA
AGCCAGAGAGTAAAATATTTGGTGA
AGCCAGAGAGTAAAATATTTGGTGAAGCAAGAGTAAAACAATTGGTGA
AGCCAGAGAGTAAATATTTGGTGA
AGCCAGAGAGTAAATAATTGGTGA
AGCCAGAGAGTAAAATAATGGTGA
AGCCAAGAGTAAAATATTGGTGA
AGCCAGAGAGTAAAACAATTTGGTGAGAGAGTAAAATTGGTGA
AGCCAGAGAGTAAAATATTTGGTGA
AGCCAGAGAGTAAAATATTTGGTGA
AGCCAGAGAGGTAAAATATTTTGGTGAAGCCCAGGAGAGTAAAATAATTGGTGA
AGCCAGAGAGTAAATATTGGTGA
AGCCAGAGAGTAAAACAATTGGTGA
AGCCAGAGAGTAAAATATTTGGTGA
AGCCAGAGAGTTAAAATAATTGGTGA
AGCCAGAGAGTAAAACTAAATTTGGTGAA
AGCCAGAGAGTAAAAATGATTTTGTGA
AGCAGAGAGTAAACAATTGGTGA
AGCCAGAGAGTAAATAATTGGTGA
AGCCAGAGAGTAAAATAATTGGTGAAG
GCCAGAGAGGTAAATATTTGGTGAA

AGCCAGAGAGTAAAATAATTGGTGA
AGCCAGAGAGTAAATATTGGTGA
AGCCAGAGAGTAAAATATTTGTGA
AGCCAGAGAGTAAAATAATTGGTGA
AGCCAGAGAGTAAAATATTTTGGTGA
AGCCAGAGAGTAAAACAATTGGTGA
AGCCAGAGAGTAAAATATTTGGTGA
AGCCAGAGAGTAAAACAATTGGTGA
AGCCCAGAGAGTAAAATATTGGTGA
AGCCAGAGAGTAAAATATTGGTGA
AGCCAGAGAGTAAAATATGGTGA
AGCCAGAGAGTAAAAATATGGTGA
AGCCAGAGAGTAAAATATTGGTGA
AGCCAGAGAGTAAAATATTGGTGA
AGCCAGAGAGTAAAATATTTGGTGA
AGCCAGAGAGTAAAATATTGGTGA
AGCCAGAGAGTAAAATTTGGTGA
AGCCAGAGAGTAAAATATTGGTGA
AGCCAGAGAGTAAAATATTGGTGA
AGCCAGAGAGTAAAATATTGGTGA
AGCCAGAGAGTAAAATATTGGTGA
AGCCAGAGAGTAAAATATTGGTGA
AGCCAGAGAGTAAAATATTGGTGA
AGCCAGAGAGTAAAATATTGGTGA
AGCCAGAGAGTAAAATATTGGTGA
AGCCAGAGAGTAAAATATTGGTGA
AGCCAGAGAGTAAAATATTGGTGA
AGCCAGAGAGTAAAATATTTGGTGA
AGCCAGAGAGTAAAATATTGGTGA
AGCCAGAGAGTAAAATATTGGTGA
AGCCAGAGAGTAAAAATTGGTGA
AGCCAGAGAGTAAAATATTGGTGA
AGCCAGAGAGTAAAAATTGTGA
AGCCAGAGAGTAAAATATTGGTGA
AGCCAGAGAGTAAAATATTGGTGA
AGCCAGAGAGTAAAATATTGGTGA
AGCCAGAGAGTAAAATATTGGTGA
AGCCAGAGAGTAAAATATTGGTGA
AGCCAGAGAGTAAAATATTGGTGA
AGCCAGAGAGTAAAATATTGGTGA
AGCCAGAGAGTAAAATATTTGGTGA
AGCCAGAGAGTAAAAATTGGTGA
AGCCAGAGAGTAAAATATTGGTGA
AGCCAGAGAGTAAAATTTGGTGA
AGCCAGAGAGTAAAATATTGGTGA
AGCCAGAGAGTAAAATATTGGTGA
AGCCAGAGAGTAAAATATTGGTGA
AGCCAGAGAGTAAAATATTGGTGA
AGCCAGAGAGTAAAATATTGGTGA
AGCCAGAGAGTAAAATATTGGTGA
AGCCAGAGAGTAAAATATTGGTGA
AGCCAGAGAGTAAAATATTTGGTGA
AGCCAGAGAGTAAAATATTTGGTGA
AGCCAGAGAGTAAAATATTGGTGA

AGCCAGAGAGTAAAAATATGGTGA
AGCCAGAGAGTAAAATATTTGGTGA
AGCCAGAGAGTAAAAATTGGTGA
AGCCAGAGAGTAAAATATTTGGTGA
AGCCAGAGAGTAAAAAATTGGTGA
AGCCAGAGAGTAAAATATTGGTGA
AGCCAGAGAGTAAAATATTGTGA
AGCCAGAGAGTAAAATATTGGTGA
AGCCAGAGAGTAAAATATTGGTGA
AGCCAGAGAGTAAAATATTGGTGA
AGCCAGAGAGTAAAATATTGGTGA
AGCCAGAGAGTAAAATATTGGTGA
AGCCAGAGAGTAAAATATTTGGTGA
AGCCAGAGAGTAAAATATTGTGA
AGCCAGAGAGTAAAATATTGGTGA
AGCCAGAGAGTAAAATATTGGTGA
AGCCAGAGAGTAAAATATTGGTGA
AGCCAGAGAGTAAAATATTTGGTGA
AGCCAGAGAGTAAAATATTGGTGA
AGCCAGAGAGTAAAAATTGGTGA
AGCCAGAGAGTAAAATATTTGGTGA
AGCCAGAGAGTAAAAATTGGTGA
AGCCAGAGAGTAAAATATTGGTGA
AGCCAGAGAGTAAAATATTGGTGA
AGCCAGAGAGTAAAATATTGGTGA
AGCCAGAGAGTAAAATATTGGTGA
AGCCAGAGAGTAAAATATTGGTGA
AGCCAGAGAGTAAAATATTGGTGA
AGCCAGAGAGTAAAATATTGGTGA
AGCCAGAGAGTAAAATATTTGGTGA
AGCCAGAGAGTAAAAAATTGGTGA
AGCCAGAGAGTAAAATATTGGTGA
AGCCAGAGAGTAAAAATTTGGTGA
AGCCAGAGAGTAAAAATTTGGTGA
AGCCAGAGAGTAAAATATTGGTGA
AGCCAGAGAGTAAAATATTGGTGA
AGCCAGAGAGTAAAATATTGGTGA
AGCCAGAGAGTAAAAAATTGGTGA
AGCCAGAGAGTAAAATATTTGTGA
AGCCAGAGAGTAAAATATTGTGA
AGCCAGAGAGTTAAAATATTGGTGA
AGCCAGAGAGTAAATATTGGTGA
AGCCAGAGAGTAAAATATTGGTGA
AGCCAGAGAGGTAAAAAATTGGTGA
AGCCAGAGAGTAAATAATTGGTGA
AGCCACAGAGAGTAAAATAATTGGTGA
AGCCACAGAGAGTAAAATAATTGGTGA
AGCCAGAGAGTAAAACAATTGGTGA
AGCCAGAGAGTGAAATATTGGTGA
AGCCAGAGAGTAAAATAATTGGTGA
AGCCAGAGAGTAAAATATTGGTGA
AGCCAGAGAGTAAAATATTTGGTGA
AGCCAGAGAGTAAAACAATTGGTGA
AGCCAGAGAGTAAAATAATTGGTGA

AGCCAGAGAGTAAAAAATTTGGTGA
AGCCAGAGAGTAAAATATATTGGTGA
AGCCAGAGAGTAAAATAATTGGTGA
AGCCAGAGAGTAAAATAATTGGTGA
AGCCAGAGAGTAAAATATTTGGTGA
AGCCAGAGAGTAAAAATATTTGGTGA
AGCCAGAGAGTAAAATATTTGGTGA
AGCCAGAGAGTAAAAAATAATTGGTGA
AGCCAGAGAGTAAACAATTGGGTGA
AGCCAGAGAGTAAAATATTTGTTGA
AGCCAAGAGAGTAAAATAATTGGTGA
AGCCAGGAGAGTAA
AGCCAGAGGTAAAATTGGGTGA
AGCCAGAGAGTAAAATATTTGGTGGA
AGCCAGAGAGTAAAATATTTGGTGA
AGCCAGAGAGTAAAATAATTGGTGA
AGCCAGAGAGTAAAATATTTGGTGA
AGCCAGAGAGTAAAACAATTGGTGA
AGCCAGAGAGTTAAAATATTTTTGGTGA
AGCCAGAGAGTAAAATAATTGGTGA
AGCCAGAGAGTAAAATATTTGGTGA
AGCCAGAGAGTAAAACAATTGGTGA
AGCCAGAGAGTAAAATATTGGTGA
AGCCAGAGAGTAAAACAATTGGTTGA
AGCCAGAGAGTAAAATATTGGTGA
AGCCAGAGAGTAAAAAATTGGTGA
AGCCAGAGAGTAAAATATTTGGTGA
AGCCAGAGAGTAAAATAATTTGGTGA
AGCCAGAGAGTAAAATATTTGGTGA
AGCCAGAGAGGTAAAATATTTGGTGAAAGCCCAGAGGAGGGTAAAAACAAATTTGGTGA
AGCCAGAGAGTAAAATATTTGGTGA
AGCCAGAGAGTAAAATAATTGGTGA
AGCCAGAGAGTAAAATATTTGGTGA
AGCCAGAGAGTAAAATATTTGGTGA
AGCCAGAGAGTAAAATATTGTGA
AGCCAGAGAGTAAAATATTGGTGA
AGCCAGAGAGTAAAATATTTGGTGA
AGCCAGAGAGTAAAATAATTGGTGA
AGCCAGAGAGTAAAATATTTGGTGA
AGCCAGAGAGTAAAATATTGGTGA
AGCAGAGAGTAAAATAATTGGTGA
AGCCAGAGAGTAAAATATTTGGTGA
AGCCAGAGAGTAAAACAATTGGTGAAGCCAGAGTAAAATATTTGGTGA
AGCCAGAGAGTAAAATATTTGGTTGA
AGCCAGAGAGTAAAACAATTGGTGA
AGCCAGAGAGTAAAATATTTGGTGA
AGCCAGAGAGTTAAAACAATTGGTGA
AGCCAGAGAGTAAAATATTTGGTGA
AGCCAGAGAGTAAAAAATTGGTGA
AGCCAGAGAGTAAATATTTGGTG
AGCCAGAGAGTAAAAAATTTGAAGCAGAGATAAAAATTTGGTGA
AGCCAGAGAGTAAAAATGGTAAGCAGAGATAAAAAATTGGTGA
AGCCAGAGAAGGTAAAAATATATTGGTGA
AGCCAGAGAGTAAAATATTTGGTGA

AGCCAGAGAGTAAAATAATTTGTGA
AGCCAGAGAGTAAAATATTTGGTGA
AGCCAGAGAGTAAAATAATTGGTGA
AGCCAGAGAGTAAAATAATTGGTGA
AGCCAGAGAGTAAAATATTTGGTGA
AGCCAGAGAGTAAAATAATTGGTGA
AGCCAGAGAGTAAAAATATTTGGTGA
AGCCAGAGAGTAAAATAATTGGTGA
AGCCAGAGAGTAAAATAATTGGTGA
AGCCAGAGAGTAAAATATTTGGTGA
AGCCAGAGAGTAAAATAATTGGTGA
AGCCAGAGAGTAAAATATTTGGTGA
AGCCAGAGAGTAAAATAATTGGTGA
AGCCAGAGAGTAAAATATTGGTGA
AGCCAGAGAGTAAAATAATTGGTGA
AGCCAGAGAGTGACAAAGTAAAATTTGGTGA
AGCCAGAGAGTAAAATATTGGTGA
AGCCAGAGAGTAAAATAATTGGTGA
AGCCAGAGAGTAAATATTGGTGA
AGCCAGAGAGTAAAATATTTGTGA
AGCCAGAGAGTAAAATAATTGGTGA
AGCCAGAGAGTGAAATAATTTGGTGA
AGCCAGAGAGTAAAATCAATGTGA
AGCCAGAGAGTAAAATATTGGTGA
AGCCAGAGAGTAAAATATTGTGCGTTGGA
AGCCAGAGAGTAAATATTTGGTGA
AGCCAGAGAGTAAAATAATTGTGA
AGCCAGAGAGTAAAATAATTGGGTGA
AGCCAGAGAGTAAATAATTGGTGA
AGCCAGAGAGTAAAATATTGGTGA
AGCCAGAGAGTAAAATAATTGGTGA
AGCCAGAGAGTAAAATAATTGGTGA
AGCCAGAGAGTAAAAAATTGGTGA
AGCCAGAGAGTAAATAATTGGTGA
AGCCAGAGAGTAAAATAATTGGTGA
AGCCAGAGAGTAAAATAATTGGTGA
AGCCAGAGAGTAAAATATTGGTGA
AGCCAGAGAGTAAAATATTTGTGA
AGCAGAGAGTAAAAAATTGGTGA
AGCCAGAGAGTAAAAATAATTGGTGA
AGCCAGAGAGTAAATAATTGGTGA
AGCCAGAGAGTAAAATATTTGGTGA
AGCCAGAGAGTTAAAATAATTGGTGA
AGCCAGAGAGTAAAATATTGGTGA
AGCCAGAGAGTAAAATATTTGGTGA
AGCCAGAGAGTAAATATTTGGTGA
AGCCAGAGAGTAAAATATTGGTGA
AGCCAGAGAGTAAAATAATTGGTGA
AGCCAGAGAGTAAAATAATTGGTGA
AGCCAGAGAGTAAAATATTTGGTGA
AGCCAGAGAGTAAAATAATTGGTGA
AGCCAGAAGAGTAAAATAATTGGTGA
AGCCAGAGAGTAAAATAATTAGGGTATTGA
AGCCAGAGAGTAAAATATTTGGTGA

AGCCAGAGAGTAAAAATATTGGTGA
AGCCAGAGAGTAAAATAATTGGGGTTGTGA
AGCCAGAGAGTAAAATATTTGGTGA
AGCCAGAGAGTAAAAAATATGGTGA
AGCCAGAGAGTAAAATATTTGGTGA
AGCCAGAGAGTAAAACAATTGGTGA
AGCCAGAGAGTAAAACAAATTGGTGA
AGCCAGAGAGTAAAATAATGGTGA
AGCCAGAGAGTAAAATAATTGGTGA
AGCCAGAGAGTAAAATATTTGGTGA
AGCCAGAGAGTAAAATAATTGGTGA
AGCCAGAGAGTAAAATAATTGGTGA
AGCCAGAGAGTAAAATAATTGGTGA
AGCCAGAGAGTAAAATATTTTGTGA
AGCCAGAGAGTTACCAAAAAATTTGGTGA
AGCCAGAGAGTAAAATAATTGGTGA
AGCCAGAGAGTAAAAATATATTGGTGA
AGCCACAGAGAGTAAAATAATTGGTGA
AGCCAGAGAGTAAAATAATTGGTGA
AGCCAGAGAGTAAAAATAATTGGTGA
AGCCAGAGAGTAAAATATTTGTGA
AGCCAGAGAGTAAAAAAATTGGTGA
AGCCAGAGAGTAAAATAATTGGTGA
AGCCAGAGAGTAAAAAATTGGTGA
AGCCAGAGAGTAAAATATTTGGTGA
AGCCAGAGAGTAAAATATTTGGTGA
AGCCAGAGAGTAAAATAATTGGTGA
AGCCAGAGAGTAAAATATTTGGTGA
AGCCAGAGAGTAAAATATTTGGTGA
AGCCAGAGAGTAAAATAATTGGTGA
AGCCAGAGAGTAAAATAATTGGTGA
AGCCAGAGAGTAAAATAAAAATATTGGTGA
AGCCAGAGAGTAAAATAATTGGTGA
AGCCAGAGAGTAAAATATTTGGTGA
AGCCAGAGAGTAAAATATTGTGA
AGCCAGAGAGTAAAATAATTGGTGA
AGCCAGAGAGTAAAATATTTGGTGA
AGCCAGAGAGTAAAAAATGTGA
AGCCAGAGAGTAAAATAATTGGTGA
AGCCAGAGAGTAAAATAATTGGTGA
AGCCAGAGAGTAAAATATTGTGA
AGCCAGAGAGTAAAAATAATTGGTGA
AGCCAGAGAGTAAAATAATTGGTGA
AGCCAGAGAGTAAAATAATTGGTGA
AGCCAGAGAGTAAAATAATTGGTGA
AGCCAGAGAGTAAAATATTTGGTGA
AGCCAGAGAGTAAAATATATGGTGA
AGCCAGAGAGTAAAATAATTGGTGA
AGCCAGAGAGTAAAATATTGGTGA
AGCCAGAGAGTAAAATAATTGGTGA
AGCCAGAGAGTAAAATAATTGGTGA
AGCCAGAGAGTAAAATAATTGGTGA
AGCCAGAGAGTAAAATATTGGTGA
AGCCAGAGAGTAAAATAATTGGTGA

AGCCAGAGAGTAAAATATTGGTGA
AGCCAGAGAGTAAAATAATTGGTGA
AGCCAGAGAGTAAAATATTTGGTGA
AGCCAGAGAGTAAAATAATTGGTGA
AGCCAGAGAGTAAAATATTTGGTGA
AGCCAGAGAGTAAAATAATTGGTGA
AGCCAGAGAGTAAAATAATTGGTGA
AGCCAGAGAGTAAAATAATTGGTGA
AGCCAGAGAGTAAAATAATTGGTGA
AGCCAGAGAGTAAAATAATTGGTGA
AGCCAGAGAGTAAAATAATTGGTGA
AGCCAGAGAGTAAAATAATTGGTGA
AGCCAGAGAGTAAAATAATTGGTGA
AGCCAGAGAGTAAAATAATTGGTGA
AGCCAGAGAGTAAAATAATTGGTGA
AGCCAGAGAGTAAAATATTTGGTGA
AGCCAGAGAGTAAATAATTGGTGA
AGCCAGAGAGTAAAATAATTGGTGA
AGCCAGAGAGTAAAATATGGTGTGA
AGCCAGAGAGTAAAATAATTGGTGA
AGCCAGAGAGTAAAAAATTGTGA
AGCCAGAGAGTAAAATATTGAAGCGAAGAAAAAATGGTGAAGCCAAAAAAAAAAAAAAAA
AGCCAGAGAGTAAAATAATTGGTGA
AGCCAGAGAGTAAAATATTTGGTGA
AGCCAGAGAGTAAAATATTCTGTGA
AGCCAGAGAGTAAAATATTTGGTGA
AGCCAGAGAGTAAAATAATTGGTGA
AGCCAGAGAGTAAAATATTTGGTGA
AGCCAGAGAGTAAAATATTTGGTGA
AGCCAGAGAGTAAAATAATTGGTGA
AGCCAGAGAGTAAAATATTTGGTGA
AGCCAGAGAGTAAAATAATTGGTGA
AGCCAGAGAGTAAAATATTTGGTGA
AGCCAGAGAGTAAAATATTGGTGA
AGCCAGAGAGTAAAATATTGTGA
AGCCAGAGAGTAAAATAATTGGTGA
AGCCAGAGAGTAAAATAATTGGTGA
AGCCAGAGAGTAAAATAATTGGTGA
AGCCAGAGAGTAAAATAATTGGTGA
AGCCAGAGAGTAAAATATTGGTGA
AGCCAGAGAGTAAAATAATTGGTGA
AGCCAGAGAGTAAAATAATTGGTGA
AGCCAGAGAGTAAAATAATTGGTGA
AGCCAGAGAGTAAAATAATTGGTGA
AGCCAGAGAGTAAAATATTTGGTGA
AGCCAGAGAGTAAAATAATTGGTGA
AGCCAGAGAGTAAAATATTGTG
AGCCAGAGAGTAAAATAATTGGTGA
AGCCAGAGAGTAAAATATTTGTGA
AGCCAGAGAGTAAAATAATTGGTGA
AGCCAGAGAGTAAAATATTTGGTGA
AGCCAGAGAGTAAAATAATTGGTGA
AGCCAGAGAGTAAAATAATTGGTGA
AGCCAGAGAGTAAAATAATTGGTGA

AGCCAGAGAGTAAAATAATTGGTGA
AGCCAGAGAGTAAAATAATTGGTGA
AGCCAGAGAGTAAAATATTGGTGA
AGCCAGAGAGTAAAAAATATGGTGA
AGCCAGAGAGTAAAATAATTGGTGA
AGCCAGAGAGTAAAATATTTGGTGA
AGCCAGAGAGTAAAATAATTGGTGA
AGCCAGAGAGTAAAATATTTGGTGA
AGCCAGAGAGTAAAATAATTTGGTGA
AGCCAGAGAGTAAAATAATTGGTGA
AGCCAGAGAGTAAAATATTTGGAAGAAAAAAAAATGA
AGCCAGAGAGTAAAATAATTGGTGA
AGCCAGAGAGTAAAATAATTGGTGA
AGCCAGAGAGTAAAATAATTGGTGA
AGCCAGAGAGTAAAATATTTGGTGA
AGCCAGAGAGTAAAATAATTGGTGA
AGCCAGAGAGTAAAATATTGGTGA
AGCCAGAGAGTAAAATAACTGTGA
AGCCAGAGAGTAAAATATTGTGA
AGCCAGAGAGTAAAATAATTGGTGA
AGCCAGAGAGTAAAATAATTGGTGA
AGCCAGAGAGTAAAATAATTGGTGA
AGCCAGAGAGTAAAATAATTGGTGA
AGCCAGAGAGTAAAATATTTGGTGA
AGCCAGAGAGTAAAAATAATTGGTGA
AGCCAGAGAGTAAAATAATTGGTGA
AGCCAGAGAGTAAAATAATTGGTGA
AGCCAGAGAGTAAAATAATTGGTGA
AGCCAGAGAGTAAAATATTTGGTGA
AGCCAGAGAGTAAAATAATTGGTGA
AGCCAGAGAGTAAAATAATTGGTGA
AGCCAGAGAGTAAAATAATTGGTGA
AGCCAGAGAGTAAAATAATTGTGA
AGCCAGAGAGTAAAATAATTGGTGA
AGCCAGAGAGTAAAATAATTGGTGA
AGCCAGAGAGTAAAATAATTGGTGA
AGCCAGAGAGTAAAATAATTGGTGA
AGCCAGAGAGTAAAATAATTGGTGA
AGCCAGAGAGTAAAATAATTGGTGA
AGCCAGAGAGTAAAATAATTGGTGA
AGCCAGAGAGTAAAATAATTGGGGA
AGCCAGAGAGTAAAATAATTGGTGA
AGCCAGAGAGTAAAATATTTGGTGA
AGCCAGAGAGTAAAATAATTGGTGA
AGCCAGAGAGTAAAATATTGGTGA
AGCCAGAGAGTAAAATAATTGGTGA
AGCCAGAGAGTAAAATAATTGGTGA
AGCCAGAGAGTAAAATATTGTGA
AGCCAGAGAGTAAAATAATTGGTGA
AGCCAGAGAGTAAAATATTTGGTGA
AGCCAGAGAGTAAAATAATTGGTGA
AGCCAGAGAGTAAAATATTTGGTGA
AGCCAGAGAGTAAAATAATTGGTGA
AGCCAGAGAGTAAAATAATTGGTGA

AGCCAGAGAGTAAAATAATTGGTGA
AGCCAGAGAGTAAAATAATTGGTGA
AGCCAGAGAGTAAAATATTGGTGA
AGCCAGAGAGTAAAATAATTGGTGA
AGCCAGAGAGTAAAAATATTGGTGA
AGCCAGAGAGTAAAATAATTGGTGA
AGCCAGAGAGTAAAATAATTGGTGA
AGCCAGAGAGTAAAATAATTGTGA
AGCCAGAGAGTAAAATATTTGGTGA
AGCCAGAGAGTAAAATAATTGGTGA
AGCCAGAGAGTAAAATATTTGGTGA
AGCCAGAGAGTAAAATATTTGGTGA
AGCCAGAGAGTAAAATATATTGTGA
AGCCAGAGAGTAAAATATTGGTGA
AGCCAGAGAGTAAAATAATTGGTGA
AGCCAGAGAGTAAAATAATTGGTGA
AGCCAGAGAGTAAAATAATTGGTGA
AGCCAGAGAGTAAAATAATTGGTGA
AGCCAGAGAGTAAAATAATTGGTGA
AGCCAGAGAGTAAAATAATTGGTGA
AGCCAGAGAGTAAAATATTTGGTGA
AGCCAGAGAGTAAAATAATTGGTGA
AGCCAGAGAGTAAAATAATTGGTGA
AGCCAGAGAGTAAAATATTTGGTGA
AGCCAGAGAGTAAAAAAAAAAAAAAAAAAAGAAGTAAAAATTGTGA
AGCCAGAGAGTAAAATAATTGGTGA
AGCCAGAGAGTAAAATATTTGGTGA
AGCCAGAGAGTAAAATAATTGGTGA
AGCCAGAGAGTAAAATAATTGGTGA
AGCCAGAGAGTAAAATAATTGGTGA
AGCCAGAGAGTAAAATAATTGGTGA
AGCCAGAGAGTAAAATATTTGGTGA
AGCCAGAGAGTAAAATAATTGGTGA
AGCCAGAGAGTAAAATAATTGGTGA
AGCCAGAGAGTAAAATATTTGGTGA
AGCCAGAGAGTAAAATAATTGGTGA
AGCCAGAGAGTAAAATAATTGGTGA
AGCCAGAGAGTAAAATATTGGTGA
AGCCAGAGAGTAAAATAATTGGTGA
AGCCAGAGAGTAAAATATTTGGTGA
AGCCAGAGAGTAAAATAATTGGTGA
AGCCAGAGAGTAAAAATAATTGGTGA
AGCCAGAGAGTAAAATATTGGTGA
AGCCAGAGAGTAAAATAATTGGTGA
AGCCAGAGAGTAAAATATTTGGTGA
AGCCAGAGAGTAAAATAATTGGTGA
AGCCAGAGAGTAAAATAATTGGTGA
AGCCAGAGAGTAAAATAATTGGTGA
AGCCAGAGAGTAAAATAATTGGTGA
AGCCAGAGAGTAAAATATTTGGTGA
AGCCAGAGAGTAAAATAATTGGTGA
AGCCAGAGAGTAAAATAATTGGTGA
AGCCAGAGAGTAAAATATTTGGTGA
AGCCAGAGAGTAAAATATATGGTGA

AGCCAGAGAGTAAAATAATTGGTGA
AGCCAGAGAGTAAAATATTTGGTGA
AGCCAGAGAGTAAAATAATTGGTGA
AGCCAGAGAGTAAAATAATTGGTGA
AGCCAGAGAGTAAAATAATTGGTGA
AGCCAGAGAGTAAAATAATTGGTGA
AGCCAGAGAGTAAAATATTTGGTGA
AGCCAGAGAGTAAAATATTGGTGA
AGCCAGAGAGTAAAATAATTGGTGA
AGCCAGAGAGTAAAATAATTGGTGA
AGCCAGAGAGTAAAATATTTGGTGA
AGCCAGAGAGTAAAATAATTGGTGA
AGCCAGAGAGTAAAATAATTGGTGA
AGCCAGAGAGTAAAATAATTGGTGA
AGCCAGAGAGTAAAATATTTGGTGA
AGCCAGAGAGTAAAATAATTGGTGA
AGCCAGAGAGTAAAATAATTGGTGA
AGCCAGAGAGTAAAATATTTGGTGA
AGCCAGAGAGTAAAATAATTGGTGA
AGCCAGAGAGTAAAATATTGGTGA
AGCCAGAGAGTAAAATAATTGGTGA
AGCCAGAGAGTAAAATAATTGGTGA
AGCCAGAGAGTAAAATATTTGGTGA
AGCCAGAGAGTAAAATATTGGTGA
AGCCAGAGAGTAAAATAATTGGTGA
AGCCAGAGAGTAAAATAATTGGTGA
AGCCAGAGAGTAAAATAATTGGTGA
AGCCAGAGAGTAAAAATTTGTGA
AGCCAGAGAGTAAAATAATTGGTGA
AGCCAGAGAGTAAAATAATTGGTGA
AGCCAGAGAGTAAAATAATTGGTGA
AGCCAGAGAGTAAAATAATTGGTGA
AGCCAGAGAGTAAAATAATTGTGA
AGCCAGAGAGTAAAATAATTGGTGA
AGCCAGAGAGTAAAATAATTGGTGA
AGCCAGAGAGTAAAATAATTGGTGA
AGCCAGAGAGTAAAATAATTGGTGA
AGCCAGAGAGTAAAATAATTGGTGA
AGCCAGAGAGTAAAAATGA
AGCCAGAGAGTAAAATAATTGGTGA
AGCCAGAGAGTAAAATAATTGGTGA
AGCCAGAGAGTAAAATATTGGTGA
AGCCAGAGAGTAAAATAATTGGTGA
AGCCAGAGAGTAAAATAATTGGTGA
AGCCAGAGAGTAAAATAATTGGTGA
AGCCAGAGAGTAAAATAATTGGTGA
AGCCAGAGAGTAAAATAATTGGTGA
AGCCAGAGAGTAAAATATTGTGA
AGCCAGAGAGTAAAATAATTGGTGA
AGCCAGAGAGTAAAATAATTGGTGA
AGCCAGAGAGTAAAATAATTGGTGA
AGCCAGAGAGTAAAATAATTGGTGA
AGCCAGAGAGTAAAATAATTGGTGA
AGCCAGAGAGTAAAATAATTGTGA

AGCCAGAGAGTAAAATAATTGGTGA
AGCCAGAGAGTAAAATATAGTGGGA
AGCCACAGAGAGTAAAATATTGGTGA
AGCCAGAGAGTAAAATAATTGGTGA
AGCCAGAGAGTAAAATATCCTGTGA
AGCCAGAGAGTAAAATAATTGGTGA
AGCCAGAGAGTAAAATAATTGGTGA
AGCCAGAGAGTAAAATATTTGGTGA
AGCCAGAGAGTAAAATATTGGTGA
AGCCAGAGAGTAAAATATTGGTGA
AGCCAGAGAGTAAAATATTGGTGA
AGCCAGAGAGTAAAAATGAAGCAGAAGTAAAATATTGGTGA
AGCCAGAGAGTAAAATATTGGTGA
AGCCAGAGAGTAAAATATTGGTGA
AGCCAGAGAGTAAAATATTGTGA
AGCCAGAGAGTAAAATATTGTGA
AGCCAGAGAGTAAAATATTGGTGA
AGCCAGAGAGTAAAATAATTGTGA
AGCCAGAGAGTAAAATAATTGGTGA
AGCCAGAGAGTAAAATAATTGGTGA
AGCCAGAGAGTAAAATAATTGGTGA
AGCCAGAGAGTAAAATATCCTGTGA
AGCCAGAGAGTAAAATATTTTGTGA
AGCCAGAGAGTAAAATAATTGGTGA
AGCCAGAGAGTAAAATATTTGGTGA
AGCCAGAGAGTAAAATAATTGGTGA
AGCCAGAGAGTAAAATAATTGGTGA
AGCCAGAGAGTAAAATAATTGGTGA
AGCCAGAGAGTAAAATATTTGGTGA
AGCCAGAGAGTAAAATAATTAGTGA
AGCCAGAGAGTAAAATATTTTGTGA
AGCCAGAGAGTAAAATAATTGGTGA
AGCCAGAGAGTAAAATATCCTGTGA
AGCCAGAGAGTAAAATAATTGGTGA
AGCCAGAGAGTAAAATATCCTGTGA
AGCCGGAGAGTAAAATATTTGGTGA
AGCCAGAGAGTAAAATAATCGGTGTACAGAGAGTAAAATTGTGA
AGCCGGAGAGTAAAATATTTGGTGA
AGCCAGAGAGTAAAATGTTTGGTGA
AGCCAGAGAGTAAAATTTTTGGTGA
AGCCAGAGAGTAAAATATCCTGTGA
AGCCAGAGAGTAAAATAATCGGTGA
AGCCAGAGAGTAAAATATTTGGTGA
AGCCAGAAGGTAAAATATTTGGTGA
AGCCAGAGAGTAAAATATCCTGTGA
AGCCGGAGAGTAAAATATTTGGTGA
AGCCAGAGAGTAAAATAATCGGTGT
AGCCAGAGAGTAAAATAATTAGTGA
Nr of selected repeats 627 Similarity 0.736670
Consensus:
AGCCAGAGAGTAAAATAaTTGGTGA
>Cele-UNSB01_2:5722242-5722548 Satlength=307 Nr of Repeats=9 RepeatLength=34
seed=GGAATTGAAA
GGAATTGAAATTTCCAACAAATCGGCAAATTGCC

GGAATTGAAATTTCCAGCAAATCGGCAAATTGCC
GGAATTGAAATTTGCGGCAAATCGGCAAATTTCC
GGAATTGAAATTTCCGGCAAATCGGCAAAATCTC
GGAATAGAAAATTCCGGCAAATCGGCAAAATCTG
GGAATTGAAATTTCCGGCAAATCGGCAAAATCTG
GGAATTGAAATTTCCGGCAAATCGGCAAATTGCC
GGAATTGAAATTTCCGGCAAATCGACAAATTGCC
GGAATTGAAATTTCCGGCAAATCGACAAATTGCC
Nr of selected repeats 9 Similarity 0.831481
Consensus:
GGAATTGAAATTTCCgGCAAATCGgCAAAtTgCc
>Cele-UNSB01_2:5970897-5971730 Satlength=834 Nr of Repeats=4 RepeatLength=203
seed=GTTCAAGCAG
GTTCAAGCAGAACAAGTACATTTTCAGTTATACCTTTTAGATCGCATTTTGAGATTGAATGGAATAAACGAGTTATATG
CAAAATTAGTAGACTTTCCAAACTTTGAAAATTAATATCTGGCTTCAAACTAACTTTTCTGAAAATCCCCGTAGCCATT
TTGTTGCAAATTTTGTGCTCTTTCAGAATTTAAAAACGGCATTCCAAACATTTCCGTATTTGCCGA
GTTCAAGCAGAACAAGTAGATCACATTTTGAGATTGAATAGAATAAACGAGTTATATGCAAAATTAGTAGACTTTCCAA
ACTTTGAAAATTAATATCTGGCTTCAAACTAACTTTTCTGAAAATCCCCGTAGCCATTTTGTTGCAAATTTTGTGCTCT
TTCAGAATTTAGAAACGGCATTCCAAACATTTCCATATTCGCCAG
GTTCAAGCAGAACAAGTAGATCACGTTTTGAGATTGAATGGAATAAACGAGTTATATGCAAAATTAGTAGACTTTCCAA
ACTTTGAAAATTAATATCTGGCTTCAAACTAACTTTTCTGAAAATCCCCGAAGCCATTTTGTTGCAAGTTTTGTGCTCT
TTCAGAATTTAAAAAGGGCATTCCAAACATTTCCATATTCGCCGA
GTTCAAGCAGAACAAGAAGATCACATTTTGAGATTGAATGGACTAAACGAGTTATATGCAAAATTAGTAGACTTTCCAA
ACTTTGAAAATTAATATCTGGCTTCAAACTAACTTTTCTGAAAATCCCCGTAGTCATTTTGTTGCAAATTTTGTGCTCT
TTCAGAACTTAAAAGCGGCACTCCAAACATTTCCATATTCGCCGA
Nr of selected repeats 3 Similarity 0.935730
Consensus:
GTTCAAGCAGAACAAGtAGATCACaTTTTGAGATTGAATgGAaTAAACGAGTTATATGCAAAATTAGTAGACTTTCCAA
ACTTTGAAAATTAATATCTGGCTTCAAACTAACTTTTCTGAAAATCCCCGtAGcCATTTTGTTGCAAaTTTTGTGCTCT
TTCAGAAtTTAaAAacGGCAtTCCAAACATTTCCATATTCGCCgA
>Cele-UNSB01_2:6168224-6172025 Satlength=3802 Nr of Repeats=21 RepeatLength=181
seed=TTTTTAACTT
TTTTTAACTTTTAAATCCACTAAAACTTGGAATGGTACAGAACATGCAAATTGTAATTTTAAGAACTGAATGTGGTTTC
GAATATTCATGGATTGCTTTATTCGAACACTGACTCTGAAATATTTTGCATCCATTTGACTATTAATTGTCATATTATA
CCGAACACTACTAACTTTACTGG
TTTTTAACTTTTAAATCCACTAAAAATTGGAATGGTACAGAACATGAAAATTGTAATTTTAAAAACTGAATGTGGTTTC
GAATATTTATGGATTCCTTTATTCTAAAACTGACTCTGAAATATTTTGCATCCATTTGACTATTAATTGCCATATTATA
CCGAACACTACTAACTTTACTGG
TTTTTAACTTTTGAATCCACTAAAACTTGGAATGGTACAGAACATGCAAATTGTAATTTCAAGAACTGAATGTAGTTTC
GAATATTTATGGATTGCTTTATTCTAAAGCTGACTCTGAAATATTTTGCATCCATTTGACTATTAATTGCCATATTATA
CCGAACACTACTAACTTTACTGG
TTTTTAACTTTTAAATCCACTAAAACTCGGAATGGTACAGAACATGCAAATTGTAATTTCAAGAACTGAATGTGGTTTC
GAATATTTATGGATTGCTTTGTTCCCACACTGACTCTGAAATATTTTGCATCCATTTGACTATTAATTGTCATATTATA
CCGAACACTACTAACTTTAGTGG
TTTTTAACTTTTAAATCCACTAAAACTCGGAATGGTACAGAACATGCAAATTGTAATTTCAAGAACTGAATGTGGTTTC
GAATATTTATGGATTGCTTTATTCTAAAACTGACTCTGAAATATTTTGCATCCATATGACTACTAATTGTCATATTATA
CCGAACACTACTAACTTTAGTGG
TTTTTAACTTTTAAATCCACTAAAACTCGGAATGGTACAGAACATGCAAATTGTAATTTCAAGAACTGAATGTGGTTTC
GAATATTTATGGATTGCTTTATTCTAAAACTGACTCTGAAATATTTTGCATCCATTTGACTATTAATTGTCATATTATA
CCGAACACTACTAACTTTAGTGG
TTTTTAACTTTTAAATCCACTAAAACTCGGAATGGTACAGAACATGCAAATTGTAATTTCAAGAACTGAATGTGGTTTC
GAATATTTATGGATTGCTTTATTCTAAAACTGACTCTGAAATATTTTGCATCCATATGACTACTAATTGTCATATTATA
CCGAACACTACTAACTTTAGTGG

TTTTTAACTTTTAAATCCACTAAAACTCGGAATGGTACAGAACATGCAAATTGTAATTTCAAGAACTGAATGTAGTTTC
GAATATTTATGGATTGCTTTATTCTAAAACTGACTCTGAAATATTTTGCATCCATTTGACTATTAATTGTCATATTATA
CCGAACACTACTAACTTTAGTGG
TTTTTAACTTTTAAATCCACTAAAACTCGGAATGGTACAGAACATGCAAATTGTAATTTCAAGAACTGAATGTGGTTTC
GAATATTTATGGATTGCTTTATTCTAAAACTGACTCTGAAATATTTTGCATCCATTTGACTATTAATTGTCATATTATA
CCGAACACTACTAACTTTAGTGG
TTTTTAACTTTTAAATCCACTAAAACTCGGAATGGTACAGAACATGCAAATTGTAATTTCAAGAACTGAATGTAGTTTC
GAATATTTATGGATTGCTTTATTCTAAAACTGACTCTGAAATATTTTGCATCCATATGACTACTAATTGTCATATTATA
CCGAACACTACTAACTTTAGTGG
TTTTTAACTTTTAAATCCACTAAAACTTGGAATGGTACAGAACATGCAAATTGTAATTTCAAGAACTGAATGTAGTTTC
GAATATTTATGGATTGCTTTATTCTAAAACTGACTCTGAAATATTTTGCATCCATATGACTACTAATTGTCATATTATA
CCGAACACTACTAACTTTAGTGG
TTTTTAACTTTTAAATCCACTAAAACTTGGAATGGTACAGAACATGCAAATTGTAATTTCAAGAACTGAATGTGGTTTC
GAATATTTATGGATTGCTTTGTTCCCACACTGACTCTGAAATATTTTGCATCCATTTGACTATTAATTGTCATATTATA
CCGAACACTACTAACTTTAGTGG
TTTTTAACTTTTAAATCCACTAAAACTTGGAATGGTACAGATTATGCAAATTGTAATTTCAAGAACTGAATGTGGTTTC
GAATATTTATGGATTGCTTTATTCTAAAACTGACTCTGAAATATTTTGCATCCATATGACTACTAATTGTCATATTATA
CCGAACACTACTAACTTTAGTGG
TTTTTAACTTTTAAATCCACTAAAACTTGGAATGGTACAGAACATGCAAATTGTAATTTCAAGAACTGAATGTAGTTTC
GAATATTTATGGATTGCTTTATTCTAAAACTGACTCTGAAATATTTTGCATCCATATGACTACTAATTGTCATATTATA
CCGAACACTACTAACTTTAGTGG
TTTTTAACTTTTAAATCCACTAAAACTTGGAATGGTACAGAACATGCAAATTGTAATTTCAAGAACTGAATGTGGTTTC
GAATATTTATGGATTGCTTTGTTCCCACACTGACTCTGAAATATTTTGCATCCATTTGACTATTAATTGTCATATTATA
CCGAACACTACTAACTTTAGTGG
TTTTTAACTTTTAAATCCACTAAAACTCGGAATGGTACAGAACATGCAAATTGTAATTTCAAGAACTGAATGTGGTTTC
GAATATTTATGGATTGCTTTATTCTAAAACTGACTCTGAAATATTTTGCATCCATTTGACTATTAATTGTCATATTATA
CCGAACACTACTAACTTTAGTGG
TTTTTAACTTTTAAATCCACTAAAACTCGGAATGGTACAGATTATGCAAATTGTAATTTCAAGAACTGAATGTGGTTTC
GAATATTTATGGATTGCTTTATTCTAAAACTGACTCTGAAATATTTTGCATCCATTTGACTACTAATTGTCATATTATA
CCGAACACTACTAACTTTAGTGG
TTTTTAACTTTTAAATCCACTAAAACTCGGAATGGTACAGAACATGCAAATTGTAATTTCAAGAACTGAATGTGGTTTC
GAATATTTATGGATTGCTTTATTCTAAAACTGACTCTGAAATATTTTGCATCCATATGACTACTAATTGTCATATTATA
CCGAACACTACTAACTTTAGTGG
TTTTTAACTTTTAAATCCACTAAAACTCGGAATGGTACAGAACATGCAAATTGTAATTTCAAGAACTGAATGTGGTTTC
GAATATTTATGGATTGCTTTATTCTAAAACTGACTCTGAAATATTTTGCATCCATTTGACTACTAATTGTCATATTATA
CCGAACACTACTAACTTTAGTGG
TTTTTAACTTTTAAATCCACTAAAACTCGGAATGGTACAGAACATGCAAATTGTAATTTCAAGAACTGAATGTGGTTTC
GAATATTTATGGATTGCTTTATTCTAAAACTGACTCTGAAATATTTTGCATCCATATGACTACTAATTGTCATATTATA
CCGAACACTACTAACTTTAGTGG
TTTTTAACTTTTAAATCCACTAAAACTCGGAATGGTACAGAACATGCAAATTGTAATTTCAAGAACTGAATGTGGTTTC
GAATATTTATGGATTGCTTTATTCTAAAACTGACTCTGAAATATTTTGCATCCATTTGACTACTAATTGTCATATTATA
CCGAACACTACTAACTTTACTGG
Nr of selected repeats 21 Similarity 0.964746
Consensus:
TTTTTAACTTTTAAATCCACTAAAACTcGGAATGGTACAGAACATGCAAATTGTAATTTCAAGAACTGAATGTgGTTTC
GAATATTTATGGATTGCTTTATTCTAAAACTGACTCTGAAATATTTTGCATCCATtTGACTAcTAATTGTCATATTATA
CCGAACACTACTAACTTTAGTGG
>Cele-UNSB01_2:6176621-6177425 Satlength=805 Nr of Repeats=18 RepeatLength=45
seed=CTGAAGGATC
CTGAAGGATCAGAACATCAGGAAGAGCCTCGTGATCCTTTGGAG
CTGAAGGATCAGACCATCAGGAGGTACCCTTACGATCCTTCTTAT
CTGAAGGACCAGAACATCAGAAGGTACCCATGTGATCCTTCGGAG
CTGAAGGATCAGAACATCAGGAAGAGCCTCGTGATCCTTTGGAG
CTGAAGGATCAGACCATCAGGAGGTACCCTTACGATCCTTCTTAT

CTGAAGGATCAGAACATCAGAAGGTACCCATGTGATCCTTCGGAG
CTGAAGGATCAGAACATCAGGAAGAGCCTCGTGATCCTTTGGAG
CTGAAGGATCAGACCATCAGGAGGTACCCTTACGATCCTTCTTAT
CTGAAGGATCAGAACATCAGAAGGTACCCATGTGATCCTTCGGAG
CTGAAGGATCAGAACATCAGGAAGAGCCTCGTGATCCTTTGGAG
CTGAAGGATCAGACCATCAGGAGGTACCCTTACGATCCTTCTTAT
CTGAAGGATCAGAACATCAGAAGGTACCCATGTGATCCTTCGGAG
CTGAAGGATCAGAACATCAGGAAGAGCCTCGTGATCCTTTGGAG
CTGAAGGATCAGACCATCAGGAGGTACCCTTACGATCCTTCTTAT
CTGAAGGATCAGAACATCAGAAGGTACCCATGTGATCCTTCGGAG
CTGAAGGATCAGAACATCAGGAAGAGCCTCGTGATCCTTTGGAG
CTGAAGGATCAGACCATCAGGAGGTACCCTTACGATCCTTCTTTT
CTGAAGGATCAGAACATCAGAAGGTACCTATGTGATCCCTCGGAG
Nr of selected repeats 12 Similarity 0.850954
Consensus:
CTGAAGGATCAGAaCATCAGaAGGTACCCaTacGATCCTTCggAg
>Cele-UNSB01_2:6280573-6282049 Satlength=1477 Nr of Repeats=96 RepeatLength=14
seed=CTATACGCTA
CTATACGCTATACG
CTATACGCTATACG
CTATACGCTATACG
CTATACGCTATACG
CTATACGCTATACG
CTATACGCTATACG
CTATACGCTATACG
CTATACGCTATACG
CTATACGCTATACG
CTATACGCTATACG
CTATACGCTATACG
CTATACGCTATACG
CTATACGCTATACG
CTATACGCTATACG
CTATACGCTATACG
CTATACGCTATACG
CTATACGCTATACG
CTATACGCTATACG
CTATACGCTATACG
CTATACGCTATACG
CTATACCTATACG
CTATACGCTATACG
CTATACGCTATACG
CTATACGCTATACG
CTATACGCTATACG
CTATACCTATACG
CTATACGCTATACG
CTATACGCTATACG
CTATACGCTATACGCTAC
CTATACGCTATACG
CTATACGCTATACG
CTATACGCTATACG
CTATACGCTATAC
CTATACGCTATACG
CTATACGCTATACG
CTATACGCTATACG

CTATACGCTATACG
CTATACGCTATACGCTATACGCCG
CTATACGCTATACG
CTATACGCTATACG
CTATACGCTATACG
CTATACGCTATACG
CTATACGCTATACG
CTATACGCTATACG
CTATACGCTATACG
CTATACGCTATACG
CTATACGCTATACG
CTATACGCTATACG
CTATACGCTATACG
CTATACGCCTATACG
CTATACGCTATACG
CTATACGCTATACG
CTATACGCTATACG
CTATACGCTATACG
CTATACGCTATACG
CTATACGCTATACCG
CTATACGCTATACG
CTATACGCTATACG
CTATACGCTATACG
CTATACGCTATACG
CTATACGCTATAC
CTATACGCTATACG
CTATACGCTATACG
CTATACGCTATACG
CTATACGCTATACG
CTATACGCTATACG
CTATACGCTATACG
CTATACGCTATACGC
CTATACGCTATACG
CTATACGCTATACG
CTATACGCTATACG
CTATACGCTATACG
CTATACGCTATACG
CTATACGCTATACGCTTTTTTTTTTTTTTTTTTTTTTTTTTTTTTTTTTTTTTTTTTTTTTTTTTTTTTTTTTTTTTTT
TTTTTTTTTTTTTTTTTTTTTTTTTTTTTTTTTTTTTTTTTTTTTACG
CTATACCTATACG
CTATACGCTATACG
CTATACGCTATACG
CTATACGCTATACG
CTATACGCTATACG
CTATACGCTATACG
CTATACGCTATACG
CTATAGCTATCTACG
CTTACGCTATACG
CTATACGCTATACG
CTATAACGCTATACG
CTATACGCTATACG
CTATACGCTATACG
CTATACGCTATACG
CTATACGCTATACG

CTATACGCTATACG
CTATACGCTTCCTTG
CTATACGCTATACG
CTATACCTATCG
CTATACGCTATACG
CTATACGCTATACG
CTATACGCTATACGCTATACG
Nr of selected repeats 79 Similarity 1.000000
Consensus:
CTATACGCTATACG
>Cele-UNSB01_2:6282823-6283646 Satlength=824 Nr of Repeats=34 RepeatLength=10
seed=TCCCGATCCA
TCCCGATCCATCCATCCATCCAATCCACCCAATCGTTGTGTCGTCGTCGTCGTTGTCTTGTCCATCCATCCAGCCACTT
CAAGTGATGTTCTGTACCGTTAGAACTGTGCAACATCACCCATTACAATATACCTATGTGGCAAGAACCGTCCGATAAG
CCCTCCTCTGTTTGACGCTATTTCCAGTCTCCTTGTTCTTATTTTCGCAGGAGTTCGCATTTCCCATTGAGAGATTAAT
CGGTTTCACTACCTCTGAGTACGGTACAAGATTTGTTTGTTTCCAAACAAATCAACTCAGGGAGTAGTAGTGAAACCGA
AAATGCAGCCATTGCCTGTTACTTTTGAACCCGGTTCAGAAACTGGACAGTGACAGATTCATGCGAACCAGTTCAGTTC
AAGCCAGGTATATGCAATGGGGACGCTACACTCACAAGAAGGAGCATTCGTCCACCCTCCGTCGTCGTCGTCGTCGTCT
CG
TCCCGATCCA
TCCCGATCCAT
TCCCGATCCA
TCCCGATCTA
TCCCGATCCAT
TCCCGATCCA
TCCCGATCCAT
TCCCGATTCA
TCCCGATCTA
TCCCGATCCAT
TCCCGATCCAT
TCCCGATCCAT
TCCCGATCCAT
TCCCGATCCA
TCCCGAACCA
TCCCGATCCA
TCCCGATCCA
TCCCGATCCA
TCCCGATCCA
TCCCGATCCA
TCCCGATCCAT
TCCGATCCATCCCGATCCA
TCCCGATCCA
TCCCGATCCA
TCCCGATCCA
TCCCGATCCA
TCCCGATCCA
TCCCGATCCA
TCCCGATCCA
TCCCGATCCA
TCCCGATCCA
TCCCGATCCATCCCGATCCA
Nr of selected repeats 22 Similarity 0.952670
Consensus:
TCCCGATCCA

>Cele-UNSB01_2:6392491-6392539 Satlength=49 Nr of Repeats=4 RepeatLength=12
seed=CCACTGACCA
CCACTGACCATC
CCACTGACCATC
CCACTGACCATC
CCACTGACCATG
Nr of selected repeats 4 Similarity 0.944444
Consensus:
CCACTGACCATc
>Cele-UNSB01_2:6392670-6395650 Satlength=2981 Nr of Repeats=44 RepeatLength=68
seed=AATAAAAATT
AATAAAAATTTAATTTTTATTAGTGTCTCTATTTCATCAAGAAAATACATTTTCAAAATAGAGACG
AATAAAAATTTAAAATTTTTATTAGTGTCTCTATTTTATCAAGAAAATACATTTTCAAAATAGAGACG
AATAAAAATTTAAAATTTTTATTAGTGTCTCTAATTTATGAAAAAAATATATTTTCAAAATAGAGACG
AATAAAAATTTAAAATTTTTATTAGTGTCTCTATTTTATCAAGAAAATATATTTTCAAAATAGAGACG
AATAAAAATTTAAAATTTTTATTAGTGTCTCTATTTTATCAAGAAAATATATTTTCAAAATAGAGACG
AATAAAAATTTAAAATTTTTATTAGTGTCTCTATTTTATCAAGAAAATATATTTTCAAAATAGAGACG
AATAAAAATTTAAAATTTTTATTAGTGTCTCTATTTTATAAAGAAAATATATTTTCAAAATAGAGACG
AATAAAAATTTAAAATTTTTATTAGTGTCTCTATTTTATCAAGAAAATATATTTTCAAAATAGAGACG
AATAAAAATTTAAAATTTTTATTAGTGTCTCTATTTTATCAAGAAAATACATTTTCAAAATAGAGACG
AATAAAAATTTAAAATTTTTATTAGTGTCTCTATTTCATCAAGAAAATACATTTTCAAAATAGAGACG
AATAAAAATTTAAAATTTTTATTAGTGTCTCTAATTTATGAAAAAAATATATTTTCAAAATAGAGACG
AATAAAAATTTAAAATTTTTATTAGTGTCTCTAATTTATGAAAAAAATATATTTTCAAAATAGAGACG
AATAAAAATTTAAAATTTTTATTAGTGTCTCTATTTTATCAAGAAAATATATTTTCAAAATAGAGACG
AATAAAAATTTAAAATTTTTATTAGTGTCTCTATTTTATCAAGAAAATACATTTTCAAAATAGAGACG
AATAAAAATTTAAAATTTTTATTAGTGTCTCTATTTCATCAAGAAAATACATTTTCAAAATAGAGACG
AATAAAAATTTAAAATTTTTATTAGTGTCTCTAATTTATGAAAAAAATATATTTTCAAAATAGAGACG
AATAAAAATTTAAAATTTTTATTAGTGTCTCTATTTTATCAAGAAAATATATTTTCAAAATAGAGACG
AATAAAAATTTAAAATTTTTATTAGTGTCTCTATTTTATCAAGAAAATATATTTTCAAAATAGAGACG
AATAAAAATTTAAAATTTTTATTAGTGTCTCTATTTTATCAAGAAAATATATTTTCAAAATAGAGACG
AATAAAAATTTAAAATTTTTATTAGTGTCTCTATTTTATAAAGAAAATATATTTTCAAAATAGAGACG
AATAAAAATTTAAAATTTTTATTAGTGTCTCTATTTTATCAAGAAAATATATTTTCAAAATAGAGACG
AATAAAAATTTAAAATTTTTATTAGTGTCTCTATTTTATCAAGAAAATACATTTTCAAAATAGAGACG
AATAAAAATTTAAAATTTTTATTAGTGTCTCTATTTCATCAAGAAAATACATTTTCAAAATAGAGACG
AATAAAAATTTAAAATTTTTATTAGTGTCTCTAATTTATGAAAAAAATATATTTTCAAAATAGAGACG
AATAAAAATTTAAAATTTTTATTAGTGTCTCTATTTTATCAAGAAAATATATTTTCAAAATAGAGACG
AATAAAAATTTAAAATTTTTATTAGTGTCTCTAATTTATGAAAAAAATACATTTTCAAAATAGAGACG
AATAAAAATTTAAAATTTTTATTAGTGTCTCTATTTTATAAAGAAAATATATTTTCAAAATAGAGACG
AATAAAAATTTAAAATTTTTATTAGTGTCTCTATTTTATAAAGAAAATACATTTTCACAATAGAGACG
AATAAAAATTTAATTTTTATTGGTGTCTCTATTTTATAAAGAAAATACATTTTCAAAATAGAGACG
AATAAAAATTTAAAATTTTTATTAGTGTCTCTAATTTATGAAAAAAATACATTTTCAAAATAGAGACG
AATAAAAATTTAAAATTTTTATTAGTGTCTCTATTTCATCAAGAAAATACATTTTCAAAATAGAGACG
AATAAAAATTTAAAATTTTTATTAGTGTCTCTAATTTATGAAAAAAATATATTTTCAAAATAGAGACG
AATAAAAATTTAAAATATTTATTAGTGTCTCTATTTTATCAAGAAAATATATTTTCAAAATAGAGACG
AATAAAAATTTAAAATTTTTATTAGTGTCTCTAATTTATGAAAAAAATACATTTTCAAAATAGAGACG
AATAAAAATTTAAAATTTTTATTAGTGTCTCTATTTTATAAAGAAAATATATTTTCAAAATAGAGACG
AATAAAAATTTAAAATTTTTATTAGTGTCTCTATTTTATAAAGAAAATACATTTTCACAATAGAGACG
AATAAAAATTTAATTTTTATTGGTGTCTCTATTTTATAAAGAAAATACATTTTCAAAATAGAGACG
AATAAAAATTTAAAATTTTTATTAGTGTCTCTAATTTATGAAAAAAATACATTTTCAAAATAGAGACG
AATAAAAATTTAAAATTTTTATTAGTGTCTCTATTTTATAAAGAAAATACATTTTCACAATAGAGACG
AATAAAAATTTAATTTTTATTGGTGTCTCTATTTTATAAAGAAAATACATTTTCAAAATAGAGACA
AATAAAAATTTAAAATTTTTATTGTGTCTCTATTTTATAAAGAAAATACATTTTCACAATAGAGACG
AATAAAAATTTAATTTTTATTGGTGTCTCTATTTTATAAAGAAAATACATTTTCAAAATAGAGACA
AATAAAAATTTAAAATTTTTATTAGTGTCTCTATTTTATAAAGAAAATACATTTTCACAATAGAGACG

AATAAAAATTTAATTTTTATTGGTGTCTCTATTTTATAAAGAAAATACATTTTCAAAATAGAGACA
Nr of selected repeats 38 Similarity 0.936325
Consensus:
AATAAAAATTTAAAATTTTTATTAGTGTCTCTAtTTTATcAAgAAAATAtATTTTCAAAATAGAGACG
>Cele-UNSB01_2:6532865-6533025 Satlength=161 Nr of Repeats=5 RepeatLength=32
seed=AAAAATTTTC
AAAAATTTTCACAAAAAAATTTAAATTCCTGCA
AAAAATTTTCACAGAAAATTTGAATTCCCGCC
AAAAATTTTCACAGAAACTTTGAATTCCCGCC
AAAAATTTTCACAGAAAATTTGAATTCCCGCC
AAAAATTTTCACAGAAAATTTGAATTCCCGCC
Nr of selected repeats 5 Similarity 0.916667
Consensus:
AAAAATTTTCACAgAAAaTTTgAATTCCcGCc
>Cele-UNSB01_2:6532865-6533618 Satlength=754 Nr of Repeats=6 RepeatLength=32
seed=AAAAATTTTC
AAAAATTTTCACAAAAAAATTTAAATTCCTGCA
AAAAATTTTCACAGAAAATTTGAATTCCCGCC
AAAAATTTTCACAGAAACTTTGAATTCCCGCC
AAAAATTTTCACAGAAAATTTGAATTCCCGCC
AAAAATTTTCACAGAAAATTTGAATTCCCGCC
AAAAATTTTCACAGAAAATTTGACACCACTAACCTCAAACCAATATCCCTTCAAAAGACGAAAAGTCAATTTTTGCCAA
AGTACAGTAATCCTACAGAACTCCTACAGTACCTCTACAGTACTACTACAGTACCCCGACCATATCCCACCACTAACCT
CAAACCAATATCTCTTCAAAAGACGAAAAGTCAATTTTTGCCAAAGTACAGTAATCCTACAGTACTCCTACAGTACCTC
TACAGTACTACTACAGTACCCCGACTATATCCCACTACTAACCGCAAACCTATATCTCTTCAAAAGACAAAAACTCAAT
TTTTCCTAAACTACAGTAATCCTACCGTACTCCTACAGTACTCCTACAGTACTACTACAGTACCCCGTCCATATCCCCT
TACTAAGCCCAAACTAATATCCCTCCATCAGCCGAAAACGCCTTGCCTTTGTAAGCTATGACGTCACTACTTAACAAAC
GGACACTATTTTTTTATATATAGGTAATGATATTGTCTGATTTCTGAAAGCAATCGAAAAAATTAACTTTTTAGCCTC
CTGCGTTTTGTTTCATTTTACACTAAAGATTAGAAAAAAT
Nr of selected repeats 5 Similarity 0.916667
Consensus:
AAAAATTTTCACAgAAAaTTTgAATTCCcGCc
>Cele-UNSB01_2:7497139-7497311 Satlength=173 Nr of Repeats=4 RepeatLength=41
seed=AAAACGGGAA
AAAACGGGAAAACGGGAATTCCCGTTCCCGTGAAAATTTTA
AAAACGGGAAAACGGGAATTCCCGTTCCCGTGAAAATTTTA
AAAACGGGAAAACGGGAATTCCCGTTCCCGTGAGAATTTTA
AAAACGGGAAAACGGGATTTCCCGTTCCCGTGAAAATTTTAAAAACGGG
Nr of selected repeats 3 Similarity 0.978320
Consensus:
AAAACGGGAAAACGGGAATTCCCGTTCCCGTGAaAATTTTA
>Cele-UNSB01_2:7497138-7497671 Satlength=534 Nr of Repeats=10 RepeatLength=41
seed=AAAAACGGGA
AAAAACGGGAAAACGGGAATTCCCGTTCCCGTGAAAATTTT
AAAAACGGGAAAACGGGAATTCCCGTTCCCGTGAAAATTTT
AAAAACGGGAAAACGGGAATTCCCGTTCCCGTGAGAATTTT
AAAAACGGGAAAACGGGATTTCCCGTTCCCGTGAAAATTTT
AAAAACGGGAAAACGGGAATTCCCGTTCCCGTGAAAATTTT
AAAAACGGGACAACGGGAATTCCCGTTTTTTTGAAAACACTCA
AAAAACGGGACAACCTGGGCGGTTTAAAAAAAAGAGTGCATAGTTGGCGTAAATTTTAGTGTATTCGAGGCAAATTATT
TAAAAAAAACTGCATTATAAACATTTTTATAGATTTTTTCAATATTAAACAGATATTTAAAAAGCCAAAAAATGAGGGA
AAATGTCCCGTTCCCGTGAAAGTTTT
AAAAACGGGACAACGGGAATTCCCGTTCCCGTGAAAATTTC
AAAAACGGGACAACGGGAATTCCCGTTCCCGTGAAAACACCCC

AAAAACGGGACAACGGG
Nr of selected repeats 6 Similarity 0.956640
Consensus:
AAAAACGGGAAAACGGGAATTCCCGTTCCCGTGAAAATTTT
>Cele-UNSB01_2:7564245-7565607 Satlength=1363 Nr of Repeats=34 RepeatLength=14
seed=GAAGGATCGG
GAAGGATCGGGTCG
GAAGGATCGGGCCG
GAAGGATCAGGCCG
GAAGGATCAGGCCGGAAAGATTGGGACGAGACGACGATGGTGAGAGGGAGTCCGGAAACGTGGACAATGGTAAGGAAAT
ATTTAAATGATGCTTCAGCTAAACTGATTTTTTACTGATCTACAATAAAGACGACGAA
GAAGGATCGGGTCG
GAAGGATCGGGCCG
GAAGGATCGGGCCG
GAAGGATCGGGTCG
GAAGGATCGGAACGAGACGACGATGGTGAGAGGGAGTCCGGAAACGTGGACAATGGTAAGAAAAGATTTAAATGATGCT
TCAGCTAAACTGATTTTTTACAGATCTAATAGAAAGACGACGAA
GAAGGATCGGGCCG
GAAGGATCGGGCCG
GAAGGATCAGGCCG
GAAAGATCGGGACGAGACGACGATGGGGAGAGGGAGTCCGGAAACGTGGGCAATGGTAAGAAAAGATTTAAATGATGCT
TCAGCTAAACTGATTTTTTACAGATCTACAATAAAGACGACGAA
GAAGGATCGGGTCG
GAAGGATCGGGTCG
GAAGGATCGGAACGAGACTACGATGGTGAGAGGGAGTCCGGAAACGTGGACAATGGTAAGAAAAGATTTAAATGATGCT
TCAGCTAAACTGATTTTTTACAGATCTAATAGAAAGACGACGAA
GAAGGATCGGGCCG
GAAGGATCGGGTCG
GAAGGATCGGAACGAGACGACGATGGTGAGAGGGAGTCCGGAAACGTGGACAATGGTAAGAAAAGATTTAAATGATGCT
TCAGCTAAACTGATTTTTTACAGATCTACAATAAAGACGACGAA
GAAGGATCGGGTCG
GAAGGATCGGGCCG
GAAGGGTCGGGCCG
GAAGGATCAGGCCG
GAAGGATCGGGACGAGACGACGATGGTGAGAGGGAGTCCGGAAACGTGGACAATGGTAAGAAAAGATTTAAATGATGCT
TCAGCTAAACTGATTTTTTACAGATCTACAATAAAGACGACGAA
GAAGGATCGGGTCG
GAAGGATCGGGTCG
GAAGGATCGGAACGAGACTACGATGGTGAGAGGGAGTCCGGAAACGTGGACAATGGTAAGAAAAGATTTAAATGATGCT
TCAGCTAAACTGATTTTTTACAGATCTACAATAAAGACGACGAA
GAAGGATCGGGTCG
GAAGGATCGGGCCG
GAAGGGTCGGGCCG
GAAGGATCAGGCCG
GAAGGATCGGGACGAGACGACGATGGGCAGAGGGAGTCCGGAAACGTGGACAATGGTAAGAAAAGATTTAAATGATGCT
TCAGCTAAACTGATTTTTTACAGATCTACAATAAAGACGACGAA
GAAGGATCGGGTCG
GAAGGATCGGGTCG
Nr of selected repeats 26 Similarity 0.910916
Consensus:
GAAGGATCGGGcCG
>Cele-UNSB01_2:7566750-7567567 Satlength=818 Nr of Repeats=42 RepeatLength=16
seed=CGGAAAAATT
CGGAAAAATTTTGCTG

CGGAAAACTTTCGCTG
CGGAAAAAATTGCGTCGT
GGAAAAAATTTTTCCGCGACAAAAATTTGCTG
CGTAAAAAATTCCGTCG
CGGAAAAAATTTTGGTGTGGAGAAAATTCCGTCG
CGGAAAAAATTTCGCCG
CGGAAAAATTTTGCTG
CGGAAAAAATTTTGTGG
CGGAGAAAATTCGTCG
CGGAAAAAATTTCGTCGCGTGGAAATTTCCTTG
CGGAAACATTTGGCTGCAGAAAAATAAATTTTGCTGCGGAAAAAAAATTGTCG
CGAAAAAAATTTCGCCG
CGGAAAAATTTTGCTGCGCGAAAATTCCGTCG
CGGAAAAAATTTAGCTG
CGGAAAAAATTTTGCTGC
CGAAAAAAATTTCGCCG
CGGAAAAATTTTGCTGCGCGAAAATTCCGTCG
CGGAAAAAATTTAGCTG
CGGAAAAAATTTCGCCG
CGGGAAAAATTTTGTGG
CGGAAAAAATTTGGCTT
CGGAAAAAATTTCGCCG
CGGAAAAAATTTACTG
CGGAAAAAATTTTGTAG
CGGAGAAATTTGGCTG
CGGAATAATTTCGCCG
CGGAAAAATTTTGCTCCGCGAAAATTCCGTCG
CGGAAAAAATTTAGCTG
CGGAAAAAATTTCGCCG
CGGGAAAAATTTTGTGG
CGGAAAAAATTTGGCTG
CGGAAAAAATTTCGCCG
CGGAAAAAATTGCGTCG
CGGAAACATTTAGCTG
CGGAAAAAATTTTGCTGC
CGAAAAAAATTTGCCG
CGGAAAAAAATTTGGCTG
CGGAAAAAATTTCGCCG
CGGAAAAATTTTGCTGCGCGAAAATTCCGTCG
CGGAAAAAATTTAGCTG
CGGAAAAAATTTTGTGG
Nr of selected repeats 27 Similarity 0.719401
Consensus:
CGGAAAAAATTTnGcnG
>Cele-UNSB01_2:7568262-7569624 Satlength=1363 Nr of Repeats=34 RepeatLength=14
seed=CCGATCCTTC
CCGATCCTTCCGAC
CCGATCCTTCCGAC
CCGATCCTTCTTCGTCGTCTTTATTGTAGATCTGTAAAAAATCAGTTTAGCTGAAGCATCATTTAAATCTTTTCTTACC
ATTGTCCACGTTTCCGGACTCCCTCTGCCCATCGTCGTCTCGTC
CCGATCCTTCCGGC
CTGATCCTTCCGGC
CCGACCCTTCCGGC
CCGATCCTTCCGAC

CCGATCCTTCTTCGTCGTCTTTATTGTAGATCTGTAAAAAATCAGTTTAGCTGAAGCATCATTTAAATCTTTTCTTACC
ATTGTCCACGTTTCCGGACTCCCTCTCACCATCGTAGTCTCGTT
CCGATCCTTCCGAC
CCGATCCTTCCGAC
CCGATCCTTCTTCGTCGTCTTTATTGTAGATCTGTAAAAAATCAGTTTAGCTGAAGCATCATTTAAATCTTTTCTTACC
ATTGTCCACGTTTCCGGACTCCCTCTCACCATCGTCGTCTCGTC
CCGATCCTTCCGGC
CTGATCCTTCCGGC
CCGACCCTTCCGGC
CCGATCCTTCCGAC
CCGATCCTTCTTCGTCGTCTTTATTGTAGATCTGTAAAAAATCAGTTTAGCTGAAGCATCATTTAAATCTTTTCTTACC
ATTGTCCACGTTTCCGGACTCCCTCTCACCATCGTCGTCTCGTT
CCGATCCTTCCGAC
CCGATCCTTCCGGC
CCGATCCTTCTTCGTCGTCTTTCTATTAGATCTGTAAAAAATCAGTTTAGCTGAAGCATCATTTAAATCTTTTCTTACC
ATTGTCCACGTTTCCGGACTCCCTCTCACCATCGTAGTCTCGTT
CCGATCCTTCCGAC
CCGATCCTTCCGAC
CCGATCCTTCTTCGTCGTCTTTATTGTAGATCTGTAAAAAATCAGTTTAGCTGAAGCATCATTTAAATCTTTTCTTACC
ATTGCCCACGTTTCCGGACTCCCTCTCACCATCGTCGTCTCGTC
CCGATCTTTCCGGC
CTGATCCTTCCGGC
CCGATCCTTCCGGC
CCGATCCTTCTTCGTCGTCTTTCTATTAGATCTGTAAAAAATCAGTTTAGCTGAAGCATCATTTAAATCTTTTCTTACC
ATTGTCCACGTTTCCGGACTCCCTCTCACCATCGTCGTCTCGTT
CCGATCCTTCCGAC
CCGATCCTTCCGGC
CCGATCCTTCCGGC
CCGATCCTTCCGAC
CCGATCCTTCTTCGTCGTCTTTATTGTAGATCAGTAAAAAATCAGTTTAGCTGAAGCATCATTTAAATATTTCCTTACC
ATTGTCCACGTTTCCGGACTCCCTCTCACCATCGTCGTCTCGTCCCAATCTTTCCGGC
CTGATCCTTCCGGC
CTGATCCTTCCGGC
CCGATCCTTCCGAC
Nr of selected repeats 26 Similarity 0.898608
Consensus:
CCGATCCTTCCGgC
>Cele-UNSB01_2:7569779-7570282 Satlength=504 Nr of Repeats=27 RepeatLength=10
seed=TCCATCCCGA
TCCATCCCGAC
TCATCCCGATGTATTCCGATTCACCCATCCAGATCTTCGTCGTCGCGTCGATCTGTAAAAAATCAGATTATCTAAAGCA
TTGTTTAAATTTTTCTCGATTCA
TCCATCCCGATCCTTTGTTGTTGTCGTTGTTGTCGTCGTCGTCGTCGTCGTCGTCGTCGTCGTCGTCGTCGTCGTAGCT
GTTCTCGCCTTTACCCGATTCCATCTA
TCCATCCCGATTCA
TCCATCCCAC
TCCATACCGATCTATCTCGA
TCCATCCCGA
TCCATCCCGA
TCCATCCCAA
TCCGTCCCGA
TCCATCCCGA
TCCATCCCGA
TCCATCCCGATCTATTCCAA

TCCATCCCGA
TCCATCCCAA
TCCATCCCAATCCGTCCCCA
TCCATCCCGA
TCCATCCCGA
TCCGTCCCGA
TCCATCCCGA
TCCGTCCCGA
TCCATCCCGA
TCCATCCCGA
TCCATCCCGA
TCCATCCCGA
TCCATCCCGA
TCCATCCCAATCCATCCCGA
Nr of selected repeats 19 Similarity 0.831650
Consensus:
TCCATCCCGA
>Cele-UNSB01_2:7594307-7594367 Satlength=61 Nr of Repeats=4 RepeatLength=15
seed=GTAGATCTTG
GTAGATCTTGTTTTG
GTAGATCTTGAAAAT
GTAGATCTTGTTTTG
GTGGATCTTGTTTTC
Nr of selected repeats 4 Similarity 0.675347
Consensus:
GTaGATCTTGtttTg
>Cele-UNSB01_2:8013550-8013642 Satlength=93 Nr of Repeats=6 RepeatLength=15
seed=CGAAAAGGGG
CGAAAAGGGGAGCAAAA
CGAAAAGGGGATCTG
CAAAAAGGGGATCTG
CGAAAAGGGGAGATA
CGAAAAGGGGAGATA
CGAAAAGGGGAGCAA
Nr of selected repeats 5 Similarity 0.768889
Consensus:
CgAAAAGGGGAgcta
>Cele-UNSB01_2:8013818-8013910 Satlength=93 Nr of Repeats=6 RepeatLength=15
seed=CCCCTTTTCG
CCCCTTTTCGTTGCT
CCCCTTTTCGTATCT
CCCCTTTTCGTATCA
CCCCTTTTCGCAGAT
CCCCTTTTTGCAGAT
CCCCTTTTCGTTCTGCT
Nr of selected repeats 5 Similarity 0.733333
Consensus:
CCCCTTTTcGtagct
>Cele-UNSB01_2:8406477-8418390 Satlength=11914 Nr of Repeats=144
RepeatLength=79 seed=TGACAGAATA
TGACAGAATACCGGCAGTATTCTGGGGTCTCATTAGGTTGTCGTTTCTTTTCTGAATCTCCACTCTTCCGAATGAGTCC
TGACAGAATACCGACAGTATTTTGGGGTCTCATTAGGTTTCCTTTTCTTTTCTGAATCTCCACTCTTCCTAATGAGTCC
TGACAGAATACCAGCAGTATTTTGGGGTCTCATTAGGTTTCCTTTTCTTTTCTGAATCTCCACTCTTCCTAATGAGTCC
TGACCAAGGGTGTCAATTCCCGTTTCCCGTTTTTCCCATTGTCCCGTTTTTGGGGTGTTTTCACGGGAACGGGAATTCC

CGTTTTCCCGTTTTTTAAATTTTCACGGGAACGGGAATTCCCGTTTTCCCGTTTTAAAAATTTTCACGGGAACGGGAAT
TCCCGCTGTCCCGTTTTTTTTATTTTCACGGGAACGGGACAATTTTCCTCATGATTGTGTTTCAAATTTTAAAGTTCTG
TTTTTGAGTTTTGTACCAAGACTTAAGTTCATGCAGATTAATGACTTACATGCAGTTTTTTTAATCAATTCGCCTCGAA
TACACTAAAATTTGCGCCAACTGTGCACTCTTTTTTTTTAAACCGCCCCGTTTGTCCCGTTTTTTGAGTGTTTCAAAAA
AACGGGAATTCCCGTTGTCCCGTTTTTAAAATTTTCACGGGAACGGGAATTCCCGTTGTCCCGTTTTTAAAATTTTCAC
GGGAACGGGAATTCCCGTTTTCCCGTTTTTGTAATTTTTACGGGACATTGACACCCTTGGTCC
TGACAGAATACCGGCAGTATTTTGGGGTCTCATTAGGTTTCCTTTTCTTTTCTGAATCTCCACTCTTCCTAATGAGTCC
TGACAGAATACCGGCAGTATTTTGGGGTCTCAATAGGTCATTGTTTCTTTTCTGAATCTCCACTCTTCCTAATGAGTCC
TGACAGAATACCGGCAGTATTTTGGTGTCTCATTAGGTTTAGTTTCGTTCCTGAATCTGCACTCTTCCTAATGAGTCC
TGACAGAATACCGGCAGTATACTGGGGTCTCATTAGGTCATTGTTTCTTTCCTGAATCTCCACTCTTCCTAATGAGTCC
TGACAGAATACCGGCAGTATTTTGGGGTCTCATTAGGTCATTGTTTCTTTTCTGAATCTCCACTCTTCCTAATGAGTCC
TGACAGAATACCGGCAGTATTTTGGGGTCTCATTAGGTTTAGTTTCGTTCCTGAATCTGCACTCTTCCTAATGAGTCC
TGACAGAATACCGGCAGTATTTTGGGGTCTCATTAGGTCATAGTTTCGTTCCTGAATCTCCACTCTTCCTAATGAGTCC
TGACAGAATACCGGCAGTATTTTGGGGTCTCATTAGGTTATAGTTTCGTTCCTGAATCTCCACTCTTCCTAATGAGTCC
TGACAGAATACCGGCAGTATTTTGGTGTCTCATTAGGTTTCCTTTTCTTTTCTGAATCTCCACTCTTCCTGATGAGTCC
TGACAGAATACCGGCAGTATTTTGGGGTCTCATTAGGTTTAGTTTCGTTCCTTAATCTGCACTCTTCCTAATGAGTCC
TGACAGAATACCGGCAGTATTTTGGGGTCACATTAGGTTTAGTTTCGTTCCTGAATCTGCACTCTTCCTAATGAGTCT
TGACAGAATACCGGCAGTATTTTGGGGTCTCATTAGGTCATAGTTTCGTTCCTGAATCTCCACTCTTCCTAATGAGTCC
TGACAGAATACCGGCAGTATTTTGGTGTCTCATTAGGTTTCCTTTTCTTTTCTGAATCTCCACTCTTCCTAATGAGTCC
TGACAGAATACCGGCAGTATTTTGGGGTCTCAATAGGTTTAGTTTCGTTCCTGAATCTCCACTCTTCCTAATGAGTCC
TGACAGAATACCGGCAGTATTTTGGGGTCTCATTAGGTCATTGTTTCTTTTCTGAATCTCCACTCTTCCTAATGAGTCC
TGACAGAATACCGGCAGTATTTTGGGGTCTCATTAGGTTTCCTTTTCTTTTCTGAATCTCCACTCTTCCTAATGAGTCC
TGACAGAATACCGGCAGTATTTTGGGGTCTCATTAGGTCATTGTTTCTTTTCTGAATCTCCACTCTTCCTAATGAGTCC
TGACAGAATACCGGCAGTATTTTGGGGTCTCATTAGGTCATAGTTTCGTTCCTGAATCTCCACTCTTCCTAATGAGTCC
TGACAGAATACCGGCAGTATTTTGGGGTCTCATTAGGTTTCCTTTTCTTTTCTGAATCTCCACTCTTCCTAATGAGTCC
TGACAGAATACCGGCAGTATTTTGGGGTCTCAATAGGTTTAGTTTCGTTCCTGAATCTCCACTCTTCCTAATGAGTCC
TGACAGAATACCGGCAGTATTTTGGGGTCTCATTAGGTCATTGTTTCTTTTCTGAATCTCCACTCTTCCTAATGAGTAC
TGACAGAATACCGGCAGTATTCTGGGGTCTCATTAGGTTTAGTTTCGTTCCTGAATCTGCACTCTTCCTAATGAGTCC
TGACAGAATACCGGCAGTATTCTGGGGTCTCATTAGGTTTTGTTTCGTTCCTGAATCTGCACTCTTCCTAATGAGTCC
TGAAAGAATACCGGCAGTATTTTGGGGTCTCATTAGGTTTCCTTTTCTTTTCTGAATCTGCACTCTTCCTAATGAGTCC
TGACAGAATACCGGCAGTATTCTGGGGTCTCATTAGGTCATTGTTTCTTTTCTGAATCTCCACTCTTCCTAATGAGTCC
TGACAGAATACCGGCAGTATTTTGGGGTCTCATTAGGTCATTGTTTCTTTTCTGAATCTCCACTCTTCCTAATGAGTCC
TGACAGAATACCGGCAGTATTTTGGGGTCTCATTAGGTTTAGTTTCGTTCCTGAATCTGCACTCTTCCTAATGAGTCC
TGACAGAATACCGGCAGTATTTTGGGGTCTCATTAGGTCATAGTTTCGTTCCTGAATCTCCACTCTTCCTAATGAGTCC
TGACAGAATACCGGCAGTATTTTGGTGTCTCATTAGGTTTCCTTTTCTTTTCTGAATCTCCACTCTTCCTGATGAGTCC
TGACAGAATACCGGCAGTATTTTGGGGTCTCATTAGGTTTAGTTTCGTTCCTTAATCTGCACTCTTCCTAATGAGTCC
TGACAGAATACCGGCAGTATTTTGGGGTCACATTAGGTTTAGTTTCGTTCCTGAATCTGCACTCTTCCTAATGAGTCC
TGACAGAATACCGGCAGTATTTTGGTGTCTCATTAGGTTTCCTTTTCTTTTCTGAATCTCCACTCTTCCTGATGAGTCC
TGACAGAATACCGGCAGTATTTTGGGGTCTCATTAGGTCATAGTTTCGTTCCTGAATCTCCACTCTTCCTAATGAGTCC
TGACAGAATACCGGCAGTATTTTGGTGTCTCATTAGGTTTCCTTTTCTTTTCTGAATCTCCACTCTTCCTAATGAGTCC
TGACAGAATACCGGCAGTATTTTGGGGTCTCATTAGGTTTAGTTTCTTTTCTGAATCTCCACTCTTCCTAATGAGTCC
TGACAGAATACCGGCAGTATTTTGGGGTCTCATTAGGTTTAGTTTCTTTTCTGAATCTCCACTCTTCCTAATGAGTCC
TGACAGAATACCGGCAGTATTTTGGGGTCTCAATAGGTTTAGTTTCGTTTCTGAATCTCCACTCTTCCTAATGAGTCC
TGACAGAATACCGGCAGTATTTTGGGGTCTCATTAGGTTTTGTTTCTTTTCTGAATCTCCACTCTTCCTAATGAGTCC
TGACAGAATACCGGCAGTATTTTGGGGTCTCATTAGGTTTAGTTTCTTTTCTGAATCTCCACTCTTCCTAATGAGTCC
TGACAGAATACCGGCAGTATTTTGGTGTCTCATTAGGTTTCCTTTTCTTTTCTGAATCTCCACTCTTCCTAATGAGTCC
TGACAGAATACCGGCAGTATTTTGGGGTCTCATTAGGTCATTGTTTCTTTTCTGAATCTCCACTCTTCCTAATGAGTCC
TGACAGAATACCGGCAGTATTTTGGGGTCTCATTAGGTTTAGTTTCGTTCCTGAATCTGCACTCTTCCTAATGAGTCC
TGACAGAATACCGGCAGTATTTTGGGGTCTCATTAGGTTTAGTTTCGTTCCTGAATCTCCACTCTTCCTAATGAGTCC
TGACAGAATACCGGCAGTATTTTGGGGTCTCATTCGGTCATTGTTTCTTTTCTGAATCTCCACTCTTCCTAATGAGTCC
TGACAGAATACCGGCAGTATTCTGGGGTCTCATTAGGTTGTCGTTTCTTTTCTGAATTTCCACTCTTCCGAATGAGTCC
TGACAGAATACCGGCAGTATTTTGGGGTCTCAATAGGTTTAGTTTCGTTCCTGAATCTCCACTCTTCCTAATGAGTCC
TGACAGAATACCAGCAGTATTTTGGGGTCTCAATAGGTTTAGTTTCGTTCTTGAATCTCCACTCTTCCTAATGAGTCC
TGACAGAATACCGGCAGTATTTTGGGGTCTCATTAGGTCATTGTTTCTTTTCTGAATCTCCACTCTTCCTAATGAGTCC

TGACAGAATACCGGCAGTATTTTGGTGTCTCATTAGGTTTCCTTTTCTTTTCTGAATCTCCACTCTTCCTAATGAGTCC
TGACAGAATACCGGCAGTATTTTGGGTTCTCATTAGGTCATTGTTTCTTTTCTGAATCTCCACTCTTCCTAATGAGTCC
TGACAGAATACCGGCAGTATTTTGGGGTCTCATTAGGTTTAGTTTCGTTCCTGAATCTGCACTCTTCCTAATGAGTCC
TGACAGAATACCGGCAGTATTTTGGGGTCTCATTAGGTCATAGTTTCGTTCCTGAATCTCCACTCTTCCTAATGAGTCC
TGACAGAATACCGGCAGTATTTTGGGGTCTCATTAGGTCATTGTTTCTTTTCTGAATCTGCACTCTTCCTAATGAGTCC
TGACAGAATACCGGCAGTATTTTGGTGTCTCATTAGGTTTCCTTTTCTTTTCTGAATCTCCACTCTTCTTAATGAGTCC
TGGCAGAATACCGGCAGTATTCTGGGGTCTCATTAGGTTGTCGTTTCTTTTCTGAATCTCCACTCTTCCGAATGAGTCC
TGACAGAATACCGGCAGTATTTTGGGGTCTCAATAGGTTTAGTTTCGTTCCTGAATCTCCACTCTTCCTAATGAGTCC
TGACAGAATACCGGCAGTATTTTGGGGTCTCAATAGGTTTAGTTTCGTTCCTGAATCTCCACTCTTCCTAATGAGTCC
TGACAGAATACCGGCAGTATTTTGGGGTCTCATTAGGTTTCCTTTTCTTTTCTGAATCTCCACTCTTCCGAATGAGTCC
TGACAGAATACCGGCAGTATTTTGGGGTCTCATTAAGTTTCCTTTTCTTTTCTGAATCTCCACTCTTCCTAACGAGTCC
TGACAGAATACCGGCAGTATTCTGGGGTCTCATTAGGTTGTCGTTTCTTTTCTGAATCTCCACTCTTCCGAATGAGTCC
TGACAGAATACCGGCAGTATTCTGGGGTCTCATTAGGTTGTCGTTTCTTTTCTGAATTTCCACTCTTCCTAATGAGTCC
TGACAGAATACCGGCAATATTCTGGTGTCTCATTAGGTTGTCGTTTCTTTTCTGAATCTCCACTCTTCCTAATGAGTCC
TGACAGAATACCGGCAGTATTTTGGTGTCTCATTAGGTTTCCTTTTCTTTTCTGAATCTCCACTCTTCCTAATGAGTCC
TGACAGAATACCGGCAGTATTTTGGGGTCTCATTAGGTCATTGTTTCTTTTCTGAATCTCCACTCTTCCTAATGAGTCC
TGACAGAATACCGGCAGTATTTTGGTGTCTCATTAGGTTTCCTTTTCTTTTCTGAATCTCCACTCTTCCTAATGAGTCC
TGACAGAATACCGGCAGTATTTTGGTGTCTCATTAGGTTTCCTTTTCTTTTCTGAATCTCCACTCTTCCTAATGAGTCC
TGACAGAATACCGGCAGTATTCTGGGGTCTCAATAGGTTTAGTTTCGTTCCTGAATCTCCACTCTTCCTAATGAGTCC
TGACAGAATACCGGCAGTATTTTGGGGTCTCAATAGGTTTAGTTTCGTTCCTGAATCTCCACTCTTCCTAATGAGTCC
TGACAGAATACCGGCAGTATTTTGGGGTCTCATTAGGTCATTGTTTCTTTTCTGAATCTCCACTCTTCCTAATGAGTCC
TGACAGAATACCGGCAGTATTTTGGGGTCTCAATAGGTTTAGTTTCGTTCCTGAATCTCCACTCTTCCTAATGAGTCC
TGACAGAATACCGGCAGTATTTTGGGGTCTCAATAGGTTTAGTTTCGTTCTTGAATCTCCACTCTTCCTAATGAGTCC
TGACAGAATACCGGCAGTATTTTGGGGTCTCAATAGGTTTAGTTTCGTTCCTGAATCTCCACTCTTCCTAATGAGTCC
TGACAGAATACCGGCAGTATTTTGGGGTCTCAATAGGTTTAGTTTCGTTCTTGAATCTCCACTCTTCCTAATGAGTCC
TGACAGAATACCGGCAGTATTCTGGGGTCTCATTAGGTTTCCTTTTCTTTTCTGAATCTCCACTCTTCCGAATGAGTCC
TGACAGAATACCGGCAGTATTTTGGGGTCTCATTAGGTTTCCTTTTCTTTTCTGAATCTCCACTCTTCCTAATGAGTCC
TGACAGAATACCGGCAGTATTTTGGGGTCTCAATAGGTTTAGTTTCGTTCTTGAATCTCCACTCTTCCTAATGAGTCC
TGACAGAATACCGGCAGTATTTTGGGGTCTCAATAGGTTTAGTTTCGTTCTTGAATCTCCACTCTTCCTAATGAGTCC
TGACAGAATACCGGCAGTATTTTGGGGTCTCTATAGGTTTAGTTTCGTTCTTGAATCTCCACTCTTCCCAATGAGTCC
TGACAGAATACCGGCAGTATTCTGGGGTCTCATTAGGTTGTCGTTTCTTTTCTGAATCTCCACTCTTCCGAATGAGTCC
TGACAGAATACCGGCAGTATTTTGGGGTCTCATTAGGTTTCCTTTTCTTTTCTGAATCTCCACTCTTCCTAATGAGTCC
TGACAGAATACCGGCAGTATTCTGGGGTCTCATTAGGTTGTCGTTTCTTTTCTGAATCTCCACTCTTCCGAATGAGTCC
TGACAGAATACCGGCAGTATTTTGGGGTCTCAATAGGTTTAGTTTCGTTCCTGAATCTGCACTCTTCCTAATGAGTCC
TGACAGAATACCAGCAGTATTTTGGGGTCTCATTAGGTTTCCTTTTCTTTTCTGAATCTCCACTCTTCCTAATGAGTCC
TGACAGAATACCGGCAGTATTCTGGGGTCTCATTAGGTTGTCGTTTCTTTTCTGAATCTCCACTCTTCCTAATGAGTCC
TGACAGAATACCAGCAGTATTTTGGGGTCTCATTAGGTTTCCTTTTCTTTTCTGAATCTCCACTCTTCCTAATGAGTCC
TGACAGAATACCGGCAGTATTCTGGGGTCTCATTAGGTTGTCGTTTCTTTTCTGAATCTCCACTCTTCCTAATGAGTCC
TGACAGAATACCAGCAGTATTTTGGGGTCTCATTAGGTTTCCTTTTCTTTTCTGAATCTCCACTCTTCCTAATGAGTCC
TGACAGAATACCGGCAGTATTTTGGGGTCTCATTAGGTTTCCTTTTCTTTTCTGAATCTCCACTCTTCCTAATGAGTCC
TGACAGAATACCAGCAGTATTTTGGGGTCTCATTAGGTCATTGTTTCTTTTCTGAATCTCCACTCTTCCTAATGAGTCC
TGACAGAATACCGGCAGTATTTTGGGGTCTCATTAGGTTTCCTTTTCTTTTCTGAATCTCCACTCTTCCGAATGAGTCC
TGACAGAATACCGGCAGTATTTTGGGGTCTCATTAGGTCATTGTTTCTTTTCTGAATCTCCACTCTTCCTAATGAGTCC
TGACAGAATACCGGCAGTATTTTGGGGTCTCATTAGGTTTCCTTTTCTTTTCTGAATCTCCACTCTTCCTAATGAGTCC
TGACAGAATACCGGCAGTATTTTGGGGTCTCATTAGGTCATTGTTTCTTTTCTGAATCTCCACTCTTCCTAATGAGTCC
TGACAGAATACCGGCAGTATTTTGGGGTCTCATTAGGTTTCCTTTTCTTTTCCGAATCTCCACTCTTCCTAATGAGTCC
TGACAGAATACCAGCAGTATTTTGGGGTCTCATTAGGTCATTGTTTCTTTTCTGAATCTCCACTCTTCCTAATGAGTCC
TGACAGAATACCAGCAGTATTTTGGGGTCTCATTAGGTCATTGTTTCTTTTCTGAATCTCCACTCTTCCTAATGAGTCC
TGACAGAATACCGGCAGTATTCTGGGGTCTCATTAGGTTTCCTTTTCTTTTCTGAATCTCCACTCTTCCTAATGAGTCC
TGACAGAATACCGGCAGTATTCTGGGGTCTCATTAGGTTGTCGTTTCTTTTCTGAATCTCCACTCTTCCGAATGAGTCC
TGACAGAATACCGGCAGTATTTTGGGGTCTCATTAGGTTTCCTTTTCTTTTCTGAATCTCCACTCTTCCTAATGAGTCC
TGACAGAATACCGGCAGTATTCTGGGGTCTCATTAGGTTGTCGTTTCTTTTCTGAATCTCCACTCTTCCGAATGAGTCC
TGACAGAATACCGGCAGTATTTTGGGGTCTCAATAGGTTTAGTTTCGTTCCTGAATCTGCACTCTTCCTAATGAGTCC
TGACAGAATACCAGCAGTATTTTGGGGTCTCATTAGGTTTCCTTTTCTTTTCTGAATCTCCACTCTTCCTAATGAGTCC

TGACAGAATACCAGCAGTATTCTGGGGTCTCATTAGGTTTCCTTTTCTTTTCTGAATCTCCACTCTTCCTAATGAGTCC
TGACAGAATACCGGCAGTATTCTGGGGTCTCATTAGGTTGTCGTTTCTTTTCTGAATCTCCACTCTTCCGAATGAGTCC
TGACAGAATACCAGCAGTATTTTGGGGTCTCATTAGGTTTCCTTTTCTTTTCTGAATCTCCACTCTTCCTAATGAGTCC
TGACAGAATACCGGCAGTATTCTGGGGTCACATTAGGTTGTCGTTTCTTTTCCGAATCTCCACTCTTCCTAATGAGTCC
TGACAGAATACCAGCAGTATTTTGGGGTCTCATTAGGTCATTGTTTCTTTTCTGAATCTCCACTCTTCCTAATGAGTCC
TGACAGAATACCAGCAGTATTTTGGGGTCTCATTAGGTTTCCTTTTCTTTTCTGAATCTCCACTCTTGCTAATGAGTCC
TGACAGAATACCGGCAGTATTTTGGGGTCTCATTAGGTCATTGTTTCTTTTCTGAATCTCCACTCTTCCTAATGAGTCC
TGACAGAATACCAGCAGTATTTTGGGGTCTCATTAGGTTTCCTTTTCTTTTCTGAATCTCCACTCTTGCTAATGAGTCC
TGACAGAATACCGGCAGTATTCTGGGGTCTCATTAGGTTGTCGTTTCTTTTCTGAGTCTCCACTCTTCCGAATGAGTCC
TGACAGAATACCGGCAGTATTTTGGGGTCTCAATAGGTTTTAGTTTCGTTCCTGAATCTGCACTCTTCCTAATGAGTCC
TGACAGAATACCAGCAGTATTTTGGGGTCTCATTAGGTTTCTTTTTCTTTTCTGAATCTCCACTCTTCCTAATGAGTCC
TGACAGAATACCAGCAGTATTTTGTGGTCTCATTAGGTTTCCTTTTCTTTTCTGAATCTCCACTCTTCCTAATGAGCCC
TGACAGAATACCGGCAGTATTTTGGGGTCTCATTAGGTTTCCTTTTCTTTTCTGAATCTCCACTCTTCGGAATGAGTCC
TGACAGATTACCGGCAGTATTTTGGGGTCTCATTAGGTTTCCTTTTCTTTTCTGAATCTCCACTCTTCCTAATGAGTCC
TGACAGAATACCGGCAGTATTTTGGGGTCTCATTAGGTTGTCGTTTCTTTTCTGAATCTCCACTCTTCCGAATGAGTCC
TGACAGAATACCGGCAGTATTTTGGGGTCTCATTAGGTTGTCGTTTCTTTTCTGAATCTCCACTCTTCCTAATGAGTCC
TGACAGAATACCGGCAGTATTTTGGGGTCTCAATAGGTTTTAGTTTCGTTCCTGAATCTGCACTCTTCCTAATGAGTCC
TGACAGAATACCAGCAGTATTTTGGGGTCTCATTAGGTTTCCTTTTCTTTTCTGAATCTCCACTCTTCCTAATGAGTCC
TGACAGAATACCGGCAGTATTCTGGGGTCTCATTAGGTTGTCGTTTCTTTTCTGAATCTCCACTCTTCCGAATGAGTCC
TGACAGAATACCGGCAGTATTTTGGGGTCTCATTAGGTTTCCTTTTCTTTTCTGAATCTCCACTCTTCCTAATGAGTCC
TGACAGAATACCGGCAGTATTCTGGGGTCTCATTAGGTTGTCGTTTCTTTTCTGAATCTCCACTCTTCCGAATGAGTCC
TGACAGAATACCGGCAGTATTTTGGGGTCTCATTAGGTTTCCTTTTCTTTTCTGAATCTCCACTCTTCCTAATGAGTCC
TGACAGAATACCGGCAGTATTCTGGGGTCTCATTAGGTTGTCGTTTCTTTTCTGAATCTCCACTCTTCCGAATGAGTCC
TGACAGAATACCGGCAGTATTTTGGGGTCTCATTAGGTTTCCTTTTCTTTTCTGAATCTCCACTCTTCCTAATGAGTCC
TGACAGAATACCGGCAGTATTTTGGGGTCTCATTAGGTTGTCGTTTCTTTTCTGAATCTCCACTCTTCCGAATGAGTCC
TGACAGAATACCGGCAGTATTTTGGGGTCTCATTAGGTTTCCTTTTCTTTTCTGAATCTCCACTCTTCCTAATGAGTCC
TGACAGAATACCGGCAGTATTCTGGGGTCTCATTAGGTTGTCGTTTCTTTTCTGAATCTCCACTCTTCCGAATGAGTCC
TGACAGAATACCGACAGTATTTTGGGGTCTCATTAGGTTTCCTTTTCTTTTCTGAATCTCCACTCTTCCTAATGAGTCC
TGACAGAATACCGGCAGTATTCTGGGGTCTCATTAGGTTGTCGTTTCTTTTCTGAATCTCCACTCTTCCGAATGAGTCC
TGACAGAATACCGGCAGTATTTTGGGGTCTCAATAGGTTTTAGTTTCGTTCCTGAATCTCCACTCTTCCTAATGAGTCC
TGACAGAATACCGGCAGTATTTTGGGGTCTCAATAGGTTTCCTTTTCTTTTCTGAATCTCCACTCTTCCTAATGAGTCC
TGACAGAATACCGGCAGTATTCTGGGGTCTCATTAGGTTTCCTTTTCTTTTCTGAATCTCCACTCTTCCTAATGAGTCC
TGACAGAATACCGGCAGTATTCTAGGGTCTCATTAGGTTTCCTTTTCTTTTCTGAATCTCCACTCTTCCTAATGAGTCC
TGACAGAATACCGGCAGTATTTTGGGGTCTCATTAGGTTGTCGTTTCTTTTCTGAATCTCCACTCTTCCGAATGAGTCC
TGACAGAATACCGACAGTATTTTGGGGTCTCATTAGGTTTCCTTTTCTTTTCTGAATCTCCACTCTTCCTAATGAGTCC
TGACAGAATACCGGCAGTATTCTGGGGTCTCATTAGGTTGTCGTTTCTTTTCTGAATCTCCACTCTTCCGAATGAGTCC
TGACAGAATACCGGCAGTATTTTGGGGTCTCAATAGGTTTTAGTTTCGTTCCTGAATCTACACTCTTCCTAATGAGTCC
TGACAGAATACCGGCAGTATTTTGGGGTCTCAATAGGTTTCCTTTTCTTTTCTGAATCTCCACTCTTCCTAATGAGTCC
TGACAGAATACCGGCAGTATTCTGGGGTCTCATTAGGTTTTAGTTTGGTTCCTGAATCTCCACTCTTCCTAATGAGTCC
Nr of selected repeats 143 Similarity 0.910204
Consensus:
TGACAGAATACCGGCAGTATTtTGGGGTCTCATTAGGTTttcgTTTCtTTtCTGAATCTCCACTCTTCCTAATGAGTCC
>Cele-UNSB01_2:8585644-8586044 Satlength=401 Nr of Repeats=10 RepeatLength=40
seed=AAAGTTCTGG
AAAGTTCTGGAACATTACAGAAATTTCTCGATTTTTCTAG
AAAGTTCTGGAACATTCCAAAAATTTCCCAATTTTTCTAG
AAAGTTCTGGAACATTACAGAAATTTCTCAATTTTTCTAG
AAAGTTCTGGAACATTCCAGACTTTTCCCAATTTTTCTAG
AAAGTTCTGGAACATTCCAGACTTTTCCCAATTTTTCTAG
AAAGTTCTGGAACATTACAGAAATTTCTCAATTTTTCTAG
AAAGTTCTGGAACATTCCAGACTTTTCCCAATTTTTCTAG
AAAGTTCTGGAACATTCCAGACTTTTCCCAATTTTTCTAG
AAAGTTCTGGAACATTCCAGAATTTTCTCGATTTTTCTAC
AAAGTTCTGGAACATTCCAGAATTTTCTCGATTTTTCTAG

Nr of selected repeats 10 Similarity 0.901482
Consensus:
AAAGTTCTGGAACATTcCAGAatTTTCcCaATTTTTCTAG
>Cele-UNSB01_2:8586336-8586411 Satlength=76 Nr of Repeats=4 RepeatLength=15
seed=GGGTCTCACC
GGGTCTCACCACGGTGGGTATTACCACGAT
GGGTCTCACCACGAC
GGGTCTCACCACGGT
GGGTCTCGCCACGAT
Nr of selected repeats 3 Similarity 0.822222
Consensus:
GGGTCTCaCCACGat
>Cele-UNSB01_2:9101090-9103530 Satlength=2441 Nr of Repeats=29 RepeatLength=86
seed=AGCCAGTATT
AGCCAGTATTTCGGGGTCTGGCAATAGCTTCTCTTTAACTTCGAACTCTTCCATCTTTTCCCTATTGCCACGACCTTTC
AAAATAC
AGCCAGTATTTCGGGGTCTGGCAATAGCTTCTCGTTAACTTCGAACTCTTCCATCTTTTCCCTATTGCCACGACCTTTC
AAAATAC
AGCCAGTATTTCGGGGTCTGGCAATAGCTTCTCTTTAACTTCGAACTCTTCCATCTTTTCCCTATTGCCACGACCTTTC
AAAATGC
AGCCAGTATTTCGGGGTCTGGCAATAGCTTCTCTTTAACTTCGAACTCTTCCATCTTTTCCCTATTGCCACGACCTTTC
AAAATGC
AGCCAGTATTTCGGGGTCTGGCAATAGCTTCTCTTTAACTTCGAACTCTTCCATCTTTTCCCTATTGCTACGACCTTTC
AAAATAC
AGCCAGTATTTCGGGGTCTGGCAATAGCTTCTCGTTAACTTCGAACTCTTCCATCTTTTCCCTATTGCCACGACCTTTC
AAAATAC
AGCCAGTATTTCGGGGTCTGGCAATAGCTTCTCGTTAACTTCGAACTCTTCCATCTTTTCCCTATTGCTACGACCTTTC
AAAATAC
AGCCAGTATTTCGGGGTCTGGCAATAGCTTCTCGTTAACTTCGAACTCTTCCATCTTTTCCCTATTGCCACGACCTTTC
AAAATAC
AGCCAGTATTTCGGGGTCTGGCAATAGCTTCTCGTTAACTTCGAACTCTTCCATCTTTTCCCTATTGCCACGACCTTTC
AAAATGC
AGCCAGTATTTCGGGGTCTGGCAATAGCTTCTCGTTAACTTCGAACTCTTCCATCTTTTCCCTATTGCCACGACCTTTC
AAAATAC
AGCCAGTATTTCGGGGTCTGGCAATAGCTTCTCGTTAACTTCGAACTCTTCCATCTTTTCCCTATTGCCACGACCTTTC
AAAATGC
AGCCAGTATTTCGGGGTCTGGCAATAGCTTCTCGTTAACTTCGAACTCTTCCATCTTTTCCCTATTGCCACGACCTTTC
AAAATAC
AGCCAGTATTTCGGGGTCTGGCAATAGCTTCTCGTTAACTTCGAACTCTTCCATCTTTTCCCTATTGCCACGACCTTTC
AAAATGC
AGCCAGTATTTCGGGGTCTGGCAATAGCTTCTCGTTAACTTCGAACTCTTCCATCTTTTCCCTATTGCCACGACCTTTC
AAAATAC
AGCCAGTATTTCGGGGTCTGGCAATAGCTTCTCGTTAACTTCGAACTCTTCCATCTTTTCCCTATTGCCACGACCTTTC
AAAATAC
AGCCAGTATTTCGGGGTCTGGCAATAGCTTCTCGTTAACTTCGAACTCTTCCATCTTTTCCCTATTGCTACGACATTTC
AAAATAC
AGCCAGTATTTCGGGGTCTGGCAATAGCTTCTCGTTAACTTCGAACTCTTCCATCTTTTCCCTATTGCCACGACCTTTC
AAAATAC
AGCCAGTATTTCGGGGTCTGGCAATAGCTTCTCGTTAACTTCGAACTCTTCCATCTTTTCCCTATTGCTACGACATTTC
AAAATAC
AGCCAGTATTTCGGGGTCTGGCAATAGCTTCTCGTTAACTTCGAACTCTTCCATCTTTTCCCTATTGCCACGACCTTTC
AAAATAC
AGCCAGTATTTCGGGGTCTGGCAATAGCTTCTCGTTAACTTCGAACTCTTCCATCTTTTCCCTATTGCTACGACATTTC
AAAATAC

AGCCAGTATTTCGGGGTCTGGCAATAGCTTCTCGTTAACTTCGAACTCTTCCATCTTTTCCCTATTGCCACGACCTTTC
AAAATAC
AGCCAGTATTTCGGGGTCTGGCAATAGCTTCTCGTTAACTTCGAACTCTTCCATCTTTTCCCTATTGCCACGACCTTTC
AAAATGC
AGCCAGTATTTCGGGGTCTGGCAATAGCTTCTCGTTAACTTCGAACTCTTCCATCTTTTCCCTATTGCCACGACCTTTC
AAAATAC
AGCCAGTATTTCGGGGTCTGGCAATAGCTTCTCGTTAACTTCGAACTCTTCCATCTTTTCCCTATTGCCACGACCTTTC
AAAATGC
AGCCAGTATTTCGGGGTCTGGCAATAGCTTCTCGTTAACTTCGAACTCTTCCATCTTTTCCCTATTGCCACGACCTTTC
AAAATAC
AGCCAGTATTTCGGGGTCTGGCAATAGCTTCTCGTTAACTTCGAACTCTTCCATCTTTTCCCTATTGCTACGACATTTC
AAAATAC
AGCCAGTATTTCGGGGTCTGGCAATAGCTTCTCGTTAACTTCGAACTCTTCCATCTTTTCCCTATTGCTACGACCTTTC
AAAATAC
AGCCAGTATTTCGGGGTCTGGCAATAGCTTCTCGTTAACTTCGAACTCTTCCATCTTTTCCCTATTGCCACGACCTTTC
AAAATAC
AGCCAGTATTTCGGGGTCTGGCAATAGTATTT
Nr of selected repeats 28 Similarity 0.980066
Consensus:
AGCCAGTATTTCGGGGTCTGGCAATAGCTTCTCGTTAACTTCGAACTCTTCCATCTTTTCCCTATTGCcACGACCTTTC
AAAATaC
>Cele-UNSB01_2:9328143-9328548 Satlength=406 Nr of Repeats=27 RepeatLength=15
seed=TGGTGAGACC
TGGTGAGACCCATCG
TGGTGAGACCCTTCG
TGGTGAGACCCTTCG
TGGTGAGACCCATCG
TGGTGAGACCCTTCG
TGGTGAGACCCTTCG
TGGTGAGACCAATCG
TGGTGAGACCTTTCG
TGGTGAGACCCTTCG
TGGTGAGACCAATCG
TGGTGAGACCCTTCG
TGGTGAGACCCATCG
TGGTGATACCCTTCG
TGGTGAGACCCTTCG
TGGTGAGACCCATCG
TGGTGAGACCCTTCG
TGGTGAGACCCTTCG
TGGTGAGACCAATCG
TGGTGAGACCTTTCG
TGGTGAGACCCGTCG
TGGTGAGACCAATCG
TGGTGAGACCCTTCG
TGGTGAGACCCTTCG
TGGTGAGACCCATCG
TGGTGAGACCCTTCT
TGGTGAGACCCATCGC
GGTGAGACCCTTCG
Nr of selected repeats 25 Similarity 0.902519
Consensus:
TGGTGAGACCctTCG

>Cele-UNSB01_2:9328575-9329101 Satlength=527 Nr of Repeats=15 RepeatLength=35
seed=ATTCAAATTT
ATTCAAATTTTTAGTGAAAAAAAATTTTGGAGGGAA
ATTCAAATTTTCAGTGAAAAAAAATTTTGGCGGGAA
ATTCAAATTTTCTGTGAAAAAAAATTTTGGCGGGAA
ATTCAAATTTTCAGTGAAAAAAATTTGGGCGGGAG
ATTCAAATTTTCAGTGAAAAAAAATTTAGCGGGAA
ATTCAAATTTTCAGTGAAAAAAATTTTTAGCGGGAA
ATTCAAATTTTCAGTAAAAAAAAATTTTGGAGGGAA
ATTCAAATTTTCAGTGAAAAAAAAATTTTGGCGGGAA
ATTAAAATTTTCAGTGAAAAAAAATTTTGGAGGGAA
ATTCAAATTTTCAGTGAAAAAAAAATTTTGGCGGGAA
ATTCAAATTTTCAGTGAAAAAAAATTTTGGAGGGAA
ATTCAAATTTTCAGTGAAAAAAAAATTTTGGCGGGAA
ATTAAAATTTTCAGTGAAAAAAAATTTTAGCGGGAA
ATTCAAATTTGCAGTGTAAAAAAATTTTGGCGGGAA
ATTCAAATTTTCTGTGAAAAAAAATTTTGGCGGGAA
Nr of selected repeats 10 Similarity 0.900952
Consensus:
ATTcAAATTTTCaGTGAAAAAAAATTTTgGcGGGAA
>Cele-UNSB01_2:9328566-9329542 Satlength=977 Nr of Repeats=16 RepeatLength=35
seed=TGGCGGGAAA
TGGCGGGAAATTCAAATTTTTAGTGAAAAAAAATTT
TGGAGGGAAATTCAAATTTTCAGTGAAAAAAAATTT
TGGCGGGAAATTCAAATTTTCTGTGAAAAAAAATTT
TGGCGGGAAATTCAAATTTTCAGTGAAAAAAATTTGGGCGGGAGATTCAAATTTTCAGTGAAAAAAATT
TAGCGGGAAATTCAAATTTTCAGTGAAAAAAATTTT
TAGCGGGAAATTCAAATTTTCAGTAAAAAAAAATTT
TGGAGGGAAATTCAAATTTTCAGTGAAAAAAAAATTT
TGGCGGGAAATTAAAATTTTCAGTGAAAAAAAATTT
TGGAGGGAAATTCAAATTTTCAGTGAAAAAAAAATTT
TGGCGGGAAATTCAAATTTTCAGTGAAAAAAAATTT
TGGAGGGAAATTCAAATTTTCAGTGAAAAAAAAATTT
TGGCGGGAAATTAAAATTTTCAGTGAAAAAAAATTT
TAGCGGGAAATTCAAATTTGCAGTGTAAAAAAATTT
TGGCGGGAAATTCAAATTTTCTGTGAAAAAAAATTT
TGGCGGGAAATTCAAATTTTCAGTGAAAAACAATTT
TGGCGGAAAATTCCAATTTCTGAGAAAAATCGAGAAATGTCTGCAATGTTCCAGAAGTTTCTAGAAAATTCGAGAAAAT
TCCGGAATGGTCCAGAATTTTCTAGAAAATTCTAGAAAGTTCTGGAATGGTCCAGAAGTTTCTAGAAAATTCGAGAAAA
TTCCGGAATGGTCCAGAATTTTCTAGAAAATCCGAGAAAGTTCTGGAATGGTCCAGAATTTTCTAGAAAATTCGGGAAA
ATTCTGGAATATTCCAGAACTTTCTAGAAAAATCGGGAAAAGTCTGCAATGTTCCAGAACTCTCTAAAAAATTCGATAA
AATTCTGAAATGTTCAATTTCGTGGAAAAATTCAAGAAACTTCTGCAAAGTTCTACACGAGGTTCTGGCTCGACCCCGC
GCTCCATTGGACACTGAAA
Nr of selected repeats 10 Similarity 0.909418
Consensus:
TgGCGGGAAATTcAAATTTTCaGTGAAAAAAAATTT
>Cele-UNSB01_2:9530992-9537371 Satlength=6380 Nr of Repeats=247 RepeatLength=25
seed=CTCTCTGGCT
CTCTCTGGCTTCACAGAATAATTTA
CTCTCCGGCTTCACAGTATATTTTA
CTCTCTGGCTTCACAGTATATTTTA
CTCTCTGGCTTCACAGAATAATTTA
CTCTCTGGCTTCACAGTAAATTTTA
CTCTCTGGCTTCACAGTATATTTTA

CTCTCTGGCTTCACAGAATAATTTA
CTCTCTGGCTTCACAGAATAATTTA
CTCTCTGGCTTCACAGTATATTTTA
CTCTCTGGCTTCACAGTATATTTTA
CTCTCTGGCTTCACAGAATAATTTA
CTCTCTGGCTTCACAGTATATTTTA
CTCTCTGGCTTCACAGTATATTTTA
CTCTCTGGCTTCACAGTATATTTTA
CTCTCTGGCTTCACAGTATATTTTA
CTCTCTGGCTTCACAGAATAATTTA
CTCTCTGGCTTCATAGTATATTTTTTTTTTTTTTTTATTTTTA
CTCTCTGGCTTCACAGTATATTTTA
CTCTCTGGCTTCACAGTATATTTTACTCTCTTCACAGTATATTTTA
CTCTCTGGCTTCACAGTATATTTTA
CTCTCTGGCTTCACAGTATATTTTA
CTCTCTGGCTTCACAGTATATTTTA
CTCTCTGGCTTCACAGTATATTTTA
CTCTCTGGCTTCACAGTATATTTTA
CTCTCTGGCTTCACAGAATAATTTA
CTCTCTGGCTTCACAGTATATTTTA
CTCTCTGGCTTCACAGTATATTTTA
CTCTCTGGCTTCACAGTATATTTTA
CTCTCTGGCTTCACAGTATATTTA
CTCTCTGGCTTCACAGTATATTTTACTCCTCTGTTCTTGCTTCACAGAATAATTTA
CTCTCTGGCTTCACAGTATATTTTA
CTCTCTGGCTTCACAGTAATTTA
CTCTCTGGCTTCACAGTATATTTTTCAAGAATTTTA
CTCTCTGGCTTCACAGTATATTTA
CTCTCTGGCTTCACAGTAATTTT
TCTCTGGCTTCACAGTATATTTTA
CTCTCTGGCTTCACAGAATATTTA
CTCTCTGGCTTCACAGTATATTTTA
CTCTCTGGCTTCACAGTATATTTTA
CTCTCTGGCTTCACAGTATATTTTA
CTCTCTGGCTCTCTGGCTTCACAGTATATTTTA
CTCTCTGGCTTCACAGTAATTTTA
CTCTCTGGCTTCACAGTAATTTA
CTCTCTGGCTTCACAGATAATTTA
CTCTCTGGCTTCACAGTAATTTTA
CTCTCTGGCTTCACAGTATATTTTA
CTCTCTGGCTTCACAGAATAATTTA
CTCTCTGGCTTCACAGTATATTTTA
CTCTCTGGCTTCACAGATATTTA
CTCTCTGGCTTCACAGTATATTTTA
CTCTCTGGCTTCACAGAATAATTTA
CTCTCTGGCTTCACAGTATATTTTA
CTCTCTGGCTTCACAGAATAATTTT
CTCTCTGGCTTCACAGTATATTTTA
CTCTCTGGCTTCACAGAATAATTTA
CTCTCTGGCTTCACAGTATATTTTA
CTCTCTGGCTTCACAGAATAATTTA
CTCTCTGGCTTCACAGTAATTTA
CTCTCTGGCTTCAATATTTTA
CTCTCTGGCTTCACAGTATATTTTA

CTCTCTGGCTTCACAGTATATTTA
CTCTCTGGCTTCACAGTATATTTTA
CTCTCTGGCTTCACAGTATATTTTA
CTCTCTGGCTTCACAGTATATTTTA
CTCTCTGGCTTCACAGATATTTTA
CTCTCTGGCTTCACAGTATATTTTA
CTCTCTGGCTTCACAGTATATTTTA
CTCTCTGGCTTCACAGAATAATTTA
CTCTCTGGCTTCACAGTATATTTTA
CTCTCTGGCTTCACAGAATTTTA
CTCTCTGGCTTCACAGTAATTTTA
CTCTCTGGCTTTTTTTTCTTCACAGTAATTTA
CTCTCTGCTTTCACAGTATATTTTA
CTCTCTGGCTTCACAGTATATTTTA
CTCTCTGGCTTCACAGAATAATTTA
CTCTCTGGCTTCACAGTATATTTTA
CTCTCTGGCTTCACAGTATATTTTA
CTCTCTGGCTTCACAGTATATTTTA
CTCTCTGGCTTCACAGTATATTTTTCTCTTTG
CTCTCTGGCTTCACAGTATATTTTA
CTCTCTGGCTTCACAGTAATTTA
CTCTCTGCTTCACAGTAATTTA
CTCTCTGGCTTCACAGTATATTTTA
CTCTCTGGCTTCACAGAATATTTTA
CTCTCTGGCTTCACAGTATATTTTA
CTCTCTGGCTTCACAGATAATTTA
CTCTCTGGCTTCACAGTATATTTTA
CTCTCTGGCTTCACAGTATATTTTA
CTCTCTGGCTTCACAGTATTTTTTTTGTTAAGTTTTTA
CTCTCTGGCTTCACAGTATATTTTA
CTCTCTGGCTTCACAGTATATTTTA
CTCTCTGGCTTCACAGAATATTTA
CTCTCTGGCTTCACAGTATATTTTA
CTCTCTGGCTTCACAGAATAATTTA
CTCTCTGGCTTCACAGTATATTTTA
CTCTCTGGCTTCACAGAATAATTTA
CTCTCTGGCTTCACAGTATATTTTA
CTCTCTGGCTTCACAGTATATTTTA
CTCTCTGGCTTCACAGTATATTTTA
CTCTCTGGCTTCACAGTATATTTTA
CTCTCTGGCTTCACAGTATATTTTA
CTCTCTGGCTTCACAGTATATTTTA
CTCTCTGGCTTCACAGTATATTTTA
CTCTCTGGCTTCACAGAATAATTTA
CTCTCTGGCTTCACAGTATATTTTA
CTCTCTGGCTTCACAGAATAATTTA
CTCTCTGGCTTCACAGTAATATTTTTA
CTCTCTGGCTTCACAGTATATTTTA
CTCTCTGGCTTCACAGTATATTTTA
CTCTCTGGCTTCACAGTATATTTTA
CTCTCTGGCTTCACAGTATATTTTA
CTCTCTGGCTTCACAGTATATTTTA
CTCTCTGGCTTCACAGATATTTTA
CTCTCTGGCTTCACAGTATATTTTA

CTCTCTGGCTTCACAGAATATTTA
CTCTCTGGCTTCACAGTATATTTTACTCTCTGTTTTTTTTTTTTGTTCACAGTATATTTTA
CTCTCTGGCTTCACAGTATTTA
CTCTCTGGCTTCACAGTATATTTTA
CTCTCTGGCTTCACAGTATATTTTA
CTCTCTGGCTTCACAGTATATTTTA
CTCTCTGGCTTCACAGTATATTTTA
CTCTCTGGCTTCACAGAATAATTTA
CTCTCTGGCTTCACAGTATATTTTA
CTCTCTGGCTTCACAGTATATTTTA
CTCTCTGGCTTCACAGTATATTTA
CTCTCTGGCTTCACAGTATATTTTA
CTCTCTGGCTTCACAGTATATTTTA
CTCTCTGGCTTCACAGAATAATTTA
CTCTCTGGCTTCACAGTATATTTTA
CTCTCTGGCTTCACAGAATATTTA
CTCTCTGGCTTCACAGTATATTTTA
CTCTCTGGCTTCACAGAATAATTTA
CTCTCTGGCTTCACAGTATATTTTA
CTCTCTGGCTTCACAGAATATTTA
CTCTCTGGCTTCACAGAATATTTTA
CTCTCTGGCTTCACAGTATATTTA
CTCTCTGGCTTCACAGTATATTTTA
CTCTCTGGCTTCACAGAATATTTA
CTCTCTGGCTTCACAGTATATTTTA
CTCTCTGGCTTCACAGAATAATTTA
CTCTCTGGCTTCACAGTATATTTA
CTCTCTGGCTTCACAGTATATTTTA
CTCTCTGGCTTCACAGTATATTTTTTTTTAATATTTTA
CTCTCTGGCTTCACAGAATAATTTA
CTCTCTGGCTTCACAGTATATTTTA
CTCTCTGCTTCACAGTATATTTTA
CTCTCTGGCTTCACAGTATATTTTA
CTCTCTGGCTTCACAGTATATTTTA
CTCTCTGGCTTCACAGTATATTTTA
CTCTCTGCTTCACAGTATTATTTTA
CTCTTGGCTTCACAGTATATTTA
CTCTCTGGCTTCACAGAATAATTTA
CTCTCTGGCTTCACAGTATATTTTA
CTCTCTGGCTTCACAGAATAATTTT
CTCTCTGGCTTCACAGTATATTTTA
CTCTCTGGCTTCACAGAATAATTTA
CTCTCTGGCTTCACAGTATATTTTA
CTCTCTGGCTTCACAGAATAATTTA
CTCTCTGGCTTCACAGTATATTTTA
CTCTCTGGCTTCACAGTATATATTTTA
CTCTCTGGCTTCACAGTATATTTTA
CTCTCTGGCTTCACAGTAATAATTTTA
CTCTCTGGCTTCACAGTATATTTTA
CTCTCTGGCTTCACAGTATATTTTA
CTCTCTGGCTCTCTGGCTTCACAGTATATTTTA
CTCTCTGGCTTCACAGTATATTTTA
CTCTCTGGCTTCACAGAATAATTTA
CTCTCTGGCTTCACAGTATATTTTA

CTCTCTGGCTTCACAGTATATTTTA
CTCTCTGGCTTCACAGTATATTTTA
CTCTCTGGCTTCACAGAATAATTTA
CTCTCTGGCTTCATAGTATATTTTA
CTCTCTGGCTTCACAGAATAATTTA
CTCTCTGGCTTCACAGTATATTTTA
CTCTCTGGCTTCACAGTATATTTTA
CTCTCTGGCTTCACAGTATATTTA
CTCTCTGGCTTCACAGAATAATTTA
CTCTCTGGCTTCACAGAATAATTTA
CTCTCTGGCTTCACAGTATATTTTA
CTCTCTGGCTTCACAGTATATTTTA
CTCTCTGGCTTCACAGAATAATTTA
CTCTCTGGCTTCAAGTATATTTTA
CTCTCTGGCTTCACAGAATAATTTA
CTCTCTGGCTTCATAGTATATTTTA
CTCTCTGGCTTCACAGAATAATTTA
CTCTCTGGCTTCATAGTATATTTTA
CTCTCTGGCTTCACAGAATAATTTA
CTCTCTGGCTTCATAGTATATTTTA
CTCTCTGGCTTCACAGAATAATTTA
CTCTCTGGCTTCATAGTATATTTTA
CTCTCTGGCTTCATAGTATATTTTA
CTCTCTGGCTTCACAGAATAATTTA
CTCTCTGGCTTCACAGTATATTTTA
CTCTCTGGCTTCACAGTATATTTTA
CTCTCTGGCTTCACAGTATATTTTA
CTCTCTGGCTTCACAGTATATTTTA
CTCTCTGGCTTCACAGTATATTTTA
CTCTCTGGCTTCACAGAATAATTTA
CTCTCTGGCTTCATAGTATATTTTA
CTCTCTGGCTTCACAGAATAATTTA
CTCTCTGGCTTCACAGTATATTTTA
CTCTCTGGCTTCACAGTATATTTTA
CTCTCTGGCTTCACAGTATATTTTA
CTCTCTGGCTTCACAGAATAATTTA
CTCTCTGGCTTCACAGTATATTTTA
CTCTCTGGCTTCACAGAATAATTTA
CTCTCCGGCTTCACAGTACATTTTA
CTCTCTGGCTTCACAGAATAATTTA
CTCTCTGGCTTCACAGTATATTTCA
CTCTCTGGCTTCACAGAATAATTTA
CTCTCTGGCTTCACAGTACATTTTACTCATGGCTTCACAGAATAATTTA
CTCTCTGGCTTCACAGTATATTTCA
CTCTCTGGCTTCACAGAATAATTTA
CTCTCTGGCTTCACAGTACATTTTACTCATGGCTTCACAGAATAATTTA
CTCTCTGGCTTCACAGTATATTTTA
CTCTCTGGCTTCACAGTATATTTTA
CTCTCTGGCTTCACAGAATAATTTA
CTCTCTGGCTTCACAGTATATTTTA
CTCTCTGGCTTCACAGAATATTTTA
CTCTCTGGCTTCACAGTATATTTTA
CTCTCTGGCTTCACAGAATAATTTA
CTCTCTGGCTTCACAGTATATTTTA

CTCTCTGGCTTCACAGAATAATTTA
CTCTCTGGCTTCACAGTATATTTTA
CTCTCTGGCTTCACAGATATTTTCTTTTTTTTTTTTTTTATTTTA
CTCTCTGGCTTCACAGAATAATTTA
CTCTCTGGCTTCACAGAATAATTTA
CTCTCTGGCTTCATAGTATATTTTA
CTCTCTGGCTTCACAGAATAATTTA
CTCTCTGGCTTCACATTTTTGTTATATTTTA
CTCTCTGGCTTCACAGAATAATTTA
CTCTCTGGCTTCACAGTAATTTTA
CTCTCTGGCTTCACAGAATAATTTA
CTCTCTGGCTTCACAGTATATTTTA
CTCTCTGGCTTCACAGTATATTTTA
CTCTCTGGCTTCACAGTATATTTTA
CTCTCTGGCTTCACAGAATAATTTA
CTCTCTGGCTTCACAGTATATTTTA
CTCTCTGGCTTCACAGAATAATTTA
CTCTCTGGCTTCACAGTATATTTTA
CTCTCTGGCTTCACAGAATAATTTA
CTCTCTGGCTTCATAGTATATTTTA
CTCTCTGGCTTCACAGTATATTTTA
CTCTCTGGCTTCACAGTACATTTTA
CTCTCTGGCTTCACAGAATAATTTA
CTCTCTGGCTTCACAGTATATTTTA
CTCTCTGGCTTCACAGTATATTTTA
Nr of selected repeats 194 Similarity 0.906588
Consensus:
CTCTCTGGCTTCACAGtATAtTTTA
>Cele-UNSB01_2:9763711-9764616 Satlength=906 Nr of Repeats=23 RepeatLength=40
seed=TTCCAGAATT
TTCCAGAATTTTCTCGATTTTTCTAGAAAGTTCTGGAACA
TTCCAGAATTTTCTCGATTTTTCTAGAAAGTTCTGGAACA
TTCCAGAATTTTCCCGATTTATCTAGAAAGTTCTGGAACA
TTCCAGAATTTTTCCGATTTTTCTAGAAAGTTCTGGAACA
TTCCAGAATTTTCTCGATTATTCTAGAAATTTCTGGAACA
TTCCAGAATTTTCCCGATTTATCTAGAAAGTTCTGGAACA
TTCCAGAATTTCCTCGATTTTTCTAGAAAGTTCTGGAACA
TTCCAGAATTTTCCCGATTTATCTAGAAAGTTCTGGAACA
TTCCAGAATTTCCTCGATTTTTCTAGAAAGTTCTGGAACA
TTCCAGAATTTTCCCGATTTATCTAGAAAGTTCTGGAACA
TTCCAGAATTTTCTCGATTTTTCTAGAAAGTTCTGGAACA
TTCCAGAATTTTCCCGATTTATCTAGAAAGTTCTGGAACA
TTCCAGAATTTTCTCGATTTTTCTAGAAAGTTCTGGAACA
TTCCAGAATTTTCTCGATTTTTCTAGAAAGTTCTGAAACA
TTCCAGAATTTTCTCGATTTTTCTAGAAAGTTCTGGAACA
TTCCAGAATTTTCCCGATTTTTCTAGAAAGTTCTGGAACA
TTCCAGAATTTTCTCGATTTTTCTAGAAAGTTCTGGAACA
TTCCAGAATTTTCCCGATTTATCTAGAAAGTTCTGGAACA
TTCCAGAGTTTTCCCGATTTTTCTAGAAAGTTCTGGAACA
TTCCAGAATTTTCTCGATTTTTCTAGAAAGTTCTGGAACA
TTCCAGAATTTTCCCGATTTTTCTAGAAAGTTCTGGAACA
TTCCAGAATTTTCCCGATTTTTCTAGAAAGTTCTGGAACA
TTCCAGAATTTTCCCGATTCTAGAT
Nr of selected repeats 22 Similarity 0.947763

Consensus:
TTCCAGAATTTTCcCGATTTtTCTAGAAAGTTCTGGAACA
>Cele-UNSB01_2:9764656-9765286 Satlength=631 Nr of Repeats=18 RepeatLength=35
seed=CCAAAATATT
CCAAAATATTTTTCTCAGAAAATTAAAATTTCCCT
CCAAAATATTTTTTTCAGAAAATTTAAATTTCCCT
CCAAAATATTTTTCTCAGAAAATTAAAACTTCCCT
CCAAAATATTTTTTTCAGAAAATTTAAATTTCCCT
CCAAAATATTTTTTTCAGAAAATTAAAATTTCCCT
CCAAAATATTTTTCTCAGAAAATTAAAATTTCCCT
CCAAAATATTTTTTTCAGAAAATTTAAATTTCCCT
CCAAAATATTTTTTTCAGAAAATTCAAATTTCCCT
CCAAAATATTTTTCTCAGAAAATTAAAATTTCCCT
CCAAAATATTTTTTTCAGAAAATTTAAATTTCCCT
CCAAAATATTTTTCTCAGAAAATTAAAATTTCCCT
CCAAAATATTTTTTTCAGAAAATTTAAATTTCCCT
CCAAAATATTTTTTTCAGAAAATTTAAATTTCCCT
CCAAAATATTTTTTTCAGAAAATTTAAATTTCCCT
CCAAAATATTTTTTTCAGAAAATTTAAATTTCCCG
CCAAAATATTTTTCACAGAAAATTTAAATTTCCCG
CCAAAATATTTTTCACAGAAAATTTAAATTTCCCG
CCAAAATTTTTTTCACAGAAAATTTAAATTTCCCG
Nr of selected repeats 18 Similarity 0.925801
Consensus:
CCAAAATATTTTTtTCAGAAAATTtAAATTTCCCt
>Cele-UNSB01_2:9765369-9765639 Satlength=271 Nr of Repeats=17 RepeatLength=15
seed=CCACGATGGG
CCACGATGGGTCTCA
CCACGAAGGGTATCA
CCACGATGGGTCTCA
CCACGATGGGTCTCA
CCTCGATGGGTCTCA
CCACGATGGGTATCA
CCACGATGGGTCTCA
CCACGACGGGTTTCACCACGGTTGGTCTCG
CCACGATGGGTCTCG
CCACGATGGGTCTCG
CCACGATGGGTCTCG
CCACGATGGGTCTCA
CCACGATGGGTCTTT
CCACGATGGGTCTCT
CCACGATGGGTCTCA
CCACGATGGGTCTCG
CCACGATGGGTCTCG
Nr of selected repeats 16 Similarity 0.891852
Consensus:
CCACGATGGGTCTCa
>Cele-UNSB01_2:9765885-9766019 Satlength=135 Nr of Repeats=5 RepeatLength=11
seed=GTACTCCTAC
GTACTCCTACA
GTACTCCTACA
GTACTACTACAGTACCCCGACCATATCCCCCTACTAACCCCAAACCTATATCTCTTCAAAAGACTAAAACACAATTTTT
CCTAAACTACA
GTAATCCTACC

GTACTCCTACA
Nr of selected repeats 4 Similarity 0.878788
Consensus:
GTAcTCCTACa
>Cele-UNSB01_2:9825153-9825520 Satlength=368 Nr of Repeats=4 RepeatLength=42
seed=CAGCCGACAG
CAGCCGACAGCCGAAAAATTGCGATTTTTCGGCTGGTAGCAC
CAGCCGACAGCCGACTGATAGTGATAGGTCGGCTGGCGTTTTGAAAAAAATAAACTGACTTTTTGGCATATTTTGAGAC
TGAGTGCAAGTTTTACAAGTGGAAAAAGTACGGAAATTTGGTTTTTTGTTGAATAAAATGATGTAATATTTTGCTATAA
TTTGTTTTTGTACTAAAAGTATACTAAATAGCTCCGAAACTTTGAAACAACTCTTAACCAAAATCTCAGTCGGCTGGTG
GCGC
CAGCCGACAGCCGAAAAACTTGGAATTTTCGGCTGGTAGCAC
CAGCCGACAGCCGACTGATAAAAATTGCTCGGCTGTCGGTGT
Nr of selected repeats 3 Similarity 0.533769
Consensus:
CAGCCGACAGCCGAAAAAttggAaTTtTCGGCTGGTaGcac
>Cele-UNSB01_2:10367690-10367782 Satlength=93 Nr of Repeats=6 RepeatLength=15
seed=TCCCCTTTTC
TCCCCTTTTCGTTGC
TCCCCTTTTCGTATC
TCCCCTTTCCGTATC
TCCCCTTTTCGCAGA
TCCCCTTTTTGCAGA
TCCCCTTTTCGTTCTGC
Nr of selected repeats 5 Similarity 0.733333
Consensus:
TCCCCTTTtcGtagc
>Cele-UNSB01_2:10468271-10470517 Satlength=2247 Nr of Repeats=10
RepeatLength=225 seed=TTTTCGAAAT
TTTTCGAAATGTCTTTCAGAAACTTTGTAGTAAATTTTAAGCTCTTTCAGATTATATTAACAATATTCCAGTAGGTACA
AGAAGCTTCAAGTAGTTACAGAAATAGTACATTTTCAGCCCTACCTTTTAGTGCGTATTTTATTATTAATGAAAACTAC
TATGTATAGGCAAAAATAGATGGATTTTACAAACTTTGAAAATTCATAAACCTCTTCAAAGTAACTG
TTTTCGAAATGTCTTTCAGAAACTTTGTAGTAAATTTTAAGCTCTTTCAGATTATATTAACAATATTCCAGTAGGTACA
AGAAGCTTCAAGTAGTTACAGAAATAGTATATTTTCAGCCCTACCTTTTAGTGCGTATTTTATTATTTTATGAAAACTA
CCATTTATAGGCAAAAAGATGGATTTTCCAATTTTTGAAAATTCATAAATCTCTTCAATGTAACTT
TTTTCGAAATGTCTTTCAGAAACTTTGTAGTAAATTTTAAGCTCTTTCAGATTATATTAACAATATTCCAGTAGGTACA
AGAAGCTTCAAGTAGTTACAGAAATAGTACATTTTCAGCCCTACCTTTTAGTGCGTATTTTATTATTAATGAAAACTAC
TATGTATAGGCAAAAATAGATGGATTTTACAAACTTTGAAAATTCATAAACCTCTTCAAAGTAACTG
TTTTCGAAATGTCTTTCAGAAACTTTGTAGTAAATTTTAAGCTCTTTCAGATTATATTAACAATATTCCAGTAGGTACA
AGAAGCTTCAAGTAGTTACAGAAATAGTATATTTTCAGCCCTACCTTTTAGTGCGTATTTTTTTATTTTATGAAAACTA
CCATTTATAGGCAAAAAGATGGATTTTCCAATTTTTGAAAATTCATAAATCTCTTCAATGTAACTT
TTTTCGAAATGTCTTTCAGAAACTTTGTAGTAAATTTTAAGCTCTTTCAGATTATATTAACAATATTCCAGTAGGTACA
AGAAGCTTCAAGTAGTTACAGAAATAGTACATTTTCAGCCCTACCTTTTAGTGCGTATTTTATTATTAATGAAAACTAC
TATGTATAGGCAAAAATAGATGGATTTTACAAACTTTGAAAATTCATAAACCTCTTCAAAGTAACTG
TTTTCGAAATGTCTTTCAGAAACTTTGTAGTAAATTTTAAGCTCTTTCAGATTATATTAACAATATTCCAGTAGGTACA
AGAAGCTTCAAGTAGTTACAGAAATAGTATATTTTCAGCCCTACCTTTTAGTGCGTATTTTTTTATTTTATGAAAACTA
CCATTTATAGGCAAAAAGATGGATTTTCCAATTTTTGAAAATTCATAAATCTCTTCAATGTAACTT
TTTTCGAAATGTCTTTCAGAAACTTTGTAGTAAATTTTAAGCTCTTTCAGATTATATTAACAATATTCCAGTAGGTACA
AGAAGCTTCAAGTAGTTACAGAAATAGTACATTTTCAGCCCTACCTTTTAGTGCGTATTTTATTATTAATGAAAACTAC
TATGTATAGGCAAAAATAGATGGATTTTACAAACTTTGAAAATTCATAAACCTCTTCAAAGTAACTG
TTTTCGAAATGTCTTTCAGAAACTTTGTAGTAAATTTTAAGCTCTTTCAGATTATATTAACAATATTCCAGTAGGTACA
AGAAGCTTCAAGTAGTTACAGAAATAGTATATTTTCAGCCCTACCTTTTAGTGCGTATTTTTTTATTTTATGAAAACTA
CCATTTATAGGCAAAAAGATGGATTTTCCAATTTTTGAAAATTCATAAATCTCTTCAATGTAACTT

TTTTCGAAATGTCTTTCAGAAACTTTGTAGTAAATTTTAAGCTCTTTCAGATTATATTAACAATATTCCAGTAGGTACA
AGAAGCTTCAAGTAGTTACAGAAATAGTACATTTTCAGCCCTACCTTTTAGTGCGTATTTTATTATTAATGAAAACTAC
TATGTATAGGCAAAAATAGATGGATTTTACAAACTTTGAAAATTCATAAACCTCTTCAAAGTAACTG
TTTTCGAAATGTCTTTCAGAAACTTTGTAGTAAATTTTAAGCTCTTTCAGATTATATTAACAATATTCCAGTAGGTACA
AGAAGCTTCAAGTAGTTACAGAAATAGTACATTTTCAGCCCTACCTTTTAGTGCGTATTTTATTATTAATGAAAACTAC
TATGTATAGGCAAAAATAGATGGATTTTACAAACTTTGAAAATTCATAAACCTCTTCAAAGTAACTG
Nr of selected repeats 6 Similarity 1.000000
Consensus:
TTTTCGAAATGTCTTTCAGAAACTTTGTAGTAAATTTTAAGCTCTTTCAGATTATATTAACAATATTCCAGTAGGTACA
AGAAGCTTCAAGTAGTTACAGAAATAGTACATTTTCAGCCCTACCTTTTAGTGCGTATTTTATTATTAATGAAAACTAC
TATGTATAGGCAAAAATAGATGGATTTTACAAACTTTGAAAATTCATAAACCTCTTCAAAGTAACTG
>Cele-UNSB01_2:10597740-10597833 Satlength=94 Nr of Repeats=5 RepeatLength=18
seed=TAAATAAGTG
TAAATAAGTGGCTATAAGT
TAAATAAGTGGCTATAAG
TAAATAAGTGGCTATAAT
TAAATAAGTGGCTATAAT
TAAATAAGTGGCTATATAAT
Nr of selected repeats 3 Similarity 0.950617
Consensus:
TAAATAAGTGGCTATAAt
>Cele-UNSB01_2:10844890-10845587 Satlength=698 Nr of Repeats=8 RepeatLength=35
seed=TTTCTGGCAA
TTTCTGGCAAATCGGCAGTTTGCCGAAAATTAAAT
TTTCTGGCAAACCGGCAATTTGCCGAAAATTAAAA
TTTCTGACAAACCGGCAAACCGGCACTTTGCCGAAAATTAAAT
TTTCTGGCAAACCGGCAATTTGCCAAAAATTAAAA
TTTCTGGCAAACCGGCAAACCGGCACTTTGCCGAAAATTAAAT
TTTCTGGCAAACCGACAATTTGCCAAAAATTAACA
TTTCTGGCAAACCGGCAATTTGCCGAAAATTAAAA
TTTCTGGCAAACCGGCCAATTGCCAGAATTGAAATTTTTCATAATTTTTCAGTATTATTCAAGTTTTTTTTTTGAGATT
TTCTTCGCTTCAACATACTTAACATCGATTTTATCCGTCGGTGCGCTGTGACATGCTTCTCCAAAATGAACAAGAGCTG
TACAATCAGCGTGAGCAGCAGCGACTTCATCAACACAGCAGCTGGAAATTTCCATTATTTTCTTTTTTTTAATGAAAAA
TCAAACCTTCTATAAGAAGTATCAGCTAGAACAAATGTCTTCTTTGCACTTTCAGATCGAAATCTGCTTTCGATGAGTT
TTGTGACTTTTTCCGAGTATGGAAGCAGAGAATCCGGGAATTGTAGAGCTATTCGTTGATGATTTCCCTGTTTAATCCA
TTCAGATGTGGCGTCAATTTCAAAAAATGAATGAATATCGT
Nr of selected repeats 5 Similarity 0.900952
Consensus:
TTTCTGGCAAAcCGgCAaTTTGCCgAAAATTAAaa
>Cele-UNSB01_2:10964073-10965096 Satlength=1024 Nr of Repeats=24
RepeatLength=11 seed=GTACTGTAGG
GTACTGTAGGA
GTACTGTAGGTATACGGTAGGGTTACTGTAGTTTAGGAAAAATTGAGTTTTTGTATACAGAAGAGGTATTGGGTTGGGA
GTTGCTGGGGGATAATGTCAAG
GTACTGTAGTA
GTACTGTAGGA
GTACTTTAGGTATACGGTAGGGTTACTGTAGTTTAGGAAAAATTGAGTTTTTGTATACAGAAGAGGTATTGGGTTGGGA
GTTGCTGGGGGATAATGTCAAG
GTACTGTAGTA
GTACTGTAGGA
GTACTGTAGGTATACGGTAGGGTTACTGTAGTTTAGGAAAATTGAGTTTTTGTCTACAGAAGAGGTATTGGGTAGAGAG
TTGCTGGGGGATAATGTCAAG
GTACTGTAGTA
GTACTGTAGGA

```
GTACTGTAGGTATACGGTAGGGTTTCTGTAGTTTAGGAAAATTGAGTTTTTGTCTACAGAAGAGGTATTGGGTAGAGAG
TTGCTGGGGGATAATGCCAAG
GTACTGTAGTA
GTACTGTAGTA
GTATTGTAGGTATACGGTAGGGTTACTGTAGTTTACGAAAAATTGAGTTTTTGTCTACAGAAGAGGTATTGGGTAGAGA
GTTGCTGGGGGATAATGTCAAG
GTACTGTAGTA
GTACTGTAGGTATACGGTAGGGTTTCTGTAGTTTAGGAAACATTGAGGTTTTGTCTACAGAAGAAGTATTGGGTTGGGA
GTTGGTGAGGGATAATGTCAAA
GTACTGTAGTG
GTACTGTAGGA
GTACTGTAGGTATACGGTAGGGTTACTGTAGTTTAGGAACAATTGAGTTTTTCTCTACAGAAGATGTATTGGGTTGGGA
GTTGGTGAGGGATAATGTCAAG
GTACTGTAGTG
GTACTGTAGGA
GTACTGTAGGTATACGGTAGGGTTACTGTAGTTTAGGAAATTTTGAGTTTAAAACATTTTGAAGAGGTGTTGGCTCGAA
GAGGTGTAGTTTTTCTCTACAGAAGATGTATTGGGTTGGGAGTTGGTGAGGGATAATGTCAAG
GTACTGTAGTG
GTACTGTAGGA
Nr of selected repeats 16 Similarity 0.896970
Consensus:
GTACTGTAGtA
>Cele-UNSB01_2:11255667-11256407 Satlength=741 Nr of Repeats=61 RepeatLength=12
seed=AAAAATAAAA
AAAAATAAAAAT
AAAAATAAAAAT
AAAAATAAAAAT
AAAAATAAAAAT
AAAAATAAAAT
AAAAATAAAAAT
AAAAATAAAAAT
AAAAATAAAAAT
AAAAATAAAAAT
AAAAATAAAAAT
AAAAATAAAAAT
AAAAATAAAAAT
AAAAATAAAAT
AAAAATAAAAAT
AAAAATAAAAAT
AAAAATAAAAAT
AAAAATAAAAAT
AAAAATAAAAAT
AAAAATAAAAAT
AAAAATAAAAAT
AAAAATAAAAAT
AAAAATAAAAAT
AAAAATAAAAAT
AAAAATAAAAAT
AAAAATAAAAAT
AAAAATAAAAAT
AAAAATAAAAAT
AAAAATAAAAAT
AAAAATAAAA
```

AAAAATAAAAAT
AAAAATAAAAAT
AAAAATAAAAAT
AAAAATAAAAAT
AAAAATAAAAAT
AAAAATAAAAAT
AAAAATAAAAATAA
AAAAAAAAAT
AAAAATAAAAAT
AAAAATAAAAAT
AAAAATAAAAAT
AAAAATAAAAAT
AAAAATAAAAAT
AAAAATAAAAATAAA
AAAAATAATAAAAAT
AAAAATAAAAAT
AAAAATAAAAAT
AAAAATAAAAAT
AAAAATAAAAAT
AAAAATAAAAAT
AAAAATAAAAATAAA
AAAAATAATAAAAAT
AAAAATAAAAAT
AAAAATAAAAAT
AAAAATAAAAAT
AAAAATAAAAAT
AAAAATAAAAAT
AAAAATAAAAAT
AAAAATAAAAAT
AAAAATAAAAAT
AAAAATAAAAAT
Nr of selected repeats 52 Similarity 1.000000
Consensus:
AAAAATAAAAAT
>Cele-UNSB01_2:11255666-11256503 Satlength=838 Nr of Repeats=64 RepeatLength=12
seed=TAAAAATAAA
TAAAAATAAAAA
TAAAAATAAAAA
TAAAAATAAAAA
TAAAAATAAAAA
TAAAAATAAAA
TAAAAATAAAAA
TAAAAATAAAAA
TAAAAATAAAAA
TAAAAATAAAAA
TAAAAATAAAAA
TAAAAATAAAAA
TAAAAATAAAAA
TAAAAATAAAA
TAAAAATAAAAA
TAAAAATAAAAA
TAAAAATAAAAA
TAAAAATAAAAA
TAAAAATAAAAA

TAAAAATAAAAA
TAAAAATAAAAA
TAAAAATAAAAA
TAAAAATAAAAA
TAAAAATAAAAA
TAAAAATAAAAA
TAAAAATAAAAA
TAAAAATAAAAA
TAAAAATAAAAA
TAAAAATAAAAA
TAAAAATAAAAA
TAAAAATAAAA
AAAAATAAAAA
TAAAAATAAAAA
TAAAAATAAAAA
TAAAAATAAAAA
TAAAAATAAAAA
TAAAAATAAAAA
TAAAAATAAAAA
TAAAAAAAAAAA
TAAAAATAAAAA
TAAAAATAAAAA
TAAAAATAAAAA
TAAAAATAAAAA
TAAAAATAAAAA
TAAAAATAAAAA
TAAAAAAAAATAA
TAAAAATAAAAA
TAAAAATAAAAA
TAAAAATAAAAA
TAAAAATAAAAA
TAAAAATAAAAA
TAAAAATAAAAA
TAAAAATAAAAAAAAATAA
TAAAAATAAAAA
TAAAAATAAAAA
TAAAAATAAAAA
TAAAAATAAAAA
TAAAAATAAAAA
TAAAAATAAAAA
TAAAAATAAAAA
TAAAAATAAAAA
TAAAAATAAAAA
TAAAAATAAAAA
TAAAAATAATTTTTATTTTTATTTTTATTTATATTTAAAAATTTTTATT
TAAAAATAAATTTTTATTTTTATTTTTATTTTTATTTTTATT
Nr of selected repeats 57 Similarity 0.988443
Consensus:
TAAAAATAAAAA
>Cele-UNSB01_2:11256637-11258512 Satlength=1876 Nr of Repeats=15
RepeatLength=124 seed=AATTTTCAAA
AATTTTCAAAGTTTGGAAAATCTATTAATTTTGCATATAATTTTTATAAATTCTGAAAGAACATAAAATTTGCTACAAA
ATGGCTACGGGAATTTCTAAAAAATTTAGTTTGAAGACAGAAATT

AATTTTCAAAGTTTGGAAAATCTATTAATTTTACATATAAATTGTGGTCTACAAGGTATGACTGAAAATTCACTTTTTC
TGCTTGAACTTGGCGGTTTCAAAATTTTTTGAAATTCTGTTTTTA
AATTTTGAAAGAACATAAAATTTGCTACAAAATGGCTCCGGGAATTTCTAAAAAATTTAGTTTGAAGCCAGAAATT
AATTTTCAAAGTTTGGAAAATCTATTAATTTTGCATATAATTTTTATAAATTCTGAAAGAACATAAAATTTGCTACATA
ATGGCTACGGGAATTTCTAAAAAAGTTAGTTTGAAGCCAGAAATT
AATTTTCAAAGTTTGGAAAATCTATTAATTTTGCATATAAATTGTGGTCTACAAGGTATGACTGAAAATTCACTTTTTC
TGCTTGAACTTGGCGGTTTCGAATTTTTTTGAAATTCTGTTTTCAAATTCTGAAAGAACATAAAATTTGCTACAAAATG
GTTACGGGAATTTTTGAAAAAGTTAGTTTGAAGCCAGAAATT
AATTTTCAAAGTTTGGAAAATCTATTAATTTTGCATATAAATTGTGGTCTACAAGGTATGACTGAAAATTCACTTTTTC
TGCTTGAACTTGGCGGTTTCAAAATCTTTTGAAATTCTGTTTTTA
AATTTTGAAAGAACATAAAATTTGCTACAAAATGGCTACGGGAATTTCTAAAAAATTTAGTTTGAAGACAGAAATT
AATTTTCAAAGTTTGGAAAATCTATTAATTTTGCATATAATTTTTATAAATTCTGAAAGAACATAAAATTTGCTACAAA
ATGGCTACGGGAATTTCTAAAAAATTTAGTTTGAAGACAGAAATT
AATTTTCAAAGTTTGGAAAATCTATTAATTTTGCATATAATTTTTATAAATTCTGAAAGAACATAAAATTTGCTACAAA
ATGGCTACGGGAATTTCTAAAAAATTTAGTTTGAAGACAGAAATT
AATTTTCAAAGTTTGGAAAATCTATTAATTTTGCATATAATTTTTATAAATTCTGAAAGAACATAAAATTTGCTACAAA
ATGGCTACGGGAATTTCTAAAAAATTTAGTTTGAAGACAGAAATT
AATTTTCAAAGTTTGGAAAATCTATTAATTTTGCATATAATTTTTATAAATTCTGAAAGAACATAAAATTTGCTACAAA
ATGGCTACGGGAATTTCTAAAAAATTTAGAAGACAGAAATT
AATTTTCAAAGTTTGGAAAATCTATTAATTTTGCATATAATTTTTATAAATTCTGAAAGAACATAAAATTTGCTACAAA
ATGGCTACGGGAATTTCTAAAAAAGTTAGTTTGAAGCCAGAAATG
AATTTTCAAAGTTTGGAAAATCTATTAATTTTGCATATAAATTGTGGTCTACAAGGTATGACTGAAAATTCACTTTTTC
TGCTTGAACTTGGCGGTTTCAAAATTTTTTGAAATTCTGTTTTTA
AATTTTGAAAGAACATAAAATTTGCTACAAAATGGCTACGGGAATTTCTAAAAAAGTTAGTTTGAAGAGATTTATG
AATTTTCAAAGTTTCGAAAATCCACCTATTTTTGCCTATTAATGGTAGTTTTCATTAAATAATAAAATACGCACTAAAA
GGTAGGGCTGAAAATGTACTATTTCTGTAACTACGTGAAGCTTCTTGTACCTTCTGTAATATTGTTGTATATTCTAAAA
GAGCTCAGAATTTACTACAAAATTAAAAAAAAATTTCTTTCAAGATATTTATG
Nr of selected repeats 9 Similarity 0.708025
Consensus:
AATTTTCAAAGTTTGGAAAATCTATTAATTTTGCATATAATTTtTAtAAatTcTGAaaGAAcATaAaaTTTgCTaCAAa
aTGGctaCgGGaaTTTCtaaAAAAtTTagTTTGAAgnCaGaaaTT
>Cele-UNSB01_2:11366885-11367108 Satlength=224 Nr of Repeats=5 RepeatLength=44
seed=AAGGATCAGA
AAGGATCAGAACATCAGAAGGTACCCATGTGATCCTTCGGATCGG
AAGGATCAGATCATCAGGCAGAGCCTCATGCTCTTTTGGAGCTG
AAGGATCAGAACATCAGGAAGAGCCTCGTGATCCTTTGGAGCTA
AAGGATCAGACCATCAGGAGGTTCCCTTACGATCCTTCTTATCTGA
AAGATCAGAACATCAGAAGGTACCCATGTGATCCTTCGGAGCTG
Nr of selected repeats 3 Similarity 0.696296
Consensus:
AAgGATCAGAaCATCAGgaaGAgCCtcgTGaTCcTTtGGAGCTg
>Cele-UNSB01_2:11443617-11443668 Satlength=52 Nr of Repeats=4 RepeatLength=12
seed=ACTATAACTA
ACTATAACTACAACT
ACTACAACTACA
ACTATAACTACA
ACTATAACTATA
Nr of selected repeats 3 Similarity 0.851852
Consensus:
ACTAtAACTAcA
>Cele-UNSB01_2:11502184-11502280 Satlength=97 Nr of Repeats=5 RepeatLength=24
seed=GCTTAGGCTT
GCTTAGGCTTAG
GCTTAGGCTTAGGCTTGGGCTCAA

GCTTAGGTTTGGGCCTAGGCCTAG
GCTCAGGCTTGGGCTTATGCTAAA
GCTTAGGCATAC
Nr of selected repeats 3 Similarity 0.648148
Consensus:
GCTtAGGcTTgGGCtTagGCtnAa
>Cele-UNSB01_2:11560093-11560292 Satlength=200 Nr of Repeats=9 RepeatLength=20 seed=AAAAAATAGA
AAAAAATAGACTGGCATACC
AAAAAATAGATTGGCATACG
AAAAAATAGATTGGCATACG
AAAAAATAGGTTGGCATACG
AAAAAATAGATTGGCATACG
AAAAAATAGATTGGCATACG
AAAAAATAAGTTGACATACC
AAAAAATAGATTGGCATACG
AAAAAATAGATTGGCCTACGATAAATAGATCGACATACC
Nr of selected repeats 8 Similarity 0.892857
Consensus:
AAAAAATAGaTTGGCATACg
>Cele-UNSB01_2:11664501-11665796 Satlength=1296 Nr of Repeats=29 RepeatLength=45 seed=TCTGATCCTT
TCTGATCCTTCAGCTCCGAAGGATCACATGGGTACCTTCTGATGT
TCTGATCCTTCAGATAAGAAGAATCGTAAGGGTACCTCCTGATGG
TCTGATCCTTCAGCTCCAAAGGATCACGAGGCTCTTCCTGATGG
TCTGATCCTTCAGCTCCGAAGGATCACATGGGTACCTTCTGATGT
TCTGATCCTTCAGATAAGAAGGATCGTAAGGGTACCTCCTGATGG
TCTGATCCTTCAGCTCCAAAGGATCACGAGGCTCTTCCTGATGT
TCTGATCCTTCAGCTCCGAAGAATCACATGGGTACCTTCTGATGT
TCTGATCCTTCAGATAAGAAGGATCGTAAGGGTACCTCCTGATGG
TCTGATCCTTCAGCTCCAAAGGATCACGAGGCTCTTCCTGATGT
TCTGATCCTTCAGCTCCGAAGGATCACATGGGTACCTTCTGATGT
TCTGATCCTTCAGATAAGAAGGATCGTAAGGGTACCTCCTGATGG
TCTGATCCTTCAGCTCCAAAGGATCACGAGGCTCTTCCTGATGT
TCTGATCCTTCAGCTCCGAAGAATCACATGGGTACCTTCTGATGT
TCTGATCCTTCAGATAAGAAGGATCGTAAGGGTACCTCCTGATGG
TCTGATCCTTCAGCTCCAAAGGATCACGAGGCTCTTCCTGATGG
TCTGATCCTTCAGCTCCGAAGGATCACATGGGTACCTTCTGATGT
TCTGATCCTTCAGATAAGAAGGATCGTAAGGGTACCTCCTGATGG
TCTGATCCTTCAGCTCCAAAGGATCACGAGGCTCTTCCTGATGT
TCTGATCCTTCAGCTCCGAAGAATCACATGGGTACCTTCTGATGT
TCTGATCCTTCAGATAAGAAGGATCGTAAGGGTACCTCCTGATGG
TCTGATCCTTCAGCTCCAAAGGATCACGAGGCTCTTCCTGATGT
TCTGATCCTTCAGCTCCGAAGGATCACATGGGTACCTTCTGATGT
TCTGATCCTTCAGATAAGAAGGATCGTAAGGGTACCTCCTGATGG
TCTGATCCTTCAGCTCCAAAGGATCACGAGGCTCTTCCTGATGT
TCTGATTCTTCAGCTCCGAAGGATCACATGGGTACCTTCTGATGT
TCTGATCCTTCAGATAAGAAGGATCGTAAGGGTACCTCCTGATGG
TCTGATACTTCAGCTCCAAAGGATCACGAGGCTCTTCCTGATGT
TCTGATCCTTCAGCTCCAAAAGAACATGAGGCTCTGCCTGATGA
TCTGATCCTTCCGATCCGAAGGATCACATGGGTACCTTCTGATGA
Nr of selected repeats 19 Similarity 0.857050
Consensus:
TCTGATCCTTCAGaTccGAAGgATCacAtGGGTACCTtCTGATGn

>Cele-UNSB01_2:11667411-11669597 Satlength=2187 Nr of Repeats=49
RepeatLength=45 seed=GAAGGATCAG
GAAGGATCAGATCATCAGAAGGTACCCATGTGATCCTTCGGATCG
GAAGGATCAGATCATCAGGCAGAGCCTCATGTTCTTTTGGAGCT
GAAGTATCAGACCATCAGGAGGTACCCTTACGATCCTTCTTATCT
GAAGGATCAGAACATCAGAAGGTACCCATGTGATCCTTCGGAGCT
GAAGGATCAGAACATCAGGAAGAGCCTCGTGATCCTTTGGAGCT
GAAGGATCAGATCATCAGAAGGTACCCATGTGATCCTTCGGAGCT
GAAGGATCAGAACATCAGGAAGAGCCTCGTGATCCTTTGGAGCT
GAAGGATCAGATCATCAGAAGGTACCCATGTGATCCTTCGGATCG
GAAGGATCAGATCATCAGGCAGAGCCTCATGTTCTTTTGGCGCT
GAAGGATCAGAACATCAGGAAGAGCCTCGTGATCCTTTGGAGCT
GAAGTATCAGACCATCAGGAGGTACCCTTACGATCCTTCTTATCT
GAAGGATCAGAACATCAGAAGGTACCCATGTGATCCTTCGGAGCT
GAAGGATCAGAACATCAGGAAGAGCCTCGTGATCCTTTGGAGCT
GAAGGATCAGACCATCAGGAGGTACCCTTACGATCCTTCTTATCT
GAAGGATCAGAACATCAGAAGGTACCCATGTGATCCTTCGGAGCT
GAAGGATCAGAACATCAGGAAGAGCCTCGTGATCCTTTGGAGCT
GAAGGATCAGATCATCAGAAGGTACCCATGTGATCCTTCGGAGCT
GAAGGATCAGAACATCAGGAAGAGCCTCGTGATCCTTTGGAGCT
GAAGGATCAGATCATCAGAAGGTACCCATGTGATCCTTCGGAGCT
GAAGGATCAGAACATCAGGAAGAGCCTCGTGATCCTTTGGAGCT
GAAGGATCAGATCATCAGAAGGTACCCATGTGATCCTTCGGATCG
GAAGGATCAGATCATCAGGCAGAGCCTCATGTTCTTTTGGCGCT
GAAGGATCAGAACATCAGGAAGAGCCTCGTGATCCTTTGGAGCT
GAAGTATCAGACCATCAGGAGGTACCCTTACGATCCTTCTTATCT
GAAGGATCAGAACATCAGAAGGTACCCATGTGATCCTTCGGAGCT
GAAGGATCAGAACATCAGGAAGAGCCTCGTGATCCTTTGGAGCT
GAAGGATCAGACCATCAGGAGGTACCCTTACGATCCTTCTTATCT
GAAGGATCAGAACATCAGAAGGTACCCATGTGATTCTTCGGAGCT
GAAGGATCAGAACATCAGGAAGAGCCTCGTGATCCTTTGGAGCT
GAAGGATCAGACCATCAGGAGGTACCCTTACGATCCTTCTTATCT
GAAGGATCAGAACATCAGAAGGTACCCATGTGATTCTTCGGAGCT
GAAGGATCAGAACATCAGGAAGAGCCTCGTGATCCTTTGGAGCT
GAAGGATCAGACCATCAGGAGGTACCCTTACGATCCTTCTTATCT
GAAGGATCAGAACATCAGAAGGTACCCATGTGATCCTTCGGAGCT
GAAGGATCAGAACATCAGGAAGAGCCTCGTGATCCTTTGGAGCT
GAAGGATCAGACCATCAGGAGGTACCCTTACGATCCTTCTTATCT
GAAGGATCAGAACATCAGAAGGTACCCATGTGATTCTTCGGAGCT
GAAGGATCAGAACATCAGGAAGAGCCTCGTGATCCTTTGGAGCT
GAAGGATCAGACCATCAGGAGGTACCCTTACGATCCTTCTTATCT
GAAGGATCAGAACATCAGAAGGTACCCATGTGATTCTTCGGAGCT
GAAGGATCAGAACATCAGGAAGAGCCTCGTGATCCTTTGGAGCT
GAAGGATCAGACCATCAGGAGGTACCCTTACGATCCTTCTTATCT
GAAGGATCAGAACATCAGAAGGTACCCATGTGATCCTTCGGAGCT
GAAGGATCAGACCATCAGGAAGAGCCTCGTGATCCTTTGGAGCT
GAAGGATCAGACCATCAGGAGGTACCCTTACGATCCTTCTTATCT
GAAGGATCAGAACATCAGAAGGTACCCATGTGATCCTTCGGAGCT
GAAGGATCAGACCATCAGGAAGAGCCTCGTGATCCTTTGGAGCT
GAAGGATCAGACCATCAGGAGGTACCCTTACGATTCTTCTTATCT
GAAGGATCAGAACATCAGAAGGTACCCATGTGATCCTTCGGAGCT
Nr of selected repeats 30 Similarity 0.857233
Consensus:
GAAGGATCAGAnCATCAGaAGGTACCCaTgtGATCCTTCggAgCT

>Cele-UNSB01_2:11673801-11673893 Satlength=93 Nr of Repeats=6 RepeatLength=15
seed=CGAAAAGGGG
CGAAAAGGGGAGCAGAA
CGAAAAGGGGATCTG
CAAAAAGGGGATCTG
CGAAAAGGGGAGATA
CGAAAAGGGGAGATA
CGAAAAGGGGAGCAA
Nr of selected repeats 5 Similarity 0.768889
Consensus:
CgAAAAGGGGAgcta
>Cele-UNSB01_2:11674068-11674143 Satlength=76 Nr of Repeats=5 RepeatLength=15
seed=TCCCCTTTTC
TCCCCTTTTCGTTGC
TCCCCTTTTCGTATC
TCCCCTTTTCGTATC
TCCCCTTTTCGCAGA
TCCCCTTTTTGCAGA
Nr of selected repeats 5 Similarity 0.768889
Consensus:
TCCCCTTTTcGtagc
>Cele-UNSB01_2:11799205-11799469 Satlength=265 Nr of Repeats=8 RepeatLength=33
seed=AATTGAAAAT
AATTGAAAATCCAGCAAATGGGCAAATTGCCGT
AATTGAAAATCCGGCAAATGGGCAAATTGCCGT
AATTGAAAATCCGGCAAATGGACAAATTGCCGG
AATTGAAAATCCGGCAAATGGGAAAAATTGCCGT
AATTGAAAATCCGGCTAATCCGCAGATTGTCGG
AATTGAAAATCCGGCAAATCAGCAAATGGCCGG
AATTGAGAATCCGGCAAATGGGCAAATTGCCGT
AATTGAAAATCCGGCTAATCGGCAGATTGTCGG
Nr of selected repeats 8 Similarity 0.834055
Consensus:
AATTGAAAATCCGGCaAATggGCAaATTGcCGg
>Cele-UNSB01_2:11872286-11872364 Satlength=79 Nr of Repeats=6 RepeatLength=12
seed=CTTAGGCTTA
CTTAGGCTTAGGGTCAGG
CTTAGGCTTAGG
CTTAGGCTTAGG
CTTAGACTTAGG
CTTAGACTTAGA
CTTAGGCTTGGG
Nr of selected repeats 5 Similarity 0.844444
Consensus:
CTTAGgCTTaGg
>Cele-UNSB01_2:12045935-12046042 Satlength=108 Nr of Repeats=8 RepeatLength=12
seed=AGGCTTAGGC
AGGCTTAGGCTT
AGGCTTAAGCTT
AGGTTTAGGCCT
AGGCTTAGGCAT
AGGCTCAGGCTT
AGACTTAGGCTC
AGGCTAGGCTC

AGGCTTAGGCTCAAGCTCAGGCTT
Nr of selected repeats 6 Similarity 0.748148
Consensus:
AGGCTTAGGCtT
>Cele-UNSB01_2:12047204-12047947 Satlength=744 Nr of Repeats=32 RepeatLength=20
seed=TAAATCTACA
TAAATCTACACGAGTTCGTG
TAAATCTACACAGGGTGGTG
TAAATCTACAGGGCCTTGCG
TAAATCTACATATCGTGACGTAAGCCTACACTGATTCTTG
TAAATCTACACTGGTTCGCG
TAAATCTACACTAGCTCGGCGCTCTCCTGCG
TAAATCTACAAAGCGTGACG
TAAATCTACACAGGCTGGTG
TAAATCTACAGGGCCTTGCG
TAAATCTACATATCGTGGCGTTAGCCTACACTGATTCTTG
TAAATCTACACTTGTTTGCG
TAAATCAACACAGGCCGGTG
TAAATCTACACTGACTTGCG
TAAATCTACACAGGGTGGTG
TAAATCTACAGGGCCTTGCG
TAAATCTACATATCGTGACGTAAGCCTACACTGATTCTTG
TAAATCTACACTGGTTCGCG
TAAATCTACACTAGCTCGGCGCTCTCCTGCG
TAAATCTACAAAGCGTGACG
TAAATCTACACAGGCTGGTG
TAAATCTACAGGGCCTTGCG
TAAATCTACATATCGTGGCGTTAGCCTACACTGATTCTTG
TAAATCTACACTTGTTTGCG
TAAATCAACACAGGCCGGTG
TAAATCTACACTGACTTGCG
TAAATCTACACAGGGTGGTG
TAAATCTACAGGGCCTTGCG
TAAATCTACACTAGCTCGCG
TAAATCAACACCTTGTTCGCG
TAAATCTACACTGCCTTGTG
TAAATCGACATTTAGTTGCG
TAAATCTACACTAGCTAGCG
Nr of selected repeats 25 Similarity 0.700222
Consensus:
TAAATCTACAcnggcTnGcG
>Cele-UNSB01_2:12070558-12070690 Satlength=133 Nr of Repeats=10 RepeatLength=12
seed=AAGCCTAAGC
AAGCCTAAGCCT
AAGCCTAACCAT
AAGCCTAAGCCTA
AACCTAAGCCT
AAGCCTAAGCCT
AAGCTTAAGCCT
AAGCCTACGCCTTAGCTT
AAGCCTAAGCCC
AAGCCCAAGCCC
AAGCCTAAGCCTAAGCCT
Nr of selected repeats 6 Similarity 0.792593

Consensus:
AAGCCTAAGCCt
>Cele-UNSB01_2:12072605-12072779 Satlength=175 Nr of Repeats=10 RepeatLength=12 seed=AGGCTTAGGC
AGGCTTAGGCCCAGGCTTTAGTTT
AGGCTTAGACTT
AGACTTAGGCTT
AGGCCTAGGCTT
AGGCATAGGCTT
AGGCTTAGGCTT
AGGCTAAGGCTTATGCTCA
AGCTTAGGCTAAGGAATATGCTTGAACTTTGGTTT
AGGTTTAGGCTT
ATGCTTAGGCTTAGTCTTAGGGTC
Nr of selected repeats 6 Similarity 0.822222
Consensus:
AGGCtTAGGCTT
>Cele-UNSB01_2:12142720-12142795 Satlength=76 Nr of Repeats=4 RepeatLength=15 seed=AAAACAAGAT
AAAACAAGATCTACG
AAAACAAGATCTACATTTTCAAGATCTACG
AAAAGAAGATCTACA
AAAACAAGATCCACC
Nr of selected repeats 3 Similarity 0.792593
Consensus:
AAAAcAAGATCtACn
>Cele-UNSB01_2:12311843-12313532 Satlength=1690 Nr of Repeats=56 RepeatLength=30 seed=CAAACTACAG
CAAACTACAGTAGCACTGTAGTACCATAAC
CAAACTACAGTAGTACTGTAGTACCATAACC
CAAACTACAGTAGCACTGTAGTACCATAAC
CAAACTACAGTAGTACTGTAGTACCATAAT
CAAACTACAGTAGTACTTTTGTTACCCTAAT
CAAACTTCAGTAGTATTGTAGTACCATAAC
CAAACTACAGTAGCACTGTAGTACCATAAC
CAAACTACAGTAGTACTGTAGTATTATAACC
CAAACTACAGTAGCACTGTAGTACCATAAC
CAAACTACAGTAGTACTGTAGTACCATAAT
CAAACTACAGTAGTACTTTTGTTACCCTAAT
CAAACTTCAGTAGTATTGTAGTACCATAAC
CAAACTACAGTAGTACTGTAGTACCATAAT
CAAACTACAGTAGTACTTTTGTTACCCTAAT
CAAACTTCAGTAGTATTGTAGTACCATAAC
CAAACTACAGTAGCACTGTAGTACCATAAC
CAAACTACAGTAGTACTGTAGTACCATAAT
CAAACTACAGTAGTACTTTTGTTACCCTAAT
CAAACTTCAGTAGTATTGTAGTACCATAAC
CAAACTATAGTAGTACTGTAGTACCATAAT
CAAACTACAGTAGTACTTTTGTTACCCTAAT
CAAACTTCAGTAGTATTGTAGTACCATAAC
CAAACTACAGTAGCACTGTAGTACCATAAC
CAAACTACAGTAGTACTGTAGTACCATAAC
CAAACTACAGTAGTACTGTAGTACCATAAT
CAAACTACAGTAGTACTTTGTTACCCTAAT

CAAACTTCAGTAGTACTGTAGTACCATAAC
CAAACTACAGTAGCACTGTAGTACCATAAT
CAAACTACAGTAGTACTTTTGTTACCCTAAT
CAAACTTCAGTAGTATTGTAGTACCATAAC
CAAACTATAGTAGTACTGTAGTACCATAAT
CAAACTACAGTAGTACTTTTGTTACCCTAAT
CAAACTTCAGTAGTATTGTAGTACCATAAC
CAAACTACAGTAGCACTGTAGTACCATAAC
CAAACTACAGTAGTACTGTAGTACCATAAC
CAAACTACAGTAGTACTGTAGTACCATAAT
CAAACTACAGTAGTACTTTGTTACCCTAAT
CAAACTTCAGTAGTACTGTAGTACCATAAC
CAAACTACAGTAGCACTGTAGTACCATAAC
CAAACTACAGTAGTACTGTAGTACCATAAT
CAAACTACAGTAGTACTTTGTTACCCTAAT
CAAACTTCAGTAGTACTGTAGTACCATAAC
CAAACTACAGTAGTACTGTAGTACCATAAC
CAAACTACAGTAGTACTTTGTTACCCTAAT
CAAACTTCAGTAGTATTGTAGTACCATAAC
CAAACTACAGTAGCACTGTAGTACCATAAC
CAAACTACAGTAGTACTGTAGTACCATAAC
CAAACTACAGTAGTACTTTGTTACCCTAAT
CAAACTTCAGTAGTATTGTAGTACCATAAC
CAAACTACAGTAGCACTGTAGTACCATAAC
CAAACTACAGTAGTACTGTAGTACCATAAC
CAAACTACAGTAGTACTTTGTTACCCTAAT
CAAACTTCAGTAGTATTGTAGTACCATAAC
CAAACTACAGTAGCACTGTAGTACCATAAC
CAAACTACAGTAGTACTGTAGTACCATAAT
CAAACTACAGTAGTACTTTGTTACCATAAC
Nr of selected repeats 47 Similarity 0.880029
Consensus:
CAAACTaCAGTAGtAcTGTAGTACCATAAc
>Cele-UNSB01_2:12381269-12381383 Satlength=115 Nr of Repeats=7 RepeatLength=12
seed=TTAGTCTTAG
TTAGTCTTAGTC
TTAGTCTTAGGC
TTAGGCTTAGCC
TTAGCCTTAGTTTTAGGCCCGGGTTCAGGC
TTAGCCTTAGGCTTAGGCTTTGGC
TTAGTCTTAGGC
TTAGTCTTAGGA
Nr of selected repeats 5 Similarity 0.833333
Consensus:
TTAGtCTTAGgc
>Cele-UNSB01_2:12388949-12389045 Satlength=97 Nr of Repeats=7 RepeatLength=12
seed=CCTAAGCCTA
CCTAAGCCTAAG
CCTAACCCTAAG
CCTAAGCCTACA
CCTAAGACTACA
CCTAAGCCTAAG
CCTAAGCCTATGCCCAAGTCTAAGA
CTAAGCCTAAG

Nr of selected repeats 5 Similarity 0.741026
Consensus:
CCTAAgcCTAAg
>Cele-UNSB01_2:12463695-12463773 Satlength=79 Nr of Repeats=5 RepeatLength=12
seed=TTAGGCTTAG
TTAGGCTTAGGT
TTAGGCTCAGGC
TTAGGCTTAGGC
TTAGGCGTAGAC
TTAGACTTAGGCTTAGGTGTAGACTTAGAC
Nr of selected repeats 4 Similarity 0.777778
Consensus:
TTAGGCttAGgc
>Cele-UNSB01_2:12477614-12477893 Satlength=280 Nr of Repeats=4 RepeatLength=69
seed=AGGTAGGCCT
AGGTAGGCCTGTAGGCATTGACTGGCAGAAAAGTGTAGGCAGGCAGGCAGGTAAAAAGATACATGCGGC
AGGTAGGCCTGTAAGCACTGACATGCGCAAAAGCGTAGACAGGTAAACAGGTAAAACGATAGTTGAGAC
AGGTAGGCCTGTAGGCATTGACTGTCAGAAAAGCGTAGACAGGCAAGCTGATAAAAAGATACATGAGGC
AGGTAGGCCTGTAGGCATAGACTAGTCACAAAAATGTAGGCAAGCGGGTGAAAAGGTACCTGGACATGCGGA
Nr of selected repeats 3 Similarity 0.747619
Consensus:
AGGTAGGCCTGTAgGCAtTGACTGnCagAAAAGcGTAGaCAGGcAagCaGgTAAAAaGATAcaTGaGgC
>Cele-UNSB01_2:12492091-12492147 Satlength=57 Nr of Repeats=5 RepeatLength=11
seed=CTACAGTACC
CTACAGTACCT
CTACAGTACCC
CTACAGTACCT
CTACAGTACCC
CTACAGTACCCC
Nr of selected repeats 4 Similarity 0.919192
Consensus:
CTACAGTACCc
>Cele-UNSB01_2:12494810-12494978 Satlength=169 Nr of Repeats=14 RepeatLength=12
seed=CCTATACCTA
CCTATACCTATG
CCTATACCTATA
CCTATACCTATA
CCTATACCTATA
CCTATACCTATA
CCTATACCTATA
CCTATACCTATA
CCTATACCTATA
CCTATACCTATA
CCTATACCTATA
CCTATACCTACA
CCTATACCTATA
CCTATACCTATA
CCTATACCTATA
Nr of selected repeats 14 Similarity 0.968254
Consensus:
CCTATACCTATA
>Cele-UNSB01_2:12522288-12522472 Satlength=185 Nr of Repeats=13 RepeatLength=12
seed=AGCCTAAGCC
AGCCTAAGCCTA

AGCCTAAGCCTA
AGCCTAAGGCTAACCCAA
AGCCTAAGCCTAAGCCT
AGCTCTAAGCCTT
AGCCTAAGCCTA
AGCCAAAGCCTA
AGCCAAAGCCTA
AGCCAAAGCCTA
AGCCTAAGTCTA
AGCCTAGCCT
AGCCTAAGCCTAAGAGTAAGCCTAACTCTA
AGCCTTAGCCTA
Nr of selected repeats 8 Similarity 0.884921
Consensus:
AGCCtAAGCCTA
>Cele-UNSB01_2:12544046-12545336 Satlength=1291 Nr of Repeats=30 RepeatLength=43 seed=TTTCCGGCAA
TTTCCGGCAAACCGGCAAACCGGTGATTTGCCGAAAATGCGAA
TTTCCGGCAAATCGGCAAACCGGCATATTGCCAAAAATAAAAA
TTTCCGGCAAATCGGCAAACCGGCAATTTGCCGGAAACAAAAA
TTTCCGGCAAATCGGCAAACCGGCGATTTGCCGAAAATGAAAG
TTTCCGGCAAATCGGCAAACCGATGATTTGCCGAAAATGAAAA
TTTCCGGCAAATCGGCAAACCGGCAATTTGTCGAAAATGAAAA
TTTCCGGCAAATCGGCAAACCGGCATTTTACCAAAAATAAAAA
TTTCCGGCAAATCGGCAAACCGGCATATTGCCAAAAATAAAAA
TTTCCGGCAAATCGGCAAACCGGCATTTTGCCGAAAATGAAAG
TTTCCGGCAAATCGGCAAACCGGTGATTTGCCGAAAATGAAAA
TTTCCGGCAAATCGGCAAACCGTCAATTTGTCGAAAATGAAAA
TTTCCGGCAAATCGGCAAACCGGCATTTTACCAAAAATAAAAA
TTTCCGGCAAATCCGCAAACCGGCATTTTGCCGAAAATGAAAG
TTTCCGGCAAATCGGCAAACCGGTGATTTGCCGAAAATGAAAA
TTTCCGGCAAATCGGCAAACCGTCAATTTGTCGAAAATGAAAA
TTTCCGGCAAATCGGCAAACCGGCGATTTGCCGAAAATGAAAG
TTTCCGGCAAATCGGCAAACCGATGATTTGCCGAAAATGAAAA
TTTCCGGCAAATCGGCAAACCGGCAATTTGTCGAAAATGAAAA
TTTCCGGCAAATCGGCAAACCGGCATTTTACCAAAAATAAAAA
TTTCCGGCAAATCGGCAAACCGGCATATTGCCAAAAATAAAAA
TTTCCGGCAAATCGGCAAACCGGCATTTTGCCGAAAATGAAAG
TTTCCGGCAAATCGGCAAACCGGTGATTTGCCGAAAATGAAAA
TTTCCGGCAAATCGGCAAACCGTCAATTTGTCGAAAATGAAAA
TTTCCGGCAAATCGGCAAACCGGCATTTTACCAAAAATAAAAA
TTTCCGGCAAATCCGCAAACCGGCATATTGCCAAAAATAAAAA
TTTCCGGCAAATCGGCAAACCGGCATTTTGCCGAAAATGAAAG
TTTCCGGCAAATCGGCAAACCGGTGATTTGCCGAAAATGAACA
TTTCCGGCAAATCGGCAAACCGGCATTTTGCCGAAAATGAAAG
TTTCCGGCAAATCGGCAAACCGGTGATTTGCCGAAAATGAACA
TTTCCGGCAAATCGGCAAACCGGCGATTTGCCGAAAATGAAAA
Nr of selected repeats 30 Similarity 0.867914
Consensus:
TTTCCGGCAAATCGGCAAACCGGcaaTTTGCCgAAAATgAAAa
>Cele-UNSB01_2:12597085-12597273 Satlength=189 Nr of Repeats=6 RepeatLength=31 seed=GACATCTCGC
GACATCTCGCAGGATCCTTACTGTTAGCGGCA
GACATCTCGCAGGACAATATAAAAAGCGACC

GACACCTCGCAGGACATTATAGGAAACGCCC
GACATCTCGCAGGGCCTTACAATTAGCGACC
GACATCTCACAGGATCCCTATTGGTAGCGGCC
GACATGTCGCAAAACTTGAAAGGTAGAGTTC
Nr of selected repeats 4 Similarity 0.584229
Consensus:
GACAtcTCGCAggaCattAtAagaAgcGacC
>Cele-UNSB01_2:12608492-12608559 Satlength=68 Nr of Repeats=5 RepeatLength=12
seed=GGCTTAGGCT
GGCTTAGGCTTA
GGCTTAGGCTTA
GGCTTAGGATTA
GGCTTAGGCTTA
GGCTTAGGCTCAGGCTTAG
Nr of selected repeats 4 Similarity 0.944444
Consensus:
GGCTTAGGcTTA
>Cele-UNSB01_2:12716681-12716766 Satlength=86 Nr of Repeats=5 RepeatLength=17
seed=ACTTGGTACA
ACTTGGTACATAGGCCA
ACTTGGTACATAGACCA
ACTTGGTACATAGGCCA
ACTTGGTACATAGACCA
ACTTGATACATAGGCCT
Nr of selected repeats 5 Similarity 0.890196
Consensus:
ACTTGgTACATAGgCCa
>Cele-UNSB01_2:12718309-12718687 Satlength=379 Nr of Repeats=8 RepeatLength=42
seed=TTGCCGAACC
TTGCCGAACCGTAAAAATTTCGGCAAACGGGAATTGCCAAAG
TTGCCGAACCGTAAAAATTTTGGCAACCTGGAACTGCCAAAG
TTGCCGAACCGTAAAAATTTCGGCAACCGGAATTTGCCGAAA
TTGCCGAATCCAAAAAATTTCGGCAACCTGGAATTGCCAAAG
TTGCCGAACCGTAAAAATTTCGGCAAACGGGAATTGCCAAAG
TTGCCGAACCCAAAAATTTTCGGCAACCGGAATTTGCCGAAA
TTGCCGAATCCAAAAAATTTCGGCAAACGGGAATTGCCAAAG
TTGCCGAACCCAAAAATTTTCGGCAACCGGCAACTGCCAAAGTTGCCGCACTGTAAAAATTTCGGCAACCGAAAATTGT
CGAAA
Nr of selected repeats 7 Similarity 0.797619
Consensus:
TTGCCGAAcCgtAAAAATTTCGGCAAcCnGGAATTGCCAAAg
>Cele-UNSB01_2:12791702-12792113 Satlength=412 Nr of Repeats=11 RepeatLength=37
seed=ATTTTGGCGG
ATTTTGGCGGGAATTCAAATTTTAATTTTTTTAAAAT
ATTTTGGCGGGAATTTAAAATTTCAATTTTTTGAAAAT
ATTTTGGCGGGAATTCAAATTTTAATTTTTTGAAAAT
ATTTTGGCGGAAATTCAAATTTTAATTTTTTGAAAAT
ATTTTGGCGGGAATTCAAATTTTAATTTTTTGAAAAT
ATTTTGGCGGGAATTTAAAATTTTAATTTTTTGAAAAT
ATTTTGGCGGGAATTCAAATTTTAATTTTTTGAAAAT
ATTTTGGCGGGAATTTAAAATTTTAATTTTTTGAAAAT
ATTTTGGCGGAAATTCAAATTTTAATTTTTTGAAAAT
ATTTTGGCGGGAATTTAAAATTTTAATTTTTTGAAAAT
ATTTTGGCGGGAATTCAAAATTTAATTTTTTGAACAT

Nr of selected repeats 7 Similarity 0.951952
Consensus:
ATTTTGGCGGgAATTCAAATTTTAATTTTTTGAAAAT
>Cele-UNSB01_2:12792220-12794215 Satlength=1996 Nr of Repeats=131
RepeatLength=15 seed=TCGTGGTGAG
TCGTGGTGAGACCCA
TCGTGGTGAGACCCA
TCGTGGTGAGACCTT
TCGTGGAGAGACCCA
TCGTGGTGAGACCCT
TCGTGGTGAGACCCA
TCGGGGTGAGACCCA
TCGTGGTGAGACCTT
TCGTGGAGAGACCCA
TCGTGGTGAGACCCT
TCGTGGTGAGACCCT
TCGTGGTGAGACCTT
TCGTGGTGAGACCCA
TCGGGGTGAGACCCA
TCGTGGTGAGACCCA
TCGTGGTGAGACCCT
TCGTGGTCAGACCCA
TCGTGGTGATACCCA
TCGTGGTGAGACCCT
TCGTGGTGAGACCCA
TCGTGGTGAGACCTT
TCGTGGTGAGACCCA
TCGTGGTGAGACCCA
TCGTGGTGAGACTCA
TCGTGGTGAGACCCT
TCGTGGTGAGACCCT
TCGTGGTCAGACCCA
TCGTGGTGAGACCCA
TCGTGGTGAGACTTT
TCGTGGTGAGACCCA
TCGTGGTGAGACCCA
TCGTGGTGAGACCTT
TCGTGGTGAGACTCA
TCGTGGTGAGACCCT
TCGTGGTGAGACCCA
TCGTGGTGAGACCCT
TCGTGGTGAGACCCA
TCGTGGTGATACCCA
TCGTGGTGAGACCTT
TCGTGGTGAGACTCA
TCGTGGTGTGACCCT
TCGTGGTGAGACCCA
TCGTGGTGAGACCCA
TCGTGGTGAGACCTT
TCGTGGTGAGACCCA
TCGTGGTGAGACCCA
TCGTGGTGAGACCCA
TCGTGGTGAGACCCA
TCGTGGTGAGACCCT

TCGTGGCGAGACCCA
TCGTGGTGAGACCTT
TCGTGGTGAGACCCA
TCGTGGTGAGACCCA
TCGTGGTGAGACTCA
TCGTGGTGAGACCCT
TCGTGGTGAGACCCA
TCGTGATGAGACCCTTAGTGGTTAGACCCA
TCGTGGTGAGACCCT
TCGTGGTGAGACCTTTCATGGAGAGACCCA
TCGTGGTGAGACCCT
TCGTGGTTAGACCCA
TCGTGGTGAGACCTT
TCGTGGTGAGACCTT
TCGTGGTGAGACCCA
TCGTGGTGAGACCTT
TCGTGGTGAGACCCA
TCGTGGTGAGACCCT
TCGTGGTCAGATCCA
TCGTGGTGAGACCCA
TCGTGGTGAGACCCA
TCGTGGTGAGACCCA
TCGTGGTGAGACTCA
TCGTGGTGAGACCCA
TCGTGGTTAGACCCA
TCGTGGTGAGATCTT
TCGTGGTGAGACCCA
TCGTGGTGAGACCCA
TCGTGGTGAGACCCA
TCGTGGTGAGATCTT
TCGTGGTGAGACCCA
TCGTGGTGAGACCCA
TCGTGGTGAGACCCT
TCGTGGTTAGACCCA
TCGTGGTGAGACCCT
TCGTGGTTAGACCCA
TCGTGGTGAGATCTT
TCGTGGTGAGACCCA
TCGTGGTGAGACCCA
TCGTGGTGAGACCCA
TCGTGGTGAGACTCA
TCGTGGTGAGACCCA
TCGTGGTGATACCCA
TCGTGGTGAGACGCA
TCGTGGTGAGACCTT
TCGTGGTGAGACCTT
TCGTGGTGAGACCCA
TCGTGGTGAGACCCA
TCGTGGTGAGACCCA
TCGTGGTGAGACTCA
TCGTGGTGAGACCCA
TCGTGGTTAGACCCA
TCGTGGTGAGATCTT
TCGTGGTGAGACCCA

TCGTGGTGAGACCCA
TCGTGGTGAGACCCA
TCGTGGTGAGACTCA
TCGTGGTGAGACCCT
TCGTGGTTAGACCCA
TCGTGGTGAGACCCT
TCGTGGTTAGACCCA
TCGTGGTGAGATCTT
TCGTGGTGAGACCCA
TCGTGGTGAGACCCA
TCGTGGTGAGACCCA
TCGTGGTGAGACTCA
TCGTGGTGAGACCCA
TCGTGGTGATACCCA
TCGTGGTGAGACGCA
TCGTGGTGAGACCTT
TCGTGGTGAGACCTT
TCGTGGTGAGACCCA
TCGTGGTGAGACCTT
TCGTGGTGAGACCCT
TCGTGGTGAGACCAT
TCGTGGTCAGACCCA
TCGTGGTGAGACCCA
TCGTGGTGAGACCTT
TCGTGGTGAGACCCA
TCGTGGTGAGACTTT
TCGTGGTGAGACCAA
TCGTGGTGAGACCTT
Nr of selected repeats 129 Similarity 0.876421
Consensus:
TCGTGGTGAGACCca
>Cele-UNSB01_2:12843731-12843956 Satlength=226 Nr of Repeats=7 RepeatLength=11
seed=CTACAGTACC
CTACAGTACCCCGGCCATATCCCCCACTAACCCCAATCCAATACCTCTTTAAAAGACGAAAAGTCTTTTTTTTCCAAAA
CTACAGTAATC
CTACAGTACTC
CTACAGTACCT
CTACAGTACTG
CTACAGTACCCCAGTCATAACCCCCCTACTAACCTCAAAGCAATATCACTTCAAAAGACGAAAAGCCGATTTTTCCAAA
A
CTACAGTACCT
CTACAGTACTA
Nr of selected repeats 5 Similarity 0.775000
Consensus:
CTACAGTACTn
>Cele-UNSB01_2:12862834-12863019 Satlength=186 Nr of Repeats=5 RepeatLength=43
seed=TATTTTCATT
TATTTTCATTTTCGGCAATTTGCCGGTTTGCCGATTTGCCGGG
TATTTTCATTTTTTGGCAATTTGCCGGA
TATTTTCATTTTCGGCAAATTGCCGGTTTGCCGATTTGCCGGG
TATTTTCATTTTTTGGCAATTTGCCGGA
TATTTTCATTTTCGGCAAATTGCCGGTTTGCCGATTTGCCGGG
Nr of selected repeats 3 Similarity 0.979328
Consensus:

TATTTTCATTTTCGGCAAaTTGCCGGTTTGCCGATTTGCCGGG
>Cele-UNSB01_2:12892893-12893074 Satlength=182 Nr of Repeats=4 RepeatLength=45
seed=ATCTGATCCT
ATCTGATCCTTCAGCTCCGAAGGATCACAGGGGGTACCTCCTGATG
ATCTGATCCTTCAGCTCCGAAGGATCAGAAGGGTACCTCCTGGTG
ATCTGATCCTTAAGCTCCGAAGGATCATAAGGGTACCTCCTGGTG
ATCTGATCCTTCAGCTCCGAAGGATCACAAGGGTACCTCCTGGTG
Nr of selected repeats 3 Similarity 0.950617
Consensus:
ATCTGATCCTTcAGCTCCGAAGGATCAnAAGGGTACCTCCTGGTG
>Cele-UNSB01_2:12900948-12901228 Satlength=281 Nr of Repeats=8 RepeatLength=35
seed=ATTTCAGGCA
ATTTCAGGCAATTTGCTGATTTGCCGGAAATTACA
ATTTCAGGCAATTTACCGATTTGCCAGAAATGTTC
ATTTCCGGCAATTTGTCGATTTGCCGGAAATTTTC
ATTTCAGGCAATTTACCGATTTGCCAGAAATGTTC
ATTTCCGGCAATTTGTCGATTTGCCAGAAATTTTC
ATTTCAGGCAACTTGCCAATTTGCTGGAAATTTTC
ATTTCAGACAATTTACCGATTTGGCAGAAATGTTC
ATTTCCGGCAATTCGCCGATTTGCCAGAAATTTTC
Nr of selected repeats 8 Similarity 0.791832
Consensus:
ATTTCaGGCAATTTgcCGATTTGCCaGAAATtTTC
>Cele-UNSB01_2:12905171-12905894 Satlength=724 Nr of Repeats=9 RepeatLength=14
seed=AACTACAAAC
AACTACAAACTACA
AACTATAAACTACA
AACTATAAACTACA
AACTACAAACTACA
AACTACAAACTATA
AACTACAAACTACC
AACTACAAACAGCA
AACTACAAACTACAATCTACAAAACTCTACCACACGGTCAAATTTTTTAAAGCTTTTTTTTTGTTAGTAAAATAAAGGA
CAGTCATTAGATGGCTGAAAAAAAATTGGGCAAAAATAAATATTTGCCTAAATTTATTGAAAACGGGTAATTCTTGCGGA
TATATGCTTAATTCAGAAAATCTAGGTTTATCTTATCAAAACTATTGAAAAGTGGCAAAATTGGGCAATTTTTGGCAAA
AATTCACAATTTTGAAACTCCTCTAACATGGTTAATTTTGTAGTTAGAGGACTCAAAATTGATCTCAGAACCCTAAAAA
TTGTCCTTTTTCAGTTATCAGTAGTTTTGTTTAAATTCGTTTTTTAAAAAATTTTTTGCCCAAAAAAGGACAGTCAAAC
GAACAAAAAGGACAGGCTGAAAAAAAATTGGGCAAAAATAAAATATTGCCTAATTGTGTTGAAAACGGGAAATTCATATA
TGCCGAATTCAGAAAATCTAGGTTTAACCCATCAAAAACTTTTAAAAAGTGGCAAAACTGAACAATTTATGGTAAAAAA
TCACAACTCTCAAAGTTTCGTTGCTGACTGTAGTGTAGATTAAACTATACA
AACTACAAACTACAAACAACA
Nr of selected repeats 7 Similarity 0.845805
Consensus:
AACTAcAAACTACA
>Cele-UNSB01_2:12906532-12906652 Satlength=121 Nr of Repeats=7 RepeatLength=15
seed=ATTTCCGGAT
ATTTCCGGATCTACG
ATTTCCGGATCTATG
ATTTCCGGATCTACA
ATTTCCGGATCTATG
ATTTCCGGATCTACG
ATTTCCGGATCTACG
ATTTCCGGATCTATGATTTCCGAGTCTACG
Nr of selected repeats 6 Similarity 0.922963

Consensus:
ATTTCCGGATCTAcG
>Cele-UNSB01_2:12914019-12914481 Satlength=463 Nr of Repeats=11 RepeatLength=35
seed=TCAATTCCGG
TCAATTCCGGCAATTTTTGGATTTGCCGGAAATTT
TCAATTCCGGCAATTTTCCGGTTTGCCGGAAAAATT
TCAATTCCGGAAATTTGCCGATTTGCCGGAAATTT
TCAATTGCGGCAATTTGCCGATTTGCCGGAAATTT
TCAATTCCGGCAATTTTCCGGTTTGCCGGGAATTT
TCAATTCCGGAAATTTGCCGATTTGCCGGAAAAATT
TCAATTCCGGAAATTTGCCGATTTGCCGCAAATTT
TCAATTCCGGCAATTTGCCGATTTGCCGGAAATTT
TCAATTCCGGCAATTTTCCGATTTGCCGAAAGTTT
TCAATTCCGGCAATTTTCCGATTCGCCGGAAATTTTCTTTTTCGGCAAATTGCCGATTTGCCGAAAATTTCAAATCCGA
CAATTCGCCGGTTTGCCGATTTTCTGGAAAGTC
TCAATTCCGGCAATTTGTTGATTTGCCGGAAATTT
Nr of selected repeats 10 Similarity 0.869630
Consensus:
TCAATTCCGGcAATTTgccGaTTTGCCGgAAAtTT
>Cele-UNSB01_2:12938825-12939101 Satlength=277 Nr of Repeats=8 RepeatLength=35
seed=TGTGTAATAA
TGTGTAATAATAAAATATCACTTTTTTTTCAGGAT
TGTGTAATAATAAAATTTCACATTTTTTCACGAA
TGTGTAATAAGAAAAGTTCGATTTTTTCACGAG
TATGTAATAAGAAAATTTCACATTTTTTCACGAG
TGTGTAATAAGAAAATTTCAAATGTTTTTCACGAG
TGTGTAATAAGAAAATTTCAAATTTTTTAAACGAA
TGTGTAATAAGAAAATTTCAAATTTTTTTCAAAAA
TATGTAATAAGAAAATTTCAAATTTTTTTCACGTA
Nr of selected repeats 5 Similarity 0.794286
Consensus:
TgTGTAATAAgAAAATtTCAaaTtTTTTtcAcgaa
>Cele-UNSB01_2:12968710-12969129 Satlength=420 Nr of Repeats=28 RepeatLength=12
seed=GGCTTAGGCT
GGCTTAGGCTTA
GGCTTAGGCTTA
GGCCTAGGCTTG
GGCTTAGGCTTGGGTTTA
GGATTAGGCTGA
GGCTTTGGCTTAGACTCA
GGCTTAGGCTAA
GGCTTAGGCTTATATTTATGTTTA
GGCTTAGGCTTAGACTCA
GGCTTAGGCTT
GGCTTAGGCTTA
GGCTGAGGCTTG
GGCTTAGACTCA
GGCTTGGGCTTA
GGCTGAGGCTGA
GACTTAGGCTTA
GGCTTGGGCTTAAGCTTG
GGCTTAGGCTTA
GGCTGAGGCTTG
GGCTTAGACTCA

GGCTAAGGCTTAGGATTAGGCGTC
GGCTTAGGCTTAA
GCTTAGGCTTA
GGCTTAGGCTTA
GGCTTAGGCTTA
GGCTTAGACTAAAGTTTAGGCATAAGTTTA
GGCTTAGGCTTA
GACTTAGGCTTATGCCTAGGCTTCTGCTTA
Nr of selected repeats 17 Similarity 0.799020
Consensus:
GGCTTAGGCTtA
>Cele-UNSB01_2:12988123-12990065 Satlength=1943 Nr of Repeats=72
RepeatLength=27 seed=GTGGGAAGCC
GTGGGAAGCCACAGAGAGTAAAATATA
GTGGGAAGCCACAGAGAGTAAAATATA
GTGGGAAGCCACAGAGAGTAAAATATA
GTGGGAAGCCACAGAGAGTAAAATATA
GTGGGAAGCCACAGAGAGTAAAATATA
GTGGGAAGCCACAGAGAGTAAAATATA
GTGGGAAGCCACAGAGAGTAAAATATA
GTGGGAAGCCACAGAGAGTAAAATATA
GTGGGAAGCCATAGAGAGTAAAATATA
GTGGGAAGCCATAGAGAGTAAAATATA
GTGGGAAGCCACAGAGAGTAAAATATA
GTGGGAAGCCACAGAGAGTAAAATATA
GTGGGAAGCCATAGAGAGTAAAATATA
GTGGGAAGCCATAGAGAGTAAAATATA
GTGGGAAGCCATAGAGAGTAAAATATA
GTGGGAAGCCACAGAGAGTAAAATATA
GTGGGAAGCCACAGAGAGTAAAATATA
GTGGGAAGCCATAGAGAGTAAAATATA
GTGGGAAGCCACAGAGAGTAAAATATA
GTGGGAAGCCATAGAGAGTAAAATATA
GTGGGAAGCCATAGAGAGTAAAATATA
GTGGGAAGCCATAGAGAGTAAAATATA
GTGGGAAGCCATAGAGAGTAAAATATA
GTGGGAAGCCATAGAGAGTAAAATATA
GTGGGAAGCCACAGAGAGTAAAATATA
GTGGGAAGCCACAGAGAGTAAAATATA
GTGGGAAGCCACAGAGAGTAAAATATA
GTGGGAAGCCATAGAGAGTAAAATATA
GTGGGAAGCCACAGAGAGTAAAATATA
GTGGGAAGCCATAGAGAGTAAAATATA
GTGGGAAGCCATAGAGAGTAAAATATA
GTGGGAAGCCATAGAGAGTAAAATATA
GTGGGAAGCCATAGAGAGTAAAATATA
GTGGGAAGCCACAGAGAGTAAAATATA
GTGGGAAGCCATAGAGAGTAAAATATA
GTGGGAAGCCATAGAGAGTAAAATATA
GTGGGAAGCCATAGAGAGTAAAATATA
GTGGGAAGCCATAGAGAGTAAAATATA
GTGGGAAGCCATAGAGAGTAAAATATA
GTGGGAAGCCATAGAGAGTAAAATATA
GTGGGAAGCCATAGAGAGTAAAATATA

GTGGGAAGCCATAGAGAGTAAAATATA
GTGGGAAGCCATAGAGAGTAAAATATA
GTGGGAAGCCATAGAGAGTAAAATATA
GTGGGAAGCCACAGAGAGTAAAATATA
GTGGGAAGCCATAGAGAGTAAAATATA
GTGGGAAGCCACAGAGAGTAAAATATA
GTGGGAAGCCATAGAGAGTAAAATATA
GTGGGAAGCCATAGAGAGTAAAATATA
GTGGGAAGCCATAGAGAGTAAAATATG
GTGGGAAGCCAGAGAGTAAAATATG
GTGGGAAGCCATAGAGAGTAAAATATA
GTGGGAAGCCATAGAGAGTAAAATATA
GTGGGAAGCCACAGAGAGTAAAATATA
GTGGGAAGCCACAGAGAGTAAAATATA
GTGGGAAGCCACAGAGAGTAAAATATA
GTGGGAAGCCATAGAGAGTAAAATATA
GTGGGAAGCCATAGAGAGTAAAATATA
GTGGGAAGCCATAGAGAGTAAAATATA
GTGGGAAGCCATAGAGAGTAAAATATA
GTGGGAAGCCATAGAGAGTAAAATATA
GTGGGAAGCCATAGAGAGTAAAATATA
GTGGGAAGCCACAGAGAGTAAAATATA
GTGGGAAGCCATAGAGAGTAAAATATA
GTGGGAAGCCATAGAGAGTAAAATATA
GTGGGAAGCCATAGAGAGTAAAATATA
GTGGGAAGCCATAGAGAGTAAAATATA
GTGGGAAGCCATAGAGAGTAAAATATA
GTGGGAAGCCATAGAGAGTAAAATATA
GTGGGAAGCCACAGAGAGTAAAATATA
GTGGGAAGCCACAGAGAGTAAAATATA
GTGGGAAGCCATAGAGAGTAAAATATA
Nr of selected repeats 71 Similarity 0.975358
Consensus:
GTGGGAAGCCAtAGAGAGTAAAATATA
>Cele-UNSB01_2:12990090-13000798 Satlength=10709 Nr of Repeats=416
RepeatLength=25 seed=TGGCTTCCCA
TGGCTTCCCACTATATTTTACTCTCTG
TGGCTTCCCACTATATTTTACTCTCTG
TGGCTTCCCACTATATTTTACTCTC
TGGCTTCCCACTATATTTTACTCTC
TGGCTTCCCACTATATTTTACTCTC
TGGCTTCCCACTATATTTTACTCTC
TGGCTTCCCACCATATTTTACTCTC
TGGCTTCCCACCATATTTTACTCTC
TGGCTTCCCACTATATTTTACTCTC
TGGCTTCCCACTATATTTTACTCTC
TGGCTTCCCACTATATTTTACTCTC
TGGCTTCCCACTATATTTTACTCTC
TGGCTTCCCACTATATTTTACTCTC
TGGCTTCCCACTATATTTTACTCTC
TGGCTTCCCACTATATTTTACTCTC
TGGCTTCCCACCATATTTTACTCTC
TGGCTTCCCACTATACTCTC
TGGCTTCCCACCATATTTTACTCTC

TGGCTTCCCACTATATTTTACTCTC
TGGCTTCCCACTATATTTTACTCTC
TGGCTTCCCACTATATTTTACTCTC
TGGCTTCCCACTATATTTTACTCTC
TGGCTTCCCACTATATTTTACTCTC
TGGCTTCCCACTATATTTTACTCTC
TGGCTTCCCACTATATTTTACTCTC
TGGCTTCCCACTATATTTTACTCTC
TGGCTTCCCACTATATTTTACTCTC
TGGCTTCCCACTATATTTTACTCTC
TGGCTTCCCACTATATTTTACTCTC
TGGCTTCCCACTATATTTTACTCTC
TGGCTTCCCACTATATTTTACTCTC
TGGCTTCCCACTATATTTTACTCTC
TGGCTTCCCACTATATTTTACTCTC
TGGCTTCCCACTATATTTTACTCTC
TGGCTTCCCACTATATTTTACTCTC
TGGCTTCCCACTATATTTTACTCTC
TGGCTTCCCACTATATTTTACTCTC
TGGCTTCCCACTATATTTTACTCTC
TGGCTTCCCACTATATTTTACTCTCTA
TGGCTTCCCACTATATTTTACTCTCTA
TGGCTTCCCACTATATTTTACTCTCT
TGGCTTCCCACTATATTTTACTCTCTA
TGGCTTCCCACTATATTTTACTCTCTA
TGGCTTCCCACTATATTTTACTCTCTA
TGGCTTCCCACTATATTTTACTCTCT
TGGCTTCCCACTATATTTTACTCTCTA
TGGCTTCCCACTATATTTTACTCTCT
TGGCTTCCCACTATATTTTACTCTCTA
TGGCTTCCCACTATATTTTACTCTCTA
TGGCTTCCCACTATATTTTACTCTCTG
TGGCTTTCCACTATATTTTACTCTC
TGGCTTCCCACTATATTTTACTCTCT
TGGCTTCCCACTATATTTTACTCTC
TGGCTTCCCACTATATTTTACTCTC
TGGCTTCCCACTATATTTTACTCTCT
TGGCTTCCCACTATATTTTACTCTCT
TGGCTTCCCACTATATTTTACTCTCT
TGGCTTCCCACTATATTTTACTCTC
TGGCTTCCACTATATTTTACTCTCTG
TGGCTTCCCACTATATTTTACTCTCTG
TGGCTTCCCACTATATTTTACTCTCTG
TGGCTTCCCACTATATTTTACTCTCTG
TGGCTTCCCACTATATTTTACTCTCTG
TGGCTTCCCACTATATTTTACTCTCTG
TGGCTTCCCACTATATTTTACTCTCTG
TGGCTTCCCACTATATTTTACTCTCT
TGGCTTCCCACTATATTTTACTCTC
TGGCTTCCCACTATATTTTACTCTCTG
TGGCTTCCCACTATATTTTACTCTC
TGGCTTCCCACTATATTTTACTCTC
TGGCTTCCCACTATATTTTACTCTC
TGGCTTCCCACTATATTTTACTCTC

TGGCTTCCCACTATATTTTACTCTC
TGGCTTCCCACTATATTTTACTCTCTG
TGGCTTCCCACTATATTTTACTCTCT
TGGCTTCCCACTATATTTTACTCTCTG
TGGCTTCCCACTATATTTTACTCTCTG
TGGCTTCCCACTATTTTATTTGGCTTATTTTACTCTC
TGGCTTCCCACTATATCTCTCTTATATTTTTACTCTC
TGGCTTCCCACTATATTTTACTCTC
TGGCTTCCCACTATATTTTACTCTC
TGGCTTCCCACTATATTTTACTCTC
TGGCTTCCCACTATATTTTACTCTC
TGGCTTCCCACTATATTTTACTCTC
TGGCTTCCCACTATATTTTACTCTC
TGGCTTCCCACTATATTTTACTCTC
TGGCTTCCCACTATATTTTACTCTC
TGGCTTCCCACTATTTTTTTTTTTTATTTTACTCTC
TGGCTTCCCACTATATTTTACTCTC
TGGCTTCCCACTATATTTTACTCTC
TGGCTTCCCACTATATTTTACTCTC
TGGCTTCCCACTATATTTTACTCTCTG
TGGCTTCCCACTATATTTTACTCTC
TGGCTTCCCACTATATTTTACTCTC
TGGCTTCCCACTATATTTTACTCTC
TGGCTTCCCACTATATTTTACTCTC
TGGCTTCCCACTATATTTTACTCTCTGTGGCTTCACCGATTATTTTACTCTC
TGGCTTCCCACTATATTTTACTCTC
TGGCTTCCCACTATATTTTACTCTC
TGGCTTCCCACTATATTTTACTCTC
TGGCTTCCCACTATATTTTACTCTC
TGGCTTCCCACTATATTTTACTCTC
TGGCTTCCCACTATATTTTACTCTC
TGGCTTCCCACTATATTTTACTCTC
TGGCTTCCCACTATATTTTACTCTC
TGGCTTCCCACTATATTTTACTCTC
TGGCTTCCCACTATATTTTACTCTC
TGGCTTCCCACTATATTTTACTCTC
TGGCTTCCCACTATATTTTACTCTC
TGGCTTCCCACTATATTTTACTCTC
TGGCTTCCCACTATATTTTACTCTC
TGGCTTCCCACTATATTTTACTCTC
TGGCTTCCCACTATATTTTACTCTC
TGGCTTCCCACTATATTTTACTCTCTG
TGGCTTCCCACTATATTTTACTCTC
TGGCTTCCCACTATATTTTACTCTC
TGGCTTCCCACTATATTTTACTCTC
TGGCTTCCCACTATATTTTACTCTCTG
TGGCTTCCCACTATATTTTACTCTCT
TGGCTTCCCACTATATTTTACTCTCT
TGGCTTCCCACTATATTTTACTCTCTG
TGGCTTCCCACTATATTTTACTCTCTG
TGGCTTCCCACTATATTTTACTCTCTG
TGGCTTCCCACTATATTTTACTCTC
TGGCTTCCCACTATATTTTACTCTC
TGGCTTCCCACTATATTTTACTCTCTG

TGGCTTCCCACTATATTTTACTCTC
TGGCTTCCCACTATATTTTACTCTCTG
TGGCTTCCCACTATATTTTACTCTCTG
TGGCTTCCCACTATATTTTACTCTC
TGGCTTCCCACTATATTTTACTCTC
TGGCTTCCCACTATATTTTACTCTC
TGGCTTCCCACTATATTTTACTCTC
TGGCTTCCCACTATATTTTACTCTC
TGGCTTCCCACTATATTTTACTCTC
TGGCTTCCCACTATATTTTACTCTCTG
TGGCTTCCCACTATATTTTACTCTCTG
TGGCTTCCCACTATATTTTACTCTCT
TGGCTTCCCACTATATTTTACTCTCT
TGGCTTCCCACTATATTTTACTCTCTG
TGGCTTCCCACTATATTTTACTCTCTG
TGGCTTCCCACTATATTTTACTCTCTG
TGGCTTCCCACTATATTTTACTCTCTG
TGGCTTCCCACTATATTTTACTCTC
TGGCTTCCCACTATATTTTACTCTCTG
TGGCTTCCCACTATATTTTACTCTCTG
TGGCTTCCCACTATATTTTACTCTCT
TGGCTTCCCACTATATTTTACTCTC
TGGCTTCCCACTATATTTTACTCTC
TGGCTTCCCACTATATTTTACTCTCTG
TGGCTTCCCACTATATTTTACTCTC
TGGCTTCCCACTATATTTTACTCTCTG
TGGCTTCCCACTATATTTTACTCTC
TGGCTTCCCACTATATTTTACTCTC
TGGCTTCCCACTATATTTTACTCTC
TGGCTTCCCACTATATTTTACTCTC
TGGCTTCCCACTATATTTTACTCTC
TGGCTTCCCACTATATTTTACTCTC
TGGCTTCCCACTATATTTTACTCTC
TGGCTTCCCACTATATTTTACTCTC
TGGCTTCCCACTATATTTTACTCTC
TGGCTTCCCACTATATTTTACTCTC
TGGCTTCCCACTATATTTTACTCTC
TGGCTTCCCACTATATTTTACTCTC
TGGCTTCCCACTATATTTTACTCTC
TGGCTTCCCACTATATTTTACTCTC
TGGCTTCCCACTATATTTTACTCTC
TGGCTTCCCACTATATTTTACTCTC
TGGCTTCCCACTATATTTTACTCTC
TGGCTTCCCACTATATTTTACTCTC
TGGCTTCCCACTATATTTTACTCTC
TGGCTTCCCACTATATTTTACTCTC
TGGCTTCCCACTATATTTTACTCTC
TGGCTTCCCACTATATTTTACTCTC
TGGCTTCCCACTATATTTTACTCTC
TGGCTTCCCACTATATTTTACTCTC
TGGCTTCCCACTATATTTTACTCTC
TGGCTTCCCACTATATTTTACTCTC
TGGCTTCCCACTATATTTTACTCTC
TGGCTTCCCACTATATTTTACTCTC

TGGCTTCCCACTATATTTTACTCTC
TGGCTTCCCACTATATTTTACTCTCTG
TGGCTTCCCACTATATTTTACTCTCTG
TGGCTTCCCACTATATTTTACTCTC
TGGCTTCCCACTATATTTTACTCTCTG
TGGCTTCCCACTATATTTTACTCTCTG
TGGCTTCCCACTATATTTTACTCTC
TGGCTTCCCACTATATTTTACTCTC
TGGCTTCCCACTATATTTTACTCTC
TGGCTTCCCACTATATTTTACTCTCTG
TGGCTTCCCACTATATTTTACTCTCTG
TGGCTTCCCACTATATTTTACTCTCTG
TGGCTTCCCACTATATTTTACTCTC
TGGCTTCCCACTATATTTTACTCTC
TGGCTTCCCACTATATTTTACTCTC
TGGCTTCCCACTATATTTTACTCTC
TGGCTTCCCACTATATTTTACTCTC
TGGCTTCCCACTATATTTTACTCTC
TGGCTTCCCACTATATTTTACTCTC
TGGCTTCCCACTATATTTTACTCTCTG
TGGCTTCCCACTATATTTTACTCTCTG
TGGCTTCCCACTATATTTTACTCTCTA
TGGCTTCCCACTATATTTTACTCTCTG
TGGCTTCCCACTATATTTTACTCTCTG
TGGCTTCCCACTATATTTTACTCTCT
TGGCTTCCCACTATATTTTACTCTCTG
TGGCTTCCCACTATATTTTACTCTC
TGGCTTCCCACTATATTTTACTCTC
TGGCTTCCCACTATATTTTACTCTCTG
TGGCTTCCCACTATATTTTACTCTCTG
TGGCTTCCCACTATATTTTACTCTCT
TGGCTTCCCACTATATTTTACTCTCT
TGGCTTCCCACTATATTTTACTCTCTA
TGGCTTCCCACTATATTTTACTCTCTA
TGGCTTCCCACTATATTTTACTCTCTG
TGGCTTCCCACTATATTTTACTCTCT
TGGCTTCCCACTATATTTTACTCTCTG
TGGCTTCCCACTATATTTTACTCTCTG
TGGCTTCCCACTATATTTTACTCTC
TGGCTTCCCACTATATTTTACTCTC
TGGCTTCCCACTATATTTTACTCTC
TGGCTTCCCACTATATTTTACTCTCTG
TGGCTTCCCACTATATTTTACTCTCTG
TGGCTTCCCACTATATTTTACTCTC
TGGCTTCCCACTATATTTTACTCTC
TGGCTTCCCACTATATTTTACTCTC
TGGCTTCCCACTATATTTTACTCTCTG
TGGCTTCCCACTATATTTTACTCTC
TGGCTTCCCACTATATTTTACTCTC
TGGCTTCCCACTATATTTTACTCTC
TGGCTTCCCACTATATTTTACTCTCT
TGGCTTCCCACTATATTTTACTCTCTA
TGGCTTCCCACTATATTTTACTCTCTG
TGGCTTCCCACTATATTTTACTCTCT

TGGCTTCCCACTATATTTTACTCTCTG
TGGCTTCCCACTATATTTTACTCTCTG
TGGCTTCCCACTATATTTTACTCTCTG
TGGCTTCCCACTATATTTTACTCTCTG
TGGCTTCCCACTATATTTTACTCTCTG
TGGCTTCCCACTATATTTTACTCTCTG
TGGCTTCCCACTATATTTTACTCTCTA
TGGCTTCCCACTATATTTTACTCTC
TGGCTTCCCACTATATTTTACTCTCTG
TGGCTTCCCACTATATTTTACTCTC
TGGCTTCCCACCATATTTTACTCTC
TGGCTTCCCACTATATTTTACTCTC
TGGCTTCCCACCATATTTTACTCTA
TGGCTTCCCACTATATTTTACTCTC
TGGCTTCCCACTATATTTTACTCTC
TGGCTTCCCACTATATTTTACTCTC
TGGCTTCCCACTATATTTTACTCTC
TGGCTTCCCACTATATTTTACTCTC
TGGCTTCCCACTATATTTTACTCTC
TGGCTTCCCACTATATTTTACTCTC
TGGCTTCCCACTATATTTTACTCTC
TGGCTTCCCACTATATTTTACTCTC
TGGCTTCCCACTATATTTTACTCTC
TGGCTTCCCACTATATTTTACTCTC
TGGCTTCCCACTATATTTTACTCTC
TGGCTTCCCACTATATTTTACTCTC
TGGCTTCCCACTATATTTTACTCTC
TGGCTTCCCACTATATTTTTTTTTACTATATTTTACTCTC
TGGCTTCCCACTATATTTTACTCTC
TGGCTTCCCACTATATTTTACTCTC
TGGCTTCCCACTATATTTTACTCTC
TGGCTTCCCACTATATTTTACTCTC
TGGCTTCCCACTATATTTTACTCTC
TGGCTTCCCACTATATTTTACTCTC
TGGCTTCCCACTATATTTTACTCTC
TGGCTTCCCACCATATTTTACTCTC
TGGCTTCCCACTATATTTTACTCTC
TGGCTTCCCACTATATTTTACTCTC
TGGCTTCCCACTATATTTTACTCTC
TGGCTTCCCACTATATTTTACTCTCTA
TGGCTTCCCACTATATTTTACTCTCT
TGGCTTCCCACTATATTTTACTCTC
TGGCTTCCCACCATATTTTACTCTCTA
TGGCTTCCCACTATATTTTACTCTCTA
TGGCTTCCCACTATATTTTACTCTC
TGGCTTCCCACTATATTTTTCTTCTTATTTTACTCTC
TGGCTTCCCACTATATTTTACTCTCT
TGGCTTCCCACTATATTTTACTCTCTA
TGGCTTCCCACTATATTTTACTCTCT
TGGCTTCCCACTATATTTTACTCTC
TGGCTTCCCACTATATTTTACTCTC
TGGCTTCCCACTATATTTTACTCTC
TGGCTTCCCACTATATTTTACTCTC
TGGCTTCCCACTATATTTTACTCTC

TGGCTTCCCACTATATTTTACTCTC
TGGCTTCCCACTATATTTTACTCTC
TGGCTTCCCACCATATTTTACTCTC
TGGCTTCCCACATATTTTACTCTCT
TGGCTTCCCACTATATTTTACTCTCTA
TGGCTTCCCACTATATTTTACTCTCTA
TGGCTTCCCACTATATTTTACTCTCTA
TGGCTTCCCACTATATTTTACTCTCTG
TGGCTTCCCACTATATTTTACTCTC
TGGCTTCCCACTATATTTTACTCTCTA
TGGCTTCCCACTATATTTTACTCTCTG
TGGCTTCCCACTATATTTTACTCTCTA
TGGCTTCCCACTATATTTTACTCTCTA
TGGCTTCCCACTATATTTTACTCTCTA
TGGCTTCCCACTATATTTTACTCTCTA
TGGCTTCCCACTATATTTTACTCTC
TGGCTTCCCACTATATTTTACTCTC
TGGCTTCCCACCATATTTTACTCTA
TGGCTTCCCACTATATTTTACTCTC
TGGCTTCCCACCATATTTTACTCTC
TGGCTTCCCACTATATTTTACTCTC
TGGCTTCCCACCTATATTTTACTCTC
TGGCTTCCCACCATATTTTACTCTC
TGGCTTCCCACTATATTTTACTCTC
TGGCTTCACCAAATATTTTACTCTC
TGGCTTCCCACTATATTTTACTCTC
TGGCTTCCCACCATATTTTACTCTC
TGGCTTCCCACTATATTTTACTCTA
TGGCTTCCCACTATATTTTACTCTC
TGGCTTCCCACCATATTTTACTCTC
TGGCTTCCCACTATATTTTACTCTC
TGGCTTCCCACCATATTTTACTCTC
TGGCTTCCCACTATATTTTACTCTC
TGGCTTCCCACCATATTTTACTCTC
TGGCTTCCCACTATATTTTACTCTC
TGGCTTCCCACTATATTTTACTCTC
TGGCTTCCCACCATATTTTACTCTC
TGGCTTCCCACCATATTTTACTCTC
TGGCTTCCCACCATATTTTACTCTC
TGGCTTCCCACCATATTTTACTCTC
TGGCTTCCCACTATATTTTACTCTC
TGGCTTCCCACTATATTTTACTCTC
TGGCTTCCCACTATATTTTACTCTC
TGGCTTCCCACTATATTTTACTCTC
TGGCTTCCCACTATATTTTACTCTC
TGGCTTCCCACTATATTTTACTCTC
TGGCTTCCCACTATATTTTACTCTC
TGGCTTCCCACTATATTTTACTCTC
TGGCTTCCCACCATATTTTACTCTC
TGGCTTCCCACCATATTTTACTCTC
TGGCTTCCCACTATATTTTACTCTC
TGGCTTCCCACTATATTTTACTCTC
TGGCTTCCCACTATATTTTACTCTC
TGGCTTCCCACTATATTTTACTCTC

TGGCTTCCCACTATATTTTACTCTC
TGGCTTCCCACTATATTTTTTTTTTTTTTTTTATTTTACTCTC
TGGCTTCCCACCATATTTTACTCTC
TGGCTTCCCACTATATTTTACTCTC
TGGCTTCCCACCATATTTTACTCTC
TGGCTTCCCACTATATTTTACTCTC
TGGCTTCCCACCATATTTTACTCTC
TGGCTTCCCACTATATTTTACTCTC
TGGCTTCCCACCATATTTTACTCTC
TGGCTTCCCACTATATTTTACTCTC
TGGCTTCCCACTATATTTTACTCTC
TGGCTTCCCACCATATTTTACTCTC
TGGCTTCCCACTATACTCTC
TGGCTTCCCACCATATTTTACTCTC
TGGCTTCCCACTATATTTTACTCTC
TGGCTTCCCACTATATTTTACTCTC
TGGCTTCCCACTATATTTTACTCTC
TGGCTTCCCACTATATTTTACTCTC
TGGCTTCCCACTATATTTTACTCTC
TGGCTTCCCACTATATTTTACTCTCTA
TGGCTTCCCACTATATTTTACTCTC
TGGCTTCCCACTATATTTTACTCTCTA
TGGCTTCCCACTATATTTTACTCTC
TGGCTTCCCACTATATTTTACTCTC
TGGCTTCCCACTATATTTTACTCTC
TGGCTTCCCACTATATTTTACTCTC
TGGCTTCCCACTATATTTTACTCTC
TGGCTTCCCACTATATTTTACTCTC
TGGCTTCCCACTATATTTTACTCTC
TGGCTTCCCACTATATTTTACTCTC
TGGCTTCCCACTATATTTTACTCTC
TGGCTTCCCACTATATTTTACTCTC
TGGCTTCCCACTATATTTTACTCTC
TGGCTTCCCACTATATTTTACTCTC
TGGCTTCCCACTATATTTTACTCTC
TGGCTTCCCACTATATTTTACTCTC
TGGCTTCCCACTATATTTTACTCTC
TGGCTTCCCACTATATTTTACTCTC
TGGCTTCCCACTATATTTTACTCTC
TGGCTTCCCACTATATTTTACTCTC
TGGCTTCCCACTATATTTTTTTATTTTACTCTC
TGGCTTCCCACTATATTTTACTCTC
TGGCTTCCCACTATATTTTACTCTC
TGGCTTCCCACTATATTTTACTCTC
TGGCTTCCCACTATATTTTACTCTC
TGGCTTCCCACTATATTTTACTCTC
TGGCTTCCCACTATATTTTACTCTC
TGGCTTCCCACTATATTTTACTCTC
TGGCTTCCCACTATATTTTACTCTC
TGGCTTCCCACTATATTTTACTCTC
TGGCTTCCCACTATATTTTACTCTC
TGGCTTCCCACTATATTTTACTCTC
TGGCTTCCCACTATATTTTACTCTC
TGGCTTCCCACTATATTTTACTCTC

TGGCTTCCCACTATATTTTACTCTC
TGGCTTCCCACTATATTTTACTCTC
TGGCTTCCCACTATATTTTACTCTC
TGGCTTCCCACTATATTTTACTCTC
TGGCTTCCCACTATATTTTACTCTC
TGGCTTCCCACTATATTTTACTCTC
TGGCTTCCCACTATATTTTACTCTC
TGGCTTCCCACTATATTTTACTCTC
TGGCTTCCCACTATATTTTACTCTC
TGGCTTCCCACTATATTTTACTCTC
TGGCTTCCCACTATATTTTACTCTC
TGGCTTCCCACCATATTTTACTCTC
TGGCTTCCCACTATATTTTACTCTC
TGGCTTCCCACTATATTTTACTCTC
TGGCTTCCCACTATATTTTACTCTC
TGGCTTCCCACTATATTTTACTCTC
TGGCTTCCCACTATATTTTACTATC
TGGCTTCCCACTATTTTTTACTCTC
TGGCTTCCCACTATATTTTACTCTC
TGGCTTCCCACTATATTTTACTCTC
Nr of selected repeats 292 Similarity 0.913730
Consensus:
TGGCTTCCCACTATATTTTACTCTC
>Cele-UNSB01_2:13043920-13044316 Satlength=397 Nr of Repeats=21 RepeatLength=18
seed=ACTACTGTAT
ACTACTGTATCTCAGGGG
ACTACTGTATCACAGGAGA
ATACTGTATCGCAGGAG
ACTACTGTATCGCAAGAT
ACTACTGTATCTCAGGAG
ACTACTGTATCACAGGAT
ACAACTGTATCACAGGAT
ACTACTGTATCACAGGAG
AGTACTGTATCGCAGGAG
ACTACTGTATCGCAAGAT
ACTACTGTATCGCAGGAG
ACTTCTGTATCGCACGAG
ACTAATGTATCACCGGAC
ACAACTGTATCACAGGAA
AGTACTGTATCGCAGGAT
ACTACGGTATCGCAGGAG
ACTACTCTATCGCAGGAG
ACTACTGTATCACAGGAG
ACTACTGTATCACAGGAT
ACTACTGTATCACAGGAGACTACGGCATCACAGGAT
ACAACTGTATCACAGGAT
Nr of selected repeats 18 Similarity 0.797628
Consensus:
ACTACTGTATCnCAGGAg
>Cele-UNSB01_2:13060029-13060418 Satlength=390 Nr of Repeats=5 RepeatLength=43
seed=GATTTGCCGG
GATTTGCCGGAAATTTTCAATCCCGGCAAATTGCAGGTTCGCC
GATTTGCCGGAAATTTTCAATCCCGGCAAATTGCAGGTTTGCC
GATTTGCCGGAAATTTTCAATTCCGGCAAATTGTCGACTTGCC

GTTTGCCGGCTATCA
GATTTGCCGGAAATATTTAGAGGGATTTTGTATAAGACGAATGCACTTAAAACTGTGCAATTTTTCTCGTTTTTTCTAC
ATATTTTCATAGAATATGCTTAATTTTCAAAATAGATTTAGGAACATTCAAAGGATGCGTTTAATTTTGCCATTTGAAA
TTGAAATTCTGAAACTTCCAAAACAAATAAGTACAAAACCACAATTTGCCGAAAATTTTAGGCAAATCGGAAATTTGCC
GATTCGCC
Nr of selected repeats 3 Similarity 0.875969
Consensus:
GATTTGCCGGAAATTTTCAATcCCGGCAAATTGcaGgtTtGCC
>Cele-UNSB01_2:13097657-13097799 Satlength=143 Nr of Repeats=5 RepeatLength=18
seed=CCTAAGCCTA
CCTAAGCCTAAG
CCTAATCCTAAGCCTAAATAGCGTCAGTAGCAAGCTCACGCTCGCCACTGACGCCAAGCATAAGTCTAGGTTTAGG
CCTGAGCCTAAGCTCAAC
CCTAAGCCTAAGACTAAA
CATAAGCCTAAGCCTAAG
Nr of selected repeats 3 Similarity 0.660819
Consensus:
CcTaAGCCTAAGnCTaAa
>Cele-UNSB01_2:13106081-13106253 Satlength=173 Nr of Repeats=4 RepeatLength=43
seed=CGGCAAATTA
CGGCAAATTACCGATTTGCCGGTTTGCCGGAAATTTTCATTTT
CGGCAAATTACCAATTTGCCGATTTGCCGGAAATTTTCATTTT
CGGCAAATTATAAGTTTGCCGGTTTGCCGAAAATTTTCAGTTC
CGGCAATTTACCGATTTGCCGATATGTCGGAAATTTTCAATTC
Nr of selected repeats 4 Similarity 0.803618
Consensus:
CGGCAAaTTAccaaTTTGCCGaTtTGcCGgAAATTTTCAtTTc
>Cele-UNSB01_2:13133064-13133208 Satlength=145 Nr of Repeats=9 RepeatLength=16
seed=GCCTACATGC
GCCTACATGCCTGCAT
GCCTACATGCCTACAT
GCCTACATGCCTACAAG
GCTACATGCCTTCAT
GCCTACATGCATACAT
GCCTAAATGCCTACAT
GCCTACATGCCTACAT
GCCTATATGCCTGCAT
GCCTGCATGCCTGCAT
Nr of selected repeats 7 Similarity 0.861111
Consensus:
GCCTAcATGCCTaCAT
>Cele-UNSB01_2:13134300-13134450 Satlength=151 Nr of Repeats=4 RepeatLength=30
seed=TTCAGGCAGG
TTCAGGCAGGCATATGTCACATCGAATGTA
TTCAGGCAGGTATAGGTTTCTTTGAAAATT
TTCAGGCAGGCATAGGTTGACTTTGAGGTGTTCAGTTAGGCAAAGAACACCTCAAAGGTA
TTCAGGCAGGCATTGATTACTTTGAAAAAT
Nr of selected repeats 3 Similarity 0.644444
Consensus:
TTCAGGCAGGcATaggTtaCtTtGAAaatt
>Cele-UNSB01_2:13161799-13161943 Satlength=145 Nr of Repeats=6 RepeatLength=19
seed=TATGGTGCAT
TATGGTGCATCTGACAAGT
TATGGTACATTTGACCAGTTACGGTGAATCTAACCAGT

TATGGTGCATTTAACTAGT
TATGGTACATTTGACCAGT
TACGGTGCATCTAACTTGA
TATGGTGCATCGACGGTGAATCTAGCCAGT
Nr of selected repeats 4 Similarity 0.707602
Consensus:
TAtGGTgCATcTaACtaGt
>Cele-UNSB01_2:13192718-13192854 Satlength=137 Nr of Repeats=4 RepeatLength=34 seed=TTCAATTCCG
TTCAATTCCGGCAATTTCCCGATTTGCCGGAAAT
TTCAATTCCGGTAACTCGTCGATTTTTCGGAAAT
TTCAATTCCGGCTTTTCGTCGATTTGCCGGACAT
TTCAATTCCGGCAATTCGTCGATTTGCCGGAAAT
Nr of selected repeats 4 Similarity 0.803922
Consensus:
TTCAATTCCGGcaatTcgtCGATTTgcCGGAaAT
>Cele-UNSB01_2:13216705-13216849 Satlength=145 Nr of Repeats=8 RepeatLength=16 seed=TAGGCACGTA
TAGGCACGTAGACATGTAGCTACG
TAGGTACGTAGGCACA
TAGGCACGTAGGCACG
TAGGCACGTAGGCACG
TAGGCACGTAGGCACG
TAGGCACATAGGCACG
TAGGCACGTAGGCACG
TAGGCACGTAGGCACGTAGGCACG
Nr of selected repeats 6 Similarity 0.916667
Consensus:
TAGGCACGTAGGCACG
>Cele-UNSB01_2:13339084-13339231 Satlength=148 Nr of Repeats=7 RepeatLength=21 seed=AATCTAGCCA
AATCTAGCCAGACGCACCCCT
AATCTAGCCAGACGCACACCT
AATCTAGCCAGCCGCACACCT
AATCTAGCCAGACGCACACCT
AATCTAGCCAGACGCACACCT
AATCTAGCCAGACGCACACCA
AATCTAGCCAGACGCACACCC
Nr of selected repeats 7 Similarity 0.930461
Consensus:
AATCTAGCCAGACGCACACCt
>Cele-UNSB01_2:13398823-13400207 Satlength=1385 Nr of Repeats=8 RepeatLength=165 seed=AAAATTCAAT
AAAATTCAATACCAATGATTAAAACAAGATTGAAGTATAAAAACTGTGGGAAATTTTTTTTGGTCGACTTCCAAAATTA
TTCCTCAGCTCCAGCCAAAATTTGAAAAGTATTATTATACATAATTTTCGGTCATTTTGGCAAGATAGGAATACTTTTT
AACATTTTTCCCACTGGCGCTGCTCAACCTTTGAGAATATTCAAACATCTAATAAAAAATCCCAAAACTTA
AAAATTCGATTTTGGACTTATCTATTCTCTCTTTTTTATAACATGTATTTCAGTTGATAGAGGACTCAAAATTATAATG
CTCGTAATTATTTATAAGTGTGTGTTGTCTCAGAATCAATTCTAAAAAACAGATTTTTTTTGAAAGTTTAAGTAATATC
CGCGGAG
AAAATTCAATTTTGGGCATTTCTATTATCTTTTTTTTTAAACATGTATTTCAGTGAATAGAAGACTCAAAATTATAATG
CTCGTAATTATTTATAAGTGTGTGTTGTCTCAGAATCAATTCTAAAAAACAGATTTTTTTCGAAAGTTTAAGTAATTTC
TGCGGAA

AAAATTCAATTTTGGACATTTCTATTATCTTTTTTTGAAAACATGTATTTCAGTGAATAGAAGACTCAAAATTATAATG
CTCGTAATTATTTATAAGTGTGTGTTGTCTCAGAATCAATTCTAAAAAACAGATTTTTTTTGAAAGTTTAAGTAATATC
CGCGGAG
AAAATTCAATTTTGGGCATTTCTATTATCTTTTTTTTTAAACATGTATTTCAGTGAATAGAAGACTCAAAATTATAATG
CTCGTAATTATTTATAAGTGTGTGTTGTCTGAGAATCAATTCTAAAAAACAGATTTTTTTCGAAAGTTTAAGTAATTTC
TGCGGAA
AAAATTCAATTTTGGACATTTCTATTATCTTTTTTTGAAAACATGTATTTCAGTGAATAGAAGACTCAAAATTATAATG
CTCGTAATTATTTATAAGTGTGTGTTGTCTCAGAATCAATTCTAAAAAACAGATTTTTTTTGAAAGTTTAAGTAATATC
CGCGGAG
AAAATTCAATTTTGGGCATTTCTATTATCTTTTTTTTTAAACATGTATTTCAGTGAATAGAAGACTCAAAATTATAATG
CTCGTAATTATTTATAAGTGTGTGTTGTCTGAGAATCAATTCTAAAAAACAGATTTTTTTCGAAAGTTTAAGTAATTTC
TGCGGAA
AAAATTCAATTTTGGACATTTCTATTATCTTTTTTTGAAAACATGTATTTCAGTGAATAGAAGACTCAAAATTATAATG
CTCGTAATTATTTATAAGTGTGTGTTGTCTCAGAATCAATTCTAAAAAACAGATTTTTTTTGAAAGTTTAAGTAATATC
CGCGGAG
Nr of selected repeats 7 Similarity 0.943050
Consensus:
AAAATTCAATTTTGGaCATTTCTATTATCTTTTTTTtaAAACATGTATTTCAGTGAATAGAAGACTCAAAATTATAATG
CTCGTAATTATTTATAAGTGTGTGTTGTCTcAGAATCAATTCTAAAAAACAGATTTTTTTtGAAAGTTTAAGTAATaTC
cGCGGAg
>Cele-UNSB01_2:13437444-13437847 Satlength=404 Nr of Repeats=10 RepeatLength=31
seed=CACGCCTATT
CACGCCTATTTTATGGGCGGAGTCTACAAGG
CACGCCTATTTTGTGGGAGGAGTCTACAAGC
CACGCCTATTTTTTGGGCGGAGTCTGCAAGT
CACGCCTATTTCATGGACGAAGACTACAAGC
CACGCCTATTTTATGGGCGGAGAGTACAAGC
CACGTCTATTTTATGGGCGGGGTCTACAAGGCGCGTCTATTTCACGGGCGGAGCCTACAAGC
CACGCCCATTTTTTGGACGGAGACTACAAGA
CACGCCTATTTCATGGGCGGAGTCTACAAGCCACGCCCGTTTTGTGGACGGAGACTACAAAT
CACGCCTATTTTATGGGCAGAGTCTGCAAGGCGCGCATATTTTATGGGCGGAGCCTGCAAGC
CACGCCTATTTTATGGGTGGAGTCTACAAGC
Nr of selected repeats 7 Similarity 0.811572
Consensus:
CACGCCTATTTTaTGGgcGGAGtCTACAAGc
>Cele-UNSB01_2:13440285-13440859 Satlength=575 Nr of Repeats=12 RepeatLength=48
seed=TGCCTACGTG
TGCCTACGTGCCTACATAAGAAGCGTAGGTAGCTTCAAAGGTGTTGCG
TGCCTACGTGCCTGCAAAACAGGCGTGGGCAGCTTTAAAGGTGATGTA
TGCCTACGTGCCTACAAAATAAGCATAGGTAGCTCTAAAGGTAATGCA
TGCCTACGTGCTTACAAAACAGGCGTGGGCAGCTTTAAAGGTGATGTA
TGCCTACGTGCTTACAAAACAGGCGAATTAGCTTTGAAGGTGATGCA
TGCCTACGTGCCTACAAAACAGGCGTGGGCAGCTTTAAAGGTGATGCA
TGCCTACGTGCCTACAAAATAAGCATAGGTAGCTCTAAAGGTAATGCA
TGCCTACGTGCTTACAAAACAGGCGAATTAGCTTTGAAGGTGATGCA
TGCCTACGTGCCTACAAAACAGGCGTGGGCAGCTTTAAAGGTGATGCA
TGCCTACGTGCCTACAAAATAAGCATAGGTAGCTCTAAAGGTAATGCA
TGCCTACGTGCCTACATAAGAGGCATAGGTAACTTCAAAAGTGTTGCG
TGCCTACGTGCCTGCAAAACAGGCGTGGGCAGCTTTAAAGGTGTTGCG
Nr of selected repeats 10 Similarity 0.812346
Consensus:
TGCCTACGTGCCTaCAaAAcAgGCgTaGGcAGCTttAAAGGTgaTGca
>Cele-UNSB01_2:13459229-13459607 Satlength=379 Nr of Repeats=13 RepeatLength=14
seed=TAGTTTGTAG

TAGTTTGTAGTTTG
TAGTTTGTAGTTTA
TAGTTTGTATT
TAGTTTATAGTTTGTATATAAATTGCATGCATGAAGTTCCAATTCCAATTTAAAAACGTTTTTTTATGATTATGACTTT
TTAATCTGTATTTTGAAGTTTA
TAGTATGTAGTATG
TAGTATGTAGTTTG
TAGTTGGTAGTTTGTAGTCTA
TGGTTTGTAGTTTG
TAGTTTGTAGCTTG
TAGTTTGTAGTTTTCTTATAGCTATGGAAATTAATTTTGTAAGAATGAAATTCCTGCTCTGCCAAAATAACCCTAGTAT
GTTTCGTAGTTTTTTATTAAAGTCTG
TAGTATGTAGTTTG
TAGTTTATAGTTTG
TAGTTTGTAGTTTATACTTTATAGTTTG
Nr of selected repeats 8 Similarity 0.829932
Consensus:
TAGTtTGTAGTTTG
>Cele-UNSB01_2:13460078-13460334 Satlength=257 Nr of Repeats=15 RepeatLength=16
seed=GGCATGTAGG
GGCATGTAGGCAGGTA
GGAATGTAGGCATGTA
GGCATGTAGGCATGTA
GGCATGTAGGCATGTAGGCATCTC
GGCATGTAGGCATCTA
GGCATATAGGCAAGTA
GGCATCTAGGCATGTA
GGCATGTAGGCATGTA
GGCATGTAGGCACTTA
GGCATGTAGGCATGTA
GGCATGTAGGCATGTA
GGCATCTAGGCATGTA
GGCATCTAGGCATATG
GGCAAGTAGGCATCTA
GGCATGTAGGCATGTAGGCATTTA
Nr of selected repeats 13 Similarity 0.840812
Consensus:
GGCATgTAGGCAtgTA
>Cele-UNSB01_2:13465788-13466109 Satlength=322 Nr of Repeats=20 RepeatLength=16
seed=GGCATGTAGG
GGCATGTAGGTAGGTA
GGCATATAGGCATGTA
GGTATGTAGGCATATA
GGCATGTAGGTATGTA
GGCATGTAGGCATGTA
GGCATGTTGGCATGTA
GGCATGTAGGTAGGTA
GGCATGTAGGCATGTT
GTCATGTAGGTATATA
GGCATATAGGTATGCA
GGCATGTTGGCATGTA
GGCATGTAGGTAGGTA
GGCATGTAGGCAGGTA
GGCACGTAGGCATCTA

GGCATGTAGGCATGTTA
GGCATTTAGGCATGTA
GGCAGGTAGGCATATA
GGCATATAGGTACGTA
GGCATATAGGCATGTA
GCCATGTAGGAATGTA
Nr of selected repeats 19 Similarity 0.773879
Consensus:
GGCATgTAGGcAtgTA
>Cele-UNSB01_2:13474627-13476516 Satlength=1890 Nr of Repeats=78
RepeatLength=20 seed=AAAATTTGAA
AAAATTTGAAATTTTGTCCAAAAATCATAGTTTTTTCCGAAAAAAAACCAGA
AAAATGTGAAAATTACCCCAAATTTCTAATTTTGAAGCGATTTTCTCCGAATTTTGAGCCCCAAACGTCGGAATTTCTC
CGCTCCCGTACCCTGTGCTCTTCCTCCCAGCAGCCGGAGCCTTCCGTTTCCGTGTCTCGAGTGCTTTTACAATTGGAAT
CTCCTGTACATCGTCCATCGCCTCTTTTTCTTCCATTTTTTCGTCATTTTCCTCTTTTGCGACAAGCTGAATTCGTTCC
AAGGCTTCACGCACCATTCCCATAAAGACTTTGTGATTTTTTCGTTGGTATTCTCTGAAA
AAAATTTGAATTTTTAAGCC
AAAATTTGAATTTTTAAGTCA
AAAATTTGAATTTTTAAGTC
AAAATTTGAATTTTTAAGTC
AAAATTTGAATTTTTAAGCC
AAAATTTGAATTTTTCAGTCA
AAAATTTGAATTTTTAAGTC
AAAATTTGAATTTTTCAGTC
AAAATTTGAATTTTTAAGTC
AAAATTTGAATTTTCTGCCA
AAAATTGAATTTTTAAGTC
AAAATTTGAATTTTCTGCCA
AAAATTGAATTTTTAAGTC
AAAATTTGAATTTTTAAGTC
AAAATTTGAATTTTTAAGCC
AAAATTTGAATTTTTCAGTCA
AAAATTTGAATTTTTAAGTC
AAAATTTGAATTTTTAAGTC
AAAATTTGAATTTTTAAGTC
AAAATTTGAATTTTCTGCCA
AAAATTGAATTTTTAAGTC
AAAATTTGAATTTTTAAGTC
AAAATTTGAATTTTTAAGCC
AAAATTTGAATTTTTCAGTCA
AAAATTTGAATTTTTAAGTC
AAAATTTGAATTTTTAAGTC
AAAATTTGAATTTTTAAGTC
AAAATTTGAATTTTCTGCCA
AAAATTGAATTTTTAAGTC
AAAATTTGAATTTTTAAGTG
AAAATTTGAATTTTTAAGTC
AAAATTTGAATTTTTCAGTCA
AAAATATGAATTTTTAAGTG
AAAATTTGAATTTGTAAGTC
AAAATTTGAATTTTTAAGTC
AAAATTTGAATTTTTCAGTC
AAAATTTGAATTTTTAAGTC
AAAATTTGAATTTTCTGCCA

AAAATTGAATTTTTAAGTC
AAAATTTGAATTTTCTGCCA
AAAATTGAATTTTTAAGTC
AAAATTTGAATTTTTAAGTC
AAAATTTGAATTTTTAAGCC
AAAATTTGAATTTTTCAGTCA
AAAATTTGAATTTTTAAGTC
AAAATTTGAATTTTTAAGTC
AAAATTTGAATTTTTAAGTC
AAAATTTGAATTTTCTGCCA
AAAATTGAATTTTTAAGTC
AAAATTTGAATTTTTAAGTC
AAAATTTGAATTTTTAAGCC
AAAATTTGAATTTTTCAGTCA
AAAATTTGAATTTTTAAGTC
AAAATTTGAATTTTTAAGTC
AAAATTTGAATTTTTAAGTC
AAAATTTGAATTTTCTGCCA
AAAATTGAATTTTTAAGTC
AAAATTTGAATTTTTAAGTG
AAAATTTGAATTTTTAAGTC
AAAATTTGAATTTTTCAGTCA
AAAATATGAATTTTTAAGTG
AAAATTTGAATTTGTAAGTC
AAAATTTGAATTTTTAAGTC
AAAATTTGAATTTTACAGCC
AAACTTTGAATTTTTCAGTC
AAAATTTGAATTTTTCAGTC
AAAATTTGAATTTTACAGCC
AAAATTTGAATTTTACAGCC
AAAATTTGAATTTTTCAGTC
AAAATTTGAATTTTTCAGTC
AAAATTTGAATTTTTCTGCTGG
AAATTTGAATTTTTAGTAAAAAATTGGGTTCTCAGTC
AAAATTTGAATTTTTCAGCC
AAAATTTGAATTTTTCAGTCA
AAAATTTGAATTTTTCTGTC
AAAATTTGAATTTTTTAGTC
Nr of selected repeats 57 Similarity 0.826829
Consensus:
AAAATTTGAATTTTTaAGtC
>Cele-UNSB01_2:13490523-13491161 Satlength=639 Nr of Repeats=5 RepeatLength=122 seed=GATCTACAAA
GATCTACAAAAAAGGCGGGAGAAGATCTTTTCGAGCCGCAGAGTTCTCAACTGATTTGGTATGGTTTGACGTCACATAT
TTTTGAGCAAAAAAATTCCCGCATTTTTTGGAGATAAAACCGTGATGGGACAGCCTGGCGCCAAGTGCTTGATCTGCGT
A
GATCTACAAAAACGCGGGAAAAGTGACGCAGAGTTTTGAACTGATTTTGCATAAAAATTCCCGCATTTTTTGTAGATCA
AACCGTTATGGGACAGCCTGGTGCCACGTGCTTGATCTACGCA
GATCTACAAAAATGCGGGAAAAGTGACACAGAGTTCTCAACTGATTTTGCATAAAAATTCCCGCATTTTTTGGAGATCA
AACCGTGATGGGACAGCCTGGCACCACTTGCTTGATCTACGTA
GATCTACAAAAATGCGGGAAAAGTGACACAACTGATTTTGCATAAAAATTCCCGCTTTTTTTGTAGATCAAACCGTTAT
GGGACAGCCTGGTGCCACGTGCTTGATCTACGTA
GATCTACAAAAATGCGGGAAAAGTGACACAGAGTTCTCAACTGATTTTGCATAAAAATTCCCGCATTTTTTGGAGATCA
AACCGTGATGGGACAGCCTGGCACCACTTGCTTGATCTACGTA

Nr of selected repeats 3 Similarity 0.927140
Consensus:
GATCTACAAAAAtGCGGGAAAAGTGACaCAGAGTTcTcAACTGATTTTGCATAAAAATTCCCGCATTTTTTGgAGATCA
AACCGTgATGGGACAGCCTGGcaCCACtTGCTTGATCTACGtA
>Cele-UNSB01_2:13499104-13499209 Satlength=106 Nr of Repeats=5 RepeatLength=21
seed=CTAGAAAAAC
CTAGAAAAACCAGGCCATCAA
CTAGAAAAACTCGGCCATCAA
CTAGAAAAACTCGGCCATCAA
CTAGAAAAACTCGTCCATCAA
CTAGAAAAACTCGGCTATCAA
Nr of selected repeats 5 Similarity 0.898413
Consensus:
CTAGAAAAACtcGgCcATCAA
>Cele-UNSB01_2:13500094-13501231 Satlength=1138 Nr of Repeats=46
RepeatLength=21 seed=TTTTCTAGTT
TTTTCTAGTTGATGGCCGAGT
TTTCCTAGTTGATAGCCGAGT
TTTTCTAGTTGATGGCCGAGT
TTTTCTAGTTGATGGCCGAGT
TTTCCTAGTTGACGGCCGATT
TTTTCTAGTTGATGTCCGAGC
TTTTCTAGTTGATGGCCTAGA
TTTTCTAGTTGCTGGCCGAAT
TTTTCTAGTTAATGTCAGAGTTTTTGGTGGCCTAGT
TTTCCTAGTTCATGGCAAAGT
TTTTCTAGTTGATGGCCGAGTTTTCTTTAGGTGACGGCCGAGT
TTTCCTAGTTGACGGCCGATT
TTTTCTAGTTGATGTCCGAGT
TTTTCTAGTTGATGGCCGAGTTTTCCTAGCTCATGGCCTAGG
TTTTCTAGTTGCTGGCCGAAT
TTTTCTAGTTGATGGCCGAGT
TTTTCTAGTTGATGGCCGAGT
TTTCCTAGTTGACGGCCGATT
TTTTCTAGTTGATGTCCGAGC
TTTTCTAGTTGATGGCCTAGA
TTTTCTAGTTGCTGGCCGAAT
TTTTCTAGTTAATGTCAGAGTTTTTGGTGGCCTAGT
TTTCCTAGTTCATGGCAAAGT
TTTTCTAGTTGATGGCCGAGTTTTCTCTAGGTGACGGCCGAGT
TTTCCTAGTTGACGGCCGATT
TTTTCTAGTTGATGTCCGAGT
TTTTCTAGTTGATGGCCGAGTTTTCCTAGCTCATGGCCAAGT
TTTTCTAGTTCATGGTCGATTT
TTTTCTTGTTGATGGCCTAGA
TTTTCTAGTTGATGGCCGAGT
TTTTCTAGTTGATGGCCGAGT
TTTCCTAGTTGACGGCCGATT
TTTTCTAGTTGATGTCCGAGC
TTTTCTAGTTGATGGCCTAGA
TTTTCTAGTTGCTGGCCGAAT
TTTTCTAGTTAATGTCAGAGTTTTTGGTGGCCTAGT
TTTCCTAGTTCATGGCAAAGT
TTTTCTAGTTGATGGCCGAGTTTTCTCTAGGTGACGGCCGAGT

TTTCCTAGTTGACGGCCGATT
TTTTCTAGTTGCTGGCCGAAT
TTTTCTAGTTAATGTCAGAGTTTTTGGTGTCCGAGC
TTTTCTAGTTGATGGCCGAGT
TTTTCTAGTTGATGGTCGATTT
TTTTCTTGTTGATGGTCGATC
TTTTCTAGTTGATGGCCGAGTT
TTCTCTAGTTGATGGCCTAGG
Nr of selected repeats 34 Similarity 0.794245
Consensus:
TTTtCTAGTTGATGGCCgAgt
>Cele-UNSB01_2:13509974-13510060 Satlength=87 Nr of Repeats=6 RepeatLength=12
seed=CTTAGGCTTA
CTTAGGCTTAAG
CTTAGGCTTAGT
CTTAGGCTTAAG
CTTAGGCTTAGTCTTTGT
CTTAGGCTTGGA
CTTAGGCTTAGGTTTAGCGC
Nr of selected repeats 4 Similarity 0.743590
Consensus:
CTTAGGCTTaaGn
>Cele-UNSB01_2:13528433-13528477 Satlength=45 Nr of Repeats=4 RepeatLength=11
seed=TGGAAAATTG
TGGAAAATTGA
TGGAAAATTGA
TGGAAAATTGA
TGGAAAATTGA
Nr of selected repeats 4 Similarity 1.000000
Consensus:
TGGAAAATTGA
>Cele-UNSB01_2:13541965-13542724 Satlength=760 Nr of Repeats=8 RepeatLength=95
seed=GGCCTGCGGC
GGCCTGCGGCCCTCAACCTGACTTATTGGGCTCAGAATAGGCTTGACGGTTGCCCAACACTTCTTCGTGTATTGCCTGT
TCCGCTGAGAATGTGGTGAGAAGTTACCTATGTGGTGAGAAGTTACCTTCCAAGGGTATTCG
GGCCTGCGGCCCTCAAACCTGGTTGGTAGGCTTGAGCGAATAACAATCTGCTTGGAATAGGATTAAAGCTGGCCCTTAT
TTTTCATGAGTATTCA
GGCCTGCGGCCCTCAAACCTGGTTGGTAGGCTTGAGTGAATAACAACCTGCTTGGAAGAGGATTAAAGCTGACCCACAG
TTTCCAAGAGTGTTCG
GGCCTGCGGCGCTCAAACCTGGTTGGTAGGCTTGAGTAAATAACAACCTGCTTGGAATAGGATTATAGCCAGCCCAGTT
TGCAAGACTAGTCG
GGCCTGCGGCCCTCAAACCTGGTTGTTAGGCTTGAGTAAATAACAACCTG
GGCCTGCGGCCCTCAAACCTGGTTGTTAGTCTTGAGTAAATAACAACCTGCTTGGAATAGGATTAAAGCTGGCCCTTAG
TTTCCATGAGTATTCG
GGCCTGCGGCCCTCAAACCTGGTTGGTATTCTTGAGCGAATAACAATCTGCTTGGAATAGGATTAAAGCTGGCCCTTAG
TTTCCATGAGTATTCG
GACCTGCGGCCCTCAAACCTGGTTGGTATGCTTGAGCGAATAACAATCTGCTTGGAATATGATTAAAACCAGCTCACAG
TTTCCAAGAGTGTTCG
Nr of selected repeats 5 Similarity 0.859649
Consensus:
GgCCTGCGGCCCTCAAACCTGGTTGgTAggCTTGAGcgAATAACAAtCTGCTTGGAAtAgGATTAAAgCtggCcCttAg
TTTcCAtGAGTaTTCg
>Cele-UNSB01_2:13551182-13551985 Satlength=804 Nr of Repeats=20 RepeatLength=35
seed=GATTTGCCGG

GATTTGCCGGAAAATTTCGATTCTGGCAATTTGCTGATTTACCAAAAATTTTCAATTCCGGCAATTTGTC
GATTTGCCGGAAATGTTCAATTCCGGCAATTTACT
GATTTGCTGGAAATTTTATATTTCGCAATTTGTC
GATTTGCCGGAAATGTTCAATTCTGACAATTTGCC
GATTTGCCGGAAAAGTTTAATTCCGGCAATTTCCC
GATTTGCCGGAAAAGTTAAATTCCGGCAACTTGCC
GATTTACCGGAAAATTTCAAATCCGCCAATTTGTCAAAAAGCAGGAATTATTCAATTCCAGCAATTTGCC
GATTTGCCGGAAATTTTCAATTCCGGCAATTTACT
GATTTGCTGGAAATTTTCAATTCCGGCAATTTGCC
GATTGGCCGGAAATTTTCAATTCCAACAATTTGCC
GATTTGCCGGAAAATTTCAATTCCAACAATTTGCC
GATTTGCCGGAAAATTTCAATTCCGACAATTTGCC
GATTTGCCGGAAAATTTCAATTCCGGCAATTTGCC
GATTTGCCGGAAAATTTCAATTCCGGCAATTTGCC
GATTTGCCGGAAAATTTCAATTCCGGCAATTTGTC
GATTTGCCGGAAATTTTCAAATCCGGCAATTTGCC
GATCTGCCGGAGAATTTCAATTCCGGCAATTTACT
GATTTGCCGGAATTATAGAATTCTGGCAATTTGCCGATCTATCGAAAGTGTTCAATTCCGGAAATTTGCC
GATTTTCCGGAAATATTCAATTCCGGCAAATTGCC
GATTTACCGGAAATTTTAATTCCGGCATTCTACT
Nr of selected repeats 15 Similarity 0.816490
Consensus:
GATTTGCCGGAAAttTTCAATTCCGgCAATTTgCc
>Cele-UNSB01_2:13571681-13571955 Satlength=275 Nr of Repeats=20 RepeatLength=11 seed=CCTACAGTAC
CCTACAGTACC
CCTTCAGTACTG
CTACAGTACACCTACAGTGTC
CCTATAGTACC
CCTATAGTACT
CCTACAGTACT
CCTACCGTACC
CCTACTGTACTT
CTACAGTACC
CATACAGTACC
CCTACAGTATC
CCTACAGTTCTCTTATAGACCAGTACAGTACTAACACAGCACC
CCTACAGTACCCCTACACTCCC
CCTACAGTACTC
CCACAGTACC
CCTACAGTACC
CCTACAGTACC
CCTACAGTACC
CCTACAGTACT
CCTACAGTACC
Nr of selected repeats 12 Similarity 0.853076
Consensus:
CCTACAGTACc
>Cele-UNSB01_2:13579229-13580664 Satlength=1436 Nr of Repeats=41 RepeatLength=35 seed=GAATTTTTGA
GAATTTTTGAAACTCCGCGATTTGCCGATTTGCCA
GAGTTTTTGATTTTCGGCAATTTGCCAATTTGCCA
GAATTTTTGAAACTCCGCGATTTGCCGATTTGCCG
GAATTTTTGATTTTCGGCAATTTGCCGATTTGCCG

GAATTTTTGATTTTCGGCAGTTTGCCGATTTGCCG
GAATTTTTGATTTTCGGCAATTTGCCGATTTGCCG
GAATTTTTGATTTTCGGCAATTTGCCGATTTGCCG
GAATTTTTGAAACTTGGCAATTTGCCGATTTGCCG
GAATTTTTGATTTTCGGCAGTTTGCCGATTTGCCG
GAATTTTTGATTTTCGGCAATTTGCCGGGTTGCCG
GAATTTTTGAAACTTGGCAATTTGCCGATTTGCCG
GATTTTTTGATTTTCGGCTATTAGCCGATTTGCCG
GAATTTTTGAAACTCCGCGATTTGCCGATTTGCCA
GAGTTTTTGATTTTCGGCAATTTGCCAATTTGCCA
GAATTTTTGAAACTCCGCGATTTGCCGATTTGCCG
GAATTTTTGATTTTCGGCAATTTGCCGATTTGCCG
GAATTTTTGATTTTCGGCAGTTTGCCGATTTGCCG
GAATTTTTGATTTTCGGCAATTTGCCGATTTGCCG
GAATTTTTGATTTTCGGCAATTTGCCGATTTGCCG
GAATTTTTGAAACTTGGCAATTTGCCGATTTGCCG
GAATTTTTGATTTTCGGCAGTTTGCCGATTTGCCG
GAATTTTTGATTTTCGGCAATTTGCCGGGTTGCCG
GAATTTTTGAAACTTGGCAATTTGCCGATTTGCCG
GATTTTTTGATTTTCGGCTATTAGCCGATTTGCCG
GAATTTTTGAAACTCCGCGATTTGCCGATTTGCCA
GAGTTTTTGATTTTCGGCAATTTGCCAATTTGCCA
GAATTTTTGAAACTCCGCGATTTGCCGATTTGCCG
GAATTTTTGATTTTCGGCAATTTGCCGATTTGCCG
GAATTTTTGATTTTCGGCAGTTTGCCGATTTGCCG
GAATTTTTGATTTTCGGCAATTTGCCGATTTGCCG
GAATTTTTGATTTTCGGCAATTTGCCGATTTGCCG
GAATTTTTGAAACTTGGCAATTTGCCGATTTGCCG
GAATTTTTGATTTTCGGCAGTTTGCCGATTTGCCG
GAATTTTTGATTTTCGGCAATTTGCCGGGTTGCCG
GAATTTTTGAAACTTGGCAATTTGCCGATTTGCCG
GATTTTTTGATTTTCGGCTATTAGCCGATTTGCCG
GAATTTTTGAAACTCGGCGATTTGCCAATTTGCCA
GAATTTTTGATTTTCGGCAATTTGCCAATTTGCCA
GAATTTTTGAAACTCGGCAATTTGCCGATTTGCCA
GAATTTTTGATTTTCGGCAATTTGCCGATTTGCCG
GAATTTTTGAAACTCGGCAATTTGCCGATTTGCCA
Nr of selected repeats 41 Similarity 0.852172
Consensus:
GAATTTTTGAtttTCGGCaATTTGCCGATTTGCCg
>Cele-UNSB01_2:13658533-13659809 Satlength=1277 Nr of Repeats=26
RepeatLength=27 seed=AGCAGCCGAC
AGCAGCCGACACTGCACGGGTCCTAAAAGATTTCGGCTGCTGTGAGACCTGTGAAGCGTCGGCTGCTAACCATTAGGCG
AATCTA
AGCGGCCGACGTTCCATAGGCCTGCAAGATGTCGGCTGCTTTACTTTGGCAGCCGATAGACGATTGTTCCAGCAAATCT
CGACCTTATTCAA
AGCAGCCGACATTTTACGGGACCCTAGGGAACCAGTGAAATGTCGGCTGCTAACTCATCTCAACAAAGTTAACCCATGT
TTTCTTACAGTAGCCTTCTGCTATTTCGACCCGGCGCCCCACGAACCGGCGTTCGTCTACCGTACCTATCACACCCTGC
GGCGGTTGAGTCGACGCAATGGAGAAGCTCGAATTCGAATGTCCATCAAATCAGAAGAGCAGGAGGAGGAGAGCAACGA
CTCGCCGGCTCAAGTGCTATTTGTGTAGAGATCGAAATATTGCTTCTTCTTCTTCTTCTACTTCTTCCTTCTATACTAG
CAACCCCTCTGTCTGTAGTGTGTGTATGAATTTTGCAAGCGCGCTTTTTGATGGGCTTTTTGAAAGGGTGAATACTCAT
TTTCTTGGTA
AGCAGCCGACATTTCGCGGGTCGGTGAAATTCTAGGAGAGCTATAT
AGCAGCCGACGCCTTGCGGGTCGCTCA

AGCAGCCGACATTTTGCAGTTTGCTTA
AGCAGCCGACATTTTGCAGGTTGTTTAAAAAGCCGACATTTTGCGGGTTTCTTG
AGCAGCCGACACCTTGCAGGTTGCTCA
AGCAGCCGACACCTTGCTGGTCGCTTA
AGCAGCCGACACCTTGATGGTTGTTCA
AGCAGCCGACATCTTGCAGGTTTCTTA
AGCAGCGGACACCTTGATGGTTGTTCA
AGCAGCCGACATCTTGCAGGTTGCTTA
AGCAGCCGACACCTTGAAGGTTTCTTG
AGCAGCCGATACCTTGCGGGTTGCTTA
AGCAGCGGACACCTTGATGGTTGCTCA
AGCAGCCGACACCTTGCTGGTCGCTTA
AGCAGCCGACACCTTGATGGTTGTTCA
AGCAGCCGACATCTTGCAGGTTTCTTA
AGCAGCGGACACCTTGATGGTTGTTCA
AGCAGCCGACATCTTGCAGGTTGCTTA
AGCAGCCGACACCTTGAAGGTTTCTTG
AGCAGCCGATACCTTGCGGGTTGCTTA
AGCAGCGGACACCTTGATGGTTGTTCA
AGCAACCGACATCTTGCAGGTTGCTTA
AGCAGCCGACACCTTACAGGTTGCATAAGCAGATGACACTTTACAGGTTGCTTA
Nr of selected repeats 20 Similarity 0.791813
Consensus:
AGCAGCcGACAcCTTGcnGGTTgcTtA
>Cele-UNSB01_2:13663688-13663784 Satlength=97 Nr of Repeats=6 RepeatLength=16
seed=ACATGCCTAC
ACATGCCTACATGACT
ACATGCCTACATGCCT
ACATGCCTACATGCCT
ACATGCCTACATGCCT
ACATGCTTACATGCCC
ACATGCCTACATACCT
Nr of selected repeats 6 Similarity 0.888889
Consensus:
ACATGCCTACATGCCT
>Cele-UNSB01_2:13678541-13678601 Satlength=61 Nr of Repeats=5 RepeatLength=12
seed=AATACCAACC
AATACCAACCAC
AATACCAACCAC
AATACCAACCAC
AATACCAACCAG
AATACCAACCAG
Nr of selected repeats 5 Similarity 0.933333
Consensus:
AATACCAACCAc
>Cele-UNSB01_2:13684820-13685366 Satlength=547 Nr of Repeats=24 RepeatLength=20
seed=GTAAATCGAC
GTAAATCGACAAACGACAACCAGC
GTAAATCGACAAAAGACATC
GTAAATCGACACACAACGTC
GTAAATCTACACACAACAGC
GTAAATCGACAAACGACAGCGCAAATAAATAAACGACGGC
GTAAATCGACACAGCACGTT
GTAAATCAACACTCCGTGTC

GTAAATCGACAAAAGCCATC
GTAAATCGACACACAACGTC
GTGAATCGACAAACGACAGCCAGC
GTCAATCGACAGACGCTATC
GTAAATCGACAAACGACAAGCAGC
GTAAATCGACATACAATGTC
GTAAATCGGCAAACGACAGC
GTAAATCGACAGGAGACATTGTTAGGAAACAGC
GTAAATCGACAAACGACAGTATGC
GTAAATCGACATACGACTGG
GTAAATCGACAGAAGACATTGCTAGACAACAGC
GTAAATCGACAGACGCTATC
GTAAATTGACACACAACGTC
GTAAATCGGCATACAACGTC
GTAAATCGACAAACGACAGCCCGC
GTAAATCGACACACAACGTC
GTAAATCCACACACTACGTC
Nr of selected repeats 16 Similarity 0.655886
Consensus:
GTAAATCGACAcACnacgTC
>Cele-UNSB01_2:13754985-13755264 Satlength=280 Nr of Repeats=7 RepeatLength=35
seed=TTGCCGGAAT
TTGCCGGAATTGAATATTTGCGGCAAATCTGTAAT
TTGCCGGAATTGAATATTTGCGGCAAATCTGTAAT
TTGCCGGAATTAAATGTTTCCGGAACATCGGCAAA
TTGCCGGTATTAAATGTTTCCGGAACATCGGCAAA
TTGCCGGAATTGAGTATTTCCGGTAATTCGGCAAA
TTGCCGGAATTGAGTATTTCCGGCAAATCGGCAAA
TTGCCGGAATTGAAATTTCCAGCAAATCGGCAAATTGCCGTAAATGATTATTTCCGGCAAATCGGCAAA
Nr of selected repeats 6 Similarity 0.784127
Consensus:
TTGCCGGAATTgAaTaTTTcCGGcAaATCgGcAAa
>Cele-UNSB01_2:13755448-13762935 Satlength=7488 Nr of Repeats=166
RepeatLength=45 seed=ACCAGGAGGT
ACCAGGAGGTACCCTTCTTATCCTTCGGAGCTGAATGATCAGATT
ACCAGGAGGTACTCTTCTTATCCTTCGGAGCTGAATGATCAGATC
ACCAGGAGGTACCCTTCTGATCCTTCGGAGCTGAAGGATCAGATC
ACCAGGAGGTACCCTTCTTATCCTTCGGAGCTGAATGATCAGATT
ACCAGGAGGTACTCTTCTTATCCTTCGGAGCTGAATGATCAGATC
ACCAGGAGGTACCCTTCTTATCCTTTGAAGCTGAAGGATCAGATC
ACCAGGAGGTACCCTTCTTATCCTTCGGAGCTGAATGATCAGATT
ACCAGGAGGTACTCTTCTTATCCTTCGGAGCTGAATGATCAGATC
ACCAGGAGGTACCCTTCTGATCCTTCGGAGCTGAAGGATCAGATC
ACCAGGAGGTACTCTTCTTATCCTTCGGAGCTGAATGATCAGATC
ACCAGGAGGTACCCTTCTTATCCTTCGGAGCTGAAGGATCAGATC
ACCAGGAGGTACCCTTCTTATCCTTCGGAGCTGAAGGATCAGATC
ACCAGGAGGTACCCTTCTTATCCTTCGGAGCTGAATGATCAGATT
ACCAGGAGGTACTCTTCTTATCCTTCGGAGCTGAATGATCAGATC
ACCAGGAGGTACCCTTCTGATCCTTCGGAGCTGAAGGATCAGATC
ACCAGGAGGTACCCTTCTTATCCTTCGGAGCTGAATGATCAGATT
ACCAGGAGGTACTCTTCTTATCCTTCGGAGCTGAAGGATCAGATC
ACCAGGAGGTACCCTTCTTATCCTTCGGAGCTGAATGATCAGATT
ACCAGGAGGTACTCTTCTTATCCTTCGGAGCTGAATGATCAGATC
ACCAGGAGGTACCCTTCTGATCCTTCGGAGCTGAATGATCAGATT

ACCAGGAGGTACTCTTCTTATCCTTCGGAGCTGAATGATCAGATC
ACCAGGAGGTACCCTTCTTATCCTTTGAAGCTGAAGGATCAGATC
ACCAGGAGGTACCCTTCTTATCCTTCGGAGCTGAATGATCAGATT
ACCAGGAGGTACTCTTCTTATCCTTCGGAGCTGAATGATCAGATC
ACCAGGAGGTACCCTTCTGATCCTTCGGAGCTGAAGGATCAGATC
ACCAGGAGGTACCCTTCTTATCCTTCGGAGCTGAATGATCAGATT
ACCAGGAGGTACTCTTCTTATCCTTCGGAGCTGAAGGATCAGATC
ACCAGGAGGTACCCTTCTTATCCTTCGGAGCTGAATGATCAGATT
ACCAGGAGGTACTCTTCTTATCCTTCGGAGCTGAATGATCAGATC
ACCAGGAGGTACCCTTCTGATCCTTCGGAGCTGAAGGATCAGATC
ACCAGGAGGTACCCTTCTTATCCTTCGGAGCTGAATGATCAGATT
ACCAGGAGGTACTCTTCTTATCCTTCGGAGCTGAATGATCAGATC
ACCAGGAGGTACCCTTCTTATCCTTTGAAGCTGAAGGATCAGATC
ACCAGGAGGTACCCTTCTTATCCTTCGGAGCTGAATGATCAGATT
ACCAGGAGGTACTCTTCTTATCCTTCGGAGCTGAATGATCAGATC
ACCAGGAGGTACCCTTCTTATCCTTTGAAGCTGAAGGATCAGATC
ACCAGGAGGTACCCTTCTTATCCTTCGGAGCTGAAGGATCAGATC
ACCAGGAGGTACCCTTCTTATCCTTTGAAGCTGAAGGATCAGATC
ACCAGGAGGTACCCTTCTTATCCTTCGGAGCTGAATGATCAGATT
ACCAGGAGGTACTCTTCTTATCCTTCGGAGCTGAATGATCAGATC
ACCAGGAGGTACCCTTCTTATCCTTTGAAGCTGAAGGATCAGATC
ACCAGGAGGTACCCTTCTTATCCTTCGGAGCTGAATGATCAGATT
ACCAGGAGGTACTCTTCTTATCCTTCGGAGCTGAATGATCAGATC
ACCAGGAGGTACCCTTCTTATCCTTTGAAGCTGAAGGATCAGATC
ACCAGGAGGTACCCTTCTTATCCTTCGGAGCTGAAGGATCAGATC
ACCAGGAGGTACCCTTCTTATCCTTTGAAGCTGAAGGATCAGATC
ACCAGGAGGTACCCTTCTTATCCTTCGGAGCTGAATGATCAGATT
ACCAGGAGGTACTCTTCTTATCCTTCGGAGCTGAATGATCAGATC
ACCAGGAGGTACCCTTCTTATCCTTTGAAGCTGAAGGATCAGATC
ACCAGGAGGTACCCTTCTTATCCTTCGGAGCTGAAGGATCAGATC
ACCAGGAGGTACCCTTCTTATCCTTCGGAGCTGAATGATCAGATT
ACCAGGAGGTACTCTTCTTATCCTTCGGAGCTGAATGATCAGATC
ACCAGGAGGTACCCTTATGATCCTTTGAAGCTGAAGGATCAGATC
ACCAGGAGGTACCCTTCTTATCCTTCGGAGCTGAATGATCAGATT
ACCAGGAGGTACTCTTCTTATCCTTCGGAGCTGAATGATCAGATC
ACCAGGAGGTACCCTTCTGATCCTTCGGAGCTGAAGGATCAGATC
ACCAGGAGGTACCCTTCTTATCCTTCGGAGCTGAATGATCAGATT
ACCAGGAGGTACTCTTCTTATCCTTCGGAGCTGAATGATCAGATC
ACCAGGAGGTACCCTTCTTATCCTTTGAAGCTGAAGGATCAGATC
ACCAGGAGGTACCCTTCTTATCCTTCGGAGCTGAAGGATCAGATC
ACCAGGAGGTACTCTTCTTATCCTTCGGAGCTGAATGATCAGATC
ACCAGGAGGTACCCTTATGATCCTTTGAAGCTGAAGGATCAGATC
ACCAGGAGGTACCCTTCTTATCCTTCGGAGCTGAATGATCAGATT
ACCAGGAGGTACTCTTCTTATCCTTCGGAGCTGAATGATCAGATC
ACCAGGAGGTACCCTTCTGATCCTTCGGAGCTGAAGGATCAGATC
ACCAGGAGGTACCCTTCTTATCCTTCGGAGCTGAATGATCAGATT
ACCAGGAGGTACTCTTCTTATCCTTCGGAGCTGAATGATCAGATC
ACCAGGAGGTACCCTTCTTATCCTTTGAAGCTGAAGGATCAGATC
ACCAGGAGGTACCCTTCTTATCCTTCGGAGCTGAAGGATCAGATC
ACCAGGAGGTACCCTTCTTATCCTTCGGAGCTGAATGATCAGATT
ACCAGGAGGTACTCTTCTTATCCTTCGGAGCTGAATGATCAGATT
ACCAGGAGGTACTCTTCTTATCCTTCGGAGCTGAATGATCAGATT
ACCAGGAGGTACTCTTCTTATCCTTCGGAGCTGAATGATCAGATC
ACCAGGAGGTACCCTTCTGATCCTTCGGAGCTGAAGGATCAGATC

ACCAGGAGGTACCCTTCTTATCCTTCGGAGCTGAATGATCAGATT
ACCAGGAGGTACTCTTCTTATCCTTCGGAGCTGAATGATCAGATC
ACCAGGAGGTACCCTTCTTATCCTTCGGAGCTGAATGATCAGATT
ACCAGGAGGTACTCTTCTTATCCTTCGGAGCTGAATGATCAGATC
ACCAGGAGGTACCCTTCTTATCCTTTGAAGCTGAAGGATCAGATC
ACCAGGAGGTACCCTTCTTATCCTTCGGAGCTGAAGGATCAGATC
ACCAGGAGGTACTCTTCTTATCCTTCGGAGCTGAATGATCAGATT
ACCAGGAGGTACTCTTCTTATCCTTCGGAGCTGAATGATCAGATC
ACCAGGAGGTACCCTTCTGATCCTTCGGAGCTGAAGGATCAGATC
ACCAGGAGGTACCCTTCTTATCCTTCGGAGCTGAATGATCAGATT
ACCAGGAGGTACTCTTCTTATCCTTCGGAGCTGAATGATCAGATC
ACCAGGAGGTACCCTTCTGATCCTTCGGAGCTGAAGGATCAGATC
ACCAGGAGGTACCCTTCTTATCCTTCGGAGCTGAATGATCAGATT
ACCAGGAGGTACTCTTCTTATCCTTCGGAGCTGAATGATCAGATC
ACCAGGAGGTACCCTTCTGATCCTTCGGAGCTGAAGGATCAGATC
ACCAGGAGGTACCCTTCTTATCCTTTGAAGCTGAAGGATCAGATC
ACCAGGAGGTACCCTTCTTATCCTTCGGAGCTGAAGGATCAGATC
ACCAGGAGGTACCCTTCTTATCCTTCGGAGCTGAATGATCAGATT
ACCAGGAGGTACTCTTCTTATCCTTCGGAGCTGAATGATCAGATC
ACCAGGAGGTACCCTTATGATCCTTTGAAGCTGAAGGATCAGATC
ACCAGGAGGTACCCTTCTTATCCTTCGGAGCTGAATGATCAGATT
ACCAGGAGGTACTCTTCTTATCCTTCGGAGCTGAATGATCAGATC
ACCAGGAGGTACCCTTCTGATCCTTCGGAGCTGAAGGATCAGATC
ACCAGGAGGTACCCTTCTTATCCTTCGGAGCTGAATGATCAGATT
ACCAGGAGGTACTCTTCTTATCCTTCGGAGCTGAATGATCAGATC
ACCAGGAGGTACCCTTCTTATCCTTTGAAGCTGAAGGATCAGATC
ACCAGGAGGTACCCTTCTTATCCTTCGGAGCTGAAGGATCAGATC
ACCAGGAGGTACTCTTCTTATCCTTCGGAGCTGAATGATCAGATC
ACCAGGAGGTACCCTTATGATCCTTTGAAGCTGAAGGATCAGATC
ACCAGGAGGTACCCTTCTTATCCTTCGGAGCTGAATGATCAGATT
ACCAGGAGGTACTCTTCTTATCCTTCGGAGCTGAATGATCAGATC
ACCAGGAGGTACCCTTCTGATCCTTCGGAGCTGAAGGATCAGATC
ACCAGGAGGTACCCTTCTTATCCTTCGGAGCTGAATGATCAGATT
ACCAGGAGGTACTCTTCTTATCCTTCGGAGCTGAATGATCAGATC
ACCAGGAGGTACCCTTCTTATCCTTTGAAGCTGAAGGATCAGATC
ACCAGGAGGTACCCTTCTTATCCTTCGGAGCTGAAGGATCAGATC
ACCAGGAGGTACCCTTCTTATCCTTCGGAGCTGAATGATCAGATT
ACCAGGAGGTACTCTTCTTATCCTTCGGAGCTGAATGATCAGATT
ACCAGGAGGTACTCTTCTTATCCTTCGGAGCTGAATGATCAGATT
ACCAGGAGGTACTCTTCTTATCCTTCGGAGCTGAATGATCAGATC
ACCAGGAGGTACCCTTCTGATCCTTCGGAGCTGAAGGATCAGATC
ACCAGGAGGTACCCTTCTTATCCTTCGGAGCTGAATGATCAGATT
ACCAGGAGGTACTCTTCTTATCCTTCGGAGCTGAATGATCAGATC
ACCAGGAGGTACCCTTCTTATCCTTCGGAGCTGAATGATCAGATT
ACCAGGAGGTACTCTTCTTATCCTTCGGAGCTGAATGATCAGATC
ACCAGGAGGTACCCTTCTTATCCTTTGAAGCTGAAGGATCAGATC
ACCAGGAGGTACCCTTCTTATCCTTCGGAGCTGAAGGATCAGATC
ACCAGGAGGTACTCTTCTTATCCTTCGGAGCTGAATGATCAGATT
ACCAGGAGGTACTCTTCTTATCCTTCGGAGCTGAATGATCAGATC
ACCAGGAGGTACCCTTCTGATCCTTCGGAGCTGAAGGATCAGATC
ACCAGGAGGTACCCTTCTTATCCTTCGGAGCTGAATGATCAGATT
ACCAGGAGGTACTCTTCTTATCCTTCGGAGCTGAATGATCAGATC
ACCAGGAGGTACCCTTCTGATCCTTCGGAGCTGAAGGATCAGATC
ACCAGGAGGTACCCTTCTTATCCTTCGGAGCTGAATGATCAGATT

ACCAGGAGGTACTCTTCTTATCCTTCGGAGCTGAATGATCAGATC
ACCAGGAGGTACCCTTCTGATCCTTCGGAGCTGAAGGATCAGATC
ACCAGGAGGTACCCTTCTTATCCTTCGGAGCTGAATGATCAGATT
ACCAGGAGGTACTCTTCTTATCCTTCGGAGCTGAATGATCAGATC
ACCAGGAGGTACCCTTCTTATCCTTTGAAGCTGAAGGATCAGATC
ACCAGGAGGTACCCTTCTTATCCTTCGGAGCTGAAGGATCAGATC
ACCAGGAGGTACTCTTCTTATCCTTCGGAGCTGAATGATCAGATC
ACCAGGAGGTACCCTTATGATCCTTTGAAGCTGAAGGATCAGATC
ACCAGGAGGTACCCTTCTTATCCTTCGGAGCTGAATGATCAGATT
ACCAGGAGGTACTCTTCTTATCCTTCGGAGCTGAATGATCAGATC
ACCAGGAGGTACCCTTCTGATCCTTCGGAGCTGAAGGATCAGATC
ACCAGGAGGTACCCTTCTTATCCTTCGGAGCTGAATGATCAGATT
ACCAGGAGGTACTCTTCTTATCCTTCGGAGCTGAATGATCAGATC
ACCAGGAGGTACCCTTCTTATCCTTTGAAGCTGAAGGATCAGATC
ACCAGGAGGTACCCTTCTTATCCTTCGGAGCTGAAGGATCAGATC
ACCAGGAGGTACCCTTCTTATCCTTCGGAGCTGAATGATCAGATT
ACCAGGAGGTACTCTTCTTATCCTTCGGAGCTGAATGATCAGATTTTTTTTTTGATCAGATT
ACCAGGAGGTACTCTTCTTATCCTTCGGAGCTGAATGATCAGATC
ACCAGGAGGTACCCTTCTGATCCTTCGGAGCTGAAGGATCAGATC
ACCAGGAGGTACCCTTCTTATCCTTCGGAGCTGAATGATCAGATT
ACCAGGAGGTACTCTTCTTATCCTTCGGAGCTGAATGATCAGATC
ACCAGGAGGTACCCTTCTTATCCTTTGAAGCTGAAGGATCAGATC
ACCAGGAGGTACCCTTCTTATCCTTCGGAGCTGAAGGATCAGATC
ACCAGGAGGTACCCTTCTTATCCTTCGGAGCTGAATGATCAGATT
ACCAGGAGGTACTCTTCTTATCCTTCGGAGCTGAATGATCAGATC
ACCAGGAGGTACCCTTCTTATCCTTTGAAGCTGAAGGATCAGATC
ACCAGGAGGTACCCTTCTTATCCTTCGGAGCTGAAGGATCAGATC
ACCAGGAGGTACCCTTCTTATCCTTCGGAGCTGAATGATCAGATT
ACCAGGAGGTACTCTTCTTATCCTTCGGAGCTGAATGATCAGATC
ACCAGGAGGTACCCTTCTTATCCTTTGAAGCTGAAGGATCAGATC
ACCAGGAGGTACCCTTCTTATCCTTCGGAGCTGAATGATCAGATT
ACCAGGAGGTACTCTTCTTATCCTTCGGAGCTGAATGATCAGATC
ACCAGGAGGTACCCTTCTTATCCTTCGGAGCTGAATGATCAGATC
ACCAGGAGGTACCCTTCTTATCCTTTGAAGCTGAAGGATCAGATC
ACCAGGAGGTACCCTTCTTATCCTTCGGAGCTGAATGATCAGATT
ACCAGGAGGTACTCTTCTTATCCTTCGGAGCTGAATGATCAGATC
ACCAGGAGGTACCCTTCTGATCCTTCGGAGCTGAAGGATCAGATC
ACCAGGAGGTACCCTTATGATCCTTTGAAGCTGAAGGATCAGATC
Nr of selected repeats 165 Similarity 0.932682
Consensus:
ACCAGGAGGTACcCTTCTTATCCTTCGGAGCTGAAtGATCAGATc
>Cele-UNSB01_2:13794379-13794568 Satlength=190 Nr of Repeats=10 RepeatLength=16
seed=CCTACATGCC
CCTACATGCCTTCATG
CCTACATGCCTACATG
CCTACATGCCTACATG
CCTACATGCCTACATG
CCTACATGCCTACATGCCTAG
CCTACATGCCTACATG
CCTACATGCCTACATG
CCTACATGCCTACATG
CCTACATGCCTACATGCCTACATGAATACATG
CCTACATGCCTACATGCCTACATG
Nr of selected repeats 7 Similarity 0.976190

Consensus:
CCTACATGCCTACATG
>Cele-UNSB01_2:13805325-13805605 Satlength=281 Nr of Repeats=7 RepeatLength=35
seed=CTTGCCGATT
CTTGCCGATTTTTCAGAAATTTTCAATTCCGGCAA
CTTGCCGATTTGCCGGAAATTCTCAATTCCGGCAATTTGTCGATTTGCCGGAAATTTTTAATTCCGCCAA
CTTGCCGATTTGCCGGAAATTTTCAATTCCGGCAA
CTTGCCGATTTGCCAGAAATTTTCATTTTCGGCAA
CTTGCCGATTTGCCAGAAATTTTCATTTTCGGCAA
CTTGCCGATTTGCCAGAAATTTTCAATTCCAGCAA
CTTGCCGATTTGCCGGAAATTCTGAATTCCGCCAA
Nr of selected repeats 6 Similarity 0.862857
Consensus:
CTTGCCGATTTGCCaGAAATTTTCAaTTcCGGCAA
>Cele-UNSB01_2:13813351-13813423 Satlength=73 Nr of Repeats=6 RepeatLength=12
seed=TAGGCTTAGG
TAGGCTTAGGCT
TAGGCTTAGGCT
TAGGCTTAGGCT
TAGGCTTAGGCT
TAGGCTTAGGCT
TAGGTTTAGGCT
Nr of selected repeats 6 Similarity 0.962963
Consensus:
TAGGCTTAGGCT
>Cele-UNSB01_2:13831609-13831953 Satlength=345 Nr of Repeats=12 RepeatLength=27
seed=TTCCGGCAAA
TTCCGGCAAAACGGCAATTGAAATT
TTCCGGCAAATCGACAAATTGCCGGAATTGAAAATTTGAAAAT
TTCCGGCAAATCGTCAAATTGCCGGAATTTAAAAT
TTCCGGCAAATGGGCAATTGTAAAC
TTCCGGCAAATCGGCAATTTTGAAAAT
TTCCGGCAAATCGGCAATTTTAAAAAT
TTCCGGCAAATCGGCATTTTTGAAAAT
TTCCGGCAAATCGGCAAATTGCAAAAT
TTCCGGCAAATCGGCAAATTGCAAAAT
TTCCGGCAAATCGGCAAATTGCAAAAT
TTCCGGCAAATCGGCAAATTGCAAAAT
TTCCGGCAAATCGGCAAATTGCAAAAT
Nr of selected repeats 8 Similarity 0.904762
Consensus:
TTCCGGCAAATCGGCAAaTTgcAAAAT
>Cele-UNSB01_2:13844810-13844989 Satlength=180 Nr of Repeats=5 RepeatLength=36
seed=TTGCCTAAAA
TTGCCTAAAATTAAAATTTCCAGCAAATCGGCAAAT
TTGCCTAAAATGAAAATTCCGGCAAATCAGCAAAA
TTGCCTAAAATTAAAATTTCCGGCAAATCAGCAAAT
TTGCCTAAAATTAAAATTACCGGCAAATCAGCAAAT
TTGCCTAAAATTAAAATTTCCGGCAAATCGGCAAAT
Nr of selected repeats 4 Similarity 0.938272
Consensus:
TTGCCTAAAATTAAAATTtCCgGCAAATCaGCAAAT
>Cele-UNSB01_2:13878619-13878763 Satlength=145 Nr of Repeats=10 RepeatLength=12
seed=GCCTAAGCCT

GCCTAAGCCTAAATAAGCGTAA
GCCGAAGCCTAA
GTCTAAGCCTAAAA
GCCTAATCCTAAATCTTA
GCCTAAGCCTAG
GCTTAAGCCTCA
GCCTAAGCCTAA
GCCTCAGCCTAA
GTCTAAGCCTCT
GCCTAAGCCTAAGCCTCA
Nr of selected repeats 6 Similarity 0.725926
Consensus:
GCCTAAGCCTaa
>Cele-UNSB01_2:13900624-13901003 Satlength=380 Nr of Repeats=15 RepeatLength=21
seed=CTCGTAAATC
CTCGTAAATCGACAGGGATCC
CTCGTAGATCGACAAGAACTC
CTCGCAAATCGACAAAAGCTC
CTCGTAAATCTACAAGAACTCCTTGTAAATTGACAATAACTC
CTCGTAAATCCACAAGACCTCTTCGTAAATAGACAAAGAATC
CTCGTAAATCTACAAGCAATC
CTCGTAAATCGACAAGAACTC
CTCGTAAATCGGCAAGAACTC
CTCGTAATTCGACAAGAACTC
CTCGTAAATCCACAAGGACTCCTTGTAAATTGACAATAACTC
CTCGTAAATCGACAAAAACTCA
TCGTAAATCGACAAGAAAAC
CTCGTAAATCGACAAAAAACTC
CTCGTAAATCGACAAGTACTC
CTCGTAAATCGACAAGGATCT
Nr of selected repeats 9 Similarity 0.754630
Consensus:
CTCGTAAATCGACAAGaAcTC
>Cele-UNSB01_2:13903082-13903160 Satlength=79 Nr of Repeats=5 RepeatLength=13
seed=GGCTTCTAGT
GGCTTCTAGTCCA
GGCTTCTAGTCTAGGCTTCGGGTCTA
GGCTTCTAGTCTA
GGCTTCTAGTCTC
GGCTTCTAGTCCA
Nr of selected repeats 4 Similarity 0.880342
Consensus:
GGCTTCTAGTCca
>Cele-UNSB01_2:13911573-13912594 Satlength=1022 Nr of Repeats=28
RepeatLength=35 seed=TTTGCCGGAA
TTTGCCGGAAATTTTGAATATTGGCATATTGCCGA
TTTGCTGGAAATTTTCAAATCCGGCAAATTGCTGC
TTTGCCGAAAATTTTGATTTCGGCATTTTGCTGT
TTTGCCGGAAAATTTTAATTCCAGCAA
TTTGCCGGAAATTTCGATTTTCGGCAACATGCCGA
TTTGCCGGAAATTTTGAATTTCGGCATTTTGTCGATTTGCCGG
TTTGCCGAAAATTTTGAATTTCGGTAATTTGTCGA
TTTGCCGGAAGTTTTGATTTTCGGCATTTTGCTGT
TTTGCCGGAAATTTTCGAATATTGGCATTTTCCCGA

TTTGCCGGAAATTTTCAATTCCGGCACATTGCCGA
TTTGTCGGAAATTTTGATTTTTGGCAACTTGCCGA
TTTGCCGGATATTTTGGTTTTCGGTAATATGCCGATTTGCCGG
TTTGCCGGAAATTTTAAAATCCGGCAAATTGCCGGTTTCCTGTAAATTTTAAAATCCGGCTAATAGCCGG
TGTGCCGGAAATTTTCAATTCCAGCAACTTGCCGAT
TTTCCGGAAATTTTAAATTTCGGTAATTTGCCGT
TTTGCCGGAAATTTTGATTTTCGGCATTTTGTCGA
TTCGCCGGAAATTTTGATTTTCGCCATTTTGCCGT
TTTGCCGGAAATTTTGATTTTCGGCATTTTGTCGA
TTTGCCGGATATTTTGAATTCCGGCAAACTTCCGG
TTTGCCGGAAACTTTGATTTTCGCCATTTTGCCGT
TTTGCCGGAAATTTTGATTTTCGGCATTTTGTCGA
TTTGCCGGATATTTTGAATTCCGGCAAACTTCCGG
TTTGCCGGAAACTTTGATTTTCGCCATTTTGTCGA
TTTGCCGGAAATTTTCAAATCCGGCAAATAGTCGA
TTCGCCGGAAATTTTGATTTCGACAATTTGCCGT
TTTGCCGGAAATTTTCAATTCCGGCAATTTGCCGA
TTTGCCGGAAATTTCAATTTCGACAATTTGCCGT
TTTGCCGGAAATTTTCAATTCCGGCAATTTGCCGT
Nr of selected repeats 18 Similarity 0.750016
Consensus:
TTTGCCGGAAATTTTgAaTTtCGGCAatTTGcCGa
>Cele-UNSB01_2:13932545-13934837 Satlength=2293 Nr of Repeats=68
RepeatLength=35 seed=CCGGAATTGA
CCGGAATTGAAATTTCCGGCGAATCGGCAAATTG
CCGGAATTGAAAATTTCTGGAAAATCGGCAAATTG
CCGGAATTGAAAATTTCTGGCATATCGACAATTTG
CCGGAATTGAAAATTTCTGGAAAATCAACAAATTG
CCGGAATTGAAAATTTCTGGAAAATTAACAAATTG
CCGGAATTGAAAATTTCTGGAAAATCAACAAATTG
CCGGAATTGAAAATTTTTGGAAAATTAACAAATTG
CCGGAATTGAAAATTTCTGGAAAATCAACAAATTG
CCGGAATTGAAAATTTCTGGAAAATTAACAAATTG
CCGGAATTGATAATTTCTGGCAAATCGGAAAACTG
CCGGAATTGACAATATCCGGCAAGCGGCAAATTG
CCGGAATTGAAATTTCCGGCGAATCGGCAAATTG
CCGGAATTGAAAATTTCTGGAAAATGGGTAAATTG
CCGGAATTGAAAATTTCTGGAAAATGGGTAAATTG
CCGGAATTGAAAATTTCTGGAAAATCAACAAATTG
CCGGAATTGAAAATTTCTGGCATATCGACAATTTG
CCGGAATTGAAAATTTCTGGAAAATCAACAAATTG
CCGGAATTGAAAATTTCTGGAAAATTAACAAATTG
CCGGAATTGAAAATTTCTGAAAAATCAACAAATTG
CCGGAATTGAAAATTTTTGGAAAATTAACAAATTG
CCGGAATTGAAAATTTCTGGAAAATCAACAAATTG
CCGGAATTGAAAATTTCTGGAAAATTAACAAATTG
CCGGAATTGAAAATTTCTGGAAAATCAACAAATTG
CCGGAATTGACAAATTG
CCGGAATTGATAATTTCTGGCAAATCGGAAAACTG
CCGGAATTGACAATATCCGGCAAGCGGCAAATTG
CCGGAATTGAAATTTCCGGCGAATCGGCAAATTG
CCGGAATTGAAAATTTCTGGAAAATGGGTAAATTG
CCGGAATTGAAAATTTCTGGAAAATGGGTAAATTG
CCGGAATTGAAAATTTCTGGAAAATCAACAAATTG

CCGGGATTGAAAATTTCTGGCATATCGACAATTTG
CCGGAATTGAAAATTTCTGGAAAATCAACAAATTG
CCGGAATTGAAAATTTCTGGAAAATTAACAAATTG
CCGGAATTGAAAATTTCTGGAAAATCAACAAATTG
CCGGAATTGATAATTTCTGGCAAATCGGAAAACTG
CCGGAATTGACAATATCCGGCAAGCGGCAAATTG
CCGGAATTGAAATTTCCGGCGAATCGGCAAATTG
CCGGAATTGAAAATTTCTGGAAAATGGGTAAATTG
CCGGAATTGAAAATTTCTGGAAAATGGGTAAATTG
CCGGAATTGACAATATCCGGCAAGCGGCAAATTG
CCGGAATTGAAAATTTCTGGCATATCGACAATTTG
CCGGAATTGAAAATTTCTGGAAAATCGGCAAATTG
CCGGAATTGAAAATTTCTGGAAAATCAACAAATTG
CCGGAATTGAAAATTTCTGGCATATCGACAAATTG
CCGGAATTGACAAATTG
CCGGAATTGATAATTTCTGGCAAATCGGAAAACTG
CCGGAATTGACAATATCCGGCAAGCGGCAAATTG
CCGGAATTGAAATTTCCAGCAAATCGGCTAATTG
CCGGAATTGAAAATTTCTGGAAAATTGATAAATTG
CCAGAATTGAAATTTCTGGAAAATTGACAAATTT
CCGGAATTGAAAATTCCCGGCAAATCGGCAATTTG
CCGGAATTGACAAATTG
CCGGAATTGATAATTTCTGGCAAATCGGAAAACTG
CCGGAATTGACAATATCCGGCAAGCGGCAAATTG
CCGGAATTGAAATTTCCGGCGAATCGGCAAATTG
CCGGAATTGAAAATTTCTGGAAAATGGGTAAATTG
CCGGAATTGAAAATTTCTGGAAAATGGGTAAATTG
CCGGAATTGAAAATTTCTGGAAAATCAACAAATTG
CCGGAATTGAAAATTTCTGGCATATCGACAATTTG
CCGGAATTGAAAATTTCTGGAAAATCGGCAAATTG
CCGGAATTGAAAATTTCTGGAAAATCAACAAATTG
CCGGAATTGAAAATTTCTGGCATATCGACAAATTG
CCGGAATTGACAAATTG
CCGGAATTGATAATTTCTGGCAAATCGGAAAACTG
CCGGAATTGACAATATCCGGCAAGCGGCAAATTG
CCGGAATTGAAATTTCCAGCAAATCGGCTAATTG
CCGGAATTGAAAATTTCTGGAAAATTGATAAATTG
CCAGAATTGAAATTTCTGGAAAATTGACAAATTT
Nr of selected repeats 48 Similarity 0.862310
Consensus:
CCGGAATTGAAAATTTCTGGaAAATcgacAAATTG
>Cele-UNSB01_2:13962674-13962948 Satlength=275 Nr of Repeats=23 RepeatLength=10
seed=CCGCTACCGC
CCGCTACCGC
CCGCCACCGCCTGCTGATGC
CCGCTACCGGCCTGCTACAGCA
CCGCTACCGCCCTGCTACAGCA
CCGCTACCGC
CCGCTACCGC
CCGCTACCGC
CCGCTACCGC
CCGCTACCGC
CCGCTACCGC
CCGCTACCGC

CCGCTACCGC
CCGCTACCGC
CCGCTACCGC
CCGCTACCGC
CCGCTACCGC
CCGCTACCGC
CCGCTACCGC
CCGCTACCGC
CCGCTACCGC
CCGCTACCGC
CCGCTACCGC
CCGCTACCGCCCGCTACCTC
Nr of selected repeats 19 Similarity 1.000000
Consensus:
CCGCTACCGC
>Cele-UNSB01_2:13967191-13967257 Satlength=67 Nr of Repeats=5 RepeatLength=11
seed=CCGCTACCGC
CCGCTACCGCC
CCGCTACCGCA
CCGCTACCGCCCCGCTTTCGCC
CCGCTACCGCC
CCGCTACCGCA
Nr of selected repeats 4 Similarity 0.919192
Consensus:
CCGCTACCGCa
>Cele-UNSB01_2:13968253-13968583 Satlength=331 Nr of Repeats=23 RepeatLength=12
seed=GCCTAAGCCT
GCCTAAGCCTAAGCTTAG
GCCTAAGCCTAA
GCCCAAGCCTAC
GCATAAGCCTAA
GCCTAAGCCTGA
GCCTAAGCTTAA
GCCTAAGCCTAA
GCCTAAGCCTAA
GACTAAGCCTAAGCCCAA
GCCCAAGCCTAC
GCCTAAGCCTAA
GCCTAAACCTGAGCCTAAGCAAAAGCCAAA
GCTTAAGCCTAAA
CCTAAGCCTAAGACTGA
GCCTTAGCCTAA
GCTTAAGCCTGA
GCCTAAGCCTAAGCCTGATCCTAG
GCCTAAGCCTAG
GCCTAAGCCTAG
GCCTAAGCCTAG
GCCTAAGACTAAACCTAAA
CCTAAGCCTAA
GCCTATGCCTAT
Nr of selected repeats 15 Similarity 0.803175
Consensus:
GCCTAAGCCTAa

>Cele-UNSB01_2:13971711-13972389 Satlength=679 Nr of Repeats=37 RepeatLength=18
seed=AATTTACCAA
AATTTACCAAAATTTGACATTTTGTTAAAATTTGGCAATTTTCCATAATTTGACAATTATTAATTTTCAA
AATTTACCAATTTACCAA
AATTTCCCAAGTTCCCAA
AATTTACCAAAATTGGGC
AATTTACCAA
AATTTACCAA
AATTTACCAATTTTCCAA
AATTTACCAATTTACCAA
AATTTACCAATTTACCAA
AATTTACCAATTTTCCGA
AATTTACCAATTTACCAA
AATTTACCAATTTACCAA
AATTTACCAATTTTCCAA
AATTTACCAATTTACCAA
AATTTACCAATTTTCCAA
AATTTACCAATTTACCAA
AATTTACCAAAATTGGGC
AATTTACCAA
AATTTACCAATTTACCAA
AATTTACCAATTTACCAA
AATTTACCAATTTTCCAA
AATTTACCAATTTACCAA
AATTTACCAATTTTCCAA
AATTTACCAATTTACCAA
AATTTACCAAAATTGGGC
AATTTACCAA
AATTTACCAATTTACCAAAATTTGGC
AATTTACCAAAACTGGGC
AATTTACCAA
AATTTACCAATTTACCAA
AATTTACCAATTTTCCAA
AATTTACCAATTTACCAA
AATTTACCAATTTTCCAA
AATTTACCAATTTACCAA
AATTTACCAAAATTGGGC
AATTTACCAA
AATTTACCAATTTACCAA
Nr of selected repeats 29 Similarity 0.745813
Consensus:
AATTTACCAATTTaCCaA
>Cele-UNSB01_2:13977826-13977988 Satlength=163 Nr of Repeats=10 RepeatLength=12
seed=GCCTAAGCCT
GCCTAAGCCTAAACTTAAGGCTAACCCTAAGTTAAG
GCCTAAGCATGAGCCTAAGAATGA
GCCTAAGCCTTA
GCCTACGCCTTA
GCCTACGCCTAG
GCCTAAGCCTAA
GCCTAAGCCTAAGCTCAA
GCCTAAGCCTAA
GCCTAAGCTCAA
GCCTAAGCCTAA

Nr of selected repeats 7 Similarity 0.798942
Consensus:
GCCTAaGCCTaA
>Cele-UNSB01_2:13981365-13981461 Satlength=97 Nr of Repeats=7 RepeatLength=12
seed=TTAGGCTTAG
TTAGGCTTAGGCTTTGGCTTCGGC
TTCGGCTTAGGA
TTAGGTTTAGGC
TTAGGCTTAGTC
TTAGGCTTCGGC
TTAGACTTAGGC
TTAGGCTTAGTC
Nr of selected repeats 6 Similarity 0.755556
Consensus:
TTAGGCTTAGgC
>Cele-UNSB01_2:14003653-14004477 Satlength=825 Nr of Repeats=18 RepeatLength=34
seed=AATTCCGGCA
AATTCCGGCAATTTGCTGATTCATCAGAAATTTC
AATTCCGGCAACTTGCCTATTTTCCGGAAATTTC
AATTCCGGCAATTTGCTGATTTATCAGAAATTTC
AATTCCGGCAACTTGCCTATTTTCCGGAAATTTC
AATTCTGGCAATTCGCTGATTTTCCGGAAATTTC
AATTCCGGCAATTTGCTGATTTATCGGAAATTTC
AATTCCGGCAATTTGCTGATTTATCAGTAACTTC
AATTCCGGCAATTTGCCTATTTTCCGGAAATTTC
AATTCCGGCAATTTGGCGAACTACCAGAAACTTTCAATTTTGCCGGTTTACCGATTTTCCGGAAATTTC
AATTCCGGCAATTTGCTGATTTATCGGAAACTTC
AATTCCGGCAATTTGGCGATTTGCCAGAAATTTTCAATTTTGCCGGTTCACCGATTTTCCGGAAACTTC
AATTCCGGCAATTTGGCGATTTGTCAGAAACTTTCAATTTTGCCGGTTTACCGATTTTCCGGAAACTTC
AATTCCGGCAATTTGCTGATTTATCGGAAATTTC
AATTCCGGCAATTTGGCGATTTGCCAGAAATTTTCAATTTTGCCGGTTCACCGATTTTCCGGAAATTTC
AATTCCGGCAATTTGGCGATTTGTCAGAAATTTTCAATTTTGCCGGTTCACCGATTTTCCGGAAATTTC
AATTCCGGCAATTTTCTGATTAGTCGGAAATTTCAATCCTCCGGCAATTTTGCCGATTTGCCAGAAATTTC
AATTCAGGCAATTTTCCAATTTGTCGGAAGTTTC
AATTCCCGCAATTTTCCGATTTGCCGGAAATTTT
Nr of selected repeats 12 Similarity 0.808081
Consensus:
AATTCCGGCAATTTGCtgATTTatCgGAAATTTC
>Cele-UNSB01_2:14078695-14078860 Satlength=166 Nr of Repeats=5 RepeatLength=33
seed=AAAATTTGAA
AAAATTTGAATTTCCCGTCAAAATTTTTCTCAG
AAAATTCGAATTTGGCGCCAAAATTTTTCACAA
AAAATCTGAATTTCTCGCCAAAATGTTTCTCGG
AAAATTTGAATTTCCCGCTAAAAATTTTCCCAG
AAAATTTGAACTTCCTGCCAAAATTGTTCTCAG
Nr of selected repeats 5 Similarity 0.749495
Consensus:
AAAATttGAAtTTcccGccAAAAtttTTCtCag
>Cele-UNSB01_2:14106829-14106943 Satlength=115 Nr of Repeats=7 RepeatLength=12
seed=AGCCTAAGCC
AGCCTAAGCCTAAACCTGAATGCGAGCCTGAGTTTGA
ACCTAAGCCTAC
ACCTAAGCCTGAACCTG
AGCCTAAGCCCA

AGCTTAAGCCTA
AGCCTAAGCCTA
AGTCTAAGCCTA
Nr of selected repeats 5 Similarity 0.733333
Consensus:
AgccTAAGCCtA
>Cele-UNSB01_2:14136989-14138428 Satlength=1440 Nr of Repeats=41
RepeatLength=14 seed=TACAAACTAC
TACAAACTACAAAC
TACAAACTACAAAC
TACAAACTACAAACTACAAATC
ACAAACTACCAACTACAACTATAAACTGCTTTAAGGTAAAATGGCATTCATGACGATTCATGTTGTACACGATTATACA
CTAGACTTTTAGCCTCTAATACTAGGAAAAAACTAGGAATGTTATAGAC
TACAACCTACAAAC
TACAATCTACAAAT
TACAAATTACAAAC
TACAAACTACAAAC
TACAAACTACAAAC
TACAAACTACAAATC
ACAAACTACCAACTACAACTATAAACTGCTTTAAGGTAAAATGGCATTCATGACGATTCATGTTGTACACGATTATACA
CTAGACTTTTAGCCTCTAATACTAGGAAAAAACTAGGAATGTTATAGAC
TACAACCTACAAAC
TACAATCTACAAAT
TACAAATTACAAAC
TACAAACTACAAAC
TACAAACTACAAAC
TACAAACTACAAATC
ACAAACTACCAACTACAACTATAAACTGCTTTAAGGTAAAATGGCATTCATGACGATTCATGTTGTACACGATTATACA
CTAGACTTTTAGCCTCTAATACTAGGAAAAAACTAGGAATGTTATAGACTACAACT
TACAAACTACAATC
TACAAATTACAAAT
TACAAACTACAAAC
TACAAACTACAAAC
TACAAACTACAAATC
ACAAACTACCAACTACAACTATAAACTGCTTTAAGGTAAAATGGCATTCATGACGATTCATGTTGTACACTATTATACA
CTAGACTTTTAGCCTCTAATACTAGGAAAAAACTAGGACTGTTATAGAC
TACAACCTACAAAC
TACAATCTACAAAT
TACAAATTACAAAC
TACAAACTACAAAC
TACAAACTACAAACTACAAATC
ACAAACTACCAACTACAACTATAAACTGCTTTAAGGTAAAATGGCATTCATGACGATTCATGTTGTACACTATTATACA
CTAGACTTTTAGCCTCTAATACTAGGAAAAAACTAGGACTGTTATAGAC
TACAACCTACAAAC
TACAATCTACAAAT
TACAAATTACAAAC
TACAAACTACAAAC
TACAAACTACAAACTACAAATC
ACAAACTACCAACTACAACTATAAACTGCTTTAAGGTAAAATGGCATTCATGACGATTCATGTTGTACACTATTATACA
CTAGACTTTTAGCCTCTAATACTAGGAAAAAACTAGGACTGTTATAGAC
TACAACCTACAAAC
TACAATCTACAAAT
TACAAATTACAAAC
TACAAACTACAAACTATAATC

TACAAACTACTTTATGAAATAAGGAAATTTTGGACTGTGCTTTTTAAAGTTTTTTTTTGGCCTTTTAGTGGCCAGGTAG
GTAATGTGACGACACCTACAGACCTATAGCAGATAGGCACGAGGTAGGCAGGTATTTTAGGAGCATACTACAACC
Nr of selected repeats 27 Similarity 0.871659
Consensus:
TACAAacTACAAAc
>Cele-UNSB01_2:14141898-14142455 Satlength=558 Nr of Repeats=7 RepeatLength=62
seed=TGCTAACTAG
TGCTAACTAGGTAGAACCCGCTCGCGAAACGGCTAATGCGCGGTCCCAGTTGGTTGTTGGCCGCTAAATATCTAGGACC
CACGTAGCTGTCGGCTGCACTTGCTGTCCCTGTTGGTTGTCGGCTGCTAGCTCGGTAGGGTTCGCTTAGGTGTCGGCTG
CATTAGCAGTCCTGGTTTAATGTCGGC
TGCTAACTAGCGCGGGCCTGCTTAGGTGCCGGCTGCGAAAGCAGTCCTGGTTTAATGTCGGC
TGCTAACTAGGTACGGCCAGCTTAAATGTCGGATGCACGAGTAGTCCTGGTTTAATGTCGGT
TGCTAACTAGCTAGGTCCCGCTTAGGTGCCGGCTGCACGAGCAGTCCTGGTTTAATGCCGGC
TGCTAACTAGGCACGGCCAGCTTAGGTGCCGGCTGCACGAGCAGTCCTGGTTTAATGTCGGT
TGCTAACTAGCTAGGTCCCGCTTAGGTGCCGGCTGCACGAGCAGTCCTGGTTTAATTCCGGC
TGCTAACTAGGCACGGCCAGCTTAGATGTCGGATGCACGAGCAGTCCTGGTTTAATGTCGGT
Nr of selected repeats 6 Similarity 0.822222
Consensus:
TGCTAACTAGctAcGgCCaGCTTAGgTGcCGGcTGCACGAGCAGTCCTGGTTTAATGtCGGc
>Cele-UNSB01_2:14148263-14148341 Satlength=79 Nr of Repeats=6 RepeatLength=12
seed=GGCTTTGGCT
GGCTTTGGCTTT
GGCTTAGGCTTATGCTTC
GGCTTTGGCTTT
GGCTTTGGCTTT
GGCTTTGGCTTT
GGCTTTGGCTTT
Nr of selected repeats 5 Similarity 1.000000
Consensus:
GGCTTTGGCTTT
>Cele-UNSB01_2:14148276-14148504 Satlength=229 Nr of Repeats=14 RepeatLength=12
seed=GCTTAGGCTT
GCTTAGGCTTAT
GCTTCGGCTTTG
GCTTTGGCTTTG
GCTTTGGCTTTG
GCTTTGGCTTTG
GCTTTGGCTTTGGCTTTGGATTTG
GCTTAGGCTTAG
GCTTAGGATTTTCCGGTTTTTTTGATTTTTCAGCGGTTCCTGTTGGCCGATTAT
GCTTATGCTTAG
GCTTAGGCTTAG
GCTTACGCTTAG
GCTTAGGCTTGA
GCTTAGTCTTAG
GCTTAAGCTTAGGCTTCG
Nr of selected repeats 11 Similarity 0.711888
Consensus:
GCTTaGGCTTaG
>Cele-UNSB01_2:14178132-14178499 Satlength=368 Nr of Repeats=11 RepeatLength=11
seed=GTACTGTAGG
GTACTGTAGGG
GTACTGTAGGA

GTACTGTAGGAGGGTTACTGTAGTTTAGGAAAAATTGACTTTTCGTCTTTTGAAGAGATATTGGTTTGGGGTTAGTGGA
AGGATATGGTCGGG
GTACTGTAGGG
GTACTATAGGA
GTACTGTAGGAGGGTTACTATAGCTTAAGAAAAATTGACTTTTAGTCTTTTGAAGAGATAATGGTTTGGGGTCAGTGGA
AGGATATGGTCGGG
GTACTGTAGGG
GTACTGTAGGA
GTACTGTAGGAGGGTTACTGTAGTTTAGGAAAAATTGACTTTTCGTCTTTTGAAGAGATATTGGTTTGGGGTTAGTGGA
GAGATATGGTCGGG
GTACTGTAGGG
GTACTATAGGA
Nr of selected repeats 8 Similarity 0.878788
Consensus:
GTACTgTAGGa
>Cele-UNSB01_2:14178691-14179588 Satlength=898 Nr of Repeats=30 RepeatLength=30
seed=TTTCTAGAAA
TTTCTAGAAAAAATCAGAAAACTTTTGGAAC
TTTCTAGAAACATTTGGAAACTTCTAGAAC
TTTCTAGAAAAAATTTGGAATCTTCTAGAAC
TTTCTAGAAAAAATCTGGAAACTTCTAGAAT
TTTCTAGGAAAATCAGAAAACTTTTGGAAC
TTTCTAGAAAAAATCTGGAAACTTCTAGAAT
TTTCTAGGAAAATTTGGAATCTTCTAGAAC
TTTCTAGAAAAAATCAGAAAACTTCTAGAAC
TTTCTAGAAAAAATTTGGAATCTTCTAGAAC
TTTCTAGAAAAAATCTGGAAACTTCTAGAAT
TTTCTAGAAAAAATCAGAAACTTCTAGAAC
TTTCTAGAAAAAATTTGGAATCTTCTAGAAC
TTTCTAGAAAAAATCTGGAAACTTCTAGAAT
TTTCTAGAAAAAATCAGAAAACTTCTAGAACTCTCTAGAACAATTTGGAATCTTCTAGAAC
TTTCTAGAAAAAATCAGAAAACTTCTAGAAC
TTTCTAGAAAAAATTTGGAATCTTCTAGAAC
TTTCTAAAAAAAATCTTGACA
TTTCTAGAAT
TTTCTAGAAAAAATCAGAAACTTCTAGAAC
TTTCTAGAAAAAATTTGGAATCTTCTAGAAC
TTTCTAGAAAAAATCTGGAAACTTCTAGAAC
TTTCTAGAAAAAATTTGGAATCTTCTAGAAC
TTTCTAGAAAAAATCTGGAAACTTCTAGAAT
TTTCTAGGAAAATCAGAAAACTTTTGGAAC
TTTCTAGAAAAAATCTGGAAACTTCTAGAAT
TTTCTAGAAAAAATCAGAAACTTCTAGAAC
TTTCTAGAAAAAATTTGGAATCTTCTAGAAC
TTTCTATAAAAAATCTGGAAACTTCTAGAAT
TTTCTAGGAAAATCAGAAAACTTTTGGAAC
TTTCTAGAAAAAATCTGGAAACTTCTAGAAC
Nr of selected repeats 24 Similarity 0.857649
Consensus:
TTTCTAGAAAAAATctGgAAaCTTCTAGAAc
>Cele-UNSB01_2:14194765-14194942 Satlength=178 Nr of Repeats=5 RepeatLength=14
seed=GTAGTTTGTA
GTAGTTTGTAGTTT
GTAGTTTGTAGCTT

GTAGTTTGTAGTCC
GTAGTTTTAGCAG
GTAGTTTGAAAGTATGCAGCTTTTTTTTGTTTAAAGTTTTGTATTCGTATACTTAAATACTTGTTAGAAATGTTCAATA
GAAATTGTTCAAAAATATACATAGTTTGTGGTCATTGTAGTTT
Nr of selected repeats 3 Similarity 0.809524
Consensus:
GTAGTTTGTAGttt
>Cele-UNSB01_2:14195501-14195591 Satlength=91 Nr of Repeats=5 RepeatLength=12
seed=TAGGCTTAGG
TAGGCTTAGGCT
TAGGCTTAGGCTTAACCG
TAGACTTAGGTTTAGGCTCCGGCTTAGGTTCAGGCT
TAGCCTTAGGCC
TAGGCTTAGGCT
Nr of selected repeats 3 Similarity 0.851852
Consensus:
TAGgCTTAGGCt
>Cele-UNSB01_2:14213145-14213210 Satlength=66 Nr of Repeats=4 RepeatLength=12
seed=TAGGCTTAGG
TAGGCTTAGGCT
TAGGCTTAGGTTCAGATTTAGACTTATAT
TAGGCTTAGGTT
TAGGCTTAGGCT
Nr of selected repeats 3 Similarity 0.925926
Consensus:
TAGGCTTAGGcT
>Cele-UNSB01_2:14289556-14289862 Satlength=307 Nr of Repeats=8 RepeatLength=34
seed=CCGTGCCTAC
CCGTGCCTACTAACCTACCGACCTTGCCTGCCTCTCGTGGCTACTTACCTACCGACTTTACCTGCCAT
CCGTGCCTACTAACCTACCGACCTTGCCTGCCTC
CCGTGCCTACTTACCTACCGACTTTACCTGTCTT
CCATGCCTACTTACCTACCGACTTTACCTGCCAT
CCGTGCCTACTTACCTACCGACCTTGTCTGCCTT
CCGTGCCTACTAACCTACCAACCCTGCCTGCCTT
CCGTGCCTACTAACCTATCGACCTTGTCTGCCTT
CCGTGCCTACTAACCTATCGACCTTGCCTGCCTC
Nr of selected repeats 7 Similarity 0.828198
Consensus:
CCGTGCCTACTaACCTAcCGACcTTgcCTGCCTt
>Cele-UNSB01_2:14294397-14294650 Satlength=254 Nr of Repeats=11 RepeatLength=18
seed=CTAGGTGCCT
CTAGGTGCCTATTCGTACCTAGGCGCATATTCATAC
CTAGGTACCTATCCATTC
CTAGGTGCCTATACATTC
CTAGGCGCCTATGCATAC
CTAGGTGCCTTTGCATAC
CTAGGTGCCTTTGCATAC
CTAGGTGCCTATTCATACCTACGTGTATAACCATAT
CTAGGTGTCTATCCATTA
CTAGGTGCCTATCCATTC
CTAGGTGCCTATCCATACCTACAGTGCCTATCCATTA
CTAGGTGCCTATCCATTC
Nr of selected repeats 8 Similarity 0.804233
Consensus:

CTAGGTGCCTaTcCATtC
>Cele-UNSB01_2:14297875-14298051 Satlength=177 Nr of Repeats=10 RepeatLength=16
seed=TAGGCATATA
TAGGCATATAGGCATA
TAGGCATATAGGCTTA
TAGGCATATAGGCTTG
TAGGCACATAGGCATA
TAGGCATATAGGCATA
TAGGCATATATGCATA
TAGGCATGTAGGCATA
TAGGGATATAGACATG
TAGGCATATAGGCATA
TAGGCATATAGGCATATAGGCAAAAAGGCATG
Nr of selected repeats 9 Similarity 0.842593
Consensus:
TAGGCATATAGGCaTa
>Cele-UNSB01_2:14303812-14304194 Satlength=383 Nr of Repeats=12 RepeatLength=22
seed=CTACAGTAAC
CTACAGTAACCATATTATTGAA
CTACAGTAACCCTAGTTTTGAA
CTACAGTAACCCAACTATTGAA
CTACAGTAACCCTAGTATTGAA
CTACAGTAACCCTAGTGTTAAA
CTACAGTAACCCTAGTATTGAA
CTACAGTAACCTTAGTGTTGAA
CTAAAGTAACCCTAGTGTTAAG
CTACAGTAACCCTGGTGTTACACAGCAACCTTGGTAGATAATAGTCACGCCGCAC
CTACAGTACTCTATACCTTACCAAATGATACATCTAGGCCACTTCACCA
CTACAGTACTTGTAAGATACAGTATACATCCTATACTCCTATAGATATTCCGTAACCGTAGCCAAGAGCAATC
CTACAGTAATCTTAACCCTCAGGCCAAAT
Nr of selected repeats 8 Similarity 0.798395
Consensus:
CTACAGTAACCcTAgTaTTgAA
>Cele-UNSB01_2:14320079-14320251 Satlength=173 Nr of Repeats=4 RepeatLength=43
seed=GAAAACTAGG
GAAAACTAGGCCACAGAGTTCGAGTTGACGGCCTAGAAATCTA
GAAAACTAGGCCACGGAGTTCAAATTGATGGCCGAGAAGTCTA
GAAAACGAGGCCACAAACTTGAAATTGGTGGCATAGAGCTCTC
GAAAACTAGGCCATGGAGTTGAAGTTCCTGGCCTAGAAATCTA
Nr of selected repeats 4 Similarity 0.715762
Consensus:
GAAAACtAGGCCAcagAgTTcaAaTTgatGGCctAGAaaTCTa
>Cele-UNSB01_2:14326178-14326688 Satlength=511 Nr of Repeats=14 RepeatLength=34
seed=CGGAATTGAA
CGGAATTGAAATTTCCGGCAAATTGGTAAAATGC
CGGAATTGAAATTTCTGGCAAATTGGTAAAATGC
CGGAATTGAAATTTCCGGCAAATTGGTAAAATGC
CGGAATTGAAATTTCCGGCAAATTGGTAAAATGC
CGGAATCGAAAATTCCGGCAAATTGGTAAAATGC
CGGAATTGAAAATTCCGGCAAATTGGTAAATTGG
CGGAATTGAAATTTCCGGCAAATTGATGAATTGC
CGGAATGGAAACTTCTGGCAAATTGGTACAATGC
CGGAATTGAAATTTCTGCCAAATTGGTAAAATGG
CGGAATCGAAAATTCCGGCAAATTGGTAAAATGC

CGGAATTGAAATTTCCGGCAAATCGGCCAGTTGGCTGAATGGAAAATACCGGCAAATTGATGAAATGC
CGGAATTGAAATTTCCCGCAAATCGGCAAGTTGG
CGGAATGGAAAATACCGGCAAATTGATGAAATGC
CGGAATTGAAACTTCCGGCAAACTGGTAAATTGG
Nr of selected repeats 13 Similarity 0.833082
Consensus:
CGGAATtGAAAtTTCcGGCAAATTGGTAAAaTGc
>Cele-UNSB01_2:14355758-14355994 Satlength=237 Nr of Repeats=18 RepeatLength=12
seed=ATAGGCATAG
ATAGGCATAGAC
ATAGGCATAGGC
ATAGGGCATAGGC
ATAGGCATAGGCATAGAC
ATACGCATAGGC
ATAGGCATAGGC
ATAGGAATAGGC
ATAGGCAAAGGC
ATAGGCATAGGCATTGTC
ATAGGCATTGTC
ATAGGCATAGGC
ATAGGGCATAGGC
ATAGGCATAGGCATAGAC
ATACGCATAGGC
ATAGGCATAGGC
ATAGGCATAGGCA
AAGGCATAGGC
ATAGGCATTGTC
Nr of selected repeats 11 Similarity 0.834343
Consensus:
ATAGGCATAGgC
>Cele-UNSB01_2:14379544-14380198 Satlength=655 Nr of Repeats=13 RepeatLength=13
seed=TATACAAAAA
TATACAAAAAATA
TATACAAAAAGTATCAAATTTGTATTTCCGGCAATTCCAGCAGTTGCCGGTTTTGTAAATTTTCGAGAATCGGCAATTC
CGGCAATTGCCCGTTTTGTGAATTTCCGGCACAATTCCGGCGATTACCGGTTT
TATACAAAATGAA
TATACAAAAAGTA
TATACAAAAAGTATCAGATTTGTATTTCCGGCAATTCCGGCGATTTTTTTTTGTGACTTTTTGGGAACCGGCAGTTTTG
GCAATCGCCGGTTTTGTGAATTTTCGGCAATCGGCAATTCCGGAAATTTCCGGTTT
TATACAAAAAGAA
TATACAAAAAGTA
TATACGAAAAAATATCAAATTTATATTTCCGGTAATGCCGGCAGTTGCCGGTTTTGTAAATTTTCGGGAATCGGCAATT
CCGGCAATTGCCGGTTTTGTGAATTTTCGGCAATCGGCAATTCCGGAAATCGCCGGTTT
TATACAAAAAGAA
TATACAAAAATTA
TATACAAAAAGTATCAAATTTGTATTTCCGGCGATTCCGGCAATTGCCGGTTTTGTAAATTTTCGAGAATCGGCAATTC
CGGCAATTGCCGGTTTTGTGAATTTCCGGCACAATTCCGGCGATTACCGGTTT
TATACAAAATGAA
TCTACAAAAAGTA
Nr of selected repeats 9 Similarity 0.837607
Consensus:
TATACAAAAagtA
>Cele-UNSB01_2:14381587-14382260 Satlength=674 Nr of Repeats=16 RepeatLength=42
seed=AAAACCGGCA

AAAACCGGCAATTGCTGGAATTGCCGATTGCCGAATATTACC
AAAACTGGCAATTGCCAAAATTGCCGATTGCCGGGAAGATTG
AAAACCGGCAATTGCTAGAATTGCCGATTGCCGAATATTACC
AAAACTGGCAATTGCCAAAATTGCCGATTGCCCGAAATATTG
AAAACCGGCAATTGCCGAAATTGCCTATTGCCGGAAATATTA
AAAACCGGCAATCGCTGAAATTTAGGCTATTTGGCTGATCTTGGCAATTACCGAAATTGCTGACTGCCGGAAATATTG
AAAACCGGCAATTGCCGGAATTGCCAAAAATTTCC
AAAACCGGCAATTGCCGGAATTGCCGATTGCCGATTATTACC
AAAACCGGCAATTGCCGGAATTGCCGGAATCGCCAAAAATTTCC
AAAACCGGCAATTGCCGGAATTGCCGAGTGCCGATTATTACC
AAAACCGGCAATTGCCGAAAATATTG
AAAACCGGCAATTGTCGCAATTGCCAAAAAATTCC
AAAACCGGCAATTACCGAAATTGCTGATTGCCGGGCATATTG
AAAACCGGCAATTGCCGGAATTGCCGAGTGCAGGAAAGATTA
AAAACCGGCAATTCCCAGAATTGCCGATTGCTGGAAATATTG
AAAACCGGCAATTGCCGGAATTGCCAAAAATTTCC
Nr of selected repeats 10 Similarity 0.749832
Consensus:
AAAACcGGCAATTgCcggAATTGCCGAtTGCcgGnAatATTa
>Cele-UNSB01_2:14401364-14401594 Satlength=231 Nr of Repeats=10 RepeatLength=21
seed=CAATTTTCTG
CAATTTTCTGTCAGACTTTGA
CAATTTTCTGTTTAACTTTGG
CAATTTTCTGTTTAACTTTGG
CAATTTTCTGTTCAACTTTGA
CAATTTCTGTTCAACTTTGA
CAATTTTCTGTTTAACTTTGA
CAATTTTCTGTTCAACTTTGA
CAATTTTCCGCAAGATTTTGGTACTTCTCTGCCAAACTTTGG
CAATTTTCTGCCAAACTTTGG
CAATTTTCTGTCAGACTTTGA
Nr of selected repeats 8 Similarity 0.841270
Consensus:
CAATTTTCTGTtnaACTTTGa
>Cele-UNSB01_2:14428274-14428974 Satlength=701 Nr of Repeats=18 RepeatLength=35
seed=GCAAATCGGC
GCAAATCGGCAAGTTGCCGGAATTGAAAATTTCAG
GCAAATCGGCAAATTGTCGGCATGGAAATTTTCCG
GCAAATCGGCAAATTGCCGGAATTGAAAATTTCAG
GCAAATCGGCAAGTTGCCGGAATTGAAAATTTCAG
GCAAATCGGCAAATTGCTGGAATTCAAAATTTCCGGCAATTCGGTTAAATTGCCGGAATTCAAAATTTCCG
GCAAATCGCCGGAATCCAAAATTTCCG
GCAAATCGTCAAATTGCCTGAATTGAAAATTTCCA
GCAAATCAGCAAATTGCCGGAATTGAAATTTACCG
GCAAATCGGCAAATGGCCGGAATTGAAAATTTCCA
GCAAATCGGCAAATTGCTGGAATTGAAAATTTCCG
GCAAATCGGCAAATGGCCGGAATTGAAAACTTCCG
GCAAATCGGCAAGTTGTCGGTATTGAAATTTTCCG
GCAAATCGGCAAATTGCCGGAATTCAAAATTTCCA
GCAAATCGGCAATTTGCCCGAATTTAAGATTTCCG
GCAAATCGGCAAATTGCCGGAATTTAAAATTTCCG
GCAAATCGGCAAATTGCCGGATTAAAAAATTTCCGAAAAATGGGCAAATTGCCGGAATTGAAAATTTCCG
GCAAATCGTCAAATTGCCTGAATTGAAAATTTCCA
GCAAATCGGGAAATTGCTGGAATTCAAAATTTCCGGCAAATG

Nr of selected repeats 14 Similarity 0.835060
Consensus:
GCAAATCGGCAAaTTGCCgGAATTgAAAaTTTCcg
>Cele-UNSB01_2:14453377-14453692 Satlength=316 Nr of Repeats=8 RepeatLength=35
seed=CGGCAAATCG
CGGCAAATCGGCAAATTGCCGGAATTGTTAATTTC
CGGCAAATCGGCAAATTGCCGGAACTGTTAATTTC
CGGCAAATCGGCAATTTACCGGAATTGCAAATTTC
CGGCAAATCGGCAAACTGTCCGAATTGAAATGTTT
CGGCAAATCGGCAAACTGCCGGAATTGCAAATTTC
CGGCAAATCGGAAAATTGCCGAAAATGAAAGCTTCCGGAAAATCAGCAAATTGCCGGAATTGTTAATTTC
CGGCAAATCGGCAATTTACCGGAATTGAAAATTTC
CGGCAAATCGGCAAACTGTCCGAATTGAAATGTTT
Nr of selected repeats 7 Similarity 0.780864
Consensus:
CGGCAAATCGGCAAatTgcCgGAATTGnaAATTTc
>Cele-UNSB01_2:14458676-14459058 Satlength=383 Nr of Repeats=11 RepeatLength=35
seed=CCGGCAAATC
CCGGCAAATCAGCAAA
CCGGCAAATGGGCAAATTGCCGAAAATGAAAGCAT
CCGGCAAATCGGCAATATGTCGGTATTGAAAATTTCAGCCAAA
CCGGCAAATCGGTAAATTGGCGGAATTGAAACTTT
CCGGCAAATCGGTAAATTGGCGGTATTGAAACTTT
CCGGCAAACCGGCAAATCGGTAAATTGGCGGAATTGAAACTTT
CCGGCAAACCGGCGAATTATCGGAATTGAAAATTT
CCGGCAAATCGGCAAAATGTCCGAATTGAAAATTT
CCGGCAAATCGGTAAATTGGCGGTATTGAAACTTT
CCGGCAAATCGGTAAATTGGCGGTATTGAAACTTT
CCGGCAAATCGACAAATTGCCGAAAATGAAAGCTT
Nr of selected repeats 8 Similarity 0.757275
Consensus:
CCGGCAAATCGGcAAATTGgCGgaAtTGAAAcTTt
>Cele-UNSB01_2:14462473-14462688 Satlength=216 Nr of Repeats=5 RepeatLength=35
seed=CGGCAAATTG
CGGCAAATTGCCGGAATTTAAATTTC
CGGCAAATCGGCTAATTGCCGAAATTGAAAATTTC
CGGCAATTTGTTGGTTTGCACTTTTTTTTGGAAATTTCAGAATTTCAATTTAAATCTGGAAAATTGCACGCATCCAAGG
AAAAC
CGGCAAATTGCCGGAATTGAAAATTTCCGACAAAT
CGGCAAATTGCCGGAATTGAAAATTTCCGGTAAAT
Nr of selected repeats 3 Similarity 0.586563
Consensus:
CGGCaAATTGCCGgAATTGAAAATTTCcgnnaaat
>Cele-UNSB01_2:14464146-14465077 Satlength=932 Nr of Repeats=26 RepeatLength=32
seed=AACCAATCAG
AACCAATCAGCGTTGAGCTCTTCCCATTTTGA
AACCAATCAGCGTTGAGCTCCTCCTACTTTTGA
AACCAATCAGCGATGGGCTCCACCCTTTTTGA
AACCAATCAGCGATCGGCTCCACCCATTTTGG
AACCAATCAGCACTCAGCTTCTCCCATTTTGA
AACCAATCAGAGCTCAGCTCCACTCATTTTGG
AACCAATCAGCGCTCAGCTCCACTCATTTTGG
AACCAATCAGCGCTCAGCTCCACTCATTTTGG
AACCAATCAGCGCTCAGCGCCGCCCATTCTGG

AACCAATCAGCGATAAGCTACACCCACTTTTGGACTTATCAGCACTCAGCTCCTCCCATTTTGG
AACCAATCAGCGCTCAGCTCCACCCATTTTGG
AACCAATCAGCACTCAGCTTCACTCATTTTGG
AACCAATCAGCGCTCAGCTCCACTCATTTTGG
AACCAATCAGAGCTCAGCTCCACTCATTTTGG
AACCAATCAGCGCTCAGCTCCACTCATTTTGG
AACCAATCAGCGCTCAGCGCCGCCCATTCTGG
AACCAATCAGCGATAAGCTACACCCACTTTTGGACTTATCAGCACTCAGCTCCTCCCATTTTGG
AACCAATCAGCGCTCAGCTCCACCCATTTTGG
AACCAATCAGCACTCAGCTTCACTCATTTTGG
AACCAATCAGCGCTCAGCTCCACTCATTTTGGG
ACCAATCAGCGCTCAGCTCCACTCATTTTGG
AACCAATCAGCGCTCAGCTCCACTCATTTTGG
AACCAATCAGGATTCAGTTCCACCCACTTTTG
AACCAATCAGAACTCAGCTCCTCCCATTTCGGGACCAATCACCGATTAGCTCCACCCACTTCTGG
AACCAATCAGCACTCAGCTCCACCCACTTTTGC
AACCAATCAGCGCTCAGCCCCTCCAATTTCGG
Nr of selected repeats 19 Similarity 0.803296
Consensus:
AACCAATCAGCgcTCAGCTCCaCcCATTTTGg
>Cele-UNSB01_2:14513028-14524678 Satlength=11651 Nr of Repeats=63
RepeatLength=185 seed=CTCGGACATA
CTCGGACATAATTGGTGTTCTGTATTCACTTATGATACGTTCGGCTGCGTACATTGGCCAAGAGTGTTCTAGTAAGAGA
ATTTCATTTAGAATATGAACAATTTGGGTAATTGGAAAGTGTCTTCGGGAATCTTTATACATCCGACAAATTCGAGTAT
GTTAGTATTCTGAATTGGATTCTTTTT
CTCGGACATAATTGGTGTTCTGTATTCACTTATGATACGTTCGGCTGCGTACGTTGGCCAAGAGTGTTCTAGTAAGAGA
ATTTCATTTAGAATATGAACAATTTGGGTAATTGGAAAGTGTCTTCGGGAATGATTATTCGTCCGATGTATTCGACTAT
GTTCATATTCTGAGTCGGATCTTTTT
CTCGGACATAATTGGTGTTCTGTATTCACTTATGATACGTTCGGCTGCGTACGTTGGCCAAGAGTGTTCTAGTAAGAGA
ATTTCATTTAGAATATGAACAATTTGGGTAATTGGAAAGTGTCTTCGGGAATCTTTATACATCCGACAAATTCGAGTAT
GTTAATATTCTGAATTGGATTCTTTTT
CTCGGACATAATTGGTGTTCTGTATTCACTTATGATACGTTCGGCTGCGTACATTGGCCAAGAGTGTTCTAGTAAGAGA
ATTTCATTTAGAATATGAACAATTTGGGTAATTGGAAAGTGTCTTCGGGAATCTTTATACATCCGACAAATTCGAGTAT
GTTAGTATTCTGAATTGGATTCTTTTT
CTCGGACATAATTGGTGTTCTGTATTCACTTATGATACGTTCGGCTGCGTACGTTGGCCAAGAGTGTTCTAGTAAGAGA
ATTTCATTTAGAATATGAACAATTTGGGTAATTGGAAAGTGTCTTCGGGAATGATTATTCGTCCGATGTATTCGACTAT
GTTCATATTCTGAGTCGGATCTTTTT
CTCGGACATAATTGGTGTTCTGTATTCACTTATGATACGTTCGGCTGCGTACATTGGCCAAGAGTGTTCTAGTAAGAGA
ATTTCATTTAGAATATGAACAATTTGGGTAATTGGAAAGTGTCTTCGGGAATCTTTATACATCCGACAAATTCGAGTAT
GTTAATATTCTGAATTGGATTCTTTTT
CTCGGACATAATTGGTGTTCTGTATTCACTTATGATACGTTCGGCTGCGTACATTGGCCAAGAGTGTTCTAGTAAGAGA
ATTTCATTTAAAATATGAACAATTTGGGTAATTGGAAAGTGTCTTCGGGAATGATTATTCGTCCGATGTATTCGACTAT
GTTCATATTCTGAGTCGGATCTTTTT
CTCGGACATAATTGGTGTTCTGTATTCACTTATGATACGTTCGGCTGCGTACATTGGCCAAGAGTGTTCTAGTAAGAGA
ATTTCATTTAGAATATGAACAATTTGGGTAATTGGAAAGTGTCTTCGGGAATCTTTATACATCCGACAAATTCGAGTAT
GTTAGTATTCTGAATTGGATTCTTTTT
CTCGGACATAATTGGTGTTCTGTATTCACTTATGATACGTTCGGCTGCGTACGTTGGCCAAGAGTGTTCTAGTAAGAGA
ATTTCATTTAGAATATGAACAATTTGGGTAATTGGAAAGTGTCTTCGGGAATCTTTATACATCCGACAAATTCGAGTAT
GTTAGTATTCTGAATTGGATTCTTTTT
CTCGGACATAATTGGTGTTCTGTATTCACTTATGATACGTTCGGCTGCGTACATTGGCCAAGAGTGTTCTAGTAAGAGA
ATTTCATTTAGAATATGAACAATTTGGGTAATTGGAAAGTGTCTTCGGGAATCTTTATACATTCGACAAATTCGAGTAT
GTTAGTATTCTGAATTGGATTCTTTTT

CTCGGACATAATTGGTGTTCTGTATTCACTTATGATACGTTCGGCTGCGTACGTTGGCCAAGAGTGTTCTAGTAAGAGA
ATTTCATTTAGAATATGAACAATTTGGGTAATTGGAAAGTGTCTTCGGGAATCTTTATACATCCGACAAATTCGAGTAT
GTTAATATTCTGAATTGGATTCTTTTT
CTCGGACATAACTGGTGTTCTGTATTCACTTATGATACGTTCGGCTGCGTACATTGGCCAAGAGTGTTCTAGTAAGAGA
ATTTCATTTAGAATATGAACAATTTGGGTAATTGGAAAGTGTCTTCGGGAATCTTTATACATCCGACAAATTCGAGTAT
GTTAGTATTCTGAATTGGATTCTTTTT
CTCGGACATAATTGGTGTTCTGTATTCACTTATGATACGTTCGGCTGCGTACGTTGGCCAAGAGTGTTCTAGTAAGAGA
ATTTCATTTAGAATATGAACAATTTGGGTAATTGGAAAGTGTCTTCGGGAATCTTTATACATCCGACAAATTCGAGTAT
GTTAATATTCTGAATCGGATTCTTTTT
CTCGGACATAACTGGTGTTCTGTATTCACTTATGATACGTTCGGCTGCGTACGTTGGCCAAGAGTGTTCTAGTAAGAGA
ATTTCATTTAGAATATGAACAATTTGGGTAATTGGAAAGTGTCTTCGGGAATCTTTATACATCCGACAAATTCGAGTAT
GTTAGTATTCTGAATTGGATTCTTTTT
CTCGGACATAATTGGTGTTCTGTATTCACTTATGATACGTTCGGCTGCGTACATTGGCCAAGAGTGTTCTAGTAAGAGA
ATTTCATTTAGAATATGAACAATTTGGGTAATTGGAAAGTGTCTTCGGGAATCTTTATACATCCGACAAATTCGAGTAT
GTTAGTATTCTGAATTGGATTCTTTTT
CTCGGACATAATTGGTGTTCTGTATTCACTTATGATACGTTCGGCTGCGTACGTTGGCCAAGAGTGTTCTAGTAAGAGA
ATTTCATTTAGAATATGAACAATTTGGGTAATTGGAAAGTGTCTTCGGGAATCTTTATACATCCGACAAATTCGAGTAT
GTTAATATTCTGAATTGGATTCTTTTT
CTCGGACATAATTGGTGTTCTGTATTCACTTATGATACGTTCGGCTGCGTACATTGGCCAAGAGTGTTCTAGTAAGAGA
ATTTCATTTAGAATATGAACAATTTGGGTAATTGGAAAGTGTCTTCGGGAATCTTTATACATCCGACAAATTCGAGTAT
GTTAATATTCTGAATTGGATTCTTTTT
CTCGGACATAACTGGTGTTCTGTATTCACTTATGATACGTTCGGCTGCGTACATTGGCCAAGAGTGTTCTAGTAAGAGA
ATTTCATTTAGAATATGAACAATTTGGGTAATTGGAAAGTGTCTTCGGGAATCTTTATACATCCGACAAATTCGAGTAT
GTTAGTATTCTGAATTGGATTCTTTTT
CTCGGACATAATTGGTGTTCTGTATTCACTTATGATACGTTCGGCTGCGTACGTTGGCCAAGAGTGTTCTAGTAAGAGA
ATTTCATTTAGAATATGAACAATTTGGGTAATTGGAAAGTGTCTTCGGGAATCTTTATACATCCGACAAATTCGAGTAT
GTTAATATTCTGAATCGGATTCTTTTT
CTCGGACATAACTGGTGTTCTGTATTCACTTATGATACGTTCGGCTGCGTACGTTGGCCAAGAGTGTTCTAGTAAGAGA
ATTTCATTTAGAATATGAACAATTTGGGTAATTGGAAAGTGTCTTCGGGAATCTTTATACATCCGACAAATTCGAGTAT
GTTAGTATTCTGAATTGGATTCTTTTT
CTCGGACATAATTGGTGTTCTGTATTCACTTATGATACGTTCGGCTGCGTACATTGGCCAAGAGTGTTCTAGTAAGAGA
ATTTCATTTAGAATATGAACAATTTGGGTAATTGGAAAGTGTCTTCGGGAATCTTTATACATCCGACAAATTCGAGTAT
GTTAATATTCTGAATTGGATTCTTTTT
CTCGGACATAATTGGTGTTCTGTATTCACTTATGATACGTTCGGCTGCGTACGTTGGCCAAGAGTGTTCTAGTAAGAGA
ATTTCATTTAGAATATGAACAATTTGGGTAATTGGAAAGTGTCTTCGGGAATCTTTATACATCCGACAAATTCGAGTAT
GTTAATATTCTGAATCGGATTCTTTTT
CTCGGACATAATTGGTGTTCTGTATTCACTTATGATACGTTCGGCTGCGTACGTTGGCCAAGAGTGTTCTAGTAAGAGA
ATTTCATTTAGAATATGAACAATTTGGGTAATTGGAAAGTGTCTTCGGGAATCTTTATACATCCGACAAATTCGAGTAT
GTTAATATTCTGAATCGGATTCTTTTT
CTCGGACATAACTGGTGTTCTGTATTCACTTATGATACGTTCGGCTGCGTACGTTGGCCAAGAGTGTTCTAGTAAGAGA
ATTTCATTTAGAATATGAACAATTTGGGTAATTGGAAAGTGTCTTCGGGAATCTTTATACATCCGACAAATTCGAGTAT
GTTAGTATTCTGAATTGGATTCTTTTT
CTCGGACATAATTGGTGTTCTGTATTCACTTATGATACGTTCGGCTGCGTACATTGGCCAAGAGTGTTCTAGTAAGAGA
ATTTCATTTAGAATATGAACAATTTGGGTAATTGGAAAGTGTCTTCGGGAATCTTTATACATCCGACAAATTCGAGTAT
GTTAATATTCTGAATTGGATTCTTTTT
CTCGGACATAATTGGTGTTCTGTATTCACTTATGATACGTTCGGCTGCGTACGTTGGCCAAGAGTGTTCTAGTAAGAGA
ATTTCATTTAGAATATGAACAATTTGGGTAATTGGAAAGTGTCTTCGGGAATCTTTATACATCCGACAAATTCGAGTAT
GTTAATATTCTGAATCGGATTCTTTTT
CTCGGACATAACTGGTGTTCTGTATTCACTTATGATACGTTCGGCTGCGTACATTGGCCAAGAGTGTTCTAGTAAGAGA
ATTTCATTTAGAATATGAACAATTTGGGTAATTGGAAAGTGTCTTCGGGAATCTTTATACATCCGACAAATTCGAGTAT
GTTAATATTCTGAATTGGATTCTTTTT
CTCGGACATAATTGGTGTTCTGTATTCACTTATGATACGTTCGGCTGCGTACATTGGCCAAGAGTGTTCTAGTAAGAGA
ATTTCATTTAGAATATGAACAATTTGGGTAATTGGAAAGTGTCTTCGGGAATCTTTATACATCCGACAAATTCGAGTAT
GTTAATATTCTGAATTGGATTCTTTTT

CTCGGACATAACTGGTGTTCTGTATTCACTTATGATACGTTTGGCTGCGTACATTGGCCAAGAGTGTTCTAGTAAGAGA
ATTTCATTTAGAATATGAACAATTTGGGTAATTGGAAAGTGTCTTCGGGAATGATTATTCATCCGACAAATTCGAGTAT
GTTAATATTCTGAATTGGATTCTTTTT
CTCGGACATAACTGGTGTTCTGTATTCACTTATGATACGTTTGGCTGCGTACATTGGCCAAGAGTGTTCTAGTAAGAGA
ATTTCATTTAGAATATGAACAATTTGGGTAATTGGAAAGTGTCTTCGGGAATGATTATTCATCCGACAAATTCGAGTAT
GTTAATATTCTGAATTGGATTCTTTTT
CTCGGACATAACTGGTGTTCTGTATTCACTTATGATACGTTTGGCTGCGTACATTGGCCAAGAGTGTTCTAGTAAGAGA
ATTTCATTTAGAATATGAACAATTTGGGTAATTGGAAAGTGTCTTCGGGAATGATTATTCATCCGACAAATTCGAGTAT
GTTAATATTCTGAATTGGATTCTTTTT
CTCGGACATAACTGGTGTTCTGTATTCACTTATGATACGTTTGGCTGCGTACATTGGCCAAGAGTGTTCTAGTAAGAGA
ATTTCATTTAGAATATGAACAATCTGGGTAATTGGAAAGTGTCTTCGGGAATCTTTATACATCCGACAAATTCGAGTAT
GTTAATATTCTGAATCGGATTCTTTTT
CTCGGACATAACTGGTGTTCTGTATTCACTTATGATACGTTCGGCTGCGTACATTGGCCAAGAGTGTTCTAGTAAGAGA
ATTTCATTTAGAATATGAACAATTTGGGTAATTGGAAAGTGTCTTCGGGAATCTTTATACATCCGACAAATTCGAGTAT
GTTAATATTCTGAATTGGATTCTTTTT
CTCGGACATAATTGGTGTTCTGTATTCACTTATGATACGTTCGGCTGCGTACATTGGCCAAGAGTGTTCTAGTAAGAGA
ATTTCATTTAGAATATGAACAATTTGGGTAATTGGAAAGTGTCTTCGGGAATCTTTATACATCCGACAAATTCGAGTAT
GTTAATATTCTGAATTGGATTCTTTTT
CTCGGACATAACTGGTGTTCTGTATTCACTTATGATACGTTCGGCTGCGTACATTGGCCAAGAGTGTTCTAGTAAGAGA
ATTTCATTTAGAATATGAACAATTTGGGTAATTGGAAAGTGTCTTCGGGAATCTTTATACATCCGACAAATTCGAGTAT
GTTAATATTCTGAATTGGATTCTTTTT
CTCGGACATAATTGGTGTTCTGTATTCACTTATGATACGTTCGGCTGCGTACATTGGCCAAGAGTGTTCTAGTAAGAGA
ATTTCATTTAGAATATGAACAATTTGGGTAATTGGAAAGTGTCTTCGGGAATCTTTATACATCCGACAAATTCGAGTAT
GTTAATATTCTGAATTGGATTCTTTTT
CTCGGACATAATTGGTGTTCTGTATTCACTTATGATACGTTCGGCTGCGTACATTGGCCAAGAGTGTTCTAGTAAGAGA
ATTTCATTTAGAATATGAACAATTTGGGTAATTGGAAAGTGTCTTCGGGAATGATTATTCATCCGACAAATTCGAGTAT
GTTAATATTCTGAATTGGATTCTTTTT
CTCGGACATAACTGGTGTTCTGTATTCACTTATGATACGTTTGGCTGCGTACATTGGCCAAGAGTGTTCTAGTAAGAGA
ATTTCATTTAGAATATGAACAATTTGGGTAATTGGAAAGTGTCTTCGGGAATCTTTATACATCCGACAAATTCGAGTAT
GTTAATATTCTGAATTGGATTCTTTTT
CTCGGACATAATTGGTGTTCTGTATTCACTTATGATACGTTCGGCTGCGTACATTGGCCAAGAGTGTTCTAGTAAGAGA
ATTTCATTTAGAATATGAACAATTTGGGTAATTGGAAAGTGTCTTCGGGAATCTTTATACATCCGACAAATTCGAGTAT
GTTAATATTCTGAATTGGATTCTTTTT
CTCGGACATAACTGGTGTTCTGTATTCACTTATGATACGTTCGGCTGCGTACATTGGCCAAGAGTGTTCTAGTAAGAGA
ATTTCATTTAGAATATGAACAATTTGGGTAATTGGAAAGTGTCTTCGGGAATCTTTATACATCCGACAAATTCGAGTAT
GTTAATATTCTGAATTGGATTCTTTTT
CTCGGACATAATTGGTGTTCTGTATTCACTTATGATACGTTCGGCTGCGTACGTTGGCCAAGAGTGTTCTAGTAAGAGA
ATTTCATTTAGAATATGAACAATTTGGGTAATTGGAAAGTGTCTTCGGGAATCTTTATACATCCGACAAATTCGAGTAT
GTTAATATTCTGAATCGGATTCTTTTT
CTCGGACATAACTGGTGTTCTGTATTCACTTATGATACGTTCGGCTGCGTACATTGGCCAAGAGTGTTCTAGTAAGAGA
ATTTCATTTAGAATATGAACAATTTGGGTAATTGGAAAGTGTCTTCGGGAATCTTTATACATCCGACAAATTCGAGTAT
GTTAATATTCTGAATTGGATTCTTTTT
CTCGGACATAATTGGTGTTCTGTATTCACTTATGATACGTTCGGCTGCGTACGTTGGCCAAGAGTGTTCTAGTAAGAGA
ATTTCATTTAGAATATGAACAATTTGGGTAATTGGAAAGTGTCTTCGGGAATCTTTATACATCCGACAAATTCGAGTAT
GTTAATATTCTGAATCGGATTCTTTTA
CTCGGACATAATTGGTGTTCTGTATTCACTTATGATACGTTCGGCTGCGTACATTGGCCAAGAGTGTTCTAGTAAGAGA
ATTTCATTTAGAATATGAACAATTTGGGTAATTGGAAAGTGTCTTCGGGAATCTTTATACATCCGACAAATTCGAGTAT
GTTAATATTCTGAATTGGATTCTTTTT
CTCGGACATAACTGGTGTTCTGTATTCACTTATGATACGTTCGGCTGCGTACATTGGCCAAGAGTGTTCTAGTAAGAGA
ATTTCATTTAGAATATGAACAATTTGGGTAATTGGAAAGTGTCTTCGGGAATCTTTATACATCCGACAAATTCGAGTAT
GTTAATATTCTGAATTGGATTCTTTTT
CTCGGACATAATTGGTGTTCTGTATTCACTTATGATACGTTCGGCTGCGTACATTGGCCAAGAGTGTTCTAGTAAGAGA
ATTTCATTTAGAATATGAACAATTTGGGTAATTGGAAAGTGTCTTCGGGAATCTTTATACATCCGACAAATTCGAGTAT
GTTAATATTCTGAATTGGATTCTTTTT

CTCGGACATAACTGGTGTTCTGTATTCACTTATGATACGTTTGGCTGCGTACATTGGCCAAGAGTGTTCTAGTAAGAGA
ATTTCATTTAGAATATGAACAATTTGGGTAATTGGAAAGTGTCTTCGGGAATGATTATTCATCCGACAAATTCGAGTAT
GTTAATATTCTGAATTGGATTCTTTTT
CTCGGACATAACTGGTGTTCTGTATTCACTTATGATACGTTTGGCTGCGTACATTGGCCAAGAGTGTTCTAGTAAGAGA
ATTTCATTTAGAATATGAACAATTTGGGTAATTGGAGAGTGTCTTCGGGAATCTTTATACATCCGACAAATTCGAGTAT
GTTAATATTCTGAATTGGATTCTTTTT
CTCGGACATAACTGGTGTTCTGTATTCACTTATGATACGTTCGGCTGCGTACATTGGCCAAGAGTGTTCTAGTAAGAGA
ATTTCATTTAGAATATGAACAATTTGGGTAATTGGAAAGTGTCTTCGGGAATCTTTATACATCCGACAAATTCGAGTAT
GTTAATATTCTGAATTGGATTCTTTTT
CTCGGACATAACTGGTGTTCTGTATTCACTTATGATACGTTCGGCTGCGTACATTGGCCAAGAGTGTTCTAGTAAGAGA
ATTTCATTTAGAATATGAACAATTTGGGTAATTGGAAAGTGTCTTCGGGAATCTTTATACATCCGACAAATTCGAGTAT
GTTAATATTCTGAATTGGATTCTTTTT
CTCGGACATAACTGGTGTTCTGTATTCACTTATGATACGTTCGGCTGCGTACATTGGCCAAGAGTGTTCTAGTAAGAGA
ATTTCATTTAGAATATGAACAATTTGGGTAATTGGAAAGTGTCTTCGGGAATCTTTATACATCCGACAAATTCGAGTAT
GTTAATATTCTGAATTGGATTCTTTTT
CTCGGACATAATTGGTGTTCTGTATTCACTTATGATACGTTCGGCTGCGTACATTGGCCAAGAGTGTTCTAGTAAGAGA
ATTTCATTTAGAATATGAACAATTTGGGTAATTGGAAAGTGTCTTCGGGAATCTTTATACATCCGACAAATTCGAGTAT
GTTAATATTCTGAATTGGATTCTTTTT
CTCGGACATAACTGGTGTTCTGTATTCACTTATGATACGTTCGGCTGCGTACATTGGCCAAGAGTGTTCTAGTAAGAGA
ATTTCATTTAGAATATGAACAATTTGGGTAATTGGAAAGTGTCTTCGGGAATGATTATTCATCCGACAAATTCGAGTAT
GTTAATATTCTGAATTGGATTCTTTTT
CTCGGACATAATTGGTGTTCTGTATTCACTTATGATACGTTCGGCTGCGTACATTGGCCAAGAGTGTTCTAGTAAGAGA
ATTTCATTTAGAATATGAACAATTTGGGTAATTGGAAAGTGTCTTCGGGAATGATTATTCGTCCGATGTATTCGACTAT
GTTCATATTCTGAGTCGGATCTTTTT
CTCGGACATAATTGGTGTTCTGTATTCACTTATGATACGTTCGGCTGCGTACATTGGCCAAGAGTGTTCTAGTAAGAGA
ATTTCATTTAGAATATGAACAATTTGGGTAATTGGAAAGTGTCTTCGGGAATCTTTATACATCCGACAAATTCGAGTAT
GTTAATATTCTGAATTGGATTCTTTTT
CTCGGACATAACTGGTGTTCTGTATTCACTTATGATACGTTCGGCTGCGTACATTGGCCAAGAGTGTTCTAGTAAGAGA
ATTTCATTTAGAATATGAACAATTTGGGTAATTGGAAAGTGTCTTCGGGAATGATTATTCATCCGACAAATTCGAGTAT
GTTAATATTCTGAATTGGATTCTTTTT
CTCGGACATAATTGGTGTTCTGTATTCACTTATGATACGTTCGGCTGCGTACATTGGCCAAGAGTGTTCTAGTAAGAGA
ATTTCATTTAGAATATGAACAATTTGGGTAATTGGAAAGTGTCTTCGGGAATCTTTATACATCCGACAAATTCGAGTAT
GTTAATATTCTGAATTGGATTCTTTTT
CTCGGACATAATTGGTGTTCTGTATTCACTTATGATACGTTCGGCTGCGTACCTTGGCCAAGAGTGTTCTAGTAAGAGA
ATTTCATTTAGAATATGAACAATTTGGGTAATTGGAGAGTGTCTTCGGGAATCTTTATACATCCGACAAATTCGAGTAT
GTTAATATTCTGAATTGGATTCTTTTT
CTCGGACATAATTGGTGTTCTGTATTCACTTATGATACGTTCGGCTGCGTACCTTGGCCAAGAGTGTTCTAGTAAGAGA
ATTTCATTTAGAATATGAACAATTTGGGTAATTGGAAAGTGTCTTCGGGAATCTTTATACATCCGACAAATTCGAGTAT
GTTAATATTCTGAATTGGATTCTTTTT
CTCGGACATAACTGGTGTTCTGTATTCACTTATGATACGTTCGGCTGCGTACATTGGCCAAGAGTGTTCTAGTAAGAGA
ATTTCATTTAGAATATGAACAATTTGGGTAATTGGAAAGTGTCTTCGGGAATGATTATTCATCCGACAAATTCGAGTAT
GTTAATATTCTGAATTGGATTCTTTTT
CTCGGACATAATTGGTGTTCTGTATTCACTTATGATACGTTCGGCTGCGTACATTGGCCAAGAGTGTTCTAGTAAGAGA
ATTTCATTTAGAATATGAACAATTTGGGTAATTGGAAAGTGTCTTCGGGAATCTTTATACATCCGACAAATTCGAGTAT
GTTAATATTCTGAATTGGATTCTTTTT
CTCGGACATAATTGGTGTTCTGTATTCACTTATGATACGTTCGGCTGCGTACATTGGCCAAGAGTGTTCTAGTAAGAGA
ATTTCATTTAGAATATGAACAATTTGGGTAATTGGAAAGTGTCTTCGGGAATCTTTATACATCCGACAAATTCGAGTAT
GTTAATATTCTGAATTGGATTCTTTTT
CTCGGACATAATTGGTGTTCTGTATTCACTTATGATACGTTCGGCTGCGTACATTGGCCAAGAGTGTTCTAGTAAGAGA
ATTTCATTTAGAATATGAACAATTTGGGTAATTGGAAAGTGTCTTCGGGAATCTTCATACATCCGACAAATTCGAGTAT
GTTTATATTCTGAATCAGATCTTTTT
Nr of selected repeats 58 Similarity 0.981369
Consensus:

CTCGGACATAAtTGGTGTTCTGTATTCACTTATGATACGTTCGGCTGCGTACaTTGGCCAAGAGTGTTCTAGTAAGAGA
ATTTCATTTAGAATATGAACAATTTGGGTAATTGGAAAGTGTCTTCGGGAATCTTTATACATCCGACAAATTCGAGTAT
GTTAATATTCTGAATTGGATTCTTTTT
>Cele-UNSB01_2:14526196-14534994 Satlength=8799 Nr of Repeats=53
RepeatLength=166 seed=TCGTAATTAT
TCGTAATTATTCATAAGTTTGTGTTGTCTCCGAGCCATGTCTGAAAAAACAGATTTTTTTCGAAAATTTTAAAAAGTT
CGTAAAAATTGGATTTTTGACAAATCTATGCTCTCTTTTTTTGTTTGAAATTTAGTTCAGTGACATACAATTTCAAAAA
TATAATGT
TCGTAATTATTCATAAGTTTGTGTTGTCTCAGAGCCATGTCTGAAAAAACAGATTTTTTTCGAAAATTTTAAAAAGTT
CGTAAAAATTGGATTTTTGACAAATCTATGCTCTCTTTTTTTGTTTGAAATTTAGTTCAGTGACATACAATTTCAAAAA
TATAATGT
TCGTAATTATTCATAAGTTTGTGTTGTCTCAGAGCCATGTCTGAAAAAACAGATTTTTTTCGAAAATTTTAAAAAGTT
CGTAAAAATTGGATTTTTGACAAATCTATGCTCTCTTTTTTTGTTTGAAATTTAGTTCAGTGACATACAATTTCAAAAA
TATAATGT
TCGTAATTATTCATAAGTTTGTGTTGTCTCAGAGCCATGTCTGAAAAAACAGATTTTTTTCGAAAATTTTAAAAAGTT
CGTAAAAATTGGATTTTTGACAAATCTATGCTCTCTTTTTTTGTTTGAAATTTAGTTCAGTGACATACAATTTCAAAAA
TATAATGT
TCGTAATTATTCATAAGTTTGTGTTGTCTCAGAGCCATGTCTGAAAAAACAGATTTTTTTCGAAAATTTTAAAAAGTT
CGTAAAAATTGGATTTTTGACAAATCTATGCTCTCTTTTTTTGTTTGAAATTTAGTTCAGTGACATACAATTTCAAAAA
TATAATGT
TCGTAATTATTCATAAGTTTGTGTTGTCTCAGAGCCATGTCTGAAAAAACAGATTTTTTTCGAAAATTTTAAAAAGTT
CGTAAAAATTGGATTTTTGACAAATCTATGCTCTCTTTTTTTGTTTGAAATTTAGTTCAGTGACATACAATTTCAAAAA
TATAATGT
TCGTAATTATTCATAAGTTTGTGTTGTCTCAGAGCCATGTCTGAAAAAACAGATTTTTTTCGAAAATTTTAAAAAGTT
CGTAAAAATTGGATTTTTGACAAATCTATGCTCTCTTTTTTTGTTTGAAATTTAGTTCAGTGACATACAATTTCAAAAA
TATAATGT
TCGTAATTATTCATAAGTTTGTGTTGTCTCAGAGCCATGTCTGAAAAAACAGATTTTTTTCGAAAATTTTAAAAAGTT
CGTAAAAATTGGATTTTTGACAAATCTATGCTCTCTTTTTTTGTTTGAAATTTAGTTCAGTGACATACAATTTCAAAAA
TATAATGT
TCGTAATTATTCATAAGTTTGTGTTGTCTCAGAGCCATGTCTGAAAAAACAGATTTTTTTCGAAAATTTTAAAAAGTT
CGTAAAAATTGGATTTTTGACAAATCTATGCTCTCTTTTTTTGTTTGAAATTTAGTTCAGTGACATACAATTTCAAAAA
TATAATGT
TCGTAATTATTCATAAGTTTGTGTTGTCTCAGAGCCATGTCTGAAAAAACAGATTTTTTTCGAAAATTTTAAAAAGTT
CGTAAAAATTGGATTTTTGACAAATCTATGCTCTCTTTTTTTGTTTGAAATTTAGTTCAGTGACATACAATTTCAAAAA
TATAATGT
TCGTAATTATTCATAAGTTTGTGTTGTCTCAGAGCCATGTCTGAAAAAACAGATTTTTTTCGAAAATTTTAAAAAGTT
CGTAAAAATTGGATTTTTGACAAATCTATGCTCTCTTTTTTTGTTTGAAATTTAGTTCAGTGACATACAATTTCAAAAA
TATAATGT
TCGTAATTATTCATAAGTTTGTGTTGTCTCAGAGCCATGTCTGAAAAAACAGATTTTTTTCGAAAATTTTAAAAAGTT
CGTAAAAATTGGATTTTTGACAAATCTATGCTCTCTTTTTTTGTTTGAAATTTAGTTCAGTGACATACAATTTCAAAAA
TATAATGT
TCGTAATTATTCATAAGTTTGTGTTGTCTCAGAGCCATGTCTGAAAAAACAGATTTTTTTCGAAAATTTTAAAAAGTT
CGTAAAAATTGGATTTTTGACAAATCTATGCTCTCTTTTTTTGTTTGAAATTTAGTTCAGTGACATACAATTTCAAAAA
TATAATGT
TCGTAATTATTCATAAGTTTGTGTTGTCTCAGAGCCATGTCTGAAAAAACAGATTTTTTTCGAAAATTTTAAAAAGTT
CGTAAAAATTGGATTTTTGACAAATCTATGCTCTCTTTTTTTGTTTGAAATTTAGTTCAGTGACATACAATTTCAAAAA
TATAATGT
TCGTAATTATTCATAAGTTTGTGTTGTCTCAGAGCCATGTCTGAAAAAACAGATTTTTTTCGAAAATTTTAAAAAGTT
CGTAAAAATTGGATTTTTGACAAATCTATGCTCTCTTTTTTTGTTTGAAATTTAGTTCAGTGACATACAATTTCAAAAA
TATAATGT
TCGTAATTATTCATAAGTTTGTGTTGTCTCAGAGCCATGTCTGAAAAAACAGATTTTTTTCGAAAATTTTAAAAAGTT
CGTAAAAATTGGATTTTTGACAAATCTATGCTCTCTTTTTGTGTTTGAAATTTAGTTCAGTGACATACAATTTCAAAAA
TATAATGT

TCGTAATTATTCATAAGTTTGTGTTGTCTCAGAGCCATGTCTGAAAAAACAGATTTTTTTCGAAAATTTTAAAAAGTT
CGTAAAAATTGGATTTTTGACAAATCTATGCTCTCTTTTTTTGTTTGAAATTTAGTTCAGTGACATACAATTTCAAAAA
TATAATGT
TCGTAATTATTCATAAGTTTGTGTTGTCTCAGAGCCATGTCTGAAAAAACAGATTTTTTTCGAAAATTTTAAAAAGTT
CGTAAAAATTGGATTTTTGACAAATCTATGCTCTCTTTTTTTGTTTGAAATTTAGTTCAGTGACATACAATTTCAAAAA
TATAATGT
TCGTAATTATTCATAAGTTTGTGTTGTCTCAGAGCCATGTCTGAAAAAACAGATTTTTTTCGAAAATTTTAAAAAGTT
CGTAAAAATTGGATTTTTGACAAATCTATGCTCTCTTTTTTTGTTTGAAATTTAGTTCAGTGACATACAATTTCAAAAA
TATAATGT
TCGTAATTATTCATAAGTTTGTGTTGTCTCAGAGCCATGTCTGAAAAAACAGATTTTTTTCGAAAATTTTAAAAAGTT
CGTAAAAATTGGATTTTTGACAAATCTATGCTCTCTTTTTTTGTTTGAAATTTAGTTCAGTGACATACAATTTCAAAAA
TATAATGT
TCGTAATTATTCATAAGTTTGTGTTGTCTCAGAGCCATGTCTGAAAAAACAGATTTTTTTCGAAAATTTTAAAAAGTT
CGTAAAAATTGGATTTTTGACAAATCTATGCTCTCTTTTTTTGTTTGAAATTTAGTTCAGTGACATACAATTTCAAAAA
TATAATGT
TCGTAATTATTCATAAGTTTGTGTTGTCTCAGAGCCATGTCTGAAAAAACAGATTTTTTTCGAAAATTTTAAAAAGTT
CGTAAAAATTGGATTTTTGACAAATCTATGCTCTCTTTTTTTGTTTGAAATTTAGTTCAGTGACATACAATTTCAAAAA
TATAATGT
TCGTAATTATTCATAAGTTTGTGTTGTCTCAGAGCCATGTCTGAAAAAACAGATTTTTTTCGAAAATTTTAAAAAGTT
CGTAAAAATTGGATTTTTGACAAATCTATGCTCTCTTTTTTTGTTTGAAATTTAGTTCAGTGACATACAATTTCAAAAA
TATAATGT
TCGTAATTATTCATAAGTTTGTGTTGTCTCAGAGCCATGTCTGAAAAAACAGATTTTTTTCGAAAATTTTAAAAAGTT
CGTAAAAATTGGATTTTTGACAAATCTATGCTCTCTTTTTTTGTTTGAAATTTAGTTCAGTGACATACAATTTCAAAAA
TATAATGT
TCGTAATTATTCATAAGTTTGTGTTGTCTCAGAGCCATGTCTGAAAAAACAGATTTTTTTCGAAAATTTTAAAAAGTT
CGTAAAAATTGGATTTTTGACAAATCTATGCTCTCTTTTTTTGTTTGAAATTTAGTTCAGTGACATACAATTTCAAAAA
TATAATGT
TCGTAATTATTCATAAGTTTGTGTTGTCTCAGAGCCATGTCTGAAAAAACAGATTTTTTTCGAAAATTTTAAAAAGTT
CGTAAAAATTGGATTTTTGACAAATCTATGCTCTCTTTTTTTGTTTGAAATTTAGTTCAGTGACATACAATTTCAAAAA
TATAATGT
TCGTAATTATTCATAAGTTTGTGTTGTCTCAGAGCCATGTCTGAAAAAACAGATTTTTTTCGAAAATTTTAAAAAGTT
CGTAAAAATTGGATTTTTGACAAATCTATGCTCTCTTTTTTTGTTTGAAATTTAGTTCAGTGACATACAATTTCAAAAA
TATAATGT
TCGTAATTATTCATAAGTTTGTGTTGTCTCAGAGCCATGTCTGAAAAAACAGATTTTTTTCGAAAATTTTAAAAAGTT
CGTAAAAATTGGATTTTTGACAAATCTATGCTCTCTTTTTTTGTTTGAAATTTAGTTCAGTGACATACAATTTCAAAAA
TATAATGT
TCGTAATTATTCATAAGTTTGTGTTGTCTCAGAGCCATGTCTGAAAAAACAGATTTTTTTCGAAAATTTTAAAAAGTT
CGTAAAAATTGGATTTTTGACAAATCTATGCTCTCTTTTTTTGTTTGAAATTTAGTTCAGTGACATACAATTTCAAAAA
TATAATGT
TCGTAATTATTCATAAGTTTGTGTTGTCTCAGAGCCATGTCTGAAAAAACAGATTTTTTTCGAAAATTTTAAAAAGTT
CGTAAAAATTGGATTTTTGACAAATCTATGCTCTCTTTTTTTGTTTGAAATTTAGTTCAGTGACATACAATTTCAAAAA
TATAATGT
TCGTAATTATTCATAAGTTTGTGTTGTCTCAGAGCCATGTCTGAAAAAACAGATTTTTTTCGAAAATTTTAAAAAGTT
CGTAAAAATTGGATTTTTGACAAATCTATGCTCTCTTTTTTTGTTTGAAATTTAGTTCAGTGACATACAATTTCAAAAA
TATAATGT
TCGTAATTATTCATAAGTTTGTGTTGTCTCAGAGCCATGTCTGAAAAAACAGATTTTTTTCGAAAATTTTAAAAAGTT
CGTAAAAATTGGATTTTTGACAAATCTATGCTCTCTTTTTTTGTTTGAAATTTAGTTCAGTGACATACAATTTCAAAAA
TATAATGT
TCGTAATTATTCATAAGTTTGTGTTGTCTCAGAGCCATGTCTGAAAAAACAGATTTTTTTCGAAAATTTTAAAAAGTT
CGTAAAAATTGGATTTTTGACAAATCTATGCTCTCTTTTTTTGTTTGAAATTTAGTTCAGTGACATACAATTTCAAAAA
TATAATGT
TCGTAATTATTCATAAGTTTGTGTTGTCTCAGAGCCATGTCTGAAAAAACAGATTTTTTTCGAAAATTTTAAAAAGTT
CGTAAAAATTGGATTTTTGACAAATCTATGCTCTCTTTTTTTGTTTGAAATTTAGTTCAGTGACATACAATTTCAAAAA
TATAATGT

TCGTAATTATTCATAAGTTTGTGTTGTCTCAGAGCCATGTCTGAAAAAACAGATTTTTTTTCGAAAATTTTAAAAAGTT
CGTAAAAATTGGATTTTTGACAAATCTATGCTCTCTTTTTTTGTTTAAAATTTAGTTCAGTGACATACAATTTCAAAAA
TATAATGT
TCGTAATTATTCATAAGTTTGTGTTGTCTCAGAGCCATGTCTGAAAAAACAGATTTTTTTTCGAAAATTTTAAAAAGTT
CGTAAAAATTGGATTTTTGACAAATCTATGCTCTCTTTTTTTGTTTGAAATTTAGTTCAGTGACATACAATTTCAAAAA
TATAATGT
TCGTAATTATTCATAAGTTTGTGTTGTCTCAGAGCCATGTCTGAAAAAACAGATTTTTTTTCGAAAATTTTAAAAAGTT
CGTAAAAATTGGATTTTTGACAAATCTATGCTCTCTTTTTTTGTTTGAAATTTAGTTCAGTGACATACAATTTCAAAAA
TATAATGT
TCGTAATTATTCATAAGTTTGTGTTGTCTCAGAGCCATGTCTGAAAAAACAGATTTTTTTTCGAAAATTTTAAAAAGTT
CGTAAAAATTGGATTTTTGACAAATCTATGCTCTCTTTTTTTGTTTGAAATTTAGTTCAGTGACATACAATTTCAAAAA
TATAATGT
TCGTAATTATTCATAAGTTTGTGTTGTCTCAGAGCCATGTCTGAAAAAACAGATTTTTTTTCGAAAATTTTAAAAAGTT
CGTAAAAATTGGATTTTTGACAAATCTATGCTCTCTTTTTTTGTTTGAAATTTAGTTCAGTGACATACAATTTCAAAAA
TATAATGT
TCGTAATTATTCATAAGTTTGTGTTGTCTCAGAGCCATGTCTGAAAAAACAGATTTTTTTTCGAAAATTTTAAAAAGTT
CGTAAAAATTGGATTTTTGACAAATCTATGCTCTCTTTTTTTGTTTGAAATTTAGTTCAGTGACATACAATTTCAAAAA
TATAATGT
TCGTAATTATTCATAAGTTTGTGTTGTCTCAGAGCCATGTCTGAAAAAACAGATTTTTTTTCGAAAATTTTAAAAAGTT
CGTAAAAATTGGATTTTTGACAAATCTATGCTCTCTTTTTTTGTTTGAAATTTAGTTCAGTGACATACAATTTCAAAAA
TATAATGT
TCGTAATTATTCATAAGTTTGTGTTGTCTCAGAGCCATGTCTGAAAAAACAGATTTTTTTTCGAAAATTTTAAAAAGTT
CGTAAAAATTGGATTTTTGACAAATCTATGCTCTCTTTTTTTGTTTGAAATTTAGTTCAGTGACATACAATTTCAAAAA
TATAATGT
TCGTAATTATTCATAAGTTTGTGTTGTCTCAGAGCCATGTCTGAAAAAACAGATTTTTTTTCGAAAATTTTAAAAAGTT
CGTAAAAATTGGATTTTTGACAAATCTATGCTCTCTTTTTTTGTTTGAAATTTAGTTCAGTGACATACAATTTCAAAAA
TATAATGT
TCGTAATTATTCATAAGTTTGTGTTGTCTCAGAGCCATGTCTGAAAAAACAGATTTTTTTTCGAAAATTTTAAAAAGTT
CGTAAAAATTGGATTTTTGACAAATCTATGCTCTCTTTTTTTGTTTGAAATTTAGTTCAGTGACATACAATTTCAAAAA
TATAATGT
TCGTAATTATTCATAAGTTTGTGTTGTCTCAGAGCCATGTCTGAAAAAACAGATTTTTTTTCGAAAATTTTAAAAAGTT
CGTAAAAATTGGATTTTTGACAAATCTATGCTCTCTTTTTTTGTTTGAAATTTAGTTCAGTGACATACAATTTCAAAAA
TATAATGT
TCGTAATTATTCATAAGTTTGTGTTGTCTCAGAGCCATGTCTGAAAAAACAGATTTTTTTTCGAAAATTTTAAAAAGTT
CGTAAAAATTGGATTTTTGACAAATCTATGCTCTCTTTTTTTGTTTGAAATTTAGTTCAGTGACATACAATTTCAAAAA
TATAATGT
TCGTAATTATTCATAAGTTTGTGTTGTCTCAGAGCCATGTCTGAAAAAACAGATTTTTTTTCGAAAATTTTAAAAAGTT
CGTAAAAATTGGATTTTTGACAAATCTATGCTCTCTTTTTTTGTTTGAAATTTAGTTCAGTGACATACAATTTCAAAAA
TATAATGT
TCGTAATTATTCATAAGTTTGTGTTGTCTCAGAGCCATGTCTGAAAAAACAGATTTTTTTTCGAAAATTTTAAAAAGTT
CGTAAAAATTGGATTTTTGACAAATCTATGCTCTCTTTTTTTGTTTGAAATTTAGTTCAGTGACATACAATTTCAAAAA
TATAATGT
TCGTAATTATTCATAAGTTTGTGTTGTCTCAGAGCCATGTCTGAAAAAACAGATTTTTTTTCGAAAATTTTAAAAAGTT
CGTAAAAATTGGATTTTTGACAAATCTATGCTCTCTTTTTTTGTTTGAAATTTAGTTCAGTGACATACAATTTCAAAAA
TATAATGT
TCGTAATTATTCATAAGTTTGTGTTGTCTCAGAGCCATGTCTGAAAAAACAGATTTTTTTTCGAAAATTTTAAAAAGTT
CGTAAAAATTGGATTTTTGACAAATCTATGCTCTCTTTTTTTGTTTGAAATTTAGTTCAGTGACATACAATTTCAAAAA
TATAATGT
TCGTAATTATTCATAAGTTTGTGTTGTCTCAGAGCCATGTCTGAAAAAACAGATTTTTTTTCGAAAATTTTAAAAAGTT
CGTAAAAATTGGATTTTTGACAAATCTATGCTCTCTTTTTTTGTTTGAAATTTAGTTCAGTGACATACAATTTCAAAAA
TATAATGT
TCGTAATTATTCATAAGTTTGTGTTGTCTCAGAGCCATGTCTGAAAAAACAGATTTTTTTTCGAAAATTTTAAAAAGTT
CGTAAAAATTGGATTTTTGACAAATCTATGCTCTCTTTTTTTGTTTGAAATTTAGTTCAGTGACATACAATTTCAAAAA
TATAATGT

TCGTAATTATTCATAAGTTTGTGTTGTCTCAGAGCCATGTCTGAAAAAACAGATTTTTTTTCGAAAATTTTAAAAAGTT
CGTAAAAATTGGATTTTTGACAAATCTATGCTCTCTTTTTTTGTTTGAAATTTAGTTCAGTGACATACAATTTCAAAAA
TATAATGT
Nr of selected repeats 53 Similarity 0.999091
Consensus:
TCGTAATTATTCATAAGTTTGTGTTGTCTCAGAGCCATGTCTGAAAAAACAGATTTTTTTTCGAAAATTTTAAAAAGTT
CGTAAAAATTGGATTTTTGACAAATCTATGCTCTCTTTTTTTGTTTGAAATTTAGTTCAGTGACATACAATTTCAAAAA
TATAATGT
>Cele-UNSB01_2:14535014-14566604 Satlength=31591 Nr of Repeats=1170
RepeatLength=27 seed=TGTGGCTTCC
TGTGGCTTCCCACTATATTTTACTCTC
TGTGGCTTCCCACTATATTTTACTCTC
TGTGGCTTCCCACTATATTTTACTCTC
TGTGGCTTCCCACTATATTTTACTCTC
TGTGGCTTCCCACTATATTTTACTCTC
TGTGGCTTCCCACTATATTTTACTCTC
TGTGGCTTCCCACTATATTTTACTCTC
TGTGGCTTCCCACTATATTTTACTCTC
TGTGGCTTCCCACTATATTTTACTCTC
TGTGGCTTCCCACTATATTTTACTCTC
TGTGGCTTCCCACTATATTTTACTCTC
TGTGGCTTCCCACTATATTTTACTCTC
TGTGGCTTCCCACTATATTTTACTCTC
TGTGGCTTCCCACTATATTTTACTCTC
TGTGGCTTCCCACTATATTTTACTCTC
TGTGGCTTCCCACTATATTTTACTCTC
TGTGGCTTCCCACTATATTTTACTCTC
TGTGGCTTCCCACTATATTTTACTCTC
TGTGGCTTCCCACTATATTTTACTCTC
TGTGGCTTCCCACTATATTTTACTCTC
TGTGGCTTCCCACTATATTTTACTCTC
TGTGGCTTCCCACTATATTTTACTCTC
TGTGGCTTCCCACTATATTTTACTCTC
TGTGGCTTCCCACTATATTTTACTCTC
TGTGGCTTCCCACTATATTTTACTCTC
TGTGGCTTCCCACTATATTTTACTCTC
TGTGGCTTCCCACTATATTTTACTCTC
TGTGGCTTCCCACTATATTTTACTCTC
TGTGGCTTCCCACTATATTTTACTCTC
TGTGGCTTCCCACTATATTTTACTCTC
TGTGGCTTCCCACTATATTTTACTCTC
TGTGGCTTCCCACTATATTTTACTCTC
TGTGGCTTCCCACTATATTTTACTCTC
TGTGGCTTCCCACTATATTTTACTCTC
TGTGGCTTCCCACTATATTTTACTCTC
TGTGGCTTCCCACTATATTTTACTCTC
TGTGGCTTCCCACTATATTTTACTCTC
TGTGGCTTCCCACTATATTTTACTCTC
TGTGGCTTCCCACTATATTTTACTCTC
TGTGGCTTCCCACTATATTTTACTCTC
TGTGGCTTCCCACTATATTTTACTCTC
TGTGGCTTCCCACTATATTTTACTCTC
TGTGGCTTCCCACTATATTTTACTCTC
TGTGGCTTCCCACTATATTTTACTCTC

TGTGGCTTCCCACTATATTTTACTCTC
TGTGGCTTCCCACTATATTTTACTCTC
TGTGGCTTCCCACTATATTTTACTCTC
TGTGGCTTCCCACTATATTTTACTCTC
TGTGGCTTCCCACTATATTTTACTCTC
TGTGGCTTCCCACTATATTTTACTCTC
TGTGGCTTCCCACTATATTTTACTCTC
TGTGGCTTCCCACTATATTTTACTCTC
TGTGGCTTCCCACTATATTTTACTCTC
TGTGGCTTCCCACTATATTTTACTCTC
TGTGGCTTCCCACTATATTTTACTCTC
TGTGGCTTCCCACTATATTTTACTCTC
TGTGGCTTCCCACTATATTTTACTCTC
TGTGGCTTCCCACTATATTTTACTCTC
TGTGGCTTCCCACTATATTTTACTCTC
TGTGGCTTCCCACTATATTTTACTCTC
TGTGGCTTCCCACTATATTTTACTCTC
TGTGGCTTCCCACTATATTTTACTCTC
TGTGGCTTCCCACTATATTTTACTCTC
TGTGGCTTCCCACTATATTTTACTCTC
TGTGGCTTCCCACTATATTTTACTCTC
TGTGGCTTCCCACTATATTTTACTCTC
TGTGGCTTCCCACTATATTTTACTCTC
TGTGGCTTCCCACTATATTTTACTCTC
TGTGGCTTCCCACTATATTTTACTCTC
TGTGGCTTCCCACTATATTTTACTCTC
TGTGGCTTCCCACTATATTTTACTCTC
TGTGGCTTCCCACTATATTTTACTCTC
TGTGGCTTCCCACTATATTTTACTCTC
TGTGGCTTCCCACTATATTTTACTCTC
TGTGGCTTCCCACTATATTTTACTCTC
TGTGGCTTCCCACTATATTTTACTCTC
TGTGGCTTCCCACTATATTTTACTCTC
TGTGGCTTCCCACTATATTTTACTCTC
TGTGGCTTCCCACTATATTTTACTCTC
TGTGGCTTCCCACTATATTTTACTCTC
TGTGGCTTCCCACTATATTTTACTCTC
TGTGGCTTCCCACTATATTTTACTCTC
TGTGGCTTCCCACTATATTTTACTCTC
TGTGGCTTCCCACTATATTTTACTCTC
TGTGGCTTCCCACTATATTTTACTCTC
TGTGGCTTCCCACTATATTTTACTCTC
TGTGGCTTCCCACTATATTTTACTCTC
TGTGGCTTCCCACTATATTTTACTCTC
TGTGGCTTCCCACTATATTTTACTCTC
TGTGGCTTCCCACTATATTTTACTCTC
TGTGGCTTCCCACTATATTTTACTCTC
TGTGGCTTCCCACTATATTTTACTCTC
TGTGGCTTCCCACTATATTTTACTCTC
TGTGGCTTCCCACTATATTTTACTCTC
TGTGGCTTCCCACTATATTTTACTCTC
TGTGGCTTCCCACTATATTTTACTCTC
TGTGGCTTCCCACTATATTTTACTCTC
TGTGGCTTCCCACTATATTTTACTCTC

TGTGGCTTCCCACTATATTTTACTCTC
TGTGGCTTCCCACTATATTTTACTCTC
TGTGGCTTCCCACTATATTTTACTCTC
TGTGGCTTCCCACTATATTTTACTCTC
TGTGGCTTCCCACTATATTTTACTCTC
TGTGGCTTCCCACTATATTTTACTCTC
TGTGGCTTCCCACTATATTTTACTCTC
TGTGGCTTCCCACTATATTTTACTCTC
TGTGGCTTCCCACTATATTTTACTCTC
TGTGGCTTCCCACTATATTTTACTCTC
TGTGGCTTCCCACTATATTTTACTCTC
TGTGGCTTCCCACTATATTTTACTCTC
TGTGGCTTCCCACTATATTTTACTCTC
TGTGGCTTCCCACTATATTTTACTCTC
TGTGGCTTCCCACTATATTTTACTCTC
TGTGGCTTCCCACTATATTTTACTCTC
TGTGGCTTCCCACTATATTTTACTCTC
TGTGGCTTCCCACTATATTTTACTCTC
TGTGGCTTCCCACTATATTTTACTCTC
TGTGGCTTCCCACTATATTTTACTCTC
TGTGGCTTCCCACTATATTTTACTCTC
TGTGGCTTCCCACTATATTTTACTCTC
TGTGGCTTCCCACTATATTTTACTCTC
TGTGGCTTCCCACTATATTTTACTCTC
TGTGGCTTCCCACTATATTTTACTCTC
TGTGGCTTCCCACTATATTTTACTCTC
TGTGGCTTCCCACTATATTTTACTCTC
TGTGGCTTCCCACTATATTTTACTCTC
TGTGGCTTCCCACTATATTTTACTCTC
TGTGGCTTCCCACTATATTTTACTCTC
TGTGGCTTCCCACTATATTTTACTCTC
TGTGGCTTCCCACTATATTTTACTCTC
TGTGGCTTCCCACTATATTTTACTCTC
TGTGGCTTCCCACTATATTTTACTCTC
TGTGGCTTCCCACTATATTTTACTCTC
TGTGGCTTCCCACTATATTTTACTCTC
TGTGGCTTCCCACTATATTTTACTCTC
TGTGGCTTCCCACTATATTTTACTCTC
TGTGGCTTCCCACTATATTTTACTCTC
TGTGGCTTCCCACTATATTTTACTCTC
TGTGGCTTCCCACTATATTTTACTCTC
TGTGGCTTCCCACTATATTTTACTCTC
TGTGGCTTCCCACTATATTTTACTCTC
TGTGGCTTCCCACTATATTTTACTCTC
TGTGGCTTCCCACTATATTTTACTCTC
TGTGGCTTCCCACTATATTTTACTCTC
TGTGGCTTCCCACTATATTTTACTCTC
TGTGGCTTCCCACTATATTTTACTCTC
TGTGGCTTCCCACTATATTTTACTCTC
TGTGGCTTCCCACTATATTTTACTCTC
TGTGGCTTCCCACTATATTTTACTCTC
TGTGGCTTCCCACTATATTTTACTCTC
TGTGGCTTCCCACTATATTTTACTCTC
TGTGGCTTCCCACTATATTTTACTCTC

TGTGGCTTCCCACTATATTTTACTCTC
TGTGGCTTCCCACTATATTTTACTCTC
TGTGGCTTCCCACTATATTTTACTCTC
TGTGGCTTCCCACTATATTTTACTCTC
TGTGGCTTCCCACTATATTTTACTCTC
TGTGGCTTCCCACTATATTTTACTCTC
TGTGGCTTCCCACTATATTTTACTCTC
TGTGGCTTCCCACTATATTTTACTCTC
TGTGGCTTCCCACTATATTTTACTCTC
TGTGGCTTCCCACTATATTTTACTCTC
TGTGGCTTCCCACTATATTTTACTCTC
TGTGGCTTCCCACTATATTTTACTCTC
TGTGGCTTCCCACTATATTTTACTCTC
TGTGGCTTCCCACTATATTTTACTCTC
TGTGGCTTCCCACTATATTTTACTCTC
TGTGGCTTCCCACTATATTTTACTCTC
TGTGGCTTCCCACTATATTTTACTCTC
TGTGGCTTCCCACTATATTTTACTCTC
TGTGGCTTCCCACTATATTTTACTCTC
TGTGGCTTCCCACTATATTTTACTCTC
TGTGGCTTCCCACTATATTTTACTCTC
TGTGGCTTCCCACTATATTTTACTCTC
TGTGGCTTCCCACTATATTTTACTCTC
TGTGGCTTCCCACTATATTTTACTCTC
TGTGGCTTCCCACTATATTTTACTCTC
TGTGGCTTCCCACTATATTTTACTCTC
TGTGGCTTCCCACTATATTTTACTCTC
TGTGGCTTCCCACTATATTTTACTCTC
TGTGGCTTCCCACTATATTTTACTCTC
TGTGGCTTCCCACTATATTTTACTCTC
TGTGGCTTCCCACTATATTTTACTCTC
TGTGGCTTCCCACTATATTTTACTCTC
TGTGGCTTCCCACTATATTTTACTCTC
TGTGGCTTCCCACTATATTTTACTCTC
TGTGGCTTCCCACTATATTTTACTCTC
TGTGGCTTCCCACTATATTTTACTCTC
TGTGGCTTCCCACTATATTTTACTCTC
TGTGGCTTCCCACTATATTTTACTCTC
TGTGGCTTCCCACTATATTTTACTCTC
TGTGGCTTCCCACTATATTTTACTCTC
TGTGGCTTCCCACTATATTTTACTCTC
TGTGGCTTCCCACTATATTTTACTCTC
TGTGGCTTCCCACTATATTTTACTCTC
TGTGGCTTCCCACTATATTTTACTCTC
TGTGGCTTCCCACTATATTTTACTCTC
TGTGGCTTCCCACTATATTTTACTCTC
TGTGGCTTCCCACTATATTTTACTCTC
TGTGGCTTCCCACTATATTTTACTCTC
TGTGGCTTCCCACTATATTTTACTCTC
TGTGGCTTCCCACTATATTTTACTCTC
TGTGGCTTCCCACTATATTTTACTCTC
TGTGGCTTCCCACTATATTTTACTCTC
TGTGGCTTCCCACTATATTTTACTCTC
TGTGGCTTCCCACTATATTTTACTCTC

TGTGGCTTCCCACTATATTTTACTCTC
TGTGGCTTCCCACTATATTTTACTCTC
TGTGGCTTCCCACTATATTTTACTCTC
TGTGGCTTCCCACTATATTTTACTCTC
TGTGGCTTCCCACTATATTTTACTCTC
TGTGGCTTCCCACTATATTTTACTCTC
TGTGGCTTCCCACTATATTTTACTCTC
TGTGGCTTCCCACTATATTTTACTCTC
TGTGGCTTCCCACTATATTTTACTCTC
TGTGGCTTCCCACTATATTTTACTCTC
TGTGGCTTCCCACTATATTTTACTCTC
TGTGGCTTCCCACTATATTTTACTCTC
TGTGGCTTCCCACTATATTTTACTCTC
TGTGGCTTCCCACTATATTTTACTCTC
TGTGGCTTCCCACTATATTTTACTCTC
TGTGGCTTCCCACTATATTTTACTCTC
TGTGGCTTCCCACTATATTTTACTCTC
TGTGGCTTCCCACTATATTTTACTCTC
TGTGGCTTCCCACTATATTTTACTCTC
TGTGGCTTCCCACTATATTTTACTCTC
TGTGGCTTCCCACTATATTTTACTCTC
TGTGGCTTCCCACTATATTTTACTCTC
TGTGGCTTCCCACTATATTTTACTCTC
TGTGGCTTCCCACTATATTTTACTCTC
TGTGGCTTCCCACTATATTTTACTCTC
TGTGGCTTCCCACTATATTTTACTCTC
TGTGGCTTCCCACTATATTTTACTCTC
TGTGGCTTCCCACTATATTTTACTCTC
TGTGGCTTCCCACTATATTTTACTCTC
TGTGGCTTCCCACTATATTTTACTCTC
TGTGGCTTCCCACTATATTTTACTCTC
TGTGGCTTCCCACTATATTTTACTCTC
TGTGGCTTCCCACTATATTTTACTCTC
TGTGGCTTCCCACTATATTTTACTCTC
TGTGGCTTCCCACTATATTTTACTCTC
TGTGGCTTCCCACTATATTTTACTCTC
TGTGGCTTCCCACTATATTTTACTCTC
TGTGGCTTCCCACTATATTTTACTCTC
TGTGGCTTCCCACTATATTTTACTCTC
TGTGGCTTCCCACTATATTTTACTCTC
TGTGGCTTCCCACTATATTTTACTCTC
TGTGGCTTCCCACTATATTTTACTCTC
TGTGGCTTCCCACTATATTTTACTCTC
TGTGGCTTCCCACTATATTTTACTCTC
TGTGGCTTCCCACTATATTTTACTCTC
TGTGGCTTCCCACTATATTTTACTCTC
TGTGGCTTCCCACTATATTTTACTCTC
TGTGGCTTCCCACTATATTTTACTCTC
TGTGGCTTCCCACTATATTTTACTCTC
TGTGGCTTCCCACTATATTTTACTCTC
TGTGGCTTCCCACTATATTTTACTCTC
TGTGGCTTCCCACTATATTTTACTCTC
TGTGGCTTCCCACTATATTTTACTCTC
TGTGGCTTCCCACTATATTTTACTCTC
TGTGGCTTCCCACTATATTTTACTCTC

TGTGGCTTCCCACTATATTTTACTCTC
TGTGGCTTCCCACTATATTTTACTCTC
TGTGGCTTCCCACTATATTTTACTCTC
TGTGGCTTCCCACTATATTTTACTCTC
TGTGGCTTCCCACTATATTTTACTCTC
TGTGGCTTCCCACTATATTTTACTCTC
TGTGGCTTCCCACTATATTTTACTCTC
TGTGGCTTCCCACTATATTTTACTCTC
TGTGGCTTCCCACTATATTTTACTCTC
TGTGGCTTCCCACTATATTTTACTCTC
TGTGGCTTCCCACTATATTTTACTCTC
TGTGGCTTCCCACTATATTTTACTCTC
TGTGGCTTCCCACTATATTTTACTCTC
TGTGGCTTCCCACTATATTTTACTCTC
TGTGGCTTCCCACTATATTTTACTCTC
TGTGGCTTCCCACTATATTTTACTCTC
TGTGGCTTCCCACTATATTTTACTCTC
TGTGGCTTCCCACTATATTTTACTCTC
TGTGGCTTCCCACTATATTTTACTCTC
TGTGGCTTCCCACTATATTTTACTCTC
TGTGGCTTCCCACTATATTTTACTCTC
TGTGGCTTCCCACTATATTTTACTCTC
TGTGGCTTCCCACTATATTTTACTCTC
TGTGGCTTCCCACTATATTTTACTCTC
TGTGGCTTCCCACTATATTTTACTCTC
TGTGGCTTCCCACTATATTTTACTCTC
TGTGGCTTCCCACTATATTTTACTCTC
TGTGGCTTCCCACTATATTTTACTCTC
TGTGGCTTCCCACTATATTTTACTCTC
TGTGGCTTCCCACTATATTTTACTCTC
TGTGGCTTCCCACTATATTTTACTCTC
TGTGGCTTCCCACTATATTTTACTCTC
TGTGGCTTCCCACTATATTTTACTCTC
TGTGGCTTCCCACTATATTTTACTCTC
TGTGGCTTCCCACTATATTTTACTCTC
TGTGGCTTCCCACTATATTTTACTCTC
TGTGGCTTCCCACTATATTTTACTCTC
TGTGGCTTCCCACTATATTTTACTCTC
TGTGGCTTCCCACTATATTTTACTCTC
TGTGGCTTCCCACTATATTTTACTCTC
TGTGGCTTCCCACTATATTTTACTCTC
TGTGGCTTCCCACTATATTTTACTCTC
TGTGGCTTCCCACTATATTTTACTCTC
TGTGGCTTCCCACTATATTTTACTCTC
TGTGGCTTCCCACTATATTTTACTCTC
TGTGGCTTCCCACTATATTTTACTCTC
TGTGGCTTCCCACTATATTTTACTCTC
TGTGGCTTCCCACTATATTTTACTCTC
TGTGGCTTCCCACTATATTTTACTCTC
TGTGGCTTCCCACTATATTTTACTCTC
TGTGGCTTCCCACTATATTTTACTCTC
TGTGGCTTCCCACTATATTTTACTCTC
TGTGGCTTCCCACTATATTTTACTCTC
TGTGGCTTCCCACTATATTTTACTCTC

TGTGGCTTCCCACTATATTTTACTCTC
TGTGGCTTCCCACTATATTTTACTCTC
TGTGGCTTCCCACTATATTTTACTCTC
TGTGGCTTCCCACTATATTTTACTCTC
TGTGGCTTCCCACTATATTTTACTCTC
TGTGGCTTCCCACTATATTTTACTCTC
TGTGGCTTCCCACTATATTTTACTCTC
TGTGGCTTCCCACTATATTTTACTCTC
TGTGGCTTCCCACTATATTTTACTCTC
TGTGGCTTCCCACTATATTTTACTCTC
TGTGGCTTCCCACTATATTTTACTCTC
TGTGGCTTCCCACTATATTTTACTCTC
TGTGGCTTCCCACTATATTTTACTCTC
TGTGGCTTCCCACTATATTTTACTCTC
TGTGGCTTCCCACTATATTTTACTCTC
TGTGGCTTCCCACTATATTTTACTCTC
TGTGGCTTCCCACTATATTTTACTCTC
TGTGGCTTCCCACTATATTTTACTCTC
TGTGGCTTCCCACTATATTTTACTCTC
TGTGGCTTCCCACTATATTTTACTCTC
TGTGGCTTCCCACTATATTTTACTCTC
TGTGGCTTCCCACTATATTTTACTCTC
TGTGGCTTCCCACTATATTTTACTCTC
TGTGGCTTCCCACTATATTTTACTCTC
TGTGGCTTCCCACTATATTTTACTCTC
TGTGGCTTCCCACTATATTTTACTCTC
TGTGGCTTCCCACTATATTTTACTCTC
TGTGGCTTCCCACTATATTTTACTCTC
TGTGGCTTCCCACTATATTTTACTCTC
TGTGGCTTCCCACTATATTTTACTCTC
TGTGGCTTCCCACTATATTTTACTCTC
TGTGGCTTCCCACTATATTTTACTCTC
TGTGGCTTCCCACTATATTTTACTCTC
TGTGGCTTCCCACTATATTTTACTCTC
TGTGGCTTCCCACTATATTTTACTCTC
TGTGGCTTCCCACTATATTTTACTCTC
TGTGGCTTCCCACTATATTTTACTCTC
TGTGGCTTCCCACTATATTTTACTCTC
TGTGGCTTCCCACTATATTTTACTCTC
TGTGGCTTCCCACTATATTTTACTCTC
TGTGGCTTCCCACTATATTTTACTCTC
TGTGGCTTCCCACTATATTTTACTCTC
TGTGGCTTCCCACTATATTTTACTCTC
TGTGGCTTCCCACTATATTTTACTCTC
TGTGGCTTCCCACTATATTTTACTCTC
TGTGGCTTCCCACTATATTTTACTCTC
TGTGGCTTCCCACTATATTTTACTCTC
TGTGGCTTCCCACTATATTTTACTCTC
TGTGGCTTCCCACTATATTTTACTCTC
TGTGGCTTCCCACTATATTTTACTCTC
TGTGGCTTCCCACTATATTTTACTCTC
TGTGGCTTCCCACTATATTTTACTCTC
TGTGGCTTCCCACTATATTTTACTCTC
TGTGGCTTCCCACTATATTTTACTCTC

TGTGGCTTCCCACTATATTTTACTCTC
TGTGGCTTCCCACTATATTTTACTCTC
TGTGGCTTCCCACTATATTTTACTCTC
TGTGGCTTCCCACTATATTTTACTCTC
TGTGGCTTCCCACTATATTTTACTCTC
TGTGGCTTCCCACTATATTTTACTCTC
TGTGGCTTCCCACTATATTTTACTCTC
TGTGGCTTCCCACTATATTTTACTCTC
TGTGGCTTCCCACTATATTTTACTCTC
TGTGGCTTCCCACTATATTTTACTCTC
TGTGGCTTCCCACTATATTTTACTCTC
TGTGGCTTCCCACTATATTTTACTCTC
TGTGGCTTCCCACTATATTTTACTCTC
TGTGGCTTCCCACTATATTTTACTCTC
TGTGGCTTCCCACTATATTTTACTCTC
TGTGGCTTCCCACTATATTTTACTCTC
TGTGGCTTCCCACTATATTTTACTCTC
TGTGGCTTCCCACTATATTTTACTCTC
TGTGGCTTCCCACTATATTTTACTCTC
TGTGGCTTCCCACTATATTTTACTCTC
TGTGGCTTCCCACTATATTTTACTCTC
TGTGGCTTCCCACTATATTTTACTCTC
TGTGGCTTCCCACTATATTTTACTCTC
TGTGGCTTCCCACTATATTTTACTCTC
TGTGGCTTCCCACTATATTTTACTCTC
TGTGGCTTCCCACTATATTTTACTCTC
TGTGGCTTCCCACTATATTTTACTCTC
TGTGGCTTCCCACTATATTTTACTCTC
TGTGGCTTCCCACTATATTTTACTCTC
TGTGGCTTCCCACTATATTTTACTCTC
TGTGGCTTCCCACTATATTTTACTCTC
TGTGGCTTCCCACTATATTTTACTCTC
TGTGGCTTCCCACTATATTTTACTCTC
TGTGGCTTCCCACTATATTTTACTCTC
TGTGGCTTCCCACTATATTTTACTCTC
TGTGGCTTCCCACTATATTTTACTCTC
TGTGGCTTCCCACTATATTTTACTCTC
TGTGGCTTCCCACTATATTTTACTCTC
TGTGGCTTCCCACTATATTTTACTCTC
TGTGGCTTCCCACTATATTTTACTCTC
TGTGGCTTCCCACTATATTTTACTCTC
TGTGGCTTCCCACTATATTTTACTCTC
TGTGGCTTCCCACTATATTTTACTCTC
TGTGGCTTCCCACTATATTTTACTCTC
TGTGGCTTCCCACTATATTTTACTCTC
TGTGGCTTCCCACTATATTTTACTCTC
TGTGGCTTCCCACTATATTTTACTCTC
TGTGGCTTCCCACTATATTTTACTCTC
TGTGGCTTCCCACTATATTTTACTCTC
TGTGGCTTCCCACTATATTTTACTCTC
TGTGGCTTCCCACTATATTTTACTCTC
TGTGGCTTCCCACTATATTTTACTCTC
TGTGGCTTCCCACTATATTTTACTCTC
TGTGGCTTCCCACTATATTTTACTCTC

TGTGGCTTCCCACTATATTTTACTCTC
TGTGGCTTCCCACTATATTTTACTCTC
TGTGGCTTCCCACTATATTTTACTCTC
TGTGGCTTCCCACTATATTTTACTCTC
TGTGGCTTCCCACTATATTTTACTCTC
TGTGGCTTCCCACTATATTTTACTCTC
TGTGGCTTCCCACTATATTTTACTCTC
TGTGGCTTCCCACTATATTTTACTCTC
TGTGGCTTCCCACTATATTTTACTCTC
TGTGGCTTCCCACTATATTTTACTCTC
TGTGGCTTCCCACTATATTTTACTCTC
TGTGGCTTCCCACTATATTTTACTCTC
TGTGGCTTCCCACTATATTTTACTCTC
TGTGGCTTCCCACTATATTTTACTCTC
TGTGGCTTCCCACTATATTTTACTCTC
TGTGGCTTCCCACTATATTTTACTCTC
TGTGGCTTCCCACTATATTTTACTCTC
TGTGGCTTCCCACTATATTTTACTCTC
TGTGGCTTCCCACTATATTTTACTCTC
TGTGGCTTCCCACTATATTTTACTCTC
TGTGGCTTCCCACTATATTTTACTCTC
TGTGGCTTCCCACTATATTTTACTCTC
TGTGGCTTCCCACTATATTTTACTCTC
TGTGGCTTCCCACTATATTTTACTCTC
TGTGGCTTCCCACTATATTTTACTCTC
TGTGGCTTCCCACTATATTTTACTCTC
TGTGGCTTCCCACTATATTTTACTCTC
TGTGGCTTCCCACTATATTTTACTCTC
TGTGGCTTCCCACTATATTTTACTCTC
TGTGGCTTCCCACTATATTTTACTCTC
TGTGGCTTCCCACTATATTTTACTCTC
TGTGGCTTCCCACTATATTTTACTCTC
TGTGGCTTCCCACTATATTTTACTCTC
TGTGGCTTCCCACTATATTTTACTCTC
TGTGGCTTCCCACTATATTTTACTCTC
TGTGGCTTCCCACTATATTTTACTCTC
TGTGGCTTCCCACTATATTTTACTCTC
TGTGGCTTCCCACTATATTTTACTCTC
TGTGGCTTCCCACTATATTTTACTCTC
TGTGGCTTCCCACTATATTTTACTCTC
TGTGGCTTCCCACTATATTTTACTCTC
TGTGGCTTCCCACTATATTTTACTCTC
TGTGGCTTCCCACTATATTTTACTCTC
TGTGGCTTCCCACTATATTTTACTCTC
TGTGGCTTCCCACTATATTTTACTCTC
TGTGGCTTCCCACTATATTTTACTCTC
TGTGGCTTCCCACTATATTTTACTCTC
TGTGGCTTCCCACTATATTTTACTCTC
TGTGGCTTCCCACTATATTTTACTCTC
TGTGGCTTCCCACTATATTTTACTCTC
TGTGGCTTCCCACTATATTTTACTCTC
TGTGGCTTCCCACTATATTTTACTCTC
TGTGGCTTCCCACTATATTTTACTCTC
TGTGGCTTCCCACTATATTTTACTCTC

TGTGGCTTCCCACTATATTTTACTCTC
TGTGGCTTCCCACTATATTTTACTCTC
TGTGGCTTCCCACTATATTTTACTCTC
TGTGGCTTCCCACTATATTTTACTCTC
TGTGGCTTCCCACTATATTTTACTCTC
TGTGGCTTCCCACTATATTTTACTCTC
TGTGGCTTCCCACTATATTTTACTCTC
TGTGGCTTCCCACTATATTTTACTCTC
TGTGGCTTCCCACTATATTTTACTCTC
TGTGGCTTCCCACTATATTTTACTCTC
TGTGGCTTCCCACTATATTTTACTCTC
TGTGGCTTCCCACTATATTTTACTCTC
TGTGGCTTCCCACTATATTTTACTCTC
TGTGGCTTCCCACTATATTTTACTCTC
TGTGGCTTCCCACTATATTTTACTCTC
TGTGGCTTCCCACTATATTTTACTCTC
TGTGGCTTCCCACTATATTTTACTCTC
TGTGGCTTCCCACTATATTTTACTCTC
TGTGGCTTCCCACTATATTTTACTCTC
TGTGGCTTCCCACTATATTTTACTCTC
TGTGGCTTCCCACTATATTTTACTCTC
TGTGGCTTCCCACTATATTTTACTCTC
TGTGGCTTCCCACTATATTTTACTCTC
TGTGGCTTCCCACTATATTTTACTCTC
TGTGGCTTCCCACTATATTTTACTCTC
TGTGGCTTCCCACTATATTTTACTCTC
TGTGGCTTCCCACTATATTTTACTCTC
TGTGGCTTCCCACTATATTTTACTCTC
TGTGGCTTCCCACTATATTTTACTCTC
TGTGGCTTCCCACTATATTTTACTCTC
TGTGGCTTCCCACTATATTTTACTCTC
TGTGGCTTCCCACTATATTTTACTCTC
TGTGGCTTCCCACTATATTTTACTCTC
TGTGGCTTCCCACTATATTTTACTCTC
TGTGGCTTCCCACTATATTTTACTCTC
TGTGGCTTCCCACTATATTTTACTCTC
TGTGGCTTCCCACTATATTTTACTCTC
TGTGGCTTCCCACTATATTTTACTCTC
TGTGGCTTCCCACTATATTTTACTCTC
TGTGGCTTCCCACTATATTTTACTCTC
TGTGGCTTCCCACTATATTTTACTCTC
TGTGGCTTCCCACTATATTTTACTCTC
TGTGGCTTCCCACTATATTTTACTCTC
TGTGGCTTCCCACTATATTTTACTCTC
TGTGGCTTCCCACTATATTTTACTCTC
TGTGGCTTCCCACTATATTTTACTCTC
TGTGGCTTCCCACTATATTTTACTCTC
TGTGGCTTCCCACTATATTTTACTCTC
TGTGGCTTCCCACTATATTTTACTCTC
TGTGGCTTCCCACTATATTTTACTCTC
TGTGGCTTCCCACTATATTTTACTCTC
TGTGGCTTCCCACTATATTTTACTCTC
TGTGGCTTCCCACTATATTTTACTCTC
TGTGGCTTCCCACTATATTTTACTCTC

TGTGGCTTCCCACTATATTTTACTCTC
TGTGGCTTCCCACTATATTTTACTCTC
TGTGGCTTCCCACTATATTTTACTCTC
TGTGGCTTCCCACTATATTTTACTCTC
TGTGGCTTCCCACTATATTTTACTCTC
TGTGGCTTCCCACTATATTTTACTCTC
TGTGGCTTCCCACTATATTTTACTCTC
TGTGGCTTCCCACTATATTTTACTCTC
TGTGGCTTCCCACTATATTTTACTCTC
TGTGGCTTCCCACTATATTTTACTCTC
TGTGGCTTCCCACTATATTTTACTCTC
TGTGGCTTCCCACTATATTTTACTCTC
TGTGGCTTCCCACTATATTTTACTCTC
TGTGGCTTCCCACTATATTTTACTCTC
TGTGGCTTCCCACTATATTTTACTCTC
TGTGGCTTCCCACTATATTTTACTCTC
TGTGGCTTCCCACTATATTTTACTCTC
TGTGGCTTCCCACTATATTTTACTCTC
TGTGGCTTCCCACTATATTTTACTCTC
TGTGGCTTCCCACTATATTTTACTCTC
TGTGGCTTCCCACTATATTTTACTCTC
TGTGGCTTCCCACTATATTTTACTCTC
TGTGGCTTCCCACTATATTTTACTCTC
TGTGGCTTCCCACTATATTTTACTCTC
TGTGGCTTCCCACTATATTTTACTCTC
TGTGGCTTCCCACTATATTTTACTCTC
TGTGGCTTCCCACTATATTTTACTCTC
TGTGGCTTCCCACTATATTTTACTCTC
TGTGGCTTCCCACTATATTTTACTCTC
TGTGGCTTCCCACTATATTTTACTCTC
TGTGGCTTCCCACTATATTTTACTCTC
TGTGGCTTCCCACTATATTTTACTCTC
TGTGGCTTCCCACTATATTTTACTCTC
TGTGGCTTCCCACTATATTTTACTCTC
TGTGGCTTCCCACTATATTTTACTCTC
TGTGGCTTCCCACTATATTTTACTCTC
TGTGGCTTCCCACTATATTTTACTCTC
TGTGGCTTCCCACTATATTTTACTCTC
TGTGGCTTCCCACTATATTTTACTCTC
TGTGGCTTCCCACTATATTTTACTCTC
TGTGGCTTCCCACTATATTTTACTCTC
TGTGGCTTCCCACTATATTTTACTCTC
TGTGGCTTCCCACTATATTTTACTCTC
TGTGGCTTCCCACTATATTTTACTCTC
TGTGGCTTCCCACTATATTTTACTCTC
TGTGGCTTCCCACTATATTTTACTCTC
TGTGGCTTCCCACTATATTTTACTCTC
TGTGGCTTCCCACTATATTTTACTCTC
TGTGGCTTCCCACTATATTTTACTCTC
TGTGGCTTCCCACTATATTTTACTCTC
TGTGGCTTCCCACTATATTTTACTCTC
TGTGGCTTCCCACTATATTTTACTCTC
TGTGGCTTCCCACTATATTTTACTCTC
TGTGGCTTCCCACTATATTTTACTCTC

TGTGGCTTCCCACTATATTTTACTCTC
TGTGGCTTCCCACTATATTTTACTCTC
TGTGGCTTCCCACTATATTTTACTCTC
TGTGGCTTCCCACTATATTTTACTCTC
TGTGGCTTCCCACTATATTTTACTCTC
TGTGGCTTCCCACTATATTTTACTCTC
TGTGGCTTCCCACTATATTTTACTCTC
TGTGGCTTCCCACTATATTTTACTCTC
TGTGGCTTCCCACTATATTTTACTCTC
TGTGGCTTCCCACTATATTTTACTCTC
TGTGGCTTCCCACTATATTTTACTCTC
TGTGGCTTCCCACTATATTTTACTCTC
TGTGGCTTCCCACTATATTTTACTCTC
TGTGGCTTCCCACTATATTTTACTCTC
TGTGGCTTCCCACTATATTTTACTCTC
TGTGGCTTCCCACTATATTTTACTCTC
TGTGGCTTCCCACTATATTTTACTCTC
TGTGGCTTCCCACTATATTTTACTCTC
TGTGGCTTCCCACTATATTTTACTCTC
TGTGGCTTCCCACTATATTTTACTCTC
TGTGGCTTCCCACTATATTTTACTCTC
TGTGGCTTCCCACTATATTTTACTCTC
TGTGGCTTCCCACTATATTTTACTCTC
TGTGGCTTCCCACTATATTTTACTCTC
TGTGGCTTCCCACTATATTTTACTCTC
TGTGGCTTCCCACTATATTTTACTCTC
TGTGGCTTCCCACTATATTTTACTCTC
TGTGGCTTCCCACTATATTTTACTCTC
TGTGGCTTCCCACTATATTTTACTCTC
TGTGGCTTCCCACTATATTTTACTCTC
TGTGGCTTCCCACTATATTTTACTCTC
TGTGGCTTCCCACTATATTTTACTCTC
TGTGGCTTCCCACTATATTTTACTCTC
TGTGGCTTCCCACTATATTTTACTCTC
TGTGGCTTCCCACTATATTTTACTCTC
TGTGGCTTCCCACTATATTTTACTCTC
TGTGGCTTCCCACTATATTTTACTCTC
TGTGGCTTCCCACTATATTTTACTCTC
TGTGGCTTCCCACTATATTTTACTCTC
TGTGGCTTCCCACTATATTTTACTCTC
TGTGGCTTCCCACTATATTTTACTCTC
TGTGGCTTCCCACTATATTTTACTCTC
TGTGGCTTCCCACTATATTTTACTCTC
TGTGGCTTCCCACTATATTTTACTCTC
TGTGGCTTCCCACTATATTTTACTCTC
TGTGGCTTCCCACTATATTTTACTCTC
TGTGGCTTCCCACTATATTTTACTCTC
TGTGGCTTCCCACTATATTTTACTCTC
TGTGGCTTCCCACTATATTTTACTCTC
TGTGGCTTCCCACTATATTTTACTCTC
TGTGGCTTCCCACTATATTTTACTCTC
TGTGGCTTCCCACTATATTTTACTCTC
TGTGGCTTCCCACTATATTTTACTCTC
TGTGGCTTCCCACTATATTTTACTCTC

TGTGGCTTCCCACTATATTTTACTCTC
TGTGGCTTCCCACTATATTTTACTCTC
TGTGGCTTCCCACTATATTTTACTCTC
TGTGGCTTCCCACTATATTTTACTCTC
TGTGGCTTCCCACTATATTTTACTCTC
TGTGGCTTCCCACTATATTTTACTCTC
TGTGGCTTCCCACTATATTTTACTCTC
TGTGGCTTCCCACTATATTTTACTCTC
TGTGGCTTCCCACTATATTTTACTCTC
TGTGGCTTCCCACTATATTTTACTCTC
TGTGGCTTCCCACTATATTTTACTCTC
TGTGGCTTCCCACTATATTTTACTCTC
TGTGGCTTCCCACTATATTTTACTCTC
TGTGGCTTCCCACTATATTTTACTCTC
TGTGGCTTCCCACTATATTTTACTCTC
TGTGGCTTCCCACTATATTTTACTCTC
TGTGGCTTCCCACTATATTTTACTCTC
TGTGGCTTCCCACTATATTTTACTCTC
TGTGGCTTCCCACTATATTTTACTCTC
TGTGGCTTCCCACTATATTTTACTCTC
TGTGGCTTCCCACTATATTTTACTCTC
TGTGGCTTCCCACTATATTTTACTCTC
TGTGGCTTCCCACTATATTTTACTCTC
TGTGGCTTCCCACTATATTTTACTCTC
TGTGGCTTCCCACTATATTTTACTCTC
TGTGGCTTCCCACTATATTTTACTCTC
TGTGGCTTCCCACTATATTTTACTCTC
TGTGGCTTCCCACTATATTTTACTCTC
TGTGGCTTCCCACTATATTTTACTCTC
TGTGGCTTCCCACTATATTTTACTCTC
TGTGGCTTCCCACTATATTTTACTCTC
TGTGGCTTCCCACTATATTTTACTCTC
TGTGGCTTCCCACTATATTTTACTCTC
TGTGGCTTCCCACTATATTTTACTCTC
TGTGGCTTCCCACTATATTTTACTCTC
TGTGGCTTCCCACTATATTTTACTCTC
TGTGGCTTCCCACTATATTTTACTCTC
TGTGGCTTCCCACTATATTTTACTCTC
TGTGGCTTCCCACTATATTTTACTCTC
TGTGGCTTCCCACTATATTTTACTCTC
TGTGGCTTCCCACTATATTTTACTCTC
TGTGGCTTCCCACTATATTTTACTCTC
TGTGGCTTCCCACTATATTTTACTCTC
TGTGGCTTCCCACTATATTTTACTCTC
TGTGGCTTCCCACTATATTTTACTCTC
TGTGGCTTCCCACTATATTTTACTCTC
TGTGGCTTCCCACTATATTTTACTCTC
TGTGGCTTCCCACTATATTTTACTCTC
TGTGGCTTCCCACTATATTTTACTCTC
TGTGGCTTCCCACTATATTTTACTCTC
TGTGGCTTCCCACTATATTTTACTCTC
TGTGGCTTCCCACTATATTTTACTCTC
TGTGGCTTCCCACTATATTTTACTCTC
TGTGGCTTCCCACTATATTTTACTCTC

TGTGGCTTCCCACTATATTTTACTCTC
TGTGGCTTCCCACTATATTTTACTCTC
TGTGGCTTCCCACTATATTTTACTCTC
TGTGGCTTCCCACTATATTTTACTCTC
TGTGGCTTCCCACTATATTTTACTCTC
TGTGGCTTCCCACTATATTTTACTCTC
TGTGGCTTCCCACTATATTTTACTCTC
TGTGGCTTCCCACTATATTTTACTCTC
TGTGGCTTCCCACTATATTTTACTCTC
TGTGGCTTCCCACTATATTTTACTCTC
TGTGGCTTCCCACTATATTTTACTCTC
TGTGGCTTCCCACTATATTTTACTCTC
TGTGGCTTCCCACTATATTTTACTCTC
TGTGGCTTCCCACTATATTTTACTCTC
TGTGGCTTCCCACTATATTTTACTCTC
TGTGGCTTCCCACTATATTTTACTCTC
TGTGGCTTCCCACTATATTTTACTCTC
TGTGGCTTCCCACTATATTTTACTCTC
TGTGGCTTCCCACTATATTTTACTCTC
TGTGGCTTCCCACTATATTTTACTCTC
TGTGGCTTCCCACTATATTTTACTCTC
TGTGGCTTCCCACTATATTTTACTCTC
TGTGGCTTCCCACTATATTTTACTCTC
TGTGGCTTCCCACTATATTTTACTCTC
TGTGGCTTCCCACTATATTTTACTCTC
TGTGGCTTCCCACTATATTTTACTCTC
TGTGGCTTCCCACTATATTTTACTCTC
TGTGGCTTCCCACTATATTTTACTCTC
TGTGGCTTCCCACTATATTTTACTCTC
TGTGGCTTCCCACTATATTTTACTCTC
TGTGGCTTCCCACTATATTTTACTCTC
TGTGGCTTCCCACTATATTTTACTCTC
TGTGGCTTCCCACTATATTTTACTCTC
TGTGGCTTCCCACTATATTTTACTCTC
TGTGGCTTCCCACTATATTTTACTCTC
TGTGGCTTCCCACTATATTTTACTCTC
TGTGGCTTCCCACTATATTTTACTCTC
TGTGGCTTCCCACTATATTTTACTCTC
TGTGGCTTCCCACTATATTTTACTCTC
TGTGGCTTCCCACTATATTTTACTCTC
TGTGGCTTCCCACTATATTTTACTCTC
TGTGGCTTCCCACTATATTTTACTCTC
TGTGGCTTCCCACTATATTTTACTCTC
TGTGGCTTCCCACTATATTTTACTCTC
TGTGGCTTCCCACTATATTTTACTCTC
TGTGGCTTCCCACTATATTTTACTCTC
TGTGGCTTCCCACTATATTTTACTCTC
TGTGGCTTCCCACTATATTTTACTCTC
TGTGGCTTCCCACTATATTTTACTCTC
TGTGGCTTCCCACTATATTTTACTCTC
TGTGGCTTCCCACTATATTTTACTCTC
TGTGGCTTCCCACTATATTTTACTCTC
TGTGGCTTCCCACTATATTTTACTCTC
TGTGGCTTCCCACTATATTTTACTCTC

TGTGGCTTCCCACTATATTTACTCTC
TGTGGCTTCCCACTATATTTACTCTC
TGTGGCTTCCCACTATATTTACTCTC
TGTGGCTTCCCACTATATTTACTCTC
TGTGGCTTCCCACTATATTTACTCTC
TGTGGCTTCCCACTATATTTACTCTC
TGTGGCTTCCCACTATATTTACTCTC
TGTGGCTTCCCACTATATTTACTCTC
TGTGGCTTCCCACTATATTTACTCTC
TGTGGCTTCCCACTATATTTACTCTC
TGTGGCTTCCCACTATATTTACTCTC
TGTGGCTTCCCACTATATTTACTCTC
TGTGGCTTCCCACTATATTTACTCTC
TGTGGCTTCCCACTATATTTACTCTC
TGTGGCTTCCCACTATATTTACTCTC
TGTGGCTTCCCACTATATTTACTCTC
TGTGGCTTCCCACTATATTTACTCTC
TGTGGCTTCCCACTATATTTACTCTC
TGTGGCTTCCCACTATATTTACTCTC
TGTGGCTTCCCACTATATTTACTCTC
TGTGGCTTCCCACTATATTTACTCTC
TGTGGCTTCCCACTATATTTACTCTC
TGTGGCTTCCCACTATATTTACTCTC
TGTGGCTTCCCACTATATTTACTCTC
TGTGGCTTCCCACTATATTTACTCTC
TGTGGCTTCCCACTATATTTACTCTC
TGTGGCTTCCCACTATATTTACTCTC
TGTGGCTTCCCACTATATTTACTCTC
TGTGGCTTCCCACTATATTTACTCTC
TGTGGCTTCCCACTATATTTACTCTC
TGTGGCTTCCCACTATATTTACTCTC
TGTGGCTTCCCACTATATTTACTCTC
TGTGGCTTCCCACTATATTTACTCTC
TGTGGCTTCCCACTATATTTACTCTC
TGTGGCTTCCCACTATATTTACTCTC
TGTGGCTTCCCACTATATTTACTCTC
TGTGGCTTCCCACTATATTTACTCTC
TGTGGCTTCCCACTATATTTACTCTC
TGTGGCTTCCCACTATATTTACTCTC
TGTGGCTTCCCACTATATTTACTCTC
TGTGGCTTCCCACTATATTTACTCTC
TGTGGCTTCCCACTATATTTACTCTC
TGTGGCTTCCCACTATATTTACTCTC
TGTGGCTTCCCACTATATTTACTCTC
TGTGGCTTCCCACTATATTTACTCTC
TGTGGCTTCCCACTATATTTACTCTC
TGTGGCTTCCCACTATATTTACTCTC
TGTGGCTTCCCACTATATTTACTCTC
TGTGGCTTCCCACTATATTTACTCTC
TGTGGCTTCCCACTATATTTACTCTC
TGTGGCTTCCCACTATATTTACTCTC
TGTGGCTTCCCACTATATTTACTCTC
TGTGGCTTCCCACTATATTTACTCTC
TGTGGCTTCCCACTATATTTACTCTC

TGTGGCTTCCCACTATATTTTACTCTC
TGTGGCTTCCCACTATATTTTACTCTC
TGTGGCTTCCCACTATATTTTACTCTC
TGTGGCTTCCCACTATATTTTACTCTC
TGTGGCTTCCCACTATATTTTACTCTC
TGTGGCTTCCCACTATATTTTACTCTC
TGTGGCTTCCCACTATATTTTACTCTC
TGTGGCTTCCCACTATATTTTACTCTC
TGTGGCTTCCCACTATATTTTACTCTC
TGTGGCTTCCCACTATATTTTACTCTC
TGTGGCTTCCCACTATATTTTACTCTC
TGTGGCTTCCCACTATATTTTACTCTC
TGTGGCTTCCCACTATATTTTACTCTC
TGTGGCTTCCCACTATATTTTACTCTC
TGTGGCTTCCCACTATATTTTACTCTC
TGTGGCTTCCCACTATATTTTACTCTC
TGTGGCTTCCCACTATATTTTACTCTC
TGTGGCTTCCCACTATATTTTACTCTC
TGTGGCTTCCCACTATATTTTACTCTC
TGTGGCTTCCCACTATATTTTACTCTC
TGTGGCTTCCCACTATATTTTACTCTC
TGTGGCTTCCCACTATATTTTACTCTC
TGTGGCTTCCCACTATATTTTACTCTC
TGTGGCTTCCCACTATATTTTACTCTC
TGTGGCTTCCCACTATATTTTACTCTC
TGTGGCTTCCCACTATATTTTACTCTC
TGTGGCTTCCCACTATATTTTACTCTC
TGTGGCTTCCCACTATATTTTACTCTC
TGTGGCTTCCCACTATATTTTACTCTC
TGTGGCTTCCCACTATATTTTACTCTC
TGTGGCTTCCCACTATATTTTACTCTC
TGTGGCTTCCCACTATATTTTACTCTC
TGTGGCTTCCCACTATATTTTACTCTC
TGTGGCTTCCCACTATATTTTACTCTC
TGTGGCTTCCCACTATATTTTACTCTC
TGTGGCTTCCCACTATATTTTACTCTC
TGTGGCTTCCCACTATATTTTACTCTC
TGTGGCTTCCCACTATATTTTACTCTC
TGTGGCTTCCCACTATATTTTACTCTC
TGTGGCTTCCCACTATATTTTACTCTC
TGTGGCTTCCCACTATATTTTACTCTC
TGTGGCTTCCCACTATATTTTACTCTC
TGTGGCTTCCCACTATATTTTACTCTC
TGTGGCTTCCCACTATATTTTACTCTC
TGTGGCTTCCCACTATATTTTACTCTC
TGTGGCTTCCCACTATATTTTACTCTC
TGTGGCTTCCCACTATATTTTACTCTC
TGTGGCTTCCCACTATATTTTACTCTC
TGTGGCTTCCCACTATATTTTACTCTC
TGTGGCTTCCCACTATATTTTACTCTC
TGTGGCTTCCCACTATATTTTACTCTC
TGTGGCTTCCCACTATATTTTACTCTC
TGTGGCTTCCCACTATATTTTACTCTC
TGTGGCTTCCCACTATATTTTACTCTC

TGTGGCTTCCCACTATATTTTACTCTC
TGTGGCTTCCCACTATATTTTACTCTC
TGTGGCTTCCCACTATATTTTACTCTC
TGTGGCTTCCCACTATATTTTACTCTC
TGTGGCTTCCCACTATATTTTACTCTC
TGTGGCTTCCCACTATATTTTACTCTC
TGTGGCTTCCCACTATATTTTACTCTC
TGTGGCTTCCCACTATATTTTACTCTC
TGTGGCTTCCCACTATATTTTACTCTC
TGTGGCTTCCCACTATATTTTACTCTC
TGTGGCTTCCCACTATATTTTACTCTC
TGTGGCTTCCCACTATATTTTACTCTC
TGTGGCTTCCCACTATATTTTACTCTC
TGTGGCTTCCCACTATATTTTACTCTC
TGTGGCTTCCCACTATATTTTACTCTC
TGTGGCTTCCCACTATATTTTACTCTC
TGTGGCTTCCCACTATATTTTACTCTC
TGTGGCTTCCCACTATATTTTACTCTC
TGTGGCTTCCCACTATATTTTACTCTC
TGTGGCTTCCCACTATATTTTACTCTC
TGTGGCTTCCCACTATATTTTACTCTC
TGTGGCTTCCCACTATATTTTACTCTC
TGTGGCTTCCCACTATATTTTACTCTC
TGTGGCTTCCCACTATATTTTACTCTC
TGTGGCTTCCCACTATATTTTACTCTC
TGTGGCTTCCCACTATATTTTACTCTC
TGTGGCTTCCCACTATATTTTACTCTC
TGTGGCTTCCCACTATATTTTACTCTC
TGTGGCTTCCCACTATATTTTACTCTC
TGTGGCTTCCCACTATATTTTACTCTC
TGTGGCTTCCCACTATATTTTACTCTC
TGTGGCTTCCCACTATATTTTACTCTC
TGTGGCTTCCCACTATATTTTACTCTC
TGTGGCTTCCCACTATATTTTACTCTC
TGTGGCTTCCCACTATATTTTACTCTC
TGTGGCTTCCCACTATATTTTACTCTC
TGTGGCTTCCCACTATATTTTACTCTC
TGTGGCTTCCCACTATATTTTACTCTC
TGTGGCTTCCCACTATATTTTACTCTC
TGTGGCTTCCCACTATATTTTACTCTC
TGTGGCTTCCCACTATATTTTACTCTC
TGTGGCTTCCCACTATATTTTACTCTC
TGTGGCTTCCCACTATATTTTACTCTC
TGTGGCTTCCCACTATATTTTACTCTC
TGTGGCTTCCCACTATATTTTACTCTC
TGTGGCTTCCCACTATATTTTACTCTC
TGTGGCTTCCCACTATATTTTACTCTC
TGTGGCTTCCCACTATATTTTACTCTC
TGTGGCTTCCCACTATATTTTACTCTC
TGTGGCTTCCCACTATATTTTACTCTC
TGTGGCTTCCCACTATATTTTACTCTC
TGTGGCTTCCCACTATATTTTACTCTC
TGTGGCTTCCCACTATATTTTACTCTC
TGTGGCTTCCCACTATATTTTACTCTC

TGTGGCTTCCCACTATATTTTACTCTC
TGTGGCTTCCCACTATATTTTACTCTC
TGTGGCTTCCCACTATATTTTACTCTC
TGTGGCTTCCCACTATATTTTACTCTC
TGTGGCTTCCCACTATATTTTACTCTC
TGTGGCTTCCCACTATATTTTACTCTC
TGTGGCTTCCCACTATATTTTACTCTC
TGTGGCTTCCCACTATATTTTACTCTC
TGTGGCTTCCCACTATATTTTACTCTC
TGTGGCTTCCCACTATATTTTACTCTC
TGTGGCTTCCCACTATATTTTACTCTC
TGTGGCTTCCCACTATATTTTACTCTC
TGTGGCTTCCCACTATATTTTACTCTC
TGTGGCTTCCCACTATATTTTACTCTC
TGTGGCTTCCCACTATATTTTACTCTC
TGTGGCTTCCCACTATATTTTACTCTC
TGTGGCTTCCCACTATATTTTACTCTC
TGTGGCTTCCCACTATATTTTACTCTC
TGTGGCTTCCCACTATATTTTACTCTC
TGTGGCTTCCCACTATATTTTACTCTC
TGTGGCTTCCCACTATATTTTACTCTC
TGTGGCTTCCCACTATATTTTACTCTC
TGTGGCTTCCCACTATATTTTACTCTC
TGTGGCTTCCCACTATATTTTACTCTC
TGTGGCTTCCCACTATATTTTACTCTC
TGTGGCTTCCCACTATATTTTACTCTC
TGTGGCTTCCCACTATATTTTACTCTC
TGTGGCTTCCCACTATATTTTACTCTC
TGTGGCTTCCCACTATATTTTACTCTC
TGTGGCTTCCCACTATATTTTACTCTC
TGTGGCTTCCCACTATATTTTACTCTC
TGTGGCTTCCCACTATATTTTACTCTC
TGTGGCTTCCCACTATATTTTACTCTC
TGTGGCTTCCCACTATATTTTACTCTC
TGTGGCTTCCCACTATATTTTACTCTC
TGTGGCTTCCCACTATATTTTACTCTC
TGTGGCTTCCCACTATATTTTACTCTC
TGTGGCTTCCCACTATATTTTACTCTC
TGTGGCTTCCCACTATATTTTACTCTC
TGTGGCTTCCCACTATATTTTACTCTC
TGTGGCTTCCCACTATATTTTACTCTC
TGTGGCTTCCCACTATATTTTACTCTC
TGTGGCTTCCCACTATATTTTACTCTC
TGTGGCTTCCCACTATATTTTACTCTC
TGTGGCTTCCCACTATATTTTACTCTC
TGTGGCTTCCCACTATATTTTACTCTC
TGTGGCTTCCCACTATATTTTACTCTC
TGTGGCTTCCCACTATATTTTACTCTC
TGTGGCTTCCCACTATATTTTACTCTC
TGTGGCTTCCCACTATATTTTACTCTC
TGTGGCTTCCCACTATATTTTACTCTC
TGTGGCTTCCCACTATATTTTACTCTC
TGTGGCTTCCCACTATATTTTACTCTC
TGTGGCTTCCCACTATATTTTACTCTC

TGTGGCTTCCCACTATATTTACTCTC
TGTGGCTTCCCACTATATTTACTCTC
TGTGGCTTCCCACTATATTTACTCTC
TGTGGCTTCCCACTATATTTACTCTC
TGTGGCTTCCCACTATATTTACTCTC
TGTGGCTTCCCACTATATTTACTCTC
TGTGGCTTCCCACTATATTTACTCTC
TGTGGCTTCCCACTATATTTACTCTC
TGTGGCTTCCCACTATATTTACTCTC
TGTGGCTTCCCACTATATTTACTCTC
TGTGGCTTCCCACTATATTTACTCTC
TGTGGCTTCCCACTATATTTACTCTC
TGTGGCTTCCCACTATATTTACTCTC
TGTGGCTTCCCACTATATTTACTCTC
TGTGGCTTCCCACTATATTTACTCTC
TGTGGCTTCCCACTATATTTACTCTC
TGTGGCTTCCCACTATATTTACTCTC
TGTGGCTTCCCACTATATTTACTCTC
TGTGGCTTCCCACTATATTTACTCTC
TGTGGCTTCCCACTATATTTACTCTC
TGTGGCTTCCCACTATATTTACTCTC
TGTGGCTTCCCACTATATTTACTCTC
TGTGGCTTCCCACTATATTTACTCTC
TGTGGCTTCCCACTATATTTACTCTC
TGTGGCTTCCCACTATATTTACTCTC
TGTGGCTTCCCACTATATTTACTCTC
TGTGGCTTCCCACTATATTTACTCTC
TGTGGCTTCCCACTATATTTACTCTC
TGTGGCTTCCCACTATATTTACTCTC
TGTGGCTTCCCACTATATTTACTCTC
TGTGGCTTCCCACTATATTTACTCTC
TGTGGCTTCCCACTATATTTACTCTC
TGTGGCTTCCCACTATATTTACTCTC
TGTGGCTTCCCACTATATTTACTCTC
TGTGGCTTCCCACTATATTTACTCTC
TGTGGCTTCCCACTATATTTACTCTC
TGTGGCTTCCCACTATATTTACTCTC
TGTGGCTTCCCACTATATTTACTCTC
TGTGGCTTCCCACTATATTTACTCTC
TGTGGCTTCCCACTATATTTACTCTC
TGTGGCTTCCCACTATATTTACTCTC
TGTGGCTTCCCACTATATTTACTCTC
TGTGGCTTCCCACTATATTTACTCTC
TGTGGCTTCCCACTATATTTACTCTC
TGTGGCTTCCCACTATATTTACTCTC
TGTGGCTTCCCACTATATTTACTCTC
TGTGGCTTCCCACTATATTTACTCTC
TGTGGCTTCCCACTATATTTACTCTC
TGTGGCTTCCCACTATATTTACTCTC
TGTGGCTTCCCACTATATTTACTCTC
TGTGGCTTCCCACTATATTTACTCTC
TGTGGCTTCCCACTATATTTACTCTC
TGTGGCTTCCCACTATATTTACTCTC
TGTGGCTTCCCACTATATTTACTCTC

TGTGGCTTCCCACTATATTTTACTCTC
TGTGGCTTCCCACTATATTTTACTCTC
TGTGGCTTCCCACTATATTTTACTCTC
TGTGGCTTCCCACTATATTTTACTCTC
TGTGGCTTCCCACTATATTTTACTCTC
TGTGGCTTCCCACTATATTTTACTCTC
TGTGGCTTCCCACTATATTTTACTCTC
TGTGGCTTCCCACTATATTTTACTCTC
TGTGGCTTCCCACTATATTTTACTCTC
TGTGGCTTCCCACTATATTTTACTCTC
TGTGGCTTCCCACTATATTTTACTCTC
TGTGGCTTCCCACTATATTTTACTCTC
TGTGGCTTCCCACTATATTTTACTCTC
TGTGGCTTCCCACTATATTTTACTCTC
TGTGGCTTCCCACTATATTTTACTCTC
TGTGGCTTCCCACTATATTTTACTCTC
TGTGGCTTCCCACTATATTTTACTCTC
TGTGGCTTCCCACTATATTTTACTCTC
TGTGGCTTCCCACTATATTTTACTCTC
TGTGGCTTCCCACTATATTTTACTCTC
TGTGGCTTCCCACTATATTTTACTCTC
TGTGGCTTCCCACTATATTTTACTCTC
TGTGGCTTCCCACTATATTTTACTCTC
TGTGGCTTCCCACTATATTTTACTCTC
TGTGGCTTCCCACTATATTTTACTCTC
TGTGGCTTCCCACTATATTTTACTCTC
TGTGGCTTCCCACTATATTTTACTCTC
TGTGGCTTCCCACTATATTTTACTCTC
TGTGGCTTCCCACTATATTTTACTCTC
TGTGGCTTCCCACTATATTTTACTCTC
TGTGGCTTCCCACTATATTTTACTCTC
TGTGGCTTCCCACTATATTTTACTCTC
TGTGGCTTCCCACTATATTTTACTCTC
TGTGGCTTCCCACTATATTTTACTCTC
TGTGGCTTCCCACTATATTTTACTCTC
TGTGGCTTCCCACTATATTTTACTCTC
TGTGGCTTCCCACTATATTTTACTCTC
TGTGGCTTCCCACTATATTTTACTCTC
TGTGGCTTCCCACTATATTTTACTCTC
TGTGGCTTCCCACTATATTTTACTCTC
TGTGGCTTCCCACTATATTTTACTCTC
TGTGGCTTCCCACTATATTTTACTCTC
TGTGGCTTCCCACTATATTTTACTCTC
TGTGGCTTCCCACTATATTTTACTCTC
TGTGGCTTCCCACTATATTTTACTCTC
TGTGGCTTCCCACTATATTTTACTCTC
TGTGGCTTCCCACTATATTTTACTCTC
TGTGGCTTCCCACTATATTTTACTCTC
TGTGGCTTCCCACTATATTTTACTCTC
TGTGGCTTCCCACTATATTTTACTCTC
TGTGGCTTCCCACTATATTTTACTCTC
TGTGGCTTCCCACTATATTTTACTCTC
TGTGGCTTCCCACTATATTTTACTCTC
TGTGGCTTCCCACTATATTTTACTCTC
TGTGGCTTCCCACTATATTTTACTCTC

TGTGGCTTCCCACTATATTTTACTCTC
TGTGGCTTCCCACTATATTTTACTCTC
TGTGGCTTCCCACTATATTTTACTCTC
TGTGGCTTCCCACTATATTTTACTCTC
TGTGGCTTCCCACTATATTTTACTCTC
TGTGGCTTCCCACTATATTTTACTCTC
TGTGGCTTCCCACTATATTTTACTCTC
TGTGGCTTCCCACTATATTTTACTCTC
TGTGGCTTCCCACTATATTTTACTCTC
TGTGGCTTCCCACTATATTTTACTCTC
TGTGGCTTCCCACTATATTTTACTCTC
TGTGGCTTCCCACTATATTTTACTCTC
TGTGGCTTCCCACTATATTTTACTCTC
TGTGGCTTCCCACTATATTTTACTCTC
TGTGGCTTCCCACTATATTTTACTCTC
TGTGGCTTCCCACTATATTTTACTCTC
TGTGGCTTCCCACTATATTTTACTCTC
TGTGGCTTCCCACTATATTTTACTCTC
TGTGGCTTCCCACTATATTTTACTCTC
TGTGGCTTCCCACTATATTTTACTCTC
TGTGGCTTCCCACTATATTTTACTCTC
TGTGGCTTCCCACTATATTTTACTCTC
TGTGGCTTCCCACTATATTTTACTCTC
TGTGGCTTCCCACTATATTTTACTCTC
TGTGGCTTCCCACTATATTTTACTCTC
TGTGGCTTCCCACTATATTTTACTCTC
TGTGGCTTCCCACTATATTTTACTCTC
TGTGGCTTCCCACTATATTTTACTCTC
TGTGGCTTCCCACTATATTTTACTCTC
TGTGGCTTCCCACTATATTTTACTCTC
TGTGGCTTCCCACTATATTTTACTCTC
TGTGGCTTCCCACTATATTTTACTCTC
TGTGGCTTCCCACTATATTTTACTCTC
TGTGGCTTCCCACTATATTTTACTCTC
TGTGGCTTCCCACTATATTTTACTCTC
TGTGGCTTCCCACTATATTTTACTCTC
TGTGGCTTCCCACTATATTTTACTCTC
TGTGGCTTCCCACTATATTTTACTCTC
TGTGGCTTCCCACTATATTTTACTCTC
TGTGGCTTCCCACTATATTTTACTCTC
TGTGGCTTCCCACTATATTTTACTCTC
TGTGGCTTCCCACTATATTTTACTCTC
TGTGGCTTCCCACTATATTTTACTCTC
TGTGGCTTCCCACTATATTTTACTCTC
TGTGGCTTCCCACTATATTTTACTCTC
TGTGGCTTCCCACTATATTTTACTCTC
TGTGGCTTCCCACTATATTTTACTCTC
TGTGGCTTCCCACTATATTTTACTCTC
TGTGGCTTCCCACTATATTTTACTCTC
TGTGGCTTCCCACTATATTTTACTCTC
TGTGGCTTCCCACTATATTTTACTCTC
TGTGGCTTCCCACTATATTTTACTCTC
TGTGGCTTCCCACTATATTTTACTCTC
TGTGGCTTCCCACTATATTTTACTCTC

TGTGGCTTCCCACTATATTTTACTCTC
TGTGGCTTCCCACTATATTTTACTCTC
TGTGGCTTCCCACTATATTTTACTCTC
TGTGGCTTCCCACTATATTTTACTCTC
TGTGGCTTCCCACTATATTTTACTCTC
TGTGGCTTCCCACTATATTTTACTCTC
TGTGGCTTCCCACTATATTTTACTCTC
TGTGGCTTCCCACTATATTTTACTCTC
TGTGGCTTCCCACTATATTTTACTCTC
TGTGGCTTCCCACTATATTTTACTCTC
TGTGGCTTCCCACTATATTTTACTCTC
TGTGGCTTCCCACTATATTTTACTCTC
TGTGGCTTCCCACTATATTTTACTCTC
TGTGGCTTCCCACTATATTTTACTCTC
TGTGGCTTCCCACTATATTTTACTCTC
TGTGGCTTCCCACTATATTTTACTCTC
TGTGGCTTCCCACTATATTTTACTCTC
TGTGGCTTCCCACTATATTTTACTCTC
TGTGGCTTCCCACTATATTTTACTCTC
TGTGGCTTCCCACTATATTTTACTCTC
TGTGGCTTCCCACTATATTTTACTCTC
TGTGGCTTCCCACTATATTTTACTCTC
TGTGGCTTCCCACTATATTTTACTCTC
TGTGGCTTCCCACTATATTTTACTCTC
TGTGGCTTCCCACTATATTTTACTCTC
TGTGGCTTCCCACTATATTTTACTCTC
TGTGGCTTCCCACTATATTTTACTCTC
TGTGGCTTCCCACTATATTTTACTCTC
TGTGGCTTCCCACTATATTTTACTCTC
TGTGGCTTCCCACTATATTTTACTCTC
TGTGGCTTCCCACTATATTTTACTCTC
TGTGGCTTCCCACTATATTTTACTCTC
TGTGGCTTCCCACTATATTTTACTCTC
TGTGGCTTCCCACTATATTTTACTCTC
TGTGGCTTCCCACTATATTTTACTCTC
TGTGGCTTCCCACTATATTTTACTCTC
TGTGGCTTCCCACTATATTTTACTCTC
TGTGGCTTCCCACTATATTTTACTCTC
TGTGGCTTCCCACTATATTTTACTCTC
TGTGGCTTCCCACTATATTTTACTCTC
TGTGGCTTCCCACTATATTTTACTCTC
TGTGGCTTCCCACTATATTTTACTCTC
TGTGGCTTCCCACTATATTTTACTCTC
TGTGGCTTCCCACTATATTTTACTCTC
TGTGGCTTCCCACTATATTTTACTCTC
TGTGGCTTCCCACTATATTTTACTTTC
Nr of selected repeats 1170 Similarity 0.999916
Consensus:
TGTGGCTTCCCACTATATTTTACTCTC
>Cele-UNSB01_2:14573634-14573711 Satlength=78 Nr of Repeats=5 RepeatLength=15
seed=CGAAAAGGGG
CGAAAAGGGGAGCAGAA
CGAAAAGGGGATCTG
CAAAAAGGGGATCTG

CGAAAAGGGGAGATA
CGAAAAGGGGAGCAA
Nr of selected repeats 4 Similarity 0.748148
Consensus:
CgAAAAGGGGAgcta
>Cele-UNSB01_2:14573886-14573978 Satlength=93 Nr of Repeats=6 RepeatLength=15
seed=TCCCCTTTTC
TCCCCTTTTCGTTGC
TCCCCTTTTCGTATC
TCCCCTTTTCGCAGA
TCCCCTTTTTGCAGA
TCCCCTTTTTGCAGA
TCCCCTTTTCGTTTTGC
Nr of selected repeats 5 Similarity 0.768889
Consensus:
TCCCCTTTTcGcaga
>Cele-UNSB01_2:14582719-14582837 Satlength=119 Nr of Repeats=5 RepeatLength=18
seed=TAGTCTTGCA
TAGTCTTGCAGCCTCTAT
TAGTCTTGCACCCCTAGAGGTCAATCGAAAAA
TAGTCTTGCAGCCTCTAT
TAGTCTTGCAGTTTCTAT
TAATCTTGCACCCCTACGGGTTGATTGAAAAT
Nr of selected repeats 3 Similarity 0.901235
Consensus:
TAGTCTTGCAGccTCTAT
>Cele-UNSB01_2:14966230-14966326 Satlength=97 Nr of Repeats=6 RepeatLength=16
seed=GCAGTTTGCC
GCAGTTTGCCGAGTTT
GCCGTTTGCCGAATTG
GCAGTTTGCCGAGTTT
GCCGTTTGCCGAATTG
GCAGTTTGCCCAGTTT
GCCGTTTGCCGAAGTG
Nr of selected repeats 6 Similarity 0.794444
Consensus:
GCaGTTTGCCGAaTTg
>Cele-UNSB01_2:15007973-15008319 Satlength=347 Nr of Repeats=4 RepeatLength=35
seed=TCCGGCAAAT
TCCGGCAAATCGGCAAATTGGTGAAATTTAAAATT
TCCGGCAAATCGGAAATTTTTTTATTTTTTCTCAACATTTTTAGATTTTTTACTACAAAAATCAAAAAAAAAAATTTCAA
AAATCAAAAAAAAATTTAATTTTTTGAAAATCAATTTTTTTTTCTCTAAAAAATCCAAAATTGCGATTAAAGATAAAAAA
TAATTTTTAAAAAAATATTTAATTTTTTTTCCGAAAAAAAATCAGCAAAAATGCTGGCTATTCAATTAGCCGGAAATTTT
CAAT
TCCGGCAATTTTCCGATTTGCCGGAAACTTTCAAT
TCCGGCAAATCGGCAAATTGCCGGAATTGAGAATC
Nr of selected repeats 3 Similarity 0.586420
Consensus:
TCCGGCAAaTcggCaAaTTGccGaAAtTtanAATn
>Cele-UNSB01_2:15030456-15030601 Satlength=146 Nr of Repeats=7 RepeatLength=21
seed=ATTGGATTAG
ATTGGATTAGATTTGTTCCAT
ATTCGATTAGATTTCGTCCAT
ATTGGATTAGAATTGTTCCAT

ATTGGATTAGATTCCGGCCAT
ATTGGATTAGATTTGTTCCAC
ATTGGATTAGATTTCAACCAT
ATTTGATTAGAAAATCCAT
Nr of selected repeats 6 Similarity 0.792593
Consensus:
ATTGGATTAGATTTcttCCAT
>Cele-UNSB01_2:15064033-15064173 Satlength=141 Nr of Repeats=5 RepeatLength=30
seed=TTCTGGAAAA
TTCTGGAAAATTCTAGAATGTTCCAGAACC
TTCTGGAAAATTCTAAAAAGCTTTAGAACC
TTCTGGAAAA
TTCTAGAAAATTCTGGAATGTTCCAGAACT
TTCTGGAAAATTCGAAAAAATTCTGGATTGTTCTAGAACC
Nr of selected repeats 3 Similarity 0.762963
Consensus:
TTCTgGAAAATTCTagAAtGtTccAGAACc
>Cele-UNSB01_2:15064232-15064367 Satlength=136 Nr of Repeats=9 RepeatLength=15
seed=AGACCCATCG
AGACCCATCGTGGCG
AGGCCCATCGTTGTG
AGACCCATCGTGGTG
AGACGCATCGAGGTG
AGACCCATCGTGGTG
AGACCCATCGTGGTG
AGACCCATCGTGGTA
AGACCCATCGTGGTG
AGACCCATCGTGGTG
Nr of selected repeats 9 Similarity 0.881481
Consensus:
AGACCCATCGTGGTG
>Cele-UNSB01_2:15064421-15065299 Satlength=879 Nr of Repeats=25 RepeatLength=35
seed=TTAAATTTTC
TTAAATTTTCTGTGAAAAATATTTTGGCGGGAAAA
TTAAATTTTCTGAAAAAAATATTTTGGAGGGAAAT
TTAAATTTTCTGTGAAAAATATTTTGGCGGGAAAT
TTAAATTTTCTGTGAAAAATATTTTGGAGGGAAAT
TTAAATTTTCTGTGAAAAATATTTTGGCGGGAAAT
TTAAATTTTCTGAAAAAAATATTTTGGCGGGAAAT
TTAAATTTTCTGAAAAAAAATATTTTGGAGGGAAGT
TTAAATTTTCTGTGAAAAATATTTTGGAGGGAAAT
TTAAATTTTCTGTGTAAAATATTTTGGCGGGAAAT
TTAAATTTTCTGAAAAAAATATTTTGGAGGGAAAT
TTAAATTTTCTGTGAAAAATATTTTGGAGGGAAAT
TTAAATTTTCTGTGTAAAATATTTTGGAGGGAAAT
TTAAATTTTCTGTGAAAAATATTTTGGAGGGAAAT
TTAAATTTTCTGTGTAAAATATTTTGGCGGGAAAT
TTAAATTTTCTGAAAAAAATATTTTGGCGGGAAAT
TTAAATTTTCTGAAAAAAAATATTTTGGCGGGAAAT
TTAAATTTTCTGTGAAAAATATTTTGGAGGGAAAT
TTAAATTTTCTGTGAAAAATATTTTGGAGGGAAAT
TTAAATTTTCTGTGAAAAATATTTTGGAGGGAAAT
TTAAATTTTCTGTGAAAAATATTTTGGAGGGAAAT
TTAAATTTTCTGTGAAAAATATTTTGGCGGGAAAT

TTAAATTTTCTGAAAAAAATATTTTGGCGGGAAAT
TTAAATTTTCTGAAAAAAAATATTTTGGCGGGAAAT
TTAAATTTTCTGTGAAAAATATTTTGGAGGGAAAT
TTAAATTTTCTGTGAAAAATATTTTGGCGGGAAAT
Nr of selected repeats 22 Similarity 0.939312
Consensus:
TTAAATTTTCTGtgAAAAATATTTTGGaGGGAAAT
>Cele-UNSB01_2:15097668-15098195 Satlength=528 Nr of Repeats=13 RepeatLength=35
seed=TTTCCGGCAA
TTTCCGGCAAATCGGCAAATTGTTGGACTTGAAAA
TTTCCGGCAAATCGGCGAATTGCATTAATTTAAAA
TTTCCGGCAAATTAGCCAATTGCCGGACTTTAGAA
TTTCCGGCAAATCGGCGAATTGCTGAAATTGAAAA
TTTCGGCAAATCGGCAAATTGCCGGAGTTGGAAA
TTTCCGGCAAATCGGCAAATTGCAGTAATTGAAAA
TTTCCGGCAAATTGGTCAATTGCCGGACTTTAGAAT
TTTCGGCAAATCGGCGAATTGCAGTAATTGAAAA
TTTCCGGCAAATTGACCAATTGCCAGAATTGAAAA
TTTCCGGCAAATCGGCAAATTGTTGGACTTGAAAA
TTTCCGGCAAATCGGCAAATTGTTGGACATGAAAA
TTTCCGGCAAATTGTGATTTTTCACATTTTTTTGATATATGAAAATATTTAAAGAAAACGGGAAAATTACAACAAAAAA
AACTCAGTGTTTTGCATTTTGA
TATCCGGCAAACGACAAACCGGCAATTTGCCAAAAATGAAAA
Nr of selected repeats 8 Similarity 0.791837
Consensus:
TTTCCGGCAAATcGGCaAATTGctggAaTTgAAAA
>Cele-UNSB01_2:15105694-15106081 Satlength=388 Nr of Repeats=11 RepeatLength=35
seed=CAGAAAATTT
CAGAAAATTTAAATTTCCCGCCAAAATATTTTTTTT
CAGAAAATTTAAATTTCCCGCCAAAATATTTTTCA
CAGAAAATTTAAATTTCCCTCCAAAATATTTTTCA
CAGAAAATTTAAATTTCCCTCCAAAATATTTTTCA
CAGAAAATTTAAATTTCCCGCCAAAATATTTTTTT
CAGAAAATTTAAATTTCCCGCCAAAATATTTTTTTT
CAGAAAATTTAAATTTCCCGCCAAAATATTTTTTT
CAGAAAATTTAAATTTCCCGCCAAAATATTTTTCA
CAGAAAATTTAAATTTCCCTCCAAAATATTTTTCA
CAGAAAATTTAAATTTCCCTCCAAAATATTTTTCA
CAGAAAATTTAAATTTCCCGCCAAAATATTTTTCA
Nr of selected repeats 9 Similarity 0.949206
Consensus:
CAGAAAATTTAAATTTCCCgCCAAAATATTTTTca
>Cele-UNSB01_2:15106134-15106254 Satlength=121 Nr of Repeats=7 RepeatLength=15
seed=CCACGATGGG
CCACGATGGGTCTCA
CCACGATGGGTCTCA
CCACGATGGGTCTTA
CCACGATGGGTCTCA
CCACGATGGGTCTCACCTCGATGCGTCTCA
CCACGATGGGTCTCA
CAACGATGGGCCTCG
Nr of selected repeats 6 Similarity 0.881481
Consensus:
CCACGATGGGTCTCA

>Cele-UNSB01_2:15106314-15106454 Satlength=141 Nr of Repeats=5 RepeatLength=30
seed=AATTTTCCAG
AATTTTCCAGAAGGTTCTAGAACAATCCAGAATTTTTTCG
AATTTTCCAGAAAGTTCTGGAACATTCCAG
AATTTTCTAG
AATTTTCCAGAAGGTTCTAAAGCTTTTCAG
AATTTTCCAGAAGGTTCTGGAACATTCTAG
Nr of selected repeats 3 Similarity 0.792593
Consensus:
AATTTTCCAGAAgGTTCTggAaCaTTccAG
>Cele-UNSB01_2:15121230-15121314 Satlength=85 Nr of Repeats=6 RepeatLength=12
seed=TTAGGCTTAG
TTAGGCTTAGGT
TTAGGCTCAGGCTTAAGT
TTAGGCTAAGGT
TTAGGCTTAGGC
TTAGGCTTAGAC
TTAGGCTTAGGCTTAGGC
Nr of selected repeats 4 Similarity 0.814815
Consensus:
TTAGGCTtAGgc
>Cele-UNSB01_2:15182818-15182878 Satlength=61 Nr of Repeats=5 RepeatLength=12
seed=AAGCCTAAGC
AAGCCTAAGCCT
AAGCCCAAGCCT
AAGCCTAAGCCC
AAGCCTAAGCCT
AAGCCCAAGCCT
Nr of selected repeats 5 Similarity 0.888889
Consensus:
AAGCCtAAGCCt
>Cele-UNSB01_2:15212191-15212515 Satlength=325 Nr of Repeats=14 RepeatLength=20
seed=TTCAGTCAAA
TTCAGTCAAAATTTAGATTT
TTCAGTCAAAAATTAAGTTTTT
TTCAGTCAAAATTTAGATTTTTCTGTATAAAATTAAGTTC
TTCAGTCAAAATTTAGATTTTTCTGTATAAAATTAAGTTC
TTCAGTCAAAATTTAGATTT
TTCTGTCAAAAATTAAGTTT
TTCAGTCGAAATTTTAATTT
TTCAGTCCAAATTTGAATTT
TTCGGTCAAAATTTAGATTT
TTCTGTCAAAAATTAAGTTT
TTCAGTCAAAATTTAGATTT
TTCAGTCAAAAATTAAGTTC
TTCAGTCAAAATTTAGATTT
TTCAGTCAAAAATTAAGTTTTT
Nr of selected repeats 10 Similarity 0.802963
Consensus:
TTCaGTCaAAAtTTaaaTTT
>Cele-UNSB01_2:15219171-15219261 Satlength=91 Nr of Repeats=6 RepeatLength=15
seed=TCGTGGCGAG
TCGTGGCGAGACCCA
TCGTTGCGAGACCCT

TCGTGGCGAGACCCA
TCGTGGCGAGACCCA
TCGTGGCGAGACCCA
TCGTGGCGAGACCCA
Nr of selected repeats 6 Similarity 0.940741
Consensus:
TCGTGGCGAGACCCA
>Cele-UNSB01_2:15219280-15219502 Satlength=223 Nr of Repeats=7 RepeatLength=32
seed=TTTTGGCGGG
TTTTGGCGGGAATTTAAATTTTCTTTGAAATT
TTTCGGCGGGAATTCAAATTTTCTGTGAAAAT
TTTTGGCGGGAATTTAAATTTTCTTTAAAATT
TTTTGGCGGGAATTCAAATTTTCTGTGAAAAA
TTTTGGCGGGAATTCAAATTTTCTGAAAAAT
TTTTGGCGGGAGTTCAAATTTTCTCTGAAAAA
TTTTGGCGGGAATTCAAATTTTCTGAAAAAT
Nr of selected repeats 5 Similarity 0.841667
Consensus:
TTTtGGCGGGAaTTcAAATTTTCTnTgAAAat
>Cele-UNSB01_2:15221118-15221238 Satlength=121 Nr of Repeats=8 RepeatLength=15
seed=GGTGAGACCC
GGTGAGACCCTTCGT
GGTGAGACCCATCGT
GGTGAGACCCTTCGT
GGTGAGACCCATCGT
GGTGAGACCCATCGT
GGTGAGACCCTTCGT
GGTGAGACCCATCGT
GGTGAGACCCTTCGC
Nr of selected repeats 8 Similarity 0.926984
Consensus:
GGTGAGACCCaTCGT
>Cele-UNSB01_2:15221118-15221591 Satlength=474 Nr of Repeats=14 RepeatLength=15
seed=GGTGAGACCC
GGTGAGACCCTTCGT
GGTGAGACCCATCGT
GGTGAGACCCTTCGT
GGTGAGACCCATCGT
GGTGAGACCCATCGT
GGTGAGACCCTTCGT
GGTGAGACCCATCGT
GGTGAGACCCTTCGC
GGTGAGACCCTTCGC
GGTGATACCCACATTTTTGGCGGGAAATTCAAATTTTCTGTGAAAACAATTTTGGCGGGAAATTCAAATTTTCAGTGAA
AAAATTTTGGCGGGAAATTCAAATTTTCAGTGAAAAAATTTTGGTGGGAAATTCAAATTTTCTATGAAAAAAATATCAT
TACTTATATATAAAAAAATACAGTTCGTCTGTCCATAGTTTGTAGTCTATGTAGTCTTTGTAGTCTGTGACGTCACACC
CAAAGTCAGTGAGAGTTGTGGGCGGGGCCTGTCACCTTCGTC
GTGAGACCCATCGT
GGTGAGACCCTTCGT
GGTGAGACCCATCGT
GGTGAGACCCTTCGC
Nr of selected repeats 12 Similarity 0.916498
Consensus:
GGTGAGACCCtTCGt

>Cele-UNSB01_2:15221267-15222837 Satlength=1571 Nr of Repeats=42
RepeatLength=32 seed=TTTTGGCGGG
TTTTGGCGGGAAATTCAAATTTTCTGTGAAAACAA
TTTTGGCGGGAAATTCAAATTTTCAGTGAAAAAA
TTTTGGCGGGAAATTCAAATTTTCAGTGAAAAAA
TTTTGGTGGGAAATTCAAATTTTCTATGAAAAAAATATCATTACTTATATATAAAAAAATACAGTTCGTCTGTCCATAG
TTTGTAGTCTATGTAGTCTTTGTAGTCTGTGACGTCACACCCAAAGTCAGTGAGAGTTGTGGGCGGGGCCTGTCACCTT
CGTCGTGAGACCCATCGTGGTGAGACCCTTCGTGGTGAGACCCATCGTGGTGAGACCCTTCGCGGTGAGACCCTTCGCG
GTGATACCCACAT
TTTTGGCGGGAAATTCAAATTTTCTGTGAAAACAA
TTTTGGCGGGAAATTCAAATTTTCAGTGAAAAAA
TTTTGGCGGGAAATTCAAATTTTCAGTGAAAAAA
TTTTGGCGTGAATTCAAATTTTCTGAAACAT
TTTTGGCGGGAATTCAAATTTTCTCTGAAAAT
TTTTGGCGGGAATTTAAATTTTCTTCAAAATT
TTTTGGCGGGAATTCAAATTTTCTTCAAAATT
TTTTGGCGGGAATTCAAATTTTCTTTGAAAAT
TTTTGGCGTGAATTCAAATTTTCTGAAACAT
TTTTGGCGGGAATTCAAATTTTCTCTGAAAAT
TTTTGGCGGGAATTTAAATTTTCTTTAAAATT
TTTTGGCGGGAATTCAAAATTTCTCTGAAAAT
TTTTGGCGGGAATTTAAATTTTCTTTAAAATT
TTTTGGCGGGAATTCAAATTTTCTGTGAAAAA
TTTTGGCGGGAATTCAAATTTTCTGAAACAT
TTTTGGCGGGAATTCAAATTTTCTCTGAAAAA
TTTTGGCGGGAATTTAAATTTCCTTCAAAATT
TTTTGGCGGGAATTCAAATTTTCTTTGAAAATT
TTTTGGCGGGAATTCAAATTTTCTGAAAAATT
TTTTGGCGGGAATTCAAATTTTCTTTAAAATT
TTTTGGCGGGAATTCAAATTTTCTCTGAAAAT
TTTTGGCGGGAATTCAAATTTTCTTCAAAATT
TTTTGGCGGGAATTCAAATTTTCTTTGAAAAT
TTTTGGCGTGAATTCAAATTTTCTGAAACAT
TTTTGGCGGGAATTCAAATTTTCTCTGAAAAT
TTTTGGCGGGAATTTAAATTTTCTTTAAAATT
TTTTGGCGGGAATTCAAAATTTCTCTGAAAAT
TTTTGGCGGGAATTTAAATTTTCTTTAAAATT
TTTTGGCGGGAATTCAAATTTTCTGTGAAAAA
TTTTGGCGGGAATTCAAATTTTCTGAAAAAT
TTTTGGCGGGAGTTCAAATTTTCTCTGAAAAA
TTTTGGCGGGAATTTAAATTTCCTTCAAAATT
TTTTGGCGGGAATTCAAATTTTCTTTGAAAAT
TTTTGGCGGGAATTCAAATTTTCTGAAAAAT
TTTTGGCGGGAATTCAAATTTTCTCTGAAAAT
TTTTGGCGGGAATTTAAATTTTCTTTAAAATT
TTTTGGCGGGAATTCAAATTTTCTGTGAAAAA
TTTTGGCGGGAATTCAAATTTTCTGAAAAAT
Nr of selected repeats 27 Similarity 0.855679
Consensus:
TTTTGGCGGGAATTcAAATTTTCTnTgAAAAT
>Cele-UNSB01_2:15224452-15224572 Satlength=121 Nr of Repeats=8 RepeatLength=15
seed=GGTGAGACCC
GGTGAGACCCTTCGT
GGTGAGACCCATCGT

GGTGAGACCCTTCGT
GGTGAGACCCATCGT
GGTGAGACCCATCGT
GGTGAGACCCTTCGT
GGTGAGACCCATCGT
GGTGAGACCCTTCGC
Nr of selected repeats 8 Similarity 0.926984
Consensus:
GGTGAGACCCaTCGT
>Cele-UNSB01_2:15224452-15225368 Satlength=917 Nr of Repeats=26 RepeatLength=15 seed=GGTGAGACCC
GGTGAGACCCTTCGT
GGTGAGACCCATCGT
GGTGAGACCCTTCGT
GGTGAGACCCATCGT
GGTGAGACCCATCGT
GGTGAGACCCTTCGT
GGTGAGACCCATCGT
GGTGAGACCCTTCGC
GGTGAGACCCTTCGC
GGTGATACCCACATTTTTGGCGGGAAATTCAAATTTTCTGTGAAAACAATTTTGGCGGGAAATTCAAATTTTCAGTGAA
AAAATTTTGGCGGGAAATTCAAATTTTCAGTGAAAAAAATTTTGGTGGGAAATTCAAATTTTCTATGAAAAAAAATATCAT
TACTTATATATAAAAAAAATACAGTTCGTCTGTCCATAGTTTGTAGTCTATGTAGTCTTTGTAGTCTGTGACGTCACACC
CAAAGTCAGTGAGAGTTGTGGGCGGGGCCTGTCACCTTCGTC
GTGAGACCCATCGT
GGTGAGACCCTTCGT
GGTGAGACCCATCGT
GGTGAGACCCTTCGC
GGTGAGACCCTTCGC
GGTGAGACCCATCGT
GGTGAGACCCTTCGT
GGTGAGACCCATCGT
GGTGAGACCCTTCGC
GGTGAGACCCTTCGC
GGTGATACCCACATTTTTGGCGGGAAATTCAAATTTTCTGTGAAAACAATTTTGGCGGGAAATTCAAATTTTCAGTGAA
AAAATTTTGGCGGGAAATTCAAATTTTCAGTGAAAAAAATTTTGGTGGGAAATTCAAATTTTCTATGAAAAAAAATATCAT
TACTTATATATAAAAAAAATACAGTTCGTCTGTCCATAGTTTGTAGTCTATGTAGTCTTTGTAGTCTGTGACGTCACACC
CAAAGTCAGTGAGAGTTGTGGGCGGGGCCTGTCACCTTCGTC
GTGAGACCCATCGT
GGTGAGACCCTTCGT
GGTGAGACCCATCGT
GGTGAGACCCTTCGC
GGTGAGACCCTTCGC
Nr of selected repeats 22 Similarity 0.913805
Consensus:
GGTGAGACCCtTCGt
>Cele-UNSB01_2:15224601-15228185 Satlength=3585 Nr of Repeats=96 RepeatLength=32 seed=TTTTGGCGGG
TTTTGGCGGGAAATTCAAATTTTCTGTGAAAACAA
TTTTGGCGGGAAATTCAAATTTTCAGTGAAAAAA
TTTTGGCGGGAAATTCAAATTTTCAGTGAAAAAA
TTTTGGTGGGAAATTCAAATTTTCTATGAAAAAAAATATCATTACTTATATATAAAAAAAATACAGTTCGTCTGTCCATAG
TTTGTAGTCTATGTAGTCTTTGTAGTCTGTGACGTCACACCCAAAGTCAGTGAGAGTTGTGGGCGGGGCCTGTCACCTT
CGTCGTGAGACCCATCGTGGTGAGACCCTTCGTGGTGAGACCCATCGTGGTGAGACCCTTCGCGGTGAGACCCTTCGCG

GTGAGACCCATCGTGGTGAGACCCTTCGTGGTGAGACCCATCGTGGTGAGACCCTTCGCGGTGAGACCCTTCGCGGTGA
TACCCACAT
TTTTGGCGGGAAATTCAAATTTTCTGTGAAAACAA
TTTTGGCGGGAAATTCAAATTTTCAGTGAAAAAA
TTTTGGCGGGAAATTCAAATTTTCAGTGAAAAAA
TTTTGGTGGGAAATTCAAATTTTCTATGAAAAAAATATCATTACTTATATATAAAAAAATACAGTTCGTCTGTCCATAG
TTTGTAGTCTATGTAGTCTTTGTAGTCTGTGACGTCACACCCAAAGTCAGTGAGAGTTGTGGGCGGGGCCTGTCACCTT
CGTCGTGAGACCCATCGTGGTGAGACCCTTCGTGGTGAGACCCATCGTGGTGAGACCCTTCGCGGTGAGACCCTTCGCG
GTGAGACCCACAT
TTTTGGCGGGAAATTCAAATTTTCTCTGAAAAT
TTTTGGCGGGAATTCAAATTTTCTGAAACAT
TTTTGGCGGGAATTTAAATTTTCTTTGAAAAT
TTTTGGCGTGAATTCAAATTTTCTGAAACAT
TTTTGGCGGGAATTCAAATTTTCTCTGAAAAT
TTTTGGCGGGAATTTAAATTTTCTTTAAAATT
TTTTGGCGGGAATTCAAAATTTCTCTGAAAAT
TTTTGGCGTGAATTCAAATTTTCTGAAACAT
TTTTGGCGGGAATTCAAATTTTCTCTGAAAAT
TTTTGGCGGGAATTCAAATTTTCTGAAACAT
TTTTGGCGAGAATTCAAATTTTCTTTGAAAAT
TTTTGGCGTGAATTCAAATTTTCTGAAACAT
TTTTGGCGGGAATTCAAATTTTCTCTGAAAAT
TTTTGGCGGGATTTTAAATTTTCTTTAAAATT
TTTTGGCGGGAGTTCAAAATTTCTCTGAAAAT
TTTTGGCGTGAATTCAAATTTTCTGAAACAT
TTTTGGCGGGAATTCAAATTTTCTCTGAAAAT
TTTTGGCGGGAATTTAAATTTTCTTTAAAATT
TTTTGGCGGGAATTCAAAATTTCTCTGAAAAT
TTTTGGCGGGAATTTAAATTTTCTTTAAAATT
TTTTGGCGGGAATTCAAATTTTCTGTGAAAAA
TTTTGGCGGGAATTCAAATTTTCTGAAAAAT
TTTTGGCGGGAGTTCAAATTTTCTCTGAAAAA
TTTTGGCGGGAATTTAAATTTCCTTCAAAATT
TTTTGGCGGGAATTCAAATTTTCTTTGAAAAT
TTTTGGCGGGAATTCAAATTTTCTGAAAAATT
TTTTGGCGGGAATTCAAATTTTCTTTGAAAAT
TTTTGGCGTGAATTCAAATTTTCTGAAACAT
TTTTGGCGGGAATTCAAATTTTCTCTGAAAAT
TTTTGGCGGGAATTTAAATTTTCTTTAAAATT
TTTTGGCGGGAATTCAAAATTTCTCTGAAAAT
TTTTGGCGGGAATTTAAATTTTCTTTAAAATT
TTTTGGCGGGAATTCAAATTTTCTGTGAAAAA
TTTTGGCGGGAATTCAAATTTTCTGAAAAAT
TTTTGGCGGGAGTTCAAATTTTCTCTGAAAAA
TTTTGGCGGGAATTTAAATTTCCTTCAAAATT
TTTTGGCGGGAATTCAAATTTTCTTTGAAAATT
TTTTGGCGGGAATTCAAATTTTCTGAAAAATT
TTTTGGCGGGAATTCAAATTTTCTTTAAAATA
TTTTGGCGGGAATTCAAATTTTCTCTGAAAAT
TTTTGGCGGGAATTCAAATTTTCTTCAAAATT
TTTTGGCGGGAATTCAAATTTTCTTTGAAAAT
TTTTGGCGTGAATTCAAATTTTCTGAAACAT
TTTTGGCGGGAATTCAAATTTTCTCTGAAAAT
TTTTGGCGGGAATTTAAATTTTCTTTAAAATT

TTTTGGCGGGAATTCAAAATTTCTCTGAAAAT
TTTTGGCGGGAATTTAAATTTTCTTTAAAATT
TTTTGGCGGGAATTCAAATTTTCTGTGAAAAA
TTTTGGCGGGAATTCAAATTTTCTGAAACAT
TTTTGGCGGGAATTCAAATTTTCTCTGAAAAA
TTTTGGCGGGAATTTAAATTTCCTTCAAAATT
TTTTGGCGGGAATTCAAATTTTCTTTGAAAATT
TTTTGGCGGGAATTCAAATTTTCTGAAAAATT
TTTTGGCGGGAATTCAAATTTTCTTTAAAATT
TTTTGGCGGGAATTCAAATTTTCTCTGAAAAT
TTTTGGCGGGAATTCAAATTTTCTTCAAAATT
TTTTGGCGGGAATTCAAATTTTCTTTGAAAAT
TTTTGGCGTGAATTCAAATTTTCTGAAACAT
TTTTGGCGGGAATTCAAATTTTCTCTGAAAAT
TTTTGGCGGGAATTTAAATTTTCTTTAAAATT
TTTTGGCGGGAATTCAAAATTTCTCTGAAAAT
TTTTGGCGGGAATTTAAATTTTCTTTAAAATT
TTTTGGCGGGAATTCAAATTTTCTGTGAAAAA
TTTTGGCGGGAATTCAAATTTTCTGAAACAT
TTTTGGCGGGAATTCAAATTTTCTCTGAAAAA
TTTTGGCGGGAATTTAAATTTCCTTCAAAATT
TTTTGGCGGGAATTCAAATTTTCTTTGAAAATT
TTTTGGCGGGAATTCAAATTTTCTGAAAAATT
TTTTGGCGGGAATTCAAATTTTCTTTAAAATT
TTTTGGCGGGAATTCAAATTTTCTCTGAAAAT
TTTTGGCGGGAATTCAAATTTTCTTCAAAATT
TTTTGGCGGGAATTCAAATTTTCTTTGAAAAT
TTTTGGCGTGAATTCAAATTTTCTGAAACAT
TTTTGGCGGGAATTCAAATTTTCTCTGAAAAT
TTTTGGCGGGAATTTAAATTTTCTTTAAAATT
TTTTGGCGGGAATTCAAAATTTCTCTGAAAAT
TTTTGGCGGGAATTTAAATTTTCTTTAAAATT
TTTTGGCGGGAATTCAAATTTTCTGTGAAAAA
TTTTGGCGGGAATTCAAATTTTCTGAAAAAT
TTTTGGCGGGAGTTCAAATTTTCTCTGAAAAA
TTTTGGCGGGAATTTAAATTTCCTTCAAAATT
TTTTGGCGGGAATTCAAATTTTCTTTGAAAAT
TTTTGGCGGGAATTCAAATTTTCTGAAAAATT
TTTTGGCGGGAATTCAAATTTTCTGTGAAAAT
TTTTGGCGGGAATTTAAATTTTCTTTAAAATT
TTTTGGCGGGAATTCAAATTTTCTGTGAAAAA
TTTTGGCGGGAATTCAAATTTTCTGAAAATT
TTTTGGCGGGAGTTCAAATTTTCTGAAAAAT
Nr of selected repeats 67 Similarity 0.850591
Consensus:
TTTTGGCGGGAATTcAAATTTTCTnTgAAAAT
>Cele-UNSB01_2:15229801-15229921 Satlength=121 Nr of Repeats=8 RepeatLength=15
seed=GGTGAGACCC
GGTGAGACCCTTCGT
GGTGAGACCCATCGT
GGTGAGACCCTTCGT
GGTGAGACCCATCGT
GGTGAGACCCATCGT
GGTGAGACCCTTCGT

GGTGAGACCCATCGT
GGTGAGACCCTTCGC
Nr of selected repeats 8 Similarity 0.926984
Consensus:
GGTGAGACCCaTCGT
>Cele-UNSB01_2:15229801-15230289 Satlength=489 Nr of Repeats=15 RepeatLength=15
seed=GGTGAGACCC
GGTGAGACCCTTCGT
GGTGAGACCCATCGT
GGTGAGACCCTTCGT
GGTGAGACCCATCGT
GGTGAGACCCATCGT
GGTGAGACCCTTCGT
GGTGAGACCCATCGT
GGTGAGACCCTTCGC
GGTGAGACCCTTCGC
GGTGATACCCACATTTTTGGCGGGAAATTCAAATTTTCTGTGAAAACAATTTTGGCGGGAAATTCAAATTTTCAGTGAA
AAAATTTTGGCGGGAAATTCAAATTTTCAGTGAAAAAATTTTGGTGGGAAATTCAAATTTTCTATGAAAAAAATATCAT
TACTTATATATAAAAAAATACAGTTCGTCTGTCCATAGTTTGTAGTCTATGTAGTCTTTGTAGTCTGTGACGTCACACC
CAAAGTCAGTGAGAGTTGTGGGCGGGGCCTGTCACCTTCGTC
GTGAGACCCATCGT
GGTGAGACCCTTCGT
GGTGAGACCCATCGT
GGTGAGACCCTTCGC
GGTGAGACCCTTCGC
Nr of selected repeats 13 Similarity 0.913390
Consensus:
GGTGAGACCCtTCGt
>Cele-UNSB01_2:15229950-15234144 Satlength=4195 Nr of Repeats=125
RepeatLength=32 seed=TTTTGGCGGG
TTTTGGCGGGAAATTCAAATTTTCTGTGAAAACAA
TTTTGGCGGGAAATTCAAATTTTCAGTGAAAAAA
TTTTGGCGGGAAATTCAAATTTTCAGTGAAAAAA
TTTTGGTGGGAAATTCAAATTTTCTATGAAAAAAATATCATTACTTATATATAAAAAAATACAGTTCGTCTGTCCATAG
TTTGTAGTCTATGTAGTCTTTGTAGTCTGTGACGTCACACCCAAAGTCAGTGAGAGTTGTGGGCGGGGCCTGTCACCTT
CGTCGTGAGACCCATCGTGGTGAGACCCTTCGTGGTGAGACCCATCGTGGTGAGACCCTTCGCGGTGAGACCCTTCGCG
GTGAGACCCACAT
TTTTGGCGGGAAATTCAAATTTTCTCTGAAAAT
TTTTGGCGGGAATTCAAATTTTCTGAAACAT
TTTTGGCGGGAATTCAAATTTTCTTTGAAAAT
TTTTGGCGTGAATTCAAATTTTCTGAAACAT
TTTTGGCGGGAATTCAAATTTTCTCTGAAAAT
TTTTGGCGGGAATTTAAATTTTCTTTAAAATT
TTTTGGCGGGAATTCAAAATTTCTCTGAAAAT
TTTTGGCGTGAATTCAAATTTTCTGAAACAT
TTTTGGCGGGAATTCAAATTTTCTCTGAAAAT
TTTTGGCGGGAATTCAAATTTTCTGAAACAT
TTTTGGCGGGAATTCAAATTTTCTTTGAAAAT
TTTTGGCGTGAATTCAAATTTTCTGAAACAT
TTTTGGCGGGAATTCAAAATTTCTCTGAAAAT
TTTTGGCGGGAATTTAAATTTTCTTTAAAATT
TTTTGGCGGGAATTCAAATTTTCTGTGAAAAA
TTTTGGCGGGAATTCAAATTTTCTGAAACAT
TTTTGGCGGGAATTCAAATTTTCTCTGAAAAA

TTTTGGCGGGAATTTAAATTTCCTTCAAAATT
TTTTGGCGGGAATTCAAATTTTCTTTGAAAATT
TTTTGGCGGGAATTCAAATTTTCTGAAAAATT
TTTTGGCGGGAATTCAAATTTTCTTTAAAATT
TTTTGGCGGGAATTCAAATTTTCTCTGAAAAT
TTTTGGCGGGAATTCAAATTTTCTTCAAAATT
TTTTGGCGGGAATTCAAATTTTCTTTGAAAAT
TTTTGGCGTGAATTCAAATTTTCTGAAACAT
TTTTGGCGGGAATTCAAATTTTCTCTGAAAAT
TTTTGGCGGGAATTTAAATTTTCTTTAAAATT
TTTTGGCGGGAATTCAAAATTTCTCTGAAAAT
TTTTGGCGGGAATTTAAATTTTCTTTAAAATT
TTTTGGCGGGAATTCAAATTTTCTGTGAAAAA
TTTTGGCGGGAATTCAAATTTTCTCTGAAAAA
TTTTGGCGGGAATTTAAATTTCCTTCAAAATT
TTTTGGCGGGAATTCAAATTTTCTTTGAAAAT
TTTTGGCGGGAATTCAAATTTTCTGAAACAT
TTTTGGCGGGAATTCAAATTTTCTCTGAAAAT
TTTTGGCGGGAATTCAAATTTTCTGAAACAT
TTTTGGCGGGAATTCAAATTTTCTTTGAAAAT
TTTTGGCGTGAATTCAAATTTTCTGAAACAT
TTTTGGCGGGAATTCAAAATTTCTCTGAAAAT
TTTTGGCGGGAATTTAAATTTTCTTTAAAATT
TTTTGGCGGGAATTCAAATTTTCTGTGAAAAA
TTTTGGCGGGAATTCAAATTTTCTGAAACAT
TTTTGGCGGGAATTCAAATTTTCTCTGAAAAA
TTTTGGCGGGAATTTAAATTTCCTTCAAAATT
TTTTGGCGGGAATTCAAATTTTCTTTGAAAATT
TTTTGGCGGGAATTCAAATTTTCTGAAAAATT
TTTTGGCGGGAATTCAAATTTTCTTTAAAATT
TTTTGGCGGGAATTCAAATTTTCTCTGAAAAT
TTTTGGCGGGAATTCAAATTTTCTTCAAAATT
TTTTGGCGGGAATTCAAATTTTCTTTGAAAAT
TTTTGGCGTGAATTCAAATTTTCTGAAACAT
TTTTGGCGGGAATTCAAATTTTCTCTGAAAAT
TTTTGGCGGGAATTTAAATTTTCTTTAAAATT
TTTTGGCGGGAATTCAAAATTTCTCTGAAAAT
TTTTGGCGGGAATTTAAATTTTCTTTAAAATT
TTTTGGCGGGAATTCAAATTTTCTGTGAAAAA
TTTTGGCGGGAATTCAAATTTTCTCTGAAAAA
TTTTGGCGGGAATTTAAATTTCCTTCAAAATT
TTTTGGCGGGAATTCAAATTTTCTTTGAAAAT
TTTTGGCGGGAATTCAAATTTTCTGAAACAT
TTTTGGCGGGAATTCAAATTTTCTCTGAAAAT
TTTTGGCGGGAATTCAAATTTTCTGAAACAT
TTTTGGCGGGAATTCAAATTTTCTTTGAAAAT
TTTTGGCGTGAATTCAAATTTTCTGAAACAT
TTTTGGCGGGAATTCAAATTTTCTCTGAAAAT
TTTTGGCGGGATTTTAAATTTTCTTTAAAATT
TTTTGGCGGGAGTTCAAAATTTCTCTGAAAAT
TTTTGGCGTGAATTCAAATTTTCTGAAACAT
TTTTGGCGGGAATTCAAATTTTCTCTGAAAAT
TTTTGGCGGGAATTTAAATTTTCTTTAAAATT
TTTTGGCGGGAATTCAAAATTTCTCTGAAAAT

TTTTGGCGGGAATTTAAATTTTCTTTAAAATT
TTTTGGCGGGAATTCAAATTTTCTGTGAAAAA
TTTTGGCGGGAATTCAAATTTTCTGAAAAATT
TTTTGGCGGGAATTCAAATTTTCTTTGAAAAT
TTTTGGCGTGATTGAAAATT
TTTTGGCGGGAATTCAAATTTTCTGAAAAATT
TTTTGGCGGGAATTCAAATTTTCTTTAAAATT
TTTTGGCGGGAATTCAAATTTTCTCTGAAAAT
TTTTGGCGGGAATTCAAATTTTCTTCAAAATT
TTTTGGCGGGAATTCAAATTTTCTTTGAAAAT
TTTTGGCGTGAATTCAAATTTTCTGAAACAT
TTTTGGCGGGAATTCAAATTTTCTCTGAAAAT
TTTTGGCGGGAATTTAAATTTTCTTTAAAATT
TTTTGGCGGGAATTCAAAATTTCTCTGAAAAT
TTTTGGCGGGAATTTAAATTTTCTTTAAAATT
TTTTGGCGGGAATTCAAATTTTCTGTGAAAAA
TTTTGGCGGGAATTCAAATTTTCTTCAAAATT
TTTTGGCGGGAATTCAAATTTTCTTTGAAAAT
TTTTGGCGTGAATTCAAATTTTCTGAAACAT
TTTTGGCGGGAATTCAAATTTTCTCTGAAAAT
TTTTGGCGGGAATTTAAATTTTCTTTAAAATT
TTTTGGCGGGAATTCAAAATTTCTCTGAAAAT
TTTTGGCGGGAATTTAAATTTTCTTTAAAATT
TTTTGGCGGGAATTCAAATTTTCTGTGAAAAA
TTTTGGCGGGAATTCAAATTTTCTGAAACAT
TTTTGGCGGGAATTCAAATTTTCTCTGAAAAA
TTTTGGCGGGAATTTAAATTTCCTTCAAAATT
TTTTGGCGGGAATTCAAATTTTCTTTGAAAATT
TTTTGGCGGGAATTCAAATTTTCTGAAAAATT
TTTTGGCGGGAATTCAAATTTTCTTTAAAATT
TTTTGGCGGGAATTCAAATTTTCTCTGAAAAT
TTTTGGCGGGAATTCAAATTTTCTTCAAAATT
TTTTGGCGGGAATTCAAATTTTCTTTGAAAAT
TTTTGGCGTGAATTCAAATTTTCTGAAACAT
TTTTGGCGGGAATTCAAATTTTCTCTGAAAAT
TTTTGGCGGGAATTTAAATTTTCTTTAAAATT
TTTTGGCGGGAATTCAAAATTTCTCTGAAAAT
TTTTGGCGGGAATTTAAATTTTCTTTAAAATT
TTTTGGCGGGAATTCAAATTTTCTGTGAAAAA
TTTTGGCGGGAATTCAAATTTTCTGAAAAAT
TTTTGGCGGGAGTTCAAATTTTCTCTGAAAAA
TTTTGGCGGGAATTTAAATTTCCTTCAAAATT
TTTTGGCGGGAATTCAAATTTTCTTTGAAAAT
TTTTGGCGGGAATTCAAATTTTCTGAAAAATT
TTTTGGCGGGAATTCAAATTTTCTTTGAAAAT
TTTTGGCGTGAATTCAAATTTTCTGAAACAT
TTTTGGCGGGAATTCAAATTTTCTCTGAAAAT
TTTTGGCGGGAATTTAAATTTTCTTTAAAATT
TTTTGGCGGGAATTCAAATTTTCTGTGAAAAA
TTTTGGCGGGAATTCAAATTTTCTGAAAAAT
Nr of selected repeats 93 Similarity 0.859221
Consensus:
TTTTGGCGGGAATTcAAATTTTCTnTgAAAAT

>Cele-UNSB01_2:15235758-15235848 Satlength=91 Nr of Repeats=6 RepeatLength=15
seed=GGTGAGACCC
GGTGAGACCCTTCGT
GGTGAGACCCATCGT
GGTGAGACCCTTCGT
GGTGAGACCCATCGT
GGTGAGACCCTTCGC
GGTGAGACCCTTCGC
Nr of selected repeats 6 Similarity 0.905185
Consensus:
GGTGAGACCCtTCGt
>Cele-UNSB01_2:15235864-15236243 Satlength=380 Nr of Repeats=11 RepeatLength=34
seed=TTGGCGGGAA
TTGGCGGGAAATTCAAATTTTCTGTGAAAAAAATT
TTGGCGGGAAATTCAAATTTTCAGTGAAAAAATT
TTGGCGGGAAATTCAAATTTTCAGTGAAAAAATT
TTGGCGGGAAATTCAAATTTTCAGTGAAAACATT
TTGGCGGGAAGTTCAATTTTTCTATGAAAAAAATA
TTGGCGGGAAATTCAAATTTTCAGTGAAAAAATT
TTGGCGGGAAATTCAAATTTTCAGTGAAAAAATT
TTGGCGGGAAATTCAAATTTTCAGTGAAAAAATT
TTGGCGGGAAATTCAAATTTTCAGTGAAAAAACAAT
TTGGCGGGAAATTCAAATTTTCAGTGAAAAAGATT
TTGACGGGAAATTCAAATTTTCTGAGGAAAACAT
Nr of selected repeats 7 Similarity 0.880511
Consensus:
TTGGCGGGAAATTCAAATTTTCAGTGAAAAaATT
>Cele-UNSB01_2:15242777-15242951 Satlength=175 Nr of Repeats=9 RepeatLength=15
seed=AGACCCATCG
AGACCCATCGTGGCG
AGACCCATCGTGGCG
AGACCCATCGTGGTG
AGACCCATCGTGGAA
AGACCCATCGTGGCG
AGACCCATCGTTGCG
AGACCCATCGTGGCGAGACCCAAAATTTTGGCGGGAAATTTAAATTTCGTGGCG
AGACCCATCGTGGTG
AGACCCATCGTGGTG
Nr of selected repeats 8 Similarity 0.895238
Consensus:
AGACCCATCGTGGcG
>Cele-UNSB01_2:15242891-15243160 Satlength=270 Nr of Repeats=5 RepeatLength=35
seed=ATTTTGGCGG
ATTTTGGCGGGAAATTTAAATTTCGTGGCGAGACCCATCGTGGTGAGACCCATCGTGGTGAGACCCATCGTGGTGAGAC
CTATCGTGGTGAGACCCGTCGTGGTGAGACCCACCGTGGTAAGACCCAAA
ATTTTGGCGGGTAATTTAAATATTCGGAGAAAAAT
ATTTTGGCGGGAAATTTAAATTTTCTGTGAAAAAT
ATTTTGGCGGGTAATTTAAATTTTCGGAGAAAAAT
ATTTTGGCGGGAAATTTAAATTTTCTGTGAAAAAT
Nr of selected repeats 4 Similarity 0.904762
Consensus:
ATTTTGGCGGGaAATTTAAATtTTCgGaGAAAAAT
>Cele-UNSB01_2:15286316-15286491 Satlength=176 Nr of Repeats=5 RepeatLength=35
seed=TTTGCCGGAA

TTTGCCGGAAATTTAAATTCAAGCAAGTTGCCGT
TTTGCCGGAAATTTTAAATTCTGGCAATTTGCCGA
TTTGCCGGAAATTTTAAATACGGGACACTTTGTCGA
TTTGCCGGAAATTTTGAATTTGGGCAGTTTGCCGA
TTTGCCGGAAATTGCTAATTCCGGCAAATTGCGGA
Nr of selected repeats 3 Similarity 0.771429
Consensus:
TTTGCCGGAAATTttnAATTcnGGCAatTTGCcGA
>Cele-UNSB01_2:15290141-15290702 Satlength=562 Nr of Repeats=16 RepeatLength=35
seed=TTTGCCGGAA
TTTGCCGGAAATTGAATATTTGGACAATTTTTCGGCAATTTCAAATTCCAGCGATTTGCCGA
TTTGCCGGAAAGTTTCAATTCAGGCAACTTGTCGT
TTTGCCGGAAATTTAAAATTCGGGCAA
TTTGCCGGAAAATTTCAATTCCGCCAACTTGCCGA
TTTGCCGGAAATTTAAAATTCGGGCAA
TTTGCCGGAAAATTTCAATTCCGCCAACTTGTTGA
TTTGTCGGAAATTTTAAATTCCGGCAATTTGCAAA
TTTCCGGAAATTTTAAATTCCAGCGATTTGCCAAT
TTTCCGGAAATTTTAAATCCAGCAACTTGCCGA
TTTGCCGGAAATTTTCAATTCCGGCAACTTGCCGA
TTTGCCGGAAATTCCCAATTCCGGCAGTTTGCCGA
TTTGCCGGAAATTTTAAATTCCAGCGAGTTGCCGG
TTTGCCGGAAGTTCCCAATTCCGGCAGTTTGCCGA
TTTGCCGGAAATTTTAAATTCCAGCGAGTTGCCGT
TTTGCCGGAAATTCCCAATTCCGGCAG
TTTGCCGGAAATTCCCAATTCCGGCAGTTTGCCGA
Nr of selected repeats 11 Similarity 0.731650
Consensus:
TTTGCCGGAAAtTttcAATTCCgGCaanTTGCCGa
>Cele-UNSB01_2:15354583-15354863 Satlength=281 Nr of Repeats=8 RepeatLength=35
seed=TTTGCCGGAA
TTTGCCGGAAATTTTCAATTCCGGCAATTTGCCGG
TTTGCCGGAAATTTTCAATTCCGGCAATATACTGA
TTTGCCGGAAAATTTCAATTCCCGCAATTTGCCGA
TTTGCCGGAAATTTTCAATTCCGGCAATTTGCCTT
TTTGCCGGAAATTTTCAATTCTGGCAATATACTGA
TTTGCCGGAAAATTTCAATTCCCGCAATTTGCCGA
TTTGCCGGAAATTTTCAATTCCGGCAATTTGCCTT
TTTGCCGGAAATTTTCAATTCTGGCAATTTGCCGG
Nr of selected repeats 8 Similarity 0.858503
Consensus:
TTTGCCGGAAAtTTTCAATTCcgGCAATtTgCcga
>Cele-UNSB01_2:15381973-15383739 Satlength=1767 Nr of Repeats=38
RepeatLength=15 seed=GGGTCTCACC
GGGTCTCACCGCGAA
GGGTCTCACCACAAC
GGGTCTCACCACGAA
GGGTCTCACCACGAT
GGGTCTCACCACGAT
GGGTCTCACCACGAA
GGGTCTCACCACGAT
GGGTCTCACGACGAAGGTGACAGGCCCCGCCCACAACTCTCACTGACTTTGGGTGTGACGTCACAGACTACAAAGACTA
CATAGACTACAAACTATGGACAGACGAACTGTATTTTTTTATATATAAGTAATGATATTTTTTTCATAGAAAATTTGAA

TTTCCCGCCAAAATTTTTTCACTGAAAATTTGAATTTCCCGCCAAAATTTTTTCACTGAAAATTTGAATTTCCCGCCAA
AATTTTTTCACTGAAAATTTGAATTTCCCGCCAAAAATGT
GGGTCTCACCGCGAA
GGATCTCACCACGAT
GGGTCTCACCACGAA
GGGTCTCACCACGAT
GGGTCTCACCACGAT
GGGTCTCACCACGAA
GGGTCTCACCACGAT
GGGTCTCACGACGAAGGTGACAGGCCCCGCCCACAACTCTCACTGACTTTGGGTGTGACGTCACAGACTACAAAGACTA
CATAGACTACAAACTATGGACAGACGAACTGTATTTTTTTATATATAAGTAATGATATTTTTTTCATAGAAAATTTGAA
TTTCCCGCCAAAATTTTTTCACTGAAAATTTGAATTTCCCGCCAAAATTTTTTCACTGAAAATTTGAATTTCCCGCCAA
AATTTTTTCACTGAAAATTTGAATTTCCCGCCAATATTTTTTTCATAGAAAAATTGAACTTCCCGCCAAAATGTTTTCA
CTGAAAATTTGAATTTCCCGCCAAAATTTTTTCACTGAAAATTTGAATTTCCCGCCAAAATTTTTTCACTGAAAATTTG
AATTTCCCACCAAAATTTTTTCACAGAAAATTTGAATTTCCCGCCAAAATTTTTTCACTGAAAATTTGAATTTCCCGCC
AAAATTTTTTCACTGAAAATTTGAATTTCCCGCCAAAATTTTTTTCACAGAAAATTTGAATTTCCCGCCAAAAATGT
GGGTCTCACCGCGAA
GGGTCTCACCGCGAA
GGGTCTCACCACGAT
GGGTCTCACCACGAA
GGGTCTCACCACGAT
GGGTCTCACCACGAT
GGGTCTCACCACGAA
GGGTCTCACCACGAT
GGGTCTCACCACGAA
GGGTCTCACCACGAT
GGGTCTCACGACGAAGGTGACAGGCCCCGCCCACAACTCTCACTGACTTTGGGTGTGACGTCACAGACTACAAAGACTA
CATAGACTACAAACTATGGACAGACGAACTGTATTTTTTTATATATAAGTAATGATATTTTTTCATAAAAAAATTTGAA
TTTCCCGCCAAAATTTTTTCACTGAAAATTTGAATTTCCCGCCAAAATTTTTTCACTGAAAATTTGAATTTCCCGCCAA
TATTTTTTCATAGAAAAATTGAACTTCCCGCCAAAATGTTTTCACTGAAAATTTGAATTTCCCGCCAAAATTTTTTCAC
TGAAAATTTGAATTTCCCGCCAAAATTTTTTCACTGAAAATTTGAATTTCCCGCCAAAATTTTTTCACAGAAAATTTGA
ATTTCCCGCCAAAAATGT
GGGTCTCACCGCGAA
GGGTCTCACCGCGAA
GGGTCTCACCACGAT
GGGTCTCACCACGAA
GGGTCTCACCACGAT
GGGTCTCACCACGAT
GGGTCTCACCACGAA
GGGTCTCACCACGAT
GGGTCTCACCACGAA
GGGTCTCACCACGAT
GGGTCTCACCACGAA
Nr of selected repeats 35 Similarity 0.915593
Consensus:
GGGTCTCACCACGAn
>Cele-UNSB01_2:15429658-15431122 Satlength=1465 Nr of Repeats=9
RepeatLength=163 seed=AAATAAAAAA
AAATAAAAAAGAATGACTCCATATGATTTTCCCATACTTTTGAATTTTCGAAAAAATCAGCTTTTATATATTTTTATCA
ACTTTTAAAAAAATACTTCCGTACAAGCTAATATACTCTGTTTGGCATTTTCTGGTAACTTTGACTATGCTGAACAGTT
TTATG
AAATAAAAAAGAATGACTCCATATGATTTTCCCATACTTTTGAATTTTTGAAAAAATCAGCTTTTATATATTTTTATCA
ATTTTTAAAAAAATACTTCCGTACAAGCTAATATACTTTGATTGGCATATTCTGGTAATTTTAACTATGCTGAACAGTT
TTATG

AAATAAAAAAGAATGACTCCATATGATTTTCCCATACTTTTGAATTTTTGAAAAAATCAGCTTTTATATATTTTTATCA
ATTTTTAAAAAAATACTTCCGTACAAGCTAATATACTTTGATTGGCATATTCTGGTAATTTTAACTATGCTGAACAGTT
TTATG
AAATAAAAAAGAATGACTCCATATGATTTTCCCATACTTTTGAATTTTTGAAAAAATCAGCTTTTATATATTTTTATCA
ATTTTTAAAAAAATACTTCCGTACAGGCTAATATACTTTGATTGGCATATTCTGGTAATTTTGACTATGCTGAACAGTT
TTATG
AAATAAAAAAGAATGACTCCATATGATTTTCCAATACTTTTGAATTTTTGAAAAAATCAGCTTTTATATATTTTTATCA
ATTTTTAAAAAAATACTTCCGTACAAGCTAATATACTTTGATTGGCATATTCTGGTAATTTTAACTATGCTGAACAGTT
TTATG
AAATAAAAAAGAATGACTCCATATGATTTTCCCATACTTTTGAATTTTTGAAAAAATCAGCTTTTATATATTTTTATCA
ATTTTTAAAAAAATACTTCCGTACAAGCTAATATACTTTGATTGGCATATTCTGGTAATTTTAACTATGCTGAACAGTT
TTATG
AAATAAAAAAGAATGACTCCATATGATTTTCCAATACTTTTGAATTTTTGAAAAAATCAGCTTTTATATATTTTTATCA
ATTTTTAAAAAAATACTTCCGTACAAGCTAATATACTTTGATTGGCATATTCTGGTATGTTTGACTATGCTGAACAGTT
TTATG
AAATAAAAAAGAATGACTCCATATGATTTTCCCATACTTTTGAATTTTTGAAAAAACCAGCTTTTATATATTTTTATCA
ATTTTTAAAAAAATACTTCCGTACAAGCTAATATACTCTGTTTGGCATATTCTGGTAATTTTGACTATGCTGAACGGTT
TTATG
AAATAAAAAAGAATGACTCCACATGATTTTCCAGTACTTTTTAATTTTTGAATTAACAGCTTTCGGATACATTCTGTTT
TTTTTTTAAATAAACTTCCGTAGAAGCTACCATTTTTTTTGGTGTTTTCTAGTATATTTGAACATGCTAAACAGTTTTG
TA
Nr of selected repeats 8 Similarity 0.966696
Consensus:
AAATAAAAAAGAATGACTCCATATGATTTTCCcATACTTTTGAATTTTTGAAAAAATCAGCTTTTATATATTTTTATCA
ATTTTTAAAAAAATACTTCCGTACAAGCTAATATACTtTGaTTGGCATATTCTGGTAAtTTTaACTATGCTGAACAGTT
TTATG
>Cele-UNSB01_2:15432049-15432695 Satlength=647 Nr of Repeats=18 RepeatLength=34
seed=TTTCAATTCC
TTTCAATTCCGGCAACTTTCCGATTTGGCGGAAA
TTTCAATTCCGGCAACTTGCCGATTTGCCAAAAAT
TTTAATTCCGACAACTTGCCGATTTGCCGGATA
TTTCAATTCCGGCAATTTGTCGATTTGCCGGAAA
TTTCAATTCCGGCAATTTACCGATTTGCCGGATA
TTTCAATTCCGGCAATTTGTCGATTTGCCGGAAA
TTTCAATTCCGGCAATTTACCGATTTGCCGGATA
TTTCAATTCCGGGACCTTGCCGATTTGCCGGAAATTTCAATGTCGGCAATTTGCCGATTTGCGGGAAA
TTTCAATTCCGGCAATTTGCCGATTTGCCGGAAA
TTTCAATTCCGGCAATTTGCCGATTTGCGGGAAA
TTTCAATTCCGGCAATTTGCCATTTTGGCAAAAAT
TTTAATTCCGGCAACTTGCCGATTTGCCGGATA
TTTCAATTCCGGCAATTTGTCGATTTGCCGGAAA
TTTCAATTCCGGCAATTTACCGATTTGCCGGATA
TTTCAATTCCGGCACCTTGCCGATTTGCCGGAAA
TTTCAATTCCGGCAATTTGCCGATTTGCGGGAAA
TTTCAATTCCGGCAATTTGCCGATTTGCCGGAAA
TTTCAATTCCGGCAATTTGCCGATTTGCGGGAAA
Nr of selected repeats 13 Similarity 0.912016
Consensus:
TTTCAATTCCGGCAATTTgcCGATTTGCcGGAaA
>Cele-UNSB01_2:15437952-15438032 Satlength=81 Nr of Repeats=4 RepeatLength=20
seed=TGTAGATTTA
TGTAGATTTACGAAGGACAA
TGTAGATTTACGCGGGATGG
TGTAGATTTACAAAGAATAA

TATAGATTTACACAGGACAG
Nr of selected repeats 4 Similarity 0.688889
Consensus:
TgTAGATTTACaaaGgAcaa
>Cele-UNSB01_2:15454038-15454614 Satlength=577 Nr of Repeats=12 RepeatLength=35
seed=CCGATTTGCC
CCGATTTGCCGGAAATTTTAAATTCCGGCAATTTTCAGATTGCCGGATAGCAGATTTCCCGGAAATTTTTAGAGCGATT
TTGTATAAGGAGGGACCCTTAAAACCGCCTTTTTGAAATTTTAGGAAATTCTGAAATTTTCATTTTAAAAAATGTGCAA
AACGACAATTTGCAGAAGATTTTCGGCAATTTG
CCGATTTTCCGGAAATTTCGAATTCCGGCAACTTC
CCGATTTGCCGAAATTTTTCAACTACGGCTATTTG
CCGATTTGCCGGAAATTTTGAATTCCGGCAATTTG
CCGATTTGCCGGAAATTTCGAATTCCGGCAACTTC
CCGATTTGCCGAAATTTTTCAACTACGGCTATTTG
CCGATTTGCCGGAAATTTTGAATTCCGGCAATTTC
CCGATTTGCCGAAATTTTTCAACTACGGCTATTTG
CCGATTTGCCGGAAATTTCGAATTCCGGCAACTTC
CCGATTTGCCGAAATTTTTCAACTACGGCTATTTG
CCGATTTGCCGGAAATTTTGAATTCCGGCAATTTC
CCGATTTGCCGAAATTTTTCAACTACGACTATTTG
Nr of selected repeats 11 Similarity 0.807446
Consensus:
CCGATTTGCCGgAAaTTTtgAAtTcCGGCaAtTTg
>Cele-UNSB01_2:15454990-15455468 Satlength=479 Nr of Repeats=9 RepeatLength=35
seed=TTGCCGGAAT
TTGCCGGAATTTAAAATTTCTGGAAAGTCGGCAAA
TTGCCAGAATTGAAAAATTCTGGCAAACCGCCAAA
TTGCCGGAATTTTTAATTTCCGGCAAATCGGCAAA
TTGCCGGAATCAGAAATTTCCGGCAAATCGGCAATTTTCCGAAAATGATAATTTCTGAAAAATCGGCAAA
TTGCCGGAATTAAAAATTTCTGGCAAACCGCCAAA
TTGCCGGAATGTTTAATTTACGGCAAATCGGCAAA
TTGCCGGAATCAGAAATTTCCGGCAAATCGGCAATTTTCCGAAAATGAGAATTTCCGGCAATTCGGCAAACCGGCAATT
TACCAAAAATGAAAATTTCGGCACACCGCCAAA
TTGCCGGAATTGAAAATTTCTGGCAAATCGGCAAATCGGCAATTTGCCGAAAATGAAAATTTCTGGCAAATCAGAAAAC
CGCCAAA
TTGTCGGAATTGAAAATTTCTGACAATCCGCCAAG
Nr of selected repeats 6 Similarity 0.740952
Consensus:
TTGCCGGAATTtaaAATTTCtGGCAAacCGcCAAA
>Cele-UNSB01_2:15522229-15522469 Satlength=241 Nr of Repeats=12 RepeatLength=23
seed=GCTACAGTAC
GCTACAGTACCCCAGCTTGACTT
GCTACAGTACTTTA
GCTACAGTACCCCAGCTTGACTT
GCTACAGTACTTTA
GCTACAGTACCCCATCTTGACAT
GCTACAGTACACTATCTTGACCA
GCTACAGTACCACAGCTTGACTT
GCTACAGTACTTTA
GCTACAGTACCACAGCTTGACTT
GCTACAGTACTTTA
GCTACAGTACCCCATCTTGACAT
GCTACAGTAACCTAGCTTGACCG
Nr of selected repeats 8 Similarity 0.821946

Consensus:
GCTACAGTACCccAgCTTGACtt
>Cele-UNSB01_2:15522855-15525136 Satlength=2282 Nr of Repeats=188
RepeatLength=12 seed=TTAGGCTTAG
TTAGGCTTAGGC
TTAGGCTTAGGC
TTAGGCTTAGGC
TTAGGCTTAGGC
TTAGGCTTAGGC
TTAGGCTTAGGC
TTAGGCTTAGGC
TTAGGCTTAGGC
TTAGGCTTAGGC
TTAGGCTTAGGC
TTAGGCTTAGGC
TTAGGCTTAGGC
TTAGGCTTAGGC
TTAGGCTTAGGC
TTAGGCTTAGGC
TTAGGCTTAGGC
TTAGGCTTAGGC
TTAGGCTTAGGC
TTAGGCTTAGGC
TAGGCTTAGGC
TTAGGCTTAGGC
TTAGGCTTAGGC
TTAGGCTTAGGC
TTAGGCTTAGGC
TTAGGCTTAGGC
TTAGGCTTAGGC
TTAGGCTTAGGC
TTAGGCTTAGGC
TTAGGCTTAGGC
TTAGGCTTAGGC
TTAGGCTTAGGC
TTAGGCTTAGGCT
TTAGGCTTAGGC
TTAGGCTTAGGC
TTAGGCTTAGGC
TTAGGCTTAGGC
TTAGGCTTAGGC
TTAGGCTTAGGC
TTAGGCTTAGGC
TTAGGCTTAGGC
TTAGGCTTAGGC
TTAGGCTTAGGGC
TTAGGCTTAGGC
TTAGGCTTAGGC
TTAGGCTTAGGC
TTAGGCTTAGGC
TTAGGCTTTAGGC
TTAGGCTTAGGC
TTAGGCTTAGGC
TTAGGCTTAGGC

TTAGGCTTAGGC
TTAGGCTTAGGC
TTAGGCTTAGGC
TTAGGCTTAGGC
TTAGGCTTAGGC
TTAGGCTTAGGC
TTAGGCTTAGGC
TTAGGCTTAGGC
TTAGGCTTAGGC
TTAGGCTTAGGC
TTAGGCTTAGGC
TTAGGCTTAGGC
TTAGGCTTAGGC
TTAGGCTTAGGC
TTAGGCTTAGGC
TTAGGCTTAGGC
TTAGGCTTAGGC
TTAGGCTTAGGC
TTAGGCTTAGGC
TTAGGCTTAGGC
TTAGGCTTATAGGGC
TTAGCTTAGGC
TTAGGCTTAGGC
TTAGGCTTAGGC
TTAGGCTTAGGC
TTAGGCTTAGGC
TTAGGCTTAGGC
TTAGGCTTAGGC
TTAGGCTTAGGC
TTAGGCTTAGGC
TTAGGCTTAGGC
TTAGGCTTAGGC
TTAGGCTTAGGC
TTAGGCTTAGGC
TTAGGCTTAGGC
TTAGGCTTAGGC
TTAGGCTTAGGC
TTAGGCTTAGGC
TTAGGCTTAGGC
TTAGGCTTAGGC
TTAGGCTTAGGC
TTAGGCTTAGGC
TTAGGCTTAGGC
TTAGGCTTAGGC
TTAGGCTTAGGC
TTAGGCTTAGGC
TTAGGCTTAGGC
TTAGGCTTAGGC
TTAGGCTTAGGC
TTAGGCTTAGGC
TTAGGCTTAGGC
TTAGGCTTAGGC
TTAGGCTTAGGC
TTAGGCTTAGGC

TTAGGCTTAGGC
TTAGGCTTAGGC
TTAGGCTTAGGC
TTAGGCTTAGGC
TTAGGCTTAGGCC
TAGGCTTAGGCTTAGCAGGC
TTAGGCTTAGGCTTAGGCGC
TTAGGCTTAGGC
TTAGGGCTTAGGC
TTAGGCTTAGGC
TTAGGCTTAGGC
TTAGGCTTAGGC
TTAGGCTTAGGC
TTAGGCTTAGGC
TTAGGCTTAGGC
TTAGGCTTAGGC
TTAGGCTTAGGC
TTAGGCTTAGGC
TTAGGCTTAGGC
TTAGGCTTAGGC
TTAGGCTTAGGC
TTAGGCTTAGGC
TTAGGCTTAGGC
TTAGGCTTAGGC
TTAGGCTTAGGC
TTAGGCTTAGGC
TTAGGCTTAGGC
TTAGGCTTAGGC
TTAGGCTTAGGC
TTAGGCTTAGGC
TTAGGCTTAGGC
TTAGGCTTAGGC
TTAGGCTTAGGC
TTAGGCTTAGGC
TTAGGCTTAGGC
TTAGGCTTAGGC
TTAGGCTTAGGC
TTAGGCTTAGGC
TTAGGCTTAGGC
TTAGGCTTAGGC
TTAGGCTTAGGC
TTAGGCTTAGGC
TTAGGCTTAGGC
TTAGCTTAGGC
TTAGGCTTAGGC
TTAGGCTTAGGC
TTAGGCTTAGGC
TTAGGCTTAGGC
TTAGGCTTAGGC
TTAGGCTTAGGC
TTAGGCTTAGGC
TTAGGCTTAGGC
TTAGGCTTAGGC
TTAGGCTTAGGC

TTAGGCTTAGGC
TTAGGCTTAGGC
TTAGGCTTAGGC
TTAGGCTTAGGC
TTAGGCTTAGGC
TTAGGCTTAGGC
TTAGGCTTAGGC
TTAGGCTTAGGC
TTAGGCTTAGGC
TTAGGCTTAGGC
TTAGGCTTAGGGC
TTAGGCTTAGGC
TTAGGCTTAGGC
TTAGGCTTAGGC
TTAGGCTTAGGC
TTAGGCTTAGGC
TTAGGCTTAGGTT
TTAGGCTTAGGC
TTAGGCTTAGGC
TTAGGCTTAGGC
TTAGGCTTAGGC
TTAGGCTTAGGC
TTAGGCTTAGGC
TTAGGCTTTAGGC
TTAGGCTTAGGC
TTAGGCTTAGGC
TTAGGCTTAGGC
TTAGGCTTAGGC
TTAGGCTTAGGC
TTAGGGCTTAGGC
Nr of selected repeats 173 Similarity 1.000000
Consensus:
TTAGGCTTAGGC
>Cele-UNSB01_3:11-3196 Satlength=3186 Nr of Repeats=261 RepeatLength=12
seed=TAAGCCTAAG
TAAGCCTAAGCC
TAAGCCTAAGCC
TAAGCCTAAGCC
TAAGCCTAAGCC
TAAGCCTAAGCC
TAAGCCTAAGCC
TAAGCCTAAGCC
TAAGCCTAAGCC
TAAGCCTAAGCC
TAAGCCTAAGCC
TAAGCCTAAGCC
TAAGCCTAAGCC
TAAGCCTAAGCC
TAAGCCTAAGCC
TAAGCCTAAGCC
TAAGCCTAAGCC
TAAGCCTAAGCC
TAAGCCTAAGCC
TAAGCCTAAGCC

TAAGCCTAAGCC
TAAGCCTAAGCC
TAAGCCTAAGCC
TAAGCCTAAGCC
TAAGCCTAAGCC
TAAGCCTAAGCC
TAAGCCTAAGCC
TAAGCCTAAGCC
TAAGCCTAAGCC
TAAGCCTAAGCC
TAAGCCTAAGCC
TAAGCCTAAGCC
TAAGCCTAAGCC
TAAGCCTAAGCC
TAAGCCTAAGCC
TAAGCCTAAGCC
TAAGCCTAAGCC
TAAGCCTAAGCC
TAAGCCTAAGCC
TAAGCCTAAGCC
TAAGCCTAAGCC
TAAGCCTAAGCC
TAAGCCTAAGCC
TAAGCCTAAGCC
TAAGCCTAAGCC
TAAGCCTAAGCC
TAAGCCTAAGCC
TAAGCCTAAGCC
TAAGCCTAAGCC
TAAGCCTAAGCC
TAAGCCTAAGCC
TAAGCCTAAGCC
TAAGCCTAAGCC
TAAGCCTAAGCC
TAAGCCTAAGCC
TAAGCCTAAGCC
TAAGCCTAAGCC
TAAGCCTAAGCC
TAAGCCTAAGCC
TAAGCCTAAGCC
TAAGCCTAAGCC
TAAGCCTAAGCC
TAAGCCTAAGCC
TAAGCCTAAGCC
TAAGCCTAAGCC
TAAGCCTAAGCC
TAAGCCTAAGCC
TAAGCCTAAGCC
TAAGCCTAAGCC
TAAGCCTAAGCC
TAAGCCTAAGCC
TAAGCCTAAGCC
TAAGCCTAAGCC
TAAGCCTAAGCC

TAAGCCTAAGCC
TAAGCCTAAGCC
TAAGCCTAAGCC
TAAGCCTAAGCC
TAAGCCTAAGCC
TAAGCCTAAGCC
TAAGCCTAAGCC
TAAGCCTAAGCC
TAAGCCTAAGCC
TAAGCCTAAGCC
TAAGCCTAAGCC
TAAGCCTAAGCC
TAAGCCTAAGCC
TAAGCCTAAGCC
TAAGCCTAAGCC
TAAGCCTAAGCC
TAAGCCTAAGCC
TAAGCCTAAGCC
TAAGCCTAAGCC
TAAGCCTAAGCC
TAAGCCTAAGCC
TAAGCCCTAAGCC
TAAGCCTAAGCC
TAAGCCTAAGCC
TAAGCCTAAGCC
TAAGCCTAAGCC
TAAGCCTAAGCC
TAAGCCTAAGCC
TAAGCCTAAGCC
TAAGCCTAAGCC
TAAGCCTAAGCC
TAAGCCTAAGCC
TAAGCCTAAGCC
TAAGCCTAAGCC
TAAGCCTAAGCC
TAAGCCTAAGCC
TAAGCCTAAGCC
TAAGCCTAAGCC
TAAGCCTAAGCC
TAAGCCTAAGCC
TAAGCCTAAGCC
TAAGCCTAAGCC
TAAGCCTAAGCC
TAAGCCTAAGCC
TAAGCCTAAGCC
TAAGCCTAAGCC
TAAGCCTAAGCC
TAAGCCTAAGCC
TAAGCCTAAGCC
TAAGCCTAAGCC
TAAGCCTAAGCC
TAAGCCTAAGCC
TAAGCCTAAGCC
TAAGCCTAAGCC

TAAGCCTAAGCC
TAAGCCTAAGCC
TAAGCCTAAGCC
TAAGCCTAAGCC
TAAGCCTAAGCC
TAAGCCTAAGCC
TAAGCCTAAGCC
TAAGCCTAAGCC
TAAGCCTAAGCC
TAAGCCTAAGCC
TAAGCCTAAGCC
TAAGCCTAAGCC
TAAGCCTAAGCC
TAAGCCTAAGCC
TAAGCCTAAGCC
TAAGCCTAAGCC
TAAGCCTAAGCC
TAAGCCTAAGCC
TAAGCCTAAGCC
TAAGCCTAAGCC
TAAGCCTAAGCC
TAAGCCTAAGCC
TAAGCCTAAGCCC
TAAGCCTAAGCC
TAAGCCTAAGCC
TAAGCCTAAGCC
TAAGCCTAAGCC
TAAGCCTAAGCC
TAAGCCTAAGCC
TAAGCCTAAGCC
TAAGCCTAAGCC
TAAGCCTAAGCC
TAAGCCTAAGCC
TAAGCTAAGCC
TAAGCCTAAGCC
TAAGCCTAGC
TAAGCCTAAGCTGCC
TAAGCCTAAGCTGC
TAAGCCTAAGCGC
TAAGCCTAAGCC
TAAGCCTAAGCC
TAAGCCTAAGCC
TAAGCCTAAGCC
TAAGCCTAAGCC
TAAGCCTAAGCC
TAAGCCTAAGCC
TAAGCCTAAGCC
TAAGCCTAAGCC
TAAGCCTAAGCC
TAAGCCTAAGCC
TAAGCCTAAGCC
TAAGCCTAAGCC
TAAGCCTAAGCC
TAAGCCTAAGCCC

TAAGCCTAAGCC
TAAGCCTAAGCC
TAAGCCTAAGCC
TAAGCCTAAGCC
TAAGCCTAAGCC
TAAGCCTAAGCC
TAAGCCTAAGCC
TAAGCCTAAGCC
TAAGCCTAAGCC
TAAGCCTAAGCC
TAAGCCTAAGCC
TAAGCCTAAGCC
TAAGCCTAAGCC
TAAGCCTAAGCC
TAAGCCTAAGCC
TAAGCCTAAGCC
TAAGCCTAAGCC
TAAGCCTAAGCC
TAAGCCTAAGCC
TAAGCCTAAGCC
TAAGCTAAGCC
TAAGCCTAAGCC
TAAGCCTAGC
TAAGCCTAAGCCTAAGCTAGC
TAAGCCTAAGCCTAAGTGCC
TAAGCCTAAGTGCC
TAAGCCTAAGGCC
TAAGCCTAAGCC
TAAGCCTAAGCC
TAAGCCTAAGCC
TAAGCCTAAGCC
TAAGCCTAAGCC
TAAGCCTAAGCC
TAAGCCTAAGCC
TAAGCCTAAGCC
TAAGCCTAAGCC
TAAGCCTAAGCC
TAAGCCTAAGCC
TAAGCCTAAGCC
TAAGCCTAAGCC
TAAGCCTAAGCC
TAAGCCTAAGCC
TAAGCCTAAGCC
TAAGCCTAAGCC
TAAGCCTAAGCC
TAAGCCTAAGCC
TAAGCCTAAGCC
TAAGCCTAAGCC
TAAGCCCTAAGCC
TAAGCCTAAGCC
TAAGCCTAAGCC
TAAGCCTAAGCC
TAAGCCTAAGCC
TAAGCCTAAGCC

TAAGCCTAAGCC
TAAGCCTAAGCC
TAAGCCTAAGCC
TAAGCCTAAGCC
TAAGCCTAAGCC
TAAGCCTAAGCC
TAAGCCTAAGCC
TAAGCCTAAGC
TAAGCCTAAGCCTAAGCCTGCC
TAAGCCTAAGCC
TAAGCCTAAGCTGCC
TAAGCCTAAGCCTAAGCCTC
TAAGCCTAAGCCTA
TAAGCCTAAGCC
TAAGCCCTAAGCC
TAAGCCTAAGCC
TAAGCCTAAGCC
TAAGCCTAAGCC
TAAGCCTAAGCC
TAAGCCTAAGCC
TAAGCCTAAGCC
TAAGCCTAAGCC
TAAGCCTAAGCC
TAAGCCTAAGCC
TAAGCCTAAGCC
TAAGCCTAAGCCTAAGCC
Nr of selected repeats 239 Similarity 1.000000
Consensus:
TAAGCCTAAGCC
>Cele-UNSB01_3:81693-82933 Satlength=1241 Nr of Repeats=39 RepeatLength=31
seed=TAAATAATTA
TAAATAATTAGCAGACCAAAGTTGGGTCTGC
TAAATAATTAGCAGACAAACGTTGGGTCTGC
TAAATAATTAGCAGATTATATTTGGGTCTGC
TAAATAATTAGCAGACCAACGTTGGGTCTGC
TAAATAATTAGCAGACTATTTTTGGGTATGC
TAAATATTTAGCAGACCAAAGTTTGGTCTGT
TAAATAATTAGCAGACCAACGTTGGGTCTGC
TAAATATTTAGCAGACCATAGTTGGGTATGC
TAAATAATTAGCAGACCAAAGTTGGGTCTGC
TAAATAATTAGCAGACCAAAGTTGGGTATGC
TAAATAATTAGCAGACCATTTTTGGGTATGC
TAAATAATTAGCAGACCAAAGTTGGGTCTGC
TAAATAATTAGCAGACCAAAGTTGGGTCTGC
TAAATAATTAGCAGACCAAAGTTGGGTCTGC
TAAATAATTAGCAGACAAACGTTGGGTCTGC
TAAATAATTAGCAGATTATATTTGGGTCTGC
TAAATAATTAGCAGACCAACGTTGGGTCTGC
TAAATAATTAGCAGACTATTTTTGGGTATGC
TAAATATTTAGCAGACCAAAGTTTGGTCTGT
TAAATAATTAGCAGACCAACGTTGGGTCTGC
TAAATATTTAGCAGACCATAGTTGGGTATGC
TAAATAATTAGCAGACCAAAGTTGGGTCTGC
TAAATAATTAGCAGACCAAAGTTGGGTATGC

TAAATAATTAGCAGACCATTTTTGGGTATGC
TAAATAATTAGCAGACCAAAGTTGGGTCTGC
TAAATAATTAGCAGACCAAAGTTGGGTCTGC
TAAATAATTAGCAGACCAAAGTTGGGTCTGC
TAAATAATTAGCAGACCAAAGTTGGGACTGC
TAAATATTTAGCAGACCAAAGTTGGGTCTGC
TAAATAATTAGCAGATTATATTTGGGTATGC
TAAATAATTAGCAGACCAAAGTTGGGTCTGC
TAAATAATTAGCAGACCAAAGTTGGGACTGCTAAATATTCAGCAGACCAACGTTGGGTCTGC
TAAATAATTAGCAGATCATATTTGGGTATGC
TAAATAATTAACTGACCACAGTTGGGTCTGC
TAAATAATTAGCAGACCATTTTTGGGTATGC
TAAATAATTAGCAGACCAAAGTTGGGTCTGC
TAAATATTTAGCAGACCATTTTTGTGTCTGC
TAAATATTTAGCAGACCATTTTTGGGTATGC
TAAATAATTAGCAGACCAAAGTTGGGTCTGC
Nr of selected repeats 38 Similarity 0.865584
Consensus:
TAAATAATTAGCAGACCAaagTTGGGTcTGC
>Cele-UNSB01_3:83964-84341 Satlength=378 Nr of Repeats=14 RepeatLength=21
seed=AAATTTGGCT
AAATTTGGCTGGAAATCGTGA
AAATTTGGCTTAAAATGTTGA
AATTTTGGCTTAAAATGGTTC
AAATTTGGCTTTAAATAGTGA
AAATTTGGCTTAAAATGTTGA
AAATTTGGCTTAAAATGGTTC
AAATTTGGCTTTAAATAGTGAAAAAATCGGCTTGAAATGGTG
AAATTTGGCTTGAAATGGTGA
AAATTTGGCTTAAAATGTTGA
AAATTTGGCTTAAAATGGTTC
AAATTTGGCTTTAAATAGTGAAAAAATCGGCTTTAAATAGTGAAAAAATCGGCTTGAAATGGTGA
AAATTTGGCTTGAAATGGTGA
AAATTTGGCTTGAAATGGTGA
AAATTTGGCTTTAAATAGTGAAAAAATCGGCTTGAAATGTTGA
Nr of selected repeats 11 Similarity 0.774105
Consensus:
AAATTTGGCTTaAAATgGtTga
>Cele-UNSB01_3:86662-86935 Satlength=274 Nr of Repeats=13 RepeatLength=21
seed=CCATTTTAAG
CCATTTTAAGCCAATTTTGGA
CCATTTTAAGCCAATTTTGGA
CCATTTTAAGCCAATTTTGGA
CCATTTTAAGCCAATTTTGGA
CCATTTTAAGCCAATTTTGGA
CCATTTTAAGCCAATTTTGGA
CCATTTTAAGCCAATTTTGGA
CCATTTTAAGCCAATTTTGGA
CCATTTTAAGCCAATTTTGGA
CCATTTTAAGCCAATTTTGGG
CCATTTTAAGCCAATTTAGGA
CCATTTTAAGACAATTTAGGA
CCATTTTAAGACAATTTAGGA
Nr of selected repeats 13 Similarity 0.947904

Consensus:
CCATTTTAAGCCAATTTtGGA
>Cele-UNSB01_3:86662-87180 Satlength=519 Nr of Repeats=25 RepeatLength=21
seed=CCATTTTAAG
CCATTTTAAGCCAATTTTGGA
CCATTTTAAGCCAATTTTGGA
CCATTTTAAGCCAATTTTGGA
CCATTTTAAGCCAATTTTGGA
CCATTTTAAGCCAATTTTGGA
CCATTTTAAGCCAATTTTGGA
CCATTTTAAGCCAATTTTGGA
CCATTTTAAGCCAATTTTGGA
CCATTTTAAGCCAATTTTGGA
CCATTTTAAGCCAATTTTGGG
CCATTTTAAGCCAATTTAGGA
CCATTTTAAGACAATTTAGGA
CCATTTTAAGACAATTTAGGA
CCATTTTAAGACAATTTAGAT
CATTTTAAGTCAATTTAGAT
CATTTTAAGTCAATTTAGAT
CATTTTAAGTCAATTTAGAT
CATTTTAAGTCAATTTAGAT
CATTTTAAGTCAATTTAGGA
CCATTTTAAGCCAATTTTGGAT
CATTTTAAGGCTATTTAGAT
CATTTTAAGGCTATTTAGAT
CATTTTAAGCCAATTTTGAA
CCATTTTAAGCCAATTTTGAA
CCATTTTAAGCCAATTTTGAA
Nr of selected repeats 16 Similarity 0.877588
Consensus:
CCATTTTAAGCCAATTTtGGA
>Cele-UNSB01_3:88388-88698 Satlength=311 Nr of Repeats=9 RepeatLength=31
seed=TGCTAAATAA
TGCTAAATAATTAGCAGACCATTTTTGGGTC
TGCTAAATAATTAGCAGACCATTTTTGGGTA
TGCTAAATAATTAGCAGACCATTTTTGGGTA
TGCTAAATAATTAGCAGACCATTTTTGGGTTTGCAAAATATTTAACAGACCAAAGTTGGGTA
TGCTAAATAATTAGCAGACCAAAGTTGGGTA
TGCTAAATAATTAGCAGACCATTTTTGGGTA
TGCTAAATAATTAGCAGACCAAAGTTGGGTA
TGCTAAATAATTAGCAGACCAAAGTTGGGTA
TGCTAAATAATTAGCAGACCAAAGTTGGGTC
Nr of selected repeats 8 Similarity 0.907834
Consensus:
TGCTAAATAATTAGCAGACCAaagTTGGGTa
>Cele-UNSB01_3:119691-119763 Satlength=73 Nr of Repeats=5 RepeatLength=12
seed=GCCTAAGCCT
GCCTAAGCCTAAGTCATG
GCCTAAGCCTAAGTCATA
GCCTAAGCCTAA
GCCTAAGCCTAG
GCCTCAGCCTAA
Nr of selected repeats 3 Similarity 0.851852

Consensus:
GCCTaAGCCTAa
>Cele-UNSB01_3:134796-135400 Satlength=605 Nr of Repeats=7 RepeatLength=34
seed=AATTCCGGCA
AATTCCGGCAATGTGTCGGTTTGCCGATTTGCCGAAAATTTCTATTTATAGGATGCGTACAATTTTGCCGATTGAAATT
TAAATTCTGAAATTTCCAAAAAAATGCGAAATCACAATTTGCTGAAAATTTTCGGAAAAAAATCGTTTGCAGCCCACCC
CAAATTATTTTTTCGACA
AATTCGGCAGATCGATAAATTGCCGGTTTATCGGTTTGCCGGAAATTTTAATTTTTGGCAAATGATTTGCCGGAAATGT
TTAGAGAGATTTTTTATAAGACGGAAACACTTACAACTGTGCTTTTTTTCATAGAATTTGCTTATTTTTCAAAATAGAT
GTACATACATAGGAACATTTCATTCATAGGATGCGTACAATTTTGCCGATTGAAATTTCCAAAAAAAAAAAGTGCAGAA
CCACAATTTGCCGGAAATTTC
AATTCCGGCAATTTACCAATTTGCCAGAAATTTC
AATTCCGGCAATTTACCAATTTGCCAAAAAATTTC
AATTCCGGCAATTTACCAATTTGCCAGAAATTTC
AATTCCGGCAATTTACCAATTTGCCAAAAAATTTC
AATTCCGGCAATTTACCAATTTGCCAAAAAATTTC
Nr of selected repeats 5 Similarity 0.976471
Consensus:
AATTCCGGCAATTTACCAATTTGCCAaAAATTTC
>Cele-UNSB01_3:192738-193322 Satlength=585 Nr of Repeats=4 RepeatLength=31
seed=TTAGCAGACC
TTAGCAGACCCAACTATGGTCTGCTAATTAT
TTAGCAGACCCAAGTATGGTCTGCTAATTAT
TTAGCAGACCCAACTATGGTCTGCTAAATAT
TTAGCAGACCCAAATTTTTATGTTTTCCAGTTATTTTTCCTTCAATTTTTCAGTATTTTCAGCCAGTACCTGAATAAAA
TCGCGGCGTTTGTAGTTTTACATCCCAGCGAAGTCGTTGTCTGAGGAATTAAATCCTTTAAATCACTGTATCCGCTCTG
AAAAGTTGAAATTTTAGCTTTTTTCCACGAATTTCCAAGCTTCAACAAACATTTATAGCTTCTCTTCGCTGCCGTTCAC
ACCTCAAGTGCGTCGCCTTCTTTCTATCCGAGCTAAATCCGCCGGAATTCGTCGCTGGCGTGTTTGGGGCCGATGGGGA
GCTCGCCGAGCCGTCCGGCGACATTAGGTCCATATCGTCCGGCTTTTGAGAGGAGCTGGCAGCTGCGGAACGGCTTCGA
GACATTTTTGGGCTGAAAAACAGAGAATTTCAGTAGAGTAATTGGGAAAAAAACGAAGAAAAACGCGGATTTTTGAAAT
TTGGGTCTGCTGATTAA
Nr of selected repeats 3 Similarity 0.942652
Consensus:
TTAGCAGACCCAAcTATGGTCTGCTAAtTAT
>Cele-UNSB01_3:194647-195253 Satlength=607 Nr of Repeats=5 RepeatLength=31
seed=TTAGCAGACC
TTAGCAGACCCAAATCTTCCCTTATTTTGTGTGTTTTTTTGCGTATTTTGCCCCAAAAATTATCTATAATTTCCCATTT
TTCAGGCCAACTCGAGTTTTGAAGCGTCAAAATTGAGAAAATTCGATGGGAAACGTGCAAAATTCGTTAATTCCCGAAT
TTTGGAGACAATTCTGACTGTTTTACACTGAAATGCACGATTTTCTCGGAAAATTATTGATTTTTGCCCTGAAATTTCA
GAATTTGGCCGATTTTCTTTAGAATTTGGGTCTGCCAACTAATTAGCAGGCTTGTAGAAACGCCACGTGGTTTTCGATG
GTCTGCTAAATAT
TTAGCAGACCCAAGTATGGTCTGCTAAATAA
TTAGCAGACCCAAGTATGGTCTGCTAAATAA
TTAGCAGACCCAAGTATGGTCTGCTAAATAT
TTAGCAGACCCTAATCCCGTATTTATCCTTTAAATTTAGTTTTTTTCGCGGAATTTACTGGTTTTTTCTTGTTTTTCAA
CCAGGAAATCGTTCAAATTCATCACTTTTTGTCCCAGAAGCCGAGAAAAATCAGTTTTTCAGCTCATATCGAAATTTGT
TTTTCAAAAAGAAATTATAAAAAAGT
Nr of selected repeats 3 Similarity 0.971326
Consensus:
TTAGCAGACCCAAGTATGGTCTGCTAAATAa
>Cele-UNSB01_3:196367-196735 Satlength=369 Nr of Repeats=7 RepeatLength=31
seed=GGTCTGCTAA
GGTCTGCTAAATATTTAGCAGACCCATTTTT
GGTCTGCTAAATATTTAGCATACCCAACTTT

GGTCTGCTAATTATTTAGCAGACCCAAGTAT
GGTCTGCTAAATATTTAGCAGACCCCAGTAT
GGTCTGCTAAATATTTAGCAGACCCCAGTAT
GGTCTGCTAAATATTTAGCATACCCAATTTT
GGTCTGCTAAACATTTTGCAGACCCAATTTCGAAAAATCTTAATTTTTTCCTCAAAAACGGAATTTTCTTGGTTTTTAC
AGCAAAAAATCGAGAATTTCCGCGATTTTCTGACTAGAAAACCTGAAAAATTCGTGAAATTCGTTGAAAAAACTCAATTT
GGAACAGAAATTCGAAAAAAAATG
Nr of selected repeats 6 Similarity 0.868100
Consensus:
GGTCTGCTAAATATTTAGCAgACCCaAgTaT
>Cele-UNSB01_3:198288-198567 Satlength=280 Nr of Repeats=9 RepeatLength=31
seed=ACCCAAGTAT
ACCCAAGTATGGTCTGCTAAATATGTAGCAG
ACCCAAGTATGGTCTGCTAATTATTTAGCAA
ACCCAAGTATGGTCTGCTAAATGATTAGCAA
ACCCAAGTATGGTCTGCTAAATGATTAGCAG
ACCCAAGTATGGTCTGCTAAATGTTTAGCAG
ACCCAAGTATGGTCTGCTCAATGTTTAGCAG
ACCCAAGTATGGTCTGCTCAATGTTTAGCAG
ACCCAAGTATGGTCTGCTCAATGTTTAGCAG
ACCCAAGTATGGTCTGCTCAATGTTTAGCAG
Nr of selected repeats 9 Similarity 0.906810
Consensus:
ACCCAAGTATGGTCTGCTaAATgtTTAGCAg
>Cele-UNSB01_3:211856-211976 Satlength=121 Nr of Repeats=7 RepeatLength=16
seed=TAGGCATCTA
TAGGCATCTATGCACT
TAGGCATCTAGGCAGG
TAGGCACCTAGGCATCTATGCACA
TAGGCACCTAGGCACC
TAGGCATCTATGCATC
TAGGCATCTATGCACC
TAGGCATCTATGCACC
Nr of selected repeats 6 Similarity 0.827778
Consensus:
TAGGCATCTAtGCAcc
>Cele-UNSB01_3:214401-214633 Satlength=233 Nr of Repeats=8 RepeatLength=12
seed=AAGCCTAAGC
AAGCCTAAGCCT
AAGCCTAAGCTCAACTATACGACTCTCTGTTAAATTTCGCTTTGATCGGCCAACGGGAACCCTTTGAAAAATGAAAAAA
AAACGCTATAAACCCAAGCCTAAATAGTAAAAAACGCTCGCCACTGACGCC
AAGCTTAAGCTTAAGCAT
AAGACTAAGCCT
AAGCCTAAGCCT
AAGCCTAAGCCG
AAGCCTAAGATT
AAGACTAAGCCTAGGGTTAAGCGT
Nr of selected repeats 5 Similarity 0.822222
Consensus:
AAGcCTAAGcct
>Cele-UNSB01_3:249570-249725 Satlength=156 Nr of Repeats=4 RepeatLength=31
seed=AGCTCAAAAT
AGCTCAAAATTCTGCAAAATGCTCATTTTCG
AGCTCAAAATCCACAAAATTACTTATTTTTGAGCTTAAGATCTACAAAACTGTTCCTTATTG

AGCTCAAAATCTGTAAAAATGCCCATTTTTG
AGATCAAAATTCACAAAAATGCTCATTTTTA
Nr of selected repeats 3 Similarity 0.711806
Consensus:
AGcTCAAAAtTCtgaAAAATGCtCATTTTTtg
>Cele-UNSB01_3:260141-260567 Satlength=427 Nr of Repeats=12 RepeatLength=30
seed=TTTTTTGAGC
TTTTTTGAGCAAAAAAATGCGAAAAATCTAA
TTTTTTGAGCTAAAAATTCAAAATCTCAGTTTT
TTTTTTGAAGCTAGAAATGCAAAAATGGAATCTTTCCAAGCTTAAAATCCAAAAAATCTTG
TGTTTTGAGCCAAAAATTCAAAAAATCTCG
TTTTTTGAGCCAAAAACTCAAAAAATCTCG
TTTTTTGAGCCAAAAACTCTAAAAAGCTCG
TTTTTTGAGCCAAAAATCCAAAAAATCTTG
TGTTTTGAGCCAAAAATTCAAAAAATCTCG
TTTTTTGAGCCAAAAATTCAAAAAATCTCG
TGTTTTGAGCAAAAAAATGCGAAAAATCTCA
TTTTTTGAGCTAAAAATTCAAAATCTCAGT
TTTTTTGAAGCTAGAAATGCAAAAATGGAATCTTTCCAAGCTTAAAATTCAAAAAATCTCGT
Nr of selected repeats 9 Similarity 0.761574
Consensus:
TtTTTTGAGCcAAAAAttCaAAAAAATCTcg
>Cele-UNSB01_3:265034-265468 Satlength=435 Nr of Repeats=11 RepeatLength=11
seed=CCTACAGTAC
CCTACAGTACC
CCTACAGTACA
CCTACAGTACCCCGACCATATCCCCCACTAACCCCAAACCAATATCCCTTCAAAAGACGAAAAGTCAATTTTTCTAAAA
CTACAGTAAT
CCTACAGTACC
CCTACAGTACC
CCTACAGTACCCCGACCATATCCCCCACTAATCCCAAACTAATATTCCTTCAAAAGACGAAAAGTCAATTTTTCTAAAA
CTACAGTAAT
CCTACAGTACC
CCTACAGTACCCCGACCATATCTCCCACTAATCCCAAACTAATATTCTTTCAAAAGACGAAAAGTCAATTTTTCTAAAA
CTACAGTAAC
CCTACAGTACC
CCTACAGTACCCCGACCATATCCCCCCACTAACCCCAAACCAATATCCCTTCAAAAGACGAAAAGTCAATTTTTCTAAA
ACTACAGTAAT
CCTACAGTACC
Nr of selected repeats 7 Similarity 0.965368
Consensus:
CCTACAGTACC
>Cele-UNSB01_3:272176-273482 Satlength=1307 Nr of Repeats=13 RepeatLength=101
seed=GGTTACTGTA
GGTTACTGTAGCCCCAGAAGTACGTAAACACTGACGAGGCACCGAATCTTACGACAAAATGCAGAAAATCGTCTAAAAA
TTGGTTTTTGGG
GGTTACTGTAGTTCCAAAAGTACGCAAACACTGACTACGGTTCCCTAATACATCAGATATTACGACAAAGTGCAGAAAA
TCGCTAAAAATGACTTTACTGG
GGTTACTGTAGCTCGAGAAATACGCAAACACCACTGACTACGGTTCCCTAATACATCAGATATTATGACAAAGTGCAGA
AAATCGCTAAAAATGACTTTACTGG
GGTTACTGTAGCTCCAAAAGTACGCAAACACTGACTACGGTTCCCTAATACATCAGATATTACGACAAAGTGCAGAAAA
TCGCTAAAAATGACTTTACTGG
GGTTACTGTAGCTCCAGAAATACGCAAACACCGACTACGGGACCCTAATACATCAGATATTATGACAAAGTGCAGAAAA
TCGCTAAAAATGACTTTACTGG

GGTTACTGTAGCTCCAAAAGTACGCAAACACTGACTACGGTTCCCTAATACATCAGATATTACGACAAAGTGCAGAAAA
TCGCTAAAAATGACTTTACTGG
GGTTACTGTAGCTCCAGAAATACGCAAACACCGACTACGGGACCCTAATACATCGGATATTACGACAAAGTGCAGAAAA
TCGCTAAAAATGACTTTACTGG
GGTTACTGTAGCTCCAGAAATACGCAAACACCGACTACGGTTCCCTAATACATCAGATATTACGACAAAGTGCAGAAAA
TCGCTAAAAATGACTTTACTGG
GGTTACTGTACCTCCAGAAATACGCAAACACCGACTACGTGACCCTAATACATCAGATATTACGACAAAGTGCAGAAAA
TCGCTAAAAATGACTTTACTGG
GGTTACTGTAGCTCCAGAAATACGCAAACACCGACTACGGTTCCCTAATACATCAGATATTACGACAAAGTGCAGAAAA
TCGCTAAAAATGACTTTACTGG
GATTACTGTACCTCCAGAAATACGCAAACACCGACTACGTGACCCTAATACATCAGATATTACGACAAAGTGCAGAAAA
TCGCTAAAAATGACTTTACTGG
GGTTACTGTAGCTCCAGAAATACGCAAACACCGACTACGGTTCCCTAATACATCAGATATTACGACAAAGTGCAGAAAA
TCGCTAAAAATGACTTTACTGG
GGTTACTGTACCTCCAGAAATACGCAAACACCGACTACGTGACCCTAATACATCAGATATTACGACAAAGTGCAGAAAT
TCTCTAAAAATGACTTTACTGG
Nr of selected repeats 11 Similarity 0.942394
Consensus:
GGTTACTGTAgCTCCAgAAaTACGCAAACACcGACTACGgttCCCTAATACATCAGATATTACGACAAAGTGCAGAAAA
TCGCTAAAAATGACTTTACTGG
>Cele-UNSB01_3:344303-344830 Satlength=528 Nr of Repeats=17 RepeatLength=31
seed=TGGTCTGCTA
TGGTCTGCTAAATAATTAGCATACCCAACTT
TGGTCTGCTAAATTTTTAGCAGACCCAACAT
TGGTCTGCTATATATTTAGCAGACCCAACAT
TGGTCTGCTAAATATTTAGCATACCCAACTG
TGGTCGGCTAAATATTTAGCATACCCAATTT
TGGTCTGCTAAATAATTAGCATACCCAACTT
TGGTCTGCTAAATAATTAGCACACCCAACTT
TGGTATGCTAAATATTTAGCAGACCCAATTT
TGGTCTGCTAAATAATTAGCATACCCAACTT
TGGTCTGCTAAATAATTAGCATACCCAACTT
TGGTATGCTAAATATTTAGCAGACCCAATTT
TGGTCTGCTAATTATTTAGCAGACCCAATTT
TGGTCTGCTAAATTTTTAGCATACCCAACTT
TGGTCTGCTAAATATTTAGCAGACCAAAGATG
GGTCTGCTAAATATTTAGTATACCCAACTG
TGGTCTGCTAAATATTTAGCATACCCAACTG
TGGTCTGCTAATTATTTAGCAGACCCAATTT
Nr of selected repeats 15 Similarity 0.868920
Consensus:
TGGTCTGCTAAATAtTTAGCAtACCCAAcTT
>Cele-UNSB01_3:347263-347821 Satlength=559 Nr of Repeats=17 RepeatLength=31
seed=TATGCTAAAT
TATGCTAAATATTTAGCAGACCAAAAATAGG
TATGCTAAATATTTAGCAGACCAAAAATAGG
TATGCTAAATATTTAGCAGACCAAAAATAGG
TATGCTAAATATGTAGCAGACCAAAAATAGG
TATGCTAAATATGTAGCAGACCCAAAGTATG
TATGCTAAATATTTAGCAGACCCAAAGTAGG
TATGCTAAATATTTAGCAGACCAAAAGTGGG
TATGCTAAATATTTAGCAGACCAAAAATAGG
TATGCTAAATGTTTAGCAGACCAAAAGTGGG
TATGCTAAATATTTAGCAGACCAAAAATAGG

TATGCTAAATGTTTAGCAGACCAAAAGTGGG
TATGCTAAATATTTAACAGACCAAAGGTGGG
TATGCTAAATATTTAGCAGACCAAAAGTGGG
TATGCTAAATATTTAACAGACCAAAAATAGG
TATGCTAAATGTTTAGCAGACCAAAGGTGGG
TATGCTAAATATTTAGCAGACCAAAGTTGGGTTTGCTAGATATTTAGCAGACCAAAGATAGG
TATGCTAGATATTTAGCAGACCAAAGTTGGG
Nr of selected repeats 16 Similarity 0.883154
Consensus:
TATGCTAAATATTTAGCAGACCAAAAgTaGG
>Cele-UNSB01_3:350896-352295 Satlength=1400 Nr of Repeats=23 RepeatLength=31
seed=TTAGCAGACC
TTAGCAGACCCAAGGTTGGTCTGCTAAATAT
TTAGCAGACCCAAAGTAGGTATGCTAAATAT
TTAGCAGACCAAAAATAGGTATGCTAAATAT
TTAGCAGACCAAAAATAGGTATGCTAAATAT
TTAGCAGACCAAAAATAGGTATGCTAAATAT
TTAGCAGACCAAAAATAGGTATGCTAAATAT
TTAGCAGACCAAAAATAGGTATGCTAAATAT
TTAGCAGACCCAAAGTAGGTATGCTAAATAT
TTAGCAGACCAAAGTTGGGTTTGCTAGATAT
TTAGCAGACCAAAAATAGGTATGCTAAATGT
TTAGCAGACCAAAGGTGGGTATGCTAAATAT
TTAGCAGACCAAAGTTGGGTTTGCTAGATAT
TTAGCAGACCCAAGGTAGGTATGCTAAATAT
TTAGCAGACCAAAGTTGGGTTTGCTAGATAT
TTAGCAGACCAAAAATAGGTATGCTAAATAT
TTAGCAGACCAAAAATAGGTATGCTAAATAT
TTAGCAGACCCAAAGTAGGTATGCTAAATAT
TTAGCAGACCCAAAGTAGGTATGCTAAATAT
TTAGCAGACCAAAAATAGGTATGCTAAATAT
TTAGCAGACCCAATGTTGGTAAGCTAAATAT
TTAGCAGACCCAATGTTGGTATGCTAAATAT
TTAGCAGACCAAAGGTGGGGATGCTAAATAT
TTAGCAGACCCAAAATTAATATCTCCCTTACCGCTGCCTCGCGGCTTCTGCCTTCTTCGCATTCGCCTGCTTCATCTGT
GTCCGTACCCCCTCCACATCATTCGTTCCATGCTCTGGATCCACGCCGAGTTGTTTCAGACGCCGTCGATGATTATAGA
CCCTCCGCTCATTTTCCGTCATCCGTTGATATCGAGACCTCGCCGCGGCCGCCCGTTTCTGTTTCGAACGTTCTTCTCG
ATATTTGTCGGCCATTTCGATTTGTTCCATATGAATCTGATCCTGAATATCTTGCATATCATCAGTTTCCTTCATCAGG
GTCTCCAAGTCTTCCTCGGCCTGCGATTTTGAAGAATGAAGAGAATTTAGAAGGTTTTGTGGTTTTGAAGGTTCTGCAG
TGTTCATAAAAGCATCTCCAAAGTAGGAATCCATAAAGGCATTCATTTGATCGGCTTGTACAATGCAGGGAAGAGTCTG
AAAATTTGAAAATTCATATGGTGGTCGTAAAAATCGCTTTTTAAATGAAAATTTATGCTTTGTAAATTTTCTCTGAATT
GATTCGTTCTAGTACAAATTAAATTAAATTCTACCCAAATTTTATTTTAACACTAAAAATTAATAAATTATACAAAAAT
TGAAAGAATTCTGCGGAGAAAATCAAGAAATTCTCGAGAAATGAATTTTAAATTGTGGTGAATTGAACTTGGGTCTGCT
AGATAT
Nr of selected repeats 22 Similarity 0.867430
Consensus:
TTAGCAGACCaAAanTaGGTATGCTAAATAT
>Cele-UNSB01_3:355335-355552 Satlength=218 Nr of Repeats=7 RepeatLength=31
seed=CTGCTAAATA
CTGCTAAATATTTAGCATACCAACTTTGGGT
CTGCTAAATATTTAGCAGACCAACTTTGGGT
CTGCTAAATATTTAGCATACCCACTTTGGGT
CTGCTAAATATTTAGCATACCCACTTTGGGT
CTGCTAAATATTTAGCATACCCACTTTGGGT

CTGCTAAATATTTAGCATACCAACTTCGGGC
CTGCTAAATATTTAGCATACCAACTTTGGGT
Nr of selected repeats 7 Similarity 0.938556
Consensus:
CTGCTAAATATTTAGCATACCaACTTTGGGT
>Cele-UNSB01_3:356762-357146 Satlength=385 Nr of Repeats=13 RepeatLength=31
seed=TTAGCAGACC
TTAGCAGACCAAAGATGGGTATGCTAAATAT
TTAGCAGACCCAAATAAATAT
TTAGCAGACCAAAGTTGGGTATGCTAAATAT
TTAGCAGACCAAAGTTGGGTATGCTAAATAT
TTAGCAGACCAAAGTTGGGTATGCTAAATAT
TTAGCAGACCAAAGTTGGGTATGCTAAATAT
TTAGCAGACCAAAGTTGGGTATGCTAAATAA
TTAGCAGACCAAAGTTGGGTATGCTAAATAT
TTAGCAGACCAAAGTTGGGTATGCTAAATAT
TTAGCAGACCAAAGTTGGGTATGCTAAATAT
TTAGCAGACCAAAGTTGGGTATGCTAAATAT
TTAGCAGACCCATTCTGAATAT
TTAGCAGACCCAAGTTTGGTATGGTAAATAT
Nr of selected repeats 11 Similarity 0.960899
Consensus:
TTAGCAGACCAAAGTTGGGTATGCTAAATAT
>Cele-UNSB01_3:415402-416126 Satlength=725 Nr of Repeats=7 RepeatLength=40
seed=TCCACGTGGA
TCCACGTGGAATTTTTAGAAATTTCTCAAAAAAATTGAAA
TCCACGTGAAGTTTCAGAATTTCCCAGGAAAATTGAAATCCCCTTAGAATTTTCTTAGGAAGTTTGAATTTCCTGTGAA
ATTTCAGAGGCACGACCGAAAGGAGAACAGGTGAAAAGGAGAATTACGAAAAGGAGAACACAAAACCCCGCCCATTTT
TCCGTGCCGTGCCCAAGTTTTTGCAGAAACTTTTGCAAATTGTAATTTTCAAACGAAACAACGAAGCTCCGAAATAACG
CATTCGTGATAAACAACAATTTTTCACCTGTTCTCCTTTCGGTGGTGTCTCTGAAATTTCAGAATTTTCACAGGAAATT
TAAAA
TCTACGTGGAATTTTCAGAATTTTCTCAGAAATTTTGATT
TCCACGTGGAACTTTCCGAATTTTCGTAGGAAATTTGAAA
TCCACGTGGAATGTTTAGAATTTTCTCAGAAAATTTGAATCAGTGGTGGGCGGCAATTGCCGTTCGGCAAATTGATTTG
TCGACAAATTCGGCAAATCGGCTATTTGCCGGTGTGCCGATTTGCCGGAAATTTTCCATTACGGCAATTTGCCGATTTG
CCGATTCGCCGGAAAAATCGCTTGCCGCCCACCCCTGCTATGAAT
TCCATGTGGAATTTTCAGAATTTTCCCAGAAATTTTCAAA
TCCACGTGGAATTTCCGGGGATTTCCCTGGAAATTTGAAA
Nr of selected repeats 5 Similarity 0.650794
Consensus:
TCcAcGTGGAATTTtcaGaAtTTtCncagaAAatTTgAaa
>Cele-UNSB01_3:421701-421887 Satlength=187 Nr of Repeats=6 RepeatLength=31
seed=TTGGGTCTGC
TTGGGTCTGCTAAATATTTAGCAGACCATAA
TTGGGTCTGCTAAATAATTAGCAGACCATAA
TTGGGTCTGCTAAATATTTAGCAGATTACAC
TTGGGTCTACTAAATAATTAGCAGGCCATAC
TTGGGTCTGCTAAATATTTAGCAGGCCATAC
TTGGGTCTGCTAAATAATTAGCAGACCATAA
Nr of selected repeats 6 Similarity 0.868100
Consensus:
TTGGGTCTGCTAAATAaTTAGCAGaCCATAa
>Cele-UNSB01_3:437143-437747 Satlength=605 Nr of Repeats=6 RepeatLength=31
seed=TATTTAGCAG

TATTTAGCAGACCCAATTATGGTCTGCTAAT
TATTTAGCAGACCCAATTATGGTCTGCTAAT
TATTTAGCAGACCCAAGTATGGTTTGCTAAT
TATTTAGCAGACCCAAATATAGTCTGCTAAA
TATTTAGCAGACCCAATTATGATCTGCTAAT
TATTTAGCAGACCCAAGCTTACTGTAAATTTTAAATTATTCCGATAATTCATTCTAGAATAAATATACTTTGAATTCGA
GAAACTATTGAAAAAAAAAACAAAAATTAGTTTTTAAAAAGTTTCAAAAAATATCATTTTGGTGAAATTTGAGATAATT
TGGAGCTTTTTGAGCGATTTTTTGGGTTTTTACGAGGATTTCCATCATTTTTAGCTTCAAAAACTGTGTAAATTCAGCT
AAACAATGATTAAAAGTGCCGGAAATTGCCGGAAACCCGGAGAAAAACTTAAAAATCAGACCAAGGGGTGCAGAAATAT
TTTTAAAATTTGGATTTTTCGTGAAAAGTTTAGTTTTTAGAGTTTTCGAGTAGTTTCTGGTACTAAAAAGTATTTAAAA
AGTATTTTATATCATTTTTTCGCTTTGAAAATTCCCAGAAAACTGAGAAAATAA
Nr of selected repeats 5 Similarity 0.901075
Consensus:
TATTTAGCAGACCCAAtTATggTcTGCTAAt
>Cele-UNSB01_3:438816-440336 Satlength=1521 Nr of Repeats=38 RepeatLength=40
seed=TAGAAAGTTC
TAGAAAGTTCTGGAACATTCCAGAATTTTCTCGATTTTTC
TAGAAAGTTCTGGAACATTCCAGAAATTTCCCGATTTTTC
TAGAAAGTTCTGGAACATTCCAGACTTTTCCCAATTTTTC
TAGAAAGTTCTTGAACATTCCAGAAATTTCTTGATTTTTC
TAGAAAGTTCTGGTACATTCCAGAATTTTTCCAATTTTTC
TAGAAAGTTCTGGAACATTCCAGAAATTTCTCGATTTTTC
TAGAAAGTTCTGGTACATTCCAGAATTTTCTCGATTTTTC
TAGAAAGTTCTGGAACATTCCAGAAATTTCCCGATTTTTC
TAGAAAGTTCTGGAACATTCCAGACTTTTCCCAATTTTTC
TAGAAAGTTCTTGAACATTCCAGAAATTTCTCGATTTTTC
TAGAAAGTTCTGGAACATTCCAGAATTTTCCCAATTTTTC
TAGAAAGTTCTTGAACATTCCAGAAATTTCTCGATTTTTC
TAGAAAGTTCTGGTACATTCCAGAATTTTTCCAATTTTTC
TAGAAAGTTCTGGAACATTCCAGAATTTTCTCGATTTTTC
TAGAAAGTTCTTGAACATTCCAGAAATTTCCCAATTTTTC
TAGAAAGTTCTTGAACATTCCAGAAATTTCCCAATTTTTC
TAGAAAGTTCTGGAACATTCCAGAAATTTCCCAATTTTTC
TAGAAAGTTCTGGAACATTCCAGAATTTTCTCGATTTTTC
TAGAAAGTTCTGGAACATTCCAGAAATTTCTCAATTTTTC
TAGAAAGTTCTGGTACATTCCAGAATTTTCTCGATTTTTT
TAGAAAGTTCTGGAACATTCCAGAATTTTCTCGATTTTTC
TAGAAAGTTCTTGAACATTCCAGACTTTTCCCAATTTTAC
TAGAAAGTTCTGGAACATTCCAGAATTTTCTCGATTTTTC
TAGAAAGTTCTGGAACATTCCAGAATCTTCTCGATTTTTC
TAGAAAGTTCTTGAACATTCCAGAAATTTCCCGATTTTTC
TAGAAAGTTCTGGAACATTCCAAAATTTTCTCGATTTTTC
TAGAAAGTTCTTGAACATTCCAGAAATTTCTCGATTTTTC
TAGAAAGTTCTGGTACATTCCAGAATTTTTCCAATTTTTC
TAGAAAGTTCTGGAACATTCCAGAATTTTCTCGATTTTTC
TAGAAAGTTCTTGAACATTCCAGAAATTTCCCGATTTTTC
TAGAAAGTTCTGGAACATTCCAGACTTTTCCCAATTTTTC
TAGAAAGTTCTGGAACATTCCAGACTTTTCCCAATTTTTC
TAGAAAGTTCTGGAACATTCCAGAAATTTCTCGATTTTTC
TAGAAAGTTCTGGAACATTCCAGACTTTTCCCAATTTTTC
TAGAAAGTTCTGGAACATTCCAGAATTTTCTCGATTTTTC
TAGAAAGTTCTTGAACATTCCAGAAATTTCCCGATTTTTC
TAGAAAGTTCTGGAACATTCCAGACTTTTTCCAATTTTTC
TAGAAAGTTCTGGAACATTCCAGAATTTTCTCGATTTTTC

Nr of selected repeats 38 Similarity 0.903556
Consensus:
TAGAAAGTTCTgGAACATTCCAGAAtTTTCcCgATTTTTC
>Cele-UNSB01_3:438790-441355 Satlength=2566 Nr of Repeats=43 RepeatLength=40
seed=ACATTCCAGA
ACATTCCAGAATTTTCTCGATTTTTCTAGAAAGTTCTGGA
ACATTCCAGAATTTTCTCGATTTTTCTAGAAAGTTCTGGA
ACATTCCAGAAATTTCCCGATTTTTCTAGAAAGTTCTGGA
ACATTCCAGACTTTTCCCAATTTTTCTAGAAAGTTCTTGA
ACATTCCAGAAATTTCTTGATTTTTCTAGAAAGTTCTGGT
ACATTCCAGAATTTTTCCAATTTTTCTAGAAAGTTCTGGA
ACATTCCAGAAATTTCTCGATTTTTCTAGAAAGTTCTGGT
ACATTCCAGAATTTTCTCGATTTTTCTAGAAAGTTCTGGA
ACATTCCAGAAATTTCCCGATTTTTCTAGAAAGTTCTGGA
ACATTCCAGACTTTTCCCAATTTTTCTAGAAAGTTCTTGA
ACATTCCAGAAATTTCTCGATTTTTCTAGAAAGTTCTGGA
ACATTCCAGAATTTTCCCAATTTTTCTAGAAAGTTCTTGA
ACATTCCAGAAATTTCTCGATTTTTCTAGAAAGTTCTGGT
ACATTCCAGAATTTTTCCAATTTTTCTAGAAAGTTCTGGA
ACATTCCAGAATTTTCTCGATTTTTCTAGAAAGTTCTTGA
ACATTCCAGAAATTTCCCAATTTTTCTAGAAAGTTCTTGA
ACATTCCAGAAATTTCCCAATTTTTCTAGAAAGTTCTGGA
ACATTCCAGAAATTTCCCAATTTTTCTAGAAAGTTCTGGA
ACATTCCAGAATTTTCTCGATTTTTCTAGAAAGTTCTGGA
ACATTCCAGAAATTTCTCAATTTTTCTAGAAAGTTCTGGT
ACATTCCAGAATTTTCTCGATTTTTTTAGAAAGTTCTGGA
ACATTCCAGAATTTTCTCGATTTTTCTAGAAAGTTCTTGA
ACATTCCAGACTTTTCCCAATTTTACTAGAAAGTTCTGGA
ACATTCCAGAATTTTCTCGATTTTTCTAGAAAGTTCTGGA
ACATTCCAGAATCTTCTCGATTTTTCTAGAAAGTTCTTGA
ACATTCCAGAAATTTCCCGATTTTTCTAGAAAGTTCTGGA
ACATTCCAAAATTTTCTCGATTTTTCTAGAAAGTTCTTGA
ACATTCCAGAAATTTCTCGATTTTTCTAGAAAGTTCTGGT
ACATTCCAGAATTTTTCCAATTTTTCTAGAAAGTTCTGGA
ACATTCCAGAATTTTCTCGATTTTTCTAGAAAGTTCTTGA
ACATTCCAGAAATTTCCCGATTTTTCTAGAAAGTTCTGGA
ACATTCCAGACTTTTCCCAATTTTTCTAGAAAGTTCTGGA
ACATTCCAGACTTTTCCCAATTTTTCTAGAAAGTTCTGGA
ACATTCCAGAAATTTCTCGATTTTTCTAGAAAGTTCTGGA
ACATTCCAGACTTTTCCCAATTTTTCTAGAAAGTTCTGGA
ACATTCCAGAATTTTCTCGATTTTTCTAGAAAGTTCTTGA
ACATTCCAGAAATTTCCCGATTTTTCTAGAAAGTTCTGGA
ACATTCCAGACTTTTTCCAATTTTTCTAGAAAGTTCTGGA
ACATTCCAGAATTTTCTCGATTTTTCTAGAAAGTTCTGGA
ACATTCGAGAATTTTTTCGAAATTTCCAGAAGATTCTAGATTTCCAGAATTTTAGAATTTTCAGAAAATTTCAATTTCC
CGCAAAAATATTTTTCACAGAAAATTTAAATTTCCCGCCAAAATATTTTTCACAGAAAATTTAAATTTCCCGCCAAAAT
ATTTTTCACAGAAAATTTAAATTTCCCTCCAAAATATTTTTCTCAGAAAATTTAAATTTCCCGCCGAAATATTTTTCAC
AGAAAATTTAAATTTCCCGCCAAAATATTTTTCTCAGAAAATTTAAATTTCCCGCCAAAATTTTGGGTCTTACCACGGT
GGGTCTCACCACGACGGGTCTCACCACGTTGGGTCTCACCACGATGGGTCTCACCACGATGGGTCTTTTCACGATGGGT
CTCACCACGGTGGGTCTCGCCACGATGGGTCTCGCCACGATGGGTCTCGCCACGAAGATCTCGCAGCAACATTTTTTTA
ATTTTCCAGAAGGTTCTAGA
ACAATCCAGAATTTTTTCGAATTTTCCAGAAGGTTCTGGA
ACATTCCAGAATTTTCTAGAATTTTCCAGAACCTTATTTCCCGCCAAAATATTTTTCACAGAAAATTTAAATTTCCCGC
CAAAATATTTTTCACAGAAAATTTAAATTTCCCTTCAAAATATTTTTCTCAGAAAATTTAAATTTCCCGCCAAAATATT

TTTCACAGAAAATTTAAATTTCCCGCCAAAATTTTGGGTCTTACCACGGTGGGTCTCACCACGACGGGTCTCACCACGA
TGGGTCTCGCCACGATGGGTCTCGCCACGATGGGTCTCGCCACGATGGGTCTCACCACGATGGGTCTCACCACGATGGG
TCTTTTCACGATGGGTCTCACCACGGTGGGTCTCGCCACGATGGGTCTCGCCACGATGGGTCTCGCCACGAAGATCTCG
CAGCAACATTTTTTTAATTTTCCAGAAGGTTCTAGA
ACAATCCAGAATTTTTTCGAATTTTCCAGAAGGTTCTGGA
Nr of selected repeats 41 Similarity 0.892358
Consensus:
ACATTCCAGAAtTTTCtCgATTTTTCTAGAAAGTTCTgGA
>Cele-UNSB01_3:441483-441987 Satlength=505 Nr of Repeats=18 RepeatLength=11
seed=CTACAGTACT
CTACAGTACTC
CTACAGTACTA
CTACAGTACCCCGACCATATTCCACTACTAACCGCAAACCTATATCTCTTCAAAAGACAAAAACACAATTTTTCATAAA
CTACAGTAATC
CTACCGTACTC
CTACAGTACTC
CTACAGTACTACTGCAGTACCCCGACCATATCCCACTACTAACCCCCAAACCTATATCTCTTCAAAAGACAAAAGCTCA
ATTTTTCCTAAA
CTACAGTAATC
CTACAGTACTC
CTACAGTACCT
CTACAGTACTA
CTACAGTACCCCGACCATATCCCACCACTAACCCCAAACCTATATCTCTTCAAAAGACAAAAACTCAATTTTTCCTAAA
CTACAGTAATC
CTACAGTACTCCTAAAGTACCT
CTACAGTACTA
CTACAGTACCCCGACCATATCCCACCACTAACCCCAAACCTATATCTCTTCAAAAGACAAAAACTCAATTTTTCCTAAA
CTACAGTAATC
CTACAGTACTCCTACAGTACCT
Nr of selected repeats 12 Similarity 0.773779
Consensus:
CTACAGTAcTc
>Cele-UNSB01_3:464201-477164 Satlength=12964 Nr of Repeats=79 RepeatLength=164
seed=ATATAATGCT
ATATAATGCTCGTAATTATTAATAAATGTGTGTTGTCTCAGAGCCACGTCTGAAAATACAGATTTTTTCTCGAAAATTT
CAAAAACTTTGTAGAAATTCGATTTTTGACATAAGTGATCTCTCTTTTTTTCTGACATGTATTTCAGTGACAACTGTAC
TCGAAA
ATATAATGCTCGTAATTATTAATAAATGTGTGTTGTCTCAGAGCCACGTCTGAAAATACAGATTTTTTCTCGAAAATTT
CAAAAACTTTGTAGAAATTCGATTTTTGACATAAATGATCGCTCTTTTTTTCTGACATGTATTTCAGTGACAACTAAAC
TAGAAA
ATATAATGCTCGTAATTATTTATAAGTGTGTGTTGTCTCAGAGCCACGTCTGAAAATACAGATTTTTTCTCGAAAATTT
CAAAAACTTTGTAGAAATTCGATTTTTGACATAAATGATCTCTCTTTTTTTCTGACATGTATTTCAGTGACAAATGGAC
TAGAAA
ATATAATGCTCGTAATTATTTATAAGTGTGTGTTGTCTCAGAGCCACGTCTGAAAATACAGATTTTTTCTCGAAAATTT
CAAAAACTTTGTAGAAATTCGATTTTTGACATAAATGATCGCTCTTTTTTTCTGACATGTATTTCAGTGACAACTAAAC
TAGAAA
ATATAATGCTCGTAATAATTTATAAGTGTGTGTTGTCTCAGAGCCACGTCTGAAAATACAGATTTTTTCTCGAAAATTT
CAAAAACTTTGTAGAAATTCGATTTTTGACATAAATGATCTCTCTTTTTCTCTGACATGTATTTCAGTGACAAATGGAC
TAGAAA
ATATAATGCTCGTAATTATTTATAAGTGTGTGTTGTCTCAGAGCCACGTCTGAAAATACAGATTTTTTCTCGAAAATTT
CAAAAACTTTGTAGAAATTCGATTTTTGACATAAATGATCTCTCTTTTTTTCTGACATGTATTTCAGTGACAACTAAAC
TAGAAA

ATATAATGCTCGTAATTATTTATAAGTGTGTGTTGTCTCAGAGCCACGTCTGAAAATACAGATTTTTTCTCGAAAATTT
CAAAAACTTTGTAGAAATTCGATTTTTGACATAAATGATCGCTCTTTTTTTCTGACATGTATTTCAGTGACAACTAAAC
TAGAAA
ATATAATGCTCGTAATTATTTATAAGTGTGTGTTGTCTCAGAGCCACGTCTGAAAATACAGATTTTTTCTCGAAATTTT
CAAAAACTTTGTAGAAATTCGATTTTTGACATAAATGATCGCTCTTTTTTTCTGACATGTATTTCAGTGACAAATGGAT
TAGAAA
ATATAATGCTCGTAATTATTTATAAGTGTGTGTTGTCTCAGAGCCACGTCTGAAAATACAGATTTTTTCTCGAAAATTT
CAAAAACTTTGTAGAAATTCGATTTTTGACATAAATGATCTCTCTTTTTTTCTGACATGTATTTCAGTGACAAATGGAT
TAGAAA
ATATAATGCTCGTAATTATTTATAAGTGTGTGTTGTCTCAGAGCCACGTCTGAAAATACAGATTTTTTCTCGAAAATTT
CAAAAACTTTGTAGAAATTCGATTTTTGACATAAATGATCTCTCTTTTTTTCTGACATGTATTTCAGTGACAAATGGAT
TAGAAA
ATATAATGCTCGTAATTATTTATAAGTGTGTGTTGTCTCAGAGCCACGTCTGAAAATACAGATTTTTTCTCGAAAATTT
CAAAAACTTTGTAGAAATTCGATTTTTGACATAAATGATCTCTCTTTTTTTCTGACATGTATTTCAGTGACAAATGGAT
TAGAAA
ATATAATGCTCGTAATTATTTATAAGTGTGTGTTGTCTCAGAGCCACGTCTGAAAATACAGATTTTTTCTCGAAAATTT
CAAAAACTTTGTAGAAATTCGATTTTTGACATAAATGATCTCTCTTTTTTTCTGACATGTATTTCAGTGACAAATGGAT
TAGAAA
ATATAATGCTCGTAATTATTTATAAGTGTGTGTTGTCTCAGAGCCACGTCTGAAAATACAGATTTTTTCTCGAAAATTT
CAAAAACTTTGTAGAAATTCGATTTTTGACATAAATGATCTCTCTTTTTTTCTGACATGTATTTCAGTGACAAATGGAT
TAGAAA
ATATAATGCTCGTAATTATTTATAAGTGTGTGTTGTCTCAGAGCCACGTCTGAAAATACAGATTTTTTCTCGAAAATTT
CAAAAACTTTGTAGAAATTCGATTTTTGACATAAATGATCGCTCTTTTTTTCTGACATGTATTTCAGTGACAAATGGAT
TAGAAA
ATATAATGCTCGTAATTATTTATAAGTGTGTGTTGTCTCAGAGCCACGTCTGAAAATACAGATTTTTTCTCGAAAATTT
CAAAAACTTTGTAGAAATTCGATTTTTGACATAAATGATCGCTCTTTTTTTCTGACATGTATTTCAGTGACAAATGGAC
TCGAAA
ATATAATGCTCGTAAATATTAATAAATGTGTGTTGTCTCAGAGCCACGTCTGAAAATACAGATTTTTTCTCGAAAATTT
CAAAAACTTTGTAGAAATTCGATTTTTGACATAAATGATCTCTCTTTTTTTCTGACATGTATTTCAGTGACAAATGGAT
TAGAAA
ATATAATGCTCGTAATTATTTATAAGTGTGTGTTGTCTCAGAGCCACGTCTGAAAATACAGATTTTTTCTCGAAAATTT
CAAAAACTTTGTAGAAATTCGATTTTTGACATAAATGATCTCTCTTTTTTTCTGACATGTATTTCAGTGACAAATGGAT
TAGAAA
ATATAATGCTCGTAATTATTTATAAGTGTGTGTTGTCTCAGAGCCACGTCTGAAAATACAGATTTTTTCTCGAAAATTT
CAAAAACTTTGTAGAAATTCGATTTTTGACATAAATGATCGCTCTTTTTTTCTGACATGTATTTCAGTGACAAATGGAT
TAGAAA
ATATAATGCTCGTAATTATTTATAAGTGTGTGTTGTCTCAGAGCCACGTCTGAAAATACAGATTTTTTCTCGAAAATTT
CAAAAACTTTGTAGAAATTCGATTTTTGACATAAATGATCGCTCTTTTTTTCTGACATGTATTTCAGTGACAACTAAAC
TAGAAA
ATATAATGCTCGTAATTATTTATAAGTGTGTGTTGTCTCAGAGCCACGTCTGAAAATACAGATTTTTTCTCGAAAATTT
CAAAAACTTTGTAGAAATTCGATTTTTGACATAAATGATCGCTCTTTTTTTCTGACATGTATTTCAGTGACAAATGGAT
TAGAAA
ATATAATGCTCGTAATTATTTATAAGTGTGTGTTGTCTCAGAGCCACGTCTGAAAATACAGATTTTTTCTCGAAAATTT
CAAAAACTTTGTAGAAATTCGATTTTTGACATAAATGATCGCTCTTTTTTTCTGACATGTATTTCAGTGACAACTAAAC
TAGAAA
ATATAATGCTCGTAATTATTTATAAGTGTGTGTTGTCTCAGAGCCACGTCTGAAAATACAGATTTTTTCTCGAAAATTT
CAAAAACTTTGTAGAAATTCGATTTTTGACATAAATGATCGCTCTTTTTTTCTGACATGTATTTCAGTGACAAATGGAT
TAGAAA
ATATAATGCTCGTAATTATTTATAAGTGTGTGTTGTCTCAGAGCCACGTCTGAAAATACAGATTTTTTCTCGAAAATTT
CAAAAACTTTGTAGAAATTCGATTTTTGACATAAATGATCTCTCTTTTTTTCTGACATGTATTTCAGTGACAAATGGAT
TAGAAA
ATATAATGCTCGTAATTATTTATAAGTGTGTGTTGTCTCAGAGCCACGTCTGAAAATACAGATTTTTTCTCGAAAATTT
CAAAAACTTTGTAGAAATTCGATTTTTGACATAAATGATCGCTCTTTTTTTCTGACATGTATTTCAGTGACAAATGGAT
TAGAAA

ATATAATGCTCGTAATTATTTATAAGTGTGTGTTGTCTCAGAGCCACGTCTGAAAATACAGATTTTTTCTCGAAAATTT
CAAAAACTTTGTAGAAATTCGATTTTTGACATAAATGATCTCTCTTTTTTTCTGACATGTATTTCAGTGACAAATGGAT
TAGAAA
ATATAATGCTCGTAATTATTTATAAGTGTGTGTTGTCTCAGAGCCACGTCTGAAAATACAGATTTTTTCTCGAAAATTT
CAAAAACTTTGTAGAAATTCGATTTTTGACATAAATGATCGCTCTTTTTTTCTGACATGTATTTCAGTGACAAATGGAT
TAGAAA
ATATAATGCTCGTAATTATTTATAAGTGTGTGTTGTCTCAGAGCCACGTCTGAAAATACAGATTTTTTCTCGAAAATTT
CAAAAACTTTGTAGAAATTCGATTTTTGACATAAATGATCGCTCTTTTTTTCTGACATGTATTTCAGTGACAACTAAAC
TAGAAA
ATATAATGCTCGTAATTATTTATAAGTGTGTGTTGTCTCAGAGCCACGTCTGAAAATACAGATTTTTTCTCGAAAATTT
CAAAAACTTTGTAGAAATTCGATTTTTGACATAAATGATCGCTCTTTTTTTCTGACATGTATTTCAGTGACAACTAAAC
TAGAAA
ATATAATGCTCGTAATTATTTATAAGTGTGTGTTGTCTCAGAGCCACGTCTGAAAATACAGATTTTTTCTCGAAAATTT
CAAAAATTTTGTAGAAATTCGATTTTTGACATAAATGATCGCTCTTTTTTTCTGACATGTATTTCAGTGACAACTAAAC
TAGAAA
ATATAATGCTCGTAATTATTTATAAGTGTGTGTTGTCTCAGAGCCACGTCTGAAAATACAGATTTTTTCTCGAAAATTT
CAAAAATTTTGTAGAAATTCGATTTTTGACATAAATGATCGCTCTTTTTTTCTGACATGTATTTCAGTGACAACTAAAC
TAGAAA
ATATAATGCTCGTAATTATTTATAAGTGTGTGTTGTCTCAGAGCCACGTCTGAAAATACAGATTTTTTCTCGAAAATTT
CAAAAATTTTGTAGAAATTCGATTTTTGACATAAATGATCGCTCTTTTTTTCTGACATGTATTTCAGTGACAACTAAAC
TAGAAA
ATATAATGCTCGTAATTATTTATAAGTGTGTGTTGTCTCAGAGCCACGTCTGAAAATACAGATTTTTTCTCGAAAATTT
CAAAAACTTTGTAGAAATTCGATTTTTGACATAAATGATCGCTCTTTTTTTCTGACATGTATTTCAGTGACAACTAAAC
TAGAAA
ATATAATGCTCGTAATTATTTATAAGTGTGTGTTGTCTCAGAGCCACGTCTGAAAATACAGATTTTTTCTCGAAAATTT
CAAAAACTTTGTAGAAATTCGATTTTTGACATAAATGATCGCTCTTTTTTTCTGACATGTATTTCAGTGACAAATGGAT
TAGAAA
ATATAATGCTCGTAATTATTTATAAGTGTGTGTTGTCTCAGAGCCACGTCTGAAAATACAGATTTTTTCTCGAAAATTT
CAAAAACTTTGTAGAAATTCGATTTTTGACATAAATGATCGCTCTTTTTTTCTGACATGTATTTCAGTGACAACTAAAC
TAGAAA
ATATAATGCTCGTAATTATTTATAAGTGTGTGTTGTCTCAGAGCCACGTCTGAAAATACAGATTTTTTCTCGAAAATTT
CAAAAACTTTGTAGAAATTCGATTTTTGACATAAATGATCGCTCTTTTTTTCTGACATGTATTTCAGTGACAACTAAAC
TAGAAA
ATATAATGCTCGTAATTATTTATAAGTGTGTGTTGTCTCAGAGCCACGTCTGAAAATACAGATTTTTTCTCGAAAATTT
CAAAAATTTTGTAGAAATTCGATTTTTGACATAAATGATCGCTCTTTTTTTCTGACATGTATTTCAGTGACAACTAAAC
TAGAAA
ATATAATGCTCGTAATTATTTATAAGTGTGTGTTGTCTCAGAGCCACGTCTGAAAATACAGATTTTTTCTCGAAAATTT
CAAAAATTTTGTAGAAATTCGATTTTTGACATAAATGATCGCTCTTTTTTTCTGACATGTATTTCAGTGACAACTAAAC
TAGAAA
ATATAATGCTCGTAATTATTTATAAGTGTGTGTTGTCTCAGAGCCACGTCTGAAAATACAGATTTTTTCTCGAAAATTT
CAAAAATTTTGTAGAAATTCGATTTTTGACATAAATGATCGCTCTTTTTTTCTGACATGTATTTCAGTGACAACTAAAC
TAGAAA
ATATAATGCTCGTAATTATTTATAAGTGTGTGTTGTCTCAGAGCCACGTCTGAAAATACAGATTTTTTCTCGAAAATTT
CAAAAATTTTGTAGAAATTCGATTTTTGACATAAATGATCGCTCTTTTTTTCTGACATGTATTTCAGTGACAACTAAAC
TAGAAA
ATATAATGCTCGTAATTATTTATAAGTGTGTGTTGTCTCAGAGCCACGTCTGAAAATACAGATTTTTTCTCGAAAATTT
CAAAAATTTTGTAGAAATTCGATTTTTGACATAAATGATCTCTCTTTTTTTCTGACATGTATTTCAGTGACAAATGGAC
TAGAAA
ATATAATGCTCGTAATAATTTATAAGTGTGTGTTGTCTCAGAGCCACGTCTGAAAATACAGATTTTTTCTCGAAAATTT
CAAAAACTTTGTAGAAATTCGATTTTTGACATAAATGATCTCTCTTTTTCTCTGACATGTATTTCAGTGACAAATGGAC
TAGAAA
ATATAATGCTCGTAATTATTTATAAGTGTGTGTTGTCTCAGAGCCACGTCTGAAAATACAGATTTTTTCTCGAAAATTT
CAAAAACTTTGTAGAAATTCGATTTTTGACATAAATGATCTCTCTTTTTTTCTGACATGTATTTCAGTGACAACTAAAC
TAGAAA

ATATAATGCTCGTAATTATTTATAAGTGTGTGTTGTCTCAGAGCCACGTCTGAAAATACAGATTTTTTCTCGAAATTTT
CAAAAACTTTGTAGAAATTCGATTTTTGACATAAATGATCGCTCTTTTTTTCTGACATGTATTTCAGTGACAAATGGAT
TAGAAA
ATATAATGCTCGTAATTATTTATAAGTGTGTGTTGTCTCAGAGCCACGTCTGAAAATACAGATTTTTTCTCGAAAATTT
CAAAAACTTTGTAGAAATTCGATTTTTGACATAAATGATCTCTCTTTTTTTCTGACATGTATTTCAGTGACAAATGGAT
TAGAAA
ATATAATGCTCGTAATTATTTATAAGTGTGTGTTGTCTCAGAGCCACGTCTGAAAATACAGATTTTTTCTCGAAAATTT
CAAAAACTTTGTAGAAATTCGATTTTTGACATAAATGATCGCTCTTTTTTTCTGACATGTATTTCAGTGACAAATGGAC
TAGAAA
ATATAATGCTCGTAATAATTTATAAGTGTGTGTTGTCTCAGAGCCACGTCTGAAAATACAGATTTTTCTCGAAAATTTC
AAAAACTTTGTAGAAATTCGATTTTTGACATAAATGATCGCTCTTTTTTTCTGACATGTATTTCAGTGACAAATGGACT
AGAAA
ATATAATGCTCGTAATTATTTATAAGTGTGTGTTGTCTCAGAGCCACGTCTGAAAATACAGATTTTTTCTCGAAAATTT
CAAAAACTTTGTAGAAATTCGATTTTTGACATAAATGATCGCTCTTTTTTTCTGACATGTATTTCAGTGACAAATGGAC
TAGAAA
ATATAATGCTCGTAATAATTTATAAGTGTGTGTTGTCTCAGAGCCACGTCTGAAAATACAGATTTTTTCTCGAAAATTT
CAAAAACTTTGTAGAAATTCGATTTTTGACATAAATGATCTCTCTTTTTCTCTGACATGTATTTCAGTGACAAATGGAC
TAGAAA
ATATAATGCTCGTAATTATTTATAAGTGTGTGTTGTCTCAGAGCCACGTCTGAAAATACAGATTTTTTCTCGAAAATTT
CAAAAACTTTGTAGAAATTCGATTTTTGACATAAATGATCTCTCTTTTTTTCTGACATGTATTTCAGTGACAAATGGAT
TAGAAA
ATATAATGCTCGTAATTATTTATAAGTGTGTGTTGTCTCAGAGCCACGTCTGAAAATACAGATTTTTTCTCGAAATTTT
CAAAAACTTTGTAGAAATTCGATTTTTGACATAAATGATCGCTCTTTTTTTCTGACATGTATTTCAGTGACAAATGGAT
TAGAAA
ATATAATGCTCGTAATTATTTATAAGTGTGTGTTGTCTCAGAGCCACGTCTGAAAATACAGATTTTTTCTCGAAAATTT
CAAAAACTTTGTAGAAATTCGATTTTTGACATAAATGATCTCTCTTTTTTTCTGACATGTATTTCAGTGACAAATGGAT
TAGAAA
ATATAATGCTCGTAATTATTTATAAGTGTGTGTTGTCTCAGAGCCACGTCTGAAAATACAGATTTTTTCTCGAAAATTT
CAAAAACTTTGTAGAAATTCGATTTTTGACATAAATGATCGCTCTTTTTTTCTGACATGTATTTCAGTGACAAATGGAT
TAGAAA
ATATAATGCTCGTAATTATTTATAAGTGTGTGTTGTCTCAGAGCCACGTCTGAAAATACAGATTTTTCTCGAAAATTTC
AAAAACTTTGTAGAAATTCGATTTTTGACATAAATGATCTCTCTTTTTTTCTGACATGTATTTCAGTGACAAATGGATT
AGAAA
ATATAATGCTCGTAATTATTTATAAGTGTGTGTTGTCTCAGAGCCACGTCTGAAAATACAGATTTTTTCTCGAAAATTT
CAAAAACTTTGTAGAAATTCGATTTTTGACATAAATGATCGCTCTTTTTTTCTGACATGTATTTCAGTGACAACTGGAC
TCGAAA
ATATAATGCTCGTAAATATTAATAAATGTGTGTTGTCTCAGAGCCACGTCTGAAAATACAGATTTTTTCTCGAAAATTT
CAAAAACTTTGTAGAAATTCGATTTTTGACATAAATGATCGCTCTTTTTTTTCTGACATGTATTTCAGTGACAACTGGA
CTCGAAA
ATATAATGCTCGTAAATATTAATAAATGTGTGTTGTCTCAGAGCCACGTCTGAAAATACAGATTTTTTCTCGAAAATTT
CAAAAACTTTGTAGAAATTCGATTTTTGACATAAATGATCGCTCTTTTTTTTCTGACATGTATTTCAGTGACTACTGGA
ATCGAAA
ATATAATGCTCGTAATTATTAATAAATGTGTGTTGTCTCAGAGCCATGTCTGAAAATACAGATTTTTTTTCGAAAATTT
CAAAAACTTTGTAGAAATTCGATTTTTGACATAAATGATCGCTCTTTTTTTTCTGACATGTATTTCAGTGACTACTGGA
ATCGAAA
ATATAATGCTCGTAATTATTAATAAATGTGTGTTGTCTCAGAGCCATGCCTGAAAATACAGATTTTTTTTTCGAAAATT
TCAAAAACTTTGTAGAAATTCGATTTTTGACATAAATGATCGCTCTTTTTTTCTGACATGTATTTCAGTGACTACTGGA
ATCGAAA
ATATAATGCTCGTAATTATTTATAAGTGTGTGTTGCCTCAGAGCCACGTCTGAAAATACAGAATTTTTCTCGAAAATTT
CAAAAATTTTGTAGAAATTCGATTTTTGACATAAATGATCGCTCTTTTTTTCTGACATGTATTTCAGTGACTACTGGAA
TCGAAA
ATATAATGCTCGTAATTATTTATAAGTGTGTGTTGTCTCAGAGCCACGTCTGAAAATACAGATTTTTTCTCGAAAATTT
CAAAAACTTTGTAGAAATTCGATTTTTGACATAAATGATCGCTCTTTTTTTCTGACATGTATTTCAGTGACAACTAAAC
TAGAAA

ATATAATGCTCGTAATTATTTATAAGTGTGTGTTGTCTCAGAGCCACGTCTGAAAATACAGATTTTTTCTCGAAATTTT
CAAAAACTTTGTAGAAATTCGATTTTTGACATAAATGATCGCTCTTTTTTTCTGACATGTATTTCAGTGACAAATGGAT
TAGAAA
ATATAATGCTCGTAATTATTTATAAGTGTGTGTTGTCTCAGAGCCACGTCTGAAAATACAGATTTTTTCTCGAAAATTT
CAAAAACTTTGTAGAAATTCGATTTTTGACATAAATGATCTCTCTTTTTTTCTGACATGTATTTCAGTGACAAATGGAT
TAGAAA
ATATAATGCTCGTAATTATTTATAAGTGTGTGTTGTCTCAGAGCCACGTCTGAAAATACAGATTTTTTCTCGAAAATTT
CAAAAACTTTGTAGAAATTCGATTTTTGACATAAATGATCGCTCTTTTTTTCTGACATGTATTTCAGTGACAAATGGAT
TAGAAA
ATATAATGCTCGTAATTATTTATAAGTGTGTGTTGTCTCAGAGCCACGTCTGAAAATACAGATTTTTTCTCGAAAATTT
CAAAAACTTTGTAGAAATTCGATTTTTGACATAAATGATCTCTCTTTTTTTCTGACATGTATTTCAGTGACAAATGGAT
TAGAAA
ATATAATGCTCGTAATTATTTATAAGTGTGTGTTGTCTCAGAGCCACGTCTGAAAATACAGATTTTTTCTCGAAAATTT
CAAAAACTTTGTAGAAATTCGATTTTTGACATAAATGATCGCTCTTTTTTTCTGACATGTATTTCAGTGACAACTGGAC
TCGAAA
ATATAATGCTCGTAAATATTAATAAATGTGTGTTGTCTCAGAGCCACGTCTGAAAATACAGATTTTTTCTCGAAAATTT
CAAAAACTTTGTAGAAATTCGATTTTTGACATAAATGATCGCTCTTTTTTTTCTGACATGTATTTCAGTGACAAATGGA
TTAGAAA
ATATAATGCTCGTAATTATTTATAAGTGTGTGTTGTCTCAGAGCCACGTCTGAAAATACAGATTTTTTCTCGAAAATTT
CAAAAACTTTGTAGAAATTCGATTTTTGACATAAATGATCTCTCTTTTTTTCTGACATGTATTTCAGTGACAAATGGAT
TAGAAA
ATATAATGCTCGTAATTATTTATAAGTGTGTGTTGTCTCAGAGCCACGTCTGAAAATACAGATTTTTTCTCGAAAATTT
CAAAAACTTTGTAGAAATTCGATTTTTGACATAAATGATCGCTCTTTTTTTCTGACATGTATTTCAGTGACAACTGGAC
TCGAAA
ATATAATGCTCGTAAATATTAATAAATGTGTGTTGTCTCAGAGCCACGTCTGAAAATACAGATTTTTTCTCGAAAATTT
CAAAAACTTTGTAGAAATTCGATTTTTGACATAAATGATCTCTCTTTTTTTCTGACATGTATTTCAGTGACAAATGGAT
TAGAAA
ATATAATGCTCGTAATTATTTATAAGTGTGTGTTGTCTCAGAGCCACGTCTGAAAATACAGATTTTTTCTCGAAAATTT
CAAAAACTTTGTAGAAATTCGATTTTTGACATAAATGATCTCTCTTTTTTTCTGACATGTATTTCAGTGACAAATGGAT
TAGAAA
ATATAATGCTCGTAATTATTTATAAGTGTGTGTTGTCTCAGAGCCACGTCTGAAAATACAGATTTTTTCTCGAAAATTT
CAAAAACTTTGTAGAAATTCGATTTTTGACATAAATGATCTCTCTTTTTTTCTGACATGTATTTCAGTGACAAATGGAT
TAGAAA
ATATAATGCTCGTAATTATTTATAAGTGTGTGTTGTCTCAGAGCCACGTCTGAAAATACAGATTTTTTCTCGAAAATTT
CAAAAACTTTGTAGAAATTCGATTTTTGACATAAATGATCGCTCTTTTTTTTCTGACATGTATTTCAGTGACAACTGGA
CTCGAAA
ATATAATGCTCGTAAATATTAATAAATGTGTGTTGTCTCAGAGCCACGTCTGAAAATACAGATTTTTTCTCGAAAATTT
CAAAAACTTTGTAGAAATTCGATTTTTGACATAAATGATCGCTCTTTTTTTTCTGACATGTATTTCAGTGACAAATGGA
TTAGAAA
ATATAATGCTCGTAATTATTTATAAGTGTGTGTTGTCTCAGAGCCACGTCTGAAAATACAGATTTTTTCTCGAAAATTT
CAAAAACTTTGTAGAAATTCGATTTTTGACATAAATGATCTCTCTTTTTTTCTGACATGTATTTCAGTGACAAATGGAT
TAGAAA
ATATAATGCTCGTAATTATTAATAAATGTGTGTTGTCTCAGAGCCATGTCTGAAAATACAGATTTTTTTTCGAAAATTT
CAAAAACTTTGTAGAAATTCGATTTTTGACATAAATGATCGCTCTTTTTTTTCTGACATGTATTTCAGTGACTACTGGA
ATCGAAA
ATATAATGCTCGTAATTATTAATAAATGTGTGTTGTCTCAGAGCCATGCCTGAAAATACAGATTTTTTTTCGAAAATT
TCAAAAACTTTTTAGAAATTCGATTTTTGACATAAATGATCGCTCTTTTTTTCTGACATGTATTTCAGTGACTACTGGA
ATCGAAA
ATATAATGCTCGTAATTATTTATAAGTGTGTGTTGTCTCAGAGCCATGCCTGAAAATACAGATTTTTTTTCGAAAATTT
CAAAAACTTTGTAGAAATTCGATTTTTGACATAAATGATCGCTCTTTTTTTCTGACATGTATTTCAGTGACTACTGGAA
TCGAAC
ATATAATGCTCGTAATTATTAATAAATGTGTGTTGTCTCAGAGCCATGCCTGAAAATACAGATTTTTTTTCGAAAATTT
CAAAAACTTTGTAGAAATTCGATTTTTGACATAAATGATCGCTCTTTTTTTCTGACATGTATTTCAGTGACTACTGGAA
TCGAAA

ATATAATGCTCGTAATTATTTATAAGTGTGTGTTGCCTCAGAGCCACGTCTGAAAATACAGATTTTTTCTCGAAAATTT
CAAAAATTTTGTAGAAATTCGATTTTTGACATAAATGATCTCTCTTTTTTTCTGACATGTATTTCAGTGACTACTGGAA
TCGAAA
Nr of selected repeats 68 Similarity 0.967912
Consensus:
ATATAATGCTCGTAATTATTTATAAGTGTGTGTTGTCTCAGAGCCACGTCTGAAAATACAGATTTTTTCTCGAAAATTT
CAAAAACTTTGTAGAAATTCGATTTTTGACATAAATGATCgCTCTTTTTTTCTGACATGTATTTCAGTGACAAaTggAn
TAGAAA
>Cele-UNSB01_3:495426-496459 Satlength=1034 Nr of Repeats=16 RepeatLength=31
seed=TTTAGCAGAC
TTTAGCAGACCAAAGTTGGGTATGCTAAATA
TTTAGCAGACCCAAACCAATA
TTTAGCAGACCAAAGTTGGGTATGCTAAATA
TTTAGCAGACCAATGCCAATA
TTTAGCAGACCCAAGTTAGGTATGCTAAATA
TTTAGCAGACCCAAGTTGGGTATGCTAAATA
TTTAGCAGACCAAAGTTGGGTCTGCTAAATA
TTTAGCAGACCATTGCCAATA
TTTAGCAGACCCAAGTTAGGTATGCTAAATA
TTTAGCAGACCAATGCCCATA
TTTAGCAGACCCAAGTTAGGTATGCTAAATA
TTTAGCAGACCAAAGTTGGGTCTGCTAAATA
TTTAGCAGACCAAAGTTGGGTCTGCTAAATA
TTTAGCAGACCAAAGTTGGGTGTGCTAAATA
TTTAGCAGACCCAAGCCAAT
TTTAGCAGACCAATAGAATTTTATAAAAGTGGAAATTTGAAAAAATTGTCAATTTTACTAGCCAATTTTTCCAAAATTT
TGAAAATTTCAATAGAATTTCCGAACAAAAACCGTCGATCCTCACAATTTTCACAGCGACCCCATCATCCTCGTCAACT
TCGAACGGAACGTCTGGAAGCTGCGGAGCAAACCGCCCGGCTGAAATTTCCTGCGCCGCCCATAATACTCCCTGTACAC
GTGGCAAATTTAGGAGCTCAACGAGTTGCTGAACCGACGAAGATTTGATATTTGAAATGTCCCAGTCGTCGAGAACGTT
CTGTGATAGTTCTTGAAATGAGATCTCTTGTTTCTGGTCGTTTGTTGAGAAATTCCGCAGAATTGATACGGCTTTTTGT
TGCTTTTTCGTTAAACTTGTTCGTGGAGGCCTTTCGTCGTCTGGAATTTCAGTAAATTGGTGTTTATTGCCACTTTTTT
TTTCAAAAGTAGCTAAAATTTGCTTAAAAACCATAAAAAACTTGATTTTTTAAGATTAAAAATGTTTAAACTAGCTTA
TTTATCTACTTAAAATTCTAAAAACCGTATTTTCAAAAAAAAAGTTATATTTGGGTCTGCGAAATA
Nr of selected repeats 10 Similarity 0.931183
Consensus:
TTTAGCAGACCaAAGTTgGGTaTGCTAAATA
>Cele-UNSB01_3:503013-503261 Satlength=249 Nr of Repeats=8 RepeatLength=31
seed=TTGGGTATGC
TTGGGTATGCTAAATATTTAGCAGACCAAGG
TTGGGTATGCTAAATATTTAGCAGACCAAAG
TTGGGTATGCTAAATATTTAGCAGACCAAGG
TTGGGTATGCTAAATATTTAGCAGACCAAAG
TTGGGTATGCTAAATATTTAGCAGACCAAGG
TTGGGTATGCTAAATATTTAGCAGACCAAAG
TTGGGTATGCTAAATATTTAGCAGACCAAAG
TTGGGTATGCTAAATATTTAGCAGACCAATG
Nr of selected repeats 8 Similarity 0.970814
Consensus:
TTGGGTATGCTAAATATTTAGCAGACCAAaG
>Cele-UNSB01_3:506857-507105 Satlength=249 Nr of Repeats=8 RepeatLength=31
seed=GTATGCTAAA
GTATGCTAAATATTTAGCAGACCAAAGTTGG
GTATGCTAAATATTTAGCAGACCAAAGTTGG
GTATGCTAAATATTTAGCAGACCAAAGTTGA

GTATGCTAAATATTTAGCAGACCAAAGTTGG
GTATGCTAAATATTTAGCAGACCAAAGTTAG
GTATGCTAAATATTTAGCAGACCAAAATTGG
GTATGCTAAATATTTAGCAGACCAAAGTTGG
GTATGCTAAATATTTAGCAGACCAAAGTTGG
Nr of selected repeats 8 Similarity 0.967742
Consensus:
GTATGCTAAATATTTAGCAGACCAAAGTTGG
>Cele-UNSB01_3:516006-516440 Satlength=435 Nr of Repeats=14 RepeatLength=31
seed=GGTCTGCTAA
GGTCTGCTAAATATTTATCATACCCAACTTT
GGTCTGCTAAATATTTAGCATACCCAACTTT
GGTCTGCTAAATATTTAGCATACCCAACTTT
GGTCTGCTAAATATTTAGTAGACCCAACTTT
GGTCTGCTAAATATTTAGCATGCCCAACTTT
GGTCTGCTAAATATTTAGCATACCCAACTTT
GGTCTGCTAAATATTTATCATACCCAACTTT
GGTCTGCTAAATATTTAGCATACCCAACTTT
GGTCTGCTAAATATTTAGCATACCCAACTTT
GGTCTGCTAAATATTTAGTAGACCCAACTTT
GGTCTGCTAAATATTTAGCATGCCCAACTTT
GGTCTGCTAAATATTTAGCATACCCAACTTT
GGTCTGCTAAATATTTAGCATACCCAACTTT
GGTCTGCTAAATATTTAGCATACCCAACTTT
Nr of selected repeats 14 Similarity 0.954626
Consensus:
GGTCTGCTAAATATTTAGCATACCCAACTTT
>Cele-UNSB01_3:517798-519152 Satlength=1355 Nr of Repeats=34 RepeatLength=31
seed=TTAGCAGACC
TTAGCAGACCCAACCTCCCGAAACTGATTTTTAAAGCTAAAAATTATTAGAATTTTCAGACGTTTCTCTCCGAAATATA
GCATGCCAATAGAAAAATTGACAAAAATCAAGGTTTTCTCATCGAAAATAGCAAAATAACTTTTAAAAATCTACAGAAA
CTCACAATTTTTGTTTAAAATGCCAACATTTATAAATTTTCTAAATATTTAGTTTTAAATTTGTAATTTTCTCACTTTT
TTCTGTGAAAAAATATGCAAAATGAAACTGAAAAATCCACTAAAACATGTTTAAAAATTTATCTTGAAGAAAAACGTAG
GAAATTTCAAATTTGCAGCTAAAAAATCAGCGAAAAAATTTTTTTTTCAAAATTTGAGGAGGTTGGGTATGCTAAATAT
TTAGCAGACCCAAGGAAAAATAT
TTAGCAGACCAAAGATAGGTATGCTAAATAC
TTAGCAGACCAAAGATAGGTATGCTAAATAT
TTAGCAGACCAAAGAAGGGTATGCTAAATAT
TTAGCAGACCCAAGGTGGGTATGCTAAATAT
TTAGCAGACCCAAGGAAAAATAT
TTAGCAGACCAAAGATAGGTATGCTAAATAT
TTAGCAGACCAAAGATGGATATGCTAAATAT
TTAGCAGACCAAAGATGGGTATGCTAAATAA
TTAGCAGACCAAAGATGGGTATGCTAAATAA
TTAGCAGACCCAAAGAAAAATAT
TTAGCAGACCAAAGTTGGGTATGCTAAATAA
TTAGCAGACCAAAGTTGGGTATGCTAAATAT
TTAGCAGACCAAAGTTGGGTATGCTAAATAT
TTAGCAGACCAAAGATGGGTATGCTAAATAA
TTAGCAGACCAAAGTTGGGTATGCTAAATAT
TTAGCAGACCCAAGGAAAAATAT
TTAGCAGACCAAAGATGGGTATGCTAAATAT
TTAGCAGACCCAAGGAAAAATAT
TTAGCAGACCAAAGATAGGTATGCTAAACAT

TTAGCAGACCCAAGGAAAAATAT
TTAGCAGACCAAAGATAGGTATGCTAAATAT
TTAGCAGACCAAAGATGGATATGCTAAATAT
TTAGCAGACCAAAGATGGGTATGCTAAATAA
TTAGCAGACCCAAAGAAAAATAT
TTAGCAGACCAAAGATAGGTATGCTAAATAT
TTAGCAGACCAAAGATGGATATGCTAAATAT
TTAGCAGACCAAAGATGGGTATGCTAAATAA
TTAGCAGACCAAAGTTGGGTATGCTAAATAT
TTAGCAGACCCAAAGAAAAATAT
TTAGCAGACCAAAGATAGGTATGCTAAATAT
TTAGCAGACCAAAGATGGGTATGCTAAATAT
TTAGCAGACCAAAGTTGGGTATGCTAAATAT
Nr of selected repeats 25 Similarity 0.924301
Consensus:
TTAGCAGACCAAAGaTgGGTATGCTAAATAt
>Cele-UNSB01_3:523916-525062 Satlength=1147 Nr of Repeats=37 RepeatLength=31
seed=GGTCTGCTAA
GGTCTGCTAAATATTTAGCATACACATTTTT
GGTCTGCTAATTATTCAGCATACCCAAATTT
GGTCTGCTAAATATTTAGCATACCCAACATT
GGTCTGCTAATTGTTTAGCATACCAAAATGT
GGTCTGCTAAAAATATTTAGCATACCCAACTTTA
GTCTGCTAAATATTTAGCATACCCAAATTT
GGTCTGCTAAAAATATTTAGCATACCGAACTTT
GGTCTGCTAATTATTTAGCATACCCAACTTT
GGTCTGCTAATTATTTAGCCTACCGAACTTT
GGTCTGCTAATTATTTAGCGTACCCAACTTT
GGTCTGCTAATTATTTAGCATACCCAAATTT
GGTCTGCTAAAAATATTTAGCATACCGAACTTT
GGTCTGCTAAATATTTAGCATACCCATTTTT
GGTCTGCTAAATATTTAGCACACCAAACTTT
GGTCTGCTAATTATTTAGCATACCTAACTTG
GGTCTGCTATATATTTAGCATACCCAACTTG
GGTCTGCTATATATTTAGCATACCCAACTTT
GGTCTGCTAATTATTTAGCATACCGAACTTT
GGTCTGCTAATTATTTAGCATACCCAACTTT
GGTCTGCTAATTATTTAGCCTACCGAACTTT
GGTCTGCTAATTATTTAGCGTACCCAACTTT
GGTCTGCTAATTATTTAGCATACCCAAATTT
GGTCTGCTAAAAATATTTAGCATACCGAACTTT
GGTCTGCTAAATATTTAGCATACCCATTTTT
GGTCTGCTAAATATTTAGCACACCAAACTTT
GGTCTGCTAATTATTTAGCATACCTAACTTG
GGTCTGCTATATATTTAGCATACCCAACTTG
GGTCTGCTATATATTTAGCATACCCAACTTT
GGTCTGCTAATTATTTAGCATACCCAACTTT
GGTCTGCTAAATATTTAGCATACCCATTTTT
GGTCTGCTAAATATTTAGCACACCCAACTTT
GGTCTGCTAATTATTTAGCATACCTAACTTG
GGTCTGCTATATATTTAGCATACCCAACTTG
GGTCTGCTATATATTTAGCATACCCAACTTT
GGTCTGCTAATTATTTAGCATACCCAACTTT
GGTCTGCTAAATATTTAGCATACCCATTTTT

GGTCTGCTAAATATTCAAATAT
Nr of selected repeats 31 Similarity 0.865048
Consensus:
GGTCTGCTAAtTATTTAGCATACCcAAcTTT
>Cele-UNSB01_3:546017-546152 Satlength=136 Nr of Repeats=6 RepeatLength=11
seed=CTACAGTACC
CTACAGTACCC
CTAGAGTACCAC
CACAGTACCTTGACATTATCCCCCACTAACTTCCAATCTAATACCTCTTCAAACGCTAAAAAATTCAAATTTTCCCCAA
CTACAGTAACC
CTACAGTACCA
CTACAGTACCA
Nr of selected repeats 4 Similarity 0.858586
Consensus:
CTACAGTAcCa
>Cele-UNSB01_3:611245-611586 Satlength=342 Nr of Repeats=11 RepeatLength=31
seed=GGTCTGCTAA
GGTCTGCTAATTATTTAGCATACCCAACTTC
GGTCTGCTAATTATTTAGCATACCGAACTTC
GGTCTGCTAATTATTTAGCATACCCAACTTC
GGTCTGCTAATTATTTAGCATACCGAACTTC
GGTCTGCTAATTATTTAGCATACCCAACTTC
GGTCTGCTAATTATTTAGCATACCCAACTTC
GGTCTGCTAATTATTTAGCATACCGAACTTC
GGTCTGCTAATTATTTAGCATACCGAACTTC
GGTCTGCTAATTATTTAGCATACCCAACTTC
GGTCTGCTAATTATTTAGCATACCGAACTTC
GGTCTGCTAATTATTTAGCATACCCAACTTT
Nr of selected repeats 11 Similarity 0.968719
Consensus:
GGTCTGCTAATTATTTAGCATACCcAACTTC
>Cele-UNSB01_3:614340-614495 Satlength=156 Nr of Repeats=5 RepeatLength=31
seed=TGGGTATGCT
TGGGTATGCTAAATAATTAGCAGACCAAAGT
TGGGTATGCTAAATAATTAGCAGACCAAAGT
TGGGTATGCTAAATAATTAGCAGACCAAAGT
TGGGTATGCTAAATAATTAGCAGACCAAAGT
TGGGTATGCTAAATAATTAGCAGACCAAAGT
Nr of selected repeats 5 Similarity 1.000000
Consensus:
TGGGTATGCTAAATAATTAGCAGACCAAAGT
>Cele-UNSB01_3:660202-660394 Satlength=193 Nr of Repeats=6 RepeatLength=31
seed=TTAGCAGACC
TTAGCAGACCAATGAGGATAGGTCTGCTAAATAT
TTAGCAGACTCATGGTAGGTATGCTAAATAT
TTAGCAGACCCATAGGTGGTCTGCTAAATAA
TTAGCAGACCCATAGTTGGTCTGCTAAATAA
TTAGCAGACCAATGGGGATGGGTCTGCTAAATAT
TTAGCAGACCCAAGTTAGGTATGCTAAATAT
Nr of selected repeats 4 Similarity 0.781250
Consensus:
TTAGCAGACcCAtaGgTaGGTaTGCTAAATAa
>Cele-UNSB01_3:662174-662284 Satlength=111 Nr of Repeats=6 RepeatLength=18
seed=TAGGCTTAGG

TAGGCTTAGGTGTGAGCT
TAGTCTTAGGCTAACCTT
TAGGCTTAAGCTTAGGTG
TAGGCTTAGGTT
TAGGCCTAGGCTTATGCG
TAGGCTTAGGCTTGTGTCTCCAGGCA
Nr of selected repeats 4 Similarity 0.527778
Consensus:
TAGgCtTAgGcTtAgctg
>Cele-UNSB01_3:665955-666358 Satlength=404 Nr of Repeats=13 RepeatLength=31
seed=TGGGTCTGCT
TGGGTCTGCTAAATATTTAGCAGACCAACTA
TGGGTCTGCTAAATATTTAGCAGACCAACTA
TGGGTCTGCTAAATATTCAGCAGACCAACTG
TGGGTCTGCTAAATATTTAGCATACCTATCG
TGGGTCTGCTAAATATTTAGCAGACCAACTG
TGGGTCTGCTAAATATTTAGCAGACCAACTA
TGGGTCTGCTAGATATTCAGTAGACCAACTG
TGGGTCTGCTAAATATTCAGCAGACCAACTG
TGGGTCTGCTAAATATTTAGCAGACCAACTG
TGGGTCTGCTAAATATTTAGCAGACCAACTG
TGGGTCTGCTAAATATTTAGCAGACCAACTA
TGGGTCTGCTAGATATTCAGCAGACCAACTG
TGGGTCTGCTAAATATTTAGCAGACCAACTA
Nr of selected repeats 13 Similarity 0.912876
Consensus:
TGGGTCTGCTAAATATTtAGCAGACCAACTg
>Cele-UNSB01_3:727287-727380 Satlength=94 Nr of Repeats=5 RepeatLength=15
seed=GGGGGCAACA
GGGGGCAACAAAAAA
GGGGGCAACATTTGAACTCTCCCT
GGGGGCAACAAATTA
GGGGGCAACATTTTCATAAATGTA
GGGGGCAGCAAAAAA
Nr of selected repeats 3 Similarity 0.822222
Consensus:
GGGGGCAaCAAAaaA
>Cele-UNSB01_3:731921-732069 Satlength=149 Nr of Repeats=8 RepeatLength=15
seed=TGTTGCCCCC
TGTTGCCCCCTAATT
TGTTGCCCCCGGGGAGAGTTCAAA
TGTTGACCCCTATTA
TGTTGCCCCCTTTTT
TGCTGCCCCCTACATTTATGAAAA
TGTTGCCCCCTAATT
TGTTGCCCCCCAGGGAGAGTTCAAA
TGTTGTCCCCTTTTT
Nr of selected repeats 5 Similarity 0.795556
Consensus:
TGTTGcCCCCTatTt
>Cele-UNSB01_3:882434-882682 Satlength=249 Nr of Repeats=7 RepeatLength=31
seed=TAAATATTTA
TAAATATTTAGCAGACCAAAGTTAGGTATGC
TAAATATTTAGTAGACCAAAGTTGGGTATGC

TAAATATTTAGCAGGCCAAAGTTAGGTATGA
TAAATATTTAGCAGACCACAGTTAGGTATGCTATATGTTTAGCAGACCAAAGTTGGGTATGC
TAAATATTTAGCAGACCAAAATTAGGTATGC
TATATATTTAACACACCAAAGTTAGGTATCC
TAAATATTTAGCAGACCAAAGTTGGGTATGC
Nr of selected repeats 6 Similarity 0.862366
Consensus:
TAAATATTTAGCAGACCAAAGTTaGGTATGC
>Cele-UNSB01_3:949283-949630 Satlength=348 Nr of Repeats=8 RepeatLength=18
seed=TGCAAGACTA
TGCAAGACTAATAGAGAC
TGCAAGACTATTAGAAGC
TGCAAGACTAATTTTCGATAGCCAAATAATTTTGGAACAGTTATAAACTTTTGTTGAAAACTTAACTTAATACCGTTTA
AGTCATAAAAAATTATTTATTTTCCAATATTTCATGAGCTATTTTATCATTTTCAAGTTCATTTTATCCAAAAAGTTAC
AAAATTTTTAAAGTTTCCTCATAGTATTGCTATCTAGGCAAAAATAGGAG
TGCAAGACTATTGGAGAC
TGCAATACTAATAGAGAC
TGCAAGACTATTTTTTGATAGACCTCTGGGGG
TGCATGACTAATAGGGG
TGCAAGACTAATAGAGGC
Nr of selected repeats 5 Similarity 0.822222
Consensus:
TGCAAgACTAaTaGAgaC
>Cele-UNSB01_3:949715-950024 Satlength=310 Nr of Repeats=5 RepeatLength=18
seed=TAGTGCTGCA
TAGTGCTGCACCAACAGGCCTTCCGAAAAT
TAGTGCTGCAGTCTCTAT
TAGTGCTGCACCCCATTTTCTACTGAAAGAGTGACTCAATAGTTTTGACATTTTGGGGAGTGTTTTTGTATATTTTTGA
AGATTTCTAGTTAATTTACAAATATAATTGGATTATTGGGTCATCTTTGTTACAAAATTTCACAATTTTAATGGATTTC
TGCTTAAAAGATGAGCATTTGTGACTAAAAACTAGTGATCTCTAATTGAAAAAAGCCGTTCGAAAAT
TAGTGCTGCAGCCTCCAG
TAGTACTGCAGTCTCTAA
Nr of selected repeats 3 Similarity 0.777778
Consensus:
TAGTgCTGCAGtCTCtAn
>Cele-UNSB01_3:958005-958109 Satlength=105 Nr of Repeats=4 RepeatLength=26
seed=CGGCCAGAGT
CGGCCAGAGTCACTTCTTATGGTGAA
CGGCCAGAGTCACTATATTTGGTGAA
CGGCCAGAGTCACTTCTTATGGTGAA
CGGCCAGAGTCACTAATTTTGGTGAA
Nr of selected repeats 4 Similarity 0.863248
Consensus:
CGGCCAGAGTCACTactTaTGGTGAA
>Cele-UNSB01_3:958146-965823 Satlength=7678 Nr of Repeats=284 RepeatLength=27
seed=AGAGAGTAAA
AGAGAGTAAAATATAGTGGGAAGCCAC
AGAGAGTAAAATATAGTGGGAAGCCAC
AGAGAGTAAAATATAGTGAGAAGCCAC
AGAGAGTAAAATATAGTGGGAAGCCAC
AGAGAGTAAAATATAGTGGGAAGCCAC
AGAGAGTAAAATATAGTGAGAAGCCAC
AGAGAGTAAAATATAGTGGGAAGCCAC
AGAGAGTAAAATATAGTGGGAAGCCAC

AGAGAGTAAAAAATAGTGGGAAGCCAC
AGAGAGTAAAATATAGTGGGAAGCCAC
AGAGAGTAAAATATAGTGGGAAGCCAC
AGAGAGTAAAATATAGTGGGAAGCCAC
AGAGAGTAAAATATAGTGAGAAGCCAC
AGAGAGTAAAATATAGTGGGAAGCCAC
AGAGAGTAAAATATAGTGAGAAGCCAC
AGAGAGTAAAATATAGTGGGAAGCCAC
AGAGAGTAAAATATAGTGGGAAGCCAC
AGAGAGTAAAATATATTGGGAAGCCAC
AGAGAGTAAAATATAGTGGGAAGCCAC
AGAGAGTAAAATATAGTGGGAAGCCAC
AGAGAGTAAAATATAGTGGGAAGCCAC
AGAGAGTAAAATATATTGGGAAGCCAC
AGAGAGTAAAATATAGTGAGAAGCCAC
AGAGAGTAAAATATAGTGAGAAGCCAC
AGAGAGTAAAATATAGTGGGAAGCCAC
AGAGAGTAAAATATAGTGAGAAGCCAC
AGAGAGTAAAATATATTGGGAAGCCAC
AGAGAGTAAAATATAGTGGGAAGCCAC
AGAGAGTAAAATATAGTGGGAAGCCAC
AGAGAGTAAAATATAGTGGGAAGCCAC
AGAGAGTAAAATATATTGGGAAGCCAC
AGAGAGTAAAATATAGTGGGAAGCCAC
AGAGAGTAAAATATAGTGGGAAGCCAC
AGAGAGTAAAATATAGTGGGAAGCCAC
AGAGAGTAAAATATAGTGGGAAGCCAC
AGAGAGTAAAATATAGTGGGAAGCCAC
AGAGAGTAAAATATAGTGGGAAGCCAC
AGAGAGTAAAAAATAGTGGGAAGCCAC
AGAGAGTAAAATATAGTGGGAAGCCAC
AGAGAGTAAAATATAGTGGGAAGCCAC
AGAGAGTAAAATATAGTGGGAAGCCAC
AGAGAGTAAAATATAGTGAGAAGCCAC
AGAGAGTAAAATATAGTGGGAAGCCAC
AGAGAGTAAAATATAGTGAGAAGCCAC
AGAGAGTAAAATATAGTGGGAAGCCAC
AGAGAGTAAAATATAGTGGGAAGCCAC
AGAGAGTAAAATATATTGGGAAGCCAC
AGAGAGTAAAATATAGTGGGAAGCCAC
AGAGAGTAAAATATAGTGGGAAGCCAC
AGAGAGTAAAATATAGTGGGAAGCCAC
AGAGAGTAAAATATATTGGGAAGCCAC
AGAGAGTAAAATATAGTGAGAAGCCAC
AGAGAGTAAAATATAGTGGGAAGCCAC
AGAGAGTAAAATATAGTGGGAAGCCAC
AGAGAGTAAAATATAGTGGGAAGCCAC
AGAGAGTAAAATATAGTGAGAAGCCAC
AGAGAGTAAAATATAGTGGGAAGCCAC
AGAGAGTAAAATATAGTGAGAAGCCAC
AGAGAGTAAAATATAGTGGGAAGCCAC
AGAGAGTAAAATATAGTGGGAAGCCAC
AGAGAGTAAAATATAGTGGGAAGCCAC
AGAGAGTAAAATATATTGGGAAGCCAC

AGAGAGTAAAATATAGTGAGAAGCCAC
AGAGAGTAAAATATAGTGGGAAGCCAC
AGAGAGTAAAATATAGTGGGAAGCCAC
AGAGAGTAAAATATATTGGGAAGCCAC
AGAGAGTAAAATATAGTGAGAAGCCAC
AGAGAGTAAAATATAGTGGGAAGCCAC
AGAGAGTAAAATATAGTGGGAAGCCAC
AGAGAGTAAAATATAGTGGGAAGCCAC
AGAGAGTAAAATATATTGGGAAGCCAC
AGAGAGTAAAATATATTGGGAAGCCAC
AGAGAGTAAAATATAGTGGGAAGCCAC
AGAGAGTAAAATATAGTGGGAAGCCAC
AGAGAGTAAAATATAGTGGGAAGCCAC
AGAGAGTAAAATATATTGGGAAGCCAC
AGAGAGTAAAATATAGTGGAAGCCAC
AGAGAGTAAAATATAGTGGGAAGCCAC
AGAGAGTAAAATATATTGGGAAGCCAC
AGAGAGTAAAATATATTGGGAAGCCAC
AGAGAGTAAAATATATTGGGAAGCCAC
AGAGAGTAAAATATATTGGGAAGCCAC
AGAGAGTAAAATATATTGGGAAGCCAC
AGAGAGTAAAATATATTGGGAAGCCAG
AGAGAGTAAAATATATTGGGAAGCCAC
AGAGAGTAAAATATAGTGGGAAGCCAC
AGAGAGTAAAATATAGTGGGAAGCCAC
AGAGAGTAAAATATAGTGAGAAGCCAC
AGAGAGTAAAATATAGTGGGAAGCCAC
AGAGAGTAAAATATAGTGGAGAAAGCCAC
AGAGAGTAAAATATATTGGGAAGCCAC
AGAGAGTAAAATATATTGGGAAGCCAC
AGAGAGTAAAATATAGTGGGAAGCCAC
AGAGAGTAAAATATATTGGGAAGCCAC
AGAGAGTAAAATATATTGGGAAGCCAC
AGAGAGTAAAATATATTGGGAAGCCAC
AGAGAGTAAAATATAGTGGGAAGCCAC
AGAGAGTAAAATATATTGGGAAGCCAC
AGAGAGTAAAATATATTGGGAAGCCAC
AGAGAGTAAAATATATTGGGAAGCCAC
AGAGAGTAAAATATATTGGGAAGCCAC
AGAGAGTAAAATATATTGGGAAGCCAC
AGAGAGTAAAATATATTGGGAAGCCAC
AGAGAGTAAAATATATTGGGAAGCCAC
AGAGAGTAAAATATATTGGGAAGCCAC
AGAGAGTAAAATATATTGGGAAGCCAC
AGAGAGTAAAATATAGTGGGAAGCCAC
AGAGAGTAAAATATATTGGGAAGCCAC
AGAGAGTAAAATATATTGGGAAGCCAC
AGAGAGTAAAATATATTGGGAAGCCAC
AGAGAGTAAAATATATTGGGAAGCCAC
AGAGAGTAAAATATATTGGGAAGCCAG
AGAGAGTAAAATATATTGGGAAGCCAC
AGAGAGTAAAATATAGTGGGAAGCCAC
AGAGAGTAAAATATAGTGGGAAGCCAC
AGAGAGTAAAATATATTGGGAAGCCAC

AGAGAGTAAAATATATTGGGAAGCCAC
AGAGAGTAAAATATAGTGGGAAGCCAC
AGAGAGTAAAATATATTGGGAAGCCAC
AGAGAGTAAAATATATTGGGAAGCCAC
AGAGAGTAAAATATATTGGGAAGCCAC
AGAGAGTAAAATATATTGGGAAGCCAC
AGAGAGTAAAATATATTGGGAAGCCAC
AGAGAGTAAAATATATTGGGAAGCCAC
AGAGAGTAAAATATATTGGGAAGCCAC
AGAGAGTAAAATCATCGGGGAAGCCAC
AGAGAGTAAAATATATTGGGAAGCCAC
AGAGAGTAAAATATATCGGGAAGCCAG
AGAGAGTAAAATATATTGGGAAGCCAC
AGAGAGTAAAATATATTGGGAAGCCAC
AGAGAGTAAAATATATTGGGAAGCCAC
AGAGAGTAAAATATATTGGGAAGCCAC
AGAGAGTAAAATATATTGGGAAGCCAC
AGAGAGTAAAATATATTGGGAAGCCAC
AGAGAGTAAAATATATTGGGAAGCCAC
AGAGAGTAAAATATATTGGGAAGCCAC
AGAGAGTAAAATATATTGGGAAGCCAC
AGAGAGTAAAATATATTGGGAAGCCAC
AGAGAGTAAAATATATTGGGAAGCCAC
AGAGAGTAAAATATATTGGGAAGCCAC
AGAGAGTAAAATATATTGGGAACCA
AGAGAGTAAAATATATGGGAAGCCAC
AGAGAGTAAAATATATTGGGAAGCCAC
AGAGAGTAAAATATATTGGGAAGCCAC
AGAGAGTAAAATATATTGGGAAGCCAC
AGAGAGTAAAATATATTGGGAAGCCAC
AGAGAGTAAAATATATTGGGAAGCCAC
AGAGAGTAAAATATATTGGGAAGCCAC
AGAGAGTAAAAATATATTGGGAAGCCAC
AGAGAGTAAAATATATTGGGAAGCCAC
AGAGAGTAAAATATATTGGGAAGCCAC
AGAGAGTAAAATATATTGGGAAGCCAC
AGAGAGTAAAATATAGTGGGAAGCCAC
AGAGAGTAAAAAAAAAAAAAAAAATATATGGAAGCCAC
AGAGAGTAAAATATATTGGGAAGCCAC
AGAGAGTAAAATATATTGGGAAGCCAC
AGAGAGTAAAATATAGTGGGAAGCCAC
AGAGAGTAAAATATATTGGGAAGCCAC
AGAGAGTAAAATATATTGGGAAGCCAC
AGAGAGTAAAATATATGGAAGCCA
AGAGAGTAAAAAAAAAAAAAATAGGGAAGCCAC
AGAGAGTAAAATATATTGGGAAGCCAC
AGAGAGTAAAATATATTGGGAAGCCAC
AGAGAGTAAAATATATTGGGAAGCCAC
AGAGAGTAAAATATATTGGGAAGCCAC
AGAGAGTAAAATATATTGGGAAGCCAC
AGAGAGTAAAATATATTGGGAAGCCAC
AGAGAGTAAAATATATGGGAAGCCAC
AGAGAGTAAAATATATTGGGAAGCCAC
AGAGAGTAAAATATATTGGGAAGCCAC

AGAGAGTAAAATATATGGGAAGCCAC
AGAGAGTAAAATATATTGGGAAGCCAC
AGAGAGTAAAATATATTGGGAAGCCAC
AGAGAGTAAAATATATTGGGAAGCCAC
AGAGAGTAAAATATATTGGGAAGCCAC
AGAGAGTAAAATATATTGGGAAGCCAC
AGAGAGTAAAATATATTGGGAAGCCAC
AGAGAGTAAAATATATTGGGAAGCCAC
AGAGAGTAAAATATATTGGGAAGCCAC
AGAGAGTAAAATATATTGGGAAGCCAC
AGAGAGTAAAATATATGGGAAGCCAC
AGAGAGTAAAAAAAAAAAAAATATATTGGGAAGCCAC
AGAGAGTAAAATATATTGGGAAGCCAC
AGAGAGTAAAATATATTGGGAAGCCAC
AGAGAGTAAAATATATTGGGAAGCCAC
AGAGAGTAAAATATATTGGGAAGCCAC
AGAGAGTAAAATATATGGGAAGCCAC
AGAGAGTAAAATATATGGGAAGCCAC
AGAGAGTAAAATATATTGGGAAGCCAC
AGAGAGTAAAATATATTGGGAAGCCAC
AGAGAGTAAAATATATTGGGAAGCCAC
AGAGAGTAAAATATATTGGGAAGCCAC
AGAGAGTAAAATATAGTGGGAAGCCAC
AGAGAGTAAAATATATTGGGAAGCCAC
AGAGAGTAAAATATATTGGGAAGCCAC
AGAGAGTAAAATATAGTGGGAAGCCAC
AGAGAGTAAAATATATTGGGAAGCCAC
AGAGAGTAAAATATATTGGGAAGCCAC
AGAGAGTAAAATATATTGGGAAGCCAC
AGAGAGTAAAATATATTGGGAAGCCAC
AGAGAGTAAAATATATTGGGAAGCCAC
AGAGAGTAAAATATATTGGGAAGCCAG
AGAGAGTAAAATATATTGGAAGCCAC
AGAGAGTAAAATATATTGGGAAGCCAC
AGAGAGTAAAATATAGTGGGAAGCCAG
AGAGAGTAAAATATATGGGAAGCCAC
AGAGAGTAAAATATAGTGGGAAGCCAC
AGAGAGTAAAATATATTGGGAAGCCAC
AGAGAGTAAAATATAATGGGAAGCCA
AGAGAGTAAAATATAAAAAAAAAAATGGGAAGCCAC
AGAGAGTAAAATATATTGGGAAGCCAG
AGAGAGTAAAATATAGTGGGAAGCCAC
AGAGAGTAAAATATAGTGGGAAGCCAG
AGAGAGTAAAATATATTGGGAAGCCAC
AGAGAGTAAAATATATGGGAAGCCAC
AGAGAGTAAAATATAGTGGGAAGCCAC
AGAGAGTAAAATATATGGGAAGCCAG
AGAGAGTAAAATATAGTGGGAAGCCAC
AGAGAGTAAAATATAGTGGGAAGCCAC
AGAGAGTAAAATATAGTGGGAAGCCAC
AGAGAGTAAAATATACGGGGAAGCCA
AGAGAGTAAAATATATGGGAAGCCAC
AGAGAGTAAAATATATGGGAAGCCAC
AGAGAGTAAAATATAGTGGAAGCCA

AGAGAGTAAAATATAGTGGGAAGCCAC
AGAGAGTAAAATATATTGGGAAGCCAC
AGAGAGTAAAATATATGGGAAGCCAC
AGAGAGTAAAATATATTGGGAAGCCAC
AGAGAGTAAAATATATTGGGAAGCCAC
AGAGAGTAAAATATATTGGGAAGCCAC
AGAGAGTAAAATATATGGGAAGCCAC
AGAGAGTAAAATATATTGGAAGCCAC
AGAGAGTAAAATATATTGGGAAGCCAC
AGAGAGTAAAATATATGGGAAGCCAG
AGAGAGTAAAATATAGTGGGAAGCCAC
AGAGAGTAAAATATATTGGGAAGCCAG
AGAGAGTAAAATATAGTGGGAAGCCAG
AGAGAGTAAAATATAGTGGGAAGCCAG
AGAGAGTTAAATATGGTGAGAAGCCAC
AGAGAGTAAAATATATTGGGAAGCCAC
AGAGAGTAAAATATAGTGGGAAGCCAG
AGAGAGTAAAATATAGTGGGAAGCCAG
AGAGAGTAAAATATAGTGGGAAGCCAC
AGAGAGTAAAATATATTGGGAAGCCAC
AGAGAGTAAAATATATTGGGAAGCCAG
AGAGAGTAAAATATAGTGGAAGCCAC
AGAGAGTAAAATATAGTGGGAAGCCAC
AGAGAGTAAAATATATTGGGGAAGATAGCCAG
AGAGAGTAAAATATAGTGGGAAGCCAG
AGAGAGTAAAATATAGTGGGAAGCCAC
AGAGAGTAAAATATATTGGGAAGCCAC
AGAGAGTAAAATATATTGGGAAGCCAC
AGAGAGTAAAATATAGTGGGAAGCCAC
AGAGAGTAAAATATAGTGGGAAGCCAC
AGAGAGTAAAATATAGTGGGAAGCCAC
AGAGAGTAAAATATATTGGGAAGCCAG
AGAGAGTAAAATATAGTGGGAAGCCAA
AGAGAGTAAAATATATGGAAGAAAGA
AGAAGTAAAATATATTGGGAAGCCAG
AGAGAGTAAAATATAGTGGGAAGCCAC
AGAGAGTAAAATATAGTGGGAAGCCAC
AGAGAGTAAAATATATTGGGAAGCCAG
AGAGAGTAAAATATAGTGGGAAGCCAC
AGAGAGTAAAATATAGTGGGAAGCCAC
AGAGAGTAAAATATATTGGGAAGCCAC
AGAGAGTAAAATATATTGGGAAGCCAC
AGAGAGTAAAATATAGTGGGAAGCCAC
AGAGAGTAAAATAAAAAAAAAAATGGAAGCA
AGAGAGTAAAATATATGGGAAGCC
AAGAGTAAAATATGATGGGAAGCC
AGAGAGTAAAATATAGTGGGAAGCCAG
AGAGAGTAAAATATAGTGGGAAGCCA
AGAGAGTAAAATATAGTGGAACCA
AGAGAGTAAAATATAGTGGGAAGCCAC
AGAGAGTAAAATATATTGGGAAGCCAG
AGAGAGTAAAATATAGTGGGAAGCCAC
AGAGAGTAAAATATATGGGAAGCCAC
AGAGAGTAAAATATATTGGGAAGCCAG

AGAGAGTAAAATATATTGGGAAGCCAG
AGAGAGTAAAATATAGTGGGAAGCCAG
AGAGAGTAAAATATAGTGGGAAGCCAC
AGAGAGTAAAATATAGTGGGAAGCCAC
AGAGAGTAAAATATAGTGGGAAGCCAC
AGAGAGTAAAATATAGTGGGAAGCCAC
Nr of selected repeats 246 Similarity 0.923613
Consensus:
AGAGAGTAAAATATAtTGGGAAGCCAC
>Cele-UNSB01_3:958162-973687 Satlength=15526 Nr of Repeats=569 RepeatLength=27
seed=TGGGAAGCCA
TGGGAAGCCACAGAGAGTAAAATATAG
TGGGAAGCCACAGAGAGTAAAATATAG
TGAGAAGCCACAGAGAGTAAAATATAG
TGGGAAGCCACAGAGAGTAAAATATAG
TGGGAAGCCACAGAGAGTAAAATATAG
TGAGAAGCCACAGAGAGTAAAATATAG
TGGGAAGCCACAGAGAGTAAAATATAG
TGGGAAGCCACAGAGAGTAAAAAATAG
TGGGAAGCCACAGAGAGTAAAATATAG
TGGGAAGCCACAGAGAGTAAAATATAG
TGGGAAGCCACAGAGAGTAAAATATAG
TGGGAAGCCACAGAGAGTAAAATATAG
TGAGAAGCCACAGAGAGTAAAATATAG
TGGGAAGCCACAGAGAGTAAAATATAG
TGAGAAGCCACAGAGAGTAAAATATAG
TGGGAAGCCACAGAGAGTAAAATATAG
TGGGAAGCCACAGAGAGTAAAATATAT
TGGGAAGCCACAGAGAGTAAAATATAG
TGGGAAGCCACAGAGAGTAAAATATAG
TGGGAAGCCACAGAGAGTAAAATATAG
TGGGAAGCCACAGAGAGTAAAATATAT
TGGGAAGCCACAGAGAGTAAAATATAG
TGAGAAGCCACAGAGAGTAAAATATAG
TGAGAAGCCACAGAGAGTAAAATATAG
TGGGAAGCCACAGAGAGTAAAATATAG
TGAGAAGCCACAGAGAGTAAAATATAT
TGGGAAGCCACAGAGAGTAAAATATAG
TGGGAAGCCACAGAGAGTAAAATATAG
TGGGAAGCCACAGAGAGTAAAATATAG
TGGGAAGCCACAGAGAGTAAAATATAT
TGGGAAGCCACAGAGAGTAAAATATAG
TGGGAAGCCACAGAGAGTAAAATATAG
TGGGAAGCCACAGAGAGTAAAATATAG
TGGGAAGCCACAGAGAGTAAAATATAG
TGGGAAGCCACAGAGAGTAAAATATAG
TGGGAAGCCACAGAGAGTAAAATATAG
TGGGAAGCCACAGAGAGTAAAAAATAG
TGGGAAGCCACAGAGAGTAAAATATAG
TGGGAAGCCACAGAGAGTAAAATATAG
TGGGAAGCCACAGAGAGTAAAATATAG
TGGGAAGCCACAGAGAGTAAAATATAG
TGAGAAGCCACAGAGAGTAAAATATAG
TGGGAAGCCACAGAGAGTAAAATATAG

TGAGAAGCCACAGAGAGTAAAATATAG
TGGGAAGCCACAGAGAGTAAAATATAG
TGGGAAGCCACAGAGAGTAAAATATAT
TGGGAAGCCACAGAGAGTAAAATATAG
TGGGAAGCCACAGAGAGTAAAATATAG
TGGGAAGCCACAGAGAGTAAAATATAG
TGGGAAGCCACAGAGAGTAAAATATAT
TGGGAAGCCACAGAGAGTAAAATATAG
TGAGAAGCCACAGAGAGTAAAATATAG
TGGGAAGCCACAGAGAGTAAAATATAG
TGGGAAGCCACAGAGAGTAAAATATAG
TGGGAAGCCACAGAGAGTAAAATATAG
TGAGAAGCCACAGAGAGTAAAATATAG
TGGGAAGCCACAGAGAGTAAAATATAG
TGAGAAGCCACAGAGAGTAAAATATAG
TGGGAAGCCACAGAGAGTAAAATATAG
TGGGAAGCCACAGAGAGTAAAATATAG
TGGGAAGCCACAGAGAGTAAAATATAT
TGGGAAGCCACAGAGAGTAAAATATAG
TGAGAAGCCACAGAGAGTAAAATATAG
TGGGAAGCCACAGAGAGTAAAATATAG
TGGGAAGCCACAGAGAGTAAAATATAT
TGGGAAGCCACAGAGAGTAAAATATAG
TGAGAAGCCACAGAGAGTAAAATATAG
TGGGAAGCCACAGAGAGTAAAATATAG
TGGGAAGCCACAGAGAGTAAAATATAG
TGGGAAGCCACAGAGAGTAAAATATAT
TGGGAAGCCACAGAGAGTAAAATATAT
TGGGAAGCCACAGAGAGTAAAATATAG
TGGGAAGCCACAGAGAGTAAAATATAG
TGGGAAGCCACAGAGAGTAAAATATAG
TGGGAAGCCACAGAGAGTAAAATATAT
TGGGAAGCCACAGAGAGTAAAATATAG
TGGAAGCCACAGAGAGTAAAATATAG
TGGGAAGCCACAGAGAGTAAAATATAT
TGGGAAGCCACAGAGAGTAAAATATAT
TGGGAAGCCACAGAGAGTAAAATATAT
TGGGAAGCCACAGAGAGTAAAATATAT
TGGGAAGCCACAGAGAGTAAAATATAT
TGGGAAGCCACAGAGAGTAAAATATAT
TGGGAAGCCAGAGAGAGTAAAATATAT
TGGGAAGCCACAGAGAGTAAAATATAG
TGGGAAGCCACAGAGAGTAAAATATAG
TGGGAAGCCACAGAGAGTAAAATATAG
TGAGAAGCCACAGAGAGTAAAATATAG
TGGGAAGCCACAGAGAGTAAAATATAGTGGAGAAAGCCACAGAGAGTAAAATATAT
TGGGAAGCCACAGAGAGTAAAATATAT
TGGGAAGCCACAGAGAGTAAAATATAG
TGGGAAGCCACAGAGAGTAAAATATAT
TGGGAAGCCACAGAGAGTAAAATATAT
TGGGAAGCCACAGAGAGTAAAATATAT
TGGGAAGCCACAGAGAGTAAAATATAG
TGGGAAGCCACAGAGAGTAAAATATAT
TGGGAAGCCACAGAGAGTAAAATATAT

TGGGAAGCCACAGAGAGTAAAATATAT
TGGGAAGCCACAGAGAGTAAAATATAT
TGGGAAGCCACAGAGAGTAAAATATAT
TGGGAAGCCACAGAGAGTAAAATATAT
TGGGAAGCCACAGAGAGTAAAATATAT
TGGGAAGCCACAGAGAGTAAAATATAT
TGGGAAGCCACAGAGAGTAAAATATAT
TGGGAAGCCACAGAGAGTAAAATATAG
TGGGAAGCCACAGAGAGTAAAATATAT
TGGGAAGCCACAGAGAGTAAAATATAT
TGGGAAGCCACAGAGAGTAAAATATAT
TGGGAAGCCACAGAGAGTAAAATATAT
TGGGAAGCCACAGAGAGTAAAATATAT
TGGGAAGCCAGAGAGAGTAAAATATAT
TGGGAAGCCACAGAGAGTAAAATATAG
TGGGAAGCCACAGAGAGTAAAATATAG
TGGGAAGCCACAGAGAGTAAAATATAT
TGGGAAGCCACAGAGAGTAAAATATAT
TGGGAAGCCACAGAGAGTAAAATATAG
TGGGAAGCCACAGAGAGTAAAATATAT
TGGGAAGCCACAGAGAGTAAAATATAT
TGGGAAGCCACAGAGAGTAAAATATAT
TGGGAAGCCACAGAGAGTAAAATATAT
TGGGAAGCCACAGAGAGTAAAATATAT
TGGGAAGCCACAGAGAGTAAAATATAT
TGGGAAGCCACAGAGAGTAAAATATAT
TGGGAAGCCACAGAGAGTAAAATCATCG
GGGAAGCCACAGAGAGTAAAATATAT
TGGGAAGCCACAGAGAGTAAAATATATC
GGGAAGCCAGAGAGAGTAAAATATAT
TGGGAAGCCACAGAGAGTAAAATATAT
TGGGAAGCCACAGAGAGTAAAATATAT
TGGGAAGCCACAGAGAGTAAAATATAT
TGGGAAGCCACAGAGAGTAAAATATAT
TGGGAAGCCACAGAGAGTAAAATATAT
TGGGAAGCCACAGAGAGTAAAATATAT
TGGGAAGCCACAGAGAGTAAAATATAT
TGGGAAGCCACAGAGAGTAAAATATAT
TGGGAAGCCACAGAGAGTAAAATATAT
TGGGAAGCCACAGAGAGTAAAATATAT
TGGGAAGCCACAGAGAGTAAAATATAT
TGGGAAGCCACAGAGAGTAAAATATAT
TGGGAACCAAGAGAGTAAAATATA
TGGGAAGCCACAGAGAGTAAAATATAT
TGGGAAGCCACAGAGAGTAAAATATAT
TGGGAAGCCACAGAGAGTAAAATATAT
TGGGAAGCCACAGAGAGTAAAATATAT
TGGGAAGCCACAGAGAGTAAAATATAT
TGGGAAGCCACAGAGAGTAAAATATAT
TGGGAAGCCACAGAGAGTAAAAATATAT
TGGGAAGCCACAGAGAGTAAAATATAT
TGGGAAGCCACAGAGAGTAAAATATAT
TGGGAAGCCACAGAGAGTAAAATATAT
TGGGAAGCCACAGAGAGTAAAATATAG

TGGGAAGCCACAGAGAGTAAAAAAAAAAAAAAAATATA
TGGGAAGCCACAGAGAGTAAAATATAT
TGGGAAGCCACAGAGAGTAAAATATAT
TGGGAAGCCACAGAGAGTAAAATATAG
TGGGAAGCCACAGAGAGTAAAATATAT
TGGGAAGCCACAGAGAGTAAAATATAT
TGGGAAGCCACAGAGAGTAAAATATA
TGGAAGCCAAGAGAGTAAAAAAAAAAAAAATA
GGGAAGCCACAGAGAGTAAAATATAT
TGGGAAGCCACAGAGAGTAAAATATAT
TGGGAAGCCACAGAGAGTAAAATATAT
TGGGAAGCCACAGAGAGTAAAATATAT
TGGGAAGCCACAGAGAGTAAAATATAT
TGGGAAGCCACAGAGAGTAAAATATAT
TGGGAAGCCACAGAGAGTAAAATATA
TGGGAAGCCACAGAGAGTAAAATATAT
TGGGAAGCCACAGAGAGTAAAATATAT
TGGGAAGCCACAGAGAGTAAAATATA
TGGGAAGCCACAGAGAGTAAAATATAT
TGGGAAGCCACAGAGAGTAAAATATAT
TGGGAAGCCACAGAGAGTAAAATATAT
TGGGAAGCCACAGAGAGTAAAATATAT
TGGGAAGCCACAGAGAGTAAAATATAT
TGGGAAGCCACAGAGAGTAAAATATAT
TGGGAAGCCACAGAGAGTAAAATATAT
TGGGAAGCCACAGAGAGTAAAATATAT
TGGGAAGCCACAGAGAGTAAAATATAT
TGGGAAGCCACAGAGAGTAAAATATA
TGGGAAGCCACAGAGAGTAAAAAAAAAAAAAATATAT
TGGGAAGCCACAGAGAGTAAAATATAT
TGGGAAGCCACAGAGAGTAAAATATAT
TGGGAAGCCACAGAGAGTAAAATATAT
TGGGAAGCCACAGAGAGTAAAATATAT
TGGGAAGCCACAGAGAGTAAAATATA
TGGGAAGCCACAGAGAGTAAAATATA
TGGGAAGCCACAGAGAGTAAAATATAT
TGGGAAGCCACAGAGAGTAAAATATAT
TGGGAAGCCACAGAGAGTAAAATATAT
TGGGAAGCCACAGAGAGTAAAATATAT
TGGGAAGCCACAGAGAGTAAAATATAG
TGGGAAGCCACAGAGAGTAAAATATAT
TGGGAAGCCACAGAGAGTAAAATATAT
TGGGAAGCCACAGAGAGTAAAATATAG
TGGGAAGCCACAGAGAGTAAAATATAT
TGGGAAGCCACAGAGAGTAAAATATAT
TGGGAAGCCACAGAGAGTAAAATATAT
TGGGAAGCCACAGAGAGTAAAATATAT
TGGGAAGCCACAGAGAGTAAAATATAT
TGGGAAGCCACAGAGAGTAAAATATAT
TGGGAAGCCAGAGAGAGTAAAATATAT
TGGAAGCCACAGAGAGTAAAATATAT
TGGGAAGCCACAGAGAGTAAAATATAG
TGGGAAGCCAGAGAGAGTAAAATATA
TGGGAAGCCACAGAGAGTAAAATATAG

TGGGAAGCCACAGAGAGTAAAATATAT
TGGGAAGCCACAGAGAGTAAAATATAA
TGGGAAGCCAAGAGAGTAAAATATAAAAAAAAAAAAA
TGGGAAGCCACAGAGAGTAAAATATAT
TGGGAAGCCAGAGAGAGTAAAATATAG
TGGGAAGCCACAGAGAGTAAAATATAG
TGGGAAGCCAGAGAGAGTAAAATATAT
TGGGAAGCCACAGAGAGTAAAATATA
TGGGAAGCCACAGAGAGTAAAATATAG
TGGGAAGCCACAGAGAGTAAAATATA
TGGGAAGCCAGAGAGAGTAAAATATAG
TGGGAAGCCACAGAGAGTAAAATATAG
TGGGAAGCCACAGAGAGTAAAATATAG
TGGGAAGCCACAGAGAGTAAAATATACG
GGGAAGCCAAGAGAGTAAAATATA
TGGGAAGCCACAGAGAGTAAAATATA
TGGGAAGCCACAGAGAGTAAAATATAG
TGGAAGCCAAGAGAGTAAAATATAG
TGGGAAGCCACAGAGAGTAAAATATAT
TGGGAAGCCACAGAGAGTAAAATATA
TGGGAAGCCACAGAGAGTAAAATATAT
TGGGAAGCCACAGAGAGTAAAATATAT
TGGGAAGCCACAGAGAGTAAAATATAT
TGGGAAGCCACAGAGAGTAAAATATA
TGGGAAGCCACAGAGAGTAAAATATAT
TGGAAGCCACAGAGAGTAAAATATAT
TGGGAAGCCACAGAGAGTAAAATATA
TGGGAAGCCAGAGAGAGTAAAATATAG
TGGGAAGCCACAGAGAGTAAAATATAT
TGGGAAGCCAGAGAGAGTAAAATATAG
TGGGAAGCCAGAGAGAGTAAAATATAG
TGGGAAGCCAGAGAGAGTTAAATATGG
TGAGAAGCCACAGAGAGTAAAATATAT
TGGGAAGCCACAGAGAGTAAAATATAG
TGGGAAGCCAGAGAGAGTAAAATATAG
TGGGAAGCCAGAGAGAGTAAAATATAG
TGGGAAGCCACAGAGAGTAAAATATAT
TGGGAAGCCACAGAGAGTAAAATATAT
TGGGAAGCCAGAGAGAGTAAAATATAG
TGGAAGCCACAGAGAGTAAAATATAG
TGGGAAGCCACAGAGAGTAAAATATATTGGGGAAGATAGCCAGAGAGAGTAAAATATAG
TGGGAAGCCAGAGAGAGTAAAATATAG
TGGGAAGCCACAGAGAGTAAAATATAT
TGGGAAGCCACAGAGAGTAAAATATAT
TGGGAAGCCACAGAGAGTAAAATATAG
TGGGAAGCCACAGAGAGTAAAATATAG
TGGGAAGCCACAGAGAGTAAAATATAG
TGGGAAGCCACAGAGAGTAAAATATAT
TGGGAAGCCAGAGAGAGTAAAATATAG
TGGGAAGCCAAAGAGAGTAAAATATATGGAAGAAAGAAGAAGTAAAATATAT
TGGGAAGCCAGAGAGAGTAAAATATAG
TGGGAAGCCACAGAGAGTAAAATATAG
TGGGAAGCCACAGAGAGTAAAATATAT
TGGGAAGCCAGAGAGAGTAAAATATAG

TGGGAAGCCACAGAGAGTAAAATATAG
TGGGAAGCCACAGAGAGTAAAATATAT
TGGGAAGCCACAGAGAGTAAAATATAT
TGGGAAGCCACAGAGAGTAAAATATAG
TGGGAAGCCACAGAGAGTAAAATAAAAAAAAAAAATGGAAGCAAGAGAGTAAAATATA
TGGGAAGCCAAGAGTAAAATATGA
TGGGAAGCCAGAGAGTAAAATATAG
TGGGAAGCCAGAGAGAGTAAAATATAG
TGGGAAGCCAAGAGAGTAAAATATAGTGGAACCAAGAGAGTAAAATATAG
TGGGAAGCCACAGAGAGTAAAATATAT
TGGGAAGCCAGAGAGAGTAAAATATAG
TGGGAAGCCACAGAGAGTAAAATATA
TGGGAAGCCACAGAGAGTAAAATATAT
TGGGAAGCCAGAGAGAGTAAAATATAT
TGGGAAGCCAGAGAGAGTAAAATATAG
TGGGAAGCCAGAGAGAGTAAAATATAG
TGGGAAGCCACAGAGAGTAAAATATAG
TGGGAAGCCACAGAGAGTAAAATATAG
TGGGAAGCCACAGAGAGTAAAATATAG
TGGGAAGCCACAGAGAGTAAAATATAT
TGGGAAGCCACAGAGATTAAAATATAT
TGGGAAGCCACAGAGATTAAAATATAT
TGGGAAGCCACAGAGATTAAAATATAT
TGGGAAGCCACAGAGATTAAAATATAT
TGGGAAGCCACAGAGAGTAAAATATAT
TGGGAAGCCACAGAGAGTAAAATATAT
TGGGAAGCCACAGAGAGTAAAATATAT
TGGGAAGCCACAGAGAGTAAAATATAG
TGGGAAGCCACAGAGAGTAAATTATAG
TGGGAAGCCACAGAGAGTAAATTATAG
TGGGAAGCCAGAGAGAGTAAAATATAG
TGGGAAGCCACAGAGAGTAAATTATAG
TGGGAAGCCAGAGAGAGTAAAATATGG
TGAGAAGCCAGAGAGAGTAAAATATAT
TGGGAAGCCAGAGAGAGTAAAATATAG
TGGGAAGCCACAGAGAGTAAATTATAG
TGGGAAGCCAGAGAGAGTAAAATATGG
TGAGAAGCCACAGAGAGTAAAATATAT
TGGGAAGCCAGAGAGAGTAAAATATAG
TGGAAAGCCAGAGAGAGTAAAATATAT
TGGGAAGCCACAGAGATTAAAATATAT
TGGGAAGCCACAGAGATTAAAATATAT
TGGGAAGCCACAGAGATTATAATATAT
TGGGAAGCCACAGAGAGTAAAATATAT
TGGGAAGCCACAGAGAGTAAAATATAT
TGGGAAGCCACAGAGAGTAAAATATAT
TGGGAAGCCACAGAGATTAAAATATAT
TGGGAAGCCACAGAGATTAAAATATAT
TGGGAAGCCACAGAGAGTAAAATATAT
TGGGAAGCCACAGAGAGTAAAATATAT
TGGGAAGCCACAGAGAGTAAAATATAT
TGGGAAGCCACAGAGATTAAAATATAT
TGGGAAGCCACAGAGATTAAAATATAG
TGGGAAGCCAGAGAGAGTAAAATATAG

TGGGAAGCCACAGAGAGTAAAATATAT
TGGGAAGCCACAGAGAGTAAAATATAT
TGGGAAGCCACAGAGATTAAAATATAG
TGGGAAGCCAGAGAGAGAGTAAAATATAG
TGGGAAGCCAGAGAGAGAGTTAAATATGG
TGAGAAGCCACAGAGAGTAAAATATAT
TGGGAAGCCACAGAGATTAAAATATAT
TGGGAAGCCACAGAGATTAAAATATAT
TGGGAAGCCAGAGAGAGAGTAAAATATAG
TGGGAAGCCACAGAGAGTAAAATATAT
TGGGAAGCCACAGAGATTAAAATATAT
TGGGAAGCCACAGAGATTAAAATATAT
TGGGAAGCCACAGAGATTAAAATATAT
TGGGAAGCCACAGAGATTAAAATATAT
TGGGAAGCCACAGAGAGTAAAATATAT
TGGGAAGCCACAGAGAGTAAAATATAT
TGGGAAGCCACAGAGAGTAAAATATAT
TGGGAAGCCACAGAGATTAAAATATAT
TGGGAAGCCACAGAGATTAAAATATAT
TGGGAAGCCACAGAGAGTAAAATATAG
TGGGAAGCCAGAGAGAGAGTTAAATATGG
TGAGAAGCCACAGAGAGTAAAATATAG
TGGGAAGCCACAGAGAGTAAAATATAT
TGGGAAGCCACAGAGATTAAAATATAT
TGGGAAGCCACAGAGATTAAAATATAT
TGGGAAGCCACAGAGAGTAAAATATAG
TGGGAAGCCAGAGAGAGAGTTAAATATGG
TGAGAAGCCACAGAGAGTAAAATATAT
TGGGAAGCCACAGAGAGTAAAATATAT
TGGGAAGCCACAGAGAGTAAAATATAT
TGGGAAGCCACAGAGAGTAAAATATAG
TGGGAAGCCAGAGAGAGAGTAAAATATAG
TGGGAAGCCAGAGAGAGAGTAAAATATAG
TGGGAAGCCAGAGAGAGAGTAAAATATAT
TGGGAAGCCACAGAGATTAAAATATAT
TGGGAAGCCACAGAGATTAAAATATAT
TGGGAAGCCACAGAGAGTAAAATATAT
TGGGAAGCCACAGAGAGTAAAATATAT
TGGGAAGCCACAGAGAGTAAAATATAG
TGGGAAGCCAGAGAGAGAGTAAAATATAG
TGGGAAGCCAGAGAGAGAGTAAAATATAT
TGGGAAGCCACAGAGAGTAAAATATAG
TGGGAAGCCACAGAGAGTAAAATATAG
TGGGAAGCCAGAGAGAGAGTAAAATATAG
TGGGAAGCCAGAGAGAGAGTAAAATATAT
TGGGAAGCCACAGAGATTAAAATATAT
TGGGAAGCCACAGAGATTAAAATATAT
TGGGAAGCCACAGAGAGTAAAATATAT
TGGGAAGCCACAGAGAGTAAAATATAT
TGGGAAGCCACAGAGAGTAAAATATAG
TGGGAAGCCAGAGAGAGAGTAAAATATAG
TGGGAAGCCAGAGAGAGAGTAAAATATGG
TGAGAAGCCACAGAGAGTAAAATATAT
TGGGAAGCCAGAGAGAGAGTAAATTATAG

TGGGAAGCCACAGAGAGTAAATTATAG
TGGGAAGCCACAGAGAGTAAATTATAG
TGGGAAGCCAGAGAGAGTAAAATATGG
TGAGAAGCCAGAGAGAGTAAAATATAT
TGGGAAGCCAGAGAGAGTAAAATATAG
TGGGAAGCCACAGAGAGTAAATTATAG
TGGGAAGCCAGAGAGAGTAAAATATGG
TGAGAAGCCACAGAGAGTAAAATATAT
TGGGAAGCCAGAGAGAGTAAAATATAG
TGGAAAGCCACAGAGAGTAAAATATAG
TGGGAAGCCACAGAGATTAAAATATAT
TGGGAAGCCACAGAGATTAAAATATAG
TGGGAAGCCACAGAGATTAAAATATAT
TGGGAAGCCACAGAGATTAAAATATAG
TGGGAAGCCACAGAGATTAAAATATAT
TGGGAAGCCAGAGAGAGTAAAATATGG
TGAGAAGCCACAGAGAGTAAAATATAG
TGGGAAGCCACAGAGATTAAAATATAT
TGGGAAGCCACAGAGATTAAAATATAG
TGGGAAGCCACAGAGATTAAAATATAT
TGGGAAGCCACAGAGAGTAAAATATAG
TGGGAAGCCACAGAGATTAAAATATAT
TGGGAAGCCACAGAGATTAAAATATAG
TGGGGAGCCACAGAGAGTAAAATATAG
TGGGAAGCCACAGAGATTAAAATATAT
TGGGAAGCCACAGAGAGTAAAATATAG
TGGGAAGCCACAGAGATTAAAATATAT
TGGGAAGCCACAGAGATTAAAATATAG
TGGGAAGCCACAGAGAGTAAAATATAA
TGGGAAGCCACAGAGAGTAAAATATAG
TGGGAAGCCACAGAGATTAAAATATAT
TGGGAAGCCACAGAGAGTAAAATATAG
TGGGAAGCCACAGAGATTAAAATATAG
TGGGAAGCCACAGAGAGTAAAATATAT
TGGGAAGCCACAGAGAGTAAAATATAG
TGGGAAGCCACAGAGATTAAAATATAG
TGGGAAGCCAGAGAGAGTAAAATATAG
TGGGAAGCCACAGAGATTAAAATATAG
TGGGAAGCCACAGAGAGTAAAATATAG
TGGGAAGCCACAGAGATTAAAATATAG
TGGGAAGCCAGAGAGAGTAAAATATAG
TGGGAAGCCACAGAGATTAAAATATAG
TGGGAAGCCAGAGAGAGTAAAATATAG
TGGGAAGCCAGAGAGAGTAAAATATAG
TGGGAAGCCACAGAGATTAAAATATAG
TGGGAAGCCAGAGAGAGTAAAATATAG
TGGGAAGCCACAGAGATTAAAATATAG
TGGGAAGCCAGAGAGAGTAAAATATAG
TGGGAAGCCACAGAGATTAAAATATAG
TGGGAAGCCACAGAGATTAAAATATAG
TGGGAAGCCAGAGAGAGTAAAATATAG
TGGGAAGCCACAGAGATTAAAATATAG
TGGGAAGCCACAGAGAGTAAAATATAG
TGGGAAGCCACAGAGAGTAAAATATAT

TGGGAAGCCACAGAGATTAAAATATAT
TGGGAAGCCACAGAGAGTAAAATATAG
TGGGAAGCCACAGAGATTAAAATATAT
TGGGAAGCCACAGAGAGTAAAATATAG
TGGGAAGCCACAGAGATTAAAATATAG
TGGGAAGCCAGAGAGAGTAAAATATGG
TGAGAAGCCACAGAGAGTAAAATATAG
TGGGAAGCCACAGAGATTAAAATATAT
TGGGAAGCCACAGAGAGTAAAATATAG
TGGGAAGCCACAGAGATTAAAATATAG
TGGGAAGCCAGAGAGAGTAAAATATAG
TGGGAAGCCACAGAGATTAAAATATAG
TGGGAAGCCAGAGAGAGTAAAATATAG
TGGGAAGCCACAGAGATTAAAATATAG
TGGGAAGCCACAGAGATTAAAATATAG
TGGGAAGCCAGAGAGAGTAAAATATAG
TGGGAAGCCACAGAGATTAAAATATAG
TGGGAAGCCACAGAGAGTAAAATATAG
TGGGAAGCCACAGAGAGTAAAATATAT
TGGGAAGCCACAGAGATTAAAATATAT
TGGGAAGCCACAGAGAGTAAAATATAG
TGGGAAGCCACAGAGATTAAAATATAT
TGGGAAGCCACAGAGAGTAAAATATAG
TGGGAAGCCACAGAGATTAAAATATAG
TGGGAAGCCAGAGAGAGTAAAATATGG
TGAGAAGCCACAGAGAGTAAAATATAG
TGGGAAGCCACAGAGATTAAAATATAT
TGGGAAGCCACAGAGAGTAAAATATAG
TGGGAAGCCACAGAGATTAAAATATAG
TGGGAAGCCAGAGAGAGTAAAATATAG
TGGGAAGCCAGAGAGAGTAAAATATGG
TGAGAAGCCACAGAGAGTAAAATATAG
TGGGAAGCCACAGAGATTAAAATATAT
TGGGAAGCCACAGAGATTAAAATATAG
TGGGAAGCCACAGAGAGTAAAATATAG
TGGGAAGCCACAGAGAGTAAAATATAT
TGGGAAGCCACAGAGATTAAAATATAT
TGGGAAGCCACAGAGAGTAAAATATAG
TGGGAAGCCACAGAGATTAAAATATAT
TGGGAAGCCACAGAGAGTAAAATATAG
TGGGAAGCCAGAGAGAGTAAAATATGG
TGAGAAGCCACAGAGAGTAAAATATAG
TGGGAAGCCACAGAGATTAAAATATAG
TGGGAAGCCAGAGAGAGTAAAATATAG
TGGGAAGCCAGAGAGAGTAAAATATGG
TGAGAAGCCACAGAGAGTAAAATATAG
TGGGAAGCCACAGAGATTAAAATATAT
TGGGAAGCCACAGAGAGTAAAATATAG
TGGGAAGCCACAGAGATTAAAATATAT
TGGGAAGCCACAGAGAGTAAAATATAG
TGGGAAGCCACAGAGATTAAAATATAT
TGGGAAGCCACAGAGAGTAAAATATAG
TGGGAAGCCACAGAGATTAAAATATAG
TGGGAAGCCAGAGAGAGTAAAATATAG

TGGGAAGCCAGAGAGAGTAAAATATGG
TGAGAAGCCACAGAGAGTAAAATATAG
TGGGAAGCCACAGAGCGTAAAATATAT
TGGGAAGCCACAGAGATTAAAATATAT
TGGGAAGCCACAGAGAGTAAAATATAG
TGGGAAGCCACAGAGAGTAAAATATAG
TGGGAAGCCAGAGAGAGTAAAATATAG
TGGGAAGCCAGAGAGAGTAAAATATGG
TGAGAAGCCACAGAGAGTAAAATATAG
TGGGAAGCCACAGAGAGTAAAATATAT
TGGGAAGCCACAGAGATTAAAATATAT
TGGGAAGCCACAGAGAGTAAAATATAG
TGGGAAGCCACAGAGATTAAAATATAT
TGGGAAGCCACAGAGAGTAAAATATAG
TGGGAAGCCAGAGAGAGTAAAATATGG
TGAGAAGCCACAGAGAGTAAAATATAG
TGGGAAGCCACAGAGAGTAAAATATAG
TGGGAAGCCAGAGAGAGTAAAATATAG
TGGGAAGCCAGAGAGAGTAAAATATGG
TGAGAAGCCACAGAGAGTAAAATATAG
TGGGAAGCCACAGAGATTAAAATATAT
TGGGAAGCCACAGAGATTAAAATATAT
TGGGAAGCCACAGAGAGTAAAATATAG
TGGGAAGCCACAGAGATTAAAATATAT
TGGGAAGCCACAGAGAGTAAAATATAG
TGGGAAGCCACAGAGATTAAAATATAG
TGGGAAGCCAGAGAGAGTAAAATATAG
TGGGAAGCCAGAGAGAGTAAAATATGG
TGAGAAGCCACAGAGAGTAAAATATAG
TGGGAAGCCACAGAGAGTAAAATATAT
TGGGAAGCCACAGAGATTAAAATATAT
TGGGAAGCCACAGAGAGTAAAATATAG
TGGGAAGCCACAGAGAGTAAAATATGG
TGAGAAGCCACAGAGAGTAAAATATAG
TGGGAAGCCACAGAGAGTAAAATATAT
TGGGAAGCCACAGAGATTAAAATATAT
TGGGAAGCCAGAGAGAGTAAAATATAA
TGGGAAGCCACAGAGAGTAAAATATATAG
TGGGAAGCCAGAGAGAGTAAAATATGG
TGGGAAGCCACAGAGAGTAAAATATAA
TGGGAAGCCACAGAGAGTAAAATATAG
TGGGAAGCCACAGAGAGTAAAATATAG
TGGGAAGCCACAGAGAGTAAAATATAG
TGGGAAGCCACAGAGAGTAAAATATAG
TGGGAAGCCAGAGAGAGTAAAATATGG
TGGGAAGCCAGAGAGAGTAAAATATAA
TGGGAAGCCACAGAGAGTAAAATATATAG
TGGGAAGCCAGAGAGAGTAAAATATGG
TGGGAAGCCACAGAGAGTAAAATATAA
TGGGAAGCCACAGAGAGTAAAATATAA
TGGGAAGCCACAGAGAGTAAAATATAA
TGGGAAGCCAGAGAGAGTAAAATATAA
TGGGAAGCCACAGAGAGTAAAATATATAG
TGGGAAGCCAGAGAGAGTAAAATATGG

TGGGAAGCCACAGAGAGTAAAATATAA
TGGGAAGCCACAGAGAGTAAAATATAA
TGGGAAGCCACAGAGAGTAAAATATAA
TGGGAAGCCACAGAGAGTAAAATATAG
TGGGAAGCCACAGAGAGTAAAATATATAG
TGGGAAGCCAGAGAGAGTAAAATATGG
TGGGAAGCCACAGAGAGTAAAATATAG
TGGGAAGCCACAGAGAGTAAAGTATAA
TGGGAAGCCACAGAGAGTAAAATATAG
TCGGAAGCCACAGAGAGTAAAATATAG
TGGGAAGCCACAGAGAGTAAAATATAG
TCGGAAGCCACAGAGAGTAAAATATAG
TGGGAAGCCACAGAGAGTAAAATATAA
TGGGAAGCCACAGAGAGTAAAATATAG
TCGGAAGCCACAGAGAGTAAAATATAG
TGGGAAGCCACAGAGAGTAAAATATAA
TGGGAAGCCACAGAGAGTAAAATATATAG
TGGGAAGCCAGAGAGAGTAAAATATGG
TGGGAAGCCACAGAGAGTAAAATATAA
TGGGAAGCCACAGAGAGTAAAATATAA
TGGGAAGCCACAGAGAGTAAAATATAA
TGGGAAGCCACAGAGAGTAAAATATAG
TGGGAAGCCACAGAGAGTAAAATATATAG
TGGGAAGCCAGAGAGAGTAAAATATGG
TGGGAAGCCACAGAGAGTAAAATATAGT
TGGAAGCCACAGAGAGTAAAATATAA
TGGGAAGCCAGAGAGAGTAAAATATATAG
TGGGAAGCCAGAGAGAGTAAAATATGG
TGGGAAGCCAGAGAGAGTAAAATATAG
TGGGAAGCCACAGAGAGTAAAATATATAG
TGGGAAGCCAGAGAGAGTAAAATATGG
TGGGAAGCCACAGAGAGTAAAATATAG
TGGGAAGCCACAGAGAGTAAAATATAA
TGGGAAGCCACAGAGAGTAAAATATAG
TGGGAAGCCAGAGAGAGTAAAATATAA
TGGGAAGCCACAGAGAGTAAAATATAG
TGGGAAGCCAGAGAGAGTAAAATATGG
TGAGAAGCCACAGAGAGTAAAATATAG
TGGGAAGCCACAGAGAGTAAAATATATAG
TGGGAAGCCAGAGAGAGTAAAATATAG
Nr of selected repeats 519 Similarity 0.929610
Consensus:
TGGGAAGCCACAGAGAGTAAAATATAg
>Cele-UNSB01_3:1041359-1041618 Satlength=260 Nr of Repeats=12 RepeatLength=22
seed=TAAATCTACA
TAAATCTACATAGAGAGTTTCG
TAAATCTACACACTTGGCCTCG
TAAATCTACATAGAGAGCCCCG
TAAATCTACACAAATAGCCCCG
TAAATCTACACACTCGGTCCCG
TAAATCTACACAGAGAGCTCCG
TAAATCTGCACACTTAGTCCCG
TAAATCTACACAAAATCCTG
TGAATCTACAGACATAGTCCTG

TAAATCTACACACTTAACCCCG
TAAATCTACACAGAGAGTTCCT
TAAATCTACACAGGACCCG
Nr of selected repeats 10 Similarity 0.692929
Consensus:
TAAATCTACAcAcataGcccCG
>Cele-UNSB01_3:1053032-1053110 Satlength=79 Nr of Repeats=5 RepeatLength=16 seed=TTATAGTGTT
TTATAGTGTTATAGTG
TTATAGTGTTATAGTG
TTATAATGTTATAGTA
TTATAGTTTATAGTA
TTATAGTTTACAGTG
Nr of selected repeats 3 Similarity 0.888889
Consensus:
TTATAgTGTTATAGTg
>Cele-UNSB01_3:1059346-1075750 Satlength=16405 Nr of Repeats=279 RepeatLength=59 seed=AACTGAGAAT
AACTGAGAATTATGGAGAATATGGAAATTTAAATGTGCACTGGTTTTCAAAATTTTTTA
AACTGAGAATTATGGAGAATATGGAAATTCATGTGTGCACTGGTTTTCAAAATTTTTTA
AACTGAGAATTATGGAGAATATGGAAATTTAAATGTGCACTGGTTTTCAAAATTTTTTA
AACTGAGAATTATGGAAAAAATGGAAATTTACATGTGCACTGGTTTTCAAAATTTTTTA
AACTGAGAATTATGGAAAAAATGGAAATTTACATGTGCACTGGTTTTCAAAATTTTTTA
AACTGAGAATGATGGAGAATATGAAAATTTAAATGTGCACTGGTTTTCAAAATTTTTTA
AACTGAGAATGATGGAGAATATGAAAATTTAAATGTGCACTGGTTTTCAAAATTTTTTA
AACTGAGAATTATGGAGAATATGGAAATATAAATGTGCACTGGTTTTCAAAATTTTTTA
AACTGAGAATTATGGAAAAAATGGAAATTTACATGTGCACTGGTTTTCAAAATTTTTTA
AACTGAGAATGATGGAGAATATGAAAATTTAAATGTGCACTGGTTTTCAAAACTTTTTA
AACTGAGAATGATGGAGAATATGAAAATTTAAATGTGCACTGGTTTTCAAAATTTTTTA
AACTGAGAATGATGGAGAATATGAAAATTTAAATGTGCACTGGTTTTCAAAATTTTTTA
AACTGAGAATTATGGAGAATATGAAAATTTAAATGTGCACTGGTTTTCAAAATTTTTTA
AACTGAGAATTATGGAGAATATGGAAATTTAAATGTGCACTGGTTTTCAAAATTTTTTA
AACTGAGAATGATGGAGAATATGAAAATTTAAATGTGCACTGGTTTTCAAAATTTTTTA
AACTGAGAATTATGGAAAAAATGGAAATTTACATGTGCACTGGTTTTCAAAATTTTTTA
AACTGAGAATGATGGAGAATATGAAAATTTAAATGTGCACTGGTTTTCAAAATTTTTTA
AACTGAGAATTATGGAGAATATGGAAATTTAAATGTGCACTGGTTTTCAAAATTTTTTA
AACTGAGAATGATGGAGAATATGGAAATTTAAATGTGCACTGGTTTTCAAAATTTTTTA
AACTGAGAATTATGGAAAAAATGGAAATTTACATGTGCACTGGTTTTCAAAATTTTTTA
AACTGAGAATGATGGAGAATATGAAAATTTAAATGTGCACTGGTTTTCAAAATTTTTTA
AACTGAGAATTATGGAGAATATGGAAATTTAAATGTGCACTGGTTTTCAAAATTTTTTA
AACTGAGAATGATGGAGAATATGAAAATTTAAATGTGCACTGGTTTTCAAAATTTTTTA
AACTGAGAATGATGGAGAATATGAAAATTTAAATGTGCACTGGTTTTCAAAATTTTTTA
AACTGAGAATTATGGAGAATATGGAAATTTAAATGTGCACTGGTTTTCAAAATTTTTTA
AACTGAGAATGATGGAGAATATGAAAATTTAAATGTGCACTGGTTTTCAAAATTTTTTA
AACTGAGAATTATGGAGAATATGAAAATTTAAATGTGCACTGGTTTTCAAAATTTTTTA
AACTGAGAATTATGGAGAATATGAAAATTTAAATGTGCACTGGTTTTCAAAATTTTTTA
AACTGAGAATTATGGAGAATACGAAAATTTAAATGTGCACTGGTTTTCAAAATTTTTTA
AACTGAGAATTATGGAGAATATGAAAATTTAAATGTGCACTGGTTTTCAAAATTTTTTA
AACTGAGAATTATGGAGAATATGGAAATTTAAATGTGCACTGGTTTTCAAAATTTTTTA
AACTGAGAATTATGGAGAATATGGAAATTCATGTGTGCACTGGTTTTCAAAATTTTTTA
AACTGAGAATTATGGAAAAAATGGAAATTTAATGTGCACTGGTTTTCAAAATTTTTTA
AACTGAGAATTATGGAGAATATGAAATTTAAATGTGCACTGGTTTTCAAAATTTTTTA
AACTGAGAATTATGGAGAATATGAAATTTAAATGTGCACTGGTTTTCAAAATTTTTTA
AACTGAGAATTATGGAGAATATGAAATTTGAAATGTGCACTGGTTTTCAAAATTTTTTA

AACTGAGAATTATGGAGAATATGGAAATTTAAATGTGCACTGGTTTTCAAAATTTTTTA
AACTGAGAATTATGGAGAATATGGAAATTTAAATGTGCACTGGTTTTCAAAATTTTTTA
AACTGAGAATTATGGAGAATATGGAAATTTAAATGTGCACTGGTTTTCAAAATTTTTTA
AACTGAGAATTATGGAGAATATGGAAATTTAAATGTGCACTGGTTTTCAAAATTTTTTA
AACTGAGAATTATGGAGAATATGGAAATTTAAATGTGCACTGGTTTTCAAAATTTTTTA
AACTGAGAATTATGGAGAATATGGAAATTTAAATGTGCACTGGTTTTCAAAATTTTTTA
AACTGAGAATTATGGAGAATATGGAAATTTAAATGTGCACTGGTTTTCAAAATTTTTTA
AACTGAGAATTATGGAGAATATGGAAATTTAAATGTGCACTGGTTTTCAAAATTTTTTA
AACTGAGAATTATGGAGAATATGGAAATTTAAATGTGCACTGGTTTTCAAAATTTTTTA
AACTGAGAATTATGGAGAATATGGAAATTTAAATGTGCACTGGTTTTCAAAATTTTTTA
AACTGAGAATTATGGAGAATATGGAAATTTAAATGTGCACTGGTTTTCAAAATTTTTTA
AACTGAGAATTATGGAGAATATGGAAATTTAAATGTGCACTGGTTTTCAAAATTTTTTA
AACTGAGAATTATGGAGAATATGGAAATTTAAATGTGCACTGGTTTTCAAAATTTTTTA
AACTGAGAATTATGGAGAATATGGAAATTTAAATGTGCACTGGTTTTCAAAATTTTTTA
AACTGAGAATTATGGAGAATATGGAAATTTAAATGTGCACTGGTTTTCAAAATTTTTTA
AACTGAGAATTATGGAGAATATGGAAATTTAAATGTGCACTGGTTTTCAAAATTTTTTA
AACTGAGAATTATGGAGAATATGGAAATTTAAATGTGCACTGGTTTTCAAAATTTTTTA
AACTGAGAATTATGGAGAATATGGAAATTTAAATGTGCACTGGTTTTCAAAATTTTTTA
AACTGAGAATTATGGAGAATATGGAAATTTAAATGTGCACTGGTTTTCAAAATTTTTTA
AACTGAGAATTATGGAGAATATGGAAATTTAAATGTGCACTGGTTTTCAAAATTTTTTA
AACTGAGAATTATGGAGAATATGGAAATTTAAATGTGCACTGGTTTTCAAAATTTTTTA
AACTGAGAATTATGGAGAATATGGAAATTTAAATGTGCACTGGTTTTCAAAATTTTTTA
AACTGAGAATTATGGAGAATATGGAAATTAATGTGCACTGGTTTTCAAAATTTTTTA
AACTGAGAATTATGGAGAATATGGAAATTTAAATGTGCACTGGTTTTCAAAATTTTTA
AACTGAGAATTATGGAGAATATGGAAATTTAAATGTGCACTGGTTTTCAAAATTTTTTA
AACTGAGAATTATGGAGAATATGGAAATTTAAATGTGCACTGGTTTTCAAAATTTTTTA
AACTGAGAATTATGGAGAATATGGAAATTTAAATGTGCACTGGTTTTCAAAATTTTTTA
AACTGAGAATTATGGAGAATATGGAAATTTAAATGTGCACTGGTTTTCAAAATTTTTTA
AACTGAGAATTATGGAGAATATGGAAATTAATGTGCACTGGTTTTCAAAATTTTTTA
AACTGAGAATTATGGAGAATATGGAAATTTAAATGTGCACTGGTTTTCAAAATTTTTTA
AACTGAGAATTATGGAGAATATGGAAATTTAAATGTGCACTGGTTTTCAAAATTTTTA
AACTGAGAATTATGGAGAATATGGAAATTTAAATGTGCACTGGTTTTCAAAATTTTTTA
AACTGAGAATTATGGAGAATATGGAAATTTAAATGTGCACTGGTTTTCAAAACTTTTTA
AACTGAGAATTATGGAGAATATGGAAATTAATGTGCACTGGTTTTCAAAATTTTTA
AACTGAGAATTATGGAGAATATGGAAATTTAAATGTGCACTGGTTTTCAAAATTTTTA
AACTGAGAATTATGGAGAATATGGAAATTTAAATGTGCACTGGTTTTCAAAATTTTTTA
AACTGAGAATTATGGAGAATATGGAAATTTAAATGTGCACTGGTTTTCAAAATTTTTA
AACTGAGAATTATGGAGAATATGGAAATTTAAATGTGCACTGGTTTTCAAAATTTTTTA
AACTGAGAATTATGGAGAATATGGAAATTTAAATGTGCACTGGTTTTCAAAATTTTTTA
AACTGAGAATTATGGAGAATATGGAAATTTAAATGTGCACTGGTTTTCAAAATTTTTTA
AACTGAGAATTATGGAAAAATGGAAATTTAAATGTGCACTGGTTTTCAAAATTTTTTA
AACTGAGAATTATGGAGAATATGGAAATTTAAATGTGCACTGGTTTTCAAAATTTTTTA
AACTGAGAATTATGGAAATATGGAAATTTAAATGTGCACTGGTTTTCAAAATTTTTTA
AACTGAGAATTATGGAGAATATGGAAATTTAAATGTGCACTGGTTTTCAAAATTTTTTA
AACTGAGAATTATGGAGAATATGGAAATTTAAATGTGCACTGGTTTTCAAAATTTTTTA
AACTGAGAATTATGGAGAATATGGAAATTTAAATGTGCACTGGTTTTCAAAATTTTTTA
AACTGAGAATTATGGAGAATATGGAAATTTAAATGTGCACTGGTTTTCAAAATTTTTTA
AACTGAGAATTATGGAGAATATGGAAATTTAAATGTGCACTGGTTTTCAAAATTTTTTA
AACTGAGAATTATGGAGAATATGGAAATTTAAATGTGCACTGGTTTTCAAAATTTTTTA
AACTGAGAATTATGGAGAATATGGAAATTTAAATGTGCACTGGTTTTCAAAATTTTTTA
AACTGAGAATTATGGAGAATATGGAAATTTAAATGTGCACTGGTTTTCAAAATTTTTA
AACTGAGAATTATGGAGAATATGGAAATTTAAATGTGCACTGGTTTTCAAAATTTTTTA
AACTGAGAATTATGGAGAATATGGAAATTTAAATGTGCACTGGTTTTCAAAATTTTTTA
AACTGAGAATTATGGAGAATATGGAAATTTAAATGTGCACTGGTTTTCAAAATTTTTTA

AACTGAGAATTATGGAAAAAATGGAAATTTAAATGTGCACTGGTTTTCAAAATTTTTTA
AACTGAGAATTATGGAGAATATGGAAATTTAAATGTGCACTGGTTTTCAAAATTTTTTA
AACTGAGAATTATGGAGAATATGGAAATTTAAATGTGCACTGGTTTTCAAAATTTTTTA
AACTGAGAATTATGGAGAATATGGAAATTTAAATGTGCACTGGTTTTCAAAATTTTTTA
AACTGAGAATTATGGAGAATATGGAAATTTAAATGTGCACTGGTTTTCAAAATTTTTTA
AACTGAGAATTATGGAGAATATGGAAATTTAAATGTGCACTGGTTTTCAAAATTTTTTA
AACTGAGAATTATGGAGAATATGGAAATTTAAATGTGCACTGGTTTTCAAAATTTTTTA
AACTGAGAATTATGGAGAATATGGAAATTTAAATGTGCACTGGTTTTCAAAATTTTTTA
AACTGAGAATTATGGAGAATATGGAAATTTAAATGTGCACTGGTTTTCAAAATTTTTTA
AACTGAGAATTATGGAGAATATGGAAATTTAAATGTGCACTGGTTTTCAAAATTTTTTA
AACTGAGAATTATGGAGAATATGGAAATTTAAATGTGCACTGGTTTTCAAAATTTTTA
AACTGAGAATTATGGAGAATATGGAAATTTAAATGTGCACTGGTTTTCAAAATTTTTA
AACTGAGAATTATGGAGAATATGGAAATTTAAATGTGCACTGGTTTTCAAAATTTTTTA
AACTGAGAATTATGGAGAATATGGAAATTTAAATGTGCACTGGTTTTCAAAATTTTTTA
AACTGAGAATTATGGAGAATATGGAAATTTAAATGTGCACTGGTTTTCAAAATTTTTA
AACTGAGAATTATGGAAAAATGGAAATTTAAATGTGCACTGGTTTTCAAAATTTTTTA
AACTGAGAATTATGGAGAATATGGAAATTAAATGTGCACTGGTTTTCAAAATTTTTA
AACTGAGAATTATGGAGAATATGGAAATTTAAATGTGCACTGGTTTTCAAAATTTTTTA
AACTGAGAATTATGGAAATATGGAAATTTAAATGTGCACTGGTTTTCAAAATTTTTA
AACTGAGAATTATGGAGAATATGGAAATTTAAATGTGCACTGGTTTTCAAAATTTTTTA
AACTGAGAATTATGGAGAATATGGAAATTTAAATGTGCACTGGTTTTCAAAATTTTTTA
AACTGAGAATTATGGAGAATATGGAAATTTAAATGTGCACTGGTTTTCAAAATTTTTA
AACTGAGAATTATGGAGAATATGGAAATTTAAATGTGCACTGGTTTTCAAAATTTTTTA
AACTGAGAATTATGGAGAATATGGAAATTTAAATGTGCACTGGTTTTCAAAATTTTTTA
AACTGAGAATTATGGAGAATATGGAAATTTAAATGTGCACTGGTTTTCAAAATTTTTTA
AACTGAGAATTATGGAGAATATGGAAATTTAAATGTGCACTGGTTTTCAAAATTTTTTA
AACTGAGAATTATGGAGAATATGGAAATTTAAATGTGCACTGGTTTTCAAAATTTTTTA
AACTGAGAATTATGGAGAATATGGAAATTTAAATGTGCACTGGTTTTCAAAATTTTTTA
AACTGAGAATTATGGAGAATATGGAAATTTAAATGTGCACTGGTTTTCAAAATTTTTA
AACTGAGAATTATGGAGAATATGGAAATTTAAATGTGCACTGGTTTTCAAAATTTTTTA
AACTGAGAATTATGGAGAATATGGAAATTTAAATGTGCACTGGTTTTCAAAATTTTTTA
AACTGAGAATTATGGAGAATATGGAAATTTAAATGTGCACTGGTTTTCAAAATTTTTTA
AACTGAGAATTATGGAGAATATGGAAATTTAAATGTGCACTGGTTTTCAAAATTTTTTA
AACTGAGAATTATGGAGAATATGGAAATTTAAATGTGCACTGGTTTTCAAAATTTTTA
AACTGAGAATTATGGAAAAATGGAAATTTAAATGTGCACTGGTTTTCAAAATTTTTTA
AACTGAGAATTATGGAGAATATGGAAATTTAAATGTGCACTGGTTTTCAAAATTTTTTA
AACTGAGAATTATGGAAAAATGGAAATTTAAATGTGCACTGGTTTTCAAAATTTTTTA
AACTGAGAATTATGGAGAATATGGAAATTTAAATGTGCACTGGTTTTCAAAATTTTTTA
AACTGAGAATTATGGAGAATATGGAAATTTAAATGTGCACTGGTTTTCAAAATTTTTTA
AACTGAGAATTATGGAGAATATGGAAATTTAAATGTGCACTGGTTTTCAAAATTTTTA
AACTGAGAATTATGGAGAATATGGAAATTTAAATGTGCACTGGTTTTCAAAATTTTTTA
AACTGAGAATTATGGAGAATATGGAAATTTAAATGTGCACTGGTTTTCAAAATTTTTTA
AACTGAGAATTATGGAGAATATGGAAATTTAAATGTGCACTGGTTTTCAAAATTTTTTA
AACTGAGAATTATGGAGAATATGGAAATTTAAATGTGCACTGGTTTTCAAAATTTTTTA
AACTGAGAATTATGGAGAATATGGAAATTTAAATGTGCACTGGTTTTCAAAATTTTTTA
AACTGAGAATTATGGAGAATATGGAAATTTAAATGTGCACTGGTTTTCAAAATTTTTTA
AACTGAGAATTATGGAGAATATGGAAATTTAAATGTGCACTGGTTTTCAAAATTTTTTA
AACTGAGAATTATGGAGAATATGGAAATTTAAATGTGCACTGGTTTTCAAAATTTTTA
AACTGAGAATTATGGAGAATATGGAAATTTAAATGTGCACTGGTTTTCAAAATTTTTTA
AACTGAGAATTATGGAGAATATGGAAATTTAAATGTGCACTGGTTTTCAAAATTTTTA
AACTGAGAATTATGGAGAATATGGAAATTTAAATGTGCACTGGTTTTCAAAATTTTTTA
AACTGAGAATTATGGAGAATATGGAAATTTAAATGTGCACTGGTTTTCAAAATTTTTTA
AACTGAGAATTATGGAGAATATGGAAATTAAATGTGCACTGGTTTTCAAAATTTTTTA
AACTGAGAATTATGGAGAATATGGAAATTTAAATGTGCACTGGTTTTCAAAATTTTTTA

AACTGAGAATTATGGAGAATATGGAAATTTAAATGTGCACTGGTTTTCAAAATTTTTTA
AACTGAGAATTATGGAGAATATGGAAATTTAATGTGCACTGGTTTTCAAAATTTTTA
AACTGAGAATTATGGAGAATATGGAAATTTAAATGTGCACTGGTTTTCAAAATTTTTTA
AACTGAGAATTATGGAGAATATGAATTATGTGTGTTTTTCAATTTTTA
AACTGAGAATTATGGAGAATATGGAAATTTACATGTGCACTGGTTTTCAAAATTTTTTA
AACTGAGAATTATGGAGAATATGGAAATTCAAATGTGCACTGGTTTTCAAAATTTTTTA
AACTGAGAATTATGGAGAATATGGAAATTTAAATGTGCACTGGTTTTCAAAATTTTTA
AACTGAGAATTATGGAGAATATGGAAGTTTAAATGTGCACTGGTTTTCAAAATTTTTA
AACTGAGAATTATGGAGAATATGGAAATTTAAATGTGCACTGGTTTTCAAAACTTTTTA
AACTGAGAATTATGGAGAATATGGAAATTTAAATGTGCACTGGTTTTCAAAACTTTTTA
AACTGAGAATTATGGAAAATGGAAATTTAAATGTGCACTGGTTTTCAAAACTTTTTA
AACTGAGAATTATGGAAAAATAAATTTAAATGTGCACTGGTTTTCAAAACTTTTTA
AACTGAGAATTATGGAAAAAAATGGAAATTTAAATGTGCACTGGTTTTCAAAACTTTTTA
AACTGAGAATTATGGAGAATATGGAAATTTAAATGTGCACTGGTTTTCAAAACTTTTTA
AACTGAGAATTATGGAAAAAAATGGAAATTTAAATGTGCACTGGTTTTCAAAACTTTTTA
AACTGAGAATTATGGAAAAAAATATAAATTTAAATGTGCACTGGTTTTCAAAACTTTTTA
AACTGAGAATTATGGAAAAAATGGAAATTTAAATGTGCACTGGTTTTCAAAACTTTTTA
AACTGAGAATTATGGAAAAAAATATAAATTTAAATGTGCACTGGTTTTCAAAACTTTTTA
AACTGAGAATTATGGAAAATATGGAAATTTAAATGTGCACTGGTTTTCAAAACTTTTTA
AACTGAGAATTATGGAAAAAAATGGAAATTTAAATGTGCACTGGTTTTCAAAACTTTTTA
AACTGAGAATTATGGAAAAAATATAAATTTAAATGTGCACTGGTTTTCAAAACTTTTTA
AACTGAGAATTATGGAAAAAAATGGAAATTTAAATGTGCACTGGTTTTCAAAACTTTTTA
AACTGAGAATTATGGAAAAAAATATAAATTTAAATGTGCACTGGTTTTCAAAACTTTTTA
AACTGAGAATTATGGAGAATATGGAAATTTAAATGTGCACTGGTTTTCAAAACTTTTTA
AACTGAGAATTATGGAAAAAAATGGAAATTTAAATGTGCACTGGTTTTCAAAACTTTTTA
AACTGAGAATTATGGAAAAAAATATAAATTTAAATGTGCACTGGTTTTCAAAACTTTTTA
AACTGAGAATTATGGAGAATATGGAAATTTAAATGTGCACTGGTTTTCAAAACTTTTTA
AACTGAGAATTATGGAAAAAAATGGAAATTTAAATGTGCACTGGTTTTCAAAACTTTTTA
AACTGAGAATTATGGAAAAAAATATAAATTTAAATGTGCACTGGTTTTCAAAACTTTTTA
AACTGAGAATTATGGAGAATATGGAAATTTAAATGTGCACTGGTTTTCAAAACTTTTTA
AACTGAGAATTATGGAAAAAAATATAAATTTAAATGTGCACTGGTTTTCAAAACTTTTTA
AACTGAGAATTATGGAGAATATGGAAATTTAAATGTGCACTGGTTTTCAAAACTTTTTA
AACTGAGAATTATGGAAAAAAATGGAAATTTAAATGTGCACTGGTTTTCAAAACTTTTTA
AACTGAGAATTATGGAAAAAAATATAAATTTAAATGTGCACTGGTTTTCAAAACTTTTTA
AACTGAGAATTATGGAAAAAAATATAAATTTAAATGTGCACTGGTTTTCAAAACTTTTTA
AACTGAGAATTATGGAAAAAAATGGAAATTTAAATGTGCACTGGTTTTCAAAACTTTTTA
AACTGAGAATTATGGAAAAAAATATAAATTTAAATGTGCACTGGTTTTCAAAACTTTTTA
AACTGAGAATTATGGAGAATATGGAAATTTAAATGTGCACTGGTTTTCAAAACTTTTTA
AACTGAGAATTATGGAAAAAAATATAAATTTAAATGTGCACTGGTTTTCAAAACTTTTTA
AACTGAGAATTATGGAAAAAAATGGAAATTTAAATGTGCACTGGTTTTCAAAACTTTTTA
AACTGAGAATTATGGAGAATATGGAAATTTAAATGTGCACTGGTTTTCAAAATTTTTA
AACTGAGAATTATGGAAAAAAATATAAATTTAAATGTGCACTGGTTTTCAAAACTTTTTA
AACTGAGAATTATGGAAAAAAATATAAATTTAAATGTGCACTGGTTTTCAAAACTTTTTA
AACTGAGAATTATGGAAAAAAATATAAATTTAAATGTGCACTGGTTTTCAAAACTTTTTA
AACTGAGAATTATGGAGAATATGGAAATTTAAATGTGCACTGGTTTTCAAAACTTTTTA
AACTGAGAATTATGGAAAAAAATATAAATTTAAATGTGCACTGGTTTTCAAAACTTTTTA
AACTGAGAATTATGGAAAAAAATATAAATTTAAATGTGCACTGGTTTTCAAAACTTTTTA
AACTGAGAATTATGGAGAATATGGAAATTTAAATGTGCACTGGTTTTCAAAACTTTTTA
AACTGAGAATTATGGAAAAAAATGGAAATTTAAATGTGCACTGGTTTTCAAAACTTTTTA
AACTGAGAATTATGGAAAAAAATATAAATTTAAATGTGCACTGGTTTTCAAAACTTTTTA
AACTGAGAATTATGGAAAAAAATATAAATTTAAATGTGCACTGGTTTTCAAAACTTTTTA
AACTGAGAATTATGGAGAATATGGAAATTTAAATGTGCACTGGTTTTCAAAACTTTTTA
AACTGAGAATTATGGAAAAAAATATAAATTTAAATGTGCACTGGTTTTCAAAACTTTTTA
AACTGAGAATTATGGAAAAAAATATAAATTTAAATGTGCACTGGTTTTCAAAACTTTTTA

AACTGAGAATTATGGAAAAAAATATAAATTTAAATGTGCACTGGTTTTCAAAACTTTTTA
AACTGAGAATTATGGAAAAAAATATAAATTTAAATGTGCACTGGTTTTCAAAACTTTTTA
AACTGAGAATTATGGAAAAAAATATAAATTTAAATGTGCACTGGTTTTCAAAACTTTTTA
AACTGAGAATTATGGAAAAAAATATAAATTTAAATGTGCACTGGTTTTCAAAACTTTTTA
AACTGAGAATTATGGAAAAAAATGGAAATTTAAATGTGCACTGGTTTTCAAAACTTTTTA
AACTGAGAATTATGGAAAAAAATATAAATTTAAATGTGCACTGGTTTTCAAAACTTTTTA
AACTGAGAATTATGGAAAAAAATATAAATTTAAATGTGCACTGGTTTTCAAAACTTTTTA
AACTGAGAATTATGGAAAAAAATATAAATTTAAATGTGCACTGGTTTTCAAAACTTTTTA
AACTGAGAATTATGGAAAAAAATATAAATTTAAATGTGCACTGGTTTTCAAAACTTTTTA
AACTGAGAATTATGGAAAAAAATGGAAATTTAAATGTGCACTGGTTTTCAAAACTTTTTA
AACTGAGAATTATGGAAAAAAATATAAATTTAAATGTGCACTGGTTTTCAAAACTTTTTA
AACTGAGAATTATGGAAAAAAATATAAATTTAAATGTGCACTGGTTTTCAAAACTTTTTA
AACTGAGAATTATGGAAAAAAATGGAAATTTAAATGTGCACTGGTTTTCAAAACTTTTTA
AACTGAGAATTATGGAAAAAAATATAAATTTAAATGTGCACTGGTTTTCAAAACTTTTTA
AACTGAGAATTATGGAGAATATGGAAATTTAAATGTGCACTGGTTTTCAAAACTTTTTA
AACTGAGAATTATGGAAAAAAATATAAATTTAAATGTGCACTGGTTTTCAAAACTTTTTA
AACTGAGAATTATGGAAAAAAATATAAATTTAAATGTGCACTGGTTTTCAAAACTTTTTA
AACTGAGAATTATGGAAAAAAATATAAATTTAAATGTGCACTGGTTTTCAAAACTTTTTA
AACTGAGAATTATGGAAAAAAATATAAATTTAAATGTGCACTGGTTTTCAAAATTTTTTA
AACTGAGAATTATGGAAAAAAATGGAAATTTAAATGTGCACTGGTTTTCAAAACTTTTTA
AACTGAGAATTATGGAAAAAAATATAAATTTAAATGTGCACTGGTTTTCAAAATTTTTTA
AACTGAGAATTATGGAAAAAAATGGAAATTTAAATGTGCACTGGTTTTCAAAACTTTTTA
AACTGAGAATTATGGAAAAAAATATAAATTTAAATGTGCACTGGTTTTCAAAATTTTTTA
AACTGAGAATTATGGAAAAAAATATAAATTTAAATGTGCACTGGTTTTCAAAACTTTTTA
AACTGAGAATTATGGAAAAAAATATAAATTTAAATGTGCACTGGTTTTCAAAACTTTTTA
AACTGAGAATTATGGAAAAAAATGGAAATTTAAATGTGCACTGGTTTTCAAAACTTTTTA
AACTGAGAATTATGGAAAAAAATATAAATTTAAATGTGCACTGGTTTTCAAAACTTTTTA
AACTGAGAATTATGGAAAAAAATATAAATTTAAATGTGCACTGGTTTTCAAAATTTTTTA
AACTGAGAATTATGGAAAAAAATGGAAATTTAAATGTGCACTGGTTTTCAAAACTTTTTA
AACTGAGAATTATGGAAAAAAATATAAATTTAAATGTGCACTGGTTTTCAAAACTTTTTA
AACTGAGAATTATGGAAAAAAATATAAATTTAAATGTGCACTGGTTTTCAAAACTTTTTA
AACTGAGAATTATGGAAAAAAATATAAATTTAAATGTGCACTGGTTTTCAAAACTTTTTA
AACTGAGAATTATGGAAAAAAATGGAAATTTAAATGTGCACTGGTTTTCAAAATTTTTTA
AACTGAGAATTATGGAAAAAAATATAAATTTAAATGTGCACTGGTTTTCAAAATTTTTTA
AACTGAGAATTATGGAAAAAAATGGAAATTTAAATGTGCACTGGTTTTCAAAACTTTTTA
AACTGAGAATTATGGAAAAAAATATAAATTTAAATGTGCACTGGTTTTCAAAATTTTTTA
AACTGAGAATTATGGAAAAAAATATAAATTTAAATGTGCACTGGTTTTCAAAATTTTTTA
AACTGAGAATTATGGAAAAAAATATAAATTTAAATGTGCACTGGTTTTCAAAATTTTTTA
AACTGAGAATTATGGAAAAAAATGGAAATTTAAATGTGCACTGGTTTTCAAAATTTTTTA
AACTGAGAATTATGGAAAAAAATATAAATTTAAATGTGCACTGGTTTTCAAAATTTTTTA
AACTGAGAATTATGGAAAAAAATATAAATTTAAATGTGCACTGGTTTTCAAAACTTTTTA
AACTGAGAATTATGGAAAAAAATGGAAATTTAAATGTGCACTGGTTTTCAAAATTTTTA
AACTGAGAATTATGGAAAAAAATATAAATTTAAATGTGCACTGGTTTTCAAAATTTTTTA
AACTGAGAATTATGGAAAAAAATGGAAATTTAAATGTGCACTGGTTTTCAAAACTTTTTA
AACTGAGAATTATGGAAAAAAATATAAATTTAAATGTGCACTGGTTTTCAAAATTTTTTA
AACTGAGAATTATGGAAAAAAATATAAATTTAAATGTGCACTGGTTTTCAAAATTTTTTA
AACTGAGAATTATGGAAAAAAATATAAATTTAAATGTGCACTGGTTTTCAAAATTTTTTA
AACTGAGAATTATGGAAAAAAATATAAATTTAAATGTGCACTGGTTTTCAAAATTTTTTA
AACTGAGAATTATGGAAAAAAATATAAATTTAAATGTGCACTGGTTTTCAAAATTTTTTA
AACTGAGAATTATGGAAAAAAATGGAAATTTAAATGTGCACTGGTTTTCAAAACTTTTTA
AACTGAGAATTATGGAAAAAAATATAAATTTAAATGTGCACTGGTTTTCAAAATTTTTTA
AACTGAGAATTATGGAAAAAAATATAAATTTAAATGTGCACTGGTTTTCAAAATTTTTTA
AACTGAGAATTATGGAAAAAAATATAAATTTAAATGTGCACTGGTTTTCAAAATTTTTTA
AACTGAGAATTATGGAAAAAAATGGAAATTTAAATGTGCACTGGTTTTCAAAATTTTTTA

AACTGAGAATTATGGAAAAAATATAAATTTAAATGTGCACTGGTTTTCAAAATTTTTA
AACTGAGAATTATGGAAAAAATATAAATTTAAATGTGCACTGGTTTTCAAAACTTTTA
AACTGAGAATTATGGAAAAAATGGAAATTTAAATGTGCACTGGTTTTCAAAATTTTTA
AACTGAGAATTATGGAAAAAATATAAATTTAAATGTGCACTGGTTTTCAAAATTTTTA
AACTGAGAATTATGGAAAAAATGGAAATTTAAATGTGCACTGGTTTTCAAAACTTTTA
AACTGAGAATTATGGAAAAAATATAAATTTAAATGTGCACTGGTTTTCAAAATTTTTA
AACTGAGAATTATGGAAAAAATATAAATTTAAATGTGCACTGGTTTTCAAAATTTTTA
AACTGAGAATTATGGAAAAAATGGAAATTTAAATGTGCACTGGTTTTCAAAATTTTTA
AACTGAGAATTATGGAAAAAATGGAAATTTAAATGTGCACTGGTTTTCAAAATTTTTA
AACTGAGAATTATGGAAAAAATATAAATTTAAATGTGCACTGGTTTTCAAAACTTTTA
AACTGAGAATTATGGAAAAAATATAAATTTAAATGTGCACTGGTTTTCAAAATTTTTA
AACTGAGAATTATGGAGAATATGGAAATTTAAATGTGCACTGGTTTTCAAAACTTTTA
AACTGAGAATTATGGAAAAAATATATATTTAAATGTGCACTGGTTTTCAAAACTTTTA
AACTGAGAATTATGGAAAAAATGGAAATTTAAATGTGCACTGGTTTTCAAAATTTTTA
AACTGAGAATTATGGAAAAAATATAAATTTAAATGTGCACTGGTTTTCAAAACTTTTA
AACTGAGAATTATGGAGAATATGGAAATTTAAATGTGCACTGGTTTTCAAAACTTTTA
AACTGAGAATTATGGAAAAAATATATATTTAAATGTGCACTGGTTTTCAAAACTTTTA
AACTGAGAATTATGGAAAAAATATATATTTAAATGTGCACTGGTTTTCAAAACTTTTA
AACTGAGAATTATGGAAAAAATATAAATTTAAATGTGCACTGGTTTTCAAAACTTTTA
AACTGAGAATTATGGAAAAAATATAAATTTAAATGTGCACTGGTTTTCAAAACTTTTA
AACTGAGAATTATGGAAAAAATATAAATTTAAATGTGCACTGGTTTTCAAAACTTTTA
AACTGAGAATTATGGAAAAAATATAAATTTAAATGTGCACTGGTTTTCAAAACTTTTA
AACTGAGAATTATGGAAAAAATATAAATTTAAATGTGCACTGGTTTTCAAAACTTTTA
AACTGAGAATTATGGAAAAAATATAAATTTAAATGTGCACTGGTTTTCAAAACTTTTA
AACTGAGAATTATGGAAAAAATATAAATTTAAATGTGCACTGGTTTTCAAAACTTTTA
AACTGAGAATTATGGAAAAAATATATAATTAAATGTGCACTGGTTTTCAAAACTTTTA
AACTGAGAATTATGGAAAAAATGGAAATTTAAATGTGCACTGGTTTTCAAAACTTTTA
Nr of selected repeats 241 Similarity 0.923220
Consensus:
AACTGAGAATTATGGAgAAtATggAAATTTAAATGTGCACTGGTTTTCAAAAtTTTTA
>Cele-UNSB01_3:1107553-1107733 Satlength=181 Nr of Repeats=10 RepeatLength=12
seed=AAGCCTAAGC
AAGCCTAAGCCT
AAGCCTAAGCCT
AAGCCTAAGCCT
AAGCCAAAGCCA
AAGCCTAAGCCT
AAGCCTGAGCAT
AAGCCTAAGCCGAAGGATCAGCAT
AAGCCTAAGCCTAAGCTTAAACCCACGCCTGAGTTTACGCCTAGGCCTAAGAATC
AGCCTAAGCCTGAGCTT
AAGCTTAAGCCT
Nr of selected repeats 7 Similarity 0.841270
Consensus:
AAGCCTAAGCCT
>Cele-UNSB01_3:1112086-1112814 Satlength=729 Nr of Repeats=14 RepeatLength=40
seed=AAAATCCACG
AAAATCCACGTAGATTATCGATTTTTCATAGAATTTTGCA
AAAATCCACGTAGATTATCGATTTTTCATAGAATTTTGCA
AAAATCCACGTGGATTATTGATTTTTCATAGAGTTTCGGA
AAAATCCACGTGGATTATTAATTTTTCATAGAATTGTGAA
AAAATCCACGTAGATTATCGCTTTTTCATAGAATTTTGTA
AAAATCCACGTGGAATATCGATTTTTTAGGAATTTTGCA
AAAATCCACGTAGATTATTGATTTTTCATGAAATTTTGCA

AAAATCCACGTAGATTATTGATTTTTCATCGAATTTTGCA
AAAATCCACGTAGATTATTGATTTTTCATGGAATTTTGCA
AAAATCCACGTAGATTATTGATTTTTTATGAAATTTTGCA
AAAATCCACGTAGATTATTGATTTTTCATGGAATTTTGCA
AAAATCCACGTAGATTATTGATTTTTCATCGAATTTTGCA
AAAATCCACGTAGATTATTGATTTTTCTTGGAATTTTGCG
AAAATCTACGCGGATTATCGATTTTTCTCAAAAATGCTTCAAAAAATCGATAAAGATCGTTACTGATTGTCGATTTTTA
AAGAATCTTGTGCTGAAATCGTGAAAATCGGTTCGATAAACAATCACCGAAAAAAATCCAAAAATCATGGTACATCGAC
GAAAATATGAGAATTCAGGCATTTTTTCCGGAAAATGCTTAGAAAATCGAT
Nr of selected repeats 12 Similarity 0.883838
Consensus:
AAAATCCACGTAGATTATtGATTTTTCATnGAATTTTGcA
>Cele-UNSB01_3:1112079-1113479 Satlength=1401 Nr of Repeats=16 RepeatLength=40
seed=TTTTGCAAAA
TTTTGCAAAAATCCACGTAGATTATCGATTTTTCATAGAA
TTTTGCAAAAATCCACGTAGATTATCGATTTTTCATAGAA
TTTTGCAAAAATCCACGTGGATTATTGATTTTTCATAGAGTTTCGGAAAAATCCACGTGGATTATTAATTTTTCATAGA
ATTGTGAAAAAATCCACGTAGATTATCGCTTTTTCATAGAA
TTTTGTAAAAATCCACGTGGAATATCGATTTTTTAGGAA
TTTTGCAAAAATCCACGTAGATTATTGATTTTTCATGAAA
TTTTGCAAAAATCCACGTAGATTATTGATTTTTCATCGAA
TTTTGCAAAAATCCACGTAGATTATTGATTTTTCATGGAA
TTTTGCAAAAATCCACGTAGATTATTGATTTTTTATGAAA
TTTTGCAAAAATCCACGTAGATTATTGATTTTTCATGGAA
TTTTGCAAAAATCCACGTAGATTATTGATTTTTCATCGAA
TTTTGCAAAAATCCACGTAGATTATTGATTTTTCTTGGAA
TTTTGCGAAAATCTACGCGGATTATCGATTTTTCTCAAAAATGCTTCAAAAAATCGATAAAGATCGTTACTGATTGTCG
ATTTTTAAAGAATCTTGTGCTGAAATCGTGAAAATCGGTTCGATAAACAATCACCGAAAAAAATCCAAAAATCATGGTA
CATCGACGAAAATATGAGAATTCAGGCATTTTTTCCGGAAAATGCTTAGAAAATCGATAAAATCCACGTCAATTATCGA
TTTTTCTCCAATTTTTCGGCATTTTTCCACTATTTCTCACCTGCAACTTGAGAATATCGTGTAAAAAGTACACAAACGG
AATTGTGGTGACATCCTGATACACATATTTCCGTGTCTTCTGACTTTGATTCGGTAAAACACGGAATTTCGTGAATCCC
GGCTCCTTCCCAATGGCAAATGGGAAGAATTTCCCGAAAGCCGAGTACATTTGACGGTTTGGCTCAATTATTGGGTCGG
CTCCGTAGAATTTTGTGTTCGGAATTGTTCGATTAAGGCGTTCCTCGGCGGTTGTGTCATGGCCAACGCCTAGAGTGAC
GAGAATGTTCTCTTCTTTCTGAAAATCGGGAAATTTCAGCGAAAATCAGTCAATTTTGTGAAAAATCAATACTTTTTTC
ATTGAT
TTTTGCAAAATTCCATGAAAAACTCGATAATTCACGTGGATTTTTTGTAAACTTCTATGAAAAATCGATAATCCACCTG
GAT
TTTTGCAAAATTCTATGAAAAATCGATAATCCACCTGGAT
TTTTGAAAAATTCTGTAAAAAACGATAATCCACGTTGATTTTTCACAGAATTCTATAAAAAATCGATAATCCACGTGGA
TT
TTTTGCAAAATTCTATGAAAAATCGATAATCCAAGTGGAT
Nr of selected repeats 11 Similarity 0.759596
Consensus:
TTTTGCAAAAATCCACGTAGATTATtGATTTTTCATgGAA
>Cele-UNSB01_3:1175770-1176177 Satlength=408 Nr of Repeats=4 RepeatLength=96
seed=GAGGGCCGCA
GAGGGCCGCAGGCCCGAACACTCTTGGAAACTTTGGGCTGGCTTTGATCCTATTCTAAGCAGATATGTTATTTACTCAA
ACCTAACAACCAGGTTC
GAGGGCCGCAGGCCCGAACACTCTTGGAAACTATGGGCTGGCTTTGATCCTATTCTAAGCAGATATGTTATTCACTCAA
GCCTAATAACCAGGTTT
GAGGGCCGCAGGCCCAAATACTCTTGAAAACTGTGGGCTGGCTTTAATCCTATTCTTAGCAGAGTAGGCTGTAGGCCTG
CGACGCGGTTGTTATTTACTCAAACCTAACAACCAGGTTC
GAGGGCCGCAGGCCCGAACACTCTTGGAAACTATGGGCTGGCTTTGATCCTATTCTAAGCAGATATGTTATTCACTCAA
GCCTAATAACCAGGTTA

Nr of selected repeats 3 Similarity 0.949074
Consensus:
GAGGGCCGCAGGCCCGAACACTCTTGGAAACTaTGGGCTGGCTTTGATCCTATTCTAAGCAGATATGTTATTcACTCAA
gCCTAAtAACCAGGTTn
>Cele-UNSB01_3:1204553-1205580 Satlength=1028 Nr of Repeats=13 RepeatLength=35
seed=TTTAAAATTT
TTTAAAATTTCCGGCAAATCGCCAAATTGCCGGAA
TTGAAAATTTCGGGCAAATTGGCAACTTGCCGGAT
TTTAAAATTTCCGGCAAATCGTAAAATTGCCGGAA
TTGAAAATTTCCGGCAAATCGGCAAATCGCCAAATTGCCGGAA
TTGAAAATTTCCGGCAAATTGGCAAATTGCCGGAA
TTAAAAATTTTGGGCAAATCGGAAAATTGCCGGGCTTGAAAATTACGGGCGAACCGCCAAATTGCTGGAATTGATAATT
TCAGGCAAATAGGAAAATTGCTGGAATTGATAATTTCAGGCAAATAGGAAAATTGCTGGAATTGATAATTTCGGGCAAA
CTGGCAACTTGCCGGAT
TTTAAAATTTCCGGCAAATCGCCAAATTGCCGGAA
TTGAAAATTTCGGGCAAATTGGCAACTTGCCGGAT
TTTAAAATTTCCGGCAAATCGTAAAATTGCCGGAA
TTGAAAATTTCCGGCAAATCGGCAAATCGCCAAATTGCCGGAA
TTGAAAATTTCCGGCAAATTGGCAAATTGCCGGAA
TTAAAAATTTTGGGCAAATCGGAAAATTGCCGGGCTTGAAAATTACGGGCGAACCGCCAAATTGCTGGAATTGATAATT
TCAGGCAAATAGGAAAATTGTCGGAATTGAAAATTACCGGCAAATCGGCAAATTGCCGGAATTGAAAATAACTAAAATT
TCGGAAAATGGGCAAACTGGCAAATTGCCGGAA
TTTAAAATTTCCGCCAAATCGATAAACCGACGAACTGCCAGCGCGGAATTCAAATTTCAACACAAAAATGGTCAAAAAT
TCAAATTTCGCGCCAAAATCCGATCACAAAAAATTGCCAAAAAGTACCAAAACGTGCCAAAAAATTGCCAAAAAAATTGC
CAAAAAGTGCCAAAAAATTGCCAAAAAGCGCCAAAAAGTGCCAAATTTTTAGGCCGGTTTTTTTCGGAATTTTTTGAAA
TTAAAAAAAAAAGCTCAAATTCAACAACTTTTTTCTTTGAAAAATAGGTGTTTTTTAA
Nr of selected repeats 8 Similarity 0.863946
Consensus:
TTgAAAATTTCcGGCAAATcGgcAAaTTGCCGGAa
>Cele-UNSB01_3:1210986-1211328 Satlength=343 Nr of Repeats=4 RepeatLength=21
seed=ACTACAGTAC
ACTACAGTACCCCGTACGACT
ACTACAGTACCCCCCACTACC
ACTACAGTACCCCCAACCACT
ACTACAGTACCCCCAATCACCACAACATATTCTGAAGACATCATTGACTCGGAAGTGACAAAATCGCCGCAGGAGCTCA
TTGCAGAGCTCAAAAAAGCTTTGAAGTCGGAAATTTCTGGAAAACCGAAAATTTTCAAGGAAGCCGAGGAGCCCGAAAT
CGATTTTGTAGCTTTTGGAACGAAAAACCGAGAGATGAACGCAATTGACACTGAAAATTCGGAGGAAATTGAAAAATTC
GAGCCAATCGGAGCGACACGGTTTGCTCAGTGCTCCGTGAGG
Nr of selected repeats 3 Similarity 0.788360
Consensus:
ACTACAGTACCCCcnACnACt
>Cele-UNSB01_3:1301641-1302992 Satlength=1352 Nr of Repeats=116 RepeatLength=11
seed=TTTTTGCACA
TTTTTGCACAT
TTTTTGCACAA
TTTTTGCACAA
TTTTTGCACA
TTTTTGCACAATATTCGCACAT
TTTTTGCACAT
TTTTTGCACAA
TTTTTGCACAA
TTTTTGCACA
TTTTTGCACAATATTCGCACAT
TTTTTGCACAT

TTTTTGCACA
TTTTTGCACAT
TTTTTGCACAA
TTTTTGCACA
TTTTTGCACAT
TTTTTGCACAA
TTTTTGCACA
TTTTTGCACAATATTCGCACAT
TTTTTGCTCA
TTTTTGCACAT
TTTTTGCACAT
TTTTTGCACAT
TTCTTGCACAT
TTCTTGCACAT
TTCTTGCACAT
TTCTTGCACAT
TTTTTGCACAA
TTTTTGCACA
TTTTTGCACAATATTCGCACAT
TTTTTGCACAT
TTTTTGCACA
TTTTTGCACAT
TTTTTGCACA
TTTTTGCACAT
TTTTTGCACAA
TTTTTGCACA
TTTTTGCACAT
TTTTTGCACAA
TTTTTGCACA
TTTTTGCACAT
TTTTTGCACAA
TTTTTGCACA
TTTTTGCACAATATTCGCACAT
TTTTTGCTCA
TTTTTGCACAT
TTTTTGCACAT
TTTTTGCACAT
TTCTTGCACAT
TTCTTGCACAT
TTCTTGCACAT
TTTTTGCACAA
TTTTTGCACA
TTTTTGCACAATATTCGCACAT
TTTTTGCACAT
TTTTTGCACA
TTTTTGCACAT
TTTTTGCACA
TTTTTGCACAT
TTTTTGCACAA
TTTTTGCACA
TTTTTGCACAT
TTTTTGCACAA
TTTTTGCACAA
TTTTAGCACAT

TTTTAGCACAT
TTTTTGCTCA
TTTTTGCACAT
TTTTTGCACAT
TTTTTGCACA
TTTTTGCACAATATTCGCACAT
TTTTTGCACAT
TTCTTGCACA
TTTTTGCACA
TTTTTGCACAATATTCGCACAT
TTTTTGCACAT
TTTTTGCACA
TTTTTGCACAT
TTTTTGCACAA
TTTTTGCACA
TTTTTGCACAT
TTTTTGCACAA
TTTTTGCACAT
TTTTAGCACA
TTTTTGCACA
TTTTTGCACAATGTTCGCACA
TTTTTGCACAT
TTTTAGCACAT
TTTTTGCACAT
TTTTTGCACG
TTTTTGCACAT
TTTTAGCACAT
TTTTTGCACAT
TTTTTGCACA
TTTTTGCACAT
TTTTTGCACAA
TTTTTGCACA
TTTTTGCACAT
TTTTTGCACAA
TTTTTGCACAT
TTTTAGCACA
TTTTTGCACA
TTTTTGCACAATGTTCGCACA
TTTTTGCACAT
TTTTAGCACAT
TTTTTGCACAT
TTTTTGCACG
TTTTTGCACAT
TTTTAGCACAT
TTTTTGCACA
TTTTTGCACAT
TTTTTGCACAA
TTTTTGCACAT
TTTTTGCACG
TTTTTGCACAT
TTTTAGCACAT
Nr of selected repeats 73 Similarity 0.910060
Consensus:
TTTTTGCACAt

>Cele-UNSB01_3:1307999-1309276 Satlength=1278 Nr of Repeats=65 RepeatLength=20
seed=AAAAATAGAT
AAAAATAGATTGACAGACGA
AAAAATAAATTGGCAGACGA
AAAAATAGATTGACAGACGA
AAAAATAGATTGACAAACG
AAAAATAGATTGACAGACG
AAAAATAGATTGACAGACGA
AAAAATAGATTGACAGACG
AACAATAGATGGACATTACAA
AAAAATAGATTGACAGACGA
AAAAATAGATTGACAGACAA
AAAAATAGATTGACAGACGA
AAAAATAGATTGACAGACGA
AAAAATAGATTGACAGACGA
AAAAATAGATTGACAGACG
AAAAATAGATTGACAGACGA
AAAAATAGATTGACAGACGA
AAAAATAGATTGACAGACGA
AAAAATAGATTGACAGACG
AAAAATAGATTGACATACCA
AAAAATAGATTGACAGACG
AAAAATAGATTGACAAACA
AAAAATAGATTGACAGACG
AAAAATAGATTGACAAACA
AAAAATAGATTGACAGACGA
AAAAATAGATTGACAGACGA
AAAAATAGATTGGCAGACG
AAAAATAGATTGACAAACA
AAAAATAGATTGACAGACGA
AAAAATAGATTGACAGACGA
AAAAATAGATTGACAAACG
AAAAATAGATTGACAGACG
AAAAATAGATTGACAGACGA
AAAAATAGATTGACAGACG
AACAATAGATGGACATTACA
AAAAATAGATTGACAGACGA
AAAAATAGATTGACAGACAA
AAAAATAGATTGACAGACGA
AAAAATAGATTGACAGACGA
AAAAATAGATTGACAGACGA
AAAAATAGATTGACAGACGA
AAAAATAGATTGACAGACGA
AAAAATAGATTGACAGACGA
AAAAATAGATTGACAAACG
AAAAATAGATTGACAGACG
AAAAATAGATTGACAAACA
AAAAATAGATTGACAGACG
AAAAATAGATTGACAAACA
AAAAATAGATTGACAGACGA
AAAAATAGATTGGCAGACGA
AAAAATAGATTGACAGACGA
AAAAATAGATTGACAAACG
AAAAATAGATTGACAGACG

AAAAATAGATTGACAGACGA
AAAAATAGATTGACAGACG
AACAATAGATGGACATTACA
AAAAATAGATTGACAGACGA
AAAAATAGATTGACAGACAA
AAAAATAGATTGACAGACGA
AAAAATAGATTGACAGACGA
AAAAATAGATTGACAGACGA
AAAAATAGATTGACAGACGA
AAAAATAGATTGACAGACGA
AAAAATAGATTGACAGACG
AAAAATAGATAGACGGACG
AAAATTAGATTGACAGACGA
Nr of selected repeats 40 Similarity 0.893203
Consensus:
AAAAATAGATTGACAGACGA
>Cele-UNSB01_3:1312329-1312425 Satlength=97 Nr of Repeats=5 RepeatLength=12
seed=AGGCTTAGGC
AGGCTTAGGCTC
AGGCTTAGGCTT
AGGCTTAGGCTC
AGGCTTAGGCTTAGGCATATGCTG
AGGCTTAGGCTAAGACTTATGAAGAGGCTCAAGCTT
Nr of selected repeats 3 Similarity 0.925926
Consensus:
AGGCTTAGGCTc
>Cele-UNSB01_3:1320709-1320805 Satlength=97 Nr of Repeats=5 RepeatLength=12
seed=AGGCTTAGGC
AGGCTTAGGCTC
AGGCTTAGGCTT
AGGCTTAGGCTC
AGGCTTAGGCTTAGGCATATGCTG
AGGCTTAGGCTAAGACTTATGAAGAGGCTCAAGCTT
Nr of selected repeats 3 Similarity 0.925926
Consensus:
AGGCTTAGGCTc
>Cele-UNSB01_3:1329089-1329185 Satlength=97 Nr of Repeats=5 RepeatLength=12
seed=AGGCTTAGGC
AGGCTTAGGCTC
AGGCTTAGGCTT
AGGCTTAGGCTC
AGGCTTAGGCTTAGGCATATGCTG
AGGCTTAGGCTAAGACTTATGAAGAGGCTCAAGCTT
Nr of selected repeats 3 Similarity 0.925926
Consensus:
AGGCTTAGGCTc
>Cele-UNSB01_3:1364177-1382615 Satlength=18439 Nr of Repeats=734
RepeatLength=25 seed=CAGAGAGTAA
CAGAGAGTAAAATAATTGGTGAAGC
CAGAGAGTAAAATAATTGGTGAAGC
CAGAGAGTAAAATAATTGGTGAAGC
CAGAGAGTAATTTATTTGGTGAAGC
CAGAGAGTAAAATAATTGGTGAAGC
CAGAGAGTAAAATAATTGGTGAAGC

CAGAGAGTAAAATAATTGGTGAAGC
CAGAGAGTAAAATAATTGGTGAAGC
CAGAGAGTAATTTATTTGGTGAAGC
CAGAGAGTAAAATAATTGGTGAAGC
CAGAGAGTAAAATAATTGGTGAAGC
CAGAGAGTAAAATAATTGGTGAAGC
CAGAGAGTAAAATAATTGGTGAAGC
CAGAGAGTAAAATAATTGGTGAAGC
CAGAGAGTAAAATAATTGGTGAAGT
CAGAGAGTAATTTATTTGGTGAAGC
CAGAGAGTAAAATAATTGGTGAAGC
CAGAGAGTAAAATAATTGGTGAAGC
CAGAGAGTAAAATAATTGGTGAAGC
CAGAGAGTAAAATAATTGGTGAAGT
CAGAGAGTAATTTATTTGGTGAAGC
CAGAGAGTAAAATAATTGGTGAAGCA
AGAGAGTAATTTATTTGGTGAAGC
CAGAGAGTAAAATAATTGGTGAAGC
CAGAGAGTAAAATAATTGGTGAAGC
CAGAGAGTAATTTATTTGGTGAAGC
CAGAGAGTAAAATAATTGGTGAAGC
CAGAGAGTAATTTATTTGGTGAAGC
CAGAGAGTAAAATAATTGGTGAAGC
CAGAGAGTAAAATAATTGGTGAGGC
CAGAGAGTAAAATAGTTGGTGAAGC
CAGAGAGTAAAATAATTGGTGAAGT
CAGAGAGTAATTTATTTGGTGAAGC
CAGAGAGTAAAATAATTGGTGAAGC
CAGAGAGTAAAATAATTGGTGAAGC
CAGAGAGTAATTTATTTGGTGAAGC
CAGAGAGTAAAATAATTGGTGAAGCA
AGAGAGTAATTTATTTGGTGAAGC
CAGAGAGTAAAATAATTGGTGAAGCA
AGAGAGTAATTTATTTGGTGAAGC
CAGAGAGTAAAATAATTGGTGAAGC
CAGAGAGTAATTTATTTGGTGAAGC
CAGAGAGTAAAATAATTGGTGAAGC
CAGAGAGTAAAATAATTGGTGAAGC
CAGAGAGTAATTTATTTGGTGAAGC
CAGAGAGTAAAATAATTGGTGAAGCA
AGAGAGTAATTTATTTGGTGAAGC
CAGAGAGTAAAATAATTGGTGAAGCA
AGAGAGTAATTTATTTGGTGAAGC
CAGAGAGTAAAATAATTGGTGAAGC
CAGAGAGTAATTTATTTGGTGAAGC
CAGAGAGTAAAATAATTGGTGAAGC
CAGAGAGTAAAATAATTGGTGAAGC
CAGAGAGTAAAATAATTGGTGAAGC
CAGAGAGTAAAATAATTGGTGAAGC
CAGAGAGTAAAATAATTGGTGAAGC
CAGAGAGTAATTTATTTGGTGAAGC
CAGAGAGTAAAATAATTGGTGAAGC
CAGAGAGTAAAATAGTTGGTGAAGC
CAGAGAGTAAAATAATTGGTGAAGC

CAGAGAGTAAAATAATTGGTGAAGC
CAGAGAGTAAAATAATTGGTGAAGT
CAGAGAGTAATTTATTTGGTGAAGC
CAGAGAGTAAAATAATTGGTGAAGCA
AGAGAGTAATTTATTTGGTGAAGC
CAGAGAGTAAAATAATTGGTGAAGC
CAGAGAGTAATTTATTTGGTGAAGC
CAGAGAGTAAAATAATTGGTGAAGC
CAGAGAGTAAAATAATTGGTGAAGC
CAGAGAGTAAAATAATTGGTGAGGC
CAGAGAGTAAAATAGTTGGTGAAGC
CAGAGAGTAAAATAATTGGTGAAGT
CAGAGAGTAATTTATTTGGTGAAGC
CAGAGAGTAAAATAATTGGTGAAGC
CAGAGAGTAAAATAATTGGTGAAGC
CAGAGAGTAAAATAATTGGTGAAGT
CAGAGAGTAATTTATTTGGTGAAGC
CAGAGAGTAAAATAATTGGTGAAGCA
AGAGAGTAAAATAATTGGTGAGGC
CAGAGAGTAAAATAGTTGGTGAAGC
CAGAGAGTAAAATAATTGGTGAAGT
CAGAGAGTAATTTATTTGGTGAAGC
CAGAGAGTAAAATAATTGGTGAAGC
CAGAGAGTAAAATAATTGGTGAAGC
CAGAGAGTAAAATAATTGGTGAAGC
CAGAGAGTAAAATAATTGGTGAAGC
CAGAGAGTAAAATAATTGGTGAAGT
CAGAGAGTAATTTATTTGGTGAAGC
CAGAGAGTAAAATAATTGGTGAAGCA
AGAGAGTAATTTATTTGGTGAAGC
CAGAGAGTAAAATAATTGGTGAAGCA
AGAGAGTAAAATAGTTGGTGAAGC
CAGAGAGTAAAATAATTGGTGAAGCA
AGAGAGTAAAATAATTGGTGAAGC
CAGAGAGTAAAATAATTGGTGAAGCA
AGAGAGTAATTTATTTGGTGAAGC
CAGAGAGTAAAATAATTGGTGAAGCA
AGAGAGTAAAATAGTTGGTGAAGC
CAGAGAGTAAAATAATTGGTGAAGC
CAGAGAGTAAAATAATTGGTGAAGC
CAGAGAGTAAAATAATTGGTGAAGT
CAGAGAGTAATTTATTTGGTGAAGC
CAGAGAGTAAAATAATTGGTGAAGCA
AGAGAGTAAAATAGTTGGTGAAGC
CAGAGAGTAAAATAATTGGTGAAGC
CAGAGAGTAAAATAATTGGTGAAGC
CAGAGAGTAAAATAATTGGTGAAGT
CAGAGAGTAATTTATTTGGTGAAGC
CAGAGAGTAAAATAATTGGTGAAGCA
AGAGAGTAATTTATTTGGTGAAGC
CAGAGAGTAAAATAATTGGTGAAGT
CAGAGAGTAATTTATTTGGTGAAGC
CAGAGAGTAAAATAATTGGTGAAGCA
AGAGAGTAAAATAATTGGTGAGGC

CAGAGAGTAAAATAGTTGGTGAAGC
CAGAGAGTAAAATAATTGGTGAAGT
CAGAGAGTAATTTATTTGGTGAAGC
CAGAGAGTAAAATAATTGGTGAAG
CAGAGAGTAAAATAATTGGTGAGGC
CAGAGAGTAAAATAGTTGGTGAAGC
CAGAGAGTAAAATAATTGGTGAAGCA
AGAGAGTAATTTATTTGGTGAAGC
CAGAGAGTAAAATAATTGGTGAAGCA
AGAGAGTAATTTATTTGGTGAAGC
CAGAGAGTAAAATAATTGGTGAAG
CAGAGAGTAAAAATTGGTGAAG
CAGAGAGTAAAATATTGGTGAAGC
CAGAGAGTAAAATAATTGGTGAAGC
CAGAGAGTAAAATAATTGGTGAAGC
CAGAGAGTAAAATAATTGGTGAAGT
CAGAGAGTAATTTATTTGGTGAAGC
CAGAGAGTAAAATAATTGGTGAAGCA
AGAGAGTAATTTATTTGGTGAAGC
CAGAGAGTAAAATAATTGGTGAAG
CAGAGAGTAATTTATTTGGTGAAGC
CAGAGAGTAAAATAATTGGTGAAG
CAGAGAGTAAAATAATTGGTGAGGC
CAGAGAGTAAAATAGTTGGTGAAGC
CAGAGAGTAAAATAATTGGTGAAGT
CAGAGAGTAATTTATTTGGTGAAGC
CAGAGAGTAAAATAATTGGTGAAGC
CAGAGAGTAAAATAATTGGTGAGC
CAGAGAGTAAAATATTGGTGAAGC
CAGAGAGTAAAATAATTGGTGAAGCA
AGAGAGTAATTTATTTGGTGAAGC
CAGAGAGTAAAATAATTGGTGAAG
CAGAGAGTAAAATAATTGGTGAAGCA
AGAGAGTAAAATATTGGTGAAGC
CAGAGAGTAAAATAATTGGTGAAG
CAGAGAGTAAAATATTGGTGAAGC
CAGAGAGTAAAATAATTGGTGAAGC
CAGAGAGTAAAATAATTGGTGAAGC
CAGAGAGTAAAATAATTGGTGAAG
CAGAGAGTAATATTGGTGAAGC
CAGAGAGTAAAATAATTGGTGAAGCA
AGAGAGTAAAATATTGGTGAAGC
CAGAGAGTAAAATAATTGGTGAAGC
CAGAGAGTAAAATAATTGGTGAAGC
CAGAGAGTAAAATAATTGGTGAAGC
CAGAGAGTAAAATAATTGGTGAAG
CAGAGAGTAATTTATTTGGTGAAGC
CAGAGAGTAAAATAATTGGTGAAGC
CAGAGAGTAAAATAATTGGTGAAGC
CAGAGAGTAAAATAATTGGTGAAGC
CAGAGAGTAAAATAATTGGTGAAGC
CAGAGAGTAAAATAATTGGTGAAG
CAGAGAGTAATTTATTTGGTGAAGC
CAGAGAGTAAAATAATTGGTGAAG

CAGAGAGTAATATTTGGTGAAGC
CAGAGAGTAAAATAATTGGTGAAGC
CAGAGAGTAAAATAATTGGTGAAGC
CAGAGAGTAAAATATTGGTGAAGC
CAGAGAGTAAAATAATTGGTGAAGC
CAGAGAGTAAAATAATTGGTGAAGC
CAGAGAGTAAAATAATTGGTGAAGC
CAGAGAGTAAAATAATTGGTGAAGT
CAGAGAGTAATTTATTTGGTGAAGC
CAGAGAGTAAAATAATTGGTGAAGC
CAGAGAGTAAAATAATTGGTGAAGC
CAGAGAGTAAAATAATTGGTGAAGC
CAGAGAGTAAAATAATTGGTGAAG
CAGAGAGTAATTTATTTGGTGAAGC
CAGAGAGTAAAATATTGGTGAAGC
CAGAGAGTAAAATAATTGGTGAAGC
CAGAGAGTAAAATAATTGGTGAAGC
CAGAGAGTAAAATAATTGGTGAAGC
CAGAGAGTAAAATAATTGGTGAAGC
CAGAGAGTAAAATAATTGGTGAAGCA
AGAGAGTAATTTATTTGGTGAAGC
CAGAGAGTAAAATAATTGGTGAAGC
CAGAGAGTAATTTATTTGGTGAAGC
CAGAGAGTAAAATAATTGGTGAAGC
CAGAGAGTAAAATAATTGGTGAAGC
CAGAGAGTAAAATAATTGGTGAAGC
CAGAGAGTAAAATAATTGGTGAAGC
CAGAGAGTAAAATAATTGGTGAAGC
CAGAGAGTAAAATAGTTGGTGAAGC
CAGAGAGTAAAATAATTGGTGAAGC
CAGAGAGTAAAATAATTGGTGAAGC
CAGAGAGTAAAATAATTGGTGAAGC
CAGAGAGTAATTTATTTGGTGAAGC
CAGAGAGTAAAATAATTGGTGAAGC
CAGAGAGTAAAATAGTTGGTGAAGC
CAGAGAGTAAAATAATTGGTGAAG
CAGAGAGTAATTTATTTGGTGAAGC
CAGAGAGTAAAATAATTGGTGAAGC
CAGAGAGTAAAATAATTGGTGAAGC
CAGAGAGTAATTTATTTGGTGAAGC
CAGAGAGTAAAATAATTGGTGAA
AGAGAGTAATATTGGTGAAGC
CAGAGAGTAAAATAATTGGTGAAGT
CAGAGAGTAATTTATTTGGTGAAGC
CAGAGAGTAAAATAATTGGTGAAGC
CAGAGAGTAAAATAATTGGTGAAG
CAGAGAGTAATTTATTTGGTGAAGC
CAGAGAGTAAAATAATTGGTGAAGC
CAGAGAGTAATTTATTTGGTGAAGC
CAGAGAGTAAAATAATTGGTGAAGC
CAGAGAGTAAAATAATTGGTGAAGC
CAGAGAGTAAAATAATTGGTGAAGC
CAGAGAGTAAAATAATTGGTGAAGC
CAGAGAGTAAAATAATTGGTGAAGC

CAGAGAGTAATTTATTTGGTGAAGC
CAGAGAGTAAAATAATTGGTGAAGC
CAGAGAGTAAAATAATTGGTGAAGC
CAGAGAGTAAAATAATTGGTGAAG
CAGAGAGTAATTTATTTGGTGAAGC
CAGAGAGTAAAATAATTGGTGAAGC
CAGAGAGTAAAATAATTGGTGAAGC
CAGAGAGTAAAATAATTGGTGAAG
CAGAGAGTAATTTATTTGGTGAAGC
CAGAGAGTAAAATAATTGGTGAAG
CAGAGAGTAATTTATTTGGTGAAGC
CAGAGAGTAAAATAATTGGTGAAGC
CAGAGAGTAAAATAATTGGTGAAGC
CAGAGAGTAAAATAATTGGTGAAGC
CAGAGAGTAAAATATTGGTGAAGC
CAGAGAGTAAAATAATTGGTGAAGC
CAGAGAGTAATTTATTTGGTGAAGC
CAGAGAGTAAAATAATTGGTGAAGC
CAGAGAGTAAAATAATTGGTGAAGC
CAGAGAGTAAAATAATTGGTGAAGC
CAGAGAGTAAATATTGGTGAAGC
CAGAGAGTAAAATAATTGGTGAAGC
CAGAGAGTAATTTATTTGGTGAAGC
CAGAGAGTAAAATAATTGGTGAAGC
CAGAGAGTAATTTATTTGGTGAAGC
CAGAGAGTAAAATAATTGGTGAAGC
CAGAGAGTAAAATAATTGGTGAAGC
CAGAGAGTAAAATAATTGGTGAAGC
CAGAGAGTAAAAATAATTGGTGAAGC
CAGAGAGTAAATATTGGTGAAGC
CAGAGAGTAAAATAATTGGTGAAGC
CAGAGAGTAAAATAATTGGTGAAGC
CAGAGAGTAATTTATTTGGTGAAGC
CAGAGAGTAAAATAATTGGTGAAGC
CAGAGAGTAAAATAATTGGTGAAGC
CAGAGAGTAAAATAATTGGTGAAG
CAGAGAGTAATTTATTTGGTGAAGC
CAGAGAGTAAAATAATTGGTGAAGC
CAGAGAGTAATTTATTTGGTGAAGC
CAGAGAGTAAAATAATTGGTGAAGC
CAGAGAGTAATTTATTTGGTGAAGC
CAGAGAGTAAAATAATTGGTGAAGC
CAGAGAGTAAAATAATTGGTGAAGC
CAGAGAGTAATTTATTTGGTGAAGC
CAGAGAGTAAAATAATTGGTGAAGC
CAGAGAGTAAAATAATTGGTGAAGC
CAGAGAGTAAAATAATTGGTGAAGC
CAGAGAGTAAAATATTGGTGAAGC
CAGAGAGTAAAATAATTGGTGAAGC
CAGAGAGTAATTTATTTGGTGAAGC
CAGAGAGTAAAATAATTGGTGAAGC
CAGAGAGTAAAATAATTGGTGAAGC
CAGAGAGTAAAATAATTGGTGAAG
CAGAGAGTAAAATTATTGGTGAAGC

CAGAGAGTAAATAATTGGTGAAGC
CAGAGAGTAATATTGGTGAAG
CAGAGAGTAATTATTTGGTGAAGC
CAGAGAGTAAAATAATTGGTGAAGC
CAGAGAGTAATTTATTTGGTGAAGC
CAGAGAGTAAAATAATTGGTGAAGC
CAGAGAGTAAAATAATTGGTGAAGC
CAGAGAGTAAAATAATTGGTGAAGC
CAGAGAGTAATTTATTTGGTGAAGC
CAGAGAGTAAAATAATTGGTGAAGC
CAGAGAGTAAAATAATTGGTGAAGC
CAGAGAGTAAAATAATTGGTGAAGC
CAGAGAGTAAAATAATTGGTGAAGC
CAGAGAGTAAAATAATTGGTGAAGC
CAGAGAGTAAAATAATTGGTGAAGC
CAGAGAGTAATTTATTTGGTGAAGC
CAGAGAGTAAAATAATTGGTGAAGC
CAGAGAGTAATTTATTTGGTGAAGC
CAGAGAGTAAAATAATTGGTGAAGC
CAGAGAGTAAAATAATTGGTGAAGC
CAGAGAGTAAAATAATTGGTGAAGCA
AGAGAGTAATTTATTTGGTGAAGC
CAGAGAGTAAAATAATTGGTGAAGC
CAGAGAGTAAAATTAATTTGGTGAAGC
CAGAGAGTAAAATAATTGGTGAAGC
CAGAGAGTAAAATAATTGGTGAAGC
CAGAGAGTAAAATATATGGTGAAGC
CAGAGAGTAAAATATATGGTGAAGC
CAGAGAGTAAAATAATTGGTGAAGC
CAGAGAGTAATTTATTGGTGAAGC
CAGAGAGTAAAATAATTGGTGAAGC
CAGAGAGTAATTATTTGGTGAAGC
CAGAGAGTAAAATAATTGGTGAAGC
CAGAGAGTAAAATAATTGGTGAAGC
CAGAGAGTAAAATAATTGGTGAAGT
CAGAGAGTAATTTATTTGGTGAAGC
CAGAGAGTAAAATAATTGGTGAAGC
CAGAGAGTAATTTATTTGGTGAAGC
CAGAGAGTAAAATAATTGGTGAAGC
CAGAGAGTAATTTATTTGGTGAAGC
CAGAGAGTAAAATAATTGGTGAAG
CAGAGAGTAATTTATTTGGTGAAGC
CAGAGAGTAAAATAATTGGTGAAGC
CAGAGAGTAAAATAATTGGTGAAGC
CAGAGAGTAAAATAATTGGTGAAGC
CAGAGAGTAAAATAATTGGTGAAG
CAGAGAGTAATTTATTTGGTGAAGC
CAGAGAGTAAAATAATTGGTGAAGC
CAGAGAGTAAAATAATTGGTGAAGC
CAGAGAGTAAAATAATTGGTGAAGC
CAGAGAGTAAAATAATTGGTGAAGC
CAGAGAGTAATTTATTTGGTGAAGC
CAGAGAGTAAAATAATTGGTGAAG
CAGAGAGTAATTTATTTGGTGAAGC

CAGAGAGTAAAATAATTGGTGAAGC
CAGAGAGTAATTTATTTGGTGAAGC
CAGAGAGTAAAATAATTGGTGAAGC
CAGAGAGTAAAATAATTGGTGAAGC
CAGAGAGTAAAATAATTGGTGAAGC
CAGAGAGTAAAATATTTGGTGAAGC
CAGAGAGTAAAATAATTGGTGAAGC
CAGAGAGTAAAATAATTGGTGAAGC
CAGAGAGTAAAATAATTGGTGAAGC
CAGAGAGTAAATAATTGGTGAAGC
CAGAGAGTAAAATAATTGGTGAAGCA
AGAGAGTAATTTATTTGGTGAAGC
CAGAGAGTAAAATAATTGGTGAAGC
CAGAGAGTAAAATAATTGGTGAAGC
CAGAGAGTAAAATAATTGGTGAAGC
CAGAGAGTAAAATAATTGGTGAAGC
CAGAGAGTAAAATAATTGGTGAAGC
CAGAGAGTAAAATAATTGGTGAAGC
CAGAGAGTAATTTATTTGGTGAAGC
CAGAGAGTAAAATAATTGGTGAAGC
CAGAGAGTAATTTATTTGGTGAAGC
CAGAGAGTAAAATAATTGGTGAAGC
CAGAGAGTAAAATAATTGGTGAAGC
CAGAGAGTAATTTATTTGGTGAAGC
CAGAGAGTAAAATAATTGGTGAAGC
CAGAGAGTAATTATTGGTGAAGC
CAGAGAGTAAATAATTGGTGAAGC
CAGAGAGTAATTTATTTGGTGAAGC
CAGAGAGTAAAATTGGTGAAGC
CAGAGAGTAATATTGGTGAAGC
CAGAGAGTAAAATTATTTGGTGAAGC
CAGAGAGTAAAATAATTGGTGAAGC
CAGAGAGTAAAATAATTGGTGAAGC
CAGAGAGTAATTTATTTGGTGAAGCAGAGAAAATAATTGGTGAAGC
CAGAGAGTAAAATAATTGGTGAAGC
CAGAGAGTAAATTATTGGTGAAGC
CAGAGAGTAAAATAATTGGTGAAGC
CAGAGAGTAAAATATTGGTGAAGC
CAGAGAGTAAAATAATTGGTGAAGC
CAGAGAGTAAAATATTGGTGAAGC
CAGAGAGTAAAATATTGGTGAAGC
CAGAGAGTAAAATAATTGGTGAAGC
CAGAGAGTAAAATATTGGTGAAGC
CAGAGAGTAAAATAATTGGTGAAGC
CAGAGAGTAAAAATTGGTGAAGC
CAGAGAGTAAAATAATTGGTGAAGC
CAGAGAGTAAAATAATTGGTGAAGC
CAGAGAGTAAAATAATTGGTGAAGC
CAGAGAGTAAAATAATTGGTGAAGC
CAGAGAGTAAAATAATGGTGAAAAAAAA
CAGAGAGAAAATAATTGGTGAAGCC
CAGAGAGTAAAATAATTGGTGAAC
CAAAGAGTAAAATAATTGGTGAAGC
CAGAGAGTAAAATTAATTGGTGAAGC

CAGAGAGTAAAAAATATGGGTATTTGGTGAAGC
CAGAGAGTAAAATAATTGGTGAAGC
CAGAGAGTAAAATAATTGGGTGAAGC
CAGAGAGTAATTTATTTGGTGAAGCC
CAGAAGAGTAAAATAATTGGTGAAGC
CAGAGAGTAAAATAATTGGTGAAG
CAGAGAGTAATTTGTTGGTGAAGC
CAGAGAGTAAAATAATTGGTGAAGC
CAGAGAGTAAAATAATTGGTGAAGC
CAGAGAGTAAAATAATGGTGAAGC
CAGAGAGTAAAATAATTGGTGAATTGGTGAAGC
CAGAGAGTAAAATAATTGGTGAAGC
CAGAGAGTAAATAATTTATTTGGTGAAGCCAGAGGAGGTAAAATAAATTGGTGAAGC
CAGAGAGTAATTTATTTGGTGAAGC
CAGAGAGTAAAATAATTGGTGAAGC
CAGAGAGTAAAATAATTGGTGAAGC
CAGAGAGTAAAATAATTGGTGAAGC
CAGAGAGTAAAATAATTGGTGAAGC
CAGAGAGTAATATAATTGGTGAAGC
CAGAGAGTAAAATAATTGGTGAAGC
CAGAGAGTAAAATAATTGGTGAAGC
CAGAGAGTAAAATAATTGGTGAAGC
CAGAGAGTAAAATAATTGGTGAAGC
CAGAGAGTAAAATAATTGGTGAAGC
CAGAGAGTAAAATAATTGGTGAAGC
CAGAGAGTAATTTGGTGAAGC
CAGAGAGTAATATAATTGGTGAAGC
CAGAGAGTAAAATAATTGGTGAAGC
CAGAGAGTAAAATAATTGGTGAAGC
CAGAGAGTAAAATAATTGGTGAAGC
CAGAGAGTAAAATAATTGGTGAAGC
CAGAGAGTAATTTTAGCAGAGTATTTGGTGAAGC
CAGAGAGTAAAATAATTGGTGAAGC
CAGAGAGTAAAATAATTGGTGAAGC
CAGAGAGTAAAATAATTGGTGAAGC
CAGAGAGTAAAAATAATTGGTGAAGC
CAGAGAGTAAAATAATTGGTGAAGC
CAGAGAGTAAAATAATTGGTGAAGC
CAGAGAGTAAATTAATTGGTGAAGC
CAGAGAGTAAAATAATTGGTGAAGC
CAGAGAGTAAATTTATTTGGTGAAGC
CAGAGAGTAAAATAATTGGTGAAGC
CAGAGAGGTAAAATAATTGGTGAAGC
CAGAGAGTAATTTATTTGGTGAAGC
CAGAGAGTAAAATAATTGGTGAAGC
CAGAGAGTAATTTAATTTGGTGAAGC
CAGAGAGTAAAATAATTGGTGAAGC
CAGAGAGTAAAATAATTGGTGAAGC
CAGAGAGTAAAATAATTGGTGAAGC
CAGAGAGTAAAATAATTGGTGAAGC
CAGAGAGTAAAATAATTGGTGAAGC
CAGAGAGTAATTTATTTGGTGAAGC
CAGAGAGTAAAATAATTGGTGAAGC
CAGAGAGTAAAATAATTGGTGAAGC

CAGAGAGTAAAATAATTTGGTGAAGC
CAGAGAGTAAAATAATTGGTGAAGC
CAGAGAGTAATAATTTAATTTGGTGAAGC
CAGAGAGTAAAATTAATATTTGGTGAAGC
CAGAGAGTAAATAATAATTGGTGAAGC
CAGAGAGTAAAATAATTGGTGAAGC
CAGAGAGTAAAATAATTGGTGAAGC
CAGAGAGTAAAATAATTGGTGAAGC
CAGAGAGTAAAATAATTGGTGAAGC
CAGAGAGTAAAATAATTGGTGAAGC
CAGAGAGTAAAATAATTGGTGAAGC
CAGAGAGTAAAATAATTGGTGAAGC
CAGAGAGTAAAATAATTGGTGAAGC
CAGAGAGTAATTTATTTGGTGAAGC
CAGAGAGTAAAATAATTGGTGAAGC
CAGAGAGTAAAATAATTGGTGAAGC
CAGAGAGTAAAATAATTGGTGAAGC
CAGAGAGTAAAATAATTGGTGAAGC
CAGAGAGTAAAATAATTGGTGAAGC
CAGAGAGTAATTTAATTGGTGAAGC
CAGAGAGTAAAATAATTGGTGAAGC
CAGAGAGTAAAATAATTGGTGAAGC
CAGAGAGTAAAATAATTGGTGAAGC
CAGAGAGTAAAATAATTGGTGAAGC
CAGAGAGTAATTTATTTGGTGAAGC
CAGAGAGTAAAATAATTGGTGAAGC
CAGAGAGTAATTTATTTGGTGAAGC
CAGAGAGTAAATTATTGGTGAAGC
CAGAGAGTAAATAATTGGTGAAG
CAGAGAGTAATTTATTTGGTGAAGC
CAGAGAGTAAAATAATTGGTGAAGC
CAGAGAGTAAAATAATTGGTGAAGC
CAGAGAGTAATTTATTTGGTGAAGC
CAGAGAGTAAAATAATTGGTGAAGC
CAGAGAGTAAATAATTGGTGAAGC
CAGAGAGTAAAATAATTGGTGAAGC
CAGAGAGTAATTTATTTGGTGAAGC
CAGAGAGTAAAATAATTGGTGAAGC
CAGAGAGTAAAATAATTGGTGAAGC
CAGAGAGTAAATAATTGGTGAAGC
CAGAGAGTAAAATAATTGGTGAAGC
CAGAGAGTAAAATAATTGGTGAAGC
CAGAGAGTAAAATAATTGGTGAAGC
CAGAGAGTAAAATAATTGGTGAAGC
CAGAGAGTAAAATAATTGGTGAAGC
CAGAGAGTAAAATAATTGGTGAAGC
CAGAGAGTAAAATAATTGGTGAAGC
CAGAGAGTAATTTATTTGGTGAAGC
CAGAGAGTAAAATAATTGGTGAAGC
CAGAGAGTAAAATAATTGGTGAAGC
CAGAGAGTAATTTATTTGGTGAAGC
CAGAGAGTAAAATAATTGGTGAAGC
CAGAGAGTAAAATAATTGGTGAAG
CAGAGAGTAATATTGGTGAAGC

CAGAGAGTAAAATAATTGGTGAAGC
CAGAGAGTAATTTATTTGGTGAAGC
CAGAGAGTAAAATAATTGGTGAAGC
CAGAGAGTAAAATAATTGGTGAAGC
CAGAGAGTAAAATAATTGGTGAAGC
CAGAGAGTAAAATAATTGGTGAAGC
CAGAGAGTAAAATAATTGGTGAAGC
CAGAGAGTAAAATAATTGGTGAAGC
CAGAGAGTAAAATAATTGGTGAAGC
CAGAGAGTAAAATAATTGGTGAAGC
CAGAGAGTAAAATAATTGGTGAAGC
CAGAGAGTAAAATAATTGGTGAAGC
CAGAGAGTAATTTATTTGGTGAAGC
CAGAGAGTAAAATAATTGGTGAAGC
CAGAGAGTAAAATAATTGGTGAAGC
CAGAGAGTAATTTATTTGGTGAAGC
CAGAGAGTAAAATAATTGGTGAAGC
CAGAGAGTAAAATAATTGGTGAAGCAGAGAATATGGTGAAGC
CAGAGAGTAAAATAATTGGTGAAGC
CAGAGAGTAAAATATTGGTGAAGC
CAGAGAGTAAAATAATTGGTGAAG
CAGAGAGTAATATTGGTGAAGC
CAGAGAGTAAAATAATTGGTGAAGC
CAGAGAGTAAAATAATTGGTGAAGC
CAGAGAGTAAAATAATTGGTGAAGC
CAGAGAGTAAAATAATTGGTGAAGC
CAGAGAGTAAAATAATTGGTGAAGC
CAGAGAGTAAAATAATTGGTGAAGC
CAGAGAGTAATTTATTTGGTGAAGC
CAGAGAGTAAAATAATTGGTGAAGC
CAGAGAGTAATTTATTTGGTGAAGC
CAGAGAGTAAAATAATTGGTGAAGC
CAGAGAGTAAAATAATTGGTGAAGC
CAGAGAGTAAAATAATTGGTGAAGC
CAGAGAGTAAATAATTGGTGAAGC
CAGAGAGTAAAATAATTGGTGAAGC
CAGAGAGTAATTTATTTGGTGAAGC
CAGAGAGTAAAATAATTGGTGAAGC
CAGAGAGTAATTTATTTGGTGAAGC
CAGAGAGTAAAATAATTGGTGAAGC
CAGAGAGTAAAATAATTGGTGAAGC
CAGAGAGTAAAAATAAAAATAAAATAATTGGTGAAGC
CAGAGAGTAATTTATTTGGTGAAGC
CAGAGAGTAAAATAATTGGTGAAGC
CAGAGAGTAATTTATTTGGTGAAGC
CAGAGAGTAAAATAATTGGTGAAGC
CAGAGAGTAATTTATTTGGTGAAGC
CAGAGAGTAAAATAATTGGTGAAGC
CAGAGAGAAAAAAAAAAAATAATTGGTGAAG
CAGAGAGTAAAATAATTGGTGAAGC
CAGAGAGTAAAATAATTGGTGAAGC
CAGAGAGTAAAATAATTGGTGAAGC
CAGAGAGTAAAATAATTGGTGAAGC
CAGAGAGTAAAATAATTGGTGAAGC

CAGAGAGTAAAATAATTGGTGAAGC
CAGAGAGTAAAATAATTGGTGAAGC
CAGAGAGTAAAATAATTGGTGAAGC
CAGAGAGTAAAATAATTGGTGAAGC
CAGAGAGTAAAATAATTGGTGAAGC
CAGAGAGTAAAATAATTGGTGAAGC
CAGAGAGTAAAATAATTGGTGAAGC
CAGAGAGTAAAATAATTGGTGAAGC
CAGAGAGTAAAATAATTGGTGAAG
CAGAGAGTAATATTGGTGAAG
CAGAGAGTAAAATAATTGGTGAAGC
CAGAGAGTAAAATAATTGGTGAAGCAAAAAAAAAAAAAAAAAAAAAAAAAAAAAAAAAAAAAAAGAGAAAATAATTGGT
GAAGC
CAGAGAGTAAAATAATTGGTGAAGC
CAGAGAGTAAAATAATTGGTGAAGC
CAGAGAGTAAAATAATTGGTGAAGC
CAGAGAGTAAAATAATTGGTGAAGC
CAGAGAGTAAAATAATTGGTGAAGC
CAGAGAGTAAAATAATTGGTGAAGC
CAGAGAGTAAAATAATTGGTGAAGC
CAGAGAGTAAAATAATTGGTGAAGC
CAGAGAGTAAAATAATTGGTGAAGC
CAGAGAGTAAAATAATTGGTGAAGC
CAGAGAGTAAAATAATTGGTGAAGC
CAGAGAGTAAAATAATTGGTGAAGC
CAGAGAGTAAAATAATTGGTGAAGC
CAGAGAGTAAAATAATTGGTGAAGC
CAGAGAGTAAAATAATTGGTGAAGC
CAGAGAGTAAAATAATTGGTGAAGC
CAGAGAGTAAAATAATTGGTGAAGC
CAGAGAGTAAAATAATTGGTGAAG
CAGAAGTAATATTGGTGAAGC
CAGAGAGTAAATATTATTGGTGAAGC
CAGAGAGTAAAATAATTGGTGAAGC
CAGAGAGTAATAATTGGTGAAGC
CAGAGAGTAAAATAATTGGTGAAGC
CAGAGAGTAAAATAATTGGTGAAGC
CAGAGAGTAAAATAATTGGTGAAGC
CAGAGAGTAAAATAATTGGTGAAGC
CAGAGAGTAAAATATTGGTGAAGC
CAGAGAGTAAAATAATTGGTGAAGC
CAGAGAGTAAAATAATTGGTGAAGC
CAGAGAGTAAAATAATTGGTGAAGT
CAGAGAGTAATTTATTTGGTGAAGC
CAGAGAGTAAAATAATTGGTGAAGC
CAGAGAGTAAAATAATTGGTGAAGC
CAGAGAGTAAAATAATTGGTGAAGC
CAGAGAGTAATTTATTTGGTGAAGC
CAGAGAGTAAAATAATTGGTGAAGC
CAGAGAGTAAAATAATTGGTGAAGC
CAGAGAGTAAAATAATTGGTGAAGC
CAGAGAGTAAAATAATTGGTGAAGT
CAGAGAGTAATTTAGTTGGTGAAGC
CAGAGAGTAAAATAATTGGTGAAGC

CAGAGAGTAAAATAGTTGGTGAAGT
CAGAGAGTAATTTATTTGGTGAAGC
CAGAGAGTAAAATAATTGGTGAAGT
CAGAGAGTAATTTAGTTGGTGAAGC
CAGAGAGTAATTTATTTGGTGAAGT
CAGAGAGTAAAATAATTGGTGAAGT
CAGAGAGTAATTTAGTTGGTGAAGC
CAGAGAGTAAAATAATTGGTGAAGC
CAGAGAGTAAAATAATTGGTGAAGT
CAGAGAGTAATTTAGTTGGTGAAGC
CAGAGAGTAAAATAATTGGTGAAGC
CAGAGAGTAAAATAGTTGGTGAAGT
CAGAGAGTAATTTATTTGGTGAAGC
CAGAGAGTAAAATAATTGGTGAAGT
CAGAGAGTAATTTAGTTGGTGAAGC
CAGAGAGTAATTTAGTTGGTGAAGC
CAGAGAGTAAAATAATTGGTGAAGC
CAGAGAGTAAAATAATTGGTGAAGT
CAGAGAGTAATTTATTTGGTGAAGC
CAGAGAGTAAAATAATTGGTGAAGC
CAGAGAGTAAAATAGTTGGTGAAGC
CAGAGAGTAAAATAATTGGTGAAGC
CAGAGAGTAAAATAATTGGTGAAGC
CAGAGAGTAAAATAATTGGTGAAGT
CAGAGAGTAATTTATTTGGTGAAGC
CAGAGAGTAAAATAATTGGTGAAGC
CAGAGAGTAAAATAATTGGTGAAGC
CAGAGAGTAAAATAATTGGTGAAGT
CAGAGAGTAATTTAGTTGGTGAAGC
CAGAGAGTAAAATAATTGGTGAAGC
CAGAGAGTAAAATAATTGGTGAAGC
CAGAGAGTAAAATAATTGGTGAAGC
CAGAGAGTAAAATAATTGGTGAAGC
CAGAGAGTAAAATAATTGGTGAAGC
CAGAGAGTAAAATAATTGGTGAAGC
CAGAGAGTAAAATAATTGGTGAAGC
CAGAGAGTAATTTATTTGGTGAAGC
CAGAGAGTAAAATAATTGGTGAAGC
CAGAGAGTAAAATAATTGGTGAAGC
CAGAGAGTAATTTATTTGGTGAAGT
CAGAGAGTAATTTATTTGGTGAAGC
CAGAGAGTAAAATAATTGGTGAAGC
CAGAGAGTAATTTATTTGGTGAAGC
CAGAGAGTAAAATAATTGGTGAAGC
CAGAGAGTAATTTATTTGGTGAAGC
CAGAGAGTAAAATAATTGGTGAAGC
CAGAGAGTAAAATAATTGGTGAAGC
CAGAGAGTAATTTATTTGGTGAAGC
CAGAGAGTAAAATAATTGGTGAAGC
CAGAGAGTAAAATAATTGGTGAAGC
CAGAGAGTAATTTATTTGGTGAAGC
CAGAGAGTAAAATAATTGGTGAAGC
CAGAGAGTAAAATAATTGGTGAAGC
CAGAGAGTAAAATAGTTGGTGAAGC

CAGAGAGTAAAATAATTGGTGAAGC
CAGAGAGTAATTTATTTGGTGAAGC
CAGAGAGTAAAATAATTGGTGAAGC
CAGAGAGTAAAATAATTGGTGAAGC
CAGAGAGTAATTTATTTGGTGAAGT
CAGAGAGTAATTTATTTGGTGAAGC
CAGAGAGTAAAATAATTGGTGAAGC
CAGAGAGTAAAATAATTGGTGAAGC
CAGAGAGTAATTTATTTGGTGAAGC
CAGAGAGTAATTTATTTGGTGAAGC
CAGAGAGTAATTTATTTGGTGAAGC
CAGAGAGTAATTTATTTGGTGAAGC
CAGAGAGTAATTTATTTGGTGAAGC
CAGAGAGTAAAATAATTGGTGAAGC
CAGAGAGTAATTTATTTGGTGAAGC
CAGAGAGTAAAATAATTGGTGAAGC
CAGAGAGTAAAATAATTGGTGAAGC
CAGAGAGTAATTTATTTGGTGAAGC
CAGAGAGTAAAATATTTGGTGAAGC
CAGAGAGTAAAATAATTGGTGAAGC
CAGAGAGTAATTTATTTGGTGAAGC
CAGAGAGTAAAATAATTGGTGAAGC
CAGAGAGTAAAATAATTGGTGAGGC
CAGAGAGTAAAATAATTAGTGAAGC
CAGAGAGTAAAATAATTGGTGAAGC
CAGAGAGTAAAATAATTGGTGAAGC
CAGAGAGTAAAATAATTGGTGAAGC
CAGAGTGTAAAATAATTGGTGAAGC
CAGAGAGTAAAATAATTGGTGAAGC
CAGAGAGTAAAATAATTGGTGAAGC
CAGAGAGTAAAATAATTGGTGAAGC
CAGAGAGTAAAATAATTGGTGAGGC
CAGAGAGTAAAATAATTAGTGAAGC
CAGAGAGTAAAATAATTAGTGAAGC
CAGAGAGTAAAATAATTGGTGAAGC
CAGAGAGTAAAATAATTAGTGAAGC
CAGAGAGTAAAATAATTGGTGAAGC
CAGAGAGTAAAATAATTGGTGAAGC
CAGAGAGTAAAATAATTGGTGAAGC
CAGAGAGTAAAATAATTGGTGAGGC
CAGAGAGTAAAATAATTAGTGAAGC
CAGAGAGTAAAATAATTGGTGAAGC
CAGAGAGTAAAATAATTGGTGAAGC
CAGAGAGTAATTTATTTGGTGAAGC
CAGAGAGTAATTTATTTGGTGAAGC
CAGAGAGTAATTTATTTGGTGAAGC
CAGAGAGTAATTTATTTGGTGAAGC
CAGAGAGTAATTTATTTGGTGAAGC
CAGAGAGTAAAATAATTGGTGAAGC
CAGAGAGTAATTTATTTGGTGAAGC
CAGAGAGTAAAATAATTGGTGAAGC
CAGAGAGTAAAATAATTGGTGAAGC
CAGAGAGTAATTTATTTGGTGAAGC
CAGAGAGTAAAATATTTGGTGAAGC

CAGAGAGTAAAATAATTGGTGAAGC
CAGAGAGTAATTTATTTGGTGAAGC
CAGAGAGTAAAATAATTGGTGAAGC
CAGAGAGTAAAATAATTGGTGAGGC
CAGAGAGTAAAATAATTAGTGAAGC
CAGAGAGTAAAATAATTGGTGAAGC
CAGAGAGTAAAATAATTGGTGAAGC
CAGAGAGTAAAATAATTGGTGAAGC
CAGAGTGTAAAATAATTGGTGAAGC
CAGAGAGTAAAATAATTGGTGAAGC
CAGAGAGTAAAATAATTGGTGAAGC
CAGAGAGTAAAATAATTGGTGAAGC
CAGAGAGTAAAATAATTGGTGAGGC
CAGAGAGTAAAATAATTAGTGAAGC
CAGAGAGTAAAATAATTAGTGAAGC
CAGAGAGTAAAATAATTGGTGAAGC
CAGAGAGTAAAATAATTAGTGAAGC
CAGAGAGTAAAATAATTGGTGAAGC
CAGAGAGTAAAATAATTGGTGAAGC
CAGAGAGTAAAATAATTGGTGAAGC
CAGAGAGTAAAATAATTGGTGAGGC
CAGAGAGTAAAATAATTAGTGAAGC
CAGAGAGTAAAATAATTAGTGAAGC
CAGAGAGTAAAATAATTAGTGAAGC
CAGAGAGTAAAATAATTGGTGAAGC
CAGAGAGTAAAATAATTAGTGAAGC
CAGAGAGTAAAATAATTAGTGAAGC
Nr of selected repeats 587 Similarity 0.896061
Consensus:
CAGAGAGTAAaaTAaTTGGTGAAGC
>Cele-UNSB01_3:1391886-1392275 Satlength=390 Nr of Repeats=16 RepeatLength=21
seed=TTTTTGGTAA
TTTTTGGTAAAATTTGGTGAAATTTGGTAAC
TTTTTGGTAAAATTTGATAAT
TTTTTGGTAAAATTTGGTAACTTTTGATAAAATTTGGTAACTTTTCGGTGAAAATTCGGTAAC
TTTTTGATAAAATTCGGTAAC
TTTTTGGTAAAATTTGGTAAT
TTTTTGGTAAAATTTAGTAAC
TTTTTGGTAAAATTCGGTAAC
TTTTTGGTAAAATTTGGTAAC
TTTTTGGTAAAATTTGATAAT
TTTTTGGTAAAATTTGGTAAT
TTTTTGGTAAAATTTGGTAAT
TTTTTGATAAAATTTAGTATT
TTTTTGGTAAAATTCGGTAAC
TTTTTGGTAAAATTTGGTAAC
TTTTTGGTAAAATTCGGTAAC
TTTTTGATAAAATTCGGTAACT
Nr of selected repeats 13 Similarity 0.873016
Consensus:
TTTTTGGTAAAATTtGGTAAc
>Cele-UNSB01_3:1394199-1394513 Satlength=315 Nr of Repeats=12 RepeatLength=21
seed=CAAAAAGTTA
CAAAAAGTTACCAAATTTTACCAAAAATATGCCAAATTTTCC

CAAAAAGTTATCAAATTTTAC
CAAAAAGTTACCAAATTTTAG
CAAAAAGTTACTAAAAATTTATTTAAAAAACCCAATTTTGCCAAAAATATACCAAGTTTTAG
CAAAAAGTTACCAAATTTTAC
CGAAAAGTTACCAAATTTTAA
CAAAAAGTTACCAAATTTTAA
CAAAAAGTTACCAAATTTTAA
CAAAAAGTTACCAAATTTTAA
CAAAAAGTTACCAAATTTTAA
CAAAAAGTTACCAAATTTTAA
CAAAAAGTTACCAAGTTTTAC
Nr of selected repeats 10 Similarity 0.923810
Consensus:
CAAAAAGTTACCAAATTTTAa
>Cele-UNSB01_3:1395088-1395322 Satlength=235 Nr of Repeats=5 RepeatLength=35
seed=CAAATCGGCA
CAAATCGGCAAATTGCCGGAATTAAAATTTTTTGG
CAAATCGGCAAATCGGCACATTACCTTAATTGAAATTTTTGA
CAAATCGACAAATTGCCGGAATTGAAAATTTCCGG
CAAACCGGCAAATCGGCAAATGACCAAATTGTTGAATTTTCCGGAAAAACGGCAACTTGCCGGAATTGAAAATTTCCTG
CAAATAAG
CAAACCGGCAAGTTACCGAAATTGAAAAGATCCGA
Nr of selected repeats 3 Similarity 0.695238
Consensus:
CAAAtCGgCAAaTTgCCGgAATTgAAAattTccGg
>Cele-UNSB01_3:1399964-1400110 Satlength=147 Nr of Repeats=5 RepeatLength=11
seed=GTACTGTAGG
GTACTGTAGGG
GTACTGTAGGAA
TACTGTAGGATTACTGTAGTTTTGGAAAAACTAACTTTTTGTCTTTTGAAGGTATATTGGGGTGGGGGTTAGTAGGGAG
ATATTGTTTGGGTACTGTTGTA
GTACTGTAGGA
GTACTGTAGGA
Nr of selected repeats 3 Similarity 0.919192
Consensus:
GTACTGTAGGa
>Cele-UNSB01_3:1400275-1400335 Satlength=61 Nr of Repeats=4 RepeatLength=15
seed=TCGTGGTGAG
TCGTGGTGAGACCCT
TCGTGGTGAGACCCA
TCGTGGTGAGACCCT
TCGTGGTGAGACCCT
Nr of selected repeats 4 Similarity 0.955556
Consensus:
TCGTGGTGAGACCCt
>Cele-UNSB01_3:1400394-1400521 Satlength=128 Nr of Repeats=4 RepeatLength=31
seed=AATTCAAATT
AATTCAAATTTATTTAAAAAAATTTGGCGGG
AATTCAAATTTCTTCAAAAAAATTTTGGCGGG
AATGCAAATTTCTTCAAAAAAATTTTGGCGGG
AATTCAAATTTTCTGAGAAAAGAATTGGGCAGGA
Nr of selected repeats 3 Similarity 0.885305
Consensus:
AATtCAAATTTcTTcAAAAAAtTTTGGCGGG

>Cele-UNSB01_3:1406406-1406896 Satlength=491 Nr of Repeats=33 RepeatLength=11
seed=GGTACTGTAG
GGTACTGTAGG
GGTCTGTAGGGGTTATGTAGGGATGCTGTAGGGGTACTGGGGGGGGGGGGGGGGGGGG
GGTACTGTAGGGGTGCTGTACG
GGTGCTGTAGG
GGTACTGTAGT
GGTTCTGTAGG
GGTACTGTAGG
GGTTCTGTAGGAATTCTGTAGG
GGTACTCTAGT
GGTACTGTAGT
GGTACTGTAGT
GGTACTGTAGG
GGTACTGTAGG
GGTACTGTTGT
GGTACTGTAGG
GGTACTGTAGG
GCTACTGTAGGGGAACAGTAGGGATATTGTAGG
GGTATTGTAGG
GGTACTGTAAG
GGTACTGTAGAGATCAAAACTATCTGTAGG
GATACTGTAGGA
GGTCTGTAGT
GGTACTGTAGG
GGTATTGTAGG
GGTACTGTAAG
GGTACTGTAGAGATCAAAACTATCTGTAGG
GATACTGTAGGA
GGTCTGTAGT
GGTACTGTAG
GGTAGTGTAGG
GGTACTGTAGG
GGTCTGTAGG
GGTTCTGTAGG
Nr of selected repeats 21 Similarity 0.844156
Consensus:
GGTACTGTAGg
>Cele-UNSB01_3:1413910-1414798 Satlength=889 Nr of Repeats=40 RepeatLength=14
seed=ATACCTAATA
ATACCTAATAACTA
ATACCTTATACCTT
ATACCTAATACCTA
ATACCTAATACCTA
ATACCTAATACCTTCTACCTCATACTTAA
AACCTAATACCTT
ATACCGAATACCTA
ATATCTAATACCTC
ATACCTAATACTTA
ATACCTAATAACTA
ATACCTTATACCTT
ATACCTTATACCTA
ATACCTAATAACTA
ATACCTAATACCTTCTACCTCATACTTAA

AACCTAATACCTT
ATACCTAATACCTA
ATATCTAATACCTTATTCCTTATATCTAATGCCGAACACCTTATATATTAAACCTAATGTTTA
ATACCTAATACCTA
ATACCTAACACCTA
ATACCTAATACCTA
ATACCTAATTCCTA
ATATCTAATACCTTACACCTTAAACCTCATACCTCATGCCTT
ATACCTAATACCTAATTGCTAATA
ATATCTAATACCTA
ATACCAATACCTA
ATACCTAATACTTA
ATACCTAATACCTA
ATACCTAATACCTA
ATACCTAATACCTA
ATACCTAAAACCTTATTCCTT
ATACCTAATACCTA
ATACCTAATACCTA
ATACCTAATACCTAATACTTTATTCCTT
ATACCTAATACCTG
ATACCTAATACCTA
ATACCTAATACCTA
ATACCTAAAGCCTA
ATACCTAATACCTA
ATACCTTATACCTTATACCTACAAGGCATTTATATCCACTTCTACAACTTCAAAAGAGGGCGCATTTATCCGAAATGGT
CTCGTCACGCGCCCGAAAATCAATGGGTCGAACCATCGCGCGTGAACACGCCCCTCTTTAAAGTTGCAGAAGTAGAAAA
AATGCGATGCAATATTTTATGCCTAGTATCGGATA
ATACCTAATACCTAATGCCTGATACCTT
Nr of selected repeats 28 Similarity 0.877047
Consensus:
ATACCTAATACCTA
>Cele-UNSB01_3:1466668-1467327 Satlength=660 Nr of Repeats=5 RepeatLength=43
seed=GGCTCTCGGC
GGCTCTCGGCTCGCGCCGAGGGCCGAGTTCCACTAAAAGTTTC
GGCTCTCGGCTTGCGCCGAGAGCCGAGGGCTGATTGCTTTATATTTCGGTATCGATTTTCGCACGCTCCCTCCGATAAT
GAATAAAAATTATAATTACAACTACGACTTTACAACTATTAAAATAAATAGTTTCGCTGCTAACAATAAGGAATTGAGT
ACTTTTCCGATTTCTGCCCCCAAAAACATTGTATGTCATGTAGGGGGTTGTAAAGATATACTCAAAAGGGTTCCCACAG
GAAAGGATAACCATAATAGATACCCCAATAACAGATCTAATATTTGATTATATGGGTCATTGGGCTGTAAATATGTGTC
TCTTTATGGGGTAGGTGGCGATGTGTTGCCAGCTAACATTAACGTGTATTAATAAGAATTAGACTATGTAATAAGAATT
AGACTATGATAGTTATGAGAGAGAATATAAGAGGAAGGTGCGAGGATTCCGAAACCAATCGGCGTGAGCCGAAAAACAG
AAAAAAAAATTTGTC
GGCTCTCGGCTCGCGCCGAGAGCCGAGCTCTATTTTCCGGTTC
GGCTCTCGGCTTACGCCGAGAGCCGAGTTTAAATTTTTTTC
GGCTCTCGGCTTGTGCCGAGAGCCGAAAAATACCCAAATTGTC
Nr of selected repeats 3 Similarity 0.627907
Consensus:
GGCTCTCGGCTcGcGCCGAGaGCCGAgntctActnaaagttTC
>Cele-UNSB01_3:1616576-1616906 Satlength=331 Nr of Repeats=16 RepeatLength=20
seed=CATAAAAATT
CATAAAAATTCTGAAAAATG
CATAAAAATTCAGAAAAATG
CATAAAAATTCTGAAAAATG
CATAAAAATTCAGAAAAATG
CATAAAAATTCTGAAAAAATCTGAAAAAAG

CATAAAAATG
CATAAAAATTCAGAAAAATA
CATAAAAATTCAGAAAAATA
CATAAAAATTCAGAAAAATA
CATAAAAATTCAGAAAAATA
CATAAAAATTCAGAAAAATG
CATAAAAATTCAGAAAAATA
CATAAAAATTCAGAAAAATG
CATAAAAATTCAGAAAAATG
CATAAAAATTCTGAAAAATTCTGAAAAATG
CATAAAAATTCAGAAAAATG
Nr of selected repeats 13 Similarity 0.947009
Consensus:
CATAAAAATTCAGAAAAATg
>Cele-UNSB01_3:1617648-1618407 Satlength=760 Nr of Repeats=37 RepeatLength=20
seed=TTTCACTAAA
TTTCACTAAAAATCCTAATT
TTTCAATAAAAGTCTCAAAATTACACAAAAATTCCTAAT
TTTCACTAAAAATTTCAAAT
TTTCACTAAACATCTCAAAA
TTTCACTAAAAATCTCAAAA
TTTCACTAAAAATCTCAAAA
TTTCACTAAACATCTCAAAA
TTTCACTAAACATCTCAAAA
TTTCACTAAAAATCTCAAAA
TTTCACTAAAAATCTCAAAA
TTTCACTAAAAATCTCAAAA
TTTCACTAAACATCTCAAAA
TTTCACTAAACATCTCAAAA
TTTCACTAAAAATCTCAAAA
TTTCACTAAAAATCTCAAAA
TTTCACTAAAAATCTCAAAA
TTTCACTAAAAATCTCAAAA
TTTCACTAAAAATCTCAAAA
TTTCACTAAACATCTCAAAA
TTTCACTAAAAATCTCAAAA
TTTCACTAAAGATCTCAAAA
TTTCACTAAAAATCTCAAAA
TTTCACTAAAAATCTCAAAA
TTTCACTAAAAATCTCAAAA
TTTCACTAAAAATCTCAAAA
TTTCACTAAACATCTCAAAA
TTTCACTAAACATCTCAAAA
TTTCACTAAAAATCTCAAAA
TTTCACTAAAAATCTCAAAA
TTTCACTAAAAATCTCAAAA
TTTCACTAAAAATCTCAAAA
TTTCACTAAACATCTCAAAA
TTTCACTAAACATCTCAAAA
TTTCACTAAAAATCTCAAAA
TTTCACTAAAAATCTCAAAA
TTTCACTAAAAATCTCAAAA
TTTCACTAAAAATCTCAAAA
Nr of selected repeats 36 Similarity 0.904019

Consensus:
TTTCACTAAAaATCTCAAAA
>Cele-UNSB01_3:1642030-1642586 Satlength=557 Nr of Repeats=7 RepeatLength=21
seed=CCTAAAAATT
CCTAAAAATTTCAATTTTTGT
CCTAAAAATTCCAAATTTTAA
CCTAAAAATTCCAAATTTTAA
CCTAAAAATTCCAAATTTTAACCTAAATTTCCAAATTTTAACTTAAAATTCCAAATTTTTA
CCTAAAAATTCCCACTTTTTTCCAGGCGGCTTCGTAGATCACCTCTCCAATTTGCTTCATTTGCTCAATGAGCCAACAG
GCTCCACCCTCACAGCTCAATCAATAGTCGAAAGTTTAAAGGCGCATGGTTGGAGTATTACTGTAGGCGTTGAACATGA
TCTCTACGAACTTTTCAATGTATTTGTAACAACTTGGGAGGATGAATTGAAGAGTTCCCGGCGTATTTTAATGAATCAA
TCGATTGAAAATTGTCATAGTAGTAGTAGTGAAGATGATGAAGATGTGGTGGTGGAATCGACGAGTTTAAGAGGATCCC
CGTCAATTATCAGAAAATTGATGAGTTTTCAGAGGTTCGGACGTGGAAAATTAGCTAAAAATCGGGTTTTTTAA
CCTAAAAATTGCCGTGTTTGA
CCTAAAAATCGCATTTTTTTG
Nr of selected repeats 5 Similarity 0.695238
Consensus:
CCTAAAAATtnCaattTTTna
>Cele-UNSB01_3:1690415-1691093 Satlength=679 Nr of Repeats=5 RepeatLength=72
seed=ATGAATTTTA
ATGAATTTTAATACAATTTTCCTGACGATGAAGTCATAAAAATTCATTTTTTGACCTTTCAACTTTAGTTTT
ATGAATTTTAACTAACAAATTCAGATTCAGCACCCAATTTTACCTCTAGACACACGGTTTTTGCAAAAATTTTGGAAAA
TTCTGCAATTTGTCGTAAGCTGTTGGGTGTTTGCGTACTTTTGAAGCTACAGTACCCCAAAATGTTTGATATTTGCGCT
GTAAACTTACAAAAAAGGTTTCCAGGGTAAAATTTAACGAGGAATTTGAATTTTTACATAAAAATCTGCAAAA
ATGAATTTTAATACAATTTTCCTGACGATAAAGTCATAAAAACTCATTTTTTGACCTTTCAACTTTAGTTTT
ATGAATTTTAACTAACAAATTCAGATTCAGCACCCAATTTTACCTCTAGACACACGGTTTTTGCAAAAATTTTGGAAAA
TTCTGCAATTTGTCGTAAGCTGTTGGGTGTTTGCGTACTTTTGAAGCTACAGTACCCCAAAATGTTTGATATTTGCGCT
GTAAACTTACAAAAAAGGTTTCCAGGGTAAAATTTAACGAGGAATTTGAATTTTTACATGAAAATCTGCAAAA
ATGAATTTTAATACAATTTTCCTGACGATGAAGTCATAAAAATTCATTTTTTGACCTTTCAACTTTAGTTTT
Nr of selected repeats 3 Similarity 0.975309
Consensus:
ATGAATTTTAATACAATTTTCCTGACGATgAAGTCATAAAAAtTCATTTTTTGACCTTTCAACTTTAGTTTT
>Cele-UNSB01_3:1715361-1716220 Satlength=860 Nr of Repeats=41 RepeatLength=11
seed=TACTGTAGGG
TACTGTAGGGG
TACTGTAGAGG
TACTGTAGGGGTTTATTGGGGGTACTGTATAATTATTGTAGAGGTAGG
TACTGTAGGGA
TACTGTAGGGGTGCTGTAGAGG
TACTGTAGGGG
TAATGTAGGGG
TACGGTAGGGA
TACTGTTGGGG
TACTGTAGGAG
TACAGTAGGGC
TACTGTAGGGGTAACTGTATGAG
TACTGCAGGGG
TACTGTAGGGGGGTGCTGTAACTTCACCGTTTAGG
TACTGTAGGTC
TACTGTAGGGG
TACTGTAGGAA
TACGGTAGGGG
TACTGTAGGGA

```
TACTGTAGAGGTACTGTAGAACACGGTACATTAGTAAAATTACCTCCAACAAATAGAAGCACTCATCATTTTTAGAAAT
TGAATGGTCAAAACACAAGCGATAAACAATGAAAAATGAGGGGCGGGGATGGATAGATCTGCCTTTTTTGCAAATTGCG
ATGCTATCTTGCACTTTTGTGCAAGATAGCAAACCTGCAAAAGAGAG
TACTGTAGGGG
TACTGTAGGGG
TACTGTAGAGG
TACTGTAGGGGTTTCTTGGGGGTACTGTATAATTATTGTAGAGGTAAG
TACTGTAGGGA
TACTGTAGGGG
TGCTGTAGGGG
TACTGTAGGGGTAATGTAAGGG
TACTATAGGGG
TACTGTAGGGG
TACTGTAGAGG
TACTGTAGGGGTTTCTTAGGGGTACTGTATGATTATTGTAAAGGTAGG
TACTGTAGGGA
TACTGTAGGGG
TGCTGTAGGGG
TAATGTAGGGG
TAATGTAGGGGTAACTGTATGAG
TACTGTAGGGGGTACTGTAAGTTTACCGTTTAGG
TACTGTAGGTC
TACTGTAGGGG
TACTGTAGGGATACGGTAAGGG
Nr of selected repeats 30 Similarity 0.811076
Consensus:
TACTGTAGGGg
>Cele-UNSB01_3:1724995-1725361 Satlength=367 Nr of Repeats=4 RepeatLength=92
seed=CTGCGGAACC
CTGCGGAACCCCGAACGTGTCGGCCGCTTCCAAAAACAACCACCTCTTCACACTTCATTGCGCACACACCAAACTACTA
GTTTCACGTCAAA
CTGCGGAACCCCGAACGTGTCGGCTGCTTCCAAACAACCACCTCTTCACACTTCATTGCGCACACACCAAACTACTAGT
TTCACGTCAAA
CTGCGGAACCCCGAACGTGTCGGCCGCTTCCAAAAACAACCACCTCTTCACACTTCATTGCGCACACACCAAACTACTA
GTTTCACGTCAAA
CTGCGGAACCCCGAACGTGTCGGCCGCTTCCAAAAACAACCACCTCTTCACACTTCATTGCGCACACACCAAACTACTA
GTTTCACGTCAAA
Nr of selected repeats 3 Similarity 1.000000
Consensus:
CTGCGGAACCCCGAACGTGTCGGCCGCTTCCAAAAACAACCACCTCTTCACACTTCATTGCGCACACACCAAACTACTA
GTTTCACGTCAAA
>Cele-UNSB01_3:1738456-1739086 Satlength=631 Nr of Repeats=52 RepeatLength=12
seed=TGAGCCTGAG
TGAGCCTGAGCC
TGAGCCTGAGCC
TGAGCCTGAGCC
TGAGCCTGAGCC
TGAGCCTGAGCC
TGAGCCTGAGCC
TGAGCCTGAGCC
TGAGCCTGAGCC
TGAGCCTGAGCC
TGAGCCTGCGCC
TGAGCCTGAGCC
```

TGAGCCTGAGCC
TGAGCCTGAGCC
TGAGCCTGAGCC
TGAGCCTGAGCC
TGAGCCTGAGCC
TGAGCCTGAGCC
TGAGCCTGAGCC
TGAGCCTGAGCA
TGAGCCTGAGCC
TGAGCCTGAGCC
TGAGCCTGAGTC
TGAGCCTGAGCC
TGAGCCTGAGCC
TGAGCCTGAGCC
TGAGCCTGAGCA
TGCGCCTGAGCC
TGAGCCTGAGCC
TGAGCCTGAGTC
TGAGCCTGAGCC
TGAGCCTGAGCC
TGAGCCTGAGCC
TGAGCCTGAGCC
TGAGCCTGAGCC
TGAGCCTGAGCC
TGAGCCTGAGCC
TGAGCCTGAGCC
TGAGCCTGAGCC
TGAGCCTGAGCC
TGAGCCTGAGCC
TGAGCCTGAGCC
TGAGCCTGAGCC
TGAGCCTGAGCC
TGAGCCTGAGCC
TGAGCCTGAGCC
TGAGCCTGAGCC
TGAGCCTGAGCC
TGAGCCTGAGCC
TGAGCCTGAGCC
TGAGCCTGAGCC
TGAGCCTGAGCC
TGAGCATGAGCC
TGAGCATGAGCCTGAGCA
Nr of selected repeats 51 Similarity 0.969848
Consensus:
TGAGCCTGAGCC
>Cele-UNSB01_3:1753459-1753580 Satlength=122 Nr of Repeats=11 RepeatLength=11
seed=GCAAAAAATG
GCAAAAAATGT
GCAAAAAATGT
GCAAAAAATGT
GCAAAAAATGT
GCAAAAAATGT
GCAAAAAATGT
GCAAAAAATGT
GCAAAAAATGT

GCAAAAAATGG
GCAAAAAATGT
GCAAAAAATGG
Nr of selected repeats 11 Similarity 0.960331
Consensus:
GCAAAAAATGT
>Cele-UNSB01_3:1796051-1796519 Satlength=469 Nr of Repeats=35 RepeatLength=12
seed=CTAAGCCTAA
CTAAGCCTAACC
CTAAGCCTGAGC
CTAAGCCTAAAC
CTAAGCCTGAGC
CTAAACCTAAAC
CTAAGCCTGACC
CTAAGCCTAAAC
CTAAGCCTAAGC
CTAAGCCTAAGC
CTAAGCCTAACC
CTAAGCCTGAGC
CTAAGCCTAAAC
CTAAGCCTGAGC
CTAAACCTAAAC
CTAAGCCTGAGC
CTAAGCCTAAAC
CTAAGCCTAAGC
CTAAGCCTAAGC
CTAAGCCTAACC
CTAATCCTAAGCCTGAAC
CTAAGCCAAAAC
CTAAGCCTAAGC
CTAAGCCTGAGC
CTAAGCCTAAGA
CTAAGCTTAAGTCTGAGCCAAAAC
CTAAGCCTAAGCT
TAAGCCTAAGC
CTAAGCCTAACC
CTAAGCCTGAGCCTAATCCTATGCCTGAGCT
TAAGCCTAAGCCCAAGCA
TAAGCCTAATT
CTAGGCCTAAGCCTAATC
CTAGGCCTAAGC
CTAAGCCAAGGT
CTAAGCCTAATC
Nr of selected repeats 27 Similarity 0.758492
Consensus:
CTAAGCCTaAgC
>Cele-UNSB01_3:1833347-1833868 Satlength=522 Nr of Repeats=14 RepeatLength=34
seed=AATTGAAATT
AATTGAAATTTCCGGCAAATCGGCAAAATGTCGG
AATTGAAATTTTCGGCAAATCGGCAAATCGACCAAATTGCCGG
AATTGAAATTTCCGGTAAATCGGAAAATTGCCGG
AATTGAAATTTCCGGCAAATCGGCAAATTGCCGG
AATTGAAATTTTCGGCAAATCGGCAAATCGACCAAATTGCCTA
AATTGAAATTTCCTGCAAATTGGGAAAATGTCGG

AATTGAAATTTCCGGCAAATCGGCAAATTCCCAA
AATTGAAATTTTCGGCAAATCGGCAAAATGTCGG
AATTGAAATTTTTGGCAAATCGGCAAATCGACCAAATTGCCTA
AATTGAGATTTCCGGCAAATCGGCAAATCGACCAAATTGCCTA
AATTGAAATTTCCTGCAAATTGGGAAAATGTCGG
AATTTAAATTTTTGGCATATCGGCAAATTCCCAA
AATTGAAATTTCCGGCAAATCGGCAAATTGCCGG
AATTGAAATTTCCGGCAAATGGGCAAATCGACCAAATTGCCTA
Nr of selected repeats 9 Similarity 0.808279
Consensus:
AATTGAAATTTcCgGCAAATcGGcAAAtTgcCgg
>Cele-UNSB01_3:1843958-1844048 Satlength=91 Nr of Repeats=6 RepeatLength=12
seed=AGGCTTAGGC
AGGCTTAGGCTC
AGGCTGAGGCTT
AGGCTCAGGCTC
AGGCTTATGCTTCGGCTT
AGGTTTAGGCAT
AGGCTTAGGCCCATCGTTGGGCTT
Nr of selected repeats 4 Similarity 0.668803
Consensus:
AGGcTtAGGCTc
>Cele-UNSB01_3:1887376-1889731 Satlength=2356 Nr of Repeats=56 RepeatLength=19
seed=AAAATCGATA
AAAATCGATATTTTATCGG
AAAATCAATATTTTATCGG
AAAATCGATACTTTATTGG
AAAATCTATATTTTTCTGG
AAAATCTATATTTTATCGGAAAGTCGGTAATTTGCCGGAAGATCTATATTTAGTTGG
AAAATCGATAATCCTTTAG
AAAATCAATAAATTATCGT
AAAATCGATAATTCATCGA
AAAATCGATAATTTATAGG
AAAATTGATAAATTGTCGA
AAGATCGATAATTTATCGGAAAATCTTTATTTTGCTGG
AAAATCGATATTTTATCGG
AAAATCGATAATTTGTCCG
AAAATCGATAGTTTTCCGG
AAAATCGAAAATTTGCCGG
AAAATCGATGAATCGCCGGAAAGTCGGTAATATGTCGGATAATAGATATTTTGTCGGAAAATCTGTAAATTACCGG
AAAATCGATAAATTACCGG
AAAATCGATAATTTGCCTG
AAAATCGATAATTGCCGA
AAAATCGATATTTTAATAG
AAAATCGATATTTTATCGG
AAAATCGATATTTTCCCGAAAACCCGATTTTCCAATAACTTTTTCTGA
AAAATCAATATTTCGAAGTTAAAAAATTCTTAGAACAGATATTTTTTTTTCAAATTTCAAGTCTCCCAATTTCCAGGTC
GAGCTATCATTCCGTCAAGGTGACATCATAATTGTGCTTGGCGATATGGACGAAGACGGCTTCTACATGGGAGAGCTGA
ACGGCCTGCGCGGCCTAGTCCCGTCCAACTTCCTCCAGCCGCAGCCCCTCAACAATCTACTTCCGTCGCAACCGACCGA
GCCAATGCGGAAAGGTGTCGCGTTCTCGGATACACAGCAAATGATGAGAAAGTGAGCGTTGAAAAAAATAATTGAA
AAAATCGATAATTGTTCGAAACATCGAATTTCAGACCAATCCGTCAATCATCACAAACCAGCGCCGGACCATCACAAGG
CTCCAGCGGAGGCCTTGCAGGCGCGGTGGCGAGCGCCGCCGCCGCCAGCGGAGGCATAAAACCGGTGGCGAAGAAGAGC
TCGGCAGGAAGCGCCGGCGCCGGTGCAAAACCTCTGACGAAGAAGACCAGCGACGTTGGAAAGGTTAGGGAACTTTGTG
GTTTAA

AAAATCGATAATTTAAATTTTCAGTCGTCTGCTCCAAACGCTCGAAAAACTAGTACAGCTGTCAAGAAGGCGGATACCG
GTGCAAAGGTGAGA
AAAATCGATAATCCGTGGGT
AAATCGATAAATCGCCGG
AAAATCGATAAATCGCCGG
AAAATCGATATTTTGTCAG
AAAATCAATAAATTTGCTGAAACTCCATACTAATATGATTTTTTTTTGCAGAAAAAAGGATAACCCGACATCTGGACAT
CCGGAAATATGGAAATCCCCTCAATTTTCACAAAATTTCACTTAAAAAACACACAACTTTTTCCCTTATTTATTATATT
TAACAATTTTCAAATTAGATAATTTTTATAATTTAATTTATTGATTTTTTATTTAAAATTTCAAAAATCCTTATTTAAA
AATCCCGCTGAAAAAAATAAAATATAGGCGAATTTCTCTTGATCTCACTCTCTTTTCCACCGAACAAAAGCTCATAATT
TGTGCATTTTTATTGATTTTTGATTTTTCCCCCCATTTTCCCTAACAAAATTTGTGATACTATGCCTAATTTATGCGCT
TTTTTGCGCAATTTTCCCATTTTGAGA
AAAATCGATATTTATTGATTTTTTAATTTATTAATTTATAAAGATTTTTAATATGATTTTACTGTTTTATAGACTGTTT
AGATTTTCATTTTGTCGGAAAATCAATGTTTTATTAG
AAATCGATATTTACTTAATCGG
AAAGTCGATAAAATGTCGG
AAAATCGATAATTTATCGG
AAAATCGATAATTTATCGGG
AAATCGATAATTCGCGG
AAAATCGATATCTTATCGG
AAAATCGATATTTTATCGGAAAATCATTATTTTGCCTG
AAAATCGATAATTTATCAG
AAAATCGATAATTTATCGGAAAATCATTATTTTGCCTG
AAAATCGATAATTTATCGG
AAAATCGATAATTTATCGG
AAAATCGATATCTTATCGG
AAAATCGATATTTTATCGG
AAAATCGATAATTTATCGG
AAAATCGATATCTTATCGG
AAAATCGATATTTTATCGG
AAAATCGATAATTCGCGG
AAAATCGATATCTTATCGG
AAAATCGATATTTTATCGG
AAAATCGATAATTCGCGG
AAAATCGATATCTTATCGG
AAAATCGATATTTTATCGGAAAATCATTATTTTGCCTG
AAAATCGATAATTTATCGG
AAAATCGATATCTTATCGG
AAAATCGATAATTTCTCGG
Nr of selected repeats 36 Similarity 0.647665
Consensus:
AAAATCGATAatTTaTCGG
>Cele-UNSB01_3:1891974-1892414 Satlength=441 Nr of Repeats=36 RepeatLength=11
seed=ATTTTTTGCA
ATTTTTTGCAC
ATTTTTTGTAC
ATTTTTTGCAT
ATTTTTTGCAA
ATTTTTTGAAC
ATTTTTTGCAC
ATTTTTTGCAC
ATTTTTTGCAC
ATTTTTTGCAC
ATTTTTTGCAA

ATTTTTTGCAC
ATTTTTTGCACATTTTTTTAAG
ATTTTTTGCAC
ATTTTTTGCAC
ATTTTTTGCAC
ATTTTTTGCAC
ATTTTTTGCAC
ATTTTTTGCAA
ATTTTTTGAAC
ATTTTTTGCACATTTTTTAAAG
ATTTTTTGCAC
ATTTTTTGCAC
ATCTTTTGCAC
ATTTTTTGCAC
ATTTTTTGCAC
ATTTTTTGCAA
ATTTTTTGAAC
ATTTTTTGCAC
ATTTTTTGCAA
ATTTTTTGCAAATTTTTTAAAG
ATTTTTTGCAC
ATTTTTTGCACATTTTTTAAAG
ATTTTTTGCAC
ATTTTTTGCAC
ATTTTTTGCAC
ATTTTTTGCAA
Nr of selected repeats 32 Similarity 0.920088
Consensus:
ATTTTTTGCAc
>Cele-UNSB01_3:1974698-1975558 Satlength=861 Nr of Repeats=44 RepeatLength=19
seed=AAAATCGATA
AAAATCGATAATTTCTCGG
AAAATCGATAATTCCTCAGAAAAACGAAAATTGCTCGG
AAAATCGATAATTCCTCAA
AAAATCGATAATTTCTCAG
AAAATCGAAAATTGCTCGG
AAAATCGATAACTCGTCGG
AAATCGATAATTCGTCGG
AAAATCGATAATTCGTCGA
AAAATCGATATTTTAACGG
AAAGTCGATAATTCGTCGG
AAAATCGATTATTCGTCGG
AAAATCGATAATTCTTCGA
AAAATCGATAATTCGTCGG
AAAATCGATAATTTCTCGG
AAAATCGATAATTTCTCAG
AAAATCGAAAATTGCTCGG
AAAATCGATAATTTCTCAG
AAAATCGAAAATTCGTCGG
AAAATCGATATTTTAACGG
AAAGTCGATAATTCGTCGG
AAAATCGATTATTCGTCGG
AAAATCGATAATTCTTCGA
AAAATCGATAATTCGTCGG

AAAATCGATAATTTCTCGG
AAAATCGATAATTTCTCAG
AAAATCGAAAATTGCTCGG
AAAATCGATAATTTCTCAG
AAAATCGATAATTCGTCGG
AAAATCGATAATTCGTCAATA
AAAATCGATAATAGGTCGG
AAAATCGATAATTTCTCGG
AAAATCGATAATTTCTCAG
AAAATCGAAAATTGCTCGG
AAAATCGATAATTTCTCAG
AAAATCGATAATTCGTCGG
AAAATCGATAATTCGTCAATA
AAAATCGATAATAGGTCGG
AAAATCGATAATTTCTCGG
AAAATCGATAATTTCTCAG
AAAATCGAAAATTGCTCGG
AAAATCGATAATTTCTCAG
AAAATCGATAATTCGTCAG
AAAATCGATAATTCGTCAATA
AAAATCGAGAATAGGTCGG
Nr of selected repeats 39 Similarity 0.805668
Consensus:
AAAATCGATAATTncTCgG
>Cele-UNSB01_3:2017089-2018496 Satlength=1408 Nr of Repeats=57 RepeatLength=21
seed=TGGAAATTTT
TGGAAATTTTAGCCCAAAAAC
TGGAAATTTTACCCCAAAAAT
TGGAAATTTTGACCCAAAAAC
TAGAAATTTTAGTCCAAAAAC
TGGAAATTTTGACCTAAAAAC
TGGGAATTTTGACCCAAAAAT
TGGAAATTTTAGCCTAAAAAC
TGGAAATTTTAACCCAAAAAT
TGGAAATTTTAACCCAAAAACTAGAATTTTTAGCCCAAAAAC
TGGAAATTTTAACCCAAAAAT
TGGAAATTTTAACCCAAAAACTAGAATTTTTAGCCCAAAAAC
TGGAAATTTTAACCCAAAAACTAGAATTTTTAGCCCAAAAAC
TGGAAATTTTACCCCAAAAAT
TGGAAATTTTGACCCAAAAAC
TAGAAATTTTAGTCCAAAAAC
TGGAAATTTTGACCTAAAAAC
TGGGAATTTTGACCCAAAAAT
TGGAAATTTTAGCCTAAAAAC
TGGAAATTTTAACCCAAAAAT
TGGAAATTTTAACCCAAAAACTAGAATTTTTAGCCCAAAAAC
TGGAAATTTTAACCCAAAAAT
TGGAAATTTTAACCCAAAAACTAGAATTTTTAGCCCAAAAAC
TGGAAATTTTAACCCAAAAACTAGAATTTTTAGCCCAAAAAC
TGGAAATTTTAACCCAAAAAC
TGGAAATTTTAGCCCAAAAAT
TGGAAATTTTGACCCAAAAAT
TGGAAATTTTAACCCAAAAAT
TGGAAATTTTGACCTAAAAAC

TAGAAATTTTAGCTCAAAAAT
TGGAAATTTTAACCCAAAAAC
TGGAAATTTTAACCCAAAAAT
TGGAAATTTTAACCCAAAAAC
TGGAAATTTTAGCCCAAAAAT
TGGAAATTTTGACCCAAAAAT
TGGAAATTTTGACCTAAAAAC
TAGAAATTTTAGCTCAAAAAT
TGGAAATTTTAACCCAAAAAT
TGGAAATTTTAACCCAAAAAT
TGGAAATTTTAACCCAAAAACTAGAATTTTTAGCCCAAAAAC
TGGAAATTTTAACCCAAAAACTAGAATTTTTAGCCCAAAAAC
TGGAAATTTTACCCCAAAAACT
TGAAATTTTAGTCCAAAAAC
TGGAAATTTTGACCCAAAAAT
TGGAAATTTTAGCCTAAAAAC
TGGAAATTTTGACCCAAAAAT
TGGAAATTTTAGCCTAAAAAC
TGGAAATTTTAACCCAAAAAT
TGGAAATTTTAACCCAAAAAG
TGGAAATTTTAGCCCAAAAAT
TGGAAATTTTAACCCAAAAAT
TGGAAATTTTAACCCAAAAAT
TGGAAATTTTGACCTAAAAAC
TAGAAATTTTAGCTCAAAAAT
TGGAAATTTTAACCCAAAAACTAGAATTTTTAGCCCAAAAAC
TGGAAATTTTAGCCCAAAAAT
TGGAAATTTTGACCCAAAAAC
TGGAAATTTTAGCCCAAAAACTAGAATTTTTAGCCCAAAAAC
Nr of selected repeats 45 Similarity 0.855059
Consensus:
TGGAAATTTTaaCCcAAAAAt
>Cele-UNSB01_3:2046603-2047209 Satlength=607 Nr of Repeats=30 RepeatLength=12
seed=TAGGCTTAGG
TAGGCTTAGGATTTTCCGGTTTTTTATTTTTGAGGGGGTTCCTGTTTGCCGATTC
AGGCTTAGGCG
TAGGCTTAGGCT
TAGGCTTAGGCT
TAGGCATAGGCT
TAGGCTTAGGCT
TAGGCTTAGGCT
TAGGCATAGGCT
TAGGCTTAGGCTTAGGCGTAAGCTT
TGGCTTAGGTTTAGACCAACACTTAGGCCTGGTCTTAGACG
TAGGCTTTGGCT
TAGGCTTAGGTT
TAGGCGTAGGCTT
TGGCTTAGGTTTAGACCAACACTTAGGCCTGGTCTTAGACGTAGGCTC
TGGCTTAGGCT
TAGGTTTAGGCGTAGGCTTTGACT
TAGGCTTAGTCT
TAGGCTTAGGAA
TAGGTTTAGGCT

TAGGCTTAGGCTTTGGCTTTGGTTTAGACCAACACATAGGCCAAGTCTTAGGCCTAGTCTTAGTTTTACGCTTAGACAA
AGACGC
AGGCTTAGGCG
TAGGCCTAGGCT
TAAGCTTAGGCT
TAGGCTCAGGCA
TAGGCTTAGGCT
TAGGCTTAGGCG
TAGGCTTTGGCTTAGGTTTAGACCAACACTTAGGCCTGGTCTTAGATG
TAGGCTTTGGCT
TAGGCTTGGGTTTAGGAA
TAGGTTTAGGCT
Nr of selected repeats 18 Similarity 0.824256
Consensus:
TAGGCTTAGGCT
>Cele-UNSB01_3:2058475-2058618 Satlength=144 Nr of Repeats=11 RepeatLength=11
seed=GTCGCGTTGC
GTCGCGTTGCG
GTCGCATTGCG
GTCGCGTTGCG
GTCGCGTTGCG
GTCGCGTTGCA
GTCGCGTTGCG
GTCGCATTGCG
GTCGCGTTGCGGTCAAATTGTG
GTCGCGTTGCA
GTCGCCTTGCG
GTCGCGTTGCGATCACACTGCG
Nr of selected repeats 9 Similarity 0.885522
Consensus:
GTCGCgTTGCg
>Cele-UNSB01_3:2068206-2068691 Satlength=486 Nr of Repeats=44 RepeatLength=11
seed=TGTGCAAAAA
TGTGCAAAAAA
TGTGCAAAAA
TGTGCAAAAAA
TGTGCAAAAAT
TGTGCAAAAA
TGTGCAAAAAA
TGTGCAAAAAA
TGTGCAAAAA
TGTGCAAAAAA
TGTGCAAAAAA
TGTGCAAAAA
TGTGCAAAAAA
TGTGCAAAAAA
TGTGCAAAAA
TGTGCAAAAAA
TGTGCAAAAAT
TGTGCAAAAA
TGTGCAAAAAA
TGTGCAAAAAA
TGGGCAAAAAA
TGTGCAAAAAA

TGTGCAAAAA
TGTGCAAAAAA
TGTGCAAAAAA
TGTGCAAAAA
TGTGCAAAAAA
TGTGCAGAAAAGGTGCAAGAAA
TGTGCAGAAAA
TGTGCAAAAAA
TGTGCAAAAAG
TGTGCAAAAAA
TGTGCAAAAAA
TGTGCAAAAAT
TATGCAAAAAA
TGTGCAAAAAA
TGTGCAAAAAA
TGTTCAAAAAA
TGTGCAGAAAAG
GTGCAAAAATGTGCAAAAAA
TGTGCAAAAAA
TGTGCAAAAAA
TGTGCAAAAA
TGTGCAAAAAA
Nr of selected repeats 31 Similarity 0.939785
Consensus:
TGTGCAAAAAA
>Cele-UNSB01_3:2092267-2093197 Satlength=931 Nr of Repeats=27 RepeatLength=31
seed=TGCGTCGGAA
TGCGTCGGAAATTTATTCCGATCATTGCCGG
TGCGTCGGAAATGCATTCTTATCATCAGCGG
TGCGTCGGAAATTTATTCCGATTATCGGCGA
TGCGTCGGAACACAACTCCAATCATTGCCGG
TGCGTCGGAAATTTATTTCGATCATCGCCGGTGCTTCGGACTTTAGTTCTGATCATCGCCGGTGCTTCGGACTTTAGTT
CTGATCATCGCCGGC
GCGTCGGAAATTCATTCCGTTTATCGCCGA
TGCGTCGGAACACAATTCCGATGATCGCCGG
TGCGTCGGGAATTTATTCCGATCATTGCCGG
TGCGTCGGAAATGCATTCTTATCATCAGCGG
TGCGTCGGAAATTTATTCCGATTATCGGCGA
TGCGTCGGAACACAACTCCAATCATTGCCGG
TGCGTCGGAAATTTATTTCGATCATCGCCGGTGCTTCGGACTTTAGTTCTGATCATCGCCGGC
GCGTCGGAAATTCATTCCGTTTATCGCCGA
TGCGTCGGAACACAATTCCGATGATCGCCGG
TGCGTCGGGAATTTATTCCGATCACCGGCGA
TGCGTCGGAACACAATTCCGATAATCGCCGG
TGCGTCGGAAAATTATTCCGATCATCGGCGA
TGCGACGGAAATTCATTCCGTTTATCGGCGA
TGCGTCGGAAAACAATTCCGATTATCACCGG
TGCGTCGGAAAATAATTCCGATCATCGACGG
TACGTCGGAAGTTTATTCCGATCAACGCCGA
TGCGTTGGAAATTTATTCCGATCACCGCCGG
TGCATCGGAAATTCATTCCGATCATCGCCGG
TGCATCGGAAATTCATTCCGATCACCGCCGG
TGCATCGGAAATTCATTCCGATCATCGCCGA
TGCGTCGGAAATTTATTCCAATCACCGGCGA

TGCGTCGGAACACAATTCCAATCATTGCCGG
Nr of selected repeats 23 Similarity 0.733775
Consensus:
TGCGTCGGAAattnATTCCgATcAtcGcCGg
>Cele-UNSB01_3:2094174-2094918 Satlength=745 Nr of Repeats=17 RepeatLength=31
seed=TCCGATGCAT
TCCGATGCATTGCCGATAATTGCCACCTGGT
TCCGATGCATTGGAAATGATCGGAAAATGGT
TCCAATGCATTGCCGATAGTCGGAACCTGTT
TCCGATGCATCGGCAATGATCGGAACTCTGC
TCCGACGCATCGGTAATGATCGGAACCTGGT
TCCGATGCATCGCCGATAGTCAGAATCCGGT
TCCGATGCATCGGAAATAATCGGAACCAACTGCCGACGTATCGCCGATTATCGGAACCTGGT
TCCGATGCATCGCCGATAGTCAGAATCCGGT
TCCGATGCATCGGCAATAATCGGAACCAACTGCCGACGTATCGCCGATTATCGGAACCTGGTTCCAACCCATCGGCAAT
GATCGGAAATTGAT
TCCGATGCATCGCCGATGATCAGAGCCAGGTTCTGATGAATCGCCTATAATCGGAAATTGGTTTCGATGTATCGCCGGT
AGTTGGAATTTAGC
TCCGATGCATTGCCGATAATCGGCACCTGGT
TCCGATGTATTGGCAATAATCGGAACCTCGC
TCCGATGCATCGCCGATGATCGGAACCTGGT
TCCGATGCATCGCAGATAGTCAGAATCCGGT
TCCGATGCATCGGCAATAATCGGAACCAACTGCCGACGTATCGCCGATTATCGGAACCTGGTTCCAACCCATCGGCAAT
GATCGGAAATTGAT
TCCGACGCATTGCCGGCAGTCATAACCTAGT
TCGGATGCATTGCCGCCAGTCATAACCTGGC
Nr of selected repeats 13 Similarity 0.625486
Consensus:
TCCGATGCATtGccgATaaTCggAAcCtGgt
>Cele-UNSB01_3:2095462-2096950 Satlength=1489 Nr of Repeats=36 RepeatLength=19
seed=TATCGATTTT
TATCGATTTTCGAAAAATCAAGTTTTCAGGATATGGACAAAGGGAGATCACACTATCGGGTAAGTTTTTTGATGCATAA
CAGGGGTAGAAAACATTGGGTAAATCAAAAAAAAAAAAGTCAATAATCACTCACTAAAAATTAAAATTCTAATCAAAAC
CAAAACAAAATTATTTTTTGAAAAAAAAAACAAATTAGGGACCCTGAAATTCAATTATGATGAAGAAAAGAAAAAAAAA
CTTTAAAAAAAAATTTTCTTGAAAATTAAAAAAAAAAGGTTTTTCCCTAAAA
TATCGATTTTTAAAA
TATTGATTTTTATAACAAAAAAGTTGATTTTTCGCTAAAA
TATCAATTTTTAGAA
TATTGATTTTTTGCTAAAA
TATTGATTTTCACGGAAA
TATAGATTTTCTGCAAAAA
TATCGATTTTTTCTAATAAAAATAAAATTAAAAATTTTTTTCACAAT
TATCGATTATTCTCGAAAT
TATCGATTTTCACTCAAACTGAACAAAAAAAATCAATAAAACCCAAAATCACAAGAAAAACGACACCTGGCGGGGGAAA
CAACACTCCATCACTACGACAGTCGGACAGGCACTGAAGGCGGAACTCTTCGAAAT
TATCGATTTTTCCGGGCAGCTGCAGAAGTTGCGGCTTCG
ATCGATTTTTCTGCTGAATCGGCGGCGGCGGTGTCAGGGTACTGAAGGGGTTAGTGGTGTTGTTGCAGGAAAACGTGTT
CAACGCGATGCACGACGTGGCACGAGCTAATTGCGAGCTACCGACGAGCACATTGTTCAACAAAAGGTGAGCCGAGTCA
GAGTGGGAGCCTAAATTTTTAGCACCTGTGGTTGTGGCGGTGGCGAGCGGCGGATCCACAGAGGCGATTTCCAGTTTGA
TGCCGGATTTTAGGGAGGTAATGACGTTGCCGTACAAACCCGCCATCACAACTAGCGGGATTACAAGTAGGACGACGGT
CATGTATACCTGAAAATATAT
TATCGATTTTAAGTGAAATTTCCGATTTTCCTGGAAAC
TATTGATTTTCCATTATGCTTCCAATTTTTGGGAAAA
TCTCGATTTTCCGTAAAAG

TATTGATTTTTAGTGAAAT
TATCGATTTTTAGTGGAAT
TATCAATTTTCCGTAGAAG
TATTGATTTTTCCTTAAAAT
TATCGATTTTTCGTAAAAA
TATTGATTTTTCGTAAAAT
TATCGATTTTCCTTAAAAT
TATCGATTTTCCGTAGAAG
TATTGATTTTTCCTTAAAAT
TATCGATTTTCCGTAAAAG
TATTGATTTTTAGTGAAAT
TATCGATTTTCCTTAAAAT
TATCGATTTTTCGTAAAAA
TATTGATTTTTAGTGAAAT
TATCGATTTTCCTTAAAAT
TATCGATTTTTCGTAAAAA
TATTGATTTTTAGTGAAAT
TATCGATTTTCCTTAAAAT
TATCGATTTTCCGTAAAAG
TATTGATTTTTAGTGAAAT
TATCGATTTTTCCATAAAAT
Nr of selected repeats 22 Similarity 0.729931
Consensus:
TATcGATTTTtcgTaAAAt
>Cele-UNSB01_3:2118334-2118593 Satlength=260 Nr of Repeats=20 RepeatLength=11
seed=CTACAGTACC
CTACAGTACCCCAAAATAGATACAGTACTTTTAGAATAACC
CTACAGTACTT
CTACAGTATCC
CTACAGTACCT
CTACAGTACCA
CTACAGTACC
CTACAGTATCC
CTACAGTACCT
CTACAGTACCA
CTACAGTACCT
CTACAGTACCA
CTACAGTACC
CTACAGTACCC
CTACAGTACCCC
CACAGTACCC
CTATAGTACCCCTACAGTATTC
CTACAGTACCC
CTACAGTACCT
CTACAGTACCC
CTACAGTACCC
Nr of selected repeats 14 Similarity 0.795788
Consensus:
CTACAGTACCn
>Cele-UNSB01_3:2118949-2119137 Satlength=189 Nr of Repeats=9 RepeatLength=20
seed=TGTAGATTTA
TGTAGATTTACGCGGTTCTG
TGTGGATTTACAGGAATCTA
TGTCGATTTACGAGGTTTTTTAAA

TGTAGATTTAAAGGATTCTA
TGTAGATTTACGGGGTACTG
TGTAGGTTTACGGGGATGTTCCT
TGTAGATTTACGGAGTTCTA
TGTAAATTTACGAGGTTTTA
TGTAAATTTACGGGGGTCATG
Nr of selected repeats 6 Similarity 0.697778
Consensus:
TGTAGATTTACggGgTTCTa
>Cele-UNSB01_3:2149341-2150059 Satlength=719 Nr of Repeats=12 RepeatLength=35 seed=TTTGCCGGAA
TTTGCCGGAATCGTGTATTGCGAGTTTTCCTAAAAGTATATCTTTACTTAAAAAGTCTTAAGAAGAAAAAAACCAACTT
GTCGTGGGTTCATAACAGCATCCTTTATTAGTGGCAATATTTTGGTAACATTTTTCATTTTATAGGTTGTGCCTGTTTC
CGATAAACCATATCGCTGGACACTGTAGATGATGGCTGGGATTTTAGATTTGTCAAACTTTGTTTTGTCAACTTATGAT
ACTTATAACAGGGATGGGCGGCAATTGCCACTCGGTAAATCGGCAAATTGGCAGCTTGCCGATTTGTCAGAAATTTTCA
ATTCCGACAATTTGCCGA
TTTGCCGGAAATTTTCAATTCCGACAATTTGCCGA
TTTGCCGGAAATTTTCAATTCCGAGAATTGGCCGA
TTTGCCGGAAATTTTCAATTCCGACAATTTGCCGA
TTTGCCGGAAATTTTCAATTCCGACAAGTTGCCGA
TTTGCCGGAAATTTTCAATTTCGACAATTTGCCGA
TTTGCCGGAATTTTTCAATTCTGACAATTTGCCGA
TTTGCCGGAAATTTTCAATTCCGACAAGTTGCCGA
TTTGCCGGAAATTTTCAATTCCGACAAGTTGCCGA
TTTGCCGGAAATTTTCAATCCCGGCAATTTGCCGA
TTTGCCGGAATTTTCAATTCCGACAATTTGCCGA
TTTGCCGGAAATTTTCAATTCCGACAAGTTGCCGA
Nr of selected repeats 10 Similarity 0.926349
Consensus:
TTTGCCGGAAATTTTCAATTCCGACAAtTTGCCGA
>Cele-UNSB01_3:2160246-2184061 Satlength=23816 Nr of Repeats=142 RepeatLength=168 seed=TTTATTTTTG
TTTATTTTTGTTTTTCTGTGCCTACATATACCTGTTGAAATTGGAATTCAAGACAAAAACCCCTGTAGCGCGTCTGAAA
ATGGATATCTAGGTACAAAAACTTACCAATAATATTTTAAAACAATCTAGAATCCAAATCTATCAAAACATTTCCGGAA
TTCCATAATG
TTTATTTTTGTTTTTTTGTGCCTACATATACCTGTTGAAATTGGAATTCAAGACAAAAACCCCTGTAGCGCGTCTGAAA
ATGGATATCTAGGTACAAAAACTTACCAATAATATTTTAAAACAATCTAGAATCCAAATCTATCAAAACATTTCCGGAA
TTCCATAATG
TTTATTTTTGTTTTTTTGTGCCTACATATACCTGTTGAAATTGGAATTCAAGACAAAAACCCCTGTAGCGCGTCTGAAA
ATGGATATCTAGGTACAAAAACTTACCAATAATATTTTAAAACAATCTAGAATCCAAATCTATCAAAACATTTCCATAA
TTCCAAAATT
TTTATTTTTGTTTTTTTGTGCCTACATATACCTGTTGAAATTGGAATTCAAGACAAAAACCCCTGTAGCGCGTCTGAAA
ATGGATATCTAGGTACAAAAACTTACCAATAATATTTTAAAACAATCTAGAATCCAAATCTATCAAAACATTTCCGGAA
TTCCATAATG
TTTATTTTTGTTTTTTTGTGCCTACATATACCTGTTGAAATTGGAATTCAAGACAAAAACCCCTGTAGCGCGTCTGAAA
ATGGATATCTAGGTACAAAAACTTACCAATAATATTTTAAAACAATCTAGAATCCAAATCTATCAAAACATTTCCGGAA
TTCCATAATG
TTTATTTTTGTTTTTTTGTGCCTACATATACCTGTTGAAATTGGAATTCAAGACAAAAACCCCTGTAGCGCGTCTGAAA
ATGGATATCTAGGTACAAAAACTTACCAATAATATTTTAAAACAATCTAGAATCCAAATCTATCAAAACATTTCCGGAA
TTCCATAATG
TTTATTTTTGTTTTTTTGTGCCTACATATACCTGTTGAAATTGGAATTCAAGACAAAAACCCCTGTAGCGCGTCTGAAA
ATGGATATCTAGGTACAAAAACTTACCAATAATATTTTAAAACAATCTAGAATCCAAATCTATCAAAACATTTCCATAA
TTCCAAAATT

TTTATTTTTGTTTTTTTGTGCCTACATATACCTGTTGAAATTGGAATTCAAGACAAAAACCCCTGTAGCGCGTCTGAAA
ATGGATATCTAGGTACAAAAACTTACCAATAATATTTTAAAACAATCTAGAATCCAAATCTATCAAAACATTTCCGGAA
TTCCATAATG
TTTATTTTTGTTTTTTTGTGCCTACATATACCTGTTGAAATTGGAATTCAAGACAAAAACCCCTGTAGCGCGTCTGAAA
ATGGATATCTAGGTACAAAAACTTACCAATAATATTTTAAAACAATCTAGAATCCAAATCTATCAAAACATTTCCGGAA
TTCCATAATG
TTTATTTTTGTTTTTTTGTGCCTACATATACCTGTTGAAATTGGAATTCAAGACAAAAACCCCTGTAGCGCGTCTGAAA
ATGGATATCTAGGTACAAAAACTTACCAATAATATTTTAAAACAATCTAGAATCCAAATCTATCAAAACATTTCCGGAA
TTCCATAATG
TTTATTTTTGTTTTTTTGTGCCTACATATACCTGTTGAAATTGGAATTCAAGACAAAAACCCCTGTAGCGCGTCTGAAA
ATGGATATCTAGGTACAAAAACTTACCAATAATATTTTAAAACAATCTAGAATCCAAATCTATCAAAACATTTCCGGAA
TTCCATAATG
TTTATTTTTGTTTTTTTGTGCCTACATATACCTGTTGAAATTGGAATTCAAGACAAAAACCCCTGTAGCGCGTCTGAAA
ATGGATATCTAGGTACAAAAACTTACCAATAATATTTTAAAACAATCTAGAATCCAAATCTATCAAAACATTTCCATAA
TTCCAAAATT
TTTATTTTTGTTTTTTTGTGCCTACATATACCTGTTGAAATTGGAATTCAAGACAAAAACCCCTGTAGCGCGTCTGAAA
ATGGATATCTAGGTACAAAAACTTACCAATAATATTTTAAAACAATCTAGAATCCAAATCTATCAAAACATTTCCGGAA
TTCCATAATG
TTTATTTTTGTTTTTTTGTGCCTACATATACCTGTTGAAATTGGAATTCAAGACAAAAACCCCTGTAGCGCGTCTGAAA
ATGGATATCTAGGTACAAAAACTTACCAATAATATTTTAAAACAATCTAGAATCCAAATCTATCAAAACATTTCCGGAA
TTCCATAATG
TTTATTTTTGTTTTTTTGTGCCTACATATACCTGTTGAAATTGGAATTCAAGACAAAAACCCCTGTAGCGCGTCTGAAA
ATGGATATCTAGGTACAAAAACTTACCAATAATATTTTAAAACAATCTAGAATCCAAATCTATCAAAACATTTCCGGAA
TTCCATAATG
TTTATTTTTGTTTTTTTGTGCCTACATATACCTGTTGAAATTGGAATTCAAGACAAAAACCCCTGTAGCGCGTCTGAAA
ATGGATATCTAGGTACAAAAACTTACCAATAATATTTTAAAACAATCTAGAATCCAAATCTATCAAAACATTTCCATAA
TTCCAAAATT
TTTATTTTTGTTTTTTTGTGCCTACATATACCTGTTGAAATTGGAATTCAAGACAAAAACCCCTGTAGCGCGTCTGAAA
ATGGATATCTAGGTACAAAAACTTACCAATAATATTTTAAAACAATCTAGAATCCAAATCTATCAAAACATTTCCGGAA
TTCCATAATG
TTTATTTTTGTTTTTTTGTGCCTACATATACCTGTTGAAATTGGAATTCAAGACAAAAACCCCTGTAGCGCGTCTGAAA
ATGGATATCTAGGTACAAAAACTTACCAATAATATTTTAAAACAATCTAGAATCCAAATCTATCAAAACATTTCCGGAA
TTCCATAATG
TTTATTTTTGTTTTTTTGTGCCTACATATACCTGTTGAAATTGGAATTCAAGACAAAAACCCCTGTAGCGCGTCTGAAA
ATGGATATCTAGGTACAAAAACTTACCAATAATATTTTAAAACAATCTAGAATCCAAATCTATCAAAACATTTCCATAA
TTCCAAAATT
TTTATTTTTGTTTTTTTGTGCCTACATATACCTGTTGAAATTGGAATTCAAGACAAAAACCCCTGTAGCGCGTCTGAAA
ATGGATATCTAGGTACAAAAACTTACCAATAATATTTTAAAACAATCTAGAATCCAAATCTATCAAAACATTTCCATAA
TTCCAAAATT
TTTATTTTTGTTTTTTTGTGCCTACATATACCTGTTGAAATTGGAATTCAAGACAAAAACCCCTGTAGCGCGTCTGAAA
ATGGATATCTAGGTACAAAAACTTACCAATAATATTTTAAAACAATCTAGAATCCAAATCTATCAAAACATTTCCATAA
TTCCAAAATT
TTTATTTTTGTTTTTTTGTGCCTACATATACCTGTTGAAATTGGAATTCAAGACAAAAACCCCTGTAGCGCGTCTGAAA
ATGGATATCTAGGTACAAAAACTTACCAATAATATTTTAAAACAATCTAGAATCCAAATCTATCAAAACATTTCCGGAA
TTCCATAATG
TTTATTTTTGTTTTTTTGTGCCTACATATACCTGTTGAAATTGGAATTCAAGACAAAAACCCCTGTAGCGCGTCTGAAA
ATGGATATCTAGGTACAAAAACTTACCAATAATATTTTAAAACAATCTAGAATCCAAATCTATCAAAACATTTCCGGAA
TTCCATAATG
TTTATTTTTGTTTTTTTGTGCCTACATATACCTGTTGAAATTGGAATTCAAGACAAAAACCCCTGTAGCGCGTCTGAAA
ATGGATATCTAGGTACAAAAACTTACCAATAATATTTTAAAACAATCTAGAATCCAAATCTATCAAAACATTTCCGGAA
TTCCATAATG
TTTATTTTTGTTTTTTTGTGCCTACATATACCTGTTGAAATTGGAATTCAAGACAAAAACCCCTGTAGCGCGTCTGAAA
ATGGATATCTAGGTACAAAAACTTACCAATAATATTTTAAAACAATCTAGAATCCAAATCTATCAAAACATTTCCATAA
TTCCAAAATT

TTTATTTTTGTTTTTTTGTGCCTACATATACCTGTTGAAATTGGAATTCAAGACAAAAACCCCTGTAGCGCGTCTGAAA
ATGGATATCTAGGTACAAAAACTTACCAATAATATTTTAAAACAATCTAGAATCCAAATCTATCAAAACATTTCCGGAA
TTCCATAATG
TTTATTTTTGTTTTTTTGTGCCTACATATACCTGTTGAAATTGGAATTCAAGACAAAAACCCCTGTAGCGCGTCTGAAA
ATGGATATCTAGGTACAAAAACTTACCAATAATATTTTAAAACAATCTAGAATCCAAATCTATCAAAACATTTCCGGAA
TTCCATAATG
TTTATTTTTGTTTTTTTGTGCCTACATATACCTGTTGAAATTGGAATTCAAGACAAAAACCCCTGTAGCGCGTCTGAAA
ATGGATATCTAGGTACAAAAACTTACCAATAATATTTTAAAACAATCTAGAATCCAAATCTATCAAAACATTTCCATAA
TTCCAAAATT
TTTATTTTTGTTTTTTTGTGCCTACATATACCTGTTGAAATTGGAATTCAAGACAAAAACCCCTGTAGCGCGTCTGAAA
ATGGATATCTAGGTACAAAAACTTACCAATAATATTTTAAAACAATCTAGAATCCAAATCTATCAAAACATTTCCATAA
TTCCAAAATT
TTTATTTTTGTTTTTTTGTGCCTACATATACCTGTTGAAATTGGAATTCAAGACAAAAACCCCTGTAGCGCGTCTGAAA
ATGGATATCTAGGTACAAAAACTTACCAATAATATTTTAAAACAATCTAGAATCCAAATCTATCAAAACATTTCCATAA
TTCCAAAATT
TTTATTTTTGTTTTTTTGTGCCTACATATACCTGTTGAAATTGGAATTCAAGACAAAAACCCCTGTAGCGCGTCTGAAA
ATGGATATCTAGGTACAAAAACTTACCAATAATATTTTAAAACAATCTAGAATCCAAATCTATCAAAACATTTCCATAA
TTCCAAAATT
TTTATTTTTGTTTTTTTGTGCCTACATATACCTGTTGAAATTGGAATTCAAGACAAAAACCGCTGTAGCGCGTCTGAAA
ATGGATATCTAGGTACAAAAACTTACCAATAATATTTTAAAACAATCTAGAATCCAAATCTATCAAAACATTTCCATAA
TTCCAAAATT
TTTATTTTTGTTTTTTTGTGCCTACATATACCTGTTGAAATTGGAATTCAAGACAAAAACCCCTGTAGCGCGTCTGAAA
ATGGATATCTAGGTACAAAAACTTACCAATAATATTTTAAAACAATCTAGAATCCAAATCTATCAAAACATTTCCATAA
TTCCAAAATT
TTTATTTTTGTTTTTTTGTGCCTACATATACCTGTTGAAATTGGAATTCAAGACAAAAACCCCTGTAGCGCGTCTGAAA
ATGGATATCTAGGTACAAAAACTTACCAATAATATTTTAAAACAATCTAGAATCCAAATCTATCAAAACATTTCCATAA
TTCCAAAATT
TTTATTTTTGTTTTTTTGTGCCTACATATACCTGTTGAAATTGGAATTCAAGACAAAAACCCCTGTAGCGCGTCTGAAA
ATGGATATCTAGGTACAAAAACTTACCAATAATATTTTAAAACAATCTAGAATCCAAATCTATCAAAACATTTCCATAA
TTCCAAAATT
TTTATTTTTGTTTTTTTGTGCCTACATATACCTGTTGAAATTGGAATTCAAGACAAAAACCCCTGTAGCGCGTCTGAAA
ATGGATATCTAGGTACAAAAACTTACCAATAATATTTTAAAACAATCTAGAATCCAAATCTATCAAAACATTTCCGGAA
TTCCATAATG
TTTATTTTTGTTTTTTTGTGCCTACATATACCTGTTGAAATTGGAATTCAAGACAAAAACCCCTGTAGCGCGTCTGAAA
ATGGATATCTAGGTACAAAAACTTACCAATAATATTTTAAAACAATCTAGAATCCAAATCTATCAAAACATTTCCGGAA
TTCCATAATG
TTTATTTTTGTTTTTTTGTGCCTACATATACCTGTTGAAATTGGAATTCAAGACAAAAACCCCTGTAGCGCGTCTGAAA
ATGGATATCTAGGTACAAAAACTTACCAATAATATTTTAAAACAATCTAGAATCCAAATCTATCAAAACATTTCCGGAA
TTCCATAATG
TTTATTTTTGTTTTTTTGTGCCTACATATACCTGTTGAAATTGGAATTCAAGACAAAAACCCCTGTAGCGCGTCTGAAA
ATGGATATCTAGGTACAAAAACTTACCAATAATATTTTAAAACAATCTAGAATCCAAATCTATCAAAACATTTCCATAA
TTCCAAAATT
TTTATTTTTGTTTTTTTGTGCCTACATATACCTGTTGAAATTGGAATTCAAGACAAAAACCCCTGTAGCGCGTCTGAAA
ATGGATATCTAGGTACAAAAACTTACCAATAATATTTTAAAACAATCTAGAATCCAAATCTATCAAAACATTTCCGGAA
TTCCATAATG
TTTATTTTTGTTTTTTTGTGCCTACATATACCTGTTGAAATTGGAATTCAAGACAAAAACCCCTGTAGCGCGTCTGAAA
ATGGATATCTAGGTACAAAAACTTACCAATAATATTTTAAAACAATCTAGAATCCAAATCTATCAAAACATTTCCGGAA
TTCCATAATG
TTTATTTTTGTTTTTTTGTGCCTACATATACCTGTTGAAATTGGAATTCAAGACAAAAACCCCTGTAGCGCGTCTGAAA
ATGGATATCTAGGTACAAAAACTTACCAATAATATTTTAAAACAATCTAGAATCCAAATCTATCAAAACATTTCCATAA
TTCCAAAATT
TTTATTTTTGTTTTTTTGTGCCTACATATACCTGTTGAAATTGGAATTCAAGACAAAAACCCCTGTAGCGCGTCTGAAA
ATGGATATCTAGGTACAAAAACTTACCAATAATATTTTAAAACAATCTAAATCCAAATCTATCAAAACATTTCCGGAAT
TCCATAATG

TTTATTTTTGTTTTTTTGTGCCTACATATACCTGTTGAAATTGGAATTCAAGACAAAAACCCCTGTAGCGCGTCTGAAA
ATGGATATCTAGGTACAAAAACTTACCAATAATATTTTAAAACAATCTAGAATCCAAATCTATCAAAACATTTCCGGAA
TTCCATAATG
TTTATTTTTGTTTTTTTGTGCCTACATATACCTGTTGAAATTGGAATTCAAGACAAAAACCCCTGTAGCGCGTCTGAAA
ATGGATATCTAGGTACAAAAACTTACCAATAATATTTTAAAACAATCTAGAATCCAAATCTATCAAAACATTTCCGGAA
TTCCATAATG
TTTATTTTTGTTTTTTTGTGCCTACATATACCTGTTGAAATTGGAATTCAAGACAAAAACCCCTGTAGCGCGTCTGAAA
ATGGATATCTAGGTACAAAAACTTACCAATAATATTTTAAAACAATCTAGAATCCAAATCTATCAAAACATTTCCATAA
TTCCAAAATT
TTTATTTTTGTTTTTTTGTGCCTACATATACCTGTTGAAATTGGAATTCAAGACAAAAACCCCTGTAGCGCGTCTGAAA
ATGGATATCTAGGTACAAAAACTTACCAATAATATTTTAAAACAATCTAGAATCCAAATCTATCAAAACATTTCCATAA
TTCCAAAATT
TTTATTTTTGTTTTTTTGTGCCTACATATACCTGTTGAAATTGGAATTCAAGACAAAAACCCCTGTAGCGCGTCTGAAA
ATGGATATCTAGGTACAAAAACTTACCAATAATATTTTAAAACAATCTAGAATCCAAATCTATCAAAACATTTCCGGAA
TTCCATAATG
TTTATTTTTGTTTTTTTGTGCCTACATATACCTGTTGAAATTGGAATTCAAGACAAAAACCCCTGTAGCGCGTCTGAAA
ATGGATATCTAGGTACAAAAACTTACCAATAATATTTTAAAACAATCTAGAATCCAAATCTATCAAAACATTTCCGGAA
TTCCATAATG
TTTATTTTTGTTTTTTTGTGCCTACATATACCTGTTGAAATTGGAATTCAAGACAAAAACCCCTGTAGCGCGTCTGAAA
ATGGATATCTAGGTACAAAAACTTACCAATAATATTTTAAAACAATCTAGAATCCAAATCTATCAAAACATTTCCGGAA
TTCCATAATG
TTTATTTTTGTTTTTTTGTGCCTACATATACCTGTTGAAATTGGAATTCAAGACAAAAACCCCTGTAGCGCGTCTGAAA
ATGGATATCTAGGTACAAAAACTTACCAATAATATTTTAAAACAATCTAGAATCCAAATCTATCAAAACATTTCCATAA
TTCCAAAATT
TTTATTTTTGTTTTTTTGTGCCTACATATACCTGTTGAAATTGGAATTCAAGACAAAAACCCCTGTAGCGCGTCTGAAA
ATGGATATCTAGGTACAAAAACTTACCAATAATATTTTAAAACAATCTAGAATCCAAATCTATCAAAACATTTCCGGAA
TTCCATAATG
TTTATTTTTGTTTTTTTGTGCCTACATATACCTGTTGAAATTGGAATTCAAGACAAAAACCCCTGTAGCGCGTCTGAAA
ATGGATATCTAGGTACAAAAACTTACCAATAATATTTTAAAACAATCTAGAATCCAAATCTATCAAAACATTTCCATAA
TTCCAAATT
TTTATTTTTGTTTTTTTGTGCCTACATATACCTGTTGAAATTGGAATTCAAGACAAAAACCCCTGTAGCGCGTCTGAAA
ATGGATATCTAGGTACAAAAACTTACCAATAATATTTTAAAACAATCTAGAATCCAAATCTATCAAAACATTTCCATAA
TTCCAAATT
TTTATTTTTGTTTTTTTGTGCCTACATATACCTGTTGAAATTGGAATTCAAGACAAAAACCCCTGTAGCGCGTCTGAAA
ATGGATATCTAGGTACAAAAACTTACCAATAATATTTTAAAACAATCTAGAATCCAAATCTATCAAAACATTTCCGGAA
TTCCATAATG
TTTATTTTTGTTTTTTTGTGCCTACATATACCTGTTGAAATTGGAATTCAAGACAAAAACCCCTGTAGCGCGTCTGAAA
ATGGATATCTAGGTACAAAAACTTACCAATAATATTTTAAAACAATCTAGAATCCAAATCTATCAAAACATTTCCGGAA
TTCCATAATG
TTTATTTTTGTTTTTTTGTGCCTACATATACCTGTTGAAATTGGAATTCAAGACAAAAACCCCTGTAGCGCGTCTGAAA
ATGGATATCTAGGTACAAAAACTTACCAATAATATTTTAAAACAATCTAGAATCCAAATCTATCAAAACATTTCCATAA
TTCCAAAT
TTTATTTTTGTTTTTTTGTGCCTACATATACCTGTTGAAATTGGAATTCAAGACAAAAACCCCTGTAGCGCGTCTGAAA
ATGGATATCTAGGTACAAAAACTTACCAATAATATTTTAAAACAATCTAGAATCCAAATCTATCAAAACATTTCCATAA
TTCCAAATT
TTTATTTTTGTTTTTTTGTGCCTACATATACCTGTTGAAATTGGAATTCAAGACAAAAACCCCTGTAGCGCGTCTGAAA
ATGGATATCTAGGTACAAAAACTTACCAATAATATTTTAAAACAATCTAGAATCCAAATCTATCAAAACATTTCCATAA
TTCCAAAATT
TTTATTTTTGTTTTTTTGTGCCTACATATACCTGTTGAAATTGGAATTCAAGACAAAAACCCCTGTAGCGCGTCTGAAA
ATGGATATCTAGGTACAAAAACTTACCAATAATATTTTAAAACAATCTAGAATCCAAATCTATCAAAACATTTCCATAA
TTCCAAAT
TTTATTTTTGTTTTTTTGTGCCTACATATACCTGTTGAAATTGGAATTCAAGACAAAAACCCCTGTAGCGCGTCTGAAA
ATGGATATCTAGGTACAAAAACTTACCAATAATATTTTAAAACAATCTAGAATCCAAATCTATCAAAACATTTCCATAA
TTCCAAAT

TTTATTTTTGTTTTTTTGTGCCTACATATACCTGTTGAAATTGGAATTCAAGACAAAAACCCCTGTAGCGCGTCTGAAA
ATGGATATCTAGGTACAAAAACTTACCAATAATATTTTAAAACAATCTAGAATCCAAATCTATCAAAACATTTCCATAA
TTCCAAAT
TTTATTTTTGTTTTTTTGTGCCTACATATACCTGTTGAAATTGGAATTCAAGACAAAAACCCCTGTAGCGCGTCTGAAA
ATGGATATCTAGGTACAAAAACTTACCAATAATATTTTAAAACAATCTAGAATCCAAATCTATCAAAACATTTCCGGAA
TTCCATAATG
TTTATTTTTGTTTTTTTGTGCCTACATATACCTGTTGAAATTGGAATTCAAGACAAAAACCCCTGTAGCGCGTCTGAAA
ATGGATATCTAGGTACAAAAACTTACCAATAATATTTTAAAACAATCTAGAATCCAAATCTATCAAAACATTTCCATAA
TTCCAAATT
TTTATTTTTGTTTTTTTGTGCCTACATATACCTGTTGAAATTGGAATTCAAGACAAAAACCCCTGTAGCGCGTCTGAAA
ATGGATATCTAGGTACAAAAACTTACCAATAATATTTTAAAACAATCTAGAATCCAAATCTATCAAAACATTTCCGGAA
TTCCATAATG
TTTATTTTTGTTTTTTTGTGCCTACATATACCTGTTGAAATTGGAATTCAAGACAAAAACCCCTGTAGCGCGTCTGAAA
ATGGATATCTAGGTACAAAAACTTACCAATAATATTTTAAAACAATCTAGAATCCAAATCTATCAAAACATTTCCATAA
TTCCAAATT
TTTATTTTTGTTTTTTTGTGCCTACATATACCTGTTGAAATTGGAATTCAAGACAAAAACCCCTGTAGCGCGTCTGAAA
ATGGATATCTAGGTACAAAAACTTACCAATAATATTTTAAAACAATCTAGAATCCAAATCTATCAAAACATTTCCATAA
TTCCAAAATT
TTTATTTTTGTTTTTTTGTGCCTACATATACCTGTTGAAATTGGAATTCAAGACAAAAACCCCTGTAGCGCGTCTGAAA
ATGGATATCTAGGTACAAAAACTTACCAATAATATTTTAAAACAATCTAGAATCCAAATCTATCAAAACATTTCCGAAT
TCCATAATG
TTTATTTTTGTTTTTTTGTGCCTACATATACCTGTTGAAATTGGAATTCAAGACAAAAACCCCTGTAGCGCGTCTGAAA
ATGGATATCTAGGTACAAAAACTTACCAATAATATTTTAAAACAATCTAGAATCCAAATCTATCAAAACATTTCCATAA
TTCCAAAT
TTTATTTTTGTTTTTTTGTGCCTACATATACCTGTTGAAATTGGAATTCAAGACAAAAACCCCTGTAGCGCGTCTGAAA
ATGGATATCTAGGTACAAAAACTTACCAATAATATTTTAAAACAATCTAGAATCCAAATCTATCAAAACATTTCCGGAA
TTCCATAATG
TTTATTTTTGTTTTTTTGTGCCTACATATACCTGTTGAAATTGGAATTCAAGACAAAAACCCCTGTAGCGCGTCTGAAA
ATGGATATCTAGGTACAAAAACTTACCAATAATATTTTAAAACAATCTAGAATCCAAATCTATCAAAACATTTCCGGAA
TTCCATAATG
TTTATTTTTGTTTTTTTGTGCCTACATATACCTGTTGAAATTGGAATTCAAGACAAAAACCCCTGTAGCGCGTCTGAAA
ATGGATATCTAGGTACAAAAACTTACCAATAATATTTTAAAACAATCTAGAATCCAAATCTATCAAAACATTTCCATAA
TTCCAAAT
TTTATTTTTGTTTTTTTGTGCCTACATATACCTGTTGAAATTGGAATTCAAGACAAAAACCCCTGTAGCGCGTCTGAAA
ATGGATATCTAGGTACAAAAACTTACCAATAATATTTTAAAACAATCTAGAATCCAAATCTATCAAAACATTTCCGGAA
TTCCATAATG
TTTATTTTTGTTTTTTTGTGCCTACATATACCTGTTGAAATTGGAATTCAAGACAAAAACCCCTGTAGCGCGTCTGAAA
ATGGATATCTAGGTACAAAAACTTACCAATAATATTTTAAAACAATCTAGAATCCAAATCTATCAAAACATTTCCGAAT
TCCATAATG
TTTATTTTTGTTTTTTTGTGCCTACATATACCTGTTGAAATTGGAATTCAAGACAAAAACCCCTGTAGCGCGTCTGAAA
ATGGATATCTAGGTACAAAAACTTACCAATAATATTTTAAAACAATCTAGAATCCAAATCTATCAAAACATTTCCATAA
TTCCAAATT
TTTATTTTTGTTTTTTTGTGCCTACATATACCTGTTGAAATTGGAATTCAAGACAAAAACCCCTGTAGCGCGTCTGAAA
ATGGATATCTAGGTACAAAAACTTACCAATAATATTTTAAAACAATCTAGAATCCAAATCTATCAAAACATTTCCATAA
TTCCAAATT
TTTATTTTTGTTTTTTTGTGCCTACATATACCTGTTGAAATTGGAATTCAAGACAAAAACCCCTGTAGCGCGTCTGAAA
ATGGATATCTAGGTACAAAAACTTACCAATAATATTTTAAAACAATCTAGAATCCAAATCTATCAAAACATTTCCGGAA
TTCCATAATG
TTTATTTTTGTTTTTTTGTGCCTACATATACCTGTTGAAATTGGAATTCAAGACAAAAACCCCTGTAGCGCGTCTGAAA
ATGGATATCTAGGTACAAAAACTTACCAATAATATTTTAAAACAATCTAGAATCCAAATCTATCAAAACATTTCCGGAA
TTCCATAATG
TTTATTTTTGTTTTTTTGTGCCTACATATACCTGTTGAAATTGGAATTCAAGACAAAAACCCCTGTAGCGCGTCTGAAA
ATGGATATCTAGGTACAAAAACTTACCAATAATATTTTAAAACAATCTAGAATCCAAATCTATCAAAACATTTCCGGAA
TTCCATAATG

TTTATTTTTGTTTTTTTGTGCCTACATATACCTGTTGAAATTGGAATTCAAGACAAAAACCCCTGTAGCGCGTCTGAAA
ATGGATATCTAGGTACAAAAACTTACCAATAATATTTTAAAACAATCTAGAATCCAAATCTATCAAAACATTTCCGGAA
TTCCATAATG
TTTATTTTTGTTTTTTTGTGCCTACATATACCTGTTGAAATTGGAATTCAAGACAAAAACCCCTGTAGCGCGTCTGAAA
ATGGATATCTAGGTACAAAAACTTACCAATAATATTTTAAAACAATCTAGAATCCAAATCTATCAAAACATTTCCGAAT
TCCATAAT
TTTATTTTTGTTTTTTTGTGCCTACATATACCTGTTGAAATTGGAATTCAAGACAAAAACCCCTGTAGCGCGTCTGAAA
ATGGATATCTAGGTACAAAAACTTACCAATAATATTTTAAAACAATCTAGAATCCAAATCTATCAAAACATTTCCGGAA
TTCCATAATG
TTTATTTTTGTTTTTTTGTGCCTACATATACCTGTTGAAATTGGAATTCAAGACAAAAACCCCTGTAGCGCGTCTGAAA
ATGGATATCTAGGTACAAAAACTTACCAATAATATTTTAAAACAATCTAGAATCCAAATCTATCAAAACATTTCCGGAA
TTCCATAATG
TTTATTTTTGTTTTTTTGTGCCTACATATACCTGTTGAAATTGGAATTCAAGACAAAAACCCCTGTAGCGCGTCTGAAA
ATGGATATCTAGGTACAAAAACTTACCAATAATATTTTAAAACAATCTAGAATCCAAATCTATCAAAACATTTCCGGAA
TTCCATAATG
TTTATTTTTGTTTTTTTGTGCCTACATATACCTGTTGAAATTGGAATTCAAGACAAAAACCCCTGTAGCGCGTCTGAAA
ATGGATATCTAGGTACAAAAACTTACCAATAATATTTTAAAACAATCTAGAATCCAAATCTATCAAAACATTTCCATAA
TTCCAAATT
TTTATTTTTGTTTTTTTGTGCCTACATATACCTGTTGAAATTGGAATTCAAGACAAAAACCCCTGTAGCGCGTCTGAAA
ATGGATATCTAGGTACAAAAACTTACCAATAATATTTTAAAACAATCTAGAATCCAAATCTATCAAAACATTTCCGGAA
TTCCATAATG
TTTATTTTTGTTTTTTTGTGCCTACATATACCTGTTGAAATTGGAATTCAAGACAAAAACCCCTGTAGCGCGTCTGAAA
ATGGATATCTAGGTACAAAAACTTACCAATAATATTTTAAAACAATCTAGAATCCAAATCTATCAAAACATTTCCATAA
TTCCAAATT
TTTATTTTTGTTTTTTTGTGCCTACATATACCTGTTGAAATTGGAATTCAAGACAAAAACCCCTGTAGCGCGTCTGAAA
ATGGATATCTAGGTACAAAAACTTACCAATAATATTTTAAAACAATCTAGAATCCAAATCTATCAAAACATTTCCATAA
TTCCAAATT
TTTATTTTTGTTTTTTTGTGCCTACATATACCTGTTGAAATTGGAATTCAAGACAAAAACCCCTGTAGCGCGTCTGAAA
ATGGATATCTAGGTACAAAAACTTACCAATAATATTTTAAAACAATCTAGAATCCAAATCTATCAAAACATTTCCGGAA
TTCCATAATG
TTTATTTTTGTTTTTTTGTGCCTACATATACCTGTTGAAATTGGAATTCAAGACAAAAACCCCTGTAGCGCGTCTGAAA
ATGGATATCTAGGTACAAAAACTTACCAATAATATTTTAAAACAATCTAGAATCCAAATCTATCAAAACATTTCCGGAA
TTCCATAATG
TTTATTTTTGTTTTTTTGTGCCTACATATACCTGTTGAAATTGGAATTCAAGACAAAAACCCCTGTAGCGCGTCTGAAA
ATGGATATCTAGGTACAAAAACTTACCAATAATATTTTAAAACAATCTAGAATCCAAATCTATCAAAACATTTCCGGAA
TTCCATAATG
TTTATTTTTGTTTTTTTGTGCCTACATATACCTGTTGAAATTGGAATTCAAGACAAAAACCCCTGTAGCGCGTCTGAAA
ATGGATATCTAGGTACAAAAACTTACCAATAATATTTTAAAACAATCTAGAATCCAAATCTATCAAAACATTTCCGGAA
TTCCATAATG
TTTATTTTTGTTTTTTTGTGCCTACATATACCTGTTGAAATTGGAATTCAAGACAAAAACCCCTGTAGCGCGTCTGAAA
ATGGATATCTAGGTACAAAAACTTACCAATAATATTTTAAAACAATCTAGAATCCAAATCTATCAAAACATTTCCATAA
TTCCAAATT
TTTATTTTTGTTTTTTTGTGCCTACATATACCTGTTGAAATTGGAATTCAAGACAAAAACCCCTGTAGCGCGTCTGAAA
ATGGATATCTAGGTACAAAAACTTACCAATAATATTTTAAAACAATCTAGAATCCAAATCTATCAAAACATTTCCGGAA
TTCCATAATG
TTTATTTTTGTTTTTTTGTGCCTACATATACCTGTTGAAATTGGAATTCAAGACAAAAACCCCTGTAGCGCGTCTGAAA
ATGGATATCTAGGTACAAAAACTTACCAATAATATTTTAAAACAATCTAGAATCCAAATCTATCAAAACATTTCCGGAA
TTCCATAATG
TTTATTTTTGTTTTTTTGTGCCTACATATACCTGTTGAAATTGGAATTCAAGACAAAAACCCCTGTAGCGCGTCTGAAA
ATGGATATCTAGGTACAAAAACTTACCAATAATATTTTAAAACAATCTAGAATCCAAATCTATCAAAACATTTCCGGAA
TTCCATAATG
TTTATTTTTGTTTTTTTGTGCCTACATATACCTGTTGAAATTGGAATTCAAGACAAAAACCCCTGTAGCGCGTCTGAAA
ATGGATATCTAGGTACAAAAACTTACCAATAATATTTTAAAACAATCTAGAATCCAAATCTATCAAAACATTTCCGAAT
TCCAAAT

TTTATTTTTGTTTTTTTGTGCCTACATATACCTGTTGAAATTGGAATTCAAGACAAAAACCCCTGTAGCGCGTCTGAAA
ATGGATATCTAGGTACAAAAACTTACCAATAATATTTTAAAACAATCTAGAATCCAAATCTATCAAAACATTTCCATAA
TTCCAAATT
TTTATTTTTGTTTTTTTGTGCCTACATATACCTGTTGAAATTGGAATTCAAGACAAAAACCCCTGTAGCGCGTCTGAAA
ATGGATATCTAGGTACAAAAACTTACCAATAATATTTTAAAACAATCTAGAATCCAAATCTATCAAAACATTTCCGGAA
TTCCATAATG
TTTATTTTTGTTTTTTTGTGCCTACATATACCTGTTGAAATTGGAATTCAAGACAAAAACCCCTGTAGCGCGTCTGAAA
ATGGATATCTAGGTACAAAAACTTACCAATAATATTTTAAAACAATCTAGAATCCAAATCTATCAAAACATTTCCGGAA
TTCCATAATG
TTTATTTTTGTTTTTTTGTGCCTACATATACCTGTTGAAATTGGAATTCAAGACAAAAACCCCTGTAGCGCGTCTGAAA
ATGGATATCTAGGTACAAAAACTTACCAATAATATTTTAAAACAATCTAGAATCCAAATCTATCAAAACATTTCCGAAT
TCCATAATG
TTTATTTTTGTTTTTTTGTGCCTACATATACCTGTTGAAATTGGAATTCAAGACAAAAACCCCTGTAGCGCGTCTGAAA
ATGGATATCTAGGTACAAAAACTTACCAATAATATTTTAAAACAATCTAGAATCCAAATCTATCAAAACATTTCCATAA
TTCCAAAATT
TTTATTTTTGTTTTTTTGTGCCTACATATACCTGTTGAAATTGGAATTCAAGACAAAAACCCCTGTAGCGCGTCTGAAA
ATGGATATCTAGGTACAAAAACTTACCAATAATATTTTAAAACAATCTAGAATCCAAATCTATCAAAACATTTCCGGAA
TTCCATAATG
TTTATTTTTGTTTTTTTGTGCCTACATATACCTGTTGAAATTGGAATTCAAGACAAAAACCCCTGTAGCGCGTCTGAAA
ATGGATATCTAGGTACAAAAACTTACCAATAATATTTTAAAACAATCTAGAATCCAAATCTATCAAAACATTTCCGGAA
TTCCATAATG
TTTATTTTTGTTTTTTTGTGCCTACATATACCTGTTGAAATTGGAATTCAAGACAAAAACCCCTGTAGCGCGTCTGAAA
ATGGATATCTAGGTACAAAAACTTACCAATAATATTTTAAAACAATCTAGAATCCAAATCTATCAAAACATTTCCGGAA
TTCCATAATG
TTTATTTTTGTTTTTTTGTGCCTACATATACCTGTTGAAATTGGAATTCAAGACAAAAACCCCTGTAGCGCGTCTGAAA
ATGGATATCTAGGTACAAAAACTTACCAATAATATTTTAAAACAATCTAGAATCCAAATCTATCAAAACATTTCCGGAA
TTCCATAATG
TTTATTTTTGTTTTTTTGTGCCTACATATACCTGTTGAAATTGGAATTCAAGACAAAAACCCCTGTAGCGCGTCTGAAA
ATGGATATCTAGGTACAAAAACTTACCAATAATATTTTAAAACAATCTAGAATCCAAATCTATCAAAACATTTCCGGAA
TTCCATAATG
TTTATTTTTGTTTTTTTGTGCCTACATATACCTGTTGAAATTGGAATTCAAGACAAAAACCCCTGTAGCGCGTCTGAAA
ATGGATATCTAGGTACAAAAACTTACCAATAATATTTTAAAACAATCTAGAATCCAAATCTATCAAAACATTTCCGGAA
TTCCATAATG
TTTATTTTTGTTTTTTTGTGCCTACATATACCTGTTGAAATTGGAATTCAAGACAAAAACCCCTGTAGCGCGTCTGAAA
ATGGATATCTAGGTACAAAAACTTACCAATAATATTTTAAAACAATCTAGAATCCAAATCTATCAAAACATTTCCGAAT
TCCATAATG
TTTATTTTTGTTTTTTTGTGCCTACATATACCTGTTGAAATTGGAATTCAAGACAAAAACCCCTGTAGCGCGTCTGAAA
ATGGATATCTAGGTACAAAAACTTACCAATAATATTTTAAAACAATCTAGAATCCAAATCTATCAAAACATTTCCGGAA
TTCCATAATG
TTTATTTTTGTTTTTTTGTGCCTACATATACCTGTTGAAATTGGAATTCAAGACAAAAACCCCTGTAGCGCGTCTGAAA
ATGGATATCTAGGTACAAAAACTTACCAATAATATTTTAAAACAATCTAGAATCCAAATCTATCAAAACATTTCCATAA
TTCCAAAATT
TTTATTTTTGTTTTTTTGTGCCTACATATACCTGTTGAAATTGGAATTCAAGACAAAAACCCCTGTAGCGCGTCTGAAA
ATGGATATCTAGGTACAAAAACTTACCAATAATATTTTAAAACAATCTAGAATCCAAATCTATCAAAACATTTCCATAA
TTCCAAATT
TTTATTTTTGTTTTTTTGTGCCTACATATACCTGTTGAAATTGGAATTCAAGACAAAAACCCCTGTAGCGCGTCTGAAA
ATGGATATCTAGGTACAAAAACTTACCAATAATATTTTAAAACAATCTAGAATCCAAATCTATCAAAACATTTCCGGAA
TTCCATAATG
TTTATTTTTGTTTTTTTGTGCCTACATATACCTGTTGAAATTGGAATTCAAGACAAAAACCCCTGTAGCGCGTCTGAAA
ATGGATATCTAGGTACAAAAACTTACCAATAATATTTTAAAACAATCTAGAATCCAAATCTATCAAAACATTTCCATAA
TTCCAAAATT
TTTATTTTTGTTTTTTTGTGCCTACATATACCTGTTGAAATTGGAATTCAAGACAAAAACCCCTGTAGCGCGTCTGAAA
ATGGATATCTAGGTACAAAAACTTACCAATAATATTTTAAAACAATCTAGAATCCAAATCTATCAAAACATTTCCATAA
TTCCAAAAT

TTTATTTTTGTTTTTTTGTGCCTACATATACCTGTTGAAATTGGAATTCAAGACAAAAACCCCTGTAGCGCGTCTGAAA
ATGGATATCTAGGTACAAAAACTTACCAATAATATTTTAAAACAATCTAGAATCCAAATCTATCAAAACATTTCCATAA
TTCCAAAATT
TTTATTTTTGTTTTTTTGTGCCTACATATACCTGTTGAAATTGGAATTCAAGACAAAAACCCCTGTAGCGCGTCTGAAA
ATGGATATCTAGGTACAAAAACTTACCAATAATATTTTAAAACAATCTAGAATCCAAATCTATCAAAACATTTCCGGAA
TTCCATAATG
TTTATTTTTGTTTTTTGTGCCTACATATACCTGTTGAAATTGGAATTCAAGACAAAAACCCCTGTAGCGCGTCTGAAAA
TGGATATCTAGGTACAAAAACTTACCAATAATATTTTAAAACAATCTAGAATCCAAATCTATCAAAACATTTCCGGAAT
TCCATAATG
TTTATTTTTGTTTTTTGTGCCTACATATACCTGTTGAAATTGGAATTCAAGACAAAAACCCCTGTAGCGCGTCTGAAAA
TGGATATCTAGGTACAAAAACTTACCAATAATATTTTAAAACAATCTAGAATCCAAATCTATCAAAACATTTCCATAAT
TCCAAAATT
TTTATTTTTGTTTTTTTGTGCCTACATATACCTGTTGAAATTGGAATTCAAGACAAAAACCCCTGTAGCGCGTCTGAAA
ATGGATATCTAGGTACAAAAACTTACCAATAATATTTTAAAACAATCTAGAATCCAAATCTATCAAAACATTTCCGAAT
TCCATAATG
TTTATTTTTGTTTTTTTGTGCCTACATATACCTGTTGAAATTGGAATTCAAGACAAAAACCCCTGTAGCGCGTCTGAAA
ATGGATATCTAGGTACAAAAACTTACCAATAATATTTTAAAACAATCTAGAATCCAAATCTATCAAAACATTTCCGGAA
TTCCATAATG
TTTATTTTTGTTTTTTTGTGCCTACATATACCTGTTGAAATTGGAATTCAAGACAAAAACCCCTGTAGCGCGTCTGAAA
ATGGATATCTAGGTACAAAAACTTACCAATAATATTTTAAAACAATCTAGAATCCAAATCTATCAAAACATTTCCGGAA
TTCCATAATG
TTTATTTTTGTTTTTTGTGCCTACATATACCTGTTGAAATTGGAATTCAAGACAAAAACCCCTGTAGCGCGTCTGAAAA
TGGATATCTAGGTACAAAAACTTACCAATAATATTTTAAAACAATCTAGAATCCAAATCTATCAAAACATTTCCATAAT
TCCAAAATT
TTTATTTTTGTTTTTTTGTGCCTACATATACCTGTTGAAATTGGAATTCAAGACAAAAACCCCTGTAGCGCGTCTGAAA
ATGGATATCTAGGTACAAAAACTTACCAATAATATTTTAAAACAATCTAGAATCCAAATCTATCAAAACATTTCCGGAA
TTCCATAATG
TTTATTTTTGTTTTTTTGTGCCTACATATACCTGTTGAAATTGGAATTCAAGACAAAAACCCCTGTAGCGCGTCTGAAA
ATGGATATCTAGGTACAAAAACTTACCAATAATATTTTAAAACAATCTAGAATCCAAATCTATCAAAACATTTCCATAA
TTCCAAAAT
TTTATTTTTGTTTTTTTGTGCCTACATATACCTGTTGAAATTGGAATTCAAGACAAAAACCCCTGTAGCGCGTCTGAAA
ATGGATATCTAGGTACAAAAACTTACCAATAATATTTTAAAACAATCTAGAATCCAAATCTATCAAAACATTTCCATAA
TTCCAAAATT
TTTATTTTTGTTTTTTTGTGCCTACATATACCTGTTGAAATTGGAATTCAAGACAAAAACCCCTGTAGCGCGTCTGAAA
ATGGATATCTAGGTACAAAAACTTACCAATAATATTTTAAAACAATCTAGAATCCAAATCTATCAAAACATTTCCGGAA
TTCCATAATG
TTTATTTTTGTTTTTTTGTGCCTACATATACCTGTTGAAATTGGAATTCAAGACAAAAACCCCTGTAGCGCGTCTGAAA
ATGGATATCTAGGTACAAAAACTTACCAATAATATTTTAAAACAATCTAGAATCCAAATCTATCAAAACATTTCCATAA
TTCCAAAATT
TTTATTTTTGTTTTTTTGTGCCTACATATACCTGTTGAAATTGGAATTCAAGACAAAAACCCCTGTAGCGCGTCTGAAA
ATGGATATCTAGGTACAAAAACTTACCAATAATATTTTAAAACAATCTAGAATCCAAATCTATCAAAACATTTCCATAA
TTCCAAAATT
TTTATTTTTGTTTTTTTGTGCCTACATATACCTGTTGAAATTGGAATTCAAGACAAAAACCCCTGTAGCGCGTCTGAAA
ATGGATATCTAGGTACAAAAACTTACCAATAATATTTTAAAACAATCTAGAATCCAAATCTATCAAAACATTTCCATAA
TTCCAAAATT
TTTATTTTTGTTTTTCTGTGCCTACATATACCTGTTGAAATTGGAATTCAAGACAAAAACCCCTGTAGCGCGTCTGAAA
ATGGATATCTAGGTACAAAAACTTACCAATAATATTTTAAAACAATCTAGAATCCAAATCTATCAAAACATTTCCATAA
TTCCAAAATT
TTTATTTTTGTTTTTTTGTGCCTACATATACCTGTTGAAATTGGAATTCAAGACAAAAACCCCTGTAGCGCGTCTGAAA
ATGGATATCTAGGTACAAAAACTTACCAATAATATTTTAAAACAATCTAGAATCCAAATCTATCAAAACATTTCCGGAA
TTCCATAATG
TTTATTTTTGTTTTTTTGTGCCTACATATACCTGTTGAAATTGGAATTCAAGACAAAAACCCCTGTAGCGCGTCTGAAA
ATGGATATCTAGGTACAAAAACTTACCAATAATATTTTAAAACAATCTAGAATCCAAATCTATCAAAACATTTCCATAA
TTCCAAAATT

TTTATTTTTGTTTTTCTGTGCCTACATATACCTGTTGAAATTGGAATTCAAGACAAAAACCCCTGTAGCGCGTCTGAAA
ATGGATATCTAGGTACAAAAACTTACCAATAATATTTTAAAACAATCTAGAATCCAAATCTATCAAAACATTTCCATAA
TTCCAAAATT
TTTATTTTTGTTTTTTTGTGCCTACATATACCTGTTGAAATTGGAATTCAAGACAAAAACCCCTGTAGCGCGTCTGAAA
ATGGATATCTAGGTACAAAAACTTACCAATAATATTTTAAAACAATCTAGAATCCAAATCTATCAAAACATTTCCGGAA
TTCCATAATG
TTTATTTTTGTTTTTTTGTGCCTACATATACCTGTTGAAATTGGAATTCAAGACAAAAACCCCTGTAGCGCGTCTGAAA
ATGGATATCTAGGTACAAAAACTTACCAATAATATTTTAAAACAATCTAGAATCCAAATCTATCAAAACATTTCCGGAA
TTCCATAATG
TTTATTTTTGTTTTTTTGTGCCTACATATACCTGTTGAAATTGGAATTCAAGACAAAAACCCCTGTAGCGCGTCTGAAA
ATGGATATCTAGGTACAAAAACTTACCAATAATATTTTAAAACAATCTAGAATCCAAATCTATCAAAACATTTCCATAA
TTCCAAAATT
TTTATTTTTGTTTTTTTGTGCCTACATATACCTGTTGAAATTGGAATTCAAGACAAAAACCCCTGTAGCGCGTCTGAAA
ATGGATATCTAGGTACAAAAACTTACCAATAATATTTTAAAACAATCTAGAATCCAAATCTATCAAAACATTTCCGGAA
TTCCATAATG
TTTATTTTTGTTTTTTTGTGCCTACATATACCTGTTGAAATTGGAATTCAAGACAAAAACCCCTGTAGCGCGTCTGAAA
ATGGATATCTAGGTACAAAAACTTACCAATAATATTTTAAAACAATCTAGAATCCAAATCTATCAAAACATTTCCATAA
TTCCAAAATT
TTTATTTTTGTTTTTTTGTGCCTACATATACCTGTTGAAATTGGAATTCAAGACAAAAACCCCTGTAGCGCGTCTGAAA
ATGGATATCTAGGTACAAAAACTTACCAATAATATTTTAAAACAATCTAGAATCCAAATCTATCAAAACATTTCCATAA
TTCCAAAATT
TTTATTTTTGTTTTTTTGTGCCTACATATACCTGTTGAAATTGGAATTCAAGACAAAAACCCCTGTAGCGCGTCTGAAA
ATGGATATCTAGGTACAAAAACTTACCAATAATATTTTAAAACAATCTAGAATCCAAATCTATCAAAACATTTCCATAA
TTCCAAAATT
TTTATTTTTGTTTTTTTGTGCCTACATATACCTGTTGAAATTGGAATTCAAGACAAAAACCCCTGTAGCGCGTCTGAAA
ATGGATATCTAGGTACAAAAACTAACCAATAATATTTTAAAACAATCTAGAATCCAAATCTATCAAAACATTTCCGGAA
TTCCATAATG
Nr of selected repeats 110 Similarity 0.984798
Consensus:
TTTATTTTTGTTTTTTTGTGCCTACATATACCTGTTGAAATTGGAATTCAAGACAAAAACCCCTGTAGCGCGTCTGAAA
ATGGATATCTAGGTACAAAAACTTACCAATAATATTTTAAAACAATCTAGAATCCAAATCTATCAAAACATTTCCggAA
TTCCAtAATg
>Cele-UNSB01_3:2204608-2205196 Satlength=589 Nr of Repeats=25 RepeatLength=21
seed=GCCTAAAAAC
GCCTAAAAACGTAAAATTTAGC
CCTAAAAACGTGAATTTTAGTCCTAAAAATGTAAATTTTAG
GCATAAAAACGTAAATTTTAG
GCATAAAAACGTAAATTTTAA
GCTTAAAAACGTGAATTTTAA
GCCTAAAACCGTGAATTTTAA
GCCTAAAACCGTGAATTTTAA
GCCTAAAAACGTGAATTTTAG
GCCTAAAAACGTAAAATTTAGC
CCTAAAAACGTGAATTTTAG
GCCTAAAAACGTAAATTTTAG
GCCTAAAAACTTAAATTTTAAGTGTGAAACCGTGAATTTTAA
GCCTAAAAACTTAAATTTTAA
GCCTAAAAACCTAAATTTTAG
GCCTGAAAACGTAAATTTTAA
GCCTAAAAACGTAAATTTTAA
GCCTAAAAATGTAAATTTTAG
GCCTAAAACCGTGAATTTTAG
GCCTAAAAATGTAACTTTTAG
GCCTAAAAACATACATTTTGG

```
GTCTAAAAACGTAAATTGTAGT
GCTAAAAACGCAAATTAAA
GCCTAAAACCTTGATTTTTAA
GCCTAGAAACTTGGATTTTAGC
CCTAAAAACGTGAATTTTAGGTCGTAAAAACGTGAAAATTAG
Nr of selected repeats 16 Similarity 0.805291
Consensus:
GCCTAAAAaCgTaAATTTTAa
>Cele-UNSB01_3:2212231-2213000 Satlength=770 Nr of Repeats=22 RepeatLength=31
seed=ATTTCCCGCC
ATTTCCCGCCAAAAAATATTTGAAAATTTGA
ATTTCCCGCCAAAAAATATTTAAAAATTTGAATTTTTGC
ATTTCCCGCCAAAAAATATTTAAAAATTTGAATTTTTGC
ATTTCCCGCCAAAAAATATTTGAAAACTTGA
ATTTCCCGCCAAAAAATATTTAAAAATTTGAATTTTTGC
ATTTCCCGCCAAAAAATATTTGAAAACTTGA
ATTTCCCGCCAAAAAAATATTTAAAAATATGAATTTTCGA
ATTTCCCGCAAAAAAATATTTGAAAATTTGA
ATTTCCCGCCAAAAAATATTTAAAAATTTGA
ATTTCCCGCCAAATAATATTTGAAAATTTGGATTTGCAGCCAAAAAGCATTTGAAAATTTTAATTTTTGA
ATTTCCCGCCAAAAAGTATTTGAAAATTTAA
ATTTCCCGCCAAAAAATATTAAAAATTTTGAATTTTTGC
ATTTCCCGCCAAAAAATATTCGAAAATTTAA
ATTTCCCGCCAAAAAATATTAGAAAATTTGA
ATTTCCCGCCAAAAAATATTTGAAAATTTGA
ATTTCCCGCAAAAAAATATTTGAAAATCTGA
ATTTCCCGCAAAAAAATATTTGAAAATTTGA
ATTTCCCGCCAAAATAATTTGAATTTTTAA
ATTTCCCGCCAAAAAATATTTGAAAATTTGA
ATTTCCCGCCAAAAAATATTTGAAAATTTGA
ATTTCCCGCCAAAAAATATTAAAAATTTTGAATTTTTGC
ATTTCCCGCCAAAAAATATTCGAAAATTTAA
Nr of selected repeats 14 Similarity 0.922486
Consensus:
ATTTCCCGCcAAAAAATATTtGAAAATTTgA
>Cele-UNSB01_3:2214780-2216279 Satlength=1500 Nr of Repeats=39 RepeatLength=39
seed=TTTTTGGCGG
TTTTTGGCGGGAAATTCAAAAATTCAAATTTTCAAATAT
TTTTTGGCGGGAAATTCAAAAATTTAAATTTTTTGAATAT
TTTTTGGCGGGAAATTTCAAAATTCAAATTTTTGAATAT
TTTTTGGCGGGAAATTTCAAAATTCAAATTTTTGAATAT
TTTTTGGCGGGAAATTCAAAAATTCAAATTTTTTGAATAT
TTTTTGGCGGGAAATTCAAAAATTTAAATTTTCAAATAT
TTTTTGGCGGGAAATTTAAAAATTCAAATTTCTGAATA
TTTTTGGCGGGAAATTTCAAAATTCAAATTTTTGAATAT
TTTTTGCCGGGAAATTCAAAAATTCAAATTTTTTGAATAT
TTTTTGGCGGGAAATTTCAAAATTCAAATTTTTGAATAT
TTTTTGGCGGGAAATTCAAAAATTAAAATTTTCAAATGT
TTTTTGGCGGGAAATTCAAAAATTCAAATTTTCAAATAT
TTTTTGGCGGGAAATTCAAAAATTCAAATTTACAAATAT
TTTTTGGCGGGAAATTTAAAAATTCAAATTTTTGAATAT
TTTTTGCCGGGAAATTCAAAAATTCAAATTTTTTGAATAT
TTTTTGGCGGGAAATTTCAAAATTCAAATTTTTGAATAT
TTTTTGGCGGGAAATTCAAAAATTAAAATTTTCAAATGT
```

TTTTTGGCGGGAAATTCAAAAATTCAAATTTTCAAATAT
TTTTTGGCGGGAAATTCAAAAATTCAAATTTACAAATAT
TTTTTGGCGGGAAATTTAAAAATTCAAATTTTCAAATAT
TTTTTGGCGGGAAATTCAAAAATTTAAATTTTCAAATAT
TTTTTGGCGGGAAATTTAAAAATTCAAATTTCTGAATA
TTTTTGGCGGGAAATTCAAAAATTCAAATTTACAAATAT
TTTTTGGCGGGAAATTCAAAAATTCAAATTTTCAAATAT
TTTTTGGCGGGAAATTCAAAAATTAAAATTTTCAAATAT
TTTTTGGCGGGAAATTCAAAAATTCAAATTTACAAATAT
TTTTTGGCGGGAAATTTAAAAATTCAAATTTTCAAATAT
TTTTTGGCGGGAAATTTAAAAATTCAAATTTTCAAATAT
TTTTTGGCGGGAAATTCAAAAATTCAAAACT
TTTTTGGCGGGAAATTCAAAAATTCAAATTTACAAATAT
TTTTTGGCGGGAAATTTAAAAATTCAAATTTTCAAATAT
TTTTTGGCGGGAAATTCAAAAATTCAAAACT
TTTTTGGCGGGAAATTCAAATTTTCAAAAA
TTTTTGGCGGGAAATTCAAAAATTGAAATTTTCAAATAT
TTTTTGGCGGGAAATTTCAAAATTCAAATTTTTGAATAT
TTTTTGGCGGGAAATTCAAAAATTCAAATTTTCAAATAT
TTTTTGGCGGGAAATTCAAAAATTCAAAATTTCAAATAT
TTTTTGGCGGGAAATTCAAAAAATTCAAATTTTAGAATAT
TTTTTGGCGGGAAATTCAAAAATTTAAATTTTCAAATAT
Nr of selected repeats 29 Similarity 0.914530
Consensus:
TTTTTGGCGGGAAATTcaAAAATTcAAATTTTcaAATAT
>Cele-UNSB01_3:2219970-2221469 Satlength=1500 Nr of Repeats=39 RepeatLength=39
seed=TTTTTGGCGG
TTTTTGGCGGGAAATTCAAAAATTCAAATTTTCAAATAT
TTTTTGGCGGGAAATTCAAAAATTTAAATTTTTTGAATAT
TTTTTGGCGGGAAATTTCAAAATTCAAATTTTTGAATAT
TTTTTGGCGGGAAATTTCAAAATTCAAATTTTTGAATAT
TTTTTGGCGGGAAATTCAAAAATTCAAATTTTTTGAATAT
TTTTTGGCGGGAAATTCAAAAATTTAAATTTTCAAATAT
TTTTTGGCGGGAAATTTAAAAATTCAAATTTCTGAATA
TTTTTGGCGGGAAATTTCAAAATTCAAATTTTTGAATAT
TTTTTGCCGGGAAATTCAAAAATTCAAATTTTTTGAATAT
TTTTTGGCGGGAAATTTCAAAATTCAAATTTTTGAATAT
TTTTTGGCGGGAAATTCAAAAATTAAAATTTTCAAATGT
TTTTTGGCGGGAAATTCAAAAATTCAAATTTTCAAATAT
TTTTTGGCGGGAAATTCAAAAATTCAAATTTACAAATAT
TTTTTGGCGGGAAATTTAAAAATTCAAATTTTTGAATAT
TTTTTGCCGGGAAATTCAAAAATTCAAATTTTTTGAATAT
TTTTTGGCGGGAAATTTCAAAATTCAAATTTTTGAATAT
TTTTTGGCGGGAAATTCAAAAATTAAAATTTTCAAATGT
TTTTTGGCGGGAAATTCAAAAATTCAAATTTTCAAATAT
TTTTTGGCGGGAAATTCAAAAATTCAAATTTACAAATAT
TTTTTGGCGGGAAATTTAAAAATTCAAATTTTCAAATAT
TTTTTGGCGGGAAATTCAAAAATTTAAATTTTCAAATAT
TTTTTGGCGGGAAATTTAAAAATTCAAATTTCTGAATA
TTTTTGGCGGGAAATTCAAAAATTCAAATTTACAAATAT
TTTTTGGCGGGAAATTCAAAAATTCAAATTTTCAAATAT
TTTTTGGCGGGAAATTCAAAAATTAAAATTTTCAAATAT
TTTTTGGCGGGAAATTCAAAAATTCAAATTTACAAATAT
TTTTTGGCGGGAAATTTAAAAATTCAAATTTTCAAATAT

TTTTTGGCGGGAAATTTAAAAATTCAAATTTTCAAATAT
TTTTTGGCGGGAAATTCAAAAATTCAAAACT
TTTTTGGCGGGAAATTCAAAAATTCAAATTTACAAATAT
TTTTTGGCGGGAAATTTAAAAATTCAAATTTTCAAATAT
TTTTTGGCGGGAAATTCAAAAATTCAAAACT
TTTTTGGCGGGAAATTCAAATTTTCAAAAA
TTTTTGGCGGGAAATTCAAAAATTGAAATTTTCAAATAT
TTTTTGGCGGGAAATTTCAAAATTCAAATTTTGAATAT
TTTTTGGCGGGAAATTCAAAAATTCAAATTTTCAAATAT
TTTTTGGCGGGAAATTCAAAAATTCAAAATTTCAAATAT
TTTTTGGCGGGAAATTCAAAAAATTCAAATTTTAGAATAT
TTTTTGGCGGGAAATTCAAAAATTTAAATTTTCAAATAT
Nr of selected repeats 29 Similarity 0.914530
Consensus:
TTTTTGGCGGGAAATTcaAAAATTcAAATTTTcaAATAT
>Cele-UNSB01_3:2223808-2223967 Satlength=160 Nr of Repeats=4 RepeatLength=40
seed=TCCCGCCAAA
TCCCGCCAAAAAATATTCAAAAATTTGAATTTTTTGAATT
TCCCGCCAAAAAATATTCAAAAAATTTGAATTTTGAAATT
TCCCGCCAAAAAATATTTGAAAATTTAAATTTTTGAATT
TCCCGCCAAAAAATATTCAAAAAATTTAAATTTTTGAATT
Nr of selected repeats 3 Similarity 0.866667
Consensus:
TCCCGCCAAAAAATATTCAAAAAaTTtaAaTTTTtgAATT
>Cele-UNSB01_3:2325106-2325938 Satlength=833 Nr of Repeats=23 RepeatLength=32
seed=CTCAGCCAAT
CTCAGCCAATCAACGTTTAACTCCGCCTATTTTTCAGCGAATCAACGTTTAGCTCCGCCTATTC
CTCAGCCAATCGACGT
CTCAGCCAATCAACGTTTTGCTCCGCCTATTTTTCAGCGAATCAACGTTTAGCTCCGCCTATTC
CTCAGCCAATCGACGT
CTCAGCCAATCAGCAATTAGCTCTGCCTATTT
CTCAGCCAATTAACGTTTATCTCCGCCTATTT
CTCAGCCAATCAACGTTTAAATCCGCCTATAT
CTCAGCCAATCAACGTTTAACTCCGCCTATTTTTCAGCGAATCAACGTTTAGCTCCGCCTATTC
CTCAGCCAATCGACGTTTAGCTCCGCCTATTT
CTCAGCCAATCAGCAATTAGCTCTGCCTATTT
CTCAGCCAATTAACGTTTATCTCCGCCTATTT
CTCAGCCAATCAACGTTTAAATCCGCCTATAT
CTCAGCCAATCAACGTTTAACTCCGCCTATTTTTCAGCGAATCAACGTTTAGCTCCGCCTATTC
CTCAGCCAATCGACGTTTAGCTCCGCCTATTT
CTCAGCCAATCAGCAATTAGCTCTGCCTATTT
CTCAGCCAATTAACGTTTATCTCCGCCTATTT
CTCAGCCAATCAACGTTTAGATCCGCCTATAT
CTCAGCCAATCAACGTTTAGCTCCGCCTATTT
CTCAGCCAATCAACGTTTAGCTCCGCCTATTT
CTCAGCCAATCAACGTTTATCTCCGCCTATTT
CTCAGCCAATCAACGTTTAGATCCGCCTATAT
CTCAGCCAATCAACGTTTAGCTCCGCCTATTT
CTCAGCCAATCAACGTTTAGCTCCGCCTATTT
Nr of selected repeats 17 Similarity 0.871936
Consensus:
CTCAGCCAATCAACGTTTAgcTCCGCCTATtT
>Cele-UNSB01_3:2342333-2342437 Satlength=105 Nr of Repeats=5 RepeatLength=21
seed=CTGACCAAAT

CTGACCAAATTTGTTTCAGCG
CTGACCAAATTTGTTTTAGCA
CTGACCAAATTTTTTTCAGCA
CTGACCAAATTTTTTCAGCA
CTGACAAAATTTGTTCCAGCA
Nr of selected repeats 4 Similarity 0.841270
Consensus:
CTGACcAAATTTgTTtcAGCa
>Cele-UNSB01_3:2351961-2352192 Satlength=232 Nr of Repeats=9 RepeatLength=21
seed=GTCAGTGCTG
GTCAGTGCTGGAGTAAATTT
GTCAGTGCTGTAACACATTTT
GTCAGTGATGTAACAAATTTT
GTCAGTGCTGGGACAAATTTT
GTCAGTGCTGGGACAAATTTT
GTCAGTGCTGGAATACATTTT
GTCAGTGCTGGAACAAATTTT
GTCAGTGCTGGAGCAAATTTTGGCCAGAGCTGGAGCAACTTTG
GTCAGTGCTGGAAAAAATTTTGTTCCAGCACTGACCAACTTTT
Nr of selected repeats 6 Similarity 0.856085
Consensus:
GTCAGTGCTGgaACAaATTTT
>Cele-UNSB01_3:2562874-2563954 Satlength=1081 Nr of Repeats=40 RepeatLength=10
seed=AGGCAGGCTT
AGGCAGGCTTAGGTAGGCAT
AGGCAGGCAT
AGGCAGGCTT
AGGTAGGCTT
AGACAGGCTT
AGGTAGGCTT
AGGCAGGCAT
AGGCAGGCAT
AGGCAGGCTTAGGCAAGCAT
AGGCAGGCTT
AGGCAGGCTT
AGGCAGGCTT
AGGCAGGCTT
AGGCAGGCTTACAGAGGTTTAGGCAGACTC
AGGCAGGCTT
AGGCAAGCTT
AGGCAGGCTT
AGGCAAGCTTGGGCAGGATTAAGCATGTGTAGGCAAGCGTAGGCAGGTGTTGGCAGGCATGGGCAGGCT
AGGTAGGCTT
AGGCAGGCTCAGAAGGCTT
AGGTAGGCTT
AGGCTGGCTT
AGGCAGGCTT
AGTCAGGCTTATGCAGGCAT
AGGCATGCTT
AGGCAGGCTT
AGGCAGGCTT
AGGCAGGCTTACAGAGGTTTAGGCAGACTC
AGGCAGGCTT
AGGCAAGCTT

AGGCAGGCTT
AGGCAAGCTTGGGCAGGATTAAGCATGTGTAGGCAAGCGTAGGCAGGTGTTGGCAGGCATGGGCAGGCT
AGGTAGGCTT
AGGCAGGCTAGGCAGGCAT
AGGCAGGCTTAGGCGAGCTT
AGGCAGGCTT
AGGCAGGCTT
AGGCAAGCTTAGGCATGCTA
AGGCAGGCTTAAGCAGGCAT
AGGCAGGCGTAGGCAGTATTTTTTGCAAATTTTCAACTTTCCCATAAGATCGTTTCCACTATTACCAAATTGAATTATC
TTCTGTAATTCGATTTGTGATCCACTATTTCCCTATTTGAGTGAATATTTTTCAATATTTTTTCCATTTTCAACAATGC
CTTCCTTTCTCCTACTTTTCGCCTTCCTAACCCCATTGGTCACAGGACTGACCCATTTCCATTTCAGTGGACAACTCAA
CTGCCACCTACCTATCTTCCACTATCAATTGGAAATTTGGGAAGAGGATTATTTATTACGATGATTTGCTAACCAAGCA
GAGGGTGATCAAGTCATATTCCCCACATAGCTTCGTGATTGACACGCAGGAGTCGGATGATGGAGTTGATTTTGGGGTG
AGTTTCGATTATCGGAAGTTAAGCCGGAATATTCCTGGCTTATGTTCTCAAGCAGGCGT
Nr of selected repeats 27 Similarity 0.883001
Consensus:
AGGCAGGCTT
>Cele-UNSB01_3:2564180-2564252 Satlength=73 Nr of Repeats=5 RepeatLength=12
seed=CCAAGCCCAA
CCAAGCCCAAGC
CCAAGCCCAAGCCTAAGT
CCAAGCCCAATC
CCAATCCCAAGC
CCAAGCCCAAGCCCAAGC
Nr of selected repeats 3 Similarity 0.851852
Consensus:
CCAAgCCCAAgC
>Cele-UNSB01_3:2578277-2578816 Satlength=540 Nr of Repeats=27 RepeatLength=20
seed=GACCCAGCCT
GACCCAGCCTACCCCAGGCA
GACCCAGCCTACCCCAGGCA
GACCCAGCCTACCCCAGGCA
GACCCAGCCTACCCCAGGCA
GACCCAGCCTACCCCAGGCA
GACCCAGCCTACCCCAGGCA
GACCCAGCCTACTCCAGGCA
GACCCAGCCTACCCCAGGCA
GACCCAGCCTACCCCAGGCA
GACCCAGCCTACCCCAGGCA
GACCCAGCCTACCCCAGGCA
GACCCAGCCTACTCCAGGCA
GACCCAGCCTACCCAGGCA
GACCCAGCCTACCCCAGGCA
GACCCAGCCTACCCCAGGCA
GACCCAGCCTACCCCAGGCA
GACCCAGCCTACCCCAGGCA
GACCCAGCCTACCCCAGGCA
GACCCAGCCTACCCCAGGCA
GACCCAGCCTACCCCAGGCA
GACCCAGCCTACCCCAGGCA
GACCCAGCCTACCCCAGGCA
GACCCAGCCTACCCCAGGCA
GACCCAGCCTACCCCAGGCA
GACCCAGCCTACCCCAGGCA

GACCCAGCCTACCCCAGGCA
GACCCAGCCTACCCCAGGTA
GACCCAGCCTACCCCAGGCA
Nr of selected repeats 26 Similarity 0.985026
Consensus:
GACCCAGCCTACCCCAGGCA
>Cele-UNSB01_3:2578282-2579101 Satlength=820 Nr of Repeats=41 RepeatLength=20
seed=AGCCTACCCC
AGCCTACCCCAGGCAGACCC
AGCCTACCCCAGGCAGACCC
AGCCTACCCCAGGCAGACCC
AGCCTACCCCAGGCAGACCC
AGCCTACCCCAGGCAGACCC
AGCCTACCCCAGGCAGACCC
AGCCTACTCCAGGCAGACCC
AGCCTACCCCAGGCAGACCC
AGCCTACCCCAGGCAGACCC
AGCCTACCCCAGGCAGACCC
AGCCTACCCCAGGCAGACCC
AGCCTACTCCAGGCAGACCC
AGCCTACCCAGGCAGACCC
AGCCTACCCCAGGCAGACCC
AGCCTACCCCAGGCAGACCC
AGCCTACCCCAGGCAGACCC
AGCCTACCCCAGGCAGACCC
AGCCTACCCCAGGCAGACCC
AGCCTACCCCAGGCAGACCC
AGCCTACCCCAGGCAGACCC
AGCCTACCCCAGGCAGACCC
AGCCTACCCCAGGCAGACCC
AGCCTACCCCAGGCAGACCC
AGCCTACCCCAGGCAGACCC
AGCCTACCCCAGGCAGACCC
AGCCTACCCCAGGTAGACCC
AGCCTACCCCAGGCAGACCC
AGCCTACCCCAGGCAGACTC
AGCCTACCCCAGACAGGCCT
AGCCTACCCCAGACAGGCCT
AGCCTACCCCAGACAGGCCT
AGCCTGCCCCAGGCAGGCCT
AGCCTACCCCAGGCAGGCCT
AGCCTACCCCAGGCAGGCAT
AGCCTACCCCAGGCAGGCCT
AGCCTACCCCAGGCAGGCCT
AGCCTACCCCAGGCAGGCCT
AGCCTACCCCAGGCAGGCCT
AGCCTACCCCAGGCAGGCCT
AGCCTACCTCAGGCAGGCCT
AGCCTACCCCAGGCAGGCCT
Nr of selected repeats 40 Similarity 0.907436
Consensus:
AGCCTACCCCAGGCAGaCCc
>Cele-UNSB01_3:2604301-2604569 Satlength=269 Nr of Repeats=6 RepeatLength=45
seed=TCTGATCCTT

TCTGATCCTTCAGCTCCGAAGGATCACATGGGTACCTTCTGATGT
TCTGATCCTTCAGCTCCAAAAGAACATGAGGCTCTGCCTGATGA
TCTGATCCTTCCGATCCGAAGGATCACATGGGTACCTCCTGATGG
TCTGATCCTTCAGCTCCAAAGGATCACGAGGCTCTTCCTGATGT
TCTGATCCTTCAACTCCGAAGGATCACATGGGTGCCTCCTGATGA
TCTGATCCTTCAGATAAGAAGGATCGTAATGGTGCCTCCTGATGG
Nr of selected repeats 4 Similarity 0.802469
Consensus:
TCTGATCCTTCagaTccGAAGGATCacAtgGGTaCCTcCTGATGg
>Cele-UNSB01_3:2604631-2620875 Satlength=16245 Nr of Repeats=650
RepeatLength=25 seed=AGAGAGTAAA
AGAGAGTAAAATATACGGTGAAGCC
AGAGAGTAAAATATATGGTGAAGCC
AGAGAGTAAAATATTCTGTGAAGCC
AGAGAGTAAAATATACGGTGAAGCC
AGAGAGTAAAATATACGGTGAAGCC
AGAGAGTAAAATATATGGTGAAGCC
AGAGAGTAAAATATACGGTGAAGCC
AGAGAGTAAAATATACGGTGAAGCC
AGAGAGTAAAATATTCTGTGAAGCC
AGAGAGTAAAATATACGGTGAAGCC
AGAGAGTAAAATATATGGTGAAGCC
AGAGAGTAAAATATTCTGTGAAGCC
AGAGAGTAAAATATATGGTGAAGCC
AGAGAGTAAAATATATGGTGAAGCC
AGAGAGTAAAATATATGGTGAAGCC
AGAGAGTAAAATATTCTGTGAAGCC
AGAGAGTAAAATATTCTGTGAAGCC
AGAGAGTAAAATATTCTGTGAAGCC
AGAGAGTAAAATATTCTGTGAAGCC
AGAGAGTAAAATATTCTGTGAAGCC
AGAGAGTAAAATATATGGTGAAGCC
AGAGAGTAAAATATATGGTGAAGCC
AGAGAGTAAAATATTCTGTGAAGCC
AGAGAGTAAAATATTCTGTGAAGCC
AGAGAGTAAAATATATGGTGAAGCC
AGAGAGTAAAATATATGGTGAAGCC
AGAGAGTAAAATATTCTGTGAAGCC
AGAGAGTAAAATATTCTGTGAAGCC
AGAGAGTAAAATATTCTGTGAAGCC
AGAGAGTAAAATATTCTGTGAAGCC
AGAGAGTAAAATATTCTGTGAAGCC
AGAGAGTAAAATATATGGTGAAGCC
AGAGAGTAAAATATATGGTGAAGCC
AGAGAGTAAAATATATGGTGAAGCC
AGAGAGTAAAATATTCTGTGAAGCC
AGAGAGTAAAATATTCTGTGAAGCC
AGAGAGTAAAATATATGGTGAAGCC
AGAGAGTAAAATATATGGTGAAGCC
AGAGAGTAAAATATATGGTGAAGCC
AGAGAGTAAAATATATGGTGAAGCC
AGAGAGTAAAATATTCTGTGAAGCC
AGAGAGTAAAATATTCTGTGAAGCC
AGAGAGTAAAAATATTCTGTGAAGCC

AGAGAGTAAAATATTCTGTGAAGCC
AGAGAGTAAAATATTCTGTGAAGCC
AGAGAGTAAAATATATGGTGAAGCC
AGAGAGTAAAATATATGGTGAAGCC
AGAGAGTAAAATATATGGTGAAGCC
AGAGAGTAAAATATTCTGTGAAGCC
AGAGAGTAAAATATTCTGTGAAGCC
AGAGAGTAAAATATATGGTGAAGCC
AGAGAGTAAAATATATGGTGAAGCC
AGAGAGTAAAATATATGGTGAAGCC
AGAGAGTAAAATATATGGTGAAGCC
AGAGAGTAAAATATATGGTGAAGCC
AGAGAGTAAAATATTCTGTGAAGCC
AGAGAGTAAAATATTCTGTGAAGCC
AGAGAGTAAAATATATGGTGAAGCC
AGAGAGTAAAATATATGGTGAAGCC
AGAGAGTAAAATATATGGTGAAGCC
AGAGAGTAAAATATATGGTGAAGCC
AGAGAGTAAAATATATGGTGAAGCC
AGAGAGTAAAATATTCTGTGAAGCC
AGAGAGTAAAATATTCTGTGAAGCC
AGAGAGTAAAATATATGGTGAAGCC
AGAGAGTAAAATATATGGTGAAGCC
AGAGAGTAAAATATATGGTGAAGCC
AGAGAGTAAAATATATGGTGAAGCC
AGAGAGTAAAATATTCTGTGAAGCC
AGAGAGTAAAATATTCTGTGAAGCC
AGAGAGTAAAATATATGGTGAAGCC
AGAGAGTAAAATATATGGTGAAGCC
AGAGAGTAAAATATATGGTGAAGCC
AGAGAGTAAAATATATGGTGAAGCC
AGAGAGTAAAATATATGGTGAAGCC
AGAGAGTAAAATATATGGTGAAGCC
AGAGAGTAAAATATATGGTGAAGCC
AGAGAGTAAAATATATGGTGAAGCC
AGAGAGTAAAATATATGGTGAAGCC
AGAGAGTAAAATATATGGTGAAGCC
AGAGAGTAAAATATTCTGTGAAGCC
AGAGAGTAAAATATTCTGTGAAGCC
AGAGAGTAAAATATTGTGAAGCC
AGAGAGTAAAATATATGGTGAAGCC
AGAGAGTAAAATATATGGTGAAGCC
AGAGAGTAAAATATTCTGTGAAGCC
AGAGAGTAAAATATTCTGTGAAGCC
AGAGAGTAAAATATATGGTGAAGCC
AGAGAGTAAAATATATGGTGAAGCC
AGAGAGTAAAATATTCTGTGAAGCC
AGAGAGTAAAATATTCTGTGAAGCC
AGAGAGTAAAATATATGGTGAAGCC
AGAGAGTAAAATATTCTGTGAAGCC
AGAGAGTAAAATATTCTGTGAAGCC
AGAGAGTAAAATATATGGTGAAGCC
AGAGAGTAAAATATAAAAAAAAAAAATGTGAAGCC
AGAGAGTAAAATATATGGTGAAGCC

AGAGAGTAAAATATTCTGTGAAGCC
AGAGAGTAAAATATTCTGTGAAGCC
AGAGAGTAAAATATATGGTGAAGCC
AGAGAGTAAAATATTCTGTGAAGCC
AGAGAGTAAAATAA
AGAGAGTAAAATATTCTGTGAAGCC
AGAGAGTAAAATATTCTGTGAAGCC
AGAGAGTAAAATATATGGTGAAGCC
AGAGAGTAAAATATATGGTGAAGCC
AGAGAGTAAAATATTCTGTGAAGCC
AGAGAGTAAAATATTCTGTGAAGCC
AGAGAGTAAAATATTCTGTGAAGCC
AGAGAGTAAAAAAAAAAAAAAAAAAAAAAAAAATATGTGAAGCC
AGAGAGTAAAATATCTGGTGAAGCC
AGAGAGTAAAATATATGGTGAAGCC
AGAGAGTAAAATATTCTGTGAAGCC
AGAGAGTAAAATATTCTGTGAAGCC
AGAGAGTAAAATATAGTGTGAAGCC
AGAGAGTAAAATATCTGTGAAGCC
AGAGAGTAAAATATTCTGTGAAGCC
AGAGAGTAAAATATATGGTGAAGCC
AGAGAGTAAAATATATGGTGAAGCC
AGAGAGTAAAATATTCTGTGAAGCC
AGAGAGTAAAATATGGTGAAGCC
AGAGAGTAAAATATTCTGTGAAGCC
AGAGAGTAAAATATTCTGTGAAGCC
AGAGAGTAAAATATATGGTGAAGCC
AGAGAGTAAAATATATGGTGAAGCC
AGAGAGTAAAATATCTGTGAAGCC
AGAGAGTAAAATATGGTGAAGCC
AGAGAGTAAAATATATGTGAAGCC
AGAGAGTAAAATATAGTGGGAAGCC
AGAGAGTAAAATATATGGGAAGCC
AGAGAGTAAAATATTGTGAAGCC
AGAGAGTAAAATATTTGTGAAGCC
AGAGAGTAAAATATTCTGTGAAGCC
AGAGAGTAAAATATATGGTGAAGCC
AGAGAGTAAAATATATGGTGAAGCC
AGAGAGTAAAATATATGGTGAAGCC
AGAGAGTAAAATATATGGTGAAGCC
AGAGAGTAAAATATATGGTGAAGCC
AGAGAGTAAAATATTCTGTGAAGCC
AGAGAGTAAAATATTGTGAAGCC
AGAGAGTAAAATATATGGTGAAGCC
AGAGAGTAAAATATTTGTGAAGCC
AGAGAGTAAAATATTGTGAAGCC
AGAGAGTAAAATATTTGTGAAGCC
AGAGAGTAAATATTGTGAAGCC
AGAGAGTAAAATATTGTGAAGCC
AGAGAGTAAAATATTTGAAGCC
AGAGAGTAAAATATTGTGAAGCC
AGAGAGTAAAATATATGGTGAAGCC
AGAGAGTAAAATATATGTGTGAAGCC
AGAGAGTAAAATATTCTGTGAAGCC

AGAGAGTAAAATATTCTGTGAAGCC
AGAGAGTAAAATATTCTGTGAAGCC
AGAGAGTAAAATATTGTGAAGCC
AGAGAGTAAAATATTGTGAAGCC
AGAGAGTAAAATATTTGTGAAGCC
AGAGAGTAAAATATATTGTGAAGCC
AGAGAGTAAAATATAGTGAAGCC
AGAGAGTAAAATATATTGTGAAGCC
AGAGAGTAAAATATATGTGAAGCC
AGAGAGTAAAATATATGTGAAGCC
AGAGAGTAAAATATAGTGTGAAGCC
AGAGAGTAAAATATTCTGTGAAGCC
AGAGAGTAAAATATTCTGTGAAGCC
AGAGAGTAAAATATTCTGTGAAGCC
AGAGAGTAAAATATAGTGTGAAGCC
AGAGAGTAAAATATTGTGAAGCC
AGAGAGTAAAATATTGGTGAAGCC
AGAGAGTAAAATATTGTGAAGCC
AGAGAGTAAAATATTGTGAAGCC
AGAGAGTAAAATATATGGTGAAGCC
AGAGAGTAAAATATATGTGAAGCC
AGAGAGTAAAATATGGTGAAGCC
AGAGAGTAAAAAATATGGTGAAGCC
AGAGAGTAAAATATTCTGTGAAGCC
AGAGAGTAAAATATTGTGAAGCC
AGAGAGTAAAATATTCTGTGAAGCC
AGAGAGTAAAATATTTGGTGAAGCC
AGAGAGTAAAATATATGGTGAAGCCAGAAAGAAGAAGAAAATATGGTGAAGCC
AGAGAGTAAAAAATATGGTGAAGCC
AGAGAGTAAAATATTCTGTGAAGCC
AGAGAGTAAAATATTGTGAAGCC
AGAGAGTAAAATATTGTGAAGCC
AGAGAGTAAAATATTGTGAAGCC
AGAGAGTAAAATATGTGAAGCC
AGAGAGTAAAATATTGTGAAGCC
AGAGAGTAAAATATATGGTGAAGCC
AGAGAGTAAAATATATGTGAAGCC
AGAGAGTAAAATATTGTGAAGCC
AGAGAGTAAATATATGGTGAAGCC
AGAGAGTAAAATATATGGTGAAGCC
AGAGAGTAAAATATTTGTGAAGCC
AGAGAGTAAAATATTGTGAAGCC
AGAGAGTAAAATATTGTGAAGCC
AGAGAGTAAAATATTGTGAAGCC
AGAGAGTAAAATATATGGTGAAGCC
AGAGAGTAAAATATGGTGAGCC
AGAGAGTAAATATATGGTGAAGCC
AGAGAGTAAAATATTATGGTGAAGCC
AGAGAGTAAAATATTTGTGAAGCC
AGAGAGTAAAATATATGGTGAAGCC
AGAGAGTAAAATATATGGTGAAGCC
AGAGAGTAAATATATGGTGAAGCC
AGAGAGTAAAATATATGGTGAAGCC
AGAGAGTAAAATATATATGGTGAAGCC

AGAGAGTAAAATATATGGTGAAGCC
AGAGAGTAAAATATTCTGTGAAGCC
AGAGAGTAAAATATATGGTGAAGCC
AGAGAGTAAAATATTCTGTGAAGCC
AGAGAGTAAAATATTCTGTGAAGCC
AGAGAGTAAAATATTCTGTGAAGCC
AGAGAGTAAAATATATGGTGAAGCC
AGAGAGTAAAATATATGGTGAAGCC
AGAGAGTAAAATATATGGTGAAGCC
AGAGAGTAAAATATATGGTGAAGCC
AGAGAGTAAAATATATGGTGAAGCC
AGAGAGTAAAATATATGGTGAAGCC
AGAGAGTAAAATATATGTGAAGCC
AGAGAGTAAAATATATGGTGAAGCC
AGAGAGTAAAAATATGGTGAAGCC
AGAGAGTAAAATATTGTGAAGCC
AGAGAGTAAATATGTGAAGCC
AGAGATAAAATATCTGTGAAGCC
AGAGAGTAAAATATACTGTGAAGCC
AGAGAGTAAAATATATGGTGAAGCC
AGAGAGTAAAATATATGTGAAGCC
AGAGAGTAAAATATATGGTGAAGCC
AGAGAGTAAAATATATGGTGAAGCC
AGAGAGTAAATATATGGTGAAGCC
AGAGAGTAAAATATAAC
AGAGAGTAAAATATATGGTGAAGCC
AGAGAGTAAAATATATGGTGAAGCC
AGAGAGTAAAATATATGGTGAAGCC
AGAGAGTAAAAAAACATGGTGAAGCC
AGAGAGTAAAATATATGGTGAAGCCAG
AGGAGTAAAATATATGGTGAACC
AGAGAGTAAAATATATGGTGAAGCCGAGAGTATATATGGTGAAGCC
AGAGAGTAAAATATATGGTGAAGCC
AGAGAGTAAAATATATGGTGAAGCC
AGAGAGTAAAATATATGGTGAAGCC
AGAGAGTAAAATATATGGTGAAGCC
AGAGAGTAAAATATTCTGTGAAGCC
AGAGAGTAAAATATATGGTGAAGCC
AGAGAGTAAAATATACTGGGAAGCC
AGAGAGTAAAATATACTGTGAAGCC
AGAGAGTAAAATATATGGTGAAGCC
AGAGAGTAAAATATATGGTGAAGCC
AGAGAGTAAAATAATATGGTGAAGCC
AGAGAGTAAAATATATGGTGAAGCC
AGAGAGTAAAATATATGGTGAAGCC
AGAGAGTAAAATATATGGTGAAGCCAGAG
AGAAGTAAAATATATATGGTGAGCC
AGAGAGTAAAATATATGGTGAAGCC
AGAGAGTAAAATATTCTGTGAAGCC
AGAGAGTAAAATATTCGGTGAAGCC
AGAGAGTAAAATATATGGTGAAGCC
AGAGAGTAAAATATTCTGTGAAGCC
AGAGAGTAAAATATTCTGTGAAGCC
AGAGAGTAAAATATTCTGTGAAGCC

AGAGAGTAAAATATATGGTGAAGCC
AGAGAGTAAAATATATGGTGAAGCC
AGAGAGTAAAATATACTGGGAAGCC
AGAGAGTAAAATATATGGTGAAGCC
AGAGAGTAAAATATGGTGAAGC
AGAGAGTAAATATATGGTGAAGCC
AGAGAGTAAAATATATGGTGAAGCC
AGAGAGTAAAATATTGGTGAAGCC
AGAGAGTAAAATATTCTGTGAAGCC
AGAGAGTAAAATATACTGTGAAGCC
AGAGAGTAAAATATACTGGGAAGCC
AGAGAGTAAAATATATGGTGAAGCC
AGAGAGTAAAATATATGGTGAAGCC
AGAGAGTAAAATATATGGTGAAGCC
AGAGAGTAAAATATATGGTGAAGCC
AGAGAGTAAAATATATGGTGAAGCC
AGAGAGTAAAATATATGGTGAAGCC
AGAGAGTAAAATATATGGTGAAGCC
AGAGAGTAAAATATATGGTGAAGCC
AGAGAGTAAAATATATGGTGAAGCC
AGAGAGTAAATATATGGTGAAGCC
AGAGAGTAAAATATATGGTGAAGCC
AGAGAGTAAAATATATGGTGAAGCC
AGAGAGTAAAATATATGGTGAAGCC
AGAGAGTAAAATATATGGTGAAGCC
AGAGAGTAAAATATATGGTGAAGCC
AGAGAGTAAAATATATGGTGAAGCC
AGAGAGTAAAATATATGGTGAAGCC
AGAGAGTAAAATATATGGTGAAGCC
AGAGAGTAAAATATATGGTGAAGCC
AGAGAGTAAAATATATGGTGAAGCC
AGAGAGTAAAATATATGGTGAAGCC
AGAGAGTAAAATATATGGTTGAAGCC
AGAGAGTAAAATATATGGTGAAGCC
AGAGAGTAAAATATATGGTGAAGCC
AGAGAGTAAAATATATGGTGAAGCC
AGAGAGTAAAATATATGGTGAAGCC
AGAGAGTAAAATATATGGTGAAGCC
AGAGAGTAAAATATATGGTGAAGCC
AGAGAGTAAAATATATGGTGAAGCC
AGAGAGTAAAATATAAAAAAAAAAATAGTGAAGCC
AGAGAGTAAAATATATGGTGAAGCC
AGAGAGTAAAATATATGTGAAGCC
AGAGAGTAAATATATGGTGAAGCC
AGAGAGTAAAATATATGGTGAAGCC
AGAGAGTAAAATATACGGTGAAGCC
AGAGAGTAAAATATATGGTGAAGCC
AGAGAGTAAAATATATGGTGAAGCC
AGAGAGTAAAATATATGGTGAAGCC
AGAGAGTAAAATATATGGTGAAGCC
AGAGAGTAAAAAAAACATGGTGAAGCC
AGAGAGTAAAATATATGGTGAAGCC
AGAGAGTAAAATATATGGTGAAGCC
AGAGAGTAAAATATATGGTGAAGCC

AGAGAGTAAAATATATGGTGAAGCC
AGAGAGTAAAATATATGGTGAAGCC
AGAGAGTAAAATATATGGTGAAGCC
AGAGAGTAAAATATATGGTGAAGCC
AGAGAGTAAAATATATGGTGAAGCC
AGAGAGTAAAATATATGGTGAAGCC
AGAGAGTAAAATATATGGTGAAGCC
AGAGAGTAAAATATACGGTGAAGCC
AGAGAGTAAAATATATGGTGAAGCC
AGAGAGTAAAATATATGTGAAGCC
AGAGAGTAAAATATATGGTGAAGCC
AGAGAGTAAAATATATGGTGAAGCC
AGAGAGTAAAATATACTGGGAAGCC
AGAGAGTAAAATATATGGTGAAGCC
AGAGAGTAAAATATACTGGTGAAGCC
AGAGAGTAAAATATATGGTGAAGCC
AGAGAGTAAAATATATGGTGAAGCC
AGAGAGTAAAAAAATATGGTGAAGCC
AGAGAGTAAAATATATGGTGAAGCC
AGAGAGTAAAATATATGGTGAAGCC
AGAGAGTAAAATATTCGGTGAAGCC
AGAGAGTAAAATATATGGTGAAGCC
AGAGAGTAAAATATATGGTGAAGCC
AGAGAGTAAAATATATGGTGAAGCC
AGAGAGTAAAATATATGGTGAAGCC
AGAGAGTAAAATATATGGTGAAGCC
AGAGAGTAAAATATTCTGTGAAGCC
AGAGAGTAAAATATTCTGTGAAGCC
AGAGAGTAAAATATTCTGTGAAGCC
AGAGAGTAAAATATTCTGTGAAGCC
AGAGAGTAAAATATTCTGGTGAAGCC
AGAGAGTAAAATATACGGTGAAGCC
AGAGAGTAAAATATTCTGTGAAGCC
AGAGAGTAAAATATATGGTGAAGCC
AGAGAGTAAAATATATGGTGAAGCC
AGAGAGTAAAATATATGGTGAAGCC
AGAGAGTAAAATATATGGTGAAGCC
AGAGAGTAAAATATATGGTGAAGCC
AGAGAGTAAAATATTGAAC
AGAGATAAAATATTGTGAAGCC
AGAGAGTAAAATATATGGTGAAGCC
AGAGAGTAAAATATATGGTGAAGCC
AGAGAGTAAAATATATGGTGAAGCC
AGAGAGTAAAATATTCTGTGAAGCC
AGAGAGTAAAATATTGTGAAGCC
AGAGAGTAAAATATATGGTGAAGCC
AGAGAGTAAAATATATGGTGAAGCC
AGAGAGTAAAATATATGGTGAAGCC
AGAGAGTAAAATATATGGTGAAGCC
AGAGAGTAAAATATATGGTGAAGCC
AGAGAGTAAAATATATGGTGAAGCC
AGAGAGTAAAATATATGGTGAAGCC
AGAGAGTAAAATATATGGTGAAGCC
AGAGAGTAAAATATATGGTGAAGCC

AGAGAGTAAAATATATGGTGAAGCC
AGAGAGTAAAATATATGGTGAAGCC
AGAGAGTAAAATATATGGTGAAGCC
AGAGAGTAAAATATATGGTGAAGCC
AGAGAGTAAAATATATGGTGAAGCC
AGAGAGTAAAATATTCTGTGAAGCC
AGAGAGTAAAATATTCTGTGAAGCC
AGAGAGTAAAATATTCGTGTGAGAGAAAATATTCTGTGAAGCC
AGAGAGTAAAATATTCTGTGAAGCC
AGAGAGTAAAATATATGGTGAAGCC
AGAGAGTAAAATATTCTGTGAAGCC
AGAGAGTAAAATATATGGTGAAGCC
AGAGAGTAAAATATATGGTGAAGCC
AGAGAGTAAAATATATGGTGAAGCCAG
AGAGGTAAAATATATGGTGAAGCC
AGAGAGTAAAATATATGGTGAAGCC
AGAGAGTAAAATATATGGTGAAGCC
AGAGAGTAAAATATATGGTGAAGCC
AGAGAGTAAAATATATGGTGAAGCC
AGAGAGTAAAATATTCTGTGAAGCC
AGAGAGTAAAATATTCTGTGAAGCC
AGAGAGTAAAATATTCTGTGAAGCC
AGAGAGTAAAATATATGGTGAAGCC
AGAGAGTAAAATATATGGTGAAGCC
AGAGAGTAAAATATATGGTGAAGCC
AGAGAGTAAAATATATGGTGAAGCC
AGAGAGTAAAATATATGGTGAAGCC
AGAGAGTAAAATATATGGTGAAGCC
AGAGAGTAAAATATATGGTGAAGCC
AGAGAGTAAAATATTCTGTGAAGCC
AGAGAGTAAAATATTCTGTGAAGCC
AGAGAGTAAAATATTCTGTGAAGCC
AGAGAGTAAAATATTCTGTGAAGCC
AGAGAGTAAAATATTCTGTGAAGCC
AGAGAGTAAAATATATGGTGAAGCC
AGAGAGTAAAATATACGGTGAAGCC
AGAGAGTAAAATATATGGTGAAGCC
AGAGAGTAAAATATATGGTGAAGCC
AGAGAGTAAAATATATGGTGAAGCC
AGAGAGTAAAATATATGGTGAAGCC
AGAGAGTAAAATATATGGTGAAGCC
AGAGAGTAAAATATATGGTGAAGCC
AGAGAGTAAAATATATGGTGAAGCC
AGAGAGTAAAATATCTGTGAAGCC
AGAGAGTAAAATATTCTGTGAAGCC
AGAGAGTAAAATATATGGTGAAGCC
AGAGAGTAAAATATATGGTGAAGCC
AGAGAGTAAAATATTCTGTGAAGCC
AGAGAGTAAAATATATGGTGAAGCC
AGAGAGTAAAATATATGGTGAAGCC
AGAGAGTAAAAAAATATGGTGAAGCC
AGAGAGTAAAATATATGGTGAAGCC
AGAGAGTAAAATATATGGTGAAGCC
AGAGAGTAAAATATATGGTGAAGCC

AGAGAGTAAAATATTCTGTGAAGCC
AGAGAGTAAAATATTCTGTGAAGCC
AGAGAGTAAAATATTCTGTGAAGCC
AGAGAGTAAAATATAGTGTGAAGCC
AGAGAGTAAAATATTCTGTGAAGCC
AGAGAGTAAAATATATGGTGAAGCC
AGAGAGTAAAATATATCGGTGAAGCC
AGAGAGTAAAATATTCTGTGAAGCC
AGAGAGTAAAATATATGGTGAAGCC
AGAGAGTAAAATATATGGTGAAGCC
AGAGAGTAAAATATATGGTGAAGCC
AGAGAGTAAAATATATGGTGAAGCC
AGAGAGTAAAATATATGGTGAAGCC
AGAGAGTAAAATATTCTGTGAAGCC
AGAGAGTAAAATATATGGTGAAGCC
AGAGAGTAAAATATATGGTGAAGCC
AGAGAGTAAAATATATGGTGAAGCC
AGAGAGTAAAATATATGGTGAAGCC
AGAGAGTAAAATATATGGTGAAGCC
AGAGAGTAAAATATATGGTGAAGCC
AGAGAGTAAAATATATGGTGAAGCC
AGAGAGTAAAATATTCTGTGAAGCC
AGAGAGTAAAATATTCTGTGAAGCC
AGAGAGTAAAATATTCTGTGAAGCC
AGAGAGTAAAATATATGGTGAAGCC
AGAGAGTAAAATATATGGTGAAGCC
AGAGAGTAAAATATATGGTGAAGCC
AGAGAGTAAAATATATGGTGAAGCC
AGAGAGTAAAATATATGGTGAAGCC
AGAGAGTAAAATATATGGTGAAGCC
AGAGAGTAAAATATATGGTGAAGCC
AGAGAGTAAAATATATGGTGAAGCC
AGAGAGTAAAATATTCTGTGAAGCC
AGAGAGTAAAATATTCTGTGAAGCC
AGAGAGTAAAATATTCTGTGAAGCC
AGAGAGTAAAATATTCTGTGAAGCC
AGAGAGTAAAATATTCTGTGAAGCC
AGAGAGTAAAATATATGGTGAAGCC
AGAGAGTAAAATATATGGTGAAGCC
AGAGAGTAAAATATATGGTGAAGCC
AGAGAGTAAAATATCTGTGAAGCC
AGAGAGTAAAATATTCTGTGAAGCC
AGAGAGTAAAATATTCTGTGAAGCC
AGAGAGTAAAATATTCTGTGAAGCC
AGAGAGTAAAATATAGTGTGAAGCC
AGAGAGTAAAATATATGGTGAAGCC
AGAGAGTAAAATATTCTGTGAAGCC
AGAGAGTAAAATATATGGTGAAGCC
AGAGAGTAAAATATATGGTGAAGCC
AGAGAGTAAAATATATGGTGAAGCC
AGAGAGTAAAATATATGGTGAAGCC
AGAGAGTAAAATATTCTGTGAAGCC
AGAGAGTAAAATATTCTGTGAAGCC
AGAGAGTAAAATATTCTGTGAAGCC

AGAGAGTAAAATATTCTGTGAAGCC
AGAGAGTAAAATATATGGTGAAGCC
AGAGAGTAAAATATATGGTGAAGCC
AGAGAGTAAAATATATGGTGAAGCC
AGAGAGTAAAATATATGGTGAAGCC
AGAGAGTAAAATATTCTGTGAAGCC
AGAGAGTAAAATATTCTGTGAAGCC
AGAGAGTAAAATATTGTGAAGCC
AGAGAGTAAAATATTCTGTGAAGCC
AGAGAGTAAAATATTCTGTGAAGCC
AGAGAGTAAAATATATGGTGAAGCC
AGAGAGTAAAATATATGGTGAAGCC
AGAGAGTAAAATATATGGTGAAGCC
AGAGAGTAAAATATATGGTGAAGCC
AGAGAGTAAAATATATGGTGAAGCC
AGAGAGTAAAATATATGGTGAAGCC
AGAGAGTAAAATATTCTGTGAAGCC
AGAGAGTAAAATATTCTGTGAAGCC
AGAGAGTAAAATATATGGTGAAGCC
AGAGAGTAAAATATATGGTGAAGCC
AGAGAGTAAAATATATGGTGAAGCC
AGAGAGTAAAATATATGGTGAAGCC
AGAGAGTAAAATATACGGTGAAGCC
AGAGAGTAAAATATATGGTGAAGCC
AGAGAGTAAAATATATGGTGAAGCC
AGAGAGTAAAATATATGGTGAAGCC
AGAGAGTAAAATATATGGTGAAGCC
AGAGAGTAAAATATATGGTGAAGCC
AGAGAGTAAAATATATGTGAAGCC
AGAGAGTAAAATATATGGTGAAGCC
AGAGAGTAAAATATATGGTGAAGCC
AGAGAGTAAAATATATGGTGAAGCC
AGAGAGTAAAATATATGGTGAAGCC
AGAGAGTAAAATATATGGTGAAGCC
AGAGAGTAAAATATTCTGTGAAGCC
AGAGAGTAAAATATATGTGAAGCC
AGAGAGTAAAATATGTGAAGCC
AGAGAGTAAAATATATGGTGAAGCC
AGAGAGTAAAATATATGGTGAAGCC
AGAGAGTAAAATATATGGTGAAGCC
AGAGAGTAAAATATATGGTGAAGCC
AGAGAGTAAAATATATGGTGAAGCC
AGAGAGTAAAATATATGGTGAAGCC
AGAGAGTAAAAAAAA
AGAGAGTAAAATATATGGTGAAGCC
AGAGAGTAAAATATATGGTGAAGCC
AGAGAGTAAAATATATGGTGAAGCC
AGAGAGTAAAATATATGTGAAGCC
AGAGAGTAAAATATTCTGTGAAGCC
AGAGAGTAAAATATTCTGTGAAGCC
AGAGAGTAAAATATATGGTGAAGCC
AGAGAGTAAAATATACTGTGAAGCC
AGAGAGTAAAATATATGGTGAAGCC
AGAGAGTAAAATATACGGTGAAGCC

AGAGAGTAAAATATATGGTGAAGCC
AGAGAGTAAAATATATGGTGAAGCC
AGAGAGTAAAATATATGGTGAAGCC
AGAGAGTAAAATATATGGTGAAGCC
AGAGAGTAAAATATATGGTGAAGCC
AGAGAGTAAAATATATGGTGAAGCC
AGAGAGTAAAATATATGGTGAAGCC
AGAGAGTAAAATATATGGTGAAGCC
AGAGAGTAAAATATATGGTGAAGCC
AGAGAGTAAAATATATGGTGAAGCC
AGAGAGTAAAATATATGGTGAAGCC
AGAGAGTAAAATATATGGTGAAGCC
AGAGAGTAAAATATATGGTGAAGCC
AGAGAGTAAAATATATGGTGAAGCC
AGAGAGTAAAATATATGGTGAAGCC
AGAGAGTAAAATATATGGTGAAGCC
AGAGAGTAAAATATATGGTGAAGCC
AGAGAGTAAAATATATGGTGAAGCC
AGAGAGTAAAATATATGGTGAAGCC
AGAGAGTAAAATATATGGTGAAGCC
AGAGAGTAAAATATATGGTGAAGCC
AGAGAGTAAAATATATGGTGAAGCC
AGAGAGTAAAATAAAAAAAAAAATATGGTGAAGCC
AGAGAGTAAAATATATGGTGAAGCC
AGAGAGTAAAATATATGGTGAAGCC
AGAGAGTAAAATATATGGTGAAGCC
AGAGAGTAAAATATATGGTGAAGCC
AGAGAGTAAAATATATGGTGAAGCC
AGAGAGTAAAATATATGGTGAAGCC
AGAGAGTAAAATATATGGTGAAGCC
AGAGAGTAAAATATATGGTGAAGCC
AGAGAGTAAAATATATGGTGAAGCC
AGAGAGTAAAATATATGGTGAAGCC
AGAGAGTAAAATATATGGTGAAGCC
AGAGAGTAAAATATATGGTGAAGCC
AGAGAGTAAAATATATGGTGAAGCC
AGAGAGTAAAATATATGGTGAAGCC
AGAGAGTAAAATATATGGTGAAGCC
AGAGAGTAAAATATATGGTGAAGCC
AGAGAGTAAAATATATGGTGAAGCC
AGAGAGTAAAATATATGGTGAAGCC
AGAGAGTAAAATATATGGTGAAGCC
AGAGAGTAAAATATATGGTGAAGCC
AGAGAGTAAAATATATGGTGAAGCC
AGAGAGTAAAATATATGGTGAAGCC
AGAGAGTAAAATAAAAAAAAAAAAAAGC
AGAGAGTAAAATATATGGTGAAGCC
AGAGAGTAAAATATATGGTGAAGCC
AGAGAGTAAAATATATGGTGAAGCC
AGAGAGTAAAATATATGGTGAAGCC
AGAGAGTAAAATATATGGTGAAGCC
AGAGAGTAAAATATATGGTGAAGCC
AGAGAGTAAAATATATGGTGAAGCC
AGAGAGTAAAATATATGGTGAAGCC
AGAGAGTAAAATATATGGTGAAGCC
AGAGAGTAAAATATATGGTGAAGCC

AGAGAGTAAAATATATGGTGAAGCC
AGAGAGTAAAATATATGTGAAGCC
AGAGAGTAAAATATATGGTGAAGCC
AGAGAGTAAAATATATGGTGAAGCC
AGAGAGTAAAATATATGGTGAAGCC
AGAGAGTAAAATATATGGTGAAGCC
AGAGAGTAAAATATATGGTGAAGCC
AGAGAGTAAAATATATGGTGAAGCC
AGAGAGTAAAATATATGGTGAAGCC
AGAGAGTAAAATATATGGTGAAGCC
AGAGAGTAAAATATATGGTGAAGCC
AGAGAGTAAAATATATGGTGAAGCC
AGAGAGTAAAATATTCTGGTGAAGCC
AGAGAGTAAAATATATGGTGAAGCC
AGAGAGTAAAATATATGGTGAAGCC
AGAGAGTAAAATATATGGTGAAGCC
AGAGAGTAAAATATATGGTGAAGCC
AGAGAGTAAAATATATGGTGAAGCC
AGAGAGTAAAATATTCTGTGAAGCC
AGAGAGTAAAATATATGGTGAAGCC
AGAGAGTAAAATATTCTGTGAAGCC
AGAGAGTAAAATATATGGTGAAGCC
AGAGAGTAAAATATATGGTGAAGCC
AGAGAGTAAAATATATGGTGAAGCC
AGAGAGTAAAATATAGTGTGAAGCC
AGAGAGTAAAATATATGGTGAAGCC
AGAGAGTAAAATATATGGTGAAGCC
AGAGAGTAAAATATATGGTGAAGCC
AGAGAGTAAAATATTCTGTGAAGCC
AGAGAGTAAAATATATGGTGAAGCC
AGAGAGTAAAATATATGGTGAAGCC
AGAGAGTAAAATATATGGTGAAGCC
AGAGAGTAAAATATTCTGTGAAGCC
AGAGAGTAAAATATATGGTGAAGCC
AGAGAGTAAAATATATGGTGAAGCC
AGAGAGTAAAATATATGGTGAAGCC
AGAGAGTAAAATATATGGTGAAGCC
AGAGAGTAAAATATATGTGAAGCC
AGAGAGTAAAATATATGGTGAAGCC
AGAGAGTAAAATATATGGTGAAGCC
AGAGAGTAAAATATTATGTGAAGCC
AGAGAGTAAAAAAAAAATAAAATATTGTGAAGCC
AGAGAGTAAAATATTCTGTGAAGCC
AGAGAGTAAAATATTCTGTGAAGCC
AGAGAGTAAAATATTCTGTGAAGCC
AGAGAGTAAAATATTCTGTGAAGCC
AGAGAGTAAAATATTCTGTGAAGCC
AGAGAGTAAAATATTCTGTGAAGCC
AGAGAGTAAAATATTCTGTGAAGCC
AGAGAGTAAAATATTCTGTGAAGCC
AGAGAGTAAAATATACTGGTGAAGCC
AGAGAGTAAAATATACGGTGAAGCC
AGAGAGTAAAATATATGGTGAAGCC
AGAGAGTAAAATATATGGTGAAGCC

AGAGAGTAAAATATATGGTGAAGCC
AGAGAGTAAAATATATGGTGAAGCC
AGAGAGTAAAATATATGGTGAAGCC
AGAGAGTAAAATATATGGTGAAGCC
AGAGAGTAAAATATATGGTGAAGCC
AGAGAGTAAAATATATGGTGAAGCC
AGAGAGTAAAATATATGGTGAAGCC
AGAGAGTAAAATATATGGTGAAGCC
AGAGAGTAAAATATATGGTGAAGCC
AGAGAGTAAAATATTCTGTGAAGCC
AGAGAGTAAAATATTCTGTGAAGCC
AGAGAGTAAAATATATGGTGAAGCC
AGAGAGTAAAATATTCTGTGAAGCC
Nr of selected repeats 546 Similarity 0.862999
Consensus:
AGAGAGTAAAATATAtgGGTGAAGCC
>Cele-UNSB01_3:2620888-2621068 Satlength=181 Nr of Repeats=5 RepeatLength=40
seed=TTCCAGAATT
TTCCAGAATTTTCCCGATTTTTCTAGAAAGTTCTGGAACG
TTCCAGAATTTTCTCGATTTTTCTAGAAAGTTCTGGAACA
TTCCAGAATTTTCCCGATTTTTCTAGAAAGTTCTGGAACA
TTCCAGAATTTTCCCGATTTTTCTAGAAAGTTCTGGAACA
TTCGAGAATTTTCTCGAAAT
Nr of selected repeats 4 Similarity 0.966667
Consensus:
TTCCAGAATTTTCcCGATTTTTCTAGAAAGTTCTGGAACa
>Cele-UNSB01_3:2621291-2621771 Satlength=481 Nr of Repeats=29 RepeatLength=15
seed=GGTCTCACCA
GGTCTCACCACGACG
GGTCTCACCACGATG
GGTCTCACCACGGTG
GGTCTCACCACGATGGGCCTCGCCACGATG
GGTCTCACCACGGTG
GGTCTCACCACGATG
GGTCTCACCACGATG
GGTCTCACCACGATG
GGTCTCGCCACGATG
GGTTTCACCACGAAG
GGTCTCACCACGGTG
GGTCTCACCACGATG
GGTCTCACCACGATG
GGTCTCACCACGATG
GGTCTCGCCACGATG
GGTCTCACCACGATGGGTCTTTCCACGATA
GGTCTCACCACGATG
GGTCTCACCACGGTG
GGTCTCACCACGATG
GGTCTCACCACGATG
GGTCTCACCACGATG
GGTCTCGCCACGATG
GGTCTCACCACGAAG
GGTCTCACCACGGTG
GGTCTCACCACGATG
GGTCTCACCACGATG

GGTCTCACCACGATG
GGTCTCGCCACGATG
GGTCTCACCACGATGGGTCTTTCCACGATA
Nr of selected repeats 26 Similarity 0.920957
Consensus:
GGTCTCACCACGATG
>Cele-UNSB01_3:2665588-2665980 Satlength=393 Nr of Repeats=11 RepeatLength=35
seed=TTTGCCGGAA
TTTGCCGGAAATTTTCAATTCCGGCAATTTGCCGA
TTTGCTGGAAATGTTCAATTGCGGCAAATTGCCGA
TTTGCCGGAAAGTTTCAATTCCGGCAATTTGCTGAA
TTGCCGGAAATTTTTAATCCCGGCAATTTGCCGA
TTTGCCGGAAATTTTCATTTCTGGCAATTTGCCGA
TTTGCCGGAAGTTTTCAGTTCCGGGAATTTTCCGATTTGCCGA
TTTGCCGGAAATTTCCAATTCCGGCGAATTGCCGA
TTTGCCGAAAATTTCAATTCCGAAAATTTGCCGA
TTTGCCGGAAATGTTCAATTCCGACAATTTTCCGA
TTTACCGGAAATTTTCAATTCTGGCAATTTGCCGG
TTTGCCGGAAAAGTTTAATTCTGGCAATTTGCTGA
Nr of selected repeats 7 Similarity 0.807710
Consensus:
TTTGCCGGAAATtTTCAATTCcGGCAAtTTGCCGA
>Cele-UNSB01_3:2911307-2911655 Satlength=349 Nr of Repeats=10 RepeatLength=35
seed=TTTGCCGGAA
TTTGCCGGAAAATTCAAATTCCGGCAATTTGCCGC
TTTGCCGGAAATTTTTAATTCTGGCAATTTGCCGA
TTTGCCGGAAATTTTTAATTCTGGCAATTTGCCAA
TTTGCCGGAAATTTTCAATTCCGGTAATTTGCCAA
TTTGCCGGAAATTTTCAATTCCGGTAATTTGCCGA
TTTGCCGGAAATTTTTAATTCCGGCAATTTGCCGC
TATGCCGGAAATTTCAATTCCGGTAATTTGCCGA
TTTGCCGGAAATTTTTAAATCTGGCAATTTGCCGA
TTTGCCGGAAATTTCAATTCCGGCAACTTGCCGT
TTTGCCGGAAATGTTTAATTACGGCAATTTGCCGA
Nr of selected repeats 8 Similarity 0.859864
Consensus:
TTTGCCGGAAATTTTtAATTCcGGcAATTTGCCga
>Cele-UNSB01_3:2955858-2957390 Satlength=1533 Nr of Repeats=44 RepeatLength=19
seed=AAAATCGATA
AAAATCGATATTTTGAATAAATTTTCTGA
AAAATCAATACTAAATTCGAATTTCGATTTTCAAATAAAATTCGATTTTGTTTCGCACAAAAGTTAATAATTTAGTCAT
AG
AAAATCGATAATTTTCATGAAATTTTTGAATA
AAAATCAATATTTTATCGATTTTTTTCCAGAAG
AAAATCGATAAACTGCAAAAAAAATATTTACGATTTCGACGACATTTTAAAAATAGAAAAAATTTCAGCATCCAATACC
CCAATTAGATTTATATTCCAGATGACGTCATCACCAGAAATTTATCACGAAAAGCAACGTCTTCAATACTGCCTTATCC
ATACGGTTAACAATATTCTACAAAAATCCGAATTTGATCCGACAAAAATGGACGAAATTTGCTATGCGTTTAATGAGAG
TAAATGGTTTAATCCACATAGGTACGCCGA
AAAATTGATAATTTCCGGA
AAAATCGATAATTTCCGGA
AAAATCGATTATTTCTGGA
AAAATCGATAATTTCCGGA
AAAATCGATAATTTCCGGA
AAAATCGATAATTTCCGGA

AAAATCGATAGTTTCCTGAAAACTATCGATATTTCCGGA
AAAATCGATAATTTCTGGA
AAAATCGATAATTTCTGGA
AAAATCGATAATTTCAGAA
AAAATTGATAATTTCCGGA
AAAATCGATAATTTCCGGA
AAAATCGATAATTTCTGGA
AAAATCGATAATTTCAGA
AAAATTGATAATTTCCGGA
AAAATCGATAATTTCTGGA
AAAATCGATAATTTCAGAAAAAAACTGATAATTTCCGGA
AAAATCGATAATTTCCGGA
AAACTCGATAATTTCCGGA
AAAATCGATAATTTCTGGA
AAAATCGATAATTTCAGA
AAAATTGATAATTTCCTGA
AAAATCGATAATTTCCGGA
AAAATCGATAATTTCTGGA
AAAATCAATAATTTCTGGA
AAAATTGATAATTTCCGGA
AAAATCGATAATTTCCGGA
AAAATCGATAATTTCTGGA
AAAATCGATAATTTCTGGA
AAAATCAATAATTTCTGGA
AAAATTGATAATTTCCGGA
AAAATCGATAATTTCCGGA
AAAATCGATAATTTCTGGA
AAAATCGATAATTTCTGGA
AAAACCGATAATTTCTTGAAA
AAAATCGATAATTTCCGGA
AAAATCGATAATTTCTTGAAAAAAATGGAAAAATTATTTATTTTTTCAAAAATCAAATTTCAACAATTTTTTTCAGATC
CTGGATAGGAACCGGAAATTACGATGCGAATATCCTGATGGCGGCGCTTCAAAAACACGATTTAAAGGTGAACAACAAC
GATGCTCCGATCTTCCGCATCTTCGA
AAAATCAATAATTATCGATTTTTTCCAGGTAATGTGGTTCGACAAGCGTGTTTCAGTCGAA
AAAATCAATATGGAAAATGTGAAAGCAGTGGTTTTTAATATTCCATCACGGACCCTCCTCACATTGTACAGGGGACGAC
ATTGGTTTGCAGTTATTCAGAAAAATGGAATGTGAGTTGCCGA
Nr of selected repeats 31 Similarity 0.912620
Consensus:
AAAATCGATAATTTCcGGA
>Cele-UNSB01_3:3099001-3099720 Satlength=720 Nr of Repeats=4 RepeatLength=180
seed=TGTCGGCCGC
TGTCGGCCGCTATATCTTTTGACTTAAACGTTCAAAAAATTTTAAACATGTTTAACTTGTAGAACATATTATGAGCAAC
AGTTGGGTAGTTGAAATTTTATTTCTAAAGTTTTTAGTTTCTGAGTTATACTCATTTCAATATCCCCCAAGTGAAATCG
CCTAAAAATGAACTCGCGAGG
TGTCGGCCGCTGTATCTTTTGCCTCAATTTTTTTTAAATTTTCAAACATACATATTCCATTAGGTAACATTATGAGCTA
CAGTTTGTTAGTTGGAATTTTATTTCTAAAATTACTAGTTTCTGAGTTATACTCATTTCAATATCCCCCAAGTGAAATC
GCCTAAAAATGAACTCGCGAGG
TGTCGGCCGCTATATCGTTTGCCTTGAATTTTTTTACAAACTGATACATTTTTAAACCTGTAGAAAATAACACGGGTTA
CCATTTGTTAGTAAAAATTTCATTTCTACAATTTTTAGTTTCTGAGTTAAACTCATTCCAACCCACCCCAAGAGAAATT
TCCTAAAAATGAACTCGCGAGG
TGTCGGCCGCTGTATCTTTTGCCTCAATTTTTTTTTAATTTTTAAACATGCATATTCCAGTAGATAACATTATGAGCTA
CAGTTTGTTAGTTAAAATTTTATTTCTAAAATTACTAGTTTCTGAGTTATACTCATTCCAACCCACCCCAAGAGAAATT
TCCTAAAAATAGACTCGCAAAG
Nr of selected repeats 3 Similarity 0.750153

Consensus:
TGTCGGCCGCTgTATCtTTTGCCTcAATTTTTTTtnAAtttTnAaACATncaTAttCCagTAGatAAcAttAtGaGcTA
CagTTTGTTAGTtaaAATTTtATTTCTAaAATTacTAGTTTCTGAGTTAtACTCATTcCAAcccaCCCCAAGaGAAATt
tCCTAAAAATgaACTCGCgAgG
>Cele-UNSB01_3:3140206-3140936 Satlength=731 Nr of Repeats=5 RepeatLength=16
seed=TGTATATATA
TGTATATATATATATA
TGTATATATATATATA
TGTATATATATATATA
TGTATATATATATATACAGTGCGTCCAACTTCTATAGCACCCCCCTCTATTTTGAGGGTTTGAGGCTTTCTGGAAATTATT
TTGAAAAAGTGTGAATGTAGTTCGATTACAAATGTTAAAAAACCGCCACACCTCGGTTTTTGTGAGAATATCTTAAAAA
CTACGGAAATTAGGTCAAAAAGTTGAAAAAACGGCCGTCCAACTTCTATAGCACCCCCTCTGATTTTTGACAATTATCG
TTAAAATAGTCAATTTTTACTGGTTTTCTTTAACACTAATTTTTTTTTCTTGGATAAAAATGTGATCTCGGTGAAGACA
TTTCAGAACAAAACGATAGGAACTTCTTAAGAATATCGGAATAATGCTAAACAAACATATTTGACACACATTTACAAGA
AAAAAAATTAGTGTTAAAGAAAACCAGTAAAAATTGACTATTTTAACGATAATTTTCAAAAATTCGAGGGGGTGCTATA
GAAGTTGGACGGCCGTTTTTTCAACTTTTTGACCTAATTTCCGTAGTTTTTAAGATATTCTCACAAAAACCGAGGTGTG
GCGGTTTTTTAACATTTGTAATCGAACTACATTCACACTTTTTCAAAATAATTTCCAGAAAGCCTCAAACCCTCAAAAT
AGAGGGGGGTGCTATAGAAGTTGGACGCAC
TGTATATATAGTGTCACTCA
Nr of selected repeats 3 Similarity 1.000000
Consensus:
TGTATATATATATATA
>Cele-UNSB01_3:3145893-3146730 Satlength=838 Nr of Repeats=18 RepeatLength=37
seed=ATTTTGGCGG
ATTTTGGCGGTGCTTCGAATCCATTTTAAACATCATTTACTATATATAAAGCGCGTGTCCTTCCGTCCGTTTGTAGTTT
GTAGTTTGATCTTTGGTCTTTGAACTCTGTAGTTTGTAGTCTCAATCACCTAAGAGGGGTCCTATAGCCCCCGCCCCTT
TTGTGACGTCATCACAAATTGGCGGGAAATTCAAATTTTCTGAGAAATC
ATTTTGGCGGGATTTCAAATTTGAATTTTTTGAAAAT
ATTTTGGCGGGAATTCAAATTTTAATTTTTTGAAAAT
ATTTTGGCGGGAATTCAAATTTTAATTTTTTGAAAAC
ATTTTGGCGGGAATTCAAAATTTAATTTTTCGAAAAC
ATTTTGGCGGGAATTCAAAATTTAATTTTTCGAAAAC
ATTTTGGCGGGAATTCAAAATTTAATTTTTTGAAAAC
ATTTTGGCGGGAATTCAAATTTTAATTTTTTGAAAAC
ATTTTGGCGGGAATTCAAAATTTAATTTTTCGAAAAC
ATTTTGGCGGGAATTCAAAATTTAATTTTTCGAAAAC
ATTTTGGCGGGAATTCAAAATTTAATTTTTTGAAAAC
ATTTTGGCGGGAATTCAAATTTTAATTTTTCGAAAAC
ATTTTGGCGGGAATTCAAAATTTAATTTTTCGAAAAC
ATTTTGGCGGGAATTCAAAATTTAATTTTTCGAAAAC
ATTTTGGCGGGAATTTAAAATTTTAATTTTTTGAAAAC
ATTTTGGCGGGAATTCAAATTTTAATTTTTCGAAAAC
ATTTTGGCGGGAATTCAAATTTTAATTTTTTGAAAAT
ATGTTGGCGGGAATCAAAATTTTAATGTTTTGAAAAT
Nr of selected repeats 16 Similarity 0.920120
Consensus:
ATTTTGGCGGGAATTCAAAaTTTAATTTTTcGAAAAc
>Cele-UNSB01_3:3152376-3152920 Satlength=545 Nr of Repeats=14 RepeatLength=35
seed=AATTGCCGGA
AATTGCCGGAATTGAAACTTTCCGGCAAATTGGAA
AATTGCCGGAATTGAAACTTTCCGGCGAATTGGAA
AATTGCCGGAATTGAAAATTACCGGCAAAACGGCA
AATTGCCGGAATTTAAAATTTCAGGAAAATTGGCA
AATTGCCGGATTTTAAAATTTCCGGCAAATTGGAA

AAGTGCCGGAATTTAAAATTTCAGGAAAATTGGCA
AATTGCCGGAATTTAAAATTTCCGGCAAATTGGAA
AATTGCCGGAATTTAAATTTTCCTGCAAATCAGAAAATTGTCATTTTGCCAGAAAACCGGCAAATCAGAAAATTTCCGG
CAAAACGGCA
AATTGCCGGAATTGAAAATTACCGGCAAAACGGCA
AATTGCCGGAATTTAAAATTTCAGGAAAATTGGCA
AATTGCCGGATTTTAAAATTTCAGGAAATTGACA
AATTGCCGGAATTTAAAATTTCAGGAAAATTGGCA
AATTGCCGGAATTTAAAATTTCCGGCAAAACGGCA
AATTGCCGGACATTTAAAACTTCCGGCAAATCAGAA
Nr of selected repeats 11 Similarity 0.843463
Consensus:
AATTGCCGGAATTtAAAATTTCcGGcAAAttGGcA
>Cele-UNSB01_3:3159908-3160529 Satlength=622 Nr of Repeats=13 RepeatLength=35
seed=AAATCGGCAA
AAATCGGCAATTTGCCATTTTTCAAATTTTCGGAAAATTTGGCATTTGCCCAAATTGAAAAATTTTAAATTTTCGGC
AAATCGGAAAATTGCCGGAATTGAAAATTTCCAGA
AAATCGGCAAATTGCAGAAATTGAAAATTTCCGGC
AAATCGGCAAATTGCGGGAATTGAAAATTTCCAGC
AAATCGGCAATTTGCCGGAATTGAAAATTTCTGGC
AAATCGGCAATTTGCCGGAATTAAAAATTTCCAGA
AAATCGGCAAATTGCCGGAATTGAAAATTTCCAGA
AAATCGGCAAATTGTCGGAATTGAAATTTCCGGCAAACTGGCAATTTCCCGAAACTGAAAATTTCCGGCAAACAGCA
AAGTCGGCAATTTGCGGTTTTCCAAATTTTTGGCAAATTCTGC
AAACCGGCAACTAGTCGATTTGCCGGAA
AAACGGCAACTGCCGAAAGTTTCGGTAAATTGTGGGTTTGCGTAATTTAAAAAATCCCCTCAAAAAATCCGGCGAATTG
ATATCCGGCAAAATTGCCGGAATTGAAAATTTCCGGC
AAATCGGCAAATTGCCGGAATTGAAAATTTCCAGC
AAATCGGCAATTTGCCGGAATTGAAAATTTCCAGA
Nr of selected repeats 8 Similarity 0.885714
Consensus:
AAATCGGCAAaTTGCcGGAATTGAAAATTTCCaGa
>Cele-UNSB01_3:3164980-3165718 Satlength=739 Nr of Repeats=38 RepeatLength=18
seed=GTCGATTTAC
GTCGATTTACGAGATTTT
GTCGATTTACGAGATTTT
GTCGATTTACGAGTTTGG
GTCGATTTACGACATTGT
GTCGATTTACGAAAGTAT
GTCGATTTACGAGAGTTT
GTCGATTTACGACATTGT
GTCGATTTACGAAATTAT
GTCGATTTACGAGAGTTT
GTCGATTTACGACTTTGT
GTCGATTTACGAGAGTTT
GTCGATTTACTAGGACTTT
TCGATTTACGAGATTTT
GTCAATTTACTAGAACGT
GTCGATTTACGAAATTGTGTCGAGTTCCGAGATTTT
GTAGATTTACGAGATTTTGTCAAGTTACTAGAATGT
GTCGATTAACGAGATTTT
GTCGATTTACGAAAGTAT
GTCGATTTACGAAATTAT
GTCGATTTACGAGAGTTT

GTCGATTTACGACTTTGT
GTCGATTTACGAGATTTT
GTCGATTTACGAAATTAT
GTCGATTTACGAGATTTG
GTTGATTTACGACATTGT
GTCGATTTACGAAAGTAT
GTCGATTTACGACTTTGT
GTCGATTTACGAGAGTTT
GTCGATTTACGACTTTGT
GTCGATTTACGAGAGTTT
GTCGATTTACTAGCACTT
GTCGATTTACGAGATTTT
GTCAATTTACTAGAACGT
GTCGATTTACGAAATTGT
GTCGAGTTACGAGATTTT
GTAGATTTACGAGAATTT
GTAGATTTACGAGATTTT
GTAGATTTACGAGATTTTGTCAAGTTACTAGAATGT
Nr of selected repeats 33 Similarity 0.778900
Consensus:
GTCGATTTACGAgAtTnT
>Cele-UNSB01_3:3200630-3201398 Satlength=769 Nr of Repeats=4 RepeatLength=151
seed=TTTAAAGGCG
TTTAAAGGCGCATAGGTAGATCAAAACTCAAAGTTGGCGGATTCTAGTCAATATGGGGCTTATTTTAAAGCTCTCGGTA
AGCTGAATGTAGTTTAGCTACTGAAAATTGAAAATTTAAACAATTTTTGGGTCTAGCAGCGAAAATTTCGAA
TTTAAAGGCGCATAGGTAGGACAAATTTCAAAATTGACGGAATCTAGTCAATATGGGGCTTATTTGAAAGCTCTCAATG
AGCTGAATCTAGTTTGGCTACTAAAAATCAAAAAATACTGAAAAATTTCGCGGCGAGACCCAAAATTCTATTTTTCATC
AGATTTTAATGAAAAAAACCCGGATTTTTTCGAATTTTTTTGATAAATTTAAAAATTAAAATTGAACCACTAATAACTT
CAAAATGGCACATCTGTCGCCTAGTTCTCGGGAATTTCTGTATATTTTGGGTCCCGCCACCAAAAAAAAAATTCTAAA
TTTAAAGGAGCATAACCCAATTTCCCTCTTCATTTTCGAGGAAATCACTTAAAAATTTGAATTCTTTGAAAAATTCCAG
TCAAAACACACTTTTATTAAGCCTCTAACGTCGATTTTTAACTATTTCGGGTCTCGCCACGAAAAATTGTAG
TTTAAAGGCGCATAGGTAGCTGAAATTTCAAAATTGACGGATTCTAGTCAATATGGGGCACATTTTGAAGCTCTCAGTG
AGCTTAATATACTGCGGCTACTAAAAATTGAATATTTTCAAATTTTTTGGGTCTCGCAGCGAAAGTTTCGAA
Nr of selected repeats 3 Similarity 0.500000
Consensus:
TTTAAAGGcGCATAggtAgnTcaaantTCAaanTtGacGgAtTCtagTcAAtATggGgcttaTTTtaAAgcTctCaGTa
AgCtnAaTnTanTtnaGCtaCTnAAaaTtGaanATTTtnAaattTTTtGGGTCTcGCagCGAAAatTTcGaA
>Cele-UNSB01_3:3207516-3208428 Satlength=913 Nr of Repeats=5 RepeatLength=151
seed=TTTAAAGGCG
TTTAAAGGCGCATAGGTAGATCAAAACTCAAAGTTGGCGGATTCTAGTCAATATGGGGCTTATTTTAAAGCTCTCGGTA
AGCTGAATGTAGTTTAGCTACTGACAATTGAAAATTTAAACAATTTTTGGGTCTAGCAGCGAAAATTTCGAA
TTTAAAGGCGCATAGGTAGGACAAATTTCAAAATTGACGGAATCTAGTCAATATGGGGCTTATTTGAAAGCTCTCAATG
AGCTGAATCTAGTTTAGCTACTAAAAATAAAAAAAATACTGAAAAATTTCGCGGCGAGACCCAAAATTCTATTTTTCAT
CAGATTTTAATGAAAAAAACCCGGATTTTTTCGAATTTTTTTGATAAATTTAAAATTTAAAATTGAACCACTAATAACT
TCAAAATGGCACATCTGTCGCCTAGTTCTCGGGAATTTCTGTATATTTTGGGTCCCGCCACGAAAAAAAAAATCTAAA
TTTAAAGGCACATGACCCAATTTCCCCCTTCATTTTCGAGGAAATCACTTAAAAATTTGAATTCTTTGAAAAATTCCAG
TCAAAACACACTTTTATTAAGCCTCTAACGTCGATTTTTAACTATTTCGGGTCTCGCCACGAAAAATTGTAG
TTTAAAGGCGCATAGGTAGTTGAAATTTCAAAATTGACGGATTCTAGTCAATATGGGGCACATTTTGAAGCTCTCAGTG
AGCTTAATATACTGCGGCTACTAAAAATTGAATATTTTCAAATTTTTTGGGTCTCGCAGCGAAAGTTTCGAA
TTTAAAGGCGCATACAGTCATTTTATGGCGGTTCTAGACAAACTTTCTGAGTAAATTTTGATTTATTTTTCAATTTCAC
TTATAAACCAGTCTCAGTCATTATATTTGACAAATTTTGGTGGGTCCCGCCACGAAAATTTCAAA
Nr of selected repeats 3 Similarity 0.500000
Consensus:

TTTAAAGGCgCATAggtAgtTcaaantTCAaanTtGacGgAtTCtagTcAAtATggGgcttaTTTtaAAgcTctCaGTa
AgCtnAaTnTanTtnaGCtaCTaAcaaTtGaanATTTtnAaattTTTtGGGTCTcGCagCGAAAatTTcGaA
>Cele-UNSB01_3:3351031-3351269 Satlength=239 Nr of Repeats=7 RepeatLength=30
seed=TAGAACTTTC
TAGAACTTTCCAAATTCTTTCAGAAAACTCTAGAAGTTTACAGAATTTTCTAAAAACTGT
TAGAACTTTTCAGTTTTTTCTAGAAAACTC
TAGAACTTTCCAAATTTCTCTTGAAAATTC
TAGAACTTTCCAAAGTTTTCTAAAAAATTC
TAGAACTTTCCAAATTTCTCTAGAAAATTC
TAGAGCTTTCCAAAGTTTTCTAGAAATTTT
TAGAACTTTCCAGATTTTTTCTAGAAAG
Nr of selected repeats 5 Similarity 0.786667
Consensus:
TAGAaCTTTcCAaatTTtTCTagAAAatTc
>Cele-UNSB01_3:3352849-3353088 Satlength=240 Nr of Repeats=8 RepeatLength=30
seed=TAGAACTTTC
TAGAACTTTCCAAATTTCTCTAGAAAATTC
TAGAACTTTCCAAAGTTTCTAAAAAAAAATTC
TAGAACTTTCCAAATTTCTCTAGAAAATTC
TAGAACTTTCCAAATTTCTCTAGAAAATTC
TAGAACTTTCCAAATTTCTCTAGAAAATTC
TAGAACTTTCCAAATTTCTCTAGAAAATTC
TAGAGCTTTCCAAAGTTTTCTAGAAATTTT
TAGAACTTTCCAGATTTTTTCTAGAAAC
Nr of selected repeats 6 Similarity 0.925926
Consensus:
TAGAACTTTCCAAATTTCTCTAGAAAATTC
>Cele-UNSB01_3:3356021-3356788 Satlength=768 Nr of Repeats=13 RepeatLength=19
seed=CTTGGCGCAA
CTTGGCGCAATTCTCGTGC
CTTGGCGCAATTCTCGTGC
CTTGGCGCATTTTTAGTGTCTTGATGCAATTTTGGTGCCTTGACGCGATTCTAGTGCA
TTGGCGCAATTTTGATGCCTAGGCGCATTTTCGGTGTCTTTGCTCAATTCTCGTGCCTGACGCGATTTTTGATC
CTTGGCGCATTTTCAGTGCT
TTGGCGCAACTCTCGATCCTTGACGCATTTCTCGATCCTTAAACATTTTTTGCAGAAAAAGCTCCACCAACTCTCCCAA
TCTCAACAGCCGAGTTTCTCCGATTCATCTGTCAAAAAACGCTCGAGTTCTATGAAAATCGCCCGTGAATCGTCGGCGT
CGTCTACCGAGTCACAACGTGGAATCTACAGTAACCAACAGAGATCGATAGGAAATAATTTGTTACAACGAGTTGTGAA
GTTGCAGGCAGAATTGATGGCAAAGGACGAAAAAGTACGTTTTGGAGTACGAAATGGGAAAAACGATTGAAAAATTTGG
AAAATTCGGGGAATTTCGGAATAGAGACAAAAACAATTGGAATTTGGAGATTGTTCGCGCATTTTTTGTGCCTAGGCGC
AGTTTCGGTGTA
TTGGCGCAATTCTCGTGCCTTGGCGCTTTTTTGGTGCACTGGGGCAGATCTTTTGC
CTTGACGCAACTTTAGTGC
CTTGGCGCAAGTTTGGTGT
CTTGGCGCAATTCTAGTGC
CTTGACGCAATTCTCGATC
CTTGGCGCAAATTTGGTGC
CTTGACGCAATTCTCGTGT
Nr of selected repeats 8 Similarity 0.764411
Consensus:
CTTGgCGCAAtTcTcGTGc
>Cele-UNSB01_3:3394846-3394930 Satlength=85 Nr of Repeats=4 RepeatLength=21
seed=TCCAGCACTG
TCCAGCACTGACAAAATTTGC
TCCAGCACTGACAAATTTTGT

TCCAGCACTGACAAAATTTGT
TCCAGCACTGACAAAATTTGT
Nr of selected repeats 4 Similarity 0.936508
Consensus:
TCCAGCACTGACAAAaTTTGt
>Cele-UNSB01_3:3456121-3456336 Satlength=216 Nr of Repeats=18 RepeatLength=12
seed=AAAATAAAAA
AAAATAAAAATA
AAAATAAAAATA
AAAATAAAAATA
AAAATAAAAACA
AAAATAAAAATA
AAAATAAAAATA
AAAATAAAAATA
AAAATAAAAATA
AAAATAAAAATA
AAAATAAAAATA
AAAATAAAAATA
AAAATAAAAATA
AAAATAAAAATA
AAAATAAAAATA
AAAATAAAAATA
AAAATAAAAATA
AAAATAAAATA
AAAATAAAAATA
Nr of selected repeats 17 Similarity 0.986928
Consensus:
AAAATAAAAATA
>Cele-UNSB01_3:3456392-3470370 Satlength=13979 Nr of Repeats=556
RepeatLength=25 seed=ACTCTCTGGC
ACTCTCTGGCTTCCCACTATATTTT
ACTCTCTGGCTTCCCACTATATTTT
ACTCTCTGGCTTCCCATCATATTTT
ACTCTCTGGCTTCCCACTATATTTT
ACTCTCTGGCTTCCCATCATATTTT
ACTCTCTGGCTTCCCATCATATTTT
ACTCTCTGGCTTCCCACTATATTTT
ACTCTCTGGCTTCCCATCATATTTT
ACTCTCTGGCTTCCCATCATATTTT
ACTCTCTGGCTTCCCATCATATTTT
ACTCTCTGGCTTCCCATCATATTTT
ACTCTCTGGCTTCCCATCATATTTT
ACTCTCTGGCTTCCCATCATATTTT
ACTCTCTGGCTTCCCACTATATTTT
ACTCTCTGGCTTCCCACTATATTTT
ACTCTCTGGCTTCCCATCATATTTT
ACTCTCTGGCTTCCCACTATATTTT
ACTCTCTGGCTTCCCATCATATTTT
ACTCTCTGGCTTCCCACTATATTTT
ACTCTCTGGCTTCCCATCATATTTT
ACTCTCTGGCTTCCCATCATATTTT
ACTCTCTGGCTTCCCATCATATTTT
ACTCTCTGGCTTCCCATCATATTTT
ACTCTCTGGCTTCCCATCATATTTT

ACTCTCTGGCTTCCCACTATATTTT
ACTCTCTGGCTTCCCATCATATTTT
ACTCTCTGGCTTCCCACTATATTTT
ACTCTCTGGCTTCCCATCATATTTT
ACTCTCTGGCTTCCCACTATATTTT
ACTCTCTGGCTTCCCATCATATTTT
ACTCTCTGGCTTCCCACTATATTTT
ACTCTCTGGCTTCCCATCATATTTT
ACTCTCTGGCTTCCCACTATATTTT
ACTCTCTGGCTTCCCATCATATTTT
ACTCTCTGGCTTCCCACTATATTTT
ACTCTCTGGCTTCCCATCATATTTT
ACTCTCTGGCTTCCCACTATATTTT
ACTCTCTGGCTTCCCACTATATTTT
ACTCTCTGGCTTCCCATCATATTTT
ACTCTCTGGCTTCCCACTATATTTT
ACTCTCTGGCTTCCCATCATATTTT
ACTCTCTGGCTTCCCACTATATTTT
ACTCTCTGGCTTCCCATCATATTTT
ACTCTCTGGCTTCCCACTATATTTT
ACTCTCTGGCTTCCCATCATATTTT
ACTCTCTGGCTTCCCACTATATTTT
ACTCTCTGGCTTCCCATCATATTTT
ACTCTCTGGCTTCCCATCATATTTT
ACTCTCTGGCTTCCCATCATATTTT
ACTCTCTGGCTTCCCACTATATTTT
ACTCTCTGGCTTCCCACTATATTTT
ACTCTCTGGCTTCCCATCATATTTT
ACTCTCTGGCTTCCCACTATATTTT
ACTCTCTGGCTTCCCATCATATTTT
ACTCTCTGGCTTCCCATCATATTTT
ACTCTCTGGCTTCCCATCATATTTT
ACTCTCTGGCTTCCCATCATATTTT
ACTCTCTGGCTTCCCATCATATTTT
ACTCTCTGGCTTCCCATCATATTTT
ACTCTCTGGCTTCCCATCATATTTT
ACTCTCTGGCTTCCCATCATATTTT
ACTCTCTGGCTTCCCATCATATTTT
ACTCTCTGGCTTCCCACTATATTTT
ACTCTCTGGCTTCCCATCATATTTT
ACTCTCTGGCTTCCCATCATATTTT
ACTCTCTGGCTTCCCATCATATTTT
ACTCTCTGGCTTCCCACTATATTTT
ACTCTCTGGCTTCCCATCATATTTT
ACTCTCTGGCTTCCCACTATATTTT
ACTCTCTGGCTTCCCATCATATTTT
ACTCTCTGGCTTCCCACTATATTTT
ACTCTCTGGCTTCCCATCATATTTT
ACTCTCTGGCTTCCCACTATATTTT
ACTCTCTGGCTTCCCATCATATTTT
ACTCTCTGGCTTCCCATCATATTTT
ACTCTCTGGCTTCCCATCATATTTT
ACTCTCTGGCTTCCCATCATATTTT
ACTCTCTGGCTTCCCATCATATTTT

ACTCTCTGGCTTCACCACATATTTT
ACTCTCTGGCTTCCCACTATATTTT
ACTCTCTGGCTTCCCATCATATTTT
ACTCTCTGGCTTCCCATCATATTTT
ACTCTCTGGCTTCCCACTATATTTT
ACTCTCTGGCTTCCCATCATATTTT
ACTCTCTGGCTTCCCATCATATTTT
ACTCTCTGGCTTCCCATCATATTTT
ACTCTCTGGCTTCCCATCATATTTT
ACTCTCTGGCTTCCCATCATATTTT
ACTCTCTGGCTTCCCACTATATTTT
ACTCTCTGGCTTCCCACTATATTTT
ACTCTCTGGCTTCCCACTATATTTT
ACTCTCTGGCTTCCCACTATATTTT
ACTCTCTGGCTTCCCACTATATTTT
ACTCTCTGGCTTCACCATATATTTT
ACTCTCTGGCTTCCCACTATATTTT
ACTCTCTGGCTTCCCACTATATTTT
ACTCTCTGGCTTCCCACTATATTTT
ACTCTCTGGCTTCCCACTATATTTT
ACTCTCTGGCTTCCCACTATATTTT
ACTCTCTGGCTTCCCATCATATTTT
ACTCTCTGGCTTCCCACTATATTTT
ACTCTCTGGCTTCCCATCATATTTT
ACTCTCTGGCTTCCCACTATATTTT
ACTCTCTGGCTTCCCACTATATTTT
ACTCTCTGGCTTCCCATCATATTTT
ACTCTCTGGCTTCCCATCATATTTT
ACTCTCTGGCTTCCCACTATATTTT
ACTCTCTGGCTTCCCATCATATTTT
ACTCTCTGGCTTCCCATCATATTTT
ACTCTCTGGCTTCCCATCATATTTT
ACTCTCTGGCTTCCCATCATATTTT
ACTCTCTGGCTTCCCATCATATTTT
ACTCTCTGGCTTCCCACTATATTTT
ACTCTCTGGCTTCCCACTATATTTT
ACTCTCTGGCTTCACCATATATTTT
ACTCTCTGGCTTCCCACTATATTTT
ACTCTCTGGCTTCCCATCATATTTT
ACTCTCTGGCTTCCCACTATATTTT
ACTCTCTGGCTTCCCATCATATTTT
ACTCTCTGGCTTCCCACTATATTTT
ACTCTCTGGCTTCCCATCATATTTT
ACTCTCTGGCTTCCCACTATATTTT
ACTCTCTGGCTTCCCATCATATTTT
ACTCTCTGGCTTCCCATCATATTTT
ACTCTCTGGCTTCCCATCATATTTT
ACTCTCTGGCTTCCCATCATATTTT
ACTCTCTGGCTTCCCATCATATTTT
ACTCTCTGGCTTCCCACTATATTTT
ACTCTCTGGCTTCCCATCATATTTT
ACTCTCTGGCTTCCCATCATATTTT
ACTCTCTGGCTTCCCACTATATTTT
ACTCTCTGGCTTCCCATCATATTTT

ACTCTCTGGCTTCCCACTATATTTT
ACTCTCTGGCTTCCCATCATATTTT
ACTCTCTGGCTTCCCATCATATTTT
ACTCTCTGGCTTCCCACTATATTTT
ACTCTCTGGCTTCCCACTATATTTT
ACTCTCTGGCTTCCCACTATATTTT
ACTCTCTGGCTTCCCACTATATTTT
ACTCTCTGGCTTCCCACTATATTTT
ACTCTCTGGCTTCCCACTATATTTT
ACTCTCTGGCTTCCCACTATATTTT
ACTCTCTGGCTTCCCACTATATTTT
ACTCTCTGGCTTCCCATCATATTTT
ACTCTCTGGCTTCCCATCATATTTT
ACTCTCTGGCTTCCCATCATATTTT
ACTCTCTGGCTTCCCACTATATTTT
ACTCTCTGGCTTCCCACTATATTTT
ACTCTCTGGCTTCCCACTATATTTT
ACTCTCTGGCTTCCCACTATATTTT
ACTCTCTGGCTTCCCACTATATTTT
ACTCTCTGGCTTCCCACTATATTTT
ACTCTCTGGCTTCCCACTATATTTT
ACTCTCTGGCTTCCCATCATATTTT
ACTCTCTGGCTTCCCATCATATTTT
ACTCTCTGGCTTCCCATCATATTTT
ACTCTCTGGCTTCCCACTATATTTT
ACTCTCTGGCTTCCCATCATATTTT
ACTCTCTGGCTTCCCACTATATTTT
ACTCTCTGGCTTCCCACTATATTTT
ACTCTCTGGCTTCCCATCATATTTT
ACTCTCTGGCTTCCCATCATATTTT
ACTCTCTGGCTTCCCATCATATTTT
ACTCTCTGGCTTCCCACTATATTTT
ACTCTCTGGCTTCCCACTATATTTT
ACTCTCTGGCTTCCCACTATATTTT
ACTCTCTGGCTTCCCACTATATTTT
ACTCTCTGGCTTCCCACTATATTTT
ACTCTCTGGCTTCCCACTATATTTT
ACTCTCTGGCTTCCCACTATATTTT
ACTCTCTGGCTTCCCATCATATTTT
ACTCTCTGGCTTCCCATCATATTTT
ACTCTCTGGCTTCCCATCATATTTT
ACTCTCTGGCTTCCCACTATATTTT
ACTCTCTGGCTTCCCATCATATTTT
ACTCTCTGGCTTCCCACTATATTTT
ACTCTCTGGCTTCCCACTATATTTT
ACTCTCTGGCTTCCCATCATATTTT
ACTCTCTGGCTTCCCACTATATTTT
ACTCTCTGGCTTCCCATCATATTTT
ACTCTCTGGCTTCACCATATATTTT
ACTCTCTGGCTTCCCACTATATTTT
ACTCTCTGGCTTCCCACTATATTTT
ACTCTCTGGCTTCCCACTATATTTT
ACTCTCTGGCTTCCCATCATATTTT
ACTCTCTGGCTTCCCACTATATTTT

ACTCTCTGGCTTCCCATCATATTTT
ACTCTCTGGCTTCCCACTATATTTT
ACTCTCTGGCTTCCCATCATATTTT
ACTCTCTGGCTTCCCACTATATTTT
ACTCTCTGGCTTCCCACTATATTTT
ACTCTCTGGCTTCCCATCATATTTT
ACTCTCTGGCTTCCCATCATATTTT
ACTCTCTGGCTTCCCACTATATTTT
ACTCTCTGGCTTCCCATCATATTTT
ACTCTCTGGCTTCCCATCATATTTT
ACTCTCTGGCTTCCCACTATATTTT
ACTCTCTGGCTTCCCACTATATTTT
ACTCTCTGGCTTCCCACTATATTTT
ACTCTCTGGCTTCCCACTATATTTT
ACTCTCTGGCTTCCCACTATATTTT
ACTCTCTGGCTTCCCACTATATTTT
ACTCTCTGGCTTCCCACTATATTTT
ACTCTCTGGCTTCCCACTATATTTT
ACTCTCTGGCTTCACCACATATTTT
ACTCTCTGGCTTCCCACTATATTTT
ACTCTCTGGCTTCCCATCATATTTT
ACTCTCTGGCTTCCCATCATATTTT
ACTCTCTGGCTTCCCATCATATTTT
ACTCTCTGGCTTCCCACTATATTTT
ACTCTCTGGCTTCCCACTATATTTT
ACTCTCTGGCTTCCCACTATATTTT
ACTCTCTGGCTTCCCACTATATTTT
ACTCTCTGGCTTCCCACTATATTTT
ACTCTCTGGCTTCCCATCATATTTT
ACTCTCTGGCTTCCCACTATATTTT
ACTCTCTGGCTTCCCATCATATTTT
ACTCTCTGGCTTCCCATCATATTTT
ACTCTCTGGCTTCCCATCATATTTT
ACTCTCTGGCTTCCCACTATATTTT
ACTCTCTGGCTTCCCATCATATTTT
ACTCTCTGGCTTCCCACTATATTTT
ACTCTCTGGCTTCCCACTATATTTT
ACTCTCTGGCTTCCCATCATATTTT
ACTCTCTGGCTTCCCACTATATTTT
ACTCTCTGGCTTCCCATCATATTTT
ACTCTCTGGCTTCCCACTATATTTT
ACTCTCTGGCTTCCCATCATATTTT
ACTCTCTGGCTTCCCACTATATTTT
ACTCTCTGGCTTCCCATCATATTTT
ACTCTCTGGCTTCCCACTATATTTT
ACTCTCTGGCTTCCCATCATATTTT
ACTCTCTGGCTTCCCATCATATTTT
ACTCTCTGGCTTCCCATCATATTTT
ACTCTCTGGCTTCCCACTATATTTT
ACTCTCTGGCTTCCCATCATATTTT
ACTCTCTGGCTTCCCACTATATTTT
ACTCTCTGGCTTCCCATCATATTTT
ACTCTCTGGCTTCCCACTATATTTT
ACTCTCTGGCTTCCCATCATATTTT

ACTCTCTGGCTTCCCACTATATTTT
ACTCTCTGGCTTCCCATCATATTTT
ACTCTCTGGCTTCCCATCATATTTT
ACTCTCTGGCTTCCCACTATATTTT
ACTCTCTGGCTTCCCATCATATTTT
ACTCTCTGGCTTCCCACTATATTTT
ACTCTCTGGCTTCCCATCATATTTT
ACTCTCTGGCTTCCCATCATATTTT
ACTCTCTGGCTTCCCATCATATTTT
ACTCTCTGGCTTCCCACTATATTTT
ACTCTCTGGCTTCCCATCATATTTT
ACTCTCTGGCTTCCCATCATATTTT
ACTCTCTGGCTTCCCATCATATTTT
ACTCTCTGGCTTCCCACTATATTTT
ACTCTCTGGCTTCCCATCATATTTT
ACTCTCTGGCTTCCCATCATATTTT
ACTCTCTGGCTTCCCATCATATTTT
ACTCTCTGGCTTCCCATCATATTTT
ACTCTCTGGCTTATATCTCTTCCCATATTTT
ACTCTCTGGCTTCCCACTATATTTT
ACTCTCTGGCTTCCCATCATATTTT
ACTCTCTGGCTTCCCACTATATTTT
ACTCTCTGGCTTCCCATCATATTTT
ACTCTCTGGCTTCCCATCATATTTT
ACTCTCTGGCTTCCCACTATATTTT
ACTCTCTGGCTTCCCATCATATTTT
ACTCTCTGGCTTCCCACTATATTTT
ACTCTCTGGCTTCCCATCATATTTT
ACTCTCTGGCTTCCCATCATATTTT
ACTCTCTGGCTTCCCATCATATTTT
ACTCTCTGGCTTCCCATCATATTTT
ACTCTCTGGCTTCCCATCATATTTT
ACTCTCTGGCTTCACCATATATTTT
ACTCTCTGGCTTCCCATCATATTTT
ACTCTCTGGCTTCCCACTATATTTT
ACTCTCTGGCTTCCCACTATATTTT
ACTCTCTGGCTTCCCATCATATTTT
ACTCTCTGGCTTCCCACTATATTTT
ACTCTCTGGCTTCCCACTATATTTT
ACTCTCTGGCTTCCCATCATATTTT
ACTCTCTGGCTTCCCATCATATTTT
ACTCTCTGGCTTCCCACTATATTTT
ACTCTCTGGCTTCCCATCATATTTT
ACTCTCTGGCTTCCCATCATATTTT
ACTCTCTGGCTTCCCATCATATTTT
ACTCTCTGGCTTCCCATCATATTTT
ACTCTCTGGCTTCCCATCATATTTT
ACTCTCTGGCTTCCCATCATATTTT
ACTCTCTGGCTTCACCAATTATTTT
ACTCTCTGGCTTCCCATCATATTTTTTTTTTATTTT
ACTCTCTGGCTTCCCATCATATTTT
ACTCTCTGGCTTCCCATCATATTTT
ACTCTCTGGCTTCCCACTATATTTT
ACTCTCTGGCTTCCCATCATATTTT

ACTCTCTGGCTTCCCATCATATTTT
ACTCTCTGGCTTCCCATCATATTTT
ACTCTCTGGCTTCCCATCATATTTT
ACTCTCTGGCTTCCCACTATATTTT
ACTCTCTGGCTTCCCATCATATTTT
ACTCTCTGGCTTCCCACTATATTTT
ACTCTCTGGCTTCCCATCATATTTT
ACTCTCTGGCTTCCCATCATATTTT
ACTCTCTGGCTTCACCATATATTTT
ACTCTCTGGCTTCCCATCATATTTT
ACTCTCTGGCTTCCCACTATATTTT
ACTCTCTGGCTTCCCATCATATTTT
ACTCTCTGGCTTCACCATATATTTT
ACTCTCTGGCTTCCCATCATATTTT
ACTCTCTGGCTTCCCACTATATTTT
ACTCTCTGGCTTCCCATCATATTTT
ACTCTCTGGCTTCCCATCATATTTT
ACTCTCTGGCTTCCCATCATATTTT
ACTCTCTGGCTTCCCACTATATTTT
ACTCTCTGGCTTCCCATCATATTTT
ACTCTCTGGCTTCCCATCATATTTT
ACTCTCTGGCTTCACCATATATTTT
ACTCTCTGGCTTCCCATCATATTTT
ACTCTCTGGCTTCCCACTATATTTT
ACTCTCTGGCTTCCCATCATATTTT
ACTCTCTGGCTTCCCACTATATTTT
ACTCTCTGGCTTCCCATCATATTTT
ACTCTCTGGCTTCCCATCATATTTT
ACTCTCTGGCTTCCCACTATATTTT
ACTCTCTGGCTTCCCATATATTTT
ACTCTCTGGCTTCCCACTATATTTT
ACTCTCTGGCTTCCCATCATATTT
ACTCTCTGGCTTCCCATCATATTTT
ACTCTCTGGCTTCCCATCATATTTT
ACTCTCTGGCTTCCCACTATATTTT
ACTCTCTGGCTTCCCATCATATTTT
ACTCTCTGGCTTCCCATCATATTTT
ACTCTCTGGCTTCCCACTATATTTT
ACTCTCTGGCTTCCCATCATATTTT
ACTCTCTGGCTTCCCATCATATTTT
ACTCTCTGGCTTCCCATCATATTTT
ACTCTCTGGCTTCCCATCATATTTT
ACTCTCTGGCTTCCCACTATATTTT
ACTCTCTGGCTTCCCATCATATTTT
ACTCTCTGGCTTCCCATCATATTTT
ACTCTCTGGCTTCACCATATATTTT
ACTCTCTGGCTTCCCATCATATTTT
ACTCTCTGGCTTCCCACTATATTTT
ACTCTCTGGCTTCCCATCATATTTT
ACTCTCTGGCTTCCCACTATATTTT
ACTCTCTGGCTTCACCATATATTTT
ACTCTCTGGCTTCCCATCATATTTT
ACTCTCTGGCTTCCCATCATATTTTTTTTTTTT
ACTCTCTGGCTTCCCACTATATTTT

ACTCTCTGGCTTCCCATCATATTTT
ACTCTCTGGCTTCCCACTATATTTT
ACTCTCTGGCTTCCCATCATATTTT
ACTCTCTGGCTTCCCACTATATTTT
ACTCTCTGGCTTCCCATCATATTTT
ACTCTCTGGCTTCCCACATATTTT
ACTCTCTGGCTTCCCATCATATTTT
ACTCTCTGGCTTCCCATCATATTTT
ACTCTCTGGCTTCCCACATATTTT
ACTCTCTGGCTTCCCATCATATTTT
ACTCTCTGGCTTCCCACTATATTTT
ACTCTCTGGCTTCCCACTCATATTTT
ACTCTCTGGCTTCCCATCATATTTT
ACTCTCTGGCTTCCCACTATATTTT
ACTCTCTGGCTTCCCATCATATTTT
ACTCTCTGGCTTCCCCACTATATTTT
ACTCTCTGGCTTCCCATCATATTTT
ACTCTCTGGCTTCCCATCATATTTT
ACTCTCTGGCTTCCCATATATTTT
ACTCTCTGGCTTCCCACTATATTTT
ACTCTCTGGCTTCCCATCATATTTT
ACTCTCTGGCTTCCCATCATATTTT
ACTCTCTGGCTTCCCATCATATTTT
ACTCTCTGGCTTCCCATATATTTT
ACTCTCTGGCTTCCCATCATATTTT
ACTCTCTGGCTTCACCATATATTTT
ACTCTCTGGCTTCCCATCATATTTT
ACTCTCTGGCTTCCCACATATTTT
ACTCTCTGGCTTCCCATCATATTTT
ACTCTCTGGCTTCCCATATATTTTTTTTTTTTTTTTTTTTTTTTT
ACTCTCTGGCTTCCCATCATATTTT
ACTCTCTGGCTTCCCATCATATTTT
ACTCTCTGGCTTCCCACTATATTTT
ACTCTCTGGCTTCCCATCATATTTT
ACTCTCTGGCTTCCCATCATATTTT
ACTCTCTGGCTTCCCATCATATTTT
ACTCTCTGGCTTCCCACTATATTTT
ACTCTCTGGCTTCCCACATATTTT
ACTCTCTGGCTTCCCATCATATTTT
ACTCTCTGGCTTCCCATCATATTTT
ACTCTCTGGCTTCCCACTATATTTT
ACTCTCTGGCTTCCCACATATTTT
ACTCTCTGGCTTCCCATATTTT
ACTCTCTGGCTTCCCATCATATTTT
ACTCTCTGGCTTCCCACTATATTTT
ACTCTCTGGCTTCACCATATATTTT
ACTCTCTGGCTTCCCACTATATTTT
ACTCTCTGGCTTCCCATCATATTTT
ACTCTCTGGCTTCCCATATATTTT
ACTCTCTGGCTTCCCATCATATTTT
ACTCTCTGGCTTCCCACTATATTTT
ACTCTCTGGCTTCACCATATATTTT
ACTCTCTGGCTTCCCATATATTTTTTTTTTTTTTTTTTTTTTTTT
ACTCTCTGGCTTCCCATCATATTTT

ACTCTCTGGCTTCCCATCATATTTT
ACTCTCTGGCTTCCCATCATATTTT
ACTCTCTGGCTTCCCATCATATTTT
ACTCTCTGGCTTCCCACTATATTTT
ACTCTCTGGCTTCCCATCATATTTT
ACTCTCTGGCTTCCCATCATATTTT
ACTCTCTGGCTTCCCATCATATTTT
ACTCTCTGGCTTCCCATCATATTTT
ACTCTCTGGCTTCCCATCATATTTT
ACTCTCTGGCTTCCCACTATATTTT
ACTCTCTGGCTTCCCATCATATTTT
ACTCTCTGGCTTCCCACTATATTTT
ACTCTCTGGCTTCCCATCATATTTT
ACTCTCTGGCTTCCCACTATATTTT
ACTCTCTGGCTTCCCATCATATTTT
ACTCTCTGGCTTCCCATCATATTTT
ACTCTCTGGCTTCCCATCATATTTT
ACTCTCTGGCTTCCCACTATATTTT
ACTCTCTGGCTTCACCATATATTTT
ACTCTCTGGCTTCCCATCATATTTT
ACTCTCTGGCTTCCCACTATATTTT
ACTCTCTGGCTTCCCATCATATTTT
ACTCTCTGGCTTCCCACTATATTTT
ACTCTCTGGCTTCCTATCATATTTT
ACTCTCTGGCTTCCCATCATATTTT
ACTCTCTGGCTTCCCATCATATTTTTTTTTTTTTTTTTTTTTTATTTT
ACTCTCTGGCTTCCCATCATATTTT
ACTCTCTGGCTTCCCATCATATTTT
ACTCTCTGGCTTCCCATCATATTTT
ACTCTCTGGCTTCCCACTATATTTT
ACTCTCTGGCTTCCCATCATATTTT
ACTCTCTGGCTTCCCATCATATTTT
ACTCTCTGGCTTCCCATCATATTTT
ACTCTCTGGCTTCCCATCATATTTT
ACTCTCTGGCTTCCCATCATATTTT
ACTCTCTGGCTTCCCACTATATTTT
ACTCTCTGGCTTCCCATCATATTTT
ACTCTCTGGCTTCCCACTATATTTT
ACTCTCTGGCTTCCCATCATATTTT
ACTCTCTGGCTTCCCACTATATTTT
ACTCTCTGGCTTCCCATCATATTTT
ACTCTCTGGCTTCCCATCATATTTT
ACTCTCTGGCTTCCCACTATATTTT
ACTCTCTGGCTTCCCACTATATTTT
ACTCTCTGGCTTCCCATCATATTTT
ACTCTCTGGCTTCCCACTATATTTT
ACTCTCTGGCTTCCCATCATATTTT
ACTCTCTGGCTTCCCATCATATTTT
ACTCTCTGGCTTCCCACTATATTTT
ACTCTCTGGCTTCCCATCATATTTT
ACTCTCTGGCTTCCCATCATATTTT
ACTCTCTGGCTTCCCACTATATTTT
ACTCTCTGGCTTCCCACTATATTTT
ACTCTCTGGCTTCCCATCATATTTT

ACTCTCTGGCTTCCCACTATATTTT
ACTCTCTGGCTTCCCACTATATTTT
ACTCTCTGGCTTCCCATCATATTTT
ACTCTCTGGCTTCCCACTATATTTT
ACTCTCTGGCTTCCCATCATATTTT
ACTCTCTGGCTTCCCACTATATTTT
ACTCTCTGGCTTCCCATCATATTTT
ACTCTCTGGCTTCCCACTATATTTT
ACTCTCTGGCTTCCCACTATATTTT
ACTCTCTGGCTTCCCATCATATTTT
ACTCTCTGGCTTCCCACTATATTTT
ACTCTCTGGCTTCCCACTATATTTT
ACTCTCTGGCTTCCCATCATATTTT
ACTCTCTGGCTTCCCACTATATTTT
ACTCTCTGGCTTCCCACTATATTTT
ACTCTCTGGCTTCCCATCATATTTT
ACTCTCTGGCTTCCCACTATATTTT
ACTCTCTGGCTTCCCATCATATTTT
ACTCTCTGGCTTCCCATCATATTTT
ACTCTCTGGCTTCCCATCATATTTT
ACTCTCTGGCTTCCCACTATATTTT
ACTCTCTGGCTTCCCATCATATTTT
ACTCTCTGGCTTCCCATCATATTTT
ACTCTCTGGCTTCCCATCATATTTT
ACTCTCTGGCTTCCCATCATATTTT
ACTCTCTGGCTTCCCATCATATTTT
ACTCTCTGGCTTCCCACTATATTTT
ACTCTCTGGCTTCCCACTATATTTT
ACTCTCTGGCTTCCCATCATATTTT
ACTCTCTGGCTTCCCACTATATTTT
ACTCTCTGGCTTCCCACTATATTTT
ACTCTCTGGCTTCCCATCATATTTT
ACTCTCTGGCTTCCCACTATATTTT
ACTCTCTGGCTTCCCATCATATTTT
ACTCTCTGGCTTCCCATCATATTTT
ACTCTCTGGCTTCCCACTATATTTT
ACTCTCTGGCTTCCCATCATATTTT
ACTCTCTGGCTTCCCATCATATTTT
ACTCTCTGGCTTCCCATCATATTTT
ACTCTCTGGCTTCCCATCATATTTT
ACTCTCTGGCTTCCCATCATATTTT
ACTCTCTGGCTTCCCACTATATTTT
ACTCTCTGGCTTCCCACTATATTTT
ACTCTCTGGCTTCCCATCATATTTT
ACTCTCTGGCTTCCCACTATATTTT
ACTCTCTGGCTTCCCACTATATTTT
ACTCTCTGGCTTCCCATCATATTTT
ACTCTCTGGCTTCCCACTATATTTT
ACTCTCTGGCTTCCCATCATATTTT
ACTCTCTGGCTTCCCACTATATTTT
ACTCTCTGGCTTCCCATCATATTTT
ACTCTCTGGCTTCCCATCATATTTT
ACTCTCTGGCTTCCCACTATATTTT
ACTCTCTGGCTTCCCATCATATTTT

ACTCTCTGGCTTCCCATCATATTTT
ACTCTCTGGCTTCCCATCATATTTT
ACTCTCTGGCTTCCCATCATATTTT
ACTCTCTGGCTTCCCATCATATTTT
ACTCTCTGGCTTCCCACTATATTTT
ACTCTCTGGCTTCCCATCATATTTT
ACTCTCTGGCTTCCCATCATATTTT
ACTCTCTGGCTTCCCATCATATTTT
ACTCTCTGGCTTCCCATCATATTTT
ACTCTCTGGCTTCCCACTATATTTT
ACTCTCTGGCTTCCCATCATATTTT
ACTCTCTGGCTTCCCACTATATTTT
ACTCTCTGGCTTCCCATCATATTTT
ACTCTCTGGCTTCCCATCATATTTT
ACTCTCTGGCTTCCCATCATATTTT
ACTCTCTGGCTTCCCATCATATTTT
ACTCTCTGGCTTCCCACTATATTTT
ACTCTCTGGCTTCCCATCATATTTT
ACTCTCTGGCTTCCCATCATATTTT
ACTCTCTGGCTTCCCACTATATTTT
ACTCTCTGGCTTCCCATCATATTTT
ACTCTCTGGCTTCCCATCATATTTT
ACTCTCTGGCTTCCCACTATATTTT
ACTCTCTGGCTTCCCATCATATTTT
ACTCTCTGGCTTCCCACTATATTTT
ACTCTCTGGCTTCCCATCATATTTT
ACTCTCTGGCTTCCCATCATATTTT
ACTCTCTGGCTTCCCACTATATTTT
ACTCTCTGGCTTCCCATCATATTTT
ACTCTCTGGCTTCCCATCATATTTT
ACTCTCTGGCTTCCCACTATATTTT
ACTCTCTGGCTTCCCATCATATTTT
ACTCTCTGGCTTCCCACTATATTTT
ACTCTCTGGCTTCCCATCATATTTT
ACTCTCTGGCTTCCCATCATATTTT
ACTCTCTGGCTTCCCATCATATTTT
ACTCTCTGGCTTCCCATCATATTTT
ACTCTCTGGCTTCCCATCATATTTT
ACTCTCTGGCTTCCCATCATATTTT
ACTCTCTGGCTTCCCATCATATTTT
ACTCTCTGGCTTCCCACCATATTTT
ACTCTCTGGCTTCCCACCATATTTT
ACTCTCTGGCTTCACCGTATTTTTA
ACTCTCTGGCTTCACGGAATGTTTT
ACTCTCTGGCTTCACCGTATATTTT
ACTCTCTGGCTTCACAGAATCTTTT
Nr of selected repeats 537 Similarity 0.870368
Consensus:
ACTCTCTGGCTTCCCAtcATATTTT
>Cele-UNSB01_3:3480039-3480224 Satlength=186 Nr of Repeats=12 RepeatLength=12
seed=TAGGCTTAGG
TAGGCTTAGGCT
TAGGTTTAGGCTTAGGCCCAGTCTTAGACG
TAGGCTCAGGCT

TAGTCTTAGGCTTCGTCT
TAGGATTAGGTT
TAGGCTTAGGCT
TAGGCTTAGGCT
TAGTCTTAGGCT
TAGGCTTAGGCT
TAGGCTTAGGCT
TAGGCTTAGGCT
TAGGCTTAGGCTTAGGCTTACCCATGACG
Nr of selected repeats 9 Similarity 0.901235
Consensus:
TAGGCTTAGGCT
>Cele-UNSB01_3:3518362-3520167 Satlength=1806 Nr of Repeats=19 RepeatLength=95
seed=TAATCCTATT
TAATCCTATTCCAAGCAGATTGTTATTCGCTCAAGCCTACAAATCAGGTTTGAGGGCCGCAGGCCCGAATACTCTTGGA
AACTGTGGGCCAGCTT
TAATCCTATTCCAATCAGATTGTTATTCGCTCAAGCCTACAAATCAGGTTTGAGGGCCGCAGGCCCGAACACTCTTGGA
AACTGTGGGCCAGCTT
TAATCCTATTCCAAGCAGATTGTTATTCGCTCAAGCCTACAAATCAGGTTTGAGGGCCGCAGGCCCGAACACTCTTGGA
AACTGTGGGCCAGCTT
TAATCCTATTCCAAGCAGATTGTTATTCGCTCAAGCCTACAAACCAGGTTTGAGGGCCGCAGGCCCGAATACTCATGGA
AACTAAGGGCCAGCTT
TAATCCTATTCCAAGCAGATTGTTATTCGCTCAAGCCTACAAACCAGGTTTGAGGGCCGCAGGCCCGAATACTCATGGA
AACTGTGGGCCAGCTT
TAATCCTATTCCAAGCAGATTGTTATTCGCTCAAGCCTACAAATCAGGTTTGAGGGCCGCAGGCCCGAACACTCTTGGA
AACTGTGGGCCAGCTT
TAATCCTATTCCAAGCAGATTGTTATTCGCTCAAGCCTACAAACCAGGTTTGAGGGCCGCAGGCCCGAATACTCATGGA
AACTAAGGGCCAGCTT
TAATCCTATTCCAAGCAGATTGTTATTCGCTCAAGCCTACAAACCAGGTTTGAGGGCCGCAGGCCCGAATACTCATGGA
AACTGTGGGCCAGCTT
TAATCCTATTCCAAGCAGATTGTTATTCGCTCAAGCCTACAAACCAGGGTTGAGGGCCGCAGGCCCGAACACTCTTGGA
AACTGTGGGCCAGCTT
TAATCCTATTCCAAGCAGATTGTTATTCGCTCAAGCCTACAAACCAGGTTTGAGGGCCGCAGGCCCGAATACTCATGGA
AACTAAGGGCCAGCTT
TAATCCTATTCCAAGCAGATTGTTATTCGCTCAAGCCTACAAACCAGGTTTGAGGGCCGCAGGCCCGAATACTCATGGA
AACTGTGGGCCAGCTT
TAATCCTATTCCAAGCAGATTGTTATTCGCTCAAGCCTACAAACCAGGGTTGAGGGCCGCAGGCCCGAACACTCTTGGA
AACTGTGGGCCAGCTT
TAATCCTATTCCAAGCAGATTGTTATTCGCTCAAGCCTACAAACCAGGTTCGAGGGCCGCAGGCCCGAATACTCATGGA
AACTAAGGGCCAGCTT
TAATCCTATTCCAAGCAGATTGTTATTCGCTCAAGCCTACAAACCAGGTTTGAGGGCCGCAGGCCCGAATACTCATGGA
AACTGTGGGCCAGCTT
TAATCCTATTCCAAGCAGATTGTTATTCGCTCAAGCCTACAAACCAGGGTTGAGGGCCGCAGGCCCGAACACTCTTGGA
AACTGTGGGCCAGCTT
TAATCCTATTCCAAGCAGATTGTTATTCGCTCAAGCCTACAAACCAGGTTTGAGGGCCGCAGGCCCGAATACTCATGGA
AACTAAGGGCCAGCTT
TAATCCTATTCCAAGCAGATTGTTATTCGCTCAAGCCTACAAACCAGGTTTGAGGGCCGCAGGCCCGAATACTCATGGA
AACTGTGGGCCAGTTT
TAATCCTATTCCAAGCAGATTGTTATTCGCTCAAGCCTACAAACCAGGGTTGAGGGCCGCAGGCCCGAACACTCTTGGA
AACTAAGGGCCAGCTT
TAATCCTATTTCAAGCAGATTGTTATTTGCTCAAGCATACCAACCAAGTTTGAGGGCCGCAGGCCCGAATACTCATGGA
AACTAAGGGCCAGCTG
Nr of selected repeats 19 Similarity 0.948948
Consensus:

TAATCCTATTCCAAGCAGATTGTTATTCGCTCAAGCCTACAAAcCAGGtTTGAGGGCCGCAGGCCCGAAtACTCaTGGA
AACTgtGGGCCAGCTT
>Cele-UNSB01_3:3579672-3580411 Satlength=740 Nr of Repeats=47 RepeatLength=15
seed=GTGGTGAGAC
GTGGTGAGACCCATC
GTGGTGAGACCCGTC
GTGGTGAGACCTTTC
GTGGTGAGACCCTTC
GTGGCGAGACCCATC
GTGGTGAGACCCATA
GTGGTGAGGCCTTTC
GTGGTGAGACCCTTC
GTGGTGAGACCATTC
GTGGTGAGATCCATC
GTGATGAGACCTTTC
GTGGCGAGACCCATA
GTGGTGAGACCTTTC
GTGGTGAGACCCTTC
GTGGTGAGACCCTTC
GTGGCGAGACCCATC
GTGGTGAGACCTTTC
GTGGTGAGACCCTTC
GTGGTGAGACCCTTC
GTGGTGAGACCTTTC
GTGGTGAGACCCTTC
GTGGCGAGACCCATC
GTGGTGAGACCTTTCTAGGTGAGACCATTC
GTGGTGAGACCCATC
GTGATGAGACCTTTC
GTGGCGAGACCCATA
GTGGTGAGACCTTTC
GTGGTGAGACCCTTC
GTGGTGAGACCTTTC
GTGGTGAGACCCTTC
GTGGCGAGACCCATC
GTGGTGAGACCATTCTAGGTGAGACCATTC
GTGGTGAGACCCATC
GTGGTGAGACCTTTC
GTGGCGAGACCCATC
GTGGTGAGACCCATA
GTGGTGAGGCCTTTC
GTGGTGAGACCCTTC
GTGGTGAGACCCTTC
GTGGCGAGACCCATC
GTGGTGAGACCCATA
GTGGTGAGAGCTTTC
GTCGTGAGACCATTC
GTGGTGAGACCTTTC
GTGGTGATACCCGTTGTTC
GTGGTGAGACCTTTC
GTGGTGAGACCTTTC
Nr of selected repeats 44 Similarity 0.837350
Consensus:
GTGGTGAGACCctTC

>Cele-UNSB01_3:3676441-3676612 Satlength=172 Nr of Repeats=5 RepeatLength=34
seed=AATTGAAATT
AATTGAAATTTCCGGCAAATCGACAAATTGCGGG
AATTGGAAATTTACGGCAAATCGGCAAGTTGCCGG
AATTGAAATTTCCTGCAAAGCGGCAAATTGCCGA
AATTGAAATTTCCGGCAAATCGGCAAGTTGTCGG
AATTGAAATTTCCTGTAAAGCGGCAAATTGTCGG
Nr of selected repeats 4 Similarity 0.800794
Consensus:
AATTGAAATTTCCgGcAAAgCGgCAAaTTGtCGg
>Cele-UNSB01_3:3780853-3780925 Satlength=73 Nr of Repeats=4 RepeatLength=16
seed=CCTACATACC
CCTACATACCTACATA
CCTACATACCTACATA
CCTACATATCTACAAA
CCTACATACCTACATACCTACATA
Nr of selected repeats 3 Similarity 0.888889
Consensus:
CCTACATAcCTACAtA
>Cele-UNSB01_3:3795055-3795458 Satlength=404 Nr of Repeats=8 RepeatLength=35
seed=TTCCGGCAAA
TTCCGGCAAATCGGCAAATTGCCGGAATTGAATTTTCCGGCACTTTGACGGGATTGAAAAT
TTCCGGCAAATCGACAATTTTCCGAAAATGAAAAT
TTCCAGCAAATCGGCAAACTGCCGGAATCGAAAAT
TTCCGGCAAATCGGCAAATTGCCGGAATTGAAAATTTATGGCAAATCGGCAAATTGCCGGAATTGAAGTTTCGGGAAAA
TCGGTAATTTTGTTGAAATGGAAAAA
TTCCGGCAAATCGGCAAGTTGCCGGAATTGAAAAT
TTCCGGCAAACCGACAAATTGCCGGAATTTAAAAG
TTCCGGCAAATCGGAAGATTGCCGGAATTTGAAAT
TTCCGGCAAATTACCGGAATTAAAATTTCGGGAAAGTCGGCAATTTTGCCGAAAAGGAAAAT
Nr of selected repeats 5 Similarity 0.760000
Consensus:
TTCCgGCAAAtCGgcAaatTgCCGgAAttgaAAAt
>Cele-UNSB01_3:3799869-3800537 Satlength=669 Nr of Repeats=5 RepeatLength=65
seed=AAGACACTAA
AAGACACTAATAAAATATTATTTTTTATTCGTCTTTTCCAAATCAGTGTGTCCATTTTTAAAGTGTCTACTGGAAGCTT
CATTTTTTGAATTCCATAAATTAAAATTTCATTTCCAAATCAGTGTGTCCACTTTTAAAGTGTCCACTGGAAGCTTCAT
TTTTTGAACTCCAGAAATTTAAGTTTCATTTCCAAATCAGTGTCCCGTGGTGGAAAAATTTTTTTTCCTTTTTTTTGG
GATGAAAAAATTCGCAAAAAAAATTTTTCATCCCAGAAAAAAAAGGGGGAAAAAAGGTTCAGAAAAAACAAACAAACAT
TTTTGTCGCTGGATGGACTTTAGTCCAACAAAGGTAACGAGGGTTTGAAATGATTTTGAAAAAAAAGTAAAATAAAATT
TTTGATGAAAATTA
AAGACACTAATAAAAATATTATTTTTATTCGTCTTTTAATTTTACAAAATTTTCAATAAAAATTT
AAGACACTAATAAAAATATTATTTTTATTCGTCTTATAATTTTAGAAAATTTTCAATAAAAATTT
AAGACACTAATAAAATATTATTTTTATTCGTCTTTTAATTTTAGAAAATTTTCAATAAAAATTA
AAGACACTAATAAAAATATGATTTTTATTCGTCTTTTAATTTTAGAAAATTTTCAATAAAAATTA
Nr of selected repeats 3 Similarity 0.945299
Consensus:
AAGACACTAATAAAAATATtATTTTTATTCGTCTTtTAATTTTAgAAAATTTTCAATAAAAATTt
>Cele-UNSB01_3:3818464-3818544 Satlength=81 Nr of Repeats=5 RepeatLength=12
seed=TAAGCCTAAG
TAAGCCTAAGCTTGTTGGAAAATATG
TAAGCCTAAGCC
TAAGCCAAAGCG
TAAGCCTAAACC

TAAGCCTAAACCTATGAC
Nr of selected repeats 3 Similarity 0.777778
Consensus:
TAAGCCtAAgCc
>Cele-UNSB01_3:3873879-3874448 Satlength=570 Nr of Repeats=9 RepeatLength=30
seed=CCTTGTAGGC
CCTTGTAGGCAAGGAGGAAGACGTAGGTCACCTTGAAAACTGCAAAAAGTAGGCCG
CCTTGTAGGTAAGGAGGTAGGTGTAGGCCG
CCTTGTAGGCAGAGAGATAGGCGTAGGTCG
CCTTGTAGGCAAGGAGTTAGGCGTAGGTCA
CCTTGTAGGCAAGGAGGTAGGAGTAGGTCGAGTTGTAGGCAAGGAGGTAGGCGTAGGTCG
CCTTGTAGGCAAGGAGGAAGGCGTAGGTCA
CCTTGTAGGCAGAGAGGTAGGCGCACGGCCCGGCAAGTGGTACATCCATGCAAATGCGCTCTACTGATAATTTGAGTGT
AGACCAGGTTTGGGCGCGTGATAACGAAAAAAGCTTTGGTCCAAAAAAATTTAGAATTTAATTTCGGACATTTTTTATA
TGCATCACAAAAAAACTGGACCAACCGTTTTTGAGATACACGCGCCCAAACGTCCAGGTATACGGTAGACAAATTGCGT
ACAGGTACCACTGCTCGGGCCGTGAGGCGTAGGTAG
CCTTGTAGGCAAGGAGGTAGGCGTAGGTCA
CCTTGTAGGCCAGGAGGTAGGCGTAGGTCG
Nr of selected repeats 6 Similarity 0.842963
Consensus:
CCTTGTAGGCAAGGAGgTAGGCGTAGGTCa
>Cele-UNSB01_3:3880760-3880928 Satlength=169 Nr of Repeats=10 RepeatLength=16
seed=TAGGCATGTA
TAGGCATGTAGGCATG
TAGACATGTAGGCATG
TAGACATGTAGGCATG
TAGGCATGAAGGTACG
TAGGCATGTAGGCATG
TAGGCATGTAGGCATG
TAGGCATGTAGGCATG
TAGGCATGTAGGCATG
TAGGCATGTAGGCATG
TAGGCATGTAGGCATGTAGGCATG
Nr of selected repeats 9 Similarity 0.912037
Consensus:
TAGgCATGTAGGCATG
>Cele-UNSB01_3:3928597-3928907 Satlength=311 Nr of Repeats=5 RepeatLength=18
seed=TGCAGCACTA
TGCAGCACTAATTTCCAGGCTCTTTTTTAA
TGCAGCACTATTAGAGAC
TGCAGCACTAATTTTCGAACGGCTATTTTCAATTGAAGATCATTAGTTTTTAGTCACACAAGCTCATCTTTTAAGCAGA
TATTCATTAAAACTGTGTACTTTTGTAACAAGAATGACCCAATAATTTAATTATATTTGTAAAATAATAAGAAATCATC
AAAAATATACAGAAAATCTCCCCAAAATGTCGAAACTATTGAGTGACTTTTTCAAAAGAAAAAGGGGG
TGCAGCACTATTAGAGAC
TGCAGTACTACTGGAGAC
Nr of selected repeats 3 Similarity 0.851852
Consensus:
TGCAGcACTAtTaGAGAC
>Cele-UNSB01_3:4029920-4030220 Satlength=301 Nr of Repeats=8 RepeatLength=40
seed=AGAACCTTCT
AGAACCTTCTGGGAATTTCTGGAAAATTCTAGAATGTTCC
AGAACCTTCTGGAAAATTCTGAAAAGCTTT
AGAACCTTCTGAAAAATTCTAGAAAATTCTGGAATGGTCC
AGAACCTTCTGGAAAATTCGAAAAAATTCTGGAATGTTCC

AGAACCTTCTGGGAATTTCTGGAAAAATTCTAGAATGTTCC
AGAACCTTCTGGAAAAATTCTGAAAAGCTTT
AGAACCTTCTGAAAAAATTCTAGAAAATTCTGGAATGGTCC
AGAACCTTCTAGAAAATTCGAAAAAATTCTGGATTGTTCT
Nr of selected repeats 6 Similarity 0.824444
Consensus:
AGAACCTTCTGgaAAaTTCtagAAAATTCTgGAATGtTCC
>Cele-UNSB01_3:4030293-4031043 Satlength=751 Nr of Repeats=50 RepeatLength=15
seed=CGTGGTGAGA
CGTGGTGAGACCCAT
CGTGGCGAGACCCAC
CGTGGTGAGACCCGT
CGTGGTGAGACCCAT
CGTGGTGAGACCCAT
CGTGGTGAGATCCAT
CGTGGTGAGACCCAT
CGTGGTGAGATCCAT
CGTGGTGAGACCCAT
CGTGGTGAGATCCAT
CGTGGTGAGACCCAT
CGTGGTGAGACCCAT
CGTGGTGAGACCCAT
CGTGGTGAGACCCAT
CGTGGTGAGACCCAT
CGTGGTGAGATCCAT
CGTGGTGAGACCCAT
CGTGGTGAGATCCAT
CGTGGTGAGACCCAT
CGTGGTGAGATCCAT
CGTGGTGAGACCCAT
CGTGCTGAGACCCAT
CGTGGTGAGACCCAT
CGTGGTGAGATCCAT
CGTGGTGAGACCCAT
CGTGGTGAGATCCAT
CGTGGTGAGACCCAT
CGTGGTGAGATCCAT
CGTGGTGAGACCCAT
CGTGGTGAGATCCAT
CGTGGTGAGACCCAT
CGTGGTGAGACCCAT
CGTGGTGAGACCCAT
CGTGGTGAGACCCAT
CGTGGTGAGACCCAT
CGTGGTGAGATCCAT
CGTGGTGAGACCCAT
CGTGGTGAGATCCAT
CGTGGTGAGACCCAT
CGTGGTGAGATCCAT
CGTGGTGAGACCCAT
CGTGCTGAGACCCAT
CGTGGTGAGACCCAT
CGTGGTGAGATCCAT
CGTGGTGAGACCCAT

CGTGGTGAGATCCAT
CGTGGTGAGACCCAT
CGTGGTGAGATCCAT
CGTGGTGAGACCCAT
CGTGGTGAGACCCGT
Nr of selected repeats 50 Similarity 0.939483
Consensus:
CGTGGTGAGAcCCAT
>Cele-UNSB01_3:4031086-4031330 Satlength=245 Nr of Repeats=7 RepeatLength=35
seed=AATTTAAATT
AATTTAAATTTTCTGTGAAAAATATTTTGGCGGGA
AATTTAAATTTTCTGTGAGAAATATTTTGGCGGGA
AATTTAAATTTTCTGAAAAAAATATTTTAACGGGA
AATTTAAATTTTCTGTAAAAAATATTTTGGCGGGT
AATTTAAATTTTCGGAGAAAAAATTTTGGAGGGA
AATTTAAATTTTCTGAAAAAAATATTTTGGTGGGA
AATTTAAATTTTCTGAGAAAAATATTTTGGCGGGA
Nr of selected repeats 6 Similarity 0.890794
Consensus:
AATTTAAATTTTCTGaaAAAAATATTTTGGCGGGA
>Cele-UNSB01_3:4031404-4032964 Satlength=1561 Nr of Repeats=39 RepeatLength=40
seed=GTTCCAGAAC
GTTCCAGAACTTTCTAGAAAAAATCGGGAAAAATTCTGGAAT
GTTCCAGAACTTTCTAGAAAAAATCGAGAAAAATTCTGGAAT
GTTCCAGAACTTTCTAGAAAAAATCGAGAAAAATTCTGGAAT
GTTCCAGAACTTTCTAGAAAAAATCGAGAAAAATTCTGGAGT
GTTCCAGAACTTTCTAAAAAAAATCGAGAAAAATTCTGGAAC
GTTCCAGAACTTTCTAGAAAAAATCGAGAAAGTTCTGGAGT
GTTCCAGAACTTTCTAGAAAAAATCGAGAAAAATTCTGGAAT
GTTCCAGAACTTTCTAGAAAAAATCGAGAAAAATTCTGGAGT
GTTCCAGAACTTTCTAGAAAAAATCGAGAAAGTTCTGGAGT
GTTCCAGAACTTTCTAGAAAAAATCGAGAAAAATTCTGGAAT
GTTCCAGAACTTTCTAGAAAAAATCGAGAAAAATTCTGGAGT
GTTCCAGAACTTTCTAGAAAAAATCGAGAAAAATTCTGGAAT
GTTCCAGAACTTTCTAGAAAAAATCGAGAAAAATTCTGGAAT
GTTCCAGAACTTTCTAGAAAAAATCGAGAAAAATTCTGGAAC
GTTCCAGAACTTTCTAGAAAAAATCGAGAAAACTCTGGAGT
GTTCCAGAACTTTCTAAAAAAAATCGAGAAAAATTCTGGAAT
GTTCCAGAATTTTCTAGAAAAAATCGAGAAAAATTCTGGAGT
GTTCCAGAACTTTCTAGAAAAAATCGAGAAAAATTCTGGAAT
GTTCCAGAACTTTCTAGAAAAAATCGAGAAAAATTCTGGAAT
GTTCCAGAACTTTCTAGAAAAAATCGAGAAAAATTCTGGAAT
GTTCCAGAACTTTCTAGAAAAAATCGAGAAAAATTCTGGAGT
GTTCCAGAACTTTCTAGAAAAAATCGAGAAAGTTCTGGAGT
GTTCCAGAACTTTCTAGAAAAAATCGAGAAAAATTCTGGAAT
GTTCCAGAACTTTCTAGAAAAAATCGAGAAAAATTCTGGAGT
GTTCCAGAACTTTCTAGAAAAAATCGAGAAAAATTCTGGAAT
GTTCCAGAACTTTCTAGAAAAAATCGAGAAAAATTCTGGAAT
GTTCCAGAACTTTCTAGAAAAAATCGAGAAAAATTCTGGAAC
GTTCCAGAACTTTCTAAAAAAAATCGAGAAAAATTCTGGAAT
GTTCCAGAACTTTCTAGAAAAAATCGAGAAAAATTCTGGAGT
GTTCCAGAACTTTCTAGAAAAAATCGAGAAAAATTCTGGAAT
GTTCCAGAACTTTCTAGAAAAAATCGAGAAAAATTCTGGAAT
GTTCCAGAACTTTCTAGAAAAAATCGAGAAAACTCTGGAGT

GTTCCAGAACTTTCTAAAAAAAATCGAGAAAGTTCTGGAAC
GTTCCAGAACTTTCTAGAAAAAATCGAGAAAAATTCTGGAAT
GTTCCAGAACTTTCTAGAAAAAATTGGGAAAAATTCTGGAAT
GTTCCTGAACTTTCTTGAAAAATCGAAAAAAATTCTCGAAT
GTTCCAGAACTTTCTAGAAAAAATTGGGAAATTTCTGGAAT
GTTCCAGAACTTTCTAGAAAAAATCGAGAAAAATTCTGGAAC
GTTCCAGAACTTTCTAGAAAAAATCGAGAAAAATTCTGGAAC
Nr of selected repeats 39 Similarity 0.942330
Consensus:
GTTCCAGAACTTTCTAGAAAAAATCGAGAAAAATTCTGGAaT
>Cele-UNSB01_3:4122367-4122965 Satlength=599 Nr of Repeats=31 RepeatLength=19
seed=AAAAATAGAT
AAAAATAGATTGTTACACGA
AAAAATAGATTGTTACACG
AAAAATAGATTGTTACACGA
AAAAATAGATTGTTACACGA
AAAAATAGATTGTTACACG
AAAAATAGATTGTTACACG
AAAAATAGATTGTTACACG
AAAAATAGATTGTTACACG
AAAAATAGATTGTTACACG
AAAAATAGATTGTTACACG
AAAAATAGATTGTTACACGA
AAAAATAGATTGTTACACG
AAAAATAGATTGTTACACG
AAAAATAGATTGTTACACG
AAAAATAGATTGTTACACG
AAAAATAGATTGTTACACG
AAAAATAGATTGTTACACG
AAAAATAGATTGTTACACG
AAAAATAGATTGTTACACG
AAAAATAGATTGTTACACGA
AAAAATAGATTGTTACACGA
AAAAATAGATTGTTACACG
AAAAATAGATTGTTACACG
AAAAATAGATTGTTACACG
AAAAATAGATTGTTACACG
AAAAATAGATTGTTACACGA
AAAAATAGATTGTTACACG
AAAAATAGATTGTTACACGA
AAAAATAGATTGTTACACG
AAAAATAGATTGTTACACG
AAAAATAGATTGTTACACGA
Nr of selected repeats 22 Similarity 1.000000
Consensus:
AAAAATAGATTGTTACACG
>Cele-UNSB01_3:4344679-4344767 Satlength=89 Nr of Repeats=4 RepeatLength=22
seed=CAAAACCCAA
CAAAACCCAAGTAGATCCCACT
CAAAACCCAAGTAGATCCCACT
CAAAACCCAAGTAGATCCCACT
CAAAACCCAAGCGGATCCCACT
Nr of selected repeats 4 Similarity 0.939394
Consensus:

CAAAACCCAAGtaGATCCCACT
>Cele-UNSB01_3:4389614-4389732 Satlength=119 Nr of Repeats=7 RepeatLength=17
seed=AGGGGGCATC
AGGGGGCATCGAGAG
AGGGGGCATCGCGTTCTA
AGGGGGCATTAGGTCAA
AGGGGGCATTTGTTCGT
AGGGGGCATCCGTTCAG
AGGGGGCATCCATTCAG
AGGGGGCATCCGTTCAG
Nr of selected repeats 5 Similarity 0.749020
Consensus:
AGGGGGCATccgtTCag
>Cele-UNSB01_3:4392502-4392620 Satlength=119 Nr of Repeats=7 RepeatLength=17
seed=GATGCCCCCT
GATGCCCCCTCTGAACG
GATGCCCCCTCTGAATG
GATGCCCCCTCTGAACG
GATGCCCCCTACGAACAA
ATGCCCCCTTTGACCTA
ATGCCCCCTTAGAACGC
GATGCCCCCTCTCTC
Nr of selected repeats 5 Similarity 0.729630
Consensus:
gATGCCCCCTctGAacg
>Cele-UNSB01_3:4778835-4779093 Satlength=259 Nr of Repeats=5 RepeatLength=17
seed=ATTATTTTCA
ATTATTTTCATTCCGTG
ATTATTTTCATTCCGTG
ATTATTTTCATTCCGTG
ATTATTTTCATTCCGTG
ATTATTTTCACTCGGGAATCGCTCGCCCACTATGGGGGAGTCTACGCAAGGACAACGCAAGGACAAGGACAACATTCTA
ATGGAATGGAAACAATTGCCCGACTGCACCAATTCTAGTTCAAGCGAACAATGATAACTTTTGTATTCTGTATTCCTTC
ACGTCTCCCAGCGAGCGTAATAAATTATTATT
Nr of selected repeats 4 Similarity 1.000000
Consensus:
ATTATTTTCATTCCGTG
>Cele-UNSB01_3:5426163-5426753 Satlength=591 Nr of Repeats=28 RepeatLength=20
seed=CTAGGTCTGC
CTAGGTCTGCCTAGGGTAGGCTATGGTAGG
CTAGGTCTGCCTAGGGTAGG
CTAGGTCTGCCCAGGGTAGA
CTAGGTCTGCCTGAGGTAGG
CTAGGTCTGCCTAGGGTAGG
CTAGGTCTGCCTAGGGTAGA
CTAGGTCTGCCTAGGGTAGA
CTAGGTCTGCCTGAGGTAGG
CTAGGTCTGCCTAGGGTAGGCTATGGTAGG
CTAGGTCTGCCTAGGGTAGG
CTAGGTCTGCCCAGGGTAGA
CTAGGTCTGCCTGAGGTAGG
CTAGGTCTGCCTAGGGTAGGCTATGGTAGG
CTAGGTCTGCCTAGGGTAGG
CTAGGTCTGCCCAGGGTAGA

CTAGGTCTGCCTGAGGTAGG
CTAGGTCTGCCTAGGGTAGG
CTAGGTCTGCCTAGGGTAGA
CTAGGTCTGCCTAGGGTAGA
CTAGGTCTGCCTGAGGTAGG
CTAGGTCTGCCTAGGGTAGG
CTAGTTCTGCCTAGGGTAGA
CTAGGTCTGCCTAGGGTAGA
CTAGGTCTGCCTGAGGTAGG
CTAGGTCTGCCTAGGGTAGG
CTAGGTCTGCCCAGAGCGGG
CTAGGTCTGCCTGGGGTAGG
CTAGGTCTGCCTGGGGTAGG
Nr of selected repeats 25 Similarity 0.872444
Consensus:
CTAGGTCTGCCTagGGTAGg
>Cele-UNSB01_3:5426187-5427065 Satlength=879 Nr of Repeats=40 RepeatLength=20
seed=GGTAGGCTAG
GGTAGGCTAGGTCTGCCTAG
GGTAGGCTAGGTCTGCCCAG
GGTAGACTAGGTCTGCCTGA
GGTAGGCTAGGTCTGCCTAG
GGTAGGCTAGGTCTGCCTAG
GGTAGACTAGGTCTGCCTAG
GGTAGACTAGGTCTGCCTGA
GGTAGGCTAGGTCTGCCTAG
GGTAGGCTAT
GGTAGGCTAGGTCTGCCTAG
GGTAGGCTAGGTCTGCCCAG
GGTAGACTAGGTCTGCCTGA
GGTAGGCTAGGTCTGCCTAG
GGTAGGCTAT
GGTAGGCTAGGTCTGCCTAG
GGTAGGCTAGGTCTGCCCAG
GGTAGACTAGGTCTGCCTGA
GGTAGGCTAGGTCTGCCTAG
GGTAGGCTAGGTCTGCCTAG
GGTAGACTAGGTCTGCCTAG
GGTAGACTAGGTCTGCCTGA
GGTAGGCTAGGTCTGCCTAG
GGTAGGCTAGTTCTGCCTAG
GGTAGACTAGGTCTGCCTAG
GGTAGACTAGGTCTGCCTGA
GGTAGGCTAGGTCTGCCTAG
GGTAGGCTAGGTCTGCCCAGAGCGGGCTAGGTCTGCCTGG
GGTAGGCTAGGTCTGCCTGG
GGTAGGCTAGGTCTGCCTGG
GGTAGGCTAGATCTGCCTAGGGCTAGCCTAG
GGTAGGCTAG
GGTAGGCTAT
GGTAGGCTAG
GGTAGGCTAGGTCTACCTGG
GGTAGGCTTGGTTTGCCTTG

GGTAGGGTAGGGCTGCCTAGGAATACCGCTGGAAGAAAATCTCTGATCTGGCGGGGAGCCCGGCTGTAGGGTCTGGGGA
GG
GGTAGACTAGGATCTGGGGTAAGGCTGTCCAG
GGAAGGCTAGGGTCAGGAGTAGGACTGCCTAG
GGTAGGCTAGGGGTAGTGCTGTCTAG
GGTAGGCTAGGGGTAGTGCTGTCTAG
Nr of selected repeats 28 Similarity 0.844293
Consensus:
GGTAGgCTAGGTCTGCCTaG
>Cele-UNSB01_3:5494321-5534864 Satlength=40544 Nr of Repeats=1542
RepeatLength=26 seed=GTTAGAGTCA
GTTAGAGTCATTATTTTAGGTGAATG
GTTAGAGTCATTATTTTAGGTGAATG
GTTAGAGTCATTATTTTAGGTGAATG
GTTAGAGTCATTATTTAGGTGAATG
GTTAGAGTCATTATTTTAGGTGAATG
GTTAGAGTCATATTTTAGGTGAATG
GTTAGAGTCATTATTTTAGGTGAATG
GTTAGAGTCATTTTTTAGGTGAATG
GTTAGAGTCATATTTTAGGTGAATG
GTTAGAGTCATTATTTTAGGTGAATG
GTTAGAGTCATTATTTTAGGTGAATG
GTTAGAGTCATATTTTTAGGTGAATG
GTTAGAGTCATTATTTTAGGTGAATG
GTTAGAGTCATTATTTTAGGTGAATG
GTTAGAGTCATTATTTTAGGTGAATG
GTTAGAGTCATTTTTTAGGTGAATG
GTTAGAGTCATATTTTTAGGTGAATG
GTTAGAGTCATTATTTTAGGTGAATG
GTTAGAGTCATTATTTTAGGTGAATG
GTTAGAGTCATTATTTAGGTGAATG
GTTAGAGTCATTATTTTTAGGTGAATG
GTTAGAGTCATTATTTTAGGTGAATG
GTTAGAGTCATTATTTTAGGTGAATG
GTTAGAGTCATTATTTTAGGTGAATG
GTTAGAGTCATTATTTTAGGTGAATG
GTTAGAGTCATTATTTTAGGTGAATG
GTTAGAGTCATTATTTTAGGTGAATG
GTTAGAGTCATATTTTAGGTGAATG
GTTAGAGTCATATTTTTAGGTGAATG
GTTAGAGTCATTATTTTAGGTGAATG
GTTAGAGTCATTATTTTAGGTGAATG
GTTAGAGTCATTATTTTAGGTGAATG
GTTAGAGTCATTATTTTAGGTGAATG
GTTAGAGTCATTATTTTAGGTGAATG
GTTAGAGTCATTATTTTAGGTGAATG
GTTAGAGTCATTATTTTAGGTGAATG
GTTAGAGTCATTATTTTAGGTGAATG
GTTAGAGTCATTATTTTAGGTGAATG
GTTAGAGTCATTATTTTAGGTGAATG
GTTAGAGTCATTATTTTAGGTGAATG
GTTAGAGTCATTATTTTAGGTGAATG
GTTAGAGTCATTATTTTAGGTGAATG
GTTAGAGTCATTATTTTAGGTGAATG

GTTAGAGTCATTATTTTAGGTGAATG
GTTAGAGTCATTATTTTAGGTGAATG
GTTAGAGTCATTATTTTAGGTGAATG
GTTAGAGTCATTATTTTAGGTGAATG
GTTAGAGTCATTATTTTAGGTGAATG
GTTAGAGTCATATTTTTAGGTGAATG
GTTAGAGTCATTATTTTAGGTGAATG
GTTAGAGTCATTATTTTAGGTGAATG
GTTAGAGTCATTATTTTAGGTGAATG
GTTAGAGTCATTATTTTAGGTGAATG
GTTAGAGTCATTATTTTAGGTGAATG
GTTAGAGTCATTATTTTAGGTGAATG
GTTAGAGTCATTATTTTAGGTGAATG
GTTAGAGTCATTATTTTAGGTGAATG
GTTAGAGTCATTATTTTAGGTGAATG
GTTAGAGTCATTATTTTAGGTGAATG
GTTAGAGTCATTATTTTAGGTGAATG
GTTAGAGTCATTATTTTAGGTGAATG
GTTAGAGTCATATTTTTAGGTGAATG
GTTAGAGTCATTATTTTAGGTGAATG
GTTAGAGTCATTATTTTAGGTGAATG
GTTAGAGTCATTATTTTAGGTGAATG
GTTAGAGTCATTATTTTAGGTGAATG
GTTAGAGTCATTATTTTAGGTGAATG
GTTAGAGTCATATTTTTAGGTGAATG
GTTAGAGTCATTATTTTAGGTGAATG
GTTAGAGTCATTATTTTAGGTGAATG
GTTAGAGTCATTATTTTAGGTGAATG
GTTAGAGTCATTATTTTAGGTGAATG
GTTAGAGTCATTATTTTAGGTGAATG
GTTAGAGTCATATTTTTAGGTGAATG
GTTAGAGTCATTATTTTAGGTGAATG
GTTAGAGTCATTATTTTAGGTGAATG
GTTAGAGTCATTATTTTAGGTGAATG
GTTAGAGTCATTATTTTAGGTGAATG
GTTAGAGTCATTATTTTAGGTGAATG
GTTAGAGTCATTATTTTAGGTGAATG
GTTAGAGTCATTATTTTAGGTGAATG
GTTAGAGTCATATTTTAGGTGAATG
GTTAGAGTCATTATTTTAGGTGAATG
GTTAGAGTCATTATTTTAGGTGAATG
GTTAGAGTCATTATTTTAGGTGAATG
GTTAGAGTCATTATTTTAGGTGAATG
GTTAGAGTCATTATTTTAGGTGAATG
GTTAGAGTCATATTTTAGGTGAATG
GTTAGAGTCATTATTTTAGGTGAATG
GTTAGAGTCATTATTTTAGGTGAATG
GTTAGAGTCATTATTTTAGGTGAATG
GTTAGAGTCATTATTTTAGGTGAATG
GTTAGAGTCATTATTTTAGGTGAATG
GTTAGAGTCATTATTTTAGGTGAATG
GTTAGAGTCATTATTTTAGGTGAATG
GTTAGAGTCATTATTTTTAGGTGAATG
GTTAGAGTCATTATTTTAGGTGAATG

GTTAGAGTCATTATTTTAGGTGAATG
GTTAGAGTCATTATTTTAGGTGAATG
GTTAGAGTCATTATTTTAGGTGAATG
GTTAGAGTCATTATTTTAGGTGAATG
GTTAGAGTCATTATTTTAGGTGAATG
GTTAGAGTCATTATTTTAGGTGAATG
GTTAGAGTCATTATTTTAGGTGAATG
GTTAGAGTCATTATTTTAGGTGAATG
GTTAGAGTCATTATTTTAGGTGAATG
GTTAGAGTCATTATTTTAGGTGAATG
GTTAGAGTCATTATTTTAGGTGAATG
GTTAGAGTCATTATTTTTAGGTGAATG
GTTAGAGTCATTATTTTAGGTGAATG
GTTAGAGTCATTATTTTAGGTGAATG
GTTAGAGTCATTATTTTAGGTGAATG
GTTAGAGTCATATTTTAGGTGAATG
GTTAGAGTCATTATTTTAGGTGAATG
GTTAGAGTCATATTTTAGGTGAATG
GTTAGAGTCATTATTTTAGGTGAATG
GTTAGAGTCATTATTTTAGGTGAATG
GTTAGAGTCATTATTTTAGGTGAATG
GTTAGAGTCATTATTTTAGGTGAATG
GTTAGAGTCATTATTTTAGGTGAATG
GTTAGAGTCATTATTTTAGGTGAATG
GTTAGAGTCATTATTTTAGGTGAATG
GTTAGAGTCATTATTTTAGGTGAATG
GTTAGAGTCATTATTTTAGGTGAATG
GTTAGAGTCATTATTTTAGGTGAATG
GTTAGAGTCATTATTTTAGGTGAATG
GTTAGAGTCATTATTTTAGGTGAATG
GTTAGAGTCATATTTTAGGTGAATG
GTTAGAGTCATTATTTTAGGTGAATG
GTTAGAGTCATTATTTTAGGTGAATG
GTTAGAGTCATTATTTTAGGTGAATG
GTTAGAGTCATTATTTTAGGTGAATG
GTTAGAGTCATTATTTTAGGTGAATG
GTTAGAGTCATTATTTTAGGTGAATG
GTTAGAGTCATATTTTTAGGTGAATG
GTTAGAGTCATATTTTAGGTGAATG
GTTAGAGTCATTATTTTAGGTGAATG
GTTAGAGTCATTATTTTAGGTGAATG
GTTAGAGTCATTATTTTAGGTGAATG
GTTAGAGTCATTATTTTAGGTGAATG
GTTAGAGTCATTATTTTAGGTGAATG
GTTAGAGTCATTATTTTAGGTGAATG
GTTAGAGTCATTATTTTAGGTGAATG
GTTAGAGTCATTATTTTAGGTGAATG
GTTAGAGTCATTATTTTAGGTGAATG
GTTAGAGTCATTATTTTAGGTGAATG
GTTAGAGTCATTATTTTAGGTGAATG
GTTAGAGTCATTATTTTAGGTGAATG
GTTAGAGTCATTATTTTAGGTGAATG
GTTAGAGTCATTATTTTAGGTGAATG
GTTAGAGTCATTATTTTAGGTGAATG

GTTAGAGTCATATTTTAGGTGAATG
GTTAGAGTCATTATTTTAGGTGAATG
GTTAGAGTCATATTTTTAGGTGAATG
GTTAGAGTCATTATTTTAGGTGAATG
GTTAGAGTCATTATTTTAGGTGAATG
GTTAGAGTCATTATTTTAGGTGAATG
GTTAGAGTCATATTTTTAGGTGAATG
GTTAGAGTCATATTTTTAGGTGAATG
GTTAGAGTCATATTTTAGGTGAATG
GTTAGAGTCATATTTTTAGGTGAATG
GTTAGAGTCATTATTTTAGGTGAATG
GTTAGAGTCATATTTTTAGGTGAATG
GTTAGAGTCATTATTTTAGGTGAATG
GTTAGAGTCATTATTTTAGGTGAATG
GTTAGAGTCATATTTTAGGTGAATG
GTTAGAGTCATTATTTTAGGTGAATG
GTTAGAGTCATTATTTTAGGTGAATG
GTTAGAGTCATATTTTAGGTGAATG
GTTAGAGTCATTATTTTAGGTGAATG
GTTAGAGTCATTATTTTAGGTGAATG
GTTAGAGTCATTATTTTAGGTGAATG
GTTAGAGTCATTATTTTAGGTGAATG
GTTAGAGTCATTATTTTAGGTGAATG
GTTAGAGTCATTATTTTAGGTGAATG
GTTAGAGTCATTATTTTAGGTGAATG
GTTAGAGTCATTATTTTAGGTGAATG
GTTAGAGTCATTATTTTAGGTGAATG
GTTAGAGTCATTATTTTAGGTGAATG
GTTAGAGTCATTATTTTAGGTGAATG
GTTAGAGTCATTATTTTAGGTGAATG
GTTAGAGTCATTATTTTAGGTGAATG
GTTAGAGTCATATTTTAGGTGAATG
GTTAGAGTCATTATTTTAGGTGAATG
GTTAGAGTCATTATTTTAGGTGAATG
GTTAGAGTCATATTTTAGGTGAATG
GTTAGAGTCATTATTTTAGGTGAATG
GTTAGAGTCATTATTTTAGGTGAATG
GTTAGAGTCATATTTTTAGGTGAATG
GTTAGAGTCATTATTTTAGGTGAATG
GTTAGAGTCATATTTTTAGGTGAATG
GTTAGAGTCATTATTTTAGGTGAATG
GTTAGAGTCATATTTTAGGTGAATG
GTTAGAGTCATTATTTTAGGTGAATG
GTTAGAGTCATTATTTTAGGTGAATG
GTTAGAGTCATATTTTTAGGTGAATG
GTTAGAGTCATTATTTTAGGTGAATG
GTTAGAGTCATTATTTTAGGTGAATG
GTTAGAGTCATTATTTTAGGTGAATG
GTTAGAGTCATTATTTTAGGTGAATG
GTTAGAGTCATTATTTTAGGTGAATG
GTTAGAGTCATTATTTTAGGTGAATG
GTTAGAGTCATATTTTAGGTGAATG
GTTAGAGTCATTATTTTAGGTGAATG
GTTAGAGTCATATTTTAGGTGAATG

GTTAGAGTCATTATTTTAGGTGAATG
GTTAGAGTCATTATTTTAGGTGAATG
GTTAGAGTCATATTTTTAGGTGAATG
GTTAGAGTCATTATTTTAGGTGAATG
GTTAGAGTCATTATTTTAGGTGAATG
GTTAGAGTCATTATTTTAGGTGAATG
GTTAGAGTCATTATTTTAGGTGAATG
GTTAGAGTCATTATTTTAGGTGAATG
GTTAGAGTCATTATTTTAGGTGAATG
GTTAGAGTCATTATTTTAGGTGAATG
GTTAGAGTCATTATTTTAGGTGAATG
GTTAGAGTCATTATTTTAGGTGAATG
GTTAGAGTCATTATTTTAGGTGAATG
GTTAGAGTCATTATTTTAGGTGAATG
GTTAGAGTCATTTTTTAGGTGAATG
GTTAGAGTCATTATTTTAGGTGAATG
GTTAGAGTCATTATTTTAGGTGAATG
GTTAGAGTCATTATTTTAGGTGAATG
GTTAGAGTCATTATTTTAGGTGAATG
GTTAGAGTCATATTTTAGGTGAATG
GTTAGAGTCATTATTTTAGGTGAATG
GTTAGAGTCATTATTTTAGGTGAATG
GTTAGAGTCATTATTTTAGGTGAATG
GTTAGAGTCATTATTTTAGGTGAATG
GTTAGAGTCATTATTTTAGGTGAATG
GTTAGAGTCATATTTTAGGTGAATG
GTTAGAGTCATTATTTTAGGTGAATG
GTTAGAGTCATATTTTAGGTGAATG
GTTAGAGTCATATTTTAGGTGAATG
GTTAGAGTCATTATTTTAGGTGAATG
GTTAGAGTCATTATTTTAGGTGAATG
GTTAGAGTCATTATTTTAGGTGAATG
GTTAGAGTCATATTTTAGGTGAATG
GTTAGAGTCATTATTTTAGGTGAATG
GTTAGAGTCATTATTTTAGGTGAATG
GTTAGAGTCATTATTTTAGGTGAATG
GTTAGAGTCATTATTTTAGGTGAATG
GTTAGAGTCATATTTTTAGGTGAATG
GTTAGAGTCATATTTTAGGTGAATG
GTTAGAGTCATTATTTTTAGGTGAATG
GTTAGAGTCATTTTTAGGTGAATG
GTTAGAGTCATTATTTTAGGTGAATG
GTTAGAGTCATTATTTTAGGTGAATG
GTTAGAGTCATATTTTAGGTGAATG
GTTAGAGTCATATTTTAGGTGAATG
GTTAGAGTCATATTTTAGGTGAATG
GTTAGAGTCATATTTTTAGGTGAATG
GTTAGAGTCATATTTTTAGGTGAATG
GTTAGAGTCATTATTTTAGGTGAATG
GTTAGAGTCATTATTTTTAGGTGAATG
GTTAGAGTCATTATTTTAGGTGAATG
GTTAGAGTCATATTTTAGGTGAATG
GTTAGAGTCATTATTTTAGGTGAATG
GTTAGAGTCATTATTTTAGGTGAATG

GTTAGAGTCATTATTTTAGGTGAATG
GTTAGAGTCATTATTTTAGGTGAATG
GTTAGAGTCATTATTTTAGGTGAATG
GTTAGAGTCATATTTTAGGTGAATG
GTTAGAGTCATATTTTAGGTGAATG
GTTAGAGTCATATTTTAGGTGAATG
GTTAGAGTCATTTTTAGGTGAATG
GTTAGAGTCATTATTTTAGGTGAATG
GTTAGAGTCATATTTTAGGTGAATG
GTTAGAGTCATTATTTTAGGTGAATG
GTTAGAGTCATTATTTTAGGTGAATG
GTTAGAGTCATATTTTAGGTGAATG
GTTAGAGTCATTATTTTAGGTGAATG
GTTAGAGTCATTATTTTAGGTGAATG
GTTAGAGTCATTATTTTAGGTGAATG
GTTAGAGTCATATTTTTAGGTGAATG
GTTAGAGTCATTATTTTAGGTGAATG
GTTAGAGTCATATTTTAGGTGAATG
GTTAGAGTCATTATTTTAGGTGAATG
GTTAGAGTCATTATTTTAGGTGAATG
GTTAGAGTCATTATTTTAGGTGAATG
GTTAGAGTCATTATTTTAGGTGAATG
GTTAGAGTCATTATTTTAGGTGAATG
GTTAGAGTCATATTTTTAGGTGAATG
GTTAGAGTCATTATTTTAGGTGAATG
GTTAGAGTCATTATTTTAGGTGAATG
GTTAGAGTCATTATTTTAGGTGAATG
GTTAGAGTCATATTTTTAGGTGAATG
GTTAGAGTCATTATTTTAGGTGAATG
GTTAGAGTCATATTTTAGGTGAATG
GTTAGAGTCATTATTTTAGGTGAATG
GTTAGAGTCATTATTTTAGGTGAATG
GTTAGAGTCATTATTTTAGGTGAATG
GTTAGAGTCATTTTTTAGGTGAATG
GTTAGAGTCATTATTTTAGGTGAATG
GTTAGAGTCATTATTTTAGGTGAATG
GTTAGAGTCATTATTTTAGGTGAATG
GTTAGAGTCATATTTTAGGTGAATG
GTTAGAGTCATTATTTTAGGTGAATG
GTTAGAGTCATTATTTTAGGTGAATG
GTTAGAGTCATTATTTTAGGTGAATG
GTTAGAGTCATATTTTAGGTGAATG
GTTAGAGTCATTATTTTAGGTGAATG
GTTAGAGTCATATTTTAGGTGAATG
GTTAGAGTCATATTTTAGGTGAATG
GTTAGAGTCATTATTTTTAGGTGAATG
GTTAGAGTCATTATTTTAGGTGAATG
GTTAGAGTCATTATTTTAGGTGAATG
GTTAGAGTCATTTTTTAGGTGAATG
GTTAGAGTCATATTTTAGGTGAATG
GTTAGAGTCATTATTTTAGGTGAATG
GTTAGAGTCATTATTTTAGGTGAATG
GTTAGAGTCATTATTTTAGGTGAATG
GTTAGAGTCATTATTTTAGGTGAATG

GTTAGAGTCATTTTTTAGGTGAATG
GTTAGAGTCATATTTTAGGTGAATG
GTTAGAGTCATATTTTAGGTGAATG
GTTAGAGTCATTATTTTAGGTGAATG
GTTAGAGTCATATTTTAGGTGAATG
GTTAGAGTCATTATTTTAGGTGAATG
GTTAGAGTCATTATTTTAGGTGAATG
GTTAGAGTCATATTTTAGGTGAATG
GTTAGAGTCATTATTTTAGGTGAATG
GTTAGAGTCATTATTTTAGGTGAATG
GTTAGAGTCATTATTTTAGGTGAATG
GTTAGAGTCATTATTTTAGGTGAATG
GTTAGAGTCATATTTTAGGTGAATG
GTTAGAGTCATTATTTTAGGTGAATG
GTTAGAGTCATTATTTTAGGTGAATG
GTTAGAGTCATATTTTTAGGTGAATG
GTTAGAGTCATTATTTTAGGTGAATG
GTTAGAGTCATATTTTAGGTGAATG
GTTAGAGTCATTATTTTAGGTGAATG
GTTAGAGTCATTATTTTAGGTGAATG
GTTAGAGTCATTATTTTAGGTGAATG
GTTAGAGTCATATTTTAGGTGAATG
GTTAGAGTCATTATTTTAGGTGAATG
GTTAGAGTCATTATTTTAGGTGAATG
GTTAGAGTCATTATTTTAGGTGAATG
GTTAGAGTCATTATTTTAGGTGAATG
GTTAGAGTCATTATTTTTAGGTGAATG
GTTAGAGTCATTATTTTAGGTGAATG
GTTAGAGTCATTATTTTAGGTGAATG
GTTAGAGTCATTATTTTAGGTGAATG
GTTAGAGTCATTATTTTAGGTGAATG
GTTAGAGTCATATTTTAGGTGAATG
GTTAGAGTCATTATTTTTAGGTGAATG
GTTAGAGTCATATTTTAGGTGAATG
GTTAGAGTCATATTTTTAGGTGAATG
GTTAGAGTCATTATTTTAGGTGAATG
GTTAGAGTCATTATTTTAGGTGAATG
GTTAGAGTCATTATTTTAGGTGAATG
GTTAGAGTCATATTTTAGGTGAATG
GTTAGAGTCATTATTTTAGGTGAATG
GTTAGAGTCATTATTTTAGGTGAATG
GTTAGAGTCATTATTTTAGGTGAATG
GTTAGAGTCATTATTTTAGGTGAATG
GTTAGAGTCATTATTTTAGGTGAATG
GTTAGAGTCATTATTTTAGGTGAATG
GTTAGAGTCATACTTTTAGGTGAATG
GTTAGAGTCATTATTTTAGGTGAATG
GTTAGAGTCATATTTTAGGTGAATG
GTTAGAGTCATATTTTAGGTGAATG
GTTAGAGTCATTATGTTAGGTGAATG
GTTAGAGTCATACTTTTAGGTGAATG
GTTAGAGTCATTATTTTAGGTGAATG
GTTAGAGTCATTATTTTAGGTGAATG
GTTAGAGTCATTTTTTTAGGTGAATG

GTTAGAGTCATTATTTTAGGTGAATG
GTTAGAGTCATTATTTTAGGTGAATG
GTTAGAGTCATTATTTTAGGTGAATG
GTTAGAGTCATTATTTTTAGGTGAATG
GTTAGAGTCATACTTTTAGGTGAATG
GTTAGAGTCATTATTTTAGGTGAATG
GTTAGAGTCATTATTTTAGGTGAATG
GTTAGAGTCATTTTTTTAGGTGAATG
GTTAGAGTCATTATTTTAGGTGAATG
GTTAGAGTCATACTTTTAGGTGAATG
GTTAGAGTCATTATTTTAGGTGAATG
GTTAGAGTCATTATTTTAGGTGAATG
GTTAGAGTCATTATTTTAGGTGAATG
GTTAGAGTCATTATTTTAGGTGAATG
GTTAGAGTCATTATTTTAGGTGAATG
GTTAGAGTCATTATTTTAGGTGAATG
GTTAGAGTCATTATTTTAGGTGAATG
GTTAGAGTCATTATTTTAGGTGAATG
GTTAGAGTCATATATTTTAGGTGAATG
GTTAGAGTCATTATTTTAGGTGAATG
GTTAGAGTCATTATTTTAGGTGAATG
GTTAGAGTCATCATATTTTAGGTGAATG
GTTAGAGTCATTATTTTAGGTGAATG
GTTAGAGTCATTATTTTAGGTGAATG
GTTAGAGTCATTATTTTAGGTGAATG
GTTAGAGTCATTATTTTAGGTGAATG
GTTAGAGTCATTATTTTAGGTGAATG
GTTAGAGTCATTATTTTTAGGTGAATG
GTTAGAGTCATATTTTAGGTGAATG
GTTAGAGTCATTATTTTAGGTGAATG
GTTAGAGTCATATTTTTAGGTGAATG
GTTAGAGTCATTTTTAGGTGAATG
GTTAGAGTCATTATTTTAGGTGAATG
GTTAGAGTCATTATTTTAGGTGAATG
GTTAGAGTCATTATTTTAGGTGAATG
GTTAGAGTCATTATTTTAGGTGAATG
GTTAGAGTCATTATTTTAGGTGAATG
GTTAGAGTCATTATTTTAGGTGAATG
GTTAGAGTCATTATTTTAGGTGAATG
GTTAGAGTCATTATTTTAGGTGAATG
GTTAGAGTCATTATTTTAGGTGAATG
GTTAGAGTCATTATTTTAGGTGAATG
GTTAGAGTCATTATTTTAGGTGAATG
GTTAGAGTCATTATTTTAGGTGAATG
GTTAGAGTCATATTTTTAGGTGAATG
GTTAGAGTCATTATTTAGGTGAATG
GTTAGAGTCATTATTTTAGGTGAATG
GTTAGAGTCATTATTTTAGGTGAATG
GTTAGAGTCATTATTTTAGGTGAATG
GTTAGAGTCATTATTTTAGGTGAATG
GTTAGAGTCATTATTTTAGGTGAATG
GTTAGAGTCATTATTTAGGTGAATG
GTTAGAGTCATTATTTAGGTGAATG
GTTAGAGTCATTATTTTAGGTGAATG

GTTAGAGTCATATTTTTAGGTGAATG
GTTAGAGTCATTATTTTAGGTGAATG
GTTAGAGTCATATTTTTAGGTGAATG
GTTAGAGTCATATTTTTAGGTGAATG
GTTAGAGTCATTATTTTAGGTGAATG
GTTAGAGTCATTATTTTAGGTGAATG
GTTAGAGTCATTTTTTAGGTGAATG
GTTAGAGTCATTATTTTAGGTGAATG
GTTAGAGTCATTATTTTTAGGTGAATG
GTTAGAGTCATTTTTAGGTGAATG
GTTAGAGTCATTATTTTAGGTGAATG
GTTAGAGTCATTATTTTAGGTGAATG
GTTAGAGTCATTATTTTAGGTGAATG
GTTAGAGTCATTATTTTAGGTGAATG
GTTAGAGTCATATTTTTTAGGTGAATG
GTTAGAGTCATATTTTAGGTGAATG
GTTAGAGTCATATTTTAGGTGAATG
GTTAGAGTCATTATTTTAGGTGAATG
GTTAGAGTCATTATTTTAGGTGAATG
GTTAGAGTCATTATTTTAGGTGAATG
GTTAGAGTCATTATTTTTAGGTGAATG
GTTAGAGTCATTATTTTAGGTGAATG
GTTAGAGTCATTATTTTAGGTGAATG
GTTAGAGTCATTATTTTAGGTGAATG
GTTAGAGTCATTATTTTAGGTGAATG
GTTAGAGTCATTATTTTAGGTGAATG
GTTAGAGTCATTATTTTTAGGTGAATG
GTTAGAGTCATATTTTAGGTGAATG
GTTAGAGTCATATTTTAGGTGAATG
GTTAGAGTCATTATTTTAGGTGAATG
GTTAGAGTCATTATTTAGGTGAATG
GTTAGAGTCATTATTTTAGGTGAATG
GTTAGAGTCATTATTTTAGGTGAATG
GTTAGAGTCATTATTTTAGGTGAATG
GTTAGAGTCATTATTTTAGGTGAATG
GTTAGAGTCATATTTTAGGTGAATG
GTTAGAGTCATTATTTTAGGTGAATG
GTTAGAGTCATTATTTTAGGTGAATG
GTTAGAGTCATTATTTTAGGTGAATG
GTTAGAGTCATTATTTTAGGTGAATG
GTTAGAGTCATTATTTTAGGTGAATG
GTTAGAGTCATTATTTTAGGTGAATG
GTTAGAGTCATTATTTTAGGTGAATG
GTTAGAGTCATTATTTTAGGTGAATG
GTTAGAGTCATTTTTTAGGTGAATG
GTTAGAGTCATTATTTTAGGTGAATG
GTTAGAGTCATTATTTTAGGTGAATG
GTTAGAGTCATTATTTTAGGTGAATG
GTTAGAGTCATTATTTTAGGTGAATG
GTTAGAGTCATTATTTTAGGTGAATG
GTTAGAGTCATATTTTTAGGTGAATG
GTTAGAGTCATTATTTAGGTGAATG
GTTAGAGTCATATTTTAGGTGAATG
GTTAGAGTCATTATTTTAGGTGAATG

GTTAGAGTCATTATTTTTAGGTGAATG
GTTAGAGTCATTATTTTAGGTGAATG
GTTAGAGTCATTATTTTAGGTGAATG
GTTAGAGTCATATTTTTAGGTGAATG
GTTAGAGTCATTTTTAGGTGAATG
GTTAGAGTCATATTTTAGGTGAATG
GTTAGAGTCATTATTTTAGGTGAATG
GTTAGAGTCATATTTTAGGTGAATG
GTTAGAGTCATATTTTAGGTGAATG
GTTAGAGTCATTATTTTAGGTGAATG
GTTAGAGTCATTATTTTAGGTGAATG
GTTAGAGTCATTATTTTAGGTGAATG
GTTAGAGTCATTATTTTAGGTGAATG
GTTAGAGTCATTATTTTAGGTGAATG
GTTAGAGTCATTATTTTAGGTGAATG
GTTAGAGTCATTATTTTAGGTGAATG
GTTAGAGTCATTATTTTAGGTGAATG
GTTAGAGTCATTATTTTAGGTGAATG
GTTAGAGTCATTATTTTAGGTGAATG
GTTAGAGTCATATTTTAGGTGAATG
GTTAGAGTCATTATTTTAGGTGAATG
GTTAGAGTCATTATTTTAGGTGAATG
GTTAGAGTCATTATTTTAGGTGAATG
GTTAGAGTCATTATTTTAGGTGAATG
GTTAGAGTCATTATTTTAGGTGAATG
GTTAGAGTCATTATTTTAGGTGAATG
GTTAGAGTCATTATTTTAGGTGAATG
GTTAGAGTCATTATTTTAGGTGAATG
GTTAGAGTCATTATTTTAGGTGAATG
GTTAGAGTCATTATTTTAGGTGAATG
GTTAGAGTCATTATTTTAGGTGAATG
GTTAGAGTCATATTTTAGGTGAATG
GTTAGAGTCATTATTTTAGGTGAATG
GTTAGAGTCATTATTTTAGGTGAATG
GTTAGAGTCATTATTTTAGGTGAATG
GTTAGAGTCATTATTTTAGGTGAATG
GTTAGAGTCATTATTTTAGGTGAATG
GTTAGAGTCATTATTTAGGTGAATG
GTTAGAGTCATTATTTTAGGTGAATG
GTTAGAGTCATTATTTAGGTGAATG
GTTAGAGTCATTTTTTAGGTGAATG
GTTAGAGTCATTATTTTAGGTGAATG
GTTAGAGTCATATTTTTAGGTGAATG
GTTAGAGTCATTATTTTAGGTGAATG
GTTAGAGTCATTATTTTAGGTGAATG
GTTAGAGTCATTATTTTAGGTGAATG
GTTAGAGTCATTATTTTAGGTGAATG
GTTAGAGTCATTATTTTAGGTGAATG
GTTAGAGTCATATTTTGGTGAATG
GTTAGAGTCATTATTTTAGGTGAATG
GTTAGAGTCATTATTTTAGGTGAATG
GTTAGAGTCATTATTTTAGGTGAATG
GTTAGAGTCATTATTTTAGGTGAATG
GTTAGAGTCATTATTTTAGGTGAATG

GTTAGAGTCATATTTTAGGTGAATG
GTTAGAGTCATTATTTTAGGTGAATG
GTTAGAGTCATTATTTTAGGTGAATG
GTTAGAGTCATTATTTTAGGTGAATG
GTTAGAGTCATTATTTTAGGTGAATG
GTTAGAGTCATTATTTTAGGTGAATG
GTTAGAGTCATTATTTTAGGTGAATG
GTTAGAGTCATTATTTTAGGTGAATG
GTTAGAGTCATATTTTAGGTGAATG
GTTAGAGTCATTATTTTAGGTGAATG
GTTAGAGTCATTATTTTAGGTGAATG
GTTAGAGTCATTATTTTAGGTGAATG
GTTAGAGTCATTATTTTAGGTGAATG
GTTAGAGTCATTATTTTAGGTGAATG
GTTAGAGTCATTATTTTAGGTGAATG
GTTAGAGTCATTATTTTAGGTGAATG
GTTAGAGTCATTATTTTAGGTGAATG
GTTAGAGTCATTATTTTAGGTGAATG
GTTAGAGTCATTATTTTAGGTGAATG
GTTAGAGTCATTATTTTAGGTGAATG
GTTAGAGTCATTATTTTAGGTGAATG
GTTAGAGTCATTATTTTAGGTGAATG
GTTAGAGTCATTATTTTAGGTGAATG
GTTAGAGTCATTATTTTAGGTGAATG
GTTAGAGTCATTATTTTAGGTGAATG
GTTAGAGTCATTATTTTAGGTGAATG
GTTAGAGTCATTATTTTAGGTGAATG
GTTAGAGTCATTATTTTAGGTGAATG
GTTAGAGTCATTATTTTAGGTGAATG
GTTAGAGTCATTATTTTAGGTGAATG
GTTAGAGTCATTATTTTAGGTGAATG
GTTAGAGTCATTATTTTAGGTGAATG
GTTAGAGTCATTATTTTAGGTGAATG
GTTAGAGTCATTATTTAGGTGAATG
GTTAGAGTCATTATTTTAGGTGAATG
GTTAGAGTCATATTTTAGGTGAATG
GTTAGAGTCATTATTTTAGGTGAATG
GTTAGAGTCATTATTTTAGGTGAATG
GTTAGAGTCATTATTTTAGGTGAATG
GTTAGAGTCATTATTTTAGGTGAATG
GTTAGAGTCATTATTTTAGGTGAATG
GTTAGAGTCATTATTTTAGGTGAATG
GTTAGAGTCATTATTTGGTGAATG
GTTAGAGTCATTATTTTAGGTGAATG
GTTAGAGTCATTATTTTAGGTGAATG
GTTAGAGTCATATTTTTAGGTGAATG
GTTAGAGTCATTATTTTAGGTGAATG
GTTAGAGTCATTATTTTAGGTGAATG
GTTAGAGTCATTATTTTAGGTGAATG
GTTAGAGTCATTATTTTAGGTGAATG
GTTAGAGTCATTATTTTAGGTGAATG
GTTAGAGTCATTATTTTAGGTGAATG
GTTAGAGTCATTATTTTAGGTGAATG
GTTAGAGTCATTATTTTAGGTGAATG

GTTAGAGTCATTATTTTAGGTGAATG
GTTAGAGTCATTATTTTAGGTGAATG
GTTAGAGTCATTATTTTAGGTGAATG
GTTAGAGTCATTATTTTTAGGTGAATG
GTTAGAGTCATATTTTAGGTGAATG
GTTAGAGTCATTATTTTAGGTGAATG
GTTAGAGTCATTTTTTTAGGTGAATG
GTTAGAGTCATTTTTTAGGTGAATG
GTTAGAGTCATTATTTTAGGTGAATG
GTTAGAGTCATATTTTTAGGTGAATG
GTTAGAGTCATTATTTTAGGTGAATG
GTTAGAGTCATTATTTTAGGTGAATG
GTTAGAGTCATTATTTTAGGTGAATG
GTTAGAGTCATTATTTAGGTGAATG
GTTAGAGTCATTATTTTAGGTGAATG
GTTAGAGTCATTATTTTAGGTGAATG
GTTAGAGTCATATTTTAGGTGAATG
GTTAGAGTCATATTTTAGGTGAATG
GTTAGAGTCATTATTTTAGGTGAATG
GTTAGAGTCATTATTTTAGGTGAATG
GTTAGAGTCATTATTTTAGGTGAATG
GTTAGAGTCATTTTTTAGGTGAATG
GTTAGAGTCATATTTTAGGTGAATG
GTTAGAGTCATATTTTAGGTGAATG
GTTAGAGTCATTATTTTAGGTGAATG
GTTAGAGTCATTTTTTAGGTGAATG
GTTAGAGTCATTATTTTAGGTGAATG
GTTAGAGTCATTATTTTAGGTGAATG
GTTAGAGTCATTATTTTAGGTGAATG
GTTAGAGTCATATTTTAGGTGAATG
GTTAGAGTCATATTTTAGGTGAATG
GTTAGAGTCATTATTTTAGGTGAATG
GTTAGAGTCATTATTTTAGGTGAATG
GTTAGAGTCATTATTTTAGGTGAATG
GTTAGAGTCATTATTTTAGGTGAATG
GTTAGAGTCATTATTTTAGGTGAATG
GTTAGAGTCATTATTTTAGGTGAATG
GTTAGAGTCATTATTTTAGGTGAATG
GTTAGAGTCATTATTTTAGGTGAATG
GTTAGAGTCATTATTTTAGGTGAATG
GTTAGAGTCATTATTTTAGGTGAATG
GTTAGAGTCATTATTTTAGGTGAATG
GTTAGAGTCATTATTTTAGGTGAATG
GTTAGAGTCATTATTTTAGGTGAATG
GTTAGAGTCATTATTTTAGGTGAATG
GTTAGAGTCATTATTTTAGGTGAATG
GTTAGAGTCATTATTTTAGGTGAATG
GTTAGAGTCATTATTTTAGGTGAATG
GTTAGAGTCATTATTTTAGGTGAATG
GTTAGAGTCATTATTTTAGGTGAATG
GTTAGAGTCATTATTTTAGGTGAATG
GTTAGAGTCATTATTTTAGGTGAATG
GTTAGAGTCATTATTTTAGGTGAATG
GTTAGAGTCATTATTTTAGGTGAATG

GTTAGAGTCATTATTTTAGGTGAATG
GTTAGAGTCATTATTTTAGGTGAATG
GTTAGAGTCATTATTTTAGGTGAATG
GTTAGAGTCATTATTTTAGGTGAATG
GTTAGAGTCATTATTTTAGGTGAATG
GTTAGAGTCATTATTTTAGGTGAATG
GTTAGAGTCATTATTTTAGGTGAATG
GTTAGAGTCATTATTTTAGGTGAATG
GTTAGAGTCATTATTTTAGGTGAATG
GTTAGAGTCATTATTTTAGGTGAATG
GTTAGAGTCATTATTTTAGGTGAATG
GTTAGAGTCATTATTTTAGGTGAATG
GTTAGAGTCATTATTTTAGGTGAATG
GTTAGAGTCATTATTTTAGGTGAATG
GTTAGAGTCATTATTTTAGGTGAATG
GTTAGAGTCATTATTTTAGGTGAATG
GTTAGAGTCATTATTTTAGGTGAATG
GTTAGAGTCATTATTTTAGGTGAATG
GTTAGAGTCATTATTTTAGGTGAATG
GTTAGAGTCATTATTTTAGGTGAATG
GTTAGAGTCATTATTTTAGGTGAATG
GTTAGAGTCATTATTTTAGGTGAATG
GTTAGAGTCATTATTTTAGGTGAATG
GTTAGAGTCATTATTTTAGGTGAATG
GTTAGAGTCATTATTTTAGGTGAATG
GTTAGAGTCATTATTTTAGGTGAATG
GTTAGAGTCATTATTTTAGGTGAATG
GTTAGAGTCATTATTTTAGGTGAATG
GTTAGAGTCATTATTTTAGGTGAATG
GTTAGAGTCATTATTTTAGGTGAATG
GTTAGAGTCATTATTTTAGGTGAATG
GTTAGAGTCATTATTTTAGGTGAATG
GTTAGAGTCATTATTTTAGGTGAATG
GTTAGAGTCATTATTTTAGGTGAATG
GTTAGAGTCATTATTTTAGGTGAATG
GTTAGAGTCATTATTTTAGGTGAATG
GTTAGAGTCATTATTTTAGGTGAATG
GTTAGAGTCATTATTTTAGGTGAATG
GTTAGAGTCATTATTTTAGGTGAATG
GTTAGAGTCATTATTTTAGGTGAATG
GTTAGAGTCATTATTTTAGGTGAATG
GTTAGAGTCATTATTTTAGGTGAATG
GTTAGAGTCATTATTTTAGGTGAATG
GTTAGAGTCATTATTTTAGGTGAATG
GTTAGAGTCATTATTTTAGGTGAATG
GTTAGAGTCATTATTTTAGGTGAATG
GTTAGAGTCATTATTTTAGGTGAATG
GTTAGAGTCATTATTTTAGGTGAATG
GTTAGAGTCATTATTTTAGGTGAATG
GTTAGAGTCATTATTTTAGGTGAATG
GTTAGAGTCATTATTTTAGGTGAATG
GTTAGAGTCATTATTTTAGGTGAATG
GTTAGAGTCATTATTTTAGGTGAATG
GTTAGAGTCATTATTTTAGGTGAATG

GTTAGAGTCATTATTTTAGGTGAATG
GTTAGAGTCATTATTTTAGGTGAATG
GTTAGAGTCATTATTTTAGGTGAATG
GTTAGAGTCATTATTTTAGGTGAATG
GTTAGAGTCATTATTTTAGGTGAATG
GTTAGAGTCATTATTTTAGGTGAATG
GTTAGAGTCATTATTTTAGGTGAATG
GTTAGAGTCATTATTTTAGGTGAATG
GTTAGAGTCATTATTTTAGGTGAATG
GTTAGAGTCATTATTTTAGGTGAATG
GTTAGAGTCATTATTTTAGGTGAATG
GTTAGAGTCATTATTTTAGGTGAATG
GTTAGAGTCATTATTTTAGGTGAATG
GTTAGAGTCATTATTTTAGGTGAATG
GTTAGAGTCATTATTTTAGGTGAATG
GTTAGAGTCATTATTTTAGGTGAATG
GTTAGAGTCATTATTTTAGGTGAATG
GTTAGAGTCATTATTTTAGGTGAATG
GTTAGAGTCATTATTTTAGGTGAATG
GTTAGAGTCATTATTTTAGGTGAATG
GTTAGAGTCATTATTTTAGGTGAATG
GTTAGAGTCATTATTTTAGGTGAATG
GTTAGAGTCATTATTTTAGGTGAATG
GTTAGAGTCATTATTTTAGGTGAATG
GTTAGAGTCATTATTTTAGGTGAATG
GTTAGAGTCATTATTTTAGGTGAATG
GTTAGAGTCATTATTTTAGGTGAATG
GTTAGAGTCATTATTTTAGGTGAATG
GTTAGAGTCATTATTTTAGGTGAATG
GTTAGAGTCATTATTTTAGGTGAATG
GTTAGAGTCATTATTTTAGGTGAATG
GTTAGAGTCATTATTTTAGGTGAATG
GTTAGAGTCATTATTTTAGGTGAATG
GTTAGAGTCATTATTTTAGGTGAATG
GTTAGAGTCATTATTTTAGGTGAATG
GTTAGAGTCATTATTTTAGGTGAATG
GTTAGAGTCATTATTTTAGGTGAATG
GTTAGAGTCATTATTTTAGGTGAATG
GTTAGAGTCATTATTTTAGGTGAATG
GTTAGAGTCATTATTTTAGGTGAATG
GTTAGAGTCATTATTTTAGGTGAATG
GTTAGAGTCATTATTTTAGGTGAATG
GTTAGAGTCATTATTTTAGGTGAATG
GTTAGAGTCATTATTTTAGGTGAATG
GTTAGAGTCATTATTTTAGGTGAATG
GTTAGAGTCATTATTTTAGGTGAATG
GTTAGAGTCATTATTTTAGGTGAATG
GTTAGAGTCATTATTTTAGGTGAATG
GTTAGAGTCATTATTTTAGGTGAATG
GTTAGAGTCATTATTTTAGGTGAATG
GTTAGAGTCATTATTTTAGGTGAATG
GTTAGAGTCATTATTTTAGGTGAATG
GTTAGAGTCATTATTTTAGGTGAATG
GTTAGAGTCATTATTTTAGGTGAATG

GTTAGAGTCATTATTTTAGGTGAATG
GTTAGAGTCATTATTTTAGGTGAATG
GTTAGAGTCATTATTTTAGGTGAATG
GTTAGAGTCATTATTTTAGGTGAATG
GTTAGAGTCATTATTTTAGGTGAATG
GTTAGAGTCATTATTTTAGGTGAATG
GTTAGAGTCATTATTTTAGGTGAATG
GTTAGAGTCATTATTTTAGGTGAATG
GTTAGAGTCATTATTTTAGGTGAATG
GTTAGAGTCATTATTTTAGGTGAATG
GTTAGAGTCATTATTTTAGGTGAATG
GTTAGAGTCATTATTTTAGGTGAATG
GTTAGAGTCATTATTTTAGGTGAATG
GTTAGAGTCATTATTTTAGGTGAATG
GTTAGAGTCATTATTTTAGGTGAATG
GTTAGAGTCATTATTTTAGGTGAATG
GTTAGAGTCATTATTTTAGGTGAATG
GTTAGAGTCATTATTTTAGGTGAATG
GTTAGAGTCATTATTTTAGGTGAATG
GTTAGAGTCATTATTTTAGGTGAATG
GTTAGAGTCATTATTTTAGGTGAATG
GTTAGAGTCATTATTTTAGGTGAATG
GTTAGAGTCATTATTTTAGGTGAATG
GTTAGAGTCATTATTTTAGGTGAATG
GTTAGAGTCATTATTTTAGGTGAATG
GTTAGAGTCATTATTTTAGGTGAATG
GTTAGAGTCATTATTTTAGGTGAATG
GTTAGAGTCATTATTTTAGGTGAATG
GTTAGAGTCATTATTTTAGGTGAATG
GTTAGAGTCATTATTTTAGGTGAATG
GTTAGAGTCATTATTTTAGGTGAATG
GTTAGAGTCATTATTTTAGGTGAATG
GTTAGAGTCATTATTTTAGGTGAATG
GTTAGAGTCATTATTTTAGGTGAATG
GTTAGAGTCATTATTTTAGGTGAATG
GTTAGAGTCATTATTTTAGGTGAATG
GTTAGAGTCATTATTTTAGGTGAATG
GTTAGAGTCATTATTTTAGGTGAATG
GTTAGAGTCATTATTTTAGGTGAATG
GTTAGAGTCATTATTTTAGGTGAATG
GTTAGAGTCATTATTTTAGGTGAATG
GTTAGAGTCATTATTTTAGGTGAATG
GTTAGAGTCATTATTTTAGGTGAATG
GTTAGAGTCATTATTTTAGGTGAATG
GTTAGAGTCATTATTTTAGGTGAATG
GTTAGAGTCATTATTTTAGGTGAATG
GTTAGAGTCATTATTTTAGGTGAATG
GTTAGAGTCATTATTTTAGGTGAATG
GTTAGAGTCATTATTTTAGGTGAATG
GTTAGAGTCATTATTTTAGGTGAATG
GTTAGAGTCATTATTTTAGGTGAATG
GTTAGAGTCATTATTTTAGGTGAATG
GTTAGAGTCATTATTTTAGGTGAATG
GTTAGAGTCATTATTTTAGGTGAATG

GTTAGAGTCATTATTTTAGGTGAATG
GTTAGAGTCATTATTTTAGGTGAATG
GTTAGAGTCATTATTTTAGGTGAATG
GTTAGAGTCATTATTTTAGGTGAATG
GTTAGAGTCATTATTTTAGGTGAATG
GTTAGAGTCATTATTTTAGGTGAATG
GTTAGAGTCATTATTTTAGGTGAATG
GTTAGAGTCATTATTTTAGGTGAATG
GTTAGAGTCATTATTTTAGGTGAATG
GTTAGAGTCATTATTTTAGGTGAATG
GTTAGAGTCATTATTTTAGGTGAATG
GTTAGAGTCATTATTTTAGGTGAATG
GTTAGAGTCATTATTTTAGGTGAATG
GTTAGAGTCATTATTTTAGGTGAATG
GTTAGAGTCATTATTTTAGGTGAATG
GTTAGAGTCATTATTTTAGGTGAATG
GTTAGAGTCATTATTTTAGGTGAATG
GTTAGAGTCATTATTTTAGGTGAATG
GTTAGAGTCATTATTTTAGGTGAATG
GTTAGAGTCATTATTTTAGGTGAATG
GTTAGAGTCATTATTTTAGGTGAATG
GTTAGAGTCATTATTTTAGGTGAATG
GTTAGAGTCATTATTTTAGGTGAATG
GTTAGAGTCATTATTTTAGGTGAATG
GTTAGAGTCATTATTTTAGGTGAATG
GTTAGAGTCATTATTTTAGGTGAATG
GTTAGAGTCATTATTTTAGGTGAATG
GTTAGAGTCATTATTTTAGGTGAATG
GTTAGAGTCATTATTTTAGGTGAATG
GTTAGAGTCATTATTTTAGGTGAATG
GTTAGAGTCATTATTTTAGGTGAATG
GTTAGAGTCATTATTTTAGGTGAATG
GTTAGAGTCATTATTTTAGGTGAATG
GTTAGAGTCATTATTTTAGGTGAATG
GTTAGAGTCATTATTTTAGGTGAATG
GTTAGAGTCATTATTTTAGGTGAATG
GTTAGAGTCATTATTTTAGGTGAATG
GTTAGAGTCATTATTTTAGGTGAATG
GTTAGAGTCATTATTTTAGGTGAATG
GTTAGAGTCATTATTTTAGGTGAATG
GTTAGAGTCATTATTTTAGGTGAATG
GTTAGAGTCATTATTTTAGGTGAATG
GTTAGAGTCATTATTTTAGGTGAATG
GTTAGAGTCATTATTTTAGGTGAATG
GTTAGAGTCATTATTTTAGGTGAATG
GTTAGAGTCATTATTTTAGGTGAATG
GTTAGAGTCATTATTTTAGGTGAATG
GTTAGAGTCATTATTTTAGGTGAATG
GTTAGAGTCATTATTTTAGGTGAATG
GTTAGAGTCATTATTTTAGGTGAATG
GTTAGAGTCATTATTTTAGGTGAATG
GTTAGAGTCATTATTTTAGGTGAATG
GTTAGAGTCATTATTTTAGGTGAATG
GTTAGAGTCATTATTTTAGGTGAATG

GTTAGAGTCATTATTTTAGGTGAATG
GTTAGAGTCATTATTTTAGGTGAATG
GTTAGAGTCATTATTTTAGGTGAATG
GTTAGAGTCATTATTTTAGGTGAATG
GTTAGAGTCATTATTTTAGGTGAATG
GTTAGAGTCATTATTTTAGGTGAATG
GTTAGAGTCATTATTTTAGGTGAATG
GTTAGAGTCATTATTTTAGGTGAATG
GTTAGAGTCATTATTTTAGGTGAATG
GTTAGAGTCATTATTTTAGGTGAATG
GTTAGAGTCATTATTTTAGGTGAATG
GTTAGAGTCATTATTTTAGGTGAATG
GTTAGAGTCATTATTTTAGGTGAATG
GTTAGAGTCATTATTTTAGGTGAATG
GTTAGAGTCATTATTTTAGGTGAATG
GTTAGAGTCATTATTTTAGGTGAATG
GTTAGAGTCATTATTTTAGGTGAATG
GTTAGAGTCATTATTTTAGGTGAATG
GTTAGAGTCATTATTTTAGGTGAATG
GTTAGAGTCATTATTTTAGGTGAATG
GTTAGAGTCATTATTTTAGGTGAATG
GTTAGAGTCATTATTTTAGGTGAATG
GTTAGAGTCATTATTTTAGGTGAATG
GTTAGAGTCATTATTTTAGGTGAATG
GTTAGAGTCATTATTTTAGGTGAATG
GTTAGAGTCATTATTTTAGGTGAATG
GTTAGAGTCATTATTTTAGGTGAATG
GTTAGAGTCATTATTTTAGGTGAATG
GTTAGAGTCATTATTTTAGGTGAATG
GTTAGAGTCATTATTTTAGGTGAATG
GTTAGAGTCATTATTTTAGGTGAATG
GTTAGAGTCATTATTTTAGGTGAATG
GTTAGAGTCATTATTTTAGGTGAATG
GTTAGAGTCATTATTTTAGGTGAATG
GTTAGAGTCATTATTTTAGGTGAATG
GTTAGAGTCATTATTTTAGGTGAATG
GTTAGAGTCATTATTTTAGGTGAATG
GTTAGAGTCATTATTTTAGGTGAATG
GTTAGAGTCATTATTTTAGGTGAATG
GTTAGAGTCATTATTTTAGGTGAATG
GTTAGAGTCATTATTTTAGGTGAATG
GTTAGAGTCATTATTTTAGGTGAATG
GTTAGAGTCATTATTTTAGGTGAATG
GTTAGAGTCATTATTTTAGGTGAATG
GTTAGAGTCATTATTTTAGGTGAATG
GTTAGAGTCATTATTTTAGGTGAATG
GTTAGAGTCATTATTTTAGGTGAATG
GTTAGAGTCATTATTTTAGGTGAATG
GTTAGAGTCATTATTTTAGGTGAATG
GTTAGAGTCATTATTTTAGGTGAATG
GTTAGAGTCATTATTTTAGGTGAATG
GTTAGAGTCATTATTTTAGGTGAATG
GTTAGAGTCATTATTTTAGGTGAATG
GTTAGAGTCATTATTTTAGGTGAATG

GTTAGAGTCATTATTTTAGGTGAATG
GTTAGAGTCATTATTTTAGGTGAATG
GTTAGAGTCATTATTTTAGGTGAATG
GTTAGAGTCATTATTTTAGGTGAATG
GTTAGAGTCATTATTTTAGGTGAATG
GTTAGAGTCATTATTTTAGGTGAATG
GTTAGAGTCATTATTTTAGGTGAATG
GTTAGAGTCATTATTTTAGGTGAATG
GTTAGAGTCATTATTTTAGGTGAATG
GTTAGAGTCATTATTTTAGGTGAATG
GTTAGAGTCATTATTTTAGGTGAATG
GTTAGAGTCATTATTTTAGGTGAATG
GTTAGAGTCATTATTTTAGGTGAATG
GTTAGAGTCATTATTTTAGGTGAATG
GTTAGAGTCATTATTTTAGGTGAATG
GTTAGAGTCATTATTTTAGGTGAATG
GTTAGAGTCATTATTTTAGGTGAATG
GTTAGAGTCATTATTTTAGGTGAATG
GTTAGAGTCATTATTTTAGGTGAATG
GTTAGAGTCATTATTTTAGGTGAATG
GTTAGAGTCATTATTTTAGGTGAATG
GTTAGAGTCATTATTTTAGGTGAATG
GTTAGAGTCATTATTTTAGGTGAATG
GTTAGAGTCATTATTTTAGGTGAATG
GTTAGAGTCATTATTTTAGGTGAATG
GTTAGAGTCATTATTTTAGGTGAATG
GTTAGAGTCATTATTTTAGGTGAATG
GTTAGAGTCATTATTTTAGGTGAATG
GTTAGAGTCATTATTTTAGGTGAATG
GTTAGAGTCATTATTTTAGGTGAATG
GTTAGAGTCATTATTTTAGGTGAATG
GTTAGAGTCATTATTTTAGGTGAATG
GTTAGAGTCATTATTTTAGGTGAATG
GTTAGAGTCATTATTTTAGGTGAATG
GTTAGAGTCATTATTTTAGGTGAATG
GTTAGAGTCATTATTTTAGGTGAATG
GTTAGAGTCATTATTTTAGGTGAATG
GTTAGAGTCATTATTTTAGGTGAATG
GTTAGAGTCATTATTTTAGGTGAATG
GTTAGAGTCATTATTTTAGGTGAATG
GTTAGAGTCATTATTTTAGGTGAATG
GTTAGAGTCATTATTTTAGGTGAATG
GTTAGAGTCATTATTTTAGGTGAATG
GTTAGAGTCATTATTTTAGGTGAATG
GTTAGAGTCATTATTTTAGGTGAATG
GTTAGAGTCATTATTTTAGGTGAATG
GTTAGAGTCATTATTTTAGGTGAATG
GTTAGAGTCATTATTTTAGGTGAATG
GTTAGAGTCATTATTTTAGGTGAATG
GTTAGAGTCATTATTTTAGGTGAATG
GTTAGAGTCATTATTTTAGGTGAATG
GTTAGAGTCATTATTTTAGGTGAATG
GTTAGAGTCATTATTTTAGGTGAATG
GTTAGAGTCATTATTTTAGGTGAATG

GTTAGAGTCATTATTTTAGGTGAATG
GTTAGAGTCATTATTTTAGGTGAATG
GTTAGAGTCATTATTTTAGGTGAATG
GTTAGAGTCATTATTTTAGGTGAATG
GTTAGAGTCATTATTTTAGGTGAATG
GTTAGAGTCATTATTTTAGGTGAATG
GTTAGAGTCATTATTTTAGGTGAATG
GTTAGAGTCATTATTTTAGGTGAATG
GTTAGAGTCATTATTTTAGGTGAATG
GTTAGAGTCATTATTTTAGGTGAATG
GTTAGAGTCATTATTTTAGGTGAATG
GTTAGAGTCATTATTTTAGGTGAATG
GTTAGAGTCATTATTTTAGGTGAATG
GTTAGAGTCATTATTTTAGGTGAATG
GTTAGAGTCATTATTTTAGGTGAATG
GTTAGAGTCATTATTTTAGGTGAATG
GTTAGAGTCATTATTTTAGGTGAATG
GTTAGAGTCATTATTTTAGGTGAATG
GTTAGAGTCATTATTTTAGGTGAATG
GTTAGAGTCATTATTTTAGGTGAATG
GTTAGAGTCATTATTTTAGGTGAATG
GTTAGAGTCATTATTTTAGGTGAATG
GTTAGAGTCATTATTTTAGGTGAATG
GTTAGAGTCATTATTTTAGGTGAATG
GTTAGAGTCATTATTTTAGGTGAATG
GTTAGAGTCATTATTTTAGGTGAATG
GTTAGAGTCATTATTTTAGGTGAATG
GTTAGAGTCATTATTTTAGGTGAATG
GTTAGAGTCATTATTTTAGGTGAATG
GTTAGAGTCATTATTTTAGGTGAATG
GTTAGAGTCATTATTTTAGGTGAATG
GTTAGAGTCATTATTTTAGGTGAATG
GTTAGAGTCATTATTTTAGGTGAATG
GTTAGAGTCATTATTTTAGGTGAATG
GTTAGAGTCATTATTTTAGGTGAATG
GTTAGAGTCATTATTTTAGGTGAATG
GTTAGAGTCATTATTTTAGGTGAATG
GTTAGAGTCATTATTTTAGGTGAATG
GTTAGAGTCATTATTTTAGGTGAATG
GTTAGAGTCATTATTTTAGGTGAATG
GTTAGAGTCATTATTTTAGGTGAATG
GTTAGAGTCATTATTTTAGGTGAATG
GTTAGAGTCATTATTTTAGGTGAATG
GTTAGAGTCATTATTTTAGGTGAATG
GTTAGAGTCATTATTTTAGGTGAATG
GTTAGAGTCATTATTTTAGGTGAATG
GTTAGAGTCATTATTTTAGGTGAATG
GTTAGAGTCATTATTTTAGGTGAATG
GTTAGAGTCATTATTTTAGGTGAATG
GTTAGAGTCATTATTTTAGGTGAATG
GTTAGAGTCATTATTTTAGGTGAATG
GTTAGAGTCATTATTTTAGGTGAATG
GTTAGAGTCATTATTTTAGGTGAATG
GTTAGAGTCATTATTTTAGGTGAATG

GTTAGAGTCATTATTTTAGGTGAATG
GTTAGAGTCATTATTTTAGGTGAATG
GTTAGAGTCATTATTTTAGGTGAATG
GTTAGAGTCATTATTTTAGGTGAATG
GTTAGAGTCATTATTTTAGGTGAATG
GTTAGAGTCATTATTTTAGGTGAATG
GTTAGAGTCATTATTTTAGGTGAATG
GTTAGAGTCATTATTTTAGGTGAATG
GTTAGAGTCATTATTTTAGGTGAATG
GTTAGAGTCATTATTTTAGGTGAATG
GTTAGAGTCATTATTTTAGGTGAATG
GTTAGAGTCATTATTTTAGGTGAATG
GTTAGAGTCATTATTTTAGGTGAATG
GTTAGAGTCATTATTTTAGGTGAATG
GTTAGAGTCATTATTTTAGGTGAATG
GTTAGAGTCATTATTTTAGGTGAATG
GTTAGAGTCATTATTTTAGGTGAATG
GTTAGAGTCATTATTTTAGGTGAATG
GTTAGAGTCATTATTTTAGGTGAATG
GTTAGAGTCATTATTTTAGGTGAATG
GTTAGAGTCATTATTTTAGGTGAATG
GTTAGAGTCATTATTTTAGGTGAATG
GTTAGAGTCATTATTTTAGGTGAATG
GTTAGAGTCATTATTTTAGGTGAATG
GTTAGAGTCATTATTTTAGGTGAATG
GTTAGAGTCATTATTTTAGGTGAATG
GTTAGAGTCATTATTTTAGGTGAATG
GTTAGAGTCATTATTTTAGGTGAATG
GTTAGAGTCATTATTTTAGGTGAATG
GTTAGAGTCATTATTTTAGGTGAATG
GTTAGAGTCATTATTTTAGGTGAATG
GTTAGAGTCATTATTTTAGGTGAATG
GTTAGAGTCATTATTTTAGGTGAATG
GTTAGAGTCATTATTTTAGGTGAATG
GTTAGAGTCATTATTTTAGGTGAATG
GTTAGAGTCATTATTTTAGGTGAATG
GTTAGAGTCATTATTTTAGGTGAATG
GTTAGAGTCATTATTTTAGGTGAATG
GTTAGAGTCATTATTTTAGGTGAATG
GTTAGAGTCATTATTTTAGGTGAATG
GTTAGAGTCATTATTTTAGGTGAATG
GTTAGAGTCATTATTTTAGGTGAATG
GTTAGAGTCATTATTTTAGGTGAATG
GTTAGAGTCATTATTTTAGGTGAATG
GTTAGAGTCATTATTTTAGGTGAATG
GTTAGAGTCATTATTTTAGGTGAATG
GTTAGAGTCATTATTTTAGGTGAATG
GTTAGAGTCATTATTTTAGGTGAATG
GTTAGAGTCATTATTTTAGGTGAATG
GTTAGAGTCATTATTTTAGGTGAATG
GTTAGAGTCATTATTTTAGGTGAATG
GTTAGAGTCATTATTTTAGGTGAATG
GTTAGAGTCATTATTTTAGGTGAATG
GTTAGAGTCATTATTTTAGGTGAATG

GTTAGAGTCATTATTTTAGGTGAATG
GTTAGAGTCATTATTTTAGGTGAATG
GTTAGAGTCATTATTTTAGGTGAATG
GTTAGAGTCATTATTTTAGGTGAATG
GTTAGAGTCATTATTTTAGGTGAATG
GTTAGAGTCATTATTTTAGGTGAATG
GTTAGAGTCATTATTTTAGGTGAATG
GTTAGAGTCATTATTTTAGGTGAATG
GTTAGAGTCATTATTTTAGGTGAATG
GTTAGAGTCATTATTTTAGGTGAATG
GTTAGAGTCATTATTTTAGGTGAATG
GTTAGAGTCATTATTTTAGGTGAATG
GTTAGAGTCATTATTTTAGGTGAATG
GTTAGAGTCATTATTTTAGGTGAATG
GTTAGAGTCATTATTTTAGGTGAATG
GTTAGAGTCATTATTTTAGGTGAATG
GTTAGAGTCATTATTTTAGGTGAATG
GTTAGAGTCATTATTTTAGGTGAATG
GTTAGAGTCATTATTTTAGGTGAATG
GTTAGAGTCATTATTTTAGGTGAATG
GTTAGAGTCATTATTTTAGGTGAATG
GTTAGAGTCATTATTTTAGGTGAATG
GTTAGAGTCATTATTTTAGGTGAATG
GTTAGAGTCATTATTTTAGGTGAATG
GTTAGAGTCATTATTTTAGGTGAATG
GTTAGAGTCATTATTTTAGGTGAATG
GTTAGAGTCATTATTTTAGGTGAATG
GTTAGAGTCATTATTTTAGGTGAATG
GTTAGAGTCATTATTTTAGGTGAATG
GTTAGAGTCATTATTTTAGGTGAATG
GTTAGAGTCATTATTTTAGGTGAATG
GTTAGAGTCATTATTTTAGGTGAATG
GTTAGAGTCATTATTTTAGGTGAATG
GTTAGAGTCATTATTTTAGGTGAATG
GTTAGAGTCATTATTTTAGGTGAATG
GTTAGAGTCATTATTTTAGGTGAATG
GTTAGAGTCATTATTTTAGGTGAATG
GTTAGAGTCATTATTTTAGGTGAATG
GTTAGAGTCATTATTTTAGGTGAATG
GTTAGAGTCATTATTTTAGGTGAATG
GTTAGAGTCATTATTTTAGGTGAATG
GTTAGAGTCATTATTTTAGGTGAATG
GTTAGAGTCATTATTTTAGGTGAATG
GTTAGAGTCATTATTTTAGGTGAATG
GTTAGAGTCATTATTTTAGGTGAATG
GTTAGAGTCATTATTTTAGGTGAATG
GTTAGAGTCATTATTTTAGGTGAATG
GTTAGAGTCATTATTTTAGGTGAATG
GTTAGAGTCATTATTTTAGGTGAATG
GTTAGAGTCATTATTTTAGGTGAATG
GTTAGAGTCATTATTTTAGGTGAATG
GTTAGAGTCATTATTTTAGGTGAATG
GTTAGAGTCATTATTTTAGGTGAATG
GTTAGAGTCATTATTTTAGGTGAATG

GTTAGAGTCATTATTTTAGGTGAATG
GTTAGAGTCATTATTTTAGGTGAATG
GTTAGAGTCATTATTTTAGGTGAATG
GTTAGAGTCATTATTTTAGGTGAATG
GTTAGAGTCATTATTTTAGGTGAATG
GTTAGAGTCATTATTTTAGGTGAATG
GTTAGAGTCATTATTTTAGGTGAATG
GTTAGAGTCATTATTTTAGGTGAATG
GTTAGAGTCATTATTTTAGGTGAATG
GTTAGAGTCATTATTTTAGGTGAATG
GTTAGAGTCATTATTTTAGGTGAATG
GTTAGAGTCATTATTTTAGGTGAATG
GTTAGAGTCATTATTTTAGGTGAATG
GTTAGAGTCATTATTTTAGGTGAATG
GTTAGAGTCATTATTTTAGGTGAATG
GTTAGAGTCATTATTTTAGGTGAATG
GTTAGAGTCATTATTTTAGGTGAATG
GTTAGAGTCATTATTTTAGGTGAATG
GTTAGAGTCATTATTTTAGGTGAATG
GTTAGAGTCATTATTTTAGGTGAATG
GTTAGAGTCATTATTTTAGGTGAATG
GTTAGAGTCATTATTTTAGGTGAATG
GTTAGAGTCATTATTTTAGGTGAATG
GTTAGAGTCATTATTTTAGGTGAATG
GTTAGAGTCATTATTTTAGGTGAATG
GTTAGAGTCATTATTTTAGGTGAATG
GTTAGAGTCATTATTTTAGGTGAATG
GTTAGAGTCATTATTTTAGGTGAATG
GTTAGAGTCATTATTTTAGGTGAATG
GTTAGAGTCATTATTTTAGGTGAATG
GTTAGAGTCATTATTTTAGGTGAATG
GTTAGAGTCATTATTTTAGGTGAATG
GTTAGAGTCATTATTTTAGGTGAATG
GTTAGAGTCATTATTTTAGGTGAATG
GTTAGAGTCATTATTTTAGGTGAATG
GTTAGAGTCATTATTTTAGGTGAATG
GTTAGAGTCATTATTTTAGGTGAATG
GTTAGAGTCATTATTTTAGGTGAATG
GTTAGAGTCATTATTTTAGGTGAATG
GTTAGAGTCATTATTTTAGGTGAATG
GTTAGAGTCATTATTTTAGGTGAATG
GTTAGAGTCATTATTTTAGGTGAATG
GTTAGAGTCATTATTTTAGGTGAATG
GTTAGAGTCATTATTTTAGGTGAATG
GTTAGAGTCATTATTTTAGGTGAATG
GTTAGAGTCATTATTTTAGGTGAATG
GTTAGAGTCATTATTTTAGGTGAATG
GTTAGAGTCATTATTTTAGGTGAATG
GTTAGAGTCATTATTTTAGGTGAATG
GTTAGAGTCATTATTTTAGGTGAATG
GTTAGAGTCATTATTTTAGGTGAATG
GTTAGAGTCATTATTTTAGGTGAATG
GTTAGAGTCATTATTTTAGGTGAATG
GTTAGAGTCATTATTTTAGGTGAATG

GTTAGAGTCATTATTTTAGGTGAATG
GTTAGAGTCATTATTTTAGGTGAATG
GTTAGAGTCATTATTTTAGGTGAATG
GTTAGAGTCATTATTTTAGGTGAATG
GTTAGAGTCATTATTTTAGGTGAATG
GTTAGAGTCATTATTTTAGGTGAATG
GTTAGAGTCATTATTTTAGGTGAATG
GTTAGAGTCATTATTTTAGGTGAATG
GTTAGAGTCATTATTTTAGGTGAATG
GTTAGAGTCATTATTTTAGGTGAATG
GTTAGAGTCATTATTTTAGGTGAATG
GTTAGAGTCATTATTTTAGGTGAATG
GTTAGAGTCATTATTTTAGGTGAATG
GTTAGAGTCATTATTTTAGGTGAATG
GTTAGAGTCATTATTTTAGGTGAATG
GTTAGAGTCATTATTTTAGGTGAATG
GTTAGAGTCATTATTTTAGGTGAATG
GTTAGAGTCATTATTTTAGGTGAATG
GTTAGAGTCATTATTTTAGGTGAATG
GTTAGAGTCATTATTTTAGGTGAATG
GTTAGAGTCATTATTTTAGGTGAATG
GTTAGAGTCATTATTTTAGGTGAATG
GTTAGAGTCATTATTTTAGGTGAATG
GTTAGAGTCATTATTTTAGGTGAATG
GTTAGAGTCATTATTTTAGGTGAATG
GTTAGAGTCATTATTTTAGGTGAATG
GTTAGAGTCATTATTTTAGGTGAATG
GTTAGAGTCATTATTTTAGGTGAATG
GTTAGAGTCATTATTTTAGGTGAATG
GTTAGAGTCATTATTTTAGGTGAATG
GTTAGAGTCATTATTTTAGGTGAATG
GTTAGAGTCATTATTTTAGGTGAATG
GTTAGAGTCATTATTTTAGGTGAATG
GTTAGAGTCATTATTTTAGGTGAATG
GTTAGAGTCATTATTTTAGGTGAATG
GTTAGAGTCATTATTTTAGGTGAATG
GTTAGAGTCATTATTTTAGGTGAATG
GTTAGAGTCATTATTTTAGGTGAATG
GTTAGAGTCATTATTTTAGGTGAATG
GTTAGAGTCATTATTTTAGGTGAATG
GTTAGAGTCATTATTTTAGGTGAATG
GTTAGAGTCATTATTTTAGGTGAATG
GTTAGAGTCATTATTTTAGGTGAATG
GTTAGAGTCATTATTTTAGGTGAATG
GTTAGAGTCATTATTTTAGGTGAATG
GTTAGAGTCATTATTTTAGGTGAATG
GTTAGAGTCATTATTTTAGGTGAATG
GTTAGAGTCATTATTTTAGGTGAATG
GTTAGAGTCATTATTTTAGGTGAATG
GTTAGAGTCATTATTTTAGGTGAATG
GTTAGAGTCATTATTTTAGGTGAATG
GTTAGAGTCATTATTTTAGGTGAATG
GTTAGAGTCATTATTTTAGGTGAATG
GTTAGAGTCATTATTTTAGGTGAATG

GTTAGAGTCATTATTTTAGGTGAATG
GTTAGAGTCATTATTTTAGGTGAATG
GTTAGAGTCATTATTTTAGGTGAATG
GTTAGAGTCATTATTTTAGGTGAATG
GTTAGAGTCATTATTTTAGGTGAATG
GTTAGAGTCATTATTTTAGGTGAATG
GTTAGAGTCATTATTTTAGGTGAATG
GTTAGAGTCATTATTTTAGGTGAATG
GTTAGAGTCATTATTTTAGGTGAATG
GTTAGAGTCATTATTTTAGGTGAATG
GTTAGAGTCATTATTTTAGGTGAATG
GTTAGAGTCATTATTTTAGGTGAATG
GTTAGAGTCATTATTTTAGGTGAATG
GTTAGAGTCATTATTTTAGGTGAATG
GTTAGAGTCATTATTTTAGGTGAATG
GTTAGAGTCATTATTTTAGGTGAATG
GTTAGAGTCATTATTTTAGGTGAATG
GTTAGAGTCATTATTTTAGGTGAATG
GTTAGAGTCATTATTTTAGGTGAATG
GTTAGAGTCATTATTTTAGGTGAATG
GTTAGAGTCATTATTTTAGGTGAATG
GTTAGAGTCATTATTTTAGGTGAATG
GTTAGAGTCATTATTTTAGGTGAATG
GTTAGAGTCATTATTTTAGGTGAATG
GTTAGAGTCATTATTTTAGGTGAATG
GTTAGAGTCATTATTTTAGGTGAATG
GTTAGAGTCATTATTTTAGGTGAATG
GTTAGAGTCATTATTTTAGGTGAATG
GTTAGAGTCATTATTTTAGGTGAATG
GTTAGAGTCATTATTTTAGGTGAATG
GTTAGAGTCATTATTTTAGGTGAATG
GTTAGAGTCATTATTTTAGGTGAATG
GTTAGAGTCATTATTTTAGGTGAATG
GTTAGAGTCATTATTTTAGGTGAATG
GTTAGAGTCATTATTTTAGGTGAATG
GTTAGAGTCATTATTTTAGGTGAATG
GTTAGAGTCATTATTTTAGGTGAATG
GTTAGAGTCATTATTTTAGGTGAATG
GTTAGAGTCATTATTTTAGGTGAATG
GTTAGAGTCATTATTTTAGGTGAATG
GTTAGAGTCATTATTTTAGGTGAATG
GTTAGAGTCATTATTTTAGGTGAATG
GTTAGAGTCATTATTTTAGGTGAATG
GTTAGAGTCATTATTTTAGGTGAATG
GTTAGAGTCATTATTTTAGGTGAATG
GTTAGAGTCATTATTTTAGGTGAATG
GTTAGAGTCATTATTTTAGGTGAATG
GTTAGAGTCATTATTTTAGGTGAATG
GTTAGAGTCATTATTTTAGGTGAATG
GTTAGAGTCATTATTTTAGGTGAATG
GTTAGAGTCATTATTTTAGGTGAATG
GTTAGAGTCATTATTTTAGGTGAATG
GTTAGAGTCATTATTTTAGGTGAATG
GTTAGAGTCATTATTTTAGGTGAATG

GTTAGAGTCATTATTTTAGGTGAATG
GTTAGAGTCATTATTTTAGGTGAATG
GTTAGAGTCATTATTTTAGGTGAATG
GTTAGAGTCATTATTTTAGGTGAATG
GTTAGAGTCATTATTTTAGGTGAATG
GTTAGAGTCATTATTTTAGGTGAATG
GTTAGAGTCATTATTTTAGGTGAATG
GTTAGAGTCATTATTTTAGGTGAATG
GTTAGAGTCATTATTTTAGGTGAATG
GTTAGAGTCATTATTTTAGGTGAATG
GTTAGAGTCATTATTTTAGGTGAATG
GTTAGAGTCATTATTTTAGGTGAATG
GTTAGAGTCATTATTTTAGGTGAATG
GTTAGAGTCATTATTTTAGGTGAATG
GTTAGAGTCATTATTTTAGGTGAATG
GTTAGAGTCATTATTTTAGGTGAATG
GTTAGAGTCATTATTTTAGGTGAATG
GTTAGAGTCATTATTTTAGGTGAATG
GTTAGAGTCATTATTTTAGGTGAATG
GTTAGAGTCATTATTTTAGGTGAATG
GTTAGAGTCATTATTTTAGGTGAATG
GTTAGAGTCATTATTTTAGGTGAATG
GTTAGAGTCATTATTTTAGGTGAATG
GTTAGAGTCATTATTTTAGGTGAATG
GTTAGAGTCATTATTTTAGGTGAATG
GTTAGAGTCATTATTTTAGGTGAATG
GTTAGAGTCATTATTTTAGGTGAATG
GTTAGAGTCATTATTTTAGGTGAATG
GTTAGAGTCATTATTTTAGGTGAATG
GTTAGAGTCATTATTTTAGGTGAATG
GTTAGAGTCATTATTTTAGGTGAATG
GTTAGAGTCATTATTTTAGGTGAATG
GTTAGAGTCATTATTTTAGGTGAATG
GTTAGAGTCATTATTTTAGGTGAATG
GTTAGAGTCATTATTTTAGGTGAATG
GTTAGAGTCATTATTTTAGGTGAATG
GTTAGAGTCATTATTTTAGGTGAATG
GTTAGAGTCATTATTTTAGGTGAATG
GTTAGAGTCATTATTTTAGGTGAATG
GTTAGAGTCATTATTTTAGGTGAATG
GTTAGAGTCATTATTTTAGGTGAATG
GTTAGAGTCATTATTTTAGGTGAATG
GTTAGAGTCATTATTTTAGGTGAATG
GTTAGAGTCATTATTTTAGGTGAATG
GTTAGAGTCATTATTTTAGGTGAATG
GTTAGAGTCATTATTTTAGGTGAATG
GTTAGAGTCATTATTTTAGGTGAATG
GTTAGAGTCATTATTTTAGGTGAATG
GTTAGAGTCATTATTTTAGGTGAATG
GTTAGAGTCATTATTTTAGGTGAATG
GTTAGAGTCATTATTTTAGGTGAATG
GTTAGAGTCATTATTTTAGGTGAATG
GTTAGAGTCATTATTTTAGGTGAATG
GTTAGAGTCATTATTTTAGGTGAATG

GTTAGAGTCATTATTTTAGGTGAATG
GTTAGAGTCATTATTTTAGGTGAATG
GTTAGAGTCATTATTTTAGGTGAATG
GTTAGAGTCATTATTTTAGGTGAATG
GTTAGAGTCATTATTTTAGGTGAATG
GTTAGAGTCATTATTTTAGGTGAATG
GTTAGAGTCATTATTTTAGGTGAATG
GTTAGAGTCATTATTTTAGGTGAATG
GTTAGAGTCATTATTTTAGGTGAATG
GTTAGAGTCATTATTTTAGGTGAATG
GTTAGAGTCATTATTTTAGGTGAATG
GTTAGAGTCATTATTTTAGGTGAATG
GTTAGAGTCATTATTTTAGGTGAATG
GTTAGAGTCATTATTTTAGGTGAATG
GTTAGAGTCATTATTTTAGGTGAATG
GTTAGAGTCATTATTTTAGGTGAATG
GTTAGAGTCATTATTTTAGGTGAATG
GTTAGAGTCATTATTTTAGGTGAATG
GTTAGAGTCATTATTTTAGGTGAATG
GTTAGAGTCATTATTTTAGGTGAATG
GTTAGAGTCATTATTTTAGGTGAATG
GTTAGAGTCATTATTTTAGGTGAATG
GTTAGAGTCATTATTTTAGGTGAATG
GTTAGAGTCATTATTTTAGGTGAATG
GTTAGAGTCATTATTTTAGGTGAATG
GTTAGAGTCATTATTTTAGGTGAATG
GTTAGAGTCATTATTTTAGGTGAATG
GTTAGAGTCATTATTTTAGGTGAATG
GTTAGAGTCATTATTTTAGGTGAATG
GTTAGAGTCATTATTTTAGGTGAATG
GTTAGAGTCATTATTTTAGGTGAATG
GTTAGAGTCATTATTTTAGGTGAATG
GTTAGAGTCATTATTTTAGGTGAATG
GTTAGAGTCATTATTTTAGGTGAATG
GTTAGAGTCATTATTTTAGGTGAATG
GTTAGAGTCATTATTTTAGGTGAATG
GTTAGAGTCATTATTTTAGGTGAATG
GTTAGAGTCATTATTTTAGGTGAATG
GTTAGAGTCATTATTTTAGGTGAATG
GTTAGAGTCATTATTTTAGGTGAATG
GTTAGAGTCATTATTTTAGGTGAATG
GTTAGAGTCATTATTTTAGGTGAATG
GTTAGAGTCATTATTTTAGGTGAATG
GTTAGAGTCATTATTTTAGGTGAATG
GTTAGAGTCATTATTTTAGGTGAATG
GTTAGAGTCATTATTTTAGGTGAATG
GTTAGAGTCATTATTTTAGGTGAATG
GTTAGAGTCATTATTTTAGGTGAATG
GTTAGAGTCATTATTTTAGGTGAATG
GTTAGAGTCATTATTTTAGGTGAATG
GTTAGAGTCATTATTTTAGGTGAATG
GTTAGAGTCATTATTTTAGGTGAATG
GTTAGAGTCATTATTTTAGGTGAATG
GTTAGAGTCATTATTTTAGGTGAATG

GTTAGAGTCATTATTTTAGGTGAATG
GTTAGAGTCATTATTTTAGGTGAATG
GTTAGAGTCATTATTTTAGGTGAATG
GTTAGAGTCATTATTTTAGGTGAATG
GTTAGAGTCATTATTTTAGGTGAATG
GTTAGAGTCATTATTTTAGGTGAATG
GTTAGAGTCATTATTTTAGGTGAATG
GTTAGAGTCATTATTTTAGGTGAATG
GTTAGAGTCATTATTTTAGGTGAATG
GTTAGAGTCATTATTTTAGGTGAATG
GTTAGAGTCATTATTTTAGGTGAATG
GTTAGAGTCATTATTTTAGGTGAATG
GTTAGAGTCATTATTTTAGGTGAATG
GTTAGAGTCATTATTTTAGGTGAATG
GTTAGAGTCATTATTTTAGGTGAATG
GTTAGAGTCATTATTTTAGGTGAATG
GTTAGAGTCATTATTTTAGGTGAATG
GTTAGAGTCATTATTTTAGGTGAATG
GTTAGAGTCATTATTTTAGGTGAATG
GTTAGAGTCATTATTTTAGGTGAATG
GTTAGAGTCATTATTTTAGGTGAATG
GTTAGAGTCATTATTTTAGGTGAATG
GTTAGAGTCATTATTTTAGGTGAATG
GTTAGAGTCATTATTTTAGGTGAATG
GTTAGAGTCATTATTTTAGGTGAATG
GTTAGAGTCATTATTTTAGGTGAATG
GTTAGAGTCATTATTTTAGGTGAATG
GTTAGAGTCATTATTTTAGGTGAATG
GTTAGAGTCATTATTTTAGGTGAATG
GTTAGAGTCATTATTTTAGGTGAATG
GTTAGAGTCATTATTTTAGGTGAATG
GTTAGAGTCATTATTTTAGGTGAATG
GTTAGAGTCATTATTTTAGGTGAATG
GTTAGAGTCATTATTTTTAGGTGAATG
GTTAGAGTCATTATTTTAGGTGAATG
GTTAGAGTCATTATTTTAGGTGAATG
GTTAGAGTCATTATTTTAGGTGAATG
GTTAGAGTCATTATTTTAGGTGAATG
GTTAGAGTCATATTTTTAGGTGAATG
GTTAGAGTCATTATTTTAGGTGAATG
GTTAGAGTCATTATTTTAGGTGAATG
GTTAGAGTCATTATTTTAGGTGAATG
GTTAGAGTCATTATTTTAGGTGAATG
GTTAGAGTCATTATTTTAGGTGAATG
GTTAGAGTCATTATTTTAGGTGAATG
GTTAGAGTCATTATTTTAGGTGAATG
GTTAGAGTCATTATTTTAGGTGAATG
GTTAGAGTCATTATTTTAGGTGAATG
GTTAGAGTCATTATTTTAGGTGAATG
GTTAGAGTCATTATTTTAGGTGAATG
GTTAGAGTCATTATTTTAGGTGAATG
GTTAGAGTCATTATTTTAGGTGAATG
GTTAGAGTCATTATTTTAGGTGAATG
GTTAGAGTCATTATTTTAGGCGAATG

GTTTGAGTCATTATTTTAGGTGAATG
GTTAGAGTCATTATTTTAGGTGAATG
GTTAGAGTCATTATTTTAGGTGAATG
GTTAGAGTCATTATTTTAGGTGAATG
GTTAGAGTCATTATTTTAGGTGAATG
GTTTGAGTCATTATTTTAGGTGAATG
GTTAGAGTCATTATTTTAGGTGAATG
GTTAGAGTCATTATTTTAGGTGAATG
GTTAGAGTCATTATTTTAGGCGAATG
GTTTGAGTCATATTTTTCGGTGAATG
GTTAGAGTCATTATTTTAGGTGAATG
GTTAGAGTCATTATTTTACGTGAATG
GTTAGAGTCATTATTTTAGGTGAATG
GTTAGAGTCATTATTTTAGGCGAATG
GTTTGAGTCATTATTTTAGGTGAATG
GTTAGAGTCATTATTTTAGGTGAATG
GTTAGACTCATTATGTTAGGTGAATG
GTTAGAGTCATTATTTTACGTGAATG
GTTAGAGTCATTATTTTAGGTGAATG
GTTAGAGTCATTATTTTAGGTGAATG
GTTAGAGTCATTATTTTAGGTGAATG
GTTAGACTCATTATGTTAGGTGAATG
GTTAGAGTCATTATTTTAGGTGAATG
GTTAGAGTCATTATTTTAGGTGAATG
GTTAGAGTCATTATTTTAGGTGAATG
GTTAGAGTCATATTTTTAGGTGAATG
GTTGAGTCATTATTTTAGGTGAAT
GTTAGAGTCATTATTTTAGGTGAATG
GTTAGAGTCATTATTTTAGGTGAATG
GTTAGAGTCATTATTTTACGTGAATG
GTTAGACTCATTATTTTAGGTGAATG
GTTAGAGTCATTATTTTACGTGAATG
GTTAGAGTCATTATTTTAGGTGAATG
GTTAGAGTCATTATTTTAGGTGAATG
GTTAGAGTCATTATTTTACGTGAATG
GTTAGAGTCATTATTTTAGGTGAATG
GTTAGAGTCATTATTTTAGGCGAATG
GTTAGAGTCATTATTTTAGTTGAATG
GTTAGAGTCATTATTTTAGGTGAATG
GTTAGAGTCATTATGTTAGGTGAATG
GTTAGAGTCATTATTTTAGGTGAATG
GTTAGAGTCATTATTTTAGGTGAATG
GTTAGAGTCATATTTTTAGGCGAATG
GTTTGAGTCATTATTTTAGGTGAATA
GTTAGAGTCATTATTTTAGGTGAATG
GTTAGAGTCATTATTTTAGGTGAATG
GTTAGAGTCATTATTTTAGGTGAATG
GTTAGAGTCATTATGTTAGGTGAATG
GTTAGAGTCATTATTTTACGTGAATG
GTTAGAGTCATTATTTTAGGTGAATG
GTTAGAGTCATTATTTTAGGCGAATG
GTTAGAGTCATTATTTTAGGTGAATG
GTTAGAGTCATTATTTTAGGCGAATG
GTTAGAGTCATTATTTTAGGTGAATG

GTTAGAGTCATTATTTTAGGTGAATG
GTTAGAGTCATTATTTTAGGTGAATG
GTTAGAGTCATTATTTTAGGAATG
GTTAGAGTCATTATTTTAGGTGAACG
GTTAGAGTCATTATTTTAGGTGAATG
GTTAGAGTCATTATTTTACGTGAATG
GTTAGAGTCATTATTTTACGTGAATG
GTTAGAGTCATTATTTTAGGTGAATG
GTTAGAGTCATTATTTTAGGTGAATG
GTTAGAGTCATTATTTTAGGTGAATG
GTTAGAGTCATTATTTTAGGTGAATG
GTTAGAGTCATTATTTTAGGTGAATG
GTTAGAGTCATTATTTTACGTGAATG
GTTAGAGTCATTATTTTACGTGAATG
GTTAGAGTCATTATTTTAGGTGAATG
GTTAGAGTCATTATTTTAGGCGAATG
GTTAGAGTCATTATTTTAGGTGAATG
GTTAGAGTCATTATTTTAGGTGAATG
GTTAGAGTCATTATTTTAGGTGAATG
GTTAGAGTCATTATTTTAGGTGAATG
GTTAGAGTCATTATTTTAGGTGAATG
GTTAGAGTCATTATTTTAGGTGAATG
GTTAGAGTCATTATTTTACGTGAATG
GTTAGAGTCATTATTTTAGGTGAATG
GTTAGAGTCATTATTTTAGGTGAATG
GTTAGAGTCATTATTTTAGGTGAATG
GTTAGAGTCATTATTTTAGGTGAATG
GTTAGAGTCATTATTTTAGGTGAATG
GTTAGAGTCATTATTTTAGGTGAATG
GTTAGAGTCATTATTTTAGGTGAATG
GTTAGAGTCATTATTTTAGGTGAATG
GTTAGAGTCATTATTTTAGGTGAATG
GTTAGAGTCATATTTTTAGGTGAATG
GTTAGAGTCATTATTTTAGGTGAATG
GTTAGAGTCATATTTTTAGGTGAATG
GTTAGAGTCATTATTTTAGGCGAATG
GTTTGAGTCATTATTTTAGGTGAATG
GTTAGAGTCATTATTTTAGGTGAATA
GTTAGAGTCATTATTTTAGGCGAATG
GTTTGAGTCATTATTTTAGGTGAACG
GTTAGAGTCATTATTTTAGGTGAATGG
Nr of selected repeats 1416 Similarity 0.990443
Consensus:
GTTAGAGTCATTATTTTAGGTGAATG
>Cele-UNSB01_3:5534873-5535003 Satlength=131 Nr of Repeats=5 RepeatLength=26
seed=ACCATTCACC
ACCATTCACCTAACATAATGACTCTA
ACCATTCACCCAAAATAATGACTCTA
ACCATTCACCTAAAAATATGACTCTA
ACCATTCACCTAAAATAATGACTCTA
ACCATTCACCTAAAATAATGACTCTA
Nr of selected repeats 5 Similarity 0.917949
Consensus:
ACCATTCACCtAAaAtaATGACTCTA

>Cele-UNSB01_3:6120416-6122897 Satlength=2482 Nr of Repeats=10 RepeatLength=228
seed=CCACCAGATC
CCACCAGATCAGCAGCGTCTCATCTTCGCCGGAAAGCAGCTCGAGGACGGCCGCACCCTGTCTGACTACAACATCCAGA
AGGAATCTACTCTTCACTTGGTTCTTCGTCTTAGAGGAGGAATGCAGATCTTTGTCAAGACTTTGACTGGAAAGACCAT
CACACTTGAAGTTGAAGCTTCCGACACGATCGAGAACGTCAAGGCCAAGATTCAAGACAAGGAGGGAATC
CCACCAGATCAGCAGCGTCTTATCTTTGCTGGTATGTTACATATAACAAATTTTGTTCATGAGAGACTAATTTTTTTCC
AGGAAAGCAATTGGAAGATGGACGCACACTCTCTGATTACAATATTCAGAAAGAGTCTACTCTCCACTTGGTGCTCCGC
CTCAGAGGAGGTATGCAGATCTTCGTCAAGACATTGACTGGAAAGACCATCACACTTGAAGTCGAAGCTTCCGACACGA
TCGAAAATGTCAAGGCTAAGATTCAAGATAAAGAAGGAATC
CCACCAGATCAGCAAAGACTTATCTTCGCCGGAAAGCAGCTCGAGGACGGCCGCACCCTTTCGGACTACAACATCCAGA
AGGAATCAACTCTTCATTTGGTTCTCCGTTTGAGAGGAGGTATGCAGATCTTCGTCAAGACATTGACCGGAAAGACCAT
CACCCTCGAAGTCGAAGCCTCCGACACCATCGAAAATGTCAAGGCCAAGATCCAAGACAAGGAAGGAATT
CCACCAGATCAGCAGAGACTTATTTTTGCTGGTACGTTGGCAAAATATCTAATATTTGACCTAAAATTTATTATATATT
TTCAGGAAAGCAACTCGAGGATGGCCGTACCCTTTCGGACTACAATATCCAGAAGGAATCAACCCTCCATTTGGTGCTC
CGCCTAAGAGGAGGAATGCAGATCTTCGTCAAGACTTTGACCGGAAAGACTATTACTCTTGAGGTTGAAGCTTCTGACA
CTATTGAGAACGTGAAGGCCAAGATCCAAGACAAGGAAGGTATC
CCACCAGATCAACAGCGTTTGATCTTTGCCGGAAAGCAACTCGAGGATGGCCGTACTCTCTCCGATTACAACATCCAAA
AGGAGTCTACTCTTCATTTGGTTCTCCGTCTCCGAGGAGGAATGCAAATCTTCGTCAAGACTCTTATCGGAAAGACCAT
CACCCTCGAAGTCGAAGCCTCCGATACCATCGAGAACGTGAAGGCCAAGATTCAGGACAAGGAAGGAATT
CCTCCAGATCAGCAACGTCTCATCTTCGCTGGAAAGCAGCTCGAAGACGGCCGCACCCTTTCGGACTACAACATCCAGA
AGGAATCAACTCTTCATTTGGTTCTCCGTTTGAGAGGAGGTATGCAAATCTTCGTGAAGACTTTGACTGGAAAGACTAT
CACCCTCGAAGTCGAAGCCTCTGATACCATCGAAAATGTGAAGGCCAAGATCCAAGACAAGGAAGGAATC
CCACCAGATCAGCAGCGTCTTATCTTTGCCGGTACGTTATATAGATATACATAACTCAAATCAACTATTATTATTTCAG
GAAAGCAATTGGAAGATGGGCGCACGCTCTCTGATTACAACATTCAGAAGGAATCTACTCTTCACTTGGTTCTCCGTCT
CCGAGGAGGAATGCAGATCTTCGTCAAGACTCTTACAGGAAAGACCATCACGCTCGAGGTGGAAGCTTCTGACACTATT
GAGAACGTGAAGGCCAAGATCCAAGACAAAGAAGGTATC
CCACCAGATCAGCAACGCTTGATCTTCGCCGGAAAGCAACTCGAGGATGGCCGTACCCTTTCGGACTACAATATCCAGA
AGGAATCAACCCTCCATTTGGTGCTCCGTCTCAGAGGAGGTATGCAGATCTTCGTCAAGACATTGACTGGAAAGACCAT
CACACTTGAAGTCGAAGCCTCTGATACCATCGAGAATGTGAAGGCCAAGATTCAAGACAAGGAAGGAATC
CCACCAGATCAGCAGAGACTCATCTTCGCCGGAAAACAACTCGAAGACGGTCGTACCCTCTCCGACTACAACATCCAAA
AGGAGTCTACTCTTCATTTGGTTCTCCGTCTGAGAGGAGGTATGCAGATCTTCGTCAAGACATTGACCGGAAAGACCAT
CACCCTCGAAGTCGAAGCCTCCGACACCATCGAAAATGTCAAGGCCAAGATCCAAGACAAGGAAGGAATT
CCACCAGATCAGCAGAGACTTATTTTCGCTGGTGAGTTCATATTGTTTTTAGAATTAAAACTAATTTTTATTGTTTTTC
AGGAAAGCAACTCGAGGATGGCCGTACCCTTTCGGACTACAATATCCAGAAGGAGTCTACTCTTCATTTGGTGCTCCGT
CTCAGAGGAGGTATGCAGATCTTCGTCAAGACTTTGACTGGAAAAACCATCACTCTCGAGGTCGAAGCTTCGGACACCA
TTGAGAATGTCAAAGCCAAAATCCAGGATAAGGAGGGAATC
Nr of selected repeats 6 Similarity 0.843765
Consensus:
CCACCAGATCAGCAacGtCTcATCTTCGCCGGAAAGCAaCTCGAgGAcGGCCGcACCCTtTCgGACTACAACATCCAgA
AGGAaTCaACTCTTCATTTGGTTCTCCGTcTgAGAGGAGGtATGCAgATCTTCGTCAAGACaTTGACcGGAAAGACCAT
CACcCTcGAAGTCGAAGCCTCcGAcACCATCGAaAAtGTcAAGGCCAAGATcCAAGACAAGGAAGGAATc
>Cele-UNSB01_3:6192343-6192405 Satlength=63 Nr of Repeats=4 RepeatLength=15
seed=CGAAAAGGGG
CGAAAAGGGGAGCAGAA
CGAAAAGGGGATCTG
CAAAAAGGGGATCTG
CGAAAAGGGGAGCAA
Nr of selected repeats 3 Similarity 0.762963
Consensus:
CgAAAAGGGGAtCtg
>Cele-UNSB01_3:6192580-6192657 Satlength=78 Nr of Repeats=5 RepeatLength=15
seed=TCCCCTTTTC
TCCCCTTTTCGTTGC
TCCCCTTTTCGTATC

TCCCCTTTTCGCAGA
TCCCCTTTTTGCAGA
TCCCCTTTTCGTTCTGC
Nr of selected repeats 4 Similarity 0.748148
Consensus:
TCCCCTTTTcGcaga
>Cele-UNSB01_3:6364525-6365839 Satlength=1315 Nr of Repeats=65 RepeatLength=19
seed=AAAAATAGAT
AAAAATAGATTGTTACACG
AAAAATAGATTGTTATACGAAAAAAATGCATTGTTACACA
AAAAATAGATTGTTACACG
AAAAATAGATTGTTATACGAAAAAAATGCATTGTTACACA
AAAAATAGATTGTTACACG
AAAAATAGATTGTTACACG
AAAAATAGATTGTTACACG
AAAAATAGATTGTTATACG
AAAAATAGATTGTTACACG
AAAAATAGATTGTTATACGAAAAAAATGCATTGTTACACA
AAAAATAGATTGTTACACG
AAAAATAGATTGTTACACG
AAAAATAGATTGTTATACG
AAAAATAGATTGTTACACG
AAAAATAGATTGTTATACGAAAAAATGCATTGTTACACA
AAAAATAGATTGTTACACG
AAAAATAGATTGTTACACG
AAAAATAGATTGTTACACG
AAAAATAGATTGTTACACA
AAAAATAGATTGTTACACG
AAAAATAGATTGTTACACG
AAAAATAGATTGTTACACG
AAAAATAGATTGTTACACG
AAAAATAGATTGTTACACA
AAAAATAGATTGTTACACG
AAAAATAGATTGTTACACG
AAAAATAGATTGTTACACG
AAAAATAGATTGTTACACG
AAAAATAGATTGTTACACA
AAAAATAGGTTGTTACACG
AAAAATAGATTGTTACACG
AAAAATAGATTGTTACACG
AAAAATAGATTGTTACACG
AAAAATAGATTGTTACACG
AAAAATAGATTGTTACACG
AAAAATAGATTGTTACACG
AAAAATAGATTGTTACACG
AAAAATAGATTGTTACACG
AAAAATAGATTGTTACACA
AAAAATAGGTTGTTACACG
AAAAATAGATTGTTACACG
AAAAATAGATTGTTACACG
AAAAATAGATTGTTACACG
AAAAATAGATTGTTACACG
AAAAATAGATTGTTACACA
AAAAATAGGTTGTTACACG

AAAAATAGATTGTTACACG
AAAAATAGATTGTTACACG
AAAAATAGATTGTTACACG
AAAAATAGATTGTTACACG
AAAAATAGATTGTTACACG
AAAAATAGATTGTTACACG
AAAAATAGATTGTTACACG
AAAAATAGATTGTTACACA
AAAAATAGATTGTTACACG
AAAAATAGATTGTTACACG
AAAAATAGATTGTTACACA
AAAAATAGGTTGTTACACG
AAAAATAGATTGTTACACA
AAAAATAGGTTGTTACACG
AAAAATAGATTGTTACACG
AAAAATAGATTGTTACACG
AAAAATAGATTGTTACACG
AAAAATAGATTGTTACACG
AAAAATAGATTGTTACACG
Nr of selected repeats 61 Similarity 0.968479
Consensus:
AAAAATAGATTGTTACACG
>Cele-UNSB01_3:6731291-6731428 Satlength=138 Nr of Repeats=9 RepeatLength=15
seed=CGAAAAGGGG
CGAAAAGGGGAGCAGAA
CGAAAAGGGGATCTG
CAAAAAGGGGATCTG
CGAAAAGGGGAGATA
CGAAAAGGGGAAATA
CGAAAAGGGGAGATA
CGAAAAGGGGAGATA
CGAAAAGGGGAGATA
CGAAAAGGGGAGATA
Nr of selected repeats 8 Similarity 0.847619
Consensus:
CGAAAAGGGGAgaTa
>Cele-UNSB01_3:6731603-6731679 Satlength=77 Nr of Repeats=5 RepeatLength=15
seed=TCCCCTTTTC
TCCCCTTTTCGTTGC
TCCCCTTTTTCGTATC
TCCCCTTTTCGTATC
TCCCCTTTTCGCAGA
TCCCCTTTTTGCAGA
Nr of selected repeats 4 Similarity 0.748148
Consensus:
TCCCCTTTTcGcaga
>Cele-UNSB01_3:6746945-6756350 Satlength=9406 Nr of Repeats=204 RepeatLength=11
seed=GTACTGTAGG
GTACTGTAGGA
GTACTGTAGGTATACGGTAGGGTTACTGTAGTTTAGGAAAATTGAGTTTTTGTCTACAGAAGAGGTATTGGGTAGAGAG
TTGCTGGGGGATAATGCCAAG
GTACTGTAGTA
GTACTGTAGTA

GTATTGTAGGTATACGGTAGGGTTACTGTAGTTTACGAAAAATTGAGTTTTTGTCTACAGAAGAGGTATTGGGTTGGGA
GTTGCTGGGGGATAATGTCAAG
GTACTGTAGTA
GTACTGTAGGA
GTACTGTAGGTATACGGTAGGGTTACTGTAGTTTAGGAAAATTGAGTTTTTGTCTACAGAAGAGGTATTGGGTAGAGAG
TTGCTGGGGGATAATGCCAAG
GTACTGTAGTA
GTACTGTAGTA
GTATTGTAGGTATACGGTAGGGTTACTGTAGTTTACGAAAAATTGAGTTTTTGTCTACAGAAGAGGTATTGGGTAGAGA
GTTGCTGGGGGATAATGTCAAG
GTACTGTAGTA
GTACTGTAGGTATACGGTAGAGTTTCTGTAGTTTAGGAAACATTGAGTTTTTGTCTACAGAAGAGGTATTGGGTTGGGA
GTTGGTGAGGGATAATGTCAAA
GTACTGTAGTG
GTACTGTAGGA
GTACTGTAGGTATACGGTAGGGTTACTGTAGTTTAGGAAAAAATGAGTTTTTCTCTACAGAAGATGTATTGGGTTGGGAG
TTGGTGAGGGATAATGTCAAG
GTACTGTAGTG
GTACTGTAGGA
GTACTGTAGGTATACGGTAGGGTTGCTGTAGTTTAGGAAAAATTGAGTTTTTGTATACAGAAGAGGTATTGGGTTGGGA
GTTGCTGGGGGATAATGTCAAG
GTACTGTAGTA
GTACTGTAGGA
GTACTGTAGGTATACGGTAGGGTTACTGTAGTTTAGGAAAATTGAGTTTTTGTCTACAGAAGAGGTATTGGGTTGGGAG
TTGCTGGGGGATAATGTCAAG
GTACTGTAGTA
GTACTGTAGGA
GTACTGTAGGTATACGGTAGGGTTACTGTAGTTTAGGAAAATTGAGTTTTTGTCTACAGAAGAGGTATTGGGTAGAGAG
TTGCTGGGGGATAATGCCAAG
GTACTGTAGTA
GTACTGTAGTA
GTATTGTAGGTATACGGTAGGGTTACTGTAGTTTAGGAAAATTGAGTTTTTGTCTACAGAAGAGGTTTTGGGTAGAGAG
TTGCTGGGGGATAATGCCAAG
GTACTGTAGTA
GTACTGTAGTA
GTATTGTAGGTATACGGTAGGGTTACTGTAGTTTACGAAAAATTGAGTTTTTGTCTACAGAAGAGGTATTGGGTAGAGA
GTTGCTGGGGGATAATGTCAAG
GTACTGTAGTA
GTACTGTAGGTATACGGTAGAGTTTCTGTAGTTTAGGAAACATTGAGTTTTTGTCTACAGAAGAGGTATTGGGTAGAGA
GTTGCTGGGGGATAATGTCAAG
GTACTGTAGTA
GTACTGTAGGTATACGGTAGAGTTTCTGTAGTTTAGGAAACATTGAGTTTTTGTCTACAGAAGAGGTATTGGGTTGGGA
GTTGGTGAGGGATAATGTCAAA
GTACTGTAGTG
GTACTGTAGGA
GTACTGTAGGTATACGGTAGGGTTACTGTAGTTTAGGAAAAAATTGAGTTTTTCTCTACAGAAGATGTATTGGGTTGGGA
GTTGGTGAGGGATAATGTCAAG
GTACTGTAGTG
GTACTGTAGGA
GTACTGTAGGTATACGGTAGGGTTACTGTAGTTTAGGAAAATTGAGTTTTTGTCTACAGAAGAGGTATTGGGTAGAGAG
TTGCTGGGGGATAATGCCAAG
GTACTGTAGTA
GTACTGTAGTA

GTATTGTAGGTATACGGTAGGGTTACTGTAGTTTAGGAAAATTGAGTTTTTGTCTACAGAAGAGGTTTTGGGTAGAGAG
TTGCTGGGGGATAATGCCAAG
GTACTGTAGTA
GTATTGTAGGTATACGGTAGGGTTACTGTAGTTTACGAAAAATTGAGTTTTTGTCTACAGAAGAGGTATTGGGTAGAGA
GTTGCTGGGGGATAATGTCAAG
GTACTGTAGTA
GTACTGTAGGTATACGGTAGAGTTTCTGTAGTTTAGGAAACATTGAGTTTTTGTTTACAGAAGAGGTATTGGGTAGAGA
GTTGCTGGGGGATAATGTCAAG
GTACTGTAGTA
GTACTGTAGGTATACGGTAGAGTTTCTGTAGTTTAGGAAAATTGAGTTTTTGTCTACAGAAGAGGTATTGGGTTGGGAG
TTGGTGAGGGATAATGTCAAA
GTACTGTAGTG
GTACTGTAGGA
GTACTGTAGGTATACGGTAGGGTTACTGTAGTTTAGGAAAAATTGAGTTTTTCTCTACAGAAGATGTATTGGGTTGGGA
GTTGCTGGGGGATAATGTCAAG
GTACTGTAGTA
GTACTGTAGGA
GTACTGTAGGTATACGGTAGGGTTACTGTAGTTTAGGAAAATTGAGTTTTTGTCTACAGAAGAGGTATTGGGTTGGGAG
TTGCTGGGGGATAATGTCAAG
GTACTGTAGTA
GTACTGTAGGA
GTACTGTAGGTATACGGTAGGGTTACTGTAGTTTAGGAAAATTGAGTTTTTGTCTACAGAAGAGGTATTGGGTAGAGAG
TTGCTGGGGGATAATGCCAAG
GTACTGTAGTA
GTACTGTAGTA
GTATTGTAGGTATACGGTAGGGTTACTGTAGTTTAGGAAAATTGAGTTTTTGTCTACAGAAGAGGTTTTGGGTAGAGAG
TTGCTGGGGGATAATGCCAAG
GTACTGTAGTA
GTACTGTAGTA
GTATTGTAGGTATACGGTAGGGTTACTGTAGTTTACGAAAAATTGAGTTTTTGTCTACAGAAGAGGTATTGGGTAGAGA
GTTGCTGGGGGATAATGTCAAG
GTACTGTAGTA
GTACTGTAGGTATACGGTAGAGTTTCTGTAGTTTAGGAAAATTGAGTTTTTGTCTACAGAAGAGGTATTGGGTTGGAGT
TGGTGAGGGATAATGTCAAA
GTACTGTAGTG
GTACTGTAGGA
GTACTGTAGGTATACGGTAGGGTTACTGTAGTTTAGGAAAAATTGAGTTTTTCTCTACAGAAGATGTATTGGGTTGGGA
GTTGGTGAGGGATAATGTCAAG
GTACTGTAGTG
GTACTGTAGGA
GTACTGTAGGTATACGGTAGGGTTGCTGTAGTTTAGGAAAAATTGAGTTTTTGTATACAGAAGAGGTATTGGGTTGGAG
TTGCTGGGGATAATGTCAAG
GTACTGTAGTA
GTACTGTAGGA
GTACTGTAGGTATACGGTAGGGTTACTGTAGTTTAGGAAAATTGAGTTTTTGTCTACAGAAGAGGTATTGGGTTGGAGT
TGCTGGGGATAATGTCAAG
GTACTGTAGTA
GTACTGTAGGTATACGGTAGAGTTTCTGTAGTTTAGGAAACATTGAGTTTTTGTCTACAGAAGAGGTATTGGGTTGAGA
GTTGCTGGGGGATAATGTCAAG
GTACTGTAGTA
GTACTGTAGGTATACGGTAGAGTTTCTGTAGTTTAGGAAACATTGAGTTTTTGTCTACAGAAGAGGTATTGGGTTGGAG
TTGCTGGGGGATAATGTCAAG
GTACTGTAGTA

GTATTGTAGGTATACGGTAGGGTTACTGTAGTTTAGGAAAAATTGAGTTTTTGTCTACAGAAGAGGTATTGGGTAGAGA
GTTGCTGGGGGATAATGTCAAG
GTACTGTAGTA
GTACTGTAGGTATACGGTAGAGTTTCTGTAGTTTAGGAAAATTGAGTTTTTGTCTACAGAAGAGGTATTGGGTTGGAGT
TGGTGAGGGATAATGTCAA
GTACTGTAGTG
GTACTGTAGGA
GTACTGTAGGTATACGGTAGGTTTCTGTAGTTTAGGAAAAATTGAGTTTTTGTCTACAGAAGAGGTATTGGGTTGGAGT
TGGTGAGGGATAATGTCAAG
GTACTGTAGTG
GTACTGTAGGA
GTACTGTAGGTATACGGTAGGGTTACTGTAGTTTAGGAAAAATTGAGTTTTTGTCTACAGAAGAGGTATTGGGTTGGAG
TTGCTGGGGATAATGTCAAG
GTACTGTAGTA
GTACTGTAGTA
GTATGTAGGTATACGGTAGGGTTACTGTAGTTTAGGAAAAATTGAGTTTTTGTCTACAGAAGAGGTATTGGGTTGGAGT
TGCTGGGGATAATGTCAAG
GTACTGTAGTA
GTACTGTAGGA
GTACTGTAGGTATACGGTAGGGTTACTGTAGTTTAGGAAAATTGAGTTTTTGTCTACAGAAGAGGTATTGGGTTGAGAG
TTGCTGGGGATAATGCCAAG
GTACTGTAGTA
GTACTGTAGTA
GTATGTAGGTATACGGTAGGGTTACTGTAGTTTAGGAAAAATTGAGTTTTTGTCTACAGAAGAGGTATTGGGTTGAGAG
TTGCTGGGGATAATGTCAAG
GTACTGTAGTA
GTACTGTAGGTATACGGTAGGTTTCTGTAGTTTAGGAAAATTGAGTTTTTGTCTACAGAAGAGGTATTGGGTTGGAGTT
GGTGAGGGATAATGTCAAG
GTACTGTAGTG
GTACTGTAGGA
GTACTGTAGGTATACGGTAGGGTTACTGTAGTTTAGGAAAAATTGAGTTTTTGTCTACAGAAGAGGTATTGGGTTGGAG
TTGGTGAGGGATAATGTCAAG
GTACTGTAGTG
GTACTGTAGGA
GTACTGTAGGTATACGGTAGGGTTACTGTAGTTTAGGAAAAATTGAGTTTTTGTCTACAGAAGAGGTATTGGGTTGGAG
TTGCTGGGGGATAATGTCAAG
GTACTGTAGTA
GTACTGTAGGA
GTACTGTAGGTATACGGTAGGGTTACTGTAGTTTAGGAAAATTGAGTTTTTGTCTACAGAAGAGGTATTGGGTTGGAGT
TGCTGGGGATAATGTCAAG
GTACTGTAGTA
GTACTGTAGGA
GTACTGTAGGTATACGGTAGGGTTACTGTAGTTTAGGAAAATTGAGTTTTTGTCTACAGAAGAGGTATTGGGTTGGAGT
TGCTGGGGATAATGCAAG
GTACTGTAGTGTACTGTAGTA
GTACTGTAGGTATACGGTAGGGTTACTGTAGTTTAGGAAAATTGAGTTTTTGTCTACAGAAGAGGTATTGGGTTGGAGT
TGCTGGGGATAATGCAAG
GTACTGTAGTA
GTACTGTAGTA
GTATGTAGGTATACGGTAGGGTTACTGTAGTTTAGGAAAAATTGAGTTTTTGTCTACAGAAGAGGTATTGGGTTGAGAG
TTGCTGGGGGATAATGTCAAG
GTACTGTAGTA
GTACTGTAGGTATACGGTAGGGTTACTGTAGTTTAGGAAAATTGAGTTTTTGTCTACAGAAGAGGTATTGGGTTGGAGT
TGCTGGGGATAATGTCAAG

GTACTGTAGTA
GTACTGTAGGTATACGGTAGGGTTACTGTAGTTTAGGAAAATTGAGTTTTTGTCTACAGAAGAGGTATTGGGTTGGAGT
TGGTGAGGGATAATGTCAAG
GTACTGTAGTG
GTACTGTAGGA
GTACTGTAGGTATACGGTAGGGTTACTGTAGTTTAGGAAAAATTGAGTTTTTCTCTACAGAAGAGGTATTGGGTTGGAG
TTGGTGAGGGATAATGTCAAG
GTACTGTAGTG
GTACTGTAGGA
GTACTGTAGGTATACGGTAGGGTTACTGTAGTTTAGGAAAAATTGAGTTTTTGTCTACAGAAGAGGTATTGGGTTGAGA
GTTGCTGGGGATAATGTCAAG
GTACTGTAGT
GTACTGTAGGA
GTACTGTAGGTATACGGTAGGGTTACTGTAGTTTAGGAAAATTGAGTTTTTGTCTACAGAAGAGGTATTGGGTAGAGAG
TTGCTGGGGATAATGCAAG
GTACTGTAGTA
GTACTGTAGGTATGTAGGTATACGGTAGGGTTACTGTAGTTTAGGAAAAATTGAGTTTTTGTCTACAGAAGAGGTATTG
GGTTGAGTTGCTGGGGATAATGTCAAG
GTACTGTAGTA
GTACTGTAGGTATACGGTAGAGTTACTGTAGTTTAGGAAAATTGAGTTTTTGTCTACAGAAGAGGTATTGGGTTGGAGT
TGCTGGGGATAATGTCAAG
GTACTGTAGTA
GTACTGTAGGTATACGGTAGGGTTACTGTAGTTTAGGAAAATTGAGTTTTTGTCTACAGAAGAGGTATTGGGTTGGAGT
TGGTGAGGATAATGTCAAG
GTACTGTAGTG
GTACTGTAGGA
GTACTGTAGGTATACGGTAGGGTTACTGTAGTTTAGGAAAAATTGAGTTTTTCTACAGAAGAGGTATTGGGTTGGAGTT
GCTGGGGATAATGTCAAG
GTACTGTAGTA
GTACTGTAGGA
GTACTGTAGGTATACGGTAGGGTTACTGTAGTTTAGGAAAATTGAGTTTTTGTCTACAGAAGAGGTATTGGGTTGGAGT
TGCTGGGGATAATGTCAAG
GTACTGTAGT
GTACTGTAGGA
GTACTGTAGGTATACGGTAGGGTTACTGTAGTTTAGGAAAATTGAGTTTTTGTCTACAGAAGAGGTATTGGGTTGAGAG
TTGCTGGGGATAATGCAAG
GTACTGTAGT
GTACTGTAGGA
GTACTGTAGGTATACGGTAGGGTTACTGTAGTTTAGGAAAATTGAGTTTTTGTCTACAGAAGAGGTATTGGGTAGAGAG
TTGCTGGGGATAATGCAAG
GTACTGTAGT
GTACTGTAGGAGTATGTAGGTATACGGTAGGGTTACTGTAGTTTAGGAAAAATTGAGTTTTTGTCTACAGAAGAGGTAT
TGGGTTGGAGTTGCTGGGGATAATGTCAAG
GTACTGTAGTA
GTACTGTAGGTATACGGTAGGTTACTGTAGTTTAGGAAAAATTGAGTTTTTGTCTACAGAAGAGGTATTGGGTTGGAGT
TGGTGAGGATAATGTCAAG
GTACTGTAGTG
GTACTGTAGGA
GTACTGTAGGTATACGGTAGGGTTACTGTAGTTTAGGAAAAATTGAGTTTTTCTACAGAAGAGGTATTGGGTTGGAGTT
GGTGAGGGATAATGTCAAG
GTACTGTAGTG
GTACTGTAGGA
GTACTGTAGGTATACGGTAGGGTTACTGTAGTTTAGGAAAAATTGAGTTTTTGTCTACAGAAGAGGTATTGGGTTGGAG
TTGCTGGGGATAATGTCAAG

GTACTGTAGTA
GTACTGTAGGA
GTACTGTAGGTATACGGTAGGGTTACTGTAGTTTAGGAAAAATTGAGTTTTTGTCTACAGAAGAGGTATTGGGTTGGGA
GTTGCTGGGGGATAATGTCAAG
GTACTGTAGTA
GTACTGTAGGTATACGGTAGAGTTTCTGTAGTTTAGGAAACATTGAGTTTTTGTCTACAGAAGAGGTATTGGGTTGAAG
TTGCTGGGGATAATGTCAAG
GTACTGTAGTA
GTACTGTAGGTATACGGTAGAGTTACTGTAGTTTAGGAAAATTGAGTTTTTGTCTACAGAAGAGGTATTGGGTTGGGAG
TTGCTGGGGGATAATGTCAAG
GTACTGTAGTA
GTATTGTAGGTATACGGTAGGGTTACTGTAGTTTACGAAAAATTGAGTTTTTGTCTACAGAAGAGGTATTGGGTTGGAG
TTGCTGGGGATAATGTCAAG
GTACTGTAGTA
GTACTGTAGGTATACGGTAGAGTTTCTGTAGTTTAGGAAACATTGAGTTTTTGTCTACAGAAGAGGTATTGGGTTGGAG
TTGGTGAGGGATAATGTCAA
GTACTGTAGTG
GTACTGTAGGA
GTACTGTAGGTATACGGTAGAGTTTCTGTAGTTTAGGAAACATTGAGTTTTTGTCTACAGAAGAGGTATTGGGTTGGGA
GTTGGTGAGGGATAATGTCAAG
GTACTGTAGTG
GTACTGTAGGA
GTACTGTAGGTATACGGTAGGGTTGCTGTAGTTTAGGAAATTTTGAGTTTTTGTCTACAGAAGAGGTATTGGGTAGAGA
GTTGCTGGGGGATAATGTCAAG
GTACTGTAGTA
GTACTGTAGGTATACGGTAGAGTTTCTGTAGTTTAGGAAACATTGAGTTTTTGTCTACAGAAGAGGTATTGGGTTGGAG
TTGGTGAGGGATAATGTCAAA
GTACTGTAGTG
GTACTGTAGGA
GTACTGTAGGTATACGGTAGAGTTTCTGTAGTTTAGGAAACATTGAGTTTTTGTCTACAGAAGAGGTATTGGGTTGGGA
GTTGGTGAGGGATAATGTCAAA
GTACTGTAGTG
GTACTGTAGGA
GTACTGTAGGTATACGGTAGAGTTTCTGTAGTTTAGGAAACATTGAGTTTTTGTCTACAGAAGAGGTATTGGGTTGGGA
GTTGCTGGGGGATAATGTCAAG
GTACTGTAGTA
GTACTGTAGGA
GTACTGTAGGTATACGGTAGGGTTTCGGACGAGACCTGGATAAGGAAAGGACAACTAACGGAGTGGAGAAAACAGGGGA
TCGGAAACGAGTCGCGAGATCAATCAATTTACTATATATAAAGCGCGTGTCCTTCCGTCCGTTTGTAGTTTGTAGTTTG
ATCTTTGATCTTTCAACTCTCTTTTCTGTAGTTGGAAGAGGTATTGGGTAGAGAGTTGCTGGGGGATAATGTCAAG
GTACTGTAGTA
GTACTGTAGGA
GTACTGTAGGTATACGGTAGGGTTACTGTAGTTTAGGAAAAATTGAGTTTTTGTCTACAGAAGAGGTATTGGGTTGGGAG
TTGCTGGGGGATAATGTCAAG
GTACTGTAGTA
GTACTGTAGGA
GTACTGTAGGTATACGGTAGGGTTTCGGACGAGACCTGGATAAGGAAAGGACAACTAACGGAGTGGAGAAAACAGGGGA
TCGGAAACGAGTCGCGAGATCAATCAATTTACTATATATAAAGCGCGTGTCCTTCCGTCCGTTTGTAGTTTGTAGTTTG
ATCTTTGATCTTTCAACTCTCTTTTCTGTAGTTGGAAGAGGTATTGGGTAGAGAGTTGCTGGGGGATAATGTCAAG
GTACTGTAGTA
GTACTGTAGGA
GTACTGTAGGTATACGGTAGGGTTACTGTAGTTTAGGAAAAATTGAGTTTTTCTCTACAGAAGATGTATTGGGTTGGGA
GTTGGTGAGGGATAATGTCAAG
GTACTGTAGTG

GTACTGTAGGA
GTACTGTAGGTATACGGTAGGGTTTCGGACGAGACCTGGATAAGGAAAGGACAACTAACGGAGTGGAGAAAACAGGGGA
TCGGAAACGAGTCGCGAGATCAATCAATTTACTATATATAAAGCGCGTGTCCTTCCGTCCGTTTGTAGTTTGTAGTTTG
ATCTTTGATCTTTCAACTCTCTTTTCTGTAGTTGGAAGAGGTATTGGGTAGAGAGTTGCTGGGGGATAATGTCAAG
GTACTGTAGTA
GTACTGTAGGA
GTACTGTAGGTATACGGTAGGGTTACTGTAGTTTAGGAAAAATTGAGTTTTTCTCTACAGAAGATGTATTGGGTTGGGA
GTTGGTGAGGGATAATGTCAAG
GTACTGTAGTG
GTACTGTAGGA
Nr of selected repeats 123 Similarity 0.909143
Consensus:
GTACTGTAGtA
>Cele-UNSB01_3:6807960-6808176 Satlength=217 Nr of Repeats=6 RepeatLength=21
seed=AAATCTAATC
AAATCTAATCCAATGTGGAAC
AAATCTAATCCAATATGGCCG
AAATCTAATCCAATATGGAAC
AAATCTAATCCAATATGGCCG
AAATCTAATCCAATATGGTCG
AAATCTAATCAAATATGGCTGAGATCGTGCCATATTGGATTAGATTTCGACCATATTGGATTAGATTTCGACCATATTA
GATTAGATTTGTCTTCTAATGAAATATGGAGG
Nr of selected repeats 5 Similarity 0.822727
Consensus:
AAATCTAATCCAATaTGGnCg
>Cele-UNSB01_3:7587437-7632311 Satlength=44875 Nr of Repeats=1482
RepeatLength=27 seed=GTGGGAAGCC
GTGGGAAGCCACAGAGAGTAAAAAATA
GTGGGAAGCCACAGAGAGTAAAATATG
GTGGGAAGCCACAGAGAGTAAAATATA
GTGGGAAGCCACAGAGAGTAAAATATA
GTGGGAAGCCACAGAGAGTAAAATATA
GTGGGAAGCCACAGAGAGTAAAATATA
GTGGGAAGCCACAGAGAGTAAAATATA
GTGGGAAGCCACAGAGAGTAAAATATA
GTGGGAAGCCACAGAGAGTAAAATATA
GTGGGAAGCCACAGAGAGTAAAATATA
GTGGGAAGCCACAGAGAGTAAAATATA
GTGGGAAGCCACAGAGAGTAAAATATA
GTGGGAAGCCACAGAGAGTAAAATATA
GTGGGAAGCCACAGAGAGTAAAATATA
GTGGGAAGCCACAGAGAGTAAAATATA
GTGGGAAGCCACAGAGAGTAAAATATA
GTGGGAAGCCACAGAGAGTAAAATATA
GTGGGAAGCCACAGAGAGTAAAATATA
GTGGGAAGCCACAGAGAGTAAAATATA
GTGGGAAGCCACAGAGAGTAAAATATA
GTGGGAAGCCACAGAGAGTAAAATATA
GTGGGAAGCCACAGAGAGTAAAATATA
GTGGGAAGCCACAGAGAGTAAAATATA
GTGGGAAGCCACAGAGAGTAAAATATA
GTGGGAAGCCACAGAGAGTAAAATATA
GTGGGAAGCCACAGAGAGTAAAATATA
GTGGGAAGCCACAGAGAGTAAAATATA

GTGGGAAGCCACAGAGAGTAAAATATA
GTGGGAAGCCACAGAGAGTAAAATATA
GTGGGAAGCCACAGAGAGTAAAATATA
GTGGGAAGCCACAGAGAGTAAAATATA
GTGGGAAGCCACAGAGAGTAAAATATA
GTGGGAAGCCACAGAGAGTAAAATATA
GTGGGAAGCCACAGAGAGTAAAATATA
GTGGGAAGCCACAGAGAGTAAAATATA
GTGGGAAGCCACAGAGAGTAAAATATA
GTGGGAAGCCACAGAGAGTAAAATATA
GTGGGAAGCCACAGAGAGTAAAATATA
GTGGGAAGCCACAGAGAGTAAAATATA
GTGGGAAGCCACAGAGAGTAAAATATA
GTGGGAAGCCACAGAGAGTAAAATATA
GTGGGAAGCCACAGAGAGTAAAATATA
GTGGGAAGCCACAGAGAGTAAAATATA
GTGGGAAGCCACAGAGAGTAAAATATA
GTGGGAAGCCACAGAGAGTAAAATATA
GTGGGAAGCCACAGAGAGTAAAATATA
GTGGGAAGCCACAGAGAGTAAAATATA
GTGGGAAGCCACAGAGAGTAAAATATA
GTGGGAAGCCACAGAGAGTAAAATATA
GTGGGAAGCCACAGAGAGTAAAATATA
GTGGGAAGCCACAGAGAGTAAAATATA
GTGGGAAGCCACAGAGAGTAAAATATA
GTGGGAAGCCACAGAGAGTAAAATATA
GTGGGAAGCCACAGAGAGTAAAATATA
GTGGGAAGCCACAGAGAGTAAAATATA
GTGGGAAGCCACAGAGAGTAAAATATA
GTGGGAAGCCACAGAGAGTAAAATATA
GTGGGAAGCCACAGAGAGTAAAATATA
GTGGGAAGCCACAGAGAGTAAAATATA
GTGGGAAGCCACAGAGAGTAAAATATA
GTGGGAAGCCACAGAGAGTAAAATATA
GTGGGAAGCCACAGAGAGTAAAATATA
GTGGGAAGCCACAGAGAGTAAAATATA
GTGGGAAGCCACAGAGAGTAAAATATA
GTGGGAAGCCACAGAGAGTAAAATATA
GTGGGAAGCCACAGAGAGTAAAATATA
GTGGGAAGCCACAGAGAGTAAAATATA
GTGGGAAGCCACAGAGAGTAAAATATA
GTGGGAAGCCACAGAGAGTAAAATATA
GTGGGAAGCCACAGAGAGTAAAATATA
GTGGGAAGCCACAGAGAGTAAAATATA
GTGGGAAGCCACAGAGAGTAAAATATA
GTGGGAAGCCACAGAGAGTAAAATATA
GTGGGAAGCCACAGAGAGTAAAATATA
GTGGGAAGCCACAGAGAGTAAAATATA
GTGGGAAGCCACAGAGAGTAAAATATA
GTGGGAAGCCACAGAGAGTAAAATATA
GTGGGAAGCCACAGAGAGTAAAATATA
GTGGGAAGCCACAGAGAGTAAAATATA
GTGGGAAGCCACAGAGAGTAAAATATA
GTGGGAAGCCACAGAGAGTAAAATATA

GTGGGAAGCCACAGAGAGTAAAATATA
GTGGGAAGCCACAGAGAGTAAAATATA
GTGGGAAGCCACAGAGAGTAAAATATA
GTGGGAAGCCACAGAGAGTAAAATATA
GTGGGAAGCCACAGAGAGTAAAATATA
GTGGGAAGCCACAGAGAGTAAAATATA
GTGGGAAGCCACAGAGAGTAAAATATA
GTGGGAAGCCACAGAGAGTAAAATATA
GTGGGAAGCCACAGAGAGTAAAATATA
GTGGGAAGCCACAGAGAGTAAAATATA
GTGGGAAGCCACAGAGAGTAAAATATA
GTGGGAAGCCACAGAGAGTAAAATATA
GTGGGAAGCCACAGAGAGTAAAATATA
GTGGGAAGCCACAGAGAGTAAAATATA
GTGGGAAGCCACAGAGAGTAAAATATA
GTGGGAAGCCACAGAGAGTAAAATATA
GTGGGAAGCCACAGAGAGTAAAATATA
GTGGGAAGCCACAGAGAGTAAAATATA
GTGGGAAGCCACAGAGAGTAAAATATA
GTGGGAAGCCACAGAGAGTAAAATATA
GTGGGAAGCCACAGAGAGTAAAATATA
GTGGGAAGCCACAGAGAGTAAAATATA
GTGGGAAGCCACAGAGAGTAAAATATA
GTGGGAAGCCACAGAGAGTAAAATATA
GTGGGAAGCCACAGAGAGTAAAATATA
GTGGGAAGCCACAGAGAGTAAAATATA
GTGGGAAGCCACAGAGAGTAAAATATA
GTGGGAAGCCACAGAGAGTAAAATATA
GTGGGAAGCCACAGAGAGTAAAATATA
GTGGGAAGCCACAGAGAGTAAAATATA
GTGGGAAGCCACAGAGAGTAAAATATA
GTGGGAAGCCACAGAGAGTAAAATATA
GTGGGAAGCCACAGAGAGTAAAATATA
GTGGGAAGCCACAGAGAGTAAAATATA
GTGGGAAGCCACAGAGAGTAAAATATA
GTGGGAAGCCACAGAGAGTAAAATATA
GTGGGAAGCCACAGAGAGTAAAATATA
GTGGGAAGCCACAGAGAGTAAAATATA
GTGGGAAGCCACAGAGAGTAAAATATA
GTGGGAAGCCACAGAGAGTAAAATATA
GTGGGAAGCCACAGAGAGTAAAATATA
GTGGGAAGCCACAGAGAGTAAAATATA
GTGGGAAGCCACAGAGAGTAAAATATA
GTGGGAAGCCACAGAGAGTAAAATATA
GTGGGAAGCCACAGAGAGTAAAATATA
GTGGGAAGCCACAGAGAGTAAAATATA
GTGGGAAGCCACAGAGAGTAAAATATA
GTGGGAAGCCACAGAGAGTAAAATATA
GTGGGAAGCCACAGAGAGTAAAATATA
GTGGGAAGCCACAGAGAGTAAAATATA
GTGGGAAGCCACAGAGAGTAAAATATA
GTGGGAAGCCACAGAGAGTAAAATATA
GTGGGAAGCCACAGAGAGTAAAATATA
GTGGGAAGCCACAGAGAGTAAAATATA

GTGGGAAGCCACAGAGAGTAAAATATA
GTGGGAAGCCACAGAGAGTAAAATATA
GTGGGAAGCCACAGAGAGTAAAATATA
GTGGGAAGCCACAGAGAGTAAAATATA
GTGGGAAGCCACAGAGAGTAAAATATA
GTGGGAAGCCACAGAGAGTAAAATATA
GTGGGAAGCCACAGAGAGTAAAATATA
GTGGGAAGCCACAGAGAGTAAAATATA
GTGGGAAGCCACAGAGAGTAAAATATA
GTGGGAAGCCACAGAGAGTAAAATATA
GTGGGAAGCCACAGAGAGTAAAATATA
GTGGGAAGCCACAGAGAGTAAAATATA
GTGGGAAGCCACAGAGAGTAAAATATA
GTGGGAAGCCACAGAGAGTAAAATATA
GTGGGAAGCCACAGAGAGTAAAATATA
GTGGGAAGCCACAGAGAGTAAAATATA
GTGGGAAGCCACAGAGAGTAAAATATA
GTGGGAAGCCACAGAGAGTAAAATATA
GTGGGAAGCCACAGAGAGTAAAATATA
GTGGGAAGCCACAGAGAGTAAAATATA
GTGGGAAGCCACAGAGAGTAAAATATA
GTGGGAAGCCACAGAGAGTAAAATATA
GTGGGAAGCCACAGAGAGTAAAATATA
GTGGGAAGCCACAGAGAGTAAAATATA
GTGGGAAGCCACAGAGAGTAAAATATA
GTGGGAAGCCACAGAGAGTAAAATATA
GTGGGAAGCCACAGAGAGTAAAATATA
GTGGGAAGCCACAGAGAGTAAAATATA
GTGGGAAGCCACAGAGAGTAAAATATA
GTGGGAAGCCACAGAGAGTAAAATATA
GTGGGAAGCCACAGAGAGTAAAATATA
GTGGGAAGCCACAGAGAGTAAAATATA
GTGGGAAGCCACAGAGAGTAAAATATA
GTGGGAAGCCACAGAGAGTAAAATATA
GTGGGAAGCCACAGAGAGTAAAATATA
GTGGGAAGCCACAGAGAGTAAAATATA
GTGGGAAGCCACAGAGAGTAAAATATA
GTGGGAAGCCACAGAGAGTAAAATATA
GTGGGAAGCCACAGAGAGTAAAATATA
GTGGGAAGCCACAGAGAGTAAAATATA
GTGGGAAGCCACAGAGAGTAAAATATA
GTGGGAAGCCACAGAGAGTAAAATATA
GTGGGAAGCCACAGAGAGTAAAATATA
GTGGGAAGCCACAGAGAGTAAAATATA
GTGGGAAGCCACAGAGAGTAAAATATA
GTGGGAAGCCACAGAGAGTAAAATATA
GTGGGAAGCCACAGAGAGTAAAATATA
GTGGGAAGCCACAGAGAGTAAAATATA
GTGGGAAGCCACAGAGAGTAAAATATA
GTGGGAAGCCACAGAGAGTAAAATATA
GTGGGAAGCCACAGAGAGTAAAATATA
GTGGGAAGCCACAGAGAGTAAAATATA
GTGGGAAGCCACAGAGAGTAAAATATA
GTGGGAAGCCACAGAGAGTAAAATATA

GTGGGAAGCCACAGAGAGTAAAATATA
GTGGGAAGCCACAGAGAGTAAAATATA
GTGGGAAGCCACAGAGAGTAAAATATA
GTGGGAAGCCACAGAGAGTAAAATATA
GTGGGAAGCCACAGAGAGTAAAATATA
GTGGGAAGCCACAGAGAGTAAAATATA
GTGGGAAGCCACAGAGAGTAAAATATA
GTGGGAAGCCACAGAGAGTAAAATATA
GTGGGAAGCCACAGAGAGTAAAATATA
GTGGGAAGCCACAGAGAGTAAAATATA
GTGGGAAGCCACAGAGAGTAAAATATA
GTGGGAAGCCACAGAGAGTAAAATATA
GTGGGAAGCCACAGAGAGTAAAATATA
GTGGGAAGCCACAGAGAGTAAAATATA
GTGGGAAGCCACAGAGAGTAAAATATA
GTGGGAAGCCACAGAGAGTAAAATATA
GTGGGAAGCCACAGAGAGTAAAATATA
GTGGGAAGCCACAGAGAGTAAAATATA
GTGGGAAGCCACAGAGAGTAAAATATA
GTGGGAAGCCACAGAGAGTAAAATATA
GTGGGAAGCCACAGAGAGTAAAATATA
GTGGGAAGCCACAGAGAGTAAAATATA
GTGGGAAGCCACAGAGAGTAAAATATA
GTGGGAAGCCACAGAGAGTAAAATATA
GTGGGAAGCCACAGAGAGTAAAATATA
GTGGGAAGCCACAGAGAGTAAAATATA
GTGGGAAGCCACAGAGAGTAAAATATA
GTGGGAAGCCACAGAGAGTAAAATATA
GTGGGAAGCCACAGAGAGTAAAATATA
GTGGGAAGCCACAGAGAGTAAAATATA
GTGGGAAGCCACAGAGAGTAAAATATA
GTGGGAAGCCACAGAGAGTAAAATATA
GTGGGAAGCCACAGAGAGTAAAATATA
GTGGGAAGCCACAGAGAGTAAAATATA
GTGGGAAGCCACAGAGAGTAAAATATA
GTGGGAAGCCACAGAGAGTAAAATATA
GTGGGAAGCCACAGAGAGTAAAATATA
GTGGGAAGCCACAGAGAGTAAAATATA
GTGGGAAGCCACAGAGAGTAAAATATA
GTGGGAAGCCACAGAGAGTAAAATATA
GTGGGAAGCCACAGAGAGTAAAATATA
GTGGGAAGCCACAGAGAGTAAAATATA
GTGGGAAGCCACAGAGAGTAAAATATA
GTGGGAAGCCACAGAGAGTAAAATATA
GTGGGAAGCCACAGAGAGTAAAATATA
GTGGGAAGCCACAGAGAGTAAAATATA
GTGGGAAGCCACAGAGAGTAAAATATA
GTGGGAAGCCACAGAGAGTAAAATATA
GTGGGAAGCCACAGAGAGTAAAATATA
GTGGGAAGCCACAGAGAGTAAAATATA
GTGGGAAGCCACAGAGAGTAAAATATA
GTGGGAAGCCACAGAGAGTAAAATATA
GTGGGAAGCCACAGAGAGTAAAATATA
GTGGGAAGCCACAGAGAGTAAAATATA

GTGGGAAGCCACAGAGAGTAAAATATA
GTGGGAAGCCACAGAGAGTAAAATATA
GTGGGAAGCCACAGAGAGTAAAATATA
GTGGGAAGCCACAGAGAGTAAAATATA
GTGGGAAGCCACAGAGAGTAAAATATA
GTGGGAAGCCACAGAGAGTAAAATATA
GTGGGAAGCCACAGAGAGTAAAATATA
GTGGGAAGCCACAGAGAGTAAAATATA
GTGGGAAGCCACAGAGAGTAAAATATA
GTGGGAAGCCACAGAGAGTAAAATATA
GTGGGAAGCCACAGAGAGTAAAATATA
GTGGGAAGCCACAGAGAGTAAAATATA
GTGGGAAGCCACAGAGAGTAAAATATA
GTGGGAAGCCACAGAGAGTAAAATATA
GTGGGAAGCCACAGAGAGTAAAATATA
GTGGGAAGCCACAGAGAGTAAAATATA
GTGGGAAGCCACAGAGAGTAAAATATA
GTGGGAAGCCACAGAGAGTAAAATATA
GTGGGAAGCCACAGAGAGTAAAATATA
GTGGGAAGCCACAGAGAGTAAAATATA
GTGGGAAGCCACAGAGAGTAAAATATA
GTGGGAAGCCACAGAGAGTAAAATATA
GTGGGAAGCCACAGAGAGTAAAATATA
GTGGGAAGCCACAGAGAGTAAAATATA
GTGGGAAGCCACAGAGAGTAAAATATA
GTGGGAAGCCACAGAGAGTAAAATATA
GTGGGAAGCCACAGAGAGTAAAATATA
GTGGGAAGCCACAGAGAGTAAAATATA
GTGGGAAGCCACAGAGAGTAAAATATA
GTGGGAAGCCACAGAGAGTAAAATATA
GTGGGAAGCCACAGAGAGTAAAATATA
GTGGGAAGCCACAGAGAGTAAAATATA
GTGGGAAGCCACAGAGAGTAAAATATA
GTGGGAAGCCACAGAGAGTAAAATATA
GTGGGAAGCCACAGAGAGTAAAATATA
GTGGGAAGCCACAGAGAGTAAAATATA
GTGGGAAGCCACAGAGAGTAAAATATA
GTGGGAAGCCACAGAGAGTAAAATATA
GTGGGAAGCCACAGAGAGTAAAATATA
GTGGGAAGCCACAGAGAGTAAAATATA
GTGGGAAGCCACAGAGAGTAAAATATA
GTGGGAAGCCACAGAGAGTAAAATATA
GTGGGAAGCCACAGAGAGTAAAATATA
GTGGGAAGCCACAGAGAGTAAAATATA
GTGGGAAGCCACAGAGAGTAAAATATA
GTGGGAAGCCACAGAGAGTAAAATATA
GTGGGAAGCCACAGAGAGTAAAATATA
GTGGGAAGCCACAGAGAGTAAAATATA
GTGGGAAGCCACAGAGAGTAAAATATA
GTGGGAAGCCACAGAGAGTAAAATATA
GTGGGAAGCCACAGAGAGTAAAATATA
GTGGGAAGCCACAGAGAGTAAAATATA
GTGGGAAGCCACAGAGAGTAAAATATA
GTGGGAAGCCACAGAGAGTAAAATATA

GTGGGAAGCCACAGAGAGTAAAATATA
GTGGGAAGCCACAGAGAGTAAAATATA
GTGGGAAGCCACAGAGAGTAAAATATA
GTGGGAAGCCACAGAGAGTAAAATATA
GTGGGAAGCCACAGAGAGTAAAATATA
GTGGGAAGCCACAGAGAGTAAAATATA
GTGGGAAGCCACAGAGAGTAAAATATA
GTGGGAAGCCACAGAGAGTAAAATATA
GTGGGAAGCCACAGAGAGTAAAATATA
GTGGGAAGCCACAGAGAGTAAAATATA
GTGGGAAGCCACAGAGAGTAAAATATA
GTGGGAAGCCACAGAGAGTAAAATATA
GTGGGAAGCCACAGAGAGTAAAATATA
GTGGGAAGCCACAGAGAGTAAAATATA
GTGGGAAGCCACAGAGAGTAAAATATA
GTGGGAAGCCACAGAGAGTAAAATATA
GTGGGAAGCCACAGAGAGTAAAATATA
GTGGGAAGCCACAGAGAGTAAAATATA
GTGGGAAGCCACAGAGAGTAAAATATA
GTGGGAAGCCACAGAGAGTAAAATATA
GTGGGAAGCCACAGAGAGTAAAATATA
GTGGGAAGCCACAGAGAGTAAAATATA
GTGGGAAGCCACAGAGAGTAAAATATA
GTGGGAAGCCACAGAGAGTAAAATATA
GTGGGAAGCCACAGAGAGTAAAATATA
GTGGGAAGCCACAGAGAGTAAAATATA
GTGGGAAGCCACAGAGAGTAAAATATA
GTGGGAAGCCACAGAGAGTAAAATATA
GTGGGAAGCCACAGAGAGTAAAATATA
GTGGGAAGCCACAGAGAGTAAAATATA
GTGGGAAGCCACAGAGAGTAAAATATA
GTGGGAAGCCACAGAGAGTAAAATATA
GTGGGAAGCCACAGAGAGTAAAATATA
GTGGGAAGCCACAGAGAGTAAAATATA
GTGGGAAGCCACAGAGAGTAAAATATA
GTGGGAAGCCACAGAGAGTAAAATATA
GTGGGAAGCCACAGAGAGTAAAATATA
GTGGGAAGCCACAGAGAGTAAAATATA
GTGGGAAGCCACAGAGAGTAAAATATA
GTGGGAAGCCACAGAGAGTAAAATATA
GTGGGAAGCCACAGAGAGTAAAATATA
GTGGGAAGCCACAGAGAGTAAAATATA
GTGGGAAGCCACAGAGAGTAAAATATA
GTGGGAAGCCACAGAGAGTAAAATATA
GTGGGAAGCCACAGAGAGTAAAATATA
GTGGGAAGCCACAGAGAGTAAAATATA
GTGGGAAGCCACAGAGAGTAAAATATA
GTGGGAAGCCACAGAGAGTAAAATATA
GTGGGAAGCCACAGAGAGTAAAATATA
GTGGGAAGCCACAGAGAGTAAAATATA
GTGGGAAGCCACAGAGAGTAAAATATA
GTGGGAAGCCACAGAGAGTAAAATATA
GTGGGAAGCCACAGAGAGTAAAATATA
GTGGGAAGCCACAGAGAGTAAAATATA

GTGGGAAGCCACAGAGAGTAAAATATA
GTGGGAAGCCACAGAGAGTAAAATATA
GTGGGAAGCCACAGAGAGTAAAATATA
GTGGGAAGCCACAGAGAGTAAAATATA
GTGGGAAGCCACAGAGAGTAAAATATA
GTGGGAAGCCACAGAGAGTAAAATATA
GTGGGAAGCCACAGAGAGTAAAATATA
GTGGGAAGCCACAGAGAGTAAAATATA
GTGGGAAGCCACAGAGAGTAAAATATA
GTGGGAAGCCACAGAGAGTAAAATATA
GTGGGAAGCCACAGAGAGTAAAATATA
GTGGGAAGCCACAGAGAGTAAAATATA
GTGGGAAGCCACAGAGAGTAAAATATA
GTGGGAAGCCACAGAGAGTAAAATATA
GTGGGAAGCCACAGAGAGTAAAATATA
GTGGGAAGCCACAGAGAGTAAAATATA
GTGGGAAGCCACAGAGAGTAAAATATA
GTGGGAAGCCACAGAGAGTAAAATATA
GTGGGAAGCCACAGAGAGTAAAATATA
GTGGGAAGCCACAGAGAGTAAAATATA
GTGGGAAGCCACAGAGAGTAAAATATA
GTGGGAAGCCACAGAGAGTAAAATATA
GTGGGAAGCCACAGAGAGTAAAATATA
GTGGGAAGCCACAGAGAGTAAAATATA
GTGGGAAGCCACAGAGAGTAAAATATA
GTGGGAAGCCACAGAGAGTAAAATATA
GTGGGAAGCCACAGAGAGTAAAATATA
GTGGGAAGCCACAGAGAGTAAAATATA
GTGGGAAGCCACAGAGAGTAAAATATA
GTGGGAAGCCACAGAGAGTAAAATATA
GTGGGAAGCCACAGAGAGTAAAATATA
GTGGGAAGCCACAGAGAGTAAAATATA
GTGGGAAGCCACAGAGAGTAAAATATA
GTGGGAAGCCACAGAGAGTAAAATATA
GTGGGAAGCCACAGAGAGTAAAATATA
GTGGGAAGCCACAGAGAGTAAAATATA
GTGGGAAGCCACAGAGAGTAAAATATA
GTGGGAAGCCACAGAGAGTAAAATATA
GTGGGAAGCCACAGAGAGTAAAATATA
GTGGGAAGCCACAGAGAGTAAAATATA
GTGGGAAGCCACAGAGAGTAAAATATA
GTGGGAAGCCACAGAGAGTAAAATATA
GTGGGAAGCCACAGAGAGTAAAATATA
GTGGGAAGCCACAGAGAGTAAAATATA
GTGGGAAGCCACAGAGAGTAAAATATA
GTGGGAAGCCACAGAGAGTAAAATATA
GTGGGAAGCCACAGAGAGTAAAATATA
GTGGGAAGCCACAGAGAGTAAAATATA
GTGGGAAGCCACAGAGAGTAAAATATA
GTGGGAAGCCACAGAGAGTAAAATATA
GTGGGAAGCCACAGAGAGTAAAATATA
GTGGGAAGCCACAGAGAGTAAAATATA
GTGGGAAGCCACAGAGAGTAAAATATA
GTGGGAAGCCACAGAGAGTAAAATATA

GTGGGAAGCCACAGAGAGTAAAATATA
GTGGGAAGCCACAGAGAGTAAAATATA
GTGGGAAGCCACAGAGAGTAAAATATA
GTGGGAAGCCACAGAGAGTAAAATATA
GTGGGAAGCCACAGAGAGTAAAATATA
GTGGGAAGCCACAGAGAGTAAAATATA
GTGGGAAGCCACAGAGAGTAAAATATA
GTGGGAAGCCACAGAGAGTAAAATATA
GTGGGAAGCCACAGAGAGTAAAATATA
GTGGGAAGCCACAGAGAGTAAAATATA
GTGGGAAGCCACAGAGAGTAAAATATA
GTGGGAAGCCACAGAGAGTAAAATATA
GTGGGAAGCCACAGAGAGTAAAATATA
GTGGGAAGCCACAGAGAGTAAAATATA
GTGGGAAGCCACAGAGAGTAAAATATA
GTGGGAAGCCACAGAGAGTAAAATATA
GTGGGAAGCCACAGAGAGTAAAATATA
GTGGGAAGCCACAGAGAGTAAAATATA
GTGGGAAGCCACAGAGAGTAAAATATA
GTGGGAAGCCACAGAGAGTAAAATATA
GTGGGAAGCCACAGAGAGTAAAATATA
GTGGGAAGCCACAGAGAGTAAAATATA
GTGGGAAGCCACAGAGAGTAAAATATA
GTGGGAAGCCACAGAGAGTAAAATATA
GTGGGAAGCCACAGAGAGTAAAATATA
GTGGGAAGCCACAGAGAGTAAAATATA
GTGGGAAGCCACAGAGAGTAAAATATA
GTGGGAAGCCACAGAGAGTAAAATATA
GTGGGAAGCCACAGAGAGTAAAATATA
GTGGGAAGCCACAGAGAGTAAAATATA
GTGGGAAGCCACAGAGAGTAAAATATA
GTGGGAAGCCACAGAGAGTAAAATATA
GTGGGAAGCCACAGAGAGTAAAATATA
GTGGGAAGCCACAGAGAGTAAAATATA
GTGGGAAGCCACAGAGAGTAAAATATA
GTGGGAAGCCACAGAGAGTAAAATATA
GTGGGAAGCCACAGAGAGTAAAATATA
GTGGGAAGCCACAGAGAGTAAAATATA
GTGGGAAGCCACAGAGAGTAAAATATA
GTGGGAAGCCACAGAGAGTAAAATATA
GTGGGAAGCCACAGAGAGTAAAATATA
GTGGGAAGCCACAGAGAGTAAAATATA
GTGGGAAGCCACAGAGAGTAAAATATA
GTGGGAAGCCACAGAGAGTAAAATATA
GTGGGAAGCCACAGAGAGTAAAATATA
GTGGGAAGCCACAGAGAGTAAAATATA
GTGGGAAGCCACAGAGAGTAAAATATA
GTGGGAAGCCACAGAGAGTAAAATATA
GTGGGAAGCCACAGAGAGTAAAATATA
GTGGGAAGCCACAGAGAGTAAAATATA
GTGGGAAGCCACAGAGAGTAAAATATA
GTGGGAAGCCACAGAGAGTAAAATATA
GTGGGAAGCCACAGAGAGTAAAATATA
GTGGGAAGCCACAGAGAGTAAAATATA

GTGGGAAGCCACAGAGAGTAAAATATA
GTGGGAAGCCACAGAGAGTAAAATATA
GTGGGAAGCCACAGAGAGTAAAATATA
GTGGGAAGCCACAGAGAGTAAAATATA
GTGGGAAGCCACAGAGAGTAAAATATA
GTGGGAAGCCACAGAGAGTAAAATATA
GTGGGAAGCCACAGAGAGTAAAATATA
GTGGGAAGCCACAGAGAGTAAAATATA
GTGGGAAGCCACAGAGAGTAAAATATA
GTGGGAAGCCACAGAGAGTAAAATATA
GTGGGAAGCCACAGAGAGTAAAATATA
GTGGGAAGCCACAGAGAGTAAAATATA
GTGGGAAGCCACAGAGAGTAAAATATA
GTGGGAAGCCACAGAGAGTAAAATATA
GTGGGAAGCCACAGAGAGTAAAATATA
GTGGGAAGCCACAGAGAGTAAAATATA
GTGGGAAGCCACAGAGAGTAAAATATA
GTGGGAAGCCACAGAGAGTAAAATATA
GTGGGAAGCCACAGAGAGTAAAATATA
GTGGGAAGCCACAGAGAGTAAAATATA
GTGGGAAGCCACAGAGAGTAAAATATA
GTGGGAAGCCACAGAGAGTAAAATATA
GTGGGAAGCCACAGAGAGTAAAATATA
GTGGGAAGCCACAGAGAGTAAAATATA
GTGGGAAGCCACAGAGAGTAAAATATA
GTGGGAAGCCACAGAGAGTAAAATATA
GTGGGAAGCCACAGAGAGTAAAATATA
GTGGGAAGCCACAGAGAGTAAAATATA
GTGGGAAGCCACAGAGAGTAAAATATA
GTGGGAAGCCACAGAGAGTAAAATATA
GTGGGAAGCCACAGAGAGTAAAATATA
GTGGGAAGCCACAGAGAGTAAAATATA
GTGGGAAGCCACAGAGAGTAAAATATA
GTGGGAAGCCACAGAGAGTAAAATATA
GTGGGAAGCCACAGAGAGTAAAATATA
GTGGGAAGCCACAGAGAGTAAAATATA
GTGGGAAGCCACAGAGAGTAAAATATA
GTGGGAAGCCACAGAGAGTAAAATATA
GTGGGAAGCCACAGAGAGTAAAATATA
GTGGGAAGCCACAGAGAGTAAAATATA
GTGGGAAGCCACAGAGAGTAAAATATA
GTGGGAAGCCACAGAGAGTAAAATATA
GTGGGAAGCCACAGAGAGTAAAATATA
GTGGGAAGCCACAGAGAGTAAAATATA
GTGGGAAGCCACAGAGAGTAAAATATA
GTGGGAAGCCACAGAGAGTAAAATATA
GTGGGAAGCCACAGAGAGTAAAATATA
GTGGGAAGCCACAGAGAGTAAAATATA
GTGGGAAGCCACAGAGAGTAAAATATA
GTGGGAAGCCACAGAGAGTAAAATATA
GTGGGAAGCCACAGAGAGTAAAATATA
GTGGGAAGCCACAGAGAGTAAAATATA
GTGGGAAGCCACAGAGAGTAAAATATA
GTGGGAAGCCACAGAGAGTAAAATATA

GTGGGAAGCCACAGAGAGTAAAATATA
GTGGGAAGCCACAGAGAGTAAAATATA
GTGGGAAGCCACAGAGAGTAAAATATA
GTGGGAAGCCACAGAGAGTAAAATATA
GTGGGAAGCCACAGAGAGTAAAATATA
GTGGGAAGCCACAGAGAGTAAAATATA
GTGGGAAGCCACAGAGAGTAAAATATA
GTGGGAAGCCACAGAGAGTAAAATATA
GTGGGAAGCCACAGAGAGTAAAATATA
GTGGGAAGCCACAGAGAGTAAAATATA
GTGGGAAGCCACAGAGAGTAAAATATA
GTGGGAAGCCACAGAGAGTAAAATATA
GTGGGAAGCCACAGAGAGTAAAATATA
GTGGGAAGCCACAGAGAGTAAAATATA
GTGGGAAGCCACAGAGAGTAAAATATA
GTGGGAAGCCACAGAGAGTAAAATATA
GTGGGAAGCCACAGAGAGTAAAATATA
GTGGGAAGCCACAGAGAGTAAAATATA
GTGGGAAGCCACAGAGAGTAAAATATA
GTGGGAAGCCACAGAGAGTAAAATATA
GTGGGAAGCCACAGAGAGTAAAATATA
GTGGGAAGCCACAGAGAGTAAAATATA
GTGGGAAGCCACAGAGAGTAAAATATA
GTGGGAAGCCACAGAGAGTAAAATATA
GTGGGAAGCCACAGAGAGTAAAATATA
GTGGGAAGCCACAGAGAGTAAAATATA
GTGGGAAGCCACAGAGAGTAAAATATA
GTGGGAAGCCACAGAGAGTAAAATATA
GTGGGAAGCCACAGAGAGTAAAATATA
GTGGGAAGCCACAGAGAGTAAAATATA
GTGGGAAGCCACAGAGAGTAAAATATA
GTGGGAAGCCACAGAGAGTAAAATATA
GTGGGAAGCCACAGAGAGTAAAATATA
GTGGGAAGCCACAGAGAGTAAAATATA
GTGGGAAGCCACAGAGAGTAAAATATA
GTGGGAAGCCACAGAGAGTAAAATATA
GTGGGAAGCCACAGAGAGTAAAATATA
GTGGGAAGCCACAGAGAGTAAAATATA
GTGGGAAGCCACAGAGAGTAAAATATA
GTGGGAAGCCACAGAGAGTAAAATATA
GTGGGAAGCCACAGAGAGTAAAATATA
GTGGGAAGCCACAGAGAGTAAAATATA
GTGGGAAGCCACAGAGAGTAAAATATA
GTGGGAAGCCACAGAGAGTAAAATATA
GTGGGAAGCCACAGAGAGTAAAATATA
GTGGGAAGCCACAGAGAGTAAAATATA
GTGGGAAGCCACAGAGAGTAAAATATA
GTGGGAAGCCACAGAGAGTAAAATATA
GTGGGAAGCCACAGAGAGTAAAATATA
GTGGGAAGCCACAGAGAGTAAAATATA
GTGGGAAGCCACAGAGAGTAAAATATA
GTGGGAAGCCACAGAGAGTAAAATATA
GTGGGAAGCCACAGAGAGTAAAATATA
GTGGGAAGCCACAGAGAGTAAAATATA

GTGGGAAGCCACAGAGAGTAAAATATA
GTGGGAAGCCACAGAGAGTAAAATATA
GTGGGAAGCCACAGAGAGTAAAATATA
GTGGGAAGCCACAGAGAGTAAAATATA
GTGGGAAGCCACAGAGAGTAAAATATA
GTGGGAAGCCACAGAGAGTAAAATATA
GTGGGAAGCCACAGAGAGTAAAATATA
GTGGGAAGCCACAGAGAGTAAAATATA
GTGGGAAGCCACAGAGAGTAAAATATA
GTGGGAAGCCACAGAGAGTAAAATATA
GTGGGAAGCCACAGAGAGTAAAATATA
GTGGGAAGCCACAGAGAGTAAAATATA
GTGGGAAGCCACAGAGAGTAAAATATA
GTGGGAAGCCACAGAGAGTAAAATATA
GTGGGAAGCCACAGAGAGTAAAATATA
GTGGGAAGCCACAGAGAGTAAAATATA
GTGGGAAGCCACAGAGAGTAAAATATA
GTGGGAAGCCACAGAGAGTAAAATATA
GTGGGAAGCCACAGAGAGTAAAATATA
GTGGGAAGCCACAGAGAGTAAAATATA
GTGGGAAGCCACAGAGAGTAAAATATA
GTGGGAAGCCACAGAGAGTAAAATATA
GTGGGAAGCCACAGAGAGTAAAATATA
GTGGGAAGCCACAGAGAGTAAAATATA
GTGGGAAGCCACAGAGAGTAAAATATA
GTGGGAAGCCACAGAGAGTAAAATATA
GTGGGAAGCCACAGAGAGTAAAATATA
GTGGGAAGCCACAGAGAGTAAAATATA
GTGGGAAGCCACAGAGAGTAAAATATA
GTGGGAAGCCACAGAGAGTAAAATATA
GTGGGAAGCCACAGAGAGTAAAATATA
GTGGGAAGCCACAGAGAGTAAAATATA
GTGGGAAGCCACAGAGAGTAAAATATA
GTGGGAAGCCACAGAGAGTAAAATATA
GTGGGAAGCCACAGAGAGTAAAATATA
GTGGGAAGCCACAGAGAGTAAAATATA
GTGGGAAGCCACAGAGAGTAAAATATA
GTGGGAAGCCACAGAGAGTAAAATATA
GTGGGAAGCCACAGAGAGTAAAATATA
GTGGGAAGCCACAGAGAGTAAAATATA
GTGGGAAGCCACAGAGAGTAAAATATA
GTGGGAAGCCACAGAGAGTAAAATATA
GTGGGAAGCCACAGAGAGTAAAATATA
GTGGGAAGCCACAGAGAGTAAAATATA
GTGGGAAGCCACAGAGAGTAAAATATA
GTGGGAAGCCACAGAGAGTAAAATATA
GTGGGAAGCCACAGAGAGTAAAATATA
GTGGGAAGCCACAGAGAGTAAAATATA
GTGGGAAGCCACAGAGAGTAAAATATA
GTGGGAAGCCACAGAGAGTAAAATATA
GTGGGAAGCCACAGAGAGTAAAATATA
GTGGGAAGCCACAGAGAGTAAAATATA
GTGGGAAGCCACAGAGAGTAAAATATA
GTGGGAAGCCACAGAGAGTAAAATATA

GTGGGAAGCCACAGAGAGTAAAATATA
GTGGGAAGCCACAGAGAGTAAAATATA
GTGGGAAGCCACAGAGAGTAAAATATA
GTGGGAAGCCACAGAGAGTAAAATATA
GTGGGAAGCCACAGAGAGTAAAATATA
GTGGGAAGCCACAGAGAGTAAAATATA
GTGGGAAGCCACAGAGAGTAAAATATA
GTGGGAAGCCACAGAGAGTAAAATATA
GTGGGAAGCCACAGAGAGTAAAATATA
GTGGGAAGCCACAGAGAGTAAAATATA
GTGGGAAGCCACAGAGAGTAAAATATA
GTGGGAAGCCACAGAGAGTAAAATATA
GTGGGAAGCCACAGAGAGTAAAATATA
GTGGGAAGCCACAGAGAGTAAAATATA
GTGGGAAGCCACAGAGAGTAAAATATA
GTGGGAAGCCACAGAGAGTAAAATATA
GTGGGAAGCCACAGAGAGTAAAATATA
GTGGGAAGCCACAGAGAGTAAAATATA
GTGGGAAGCCACAGAGAGTAAAATATA
GTGGGAAGCCACAGAGAGTAAAATATA
GTGGGAAGCCACAGAGAGTAAAATATA
GTGGGAAGCCACAGAGAGTAAAATATA
GTGGGAAGCCACAGAGAGTAAAATATA
GTGGGAAGCCACAGAGAGTAAAATATA
GTGGGAAGCCACAGAGAGTAAAATATA
GTGGGAAGCCACAGAGAGTAAAATATA
GTGGGAAGCCACAGAGAGTAAAATATA
GTGGGAAGCCACAGAGAGTAAAATATA
GTGGGAAGCCACAGAGAGTAAAATATA
GTGGGAAGCCACAGAGAGTAAAATATA
GTGGGAAGCCACAGAGAGTAAAATATA
GTGGGAAGCCACAGAGAGTAAAATATA
GTGGGAAGCCACAGAGAGTAAAATATA
GTGGGAAGCCACAGAGAGTAAAATATA
GTGGGAAGCCACAGAGAGTAAAATATA
GTGGGAAGCCACAGAGAGTAAAATATA
GTGGGAAGCCACAGAGAGTAAAATATA
GTGGGAAGCCACAGAGAGTAAAATATA
GTGGGAAGCCACAGAGAGTAAAATATA
GTGGGAAGCCACAGAGAGTAAAATATA
GTGGGAAGCCACAGAGAGTAAAATATA
GTGGGAAGCCACAGAGAGTAAAATATA
GTGGGAAGCCACAGAGAGTAAAATATA
GTGGGAAGCCACAGAGAGTAAAATATA
GTGGGAAGCCACAGAGAGTAAAATATA
GTGGGAAGCCACAGAGAGTAAAATATA
GTGGGAAGCCACAGAGAGTAAAATATA
GTGGGAAGCCACAGAGAGTAAAATATA
GTGGGAAGCCACAGAGAGTAAAATATA
GTGGGAAGCCACAGAGAGTAAAATATA
GTGGGAAGCCACAGAGAGTAAAATATA
GTGGGAAGCCACAGAGAGTAAAATATA
GTGGGAAGCCACAGAGAGTAAAATATA
GTGGGAAGCCACAGAGAGTAAAATATA

GTGGGAAGCCACAGAGAGTAAAATATA
GTGGGAAGCCACAGAGAGTAAAATATA
GTGGGAAGCCACAGAGAGTAAAATATA
GTGGGAAGCCACAGAGAGTAAAATATA
GTGGGAAGCCACAGAGAGTAAAATATA
GTGGGAAGCCACAGAGAGTAAAATATA
GTGGGAAGCCACAGAGAGTAAAATATA
GTGGGAAGCCACAGAGAGTAAAATATA
GTGGGAAGCCACAGAGAGTAAAATATA
GTGGGAAGCCACAGAGAGTAAAATATA
GTGGGAAGCCACAGAGAGTAAAATATA
GTGGGAAGCCACAGAGAGTAAAATATA
GTGGGAAGCCACAGAGAGTAAAATATA
GTGGGAAGCCACAGAGAGTAAAATATA
GTGGGAAGCCACAGAGAGTAAAATATA
GTGGGAAGCCACAGAGAGTAAAATATA
GTGGGAAGCCACAGAGAGTAAAATATA
GTGGGAAGCCACAGAGAGTAAAATATA
GTGGGAAGCCACAGAGAGTAAAATATA
GTGGGAAGCCACAGAGAGTAAAATATA
GTGGGAAGCCACAGAGAGTAAAATATA
GTGGGAAGCCACAGAGAGTAAAATATA
GTGGGAAGCCACAGAGAGTAAAATATA
GTGGGAAGCCACAGAGAGTAAAATATA
GTGGGAAGCCACAGAGAGTAAAATATA
GTGGGAAGCCACAGAGAGTAAAATATA
GTGGGAAGCCACAGAGAGTAAAATATA
GTGGGAAGCCACAGAGAGTAAAATATA
GTGGGAAGCCACAGAGAGTAAAATATA
GTGGGAAGCCACAGAGAGTAAAATATA
GTGGGAAGCCACAGAGAGTAAAATATA
GTGGGAAGCCACAGAGAGTAAAATATA
GTGGGAAGCCACAGAGAGTAAAATATA
GTGGGAAGCCACAGAGAGTAAAATATA
GTGGGAAGCCACAGAGAGTAAAATATA
GTGGGAAGCCACAGAGAGTAAAATATA
GTGGGAAGCCACAGAGAGTAAAATATA
GTGGGAAGCCACAGAGAGTAAAATATA
GTGGGAAGCCACAGAGAGTAAAATATA
GTGGGAAGCCACAGAGAGTAAAATATA
GTGGGAAGCCACAGAGAGTAAAATATA
GTGGGAAGCCACAGAGAGTAAAATATA
GTGGGAAGCCACAGAGAGTAAAATATA
GTGGGAAGCCACAGAGAGTAAAATATA
GTGGGAAGCCACAGAGAGTAAAATATA
GTGGGAAGCCACAGAGAGTAAAATATA
GTGGGAAGCCACAGAGAGTAAAATATA
GTGGGAAGCCACAGAGAGTAAAATATA
GTGGGAAGCCACAGAGAGTAAAATATA
GTGGGAAGCCACAGAGAGTAAAATATA
GTGGGAAGCCACAGAGAGTAAAATATA
GTGGGAAGCCACAGAGAGTAAAATATA
GTGGGAAGCCACAGAGAGTAAAATATA
GTGGGAAGCCACAGAGAGTAAAATATA

GTGGGAAGCCACAGAGAGTAAAATATA
GTGGGAAGCCACAGAGAGTAAAATATA
GTGGGAAGCCACAGAGAGTAAAATATA
GTGGGAAGCCACAGAGAGTAAAATATA
GTGGGAAGCCACAGAGAGTAAAATATA
GTGGGAAGCCACAGAGAGTAAAATATA
GTGGGAAGCCACAGAGAGTAAAATATA
GTGGGAAGCCACAGAGAGTAAAATATA
GTGGGAAGCCACAGAGAGTAAAATATA
GTGGGAAGCCACAGAGAGTAAAATATA
GTGGGAAGCCACAGAGAGTAAAATATA
GTGGGAAGCCACAGAGAGTAAAATATA
GTGGGAAGCCACAGAGAGTAAAATATA
GTGGGAAGCCACAGAGAGTAAAATATA
GTGGGAAGCCACAGAGAGTAAAATATA
GTGGGAAGCCACAGAGAGTAAAATATA
GTGGGAAGCCACAGAGAGTAAAATATA
GTGGGAAGCCACAGAGAGTAAAATATA
GTGGGAAGCCACAGAGAGTAAAATATA
GTGGGAAGCCACAGAGAGTAAAATATA
GTGGGAAGCCACAGAGAGTAAAATATA
GTGGGAAGCCACAGAGAGTAAAATATA
GTGGGAAGCCACAGAGAGTAAAATATA
GTGGGAAGCCACAGAGAGTAAAATATA
GTGGGAAGCCACAGAGAGTAAAATATA
GTGGGAAGCCACAGAGAGTAAAATATA
GTGGGAAGCCACAGAGAGTAAAATATA
GTGGGAAGCCACAGAGAGTAAAATATA
GTGGGAAGCCACAGAGAGTAAAATATA
GTGGGAAGCCACAGAGAGTAAAATATA
GTGGGAAGCCACAGAGAGTAAAATATA
GTGGGAAGCCACAGAGAGTAAAATATA
GTGGGAAGCCACAGAGAGTAAAATATA
GTGGGAAGCCACAGAGAGTAAAATATA
GTGGGAAGCCACAGAGAGTAAAATATA
GTGGGAAGCCACAGAGAGTAAAATATA
GTGGGAAGCCACAGAGAGTAAAATATA
GTGGGAAGCCACAGAGAGTAAAATATA
GTGGGAAGCCACAGAGAGTAAAATATA
GTGGGAAGCCACAGAGAGTAAAATATA
GTGGGAAGCCACAGAGAGTAAAATATA
GTGGGAAGCCACAGAGAGTAAAATATA
GTGGGAAGCCACAGAGAGTAAAATATA
GTGGGAAGCCACAGAGAGTAAAATATA
GTGGGAAGCCACAGAGAGTAAAATATA
GTGGGAAGCCACAGAGAGTAAAATATA
GTGGGAAGCCACAGAGAGTAAAATATA
GTGGGAAGCCACAGAGAGTAAAATATA
GTGGGAAGCCACAGAGAGTAAAATATA
GTGGGAAGCCACAGAGAGTAAAATATA
GTGGGAAGCCACAGAGAGTAAAATATA
GTGGGAAGCCACAGAGAGTAAAATATA
GTGGGAAGCCACAGAGAGTAAAATATA
GTGGGAAGCCACAGAGAGTAAAATATA

GTGGGAAGCCACAGAGAGTAAAATATA
GTGGGAAGCCACAGAGAGTAAAATATA
GTGGGAAGCCACAGAGAGTAAAATATA
GTGGGAAGCCACAGAGAGTAAAATATA
GTGGGAAGCCACAGAGAGTAAAATATA
GTGGGAAGCCACAGAGAGTAAAATATA
GTGGGAAGCCACAGAGAGTAAAATATA
GTGGGAAGCCACAGAGAGTAAAATATA
GTGGGAAGCCACAGAGAGTAAAATATA
GTGGGAAGCCACAGAGAGTAAAATATA
GTGGGAAGCCACAGAGAGTAAAATATA
GTGGGAAGCCACAGAGAGTAAAATATA
GTGGGAAGCCACAGAGAGTAAAATATA
GTGGGAAGCCACAGAGAGTAAAATATA
GTGGGAAGCCACAGAGAGTAAAATATA
GTGGGAAGCCACAGAGAGTAAAATATA
GTGGGAAGCCACAGAGAGTAAAATATA
GTGGGAAGCCACAGAGAGTAAAATATA
GTGGGAAGCCACAGAGAGTAAAATATA
GTGGGAAGCCACAGAGAGTAAAATATA
GTGGGAAGCCACAGAGAGTAAAATATA
GTGGGAAGCCACAGAGAGTAAAATATA
GTGGGAAGCCACAGAGAGTAAAATATA
GTGGGAAGCCACAGAGAGTAAAATATA
GTGGGAAGCCACAGAGAGTAAAATATA
GTGGGAAGCCACAGAGAGTAAAATATA
GTGGGAAGCCACAGAGAGTAAAATATA
GTGGGAAGCCACAGAGAGTAAAATATA
GTGGGAAGCCACAGAGAGTAAAATATA
GTGGGAAGCCACAGAGAGTAAAATATA
GTGGGAAGCCACAGAGAGTAAAATATA
GTGGGAAGCCACAGAGAGTAAAATATA
GTGGGAAGCCACAGAGAGTAAAATATA
GTGGGAAGCCACAGAGAGTAAAATATA
GTGGGAAGCCACAGAGAGTAAAATATA
GTGGGAAGCCACAGAGAGTAAAATATA
GTGGGAAGCCACAGAGAGTAAAATATA
GTGGGAAGCCACAGAGAGTAAAATATA
GTGGGAAGCCACAGAGAGTAAAATATA
GTGGGAAGCCACAGAGAGTAAAATATA
GTGGGAAGCCACAGAGAGTAAAATATA
GTGGGAAGCCACAGAGAGTAAAATATA
GTGGGAAGCCACAGAGAGTAAAATATA
GTGGGAAGCCACAGAGAGTAAAATATA
GTGGGAAGCCACAGAGAGTAAAATATA
GTGGGAAGCCACAGAGAGTAAAATATA
GTGGGAAGCCACAGAGAGTAAAATATA
GTGGGAAGCCACAGAGAGTAAAATATA
GTGGGAAGCCACAGAGAGTAAAATATA
GTGGGAAGCCACAGAGAGTAAAATATA
GTGGGAAGCCACAGAGAGTAAAATATA
GTGGGAAGCCACAGAGAGTAAAATATA
GTGGGAAGCCACAGAGAGTAAAATATA
GTGGGAAGCCACAGAGAGTAAAATATA

GTGGGAAGCCACAGAGAGTAAAATATA
GTGGGAAGCCACAGAGAGTAAAATATA
GTGGGAAGCCACAGAGAGTAAAATATA
GTGGGAAGCCACAGAGAGTAAAATATA
GTGGGAAGCCACAGAGAGTAAAATATA
GTGGGAAGCCACAGAGAGTAAAATATA
GTGGGAAGCCACAGAGAGTAAAATATA
GTGGGAAGCCACAGAGAGTAAAATATA
GTGGGAAGCCACAGAGAGTAAAATATA
GTGGGAAGCCACAGAGAGTAAAATATA
GTGGGAAGCCACAGAGAGTAAAATATA
GTGGGAAGCCACAGAGAGTAAAATATA
GTGGGAAGCCACAGAGAGTAAAATATA
GTGGGAAGCCACAGAGAGTAAAATATA
GTGGGAAGCCACAGAGAGTAAAATATA
GTGGGAAGCCACAGAGAGTAAAATATA
GTGGGAAGCCACAGAGAGTAAAATATA
GTGGGAAGCCACAGAGAGTAAAATATA
GTGGGAAGCCACAGAGAGTAAAATATA
GTGGGAAGCCACAGAGAGTAAAATATA
GTGGGAAGCCACAGAGAGTAAAATATA
GTGGGAAGCCACAGAGAGTAAAATATA
GTGGGAAGCCACAGAGAGTAAAATATA
GTGGGAAGCCACAGAGAGTAAAATATA
GTGGGAAGCCACAGAGAGTAAAATATA
GTGGGAAGCCACAGAGAGTAAAATATA
GTGGGAAGCCACAGAGAGTAAAATATA
GTGGGAAGCCACAGAGAGTAAAATATA
GTGGGAAGCCACAGAGAGTAAAATATA
GTGGGAAGCCACAGAGAGTAAAATATA
GTGGGAAGCCACAGAGAGTAAAATATA
GTGGGAAGCCACAGAGAGTAAAATATA
GTGGGAAGCCACAGAGAGTAAAATATA
GTGGGAAGCCACAGAGAGTAAAATATA
GTGGGAAGCCACAGAGAGTAAAATATA
GTGGGAAGCCACAGAGAGTAAAATATA
GTGGGAAGCCACAGAGAGTAAAATATA
GTGGGAAGCCACAGAGAGTAAAATATA
GTGGGAAGCCACAGAGAGTAAAATATA
GTGGGAAGCCACAGAGAGTAAAATATA
GTGGGAAGCCACAGAGAGTAAAATATA
GTGGGAAGCCACAGAGAGTAAAATATA
GTGGGAAGCCACAGAGAGTAAAATATA
GTGGGAAGCCACAGAGAGTAAAATATA
GTGGGAAGCCACAGAGAGTAAAATATA
GTGGGAAGCCACAGAGAGTAAAATATA
GTGGGAAGCCACAGAGAGTAAAATATA
GTGGGAAGCCACAGAGAGTAAAATATA
GTGGGAAGCCACAGAGAGTAAAATATA
GTGGGAAGCCACAGAGAGTAAAATATA
GTGGGAAGCCACAGAGAGTAAAATATA
GTGGGAAGCCACAGAGAGTAAAATATA
GTGGGAAGCCACAGAGAGTAAAATATA
GTGGGAAGCCACAGAGAGTAAAATATA

GTGGGAAGCCACAGAGAGTAAAATATA
GTGGGAAGCCACAGAGAGTAAAATATA
GTGGGAAGCCACAGAGAGTAAAATATA
GTGGGAAGCCACAGAGAGTAAAATATA
GTGGGAAGCCACAGAGAGTAAAATATA
GTGGGAAGCCACAGAGAGTAAAATATA
GTGGGAAGCCACAGAGAGTAAAATATA
GTGGGAAGCCACAGAGAGTAAAATATA
GTGGGAAGCCACAGAGAGTAAAATATA
GTGGGAAGCCACAGAGAGTAAAATATA
GTGGGAAGCCACAGAGAGTAAAATATA
GTGGGAAGCCACAGAGAGTAAAATATA
GTGGGAAGCCACAGAGAGTAAAATATA
GTGGGAAGCCACAGAGAGTAAAATATA
GTGGGAAGCCACAGAGAGTAAAATATA
GTGGGAAGCCACAGAGAGTAAAATATA
GTGGGAAGCCACAGAGAGTAAAATATA
GTGGGAAGCCACAGAGAGTAAAATATA
GTGGGAAGCCACAGAGAGTAAAATATA
GTGGGAAGCCACAGAGAGTAAAATATA
GTGGGAAGCCACAGAGAGTAAAATATA
GTGGGAAGCCACAGAGAGTAAAATATA
GTGGGAAGCCACAGAGAGTAAAATATA
GTGGGAAGCCACAGAGAGTAAAATATA
GTGGGAAGCCACAGAGAGTAAAATATA
GTGGGAAGCCACAGAGAGTAAAATATA
GTGGGAAGCCACAGAGAGTAAAATATA
GTGGGAAGCCACAGAGAGTAAAATATA
GTGGGAAGCCACAGAGAGTAAAATATA
GTGGGAAGCCACAGAGAGTAAAATATA
GTGGGAAGCCACAGAGAGTAAAATATA
GTGGGAAGCCACAGAGAGTAAAATATA
GTGGGAAGCCACAGAGAGTAAAATATA
GTGGGAAGCCACAGAGAGTAAAATATA
GTGGGAAGCCACAGAGAGTAAAATATA
GTGGGAAGCCACAGAGAGTAAAATATA
GTGGGAAGCCACAGAGAGTAAAATATA
GTGGGAAGCCACAGAGAGTAAAATATA
GTGGGAAGCCACAGAGAGTAAAATATA
GTGGGAAGCCACAGAGAGTAAAATATA
GTGGGAAGCCACAGAGAGTAAAATATA
GTGGGAAGCCACAGAGAGTAAAATATA
GTGGGAAGCCACAGAGAGTAAAATATA
GTGGGAAGCCACAGAGAGTAAAATATA
GTGGGAAGCCACAGAGAGTAAAATATA
GTGGGAAGCCACAGAGAGTAAAATATA
GTGGGAAGCCACAGAGAGTAAAATATA
GTGGGAAGCCACAGAGAGTAAAATATA
GTGGGAAGCCACAGAGAGTAAAATATA
GTGGGAAGCCACAGAGAGTAAAATATA
GTGGGAAGCCACAGAGAGTAAAATATA
GTGGGAAGCCACAGAGAGTAAAATATA
GTGGGAAGCCACAGAGAGTAAAATATA
GTGGGAAGCCACAGAGAGTAAAATATA

GTGGGAAGCCACAGAGAGTAAAATATA
GTGGGAAGCCACAGAGAGTAAAATATA
GTGGGAAGCCACAGAGAGTAAAATATA
GTGGGAAGCCACAGAGAGTAAAATATA
GTGGGAAGCCACAGAGAGTAAAATATA
GTGGGAAGCCACAGAGAGTAAAATATA
GTGGGAAGCCACAGAGAGTAAAATATA
GTGGGAAGCCACAGAGAGTAAAATATA
GTGGGAAGCCACAGAGAGTAAAATATA
GTGGGAAGCCACAGAGAGTAAAATATA
GTGGGAAGCCACAGAGAGTAAAATATA
GTGGGAAGCCACAGAGAGTAAAATATA
GTGGGAAGCCACAGAGAGTAAAATATA
GTGGGAAGCCACAGAGAGTAAAATATA
GTGGGAAGCCACAGAGAGTAAAATATA
GTGGGAAGCCACAGAGAGTAAAATATA
GTGGGAAGCCACAGAGAGTAAAATATA
GTGGGAAGCCACAGAGAGTAAAATATA
GTGGGAAGCCACAGAGAGTAAAATATA
GTGGGAAGCCACAGAGAGTAAAATATA
GTGGGAAGCCACAGAGAGTAAAATATA
GTGGGAAGCCACAGAGAGTAAAATATA
GTGGGAAGCCACAGAGAGTAAAATATA
GTGGGAAGCCACAGAGAGTAAAATATA
GTGGGAAGCCACAGAGAGTAAAATATA
GTGGGAAGCCACAGAGAGTAAAATATA
GTGGGAAGCCACAGAGAGTAAAATATA
GTGGGAAGCCACAGAGAGTAAAATATA
GTGGGAAGCCACAGAGAGTAAAATATA
GTGGGAAGCCACAGAGAGTAAAATATA
GTGGGAAGCCACAGAGAGTAAAATATA
GTGGGAAGCCACAGAGAGTAAAATATA
GTGGGAAGCCACAGAGAGTAAAATATA
GTGGGAAGCCACAGAGAGTAAAATATA
GTGGGAAGCCACAGAGAGTAAAATATA
GTGGGAAGCCACAGAGAGTAAAATATA
GTGGGAAGCCACAGAGAGTAAAATATA
GTGGGAAGCCACAGAGAGTAAAATATA
GTGGGAAGCCACAGAGAGTAAAATATA
GTGGGAAGCCACAGAGAGTAAAATATA
GTGGGAAGCCACAGAGAGTAAAATATA
GTGGGAAGCCACAGAGAGTAAAATATA
GTGGGAAGCCACAGAGAGTAAAATATA
GTGGGAAGCCACAGAGAGTAAAATATA
GTGGGAAGCCACAGAGAGTAAAATATA
GTGGGAAGCCACAGAGAGTAAAATATA
GTGGGAAGCCACAGAGAGTAAAATATA
GTGGGAAGCCACAGAGAGTAAAATATA
GTGGGAAGCCACAGAGAGTAAAATATA
GTGGGAAGCCACAGAGAGTAAAATATA
GTGGGAAGCCACAGAGAGTAAAATATA
GTGGGAAGCCACAGAGAGTAAAATATA
GTGGGAAGCCACAGAGAGTAAAATATA
GTGGGAAGCCACAGAGAGTAAAATATA

GTGGGAAGCCACAGAGAGTAAAATATA
GTGGGAAGCCACAGAGAGTAAAATATA
GTGGGAAGCCACAGAGAGTAAAATATA
GTGGGAAGCCACAGAGAGTAAAATATA
GTGGGAAGCCACAGAGAGTAAAATATA
GTGGGAAGCCACAGAGAGTAAAATATA
GTGGGAAGCCACAGAGAGTAAAATATA
GTGGGAAGCCACAGAGAGTAAAATATA
GTGGGAAGCCACAGAGAGTAAAATATA
GTGGGAAGCCACAGAGAGTAAAATATA
GTGGGAAGCCACAGAGAGTAAAATATA
GTGGGAAGCCACAGAGAGTAAAATATA
GTGGGAAGCCACAGAGAGTAAAATATA
GTGGGAAGCCACAGAGAGTAAAATATA
GTGGGAAGCCACAGAGAGTAAAATATA
GTGGGAAGCCACAGAGAGTAAAATATA
GTGGGAAGCCACAGAGAGTAAAATATA
GTGGGAAGCCACAGAGAGTAAAATATA
GTGGGAAGCCACAGAGAGTAAAATATA
GTGGGAAGCCACAGAGAGTAAAATATA
GTGGGAAGCCACAGAGAGTAAAATATA
GTGGGAAGCCACAGAGAGTAAAATATA
GTGGGAAGCCACAGAGAGTAAAATATA
GTGGGAAGCCACAGAGAGTAAAATATA
GTGGGAAGCCACAGAGAGTAAAATATA
GTGGGAAGCCACAGAGAGTAAAATATA
GTGGGAAGCCACAGAGAGTAAAATATA
GTGGGAAGCCACAGAGAGTAAAATATA
GTGGGAAGCCACAGAGAGTAAAATATA
GTGGGAAGCCACAGAGAGTAAAATATA
GTGGGAAGCCACAGAGAGTAAAATATA
GTGGGAAGCCACAGAGAGTAAAATATA
GTGGGAAGCCACAGAGAGTAAAATATA
GTGGGAAGCCACAGAGAGTAAAATATA
GTGGGAAGCCACAGAGAGTAAAATATA
GTGGGAAGCCACAGAGAGTAAAATATA
GTGGGAAGCCACAGAGAGTAAAATATA
GTGGGAAGCCACAGAGAGTAAAATATA
GTGGGAAGCCACAGAGAGTAAAATATA
GTGGGAAGCCACAGAGAGTAAAATATA
GTGGGAAGCCACAGAGAGTAAAATATA
GTGGGAAGCCACAGAGAGTAAAATATA
GTGGGAAGCCACAGAGAGTAAAATATA
GTGGGAAGCCACAGAGAGTAAAATATA
GTGGGAAGCCACAGAGAGTAAAATATA
GTGGGAAGCCACAGAGAGTAAAATATA
GTGGGAAGCCACAGAGAGTAAAATATA
GTGGGAAGCCACAGAGAGTAAAATATA
GTGGGAAGCCACAGAGAGTAAAATATA
GTGGGAAGCCACAGAGAGTAAAATATA
GTGGGAAGCCACAGAGAGTAAAATATA
GTGGGAAGCCACAGAGAGTAAAATATA
GTGGGAAGCCACAGAGAGTAAAATATA
GTGGGAAGCCACAGAGAGTAAAATATA

GTGGGAAGCCACAGAGAGTAAAATATA
GTGGGAAGCCACAGAGAGTAAAATATA
GTGGGAAGCCACAGAGAGTAAAATATA
GTGGGAAGCCACAGAGAGTAAAATATA
GTGGGAAGCCACAGAGAGTAAAATATA
GTGGGAAGCCACAGAGAGTAAAATATA
GTGGGAAGCCACAGAGAGTAAAATATA
GTGGGAAGCCACAGAGAGTAAAATATA
GTGGGAAGCCACAGAGAGTAAAATATA
GTGGGAAGCCACAGAGAGTAAAATATA
GTGGGAAGCCACAGAGAGTAAAATATA
GTGGGAAGCCACAGAGAGTAAAATATA
GTGGGAAGCCACAGAGAGTAAAATATA
GTGGGAAGCCACAGAGAGTAAAATATA
GTGGGAAGCCACAGAGAGTAAAATATA
GTGGGAAGCCACAGAGAGTAAAATATA
GTGGGAAGCCACAGAGAGTAAAATATA
GTGGGAAGCCACAGAGAGTAAAATATA
GTGGGAAGCCACAGAGAGTAAAATATA
GTGGGAAGCCACAGAGAGTAAAATATA
GTGGGAAGCCACAGAGAGTAAAATATA
GTGGGAAGCCACAGAGAGTAAAATATA
GTGGGAAGCCACAGAGAGTAAAATATA
GTGGGAAGCCACAGAGAGTAAAATATA
GTGGGAAGCCACAGAGAGTAAAATATA
GTGGGAAGCCACAGAGAGTAAAATATA
GTGGGAAGCCACAGAGAGTAAAATATA
GTGGGAAGCCACAGAGAGTAAAATATA
GTGGGAAGCCACAGAGAGTAAAATATA
GTGGGAAGCCACAGAGAGTAAAATATA
GTGGGAAGCCACAGAGAGTAAAATATA
GTGGGAAGCCACAGAGAGTAAAATATA
GTGGGAAGCCACAGAGAGTAAAATATA
GTGGGAAGCCACAGAGAGTAAAATATA
GTGGGAAGCCACAGAGAGTAAAATATA
GTGGGAAGCCACAGAGAGTAAAATATA
GTGGGAAGCCACAGAGAGTAAAATATA
GTGGGAAGCCACAGAGAGTAAAATATA
GTGGGAAGCCACAGAGAGTAAAATATA
GTGGGAAGCCACAGAGAGTAAAATATA
GTGGGAAGCCACAGAGAGTAAAATATA
GTGGGAAGCCACAGAGAGTAAAATATA
GTGGGAAGCCACAGAGAGTAAAATATA
GTGGGAAGCCACAGAGAGTAAAATATA
GTGGGAAGCCACAGAGAGTAAAATATA
GTGGGAAGCCACAGAGAGTAAAATATA
GTGGGAAGCCACAGAGAGTAAAATATA
GTGGGAAGCCACAGAGAGTAAAATATA
GTGGGAAGCCACAGAGAGTAAAATATA
GTGGGAAGCCACAGAGAGTAAAATATA
GTGGGAAGCCACAGAGAGTAAAATATA
GTGGGAAGCCACAGAGAGTAAAATATA
GTGGGAAGCCACAGAGAGTAAAATATA
GTGGGAAGCCACAGAGAGTAAAATATA

GTGGGAAGCCACAGAGAGTAAAATATA
GTGGGAAGCCACAGAGAGTAAAATATA
GTGGGAAGCCACAGAGAGTAAAATATA
GTGGGAAGCCACAGAGAGTAAAATATA
GTGGGAAGCCACAGAGAGTAAAATATA
GTGGGAAGCCACAGAGAGTAAAATATA
GTGGGAAGCCACAGAGAGTAAAATATA
GTGGGAAGCCACAGAGAGTAAAATATA
GTGGGAAGCCACAGAGAGTAAAATATA
GTGGGAAGCCACAGAGAGTAAAATATA
GTGGGAAGCCACAGAGAGTAAAATATA
GTGGGAAGCCACAGAGAGTAAAATATA
GTGGGAAGCCACAGAGAGTAAAATATA
GTGGGAAGCCACAGAGAGTAAAATATA
GTGGGAAGCCACAGAGAGTAAAATATA
GTGGGAAGCCACAGAGAGTAAAATATA
GTGGGAAGCCACAGAGAGTAAAATATA
GTGGGAAGCCACAGAGAGTAAAATATA
GTGGGAAGCCACAGAGAGTAAAATATA
GTGGGAAGCCACAGAGAGTAAAATATA
GTGGGAAGCCACAGAGAGTAAAATATA
GTGGGAAGCCACAGAGAGTAAAATATA
GTGGGAAGCCACAGAGAGTAAAATATA
GTGGGAAGCCACAGAGAGTAAAATATA
GTGGGAAGCCACAGAGAGTAAAATATA
GTGGGAAGCCACAGAGAGTAAAATATA
GTGGGAAGCCACAGAGAGTAAAATATA
GTGGGAAGCCACAGAGAGTAAAATATA
GTGGGAAGCCACAGAGAGTAAAATATA
GTGGGAAGCCACAGAGAGTAAAATATA
GTGGGAAGCCACAGAGAGTAAAATATA
GTGGGAAGCCACAGAGAGTAAAATATA
GTGGGAAGCCACAGAGAGTAAAATATA
GTGGGAAGCCACAGAGAGTAAAATATA
GTGGGAAGCCACAGAGAGTAAAATATA
GTGGGAAGCCACAGAGAGTAAAATATA
GTGGGAAGCCACAGAGAGTAAAATATA
GTGGGAAGCCACAGAGAGTAAAATATA
GTGGGAAGCCACAGAGAGTAAAATATA
GTGGGAAGCCACAGAGAGTAAAATATA
GTGGGAAGCCACAGAGAGTAAAATATA
GTGGGAAGCCACAGAGAGTAAAATATA
GTGGGAAGCCACAGAGAGTAAAATATA
GTGGGAAGCCACAGAGAGTAAAATATA
GTGGGAAGCCACAGAGAGTAAAATATA
GTGGGAAGCCACAGAGAGTAAAATATA
GTGGGAAGCCACAGAGAGTAAAATATA
GTGGGAAGCCACAGAGAGTAAAATATA
GTGGGAAGCCACAGAGAGTAAAATATA
GTGGGAAGCCACAGAGAGTAAAATATA
GTGGGAAGCCACAGAGAGTAAAATATA
GTGGGAAGCCACAGAGAGTAAAATATA
GTGGGAAGCCACAGAGAGTAAAATATA
GTGGGAAGCCACAGAGAGTAAAATATA

GTGGGAAGCCACAGAGAGTAAAATATA
GTGGGAAGCCACAGAGAGTAAAATATA
GTGGGAAGCCACAGAGAGTAAAATATA
GTGGGAAGCCACAGAGAGTAAAATATA
GTGGGAAGCCACAGAGAGTAAAATATA
GTGGGAAGCCACAGAGAGTAAAATATA
GTGGGAAGCCACAGAGAGTAAAATATA
GTGGGAAGCCACAGAGAGTAAAATATA
GTGGGAAGCCACAGAGAGTAAAATATA
GTGGGAAGCCACAGAGAGTAAAATATA
GTGGGAAGCCACAGAGAGTAAAATATA
GTGGGAAGCCACAGAGAGTAAAATATA
GTGGGAAGCCACAGAGAGTAAAATATA
GTGGGAAGCCACAGAGAGTAAAATATA
GTGGGAAGCCACAGAGAGTAAAATATA
GTGGGAAGCCACAGAGAGTAAAATATA
GTGGGAAGCCACAGAGAGTAAAATATA
GTGGGAAGCCACAGAGAGTAAAATATA
GTGGGAAGCCACAGAGAGTAAAATATA
GTGGGAAGCCACAGAGAGTAAAATATA
GTGGGAAGCCACAGAGAGTAAAATATA
GTGGGAAGCCACAGAGAGTAAAATATA
GTGGGAAGCCACAGAGAGTAAAATATA
GTGGGAAGCCACAGAGAGTAAAATATA
GTGGGAAGCCACAGAGAGTAAAATATA
GTGGGAAGCCACAGAGAGTAAAATATA
GTGGGAAGCCACAGAGAGTAAAATATA
GTGGGAAGCCACAGAGAGTAAAATATA
GTGGGAAGCCACAGAGAGTAAAATATA
GTGGGAAGCCACAGAGAGTAAAATATA
GTGGGAAGCCACAGAGAGTAAAATATA
GTGGGAAGCCACAGAGAGTAAAATATA
GTGGGAAGCCACAGAGAGTAAAATATA
GTGGGAAGCCACAGAGAGTAAAATATA
GTGGGAAGCCACAGAGAGTAAAATATA
GTGGGAAGCCACAGAGAGTAAAATATA
GTGGGAAGCCACAGAGAGTAAAATATA
GTGGGAAGCCACAGAGAGTAAAATATA
GTGGGAAGCCACAGAGAGTAAAATATA
GTGGGAAGCCACAGAGAGTAAAATATA
GTGGGAAGCCACAGAGAGTAAAATATA
GTGGGAAGCCACAGAGAGTAAAATATA
GTGGGAAGCCACAGAGAGTAAAATATA
GTGGGAAGCCACAGAGAGTAAAATATA
GTGGGAAGCCACAGAGAGTAAAATATA
GTGGGAAGCCACAGAGAGTAAAATATA
GTGGGAAGCCACAGAGAGTAAAATATA
GTGGGAAGCCACAGAGAGTAAAATATA
GTGGGAAGCCACAGAGAGTAAAATATA
GTGGGAAGCCACAGAGAGTAAAATATA
GTGGGAAGCCACAGAGAGTAAAATATA
GTGGGAAGCCACAGAGAGTAAAATATA
GTGGGAAGCCACAGAGAGTAAAATATA
GTGGGAAGCCACAGAGAGTAAAATATA

GTGGGAAGCCACAGAGAGTAAAATATA
GTGGGAAGCCACAGAGAGTAAAATATA
GTGGGAAGCCACAGAGAGTAAAATATA
GTGGGAAGCCACAGAGAGTAAAATATA
GTGGGAAGCCACAGAGAGTAAAATATA
GTGGGAAGCCACAGAGAGTAAAATATA
GTGGGAAGCCACAGAGAGTAAAATATA
GTGGGAAGCCACAGAGAGTAAAATATA
GTGGGAAGCCACAGAGAGTAAAATATA
GTGGGAAGCCACAGAGAGTAAAATATA
GTGGGAAGCCACAGAGAGTAAAATATA
GTGGGAAGCCACAGAGAGTAAAATATA
GTGGGAAGCCACAGAGAGTAAAATATA
GTGGGAAGCCACAGAGAGTAAAATATA
GTGGGAAGCCACAGAGAGTAAAATATA
GTGGGAAGCCACAGAGAGTAAAATATA
GTGGGAAGCCACAGAGAGTAAAATATA
GTGGGAAGCCACAGAGAGTAAAATATA
GTGGGAAGCCACAGAGAGTAAAATATA
GTGGGAAGCCACAGAGAGTAAAATATA
GTGGGAAGCCACAGAGAGTAAAATATA
GTGGGAAGCCACAGAGAGTAAAATATA
GTGGGAAGCCACAGAGAGTAAAATATA
GTGGGAAGCCACAGAGAGTAAAATATA
GTGGGAAGCCACAGAGAGTAAAATATA
GTGGGAAGCCACAGAGAGTAAAATATA
GTGGGAAGCCACAGAGAGTAAAATATA
GTGGGAAGCCACAGAGAGTAAAATATA
GTGGGAAGCCACAGAGAGTAAAATATA
GTGGGAAGCCACAGAGAGTAAAATATA
GTGGGAAGCCACAGAGAGTAAAATATA
GTGGGAAGCCACAGAGAGTAAAATATA
GTGGGAAGCCACAGAGAGTAAAATATA
GTGGGAAGCCACAGAGAGTAAAATATA
GTGGGAAGCCACAGAGAGTAAAATATA
GTGGGAAGCCACAGAGAGTAAAATATA
GTGGGAAGCCACAGAGAGTAAAATATA
GTGGGAAGCCACAGAGAGTAAAATATA
GTGGGAAGCCACAGAGAGTAAAATATA
GTGGGAAGCCACAGAGAGTAAAATATA
GTGGGAAGCCACAGAGAGTAAAATATA
GTGGGAAGCCACAGAGAGTAAAATATA
GTGGGAAGCCACAGAGAGTAAAATATA
GTGGGAAGCCACAGAGAGTAAAATATA
GTGGGAAGCCACAGAGAGTAAAATATA
GTGGGAAGCCACAGAGAGTAAAATATA
GTGGGAAGCCACAGAGAGTAAAATATA
GTGGGAAGCCACAGAGAGTAAAATATA
GTGGGAAGCCACAGAGAGTAAAATATA
GTGGGAAGCCACAGAGAGTAAAATATA
GTGGGAAGCCACAGAGAGTAAAATATA
GTGGGAAGCCACAGAGAGTAAAATATA
GTGGGAAGCCACAGAGAGTAAAATATA
GTGGGAAGCCACAGAGAGTAAAATATA

GTGGGAAGCCACAGAGAGTAAAATATA
GTGGGAAGCCACAGAGAGTAAAATATA
GTGGGAAGCCACAGAGAGTAAAATATA
GTGGGAAGCCACAGAGAGTAAAATATA
GTGGGAAGCCACAGAGAGTAAAATATA
GTGGGAAGCCACAGAGAGTAAAATATA
GTGGGAAGCCACAGAGAGTAAAATATA
GTGGGAAGCCACAGAGAGTAAAATATA
GTGGGAAGCCACAGAGAGTAAAATATA
GTGGGAAGCCACAGAGAGTAAAATATA
GTGGGAAGCCACAGAGAGTAAAATATA
GTGGGAAGCCACAGAGAGTAAAATATA
GTGGGAAGCCACAGAGAGTAAAATATA
GTGGGAAGCCACAGAGAGTAAAATATA
GTGGGAAGCCACAGAGAGTAAAATATA
GTGGGAAGCCACAGAGAGTAAAATATA
GTGGGAAGCCACAGAGAGTAAAATATA
GTGGGAAGCCACAGAGAGTAAAATATA
GTGGGAAGCCACAGAGAGTAAAATATA
GTGGGAAGCCACAGAGAGTAAAATATA
GTGGGAAGCCACAGAGAGTAAAATATA
GTGGGAAGCCACAGAGAGTAAAATATA
GTGGGAAGCCACAGAGAGTAAAATATA
GTGGGAAGCCACAGAGAGTAAAATATA
GTGGGAAGCCACAGAGAGTAAAATATA
GTGGGAAGCCACAGAGAGTAAAATATA
GTGGGAAGCCACAGAGAGTAAAATATA
GTGGGAAGCCACAGAGAGTAAAATATA
GTGGGAAGCCACAGAGAGTAAAATATA
GTGGGAAGCCACAGAGAGTAAAATATA
GTGGGAAGCCACAGAGAGTAAAATATA
GTGGGAAGCCACAGAGAGTAAAATATA
GTGGGAAGCCACAGAGAGTAAAATATA
GTGGGAAGCCACAGAGAGTAAAATATA
GTGGGAAGCCACAGAGAGTAAAATATA
GTGGGAAGCCACAGAGAGTAAAATATA
GTGGGAAGCCACAGAGAGTAAAATATA
GTGGGAAGCCACAGAGAGTAAAATATA
GTGGGAAGCCACAGAGAGTAAAATATA
GTGGGAAGCCACAGAGAGTAAAATATA
GTGGGAAGCCACAGAGAGTAAAATATA
GTGGGAAGCCACAGAGAGTAAAATATA
GTGGGAAGCCACAGAGAGTAAAATATA
GTGGGAAGCCACAGAGAGTAAAATATA
GTGGGAAGCCACAGAGAGTAAAATATA
GTGGGAAGCCACAGAGAGTAAAATATA
GTGGGAAGCCACAGAGAGTAAAATATA
GTGGGAAGCCACAGAGAGTAAAATATA
GTGGGAAGCCACAGAGAGTAAAATATA
GTGGGAAGCCACAGAGAGTAAAATATA
GTGGGAAGCCACAGAGAGTAAAATATA
GTGGGAAGCCACAGAGAGTAAAATATA
GTGGGAAGCCACAGAGAGTAAAATATA
GTGGGAAGCCACAGAGAGTAAAATATA

GTGGGAAGCCACAGAGAGTAAAATATA
GTGGGAAGCCACAGAGAGTAAAATATA
GTGGGAAGCCACAGAGAGTAAAATATA
GTGGGAAGCCACAGAGAGTAAAATATA
GTGGGAAGCCACAGAGAGTAAAATATA
GTGGGAAGCCACAGAGAGTAAAATATA
GTGGGAAGCCACAGAGAGTAAAATATA
GTGGGAAGCCACAGAGAGTAAAATATA
GTGGGAAGCCACAGAGAGTAAAATATA
GTGGGAAGCCACAGAGAGTAAAATATA
GTGGGAAGCCACAGAGAGTAAAATATA
GTGGGAAGCCACAGAGAGTAAAATATA
GTGGGAAGCCACAGAGAGTAAAATATA
GTGGGAAGCCACAGAGAGTAAAATATA
GTGGGAAGCCACAGAGAGTAAAATATA
GTGGGAAGCCACAGAGAGTAAAATATA
GTGGGAAGCCACAGAGAGTAAAATATA
GTGGGAAGCCACAGAGAGTAAAATATA
GTGGGAAGCCACAGAGAGTAAAATATA
GTGGGAAGCCACAGAGAGTAAAATATA
GTGGGAAGCCACAGAGAGTAAAATATA
GTGGGAAGCCACAGAGAGTAAAATATA
GTGGGAAGCCACAGAGAGTAAAATATA
GTGGGAAGCCACAGAGAGTAAAATATA
GTGGGAAGCCACAGAGAGTAAAATATA
GTGGGAAGCCACAGAGAGTAAAATATA
GTGGGAAGCCACAGAGAGTAAAATATA
GTGGGAAGCCACAGAGAGTAAAATATA
GTGGGAAGCCACAGAGAGTAAAATATA
GTGGGAAGCCACAGAGAGTAAAATATA
GTGGGAAGCCACAGAGAGTAAAATATA
GTGGGAAGCCACAGAGAGTAAAATATA
GTGGGAAGCCACAGAGAGTAAAATATA
GTGGGAAGCCACAGAGAGTAAAATATA
GTGGGAAGCCACAGAGAGTAAAATATA
GTGGGAAGCCACAGAGAGTAAAATATA
GTGGGAAGCCACAGAGAGTAAAATATA
GTGGGAAGCCACAGAGAGTAAAATATA
GTGGGAAGCCACAGAGAGTAAAATATA
GTGGGAAGCCACAGAGAGTAAAATATA
GTGGGAAGCCACAGAGAGTAAAATATA
GTGGGAAGCCACAGAGAGTAAAATATA
GTGGGAAGCCACAGAGAGTAAAATATA
GTGGGAAGCCACAGAGAGTAAAATATA
GTGGGAAGCCACAGAGAGTAAAATATA
GTGGGAAGCCACAGAGAGTAAAATATA
GTGGGAAGCCACAGAGAGTAAAATATA
GTGGGAAGCCACAGAGAGTAAAATATA
GTGGGAAGCCACAGAGAGTAAAATATA
GTGGGAAGCCACAGAGAGTAAAATATA
GTGGGAAGCCACAGAGAGTAAAATATA
GTGGGAAGCCACAGAGAGTAAAATATA
GTGGGAAGCCACAGAGAGTAAAATATA
GTGGGAAGCCACAGAGAGTAAAATATA

GTGGGAAGCCACAGAGAGTAAAATATA
GTGGGAAGCCACAGAGAGTAAAATATA
GTGGGAAGCCACAGAGAGTAAAATATA
GTGGGAAGCCACAGAGAGTAAAATATA
GTGGGAAGCCACAGAGAGTAAAATATA
GTGGGAAGCCACAGAGAGTAAAATATA
GTGGGAAGCCACAGAGAGTAAAATATA
GTGGGAAGCCACAGAGAGTAAAATATA
GTGGGAAGCCACAGAGAGTAAAATATA
GTGGGAAGCCACAGAGAGTAAAATATA
GTGGGAAGCCACAGAGAGTAAAATATA
GTGGGAAGCCACAGAGAGTAAAATATA
GTGGGAAGCCACAGAGAGTAAAATATA
GTGGGAAGCCACAGAGAGTAAAATATA
GTGGGAAGCCACAGAGAGTAAAATATA
GTGGGAAGCCACAGAGAGTAAAATATA
GTGGGAAGCCACAGAGAGTAAAATATA
GTGGGAAGCCACAGAGAGTAAAATATA
GTGGGAAGCCACAGAGAGTAAAATATA
GTGGGAAGCCACAGAGAGTAAAATATA
GTGGGAAGCCACAGAGAGTAAAATATA
GTGGGAAGCCACAGAGAGTAAAATATA
GTGGGAAGCCACAGAGAGTAAAATATA
GTGGGAAGCCACAGAGAGTAAAATATA
GTGGGAAGCCACAGAGAGTAAAATATA
GTGGGAAGCCACAGAGAGTAAAATATA
GTGGGAAGCCACAGAGAGTAAAATATA
GTGGGAAGCCACAGAGAGTAAAATATA
GTGGGAAGCCACAGAGAGTAAAATATA
GTGGGAAGCCACAGAGAGTAAAATATA
GTGGGAAGCCACAGAGAGTAAAATATA
GTGGGAAGCCACAGAGAGTAAAATATA
GTGGGAAGCCACAGAGAGTAAAATATA
GTGGGAAGCCACAGAGAGTAAAATATA
GTGGGAAGCCACAGAGAGTAAAATATA
GTGGGAAGCCACAGAGAGTAAAATATA
GTGGGAAGCCACAGAGAGTAAAATATA
GTGGGAAGCCACAGAGAGTAAAATATA
GTGGGAAGCCACAGAGAGTAAAATATA
GTGGGAAGCCACAGAGAGTAAAATATA
GTGGGAAGCCACAGAGAGTAAAATATA
GTGGGAAGCCACAGAGAGTAAAATATA
GTGGGAAGCCACAGAGAGTAAAATATA
GTGGGAAGCCACAGAGAGTAAAATATA
GTGGGAAGCCACAGAGAGTAAAATATA
GTGGGAAGCCACAGAGAGTAAAATATA
GTGGGAAGCCACAGAGAGTAAAATATA
GTGGGAAGCCACAGAGAGTAAAATATA
GTGGGAAGCCACAGAGAGTAAAATATA
GTGGGAAGCCACAGAGAGTAAAATATA
GTGGGAAGCCACAGAGAGTAAAATATA
GTGGGAAGCCACAGAGAGTAAAATATA
GTGGGAAGCCACAGAGAGTAAAATATA
GTGGGAAGCCACAGAGAGTAAAATATA

GTGGGAAGCCACAGAGAGTAAAATATA
GTGGGAAGCCACAGAGAGTAAAATATA
GTGGGAAGCCACAGAGAGTAAAATATA
GTGGGAAGCCACAGAGAGTAAAATATA
GTGGGAAGCCACAGAGAGTAAAATATA
GTGGGAAGCCACAGAGAGTAAAATATA
GTGGGAAGCCACAGAGAGTAAAATATA
GTGGGAAGCCACAGAGAGTAAAATATA
GTGGGAAGCCACAGAGAGTAAAATATA
GTGGGAAGCCACAGAGAGTAAAATATA
GTGGGAAGCCACAGAGAGTAAAATATA
GTGGGAAGCCACAGAGAGTAAAATATA
GTGGGAAGCCACAGAGAGTAAAATATA
GTGGGAAGCCACAGAGAGTAAAATATA
GTGGGAAGCCACAGAGAGTAAAATATA
GTGGGAAGCCACAGAGAGTAAAATATA
GTGGGAAGCCACAGAGAGTAAAATATA
GTGGGAAGCCACAGAGAGTAAAATATA
GTGGGAAGCCACAGAGAGTAAAATATA
GTGGGAAGCCACAGAGAGTAAAATATA
GTGGGAAGCCACAGAGAGTAAAATATA
GTGGGAAGCCACAGAGAGTAAAATATA
GTGGGAAGCCACAGAGAGTAAAATATA
GTGGGAAGCCACAGAGAGTAAAATATA
GTGGGAAGCCACAGAGAGTAAAATATA
GTGGGAAGCCACAGAGAGTAAAATATA
GTGGGAAGCCACAGAGAGTAAAATATA
GTGGGAAGCCACAGAGAGTAAAATATA
GTGGGAAGCCACAGAGAGTAAAATATA
GTGGGAAGCCACAGAGAGTAAAATATA
GTGGGAAGCCACAGAGAGTAAAATATA
GTGGGAAGCCACAGAGAGTAAAATATA
GTGGGAAGCCACAGAGAGTAAAATATA
GTGGGAAGCCACAGAGAGTAAAATATA
GTGGGAAGCCACAGAGAGTAAAATATA
GTGGGAAGCCACAGAGAGTAAAATATA
GTGGGAAGCCACAGAGAGTAAAATATA
GTGGGAAGCCACAGAGAGTAAAATATA
GTGGGAAGCCACAGAGAGTAAAATATA
GTGGGAAGCCACAGAGAGTAAAATATA
GTGGGAAGCCACAGAGAGTAAAATATA
GTGGGAAGCCACAGAGAGTAAAATATA
GTGGGAAGCCACAGAGAGTAAAATATA
GTGGGAAGCCACAGAGAGTAAAATATA
GTGGGAAGCCACAGAGAGTAAAATATA
GTGGGAAGCCACAGAGAGTAAAATATA
GTGGGAAGCCACAGAGAGTAAAATATA
GTGGGAAGCCACAGAGAGTAAAATATA
GTGGGAAGCCACAGAGAGTAAAATATA
GTGGGAAGCCACAGAGAGTAAAATATA
GTGGGAAGCCA
Nr of selected repeats 1481 Similarity 0.999867
Consensus:
GTGGGAAGCCACAGAGAGTAAAATATA

>Cele-UNSB01_3:7632425-7683091 Satlength=50667 Nr of Repeats=426
RepeatLength=94 seed=AAAGCACCCA
AAAGCACCCAATATTTAGAGAACAGAAGATTTTGAGAATTACTGCCTCCAGAAATTGATGATTTTCCCATTGATTTGTC
TACATAGGGCATCGA
AAAGCACCCAATATTTAGAGAACAGAAGATTTTGAGAATTACTGCCTCCAGAAATTGATGATTTTCCCATTGATTTGTC
TACATAGGGCATCGA
AAAGCACCCAATATTTAGAGAACAGAAGATTTTGAGAATTACTGCCTCCAGAAATTGATGATTTTCCCATTGATTTGTC
TACATAGGGCATCGA
AAAGCACCCAATATTTAGAGAACAGAAGATTTTGAGAATTACTGCCTCCAGAAATTGATGATTTTCCCATTGATTTGTC
TACATAGGGCATCGA
AAAGCACCCAATATTTAGAGAACAGAAGATTTTGAGAATTACTGCCTCCAGAAATTGATGATTTTCCCATTGATTTGTC
TACATAGGGCATCGA
AAAGCACCCAATATTTAGAGAACAGAAGATTTTGAGAATTACTGCCTCCAGAAATTGATGATTTTCCCATTGATTTGTC
TACATAGGGCATCGA
AAAGCACCCAATATTTAGAGAACAGAAGATTTTGAGAATTACTGCCTCCAGAAATTGATGATTTTCCCATTGATTTGTC
TACATAGGGCATCGA
AAAGCACCCAATATTTAGAGAACAGAAGATTTTGAGAATTACTGCCTCCAGAAATTGATGATTTTCCCATTGATTTGTC
TACATAGGGCATCGA
AAAGCACCCAATATTTAGAGAACAGAAGATTTTGAGAATTACTGCCTCCAGAAATTGATGATTTTCCCATTGATTTGTC
TACATAGGGCATCGA
AAAGCACCCAATATTTAGAGAACAGAAGATTTTGAGAATTACTGCCTCCAGAAATTGATGATTTTCCCATTGATTTGTC
TACATAGGGCATCGA
AAAGCACCCAATATTTAGAGAACAGAAGATTTTGAGAATTACTGCCTCCAGAAATTGATGATTTTCCCATTGATTTGTC
TACATAGGGCATCGA
AAAGCACCCAATATTTAGAGAACAGAAGATTTTGAGAATTACTGCCTCCAGAAATTGATGATTTTCCCATTGATTTGTC
TACATAGGGCATCGA
AAAGCACCCAATATTTAGAGAACAGAAGATTTTGAGAATTACTGCCTCCAGAAATTGATGATTTTCCCATTGATTTGTC
TACATAGGGCATCGA
AAAGCACCCAATATTTAGAGAACAGAAGATTTTGAGAATTACTGCCTCCAGAAATTGATGATTTTCCCATTGATTTGTC
TACATAGGGCATCGA
AAAGCACCCAATATTTAGAGAACAGAAGATTTTGAGAATTACTGCCTCCAGAAATTGATGATTTTCCCATTGATTTGTC
TACATAGGGCATCGA
AAAGCACCCAATATTTAGAGAACAGAAGATTTTGAGAATTACTGCCTCCAGAAATTGATGATTTTCCCATTGATTTGTC
TACATAGGGCATCGA
AAAGCACCCAATATTTAGAGAACAGAAGATTTTGAGAATTACTGCCTCCAGAAATTGATGATTTTCCCATTGATTTGTC
TACATAGGGCATCGA
AAAGCACCCAATATTTAGAGAACAGAAGATTTTGAGAATTACTGCCTCCAGAAATTGATGATTTTCCCATTGATTTGTC
TACATAGGGCATCGA
AAAGCACCCAATATTTAGAGAACAGAAGATTTTGAGAATTACTGCCTCCAGAAATTGATGATTTTCCCATTGATTTGTC
TACATAGGGCATCGA
AAAGCACCCAATATTTAGAGAACAGAAGATTTTGAGAATTACTGCCTCCAGAAATTGATGATTTTCCCATTGATTTGTC
TACATAGGGCATCGA
AAAGCACCCAATATTTAGAGAACAGAAGATTTTGAGAATTACTGCCTCCAGAAATTGATGATTTTCCCATTGATTTGTC
TACATAGGGCATCGA
AAAGCACCCAATATTTAGAGAACAGAAGATTTTGAGAATTACTGCCTCCAGAAATTGATGATTTTCCCATTGATTTGTC
TACATAGGGCATCGA
AAAGCACCCAATATTTAGAGAACAGAAGATTTTGAGAATTACTGCCTCCAGAAATTGATGATTTTCCCATTGATTTGTC
TACATAGGGCATCGA
AAAGCACCCAATATTTAGAGAACAGAAGATTTTGAGAATTACTGCCTCCAGAAATTGATGATTTTCCCATTGATTTGTC
TACATAGGGCATCGA
AAAGCACCCAATATTTAGAGAACAGAAGATTTTGAGAATTACTGCCTCCAGAAATTGATGATTTTCCCATTGATTTGTC
TACATAGGGCATCGA
AAAGCACCCAATATTTAGAGAACAGAAGATTTTGAGAATTACTGCCTCCAGAAATTGATGATTTTCCCATTGATTTGTC
TACATAGGGCATCGA

AAAGCACCCAATATTTAGAGAACAGAAGATTTTGAGAATTACTGCCTCCAGAAATTGATGATTTTCCCATTGATTTGTC
TACATAGGGCATCGA
AAAGCACCCAATATTTAGAGAACAGAAGATTTTGAGAATTACTGCCTCCAGAAATTGATGATTTTCCCATTGATTTGTC
TACATAGGGCATCGA
AAAGCACCCAATATTTAGAGAACAGAAGATTTTGAGAATTACTGCCTCCAGAAATTGATGATTTTCCCATTGATTTGTC
TACATAGGGCATCGA
AAAGCACCCAATATTTAGAGAACAGAAGATTTTGAGAATTACTGCCTCCAGAAATTGATGATTTTCCCATTGATTTGTC
TACATAGGGCATCGA
AAAGCACCCAATATTTAGAGAACAGAAGATTTTGAGAATTACTGCCTCCAGAAATTGATGATTTTCCCATTGATTTGTC
TACATAGGGCATCGA
AAAGCACCCAATATTTAGAGAACAGAAGATTTTGAGAATTACTGCCTCCAGAAATTGATGATTTTCCCATTGATTTGTC
TACATAGGGCATCGA
AAAGCACCCAATATTTAGAGAACAGAAGATTTTGAGAATTACTGCCTCCAGAAATTGATGATTTTCCCATTGATTTGTC
TACATAGGGCATCGA
AAAGCACCCAATATTTAGAGAACAGAAGATTTTGAGAATTACTGCCTCCAGAAATTGATGATTTTCCCATTGATTTGTC
TACATAGGGCATCGA
AAAGCACCCAATATTTAGAGAACAGAAGATTTTGAGAATTACTGCCTCCAGAAATTGATGATTTTCCCATTGATTTGTC
TACATAGGGCATCGA
AAAGCACCCAATATTTAGAGAACAGAAGATTTTGAGAATTACTGCCTCCAGAAATTGATGATTTTCCCATTGATTTGTC
TACATAGGGCATCGA
AAAGCACCCAATATTTAGAGAACAGAAGATTTTGAGAATTACTGCCTCCAGAAATTGATGATTTTCCCATTGATTTGTC
TACATAGGGCATCGA
AAAGCACCCAATATTTAGAGAACAGAAGATTTTGAGAATTACTGCCTCCAGAAATTGATGATTTTCCCATTGATTTGTC
TACATAGGGCATCGA
AAAGCACCCAATATTTAGAGAACAGAAGATTTTGAGAATTACTGCCTCCAGAAATTGATGATTTTCCCATTGATTTGTC
TACATAGGGCATCGA
AAAGCACCCAATATTTAGAGAACAGAAGATTTTGAGAATTACTGCCTCCAGAAATTGATGATTTTCCCATTGATTTGTC
TACATAGGGCATCGA
AAAGCACCCAATATTTAGAGAACAGAAGATTTTGAGAATTACTGCCTCCAGAAATTGATGATTTTCCCATTGATTTGTC
TACATAGGGCATCGA
AAAGCACCCAATATTTAGAGAACAGAAGATTTTGAGAATTACTGCCTCCAGAAATTGATGATTTTCCCATTGATTTGTC
TACATAGGGCATCGA
AAAGCACCCAATATTTAGAGAACAGAAGATTTTGAGAATTACTGCCTCCAGAAATTGATGATTTTCCCATTGATTTGTC
TACATAGGGCATCGA
AAAGCACCCAATATTTAGAGAACAGAAGATTTTGAGAATTACTGCCTCCAGAAATTGATGATTTTCCCATTGATTTGTC
TACATAGGGCATCGA
AAAGCACCCAATATTTAGAGAACAGAAGATTTTGAGAATTACTGCCTCCAGAAATTGATGATTTTCCCATTGATTTGTC
TACATAGGGCATCGA
AAAGCACCCAATATTTAGAGAACAGAAGATTTTGAGAATTACTGCCTCCAGAAATTGATGATTTTCCCATTGATTTGTC
TACATAGGGCATCGA
AAAGCACCCAATATTTAGAGAACAGAAGATTTTGAGAATTACTGCCTCCAGAAATTGATGATTTTCCCATTGATTTGTC
TACATAGGGCATCGA
AAAGCACCCAATATTTAGAGAACAGAAGATTTTGAGAATTACTGCCTCCAGAAATTGATGATTTTCCCATTGATTTGTC
TACATAGGGCATCGA
AAAGCACCCAATATTTAGAGAACAGAAGATTTTGAGAATTACTGCCTCCAGAAATTGATGATTTTCCCATTGATTTGTC
TACATAGGGCATCGA
AAAGCACCCAATATTTAGAGAACAGAAGATTTTGAGAATTACTGCCTCCAGAAATTGATGATTTTCCCATTGATTTGTC
TACATAGGGCATCGA
AAAGCACCCAATATTTAGAGAACAGAAGATTTTGAGAATTACTGCCTCCAGAAATTGATGATTTTCCCATTGATTTGTC
TACATAGGGCATCGA
AAAGCACCCAATATTTAGAGAACAGAAGATTTTGAGAATTACTGCCTCCAGAAATTGATGATTTTCCCATTGATTTGTC
TACATAGGGCATCGA
AAAGCACCCAATATTTAGAGAACAGAAGATTTTGAGAATTACTGCCTCCAGAAATTGATGATTTTCCCATTGATTTGTC
TACATAGGGCATCGA

AAAGCACCCAATATTTAGAGAACAGAAGATTTTGAGAATTACTGCCTCCAGAAATTGATGATTTTCCCATTGATTTGTC
TACATAGGGCATCGA
AAAGCACCCAATATTTAGAGAACAGAAGATTTTGAGAATTACTGCCTCCAGAAATTGATGATTTTCCCATTGATTTGTC
TACATAGGGCATCGA
AAAGCACCCAATATTTAGAGAACAGAAGATTTTGAGAATTACTGCCTCCAGAAATTGATGATTTTCCCATTGATTTGTC
TACATAGGGCATCGA
AAAGCACCCAATATTTAGAGAACAGAAGATTTTGAGAATTACTGCCTCCAGAAATTGATGATTTTCCCATTGATTTGTC
TACATAGGGCATCGA
AAAGCACCCAATATTTAGAGAACAGAAGATTTTGAGAATTACTGCCTCCAGAAATTGATGATTTTCCCATTGATTTGTC
TACATAGGGCATCGA
AAAGCACCCAATATTTAGAGAACAGAAGATTTTGAGAATTACTGCCTCCAGAAATTGATGATTTTCCCATTGATTTGTC
TACATAGGGCATCGA
AAAGCACCCAATATTTAGAGAACAGAAGATTTTGAGAATTACTGCCTCCAGAAATTGATGATTTTCCCATTGATTTGTC
TACATAGGGCATCGA
AAAGCACCCAATATTTAGAGAACAGAAGATTTTGAGAATTACTGCCTCCAGAAATTGATGATTTTCCCATTGATTTGTC
TACATAGGGCATCGA
AAAGCACCCAATATTTAGAGAACAGAAGATTTTGAGAATTACTGCCTCCAGAAATTGATGATTTTCCCATTGATTTGTC
TACATAGGGCATCGA
AAAGCACCCAATATTTAGAGAACAGAAGATTTTGAGAATTACTGCCTCCAGAAATTGATGATTTTCCCATTGATTTGTC
TACATAGGGCATCGA
AAAGCACCCAATATTTAGAGAACAGAAGATTTTGAGAATTACTGCCTCCAGAAATTGATGATTTTCCCATTGATTTGTC
TACATAGGGCATCGA
AAAGCACCCAATATTTAGAGAACAGAAGATTTTGAGAATTACTGCCTCCAGAAATTGATGATTTTCCCATTGATTTGTC
TACATAGGGCATCGA
AAAGCACCCAATATTTAGAGAACAGAAGATTTTGAGAATTACTGCCTCCAGAAATTGATGATTTTCCCATTGATTTGTC
TACATAGGGCATCGA
AAAGCACCCAATATTTAGAGAACAGAAGATTTTGAGAATTACTGCCTCCAGAAATTGATGATTTTCCCATTGATTTGTC
TACATAGGGCATCGA
AAAGCACCCAATATTTAGAGAACAGAAGATTTTGAGAATTACTGCCTCCAGAAATTGATGATTTTCCCATTGATTTGTC
TACATAGGGCATCGA
AAAGCACCCAATATTTAGAGAACAGAAGATTTTGAGAATTACTGCCTCCAGAAATTGATGATTTTCCCATTGATTTGTC
TACATAGGGCATCGA
AAAGCACCCAATATTTAGAGAACAGAAGATTTTGAGAATTACTGCCTCCAGAAATTGATGATTTTCCCATTGATTTGTC
TACATAGGGCATCGA
AAAGCACCCAATATTTAGAGAACAGAAGATTTTGAGAATTACTGCCTCCAGAAATTGATGATTTTCCCATTGATTTGTC
TACATAGGGCATCGA
AAAGCACCCAATATTTAGAGAACAGAAGATTTTGAGAATTACTGCCTCCAGAAATTGATGATTTTCCCATTGATTTGTC
TACATAGGGCATCGA
AAAGCACCCAATATTTAGAGAACAGAAGATTTTGAGAATTACTGCCTCCAGAAATTGATGATTTTCCCATTGATTTGTC
TACATAGGGCATCGA
AAAGCACCCAATATTTAGAGAACAGAAGATTTTGAGAATTACTGCCTCCAGAAATTGATGATTTTCCCATTGATTTGTC
TACATAGGGCATCGA
AAAGCACCCAATATTTAGAGAACAGAAGATTTTGAGAATTACTGCCTCCAGAAATTGATGATTTTCCCATTGATTTGTC
TACATAGGGCATCGA
AAAGCACCCAATATTTAGAGAACAGAAGATTTTGAGAATTACTGCCTCCAGAAATTGATGATTTTCCCATTGATTTGTC
TACATAGGGCATCGA
AAAGCACCCAATATTTAGAGAACAGAAGATTTTGAGAATTACTGCCTCCAGAAATTGATGATTTTCCCATTGATTTGTC
TACATAGGGCATCGA
AAAGCACCCAATATTTAGAGAACAGAAGATTTTGAGAATTACTGCCTCCAGAAATTGATGATTTTCCCATTGATTTGTC
TACATAGGGCATCGA
AAAGCACCCAATATTTAGAGAACAGAAGATTTTGAGAATTACTGCCTCCAGAAATTGATGATTTTCCCATTGATTTGTC
TACATAGGGCATCGA
AAAGCACCCAATATTTAGAGAACAGAAGATTTTGAGAATTACTGCCTCCAGAAATTGATGATTTTCCCATTGATTTGTC
TACATAGGGCATCGA

AAAGCACCCAATATTTAGAGAACAGAAGATTTTGAGAATTACTGCCTCCAGAAATTGATGATTTTCCCATTGATTTGTC
TACATAGGGCATCGA
AAAGCACCCAATATTTAGAGAACAGAAGATTTTGAGAATTACTGCCTCCAGAAATTGATGATTTTCCCATTGATTTGTC
TACATAGGGCATCGA
AAAGCACCCAATATTTAGAGAACAGAAGATTTTGAGAATTACTGCCTCCAGAAATTGATGATTTTCCCATTGATTTGTC
TACATAGGGCATCGA
AAAGCACCCAATATTTAGAGAACAGAAGATTTTGAGAATTACTGCCTCCAGAAATTGATGATTTTCCCATTGATTTGTC
TACATAGGGCATCGA
AAAGCACCCAATATTTAGAGAACAGAAGATTTTGAGAATTACTGCCTCCAGAAATTGATGATTTTCCCATTGATTTGTC
TACATAGGGCATCGA
AAAGCACCCAATATTTAGAGAACAGAAGATTTTGAGAATTACTGCCTCCAGAAATTGATGATTTTCCCATTGATTTGTC
TACATAGGGCATCGA
AAAGCACCCAATATTTAGAGAACAGAAGATTTTGAGAATTACTGCCTCCAGAAATTGATGATTTTCCCATTGATTTGTC
TACATAGGGCATCGA
AAAGCACCCAATATTTAGAGAACAGAAGATTTTGAGAATTACTGCCTCCAGAAATTGATGATTTTCCCATTGATTTGTC
TACATAGGGCATCGA
AAAGCACCCAATATTTAGAGAACAGAAGATTTTGAGAATTACTGCCTCCAGAAATTGATGATTTTCCCATTGATTTGTC
TACATAGGGCATCGA
AAAGCACCCAATATTTAGAGAACAGAAGATTTTGAGAATTACTGCCTCCAGAAATTGATGATTTTCCCATTGATTTGTC
TACATAGGGCATCGA
AAAGCACCCAATATTTAGAGAACAGAAGATTTTGAGAATTACTGCCTCCAGAAATTGATGATTTTCCCATTGATTTGTC
TACATAGGGCATCGA
AAAGCACCCAATATTTAGAGAACAGAAGATTTTGAGAATTACTGCCTCCAGAAATTGATGATTTTCCCATTGATTTGTC
TACATAGGGCATCGA
AAAGCACCCAATATTTAGAGAACAGAAGATTTTGAGAATTACTGCCTCCAGAAATTGATGATTTTCCCATTGATTTGTC
TACATAGGGCATCGA
AAAGCACCCAATATTTAGAGAACAGAAGATTTTGAGAATTACTGCCTCCAGAAATTGATGATTTTCCCATTGATTTGTC
TACATAGGGCATCGA
AAAGCACCCAATATTTAGAGAACAGAAGATTTTGAGAATTACTGCCTCCAGAAATTGATGATTTTCCCATTGATTTGTC
TACATAGGGCATCGA
AAAGCACCCAATATTTAGAGAACAGAAGATTTTGAGAATTACTGCCTCCAGAAATTGATGATTTTCCCATTGATTTGTC
TACATAGGGCATCGA
AAAGCACCCAATATTTAGAGAACAGAAGATTTTGAGAATTACTGCCTCCAGAAATTGATGATTTTCCCATTGATTTGTC
TACATAGGGCATCGA
AAAGCACCCAATATTTAGAGAACAGAAGATTTTGAGAATTACTGCCTCCAGAAATTGATGATTTTCCCATTGATTTGTC
TACATAGGGCATCGA
AAAGCACCCAATATTTAGAGAACAGAAGATTTTGAGAATTACTGCCTCCAGAAATTGATGATTTTCCCATTGATTTGTC
TACATAGGGCATCGA
AAAGCACCCAATATTTAGAGAACAGAAGATTTTGAGAATTACTGCCTCCAGAAATTGATGATTTTCCCATTGATTTGTC
TACATAGGGCATCGA
AAAGCACCCAATATTTAGAGAACAGAAGATTTTGAGAATTACTGCCTCCAGAAATTGATGATTTTCCCATTGATTTGTC
TACATAGGGCATCGA
AAAGCACCCAATATTTAGAGAACAGAAGATTTTGAGAATTACTGCCTCCAGAAATTGATGATTTTCCCATTGATTTGTC
TACATAGGGCATCGA
AAAGCACCCAATATTTAGAGAACAGAAGATTTTGAGAATTACTGCCTCCAGAAATTGATGATTTTCCCATTGATTTGTC
TACATAGGGCATCGA
AAAGCACCCAATATTTAGAGAACAGAAGATTTTGAGAATTACTGCCTCCAGAAATTGATGATTTTCCCATTGATTTGTC
TACATAGGGCATCGA
AAAGCACCCAATATTTAGAGAACAGAAGATTTTGAGAATTACTGCCTCCAGAAATTGATGATTTTCCCATTGATTTGTC
TACATAGGGCATCGA
AAAGCACCCAATATTTAGAGAACAGAAGATTTTGAGAATTACTGCCTCCAGAAATTGATGATTTTCCCATTGATTTGTC
TACATAGGGCATCGA
AAAGCACCCAATATTTAGAGAACAGAAGATTTTGAGAATTACTGCCTCCAGAAATTGATGATTTTCCCATTGATTTGTC
TACATAGGGCATCGA

AAAGCACCCAATATTTAGAGAACAGAAGATTTTGAGAATTACTGCCTCCAGAAATTGATGATTTTCCCATTGATTTGTC
TACATAGGGCATCGA
AAAGCACCCAATATTTAGAGAACAGAAGATTTTGAGAATTACTGCCTCCAGAAATTGATGATTTTCCCATTGATTTGTC
TACATAGGGCATCGA
AAAGCACCCAATATTTAGAGAACAGAAGATTTTGAGAATTACTGCCTCCAGAAATTGATGATTTTCCCATTGATTTGTC
TACATAGGGCATCGA
AAAGCACCCAATATTTAGAGAACAGAAGATTTTGAGAATTACTGCCTCCAGAAATTGATGATTTTCCCATTGATTTGTC
TACATAGGGCATCGA
AAAGCACCCAATATTTAGAGAACAGAAGATTTTGAGAATTACTGCCTCCAGAAATTGATGATTTTCCCATTGATTTGTC
TACATAGGGCATCGA
AAAGCACCCAATATTTAGAGAACAGAAGATTTTGAGAATTACTGCCTCCAGAAATTGATGATTTTCCCATTGATTTGTC
TACATAGGGCATCGA
AAAGCACCCAATATTTAGAGAACAGAAGATTTTGAGAATTACTGCCTCCAGAAATTGATGATTTTCCCATTGATTTGTC
TACATAGGGCATCGA
AAAGCACCCAATATTTAGAGAACAGAAGATTTTGAGAATTACTGCCTCCAGAAATTGATGATTTTCCCATTGATTTGTC
TACATAGGGCATCGA
AAAGCACCCAATATTTAGAGAACAGAAGATTTTGAGAATTACTGCCTCCAGAAATTGATGATTTTCCCATTGATTTGTC
TACATAGGGCATCGA
AAAGCACCCAATATTTAGAGAACAGAAGATTTTGAGAATTACTGCCTCCAGAAATTGATGATTTTCCCATTGATTTGTC
TACATAGGGCATCGA
AAAGCACCCAATATTTAGAGAACAGAAGATTTTGAGAATTACTGCCTCCAGAAATTGATGATTTTCCCATTGATTTGTC
TACATAGGGCATCGA
AAAGCACCCAATATTTAGAGAACAGAAGATTTTGAGAATTACTGCCTCCAGAAATTGATGATTTTCCCATTGATTTGTC
TACATAGGGCATCGA
AAAGCACCCAATATTTAGAGAACAGAAGATTTTGAGAATTACTGCCTCCAGAAATTGATGATTTTCCCATTGATTTGTC
TACATAGGGCATCGA
AAAGCACCCAATATTTAGAGAACAGAAGATTTTGAGAATTACTGCCTCCAGAAATTGATGATTTTCCCATTGATTTGTC
TACATAGGGCATCGA
AAAGCACCCAATATTTAGAGAACAGAAGATTTTGAGAATTACTGCCTCCAGAAATTGATGATTTTCCCATTGATTTGTC
TACATAGGGCATCGA
AAAGCACCCAATATTTAGAGAACAGAAGATTTTGAGAATTACTGCCTCCAGAAATTGATGATTTTCCCATTGATTTGTC
TACATAGGGCATCGA
AAAGCACCCAATATTTAGAGAACAGAAGATTTTGAGAATTACTGCCTCCAGAAATTGATGATTTTCCCATTGATTTGTC
TACATAGGGCATCGA
AAAGCACCCAATATTTAGAGAACAGAAGATTTTGAGAATTACTGCCTCCAGAAATTGATGATTTTCCCATTGATTTGTC
TACATAGGGCATCGA
AAAGCACCCAATATTTAGAGAACAGAAGATTTTGAGAATTACTGCCTCCAGAAATTGATGATTTTCCCATTGATTTGTC
TACATAGGGCATCGA
AAAGCACCCAATATTTAGAGAACAGAAGATTTTGAGAATTACTGCCTCCAGAAATTGATGATTTTCCCATTGATTTGTC
TACATAGGGCATCGA
AAAGCACCCAATATTTAGAGAACAGAAGATTTTGAGAATTACTGCCTCCAGAAATTGATGATTTTCCCATTGATTTGTC
TACATAGGGCATCGA
AAAGCACCCAATATTTAGAGAACAGAAGATTTTGAGAATTACTGCCTCCAGAAATTGATGATTTTCCCATTGATTTGTC
TACATAGGGCATCGA
AAAGCACCCAATATTTAGAGAACAGAAGATTTTGAGAATTACTGCCTCCAGAAATTGATGATTTTCCCATTGATTTGTC
TACATAGGGCATCGA
AAAGCACCCAATATTTAGAGAACAGAAGATTTTGAGAATTACTGCCTCCAGAAATTGATGATTTTCCCATTGATTTGTC
TACATAGGGCATCGA
AAAGCACCCAATATTTAGAGAACAGAAGATTTTGAGAATTACTGCCTCCAGAAATTGATGATTTTCCCATTGATTTGTC
TACATAGGGCATCGA

AAAGCACCCAATATTTAGAGAACAGAAGATTTTGAGAATTACTGCCTCCAGAAATTGATGATTTTCCCATTGATTTGTC
TACATAGGGCATCGA
AAAGCACCCAATATTTAGAGAACAGAAGATTTTGAGAATTACTGCCTCCAGAAATTGATGATTTTCCCATTGATTTGTC
TACATAGGGCATCGA
AAAGCACCCAATATTTAGAGAACAGAAGATTTTGAGAATTACTGCCTCCAGAAATTGATGATTTTCCCATTGATTTGTC
TACATAGGGCATCGA
AAAGCACCCAATATTTAGAGAACAGAAGATTTTGAGAATTACTGCCTCCAGAAATTGATGATTTTCCCATTGATTTGTC
TACATAGGGCATCGA
AAAGCACCCAATATTTAGAGAACAGAAGATTTTGAGAATTACTGCCTCCAGAAATTGATGATTTTCCCATTGATTTGTC
TACATAGGGCATCGA
AAAGCACCCAATATTTAGAGAACAGAAGATTTTGAGAATTACTGCCTCCAGAAATTGATGATTTTCCCATTGATTTGTC
TACATAGGGCATCGA
AAAGCACCCAATATTTAGAGAACAGAAGATTTTGAGAATTACTGCCTCCAGAAATTGATGATTTTCCCATTGATTTGTC
TACATAGGGCATCGA
AAAGCACCCAATATTTAGAGAACAGAAGATTTTGAGAATTACTGCCTCCAGAAATTGATGATTTTCCCATTGATTTGTC
TACATAGGGCATCGA
AAAGCACCCAATATTTAGAGAACAGAAGATTTTGAGAATTACTGCCTCCAGAAATTGATGATTTTCCCATTGATTTGTC
TACATAGGGCATCGA
AAAGCACCCAATATTTAGAGAACAGAAGATTTTGAGAATTACTGCCTCCAGAAATTGATGATTTTCCCATTGATTTGTC
TACATAGGGCATCGA
AAAGCACCCAATATTTAGAGAACAGAAGATTTTGAGAATTACTGCCTCCAGAAATTGATGATTTTCCCATTGATTTGTC
TACATAGGGCATCGA
AAAGCACCCAATATTTAGAGAACAGAAGATTTTGAGAATTACTGCCTCCAGAAATTGATGATTTTCCCATTGATTTGTC
TACATAGGGCATCGA
AAAGCACCCAATATTTAGAGAACAGAAGATTTTGAGAATTACTGCCTCCAGAAATTGATGATTTTCCCATTGATTTGTC
TACATAGGGCATCGA
AAAGCACCCAATATTTAGAGAACAGAAGATTTTGAGAATTACTGCCTCCAGAAATTGATGATTTTCCCATTGATTTGTC
TACATAGGGCATCGA
AAAGCACCCAATATTTAGAGAACAGAAGATTTTGAGAATTACTGCCTCCAGAAATTGATGATTTTCCCATTGATTTGTC
TACATAGGGCATCGA
AAAGCACCCAATATTTAGAGAACAGAAGATTTTGAGAATTACTGCCTCCAGAAATTGATGATTTTCCCATTGATTTGTC
TACATAGGGCATCGA
AAAGCACCCAATATTTAGAGAACAGAAGATTTTGAGAATTACTGCCTCCAGAAATTGATGATTTTCCCATTGATTTGTC
TACATAGGGCATCGA
AAAGCACCCAATATTTAGAGAACAGAAGATTTTGAGAATTACTGCCTCCAGAAATTGATGATTTTCCCATTGATTTGTC
TACATAGGGCATCGA
AAAGCACCCAATATTTAGAGAACAGAAGATTTTGAGAATTACTGCCTCCAGAAATTGATGATTTTCCCATTGATTTGTC
TACATAGGGCATCGA
AAAGCACCCAATATTTAGAGAACAGAAGATTTTGAGAATTACTGCCTCCAGAAATTGATGATTTTCCCATTGATTTGTC
TACATAGGGCATCGA
AAAGCACCCAATATTTAGAGAACAGAAGATTTTGAGAATTACTGCCTCCAGAAATTGATGATTTTCCCATTGATTTGTC
TACATAGGGCATCGA
AAAGCACCCAATATTTAGAGAACAGAAGATTTTGAGAATTACTGCCTCCAGAAATTGATGATTTTCCCATTGATTTGTC
TACATAGGGCATCGA
AAAGCACCCAATATTTAGAGAACAGAAGATTTTGAGAATTACTGCCTCCAGAAATTGATGATTTTCCCATTGATTTGTC
TACATAGGGCATCGA
AAAGCACCCAATATTTAGAGAACAGAAGATTTTGAGAATTACTGCCTCCAGAAATTGATGATTTTCCCATTGATTTGTC
TACATAGGGCATCGA
AAAGCACCCAATATTTAGAGAACAGAAGATTTTGAGAATTACTGCCTCCAGAAATTGATGATTTTCCCATTGATTTGTC
TACATAGGGCATCGA
AAAGCACCCAATATTTAGAGAACAGAAGATTTTGAGAATTACTGCCTCCAGAAATTGATGATTTTCCCATTGATTTGTC
TACATAGGGCATCGA
AAAGCACCCAATATTTAGAGAACAGAAGATTTTGAGAATTACTGCCTCCAGAAATTGATGATTTTCCCATTGATTTGTC
TACATAGGGCATCGA

AAAGCACCCAATATTTAGAGAACAGAAGATTTTGAGAATTACTGCCTCCAGAAATTGATGATTTTCCCATTGATTTGTC
TACATAGGGCATCGA
AAAGCACCCAATATTTAGAGAACAGAAGATTTTGAGAATTACTGCCTCCAGAAATTGATGATTTTCCCATTGATTTGTC
TACATAGGGCATCGA
AAAGCACCCAATATTTAGAGAACAGAAGATTTTGAGAATTACTGCCTCCAGAAATTGATGATTTTCCCATTGATTTGTC
TACATAGGGCATCGA
AAAGCACCCAATATTTAGAGAACAGAAGATTTTGAGAATTACTGCCTCCAGAAATTGATGATTTTCCCATTGATTTGTC
TACATAGGGCATCGA
AAAGCACCCAATATTTAGAGAACAGAAGATTTTGAGAATTACTGCCTCCAGAAATTGATGATTTTCCCATTGATTTGTC
TACATAGGGCATCGA
AAAGCACCCAATATTTAGAGAACAGAAGATTTTGAGAATTACTGCCTCCAGAAATTGATGATTTTCCCATTGATTTGTC
TACATAGGGCATCGA
AAAGCACCCAATATTTAGAGAACAGAAGATTTTGAGAATTACTGCCTCCAGAAATTGATGATTTTCCCATTGATTTGTC
TACATAGGGCATCGA
AAAGCACCCAATATTTAGAGAACAGAAGATTTTGAGAATTACTGCCTCCAGAAATTGATGATTTTCCCATTGATTTGTC
TACATAGGGCATCGA
AAAGCACCCAATATTTAGAGAACAGAAGATTTTGAGAATTACTGCCTCCAGAAATTGATGATTTTCCCATTGATTTGTC
TACATAGGGCATCGA
AAAGCACCCAATATTTAGAGAACAGAAGATTTTGAGAATTACTGCCTCCAGAAATTGATGATTTTCCCATTGATTTGTC
TACATAGGGCATCGA
AAAGCACCCAATATTTAGAGAACAGAAGATTTTGAGAATTACTGCCTCCAGAAATTGATGATTTTCCCATTGATTTGTC
TACATAGGGCATCGA
AAAGCACCCAATATTTAGAGAACAGAAGATTTTGAGAATTACTGCCTCCAGAAATTGATGATTTTCCCATTGATTTGTC
TACATAGGGCATCGA
AAAGCACCCAATATTTAGAGAACAGAAGATTTTGAGAATTACTGCCTCCAGAAATTGATGATTTTCCCATTGATTTGTC
TACATAGGGCATCGA
AAAGCACCCAATATTTAGAGAACAGAAGATTTTGAGAATTACTGCCTCCAGAAATTGATGATTTTCCCATTGATTTGTC
TACATAGGGCATCGA
AAAGCACCCAATATTTAGAGAACAGAAGATTTTGAGAATTACTGCCTCCAGAAATTGATGATTTTCCCATTGATTTGTC
TACATAGGGCATCGA
AAAGCACCCAATATTTAGAGAACAGAAGATTTTGAGAATTACTGCCTCCAGAAATTGATGATTTTCCCATTGATTTGTC
TACATAGGGCATCGA
AAAGCACCCAATATTTAGAGAACAGAAGATTTTGAGAATTACTGCCTCCAGAAATTGATGATTTTCCCATTGATTTGTC
TACATAGGGCATCGA
AAAGCACCCAATATTTAGAGAACAGAAGATTTTGAGAATTACTGCCTCCAGAAATTGATGATTTTCCCATTGATTTGTC
TACATAGGGCATCGA
AAAGCACCCAATATTTAGAGAACAGAAGATTTTGAGAATTACTGCCTCCAGAAATTGATGATTTTCCCATTGATTTGTC
TACATAGGGCATCGA
AAAGCACCCAATATTTAGAGAACAGAAGATTTTGAGAATTACTGCCTCCAGAAATTGATGATTTTCCCATTGATTTGTC
TACATAGGGCATCGA
AAAGCACCCAATATTTAGAGAACAGAAGATTTTGAGAATTACTGCCTCCAGAAATTGATGATTTTCCCATTGATTTGTC
TACATAGGGCATCGA
AAAGCACCCAATATTTAGAGAACAGAAGATTTTGAGAATTACTGCCTCCAGAAATTGATGATTTTCCCATTGATTTGTC
TACATAGGGCATCGA
AAAGCACCCAATATTTAGAGAACAGAAGATTTTGAGAATTACTGCCTCCAGAAATTGATGATTTTCCCATTGATTTGTC
TACATAGGGCATCGA
AAAGCACCCAATATTTAGAGAACAGAAGATTTTGAGAATTACTGCCTCCAGAAATTGATGATTTTCCCATTGATTTGTC
TACATAGGGCATCGA
AAAGCACCCAATATTTAGAGAACAGAAGATTTTGAGAATTACTGCCTCCAGAAATTGATGATTTTCCCATTGATTTGTC
TACATAGGGCATCGA
AAAGCACCCAATATTTAGAGAACAGAAGATTTTGAGAATTACTGCCTCCAGAAATTGATGATTTTCCCATTGATTTGTC
TACATAGGGCATCGA
AAAGCACCCAATATTTAGAGAACAGAAGATTTTGAGAATTACTGCCTCCAGAAATTGATGATTTTCCCATTGATTTGTC
TACATAGGGCATCGA

AAAGCACCCAATATTTAGAGAACAGAAGATTTTGAGAATTACTGCCTCCAGAAATTGATGATTTTCCCATTGATTTGTC
TACATAGGGCATCGA
AAAGCACCCAATATTTAGAGAACAGAAGATTTTGAGAATTACTGCCTCCAGAAATTGATGATTTTCCCATTGATTTGTC
TACATAGGGCATCGA
AAAGCACCCAATATTTAGAGAACAGAAGATTTTGAGAATTACTGCCTCCAGAAATTGATGATTTTCCCATTGATTTGTC
TACATAGGGCATCGA
AAAGCACCCAATATTTAGAGAACAGAAGATTTTGAGAATTACTGCCTCCAGAAATTGATGATTTTCCCATTGATTTGTC
TACATAGGGCATCGA
AAAGCACCCAATATTTAGAGAACAGAAGATTTTGAGAATTACTGCCTCCAGAAATTGATGATTTTCCCATTGATTTGTC
TACATAGGGCATCGA
AAAGCACCCAATATTTAGAGAACAGAAGATTTTGAGAATTACTGCCTCCAGAAATTGATGATTTTCCCATTGATTTGTC
TACATAGGGCATCGA
AAAGCACCCAATATTTAGAGAACAGAAGATTTTGAGAATTACTGCCTCCAGAAATTGATGATTTTCCCATTGATTTGTC
TACATAGGGCATCGA
AAAGCACCCAATATTTAGAGAACAGAAGATTTTGAGAATTACTGCCTCCAGAAATTGATGATTTTCCCATTGATTTGTC
TACATAGGGCATCGA
AAAGCACCCAATATTTAGAGAACAGAAGATTTTGAGAATTACTGCCTCCAGAAATTGATGATTTTCCCATTGATTTGTC
TACATAGGGCATCGA
AAAGCACCCAATATTTAGAGAACAGAAGATTTTGAGAATTACTGCCTCCAGAAATTGATGATTTTCCCATTGATTTGTC
TACATAGGGCATCGA
AAAGCACCCAATATTTAGAGAACAGAAGATTTTGAGAATTACTGCCTCCAGAAATTGATGATTTTCCCATTGATTTGTC
TACATAGGGCATCGA
AAAGCACCCAATATTTAGAGAACAGAAGATTTTGAGAATTACTGCCTCCAGAAATTGATGATTTTCCCATTGATTTGTC
TACATAGGGCATCGA
AAAGCACCCAATATTTAGAGAACAGAAGATTTTGAGAATTACTGCCTCCAGAAATTGATGATTTTCCCATTGATTTGTC
TACATAGGGCATCGA
AAAGCACCCAATATTTAGAGAACAGAAGATTTTGAGAATTACTGCCTCCAGAAATTGATGATTTTCCCATTGATTTGTC
TACATAGGGCATCGA
AAAGCACCCAATATTTAGAGAACAGAAGATTTTGAGAATTACTGCCTCCAGAAATTGATGATTTTCCCATTGATTTGTC
TACATAGGGCATCGA
AAAGCACCCAATATTTAGAGAACAGAAGATTTTGAGAATTACTGCCTCCAGAAATTGATGATTTTCCCATTGATTTGTC
TACATAGGGCATCGA
AAAGCACCCAATATTTAGAGAACAGAAGATTTTGAGAATTACTGCCTCCAGAAATTGATGATTTTCCCATTGATTTGTC
TACATAGGGCATCGA
AAAGCACCCAATATTTAGAGAACAGAAGATTTTGAGAATTACTGCCTCCAGAAATTGATGATTTTCCCATTGATTTGTC
TACATAGGGCATCGA
AAAGCACCCAATATTTAGAGAACAGAAGATTTTGAGAATTACTGCCTCCAGAAATTGATGATTTTCCCATTGATTTGTC
TACATAGGGCATCGA
AAAGCACCCAATATTTAGAGAACAGAAGATTTTGAGAATTACTGCCTCCAGAAATTGATGATTTTCCCATTGATTTGTC
TACATAGGGCATCGA
AAAGCACCCAATATTTAGAGAACAGAAGATTTTGAGAATTACTGCCTCCAGAAATTGATGATTTTCCCATTGATTTGTC
TACATAGGGCATCGA
AAAGCACCCAATATTTAGAGAACAGAAGATTTTGAGAATTACTGCCTCCAGAAATTGATGATTTTCCCATTGATTTGTC
TACATAGGGCATCGA
AAAGCACCCAATATTTAGAGAACAGAAGATTTTGAGAATTACTGCCTCCAGAAATTGATGATTTTCCCATTGATTTGTC
TACATAGGGCATCGA
AAAGCACCCAATATTTAGAGAACAGAAGATTTTGAGAATTACTGCCTCCAGAAATTGATGATTTTCCCATTGATTTGTC
TACATAGGGCATCGA
AAAGCACCCAATATTTAGAGAACAGAAGATTTTGAGAATTACTGCCTCCAGAAATTGATGATTTTCCCATTGATTTGTC
TACATAGGGCATCGA
AAAGCACCCAATATTTAGAGAACAGAAGATTTTGAGAATTACTGCCTCCAGAAATTGATGATTTTCCCATTGATTTGTC
TACATAGGGCATCGA
AAAGCACCCAATATTTAGAGAACAGAAGATTTTGAGAATTACTGCCTCCAGAAATTGATGATTTTCCCATTGATTTGTC
TACATAGGGCATCGA

AAAGCACCCAATATTTAGAGAACAGAAGATTTTGAGAATTACTGCCTCCAGAAATTGATGATTTTCCCATTGATTTGTC
TACATAGGGCATCGA
AAAGCACCCAATATTTAGAGAACAGAAGATTTTGAGAATTACTGCCTCCAGAAATTGATGATTTTCCCATTGATTTGTC
TACATAGGGCATCGA
AAAGCACCCAATATTTAGAGAACAGAAGATTTTGAGAATTACTGCCTCCAGAAATTGATGATTTTCCCATTGATTTGTC
TACATAGGGCATCGA
AAAGCACCCAATATTTAGAGAACAGAAGATTTTGAGAATTACTGCCTCCAGAAATTGATGATTTTCCCATTGATTTGTC
TACATAGGGCATCGA
AAAGCACCCAATATTTAGAGAACAGAAGATTTTGAGAATTACTGCCTCCAGAAATTGATGATTTTCCCATTGATTTGTC
TACATAGGGCATCGA
AAAGCACCCAATATTTAGAGAACAGAAGATTTTGAGAATTACTGCCTCCAGAAATTGATGATTTTCCCATTGATTTGTC
TACATAGGGCATCGA
AAAGCACCCAATATTTAGAGAACAGAAGATTTTGAGAATTACTGCCTCCAGAAATTGATGATTTTCCCATTGATTTGTC
TACATAGGGCATCGA
AAAGCACCCAATATTTAGAGAACAGAAGATTTTGAGAATTACTGCCTCCAGAAATTGATGATTTTCCCATTGATTTGTC
TACATAGGGCATCGA
AAAGCACCCAATATTTAGAGAACAGAAGATTTTGAGAATTACTGCCTCCAGAAATTGATGATTTTCCCATTGATTTGTC
TACATAGGGCATCGA
AAAGCACCCAATATTTAGAGAACAGAAGATTTTGAGAATTACTGCCTCCAGAAATTGATGATTTTCCCATTGATTTGTC
TACATAGGGCATCGA
AAAGCACCCAATATTTAGAGAACAGAAGATTTTGAGAATTACTGCCTCCAGAAATTGATGATTTTCCCATTGATTTGTC
TACATAGGGCATCGA
AAAGCACCCAATATTTAGAGAACAGAAGATTTTGAGAATTACTGCCTCCAGAAATTGATGATTTTCCCATTGATTTGTC
TACATAGGGCATCGA
AAAGCACCCAATATTTAGAGAACAGAAGATTTTGAGAATTACTGCCTCCAGAAATTGATGATTTTCCCATTGATTTGTC
TACATAGGGCATCGA
AAAGCACCCAATATTTAGAGAACAGAAGATTTTGAGAATTACTGCCTCCAGAAATTGATGATTTTCCCATTGATTTGTC
TACATAGGGCATCGA
AAAGCACCCAATATTTAGAGAACAGAAGATTTTGAGAATTACTGCCTCCAGAAATTGATGATTTTCCCATTGATTTGTC
TACATAGGGCATCGA
AAAGCACCCAATATTTAGAGAACAGAAGATTTTGAGAATTACTGCCTCCAGAAATTGATGATTTTCCCATTGATTTGTC
TACATAGGGCATCGA
AAAGCACCCAATATTTAGAGAACAGAAGATTTTGAGAATTACTGCCTCCAGAAATTGATGATTTTCCCATTGATTTGTC
TACATAGGGCATCGA
AAAGCACCCAATATTTAGAGAACAGAAGATTTTGAGAATTACTGCCTCCAGAAATTGATGATTTTCCCATTGATTTGTC
TACATAGGGCATCGA
AAAGCACCCAATATTTAGAGAACAGAAGATTTTGAGAATTACTGCCTCCAGAAATTGATGATTTTCCCATTGATTTGTC
TACATAGGGCATCGA
AAAGCACCCAATATTTAGAGAACAGAAGATTTTGAGAATTACTGCCTCCAGAAATTGATGATTTTCCCATTGATTTGTC
TACATAGGGCATCGA
AAAGCACCCAATATTTAGAGAACAGAAGATTTTGAGAATTACTGCCTCCAGAAATTGATGATTTTCCCATTGATTTGTC
TACATAGGGCATCGA
AAAGCACCCAATATTTAGAGAACAGAAGATTTTGAGAATTACTGCCTCCAGAAATTGATGATTTTCCCATTGATTTGTC
TACATAGGGCATCGA
AAAGCACCCAATATTTAGAGAACAGAAGATTTTGAGAATTACTGCCTCCAGAAATTGATGATTTTCCCATTGATTTGTC
TACATAGGGCATCGA
AAAGCACCCAATATTTAGAGAACAGAAGATTTTGAGAATTACTGCCTCCAGAAATTGATGATTTTCCCATTGATTTGTC
TACATAGGGCATCGA
AAAGCACCCAATATTTAGAGAACAGAAGATTTTGAGAATTACTGCCTCCAGAAATTGATGATTTTCCCATTGATTTGTC
TACATAGGGCATCGA
AAAGCACCCAATATTTAGAGAACAGAAGATTTTGAGAATTACTGCCTCCAGAAATTGATGATTTTCCCATTGATTTGTC
TACATAGGGCATCGA
AAAGCACCCAATATTTAGAGAACAGAAGATTTTGAGAATTACTGCCTCCAGAAATTGATGATTTTCCCATTGATTTGTC
TACATAGGGCATCGA

AAAGCACCCAATATTTAGAGAACAGAAGATTTTGAGAATTACTGCCTCCAGAAATTGATGATTTTCCCATTGATTTGTC
TACATAGGGCATCGA
AAAGCACCCAATATTTAGAGAACAGAAGATTTTGAGAATTACTGCCTCCAGAAATTGATGATTTTCCCATTGATTTGTC
TACATAGGGCATCGA
AAAGCACCCAATATTTAGAGAACAGAAGATTTTGAGAATTACTGCCTCCAGAAATTGATGATTTTCCCATTGATTTGTC
TACATAGGGCATCGA
AAAGCACCCAATATTTAGAGAACAGAAGATTTTGAGAATTACTGCCTCCAGAAATTGATGATTTTCCCATTGATTTGTC
TACATAGGGCATCGA
AAAGCACCCAATATTTAGAGAACAGAAGATTTTGAGAATTACTGCCTCCAGAAATTGATGATTTTCCCATTGATTTGTC
TACATAGGGCATCGA
AAAGCACCCAATATTTAGAGAACAGAAGATTTTGAGAATTACTGCCTCCAGAAATTGATGATTTTCCCATTGATTTGTC
TACATAGGGCATCGA
AAAGCACCCAATATTTAGAGAACAGAAGATTTTGAGAATTACTGCCTCCAGAAATTGATGATTTTCCCATTGATTTGTC
TACATAGGGCATCGA
AAAGCACCCAATATTTAGAGAACAGAAGATTTTGAGAATTACTGCCTCCAGAAATTGATGATTTTCCCATTGATTTGTC
TACATAGGGCATCGA
AAAGCACCCAATATTTAGAGAACAGAAGATTTTGAGAATTACTGCCTCCAGAAATTGATGATTTTCCCATTGATTTGTC
TACATAGGGCATCGA
AAAGCACCCAATATTTAGAGAACAGAAGATTTTGAGAATTACTGCCTCCAGAAATTGATGATTTTCCCATTGATTTGTC
TACATAGGGCATCGA
AAAGCACCCAATATTTAGAGAACAGAAGATTTTGAGAATTACTGCCTCCAGAAATTGATGATTTTCCCATTGATTTGTC
TACATAGGGCATCGA
AAAGCACCCAATATTTAGAGAACAGAAGATTTTGAGAATTACTGCCTCCAGAAATTGATGATTTTCCCATTGATTTGTC
TACATAGGGCATCGA
AAAGCACCCAATATTTAGAGAACAGAAGATTTTGAGAATTACTGCCTCCAGAAATTGATGATTTTCCCATTGATTTGTC
TACATAGGGCATCGA
AAAGCACCCAATATTTAGAGAACAGAAGATTTTGAGAATTACTGCCTCCAGAAATTGATGATTTTCCCATTGATTTGTC
TACATAGGGCATCGA
AAAGCACCCAATATTTAGAGAACAGAAGATTTTGAGAATTACTGCCTCCAGAAATTGATGATTTTCCCATTGATTTGTC
TACATAGGGCATCGA
AAAGCACCCAATATTTAGAGAACAGAAGATTTTGAGAATTACTGCCTCCAGAAATTGATGATTTTCCCATTGATTTGTC
TACATAGGGCATCGA
AAAGCACCCAATATTTAGAGAACAGAAGATTTTGAGAATTACTGCCTCCAGAAATTGATGATTTTCCCATTGATTTGTC
TACATAGGGCATCGA
AAAGCACCCAATATTTAGAGAACAGAAGATTTTGAGAATTACTGCCTCCAGAAATTGATGATTTTCCCATTGATTTGTC
TACATAGGGCATCGA
AAAGCACCCAATATTTAGAGAACAGAAGATTTTGAGAATTACTGCCTCCAGAAATTGATGATTTTCCCATTGATTTGTC
TACATAGGGCATCGA
AAAGCACCCAATATTTAGAGAACAGAAGATTTTGAGAATTACTGCCTCCAGAAATTGATGATTTTCCCATTGATTTGTC
TACATAGGGCATCGA
AAAGCACCCAATATTTAGAGAACAGAAGATTTTGAGAATTACTGCCTCCAGAAATTGATGATTTTCCCATTGATTTGTC
TACATAGGGCATCGA
AAAGCACCCAATATTTAGAGAACAGAAGATTTTGAGAATTACTGCCTCCAGAAATTGATGATTTTCCCATTGATTTGTC
TACATAGGGCATCGA
AAAGCACCCAATATTTAGAGAACAGAAGATTTTGAGAATTACTGCCTCCAGAAATTGATGATTTTCCCATTGATTTGTC
TACATAGGGCATCGA
AAAGCACCCAATATTTAGAGAACAGAAGATTTTGAGAATTACTGCCTCCAGAAATTGATGATTTTCCCATTGATTTGTC
TACATAGGGCATCGA
AAAGCACCCAATATTTAGAGAACAGAAGATTTTGAGAATTACTGCCTCCAGAAATTGATGATTTTCCCATTGATTTGTC
TACATAGGGCATCGA
AAAGCACCCAATATTTAGAGAACAGAAGATTTTGAGAATTACTGCCTCCAGAAATTGATGATTTTCCCATTGATTTGTC
TACATAGGGCATCGA
AAAGCACCCAATATTTAGAGAACAGAAGATTTTGAGAATTACTGCCTCCAGAAATTGATGATTTTCCCATTGATTTGTC
TACATAGGGCATCGA

AAAGCACCCAATATTTAGAGAACAGAAGATTTTGAGAATTACTGCCTCCAGAAATTGATGATTTTCCCATTGATTTGTC
TACATAGGGCATCGA
AAAGCACCCAATATTTAGAGAACAGAAGATTTTGAGAATTACTGCCTCCAGAAATTGATGATTTTCCCATTGATTTGTC
TACATAGGGCATCGA
AAAGCACCCAATATTTAGAGAACAGAAGATTTTGAGAATTACTGCCTCCAGAAATTGATGATTTTCCCATTGATTTGTC
TACATAGGGCATCGA
AAAGCACCCAATATTTAGAGAACAGAAGATTTTGAGAATTACTGCCTCCAGAAATTGATGATTTTCCCATTGATTTGTC
TACATAGGGCATCGA
AAAGCACCCAATATTTAGAGAACAGAAGATTTTGAGAATTACTGCCTCCAGAAATTGATGATTTTCCCATTGATTTGTC
TACATAGGGCATCGA
AAAGCACCCAATATTTAGAGAACAGAAGATTTTGAGAATTACTGCCTCCAGAAATTGATGATTTTCCCATTGATTTGTC
TACATAGGGCATCGA
AAAGCACCCAATATTTAGAGAACAGAAGATTTTGAGAATTACTGCCTCCAGAAATTGATGATTTTCCCATTGATTTGTC
TACATAGGGCATCGA
AAAGCACCCAATATTTAGAGAACAGAAGATTTTGAGAATTACTGCCTCCAGAAATTGATGATTTTCCCATTGATTTGTC
TACATAGGGCATCGA
AAAGCACCCAATATTTAGAGAACAGAAGATTTTGAGAATTACTGCCTCCAGAAATTGATGATTTTCCCATTGATTTGTC
TACATAGGGCATCGA
AAAGCACCCAATATTTAGAGAACAGAAGATTTTGAGAATTACTGCCTCCAGAAATTGATGATTTTCCCATTGATTTGTC
TACATAGGGCATCGA
AAAGCACCCAATATTTAGAGAACAGAAGATTTTGAGAATTACTGCCTCCAGAAATTGATGATTTTCCCATTGATTTGTC
TACATAGGGCATCGA
AAAGCACCCAATATTTAGAGAACAGAAGATTTTGAGAATTACTGCCTCCAGAAATTGATGATTTTCCCATTGATTTGTC
TACATAGGGCATCGA
AAAGCACCCAATATTTAGAGAACAGAAGATTTTGAGAATTACTGCCTCCAGAAATTGATGATTTTCCCATTGATTTGTC
TACATAGGGCATCGA
AAAGCACCCAATATTTAGAGAACAGAAGATTTTGAGAATTACTGCCTCCAGAAATTGATGATTTTCCCATTGATTTGTC
TACATAGGGCATCGA
AAAGCACCCAATATTTAGAGAACAGAAGATTTTGAGAATTACTGCCTCCAGAAATTGATGATTTTCCCATTGATTTGTC
TACATAGGGCATCGA
AAAGCACCCAATATTTAGAGAACAGAAGATTTTGAGAATTACTGCCTCCAGAAATTGATGATTTTCCCATTGATTTGTC
TACATAGGGCATCGA
AAAGCACCCAATATTTAGAGAACAGAAGATTTTGAGAATTACTGCCTCCAGAAATTGATGATTTTCCCATTGATTTGTC
TACATAGGGCATCGA
AAAGCACCCAATATTTAGAGAACAGAAGATTTTGAGAATTACTGCCTCCAGAAATTGATGATTTTCCCATTGATTTGTC
TACATAGGGCATCGA
AAAGCACCCAATATTTAGAGAACAGAAGATTTTGAGAATTACTGCCTCCAGAAATTGATGATTTTCCCATTGATTTGTC
TACATAGGGCATCGA
AAAGCACCCAATATTTAGAGAACAGAAGATTTTGAGAATTACTGCCTCCAGAAATTGATGATTTTCCCATTGATTTGTC
TACATAGGGCATCGA
AAAGCACCCAATATTTAGAGAACAGAAGATTTTGAGAATTACTGCCTCCAGAAATTGATGATTTTCCCATTGATTTGTC
TACATAGGGCATCGA
AAAGCACCCAATATTTAGAGAACAGAAGATTTTGAGAATTACTGCCTCCAGAAATTGATGATTTTCCCATTGATTTGTC
TACATAGGGCATCGA
AAAGCACCCAATATTTAGAGAACAGAAGATTTTGAGAATTACTGCCTCCAGAAATTGATGATTTTCCCATTGATTTGTC
TACATAGGGCATCGA
AAAGCACCCAATATTTAGAGAACAGAAGATTTTGAGAATTACTGCCTCCAGAAATTGATGATTTTCCCATTGATTTGTC
TACATAGGGCATCGA
AAAGCACCCAATATTTAGAGAACAGAAGATTTTGAGAATTACTGCCTCCAGAAATTGATGATTTTCCCATTGATTTGTC
TACATAGGGCATCGA
AAAGCACCCAATATTTAGAGAACAGAAGATTTTGAGAATTACTGCCTCCAGAAATTGATGATTTTCCCATTGATTTGTC
TACATAGGGCATCGA
AAAGCACCCAATATTTAGAGAACAGAAGATTTTGAGAATTACTGCCTCCAGAAATTGATGATTTTCCCATTGATTTGTC
TACATAGGGCATCGA

AAAGCACCCAATATTTAGAGAACAGAAGATTTTGAGAATTACTGCCTCCAGAAATTGATGATTTTCCCATTGATTTGTC
TACATAGGGCATCGA
AAAGCACCCAATATTTAGAGAACAGAAGATTTTGAGAATTACTGCCTCCAGAAATTGATGATTTTCCCATTGATTTGTC
TACATAGGGCATCGA
AAAGCACCCAATATTTAGAGAACAGAAGATTTTGAGAATTACTGCCTCCAGAAATTGATGATTTTCCCATTGATTTGTC
TACATAGGGCATCGA
AAAGCACCCAATATTTAGAGAACAGAAGATTTTGAGAATTACTGCCTCCAGAAATTGATGATTTTCCCATTGATTTGTC
TACATAGGGCATCGA
AAAGCACCCAATATTTAGAGAACAGAAGATTTTGAGAATTACTGCCTCCAGAAATTGATGATTTTCCCATTGATTTGTC
TACATAGGGCATCGA
AAAGCACCCAATATTTAGAGAACAGAAGATTTTGAGAATTACTGCCTCCAGAAATTGATGATTTTCCCATTGATTTGTC
TACATAGGGCATCGA
AAAGCACCCAATATTTAGAGAACAGAAGATTTTGAGAATTACTGCCTCCAGAAATTGATGATTTTCCCATTGATTTGTC
TACATAGGGCATCGA
AAAGCACCCAATATTTAGAGAACAGAAGATTTTGAGAATTACTGCCTCCAGAAATTGATGATTTTCCCATTGATTTGTC
TACATAGGGCATCGA
AAAGCACCCAATATTTAGAGAACAGAAGATTTTGAGAATTACTGCCTCCAGAAATTGATGATTTTCCCATTGATTTGTC
TACATAGGGCATCGA
AAAGCACCCAATATTTAGAGAACAGAAGATTTTGAGAATTACTGCCTCCAGAAATTGATGATTTTCCCATTGATTTGTC
TACATAGGGCATCGA
AAAGCACCCAATATTTAGAGAACAGAAGATTTTGAGAATTACTGCCTCCAGAAATTGATGATTTTCCCATTGATTTGTC
TACATAGGGCATCGA
AAAGCACCCAATATTTAGAGAACAGAAGATTTTGAGAATTACTGCCTCCAGAAATTGATGATTTTCCCATTGATTTGTC
TACATAGGGCATCGA
AAAGCACCCAATATTTAGAGAACAGAAGATTTTGAGAATTACTGCCTCCAGAAATTGATGATTTTCCCATTGATTTGTC
TACATAGGGCATCGA
AAAGCACCCAATATTTAGAGAACAGAAGATTTTGAGAATTACTGCCTCCAGAAATTGATGATTTTCCCATTGATTTGTC
TACATAGGGCATCGA
AAAGCACCCAATATTTAGAGAACAGAAGATTTTGAGAATTACTGCCTCCAGAAATTGATGATTTTCCCATTGATTTGTC
TACATAGGGCATCGA
AAAGCACCCAATATTTAGAGAACAGAAGATTTTGAGAATTACTGCCTCCAGAAATTGATGATTTTCCCATTGATTTGTC
TACATAGGGCATCGA
AAAGCACCCAATATTTAGAGAACAGAAGATTTTGAGAATTACTGCCTCCAGAAATTGATGATTTTCCCATTGATTTGTC
TACATAGGGCATCGA
AAAGCACCCAATATTTAGAGAACAGAAGATTTTGAGAATTACTGCCTCCAGAAATTGATGATTTTCCCATTGATTTGTC
TACATAGGGCATCGA
AAAGCACCCAATATTTAGAGAACAGAAGATTTTGAGAATTACTGCCTCCAGAAATTGATGATTTTCCCATTGATTTGTC
TACATAGGGCATCGA
AAAGCACCCAATATTTAGAGAACAGAAGATTTTGAGAATTACTGCCTCCAGAAATTGATGATTTTCCCATTGATTTGTC
TACATAGGGCATCGA
AAAGCACCCAATATTTAGAGAACAGAAGATTTTGAGAATTACTGCCTCCAGAAATTGATGATTTTCCCATTGATTTGTC
TACATAGGGCATCGA
AAAGCACCCAATATTTAGAGAACAGAAGATTTTGAGAATTACTGCCTCCAGAAATTGATGATTTTCCCATTGATTTGTC
TACATAGGGCATCGA
AAAGCACCCAATATTTAGAGAACAGAAGATTTTGAGAATTACTGCCTCCAGAAATTGATGATTTTCCCATTGATTTGTC
TACATAGGGCATCGA
AAAGCACCCAATATTTAGAGAACAGAAGATTTTGAGAATTACTGCCTCCAGAAATTGATGATTTTCCCATTGATTTGTC
TACATAGGGCATCGA
AAAGCACCCAATATTTAGAGAACAGAAGATTTTGAGAATTACTGCCTCCAGAAATTGATGATTTTCCCATTGATTTGTC
TACATAGGGCATCGA
AAAGCACCCAATATTTAGAGAACAGAAGATTTTGAGAATTACTGCCTCCAGAAATTGATGATTTTCCCATTGATTTGTC
TACATAGGGCATCGA
AAAGCACCCAATATTTAGAGAACAGAAGATTTTGAGAATTACTGCCTCCAGAAATTGATGATTTTCCCATTGATTTGTC
TACATAGGGCATCGA

AAAGCACCCAATATTTAGAGAACAGAAGATTTTGAGAATTACTGCCTCCAGAAATTGATGATTTTCCCATTGATTTGTC
TACATAGGGCATCGA
AAAGCACCCAATATTTAGAGAACAGAAGATTTTGAGAATTACTGCCTCCAGAAATTGATGATTTTCCCATTGATTTGTC
TACATAGGGCATCGA
AAAGCACCCAATATTTAGAGAACAGAAGATTTTGAGAATTACTGCCTCCAGAAATTGATGATTTTCCCATTGATTTGTC
TACATAGGGCATCGA
AAAGCACCCAATATTTAGAGAACAGAAGATTTTGAGAATTACTGCCTCCAGAAATTGATGATTTTCCCATTGATTTGTC
TACATAGGGCATCGA
AAAGCACCCAATATTTAGAGAACAGAAGATTTTGAGAATTACTGCCTCCAGAAATTGATGATTTTCCCATTGATTTGTC
TACATAGGGCATCGA
AAAGCACCCAATATTTAGAGAACAGAAGATTTTGAGAATTACTGCCTCCAGAAATTGATGATTTTCCCATTGATTTGTC
TACATAGGGCATCGA
AAAGCACCCAATATTTAGAGAACAGAAGATTTTGAGAATTACTGCCTCCAGAAATTGATGATTTTCCCATTGATTTGTC
TACATAGGGCATCGA
AAAGCACCCAATATTTAGAGAACAGAAGATTTTGAGAATTACTGCCTCCAGAAATTGATGATTTTCCCATTGATTTGTC
TACATAGGGCATCGA
AAAGCACCCAATATTTAGAGAACAGAAGATTTTGAGAATTACTGCCTCCAGAAATTGATGATTTTCCCATTGATTTGTC
TACATAGGGCATCGA
AAAGCACCCAATATTTAGAGAACAGAAGATTTTGAGAATTACTGCCTCCAGAAATTGATGATTTTCCCATTGATTTGTC
TACATAGGGCATCGA
AAAGCACCCAATATTTAGAGAACAGAAGATTTTGAGAATTACTGCCTCCAGAAATTGATGATTTTCCCATTGATTTGTC
TACATAGGGCATCGA
AAAGCACCCAATATTTAGAGAACAGAAGATTTTGAGAATTACTGCCTCCAGAAATTGATGATTTTCCCATTGATTTGTC
TACATAGGGCATCGA
AAAGCACCCAATATTTAGAGAACAGAAGATTTTGAGAATTACTGCCTCCAGAAATTGATGATTTTCCCATTGATTTGTC
TACATAGGGCATCGA
AAAGCACCCAATATTTAGAGAACAGAAGATTTTGAGAATTACTGCCTCCAGAAATTGATGATTTTCCCATTGATTTGTC
TACATAGGGCATCGA
AAAGCACCCAATATTTAGAGAACAGAAGATTTTGAGAATTACTGCCTCCAGAAATTGATGATTTTCCCATTGATTTGTC
TACATAGGGCATCGA
AAAGCACCCAATATTTAGAGAACAGAAGATTTTGAGAATTACTGCCTCCAGAAATTGATGATTTTCCCATTGATTTGTC
TACATAGGGCATCGA
AAAGCACCCAATATTTAGAGAACAGAAGATTTTGAGAATTACTGCCTCCAGAAATTGATGATTTTCCCATTGATTTGTC
TACATAGGGCATCGA
AAAGCACCCAATATTTAGAGAACAGAAGATTTTGAGAATTACTGCCTCCAGAAATTGATGATTTTCCCATTGATTTGTC
TACATAGGGCATCGA
AAAGCACCCAATATTTAGAGAACAGAAGATTTTGAGAATTACTGCCTCCAGAAATTGATGATTTTCCCATTGATTTGTC
TACATAGGGCATCGA
AAAGCACCCAATATTTAGAGAACAGAAGATTTTGAGAATTACTGCCTCCAGAAATTGATGATTTTCCCATTGATTTGTC
TACATAGGGCATCGA
AAAGCACCCAATATTTAGAGAACAGAAGATTTTGAGAATTACTGCCTCCAGAAATTGATGATTTTCCCATTGATTTGTC
TACATAGGGCATCGA
AAAGCACCCAATATTTAGAGAACAGAAGATTTTGAGAATTACTGCCTCCAGAAATTGATGATTTTCCCATTGATTTGTC
TACATAGGGCATCGA
AAAGCACCCAATATTTAGAGAACAGAAGATTTTGAGAATTACTGCCTCCAGAAATTGATGATTTTCCCATTGATTTGTC
TACATAGGGCATCGA
AAAGCACCCAATATTTAGAGAACAGAAGATTTTGAGAATTACTGCCTCCAGAAATTGATGATTTTCCCATTGATTTGTC
TACATAGGGCATCGA
AAAGCACCCAATATTTAGAGAACAGAAGATTTTGAGAATTACTGCCTCCAGAAATTGATGATTTTCCCATTGATTTGTC
TACATAGGGCATCGA
AAAGCACCCAATATTTAGAGAACAGAAGATTTTGAGAATTACTGCCTCCAGAAATTGATGATTTTCCCATTGATTTGTC
TACATAGGGCATCGA
AAAGCACCCAATATTTAGAGAACAGAAGATTTTGAGAATTACTGCCTCCAGAAATTGATGATTTTCCCATTGATTTGTC
TACATAGGGCATCGA

AAAGCACCCAATATTTAGAGAACAGAAGATTTTGAGAATTACTGCCTCCAGAAATTGATGATTTTCCCATTGATTTGTC
TACATAGGGCATCGA
AAAGCACCCAATATTTAGAGAACAGAAGATTTTGAGAATTACTGCCTCCAGAAATTGATGATTTTCCCATTGATTTGTC
TACATAGGGCATCGA
AAAGCACCCAATATTTAGAGAACAGAAGATTTTGAGAATTACTGCCTCCAGAAATTGATGATTTTCCCATTGATTTGTC
TACATAGGGCATCGA
AAAGCACCCAATATTTAGAGAACAGAAGATTTTGAGAATTACTGCCTCCAGAAATTGATGATTTTCCCATTGATTTGTC
TACATAGGGCATCGA
AAAGCACCCAATATTTAGAGAACAGAAGATTTTGAGAATTACTGCCTCCAGAAATTGATGATTTTCCCATTGATTTGTC
TACATAGGGCATCGA
AAAGCACCCAATATTTAGAGAACAGAAGATTTTGAGAATTACTGCCTCCAGAAATTGATGATTTTCCCATTGATTTGTC
TACATAGGGCATCGA
AAAGCACCCAATATTTAGAGAACAGAAGATTTTGAGAATTACTGCCTCCAGAAATTGATGATTTTCCCATTGATTTGTC
TACATAGGGCATCGA
AAAGCACCCAATATTTAGAGAACAGAAGATTTTGAGAATTACTGCCTCCAGAAATTGATGATTTTCCCATTGATTTGTC
TACATAGGGCATCGA
AAAGCACCCAATATTTAGAGAACAGAAGATTTTGAGAATTACTGCCTCCAGAAATTGATGATTTTCCCATTGATTTGTC
TACATAGGGCATCGA
AAAGCACCCAATATTTAGAGAACAGAAGATTTTGAGAATTACTGCCTCCAGAAATTGATGATTTTCCCATTGATTTGTC
TACATAGGGCATCGA
AAAGCACCCAATATTTAGAGAACAGAAGATTTTGAGAATTACTGCCTCCAGAAATTGATGATTTTCCCATTGATTTGTC
TACATAGGGCATCGA
AAAGCACCCAATATTTAGAGAACAGAAGATTTTGAGAATTACTGCCTCCAGAAATTGATGATTTTCCCATTGATTTGTC
TACATAGGGCATCGA
AAAGCACCCAATATTTAGAGAACAGAAGATTTTGAGAATTACTGCCTCCAGAAATTGATGATTTTCCCATTGATTTGTC
TACATAGGGCATCGA
AAAGCACCCAATATTTAGAGAACAGAAGATTTTGAGAATTACTGCCTCCAGAAATTGATGATTTTCCCATTGATTTGTC
TACATAGGGCATCGA
AAAGCACCCAATATTTAGAGAACAGAAGATTTTGAGAATTACTGCCTCCAGAAATTGATGATTTTCCCATTGATTTGTC
TACATAGGGCATCGA
AAAGCACCCAATATTTAGAGAACAGAAGATTTTGAGAATTACTGCCTCCAGAAATTGATGATTTTCCCATTGATTTGTC
TACATAGGGCATCGA
AAAGCACCCAATATTTAGAGAACAGAAGATTTTGAGAATTACTGCCTCCAGAAATTGATGATTTTCCCATTGATTTGTC
TACATAGGGCATCGA
AAAGCACCCAATATTTAGAGAACAGAAGATTTTGAGAATTACTGCCTCCAGAAATTGATGATTTTCCCATTGATTTGTC
TACATAGGGCATCGA
AAAGCACCCAATATTTAGAGAACAGAAGATTTTGAGAATTACTGCCTCCAGAAATTGATGATTTTCCCATTGATTTGTC
TACATAGGGCATCGA
AAAGCACCCAATATTTAGAGAACAGAAGATTTTGAGAATTACTGCCTCCAGAAATTGATGATTTTCCCATTGATTTGTC
TACATAGGGCATCGA
AAAGCACCCAATATTTAGAGAACAGAAGATTTTGAGAATTACTGCCTCCAGAAATTGATGATTTTCCCATTGATTTGTC
TACATAGGGCATCGA
AAAGCACCCAATATTTAGAGAACAGAAGATTTTGAGAATTACTGCCTCCAGAAATTGATGATTTTCCCATTGATTTGTC
TACATAGGGCATCGA
AAAGCACCCAATATTTAGAGAACAGAAGATTTTGAGAATTACTGCCTCCAGAAATTGATGATTTTCCCATTGATTTGTC
TACATAGGGCATCGA
AAAGCACCCAATATTTAGAGAACAGAAGATTTTGAGAATTACTGCCTCCAGAAATTGATGATTTTCCCATTGATTTGTC
TACATAGGGCATCGA
AAAGCACCCAATATTTAGAGAACAGAAGATTTTGAGAATTACTGCCTCCAGAAATTGATGATTTTCCCATTGATTTGTC
TACATAGGGCATCGA
AAAGCACCCAATATTTAGAGAACAGAAGATTTTGAGAATTACTGCCTCCAGAAATTGATGATTTTCCCATTGATTTGTC
TACATAGGGCATCGA
AAAGCACCCAATATTTAGAGAACAGAAGATTTTGAGAATTACTGCCTCCAGAAATTGATGATTTTCCCATTGATTTGTC
TACATAGGGCATCGA

AAAGCACCCAATATTTAGAGAACAGAAGATTTTGAGAATTACTGCCTCCAGAAATTGATGATTTTCCCATTGATTTGTC
TACATAGGGCATCGA
AAAGCACCCAATATTTAGAGAACAGAAGATTTTGAGAATTACTGCCTCCAGAAATTGATGATTTTCCCATTGATTTGTC
TACATAGGGCATCGA
AAAGCACCCAATATTTAGAGAACAGAAGATTTTGAGAATTACTGCCTCCAGAAATTGATGATTTTCCCATTGATTTGTC
TACATAGGGCATCGA
AAAGCACCCAATATTTAGAGAACAGAAGATTTTGAGAATTACTGCCTCCAGAAATTGATGATTTTCCCATTGATTTGTC
TACATAGGGCATCGA
AAAGCACCCAATATTTAGAGAACAGAAGATTTTGAGAATTACTGCCTCCAGAAATTGATGATTTTCCCATTGATTTGTC
TACATAGGGCATCGA
AAAGCACCCAATATTTAGAGAACAGAAGATTTTGAGAATTACTGCCTCCAGAAATTGATGATTTTCCCATTGATTTGTC
TACATAGGGCATCGA
AAAGCACCCAATATTTAGAGAACAGAAGATTTTGAGAATTACTGCCTCCAGAAATTGATGATTTTCCCATTGATTTGTC
TACATAGGGCATCGA
AAAGCACCCAATATTTAGAGAACAGAAGATTTTGAGAATTACTGCCTCCAGAAATTGATGATTTTCCCATTGATTTGTC
TACATAGGGCATCGA
AAAGCACCCAATATTTAGAGAACAGAAGATTTTGAGAATTACTGCCTCCAGAAATTGATGATTTTCCCATTGATTTGTC
TACATAGGGCATCGA
AAAGCACCCAATATTTAGAGAACAGAAGATTTTGAGAATTACTGCCTCCAGAAATTGATGATTTTCCCATTGATTTGTC
TACATAGGGCATCGA
AAAGCACCCAATATTTAGAGAACAGAAGATTTTGAGAATTACTGCCTCCAGAAATTGATGATTTTCCCATTGATTTGTC
TACATAGGGCATCGA
AAAGCACCCAATATTTAGAGAACAGAAGATTTTGAGAATTACTGCCTCCAGAAATTGATGATTTTCCCATTGATTTGTC
TACATAGGGCATCGA
AAAGCACCCAATATTTAGAGAACAGAAGATTTTGAGAATTACTGCCTCCAGAAATTGATGATTTTCCCATTGATTTGTC
TACATAGGGCATCGA
AAAGCACCCAATATTTAGAGAACAGAAGATTTTGAGAATTACTGCCTCCAGAAATTGATGATTTTCCCATTGATTTGTC
TACATAGGGCATCGA
AAAGCACCCAATATTTAGAGAACAGAAGATTTTGAGAATTACTGCCTCCAGAAATTGATGATTTTCCCATTGATTTGTC
TACATAGGGCATCGA
AAAGCACCCAATATTTAGAGAACAGAAGATTTTGAGAATTACTGCCTCCAGAAATTGATGATTTTCCCATTGATTTGTC
TACATAGGGCATCGA
AAAGCACCCAATATTTAGAGAACAGAAGATTTTGAGAATTACTGCCTCCAGAAATTGATGATTTTCCCATTGATTTGTC
TACATAGGGCATCGA
AAAGCACCCAATATTTAGAGAACAGAAGATTTTGAGAATTACTGCCTCCAGAAATTGATGATTTTCCCATTGATTTGTC
TACATAGGGCATCGA
AAAGCACCCAATATTTAGAGAACAGAAGATTTTGAGAATTACTGCCTCCAGAAATTGATGATTTTCCCATTGATTTGTC
TACATAGGGCATCGA
AAAGCACCCAATATTTAGAGAACAGAAGATTTTGAGAATTACTGCCTCCAGAAATTGATGATTTTCCCATTGATTTGTC
TACATAGGGCATCGA
AAAGCACCCAATATTTAGAGAACAGAAGATTTTGAGAATTACTGCCTCCAGAAATTGATGATTTTCCCATTGATTTGTC
TACATAGGGCATCGA
AAAGCACCCAATATTTAGAGAACAGAAGATTTTGAGAATTACTGCCTCCAGAAATTGATGATTTTCCCATTGATTTGTC
TACATAGGGCATCGA
AAAGCACCCAATATTTAGAGAACAGAAGATTTTGAGAATTACTGCCTCCAGAAATTGATGATTTTCCCATTGATTTGTC
TACATAGGGCATCGA
AAAGCACCCAATATTTAGAGAACAGAAGATTTTGAGAATTACTGCCTCCAGAAATTGATGATTTTCCCATTGATTTGTC
TACATAGGGCATCGA
AAAGCACCCAATATTTAGAGAACAGAAGATTTTGAGAATTACTGCCTCCAGAAATTGATGATTTTCCCATTGATTTGTC
TACATAGGGCATCGA
AAAGCACCCAATATTTAGAGAACAGAAGATTTTGAGAATTACTGCCTCCAGAAATTGATGATTTTCCCATTGATTTGTC
TACATAGGGCATCGA
AAAGCACCCAATATTTAGAGAACAGAAGATTTTGAGAATTACTGCCTCCAGAAATTGATGATTTTCCCATTGATTTGTC
TACATAGGGCATCGA

AAAGCACCCAATATTTAGAGAACAGAAGATTTTGAGAATTACTGCCTCCAGAAATTGATGATTTTCCCATTGATTTGTC
TACATAGGGCATCGA
AAAGCACCCAATATTTAGAGAACAGAAGATTTTGAGAATTACTGCCTCCAGAAATTGATGATTTTCCCATTGATTTGTC
TACATAGGGCATCGA
AAAGCACCCAATATTTAGAGAACAGAAGATTTTGAGAATTACTGCCTCCAGAAATTGATGATTTTCCCATTGATTTGTC
TACATAGGGCATCGA
AAAGCACCCAATATTTAGAGAACAGAAGATTTTGAGAATTACTGCCTCCAGAAATTGATGATTTTCCCATTGATTTGTC
TACATAGGGCATCGA
AAAGCACCCAATATTTAGAGAACAGAAGATTTTGAGAATTACTGCCTCCAGAAATTGATGATTTTCCCATTGATTTGTC
TACATAGGGCATCGA
AAAGCACCCAATATTTAGAGAACAGAAGATTTTGAGAATTACTGCCTCCAGAAATTGATGATTTTCCCATTGATTTGTC
TACATAGGGCATCGA
AAAGCACCCAATATTTAGAGAACAGAAGATTTTGAGAATTACTGCCTCCAGAAATTGATGATTTTCCCATTGATTTGTC
TACATAGGGCATCGA
AAAGCACCCAATATTTAGAGAACAGAAGATTTTGAGAATTACTGCCTCCAGAAATTGATGATTTTCCCATTGATTTGTC
TACATAGGGCATCGA
AAAGCACCCAATATTTAGAGAACAGAAGATTTTGAGAATTACTGCCTCCAGAAATTGATGATTTTCCCATTGATTTGTC
TACATAGGGCATCGA
AAAGCACCCAATATTTAGAGAACAGAAGATTTTGAGAATTACTGCCTCCAGAAATTGATGATTTTCCCATTGATTTGTC
TACATAGGGCATCGA
AAAGCACCCAATATTTAGAGAACAGAAGATTTTGAGAATTACTGCCTCCAGAAATTGATGATTTTCCCATTGATTTGTC
TACATAGGGCATCGA
AAAGCACCCAATATTTAGAGAACAGAAGATTTTGAGAATTACTGCCTCCAGAAATTGATGATTTTCCCATTGATTTGTC
TACATAGGGCATCGA
AAAGCACCCAATATTTAGAGAACAGAAGATTTTGAGAATTACTGCCTCCAGAAATTGATGATTTTCCCATTGATTTGTC
TACATAGGGCATCGA
AAAGCACCCAATATTTAGAGAACAGAAGATTTTGAGAATTACTGCCTCCAGAAATTGATGATTTTCCCATTGATTTGTC
TACATAGGGCATCGA
AAAGCACCCAATATTTAGAGAACAGAAGATTTTGAGAATTACTGCCTCCAGAAATTGATGATTTTCCCATTGATTTGTC
TACATAGGGCATCGA
AAAGCACCCAATATTTAGAGAACAGAAGATTTTGAGAATTACTGCCTCCAGAAATTGATGATTTTCCCATTGATTTGTC
TACATAGGGCATCGA
AAAGCACCCAATATTTAGAGAACAGAAGATTTTGAGAATTACTGCCTCCAGAAATTGATGATTTTCCCATTGATTTGTC
TACATAGGGCATCGA
AAAGCACCCAATATTTAGAGAACAGAAGATTTTGAGAATTACTGCCTCCAGAAATTGATGATTTTCCCATTGATTTGTC
TACATAGGGCATCGA
AAAGCACCCAATATTTAGAGAACAGAAGATTTTGAGAATTACTGCCTCCAGAAATTGATGATTTTCCCATTGATTTGTC
TACATAGGGCATCGA
AAAGCACCCAATATTTAGAGAACAGAAGATTTTGAGAATTACTGCCTCCAGAAATTGATGATTTTCCCATTGATTTGTC
TACATAGGGCATCGA
AAAGCACCCAATATTTAGAGAACAGAAGATTTTGAGAATTACTGCCTCCAGAAATTGATGATTTTCCCATTGATTTGTC
TACATAGGGCATCGA
AAAGCACCCAATATTTAGAGAACAGAAGATTTTGAGAATTACTGCCTC
Nr of selected repeats 425 Similarity 1.000000
Consensus:
AAAGCACCCAATATTTAGAGAACAGAAGATTTTGAGAATTACTGCCTCCAGAAATTGATGATTTTCCCATTGATTTGTC
TACATAGGGCATCGA
>Cele-UNSB01_3:7683133-7684661 Satlength=1529 Nr of Repeats=131 RepeatLength=11
seed=TGGAGCGGTT
TGGAGCGGTTT
TGGAGCGGTTT
TGGAGCGGTTA
TGGAGCGGTTT
TGGAGCGGTAT

TGGAGCGGTAT
TGGAGCGGTTT
TGGAGCGGTTT
TGGAGCGGTTA
TGGAGCGGTTT
TGGAGCGGTAT
TGGAGCGGTTA
TGGAGCGGTGAAGGAGCGGTGAA
GGAGCGGTTTTGTAGCCGTTG
TGGAGCGGTTT
TGGAGCGGTTT
TGGAGCGGTTA
TGGAGCGGTTT
TGGAGCGGTGA
TGGAGCGGTTT
TGGAGCCGTTG
TGGAGCGGTTA
TGGAGCGGTTT
TGGAGCGGTTT
TGGAGCGGTTG
TGGAGCGGTTT
TGGAGCGGTTT
TGGAGCGGTTT
TGGAGCGGTTA
TGGAGCGGTTTTGCAGCGGTAT
TGGAGCGGTAT
TGGAGCGGTTT
TGGAGCGGTTT
TGGAGCGGTAT
TGGAGCGGTTT
TGGAGCGGTTT
TGGAGCGGTGT
TGGAGCGGTTT
TGGAGCGGTGA
TGAAGCGGTTT
TGGAGCCGTTG
TGGAGCGGTTT
TGGAGCGGTTT
TGGAGCGGTTT
TGGAGCGGTTT
TGGAGCGATTT
TGGAGCGGTTA
TGGAGCGGTAT
TGGAGCGGTTT
TGGAGCGGTTT
TGGAGCGGATA
TGGAGCGGTTT
TGGAGCGGTGA
TGAAGCGGTTT
TGGAGCCGTTG
TGGAGCGGTTTC
GGAGCGGTTATGAGCGGTGA
TGGAGCGGTTT
TGGAGCGGTTG

TGGAGCGGTTT
TGGAGCGGTTG
TGGAGCGGTTT
TGGAGCGGTTG
TGGAGCGGTTT
TGGAGCGGTTT
TGGAGCGGTTT
TGGAGCGGTAT
TGGAGCGGTTT
TGGAGCGGTTT
TGGAGCGGTTG
TGGAGCGGTTT
TGGAGCGGTGA
TGGAGCGGTTT
TGGAGCGGTTG
TGGAGCGGTTT
TGGAGCGGTTT
TGGAGCGGTTT
TGGAGCGGTTT
TGGAGCGGTGA
TGGAGCGGTTT
TGGAGCGGTTG
TGGAGCGGTTT
TGGAGCGGTTT
TGGAGCGGTTTTGGAGCTGTAT
TGGAGCGGTTT
TGGAGCGGTAT
TGGAGCGGGTT
TGGAGCGGTAT
TGGAGCGGTTA
TGGAGCGGTTA
TGGAGTGGTTT
TGGAGCGGTTA
TGGAGCGGTAT
TGGAGCGGTTTTGTAGCCGTTA
TGGAGCGGTTT
TGGAGCGGTTT
TGGAGCGGTTA
TGGAGCGGTTT
TGGAGCGGTTA
TGGAGCGGTTT
TGGAGCGGTTA
TGGAGCGGTAT
TGGAGCGGTTT
TGGAGCCGTTT
TGTAGCGGTTATGAAGCGGTAT
TGGAGCGGTTA
TGGAGCGGTAT
TGGAGCGGTTT
TGGAGCGGTTT
TGGAGCGGTTTTGGAGCTGTAT
TGGAGCGGTTT
TGGAGCGGTTT
TGGAGCGGTTA

TGGAGCGGTTT
TGGAGCGGTTT
TGGAGCGGTTT
TGGAGCGGTGA
TGGAGCGGTTT
TGGAGCGGTTG
TGGAGCGGTTT
TGGAGCGGTAT
TGGAGCGGTTT
TGGAGCGGTTT
TGGAGCGGTTA
TGGAGCGGTTT
TGGAGCGGTAT
TGGAGCGGGTT
TGGAGCGGTAT
TGGAGCGGTTA
TGGAGCGGTAT
TGGAGCGGTTT
Nr of selected repeats 122 Similarity 0.884864
Consensus:
TGGAGCGGTTt
>Cele-UNSB01_3:7825014-7825135 Satlength=122 Nr of Repeats=11 RepeatLength=11 seed=CGGATAATTA
CGGATAATTAA
CGGATAATTAA
CGGATAATTAA
CGGATAATTAA
CGGATAATTAA
CGGATAATTAA
CGGATAATTAA
CGGATAATTAA
CGGATAATTAA
CGGATAATTAA
CGGATAATTAA
Nr of selected repeats 11 Similarity 1.000000
Consensus:
CGGATAATTAA
>Cele-UNSB01_3:7826007-7841897 Satlength=15891 Nr of Repeats=610 RepeatLength=26 seed=CATTAGATGA
CATTAGATGAAATTCCAAAATTAGAC
CATTAGATGAATTTCCAAAGTTAGAC
CATTAGATGAAATTCCAAAATTAGAC
CATTAGATGAATTTCCAAAGTTAGAC
CATTAGATGAATTCCCAAAGTTAGAC
CATTAGATAAATTCCCAAAGTTAGAC
CATTAGATGAAATTCCAAAATTAGAC
CATTAGATGAATTTCCAAAGTTAGAA
CATTAGATGAAATTCCAAAATTAGAC
CATTAGATGAATTTCCAAAGTTAGAC
CATTAGATGAATTCCCAAAGTTAGACAATTAGATAAATTTCCAAAGTTAGAC
CATTAGATGAATTTCCAAAGTTAGAC
CATTAAATGAATTTCCAAAGTTAGAC
CATTAGATGAATTTCCAAAGTTAGAC
CATTAGATGAATTCCCAAAGTTAGAC

CATTAGATGAATTTCCAAAGTTAGAC
CATTAGATGAATTTCCAAAGTTAGAC
CATTAGATGAAATTCAAAAATTAAAC
CATTAGATGAAATTCCAAAGTTAGAC
CATTAGATGAATTCCCAAAGTTAGAC
CATTAAATGAATTCCCAAAGTTAGAC
CATTAGATGAAATTCCAAAATTAGAC
CATTAGATGAATTTCCAAAGTTAGAAC
CATTAGATGAAATTCCAAAATTAGAC
CATTAGATGAATTTCCAAAGTTAGAC
CATTAGATGAATTCCCAAAGTTAGAC
CATTAGATGAATTTCCAAAGTTAGAC
CATTAGATGAATTTCCAAAGTTAGAC
CATTAGATGAATTCCCAAAGTTAGAC
CATTAGATGAATTCCCAAAGTTAGAC
CATTAGATGAATTCCCAAAGTTAGAC
CATTAGATGAATTTCCAAAGTTAGAC
CATTAGATGAATTTCCAAAGTTAGAC
CATTAGATGAATTTCCAAAGTTAGAC
CATTAGATGAATTCCCAAAGTTAGAC
CATTAGATGAATTCCCAAAGTTAGAC
CATTAGATGAATTCCCAAAGTTAGAC
CATTAGATGAATTCCCAAAGTTAGAC
CATTAGATGAATTCCAAAGTTAGAC
CATTAGATGAATTCCCAAAGTTAGAC
CATTAGATGAATTTCCAAAGTTAGAC
CATTAGATGAATTCCAAAGTTAGAC
CATTAGATGAATTCCCAAAGTTAGAC
CATTAGATGAATTCCCAAAGTTAGAC
CATTAGATGAATTCCCAAAGTTAGAC
CATTAGATGAAATTCCAAAGTTAGAC
CATTAGATGAATTTCCAAAGTTAGAC
CATTAGATGAATTCCCAAAGTTAGAC
CATTAGATGAATTTCCAAAGTTAGAC
CATTAGATGAATTCCCAAAGTTAGAC
CATTAGATGAATTTCCAAAGTTAGAC
CATTAGATGAATTCCCAAAGTTAGAC
CATTAGATGAATTCCAAAGTTAGAC
CATTAGATGAATTCCCAAAGTTAGA
CATTAGATGAATTCCAAAGTTAGA
CATTAGATGAATTCCAAAGTTAGAC
CATTAGATGAAATTCCAAAGTTAGAC
CATTAGATGAATTCCCAAAGTTAGAC
CATTAGATGAATTCCCAAAGTTAGAC
CATTAGATGAATTCCCAAAGTTAGAA
CATTAGATGAATTCCCAAAGTTAGAA
CATTAGATGAATTTCCAAAGTTAGAC
CATTAGATGAAATTCCAAAGTTAGAC
CATTAGATGAAATTCCAAAGTTAGAC
CATTAGATGAAATTCCAAAGTTAGAC
CATTAGATGAATTCCCAAAGTTAGAA
CATTAGATGAATTCCCAAAGTTAGAC
CATTAGATGAAATTCCAAAGTTAGAC
CATTAGATGAAATTCCAAAGTTAGAC

CATTAGATGAATTCCCAAAGTTAGAC
CATTAGATGAATTCCCAAAGTTAGAC
CATTAGATGAATTCCAAAGTTAGAC
CATTAGATGAATTCCCAAAGTTAGAC
CATTAGATGAATTCAAATTAA
CATTAATGAATTCCAAAGTTAGAC
CATTAGATGAAATTCCAAAGTTAGAC
CATTAGATGAAATTCCAAAGTTAGAC
CATTAGATGAATTCCCAAAGTTAGAC
CATTAGATGAAATTCCAAAGTTAGAC
CATTAGATGAATTCCCAAAGTTAGAC
CATTAGATGAATTCCCAAAGTTAGAC
CATTAGATGAAATTCCAAAGTTAGAC
CATTAGATGAAATTCCAAAGTTAGAC
CATTAGATGAAATTCCAAAGTTAGAC
CATTAGATGAATTCCCAAAGTTAGAC
CATTAGATGAATTCCCAAAGTTAGAC
CATTAGATGAATTCCCAAAGTTAGAC
CATTAGATGAATTCCCAAAGTTAGAC
CATTAGATGAAATTCCAAAGTTAGAC
CATTAGATAAAAATAGA
CATTAGATGAATTCCCAAAGTTAGAC
CATTAGATGAATTCCCAAAGTTAGAC
CATTAGATGAATTCCCAAAGTTAGAC
CATTAGATGAAATTCCAAAGTTAGAC
CATTAGATGAATTCCCAAAGTTAGAC
CATTAGATGAATTCCCAAAGTTAGAC
CATTAGATGAATTCCCAAAGTTAGAC
CATTAGATGAATTCCCAAAGTTAGAC
CATTAGATGAAATTCCAAAGTTAGAC
CATTAGATGAATTCCCAAAGTTAGAC
CATTAGATGAATTCCCAAAGTTAGAC
CATTAGATGAATTCCCAAAGTTAGAC
CATTAGATGAAATTCCAAAGTTAGAC
CATTAGATGAATTCCCAAAGTTAGAC
CATTAGATGAATTCCAAAGTTAGA
CATTAGATGAATTCCAAAGTTAGAC
CATTAGATGAATTCCAAAGTTAGAC
CATTAGATGAATTCCAAATAGA
CATTAGATGAATTCCAAAGTTAGA
CATTAGATGAATTCCCAAAGTTAGAC
CATTAGATGAATTCCAAAGTTAGAC
CATTAGATGAATTCCCAAAGTTAGA
CATTAGATGAATTCCAAAGTTAGAC
CATTAGATGAATTCCAAAGTTAGAC
CATTAGATGAATTCCAATAGAC
CATTAGATGAATTCCCAAAGTTAGAC
CATTAGATGAATTCCCAAAGTTAGAC
CATTAGATGAATTCCCAAAGTTAGAC
CATTAGATGAATTCCCAAAGTTAGAC
CATTAGATGAATTCCCAAAGTTAGAC
CATTAGATGAAAAAGAAAATTCCAAAGTTAGAC
CATTAGATGAATTCCCAAAGTTAGAC
CATTAGATGAATTCCCAAAGTTAGAC

CATTAGATGAATTCCCAAAGTTAGAC
CATTAGATGAATTCCCAAAGTTAGAC
CATTAGATGAATTCCCAAAGTTAGAC
CATTAGATGAATTCCCAAAGTTAGAC
CATTAGATGAATTCCAAAGTTAGAC
CATTAGATGAATTCCCAAAGTTAGAC
CATTAGATGAATTCCAAAGTTAGAC
CATTAGATGAATTCCCAAAGTTAGA
CATTAGATGAATTCCAAAGTTAGAC
CATTAGATGAATTCCCAAAGTTAGAC
CATTAGATGAATTCCCAAAGTTAGA
CATTAGATGAATTCCCAAAGTTAGAC
CATTAGATGAATTCCCAAAGTTAGAC
CATTAGATGAATTCCCAAAGTTAGAC
CATTAGATGAATTCCAAAGTTAGAC
CATTAGATGAATTCCCAAAGTTAGAC
CATTAGATGAATTCCAAAGTTAGAC
CATTAGATGAATTCCAAAGTTAGAC
CATTAGATGAATTCCCAAAGTTAGAC
CATTAGATGAATTCCCAAAGTTAGAC
CATTAGATGAATTCCAAAGTTAGAC
CATTAGATGAATTCCAAAGTTAGAC
CATTAGATGAATTCCCAAAGTTAGAC
CATTAGATGAATTCCCAAAGTTAGAC
CATTAGATGAATTCCCAAAGTTAGAC
CATTAGATGAATTCCCAAAGTTAGAC
CATTAGATGAATTCAAGTAGAC
CATTAGATGAATTCCAAAGTTAGAC
CATTAGATGAATTCCCAAAGTTAGAC
CATTAGATGAATTCCCAAAGTTAGAC
CATTAGATGAATTCCCAAAGTTAGAC
CATTAGATGAATTCCCAAAGTTAGAC
CATTAGATGAATTCCAAAGTTAGAC
CATTAGATGAATTCCCAAAGTTAGAC
CATTAGATGAAATTCCAAAGTTAGAC
CATTAGATGAATTCCCAAAGTTAGAC
CATTAGATGAAATTCCAAAGTTAGAC
CATTAGATGAATTCCAAAGTTAGAC
CATTAGATGAATTCCCAAAGTTAGAC
CATTAGATGAAATTCCAAAGTTAGAC
CATTAGATGAATTCCCAAAGTTAGAC
CATTAGATGAAATTCCAAAGTTAGAC
CATTAGATGAATTCCCAAAGTTAGAC
CATTAGATGAATTCCCAAAGTTAGAC
CATTAGATGAATTCCCAAAGTTAGAC
CATTAGATGAAATTCCAAAGTTAGAC
CATTAGATGAATTCCCAAAGTTAGAC
CATTAGATGAATTCCCAAAGTTAGAC
CATTAGATGAATTCCCAAAGTTAGAC
CATTAGATGAATTCCCAAAGTTAGAC
CATTAGATGAATTCCCAAAGTTAGAC
CATTAGATGAATTCCCAAAGTTAGAC
CATTAGATGAATTCCCAAAGTTAGAC
CATTAGATGAATTCCAAAGTTATTAGAC

CATTAGATGAATTCCCAAAGTTAGAC
CATTAGATGAATTCCCAAAGTTAGAC
CATTAGATGAATTCCCAAAGTTAGA
CATTAGATGAATTCCAAAGTTAGAC
CATTAGATGAATTCCCAAAGTTAGAC
CATTAGATGAATTCCCAAAGTTAGAC
CATTAGATGAATTCCCAAAGTTAGAC
CATTAGATGAATTCCCAAAGTTAGAC
CATTAGATGAATTCCAAAGTTAGAC
CATTAGATGAATTCCCAAAGTTAGAC
CATTAGATGAATTCCCAAAGTTAGAC
CATTAGATGAATTCCCAAAGTTAGAC
CATTAGATGAATTCCCAAAGTTAGA
CATTAGATGAATTCCAAAGTTAGAC
CATTAGATGAATTCCAAAGTTAGAC
CATTAGATGAATTCCCAAAGTTAGAC
CATTAGATGAATTCCCAAAGTTAGAC
CATTAGATGAATTCCCAAAGTTAGAA
CATTAGATGAATTCCCAAAGTTAGAC
CATTAGATGAATTCCCAAAGTTAGA
CATTAGATGAATTCCAAAGTTAGAC
CATTAGATGAATTCCCAAAGTTAGAC
CATTAGATGAATTCCAAAGTTAGAC
CATTAGATGAATTCCAAAGTTAGAC
CATTAGATGAATTCCAAAGTTAGAC
CATTAGATGAATTCCCAAAGTTAGAC
CATTAGATGAATTCCAAAGTTAGAC
CATTAGATGAATTCCAAAGTTAGAC
CATTAGATGAATTCCCAAAGTTAGAC
CATTAGATGAATTCCCAAAGTTAGA
CATTAGATGAATTCCCAAAGTTAGAC
CATTAGATGAATTCCAAAGTTAGAC
CATTAGATGAATTCCAAAGTTAGAC
CATTAGATGAATTCCAAAGTTAGAC
CATTAGATGAATTCCCAAAGTTAGAC
CATTAGATGAATTCCCAAAGTTAGAC
CATTAGATGAATTCCCAAAGTTAGAC
CATTAGATGAAATTCCAAAGTTAGAC
CATTAGATGAATTCCAAAGTTAGAC
CATTAGATGAATTCCCAAAGTTAGAC
CATTAGATGAATTCCCAAAGTTAGAA
CATTAGATGAATTCCAAAGTTAGAC
CATTAGATGAATTCCCAAAGTTAGAC
CATTAGATGAATTCCAAAGTTAGAC
CATTAGATGAATTCCCAAAGTTAGAC
CATTAGATGAATTCCCAAAGTTAGAC
CATTAGATGAATTTCCCAAAGTTAGAC
CATTAGATGAATTCCCAAAGTTAGAC
CATTAGATGAATTCCCAAAGTTAGAC
CATTAGATGAATTCCCAAAGTTAGAC
CATTAGATGAATTCCCAAAGTTAGAC
CATTAGATGAAATTCCAAAGTTAGAC
CATTAGATGAATTCCCAAAGTTAGAC
CATTAGATGAATTCCCAAAGTTAGAC

CATTAGATGAATTCCCAAAGTTAGAC
CATTAGATGAATTCCCAAAGTTAGAC
CATTAGATGAAATTCCAAAGTTAGAC
CATTAGATGAATTCCCAAAGTTAGAC
CATTAGATGAAATTCCAAAGTTAGAC
CATTAGATGAATTCCCAAAGTTAGA
CATTAGATGAATTC
CATTAGATGAATTCCCAAAGTTAGAC
CATTAGATGAATTCCCAAAGTTAGAC
CATTAGATGAATTCCCAAAGTTAGAC
CATTAGATGAATTCCCAAAGTTAGA
CATTAGATGAATTTCCCAAAGTTAGAC
CATTAGATGAATTCCCAAAGTTAGAC
CATTAGATGAATTCCAAAGTTAGAC
CATTAGATGAATTCCCAAAGTTAGAC
CATTAGATGAATTCCCAAAGTTAGAC
CATTAGATGAATTCCCAAAGTTAGAC
CATTAGATGAATTCCCAAAGTTAGAC
CATTAGATGAATTCCCAAAGTTAGAC
CATTAGATGAATTCCCAAAGTTAGA
CATTAGATGAATTAAGTAGA
CATTAGATGAATTCCCAAAGTAGAAAAAAAATAGA
CATTAGATGAATTCCCAAAGTTAGAC
CATTAGATGAAATTCCAAAGTTAGAC
CATTAGATGAATTCCCAAAGTTAGAC
CATTAGATGAATTTCCAAAGTTAGAC
CATTAGATGAAATTCCAAAGTTAGAC
CATTAGATGAAATTCCAAAGTTAGAC
CATTAGATGAATTCCCAAAGTTAGAC
CATTAGATGAATTCCCAAAGTTAGAC
CATTAGATGAATTCCCAAAGTTAGAC
CATTAGATGAATTCCCAAAGTTAGAC
CATTAGATGAATTCCCAAAGTTAGAA
CATTAGATGAATTTCCAAAGTTAGAC
CATTAGATGAATTTCCAAAGTTAGACATAGAGAATTCCAAAGTAGAC
CATTAGATGAAATTCCAAAGTTAGAC
CATTAGATGAATTCCCAAAGTTAGAA
CATTAGATGAATTTCCAAAGTTAGAC
CATTAGATGAATTCCCAAAGTTAGAC
CATTAGATGAAATTCCAAAGTTAGAC
CATTAGATGAAATTCCAAAGTTAGAC
CATTAGATGAATTCCCAAAGTTAGAC
CATTAGATGAAATTCCAAAGTTAGAC
CATTAGATGAATTCCCAAAGTTAGAC
CATTAGATGAATTCCCAAAGTTAGAC
CATTAGATGAATTCCCAAAGTTAGAC
CATTAGATGAAATTCCCAAAGTTAGAC
CATTAGATGAAATTCCAAAGTTAGAC
CATTAGATGAATTCCCAAAGTTAGAC
CATTAGATGAATTCCCAAAGTTAGAC
CATTAGATGAATTCCAAAGTTAGAC
CATTAGATGAATTCCAAAGTTAGAC
CATTAGATGAAATTCCCAAAGTTAGAC
CATTAGATGAATTCCCAAAGTTAGAA

CATTAGATGAATTCCCAAAGTTAGAATAGAACATTAGACC
CATTAGATGAATTCCCAAAGTTAGAC
CATTAGATGAATTCCCAAAGTAAATAGAC
CATGTAGATGAATTCCCAAGAGTTAGAACCATGAATTCCCCAAAATGTTAGAGAC
CATTAGATGAATTCCCAAAGTTAGAC
CATTAGATGAATTCCCAAAGTTAGACCATTAGGACTTGAAATTCCCAAAGTTAGAC
CATTAGATGAATTCCCAAAGTTAGAC
CATTAGATGAATTCCAAAGTTAGAC
CATTAGATGAATTAAAGTTAGAC
CATTAGATGAATTCCAAAGTTAGAC
CATTAGATGAATTCCAAAGTTAGAC
CATTAGATGAATTCCAAAGTTAGAC
CATTAGATGAATTCCCAAAGTTAGAC
CATTAGATGAATTCCAAAGTTAGAC
CATTAGATGAATTCCAAAGTTAGAC
CATTAGATGAATTTCCAAAGTTAGAC
CATTAGATGAATTCCAAAGTTAGAC
CATTAGATGAATTCCAAAGTTAGAC
CATTAGATGAATTCCAAAGTTAGAC
CATTAGATGAATTTCCAAAGTTAGAC
CATTAGATGAATTCCAAAGTTAGAC
CATTAGATGAATTCCAAAGTTAGAC
CATTAGATGAATTCCCAAAGTTAGAC
CATTAGATGAATTTCCAAAGTTAGAC
CATTAGATGAATTCCAAAGTTAGAC
CATTAGATGAATTCCAAAGTTAGAC
CATTAGATGAATTTCCAAAGTTAGAC
CATTAGATGAATTCCAAAGTTAGAC
CATTAGATGAATTCCAAAGTTAGAC
CATTAGATGAATTCCAAAGTTAGAC
CATTAGATGAATTTCCAAAGTTAGAC
CATTAGATGAATTCCAAAGTTAGAC
CATTAGATGAATTTCCAAAGTTAGAC
CATTAGATGAATTCCAAAGTTAGAC
CATTAGATGAATTCCAAAGTTAGAC
CATTAGATGAATTCCAAAGTTAGAC
CATTAGATGAATTCCAAAGTTAGAC
CATTAGATGAATTCCAAAGTTAGAC
CATTAGATGAATTCCAAAGTTAGAC
CATTAGATGAATTCCAAAGTTAGAC
CATTAGATGAATTCCAAAGTTAGAC
CATTAGATGAATTCCAAAGTTAGAC
CATTAGATGAATTCCCAAAGTTAGAC
CATTAGATGAATTCCAAAGTTAGAC
CATTAGATGAATTCCAAAGTTAGAC
CATTAGATGAATTCCCAAAGTTAGAC
CATTAGATGAATTTCCAAAGTTAGAC
CATTAGATGAATTTCCAAAGTTAGAC
CATTAGATGAATTCCAAAGTTAGAC
CATTAGATGAATTCCAAAGTTAGAC
CATTAGATGAATTCCAAAGTTAGAC
CATTAGATGAATTTCCAAAGTTAGAC
CATTAGATGAATTCCAAAGTTAGAC
CATTAGATGAATTCCAAAGTTAGAC

CATTAGATGAATTCCAAAGTTAGAC
CATTAGATGAATTCCAAAGTTAGAC
CATTAGATGAATTCCAAAGTTAGAC
CATTAGATGAATTCCAAAGTTAGAC
CATTAGATGAATTCCAAAGTTAGAC
CATTAGATGAATTCCAAAGTTAGAC
CATTAGATGAATTCCCAAAGTTAGAC
CATTAGATGAATTCCAAAGTTAGAC
CATTAGATGAATTCCAAAGTTAGAC
CATTAGATGAATTCCAAAGTTAGAC
CATTAGATGAATTCCAAAGTTAGAC
CATTAGATGAATTCCAAAGTTAGAC
CATTAGATGAATTCCCAAAGTTAGAC
CATTAGATGAATTTCCCAAAGTTAGAC
CATTAGATGAATTTCCCAAAGTTAGAC
CATTAGATGAATTTCCAAAGTTAGAC
CATTAGATGAATTCCAAAGATTAGACC
CATTAGATGAATTCCCCAAAGTTAGAC
CATTAGATGAATTCCCAAAGTTAGAC
CATTAGATGAATTCCAAAGTTAGAC
CATTAGATGAATTCCCAAAGTTAGAC
CATTAGATGAATTTCCAAAGTTAGAC
CATTAGATGAATTCCCAAAGTTAGAC
CATTAGATGAATTCCCAAAGTTAGAC
CATTAGATGAATTCCCAAAGTTAGAC
CATTAGATGAATTTCCCAAAGTTAGAC
CATTAGATGAATAAAGTTAGACCATAATGAATCCCAAAGTAGAC
CATTAGATGAATTCCAAAGTTAGAC
CATTAGATGAATTCCCAAGTTAGAC
CATTAGATGAATTTCCAAATTAGAC
CATTAGATGAATTCCAAAGTTAGAC
CATTAGATGAATTCCCAAAGTTAGAC
CATTAGATGAATTTCCAAAGTTAGAC
CATTAGATGAATTCCCAAAGTTAGAC
CATTAGATGAATTTCCCAAAGTTAGAC
CATTAGATGAATTCCCAAAGTTAGAC
CATTAGATGAATTCCCAAAAGTTAGAC
CATTAGATGATTTCCAAAGTTAGGAC
CATTAGATGAATTCCAAAGTTAGAC
CATTAGATGAATTCCCAAAGTTAGAC
CATTAGATGAATTTCCCAAAGTCTAGAC
CATTAGATGAATTCCAAAGTTAGAC
CATTAGATGAATTCCCAAAGTTAGAC
CATTAGATGAATTCCCAAAGTTAGAC
CATTAGATGAATTCCCAAAGTTAGAC
CATTAGATGAATTCCCAAAGTTAGAC
CATTAGATGAATTCCCAAAGTTAGAC
CATTAGATGAATTTCCAAGTTAGAC
CATTAGATGAATTCCCAAAGTTAGAC
CATTAGATGAATTCCCAAAGTTAGAC
CATTAGATGAATTCCAAAGAATTCCAAAGTAGAC
CATTAGATGAATTTCCAAAGTTAGAC
CATTAGATGAATTCCCAAAGTGAATAGAGAATCAAATTAGAC
CATTAGATGAATTCCCAAAGTTAGAC

CATTAGATGAATTTCCAAAGTTAGAC
CATTAGATGAATTCCCAAAGTTAGAC
CATTAGATGAATTTCCAAAGTTAGA
CATTAGATGAATTCCCAAAGTTAGAC
CATTAGATGAATTCCCAAAGTTAGAC
CATTAGATGAATTTCCAAAGTTAGAC
CATTAGATGAATCCAAAGTTAGA
CATAGATGAAAAAAAAAAAATTTCCAAAGTTAGAC
CATTAGATGAATCCAAAGTTAGA
CATTAGATGAATTTCCAAAGTTAGAC
CATTAGATGAATTTCCAAAGTTAGAC
CATTAGATGAATTCCCAAAGTTAGAC
CATTAGATGAATTTCCAAAGTTAGAC
CATTAGATGAATTCCCAAAGTTAGAC
CATTAGATGAATTTCCAAAGTTAGAC
CATTAGATGAATTCCCCAAATTAGAC
CATTAGATGAATTTTCCAAAGTTAGAC
CATTAGATGAATTCCCAAAGTTTAGGAC
CATTAGATGAATTTCCCAAAGTTAGAC
CATTAGATGAATTTCCAAAGTTAGAC
CATTAGATGAATTCCCAAAGTTAGAC
CATTAGATGAATTTCCAAAGTTAGAC
CATTAGATGAATTTCCAAAGTTAGAC
CATTAGATGAATTCCAAAGTTAGAC
CATTAGATGAATTTCCAAAGTTAGAC
CATTAGATGAATTCCCAAAGTTAGAC
CATTAGATGAATTCCCAAAGTTAGAC
CATTAGATGAATTCCCAAAGTTAGAC
CATTAGATGAATTCCAAAGTTAGAC
CATTAGATGAATTCCCAAAGTTAGAC
CATTAGATGAATTCCCAAAGTTAGAC
CATTAGATGAATTCCCAAAGTTAGAC
CATTAGATGAATTTCCAAAGTTAGAC
CATTAGATGAATTCCCAAAGTTAGAC
CATTAGATGAATTTCCAAAGTTAGAC
CATTAGATGAATTCCCAAAGTTAGAC
CATTAGATGAATTCCAAAGTTAGAC
CATTAGATGAATTCCAAAGTTAGAC
CATTAGATGAATTCCAAAGTTAGAC
CATTAGATGAATTCCAAAGTTAAC
CATTAGATGAATTCCCAAAGTTAGAC
CATTAGATGAATTCCCAAAGTTAGAC
CATTAGATGAATTCCCAAAGTTAGAC
CATTAGATGAATTCCAAAGTTAGAC
CATTAGATGAATTTCCAAAGTTAGAC
CATTAGATGAATTCCCAAAGTTAGAC
CATTAGATGAATTTCCAAAGTTAGAC
CATTAGATGAATTTCCAAAGTTAGAC
CATTAGATGAATTCCAAAGTTAGAC
CATTAGATGAATTCCCAAAGTTAGAC
CATTAGATGAATTCCAAAGTTAGAC
CATTAGATGAATTTCCAAAGTTAGAC
CATTAGATGAATTCCCAAAGTTAGAC
CATTAGATGAATTTCCAAAGTTAGAC

CATTAGATGAATTCCCAAAGTTAGAC
CATTAGATGAATTTCCCAAAGTTAGAC
CATTAGATGAATTTCCAAAGTTAGAC
CATTAGATGAATTTCCAAAGTTAGAC
CATTAGATGAATTTCCAAAGTTAGAC
CATTAGATGAATTTCCAAAGTTAGAC
CATTAGATGAATTCCCAAAGTTAGAC
CATTAGATGAATTTCCAAAGTTAGAC
CATTAGATGAATTTCCAAAGTTAGAC
CATTAGATGAATTCCAAAGTTAGAC
CATTAGATGAATTTCCAAAGTTAGAC
CATTAGATGAATTCCCAAAGTTAGAC
CATTAGATGAATTCCCAAAGTTAGAC
CATTAGATGAATTCCCAAAGTTAGAC
CATTAGATGAATTCCAAAGTTAGAC
CATTAGATGAATTTCCAAAGTTAGAC
CATTAGATGAATTCCCAAAGTTAGAC
CATTAGATGAATTTCCAAAGTTAGAC
CATTAGATGAATTTCCAAAGTTAGAC
CATTAGATGAATTCCCAAAGTTAGAC
CATTAGATGAATTTCCAAAGTTAGAC
CATTAGATGAATTCCAAAGTTAGAC
CATTAGATGAATTCCCAAAGTTAGAC
CATTAGATGAATTTCCAAAGTTAGAC
CATTAGATGAATTTCCAAAGTTAGAC
CATTAGATGAATTCCAAAGTTAGAC
CATTAGATGAATTCCAAAGTTAGAC
CATTAGATGAATTTCCAAAGTTAGAC
CATTAGATGAAATTCCAAAGTTAGAC
CATTAGATGAATTTCCAAAGTTAGAC
CATTAGATGAATTCCAAAGTTAGAC
CATTAGATGAATTCCCAAAGTTAGAC
CATTAGATGAATTTCCAAAGTTAGAC
CATTAGATGAATTTCCAAAGTTAGAC
CATTAGATGAATTCCAAAGTTAGAC
CATTAGATGAATTTCCAAAGTTAGAC
CATTAGATGAATTTCCAAAGTTAGAC
CATTAGATGAATTCCCAAAGTTAGAC
CATTAGATGAATTTCCAAAGTTAGAC
CATTAGATGAATTCCCAAAGTTAGAC
CATTAGATGAATTTCCAAAGTTAGAC
CATTAGATGAATTTCCAAAGTTAGAC
CATTAGATGAATTCCCAAAGTTAGAC
CATTAGATGAATTCCAAAGTTAGAC
CATTAGATGAATTCCAAAGTTAGAC
CATTAGATGAATTCCCAAAGTTAGAC
CATTAGATGAATTCCCAAAGTTAGAC
CATTAGATGAATTTCCAAAGTTAGAC
CATTAGATGAATTCCCAAAGTTAGAC
CATTAGATGAATTCCCAAAGTTAGAC
CATTAGATGAATTCCAAAGTTAGAC
CATTAGATGAATTCCAAAGTTAGAC
CATTAGATGAATTCCAAAGTTAGAC
CATTAGATGAATTTCCAAAGTTAGAC

CATTAGATGAATTCCCAAAGTTAGAC
CATTAGATGAATTCCCAAAGTTAGAC
CATTAGATGAATTTCCAAAGTTAGAC
CATTAGATGAATTCCCAAAGTTAGAC
CATTAGATGAATTTCCAAAGTTAGAC
CATTAGATGAATTCCCAAAGTTAGAC
CATTAGATGAATTCCAAAGTTAGAC
CATTAGATGAATTCCCAAAGTTAGAC
CATTAGATGAATTCCCAAAGTTAGAC
CATTAGATGAATTCCAAAGTTAGAC
CATTAGATGAATTCCCAAAGTTAGAC
CATTAGATGAATTTCCAAAGTTAGAC
CATTAATGAATCCAAAGTTAACCATAGATAATTCCAAAGTTAGAC
CATTAGATGAATTCCAAAGTTAGAC
CATTAGATGAATTCCAAAGTTAGAC
CATTAGATGAATTCCCAAAGTTAGAC
CATTAGATGAATTTCCAAAGTTAGAC
CATTAGATGAATTTCCAAAGTTAGAC
CATTAGATGAATTCCCAAAGTTAGAC
CATTAGATGAATTTCCAAAGTTAGAC
CATTAGATGAATTCCCAAAGTTAGAC
CATTAGATGAATTTCCCAAAGTTAGAC
CATTAGATGAATTCCAAAGTTAGAC
CATTAGATGAATTTCCAAAGTTAGAC
CATTAGATGAATTCCAAAGTTAGAC
CATTAGATGAATTCCAAAGTTAGAC
CATTAGATGAATTCCAAAGTTAGAC
CATTAGATGAATTCCAAAGTTAGAC
CATTAGATGAATTTCCAAAGTTAGAC
CATTAGATGAATTCCAAAGTTAGAC
CATTAGATGAATTCCCAAAGTTAGAC
CATTAGATGAATTCCCAAAGTTAGAC
CATTAAATGAATTCCCAAAGTTAGAC
CATTAGATGAATTCCCAAAGTTAGAC
CATTAGATGAATTCCAAAGTTAGAC
CATTAGATGAATTCCCAAAGTTAGAC
CATTAGATGAATTTCCAAAGTTAGAC
CATTAGATGAATTCCCAAAGTTAGAC
CATTAGATGAATTTCCAAAGTTAGAC
CATTAGATGAATTCCCAAAGTTAGAC
CATTAGATGAATTTCCAAAGTTAGAC
CATTAGATGAATTCCCAAAGTTAGAC
CATTAGATGAATTTCCAAAGTTAGAC
CATTAGATGAATTCCCAAAGTTAGAC
CATTAGATGAATTTCCAAAGTTAGAC
CATTAGATGAATTCCCAAAGTTAGAC
CATTAGATGAATTCCAAAGTTAGAC
CATTAGATGAATTCCCAAAGTTAGAC
CATTAAATGAATTCCCAAAGTTAGAC
CATTAGATGAATTCCCAAAGTTAGAC
CATTAGATGAATTTCCAAAGTTAGAC
CATTAGATGAATTTCCAAAGTTAGAC
CATTAGATGAATTCCCAAAGTTAGAC
CATTAGATGAATTCCCAAAGTTAGAC

CATTAAATGAATTCCCAAAGTTAGAC
CATTAGATGAATTCCCAAAGTTAGAC
CATTAGATGAATTCCCAAAGTTAGAC
CATTAGATGAATTCCCAAAGTTAGAC
CATTAAATGAATTCCCAAAGTTAGAC
CATTAGATGAATTCCCAAAGTTAGAC
CATTAGATGAATTCCCAAAGTTAGAC
CATTAGATGAATTCCAAAGTTAGAC
CATTAGATGAATTTCCAAAGTTAGAC
CATTAGATGAATTCCCAAAGTTAGAC
CATTAGATGAATTTCCAAAGTTAGAC
CATTAAATGAATTCCCAAAGTTAGAC
CATTAGATGAATTCCAAAGTTAGAC
CATTAGATGAATTCCCAAAGTTAGAC
CATTAGATGAATTTCCAAAGTTAGAC
CATTAGATGAATTCCCAAAGTTAGAC
CATTAGATGAATTCCCAAAGTTAGAC
CATTAGATGAATTCCCAAAGTTAGAC
CATTAGATGAATTCCAAAGTTAGAC
CATTAGATGAATTCCCAAAGTTAGAC
CATTAGATGAATTCCCAAAGTTAGAC
CATTAGATGAATTTCCCAAAGTTAGAC
CATTAGATGAATTTCCCAAAGTTAGAC
CATTAGATGAATTTCCCAAAGTTAGAC
CATTAGATGAATTCCCAAAGTTAGAC
CATTAGATGAATTTCCAAAGTTAGAC
CATTAGATGAATTCCCAAAGTTAGAC
CATTAGATGAATTCCAAAGTTAGAC
CATTAGATGAATTCCAAAGTTAGAC
CATTAAATGAATTCCCAAAGTTAGAC
CATTAGATGAATTCCCAAAGTTAGAC
CATTAGATGAATTTCCAAAGTTAGAC
CATTAGATGAATTCCCAAAGTTAGAC
CATTAGATGAATTCCCAAAGTTAGAC
CATTAGATGAATTTCCAAAGTTAGAC
CATTAGATGAATTCCCAAAGTTAGAC
CATTAGATGAATTCCCAAAGTTAGAC
CATTAGATGAATTCCCAAAGTTAGAC
CATTAGATGAATTCCCAAAGTTAGAC
CATTAGATGAATTTCCCAAAGTTAGAC
CATTAGATGAATTTCCCAAAGTTAGAC
CATTAGATGAATTCCCAAAGTTAGAC
CATTAGATGAATTTCCAAAGTTAGAC
CATTAAATGAATTCCCAAAGTTAGAC
CATTAGATGAATTTCCAAAGTTAGAC
CATTAGATGAAATTCCAAAGTTAGAC
CATTAGATGAATTCCCAAAGTTAGAC
CATTAGATGAATTTCCAAAGTTAGAC
CATTAGATGAATTCCCAAAGTTAGAC
CATTAGATGAATTTCCAAAGTTAGAC
CATTAGATGAATTCCCAAAGTTAGAC
CATTAGATGAATTTCCAAAGTTAGAC
CATTAGATGAATTCCCAAAGTTAGAC
CATTAGATGAATTTCCAAAGTTAGAC

CATTAGATGAATTTCCAAAGTTAGAC
Nr of selected repeats 417 Similarity 0.923292
Consensus:
CATTAGATGAATTcCCAAAGTTAGAC
>Cele-UNSB01_3:7846966-7847368 Satlength=403 Nr of Repeats=9 RepeatLength=45
seed=TCTGATCCTT
TCTGATCCTTCAGCTCCGAAGGATCACATGGGTACCTTCTGATGT
TCTGATCCTTCAGATAAGAAGGATCGTAAGGGTACCTCCTGATGG
TCTGATCCTTTAGCTCCAAAGGATCACGAGGCTCTTCCTGATGT
TCTGATCCTTCAGCTCCGAAGGATCACATGGGTACCTTCTGATGT
TCTGATCCTTCAGATAAGAAGGATCGTAAGGGTACCTCCTGATGG
TCTGATCCTTTAGCTCCAAAGGATCACGAGGCTCTTCCTGATGT
TCTGATCCTTCAGCTCCGAAGGATCACATGGGTACCTTCTGATGT
TCTGATCCTTCAGATAAGAAGGATCGTAAGGGTACCTCCTGATGG
TCTGATCCTTTAGCTCCAAAGGATCACGAGGCTCTTCCTGATGT
Nr of selected repeats 6 Similarity 0.857778
Consensus:
TCTGATCCTTCAGaTaaGAAGGATCacAaGGGTACCTcCTGATGg
>Cele-UNSB01_3:7847664-7848155 Satlength=492 Nr of Repeats=11 RepeatLength=45
seed=GAAGGATCAG
GAAGGATCAGAACATCAGGAGGTACCCATGTGATCATTCGGCTCT
GAAAGATCAGATCATCAGGCAGAGCCTCATGTTCTTTTGGAGCT
GAAGGATCAGACCATCAGGAGGTACCCTTACGATCCTTCTTATCT
GAAGGATCAGATCATCAGAAGGTACCCATGTGATCCTTCGGATCG
GAAGGATCAGATCATCAGGCAGAGCCTCATGTTCTTTTAGAGCT
GAAGGATCAGAACATCAGGAAGAGCCTCGTGATCCTTTGGAGCT
GAAGGATCAGACCATCAGGAGGTACCCTTACGATCCTTCTTATCT
GAAGGATCAGAACATCAGAAGGTATCCATGTGATCCTTCGGAGCT
GAAGGTTCAGAACATCAGGAAGAGCCTCGTGATCCTTTGGAGCT
GAAGTATCAGACCATCAGGAGGTACCCTTACGATCCTTCTTATCT
GAAGGATCAGAACATCAGAAGGTACCCATGTGATCCTTCGGAGCT
Nr of selected repeats 7 Similarity 0.820811
Consensus:
GAAGGATCAGAnCATCAGgAGGTACCCaTgtGATCCTTCggAtCT
>Cele-UNSB01_3:7913456-7913988 Satlength=533 Nr of Repeats=28 RepeatLength=19
seed=AATCTATTTT
AATCTATTTTTCGTGTAAC
AATCTATTTTTCGTGTAAC
AATCTATTTTTCGTGTAAC
AATCTATTTTTCGTGTAAC
AATCTATTTTTCGTGTAAC
AATCTATTTTTCGTGTAAC
AATCTATTTTTCGTGTAAC
AATCTATTTTTCGTGTAAC
AATCTATTTTTCGTGTAAC
AATCTATTTTTCGTGTAAC
AATCTATTTTTCGTGGAAC
AATCTATTTTTCGTGTAAC
AATCTATTTTTCGTGCAAC
AATCTATTTTTCGTGTAAC
AATCTATTTTTCGTGCAGC
AATCTATTTTTCGTGTAAC
AATCTATTTTTCGTGTATA
AATCTATTTTTCGTGTAAC

AATCTATTTTTCGTGTAAC
AATCTATTTTTCGTGTAAC
AATCTATTTTTCGTGTAAC
AATCTATTTTTCGTGCAAC
AATCTATTTTTCGTGTAAC
AATCTATTTTTCGTGTAAC
AATCTATTTTTCGTGTAAC
AATCTATTTTTCGTGTAAC
AATCTATTTTTCGTGTAAC
AATCTATTTTTCGTGTAAC
Nr of selected repeats 28 Similarity 0.920040
Consensus:
AATCTATTTTTCGTGTAAC
>Cele-UNSB01_3:7922958-7923224 Satlength=267 Nr of Repeats=14 RepeatLength=19
seed=CAATCTATTT
CAATCTATTTTTCGTGTAA
CAATCTATTTTTCGTGGAA
CAATCTATTTTTCGTGTAA
CAATCTATTTTTCGTGTAA
CAATCTATTTTTCGTGTAA
CAATCTATTTTTCGTGTAA
CAATCTATTTTTCGTGTAA
CAATCTATTTTTCGTGGAA
CAATCTATTTTTCGTGTAA
CAATCTATTTTTCGTGGAA
CAATCTATTTTTCGTGTAA
CAATCTATTTTTCGTGTAA
CAATCTATTTTTCGTGTAA
CAATCTATTTTTCGTGTAA
Nr of selected repeats 14 Similarity 0.974552
Consensus:
CAATCTATTTTTCGTGtAA
>Cele-UNSB01_3:7953449-7953511 Satlength=63 Nr of Repeats=4 RepeatLength=15
seed=CGAAAAGGGG
CGAAAAGGGGAGCAGAA
CGAAAAGGGGATCTG
CAAAAAGGGGATCTG
CGAAAAGGGGAGATA
Nr of selected repeats 3 Similarity 0.762963
Consensus:
CgAAAAGGGGAtcTg
>Cele-UNSB01_3:7953695-7953787 Satlength=93 Nr of Repeats=6 RepeatLength=15
seed=TCCCCTTTTC
TCCCCTTTTCGTTGC
TCCCCTTTTCGTATC
TCCCCTTTTCGTATC
TCCCCTTTTCGCAGA
TCCCCTTTTTGCAGA
TCCCCTTTTCGTTCTGC
Nr of selected repeats 5 Similarity 0.768889
Consensus:
TCCCCTTTTcGtagc
>Cele-UNSB01_3:8499608-8499713 Satlength=106 Nr of Repeats=5 RepeatLength=21
seed=AAGTTTTACT

AAGTTTTACTTTGATTTTTTT
AAGTTTTACTTTGATTTTTTT
AAGTTTTACTTTGATTTTTTT
AAGTTTTACTTTGATTTTTTT
AAGTTTTACTTTGATTTTTTA
Nr of selected repeats 5 Similarity 0.974603
Consensus:
AAGTTTTACTTTGATTTTTTt
>Cele-UNSB01_3:8579889-8580089 Satlength=201 Nr of Repeats=12 RepeatLength=16
seed=AGGCACGTAG
AGGCACGTAGGCACGT
AGGAACGTAGGCACGT
AGGCACGTAGGCACAC
AGGCACGTAGGCACGT
AGCCACGTAGCCACGT
AGGCACGTAGGCACGT
AGGCACGTAGGCACGT
AGGCACGTAGGCACGT
AGGCACGTAGGCACAC
AGGCACGTAGGCACGT
AGCCACGTAGGCACGT
AGCCACGTAGGCACGTAGCCACGT
Nr of selected repeats 11 Similarity 0.887879
Consensus:
AGGCACGTAGGCACGT
>Cele-UNSB01_3:8580701-8581151 Satlength=451 Nr of Repeats=29 RepeatLength=15
seed=ACCCATCGTG
ACCCATCGTGGCGAG
ACCCATCGTGGCGAG
ACCCATCGTGGCGAG
ACCCATCGTGGTGAG
ACCCATCGTGGTGAG
ACCCATCGTGGCGAG
ACCCACCGTGGTGAG
ATCCATCGTGGCGAG
ACCCATCGTGGCGAG
ACCCATCGTGGCGAG
ACCCATCGTGGCGAG
ACCCATCGTGGTGAG
ACCCATCGTGGTGAG
ACCCATCGTGGCGAG
ACCCACCGTGGTGAG
ACCCATCGTGGTGAG
ACCCATCGTGGTGAG
ACCCATCGTGGTGAG
ACCCATCGTGGCGAG
ACCCATCGTGGTGAG
ACCCATCGTGGTGAG
ACCCATCGTGGCGAG
ACCCACCGTGGTGAG
ACCCATCGTGGCGAG
ACCCATCGTGGCGAG
ACCCACCGTGGTGAA
ACCCACCGTGGGGAG

ACCCGTCGTGGTGAGACGCACCGTGGTAAG
ACCCATCGTGGTAAG
Nr of selected repeats 28 Similarity 0.904762
Consensus:
ACCCATCGTGGtGAG
>Cele-UNSB01_3:8581179-8581353 Satlength=175 Nr of Repeats=5 RepeatLength=35
seed=GGGAAATTTA
GGGAAATTTAAATTTTCTGTGAAAAATATTTTGGA
GGGAAATTTAAATTTTCTGTGAAAAATATTTTGGC
GGGAAATTTAAATTTTCTGTGAAAAATATTTTGGA
GGGAAATTTAAATTTTCTGTGAAAAATATTTTGGC
GGGAAATTTAAATTTTCTGAAAAAATATTTTGGC
Nr of selected repeats 4 Similarity 0.974603
Consensus:
GGGAAATTTAAATTTTCTGTGAAAAATATTTTGGa
>Cele-UNSB01_3:8581474-8581754 Satlength=281 Nr of Repeats=7 RepeatLength=40
seed=TCTGGAATGT
TCTGGAATGTTCCAGAACTTTCTAGAAAAAATTGGGAAAAG
TCTGGAATGTTCCAGAACTTTCTAGAAAAAATTGGGAAAAG
TCTGGAATGTTCCAGAACTTTCTAGAAAAAATTGAGAAAAG
TCTGGAATGTTCCAGAACTTTCTAGAAAAAATTGGGAAAAT
TCTGGAATGTTCCAAAACTTTCTAGAAAAAATTGGGAAAAG
TCTGGAATGTTCCAGAACTTTCTAGAAAAAATTGGGAAAAG
TCTGGAATGTTCCAGAACTTTCTAGAAAAAATTGGGAAAAG
Nr of selected repeats 7 Similarity 0.971429
Consensus:
TCTGGAATGTTCCAGAACTTTCTAGAAAAAATTGGGAAAAG
>Cele-UNSB01_3:8589669-8589949 Satlength=281 Nr of Repeats=7 RepeatLength=40
seed=TCTGGAATGT
TCTGGAATGTTCCAGAACTTTCTAGAAAAAATTGGGAAAAG
TCTGGAATGTTCCAGAACTTTCTAGAAAAAATTGGGAAAAG
TCTGGAATGTTCCAGAACTTTCTAGAAAAAATTGAGAAAAG
TCTGGAATGTTCCAGAACTTTCTAGAAAAAATTGGGAAAAT
TCTGGAATGTTCCAAAACTTTCTAGAAAAAATTGGGAAAAG
TCTGGAATGTTCCAGAACTTTCTAGAAAAAATTGGGAAAAG
TCTGGAATGTTCCAGAACTTTCTAGAAAAAATTGGGAAAAG
Nr of selected repeats 7 Similarity 0.971429
Consensus:
TCTGGAATGTTCCAGAACTTTCTAGAAAAAATTGGGAAAAG
>Cele-UNSB01_3:8597864-8598144 Satlength=281 Nr of Repeats=7 RepeatLength=40
seed=TCTGGAATGT
TCTGGAATGTTCCAGAACTTTCTAGAAAAAATTGGGAAAAG
TCTGGAATGTTCCAGAACTTTCTAGAAAAAATTGGGAAAAG
TCTGGAATGTTCCAGAACTTTCTAGAAAAAATTGAGAAAAG
TCTGGAATGTTCCAGAACTTTCTAGAAAAAATTGGGAAAAT
TCTGGAATGTTCCAAAACTTTCTAGAAAAAATTGGGAAAAG
TCTGGAATGTTCCAGAACTTTCTAGAAAAAATTGGGAAAAG
TCTGGAATGTTCCAGAACTTTCTAGAAAAAATTGGGAAAAG
Nr of selected repeats 7 Similarity 0.971429
Consensus:
TCTGGAATGTTCCAGAACTTTCTAGAAAAAATTGGGAAAAG
>Cele-UNSB01_3:8606059-8606339 Satlength=281 Nr of Repeats=7 RepeatLength=40
seed=TCTGGAATGT
TCTGGAATGTTCCAGAACTTTCTAGAAAAAATTGGGAAAAG

TCTGGAATGTTCCAGAACTTTCTAGAAAAATTGGGAAAAG
TCTGGAATGTTCCAGAACTTTCTAGAAAAATTGAGAAAAG
TCTGGAATGTTCCAGAACTTTCTAGAAAAATTGGGAAAAT
TCTGGAATGTTCCAAAACTTTCTAGAAAAATTGGGAAAAG
TCTGGAATGTTCCAGAACTTTCTAGAAAAATTGGGAAAAG
TCTGGAATGTTCCAGAACTTTCTAGAAAAATTGGGAAAAG
Nr of selected repeats 7 Similarity 0.971429
Consensus:
TCTGGAATGTTCCAGAACTTTCTAGAAAAATTGGGAAAAG
>Cele-UNSB01_3:9173467-9173588 Satlength=122 Nr of Repeats=11 RepeatLength=11
seed=ATTTGGAATA
ATTTGGAATAT
ATTTGGAATAT
ATTTGAAATAT
ATTTGGAATAC
ATTTGGAATAG
ATTTGGAATAT
ATTTGGAATAG
ATTTGGAATAT
ATTTGGAATAT
ATTTGGAATAT
ATTTGAAATAT
Nr of selected repeats 11 Similarity 0.903030
Consensus:
ATTTGGAATAt
>Cele-UNSB01_3:9234667-9236218 Satlength=1552 Nr of Repeats=21 RepeatLength=59
seed=TTCAAAACTT
TTCAAAACTTCAAAACTCGCAAAAAAGAACCGAAGAGATCGTTGCGAGACCAACCGAAAGAGTATACATGTGCCTTTAA
ACCGAAGTTTCCCGGTGTAACGATGATAGTATCTTTTGAAACATTATTTTCAGATATAACACACATTTTTCTACATTTC
TCTGTATTCAATGTATTAATTCATTTCTATGTATTAATTCAAAGAGTTCGCCAAAAACAATAATTCCTTGTTATTAGTT
TTATTCAATGCTACCGTACACCTAGAATCATTTTGAACGTCGAATTATAGAAATTGGTAAGATCCTCAAAAATACTGCC
ACATCTATCTTCTCGTTTCAAAAGTGCACAATCCTCAGCTACACTGAATATGGTT
TTCAAAACTTTTTAAACTTAGATTTATGGAGAATATGGAATTTCAAATGTGCACTGGTT
TTCAAAACTTTTTAAACTGAGATTTATGGAGAATATGGAATTTCAAATGTGTACTGGTT
TTCAAAACTTTTAAAACTGAGATTTATGGAGAATATGGAATTTCAAATGTGCACTGGTT
TTCAAAACTTTTAAAACTGAGATTTATGGAGAATATGGAATTTCAAATGTGCACTGGTT
TTCAAAACTTTTTAAACTGAGATTTATGGAGAATATGGAATTTCAAATGTGCACTGGTT
TTCAAAACTTTTTAAACTGAGATTTATGGAGAATATGGAATTTCAAATGTGCACTGGTT
TTCAAAACTTTTTAAACTGAGATTTATGGAGAATATGGATTTTCAAATGTGCACTGGTT
TTCAAAACTTTTTAAACTGAGATTTATGGAGAATATGGAATTTCAAATGTGTACTGGTT
TTCAAAACTTTTTAAACTGAGATTTATGGAGAATATGGAATTTCAAATGTGCACTGGTT
TTCAAAACTTTTAAAACTGAGATTTATGGAGAATATGGAATTTCAAATGTGCACTGGTT
TTCAAAACTTTTAAAACTGAGATTTATGGAGAATATGGAATTTCAAATGTGCACTGGTT
TTCAAAACTTTTAAAACTGAGATTTATGGAGAATATGGAATTTCAAATGTGTACTGGTT
TTCAAAACTTTTTAAACTGAGATTTATGGAGAATATGGAATTTCAAATGTGCACTGGTT
TTCAAAACTTTTAAAACTGAGATTTATGGAGAATATGGAATTTCAAATGTGCACTGGTT
TTCAAAACTTTTAAAACTGAGATTTATGGAGAATATGGAATTTCAAATGTGCACTGGTT
TTCAAAACTTTTAAAACTGAGATTTATGGAGAATATGGAATTTCAAATGTGCACTGGTT
TTCAAAACTTTTTAAACTGAGATTTATGGAGAATATGGAATTTCAAATGTGCACTGGTT
TTCAAAACTTTTTAAACTGAGATTTATGGAGAATATGGATTTTCAAATGTGCACTGGTT
TTCAAAACTTTTTAAACTGAGATTTATGGAGAATATGGATTTTCAAATGTGCACTGGTT
TTCAAAACTTTTTAAACTGAGATTTATGGAGAATATGGATTTTCAAATGTGCACTGGTT
Nr of selected repeats 20 Similarity 0.972643
Consensus:

TTCAAAACTTTTtAAACTGAGATTTATGGAGAATATGGAaTTTCAAATGTGCACTGGTT
>Cele-UNSB01_3:9809905-9810297 Satlength=393 Nr of Repeats=13 RepeatLength=11
seed=GTACTGTAGG
GTACTGTAGGG
GTACTGTAGGGT
TACTGTAGGATTACTGTAGTTTTGGAAAAATTGACTTTTTGTCTTTTGAAGTGATATTGGTTTGAGGTTAGTGGTGGGA
TATGGTCGGGATACTGTAGTA
GTACTGTAGGG
GTACTGTAGGGT
TACTGTAGGATTACTGTAGTTTTGGAAAAATTGACTTTTTGTCTTTTGAAGTGATATTGGTTTGAGGTTAGTGGTGGGA
TATGGTCGGG
GTACTGTAGCA
GTACTGTAGTA
GTACTGTAGGA
GTACTGTAGGATTTCTGTATTTTTGAAAAAAATTGGCTTTTCGTCTTTTGAAGTGATATTGCTTTTGGGTTAGTGTCGG
GATATGGTTGGG
GTACTGTAGTT
GTACTGTAGAG
GTATTGTAGGA
Nr of selected repeats 8 Similarity 0.796537
Consensus:
GTACTGTAGGga
>Cele-UNSB01_3:9810383-9810703 Satlength=321 Nr of Repeats=8 RepeatLength=40
seed=TCCAGAACCT
TCCAGAACCTTCTGGAAAATTCGAGAAAATTCTGGAATGT
TCCAGAACCTTCTGGAAAATCCGAGAAAATTCTGGAATGT
TCCAGAACCTTCTGGAAAATTTGAGAAAATGCTGGAATGT
TCCAGAACCTTCTGGAAAATTTGAGAAAATTCTGGAATGT
TCCAGAACCTTCTGGAAAATCCGAGAAAATGCTGGAATGT
TCCAGAACCTTCTGGAAAATTTGAGAAAATTCTGGAATGT
TCCAGAACCTTCTGGAAAATCCGAGAAAATTCTGGAATGT
TCCAGAACCTTCTGGAAAATTTGAGAAAATGCTGGAATGT
Nr of selected repeats 8 Similarity 0.945238
Consensus:
TCCAGAACCTTCTGGAAAATtcGAGAAAATtCTGGAATGT
>Cele-UNSB01_3:9810418-9811039 Satlength=622 Nr of Repeats=9 RepeatLength=40
seed=AATGTTCCAG
AATGTTCCAGAACCTTCTGGAAAATCCGAGAAAATTCTGG
AATGTTCCAGAACCTTCTGGAAAATTTGAGAAAATGCTGG
AATGTTCCAGAACCTTCTGGAAAATTTGAGAAAATTCTGG
AATGTTCCAGAACCTTCTGGAAAATCCGAGAAAATGCTGG
AATGTTCCAGAACCTTCTGGAAAATTTGAGAAAATTCTGG
AATGTTCCAGAACCTTCTGGAAAATCCGAGAAAATTCTGG
AATGTTCCAGAACCTTCTGGAAAATTTGAGAAAATGCTGG
AATGTTCCAGAACCTTCTGGAAAATTCGAGAAAATTCGTGGTGAGACCCTTCGTGGTGAGACCCATCTTGGTGAGACCC
ATCTTGGCGAGACCCATAAGTTTTGGCGGGAAATTCAAATTTTCTGATAAAAATTTTTGGCGGGTAATTCAAATTTTCT
GAGAAAAAATTTGGCGGGAATTCAAATTTTCTGTGAAAAATTTTGGCGGGAATTCAAAATTTCTTTGAAAAATTTTGGC
GGGAATTCAAAATTTCTTTGAAACATTTTGGCGGGAATTCAAAGTTTCTGAAAAAAAATTCTGG
AATGTTCCAGAAGTTTCTAGAAAATTCGAGAAAAGTCTGT
Nr of selected repeats 8 Similarity 0.903571
Consensus:
AATGTTCCAGAACCTTCTGGAAAATtcGAGAAAATtCTGG
>Cele-UNSB01_3:10130069-10130193 Satlength=125 Nr of Repeats=4 RepeatLength=31
seed=TCTGCTAAAT

TCTGCTAAATAATTAGAAGACCAATCTTGGG
TCTGCTAAATAATTAGCAGACCATACTTGAT
TCTGCTAAATAATTAGCAGACCATACTTGAT
TCTGCTAAATAATTAGTAGACCATACTTGAT
Nr of selected repeats 4 Similarity 0.878136
Consensus:
TCTGCTAAATAATTAGcAGACCAtaCTTGat
>Cele-UNSB01_3:10140093-10140163 Satlength=71 Nr of Repeats=4 RepeatLength=19
seed=TGGTTGAGGC
TGGTTGAGGCTCTCGATTT
TGGTTGAGGCTCT
TGGTTGAGGCTCTAGATTT
TGGGTGAGGCTCTAGATTT
Nr of selected repeats 3 Similarity 0.906433
Consensus:
TGGtTGAGGCTCTaGATTT
>Cele-UNSB01_3:10146078-10146173 Satlength=96 Nr of Repeats=5 RepeatLength=19
seed=ATTTTGGTTG
ATTTTGGTTGAGGCTCTAG
ATTTTGGTTGAGGTTGAGG
ATTTTGGTTGAGGCTGAGG
ATTTTGGTTGAGGCTCTAG
ATTTTGGTTGAGGCTCAGG
Nr of selected repeats 5 Similarity 0.818333
Consensus:
ATTTTGGTTGAGGcTcAGg
>Cele-UNSB01_3:10214948-10215134 Satlength=187 Nr of Repeats=6 RepeatLength=31
seed=TCTGCTAAAT
TCTGCTAAATATTTAGCATACCCACATTGGG
TCTGCTAAATATTTAGCATACCCACATTGGG
TCTGCTAAATATTTAGCATACCCACATTGGG
TCTGCTAAATATTTAGCATACCCACCTTGGG
TCTGCTAAATATTTAGCATACCCACATTGGG
TCTGCTAAATATTTAGCATACCCATCTTGGG
Nr of selected repeats 6 Similarity 0.962724
Consensus:
TCTGCTAAATATTTAGCATACCCACaTTGGG
>Cele-UNSB01_3:10216874-10217114 Satlength=241 Nr of Repeats=12 RepeatLength=20
seed=AAAAATAGAT
AAAAATAGATTGACATACCAA
AAAAATAGATTGACATACCA
AAAAATAGATTGACATACCA
AAAAATAGATTGACATACCA
AAAAATAGATTGACATACCA
AAATATAGATTGACATACCA
AAAAATAGATTGACATACC
AAAAATAGATTGACATACC
AAAAATAGATTGACATACAAA
AAAAATAGATTGACATACCA
AAAAATAGATTGGCATACCA
AAAAATAGATTGACATACAA
Nr of selected repeats 8 Similarity 0.950000
Consensus:
AAAAATAGATTGACATACCA

>Cele-UNSB01_3:10234987-10235185 Satlength=199 Nr of Repeats=4 RepeatLength=31
seed=TATGCTAAAT
TATGCTAAATAGCTAGCAGACCCAAGGTGGC
TATGCTAAATATTTAGCAGACCCAACGTTGG
TATGCTAAATATTTAGCAGACCCAATGTGGA
TATGCTAAATATTTCGCAGACCCAAGGGTGGTAGGGCTGAAAACATGATTTTTCAAAAAATGGGCCGATTTTGCGTGTT
TTGAGGGAAAATTTAGGCTTTTTGTG
Nr of selected repeats 3 Similarity 0.812500
Consensus:
TATGCTAAATAttTAGCAGACCCAAnGTGGn
>Cele-UNSB01_3:10399868-10399945 Satlength=78 Nr of Repeats=5 RepeatLength=15
seed=CGAAAAGGGG
CGAAAAGGGGAGCAGAA
CGAAAAGGGGATCTG
CAAAAAGGGGATCTG
CGAAAAGGGGAGATA
CGAAAAGGGGAGCAA
Nr of selected repeats 4 Similarity 0.748148
Consensus:
CgAAAAGGGGAgcta
>Cele-UNSB01_3:10400120-10400212 Satlength=93 Nr of Repeats=5 RepeatLength=15
seed=TCCCCTTTTC
TCCCCTTTTCGTTGC
TCCCCTTTTCGTATCTCCATTTTTCGTATC
TCCCCTTTTCGCAGA
TCCCCTTTTTGCAGA
TCCCCTTTTCGTTCTGC
Nr of selected repeats 3 Similarity 0.762963
Consensus:
TCCCCTTTTcGcaGa
>Cele-UNSB01_3:10519446-10519548 Satlength=103 Nr of Repeats=8 RepeatLength=12
seed=AAAACTAAAA
AAAACTAAAAAT
AAAACTAAAAAT
AAAACTAAAACTA
AAAATAAAACTA
AAAATAAAACT
AAAACTAAAAAT
AAAACTAAAAAT
AAAACTAAAACTAAAAAT
Nr of selected repeats 5 Similarity 0.815385
Consensus:
AAAAcTAAAAaT
>Cele-UNSB01_3:10519570-10525291 Satlength=5722 Nr of Repeats=35
RepeatLength=164 seed=ATGCCAATCA
ATGCCAATCAAAGTATAATAGCTTATACGGAAGTATTTTTTTAAAAACTGATAAAAAATATATAAAAGCTGATTTTTTC
AAAAATTCAAAAGTATGGGAAAATCATATGGAGTCATTCTTTTTTATTGCATAAAACTGTTCAGCGTAGTCAAAAATAC
CAGAAT
ATGCCAATCAAAGTATAATAGCTTATACGGAAGTATTTTTTTAAAAACTGATAAAAAATATATAAAAGCTGATTTTTTC
AAAAATTCAAAAGTATGGGAAAATCATATGGAGTCATTCTTTTTTATTGCATAAAACTGTTCAGCTAGTCAAAAATACC
AGAAT
ATGCCAATCAAAGTATAATAGCTTGTACGGAAGTATTTTTTTAAAAATTGATAAAAAATATATAAAAGCTGATTTTTTC
AAAAATTCAAAAGTATGGGAAAATCATATGGAGTCATTCTTTTTTATTGCATAAAACTGTTCAGTATAGTCAAAAATAC
CAGAAT

ATGCCAATCAAAGTATAATAGCTTGTACGGAAGTATTTTTTTAAAAATTGATAAAAAATATATAAAAGCTGATTTTTTC
AAAAATTCAAAAGTATGGGAAAATCATATGAGTCATTCTTTTTTATTGCATAAAACTGTTCAGTATAGTCAAAAATACC
AGAAT
ATGCCAATCAAAGTATAATAGCTTGTACGGAAGTATTTTTTTAAAAATTGATAAAAAATATATAAAAGCTGATTTTTTC
AAAATTCAAAAGTATGGGAAAATCATATGGAGTCATTCTTTTTTATTGCATAAAACTGTTCAGTATAGTCAAAAATACC
AGAAT
ATGCCAATCAAAGTATAATAGCTTGTACGGAAGTATTTTTTTAAAAATGATAAAAAATATATAAAAGCTGATTTTTTCA
AAAATTCAAAAGTATGGGAAAATCATATGGAGTCATTCTTTTTTATTGCATAAAACTGTTCAGTATAGTCAAAAATACC
AGAAT
ATGCCAATCAAAGTATAATAGCTTGTACGGAAGTATTTTTTTAAAAATGATAAAAAATATATAAAAGCTGATTTTTTCA
AAATTCAAAAGTATGGGAAAATCATATGGAGTCATTCTTTTTTATTGCATAAAACTGTTCAGTAGTCAAAAATACCAGA
AT
ATGCCAATCAAAGTATAATAGCTTATACGGAAGTATTTTTTTAAAAACTGATAAAAAATATATAAAAGCTGATTTTTTC
AAAAATTCAAAAGTATGGGAAAATCATATGGAGTCATTCTTTTTTATTGCATAAAACTGTTCAGTATAGTCAAAAATAC
CAGAAT
ATGCCAATCAAAGTATAATAGCTTATACGGAAGTATTTTTTTAAAAACTGATAAAAAATATATAAAAGCTGATTTTTTC
AAAAATTCAAAAGTATGGGAAAATCATATGGAGTCATTCTTTTTTATTGCATAAAACTGTTCAGCGTAGTCAAAAATAC
CAGAAT
ATGCCAATCAAAGTATAATAGCTTGTACGGAAGTATTTTTTTAAAAATTGATAAAAAATATATAAAAGCTGATTTTTTC
AAAAATTCAAAAGTATGGGAAAATCATATGGAGTCATTCTTTTTTATTGCATAAAACTGTTCAGTATAGTCAAAAATAC
CAGAAT
ATGCCAATCAAAGTATAATAGCTTGTACGGAAGTTTTTTTTTTTAAAATTGATAAAAAATATATAAAAGCTGATTTTTT
CAAAAATTCAAAAGTATGGGAAAATCATATGGAGTCATTCTTTTTTATTGCATAAAACTGTTCAGTATAGTCAAAAATA
CCAGAAT
ATGCCAATCAAAGTATAATAGCTTGTACGGAAGTATTTTTTTAAAAATTGATAAAAAATATATAAAAGCTGATTTTTTC
AAATATTCAAAAGTATGGGAAAATCATATGGAGTCATTCTTTTTTATTGCATAAAACTGTTCAGTATAGTCAAAAATAC
CAGAAT
ATGCCAATCAAAGTATAATAGCTTGTACGGAAGTATTTTTTTTAAAATTGATAAAAAATATATAAAAGCTGATTTTTTC
AAAAATTCAAAAGTATGGGAAAATCATATGGAGTCATTCTTTTTTATTGCATAAAACTGTTCAGTATAGTCAAAAATAC
CAGAAT
ATGCCAATCAAAGTATAATAGCTTGTACGGAAGTATTTTTTTTTAATTGATAAAAAATATATAAAAGCTGATTTTTTCA
AAAATTCAAAAGTATGGGAAAATCATATGAAGTCATTCTTTTTTATTGCATAAAACTGTTCAGTATAGTCAAAAATACC
AGAAT
ATGCCAATCAAAGTATAATAGCTTGTACGGAAGTTTTTTTTAAAAATTGATAAAAAATATATAAAAGCTGATTTTTTC
AAAAATTCAAAAGTATGGGAAAATCATATGAAGTCATTCTTTTTTATTGCATAAAACTGTTCAGTATAGTCAAAAATAC
CAGAAT
ATGCCAATCAAAGTATAATAGCTTGTACGGAAGTTTTTTTAAAAATTGATAAAAAATATATAAAAGCTGATTTTTTCA
AAATTCAAAAGTATGGGAAAATATATGAGTCATTCTTTTTTATTGCATAAAACTGTTCAGATAGTCAAAAATACCAGAA
T
ATGCCAATCAAAGTATAATAGCTTGTACGGAAGTTTTTTTAAAAATTGATAAAAAATATATAAAAGCTGATTTTTTCA
AAAATTCAAAAGTATGGGAAAATCATATGGAGTCATTCTTTTTTATTGCATAAAACTGTTCAGTATAGTCAAAAATACC
AGAAT
ATGCCAATCAAAGTATAATAGCTTGTACGGAAGTATTTTTTTAAAAATTGATAAAAAATATATAAAAGCTGATTTTTTC
ATATATTCAAAAGTATGGGAAAATTATATGGAGTCATTCTTTTTTATTGCATAAAACTGTTCAGCATAGTCAAAAATAC
CAGAAT
ATGCCAATCACAGTATAATAGCTTATACGGAAGTATTTTTTTAAAAATTGATAAAAAATATATAAAAGCTGATTTTTTC
AAAAATTCAAAAGTATGGGAAAATCATATGGAGTCATTCTTTTTTATTGCATAAAACTGTTCAGTATAGTCAAAAATAC
CAGAAT
ATGCCAATCAAAGTATAATAGCTTGTACGGAAGTATTTTTTTAAAAATTGATAAAAAATATATAAAAGCTGATTTTTTC
AAAAATTCAAAAGTATGGGAAAATCATATGGAGTCATTCTTTTTTATTGCATAAAACTGTTCAGTATAGTCAAAAATAC
CAGAAT
ATGCCAATCAAAGTATAATAGCTTGTACGGAAGTATTTTTTTAAAAATTGATAAAAAATATATAAAAGCTGATTTTTTC
ACATATTCAAAAGTATGGGAAAATTATATGGAGTCATTCTTTTTTATTGCATAAAACTGTTCAGCATAGTCAAAAATAC
CAGAAT

ATGCCAATCAAAGTATAATAGCTTGTACGGAAGTTTTTTTTTAAAAATTGATAAAAAATATATAAAAGCTGATTTTTTC
AAAAATTCAAAAGTATGGGAAAATCATATGGAGTCATTCTTTTTTATTGCATAAAACTGTTCAGTATAGTCAAAAATAC
CAGAAT
ATGCCAATCAAAGTATAATAGCTTGTACGGAAGTATTTTTTTAAAAATTGATAAAAAATATATAAAAGCTGATTTTTTC
AATATTCAAAAGTATGGGAAAATTATATGGAGTCATTCTTTTTTATTGCATAAAACTGTTCAGCATAGTCAAAAATACC
AGAAT
ATGCCAATCAAGTATAATAGCTTTACGGAAGTATTTTTTTAAAAATTGATAAAAAATATATAAAAGCTGATTTTTTCAA
TATTCAAAAGTATGGGAAAATTATATGGAGTCATTCTTTTTTATTGCATAAAACTGTTCAGCATAGTCAAAAATACCAG
AAT
ATGCCAATCACAGTATAATAGCTTATACGGAAGTATTTTTTTAAAAATTGATAAAAAATATATAAAAGCTGATTTTTTC
AAAAATTCAAAAGTATGGGAAAATCATATGGAGTCATTCTTTTTTATTGCATAAAACTGTTCAGTATAGTCAAAAATAC
CAGAAT
ATGCCAATCAAAGTATAATAGCTTGTACGGAAGTATTTTTTTAAAAATTGATAAAAAATATATAAAAGCTGATTTTTTC
AAAAATTCAAAAGTATGGGAAAATCATATGGAGTCATTCTTTTTTATTGCATAAAACTGTTCAGTATAGTCAAAAATAC
CAGAAT
ATGCCAATCAAAGTATAATAGCTTGTACGGAAGTATTTTTTTAAAAATTGATAAAAAATATATAAAAGCTGATTTTTTC
ATATATTCAAAAGTATGGGAAAATTATATGGAGTCATTCTTTTTTATTGCATAAAACTGTTCAGCATAGTCAAAAATAC
CAGAAT
ATGCCAATCACAGTATAATAGCTTATACGGAAGTATTTTTTTAAAAATTGATAAAAAATATATAAAAGCTGATTTTTTC
AAAAATTCAAAAGTATGGGAAAATCATATGGAGTCATTCTTTTTTATTGCATAAAACTGTTCAGTATAGTCAAAAATAC
CAGAAT
ATGCCAATCAAAGTATAATAGCTTGTACGGAAGTATTTTTTTAAAAATTGATAAAAAATATATAAAAGCTGATTTTTTC
AAAAATTCAAAAGTATGGGAAAATCATATGGAGTCATTCTTTTTTATTGCATAAAACTGTTCAGTATAGTCAAAAATAC
CAGAAT
ATGCCAATCAAAGTATAATAGCTTGTACGGAAGTATTTTTTTAAAAATTGATAAAAAATATATAAAAGCTGATTTTTTC
ACATATTCAAAAGTATGGGAAAATTATATGGAGTCATTCTTTTTTATTGCATAAAACTGTTCAGCATAGTCAAAAATAC
CAGAAT
ATGCCAATCAAAGTATAATAGCTTGTACGGAAGTTTTTTTTTAAAAATTGATAAAAAATATATAAAAGCTGATTTTTTC
AAAAATTCAAAAGTATGGGAAAATCATATGGAGTCATTCTTTTTTATTGCATAAAACTGTTCAGTATAGTCAAAAATAC
CAGAAT
ATGCCAATCAAAGTATAATAGCTTGTACGGAAGTATTTTTTTAAAAATTGATAAAAAATATATAAAAGCTGATTTTTTC
AATATTCAAAAGTATGGGAAAATTATATGGAGTCATTCTTTTTTATTGCATAAAACTGTTCAGCATAGTCAAAAATACC
AGAAT
ATGCCAATCAAAGTATAATAGCTTGTACGGAAGTTTTTTTTTTAAAATTGATAAAAAATATATAAAAGCTGATTTTTTC
AAAAATTCAAAAGTATGGGAAAATCATATGGAGTCATTCTTTTTTATTGCATAAAACTGTTCAGTATAGTCAAAAATAC
CAGAAT
ATGCCAATCAAAGTATAATAGCTTGTACGGAAGTATTTTTTTAAAAATTGATAAAAAATATATAAAAGCTGATTTTTTC
ATATATTCAAAAGTATGGGAAAATTATATGGAGTCATTCTTTTTTATTGCATAAAACTGTTCAGCATAGTCAAAAATAC
CAGAAT
ATGCCAATCACAGTATAATAGCTTATACGGAAGTATTTTTTTAAAAATTGATAAAAAATATATAAAAGCTGATTTTTTC
AAAAATTCAAAAGTGTGGGAAAATCATATGGAGTCATTCTTTTTTATTGCATAAAACTGTTCAGTATAGTCAAAAATAC
CAGAAT
Nr of selected repeats 23 Similarity 0.972621
Consensus:
ATGCCAATCAAAGTATAATAGCTTgTACGGAAGTATTTTTTTAAAAATTGATAAAAAATATATAAAAGCTGATTTTTTC
AaAaATTCAAAAGTATGGGAAAATcATATGGAGTCATTCTTTTTTATTGCATAAAACTGTTCAGtATAGTCAAAAATAC
CAGAAT
>Cele-UNSB01_3:10614332-10615157 Satlength=826 Nr of Repeats=20 RepeatLength=40
seed=AAAAGTAGGT
AAAAGTAGGTCATGACCTAGTTTTTCATGTGACCTACTTC
AAAAGTAGGTCATGACCTAGTTTTTCATGTGACCTACTTCA
AAAAGTAGGTTTTTAACGTGACCTTCTTA
AAAAGTAGGTCATGGCCTAGTTTTTAACGTGACCTCCTTA
AAAAGTAGGTCATGACCTAGTTTTTAACGTGACCTCCTTA

AAAAGTAGGTCATGACCTAGTTTTTAACGTGACCTCCTTA
AAAAGTAGGTCAGGACCTAGTTTTTAACGTGACCTCCTTA
AAAAGTAGGTCAGGACCTAGTTTTTAACGTGACCTCCTTA
AAAAGTAGGTCATGACCTAGTTTTTAACGTGACCTCCTTA
AAAAGTAGGTCATGACCTAGTTTTTAACGTGACCTCCTTA
AAAAGTAGGTCAGGACCTAGTTTTTAACTTGACCTCCTCA
AAAAGTAGGTCAGGACCTAGTTTTTAACGTGACCTCCTCA
AAAAGTAGGTCATGACCTAGTTTTTAACGTGACCTCCTTA
AAAAGTAGGTCAGGACCTAGTTTTTTTTTTTTTTTTTTTTTTTTTTTTTTTTTTTTTTTTTTAACGTGACCTCCTTA
AAAAGTAGGTCATGACCTAGTTTTTAACGTGACCTCCTTA
AAAAGTAGGTCAGACCTAGTTTTTAACGTGACCTCCTTA
AAAAGTAGGTCATGACCTAGTTTTTAACGTGACCTCCTTA
AAAAGTAGGTCATGACCTAGTTTTTAACGTGACCTCCTTA
AAAAGTAGGTCAGGACCTAGTTTTTAACGTGACCTCCTTA
AAAAGTAGGTCATGACCTAGTTTTTAACGTGACCTCCTTA
Nr of selected repeats 16 Similarity 0.951944
Consensus:
AAAAGTAGGTCAtGACCTAGTTTTTAACGTGACCTCCTTA
>Cele-UNSB01_3:10733639-10733701 Satlength=63 Nr of Repeats=4 RepeatLength=15
seed=CGAAAAGGGG
CGAAAAGGGGAGCAGAA
CGAAAAGGGGATCTG
CAAAAAGGGGATCTG
CGAAAAGGGGAGCAA
Nr of selected repeats 3 Similarity 0.762963
Consensus:
CgAAAAGGGGAtCtg
>Cele-UNSB01_3:10733861-10733953 Satlength=93 Nr of Repeats=6 RepeatLength=15
seed=TCCCCTTTTC
TCCCCTTTTCGTTGC
TCCCCTTTTCGTATC
TCCCCTTTTCGTATC
TCCCCTTTTCGCAGA
TCCCCTTTTTGCAGA
TCCCCTTTTCGTTCTGC
Nr of selected repeats 5 Similarity 0.768889
Consensus:
TCCCCTTTTcGtagc
>Cele-UNSB01_3:10778310-10778713 Satlength=404 Nr of Repeats=14 RepeatLength=16
seed=GTAGGCACGT
GTAGGCACGTGAGAAGGTAGACATGTAGGTAGGCAGGCCTAAGATAGGTAG
GTAGGCACGTAGAATGTTAATAAGCAGACAGGCATGCCGTTGGCGGGCTGGCTTGAGGTAGGTAGGCATAAGTCATGTA
GACGGTTCATAGGCAGGCAGGCTAAAGAAGAATTTTAAAAGACAATCAGGCTAAACGTAGGTACGGCAGGCAT
GTAGGCACGTAGGCACA
TAGGCACGTAGGCAC
GTAGGCAGGTAGGCAC
GTAGGCACGTAGGCAC
GTAGGCACGTAGGCAC
GTAGGCACGTAGGCAC
GTAGGCACGTAGGCAC
GTAGGCACGTAGGCAC
GTAGGCACGTAGGCAC
GTAGGCACGTAGGCAC
GTAGGCACGTAGGCAC

GTAGGCACGTAGGCACGTAGGCAC
Nr of selected repeats 9 Similarity 0.981481
Consensus:
GTAGGCACGTAGGCAC
>Cele-UNSB01_3:10787273-10787444 Satlength=172 Nr of Repeats=5 RepeatLength=34
seed=TTTCCGGCAA
TTTCCGGCAAACTGGCAAATTGCTGGAATTTAAAA
TTTCCGGCAAATCGGCGAATTGCCGGAATTGAAT
TTTCCGGCAAATTGACAAATTGTCGGAATCAAAA
TTTCCGGCAAATTGACAAATTGCCGGAATCAGAA
TTTCCGGCAAATTGACAAATTGCCGGAATCAAAA
Nr of selected repeats 4 Similarity 0.819841
Consensus:
TTTCCGGCAAATtGaCaAATTGcCGGAATcaaAA
>Cele-UNSB01_3:10934450-10934819 Satlength=370 Nr of Repeats=11 RepeatLength=29
seed=GGCCTAGAAA
GGCCTAGAAATCCTACTGGGTTACCTTTTTCTAAGTTGGT
GGCCTAGAAAACCCATTTTTAAAGTTGGT
GACCTAGAAACTTCTATTTGAAAGTAGAT
GGCATAGAAATTTCAAGTTGAAATTTGGT
GGCCTAGAAACTTCTACTTGAAAGTTGAT
GGCATAGAAATTTCAATTTGAAGTTGGTGGCCTAACAACTTCTATTTGAAAGTTGGT
GGCCTAGAAAACCCATTTTTAAAGTTGAT
GACCTAGAAACTTCTATTTGAAAGTAGAT
GGCCTATAAATTTCTACTTGAAAATTGGT
GACCTAGAAAACTCCATTTCAACAGCTGAT
GGCCTAGAAACTTCTATTCGATTCGAAAGTTGATATGGC
Nr of selected repeats 7 Similarity 0.700055
Consensus:
GgCCTAGAAAnttCtatTTgAAAgTtGaT
>Cele-UNSB01_3:10975904-10976322 Satlength=419 Nr of Repeats=11 RepeatLength=35
seed=CCGCCAAAAT
CCGCCAAAATATTTTTCACTAAAAATTTGAATTTC
CCGCCAAAATATTTTTCACTAAAAATTTGAATTTC
CCGCCAAAATATTTTTCACTAAAAATTTTAATTTC
CCGCCAAAATATTTTTCACAAAAAAATTTGAATTTT
CCGCCAAAATATTTTTCACAAAAAAATTTGAATTTC
CCGCCAAAATATTTTTCACTAAAAATTTGAATTTT
CCGCCAAAATATTTTTCACTAAAAATTTGAATTTC
CCGCCAAAATATTTTTCACTAAAATTTGAATTTCCCGCTATAATTTTTTCACAAAAAAATTTGAATTTC
CCGCCAAAATATTTTTCACAAAAAAATTTGAATTTT
CCGCCAAAATTTTTTTCACTAAAAATTTGAATTTC
CCGCCAAAATATTTTTCACTAAAAATTTTAATTTC
Nr of selected repeats 10 Similarity 0.943280
Consensus:
CCGCCAAAATATTTTTCACtAAAAATTTgAATTTc
>Cele-UNSB01_3:10980699-10982201 Satlength=1503 Nr of Repeats=43
RepeatLength=35 seed=AAATTCAAAT
AAATTCAAATTTTTTGTGAAAAATACTTTGGCGGG
AAATTCAAATTTTTAGTGAAAAATATTTTGGCGGA
AAATTCAAATTTTTAGTGAAAAATATTTTGGCGGG
AAATTCAAATTTTTTTGTGAAAAAATTATAGCGGG
AAATTCAAATTTTTTGTGAAAAATATTTTGGCGGA
AAATTCAAATTTTTAGTGAAAAATATTTTGGCGGG

AAATTCAAATTTTTTGTGAAAAATATTTTGGCGGA
AAATTCAAATTTTTAGTGAAAAATATTTTGGCGGG
AAATTCAAATTTTTAGTGAAAAATATTTTGGCGGG
AAATTCAAATTTTTAGTGAAAAATATTTTGGCGGG
AAATTTAAATTTTCTAAAAAAAAATATTTTGGCGGA
AAATTCAAATTTTTAGTGAAAAATATTTTGGCGGA
AAATTCAAATTTTTAGTGAAAAATATTTTGGCGGG
AAATTCAAATTTTTTGTGAAAAATATTTTGGCGGG
AAATTCAAATTTTTTGTGAAAAAATTATAGCGGG
AAATTCAAATTTTTTGTGAAAAATATTTTGGCGGG
AAATTCAAATTTTTAGTGAAAAATATTTTGGCGGG
AAATTCAAATTTTTAGTGAAAAATATTTTGGCGGG
AAATTCAAATTTTTAGTGAAAAATATTTTGGCGGG
AAATTCAAATTTTTAGTGAAAAATATTTTGGCGGG
AAATTCAAATTTTTAGTGAAAAATATTTTGGCGGG
AAATTCAAATTTTTAGTGAAAAATATTTTGGCGGG
AAATTCAAATTTTTAGTGAAAAATATTTTGGCGGG
AAATTTAAATTTTCTAAAAAAAAATATTTTGGCGGA
AAATTCAAATTTTTAGTGAAAAATATTTTGGCGGA
AAATTCAAATTTTTAGTGAAAAATATTTTGGCGGG
AAATTCAAATTTTTTGTGAAAAATATTTTGGCGGG
AAATTCAAATTTTTTGTGAAAAAATTATAGCGGG
AAATTCAAATTTTTTGTGAAAAATATTTTGGCGGG
AAATTCAAATTTTTAGTGAAAAATATTTTGGCGGG
AAATTCAAATTTTTAGTGAAAAATATTTTGGCGGG
AAATTCAAATTTTTAGTGAAAAATATTTTGGCGGG
AAATTCAAATTTTTAGTGAAAAATATTTTGGCGGG
AAATTCAAATTTTTAGTGAAAAATATTTTGGCGGG
AAATTCAAATTTTTAGTGAAAAATATTTTGGCGGG
AAATTCAAATTTTTTGTGAAAAATATTTTGGCGGG
AAATTCAAATTTTTTGTGAAAAAATTATAGCGGG
AAATTCAAATTTTTTGTGAAAAATATTTTGGCGGG
AAATTCAAATTTTTAGTGAAAAATATTTTGGCGGG
AAATTCAAATTTTTAGTGAAAAATATTTTGGCGGG
AAATTCAAATTTTTAGTGAAAAATATTTTGGCGGG
AAATTCAAATTTTTAGTGAAAAATATTTTGGCGGG
AAATTCAAATTTTTAGTGAAAAATATTTTGGCGGG
Nr of selected repeats 40 Similarity 0.893520
Consensus:
AAATTCAAATTTTTaGTGAAAAATATTTTGGCGGG
>Cele-UNSB01_3:11090269-11090626 Satlength=358 Nr of Repeats=8 RepeatLength=45
seed=TCTGATCCTT
TCTGATCCTTCAGCTCCGAAGGATCACATGGGTACCTTCTGATGT
TCTGATCCTTCAGATAAGAAAGATCGTAAGGGTACCTCCTGATGG
TCTGATACTTCAGCTCCAAAAGAACATGAGGCTCTGCCTGATGA
TCTGATCCTTCAGCTCTAAAAGAACATGAGGCTCTGCCTGATGA
TCTGATCCTTCCGATCCGAAGGATCACATGGGTACCTTCTGATGA
TCTGATCCTTCAGATAAGAAGGATCGTAAGGGTACCTCCTGATGG
TCTGATCCTTCAGCTCCAAAAGAACATGAGGCTCTGCCTGATGA
TCTGATCTTTCAGAGCCGAATGATCACATGGGTACCTCCTGATGT
Nr of selected repeats 5 Similarity 0.801481
Consensus:
TCTGATCcTTCaGatccGAAgGATCacAtGGGTACCTcCTGATGn

>Cele-UNSB01_3:11094399-11094498 Satlength=100 Nr of Repeats=5 RepeatLength=20
seed=GTTACACGAA
GTTACACGAAAAATAGATT
GTTACACGAAAAAATAGATT
GTTACACGAAAAAATAGATT
GTTACACGAAAAAATAGATT
GTTACACGAAAAAATAGATT
Nr of selected repeats 4 Similarity 1.000000
Consensus:
GTTACACGAAAAAATAGATT
>Cele-UNSB01_3:11109268-11109343 Satlength=76 Nr of Repeats=5 RepeatLength=15
seed=TCCCCTTTTC
TCCCCTTTTCGTATC
TCCCCTTTTCATATC
TCCCCTTTTCGCAGA
TCCCCTTTTTGCAGA
TCCCCTTTTTGCAGA
Nr of selected repeats 5 Similarity 0.751111
Consensus:
TCCCCTTTTcgcAga
>Cele-UNSB01_3:11222702-11222792 Satlength=91 Nr of Repeats=6 RepeatLength=12
seed=GGCTTAGGCT
GGCTTAGGCTTG
GGCTTAGGCTTA
GGCTTAGGTTTA
GACTTAGGCTTA
GGCTTAGGCTAA
GGCTTAGGCTTAGTCTTAGTCTAAGGCTTA
Nr of selected repeats 5 Similarity 0.822222
Consensus:
GgCTTAGGcTta
>Cele-UNSB01_3:11241850-11242636 Satlength=787 Nr of Repeats=15 RepeatLength=37
seed=TTGGCGGGAA
TTGGCGGGAAATTCAAATTTTCTGAGAAATCATT
TTGGCAGGAATTCAAATTTTAATTTTTTTAAAATATT
TTGGCGGGAATTCAAATTTTAATTTTTTGAAAATATT
TTGGCGGGAATTCAAAATTTAATTTTTCGAAAACATT
TTGGCGGGAATTCAAATTTTAATTTTTTAAAACCATT
TTGGCGGGAATTCAAATTTTAATTTTTTGAAACCATT
TTGGCGGGAATTCAAAATTTTAATTTTTTGAAAATATT
TTGGCGGGAATTCAAATTTTAATTTTTTGAAAATATT
TTGGCGGGAATTCAAATTTTAATTTTTTGAAAATATT
TTGGCGGGAATTCAAATTTTAATTTTTTGAAAATATT
TTGGCGGGAATTCAAATTTTAATTTTTTGAAAATATT
TTGGCGGGAATTCAAAATTTTAATTTTTTGAAACCATT
TTGGCGGGAATTCAAAATTTTAATTTTTTGAAAATATT
TTGGCGGGAATTCAAATTTTAATTTTTTGAAAATATT
TTGGCGGGAATTCAAATTTTAATTGAAATGTGCCATGTCAATTTAAAATGTCAAGGTACTGTAGTGGTACTGTAAGGGT
ACTGTAGGATTACTGTAGGGTTATTGTAGTTTAGTAAAAATCGAATTTTAAACATTTTAAGAGATATTGGCTTGAAAGT
TGGTGGAAGATAATGTCAAGGCACTGTAATGGTACTGTAGGAGTACTGTAGGATTATGGTAGGGTTACTGTAGTTAGGA
AAACTTTGAATTTTAAGCATTTGAAAACATT
Nr of selected repeats 10 Similarity 0.935135
Consensus:
TTGGCGGGAATTCAAATTTTAATTTTTTgAAAatATT

>Cele-UNSB01_3:11273157-11273241 Satlength=85 Nr of Repeats=5 RepeatLength=12
seed=GGCTTAGGCT
GGCTTAGGCTTA
GGCTTAAGCTCA
GGCTCAGGCTTA
GGCTTCGGCTTAAGCGTA
GGCTCAGGCTTAGGGTTAGGGTTAGGGTTA
Nr of selected repeats 3 Similarity 0.777778
Consensus:
GGCTtAgGCTtA
>Cele-UNSB01_3:11300832-11300986 Satlength=155 Nr of Repeats=5 RepeatLength=31
seed=GCAGCCGACG
GCAGCCGACGATATACGCGTTCGCTGGAGTA
GCAGCCGACGCTCTGCGAGGTTTGTAGAATG
GCGGCCGACGCTATATGAGTCTGATGGGAAA
GCAGACGACGCTTTCCGGGTTGTTTGGATT
GCAGCCGACGCCTTGTGGGTCTGATGGAGTT
Nr of selected repeats 4 Similarity 0.524411
Consensus:
GCaGCCGACGctaTacGgGTctGaTgGagta
>Cele-UNSB01_3:11302620-11306800 Satlength=4181 Nr of Repeats=209
RepeatLength=20 seed=TCTATTTTTT
TCTATTTTTTGGTATGCCAA
TCTATTTTTTGGTATGTCAA
TCTATTTTTTGGTATGTCAA
TCTATTTTTTGGTATGTCAG
TCTATTTTTTGGTATGTCAA
TCTATTTTTTGGTATGTCAG
TCTATTTTTTGGTATGTCAA
TCTATTTTTTGGTATGTCAA
TCTATTTTTTGGTATGTCAA
TCTATTTTTTGGTATGTCAG
TCTATTTTTTGGTATGCCAA
TCTATTTTTTGGTATGTCAA
TCTATTTTTTGGTATGTCAG
TCTATTTTTTGGTATGTCAA
TCTATTTTTTGGTATGTCAA
TCTATTTTTTGGTATGTCAG
TCTATTTTTTGGTATGTCAA
TCTATTTTTTGGTATGTCAA
TCTATTTTTTGGTATGTCAA
TCTATTTTTTGGTATGTCAG
TCTATTTTTTGGTATGCCAA
TCTATTTTTTGGTATGTCAA
TCTATTTTTTGGTATGTCAG
TCTATTTTTTGGTATGTCAA
TCTATTTTTTGGTATGTCAG
TCTATTTTTTGGTATGTCAA
TCTATTTTTTGGTATGTCAA
TCTATTTTTTGGTATGTCAA
TCTATTTTTTGGTATGTCAG
TCTATTTTTTGGTATGTCAA
TCTATTTTTTGGTATGTCAA
TCTATTTTTTAGTATGTCAA

TCTATTTTTTGGTATGTCAA
TCTATTTTTTGGTATGTCAA
TCTATTTTTTGGTATGTCAG
TCTATTTTTTGGTATGTCAA
TCTATTTTTTGGTATGTCAG
TCTATTTTTCGGTATGTCAA
TCTATTTTTTGGTATGTCAA
TCTATTTTTTGGTATGCCAA
TCTATTTTTTGGTATGTCAA
TCTATTTTTTGGTATGTCAA
TCTATTTTTTGGTATGCCAA
TCTATTTTTTGGTATGCCAA
TCTATTTTTTAGTATGTCAA
TCTATTTTTTGGTATGTCAA
TCTATTTTTTGGTATGCCAA
TCTATTTTTTAGTATGTCAA
TCTATTTTTTGGTATGTCAA
TCTATTTTTTGGTATGTCAA
TCTATTTTTTAGTATGTCAA
TCTATTTTTTGGTATGTCAA
TCTATTTTTTAGTATGTCAA
TCTATTTTTTTGTATGTCAA
TCTATTTTTTGGTATGTCAA
TCTATTTTTTAGTATGTCAA
TCTATTTTTTGGTATGTCAA
TCTATTTTTTGGTATGTCAA
TCTATTTTTTGGTATGTCAA
TCTATTTTTTGGTATGTCAA
TCTATTTTTTGGTATGTCAA
TCTATTTTTTAGTATGTCAA
TCTATTTTTTGGTATGTCAA
TCTATTTTTTAGTATGTCAA
TCTATTTTTTGGTATGTCAG
TCTATTTTTTGGTATGTCAA
TCTATTTTTTGGTATGTCAG
TCTATTTTTTGGTATGTCAA
TCTATTTTTTGGTATGCCAA
TCTATTTTTTAGTATGTCAA
TCTATTTTTTGGTATGTCAA
TCTATTTTTTGGTATGCCAA
TCTATTTTTTAGTATGTCAA
TCTATTTTTTGGTATGCCAA
TCTATTTTTTAGTATGTCAA
TCTATTTTTTGGTATGTCAA
TCTATTTTTTGGTATGTCAA
TCTATTTTTTAGTATGTCAA
TCTATTTTTTGGTATGTCAA
TCTATTTTTTAGTATGTCAA
TCTATTTTTTGGTATGTCAA
TCTATTTTTTGGTATGTCAA
TCTATTTTTTAGTATGTCAA
TCTATTTTTTGGTATGTCAA
TCTATTTTTTGGTATGTCAA
TCTATTTTTTGGTATGTCAA

TCTATTTTTTAGTATGTCAA
TCTATTTTTTGGTATGTCAA
TCTATTTTTTGGTATGCCAA
TCTATTTTTTAGTATGTCAA
TCTATTTTTTGGTATGTCAA
TCTATTTTTTGGTATGCCAA
TCTATTTTTTAGTATGTCAA
TCTATTTTTTGGTATGCCAA
TCTATTTTTTAGTATGTCAA
TCTATTTTTTGGTATGTCAA
TCTATTTTTTGGTATGCCAA
TCTATTTTTTAGTATGTCAA
TCTATTTTTTGGTATGTCAA
TCTATTTTTTGGTATGCCAA
TCTATTTTTTAGTATGTCAA
TCTATTTTTTGGTATGTCAA
TCTATTTTTTGGTATGTCAA
TCTATTTTTTAGTATGTCAA
TCTATTTTTTGGTATGTCAA
TCTATTTTTTAGTATGTCAA
TCTATTTTTTGGTATGTCAA
TCTATTTTTTGGTATGTCAA
TCTATTTTTTAGTATGTCAA
TCTATTTTTTGGTATGTCAA
TCTATTTTTTAGTATGTCAA
TCTATTTTTTGGTATGTCAA
TCTATTTTTTGGTATGCCAA
TCTATTTTTTAGTATGTCAA
TCTATTTTTTGGTATGTCAA
TCTATTTTTTGGTATGTCAA
TCTATTTTTTAGTATGTCAA
TCTATTTTTTGGTATGTCAA
TCTATTTTTTAGTATGTCAA
TCTATTTTTTGGTATGTCAA
TCTATTTTTTGGTATGTCAA
TCTATTTTTTAGTATGTCAA
TCTATTTTTTGGTATGTCAA
TCTATTTTTTGGTATGTCAA
TCTATTTTTTGGTATGTCAA
TCTATTTTTTAGTATGTCAA
TCTATTTTTTGGTATGTCAA
TCTATTTTTTGGTATGCCAA
TCTATTTTTTAGTATGTCAA
TCTATTTTTTGGTATGTCAA
TCTATTTTTTAGTATGTCAA
TCTATTTTTTGGTATGTCAA
TCTATTTTTTAGTATGTCAA
TCTATTTTTTGGTATGTCAA
TCTATTTTTTGGTATGTCAA
TCTATTTTTTGGTATGTCAA
TCTATTTTTTGGTATGTCAA
TCTATTTTTTGGTATGTCAA
TCTATTTTTTAGTATGTCAA
TCTATTTTTTGGTATGTCAA

TCTATTTTTTGGTATGCCAA
TCTATTTTTTAGTATGTCAA
TCTATTTTTTGGTATGTCAG
TCTATTTTTTGGTATGTCAA
TCTATTTTTTGGTATGTCAA
TCTATTTTTTGGTATGTCAA
TCTATTTTTTGGTATGTCAA
TCTATTTTTTAGTATGTCAA
TCTATTTTTTGGTATGTCAA
TCTATTTTTTAGTATGTCAA
TCTATTTTTTGGTATGTCAA
TCTATTTTTTGGTATGTCAA
TCTATTTTTTAGTATGTCAA
TCTATTTTTTGGTATGTCAA
TCTATTTTTTGGTATGTCAA
TCTATTTTTTGGTATGTCAA
TCTATTTTTTGGTATGTCAA
TCTATTTTTTGGTATGTCAA
TCTATTTTTTGGTATGTCAA
TCTATTTTTTAGTATGTCAA
TCTATTTTTTGGTATGTCAG
TCTATTTTTTGGTATGTCAA
TCTATTTTTTGGTATGTCAA
TCTATTTTTTGGTATGTCAA
TCTATTTTTTGGTATGTCAA
TCTATTTTTTAGTATGTCAA
TCTATTTTTTGGTATGTCAA
TCTATTTTTTAGTATGTCAA
TCTATTTTTTGGTATGTCAA
TCTATTTTTTGGTATGTCAA
TCTATTTTTTAGTATGTCAA
TCTATTTTTTGGTATGTCAA
TCTATTTTTTGGTATGTCAA
TCTATTTTTTGGTATGTCAA
TCTATTTTTTGGTATGTCAA
TCTATTTTTTGGTATGTCAA
TCTATTTTTTGGTATGTCAA
TCTATTTTTTAGTATGTCAA
TCTATTTTTTGGTATGTCAG
TCTATTTTTTGGTATGTCAA
TCTATTTTTTGGTATGTCAA
TCTATTTTTTGGTATGTCAA
TCTATTTTTTGGTATGTCAA
TCTATTTTTTAGTATGTCAA
TCTATTTTTTGGTATGTCAA
TCTATTTTTTGGTATGTCAA
TCTATTTTTTAGTATGTCAA
TCTATTTTTTGGTATGTCAA
TCTATTTTTTGGTATGTCAA
TCTATTTTTTGGTATGTCAA
TCTATTTTTTGGTATGTCAA
TCTATTTTTTGGTATGTCAA
TCTATTTTTTGGTATGTCAA
TCTATTTTTTAGTATGTCAA

TCTATTTTTTGGTATGTCAA
TCTATTTTTTGGTATGCCAA
TCTATTTTTTGGTATGTCAA
TCTATTTTTTGGTATGCCAA
TCTATTTTTTGGTATGTCAA
TCTATTTTTTGGTATGTCAA
TCTATTTTTTGGTATGCCAA
TCTATTTTTTGGTATGCCAA
TCTATTTTTTGGTATGTCAA
TCTATTTTTTGGTATGCCAA
TCTATTTTTTGGTATGCCAA
TCTATTTTTTGGTATGCCAA
TCTATTTTTTGGTATGTCAA
TCTATTTTTTGGTATGTCAA
TCTATTTTTTGGTATGTCAA
Nr of selected repeats 209 Similarity 0.952647
Consensus:
TCTATTTTTTgGTATGTCAA
>Cele-UNSB01_3:11327560-11327604 Satlength=45 Nr of Repeats=4 RepeatLength=11
seed=AAACTTGGGC
AAACTTGGGCA
AAACTTGGGTA
AAACTTGGGCT
AAACTTGGGCT
Nr of selected repeats 4 Similarity 0.858586
Consensus:
AAACTTGGGca
>Cele-UNSB01_3:11452503-11452739 Satlength=237 Nr of Repeats=6 RepeatLength=35
seed=AATTGCCGGA
AATTGCCGGAATTATACATTTCTGGCAAATTGGCAAATTTTCAATTCCGAGAATTTGCCA
ATTTGCCGGAACTCGAAAGTTCCTTGCAAATCGGCA
AATTGCCGGAATTAAAGATTTCCGGCAAATTGGCC
AATTGACGGAGTTCAAAATTTCTGGCAAATTGGAA
AATTGCCGGAATTGAAAATTTTCGGCAAGTTGTCA
AATTGCCGGAATTATAAATTTCCGGCAAACCAGCA
Nr of selected repeats 4 Similarity 0.720635
Consensus:
AATTGcCGGAaTTaaAaATTTccGGCAAattggca
>Cele-UNSB01_3:11461018-11461218 Satlength=201 Nr of Repeats=5 RepeatLength=40
seed=TCCAGAACCT
TCCAGAACCTTCTGGAAAATTCGAGAAAATTCTGGAATGT
TCCAGAACCTTCTGGAAAATTTGAGAAAATGCTGGAATGT
TCCAGAACCTTCTGGAAAATTTGAGAAAATGCTGGAATGT
TCCAGAACCTTCTGGAAAATTTGAGAAAATGCTGGAATGT
TCCAGAACCTTCTGGAAAATTCGAGAAAATTCTGGAATGT
Nr of selected repeats 5 Similarity 0.960000
Consensus:
TCCAGAACCTTCTGGAAAATTtGAGAAAATgCTGGAATGT
>Cele-UNSB01_3:11461401-11461721 Satlength=321 Nr of Repeats=10 RepeatLength=32
seed=TTTCTGTGAA
TTTCTGTGAAAAAATTATAACGGGAATTCAAGT
TTTCTGTGAAAAAATTATAACGGGAATTCAAAA
TTTCTGTGAAAAAATTTTGGCGGGAATTCAAAA
TTTCTGTGAAAAAATTTTGGCGGGAATTCAAAA

TTTCTGTGAAAAATTTTGGCGGGAATTCAAAA
TTTCTGTGAAAAATTTTGGCGGGAATTCAAAA
TTTCTGTGAAAAATTTTGGCGGGAATTCAAAT
TTTCTGTGAAAAATTTTGGCGGGAATACAAAT
TTTCTGTGAAAAATTATAACGGGAATTCAAGT
TTTCTGTGAAAAATTTTGGCGGGAATTCAAAA
Nr of selected repeats 10 Similarity 0.896296
Consensus:
TTTCTGTGAAAAATTtTggCGGGAATTCAAaa
>Cele-UNSB01_3:11489719-11490125 Satlength=407 Nr of Repeats=12 RepeatLength=31
seed=TCTGAGCTAC
TCTGAGCTACAGTACTCCTGTCAAGAACAAT
TCTGAGCTACAGTACTCCTGTCAAGAACAGT
TCTGAGCTACAGTACTCCTGTCAAGAACGAT
TCTGAGCTACAGTGCCCCTGCCAAGAGCGAT
TCTGAGCTACAGTACTCCTGTCAAGAACAAT
TCTGAGCTACAGTACTCCTGTCAAGAACAGT
TCTGAGCTACAGTACTCCTGTCAAGAACGAT
TCTGAGCTACAGTGCCCCTGCCAAGAACAAT
TCTGAGCTACAGTACCCCTGCCAAGAGCGAT
TCTGAGCTACAGTACTTCTGTCAAGAGCGAT
TCTGAGCTACAGTACTCCTGTCAAGAGCGATTTTGGGCTACAGTACCCCCGGTGCCAAGAGCGAT
TCTGAGCTACAGTACCTCCAGGAGCCAGAAT
Nr of selected repeats 11 Similarity 0.755258
Consensus:
TCTGAGCTACAGTACtCCTGtCAAGAaCaAT
>Cele-UNSB01_3:11508351-11508507 Satlength=157 Nr of Repeats=10 RepeatLength=12
seed=GCTTAGGCTT
GCTTAGGCTTAG
GCCTAGGCTTAGCGTTGAGCCAAG
GCTTAGGATTAGGCCTAT
GCTTAGGCTTGGGATTAG
GCCTAGGCTTAG
GCTTAGGCTTAT
GCTTCGGCTTAT
GCTTAGGATTAGGCCTATGTTTAG
GATTAGGCTTAG
GCTTATGCTTCG
Nr of selected repeats 6 Similarity 0.755556
Consensus:
GCTTAGGCTTAg
>Cele-UNSB01_3:11522131-11522299 Satlength=169 Nr of Repeats=11 RepeatLength=14
seed=GATAATTGAA
GATAATTGAAAATC
GATAATTGAAAATC
GATAATTGAAAATC
GATAATTGAAAATCGATAAATGGAAATC
GATAATTGAAAATC
GATAATTGAAAATC
GATAATTGAAAATC
GATAATTGAAAATA
GATAATTGAAAATC
GATAATTGAAAATC
GATAATTGAAAATC

Nr of selected repeats 10 Similarity 0.980952
Consensus:
GATAATTGAAAATC
>Cele-UNSB01_3:11543247-11543687 Satlength=441 Nr of Repeats=21 RepeatLength=21
seed=GGCAATTTTC
GGCAATTTTCCAGTGTAATTC
GGCAATTTTCCAGTGCAATTC
GGCAATTTTCCCGTGCAATTT
GGCAATTTTCCAGTGCAATTC
GGCAATTTTCCCGTGCAATTT
GGCAATTTTCCAGTGCAATTC
GGCAATTTTCCCGTGCAATTT
GGCAATTTTCCAGTGCAATTC
GGAAATTTTCCATTGCAATTC
GGCAATTTTCCAGTGCAATTC
GGCAATTTTCCAGTGTAATTC
GGCAATTTTCCCGTGCAATTC
GGCAATTTCCAGTGCAATTC
GGCAATTTTCCCGTGCAATTT
GGCAATTTTCCAGTGCAATTC
GGCAATTTTCCCGTGCAATTT
GGCAATTTTCCAGTGCAATTC
GGAAATTTTCCATTGCAATTC
GGCAATTTTCCAGTGCAATTC
GGCAATTTTCCAGTGTAATTC
GGCAATTTTCCCGTGCAATTC
Nr of selected repeats 20 Similarity 0.903425
Consensus:
GGCAATTTTCCaGTGCAATTc
>Cele-UNSB01_3:11612201-11612410 Satlength=210 Nr of Repeats=6 RepeatLength=35
seed=GATTTGCCGG
GATTTGCCGGAAATATTCAAATCCGGCAAATTGCA
GATTTGCCGGAAATATTAAAATCCGGCAATTTATC
GATTTGCCGGAAATATTCAAATCCGGCAAGTTGCA
GATTTGCCGAAAATATTCAAACCGGCAAACTGTA
GATTTGCCGGAAATACTAAAATCCGGCAATTTATC
GATTTGCCGGAAATATTAAAATCCGGCAATTTATC
Nr of selected repeats 5 Similarity 0.850926
Consensus:
GATTTGCCGGAAATAtTaAAATCCGGCAAtTTatC
>Cele-UNSB01_3:11614727-11615007 Satlength=281 Nr of Repeats=6 RepeatLength=35
seed=TTGCCGGATT
TTGCCGGATTGTAATATTTTCGGCAAATCGGTAAA
TTGCCGGATTTGAATATTTTCGGCAAATCTGCAGT
TTGCCGGATTGTAATATTTTCGGCAAACCGGCAAT
TTGCCGGATTTGAATATATTCGGCAAATCTGCACT
TTGCCGGATTTGAATAATTTGGGCAAATCTGCAATTTCCCAAAAATTAACATTTCCGGCAAATCTGCACT
TTGCCGGATTTGAATAATTTGGGCAAATCTGCAATTTCCCAAAAATGAACATTTCCGGCAAAACGGCACT
Nr of selected repeats 4 Similarity 0.815873
Consensus:
TTGCCGGATTggAATATtTTCGGCAAAtCgGcAat
>Cele-UNSB01_3:11631810-11631935 Satlength=126 Nr of Repeats=4 RepeatLength=11
seed=CTACAGTACT
CTACAGTACTT

CTACAGTACTC
CTACAGTACCT
CTACAGTACTACTTCAGTGCCCCGACCATATTCCCCCACTAACCTCAAACCATTAACTCTTCAAAAGACAAAAACTCAA
TTTTTTTTCTAAA
Nr of selected repeats 3 Similarity 0.838384
Consensus:
CTACAGTACtt
>Cele-UNSB01_3:11700261-11701084 Satlength=824 Nr of Repeats=59 RepeatLength=14
seed=AATTATAAAT
AATTATAAATTATA
AATTATAAATTAGA
AATTATAAATTATA
AATTATAAATTATA
AATTATAAATTATA
AATTATAAATTATA
AATTATAAATTATA
AATTATAAATTATA
AATTATAAATTATA
AATTATAAATTATA
AATTATAAATTATA
AATTATAAATTATA
AATTATAAATTATA
AATTATAAATTATA
AATTATAAATTATA
AATTATAAATTATA
AATTATAAATTATA
AATTATAAATTATA
AATTATAAATTATA
AATTATAAATTATA
AATTATAAATTATA
AATTATAAATTATA
AATTTAAATTATA
AATTATAAATTATA
AATTATAAATTATA
AATTTAAATTATA
AATTATAAATTATA
AATTATAAATTATA
AATTATAAATTATA
AATTATAAATTATA
AATTATAAATTGTA
AATTATAAATTATA
AATTATAAATTATA
AATTATAAATTGTA
AATTATAAATTATA
AATTATAAATTATA
AATTATAAATTATA
AATTATAAATTATA
AATTATAAATTATA
AATTATAAATTATA
AATTATAAATTATA
AATTATAAATTATA
AATTATAAATTGTA
AATTATAAATTATA
AATTATAAATTATA

AATTATAAATTGTA
AATTATAAATTATA
AATTATAAATTATA
AATTATAAATTATA
AATTATAAATTATA
AATTATAAATTATA
AATTTAAATTATA
AATTATAAATTATA
AATTATAAATTATA
AATTGTAAATTATA
AATTATAAATTATA
AATTATAAATTATA
AATTATAAATTATA
AATTATAAATTATA
Nr of selected repeats 56 Similarity 0.980334
Consensus:
AATTATAAATTATA
>Cele-UNSB01_3:11723112-11723392 Satlength=281 Nr of Repeats=6 RepeatLength=35
seed=TTTGCCGGAA
TTTGCCGGAAATTTAGATTTTTGGCAAATTGCCGA
TTTGCCGGAAATTTTTATTTTTGGCGATTTGCCAATTTTCCCAAAATTTTGATTTTCGGCACATTGCCGA
TTTGCCGGAAATTTTTATTTTTGTCGATTTGCCAATTTTCCCAAAATTTTGATTTTCGGCACATTGCCGA
TTTGCCGGAAATTTTTATTTCTGGCAATTTGACGA
TTTGCCGGAAATTATTATTTCTTGCAATTTGCCGA
TTTGCCGGAAATTTTGATTTTCGGTAAATGGCCGA
Nr of selected repeats 4 Similarity 0.790476
Consensus:
TTTGCCGGAAATTttgATTTctgGcAAaTtGcCGA
>Cele-UNSB01_3:11725694-11725970 Satlength=277 Nr of Repeats=12 RepeatLength=12
seed=TTAGGCTTAG
TTAGGCTTAGGCTTAGCTTCAGGCTTAAGCTTCGGCTTAAACTTCTGCGTAGTC
TTAGGCTTAGGC
TTAGGCTTAGGC
TTAGGCATAGGC
TTAGGCTTAGGCTTTGGCTTAAGCTTCTGTGTAGTCTTACAA
TTAGGCTTAAACTTAGATTTAGGCTGATGCTCAGGCTTATGCTCGGGC
TTAGGCTTAGGC
TTAGGCTTAGGC
TTAGGCTTTGGC
TTAGGTTTAGGC
TTAGGCTTAGGC
TTAGGCTTAGGCTTAGGCTCATGCTCAAATTTAGGT
Nr of selected repeats 8 Similarity 0.916667
Consensus:
TTAGGCTTAGGC
>Cele-UNSB01_3:11779688-11779808 Satlength=121 Nr of Repeats=10 RepeatLength=12
seed=CTTAGGCTTA
CTTAGGCTTAGT
CTTAGACTTATG
CTCAGGCTTAGGA
TTAGGCTTAGT
CTTAGGCTTAGG
CTTAGTCTTAGG
CTTAGGCTTAAC

CTTAGGCTTAGG
CTTAGGCTTAGA
CTTAGGCATAGG
Nr of selected repeats 8 Similarity 0.797619
Consensus:
CTTAGgCTTAgg
>Cele-UNSB01_3:11779996-11780140 Satlength=145 Nr of Repeats=7 RepeatLength=16
seed=TACAAGCCTA
TACAAGCCTACTAGCC
TACACGCCTACAAGCC
TACAAACCTACAGATCTACAAATA
TACAAGCCTATAAGCC
TACAAGCCTACAAGCA
TACAAGTCTACAAGCCTACAAGTGTATAAGCCAACAAGTC
TACAAGCCTACAAGCC
Nr of selected repeats 5 Similarity 0.866667
Consensus:
TACAaGCCTAcaAGCc
>Cele-UNSB01_3:11800186-11800737 Satlength=552 Nr of Repeats=15 RepeatLength=34
seed=AAATTTCAAT
AAATTTCAATTCCGCTAATTTGCCGATTTGCCGG
AAATTTCAATTCCGCTAATTTGCCGATTTGCTGG
AAATTTCAATTCCGCTAATTTGCCGATTTGCCGG
AAATTTCGATTCCGGCAATTTGCCGATTTGCCGG
AAATTTCAAATCCGGCAATTTGGCGATTCGCCGG
AAATCTCAATTCCGGCAATTTGCCGATTTGCTGGA
AAATTTCAATTCCGACAATTTTTATAATTTTGCCGA
AAATTTCAATTCCGGCAATTTGCCAATTTGCTGGA
AAATTTCAATTCCGCTAATTTGTCGATTTGCCGG
AAATTTCGATTCTGGCAATTTGCCGATTTGCCGG
AAATTTCAAATCCGGCAATTTGGCGATTTTCCGG
AAATTTCAATTCCGGCAATTTGCCGATTTTCCGGA
AAATTTCAATTCCAGCAATTTGCATATTTGTGCCGGAAGTTTCAAATCCGGCAACTTGCCGATTTGACGA
AAATTTCAATTCTGGCAATTTTCCGATTTGCCGG
AAATTCCAATTCCGGCAATTTGCTGACTTGCCGG
Nr of selected repeats 10 Similarity 0.841394
Consensus:
AAATTTCaAtTCcGgcAATTTGcCGATTTGCCGG
>Cele-UNSB01_3:11834269-11834616 Satlength=348 Nr of Repeats=10 RepeatLength=34
seed=TTTTCGATTT
TTTTCGATTTTTCAATTTTTGAAAAATCGAGAA
TTTTCGATTTTTCGATTTTTTGAAAAATCGGGAA
TTTTCGATTTTTCAATTTTTTGAAAAATCGGGAA
TTTTCGATTTTTCAATTTTTTGAAAAATCGGGAA
TTTTCGATTTTTCAATTTTTTGAAAAATCGAGAA
TTTTCGATTTTTCGATTTTTTGAATAATCGGGAA
TTTTCGATTTTTCAATTTTTTGAAAAATCGGGAA
TTTTCGATTTTTCAATTTTTTGAAAAATCGAGAA
TTTTCTATTTTTCGATTTTTTGAATAATCGGGAA
TTTTCGATTTTTCAATTTTTTGAAAAATCGAGAATTTTCGAT
Nr of selected repeats 8 Similarity 0.935574
Consensus:
TTTTCGATTTTTCaATTTTTTGAAaAATCGgGAA

>Cele-UNSB01_3:11851795-11852785 Satlength=991 Nr of Repeats=24 RepeatLength=35 seed=CGGCAAATCG
CGGCAAATCGGTAAATTGCCGAAACTGAATATTTC
CGGCAAATCGGTAAAC
CGGCAAATTGCCGGAATTGAAAATTTCTGGTAAATCGGCAATTTGACGAAACTGAAACTTTC
CGGCAAATCGGCAAGTTGCCAAAACTGAAAATTCC
CGGCAAATCGGCAACTTGCCGGAATTGAAATTTTC
CGGCAAATCGATATATTGCCAAAACTGAAAATTCC
CGGCAAATCGGCAACTTGCCGGAATTGAAATTTTC
CGGCAAATCGATATATTGCCAAAACTGAAAATTCC
CGGCAAATCGGCAACTTGCCGGAATTGAAATTTTC
CGGCAAATCGATATATTGCCAAAACTGAAAATTTC
CGGCAAATCGGCAAATTGTGGGAACTGAAAATTTTCTGCAATATGGCAATATGCCGAAACTGAATATTTC
CGGCAAATCGGTAAAC
CGGCAAATTGCCGGAATTGAAAATTTCTGGTAAATCGGCAATTTGACGAAACTGAAACTTTC
CGGCAAATCGGCAAGTTGCCAAAACTGAAAATTCC
CGGCAAATCGGCAACTTGCCGGAATTGAAATTTTC
CGGCAAATCGATATATTGCCAAAACTGAAAATTCC
CGGCAAATCGGCAACTTGCCGGAATTGAAATTTTC
CGGCAAATCGATATATTGCCAAAACTGAAAATTCC
CGGCAAATCGGCAACTTGCCGGAATTGAAATTTTC
CGGCAAATCGATATATTGCCAAAACTGAAAATTTC
CGGCAAATCGGCAAATTGTGGGAACTGAAAATTTTCTGCAATATGGCAATATGCCGAAACTGAAAATTTC
CGGCAAATCGGCAAATCGCCGGAATTGAAAATTTCC
CGCAAATCGACAATTCATCGATAACCATCATCGATAATCATCGATTGAAAATTTCTGGCAAAGCTGCAACTCGTCAATTTGCAGAAAATTAAAATTTC
CGGCAAATCGGTAAATTGCCGGAATTGGAAATTTC
Nr of selected repeats 16 Similarity 0.809524
Consensus:
CGGCAAATCGgcAaaTTGCCaaAAcTGAAAaTTtC
>Cele-UNSB01_3:11898352-11906615 Satlength=8264 Nr of Repeats=45 RepeatLength=184 seed=TACGCAGCCG
TACGCAGCCGAACGAATTATGAAAGAGTACAGTAGACTAATCATGATCAAGCGAAAAGATCCGACTCAGAATATGAACATATTCGAATACATCGGAGGTATATAGATTCCCGAAGACACTTTCCAATTAACCAAACGGTTCAAATTCTAGCAAACATCTCCTTACTTGAACACTCTTGGCCAATG
TACGCAGCCGAACGTATCATAAGTGAATACAGAACACCAATTATGTCCGAGAAAAAGATCCTACTCAGAATATGAACATATTCAAAAATATCGGAGGTATATAGATTCCCGAAGAGCCTTTCCAATTAACCAAACGGTTCAAATTCTAGCAAACATCTCCTTACTTGAACACTCTTGGCCAATG
TACGCAGCCGAACGTATCATAAGTGAATACAGAACACCAATTGTGTCCGAGAAAAAGATCCTACTCAGAATATGAACATATTCGAATACATCAGAGGTATATAGATTCCCGAAGAGCCTTTCCAATTAACCAAACGGTTCAAATTCTAGCAAACATCTCCTTACTTGAACACTCTTGGCCAATG
TACGCAGCCGAACGTATCATAAGTGAATACAGAACACCAATTGTGTCCGAGAAAAAGATCCTACTCAGAATATGAACATATTCAAAAATATCGGAGGTATATAGATTCCCGAAGAGCCTTTCCAATTAACCAAACGGTTCAAATTCTAGCAAACATCTCCTTACTTGAACACTCTTGGCCAATG
TACGCAGCCGAACGTATCATAAGTGAATACAGAACACCAATTATGTCCGAGAAAAAGATCCTACTCAGAATATGAACATATTCGAATACATCGGAGGTATATAGATTCCCGAAGACACTTTCCAATTAACCAAACGGTTCAAATTCTAGCAAACATCTCCTTACTTGAATACTCTTGGCCAATT
TACGCAGCCGAACGTATCATAAGTGAATACAGAACACCAATTATGTCCGAGAAAAAGATCCTACTCAGAATATGAACATATTCGAATACATCGGAGGTATATAGATTCCCGAAGAGCCTTTCCAATTAACCAAACGGTTCAAATTCTAGCAAACATCTCCTTACTTGAATACTCTTGGCCAATT
TACGCAGCCGAACGTATCATAAGTGAATACAGAACACCAATTATGTCCGAGAAAAAGATCCTACTCAGAATATGAACATATTCGAATACATCGGAGGTATATAGATTCCCGAAGAGCCTTTCCAATTAACCAAACGGTTCAAATTCTAGCAAACATCTCCTTACTTGAACACTCTTGGCCAATG

TACGCAGCCGAACGTATCATAAGTGAATACAGAACACCAATTATGTCCGAGAAAAAGATCCTACTCAGAATATGAACAT
ATTCAAAAATATCGGAGGTATATAGATTCCCGAAGAGCCTTTCCAATTAACCAAACGGTTCAAATTCTAGCAAACATCT
CCTTACTTGAACACTCTTGGCCAATG
TACGCAGCCGAACGTATCATAAGTGAATACAGAACACCAATTATGTCCGAGAAAAAGATCCTACTCAGAATATGAACAT
ATTCGAATACATCAGAGGTATATAGAGTCCCGAAGAGCCTTTCCAATTAACCAAACGGTTCAAATTCTAGCAAACATCT
CCTTACTTGAACACTCTTGGCCAATG
TACGCAGCCGAACGTATCATAAGTGAATACAGAACACCAATTATGTCCGAGAAAAAGATCCTACTCAGAATATGAACAT
ATTCGAATACATCAGAGGTATATAGAGTCCCGAAGAGCCTTTCCAATTAACCAAACGGTTCAAATTCTAGCAAACATCT
CCTTACTTGAACACTCTTGGCCAATG
TACGCAGCCGAACGTATCATAAGTGAATACAGAACACCAATTATGTCCGAGAAAAAGATCCTACTCAGAATATGAACAT
ATTCGAATACATCGGAGGTATATAGATTCCCGAAGACACTTTCCAATTAACCAAACGGTTCAAATTCTAGCAAACATCT
CCTTACTTGAACACTCTTGGCCAATG
TACGCAGCCGAACGTATCATAAGTGAATACAGAACACCAATTATGTCCGAGAAAAAGATCCTACTCAGAATATGAACAT
ATTCAAAAATATCGGAGGTATATAGATTCCCGAAGAGCCTTTCCAATTAACCAAACGGTTCAAATTCTAGCAAACATCT
CCTTACTTGAACACTCTTGGCCAATG
TACGCAGCCGAACGTATCATAAGTGAATACAGAACACCAATTGTGTCCGAGAAAAAGATCCTACTCAGAATATGAACAT
ATTCGAATACATCAGAGGTATATAGATTCCCGAAGAGCCTTTCCAATTAACCAAACGGTTCAAATTCTAGCAAACATCT
CCTTACTTGAACACTCTTGGCCAATG
TACGCAGCCGAACGTATCATAAGTGAATACAGAACACCAATTGTGTCCGAGAAAAAGATCCTACTCAGAATATGAACAT
ATTCAAAAATATCGGAGGTATATAGATTCCCGAAGAGCCTTTCCAATTAACCAAACGGTTCAAATTCTAGCAAACATCT
CCTTACTTGAACACTCTTGGCCAATG
TACGCAGCCGAACGTATCATAAGTGAATACAGAACACCAATTATGTCCGAGAAAAAGATCCTACTCAGAATATGAACAT
ATTCGAATACATCGGAGGTATATAGATTCCCGAAGACACTTTCCAATTAACCAAACGGTTCAAATTCTAGCAAACATCT
CCTTACTTGAATACTCTTGGCCAATT
TACGCAGCCGAACGTATCATAAGTGAATACAGAACACCAATTATGTCCGAGAAAAAGATCCTACTCAGAATATGAACAT
ATTCGAATACATCGGAGGTATATAGATTCCCGAAGAGCCTTTCCAATTAACCAAACGGTTCAAATTCTAGCAAACATCT
CCTTACTTGAACACTCTTGGCCAATG
TACGCAGCCGAACGTATCATAAGTGAATACAGAACACCAATTATGTCCGAGAAAAAGATCCTACTCAGAATATGAACAT
ATTCAAAAATATCGGAGGTATATAGATTCCCGAAGAGCCTTTCCAATTAACCAAACGGTTCAAATTCTAGCAAACATCT
CCTTACTTGAATACTCTTGGCCAATG
TACGCAGCCGAACGTATCATAAGTGAATACAGAACACCAATTATGTCCGAGAAAAAGATCCTACTCAGAATATGAACAT
ATTCGAATACATCGGAGGTATATAGATTCCCGAAGACACTTTCCAATTAACCAAACGGTTCAAATTCTAGCAAACATCT
CCTTACTTGAATACTCTTGGCCAATT
TACGCAGCCGAACGTATCATAAGTGAATACAGAACACCAATTATGTCCGAGAAAAAGATCCTACTCAGAATATGAACAT
ATTCGAATACATCGGAGGTATATAGATTCCCGAAGACACTTTCCAATTAACCAAACGGTTCAAATTCTAGCAAACATCT
CCTTACTTGAATACTCTTGGCCAATT
TACGCAGCCGAACGTATCATAAGTGAATACAGAACACCAATTATGTCCGAGAAAAAGATCCTACTCAGAATATGAACAT
ATTCGAATACATCGGAGGTATATAGATTCCCGAAGAGCCTTTCCAATTAACCAAACGGTTCAAATTCTAGCAAACATCT
CCTTACTTGAATACTCTTCCAATT
TACGCAGCCGAACGTATCATAAGTGAATACAGAACACCAATTATGTCCGAGAAAAAGATCCTACTCAGAATATGAACAT
ATTCGAATACATCGGAGGTATATAGATTCCCGAAGACACTTTCCAATTAACCAAACGGTTCAAATACTAGCAAACATCT
CCTTACTTGAATACTCTTGGCCAATT
TACGCAGCCGAACGTATCATAAGTGAATACAGAACACCAATTATGTCCGAGAAAAAGATCCTACTCAGAATATGAACAT
ATTCAAAAATATCGGAGGTATATAGATTCCCGAAGAGCCTTTCCAATTAACCAAACGGTTCAAATTCTAGCAAACATCT
CCTTACTTGAACACTCTTGGCCAATG
TACGCAGCCGAACGTATCATAAGTGAATACAGAACACCAATTGTGTCCGAGAAAAAGATCCTAATGAACATATTCAAAA
ATATCGGAGGTATATAGATTCCCGAAGAGCCTTTCCAATTAACCAAACGGTTCAAATTCTAGCAAACATCTCCTTACTT
GAACACTCTTGGCCAATG
TACGCAGCCGAACGTATCATAAGTGAATACAGAACACCAATTATGTCCGAGAAAAAGATCCTACTCAGAATATGAACAT
ATTCGAATACATCGGAGGTATATAGATTCCCGAAGACACTTTCCAATTAACCAAACGGTTCAAATTCTAGCAAACATCT
CCTTACTTGAATACTCTTGGCCAATG
TACGCAGCCGAACGTATCATAAGTGAATACAGAACACCAATTATGTCCGAGAAAAAGATCCTACTCAGAATATGAACAT
ATTCAAAAATATCGGAGGTATATAGATTCCCGAAGAGCCTTTCCAATTAACCAAACGGTTCAAATTCTAGCAAACATCT
CCTTACTTGAACACTCTTGGCCAATG

TACGCAGCCGAACGTATCATAAGTGAATACAGAACACCAATTATGTCCGAGAAAAAGATCCTACTCAGAATATGAACAT
ATTCGAATACATCGGAGGTATATAGATTCCCGAAGACACTTTCCAATTAACCAAACGGTTCAAATTCTAGCAAACATCT
CCTTACTTGAATACTCTTGGCCAATT
TACGCAGCCGAACGTATCATAAGTGAATACAGAACACCAATTATGTCCGAGAAAAAGATCCTACTCAGAATATGAACAT
ATTCAAAAATATCGGAGGTATATAGATTCCCGAAGAGCCTTTCCAATTAACCAAACGGTTCAAATTCTAGCAAACATCT
CCTTACTTGAACACTCTTGGCCAATG
TACGCAGCCGAACGTATCATAAGTGAATACAGAACACCAATTGTGTCCGAGAAAAAGATCCTACTCAGAATATGAACAT
ATTCGAATACATCGGAGGTATATAGATTCCCGAAGAGCCTTTCCAATTAACCAAACGGTTCAAATTCTAGCAAACATCT
CCTTACTTGAACACTCTTGGCCAATG
TACGCAGCCGAACGTATCATAAGTGAATACAGAACACCAATTATGTCCGAGAAAAAGATCCTACTCAGAATATGAACAT
ATTCGAATACATCGGAGGTATATAGATTCCCGAAGACACTTTCCAATTAACCAAACGGTTCAAATTCTAGCAAACATCT
CCTTACTTGAATACTCTTGGCCAATT
TACGCAGCCGAACGTATCATAAGTGAATACAGAACACCAATTATGTCCGAGAAAAAGATCCTACTCAGAATATGAACAT
ATTCAAAAATATCGGAGGTATATAGATTCCCGAAGAGCCTTTCCAATTAACCAAACGGTTCAAATTCTAGCAAACATCT
CCTTACTTGAACACTCTTGGCCAATG
TACGCAGCCGAACGTATCATAAGTGAATACAGAACACCAATTGTGTCCGAGAAAAAGATCCTAATGAACATATTCAAAA
ATATCGGAGGTATATAGATTCCCGAAGAGCCTTTCCAATTAACCAAACGGTTCAAATTCTAGCAAACATCTCCTTACTT
GAACACTCTTGGCCAATG
TACGCAGCCGAACGTATCATAAGTGAATACAGAACACCAATTATGTCCGAGAAAAAGATCCTACTCAGAATATGAACAT
ATTCGAATACATCGGAGGTATATAGATTCCCGAAGACACTTTCCAATTAACCAAACGGTTCAAATTCTAGCAAACATCT
CCTTACTTGAATACTCTTGGCCAATT
TACGCAGCCGAACGTATCATAAGTGAATACAGAACACCAATTATGTCCGAGAAAAAGATCCTACTCAGAATATGAACAT
ATTCAAAAATATCGGAGGTATATAGATTCCCGAAGACACTTTCCAATTAACCAAACGGTTCAAATTCTAGCAAACATCT
CCTTACTTGAATACTCTTGGCCAATT
TACGCAGCCGAACGTATCATAAGTGAATACAGAACACCAATTATGTCCGAGAAAAAGATCCTACTCAGAATATGAACAT
ATTCAAAAATATCGGAGGTATATAGATTCCCGAAGACACTTTCCAATTAACCAAACGGTTCAAATTCTAGCAAACATCT
CCTTACTTGAATACTCTTGGCCAATT
TACGCAGCCGAACGTATCATAAGTGAATACAGAACACCAATTATGTCCGAGAAAAAGATCCTACTCAGAATATGAACAT
ATTCGAATACATCGGAGGTATATAGATTCCCGAAGAGCCTTTCCAATTAACCAAACGGTTCAAATTCTAGCAAACATCT
CCTTACTTGAATACTCTTGGCCAATT
TACGCAGCCGAACGTATCATAAGTGAATACAGAACACCAATTATGTCCGAGAAAAAGATCCTACTCAGAATATGAACAT
ATTCGAATACATCGGAGGTATATAGATTCCCGAAGACACTTTCCAATTAACCAAACGGTTCAAATTCTAGCAAACATCT
CCTTACTTGAACACTCTTGGCCAATG
TACGCAGCCGAACGTATCATAAGTGAATACAGAACACCAATTGTGTCCGAGAAAAAGATCCTACTCAGAATATGAACAT
ATTCGAATACATCGGAGGTATATAGATTCCCGAAGAGCCTTTCCAATTAACCAAACGGTTCAAATTCTAGCAAACATCT
CCTTACTTGAACACTCTTGGCCAATG
TACGCAGCCGAACGTATCATAAGTGAATACAGAACACCAATTGTGTCCGAGAAAAAGATCCTACTCAGAATATGAACAT
ATTCAAAAATATCGGAGGTATATAGATTCCCGAAGAGCCTTTCCAATTAACCAAACGGTTCAAATTCTAGCAAACATCT
CCTTACTTGAATACTCTTGGCCAATT
TACGCAGCCGAACGTATCATAAGTGAATACAGAACACCAATTATGTCCGAGAAAAAGATCCTACTCAGAATATGAACAT
ATTCGAATACATCGGAGGTATATAGATTCCCGAAGAGCCTTTCCAATTAACCAAACGGTTCAAATTCTAGCAAACATCT
CCTTACTTGAACACTCTTGGCCAATG
TACGCAGCCGAACGTATCATAAGTGAATACAGAACACCAATTGTGTCCGAGAAAAAGATCCTACTCAGAATATGAACAT
ATTCGAATACATCGGAGGTATATAGATTCCCGAAGAGCCTTTCCAATTAACCAAACGGTTCAAATTCTAGCAAACATCT
CCTTACTTGAACACTCTTGGCCAATG
TACGCAGCCGAACGTATCATAAGTGAATACAGAACACCAATTGTGTCCGAGAAAAAGATCCTACTCAGAATATGAACAT
ATTCGAATACATCGGAGGTATATAGATTCCCGAAGAGCCTTTCCAATTAACCAAACGGTTCAAATTCTAGCAAACATCT
CCTTACTTGAACACTCTTGGCCAATG
TACGCAGCCGAACGTATCATAAGTGAATACAGAACACCAATTGTGTCCGAGAAAAAGATCCTACTCAGAATATGAACAT
ATTCAAAAATATCGGAGGTATATAGATTCCCGAAGAGCCTTTCCAATTAACCAAACGGTTCAAATTCTAGCAAACATCT
CCTTACTTGAATACTCTTGGCCAATT
TACGCAGCCGAACGTATCATAAGTGAATACAGAACACCAATTATGTCCGAGAAGAAGATCCTACTCAGAATATGAACAT
ATTCGAATACATCGGAGGTATATAGATTCCCGAAGAGCCTTTCCAATTAACCAAACGGTTCAAATTCTAGCAAACATCT
CCTTACTTGAACACTCTTGGCCAATG

TACGCAGCCGAACGTATCATAAGTGAATACAGAACACCAATTGTGTCCGAGAAAAAGATCCTACTCAGAATATGAACAT
ATTCGAATACATCGGAGGTATATAGATTCCCGAAGAGCCTTTCCAATTAACCAAACGGTTCAAATTCTAGCAAACATCT
CCTTACTTGAACACTCTTGGCCAATG
TACGCAGCCGAACGTATCATAAGTGAATACAGAACACCAATTGTGTCCGAGAAAAAGATCCTACTCAGAATATGAACAT
ATTCGAATACATCGGAGGTATATAGATTCCCGAAGAGCCTTTCCAATTAACCAAACGGTTCAAATTCTAGCAAACATCT
CCTTACTTGAACACTCTTGGCCAATG
Nr of selected repeats 41 Similarity 0.970891
Consensus:
TACGCAGCCGAACGTATCATAAGTGAATACAGAACACCAATTaTGTCCGAGAAAAAGATCCTACTCAGAATATGAACAT
ATTCgAAtAcATCGGAGGTATATAGATTCCCGAAGAgcCTTTCCAATTAACCAAACGGTTCAAATTCTAGCAAACATCT
CCTTACTTGAAcACTCTTGGCCAATg
>Cele-UNSB01_3:11990778-11991299 Satlength=522 Nr of Repeats=14 RepeatLength=35
seed=CCGATTTGCC
CCGATTTGCCGGAAATTTTTAATTCCGACAATTTT
CCGATTTGCCGGAAATTTTCAAATCCGGCAATTTT
CCGATTTGCCAGAAATTTTCAATTCCGGCAATTTG
CCGATTTGCCGGAAATTTTCAAATCCGGCAAATTGG
CGATTTGCCGGAAATTTTCAATTCCGGCAATTTG
CCGATTTGCCGGAAATTTTCGATTCCGGCAATTTG
CCGATTTGCCGGAAATTTTCAATTCCGGCAATTTGCCTTTGCCGAAAATTTTCGATTCCGGCAATTTG
CCGATTTGCCGAAATCTTTCAATTCCGAAAAATTG
CCGATTTGCCGGAAATTTTCAATTCCGGCAATTTG
CCGATTTGCCAGAAATTTTCAATTCCATCAATTTG
CCGATTTGCCGAAATCTTTCAATTCCGAAAAATTG
CCGATTTGCCAGAAATTTTCAATTCCGGCAATTTA
CCGATTTGCCTAAATCTTTATTCCGGCAATTTG
CCGATTTGTCGAAATCTTTCAATTCCGAAAAATTG
Nr of selected repeats 10 Similarity 0.809053
Consensus:
CCGATTTGCCnGAAATTTTCAATTCCGgcAAtTTg
>Cele-UNSB01_3:12003957-12004292 Satlength=336 Nr of Repeats=7 RepeatLength=11
seed=GTACTGTAGA
GTACTGTAGAG
GTACTGTAGAG
GTACTGTAGAA
GTACTGTAGAC
GTACTGTGGAA
GTGCTGTAGAAGTGCGTTAGGATTACTGTAGTTTAAAAAAAGTTGTTGTGTTTTGAAGGGAGATTGAGTTAGGGGTAGT
GTAGGAGTATGGTTGTGGAACTTTAGTA
GTACTGTAGGTGTATTGTATTGGAATTGTAGGGGTACGTTAGGGTTACTGTGTTTCTGTAGGAACACTCCTGGATTACT
CCAGTAGTGCTGTAGGGGTACCGTATTGGTACTGTATGGGTACTATATAGGAACTATAGGGGTACTGTATTGGTACCGT
AGGAGGTACTATAGG
Nr of selected repeats 5 Similarity 0.854545
Consensus:
GTACTGTaGAn
>Cele-UNSB01_3:12010851-12011440 Satlength=590 Nr of Repeats=30 RepeatLength=12
seed=GGCTTAGGCT
GGCTTAGGCTTA
GGCTTAGGTATA
GGCTTAGGCTTAGTCTTAGGCCTA
GGTTTAGGCTTA
GGCTTTGGCTTA
GGCTAAGGCTTA
GGCTCAGGCTTAGGTCTA

GGTTTAGGCTTA
GGCTCAGGCTGAATCTTA
GGCTTAGGTATA
GGCTTAGGCTTAGTCTTAGGCCTA
GGTTTAGGCTTA
GGCTTTGGCTTA
GGCTAAGGCTTA
GGCTCAGGCTTAGGTCTA
GGTTTAGGCTTA
GGCTCAGGCTGAATCTTA
GGCTTAGGTATA
GGCTTAGGCTTAGTCTTAGGCCTA
GGTTTAGGCTTA
GGCTTTGGCTTA
GGCTAAGGCTTA
GGCTCAGGCTTAGGCCTA
GGTTTAGGCTTA
GGCTCAGGCTGA
GGCTTAGGCTCA
GGCTGAGGCTTA
GGCTCAGGCTGA
GGCTTAGGCTTTAAAAAAATTGTTTCCAAAAACTTATTTTTAGTTGGCATACTGAAAAAAAAATTGAATTTTTTTGTAGT
TTTTTTGTCTTTTTTTTTTTTGGTTTTTTTGGAGAAATTAGAATTTTTGCTTTTCGTTTTTTGGTCAAAATTTTTTGCAA
CAGCCCTGTTA
GGCTTGGGCTTAGGCTTA
Nr of selected repeats 20 Similarity 0.774854
Consensus:
GGcTtAGGCTTA
>Cele-UNSB01_3:12025839-12025976 Satlength=138 Nr of Repeats=8 RepeatLength=12
seed=TAAGCCTAAG
TAAGCCTAAGACTAAGCCTATACG
TAAGCCTAAACC
TAAGCCTAAGCC
TAAGCCTGAGCC
TAAGCCTAAGCATAACCATAACCCGAAGCA
TAAGCCTAAGCC
TAAGCCTAGGCA
TAAGCCTAATCCTACCCGAAGCG
Nr of selected repeats 5 Similarity 0.822222
Consensus:
TAAGCCTaagCc
>Cele-UNSB01_3:12040777-12040867 Satlength=91 Nr of Repeats=6 RepeatLength=12
seed=CCTAAGCCTA
CCTAAGCCTAAGCCTGAGACTAAGCTCTAC
CCTAACCCTAAC
CCTAAGCCTAAG
CCTACGCCTAAG
CCTAGGCCTAAG
CCTAAGCCTAAG
Nr of selected repeats 5 Similarity 0.833333
Consensus:
CCTAagCCTAAg
>Cele-UNSB01_3:12087067-12087157 Satlength=91 Nr of Repeats=4 RepeatLength=12
seed=AGCCTGAGCC

AGCCTGAGCCCG
AGCCTAAGCCTGAGCCTAGGTCTAAGCCTAAACCTAAACCTAAGACTAACCCTA
AGCCTGAGCCTC
AGCCTGAGCCTG
Nr of selected repeats 3 Similarity 0.851852
Consensus:
AGCCTGAGCCtg
>Cele-UNSB01_3:12088649-12088899 Satlength=251 Nr of Repeats=9 RepeatLength=12
seed=TAAGCCTAAG
TAAGCCTAAGAC
TAAGCCTAAGCC
TAAGCCTAAGCC
TAAGCCTAAGCC
TAAGCCTAAGCC
TAAGCCCAAGCCCAAGCCCAAGCCTAGGGCTAATCAGTGATGGGCAGCGAACATGCTTTCTTGGCGAACGACGAATTCG
GCAAATTTGTCTATGCAGTTTTTAAGTTTTCCAGGTTTTTCTGAAGGTTTTTCCTGTATAATGGCG
TAAGCCTAAGCC
TAAGTCTAAGCC
TAAGCCTGAGCCTAATCC
Nr of selected repeats 7 Similarity 0.936508
Consensus:
TAAGCCTAAGCC
>Cele-UNSB01_3:12102276-12102493 Satlength=218 Nr of Repeats=6 RepeatLength=34
seed=TTGCCGAATT
TTGCCGAATTTTCCGTTTGCCGAGCTCGTCAAAC
TTGCCGAATTTGCTATTTGCCGAGCTTGTCAAAT
TTGCCGAATTTGCTATTTGCCGAGCTCGTCAAAC
TTGCCGAATTTGCCGTTTGCCGAGCTTGTCAAAT
TTGCCGAACTTGCCGTTCAATTTTGCCGTTTGTCGAGTTCGTCAAAA
TTACCGAATTTGCCGTTTGCCTAGCACGGCAGAT
Nr of selected repeats 5 Similarity 0.811765
Consensus:
TTgCCGAATTTgCcgTTTGCCgAGCtcGtCAaAt
>Cele-UNSB01_3:12120993-12121420 Satlength=428 Nr of Repeats=12 RepeatLength=35
seed=AATTGCCGGA
AATTGCCGGAAACAAAAACTTCCGGCAAATCGGCA
AATTGCTGGAATTGAAATTTTCCGGAAAATCGGCA
AATTGCCGGAATTGAAAATTTCTGGCAAATCGGCA
AATTGCTGGAATTGAAATTTTCCGGAAAATCGGCA
AATTGCCGGAATTGAGAATTTCCGGCAAATCGTCA
AATTGCTGGAATTGAAATTTTCCGGAATATCGGCA
AATTGCCGGAATTGAAAATTTCCGGCAAATCGGCA
AATTGCCGGAATTGAAAATTTTCAGCACCTCGGCA
AATTGCCGGAATTGAAAATTTTCGCAAATCAACA
AATTGCCGGAATTGAAAATTTCCGGCAAATCGGTA
AATTGCCGGAATTGGAAATTTCCGACAAATCGACAAACCGGCG
AATTGCCGGAATTGAAAATTTCCGGCAAATCGGCG
Nr of selected repeats 10 Similarity 0.840847
Consensus:
AATTGCcGGAATTGAAAaTTTCCGGcAaATCGGCA
>Cele-UNSB01_3:12166501-12166666 Satlength=166 Nr of Repeats=15 RepeatLength=11
seed=ACATTTTTTG
ACATTTTTTGC
ACATTTTTTGA

ACATTTTTTGT
ACATTTTTTGC
ACATTTTTTGA
ACATTTTTTGC
ACATTTTTTGA
ACATTTTTTGA
ACATTTTTTGT
ACATTTTTTGC
ACATTTTTTGA
ACATTTTTTGC
ACATTTTTTGC
ACATTTTTTGCG
CATTTTTTGC
Nr of selected repeats 13 Similarity 0.919192
Consensus:
ACATTTTTTGn
>Cele-UNSB01_3:12168292-12168722 Satlength=431 Nr of Repeats=13 RepeatLength=34
seed=AAGTATTTTT
AAGTATTTTTGCCGGTTTTTTTGCCGAAATTTCC
AAGTATTTTTGCCGGTTTTTTTGCCTAAATTTCC
AAGTATTTTTGCCGGCTTTTTTGCCGAAATTTCC
AAGTATTTTTGCCGATTTTTTTGCCGGAATTTCC
AAGTATTTTTGCCGGTTTTTTTGCCGAAATTTCC
AAGTATTTTTGCCGGTTTTTTTGCCTAAATTTCC
AAGTATTTTTGCCGGTTTTTTTGCCGAAATTTCC
AAGTATTTTTGCCGGTTTTTTTGCCGAAATTTCC
AAGTATTTTTGCCGGTTTTTTTGCCTAAATTTCC
AAGTATTTTTGCCGGTTTTTTTGCCGAAATTTCC
AAGTATTTTTGCCGGTTTTTTTGCCTAAATTTCC
AAGTATTTTTGCCGAAATTTTC
AAGTATTTTTGCCGGTTTTTTTGCCGAAATTTCT
Nr of selected repeats 12 Similarity 0.954843
Consensus:
AAGTATTTTTGCCGGTTTTTTTGCCgAAATTTCC
>Cele-UNSB01_3:12227970-12228196 Satlength=227 Nr of Repeats=6 RepeatLength=35
seed=TTCCGGCAAA
TTCCGGCAAATTGGCAAATTGCTGGAAATTAAAAT
TTCTGGCAAATCAGCAAACCGGCAATTTGGCGGCAATGAAAAT
TTCTGGCAAATTCACAAATTGCCGGAACTAAAAAA
TTCCGGCAAATTGGCAAGTTGCCGCTTTTGAACAT
TTCCGGCAAATCGACAAACCGGCAATTTGTCGAAAATGAAAAT
TTCCGGCAAATTGGCAAGTTGCCGTTATGGAGCAT
Nr of selected repeats 4 Similarity 0.676190
Consensus:
TTCcGGCAAATTggCAAaTTGCcGgaattgAaaAt
>Cele-UNSB01_3:12229422-12230200 Satlength=779 Nr of Repeats=21 RepeatLength=35
seed=TTCAATTCCG
TTCAATTCCGGCAATTTGCCGATTTGCCAGAAATT
TTCAATTCCGGCAATTTGACCAATTTTCCGGAAATT
TTCAATTCCGCAATTTGGCCAATTTGCCGGAAATT
TTCAATTCCGGCAATTTGTCGATTGGCCATGAATT
TTCAATTCCGGCAATTTGTCGATTTGCCAGAAATT
TTCAATTCCGACAATTTGCCGATTTGCCAGAAATT
TTCAATTCCGGCAATTTGTCGATTTGCCAGAAATG

TTCAATTCCGACAATTTGCCGATTTGCCAGAAATT
TTCAATTCCGGCAATTTGGCCAATTTTCCAGAAATT
TTCAATTCCGGCAATTTGTTAATTTGCCGGAAATTC
TCAATTCCGGCAATTTAGCCAATTTGTCAGAAATT
TTCAATTCCGGCAATTTGGCCAATTTGCCAGAAATT
TTCAATTCCGGCAATTTGTTAATTTGCCGGAAATT
TTCAATTCCGACAATTTGTCGATTTGCCAGAAATT
TTCAATTCCGGCAATTTGCCGATTTGCCAGAAATT
TTCAATTCCGGCAATTTGTCGATTTGCCAGAAATT
TTCAATTCCGACAATTTGCCGATTTGCCAGAAATT
TTCAATTCCGGCAATTTGTTAATTTGCCGGAAATT
TTCAATTCCGACAATTTGTCGATTTGCCAGAAATT
TTCAATTCCGGCAATTTGCCGATTTGCCCAGAAATTTTCCAATTCCCGGCAATTTGCCGATTTGCCAGAAATT
TTCAATTCCGGCAATTTGCCGATTTGCCCAGAAATT
Nr of selected repeats 15 Similarity 0.834492
Consensus:
TTCAATTCCGgCAATTTGtCgATTTGCCaGAAATT
>Cele-UNSB01_3:12305276-12305678 Satlength=403 Nr of Repeats=10 RepeatLength=40
seed=ATTCCAGAAT
ATTCCAGAATTTTTTCGAATTTTCCAGAAAGTTCTAGAAC
ATTCCAGAATTTCTTGGAATTTTCCAAAAGGTTCTAGAAC
ATTCCAGAATTTCTTGGAATTTTCCAAAAGGTTCTAGAAC
ATTCCAGAATTTTTTTTAAATTTTCCAGAAGGTTCTAGAAC
ATTCCAGAATTTTTTAAAATTTTCCAGAAGGTCCTAGAAT
ATTCCAGAATTTTTTCAAATTTTCCAGAAGGTTCTAGAAC
ATTCCAGAATTTTTTAAAATTTTCGAGAAGGTCCTAGAAT
ATTCCAGAATTTTTTTCAAATTTTCCAGAAGGTTCTAGAAC
ATTCCAGAATTTTTAAAAATTTTCCAGAAGCTTCTAGAAC
ATTCCAGAATTTTTTAAAATTTTCCAGAAGGTCCTAGAAT
Nr of selected repeats 8 Similarity 0.860714
Consensus:
ATTCCAGAATTTtTTaaAATTTTCCAgAAGGTtCTAGAAc
>Cele-UNSB01_3:12308090-12308555 Satlength=466 Nr of Repeats=12 RepeatLength=35
seed=AAAATTTCCG
AAAATTTCCGGCAAATCAGCTAATTGCCAGAATTG
AAAATTTCCGGTAAATCGGCAAATAGCCGGAATTG
AAAATTTCCGGCAAATCAGCTAATTGCCAGAATTG
AAAATTTCCGGTAAATCGGCAAATAGCCGGAATTG
AAAATGTCCGGCAAATCAGCTAATTGCCAGAATTG
AAAATTTCCGGTAAATCGGCAAATTGCCGGAATTG
AAAATTTCCGGCAAATCAGCTAATTGCCAGAATTG
AAAATTTCCGGAAAATCGGCGAATTGCCGGAAGTG
AAAATTTCCGGCAAATCAGCTAATTGCCAAAATTG
AAAATTTCCGGTAAATAGGCAAATTGCCGGAATTGA
AAAATTTCTGGCAAATCGGCAAAATGCCAAATTACCGTAAATTAAAATTTCTAACAATTTGCCAAAATTGA
AAAATTTTCGGTAAATCGGCAAATCAGCAATTTCCCAAAAATT
Nr of selected repeats 9 Similarity 0.868783
Consensus:
AAAATTTCCGGcAAATCaGCtAATtGCCaGAATTG
>Cele-UNSB01_3:12309471-12309813 Satlength=343 Nr of Repeats=8 RepeatLength=35
seed=GGAATTGAAA
GGAATTGAAAATTTCCGGGAAATCGGCAAGTTGGCGGAAATTAAAATTCCCGGGAAATCGGCAAATTGGC
GGAATTGAAAATTTCCGGCTAATTGGCAAATTGCCGGAAATTAAAATTTCCGAGAAATCGGCAAATTGGC
GGAATTGAAAACTTCCGGGAAATCGGCAAATTGGC

GGAATTGAAAATTTCCGGCAAAGTGGCAAGTTGGC
GGAATTGAAAATGTCCGGCAAATCGGCAAATTGGC
GAAATTGAAAATGTCCGGCAAATCGGCAAATTGGC
GGAATTGAAAATTTCCTGCAAACCGGCAAATTGCC
GGAATTGAAAATTTCCGGCAAATTGGA
Nr of selected repeats 5 Similarity 0.843810
Consensus:
GgAATTGAAAAttTCCgGcAAAtcGGCAAaTTGgC
>Cele-UNSB01_3:12312970-12313186 Satlength=217 Nr of Repeats=11 RepeatLength=20
seed=AAATAGATTG
AAATAGATTGACAGACGGAA
AAATAGATTGACAGACGAA
AAATAGATTGACAGACGAA
AAATAGATTGACAGACGGAA
AAATAGATTGACAGACGAA
AAATAGATTGACAGACGGAA
AAATAGATTGTCAGACGAAA
AAATAGATTGACAGACAAAA
AAATAGATTGACAGACGAA
AAATAGATTGACAGACGAAC
AAATAGATTGACAGACGAAA
Nr of selected repeats 7 Similarity 0.904762
Consensus:
AAATAGATTGACAGACGaAA
>Cele-UNSB01_3:12321003-12321555 Satlength=553 Nr of Repeats=10 RepeatLength=43
seed=AATTGCCGGT
AATTGCCGGTTTACCGATTTGCCGGAAATTTTTATTTTCAGAC
AATTGCCGGTTTGCCGATTTGCCAGAAATTTCGGTTTTCGGCA
AACTGCCGGTTTGCCGATTGGCCGAAAGTTTTCATTTTCGGCG
AATTGCCGGTTCGCCGATTTGCCGGAAATTTTCATTTTCGGCGAATAGGCGGTTTGCCGATTTGCCGGAATTTTTAGTT
TTCGACAAATTTTCCGTTTACCGGTTTGCTAGAAATTTTCAATTCGGACAGATAGCTGGTTTGCCGATATGCCGGAAAT
TTTCATTTTCGGAC
AATTGCCGGTTTGCCGATTTGCCAGAAATTTCAGTTTTCGGCA
AATTGCCGGTTCGACGATTTGCCGGAAATTTTCATTTTGGACA
AATTGCCGGTTTTACCGATTTGCCGGAAATTTTCATTTTCGGAC
AATTGCCGGTTTGCCGATTAGCCGGAAAATTTCATTTTCGGCG
AATTGCCGGTTTGCCGATTCGCCGGAAAATCTCATTTTCGGCA
AGTTGCCGGTTTCCCAGTAATTTTTATTTTCGGCA
Nr of selected repeats 7 Similarity 0.748854
Consensus:
AATTGCCGGTTTGCCGATTtGCCgGAAaaTTTCATTTTCGGCa
>Cele-UNSB01_3:12376126-12377123 Satlength=998 Nr of Repeats=30 RepeatLength=32
seed=AAATTTGTGA
AAATTTGTGAAAAATTTAGATCCATTTTGCTAACAAAATGTGTGCCATTTAGGCCCATTTTGTTCA
AAATTTATGAAAATTTAGGCCCATTTTGTCA
AAATTTGTGAAATTTCAGGCCCATTTTGCCA
AAATTTGTGAAAATTTAGGCCCATTTGCCA
AAATTTGTGAAAATTTAGGCCCGTTTTACTAAA
AAATTTGTGAAAATTTAGGCCCATTTTGCTCA
AAATTTGTGAAAATTTAGGCCCGTTTGCTCA
AAATTTGTGAAAATTTAGGCCCATTTTGCCA
AAATTTGTGAAATTTAGGCCCATTTTGTTCA
AAATTTGTGAAAATTCAGGCCCATTTTGCTCA
AAATTTGTGAAAATTTAGGCCCATTTTGCCCA

AAATTTGTGAATATTCAGGCCCATTTTGTTCA
AAATTTGTGAAAATTCAGGCCCATTTTGCTCA
AAATTTGTGAAAATTTAGGCCCATTATGCCAG
AAATTTGTGAAAATTTAGGCCCGTTTTACTAA
AAATTTGTGAAAATTTAGGCCCATTTTGCTCA
AAATTTGTGAATATTTAGGCCCGTTTTGCTCA
AAATTTGTGAAAATTTAGGCCCCATTTTTGGCCCCA
AAATTTGTGAATATTCAGGCCCCATTTTGGCCCCCA
AAATTTGTGAATATTCAGGCCCATTTTGCTCA
AAATTTGTGAAAATTTAGGCCCATTATGACCAG
AAATTTGTGAAAATTTAGGCCCGTTTTACTAAA
AAATTTGTGAAAATTTAGGCCCATTTTGTTCA
AAATTTGTGAAAATTTAGGCCCATTTTGCTCA
AAATTTGTGAATATTTAGGCCCGTTTTGCTCA
AAATTTGTGAAAATTTAGGCCCATTTTGCCCA
AAATTTGTGAATATTAAGGCCCATTTTGTTCA
AAATTTGTGAAAATTCAGGCCCATTTTGCTCA
AAATTTGTGAAAATTTAGGCCCATTTTGCTCA
AAATTTGTGAATATTTAGGCCCATTTTGTTCA
Nr of selected repeats 18 Similarity 0.874992
Consensus:
AAATTTGTGAAaATTtAGGCCCATTTTGcTCA
>Cele-UNSB01_3:12433825-12433951 Satlength=127 Nr of Repeats=9 RepeatLength=12
seed=TAAGCCTAAG
TAAGCCTAAGCC
TAAGCCTTAGCTTAGGAT
TAAGTCTAAGCC
TAAGCATAAGCC
TAAGCCTAAGTA
TAAGCCTAAGCCTAAGTA
TAAGCCTAAGCC
TAAGCCTAAGCCTAAGAC
TGAGCCTAAGCC
Nr of selected repeats 6 Similarity 0.814815
Consensus:
TAAGCCTAAGCC
>Cele-UNSB01_3:12445241-12445717 Satlength=477 Nr of Repeats=10 RepeatLength=32
seed=ATTCAAATTT
ATTCAAATTTCATTGGAATAATTTTGGCGGGA
ATTCAAATTTTTTAGAAAAATTTTGCCGGTAACTCCAATTTCTTCAGAAAAAAAATTTTGGCGGAAATTCTCATTTCTAT
AGAAAAAATATTGGCTGGATATCAATTTTTTGGAACATTTTTTGACGGGA
ATTCAATTTTTTACAGATACATTTTGGCGAAA
ATTCAAATTTCTTCAGAAAAGTGTTGGCGGGG
ATTCAAATTTTTTCTTAAAATTTTTGGCGAAA
ATTCAAATTTTGTCTTAAAATTTTTGGCGGTA
ATTCAAATTTCTACAGAAAAAAAATTGGCGGGAATTCAAGCTTCTTCAGAAAAATATTTGCGGAA
ATTCAAATTCTTCAAAAAAATTGGTGGGA
ATTTAAATTTCTTCATAAAACATTGCCAGAAA
ATCAAATTTCTTCGGAAAAAAAAATTGGCGAGAATCAAAATTTCTTCAAAACATTTTGGCGGGA
Nr of selected repeats 6 Similarity 0.602694
Consensus:
ATTCAAATTTctTCagAAAAnTtTTGGCGaaA
>Cele-UNSB01_3:12447381-12447465 Satlength=85 Nr of Repeats=7 RepeatLength=12
seed=GGCTTAGGCT

GGCTTAGGCTTA
GGCTTAGGCTTA
GGCTTAGGCTTA
GGCTTAGGCTTA
GGCTTAGGCTTA
GGCTTAGGCTTA
GGCTTAGGCTTT
Nr of selected repeats 7 Similarity 0.968254
Consensus:
GGCTTAGGCTTA
>Cele-UNSB01_3:12475925-12476790 Satlength=866 Nr of Repeats=62 RepeatLength=11
seed=TTTTTGCACA
TTTTTGCACAT
TTTTTGCACAT
TTTTTGCACATTGTATGCACATTTTCTGTACAT
TTTTTGCACAT
TTTTTGCACAT
TTTTTGCACAT
TTTTTCACAT
TTTTTGCACATT
TTTTTGCACAT
TTTTTGCACAT
TTTTTGCACAT
TTTTTGCACAT
TTTTTGCACAT
TTTTTGCACATTGTATGCACATTTTCTGTACAT
TTTTTGCACAT
TTTTTGCACATTTTTTCAAAT
TTTTTGCACAT
TTTTTGCACATTTTCTGTACAT
TTTTTGCACAT
TTTTTGCACAT
TTTTTGCACAT
TTTTTGCACAT
TTTTTGCACAT
TTTTTGCACATTTTCTGTACAT
TTTTTGCACAT
TTTTTGCACAT
TTTTTGCACAT
TTTTTGCACAT
TTTTTGCACAT
TTTTTGCACAT
TTTTTGCACAT
TTTTTGCACATTTTCTGTACAT
TTTTTGCACAT
TTTTTGCACAT
TTTTTGCACAT
TTTTTGCACATTTTTTCAAAT
TTTTTGCACAT
TTTTTGCACATTTTCTGTACAT
TTTTTGCACAT
TTTTTGCACAT
TTTTTGCACAT
TTTTTGCACAT

TTTTTGCACAT
TTTTTGCACAT
TTTTTGCACATTTTCTGTACAT
TTTTTGCACAT
TTTTTGCACAT
TTTTTGCACAT
TTTTTGCACAT
TTTTTGCACAT
TTTTTCACAT
TTTTTGCACAT
TTTTTGCACAT
TTTTTGCACATTGTATGCACATTTTCTGTACAT
TTTTTGCACAT
TTTTTGCACAT
TTTTTGCACATTGTATGCACATTTTCTGTACAT
TTTTTGCACAT
TTTTTGCACAT
TTTTTGCACAT
TTTTTCACAT
TTTTTGCACATTGTATGCACATTTTCTGTACAT
Nr of selected repeats 46 Similarity 1.000000
Consensus:
TTTTTGCACAT
>Cele-UNSB01_3:12492090-12492261 Satlength=172 Nr of Repeats=7 RepeatLength=19
seed=CAATTTACCA
CAATTTACCAAAAAATTTGTCAATTTATCGAAAATTTAG
CAATTTACCAAAAAATTTGT
CAATTTACCAAAAAATTTAG
CAATTTACCAGAATTTTTACAATTGTCCAAAAACTTGG
CAATTTATCAAAAAATTTAG
CAATTTACCAGAACTTTGA
CAATTTACCAAAAAATTTGT
Nr of selected repeats 5 Similarity 0.791667
Consensus:
CAATTTAcCAaAAaTTTGn
>Cele-UNSB01_3:12493882-12494280 Satlength=399 Nr of Repeats=13 RepeatLength=16
seed=ACGTAGGCAC
ACGTAGGCACGTAGGC
ACGCAGGCACGTAGGC
ACGTAGGCACGTAGGC
ACGTAGGCACATAGGC
ACGTAGGCACATAGGCACGCAGGT
ACGTAGGCACCAGGGCTGGGACTTTTTTGGTTTTTTTTTGTTGTTGGTTTTTTGGTATTTTGACGATTTTTCACGGTTA
GAAATAAATTCAAGCATCAAAATTTTTTGTTTTTTTTGTTTTTGCATTTTCGAAAACTTAATTTTTTTGTTTTTTGGTC
CAAATTGTTTTTTCGTCCCAGTCCTGGTAGGC
ACGTAGGCACGTAGGC
ACGTAGGCACGTAAAC
ACGTATGCACGTAGGC
ACGTAGGCACGTAGGC
ACGTAGGCACGTAGGC
ACGTAGGCACGTAGGC
ACGTAGGCACGTAGGCACGTAGGC
Nr of selected repeats 10 Similarity 0.916667
Consensus:

ACGTAGGCACGTAGGC
>Cele-UNSB01_3:12499748-12499928 Satlength=181 Nr of Repeats=14 RepeatLength=12 seed=CTTAGACTTA
CTTAGACTTAGA
CTTAGGCTTAGGATAAGG
CTTAGGCTTAGG
CTTAGGCTTAGA
CTTAGACTTAGA
CTTAGACTTAGA
CTTAGACTTAGA
CTTAGACTTAGA
CTTAGACTTAGA
CTTAGACTTAGA
CTTAGACTTAGA
CTTAGACTTAGA
CTTATACTTAGA
CTTAGACTTAGACTTAGA
Nr of selected repeats 12 Similarity 0.929293
Consensus:
CTTAGACTTAGA
>Cele-UNSB01_3:12542724-12542833 Satlength=110 Nr of Repeats=5 RepeatLength=15 seed=CGACCTGCGA
CGACCTGCGACCTAGAGGGTTAGGTCGCATTG
CGACCTGCGACCTAG
CGACCTGCGACCTTG
CGACCTGCGACCTAG
CGACCTGCGACCTTGCGACCTAGGTCGCATTG
Nr of selected repeats 3 Similarity 0.940741
Consensus:
CGACCTGCGACCTaG
>Cele-UNSB01_3:12547936-12548181 Satlength=246 Nr of Repeats=7 RepeatLength=35 seed=AATTGCCGGA
AATTGCCGGAATTCAAAATTTCCGGCAAATCGGAA
AATTGCCGGAATTCAAAATTTCCGGCTAATCGGCA
AATTGCCGGAATTCAAAATTTCCGGCAAACCGGCA
AATTGCCGGAACTCAAAATTTCCGGCAAATCGGCA
AATTGCCGGAATTTAAAATTTCCGGCAAACCGGCA
AATTGCCGGAATTCAAAATTTTCGGCAAACCGGCA
AATTGCCGGAATTCAAAATTTCCGGCAAATTGGCA
Nr of selected repeats 7 Similarity 0.912925
Consensus:
AATTGCCGGAATTCAAAATTTCCGGCAAAtCGGCA
>Cele-UNSB01_3:12593157-12594697 Satlength=1541 Nr of Repeats=44 RepeatLength=35 seed=TTGCCAGAAT
TTGCCAGAATTGAAAAATTCCGACAAATCGGCAAG
TTGCCAGAATTAAAAATTTCCGGAAAATCGGCAAG
TTGCCAGAATTAAAAATTTCCGGTAAATCGACAAA
TTGACAGAATTGAAAATTTCCGGCAAATCGGTAAG
TTACCAGAATTGAAAATTTCCGGTAAATTGGCAAA
TTGCCAGAAATGAAATATTCCGGGAAATCGGCAAA
TTGCCAGAATTGGAGTATTTTGGAAAATCGGCAAA
TTGCCAGAATTGAAGTATTTTGGAAAATCGGCGAA
TTGCCAGAATTGAAAAATTCCGACAAATCGGCAAG
TTGCCAGAATTAAAAATTTCCGGAAAATCGGCAAA

TTGCCAGAATTGAAGTATTTTGGAAAATCGGCAAA
TTGCCAGAATTGAAGTATTTTGGAAAATCGGCAAA
TTGCCAGAAATGAAATATTCCGGGAAATCGGCAAA
TTGCCAGAATTAAAAATTTCCGGAAAATCGGCAAA
TTGCCAGAAATGAAATATTCCGGGAAATCGGCAAA
TTGCCAGAATTGGAGTATTTTGGAAAATCGGCAAA
TTGCCAGAATTGAAAATTTTCGGGAAATCGGCAAA
TTGCCAGAATTGAAAATTTCCGGTAAATTGGCAAA
TTGCCAGAATTGGAGTATTTTGGAAAATCGGCAAA
TTGCCAGAATTGAAAATTTTCGGGAAATCGGCAAA
TTGCCAGAATTGAAAATTTCCGGTAAATTGGCAAA
TTGCCAGAAATGAAATATTCCGGGAAATCGGCAAA
TTGCCAGAATTGGAGTATTTTGGAAAATCGGCAAA
TTGCCAGAATTGGAGTATTTTGGAAAATCGGCGAA
TTGCCAGAATTGAAAAATTCCGACAAATCGGCAAG
TTGCCAGAATTAAAAATTTCCGGAAAATCGGCAAA
TTGCCAGAATTGAAGTATTTTGGAAAATCGGCAAA
TTGCCAGAAATGAAGTATTTTGGAAAATCGGCAAA
TTGCCAGAATTGAAAATTTTCGGGAAATCGGCAAA
TTGCCAGAATTGAAAATTTTCGGGAAATCGGCAGA
TTGCCAGAATTGAACATTTCCGGCAAACCGGCAAA
TTGTCAGAATTGAAATATTCAGGCGAATAGGTAAA
TTGCCAGAATTGAAAATTTTCGGGAAATCGGCAGA
TTGCCAGAATTGAACATTTCCGGCAAACCGGCAAA
TTGTCAGAATTGAAATATTCAGGCGAATAGGTAAA
TTGCCAGAATTGAAAATTTCCAGTAAATCGACAAA
TTGACAGAATTGAAGTATTTTGGAAAATCGGCAAG
TTGCCAGAATTGAAAATTTCCGGCAAATCGGTAAG
TTGCCAGAATTGAAAATTTCCGGCAAATCGGCAAA
TTGCCAGAATTGAAAATTTCCGGTAAATTGGCAAA
TTGCCAGAAATGAAATATTCCGGGAAATCGGCAAA
TTGCCAGAATTTAAAATTTCCGGGAAATCGGCAAG
TTGCCAGAATTGAAAATTTCCGGAAAATCGGCAAA
TTGCCAGAATGGGAAAATTCCGGAAAATCGGCAAG
Nr of selected repeats 44 Similarity 0.791362
Consensus:
TTGCCAGAATTGAAaaaTTccGGnAAATCGGCAAa
>Cele-UNSB01_3:12605711-12605849 Satlength=139 Nr of Repeats=9 RepeatLength=12
seed=TAGGCATAGG
TAGGCATAGGCA
TAGGCTTAGGCTTAGGACCAGGCT
TAGGCATAGACT
TAGGCTTAGGCTCAGGCTTAGTCT
TAGGCATAGGCA
TAGACATAGGCA
TAGGCATAGGCA
TAGGCATAGGCAAATGTT
TAGGCTTAGGCT
Nr of selected repeats 6 Similarity 0.829630
Consensus:
TAGGCATAGGCa
>Cele-UNSB01_3:12608882-12609486 Satlength=605 Nr of Repeats=14 RepeatLength=43
seed=AGCAGCCGAC
AGCAGCCGACAATTGCCGGTTGCCTTAAAATTGAAGTAAATCC

AGCAGCTGACAGCTAGCGGTTGCAGGAAAAAAATTCAGTAATTTC
AGCAGCCGTCAGTTGCCGGTTGCCGAAAAAATGCAGTAATTTCT
ACAGCCGACAGTTCTCGGTTGCCGAAAAAATGCAGTAAATCC
AGCAGCTGACAGTCACCGGTTGCCGAAAAATTGCAATAATTTC
AGCAGACGACAGTTGCCGGTTGCCGAAAAATTGCGATGATTTA
AGCAGCCGACAGTTGCCTGTTACCGAAGAATTGCAGTAATCTC
AGCAGCCGACAGTTGCCGGTTGCCGAAAAATTGCGATGATTTA
AGCAGCCGACAGTTGCCTGTTACCGAAGAGTTGCAGTAATCTC
AGCAGCCGACAGTTGCCGGTTGCCGAAAAATTGCGATGATTTA
AGCAGCCGACAGTTGCCTGTTACCAAAGAATTGCAGTAATCTC
AGCAGCCGACAGTTGCCGGTTGCCGAAGAATTGCAGTAATCTC
AGCAGCCGACAGTTGCCGGTTGCCGAAAAATTGCAGTAACTTC
AGCAGCCGACAGTTGCCCGTTGCCTTAAAATTGCAGTAATTTC
Nr of selected repeats 11 Similarity 0.800423
Consensus:
AGCAGCCGACAGTTGCCgGTTgCCgAAaAATTGCagTaATtTc
>Cele-UNSB01_3:12626974-12627042 Satlength=69 Nr of Repeats=4 RepeatLength=17
seed=TGAAAATAAT
TGAAAATAATCACGGAA
TGAAAATAATCACGGAA
TGAAAATAATCACGGAA
TGAAAATAATCACGGAA
Nr of selected repeats 4 Similarity 1.000000
Consensus:
TGAAAATAATCACGGAA
>Cele-UNSB01_3:12644803-12645326 Satlength=524 Nr of Repeats=7 RepeatLength=35
seed=TTGCCGGAAT
TTGCCGGAATTGAAAAATTCTGGCAAATCGGCAAATTGACGAAATTAAATAATTTTCCCGTTGCCTACCCGCCTGCCTA
CAGGCATTGCATTAAAAAGGAAAAAAAACTAATTTTTTGCGTGTTGGTTTTCTTGGAAGATTTGAATTTCCGCCAAAAT
TTTTTTGCCAAAAAATTTGAATTTCCCGCCAAAAAAAAATTTTCAAAAACAAAAAAAAATTGAAAATTTCTGGCAAATC
GGCAAT
TTGCCGGAATTAAAAATTTCCGGCAAATCAGCAAATTGGCGGAACTGAAAAATTCAAGCAAATTGTCAAATTCGCGGAA
CTAAACATTTCCGGCAAATCGGCAAA
TTGGCGGAATTGGAAATTTCCGCCAAACCGGCAAA
TTGCCGGAATTGAACATTTCTGGCAAACCGGAAAT
TTGCCGGAATTGGAAACTTCTGGCAAACCGGCAAA
TTGCCGGAATTGAACATTTCCGGCAAATCGGCAAT
TTGCCGGAATTGAAATTTTTGACAGATCGGCACTA
Nr of selected repeats 5 Similarity 0.729630
Consensus:
TTGcCGGAATTGnAaAtTTctGgCAaAcCGGcAat
>Cele-UNSB01_3:12692847-12693292 Satlength=446 Nr of Repeats=15 RepeatLength=17
seed=ATTTTTTCCG
ATTTTTTCCGCAAATAG
ATTTTTCCGCCGGTTCG
AGTTTTTCCGCCGGTAG
ATTTTTCCGCCGGTTCG
AGTTTTTCCGCAAGTAT
ATTTTTTCCGCAAGTAG
ATTTTTTCCGCAAGTAA
ATTTTTCCGCCGGTTCGAGTTTTTCGGCGAGTAG
ATTTTTTCCGCAAGTAG
ATTTTTCCGCCGATAGATTTTTCTGCCGGTAG
ATTTTTTCCGCAAGTAG

ATTTTTCCGCCGGTTCA
ATTTTTCCGCAAGTAA
ATTTTTCCGCCGGTTCGAGTTTTTCGGCAAGTAA
ATTTTTTCGCGCTATACTCTATACGCTATACGCTATACGCTATACGCTAATTTTGAAGTTTTCAAAAAAAAATAAATTT
TGGAAAATTTTATTAGATAGAGGTAGTCAAAGAGCATTTGAAGGTAGTCAATGAGCATTTATACATCAGATCTCATCTC
C
Nr of selected repeats 10 Similarity 0.687449
Consensus:
AtTTTTTCCGCaaGTag
>Cele-UNSB01_3:12700775-12700871 Satlength=97 Nr of Repeats=4 RepeatLength=24
seed=AAGCACCACC
AAGCACCACCTCCTCCGACTACAA
AAGCACCACCTCCTCCAACTACAA
AAGCACCACCTCCTCCAACTACAA
AAGCACCACCTCCTCCAACTACAA
Nr of selected repeats 4 Similarity 0.972222
Consensus:
AAGCACCACCTCCTCCaACTACAA
>Cele-UNSB01_3:12712606-12713078 Satlength=473 Nr of Repeats=10 RepeatLength=35
seed=GATTTGCCGG
GATTTGCCGGAAATTTTCGATTCCGGCAATTTGCC
GATTTGCCGGAAATTTTCGATTCCGGCAAATTGCC
GATTTGCCGGAAATTTTAAATCCGGCAAATTGCCGATTTGTCAGAAATTATCGATTCCGGCAAATTGTCGATTTTTCGG
AAATTTGCAATTCCGGCAACTTGCC
GATTTGCCGGAAATTTTCAATTCTGGCAATTTTCC
GATTTGCCGGAAATTTGTAACTCCGGCAATTTGCC
GATTTGCCGGAAACTTTCAATTCCGGCAATTTGCT
GATTTGCCGGAAATTGCGTTTTCCGGCAATTTGCC
GATTTCCGGCAAGTTGCC
GATTTGCCGGAAATTCTCAATTATGGCAATTTTCC
GATTTGCCGGAAATTTGCAATTCCGGCAATTTGCCGATCTGTCGGAAAATTTCAATTCTGGCAATTTGCCGATTTGTCA
GAAATTATCGATTCCGGCAATTTGCC
Nr of selected repeats 7 Similarity 0.791446
Consensus:
GATTTGCCGGAAAtTTtCaATTCcGGCAATTTgCC
>Cele-UNSB01_3:12784240-12784411 Satlength=172 Nr of Repeats=5 RepeatLength=35
seed=AATTTGAATT
AATTTGAATTTTCCGCCAAAATCCTTTTCTGAGAT
AATTTGAATTTCCCGCCAAAAAAATTCGGAGAA
AATTTGAATTTCCCGCCAAAATCATTTTCTGAGAA
AATTTGAATTTCCCGCCAAAAAAATTCGGAAAA
AATTTGAATTTCCCGCCAAAATCATTTTCTGAGAA
Nr of selected repeats 3 Similarity 0.923810
Consensus:
AATTTGAATTTcCCGCCAAAATCaTTTTCTGAGAa
>Cele-UNSB01_3:12788650-12788963 Satlength=314 Nr of Repeats=8 RepeatLength=35
seed=TTCAAATTTT
TTCAAATTTTATGTGAAAGACATTTTGGCGGTAAA
TTCAAATTTTCTGTGAAAGACTTTTTGGCGGTAAA
TTCAAATTTTTTGTGAAAGTCATTTTGGCGGGAAA
TTCAAGTTTTCCGAGAAAATTTTTTGGCGGGAAA
TTCAAATTTCTCACAATAATAATTTTGGCGGGAAT
TTCAAATTTTCTGAGAAAACCATTTTTCCGGGAAGTTTAATTTTTCTGTGAAAGACATTTTGGCGGGAAA
TTCAAGTTTTCACAAAAAACATTGTTGGAGGAAA

TTCAAATTTTCACAAAAAAAAGTTTTGGCGGAAAT
Nr of selected repeats 5 Similarity 0.658559
Consensus:
TTCAAATTTTctgtgaAAgacaTTTTGGCGGnAAa
>Cele-UNSB01_3:12817254-12818910 Satlength=1657 Nr of Repeats=46
RepeatLength=36 seed=GATATTTTCA
GATATTTTCATGTTCGGCAAAAATGCCGATTTGCCG
GATATTTTCATGTTCGGCAAAAGTACCGATTTGCCA
GATATTTTCATGTTCGGCAAAAGTACCGATTTGCCA
GATATTTTCATGTTCGGCAAAAGTACCGATTTGCCA
GATATTTTCATGTTCGGCAAAAGTACCGATTTGCCA
GATATTTTCATGTTCGGCAAAAGTACCGATTTGCCA
GATATTTTCATGTTCGGCAAAAGTACCGATTTGCCA
GATATTTTCATGTTCGGCAAAAGTACCGATTTGCCA
GATATTTTCATGTTCGGCAAAAGTACCGATTTGCCA
GATATTTTCATGTTCGGCAAAAGTACCGATTTGCCG
GATATTTTCATGTTCGGCAAAAGTACCGATTTGCCG
GATATTTTCATGTTCGGCAAAAGTACCGATTTGCCA
GATATTTTCATGTTCGGCAAAAGTACCGATTTGCCG
GATATTTTCATGTTCGGCAAAAATGCCGATTTGCCG
GATATTTTCATGTTCTGCAAAAGTACCGATTTGCCG
GATATTTTCATGTTCGGCAAAAGTACCGATTTGCCA
GATATTTTCATGTTCGGCAAAAGTACCGATTTGCCG
GATATTTTCATGTTCGGCAAAAATGCCGATTTGCCG
GATATTTTCATGTTCGGCAAAAGTACCGATTTGCCG
GATATTTTCATGTTCGGCAAAAGTACCGATTTGCCA
GATATTTTCATGTTCGGCAAAAGTACCGATTTGCCA
GATATTTTCATGTTCGGCAAAAGTACCGATTTGCCA
GATATTTTCATGTTCGGCAAAAGTACCGATTTGCCA
GATATTTTCATGTTCGGCAAAAGTACCGATTTGCCA
GATATTTTCATGTTTGGCAAAAGTACCGATTTGCTA
GATATTTTCATGTTCGGCAAAAGTACCGATTTGCCA
GATATTTTCATGTTTGGCAAAAGTACCGATTTGCCA
GATATTTTCATGTTTGGCAAAAGTACCGATTTGCCG
GATATTTTCATGTTCGGCAAAAGTACCGATTTGCCG
GATATTTTCATGTTCGGCAAAAGTACCGATTTGCCA
GATATTTTCATGTTCGGCAAAAGTACCGATTTGCCG
GATATTTTCATGTTCGGCAAAAATGCCGATTTGCCG
GATATTTTCATGTTCTGCAAAAGTACCGATTTGCCG
GATATTTTCATGTTCGGCAAAAGTACCGATTTGCCA
GATATTTTCATGTTCGGCAAAAGTACCGATTTGCCG
GATATTTTCATGTTCGGCAAAAATGCCGATTTGCCG
GATATTTTCATGTTCGGCAAAAGTACCGATTTGCCG
GATATTTTCATGTTCGGCAAAAGTACCGATTTGCCA
GATATTTTCATGTTCGGCAAAAGTACCGATTTGCCA
GATATTTTCATGTTCGGCAAAAGTACCGATTTGCCA
GATATTTTCATGTTCGGCAAAAGTACCGATTTGCCA
GATATTTTCATGTTCGGCAAAAGTACCGATTTGCCA
GATATTTTCATGTTTGGCAAAAGTACCGATTTGCTA
GATATTTTCATGTTCGGCAAAAGTACCGATTTGCCA
GATATTTTCATGTTTGGCAAAAGTACCGATTTGCCA
GATATTTTCATGTTTGGCAAAAGTACCGATTTGCCA
Nr of selected repeats 46 Similarity 0.952800
Consensus:

GATATTTTCATGTTCGGCAAAAGTACCGATTTGCCa
>Cele-UNSB01_3:12834918-12835593 Satlength=676 Nr of Repeats=18 RepeatLength=35
seed=TTTCCGGCAA
TTTCCGGCAAACCGGCAAATTGCTGGAACTGAGCA
TTTCCGGCAAAGCGGTAAATTGCCGGAATTGAAAA
TTTCCGGCAAATCACCAAACCGGCAGTTTGCCGAAAATCAAAAT
TTTCCGGCAAATCGATAAATTGCCGGAATTGAACA
TTTCCGGCAAATCGGTAAATTGCTGGAACTGAACA
TTTCCGGCAAATCGGCAAATTGCCGGAATTGAACA
TTTCCGGAAAATCGGCAAACCGGCAATTTGCCGAAAATCAAAAT
TTTCCGGCAAATCGGCAAATTGTCGGAATTGAAAA
TTTCCGGCAAATCGGTAAATTGCTGGAACTGAACA
TTTCCGGCAAAGCGGTAAATTGCCGGAATTGAAAA
TTTCCGGCAAATCACCAAACCGGCAGTTTGCCGAAAATCAAAAT
TTTCCGGCAAATCGATAAATTGCCGGAATTGAACA
TTTCCGGCAAATCGGTAAATTGCTGGAACTGAACA
TTTCCGGCAAATCGGCAAATTGCCGGAATTGAAAA
TTTCCGGCAAATCACCAAACCGACAGTTTGTCGAAAATCAAAAT
TTTCCGGCAAATCGATAAATTGCCGGAATTGAACA
TTTCCGGAAAATCGGCAAACCGGCAATTTGCCGAAAATCAAAAT
TTTCCGGCAAATCGGCAAATTGTCGGAATTGAAAA
Nr of selected repeats 13 Similarity 0.878877
Consensus:
TTTCCGGCAAAtCGgtAAATTGCcGGAAtTGAAcA
>Cele-UNSB01_3:12836433-12837192 Satlength=760 Nr of Repeats=21 RepeatLength=34
seed=TTTCAATTCC
TTTCAATTCCGGCATTTTACCAATTTGCCGGAAT
TTTCAATTCCGCCAATTTACCAATTTGCCGGAAGTTTCAAATTTGGCAATTTGCCGGTTTGCCGGATA
TTTCAATTCCGCCAATATACCAATTTGCCGGAAG
TTTCAATTACGGCAATTCGCCGATTTGCTGGAAT
TTTCAATTCCGCCAATTTGCCGGTTTGCCGGAAATTCCATTTCCGGCATTTTACCAATTTGCCGGTAT
TTTCCATTCCGCCAACTTGCCGATTTGAGAGAAA
TTTCAATTCCGGAAATTTGCCGATTTGCCGGAAT
TTTCAATTCCGGAAT
TTTCAATTCCGCCAATTTGCCGGTTTGCTAGAAA
TTTCAATTCCGGCATTTTACCAATTTGTCGGAAG
TTTCACTTCCGCCAATATACCAATTTGCCGGAAA
TTTCAATTCCGGAAATTTGCCGGTTTGCCGGATA
TTTCAATTCCGCCAATTTACCACTTTGCCGGAAT
TTTAAATTCCGGCATTTTACCAATTTGCCGGAAG
TTTCAATTTCGGCAATTTATTAATTTGCCGGAAG
TTTCAATTTCGGCAATTTGCCGATTTGCCGGAAT
TTTCAATTCCGGAAA
TTTCAATTCCGGCATTTTACCAATTTGCCGGAAT
TTTCAATTCCGCCAAATTATCAATTTGCCGGAAGTTTAAATTTTGGCGATTTGCCGGTTTGCCGGATA
TTTCAATTCCGCCAATTTACCACTTTGCCGGAAT
TTTCAATTCCGGAAA
Nr of selected repeats 15 Similarity 0.747664
Consensus:
TTTCAATTcCGgCAaTTTaCCaaTTTGCcGGAAn
>Cele-UNSB01_3:12842209-12842347 Satlength=139 Nr of Repeats=11 RepeatLength=12
seed=TATACATATA
TATACATATACA
TATACATATACA

TATACATATACA
TATACATATACA
TATACATATACA
TATACATATACA
TATACATATACA
TATACATATACA
TATACATATACA
TATACATATACA
TATACATATACATATACA
Nr of selected repeats 10 Similarity 1.000000
Consensus:
TATACATATACA
>Cele-UNSB01_3:12885748-12886017 Satlength=270 Nr of Repeats=5 RepeatLength=43
seed=GGCAAATCGG
GGCAAATCGGCAAACCGGCAATTTGTCAAATATTCAAATTTCC
GGCCAATCGGCAAATCGGGAAATTGGTGGAATTGAAAATTTCCGGAAAATTGGCAAATTGCCGGAAATTTCAATTTGCC
AAAAATGTAAATTTCCG
GGCAAATCGGCAATCCGGCAAACTGGTGGAATTGAAAATTTTCG
GGCAAATCGGCAAACCGGGGAATTATTGGAATTGAAAATTTCC
GGCAAATCGGCAAACCGGCACTTTGCAAAAAATAAAAATTTCT
Nr of selected repeats 3 Similarity 0.689394
Consensus:
GGCAAATCGGCAAACCGGcAaTTtgtnaAAtaTnaAAATTTCc
>Cele-UNSB01_3:12889217-12889593 Satlength=377 Nr of Repeats=11 RepeatLength=34
seed=TTTCAATTCC
TTTCAATTCCGGCAAATTGGCGATTTACCGGAAA
TTTCAATTCCGGCAATTTGCCGACTTGCCGGAAA
TTTCAATTCCGGCAATATGTCGATTTACCAAAAT
TTTCAATTCCGGCAATATGTCAATTTACCAAAAA
TTTCAATTCCGGCAATTTGCAAATTTACCGGAAG
TTTCAGTTCCAGCATTTTGCCTATTTGCCAGAAAT
TTTCAATTCCGGCAATTTGCCGATTTACCTGAAA
TTTCAATTACGGCAATTTTCCGGTTTGCCGGAAAT
TTTCAATTCGGGCAATTTGCCTTTTTGCCGGAAA
TTTCAATTCCGGCAAATTGCCGATTTGCCGGAAAT
TTTAATTCCGACAATTTGCCGATTTGCCGAAAA
Nr of selected repeats 7 Similarity 0.788982
Consensus:
TTTCAATTCCGGCAATtTGcCgATTTaCCggAAa
>Cele-UNSB01_3:12934390-12934631 Satlength=242 Nr of Repeats=8 RepeatLength=35
seed=TTTTAATTCC
TTTTAATTCCTGCAATTTGCCGGATTGTCGGAAAT
TTTTAATTCCGGCAATTTGCCGGATTGTCGGAAAT
TTTTATTTCCGGAAAT
TTGTAATTCCGGCAATTTACCGGTTTGCCGGAAAA
TTTTAATTCCGGCAATTTGCCGGTTTGCCGGGAAT
TTTTAATTCCGGCAATTTGCCGGTTTGCCGGGAAT
TTTTAATTCCGCAAT
TTTTAATTCCGGCAATTTGCCGGTTTGCCGGAAAT
Nr of selected repeats 6 Similarity 0.888254
Consensus:
TTTTAATTCCGGCAATTTGCCGGtTTGcCGGaAAT
>Cele-UNSB01_3:12996870-12997291 Satlength=422 Nr of Repeats=8 RepeatLength=35
seed=GGAAATTTTA

GGAAATTTTAAATTTCGGCAATTTGCCGGTTTGCCGGAAACTTTCAATTCCGGCAACTTGCCGATTTGCTGGAAGTCTT
AAATTCTGGCATTTTGCCAGTTTGCC
GGAAATTTTAAAATTCCGGAAGTTTGCCGTTTTGCC
GGAAATTTTAAATTCCGGCAATTTGCCGGTTAGCC
GGAAATTTTAAATTCCTGCAATCTTCCGCTTTGCT
GGAAATTTTAAATTCCGGCAACTTGCCGGTTTGAC
GGAAATTTTCATTTTCGGTAATTTGCCGGTTTGCTGAAAGTTTTAAATTCCGGCAATTTGCTGATTTGTCGAAAATTTT
CATTTTCGGCAATCTGCCAGTTTGCT
GGAAATTTTAAATTCCGGTAATTTGCCGGTTTGCC
GGAAATTTTAAATTCCGGTAATTCGCCGATTTGCT
Nr of selected repeats 5 Similarity 0.820952
Consensus:
GGAAATTTTAAATTCCgGcAAtttgCCGgTTtGcc
>Cele-UNSB01_3:13000875-13002076 Satlength=1202 Nr of Repeats=5 RepeatLength=32
seed=GACCAATCAG
GACCAATCAGCGACTCGCTCCGCCCATTTTCG
GACCAATCAGCGTCATCGAGCTCCATTTAACCCAAATTTTATAGATTAAAGATGAACGTAAAAATATAGAAAAATATTC
AATTAGTTTGAAAATAAAATTTATTTTTTAACAATTTTATTTTCAAACTAATCGAATATTTCTATATATTTCTACATTC
ATCTTCAATCTATAAAATTTGGGTCAATACGGAGCTCAAAGACGCTGATTGGTCAGAGAATGGGCGGGGCGAATCGCTG
ATTGGTCGGAGAGTTCTTAGTGATTTTCTGGTTTTTATAGAAAATTCCGATAATTTTTGGGATTTCGATTGTTCAAAAA
AAAATCCAAAATAGTCTCAAATTCAGAAAAATCACAAAAAAACTTTAAAAAATCGCGAAAACTCAAATTTTCAGACCCC
AGACGACATTGAAACACTCGCTTCCGTAGACGAAGATGGTATTAATCTAGAAGAAGACGCATGTACAATTAAATTCGAT
TCGATTCGCCGGAAAAATGACGATAATTCCGTGGAAAACGGGCAAAATGCCGCCGATTTATCCGATGAAACGGATGAAT
GGGATGTACAGAGACCAGATTCAGGAGATGATGATCCTTGAGTTCGTTTTAGACGTCGTCGTCGTGATGGTTGTGTGTG
TGTGTGTAGAATCGTTATTTCCGTTGTTGTAGCTGTTTCTCATAATTTTTAAATTTTTTTCCCAGCAAATTTGGGTTA
TTTTCGATAATTTTAACAATTTTTGCTGTAAAAATTATCTAAAATTCAGAAAAACCGCTAAAAACTTCCC
GACCAATCAGCGATTCGCCCCGCCCACTCTCT
GACCAATCAGCGTCTTCGAACTCCATATTGATACAAATTTTATAGATTGGAGATAAATGTAGAAATATAGAGAAATATT
CGATTAGTTTGAATTTTTTTTAGATTCGATGTTTTTACATACATTTTTGATTTTTAAAATTTGGGTTAATATGGAGTTC
GAAGACGCTGCTTGGTCTGAAATGGGCGGAGCAAGTCGCTGATTGGTCGGGGAGTTTTCGCGATTTTTCTGAATTTGAG
ACTATTTTGGATTTTTTTTCAAACAATCGAAATCCCAAAAATTATCGGAACTTTCTATAAAAACCAGAAAATCACTAAG
AACTCTCC
GACCAATCAGCGATTCGCCCCGCCCACTCTCT
Nr of selected repeats 3 Similarity 0.861111
Consensus:
GACCAATCAGCGAtTCGCcCCGCCCAcTcTCt
>Cele-UNSB01_3:13002319-13002507 Satlength=189 Nr of Repeats=5 RepeatLength=38
seed=TTTTGGCGGG
TTTTGGCGGGATTTCAAATTTGAATTTTTTGAAAATA
TTTTGGCGGGAATTCAAATTTTAATTTTTTTGAAAATA
TTTTGGCGGGAATTCAAAATTTAATTTTTTGAAAATA
TTTTGGCGGGAATTCAAAATTTAATTTTTCGAAAACAT
TTTTGGCGGGAATTTAAATTTTTAATTTTTTGAAAATA
Nr of selected repeats 3 Similarity 0.811966
Consensus:
TTTTGGCGGGAATTcAAAtTTTnAaTTTTTtGAAAAtA
>Cele-UNSB01_3:13026111-13026534 Satlength=424 Nr of Repeats=13 RepeatLength=35
seed=AAATTTTGAT
AAATTTTGATTTTTGGCGATTTGCCGA
AAATTTTGATTTTTGGCGATTTGCCAG
AAATTTTGATTTTTGGCAATTATCCGATTTGCCGG
AAATTTTGATTTTTGGCGATTTGCCAG
AAATTTTGATTTTTGGCAATTATCCGATTTGCCGG
AAATTTTGAATTTTGGCAATTTTCCGATTTGCCGG

AAATTTTGATTTTTGGCAATTTGCCGAATTGCCGG
AAATTTTGATTTTTGGCAATTTGCCGAATTGCCGG
AAATTTTGATTTTTGGGGATTTGCCGG
AAATTTTGATTTTTGGCAATTTGCCTATTTGTCGG
AAATTTTGATTTTTGGCAATTTGCCGATTTGTCGG
AAATTTTGATTTTTGGCAATTTGCCGATTTGCCGG
AAATTTTGATTTTTGGCAATTTTCCGATTTGCCAA
Nr of selected repeats 9 Similarity 0.900529
Consensus:
AAATTTTGATTTTTGGCAATTtgCCGAtTTGcCGG
>Cele-UNSB01_3:13035804-13036305 Satlength=502 Nr of Repeats=16 RepeatLength=35
seed=ATTTGCCGGA
ATTTGCCGGAAATTTTGATTTTCGGCAATATACCG
ATTTGCCGGAACATTTGATTTTCTGGAATATACCG
ATTTGCCGGAATTTTTGGTTTTCGGAA
ATTTGCCGGAAATTTAGAATTCCGGCAATATGCCG
ATTTGCCGGAAATTTTGATTTTCGGCAATATGCCG
ATTTGCCGGAAATTTTGATTTTCGGCAATATACCG
ATTTGCCGGAACATTTGATTTCCGGCAATATGCCG
ATTTGCCGGAATTTTTGATTTCCGGCAATATGCCG
ATTTGCCGGAAATTTTGATTTTCGGCAATATACCG
ATTTGCCGGAACATTTGGTTTCCGGCAATATGCCG
ATTTGCCGGAATTTTTGGTTTTCGGAA
ATTTGCCGGAAATTTAGAATTCCGGCA
ATTTGCCGATTTGCCGGAAATTTTGATTTCCGGCAATATGCCG
ATTTGCCGGAAATTTTGGTTTTCGGAA
ATTTGCCGGAAATTTAGAATTCCGGCAATATGCCG
Nr of selected repeats 10 Similarity 0.867936
Consensus:
ATTTGCCGGAAatTTtGAtTTcCGGCAATATgCCG
>Cele-UNSB01_3:13044209-13044762 Satlength=554 Nr of Repeats=5 RepeatLength=32
seed=ACCAATCAGC
ACCAATCAGCGATTTGCTCCGCCCACTTTCGG
ACCAATCCGCTGACCGAGGCATTTGATTGGTTTGAAATTGGGCGGAGCAGCGAATTGCTGATGCGAAATACGGGAAGTT
CTCATTTTGATGGAAATTCTGCAAAATTCTTTAAAAAAAACAAAATCTTCTCAAATTCGGAAAAAATCACAAAGGAAAT
CGAAGAAAATCGCGATTTTTGATTCCCCG
ACCAATCAGCGATTTGCTCCGCCCACTTTTGA
ACCAATCAGCGTTCGAGGCATTTGATTGGTTCAAAACTGGGCGGAGCAGCGAGTTGCTGATTGGATTTTTCAGTTTTTA
AATTTTTAAAGCTTTTTTTAACGGAAAAATTCGAGAAAACCATAGATTTTGATGAGAAATGATGAAAATTTTCATGAAA
AAATGGAAAAATGATTGGAAATTAATCAAAAAATCTTGAAAAAAAATTTTTTTCAGAGAAAATGCTTCATTTTTGGCT
CTGAAACGCCTCTTTTTTATTTGTGCCTCCCCG
ACCAATCAGCAATTTGCTCCGCCCACTTTTGA
Nr of selected repeats 3 Similarity 0.916667
Consensus:
ACCAATCAGCgATTTGCTCCGCCCACTTTtGa
>Cele-UNSB01_3:13065987-13066581 Satlength=595 Nr of Repeats=17 RepeatLength=35
seed=AAATTCAAAT
AAATTCAAATTATTTGAGAAATATTTTATGACGGG
AAATTCAAATTTTCTGATAAATATTTTATGGCGGT
AAATTCAAATTATTTGCGAGATATTTTTTGGCGGA
AAATTCAAATTATTGGAGAAATATTTTTTGGCTGG
AAATTCAAATTTTCTGAGAAATATTTTTTGGCGGG
AAATTCAAATTTTTTAAGAAAATATTTTGGCGGG
AAATTCAAAATATTTGAGAAATATTTTTTGGCAGG

AAATTCAAATTATTTGAGAAATATTTTATGGCGGA
AAATTCAAATTATTTGCGAGATATTTTTTGGCGGG
AAATTCAAATTATTTGAGAAATATTTTATGGCGGG
AAATTCAAATTTTCTGAGAAATATTTTATGGCGGG
AAATTCAAATTTTCTGAGAAATATTTTATGGCGGT
AAATTCAAATTATTTGCGAGATATTTTTTGGCGGA
AAATTCAAATTATTGGAGAAATATTTTTTGGCTGG
AAATTCAAATTTTCTGAGAAATATTTTATGGCGGA
AAATTCAAATTATTTGCGAGATATTTTTTAGCGGG
AAATTCAAATTATTTGAGAAATATTTTATGGCGGG
Nr of selected repeats 16 Similarity 0.851746
Consensus:
AAATTCAAATTaTtTGaGAaATATTTTaTGGCGGg
>Cele-UNSB01_3:13121321-13121505 Satlength=185 Nr of Repeats=5 RepeatLength=31
seed=GATTTTTTCA
GATTTTTTCAGTGAAAATCGAAAAATTTTCG
GATTTTTTCAGGGAAAACCAGAAAAATTTTGGAATTTTCAGGGAATATTAAACAATTTTTA
GATTTTTTTCAGGGAAAAACCAAAAAAATTC
GATTTTTTCAGAGAAAACCTTAAAATTTGTG
GATTTTTCAGAGAAAATTGAAAGAATTTGG
Nr of selected repeats 3 Similarity 0.659722
Consensus:
GATTTTTTCAGnGAAAAnCnaAAAAttTtcg
>Cele-UNSB01_3:13127780-13127920 Satlength=141 Nr of Repeats=4 RepeatLength=35
seed=CGGCAATTTG
CGGCAATTTGCCAAAAATGAAAATTTCCGGCAAAT
CGGCAATTTGCCAAAAATGAAAATTTCCGGCAAAT
CGGCAATTTGCCAAAAATCAAAATTTCCGGCAAAT
CGGCAATTTGCCTAAAATCAAAATTTCCGGCAAAC
Nr of selected repeats 4 Similarity 0.936508
Consensus:
CGGCAATTTGCCaAAAATcAAAATTTCCGGCAAAt
>Cele-UNSB01_3:13139705-13140905 Satlength=1201 Nr of Repeats=30
RepeatLength=20 seed=TGTAGATTTA
TGTAGATTTACAAAGGTCTA
TATAGATTTACAAAGTTCTATGTAAATCTACAACGTTCTATGTGAATTTACGGAAGCCTT
TGTAGATTTACGAAATTCTA
TGTAAATTTACAAAGTTCTA
TGTGGATTTACAAAAGTCTA
TGTAGATTTTCAAAGTTCTA
TGTAGATTTACAAAGGTCTA
TATAGATTTACAAAGTTCTATGTAAATCTACAACGTTCTATGTGAATTTACGGAAGCCTT
TGTAGATTTACGAAATTCTA
TGTAAATTTACAAAGTTCTA
TGTGGATTTACAAAAGTCTA
TGTAGATTTTCAAAGTTCTA
TGTAGATTTACAAAGGTCTATATAAATTTACAAAGTTCTATGTAAATCTACAACGTTCTATGTGAATTTACGGAAGCCT
AC
GTAGATTTACAAAATTTGA
TGTAGATTTACGAAGCCCTCTGTGGTCTTACACAGTTTTTTTGGATAATCAAAAAAATTCCTACCATTTCTTGCTCAT
TTTCAAAAGGTTACACGGTAAGGGATCAGTGATCAACTTCCAGAAACTTCTCTCTCTCCTTCCATCTCTCTCAAAAGTC
TAGTCACGTTCCCGTTCAGAAACCACAAAGTCATAAAATTTGAACGACCAGATTTTTTTTTGCCGTTTGCAAACCAGT
AACCATCCTTGATTCATCATGATTTTGATGATCACTATTGGAAAAAATCACTTTGATCAAAAAAAAATTCAAAATTGAC
GTGTTACGAACGTCTATTTTTGATTTAAAAAAGTTCACATGGGCAATTGGGCTTTGATCATTAAAAAATGTTTTCTA

TGTAGATTTACGAAGTTGTA
TGTAGATTTACGGAGCTCCA
TGTAGATTTAAGTAGAACTACGTAGATTGACAATGTAAGAAGTTCTATGTAAATTTGCAATGTTCCA
TGTAGATTTACAAAGTTATATGTTAGTTTACAAAGTCCTG
TGTAGATTTACGAAACACTA
TGTACATTTACTGCACCTTG
TGTAAATTTACGGAGCTCAA
TGTAGATTTACGGAGCTCTG
TGTGGATTTACAAAGCTCAA
TGTAGATTTACGGAGATCTATGTTCATTTACTAAACTCCT
TATAGATTTACAGAGCTCAA
TGTAGATTAACGGAGCTCAA
TGTAGATTTACGGAGCTCTG
TGTGGATTTACGGATCTCAA
TGTAGATTTACGGCGTTCTA
Nr of selected repeats 22 Similarity 0.674981
Consensus:
TGTAGATTTACgaAgnTCtA
>Cele-UNSB01_3:13143562-13144065 Satlength=504 Nr of Repeats=16 RepeatLength=19
seed=TTGGCAATTT
TTGGCAATTTACCAAAAATTTGGCAGTTGACTAAAATGTTGGCAGCTAACAAAAAAAAT
TTGGCATTTGACCAAAAAT
TTGCCAATTTATCAAAAAT
TTGGCAATTTACTGAAACT
TTGGCAGTTTACCAAAAAG
TTAGCAATTTACTAAAAAT
TTGGCAATTTACTACAAAT
TTGCCAATTTACCAAAAAGATGGTAACTTTTTTAAAATTGATCAAAAATATGGAAATCTGCTAAAAAT
TTGGCAATTTACTAAAAACTTGGCAGATGACTAAAATG
TTGGCAATTTACCAAAAAT
TTGGCAATTTACCAAAAAC
TTGGCAATTTACTAAAAAT
TTGCCAATTTACCAAAAAGATGGTAATTTTTTTTAATTTGAAAATTGATCAAAAATTTGGTATTTTACAGAAAAT
TTGGCAATTTACTAAAAAT
TTGGCAATGTACCAAAAATGGCATTTACTACAAATTTGGTAGTTTACCAAAAGC
TTGGCAATTTACAAAAAT
Nr of selected repeats 10 Similarity 0.809747
Consensus:
TTGGCAaTTTACcAAAAAt
>Cele-UNSB01_3:13181955-13182047 Satlength=93 Nr of Repeats=5 RepeatLength=15
seed=CGAAAAGGGG
CGAAAAGGGGAGCAGAA
CGAAAAGGGGAACTGCAAAAAAGGGATCTG
CGAAAAGGGGAGATA
CGAAAAGGGGAGATA
CGAAAAGGGGAGCAA
Nr of selected repeats 3 Similarity 0.881481
Consensus:
CGAAAAGGGGAGatA
>Cele-UNSB01_3:13182222-13182299 Satlength=78 Nr of Repeats=5 RepeatLength=15
seed=TCCCCTTTTC
TCCCCTTTTCGTATC
TCCCCTTTTCGTATC
TCCCCTTTTCGCAGA

TCCCCTTTTTGCAGA
TCCCCTTTTCGTTCTGC
Nr of selected repeats 4 Similarity 0.777778
Consensus:
TCCCCTTTTcGcAga
>Cele-UNSB01_3:13188476-13189044 Satlength=569 Nr of Repeats=13 RepeatLength=35
seed=TGCCGGAATT
TGCCGGAATTGAAATTTTTCGGCAAATCGGCAAACCGGTAAAATTTAACATTTTCTCTTTTACAAGGCATAGAAATCTA
GAAAAT
TGCCGGAATTTAGAATTTTCGACAAAGTGGCAAAT
TGCCGGAATTTAAATTTTCCGGCAAATCGGCAAAT
TGCCGGAATTGAAAATTTCCGGCAAATCGGCAAAT
TGCCGGAATTTAGAATTTTCAGCAATA
TGCCGGAATTTAAATTTTCCGGCAAATCGGCAAAT
TGCCGGAATTGAAAATTTCCGGCAAATCGGCAAAT
TGCCGGAATTTAACATTTCCGGTAAATAGGCAAAT
TGCAGGAATTTAAAATTTTCGGCAAAGCGGTAAGT
TGCCGGAATTGAAATTTCGGCAACTTTCCGGCAAATTGAAAATTTTCGGCAAATCGGCAAAT
TGCCGGAATTTAAAATTTTCGGCAAATCGGCAATTTGACAAATT
TGCCGGAATTGAAAATTTCTAGCTAATCGGCAAAT
TGCCAGAATTTAGAATTTTCGGCAAATTGCCGAAAATCAAAATTTTCCGGTAGTTCTCAAATCGGCAAAT
Nr of selected repeats 8 Similarity 0.817687
Consensus:
TGCCGGAATTtAAAaTTTcCGGCAAAtcGGCAAAT
>Cele-UNSB01_3:13248196-13248346 Satlength=151 Nr of Repeats=9 RepeatLength=12
seed=TAAGCCTAAG
TAAGCCTAAGCC
TGAGCCTAAGCA
TAAGCCTAAGCC
TAAGCCTAAGCA
TAAGCCTAAGCC
TGAGCCTAAGCCTAAGAC
TAAACCTAAGCC
TAAACCTAAGAC
TAAGCCTGAGCCTCAGCCTAGGCTTAATCATATGACGCAGCCTAATCC
Nr of selected repeats 7 Similarity 0.830688
Consensus:
TAAgCCTAAGCc
>Cele-UNSB01_3:13289132-13289912 Satlength=781 Nr of Repeats=10 RepeatLength=78
seed=CGAAGTTGCT
CGAAGTTGCTGGATCAACGATCACAACAGGCGGATTTGTCTGCACAACCGGATCAGAAGTCGTCGTACTTGGATCTTT
CGCAGTTGCTGGTCCAGAGATCACAACAGGCGGATTTGGCGGCATAACCTGAGCAACGGTAGTGTCATTTGGTTCTTC
CGAAGTTGCTGGATCAACGATCACAACAGGCGGATTTGGCGGCATAACCTGAGCAACGGTAGTGTCATTTGGTTCTTC
CGAAGTTGCTGGATCAACGATCACAACAGGCGGATTTGGCGGCATAACCTGAGCAACGGTAGTGTTGTTTGGTTCTTC
CGAAGTTGCTGGTCCAGAGATCACAACAGGCGGATTTGGCGGCATAACCGGAGCAACAGTAGTGTCATTTGGTTCTTC
CGAAGTTGCTGGATCAACGATCACAACAGGCGGATTTGGCGGCATAACCTGAGCAACGGTAGTGTTGTTTGGTTCTTC
CGAAGTTGCTGGTCCAGAGATCACAACAGGCGGATTTGGCGGCATAATCGGAGCAGCAGTAGTGTCATTTGGTTCTTC
CGAAGTTGCTGGTCCAGAGATCACAACAGGCGGATTTGGCGGCATAACCGGAGCAGCAGTAGTGTCATTTGGTTCTTG
CGAAGTTGCTGGATCAACGAACACAACAGGCAGATCTGTAGGCACAGTCGGAGCAGCAGTAGTGTTGTTTGGTTCTTC
CGAAGTTGCTGGTCCAGAGATCCCAACAGGCGGATTTGGCGGCATAACCGGAGCAGCAGTAGTGTCATTTGGTTCTTC
Nr of selected repeats 10 Similarity 0.831974
Consensus:
CGAAGTTGCTGGacCAaaGATCACAACAGGCGGATTTGgCGGCAtAAcCgGAGCAaCaGTAGTGTcaTTTGGTTCTTc

>Cele-UNSB01_3:13290888-13291020 Satlength=133 Nr of Repeats=7 RepeatLength=12 seed=CGCGACACGC
CGCGACACGCAA
CGCGACACGCAA
CGCGATACGCAACGCAATACGCGACGTGATACGCGA
CGCGACACGCAACGCAATACGCGACGCAATACGCAA
CGCGACACGCGA
CGCAACACGCAA
CGCGATACGCAC
Nr of selected repeats 5 Similarity 0.822222
Consensus:
CGCgAcACGCaa
>Cele-UNSB01_3:13335994-13337798 Satlength=1805 Nr of Repeats=147 RepeatLength=10 seed=GCTACCGCCC
GCTACCGCCC
GCTACCGCCC
GCTACCACCC
GCTACCGCCC
GCTACCGCCC
GCTACCACCC
GCTACCGCCC
GCTACCACCC
GCTACCGCCC
GCTACCGCCC
GCTACCGCCC
GCTACCGCCC
GCTACCGCCC
GCTACCGCCC
GCTACCGCCC
GATACCGCCC
GCTACCGCCC
GCTACCACCC
GCTACCGCCC
GATACCGCCC
GCTACCGCCC
GCTACCGCCC
GCTACCGCCC
GCTACCGCCC
GCTACCGCCC
GCTACCGTCC
GCTACCTCCC
GCTACCGCTCCGTTATGTCC
GCTACCGCCCGCTGTCGCAT
GCTACCGCCCA
CTACCGCCCGCTACCACCT
GCTACCGCCC
GCTACCGCCC
GCTACCGCCC
GCTACCGCCC
GCTACCGCCC
GCTACCGCCC
GCTACCGCCC
GCTACCACCC
GCTACCGCCC

GCTACCGCCC
GCTACCACCC
GCTACCGCCC
GCTACCACCC
GCTACCGCCC
GATACCGCCC
GCTACCGCCC
GCTACCGCCC
GCTACCGTCC
GCTACCTCCC
GCTACCGCTCCGTTATGTCC
GCTACCGCCCGCTGTCGCAT
GCTACCGCCCA
CTACCGCCCGCTACCACCT
GCTACCGCCC
GCTACCGCCC
GCTACCGCCC
GCTACCGCCC
GCTACCGCCCGCTGTCGCAT
GCTACCGCCCA
CTACCGCCCGCTACCACCT
GCTACCGCCC
GCTATCGCCC
GCTACCGTCC
GCTACCTCCC
GCTACCGCTCCGTTATGTCC
GCTACCGCCC
GCTACCGCCC
GCTACCGCCC
GCTACCGCCC
GCTACCGCCCACTACCACC
GCTACCGCCCGCTACCACC
GCTACCGCCC
GCTACCGCCC
GATACCGCCC
GCTACCGCCC
GCTACCGCCC
GCTACCGCCC
GCTACCGCCC
GCTACCGTCC
GCTACCTCCC
GCTACCGCTCCGTTATGTCC
GCTACCGCCC
GCTACCACCC
GCTACCGCCC
GATACCGCCC
GCTACCGCCC
GCTACCGTCC
GCTACCTCCC
GCTACCGCTCCGTTATGTCC
GCTACCGCCCGCTGTCGCAT
GCTACCGCCCA
CTACCGCCCGCTACCACCT
GCTACCACCC

GCTACCGCCCGCTGTCGCAT
GCTACCGCCCA
CTACCGCCCGCTACCACCT
GCTACCACCC
GCTACCGCCCGCTGTCGCAT
GCTACCGCCCA
CTACCGCCCGCTACCGCCC
GCTACCGCCC
GCTACCGCCC
GCTACCGCCCA
CTACCGCCCGCTACCACCT
GCTACCGCCC
GCTACCGCCC
GCTACCGCCC
GCTACCGCCC
GCTACCGCCC
GCTACCGCCC
GCTACCGCCC
GCTACCGCCC
GCTACCGCCC
GCTACCGTCC
GCTACCTCCC
GCTACCGCTCCGTTATGTCC
GCTACCGCCCGCTGTCGCAT
GCTACCGCCCA
CTACCGCCCGCTACCACCT
GCTACCGCCC
GCTACCGCCC
GCTACCGCCC
GCTACCGCCCGCTGTCGCAT
GCTACCGCCCA
CTACCGCCCGCTACCACCCA
CTACCGCCCGCTACCACCT
GCTACCGCCC
GCTATCGCCC
GCTACCGTCC
GCTACCTCCC
GCTACCGCTCCGTTATGTCC
GCTACCGCCC
GCTACCGCCCACTACCACC
GCTACCGCCCGCTACCACC
GCTACCGCCC
GCTACCGCCC
GCTACCACCC
GCTACCGCCGCTACCGCCCGCTACCACC
GCTACCGCCC
GCTACCGTCCGCTACCAACC
GCTACCGCTCCGTTATGCCC
GCTACCGCCCGCTACCAACC
GCTACCGCTCCGTTATGCCC
GCTACCGCCCGCTGTCGCAT
Nr of selected repeats 101 Similarity 0.923248
Consensus:
GCTACCGCCC

>Cele-UNSB01_3:13409998-13411238 Satlength=1241 Nr of Repeats=25 RepeatLength=43 seed=CGGAAATTTT
CGGAAATTTTGATTTTCGGTAATTTGCCGGTTTACTGGTTTGC
CGGAAATTTTCAATTCCGGCAACTTGCCAGTTTGCCGGTTTGC
CGGAAATTTTCATTTTCAAAAATTTGCCAGTTTGCTGATTTGCCGAACATCAGTTTGC
CGGAAGTTTTCAGAGGGATTTTTAATAAGACGGAAAAATTTAGAACTGTGAAAATTTTGGCAAATCGGAAAATTGCCAA
ATTGCCGATTTGC
CGGAAATTTTTATTTTCGGCAATTTGCCGGTTTGCCGATTTTC
CGGAAATTTTGATTTTCGGAAAATTGCCAGTTTGCCGATTTGC
CGGAAATTTTTATTTTCGACAATTTGCCGATTTGC
CGAAAATTTTCATTTTCCACAATTTGCCAGTTTGCCGATTTGC
CGGAAATTTTCAATTCCGGCAATTTGCCGATTTGCCGATTTGCAGGAAATATTTATTTTCGACAATTTGCCAGTTTGCA
CGATTTGC
CGGAAATTTTTATTTTCGGCAATTTGCCGGTTTGCCGATTTTC
CGGAAATTTTGATTTTCGGAAAATTGCCAGTTTGCCGACTTGT
CGGAAATTTTTATTTTCGACAATTGGCCGATTTACCGATTTGC
CGGAAATTTTGGTTTTCAAAAATTTGCCGGTTTGTCGATTTGC
CGGAAATTTTAAATTCCGGCAAAGTGCCGATTTGC
CGGAAAAATTTCAATTCCGGCAATTCGCCAGTTTGCCGATTTGC
CGAAAATTTTCATTTTTTAAAAATTGCCGGTTTGTCGATTTGCTAGAAATTTTCATTTTCGACAATTTGCCGGTTTGC
CGGAAATTTTCATTCTCCACAATTTGCCAGTTTTCCGATTTGC
CGGAAAGTTTCAATTCCGGCAATTTGCCGGTTTGCCAATTTGC
CGGAAATTTTCATTTTCAAAAATTTGCCGGTTCGCCGATTTGC
CGGAAATTTTCATTTTCAAAAATTTGCCAGTTTGCCGATTTGC
CGGAAGTTTTTAGAGGGATTTTTGATAAGACGGAACAATTTACTGTGAAAATTTTGGCAAATCGGAAAATTGCCAGTTT
GCCGATTTGC
CGGAAATTTTTATTTTCGGCAATTTGCCGGTTTGCCGATTTTC
CGGAAATTTTGATTTTCGGAAAATTGCCAGTTTGCCGATTTGC
CGGAAATTTTTATTTTCGACAATTTGCCGATTTGC
CGAAAATTTTCAATTCCGGCAAATATCTTGTTTGCCGGTTTTT
Nr of selected repeats 17 Similarity 0.776106
Consensus:
CGGAAATTTTnAtTTtCggcAAtTTGCCnGTTTGCCGATTTgC
>Cele-UNSB01_3:13415830-13416475 Satlength=646 Nr of Repeats=18 RepeatLength=34 seed=CGGAATTGAA
CGGAATTGAAATTTCCGGCAAATCGACAACTTGC
CGGAATTGAAATTTCCGACAAATCGGCAAATTAT
CGGAATTGAAATTTCTGTAAACCGGCAAATCGC
CGGAATTGAAATTTCGGGCAAATCGACAACTTGC
CGGAATTGAAATTTCCGGCAAATCGACAACTTGC
CGGAATTGAAATTTCCGACAAATCGGCAAATTAC
CGGAATTGAAATTTCCGGCAAATTGGCAGATTAC
CGGAATTGAAATTTCCGGCAAATGGGCAAATTGC
CGGAATTGAAATTTCGGGCAAATCGACAACTTGC
CGGAATTGAAATTTCGGGCAAATCGACAACTTGC
CGGAATTGAAATTTCGGGCAAATCGACAACTTGC
CGGAATTGAAATTTCGGGCAAATCGACAACTTGT
CGGAATTGAAACTTTCGGTAAATTGTCAGACTTGAAATTTGCGGCAAATCGGCAAATGGT
CGGAATTGAAGTGTCCGGCAAATCGGCAAATCGGCAAATTTC
CGGAATTTAAATTTCCGGCAAATGGGCAAATCGC
CGGAATTTAAATTTCCGGCAAATGGGCAAATTGC
CGGAATTGAAATTTCCGGCAAATCGGCAAATTGC
CGGAATTGAAATTTCCGACAAATCAGAAAATTGC
Nr of selected repeats 15 Similarity 0.854715

Consensus:
CGGAATTGAAATTTCcGgCAAATcGgCAAaTTgC
>Cele-UNSB01_3:13432209-13432275 Satlength=67 Nr of Repeats=5 RepeatLength=11
seed=AAAAATGTGC
AAAAATGTGCA
AAAAATGTGCAAAAAATTGTGAA
AAAAATGTGCA
AAAAATGTGCA
AAAAATGTGCA
Nr of selected repeats 4 Similarity 1.000000
Consensus:
AAAAATGTGCA
>Cele-UNSB01_3:13445252-13445836 Satlength=585 Nr of Repeats=45 RepeatLength=12
seed=AGTTATATAG
AGTTATATAGTA
AGTTATATAGTA
AGTTATATAGTT
AGTTATATAGTA
AGTTATATAGTA
AGTTATATAGTT
AGTTATATAGTTAATTGTATAGTA
AGTTATATAGTT
AGTTATATAGTA
AGTTATATAGTA
AGTTATATAGTT
AGTTATATAGTT
AGTTATATAGTT
AGTTATATAGTA
AGTTGTATAGTA
AGTTATATAGTA
AGTTATATAGTA
AGTTATATAGTA
AGTTATATAGTT
AGTTATATAGTT
AGTTATATAGTA
AGTTGTATAGTA
AGTTATATAGTA
AGTTATATAGTT
AGTTATATAGTT
AGTTATATAGTA
AGTTGTATAGTA
AGTTATATAGTA
AGTTATATAGTT
AGTTATATAGTT
AGTTATATAGTA
AGTTATATAGTT
AGTTATATAGTA
AGTTATATAGTA
AGTTATATAGTA
AGTTATATAGTTAATTGTATAGTA
AGTTATATAGTT
AGTTATATAGTA
AGTTATATAGTAAGTTATAGTT
AGTTATATAGTA

AGTTATTTAGTAAGTTATAGTA
AGTTATATAGTA
AGTTATATAGTT
AGTTATATAGTA
AGTTATATAGTA
Nr of selected repeats 41 Similarity 0.931707
Consensus:
AGTTATATAGTa
>Cele-UNSB01_3:13483516-13483661 Satlength=146 Nr of Repeats=5 RepeatLength=31 seed=CCAAAAATGA
CCAAAAATGACTGAAAATTGG
CCAAAAATGACTGAAATTAGTCAAAAATTGG
CTAAAAATGACTGAAAATATTCAAAAATTGG
CCAAAAATGACCGAAAATAGTCAAAAATTAG
CCAAAAATGACTGAAAATAGTCAAAAAATAG
Nr of selected repeats 4 Similarity 0.863799
Consensus:
CcAAAAATGACtGAAAaTAgTCAAAAAtTaG
>Cele-UNSB01_3:13559329-13561136 Satlength=1808 Nr of Repeats=95 RepeatLength=19 seed=AAAAATAGAT
AAAAATAGATTGGCATACGA
AAAAATAGCTTGGCATACGA
AAAAATAGATTGGCATACG
AAAAATAGATTGACAGACG
AAAAATAGATTGACAGACG
AAAAATAGATTGACAGACA
AAAAATAGAGTGACAGACG
AAAAATAGATTGGCAGACG
AAAAATAGATTGACAGACA
AAAAATAGATTGACAGACG
AAAAATAGATTGTTACAAG
AAAAATAGATTCTTACACG
AAAAATAGATTGTTACACG
AAAAATAGATTGTTACACG
AAAAATAGATTGTTACACG
AAAAATAGATTGTTACACG
AAAAATAGATTGTTACACG
AAAAATAGATTGGTACACG
AAAAATAGATTGTTACACG
AAAAATAGATTGTTACACG
AAAAATAGATTGGTACACG
AAAAATAGATTGTTACACG
AAAAATAGATTGTTACACG
AAAAATAGATTGTTACACG
AAAAATAGATTGTTACACG
AAAAATAGATTGTTACACG
AAAAATAGATTGGTACACG
AAAAATAGATTGTTACACG
AAAAATAGATTGTTACACG
AAAAATAGATTGTTACACG
AAAAATAGATTGTTACACG
AAAAATAGATTGTTACACG
AAAAATAGATTGTTACACG
AAAAATAGATTGGTACACG

AAAAATAGATTGTTACACG
AAAAATAGATTGGTACACG
AAAAATAGATTGTTACACG
AAAAATAGATTGGTACACG
AAAAATAGATTGTTACACG
AAAAATAGATTGGTACACG
AAAAATAGATTGTTACACG
AAAAATAGATTGTTACACG
AAAAATAGATTGGTACACG
AAAAATAGATTGTTACACG
AAAAATAGATTGTTACACG
AAAAATAGATTGTTACACG
AAAAATAGATTGTTACACG
AAAAATAGATTGTTACACG
AAAAATAGATTGTTACACG
AAAAATAGATTGTTACACG
AAAAATAGATTGTTACACG
AAAAATAGATTGTTACACG
AAAAATAGATTGTTACACG
AAAAATAGATTGTTACACG
AAAAATAGATTGTTACACG
AAAAATAGATTGTTACACG
AAAAATAGATTGTTACACG
AAAAATAGATTGTTACACG
AAAAATAGATTGTTACACG
AAAAATAGATTGTTACACG
AAAAATAGATTGTTACACG
AAAAATAGATTGTTACACG
AAAAATAGATTGTTACACG
AAAAATAGATTGTTACACG
AAAAATAGATTGTTACACG
AAAAATAGATTGTTACACG
AAAAATAGATTGTTACACG
AAAAATAGATTGTTACACG
AAAAATAGATTGTTACACG
AAAAATAGATTGTTACACG
AAAAATAGATTGTTACACG
AAAAATAGATTGTTACACG
AAAAATAGATTGTTACACG
AAAAATAGATTGGTACACG
AAAAATAGATTGTTACACG
AAAAATAGATTGGTACACG
AAAAATAGATTGTTACACG
AAAAATAGATTGGTACACG
AAAAATAGATTGTTACACG
AAAAATAGATTGGTACACG
AAAAATAGATTGTTACACG
AAAAATAGATTGTTACACG
AAAAATAGATTGGTACACG
AAAAATAGATTGTTACACG
AAAAATAGATTGTTACACG
AAAAATAGATTGTTACACG
AAAAATAGATTGTTACACG
AAAAATAGATTGGTACACG

AAAAATAGATTGTTACACG
AAAAATAGATTGTTACACG
AAAAATAGATTGGTACACG
AAAAATAGATTGTTACACG
AAAAATAGATTGGTACACG
AAAAATAGATTGTTACACG
AAAAATAGATTGGTACACG
Nr of selected repeats 93 Similarity 0.940307
Consensus:
AAAAATAGATTGtTACACG
>Cele-UNSB01_3:13583922-13584205 Satlength=284 Nr of Repeats=8 RepeatLength=30 seed=TTTCAGGCCA
TTTCAGGCCAATTGCAGTCATTTTCGAGCCGATTTCAGTCATTGTTGAACA
TTTCAGGCCAATTTCGGTCAATTTTGGACA
TTTCAGGCCAATTCCGGTCATTTTTGGACA
TTTCAGGCGAATTTTGGCCATTTTTGGATATTTCAGGGAAATTTCTGTCAT
TTTCAGGCCAATTTCAGTCATTCCTGAACA
TTTCTGGCCAATTTTCGTCATTTTTAGACA
TTTCAGGCCAATTTCAGTCATTTTTGGACA
TTTCAGGCCAATTTCAGTTATTTTTTAGACA
Nr of selected repeats 5 Similarity 0.822222
Consensus:
TTTCaGGCCAATTtcnGTCAtTttTggACA
>Cele-UNSB01_3:13762939-13765339 Satlength=2401 Nr of Repeats=60 RepeatLength=40 seed=ATTTTTCTAG
ATTTTTCTAGAAAGTTCTAGAACATTCCAGAATTTTCCCG
ATTTTTCTAGAAAGTTCTGGAACATTCCAGAATTTTCCCA
ATTTTTCTAGAAAGTTGTGAAACATTCCAGAATTTTCTCG
ATTTTTCTAGAAAGTTCTGGAACATTCCAGAATTTTCCCA
ATTTTTCTAGAAAGTTGTGAAACATTCCAGAATTTTCTCG
ATTTTTCTAGAAAGTTCTGGAACATTCCAGAAATTTCCCG
ATTTTTCTAGAAAGTTCTGGAACATTCCAGAATTTTCCCA
ATTTTTCTAGAAAGTTGTGAAACATTCCAGAATTTTCTCG
ATTTTTCTAGAAAGTTCTGGAACATTCCAGACTTTTTCCG
ATTTTTCTAGAAAGTTCTGGAACATTCCAGACTTTTCCCA
ATTTTTCTAGAAAGTTCTGGAACATTCCAGAAATTTCCCA
ATTTTTCTAGAAAGTTCTGGAACATTCCAGACTTTTTCCG
ATTTTTCTAGAAAGTTCTGGAACATTCCAGAAATTTCGCG
ATTTTTCTAGAAAGTTCAGGAACATTCCAGAATTTTCTCG
ATTTTTCTAGAAAGTTCTGGAACATTCCAGACATTTCCCA
ATTTTTCTAGAAAGTTCTGGAACATTCCAGAAATTTCCCA
ATTTTTCTAGAAAGTTCTGGAACATTCCAGACTTTTCCCA
ATTTTTCTAGAAAGTTCTGGAACATTCCAGACTTTTCCCA
ATTTTTCTAGAAAGTTCTGGAACATTCTAGAAATTTCCCA
ATTTTTCTAGAAAGTTCTGGAACATTCCAGACTTTTCCCA
ATTTTTCTAGAAAGTTCTGGAACATTCCAGACTTTTCCCA
ATTTTTCTAGAAAGTTCTGGAACATTCCAGACTTTTCCCA
ATTTTTCTAGAAAGTTCTGGAACATTCCAGAAATTTCCCG
ATTTTTCTAGAAAGTTCTGGAACATTCCAGAATTTTCTCG
ATTTTTCTAGAAAGTTCTGGAACATTCCAGAATTTTCTCG
ATTTTTCTAGACAGTTCTGGAACATTCCAGAAATTTCCCA
ATTTTTCTAGAAAGTTCTGGAACATTCCAGACTTTTCCCA
ATTTTTCTAGAAAGTTCTGGAACATTCTAGAAATTTCCCA
ATTTTTCTAGAAAGTTCAGGAACATTCCAAAATTTTCTCG

ATTTTTCTAGAAAGTTCTAGAAAATTCCAGAAATTTCCCG
ATTTTTCTAGAAAGTTCTGGAACATTACAGAAATTCCCCG
ATTTTTCTAGAAAGTTCTGGAACATTCCAGAATTTTCTCG
ATTTTTCTAGACAGTTCTGGAACATTCCAGAATTTTCCCA
ATTTTTCTAGAAAGTTCTGGAACATTCCGGAATTTTCCCA
ATTTTTCTAGAAAGTTCAGGAACATTCCAGAATTTTCTCG
ATTTTTCTAGAAAGTTCTGGAACATTCCAGAAATTTCCCA
ATTTTTCTAGAAAGTTCTGGAACATTCCAGACTTTTTCCG
ATTTTTCTAGAAAGTTCTGGAACATTCCAAAAATTTCGCG
ATTTTTCTAGAAAGTTCAGGAACATTCCAGAATTTTCTCG
ATTTTTCTAGAAAGTTCTGGAACATTCCAGACATTTCCCA
ATTTTTCTAGAAAGTTCTGGAACATTCCAGAAATTTCCCA
ATTTTTCTAGAAAGTTCTGGAACATTCCAGACTTTTCCCA
ATTTTTCTAGAAAGTTCTGGAACATTCCAGACTTTTCCCA
ATTTTTCTAGAAAGTTCTGGAACATTCCAGACTTTTCCCA
ATTTTTCTAGAAAGTTCTGGAACATTCCAGAAATTTCCCG
ATTTTTCTAGAAAGTTCTGGAACATTCCAGAATTTTCCCA
ATTTTTCTAGAAAGTTCAGGAACATTCCAGAATTTTCTCG
ATTTTTCTAGAAAGTTCTGGAACATTCCAGACATTTCCCA
ATTTTTCTAGAAAGTTCTGGAACATTCCAGACTTTTCCCA
ATTTTTCTAGAAAGTTCTGGAACATTCCAGAAATTTCCCA
ATTTTTCTAGAAAGTTCTGGAACATTCCAGACTTTTCCCA
ATTTTTCTAGAAAGTTCTGGAACATTCTAGAAATTTCCCA
ATTTTTCTAGAAAGTTCTGGAACATTCCAGAAATTTCCCA
ATTTTTCTAGAAAGTTCTGGAACATTCCAGACTTTTCTCA
ATTTTTCTAGAAAGTTCTGGAACATTCCAGACTTTTCCCA
ATTTTTCTAGAAAGTTCTGGAACATTCCAGAAATTTCCCA
ATTTTTCTAGAAAATTCTGGAACATTCCAGACTTTTCCCA
ATTTTTCTAGAAAGTTCTGGAACATTCCAGAAATTTCCCG
ATTTTTCTAGAAAGTTCTGGAACATTCCAGACTTTTCCCA
ATTTTTCTAGAAAGTTCTAGAACATTCCAGACTTTTCCCA
Nr of selected repeats 60 Similarity 0.908418
Consensus:
ATTTTTCTAGAAAGTTCTGGAACATTCCAGAatTTTCcCa
>Cele-UNSB01_3:13765752-13765977 Satlength=226 Nr of Repeats=15 RepeatLength=15
seed=CCACGATGGG
CCACGATGGGTCTCA
CCACGATGGGTCTCA
CCACGATGGGTCTCA
CCACGGTGGGTCTCA
CCACGATGGGTCTCA
CCACGATGGGTCTCG
CCACGATGGGTCTCG
CCACGATGGGTCTCA
CCACGATGGGTCTTA
CCACGATGGGTCTCA
CCACGATGGGTCTCG
CCACGATGGGTCTCG
CCACGATGGGTCTCA
CCACGATGGGTCTCG
CCACGATGGGTCTCG
Nr of selected repeats 15 Similarity 0.930582
Consensus:
CCACGATGGGTCTCa

>Cele-UNSB01_3:13900788-13900959 Satlength=172 Nr of Repeats=7 RepeatLength=24
seed=AAGAAGGATG
AAGAAGGATGCCGAAGAGAAGAAG
AAGAAAGATGCCGAAGAGAAGAAG
AAGAAGGATGCTGAAGATGAGAAG
AAGAAGGATGCAGGAGATGAGAAG
AAGAAGGATGCTGAAGATGAGAAG
AAGAAGGATGCTGGAGATGAGAAGAAG
AAGAAGGATGCTGGAGAGAAGAAA
Nr of selected repeats 6 Similarity 0.825926
Consensus:
AAGAAGGATGCtGaAGAgaAGAAG
>Cele-UNSB01_3:14059760-14060650 Satlength=891 Nr of Repeats=57 RepeatLength=15
seed=TCGTGGTGAG
TCGTGGTGAGACCCT
TCGTGGTGAGACCCA
TCGTGGTGAGACCCT
TCGTGGTGAGACCCT
TCGTGGTGAGACCTT
TCGTGGTGAGACCCA
TCGTGGTGAGACCCA
TCGTGGTGAGACTCA
TCGTGGTGAGACCCT
TCGTGGTGATACCCA
TCGTGGTAAGACCTT
TCGTGGTGAGACCCA
TCGTGGTGAGACCCA
TCGTGGTGAGACTCA
TCGTGGTGAGACCCT
TCGTGGTGAGACCCT
TCGTGGTGAGACCTT
TCGTGGTGAGACCCA
TCGTGGTGAGACCTT
TCGTGGTGAGACCCA
TCGTGGTGAGACCTT
TCGTGGTGAGAACTT
TCGTGGTGAGACCCA
TCGTGGTGAGACCTT
TCGTGGTGAGACCCA
TCGTGGTGAGACCTT
TCGTGGTGAGACCTT
TCGTGGTGAGACCTT
TCGTGGTGAGACCCA
TCGTGGTGAGACCCT
TCGTGGTGAGACCCA
TCGTGGTGAGACCCA
TCGTGGTGAGACTCA
TCGTGGTGAGACCCT
TCGTGGTGAGACCCA
TCGTGGTGAGACCCA
TCGTGGTGAGACCCTTCATGGTTAGACCCA
TCGTGGTGAGACCCT
TCGTGCTGAGACCATTCATGGAGAGACCCA
TCGTGGTGAGACCCT

TCGTGGTTAGACCCA
TCGTGGTGAGATCTT
TCGTGGTGAGACCCA
TCGTGGTGAGACTCA
TCGTGGTGAGACCCT
TCGTGGGGAGACTCT
TCGTGGTGAGACCCT
TCGTGGTTAGACCCGACCCA
TCGTGGTTAGACCCA
TCGTGGTGAGATCTT
TCGTGGTGAGCCCCA
TCGTGGTGAGACCCA
TCGTGGTGAGACTTT
TCGTGGTGAGACCCA
TCGTGGTGAGACTTT
TCGTGGTGAGACCCA
TCGTGGTGAGACCTT
Nr of selected repeats 54 Similarity 0.868872
Consensus:
TCGTGGTGAGACCct
>Cele-UNSB01_3:14063373-14064273 Satlength=901 Nr of Repeats=60 RepeatLength=15
seed=TCGTGGTGAG
TCGTGGTGAGACCCT
TCGTGGTGAGACCCA
TCGTGGTGAGACCCA
TCGTGGTGAGACCCT
TCGTGGTGAGACCCA
TCGTGGTGAGACCCA
TCGTGGTGAGACTCA
TCGTGGTGAGACCCT
TCGTGGTGAGACCTT
TCGTGGTGAGACCCA
TCGTGGTGAGACCTT
TCGTGGTGAGACCCA
TCGTGGTGAGACCTT
TCGTGGTGAGACCCA
TCGTGGTGAGACCTT
TCGTGGTGAGAACTT
TCGTGGTGAGACCCA
TCGTGGTGAGACCTT
TCGTGGTGAGACCCA
TCGTGGTGAGACCTT
TCGTGGTGAGACCTT
TCGTGGTGAGACCTT
TCGTGGTGAGACCCA
TCGTGGTGAGACCCT
TCGTGGTGAGACCCA
TCGTGGTGAGACCCA
TCGTGGTGAGACTCA
TCGTGGTGAGACCCT
TCGTGGTGAGACCCA
TCGTGGTGAGACCTT
TCGTGGTGAGACCCA
TCGTGGTGAGACCTT

TCGTGGTGAGACCCA
TCGTGGTGAGACCTT
TCGTGGTGAGACCCA
TCGTGGTGAGACCTT
TCGTGGTGAGACCTT
TCGTGGTGAGACCTT
TCGTGGTGAGACCCA
TCGTGGTGAGACCCT
TCGTGGTGAGACCCA
TCGTGGTGAGACCCT
TCGTGGTGAGACCCA
TCGTGGTGAGACCCA
TCGTGGTGAGACTCA
TCGTGGTGAGACCCT
TCGTGGTTAGACCCA
TCGTGGTTAGACCCA
TCGTGGTGAGATCTT
TCGTGGTGAGACCCA
TCGTGGTGAGACCCA
TCGTGGTTAGACCCA
TCGTGGTGAGATCTT
TCGTGGTGAGCCCCA
TCGTGGTGAGACCCA
TCGTGGTGAGACTTT
TCGTGGTGAGACCCA
TCGTGGTGAGACTTT
TCGTGGTGAGACCCA
TCGTGGTGAGACCTT
Nr of selected repeats 60 Similarity 0.880778
Consensus:
TCGTGGTGAGACCca
>Cele-UNSB01_4:1-5637 Satlength=5637 Nr of Repeats=464 RepeatLength=12
seed=AAGCCTAAGC
AAGCCTAAGCCT
AAGCCTAAGCCT
AAGCCTAAGCCT
AAGCCTAAGCCT
AAGCTAAGCCT
AAGCCTAAGCCT
AAGCCTAAGCCTAAGCTAAT
AAGCCTAAGCCT
AAGCCTAAGCCT
AAGCCTAAGCCCT
AAGCCTAAAGCCT
AAGCCTAAGCCT
AAGCCTAAGCCT
AAGCCTAAGCCT
AAGCCTAACCT
AAGCCTAAGCCT
AAGCCTAAGCCT
AAGCCTAAGCCT
AAGCCTAAGCCT
AAGCCTAAGCT
AAGCCTAAGCT

AAGCCTAGCCT
AAGCCTAAGCCT
AAGCCTAAGCCT
AAGCCTGAAGCT
AAGCCTAAGCCT
AAGCCTAAGCCT
AAGCCTAAGCCT
AAGCCTAAGCT
AAGCTAAGCCT
AAGCCTAAGCCT
AAGCCTAAGCCT
AAGCCTAAGCCT
AAGCCCTAAGCCT
AAGCCTAAAGCCT
AAGCCTAAGCCT
AAGCCTAAGCCT
AAGCCTAAGCCT
AAGCCTAAGCCT
AAGCCTAAGCCT
AAGCCTAAGCCT
AAGCCTAAGCCT
AAGCCTAAGCCT
AAGCCTAAGCCT
AAGCCTAAGCCT
AAGCCTAACCT
AAGCCTAAGCCTA
AAGCCTAAGCCT
AAGCCTAAGCCT
AAGCCTAAGCCT
AAGCCTAAGCCT
AAGCCTAAGCCT
AAGCCTAGCCT
AAGCCTAAGCCT
AAGCCTAAGCCT
AAGCCTAAGCCT
AAGCCTAAGCCT
AAGCCTAGCCT
AAGCCTAAGCCT
AAGCCAAGCCT
AAGCCTAAGCCT
AAGCCTAAGCCTAAGCAAGCCACCTAAGCCTAA
AAGCCTAAGCCT
AAGCCTAAGCCT
AAGCCTAAGCCCT
AAGCCTAAGCCT
AAGCCTAAGCCT
AAGCCTAAGCCT
AAGCCTAAGCCT
AAGCCTAAGCCT
AAGCCTAAGCCT
AAGCCTAAGCCT
AAGCCTAAGCCT
AAGCCTAAGCCT
AAGCCTAAGCCT

AAGCCTAAGCCT
AAGCCTAAGCT
AAGCCTAAGCCT
AAGCCTAAGCCT
AAGCCTAAGCCT
AAGCCTAAGCCT
AAGCCTAAGCCT
AAGCCTAAGCCT
AAGCCTAAGCCT
AAGCCTAAGCCT
AAGCCTAAGCCT
AAGCCTAAGCCTA
AGGCCTAAGCCT
AAGCCTAGCCT
AAGCCTAAGCCT
AAGCCTAAGCCT
AAGCCTAAGCGCTT
AAGCCTAAGCCT
AAGCCTAAGCCT
AAGCCTAAGCCT
AAGCCTAAGCCT
AAGCCTAAGCCT
AAGCTAAGCCT
AAGCCTAAGCCT
AAGCCTAAGCCT
AAGCTAAGCCT
AAGCCTAAGCCT
AAGCCTAAGCCT
AAGCCTAAGCCT
AAGCCTAAGCCT
AAGCCTAAGCCT
AAGCCTAAGCCT
AAGCCTAAGCCT
AAGCCTAAGCCT
AAGCCTAAGCCT
AAGCCTAAGCCT
AGGCCTAAGCCT
AAGCCTAAGCCT
AAGCCTAAGCCT
AAGCCTAAGCCTAAT
AAGCCTAAGCCTA
AAGCCTAAGCCT
AAGCCTAAGCCT
AAGCCTAAGCCT
AAGCCTAAGCCT
AAGCCTAAGCCT
AAGCCTAAGCCT
AAGCCTAAGCCT
AAGCCTAAGCCT
AAGCCTAAGCCT
AAGCCTAAGCCT
AAGCCTAAGCCT
AAGCCTAAGCCT
AAGCCTAAGCCT

AAGCCTAAGCCT
AAGCCTAAGCCT
AAGCCTAAGCCT
AAGCCTAAGCCT
AAGCCTAAGCCT
AAGCCTAAGCCT
AAGCCTAAGCCT
AAGCCTAAGCCT
AAGCCTAAGCCT
AAGCCTAAGCCT
AAGCCTAAGCCT
AAGCCTAAGCCT
AAGCCTAAGCCT
AAGCCTAAGCCT
AAGCCTAAGCCT
AAGCCTAAGCCT
AAGCCTAAGCCT
AAGCCTAAGCCT
AAGCCTAAGCCT
AAGCCTAAGCCT
AAGCCTAAGCCT
AAGCCTAAGCCT
AAGCCTAAGCCT
AAGCCTAAGCCT
AAGCCTAAGCCT
AAGCCTAAGCCT
AAGCCTAAGCCT
AAGCCTAAGCCT
AAGCCTAAGCCT
AAGCCTAAGCCT
AAGCCTAAGCCT
AAGCCTAAGCCT
AAGCCTAAGCCT
AAGCCTAAGCCT
AAGCCTAAGCCT
AAGCCTAAGCCT
AAGCCTAAGCCT
AAGCCTAAGCCT
AAGCCTAAGCCT
AAGCCTAAGCCT
AAGCCTAAGCCT
AAGCCTAAGCCT
AAGCCTAAGCCT
AAGCCTAAGCCT
AAGCCTAAGCCT
AAGCCTAAGCCT
AAGCCTAAGCCT
AAGCCTAAGCCT
AAGCCTAAGCCT
AAGCCTAAGCCT
AAGCCTAAGCCT
AAGCCTAAGCCT
AAGCCTAAGCCT
AAGCCTAAGCCT

AAGCCTAAGCCT
AAGCCTAAGCCT
AAGCCTAAGCCT
AAGCCTAAGCCT
AAGCCTAAGCCT
AAGCCTAAGCCT
AAGCCTAAGCCT
AAGCCTAAGCCT
AAGCCTAAGCCT
AAGCCTAAGCCT
AAGCCTAAGCCT
AAGCCTAAGCCT
AAGCCTAAGCCT
AAGCCTAAGCCT
AAGCCTAAGCCT
AAGCCTAAGCCT
AAGCCTAAGCCT
AAGCCTAAGCCT
AAGCCTAAGCCT
AAGCCTAAGCCT
AAGCCTAAGCCT
AAGCCTAAGCCT
AAGCCTAAGCCT
AAGCCTAAGCCT
AAGCCTAAGCCT
AAGCCTAAGCCT
AAGCCTAAGCCT
AAGCCTAAGCCT
AAGCCTAAGCCT
AAGCCTAAGCCT
AAGCCTAAGCCT
AAGCCTAAGCCT
AAGCCTAAGCCT
AAGCCTAAGCCT
AAGCCTAAGCCT
AAGCCTAAGCCT
AAGCCTAAGCCT
AAGCCTAAGCCT
AAGCCTAAGCCT
AAGCCTAAGCCT
AAGCCTAAGCCT
AAGCCTAAGCCT
AAGCCTAAGCCT
AAGCCTAAGCCT
AAGCCTAAGCCT
AAGCCTAAGCCT
AAGCCTAAGCCT
AAGCCTAAGCCT
AAGCCTAAGCCT
AAGCCTAAGCCT
AAGCCTAAGCCT
AAGCCTAAGCCT
AAGCCTAAGCCT
AAGCCTAAGCCT

AAGCCTAAGCCT
AAGCCTAAGCCT
AAGCCTAAGCCT
AAGCCTAAGCCT
AAGCCTAAGCCT
AAGCCTAAGCCT
AAGCCTAAGCCT
AAGCCTAAGCCT
AAGCCTAAGCCT
AAGCCTAAGCCT
AAGCCTAAGCCT
AAGCCTAAGCCT
AAGCCTAAGCCT
AAGCCTAAGCCT
AAGCCTAAGCCT
AAGCCTAAGCCT
AAGCCTAAGCCT
AAGCCTAAGCCT
AAGCCTAAGCCT
AAGCTAAGCCTAAGCCACCTA
AGGCCTAAGCCT
AAGCCTAAGCCTAAGC
AAGCCTAAGCCT
AAGCCTAGCCT
AAGCCTAAGCCT
AAGCTAAGCCT
AAGCCTAAGCCT
AAGCCTAAGCCT
AAGCCTAAGCCT
AAGCCTAAGCCT
AAGCCTAAGCCT
AAGCCTAAGCCT
AAGCCTAAGCCT
AAGCCTAAGCCT
AAGCCTAAGCCT
AAGCCTAAGCCT
AAGCCTAAGCCT
AAGCCTAAGCCT
AAGCCTAAGCCT
AAGCCTAAGCT
AAGCCTAAGCCT
AAGCCTAAGCCT
AAGCCTAAGCCT
AAGCCTAAGCCTAAGCT
AGCCTAAGCCT
AAGCCTAAGCCT
AAGCCTAAGCCT
AAGCCTAAGCCT
AAGCCTAAGCCT
AAGCCTAAGCCT
AAGCCTAAGCCT
AAGCCTAAAGCCT
AAGCCTAGCCT
AAGCCTAAGCCT

AAGCCTAAGCCT
AAGCCTAAGCCT
AAGCCTAAGCCT
AAGCCTAAGCCT
AAGCCTAAGCC
AGCCTAAGCCT
AAGCCTAAGCCT
AAGCCTAAGCGCCT
AAGCCTAAGCCT
AAGCCTAAGCCT
AAGCCTAAGCCT
AAGCCTAAAGCCT
AAGCCTAAGCCT
AAGCCTAAGCCT
AAGCCTAAGCCT
AAGCCTAAGCCT
AAGCCTAAGCCT
AAGCCTAAGCCT
AAGCCTAAGCCT
AAGCCTAAGCCT
AAGCCTAAGCCT
AAGCCTAAGCCT
AAGCCTAAGCCT
AAGCCTAAGCCT
AAGCCTAAGCCT
AAGCCTAAGCCT
AAGCCTAAGCCT
AAGCCTAAGCCT
AAGCCTAAGCCT
AAGCCTAAGCCT
AAGCCTAAGCCT
AAGCCTAAGCCT
AAGCCTAAGCCT
AAGCCTAAGCCT
AAGCCTAAGCCT
AAGCCTAAGCCT
AAGCCTAAGCCT
AAGCCTAAGCT
AAGCCTAAGCC
AGCCTAAGCCT
AAGCCTAAGCCT
AAGCCTAAGCCT
AAGCCTAAGCCT
AAGCCTAAGCCT
AAGCCTAAGCCT
AAGCCTAAGCCT
AAGCCTAAGCCT
AAGCCTAAGCCT
AAGCTAAGCCT
AAGCCTAAGCTAGCT
AAGCCTAAGCGCCT
AAGCCTAAGCCCT
AAGCCTAAGCCT
AAGCCTAAGCCT

AAGCCTAAGCCT
AAGCCTAAGCCT
AAGCCTAAGCCT
AAGCCTAAGCCT
AAGCCTAAGCCT
AAGCCTAAGCCT
AAGCCTAAGCCT
AAGCCTAAGCCT
AAGCCTAAGCCT
AAGCCTAAGCCT
AAGCCTAAGCCT
AAGCCTAAGCCT
AAGCCTAAGCCT
AAGCCTAAGCCT
AAGCCTAAGCCT
AAGCCTAAGCCT
AAGCCTAAGCCT
AAGCCTAAGCCT
AAGCCTAAGCCT
AAGCCTAAGCCT
AAGCCTAAGCCT
AAGCCTAAGCCT
AAGCCTAAGCCT
AAGCCTAAGCCT
AAGCCTAAGCCT
AAGCCTAAGCCT
AAGCCTAAGCCT
AAGCCTAAGCCT
AAGCCTAAGCCT
AAGCCTAAGCCT
AAGCCTAAGCCT
AAGCCTAAGCCT
AAGCCTAAGCCT
AAGCCTAAGCT
AAGCCTAAGCCTAAGC
AGCCTAAGCCT
AAGCCTAAGCTAGCT
AAGCCTAAGCCTAAGCTGCT
AAGCCTAAGCCCTAAG
AGCCTAAGCCT
AAGCCTAAGCCT
AAGCCTAAGCCT
AAGCCTAAGCCT
AAGCCTAAGCCT
AAGCCTAAGCCT
AAGCCTAAGCCT
AAGCCTAAGCCT
AAGCCTAAGCCT
AAGCCTAAGCCT
AAGCCTAAGCCT
AAGCCTAAGCCT
AAGCCTAAGCCT
AAGCCTAAGCCT
AAGCCTAAGCCT

AAGCCTAAGCCT
AAGCCTAAGCCT
AAGCCTAAGCCT
AAGCCTAAGCCT
AAGCCTAAGCCT
AAGCCTAAGCCT
AAGCCTAAGCCT
AAGCCTAAGCCT
AAGCCTAAGCCT
AAGCCTAAGCCT
AAGCCTAAGCCT
AAGCCTAAGCT
AAGCCTAAGCCT
AAGCCTAAGCCT
AAGCCTAAGCCT
AAGCCTAAGCCT
AAGCCTAAGCCT
AAGCCTAAGCCT
AAGCCTAAGCCT
AAGCCTAAGCCT
AAGCCTAAGCCT
AAGCCTAAGCCT
AAGCCTAAGCCT
AAGCCTAGCCT
AAGCCTAGCT
AAGCCTAAGCTAGCTA
AAGCCTAAGCCT
AAGCCCTAAGCCT
AAGCCCTAAGCCT
AAGCCTAAGCCT
AAGCCTAAGCCT
AAGCCTAAGCCT
AAGCCTAAGCCT
AAGCCTAAGCCT
AAGCCTAAGCCT
AAGCCTAAGCCT
AAGCCTAAGCCT
AAGCCTAAGCCT
AAGCCTAAGCCT
AAGCCTAAGCCT
AAGCCTAAGCCT
AAGCCTAAGCCT
AAGCCTAAGCCT
AAGCCTAAGCCT
AAGCCTAAGCCT
AAGCCTAAGCCT
AAGCCTAAGCCT
AAGCCTAAGCCT
AAGCCTAAGCCT
AAGCCTAAGCCT
AAGCCTAAGCCT
AAGCCTAAGCCT
AAGCCTAAGCCT

AAGCCTAAGCCT
AAGCCTAAGCCT
AAGCCTAAGCCT
AAGCCTAAGCCT
AAGCCTAAGCCT
AAGCCTAAGCCT
AAGCCTAAGCCT
AAGCCTAAGCCT
AAGCCTAAGCCT
AAGCCTAAGCCT
AAGCCTAAGCCTAAGCCT
Nr of selected repeats 403 Similarity 0.920412
Consensus:
AAGCCTAAGCCT
>Cele-UNSB01_4:125359-125607 Satlength=249 Nr of Repeats=13 RepeatLength=19
seed=TGGTTGAGGC
TGGTTGAGGCTCACAATTT
TGGTTGAGGCTCTCTATTT
TGGTTGAGGCTCACAGTTT
TGGTTGAGGCTCAGGATTTT
TGGTTGAGGCTCTCTATTA
TGGTTGAGGCTCTCTATTT
TGGTTGAGGCTCTCTATTT
TGGTTGAGGCTCTTTATTT
TGGTTGAGGCTCTTTATTT
TGGTTGAGGCTCACAGTTT
TGGTTGAGGCTCTCTATTT
TGGTTGAGGCTCTCTATTT
TGGTTGAGGCTCTTTATTT
Nr of selected repeats 12 Similarity 0.880914
Consensus:
TGGTTGAGGCTCtctATTT
>Cele-UNSB01_4:128195-129066 Satlength=872 Nr of Repeats=28 RepeatLength=19
seed=TTGGTTGAGG
TTGGTTGAGGCTCACAATT
TTGGTTGAGGCTCAAAATT
TTGGTTGAGGCTAACAATT
TTGGTTGAGGCAAAAAATT
TTGGTTGAGGCTAAAAATT
TTGGTTGAGGCTAAAAATT
TTGGTTGAGGCTAACAATT
TTGGTTGAGGCTCTCGATG
TTGGTTGAGGCTCGCGATG
TTGGTTGAGGCTTTGATT
TTGGTTGAGGCTCTCAGGAATTT
TTGGGTGAGGCTCTCAGGATT
TTGGTTGAGGCTCTCGTT
TTGGGTGAGGCTCAGGATT
TTGGTTGAGGCTCTAGATTTTCTTTTTTTTTTGCTCTAGATTTTTTTTTTTTTTTTGGGGGGGGGGGGGGGGGGGGGGCA
GATAGCTCAGTCGGTAGTGGTGGCCGCTAGCAGTCTGGAGGTCACGAGTTCAAGCCCGGCCTCACCCCCTAGGTTCACC
CAGCCTCTATTGGGAAGTGGAGCAATCCACGACTGGATTATCGGCCACAGTCCCCGGCTAGGACGTGGCTTAAATTACA
GCCCAGAGGGATCACCACCAGGCAGTGTACCTGAATCCCAGATCCGCAGTGCATAGCACTTGAAGAACGGATCGTCCTT
TAATCCTTTAATCCTTTTTTAAAGGCTAAAAATT
TTGGTTGAGGCTAACAATT

TTGGTTGAGGCTAACAATT
TTGGTTGAGGCTAAAAATT
TTGGTTGAGGCTAAAAATT
TTGGTTGAGGCTAACAATT
TTGGTTGAGGCTCTCGATG
TTGGTTGAGGCTCTCGATG
TTGGTTGAGGCTTTAATT
TTGGTTGAGGCTCTCAGGAATTT
TTGGGTGAGGCTCTTAGGATT
TTGGTTGAGGCTCTCGTT
TTGGGTGAGGCTCAGGATT
TTGGTTGAGGCTCTAGATT
Nr of selected repeats 19 Similarity 0.809582
Consensus:
TTGGTTGAGGCTaacaATt
>Cele-UNSB01_4:129890-130903 Satlength=1014 Nr of Repeats=21 RepeatLength=22
seed=TTGGTTGAGG
TTGGTTGAGGCTCAGGATTTTGAGTGAGGCTAGGGCGAGTTCTTGTTAAAATTCTATGAAGCCGCGAAAAAAATTCGGT
CACGAACCTGAAACATGACAAAAGCGTATGCGGCGTCCGTATTGATTGGCGTCTTTATATCGACTTCTTCCACTTTCCC
GTGCTCCTCAAAAACGTGACGAATCTCTCGTTCCTTCACATCCGACGGCATATTTCCGACGAAAAGCGTCCTCGTCGCC
TCGTCGTCGTCCTGTTTTAGCCGCCACGTGGATGGTTCCTTAGTCGGGCGGCCCCGATTCCCGCTTCCACTTCCGCCTT
CTCGTTTACTGTCGGCTCCGTGTTGAGCTCGATTAAATCGATCCGGAATGTATTTGCCCTCCTGATCACGTAGAATTCC
AGCTGGATCACATTGAACACGCTTGCCGAGCACTGATTTTAGACGGTCCATTAAGGTGTATCTGACCTTCCGAGCACAA
TCATTTCTCTCGAAATTCACGTAGGCTAGGCGTTCGTCTTCTTCCGGGTTTTTGACCACTTTTATCTGAAAAAATTGGT
ATAAAATTGCGGAAATCTCAGGATTTT
TTGGTTGAGGCTCAGGATTTT
TTGGTTGAGGCTCTCTGGATTT
TTGGTTGAGGCTTTCTGGATTT
TTAGTTGAGGCTCTCTGGATTT
TTGGTTGAGGCTCTCAGGATTT
TTGGTTGAGGCTTTCTGGATTT
TTGGGTGAGGCTCTCTGGATTT
TTGGTTGAGGCTCAGGATTT
TTGGTTGAGGCTTTCTGGTTTT
TTGGTTGAGGCTCTCTGGATTT
TTGGTTGAGGCTCTCAGGATTT
TTGGTTGAGGCTTTCTGGATTT
TTGGGTGAGGCTCTCTGGATTT
TTGGTTGAGGCTCAGGATTT
TTGGTTGAGGCTTTCTGGATTT
TTGGTTGAGGCTCTCTGGATTT
TTGGTTGAGGCTCTCAGGATTT
TTGGTTGAGGCTTTCTGGATTT
TTGGGTGAGGCTCTCTGGATTT
TTGGTTGAGGCTCAGGATTT
Nr of selected repeats 16 Similarity 0.915152
Consensus:
TTGGTTGAGGCTcTCTGGATTT
>Cele-UNSB01_4:160082-160208 Satlength=127 Nr of Repeats=8 RepeatLength=14
seed=CCTAAGCCTA
CCTAAGCCTAAAGCCCTAAGCACAAGGT
CCTAAGCCTAAGGT
CCTAAGCCTAAGGC
CCTAAGCCTAATGC

CCTAGGCCTAAGGC
CCTAAGCCTAAGGC
CCTAAGCCTAAGGC
CCTAAGCCTAAGGC
Nr of selected repeats 7 Similarity 0.918367
Consensus:
CCTAAGCCTAAGGC
>Cele-UNSB01_4:186112-186245 Satlength=134 Nr of Repeats=7 RepeatLength=19
seed=CCTCAACCAA
CCTCAACCAAAATCTAGAG
CCTCAACCAAAATTCTGAG
CCTCAACCAAAATCGAGAG
CCTCAACCAAAATCTAGAG
CCTCAACCAAAATTCTGTG
CCTCAACCAAAATCGAGAG
CCTCACCCAAAATTTTGAG
Nr of selected repeats 7 Similarity 0.826232
Consensus:
CCTCAACCAAAATcnaGAG
>Cele-UNSB01_4:201016-202120 Satlength=1105 Nr of Repeats=53 RepeatLength=20
seed=CTCAACCAAA
CTCAACCAAAAAATCATGGTAGC
CTCAACCAAAAATCATGGTAGC
CTCAACCAAAATTCCGGAGAGC
CTCACCCAAAAATCCAGAAAGC
CTCAACCAAAAATCCTTAGC
CTCAACCAAAAATCCTGAGC
CTCAACCAAAAAATCATGGTAGC
CTCAACCAAAAATCCTGAAC
CTCAACCAAAAATCCTGAAC
CTCAATCAAAAATCCTGAGC
CTCAACCAAAAATCATGGTAGC
CTCAACCAAAAATCCTGAGC
CTCAATCAAAAATCCTTAGC
CTCAACCAAAAATCCTTAGC
CTCAACCAAAAATCATGGTAGC
CTCAACGAAATATCCTGAGC
CTCAACCAAAAATCCTGAGC
CTCAACCAAAAATTCTTAGC
CTCAACCAAAAATCATGGTAGC
CTCAACCAAAAATCCTGAGC
CTCAATCAAAAATCCTGAGC
CTCAATCAAAAATCATGGTAGC
CTCAACCAAAAATCCTGAGC
CTCAATCAAAAATCCTTAGC
CTCAACCAAAAATCATGGTAGC
CTCAACGAAATATCCTGAGC
CTCAACCAAAAATCCTGAGAGC
CTCAACCAAAAATCCTGGTAGC
CTCAACCAAAAATCCTGAGC
CTCAACCAAAAATTCTTAGC
CTCAACCAAAAATCATGGTAGC
CTCAACCAAAAATCCTGAGT
CTCAACCAAAAATCCTGAGAGT

CTCAACCAAAAATCATGGTAGC
CTCAACGAAATATCCTGAGC
CTCAACCAAAAATCCTGAGAGC
CTCAACCAAAAATCCTGAGC
CTCAATCAAAAATCCTGAGC
CTCAACCAAAAATCATGGTAGC
CTCAACCAAAAATCCTGAGC
CTCAATCAAAAATCCTTAGC
CTCAACCAAAAATCCTGGTAGC
CTCAACCAAAAATCCTGAGC
CTCAATCAAAAATCCTGAGC
CTCAATCAAAAATCATGGTAGC
CTCAACCAAAAATCCTGAGC
CTCAATCAAAAATCCTTAGC
CTCAACCAAAAATCCTGGTAGC
CTCAACCAAAAATCCTGAGC
CTCAATCAAAAATCCTTAGC
CTCAACCAAAAATCATGGTAGC
CTCAACGAAATATCCTGATC
CTCACCCAAAAATCCTAAGC
Nr of selected repeats 32 Similarity 0.882930
Consensus:
CTCAAcCAAAAATCCTgAGC
>Cele-UNSB01_4:221202-221862 Satlength=661 Nr of Repeats=32 RepeatLength=20
seed=CTCAGCCTCA
CTCAGCCTCACCCAAAATCTTTAGCCTCAACCAAAAATC
CTCAGCCTCAATCAAAAATT
CTCAGCCTCAACCAAAAATT
CTCAGCCTCAACCTAAAATT
CTCAGCCTCAACCAAAAATT
CTGAGCCTCAACCAAAAATT
CTCAGCCTCAATCAAAAATT
CTCAGCCTCAACCAAAAATT
CTCAGCCTCAACCAAAAATT
CTCAGCCTCAACCTAAAATT
CTCAGCCTCAACCAAAAATC
CTCAGCCTCAATCAAAAATC
CTCAGCCTCAACCAAAAATT
CTCAGCCTCAATCAAAAATT
CTCAGCCTCAACCAAAAATT
CTCAGCCTCAACCAAAAATT
CTCAGCCTCAACCTAAAAATT
CTCAGCCTCAACCTAAAATT
CTCAGCCTCAACCAAAAATT
CTGAGCCTCAACCAAAAATT
CTCAGCCTCAACCAAAAATT
CTCAGCCTCAACCAAAAATT
CTCAGCTTCAACCAAAAATC
CTCAGCCTCAACCAAAAATT
CTCAGCCTCAACCAAAAATT
CTCAGCCTCAACCAAAAATT
CTCAGCCTCAACCAAAAATT
CTCAGCCTCAACCAAAAATT
CTCAGCCTCAACCAAAAATT

CTCAGCCTCAACCAAAAATT
CTGAGCCTCAACCAAAAATT
CTCAGCTTCAACCAAAAATC
Nr of selected repeats 30 Similarity 0.934713
Consensus:
CTCAGCCTCAACCAAAAATT
>Cele-UNSB01_4:236013-237239 Satlength=1227 Nr of Repeats=63 RepeatLength=18
seed=CTCAACCAAA
CTCAACCAAACTCTTAGT
CTCAACCAAACTCTTAGC
CTCAACCAAACTCTTCAGC
CTCAACCAAACTCTTAGC
CTCAACCAAACTCTTCAGC
CTCAACCAAACTCTTAGC
CTCAACCAAACTCTTAGC
CTCAACCAAACTCTTAGC
CTCAACCAAACTCTTCAGC
CTCACCCAAATTCTCAGC
CTCACCCAAATTCTCAGC
CTCAACCAAACTCTTAGT
CTCAACCAAACTCTTCAGCCTTACCCAAATTCTCAGC
CTCACCCAAATTCTCAGC
CTCAACCAAACTCTTAGC
CTCAACCAAACTCTTCAGC
CTCAACCAAACTCTTAGC
CTCAACCAAACTCTTAGC
CTCAACCAAACTCTTAGC
CTCAACCAAACTCTTCAGC
CTCAACCAAACTCTTAGC
CTCAACCAAACTCTTAGT
CTCAACCAAACTCTTAGT
CTCAACCAAACTCTTAGC
CTCAACCAAACTCTTCAGC
CTCAACCAAACTCTTAGC
CTCAACCAAACTCTTCAGC
CTCAACCAAACTCTTAGC
CTCAACCAAACTCTTAGC
CTCAACCAAACTCTTAGC
CTCAACCAAACTCTTCAGC
CTCACCCAAATTCTCAGC
CTCACCCAAATTCTCAGC
CTCAACCAAACTCTTAGT
CTCAACCAAACTCTTCAGC
CTCACCCAAATTCTCAGC
CTCAACCAAACTCTTAGC
CTCAACCAAACTCTTCAGC
CTCAACCAAACTCTTAGC
CTCAACCAAACTCTTAGC
CTCAACCAAACTCTTAGC
CTCAACCAAACTCTTCAGC
CTCAACCAAACTCTTAGC
CTCAACCAAACTCTTAGC
CTCAACCAAACTCTTAGC
CTCAACCAAACTCTTCAGC

CTCAACCAAACTCTTAGC
CTCAACCAAACTCTTAGC
CTCAACCAAACTCTTCAGC
CTCACCCAAATTCTCAGC
CTCACCCAAATTCTCAGC
CTCAACCAAACTCTTAGT
CTCAACCAAACTCTTCAGCCTTACCCAAAATCTCAGC
CTCAACCAAACTCTTTAGC
CTCAACCAAATTCTCAGC
CTCAGCCAAACTCTTCAGCCTCACCCTAACTCTTAGC
CTCAACCAAATTCTTCAGT
CTCAACCAAATTCTCAGC
CTCACCCAAATTCTCAGC
CTCAACCAAACTCTTCAGC
CTCAACCAAACTCTTAGC
CTCAACCAAACTCTTAGC
CTCAACCAAATTCTCAGCCTCACCTAAACTCTTCAGC
Nr of selected repeats 43 Similarity 0.898938
Consensus:
CTCAaCCAAAcTCTtAGC
>Cele-UNSB01_4:242271-242555 Satlength=285 Nr of Repeats=12 RepeatLength=22
seed=TGAGGCTCTC
TGAGGCTCTCTAGATTTTTGGC
TGAGGCTCTCTAGATTTTTGGT
TGAGGCTCTCAGGATTTTTGGT
TGAGGCTCTCTTGATTTTTGAT
TGAGGCTCACTGGATTTTTGAT
TGAGGCTCTCTGGATTTTTGGGTGAGGCTCAGAATTTTCGGT
TGAGGCTCTCTTGATTTTTGGT
TGAGGCTCTCTGGATTTTTGAT
TGAGGCTCTCTGGATTTTTGGG
TGAGGCTCTCAGGATTTTTGGT
TGAGGCTCTCTGGATTTTTGGT
TGAGGCTCTCTGGATTTTTGGT
Nr of selected repeats 11 Similarity 0.886501
Consensus:
TGAGGCTCTCTgGATTTTTGgT
>Cele-UNSB01_4:244645-245490 Satlength=846 Nr of Repeats=8 RepeatLength=18
seed=AGCCTCAACC
AGCCTCAACCAAACTCTTACAAGGAAAGGAAGTCAATCTATCTATGCTCCAGTGCATTTTTTCGCTGACTTCTATATCA
AGTAAATGGCCTCAAAAGGGGTTCTGAGAGATTAGAAATTGTTTTTGGGGGTTTTTAGGGTCTAACAAACAATGTGGGA
GCTTTTTGGAAATTTTTATTTTTTTGAAAAAAATCTGAAATTTTTTCGATCCATTGGACACCAAATTTTTGGTCAAAAA
TTTTTTTTTTTGAAAAAATTCCAAAACTATTTTTTTGTATCCGGGCATGCAAAAAGCTACATATTTCTTCGAATTGAGC
CTTTTTACAATTTTTTACAATTTTTTACAAAGGGCCCGATTGCAGTTTTTAAAAGTTGAAGAAAAAAAATTATTTCCACT
GAAAGAATCGAACACGAGACCTTTATTTTATTATTCCAAGCGTTTACCATTCGGCCATCAACCGTTGTTTTTTATTGTT
TTCCATGTTTGTAACCATTTCATAATGATTGAAAATCATTTTTTGTATAAGTAAAATGAATAAGTACGAAATAGTATTG
AAAACTAGGAGGGCGCAGAGAAAAATTTTCAGATGGCGATTTCAGGGGTTCAGAGTTGAGTTCCGCCGCTGAAATTTTT
GTGGTGATCTAGTCTAAACATAAGCTTTCAAATGACATACGTCTCAATTTAAAGTCCGATACTAGATTGACTTCCTTTC
CTTGTT
AGCCTCAACCAAACTCTTC
AGCCTCAACCAAATTCTT
AGCCTCAACCAAACGCTT
AGCCTCAACCAAATTCTT
AGCCTCAACCAAACTCTT

AGCCTCAACCAAACTCTT
AGCCTCACCCAAACTCTTC
Nr of selected repeats 5 Similarity 0.925926
Consensus:
AGCCTCAACCAAActCTT
>Cele-UNSB01_4:286805-287284 Satlength=480 Nr of Repeats=13 RepeatLength=37
seed=TTGGCGGGAA
TTGGCGGGAATTCAAATTTCTGAGAAAATATT
TTGGCGGGAATTCAAAATTTAATTTTTTGAACATATT
TTGGCGGGAATTCAAAATTTAATTTTTCGAAAACATT
TTGGCGGGAATTTAAAATTTTAATTTTTTGAAAATATT
TTGGCGGGAATTCAAATTTTAATTTTTTGAAAATATT
TTGGCGGGAATTCAAATTTTAATTTTTTGAAAATATT
TTGGCGGGAATTCAAATTTTAATTTTTTGAAAATATT
TTGGCGGGAATTTAAAGTTTTAATTTTTTGAAAATATT
TTGCCGGGAATTCAAATTTTTATTTTTTGAAAATATT
TTGGCGGGAATTTAAAATTTTAATTTTTTGAAAATATT
TTGCCGGGAATTCAAATTTTTATTTTTTGAAAATATT
TTGGCGGGAATTCAAATTTTAATTTTTTGAAAATATT
TTGGCGGGAATTCAAATTTTAATTTTTTGAAAATATT
Nr of selected repeats 9 Similarity 0.933934
Consensus:
TTGgCGGGAATTCAAAtTTTaATTTTTTGAAAATATT
>Cele-UNSB01_4:287372-287852 Satlength=481 Nr of Repeats=32 RepeatLength=15
seed=TCGTGGTGAG
TCGTGGTGAGAGCTT
TCGTGGAGAGACCCT
TCTTGGTGAGACCCA
TCGTGGTGAGACCCT
TCGTGGTGAGACCCA
TCGTGGTGAGACCCT
TCGTGGTGAGACCCA
TCGTGGTGAGACCCT
TCGTGGTGAGACCCA
TCGTGGTGAGACCCT
TCGTGGTGAGACCCT
TCGTGGTGAGACCCT
TCGTGGTGAGACCCA
TCGGGGTGAGACCCA
TCGTGGTGAGACCCA
TCGAGGTGAGACCCA
TCGTGGTGAGACCTT
TCGTGGTGAGACCCT
TCGTGGTGAGACCCA
TCGTTGTGAGACCCT
TCGTGGTGAGACCCA
TCGTGGTGAGACCTT
TCGTGGTGAGACCCA
TCGTGGTGAGACCTT
TCGTGGAGAGACCCA
TCGTGGTGAGACCCT
TCGTGGTGAGACCCA
TCGTGGTGAGACCCA
TCGTGGTGAGACCTT

TCGTGGTGAGACCTT
TCGTGGTGAGACCCA
TCGTGGTGAGACGCA
Nr of selected repeats 32 Similarity 0.882258
Consensus:
TCGTGGTGAGACCCa
>Cele-UNSB01_4:355820-356311 Satlength=492 Nr of Repeats=11 RepeatLength=45
seed=GAAGGATCAG
GAAGGATCAGAACATCAGGAGGTACCCATGTGATCATTCGGCTCT
GAAAGATCAGATCATCAGGCAGAGCCTCATGTTCTTTTGGAGCT
GAAGGATCAGACCATCAGGAGGTACCCTTACGATCCTTCTTATCT
GAAGGATCAGATCATCAGAAGGTACCCATGTGATCCTTCGGATCG
GAAGGATCAGATCATCAGGCAGAGCCTCATGTTCTTTTAGAGCT
GAAGGATCAGAACATCAGGAAGAGCCTCGTGATCCTTTGGAGCT
GAAGGATCAGACCATCAGGAGGTACCCGTACGATCCTTCTTATCT
GAAGGATCAGAACATCAGAAGGTACCCATGTGATCCTTCGGAGCT
GAAGGTTCAGAACATCAGGAAGAGCCTCGTGATCCTTTGGAGCT
GAAGTATCAGACCATCAGGAGGTACCCTTACGATCCTTCTTATCT
GAAGGATCAGAACATCAGAAGGTACCCATGTGATCCTTCGGAGCT
Nr of selected repeats 7 Similarity 0.826455
Consensus:
GAAGGATCAGAnCATCAGgAGGTACCCaTgtGATCCTTCggAtCT
>Cele-UNSB01_4:388221-388493 Satlength=273 Nr of Repeats=8 RepeatLength=34
seed=TTGCAGGAAA
TTGCAGGAAATTTCAATTACCGCAATTTGCCGGT
TTGCAGGAAATTTCAATTCAGCCGATTTGCCGGT
TTGCCGGAAATTTCAATTCCGGCAATTTGCCGGT
TTGCAGGAAATTTCAATTACGGCAATTTGCCGGT
TTGCAGGAAATTTCAATTCCGCCAATTCGCCGGT
TTGCAGGAAATTTTAATTACGGAAATTTGTCGGT
TTGCCGGAAATTTCAATTCCGTCAATTTGCCAAC
TTGCAGGAAATTTCCATTCCGGCAATTTGCCGAT
Nr of selected repeats 8 Similarity 0.823529
Consensus:
TTGCaGGAAATTTCAATTcCGgCAATTTGCCGgT
>Cele-UNSB01_4:391568-391630 Satlength=63 Nr of Repeats=4 RepeatLength=15
seed=TCCCCTTTTC
TCCCCTTTTCGTATC
TCCCCTTTTCGCAGA
TCCCCTTTTTGCAGA
TCCCCTTTTCGTTCTGC
Nr of selected repeats 3 Similarity 0.762963
Consensus:
TCCCCTTTTcGcAga
>Cele-UNSB01_4:552425-552551 Satlength=127 Nr of Repeats=9 RepeatLength=12
seed=TAGGCTTAGG
TAGGCTTAGGTT
TAGGCTAAGGCT
TAGGGTTAGGCTGAGGCT
TAGTCTTAGGCC
TAGGTTTAGGCT
TAGGCTTAGGCT
TAGGCTTAAGCTCAGGTTCAGACT
TAGGCTTAGGAT

TAGGATTAGGCT
Nr of selected repeats 7 Similarity 0.788360
Consensus:
TAGGcTTAGGcT
>Cele-UNSB01_4:600866-601084 Satlength=219 Nr of Repeats=10 RepeatLength=16
seed=AGGCATGTAG
AGGCATGTAGGCATGT
AGGCATGTAGGCATGT
AGGCATGTAGGCATGA
AGGCGTGTAGGCATGT
AGGCATGTAGGCATGT
AGCCATGTAGCAATGC
AGGCATGTAGCCATGT
AGGCACGTAGGCAGGT
ATGCATGTAGGCATGTAGACATTTCATGTCATCAATTTAGGTTTCCAAGCTAAAACTCCTAAATCTTGGAATGT
AGGCGTGTAGGCGTGC
Nr of selected repeats 9 Similarity 0.796296
Consensus:
AGGCaTGTAGgCATGt
>Cele-UNSB01_4:634851-634943 Satlength=93 Nr of Repeats=6 RepeatLength=15
seed=TCCCCTTTTC
TCCCCTTTTCGTTGC
TCCCCTTTTCGTATC
TCCCCTTTTCGTATC
TCCCCTTTTCGCAGA
TCCCCTTTTTGCAGA
TCCCCTTTTCGTTCTGC
Nr of selected repeats 5 Similarity 0.768889
Consensus:
TCCCCTTTTcGtagc
>Cele-UNSB01_4:646705-646786 Satlength=82 Nr of Repeats=4 RepeatLength=15
seed=TAAGCCTAAG
TAAGCCTAAGCCCAC
TAAGCCTAAGCCCAC
TAAGCCTAAGCCCAC
TAAGCCTAAGCCCACTTAGGCTAAGCCCATTAAGCC
Nr of selected repeats 3 Similarity 1.000000
Consensus:
TAAGCCTAAGCCCAC
>Cele-UNSB01_4:695215-705007 Satlength=9793 Nr of Repeats=96 RepeatLength=102
seed=TGATATGTCG
TGATATGTCGAACGATTGATAGTTTGGGAATCAAGAGAATCAGTTGGACGATCAATTCCATCTTTGGGTCTTGCTGCAG
CATATCTTTCTCCAGTACCACCT
TGATATGTCGAACGATTGATAGTTTGAGAATCAAGAGAATCAGTTGGACGATCAATTCCATCTTTGGGTCTTGCTGCAG
CATATCTTTCTCCAGTACCTCCC
TGATATGTCGAACGATTGATAGTTTGAGAATCAAGAGAATCAGTTGGACGATCAATTCCATCTTTGGGTCTTGCTGCAG
CATATCTTTCTCCAGTACCTCCC
TGATATGTCGAACGATTGATAGTTTGAGAATCAAGAGAATCAGTTGGACGATCAATTCCATCTTTGGGTCTTGCTGCAG
CATATCTTTCTCCAGTACCTCCC
TGATATGTCGAACGATTGATAGTTTGAGAATCAAGAGAATCAGTTGGACGATCAATTCCATCTTTGGGTCTTGCTGCAG
CATATCTTTCTCCAGTACCTCCC
TGATATGTCGAACGATTGATAGTTTGAGAATCAAGAGAATCAGTTGGACGATCAATTCCATCTTTGGGTCTTGCTGCAG
CATATCTTTCTCCAGTACCACCT

TGATATGTCGAACGATTGATAGTTTGAGAATCAAGAGAATCTGTTGGACGATCAATTCCATCTTTGGGTCTTGCTGCAG
CATATCTTTCTCCAGTACCACCT
TGATATGTCGAACGATTGATAGTTTGAGAATCAAGAGAATCTGTTGGACGATCAATTCCATCTTTGGGTCTTGCTGCAG
CATATCTTTCTCCAGTACCACCT
TGATATGTCGAACGATTGATAGTTTGAGAATCAAGAGAATCTGTTGGACGATCAATTCCATCTTTGGGTCTTGCTGCAG
CATATCTTTCTCCAGTACCACCT
TGATATGTCGAACGATTGATAGTTTGAGAATCAAGAGAATCAGTTGGACGATCAATTCCATCTTTGGGTCTTGCTGCAG
CATATCTTTCTCCAGTACCACCT
TGATATGTCGAACGATTGATAGTTTGAGAATCAAGAGAATCAGTTGGACGATCAATTCCATCTTTGGGTCTTGCTGCAG
CATATCTTTCTCCAGTACCACCT
TGATATGTCGAACGATTGATAGTTTGAGAATCAAGAGAATCAGTTGGACGATCAATTCCATCTTTGGGTCTTGCTGCAG
CATATCTTTCTCCAGTACCACCT
TGATATGTCGAACGATTGATAGTTTGAGAATCAAGAGAATCTGTTGGACGATCAATTCCATCTTTGGGTCTTGCTGCAG
CATATCTTTCTCCAGTACCTCCC
TGATATGTCGAACGATTGATAGTTTGAGAATCAAGAGAATCTGTTGGACGATCAATTCCATCTTTGGGTCTTGCTGCAG
CATATCTTTCTCCAGTACTTCCC
TGATATGTCGAACGATTGATAGTTTGAGAATCAAGAGAATCTGTTGGACGATCAATTCCATCTTTGGGTCTTGCTGCAG
CATATCTTTCTCCAGTACCACCT
TGATATGTCGAACGATTGATAGTTTGAGAATCAAGAGAATCTGTTGGACGATCAATTCCATCTTTGGGTCTTGCTGCAG
CATATCTTTCTCCAGTACTTCCC
TGATATGTCGAACGATTGATAGTTTGAGAATCAAGAGAATCAGTTGGACGATCAATTCCATCTTTGGGTCTTGCTGCAG
CATATCTTTCTCCAGTACCACCT
TGATATGTCGAACGATTGATAGTTTGAGAATCAAGAGAATCTGTTGGACGATCAATTCCATCTTTGGGTCTTGCTGCAG
CATATCTTTCTCCAGTACTTCCC
TGATATGTCGAACGATTGATAGTTTGAGAATCAAGAGAATCTGTTGGACGATCAATTCCATCTTTGGGTCTTGCTGCAG
CATATCTTTCTCCAGTACCACCT
TGATATGTCGAACGATTGATAGTTTGGGAATCAAGAGAATCTGTTGGACGATCAATTCCATCTTTGGGTCTTGCTGCAG
CATATCTTTCTCCAGTACTTCCC
TGATATGTCGAACGATTGATAGTTTGAGAATCAAGAGAATCTGTTGGACGATCAATTCCATCTTTGGGTCTTGCTGCAG
CATATCTTTCTCCAGTACTTCCC
TGATATGTCGAACGATTGATAGTTTGAGAATCAAGAGAATCTGTTGGACGATCAATTCCATCTTTGGGTCTTGCTGCAG
CATATCTTTCTCCAGTACCACCT
TGATATGTCGAACGATTGATAGTTTGAGAATCAAGAGAATCTGTTGGACGATCAATTCCATCTTTGGGTCTTGCTGCAG
CATATCTTTCTCCAGTACCACCT
TGATATGTCGAACGATTGATAGTTTGAGAATCAAGAGAATCAGTTGGACGATCAATTCCATCTTTGGGTCTTGCTGCAG
CATATCTTTCTCCAGTACCACCT
TGATATGTCGAACGATTGATAGTTTGAGAATCAAGAGAATCAGTTGGACGATCAATTCCATCTTTGGGTCTTGCTGCAG
CATATCTTTCTCCAGTACCACCT
TGATATGTCGAACGATTGATAGTTTGAGAATCAAGAGAATCAGTTGGACGATCAATTCCATCTTTGGGTCTTGCTGCAG
CATATCTTTCTCCAGTACCACCT
TGATATGTCGAACGATTGATAGTTTGAGAATCAAGAGAATCTGTTGGACGATCAATTCCATCTTTGGGTCTTGCTGCAG
CATATCTTTCTCCAGTACCACCT
TGATATGTCGAACGATTGATAGTTTGAGAATCAAGAGAATCTGTTGGACGATCAATTCCATCTTTGGGTCTTGCTGCAG
CATATCTTTCTCCAGTACCACCT
TGATATGTCGAACGATTGATAGTTTGAGAATCAAGAGAATCTGTTGGACGATCAATTCCATCTTTGGGTCTTGCTGCAG
CATATCTTTCTCCAGTACCACCT
TGATATGTCGAACGATTGATAGTTTGGGAATCAAGAGAATCTGTTGGACGATCAATTCCATCTTTGGGTCTTGCTGCAG
CATATCTTTCTCCAGTACCACCT
TGATATGTCGAACGATTGATAGTTTGAGAATCAAGAGAATCAGTTGGACGATCAATTCCATCTTTGGGTCTTGCTGCAG
CATATCTTTCTCCAGTACCACCT
TGATATGTCGAACGATTGATAGTTTGAGAATCAAGAGAATCAGTTGGACGATCAATTCCATCTTTGGGTCTTGCTGCAG
CATATCTTTCTCCAGTACCACCT
TGATATGTCGAACGATTGATAGTTTGGGAATCAAGAGAATCTGTTGGACGATCAATTCCATCTTTGGGTCTTGCTGCAG
CATATCTTTCTCCAGTACCTCCC

TGATATGTCGAACGATTGATAGTTTGAGAATCAAGAGAATCAGTTGGACGATCAATTCCATCTTTGGGTCTTGCTGCAG
CATATCTTTCTCCAGTACCTCCC
TGATATGTCGAACGATTGATAGTTTGGGAATCAAGAGAATCTGTTGGACGATCAATTCCATCTCTGGGTCTTGCTGCAG
CATATCTTTCTCCAGTACCTCCC
TGATATGTCGAACGATTGATAGTTTGAGAATCAAGAGAATCAGTTGGACGATCAATTCCATCTTTGGGTCTTGCTGCAG
CATATCTTTCTCCAGTACTTCCC
TGATATGTCGAACGATTGATAGTTTGAGAATCAAGAGAATCTGTTGGACGATCAATTCCATCTTTGGGTCTTGCTGCAG
CATATCTTTCTCCAGTACTTCCC
TGATATGTCGAACGATTGATAGTTTGAGAATCAAGAGAATCTGTTGGACGATCAATTCCATCTTTGGGTCTTGCTGCAG
CATATCTTTCTCCAGTACTTCCC
TGATATGTCGAACGATTGATAGTTTGAGAATCAAGAGAATCAGTTGGACGATCAATTCCATCTTTGGGTCTTGCTGCAG
CATATCTTTCTCCAGTACCTCCC
TGATATGTCGAACGATTGATAGTTTGAGAATCAAGAGAATCAGTTGGACGATCAATTCCATCTTTGGGTCTTGCTGCAG
CATATCTTTCTCCAGTACCACCT
TGATATGTCGAACGATTGATAGTTTGAGAATCAAGAGAATCTGTTGGACGATCAATTCCATCTTTGGGTCTTGCTGCAG
CATATCTTTCTCCAGTACCACCT
TGATATGTCGAACGATTGATAGTTTGAGAATCAAGAGAATCTGTTGGACGATCAATTCCATCTTTGGGTCTTGCTGCAG
CATATCTTTCTCCAGTACCACCT
TGATATGTCGAACGATTGATAGTTTGAGAATCAAGAGAATCTGTTGGACGATCAATTCCATCTTTGGGTCTTGCTGCAG
CATATCTTTCTCCAGTACCACCT
TGATATGTCGAACGATTGATAGTTTGAGAATCAAGAGAATCAGTTGGACGATCAATTCCATCTTTGGGTCTTGCTGCAG
CATATCTTTCTCCAGTACCACCT
TGATATGTCGAACGATTGATAGTTTGAGAATCAAGAGAATCAGTTGGACGATCAATTCCATCTTTGGGTCTTGCTGCAG
CATATCTTTCTCCAGTACCACCT
TGATATGTCGAACGATTGATAGTTTGGGAATCAAGAGAATCTGTTGGACGATCAATTCCATCTTTGGGTCTTGCTGCAG
CATATCTTTCTCCAGTACCTCCC
TGATATGTCGAACGATTGATAGTTTGGGAATCAAGAGAATCTGTTGGACGATCAATTCCATCTGTGGGTCTTGCTGCAG
CATATCTTTCTCCAGTACCTCCC
TGATATGTCGAACGATTGATAGTTTGAGAATCAAGAGAATCAGTTGGACGATCAATTCCATCTTTGGGTCTTGCTGCAG
CATATCTTTCTCCAGTACCACCT
TGATATGTCGAACGATTGATAGTTTGAGAATCAAGAGAATCTGTTGGACGATCAATTCCATCTTTGGGTCTTGCTGCAG
CATATCTTTCTCCAGTACTTCCC
TGATATGTCGAACGATTGATAGTTTGAGAATCAAGAGAATCTGTTGGACGATCAATTCCATCTTTGGGTCTTGCTGCAG
CATATCTTTCTCCAGTACTTCCC
TGATATGTCGAACGATTGATAGTTTGAGAATCAAGAGAATCAGTTGGACGATCAATTCCATCTTTGGGTCTTGCTGCAG
CATATCTTTCTCCAGTACCACCT
TGATATGTCGAACGATTGATAGTTTGGGAATCAAGAGAATCAGTTGGACGATCAATTCCATCTTTGGGTCTTGCTGCAG
CATATCTTTCTCCAGTACTTCCC
TGATATGTCGAACGATTGATAGTTTGAGAATCAAGAGAATCAGTTGGACGATCAATTCCATCTTTGGGTCTTGCTGCAG
CATATCTTTCTCCAGTACTTCCC
TGATATGTCGAACGATTGATAGTTTGAGAATCAAGAGAATCAGTTGGACGATCAATTCCATCTTTGGGTCTTGCTGCAG
CATATCTTTCTCCAGTACCACCT
TGATATGTCGAACGATTGATAGTTTGAGAATCAAGAGAATCAGTTGGACGATCAATTCCATCTTTGGGTCTTGCTGCAG
CATATCTTTCTCCAGTACCACCT
TGATATGTCGAACGATTGATAGTTTGAGAATCAAGAGAATCTGTTGGACGATCAATTCCATCTTTGGGTCTTGCTGCAG
CATATCTTTCTCCAGTACCTCCC
TGATATGTCGAACGATTGATAGTTTGAGAATCAAGAGAATCTGTTGGACGATCAATTCCATCTTTGGGTCTTGCTGCAG
CATATCTTTCTCCAGTACCTCCC
TGATATGTCGAACGATTGATAGTTTGAGAATCAAGAGAATCTGTTGGACGATCAATTCCATCTTTGGGTCTTGCTGCAG
CATATCTTTCTCCAGTACCACCT
TGATATGTCGAACGATTGATAGTTTGAGAATCAAGAGAATCAGTTGGACGATCAATTCCATCTTTGGGTCTTGCTGCAG
CATATCTTTCTCCAGTACCACCT
TGATATGTCGAACGATTGATAGTTTGGGAATCAAGAGAATCAGTTGGACGATCAATTCCATCTTTGGGTCTTGCTGCAG
CATATCTTTCTCCAGTACCACCT

TGATATGTCGAACGATTGATAGTTTGAGAATCAAGAGAATCTGTTGGACGATCAATTCCATCTTTGGGTCTTGCTGCAG
CATATCTTTCTCCAGTACCTCCC
TGATATGTCGAACGATTGATAGTTTGGGAATCAAGAGAATCTGTTGGACGATCAATTCCATCTGTGGGTCTTGCTGCAG
CATATCTTTCTCCAGTACCTCCC
TGATATGTCGAACGATTGATAGTTTGAGAATCAAGAGAATCAGTTGGACGATCAATTCCATCTTTGGGTCTTGCTGCAG
CATATCTTTCTCCAGTACCACCT
TGATATGTCGAACGATTGATAGTTTGGGAATCAAGAGAATCAGTTGGACGATCAATTCCATCTTTGGGTCTTGCTGCAG
CATATCTTTCTCCAGTACTTCCC
TGATATGTCGAACGATTGATAGTTTGAGAATCAAGAGAATCAGTTGGACGATCAATTCCATCTTTGGGTCTTGCTGCAG
CATATCTTTCTCCAGTACCACCT
TGATATGTCGAACGATTGATAGTTTGAGAATCAAGAGAATCAGTTGGACGATCAATTCCATCTTTGGGTCTTGCTGCAG
CATATCTTTCTCCAGTACCACCT
TGATATGTCGAACGATTGATAGTTTGGGAATCAAGAGAATCAGTTGGACGATCAATTCCATCTTTGGGTCTTGCTGCAG
CATATCTTTCTCCAGTACTTCCC
TGATATGTCGAACGATTGATAGTTTGAGAATCAAGAGAATCAGTTGGACGATCAATTCCATCTTTGGGTCTTGCTGCAG
CATATCTTTCTCCAGTACTTCCC
TGATATGTCGAACGATTGATAGTTTGGGAATCAAGAGAATCTGTTGGACGATCAATTCCATCTTTGGGTCTTGCTGCAG
CATATCTTTCTCCAGTACCTCCC
TGATATGTCGAACGATTGATAGTTTGAGAATCAAGAGAATCTGTTGGACGATCAATTCCATCTTTGGGTCTTGCTGCAG
CATATCTTTCTCCAGTACCACCT
TGATATGTCGAACGATTGATAGTTTGAGAATCAAGAGAATCTGTTGGACGATCAATTCCATCTTTGGGTCTTGCTGCAG
CATATCTTTCTCCAGTACCACCT
TGATATGTCGAACGATTGATAGTTTGGGAATCAAGAGAATCTGTTGGACGATCAATTCCATCTTTGGGTCTTGCTGCAG
CATATCTTTCTCCAGTACCACCT
TGATATGTCGAACGATTGATAGTTTGGGAATCAAGAGAATCTGTTGGACGATCAATTCCATCTTTGGGTCTTGCTGCAG
CATATCTTTCTCCAGTACCACCT
TGATATGTCGAACGATTGATAGTTTGAGAATCAAGAGAATCTGTTGGACGATCAATTCCATCTTTGGGTCTTGCTGCAG
CATATCTTTCTCCAGTACCACCT
TGATATGTCGAACGATTGATAGTTTGAGAATCAAGAGAATCTGTTGGACGATCAATTCCATCTTTGGGTCTTGCTGCAG
CATATCTTTCTCCAGTACCACCT
TGATATGTCGAACGATTGATAGTTTGAGAATCAAGAGAATCTGTTGGACGATCAATTCCATCTTTGGGTCTTGCTGCAG
CATATCTTTCTCCAGTACCACCT
TGATATGTCGAACGATTGATAGTTTGAGAATCAAGAGAATCTGTTGGACGATCAATTCCATCTTTGGGTCTTGCTGCAG
CATATCTTTCTCCAGTACCACCT
TGATATGTCGAACGATTGATAGTTTGAGAATCAAGAGAATCAGTTGGACGATCAATTCCATCTTTGGGTCTTGCTGCAG
CATATCTTTCTCCAGTACCACCT
TGATATGTCGAACGATTGATAGTTTGAGAATCAAGAGAATCTGTTGGACGATCAATTCCATCTTTGGGTCTTGCTGCAG
CATATCTTTCTCCAGTACCACCT
TGATATGTCGAACGATTGATAGTTTGAGAATCAAGAGAATCTGTTGGACGATCAATTCCATCTTTGGGTCTTGCTGCAG
CATATCTTTCTCCAGTACCACCT
TGATATGTCGAACGATTGATAGTTTGAGAATCAAGAGAATCAGTTGGACGATCAATTCCATCTTTGGGTCTTGCTGCAG
CATATCTTTCTCCAGTACCACCT
TGATATGTCGAACGATTGATAGTTTGAGAATCAAGAGAATCTGTTGGACGATCAATTCCATCTTTGGGTCTTGCTGCAG
CATATCTTTCTCCAGTACCACCT
TGATATGTCGAACGATTGATAGTTTGAGAATCAAGAGAATCAGTTGGACGATCAATTCCATCTTTGGGTCTTGCTGCAG
CATATCTTTCTCCAGTACCACCT
TGATATGTCGAACGATTGATAGTTTGAGAATCAAGAGAATCAGTTGGACGATCAATTCCATCTTTGGGTCTTGCTGCAG
CATATCTTTCTCCAGTACCACCT
TGATATGTCGAACGATTGATAGTTTGAGAATCAAGAGAATCTGTTGGACGATCAATTCCATCTTTGGGTCTTGCTGCAG
CATATCTTTCTCCAGTACCACCT
TGATATGTCGAACGATTGATAGTTTGAGAATCAAGAGAATCAGTTGGACGATCAATTCCATCTTTGGGTCTTGCTGCAG
CATATCTTTCTCCAGTACCACCT
TGATATGTCGAACGATTGATAGTTTGAGAATCAAGAGAATCTGTTGGACGATCAATTCCATCTTTGGGTCTTGCTGCAG
CATATCTTTCTCCAGTACCACCT

TGATATGTCGAACGATTGATAGTTTGGGAATCAAGAGAATCTGTTGGACGATCAATTCCATCTTTGGGTCTTGCTGCAG
CATATCTTTCTCCAGTACTTCCC
TGATATGTCGAACGATTGATAGTTTGAGAATCAAGAGAATCAGTTGGACGATCAATTCTATCTTTGGGTCTTGCTGCAG
CATATCTTTCTCCAGTACCACCT
TGATATGTCGAACGATTGATAGTTTGAGAATCAAGAGAATCTGTTGGACGATCAATTCCATCTTTGGGTCTTGCTGCAG
CATATCTTTCTCCAGTACCACCT
TGATATGTCGAACGATTGATAGTTTGGGAATCAAGAGAATCTGTTGGACGATCAATTCCATCTTTGGGTCTTGCTGCAG
CATATCTTTCTCCAGTACCTCCC
TGATATGTCGAACGATTGATAGTTTGAGAATCAAGAGAATCTGTTGGACGATCAATTCCATCTTTGGGTCTTGCTGCAG
CATATCTTTCTCCAGTACCTCCC
TGATATGTCGAACGATTGATAGTTTGAGAATCAAGAGAATCTGTTGGACGATCAATTCCATCTTTGGGTCTTGCTGCAG
CATATCTTTCTCCAGTACCACCT
TGATATGTCGAACGATTGATAGTTTGGGAATCAAGAGAATCTGTTGGACGATCAATTCCATCTTTGGGTCTTGCTGCAG
CATATCTTTCTCCAGTACCACCT
TGATATGTCGAACGATTGATAGTTTGAGAATCAAGAGAATCAGTTGGACGATCAATTCCATCTTTGGGTCTTGCTGCAG
CATATCTTTCTCCAGTACCACCT
TGATATGTCGAACGATTGATAGTTTGAGAATCAAGAGAATCTGTTGGACGATCAATTCCATCTTTGGGTCTTGCTGCAG
CATATCTTTCTCCAGTACCACCT
Nr of selected repeats 96 Similarity 0.972641
Consensus:
TGATATGTCGAACGATTGATAGTTTGAGAATCAAGAGAATCtGTTGGACGATCAATTCCATCTTTGGGTCTTGCTGCAG
CATATCTTTCTCCAGTACCaCCt
>Cele-UNSB01_4:782171-782295 Satlength=125 Nr of Repeats=4 RepeatLength=31
seed=CAAAAATCGG
CAAAAATCGGGAAATTTTCACAAAAAAATGT
CAAAAATCGGGAATTTTTCACAAAAAAATGT
CAAAAATCGGGAAATTTTCACAAAAAAATGC
CAAAAATCGGGAAATTTTCACAAAAAAATGC
Nr of selected repeats 4 Similarity 0.949821
Consensus:
CAAAAATCGGGAAaTTTTCACAAAAAAATGc
>Cele-UNSB01_4:984710-985413 Satlength=704 Nr of Repeats=35 RepeatLength=19
seed=CAATAATTTC
CAATAATTTCCCAAAAAAT
CAATAATTTCATAAAAAAT
CAATAATTTCCCAAAAAAT
CAATAATTTCCCAAAAAAT
CAATAATTTCATTAAAAAAAT
CAATAATTTCATAAAAAAT
CAATAATTTCCCAAAAAAT
CAATAATTTCCCAAAAAAT
CAATAATTTCATTAAAAAAAT
CAATAATTTCCCAAAAAAT
CAATAATTTCCCAAAAAAAT
CAATAATTTCCCAAAAAAT
CAATAATTTCATAAAAAAT
CATTAATTTCACAAAAAAT
CATTAATTTCATTTAAAAAAAT
CAATAATTTCCCAAAAAAT
CAATAATTTCACAAAAAAA
CAATAATTTCACAAAAAAT
CAATAATTTCACAAAACAT
CGATAATTTCCGAAAAATT
CAATAATTTCCCAAAAAAT

CATTAATTTCATTTAAAAAAAT
CAATAATTTCCCAAAAAAT
CAATAATTTCACAAAAACAT
CAATAATTTCACAAAACAT
CGATAATTTCCGAAAAAAT
CAATAATTTCATAAAAAAT
CAATAATTTCCCAAAAAAT
CATTAATTTCATTTTAAAAAAAT
CAATAATTTCCCAAAAAAT
CAATAATTTCCCAAAAAATTATTAATTTCATTTAAAAAAAT
CAATAATTTCCCAAAAAAT
CAATAATTTCCCAAAAAAT
CAATAATTTCCCAAAAAAT
CAATAATTTCCCAAAAAAT
Nr of selected repeats 27 Similarity 0.905233
Consensus:
CAATAATTTCccAAAAAAT
>Cele-UNSB01_4:1076311-1076365 Satlength=55 Nr of Repeats=4 RepeatLength=12
seed=AGCCTAAGCC
AGCCTAAGCCTA
AGCCTAAGCCTA
AGCCTAATCCTT
AGACTAAGCCTAAGCCTG
Nr of selected repeats 3 Similarity 0.851852
Consensus:
AGCCTAAgCCTa
>Cele-UNSB01_4:1078257-1078305 Satlength=49 Nr of Repeats=4 RepeatLength=12
seed=AGGCTTAGGC
AGGCTTAGGCTT
AGGCTTAGGCTT
AGGCTTAGGCTT
AGGCATAGGCTC
Nr of selected repeats 4 Similarity 0.888889
Consensus:
AGGCtTAGGCTt
>Cele-UNSB01_4:1160688-1160967 Satlength=280 Nr of Repeats=7 RepeatLength=40
seed=ATTTTTCTAG
ATTTTTCTAGAAAGTTCTGGAACATTCCAGACTTTTCCCA
ATTTTTCTAGAAAGTTCTGGAACATTTCAGACTTTTCCCA
ATTTTTCTAGAAAGTTCTGGAACAATCCAGACTTTTCCCA
ATTTCTCTAGAAAGTTCTGGAACATTCCAGAATTTTCTCG
ATTTTTCTAGAAAGTTCTGAACATTCCAGACTTTTCCCA
ATTTTTCTAGAGAGTTCTGGAACATTCCAGACTTTTCCCA
ATTTTTCTAGAAAGTTCTGGAACATCCCAGACTTTTCCCA
Nr of selected repeats 6 Similarity 0.911111
Consensus:
ATTTTTCTAGAAAGTTCTGGAACATTCCAGACTTTTCCCA
>Cele-UNSB01_4:1160589-1161121 Satlength=533 Nr of Repeats=4 RepeatLength=35
seed=AAAATATTTT
AAAATATTTTTGAACCAGCCGCTGACCGCGCCTACGGCGCGGGCAACGACTGGCACCATTAAAAGTATTTGACACACAT
ACACTTTCAGAATTTTCTCGATTTTTCTAGAAAGTTCTGGAACATTCCAGACTTTTCCCAATTTTTCTAGAAAGTTCTG
GAACATTTCAGACTTTTCCCAATTTTTCTAGAAAGTTCTGGAACAATCCAGACTTTTCCCAATTTCTCTAGAAAGTTCT
GGAACATTCCAGAATTTTCTCGATTTTTCTAGAAAGTTCTGAACATTCCAGACTTTTCCCAATTTTTCTAGAGAGTTCT

GGAACATTCCAGACTTTTCCCAATTTTTCTAGAAAGTTCTGGAACATCCCAGACTTTTCCCAATTTTTCTAGAAAGTTC
TGGAACATTCCAGACTTTTTTCCAGATTTTCC
AAAATATTTTTCACAGAAAATTTAAATTTCCCTCC
AAAATATTTTTTTCGGAAAATTTAAATTTCCCTCC
AAAATATTTTTTTCAGAAAATTTAAATTTCCCGCC
Nr of selected repeats 3 Similarity 0.898413
Consensus:
AAAATATTTTTttCaGAAAATTTAAATTTCCCtCC
>Cele-UNSB01_4:1161180-1161420 Satlength=241 Nr of Repeats=16 RepeatLength=15
seed=GGTCTCACCA
GGTCTCACCACGACA
GGTCTCACCACGATG
GGTCTCACCACGATG
GGTCTCACCACGACA
GGTCTCACCACGGTG
GGTATCACCACGACG
GGTCTCACCACGATG
GGTCTCACCACGATG
GGTCTCACCACGATG
GGTCTCACCACGATG
GGTCTCACCACGATG
GGTTTCACCACGACG
GGTCTCACCACGGTG
GGTCTCGCCACGATG
GGTCTCACCACGATG
GGTCTCGCCACGATG
Nr of selected repeats 16 Similarity 0.880741
Consensus:
GGTCTCACCACGAtG
>Cele-UNSB01_4:1160701-1161595 Satlength=895 Nr of Repeats=10 RepeatLength=40
seed=GTTCTGGAAC
GTTCTGGAACATTCCAGACTTTTCCCAATTTTTCTAGAAA
GTTCTGGAACATTTCAGACTTTTCCCAATTTTTCTAGAAA
GTTCTGGAACAATCCAGACTTTTCCCAATTTCTCTAGAAA
GTTCTGGAACATTCCAGAATTTTCTCGATTTTTCTAGAAA
GTTCTGAACATTCCAGACTTTTCCCAATTTTTCTAGAGA
GTTCTGGAACATTCCAGACTTTTCCCAATTTTTCTAGAAA
GTTCTGGAACATCCCAGACTTTTCCCAATTTTTCTAGAAA
GTTCTGGAACATTCCAGACTTTTTTCCAGATTTTCCAAAATATTTTTCACAGAAAATTTAAATTTCCCTCCAAAATATT
TTTTTCGGAAAATTTAAATTTCCCTCCAAAATATTTTTTTCAGAAAATTTAAATTTCCCGCCAAAATATTTTTCACAGA
AAATTTAAATTTCCCGCCAAAATTTTGGGTCTTACCACGGTGGGTCTCACCACGACAGGTCTCACCACGATGGGTCTCA
CCACGATGGGTCTCACCACGACAGGTCTCACCACGGTGGGTATCACCACGACGGGTCTCACCACGATGGGTCTCACCAC
GATGGGTCTCACCACGATGGGTCTCACCACGATGGGTCTCACCACGATGGGTTTCACCACGACGGGTCTCACCACGGTG
GGTCTCGCCACGATGGGTCTCACCACGATGGGTCTCGCCACGATGGGTCTCACCACGATGGGTCTCGCCACGAAGATCT
CGCAGCAACATTTTTTTAAATTTTCCAGAAG
GTTCTAGAACAATCCAGAATTTTTTCGAATTTTCCAGAAGGTTCTAAAGCTTTTCAGAATTTTCCAGAAG
GTTCTGTAACATTCTAGAATTTTCCAGAAATTCCCAGAAG
Nr of selected repeats 7 Similarity 0.844444
Consensus:
GTTCTGGAACATTCCAGAcTTTTCCCaATTTTTCTAGAAA
>Cele-UNSB01_4:1161685-1161942 Satlength=258 Nr of Repeats=8 RepeatLength=11
seed=GTACTCCTAC
GTACTCCTACAGTACCTCTACA

GTACTACTACAGTACCCCGACCATATCCCCCTACTAACCCCAAACCTATATTTCTTCAAAAGACTAAAACACAATTTTT
CATAAACTACA
GTAATCCTACC
GTACTCCTACA
GTACTCCTACA
GTACTACTACAGTACCCCGACCATATCCCACTAATAACTCCAAACCTATATCTCTTCAAAAGACAAAAACACAATTTTT
CATAAACTACA
GTAATCCTACC
GTACTCCTACA
Nr of selected repeats 5 Similarity 0.854545
Consensus:
GTAcTCCTACa
>Cele-UNSB01_4:1309063-1309423 Satlength=361 Nr of Repeats=9 RepeatLength=36
seed=TTATTTTCTG
TTATTTTCTGGCAGATTGCCCATTTGCCGGGAATTT
TTATTTTCTGACAAATTGCCGGTTTGCCGGAAATTT
TTATTTCCTGGCAAATTGCCGATTTGCCGGAAATTTTTATTCTCTAGCAAATTGGCGATTTGCCGGAAATAT
TTATTTTCTGGCAGATTGCCCATTTGCCGGGAATTT
TTATTTTCTGACAAATTGCCCATTTGCCGGAAATTT
TTATTTCCTGGCAAATTGCCGGTTTGCCGGAAATAT
TTATTTTCTGGCAGATTGGCGATTTGCCGGGAATTT
TTATTTTCTGAGAAATTGCCGATTTGACGGAAATTC
TTATTTTCTGAGAAATTGCCCATTTGCCGGGAATTT
Nr of selected repeats 8 Similarity 0.838624
Consensus:
TTATTTTCTGacAaATTGCCcaTTTGCCGGaAATTT
>Cele-UNSB01_4:1313914-1314237 Satlength=324 Nr of Repeats=8 RepeatLength=36
seed=TTTGCCGGAA
TTTGCCGGAATTTTTTTGGAAAAATTACCGA
TTTGTCGGAAATTTTTATTTTCTGACAAATTGCCGG
TTTGCCGGAAATATTTATTTCCTGGCAAATTGCCGA
TTTGCCGGAAATTTTTATTTTCTGACAAATTGCCGG
TTTGCCGGAAATTTTTATTTTCTGACAAATTGCCCA
TTTGCCGGAATGCTTTTTTCGGCAAATTGCCGATTTGACTAAAATTTTTATTTTCTGACAAATTGCCGA
TTTGCCGGAAATTTTTATTATCTAGCAAATTGCCCA
TTTGCCGGAAATTGTCAGTTCAGGCAATTTGCCGATCTGCTGA
Nr of selected repeats 5 Similarity 0.859259
Consensus:
TTTGcCGGAAATtTTTATTttCTgaCAAATTGCCga
>Cele-UNSB01_4:1358775-1359527 Satlength=753 Nr of Repeats=22 RepeatLength=34
seed=ATTTCCCGCC
ATTTCCCGCCAAAATTTTTCTCACTGAAAATTTGA
ATTTCCCGCCAAAATTTTTTCACTGAAAATTTGA
ATTTCCCGCCAAAATTTTTTCACTGAAAATTTGA
ATTTCCCGCCAAAATTTTTTCACTGAAAATTTGA
ATTTCCCGCCAAAATTTTTTCACTGAAAATTTGA
ATTTCCCGCCAAAATTTTTTCACTGAAAATTTGA
ATTTCCCGCCAAAATTTTTTCACTGAAAATTTGA
ATTTCCCGCCAAAATTTTTTCACTGAAAATTTGA
ATTTCCCGCCAAAATTTTTTCACTGAAAATTTGA
ATTTCCCGCCAAAATTTTTTCACTGAAAATTTGA
ATTTCCCGCCAAAATTTTTTCACTGAAAATTTGA
ATTTCCCGCCAAAATTTTTTCACTGAAAATTTGA
ATTTCCCGCCAAAATTTTTTCACTGAAAATTTGA

ATTTCCCGCCAAAATTTTTTCACTGAAAATTTGA
ATTTCCCGCCAAAATTTTTTCACTGAAAATTTGA
ATTTCCCGCCAAAATTTTTTCACTGAAAATTTGA
ATTTCCCGCCAAAATTTTTTCACTGAAAATTTGA
ATTTCCCGCCAAAATTTTTTCACTGAAAATTTGA
ATTTCCCGCCAAAATTTTTTCACTGAAAATTTAA
ATTTCCCTCCAAAATATTTTTCTCAGAAAATTTGA
ATTTCCCGCCAAAATATTTTTCACAGAAAATTTAA
ATTTCCCGCCAAAATATTTTTCTCAGAAAATTTAA
Nr of selected repeats 18 Similarity 0.995643
Consensus:
ATTTCCCGCCAAAATTTTTTCACTGAAAATTTGA
>Cele-UNSB01_4:1359612-1359777 Satlength=166 Nr of Repeats=11 RepeatLength=15
seed=CCACGATGGG
CCACGATGGGTCTCA
CCACGATGGGTCTCG
CCACGATGGGTCTCG
CCACGATGGGTCTCA
CCACGATGGGTCTTT
CCACGATGGGTCTCA
CCACGGTGGGTCTCG
CCACGATGGGTCTCA
CCACGATGGGTCTTT
CCACGATGGGTCTCA
CCACGGTGGGTCTCG
Nr of selected repeats 11 Similarity 0.880404
Consensus:
CCACGATGGGTCTCn
>Cele-UNSB01_4:1362949-1363282 Satlength=334 Nr of Repeats=9 RepeatLength=34
seed=ATTGCCGGAA
ATTGCCGGAATTGAAATTTCTGGCGA
ATTGCCGGAATTGAAATTTCCGGCAAATTGGCATATTGCTGGTTTTGAAAATTTCTAGCAAATCGACAA
ATTGCCGGAATTCAAATTCCTAGCAAATCGGCAA
ATTGCCGGAATTGAAATTTCTGGCAAATTGGCAA
ATTGCCGGAATCGAAATTTCTGGCAAATTGACAA
ATTGCCGGAATTGTAATTTCTGGCAAATTGGCAA
ATTGCCGGAATTGAAATTTCTGGCAAATCGACAT
ATTGCCGGAATTAAAATTTCTGGCAAATTGGCAA
ATTGCCGGAATTGAAATTTCTGGCAAATTGGCAA
Nr of selected repeats 7 Similarity 0.886088
Consensus:
ATTGCCGGAATTgAAATTTCTGGCAAATtGgCAA
>Cele-UNSB01_4:1370044-1370460 Satlength=417 Nr of Repeats=8 RepeatLength=12
seed=TAGGCTTAGG
TAGGCTTAGGCT
TAGGCTTAGGCG
TAGACTTAGGTT
TAGGCTTAGGTTTAGGCTTATTTTTGGGCTTATGCTTATTCT
TAGGCTTAGGCT
TAGGCTTAGGCTTAGGCTTACTTTTTTAAAAA
TAGGCTTAGGCTTGGCGTCAGTGGCGAGCGTTAGATGCTTTTTGTGCTATTACGATAATCCAGAAATAAATAATTCGGT
CATGAATTTTTTGCTTCTCAAAATCTCGAAAAATTTGAATTATGAATTATGAATATTTTTTAAAGTGCTCAACTGTTAC
TATGCAAAAATATGACATTTAATTCCAATTTTGACATAAACATGTCGAGTTTGAACTATCTAAGTACCATTTAATATTT
TTTTCTAGTTAATTGGGCTTTTGAGCTTGTTTAAAAAAATATATT

TAGGCTTAGTCT
Nr of selected repeats 5 Similarity 0.822222
Consensus:
TAGgCTTAGgct
>Cele-UNSB01_4:1429332-1429629 Satlength=298 Nr of Repeats=15 RepeatLength=21
seed=AAAATTGACT
AAAATTGACTAAAATCGTCA
AAAATTGACTAGAATCGTCA
AAAATCGACTAAAAATCGTCA
AAAATTGACTAGAATCGTCA
AAAATCGACTAAAAATCGTCA
AAATTTGACTAAAAATCGTCA
AAAATTGACTAAAAATCGTCA
AAAATTGACTAAAAAAACGTCA
AAAATTGACTAAAAAAACGTCA
AAAATTGACTAAAAATCGTTA
AAAATTGACTAAAAATCGTTA
AAAATTGACTAAAAATCGTTA
AAAATTGACTAAAAATCGTTA
AAAATTGACTCAAAAA
AAAATTGACTT
Nr of selected repeats 10 Similarity 0.908289
Consensus:
AAAATtGACTAAAAAtCGTcA
>Cele-UNSB01_4:1444044-1444098 Satlength=55 Nr of Repeats=4 RepeatLength=12
seed=CATAGGCATA
CATAGGCATAGG
CATAGGCATAGA
CATAGGCATAGG
CATAGGCATAGGCATAGG
Nr of selected repeats 3 Similarity 0.925926
Consensus:
CATAGGCATAGg
>Cele-UNSB01_4:1452742-1452796 Satlength=55 Nr of Repeats=4 RepeatLength=12
seed=TATATGTATA
TATATGTATATG
TATATGTATATG
TATATGTATATG
TATATGTATATGTATATG
Nr of selected repeats 3 Similarity 1.000000
Consensus:
TATATGTATATG
>Cele-UNSB01_4:1454445-1454610 Satlength=166 Nr of Repeats=11 RepeatLength=15
seed=AGACCCATCG
AGACCCATCGTGAAA
AGACCCATCGTGGTG
AGACCCATCGTGGCG
AGACCCACCGTGGTG
AGACCCATCGTGGAA
AGACCCATCGTGGTG
AGACCCATCGTGGCG
AGACCCATCGTGGCG
AGACCCATCGTGGTG
AGACCCATCGTGGTG

AGACCCATCGTGGTG
Nr of selected repeats 11 Similarity 0.880404
Consensus:
AGACCCATCGTGGtG
>Cele-UNSB01_4:1461281-1461373 Satlength=93 Nr of Repeats=6 RepeatLength=15
seed=TCCCCTTTTC
TCCCCTTTTCGTTGC
TCCCCTTTTCGTATC
TCCCCTTTTCGTATC
TCCCCTTTTCGTATC
TCCCCTTTTCGCAGA
TCCCCTTTTCGCTCTGC
Nr of selected repeats 5 Similarity 0.840000
Consensus:
TCCCCTTTTCGtatc
>Cele-UNSB01_4:1490085-1491772 Satlength=1688 Nr of Repeats=46 RepeatLength=35
seed=AAAAAATTTT
AAAAAATTTTGTTTGAAAAGTTAAAAAAATATATATCCTTACCAGCCGATGACCGCGCCTCTGGCGCGGCCAACGACT
GGCGGAGTTTAAAATGTATAACAAATAGATGTCCTTAA
AAAAAATTTTGGCGGGAAATTCAAATTTTCAGTGA
AAAAAATTTTGGCGGGAAATTCAAATTTTCAGTG
AAAAAATTTTGGCGGGAAATTCAAATTTTCAGTG
AAAAAATTTTGGCGGGAAATTCAAATTTTCAGTGA
AAAAAATTTTGGCGGGAAATTCAAATTTTCAGTGA
AAAAAATTTTTGGCGGGAAATTCAAATTTTCAGTGA
AAAAAATTTTGGCGGGAAATTCAAATTTTCAGTG
AAAAAATTTTGGCGGGAAATTCAAATTTTCAGTGA
AAAAAATTTTGGCGGGAAATTCAAATTTTCAGTGA
AAAAAATTTTTGGCGGGAAATTCAAATTTTCAGTGA
AAAAAATTTTGGCGGGAAATTCAAATTTTCAGTGA
AAAAAATTTTGGCGGGAAATTCAAATTTTCAGTGA
AAAAAATTTTGGCGGGAAATTCAAATTTTCAGTGA
AAAAAATTTTGGCGGGAAATTCAAATTTTCAGTGA
AAAAAATTTTGGCGGGAAATTCAAATTTTCAGTG
AAAAAATTTTGGCGGGAAATTCAAATTTTCAGTG
AAAAAATTTTGGCGGGAAATTCAAATTTTCAGTGA
AAAAAATTTTGGCGGGAAATTCAAATTTTCAGTGA
AAAAAATTTTAGCGGGAAATTCAAATTTTCAGTGA
AAAAAATTTTGGCGGGAAATTCAAATTTTCAGTGA
AAAAAATTTTGGCGGGAAATTCAAATTTTCAGTG
AAAAAATTTTGGCGGGAAATTCAAATTTTCAGTGA
AAAAAATTTTGGCGGGAAATTCAAATTTTCAGTGA
AAAAAATTTTTGGCGGGAAATTCAAATTTTCAGTGA
AAAAAATTTTGGCGGGAAATTCAAATTTTCAGTGA
AAAAAATTTTGGCGGGAAATTCAAATTTTCAGTGA
AAAAAATTTTGGCGGGAAATTCAAATTTTCAGTG
AAAAAATTTTGGCGGGAAATTCAAATTTTCAGTG
AAAAAATTTTGGCGGGAAATTCAAATTTTCAGTGA
AAAAAATTTTGGCGGGAAATTCAAATTTTCAGTGA
AAAAAATTTTAGCGGGAAATTCAAATTTTCAGTGA
AAAAAATTTTGGCGGGAAATTCAAATTTTCAGTGA
AAAAAATTTTGGCGGGAAATTCAAATTTTCAGTG
AAAAAATTTTGGCGGGAAATTCAAATTTTCAGTGA
AAAAAATTTTGGCGGGAAATTCAAATTTTCAGTGA

AAAAAATTTTGGCGGGAAATTCAAATTTTCAGTG
AAAAAATTTTGGCGGGAAATTCAAATTTTCAGTGA
AAAAAATTTTGGCGGGAAATTCAAATTTTCAGTGA
AAAAAATTTTTGGCGGGAAATTCAAATTTTCAGTGA
AAAAAATTTTGGCGGGAAATTCAAATTTTCAGTG
AAAAAATTTTGGCGGGAAATTCAAATTTTCAGTGA
AAAAAATTTTGGCGGGAAATTCAAATTTTCAGTGA
AAAAAATTTTTGGCGGGAAATTCAAATTTTCAGTGA
AAAAAATTTTGGCGGGAAATTCAAATTTTCAGTGA
AAAAAATTTTGGCGGGAAATTCAAATTTTCAGTGA
Nr of selected repeats 30 Similarity 0.963442
Consensus:
AAAAAATTTTGGCGGGAAATTCAAATTTTCAGTGA
>Cele-UNSB01_4:1491894-1492067 Satlength=174 Nr of Repeats=5 RepeatLength=35
seed=GAAAATTTGA
GAAAATTTGAATTTCCCGCCAATATTTCTTCATA
GAAAATTTGAATTTCCCGCCAAAATTTTTTTCACT
GAAAATTTGAATTTCCCGCCAAAATTTTTTTCACT
GAAAATTTGAATTTCCCGCTAAAATTTTTTTACT
GAAAATTTGAATTTCCCGCTAAAATTTTTTACACT
Nr of selected repeats 3 Similarity 0.949206
Consensus:
GAAAATTTGAATTTCCCGCcAAAATTTTTTtCACT
>Cele-UNSB01_4:1492099-1492204 Satlength=106 Nr of Repeats=7 RepeatLength=15
seed=CTCACCACGA
CTCACCACGAAGGGG
CTCACCACGATGGGT
CTCACCACGAAGGGT
CTCACCACGATGAGT
CTCACCACGAAGGGT
CTCACCACGAAAGGT
CTCACCACGATGGGT
Nr of selected repeats 7 Similarity 0.873016
Consensus:
CTCACCACGAaGGGT
>Cele-UNSB01_4:1508767-1509123 Satlength=357 Nr of Repeats=5 RepeatLength=12
seed=AGGCTTAGGC
AGGCTTAGGCTA
AGGCTTAGGCCTTAAAAAAAATCCGAATTTAATTTTTATTTTGCGAATTTTTTCATCCCAGAAAAACTGACTTTTTCAG
GGGAAAAAATCTCAGAAATTCTTGTGATTTTAGCAAAAAATCAGGTAATTTTGAGGGAAAAATTTGAATTTTTCAAAAAT
TTCCGGTAAGAACTGCACTAATTTGTGATTTTTAAACAAATTTTGGATCAAAAATACAACTTTTTTGGGAAAATTTCAT
AAAATCTGCCAATTTTGCTGAAAAAAAAGTAAAATTTTACGACTTTTTTTCCGAGAAGTTTTGGCAATTTG
AGGCTTAGGCCT
AGGCGTAGGCGT
AGGCTTAAGCTT
Nr of selected repeats 4 Similarity 0.740741
Consensus:
AGGCtTAgGCtt
>Cele-UNSB01_4:1530750-1530982 Satlength=233 Nr of Repeats=8 RepeatLength=29
seed=CCACCCGGGA
CCACCCGGGAGATGTCGGCTGTTCTAAAT
CCACCCGGGAGGCGTCGGCTGCTCTAAGC
CCACCCGGGAGTTGTCGGCTGCTCTATGC
CCACCCGGGAGATGTCGGCTGTTCTAAAT

CCACCCGGGAGGCGTCGGCTGCTCTTAAC
CCACCCGGGAGATGTCGGTTGTTCTAAAC
CCACCCGGGAGGTGTCGGCTGCTCTAAAC
CCACCCGGGAGGTGTCGGCTGCTCTAAAC
Nr of selected repeats 8 Similarity 0.850575
Consensus:
CCACCCGGGAGgtGTCGGCTGcTCTAAac
>Cele-UNSB01_4:1558589-1558964 Satlength=376 Nr of Repeats=4 RepeatLength=94
seed=TCCCATTGAT
TCCCATTGATTTGTCTACAAAGGGCATCGAAAAGCACCCAATATTTGGAGAACAGAAGATTTTGAGAATTACTGCCTTC
AGAAATGGACAATTT
TCCCATTGATTTGTCTACATAGGGCATTGAAAAGCACCCAATATTTAGAGAACAGAAGATTTTGAGAATTACTGCCTTC
AGAAATTGATGATTT
TCCCATTGATTTGTCTGCAAAGAGCATCGAAAAGCACCCAATATTTAGAGAACAGAAGATTTTGAGAATTACTGCCTTC
AGAAATGGACAATTT
TCCCATTGATTTGTCTACATAGGGCATTGAAAAGCACCCAATATTTAGAGAACAGAAGATTTTGAGAATTACTGCCTTC
AGAATTGATGATTT
Nr of selected repeats 3 Similarity 0.924350
Consensus:
TCCCATTGATTTGTCTaCAaAGgGCATcGAAAAGCACCCAATATTTaGAGAACAGAAGATTTTGAGAATTACTGCCTTC
AGAAATgGAcaATTT
>Cele-UNSB01_4:1580035-1580182 Satlength=148 Nr of Repeats=7 RepeatLength=21
seed=CCATAGGTGG
CCATAGGTGGAAGATCTCATG
CCATAGGTGGATGATCAGATA
CCATAGGTGGAAGATCAGATA
CCAAAGGTGGAAGCTCTTATA
CCATAGGTGGAAGCTTTTATA
CCATAGGTGGATGATCTCATA
CCATAGGTGGATGATCAGATA
Nr of selected repeats 7 Similarity 0.794407
Consensus:
CCATAGGTGGAaGaTCtnATA
>Cele-UNSB01_4:1584655-1585480 Satlength=826 Nr of Repeats=7 RepeatLength=101
seed=TTGATTTTTC
TTGATTTTTCGGGGGGCACTTTTGCTATCATTACTTATATATAAAAAATACTGTCCGTCTGTCCATAGTGTGTAGTCTA
CGTAGTCTTTGTAGTGTACAAAGTTTTGGCTTATGAAGGAATATTGGTTTCGGGTTAGTGGGCGGATATGGTCGGGGTA
CTGTAGGTATACGGTAGGGTTACTGTAGTTTTCGAAAAA
TTGATTTTTCGTCTTTTGAAGGGATATTGGTTTCGGGGTAGTGGGCGGATATGGTCGGGGTACTGTAGGTATACGGTAG
GGTTACTGTAGTTTTGGAAATT
TTGATTTTTCGTCTTATGAAGGAATATTGGTTTCGGGGTAGTGGGCGGATATGGTCGGGGTATTGTAGGTATACGGTAG
GGTTACTGTAGTTTTGGAAATT
TTGATTTTTCGTCTTATGAAGGAATATTGGTTTCGGGTTAGTGGGGGGATATGGTAGGGGTACTGTAGGTATACGGTAG
GGTTACTGTAGTTTTGGAAATT
TTGATTTTTCGTCTTATGAAGGAATATTGGTTTCGGGGTAGTGGGCGGATATGGTCGGGGTATTGTAGGTATACGGTAG
GGTTACTGTAGTTTTGGAAATT
TTGATTTTTCGTCTTATGAAGGAATATTGGTTTCGGGTTAGTGGGGGGATATGGTAGGGGTACTGTAGGTATACGGTAG
GGTTACTGTAGTTTTGGAAAAT
TTGATTTTTCGTCTTTTGAAGGGATATTGGTTTCGGGTTAGTGGGCGGATATGGTCGGGGTACTGTAGTAGTACTGTAA
GGGTACTGTAGGTATACGGTAGGGTTACTGTAGTTTTGGAAATT
Nr of selected repeats 5 Similarity 0.952475
Consensus:
TTGATTTTTCGTCTTaTGAAGGaATATTGGTTTCGGGgTAGTGGGcGGATATGGTcGGGGTAcTGTAGGTATACGGTAG
GGTTACTGTAGTTTTGGAAAtT

>Cele-UNSB01_4:1639262-1639353 Satlength=92 Nr of Repeats=6 RepeatLength=14
seed=ACTACACACT
ACTACACACTACAC
ACTACACACTACAC
ACTACACACTACAC
ACTACACACTACAC
ACTACACACTACAC
ACTACACACTACAAACTACAC
Nr of selected repeats 5 Similarity 1.000000
Consensus:
ACTACACACTACAC
>Cele-UNSB01_4:1643155-1643244 Satlength=90 Nr of Repeats=6 RepeatLength=12
seed=TTAGGCTTAG
TTAGGCTTAGCATTAGGATCAGTC
TTAGGCTTCGGC
TTAGGCTTAAGCTGGGC
TTAGGCTTAGGC
TTAGGCTTAGGC
TTAGGTTTAGGC
Nr of selected repeats 4 Similarity 0.888889
Consensus:
TTAGGcTTaGGC
>Cele-UNSB01_4:1647487-1647595 Satlength=109 Nr of Repeats=9 RepeatLength=12
seed=CTTAAGCTTA
CTTAAGCTTAGA
CTTAAGCTTAGG
CTTAAGCTTAGG
CTTAAGCTTAGG
CTTAAGCTTAGG
CTTAAGCTTAGG
CTTAAGCTTAGG
CTTAAGCTTAGG
CTTAAGCTTAGG
Nr of selected repeats 9 Similarity 0.975309
Consensus:
CTTAAGCTTAGG
>Cele-UNSB01_4:1654144-1654247 Satlength=104 Nr of Repeats=5 RepeatLength=23
seed=ATTTAATTAA
ATTTAATTAATATTT
ATTTATTTAATTAATATGT
ATTTATTTAATTAATTAATATTT
ATTTATTTAATTAATATTTATTC
ATTTAATTAATAATTATTTATTT
Nr of selected repeats 3 Similarity 0.729469
Consensus:
ATTTAtTTAATtAaTattTATTt
>Cele-UNSB01_4:1669669-1669879 Satlength=211 Nr of Repeats=6 RepeatLength=35
seed=ATTTCCGGCA
ATTTCCGGCAAATCGGCAATACGCCGTAAATTAAA
ATTTCCGGCAAATCGGCAATATGCCAAAAAATGGAT
ATTTCCGGCAAATCGGCGATTTGCCGAAAATGGCA
ATTTCCGGCAAGTCGGCAATATGCCGAAAATAAAC
ATTTCCGGCAAATCGGCAATACGCCGTAAATTAAA
ATTTCCGGCAAATCGGCAATATGCCGAAAATGAAC

Nr of selected repeats 6 Similarity 0.819683
Consensus:
ATTTCCGGCAAATCGGCAATAtGCCGaAAATgaAa
>Cele-UNSB01_4:1695920-1696142 Satlength=223 Nr of Repeats=6 RepeatLength=37
seed=CGCCAAAATG
CGCCAAAATGTTTTCAAAAAATTAAATTTTGAATTCC
CGCCAAAATGATTCCAAAAAATTAAAATTTGAATTCC
CGCCAAAATGTTTTCAAAAAATTAAATTTTGAATTCT
CGCCAAAATGTTTTCAAAAAATTAAAATTTGAATTCC
CGCCAAAATGTTTTCAAAAAATTAAATTTTGAATTCC
CGCCAAAATGTTTTTAAAAAATTTTAATTTTAATTCC
Nr of selected repeats 6 Similarity 0.894294
Consensus:
CGCCAAAATGTTTTCAAAAAATTAAAaTTTGAATTCC
>Cele-UNSB01_4:1703835-1704045 Satlength=211 Nr of Repeats=11 RepeatLength=12
seed=CTAAGCCTAA
CTAAGCCTAAGCCTAATACTAAGTCTGAGCCTGCGC
CTAAGCATAAGCTTAAGCTTAAGT
CTAAGCCTAAGCCTCAACCTGTGCCTAAGCATTACC
CTAAGCCTAATA
CTAAGCCTAAGC
CTGAGCCTAAAC
CTAAGCCTAATA
CTAAGCCTAAGC
CTAAGCCTAAGCCTAAACCTCAACCTGTGT
CTAAGCATAAGC
CTAAGCCTGAGC
Nr of selected repeats 7 Similarity 0.777778
Consensus:
CTAAGCCTAAgc
>Cele-UNSB01_4:1729821-1729883 Satlength=63 Nr of Repeats=4 RepeatLength=15
seed=CGAAAAGGGG
CGAAAAGGGGAGCAGAA
CGAAAAGGGGATCTG
CAAAAAGGGGTTCTG
CGAAAAGGGGAGCAA
Nr of selected repeats 3 Similarity 0.703704
Consensus:
CgAAAAGGGGatCtg
>Cele-UNSB01_4:1755267-1755667 Satlength=401 Nr of Repeats=20 RepeatLength=20
seed=CAGAAAAATC
CAGAAAAATCAATAAATGCT
CAGAAAAATCAATAAATTCT
CAGAAAAATCAATAAATTCT
CAGAAAAATCAATAAATTCT
CAGAAAATTCGATAAACTCT
CAGAAAAATCAATAAATTCT
CAGAAAAATCAATACATTCT
CAAAAAAATCGATAAACTCT
CAGAAAAATCAATAAATGCT
CAGAAAAATCAATAAATTCT
CAGAAAAATCAATAAATTCT
CAGAAAATTCGATAAACTCT
CACAAAAATCGATAAATTCT

CAGAAAATTCGATAAAGTCT
CAGAAAAGTCGATAATTTAT
CTGAAAAATCGATAAATTTT
CAGAAAAATCAATAAATTCC
CAGAAAAATCGATAAATTCC
CAGAAAAATGAATAAATTCT
CAGAAAAATCGATAAATTCC
Nr of selected repeats 20 Similarity 0.835088
Consensus:
CAGAAAAaTCaATAAAtTCT
>Cele-UNSB01_4:1766949-1767039 Satlength=91 Nr of Repeats=5 RepeatLength=12
seed=TAAGCCTAAG
TAAGCCTAAGCC
TAAGCCTAAGAC
TAAGCTTAAGCC
TAAGACTAAGGC
TAAGCCTAAACCTAAACCCAATCCAAAGCCTATGTTTAAGCC
Nr of selected repeats 4 Similarity 0.796296
Consensus:
TAAGccTAAGcC
>Cele-UNSB01_4:1777357-1777453 Satlength=97 Nr of Repeats=5 RepeatLength=16
seed=CACGTAGGCA
CACGTAGGCACGTAGG
CACGTAAGCACGTAGG
CACGTAGGCACGTAGG
CACGTAGGTACCCAGGCACCTATGCACATTGT
CACGTAGGCACTTAGG
Nr of selected repeats 4 Similarity 0.916667
Consensus:
CACGTAgGCACgTAGG
>Cele-UNSB01_4:1800043-1800133 Satlength=91 Nr of Repeats=7 RepeatLength=12
seed=TTAGGATTAG
TTAGGATTAGGA
TTAGGATTAGGA
TTATGATTAGGA
TTAGGATTAGGATTAGTC
TTAGGATTAGGA
TTAGGATTAGGA
TTAGGATTAGGA
Nr of selected repeats 6 Similarity 0.962963
Consensus:
TTAGGATTAGGA
>Cele-UNSB01_4:1802551-1803063 Satlength=513 Nr of Repeats=30 RepeatLength=16
seed=TAGGCACGTA
TAGGCACGTAGGCACGTAGATACC
TAGGCACGTAGTCGCCTAAGAACG
TAGGTACGTAGGCACG
TAGACACGTAGCTTCCAAGAAACC
TAGCCACGTAGGTACG
TAGGCACGTAGGCACG
TAGGTACGTAGGTACG
TAGGCACGTAGGCACG
TAGGCACGTAGGCACG
TAGGCACGTAGGCACG

TAGGCACGTAGGCACG
TAGGCACGTAGGCACG
TAGGCACGTAGGCACG
TAGGCACGTAGGCACG
TAGGCACGTAGGCACG
TAGGCACGTAGGCACG
TAGGCACGTAGGCACG
TAGGCACGTAGGCACG
TAGGCACGTAGGCACG
TAGGCACGTAGGCACG
TAGGCACGTAGGCACG
TAGGCACGTAGGCACG
TAGGCACGTAGGCACG
TAGGCACGTAGGCACG
TAGGCACGTAGGCACG
TAGGCACGTAGGCACG
TAGGCACGTAGGCACG
TAGGCACGTAGGCACG
TAGGCACGTAGGCGCG
TAGTCACGTAGGCACGTAGTCACG
Nr of selected repeats 26 Similarity 0.962564
Consensus:
TAGGCACGTAGGCACG
>Cele-UNSB01_4:1866819-1867315 Satlength=497 Nr of Repeats=18 RepeatLength=21
seed=TGTCGATTTA
TGTCGATTTACGAGACTGTAA
TGTCGATTTACGGGGGTTCAG
TGTCGATTTACAAGACTGTAA
TGTGGATTTACGGGGGTTCAG
TGTCGATTTACGAGACTGTAA
TGTCGATTTACGGGGGTTCAG
TGTCGATTTACGAGACTGTAA
TGTCGATTTACGGGGGTTCAG
TGTCGATTTACGAGACTGTAA
TGTCGATTTACGGGAGTTCAG
TGTCGATTTACGAGGGCTGTAA
TGTCGATTTACGGGGGTTCGG
TGTCGATTTACGAGACTGTAA
TGTCGATTTACGGGAGTTCAG
TGTCGATTTACGAGGGCTGTAA
TGTCGATTTACGAGGCTTTAA
TGTCGATTTACAGGGGTTCAG
TGTCGATTTACGGGATAACATTTTCCGAAATTTTACTGAAATTCGACTAGTTTTGGAATGAAATCCTTCAATTTTGCAA
AATCTTTCCAGGATTATTTGGTGTTTTCCGCAGCTCCGTGTATATTTACGGGGGCCTG
Nr of selected repeats 15 Similarity 0.738095
Consensus:
TGTCGATTTACGgGagTTcAg
>Cele-UNSB01_4:1908625-1908709 Satlength=85 Nr of Repeats=5 RepeatLength=12
seed=TAAGCCTAAG
TAAGCCTAAGCT
TAAGTCTAAGCT
TAAGCCTAAGCGAAAACCTAAGTTCAAACT
TAAGCCTAAGCC
TAAGTCTAAGCCTAGGCC

Nr of selected repeats 3 Similarity 0.851852
Consensus:
TAAGcCTAAGCt
>Cele-UNSB01_4:1946817-1947132 Satlength=316 Nr of Repeats=8 RepeatLength=35
seed=GAAATTTTCA
GAAATTTTCAATTCCGGCAATTCGCCGATTTGACG
GAAATTTTCAATTTCAACGAATTACCGATTTGCCGGAAAATGTCATTTTCAGCAAACTGCCGATTTGCCG
GAAATTTTCAATTCCGGCAGTTTGCCGATTTGCCG
GAAATTTTCAATTCCGGCAATTTGCCGATTTGACG
GAAATTTTCAATTCCGGCAATTTGCCGATTTGCCG
GAAATTTTCAATTCCGGCAGTTTGCCGATTTGCCG
GAAATTTTCAATTCCGGCAATTTGCCAATTTGACG
GAAATTTTCAATTCCGGCAATCTGCCGATTTGACG
Nr of selected repeats 7 Similarity 0.927438
Consensus:
GAAATTTTCAATTCCGGCAaTTTGCCGATTTGaCG
>Cele-UNSB01_4:1949645-1950620 Satlength=976 Nr of Repeats=28 RepeatLength=35
seed=CATTTTCGCC
CATTTTCGCCGAATTGCCGGTTTGCCGGAAATTTC
CATTTTCGCCGAATTGCCGATTTGCCGGAAATTTT
CATTTTCGCCGGTTTGCCGGAAATTTC
CATTTTCGCCGAATTGCCGATTTGCCGGAAATTTC
CATTTTCGCCGAATTGCCGGTTTGCCGGAAATTTC
CATTTTCGCCGAATTGCCGGTTTGCCGGAAATTTC
CATTTTCGCCGAATTGCCGGTTTTCTGGAAATTTC
CATTTTCGCCGAATTGCCGATTTGCCGGAAATTTT
CATTTTCGCCGGTTTGCCGGAAATTTC
CATTTTCGCCGAATTGCCGGTTTTCTGGAAATTTC
CATTTTCGCCGAATTGCCGATTTGCCGGAAATTTT
CATTTTCGCCGGTTTGCCGGAAATTTC
CATTTTCGCCGAATTGCCGATTTGCCGGAAATTTC
CATTTTCGCCGAATTGCCGATTTGCCGGAAATTTC
CATTTTCGCCGAATTGCCGATTTGCTGGAAATTTTCAATTTTGGCAATTCACCGGTTTGCCGGAAATTTT
CATTTTCGCCGAATTGCCGATTTGCCGGAAATTTT
CATTTTCGCCGGTTTGCCGGAAATTTC
CATTTTCGCCGAATTGCCGGAAATTTC
CATTTTCGCCGAATTGCCGATTTGCCGGAAATTTC
CATTTTCGCCGAATTGCCGGTTTGCCGGAAATTTC
CATTTTCGCCGAATTGCCGATTTGCCGGAAATTTC
CATTTTCGCCGAATTGCCGGTTTGCCGGAAATTTC
CATTTTCGCCGAATTGCCGGTTTGCCGGAAATTTC
CATTTTCGCCGAATTGCCGGTTTGCCGGAAATTTC
CATTTTCGCCGAATTGCCGGTTTTCTGGAAATTTC
CATTTTCGCCGAATTGCCGGTTTGCCGGAAATTTC
CATTTTCGCCGAATTGCCGGTTTTCTGGAAATTTC
CATTTTCGCCGAATTGCCGGTTTGCCGGAAATTTC
Nr of selected repeats 22 Similarity 0.945083
Consensus:
CATTTTCGCCGAATTGCCGgTTTGCCGGAAATTTC
>Cele-UNSB01_4:1966969-1971964 Satlength=4996 Nr of Repeats=213 RepeatLength=21
seed=GTAAATCTAC
GTAAATCTACAGTAATCCCTC
GTAAATCTACAAGAAACCCCC
GTAAATCTACAGTAATCCCCC

GTAAATTTACAGTAATACCCC
GTAAATTTACAGTAATACCCC
GTAAATCTACAGTAATCCCCCGTAAGTTTACAGTAATACCCC
GTAAATCTACAGTAATACCCC
GTAAATCTACAGTAATCCCCC
GTAAATCTACAGTAATACCCC
GTAAATCTACACAAAACTCC
GTAAATCTACAGTAATCCCTC
GTAAATCTACAGTAATCCCCC
GTAAATCTACAGTAATACCCC
GTAAATCGACAGTAATCCCCC
GTAAATTTACAGTAATACCCC
GTAAATCTACAGTAATCCCCCGTAAGTTTACAGTAATACCCC
GTAAATCTACAGTAATCCCCC
GTAAATCTACAGTAATCCACC
GTAAATTTACAGTAATACCCC
GTAAATTTACAGTAATACCCC
GTAAATCTACACAAAACTCC
GTAAATCTACAGTAATCCCTC
GTAAATCTACACAATGAGCCTCC
GTAAATCTACAGTAATACCCC
GTAAATCGACAGTAATCCCCC
GTAAATTTACAGTAATACCCC
GTAAATCTACACAAAACC
GTAAATCTACAGTAATCCCCC
GTAAATCTACAGTAATCCCCC
GTAAATTTACAGTAATACCCC
GTAAATTTACAGTAATACCCC
GTAAATCTACAGTAATACCCC
GTAAATCTACAGTAATCCCCCGTAAGTTTACAGTAATACCCC
GTAAATCTACAGTAATCCCCC
GTAAATCTACAGTAATCCCCCGTAAGTTTACAGTAATACCCC
GTAAATCTACAGTAATACCCC
GTAAATCTACAGTAATCCCCC
GTAAATCTACAGTAATACCCC
GTAAATCTACACAAAACTCC
GTAAATCTACAGTAATCCCTC
GTAAATCTACAGTAATCCCCC
GTAAATCTACAGTAATACCCC
GTAAATCGACAGTAATCCCCC
GTAAATTTACAGTAATACCCC
GTAAATCTACAGTAATACCCC
GTAAATCTACAGTAATCCCCCGTAAGTTTACAGTAATACCCC
GTAAATCTACAGTAATACCCC
GTAAATCTACAGTAATCCCCC
GTAAATTTACAGTAATACCCC
GTAAATCTACAGTAATACCCC
GTAAATCTACAGTAATCCCCCGTAAGTTTACAGTAATACCCC
GTAAATCTACAGTAATACCCC
GTAAATCTACAGTAATCCCCC
GTAAATCTACAGTAATACCCC
GTAAATCTACACAAAACTCC
GTAAATCTACAGTAATCCCTC
GTAAATCTACAGTAATCCCCC

GTAAATCTACAGTAATACCCC
GTAAATCGACAGTAATCCCCC
GTAAATTTACAGTAATACCCC
GTAAATCTACACAAAACC
GTAAATCTACAGTAATCCCCC
GTAAATCTACAGTAATACCCC
GTAAATCTACAGTAATCCCCCGTAAGTTTACAGTAATACCCC
GTAAATCTACAGTAATCCCCC
GTAAATCTACAGTAATCCCCCGTAAGTTTACAGTAATACCCC
GTAAATCTACAGTAATACCCC
GTAAATCTACAGTAATCCCCC
GTAAATCTACAGTAATACCCC
GTAAATCTACAGTAATCCCCCGTAAGTTTACAGTAATACCCC
GTAAATCTACAGTAATCCCCC
GTAAATCTACAGTAATCCCTC
GTAAATCTACAGTAATCCCCC
GTAAATCTACAGTAATACCCC
GTAAATCTACAGTAATCCCCC
GTAAATCTACAGTAATCCCCCGTAAGTTTACAGTAATACCCC
GTAAATCTACAGTAATCCCCC
GTAAATCTACAGTAATCCCCCGTAAGTTTACAGTAATACCCC
GTAAATCTACAGTAATACCCC
GTAAATCTACAGTAATCCCCC
GTAAATCTACAGTAATACCCC
GTAAATCTACAGTAATCCCCCGTAAGTTTACAGTAATACCCC
GTAAATCTACAGTAATCCCCC
GTAAATCTACAGTAATCCCTC
GTAAATCTACAGTAATCCCCC
GTAAATCTACAGTAATACCCC
GTAAATCTACAGTAATCCCCC
GTAAATCTACAGTAATCCCCC
GTAAATTTACAGTAATACCCC
GTAAATTTACAGTAATACCCC
GTAAATCTACAGTAATACCCC
GTAAATCTACAGTAATCCCCCGTAAGTTTACAGTAATACCCC
GTAAATCTACAGTAATACCCC
GTAAATCTACAGTAATCCCCCGTAAGTTTACAGTAATACCCC
GTAAATCTACAGTAATACCCC
GTAAATCTACAGTAATCCCCCGTAAGTTTACAGTAATACCCC
GTAAATCTACAGTAATACCCC
GTAAATCTACAGTAATCCCCC
GTAAATTTACAGTAATACCCC
GTAAATCTACAGTAATACCCC
GTAAATCTACAGTAATCCCCCGTAAGTTTACAGTAATACCCC
GTAAATCTACAGTAATACCCC
GTAAATCTACAGTAATCCCCC
GTAAATCTACAGTAATACCCC
GTAAATCTACACAAAACTCC
GTAAATCTACAGTAATCCCTC
GTAAATCTACAGTAATCCCCC
GTAAATCTACAGTAATACCCC
GTAAATCTACAGTAATCCCCCGTAAGTTTACAGTAATACCCC
GTAAATCTACAGTAATCCCCC
GTAAATCTACAGTAATCCCTC

GTAAATCTACAGTAATCCCCC
GTAAATCTACAGTAATACCCC
GTAAATCTACAGTAATCCCCC
GTAAATCTACAGTAATCCCCCGTAAGTTTACAGTAATACCCC
GTAAATCTACAGTAATCCCCC
GTAAATCTACAGTAATCCCCCGTAAGTTTACAGTAATACCCC
GTAAATCTACAGTAATACCCC
GTAAATCTACAGTAATCCCCC
GTAAATCTACAGTAATACCCC
GTAAATCTACAGTAATCCCCCGTAAGTTTACAGTAATACCCC
GTAAATCTACAGTAATCCCCC
GTAAATCTACAGTAATCCCTC
GTAAATCTACAGTAATCCCCC
GTAAATCTACAGTAATACCCC
GTAAATCTACAGTAATCCCCC
GTAAATCTACAGTAATCCCCC
GTAAATTTACAGTAATACCCC
GTAAATTTACAGTAATACCCC
GTAAATCTACAGTAATACCCC
GTAAATCTACAGTAATCCCCCGTAAGTTTACAGTAATACCCC
GTAAATCTACAGTAATACCCC
GTAAATCTACAGTAATCCCCCGTAAGTTTACAGTAATACCCC
GTAAATCTACAGTAATACCCC
GTAAATCTACAGTAATCCCCC
GTAAATCTACAGTAATACCCC
GTAAATCTACAGTAATACCCC
GTAAATCTACAGTAATCCCCCGTAAGTTTACAGTAATACCCC
GTAAATCTACAGTAATACCCC
GTAAATCTACAGTAATCCCCC
GTAAATCTACAGTAATACCCC
GTAAATCTACACAAAACTCC
GTAAATCTACAGTAATCCCTC
GTAAATCTACAGTAATCCCCC
GTAAATCTACAGTAATACCCC
GTAAATCGACAGTAATCCCCC
GTAAATTTACAGTAATACCCC
GTAAATCTACACAAAACC
GTAAATCTACAGTAATCCCCC
GTAAATCTACAGTAATCCCCC
GTAAATTTACAGTAATACCCC
GTAAATTTACAGTAATACCCC
GTAAATCTACAGTAATACCCC
GTAAATCTACAGTAATCCCCCGTAAGTTTACAGTAATACCCC
GTAAATCTACAGTAATACCCC
GTAAATCTACAGTAATCCCCC
GTAAATCTACAGTAATACCCC
GTAAATCTACACAAAACTCC
GTAAATCTACAGTAATCCCTC
GTAAATCTACAGTAATCCCCC
GTAAATCTACAGTAATACCCC
GTAAATCGACAGTAATCCCCC
GTAAATTTACAGTAATACCCC
GTAAATCTACACAAAACC
GTAAATCTACAGTAATCCCCC

GTAAATCTACAGTAATCCCCC
GTAAATCTACAGTAATACCCC
GTAAATTTACAGTAATACCCC
GTAAATCTACACAATGAGCCTCC
GTAAATCTACAGTAATACCCT
GTAAATCTACAGTAATCCATC
GTAAATCTACAATAAACCCCC
GTAAATCTACATAATGAGCCTCC
GTAAATCTACATTAACTCCC
GTAAATCTACAACATGCTTCC
GTAAATCTACACTAGTTCCCC
GTAAATCTACACAATCATCCCCC
GTAAATCTACAATAAGCTCCC
GTAAATCTGCAGTAATACCCC
GTAAATTTACAGTAATACCCC
GTAAATCTACACAATGAGCCTCC
GTAAATCTACATTAACCCCC
GTAAATCTACAAAATGCTTCC
GTAAATCTTCACAATCATCCCCC
GTAAATCTACAATAAGCTCCC
GTAAATCTACAGTAATACCCC
GTAAATTTACAGTAATACCCC
GTAAATTTACAGTAATCCCCC
GTAAATCTACACAAAACTCC
GTAAATCTACAGTAATCCCTC
GTAAATCTACACAATGAGCCTCC
GTAAATCTACATTAACCCCC
GTAAAACTACAAAATGCTTCC
GTAAATCTTCACAATCATCCCCC
GTAAATCTACAATAAGCTCCC
GTAAATCTACAGTAATACCCC
GTAAATTTACAGTAATACCCC
GTAAATTTACAGTAATCCCCC
GTAAATCTACACAAAACTCC
GTAAATCTACAGTAATCCCTC
GTAAATCTACACAATGAGCCTCC
GTAAATCTACATTAACCCCCGTAAAACTAAAAAATGCTTCC
GTAAATCTTCACAATCATCCCCC
GTAAATCTACAATAAGCTCCC
GTAAATCTACAGTAATACCCC
GTAAATTTACAGTAATACCCC
GTAAATTTACAGTAATCCCCC
GTAAATCTACACAAAACTCC
GTAAATCTACAGTAATCCCTC
GTAAATCTACACAATGAGCCTCC
GTAAATCTACATTAACCCCC
GTAAAACTACAAAATGCTTCC
GTAAATCTTCACAATCATCCCCC
Nr of selected repeats 158 Similarity 0.856783
Consensus:
GTAAATCTACAGTAATnCCCC
>Cele-UNSB01_4:1985632-1987358 Satlength=1727 Nr of Repeats=15 RepeatLength=80
seed=AATTTCTCAA

AATTTCTCAATGGAGTGCATTTGCATCGGCGCGAAATTCGAATTTTCAGGCGAATTTCACATAAAAGTCCCGAAAATGC
ACAAAAAAGAGCATTTCTCGAATTTGGACATGATTACGGAGCTGCTAACGCCTAGTACAACACACCACCAACCACACGG
AAATCAACTTGTCGAGGAAATGGACGATTCTGAAGACGATGATGAAGGTGATGGCTCGGAATTTCTAGTGGAACAGCAG
CCAGCAGCAGAGCCCGAAGAGCCGAAAAGTGATGGAAAAATCGAAAAATTCGGCTATGGTTTCGGTTGGAGCAAGTTTG
GAGTTATTGAGAGGCTCAGAGATGAAATCGGCAAAATTGTGGATATTTTGGAGCCCGAAAATGTGGAAATTGAGAAAAG
AGCCGACAAATTGATGGAATTCGATTGGGAAAACTTTGATGAAGGCAGATATTTGTAAGAAATCGAATTTCCCGCCAAA
AATCACTCAGAAATTGCGCTCCATTGAGTAAATGCGTGAAAAAATAAAATTTTCCGACATTTTTGTGTCAGTAGAGCAC
AGTTGCAACGCAAAATTGTTCATTTTTTAAATTTGAAATCTCGAAAACTCGCA
AATTTCTCAATAGCGCGCGCTTGCAACATCCGAACGGCGCGAAATTTGAGTTTTTGGCCATAAAATAACGAAAATCGGC
A
AATTTCTCAATAGGGCGCGCTTGCAACATTTAGTCAGCGCTAAATTTGAATTTTTGGCTATAAAATAACAAAAACTAAC
T
AATTTCTCAATAGGGCACGCTTGCAACATCTGAACGGCGCGAAATTTGAGTTTTTGGCCATAAAATAACGAAAATCGGC
A
AATTTCTCAATAGGGCGCGCTTGCAACATTTAGTCAGCGCTAAATTTGAATTTTTGGCTATAAAATAACAAAAACTAAC
T
AATTTCTCAATAGGGCACGCTTGCAACATCTGAACGGCGCGAAATTTGAATTTTTGGCCATAAAATAACGAAAATCGGC
A
AATTTCTCAATAGGGCGCGCTTGCAACATTTAGTCAGCGCTAAATTTGAATTTTTGGCTATAAAATAACAAAAACTAAC
T
AATTTCTCAATAGGGCACGCTTGCAACATCTGAACGGCGCGAAATTTGAATTTTTGGCCATAAAATAACGAAAATCGGC
A
AATTTCTCAATAGGGCGCGCTTGCAACATTTAGTCAGCGCTAAATTTGAATTTTTGGCTATAAAATAACAAAAACTAAC
T
AATTTCTCAATAGGGCACGCTTGCAACATCTGAACGGCGCGAAATTTGAATTTTTGGCCATAAAATAACGAAAATCGGC
A
AATTTCTTAATAGGGCGCGCTTGCAACATTTAGTCAGCGCTAAATTTGAATTTTTGGCTAAAAAATAACAAAAACTAAC
T
AATTTCTCAATAGGGCACGCTTGCAACATCTGAACGGCGCGAAATTTAAATTTTTGGTCATAAAATAACGAAAACTAAC
T
AATTTCTCAATAGGGCGCGCTTGCAACATCTGAACGGCGCGAAATTTAAATTTTTGGTCATAAAATAACGAAAACTAAC
T
AATTTCTCAATAGGGCGCGCTTGCAACATTTAGTCAGCGCTAAATTTGAATTTTTGGCTATAAAATAACAAAAACTAAC
T
AATTTCTCAATAGGGCACGCTTGCAACATCTGAACGGCGCGAAATTTGAATTTTTGGCCATAAAATAACGAAAATCGGC
A
Nr of selected repeats 14 Similarity 0.854212
Consensus:
AATTTCTCAATAGGGCgCGCTTGCAACATcTgaaCgGCGCgAAATTTGAATTTTTGGCcATAAAATAACgAAAActaaC
t
>Cele-UNSB01_4:2119001-2119076 Satlength=76 Nr of Repeats=5 RepeatLength=15
seed=GGGTCTCACC
GGGTCTCACCACGAT
GGGTCTCGCCACGAT
GGGTCTCACCACGAC
GGGTCTCACCACTGT
GGGTCTCGCCACGAT
Nr of selected repeats 5 Similarity 0.840000
Consensus:
GGGTCTCaCCACgat
>Cele-UNSB01_4:2158460-2158710 Satlength=251 Nr of Repeats=6 RepeatLength=40
seed=TTCCAGAATT
TTCCAGAATTTTCACGAATTTTCTCGAATTTTCCAGAAGGTTCTAGAACA
TTCCAGAATTTTCTCGAATATTCCAGAAGCTTCTAGAACA

TTCCAGAATTTTCTTGAACTTTCCAGAAGGTTCTAGAACA
TTCCAGAATTTTCTCGAATTTTTCAGAAGGTTCTAGAGCT
TTCCAGAACTTTCTCGAACTTTCCAGAAGGTTCTAGAACA
TTCCAGAATTTTCTCGAATTTTTCAGAAGGTTCTAGAGCT
Nr of selected repeats 5 Similarity 0.866667
Consensus:
TTCCAGAAtTTTCTcGAAttTTcCAGAAGgTTCTAGAaCa
>Cele-UNSB01_4:2158825-2158968 Satlength=144 Nr of Repeats=5 RepeatLength=11
seed=CTACAGTACC
CTACAGTACCT
CTACAGTACTA
CTACAGTACCCCGGCCATATCCCACCACTAACCCCAAACCTATATCTTCAAAAGACAAAAACTCATTTTTTCGTAAACT
ACAGTAATTCTACAATACTC
CTACAGTACCT
CTACAGTACTA
Nr of selected repeats 4 Similarity 0.796296
Consensus:
CTACAGTAcCTa
>Cele-UNSB01_4:2163715-2164250 Satlength=536 Nr of Repeats=8 RepeatLength=43
seed=TTGCCGGAAA
TTGCCGGAAATGTTTAGAGAGATTTTTTGAATGACGGAAACACTTAAAACTGTGCTTTTTTGAAATCTTTATCCCGTTT
TTAAAAAAAGATATTTTCATAGAATTTATTGACTTTTAAAGATCATTCATAGGATGCGTACAATTTTGCCGATTAAATT
GAAATTTGAAATTTCCAAAAAAAATGTGCAAGAACACAATTTGCCAAAAAATTTCGGCGAATCGGGAATTTTCCGGTTT
GCCGAA
TTGCCGGAAATTTTCAATCGCGGCATTTTGCCGGTTTGCTCAT
TTGCCGGAAATTTTCAATTCCGGCACTTTGCCGATTTGCTGT
TTGCCGGAAATTTTCAATTCCGGTTTTTTTCCGGA
TTGCCGGAAATTTTCAATCCCGGCATTTTGCCGGTTTGCCGGA
TTGCCGGAAATATTCAATCCCGGCATTTTGCCGGTTTGCCGAT
TTGCCGGAAATTTTCAATCCCTGCATTTTGCCGGTTTGCCGAT
TTGCCGGAAATTTTCAATTCCGGCATTTTGCCGGTTTGCCGAT
Nr of selected repeats 5 Similarity 0.868148
Consensus:
TTGCCGGAAATtTTCAATccCgGCATTTTGCCGGTTTGCCgAt
>Cele-UNSB01_4:2179058-2179920 Satlength=863 Nr of Repeats=38 RepeatLength=21
seed=GATTTACGGG
GATTTACGGGAGTCAAATGTA
GATTTACGGGGATTACTGTGGA
GATTTACGGGATGAAAATGTG
GATTTACGGGAGCACTGTGTA
GATTTACGGGACGAACATGTG
GATTTACGGAGTGAACATGTG
GATTTACGGGGTGAACATGTA
GATTTACGGCGTAAACTTGTG
GATTTACGGGATGAAAATGTGAATTTACCGGATGAACATGTA
GATTTACGGGAGCACTATGTAGATTTACAAGGTGAACATGTG
GATGTACGGGAGCACTATGTA
GATTTACAGGGTGAACATGTA
GATTTACGGGGTGAGCATGTAC
ATTTACGGGAGCACTATGTAGATTTACAAGGTGAACATGTG
GATGTACGGGAGCACTATGTA
GATTTACAGGGTGAACATGTA
GATTTACGGGGTGAGCATGTAC
ATTTACGGGAGCACTATGTA

GATTTACGGGGTGAAAATGTG
GATTTACGGGGTGAAAATGTG
GATTTACGGGGTGAACGTGTG
GATTTACGGGGTGAGCAAGTG
GATTTACGGGAGAACTATGTA
GATTTACGGGGTGAAAATGTG
GATTTACCGGATGAACATGTA
GATTTACGGGATGAACTTGTG
GATTTACGGGGTGAACGTGTG
GATTTACGGGGTGAGCAAGTG
GATTTACGGGAGCACTATGTA
GATTTACAGGGTGAAAATGTG
GATTTACGGGATGAAAATGTG
GATTTACGGGATGAACATGTA
GATTTACGGGATGGAAATGTG
GATTTACCGGATGAACATGTA
GATTTACGGGAGCACTATGTA
GATTTACAGGGTGAACATGTA
GATTTACAGGGTGAACATGTA
GATTTACAGGGTGAACATGTG
Nr of selected repeats 31 Similarity 0.686520
Consensus:
GATTTACgGGgtGAAcATGTg
>Cele-UNSB01_4:2205280-2205605 Satlength=326 Nr of Repeats=13 RepeatLength=21
seed=AAAATAGCTT
AAAATAGCTTAAAATTGATTA
AAAATAGCTGAAAATTCACTA
AAACTAGCTTAAAATAGACGA
AAAATAGCTTTAAAATTCACT
AAAATAGCTTAAAATTGACTG
AAAATAGCTTAAACTTGACTGAACATTCAAAAA
AAAATAGCTTAAAATTTAAGAAAATTGTCTGAAAGTAGCTGAAAATAGACTA
AAAATAGCTTAAAATTGACTG
AAAATAGCTGAAAATTCACTA
AAACTAGCTTAAAATAGACTA
AAAATAGCTTAAAATTCACT
AAAATAGCTTTAAATTGACTG
AAAATAGCTTAAACTTGACTGAAAATTCACT
Nr of selected repeats 9 Similarity 0.765993
Consensus:
AAAaTAGCTtAAAATtgACTa
>Cele-UNSB01_4:2267683-2267908 Satlength=226 Nr of Repeats=15 RepeatLength=15
seed=CGATGGGTCT
CGATGGGTCTCACCA
CGATGGGTCTCACCA
CGATGGGTCTCACCA
CGATGGGTCTCACCA
CGATGGGTCTCACCT
CGATGGGTCTCACCA
CGATGGGTCTCACCA
CGATGGGTCTCACCA
CGATGGGTCTCACCT
CGATGGGTCTCACCA
CGATGGGTCTCGCCA

CGATGGGTCTTTCCA
CGATGGGTCTCACCA
CGATGGGTCTCGCCA
CGATGGGTTTCGCCA
Nr of selected repeats 15 Similarity 0.914497
Consensus:
CGATGGGTCTCaCCA
>Cele-UNSB01_4:2268162-2268293 Satlength=132 Nr of Repeats=4 RepeatLength=11
seed=GTACTCCTAC
GTACTCCTACA
GTACTCCTACAGTACAACAGTACCCCGACCATATCCCACTACTAACCCCAAATCTATATCTCTTCAAAAGACTAAAACA
CAATTTTTCCTAAACTACA
GTAATCCTACC
GTACTCCTACA
Nr of selected repeats 3 Similarity 0.838384
Consensus:
GTAcTCCTACa
>Cele-UNSB01_4:2419217-2421562 Satlength=2346 Nr of Repeats=23 RepeatLength=70
seed=AAAAATCGAT
AAAAATCGATAAGTTCGCCGAAAATTTGAACTTGGCGCTGTTTTATCGATGGAGCGCATTTTTAACGGCGAAAGATTGA
ATTTTCCGCC
AAAAATCGATGTTTTGAAGTTTTTCTCAATAGAGCGCACTTTCAACAGCGAGAAATTTGAATTTTCCACC
AAAAATCGATTTTTAAAAGTTTTTCTCAATAGAGCGCACTTTCAACAGCGAAAAAATTGAATTTTCCACC
AAAAATCGATTTTTTAAAGTTTTTCTCAATAGAGCGCACTTTCAACAGCGAAAAAATTGAATTTTCCACC
AAAAATCGATTTTTTAAAGTTGTTCTCAATAGAGCGCATTTTCTACAGCGAAAAATTTGATTTTTCGTC
AAAAATCGATGTTTTTGCTTTTTTCCCAATAGAGCGCATTTTCAACAGCGAAAAAATTGAATTTTCTACC
AAAAATCGATTTTTTAAAGTTTTTCTCAATAGAGCGCATTTTCTACAGCGAAAAATTTGAATTTTCGCC
AAAAATCGATTTTTTAAAGTTTTTCTCAATAGAGCGCACTTTCAACAGTGAAAAATTGAATTTTCCGCT
AAAAATCGATGTTTTTGCTTTTTTCTCAATAGAGCGCATTTTCAACAGCGAAAAATTTGAATTTTCCGCA
AAAAATCGATGTTTTGAAGTTTTTCTCAATAGAGCGCATTTTCACCAGCGAAAAATTTGAATTTTTCGCC
AAAAATCGATTTTTTGAAGTTTTTCTCAATAGAGCGCATTTTCAACAGCGAAAAAATTGAATTTTTCGCC
AAAAATCGATTTTTTGAAGTTTTTCTCAACAGAGCGCGATTACACCAGCGAAAAATTTGAATTTTCCGCA
AAAAATCGATGTTTTGAAGTTTTTCTCAATAGAGCGCATTTTCACCAGCGAAAAATTTGAATTTTTCGCC
AAAAATCGATTTTTTGAAGTTTTTCTCAATAGAGCGCATTTTCAACAGCGAAAAAATTGAATTTTTCGCC
AAAAATCGATTTTTTGAAGTTTTTCTCAACAGAGCGCGATTACACCAGCGAAAAATTTGAATTTTCCGCA
AAAAATCGATGTTTTGAAGTTTTTCTCAATAGAGCGCATTTTCACCAGCGAAAAATTTGAATTTTTCGCC
AAAAATCGATTTTTTGAAGTTTTTCTCAATAGAGCGCATTTTCAACAGCGAAAAAATTGAATTTTCCGCC
AAAAATCGATTTTTTGAAGTTTTTCTCAATAGAGCGCATTTTCAACAGCGAAAAAATTGAATTTTCCGCC
AAAAATCGATTTTTTGAAGTTTTTCTCAATAGAGCGCATTTTCACCAGCG
AAAAACCGATTTTTCTATTTAAATTTTTTCCAGAAAATTCGCCCTCCAAACCGGCCGAACCTACGTGATGGCAACCATC
AAAAATCGCC
AAAAATCGTCGGCTCAATGGCTCCGTTGCGAGATTATCGATTTTTTGCCGAACGCCAACGTGGCTCTTCGCTACGTGGA
TCTGGGAACACGTGGCATCCTGAAGCTCAAAAATTTGCACCGAATGCACATTGAGCACACAAAAATCGCGCCGGCTTGC
ATTGAAATTGGCCGATTTCTCGACGACGATTTATCGATGGCCGACAGCGAAATGGAGTGGAATACACATTTTTGGCGTG
AAATTGTACCCTATGACGTGCCGATCGTTGTCGGACCCGATATGGGTGAGAAATTTACGGATTTCTCTCTGGAAAAGCG
ACAAAAATGCAATTTTTGCATGTTCCTGTGCGCTCCATTGATAATTTTACCCGAAATCACAGAATTTTACCAAAACTTC
ACTATTTAACTCAAAAATTCAAATTTCGCGCCGAGAACACCTGAAATTTTGGTTTTTTAACTGAAAAATTCGAAATTTT
CATGGGATTTCGCCGATTTTGCAAAATTTCAAATTTCCCGCGCCGGCGGCCTCGTGTCCCAAATGCGCTCCATTGACAA
AAATTCTCCTGCGGAACAATTCGATCGATTTTCAGGGGAAAATGCGCTCCATAGCTAATTCGCGCGAATTTTCGTC
AAAAATCGATAATTCCCATGTGCGGAATATATCGGAGCATCGT
AAAAATCAATAATTTTCCAATCATTTCAGAATTCCTGGAAACCGGCAAACTGCAATTCTCGCAGATTCGGGTCGCCGGC
GACGAAGACGACGAAAATTTGCTCGATAAAATCCCGTCGCCGTCGCCGTTTTTCACTGAACGATCCGACGATTTACGTA
CCCAGAAAGAGGATGATGATGATGGAAATGTTAGCGACGATAAGGATAGCGGATGATTTTATTGATTTTTTCGCATTTC
TCCCGTGAAAAATTGCAAAAA

Nr of selected repeats 14 Similarity 0.884668
Consensus:
AAAAATCGATtTTTTgAAGTTTTTCTCAATAGAGCGCAtTTTCAaCAGCGAAAAAaTTGAATTTTcCgCc
>Cele-UNSB01_4:2422782-2423615 Satlength=834 Nr of Repeats=8 RepeatLength=104
seed=CCTGCGAAAG
CCTGCGAAAGCGAATCTCTGGCCTGGCGGAGGGCCAACTAACTCTCTCCCTCAAGCCTTCGGGTGCTTCCTGGGTTATC
GGTGAATTTTTGGGATCTGCCAGGGA
CCTGCGAAAGCAAATCTCTGGCCTGACGGAGGGCCAGCTAACTCTCTCCCTTAAGCCTTCGGGTGCTTCCTGGGTTATC
GGTGAATTTTTGGGTCTGTCAGGGT
CCTGCGAAAGCGAATCTCTGGCCTGGCGGAGGGCCAACTAACTCTCTCCCTCAAGCCTTCGGGTGCTTCCTGGGTTATC
GGTGAATTTTTGGGTCTGTCAGGGT
CCTGCGAAAGCGAATCTCTGGCCTGGCGGAGGGCCAACTAACTCTCTCCCTCAAGCCTTCGGGTGCTTCCTGGGTTATC
GGTGAATTTTTGGGTCTGTCAGGGT
CCTGCGAAAGCGAATCTCTGGCCTGGCGGAGGGCCAACTAACTCTCTCCCTCAAGCCTTCGGGTGCTTCCTGGGTTATC
GGTGAATTTTTGGGTCTGTCAGGGT
CCTGCGAAAGCGAATCTCTGGCCTGGCGGAGGGCCAACTAACTCTCTCCCTCAAGCCTTCGGGTGCTTCCTGGGTTATC
GGTGAATTTTTGGGTCTGTCAGGGT
CCTGCGAAAGCGAATCTCTGGCCTGGCGGAGGGCCAACTAACTCTCTCCCTCAAGCCTTCGGGTGCTTCCTGGGTTATC
GGTGAATTTTTGGGTCTGTCAGGGT
CCTGCGAAAGCGAATCTCTGGCCTGGCGGAGGGCCAACTAACTCTCTCCCTCAAGCCTTCGGGTGCTTCCTGGGTTATC
GGTGAATTTTTGGGTCTGTCAGGGT
Nr of selected repeats 7 Similarity 0.985348
Consensus:
CCTGCGAAAGCGAATCTCTGGCCTGGCGGAGGGCCAACTAACTCTCTCCCTCAAGCCTTCGGGTGCTTCCTGGGTTATC
GGTGAATTTTTGGGTCTGTCAGGGT
>Cele-UNSB01_4:2456640-2457356 Satlength=717 Nr of Repeats=5 RepeatLength=144
seed=TTCTAGGCCA
TTCTAGGCCACCAATTGGAAATCATCATAACTTGGCTTAAAATCCAGATTTTTGGAGTCTGGTACCTGCAACATGTTGC
CCTGTAGTTTTTCTACAAGTTAGGATATATTAATTTTTGGTGGCCTAAAAGTCCAAATTATGGAT
TTCTAGGCCACCAATTGGAAATCATCATAACTTGGCTTAAAATCCAGATTTTTGGAGTCTGGTAACTGCAACATGTTGC
CCTGTAGTTTTTCTACAAGTTAGGATATATTGATTTTTGGTGGCCTAAAAGTCCAAATTATGGAT
TTCTAGGCCACCAATTGGAAATCATCATAACTTGGCTTAAAATCCAGATTTTTGGAGTCTGGTAACTGCAACATGTTGC
CCTGTAGTTTTTCTACAAGTTAGGATATATTAATTTTTGGTGGCCGAGAAGTCCGAATTTTGGAT
TTCTAGGCCAACAATTCGAAATCATCATAACTTGGCTTAAAATCCAGATTTTTGGAGTCCGGTAACTGCAACATGTTGC
CCTGTAGTTTTTCTACAAGTTAGGATATATTAATTTTTGGTGGTCTAGAAACTCAAAACTGGGAA
TTCTAGGCCACCAACTTGTAATCGCTAGATCTCGGCTTAAAATCAAAGTTTTTTGAAATGTAAAAATTGCACCATGTTG
CCCGAAATTTGCTCTAGAAACAGCGTTAATTTTCAATGGCCTAGAAAGTCAAAACTGGGAT
Nr of selected repeats 4 Similarity 0.915326
Consensus:
TTCTAGGCCAcCAATTgGAAATCATCATAACTTGGCTTAAAATCCAGATTTTTGGAGTCtGGTAaCTGCAACATGTTGC
CCTGTAGTTTTTCTACAAGTTAGGATATATTaATTTTTGGTGGcCtAaAAgTCcaAAtTatGGAt
>Cele-UNSB01_4:2599504-2602273 Satlength=2770 Nr of Repeats=17 RepeatLength=164
seed=CTTTTTTCTT
CTTTTTTCTTTTAAAAAACTATTCAGCATAGTCATATTTATCAAAAAAATTATTTTTTTAAAGATTATTTTTAAGAAAT
TCAGAAAATGCATCTAAAGCTTCTTTTCAAAAATTCAAAAGTACCGGAAAATCATATAAAGTGCTT
CTTTTTTCTTTTATAAAACTGTTTAGCATAGTCGAATATACCAGAAAATACCAAACAAAGTATGCTATCTTGTACGAAA
GATTATTTTAAAAAATTCAGAAAATGCATCTAAAGCGGTTTTTTTTCAAAAATTCAAAAGTACCGGAAAATCATAAAAA
GTGCTT
CTTTTTTCTTTTATAACACTGTTTAGCATAGTCAAATATACCAGAAAATACCAAACAAAGTATGCTATCTTGTACGAAA
GATTATTTTAAAAAATTCAGAAAATGCATCTAAAGCGGTTTTTTTTCAAAAATTCAAAAGTACCGGAAAATCATATAAA
GTGCTT
CTTTTTTCTTTTATAAAACTGTTTAGCATAGTCGAATATACCAGAAAATACCAAACAAAGTATGCTATCTTGTACGAAA
GATTATTTTAAAAAATTCAGAAAATGCATCTAAAGCGGTTTTTTTTCAAAAATTCAAAAGTACCGGAAAATCATAAAAA
GTGCTT

CTTTTTTCTTTTATAACACTGTTTAGCATAGTCAAATATACCAGAAAATACCAAACAAAGTATGCTATCTTGTACGAAA
GATTATTTTAAAAAATTCAGAAAATGCATCTAAAGCGGTTTTTTTTCAAAAATTCAAAAGTACCGGAAAATCATATAAA
GTGCTT
CTTTTTTCTTTTATAAAACTGTTTAGCATAGTCGAATATACCAGAAAATACCAAACAAAGTATGCTATCTTGTACGAAA
GATTATTTTAAAAAATTCAGAAAATGCATCTAAAGCGGTTTTTTTTCAAAAATTCAAAAGTACCGGAAAATCATAAAAA
GTGCTT
CTTTTTTCTTTTATAACACTGTTTAGCATAGTCAAATATACCAGAAAATACCAAACAAAGTATGCTATCTTGTACGAAA
GATTATTTTAAAAAATTCAGAAAATGCATCTAAAGCGGTTTTTTTTCAAAAATTCAAAAGTACCGGAAAATCATAAAAA
GTGCTT
CTTTTTTCTTTTATAACACTGTTTAGCATAGTCAAATATACCAGAAAATACCAAACAAAGTATGCTATCTTGTACGAAA
GATTATTTTAAAAAATTCAGAAAATGCATCTAAAGCGGTTTTTTTTCAAAAATTCAAAAGTACCGGAAAATCATAAAAA
GTGCTT
CTTTTTTCTTTTATAACACTGTTTAGCATAGTCAAATATACCAGAAAATACCAAACAAAGTATGCTATCTTGTACGAAA
GATTATTTTAAAAAATTCAGAAAATGCATCTAAAGCGGTTTTTTTTCAAAAATTCAAAAGTACCGGAAAATCATAAAAA
GTGCTT
CTTTTTTCTTTTATAACACTGTTTAGCATAGTCAAATATACCAGAAAATACCAAACAAAGTATGCTATCTTGTACGAAA
GATTATTTTAAAAAATTCAGAAAATGCATCTAAAGCGGTTTTTTTTCAAAAATTCAAAAGTACCGGAAAATCATAAAAA
GTGCTT
CTTTTTTCTTTTATAACACTGTTTAGCATAGTCAAATATACCAGAAAATACCAAACAAAGTATGCTATCTTGTACGAAA
GATTATTTTAAAAAATTCAGAAAATGCATCTAAAGCGGTTTTTTTTCAAAAATTCAAAAGTACCGGAAAATCATATAAA
GTGCTT
CTTTTTTCTTTTATAAAACTGTTTAGCATAGTCGAATATACCAGAAAATACCAAACAAAGTATGCTATCTTGTACGAAA
GATTATTTTAAAAAATTCAGAAAATGCATCTAAAGCGGTTTTTTTTCAAAAATTCAAAAGTACCGGAAAATCATAAAAA
GTGCTT
CTTTTTTCTTTTATAACACTGTTTAGCATAGTCAAATATACCAGAAAATACCAAACAAAGTATGCTATCTTGTACGGAA
GATTATTTTAAAAAATTCAGAAAATGCATCTAAAGCGGTTTTTTTTCAAAAATTCAAAAGTACCGGAAAATCATATGAA
GTGCTT
CTTTTTTCTTTTATAAAACTGTTTAGCATAGTCGAATATACCAGAAAATACCAAACAAAGTATGCTATCTTGTACGAAA
GATTATTTTAAAAAATTCAGAAAATGCATCTAAAGCGGTTTTTTTTCAAAAATTCAAAAGTACCGGAAAATCATAAAAA
GTGCTT
CTTTTTTCTTTTATAACACTGTTTAGCATAGTCAAATATACCAGAAAATACCAAACAAAGTATGCTATCTTGTACGGAA
GATTATTTTAAAAAATTCAGAAAATGCATCTAAAGCGGTTTTTTTTCAAAAATTCAAAAGTACCGGAAAATCATATGAA
GTGCTT
CTTTTTTCTTTTATAAAACTGTTTAGCATAGTCGAATATACCAGAAAATACCAAACAAAGTATGCTATCTTGTACGAAA
GATTATTTTAAAAAATTCAGAAAATGCATCTAAAGCGGTTTTTTTTCAAAAATTCAAAAGTACCGGAAAATCATAAAAA
GTGCTT
CTTTTTTCTTTTATAACACTGTTTAGCATAGTCAAATATACCAGAAAATACCAAACAAAGTATGCTATCTTGTACGGAA
GATTATTTTAAAAAATTCAGAAAATGCATCTAAAGCGGTTTTTTTTCAAAAATTCAAAAGTACCGGAAAATCATATGAA
GTGCTT
Nr of selected repeats 16 Similarity 0.982520
Consensus:
CTTTTTTCTTTTATAAcACTGTTTAGCATAGTCaAATATACCAGAAAATACCAAACAAAGTATGCTATCTTGTACGAAA
GATTATTTTAAAAAATTCAGAAAATGCATCTAAAGCGGTTTTTTTTCAAAAATTCAAAAGTACCGGAAAATCATAaAAA
GTGCTT
>Cele-UNSB01_4:2676914-2677180 Satlength=267 Nr of Repeats=11 RepeatLength=19
seed=CGCCAAGTAT
CGCCAAGTATCGAGAAAAG
CGCCAAGTGTCCGAAAATA
CGCCAAGGATCGAGAAATGCGCCAAGGCTCCAGAAATG
CGTCAAGTATCAAGAAAAG
CGCCAAGTATCGTGAAAAGCACCTAGGCGCCAGAAATG
CGTCAAGTATCGAGAAAGG
CGCCAAGTATCCAAAAATG
CGCCAAGTATCGTGAAAAT

CGCCAAGTATCCAAAAATGCGCCATGTTTCGCGAAAAG
CGCTAAGTATCCAAAAATA
CGCCAAGTATCGAGAAAAG
Nr of selected repeats 8 Similarity 0.694345
Consensus:
CGcCAAGTATCgagAAAAg
>Cele-UNSB01_4:2701520-2702341 Satlength=822 Nr of Repeats=4 RepeatLength=175
seed=AACTTGTGAC
AACTTGTGACGGTGGGTATACATTTATGTTTTAAATTGTTCGAAAACCTTCATTTCGTCAATTGGTGAAGTTTCAAA
AACTTTGTGAATCTAGTTTTTGAAAAAATTTAAAATTTGTGGCAATTTCACCGATTTTTCGTGAATTTCCGAGCTAAAA
TTGAAAAAATTCATTGTG
AACTTGTGACGGTGGGTATGGATTTTATGCACATATTTTGTTTGAAATAGTCTACCAAGTAAACTGGTGAAGTTTCAAA
AACTTTGTGGATCTAGTTTTTGAAAAAAGTTCAAAATTGCGCCAATTTTGACCAATTTTGGTGGTTTTCTGAGCTAAAA
TTGAAAAGTTGGGAATG
AACTTGTGACGGTGGGTATGGATTTTTTTCACATAATTCGTTCTAAATAGTCTACCGAGGAAACTGGTAAAGTTTCAGA
AGCTTTGTGGCCCTAGTTTTCGAAAAAAGTTCCAAAATGTGCCAATTTTGACCAATTTTGGTGGTTTTCTGAGCTAAAA
TTGAAAAGTTGGGAATG
AACTTGTGACGGTGGGTATGGATTTTTTTCACATAATTCGTTCTAAATAGTCTACCGAGGAAACTGGTAAAGTTTCAGAA
ACTTTGTGGCCCTAGTTTTCGAAAAAAGTTCCAAAATGTGCTAATTTTCACGCATTTCTGTAATAATTGCCTAATAAAT
CAAATTTCCATTTTTATTTAGGAAAATAATCGTTTTTTAATGTGATATATTTATTTAAAAAGCAATTTTCGTTCATATT
CTGTTTGTTTTGATCGAATTTTAGTGGTTTTCGGAGCAAAAATAGAAAAATTCAATATG
Nr of selected repeats 3 Similarity 0.715980
Consensus:
AACTTGTGACGGTGGGTATggATTTTaTgcacaTAatTtGTTcgAAAtagTctAccnaGtaAAcTGGTgAAGTTTCAaA
AaCTTTGTGgatCTAGTTTTtGAAAAAAgTTcaAAatTGtGcCAAtTTTgACCAaTTTTgGTGgtTTTCtGAGCTAAAA
TTGAAAAgTTgggAaTG
>Cele-UNSB01_4:2721172-2721362 Satlength=191 Nr of Repeats=5 RepeatLength=40
seed=GAATTTTCCA
GAATTTTCCAGAAAGTTCTAGAAAATTCCAGAATTTTTTC
GAATTTTTCAGAAAGTTCTAGAACATTCCAGAATTTTTTC
GAATTTTCCAGAAATTTCTAGAAAATTCCAGAACGTTTTC
GAATTTTCCAGAAGGTTCTGGAATATGCCAGATTTTTCTC
GAATTTTCCAGAACGTTCTAGAACATTCCA
Nr of selected repeats 4 Similarity 0.822222
Consensus:
GAATTTTcCAGAAagTTCTaGAAaATtCCAGAattTTtTC
>Cele-UNSB01_4:2728247-2729274 Satlength=1028 Nr of Repeats=42 RepeatLength=21
seed=TTTGGATTTT
TTTGGATTTTTAGGCTTTAAA
TTTGGATTTTTTGGCTTAAAACTGGATTTGTAGGCTTAAAA
TTCGGATTTTTAGGCTTAAAA
TTTAGATTTTTAAGCTTAAAA
TTTGGATTTTTTGGCTTAAAACTGGATTTGTAGGCTTAAAA
TTCGGATTTTTAGGCTTAAAA
TTTAGATTTTTAAGCTTAAAA
TTTGGATTTTTGAGCTTAAAAATTGGATTTGGAAGCTTAAAA
TTTGGATTTTTAGGCTTAAAG
TTTGGATTTTTAGGCTTTAAA
TTTGGATTTTTTGGCTTTAAA
TTTGGATTTTTAGGCTTAAAA
TTTGGATTTTTGAGCTTAAAAATTGGATTTGGAAGCTTAAAA
TTTGGATTTTTAGGCTTAAAG
TTTGGATTTTTAGGCTTTAAA
TTTGGATTTTTTGGCTTTAAA

TTTGGATTTTTAGGCTTAAAA
TTTGGATTTTTAAGCTTAAAA
TATGGATTTTTAAGCTTAAAA
TATGGATTTTTAGGCTTTAAA
TTTGGATTTTTAGGCTTTAAA
TTTGGATTTTTTGGCTTAAAA
TTTGGATTTTTTGGCTTAAAATTTGGAAATTTGAGCTTAAAA
TTTGGATTTTTAGGCTTAAAG
TTTGCATTTTTAAGCCTAAAA
TTTGGATTTTGAAGCTTAAAA
TTTGGATTTCTAAGCTTAAAA
TTTGGATTTTTAGGCTTAAAA
TTTGGATTTTTAAGCTTAAAA
TATGGATTTTTAAGCTTAAAA
TATGGATTTTTAGGCTTTAAA
TTTGGATTTTTAGGCTTTAAA
TTTGGATTTTTTGGCTTAAAA
TTTGGATTTTTTGGCTTAAAATTTGGAAATTTGAGCTTAAAA
TTTGGATTTTTAGGCTTAAAG
TTTGGATTTTTAAGCTTAAAA
TTTGCATTTTTAAGCCTAAAA
TTTGGATTTTGAAGCTTAAAA
TTTGGATTTCTAAGCTTAAAA
TTTGGATTTTTAGGCTTAAAATTTGAATTTCTAGGCTTCAAA
TTTGGATTTTTAGGCTTGAAA
TTTGGATTTTTAAGCTTAAAA
Nr of selected repeats 35 Similarity 0.859037
Consensus:
TTTGGATTTTTAgGCTTaAAA
>Cele-UNSB01_4:2780831-2781210 Satlength=380 Nr of Repeats=12 RepeatLength=11
seed=TACTGTAGGA
TACTGTAGGAG
TACTGTAGGATTACTGTAGTTTGGGAAAAATTGACTTTTTGTCTTATGAAGGAATATTGGTTTGAGGTTAGTGGAGGGA
TATGGTCGGGG
TACTGTAGAGA
TACTGTAGGAG
TACTGTAGGAT
TACTGTAGGATTACTGTAGTTTGGAAAAAATTGACGTTTCGTCTTTTGAAGGAATATTGGTTTGAGATTAGTGATGGGA
TATGGACGGG
TACTGTAGTAG
TACTGTAGAGG
TACTGTAGGAG
TACTGTAGGATTACTGTAGTTTGGGAAAAATTGACTTTTCATCTTTTGAAGGAATATTAGTTTGGGGTTAGTGGAGGGA
TATGGTCGGGG
TACTGTAGTAGTACAATAGAGA
TACTGTAGGAG
Nr of selected repeats 8 Similarity 0.787698
Consensus:
TACTGTAGgAG
>Cele-UNSB01_4:2843789-2844131 Satlength=343 Nr of Repeats=4 RepeatLength=87
seed=TGAAGTCGTA
TGAAGTCGTAGGATTTGTGACTGGTGTCTTTGCACTCTGTGGAGAAGACGTAGCTTCATCCGTTGTCTCATTCTTTTGG
TT

TGAAGTCGTAGGATTCTGTGTAGCGACTGGTGTCGTTGTACTCTGTGAAGGGGACGTGGCTTCTTCTGTTGAGTCAGGC
TTCTGAGG
TGAAGTCGTAGGCTTCAGTGTGGTGGTTGGTGTCGTTGTCGTAGGTGGAGAAGACGTAGCTTCATCCGTTGTCTCATTC
TTCTGGTT
TGAAGTCGTAGGATTCTGTGTGGTGACTGATGTCGTTTCATTCTGTGAAGGGGACGTGGCTTCTTCCGTTGTGCTATGC
TTCTCGGG
Nr of selected repeats 3 Similarity 0.685393
Consensus:
TGAAGTCGTAGGaTTCtGTGTgGtGacTGgTGTCGTTgtanTctGTGaAGggGACGTgGCTTCtTCcGTTGtgtcAtgC
TTCTnnGG
>Cele-UNSB01_4:2889237-2889839 Satlength=603 Nr of Repeats=7 RepeatLength=35
seed=TTTTCAATTC
TTTTCAATTCCAGGAATTTGACGGTTTGTTGATTTGCCGGAAATTTTTTTCGGCAAATTACCGGTTTGCCGAAAGTCA
ATTTGCCGGAAGTTTTTAGAGGTTTTTTTATAAGACGGAAACACTTGAAACAGTGCCTTTTTGATTTTTTTTCCGTAT
TTTTTTAGATACTTTCATAGAAATTTCCTACTTTTCAAAATAAGTGTAGGAACATTCATAGGATGCATAAAATTTTACC
GATTAAAATTGAAATTCTGAAACTTCCAAAACAAATGTGCAAATCTGCAATTTGCCGAAAACCGGCAAACTCGGCAAAT
CTGCAATTCGCCGACTTGCCAGTCTGCCTGAAA
TTTTCAATTCCGGCAATTTGCCGATTTGCCAATTTGCCTGAAAATTTTAAATCCGGAAATTTGCCGTTTTGCCTGCAT
TTTTCAATTCAAGCAATTTGCAGTTTTGCCTGAAT
TTTTCAATTCCGGCAATTTGCAGTTTTGCCTGAAT
TTTTCAATTCAAGCAATTTTCCGATTTGCCTGAAT
TCTTCAATTCCGGCAACTCGCCAATTTGCTTGAAT
TTTTCAATTCAAGCAATTTGCAGATTTGCCTGTAT
Nr of selected repeats 5 Similarity 0.801905
Consensus:
TtTTCAATTCaaGCAAtTtgCagaTTTGCcTGaAT
>Cele-UNSB01_4:2892401-2892617 Satlength=217 Nr of Repeats=5 RepeatLength=24
seed=GCTTCTGGAG
GCTTCTGGAGCTACCGGTTACCCG
GCTTCTGGAGCCACAGGTTATCCA
GTTTCTGGAGCCACAGGTTATCCTGTCTCTGGGGCTACCGGCTATCCAGCCTCTTCCATGACCGACTCACCG
GCTTCTGGAGCTACAGACTATCCA
GCCTCTGGAGCCACAGGATATCCAGCCTCGGGAGCCACCGGTTATCCGGCTTCTTCTATGACGGACTCACCG
Nr of selected repeats 3 Similarity 0.777778
Consensus:
GCTTCTGGAGCtACaGgtTAtCCa
>Cele-UNSB01_4:2898426-2898868 Satlength=443 Nr of Repeats=12 RepeatLength=19
seed=ACCTAGGCAT
ACCTAGGCATAAATTAGGC
ACCTATGCATAAGGCAGGC
ACCTAGGCATTATTCAGGC
ACCTATGCATGAATTAGGC
ACCTAGGCATAAGATAGGC
ACCTAGGCATAATTTAGGCACCGATGCATGAGGTAGGC
ACCTGGGCATAAAGTAGGC
ACCTAGGCATAAGTTAGGTACATAGTCATAAGCTAGGCACCGATACAAAAAGTACGCATATAGGCTTAAAGTAGGCACA
TAGGAATCTAGGTTTAAGATAGGTACCTACGCACAAGTTAGGCTCATAGGCATAAGGTAGGCACGTAGGCACGTGGGTA
TAAGATACGC
ACCTAGACATAAGGTAGGTATCTAGTCATAAGGTAGGCACAAAAAC
ACCTAGGCATAAATTAGGCACCGATGCATAAGGTAGGC
ATCTAGGCATAAAGCAGGC
ACCTAGGCATAAGGTAGGT
Nr of selected repeats 8 Similarity 0.751880
Consensus:

ACCTAgGCATaAagtAGGC
>Cele-UNSB01_4:2909681-2910188 Satlength=508 Nr of Repeats=14 RepeatLength=35
seed=ATTCCGGCAA
ATTCCGGCAATTTGCCGATTTGCCGAAAATTTTCA
ATTCCGGCAAATTACCGATTTGCCGAAAATTTTCA
ATTCCTGCAATTTGCCGATTTGCCGAATATTTTCA
ATTCCGGCAATTTGCCGATTTGCCGGAAAAGTTCA
ATTCGGGCAATTTGTCGATTTGCCGAAGCTGTTCA
ATTCCGGCAATTTGTCGATTTGCCTAAAATTTTCA
ATTCCGGCAATTTCCGAAACTGTTAA
ATTCCGGCAAATTGCCGATTTGCCGAAAATTTTCA
ATTCCGGCAATTTGCTGATTTTCGAAAATTTTAA
ATTCGGGCAATTTGCCAATTTGCCGAAACTGTTAA
ATTCCGGCAATTTGCCGATTTGCCGGAAAAGTTCA
ATTCGGGCAATTTGTCGATTTTCCGAAACTGTTAA
ATTCCGGCAATTTGCCAATTTGCCGAAAATTTTTA
ATTCCGGCAAATTGCCGATTTGCCGAAAATATTCAATATCGGCAATTTTCCGAAATTTTTTT
Nr of selected repeats 11 Similarity 0.820606
Consensus:
ATTCcGGCAATTTGcCGATTTGCCGAAAaTtTTcA
>Cele-UNSB01_4:2933784-2933839 Satlength=56 Nr of Repeats=5 RepeatLength=11
seed=TTGTTTACAA
TTGTTTACAAG
TTGTTTACAAG
TTGTTTACAAG
TTGTTTACAAG
TTGTTTACAAG
Nr of selected repeats 5 Similarity 1.000000
Consensus:
TTGTTTACAAG
>Cele-UNSB01_4:2990271-2990613 Satlength=343 Nr of Repeats=19 RepeatLength=16
seed=TAGGCATGTA
TAGGCATGTAGACATGTAGGTTTG
TAGGCATGTAGGAATG
TAGGCATATAGGTGTG
TAGGCATGTAGGCATG
TAGGTATGTAGGCATA
TAGGCATGTAGGCATGTAGGCATTCGGCTTG
TAGGCATTTAGGCATG
TAGGCATGTAGGCATG
TAGGAATGTAGGCATG
TAGCCATGTAGCCATG
TAGGCATGTAAGCATG
TAGGCATGCAGGCATG
TAGGCATGTAGGCATG
TAGGCATGTAGGCATG
TAGGCATGTAGGCATG
TAGGCATGTAGGCATG
TAGCCATGTAGGCATG
TAGGCATGTAGCCATG
TAGGCATGTAGGCATGTAGGCATTCGGCTTG
Nr of selected repeats 16 Similarity 0.859028
Consensus:
TAGGCATGTAGGCATG

>Cele-UNSB01_4:3002005-3002220 Satlength=216 Nr of Repeats=4 RepeatLength=43
seed=TTTGCCGGAA
TTTGCCGGAATTTAAAATTTCTGGCAAACTGGCAAACCGGTAAGTTGCCGCAATTGAAAATTTCCGGCAAATCGATAAA
TCGGCTA
TTTGCCGGAATTGAAAACTACCGGCAAATCTGCAAACTGGAAA
TTTGCCGGAATTGAAAATTTCCTGTAAATCTGCAAACCGGCAG
TTTGCCGGAATTGAAAATTTCTGGCAATTTGGCAAACCGGCAG
Nr of selected repeats 3 Similarity 0.739899
Consensus:
TTTGCCGGAATTGAAAAtTtcCtGnaAATcTGCAAACcGGcAg
>Cele-UNSB01_4:3004201-3004345 Satlength=145 Nr of Repeats=6 RepeatLength=24
seed=GCGGCCGACA
GCGGCCGACACTTTGCGGGTTGCT
GCGGCCGACACCTTACGGGTTGTA
GCGGCCGACACCTTGCGGGTTGTT
GCGGCCGACACGTTACGGGTTGTA
GCGGCCGACACCTTACGGGTTGTA
GCGGCCGACACTTTGCAGGTTGTT
Nr of selected repeats 6 Similarity 0.855556
Consensus:
GCGGCCGACACcTTaCGGGTTGTa
>Cele-UNSB01_4:3018891-3018971 Satlength=81 Nr of Repeats=4 RepeatLength=16
seed=AGGTAGGAAG
AGGTAGGAAGGAAGGT
AGATAGGAAGGTAGGA
AGGTAGGTAGGTATGT
AGGTAGGTAGGTAGGAAGGCAGGTAGGTACGC
Nr of selected repeats 3 Similarity 0.722222
Consensus:
AGgTAGGaAGGtAgGt
>Cele-UNSB01_4:3087838-3087930 Satlength=93 Nr of Repeats=6 RepeatLength=15
seed=CGAAAAGGGG
CGAAAAGGGGAGCAGAA
CGAAAAGGGGATCTG
CAAAAAGGGGATCTG
CGAAAAGGGGAGATA
CGAAAAGGGGAGTTA
CGAAAAGGGGAGCAA
Nr of selected repeats 5 Similarity 0.760000
Consensus:
CgAAAAGGGGAgcta
>Cele-UNSB01_4:3088105-3088197 Satlength=93 Nr of Repeats=6 RepeatLength=15
seed=TCCCCTTTTC
TCCCCTTTTCGTTGC
TCCCCTTTTCGTATC
TCCCCTTTTCGTATC
TCCCCTTTTCGCAGA
TCCCCTTTTTGCAGA
TCCCCTTTTCGTTCTGC
Nr of selected repeats 5 Similarity 0.768889
Consensus:
TCCCCTTTTcGtagc
>Cele-UNSB01_4:3090317-3090467 Satlength=151 Nr of Repeats=8 RepeatLength=15
seed=CACGATGGGT

CACGATGGGTCTCGG
CACGATGGGTCTCAT
CACGATGGATCTCAC
CACGATGGGTCTCAC
CACGATGGGTCACAC
CACGATGGGTCTCGC
CACGATGGATCTCAC
CACGATGGGTCTCACCGCGATGGGCCTCACCACGAAGGCTCACAC
Nr of selected repeats 7 Similarity 0.843386
Consensus:
CACGATGGgTCTCac
>Cele-UNSB01_4:3135501-3135649 Satlength=149 Nr of Repeats=4 RepeatLength=37
seed=TGGCGGGAAT
TGGCGGGAATTCAAATTTTAATTTTCCGAAAACATTT
TGGCGGGAATTCAAATTTCAATTTTTAGAACTCATTT
TGGCGGGAATTCAAATTTTAATTTTTCGAAAACATTC
TGGCGGGAATTCAAATGTTAATTTTCCGAAAACATTT
Nr of selected repeats 4 Similarity 0.867868
Consensus:
TGGCGGGAATTCAAATtTtAATTTTccGAAaaCATTt
>Cele-UNSB01_4:3176916-3177039 Satlength=124 Nr of Repeats=7 RepeatLength=12
seed=TAGGCTTAGG
TAGGCTTAGGCT
TAGGCGTAGGCA
TAGGCTTAGGCT
TAGGCTTAGGCATATGCA
TAGGCATAGGAATATACATAGGCATCCAAGGCCTTGATCCAGCCC
TAGACTTAGGTT
TAGGCTTAAGTT
Nr of selected repeats 5 Similarity 0.755556
Consensus:
TAGgCtTAgGct
>Cele-UNSB01_4:3192518-3194426 Satlength=1909 Nr of Repeats=53 RepeatLength=35
seed=CGGCAATTTG
CGGCAATTTGCCGATTTGCCGGAAATTTTCAGTTC
CAGCAATTTGCCGATTTGCCGGAAATTTTCAGTTC
CGGCAATTTGCCTATTTGCCGGAAATGTTTAATTT
CGGCAATTTGTCTATTTGCCGGAAATGTTCAATTC
CGGCAATTTGCCGATTTGCCGGAAATGTTCAATTC
CGGCAATTTGCCGATTTGCCGGAAATGTTCAATTT
CGGCAATTTGCCTATTTGCCGGAAATGTTTAATTT
CGGCAATTTGTCTATTTGCCGGAAATGTTCAATTC
CGGCAATTTGCCGATTTGCCGGAAATGTTCAATTC
CGGCAATTTGCCGATTTGCCGGAAATGTTCAATTC
CGGCAATTTGCCGATTTGCCGGAAATGTTCAATTT
CGGCAATTTGCCTATTTGCCGGAAATGTTTAATTT
CGGAAATTTGCCTATTTGCCGGAAATGTTCAATTC
CGGCAATTTCCCAATTCGCCGGAACTTTTGAACTTTTCAATTC
CGGCAATTTGCCTATTTGCCGGAAATGTTCAATTT
CGGCAATTTGCCGATTTGCCGGAAATTTTCAATTT
CGGCAATTGCCTATTTGCCGGAAATGTTTAATTT
CGGCAATTTGCCTATTTGCCGGAAATGTTTAATTT
CGGCAATTTGCCTATTTGCCGGAAATGTTCAATTC
CGGCAATTTGCCAATTCGCCGGAACTTTTCAATTC

CAGCAATTTGCCAATTCGCCGGAACTTTTCAATTC
CGGCAATTTGCCAATTCGCCGGAACTTTTGAACTTTTCAATTC
CGGCAATTTGCCAATTCGCAGGAAATTTTCAATTT
CGGCAATTTGCCTATTTGCCGGAAATGTTTAATTT
CGGTAATTTGCCTATTTGCCGGAAATGTTCAATTC
CGGCAATTTGCCAATTCGCCGGAACTTTTGAACTTTTCAATTC
CGGCAATTTGCCAATTCGCAGGAAATTTTCAATTT
CGGCAATTTGCCTATTTGCCGGAAATGTTCAATTT
CGGCAATTTGCCGATTTGCCGGAAATGTTTAATTT
CGGCAATTTGCCTATTTGCCGGAAATTTTTAATTT
CGGCAATTTGCCTATTTGCCGGAAATGTTCAATTC
CGGCAATTTGCCAATTCGCCGGAACTTTTCAATTC
CGGCAATTTGCCAATTCGCCGGAACTTTTGAACTTTTCAATTC
CGGCAATTTGCCAATTCGCAGGAAATTTTCAATTT
CGGCAATTTGCCAATTCGCCGGAACTTTTCAATTC
CGGCAAATTGCCAATTCGCCGGAACTTTTGAACTTTTCAATTC
CGGCAATTTGCCTATTTGCCGGAAATGTTCAATTC
CGGCAATTTGCCAATTCGCCGGAACTTTTCAATTC
CGGCAATTTGCCAATTCGCCGGAACTTTTGAACTTTTCAATTC
CGGCAATTTGCCAATTCGCAGGAAATTTTCAATTT
CGGCAATTTGTCTATTTGCCGGAAATGTTCAATTT
CGGCAATTTGCCGATTTGCCGGAAATGTTTAATTT
CGGCAATTTGCCTATTTGCCGGAAATGTTTAATTT
CGGCAATTTGCCTATTTGCCGGAAATGTTCAATTC
CGGCAATTTGCCAATTCGCCGGAACTTTTCAATTC
CAGCAATTTGCCAATTCGCCGGAACTTTTCAATTC
CGGCAATTTGCCAATTCGCCGGAACTTTTGAACTTTTCAATTC
CGGCAATTTGCCAATTCGCAGGAAATTTTCAATTT
CGGCAATTTGCCTATTTGCCGGAAATGTTTAATTT
CGGTAATTTGCCTATTTGCCGGAAATGTTCAATTC
CGGCAATTTGCCAATTCGCAGGAACTTTCAATTC
CGGCAATTTTCCAATTCGCAGGAACTTTCAATTT
CGGCAATTTGCCTATTTGCCGGAAATGTTCAATTC
Nr of selected repeats 43 Similarity 0.870738
Consensus:
CGGCAATTTGCCnATTtGCCGGAAATgTTcAATTc
>Cele-UNSB01_4:3222029-3222419 Satlength=391 Nr of Repeats=5 RepeatLength=16
seed=TACATGCCTA
TACATGCCTACATGCC
TACATGCCTACATGCC
TACATGCCTACATGCCTACCAAGGATGTCGGTTTTTTGATTTTTCTGATATTGTGAAAATGGAAAAATGGAAAAATTTC
GTTTTTCGTTTTTTCGTTTTTTCGATTTTTCGAAAATAAGCTTGATATTTTGGGCATTATTCTTCTTTTTCGAAGAAAT
TTGTATGTTTTTTCTTTAAAAATATAAACAAAATTAGATTAAAAAGTCATATTTTATGGTTAATTTTCTGAACATTTTT
TTTTCGATTTTTCGGTTTTTGAAAATCGAAAAATCGAAAATTTGGGGGTTTTTCGATTTTTCGATTTTCACTGAAAAAT
CGAAAAAAAA
TACATGCCTATATGCC
TACATGCCTACATGCC
Nr of selected repeats 4 Similarity 0.958333
Consensus:
TACATGCCTAcATGCC
>Cele-UNSB01_4:3226485-3226669 Satlength=185 Nr of Repeats=11 RepeatLength=16
seed=CATGCCTACA
CATGCCTACACGCCTA
CATGCCTACATACATA

CATGCCTACATGCCTG
CATGCCTACATTCCTA
CATTCCTACATGCCTG
CATGCCTACATGTCTG
CATGCCTACATGCCTA
CATGCCTACATGCCTA
CATGCCCACATGTCTA
CATGCTTACATGCCTA
CATGCCTACATGCCTATATGCATA
Nr of selected repeats 10 Similarity 0.816667
Consensus:
CATGCCTACATgcCTa
>Cele-UNSB01_4:3230422-3232144 Satlength=1723 Nr of Repeats=67 RepeatLength=16
seed=ATGCCTACAT
ATGCCTACATGCCTAC
ATGCCTACATGGCTAG
ATGCCTAGATGCCTAG
ATGCCTAGATGCCTAGATATCTAG
ATGCCTAGATGCCTAG
ATGCCTACATGCCTACATGGCTAG
ATGCCTACAAGCCTAC
ATGCCTACAATGCCTAC
ATGCCTACATGCCTACATACCTACGTGATGCACGCCTAC
ACGCCTACATGCCTACA
ACGCCTACATGACTAC
ATGACTACATGCCTGG
ATGCCTACATGCCTAC
ATGCCTACATTCCTAC
ATGCCTACATGCCTACATGGCTAG
ATGCCTAGATGCCTAG
ATGCCTAGATGGCTAG
ATGCCTAGATGCTTAG
ATGCCTAGATGCCTAGATGTCTAG
ATGCCTAGATGCCTAC
ATGCCTACATGGCTAG
ATGCCTACATGCCTAC
ATGCCTACATGCCTAC
ATGCCTACATTTCTAC
ATGCCTACAATGCCTAC
ATGCCTACATGCCTACATACCTACGTGATGCACGCCTAC
ACGCCTACATGCCTACA
ACGCCTACATGACTAC
ATGACTACATGCCTGG
ATGCCTACATGCCTAC
ATGCCTACATTCCTAC
ATGCCTACATGCCTACATGGCTAG
ATGCCTAGATGCCTAG
ATGCCTAGATGGCTAG
ATGCCTAGATGCTTAG
ATGCCTAGATGCCTAGATGTCTAG
ATGCCTAGATGCCTAC
ATGCCTACATGGCTAG
ATGCCTACATGCCTAC
ATGCCTACATGCCTAC

ATGCCTACATTTCTAC
ATGCCTACAATGCCTAC
ATGCCTACATGCCTACATACCTACGTGATGCACGCCTAC
ACGCCTACATTTCTAC
ATGCCTACATGTCTAC
ATGCCTACATGCCTAC
ATGCCTACATGCCTACATTTCTAC
ATGCCTACATGCCTACATGGCTAA
ATCCCTACATGCCTAC
ATGCCTACATGGCTAG
ATGCCTAGATGCCTAG
ATGCCTAGATGCCTAG
ATGCCTAGATGTCTAG
ATGCCTAGATGCCTAC
ATGCCTACATGGCTAG
ATGCCTACATGCCTAC
ATGCCTACATGCCTAC
ATGCCTACATTTCTAC
ATGCCTACAATGCCTAC
ATGCCTACATTCCTAC
ATGCCTACATGCCTAC
ATGCCTACATGCCTAC
ATGCCTACTTCCCTACA
AGCCTACATGCCTAC
ATGCCTACCTCAAAAGGCAATTTAAAGGAAAATTCCCTCATTGGTCAAATGTGAGCGGAGCTAATCGGTGATTGGTCAG
AATTTCATCGATTTGAATTTTTTTTGTGGAATTTTGCAGAAAGCTTAAAAATTTATCGTGAGATCAAATGCCAAATGTT
CTTGACAGGAAAAAAAAAACGTCATAGTTTTCCTGAACTTTTTTCTCGCCGGCAATACTCATAACAAAAACAACAAAAAA
ATGGGAGAATGAGATCAAAACCTCACAGGAACAAGTTGAAAATCAAATACTATATAGGTAAGTTTTCAAGTGTGCAGGC
TTGCCTATGTGCCTACCTTCAGCCTATTCCGTGCTGTTTAATATATCTACCTGCAAGCCTATGTGCCTGCCTACTTGCC
TATGCTTTGTGAGCTTTTTGAAAAAAAAGACCTTTCTTGAAAAACAAAAAAAATTGGATGGAACAATAAAAAAAAATGT
TTCCAAATATTTTTTGCCAAATATTTAAACAAAGTTCTAAATTGC
ATGCCTACATGCCTAC
ACGCCTACATGCTTACATGCCTAC
Nr of selected repeats 46 Similarity 0.819565
Consensus:
ATGCCTAcATGcCTAc
>Cele-UNSB01_4:3232737-3232899 Satlength=163 Nr of Repeats=6 RepeatLength=18 seed=CCTTCGCCTA
CCTTCGCCTACCTACATACCTTCGCAAACCTGCGTGACTTTGTCTCCCTACGTA
CCTTCGCCTACCTGCATG
CCTTCGCCTACCTACGTG
CTTTCGCCTACATGTGTG
CCTTCGCCTACCTACGTA
CCTTCACCTACCTACGTGCTTTTGACTACATGCGTG
Nr of selected repeats 4 Similarity 0.765432
Consensus:
CcTTCGCCTACcTacgTg
>Cele-UNSB01_4:3243719-3271867 Satlength=28149 Nr of Repeats=1487 RepeatLength=19 seed=CCTCACTCAA
CCTCACTCAAAATCCTCAG
CCTCACTCAAAATCCTCAG
CCTCACTTAAACTCTTCAG
CCTCACTCAAATTCTTCAA
CCTCACTCATAATCCTGAG

CCTCACTCAAAATCCTGAG
CCTCACTCAAAATCCTCAG
CCTCACTTAAACTCTTCAG
CCTCACTCAAATTCTTCAA
CCTCACTCAAAATCCTGAG
CCTCACTCATAATCCTGAG
CCTCACTCAAAATCCTGAG
CCTCACTCAAAATCCTCAG
CCTCACTTAAACTCTTCAG
CCTCACTCAAATTCTTCAA
CCTCACTCATAATCCTGAG
CCTCACTCAAAATCCTGAG
CCTCACTCAAAATCCTGAG
CCTCACTCAAAATCCTGAG
CCTCACTCAAAATCCTGAG
CCTCACTCAAAATCCTCAG
CCTCACTTAAACTCTTCAG
CCTCACTCAAATTCTTCAA
CCTCACTCATAATCCTGAG
CCTCACTCAAAATCCTGAG
CCTCACTCAAAATCCTCAG
CCTCACTTAAACTCTTCAG
CCTCACTCAAATTCTTCAA
CCTCACTCATAATCCTGAG
CCTCACTCAAAATCCTGAG
CCTCACTCAAAATCCTCAG
CCTCACTTAAACTCTTCAG
CCTCACTCAAATTCTTCAA
CCTCACTCAAATTCTTCAA
CCTCACTCATAATCCTGAG
CCTCACTCAAAATCCTGAG
CCTCACTTAAACTCTTCAG
CCTCACTCAAATTCTTCAA
CCTCACTCATAATCCTGAG
CCTCACTCAAAATCCTGAG
CCTCACTTAAACTCTTCAG
CCTCACTCAAATTCTTCAA
CCTCACTCATAATCCTGAG
CCTCACTCAAAATCCTGAG
CCTCACTCAAAATCCTCAG
CCTCACTTAAACTCTTCAG
CCTCACTCAAATTCTTCAA
CCTCACTCAAAATCCTGAG
CCTCACTCATAATCCTGAG
CCTCACTCAAAATCCTGAG
CCTCACTTAAACTCTTCAG
CCTCACTCAAATTCTTCAA
CCTCACTCATAATCCTGAG
CCTCACTCAAAATCCTGAG
CCTCACTCAAAATCCTCAG
CCTCACTTAAACTCTTCAG
CCTCACTCAAATTCTTCAA
CCTCACTCATAATCCTGAG
CCTCACTCAAAATCCTGAG

CCTCACTCAAAATCCTCAG
CCTCACTTAAACTCTTCAG
CCTCACTCAAATTCTTCAA
CCTCACTCAAAATCCTGAG
CCTCACTCATAATCCTGAG
CCTCACTCAAAATCCTGAG
CCTCACTTAAACTCTTCAG
CCTCACTCAAATTCTTCAA
CCTCACTCATAATCCTGAG
CCTCACTCAAAATCCTGAG
CCTCACTCAAAATCCTCAG
CCTCACTTAAACTCTTCAG
CCTCACTCAAATTCTTCAA
CCTCACTCATAATCCTGAG
CCTCACTCAAAACCCTGAG
CCTCACTCAAAATCCTGAG
CCTCACTCAAAATCCTGAG
CCTCACTCAAAATCCTGAG
CCTCACTCAAAATCCTGAG
CCTCACTCAAAATCCTGAG
CCTCACTCAAAATCCTGAG
CCTCACTCAAAATCCTGAG
CCTCACTCAAAATCCTGAG
CCTCACTCAAAATCCTGAG
CCTCACTCAAAATCCTGAG
CCTCACTCAAAATCCTGAG
CCTCACTCAAAATCCTGAG
CCTCACTCAAAATCCTGAG
CCTCACTCAAAATCCTGAG
CCTCACTCAAAATCCTGAG
CCTCACTCAAAATCCTGAG
CCTCACTCAAAATCCTGAG
CCTCACTCAAAATCCTCAG
CCTCACTTAAACTCTTCAG
CCTCACTCAAATTCTTCAA
CCTCACTCATAATCCTGAG
CCTCACTCAAAATCCTGAG
CCTCACTCAAAATCCTCAG
CCTCACTTAAACTCTTCAG
CCTCACTCAAATTCTTCAA
CCTCACTCAAAATCCTGAG
CCTCACTCAAAATCCTGAG
CCTCACTCAAAATCCTGAG
CCTCACTCAAAATCCTGAG
CCTCACTCAAAATCCTCAG
CCTCACTTAAACTCTTCAG
CCTCACTCAAATTCTTCAA
CCTCACTCATAATCCTGAG
CCTCACTCAAAATCCTGAG
CCTCACTCAAAATCCTGAG
CCTCACTCAAAATCCTGAG
CCTCACTCAAAATCCTGAG
CCTCACTCAAAATCCTCAG
CCTCACTTAAACTCTTCAG

CCTCACTCAAATTCTTCAA
CCTCACTCATAATCCTGAG
CCTCACTCAAAATCCTGAG
CCTCACTTAAACTCTTCAG
CCTCACTCAAATTCTTCAA
CCTCACTCATAATCCTGAG
CCTCACTCAAAATCCTGAG
CCTCACTTAAACTCTTCAG
CCTCACTCAAATTCTTCAA
CCTCACTCATAATCCTGAG
CCTCACTCAAAATCCTGAG
CCTCACTCAAAATCCTCAG
CCTCACTTAAACTCTTCAG
CCTCACTCAAATTCTTCAA
CCTCACTCAAATTCTTCAA
CCTCACTCATAATCCTGAG
CCTCACTCAAAATCCTGAG
CCTCACTTAAACTCTTCAG
CCTCACTCAAATTCTTCAA
CCTCACTCATAATCCTGAG
CCTCACTCAAAATCCTGAG
CCTCACTCAAAATCCTCAG
CCTCACTCAAAATCCTGAG
CCTCACTTAAACTCTTCAG
CCTCACTCAAATTCTTCAA
CCTCACTCATAATCCTGAG
CCTCACTCAAAATCCTGAG
CCTCACTTAAACTCTTCAG
CCTCACTCAAATTCTTCAA
CCTCACTCATAATCCTGAG
CCTCACTCAAAATCCTGAG
CCTCACTCAAAATCCTCAG
CCTCACTTAAACTCTTCAG
CCTCACTCAAATTCTTCAA
CCTCACTCATAATCCTGAG
CCTCACTCAAAATCCTGAG
CCTCACTCAAAATCCTCAG
CCTCACTTAAACTCTTCAG
CCTCACTCAAATTCTTCAA
CCTCACTCATAATCCTGAG
CCTCACTCAAAATCCTGAG
CCTCACTCAAAATCCTCAG
CCTCACTTAAACTCTTCAG
CCTCACTCAAATTCTTCAA
CCTCACTCAAAATCCTGAG
CCTCACTCAAAATCCTGAG
CCTCACTCAAAATCCTGAG
CCTCGCTCAAAATCCTCAG
CCTCACTTAAACTCTTCAG
CCTCACTCAAATTCTTCAA
CCTCACTCAAAATCCTGAG
CCTCACTCAAAATCCTGAG
CCTCACTCAAAATCCTGAG
CCTCACTCAAAATCCTCAG

CCTCACTTAAACTCTTCAG
CCTCACTCAAATTCTTCAA
CCTCACTCAAAATCCTGAG
CCTCACTCAAATCCTGAG
CCTCACTCAAAATCCTGAG
CCTCACTTAAACTCTTCAG
CCTCACTCAAATTCTTCAA
CCTCACTCATAATCCTGAG
CCTCACTCAAAATCCTGAG
CCTCGCTCAAAATCCTCAG
CCTCACTTAAACTCTTCAG
CCTCACTCAAATTCTTCAA
CCTCACTCATAATCCTGAG
CCTCACTCAAAATCCTGAG
CCTCACTCAAAATCCTGAG
CCTCACTCAAAATCCTGAG
CCTCACTCAAAATCCTGAG
CCTCACTCAAAATCCTGAG
CCTCACTCAAAATCCTGAG
CCTCACTCAAAATCCTGAG
CCTCACTCAAAATCCTGAG
CCTCACTCAAAATCCTGAG
CCTCACTCAAAATCCTGAG
CCTCACTCAAAATCCTGAG
CCTCACTCAAAATCCTGAG
CCTCACTCAAAATCCTGAG
CCTCACTCAAAATCCTGAG
CCTCACTCAAAATCCTGAG
CCTCACTCAAAATCCTGAG
CCTCACTCAAAATCCTCAG
CCTCACTTAAACTCTTCAG
CCTCACTCAAATTCTTCAA
CCTCACTCATAATCCTGAG
CCTCACTCAAAATCCTGAG
CCTCACTCAAAATCCTGAG
CCTCACTCAAAATCCTGAG
CCTCACTCAAAATCCTGAG
CCTCACTCAAAATCCTGAG
CCTCACTCAAAATCCTCAG
CCTCACTTAAACTCTTCAG
CCTCACTCAAATTCTTCAA
CCTCACTCATAATCCTGAG
CCTCACTCAAAATCCTGAG
CCTCACTCAAAATCCTGAG
CCTCACTCAAAATCCTCAG
CCTCACTTAAACTCTTCAG
CCTCACTCAAATTCTTCAA
CCTCACTCATAATCCTGAG
CCTCACTCAAAATCCTGAG
CCTCACTCAAAATCCTGAG
CCTCACTCAAAATCCTGAG
CCTCACTCAAAATCCTGAG
CCTCACTCAAAATCCTGAG
CCTCACTCAAAATCCTGAG

CCTCACTCAAAATCCTGAG
CCTCACTCAAAATCCTGAG
CCTCACTCAAAATCCTCAG
CCTCACTCAAAATCCTGAG
CCTCACTCAAAATCCTCAG
CCTCACTTAAACTCTTCAG
CCTCACTCAAATTCTTCAA
CCTCACTCATAATCCTGAG
CCTCACTCAAAATCCTGAG
CCTCACTCAAAATCCTGAG
CCTCACTCAAAATCCTGAG
CCTCACTCAAAATCCTGAG
CCTCACTCAAAATCCTGAG
CCTCACTCAAAATCCTGAG
CCTCACTCAAAATCCTGAG
CCTCACTCAAAATCCTGAG
CCTCACTCAAAATCCTGAG
CCTCACTCAAAATCCTGAG
CCTCACTCAAAATCCTGAG
CCTCACTCAAAATCCTGAG
CCTCACTCAAAATCCTGAG
CCTCACTCAAAATCCTGAG
CCTCACTCAAAATCCTGAG
CCTCACTCAAAATCCTGAG
CCTCACTCAAAATCCTGAG
CCTCACTCAAAATCCTGAG
CCTCACTCAAAATCCTCAG
CCTCACTTAAACTCTTCAG
CCTCACTCAAATTCTTCAA
CCTCACTCATAATCCTGAG
CCTCACTCATAATCCTGAG
CCTCACTCAAAATCCTGAG
CCTCACTCAAAATCCTGAG
CCTCACTCAAAATCCTGAG
CCTCACTCAAAATCCTCAG
CCTCACTTAAACTCTTCAG
CCTCACTCAAATTCTTCAA
CCTCACTCATAATCCTGAG
CCTCACTCATAATCCTGAG
CCTCACTCAAAATCCTGAG
CCTCACTCAAAATCCTGAG
CCTCACTCAAAATCCTCAG
CCTCACTTAAACTCTTCAG
CCTCACTCAAATTCTTCAA
CCTCACTCATAATCCTGAG
CCTCACTCATAATCCTGAG
CCTCACTCAAAATCCTGAG
CCTCACTCAAAATCCTGAG
CCTCACTCAAAATCCTGAG
CCTCACTCAAAATCCTGAG
CCTCACTCAAAATCCTGAG
CCTCACTCAAAATCCTGAG
CCTCACTCAAAATCCTCAG
CCTCACTCAAAATCCTGAG

CCTCACTCAAAATCCTGAG
CCTCACTCAAAATCCTGAG
CCTCACTCAAAATCCTGAG
CCTCACTCAAAATCCTGAG
CCTCACTCAAAATCCTGAG
CCTCACTCAAAATCCTGAG
CCTCACTCAAAATCCTGAG
CCTCACTCAAAATCCTCAG
CCTCACTCAAATTCTTCAA
CCTCACTCATAATCCTGAG
CCTCACTCAAAATCCTGAG
CCTCACTCAAAATCCTGAG
CCTCACTCAAAATCCTGAG
CCTCACTCAAAATCCTGAG
CCTCACTCAAAATCCTGAG
CCTCACTCAAAATCCTGAG
CCTCACTCAAAATCCTGAG
CCTCACTCAAAATCCTGAG
CCTCACTCAAAATCCTGAG
CCTCACTCAAAATCCTCAG
CCTCACTTAAACTCTTCAG
CCTCACTCAAATTCTTCAA
CCTCACTCATAATCCTGAG
CCTCACTCAAAATCCTGAG
CCTCACTCAAAATCCTGAG
CCTCACTCAAAATCCTGAG
CCTCACTCAAAATCCTGAG
CCTCACTCAAAATCCTCAG
CCTCACTTAAACTCTTCAG
CCTCACTCAAATTCTTCAA
CCTCACTCATAATCCTGAG
CCTCACTCAAAATCCTGAG
CCTCACTCAAAATCCTGAG
CCTCACTCAAAATCCTGAG
CCTCACTCAAAATCCTCAG
CCTCACTTAAACTCTTCAG
CCTCACTCAAATTCTTCAA
CCTCACTCATAATCCTGAG
CCTCACTCAAAATCCTGAG
CCTCACTCAAAATCCTGAG
CCTCACTCAAAATCCTCAG
CCTCACTTAAACTCTTCAG
CCTCACTCAAATTCTTCAA
CCTCACTCATAATCCTGAG
CCTCACTCAAAATCCTGAG
CCTCACTCAAAATCCTGAG
CCTCACTCAAAATCCTGAG
CCTCACTCAAAATCCTCAG
CCTCACTTAAACTCTTCAG
CCTCACTCAAATTCTTCAA
CCTCACTCATAATCCTGAG
CCTCAATCAAAATCCTGAG
CCTCACTCAAAATCCTGAG
CCTCACTCAAAATCCTCAG

CCTCACTCAAAATCCTCAG
CCTCACTTAAACTCTTCAG
CCTCACTCAAATTCTTCAA
CCTCACTCAAAATCCTCAG
CCTCACTCAAAATCCTGAG
CCTCACTCAAAATCCTGAG
CCTCACTCAAAATCCTGAG
CCTCACTCAAAATCCTGAG
CCTCACTCATAATCCTGAG
CCTCACTTAAACTCTTCAG
CCTCACTCAAAATCCTGAG
CCTCACTCAAAATCCTGAG
CCTCACTCAAAATCCTGAG
CCTCACTCAAAATCCTGAG
CCTCACTCAAAATCCTCAG
CCTCACTTAAACTCTTCAG
CCTCACTCAAATTCTTCAA
CCTCACTCATAATCCTGAG
CCTCACTCAAAATCCTGAG
CCTCACTCAAAATCCTGAG
CCTCACTCAAAATCCTCAG
CCTCACTCAAAATCCTGAG
CCTCACTCATAATCCTGAG
CCTCACTTAAACTCTTCAG
CCTCACTCAAAATCCTGAG
CCTCACTCAAAATCCTGAG
CCTCACTCATAATCCTGAG
CCTCACTCAAAATCCTGAG
CCTCACTTAAACTCTTCAG
CCTCACTCAAATTCTTCAA
CCTCACTCAAAATCCTGAG
CCTCACTCATAATCCTGAG
CCTCACTCAAAATCCTGAG
CCTCACTCAAAATCCTGAG
CCTCACTCAAAATCCTGAG
CCTCACTCAAAATCCTGAG
CCTCACTCAAAATCCTGAG
CCTCACTCAAAATCCTGAG
CCTCACTCAAAATCCTGAG
CCTCACTCAAAATCCTGAG
CCTCACTCAAAATCCTCAG
CCTCACTTAAACTCTTCAG
CCTCACTCAAATTCTTCAA
CCTCACTCATAATCCTGAG
CCTCACTCAAAATCCTGAG
CCTCACTCAAAATCCTGAG
CCTCACTCAAAATCCTCAG
CCTCACTCAAAATCCTGAG
CCTCACTCAAAATCCTGAG
CCTCACTCAAAATCCTGAG
CCTCACTCAAAATCCTGAG
CCTCACTCAAAATCCTGAG
CCTCACTCAAAATCCTCAG
CCTCACTTAAACTCTTCAG

CCTCACTCAAATTCTTCAA
CCTCACTCATAATCCTGAG
CCTCACTCAAAATCCTGAG
CCTCACTCAAAATCCTGAG
CCTCACTCAAAATCCTGAG
CCTCACTCAAAATCCTGAG
CCTCACTCAAAATCCTCAG
CCTCACTTAAACTCTTCAG
CCTCACTCAAATTCTTCAA
CCTCACTCAAATCCTGAG
CCTCACTCATAATCCTGAG
CCTCACTCAAAATCCTGAG
CCTCACTCAAAATCCTCAG
CCTCACTTAAACTCTTCAG
CCTCACTCAAATTCTTCAA
CCTCACTCATAATCCTGAG
CCTCACTCAAAATCCTGAG
CCTCACTCAAAATCCTGAG
CCTCACTCAAAATCCTGAG
CCTCACTCAAAATTCTCAG
CCTCACTTAAACTCTTCAG
CCTCACTCAAATTCTTCAA
CCTCACTCAAAATCCTGAG
CCTCACTCAAAATCCTGAG
CCTCACTCAAATTCAG
CCTCACTTAAACTCTTCAG
CCTCACTCAAATTCTTCAA
CCTCACTCAAAATCCTGAG
CCTCACTCAAAATCCTGAG
CCTCACTCAAAATCCTGAG
CCTCACTCAAAATCCTGAG
CCTCACTCAAAATCCTGAG
CCTCACTCAAAATCCTGAG
CCTCACTCAAATCCTGAA
CCTCACTCAAAATCCTGAG
CCTCACTCAAAATCCTGAG
CCTCACTCAAAATCCTGAG
CCTCACTCAAAATCCTCAG
CCTCACTTAAACTCTTCAG
CCTCACTCAAATTCTTCAA
CCTCACTCATAATCCTGAG
CCTCACTCAAAATCCTCAG
CCTCACTTAAACTCTTCAA
CCTCACTCAAAATCCTGAG
CCTCACTCAAAATCCTGAG
CCTCACTCAAAATCCTGAA
CCTCACTCAAAATCCTGAG
CCTCACTCAAAATCCTGAG
CCTCACTCAAAATCCTGAG
CCTCACTCAAAATCCTCAG
CCTCACTTAAACTCTTCAG
CCTCACTCAAATTCTTCAA
CCTCACTCATAATCCTGAG
CCTCACTCAAAATCCTGAG

CCTCACTCAAAATCCTCAG
CCTCACTTAAACTCTTCAG
CCTCACTCAAATTCTTCAA
CCTCACTCATAATCCTGAG
CCTCACTCAAAATCCTGAG
CCTCACTCAAAATCCTGAG
CCTCACTCAAAATCCTGAG
CCTCACTCAAAATCGAG
CCTCACTCAAAATCCTCAG
CCTCACTCAAAATCCTGAG
CTCACTCAAAATCCTGAG
CCTCACTCAAAATCCTCAG
CCTCACTCAAAATCCTGAG
CCTCACTCAAAATCCTCAG
CCTCACTCAAAATCCTGAG
CCTCACTCAAAATCCTGAG
CCTCACTCAAAATCCTGAG
CCTCACTCAAAATCCTGAG
CCTCACTCAAAATCCTCAG
CCTCACTTAAACTCTTCAG
CCTCACTCAAATTCTTCAA
CCTCACTCAAAATCCTGAG
CCTCACTCAAAATCCTGAG
CCTCACTCAAAATTCTCAG
CCTCACTTAAACTCTTCAG
CCTCACTCAAATTCTTCAA
CCTCACTCAAAATCCTGAG
CCTCACTCAAAATCCTGAG
CCTCACTCAAAATCCTGAG
CCTCACTCAAAATCCTGAG
CCTCACTCAAAATCCTGAG
CCTCACTCAATCCTGAG
CCTCACTCAAAATCCTGAG
CCTCACTCAAAATTCTCAG
CCTCACTTAAACTCTTCAG
CCTCACTCAAATTCTTCAA
CCTCACTCAAAATCCTGAG
CCTCACTCAAAATCCTGAG
CCTCACTCAAAATCCTGAA
CCTCACTCAAAATCCTGAG
CCTCACTCAAAATCCTGAG
CCTCACTCAAAATCCTGAG
CCTCACTCAAAATCCTCAG
CCTCACTTAAACTCTTCAG
CCTCACTCAAATTCTTCAA
CCTCACTCATAATCCTCAG
CCTCACTCAAAATCCTGAG
CCTCACTCAAAATCCTGAG
CCTCACTTAAACTCTTCAG
CCTCACTCAAATTCTTCAA
CCTCACTCATAATCCTGAG
CCTCACTCAAAATCCTGAG
CCTCACTCAAAATCCTGAG
CCTCACTCAAAATCCTGAG

CCTCACTCAAAATCCTGAG
CCTCACTCAAAATCCTCAG
CCTCACTAAACTCTTCAG
CCTCACTCAAATTCTTCAA
CCTCACTCATAATCCTGAG
CCTCACTCAAAATCCTGAG
CCTCACTCAAAATCCTGAG
CCTCACTCAAAATCCTGAG
CCTCACTCAAAATCCTGAG
CCTCACTCAAAATCCTGAG
CCTCACTCAAAATCCTCAG
CCTCACTTAAACTCTTCAG
CCTCACTCAAATTCTTCAA
CCTCACTCATAATCCTGAG
CCTCACTCAAAATCCTGAG
CCTCACTCAAAATCCTGAG
CCTCACTCAAAATCCTGAG
CCTCACTCAAAATCCTGAG
CCTCACTCAAAATCCTGAG
CCTCACTCAAAATCCTGAG
CCTCACTCAAAATCCTGAG
CCTCACTCAAAATCCTGAG
CCTCACTCAAAATCCTGAG
CCTCACTCAAAATCCTGAG
CCTCACTCAAAATCCTCAG
CCTCACTTAAACTCTTCAG
CCTCACTCAAATTCTTCAA
CCTCACTCATAATCCTGAG
CCTCACTCAAAATCCTCAG
CCTCACTCAAAATCCTGAG
CCTCACTCAAAATCCTGAG
CCTCACTCAAAATCCTGAG
CCTCACTCAAAATCCTGAG
CCTCACTCAAAATCCTGAG
CCTCACTCAAAATCCTGAG
CCTCACTCAAAATCCTCAG
CCTCACTCAAAATCCTCAG
CCTCACTCAAAATCCTGAG
CCTCACTCAAAATCCTCAG
CCTCACTCAAAATCCTGAG
CCTCACTCAAAATCCTCAG
CCTCACTCAAAATCCTGAG
CCTCACTCAAAATCCTGAG
CCTCACTCAAAATCCTCAG
CCTCACTTAAACTCTTCAG
CCTCACTCAAATTCTTCAA
CCTCACTCATAATCCTCAG
CCTCACTCAAAATCCTCAG
CCTCACTCAAAATCCTCAG
CCTCACTTAAACTCTTCAG
CCTCACTCAAATTCTTCAA
CCTCACTCATAATCCTGAG
CCTCACTCAAATCCTGAG
CCTCACTCAAAATCCTCAG

CCTCACTCAAACTCTTCAG
CCTCACTCAAATTCTTCAA
CCTCACTCATAATCCTCAG
CCTCACTCAAAATCCTGAG
CCTCACTCAAAATCCTCAG
CCTCACTCAAATCCTTCAG
CCTCACTCAAATTCTTCAA
CCTCACTCATAATCCTCAG
CCTCACTCAAAATCCTGAG
CCTCACTCAAAATCCTCAG
CCTCACTTAAACTCTTCAG
CCTCACTCAAATTCTTCAA
CCTCACTCATAATCCTGAG
CCTCACTCAAAATCCTGAG
CCTCACTCAAAATCCTGAG
CCTCACTCAAAATCCTGAG
CCTCACTCAAAATCCTCAG
CCTCACTCAAAATCCTGAG
CCTCACTCAAAATCCTGAG
CCTCACTCAAAATCCTCAG
CCTCACTCAAAATCCTGAG
CCTCACTCAAAATCCTCAG
CCTCACTCAAAATCCTGAG
CCTCACTCAAAATCCTGAG
CCTCACTCAAAATCCTGAG
CCTCACTCAAAATCCTCAG
CCTCACTTAAATCTTCAG
CCTCACTCAAATTCTTCAA
CCTCACTCATAATCCTGAG
CCTCACTCAAAATCCTGAG
CCTCACTCAAAATCCTGAG
CCTCACTCAAAATCCTGAG
CCTCACTCAAAATCCTCAG
CCTCACTTAAACTCTTCAG
CCTCACTCAAATTCTTCAA
CCTCACTCATCTCTCAG
CCTCACTCAAATCCTGAG
CCTCACTCAAAATCCTGAG
CCTCACTCAAAATCCTGAG
CCTCACTCAAAATCCTCAG
CCTCACTCAAAATCCTCAG
CCTCACTCAAAACCTCAG
CCTCACTCAAACTCTTCAG
CCTCACTCAAATCTTCAA
CCTCACTCAAATCCTGCAG
CCTCACTCAAAATCCTGAG
CCTCACTCAAAATCCTCAG
CCTCACTTAAACTCTTCAG
CCTCACTCAAATTCTTCAA
CCTCACTCATAATCCTGAG
CCTCACTCAAAATCCTGAG
CCTCACTCAAAATCCTCAG
CCTCACTCAAACTCTCAG
CCTCACTCAAATTCTTCAG

CCTCACTCAAATCCTGAG
CCTCACTCAAAATCCTGAG
CCTCACTCAAAATCCTGAG
CCTCACTCAAATCTGAG
CCTCACTCAAAATTCAG
CCTCACTCAAATCCTGAG
CCTCACTCAAAATCCTGAG
CCTCACTCAAAATCCTCAG
CCTCACTCAAAATCCTGAG
CCTCACTCAAAATCCTCAG
CCTCACTCAAAATCCTGAG
CCTCACTCAAAATCCTGAG
CCTCACTCAAAATCCTGAG
CCTCACTCAAAATCCTCAG
CCTCACTTAAACTCTTCAG
CCTCACTCAAATTCTTAA
CCTCACTCAAATCCTGAG
CCTCACTCAAAATCCTGAG
CCTCACTCAAAATCCTGAG
CCTCACTCAAAATCCTGAG
CCTCACTCAAAATCCTCAG
CCTCACTTAAACTCTTCAG
CCTCACTCAAATTCTCACTCATAATCCTGAG
CCTCACTCAAAATCCTGAG
CCTCACTCAAATCCTGAG
CCTCACTCAAAATCCTCAG
CCTCACTAAACTCTCAG
CCTCACTCAAATTCTTCAA
CCTCACTCAATCCTGAG
CCTCACTCAAATCTGAG
CCTCACTCAAATCCTGAG
CCTCACTCAAAATCCTGAG
CCTCACTCAAAATCCTGAG
CCTCACTCAAAATCCTCAG
CCTCACTTAAACTCTTCAG
CCTCACTCAAATCTTCAA
CCTCACTCAAATCCTGAG
CCTCACTCAATCCTGAG
CCTCACTCAAAATCCTGAG
CCTCACTCAAAATCCTCAG
CCTCACTTAAACTCTTCAG
CCTCACTCAAATTCTTCAA
CCTCACTCATAATCCTGAG
CCTCACTCAAAATCCTGAG
CCTCACTCAAAATCCTGAG
CCTCACTCAAAATCCTGAG
CCTCACTCAAAATCCTCAG
CCTCACTCAAAATCCTGAG
CCTCACTCAAAATCCTGAG
CCTCACTCAAAATCCTGAG
CCTCACTCAATCCTGAG
CCTCACTCAAAATCCTGAG
CCTCACTCAAAATCCTCAG
CCTCACTCAAAATCCTCAG

CCTCACTCAAAATCCTCAAG
CCTCACTCAAAATCCTGAG
CCTCACTCAAAATCCTGAG
CCTCACTCAAAATCCTGAG
CCTCACTCAAAATCCTGAG
CCTCATTCAAAATCCTGAG
CCTCACTCAAAATCCTCAG
CCTCACTCAAAATCCTCG
CCTCACTCAAAATCCAG
CCTCACTCAAATTCTCCA
CCTCACTCAATCCTGAG
CCTCACTCAAAATCCTCAG
CCTCACTCAAAATCCTCAG
CCTCACTCAAATCCTTCAG
CCTCACTCAAAATCCTCAG
CCTCACTCAAATCCTCAG
CCTCACTCAAATTCTTCAA
CCTCACTCAAATCCTGAG
CCTCACTCAAAATCCTGAG
CCTCACTCAAAATCCTCAG
CCTCACTCAAATCCTCAG
CCTCACTCAAAATCCTGAG
CCTCACTCAAAATCCTGAG
CCTCACTCAAAATCCTGAG
CCTCACTCAAAAATCCTCAG
CCTCACTTAAACTCTTCAG
CCTCACTCAAATCTCA
CCTCACTCATAATCCTGAG
CCTCACTCAAAATCCTGA
CCTCACTCAAAATCCTGAG
CCTCACTCAAATCCTGAG
CCTCACTCAAAATCCTCAG
CCTCACTCAAAATCCTGAG
CTCACTCAAAATCCTGAG
CCTCACTCAAAATCCTCAG
CCTCACTCAAAATCCTGAG
CCTCACTCAAAATCCTCAG
CCTCACTCAAAATCCTGAG
CCTCACTCAAATCCTGAG
CCTCACTCAAAATCCTGAG
CCTCACTCAAAATCCTCAG
CCTCACTTAAACTCTTCAG
CTCACTCAAATCTTCAA
CCTCACTCATAATCTGAG
CCTCACTCATAATCCTGAG
CCTCACTCAAAATCCTGAG
CCTCACTCAAAATCCTCAG
CCTCACTCAAAATCCTCAG
CCTCACTCAAAACTCTTCAG
CCTCACTCAAATTCTTCAA
CCTCACTCATAATCCTCAG
CCTCACTCATAATCCTGAG
CCTCACTCATAATCCTGAG
CCTCACTCAAAATCCTGAG

CCTCACTCAAATCCTCAG
CCTCACTCAAAATCCTCAG
CCTCACTCAAAATCCTCAG
CCTCACTTAAACTCTTCAG
CCTCACTCAAATTCTTCAA
CCTCACTCATAATCCTGAG
CCTCACTCAAAATCCTGAG
CCTCACTCAAAATCCTCAG
CCTCACTTAAACTCTTCAG
CCTCACTCAAATTCTTCAACCTACTCATAATCCTGAG
CCTCACTCAAAATCCTGAG
CCTCACTCAAAATCCTCAG
CCTCACTTAAACTCTTCAG
CCTCACTCAAATTCTTCAA
CCTCACTCAATAATCCTGAG
CCTCACTCAAAAATCCTGAG
CCTCACTCAAAATCCTGAG
CCTCACTCAAATCCTGAG
CCTCACTCAAAATCCTCAG
CCTCACTCAAAATCCTGAG
CCTCACTCAAAATCCTGAG
CCTCACTCAAAATCCTCAG
CCTCACTCAAAATCCTCAG
CCTCACTCAAATTCTTCAA
CCTCACTCAAAATCCTGAG
CCTCACTCAAAATCCTGAG
CCTCACTCAAAATCCTGAG
CCTCACTCAAAATCCTCAG
CCTCACTTAAACTCTTCAG
CCTCACTCAAATTCTTCAA
CCTCACTCAAATCCTGAG
CCTCACTCAAATCCTGAG
CCTCACTCAAAATCCTGAG
CCTCACTCAAAATCCTGAG
CCTCACTCAAAATCCTCAG
CCTCACTAAATCTTCAG
CCTCACTCAAATCTTCA
CCTCACTCATAATCCTGAG
CCTCACTCAAATCCTGAG
CCTCACTCAAAATCCTGAG
CCTCACTCAAAATCCTGAG
CCTCACTCAAAATCCTGAG
CCTCACTCAAAATCCTCAG
CCTCACTTAAACTCTTCAG
CCTCACTCAAATTCTTCAA
CCTCACTCATAATCCTGAG
CCTCACTCAAATCCTGAG
CCTCACTCAAAATCCTGAG
CCTCACTCAAAATCCTGAG
CCTCACTAAATCTCAG
CCTCACTCAAATCTTCAA
CCTCACTCAAAATCCTGAG
CTCACTCAAAATCCTGAG
CCTCACTCAAAATCCTGAG

CCTCACTCAAAATCCTCAG
CCTCACTCAAAATCCTCG
CCTCACTCAAAATCCTCAG
CCTCACTCAAAATCCTGAG
CCTCACTCAAAATCCTGAG
CCTCACTCAAATCCTCAG
CCTCACTCAAATCCTCAG
CCTCACTCAAAATCCTCAG
CCTCACTCAAAATCCTCAG
CCTCACTCAAATCCTCAG
CCTCACTCAAAATCCTCAG
CCTCACTCAAAATCCTGAG
CCTCACTCAAAATCCTCAG
CCTCACTCAAAATCCTCAG
CCTCACTTCAAAATCCTCAG
CCTCACTCAAAATCCTCAG
CCTCACTCAAAATCCTGAG
CCTCACTCAAAATCCTCAG
CCTCACTCAAATCCAG
CCTCACTCAAATCCTCAG
CCTCACTCAAAATCCTCAG
CCTCACTCAAATCCTCAG
CCTCACTCAAAATCCTGAG
CCTCACTCAAAATCCTCAG
CCTCACTCAAATCCTCAG
CCTCACTCAAAATCCTCAA
CCTCACTCAAATCCTGAG
CCTCACTCAAAATCCTGAG
CCTCACTCAAAATCCTGAG
CCTCACTCAAAATCCTCAG
CCTCACTCAAAATCCTCAG
CCTCACTCAAAATCCTCAAG
CCTCACTCAAAATCCTCAG
CCTCACTCAAAATCCTCAG
CCTCACTAAACTCTCAG
CCTCACTCAAATTCTTCAA
CCTCACTCAATCCTGAG
CCTCACTCAAAATCCTGAG
CCTCACTCAAAATCCTGAG
CCTCACTCAAAATCCTGAG
CCTCACTCAAAATCCTGAG
CCTCACTCAAAATCCTGAG
CCTCACTCAAAATCCTGAG
CCTCACTCAAATCCTGAG
CCTCACTCAAAATCCTGAG
CCTCACTCAAAATCCTGAG
CCTCACTCAAATCCTGAG
CCTCACTCAAAATCCTGAG
CCTCACTCAAAATCCTCAG
CCTCACTCAAAATCCTGAG
CCTCACTCAAAATCCTGAG
CCTCACTCAAAATCCTGAG
CCTCACTCAAAATCCTCAG
CCTCACTCAAAATCCTGAG

CCTCACTCAAAATCCTGAG
CCTCACTCAAAATCCTCAG
CCTCACTTAAACTCTTCAG
CCTCACTCAAATTCTCAA
CCTCACTCAAAATCCTGAG
CCTCACTCAAAATCCTGAG
CCTCACTCAAAATCCTGAG
CCTCACTCAAAATCCTCAG
CCTCACTCAAAATCTCAAG
CCTCACTCAAATCCTGAG
CCTCACTCAAAATCCTGAG
CCTCACTCAAAATCCTGAG
CCTCACTCAAAATCCTGAG
CCTCACTCAAAATCCTGAG
CCTCACTCAAATCTGAG
CCTCACTCAAAATCCTGAG
CCTCACTCAAAATCCTGAG
CCTCACTCAAAATCCTGAG
CCTCACTCAAAATCCTGAG
CCTCACTCAAAATCCTGAG
CCTCACTCAAAATCCTGAG
CCTCACTCAAAATCCTCAG
CCTCACTCAAAATCCTGAG
CCTCACTCAAAATCCTGAG
CCTCACTCAAAATCCTGAG
CCTCACTCAAAATCCTGAG
CCTCACTCAAAATCCTCAG
CCTCACTTAAACTCTCAG
CCTCACTCAAATTCTTCAA
CCTCACTCAAAATCCTGAG
CCTCACTCAAAATCCTGAG
CCTCACTCAAAATCCTGAG
CCTCACTAAATCCTGAG
CCTCACTCAAAATCCTCAG
CCTCACTCAAAATCCTGAG
CCTCACTCAAAATCCTGAG
CCTCACTCAAAATCCTCAG
CCTCACTCAAAATCCTCAG
CCTCACTCAAAATCCTGAG
CCTCACTCAAAATCCTGAG
CCTCACTCAAAATCCTGAG
CCTCACTCAAATCCTGAG
CCTCACTCAAAATCCTGAG
CCTCACTCAAAATCCTGAG
CCTCACTCAAAATCCTGAG
CCTCACTCAAAATCCTGAG
CCTCACTCAAAATCCTCAG
CCTCACTTAAACTCTTCAG
CCTCACTCAAATCTTCAA
CCTCACTCATAATCCTGAG
CCTCACTCAAAATCCTGAG
CCTCACTCAAAATCCTGAG
CCTCACTCAAAATCCTGAG
CCTCACTCAAAATCCTGAG

CCTCACTCAAATCCTCAG
CCTCACTCAAAATCCTGAG
CCTCACTCAAAATCCTGAG
CCTCACTCAAAATCCTCAG
CCTCACTCAAAATCCTGAG
CCTCACTCAAAATCCTGAG
CCTCACTCAAAATCCTGAG
CCTCACTCAAAATCCTGAG
CCTCACTCAAAATCCTCAG
CCTCACTCAAAATCCTGAG
CCTCACTCAAAATCCTGAG
CCTCACTCAAAATCCTGAG
CCTCACTCAAAATCCTGAG
CCTCACTCAAAATCCTCAG
CCTCACTCAAACTCTTCAG
CCTCACTCAAAATCTTCAG
CCTCACTCATAATCCTGAG
CCTCACTCAAAATCCTGAG
CCTCACTCAAAATCCTCAG
CCTCACTTAAACTCTTAG
CCTCACTCAAATCTC
CCTCACTCATAAATCCTGAG
CCTCACTCAAATCCTGAG
CCTCACTCAAAATCCTGAG
CCTCACTCAAAATCCTGAG
CCTCACTCAAAATCCTGAG
CCTCACTCAAAATCCTCAG
CCTCACTTAAACTCTTCAG
CCTCACTCAAATTCTTCAA
CCTCACTCATAATCCTGAG
CCTCACTCAAATCCTGAG
CCTCACTCAAAATCCTGAG
CCTCACTCAAAATCCTCAG
CCTCACTTAAACTCTTCAG
CCTCACTCAAATTCTTCAA
CCTCACTCATAATCCTGAG
CCTCACTCAAAATCCTGAG
CCTCACTCAAAATCCTGAG
CCTCACTCAAAATCCTCAG
CCTCACTTAAACTCTTCAG
CCTCACTCAAATTCTTCAA
CCTCACTCATAATCCTGAG
CCTCACTCAAATCCTGAG
CCTCACTCAAAATCCTGAG
CCTCACTCAAAATCCTCAG
CCTCACTCAAATCCTGAAG
CCTCACTCAAATCCTGAG
CCTCACTCAAAATCCTGAG
CCTCACTCAAAATCCTCAG
CCTCACTTAAACTCTTCAG
CCTCACTCAAATTCTTCAA
CCTCACTCATAATCCTGAG
CCTCACTCAAATCCTGAG
CCTCACTCAAAATCCTGAG

CCTCACTCAAAATCCTCAG
CCTCACTTAAACTCTTCAG
CCTCACTCAAATTCTTCAA
CCTCACTCAAAATCCTGAG
CCTCACTCAAAATCCTGAG
CCTCACTCAAAATCCTGAG
CCTCACTCAAAATCCTCAG
CCTCACTCAAAATCCTCAG
CCTCACTTAAACTCTTCAG
CCTCACTCAAATTCTTCAA
CCTCACTCATAATCCTGAG
CCTCACTCAAAATCCTGAG
CCTCACTCAAAATCCTCAG
CCTCACTTAAACTCTTCAG
CCTCACTCAAATTCTTCAA
CCTCACTCATAATCCTGAG
CCTCACTCAAAATCCTGAG
CCTCACTCAAAATCCTCAG
CCTCACTTAAACTCTTCAG
CCTCACTCAAATTCTTCAA
CCTCACTCATAATCCTGAG
CCTCACTCAATCCTGAG
CCTCACTCAAAATCCTGAG
CCTCACTCAAAATCCTGAG
CCTCACTCAAAATCCTGAG
CCTCACTCAAAATCCTCAG
CCTCACTTAAACTCTTCAG
CCTCACTCAAATTCTTCAA
CCTCACTCAAATCCTGAG
CCTCACTCAAATCCTGAG
CCTCACTCAAAATCCTGAG
CCTCACTCAAAATCCTCAG
CCTCACTTAAACTCTTCAG
CCTCACTCAAATTCTTCAA
CCTCACTCATAATCCTGAG
CCTCACTCATAATCCTGAG
CCTCACTCAAAATCCTGAG
CCTCACTCAAAATCCTCAG
CCTCACTCAAAATCCTGAG
CCTCACTCAAATCCTGAG
CCTCACTCAAAATCCTGAG
CCTCACTCAAAATCCTCAG
CCTCACTTAAACTCTTCAG
CCTCACTCAAATTCTTCAA
CCTCACTCATAATCCTGAG
CCTCACTCATAATCCTGAG
CCTCACTCAAAATCCTGAG
CCTCACTCAAAATCCTCAG
CCTCACTTAAACTCTTCAG
CCTCACTCAAATTCTTCAA
CCTCACTCATAATCCTGAG
CCTCACTCATAATCCTGAG
CCTCACTCAAAATCCTGAG
CCTCACTCAAAATCCTCAG

CCTCACTCAAAATCCTCAG
CCTCACTCAAAATCCTCAG
CCTCACTCAAAATCCTCAG
CCTCACTCAAAATCCTCAG
CCTCACTCAAAATCCTGAG
CCTCACTCAAAATCCTCAG
CCTCACTCAAAATCCTCAG
CCTCACTCAAATCCTCAG
CCTCACTCAAAATCCTCAG
CCTCACTCAAAATCCTGAG
CCTCACTCAAAATCCTGAG
CCTCACTCAAAATCCTCAG
CCTCACTCAAAATCCTCAG
CCTCACTCAAAATCCTCAG
CCTCACTCAAAATCCTCAG
CCTCACTCAAAATCCTCAG
CCTCACTCAAATCCTCAG
CCTCACTCAAATCCTCAG
CCTCACTCAAAATCCTCAG
CCTCACTCAAAATCCTGAG
CCTCACTCAAAATCCTCAG
CCTCACTTAAACTCTTCAG
CCTCACTCAAATTCTTCAA
CCTCACTCAAAATCCTGAG
CCTCACTCAAAATCCTGAG
CCTCACTCAAAATCCTCAG
CCTCACTCAAAATCCTCAG
CCTCACTCAAAATCCTGAG
CCTCACTCAAAATCCTCAG
CCTCACTCAAAATCCTCAG
CCTCACTCAAAATCCTCAG
CCTCACTCAAAATCCTCAG
CCTCACTCAAAATCCTCAG
CCTCACTCAAAATCCTCAG
CCTCACTCAAAATCCTCAG
CCTCACTCAAAATCCTCAG
CCTCACTCAAATCCTGAG
CCTCACTCAAAATCCTGAG
CCTCACTCAAAATCCTCAG
CCTCACTCAAAATCCTCAG
CCTCACTCAAAATCCTCAG
CCTCACTCAAAATCCTGAG
CCTCACTCAAAATCCTCAG
CCTCACTCAAATCCTCAG
CCTCACTCAAATCTCAG
CCTCACTCAAATCCTCAG
CCTCACTCAAAATCCTCAG
CCTCACTCAAAATCCTCAG
CCTCACTCAAAATCCTCAG
CCTCACTCAAAATCCTCAG
CCTCACTCAAAATCCTCAG
CCTCACTCAAAATCCTCAGG
CCTCACTCAAATCCTCAG
CCTCACTCAAATTCTTCAA

CCTCACTCAAAATCCTGAG
CCTCACTCAAAATCCTGAG
CCTCACTCAAAATCCTCAG
CCTCACTCAAAATCCTCAG
CCTCACTCAAAATCCTGAG
CCTCACTCAAAATCCTCAG
CCTCACTTAAACTCTTCAG
CCTCACTCAAATCTTCAA
CCTCACTCATAATCCTGAG
CCTCACTCAAAATCCTGAG
CCTCACTCAAAATCCTGAG
CCTCACTCAAAATCCTGAG
CCTCACTCAAAATCCTGAG
CCTCACTCAAAATCCTGAG
CCTCACTCAAAATCCTGAG
CCTCACTCAAAATCCTGAG
CCTCACTCAAAATCCTCAG
CCTCACTCAAAATCCTCAG
CCTCACTCAAAATCCTCAG
CCTCACTTAAACTCTTCAG
CCTCACTCAAATTCTTCAA
CCTCACTCATAATCCTGAG
CCTCACTCAAAATCCTGAG
CCTCACTCAAAATCCTGAG
CCTCACTCAAAATCCTCAG
CCTCACTCAAACTCTTCAG
CCTCACTCAAATTCTCAA
CCTCACTCAAAATCCTGAG
CCTCACTCAAATCCTGAG
CCTCACTCAAAATCCTGAG
CCTCACTCAAAATCCTCAG
CCTCACTCAAATCCTCAG
CCTCACTCAAAATCCTCAG
CCTCACTCAAAATCCTCAG
CCTCACTCAAAATCCTGAG
CCTCACTCAAAATCCTGAG
CCTCACTCAAAATCCTGAG
CCTCACTCAAAATCCTGAG
CCTCACTCAAAATCCTCAG
CCTCACTTAAACTCTTCAG
CCTCACTCAAATTCTTCAA
CCTCACTCATAATCCTGAG
CCTCACTCAAAATCCTGAG
CCTCACTCAAAATCCTCAG
CCTCACTCAAAATCCTGAG
CCTCACTCAAAATCCTGAG
CCTCACTCAAAATCCTCAG
CCTCACTAAACTCTTCAG
CCTCACTCAAATCTCAA
CCTCACTCATAATCCTGAG
CCTCACTCAAATCCTGAG
CCTCACTCAAAATCCTGAG
CCTCACTCAAAATCCTGAG
CCTCACTCAAAATCCTGAG

CCTCACTCAAAATCCTCAG
CCTCACTTAAACTCTTCAG
CCTCACTCAAATTCTTCAA
CCTCACTCATAATCCTGAG
CCTCACTCAAAATCCTGAG
CCTCACTCAAAATCCTGAG
CCTCACTCAAAATCCTCAG
CCTCACTTAAACTTTCAG
CCTCACTCAAATTCTTCAA
CCTCACTCATAATCCTGAG
CCTCACTCAAAATCCTGAG
CCTCACTCAAAATCCTGAG
CCTCACTCAAAATCCTCAG
CCTCACTCAAAATCCTCAG
CCTCACTCAAAATCCTCAG
CCTCACTCAAAATCCTCAG
CCTCACTCAAATCCTCAG
CCTCACTCAAAATCCTGAG
CCTCACTCAAAATCCTCAG
CCTCACTCAAAATCCTCAG
CCTCACTCAAAATCCTGAG
CCTCACTCAAAATCCTGAG
CCTCACTCAAAATCCTGAG
CCTCACTCAAAATCCTGAG
CCTCACTCAAAATCCTCAG
CCTCACTTAAACTCTTCAG
CCTCACTCAAATTCTTCAA
CCTCACTCATAATCCTGAG
CCTCACTCAAAATCCTCAG
CCTCACTCAAAATCCTGAG
CCTCACTCAAAATCCTGAG
CCTCACTCAAAATCCTCAG
CCTCACTTAAACTCTTCAG
CCTCACTCAAATTCTTCAA
CCTCACTCATAATCCTGAG
CCTCACTCAAAATCCTGAG
CCTCACTCAAAATCCTGAG
CCTCACTCAAAATCCTGAG
CCTCACTCAAAATCCTGAG
CCTCACTCAAAATCCTCAG
CCTCACTTAAACTCTTCAG
CCTCACTCAAATTCTTCAA
CCTCACTCATAATCCTGAG
CCTCACTCAAAATCCTGAG
CCTCACTCAAAATCCTCAG
CCTCACTTAAACTCTTCAG
CCTCACTCAAATTCTTCAA
CCTCACTCATAATCCTGAG
CCTCACTCAAAATCCTGAG
CCTCACTCAAAATCCTCAG
CCTCACTTAAATCTTCAG
CCTCACTCAAATTCTTCAA
CCTCACTCATAATCCTGAG
CCTCACTCAAAATCCTGAG

CCTCACTCAAAATCCTCAG
CCTCACTCAAAATCCTGAG
CCTCACTCAAAATCCTGAG
CCTCACTCAAAATCCTGAG
CCTCACTCAAAATCCTGAG
CCTCACTCAAAATCCTCAG
CCTCACTTAAACTCTTCAG
CCTCACTCAAATTCTCAA
CCTCACTCAAATCCTGAG
CCTCACTCAAAATCCTGAG
CCTCACTCAAAATCCTGAG
CCTCACTCAAAATCCTCAG
CCTCACTTAAACTCTTCAG
CCTCACTCAAATTCTTCAA
CCTCACTCATAATCCTGAG
CCTCACTCAAAATCCTGAG
CCTCACTCAAAATCCTCAG
CCTCACTCAAAATCCTGAG
CCTCACTCAAAATCCTCAG
CCTCACTCAAAATCCTCAG
CCTCACTCAAAATCCTGAG
CCTCACTCAAATCCTCAG
CCTCACTCAAAATCCTCAG
CCTCACTCAAAATCCTCAG
CCTCACTCAAAATCCTCAG
CCTCACTCAAAATCCTCAG
CCTCACTCAAAATCCTCAG
CCTCACTCAAAATCCTCGG
CCTCACTCAAAATCCTCGG
CCTCACTCAAATTCTTCAA
CCTCACTCAAAATCCTGAG
CCTCACTCAAAATCCTGAG
CCTCACTCAAAATCCTCAG
CCTCACTCAAAATCCTCAG
CCTCACTCAAAATCCTCAG
CCTCACTCAAAATCCTCAG
CCTCACTCAAATCCTGAG
CCTCACTCAAAATCCTGAG
CCTCACTCAAAATCCTGAG
CCTCACTCAAAATCCTCAG
CCTCACTCAAAATCCTGAG
CCTCACTCAAAATCCTGAG
CCTCACTCAAAATCCTCAG
CCTCACTTAAACTCTTCAG
CCTCACTCAAATTCTTCAA
CCTCACTCATAATCCTGAG
CCTCACTCAAAATCCTGAG
CCTCACTCAAAATCCTCAG
CCTCACTCAAAATCCTGAG
CCTCACTCAAAATCCTGAG
CCTCACTCAAAATCCTCAG
CCTCACTTAAACTCTTCAG
CCTCACTCAAATTCTTCAA
CCTCACTCATAATCCTGAG

CCTCACTCAAAATCCTGAG
CCTCACTCAAAATCCTGAG
CCTCACTCAAAATCCTGAG
CCTCACTCAAAATCCTGAG
CCTCACTCAAATCCTGAG
CCTCACTCAAAATCCTCAG
CCTCACTTAAACTCTTCAG
CCTCACTCAAATTCTTCAA
CCTCACTCATAATCCTGAG
CCTCACTCAAAATCCTGAG
CCTCACTCAAAATCCTGAG
CCTCACTCAAAATCCTGAG
CCTCACTCAAAATCCTGAG
CCTCACTCAAAATCCTGAG
CCTCACTCAAAATCCTCAG
CCTCACTTAAACTCTTCAG
CCTCACTCAAATTCTTCAA
CCTCACTCATAATCCTGAG
CCTCACTCAAAATCCTGAG
CCTCACTCAAAATCCTGAG
CCTCACTCAAAATCCTCAG
CCTCACTTAAACTCTTCAG
CCTCACTCAAATTCTTCAA
CCTCACTTAAACTCTTCAG
CCTCACTCAAATTCTTCAA
CCTCACTCATAATCCTGAG
CCTCACTCAAAATCCTGAG
CCTCACTCAAAATCCTCAG
CCTCACTCAAAATCCTGAG
CCTCACTCAAAATCCTCAG
CCTCACTTAAACTCTTCAG
CCTCACTCAAATTCTTCAA
CCTCACTTAAACTCTTCAG
CCTCACTCAAATTCTTCAA
CCTCACTTAAACTCTTCAG
CCTCACTCAAATTCTTCAA
CCTCACTTAAACTCTTCAG
CCTCACTCAAATTCTTCAA
CCTCACTCATAATCCTGAG
CCTCACTCAAAATCCTGAG
CCTCACTCAAATCCTCAG
CCTCACTCAAAATCCTGAG
CCTCACTCAAAATCCTGAG
CCTCACTCAAAATCCTGAG
CCTCACTCAAAATCCTGAG
CCTCACTCAAAATCCTCAG
CCTCACTTAAACTCTTCAG
CCTCACTCAAATTCTTCAA
CCTCACTTAAACTCTTCAG
CCTCACTCAAATTCTTCAA
CCTCACTCATAATCCTGAG
CCTCACTCAAAATCCTCAG
CCTCACTTAAACTCTTCAG
CCTCACTCAAATTCTTCAA

CCTCACTCATAATCCTGAG
CCTCACTCAAAATCCTGAG
CCTCACTCAAAATCCTCAG
CCTCACTCAAAATCCTGAG
CCTCACTCAAAATCCTGAG
CCTCACTCAAAATCCTGAG
CCTCACTCAAAATCCTGAG
CCTCACTCAAAATCCTCAG
CCTCACTTAAACTCTTCAG
CCTCACTCAAATTCTTCAA
CCTCACTTAAACTCTTCAG
CCTCACTCAAATTCTTCAA
CCTCACTCATAATCCTGAG
CCTCACTCAAAATCCTGAG
CCTCACTCAAAATCCTGAG
CCTCACTCAAAATCCTCAG
CCTCACTTAAACTCTTCAG
CCTCACTCAAATTCTTCAA
CCTCACTCATAATCCTGAG
CCTCACTCAAAATCCTGAG
CCTCACTCAAAATCCTCAG
CCTCACTCAAAATCCTGAG
CCTCACTCAAAATCCTGAG
CCTCACTCAAAATCCTCAG
CCTCACTTAAACTCTTCAG
CCTCACTCAAATTCTTCAA
CCTCACTCATAATCCTGAG
CCTCACTCAAAATCCTGAG
CCTCACTCAAAATCCTGAG
CCTCACTCAAAATCCTGAG
CCTCACTCAAAATCCTGAG
CCTCACTCAAAATCCTCAG
CCTCACTTAAACTCTTCAG
CCTCGCTCAAATTCTTCAA
CCTCACTCATAATCCTGAG
CCTCACTCAAAATCCTGAG
CCTCACTCAAAATCCTGAG
CCTCACTCAAAATCCTCAG
CCTCACTTAAACTCTTCAG
CCTCACTCAAATTCTTCAA
CCTCACTCATAATCCTGAG
CCTCACTCAAAATCCTGAG
CCTCACTCAAAATCCTCAG
CCTCACTCAAAATCCTGAG
CCTCACTCAAAATCCTCAG
CCTCACTTAAACTCTTCAG
CCTCACTCAAATTCTTCAA
CCTCACTTAAACTCTTCAG
CCTCACTCAAATTCTTCAA
CCTCACTCATAATCCTGAG
CCTCACTCAAAATCCTGAG
CCTCACTCAAAATCCTGAG
CCTCACTCAAAATCCTCAG
CCTCACTTAAACTCTTCAG

CCTCGCTCAAATTCTTCAA
CCTCACTCATAATCCTGAG
CCTCACTCAAAATCCTGAG
CCTCACTCAAAATCCTGAG
CCTCACTCAAAATCCTGAG
CCTCACTCAAAATCCTCAG
CCTCACTTAAACTCTTCAG
CCTCACTCAAATTCTTCAA
CCTCACTCATAATCCTGAG
CCTCACTCAAAATCCTGAG
CCTCACTCAAAATCCTGAG
CCTCACTCAAAATCCTGAG
CCTCACTCAAAATCCTCAG
CCTCACTTAAACTCTTCAG
CCTCACTCAAATTCTTCAA
CCTCACTCATAATCCTGAG
CCTCACTCAAAATCCTGAG
CCTCACTCAAAATCCTGAG
CCTCACTCAAAATCCTGAG
CCTCACTCAAAATCCTGAG
CCTCACTCAAAATCCTGAG
CCTCACTCAAAATCCTCAG
CCTCACTTAAACTCTTCAG
CCTCACTCAAATTCTTCAA
CCTCACTCAAAATCCTCAG
CCTCACTCAAAATCCTGAG
CCTCACTCAAAATCCTCAG
CCTCACTTAAACTCTTCAG
CCTCACTCAAATTCTTCAA
CCTCACTCATAATCCTGAG
CCTCACTCAAAATCCTGAG
CCTCACTCAAAATCCTGAG
CCTCACTCAAAATCCTGAG
CCTCACTCAAAATCCTGAG
CCTCACTCAAAATCCTGAG
CCTCACTCAAAATCCTCAG
CCTCACTTAAACTCTTCAG
CCTCACTCAAATTCTTCAA
CCTCACTCATAATCCTGAG
CCTCACTCAAAATCCTGAG
CCTCACTCAAAATCCTCAG
CCTCACTCAAAATCCTGAG
CCTCACTCAAAATCCTGAG
CCTCACTCAAAATCCTCAG
CCTCACTTAAACTCTTCAG
CCTCACTCAAATTCTTCAA
CCTCACTCAAAATCCTCAG
CCTCACTCAAAATCCTCAG
CCTCACTCAAAATCCTCAG
CCTCACTCAAAATCCTCAG
CCTCACTCAAAATCCTCAG
CCTCACTCAAAATCCTCAG
CCTCACTCAAAATCCTCAG
CCTCACTCAAAATCCTCAG

CCTCACTCAAAATCCTCAG
CCTCACTCAAAATCCTGAG
CCTCACTCAAAATCCTGAG
CCTCACTCAAAATCCTCAG
CCTCACTTAAACTCTTCAG
CCTCACTCAAATTCTTCAA
CCTCACTCAAAATCCTCAG
CCTCACTCAAAATCCTCAG
CCTCACTCAAAATCCTCAG
CCTCACTCAAAATCCTCAG
CCTCACTCAAAATCCTCAG
CCTCACTCAAAATCCTGAG
CCTCACTCAAAATCCTCAG
CCTCACTTAAACTCTTCAG
CCTCACTCAAATTCTTCAA
CCTCACTCAAAATCCTCAG
CCTCACTCAAAATCCTGAG
CCTCACTCAAAATCCTCAG
CCTCACTTAAACTCTTCAG
CCTCACTCAAATTCTTCAA
CCTCACTCATAATCCTGAG
CCTCACTCAAAATCCTGAG
CCTCACTCAAAATCCTGAG
CCTCACTCAAAATCCTGAG
CCTCACTCAAAATCCTGAG
CCTCACTCAAAATCCTGAG
CCTCACTCAAAATCCTCAG
CCTCACTTAAACTCTTCAG
CCTCACTCAAATTCTTCAA
CCTCACTCATAATCCTGAG
CCTCACTCAAAATCCTGAG
CCTCACTCAAAATCCTCAG
CCTCACTCAAAATCCTGAG
CCTCACTCAAAATCCTGAG
CCTCACTCAAAATCCTCAG
CCTCACTTAAACTCTTCAG
CCTCACTCAAATTCTTCAA
CCTCACTCAAAATCCTCAG
CCTCACTCAAAATCCTCAG
CCTCACTCAAAATCCTCAG
CCTCACTCAAAATCCTCAG
CCTCACTCAAAATCCTCAG
CCTCACTCAAAATCCTCAG
CCTCACTCAAAATCCTCAG
CCTCACTCAAAATCCTCAG
CCTCACTCAAAATCCTCAG
CCTCACTCAAAATCCTGAG
CCTCACTCAAAATCCTGAG
CCTCACTCAAAATCCTCAG
CCTCACTTAAACTCTTCAG
CCTCACTCAAATTCTTCAA
CCTCACTCAAAATCCTCAG
CCTCACTCAAAATCCTCAG
CCTCACTCAAAATCCTCAG

CCTCACTCAAAATCCTCAG
CCTCACTCAAAATCCTCAG
CCTCACTCAAAATCCTCAG
CCTCACTCAAAATCCTCAG
CCTCACTCAAAATCCTCAG
CCTCACTCAAAATCCTCAG
CCTCACTCAAAATCCTCAG
CCTCACTCAAAATCCTGAG
CCTCACTCAAAATCCTGAG
CCTCACTCAAAATCCTGAG
CCTCACTCAAAATCCTGAG
CCTCACTCAAAATCCTGAG
CCTCACTCAAAATCCTGAG
CCTCACTCAAAATCCTCAG
CCTCACTTAAACTCTTCAG
CCTCACTCAAATTCTTCAA
CCTCACTCATAATCCTGAG
CCTCACTCAAAATCCTGAG
CCTCACTCAAAATCCTCAG
CCTCACTTAAACTCTTCAG
CCTCACTCAAATTCTTCAA
CCTCACTCAAAATCCTGAG
CCTCACTCAAAATCCTGAG
CCTCACTCAAAATCCTGAG
CCTCACTCAAAATCCTGAG
CCTCACTCAAAATCCTCAG
CCTCACTTAAACTCTTCAG
CCTCACTCAAATTCTTCAA
CCTCACTCATAATCCTGAG
CCTCACTCAAAATCCTGAG
CCTCACTCAAAATCCTGAG
CCTCACTCAAAATCCTGAG
CCTCACTCAAAATCCTGAG
CCTCACTCAAAATCCTCAG
CCTCACTTAAACTCTTCAG
CCTCACTCAAATTCTTCAA
CCTCACTCATAATCCTGAG
CCTCACTCAAAATCCTGAG
CCTCACTCAAAATCCTGAG
CCTCACTCAAAATCCTGAG
CCTCACTCAAAATCCTGAG
CCTCACTCAAAATCCTCAG
CCTCACTTAAACTCTTCAG
CCTCACTCAAATTCTTCAA
CCTCACTCATAATCCTGAG
CCTCACTCAAAATCCTGAG
CCTCACTTAAACTCTTCAG
CCTCACTCAAATTCTTCAA
CCTCACTCATAATCCTGAG
CCTCACTCAAAATCCTGAG
CCTCACTCAAAATCCTCAG
CCTCACTCAAAATCCTGAG
CCTCACTTAAACTCTTCAG
CCTCACTCAAATTCTTCAA

CCTCACTCATAATCCTGAG
CCTCACTCAAAATCCTGAG
CCTCACTTAAACTCTTCAG
CCTCACTCAAATTCTTCAA
CCTCACTCAAATTCTTCAA
CCTCACTCATAATCCTGAG
CCTCACTCAAAATCCTCAG
CCTCACTCAAAATCCTCAG
CCTCACTCAAAATCCTCAG
CCTCACTCAAAATCCTCAG
CCTCACTCAAAATCCTCAG
CCTCACTCAAAATCCTCAG
CCTCACTCAAAATCCTCAG
CCTCACTCAAAATCCTCAG
CCTCACTCAAAATCCTCAG
CCTCACTCAAAATCCTCAG
CCTCACTCAAAATCCTCGG
CCTCACTCAAAATCCTCAG
CCTCACTCAAAATCCTCAG
CCTCACTCAAAATCCTCAG
CCTCACTCAAAATCCTCGG
CCTCACTCAAAATCCTCAG
CCTCACTCAAAATCCTCAG
CCTCACTCAAAATCCTCGG
Nr of selected repeats 1365 Similarity 0.827742
Consensus:
CCTCACTCAAAaTCcTgAG
>Cele-UNSB01_4:3274030-3274126 Satlength=97 Nr of Repeats=6 RepeatLength=16
seed=AGGCATGTAG
AGGCATGTAGGCATGT
AGGCATGTAGGCATGC
AGGCATGTAGGCATGT
AGGCATGTAGACATGT
AGGAATGTAGGCATGT
AGGCATGTAGACATAT
Nr of selected repeats 6 Similarity 0.872222
Consensus:
AGGCATGTAGgCATGT
>Cele-UNSB01_4:3353341-3353481 Satlength=141 Nr of Repeats=5 RepeatLength=33
seed=CGGCATATGC
CGGCATATGCCGTGAATTTTAAAATCGGCATAA
CGGCATATGCCGTGAATTTTAAAATCGGCATAA
CGGCATGTGCCATGAATTTTAAAATCGGCATAA
CGGCATATGCCGCGCTCCAAAAAC
CGGCATTATGCCGGCAT
Nr of selected repeats 3 Similarity 0.946128
Consensus:
CGGCATaTGCCgTGAATTTTAAAATCGGCATAA
>Cele-UNSB01_4:3371888-3372867 Satlength=980 Nr of Repeats=27 RepeatLength=35
seed=AATCGGCAAG
AATCGGCAAGTTGCCGGAATTGAAATTTCCGGGA
AATCGGCAAGTTACCGAAATTGAAAATTTCCGGCA
AATCGGCAAGTTACCGGAATTGAAAATTACCGGCA
AATCGGCAAGTTACAGGAATTGAAAGCTTCAGGCA

AATCGGCAAGTTACAGGAATTGAAAGCTTCAGGCA
AATCGGCAAGTTACAGGAATTGAAAGCTTCAGGCA
AATCGGCAAGTTGCCGGAATTGAAAATTTCCGGCA
AATCAGCAAGTTACAGAAATTGAAAGCTTCAGGCA
AATCGGCAAGTTGCCGGAATTGAAAATTTCCGGCA
AATCGGCAAGTTACCGAAATTGAAAATTTCCGGCA
AATTGGCAAGCTGCCGGAATTGAAAATTACCGGCA
AATCGGCAAGTTACCGGAATTGAAAATTTCCGGCG
AATCGGCAAGTTACCGGAATTGAAAATTTCCGGTG
AATTGGCAAGTTGCCGAAATTGAAAATTTCCGGCG
AATCGGCAAGTTACCGAAATTGAAAATTTCCGGCA
AATCGGCAAGTTACCGGAATTGAAAATTACCGGCA
AATCGGCAAGTTACAGGAATTGAAAACTTCAGGCA
AATCGGCAAGTTACCAGAATTGAAAATTTCCGGCAAATTGACAAGTTACCGGAATTGAAAATTTCCGGCA
AATCGGCAAGTTGCCGGAATTGAAAATTTCCGGGA
AATCGGCAAGTTACCGGAATTGAAAGCTTCAGGCT
AATCGGCAAGCTGCCGGAATTGAAAATAACCGGCA
AATCGGCAAGTTACCGAAATTGAAAATTTCCGGCA
AATCGGCAAGTTACCGAAATTGAAAATTTCCGGGA
AATCGGCAAGTTACCGAAATTGAAGATTTCCGGCA
AATCGGCAAGTTACCGAAATTGAAGATTTCCGGCA
AATCGGCAAGTTACCGAAATTGAAAATTTCCGGGA
AATCGGCAAGTTACCGAAATTGAAGATTTCCGGCA
Nr of selected repeats 25 Similarity 0.852698
Consensus:
AATCGGCAAGTTaCcGgAATTGAAAatTTCcGGCA
>Cele-UNSB01_4:3428944-3430273 Satlength=1330 Nr of Repeats=24 RepeatLength=45
seed=TCTGATCCTT
TCTGATCCTTAAGCTCCGAAGGATCACAGGGGGTACCTCCTGATGA
TCTGATCCTTCAGCTCCAAAGGATCAGAAGGGTACCTCCTGGTGA
TCTGATCCTTAAGCTCCGAAGGATCACAGGGGGTACCTCCTGGTGA
TCTGATCCTTCAGCTTCAAAGGATCATAAGGGTACCTCCTGGTGA
TCTGATCCTTCAGCTTCAAAGGATCAGAAGGGTACCTCCTGGTGA
TCTGATCCTTAAGCTCCGAAGGATCACAGGGGGTACCTCCTGGTGA
TCTGATCCTTCAGCTTCAAAGGATCATAAGGGTACCTCCTGGTGA
TCTGATCCTTCAGCTTCAAAGGATCAGAAGGGTACCTCCTGGTGA
TCTGATCCTTCAGCTTCAAAGGATCAGAAGGGTACCTCCTGGTGA
TCTGATCCTTCAGCTCCGAAGGATCAGAAGGGTACCTCCTGATGA
TCTGATCCTTCAGCTTCAAAGGATCATAAGGGGTATAAATGCAATAAATCAAGCAAAGTTGATACAGGTCCCAGGTTCG
AACTTTGTCTGACAAACTTTTTTTACTTTGACGGACAGTCCTGAATACACCTATTGAACATTTCTAATAGTCCATAGG
ATTCCATTTCACCATGCTTTATATCAGTTTCCGGTAATTTTTGTTTGTCCGCTAAGATACTTTGTCACCCAAAAGTTTG
TCATCACGGAGAATTGATCAACTTTGCTTGATTTATTGCTTTTATACCCCT
TATGATCCTTTGAAGCTGAAGGATCAGATCACCAGGAGATACCCCC
TGTGATCCTTTGAAGCTGAAGGATCAGATCATCAGGAGATACCCCC
TGTGATCCTTTGAAGCTGAAGGATCAGATCATCAGGAGGTACCCT
TCTGATCCTTCGGAGCTGAAGGATCAGATCACCAGGAGGTACCCT
TCTGATCCTTTGAAGCTGAAGGACCAGATCACCAGGAGGTACCCT
TATGATCCTTTGAAGCTGAAGGATCAGATCACCAGGAGGTACCCCC
TGTGATCCTTCGGAGCTTAAAGATCAGATCACCAGGAGGTACCTT
TATGATCCTTTGAAGCTGAAGGATCAGATCATCAGGAGGTACCCT
TCTGATCCTTTGAAGCTGAAGGATCAGATCATCAGGAGGTACCCT
TCTGATCCTTTGAAGCTGAAGGATCAGGTCACCAGGAGGTACCCT
TCTGATCCTTCGGAGCTGAAGGATCAGATCATCAGGAGGTACCCT
TCTGATCCTTTGAAGCTGAAGGATCAGATCATCAGGAGGTACCCT

TCTGATCCTTTGAAGCTGAAGGATCAGATCATCAGGAGGTACCCT
Nr of selected repeats 17 Similarity 0.598811
Consensus:
TCTGATCCTTcgnAGCTgAAGGATCAGatcAncAGGagGTACCCT
>Cele-UNSB01_4:3517707-3518097 Satlength=391 Nr of Repeats=27 RepeatLength=12
seed=TAAGCCTAAG
TAAGCCTAAGCT
TAAGCCTAAGCT
TAAGCCTAAGCT
TAAGCCTAAGCT
TAAGCCTAAGCT
TAAGCCTAAGCT
TAAGCCTAAGCT
TAAGCCTAAGCT
TAAGCCTAAGCT
TAAGCCTAAGCT
TAAGCCTAAGCT
TAAGCCTAAGCT
TAAGCCTAAGAT
TAAGCCTAAGCCTAAGAT
TAAGCCTAAGCC
TAAGCTTAAGCC
TAAGCCTAAGCCTAAGAA
TAAGCCTAAACC
TAAGCCTATGCC
TATGCCTAAGCC
TAAGCCTAAGTCTTAGCCTTGGCT
TAAGCCTAAGCCAGAGCTTAAGCTCAAGCCCAAAAT
TAAGCCTAAGCC
TAAGCCTCAGCCTAGGTC
TAAGCCTAGGTC
TAAGCCTAAGCT
TAAGCCTAAAACTAGGCCCAAGCC
Nr of selected repeats 21 Similarity 0.808059
Consensus:
TAAGCCTAAGCt
>Cele-UNSB01_4:3630301-3630781 Satlength=481 Nr of Repeats=28 RepeatLength=16
seed=TGCCTACATG
TGCCTACATGCCTTCA
TGCCTACATGCCCACA
TGCCTACATGCCTACA
TGCCTACTTGCCAACA
TGCCTACATGCCCACA
TGCCTACATGCCTACA
TGCCTACATGCCTACA
TGCCTACATGCCTACA
TGCCTACATGCCTACA
TGCCTACATGTCTACA
TGCCTACATGCCTACA
TGCCTACATGCCTACA
TGCCTACATGCCTACA
TGCCTACATGCCTATA
TGCATACATGTTTGCATTCCTTCA
TGCCTACATGCCTACA

TGCCTACATGACCACA
TGCCTACATGCCTACA
TGCCTACTTGCCAACA
TGCCTACATGCCCACA
TGCCTACATGCCTACA
TGCCTACATGCCTACA
TGCCTACATGCCTACA
TGCCTATATGCATACATGTTTGCA
TGCCTTCATGCCTACA
TGCCTACATGCCTTCAT
TCCTACATGTCTACA
TGCCTACATGCATACATGTTTGCATGCCTACA
Nr of selected repeats 23 Similarity 0.914032
Consensus:
TGCCTACATGCCtACA
>Cele-UNSB01_4:3670951-3671065 Satlength=115 Nr of Repeats=7 RepeatLength=12
seed=TTAGGCTTAG
TTAGGCTTAGGCATAGGCTCAGGCTTTGGCTCAGGA
TTAGGCTTAGGCTTAAAC
TTAGGATTAGGA
TTAGGCTTAGGC
TTAGGATTAGGC
TTAGGCTTAGGA
TTAGGCTTAGGA
Nr of selected repeats 5 Similarity 0.866667
Consensus:
TTAGGcTTAGGa
>Cele-UNSB01_4:3714325-3715225 Satlength=901 Nr of Repeats=15 RepeatLength=21
seed=TGTAGATTTA
TGTAGATTTATGGGAACCTGC
TGTAGATTTATAAAAACCTGGTGTGCATTTACGGGCATATATTGTGAACTAATGGGAACCTAATCCGGATTTAAGCAAA
TCTGG
TGTAGATTTACGGGGGCCTACTATACATTTACAGAAGTATAT
TGTAGATATACGGGAATCTTG
TGTAGATTTTCGTGAATAGTGAGTTGTGATCCAACAACTTTCAATTTTCAAAAAATCGGGCGTCAAAGTACTCAAGCAC
CTGCAAATAGAAAAACCCAGCTGACGCTTAATACTCCCCGTCATCAGAGAGCAAATTGCATCAGATACAGTAAGGAGGG
GTACACATGTGGATCTCACATGTGAAAAGTTCAGCAGATTCATGTGAATTATGTGTTTTTTTTTGTTGCGGTTTCAGTT
TTTCTTTCAGCGCGCGCGCCCGAAATAAATGTGATGCGACACACAGCGGGCCGACAAAGGGAGAAAGCCCCCGGCCGGC
GTAATTGGATGAAATTAGTGGTGATATGGAAGGATTGAGATGGGAGATTATATGATTAATGTTGTTAAACACAAACACT
TGCTGGTGAGGATGGTATGATTTTGGGGGAGAGAGAGAAAATCGGATTTTTGGAAGGATGATGTAGGATGACGGAGTGA
TAACAGTGATACGGTACTTGTTTTGGTTTGGAAATTGTTGGGTTAG
TGTGGATTTACGGGAGGCATTG
TGTAGATTTACGGAAGCCTAG
TGTAGAATTACGGGAGCATAG
TGTATATTTACGGGAGTCTAT
TGTAGAATTACGGGAGCATAG
TGTAGATTTATGCGAGTATAT
TGTAGATTTACGGGAGCATAG
TGTAGATCTACGGGAGGCTAT
TGTAGATTTACGGGAGGTATTG
TGTAGATTTACGAGAGTCTAT
Nr of selected repeats 10 Similarity 0.670875
Consensus:
TGTAGAttTAcGgGAgccTan

>Cele-UNSB01_4:3733029-3733089 Satlength=61 Nr of Repeats=4 RepeatLength=15
seed=TCGTGGTGAG
TCGTGGTGAGACCCA
TCGTGGTGAGACCCT
TCGTGGTGAGACCCA
TCGTGGTGAGACCCT
Nr of selected repeats 4 Similarity 0.940741
Consensus:
TCGTGGTGAGACCCa
>Cele-UNSB01_4:3733495-3734015 Satlength=521 Nr of Repeats=15 RepeatLength=40
seed=TTTCTAGAAA
TTTCTAGAAAATTCGAGAAAATTCCGGATTGGTCCAGAAT
TTTCTAGAAAA
TTCTAGAAAGTTGTGGAATGGTCCAGAAT
TTTCTAGAAAATTCGGGAAAACTCTGGAATATTCCAGAAC
TTTCTAGAAAAAATCGAGAAATGTCTGCAATGTTCTAGAAC
TTTCTAGAAAATTCGAGAAAATTCCGGAATGGTCCAGAAT
TTTCTAGAAAATTCGAGAAAATTCCGGAATGGTCCAGAAT
TTTCTAGAAAATTCGGTAAAATTCTGGAATATTCCAGAAC
TTTCTAGAAAAAATCGGGAAAAGTCGGCAATGTTCTAGAAC
TTTCTAGAAAATTCGAGAAAATTCCGGAATGGTCCAGAAT
TTTCTAGAAAA
TTCTAGAAAGTTCTGGAATGGTCCAGAAG
TTTCTAGAAAGTTCGAGAAAATTCCGGAATGGTCCAGAAT
TTTCTAGAAAATCCGAGAAAGTTCTGGAATGGTCCAGAAT
TTTCTAGAAAATTTGGGAAAATTCTGGAATATTCCAGAAC
Nr of selected repeats 11 Similarity 0.820606
Consensus:
TTTCTAGAAAATTCGaGAAAAtTCnGGAATggTCCAGAAt
>Cele-UNSB01_4:3758727-3758973 Satlength=247 Nr of Repeats=6 RepeatLength=41
seed=AAATTTTTAA
AAATTTTTAACTAGAAGTGGGCGAAAATCATTCAAAAATTC
AAATTTTTAACTAAAAGTGGGCGAAAATCATTCAAAAATTC
AAATTTTTAACTAAAAGTGGGCGAAAATCATTCAAAAATTC
AAATTTTTAACGAGAAGTGGGCGAAAATCATTCAAAAATTG
AAATTTTTAACGAGAAGTGGGCGAAAATCATTCAAAAATTG
AAATTTTTAACGAGAAGTGGGCGAAAATCATTCAAAAATTC
Nr of selected repeats 6 Similarity 0.945799
Consensus:
AAATTTTTAACgAgAAGTGGGCGAAAATCATTCAAAAATTc
>Cele-UNSB01_4:3817232-3817391 Satlength=160 Nr of Repeats=5 RepeatLength=21
seed=TAGTTTGTAG
TAGTTTGTAGTGTGCAGTGTG
TAGTTTGTAGATTGCAGTTCCAAAAAACCACAATCAAAAATATTAGAGAAATGGAAATTATTTCAAGTTTTTGCTAATA
TTGC
AGTTTGTAGTTTG
TAGTTTATAGTTCACAGTTTA
TAGTTTATAGTTTGTACTTTG
Nr of selected repeats 3 Similarity 0.661376
Consensus:
TAGTTTaTAGTttgcAgTtTg
>Cele-UNSB01_4:3888107-3888300 Satlength=194 Nr of Repeats=12 RepeatLength=16
seed=TAGGCATGTA
TAGGCATGTA

TAGGCATGTAGGCATG
TAGGCATGTAGGCATG
TAGGCATGTAGGCATG
TAGGCATGTAGGCATG
TAGCATGTAGGCATG
TAGGCATGTAGGCATG
TAGGCATGTAGGCATG
TAGGCATGTAGGCATG
TAGGCATGTAGGAATG
TAGGCATGTAGGCATG
TAGGAATGTAGACATGTAGGGATG
Nr of selected repeats 9 Similarity 0.981481
Consensus:
TAGGCATGTAGGCATG
>Cele-UNSB01_4:3896416-3896737 Satlength=322 Nr of Repeats=5 RepeatLength=18
seed=ATTAGTATTG
ATTAGTATTGCCCCTCGGAGACCTTCCGAAA
ATTAGTATTGCCCTACCT
ATTAGTCTTGCACTCTCTAATAGTCTTGCGTTAACTTTTTTGTATTGCAAACCCGTCTCGCTAATTTCAGGATTTCAAC
AACAATTTTTGCCTATTTTGCAACAGTTTTCACATCATTTCTACGAGATTGAAGTTTTTGAACTTATATGAAAATCAAA
TTTTCTGAATTTTTACATATGATTCGAGTTTTTTCCAGGAAAAAACTAGTAAGCGCAAAGTCAGAAAATGTTCTGAAA
ATTAGTATTGCACTCCTT
ATTAGTCTTGCACTCCCT
Nr of selected repeats 3 Similarity 0.802469
Consensus:
ATTAGTaTTGCaCTcCcT
>Cele-UNSB01_4:4053946-4054647 Satlength=702 Nr of Repeats=6 RepeatLength=12
seed=TAAGCCTAAG
TAAGCCTAAGTC
TAAGCCTATGCC
TAAACCTAAGCC
TAAGCCTCAGTC
TAAGCCTATGCCTAAACC
TAAACCTAAGCCTAAGCTAAAGCCTGATATCTATTTTTCAGTCCCATTCACCTGTGGAGGCCACTTTACCGCCCAGGCC
TACATCCAATCCTTTGTCTCTCCCGGCTACCCAAAGACCTTTGCAAATGGCGCCGAATGTGTGTGGACTGTGGAATCTA
CGCCAGGACAAGTGGTTTCTTTGATTGTGAGTTTTTTAATTTTTTTTTGAATGTACTGGGTATGGGTATTATTATTTTA
GATGAAAAGCGGCAAAACATGGGGATAAATTCAAAGAAAAAATGAAAATTTCAGCAGAATTTGTTTTATCCGGTAAAAA
ACCGGATTTAACAACAACTCTTTGTTTTATAAGAATTTTCATTTGTTCCACAACATTAAATTAACATTAAATTAACATT
AGTTCTCGTTAGGACCTTCACACACCTTCGCAAGACCATTTTCATAAGCACGGATGTTGAAATCGATGGAATGTCTGAA
AATTAATAATAACTAAATCGTTTTAGCGAACAATCTAACTTACTCTCCAAATGTCCATACATTGGTTTTTTCGTTAGGT
CGTAGGCATCGATCCATTCCTTCGGCAGTGCAATTATGGTGGAGTACGTAGGCAAACTCGTAGTGCTGAGTTTAGGATA
GTT
Nr of selected repeats 4 Similarity 0.759259
Consensus:
TAAgCCTaaGcC
>Cele-UNSB01_4:4229494-4229583 Satlength=90 Nr of Repeats=7 RepeatLength=12
seed=GTTGCGTGTC
GTTGCGTGTCGC
GTTGCGTGTCGC
GTTGCGTGTCGC
GTTGCGTGTCGTGTCGC
GTTGCGTGTCGC
GTTGCGTGTCGA
GTTGCGTGTCGC

Nr of selected repeats 6 Similarity 0.962963
Consensus:
GTTGCGTGTCGC
>Cele-UNSB01_4:4229497-4230359 Satlength=863 Nr of Repeats=71 RepeatLength=12
seed=GCGTGTCGCG
GCGTGTCGCGTT
GCGTGTCGCGTT
GCGTGTCGCGTT
GCGTGTCGTGTCGCGTT
GCGTGTCGCGTT
GCGTGTCGAGTT
GCGTGTCGCGTT
GCGTGTCGCGTCGAGTC
GAGTGTCGCGTC
GAGTGTCGCGTC
GAGTGTCGCGTC
GAGTGTCGCGTC
GAGTGTCGCGTC
GAGTGTCGCGTC
GCGTGTCGCGTT
GCGTGTCGCGTC
GAGTGTCGCGTC
GCGTGTCGCGTT
GCATGTCGCGTT
GCGTGTCGCGTT
GCGTGTCGCGTC
GCGTGTCGCGTT
GCGTGTCGCGTT
GCGTGTCGCGTT
GCGTGTCGCGTT
GCGTGTCGCGTC
GAGTGTCGCGTC
GAGTGTCGCGTC
GAGTGTCGCGTC
GAGTGTCGCGTC
GAGTGTCGCGTC
GCGTGTCGCGTT
GCGTGTCGCGTT
GCGTGTCGCGTT
GCGTGTCGCGTT
GCGTGTCGCGTT
GCGTGTCGCGTT
GCGTGTCGCGTT
GCGTGTCGCGTT
GCGTGTCGCGTT
GCGTGTCGCGTT
GCGTGTCGCGTT
GCGTGTCGCGTT
GCGTGTCGCGTT
GCGTGTCGCGTT
GCGTGTCGCGTT
GCGTGTCGCGTT
GCGTGTCGCGTT
GCGTGTCGCGAT

GCGTGTCGCGTT
GCGTGTCGCGTT
GCGTGTCGCGTT
GCGTGTCGCGTT
GCGTGTCGCGTT
GCGTGTCGCGTT
GCGTGTCGCGTT
GCGTGTCGCGTT
GCGTGTCGCGTT
GCGTGTCGCGTT
GCGTGTCGCGTT
GCGTGTCGCGTT
GCGTGTCGCGGT
GCGTCTCGCGTT
GCGTGTCGCGTT
GTGTGTCGCGTT
GCGTGTCGCGTT
GCGTATCGCGTT
GCGTATCGCGTT
GCGTGTCGCGTT
GCGTATCGCGTT
GCGTGTCGCGTT
Nr of selected repeats 69 Similarity 0.901298
Consensus:
GCGTGTCGCGTt
>Cele-UNSB01_4:4261575-4261676 Satlength=102 Nr of Repeats=6 RepeatLength=17
seed=GATGCCCCCT
GATGCCCCCTCTGAACG
GATGCCCCCTCTGAACG
GATGCCCCCTACGAACAA
ATGCCCCCTTTGACCTA
ATGCCCCCTTAGAACGC
GATGCCCCCTCTCTC
Nr of selected repeats 4 Similarity 0.722222
Consensus:
gATGCCCCCTctGAaCgn
>Cele-UNSB01_4:4368763-4369020 Satlength=258 Nr of Repeats=5 RepeatLength=11
seed=GTACTGTAGG
GTACTGTAGGA
GTACTGTAGGTATACGGTAGGGTTACTGTAGTTTAGGAAAAATTGAGTTTTTGTCTACAGAAGAGGTATTGGGTTGGGA
GTTGGTGGGAGATAATGCCAAGCTACTGTAGTG
GTACTGTAGGA
GTACTGTAGGTATACGGTAGGGTTACCGTAGTTTAGGAAAAATTGAGTTTTTGTCTACAGAAGAGGTATTGGGTTGGGA
GTTGGTGGGAGATAATGCCAAGCTACTGTAGTG
GTACTGTAGGA
Nr of selected repeats 3 Similarity 1.000000
Consensus:
GTACTGTAGGA
>Cele-UNSB01_4:4388126-4388633 Satlength=508 Nr of Repeats=13 RepeatLength=11
seed=CTACAGTACT
CTACAGTACTC
CTACAGTACTA
CTACAGTACCTTGACATTATCCCCCAGCAACTCCCAACCCAAAACCTCTTCTGTAGACAAAAACTCAATTTTTCCCAGA
CTACAGTAACCCTACCGTATGC

CTACAGTACTC
CTACAGTACTA
CTACAGTACCTTGACATTATCCCCCAGCAACTCCCAACCCAATACCTCTTCTGTAGACAAAAACTCAATTTTTCCCAAA
CTACAGTAACCCTACCGTATAC
CTACAGTTCTC
CTACAATACTA
CTACAGTACCTTGACATTATCCCCCCCACCAACTCCCAACCCAATACCTCTTCTGTAGACAAAACCTCAATTTTTCCTA
AACTACAGTAACCCTACCGTATAC
CTACAGTACTC
CTACAGTACTA
CTACAGTACCTTGACATTATCCCCCCTACCCACTCCCAACCCAATACCTCTTCTGTAGACAAAAACCCAATTTTTCCCA
AACTACAGTAACCCTACCGTATAC
CTACAGTACTC
Nr of selected repeats 9 Similarity 0.878788
Consensus:
CTACAGTACTc
>Cele-UNSB01_4:4398109-4398181 Satlength=73 Nr of Repeats=4 RepeatLength=18
seed=TTTTTTGATA
TTTTTTGATAATTCTTGT
TTTTTTGATAATTCTTGA
TTTTTTGATAATTCTTGA
TTTTTTGATAATTCTTGA
Nr of selected repeats 4 Similarity 0.962963
Consensus:
TTTTTTGATAATTCTTGa
>Cele-UNSB01_4:4418485-4418703 Satlength=219 Nr of Repeats=5 RepeatLength=35
seed=TTCCGGCAAA
TTCCGGCAAATCGACAATTTGCCGAAAAACAGAGT
TTCCGGCAAATCGGCATATTAGCAAATTGCCGGAATTGAAAA
TTCCTGGCAAACCGGCAATTTGGGTTTGCACTTTTCTTATTGGAAATTTCAGAATTTCAATTTTTGAAAAT
TTCAGGCAAATCGGCAAATTGCCGGAATTGAATAC
TTCCGGCAAATCGACAATTGGCCGAAAATCAGAGT
Nr of selected repeats 3 Similarity 0.675676
Consensus:
TTCcGGCAAATCGaCAAtTtGCCGaaAAtcaGAgT
>Cele-UNSB01_4:4464716-4464793 Satlength=78 Nr of Repeats=5 RepeatLength=15
seed=CGAAAAGGGG
CGAAAAGGGGAGCAGAA
CGAAAAGGGGATCTG
CAAAAAGGGGATCTG
CGAAAAGGGGAGATA
CGAAAAGGGGAGCAA
Nr of selected repeats 4 Similarity 0.748148
Consensus:
CgAAAAGGGGAgcta
>Cele-UNSB01_4:4479166-4488089 Satlength=8924 Nr of Repeats=327 RepeatLength=26
seed=GTCTAACTTT
GTCTAACTTTAAACAATTAATCTACCGTTCTAACATTTGAAAATTAATCTACCGTTCTAACTCTTAAAAATCTACAAGA
AGATCTTTCCTTGCGCTTGAAGCGCAAAAGAAAAGAAAAAGGACTATTTAGACTTAGGCACATACTAGGATTCCCAACT
GGAATAAAATATTGGAAATCCTTATGACACACCGGCGGTATGGCGCGGCTTAAGCCTAAATAGCCACTTTTATCAAAAT
ACATTTGAGCGAGGCGGTTGTAAACTATTCGTTCTTTAGCAAAAATTAAAAAAAAAAAAATATAACTTTATTTAAATTT
AAAAATCGGAACGGTATTTAATAACTTTGGAAATTCATCTACTG
GTCTAACTTTGGAAATTCATCTAATG
GTCTAACTTTGGAAATTCATCTAATG

GTCTAACTTTGGAAATTCATCTAATG
GTCTAACTTTGGAAATTCATCTAATG
GTCTAACTTTGGAAATTCATCTAATG
GTCTAACTTTGGAAATTCATCTAATG
GTCTAACTTTGGAAATTCATCTAATG
GTCTAACTTTGGAAATTCATCTAATG
GTATAACTTTGAAAATTCATCTAATG
GTCTAACTTTGGAAATTCATCTAATG
GTCTAACTTTGGAAATTCATCTAATG
GTCTAACTTTGGAAATTCATCTAATG
GTATAACTTTGAAAATTCATCTAATG
GTCTAACTTTGGAAATTCATCTAATG
GTCTAACTTTGGAAATTCATCTAATG
GTCTAACTTTGGAAATTCATCTAATG
GTCTAACTTTGGAAATTCATCTAATG
GTCTAACTTTGGAAATTCATCTAATG
GTCTAACTTTGGAAATTCATCTAATG
GTCTAACTTTGGAAATTCATCTAATG
GTCTAACTTTGGAAATTCATCTAATG
GTCTAACTTTGGAAATTCATCTAATG
GTCTAACTTTGGAAATTCATCTAATG
GTCTAACTTTGGAAATTCATCTAATG
GTCTAACTTTGGAAATTCATCTAATG
GTCTAACTTTGGAAATTCATCTAATG
GTCTAACTTTGGAAATTCATCTAATG
GTCTAACTTTGGAAATTCATCTAATG
GTCTAACTTTGGAAATTCATCTAATG
GTCTAACTTTGGAAATTCATCTAATG
GTCTAACTTTGGAAATTCATCTAATG
GTCTAACTTTGGAAATTCATCTAATG
GTCTAACTTTGGGAATTCATCTAATG
GTCTAACTTTGGAAATTCATCTAATG
GTCTAACTTTGGAAATTCATCTAATG
GTCTAACTTTGGAAATTCATCTAATG
GTCTAACTTTGGAAATTCATCTAATG
GTCTAACTTTGGAAATTCATCTAATG
GTCTAACTTTGGAAATTCATCTAATG
GTCTAACTTTGGAAATTCATCTAATG
GTCTAACTTTGGAAATTCATCTAATG
GTCTAACTTTGGAAATTCATCTAATG
GTCTAACTTTGGAAATTCATCTAATG
GTCTAACTTTGGAAATTCATCTAATG
GTCTAACTTTGGAAATTCATCTAATG
GTCTAACTTTGGAAATTCATCTAATG
GTCTAACTTTGGAAATTCATCTAATG
GTATAACTTTGAAAATTCATCTAATG
GTCTAACTTTGGAAATTCATCTAATG
GTCTAACTTTGGAAATTCATCTAATG
GTCTAACTTTGGAAATTCATCTAATG
GTCTAACTTTGGAAATTCATCTAATG
GTCTAACTTTGGAAATTCATCTAATG
GTCTAACTTTGGAAATTCATCTAATG
GTCTAACTTTGGAAATTCATCTAATG
GTCTAACTTTGGAAATTCATCTAATG

GTCTAACTTTGGAAATTCATCTAATG
GTCTAACTTTGGAAATTCATCTAATG
GTCTAACTTTGGAAATTCATCTAATGGTCTATAACTTTGAAAATTCATCTAATG
GTCTAACTTTGGAAATTCATCTAATG
GTCTAACTTTGGAAATTCATCTAATG
GTCTAACTTTGGAAATTCATCTAATG
GTCTAACTTTGGAAATTCATCTAATG
GTCTAACTTTGGAAATTCATCTAATG
GTCTAACTTTGGAAATTCATCTAATG
GTCTAACTTTGGAAATTCATCTAATG
GTCTAACTTTGAAAATTCATCTAATG
GTATAACTTTGAAAATTCATCTAATG
GTCTAACTTTGGAAATTCATCAACTG
GTCTAACTTTGGGAATTCATCTAATG
GTCTAACTTTGGAAATTCATCAACTG
GTCTAACTTTGGAAATTCATCTAATG
GTCTAACTTTGGAAATTCATCAACTG
GTCTAACTTTGGGAATTCATCTAATG
GTCTAACTTTGGAAATTCATCAACTG
GTCTAACTTTGGGAATTCATCTAATG
GTCTAACTTTGGAAATTCATCAACTG
GTCTAACTTTGGAAATTCATCTAATG
GTCTAACTTTGGGAATTCATCTAATG
GTCTAACTTTGGAAATTCATCTAATG
GTCTAACTTTGAAAATTCATCTAGTG
GTCTAACTTTGGGAATTCATCTAATG
GTCTAACTTTGGAAATTCATCAACTG
GTCTAACTTTGGGAATTCATCTAATG
GTCTAACTTTGAAAATTCATCTAATG
GTCTAACTTTGGAAATTCATCAACTG
GTCTAACTTTGGGAATTCATCTAATG
GTCTAACTTTGGAAATTCATCTAATG
GTCTAACTTTGGAAATTCATCTAATG
GTCTAACTTTGGAAATTCATCTAATG
GTCTAACTTTGAAAATTCATCTAGTG
GTCTAACTTTGGAAATTCATCAACTG
GTCTAACTTTGGGAATTCATCTAATG
GTCTAACTTTGGAAATTCATCTAATG
GTCTAACTTTGGAAATTCATCAACTG
GTCTAACTTTGGGAATTCATCTAATG
GTCTAACTTTGAAAATTCATCTAATG
GTCTAACTTTGGAAATTCATCAACTG
GTCTAACTTTGGGAATTCATCTAATG
GTCTAACTTTGAAAATTCATCTAATG
GTCTAACTTTGGAAATTCATCAACTG
GTCTAACTTTGGGAATTCATCTAATG
GTCTAACTTTGGAAATTCATCTAATG
GTCTAACTTTGGAAATTCATCTAATG
GTCTAACTTTGGGAATTCATCTAATG
GTATAACTTTGAAAATTCATCTAATG
GTCTAACTTTGGAAATTCATCAACTG
GTCTAACTTTGGGAATTCATCTAATG
GTCTAACTTTGGAAATTCATCTAATG
GTCTAACTTTGGAAATTCATCTAATG

GTCTAACTTTGGAAATTCATCTAATG
GTCTAACTTTGGAAATTCATCTAATG
GTCTAACTTTGGGAATTCATCTAATG
GTATAACTTTGAAAATTCATCTAATG
GTCTAACTTTGGAAATTCATCAACTG
GTCTAACTTTGGGAATTCATCTAATG
GTCTAACTTTGGAAATTCATCTAATG
GTCTAACTTTGGAAATTCATCTAATG
GTCTAACTTTGGAAATTCATCTAATG
GTCTAACTTTGGGAATTCATCTAATG
GTATAACTTTGAAAATTCATCTAATG
GTCTAACTTTGGAAATTCATCAACTG
GTCTAACTTTGGGAATTCATCTAATG
GTCTAACTTTGGAAATTCATCTAATG
GTCTAACTTTGGAAATTCATCTAATG
GTCTAACTTTGGAAATTCATCTAATG
GTCTAACTTTGAAAATTCATCTAGTG
GTCTAACTTTGGAAATTCATCAACTG
GTCTAACTTTGGGAATTCATCTAATG
GTCTAACTTTGGGAATTCATCTAATG
GTCTAACTTTGGAAATTCATCTAATG
GTCTAACTTTGGAAATTCATCTAATG
GTCTAACTTTGGAAATTCATCTAATG
GTCTAACTTTGAAATTCATCAACTG
GTCTAACTTTGGAAATTCATCTAATG
GTCTAACTTTGGAAATTCATCTAATG
GTCTAACTTTGGAAATTCATCTAATG
GTCTAACTTTGGAAATTCATCTAATG
GTCTAACTTTGGAAATTCATCAACTG
GTCTAACTTTGGGAATTCATCTAATG
GTATAACTTTGAAAATTCATCTAATG
GTCTAACTTTGGAAATTCATCTAATG
GTCTAACTTTGGAAATTCATCTAATG
GTCTAACTTTGGGAATTCATCTAATG
GTCTAACTTTGGAAATTCATCTAATG
GTCTAACTTTGGAAATTCATCTAATG
GTCTAACTTTGGGAATTCATCTAATG
GTATAACTTTGAAAATTCATCTAATG
GTCTAACTTTGGAAATTCATCAACTG
GTCTAACTTTGGGAATTCATCTAGTG
GTCTAACTTTGGGAATTCATCTAATG
GTATAACTTTGAAAATTCATCTAGTG
GTCTAACTTTGGGAATTCATCTAATG
GTATAACTTTGAAAATTCATCTAGTG
GTCTAACTTTGGGAATTCATCTAATG
GTATAACTTTGAAAATTCATCTAGTG
GTCTAACTTTGGGAATTCATCTAATG
GTATAACTTTGAAAATTCATCTAATG
GTCTAACTTTGGAAATTCATCTAATG
GTCTAACTTTGGAAATTCATCTAATG
GTCTAACTTTGGAAATTCATCTAATG
GTCTAACTTTGGAAATTCATCTAATG
GTCTAACTTTGGAAATTCATCTAATG
GTCTAACTTTGGAAATTCATCTAATG

GTCTAACTTTGGAAATTCATCTAATG
GTCTAACTTTGGAAATTCATCTAATG
GTCTAACTTTGGAAATTCATCTAATG
GTCTAACTTTGGAAATTCATCTAATG
GTCTAACTTTGGAAATTCATCTAATG
GTCTAACTTTGGAAATTCATCTAATG
GTCTAACTTTGGAAATTCATCTAATG
GTCTAACTTTGGAAATTCATCTAATG
GTCTAACTTTGGAAATTCATCTAATG
GTCTAACTTTGGAATTCATCTAATG
GTCTAACTTTGGAAATTCATCTAATG
GTCTAACTTTGGAAATTCATCTAATG
GTCTAACTTTGGAAATTCATCTAATG
GTCTAACTTTTTTTTTTTTTTTTTTTTTTTTTTTTTTTCTTTGGAATTCATCTAATG
GTCTAACTTTGGAAATTCATCTAATG
GTCTAACTTTGGAAATTCATCTAATG
GTCTAACTTTGGAAATTCATCTAATG
GTCTAACTTTGGAAATTCATCTAATG
GTCTAACTTTGGAAATTCATCTAATG
GTCTAACTTTGGAAATTCATCTAATG
GTCTAACTTTGGAAATTCATCTAATG
GTCTAACTTTGGAATTCATCTAATG
GTCTAACTTTGGAAATTCATCTAATG
GTCTAACTTTGGAAATTCATCTAATG
GTCTAACTTTGGAATTCATCTAATG
GTCTAACTTTGGAAATTCATCTAATG
GTCTAACTTTGGAAATTCATCTAATG
GTCTAACTTTGGAAATTCATCTAATG
GTCTAACTTTGGAAATTCATCTAATG
GTCTAACTTTGGAAATTCATCTAATG
GTCTAACTTTGGAAATTCATCTAATG
GTCTAACTTTGAAATTCATCTAATG
GTCTAACTTTGGAAATTCATCTAATG
GTCTAACTTTGGAATTCATCTAATG
GTCTAACTTTGGAAATTCATCTAATG
GTCTAACTTTGAAATTCATCTAATG
GTCTAACTTTGGAAATTCATCTAATG
GTCTAACTTTGGAAATTCATCTAATG
GTCTAACTTTGGAAATTCATCTAATG
GTCTAACTTTGGAAATTCATCTAATG
GTCTAACTTTGGAAATTCATCTAATG
GTCTAACTTTGGAAATTCATCTAATG
GTCTAACTTTGGAAATTCATCTAATG
GTCTAACTTTGGAAATTCATCTAATG
GTCTAACTTTGAAATTCATCTAATG
GTCTAACTTTGGAAATTCATCTAATG
GTCTAACTTTGGAAATTCATCTAATG
GTCTAACTTTGGAAATTCATCTAATG
GTCTAACTTTGAAATTCATCTATTTTTTTTTTTTTTTTTTTTTTTTTTTTTTTCTAATG
GTTAACTTTGAAATTCATCTAATG
GTCTAACTTTGGAAATTCATCTAATG
GTCTAACTTTGGAAATTCATCAATG
GTCTAACTTTGGAATTCATCTAATG
GTCTAACTTTGGAAATTCATCTAATG

GTCTAACTTTGGAAATTCATCTAATG
GTCTAACTTTGGAAATTCATCTAATG
GTCTAACTTTGGAAATTCATCTAATG
GTCTAACTTTGGAAATTCATCTAATG
GTCTAACTTTGGAAATTCATCTAATG
GTCTAACTTTGGAAATTCATCTAATG
GTCTAACTTTGGAAATTCATCTAATG
GTCTAACTTTGGAAATTCATCAACTG
GTCTAACTTTGGAAATTCATCTAATG
GTCTAACTTTGGAAATTCATCTAATG
GTCTAACTTTGGAAATTCATCTAATG
GTCTAACTTTGGAAATTCATCTAATG
GTCTAACTTTGGAAATTCATCTAATG
GTCTAACTTTGGAAATTCATCTAATG
GTCTAACTTTGGAAATTCATCTAATG
GTCTAACTTTGGAAATTCATCAACTG
GTCTAACTTTGGAAATTCATCTAATG
GTCTAACTTTGGAAATTCATCTAATG
GTCTAACTTTGGAAATTCATCTAATG
GTCTAACTTTGGAAATTCATCTAATG
GTCTAACTTTGGAAATTCATCTAATG
GTCTAACTTTGGAAATTCATCTAATG
GTCTAACTTTGGAAATTCATCTAATG
GTCTAACTTTGGAAATTCATCTAATG
GTCTAACTTTGGAAATTCATCTAATG
GTCTAACTTTGGAAATTCATCTAATG
GTCTAACTTTGGAAATTCATCTAATG
GTCTAACTTTGGAAATTCATCTAATG
GTCTAACTTTGGAAATTCATCTAATG
GTCTAACTTTGGAAATTCATCTAATG
GTCTAACTTTGGAAATTCATCTAATG
GTCTAACTTTGGAAATTCATCTAATG
GTCTAACTTTGGAAATTCATCTAATG
GTCTAACTTTGGAAATTCATCTAATG
GTCTAACTTTGGAAATTCATCTAATG
GTCTAACTTTGGAAATTCATCTAATG
GTCTAACTTTGGAAATTCATCTAATG
GTCTAACTTTGAAATTCATCTAATG
GTCTAACTTTGGAAATTCATCTAATG
GTCTAACTTTGGAAATTCATCTAATG
GTCTAACTTTGGAAATTCATCTAATG
GTCTAACTTTGGAAATTCATCTAATG
GTCTAACTTTGGAAATTCATCTAATG
GTCTAACTTTGGAAATTCATCTAATG
GTCTAACTTTGGAAATTCATCTAATG
GTCTAACTTTGGAAATTCATCTAATG
GTCTAACTTTGGAAATTCATCTAATG
GTCTAACTTTGGAAATTCATCTAATG
GTCTAACTTTGGAAATTCATCTATG
GTCTAACTTTTTTTTG
GTCTAACTTTGGAAATTCATCTAATG
GTCTAACTTTGGAAATTCATCTAATG
GTCTAACTTTGGAAATTCATCTAATG
GTCTAACTTTGGAAATTCATCTAATG

GTCTAACTTTGGAAATTCATCTAATG
GTCTAACTTTGGAAATTCATCTAATG
GTCTAACTTTGGAAATTCATCTAATG
GTCTAACTTTGGAAATTCATCTAATG
GTCTAACTTTGGAAATTCATCTAATG
GTCTAACTTTGGAAATTCATCTAATG
GTCTAACTTTGGAAATTCATCTAATG
GTCTAACTTTGGAAATTCATCTAATG
GTCTAACTTTGGAAATTCATCTAATG
GTCTAACTTTGGAAATTCATCTAATG
GTCTAACTTTGGAAATTCATCTAATG
GTCTAACTTTGGAAATTCATCTAATG
GTCTAACTTTGGAAATTCATCTAATG
GTCTAACTTTGGAAATTCATCTAATG
GTCTAACTTTGGAAATTCATCTAATG
GTCTAACTTTGGAAATTCATCTAATG
GTCTAACTTTGGAAATTCATCTAATG
GTCTAACTTTGGAAATTCATCTAATG
GTCTAACTTTGGAAATTCATCTAATG
GTCTAACTTTGGAAATTCATCTAATG
GTCTAACTTTGAATTTTTTCTTTAATTCATTAATG
GTCTAACTTTGAAATTCATCTAATG
GTCTAACTTTGGAAATTCATCTAATG
GTCTAACTTTGGAAATTCATCTAATG
GTCTAACTTTGGAAATTCATCTAATG
GTCTAACTTTGGAAATTCATCTAATG
GTCTAACTTTGGAAATTCATCTAATG
GTCTAACTTTGGAAATTCATCTAATG
GTCTAACTTTGGAAATTCATCTAATG
GTCTAACTTTGGAAATTCATCAACTG
GTCTAACTTTGGAAATTCATCTAATG
GTCTAACTTTGGAAATTCATCTAATG
GTCTAACTTTGGAAATTCATCTAATG
GTCTAACTTTGGAAATTCATCTAATG
GTCTAACTTTGGAAATTCATCTAATG
GTCTAACTTTGGAAATTCATCTAATG
GTCTAACTTTGGAAATTCATCTAATG
GTCTAACTTTGGAAATTCATCTAATG
GTCTAACTTTGGAAATTCATCTAATG
GTCTAACTTTGGAAATTCATCTAATG
GTCTAACTTTGGAAATTCATCTAATG
GTCTAACTTTGGAAATTCATCTAATG
GTCTAACTTTTTTTTTTTTTTTTTTTTTTTTGAATTCATCTAATG
GTCTAACTTTGGAAATTCATCTAATG
GTCTAACTTTGGAAATTCATCTAATG
GTCTAACTTTGAAATTCATCTAATG
GTCTAACTTTGGAAATTCATCTAATG
GTCTAACTTTGGAAATTCATCTAATG
GTCTAACTTTGAAAATTCATCTAATG
GTCTAACTTTGGAAATTCATCTAATG
GTCTAACTTTGGAAATTCATCTAATG
GTCTAACTTTGAAAATTCATCTAATG
GTCTAACTTTGGAAATTCATCTAATG
GTCTAACTTTGGGAATTCATCTAATG

Nr of selected repeats 305 Similarity 0.965297
Consensus:
GTCTAACTTTGGAAATTCATCTAATG
>Cele-UNSB01_4:4525521-4525683 Satlength=163 Nr of Repeats=12 RepeatLength=12
seed=GCCTAAGCCT
GCCTAAGCCTAA
GCCTAAGACTAC
GCCTACGCCTAA
GCTTAAGCCTCC
GCCTAAGCCTCC
GCATAAGCCTAAGCCTAAACATAAGTTTAA
GCCTAAGCCTAC
GCTTAAGCCTAC
GCCTAAACCTTA
GCCTAAGGCTAA
GCCTAAGCCTAC
GCCCAAGCCTAA
Nr of selected repeats 11 Similarity 0.751515
Consensus:
GCCTAAGCCTac
>Cele-UNSB01_4:4598852-4599551 Satlength=700 Nr of Repeats=6 RepeatLength=11
seed=GCAGTAGGCA
GCAGTAGGCAG
GCAGTAGGAAG
GCAGTAGGCAA
GCAGTAGGCAA
GCAGTAGGCAG
GCAGTAGGCAAGCGGTGGGCAGTCAGAAGGCAGGCTGTAGGGAAATGCACTTGACGAAAATAGCTTACCCCACAGATCC
ATGCGTAATTTGTGATTTTATCTTGATGTAGTATAATGGCAGTGAGAATCGCAAAAACTGCGGCAAACAACGGAGGAAC
AAATCCAACCCAGAAGAGGAGTGTTGTGTTTAATTGGAATAATTCCTTCAAAAAATTAAATTTTGATCTCTAGATAATT
GAAAAGAAAACTCACAACCGCTTGAGGTCTCTTTTTGTGAACACTCTCGAAACGTTTTTCCATCAACATTTTTGCCTGA
ATCTCTGTTCCTCAAGAGGATGGACAACAAGTAGTAAGAAAGTAGGACAAGCTTAATGATGCCGACGATGAGAAATGAA
AGACGGGTTTGACTTGTTCCATATTGGGGAAAAACAGAGGAGGAGTGGATTCAAAGATTACTACAAATGTTTCCAAATT
GACTCATTCATTTGGTGTAAAAGTTATTGCTAAATGTAGTACGGGGTGTCAACACCCTGATATTATTATAAATGTAAAC
ATTTCACGGGGAAAGAAGCTATAATTTGGGAAATGTGCAGCCGACAAATTCCTGGTTCACAATAGTCATCATCAGAAGC
TGTCGCAGGCAG
Nr of selected repeats 5 Similarity 0.878788
Consensus:
GCAGTAGGcAg
>Cele-UNSB01_4:4620647-4621153 Satlength=507 Nr of Repeats=13 RepeatLength=11
seed=CTACAGTACT
CTACAGTACTC
CTACAGTACTA
CTACAGTACCTTGACATTATCCCCCAGCAACTCCCAACCCAAAACCTCTTCTGTAGACAAAAACTCAATTTTTCCCAGA
CTACAGTAACCCTACCGTATGC
CTACAGTACTC
CTACAGTACTA
CTACAGTACCTTGACATTATCCCCCAGCAACTCCCAACCCAATACCTCTTCTGTAGACAAAAACTCAATTTTTCCCAAA
CTACAGTAACCCTACCGTATAC
CTACAGTTCTC
CTACAATACTA
CTACAGTACCTTGACATTATCCCCCCCACCAACTCCCAACCCAATACCTCTTCTGTAGACAAAACCTCAATTTTCCTAA
ACTACAGTAACCCTACCGTATAC
CTACAGTACTC

CTACAGTACTA
CTACAGTACCTTGACATTATCCCCCCCACCCACTCCCAACCCAATACATCTTCTGTAGACAAAAACCCAATTTTTCCCA
AACTACAGTAACCCTACCGTATAC
CTACAGTACTC
Nr of selected repeats 9 Similarity 0.878788
Consensus:
CTACAGTACTc
>Cele-UNSB01_4:4665165-4665209 Satlength=45 Nr of Repeats=4 RepeatLength=11
seed=TGACAACTTT
TGACAACTTTG
TGACAACTTTG
TGACAACTTTG
TGACAACTTTG
Nr of selected repeats 4 Similarity 1.000000
Consensus:
TGACAACTTTG
>Cele-UNSB01_4:4768182-4768259 Satlength=78 Nr of Repeats=5 RepeatLength=15
seed=CGAAAAGGGG
CGAAAAGGGGAGCAGAA
CGAAAAGGGGATCTG
CAAAAAGGGGATCTG
CGAAAAGGGGAGATA
CGAAAAGGGGAGATA
Nr of selected repeats 4 Similarity 0.777778
Consensus:
CgAAAAGGGGAgaTa
>Cele-UNSB01_4:4768434-4768526 Satlength=93 Nr of Repeats=6 RepeatLength=15
seed=TCCCCTTTTC
TCCCCTTTTCGTTGC
TCCCCTTTTCGTATC
TCCCCTTTTCGTATC
TCCCCTTTTCGCAGA
TCCCCTTTTTGCAGA
TCCCCTTTTCGTTCTGC
Nr of selected repeats 5 Similarity 0.768889
Consensus:
TCCCCTTTTcGtagc
>Cele-UNSB01_4:4858228-4858874 Satlength=647 Nr of Repeats=17 RepeatLength=35
seed=TTGCCGGAAA
TTGCCGGAAATTTTGGTTTCCAGCAAGTTGCCGAA
TTGCCGGAAATTTTGAGTTGCCTAT
TTGCCGGAAATTTTGAGTTGCCTATTTGGCAGAAATTTTGGTTTCCGGCAAGTTTCCGAT
TTGCCGGAAATTTTGGTTTCCGGCAATTTGCCTATTTGCGGGATATTTTGGTTTCCAGCAAGTTGCCGAA
TTGCCGGAAATTTTGGTTTCCGGCAAGTTTCCGAT
TTGCCGGAAATTTAGGTCTCCGGCAAGTTACCTAT
TTGCCGGAAATTTTGGTTTCCGGCAAGTTTCCGAT
TTGCCGGAAATTTTGGTTTCCGGCAATTTGCCGAT
TTGCCGGAAATTTTGGTTTCCGGCAAGTTTCCGAT
TTGCCGGAAATTTTGGTCTCCGGCAAGTTGCCTAT
TTGCCGGAAATTTTGGTTTCCGGCAAGTTTCCGAT
TTGCCGGAAATTTTGGTCTCCGACAAGTTGCCGAT
TTGCCGGAAATTTTGGTTTCCTGCAAGTTTCCGAT
TTGCCGGAAATTTTAGTCTCCGGCAAGTTTCCGAT
TTGCCGGAAATTTTTGGTCTCCGGCAAGTTGCCGAT

TTGCCGGAAATTTTGGTTTCCGGCAAGTTTCCGAT
TTGCCGGAAATTTTGGTTTCCGGCAAGTTTCCGAT
Nr of selected repeats 13 Similarity 0.909646
Consensus:
TTGCCGGAAATTTTGGTtTCCGGCAAGTTtCCGAT
>Cele-UNSB01_4:4963981-4964546 Satlength=566 Nr of Repeats=18 RepeatLength=15
seed=GAGACCCATC
GAGACCCATCGTGGT
GAGACCCGTCGTGGT
GAGATCCATCGTGGC
GAGACCCACCGTGGTGTGACACATCGTGGT
GAGACCCATCGAGGT
GAGACCCATCGTGGT
GAGACCCATCGTGGT
GAGACCCATCGTGGT
GAGACCCATCGTGGTAAGACCCAAAATTTTGGCGGGAAATTTTAATTTTCTGTGAAAAATATTTTGGAGGGAAATTTAA
ATTTTCTGAAAATTCTAAAACTCTGGAAATCTAGAATCTTCTGGAAATTTCGAAAAAATTCTCGAATGTTCCAGAACTT
TCTAGAAAAATCGAGAAAATTCTGGAATGTTCCAGAACCTTCTGGAAAATTCGAAAAAATTCTGGATTGTTCTAGAACC
TGCTGGAAAATTTTAAAAAATGTTGTTCCGAGATCTTCGTGGA
GAGACCCATCGTGGC
GAGACCCATCGTGGT
GAGACCCGTCGTGGT
GAGATCCATCGTGGC
GAGACCCACCGTGGTGTGACACATCGTGGT
GAGACCCATCGAGGT
GAGACCCATCGTGGT
GAGACCCATCGTGGT
GAGACCCATCGTGGT
Nr of selected repeats 15 Similarity 0.903492
Consensus:
GAGACCCATCGTGGt
>Cele-UNSB01_4:4986658-4986858 Satlength=201 Nr of Repeats=5 RepeatLength=40
seed=TTCTAGAAAG
TTCTAGAAAGTTCTGGAACATTCTAGAATTTTCTCGATTT
TTCTAGAAAGTTCTGGAACATTCTAGAATTTTCTCGATTT
TTCTAGAAAGTTCTGGAACATTCCAGAATTTTCCCGATTT
TTCTAGAAAGTTCTGGAACATTCGAGACTTTTCCCAATTT
TTCTAGAAAGTTCTGGAGCATTCCAGAATTTTCCCGATTT
Nr of selected repeats 5 Similarity 0.913333
Consensus:
TTCTAGAAAGTTCTGGAaCATTCnAGAaTTTTCcCgATTT
>Cele-UNSB01_4:4986914-4987437 Satlength=524 Nr of Repeats=15 RepeatLength=35
seed=TTTTCAGAAA
TTTTCAGAAAATTTAAATTTCCCTCCAAAATATTT
TTTTCAGAAAATTAAAATTTCCCTCCAAAATATT
TTTTCAGAAAATTAAAATTTCCCTCCAAAATATTT
TTTTCAGAAAATTAAAATTTCCCTCCAAAATATTT
TTTTCAGAAAATTTAAATTTCCCTCCAAAATATTT
TTTTCAGAAAATTAAAATTTCCCTCCAAAATATTT
TTTTCAGAAAATTAAAATTTCCCTCCAAAATATTT
TTTTCAGAAAATTTAAATTTCCCTCCAAAATATTT
TTTTCAGAAAATTTAAATTTCCCTCCAAAATATTT
TTTTCAGAAAATTAAAATTTCCCTCCAAAATATTT
TTTTCAGAAAATTAAAATTTCCCTCCAAAATATTT

TTTTCAGAAAATTAAAATTTCCCTCCAAAATATTT
TTTTCAGAAAATTTAAATTTCTCTCCAAAATATTT
TTTTCAGAAAATTTAAATTTCCCTCCAAAATATTT
TTTTCAGAAAATTTAAATTTCCCTCCAAAATATT
Nr of selected repeats 13 Similarity 0.973626
Consensus:
TTTTCAGAAAATTaAAATTTCCCTCCAAAATATTT
>Cele-UNSB01_4:4987504-4988044 Satlength=541 Nr of Repeats=32 RepeatLength=15
seed=GGGTCTCACC
GGGTCTCACCACGAC
GGGTCTCACCACGAT
GGGTCTCACCTCGAT
GGGTCTCACCACGAT
GGGTCTCACCACGAT
GGGTCTCACCACGAT
GGGTCTCACCACGAT
GGGTCTCACCACGGT
GGGTCTCACCACGATGGGCCTCGCCGCGAT
GGGTCTCACCACGGT
GGGTCTCACCACGAT
GGGTCTCACCACGAT
GGGTCTCACCACGGT
GGGTCTCACCACGATGGGCCTCGCCGCGAT
GGGTCTCACCACGGT
GGGTCTCACCACGAT
GGGTCTCACCACGAT
GGGTCTCACCACGAT
GGGTCTCACCACGATGGGCCTCGCCGCGAT
GGGTCTCACCACGGT
GGGTCTCACCACGAT
GGGTCTCACCACGAT
GGGTCTCACCACGAT
GGGTCTCACCACGAT
GGGTCTCGCCACGAT
GGGTCTCACCACGGT
GGGTCTCACCACGATGGGCCTCGCCGCGAT
GGGTCTCACCACGGT
GGGTCTCACCTCGAT
GGGTCTCACCACGAT
GGGTCTCACCACGAT
GGGTCTCACCACGAT
Nr of selected repeats 28 Similarity 0.940506
Consensus:
GGGTCTCACCACGaT
>Cele-UNSB01_4:4988071-4988281 Satlength=211 Nr of Repeats=14 RepeatLength=15
seed=ACCCATCGTG
ACCCATCGTGGTGAG
ACCCATCGTGGCGAG
ACCCATCGTGGTGAG
ACCCATCGTGGTGAG
ACCCATCGTGGCGAG
ACCCATCGTGGTGAG
ACCCATCGTGGTGAG
ACCCATCGTGGAAAT

ACCCATCGTGGTGAG
ACCCACCGTGGTGAG
ACCCATCGTGGTGAG
ACCCATCGTGGTGAG
ACCCATCGTGGTGAG
ACCCACCGTGGTGAG
Nr of selected repeats 14 Similarity 0.916972
Consensus:
ACCCATCGTGGtGAG
>Cele-UNSB01_4:4987504-4989222 Satlength=1719 Nr of Repeats=80 RepeatLength=15
seed=GGGTCTCACC
GGGTCTCACCACGAC
GGGTCTCACCACGAT
GGGTCTCACCTCGAT
GGGTCTCACCACGAT
GGGTCTCACCACGAT
GGGTCTCACCACGAT
GGGTCTCACCACGAT
GGGTCTCACCACGGT
GGGTCTCACCACGATGGGCCTCGCCGCGAT
GGGTCTCACCACGGT
GGGTCTCACCACGAT
GGGTCTCACCACGAT
GGGTCTCACCACGGT
GGGTCTCACCACGATGGGCCTCGCCGCGAT
GGGTCTCACCACGGT
GGGTCTCACCACGAT
GGGTCTCACCACGAT
GGGTCTCACCACGAT
GGGTCTCACCACGATGGGCCTCGCCGCGAT
GGGTCTCACCACGGT
GGGTCTCACCACGAT
GGGTCTCACCACGAT
GGGTCTCACCACGAT
GGGTCTCACCACGAT
GGGTCTCGCCACGAT
GGGTCTCACCACGGT
GGGTCTCACCACGATGGGCCTCGCCGCGAT
GGGTCTCACCACGGT
GGGTCTCACCTCGAT
GGGTCTCACCACGAT
GGGTCTCACCACGAT
GGGTCTCACCACGAT
GGGTCTCACCACGATGGGCGTGGAAAGACCCATCGTGGTGAGACCCATCGTGGCGAGACCCATCGTGGTGAGACCCATC
GTGGTGAGACCCATCGTGGCGAGACCCATCGTGGTGAGACCCATCGTGGTGAGACCCATCGTGGAAATACCCATCGTGG
TGAGACCCACCGTGGTGAGACCCATCGTGGTGAGACCCATCGTGGTGAGACCCATCGTGGTGAGACCCACCGTGGTGAG
ACCCATCGTGGTGACAGAAAATTTAAATTTCCCTCCAAAATATTTTTTTCAGAAAATTTAAATTTCCCTCCAAAATATT
TTTTCAGAAAATTTAAATTTCCCGCCAAAATATTTTTCACAGAAAATTTAAATTTCCCGCCAAAATT
GGGTCTCACCACGAC
GGGTCTCACCACGAT
GGGTCTCACCTCGAT
GGGTCTCACCACGAT
GGGTCTCACCACGAT
GGGTCTCACCACGAT

GGGTCTCACCACGAT
GGGTCTCACCACGGT
GGGTCTCACCACGATGGGCCTCGCCGCGAT
GGGTCTCACCACGGT
GGGTCTCACCACGAT
GGGTCTCACCACGAT
GGGTCTCACCACGGT
GGGTCTCACCACGATGGGCCTCGCCGCGAT
GGGTCTCACCACGGT
GGGTCTCACCACGAT
GGGTCTCACCACGAT
GGGTCTCACCACGAT
GGGTCTCACCACGATGGGCCTCGCCGCGAT
GGGTCTCACCACGGT
GGGTCTCACCACGAT
GGGTCTCACCACGAT
GGGTCTCACCACGAT
GGGTCTCACCACGAT
GGGTCTCGCCACGAT
GGGTCTCACCACGGT
GGGTCTCACCACGATGGGCCTCGCCGCGAT
GGGTCTCACCACGGT
GGGTCTCACCTCGAT
GGGTCTCACCACGAT
GGGTCTCACCACGAT
GGGTCTCACCACGAT
GGGTCTCACCACGAT
GGGTCTCACCACGAT
GGGTCTCACCACGGT
GGGTCTCACCACGAT
GGGTCTCACCACGAT
GGGTCTCACCACGAT
GGGTCTCACCACGGT
GGGTCTCACCACGATGGGTATTTCCACGAT
GGGTCTCACCACGAT
GGGTCTCACCACGAT
GGGTCTCGCCACGAT
GGGTCTCACCACGAT
GGGTCTCACCACGAT
GGGTCTCGCCACGAT
GGGTCTCACCACGATGGGTCTTTCCACGAT
Nr of selected repeats 69 Similarity 0.943090
Consensus:
GGGTCTCACCACGaT
>Cele-UNSB01_4:4990413-4990623 Satlength=211 Nr of Repeats=6 RepeatLength=35
seed=AATATTTTTT
AATATTTTTTTCAGAAAATTAAAATTTCCCTCCAA
AATATTTTTTTCAGAAAATTAAAATTTCCCTCCAA
AATATTTTTTTCAGAAAATTAAAATTTCCCTCCAA
AATATTTTTTTCAGAAAATTTAAATTTCTCTCCAA
AATATTTTTTTCAGAAAATTTAAATTTCCCTCCAA
AATATTTTTTTCAGAAAATTTAAATTTCCCTCCAA
Nr of selected repeats 6 Similarity 0.964444
Consensus:

AATATTTTTTTCAGAAAATTaAAATTTCCCTCCAA
>Cele-UNSB01_4:4990696-4991348 Satlength=653 Nr of Repeats=40 RepeatLength=15
seed=GGGTCTCACC
GGGTCTCACCACGAC
GGGTCTCACCACGAT
GGGTCTCACCTCGAT
GGGTCTCACCACGAT
GGGTCTCACCACGAT
GGGTCTCACCACGAT
GGGTCTCACCACGAT
GGGTCTCACCACGGT
GGGTCTCACCACGATGGGCTCGCCGAT
GGGTCTCACCACGGT
GGGTCTCACCACGAT
GGGTCTCACCACGAT
GGGTCTCACCACGGT
GGGTCTCACCACGAT
GGGTCTCACCACGAT
GGGTCTCACCACGAT
GGGTCTCACCACGAT
GGGTCTCCCACGAT
GGGTCTCACCACGT
GGTCTCACCACGATGGGCCTCGCCCGAT
GGGTCTCACCACGGT
GGGTCTCACCCGAT
GGGTCTCACCACGAT
GGGTCTCACCACGAT
GGGTCTCACCACGAT
GGGTCTCACCACGAT
GGGTCTCACCACGAT
GGGTCTCACCACGGT
GGGTCTCACCACGAT
GGGTCTCACCACGAT
GGGTCTCACCACGAT
GGGTCTCACCACGGT
GGGTCTCACCACGATGGGTATTTCCACGAT
GGGTCTCACCACGAT
GGGTCTCACCACGAT
GGGTCTCGCCACGAT
GGGTCTCACCACGAT
GGGTCTCACCACGAT
GGGTCTCGCCACGAT
GGGTCTCACCACGATGGGTCTTTCCACGAT
Nr of selected repeats 33 Similarity 0.951515
Consensus:
GGGTCTCACCACGAT
>Cele-UNSB01_4:5061085-5061475 Satlength=391 Nr of Repeats=8 RepeatLength=39
seed=CCAACTGGAG
CCAACTGGAGCCAGCACTATGACTGCTGTCGGAGGAGCA
CCAACTGGAGCCAGCCCTATGACGGCCTTTGGAGGAGCA
CCAACTGGAGCCAGCACAATGACCGCTGTCGGCGGAGCA
CCAACTGGAGCCAGCACTATGACGGCCGTTGGAGGAGCA
CCAACTGGAGCGAGCACAATGACCGCTGTTGGTGGAGCA
CCAACTGGAGCAAGCACCATGACCGCCGTCGGAGGAGCC

CCAACCGGAGCAAGTACAATGACCGCTGTTGGTGGAGTC
CCAACTGGAGCGTCTACAATGACTGCTATCGGAGGAGCCCCATCTGGTGCTTCCACTATGACCGCTGTGGGAGGTGCTC
CGAGAGGAGCTTCAACCATGACTGCTGTCGGTGGAGCA
Nr of selected repeats 7 Similarity 0.784921
Consensus:
CCAACTGGAGCcAGCACnATGACcGCtGTtGGaGGAGCa
>Cele-UNSB01_4:5124505-5124705 Satlength=201 Nr of Repeats=12 RepeatLength=16
seed=CTACGTGCCT
CTACGTGCCTACGTGC
CTACGTGTCTACGTGC
CTACGTGCCTACGTGC
CTACGTGCCTACGTGC
CTACGTGCCTACGTGC
CTACGTGCCTACGTGC
CTACGTGCCTACGTGC
CTACGTGCCTACGTGC
CTACGTGCCTACGTTC
CTACGTGCCTACGTGC
CTACGTGCCTACGTGC
CTACGTTCCTACGTGCCTACGTGC
Nr of selected repeats 11 Similarity 0.969697
Consensus:
CTACGTGCCTACGTGC
>Cele-UNSB01_4:5337721-5338093 Satlength=373 Nr of Repeats=18 RepeatLength=12
seed=AGAAGGAGGA
AGAAGGAGGAGA
AGAAAGAGGAGA
AGAAGGAGGAGAAGGAGGATAA
AAAGGAGGAGA
AGAAGGAGGAAA
AGAAGGAAGACGATAAGAAAGATGAGAAAAAGGATGAGAAAAAGGATGAAAAGGAAGAAGATAAGAAGAGTGAAGATA
AGAAGGAGGATGAAAAAGAAAAAGATGACGATA
AGAAGGAAGACA
AGAAGGATGATAAGAAGAGTGATAATAAGGATGATAAAGATGATGAGAAGAAAGAAGAGAAGAAAGATGATAAAGAGGA
AA
AGAAGGAGGAAA
AGAAGGAGGAAA
AGAAGGAGGAAA
AGAAGGAGGAAA
AGAAGGAGGAAA
AGAAGGAGGAGGAAA
AGAAGGAAGAGA
AGAAAGAGGAAA
Nr of selected repeats 11 Similarity 0.864646
Consensus:
AGAAGGAGGAaA
>Cele-UNSB01_4:5363528-5363903 Satlength=376 Nr of Repeats=15 RepeatLength=25
seed=TTTACTCTCT
TTTACTCTCTGTCTTCACAGTATAT
TTTACTCTCTTGCTTCACAGAATAA
TTTACTCTCTGGCTTCACAGAATAT
TTTACTCACTGGCTTCACAGAATAT
TTTACTCTCTGGCTTCACAGTATAT
TTTACTCTCTGGCTTCAGAGTATAT

TTTACTCTCTGGCTTCACAGAATAA
TTTACTCTCTGGCTTCACAGTATAT
TTTACTCTCTGGCTTCACAGTACAT
TTTACTCTCTGGCTTCACAGAATAA
TTTACTCTCTGGCTTCACAATATAT
TTTACTCTCTGGCTTCACAGAATAA
TTTACTCTCTGGCTTCACAGAATAA
TTTACTCTCTGGATTCACAGTATAT
TTTACTCTCTGGCTTCACAGTACAT
Nr of selected repeats 15 Similarity 0.890286
Consensus:
TTTACTCTCTGGCTTCACAGtATAt
>Cele-UNSB01_4:5366596-5366788 Satlength=193 Nr of Repeats=10 RepeatLength=12
seed=TCCTCTTTCT
TCCTCTTTCTTTTCATCCTTCTTTTCTTCCTTCTTTTCTTCCTTATTCTCATCGTTTTTC
TCCTCTTTCTTC
TCCTCTTTCTTC
TCCTCTTTTTTC
TCCTCTTTCTTC
TCCTCTTTCTTC
TCTTCTTTCTTC
TCTTCTTTCTTTGCTTCCTTTTTGTCATCCTTTTTG
TCTTCTTTCTTT
TCCTCTTTCTTC
Nr of selected repeats 8 Similarity 0.896825
Consensus:
TCcTCTTTCTTC
>Cele-UNSB01_4:5521070-5521205 Satlength=136 Nr of Repeats=9 RepeatLength=15
seed=CCACGATGGG
CCACGATGGGTCTCG
CCACGATGGGTCTCG
CCACGATGGGTCTCAA
CACGATGGGTCTCA
CCACGATGGGTCTCA
CCACGATGGGCCTCG
CCACGATGAGTCTCA
CCACGATGGGTCTCA
CCACGATGGGTCTCA
Nr of selected repeats 7 Similarity 0.898413
Consensus:
CCACGATGGGTCTCa
>Cele-UNSB01_4:5529418-5534698 Satlength=5281 Nr of Repeats=110 RepeatLength=48
seed=TTGCCCGTAA
TTGCCCGTAATTTATATTTTTCTGAAGATGAGCAATTCAATTTTTGAT
TTGCCTGTAATTTATATTTTTCTGAAGATGAGCAATTCAATTTTTGAT
TTGCCCGTAATTTATATTTTTCTGAAGATGAACAATTCAATTTTTGAT
TTGCCCGTAATTTATATTTTTCTGAAGATGAACAATTCAATTTTTGAT
TTGCCCGTAATTTATATTTTTCTGAAGATGAACAATTCAATTTTTGAT
TTGCCCGTAATTTATATTTTTCTGAAGATGAGCAATTCAATTTTTAAA
TTGCCCGTAATTTACATTTTTCTGAAGATGAGCAATTCAATTTTTGAT
TTGCCCGTAATTTATATTTTTCTGAAGATGAGCAATTCAATTTTTGAT
TTGCCCGTAATTTATATTTTTCTGAAGATGAGCAATTCAATTTTTAAA
TTGCCCGTAATTTACATTTTTCTGAAGATGAGCAATTCAATTTTTGAT
TTGCCCGTAATTTATATTTTTCTGAAGATGAACAATTCAATTTTTGAT

TTGCCCGTAATTTATATTTTTCTGAAGATGAGCAATTCAATTTTTGAT
TTGCCCGTAATTTATATTTTTCTGAAGATGAGCAATTCAATTTTTAAA
TTGCCCGTAATTTACATTTTTCTGAAGATGAGCAATTCAATTTTTGAT
TTGCCCGTAATTTATATTTTTCTGAAGATGAGCAATTCAATTTTTGAT
TTGCCCGTAATTTATATTTTTCTGAAGATGAACAATTCAATTTTTGAT
TTGCCCGTAATTTATATTTTTCTGAAGATGAGCAATTCAATTTTTGAT
TTGCCCGTAATTTATATTTTTCTGAAGATGAACAATTCAATTTTTGAT
TTGCCCGTAATTTATATTTTTCTGAAGATGAGCAATTCAATTTTTGAT
TTGCCCGTAATTTATATTTTTCTGAAGATGAGCAATTCAATTTTTGAT
TTGCCCGTAATTTATATTTTTCTGAAGATGAGCAATTCAATTTTTAAA
TTGCCCGTAATTTATATTTTTCTGAAGATGAACAATTCAATTTTTGAT
TTGCCCGTAATTTATATTTTTCTGAAGATGAACAATTCAATTTTTGAT
TTGCCCGTAATTTATATTTTTCTGAAGATGAGCAATTCAATTTTTGAT
TTGCCCGTAATTTATATTTTTCTGAAGATGAGCAATTCAATTTTTGAT
TTGCCCGTAATTTATATTTTTCTGAAGATGAGCAATTCAATTTTTAAA
TTGCCCGTAATTTATATTTTTCTGAAGATGAACAATTCAATTTTTGAT
TTGCCCGTAATTTATATTTTTCTGAAGATGAGCAATTCAATTTTTGAT
TTGCCCGTAATTTATATTTTTCTGAAGATGAACAATTCAATTTTTGAT
TTGCCCGTAATTTATATTTTTCTGAAGATGAGCAATTCAATTTTTGAT
TTGCCCGTAATTTATATTTTTCTGAAGATGAACAATTCAATTTTTGAT
TTGCCCGTAATTTATATTTTTCTGAAGATGAGCAATTCAATTTTTGAT
TTGCCCGTAATTTATATTTTTCTGAAGATGAGCAATTCAATTTTTGAT
TTGCCCGTAATTTATATTTTTCTGAAGATGAGCAATTCAATTTTTAAA
TTGCCCGTAATTTATATTTTTCTGAAGATGAACAATTCAATTTTTGAT
TTGCCCGTAATTTATATTTTTCTGAAGATGAACAATTCAATTTTTGAT
TTGCCCGTAATTTATATTTTTCTGAAGATGAGCAATTCAATTTTTGAT
TTGCCCGTAATTTATATTTTTCTGAAGATGAGCAATTCAATTTTTGAT
TTGCCCGTAATTTATATTTTTCTGAAGATGAGCAATTCAATTTTTGAT
TTGCCCGTAATTTATATTTTTCTGAAGATGAACAATTCAATTTTTGAT
TTGCCCGTAATTTATATTTTTCTGAAGATGAGCAATTCAATTTTTGAT
TTGCCCGTAATTTATATTTTTCTGAAGATGAGCAATTCAATTTTTAAA
TTGCCCGTAATTTATATTTTTCTGAAGATGAACAATTCAATTTTTGAT
TTGCCCGTAATTTATATTTTTCTGAAGATGAACAATTCAATTTTTGAT
TTGCCCGTAATTTATATTTTTCTGAAGATGAGCAATTCAATTTTTGAT
TTGCCCGTAATTTATATTTTTCTGAAGATGAGCAATTCAATTTTTGAT
TTGCCCGTAATTTATATTTTTCTGAAGATGAGCAATTCAATTTTTGAT
TTGCCCGTAATTTATATTTTTCTGAAGATGAACAATTCAATTTTTGAT
TTGCCCGTAATTTATATTTTTCTGAAGATGAACAATTCAATTTTTGAT
TTGCCCGTAATTTATATTTTTCTGAAGATGAGCAATTCAATTTTTGAT
TTGCCCGTAATTTATATTTTTCTGAAGATGAACAATTCAATTTTTGAT
TTGCCCGTAATTTATATTTTTCTGAAGATGAGCAATTCAATTTTTAAA
TTGCCCGTAATTTACATTTTTCTGAAGATGAGCAATTCAATTTTTGAT
TTGCCCGTAATTTATATTTTTCTGAAGATGAGCAATTCAATTTTTGAT
TTGCCCGTAATTTATATTTTTCTGAAGATGAGCAATTCAATTTTTGAT
TTGCCCGTAATTTATATTTTTCTGAAGATGAGCAATTCAATTTTTAAA
TTGCCCGTAATTTACATTTTTCTGAAGATGAGCAATTCAATTTTTGAT
TTGCCCGTAATTTATATTTTTCTGAAGATGAACAATTCAATTTTTGAT
TTGCCCGTAATTTATATTTTTCTGAAGATGAGCAATTCAATTTTTGAT
TTGCCCGTAATTTATATTTTTCTGAAGATGAGCAATTCAATTTTTGAT
TTGCCCGTAATTTATATTTTTCTGAAGATGAGCAATTCAATTTTTGAT
TTGCCCGTAATTTATATTTTTCTGAAGATGAACAATTCAATTTTTGAT
TTGCCCGTAATTTATATTTTTCTGAAGATGAGCAATTCAATTTTTGAT
TTGCCCGTAATTTATATTTTTCTGAAGATGAAAAATTCAATTTTTGAT
TTGCCCGTAATTTATATTTTTCTGAAGATGAGCAATTCAATTTTTGAT

TTGCCCGTAATTTATATTTTTCTGAAGATGAGCAATTCAATTTTTGAT
TTGCCCGTAATTTATATTTTTCTGAAGATGAGCAATTCAATTTTTGAT
TTGCCCGTAATTTATATTTTTCTGAAGATGAGCAATTCAATTTTTGAT
TTGCCCGTAATTTATATTTTTCTGAAGATGAACAATTCAATTTTTGAT
TTGCCCGTAATTTATATTTTTCTGAAGATGAGCAATTCAATTTTTGAT
TTGCCCGTAATTTATATTTTTCTGAAGATGAACAATTCAATTTTTGAT
TTGCCCGTAATTTATATTTTTCTGAAGATGAACAATTCAATTTTTGAT
TTGCCCGTAATTTATATTTTTCTGAAGATGAGCAATTCAATTTTTGAT
TTGCCCGTAATTTATATTTTTCTGAAGATGAGCAATTCAATTTTTGAT
TTGCCCGTAATTTATATTTTTCTGAAGATGAGCAATTCAATTTTTAAA
TTGCCCGTAATTTAAATTTTTCTGAAGATGAGCAATTCAATTTTTAAA
TTGCCCGTAATTTACATTTTTCTGAAGATGAGCAATTCAATTTTTAAA
TTGCCCGTAATTTACATTTTTCTGAAGATGAGCAATTCAATTTTTGAT
TTGCCCGTAATTTATATTTTTCTGAAGATGAGCAATTCAATTTTTAAA
TTGCCCGTAATTTACATTTTTCTGAAGATGAGCAATTCAATTTTTGAT
TTGCCCGTAATTTATATTTTTCTGAAGATGAGCAATTCAATTTTTAAA
TTGCCCGTAATTTACATTTTTCTGAAGATGAGCAATTCAATTTTTAAA
TTGCCCGTAATTTACATTTTTCTGAAGATGAGCAATTCAATTTTTAAA
TTGCCCGTAATTTACATTTTTCTGAAGATGAGCAATTCAATTTTTGAT
TTGCCCGTAATTTATATTTTTCTGAAGATGAGCAATTCAATTTTTAAA
TTGCCCGTAATTTACATTTTTCTGAAGATGAGCAATTCAATTTTTGAT
TTGCCCGTAATTTATATTTTTCTGAAGATGAACAATTCAATTTTTGAT
TTGCCCGTAATTTATATTTTTCTGAAGATGAACAATTCAATTTTTGAT
TTGCCCGTAATTTATATTTTTCTGAAGATGAACAATTCAATTTTTGAT
TTGCCCGTAATTTATATTTTTCTGAAGATGAGCAATTCAATTTTTGAT
TTGCCCGTAATTTATATTTTTCTGAAGATGAGCAATTCAATTTTTGAT
TTGCCCGTAATTTATATTTTTCTGAAGATGAGCAATTCAATTTTTGAT
TTGCCCGTAATTTATATTTTTCTGAAGATGAGCAATTCAATTTTTGAT
TTGCCCGTAATTTATATTTTTCTGAAGATGAGCAATTCAATGTTTGAT
TTGCCCGTAATTTATATTTTTCTGAAGATGAGCAATTCAATTTTTGAT
TTGCCCGTAATTTATATTTTTCTGAAGATGAGCAATTCAATTTTTGAT
TTGCCCGTAATTTATATTTTTCTGAAGATGAGCAATTCAATTTTTGAT
TTGCCCGTAATTTATATTTTTCTGAAGATGAGCAATTCAATTTTTGAT
TTGCCCGTAATTTATATTTTTCTGAAGATGAGCAATTCAATTTTTGAT
TTGCCCGTAATTTATATTTTTCTGAAGATGAGCAATTCAATTTTTGAT
TTGCCCGTAATTTATATTTTTCTGAAGATGAGCAATTCAATTTTTGAT
TTGCCCGTAATTTATATTTTTCTGAAGATGAGCAATTCAATTTTTAAA
TTGCCCGTAATTTACATTTTTCTGAAGATGAACAATTCAATTTTTGAT
TTGCCCGTAATTTATATTTTTCTGAAGATGAGCAATTCAATTTTTGAT
TTGCCCGTAATTTATATTTTTCTGAAGATGAACAATTCAATTTTTGAT
TTGCCCGTAATTTATATTTTTCTGGAGATGAGCAATTCAATTTTTGAT
TTGCCCGTAATTTATATTTTTCTGAAGATGAGCAATTCAATTTTTGAT
TTGCCCGTAATTTATATTTTTCTGAAGATGAGCAATTCAATTTTTGAT
TTGCCCGTAATTTATATTTTTCTGAAGATGAGCAATTCAATTTTTAAA
TTGCCCGTAATTTACATTTTTCTGAAGATGAGCAATTCAATTTTTGAG
Nr of selected repeats 110 Similarity 0.963752
Consensus:
TTGCCCGTAATTTATATTTTTCTGAAGATGAgCAATTCAATTTTTGAT
>Cele-UNSB01_4:5758139-5758194 Satlength=56 Nr of Repeats=5 RepeatLength=11
seed=TCCACTGACC
TCCACTGACCG
TCCACTGACCA
TCCACTGACCA
TCCACTGACCA

TCCACTGACCA
Nr of selected repeats 5 Similarity 0.951515
Consensus:
TCCACTGACCa
>Cele-UNSB01_4:5945791-5945941 Satlength=151 Nr of Repeats=6 RepeatLength=25
seed=CCAGAGAGTA
CCAGAGAGTAAAGTATACTGGGAAG
CCAGAGAGTAAAATATACTGGGAAG
CCAGAGAGTAAAATATACTGGGAAA
CCAGAGAGTAAAATATAGTGGGAAA
CCAGAGAGTAAAAAATAGTGGGATA
CCAGAGAGTAAAATATAGTGGGATA
Nr of selected repeats 6 Similarity 0.875556
Consensus:
CCAGAGAGTAAAATATAcTGGGAaa
>Cele-UNSB01_4:6076861-6077186 Satlength=326 Nr of Repeats=6 RepeatLength=55
seed=AAGAGTCCCC
AAGAGTCCCCGTACAAATGAAAGGTTTTAAGGATTGGATACCTAGGGTT
AAGAGTCCCCACACACTGAAAGGTTTTAAGGAATTTTTTGAGGATTCCTAGGGTT
AAGAGTCCCCACACACTGAAAGGTTTTAAGGAATTTTTTGAGGATTCCTAGGGTT
AAGAGTCCCCACACACTGAAAGGTTTTAAGGAATTTTTTGAGGATTCCTAGGGTT
AAGAGTCCCCACACACTGAAAGGTTTTAAGGAATTTTTTGAGGATTCCTAGGGTT
AAGAGTCCCCACACACTGAAAGGTTTTAAGGAATTTTTTTGAGGATTCCTAGGGTT
Nr of selected repeats 4 Similarity 1.000000
Consensus:
AAGAGTCCCCACACACTGAAAGGTTTTAAGGAATTTTTTGAGGATTCCTAGGGTT
>Cele-UNSB01_4:6158202-6158352 Satlength=151 Nr of Repeats=6 RepeatLength=25
seed=TTTACTCTCT
TTTACTCTCTGGCTTCACAGAATAA
TTTACTCTCTGGCTTCACAGTATAT
TTAACTCTCTGGCTTCACAGTATAA
TTTACTCTCTGGTTTTACAGTATAT
TTTACTCTCTGGCTTCACAGAATAA
TTTACTCTCTGGCTTCACAGAATAT
Nr of selected repeats 6 Similarity 0.882667
Consensus:
TTTACTCTCTGGCTTCACAGaATAa
>Cele-UNSB01_4:6277867-6278002 Satlength=136 Nr of Repeats=7 RepeatLength=19
seed=ATTGACAGAC
ATTGACAGACGAAAAAATAG
ATTGACAGACGAAAAATAG
ATTGACAGACGAAAAATAG
ATTGACAGACGAAAAATAG
ATTGACAGACGAAAAAATAG
ATTGACAGACGAAAAATAT
ATTGACAGACGAAAAATAG
Nr of selected repeats 5 Similarity 0.971930
Consensus:
ATTGACAGACGAAAAATAg
>Cele-UNSB01_4:6359332-6359386 Satlength=55 Nr of Repeats=4 RepeatLength=13
seed=TTTCATTTGT
TTTCATTTGTTATTG
TTTCATTTGTAAT
TTTCATTTGTAGT

TTTCATTTGTAAT
Nr of selected repeats 3 Similarity 0.931624
Consensus:
TTTCATTTGTAaT
>Cele-UNSB01_4:6425603-6430447 Satlength=4845 Nr of Repeats=192 RepeatLength=25
seed=TTAGAGTAAA
TTAGAGTAAATAAATTGGCAGAACA
TTAGAGTAAATAAATTAGCAGAACA
TTAGAGTAAATAAATTAGCAGAACA
TTAGAGTAAATAAATTAGCAGAACA
TTAGAGTAAATAAATTAGCAGAACA
TTAGAGTAAATAAATTAGCAGAACA
TTAGAGTAAATAAATTAGCAGAACA
TTAGAGTAAATAAATTAGCAGAACA
TTAGAGTAAATAAATTAGCAGAACA
TTAGAGTAAATAAATTAGCAGAACA
TTAGAATAAATAAATTAGGAGAACA
TTAGAGTAAATAAATTAGCAGAACA
TTAGAGTAAATAAATTAGCAGAACA
TTAGAGTAAATGTATTGGCAGAACA
TTAGAGTAAATATATTGGCAGAACA
TTAGAGTAAATAAATTAGCAGAACA
TTAGAATAAATAAATTAGGAGAACA
TTAGAGTAAATAAATTGGCAGAACA
TTAGAGTAAATATATTGGCAGAACA
TTAGAGTAAATATATTGGCAGAACA
TTAGAGTAAATAAATTAGCAGGATG
TTAGAGTAAATAAATTAGCAGAACA
TTAGAATAAATAAATTAGGAGAACA
TTAGAGTCAATAAATTAGCAGAACA
TTAGAGTAAATAAATTGGCAGAACA
TTAGAGTAAATAAATTGGCAGAACA
TTAGAGTAAATAAATTAGCAGAACA
TTAGAGTAAATAAATTAGCAGAACA
TTAGAGTAAATAAATTAGCAGAACA
TTAGAGTAAATAAATTAGCAGAACA
TTAGAGTAAATAAATTAGCAGAACA
TTAGAGTAAATAAATTAGCAGAACA
TTAGAGTAAATAAATTAGCAGAACA
TTAGAGTAAATAAATTAGCAGAACA
TTAGAGTAAATAAATTAGGAGAACA
TTAGAGTAAATAAATTAGCAGAACA
TTAGAGTAAATAAATTAGCAGAACA
TTAGAGTAAATGTATTGGCAGAACA
TTAGAGTAAATATATTGGCAGAACA
TTAGAGTAAATAAATTAGCAGAACA
TTAGAATAAATAAATTAGGAGAACA
TTAGAGTAAATAAATTGGCAGAACA
TTAGAGTAAATATATTGGCAGAACA
TTAGAGTAAATATATTGGCAGAACA
TTAGAGTAAATAAATTAGCAGGATG
TTAGAGTAAATAAATTAGCAGAACA
TTAGAATAAATAAATTAGGAGAACA
TTAGAGTAAATATATTGGCAGAACA

TTAGAGTAAATAAATTAGCAGAACA
TTAGAATAAATAAATTAGGAGAACA
TTAGAGTAAATAAATTGGCAGAACA
TTAGAGTAAATATATTGGCAGAACA
TTAGAGTAAATATATTGGCAGAACA
TTAGAGTAAATAAATTAGCAGGATG
TTAGAGTAAATAAATTAGCAGAACA
TTAGAGTAAATAAATTAGGAGAACA
TTAGAGTAAATAAATTAGCAGAACA
TTAGAGTAAATAAATTAGCAGAACA
TTAGAGTAAATAAATTAGCAGAACA
TTAGAGTAAATATATTGGCAGAACA
TTAGAGTCAATAAATTAGCAGAACA
TTAGAATAAATAAATTAGCAGAACA
TTAGAATAAATAAATTAGGAGAACA
TTAGAGTAAATAAATTAGCAGTACA
TTAGAGTAAATAAATTAGCAGAACA
TTAGAGTAAATAAATTAGCAGAACA
TTAGAGTAAATATATTGGCAGAACA
TTAGAGTCAATAAATTAGCAGAACA
TTAGAATAAATAAATTAGCAGAACA
TTAGAGTAAATAAATTAGCAGAACA
TTAGAGTAAATAAATTAGGAGAACA
TTAGAGTAAATAAATTAGCAGTACA
TTAGAGTAAATAAATTAGCAGAACA
TTAGAGTAAATAAATTAGCAGAACA
TTAGAGTAAATAAATTAGCAGTACA
TTAGAGTAAATAAATTAGGCAGAACA
TTAGAGTAAATAAATTAGCAGAACA
TTAGAGTAAATAAATTAGCAGAACA
TTAGAGTAAATAAATTAGCAGAACA
TTAGAGTAAATAAATTAGCAGTACA
TTAGAGTAAATAAATTAGCAGAACA
TTAGAGTAAATAAATTAGCAGAACA
TTAGAGTAAATAAATTAGCAGAACA
TTAGAGTAAATAAATTAGCAGTACA
TTAGAGTAAATAAATTAGGAGAACA
TTAGAGTAAATAAATTAGCAGAACA
TTAGAGTAAATAAATTAGCAGAACA
TTAGAGTAAATAAATTAGCAGAACA
TTAGAGTAAATAAATTGGCAGAACA
TTAGAGTAAATAAATTAGGCAGAACA
TTAGAGTAAATAAATTAGGCAGAACA
TTAGAGTAAATAAATTAGCAGAACA
TTAGAGTAAATAAATTAGCAGAACA
TTAGAGTAAATAAATTAGCAGAACA
TTAGAATAAATAAATTAGAGAACA
TTAGAGTAAATAAATTAGCAGAACA
TTAGAGTAAATAAATTAGCAGAACA
TTAGAGTAAATAAATTAGCAGAACA
TTAGAGTAAATAAATTAGCAGACA
TTAGAGTAAATAAATTAGCAGAACA
TTAGAGTAAATAAATTGCAGAACA
TTAGAGTAAATAAATTAGGCAGAACA

TTAGAGTAAATAAATTAGCAGAACA
TTAGAGTAAATAAATTAGCAGAACA
TTAGAGTAAATAAATTAGCAGAACA
TTAGAGTAAATAAATTAGCAGAACA
TTAGAGTAAATAAATTAGCAGAACA
TTAGAGTAAATAATTAGCAGAAA
TTAGAGTAAATAAATTAGGAGAACA
TTAGAGTAAATAAATTAGCAGTACA
TTAGAGTAAATAAATTAGCAGAACA
TTAGAGTAAATAAATTAGCAGAACA
TTAGAGTAAATAATTGCAGAATA
TTAGAGTAAATAAATTAGAAGAACA
TTAGAGTAAATAAATTAGCAGTACA
TTAGAGTAAATAAATTAGCAGAACA
TTAGAGTAAATAAATTAGCAGAACA
TTAGAGTAAATAAATTAGCAGAACA
TTAGAGTAAATAAATTAGCAGAACA
TTAGAGTAAATAAATTAGCAGAACA
TTAGAGTAAATAAATTAGCAGAACA
TTAGAGTAAATAAATTAGGCAGAACA
TTAGAGTAAATAAATTAGCAGAACA
TTAGAGTAAATAAATTAGCAGAACA
TTAGAGTAAATAAATTAGCAGAACA
TTAGAGTAAATAAATTAGCAGAACA
TTAGAGTAAATAAATTAGCAGAACA
TTAGAGTAAATAAAAAAAAAAAAAAAAAAAAAAAAAATTAGCAGAACA
TTAGAGTAAATAAATTAGCAGAACA
TTAGAGTAAATAAATTAGCAGAACA
TTAGAGTAAATAAATTAGCAGAACA
TTAGAGTAAATAAATTAGCAGAACA
TTAGAGTAAATAAATTAGCAGAACA
TTAGAGTAAATAAATTAGGCAGAAA
TTAGAGTAAATAAATTAGCAGAACA
TTAGAGTAAATAAATTAGCAGAACA
TTAGAGTAAATAAATTAGCAGAACA
TTAGAGTAAATAAATTAGCAGAACA
TTAGAGTAAATAAATTAGCAGAACA
TTAGAGTAAATAAATTAGCAGAACA
TTAGAGTAAATAAATTAGGAGAACA
TTAGAGTAAATAAATTAGCAGAACA
TTAGAGTAAATAAATTAGCAGAACA
TTAGAGTAAATAAATTAGCAGAACA
TTAGAGTAAATAAATTAGCAGAACA
TTAGAGTAAATAAATTAGCAGAACA
TTAGAGTAAATAAATTAGCAGAACA
TTAGAGTAAAAAAAAAAAGAGTAAATAAATTAGCAGAACA
TTAGAGTAAATAAATTAGGAGAACA
TTAGAGTAAATAAATTAGCAGAACA
TTAGAGTAAATAAATTAGCAGAACA
TTAGAGTAAATATATTGGCAGAATA
TTAGAGTAAATAAATTAGCAGAACA
TTAGAGTAAATAAATTAGGAGAACA
TTAGAGTAAATAAATTAGCAGAACA
TTAGAGTAAATAAATTAGAGAACA

TTAGAGTAAATAAATTAGCAGAACA
TTAGAGTAAATAAATTAGCAGAACA
TTAGAGTCAATATATTGGCAGAATA
TTAGAGTAAATAAATTAGCAGAAAAAACAAA
TTAGAGTAAATATAATTAGGCAGAACA
TTAGAGTAAATAAATTAGCAGAACA
TTAGAGTAAATAAATTAGGAGAACA
TTAGAGTAAATAAATTAGCAGAACA
TTAGAGTAAATAAATTAGCAGAACA
TTAGAGTAAATAAATTAGGAGAACA
TTAGAGTAAATATATTGGCAGAACA
TTAGAGTAAATAAATTAGCAGAACA
TTAGAGTAAATAAATTAGCAGAACA
TTAGAGTAAATAAATTAGCAGAACA
TTAGAGTAAATAAATTAGCAGAACA
TTAGAGTAAATAAATTAGCAGAACA
TTAGAGTAAATAAATTAGCAGAACA
TTAGAGTAAATAAATTAGCAGTACA
TTAGAGTAAATAAATTAGCAGAACA
TTAGAGTAAATAAATTAGCAGAACA
TTAGAGTAAATAAATTAGCAGAACA
TTAGAGTAAATAAATTAGCAGAACA
TTAGAGTAAATAAATTAGCAGAACA
TTAGAGTAAATAAATTAGCAGAACA
TTAGAGTAAATAAATTAGGAGAACA
TTAGAGTAAATATATTGGCAGAACA
TTAGAGTAAATAAATTAGCAGAACA
TTAGAGTAAATAAATTAGCAGAACA
TTAGAGTAAATAAATTAGCAGAACA
TTAGAGTAAATAAATTAGCAGAACA
TTAGAGTAAATAAATTAGCAGAACA
TTAGAGTAAATAAATTAGCAGAACA
TTAGAGTAAATAAATTAGCAGAACA
TTAGAGTAAATAAATTAGGAGAACA
TTAGAGTAAATATATTGGCAGAACA
TTAGAGTAAATAAATTAGCAGAACA
Nr of selected repeats 177 Similarity 0.908867
Consensus:
TTAGAGTAAATAAATTAGCAGAACA
>Cele-UNSB01_4:6539759-6539908 Satlength=150 Nr of Repeats=10 RepeatLength=12
seed=CAGAATATAT
CAGAATATATTC
CAGAATATATTC
CAGAATATATTT
CAGAATATATTC
CAGAATATATTTCAGATATTC
CAGAATATATTT
CAGAATATATTC
CAAAATATATTC
CAGAATATATTTCAGATATTC
CAGAATATATTTCAAATGTATTC
Nr of selected repeats 7 Similarity 0.915344
Consensus:
CAGAATATATTc

>Cele-UNSB01_4:6669007-6669090 Satlength=84 Nr of Repeats=4 RepeatLength=22
seed=AAGCGGTAAC
AAGCGGTAACGTCGTCTATATA
AAGCGGTAACGTCGTCTATATA
AAGCGGTAACGTCGTCTATATA
AAGCGGTAACGTTATAT
Nr of selected repeats 3 Similarity 1.000000
Consensus:
AAGCGGTAACGTCGTCTATATA
>Cele-UNSB01_4:6734929-6735237 Satlength=309 Nr of Repeats=9 RepeatLength=31
seed=CCCGCCAAAA
CCCGCCAAAATTTTTGAAAAAAATTTAATT
CCCGCCAAAATTTTTGAAAAATTTGAATT
CCCGCCAAAATTTTTTGAAGAAATTCGGATT
CCCGCCAAAATTTTTTGAAAAAATTTGAATT
CCCGCCAAAATTTTTTGAAAAAATTTAAATT
CCCGCCAAAATTTTTTGAAAAAATTTGAATT
CCCGCCAAAAAATTTTTGAAAAAATTTAAATTCCCGCGTAAATTTTTTGAAAAAATTTGAATT
CCCGCCAAAATGTTTTGAAAAAATTTAAATT
CCCGCCAAAATTTTTTAAAAAAATTTGAATT
Nr of selected repeats 7 Similarity 0.897593
Consensus:
CCCGCCAAAATTTTTTGAAAAAATTTgAATT
>Cele-UNSB01_4:6945378-6946401 Satlength=1024 Nr of Repeats=11 RepeatLength=93
seed=TGCTTCTCGT
TGCTTCTCGTCGTCCACCACCACCACCGAAGGGAACTGGAACTCCACCACCACCACCAACTGGAGAACCACAGGATCTT
TCTGGAGAAGGTAA
TGCTTCTCGTCGTCCACCACCACCACCGAAGGGAACTGGAACCCCACCACCACCACCAACTGGAGAACCACAGGATCTT
TCTGCAGAAGGTAA
TGCTTCTCGTCGTCCACCACCGCCACCGAAGGGAACTGGAACCCCACCACCACCACCAACTGGAGAACCACAGGATCTT
TCTACAGAAGGTAA
TGCTTCTCGTCGTCCACCACCACCACCGAAGGGAACTGGAAGCCCAACACCACCACCAACTGGAGAACCACAGGATCTT
TCTGGAGAAGGTAA
TGCTTCTCGTCGTCCACCACCACCACCGAAGGGAACTGGAAGCCCACCACCACCACCAACTGGAGAACCACAGGATCTT
TCTACAGAAGGTAA
TGCTTCTCGTCGTCCACCACCGCCACCGAAGGGAACTGGAACCCCACCACCACCACCAACTGGAGAACCACAGGATCTT
TCTGGAGAAGGTAA
TGCTTCTCGTCGTCCACCACCACCACCGAAGGGAACTGGAAGCCCACCACCACCACCAACTGGAGAACCACAGGATCTT
TCTGGAGAAGGTAA
TGCTTCTCGTCGTCCACCACCACCACCGAAGGGAACTGGAAGCCCACCACCACCACCAACTGGAGAACCACAGGATCTT
TCTACAGAAGGTAA
TGCTTCTCGTCGTCCACCACCGCCACCGAAGGGAACTGGAACCCCACCACCACCACCAACTGGAGAACCACAGGATCTT
TCTGCAGAAGGTTA
TGCTTCTCGTCGTCCACCACCACCACCGAAGGGAACTGGAAGCCCAACACCACCACCAACTGGAGAACCACAGGATCTT
TCTGGAGAAGGTAA
TGCTTCTCGTCGTCCACCACCACCACCGAAGGGAACTGGAAGCCCACCACCACCACCAACTGGAGAACCACAGGATCTT
TCTGGAGAAGGTAA
Nr of selected repeats 11 Similarity 0.961942
Consensus:
TGCTTCTCGTCGTCCACCACCaCCACCGAAGGGAACTGGAAgCCCACCACCACCACCAACTGGAGAACCACAGGATCTT
TCTggAGAAGGTAA
>Cele-UNSB01_4:7151737-7153750 Satlength=2014 Nr of Repeats=178 RepeatLength=11
seed=TGACAACTTC
TGACAACTTCG

TGACAACTTCA
TGACAACTTCG
TGACAACTTCG
TGACAACTTCA
TGACAACTTCG
TGACAACTTCG
TGACAACTTCG
TGACAACTTCG
TGACAACTTCA
TGACAACTTTG
TGACAACTTCG
TAACAACTTCGTGAAAACCTCA
TGACAACTTCG
TGACAACTTCG
TGACAACTTTGG
GACAACTTCG
TGGCAACTTCG
TGACAACTTCGG
GACAACTTCG
TAACAACTTCGTGAAAACCTCA
TGACAACTTCG
TGACAACTTCG
TGACAACTTCGG
GACAACTTCG
TGGCAACTTCGG
GACAACTTCGGGACAATTTCGG
GACAACTTCG
TGACAACTTCA
TGACAACTTCA
TGACAACTTCGA
GACAACTTCA
TGACAACTTCA
TGACAACTTCA
TGATAACTTCGG
GACAACTTCGG
GACAACTTCG
TGACAACTTCG
TGACAACTTCG
TGACAACTTCA
TGACAACTTTG
TGACAACTTCA
TGATAACTTCGG
GACAACTTCGA
GACAACTTCG
TGGCAACTTCA
TGACAACTTTG
TGACAACTTCA
TGATAACTTCGA
GACAACTTCGG
GACAACTTCG
TAACAACTTCGTGAAAACCTCA
TGACAACTTCG
TGACAACTTCG
TGACAACTTCGG

GACAACTTCG
TGGCAACTTCGG
GACAACTTCGGGACAATTTCGG
GACAACTTCG
TGACAACTTCA
TGACAACTTCA
TGACAACTTCGA
GACAACTTCA
TGACAACTTCA
TGACAACTTCA
TGATAACTTCGG
GACAACTTCGG
GACAACTTCG
TGACAACTTCG
TGACAACTTCG
TGACAACTTCA
TGACAACTTTG
TGACAACTTCA
TGATAACTTCGG
GACAACTTCGG
GACAACTTCG
TGGCAACTTCA
TGACAACTTTG
TGACAACTTCA
TGATAACTTCGG
GACAACTTCGG
GACAACTTCG
TGACAACTTCG
TGACAACTTCG
TGACAACTTCA
TGACAACTTTG
TGACAACTTCA
TGATAACTTCGG
GACAACTTCGG
GACAACTTCG
TGACAACTTCG
TGACAACTTCG
TGACAACTTCA
TGACAACTTTG
TGACAACTTCA
TGATAACTTCG
TGACAACTTCG
TGACAACTTCG
TGACAACTTCA
TGACAACTTTG
TGACAACTTCA
TGATAACTTCG
TGACAACTTCG
TGACAACTTCA
TGACAACTTTG
TGACAACTTCA
TGATAACTTCGG
GACAACTTCG
TGACAACTTCA

TGACAACTTCG
TGACAACTTCG
TGACAACTTCG
TGACAACTTCA
TGACAACTTTG
TGACAACTTCA
TGATAACTTCG
TGACAACTTCG
TGACAACTTCG
TGACAACTTCA
TGACAACTTTG
TGACAACTTCA
TGATAACTTCG
TGACAACTTCG
TGACAACTTCA
TGACAACTTTG
TGACAACTTCA
TGATAACTTCGG
GACAACTTCG
TGACAACTTCA
TGACAACTTTG
TGACAACTTCA
TGATAACTTCGG
GACAACTTCGG
GACAACTTCG
TGACAACTTCA
TGACAACTTTG
TGACAACTTCA
TGATAACTTCG
TGACAACTTCG
TGACAACTTCG
TGACAACTTCA
TGACAACTTTG
TGACAACTTCA
TGATAACTTCG
TGACAACTTCG
TGACAACTTCA
TGACAACTTTG
TGACAACTTCA
TGATAACTTCGG
GACAACTTCG
TGACAACTTCA
TGACAACTTCG
TGACAACTTCG
TGACAACTTCG
TGACAACTTCG
TGACAACTTCA
TGACAACTTTG
TGACAACTTCA
TGATAACTTCG
TGACAACTTCG
TGACAACTTCA
TGACAACTTTG
TGACAACTTCA

TGATAACTTCGG
GACAACTTCG
TGACAACTTCA
TGACAACTTCG
TGACAACTTCG
TGACAACTTCA
TGACAACTTTG
TGACAACGTCA
TGATAACTTCGG
GACAACTTCGG
GACAACTTCG
TGACAACTTCG
TGACAACTTCG
TGACAACTTCA
TGACAACTTTG
Nr of selected repeats 131 Similarity 0.801993
Consensus:
TGACAACTTCg
>Cele-UNSB01_4:7464716-7464914 Satlength=199 Nr of Repeats=5 RepeatLength=40
seed=TCCAGAATTT
TCCAGAATTTTCTCGATTTTTCTAGAAAGTTCTGGAACAT
TCCAGAATTTTCCCGATTTTTCTAGAAAGTTCTGGAACAC
TCCAGAATTTTCTCGATTTTTCTAGAAAGTTCTGGAACAT
TCCAGAATTTTCTCGATTTTTCTAGAAAGTTCTGGAACAT
TCCAGAATTTTTTCGAAATTTCCAGAAGATTCTAGATT
Nr of selected repeats 4 Similarity 0.966667
Consensus:
TCCAGAATTTTCtCGATTTTTCTAGAAAGTTCTGGAACAt
>Cele-UNSB01_4:7464933-7465143 Satlength=211 Nr of Repeats=6 RepeatLength=35
seed=CAGAAAATTT
CAGAAAATTTAAATTTCCCGCCAAAATATTTTTCT
CAGAAAATTTAAATTTCCCGCCAAAATATTTTTCA
CAGAAAATTTAAATTTCCCGCCAAAATATTTTTCT
CAGAAAATTTAAATTTCCCTCCAAAATATTTTTTT
CAGAAAATTTAAATTTCCCTCAAAAATATTTTTTT
CAGAAAATTTAAATTTCCCGCCAAAATATTTTTCA
Nr of selected repeats 6 Similarity 0.926349
Consensus:
CAGAAAATTTAAATTTCCCgCCAAAATATTTTTct
>Cele-UNSB01_4:7464716-7465331 Satlength=616 Nr of Repeats=6 RepeatLength=40
seed=TCCAGAATTT
TCCAGAATTTTCTCGATTTTTCTAGAAAGTTCTGGAACAT
TCCAGAATTTTCCCGATTTTTCTAGAAAGTTCTGGAACAC
TCCAGAATTTTCTCGATTTTTCTAGAAAGTTCTGGAACAT
TCCAGAATTTTCTCGATTTTTCTAGAAAGTTCTGGAACAT
TCCAGAATTTTTTCGAAATTTCCAGAAGATTCTAGATT
TCCAGAATTTTAGAATTTTCAGAAAATTTAAATTTCCCGCCAAAATATTTTTCTCAGAAAATTTAAATTTCCCGCCAAA
ATATTTTTCACAGAAAATTTAAATTTCCCGCCAAAATATTTTTCTCAGAAAATTTAAATTTCCCTCCAAAATATTTTTT
TCAGAAAATTTAAATTTCCCTCAAAAATATTTTTTTCAGAAAATTTAAATTTCCCGCCAAAATATTTTTCACAGAAAAT
TTAAATTTCCCGTCAAAATTTTGGGTCTTACCACGATGGGTCTCACCACGACGGGTCTCACCACGATGGGTCTCACCTC
GATGGGTCTCACCACGATGGGTCTCACCACGATGGGTCTCGCCACGAAGATCTCGCAGCAACATTTTTTTATTTTTGAT
TTTTCAGAAGGTTCTAGAATAA
Nr of selected repeats 4 Similarity 0.966667
Consensus:

TCCAGAATTTTCtCGATTTTTCTAGAAAGTTCTGGAACAt
>Cele-UNSB01_4:7715733-7715854 Satlength=122 Nr of Repeats=6 RepeatLength=17
seed=CTCATTGCCT
CTCATTGCCTACCCTTG
CTCATTGACTACCCTTG
CTCTTTGCCTACCTCTTT
CTCATTGACTACCTGTG
CTCATTGCCTTCCTGTT
CTCATTGCCTACTTGTTGCTCGTTGACTACCTTTT
Nr of selected repeats 4 Similarity 0.764706
Consensus:
CTCATTGaCTaCCcgTg
>Cele-UNSB01_4:7767635-7768760 Satlength=1126 Nr of Repeats=25 RepeatLength=45
seed=AGCACCACCA
AGCACCACCATCAGGCGGACCACCAGGACCATTTGATCCATCAGG
AGCACCACCATCAGGCGGACCACCAGGACTATTTGATCCATCAGG
AGCACCACCATCAGGCGGACCACCAGGACCATTTGGTCCTTCAGG
AGCACCACCATCAGGCGGACCACCAGGACCATTTAATCCATCAGA
AGCACCACCATCAGGCGGACCAACAGGACCATTTGATCCTTCAGG
AGCACCACCATCAGGCGGACCACCAGGACCATTTAATCCATCAGG
AGCACCACCATCAGGCGGACCACCAGGACCATTTGATCCATCAGG
AGCACCACCATCAGGCGGACCACCAGGACCATTTAATCCATCAGG
AGCACCACCATCAGGCGGACCACCAGGACCATTTGATCCTTCAGG
AGCACCACCATCAGGCGGACCACCAGGACCATTTGATCCATCAGG
AGCACCACCATCAGGCGGACCACCAGGACCATTTGATCCATCAGG
AGCACCACCATCAGGCGGACCACCAGGACCATTTAATCCATCAGG
AGCACCACCATCAGGCGGACCACCAGGACCATTTGATCCTTCAGG
AGCACCACCATCAGGCGGACCACCAGGACCATTTAATCCATCAGG
AGCACCACCATCAGGCGGACCACCAGGACCATTTGATCCATCAGG
AGCACCACCATCAGGCGGACCACCAGGACCATTTGATCCATCAGG
AGCACCACCATCAGGCGGACCACCAGGACCATTTGATCCATCAGG
AGCACCACCATCAGGCGGACCACCAGGACCATTTAATCCATCAGG
AGCACCACCATCAGGCGGACCACCAGGACCATTTAATCCATCAGG
AGCACCACCATCAGGCGGACCACCAGGACCATTTAATCCATCAGG
AGCACCACCATCAGGCGGACCACCAGGACCATTTAATCCATCAGG
AGCACCACCATCAGGCGGACCACCAGGACCATTTAATCCATCAGG
AGCACCACCATCAGGCGGACCACCAGGACCATTTGATCCATCAGG
AGCACCACCATCAGGCGGACCACCAGGACCATTTAATCCATCAGG
AGCACCACCATCAGGCGGACCACCAGGACCATTTGATCCTTCAGG
Nr of selected repeats 25 Similarity 0.965432
Consensus:
AGCACCACCATCAGGCGGACCACCAGGACCATTTgATCCaTCAGG
>Cele-UNSB01_4:8048486-8048578 Satlength=93 Nr of Repeats=6 RepeatLength=15
seed=CGAAAAGGGG
CGAAAAGGGGAGCAGAA
CGAAAAGGGGATCTG
CAAAAAGGGGATCTG
CGAAAAGGGGAGATA
CGAAAAGGGGAGATA
CGAAAAGGGGAGCAA
Nr of selected repeats 5 Similarity 0.768889
Consensus:
CgAAAAGGGGAgcta

>Cele-UNSB01_4:8048753-8048845 Satlength=93 Nr of Repeats=6 RepeatLength=15
seed=TCCCCTTTTC
TCCCCTTTTCGTTGC
TCCCCTTTTCGTATC
TCCCCTTTTCGTATC
TCCCCTTTTCGCAGA
TCCCCTTTTTGCAGA
TCCCCTTTTCGTTCTGC
Nr of selected repeats 5 Similarity 0.768889
Consensus:
TCCCCTTTTcGtagc
>Cele-UNSB01_4:8107145-8107415 Satlength=271 Nr of Repeats=18 RepeatLength=15
seed=CTCACCACGA
CTCACCACGAAGGGT
CTCACCACGATGGGT
CTCGCCACGATGGGT
CTCGCCACGATGGGT
CTCGCCACGATGGGT
CTCACCACGAAGGGT
CTCACCACGATGGGT
CTCATCACGAAGGGT
CTCACCACGATGGGT
CTCATCACGAAGGGT
CTCACCACGATGGGT
CTCACCACGATGGGT
CTCATCACGAAGGGT
CTCACCACGATGGGT
CTCACCACGAAGGGT
CTCACCACGATGGGT
CTCACCACGAAGGGT
CTCACCACGATGGGT
Nr of selected repeats 18 Similarity 0.902977
Consensus:
CTCACCACGAtGGGT
>Cele-UNSB01_4:8289359-8289627 Satlength=269 Nr of Repeats=6 RepeatLength=45
seed=TCTGATCCTT
TCTGATCCTTCAGCTCCGAAGGATCGTAAGGGTACCTCCTGATGG
TCTGATCCTTCAGCTCCAAAGGATCACGAGGCTCTTCCTGATGT
TCTGATCCTTCAGCTCCGAAGGATCACATGGGCACCTTCTGATGA
TCTGATCCTTCAGATAAGAAGGATCGTAAGGGTGCCTCCTGATGG
TCTGATCCTTCAGCTCCGAAGGATCGTAAGGGTACCTCCTGATGG
TCTGATCCTTCAGCTCCAAAGGATCACGAGGCTCTTCCTGATGT
Nr of selected repeats 4 Similarity 0.851852
Consensus:
TCTGATCCTTCAGcTccGAAGGATCgtAaGGGtaCCTcCTGATGg
>Cele-UNSB01_4:8500583-8501255 Satlength=673 Nr of Repeats=21 RepeatLength=32
seed=TTTTCACAGA
TTTTCACAGAAATTTTGAATTCCCGCCACAAT
TTTTCACAGAAAATTTGAATTCCCGCCAAAAT
TTTTCACAGAAAACTTGAATTCCCGTTAAAAT
TTTTCACAGAAATTTTGAATTCCCGCCAAAAT
TTTTCACAGAAAATGTGAATTCCCGCCAAAAT
TTTTCACAGAAATTCTGAATTCCCGCCAAAAT
TTTTCACAGAAATTTTGAATTCCCGCCAAAAT

TTTTCACAGAAAATTTGAATTCTCGCCAAAAT
TTTTCACAGAAATTCTGAATTCCCGCCAAATT
TTTTCACAGAAATTTTGAATTCCCGCCAAAAT
TTTTCACAGAAAATTTGAATTCCCGCTAAAAA
TTTTCACAGAAATTTTGAATTCCCGCTAAAAT
TTTTCACAGAAAATTTGAATTCCCGCCAAAAT
TTTTCACAGAAATTCTGAATTCCCGCCAAAAT
TTTTCACAGAAATTTTGAATTCCCGCCAAAAT
TTTTCACAGAAAATTTGAATTCCCGCCAAAAT
TTTTCACAGAAATTCTGAATTCCCGCCAAATT
TTTTCACAGAAATTTTGAATTCCCGCCAAAAT
TTTTCACAGAAAATTTGAATTCCCGTTAAAAA
TTTTCACAGAAATTTTGAATTCCCGCCAAAAT
TTTTCACAGATATTTTGAATTCTCGCCAAAGT
Nr of selected repeats 21 Similarity 0.903571
Consensus:
TTTTCACAGAAAtTtTGAATTCCCGCCAAAAT
>Cele-UNSB01_4:8501471-8501871 Satlength=401 Nr of Repeats=10 RepeatLength=40
seed=AGAAGGTTCT
AGAAGGTTCTGGAACATTCCAGCATTTTCTCAAATTTTCC
AGAAGGTTCTGGAACATTCCAGAATTTTCTCAAATTTTCC
AGAAGGTTCTGGAACATTCCAGAATTTTCTCGGATTTTCC
AGAAGGTTCTGAAACATTCCAGAATTTTCTCAAATTTTCC
AGAAGGTTCTGGAACATTCCAGAATTTTCTCAAATTTTCC
AGAAGGTTCTGGAACATTCCAGAATTTTCTCGGATTTTCC
AGAAGGTTCTGGAACATTCCAGAATTTTCTCAAATTTTCC
AGAAGGTTCTGGAACATTCCAGAATTTTCTCAAATTTTCC
AGAAGGTTCTGGAACATTCCAGAATTTTCTCGGATTTTCC
AGAAGGTTCTGGAACATTCCAGAATTTTCTCGAATTTTCC
Nr of selected repeats 10 Similarity 0.953333
Consensus:
AGAAGGTTCTGGAACATTCCAGAATTTTCTCaaATTTTCC
>Cele-UNSB01_4:8501476-8504168 Satlength=2693 Nr of Repeats=54 RepeatLength=40
seed=GTTCTGGAAC
GTTCTGGAACATTCCAGCATTTTCTCAAATTTTCCAGAAG
GTTCTGGAACATTCCAGAATTTTCTCAAATTTTCCAGAAG
GTTCTGGAACATTCCAGAATTTTCTCGGATTTTCCAGAAG
GTTCTGAAACATTCCAGAATTTTCTCAAATTTTCCAGAAG
GTTCTGGAACATTCCAGAATTTTCTCAAATTTTCCAGAAG
GTTCTGGAACATTCCAGAATTTTCTCGGATTTTCCAGAAG
GTTCTGGAACATTCCAGAATTTTCTCAAATTTTCCAGAAG
GTTCTGGAACATTCCAGAATTTTCTCAAATTTTCCAGAAG
GTTCTGGAACATTCCAGAATTTTCTCGGATTTTCCAGAAG
GTTCTGGAACATTCCAGAATTTTCTCGAATTTTCCAGAAG
GTTCTGGAGCATTCCGGAATTTTCTCAAAGTTTCCAATATCCGTTCAATAGACGAAAAGTCAATTTTTCCCAAACTACA
GTAATCCTACAGTACTCCTACAGTACCTCTACAGTACAACTACAGTACCCCAACCATATCCCGACACTAACCCCAAAGC
AATATCACTTCAAAAGACGAAAAGCCAATTTTTTTCAAAAATACAGTAATCCTACAGTACTCCTACAGTACTACTACAG
TATCCCGACCATATCCCACCACTAACCCCAACCCACAATCCCTTCAACAGCCGGAAACACCTTGCAAGTGTGACGTCAC
AGACTACAAACTACAATTGTAACATCCCTCATGTATTTTTTTATATATAAGTAATGATATACGTAGGCACAGCTTACTT
TCAAGATGGCCTACTTTGTGTCTACACTCGTTTCAGGGGTCAATGTATATGTTAACTTTATTTCAAAATATTATGTTTG
AACCAGCCGCTGACCGCGCCTACTGCGCGGGCAACGACTGGCACCATTAAAAGTATTTGACACACATACACTTCCAGAA
TTTTCCCGATTTTTCTAGAAA
GTTCTGGAACGTTCCAGAATTTTCTCGATTTTTCTAGAAA
GTTCTGGAACGTTCCAGAATTTTCTCGATTTTTCTAGAAA

GTTCTGGAACATTCCAGAATTTTCCCAATTTTTCTAGAAA
GTTCTGGAACATTCCAGAACTTTCCCAATTTTTCTAGAAA
GTTCTGGAACGTTCCAGAATTTTCTCGATTTTTCTAGAAA
GTTCTGGAACGTTCCAGAATTTTCTCGATTTTTCTAGAAA
GTTCTGGAACGTTCCAGAATTTTCTCGATTTTTCTAGAAA
GTTCTGGAACATTCCAGAATTTTCTCAAATTTTCCAGAAG
GTTCTGGAACGTTCCAGAATTTTCTCGATTTTTCTAGAAA
GTTCTGGAACATTCGAGAATTTTCCCAATTTTTCTAGAAA
GTTCTGGAACATTCCAGAATTTTCTCGATTTTTCTAGAAA
GTTCTGGAACACTCCAGAATTTTCTCGATTTTTCTAGAAA
GTTCTGGAACGTTCCAGAATTTTCTCGATTTTTCTAGAAA
GTTCTGGAACGTTCCAGAATTTTCTCGATTTTTCTAGAAA
GTTCCGGAACATTCCGGAATTTTCCCATTTTTTTTAGAAA
GTTCTGGAACGTTCCAGAATTTTCTCGATTTTTCTAGAAA
GTTCTGGAACATTCCAGAATTTTCCCAATTTTTCTAGAAA
GTTCTGGAACATTCCAGAATTTTCCCAATTTTTTTAGAAA
GTTCTGGAACACTCCAGAATTTTCTCGATTTCTCTAGAAA
GTTCTGGAACACTCCAGAATTTTCTCGATTTTTCTAGAAA
GTTCTGGAACGTTCCAGAATTTTCTCGATTTTTCTAGAAA
GTTCTGGAACATTCCAGAATTTTCCCAATTTTTCTAGAAA
GTTCTGGAACATTCCAGAATTTTCCCATTTTTTCTAGAAA
GTTCTGGAACATTCCAGAATTTTCCCAATTTTTCTAGAAA
GTTCTGGAACATTCGAGAATTTTTTCGATTTTTCTAGAAA
GTTCTGGAACACTCCAGAACTTTCTCGATTTTTCTAGAAA
GTTCTGGAACATTCCAGAATTTTCCCGATTTTTCTAGAAA
GTTCTGGAACATTCCAGAATTTTCCCAATTTTTCTAGAAA
GTTCTGGAACATTCCAGAATTTTCCCAATTTTTCTAGAAA
GTTCTGGAACATTCCAGAATTTTCCCAATTTTTCTAGAAA
GTTCTGGAACATTCCAGAATTTTCCCAATTTTTCTAGAAA
GTTCTGGAACATTCCAGAATTTTCCCAATTTTTCTAGAAA
GTTCTGGAACATTCGAGAATTTTTTCGATTTTTCTAGAAA
GTTCCGGAACACTCCAGAACTTTCTCGATTTTTCTAGAAA
GTTCTGGAACATTCCAGAATTTTCCCGATTTTTCTAGAAA
GTTCTGGAACGTTCCAGAATTTTCTCGATTTTTTTAGAAA
GTTCTGGAACACTCCAGAATTTTCCCGATTTTTTTAGAAA
GTTCTGGAACACTCCAGAATTTTCCCGATTTTTCTAGAAA
GTCTGGAACGTTCCAGAATTTTCTCGATTTTTTTAGAAA
GTTCTGGAACACTCCAGAATTTTCTCGATTTTCTAGAAA
GTTCTGGAACATTCCAGAATTTTCTCGATTTTTCTAGAAA
GTTCTGGAACATTCCAGAATTTTCCCAATTTTTCTAGAAA
GTTCTGGAACATTCCAGAATTTTCCCGATTTTTCTAGAAA
Nr of selected repeats 51 Similarity 0.884078
Consensus:
GTTCTGGAACaTTCCAGAATTTTCtCgAtTTTTCtAGAAa
>Cele-UNSB01_4:8504239-8504449 Satlength=211 Nr of Repeats=6 RepeatLength=35
seed=GAAAATTTAA
GAAAATTTAAATTTCCCGCCAAAATATTTTTCTCA
GAAAATTTAAATTTCCCACCAAAATATTTTTTTCA
GAAAATTTAAATTTCCCTCCAAAATTTTTTTCTCC
GAAAATTTAAATTTCCCTCCAAAATATTTTTTTCA
GAAAATTTAAATTTCCCTCCAAAATATTTTTTTCA
GAAAATTTAAATTCCCCGCCAAAATATTTTTCACA
Nr of selected repeats 6 Similarity 0.898413
Consensus:

GAAAATTTAAATTTCCCtCCAAAATATTTTTcTCA
>Cele-UNSB01_4:8504495-8505035 Satlength=541 Nr of Repeats=35 RepeatLength=15
seed=TCTCACCACG
TCTCACCACGACGGG
TCTCACCACGATGGG
TCTCACCACGATGGA
TCTCACCACGATGGG
TCTCACCACGATGGG
TCTCACCACGATGGG
TCTCACCACGATGGG
TCTCACCACGATGGG
TCTCACCACGATGGG
TCTCACCTCGATGGG
TCTCACCACGATGGG
TCTCACCACGATGGG
TCTCACCACGATGGG
TCTCACCACGATGGG
TCTCACCAAGATGGATACCACCACGATGAG
TCTCACCACGATGGG
TCTCACCACGATGGG
TCTCACCACGATGGG
TCTCACCACGATGGG
TCTCACCACGATGGG
TCTCACCTCGATGGG
TCTCACCACGATGGA
TCTCACCACGATGGG
TCTCACCACGATGGG
TCTCACCACGATGGG
TCTCACCACGATGGA
TCTCACCACGATGGG
TCTCACCACGATGGG
TCTCACCACGATGGG
TCTCACCACGATGGA
TCTCACCACGATGGG
TCTCACCACGACGGG
TCTCACCACGATGGG
TCTCACCACGATGGA
TCTCACCACGATGGG
Nr of selected repeats 34 Similarity 0.956744
Consensus:
TCTCACCACGATGGG
>Cele-UNSB01_4:8600392-8600896 Satlength=505 Nr of Repeats=13 RepeatLength=11
seed=CTACAGTACT
CTACAGTACTC
CTACAGTACTA
CTACAGTACCTTGACATTATCCCCCCAGCAACTCCCAACCCAATACCTCTTCTGTAGACAAAAACTCAATTTTTCCTAA
ACTACAGTAACCCTACCGTGTAT
CTACAGTACTT
CTACAGTACTA
CTACAGTACCTTGACATTATCCCCCCAGCAACTCCCAACCCAATACCTCTTCTGTAGACAAAAACTCAATTTTTCCTAA
ACTACAGTAACGCTACCGTATAC
CTACAGTACTT
CTACAGTACCA

CTACAGTACCTTGACATTATCCCCCAGCAACTCCCAACCCAATACCTCTTCTGTAGACAAAAACTCATTTTTCCTAATC
TACAGTAACCCTACCGTATAC
CTACAGTACTC
CTACAGTACCA
CTACAGTACCTTGACATTATCCCTCACCAACTCCCAACCCAAAACCTCTTCTGTAGACAAAAACTCAATTTTTCCCAAA
CTACAGTAACCCTACCGTATAC
CTACAGTACTC
Nr of selected repeats 9 Similarity 0.865320
Consensus:
CTACAGTACtn
>Cele-UNSB01_4:8607400-8623699 Satlength=16300 Nr of Repeats=831
RepeatLength=20 seed=ATACGATATA
ATACGATATAGCTATAAACG
ATACGATATAGCTATAAACA
ATACGATATAGCTATAAACG
ATACGATATAGCTGTTTACG
ATACGATATAGCTATAAACG
ATACGATATAGCTGTTTACG
ATACGATATAGCTATAAACG
ATACGATATAGCTGTTTACG
AGACGATATAGCTATAAACG
ATACGATATAGCTGTTTACG
ATACGATATAGCTATAAACG
ATACGATATAGCTGTTTACG
ATACGATATAGCTATAAACG
ATACGAAATAGCTATAAACG
ATACGATATAGCTGTTTACG
ATACGATATAGCTATAAACG
ATACGATATAGCTGTTTACG
ATACGATATAGCTATAAACG
ATACGATATAGCTGTTTACG
AGACGATATAGCTATAAACG
ATACGATATAGCTATAAACA
ATACGAAATAGCTATAAACG
ATACGATATAGCTATAAACA
ATACGATATAGCTATAAACG
ATACGATATAGCTATAAACG
ATACGATATAGCTGTTTACG
ATACGATATAGCTATAAACA
ATACGATATAGCTATAAACG
ATACGATATAGCTGTTTACG
ATACGATATATACG
ATACGATATAGCT
ATACGATATAGCTATTTACG
ATACGATATAGCTATTTACG
ATACGATATAGCTACTTACG
ATACGATATAGCTTTTTACG
ATACGATATAGCTATTTACG
ATACGATATAGCTATAAACG
ATACGATATAGCTGTTTACG
ATACGATATAGCTATAAACG
ATACGATATAGCTGTTTACG
ATACGATATAGCTACTAACG
ATACGATATAGCTTTTTACG

ATACGATATAGCTATAAACG
ATACGATATAGCTTTTTACG
ATACGATATAGCTATAACG
ATACGATATAGCTGTTTACG
ATACGATATATACG
ATACGATATAGCTATAAACG
ATACGATATAGCTATTTACG
ATACGATATAGCTATAAACG
ATACGATATAGCTGTTTACG
ATACGATATAGCTAGTAACG
ATACGATATAGCTGTTTACG
ATACGATATAGCTATAACG
ATACGATATAGCTGTTAACG
ATACGATATAGCTGTTTACG
ATACGATATAGCTATAAACG
ATACGATATAGCTGTTTACG
ATACGATATAGCTATTTACG
ATACGATATAGCTACTTACG
ATACGATATAGCTGTTAACG
ATACGATATAGCTGTTTACG
ATACGATATAGCTGTTAACG
ATACGATATAGCTGTTTACG
ATACGATATAGCTATAAACG
ATACGATATAGCTGTTTACG
AGACGATATAGCTATAAACG
ATACGATATAGCTGTTTACG
ATACGATATAGCTATACG
ATACGATATAGCTATAAACG
ATACGATATAGCTGTTTACG
ATACGATATAGCTATACG
ATACGATATATACG
ATACGATATAGCTATAACG
ATACGATATAGCTATT
TACGATATAGCTATAACG
ATACGATATAGCTGTTTACG
ATACGATATAGCTATAAACG
ATACGATATAGCTGTTTACG
ATACGATATACTATAACG
ATACGATATAGCTGTTTACG
ATACGATATAGCTATAAACG
ATACGATATAGCTGTTTACG
ATACGATATAGCTACTTACG
ATACGATATAGCTGTTTACG
ATACGATATAGCTATAAACG
ATACGATATAGCTGTTTACG
ATACGATATAGCTATTACG
ATACGATATAGCTGTTTACG
AGACGATATAGCTATAAACG
ATACGATATAGCTGTTTACG
ATACGATATAGCTACTTACG
ATACGATATAGCTATTTACG
ATACGATATAGCTATAAACG
ATACAATATAGCTGTTTACG
ATACGATATAGCTATAAACG

ATACGATATAGCTGTTTACG
ATACGATATAGCTATAAACG
ATACGATATAGCTATTTACG
ATACGATATAGCTATAAACG
ATACAATATAGCTGTTTACG
ATACGATATAGCTACTTACG
ATACGATATAGCTACTTACG
ATACGATATAGCTGTTAACG
ATACGATATAGCTGTTAACG
ATACGATATAGCTATTTACG
ATACGATATAGCTATTTACG
ATACGATATAGCTATAAACA
ATACAATATAGCTGTTTACG
ATACGATATAGCTATTTACG
ATACGATATATACG
ATACGATATAGCTATAAACG
ATACGATATAGCTATTTACG
ATACGATATAGCTATAAACG
ATACGATATAGCTGTTTACG
ATACGATATAGCTGTTAACG
ATACGATATAGCTATTTACG
ATACGATATAGCTATTTACG
ATACGATATAGCTATAAACG
ATACGATATAGCTGTTTACG
ATACGATATAGCTATTTACG
ATACGATATATACG
ATACGATATAGCTATAAACG
ATACGATATAGCTATTTACG
ATACGATATAGCTATAAACG
ATACGATATAGCTGTTTACG
ATACGATATAGCTATTTACG
ATACGATATATACG
ATACGATATAGCTGTTAACG
ATACGATATAGCTGTTAACG
ATACGATATAGCTATTTACG
ATACGATATAGCTATTTACG
ATACGATATAGCTATAAACA
ATACAATATAGCTGTTTACG
ATACGATATAGCTATTTACG
ATACGATATATACG
ATACGATATAGCTATAAACG
ATACGATATAGCTATTTACG
ATACGATATAGCTATAAACG
ATACAATATAGCTGTTTACG
ATACGATATAGCTATTTACG
ATACGATATATACG
ATACGATATAGCTGTTAACG
ATACGATATAGCTGTTAACG
ATACGATATAGCTGTTTACG
ATACGATATAGCTATAAACG
ATACGATATAGCTGTTTACG
ATACGATATAGCTGTTTACG
ATACGATATATACG
ATACGATATAGCTGTTAACG

ATACGATATAGCTATTTACG
ATACGATATAGCTATAAACG
ATACGATATAGCTGTTTACG
ATACGATATAGCTATTTACG
ATACGATATATACG
ATACGATATAGCTGTTTACG
ATACGATATAGCTTTACG
ATACGATATAGCTATAAACG
ATACGATATAGCTATTTACG
ATACGATATAGCTATAAACG
ATACAATATAGCTGTTTACG
ATACGATATAGCTATTTACG
ATACGATATATACG
ATACGATATAGCTGTTAACG
ATACGATATAGCTGTTAACG
ATACGATATAGCTGTTTACG
ATACGATATAGCTATAAACG
ATACGATATAGCTGTTTACG
ATACGATATAGCTATTTACG
ATACGATATAGCTATAAACG
ATACGATATAGCTTTACG
ATACGATATAGCTGTTAACG
ATACGATATAGCTGTTTACG
ATACGATATAGCTGTTTACG
ATACGATATAGCTATTTACG
ATACGATATAGCTATAAACG
ATACAATATAGCTGTTTACG
ATACGATATAGCTGTTAACG
ATACGATATAGCTATTTACG
ATACGATATAGCTATAAACG
ATACGATATAGCTATAAACG
ATACGATATATACG
ATACGATATAGCTGTTAACG
ATACGATATAGCTGTTTACG
ATACGATATAGCTATAAACG
ATACGATATAGCTGTTTACG
ATACGATATAGCTATAAACG
ATACGATATAGCTGTTTACG
ATACGATATAGCTATAAACG
ATACGATATATACG
ATACGATATAGCTGTTAACG
ATACGATATAGCTATAAACG
ATACAATATAGCTGTTTACG
ATACGATATAGCTATAAACG
ATACGATATATACG
ATACGATATAGCTATTTACG
ATACGATATAGCTATAAACG
ATACGATATATACG
ATACGATATAGCTGTTAACG
ATACGATATAGCTGTTTACG
ATACGATATAGCTGTTTACG
ATACGATATAGCTATTTACG
ATACGATATAGCTATAAACG
ATACAATATAGCTGTTTACG

ATACGATATAGCTGTTAACG
ATACGATATAGCTATTTACG
ATACGATATAGCTATAAACG
ATACGATATAGCTGTTTACG
ATACGATATATACG
ATACGATATAGCTGTTAACG
ATACGATATAGCTGTTTACG
ATACGATATAGCTATAAACG
ATACGATATAGCTGTTTACG
ATACGATATAGCTATAAACG
ATACGATATAGCTGTTTACG
ATACGATATAGCTATAAACG
ATACGATATATACG
ATACGATATAGCTGTTAACG
ATACGATATAGCTATAAACG
ATACAATATAGCTGTTTACG
ATACGATATAGCTATAAACG
ATACGATATAGCTGTTTACG
ATACGATATAGCTGTTTACG
AGACGATATAGCTATAAACGG
TACGATATAGCTGTTTACG
ATACGATATAGCTATTTACG
ATACGATATATACG
ATACGATATAGCTATAAACG
ATACGATATAGCTATTTACG
ATACGATATAGCTATAAACG
ATACAATATAGCTGTTTACG
ATACGATATAGCTGTTAACG
ATACGATATAGCTATTTACG
ATACGATATAGCTATTTACG
ATACGATATAGCTATAAACG
ATACAATATAGCTGTTTACG
ATACGATATAGCTATTTACG
ATACGATATATACG
ATACGATATAGCTATAAACG
ATACGATATAGCTATTTACG
ATACGATATAGCTATAAACG
ATACAATATAGCTGTTTACG
ATACGATATAGCTATTTACG
ATACGATATATACG
ATACGATATAGCTGTTAACG
ATACGATATAGCTGTTAACG
ATACGATAAAGCTATTTACG
ATACGATATAGCTATTTACG
ATACGATATAGCTATAAACA
ATACAATATAGCTGTTTACG
ATACGATATAGCTATTTACG
ATACGATATATACG
ATACGATATAGCTATAAACG
ATACGATATAGCTATTTACG
ATACGATATAGCTATAAACG
ATACAATATAGCTGTTTACG
ATACGATATAGCTATTTACG
ATACGATATATACG

ATACGATATAGCTGTTAACG
ATACGATATAGCTGTTAACG
ATACGATATAGCTGTTTACG
ATACGATATTGCTATAAACG
ATACGATATAGCTGTTTACG
ATACGATATAGCTATTTACG
ATACGATATAGCTATAAACG
ATACGATATATACG
ATACGATATAGCTGTTAACG
ATACGATATAGCTGTTAACG
ATACGATATAGCTGTTTACG
ATACGATATAGCTGTTTACG
ATACGATATAGCTATTTACG
ATACGATATAGCTATAAACG
ATACGATATATACG
ATACGATATAGCTGTTAACG
ATACGATATAGCTATTTACG
ATACGATATAGCTATAAACG
ATACGATATAGCTGTTTACG
ATACGATATAGCTATTTACG
ATACGATATATACG
ATACGATATAGCTGTTTACG
ATACGATATATACG
ATACGATATAGCTATAAACG
ATACGATATAGCTATTTACG
ATACGATATAGCTGTTAACG
ATACGATATAGCTGTTAACG
ATACGATATAGCTGTTTACG
ATACGATATTGCTATAAACG
ATACGATATAGCTGTTTACG
ATACGATATAGCTATTTACG
ATACGATATAGCTATAAACG
ATACGATATATACG
ATACGATATAGCTGTTAACG
ATACGATATAGCTGTTAACG
ATACGATATAGCTGTTTACG
ATACGATATAGCTATAAACG
ATACAATATAGCTGTTTACG
ATACGATATAGCTATAAACG
ATACGATATAGCTGTTTACG
ATACGATATATACG
ATACGATATAGCTGTTAACG
ATACGATATAGCTGTTTACG
ATACGATATAGCTATAAACG
ATACGATATAGCTGTTTACG
ATACGATATAGCTATTTACG
ATACGATATAGCTATAAACG
ATACGATATAGCTGTTTACG
ATACGATATATATACG
ATACGATATAGCTGTTAACG
ATACGATATAGCTGTTTACG
ATACGATATAGCTATAAACG
ATACGATATAGCTGTTTACG
ATACGATATAGCTATTTACG

ATACGATATAGCTATTTACG
ATACGATATAGCTATAAACG
ATACGATATAGCTGTTTACG
ATACGATATATACG
ATACGATATAGCTGTTAACG
ATACGATATAGCTGTTTACG
ATACGATATAGCTATAAACG
ATACGATATAGCTGTTTACG
ATACGATATAGCTATTTACG
ATACGATATAGCTATTTACG
ATACGATATAGCTATAAACG
ATACGATATAGCTGTTTACG
ATACGATATAGCTATAAACG
ATACGATATAGCTGTTTAACG
ATACGATATAGCTATAAACG
ATACGATATAGCTGTTTACG
ATACGATATAGCTGTTTACG
ATACGATATAGCTATAAACG
ATACGATATAGCTATAAACG
ATACGATATAGCTGTTTACG
ATACGATATAGCTATAAACG
ATACGATATAGCTATAAACG
ATACGATATAGCTATAAACG
ATACGATATAGCTATAAACG
ATACGATATAGCTATAAACG
ATACGATATAGCTGATATATACG
ATACGATATAGCTATAAACG
ATACGATATAGCTATAAACG
ATACGATATAGCTGTTTACG
ATACGATATAGCTATAAACG
ATACGATATAGCTATAAACG
ATACGATATAGCTATAAACG
ATACGATATAGCTATAAACG
ATACGATATAGCTATAAACG
ATACGATATAGCTAGCTGTTTACG
ATACGATATAGCTATAAACG
ATACGATATAGCTGTTTACG
ATACGATATAGCTATAAACG
ATACGATATAGCTATAAACG
ATACGATATAGCTGTTTACG
ATACGATATAGCTATAAACG
ATACGATATAGCTGTTTACG
ATACGATATAGCTATAAACG
ATACGATATAGCTGTTTACG
ATACGATATAGCTATAAACG
ATACGATATAGCTGTTTACG
ATACGATATAGCTATAAACG
ATACGATATAGCTATAAACG
ATACGATATAGCTGTTTACG
ATACGATATAGCTATTTACG
ATACGATATAGCTATAAACG
ATACGATATAGCTGTTTACG
ATACGATATAGCTATTTACG
ATACGATATAGCTATAAACG

ATACGATATAGCTGTTTACG
ATACGATATAGCTATACG
ATACGATATAGCTATAAACG
ATACGATATAGCTGTTTACG
ATACGATATAGCTATTTACG
ATACGATATAGCTATAAACG
ATACGATATAGCTGTTTACG
ATACGATATAGCTATTTACG
ATACGATATATACG
ATACGATATAGCTGTTAACG
ATACGATATAGCTATTTACG
ATACGATATAGCTATAAACG
ATACAATATAGCTGTTTACG
ATACGATATAGCTGTTAACG
ATACGATATAGCTATTTACG
ATACGATATAGCTATTTACG
ATACGATATAGCTATAAACA
ATACAATATAGCTGATTACG
ATACGATATAGCTATTTACG
ATACGATATATACG
ATACGATATAGCTATAAACG
ATACGATATAGCTATTTACG
ATACGATATAGCTATAAACG
ATACAATATAGCTGTTTACG
ATACGATATAGCTATTTACG
ATACGATATATACG
ATACGATATAGCTGTTAACG
ATACGATATAGCTGTTAACG
ATACGATAAAGCTATTTACG
ATACGATATAGCTATAAACA
ATACAATATAGCTGTTTACG
ATACGATATAGCTATTTACG
ATACGATATAGCTATAAACA
ATACAATATAGCTGTTTACG
ATACGATATAGCTATTTACG
ATACGATATATACG
ATACGATATAGCTATAAACG
ATACGATATAGCTATTTACG
ATACGATATAGCTATAAACG
ATACAATATAGCTGTTTACG
ATACGATATAGCTATTTACG
ATACGATATAGCTGTTAACG
ATACGATATAGCTGTTAACG
ATACGATATAGCTGTTTACG
ATACGATATAGCTGTTAACG
ATACGATATAGCTGTTTACG
ATACGATATTGCTATAAACG
ATACGATATAGCTGTTTACG
ATACGATATAGCTATTTACG
ATACGATATATACG
ATACGATATAGCTGTTAACG
ATACGATATAGCTGTTTACG
ATACGATATAGCTATAAACG
ATACGATATAGCTGTTTACG

ATACGATATAGCTGTTTACG
AGACGATATAGCTGTTTACG
ATACGATATAGCTATAAACG
ATACGATATAGCTGTTTACG
AGACGATATAGCTATAAACG
ATACGATATAGCTGTTTACG
ATACGATATAGCTATAAACG
ATACGATATAGCTGTTTACG
ATACGATATAGCTATAAACG
ACACGATATAGCTATAAACG
ATACGATATAGCTGTTAACG
ATACGATATAGCTATTTACG
ATACGATATAGCTATAAACG
ATACGATATAGCTGTTTACG
ATACGATATAGCTATTTACG
ATACGATATAGCTGTTTACG
AGACGATATAGCTATAAACGG
TACGATATAGCTGTTTACG
ATACGATATAGCTATTTACG
ATACGATATATACG
ATACGATATAGCTATAAACG
ATACGATATAGCTATTTACG
ATACGATATAGCTATAAACG
ATACAATATAGCTGTTTACG
ATACGATATAGCTGTTAACG
ATACGATATAGCTATTTACG
ATACGATATAGCTATTTACG
ATACGATATAGCTATAAACA
ATACAATATAGCTGTTTACG
ATACGATATAGCTATTTACG
ATACGATATATACG
ATACGATATAGCTATAAACG
ATAGGATATAGCTATTTACG
ATACGATATAGCTATAAACG
ATACAATATAGCTGTTTACG
ATACGATATAGCTATTTACG
ATACGATATATACG
ATACGATATAGCTGTTAACG
ATACGATATAGCTGTTAACG
ATACGATAAAGCTATTTACG
ATACGATATAGCTATTTACG
ATACGATATAGCTATAAACA
ATACAATATAGCTGTTTACG
ATACGATATAGCTATTTACG
ATACGATATAGCTATAAACA
ATACAATATAGCTGTTTACG
ATACGATATAGCTATTTACG
ATACGATATATACG
ATACGATATAGCTATTTACG
ATACGATATAGCTATAAACG
ATACAATATAGCTGTTTACG
ATACGATATAGCTATTTACG
ATACGATATAGCTGTTAACG
ATACGATATAGCTGTTAACG

ATACGATATAGCTGTTTACG
ATACGATATAGCTATTTACG
ATACGATATATACG
ATACGATATAGCTGTTAACG
ATACGATATAGCTGTTAACG
ATACGATATAGCTGTTTACG
ATACGATATAGCTGTTAACG
ATACGATATAGCTGTTTACG
ATACGATATAGCTATTTACG
ATACGATATATACG
ATACGATATAGCTATTTACG
ATACGATATAGCTATAAACG
ATACAATATAGCTGTTTACG
ATACGATATAGCTATTTACG
ATACGATATATACG
ATACGATATAGCTGTTAACG
ATACGATATAGCTGTTAACG
ATACGATATAGCTGTTTACG
ATACGATATTGCTATAAACG
ATACGATATAGCTGTTTACG
ATACGATATAGCTATTTACG
ATACGATATAGCTATAAACG
ATACGATATATACG
ATACGATATAGCTGTTAACG
ATACGATATAGCTGTTAACG
ATACGATATAGCTGTTTACG
ATACGATATAGCTATAAACG
ATACAATATAGCTGTTTACG
ATACGATATAGCTGTTAACG
ATACGATATAGCTATTTACA
ATACGATATAGCTATAAACG
ATACGATATAGCTGTTTACG
ATACGATATATACG
ATACGATATAGCTGTTAACG
ATACGATATAGCTGTTTACG
ATACAATATAGCTATAAACG
ATACGATATAGCTGTTTACG
ATACGATATAGCTATTTACG
ATACGATATAGCTATTTACG
ATACGATATAGCTATAAACG
ATACGATATAGCTGTTTACG
ATACGATATATACG
ATACGATATAGCTGTTAACG
ATACGATATAGCTATTTACG
ATACGATATAGCTATTTACG
ATACGATATAGCTATTTACG
ATACGATATAGCTATAAACG
ATACGATATAGCTGTTTACG
ATACTATATAGCTATTTACG
ATACGATATAGCTATAAACG
ATACGATATAGCTATAAACG
ATACGATATAGCTGTTTACG
ATACGATATAGCTATAAACGA
AACGATATAGCTATAAACG

ATACGATATAGCTATAAACG
ATACGATATAGCTATAAACG
ATACGATATAGCTGTTTACGT
TACGATATAGCTATAAACG
ATACGATATAGCTATAAACG
ATACGATATAGCTGTTTACG
ATACGATATAGCTATAAACG
ATACGATATAGCTATAAACG
ATACGATATAGCTATAAACG
ATACGATATAGCTATAAACG
ATACGATATAGCTGTTTACGT
TACGATATAGCTATAAACG
ATACGATATAGCTATAAACG
ATACGATATAGCTGTTTACG
ATACGATATAGCTATAAACG
ATACGAGATAGCTATAAACG
ATACGATATAGCTGTTTACG
ATACGATATAGCTATAAACG
ATACGATATAGCTATTTACG
ATACGATATAGCTATAAACT
ATACGATATAGCTATTTACG
ATACGAAATAGCTATAAACG
ATTCGATATAGCTGTTTACG
ATACGATATAGCTATAAACG
ATACGATATAGCTGTTTACG
ATACGATATAGCTATAAACG
ATACGATATAGCTGTTTACG
ATACGATATAGCTGTTTACG
AGACGATATAGCTGTTTACG
ATACGATATAGCTATAAACG
ATACGATATAGCTGTTTACG
AGACGATATAGCTATAAACG
ATACGATATAGCTGTTTACG
ATACGATATAGCTATAAACG
ATACGATATAGCTGTTTTCG
ATACGATATAGCTGTTTACG
ATACGGTATAGCTATAAACG
ATACGATATAGCTATTTACGG
TACGATATAGCTATAAACG
ATACGATATAGCTGTTAACG
ATACGATATAGCTGTTTACG
ATACAATATAGCTATAAACG
ATACGATATAGCTGTTTACG
ATACGATATAGCTATTTACG
ATACGATATAGCTATTTACG
ATACGATATAGCTATAAACG
ATACGATATAGCTGTTTACG
ATACGATATATACG
ATACGATATAGCTGTTAACG
ATACGATATAGCTATTTACG
ATACGATATAGCTATTTACG
ATACGATATATACG
ATACGATATAGCTATTTACG
ATACGATATAGCTATAAACG

ATACAATATAGCTGTTTACG
ATACGATATAGCTATTTACG
ATACGATATAGCTGTTAACG
ATACGATATAGCTGTTAACG
ATACGATATAGCTGTTTACG
ATACGATATAGCTGTTAACG
ATACGATATAGCTGTTTACG
ATACGATATTGCTATAAACG
ATACGATATAGCTGTTTACG
ATACGATATAGCTATTTACG
ATACGATATATACG
ATACGATATAGCTGTTAACG
ATACGATATAGCTGTTAACG
ATACGATATAGCTGTTTACG
ATACGATATAGCTGTTAACG
ATACGATATAGCTGTTTACG
ATACGATATAGCTATAAACG
ATACGATATAGCTGTTTACG
ATACGATATAGCTATTTACG
ATACGATATATACG
ATACGATATAGCTGTTTACG
ATACGATATATACG
ATACGATATAGCTATAAACG
ATACGATATAGCTATTTACG
ATACGATATAGCTATAAACG
ATACAATATAGCTGTTTACG
ATACGATATAGCTATTTACG
ATACGATATATACG
ATACGATATAGCTGTTAACG
ATACGATATAGCTGTTAACG
ATACGATATAGCTGTTTACG
ATACGATATTGCTATAAACG
ATACGATATAGCTGTTTACG
ATACGATATAGCTATTTACG
ATACGATATAGCTATAAACG
ATACGATATATACG
ATACGATATAGCTGTTAACG
ATACGATATAGCTGTTAACG
ATACGATATAGCTGTTTACG
ATACGATATAGCTATAAACG
ATACAATATAGCTGTTTACG
ATACGATATAGCTGTTAACG
ATACGATATAGCTATTTACA
ATACGATATAGCTATAAACG
ATACGATATAGCTGTTTACG
ATACGATATATACG
ATACGATATAGCTGTTAACG
ATACGATATAGCTGTTTACG
ATACAATATAGCTATAAACG
ATACGATATAGCTGTTTACG
ATACGATATAGCTATTTACG
ATACGATATAGCTATTTACG
ATACGATATAGCTATAAACG
ATACGATATAGCTGTTTACG

ATACGATATATACG
ATACGATATAGCTGTTAACG
ATACGATATAGCTATTTACG
ATACGATATAGCTATTTACG
ATACGATATAGCTATTTACG
ATACGATATAGCTATAAACG
ATACGATATAGCTGTTTACG
ATACTATATAGCTATTTACG
ATACGATATAGCTATAAACG
ATACGATATAGCTATAAACG
ATACGATATAGCTGTTTACG
ATACGATATAGCTATAAACGA
AACGATATAGCTATAAACG
ATACGATATAGCTATAAACG
ATACGATATAGCTATAAACG
ATACGATATAGCTGTTTACGT
TACGATATAGCTATAAACG
ATACGATATAGCTATAAACG
ATACGATATAGCTGTTTACG
ATACGATATAGCTATAAACG
ATACGATATAGCTATAAACG
ATACGATATAGCTATAAACG
ATACGATATAGCTATAAACG
ATACGATATAGCTGTTTACGT
TACGATATAGCTATAAACG
ATACGATATAGCTATAAACG
ATACGATATAGCTGTTTACG
ATACGATATAGCTATAAACG
ATACGAGATAGCTATAAACG
ATACGATATAGCTGTTTACG
ATACGATATAGCTATAAACG
ATACGATATAGCTATTTACG
ATACGATATAGCTATAAACT
ATACGATATAGCTATTTACG
ATACGAAATAGCTATAAACG
ATTCGATATAGCTGTTTACG
ATACGATATAGCTATAAACG
ATACGATATAGCTGTTTACG
ATACGATATAGCTATAAACG
ATACGATATAGCTGTTTACG
ATACGATATAGCTGTTTACG
AGACGATATAGCTGTTTACG
ATACGATATAGCTATAAACG
ATACGATATAGCTGTTTACG
AGACGATATAGCTATAAACG
ATACGATATAGCTGTTTACG
ATACGATATAGCTATAAACG
ATACGATATAGCTGTTTTCG
ATACGATATAGCTGTTTACG
ATACGGTATAGCTATAAACG
ATACGATATAGCTATTTACGG
TACGATATAGCTATAAACG
ATACGATATAGCTGTTAACG
ATACGATATAGCTGTTTACG

ATACAATATAGCTATAAACG
ATACGATATAGCTGTTTACG
ATACGATATAGCTATTTACG
ATACGATATAGCTATTTACG
ATACGATATAGCTATAAACG
ATACGATATAGCTGTTTACG
ATACGATATATACG
ATACGATATAGCTGTTAACG
ATACGATATAGCTATTTACG
ATACGATATAGCTATTTACG
ATACGATATAGCTATTTACG
ATACGATATAGCTATAAACG
ATACGATATAGCTGTTTACG
ATACTATATAGCTATTTACG
ATACGATATAGCTATAAACG
ATACGATATAGCTATAAACG
ATACGATATAGCTGTTTACG
ATACGATATAGCTATAAACGA
AACGATATAGCTATAAACG
ATACGATATAGCTATAAACG
ATACGATATAGCTATAAACG
ATACGATATAGCTGTTTACGT
TACGATATAGCTATAAACG
ATACGATATAGCTATAAACG
ATACGATATAGCTGTTTACG
ATACGATATAGCTATAAACG
ATACGATATAGCTATAAACG
ATACGATATAGCTATAAACG
ATACGATATAGCTATAAACG
ATACGATATAGCTGTTTACGT
TACGATATAGCTATAAACG
ATACGATATAGCTATAAACG
ATACGATATAGCTGTTTACG
ATACGATATAGCTATAAACG
ATACGAGATAGCTATAAACG
ATACGATATAGCTGTTTACG
ATACGATATAGCTATAAACG
ATACGATATAGCTATTTACG
ATACGATATAGCTATAAACT
ATACGATATAGCTATTTACG
ATACGAAATAGCTATAAACG
ATTCGATATAGCTGTTTACG
ATACGATATAGCTATAAACG
ATACGATATAGCTGTTTACG
ATACGATATAGCTATAAACG
ATACGATATAGCTGTTTACG
ATACGATATAGCTGTTTACG
AGACGATATAGCTGTTTACG
ATACGATATAGCTATAAACG
ATACGATATAGCTGTTTACG
AGACGATATAGCTATAAACG
ATACGATATAGCTGTTTACG
ATACGATATAGCTATAAACG
ATACGATATAGCTGTTTTCG

ATACGATATAGCTGTTTACG
ATACGGTATAGCTATAAACG
ATACGATATAGCTATTTACGG
TACGATATAGCTATAAACG
ATACGATATAGCTGTTTACG
ATACGATATAGCTATAAACG
ATACGATATAGCTATTTACGG
TACGATATAGCTATAAACG
ATACGATATAGCTGTTTACG
ATACTATATAGCTATTTACG
ATACGATATAGCTATAAACG
ATACGATATAGCTATAAACG
ATACGATATAGCTGTTTACG
ATACGATATAGCTATAAACG
ATACGATATAGCTATAAACG
ATACGATATAGCTATAAACG
ATACGATATAGCTGTTTACG
ATACTATATAGCTATTTACG
ATACGATATAGCTATAAACG
ATACGATATAGCTATAAACG
ATACGATATAGCTGTTTACG
ATACGATATAGCTATAAACG
ATACGATATAGCTATAAACG
ATACGATATAGCTATAAACG
ATACGATATAGCTGTTTACGT
TACGATATAGCTATAAACG
ATACGATATAGCTATAAACG
ATACGATATAGCTGTTTACG
ATACGATATAGCTATAAACG
ATACGATATAGCTATAAACG
ATACGATATAGCTGTTTACG
ATACGATATAGCTATAAACG
ATACGATATAGCTATAAACG
ATACGATATAGCTATAAACG
ATACGATATAGCTGTTTACGT
TACGATATAGCTATAAACG
ATACGATATAGCTATAAACG
ATACGATATAGCTGTTTACG
ATACGATATAGCTATAAACG
ATACGATATAGCTATAAACG
ATACGATATAGCTGTTTACG
ATACGATATAGCTATAAACG
ATACGATATAGCTATTTACG
ATACGATATAGCTATAAACT
ATACGATATAGCTATTTACG
ATACGAAATAGCTATAAACG
ATTCGATATAGCTGTTTACG
ATACGATATAGCTATAAACG
ATACGATATAGCTGTTTACG
ATACGATATAGCTATAAACG
ATACGATATAGCTGTTTACG
ATACGATATAGCTGTTTACG
AGACGATATAGCTGTTTACG
ATACGATATAGCTATAAACG

ATACGATATAGCTATAAACG
ATACGATATAGCTGTTTACG
AGACGATATAGCTATAAACG
ATACGATATAGCTGTTTACG
ATACGATATAGCTATAAACG
ACACGATATAGCTATAAACG
ATACGATATAGCTGTTTACG
ATACGATATAGCTATAAACG
ATACGATATAGCTATAAACT
ATACGATATAGCTGTTTACG
ATACGATATAGCTATAAACG
ATACGATATAGCTATAAACT
ATACGATATAGCTGTTTACG
ATACGATATAGCTGTTTACG
ATACGATATAGCTATAAACG
ATACGATATAGCTGTTTACG
ATACGATATAGCTATAAACG
ATACGATATAGCTATTTACGG
TACGATATAGCTATAAACG
ATACGATATAGCTATTTACG
ATACGATATAGCTATAAACG
ATACGATATAGCTGTTTACG
ATACGATATAGCTATAAACG
ATACGATGTAGCTATTTACGGTATGATATAGCTATAAACGAGAGGATATAGCTGTTAACG
ATACGATATAGCTATTTACG
ACACGATATAGCTATAAACG
ATACGATATAGCTGTTTACG
ATACGATATATACG
ATACGATATAGCTGTTTACG
ATACGATATATACG
ATACGATATAGCTATTTACG
ATACGATATAGCTATAAACG
ATACGATATAGCTGTTTACG
Nr of selected repeats 721 Similarity 0.881205
Consensus:
ATACGATATAGCTaTttACG
>Cele-UNSB01_4:8831351-8831453 Satlength=103 Nr of Repeats=6 RepeatLength=17
seed=TTCATTCCGT
TTCATTCCGTGATTATT
TTCATTCCGTGATTATT
TTCATTCCGTGATTATT
TTCATTCCGTGATTATT
TTCATTCCGTGATTATT
TTCATTCCGTGATTATT
Nr of selected repeats 6 Similarity 1.000000
Consensus:
TTCATTCCGTGATTATT
>Cele-UNSB01_4:9041393-9041548 Satlength=156 Nr of Repeats=8 RepeatLength=17
seed=AGGCAATGAG
AGGCAATGAGAAAGAGT
AGGCAATGAGAAAGAGT
AGGCAATGAGAAAGAGT
AGGCAATGAGAAAAGGTAGTCAACGAGCAACAAGTG
GGCAATGAGAACAGGA

AGGCAATGAGCACAGGT
AGTCAATGAGAAAGAGGT
AGGCAAAGAGCAAGGGT
Nr of selected repeats 5 Similarity 0.781482
Consensus:
AGGCAAtGAGaAAGaGT
>Cele-UNSB01_4:9095021-9107795 Satlength=12775 Nr of Repeats=451
RepeatLength=27 seed=ATAGTGGGAA
ATAGTGGGAAGCCACAGAGAGTAAATA
ATAGTGGGAAGCCACAAAGAGAGTAGAAGTAAATATTGTGAAGCCAAGAAGAAATAAGGGGCCAGAGAGTAAATAATAG
TGAAGCCGAAGTAAAATATTCTGTGAAGCCAGAGAGTAAAATATACTGTGAAGCCAGAGAGTAAATATTCTGTGAAGCC
AGAGAGTAAAATATTCTGTGAAGCAGAAAAAATAAGAAAGAAAGAGTAAAAT
ATGTGGGAAGCCACAGAGAGTAAAT
ATAGTGGGAAGAGAGTAAATA
ATAGTGGGAAGCCACAGAGAGTAAATA
ATAGTGGGAAGCCACAGAGAGTAAAATATGTGGAAGCCACAGAGAGTAAAAATAAC
TAGTGGGAAGCCACAGAGAGTAAATA
ATAGTGGGAAGCCACAGAGAGTAAAT
ATAGTGGGAAGCCACAGAGAGTAAAAT
ATAGTGGGAAGCCACAGAGAGTAAAAT
ATAGTGGGAAGCCACAGAGAGTAAAT
ATAGTGGGAAGCAAGAGAGTAAAAT
ATAGTGGGAAGCCACAGAGAGTAAAAT
ATGGTGGGAAGCCACAGAGAGTAAATA
ATAGTGGGAAGCCACAGAGAGTAAAAT
ATGGTGGGAAGCCACAGAGAGTAAAA
ATAGTGGGAAGCCACAGAGAGTAAAAT
ATAGGTGGGAAGCCACAGAGAGTAAAATA
ATAGTGGGAAGCCACAGAGAGTAAATA
ATAGTGGGAAGCCACAGAGAGTAAAAT
ATGGTGGGAAGCCACAGAGAGTAAATA
ATAGTGGGAAGCCACAGAGAGTAAATA
ATAGTGGGAAGCCACAGAGAGTAAAAT
ATAGTGGGAAGCCACAGAGAGTAAAAT
ATGGTGGGAAGCCACAGAGAGTAAAAT
ATGGTGGGAAGTACAGAGAGTAAA
ATAGTGGGAAGCCACAGAGAGTAAATA
ATAGTGGGAAGCCACAGAGAGTAAAAT
ATAGTGGGAAGCCACAGAGAGTAAAAT
ATGGTGGGAAGCCACAGAGAGTAAAAT
ATAGTGGGAAGCCACAGAGAGTAAAT
ATAGTGGGAAGCCACAGAGAGTAAAAT
ATGTGGGAAGCCACAGAGAGTAAAAT
ATGTGGGAAGCACAGAGAGTAAA
ATAGTGGGAAGCCACAGAGAGTAAAAT
ATGGTGGGAAGCCACAGAGAGTAAATA
ATAGTGGGAAGCCACAGAGAGTAAATA
ATAGTGGGAAGCCACAGAGAGTAAATA
ATAGTGGGAAGCCACAGAGAGTAAATA
ATAGTGGGAAGCCACAGAGAGTAAATA
ATAGTGGGAAGCCACAGAGAGTAAAAT
ATGGTGGGAAGAAGGAATATATGGAACCACAGAGAGTAAAATATGTGATCGGGTAAATAAAGCCACAGAGAGTAAAATA
ATAGTGGGAAGCCACAGAGAGTAAATAA
ATAGTGGGAAGCACAGAGAGTAAATA

ATAGTGGGAAGCCACAGAGAGTAAAATA
ATAGTGGGAAGCCACAGAGAGTAAATA
ATAGTGGGAAGCCACAGAGAGTAAATA
ATAGTGGGAAGCCACAGAGAGTAAAAT
ATGTGGGAAGCCACAGAGAGTAAAAT
ATGGTGGGAAGCCACAGAGAGTAAAAT
ATGTGGGAAGTCACAGAGAGTAAATA
ATAGTGGGAAGCCACAGAGAGTAAATA
ATAGTGGGAAGCCACAGAGAGTAAATA
ATAGTGGGAAGCCACAGAGAGTAAATA
ATAGTGGGAAGCCACAGAGAGTAAAAT
ATAGTGGGAAGCCACAGAGAGTAAAATATACAGAGAGTAAAAT
ATGGTGGGAAGCCACAGAGAGTAAAT
ATAGTGGGAAGCCACAGAGAGTAAATA
ATAGTGGGAAGCCACAGAGAGTAAAAT
ATAGTGGGAAGCCACAGAGAGTAAAAT
ATAGTGGGAAGCCACAGAGAGTAAAAT
ATGGTGGGAAGCCACAGAGAGTAAAAT
ATGGTGGGAAGCCACAGAGAGTAAAAT
ATAGTGGGAAGCCACAGAGAGTAAAAT
ATAGTGGGAAGCCACAGAGAGTAAAAT
ATAGTGGGAAGCCACAGAGAGTAAAAT
ATAGTGGGAAGCCACAGAGAGTAAAT
ATAGTGGGAAGCCACAGAGAGTAAAAT
ATAGTGGGAAGCCACAGAGAGTAAAT
ATAGTGGGAAGCCACAGAGAGTAAAAT
ATAGTGGGAAGCCACAGAGAGTAAAAT
ATAGTGGGAAGCCACAGAGAGTAAAAT
ATGTGGGAAGCCACAGAGAGTAAAAT
ATAGTGGGAAGCCACAGAGAGTAAAT
ATAGTGGGAAGCCAGAGAGAGTAGAAT
ATAGTGGGAAGCCACAGAGTAAAAT
ATAGTGGGAAGCCACAGAGAGTAAAAT
ATAGTGGGAAGCCACAGAGAGTAAAAT
ATAGTGGGAAGCCACAGAGAGTAAAAT
ATAGTGGGAAGCCACAGAGAGTAAAAT
ATAGTGGGAAGCCACAGAGAGTAAAAT
ATAGTGGGAAGCCACAGAGAGTAAAAT
ATAGTGGGAAGCCACAGAGAGTAAAATATGATGGGAAGCCACAGAGAGTAAAAT
ATAGTGGGAAGCCACAGAGAGTAAAAT
ATAGTGGGAAGCCACAGAGAGTAAAT
ATAGTGGGAAGCCACAGAGAGTAAAAT
ATAGTGGGAAGCCACAGAGAGTAAAATAATAGGTGAAGCCACAGAGAGTAAAAT
ATAGTGGGAAGCCACAGAGAGTAAAAT
ATAGTGGGAAGCCACAGAGAGTAAAAT
ATAGTGGGAAGCCACAGAGAGTAAAAT
ATAGTGGGAAGCCACAGAGAGTATAGTAAAAATAAT
ATAGTGGGAAGCCACAGAGAGTAAAAT
ATAGTGGGAAGCCACAGAGAGTAAAAT
ATAGTGGGAAGCCACAGAGAGTAAAAT
ATAGTGGGAAGCCACAGAGAGTAAAAT
ATAGTGGGAAGCCACAGAGAGTAAAAT
ATAGTGGGAAGCCACAGAGAGTAAAAAT
ATAGTGGGAAGCCACAGAGAGTAAAAT

ATAGTGGGAAGCCACAGAGAGTAAAAT
ATAGTGGGAAGCCACAGAGAGTAAAAT
ATAGTGGGAAGCCACAGAGAGTAAAAT
ATAGTGGGAAGCCACAGAGAGTAAAAT
ATAGTGGGAAGCCACAGAGAGTAAAAT
ATAGTGGGAAGCCACAGAGAGTAAAAT
ATAGTGGGAAGCCACAGAGAGTAAAAT
ATAGTGGGAAGCCACAGAGAGTAAAAT
ATAGTGGGAAGCCACAGAGAGTAAAAT
ATAGTGGAAGCCACAGAGAGTAAAT
ATAGTGGGAAGCCACAGAGAGTAAAAT
ATAGTGGGAAGCCACAGAGAT
ATAGTGGGAAGCCACAGAGAGTAAAAT
ATAGTGGGAAGCCACAGAGAGTAAAAT
ATAGTGGGAAGCCACAGAGAGTAAAAT
ATAGTGGGAAGCCACAGAGAGTAAAAT
ATAGTGGGAAGCCACAGAGAGTAAAAT
ATAGTGGGAAGCCACAGAGAGTAAAAT
ATAGTGGGAAGCCACAGAGAGTAAAAT
ATAGTGGGAAGCCACAGAGAGTAAAAT
ATAGTGGGAAGCCACAGAGAGTAAAAT
ATAGTGGGAAGCCACAGAGAGTAAAAT
ATAGTGGGAAGCCACAGAGAGTAAAAT
ATAGTGGGAAGCCACAGAGAGTAAAAT
ATAGTGGGAAGCCACAGAGAGTAAAAT
ATAGTGGGAAGCCACAGAGAGTAAAAT
ATAGTGGGAAGCCACAGAGAGTAAAAT
ATAGTGGGAAGCCACAGAGAGTAAAAT
ATAGTGGGAAGCCACAGAGAGTAAAAT
ATAGTGGGAAGCCACAGAGTAAAAT
ATGGTGGGAAGCCACAGAGAGTAAAAT
ATAGTGGGAAGCCACAGAGAGTAAAAT
ATAGTGGGAAGCCACAGAGAGTAAAAT
ATAGTGGGAAGCCACAGAGAGTAAAT
ATAGTGGGAAGCCACAGAGAGTAAAAT
ATAGTGGGAAGCCACAGAGAGTAAAAT
ATAGTGGGAAGCCACAGAGAGTAAAAT
ATAGTGGGAAGCCACAGAGAGTAAAAT
ATAGTGGGAAGCCACAGAGAGTAAAAT
ATAGTGGGAAGCCACAGAGAGTAAAAT
ATAGTGGGAAGCCACAGAGAGTAAAAT
ATAGTGGGAAGCCACAGAGAGTAAAAT
ATAGTGGGAAGCCACAGAGAGTAAAAT
ATAGTGGGAAGCCACAGAGAGTAAAAT
ATAGTGGGAAGCCACAGAGAGTAAAAT
ATAGTGGGAAGCCACAGAGAGTAAA
ATATTGGGAAGCCACAGAGAGTAAAATAATCGGTGTACAGAGAGTAAAAT
ATAGTGGGAAGCCACAGAGAGTAAAAT
ATAGTGGGAAGCCACAGAGAGTAAAAT
ATAGTGGGAAGCCACAGAGAGTAAAAT
ATAGTGGGAAGCCACAGAGAGTAAAAT
ATAGTGGGAAGCCACAGAGAGTAAAAT
ATAGTGGGAAGCCACAGAGAGTAAAAT

ATAGTGGGAAGCCACAGAGAGTAAAAT
ATAGTGGGAAGCCACAGAGAGTAAAAT
ATAGTGGGAAGCCACAGAGAGTAAAAT
ATAGTGGGAAGCCACAGAGAGTAAAAT
ATAGTGGGAAGCCACAGAGAGTAAAAT
ATAGTGGGAAGCCACAGAGAGTAAAAT
ATAGTGGGAAGCCACAGAGAGTAAAAT
ATAGTGGGAAGCCACAGAGAGTAAAAT
ATAGTGGGAAGCCACAGAGAGTAAAAT
ATAGTGGGAAGCCACAGAGAGTAAAAT
ATAGTGGGAAGCCACAGAGAGTAAAATATACAGAGAGTAAAAT
ATAGTGGGAAGCCACAGAGAGTAAAAT
ATAGTGGGAAGCCACAGAGAGTAAAAT
ATAGTGGGAAGCCACAGAGAGTAAAAT
ATAGTGGGAAGCCACAGAGAGTAAAAT
ATAGTGGGAAGCCACAGAGAGTAAAAT
ATAGTGGGAAGCCACAGAGAGTAAAAT
ATAGTGGGAAGCCACAGAGAGTAAAAT
ATAGTGGGAAGCCACAGAGAGTAAAT
ATAGTGGGAAGCCACAGAGAGTAAAAT
ATAGTGGGAAGCCACAGAGAGTAAAAT
ATAGTGGGAAGCCACAGAGAGTAAAAT
ATAGTGGGAAGCCACAGAGAGTAAAAT
ATAGTGGGAAGCCACAGAGAGTAAAAT
ATAGTGGGAAGCCACAGAGAGTAAAAT
ATAGTGGGAAGCCACAGAGAGTAAAAT
ATAGTGGGAAGCCAAGAAAAAAAGAAACAGAGAGTAAAAT
ATAGTGGGAAGCCACAGAGAGTAAAAT
ATAGTGGGAAGCCACAGAGAGTAAAAT
ATAGTGGGAAGCCACAGAGAGTAAAAT
ATAGTGGGAAGCCACAGAGAGTAAAAT
ATAGTGGGAAGCCACAGAGAGTAAAAT
ATAGTGGGAAGCCACAGAGAGTAAAA
ATAGTGGGAAGCCACAGAGAGTAAAT
ATAGTGGGAAGCCACAGAGAGTAAAAT
ATAGTGGGAAGCCACAGAGAGTAAAAT
ATAGTGGGAAGCCACAGAGAGTAAAT
ATAGTGGGAAGCCACAGAGAGTAAAA
ATAGTGGGAAGCACAGAGAGTAAAT
ATAGTGGGAAGCCACAGAGAGTAAAAT
ATAGTGGGAAGCCACAGAGAGTAAAAT
ATAGTGGGAAGCCACAGAGAGTAAAAT
ATAGTGGGAAGCCACAGAGAGTAAAT
ATAGTGGGAAGCCACAGAGAGTAAAAT
ATAGTGGGAAGCCACAGAGAGTAAAT
ATAGTGGGAAGCCACAGAGAGTAAAA
ATAGTGGGAAGCCACAGAGAGTAAAAT
ATAGTGGGAAGCCACAGAGAGTAAAA
ATAGTGGGAAGCCACAGAGAGTAAAAT
ATAGTGGGAAGCCACAGAGAGTAAA
ATATGGGAAGCCACAGAGAGTAAAAT
ATGGTGGGAAGCCACAGAGAGTAAATAG
TAGTGGGAAGCCACAGAGAGTAAAT
ATAGTGGGAAGCCACAGAGAGTAAAAT

ATAGTGGGAAGCCACAGAGAGTAAAAT
ATAGTGGGAAGCCACAGAGAGTAAAAT
ATAGTGGGAAGCCACAGAGAGTAAAAT
ATAGTGGGAAGCCACAGAGAGTAAAAT
ATAGTGGGAAGCCACAGAGAGTAAAATATACAGAAATA
AAGTGGGAAGCCACAGAGAGTAAAAT
ATAGTGGGAAGCCACAGAGAGTAAAAT
ATAGTGGGAAGCCACAGAGAGTAAAA
ATAGTGGGAAGCCACAGAGAGTAAAAT
ATAGTGGGAAGCCACAGAGAGTAAAAT
ATAGTGGGAAGCCACAGAGAGTAAATAG
TAGTGGGAAGCCAAAGAGAGTAAAT
ATAGTGGGAAGCCACAGAGAGTAAAAT
ATAGTGGGAAGCCACAGAGAGTAAAATA
ATAGTGGGAAGCCACAGAGAGTAAAAT
ATAGTGGGAAGCCACAGAGAGTAAATA
ATAGTGGGAAGCCACAGAGAGTAAA
ATAGTGGGAAGCCACAGAGAGTAAAT
ATAGTGGGAAGCCACAGAGAGTAAAAT
ATAGTGGGAAGCCACAGAGAGTAAAA
ATAGTGGGAAGCCACAGAGAGTAAAAT
ATAGTGGGAAGTCACAGAGAGTAAATA
ATAGTGGGAAGCCACAGAGAGTAAATA
ATAGTGGGAAGCCACAGAGAGTAAAT
ATAGTGGGAAGCCACAGAGAGTAAATA
ATAGTGGGAAGCCACAGAGAGTAAAATA
ATAGTGGGAAGCCACAGAGAGTAAAT
ATAGTGGGAAGCCACAGAGAGTAAAAT
ATAGTGGGAAGCCACAGAGAGTAAAAT
ATAGTGGAAGCCACAGAGAGTAAAT
ATAGTGGGAAGCCACAGAGAGTAAATA
ATAGTGGGAAGCCACAGAGAGTAAAAT
ATAGTGGGAAGCCACAGAGAGTAAAAT
ATAGTGGGAAGCCACAGAGAGTAAA
ATAGTGGGAAGCCACAGAGAGTAAAAT
ATAGTGGGAAGCCACAGAGAGTAAAAT
ATAGTGGGAAGCCACAGAGAGTAAAAT
ATAGTGGGAAGCCACAGAGAGTAAAT
ATAGTGGGAAGCCACAGAGAGTAAAT
ATAGTGGGAAGCCACAGAGAGTAAATA
ATAGTGGGAAGCCACAGAGAGTAAAAT
ATAGTGGGAAGCCACAGAGAGTAAATA
ATAGTGGGAAGCCACAGAGAGTAAAAT
ATAGTGGGAAGCCACAGAGAGTAAAAT
ATGGTGGGAAGTCACAGAGAGTAAATA
ATAGTGGGAAGCCACAGAGAGTAAAAT
ATAGTGGGAAGCCACAGAGAGTAAATA
ATAGTGGGAAGCACAGAGAGTAAAA
ATAGTGGGAAGCCACAGAGAGTAAATA
ATAGTGGGAAGCCACAGAGAGTAAATA
ATAGTGGGAAGCCACAGAGAGTAAAAT
ATAGTGGGAAGCCACAGAGAGTAAATA
ATAGTGGGAAGCCACAGAGAGTAAATAG
TAGTGGGAAGCCACAGAGAGTAAATA

ATAGTGGGAAGCCACAGAGAGTAAATA
ATAGTGGGAAGCCACAGAGAGTAAAAAT
ATAGTGGGAAGCCACAGAGAGTAAAAT
ATAGTGGGAAGCCACAGAGAGTAAAA
ATAGTGGGAAGCCACAGAGAGTAAATA
ATAGTGGGAAGCCACAGAGAGTAAAAT
ATAGTGGGAAGCCACAGAGAGTAAAAT
ATAGTGGGAAGCCACAGAGAGTAAATAG
TAGTGGGAAGCCACAGAGAGTAAAAT
ATAGTGGGAAGCCACAGAGAGTAAAAT
ATAGTGGGAAGCCACAGAGAGTAAAAT
ATAGTGGGAAGCCACAGAGAGTAAATA
ATAGTGGAAGCCAAAAAAA
AAGTGGGAAGCCACAGAGAGTAAAAAT
ATAGTGGGAAGCCACAGAGAGTAAATA
ATAGTGGGAAGCCACAGAGAGTAAAAT
ATAGTGGGAAGCCACAGAGAGTAAAAT
ATAGTGGGAAGCCACAGAGAGTAAAAT
ATAGTGGGAAGCCACAGAGAGTAAAAT
ATAGTGGGAAGCCACAGAGAGTAAAAT
ATAGTGGGAAGCCACAGAGAGTAAAA
ATAGTGGGAAGCCACAGAGAGTAAAATAGTGAAGCACAGAGAGAAA
ATAGTGGGAAGCCACAGAGAGTAAAAT
ATAGTGGGAAGCCACAGAGAGTAAATA
ATAGTGGGAAGCCACAGAGAGTAAAAT
ATAGTGGGAAGCCACAGAGAGTAAAAT
ATAGTGGGAAGCCACAGAGAGTAAAAT
ATAGTGGGAAGCCACAGAGAGTAAAAT
ATAGTGGGAAGCCACAGAGAGTAAAAT
ATAGTGGGAAGCCACAGAGAGTAAAAT
ATAGTGGGAAGCCACAGAGAGTAAATAG
TAGTGGGAAGCCACAGAGAGTAAATA
ATAGTGGGAAGCCACAGAGAGTAAAAT
ATAGTGGGAAGCCACAGAGAGTAAAAT
ATAGTGGGAAGCCACAGAGAGTAAATAT
ATAGTGGGAAGCCACAGAGAGTAAAAT
ATAGTGGGAAGCCAGAGAGTAAAATA
ATAGGGGAAGCCACAGAGAGTAAAAT
ATAGTGGGAAGCCACAGAGAGTAAATA
ATAGTGGGAAGCCACAGAGAGTAAATA
ATAGTGGGAAGCCACAGAGAGTAAATA
ATAGTGGGAAGCCACAGAGAGTAAATA
ATAGTGGGAAGCCACAGAGAGTAAAT
ATAGTGGGAAGCCACAGAGAGTAAATA
ATAGTGGGAAGCCACAGAGAGTAAATA
ATAGTGGGAAGCCACAGAGAGTAAAAT
ATAGTGGGAAGCCACAGAGAGTAAAAT
ATAGTGGGAAGCCACAGAGAGTAAATA
ATAGTGGGAAGCCACAGAGAGTAAAT
ATAGTGGGAAGCCACAGAGAGTAAATA
ATAGTGGGAAGCCACAGAGAGTAAAAT
ATGGTGGGAAGCCACAGAGAGTAAATA
ATAGTGGGAAGCCACAGAGAGTAAATA
ATAGTGGGAAGCCACAGAGAGTAAAAT

ATGGTGGGAAGCCACAGAGAGTAAATA
ATAGTGGGAAGCCACAGAGAGTAAAATA
ATAGTGGGAAGCCACAGAGAGTAAATA
ATAGTGGGAAGCCACAGAGAGTAAATA
ATAGTGGGAAGCCACAGAGAGTAAATA
ATAGTGGGAAGCCACAGAGAGTAAATA
ATAGTGGGAAGCCACAGAGAGTAAATA
ATAGTGGGAAGCCACAGAGAGTAAAT
ATGTGGGAAGCCACAGAGAGTAAATA
ATAGTGGGAAGCACAGAGATAAAAATATGGAAGCCACAGAGAGTAAAA
ATAGTGGGAAGCCACAGAGAGTAAATA
ATAGTGGGAAGCCACAGAGAGTAAATAATATGAAGCACAGAGAGTAAAA
ATAGTGGGAAGCCACAGAGAGTAAATA
ATAGTGGGAAGCCACAGAGAGTAAATA
ATAGTGGGAAGCCACAGAGAGTAAATA
ATAGTGGGAAGCCACAGAGAGTAAATA
ATAGTGGGAAGCCACAGAGAGTAAATA
ATAGTGGGAAGCCACAGAGAGTAAATA
ATAGTGGGAAGCCACAGAGAGTAAAAT
ATGGTGGGAAGCCACAGAGAGTAAATA
ATAGTGGGAAGCCACAGAGAGTAAATA
ATAGTGGGAAGCCACAGAGAGTAAATA
ATAGTGGGAAGCCACAGAGAGTAAATA
ATAGTGGGAAGCCACAGAGAGTAAATA
ATAGTGGGAAGCCACAGAGAGTAAAATAAAAAAATA
ATAGTGGGAAGCCACAGAGAGTAAATA
ATAGTGGGAAGCCACAGAGAGTAAAAT
ATGGTGGGAAGCCACAGAGAGTAAATA
ATAGTGGGAAGCCACAGAGAGTAAAAT
ATGGTGGGAAGCCACAGAGAGTAAATA
ATAGTGGGAAGCCACAGAGAGTAAA
ATAGTGGGAAGCCACAGAGAGTAAATA
ATAGTGGGAAGCCACAGAGAGTAAATA
ATAGTGGGAAGCCACAGAGAGTAAATA
ATAGTGGGAAGCCACAGAGAGTAAATA
ATAGTGGGAAGCCACAGAGAGTAAATA
ATAGTGGGAAGCCACAGAGAGTAAAAT
ATGGTGGGAAGCCACAGAGAGTAAAAT
ATGGTGGGAAGCCACAGAGAGTAAATA
ATAGTAGGAAGCCACAGAGAGTAAA
ATATGGGAAGCCACAGAGAGTAAAAT
ATAGTGGGAAGCCACAGAGAGTAAAAT
ATAGTGGGAAGCCACAGAGAGTAAATA
ATAGTGGGAAGCCACAGAGAGTAAAT
ATAGTGGGAAGCCACAGAGAGTAAAAT
ATAGTGGGAAGCCACAGAGAGTAAATAG
TAGTGGGAAGCCACAGAGAGTAAATA
ATAGTGGGAAGCCACAGAGAGTAAAAT
ATGGTGGGAAGCCACAGAGAGTAAATA
ATAGTGGGAAGCCACAGAGAGTAAAA
ATAGTGGGAAGCCACAGAGAGTAAA
ATAGTGGGAAGCCACAGAGAGTAAATA
ATAGTGGGAAGCCACAGAGAGTAAATA
ATAGTGGGAAGCCACAGAGAGTAAATA

ATAGTGGGAAGCCACAGAGAGTAAAAT
ATGGTGGGAAGCCACAGAGAGTAAAAT
ATAGTGGGAAGCCACAGAGAGTAAAAAAGTAAAAAAAAA
ATAGTGGAAGCCACAGAGAGTAAATA
ATAGTGGGAAGCCACAGAGAGTAAAA
ATAGTGGGAAGCCACAGAGAGTAAATA
ATAGTGGGAAGCCACAGAGAGTAAATA
ATAGTGGGAAGCCACAGAGAGTAAAT
ATGGTGGGAAGCCACAGAGAGTAAAAT
ATAGTGGGAAGCCACAGAGAGTAAAAT
ATAGTGGGAAGCCACAGAGAGTAAAATAATCGGTGAAGCCACAGAGAGTAAAAT
ATAGTGGGAAGCCACAGAGAGTAAATA
ATAGTGGGAAGCCACAGAGAGTAAATA
ATAGTGGGAAGCCACAGAGAGTAAATA
ATAGTGGGAAGCCACAGAGAGTAAATA
ATAGTGGAAGCCACAGAGAGTAAAAATAAAAAATA
ATAGTGGGAAGCCACAGAGAGTAAATA
ATAGTGGGAAGCCACAGAGAGTAAATA
ATAGTGGAAGCCACAGAGAGTAAAAT
ATGGTGGGAAGCCACAGAGAGTAAATA
ATAGTGGGAAGCCACAGAGAGTAAAT
ATGTGGGAAGCCACAGAGAGTAAATA
ATAGTGGGAAGCCACAGAGAGTAAATA
ATAGTGGGAAGCCACAGAGAGTAAATA
ATAGTGGGAAGCCACAGAGAGTAAAAT
ATGGTGGGAAGCCACAGAGAGTAAATA
ATAGTGGGAAGCCACAGAGAGTAAAAT
ATGGTGGGAAGCCACAGAGAGTAAATA
ATAGTGGGAAGCCACAGAGAGTAAAAT
ATGGTGGGAAGCCACAGAGAGTAAATA
ATAGTGGGAAGCCACAGAGAGTAAATA
ATAGTGGGAAGCCACAGAGAGTAAATA
ATAGTGGGAAGCCACAGAGAGTAAATA
ATAGTGGGAAGCCACAGAGAGTAAATA
ATAGTGGGAAGCCACAGAGAGTAAATA
ATAGTGGGAAGCCACAGAGAGTAAAAT
ATGGTGGGAAGCCACAGAGAGTAAATA
ATAGTGGGAAGCCACAGAGAGTAAAAT
ATGGTGGGAAGCCACAGAGAGTAAAATATACAGAGAGTAAAAT
ATAGTGGGAAGCCACAGAGAGTAAAAT
ATGGTGGGAAGCCACAGAGAGTAAATA
ATAGTGGGAAGCCACAGAGAGTAAAAT
ATGGTGGGAAGCCACAGAGAGTAAATA
ATAGTGGGAAGCCACAGAGAGTAAATA
ATAGTGGGAAGCCACAGAGAGTAAATA
ATAGTGGGAAGCCACAGAGAGTAAATA
ATAGTGGGAAGCCACAGAGAGTAAATA
ATAGTGGGAAGCCACAGAGAGTAAATA
ATAGTGGGAAGCCACAGAGAGTAAATA
ATAGTGGGAAGCCACAGAGAGTAAAATA
ATAGTGGGAAGCCACAGAGAGTAAAAT
ATGTGGGAAGCCACAGAGAGTAAATA
ATAGTGGGAAGCCACAGAGAGTAAAAT
ATGGTGGGAAGCCACAGAGAGTAAATA

ATAGTGGGAAGCCACAGAGAGTAAAA
ATAGTGGGAAGCCACAGAGAGTAAAAAAAAAAAAAAATA
ATAGTGGGAAGCCACAGAGAGTAAAAT
ATGGTGGGAAGCCACAGAGAGTAAATA
ATAGTAGGAAGCCACAGAGAGTAAAAT
ATGGTGGGAAGCCACAGAGAGTAAATA
ATAGTGGGAAGCCACAGAGAGTAAATA
ATAGTGGGAAGCCACAGAGAGTAAAAT
ATGGTGGGAAGCCACAGAGAGTAAATA
ATAGTAGGAAGCCACAGAGAGTAAAAT
ATGGTGGGAAGCCACAGAGAGTAAATA
ATAGTGGGAAGCCACAGAGAGTAAAAT
ATGGTGGGAAGCCACAGAGAGTAAAATATACAGAGAGTAAAAA
ATAGTGGGAAGCCACAGAGAGTAAATA
ATAGTGGGAAGCCACAGAGAGTAAAAT
ATGGTGGGAAGCCACAGAGAGTAAATA
ATAGTGGGAAGCCACAGAGAGTAAATA
ATAGTGGGAAGCCACAGAGAGTAAAAT
ATGGTGGGAAGTCACAGAGAGTAAATA
ATAGTGGGAAGCCACAGAGAGTAAATA
ATAGTGGGAAGCCACAGAGAGTAAAAT
ATAGTGGGAAGCCACAGAGAGTAAAAT
ATAGTGGGAAGCCACAGAGAGTAAAAT
ATGGTGGGAAGCCACAGAGAGTAAAAT
ATAGTGGGAAGCCACAGAGAGTAAAAT
ATGGTGGGAAGCCACAGAGAGTAAATT
ATGGTGGGAAGCCACAGAGAGTAAATA
ATAGTGGGAAGCCACAGAGAGTAAAAT
ATAGTGGGAAGCCACAGAGAGTTACATTGTAAGGATTTCCCACTATGTCGATACATTGTAAGGATTTCATACTATGTCG
ATACATTGTGGGGATTTCCCACTATGTCGATACATTGTAAGGATTTCCCACTATGTCGATACATTGTGGGGATTTCGAC
Nr of selected repeats 334 Similarity 0.885216
Consensus:
ATAGTGGGAAGCCACAGAGAGTAaAAT
>Cele-UNSB01_4:9125039-9126314 Satlength=1276 Nr of Repeats=76 RepeatLength=15
seed=CTCACCACGA
CTCACCACGATGGGT
CTCACCACGAAGGGT
CTCACCACGATGGGC
CTCACCACGAAGGGT
CTCACCACGAAGGGT
CTCACCACGATGGGT
CTCACCACGATGGGT
CTCACCACGATGGGT
CTCACCACGAAAGGTCTCGCCACAATGGGT
CTCACCACGATGGGT
CTCACCACGAAAGGTCTCAATACGAAGGGT
CTCACCACGAAGGGT
CTCACCACGATGGGT
CTCACCACGATGGGT
CTCACCACGAAGGGTG
TCACCACGATGGGT
CTCACCACGATGGGT
CTCACCACGATGGGT
CTCACCACGAAGGGTCTCGCCACAATGGGT

CTCACCACGATGGGT
CTCACCACGATGGGT
CTCACCACGATGGGT
CTCACCACGAAGGGT
CTCACCACGATGGGC
CTCACCACGAAGGGT
CTCACCACGAAGGGT
CTCACCACGATGGGT
CTCACCACGATGGGT
CTCACCACGATGGGT
CTCACCACGAAAGGTCTCGCCACAATGGGT
CTCACCACGATGGGT
CTCACCACGAAAGGTCTCAATACGAAGGGT
CTCACCACGAAGGGT
CTCACCACGATGGGT
CTCACCACGATGGGT
CTCACCACGAAGGGTG
TCACCACGATGGGT
CTCACCACGATGGGT
CTCACCACGATGGGT
CTCACCACGAAGGGTCTCGCCACAATGGGT
CTCACCACGATGGGT
CTCACCACGATGGGT
CTCACCACGAAGGTTG
TCACCACGAAGGGT
CTCACCACGATGGGT
CTCACCACGATGGGT
CTCACCACGATGGGT
CTCACCACGAAGGGT
CTCACCACGAAGGGT
CTCACCACGATGGGT
CTCACCACGAAAGGTCTCGCCACAATGGGT
CTCACCACGATGGGT
CTCACCACGAAAGGT
CTCACCACGAAGGGT
CTCACCACGATGGGT
CTCACCACGATGGGTT
TCACCACGAAGGGTG
TCACCACGATGGGT
CTCACCACGATGGGT
CTCACCACGAAAGGTCTCGCCACAATGGGT
CTCACCACGATGGGT
CTCACCACGAAGGGTT
TCACCACGATGGGT
CTCACCACGAAGGGT
CTCACCACGAAGGGTT
TCACCACGATGGGT
CTCACCACGATGGGT
CTCACCACGATGGGT
CTCACCACGAAGGGT
CTCACCACGATGGGT
CTCACCACGATGGGT
CTCACCACGAAGGGT
CTCACCACGAAGGGT

CTCACCACGAAAGGTCTCGCCACAATGGGT
CTCACCACGATGGGT
CTCACCACGATGGGT
Nr of selected repeats 55 Similarity 0.889240
Consensus:
CTCACCACGAtGGGT
>Cele-UNSB01_4:9469814-9470654 Satlength=841 Nr of Repeats=56 RepeatLength=15
seed=ACGATGGGTC
ACGATGGGTCTCACC
ACGATGGGTCTCACC
ACGATGGGTCTCACC
ACGATGGGTCTCACC
ACGATGGGTCTCGCC
ACGATGGGTCTCGCC
ACGATGGGTCTCGCT
ACGATGGGTCTCACC
ACGATGGGTCTTTCC
ACGATGGGTCTCACC
ACGGTGGGTCTCGGC
ACGATGGGTCTCACC
ACGATGGGTCTCACC
ACGATGGGTCTTTCC
ACGATGGGTCTCACC
ACGGTGGGTCTCGCC
ACGATGGGTCTCGCC
ACGATGGCTCTCGCC
ACGATGGGTCTCACC
ACGATGGGTCTCACC
ACGATGGGTCTCGCC
ACGATGGGTCTCGCC
ACGATGGGTCTCGCC
ACGATGGGTCTCACC
ACGATGGGTCTCGCC
ACGATGGGTCTCGCC
ACGATGGGTCTCGCC
ACGATGGGTCTCACC
ACGATGGGTCTCGCC
ACGATGGGTCTCGCC
ACGATGGGTCTCGCC
ACGATGGGTCTCACC
ACGATGGGTCTCGCC
ACGATGGGTCTCGCC
ACGATGGGTCTCGCC
ACGATGGGTCTCACC
ACGATGGGTCTTTCC
ACGATGGGTCTCACC
ACGGTGGGTCTCGCC
ACGATGGGTCTCGCC
ACGATGGCTCTCGCC
ACGATGGGTCTCGCC
ACGATGGGTCTCGCC
ACGATGGGTCTCGCC
ACGATGGGTCTCGCC
ACGATGGGTCTCACC

ACGATGGGTCTTTCC
ACGATGGGTCTCACC
ACGGTGGGTCTCACC
ACGACGGGTCTCACC
ACGATGGGTCTCACC
ACGATGGGTCTCACC
ACGATGGGTCTCACC
ACGATGGGTCTTTCC
ACGATGGGTCTCACC
ACGGTGGGTCTCGCC
Nr of selected repeats 56 Similarity 0.902684
Consensus:
ACGATGGGTCTCnCC
>Cele-UNSB01_4:9470711-9470851 Satlength=141 Nr of Repeats=5 RepeatLength=30
seed=AATTTTCCAG
AATTTTCCAGAAGGTTCTAGAACAATCCAGAATTTTTTCG
AATTTTCCAGAAGGTTCTGGAACATTCCAG
AATTTTCTAG
AATTTTCCAGAAGGTTCTAAAGCTTTTCAG
AATTTTCCAGAAGGTTCTGGAACATTCTAG
Nr of selected repeats 3 Similarity 0.822222
Consensus:
AATTTTCCAGAAGGTTCTggAaCaTTccAG
>Cele-UNSB01_4:9470964-9471243 Satlength=280 Nr of Repeats=10 RepeatLength=11
seed=GTACTCCTAC
GTACTCCTACC
GTACTCCTACA
GTACTCCTACAGTACTATTACAGTACCCCGACCATATTCCACTACTAACCGCAAACCTATATCTCTTCAAAAGACAAAA
ACACAATTTTTCATAAACTACA
GTAATCCTACC
GTACTCCTACA
GTACTCCTACA
GTACTCCTACA
GTACTACTACAGTACCCCGACCATATCCCACTACTAACCTCAAACCTATATCTCTTCAAAAGACAAAAACACAATTTTT
CCCAAACTACA
GTAATCCTACC
GTACTCCTACA
Nr of selected repeats 8 Similarity 0.883117
Consensus:
GTAcTCCTACa
>Cele-UNSB01_4:9493015-9493149 Satlength=135 Nr of Repeats=4 RepeatLength=11
seed=CCCTACAGTA
CCCTACAGTAC
CCCTACAGTACTACTACAGTACCTTGACATTATCCATCACCAACTCCCAACGCAATATCCCTTCAAAAGCTGAACAACC
AAAATTTCCTAAACTACAGTAA
CCCTACAGTAA
CCCTACAGTAC
Nr of selected repeats 3 Similarity 0.919192
Consensus:
CCCTACAGTAc
>Cele-UNSB01_4:9825630-9825857 Satlength=228 Nr of Repeats=5 RepeatLength=45
seed=GGGTACCTCC
GGGTACCTCCTGATGATCTGATCCTTCAGCTTCAAAGGATCAGAA
GGGTACCTCCTGGTGATCTGATCCTTAAGCTCCGAAGGATCACAGG

GGGTACCTCCTGGTGATCTGATCCTTCAGCTCCGAAAGATCAGAA
GGGTACCTCCTGGTGATCTGATCCTTAAGCTCCGAAGGATCACAGG
GGGTACCTCCTGGTGATCTGATCCTTCAGCTCCGAAAGATCAGAA
Nr of selected repeats 3 Similarity 0.920988
Consensus:
GGGTACCTCCTGgTGATCTGATCCTTCAGCTcCgAAaGATCAGAA
>Cele-UNSB01_4:10086561-10087053 Satlength=493 Nr of Repeats=10 RepeatLength=11
seed=TACTGTAGGG
TACTGTAGGGT
TACTGTAGGGTTACTGTAGTTTAGGAAATTTTGATTTTTCAGCTTTTGAAGGGATATTGCGTTGGGAGTTGGTGATGGA
TAATGTCAAGGTACTGTAGTAGTACTGTCGGTG
TACTGTAGGGTTACTGTAGTTTAGGAAATTTTGATTTTTCAGCTTATGAATGGATATTGCGTTGGGAGTTGGTGATGGA
TAATGTCAAGGTACTGTAGTAG
TACTGTAGGGG
TACTGTAGGGT
TACTGTAGGGTTACTGTAGTTTGGCAAATTTTGATTTTTCAGCTTTTGAAGGGATATTGGGTTGGGAGTTGGTGATGAA
TAATGTCAAGGTACTGTAGTAG
TACTGTAGGGG
TACTGTAGGGT
TACTGTAGGGT
TACTATAGGGTTACTGTAGTTTAGGGAATTTTGATTTTTCAGCTTTTGAAGGGATATTGCGTTGGGAGTTGGTGATGGA
TAATGTCAAGGTACTGTAGTAGTACTGTAGTAG
Nr of selected repeats 6 Similarity 0.935354
Consensus:
TACTGTAGGGt
>Cele-UNSB01_4:10323949-10324337 Satlength=389 Nr of Repeats=26 RepeatLength=15
seed=GACCCATCGT
GACCCATCGTGACGA
GACCCATCGTGGTGA
GACCCATCGTGGTGA
GACCCATCGTGGTGA
GACCCATCGTGGTGA
GACCCATCGTGGTAA
GACCCATCGTGGTGA
GACCCATCGTGGCGA
GACCCATCGTGACGA
GACCCATCGTGGTGA
GACCCATCGTGGTGA
GACCCATCGTGGTGA
GACCCATCGTGACGA
GACCCATCGTGACGA
GACCCATCGTGGTGA
GACCCATCGTGGTGA
GACCCATCGTGGTGA
GACCCATCGTGACGA
GACCCATCGTGGTGA
GACCCATCGTGGT
GACCCATCGTGGTGA
GACCCGTCGTGCTGA
GACCCATCGTGGTGA
GACCCGTCGTGCTGA
GACCCATCGTGGTGA
GACCAATCGTGGTGA
Nr of selected repeats 25 Similarity 0.898074

Consensus:
GACCCATCGTGgtGA
>Cele-UNSB01_4:10324370-10326780 Satlength=2411 Nr of Repeats=67
RepeatLength=35 seed=GAAATTCAAA
GAAATTCAAATTTTCAGTGAAAAAAATTTTGGCGG
GAAATTCAAATTTTCAGTGAAAAATTTTGGCGG
GAAATTCAAATTTCAGTGAAAAAAATTTTTAGCGG
GAAATTCAAATTTTCAGTGAAAAAATTTTGGCGG
GAAATTCAAATTTTCTGTGAAAAAATTTTTGGCGG
GAAATTCAAATTTTCTGTGAGAAAAATTTTGGAGG
GAAATTCAAATTTTCAGTGAAAAAATTTTGGCGG
GAAATTCAAATTTTCAGTGAAAAAAAATTTTAGCGG
GAAATTCAAATGTTCAGTGAAAAAATTTTGGCGG
GAAATTCAAATTTTCAGTGAAAAAATTTTTGGCGG
GAAATTCAAATTTTCAGTGAAAAAAATTTTGGCGG
GAAATTCAAATTTTCTGTGAGAAAAATTTTGGCGG
GAAATTCAAATTTTCAGTGAAAAATTTTGGCGG
GAAATTCAAATTTCAGTGAAAAACATTTTGGCGG
GAAATTCAAATTTTCTGAGAAAAAAATTTTGGCGG
GAAATTCAAATTTTCAGTGAAAAATTTTGGCGG
GAAATTCAAATTTCAGTGAAAAAAATTTTTAGCGG
GAAATTCAAATTTTCAGTGAAAAAATTTTGGCGG
GAAATTCAAATTTTCTGTGAAAAAATTTTTGGCGG
GAAATTCAAATTTTCAGTGAAAAAATTTTTGGCGG
GAAATTCAAATTTTCAGTGAAAAAAACTTTGGCGG
GAAATTCAAATTTTCTGTGAGAAAAATTTTGGGGG
GAAATTCAAATTTTCAGTGAAAAAATGTTGGCGG
GAAATTCAAATTTTCAGTGAAAAATTTTAGCGG
GAAATTCAAATTTTCAGTGAAAAAATTTTGGCGG
GAAATTCAAATTTTCAGTGAAAAAATTTTTGGCGG
GAAATTCAAATTTTCAGTGAAAAAATTTTGGCAG
GAAATTCAAATTTTCAGTGAAAAAATTTTTGGCGG
GAAATTCAAATTTTCAGTGAAAAAAATTTTGGCGG
GAAATTCAAATTTTCTGTGAGAAAAATTTTGGCGG
GAAATTCAAATTTTCAGTGAAAAATTTTGGCGG
GAAATTCAAATTTCAGTGAAAAAAAATTTTAGCGG
GAAATTCAAATTTTCAGTGAAAAAATTTTGGCAG
GAAATTCAAATTTTCAGTGAAAAAATTTTTGGCGG
GAAATTCAAATTTTCAGTGAAAAAAATTTTGGCGG
GAAATTCAAATTTTCTGTGAGAAAAATTTTGGCGG
GAAATTCAAATTTTCAGTGAAAAATTTTGGCGG
GAAATTCAAATTTCAGTGAAAAAAAATTTTAGCGG
GAAATTCAAATGTTCAGTGAAAAAATTTTGGCGG
GAAATTCAAATTTTCAGTGAAAAAATTTTTGGCGG
GAAATTCAAATTTTCTGTGAGAAAAATTTTGGGGG
GAAATTCAAATTTTCTGTGAAAAAAATTTTGGCGG
GAAATTCAAATTTTCAGTGAAAAACATTTTGGCGG
GAAATTCAAATTTTCAGTGAAAAACATTTTGGCGG
GAAATTCAAATTTTCAGTGAAAAAATTTTTGGCGG
GAAATTCAAATTTTCTGTGAGAAAAATTTTGGGGG
GAAATTCAAATTTTCTGTGAAAAAAATTTTGGCGG
GAAATTCAAATTTTCAGTGAAAAACATTTTGGCGG
GAAATTCAAATTTTCAGTGAAAAACATTTTGGCGG
GAAATTCAAATTTTCTGTGAGAAAAATTTTGGGGG

GAAATTCAAATTTTCAGTGAAAAAATTTTTGGCGG
GAAATTCAAATTTTCAGTGAAAAAAATTTTGGCGG
GAAATTCAAATTTTCTGTGAGAAAAATTTTGGCGG
GAAATTCAAATTTTCAGTGAAAAATTTTGGCGG
GAAATTCAAATTTCAGTGAAAAAAAATTTTAGCGG
GAAATTCAAATGTTCAGTGAAAAAAATTTTGGCGG
GAAATTCAAATTTTCAGTGAAAAAATTTTTGGCGG
GAAATTCAAATTTTCTGTGAGAAAAATTTTGGGGG
GAAATTCAAATTTTCTGTGAAAAAATTTTTGGCGG
GAAATTCAAATTTTCAGTGAAAAACATTTTGGCGG
GAAATTCAAATTTTCAGTGAAAAACATTTTGGCGG
GAAATTCAAATTTTCTGAGAAAAAAATTTTTGGCGG
GAAATTCAAATTTTCTGAGAAACTTTTTGTTAGCTTAAGTACCACCTGGAACTGGCGAGACCCATCGTGGTGAGACCCT
TAAAAATATGGGCGGGGACTTCAAAATTTATGAGAAAAGAAATTTTGGCGA
GAATTCAAATTTTCTTTGAAAAATTTTGGCGG
GAATTCAAATTTTCTGAGAAAAATTTTGGCGG
GAATTCAAATTTTCTGGGAAAAAAATTTTTTGCGG
GAAATTCAGATTTTCTGAGAAAAAAGATTTTGGCGG
Nr of selected repeats 43 Similarity 0.847276
Consensus:
GAAATTCAAATTTTCaGTGAaAAAAaTTTTGGCGG
>Cele-UNSB01_4:10610325-10610863 Satlength=539 Nr of Repeats=7 RepeatLength=77
seed=GGAAGCTTCA
GGAAGCTTCATCTTTTGAATTATAAAAAATAAATTTCATTTCCAAATCAGTGTGTGCACTATCAAAAGTGTCCACT
GGAAGCTTCATCTTTTGAATTATAAAAAAATAAATTTCATTTCAAAATCAGTGTGTGCACTATCAAAAGTGTCCACT
GGAAGCTTCATCTTTTGAATTATAAAAAAATAAATTTCATTTCAAAATCAGTGTGTGCACTATCAAAAGTGTCCACT
GGAAGCTTCATCTTTTGAATTATAAAAAAACAAATTTCATTTCAAAATCAGTGTGTGCACTATCAAAAGTGTCCACT
GGAAGCTTCATCTTTTGAATTATAAAAAAATAAATTTCATTTCAAAATCAGTGTGTGCACTATCAAAAGTGTCCACT
GGAAGCTTCATCTTTTGAATTATAAAAAAACAAATTTCATTTCAAAATCAGTGTGTGCACTATCAAAAGTGTCCACT
GGAAGCTTCATCTTTTGAATTATAAAAAAATAAATTTCATTTCAAAATCAGTGTGTGCACTATCAAAAGTGACCACT
Nr of selected repeats 6 Similarity 0.984993
Consensus:
GGAAGCTTCATCTTTTGAATTATAAAAAAAtAAATTTCATTTCAAAATCAGTGTGTGCACTATCAAAAGTGTCCACT
>Cele-UNSB01_4:10751931-10752311 Satlength=381 Nr of Repeats=20 RepeatLength=19
seed=ATTGTTACAC
ATTGTTACACGAAAAATAG
ATTGTTACACGAAAAATAG
ATTGTTACACGAAAAATAG
ATTGTTACACGAAAAATAG
ATTGTTACACGAAAAATAG
ATTGTTACACGAAAAATAG
ATTGTTACAAGAAAAATAG
ATTGTTACACGAAAAATAG
ATTGTTACACGAAAAATAT
ATTGTTACACGAAAAATAG
ATTGTTACACGAAAAATAT
ATTGTTACACGAAAAATAG
ATTGTTACACGAAAAATAG
ATTGTTACAAGAAAAATAG
ATTGTTACACGAAAAATAT
ATTGTTACACGAAAAATAG
ATTGTTACACGAAAAATAG
ATTGTTACACGAAAAATAG

ATTGTTACACGAAAAATAG
Nr of selected repeats 20 Similarity 0.967867
Consensus:
ATTGTTACACGAAAAATAG
>Cele-UNSB01_4:11013752-11031328 Satlength=17577 Nr of Repeats=392
RepeatLength=45 seed=TCCTGGTGAT
TCCTGGTGATCTGATCCTTAAGCTCCGAAGGATCAGAAGGGTACC
TCCTGGTGATGTGATCCTTAAGCTCCGAAGGATCACAGGGGTACC
TCCTGGTGATCTGATCCTTAAGCTCCGAAGGATCACAGGGGTACC
TCCTGGTGATCTGATCCTTAAGCTCCGAAGGATCAGAAGGGTACC
TCCTGGTGATCTGATCCTTAAGCTCCGAAGGATCACAGGGTACC
TCCTGGTGATCTGATCCTTAAGCTCCGAAGGATCAGAAGGGTACC
TCCTGGTGATCTGATCCTTAGCTCGAAGGATCAAAGGGTACC
TCCTGGTGATCTGATCCTTCAGCTCCGAAGGATCACAAGGGTACC
TCCTGGTGATCTGATCCTTAGCTCCGAAGGATCACAAGGGTACC
TCCTGGTGATCTGATCCTTAAGCTCCGAAGGATCAGAAGGGTACC
TCCTGGTGATCTGATCCTTAAGCTCCGAAGGATCACAGGGGTACC
TCCTGGTGATCTGATCCTTAAGCTCCGAAGGATCAGAAGGGTACC
TCCTGGTGATCTGATCCTTCAGCTTCAAAGGATCAAAGGGTACC
TCCTGGTGATCTGATCCTTAAGCTCCGAAGGATCAAAGGGTACC
TCCTGGTGATCTGATCCTTAAGCTCCGAAGGATCAGAAGGGTACC
TCCTGGTGATCTGATCCTTAAGCTCCGAAGGATCAGAAGGGTACC
TCCTGGTGATCTGATCCTTAAGCTCCGAAGGATCACAGGGGTACC
TCCTGGTGATCTGATCCTTCAGCTCCGAAGGATCAGAAGGGTACC
TCCTGGTGATCTGATCCTTCAGCTCCGAAGGATCAGAAGGGTACC
TCCTGGTGATCTGATCCTTAAGCTCCGAAGGATCAGAAGGGTACC
TCCTGGTGATCTGATCCTTAAGCTCCGAAGGATCAGAAGGGTACC
TCCTGGTGATCTGATCCTTCAGCTCCGAAGGATCAGAAGGTACC
TCCTGGTGATCTGATCCTTAAGCTCCGAAGGATCACAAGGGTACC
TCCTGGTGATCTGATCCTTAAGCTCCGAAGGATCAGAAGGGTAC
TCCTGGTGATCTGATCCTTAGCTCGAAGGATCAGAAGGGTACC
TCCTGGTGATCTGATCCTTCAGCTTCAAAGGATCAGAAGGGTACC
TCCTGGTGATCTGATCCTTCAGCTCCGAAGGATCAGAAGGGTACC
TCCTGGTGATCTGATCCTTAAGCTCCGAAGGATCAGAAGGGTACC
TCCTGGTGATCTGATCCTTAAGCTCCGAAGGATCACAGGGGTACC
TCCTGGTGATCTGATCCTTCAGCTCCGAAGGATCAGAAGGGTACC
TCCTGGTGATCTGATCCTTCAGCTCCGAAGGATCAAAGGGTACC
TCCTGGTGATCTGATCCTTAAGCTCCGAAGGATCAGAAGGGTACC
TCCTGGTGATCTGATCCTTAAGCTCCGAAGGATCAGAAGGGTACC
TCCTGGTGATCTGATCCTTAAGCTCCGAAGGATCACAGGGGTACC
TCCTGGTGATCTGATCCTTCAGCTTCAAAGGATCAGAAGGGTACC
TCCTGGTGATCTGATCCTTCAGCTCCGAAGGATCAGAAGGGTACC
TCCTGGTGATCTGATCCTTAGCTCCGAAGGATCAGAAGGGTACC
TCCTGGTGATCTGATCCTTAAGCTCCGAAGGATCAGAAGGGTACC
TCCTGGTGATCTGATCCTTAAGCTCCGAAGGATCACAGGGGTACC
TCCTGGTGATCTGATCCTTCAGCTTCAAAGGATCAGAAGGGTACC
TCCTGGTGATCTGATCCTTCAGCTCCGAAGGATCAAAGGGTACC
TCCTGGTGATCTGATCCTTAAGCTCCGAAGGATCAGAAGGGTACC
TCCTGGTGATCTGATCCTTAGCTCCGAAGGATCAGAAGGGTACC
TCCTGGTGATCTGATCCTTCAGCTTCAAAGGATCAGAAGGGTACC
TCCTGGTGATCTGATCCTTCAGCTCCGAAGGATCAGAAGGGTACC
TCCTGGTGATCTGATCCTTAAGCTCCGAAGGATCAGAAGGGTACC
TCCTGGTGATCTGATCCTTAAGCTCCGAAGGATCACAGGGGTACC
TCCTGGTGATCTGACCTTCAGCTTCAAAGGATCAGAAGGGTACC

TCCTGGTGATCTGATCCTTCAGCTCCGAAGGATCAGAAGGGTACC
TCCTGGTGATCTGATCCTTAAGCTCCGAAGGATCACAGGGGTACC
TCCTGGTGATCTGATCCTTAAGCTCCGAAGGATCAGAAGGGTACC
TCCTGGTGATCTGATCCTTAAGCTCCGAAGGATCAGAAGGGTACC
TCCTGGTGATCTGATCCTTAAGCTCCGAAGGATCAGAAGGGTACC
TCCTGGTGATCTGATCCTTAAGCTCCGAAGGATCACAGGGGTACC
TCCTGGTGATCTGATCCTTAAGCTCCGAAGGATCAGAAGGGTACC
TCCTGGTGATCTGATCCTTCAGCTTCAAAGATCAGAAGGGTACC
TCCTGGTGATCTGATCCTTCAGCTCCGAAGGATCAGAAGGGTACC
TCCTGGTGATCTGATCCTTAAGCTCCGAAGGATCACAGGGATACC
TCCTGGTGATCTGATCCTTCAGCTTCAAAGGATCATAAGGGTACC
TCCTGGTGATCTGATCCTTAAGCTCCGAAGGATCACAAGGGTACC
TCCTGGTGATCTGATCCTTAAGCTCCGAAGGATCAGAAGGGTACC
TCCTGGTGATCTGATCCTTAAGCTCCGAAGGATCAGAAGGGTACC
TCCTGGTGATCTGATCCTTAAGCTCCGAAGGATCAGAAGGGTACC
TCCTGGTGATCTGATCCTTAAGCTCCGAAGGATCAGAAGGGTACC
TCCTGGTGATCTGATCCTTAAGCTCCGAAGGATCACAGGGGTACC
TCCTGGTGATCTGATCCTTCAGCTTCAAAGGATCAGAAGGGTACC
TCCTGGTGATCTGATCCTTCAGCTTCAAAGGATCAGAAGGGTACC
TCCTGGTGATCTGATCCTTCAGCTCCGAAGGATCAGAAGGGTACC
TCCTGGTGATCTGATCCTTAAGCTCCGAAGGATCACAGGGGTACC
TCCTGGTGATCTGACCCTTCAGCTTCAAAGGATCAGAAGGGTACC
TCCTGGTGATCTGATCCTTCAGCTCCGAAGGATCAGAAGGGTACC
TCCTGGTGATCTGATCCTTAAGCTCCGAAGGATCACAGGGGTACC
TCCTGGTGATCTGATCCTTAAGCTCCGAAGGATCACAGGGGTACC
TCCTGGTGATCTGATCCTTCAGCTTCAAAGGATCACAAGGGTACC
TCCTGGTGATCTGATCCTTCAGCTCCGAAGGATCAGAAGGGTACC
TCCTGGTGATCTGATCCTTAAGCTCCGAAGGATCAAAGGGTACC
TCCTGGTGATCTGATCCTTAAGCTCCGAAGGATCAGAAGGGTACC
TCCTGGTGATCTGATCCTTAAGCTCCGAAGGATCAGAAGGGTACC
TCCTGGTGATCTGATCCTTAAGCTCCGAAGGATCACAGGGGTACC
TCCTGGTGATCTGATCCTTCAGCTTCAAAGGATCAGAAGGGTACC
TCCTGGTGATCTGATCCTTCAGCTCCGAAGGATCAGAAGGGTACC
TCCTGGTGATCTGATCCTTCAGCTTCAAAGGATCAGAAGGGTACC
TCCTGGTGATCTGATCCTTCAGCTTCAAAGGATCAGAAGGGTACC
TCCTGGTGATCTGATCCTTCAGCTCCGAAGGATCAGAAGGGTACC
TCCTGGTGATCTGATCCTTAAGCTCCGAAGGATCACAGGGTACC
TCCTGGTGATCTGATCCTTAGCTCCGAAGGATCAAAGGGTACC
TCCTGGTGATCTGATCCTTAAGCTCCGAAGGATCACAAGGGTACC
TCCTGGTGATCTGATCCTTAAGCTCCGAAGGATCAGAAGGGTACC
TCCTGGTGATCTGATCCTTCAGCTTCAAAGGATCAAAGGGTACC
TCCTGGTGATCTGATCCTTAAGCTCCGAAGGATCAGAAGGGTACC
TCCTGGTGATCTGATCCTTAAGCTCCGAAGGATCAGAAGGGTACC
TCCTGGTGATCTGATCCTTCAGCTTCAAAGGATCACAAGGGTACC
TCCTGGTGATCTGACCTTCAGCTCCGAAGGATCAGAAGGGTACC
TCCTGGTGATCTGATCCTTAAGCTCCGAAGGATCAGAAGGGTACC
TCCTGGTGATCTGATCCTTAAGCTCCGAAGGATCACAGGGGTACC
TCCTGGTGATCTGACCTTAGCTCAAGGATCAGAAGGGTACC
TCCTGGTGATCTGATCCTTCAGCTCCGAAGGATCAAAGGGTACC
TCCTGGTGATCTGATCCTTCAGCTCCGAAGGATCAGAAGGGTACC
TCCTGGTGATCTGATCCTTCAGCTTCAAAGGATCAGAAGGGTACC
TCCTGGTGATCTGATCCTTAAGCTCCGAAGGATCAGAAGGGTACC
TCCTGGTGATCTGATCCTTAAGCTCCGAAGGATCAGAAGGGTACC
TCCTGGTGATCTGATCCTTAAGCTCCGAAGGATCACAGGGGTACC

TCCTGGTGATCTGACCTTCAGCTCAAGGATCAGAAGGGTACC
TCCTGGTGATCTGATCCTTAAGCTCCGAAGGATCAGAAGGGTACC
TCCTGGTGATCTGATCCTTAAGCTCCGAAGGATCAGAAGGGTACC
TCCTGGTGATCTGATCCTTAAGCTCCGAAGAATCAGAAGGGTACC
TCCTGGTGATCTGATCCTTAAGCTCCGAAGGATCAGAAGGGTACC
TCCTGGTGATCTGATCCTTAAGCTCCGAAGGATCACAGGGGTACC
TCCTGGTGATCTGACCCTTCAGCTTCAAAGGATCAGAAGGGTACC
TCCTGGTGATCTGATCCTTCAGCTTCAAAGGATCAGAAGGGTACC
TCCTGGTGATCTGATCCTTCAGCTCCGAAGGATCAGAAGGGTACC
TCCTGGTGATCTGATCCTTAAGCTCCGAAGGATCAGAAGGGTACC
TCCTGGTGATCTGATCCTTAAGCTCCGAAGGATCACAGGGGTACC
TCCTGGTGATCTGACCCTTCAGCTTCAAAGGATCAGAAGGGTACC
TCCTGGTGATCTGATCCTTCAGCTCCGAAGGATCAGAAGGGTACC
TCCTGGTGATCTGATCCTTAAGCTCCGAAGGATCACAGGGGTACC
TCCTGGTGATCTGATCCTTCAGCTCAAAGGATCAGAAGGGTACC
TCCTGGTGATCTGATCCTTCAGCTCAAGGATCAAAGGGTACC
TCCTGGTGATCTGATCCTTCAGCTCCGAAGGATCACAAGGGTACC
TCCTGGTGATCTGATCCTTCAGCTTCAAAGGATCATAAGGGTACC
TCCTGGTGATCTGATCCTTCAGCTTCAAAGGATCAGAAGGGTACC
TCCTGGTGATCTGATCCTTCAGCTCCGAAGGATCAGAAGGGTACC
TCCTGGTGATCTGATCCTTAAGCTCCGAAGGATCAGAAGGGTACC
TCCTGGTGATCTGATCCTTAAGCTCCGAAGGATCACAGGGGTACC
TCCTGGTGATCTGATCCTTCAGCTTCAAAGGATCAGAAGGGTACC
TCCTGGTGATCTGATCCTTCAGCTCCGAAGGATCAGAAGGGTACC
TCCTGGTGATCTGATCCTTCAGCTTCAAAGGATCAGAAGGGTACC
TCCTGGTGATCTGATCCTTCAGCTTCAAAGGATCAGAAGGGTACC
TCCTGGTGATCTGATCCTTCAGCTCCGAAGGATCAAAGGGTACC
TCCTGGTGATCTGATCCTTAAGCTCCGAAGGATCACAGGGTACC
TCCTGGTGATCTGATCCTTCAGCTTCAAAGGATCATAAGGGTACC
TCCTGGTGATCTGATCCTTCAGCTCCGAAGGATCAGAAGGGTACC
TCCTGGTGATCTGATCCTTAAGCTCCGAAGGATCAGAAGGGTACC
TCCTGGTGATCTGATCCTTCAGCTTCAAAGGATCAGAAGGGTACC
TCCTGGTGATCTGATCCTTCAGCTCCGAAGGATCAGAAGGGTACC
TCCTGGTGATCTGATCCTTAAGCTCCGAAGGATCAGAAGGGTACC
TCCTGGTGATCTGATCCTTCAGCTTCAAAGGATCAAAGGGTACC
TCCTGGTGATCTGACCTTAAGCTCCGAAGGATCAGAAGGGTACC
TCCTGGTGATCTGATCCTTAAGCTCCGAAGGATCAGAAGGGTACC
TCCTGGTGATCTGATCCTTAAGCTCCGAAGGATCACAGGGGTACC
TCCTGGTGATCTGATCCTTCAGCTCCGAAGGATCAGAAGGGTACC
TCCTGGTGATCTGATCCTTAAGCTCCGAAGGATCAGAAGGGTACC
TCCTGGTGATCTGATCCTTCAGCTCAAGGATCAAAGGGTACC
TCCTGGTGATCTGATCCTTAAGCTCCGAAGGATCAGAAGGGTACC
TCCTGGTGATCTGATCCTTAAGCTCCGAAGGATCAGAAGGGTACC
TCCTGGTGATCTGATCCTTAAGCTCCGAAGGATCAGAAGGGTACC
TCCTGGTGATCTGATCCTTAGCTCCGAAGGATCAAGGGTACC
TCCTGGTGATCTGACCTTCAGCTTCAAAGGATCAGAAGGGTACC
TCCTGGTGATCTGATCCTTAAGCTCCGAAGGATCAGAAGGGTACC
TCCTGGTGATCTGATCCTTAAGCTCCGAAGGATCAGAAGGGTACC
TCCTGGTGATCTGATCCTTAAGCTCCGAAGGATCAGAAGGGTACC
TCCTGGTGATCTGATCCTTAAGCTCCGAAGGATCACAGGGGTACC
TCCTGGTGATCTGATCCTTAAGCTCCGAAGGATCAGAAGGGTACC
TCCTGGTGATCTGATCCTTAAGCTCCGAAGGATCAGAAGGGTACC
TCCTGGTGATCTGATCCTTAGCTCAAGGATCAGGGTACC
TCCTGGTGATCTGATCCTTAAGCTCCGAAGGATCACAGGGTACC

TCCTGGTGATCTGATCCTTCAGCTTCAAAGGATCAAAGGGTACC
TCCTGGTGATCTGATCCTTCAGCTCCGAAGGATCAAAGGGTACC
TCCTGGTGATCTGATCCTTAAGCTCCGAAGGATCACAGGGTACC
TCCTGGTGATCTGATCCTTCAGCTTCAAAGGATCAGAAGGGTACC
TCCTGGTGATCTGATCCTTCAGCTTCAAAGGATCAGAAGGGTACC
TCCTGGTGATCTGATCCTTCAGCTCCGAAGGATCAGAAGGGTACC
TCCTGGTGATCTGATCCTTAAGCTCCGAAGGATCACAGGGTACC
TCCTGGTGATCTGATCCTTCAGCTTCAAAGGATCAGAAGGGTACC
TCCTGGTGATCTGATCCTTCAGCTCCGAAGGATCAGAAGGGTACC
TCCTGGTGATCTGATCCTTAAGCTCCGAAGGATCACAGGGGTACC
TCCTGGTGATCTGATCCTTCAGCTTCAAAGGATCACAAGGGTACC
TCCTGGTGATCTGACCCTTCAGCTTCAAAGGATCAGAAGGGTACC
TCCTGGTGATCTGATCCTTCAGCTTCAAAGGATCAGAAGGGTACC
TCCTGGTGATCTGATCCTTCAGCTCCGAAGGATCAGAAGGGTACC
TCCTGGTGATCTGATCCTTAAGCTCCGAAGGATCACAGGGGTACC
TCCTGGTGATCTGACCCTTCAGCTTCAAAGGATCAGAAGGGTACC
TCCTGGTGATCTGATCCTTCAGCTCCGAAGGATCAGAAGGGTACC
TCCTGGTGATCTGATCCTTAAGCTCCGAAGGATCAGAAGGGTACC
TCCTGGTGATCTGATCCTTAAGCTCCGAAGGATCACAGGGGTACC
TCCTGGTGATCTGATCCTTCAGCTTCAAAGGATCACAAGGGTACC
TCCTGGTGATCTGATCCTTCAGCTCCGAAGGATCAGAAGGGTACC
TCCTGGTGATCTGATCCTTAAGCTCCGAAGGATCAGAAGGGTACC
TCCTGGTGATCTGATCCTTAAGCTCCGAAGGATCAGAAGGGTACC
TCCTGGTGATCTGATCCTTAAGCTCCGAAGGATCACAGGGGTACC
TCCTGGTGATCTGATCCTTAAGCTCCGAAGGATCAGAAGGGTACC
TCCTGGTGATCTGATCCTTCAGCTTCAAAGGATCAGAAGGGTACC
TCCTGGTGATCTGATCCTTCAGCTCCGAAGGATCACAAGGGTACC
TCCTGGTGATCTGATCCTTAAGCTCCGAAGGATCACAGGGATACC
TCCTGGTGATCTGATCCTTCAGCTTCAAAGGATCAGAAGGGTACC
TCCTGGTGATCTGATCCTTCAGCTCCGAAGGATCACAAGGGTACC
TCCTGGTGATCTGATCCTTAAGCTCCGAAGGATCAGAAGGGTACC
TCCTGGTGATCTGATCCTTCAGCTTCAAAGGATCATAAGGGTACC
TCCTGGTGATCTGAACCTTAAGCTCCGAAGGATCAGAAGGGTACC
TCCTGGTGATCTGATCCTTAAGCTCCGAAGGATCAGAAGGGTACC
TCCTGGTGATCTGATCCTTAAGCTCCGAAGGATCACAGGGTACC
TCCTGGTGATCTGATCCTTAAGCTCCGAAGGATCAGAAGGGTACC
TCCTGGTGATCTGATCCTTCAGCTTCAAAGGATCAGAAGGGTACC
TCCTGGTGATCTGATCCTTAAGCTCCGAAGGATCAGAAGGGTACC
TCCTGGTGATCTGATCCTTAAGCTCCGAAGGATCAGAAGGGTACC
TCCTGGTGATCTGATCCTTAAGCTCCGAAGGATCACAGGGTACC
TCCTGGTGATCTGACCCTTCAGCTTCAAAGGATCAGAAGGGTACC
TCCTGGTGATCTGATCCTTCAGCTTCAAAGGATCAGAAGGGTACC
TCCTGGTGATCTGATCCTTCAGCTCCGAAGGATCAGAAGGGTACC
TCCTGGTGATCTGATCCTTAAGCTCCGAAGGATCAGAAGGGTACC
TCCTGGTGATCTGATCCTTAAGCTCCGAAGGATCACAGGGGTACC
TCCTGGTGATCTGACCCTTCAGCTTCAAAGGATCAGAAGGGTACC
TCCTGGTGATCTGATCCTTCAGCTCCGAAGGATCAGAAGGGTACC
TCCTGGTGATCTGATCCTTAAGCTCCGAAGGATCACAGGGGTACC
TCCTGGTGATCTGATCCTTAAGCTCCGAAGGATCAGAAGGGTACC
TCCTGGTGATCTGATCCTTAAGCTCCGAAGGATCAGAAGGGTACC
TCCTGGTGATCTGATCCTTAAGCTCCGAAGGATCACAGGGGTACC
TCCTGGTGATCTGATCCTTAAGCTCCGAAGGATCAGAAGGGTACC
TCCTGGTGATCTGATCCTTCAGCTTCAAAGATCAAAGGGTACC
TCCTGGTGATCTGATCCTTCAGCTCCGAAGGATCAGAAGGGTACC

TCCTGGTGATCTGATCCTTAAGCTCCGAAGGATCAGAAGGGTACC
TCCTGGTGATCTGATCCTTAAGCTCCGAAGGATCAGAAGGGTACC
TCCTGGTGATCTGATCCTTAAGCTCCGAAGGATCAGAAGGGTACC
TCCTGGTGATCTGATCCTTAAGCTCCGAAGGATCACAGGGGTACC
TCCTGGTGATCTGACCCTTCAGCTTCAAAGGATCAGAAGGGTACC
TCCTGGTGATCTGATCCTTCAGCTCCGAAGGATCAGAAGGGTACC
TCCTGGTGATCTGATCCTTAAGCTCCGAAGGATCAGAAGGGTACC
TCCTGGTGATCTGATCCTTCAGCTCCGAAGGATCAGAAGGGTACC
TCCTGGTGATCTGATCCTTCAGCTCCGAAGGATCAGAAGGGTACC
TCCTGGTGATCTGATCCTTAAGCTCCGAAGGATCACAGGGGTACC
TCCTGGTGATCTGACCCTTCAGCTTCAAAGGATCAGAAGGGTACC
TCCTGGTGATCTGATCCTTCAGCTTCAAAGGATCAGAAGGGTACC
TCCTGGTGATCTGATCCTTCAGCTCCGAAGGATCAGAAGGGTACC
TCCTGGTGATCTGATCCTTAAGCTCCGAAGGATCAGAAGGGTACC
TCCTGGTGATCTGACCCTTCAGCTTCAAAGGATCAGAAGGGTACC
TCCTGGTGATCTGATCCTTCAGCTCCGAAGGATCAGAAGGGTACC
TCCTGGTGATCTGATCCTTAAGCTCCGAAGGATCACAGGGGTACC
TCCTGGTGATCTGATCCTTCAGCTTCAAAGGATCAGAAGGGTACC
TCCTGGTGATCTGATCCTTCAGCTTCAAAGGATCAGAAGGGTACC
TCCTGGTGATCTGATCCTTCAGCTTCAAAGGATCAGAAGGGTACC
TCCTGGTGATCTGATCCTTCAGCTCCGAAGGATCAGAAGGGTACC
TCCTGGTGATCTGATCCTTCAGCTCCGAAGGATCAGAAGGGTACC
TCCTGGTGATCTGATCCTTAAGCTCCGAAGGATCACAGGGGTACC
TCCTGGTGATCTGATCCTTCAGCTTCAAAGGATCAGAAGGGTACC
TCCTGGTGATCTGATCCTTAAGCTCCGAAGGATCAGAAGGGTACC
TCCTGGTGATCTGATCCTTAAGCTCCGAAGGATCAGAAGGGTACC
TCCTGGTGATCTGATCCTTAAGCTCCGAAGGATCAGAAGGGTACC
TCCTGGTGATCTGATCCTTAAGCTCCGAAGGATCAGAAGGGTACC
TCCTGGTGATCTGATCCTTAAGCTCCGAAGGATCAGAAGGGTACC
TCCTGGTGATCTGATCCTTCAGCTTCAAAGGATCAGAAGGGTACC
TCCTGGTGATCTGATCCTTCAGCTCCGAAGGATCAGAAGGGTACC
TCCTGGTGATCTGATCCTTAAGCTCCGAAGGATCACAGGGTACC
TCCTGGTGATCTGATCCTTCAGCTTCAAAGGATCAGAAGGGTACC
TCCTGGTGATCTGATCCTTCAGCTCCGAAGGATCACAAGGGTACC
TCCTGGTGATCTGATCCTTAAGCTCCGAAGGATCAGAAGGGTACC
TCCTGGTGATCTGATCCTTCAGCTTCAAAGGATCATAAGGGTACC
TCCTGGTGATCTGATCCTTAAGCTCCGAAGGATCAGAAGGGTACC
TCCTGGTGATCTGATCCTTAAGCTCCGAAGGATCAGAAGGGTACC
TCCTGGTGATCTGATCCTTAAGCTCCGAAGGATCACAGGGTACC
TCCTGGTGATCTGATCCTTAAGCTCCGAAGGATCAGAAGGGTACC
TCCTGGTGATCTGATCCTTCAGCTTCAAAGGATCATAAGGGTACC
TCCTGGTGATCTGATCCTTAAGCTCCGAAGGATCAGAAGGGTACC
TCCTGGTGATCTGATCCTTAAGCTCCGAAGGATCAGAAGGGTACC
TCCTGGTGATCTGATCCTTAAGCTCCGAAGGATCACAGGGTACC
TCCTGGTGATCTGACCCTTCAGCTTCAAAGGATCAGAAGGGTACC
TCCTGGTGATCTGATCCTTCAGCTTCAAAGGATCAGAAGGGTACC
TCCTGGTGATCTGATCCTTCAGCTCCGAAGGATCAGAAGGGTACC
TCCTGGTGATCTGATCCTTAAGCTCCGAAGGATCAGAAGGGTACC
TCCTGGTGATCTGATCCTTAAGCTCCGAAGGATCACAGGGGTACC
TCCTGGTGATCTGACCCTTCAGCTTCAAAGGATCAGAAGGGTACC
TCCTGGTGATCTGATCCTTCAGCTCCGAAGGATCAGAAGGGTACC
TCCTGGTGATGTGATCCTTAAGCTCCGAAGGATCACAGGGGTACC
TCCTGGTGATCTGATCCTTAAGCTCCGAAGGATCAGAAGGGTACC
TCCTGGTGATCTGATCCTTAAGCTCCGAAGAATCAGAAGGGTACC

TCCTGGTGATCTGATCCTTAAGCTCCGAAGGATCACAGGGGTACC
TCCTGGTGATCTGATCCTTAAGCTCCGAAGGATCAGAAGGGTACC
TCCTGGTGATCTGATCCTTCAGCTTCAAACGATCATAAGGGTACC
TCCTGGTGATCTGATCCTTCAGCTCCGAAGGATCACAAGGGTACC
TCCTGGTGATCTGATCCTTAAGCTCCGAAGGATCAGAAGGGTACC
TCCTGGTGATCTGATCCTTAAGCTCCGAAGGATCACAGGGATACC
TCCTGGTGATCTGATCCTTAAGCTCCGAAGGATCAGAAGGGTACC
TCCTGGTGATCTGATCCTTAAGCTCCGAAGGATCACAGGGGTACC
TCCTGGTGATCTGATCCTTCAGCTTCAAAGGATCAGAAGGGTACC
TCCTGGTGATCTGATCCTTCAGCTCCGAAGGATCAGAAGGGTACC
TCCTGGTGATCTGATCCTTAAGCTCCGAAGGATCAGAAGGGTACC
TCCTGGTGATCTGATCCTTCAGCTTCAAAGGATCATAAGGGTACC
TCCTGGTGATCTGATCCTTCAGCTCCGAAGGATCAGAAGGGTACC
TCCTGGTGATCTGATCCTTAAGCTCCGAAGGATCACAGGGGTACC
TCCTGGTGATCTGATCCTTAAGCTCCGAAGGATCACAGGGGTACC
TCCTGGTGATCTGATCCTTAAGCTCCGAAGGATCAGAAGGGTACC
TCCTGGTGATCTGATCCTTCAGCTTCAAAGGATCAGAAGGGTACC
TCCTGGTGATCTGATCCTTCAGCTTCAAAGGATCATAAGGGTACC
TCCTGGTGATCTGATCCTTCAACTCCGAAGGATCACAAGGGTACC
TCCTGGTGATCTGATCCTTCAGCTTCAAAGGATCACAAGGGTACC
TCCTGGTGATCTGACCCTTCAGCTTCAAAGGATCAGAAGGGTACC
TCCTGGTGATCTGATCCTTCAGCTCCGAAGGATCAGAAGGGTACC
TCCTGGTGATCTGATCCTTAAGCTCCGAAGGATCACAGGGGTACC
TCCTGGTGATCTGATCCTTAAGCTCCGAAGGATCAGAAGGGTACC
TCCTGGTGATCTGATCCTTAAGCTCCGAAGAATCAGAAGGGTACC
TCCTGGTGATCTGATCCTTAAGCTCCGAAGGATCACAGGGGTACC
TCCTGGTGATCTGATCCTTAAGCTCCGAAGGATCAGAAGGGTACC
TCCTGGTGATCTGATCCTTAAGCTCCGAAGGATCAGAAGGGTACC
TCCTGGTGATCTGATCCTTAAGCTCCGAAGGATCACAGGGGTACC
TCCTGGTGATCTGATCCTTAAGCTCCGAAGGATCAGAAGGGTACC
TCCTGGTGATCTGATCCTTCAGCTTCAAAGGATCATAAGGGTACC
TCCTGGTGATCTGACCCTTCAGCTTCAAAGGATCAGAAGGGTACC
TCCTGGTGATCTGATCCTTAAGCTCCGAAGGATCACAGGGGTACC
TCCTGGTGATCTGATCCTTAAGCTCCGAAGGATCAGAAGGGTACC
TCCTGGTGATCTGATCCTTCAGCTCCGAAGGATCAGAAGGGTACC
TCCTGGTGATGTGATCCTTAAGCTCCGAAGGATCACAGGGGTACC
TCCTGGTGATCTGATCCTTAAGCTTCAAAGGATCAGAAGGGTACC
TCCTGGTGATCTGATCCTTCAGCTTCAAAGGATCATAAGGGTACC
TCCTGGTGATCTGATCCTTCAACTCCGAAGGATCAGAAGGGTACC
TCCTGGTGATCTGATCCTTCAGCTTCAAAGGATCAGAAGGGTACC
TCCTGGTGATCTGATCCTTCAGCTTCAAAGGATCATAAGGGTACC
TCCTGGTGATCTGATCCTTCAACTCCGAAGGATCACAAGGGTACC
TCCTGGTGATCTGATCCTTCAGCTTCAAAGGATCACAGGGGTACC
TCCTGGTGATCTGACCCTTCAGCTTCAAAGGATCAGAAGGGTACC
TCCTGGTGATCTGATCCTTCAGCTTCAAAGGATCAGAAGGGTACC
TCCTGGTGATCTGATCCTTCAGCTCCGAAGGATCAGAAGGGTACC
TCCTGGTGATCTGATCCTTAAGCTCCGAAGGATCAGAAGGGTACC
TCCTGGTGATCTGATCCTTAAGCTCCGAAGGATCACAGGGATACC
TCCTGGTGATCTGATCCTTAAGCCCCGAAGGATCAGAAGGGTACC
TCCTGGTGATCTGATCCTTAAGCTCCGAAGGATCACAGGGGTACC
TCCTGGTGATCTGACCCTTCAGCTTCAAAGGATCAGAAGGGTACC
TCCTGGTGATCTGATCCTTCAGCTTCAAAGGATCAGAAGGGTACC
TCCTGGTGATCTGATCCTTCAGCTCCGAAGGATCAGAAGGGTACC
TCCTGGTGATCTGATCCTTAAGCTCCGAAGGATCACAGGGGTACC

TCCTGGTGATCTGACCCTTCAGCTTCAAAGGATCAGAAGGGTACC
TCCTGGTGATCTGATCCTTAAGCTCCGAAGGATCACAGGGGTACC
TCCTGGTGATCTGATCCTTAAGCTCCGAAGGATCAGAAGGGTACC
TCCTGGTGATCTGATCCTTCAGCTTCAAAGGATCATAAGGGTACC
TCCTGGTGATCTGATCCTTCAACTCCGAAGGATCACAAGGGTACC
TCCTGGTGATCTGATCCTTCAGCTTCAAAGGATCATAAGGGTACC
TCCTGGTGATCTGAACCTTAAGCTCCGAAGGATCAGAAGGGTACC
TCCTGGTGATCTGATCCTTAAGCTCCGAAGGATCAGAAGGGTACC
TCCTGGTGATCTGATCCTTAAGCTCCGAAGGATCACAGGGGTACC
TCCTGGTGATCTGACCCTTCAGCTTCAAAGGATCAGAAGGGTACC
TCCTGGTGATCTGATCCTTCAGCTTCAAAGGATCAGAAGGGTACC
TCCTGGTGATCTGATCCTTCAGCTCCGAAGGATCAGAAGGGTACC
TCCTGGTGATCTGATCCTTAAGCTCCGAAGGATCAGAAGGGTACC
TCCTGGTGATCTGATCCTTAAGCTCCGAAGGATCACAGGGGTACC
TCCTGGTGATCTGACCCTTCAGCTTCAAAGGATCAGAAGGGTACC
TCCTGGTGATCTGATCCTTCAGCTCCGAAGGATCAGAAGGGTACC
TCCTGGTGATGTGATCCTTAAGCTCCGAAGGATCACAGGGGTACC
TCCTGGTGATCTGATCCTTAAGCTCCGAAGGATCAGAAGGGTACC
TCCTGGTGATCTGATCCTTAAGCTCCGAAGAATCAGAAGGGTACC
TCCTGGTGATCTGATCCTTAAGCTCCGAAGGATCACAGGGGTACC
TCCTGGTGATCTGATCCTTAAGCTCCGAAGGATCAGAAGGGTACC
TCCTGGTGATCTGATCCTTAAGCTCCGAAGGATCAGAAGGGTACC
TCCTGGTGATCTGATCCTTAAGCTCCGAAGGATCACAGGGGTACC
TCCTGGTGATCTGATCCTTAAGCTCCGAAGGATCAGAAGGGTACC
TCCTGGTGATCTGATCCTTCAGCTTCAAAGGATCATAAGGGTACC
TCCTGGTGATCTGATCCTTCAACTCCGAAGGATCACAAGGGTACC
TCCTGGTGATCTGATCCTTAAGCTCCGAAGGATCAGAAGGGTACC
TCCTGGTGATCTGATCCTTCAGCTTCAAAGGATCATAAGGGTACC
TCCTGGTGATCTGAACCTTAAGCTCCGAAGGATCAGAAGGGTACC
TCCTGGTGATCTGATCCTTAAGCTCCGAAGGATCAGAAGGGTACC
TCCTGGTGATCTGATCCTTAAGCTCCGAAGGATCACAGGGGTACC
TCCTGGTGATCTGACCCTTCAGCTTCAAAGGATCAGAGGGGTACA
TCCTGGTGATCTGATCCTTCAGCTTCAAAGGATCAGAAGGGTACC
TCCTGGTGATCTGATCCTTCAGCTCCGAAGGATCAGAAGGGTACCA
CCTGGTGATCTGATCCTTAAGCTCCGAAGGATCAGAAGGGTACC
TCCTGGTGATCTGATCCTTAAGCTCCGAAGGATCACAGGGGTACC
TCCTGGTGATCTGACCCTTCAGCTTCAAAGGATCAGAAGGGTACC
TCCTGGTGATCTGATCCTTCAGCTTCAAACGATCATAAGGGTACC
TCCTGGTGATCTGATCCTTCAGCTTCAAAGGATCAGAAGGGTACC
TCCTGGTGATCTGATCCTTAAGCTCCGAAGGATCAGAAGGGTACC
TCCTGGTGATCTGATCCTTAAGCTCCGAAGAATCAGAAGGGTACC
TCCTGGTGATCTGATCCTTAAGCTCCGAAGGATCACAGGGGTACC
TCCTGGTGATCTGATCCTTAAGCTCCGAAGGATCAGAAGGGTACC
TCCTGGTGATCTGATCCTTCAGCTCCGAAGGATCACAAGGGTACC
TCCTGGTGATCTGATCCTTAAGCTCCGAAGGATCAGAAGGGTACC
TCCTGGTGATCTGATCCTTAAGCTCCGAAGGATCACAGGGATACC
TCCTGGTGATCTGATCCTTAAGCCCCGAAGGATCAGAAGGGTACC
TCCTGGTGATCTGATCCTTAAGCTCCGAAGGATCACAGGGGTACC
TCCTGGTGATCTGACCCTTCAGCTTCAAAGGATCAGAAGGGTACC
TCCTGGTGATCTGATCCTTCAGCTTCAAAGGATCAGAAGGGTACC
TCCTGGTGATCTGATCCTTCAGCTCCGAAGGATCAGAAGGGTACC
TCCTTGTGATCTGATCCTTAAGCTCCGAAGGATCACAGGGATACC
TCCTGGTGATCTGATCCTTAAGCCCCGAAGGATCAGAAGGGTACC
TCCTGGTGATCTGATCCTTCAGCTCCGAAGGATCAGAAGGGTACC

TCCTGGTGATGTGATCCTTAAGCTCCGAAGGATCACAGGGGTACC
TCCTGGTGATCTGATCCTTCAGCTTCAAAGGATCAGAAGGGTACC
TCCTGGTGATCTGATCCTTCAGCTCCGAAGGATCAGAAGGGTACC
TCCTGGTGATCTGATCCTTAAGCTCCGAAGGATCAGAAGGGTACC
TCCTGGTGATCTGATCCTTAAGCTCCGAAGGATCACAGGGGTACC
TCCTGGTGATCTGACCCTTCAGCTTCATAGGATCAGAAGGGTACC
TCCTGGTGATCTGATCCTTCAGCTCCGAAGGATCAGAAGGGTACC
TCCTGGTGATGTGATCCTTAAGCTCCGAAGGATCACAGGGGTACC
TCCTGGTGATCTGATCCTTAAGCTCCGAAGGATCAGAAGGGTACC
TCCTGGTGATCTGATCCTTAAGCTCCGAAGAATCAGAAGGGTACC
TCCTGGTGATCTGATCCTTAAGCTCCGAAGAATCAGAAGGGTACC
TCCTGGTGATCTGATCCTTAAGCTCCGAAGGATCACAGGGGTACC
TCCTGGTGATCTGACCCTTCAGCTTCATAGGATCAGAAGGGTACC
TCCTGGTGATCTGATCCTTCAGCTCCGAAGGATCAGAAGGGTACC
TCCTGGTGATGTGATCCTTAAGCTCCGAAGGATCACAGGGGTACC
TCCTGGTGATCTGATCCTTAAGCTCCGAAGGATCAGAAGGGTACC
TCCTGGTGATCTGATCCTTAAGCTCCGAAGAATCAGAAGGGTACC
TCCTGGTGATCTGATCCTTAAGCTCCGAAGGATCACAGGGGTACC
TCCTGGTGATGTGATCCTTAAGCTCCGAAGGATCACAGGGGTACC
TCCTGGTGATCTGATCCTTCAGCTCCGAAGGATCAGAAGGGTATC
Nr of selected repeats 347 Similarity 0.928761
Consensus:
TCCTGGTGATCTGATCCTTaAGCTcCgAAGGATCAgAaGGGTACC
>Cele-UNSB01_4:11032975-11034460 Satlength=1486 Nr of Repeats=33
RepeatLength=45 seed=TCACCAGGAG
TCACCAGGAGGTACCCTTCTGATCCTTCGGAGCTTAAGGTTCAGA
TCACCAGGAGGTACCCTTGTGATCCTTCGGAGCTGAAGGATCAGA
TCACCAGGAGGTACCCTTCTGATCCTTCGGAGCTTAAGGATCAGA
TCACCAGGAGGTACCCCTGTGATCCTTCGGAGCTTAAGGATCAGA
TCACCAGGAGGTACCCTTCTGATTCTTCGGAGCTTAAGGATCAGA
TCACCAGGAGGTACCCTTCTGATCCTTCGGAGCTTAAGGATCAGA
TCACCAGGAGGTACCCTTCTGATCCTTTGAAGCTGAAGGATCAGA
TCACCAGGAGGTACCCTTATGATCGTTTGAAGCTGAAGGATCAGA
TCACCAGGAGGTACCCTTCTGATCCTTTGAAGCTGAAGGGTCAGA
TCACCAGGAGGTACCCCTGTGATCCTTCGGAGCTTAAGGATCAGA
TCACCAGGAGGTACCCTTCTGATCCTTCGGAGCTTAAGGATCAGA
TCACCAGGAGGTACCCTTCTGATCCTTCGGAGCTTAAGGTTCAGA
TCACCAGGAGGTACCCTTATGATCCTTTGAAGCTGAAGGGTCAGA
TCACCAGGAGGTACCCTTGTGATCCTTTGAAGCTGAAAGATCAGA
TCACCAGGAGATACCCTTCTGATCCTTCGGAGCTGAAGGATCAGA
TCACCAGGAGGTACCCTTCTGATCCTTTGAAGCTGAAGGGTCAGA
TCACCAGGAGGTACCCCTGTGATCCTTCGGAGCTTAAGGATCAGA
TCACCAGGAGGTACCCTTCTGATCCTTCGGAGCTTAAGGTTCAGA
TCACCAGGAGGTACCCTTGTGATCCTTTGAAGCTGAAGGATCAGA
TCACCAGGAGGTACCCTTCTGATCCTTCGGAGCTTAAGGATCAGT
TCACCAGGAGGTACCCCTGTGATCCTTCGGAGCTTAAGGATCAGA
TCACCAGGAGGTACCCTTCTGATCCTTCGGAGCTTAAGGATCAGA
TCACCAGGAGGTACCCTTGTGATCCTTCGGAGCTGAAGGATCAGA
TCACCAGGAGGTACCCTTATGATCCTTTGAAGCTGAAGGATCAGA
TCACCAGGAGGTACCCTTCTGATCCTTCGGAGCTTAAGGATCAGA
TCACCAGGAGGTACCCCTGTGATCCTTCGGAGCCTAAGGATCAGA
TCACCAGGAGGTACCCTTCTGATCCTTCGGAGCTTAAGGATCAGA
TCACCAGGAGGTACCCTTGTGATCCTTCGGAGCTGAAGGATCAGA
TCACCAGGAGGTACCCTTATGATCCTTTGAAGCTGAAGGATCAGA

TCACCAGGAGGTACCCTTCTGATCCTTCGGAGCTTAAGGATCAGA
TCACCAGGAGGTATCCCTGTGATCCTTCGGAGCTTAAGGATCAGA
TCACCAGGAGGTACCCTTCTGATCCTTCGGAGCTTAAGGATCAGA
TCACCAGGAGGTACCCTTGTGATCCTTCGGAGCTGAAGGATCAGA
Nr of selected repeats 33 Similarity 0.911616
Consensus:
TCACCAGGAGGTACCCTTcTGATCCTTcGgAGCTtAAGGATCAGA
>Cele-UNSB01_4:11073363-11083545 Satlength=10183 Nr of Repeats=395
RepeatLength=26 seed=GTTCACCAAA
GTTCACCAAATTTAGTGACTCTGGCC
GTTCACCAAATTTAGTGACTCTGGCC
GTTCACCAAAAATAGTGACTCTGGCC
GTTCACCAAATATAGTGACTCTGGCC
GTTCACCAAATTTAGTGACTCTGGCC
GTTCACCAAATTTAGTGACTCTGCC
GTTCACCAAATTTAGTGACTCTGGCC
GTTCACCAAATTTAGTGACTCTGGCC
GTTCACCAAATTTAGTGACTCTGGCC
GTTCACCAAATTAGTGACTCTGGCC
GTTCACCAAATTTAGTGACTCTGGCC
GTTCACCAAATTTAGTGACTCTGGCC
GTTCACCAAATTTAGTGACTCTGGCC
GTTCACCAAATTTAGTGACTCTGGCC
GTTCACCAAATTTAGTGACTCTGGCC
GTTCACCAAATTTAGTGACTCTGGCC
GTTCACCAAATTGGCC
GTTCACCAAATTTAGTGACTCTGGCC
GTTCACCAAATTTAGTGACTCTGGCC
GTTCACCAAATTTAGTGACTCTGGCC
GTTCACCAAATTTAGTGACTCTGGCC
GTTCACCAAATTTAGTGACTCTGGCC
GTTCACCAAATTTAGTGACTCTGGCC
GTTCACCAAATTTAGTGACTCTGGCC
GTTCACCAAATTTAGTGACTCTGGCC
GTTCACCAAATTTAGTGACTCTGGCC
GTTCACCAAATTTAGTGACTCTGGCC
GTTCACCAAATGACCAAAATAGTGACTCTGGCC
GTTCACCAAATTTAGTGACTCTGGCC
GTTCACCAAATTTAGTGACTCTGGCC
GTTCACCAAATTTAGTGACTCTGGCC
GTTCACCAAATTTAGTGACTCTGGCC
GTTCACCAAATTTAGTGACTCTGGCC
GTTCACCAAATTTAGTGACTCTGGCC
GTTCACCAAATTTAGTGACTCTGGCC
GTTCACCAAATTTAGTGACTCTGGCC
GTTCACCAAATTTAGTGACTCTGGCC
GTTCACCAAATTTAGTGACTCTGGCC
GTTCACCAAATTTAGTGACTCTGGCC
GTTCACCAAATTTAGTGACTCTGGCC
GTTCACCAAATTTAGTGACTCTGGCC
GTTCACCAAATAGTGACTCTGCC
GTTCACCAAATTTAGTGACTCTGGCC
GTTCACCAAATTTAGTGACTCTGGCC
GTTCACCAAATTTAGTGACTCTGGCC

GTTCACCAAATTTAGTGACTCTGGCC
GTTCACCAAAATAGTGACTCTGGCC
GTTCACCAAATAGTGACTCTGGCC
GTTCACCAAATTTAGTGACTCTGGCC
GTTCACCAAATTTAGTGACTCTGCC
GTTCACCAAATAGTGACTCTGCC
GTTCACCAAATTTAGTGACTCTGGCC
GTTCACCAAATTTAGTGACTCTGGCC
GTTCACCAAATTTAGTGACTCTGGCC
GTTCACCAAATAGTGACTCTGGCC
GTTCACCAAATTTAGTGACTCTGGCC
GTTCACCAAAAATAGTGACTCTGGCC
GTTCACCAAATTTAGTGACTCTGGCC
GTTCACCAAATTTAGTGACTCTGGCC
GTTCACCAAATTTAGTGACTCTGGCC
GTTCACCAAAAATAGTGACTCTGGCC
GTTCACCAAAAATAGTGACTCTGCC
GTTCACCAAAAATAGTGACTCTGGCC
GTTCACCAAATTTAGTGACTCTGGCC
GTTCACCAAATTTAGTGACTCTGGCC
GTTCACCAAATTTAGTGACTCTGGCC
GTTCACCAAAAATAGTGACTCTGGCC
GTTCACCAAAAGTGACTCTGGCC
GTTCACCAAATAGTGACTCTGGCC
GTTCACCAAATAGTGACTCTGGCC
GTTCACCAAATTTAGTGACTCTGGCC
GTTCACCAAAAATAGTGACTCTGGCC
GTTCACCAAAAATAGTGACTCTGGCC
GTTCACCAAAAATAGTGACTCTGGCC
GTTCACCAAATTAGTGACTCTGGCC
GTTCACCAAAAATAGTGACTCTGGCC
GTTCACCAAAAATAGTGACTCTGGCC
GTTCACCAAAATAGTGACTCTGGCC
GTTCACCAAATTTAGTGACTCTGGCC
GTTCACCAAATTTAGTGACTCTGGCC
GTTCACCAAAATTTAGTGACTCTGGCC
GTTCACCAAATTTAGTGACTCTGGCC
GTTCACCAAATTTAGTGACTCTGGCC
GTTCACCAAATTTAGTGACTCTGGCC
GTTCACCAAAAATAGTGACTCTGGCC
GTTCACCAAATTTAGTGACTCTGGCC
GTTCACCAAATTTAGTGACTCTGGCC
GTTCACCAAAAATAGTGACTCTGGCC
GTTCACCAAAATAGTGACTCTGGCC
GTTCACCAAAAATAGTGACTCTGACC
GTTCACCAAATAGTGACTCTGACC
GTTCACCAAATTTAGTGACTCTGGCC
GTTCACCAAAAATAGTGACTCTGGCC
GTTCACCAAATTAGTGACTCTGGCC
GTTCACCAAATTTAGTGACTCTGGCC
GTTCACCAAATTTAGTGACTCTGGCC
GTTCACCAAATTTAGTGACTCTGGCC
GTTCACCAAATTTAGTGACTCTGGCC
GTTCACCAAATATAGTGACTCTGGCC

GTTCACCAAATTTAGTGACTCTGGCC
GTTCACCAAAATAGTGACTCTGGCC
GTTCACCAAATTTAGTGACTCTGGCC
GTTCACCAAATTTAGTGACTCTGGCC
GTTCACCAAAAATAGTGACTCTGGCC
GTTCACCAAATTTAGTGACTCTGGCC
GTTCACCAAAATAGTGACTCTGGCC
GTTCACCAAATTTAGTGACTCTGGCC
GTTCACCAAATTAGTGACTCTGGCC
GTTCACCAAATTTAGTGACTCTGGCC
GTTCACCAAATTTAGTGACTCTGGCC
GTTCACCAAATTTAGTGACTCTGGCC
GTTCACCAAATTTAGTGACTCTGGCC
GTTCACCAAAAATTTAGTGACTCTGACC
GTTCACCAAATTTAGTGACTCTGGCC
GTTCACCAAATTTAGTGACTCTGGCC
GTTCACCAAAATAGTGACTCTGGCC
GTTTACCAAAATAGTGACTCTGGCC
GTTCACCAAATTTAGTGACTCTGGCC
GTTCACCAAAATAGTGACTCTGGCC
GTTCACCAAAAATAGTGACTCTGGCC
GTTCACCAAAATAGTGACTCTGGCC
GTTCACCAAATTAGTGACTCTGGCC
GTTCACCAAATTTAGTGACTCTGGCC
GTTCACCAAATTTAGTGACTCTGGCC
GTTCACCAAAATAGTGACTCTGGCC
GTTCACCAAAATAGTGACTCTGGCC
GTTCACCAAAAATAGTGACTCTGGCC
GTTCACCAAAATAGTGACTCTGGCC
GTTCACCAAATTTAGTGACTCTGGCC
GTTCACCAAATTAGTGACTCTGGCC
GTTCACCAAATTTAGTGACTCTGGCC
GTTCACCAAATAGTGACTCTGGCC
GTTCACCAAAAAGTGACTCTCGTTCCATAGTGACTCTGGCC
GTTCACCAAATAGTGACTCTGACC
GTTCACCAAATTTAGTGACTCTGGCC
GTTCACCAAAATAGTGACTCTGGCC
GTTTACCAAAATAGTGACTCTGGCC
GTTCACCAAATTTAGTGACTCTGGCC
GTTCACCAAATTTAGTGACTCTGGCC
GTTCACCAAATTAGTGACTCTGCC
GTTCACCAAATTTAGTGACTCTGGCC
GTTCACCAAATTTAGTGACTCTGGCC
GTTCACCAAATTTAGTGACTCTGGCC
GTTCACCAAATTTAGTGACTCTGGCC
GTTCACCAAAAATAGTGACTCTGGCC
GTTCACCAAAAATAGTGACTCTGGCC
GTTCACCAAATTTAGTGACTCTGACC
GTTCACCAAATTTAGTGACTCTGGCC
GTTCACCAAATTTAGTGACTCTGGCC
GTTCACCAAAAATAGTGACTCTGGCC
GTTCACCAAAAATAGTGACTCTGACC
GTTCACCAAATTTAGTGACTCTGGCC
GTTCACCAAATTTAGTGACTCTGGCC

GTTCACCAAATTTAGTGACTCTGGCC
GTTCACCAAAAATAGTGACTCTGGCC
GTTCACCAAATTTAGTGACTCTGGCC
GTTCACCAAATTTAGTGACTCTGACC
GTTCACCAAATTTAGTGACTCTGGCC
GTTCACCAAATTTAGTGACTCTGGCC
GTTCACCAAATATAGTGACTCTGACC
GTTCACCAACTGACTCTGACC
GTTCACCAAATTTAGTGACTCTGACC
GTTCACCAAAAATGACTCTGGCC
GTTCACCAAATTTAGTGACTCTGGCC
GTTCACCAAAATTTAGTGACTCTGACC
GTTCACCAAATTTAGTGACTCTGGCC
GTTCACCAAATTTAGTGACTCTGGCT
GTTCACCAAAAATAGTGACTCTGACC
GTTCACCAAATTTAGTGACTCTGACC
GTTCACCAAAAATGACTCTGACC
GTTCACCAAATTTAGTGACTCTGACC
GTTCACCAAAAATGACTCTGACC
GTTCACCAAATTTAGTGACTCTGGCC
GTTCACCAAAAATAGTGACTCTGATC
GTTCACCAAATTTAGTGACTCTGGCC
GTTCACCAAAAATTGTGACTCTGATC
GTTCACCAAATTTAGTGACTCTGACC
GTTCACCAAAAATGACTCTGACC
GTTCACCAAATTTAGTGACTCTGACC
GTTCACCAAAAATGACTCTGACC
GTTCACCAAATTTAGTGACTCTGGCC
GTTCACCAAAAATAGTGACTCTGATC
GTTCACCAAATTTAGTGACTCTGGCC
GTTCACCAAAAATTGTGACTCTGATC
GTTCACCAAATTTAGTGACTCTGGCC
GTTCACCAAAAATAGTGACTCTGATC
GTTCACCAAAAATAGTGACTCTGACC
GTTCACCAAATTTAGTGACTCTGGCC
GTTCACCAAATTCAGTGACTCTGGCC
GTTCACCAAAAATAGTGGCTCTGACC
GTTCACCAAATTTAGTGACTCTGGCC
GTTCACCAAATTTAGTGACTCTGGCC
GTTCACCAAAAATAGTGACTCTGGCC
GTTCACCAAAAATAGTGACTCTGACC
GTTCACCAAATTTAGTGACTCTGACC
GTTCACCAAATTTAGTGACTCTGGCC
GTTCACCAAATTTAGTGACTCTGGCC
GTTCACCAAATTTAGTGACTCTGACC
GTTCACCAAATTTAGTGACTCTGGCC
GTTCACCAAATGTAGTGACTCTGGCT
GTTCACCAAAAATAGTGACTCTGACC
GTTCACCAAATTTAGTGACTCTGACC
GTTCACCAAATTTAGTGACTCTGGCC
GTTCACCAAATTTAGTGACTCTGGCC
GTTCACCAAATTTAGTGACTCTGGCC
GTTCACCAAAAAAAAAATGACTCTGACC
GTTCACCAAATTTAGTGACTCTGGCC

GTTCACCAAATTTAGTGACTCTGGCC
GTTCACCAGAAATTGTGACTCTGATC
GTTCACCAAATTTAGTGACTCTGACC
GTTCACCAAAAATGACTCTGACC
GTTCACCAAATTTAGTGACTCTGACC
GTTCACCAAAAATTGTGACTCTGATC
GTTCACCAAATTTAGTGACTCTGACC
GTTCACCAAAAATGACTCTGACC
GTTCACCAAATTTAGTGACTCTGGCC
GTTCACCAAAAATAGTGACTCTGATC
GTTCACCAAATTTAGTGACTCTGGCC
GTTCACCAAAAATTGTGACTCTGATC
GTTCACCAAATTTAGTGACTCTGACC
GTTCACCAAAAATGACTCTGACC
GTTCACCAAATTTAGTGACTCTGGCC
GTTCACCAAAAATAGTGACTCTGATC
GTTCACCAAAAATAGTGACTCTGACC
GTTCACCAAATTTAGTGACTCTGGCC
GTTCACCAAATTCAGTGACTCTTGCC
GTTCACCAAAAATAGTGGCTCTGACC
GTTCACCAAATTTAGTGACTCTGGCC
GTTCACCAAATTTAGTGACTCTGGCC
GTTCACCAAAAATAGTGACTCTGGCC
GTTCACCAAAAATAGTGACTCTGGCC
GTTCACCAAATTTAGTGACTCTGGCC
GTTCACCAAATTCAGTGACTCTGGCC
GTTCACCAAAAAATAGTGACTCTGACC
GTTCACCAAATTTAGTGACTCTGGCC
GTTCACCAAATTCAGTGACTCTGGCC
GTTCACCAAAAATAGTGACTCTGACC
GTTCACCAAATTTAGTGACTCTGGTC
GTTCACTAAATTTAGTGACTTTGGCC
GTTCACCAAAAATAGTGACTCTGGCC
GTTCACCAAATTTAGTGACTCTGACC
GTTCACCAAATTTAGTGACTCTGGCC
GTTCACCAAATTTAGTGACTCTGACC
GTTCACCAAATTTAGTGACTCTGGCC
GTTCACCAAATTTAGTGACTCTGGCT
GTTCACCAAAAATAGTTACTCTGATC
GTTCACCAAAAATAGTGACTCTGACC
GTTCACCAAAAATGACTCTGACC
GTTCACCAAATTTAGTGACTCTGGTC
GTTCACTAAATTTAGTGACTTTGGCC
GTTCACCAAAATAGTGACTCTGGCC
GTTCACCAAATTTAGTGACTCTGACC
GTTCACCAAATTTAATGACTCTGGCC
GTTCACCAAATTTAGTGACTCTGGCC
GTTCACCAAAAATAGTGACTCTGACC
GTTCACCAAATTTAGTGACTCTGGCC
GTTCACCAAATTTAGTGACTCTGGCC
GTTCACCAAAAATAGTGACTCTGACC
GTTCACCAAATTTAGTGACTCTGACC
GTTCACCAAATTTAGTGACTCTGGCC
GTTCACCAAATTTAGTGACTCTGGCC

GTTCACCAAATTTAGTGACTCTGACC
GTTCACCAAATTTAGTGACTCTGGCC
GTTCACCAAATTTAGTGACTCTGGCT
GTTCACCAAAAATGACTCTGACC
GTTCACCAAATTTAGTGACTCTGACC
GTTCACCAAAAATGACTCTGACC
GTTCACCAAATTTAGTGACTCTGACC
GTTCACCAAAAATGACTCTGACC
GTTCACCAAATTTAGTGACTCTGACC
GTTCACCAAATTTAGTGACTCTGGCC
GTTCACCAAATTTAGTGACTCTGGCC
GTTCACCAAAAATAGTGACTCTGACC
GTTCACCAAATTTAGTGACTCTGACC
GTTCACCAAATTTAGTGACTCTGGCC
GTTCACCAAATTTAGTGACTCTGGCC
GTTCACCAAATTTAGTGACTCTGACC
GTTCACCAAATTTAGTGACTCTGGCC
GTTCACCAAATTTAGTGACTCTGGCT
GTTCACCAAAAATGACTCTGACC
GTTCACCAAATTTAGTGACTCTGACC
GTTCACCAAAAATGACTCTGACC
GTTCACCAAATTTAGTGACTCTGACC
GTTCACCAAAAATGACTCTGACC
GTTCACCAAATTTAGTGACTCTGGCC
GTTCACCAAAAATAGTGACTCTGATC
GTTCACCAAATTTAGTGACTCTGGCC
GTTCACCAAAAATTGTGACTCTGATC
GTTCACCAAATTTAGTGACTCTGACC
GTTCACCAAAAATGACTCTGACC
GTTCACCAAATTTAGTGACTCTGACC
GTTCACCAAAAATGACTCTGACC
GTTCACCAAATTTAGTGACTCTGATC
GTTCACCAAATTTAGTGACTCTGGCC
GTTCACCAAAAATTGTGACTCTGATC
GTTCACCAAATTTAGTGACTCTGGCC
GTTCACCAAAAATAGTGACTCTGATC
GTTCACCAAAAATAGTGACTCTGACC
GTTCACCAAATTTAGTGACTCTGGCC
GTTCACCAAATTCAGTGACTCTGGCC
GTTCACCAAAAATAGTGGCTCTGACC
GTTCACCAAATTTAGTGACTCTGGCC
GTTCACCAAATTTAGTGACTCTGGCC
GTTCACCAAAAATAGTGACTCTGGCC
GTTCACCAAAAATAGTGACTCTGACC
GTTCACCAAATTTAGTGACTCTGGCC
GTTCACCAAATTCAGTGACTCTGGCC
GTTCACCAAAAAATAGTGACTCTGACC
GTTCACCAAATTTAGTGACTCTGGCC
GTTCACCAAATTCAGTGACTCTGGCC
GTTCACCAAAAATAGTGACTCTGACC
GTTCACCAAATTTAGTGACTCTGGTC
GTTCACTAAATTTAGTGACTTTGGCC
GTTCACCAAAAATAGTGACTCTGACC
GTTCACCAAATTTAGTGACTCTGGTC

GTTCACTAAATTTAGTGACTTTGGCC
GTTCACCAAAAATAGTGACTCTGGCC
GTTCACCAAATTTAGTGACTCTGACC
GTTCACCAAATTTAGTGACTCTGGCC
GTTCACCAAATTTAGTGACTCTGACC
GTTCACCAAATTTAGTGACTCTGGCC
GTTCACCAAATTTAGTGACTCTGGCT
GTTCACCAAAAATAGTGACTCTGACC
GTTCACCAAATTTAGTGACTCTGGTC
GTTCACTAAATTTAGTGACTTTGGCC
GTTCACCAAAAATAGTGACTCTGGCC
GTTCACCAAATTTAGTGACTCTGACC
GTTCACCAAATTTAGTGACTCTGGCC
GTTCACCAAATTTAGTGACTCTGACC
GTTCACCAAATTTAGTGACTCTGGCC
GTTCACCAAATTTAGTGACTCTGGCT
GTTCACCAAAAATAGTGACTCTGATC
GTTCACCAAAAATAGTGACTCTGACC
GTTCACCAAAAATGACTCTGACC
GTTCACCAAATTTAGTGACTCTGGTC
GTTCACTAAATTTAGTGACTTTGGCC
GTTCACCAAAAATAGTGACTCTGGCC
GTTCACCAAATTTAGTGACTCTGACC
GTTCACCAAATTTAGTGACTCTGGCC
GTTCACCAAATTTAGTGACTCTGGCC
GTTCACCAAAAATAGTGACTCTGACC
GTTCACCAAATTTAGTGACTCTGGCC
GTTCACCAAATTTAGTGACTCTGGCC
GTTCACCAAAAATAGTGACTCTGACC
GTTCACCAAATTTAGTGACTCTGACC
GTTCACCAAATTTAGTGACTCTGGCC
GTTCACCAAATTTAGTGACTCTGGCC
GTTCACCAAATTTAGTGACTCTGACC
GTTCACCAAATTTAGTGACTCTGGCC
GTTCACCAAATTTAGTGACTCTGGCT
GTTCACCAAAAATAGTGACTCTGATC
GTTCACCAAAAATAGTGACTCTGACC
GTTCACCAAATTTAGTGACTCTGGCC
GTTCACCAAATTCAGTGACTCTGGCC
GTTCACCAAAAATAGTGGCTCTGACC
GTTCACCAAATTTAGTGACTCTGGCC
GTTCACCAAATTTAGTGACTCTGGCC
GTTCACCAAAAATAGTGACTCTGGCC
GTTCACCAAAAATAGTGACTCTGACC
GTTCACCAAATTTAGTGACTCTGGCC
GTTCACCAAATTCAGTGACTCTGGCC
GTTCACCAAAAAATAGTGACTCTGACC
GTTCACCAAATTTAGTGACTCTGGCC
GTTCACCAAATTCAGTGACTCTGGCC
GTTCACCAAAAATAGTGACTCTGACC
GTTCACCAAATTTAGTGACTCTGGTC
GTTCACTAAATTTAGTGACTTTGGCC
GTTCACCAAAAATAGTGACTCTGGCC
GTTCACCAAATTTAGTGACTCTGACC

GTTCACCAAATTTAGTGACTCTGGCC
GTTCACCAAATTTAGTGACTCTGGCC
GTTCACCAAAAATAGTGACTCTGACC
GTTCACCAAATTTAGTGACTCTGGCC
GTTCACCAAATTTAGTGACTCTGGCC
GTTCACCAAAAATAGTGACTCTGACC
GTTCACCAAATTTAGTGACTCTGACC
GTTCACCAAATTTAGTGACTCTGGCC
GTTCACCAAATTTAGTGACTCTGGCC
GTTCACCAAATTTAGTGACTCTGACC
GTTCACCAAATTTAGTGACTCTGGCC
GTTCACCAAATTTAGTGACTCTGGCT
GTTCACCAAAAATAGTGACTCTGACC
GTTCACCAAATTTAGTGACTCTGACC
GTTCACCAAATTTAGTGACTCTGGCC
GTTCACCAAATTTAGTGACTCTGGCC
GTTCACCAAATTTAGTGACTCTGACC
GTTCACCAAATTTAGTGACTCTGGCC
GTTCACCAAATTTAGTGACTCTGGCC
GTTCACCAAATTTAGTGACTCTGACC
GTTCACCAAAAATAGTGACTCTGACC
GTTCACCAAAAATAGTGACTCTGGCC
GTTCACCAAATTTAGTGACTCTGACC
GTTCACCAAATTTAGTGACTCTGGCC
GTTCACCAAATTTAGTGACTCTGGCC
GTTCACCAAATTTAGTGACTCTGGCC
Nr of selected repeats 331 Similarity 0.919481
Consensus:
GTTCACCAAAttTAGTGACTCTGgCC
>Cele-UNSB01_4:11083607-11084360 Satlength=754 Nr of Repeats=4 RepeatLength=11
seed=TTTGAAATAT
TTTGAAATATTCATGAACGAATGTCTGGTTTTAATTTTCCAAAGCTGAGAAATTACATTTAAAATTCTATAAAAATTAT
TCAACTTTATTTTTTTTTTTCAGTTGCCCGTAGAAAAAGCAGTGACACTTGGTCTCATCAAGGCTCAAAATGTCACAAA
AGTAGCCCCAATTGGACACGGAAGACCTCCAGCACGGAAACACGAAGAGATATCACGTCGTCATTGATTTTATTGACAA
TGAAACAAAATAACATTCTCTAGTCGATATAGGAACCCATTTGTCCATCCTATTTGTATCATATTGTTTTCTTCACAAA
ATTTCTGCTAGATTTCTATGAAAATCAGAGAGCTTTCAATTCTTCTCCACTTTTTCTATTTTGTGTGTACATGTCGTTC
CTAAACCATTTTCCCTTTCCCATCCCACACTTTCCATGCCGCCCGACCACTTTCTCAACTGTCTCCAGGTACTCTGTAA
ATTTCTGAATTTATTCATCCTGAAAAACTCTTTCTATATATTTGATGATCACTTTTATTTCTTCTTCATCTCTATACTC
TTTGTCTGTGTAAAAATTTTATAATTGTTTTCTTTTCACCATTCACACCCCGGTCTTGTAATAATAAGTGTAGGTTTCA
AGAGAGTAATAAAAGTGTATTGAGAATAAAACTTTAATTATATAGAAATCTTAAACACACCCACTTAGAAGTCTTGTTT
TTTTTTCTA
TTTGAAATATA
TTTGAAATATA
TTTGAAATATA
Nr of selected repeats 3 Similarity 1.000000
Consensus:
TTTGAAATATA
>Cele-UNSB01_4:11084695-11084799 Satlength=105 Nr of Repeats=4 RepeatLength=26
seed=CCAGAGTCAC
CCAGAGTCACTATGTTTGGTGAACTG
CCAGAGTCACAATTTTTGGTGAACAA
CCAGAGTCACTATTTTTAGTGAACTG
CCAGAGTCACTATTTTTAGTGAACTG
Nr of selected repeats 4 Similarity 0.863248

Consensus:
CCAGAGTCACtATtTTTaGTGAACtg
>Cele-UNSB01_4:11101964-11102048 Satlength=85 Nr of Repeats=4 RepeatLength=21
seed=AAATCTAATC
AAATCTAATCCAATGTGGAAC
AAATCTAATCCAATATGGCCG
AAATCTAATCCAATATGGAAC
AAATCTAATCAAATATGGTCG
Nr of selected repeats 4 Similarity 0.777778
Consensus:
AAATCTAATCcAATaTGGaaCg
>Cele-UNSB01_4:11132963-11135728 Satlength=2766 Nr of Repeats=35
RepeatLength=79 seed=GAAACTAAAA
GAAACTAAAACCTAATGAGACCCCAGAATACTGCCGGTATTCTGTCAGGACTCATTAGGTAGAGAGGAGATTCACAGAA
GAAACTAAAACCTAATGAGACCCCAGAATACTGCCGGTATTCTGTCAGGACTCATTAGGTAGAGAGGAGACTCACAGAA
GAAACTAAAACCTAATGAGACCCCAGAATACTGCCGGTATTCTGTCAGGACTCATTAGGTAGAGAGGAGATTCACAGAA
GAAACTAAAACCTAATGAGACCCCAGAATACTGCCGGTATTCTGTCAGGACTCATTAGGTAGAGAGGAGATTCACAGAA
GAAACTAAAACCTACTGAGACCCCAGAATACTGCCGGTATTCTGTCAGGACTCATTAGGTAGAGAGGAGACTCACAGAA
GAAACTAAAACCTAATGAGACCCCAGAATACTGCCGATATTCTGTCAGGACTCATCAGGTAGAGAGGAGATTCACAGAA
GAAACTAAAACCTACTGAGACCCCAGAATACTGCCGGTATTCTGTCAGGACTCATTAGGTAGAGTGGAGAGTTGGAAAT
GAAACTAAAACCTAATGAGACCCCAGAATACTGCCGGTATTCTGTCAGGACTCATTAGGTAGAGAGGAGATTCACAGAA
GAAACTAAAACCTACTGAGACCCCAGAATACTGCCGATATTCTGTCAGGACTCATTAGTTAGAGTGGAGATTCACAGAA
GAAACTAAAACCTACTGAGACCCCAGAATACTGCCGATATTCTGTCAGGACTCATTAGGTAGAGATGAGACTCACAGAA
GAAACTAAAACCTAATGAGACCCCAGAATACTGTCGGTATTCTGTCAGGACTCATTAGGTAGAGTGGAGAGTTGGAAAT
GAAACTAAAACCTAATGAGACCCCAGAATACTGCCGGTATTCTGTCAGGACTCATTAGGTAGAGAGGAGATTCACAGAA
GAAACTAAAACCTACTGAGACCCCAGAATACTGCCGGTATTCTGTCAGGACTCATTAGGTTTAGTGGAGAGTTGGAAAT
GAAACTAAAACCTAATGAGACCCCAGAATACTGCCGGTATTCTGTCAGGACTCATTAGGTAGAGTGGAGATTCACAGAA
GAAACTAAAACCTAATGAGACCCCAGAATACTGCCGGTATTCTGTCAGGACTCATTAGGTAGAGAGGAGACTCACAGAA
GAAACTAAAACCTAATGAGACCCCAGAATACTGCCGATATTCTGTCAGGACTCATCAGGTAGAGAGGAGATTCACAGAA
GAAACTAAAACCTACTGAGACCCCAGAATACTGCCGGTATTCTGTCAGGACTCATTAGGTAGAGTGGAGAGTTGGAAAT
GAAACTAAAACCTAATGAGACCCCAGAATACTGCCGGTATTCTGTCAGGACTCATTAGGTAGAGAGGAGATTCACAGAA
GAAACTAAAACCTACTGAGACCCCAGAATACTGCCGATATTCTGTCAGGACTCATTAGTTAGAGTGGAGATTCACAGAA
GAAACTAAAACCTACTGAGACCCCAGAATACTGCCGATATTCTGTCAGGACTCATTAGGTAGAGATGAGACTCACAGAA
GAAACTAAAACCTAATGAGACCCCAGAATACTGTCGGTATTCTGTCAGGACTCATTAGGTAGAGTGGAGAGTTGGAAAT
GAAACTAAAACCTAATGAGACCCCAGAATACTGCCGGTATTCTGTCAGGACTCATTAGGTAGAGAGGAGATTCACAGAA
GAAACTAAAACCTACTGAGACCCCAGAATACTGCCGGTATTCTGTCAGGACTCATTAGTTAGAGTGGAGATTCACAGAA
GAAACTAAAACCTAATGAGACCCCAGAATACTGCCGGTATTCTGTCAGGACTCATTAGGTAGAGAGGAGATTCACAGAA
GAAACTAAAACCTAATGAGACCCCAGAATACTGCCGGTATTCTGTCAGGACTCATTAGGTAAAGAGGAGACTCACAGAA
GAAACTAAAACCTAATGAGACCCCAGAATACTGCCGGTATTCTGCCAGGACTCATTAGGTAGAGAGGAGACTCACAGAA
GAAACTAAAACCTAATGAGACCCCAGAATACTGCCGGTATTCTGTCAGGACTCATTAGGTAGAGAGGAGATTCACAGAA
GAAACTAAAACCTAATGAGACCCCAGAATACTGCCGGTATTCTGTCAGGACTCATTAGTTAGAGTGGAGATTCACAGAA
GAAACTAAAACCTAATGAGACCCCAGAATACTGCCGGTATTCTGTCAGGACTCATTAGGTAGAGATGAGACTCACAGAA
GAAACTAAAACCTAATGAGACCCCAGAATACTGCCGGTATTCTGTCAGGACTCATTAGGTAGAGAGGAGATTCGCAGAA
GAAACTAAAACCTACTGAGACCCCAGAATACTGCCGGTATTCTGTCAGGACTCATTAGGTTTAGTGGAGAGTTGGAAAT
GAAACTAAAACCTAATGAGACCCCAGAATACTGCCGGTATTCTGTCAGGACTCATTAGGTAGAGTGGAGATTCACAGAA
GAAACTAAAACCTAATGAGACCCCAGAATACTGCCGGTATTCTGTCAGGACTCATTAGGTAGAGAGGAGATTCACAGAA
GAAACTAAAACCTACTGAGACCCCAGAATACTGCCGGTATTCTGTCAGGACTCATTAGTTAGAGTGGAGATTCACAGAA
GAAACTAAAACCTAATGAGACCCCAGAATACTGCCGGTATTCTGTCAGGACTCATTAGGTAGAGAGGAGATTCACAGAA
Nr of selected repeats 35 Similarity 0.918347
Consensus:
GAAACTAAAACCTAaTGAGACCCCAGAATACTGCCGGTATTCTGTCAGGACTCATTAGGTAGAGaGGAGAtTCaCAGAA
>Cele-UNSB01_4:11180885-11199179 Satlength=18295 Nr of Repeats=470
RepeatLength=39 seed=TTTATGGAAA
TTTATGGAAAAGTGGCTCTAGAGCCAGAAAACCAG

TTTATGGAAAGTGGCTCTAGAGCCACTAGAAATACCAG
TTTATGGAAAAGTGGCTCTAGAGCCACTAGAAATACCAG
TTTATGGAAAAGTGGCTCTAGAGCCACAAGAAATACCAG
TTTATGGAAAAGTGGCTCTAGAGCCACAGAAATACCAG
TTTATGGAAAAGTGGCTCTAGAGCCACTAGAAATACCAG
TTTATGGAAAAGTGGCTCTAGAGCCACTAGAAATACCAG
TTTATGGAAAAGTGGCTCTAGAGCCACAAGAAATACAG
TTTATGGAAAAGTGGCTCTAGAGCCACTAGAAATACCAG
TTTATGGAAAAGTGGCTCTAGAGCCACAAGAAATACCAG
TTTATGGAAAAGTGGCTCTAGAGCCACAGAAATACCAG
TTTATGGAAAAGTGGCTCTAAGCCACAGAAATACCAG
TTTATGGAAAAGTGGCTCTAGAGCCACAGAAATACCAG
TTTATGGAAAAGTGGCTCTAGAGCCACAGAAATACCAG
TTTATGGAAAAGTGGCTCTAGAGCCACTAGAAATACCAG
TTTATGGAAAAGTGGCTCTAGAGCCACAAGAAATACCAG
TTTATGGAAAAGTGGCTCTAGAGCCACAAGAAATACCAG
TTTATGGAAAAGTGGCTCTAGAGCCACTAGAAATACCAG
TTTATGGAAAAGTGGCTCTAGAGCCACTAGAAATACCAG
TTTATGGAAAAGTGGCTCTAGAGCCACTAGAAATACCAG
TTTATGGAAAAGTGGCTCTAGAGCCACTAGAAATACCAG
TTTATGGAAAAGTGGCTCTAGAGCCACAAGAAATACAG
TTTATGGAAAAGTGGCTCTAGAGCCACAAGAAATACCAG
TTTATGGAAAAGTGGCTCTAGAGCCACAGAAATACCAG
TTTATGGAAAAGTGGCTCTAGAGCCACTAGAAATACCAG
TTTATGGAAAGTGGCTCTAGAGCCACTAGAAATACCAG
TTTATGGAAAAGTGGCTCTAGAGCCACTAGAAATACCAGT
TTTATGGAAAAGTGGCTCTAGAGCCACAAGAAATACCAG
TTTATGGAAAAGTGGCTCTAGAGCCACTAGAAATACCAG
TTTATGGAAAAGTGGCTCTAGAGCCACAAGAAATACCAG
TTTATGGAAAAGTGGCTCTAGAGCCACTAGAAATACCAG
TTTATGGAAAAGTGGCTCTAGAGCCACAGAAATACCAG
TTTATGGAAAAGTGGCTCTAGAGCCACAAGAAATACCAG
TTTATGGAAAAGTGGCTCTAGAGCCACAGAAATACCAG
TTTATGGAAAAGTGGCTCTAGAGCCACTAGAAATACCAG
TTTATGGAAAAGTGGCTCTAGAGCCACAAGAAATACCAG
TTTATGGAAAAGTGGCTCTAGAGCCACTAGAAATACCAG
TTTATGGAAAAGTGGCTCTAGAGCCACAGAAATACCAG
TTTATGGAAAAGTGGCTCTAGAGCCACTAGAAATACCAG
TTTATGGAAAAGTGGCTCTAGAGCCACTAGAAATACCAG
TTTATGGAAAAGTGGCTCTAGAGCCACATAGAAATACCAG
TTTATGGAAAAGTGGCTCTAGAGCCACTAGAAATACCAG
TTTATGGAAAAGTGGCTCTAGAGCCACAAGAAATACCAG
TTTATGGAAAAGTGGCCACAAGAAATACCAG
TTTATGGAAAAGTGGCTCTAGAGCCACAAGAAATACCAG
TTTATGGAAAAGTGGCTCTAGAGCCACTAGAAATACCAG
TTTATGGAAAAGTGGCTCTAGAGCCACAAGAAATACCAG
TTTATGGAAAAGTGGCTCTAGAGCCACAAGAAATACCAG
TTTATGGAAAAGTGGCTCTAGAGCCACTAGAAATACCAG
TTTATGGAAAAGTGGCTCTAGAGCCACTAGAAATACCAG
TTTATGGAAAAGTGGCTCTAGAGCCACAAGAAATACCAG
TTTATGGAAAAGTGGCTCTAGAGCCACAAGAAATACCAG
TTTATGGAAAAGTGGCTCTAGAGCCACTAGAAATACCAG
TTTATGGAAAAGTGGCTCTAGAGCCACTAGAAATACCAG
TTTATGGAAAAGTGGCTCTAGAGCCACAAGAAATACCAG

TTTATGGAAAAGTGGCTCTAGAGCCACAAGAAATACCAG
TTTATGGAAAAGTGGCTCTAGAGCCACAAGAAATACCAG
TTTATGGAAAAGTGGCTCTAGAGCCACAGAAATACCAG
TTTATGGAAAAGTGGCTCTAGAGCCACTAAGAAATACCAG
TTTATGGAAAAGTGGCTCTAGAGCCACTAGAAATACCAG
TTTATGGAAAAGTGGCTCTAGAGCCACTAGAAATACCAG
TTTATGGAAAAGTGGCTCTAAGCCACAGAAATACAG
TTTATGGAAAAGTGGCTCTAGAGCCACAGAAATACCAG
TTTATGGAAAAGTGGCTCTAGAGCCACTAGAAATACCAG
TTTATGGAAAAGTGGCTCTAGAGCCACTAGAAATACCAG
TTTATGGAAAATGGAAAAGTGGCTCTAGAGCCACAAGAAATACCAG
TTTATGGAAAAGTGGCTCTAGAGCCACAAGAAATACCAG
TTTATGGAAAAGTGGCTCTAGAGCCACTAGAAATACCAG
TTTATGGAAAAGTGGTCATAGAGCCACTAGAAATACCAG
TTTATGGAAAAGTGGCTCTAGAGCCACTAGAAATACCAG
TTTATGGAAAAGTGGCTCTAGAGCCACTAGAAATACCAG
TTTATGGAAAGTGGCTCTAGAGCCACAAGAAATACCAG
TTTATGGAAAAGTGGCTCTAGAGCCACAAGAAATACCAG
TTTATGGAAAAGTGGCTCTAGAGCCACAAGAAATACCAG
TTTATGGAAAAGTGGCTCTAGAGCCACTAGAAATACCAG
TTTATGGAAAAGTGGCTCTAGAGCCACAAGAAATACCAG
TTTATGGAAAAGTGGCTCTATAGCCACAAGAAATACCAG
TTTATGGAAAAGTGGCTCTAGAGCCACTAGAAATACCAG
TTTATGGAAAAGTGGCTCTAGAGCCACTAGAAATACCAG
TTTATGGAAAAGTGGCTCTAGAGCCACAAGAAATACCAG
TTTATGGAAAAGTGGCTCTAGAGCCACTAGAAATACCAG
TTTATGGAAAAGTGGCTCTAGAGCCACAAGAAATACCAG
TTTATGGAAAAGTGGCTCTAGAGCCACAAGAAATACCAG
TTTATGGAAAAGTGGCTCTAGAGCCACAAGAAATACCAG
TTTATGGAAAAGTGGCTCTATAGCCACAAGAAATACCAG
TTTATGGAAAAGTGGCTCTAGAGCCACTAGAAATACCAG
TTTATGGAAAAGTGGCTCTAGAGCCACAAGAAATACAGTATGAAAAGTGCCTAAGCCAAGAAATACCAG
TTTATGGAAAAGTGGCTCTAGAGCCACAAGAAATACCAG
TTTATGGAAAAGTGGCTCTAGAGCCACTAGAAATACCAG
TTTATGGAAAAGTGGCTCTAGAGCCACAAGAAATACCAG
TTTATGGAAAAGTGGCTCTAGAGCCACAAGAAATACCAG
TTTATGGAAAAGTGGCTCTAGAGCCACAAGAAATACCAG
TTTATGGAAAAGTGGCTCTAGAGCCACTAGAAATACCAG
TTTATGGAAAAGTGGCTCTAGAGCCACTAGAAATACCAG
TTTATGGAAAAGTGGCTCTAGAGCCACTAGAAATACCAG
TTTATGGAAAAGTGGCTCTAGAGCCACTAGAAATACCAG
TTTATGGAAAAGTGGCTCTAGAGCCACAAGAAATACCAG
TTTATGGAAAAGTGGCTCTAGAGCCACTAGAAATACCAG
TTTATGGAAAAGTGGCTCTAGAGCCACTAGAAATACCAG
TTTATGGAAAAGTGGCTCTAGAGCCACAAGAAATACCAG
TTTATGGAAAAGTGGCTCTAGAGCCACAAGAAATACCAG
TTTATGGAAAAGTGGCTCTAGAGCCACTAGAAATACCAG
TTTATGGAAAAGTGGCTCTAGAGCCACAAGAAATACCAG
TTTATGGAAAAGTGGCTCTAGAGCCACTAGAAATACCAG
TTTATGGAAAAGTGGCTCTAGAGCCACTAGAAATACCAG
TTTATGGAAAAGTGGCTCTAGAGCCACTAGAAATACCAG
TTTATGGAAAAGTGGCTCTAGAGCCACAAGAAATACCAG
TTTATGGAAAAGTGGCTCTAGAGCCACTAGAAATACCAG
TTTATGGAAAAGTGGCTCTAAGCCACAAGAAATACCAG

TTTATGGAAAAGTGGCTCTAGAGCCACAAGAAATACCAG
TTTATGGAAAAGTGGCTCTAGAGCCACAAGAAATACCAG
TTTATGGAAAAGTGGCTCTAGAGCCACTAGAAATACCAG
TTTATGGAAAAGTGGCTCTAAGCCACAGAAATACCAG
TTTATGGAAAAGTGGCTCTAGAGCCACAAGAAATACCAG
TTTATGGAAAAGTGGCTCTAGAGCCACTAGAAATACCAG
TTTATGGAAAAGTGGCTCTAGAGCCACAAGAAATACCAG
TTTATGGAAAAGTGGCTCTAGAGCCACAAGAAATACCAG
TTTATGGAAAAGTGGCTCTAGAGCCACAGAAATACCAG
TTTATGGAAAAGTGGCTCTAGAGCCACAGAAATACCAG
TTTATGGAAAAGTGGCTCTATAGCCACAAGAAATACCAG
TTTATGGAAAAGTGGCTCTAGAGCCACTAGAAATACAG
TTTATGGAAAAGTGGCTCTAGAGCCACAAGAAATACCAG
TTTATGGAAAAGTGGCTCTAGAGCCACTAGAAATACCAG
TTTATGGAAAAGTGGCTCTAGAGCCACTAGAAATACCAG
TTTATGGAAAAGTGGCTCTAGAGCCACAGAAATACCAG
TTTATGGAAAAGTGGCTCTAGAGCCACAGAAATACCAG
TTTATGGAAAAGTGGCTCTAGAGCCACAAGAAATACCAG
TTTATGGAAAAGTGGCTCTAGAGCCACAAGAAATACCAG
TTTATGGAAAAGTGGCTCTAGAGCCACAGAAATACCAG
TTTATGGAAAAGTGGCTCTAGAGCCACAAGAAATACCAG
TTTATGGAAAAGTGGCTCTAGAGCCACTAGAAATACCAG
TTTATGGAAAAGTGGCTCTAGAGCCACTAGAAATACCAG
TTTATGGAAAAGTGGCTCTAGAGCCACAAGAAATACCAG
TTTATGGAAAAGTGGCTCTAAGCCACAAAAATACCAG
TTTATGGAAAAGTGGCTCTAGAGCCACTAGAAATACCAG
TTTATGGAAAAGTGGCTCTAGAGCCACTAGAAATACCAG
TTTATGGAAAAGTGGCTCTAGAGCCACAAGAAATACCAG
TTTATGGAAAAGTGGCTCTAGAGCCACAAGAAATACCAG
TTTATGGAAAAGTGGCTCTAGAGCCACTAGAAATACCAG
TTTATGGAAAAGTGGCTCTAGAGCCACTAGAAATACCAG
TTTATGGAAAAGTGGCTCTAGAGCCACAAGAAATACCAG
TTTATGGAAAAGTGGCTCTAGAGCCACAAGAAATACCAG
TTTATGGAAAAGTGGCTCTAGAGCCACAGAAATACCAG
TTTATGGAAAAGTGGCTCTAGAGCCACTAGAAATACCAG
TTTATGGAAAGTGGCTCTAAGCAAAGAAATACCAG
TTTATGGAAAAGTGGCTCTAGAGCCACTAGAAATACCAG
TTTATGGAAAAGTGGCTCTAGAGCCACTAGAAATACAG
TTTATGGAAAAGTGGCTCTAAGCCACAGAAATACCAG
TTTATGGAAAAGTGGCTCTAGAGCCACAAGAAATACCAG
TTTATGGAAAAGTGGCTCTATAGCCACAAGAAATACCAG
TTTATGGAAAAGTGGCTCTAGAGCCACAGAAATACCAG
TTTATGGAAAAGTGGCTCTAGAGCCACAAGAAATACCAG
TTTATGGAAAAGTGGCTCTAGAGCCACAAGAAATACCAG
TTTATGGAAAAGTGGCTCTAGAGCCACTAGAAATACCAG
TTTATGGAAAAGTGGCTCTAGAGCCACAGAAATACCAG
TTTATGGAAAAGTGGCTCTAGAGCCACAAGAAATACCAG
TTTATGGAAAAGTGGCTCTAGAGCCACAAGAAATACCAG
TTTATGGAAAAGTGGCTCTAGAGCCACTAGAAATACCAG
TTTATGGAAAAGTGGCTCTAGAGCCACTAGAAATACCAG
TTTATGGAAAAGTGGCTCTAGAGCCACTAGAAATACCAG
TTTATGGAAAAGTGGCTCTAGAGCCACAAGAAATACCAG
TTTATGGAAAAGTGGCTCTATAGCCACAAGAAATACCAG
TTTATGGAAAAGTGGCTCTAGAGCCACTAGAAATACCAG

TTTATGGAAAAGTGGCTCTAGAGCCACTAGAAATACCAG
TTTATGGAAAAGTGGCTCTAGAGCCACAAGAAATACCAG
TTTATGGAAAAGTGGCTCTAGAGCCACTAGAAATACCAG
TTTATGGAAAAGTGGCTCTAGAGCCACTAGAAATACCAG
TTTATGGAAAAGTGGCTCTAGAGCCACTAGAAATACCAG
TTTATGGAAAAGTGGCTCTAGAGCCACTAGAAATACCAG
TTTATGGAAAAGTGGCTCTAGAGCCACTAGAAATACCAG
TTTATGGAAAAGTGGCTCTAGAGCCACTAGAAATACCAG
TTTATGGAAAAGTGGCTCTAGAGCCACTAGAAATACCAG
TTTATGGAAAAGTGGCTCTAGAGCCACTAGAAATACCAG
TTTATGGAAAAGTGGCTCTAGAGCCACTAGAAATACCAG
TTTATGGAAAAGTGGCTCTAGAGCCACAAGAAATACCAG
TTTATGGAAAAGTGGCTCTAGAGCCACAAGAAATACCAG
TTTATGGAAAAGTGGCTCTAGAGCCACTAGAAATACCAG
TTTATGGAAAAGTGGCTCTAGAGCCACAAGAAATACCAG
TTTATGGAAAAGTGGCTCTAGAGCCACTAGAAATACCAG
TTTATGGAAAAGTGGCTCTAGAGCCACTAGAAATACCAG
TTTATGGAAAAGTGGCTCTAGAGCCACAGAAATACCAG
TTTATGGAAAAGTGGCTCTAGAGCCACTAGAAATACCAG
TTTATGGAAAAGTGGCTCTAGAGCCACTAGAAATACCAG
TTTATGGAAAAGTGGCTCTAGAGCCACTAGAAATACCAG
TTTATGGAAAAGTGGCTCTAGAGCCACTAGAAATACCAG
TTTATGGAAAAGTGGCTCTAGAGCCACTAGAAATACCAG
TTTATGGAAAAGTGGCTCTAGAGCCACTAGAAATACCAG
TTTATGGAAAAGTGGCTCTAGAGCCACTAGAAATACCAG
TTTATGGAAAAGTGGCTCTAGAGCCACTAGAAATACCAG
TTTATGGAAAAGTGGCTCTAGAGCCACTAGAAATACCAG
TTTATGGAAAAGTGGCTCTAGAGCCACTAGAAATACCAG
TTTATGGAAAAGTGGCTCTAGAGCCACTAGAAATACCAG
TTTATGGAAAAGTGGCTCTAGAGCCACTAGAAATACCAG
TTTATGGAAAAGTGGCTCTAGAGCCACTAGAAATACCAG
TTTATGGAAAAGTGGCTCTAGAGCCACTAGAAATACCAG
TTTATGGAAAAGTGGCTCTAGAGCCACTAGAAATACCAG
TTTATGGAAAAGTGGCTCTAGAGCCACTAGAAATACCAG
TTTATGGAAAAGTGGCTCTAGAGCCACTAGAAATACCAG
TTTATGGAAAAGTGGCTCTAGAGCCACTAGAAATACCAG
TTTATGGAAAAGTGGCTCTAGAGCCACTAGAAATACCAG
TTTATGGAAAAGTGGCTCTAGAGCCACTAGAAATACCAG
TTTATGGAAAAGTGGCTCTAGAGCCACAAGAAATACCAG
TTTATGGAAAAGTGGCTCTAGAGCCACTAGAAATACCAG
TTTATGGAAAAGTGGCTCTAGAGCCACTAGAAATACCAG
TTTATGGAAAAGTGGCTCTAGAGCCACAAGAAATACCAG
TTTATGGAAAAGTGGCTCTAGAGCCACTAGAAATACCAG
TTTATGGAAAAGTGGCTCTAGAGCCACTAGAAATACCAG
TTTATGGAAAAGTGGCTCTAGAGCCACTAGAAATACCAG
TTTATGGAAAAGTGGCTCTAGAGCCACAAGAAATACCAG
TTTATGGAAAAGTGGCTCTAGAGCCACAAGAAATACCAG
TTTATGGAAAAGTGGCTCTAGAGCCACAAGAAATACCAG
TTTATGGAAAAGTGGCTCTAGAGCCACAGAAATACCAG
TTTATGGAAAAGTGGCTCTAGAGCCACTAGAAATACCAG
TTTATGGAAAAGTGGCTCTAGAGCCACAAGAAATACCAG
TTTATGGAAAAGTGGCTCTAGAGCCACAAGAAATACCAG
TTTATGGAAAAGTGGCTCTAGAGCCACTAGAAATACCAG
TTTATGGAAAAGTGGCTCTAGAGCCACTAGAAATACCAG

TTTATGGAAAAGTGGCTCTAGAGCCACAAGAAATACCAG
TTTATGGAAAAGTGGCTCTAGAGCCACAGAAATACCAG
TTTATGGAAAAGTGGCTCTAGAGCCACTAGAAATACCAG
TTTATGGAAAAGTGGCTCTAGAGCCACTAGAAATACCAG
TTTATGGAAAAGTGGCTCTAGAGCCACAAGAAATACCAG
TTTATGGAAAAGTGGCTCTAGAGCCACAAGAAATACCAG
TTTATGGAAAAGTGGCTCTAGAGCCACTAGAAATACCAG
TTTATGGAAAAGTGGCTCTAGAGCCACTAGAAATACCAG
TTTATGGAAAAGTGGCTCTAGAGCCACAAGAAATACCAG
TTTATGGAAAAGTGGCTCTAGAGCCACAAGAAATACCAG
TTTATGGAAAAGTGGCTCTAGAGCCACAAGAAATACCAG
TTTATGGAAAAGTGGCTCTAGAGCCACTAGAAATACCAG
TTTATGGAAAAGTGGCTCTAGAGCCACAAGAAATACCAG
TTTATGGAAAAGTGGCTCTAGAGCCACAAGAAATACCAG
TTTATGGAAAAGTGGCTCTAGAGCCACAAGAAATACCAG
TTTATGGAAAAGTGGCTCTAGAGCCACAAGAAATACCAG
TTTATGGAAAAGTGGCTCTAGAGCCACAAGAAATACCAG
TTTATGGAAAAGTGGCTCTAGAGCCACAAGAAATACCAG
TTTATGGAAAAGTGGCTCTAGAGCCACTAGAAATACCAG
TTTATGGAAAAGTGGCTCTAGAGCCACTAGAAATACCAG
TTTATGGAAAAGTGGCTCTAGAGCCACTAGAAATACCAG
TTTATGGAAAAGTGGCTCTAGAGCCACAAGAAATACCAG
TTTATGGAAAAGTGGCTCTAGAGCCACAAGAAATACCAG
TTTATGGAAAAGTGGCTCTAGAGCCACAAGAAATACCAG
TTTATGGAAAAGTGGCTCTAGAGCCACTAGAAATACCAG
TTTATGGAAAAGTGGCTCTAGAGCCACAAGAAATACCAG
TTTATGGAAAAGTGGCTCTAGAGCCACAAGAAATACCAG
TTTATGGAAAAGTGGCTCTAGAGCCACTAGAAATACCAG
TTTATGGAAAAGTGGCTCTAGAGCCACTAGAAATACCAG
TTTATGGAAAAGTGGCTCTAGAGCCACAAGAAATACCAG
TTTATGGAAAAGTGGCTCTAGAGCCACAAGAAATACCAG
TTTATGGAAAAGTGGCTCTAGAGCCACTAGAAATACCAG
TTTATGGAAAAGTGGCTCTAGAGCCACTAGAAATACCAG
TTTATGGAAAAGTGGCTCTAGAGCCACAAGAAATACCAG
TTTATGGAAAAGTGGCTCTAGAGCCACAAGAAATACCAG
TTTATGGAAAAGTGGCTCTAGAGCCACAGAAATACCAG
TTTATGGAAAAGTGGCTCTAGAGCCACAAGAAATACCAG
TTTATGGAAAAGTGGCTCTAGAGCCACAAGAAATACCAG
TTTATGGAAAAGTGGCTCTAGAGCCACTAGAAATACCAG
TTTATGGAAAAGTGGCTCTAGAGCCACAAGAAATACCAG
TTTATGGAAAAGTGGCTCTAGAGCCACAAGAAATACCAG
TTTATGGAAAAGTGGCTCTAGAGCCACAAGAAATACCAG
TTTATGGAAAAGTGGCTCTAGAGCCACTAGAAATACCAG
TTTATGGAAAAGTGGCTCTAGAGCCACAAGAAATACCAG
TTTATGGAAAAGTGGCTCTAGAGCCACAAGAAATACCAG
TTTATGGAAAAGTGGCTCTAGAGCCACAAGAAATACCAG
TTTATGGAAAAGTGGCTCTAGAGCCACAAGAAATACCAG
TTTATGGAAAAGTGGCTCTAGAGCCACAAGAAATACCAG
TTTATGGAAAAGTGGCTCTAGAGCCACAAGAAATACCAG
TTTATGGAAAAGTGGCTCTAGAGCCACAAGAAATACCAG
TTTATGGAAAAGTGGCTCTAGAGCCACAAGAAATACCAG
TTTATGGAAAAGTGGCTCTAGAGCCACAAGAAATACCAG
TTTATGGAAAAGTGGCTCTAGAGCCACTAGAAATACCAG
TTTATGGAAAAGTGGCTCTAGAGCCACAAGAAATACCAG

TTTATGGAAAAGTGGCTCTAGAGCCACAAGAAATACCAG
TTTATGGAAAAGTGGCTCTAGAGCCACAAGAAATACCAG
TTTATGGAAAAGTGGCTCTAGAGCCACAAGAAATACCAG
TTTATGGAAAAGTGGCTCTAGAGCCACAAGAAATACCAG
TTTATGGAAAAGTGGCTCTAGAGCCACTAGAAATACCAG
TTTATGGAAAAGTGGCTCTAGAGCCACTAGAAATACCAG
TTTATGGAAAAGTGGCTCTAGAGCCACAGAAATACCAG
TTTATGGAAAAGTGGCTCTAAGCCACAAGAAATACCAG
TTTATGGAAAAGTGGCTCTAGAGCCACAAGAAATACCAG
TTTATGGAAAAGTGGCTCTAGAGCCACTAGAAATACCAG
TTTATGGAAAAGTGGCTCTAGAGCCACTAGAAATACCAG
TTTATGGAAAAGTGGCTCTAGAGCCACAAGAAATACCAG
TTTATGGAAAAGTGGCTCTAGAGCCACAAGAAATACCAG
TTTATGGAAAAGTGGCTCTAGAGCCACAAGAAATACCAG
TTTATGGAAAAGTGGCTCTAGAGCCACAAGAAATACCAG
TTTATGGAAAAGTGGCTCTAGAGCCACTAGAAATACCAG
TTTATGGAAAAGTGGCTCTAGAGCCACTAGAAATACCAG
TTTATGGAAAAGTGGCTCTATAGCCACAAGAAATACCAG
TTTATGGAAAAGTGGCTCTAGAGCCACAAGAAATACCAG
TTTATGGAAAAGTGGCTCTATAGCCACAAGAAATACCAG
TTTATGGAAAAGTGGCTCTAGAGCCACAAGAAATACCAG
TTTATGGAAAAGTGGCTCTAGAGCCACAAGAAATACCAG
TTTATGGAAAAGTGGCTCTATAGCCACAAGAAATACCAG
TTTATGGAAAAGTGGCTCTAGAGCCACAAGAAATACCAG
TTTATGGAAAAGTGGCTCTATAGCCACAAGAAATACCAG
TTTATGGAAAAGTGGCTCTATAGCCACAAGAAATACCAG
TTTATGGAAAAGTGGCTCTAGAGCCACTAGAAATACCAG
TTTATGGAAAAGTGGCTCTAGAGCCACAGAAATACCAG
TTTATGGAAAAGTGGCTCTAGAGCCACAAGAAATACCAG
TTTATGGAAAAGTGGCTCTATAGCCACAAGAAATACCAG
TTTATGGAAAAGTGGCTCTATAGCCACAAGAAATACCAG
TTTATGGAAAAGTGGCTCTATAGCCACAAGAAATACCAG
TTTATGGAAAAGTGGCTCTATAGCCACAAGAAATACCAG
TTTATGGAAAAGTGGCTCTATAGCCACAAGAAATACCAG
TTTATGGAAAAGTGGCTCTATAGCCACAAGAAATACCAG
TTTATGGAAAAGTGGCTCTATAGCCACAAGAAATACCAG
TTTATGGAAAAGTGGCTCTATAGCCACAAGAAATACCAG
TTTATGGAAAAGTGGCTCTAGAGCCACAAGAAATACCAG
TTTATGGAAAAGTGGCTCTAGAGCCACAAGAAATACCAG
TTTATGGAAAAGTGGCTCTATAGCCACAAGAAATACCAG
TTTATGGAAAAGTGGCTCTATAGCCACAAGAAATACCAG
TTTATGGAAAAGTGGCTCTATAGCCACAAGAAATACCAG
TTTATGGAAAAGTGGCTCTAGAGCCACTAGAAATACCAG
TTTATGGAAAAGTGGCTCTAGAGCCACAAGAAATACCAG
TTTATGGAAAAGTGGCTCTATAGCCACAAGAAATACCAG
TTTATGGAAAAGTGGCTCTATAGCCACAAGAAATACCAG
TTTATGGAAAAGTGGCTCTATAGCCACAAGAAATACCAG
TTTATGGAAAAGTGGCTCTATAGCCACAAGAAATACCAG
TTTATGGAAAAGTGGCTCTAGAGCCACAAGAAATACCAG
TTTATGGAAAAGTGGCTCTAGAGCCACAAGAAATACCAG
TTTATGGAAAAGTGGCTCTATAGCCACAAGAAATACCAG
TTTATGGAAAAGTGGCTCTAGAGCCACTAGAAATACCAG
TTTATGGAAAAGTGGCTCTAGAGCCACAAGAAATACCAG
TTTATGGAAAAGTGGCTCTATAGCCACAAGAAATACCAG

TTTATGGAAAAGTGGCTCTAGAGCCACTAGAAATACCAG
TTTATGGAAAAGTGGCTCTAGAGCCACTAGAAATACCAG
TTTATGGAAAAGTGGCTCTAGAGCCACAAGAAATACCAG
TTTATGGAAAAGTGGCTCTAGAGCCACAAGAAATACCAG
TTTATGGAAAAGTGGCTCTAGAGCCACAAGAAATACCAG
TTTATGGAAAAGTGGCTCTAGAGCCACAAGAAATACCAG
TTTATGGAAAAGTGGCTCTAGAGCCACTAGAAATACCAG
TTTATGGAAAAGTGGCTCTAGAGCCACTAGAAATACCAG
TTTATGGAAAAGTGGCTCTAGAGCCACAAGAAATACCAG
TTTATGGAAAAGTGGCTCTAGAGCCACTAGAAATACCAG
TTTATGGAAAAGTGGCTCTAGAGCCACTAGAAATACCAG
TTTATGGAAAAGTGGCTCTAGAGCCACAAGAAATACCAG
TTTATGGAAAAGTGGCTCTAGAGCCACTAGAAATACCAG
TTTATGGAAAAGTGGCTCTAGAGCCACTAGAAATACCAG
TTTATGGAAAAGTGGCTCTAGAGCCACTAGAAATACCAG
TTTATGGAAAAGTGGCTCTAGAGCCACTAGAAATACCAG
TTTATGGAAAAGTGGCTCTAGAGCCACAAGAAATACCAG
TTTATGGAAAAGTGGCTCTAGAGCCACAAGAAATACCAG
TTTATGGAAAAGTGGCTCTAGAGCCACTAGAAATACCAG
TTTATGGAAAAGTGGCTCTAGAGCCACTAGAAATACCAG
TTTATGGAAAAGTGGCTCTAGAGCCACAAGAAATACCAG
TTTATGGAAAAGTGGCTCTAGAGCCACAAGAAATACCAG
TTTATGGAAAAGTGGCTCTAGAGCCACAAGAAATACCAG
TTTATGGAAAAGTGGCTCTAGAGCCACAAGAAATACCAG
TTTATGGAAAAGTGGCTCTAGAGCCACTAGAAATACCAG
TTTATGGAAAAGTGGCTCTAGAGCCACAAGAAATACCAG
TTTATGGAAAAGTGGCTCTAGAGCCACAAGAAATACCAG
TTTATGGAAAAGTGGCTCTAGAGCCACAAGAAATACCAG
TTTATGGAAAAGTGGCTCTAGAGCCACAAGAAATACCAG
TTTATGGAAAAGTGGCTCTAGAGCCACAAGAAATACCAG
TTTATGGAAAAGTGGCTCTAGAGCCACAAGAAATACCAG
TTTATGGAAAAGTGGCTCTAGAGCCACAAGAAATACCAG
TTTATGGAAAAGTGGCTCTAGAGCCACAAGAAATACCAG
TTTATGGAAAAGTGGCTCTAGAGCCACAAGAAATACCAG
TTTATGGAAAAGTGGCTCTAGAGCCACAAGAAATACCAG
TTTATGGAAAAGTGGCTCTAGAGCCACAAGAAATACCAG
TTTATGGAAAAGTGGCTCTAGAGCCACAAGAAATACCAG
TTTATGGAAAAGTGGCTCTAGAGCCACTAGAAATACCAG
TTTATGGAAAAGTGGCTCTAGAGCCACAAGAAATACCAG
TTTATGGAAAAGTGGCTCTATAGCCACAAGAAATACCAG
TTTATGGAAAAGTGGCTCTATAGCCACAAGAAATACCAG
TTTATGGAAAAGTGGCTCTAGAGCCACTAGAAATACCAG
TTTATGGAAAAGTGGCTCTATAGCCACAAGAAATACCAG
TTTATGGAAAAGTGGCTCTAGAGCCACAAGAAATACCAG
TTTATGGAAAAGTGGCTCTAGAGCCACAAGAAATACCAG
TTTATGGAAAAGTGGCTCTAGAGCCACTAGAAATACCAG
TTTATGGAAAAGTGGCTCTAGAGCCACAAGAAATACCAG
TTTATGGAAAAGTGGCTCTATAGCCACAAGAAATACCAG
TTTATGGAAAAGTGGCTCTAGAGCCACTAGAAATACCAG
TTTATGGAAAAGTGGCTCTAGAGCCACAAGAAATACCAG
TTTATGGAAAAGTGGCTCTAGAGCCACAAGAAATACCAG
TTTATGGAAAAGTGGCTCTAGAGCCACAAGAAATACCAG
TTTATGGAAAAGTGGCTCTAGAGCCACAAGAAATACCAG
TTTATGGAAAAGTGGCTCTAGAGCCACAAGAAATACCAG

TTTATGGAAAAGTGGCTCTAGAGCCACTAGAAATACCAG
TTTATGGAAAAGTGGCTCTAGAGCCACTAGAAATACCAG
TTTATGGAAAAGTGGCTCTAGAGCCACAAGAAATACCAG
TTTATGGAAAAGTGGCTCTAGAGCCACAAGAAATACCAG
TTTATGGAAAAGTGGCTCTAGAGCCACAAGAAATACCAG
TTTATGGAAAAGTGGCTCTAGAGCCACAAGAAATACCAG
TTTATGGAAAAGTGGCTCTAGAGCCACTAGAAATACAG
TTTATGGAAAAGTGGCTCTAGAGCCACAAGAAATACCAG
TTTATGGAAAAGTGGCTCTAGAGCCACAAGAAATACCAG
TTTATGGAAAAGTGGCTCTAGAGCCACAAGAAATACCAG
TTTATGGAAAAGTGGCTCTAGAGCCACAAGAAATACCAG
TTTATGGAAAAGTGGCTCTAGAGCCACAAGAAATACCAG
TTTATGGAAAAGTGGCTCTAGAGCCACAAGAAATACCAG
TTTATGGAAAAGTGGCTCTAGAGCCACAAGAAATACCAG
TTTATGGAAAAGTGGCTCTAGAGCCACAAGAAATACCAG
TTTATGGAAAAGTGGCTCTAGAGCCACAAGAAATACCAG
TTTATGGAAAAGTGGCTCTAGAGCCACAAGAAATACCAG
TTTATGGAAAAGTGGCTCTAGAGCCACAAGAAATACCAG
TTTATGGAAAAGTGGCTCTAGAGCCACAAGAAATACCAG
TTTATGGAAAAGTGGCTCTAGAGCCACAAGAAATACCAG
TTTATGGAAAAGTGGCTCTAGAGCCACAAGAAATACCAG
TTTATGGAAAAGTGGCTCTAGAGCCACAAGAAATACCAG
TTTATGGAAAAGTGGCTCTAGAGCCACAAGAAATACCAG
TTTATGGAAAAGTGGCTCTAGAGCCACAAGAAATACCAG
TTTATGGAAAAGTGGCTCTAGAGCCACAAGAAATACCAG
TTTATGGAAAAGTGGCTCTAGAGCCACAAGAAATACCAG
TTTATGGAAAAGTGGCTCTAGAGCCACAAGAAATACCAG
TTTATGGAAAAGTGGCTCTAGAGCCACAAGAAATACCAG
TTTATGGAAAAGTGGCTCTAGAGCCACAAGAAATACCAG
TTTATGGAAAAGTGGCTCTAGAGCCACAAGAAATACCAG
TTTATGGAAAAGTGGCTCTAGAGCCACAAGAAATACCAG
TTTATGGAAAAGTGGCTCTAGAGCCACAGAAATACCAG
TTTATGGAAAAGTGGCTCTAGAGCCACAAGAAATACCAG
TTTATGGAAAAGTGGCTCTAGAGCCACAAGAAATACCAG
TTTATGGAAAAGTGGCTCTAGAGCCACAAGAAATACCAG
TTTATGGAAAAGTGGCTCTAGAGCCACAAGAAATACCAG
TTTATGGAAAAGTGGCTCTAGAGCCACAGAAATACCAG
TTTATGGAAAAGTGGCTCTAGAGCCACAGAAATACCAG
TTTATGGAAAAGTGGCTCTAGAGCCACAGAAATACCAG
TTTATGGAAAAGTGGCTCTAGAGCCACAGAAATACCAG
TTTATGGAAAAGTGGCTCTAGAGCCACAAGAAATACCAG
TTTATGGAAAAGTGGCTCTAGAGCCACAAGAAATACCAG
TTTATGGAAAAGTGGCTCTAGAGCCACAAGAAATACCAG
TTTATGGAAAAGTGGCTCTAGAGCCACAGAAATACCAG
TTTATGGAAAAGTGGCTCTAGAGCCACAAGAAATACCAG
TTTATGGAAAAGTGGCTCTAAGCCACAAGAAATACCAG
TTTATGGAAAAGTGGCTCTAGAGCCACTAGAAATACAG
TTTATGGAAAAGTGGCTCTAGAGCCACTAGAAATACCAG
TTTATGGAAAAGTGGCTCTATAGCCACAAGAAATACCAG
TTTATGGAAAAGTGGCTCTAGAGCCACAAGAAATACCAG
TTTATGGAAAAGTGGCTCTAGAGCCACAAGAAATACCAG
TTTATGGAAAAGTGGCTCTAAGCCACAAGAAATACCAG
TTTATGGAAAAGTGGCTCTAGAGCCACTAGAAATACAG
TTTATGGAAAAGTGGCTCTAGAGCCACAGAAATACCAG

TTTATGGAAAAGTGGCTCTAAGCCACAAGAAATACCAG
TTTATGGAAAAGTGGCTCTAGAGCCACAAGAAATACCAG
TTTATGGAAAAGTGGCTCTAGAGCCACAAGAAATACCAG
TTTATGGAAAAGTGGCTCTAAGCCACAAGAAATACCAG
TTTATGGAAAAGTGGCTCTAGAGCCACTAGAAATACCAG
TTTATGGAAAAGTGGCTCTAGAGCCACAAGAAATACCAG
TTTATGGAAAAGTGGCTCTAGAGCCACAAGAAATACCAG
TTTATGGAAAAGTGGCTCTAGAGCCACAAGAAATACCAG
TTTATGGAAAAGTGGCTCTAAGCCACAAGAAATACCAG
TTTATGGAAAAGTGGCTCTAGAGCCACAGAAATACCAG
TTTATGGAAAAGTGGCTCTAGAGCCACAAGAAATACCAG
TTTATGGAAAAGTGGCTCTAGAGCCACAAGAAATACCAG
TTTATGGAAAAGTGGCTCTAGAGCCACAAGAAATACCAG
TTTATGGAAAAGTGGCTCTAGAGCCACAAGAAATACCAG
TTTATGGAAAAGTGGCTCTAGAGCCACAAGAAATACCAG
TTTATGGAAAAGTGGCTCTAGAGCCACAAGAAATACCAG
TTTATGGAAAAGTGGCTCTAGAGCCACAAGAAATACCAG
TTTATGGAAAAGTGGCTCTAGAGCCACAAGAAATACCAG
TTTATGGAAAAGTGGCTCTAGAGCCACAAGAAATACCAG
TTTATGGAAAAGTGGCTCTAGAGCCACAAGAAATACCAG
TTTATGGAAAAGTGGCTCTAGAGCCACAAGAAATACCAG
TTTATGGAAAAGTGGCTCTAGAGCCACAAGAAATACCAG
TTTATGGAAAAGTGGCTCTAGAGCCACAAGAAATACCAG
TTTATGGAAAAGTGGCTCTAGAGCCACAAGAAATACCAG
TTTATGGAAAAGTGGCTCTAGAGCCACAAGAAATACCAG
TTTATGGAAAAGTGGCTCTAGAGCCACTAGAAATAACAG
TTTATGGAAAAGTGGCTCTAGAGCCACAAGAAATACCAG
TTTATGGAAAAGTGGCTCTAGAGCCACAAGAAATACCAG
TTTATGGAAAAGTGGCTCTAGAGCCACAAGAAATACCAG
TTTATGGAAAAGTGGCTCTAGAGCCACTAGAAATAACAG
TTTATGGAAAAGTGGCTCTAGAGCCACAAGAAATACCAG
TTTATGGAAAAGTGGCTCTAGAGCCACAAGAAATACCAG
TTTATGGAAAAGTGGCTCTAGAGCCACAAGAAATACCAG
TTTATGGAAAAGTGTCTCTAGAGCCACTAGAAATAACAG
TTTATGGAAAAGTGGCTCTAGAGCCACTAGAAATAACAG
TTTATGGAAAAGTGGCTCTAGAGCCACCAGAAATACCAG
TTTATGGAAAAGTGGCTCTAGAGCCACTAGAAATACCAG
Nr of selected repeats 408 Similarity 0.953106
Consensus:
TTTATGGAAAAGTGGCTCTAGAGCCACaAGAAATACCAG
>Cele-UNSB01_4:11302758-11302850 Satlength=93 Nr of Repeats=6 RepeatLength=15
seed=CGAAAAGGGG
CGAAAAGGGGAGCAGAA
CGAAAAGGGGATCTG
CAAAAAGGGGATCTG
CGAAAAGGGGAGATA
CGAAAAGGGGAGATA
CGAAAAGGGGAGCAA
Nr of selected repeats 5 Similarity 0.768889
Consensus:
CgAAAAGGGGAgcta
>Cele-UNSB01_4:11303025-11303087 Satlength=63 Nr of Repeats=4 RepeatLength=15
seed=TCCCCTTTTC
TCCCCTTTTCGTATC

TCCCCTTTTCGCAGA
TCCCCTTTTTGCAGA
TCCCCTTTTCGTTCTGC
Nr of selected repeats 3 Similarity 0.762963
Consensus:
TCCCCTTTTcGcAga
>Cele-UNSB01_4:11734646-11742696 Satlength=8051 Nr of Repeats=322
RepeatLength=25 seed=ATTACGGCAG
ATTACGGCAGATTCTATAAATTGGC
ATTACGGCAGATTCTATAAATTTGC
ATTACGGCAGATTCTAAAAATTGGC
ATTACGGCAGATTCTATAAATTTGC
ATTACGGCAGATTCTAAAAATTGGC
ATTACGGCAGATTCTAAAAATTGGC
ATTACGGCAGATTCTAAAAATTGGC
ATTACGGCAGATTCTATAAATTGGC
ATTACGGCAGATTCTATAAATTGGC
ATTACGGCAGATTCTATAAATTGGC
ATTACGGCAGATTCTATAAATTGGC
ATTACGGCAGATTCTATAAATTGGC
ATTACGGCAGATTCTATAAATTGGC
ATTACGGCAGATTCTATAAATTTGC
ATTACGGCAGATTCTAAAAATTGGC
ATTACGGCAGATTCTAAAAATTGGC
ATTACGGCAGATTCTATAAATTTGC
ATTACGGCAGATTCTAAAAATTGGC
ATTACGGCAGATTCTAAAAATTGGC
ATTACGGCAGATTCTATAAATTTGC
ATTACGGCAGATTCTAAAAATTGGC
ATTACGGCAGATTCTATAAATTTGC
ATTACGGCAGATTCTAAAAATTGGC
ATTACGGCAGATTCTATAAATTGGC
ATTACGGCAGATTCTAAAAATTGGC
ATTACGGCAGATTCTAAAAATTGGC
ATTACGGCAGATTCTAAAAATTGGC
ATTACGGCAGATTCTAAAAATTGGC
ATTACGGCAGATTCTATAAATTTGC
ATTACGGCAGATTCTAAAAATTGGC
ATTACGGCAGATTCTATAAATTGGC
ATTACGGCAGATTCTAAAAATTGGC
ATTACGGCAGATTCTATAAATTGGC
ATTACGGCAGATTCTAAAAATTGGC
ATTACGGCAGATTCTAAAAATTGGC
ATTACGGCAGATTCTATAAATTTGC
ATTACGGCAGATTCTAAAAATTGGC
ATTACGGCAGATTCTATAAATTTGC
ATTACGGCAGATTCTAAAAATTGGC
ATTACGGCAGATTCTATAAATTGGC
ATTACGGCAGATTCTATAAATTGGC
ATTACGGCAGATTCTATAAATTGGC
ATTACGGCAGATTCTATAAATTGGC
ATTACGGCAGATTCTATAAATTGGC
ATTACGGCAGATTCTAAAAATTGGC
ATTACGGCAGATTCTAAAAATTGGC

ATTACGGCAGATTCTAAAAATTGGC
ATTACGGCAGATTCTATAAATTGGC
ATTACGGCAGATTCTATAAATTGGC
ATTACGGCAGATTCTATAAATTGGC
ATTACGGCAGATTCTATAAATTGGC
ATTACGGCAGATTCTATAAATTGGC
ATTACGGCAGATTCTATAAATTTGC
ATTACGGCAGATTCTAAAAATTGGC
ATTACGGCAGATTCTATAAATTGGC
ATTACGGCAGATTCTATAAATTGTC
ATTACGGCAGATTCTAAAAATTGGC
ATTACGGCAGATTCTATAAATTGTC
ATTACGGCAGATTCTATAAATTTGC
ATTACGGCAGATTCTAAAAATTGGC
ATTACGGCAGATTCTATAAATTGGC
ATTACGGCAGATTCTATAAATTGTC
ATTACGGCAGATTCTATAAATTGGC
ATTACGGCAGATTCTATAAATTGGC
ATTACGGCAGATTCTATAAATTGGC
ATTACGGCAGATTCTATAAATTGTC
ATTACGGCAGATTCTATAAATTGTC
ATTACGGCAGATTCTAAAAATTGGC
ATTACGGCAGATTCTAAAAATTTGC
ATTACGGCAGATTCTAAAAATTTGC
ATTACGGCAGATTCTAAAAATTGGC
ATTACGGCAGATTCTATAAATTGGC
ATTACGGCAGATTCTATAAATTTGC
ATTACGGCAGATTCTAAAAATTGGC
ATTACGGCAGATTCTATAAATTGGC
ATTACGGCAGATTCTATAAATTGGC
ATTACGGCAGATTCTATAAATTGTC
ATTACGGCAGATTCTAAAAATTGGC
ATTACGGCAGATTCTAAAAATTTGC
ATTACGGCAGATTCTAAAAATTTGC
ATTACGGCAGATTCTAAAAATTGGC
ATTACGGCAGATTCTATAAATTGGC
ATTACGGCAGATTCTAAAAATTGGC
ATTACGGCAGATTCTATAAATTTGC
ATTACGGCAGATTCTAAAAATTGGC
ATTACGGCAGATTCTATAAATTGTC
ATTACGGCAGATTCTATAAATTGTC
ATTACGGCAGATTCTATAAATTTGC
ATTACGGCAGATTCTAAAAATTGGC
ATTACGGCAGATTCTATAAATTGTC
ATTACGGCAGATTCTATAAATTGTC
ATTACGGCAGATTCTATAAATTGGC
ATTACGGCAGATTCTATAAATTGTC
ATTACGGCAGATTCTAAAAATTGGC
ATTACGGCAGATTCTAAAAATTTGC
ATTACGGCAGATTCTATAAATTGGC
ATTACGGCAGATTCTATAAATTGGC
ATTACGGCAGATTCTATAAATTGTC
ATTACGGCAGATTCTATAAATTGTC
ATTACGGCAGATTCTAAAAATTGGC

ATTACGGCAGATTCTAAAAATTTGC
ATTACGGCAGATTCTAAAAATTTGC
ATTACGGCAGATTCTAAAAATTGGC
ATTACGGCAGATTCTATAAATTGGC
ATTACGGCAGATTCTATAAATTTGC
ATTACGGCAGATTCTAAAAATTGGC
ATTACGGCAGATTCTATAAATTGGC
ATTACGGCAGATTCTATAAATTGGC
ATTACGGCAGATTCTATAAATTGTC
ATTACGGCAGATTCTAAAAATTGGC
ATTACGGCAGATTCTAAAAATTTGC
ATTACGGCAGATTCTAAAAATTTGC
ATTACGGCAGATTCTAAAAATTGGC
ATTACGGCAGATTCTATAAATTGGC
ATTACGGCAGATTCTATAAATTGGC
ATTACGGCAGATTCTAAAAATTGGC
ATTACGGCAGATTCTATAAATTTGC
ATTACGGCAGATTCTAAAAATTGGC
ATTACGGCAGATTCTATAAATTGGC
ATTACGGCAGATTCTATAAATTGGC
ATTACGGCAGATTCTATAAATTGGC
ATTACGGCAGATTCTATAAATTGGC
ATTACGGCAGATTCTATAAATTGGC
ATTACGGCAGATTCTATAAATTGGC
ATTACGGCAGATTCTAAAAATTGGC
ATTACGGCAGATTCTAAAAATTGGC
ATTACGGCAGATTCTAAAAATTGGC
ATTACGGCAGATTCTATAAATTGGC
ATTACGGCAGATTCTATAAATTGGC
ATTACGGCAGATTCTATAAATTGGC
ATTACGGCAGATTCTATAAATTGGC
ATTACGGCAGATTCTATAAATTGGC
ATTACGGCAGATTCTATAAATTTGC
ATTACGGCAGATTCTAAAAATTGGC
ATTACGGCAGATTCTATAAATTGGC
ATTACGGCAGATTCTATAAATTGTC
ATTACGGCAGATTCTAAAAATTGGC
ATTACGGCAGATTCTAAAAATTGGC
ATTACGGCAGATTCTAAAAATTTGC
ATTACGGCAGATTCTATAAATTGGC
ATTACGGCAGATTCTATAAATTGGC
ATTACGGCAGATTCTATAAATTGTC
ATTACGGCAGATTCTATAAATTGTC
ATTACGGCAGATTCTAAAAATTGGC
ATTACGGCAGATTCTAAAAATTTGC
ATTACGGCAGATTCTAAAAATTTGC
ATTACGGCAGATTCTAAAAATTGGC
ATTACGGCAGATTCTATAAATTGGC
ATTACGGCAGATTCTATAAATTTGC
ATTACGGCAGATTCTAAAAATTGGC
ATTACGGCAGATTCTATAAATTGGC
ATTACGGCAGATTCTATAAATTGGC
ATTACGGCAGATTCTATAAATTGTC
ATTACGGCAGATTCTAAAAATTGGC

ATTACGGCAGATTCTAAAAATTTGC
ATTACGGCAGATTCTAAAAATTTGC
ATTACGGCAGATTCTAAAAATTGGC
ATTACGGCAGATTCTATAAATTGGC
ATTACGGCAGATTCTATAAATTTGC
ATTACGGCAGATTCTAAAAATTGGC
ATTACGGCAGATTCTATAAATTGGC
ATTACGGCAGATTCTATAAATTGGC
ATTACGGCAGATTCTATAAATTGGC
ATTACGGCAGATTCTATAAATTGTC
ATTACGGCAGATTCTAAAAATTGGC
ATTACGGCAGATTCTAAAAATTTGC
ATTACGGCAGATTCTAAAAATTTGC
ATTACGGCAGATTCTAAAAATTGGC
ATTACGGCAGATTCTATAAATTGGC
ATTACGGCAGATTCTATAAATTTGC
ATTACGGCAGATTCTAAAAATTGGC
ATTACGGCAGATTCTATAAATTGGC
ATTACGGCAGATTCTATAAATTGGC
ATTACGGCAGATTCTAAAAATTGGC
ATTACGGCAGATTCTAAAAATTGGC
ATTACGGCAGATTCTATAAATTGGC
ATTACGGCAGATTCTATAAATTGGC
ATTACGGCAGATTCTATAAATTGGC
ATTACGGCAGATTCTATAAATTGGC
ATTACGGCAGATTCTATAAATTGGC
ATTACGGCAGATTCTATAAATTGGC
ATTACGGCAGATTCTAAAAATTGGC
ATTACGGCAGATTCTAAAAATTGGC
ATTACGGCAGATTCTAAAAATTGGC
ATTACGGCAGATTCTAAAAATTGGC
ATTACGGCAGATTCTAAAAATTGGC
ATTACGGCAGATTCTATAAATTTGC
ATTACGGCAGATTCTAAAAATTGGC
ATTACGGCAGATTCTATAAATTGGC
ATTACGGCAGATTCTATAAATTGTC
ATTACGGCAGATTCTAAAAATTGGC
ATTACGGCAGATTCTATAAATTGGC
ATTACGGCAGATTCTATAAATTTGC
ATTACGGCAGATTCTAAAAATTGGC
ATTACGGCAGATTCTATAAATTGGG
ATTACGGCAGATTCTAAAAATTTGC
ATTACGGCAGATTCTATAAATTTGC
ATTACGGCAGATTCTAAAAATTGGC
ATTACGGCAGATTCTAAAAATTGGC
ATTACGGCAGATTCTAAAAATTGGC
ATTACGGCAGATTCTATAAATTTGC
ATTACGGCAGATTCTATAAATTGGC
ATTACGGCAGATTCTATAAATTGGC
ATTACGGCAGATTCTAAAAATTGGC
ATTACGGCAGATTCTATAAATTGGC
ATTACGGCAGATTCTATAAATTGGC
ATTACGGCAGATTCTATAAATTGGC
ATTACGGCAGATTCTATAAATTGGC

ATTACGGCAGATTCTATAAATTGGC
ATTACGGCAGATTCTATAAATTGTC
ATTACGGCAGATTCTATAAATTGTC
ATTACGGCAGATTCTAAAAATTGGC
ATTACGGCAGATTCTAAAAATTTGC
ATTACGGCAGATTCTAAAAATTTGC
ATTACGGCAGATTCTAAAAATTGGC
ATTACGGCAGATTCTATAAATTGGC
ATTACGGCAGATTCTATAAATTTGC
ATTACGGCAGATTCTAAAAATTGGC
ATTACGGCAGATTCTATAAATTGGC
ATTACGGCAGATTCTATAAATTGGC
ATTACGGCAGATTCTATAAATTGTC
ATTACGGCAGATTCTAAAAATTGGC
ATTACGGCAGATTCTAAAAATTTGC
ATTACGGCAGATTCTAAAAATTTGC
ATTACGGCAGATTCTAAAAATTGGC
ATTACGGCAGATTCTATAAATTGGC
ATTACGGCAGATTCTATAAATTGGC
ATTACGGCAGATTCTAAAAATTGGC
ATTACGGCAGATTCTATAAATTTGC
ATTACGGCAGATTCTAAAAATTGGC
ATTACGGCAGATTCTATAAATTGGC
ATTACGGCAGATTCTATAAATTGGC
ATTACGGCAGATTCTATAAATTGGC
ATTACGGCAGATTCTATAAATTGGC
ATTACGGCAGATTCTATAAATTGGC
ATTACGGCAGATTCTATAAATTGGC
ATTACGGCAGATTCTAAAAATTGGC
ATTACGGCAGATTCTAAAAATTGGC
ATTACGGCAGATTCTAAAAATTGGC
ATTACGGCAGATTCTATAAATTGGC
ATTACGGCAGATTCTATAAATTGGC
ATTACGGCAGATTCTATAAATTGGC
ATTACGGCAGATTCTATAAATTGGC
ATTACGGCAGATTCTATAAATTGGC
ATTACGGCAGATTCTATAAATTTGC
ATTACGGCAGATTCTAAAAATTGGC
ATTACGGCAGATTCTATAAATTGGC
ATTACGGCAGATTCTATAAATTGTC
ATTACGGCAGATTCTAAAAATTGGC
ATTACGGCAGATTCTAAAAATTGGC
ATTACGGCAGATTCTAAAAATTTGC
ATTACGGCAGATTCTATAAATTGGC
ATTACGGCAGATTCTATAAATTGGC
ATTACGGCAGATTCTATAAATTGTC
ATTACGGCAGATTCTATAAATTGTC
ATTACGGCAGATTCTAAAAATTGGC
ATTACGGCAGATTCTAAAAATTTGC
ATTACGGCAGATTCTAAAAATTTGC
ATTACGGCAGATTCTAAAAATTGGC
ATTACGGCAGATTCTATAAATTGGC
ATTACGGCAGATTCTATAAATTTGC
ATTACGGCAGATTCTAAAAATTGGC

ATTACGGCAGATTCTATAAATTGGC
ATTACGGCAGATTCTATAAATTGGC
ATTACGGCAGATTCTATAAATTGTC
ATTACGGCAGATTCTAAAAATTGGC
ATTACGGCAGATTCTAAAAATTTGC
ATTACGGCAGATTCTAAAAATTTGC
ATTACGGCAGATTCTAAAAATTGGC
ATTACGGCAGATTCTATAAATTGGC
ATTACGGCAGATTCTATAAATTTGC
ATTACGGCAGATTCTAAAAATTGGC
ATTACGGCAGATTCTATAAATTGGC
ATTACGGCAGATTCTATAAATTGGC
ATTACGGCAGATTCTATAAATTGGC
ATTACGGCAGATTCTATAAATTGTC
ATTACGGCAGATTCTAAAAATTGGC
ATTACGGCAGATTCTAAAAATTTGC
ATTACGGCAGATTCTAAAAATTTGC
ATTACGGCAGATTCTAAAAATTGGC
ATTACGGCAGATTCTATAAATTGGC
ATTACGGCAGATTCTATAAATTTGC
ATTACGGCAGATTCTAAAAATTGGC
ATTACGGCAGATTCTATAAATTGGC
ATTACGGCAGATTCTATAAATTGGC
ATTACGGCAGATTCTAAAAATTGGC
ATTACGGCAGATTCTAAAAATTGGC
ATTACGGCAGATTCTATAAATTGGC
ATTACGGCAGATTCTATAAATTGGC
ATTACGGCAGATTCTATAAATTGGC
ATTACGGCAGATTCTATAAATTGGC
ATTACGGCAGATTCTATAAATTGGC
ATTACGGCAGATTCTATAAATTGGC
ATTACGGCAGATTCTAAAAATTGGC
ATTACGGCAGATTCTAAAAATTGGC
ATTACGGCAGATTCTAAAAATTGGC
ATTACGGCAGATTCTAAAAATTGGC
ATTACGGCAGATTCTAAAAATTGGC
ATTACGGCAGATTCTATAAATTTGC
ATTACGGCAGATTCTAAAAATTGGC
ATTACGGCAGATTCTATAAATTGGC
ATTACGGCAGATTCTATAAATTGTC
ATTACGGCAGATTCTAAAAATTGGC
ATTACGGCAGATTCTATAAATTGGC
ATTACGGCAGATTCTATAAATTTGC
ATTACGGCAGATTCTAAAAATTGGC
ATTACGGCAGATTCTATAAATTGGG
ATTACGGCAGATTCTAAAAATTTGC
ATTACGGCAGATTCTATAAATTTGC
ATTACGGCAGATTCTAAAAATTGGC
ATTACGGCAGATTCTAAAAATTGGC
ATTACGGCAGATTCTAAAAATTGGC
ATTACGGCAGATTCTATAAATTTGC
ATTACGGCAGATTCTATAAATTGGC
ATTACGGCAGATTCTATAAATTGGC
ATTACGGCAGATTCTAAAAATTGGC

ATTACGGCAGATTCTATAAATTGGC
ATTACGGCAGATTCTATAAATTGGC
ATTACGGCAGATTCTATAAATTGGC
ATTACGGCAGATTCTATAAATTGGC
ATTACGGCAGATTCTATAAATTGGC
ATTACGGCAGATTCTATAAATTGGC
Nr of selected repeats 322 Similarity 0.947627
Consensus:
ATTACGGCAGATTCTAtAAATTGGC
>Cele-UNSB01_4:11742877-11743140 Satlength=264 Nr of Repeats=13 RepeatLength=17
seed=AAAATCAAAC
AAAATCAAACCGGCGGA
AAAATCAAACCGGAGAC
AAAATCAAACTGGCTGA
AAAATCGAACCGGCGAGAAAAAATTAAAACCGGCGGA
AAAATCAAACCGGCGGA
AAAATCAAACCGGCGGA
AAAATCAAACCGGAGAC
AAAATCAAACTGGCGGA
AAAATCGAACCGGCGAGAAAAAATTAAAACCGGCGGA
AAAATCAAACCGGCGGA
AAAATCAAACCGGAGAC
AAAATCAAACTGGCGGA
AAAATCGAACCGGCGAGAA
Nr of selected repeats 10 Similarity 0.809259
Consensus:
AAAATCAAACcGGcgGA
>Cele-UNSB01_4:12149712-12149981 Satlength=270 Nr of Repeats=10 RepeatLength=11
seed=GTACTGTAGG
GTACTGTAGGG
GTACTGTAGGGT
TACTGTAGGATTACTGTAGTTTTGGAAAAATTGACTTTTTGTCTTTTGAAGTGATATTGGTTTGAGGTTAGTGGTGGGA
TATGGTCGGG
GTACTGTAGTA
GTACTGTAGTA
GTACTGTAGGA
GTACTGTAGGATTACTGTATTTTTGAAAAAAATTGGTTTTTCGTCTTTTGAAGTGATATTGCTTTTGGGTTAGTGTCGG
GATATGGTTGGG
GTACTGTAGTT
GTACTGTAGAG
GTACTGTAGGA
Nr of selected repeats 7 Similarity 0.832612
Consensus:
GTACTGTAGna
>Cele-UNSB01_4:12150067-12150386 Satlength=320 Nr of Repeats=8 RepeatLength=40
seed=TCCAGAACCT
TCCAGAACCTTCTGGAAAATTCGAGAAAATTCTGGAATGT
TCCAGAACCTTCTGGAAATCCGAGAAAATTCTGGAATGT
TCCAGAACCTTCTGGAAAATTCGAGAAAATTCTGGAATGT
TCCAGAACCTTCTGGAAAATCCGAGAAAATTCTGGAATGT
TCCAGAACCTTCTGGAAAATTTGAGAAAATGCTGGAATGT
TCCAGAACCTTCTGGAAAATTTGTGAAAATTCTGGAATGT
TCCAGAACCTTCTGGAAAATTCGAGAAAATTCTGGAATGT
TCCAGAACCTTCTGGAAAATTTGAGAAAATGCTGGAATGT

Nr of selected repeats 7 Similarity 0.946032
Consensus:
TCCAGAACCTTCTGGAAAATTcGAGAAAATtCTGGAATGT
>Cele-UNSB01_4:12150586-12159522 Satlength=8937 Nr of Repeats=274
RepeatLength=32 seed=CGGGAATTCA
CGGGAATTCAAATTTTCTGTGAAAAATTTTGG
CGGGAATTCAAAATTTCTGTGAAAAATTTTGG
CGGGAATTCAAAATTTCTTTGAAAAATTTGGG
CGGGAATTCAAAATTTTTGTGAAAAATTTTGG
CGGGAATTCAAATTTTCTGTGAAAAATTTTGG
CGGGAATTCAAAATTTCTGTGAAAAATTTTGG
CGGGAATTCAAAATTTCTGTGAAAAATTTTGG
CGGGAATTCAAAATTTCTATGAAAAATTTTGG
CGGGAATTCAAATTTTCTGTGAAAAATTTTAA
CGGGAATTCAAGTTTTCTGTGAAAAATTTTGG
CGGGAATTCAAATTTTCTGTGAAAAATTTTGG
CGGGAATTCAAAATTTCTTTGAAAAATTTGGG
CGGGAATTCAAAATTTTTGTGAAAAATTTTGG
CGGGAATTCAAATTTTCTGTGAAAAATTTTGG
CGGGAATTCAAAATTTCTGTGAAAAATTTTGG
CGGGAATTCAAAATTTCTGTGAAAAATTTTGG
CGGGAATTCAAAATTTCTATGAAAAATTTTGG
CGGGAATTCAAATTTTCTGTGAAAAATTTTAA
CGGGAATTCAAGTTTTCTGTGAAAAATTTTGG
CGGGAATTCAAATTTTCTGTGAAAAATTTTGG
CGGGAATTCAAAATTTCTTTGAAAAATTTGGG
CGGGAATTCAAAATTTTTGTGAAAAATTTTGG
CGGGAATTCAAATTTTCTGTGAAAAATTTTGG
CGGGAATTCAAAATTTCTGTGAAAAATTTTGG
CGGGAATTCAAAATTTCTGTGAAAAATTTTGG
CGGGAATTCAAAATTTCTATGAAAAATTTTGG
CGGGAATTCAAATTTTCTGTGAAAAATTTTAA
CGGGAATTCAAGTTTTCTGTGAAAAATTTTGG
CGGGAATTCAAATTTTCTGTGAAAAATTGTGG
CGGGAATTCAAAATTTCTGTGAAAAATTTTGG
CGGGAATTCAAAATTTCTATGAAAAATTTTGG
CGGGAATTCAAATTTTCTGTGAAAAATTTTAA
CGGGAATTCAAGTTTTCTGTGAAAAATTTTGG
CGGGAATTCAAATTTTCTGTGAAAAATTTTGG
CGGGAATTCAAAATTTCTTTGAAAAATTTGGG
CGGGAATTCAAATTTTCTGTGAAAAATTTTAA
CGGGAATTCAAGTTTTCTGTGAAAAATTTTGG
CGGGAATTCAAATTTTCTGTGAAAAATTTTGG
CGGGAATTCAAAATTTCTGTGAAAAATTTTGG
CGGGAATTCAGAATTTCTGTGAAAAATTTTGG
CGGGAATTCAAAATTTCTGTGAAAAATTTTAA
CGGGAATTCAAAATTTCTGTGAAAAATTTTGG
CGGGAATTCAAAATTTCTATGAAAAATTTTGG
CGGGAATTCAAAATTTCTGTGAAAAATTTTGG
CGGGAATTCAAAATTTCTGTGAAAAATTTTGG
CGGGAATTCAAAATTTCTATGAAAAATTTTGG
CGGGAATTCAAATTTTCTGTGAAAAATTTTAA
CGGGAATTCAAGTTTTCTGTGAAAAATTTTGG
CGGGAATTCAAATTTTCTGTGAAAAATTTTGG

CGGGAATTCAAATTTTCTGTGAAAAATTTTAA
CGGGAATTCAAAATTTCTATGAAAAATTTTGG
CGGGAATTCAAAATTTCTATGAAAAATTTTGG
CGGGAATTCAAAATTTCTGTGAAAAATTTTGG
CGGGAATTCAAAATTTCTGTGAAAAATTTTGG
CGGGAATTCAAAATTTCTATGAAAAATTTTGG
CGGGAATTCAAATTTTCTGTGAAAAATTTTAA
CGGGAATTCAAGTTTTCTGTGAAAAATTTTGG
CGGGAATTCAAATTTTCTGTGAAAAATTTTGG
CGGGAATTCAAAATTTCTGTGAAAAATTTTGG
CGGGAATTCAAATTTTCTGTGAAAAATTTTGG
CGGGAATTCAAAATTTCTGTGAAAAATTTTGG
CGGGAATTCAAAATTTCTGTGAAAAATTTTGG
CGGGAATTCAAAATTTCTATGAAAAATTTTGG
CGGGAATTCAAATTTTCTGTGAAAAATTTTAA
CGGGAATTCAAATTTACTGAGAAAAACTTTTTGGTGGGAAATTCAAATTTTCTGTGAAAAATTTTGG
CGGGAATTCAAATTTTCTGTGAAAAATTTTGG
CGGGAATTCAAAATTTCTGTGAAAAATTTTGG
CGGGAATTCAAAATTTCTGTGAAAATTTTTAG
CGGGAATTCAAATTTTCTGTGAAAAATTTTGG
CGGGAATTCAAAATTTCTGTGAAAAAATTTGG
CGGGAATTCAGAATTTCTGTGAAAAATTTTGG
CGGGAATTCAAATTTTCTGTGAAAAATTTTGG
CGGGAATTCAAAATTTCTTTGAAAAATTTGGG
CGGGAATTCAAATTTTCTGTGAAAAATTTTAA
CGGGAATTCAAGTTTTCTGTGAAAAATTTTGG
CGGGAATTCAAATTTTCTGTGAAAAATTTTGG
CGGGAATTCAAAATTTCTGTGAAAAATTTTGG
CGGGAATTCAGAATTTCTGTGAAAAATTTTGG
CGGGAATTCAAAATTTCTGTGAAAAATTTTAA
CGGGAATTCAAAATTTCTGTGAAAAATTTTGG
CGGGAATTCAGAATTTCTGTGAAAAATTTTGG
CGGGAATACAAATTTTCTGTGAAAAATTTTGG
CGGAATTCAAAATTTCTGTGAAAAATTTTGG
CGGGAATTCAAATTTTCTGTGAAAAATTTTGG
CGGGAATTCGAAATTTCTGTGAAAAATTTTGG
CGGGAATTCAAAATTTCTGTGAAAAATTTTGG
CGGGAATTCAAAATTTCTATGAAAAATTTTGG
CGGGAATTCAAAATTTCTGTGAAAAATTTTGG
CGGGAATTCAAATTTTCTGTGAAAAATTTTGG
CGGGAATTCGAAATTTCTGTGAAAAATTTTGG
CGGGAATTCAAAATTTCTGTGAAAAATTTTGG
CGGGAATTCAAAATTTCTATGAAAAATTTTGG
CGGGAATTCAAAATTTCTATGAAAAATTTTGG
CGGGAATTCAAAATTTCTGTGAAAAATTTTGG
CGGGAATTCAAAATTTCTGTGAAAAATTTTGG
CGGGAATTCAAAATTTCTATGAAAAATTTTGG
CGGGAATTCAAATTTTCTGTGAAAAATTTTAA
CGGGAATTCAAGTTTTCTGTGAAAAATTTTGG
CGGGAATTCAAATTTTCTGTGAAAAATTTTAA
CGGGAATTCAAATTTACTGAGAAAAACTTTTTGGTGGGAAATTCAAATTTTCTGTGAAAAATTTTGG
CGGGAATTCAAATTTTCTGTGAAAAATTTTGG
CGGGAATTCAAAATTTCTGTGAAAAATTTTGG
CGGGAATTCAAAATTTCTGTGAAAATTTTTAG

CGGGAATTCAAATTTTCTGTGAAAAATTTTGG
CGGGAATTCAAAATTTCTGTGAAAAAATTTGG
CGGGAATTCAGAATTTCTGTGAAAAATTTTGG
CGGGAATTCAAATTTTCTGTGAAAAATTTTGG
CGGGAATTCAAAATTTCTTTGAAAAATTTGGG
CGGGAATTCAAATTTTCTGTGAAAAATTTTAA
CGGGAATTCAAGTTTTCTGTGAAAAATTTTGG
CGGGAATTCAAATTTTCTGTGAAAAATTTTGG
CGGGAATTCAAAATTTCTGTGAAAAATTTTGG
CGGGAATTCAGAATTTCTGTGAAAAATTTTGG
CGGGAATTCAAAATTTCTGTGAAAAATTTTAA
CGGGAATTCAAAATTTCTGTGAAAAATTTTGG
CGGGAATTCAGAATTTCTGTGAAAAATTTTGG
CGGGAATACAAATTTTCTGTGAAAAATTTTGG
CGGAATTCAAAATTTCTGTGAAAAATTTTGG
CGGGAATTCAAATTTTCTGTGAAAAATTTTGG
CGGGAATTCGAAATTTCTGTGAAAAATTTTGG
CGGGAATTCAAAATTTCTGTGAAAAATTTTGG
CGGGAATTCAAAATTTCTATGAAAAATTTTGG
CGGGAATTCAAAATTTCTGTGAAAAATTTTGG
CGGGAATTCAAATTTTCTGTGAAAAATTTTGG
CGGGAATTCGAAATTTCTGTGAAAAATTTTGG
CGGGAATTCAAAATTTCTGTGAAAAATTTTGG
CGGGAATTCAAAATTTCTATGAAAAATTTTGG
CGGGAATTCAAAATTTCTATGAAAAATTTTGG
CGGGAATTCAAAATTTCTGTGAAAAATTTTGG
CGGGAATTCAAAATTTCTGTGAAAAATTTTGG
CGGGAATTCAAAATTTCTATGAAAAATTTTGG
CGGGAATTCAAATTTTCTGTGAAAAATTTTAA
CGGGAATTCAAGTTTTCTGTGAAAAATTTTGG
CGGGAATTCAAATTTTCTGTGAAAAATTTTGG
CGGGAATTCAAAATTTCTGTGAAAAATTTTGG
CGGGAATTCAAATTTTCTGTGAAAAATTTTGG
CGGGAATTCAAAATTTCTGTGAAAAATTTTGG
CGGGAATTCAAAATTTCTGTGAAAAATTTTGG
CGGGAATTCAAAATTTCTATGAAAAATTTTGG
CGGGAATTCAAATTTTCTGTGAAAAATTTTAA
CGGGAATTCAAATTTACTGAGAAAAACTTTTTGGTGGGAAATTCAAATTTTCTGTGAAAAATTTTGG
CGGGAATTCAAATTTTCTGTGAAAAATTTTGG
CGGGAATTCAAAATTTCTGTGAAAAATTTTGG
CGGGAATTCAAAATTTCTGTGAAAATTTTTAG
CGGGAATTCAAATTTTCTGTGAAAAATTTTGG
CGGGAATTCAAAATTTCTGTGAAAAAATTTGG
CGGGAATTCAGAATTTCTGTGAAAAATTTTGG
CGGGAATTCAAAATTTCTGTGAAAAAATTTGG
CGGGAATTCAGAATTTCTGTGAAAAATTTTGG
CGGGAATTCAAAATTTCTGTGAAAAAATTTGG
CGGGAATTCAAAATTTCTGTGAAAAATTTTGG
CGGGAATTCAAAATTTCTGTGAAAAATTTTGG
CGGGAATTCAAAATTTCTGTGAAAAATTTTGG
CGGGAATTCAAAATTTCTGTGAAAAAATTTGG
CGGGAATTCAAAATTTTGTGAAAAATTGTGG
CGGGAATTCAAAATTTCTGTGAAAAATTTTGG
CGGGAATTCAAAATTTCTGTGAAAATTTTTAG

CGGGAATTCAAATTTTCTGTGAAAAATTTTGG
CGGGAATTCAAAATTTCTGTGAAAAATTTTGG
CGGGAATTCAAAATTTCTGTGAAAAATTTTGG
CGGGAATTCAAAATTTCTGTGAAAAATTTTGG
CGGGAATTCAAAATTTCTATGAAAAATTTTGG
CGGGAATTCAAATTTTCTGTGAAAAATTTTAA
CGGGAATTCAAATTTACTGAGAAAAACTTTTTGGTGGGAAATTCAAATTTTCTGTGAAAAATTTTGG
CGGGAATTCAAAATTTCTGTGAAAAATTTTGG
CGGGAATTCAAAATTTCTGTGAAAATTTTTAA
CGGGAATTCAAATTTACTGAGAAAAACTTTTTGGTGGGAAATTCAAATTTTCTGTGAAAAATTTTGG
CGGGAATTCAAAATTTCTGTGAAAAATTTTGG
CGGGAATTCAAAATTTCTGTGAAAATTTTTAG
CGGGAATTCAAATTTTCTGTGAAAAATTTTGG
CGGGAATTCAAAATTTCTGTGAAAAAATTTGG
CGGGAATTCAGAATTTCTGTGAAAAATTTTGG
CGGGAATTCAAAATTTCTGTGAAAAAATTTGG
CGGGAATTCAAAATTTCTGTGAAAAAATTTGG
CGGGAATTCAGAATTTCTGTGAAAAATTTTGG
CGGGAATTCAAATTTTCTGTGAAAAATTTTAA
CGGGAATTCAAGTTTTCTGTGAAAAATTTTGG
CGGGAATTCAAATTTTCTGTGAAAAATTGTGG
CGGGAATTCAAAATTTCTGTGAAAAATTTTGG
CGGGAATTCAAAATTTCTGTGAAAATTTTTAG
CGGGAATTCAAATTTTCTGTGAAAAATTTTGG
CGGGAATTCAAAATTTCTGTGAAAAATTTTGG
CGGGAATTCAGAATTTCTGTGAAAAATTTTGG
CGGGAATTCAGAATTTCTGTGAAAAAATTTGG
CGGGAATTCAGAATTTCTGTGAAAAATTTTGG
CGGGAATTCAAAATTTCTGTGAAAAAATTTGG
CGGGAATTCAAAATTTCTGTGAAAAAATTTGG
CGGGAATTCAAAATTTTGTGAAAAATTGTGG
CGGGAATTCAAAATTTCTGTGAAAAATTTTGG
CGGGAATTCAAAATTTCTGTGAAAATTTTTAG
CGGGAATTCAAATTTTCTGTGAAAAATTTTGG
CGGGAATTCAAAATTTCTGTGAAAAATTTTGG
CGGGAATTCAAAATTTCTGTGAAAATTTTTAG
CGGGAATTCAAATTTTCTGTGAAAAATTTTGG
CGGGAATTCAAAATTTCTGTGAAAAATTTTGG
CGGGAATTCAAAATTTCTGTGAAAAATTTTGG
CGGGAATTCAAAATTTCTGTGAAAAATTTTGG
CGGGAATTCAAATTTTCTGTGAAAAATTTTAA
CGGGAATACAAATTTTCTGTGAAAAATTTTGG
CGGGAATTCAAATTTTCTGTGAAAAATTTTGG
CGGGAATTCAAAATTTCTTTGAAAAATTTGGG
CGGGAATTCAAAATTTTTGTGAAAAATTTTGG
CGGGAATTCAAATTTTCTGTGAAAAATTTTGG
CGGGAATTCAAAATTTCTTTGAAAAATTTGGG
CGGGAATTCAAAATTTTTGTGAAAAATTTTGG
CGGGAATTCAAATTTTCTGTGAAAAATTTTGG
CGGGAATTCAAAATTTCTGTGAAAAATTTTGG
CGGGAATTCAAAATTTCTGTGAAAAATTTTGG
CGGGAATTCAAAATTTCTATGAAAAATTTTGG
CGGGAATTCAAATTTTCTGTGAAAAATTTTAA
CGGGAATTCAAGTTTTCTGTGAAAAATTTTGG

CGGGAATTCAAATTTTCTGTGAAAAATTGTGG
CGGGAATTCAAAATTTCTGTGAAAAATTTTGG
CGGGAATTCAAAATTTCTGTGAAAATTTTTAG
CGGGAATTCGAATTTTCTGTGAAAAATTTTGG
CGGGAATTCAAAATTTCTGTGAAAAATTTTGG
CGGGAATTCAGAATTTCTGTGAAAAATTTTGG
CGGGAATTCAGAATTTCTGTGAAAAAATTTGG
CGGGAATTCAGAATTTCTGTGAAAAATTTTGG
CGGGAATTCAAAATTTCTGTGAAAAAATTTGG
CGGGAATTCAAAATTTCTGTGAAAAAATTTGG
CGGGAATTCAAAATTTTGTGAAAAATTGTGG
CGGGAATTCAAAATTTCTGTGAAAAATTTTGG
CGGGAATTCAAAATTTCTGTGAAAATTTTTAG
CGGGAATTCAAATTTTCTGTGAAAAATTTTGG
CGGGAATTCAAAATTTCTGTGAAAAATTTTGG
CGGGAATTCAAAATTTCTGTGAAAATTTTTAG
CGGGAATTCAAATTTTCTGTGAAAAATTTTGG
CGGGAATTCAAAATTTCTGTGAAAAATTTTGG
CGGGAATTCAAAATTTCTGTGAAAAATTTTGG
CGGGAATTCAAAATTTCTATGAAAAATTTTGG
CGGGAATTCAAATTTTCTGTGAAAAATTGTGG
CGGGAATTCAAAATTTCTGTGAAAAATTTTGG
CGGGAATTCAAAATTTCTGTGAAAATTTTTAG
CGGGAATTCAAATTTTCTGTGAAAAATTTTGG
CGGGAATTCAGAATTTCTGTGAAAAAATTTGG
CGGGAATTCAGAATTTCTGTGAAAAATTTTGG
CGGGAATTCAAAATTTCTGTGAAAAAATTTGG
CGGGAATTCAAAATTTCTGTGAAAAAATTTGG
CGGGAATTCAAAATTTTGTGAAAAATTGTGG
CGGGAATTCAAAATTTCTGTGAAAAATTTTGG
CGGGAATTCAAAATTTCTGTGAAAATTTTTAG
CGGGAATTCAAATTTTCTGTGAAAAATTTTGG
CGGGAATTCAAAATTTCTGTGAAAAATTTTGG
CGGGAATTCAAAATTTCTGTGAAAATTTTTAG
CGGGAATTCAAATTTTCTGTGAAAAATTTTGG
CGGGAATTCAAAATTTCTGTGAAAAATTTTGG
CGGGAATTCAAAATTTCTGTGAAAAATTTTGG
CGGGAATTCAAAATTTCTATGAAAAATTTTGG
CGGGAATTCAAATTTTCTGTGAAAAATTGTGG
CGGGAATTCAAAATTTCTGTGAAAAATTTTGG
CGGGAATTCAAAATTTCTGTGAAAATTTTTAG
CGGGAATTCAAATTTTCTGTGAAAAATTTTGG
CGGGAATTCAAAATTTCTGTGAAAAATTTTGG
CGGGAATTCAGAATTTCTGTGAAAAATTTTGG
CGGGAATTCAAATTTTCTGTGAAAAATTGTGG
CGGGAATTCAAAATTTCTGTGAAAAATTTTGG
CGGGAATTCAAAATTTCTGTGAAAATTTTTAG
CGGGAATTCAAATTTTCTGTGAAAAATTTTGG
CGGGAATTCAAAATTTCTGTGAAAAATTTTGG
CGGGAATTCAGAATTTCTGTGAAAAATTTTGG
CGGGAATACAAATTTTCTGTGAAAAATTTTGG
CGGAATTCAAAATTTCTGTGAAAAATTTTGG
CGGGAATTCAAAATTTCTGTGAAAAATTTTGG
CGGGAATTCAAATTTTCTGTGAAAAGTTTTGG

CGGGAATTCAAAATTTCTGTGAAAAAATTTTGG
CGGGAATTCAAATTTTCTGTGAAAAGTTTTGG
CGGGAATTCAAAATTTCTGTGAAAAAATTTTGG
CGGGAATTCAGAATTTCTGTGAAAAAATTTTGG
CGGGAATTCAGAATTTCTGTGAAAATTTTTGG
CGGGAATTCAAATTTTCTGTGAAAAAATTTTGG
CGGGAATTCAAAATTTCTGTGAAAAAATTTTGG
CGGGAATTCAAATTTTCTGTGAAAAAATTTTGG
CGGGAATTCAAAATTTCTTTGAAAAAATTTGGG
Nr of selected repeats 262 Similarity 0.923743
Consensus:
CGGGAATTCAAAaTTTCTGTGAAAAAATTTTGG
>Cele-UNSB01_4:12289740-12290350 Satlength=611 Nr of Repeats=8 RepeatLength=35
seed=AAATTTGAAT
AAATTTGAATTTCCCGTCAAAATCTTTTTCTCAGA
AAATTTGAATTTCCCGCCAAAATTTTTCTTGG
AAATTTGAATTCCCGCCAAAATTTCTTTTCTCATAAATTTTGAAGTTCTCGCCCATATTTTTAAGGGTCCCACCACGAT
GGGTCTCGCCAGTTCGAGGTGGTACTTAAGCTAACAAAAAGTTTCTCAGA
AAATTTGAATTTCCCGCCAAAAAATTTTTTCTCAGAAATTTTGAGTTTTCGGTCAAAATTTATGGGTCTCACCACGAATG
TTCCAGAATTTTTTTCGAATTTTCCAGAAGGTTCTGGAACTTTCCAGAAGGTTCTGGAACTTTCCAGAAAGTTCTGGAA
CATTCCAGAATTTTCTCGAATTTTCCAGAAGTTCTAGAACATTATAGAATTTTCTGGAATTTTCCAGATGATTCCAAAA
CCAAAATTGAAATTCCCGCCAAAATCTTTTTCTCAGA
AAATTTGAATTTCCCTCCAAAAATTTTTTTACTGA
AAATTTGAATTTCCTGCTAAAATTTTTTTCACTGA
AAATTTGAATTTCCCGCCAAAATTTTTTTCACTGA
AAATTTGAATTTCCCGCCAAAATTTTTTTCACTTA
Nr of selected repeats 5 Similarity 0.847619
Consensus:
AAATTTGAATTTCCcgccAAAAttTTTTTcaCtgA
>Cele-UNSB01_4:12303349-12310067 Satlength=6719 Nr of Repeats=128
RepeatLength=59 seed=TCCATAATTC
TCCATAATTCTCAGTTTTAAAAATTTTGAAAACCAGTGAAGTCTTGATAATCCATATTT
TCCATAATTCTCAGTTTTAAAAATTTTGAAAACCAGTGAAGTCTTAATAT
TCCATATTCTCCATAATTCTCGGTTTTAAAAATTTTGAAAACCAGTGAAGTCTTGATAT
TCCATATTCTCCATAATTCTCGGTTTTAAAAATTTTGAAAACCAGTGAAGTCTTGATATTCCATATTT
TCCATAATTCTCGGTTTTAAAAATTTTGAAAACCAGTGAAGTCTTGATAT
TCCATATTCTCCATAATTCTCGGTTTTAAAAATTTTGAAAACCAGTGAAGTCTTGATAT
TCCATATTCTCCATAATTCTCAGTTTTAAAAATATTGAAAACCAGTGAAGTCTTGATAT
TCCATATTCTCCATAATTCTCGGTTTTAAAAATTTTGAAAACCAGTGAAGTCTTGATAT
TCCATATTCTCCATAATTCTCGGTTTTAAAAATTTTGAAAACCAGTGAAGTCTTGATAT
TCCATATTCTCCATAATTCTCAGTTTTAAAAATTTTGAAAACCAGTGAAGTCTTAATAT
TCCATATTCTCCATAATTCTCGGTTTTAAAAATTTTGAAAACCAGTGAAGTCTTGATAT
TCCATATTCTCCATAATTCTCGGTTTTAAAAATTTTGAAAACCAGTGAAGTCTTGATATTCCATATTT
TCCATAATTCTCAGTTTTAAAAATATTGAAAACCAGTGAAGTCTTGATAT
TCCATATTCTCCATAATTCTCGGTTTTAAAAATTTTGAAAACCAGTGAAGTCTTGATATTCCATATTT
TCCATAATTCTCAGTTTTAAAAATTTTGAAAACCAGTGAAGTCTTGATAT
TCCATATTCTCCATAATTCTCAGTTTTAAAAATTTTGAAAACCAGTGAAGTCTTGATATTCCATATTT
TCCATAATTCTCAGTTTTAAAAATTTTGAAAACCAGTGAAGTCTTGATATTCCATATTT
TCCATAATTCTCGGTTTTAAAAATATTGAAAACCAGTGAAGTCTTGATAT
TCCATATTCTCCATAATTCTCAGTTTTAAAAATTTTGAAAACCAGTGAAGTCTTGATATTCCATATTT
TCCATAATTCTCAGTTTTAAAAATTTTGAAAACCAGTGAAGTCTTGATAT
TCCATATTCTCCATAATTCTCGGTTTTAAAAATTTTGAAAACCAGTGAAGTCTTGATATTCCATATTT
TCCATAATTCTCAGTTTTAAAAATTTTGAAAACCAGTGAAGTCTTGATAT
TCCATATTCTCCATAATTCTCGGTTTTAAAAATTTTGAAAACCAGTGAAGTCTTGATATTCCATATTT

TCCATAATTCTCAGTTTTAAAAATTTTGAAAACCAGTGAAGTCTTGATAT
TCCATATTTCCATAATTCTCAGTTTTAAAAATTTTGAAAACCAGTGAAGTCTTGATATTCCATATTT
TCCATAATTCTCAGTTTTAAAAATTTTGAAAACCAGTGAAGTCTTGATATTCCATATTT
TCCATAATTCTCAGTTTTAAAAATTTTGAAAACCAGTGAAGTCTTGATATTCCATATTT
TCCATAATTCTCAGTTTTAAAAATTTTGAAAACCAGTGAAGTCTTGATATTCCATATTT
TCCATAATTCTCAGTTTTAAAAATTTTGAAAACCAGTGAAGTCTTGATATTCCATATTT
TCCATAATTCTCAGTTTTAAAAATTTTGAAAACCAGTGAAGTCTTGATATTCCATATTT
TCCATAATTCTCAGTTTTAAAAATTTTGAAAACCAGTGAAGTCTTGATATTCCATATTT
TCCATAATTCTCAGTTTTAAAAATTTTGAAAACCAGTGAAGTCTTGATATTCCATATTT
TCCATAATTCTCAGTTTTAAAAATTTTGAAAACCAGTGAAGTCTTGATATTCCATATTT
TCCATAATTCTCAGTTTTAAAAATTTTGAAAACCAGTGAAGTCTTGATATTCCATATTT
TCCATAATTCTCAGTTTTAAAAATTTTGAAAACCAGTGAAGTCTTGATATTCCATATTT
TCCATAATTCTCAGTTTTAAAAATTTTGAAAACCAGTGAAGTCTTGATATTCCATATTT
TCCATAATTCTCAGTTTTAAAAATTTTGAAAACCAGTGAAGTCTTGATATTCCATATTT
TCCATAATTCTCAGTTTTAAAAATTTTGAAAACCAGTGAAGTCTTGATATTCCATATTT
TCCATAATTCTCAGTTTTAAAAATTTTGAAAACCAGTGAAGTCTTGATATTCCATATTT
TCCATAATTCTCAGTTTTAAAAATTTTGAAAACCAGTGAAGTCTTGATATTCCATATTT
TCCATAATTCTCAGTTTTAAAAATTTTGAAAACCAGTGAAGTCTTGATATTCCATATTT
TCCATAATTCTCAGTTTTAAAAATTTTGAAAACCAGTGAAGTCTTGATATTCCATATTT
TCCATAATTCTCAGTTTTAAAAATTTTGAAAACCAGTGAAGTCTTGATATTCCATATTT
TCCATAATTCTCAGTTTTAAAAATTTTGAAAACCAGTGAAGTCTTGATATTCCATATTT
TCCATAATTCTCAGTTTTAAAAATTTTGAAAACCAGTGAAGTCTTGATATTCCATATTT
TCCATAATTCTCAGTTTTAAAAATTTTGAAAACCAGTGAAGTCTTGATATTCCATATTT
TCCATAATTCTCAGTTTTAAAAATTTTGAAAACCAGTGAAGTCTTGATATTCCATATTT
TCCATAATTCTCAGTTTTAAAAATTTTGAAAACCAGTGAAGTCTTGATATTCCATATTT
TCCATAATTCTCAGTTTTAAAAATTTTGAAAACCAGTGAAGTCTTGATATTCCATATTT
TCCATAATTCTCAGTTTTAAAAATTTTGAAAACCAGTGAAGTCTTGATATTCCATATTT
TCCATAATTCTCAGTTTTAAAAATTTTGAAAACCAGTGAAGTCTTGATATTCCATATTT
TCCATAATTCTCAGTTTTAAAAATTTTGAAAACCAGTGAAGTCTTGATATTCCATATTT
TCCATAATTCTCAGTTTTAAAAATTTTGAAAACCAGTGAAGTCTTGATATTCCATATTT
TCCATAATTCTCAGTTTTAAAAATTTTGAAAACCAGTGAAGTCTTGATATTCCATATTT
TCCATAATTCTCAGTTTTAAAAATTTTGAAAACCAGTGAAGTCTTGATATTCCATATTT
TCCATAATTCTCAGTTTTAAAAATTTTGAAAACCAGTGAAGTCTTGATATTCCATATTT
TCCATAATTCTCAGTTTTAAAAATTTTGAAAACCAGTGAAGTCTTGATATTCCATATTT
TCCATAATTCTCAGTTTTAAAAATTTTGAAAACCAGTGAAGTCTTGATATTCCATATTT
TCCATAATTCTCAGTTTTAAAAATTTTGAAAACCAGTGAAGTCTTGATATTCCATATTT
TCCATAATTCTCAGTTTTAAAAATTTTGAAAACCAGTGAAGTCTTGATATTCCATATTT
TCCATAATTCTCGGTTTTAAAAATTTTGAAAACCAGTGAAGTCTTGATATTCCATATTT
TCCATAATTCTCAGTTTTAAAAATTTTGAAAACCAGTGAAGTCTTGATATTCCATATTT
TCCATAATTCTCAGTTTTAAAAATTTTGAAAACCAGTGAAGTCTTGATATTCCATATTT
TCCATAATTCTCAGTTTTAAAAATTTTGAAAACCAGTGAAGTCTTGATATTCCATATTT
TCCATAATTCTCAGTTTTAAAAATTTTGAAAACCAGTGAAGTCTTGATATTCCATATTT
TCCATAATTCTCAGTTTTAAAAATTTTGAAAACCAGTGAAGTCTTGATATTCCATATTT
TCCATAATTCTCAGTTTTAAAAATTTTGAAAACCAGTGAAGTCTTGATATTCCATATTT
TCCATAATTCTCAGTTTTAAAAATTTTGAAAACCAGTGAAGTCTTGATATTCCATATTT
TCCATAATTCTCAGTTTTAAAAATTTTGAAAACCAGTGAAGTCTTGATATTCCATATTT
TCCATAATTCTCAGTTTTAAAAATTTTGAAAACCAGTGAAGTCTTGATATTCCATATTT
TCCATAATTCTCAGTTTTAAAAATTTTGAAAACCAGTGAAGTCTTGATATTCCATATTT
TCCATAATTCTCAGTTTTAAAAATTTTGAAAACCAGTGAAGTCTTGATATTCCATATTT
TCCATAATTCTCAGTTTTAAAAATTTTGAAAACCAGTGAAGTCTTGATATTCCATATTT
TCCATAATTCTCAGTTTTAAAAATTTTGAAAACCAGTGAAGTCTTGATATTCCATATTT
TCCATAATTCTCAGTTTTAAAAATTTTGAAAACCAGTGAAGTCTTGATATTCCATATTT
TCCATAATTCTCAGTTTTAAAAATTTTGAAAACCAGTGAAGTCTTGATATTCCATATTT
TCCATAATTCTCAGTTTTAAAAATTTTGAAAACCAGTGAAGTCTTGATATTCCATATTT

TCCATAATTCTCAGTTTTAAAAATTTTGAAAACCAGTGAAGTCTTGATATTCCATATTT
TCCATAATTCTCAGTTTTAAAAATTTTGAAAACCAGTGAAGTCTTGATATTCCATATTT
TCCATAATTCTCAGTTTTAAAAATTTTGAAAACCAGTGAAGTCTTGATATTCCATATTT
TCCATAATTCTCAGTTTTAAAAATTTTGAAAACCAGTGAAGTCTTGATATTCCATATTT
TCCATAATTCTCAGTTTTAAAAATTTTGAAAACCAGTGAAGTCTTGATATTCCATATTT
TCCATAATTCTCAGTTTTAAAAATTTTGAAAACCAGTGAAGTCTTGATATTCCATATTT
TCCATAATTCTCAGTTTTAAAAATTTTGAAAACCAGTGAAGTCTTGATATTCCATATTT
TCCATAATTCTCAGTTTTAAAAATTTTGAAAACCAGTGAAGTCTTGATATTCCATATTT
TCCATAATTCTCAGTTTTAAAAATTTTGAAAACCAGTGAAGTCTTGATATTCCATATTT
TCCATAATTCTCAGTTTTAAAAATTTTGAAAACCAGTGAAGTCTTGATATTCCATATTT
TCCATAATTCTCAGTTTTAAAAATTTTGAAAACCAGTGAAGTCTTGATATTCCATATTT
TCCATAATTCTCAGTTTTAAAAATTTTGAAAACCAGTGAAGTCTTGATATTCCATATTT
TCCATAATTCTCAGTTTTAAAAATTTTGAAAACCAGTGAAGTCTTGATATTCCATATTT
TCCATAATTCTCGTTTTAAAAATTTTGAAAACCAGTGAAGTCTTGATATTCCATATTT
TCCATAATTCTCGTTTTAAAAATTTTGAAAACCAGTGAAGTCTTGATATTCCATATTT
TCCATAATTCTCAGTTTTAAAAATTTTGAAAACCAGTGAAGTCTTGATATTCCATATTT
TCCATAATTCTCAGTTTTAAAAATTTTGAAAACCAGTGAAGTCTTGATATTCCATATTT
TCCATAATTCTCGTTTTAAAAATTTTGAAAACCAGTGAAGTCTTGATATTCCATATTT
TCCATAATTCTCAGTTTTAAAAATTTTGAAAACCAGTGAAGTCTTGATATTCCATATTT
TCCATAATTCTCAGTTTTAAAAATTTTGAAAACCAGTGAAGTCTTGATATTCCATATTT
TCCATAATTCTCAGTTTTAAAAATTTTGAAAACCAGTGAAGTCTTGATATTCCATATTT
TCCATAATTCTCAGTTTTAAAAATTTTGAAAACCAGTGAAGTCTTGATATTCCATATTT
TCCATAATTCTCAGTTTTAAAAATTTTGAAAACCAGTGAAGTCTTGATATTCCATATTT
TCCATAATTCTCAGTTTTAAAAATTTTGAAAACCAGTGAAGTCTTGATATTCCATATTT
TCCATAATTCTCAGTTTTAAAAATTTTGAAAACCAGTGAAGTCTTGATATTCCATATTT
TCCATAATTCTCAGTTTTAAAAATTTTGAAAACCAGTGAAGTCTTGATATTCCATATTT
TCCATAATTCTCAGTTTTAAAAATTTTGAAAACCAGTGAAGTCTTGATATTCCATATTT
TCCATAATTCTCAGTTTTAAAAATTTTGAAAACCAGTGAAGTCTTGATATTCCATATTT
TCCATAATTCTCAGTTTTAAAAATTTTGAAAACCAGTGAAGTCTTGATATTCCATATTT
TCCATAATTCTCAGTTTTAAAAATTTTGAAAACCAGTGAAGTCTTGATATTCCATATTT
TCCATAATTCTCGGTTTTAAAAATTTTGAAAACCAGTGAAGTCTTGATATTCCATATTT
TCCATAATTCTCAGTTTTAAAAATTTTGAAAACCAGTGAAGTCTTGATAT
TCCATATTTCCATAATTCTCAGTTTTAAAAATTTTGAAAACCAGTGAAGTCTTGATATTCCATATTT
TCCATAATTCTCGGTTTTAAAAATTTTGAAAACCAGTGAAGTCTTGATATTCCATTT
TCCATAATTCTCGGTTTAAAAATTTTGAAAACCAGTGAAGTCTTGATATTCCATATTT
TCCATAATTCTCGGTTTTAAAAATTTTGAAAACCAGTGAAGTCTTGATATTCCATATTT
TCCATAATTCTCGGTTTTAAAAATTTTGAAAACCAGTGAAGTCTTGATATTCCATATTT
Nr of selected repeats 91 Similarity 0.837481
Consensus:
TCCATAATTCTCAGTTTTAAAAATTTTGAAAACCAGTGAAGTCTTGATATTCCATATTT
>Cele-UNSB01_4:12453898-12454471 Satlength=574 Nr of Repeats=6 RepeatLength=96
seed=GGTTGATTTG
GGTTGATTTGCGGCACTCTACAGATAGTGGATCTGCTTCTTACCAATCTTCCCGTTACCGCCACGCACTAAAGCTCCAA
ACTATTTTATTTTTTTG
GGTTGATTTGCGGCACTCTACAGATAGTGGATCTGCTTCTTACCAATCTTCCCGTTACCGCCACGCACTAAAGCTCCAA
ACTATTTTATTTTTTTG
GGTTGATTTGCGGCACTCTACAGATAGTGGATCTGCTTCTTACCAATCTTCCCGTTACCGCCACGCACTTAAGCTCCAA
ACTATTTTATTTTTTTG
GGTTGATTTGCGGCACTCTACAGATAGTGGATCTGCTTCTTACCAATCTTCCCGTTACCGCCACGCACTAAAGCTCCAA
AATGTTTTTTTTTG
GGTTGATTTGCGGCACTCTACAGATAGTGGATCTGCTTCTTACCAATCTTCCCGTTACCGCCACGCACTTAAGCTCCAA
ACTATTTTATTTTTTTG
GGTTGATTTGCGGCACTCTACAGATAGTGGATCTGCTTCTTACCAATCTTCCCGTTACCGCCACGCACTTAAGCTCCAA
ACTATTTTATTTTTTTG

Nr of selected repeats 5 Similarity 0.991667
Consensus:
GGTTGATTTGCGGCACTCTACAGATAGTGGATCTGCTTCTTACCAATCTTCCCGTTACCGCCACGCACTtAAGCTCCAA
ACTATTTTATTTTTTTG
>Cele-UNSB01_4:12476016-12476336 Satlength=321 Nr of Repeats=18 RepeatLength=16
seed=GTGCCTACGT
GTGCCTACGTGCCTAC
GTGCCTACGTGCCTAC
GTGTCTACGTGCCTAC
GTGCCTACGTGCCTAC
GTTCCTACGTGCCTGC
GTGCCTGCGTGCCTACGTACCTTC
GTGCCTACGTGCCTACGTACCTTC
GTGCCTACGTGCCTAC
GTGCCTATGTGCCTAC
GTGCCTACGTGCCTAC
GTCCCTACGTTCCTAC
GTGCCTACGTGCCTAC
GTGCCTACGTGCCTACGCGCCTAT
GTGCCTACGTGCCTAT
GTGCCTACGTCCCTAC
GTTCCTACGTGCCTAC
GTGCCTACGTGCCTACGCGCCTAT
GTGCCTACGTGCCTAT
Nr of selected repeats 14 Similarity 0.887363
Consensus:
GTgCCTACGTGCCTAC
>Cele-UNSB01_4:12649750-12649885 Satlength=136 Nr of Repeats=5 RepeatLength=27
seed=ACAATAAAGT
ACAATAAAGTACTATGTATTACCGTAT
ACAATAAAGTACTATGTATTACCGTAT
ACAATAAAGTACTATGTATTACCGTAT
ACAATAAAGTACTATGTATTACCGTAT
ACAATAAAGTACTATGTATTACCGTAT
Nr of selected repeats 5 Similarity 1.000000
Consensus:
ACAATAAAGTACTATGTATTACCGTAT
>Cele-UNSB01_4:12734217-12734312 Satlength=96 Nr of Repeats=5 RepeatLength=19
seed=AAAAATAGAT
AAAAATAGATTGGCAGACG
AAAAATAGATTGACAGACG
AAAAATAGATTGACAGACG
AAAAATAGATTGACAGACG
AAAAATAGATTGACAGACG
Nr of selected repeats 5 Similarity 0.971930
Consensus:
AAAAATAGATTGaCAGACG
>Cele-UNSB01_4:12770724-12770816 Satlength=93 Nr of Repeats=6 RepeatLength=15
seed=CGAAAAGGGG
CGAAAAGGGGAGCAGAA
CGAAAAGGGGATCTG
CAAAAAGGGGATCTG
CGAAAAGGGGAGATA
CGAAAAGGGGAGATA

CGAAAAGGGGAGCAA
Nr of selected repeats 5 Similarity 0.768889
Consensus:
CgAAAAGGGGAgcta
>Cele-UNSB01_4:12770991-12771083 Satlength=93 Nr of Repeats=5 RepeatLength=15 seed=TCCCCTTTTC
TCCCCTTTTCGTTGC
TCCCCTTTTCGTATC
TCCCCTTTTCGTATC
TCCCCTTTTCGCAGATTCCCTTTTTGCAGA
TCCCCTTTTCGTTCTGC
Nr of selected repeats 3 Similarity 0.881481
Consensus:
TCCCCTTTTCGTatC
>Cele-UNSB01_4:12787287-12803219 Satlength=15933 Nr of Repeats=166 RepeatLength=96 seed=GTTCCCATAA
GTTCCCATAATTGGGTTAAATACCACTCATAAGTAACTAGTATGGGACTGAAAAGATACTAAATGAGCTTATTCTAAGGGTGAAGCTTCTGGTAAT
GTTCCCATAATTGGGTTAGCTACCTCTCATAAGTAACTAGTATGGGACTGAAAAGATACTAAATGAGCTTATTCTAAGGGTGAAGCTTCTGGTAAT
GTTCCCATAATTGGGTTAGCTACCTCTCATAAGTAACTAGTATGGGACTGAAAAGATACTAAATGAGCTTATTCTAAGGGTGAAGCTTCTGGTAAT
GTTCCCATAATTGGGTTAGCTACCTCTCATAAGTAACTAGTATGGGACTAAAAGATACTAAATGAGCTTATTCTAAGGGTGAAGCTTCTGGTAAT
GTTCCCATAATTGGGTTAAATACCACTCATAAGTAACTAGTATGGGACTGAAAAGATACTAAATGAGCTTATTCTAAGGGTGAAGCTTCTGGTAAT
GTTCCCATAATTGGGTTAGCTACCTCTCATAAGTAACTAGTATGGGACTAAAAGATACTAAATGAGCTTATTCTAAGGGTGAAGCTTCTGGTAAT
GTTCCCATAATTGGGTTAAATACCACTCATAAGTAACTAGTATGGGACTGAAAAGATACTAAATGAGCTTATTCTAAGGGTGAAGCTTCTGGTAAT
GTTCCCATAATTGGGGTAAATACCACTCATAAGTAACTAGTATGGGACTGAAAAGATACTAAATGAGCTTATTCTAAGGGTGAAGCTTCTGGTAAT
GTTCCCATAATTGGGTTAGCTACCTCTCATAAGTAACTAGTATGGGACTGAAAAGATACTAAATGAGCTTATTCTAAGGGTGAAGCTTCTGGTAAT
GTTCCCATAATTGGGTTAGCTACCTCTCATAAGTAACTAGTATGGGACTGAAAAGATACTAAATGAGCTTATTCTAAGGGTGAAGCTTCTGGTAAT
GTTCCCATAATTGGGTTAGCTACCTCTCATAAGTAACTAGTATGGGACTAAAAGATACTAAATGAGCTTATTCTAAGGGTGAAGCTTCTGGTAAT
GTTCCCATAATTGGGTTAAATACCACTCATAAGTAACTAGTATGGGACTGAAAAGATACTAAATGAGCTTATTCTAAGGGTGAAGCTTCTGGTAAT
GTTCCCATAATTGGGTTAGCTACCTCTCATAAGTAACTAGTATGGGACTAAAAGATACTAAATGAGCTTATTCTAAGGGTGAAGCTTCTGGTAAT
GTTCCCATAATTGGGTTAAATACCACTCATAAGTAACTAGTATGGGACTGAAAAGATACTAAATGAGCTTATTCTAAGGGTGAAGCTTCTGGTAAT
GTTCCCATAATTGGGGTAAATACCACTCATAAGTAACTAGTATGGGACTGAAAAGATACTAAATGAGCTTATTCTAAGGGTGAAGCTTCTGGTAAT
GTTCCCATAATTGGGTTAAATACCACTCATAAGTAACTAGTATGGGACTGAAAAGATACTAAATGAGCTTATTCTAAGGGTGAAGCTTCTGGTAAT
GTTCCCATAATTGGGTTAAATACCACTCATAAGTAACTAGTATGGGACTGAAAAGATACTAAATGAGCTTATTCTAAGGGTGAAGCTTCTGGTAAT
GTTCCCATAATTGGGGTAAATACCACTCATAAGTAACTAGTATGGGACTGAAAAGATACTAAATGAGCTTATTCTAAGGGTGAAGCTTCTGGTAAT
GTTCCCATAATTGGGGTAAATACCACTCATAAGTAACTAGTATGGGACAGAAAAGATACTAAATGAGCTTATTCTAAGGGTGAAGCTTCTGGTAAT

GTTCCCATAATTGGGTTAAATACCACTCATAAGTAACTAGTATGGGACTGAAAAGATACTAAATGAGCTTATTCTAAGG
GTGAAGCTTCTGGTAAT
GTTCCCATAATTGGGGTAAATACCACTCATAAGTACCTAGTATGGGACTGAAAAGATACTAAATGAGCTTATTCTAAGG
GTGAAGCTTCTGGTAAT
GTTCCCATAATTGGGTTAAATACCACTCATAAGTAACTAGTATGGGACTGAAAAGATACTAAATGAGCTTATTCTAAGG
GTGAAGCTTCTGGTAAT
GTTCCCATAATTGGGGTAAATACCACTCATAAGTAACTAGTATGGGACTGAAAAGATACTAAATGAGCTTATTCTAAGG
GTGAAGCTTCTGGTAAT
GTTCCCATAATTGGGTTAAATACCACTCATAAGTAACTAGTATGGGACAGAAAAGATACTAAATGAGCTTATTCTAAGG
GTGAAGCTTCTGGTAAT
GTTCCCATAATTGGGTTAAATACCACTCATAAGTAACTAGTATAGGACAGAAAAGATACTAAATGAGCTTATTCTAAGG
GTGAAGCTTCTGGTAAT
GTTCCCATAATTGGGTTAAATACCACTCATAAGTAACTAGTATGGGACAGAAAAGATACTAAATGAGCTTATTCTAAGG
GTGAAGCTTCTGGTAAT
GTTCCCATAATTGGGTTAAATACCACTCATAAGTAACTAGTATGGGACAGAAAAGATACTAAATGAGCTTATTCTAAGG
GTGAAGCTTCTAGTAAT
GTTCCCATAATTGGGTTAAATACCACTCATAAGTAACTAGTATGGGACTGAAAAGATACTAAATGAGCTTATTCTAAGG
GTGAAGCTTCTGGTAAT
GTTCCCATAATTGGGTTAGCTACCACTCATAAGTAACTAGTATGGGACTGAAAAGATACTAAATGAGCTTATTCTAAGG
GTGAAGCTTCTGGTAAT
GTTCCCATAATTGGGTTAAATACCACTCATAAGTAACTAGTATGGGACTGAAAAGATACTAAATGAGCTTATTCTAAGG
GTGAAGCTTCTGGTAAT
GTTCCCATAATTGGGGTAAATACCACTCATAAGTAACTAGTATGGGACTGAAAAGATACTAAATGAGCTTATTCTAAGG
GTGAAGCTTCTGGTAAT
GTTCCCATAATTGGGGTAAATACCACTCATAAGTAACTAGTATGGGACAGAAAAGATACTAAATGAGCTTATTCTAAGG
GTGAAGCTTCTGGTAAT
GTTCCCATAATTGGGTTAAATACCACTCATAAGTAACTAGTATGGGACTGAAAAGATACTAAATGAGCTTATTCTAAGG
GTGAAGCTTCTGGTAAT
GTTCCCATAATTGGGGTAAATACCACTCATAAGTACCTAGTATGGGACTGAAAAGATACTAAATGAGCTTATTCTAAGG
GTGAAGCTTCTGGTAAT
GTTCCCATAATTGGGTTAAATACCACTCATAAGTAACTAGTATGGGACTGAAAAGATACTAAATGAGCTTATTCTAAGG
GTGAAGCTTCTGGTAAT
GTTCCCATAATTGGGGTAAATACCACTCATAAGTAACTAGTATGGGACTGAAAAGATACTAAATGAGCTTATTCTAAGG
GTGAAGCTTCTGGTAAT
GTTCCCATAATTGGGTTAAATACCACTCATAAGTAACTAGTATGGGACAGAAAAGATACTAAATGAGCTTATTCTAAGG
GTGAAGCTTCTGGTAAT
GTTCCCATAATTGGGTTAAATACCACTCATAAGTAACTAGTATAGGACAGAAAAGATACTAAATGAGCTTATTCTAAGG
GTGAAGCTTCTGGTAAT
GTTCCCATAATTGGGTTAAATACCACTCATAAGTAACTAGTATGGGACAGAAAAGATACTAAATGAGCTTATTCTAAGG
GTGAAGCTTCTGGTAAT
GTTCCCATAATTGGGTTAAATACCACTCATAAGTAACTAGTATGGGACAGAAAAGATACTAAATGAGCTTATTCTAAGG
GTGAAGCTTCTAGTAAT
GTTCCCATAATTGGGTTAAATACCACTCATAAGTAACTAGTATGGGACTGAAAAGATACTAAATGAGCTTATTCTAAGG
GTGAAGCTTCTGGTAAT
GTTCCCATAATTGGGTTAGCTACCTCTCATAAGTAACTAGTATGGGACTGAAAAGATACTAAATGAGCTTATTCTAAGG
GTGAAGCTTCTGGTAAT
GTTCCCATAATTGGGTTAGCTACCTCTCATAAGTAACTAGTATGGGACTGAAAAGATACTAAATGAGCTTATTCTAAGG
GTGAAGCTTCTGGTAAT
GTTCCCATAATTGGGTTAAATACCACTCATAAGTAACTAGTATGGGACTGAAAAGATACTAAATGAGCTTATTCTAAGG
GTGAAGCTTCTGGTAAT
GTTCCCATAATTGGGTTAAATACCACTCATAAGTAACTAGTATGGGACTGAAAAGATACTAAATGAGCTTATTCTAAGG
GTGAAGCTTCTGGTAAT
GTTCCCATAATTGGGTTAAATACCACTCATAAGTAACTAGTATGGGACTGAAAAGATACTAAATGAGCTTATTCTAAGG
GTGAAGCTTCTGGTAAT

GTTCCCATAATTGGGGTAAATACCACTCATAAGTAACTAGTATGGGACTGAAAAGATACTAAATGAGCTTATTCTAAGG
GTGAAGCTTCTAGTAAT
GTTCCCATAATTGGGTTAAATACCACTCATAAGTAACTAGTATGGGACTGAAAAGATACTAAATGAGCTTATTCTAAGG
GTGAAGCTTCTGGTAAT
GTTCCCATAATTGGGTTAGCTACCTCTCATAAGTAACTAGTATGGGACTGAAAAGATACTAAATGAGCTTATTCTAAGG
GTGAAGCTTCTGGTAAT
GTTCCCATAATTGGGTTAAATACCACTCATAAGTAACTAGTATGGGACTGAAAAGATACTAAATGAGCTTATTCTAAGG
GTGAAGCTTCTGGTAAT
GTTCCCATAATTGGGTTAAATACCACTCATAAGTAACTAGTATGGGACTGAAAAGATACTAAATGAGCTTATTCTAAGG
GTGAAGCTTCTGGTAAT
GTTCCCATAATTGGGTTAAATACCACTCATAAGTAACTAGTATGGGACTGAAAAGATACTAAATGAGCTTATTCTAAGG
GTGAAGCTTCTGGTAAT
GTTCCCATAATTGGGTTAGCTACCTCTCATAAGTAACTAGTATGGGACTGAAAAGATACTAAATGAGCTTATTCTAAGG
GTGAAGCTTCTGGTAAT
GTTCCCATAATTGGGTTAGCTACCTCTCATAAGTAACTAGTATGGGACTGAAAAGATACTAAATGAGCTTATTCTAAGG
GTGAAGCTTCTGGTAAT
GTTCCCATAATTAGGGTAAATACCACTCATAAGTAACTAGTATGGGACTGAAAAGATACTAAATGAGCTTATTCTAAGG
GTGAAGCTTCTGGTAAT
GTTCCCATAATTGGGTTAGCTACCTCTCATAAGTAACTAGTATGGGACTGAAAAGATACTAAATGAGCTTATTCTAAGG
GTGAAGCTTCTGGTAAT
GTTCCCATAATTGGGTTAAATACCACTCATAAGTAACTAGTATGGGACTGAAAAGATACTAAATGAGCTTATTCTAAGG
GTGAAGCTTCTGGTAAT
GTTCCCATAATTGGGTTAGCTACCTCTCATAAGTAACTAGTATGGGACTGAAAAGATACTAAATGAGCTTATTCTAAGG
GTGAAGCTTCTGGTAAT
GTTCCCATAATTGGGTTAAATACCACTCATAAGTAACTAGTATGGGACTGAAAAGATACTAAATGAGCTTATTCTAAGG
GTGAAGCTTCTGGTAAT
GTTCCCATAATTGGGTTAAATACCACTCATAAGTAACTAGTATGGGACTGAAAAGATACTAAATGAGCTTATTCTAAGG
GTGAAGCTTCTGGTAAT
GTTCCCATAATTGGGTTAAATACCACTCATAAGTAACTAGTATGGGACTGAAAAGATACTAAATGAGCTTATTCTAAGG
GTGAAGCTTCTGGTAAT
GTTCCCATAATTGGGTTAAATACCACTCATAAGTAACTAGTATGGGACTGAAAAGATACTAAATGAGCTTATTCTAAGG
GTGAAGCTTCTGGTAAT
GTTCCCATAATTGGGTTAGCTACCTCTCATAAGTAACTAGTATGGGACTGAAAAGATACTAAATGAGCTTATTCTAAGG
GTGAAGCTTCTGGTAAT
GTTCCCATAATTGGGTTAGCTACCTCTCATAAGTAACTAGTATGGGACTGAAAAGATACTAAATGAGCTTATTCTAAGG
GTGAAGCTTCTGGTAAT
GTTCCCATAATTAGGGTAAATACCACTCATAAGTAACTAGTATGGGACTGAAAAGATACTAAATGAGCTTATTCTAAGG
GTGAAGCTTCTGGTAAT
GTTCCCATAATTGGGTTAGCTACCTCTCATAAGTAACTAGTATGGGACTGAAAAGATACTAAATGAGCTTATTCTAAGG
GTGAAGCTTCTGGTAAT
GTTCCCATAATTGGGTTAAATACCACTCATAAGTAACTAGTATGGGACTGAAAAGATACTAAATGAGCTTATTCTAAGG
GTGAAGCTTCTGGTAAT
GTTCCCATAATTGGGGTAAATACCACTCATAAGTAACTAGTATGGGACTGAAAAGATACTAAATGAGCTTATTCTAAGG
GTGAAGCTTCTGGTAAT
GTTCCCATAATTGGGGTAAATACCACTCATAAGTAACTAGTATGGGACTGAAAAGATACTAAATGAGCTTATTCTAAGG
GTGAAGCTTCTGGTAAT
GTTCCCATAATTGGGTTAGCTACCTCTCATAAGTAACTAGTATGGGACTGAAAAGATACTAAATGAGCTTATTCTAAGG
GTGAAGCTTCTAGTAAT
GTTCCCATAATTGGGTTAGCTACCTCTCATAAGTAACTAGTATGGGACTGAAAAGATACTAAATGAGCTTATTCTAAGG
GTGAAGCTTCTGGTAAT
GTTCCCATAATTGGGTTAGCTACCTCTCATAAGTAACTAGTATGGGACTGAAAAGATACTAAATGAGCTTATTCTAAGG
GTGAAGCTTCTAGTAAT
GTTCCCATAATTGGGTTAGCTACCTCTCATAAGTAACTAGTATGGGACTGAAAAGATACTAAATGAGCTTATTCTAAGG
GTGAAGCTTCTGGTAAT

GTTCCCATAATTGGGTTAGCTACCTCTCATAAGTAACTAGTATGGGACTGAAAAGATACTAAATGAGCTTATTCTAAGG
GTGAAGCTTCTGGTAAT
GTTCCCATAATTGGGTTAGCTACCTCTCATAAGTAACTAGTATGAGACTGAAAAGATACTAAATGAGCTTATTCTAAGG
GTGAAGCTTCTGGTAAT
GTTCCCATAATTGGGGTAAATACCACTCATAAGTAACTAGTATGGGACTGAAAAGATACTAAATGAGCTTATTCTAAGG
GTGAAGCTTCTGGTAAT
GTTCCCATAATTGGGTTAGCTACCTCTCATAAGTAACTAGTATGGGACTGAAAAGATACTAAATGAGCTTATTCTAAGG
GTGAAGCTTCTGGTAAT
GTTCCCATAATTGGGTTAAATACCACTCATAAGTAACTAGTATGGGACTGAAAAGATACTAAATGAGCTTATTCTAAGG
GTGAAGCTTCTGGTAAT
GTTCCCATAATTGGGGTAAATACCACTCATAAGTAACTAGTATGGGACTGAAAAGATACTAAATGAGCTTATTCTAAGG
GTGAAGCTTCTGGTAAT
GTTCCCATAATTGGGGTAAATACCACTCATAAGTAACTAGTATGGGACTGAAAAGATACTAAATGAGCTTATTCTAAGG
GTGAAGCTTCTGGTAAT
GTTCCCATAATTGGGGTAAATACCACTCATAAGTAACTAGTATGGGACTGAAAAGATACTAAATGAGCTTATTCTAAGG
GTGAAGCTTCTGGTAAT
GTTCCCATAATTGGGGTAAATACCACTCATAAGTAACTAGTATGGGACTGAAAAGATACTAAATGAGCTTATTCTAAGG
GTGAAGCTTCTGGTAAT
GTTCCCATAATTGGGTTAAATACCACTCATAAATAACTAGTATGGGACTGAAAAGATACTAAATGAGCTTATTCTAAGG
GTGAAGCTTCTGGTAAT
GTTCCCATAATTGGGTTAGCTACCTCTCATAAGTAACTAGTATGGGACTGAAAAGATACTAAATGAGCTTATTCTAAGG
GTGAAGCTTCTGGTAAT
GTTCCCATAATTGGGTTAAATACCACTCATAAGTAACTAGTATGGGACTGAAAAGATACTAAATGAGCTTATTCTAAGG
GTGAAGCTTCTGGTAAT
GTTCCCATAATTGGGGTAAATACCACTCATAAGTAACTAGTATGGGACTGAAAAGATACTAAATGAGCTTATTCTAAGG
GTGAAGCTTCTGGTAAT
GTTCCCATAATTGGGGTAAATACCACTCATAAGTAACTAGTATGGGACTGAAAAGATACTAAATGAGCTTATTCTAAGG
GTGAAGCTTCTGGTAAT
GTTCCCATAATTGGGGTAAATACCACTCATAAGTAACTAGTATGGGACTGAAAAGATACTAAATGAGCTTATTCTAAGG
GTGAAGCTTCTGGTAAT
GTTCCCATAATTGGGGTAAATACCACTCATAAGTAACTAGTATGGGACTGAAAAGATACTAAATGAGCTTATTCTAAGG
GTGAAGCTTCTGGTAAT
GTTCCCATAATTGGGGTAAATACCACTCATAAGTAACTAGTATGGGACTGAAAAGATACTAAATGAGCTTATTCTAAGG
GTGAAGCTTCTGGTAAT
GTTCCCATAATTGGGGTAAATACCACTCATAAGTAACTAGTATGGGACTGAAAAGATACTAAATGAGCTTATTCTAAGG
GTGAAGCTTCTGGTAAT
GTTCCCATAATTGGGGTAAATACCACTCATAAGTAACTAGTATGGGACTGAAAAGATACTAAATGAGCTTATTCTAAGG
GTGAAGCTTCTGGTAAT
GTTCCCATAATTGGGGTAAATACCACTCATAAGTAACTAGTATGGGACTGAAAAGATACTAAATGAGCTTATTCTAAGG
GTGAAGCTTCTGGTAAT
GTTCCCATAATTGGGTTAGCTACCTCTCATAAGTAACTAGTATGGGACTGAAAAGATACTAAATGAGCTTATTCTAAGG
GTGAAGCTTCTGGTAAT
GTTCCCATAATTGGGTTAAATACCACTCATAAGTAACTAGTATGGGACTGAAAAGATACTAAATGAGCTTATTCTAAGG
GTGAAGCTTCTGGTAAT
GTTCCCATAATTGGGTTAAATACCACTCATAAGTAACTAGTATGGGACAGAAAAGATACTAAATGAGCTTATTCTAAGG
GTGAAGCTTCTAGTAAT
GTTCCCATAATTGGGTTAAATACCACTCATAAGTAACTAGTATGGGACTGAAAAGATACTAAATGAGCTTATTCTAAGG
GTGAAGCTTCTGGTAAT
GTTCCCATAATTGGGGTAAATACCACTCATAAGTAACTAGTATGGGACAGAAAAGATACTAAATGAGCTTATTCTAAGG
GTGAAGCTTCTGGTAAT
GTTCCCATAATTGGGTTAAATACCACTCATAAGTAACTAGTATAGGACAGAAAAGATACTAAATGAGCTTATTCTAAGG
GTGAAGCTTCTGGTAAT
GTTCCCATAATTGGGTTAAATACCACTCATAAGTAACTAGTATGGGACAGAAAAGATACTAAATGAGCTTATTCTAAGG
GTGAAGCTTCTGGTAAT

GTTCCCATAATTGGGTTAAATACCACTCATAAGTAACTAGTATGGGACAGAAAAGATACTAAATGAGCTTATTCTAAGG
GTGAAGCTTCTGGTAAT
GTTCCCATAATTGGGTTAAATACCACTCATAAGTAACTAGTATGGGACTGAAAAGATACTAAATGAGCTTATTCTAAGG
GTGAAGCTTCTGGTAAT
GTTCCCATAATTGGGGTAAATACCACTCATAAGTAACTAGTATGGGACTGAAAAGATACTAAATGAGCTTATTCTAAGG
GTGAAGCTTCTGGTAAT
GTTCCCATAATTGGGTTAAATACCACTCATAAGTAACTAGTATGGGACAGAAAAGATACTAAATGAGCTTATTCTAAGG
GTGAAGCTTCTGGTAAT
GTTCCCATAATTGGGTTAGCTACCTCTCATAAGTAACTAGTATGGGACTGAAAAGATACTAAATGAGCTTATTCTAAGG
GTGAAGCTTCTGGTAAT
GTTCCCATAATTGGGGTAAATACCACTCATAAGTAACTAGTATGGGACAGAAAAGATACTAAATGAGCTTATTCTAAGG
GTGAAGCTTCTGGTAAT
GTTCCCATAATTGGGTTAAATACCACTCATAAGTAACTAGTATAGGACAGAAAAGATACTAAATGAGCTTATTCTAAGG
GTGAAGCTTCTGGTAAT
GTTCCCATAATTGGGTTAAATACCACTCATAAGTAACTAGTATGGGACTGAAAAGATACTAAATGAGCTTATTCTAAGG
GTGAAGCTTCTGGTAAT
GTTCCCATAATTGGGTTAAATACCACTCATAAGTAACTAGTATGGGACAGAAAAGATACTAAATGAGCTTATTCTAAGG
GTGAAGCTTCTAGTAAT
GTTCCCATAATTGGGTTAAATACCACTCATAAGTAACTAGTATGGGACTGAAAAGATACTAAATGAGCTTATTCTAAGG
GTGAAGCTTCTGGTAAT
GTTCCCATAATTGGGTTAAATACCACTCATAAGTAACTAGTATGGGACTGAAAAGATACTAAATGAGCTTATTCTAAGG
GTGAAGCTTCTGGTAAT
GTTCCCATAATTGGGGTAAATACCACTCATAAGTAACTAGTATGGGACTGAAAAGATACTAAATGAGCTTATTCTAAGG
GTGAAGCTTCTGGTAAT
GTTCCCATAATTGGGGTAAATACCACTCATAAGTAACTAGTATGGGACTGAAAAGATACTAAATGAGCTTATTCTAAGG
GTGAAGCTTCTGGTAAT
GTTCCCATAATTGGGGTAAATACCACTCATAAGTAACTAGTATGGGACTGAAAAGATACTAAATGAGCTTATTCTAAGG
GTGAAGCTTCTGGTAAT
GTTCCCATAATTGGGGTAAATACCACTCATAAGTAACTAGTATGGGACAGAAAAGATACTAAATGAGCTTATTCTAAGG
GTGAAGCTTCTGGTAAT
GTTCCCATAATTGGGTTAAATACCACTCATAAGTAACTAGTATAGGACAGAAAAGATACTAAATGAGCTTATTCTAAGG
GTGAAGCTTCTGGTAAT
GTTCCCATAATTGGGTTAAATACCACTCATAAGTAACTAGTATGGGACTGAAAAGATACTAAATGAGCTTATTCTAAGG
GTGAAGCTTCTGGTAAT
GTTCCCATAATTGGGTTAAATACCACTCATAAGTAACTAGTATGGGACAGAAAAGATACTAAATGAGCTTATTCTAAGG
GTGAAGCTTCTAGTAAT
GTTCCCATAATTGGGTTAAATACCACTCATAAGTAACTAGTATGGGACTGAAAAGATACTAAATGAGCTTATTCTAAGG
GTGAAGCTTCTGGTAAT
GTTCCCATAATTGGGTTAAATACCACTCATAAGTAACTAGTATGGGACTGAAAAGATACTAAATGAGCTTATTCTAAGG
GTGAAGCTTCTGGTAAT
GTTCCCATAATTGGGGTAAATACCACTCATAAGTAACTAGTATGGGACTGAAAAGATACTAAATGAGCTTATTCTAAGG
GTGAAGCTTCTGGTAAT
GTTCCCATAATTGGGGTAAATACCACTCATAAGTAACTAGTATGGGACTGAAAAGATACTAAATGAGCTTATTCTAAGG
GTGAAGCTTCTGGTAAT
GTTCCCATAATTGGGGTAAATACCACTCATAAGTAACTAGTATGGGACTGAAAAGATACTAAATGAGCTTATTCTAAGG
GTGAAGCTTCTGGTAAT
GTTCCCATAATTGGGGTAAATACCACTCATAAGTAACTAGTATGGGACTGAAAAGATACTAAATGAGCTTATTCTAAGG
GTGAAGCTTCTGGTAAT
GTTCCCATAATTGGGGTAAATACCACTCATAAGTAACTAGTATGGGACTGAAAAGATACTAAATGAGCTTATTCTAAGG
GTGAAGCTTCTGGTAAT
GTTCCCATAATTGGGGTAAATACCACTCATAAGTAACTAGTATGGGACTGAAAAGATACTAAATGAGCTTATTCTAAGG
GTGAAGCTTCTGGTAAT
GTTCCCATAATTGGGGTAAATACCACTCATAAGTAACTAGTATGGGACTGAAAAGATACTAAATGAGCTTATTCTAAGG
GTGAAGCTTCTGGTAAT

GTTCCCATAATTGGGTTAGCTACCTCTCATAAGTAACTAGTATGGGACTGAAAAGATACTAAATGAGCTTATTCTAAGG
GTGAAGCTTCTGGTAAT
GTTCCCATAATTGGGTTAAATACCACTCATAAGTAACTAGTATGGGACTGAAAAGATACTAAATGAGCTTATTCTAAGG
GTGAAGCTTCTGGTAAT
GTTCCCATAATTGGGGTAAATACCACTCATAAGTAACTAGTATGGGACAGAAAAGATACTAAATGAGCTTATTCTAAGG
GTGAAGCTTCTGGTAAT
GTTCCCATAATTGGGTTAAATACCACTCATAAGTAACTAGTATAGGACAGAAAAGATACTAAATGAGCTTATTCTAAGG
GTGAAGCTTCTGGTAAT
GTTCCCATAATTGGGTTAAATACCACTCATAAGTAACTAGTATGGGACTGAAAAGATACTAAATGAGCTTATTCTAAGG
GTGAAGCTTCTGGTAAT
GTTCCCATAATTGGGTTAAATACCACTCATAAGTAACTAGTATGGGACAGAAAAGATACTAAATGAGCTTATTCTAAGG
GTGAAGCTTCTAGTAAT
GTTCCCATAATTGGGTTAAATACCACTCATAAGTAACTAGTATGGGACTGAAAAGATACTAAATGAGCTTATTCTAAGG
GTGAAGCTTCTGGTAAT
GTTCCCATAATTGGGTTAGCTACCTCTCATAAGTAACTAGTATGGGACTGAAAAGATACTAAATGAGCTTATTCTAAGG
GTGAAGCTTCTGGTAAT
GTTCCCATAATTGGGTTAGCTACCTCTCATAAGTAACTAGTATGGGACTGAAAAGATACTAAATGAGCTTATTCTAAGG
GTGAAGCTTCTGGTAAT
GTTCCCATAATTGGGTTAGCTACCTCTCATAAGTAACTAGTATGGGACTGAAAAGATACTAAATGAGCTTATTCTAAGG
GTGAAGCTTCTGGTAAT
GTTCCCATAATTGGGTTAGCTACCTCTCATAAGTAACTAGTATGGGACTGAAAAGATACTAAATGAGCTTATTCTAAGG
GTGAAGCTTCTGGTAAT
GTTCCCATAATTGGGGTAAATACCACTCATAAGTAACTAGTATGGGACTGAAAAGATACTAAATGAGCTTATTCTAAGG
GTGAAGCTTCTGGTAAT
GTTCCCATAATTGGGTTAAATACCACTCATAAGTAACTAGTATGGGACTGAAAAGATACTAAATGAGCTTATTCTAAGG
GTGAAGCTTCTGGTAAT
GTTCCCATAATTGGGTTAAATACCACTCATAAGTAACTAGTATGGGACTGAAAAGATACTAAATGAGCTTATTCTAAGG
GTGAAGCTTCTGGTAAT
GTTCCCATAATTGGGTTAGCTACCTCTCATAAGTAACTAGTATGGGACTGAAAAGATACTAAATGAGCTTATTCTAAGG
GTGAAGCTTCTGGTAAT
GTTCCCATAATTAGGGTAAATACCACTCATAAGTAACTAGTATGGGACTGAAAAGATACTAAATGAGCTTATTCTAAGG
GTGAAGCTTCTGGTAAT
GTTCCCATAATTGGGTTAAATACCACTCATAAGTAACTAGTATGGGACTGAAAAGATACTAAATGAGCTTATTCTAAGG
GTGAAGCTTCTGGTAAT
GTTCCCATAATTGGGTTAAATACCACTCATAAGTAACTAGTATGGGACTGAAAAGATACTAAATGAGCTTATTCTAAGG
GTGAAGCTTCTGGTAAT
GTTCCCATAATTGGGTTAAATACCACTCATAAGTAACTAGTATGGGACTGAAAAGATACTAAATGAGCTTATTCTAAGG
GTGAAGCTTCTGGTAAT
GTTCCCATAATTGGGGTAAATACCACTCATAAGTAACTAGTATGGGACTGAAAAGATACTAAATGAGCTTATTCTAAGG
GTGAAGCTTCTGGTAAT
GTTCCCATAATTGGGGTAAATACCACTCATAAGTAACTAGTATGGGACTGAAAAGATACTAAATGAGCTTATTCTAAGG
GTGAAGCTTCTGGTAAT
GTTCCCATAATTGGGTTAGCTACCTCTCATAAGTAACTAGTATGGGACTGAAAAGATACTAAATGAGCTTATTCTAAGG
GTGAAGCTTCTGGTAAT
GTTCCCATAATTGGGGTAAATACCACTCATAAGTAACTAGTATGGGACTGAAAAGATACTAAATGAGCTTATTCTAAGG
GTGAAGCTTCTGGTAAT
GTTCCCATAATTGGGTTAGCTACCTCTCATAAGTAACTAGTATGGGACTGAAAAGATACTAAATGAGCTTATTCTAAGG
GTGAAGCTTCTAGTAAT
GTTCCCATAATTGGGTTAGCTACCTCTCATAAGTAACTAGTATGGGACTGAAAAGATACTAAATGAGCTTATTCTAAGG
GTGAAGCTTCTGGTAAT
GTTCCCATAATTGGGTTAGCTACCTCTCTTAAGTAACTAGTATGGGACTGAAAAGATACTAAATGAGCTTATTCTAAGG
GTGAAGCTTCTAGTAAT
GTTCCCATAATTGGGTTAGCTACCTCTCATAAGTAACTAGTATGGGACTGAAAAGATACTAAATGAGCTTATTCTAAGG
GTGAAGCTTCTGGTAAT

GTTCCCATAATTGGGTTAGCTACCTCTCTTAAGTAACTAGTATGGGACTGAAAAGATACTAAATGAGCTTATTCTAAGG
GTGAAGCTTCTAGTAAT
GTTCCCATAATTGGGTTAGCTACCTCTCATAAGTAACTAGTATGGGACTGAAAAGATACTAAATGAGCTTATTCTAAGG
GTGAAGCTTCTGGTAAT
GTTCCCATAATTGGGTTAAATACCACTCATAAATAACTAGTATGGGACTGAAAAGATACTAAATGAGCTTATTCTAAGG
GTGAAGCTTCTGGTAAT
GTTCCCATAATTGGGTTAGCTACCTCTCATAAGTAACTAGTATGGGACTGAAAAGATACTAAATGAGCTTATTCTAAGG
GTGAAGCTTCTGGTAAT
GTTCCCATAATTGGGTTAGCTACCTCTCATAAGTAACTAGTATGGGACTGAAAAGATACTAAATGAGCTTATTCTAAGG
GTGAAGCTTCTGGTAAT
GTTCCCATAATTGGGTTAAATACCACTCATAAGTAACTAGTATGGGACTGAAAAGATACTAAATGAGCTTATTCTAAGG
GTGAAGCTTCTGGTAAT
GTTCCCATAATTGGGGTAAATACCACTCATAAGTAACTAGTATGGGACTGAAAAGATACTAAATGAGCTTATTCTAAGG
GTGAAGCTTCTGGTAAT
GTTCCCATAATTGGGGTAAATACCACTCATAAGTAACTAGTATGGGACTGAAAAGATACTAAATGAGCTTATTCTAAGG
GTGAAGCTTCTGGTAAT
GTTCCCATAATTGGGGTAAATACCACTCATAAGTAACTAGTATGGGACTGAAAAGATACTAAATGAGCTTATTCTAAGG
GTGAAGCTTCTGGTAAT
GTTCCCATAATTGGGGTAAATACCACTCATAAGTAACTAGTATGGGACTGAAAAGATACTAAATGAGCTTATTCTAAGG
GTGAAGCTTCTGGTAAT
GTTCCCATAATTGGGGTAAATACCACTCATAAGTAACTAGTATGGGACTGAAAAGATACTAAATGAGCTTATTCTAAGG
GTGAAGCTTCTGGTAAT
GTTCCCATAATTGGGTTAAATACCACTCATAAGTAACTAGCATGGGACTGAAAAGTGACTAAATGAGCTTATTCTAAGG
ATGAAGCTTCTGGTAAT
Nr of selected repeats 162 Similarity 0.969395
Consensus:
GTTCCCATAATTGGGtTAaaTACCaCTCATAAGTAACTAGTATGGGACTGAAAAGATACTAAATGAGCTTATTCTAAGG
GTGAAGCTTCTGGTAAT
>Cele-UNSB01_4:12842983-12843155 Satlength=173 Nr of Repeats=5 RepeatLength=35
seed=AAACCGGCAA
AAACCGGCAATTTAAAAAATGCCGGAAGTTTTGA
AAACCGGCAATTGCCGAAATTGCCGAAAATTCTCG
AAACCGGCAATTGCCGATATTGCCAGAAATCTTAA
AAACCGGCAATTGCCGTAATTGCCGAAAATTCACA
AAACCGGCAATTGCCAATTGCCGGAAATTCACA
Nr of selected repeats 3 Similarity 0.759259
Consensus:
AAACCGGCAATTGCCGaaATTGCCgAaAAtTCtca
>Cele-UNSB01_4:12895275-12902336 Satlength=7062 Nr of Repeats=272
RepeatLength=26 seed=CATTGCGGCG
CATTGCGGCGGATTTTTGAAAAATGG
CATTGCGGCGGATTTTTGAAAAATGA
CATTGCGGCGGATTTCAGAAAAATGA
CATTGCGGCGGATTTTTGAAAAATGA
CATTGCGGCGGATTTTTGAAAAATGG
CATTGCGGCGGATTTTTGAAAAATGA
CATTGCGGCGGATTTCAGAAAAATGG
CATTGCGGCGGATTTCAGAAAAATGG
CATTGCGGCGGATTTTTGAAAAATGA
CATTGCGGCGGATTTCAGAAAAATGG
CATTGCGGCGGATTTTTGAAAAATGG
CATTGCGGCGGATTTTTGAAAAATGA
CATTGCGGCGGATTTCAGAAAAATGA
CATTGCGGCGGATTTTTGAAAAATGA

CATTGCGGCGGATTTTTGAAAAATGG
CATTGCGGCGGATTTTTGAAAAATGA
CATTGCGGCGGATTTCAGAAAAATGA
CATTGCGGCGGATTTTGAAAAATGG
CATTGCGGCGGATTTTTGAAAAATGA
CATTGCGGCGGATTTCAGAAAAATGG
CATTGCGGCGGATTTCAGAAAAATGG
CATTGCGGCGGATTTTTGAAAAATGA
CATTGCGGCGGATTTCAGAAAAATGG
CATTGCGGCGGATTTTTGAAAAATGG
CATTGCGGCGGATTTTTGAAAAATGA
CATTGCGGCGGATTTCAGAAAAATGA
CATTGCGGCGGATTTTGAAAAATGG
CATTGCGGCGGATTTTTGAAAAATGA
CATTGCGGCGGATTTCAGAAAAATGG
CATTGCGGCGGATTTCAGAAAAATGA
CATTGCGGCGGATTTTGAAAAATGG
CATTGCGGCGGATTTCAGAAAAATGG
CATTGCGGCGGATTTCAGAAAAATGG
CATTGCGGCGGATTTTTGAAAAATGA
CATTGCGGCGGATTTTTGAAAAATGA
CATTGCGGCGGATTTCAGAAAAATGG
CATTGCGGCGGATTTCAGAAAAATGG
CATTGCGGCGGATTTTTGAAAAATGA
CATTGCGGCGGATTTCAGAAAAATGG
CATTGCGGCGGATTTTCGAAAAATGA
CATTGCGGCGGATTTCAGAAAAATGA
CATTGCGGCGGATTTTCGAAAAATGG
CATTGCGGCGGATTTTTGAAAAATGA
CATTGCGGCGGATTTCAGAAAAATGG
CATTGCGGCGGATTTTTGAAAAATGG
CATTGCGGCGGATTTTTGAAAAATGA
CATTGCGGCGGATTTCAGAAAAATGA
CATTGCGGCGGATTTTGAAAAATGG
CATTGCGGCGGATTTCAGAAAAATGG
CATTGCGGCGGATTTCAGAAAAATGG
CATTGCGGCGGATTTTTGAAAAATGA
CATTGCGGCGGATTTCAGAAAAATGG
CATTGCGGCGGATTTCAGAAAAATGG
CATTGCGGCGGATTTTTGAAAAATGA
CATTGCGGCGGATTTTCGAAAAATGG
CATTGCGGCGGATTTTCGAAAAATGA
CATTGCGGCGGATTTCAGAAAAATGA
CATTGCGGCGGATTTTCGAAAAATGG
CATTGCGGCGGATTTTTGAAAAATGA
CATTGCGGCGGATTTCAGAAAAATGG
CATTGCGGCGGATTTTTGAAAAATGG
CATTGCGGCGGATTTTTGAAAAATGA
CATTGCGGCGGATTTCAGAAAAATGA
CATTGCGGCGGATTTTGAAAAATGG
CATTGCGGCGGATTTTTGAAAAATGA
CATTGCGGCGGATTTCAGAAAAATGG
CATTGCGGCGGATTTCAGAAAAATGA
CATTGCGGCGGATTTTGAAAAATGG

CATTGCGGCGGATTTCAGAAAAATGG
CATTGCGGCGGATTTCAGAAAAATGG
CATTGCGGCGGATTTCAGAAAAATGG
CATTGCGGCGGATTTTTGAAAAATGA
CATTGCGGCGGATTTCAGAAAAATGG
CATTGCGGCGGATTTTCGAAAAATGG
CATTGCGGCGGATTTTCGAAAAATGA
CATTGCGGCGGATTTCAGAAAAATGA
CATTGCGGCGGATTTTCGAAAAATGG
CATTGCGGCGGATTTTTGAAAAATGA
CATTGCGGCGGATTTCAGAAAAATGG
CATTGCGGCGGATTTTTGAAAAATGG
CATTGCGGCGGATTTTTGAAAAATGA
CATTGCGGCGGATTTCAGAAAAATGA
CATTGCGGCGGATTTTGAAAAATGG
CATTGCGGCGGATTTTTGAAAAATGA
CATTGCGGCGGATTTCAGAAAAATGG
CATTGCGGCGGATTTCAGAAAAATGA
CATTGCGGCGGATTTTGAAAAATGG
CATTGCGGCGGATTTCAGAAAAATGG
CATTGCGGCGGATTTCAGAAAAATGG
CATTGCGGCGGATTTTTGAAAAATGA
CATTGCGGCGGATTTCAGAAAAATGG
CATTGCGGCGGATTTTCGAAAAATGG
CATTGCGGCGGATTTTCGAAAAATGA
CATTGCGGCGGATTTCAGAAAAATGA
CATTGCGGCGGATTTTCGAAAAATGG
CATTGCGGCGGATTTTTGAAAAATGA
CATTGCGGCGGATTTCAGAAAAATGG
CATTGCGGCGGATTTCAGAAAAATGG
CATTGCGGCGGATTTTTGAAAAATGA
CATTGCGGCGGATTTCAGAAAAATGG
CATTGCGGCGGATTTTCGAAAAATGG
CATTGCGGCGGATTTTCGAAAAATGA
CATTGCGGCGGATTTCAGAAAAATGA
CATTGCGGCGGATTTTCGAAAAATGG
CATTGCGGCGGATTTTTGAAAAATGA
CATTGCGGCGGATTTCAGAAAAATGG
CATTGCGGCGGATTTCAGAAAAATGG
CATTGCGGCGGATTTCAGAAAAATGG
CATTGCGGCGGATTTTCGAAAAATGG
CATTGCGGCGGATTTTTGAAAAATGG
CATTGCGGCGGATTTCAGAAAAATGG
CATTGCGGCGGATTTTCGAAAAATGG
CATTGCGGCGGATTTTCGAAAAATGG
CATTGCGGCGGATTTTTGAAAAATGA
CATTGCGGCGGATTTCAGAAAAATGA
CATTGCGGCGGATTTTTGAAAAATGG
CATTGCGGCGGATTTTTGAAAAATGA
CATTGCGGCGGATTTCAGAAAAATGG
CATTGCGGCGGATTTTTGAAAAATGG
CATTGCGGCGGATTTTTGAAAAATGA
CATTGCGGCGGATTTCAGAAAAATGA
CATTGCGGCGGATTTTTGAAAAATGG

CATTGCGGCGGATTTTTGAAAAATGA
CATTGCGGCGGATTTCAGAAAAATGG
CATTGCGGCGGATTTCAGAAAAATGG
CATTGCGGCGGATTTCAGAAAAATGG
CATTGCGGCGGATTTCAGAAAAATGA
CATTGCGGCGGATTTTTGAAAAATGG
CATTGCGGCGGATTTCAGAAAAATGG
CATTGCGGCGGATTTTTGAAAAATGA
CATTGCGGCGGATTTTTGAAAAATGA
CATTGCGGCGGATTTCAGAAAAATGG
CATTGCGGCGGATTTTTGAAAAATGG
CATTGCGGCGGATTTTTGAAAAATGA
CATTGCGGCGGATTTCAGAAAAATGA
CATTGCGGCGGATTTTTGAAAAATGG
CATTGCGGCGGATTTTTGAAAAATGA
CATTGCGGCGGATTTCAGAAAAATGG
CATTGCGGCGGATTTCAGAAAAATGG
CATTGCGGCGGATTTCAGAAAAATGG
CATTGCGGCGGATTTCAGAAAAATGG
CATTGCGGCGGATTTCAGAAAAATGG
CATTGCGGCGGATTTTTGAAAAATGA
CATTGCGGCGGATTTTTGAAAAATGA
CATTGCGGCGGATTTCAGAAAAATGG
CATTGCGGCGGATTTTTGAAAAATGA
CATTGCGGCGGATTTCAGAAAAATGG
CATTGCGGCGGATTTCAGAAAAATGG
CATTGCGGCGGATTTCAGAAAAATGG
CATTGCGGCGGATTTCAGAAAAATGG
CATTGCGGCGGATTTCAGAAAAATGG
CATTGCGGCGGATTTCAGAAAAATGG
CATTGCGGCGGATTTTTGAAAAATGA
CATTGCGGCGGATTTTTGAAAAATGA
CATTGCGGCGGATTTCAGAAAAATGG
CATTGCGGCGGATTTTTGAAAATGG
CATTGCGGCGGATTTCAGAAAAATGA
CATTGCGGCGGATTTTTGAAAAATGA
CATTGCGGCGGATTTTTGAAAAATGG
CATTGCGGCGGATTTCAGAAAAATGA
CATTGCGGCGGATTTTTGAAAAATGG
CATTGCGGCGGATTTTTGAAAAATGA
CATTGCGGCGGATTTCAGAAAAATGG
CATTGCGGCGGATTTCAGAAAAATGA
CATTGCGGCGGATTTTTGAAAAATGA
CATTGCGGCGGATTTTTGAAAAATGG
CATTGCGGCGGATTTTTGAAAAATGG
CATTGCGGCGGATTTTTGAAAAATGA
CATTGCGGCGGATTTTTGAAAAATGA
CATTGCGGCGGATTTCAGAAAAATGG
CATTGCGGCGGATTTCAGAAAAATGA
CATTGCGGCGGATTTTTGAAAAATGA
CATTGCGGCGGATTTTTGAAAAATGG
CATTGCGGCGGATTTTTGAAAAATGA
CATTGCGGCGGATTTTTGAAAAATGA
CATTGCGGCGGATTTTTGAAAAATGA

CATTGCGGCGGATTTTTGAAAAATGA
CATTGCGGCGGATTTCAGAAAAATGA
CATTGCGGCGGATTTTTGAAAAATGG
CATTGCGGCGGATTTTTGAAAAATGA
CATTGCGGCGGATTTTGAAAAATGG
CATTGCGGCGGATTTCAGAAAAATGG
CATTGCGGCGGATTTTTGAAAAATGG
CATTGCGGCGGATTTTTGAAAAATGA
CATTGCGGCGGATTTCAGAAAAATGA
CATTGCGGCGGATTTTTGAAAAATGA
CATTGCGGCGGATTTTTGAAAAATGG
CATTGCGGCGGATTTTTGAAAAATGA
CATTGCGGCGGATTTTTGAAAAATGA
CATTGCGGCGGATTTCAGAAAAATGG
CATTGCGGCGGATTTCAGAAAAATGG
CATTGCGGCGGATTTTTGAAAAATGG
CATTGCGGCGGATTTTTGAAAAATGA
CATTGCGGCGGATTTTTGAAAAATGA
CATTGCGGCGGATTTTTGAAAAATGG
CATTGCGGCGGATTTTTGAAAAATGA
CATTGCGGCGGATTTCAGAAAAATGA
CATTGCGGCGGATTTTTGAAAAATGG
CATTGCGGCGGATTTTTGAAAAATGA
CATTGCGGCGGATTTTTGAAAAATGG
CATTGCGGCGGATTTTTGAAAAATGA
CATTGCGGCGGATTTTTGAAAAATGA
CATTGCGGCGGATTTCAGAAAAATGG
CATTGCGGCGGATTTTTGAAAAATGG
CATTGCGGCGGATTTTTGAAAAATGA
CATTGCGGCGGATTTCAGAAAAATGA
CATTGCGGCGGATTTTTGAAAAATGG
CATTGCGGCGGATTTTTGAAAAATGG
CATTGCGGCGGATTTCAGAAAAATGG
CATTGCGGCGGATTTCAGAAAAATGG
CATTGCGGCGGATTTTTGAAAAATGA
CATTGCGGCGGATTTCAGAAAAATGG
CATTGCGGCGGATTTCAGAAAAATGG
CATTGCGGCGGATTTCAGAAAAATGG
CATTGCGGCGGATTTTTGAAAAATGG
CATTGCGGCGGATTTCAGAAAAATGA
CATTGCGGCGGATTTTAGAAAAATGG
CATTGCGGCGGATTTTAGAAAAATGG
CATTGCGGCGGATTTTTGAAAAATGG
CATTGCGGCGGATTTTTGAAAAATGA
CATTGCGGCGGATTTCAGAAAAATGG
CATTGCGGCGGATTTCAGAAAAATGG
CATTGCGGCGGATTTTCGAAAAATGG
CATTGCGGCGGATTTTCGAAAAATGG
CATTGCGGCGGATTTTTGAAAAATGG
CATTGCGGCGGATTTTTGAAAAATGA
CATTGCGGCGGATTTCAGAAAAATGG
CATTGCGGCGGATTTCAGAAAAATGG
CATTGCGGCGGATTTCAGAAAAATGG
CATTGCGGCGGATTTTTGAAAAATGG

CATTGCGGCGGATTTCAGAAAAATGA
CATTGCGGCGGATTTTTGAAAAATGG
CATTGCGGCGGATTTCAGAAAAATGA
CATTGCGGCGGATTTCAGAAAAATGG
CATTGCGGCGGATTTTTGAAAAATGA
CATTGCGGCGGATTTCAGAAAAATGG
CATTGCGGCGGATTTCAGAAAAATGA
CATTGCGGCGGATTTTGAAAAATGG
CATTGCGGCGGATTTTTGAAAAATGA
CATTGCGGCGGATTTCAGAAAAATGG
CATTGCGGCGGATTTCAGAAAAATGG
CATTGCGGCGGATTTCAGAAAAATGG
CATTGCGGCGGATTTCAGAAAAATGG
CATTGCGGCGGATTTTTGAAAAATGA
CATTGCGGCGGATTTTTGAAAAATGG
CATTGCGGCGGATTTTTGAAAAATGA
CATTGCGGCGGATTTCAGAAAAATGG
CATTGCGGCGGATTTTCGAAAAATGG
CATTGCGGCGGATTTTCGAAAAATGG
CATTGCGGCGGATTTTTGAAAAATGA
CATTGCGGCGGATTTTTGAAAAATGA
CATTGCGGCGGATTTTTGAAAAATGA
CATTGCGGCGGATTTTTGAAAAATGG
CATTGCGGCGGATTTTCGAAAAATGG
CATTGCGGCGGATTTTCGAAAAATGG
CATTGCGGCGGATTTTCGAAAAATGG
CATTGCGGCGGATTTTCGAAAAATGG
CATTGCGGCGGATTTTTGAAAAATGA
CATTGCGGCGGATTTTTGAAAAATGA
CATTGCGGCGGATTTTTGAAAAATGA
CATTGCGGCGGATTTTTGAAAAATGA
CATTGCGGCGGATTTTTGAAAAATGA
CATTGCGGCGGATTTTTGAAAAATGA
CATTGCGGCGGATTTTTGAAAAATGA
CATTGCGGCGGATTTCAGAAAAATGG
CATTGCGGCGGATTTTTGAAAAATGA
CATTGCGGCGGATTTCAGAAAAATGA
CATTGCGGCGGATTTTTGAAAAATGG
CATTGCGGCGGATTTTTGAAAAATGA
CATTGCGGCGGATTTCAGAAAAATGG
CATTGCGGCGGATTTCAGAAAAATGG
CATTGCGGCGGATTTTTGAAAAATGA
Nr of selected repeats 261 Similarity 0.919236
Consensus:
CATTGCGGCGGATTTtnGAAAAATGg
>Cele-UNSB01_4:13061036-13070540 Satlength=9505 Nr of Repeats=198
RepeatLength=48 seed=ACGGGCAAAT
ACGGGCAAATCAAAAATTAAATTGCTCATCTTCAGAAAATTATAAATT
ACGGGCAAATCAAAAATTAAATTGCTCATCTTCAGAAAAATATAAATT
ACGGGCAAATCAAAAATTAAATTGCTCATCTTCAGAAAAATATAAATT
ACGGGCAAATCAAAAATTAAATTGCTCATCTTCAGAAAATTATAAATT
ACGGGCAAATCAAAAATTAAATTGCTCATCTTCAGAAAATTATAAATT
ACGGGCAAATCAAAAATTAATTTGCTCATCTTCAGAAAATTATAAATT
ACGGGCAAATCAAAAATTAAATTGCTCATCTTCAGAAAAATATAAATT

ACGGGCAAATCAAAAATTAAATTGCTCATCTTCAGAAAATTATAAATT
ACGGGCAAATCAAAAATTAAATTGCTCATCTTCAGAAAAATATAAATT
ACGGGCAAATCAAAAATTAAATTGCTCATCTTCAGAAAATTATAAATT
ACGGGCAAATCAAAAATTAAATTGCTCATCTTCAGAAAATTATAAATT
ACGGGCAAATCAAAAATTAAATTGCTCATCTTCAGAAAAATATAAATT
ACGGGCAAATCAAAAATTAAATTGCTCATCTTCAGCAAATTATAAATC
ACGGGCAAATCAAAAATTAAATTGCTCATCTTCAGAAAAATATAAATT
ACGGGCAAATCAAAAATTAAATTGCTCATCTTCAGAAAATTATAAATT
ACGGGCAAATCAAAAATTAAATTGCTCATCTTCAGCAAATTATAAATT
ACGGGCAAATCATAAATTAAATTGCTCATCTTCAGAAAAATATAAATT
ACGGGCAAATCAAAAATTAAATTGCTCATCTTCAGAAAAATATAAATT
ACGGGCAAATCATAAATTAAATTGCTCATCTTCAGAAAAATATAAATT
ACGGGCAAATCAAAAATTAAATTGCTCATCTTCAGAAAAATATAAATT
ACGGGCAAATCAAAAATTAAATTGCTCATCTTCAGAAAATTATAAATT
ACGGGCAAATCAAAAATTAAATTGCTCATCTTCAGAAAAATATAAATT
ACGGGCAAATCAAAAATTAATTTGCTCATCTTCAGAAAATTATAAATT
ACGGGCAAATCATAAATTAAATTGCTCATCTTCAGAAAATTATAAATT
ACGGGCAAATCAAAAATTAAATTGCTCATCTTCAGAAAATTATAAATT
ACGGGCAAATCAAAAATTAAATTGCTCATCTTCAGAAAAATATAAATT
ACGGGCAAATCAAAAATTAATTTGCTCATCTTCAGAAAATTATAAATT
ACGGGCAAATCAAAAATTAAATTGCTCATCTTCAGAAAAATATAAATT
ACGGGCAAATCAAAAATTAATTTGCTCATCTTCAGAAAATTATAAATT
ACGGGCAAATCATAAATTAAATTGCTCATCTTCAGAAAAATATAAATT
ACGGGCAAATCAAAAATTAAATTGCTCATCTTCAGCAAATTATAAATC
ACGGGCAAATCAAAAATTAAATTGCTCATCTTCAGAAAAATATAAATT
ACGGGCAAATCAAAAATTAATTTGCTCATCTTCAGAAAATTATAAATT
ACGGGCAAATCAAAAATTAAATTGCTCATCTTCAGAAAAATATAAATT
ACGGGCAAATCAAAAATTAATTTGCTCATCTTCAGAAAATTATAAATT
ACGGGCAAATCAAAAATTAAATTGCTCATCTTCAGAAAAATATAAATT
ACGGGCAAATCAAAAATTAATTTGCTCATCTTCAGAAAAATATAAATT
ACGGGCAAATCAAAAATTAAATTGCTCATCTTCAGAAAAATATAAATT
ACGGGCAAATCAAAAATTAAATTGCTCATCTTCAGAAAATTATAAATT
ACGGGCAAATCAAAAATTAAATTGCTCATCTTCAGAAAATTATAAATT
ACGGGCAAATCAAAAATTAAATTGCTCATCTTCAGAAAATTATAAATT
ACGGGCAAATCAAAAATTAAATTGCTCATCTTCAGAAAAATATAAATT
ACGGGCAAATCAAAAATTAAATTGCTCATCTTCAGAAAAATATAAATT
ACGGGCAAATCAAAAATTAAATTGCTCATCTTCAGAAAAATATAAATT
ACGGGCAAATCAAAAATTAAATTGCTCATCTTCAGAAAATTATAAATT
ACGGGCAAATCAAAAATTAATTTGCTCATCTTCAGAAAAATATAAATT
ACGGGCAAATCAAAAATTAAATTGCTCATCTTCAGAAAAATATAAATT
ACGGGCAAATCAAAAATTAAATTGCTCATCTTCAGAAAATTATAAATT
ACGGGCAAATCAAAAATTAAATTGCTCATCTTCAGAAAATTATAAATC
ACGGGCAAATCAAAAATTAAATTGCTCATCTTCAGAAAAATATAAATT
ACGGGCAAATCAAAAATTAAATTGCTCATCTTCAGAAAAATATAAATT
ACGGGCAAATCAAAAATTAAATTGCTCATCTTCAGAAAAATATAAATT
ACGGGCAAATCAAAAATTTAATTGCTCATCTTCAGAAAATTATAAATT
ACGGGCAAATCAAAAATTAAATTGCTCATCTTCAGAAAATTATAAATT
ACGGGCAAATCAAAAATTAAATTGCTCATCTTCAGAAAAATATAAATT
ACGGGCAAATCAAAAATTAAATTGCTCATCTTCAGAAAAATATAAATT
ACGGGCAAATCAAAAATTAAATTGCTCATCTTCAGAAAATTATAAATT
ACGGGCAAATCAAAAATTAAATTGCTCATCTTCAGCAAATTATAAATC
ACGGGCAAATCAAAAATTAAATTGCTCATCTTCAGAAAAATATAAATT
ACGGGCAAATCAAAAATTAATTTGCTCATCTTCAGAAAAATATAAATT
ACGGGCAAATCAAAAATTAAATTGCTCATCTTCAGAAAAATATAAATT

ACGGGCAAATCAAAAATTAAATTGCTCATCTTCAGAAAAATATAAATT
ACGGGCAAATCAAAAATTTAATTGCTCATCTTCAGAAAATTATAAATT
ACGGGCAAATCAAAAATTAAATTGCTCATCTTCAGAAAAATATAAATT
ACGGGCAAATCAAAAATTAAATTGCTCATCTTCAGAAAAATATAAATT
ACGGGCAAATCAAAAATTAAATTGCTCATCTTCAGAAAATTATAAATT
ACGGGCAAATCAAAAATTAAATTGCTCATCTTCAGAAAAATATAAATT
ACGGGCAAATCAAAAATTAAATTGCTCATCTTCAGAAAATTATAAATT
ACGGGCAAATCAAAAATTAAATTGCTCATCTTCAGAAAAATATAAATT
ACGGGCAAATCAAAAATTAAATTGCTCATCTTCAGAAAATTATAAATT
ACGGGCAAATCAAAAATTAAATTGCTCATCTTCAGAAAATTATAAATT
ACGGGCAAATCAAAAATTAAATTGCTCATCTTCAGAAAATTATAAATT
ACGGGCAAATCAAAAATTAAATTGCTCATCTTCAGCAAATTATAAATC
ACGGGCAAATCAAAAATTAAATTGCTCATCTTCAGAAAAATATAAATT
ACGGGCAAATCAAAAATTAAATTGCTCATCTTCAGCAAATTATAAATC
ACGGGCAAATCAAAAATTAAATTGCTCATCTTCAGAAAAATATAAATT
ACGGGCAAATCAAAAATTAAATTGCTCATCTTCAGAAAATTATAAATT
ACGGGCAAATCAAAAATTAAATTGCTCATCTTCAGCAAATTATAAATC
ACGGGCAAATCAAAAATTTAATTGCTCATCTTCAGAAAATTATAAATT
ACGGGCAAATCAAAAATTAAATTGCTCATCTTCAGAAAATTATAAATT
ACGGGCAAATCAAAAATTTAATTGCTCATCTTCAGAAAATTATAAATT
ACGGGCAAATCAAAAATTAAATTGCTCATCTTCAGAAAATTATAAATT
ACGGGCAAATCAAAAATTAAATTGCTCATCTTCAGAAAATTATAAATT
ACGGGCAAATCAAAAATTTAATTGCTCATCTTCAGAAAAATATAAATT
ACGGGCAAATCAAAAATTAAATTGCTCATCTTCAGAAAATTATAAATT
ACGGGCAAATCAAAAATTAAATTGTTCATCTTCAGAAAATTATAAATT
ACGGGCAAATCAAAAATTAAATTGCTCATCTTCAGAAAAATATAAATT
ACGGGCAAATCAAAAATTAAATTGCTCATCTTCAGAAAAATATAAATT
ACGGGCAAATCAAAAATTAAATTGCTCATCTTCAGAAAAATATAAATT
ACGGGCAAATCAAAAATTAAATTGCTCATCTTCAGAAAAATATAAATT
ACGGGCAAATCAAAAATTAAATTGCTCATCTTCAGAAAATTATAAATT
ACGGGCAAATCAAAAATTAAATTGTTCATCTTCAGAAAATTATAAATT
ACGGGCAAATCAAAAATTAAATTGCTCATCTTCAGAAAAATATAAATT
ACGGGCAAATCAAAAATTAAATTGCTCATCTTCAGAAAAATATAAATT
ACGGGCAAATCAAAAATTTAATTGCTCATCTTCAGAAAATTATAAATT
ACGGGCAAATCAAAAATTAAATTGCTCATCTCCAGAAAATTATAAATT
ACGGGCAAATCAAAAATTTAATTGCTCATCTTCAGAAAAATATAAATT
ACGGGCAAATCAAAAATTAAATTGCTCATCTTCAGAAAATTATAAATT
ACGGGCAAATCAAAAATTAAATTGTTCATCTTCAGAAAATTATAAATT
ACGGGCAAATCAAAAATTAAATTGCTCATCTTCAGAAAAATATAAATT
ACGGGCAAATCAAAAATTAAATTGCTCATCTTCAGAAAAATATAAATT
ACGGGCAAATCAAAAATTAAATTGCTCATCTTCAGAAAAATATAAATT
ACGGGCAAATCAAAAATTAAATTGCTCATCTTCAGAAAATTATAAATT
ACGGGCAAATCAAAAATTAAATTGCTCATCTTCAGAAAATTATAAATC
ACGGGCAAATCAAAAATTAAATTGCTCATCTTCAGAAAAATATAAATT
ACGGGCAAATCAAAAATTAAATTGCTCATCTTCAGAAAAATATAAATT
ACGGGCAAATCAAAAATTAAATTGCTCATCTTCAGAAAAATATAAATT
ACGGGCAAATCAAAAATTTAATTGCTCATCTTCAGAAAATTATAAATT
ACGGGCAAATCAAAAATTTAATTGCTCATCTTCAGAAAATTATAAATT
ACGGGCAAATCAAAAATTTAATTGCTCATCTTCAGAAAATTATAAATT
ACGGGCAAATCAAAAATTAAATTGCTCATCTTCAGAAAAATATAAATT
ACGGGCAAATCAAAAATTAAATTGCTCATCTTCAGCAAATTATAAATT
ACGGGCAAATCATAAATTAAATTGCTCATCTTCAGAAAATTATAAATT
ACGGGCAAATCAAAAATTAAATTGCTCATCTTCAGAAAAATATAAATT

ACGGGCAAATCAAAAATTAAATTGCTCATCTTCAGAAAAATATAAATT
ACGGGCAAATCAAAAATTTAATTGCTCATCTTCAGAAAATTATAAATT
ACGGGCAAATCAAAAATTTAATTGCTCATCTTCAGAAAATTATAAATT
ACGGGCAAATCAAAAATTTAATTGCTCATCTTCAGAAAATTATAAATT
ACGGGCAAATCAAAAATTAAATTGCTCATCTTCAGAAAAATATAAATT
ACGGGCAAATCAAAAATTAAATTGCTCATCTTCAGCAAATTATAAATT
ACGGGCAAATCATAAATTAAATTGCTCATCTTCAGAAAATTATAAATT
ACGGGCAAATCAAAAATTAAATTGCTCATCTTCAGAAAATTATAAATT
ACGGGCAAATCAAAAATTTAATTGCTCATCTTCAGAAAAATATAAATT
ACGGGCAAATCAAAAATTAAATTGCTCATCTTCAGAAAATTATAAATT
ACGGGCAAATCAAAAATTAAATTGTTCATCTTCAGAAAATTATAAATT
ACGGGCAAATCAAAAATTAAATTGCTCATCTTCAGAAAAATATAAATT
ACGGGCAAATCAAAAATTAAATTGCTCATCTTCAGAAAAATATAAATT
ACGGGCAAATCAAAAATTAAATTGCTCATCTTCAGAAAAATATAAATT
ACGGGCAAATCAAAAATTAAATTGCTCATCTTCAGAAAAATATAAATT
ACGGGCAAATCAAAAATTTAATTGCTCATCTTCAGAAAATTATAAATT
ACGGGCAAATCAAAAATTAAATTGCTCATCTCCAGAAAATTATAAATT
ACGGGCAAATCAAAAATTTAATTGCTCATCTTCAGAAAAATATAAATT
ACGGGCAAATCAAAAATTAAATTGCTCATCTTCAGAAAATTATAAATT
ACGGGCAAATCAAAAATTAAATTGTTCATCTTCAGAAAATTATAAATT
ACGGGCAAATCAAAAATTAAATTGCTCATCTTCAGAAAAATATAAATT
ACGGGCAAATCAAAAATTAAATTGCTCATCTTCAGAAAAATATAAATT
ACGGGCAAATCAAAAATTAAATTGCTCATCTTCAGAAAATTATAAATT
ACGGGCAAATCAAAAATTAAATTGCTCATCTTCAGAAAAATATAAATT
ACGGGCAAATCAAAAATTAAATTGCTCATCTTCAGAAAAATATAAATT
ACGGGCAAATCAAAAATTAAATTGCTCATCTTCAGAAAAATATAAATT
ACGGGCAAATCAAAAATTAAATTGCTCATCTTCAGAAAAATATAAATT
ACGGGCAAATCAAAAATTAAATTGCTCATCTTCAGAAAAATATAAATT
ACGGGCAAATCAAAAATTAAATTGTTCATCTTCAGAAAATTATAAATC
ACGGGCAAATCAAAAATTAAATTGCTCATCTTCAGAAAAATATAAATT
ACGGGCAAATCAAAAATTAAATTGCTCATCTTCAGAAAAATATAAATT
ACGGGCAAATCAAAAATTAAATTGCTCATCTTCAGAAAAATATAAATT
ACGGGCAAATCAAAAATTTAATTGCTCATCTTCAGAAAATTATAAATT
ACGGGCAAATCAAAAATTTAATTGCTCATCTTCAGAAAATTATAAATT
ACGGGCAAATCAAAAATTTAATTGCTCATCTTCAGAAAATTATAAATT
ACGGGCAAATCAAAAATTAAATTGCTCATCTTCAGAAAAATATAAATT
ACGGGCAAATCAAAAATTAAATTGCTCATCTTCAGCAAATTATAAATT
ACGGGCAAATCATAAATTAAATTGCTCATCTTCAGAAAATTATAAATT
ACGGGCAAATCAAAAATTAAATTGCTCATCTTCAGAAAATTATAAATT
ACGGGCAAATCAAAAATTAAATTGCTCATCTTCAGAAAAATATAAATT
ACGGGCAAATCAAAAATTAAATTGCTCATCTTCAGAAAAATATAAATT
ACGGGCAAATCAAAAATTAAATTGCTCATCTTCAGAAAATTATAAATT
ACGGGCAAATCAAAAATTAAATTGCTCATCTTCAGAAAATTATAAATT
ACGGGCAAATCAAAAATTAAATTGCTCATCTTCAGCAAATTATAAATC
ACGGGCAAATCAAAAATTAAATTGCTCATCTTCAGAAAAATATAAATT
ACGGGCAAATCAAAAATTAAATTGCTCATCTTCAGAAAATTATAAATT
ACGGGCAAATCAAAAATTTAATTGCTCATCTTCAGAAAATTATAAATT
ACGGGCAAATCAAAAATTAAATTGCTCATCTTCAGAAAAATATAAATT
ACGGGCAAATCAAAAATTAAATTGCTCATCTTCAGAAAATTATAAATT
ACGGGCAAATCAAAAATTAAATTGCTCATCTTCAGAAAATTATAAATT
ACGGGCAAATCAAAAATTAAATTGCTCATCTTCAGCAAATTATAAATC
ACGGGCAAATCAAAAATTAAATTGCTCATCTTCAGAAAAATATAAATT
ACGGGCAAATCAAAAATTAAATTGCTCATCTTCAGAAAATTATAAATT
ACGGGCAAATCAAAAATTAAATTGCTCATCTTCAGAAAAATATAAATT

ACGGGCAAATCAAAAATTAAATTGCTCATCTTCAGAAAAATATAAATT
ACGGGCAAATCAAAAATTAAATTGCTCATCTTCAGAAAATTATAAATT
ACGGGCAAATCAAAAATTAAATTGCTCATCTTCAGAAAAATATAAATT
ACGGGCAAATCAAAAATTAAATTGCTCATCTTCAGAAAAATATAAATT
ACGGGCAAATCAAAAATTAAATTGCTCATCTTCAGAAAATTATAAATT
ACGGGCAAATCAAAAATTAAATTGCTCATCTTCAGAAAAATATAAATT
ACGGGCAAATCAAAAATTAAATTGCTCATCTTCAGAAAAATATAAATT
ACGGGCAAATCAAAAATTAAATTGCTCATCTTCAGAAAAATATAAATT
ACGGGCAAATCAAAAATTAAATTGCTCATCTTCAGAAAATTATAAATT
ACGGGCAAATCAAAAATTAAATTGCTCATCTTCAGAAAAATATAAATT
ACGGGCAAATCAAAAATTAAATTGCTCATCTTCAGAAAAATATAAATT
ACGGGCAAATCAAAAATTAAATTGCTCATCTTCAGCAAATTATAAATC
ACGGGCAAATCAAAAATTAAATTGCTCATCTTCAGAAAATTATAAATT
ACGGGCAAATCAAAAATTAAATTGCTCATCTTCAGAAAAATATAAATT
ACGGGCAAATCAAAAATTTAATTGCTCATCTTCAGAAAATTATAAATT
ACGGGCAAATCAAAAATTTAATTGCTCATCTTCAGAAAATTATAAATT
ACGGGCAAATCAAAAATTAAATTGCTCATCTTCAGAAAATTATAAATT
ACGGGCAAATCAAAAATTTAATTGCTCATCTTCAGAAAATTATAAATT
ACGGGCAAATCAAAAATTAAATTGCTCATCTTCAGAAAATTATAAATT
ACGGGCAAATCAAAAATTAAATTGCTCATCTTCAGAAAAATATAAATT
ACGGGCAAATCAAAAATTAAATTGCTCATCTTCAGAAAATTATAAATT
ACGGGCAAATCAAAAATTAAATTGCTCATCTTCAGAAAATTATAAATT
ACGGGCAAATCAAAAATTAAATTGCTCATCTTCAGAAAAATATAAATT
ACGGGCAAATCAAAAATTAAATTGCTCATCTTCAGAAAAATATAAATT
ACGGGCAAATCAAAAATTAAATTGCTCATCTTCAGAAAAATATAAATT
ACGGGCAAATCAAAAATTAAATTGCTCATCTTCAGCAAATTATAAATC
ACGGGCAAATCAAAAATTAAATTGCTCATCTTCAGAAAATTATAAATT
ACGGGCAAATCAAAAATTAAATTGCTCATCTTCAGCAAATTATAAATC
ACGGGCAAATCAAAAATTTAATTGCTCATCTTCAGAAAATTATAAATT
Nr of selected repeats 198 Similarity 0.966024
Consensus:
ACGGGCAAATCAAAAATTAAATTGCTCATCTTCAGAAAAtTATAAATT
>Cele-UNSB01_4:13110533-13113073 Satlength=2541 Nr of Repeats=14
RepeatLength=168 seed=TTTTTAAAAA
TTTTTAAAAATATTATTGGTAAGTTTGTGGGCCAGGAAAGTAAATTTAAGCCGCGCTAGAGAGGTTTTTGTGTCGAGTT
CCAATTTTCACAGGTACTAACTAGTAGGCGCAAAAAAACAAAAATAAAAATTATCGAATTCCGGAAATGTTTTGATAGA
TTTGGATTCTAGAT
TTTTTAAAAATATTATTGGTAAGTTTGTGGGCCAGGAAAGTAAATTTAAGCCGTCAAACTAACTCTAAAGCATGATAAT
TTTTATAGGCTTTTCGTTGATTTTCTCGATTTTTATCTGCAAATTATAAAAAATGTGAAAAAGAAAAATATGTTGTGCG
GAAAGCTCACAAAATCACGCAAAATAAGCGGTAATCGTGAAAAAAACGAGAAAAATTGCAAAAAATGGAAAAAAAATTG
AATTTTTTTTTGTTTTTTTGCGCCTACTAGTACCTGTGAAAATTGGAACTCGACACAAAAACCTCTCTAGCGCGGCTTA
AATTTACTTTCCTGGCCCACAAACTTACCAATAATA
TTTTTAAAAAATCTAGAATCCAAATCTATCAAAACATTTCCGGAATTCGATAATTTTTATTTTTGTTTTTTTGAGCCTA
CTAGTACCTGTGAAAATTGGAACTCGACACAAAAACCTCTCTAGCGCGGCTTAAATTTACTTTCCTGGCCCACAAACTT
ACCAATAATA
TTTTTAAAAAATCTAGAATCCAAATCTATCAAAACATTTCCGGAATTCGATAATTTTTATTTTTGTTTTTTTGAGCCTA
CTAGTACCTGTGAAAATTGGAACTCGACACAAAAACCTCTCTAGCGCGGCTTAAATTTACTTTCCTGGCCCACAAACTT
ACCAATAATA
TTTTTAAAAAATCTAGAATCCAAATCTATCAAAACATTTCCGGAATTCGATAATTTTTATTTTTGTTTTTTTGCGCCTA
CTAGTACCTGTGAAAATTGGAACTCGACACAAAAACCTCTCTAGCGCGGCTTAAATTTACTTTCCTGGCCCACAAACTT
ACCAATAATA
TTTTTAAAAAATCTAGAATCCAAATCTATCAAAACATTTCCGGAATTCGATAATTTTTATTTTTGTTTTTTTGAGCCTA
CTAGTACCTGTGAAAATTGGAACTCGACACAAAAACCTCTCTAGCGCGGCTTAAATTTACTTTCCTGGCCCACAAACTT
ACCAATAATA

TTTTTAAAAAATCTAGAATCCAAATCTATCAAAACATTTCCGGAATTCGATAATTTTTATTTTTGTTTTTTTGCGCCTA
CTAGTACCTGTGAAAATTGGAACTCGACACAAAAACCTCTCTAGCGCGGCTTAAATTTACTTTCCTGGCCCACAAACTT
ACCAATAATA
TTTTTAAAAAATCTAGAATCCAAATCTATCAAAACATTTCCGGAATTCGATAATTTTTATTTTTGTTTTTTTGCGCCTA
CTAGTACCTGTGAAAATTGGAACTCGACACAAAAACCTCTCTAGCGCGGCTTAAATTTACTTTCCTGGCCCACAAACTT
ACCAATAATA
TTTTTAAAAAATCTAGAATCCAAATCTATCAAAACATTTCCGGAATTCGATAATTTTTATTTTTGTTTTTTTGAGCCTA
CTAGTACCTGTGAAAATTGGAACTCGACACAAAAACCTCTCTAGCGCGGCTTAAATTTACTTTCCTGGCCCACAAACTT
ACCAATAATA
TTTTTAAAAAATCTAGAATCCAAATCTATCAAAACATTTCCGGAATTCGATAATTTTTATTTTTGTTTTTTTGAGCCTA
CTAGTACCTGTGAAAATTGGAACTCGACACAAAAACCTCTCTAGCGCGGCTTAAATTTACTTTCCTGGCCCACAAACTT
ACCAATAATA
TTTTTAAAAAATCTAGAATCCAAATCTATCAAAACATTTCCGGAATTCGATAATTTTTATTTTTGTTTTTTTGCGCCTA
CTAGTACCTGTGAAAATTGGAACTCGACACAAAAACCTCTCTAGCGCGGCTTAAATTTACTTTCCTGGCCCACAAACTT
ACCAATAATA
TTTTTAAAAAATCTAGAATCCAAATCTATCAAAACATTTCCGGAATTCGATAATTTTTATTTTTGTTTTTTTGAGCCTA
CTAGTACCTGTGAAAATTGGAACTCGACACAAAAACCTCTCTAGCGCGGCTTAAATTTACTTTCCTGGCCCACAAACTT
ACCAATAATA
TTTTTAAAAAATCTAGAATCCAAATCTATCAAAACATTTCCGGAATTCGATAATTTTTATTTTTGTTTTTTTGCGCCTA
CTAGTACCTGTGAAAATTGGAACTCGACACAAAAACCTCTCTAGCGCGGCTTAAATTTACTTTCCTGGCCCACAAACTT
ACCAATAATA
TTTTTAAAAAATCTAGAATCCAAATCTATCAAAACATTTCCGGAATTCGATAATTTTTATTTTTGTTTTTTTGAGCCTA
CTAGTACCTGTGAAAATTGGAACTCGACACAAAAACCTCTCTAGCGCGGCTTAAATTTACTTTCCGTGCCCACAAACTT
ACCAATAATA
Nr of selected repeats 12 Similarity 0.993146
Consensus:
TTTTTAAAAAATCTAGAATCCAAATCTATCAAAACATTTCCGGAATTCGATAATTTTTATTTTTGTTTTTTTGaGCCTA
CTAGTACCTGTGAAAATTGGAACTCGACACAAAAACCTCTCTAGCGCGGCTTAAATTTACTTTCCTGGCCCACAAACTT
ACCAATAATA
>Cele-UNSB01_4:13187743-13188346 Satlength=604 Nr of Repeats=12 RepeatLength=12
seed=GCTTGGGTTT
GCTTGGGTTTAG
GCTTGTGTTTAGGATTGGATTCAG
GCTTGGGTTTAGTTTTTTTTGTGCTTGCCTACCACATATTTAGTTAATTTTAGCAGAAGCTCTGGTTTTCATTTAGGCTT
GCTTTTGCCAAGCAAATATGCCAATTTTCCTCGAGTCTACATACTATGCTAATTGGAAAATTTTTCAAAAAGTAACTTT
GCGTGAAACCCAAAATAAACTTACACGGTCATCCACGTGGTATGAAATAAAAATAAATTGCCTTTGTTCAATTATTTTA
TTACTTTCAATTCTTCTCACTTTTTGCACATTTCGTTAACCGTCTCACAATCAGGAATCCAATGCCAGATAAGTTATTT
GTACACAATTAAGCTTCAATAAGAGAGAATTCTCAGGTTTAATCTTAG
GCTAGGGTTTAGGTTGCTATAGGATTTTAGATTTGCTATGGGCTTAAGCTTTTGTTTATGATTGGTGTTAGGCTAAGGT
TCAAGATAGGGTTGGATTTAGGCTTGAG
GCTTGGGGTTAG
GCTTGGGTTTAA
GTTTGGGTTTAG
GCTTGGGTTTAA
GCTTGGGTTTAG
GCTTGGGTTTAG
GCTTGGGTTTAG
GCTTGGGTTTAG
Nr of selected repeats 9 Similarity 0.907407
Consensus:
GCTTGGGTTTAg
>Cele-UNSB01_4:13187743-13188478 Satlength=736 Nr of Repeats=17 RepeatLength=12
seed=GCTTGGGTTT
GCTTGGGTTTAG

GCTTGTGTTTAGGATTGGATTCAG
GCTTGGGTTTAGTTTTTTTGTGCTTGCCTACCACATATTTAGTTAATTTTAGCAGAAGCTCTGGTTTTCATTTAGGCTT
GCTTTTGCCAAGCAAATATGCCAATTTTCCTCGAGTCTACATACTATGCTAATTGGAAAATTTTTCAAAAAGTAACTTT
GCGTGAAACCCAAAATAAACTTACACGGTCATCCACGTGGTATGAAATAAAAATAAATTGCCTTTGTTCAATTATTTTA
TTACTTTCAATTCTTCTCACTTTTTGCACATTTCGTTAACCGTCTCACAATCAGGAATCCAATGCCAGATAAGTTATTT
GTACACAATTAAGCTTCAATAAGAGAGAATTCTCAGGTTTAATCTTAG
GCTAGGGTTTAGGTTGCTATAGGATTTTAGATTTGCTATGGGCTTAAGCTTTTGTTTATGATTGGTGTTAGGCTAAGGT
TCAAGATAGGGTTGGATTTAGGCTTGAG
GCTTGGGGTTAG
GCTTGGGTTTAA
GTTTGGGTTTAG
GCTTGGGTTTAA
GCTTGGGTTTAG
GCTTGGGTTTAG
GCTTGGGTTTAG
GCTTGGGTTTAG
GCTTGGGTTTAGGTTCAGGTTTAGGCTCAGGTTTAGGCTCAGGTTTAGCCTCAGGTTTAG
GCTTGGGCTTAACTTTGAGTTTAGA
CTTGGGTTTAAGTTTGAGTTTAG
GCTTGGGTTTGG
GCTTGGGTTTAG
Nr of selected repeats 11 Similarity 0.903030
Consensus:
GCTTGGGTTTAG
>Cele-UNSB01_4:13295662-13296286 Satlength=625 Nr of Repeats=24 RepeatLength=26
seed=ATTAGATGAA
ATTAGATGAATTTCCAAAGTTAGACC
ATTAGATGAATTTCCAAAGTTAGACC
ATTAGATGAATTTCCAAAGTTAGACC
ATTAGATGAAATTCCAAAGTTAGACC
ATTAGATGAATTTCCAAAGTTAGACA
ATTAGATGAATTTCCAAAGTTAGACC
ATTAGATGAATTTCCAAAGTTAGACC
ATTAGATGAATTTCCAAAGTTAGACA
ATTAGATGAATTTCCAAAGTTAGACC
ATTAGATGAATTTCCAAAGTTAGACC
ATTAGATGAATTTCCAAAGTTAGACA
ATTAGATGAATTTCCAAAGTTAGACC
ATTAGATGAATTTCCAAAGTTAGACC
ATTAGATGAATTTCCAAAGTTAGACC
ATTAGATGAATTTCCAAAGTTAGACC
ATTAGATGAAATTCCAAAGTTAGACC
ATTAGATGAATTTCCAAAGTTAGACC
ATTAGATGAATTTCCAAAGTTAGACA
ATTAGATGAATTTCCAAAGTTAGACC
ATTAGATGAATTTCCAAAGTTAGACC
ATTAGATGAATTTCCAAAGTTAGACC
ATTAGATGAATTTCCAAAGTTAGACA
ATTAGATGAATTTCCAAAGTTAGACC
ATTAGATGAATTTCCAAAGTTAGACC
Nr of selected repeats 24 Similarity 0.974173
Consensus:
ATTAGATGAATTTCCAAAGTTAGACc

>Cele-UNSB01_4:13402475-13402619 Satlength=145 Nr of Repeats=12 RepeatLength=12 seed=TATACCTATA
TATACCTATACC
TATACCCATACC
TATACCTATACC
TATACCTATACC
TATACATATACC
TATACCTATACC
TATATCTATACC
TATACCTATACC
TATACCTATACC
TATACCTATACC
TATACCTATACC
TATACCTATACC
Nr of selected repeats 12 Similarity 0.944444
Consensus:
TATACCTATACC
>Cele-UNSB01_4:13540735-13544977 Satlength=4243 Nr of Repeats=166 RepeatLength=25 seed=TTTACTCTCT
TTTACTCTCTGGCTTCACCATATTTC
TTTACTCTCTGGCTTCACAGAATATTATACTTCTGGCTTCACCATATT
TTTACTCTCTGCTTCACCATATTT
TTTACTCTCTGCTTCACAGAATAT
TTTACTCTTGCTTCACAGAATAT
TTTACTCCTCTGCTTCACAGAATAA
TTTACCTCTGCTTCACAAATA
TTTACTCTCTGCTTTCACAGAATATT
TTTACTCTCGCTTCACAAATAT
TTTACTCTCTGCTTCACAGAATCA
TTTACCTCTGGCTTCACCATATTT
TTTACTCTCTGCCTTCACAAATAT
TTTACTTTCTCTTGCTTCACAGAATATTTTACCTCGGTTCACAGAATA
TTTACTCTCTGCTTCACACTTAT
TTTACTCTCTGGCTTCACAGAATAT
TTTACTCTCTGGCTTCACAGAATAT
TTTACTCTCTGGCTTCACAGAATAT
TTTACTCTCTGGTTCACATAT
TTTACTCTCTGGCTTCACAGAATAT
TTTACTCTCTGGCTTCACAGAATAT
TTTACTCTCTGGCTTCACAGAATAT
TTTACTCTCTGGCTTCACAGAATAT
TTTACTCTCTGGCTTCACAGAATAT
TTTACTCTCTGGCTTCACAGAATAT
TTTACTCTCTGGCTTCACAGAATAT
TTTACTCTCTGGCTTCACAGAATAT
TTTACTCTCTGTTTTTTTTGCTTCACAGAATAT
TTTACTCTCTGGCTTCACAGAATAT
TTTACTCTCTGGCTTCACAGATAT
TTTACTCTCTGGCTTCACAGAATAT
TTTACTCTCTGGCTTCACAGAATAT
TTTACTCTCTGGCTTCACAGAATAT
TTTACTCTCTGGCTTCACAGAATAT
TTTACTCTCTGGCTTCACCATATTT
TTTACTCTCTGGCTTCACAGAATAT

TTTACTCTCTGGCTTCACCATATTT
TTTACTCTCTGGCTTCACAGAATAT
TTTACTCTCTGGCTTCACAGAATAT
TTTACTCTCTGGCTTCACAGAATATT
TTTACTCTCTGGCTTCACAGAATAT
TTTACTCTCTGGTTTCACCATATTT
TTTACTCTCTGGCTTCACAGAATAT
TTTACTCTCTGGCTTCACCATATTT
TTTACTCTCTGGCTTCACAGAATAT
TTTACTCTCTGGCTTCACAGAATAT
TTTACTCTCTGGCTTCACAGAATATTTTACTTTTTTTTTTTCTCTGGCTTCACATAT
TTTACTCTCTGGCTTCACAGAATAT
TTTACTCTCTGGCTTCACAGAATAT
TTTACTCTCTGGCTTCACAGAATAT
TTTACTCTCTGGCTTCACAGAATAT
TTTACTCTCTGGCTTCACATAT
TTTACTCTCTGGCTTCACCATATTT
TTTACTCTCTGGCTTCACAGAATAT
TTTACTCTCTGGCTTCACAGAATAT
TTTACTCTCTGGCTTCACCATATTT
TTTACTCTCTGGCTTCACAGAATAT
TTTACTCTCTGGCTTCACCATATTT
TTTACTCTCTGGCTTCACATAT
TTTACTCTCTGGCTTCACCATATT
TTTACTCTCTGGCTTCACAGAATAT
TTTACTCTCTGGCTTCACCATATT
TTTACTCTCTGGCTTCACAGAATAT
TTTACTCTCTGGCTTACATATT
TTTACTCTCTGGCTTCACAATAT
TTTACTCTCTGGCTTCACAGAATAT
TTTACTCTCTGGCTTCACACTATAT
TTTACTCTCTGGCTTCACAGAATAT
TTTACTCTCTGGCTTCACCATATTT
TTTACTCTCTGGCTTCACAGAATAT
TTTACTCTCTGGCTTCACACATAT
TTTACTCTCTGGCTTCACAGAATAT
TTTACTCTCTGGCTTCACACTATAT
TTTACTCTCTGGCTTCACAGAATAT
TTTACTCTCTGGCTTCACAGAATAT
TTTACTCTCTGGTTTCACCATATTT
TTTACTCTCTGGCTTCACCATATTTTTTTTTTTTTTTTTTTCTGCTTACATATTTTTTTTTTTTCACATAT
TTTACTCTCTGGCTTCACACTATCT
TTTACTCTCTGGCTTCACACTATAT
TTTACTCTCTGGCTTCACACATATT
TTTACTCTCTGGCTTCACAGAATAT
TTTACTCTCTGGCTTCACATATAT
TTTACTCTCTGGCTTCACCATATTT
TTTACTCTCTGGCTTCACCATATTT
TTTACTCTCTGGCTTCACAGAATAT
TTTACTCTCTGGCTTCACACTATCT
TTTACTCTCTGGCTTCACAGAATAT
TTTACTCTCTGGCTTCACACTATAT
TTTACTCTCTGGCTTCACAGAATAT
TTTACTCTCTGGCTTCACACTATAT

TTTACTCTCTGGCTTCACAGAATAT
TTTACTCTCTGGCTTCACATATTT
TTTACTCTCTGGCTTCACAGAATAT
TTTACTCTCTGGCTTCACACATAT
TTTACTCTCTGGCTTCACAGAATAT
TTTACTCTCTGGCTTCACACAATATT
TTTACTCTCTGGCTTCACCATATTT
TTTACTCTCTGGCTTCACAGAATAT
TTTACTCTCTGGCTTCACACTATAT
TTTACTCTCTGGCTTCACACTATCT
TTTACTCTCTGGCTTCACAGAATAT
TTTACTCTCTGGCTTCACACTATT
TTTACTCTCTGGCTTCACAGAATAT
TTTACTCTCTGGCTTCACAGAATAT
TTTACTCTCTGGCTTCACAGAATAT
TTTACTCTCTGGCTTCACAGAATAT
TTTACTCTCTGGCTTCACCATATTT
TTTACTCTCTGGCTTCACAGAATAT
TTTACTCTCTGGCTTCACAGTATAT
TTTACTCTCTGGCTTCACACTATAT
TTTACTCTCTGGCTTCACACTATCT
TTTACTCTCTGGCTTCACAGAATAT
TTTACTCTCTGGCTTCACAGAATAT
TTTACTCTCTGGCTTCACAGAATAT
TTTACTCTCTGGCTTCACAGAATAT
TTTACTCTCTGGCTTCACCATATTT
TTTACTCTCTGGCTTCACCATATTT
TTTACTCTCTGGCTTCACATATAT
TTTACTCTCTGGCTTCACACTATAT
TTTACTCTCTGGCTTCACCATATTT
TTTACTCTCTGGCTTCACAGAATAT
TTTACTCTCTGGCTTCACAGAATAT
TTTACTCTCTGGCTTCACAGAATAT
TTTACTCTCTGGCTTCACAGAATAT
TTTACTCTCTGGCTTCACACTATAT
TTTACTCTCTGGCTTCACAGAATAT
TTTACTCTCTGGCTTCACCATATTT
TTTACTCTCTGGCTTCACAGAATAT
TTTACTCTCTGGCTTCACACTATAT
TTTACTCTCTGGCTTCACAGAATAT
TTTACTCTCTGGCTTCACAGAATAT
TTTACTCTCTGGCTTCACAGAATAT
TTTACTCTCTGGCTTCACACTATAT
TTTACTCTCTGGCTTCACAGAATAT
TTTACTCTCTGGCTTCACACTATAT
TTTACTCTCTGGCTTCACAGAATAT
TTTACTCTCTGGCTTCACAGAATAT
TTTACTCTCTGGCTTCACAGAATAT
TTTACTCTCTGGCTTCACACTATCT
TTTACTCTCTGGCTTCACAGAATAT
TTTACTCTCTGGCTTCACAGAATAT
TTTACTCTCTGGCTTCACAATAT
TTTACTCTCTGGCTTCACAGAATAT
TTTACTCTCTGGCTTCACAGAATAT

TTTACTCTCTGGCTTCACACTATCT
TTTACTCTCTGGCTTCACAGTAT
TTTACTCTCTGGCTTCACACTATT
TTTACTCTCTGGCTTCACAGAATAT
TTTACTCTCTGGCTTCACACTATCT
TTTACTCTCTGGCTTCACAGAATAT
TTTACTCTCTGGCTTCACACTATCT
TTTACTCTCTGGCTTCACAGAATAT
TTTACTCTCTGGCTTCACAGAATAT
TTTACTCTCTGGCTTCACACTATAT
TTTACTCTCTGGCTTCACAGAATAT
TTTACTCTCTGGCTTCACACTATCT
TTTACTCTCTGGCTTCACAGAATAT
TTTACTCTCTGGCTTCACACTATAT
TTTACTCTCTGGCTTCACAGAATAT
TTTACTCTCTGGCTTCACACTATCT
TTTACTCTCTGGCTTCACAGAATAT
TTTACTCTCTGGCTTCACACTATAT
TTTACTCTCTGGCTTCACAGAATAT
TTTACTCTCTGGCTTCACACTATCT
TTTACTCTCTGGCTTCACAGAATAT
TTTACTCCCTGGCTTCACCATGTTT
TTTACTCTCTGGCTTCACACTATAT
Nr of selected repeats 131 Similarity 0.786650
Consensus:
TTTACTCTCTGGCTTCACAgaATAT
>Cele-UNSB01_4:13614335-13614410 Satlength=76 Nr of Repeats=5 RepeatLength=15
seed=CACAGGAGGC
CACAGGAGGCTGGGA
CACAGGAGGCTGGGA
CACAGGAGGCTGGGA
CACAGGAGGCTGGGA
CACAGGAGGCTGGGA
Nr of selected repeats 5 Similarity 1.000000
Consensus:
CACAGGAGGCTGGGA
>Cele-UNSB01_4:13645449-13645792 Satlength=344 Nr of Repeats=22 RepeatLength=15
seed=ACCCATCGTG
ACCCATCGTGGCGAG
ACCCACCGTGGTGAG
ACCCGTCGTGGTGAG
ACTCATCGTGGCGAG
ACCCATCGTGGTGAG
ACTCATCGTGGTGAG
ACCCATCGTGGCGAG
ACCCATCGTGGTGAG
ACCCATCGTGGTGAG
ACCCATCGTGGTGAT
ACCATCGTGGCGAG
ACCCATCGTGGTGAG
ACCCATCGTGGCGAG
ACCCATCGTGGTGAG
ACCCATCGTGGCGAG
ACCCATCGTGGTGAT

ACCCATCGTGGCGAG
ACCCATCGTGGTGAT
ACCATCGTGGCGAG
ACCCATCGTGGTGAG
ACCCATCGTGGCGAG
ACCCACCGTGGTGAGACCCGTCATGGTGAG
Nr of selected repeats 19 Similarity 0.894997
Consensus:
ACCCATCGTGGtGAG
>Cele-UNSB01_4:13645884-13646555 Satlength=672 Nr of Repeats=18 RepeatLength=35 seed=GAAATTTAAA
GAAATTTAAATTTTCTGAGAAAAATATTTTGGCGGGAATTTTTAATTTTCTGAGAAAAATATTTTGGCGG
GAAATTTAAATTTTCTGTGAAAAATATTTTGGCGG
GAAATTTAAATTTTCTGAAAAAAAAATTATTTTGGCGG
GAAATTTAAATTTTCTGAAAAAAAAATTATTTTGGCGG
GAAATTTAAATTTTCTGAGAAAAATATTTTGGCGG
GAAATTTAAATTTCCTGAGAAAAATATTTTGGCGG
GAAATTTAAATTTTCTGTGAAAAATATTTTGGCGG
GAAATTTAAATTTTCTGTGAAAAATATTTTGGCGG
GAAATTTAAATTTTCTGAGAAAAATATTTTGGCGG
GAAATTTAAATTTTCTGTGAAAAATATTTTGGCGG
GAAATTTAAATTTTCTGTGAAAAATATTTTGGCGG
GAAATTTAAATTTTCTGTGAAAAATATTTTGGCGG
GAAATTTAAATTTTCTGAGAAAAATATTTTGGAGA
GAAATTTAAATTTTCTGTGAAAAATATTTTGGCGG
GAAATTTAAATTTTCTGTGAAAAATATTTTTGCGG
GAAATTTAAATTTTCTGAGAAAAATATTTTGGAGA
GAAATTTAAATTTTCTGTGAAAAATATTTTGGCGG
GAAATTTAAATTTTCTGTGAAAAATATTTTTGCGG
Nr of selected repeats 15 Similarity 0.948481
Consensus:
GAAATTTAAATTTTCTGtGAAAAATATTTTGGCGG
>Cele-UNSB01_4:13646609-13646697 Satlength=89 Nr of Repeats=4 RepeatLength=23 seed=AATATTTATT
AATATTTATTTATTTATTTAATA
AATATTTATTTATTTATTTAATA
AATATTTATTTATTTATTTAATT
AATATTTATTTATTTATTT
Nr of selected repeats 3 Similarity 0.961353
Consensus:
AATATTTATTTATTTATTTAATa
>Cele-UNSB01_4:13645885-13647427 Satlength=1543 Nr of Repeats=25 RepeatLength=35 seed=AAATTTAAAT
AAATTTAAATTTTCTGAGAAAAATATTTTGGCGGGAATTTTTAATTTTCTGAGAAAAATATTTTGGCGGG
AAATTTAAATTTTCTGTGAAAAATATTTTGGCGGG
AAATTTAAATTTTCTGAAAAAAAAATTATTTTGGCGGG
AAATTTAAATTTTCTGAAAAAAAAATTATTTTGGCGGG
AAATTTAAATTTTCTGAGAAAAATATTTTGGCGGG
AAATTTAAATTTCCTGAGAAAAATATTTTGGCGGG
AAATTTAAATTTTCTGTGAAAAATATTTTGGCGGG
AAATTTAAATTTTCTGTGAAAAATATTTTGGCGGG
AAATTTAAATTTTCTGAGAAAAATATTTTGGCGGG
AAATTTAAATTTTCTGTGAAAAATATTTTGGCGGG
AAATTTAAATTTTCTGTGAAAAATATTTTGGCGGG

AAATTTAAATTTTCTGTGAAAAATATTTTGGCGGG
AAATTTAAATTTTCTGAGAAAAATATTTTGGAGAG
AAATTTAAATTTTCTGTGAAAAATATTTTGGCGGG
AAATTTAAATTTTCTGTGAAAAATATTTTTGCGGG
AAATTTAAATTTTCTGAGAAAAATATTTTGGAGAG
AAATTTAAATTTTCTGTGAAAAATATTTTGGCGGG
AAATTTAAATTTTCTGTGAAAAATATTTTTGCGGG
AAATTTAAATTTTCTGAAAATCCCAAAATTCTGG
AAATCTAAATTAATTAATTAATATTTATTTATTTATTTAATAAATATTTATTTATTTATTTAATAAATATTTATTTATT
TATTTAATTAATATTTATTTATTTATTTAATATTTATTTATTTATTTAATTAACCAGCCGCTGACCGCGCCTACGGCGC
GGGCAACGACTGGCACCATTAAAAGTATTTGACACACATACACTTCCAGAATTTTCTCGATTTTTCTAGAAAGTTCTGG
AACACTCCAGAATTTTCCCGATTTTTCTAGAAAGTTCTGGAACATTCCAGTATTTTCTCGATTTTTCTAGAAAGTTCTG
GAACATTCCAGTATTTTCTCGATTTTTCTAGAAAGTTCTGGAACATTCCAGTATTTTCTCGATTTTTCTAGAAAGTTCT
GGAACATTCCAGTATTTTCTCGATTTTTCTAGAAAGTTCTGGAACATTCCAGAATTTTCTCGATTTTTCTAGAAAGTTC
TGGAACATTCCAGAATTTTCTCGATTTTTCTAGAAAGTTCTGGAACATTCCAAAATTTTCTCGATTTTTCTAGAAAGTT
CTGGAACATTCGAGAATTTTTTCGATTTTTCTAGAAAGTTCGGGAACATTCGAGAATTTTTTCGAAATTTCCAGAAGAT
TTTAGATTTCCAGAATTTTAGAATTTTCAGA
AAATTTAAATTACCCGCCAAAATATTTTTCACAGA
AAATTAAAATTTCCCTCCAAAATATTTTTTTCAGA
AAATTCAAATTTCCCGCCAAAATATTTTTCACAGA
AAATTTAAATTTCCCGCAAAATATTTTTCACAGA
AAATTTAAATTTCCCGCCAAAATATTTTTCTCAGA
Nr of selected repeats 19 Similarity 0.791694
Consensus:
AAATTTAAATTTtCtGtgaAAAATATTTTggcggG
>Cele-UNSB01_4:13647491-13647701 Satlength=211 Nr of Repeats=14 RepeatLength=15
seed=CCACGATGGG
CCACGATGGGTCTCA
CCACGATGGGTCTCG
CCACGATGGGTCTCT
CCACGATGGGTCTCT
CCACGATGGGTCTCA
CCACGGTGGGTCTCG
CCACGGTGGGTCTCA
CCACGATGGGTCTCA
CCACGATGGGTCTCG
CCACGATGGGTCTCT
CCACGATGGGTCTCT
CCACGATGGGTCTCT
CCACGATGGGTCTTT
CCACGATGGGTCTCA
Nr of selected repeats 14 Similarity 0.902320
Consensus:
CCACGATGGGTCTCn
>Cele-UNSB01_4:13646820-13647869 Satlength=1050 Nr of Repeats=12
RepeatLength=40 seed=GTTCTGGAAC
GTTCTGGAACACTCCAGAATTTTCCCGATTTTTCTAGAAA
GTTCTGGAACATTCCAGTATTTTCTCGATTTTTCTAGAAA
GTTCTGGAACATTCCAGTATTTTCTCGATTTTTCTAGAAA
GTTCTGGAACATTCCAGTATTTTCTCGATTTTTCTAGAAA
GTTCTGGAACATTCCAGTATTTTCTCGATTTTTCTAGAAA
GTTCTGGAACATTCCAGAATTTTCTCGATTTTTCTAGAAA
GTTCTGGAACATTCCAGAATTTTCTCGATTTTTCTAGAAA
GTTCTGGAACATTCCAAAATTTTCTCGATTTTTCTAGAAA

GTTCTGGAACATTCGAGAATTTTTTCGATTTTTCTAGAAA
GTTCGGGAACATTCGAGAATTTTTTCGAAATTTCCAGAAGATTTTAGATTTCCAGAATTTTAGAATTTTCAGAAAATTT
AAATTACCCGCCAAAATATTTTTCACAGAAAATTAAAATTTCCCTCCAAAATATTTTTTTCAGAAAATTCAAATTTCCC
GCCAAAATATTTTTCACAGAAAATTTAAATTTCCCGCAAAATATTTTTCACAGAAAATTTAAATTTCCCGCCAAAATAT
TTTTCTCAGAAAATTTAAATTTCCCGCCAAAATTTTGGGTCTTACCACGGTGGGTCTTACCACGACGGGTCTCACCACG
ATGGGTCTCACCACGATGGGTCTCGCCACGATGGGTCTCTCCACGATGGGTCTCTCCACGATGGGTCTCACCACGGTGG
GTCTCGCCACGGTGGGTCTCACCACGATGGGTCTCACCACGATGGGTCTCGCCACGATGGGTCTCTCCACGATGGGTCT
CTCCACGATGGGTCTCTCCACGATGGGTCTTTCCACGATGGGTCTCACCACGATGGGTCTCGCCACGAAGATCTCGCAG
CAACATTTTTTAAAATTTTCCAGAAG
GTTTTGGAACATTCCAGAATTTTCTAGAATTTTCCAGAAGGTTCTAAAGCTTTTCAGCATTTTCCAGAAG
GTTCTGGAACATTCTAGAAATTTCCAGAAATTCCCAGAAG
Nr of selected repeats 10 Similarity 0.891111
Consensus:
GTTCTGGAACATTCcAGaATTTTCtCGATTTTTCTAGAAA
>Cele-UNSB01_4:13647739-13648797 Satlength=1059 Nr of Repeats=13
RepeatLength=27 seed=TTTTTAAAAT
TTTTTAAAATTTTCCAGAAGGTTTTGGAACATTCCAGAATTTTCTAGAATTTTCCAGAAGGTTCTAAAGCTTTTCAGCA
TTTTCCAGAAGGTTCTGGAACATTCTAGAAATTTCCAGAAATTCCCAGAAGGTTCTGGAACATTTCAGAATTTTCCCGA
AGTTTCCAATTTCCCTTCCAAAGACAAAAACACAATTTTTCCCAAACTACAGTAATCCTACCCTACTCCTACAGTACTA
CTACAGTACCCCGCCCATATCCCCCTACTAACCCCAAACTAATATCCCTCCAACAGCCGAAAACGCCTGCCTTTGTAAG
CTATGACGTCACTTCTTAACAAACGGACACTATTTTTTTATATATAGGTAATGATAAGACAAAAAGTCAATTTTTCCCA
AACTACAGTAATCTTACAGTACTCCTACAGTACCTCTACAGTATTACTACAGTACCCGTCCATATCCCACCACTAATCT
CAAACCAATATTCCTTCAAAAGACAAAACGTCAATTTTTCCAAACTACAGTAATCCTACCGTACTACTACAGTACCCCG
TCCATATCCCCCTACTAACCCCAAACTAATATCCCTCCAACAGCCGAAAACGCCTGCCTTTATAAGCTATGACGTCACA
GACTACAAACTACGGAACAAACGGAATTATTTTTTTATATATAGGTAATGATATTTATTTATTTAATTAATATTTATTT
ATTTATTTAATTAATATTTATTTATTTATTTAAC
TTTTTAAACTTCTAAC
TTTTTAAAATTCATATACCGTTCTAAC
TATTTAAAATTCATATACCATTCTAAT
TTTTTGAAATTCACATACCGTTCTAAC
TTTTCAAAATTCATATACCGTTCTAAC
TTTTTAAAATTCATGTACCATTCTAAT
TTTTTGAAATTCATGTACCGTTCTAAC
TTTTTAAAATTCATATACCGTTCTAAC
TTTTTAAAATTCATATACCGTTCTAAC
TTTTTAAAATTCATATACCGTTCTAAC
TTTTTAAAATTCATGTACCATTCTAAT
TTTTTAAAATTCATATACCGTTCTAAC
Nr of selected repeats 11 Similarity 0.892256
Consensus:
TTTTTAAAATTCATaTACCgTTCTAAc
>Cele-UNSB01_4:13661650-13661868 Satlength=219 Nr of Repeats=6 RepeatLength=31
seed=AATTCAAATT
AATTCAAATTTCTTCAAAAAATTTTGGCGGG
AATTCAAATTTTCTCAAAAAATTTTGGCGGG
AATTCAAATTTCTTCAAAAAATTTTGGCGGG
AATTCAAATTTTCTCAAAAAATTTTGGCGGG
AATTCAAATTTCTTCACAAAATTTTGGCGGGAATCCGAATTTCTTCAAAAAATTTTGGCGGG
AATTCAAATTTATTTAAAAAATTTAAGCCAAA
Nr of selected repeats 4 Similarity 0.942652
Consensus:
AATTCAAATTTccTCAAAAAATTTTGGCGGG
>Cele-UNSB01_4:13730774-13730818 Satlength=45 Nr of Repeats=4 RepeatLength=11
seed=CACATTTTTT

CACATTTTTTG
CACATTTTTTC
CACATTTTTTG
CACATTTTTTC
Nr of selected repeats 4 Similarity 0.919192
Consensus:
CACATTTTTTc
>Cele-UNSB01_4:13731432-13732897 Satlength=1466 Nr of Repeats=8
RepeatLength=164 seed=AAAATTCAAA
AAAATTCAAATGATTTGAAAATGAAAGAGCCCGAAACCGAAGAAATTGTATCTCTTGATTTTTATAATTTTTTCACGGA
ACTTTTTGTGCATTTTTTGCTCAGCAAGTGGATGTAGATTGCACAATTTTTTGATCTTGCATTAAAAAAAAATGTAATG
TCTTTATTCTATATATTTACTCTAATGTTCTGCCATTATGAGTAAATATAAAACTCTTCAGCATAGTCAAAAATACCAG
AAAATGACAATCAAAGTATAATAGCTTGTACGGAAGTATTTTTTTAAAAATTGATAAAAATAAATAAAAGCTGATTTTT
TCA
AAAATTCAAAAGTATGGGAAAATCATATGGAGTCATTCTTTTTTATTTCATAAAACTCTTCAGCATAGTCAAAAATACC
AGAAAATGCCAATCAAAGTATAATAGCTTGTACGGAAGTATTTTTTTAAAAATTGATAAAAATATATAAAAGCTGATTT
TTTCT
AAAATTCAAAAGTATGGGAAAATCATATGGAGTCATTCTTTTTTATTTCATAAAACTCTTCAGCATAGTCAAAAATACC
AGAAAATGCCAATCAAAGTATAATAGCTTGTACGGAAGTATTTTTTTAAAAATTGATAAAAATATATAAAAGCTGATTT
TTTTCA
AAAATTCAAAAGTATGGGAAAATCATATGGAGTCATTCTTTTTTATTTCATAAAACTCTTCAGCATAGTCAAAAATACC
AGAAAATGCCAATCAAAGTATAATAGCTTGTACGGAAGTATTTTTTTAAAAATTGATAAAAATATATAAAAGCTGATTT
TTTTCA
AAAATTCAAAAGTATGGGAAAATCATATGGAGTCATTCTTTTTTATTTCATAAAACTCTTCAGCATAGTCAAAAATACC
AGAAAATGCCAATCAAAGTATAATAGCTTGTACGGAAGTATTTTTTTAAAAATTGATAAAAATATATAAAAGCTGATTT
TTTTCA
AAAATTCAAAAGTATGGGAAAATCATATGGAGTCATTCTTTTTTATTTCATAAAACTCTTCAGCATAGTCAAAAATACC
AGAAAATGCCAATCAAAGTATAATAGCTTGTACGGAAGTATTTTTTTAAAAATTGATAAAAATATATAAAAGCTGATTT
TTTTCA
AAAATTCAAAAGTATGGGAAAATCATATGGAGTCATTCTTTTTTATTTCATAAAACTCTTCAGCATAGTCAAAAATACC
AGAAAATGCCAATCAAAGTATAATAGCTTGTACGGAAGTATTTTTTTAAAAATTGATAAAAATATATAAAAGCTGATTT
TTTTCA
AAAATTCAAAAGTATGGGAAAATCATATGGAGTCATTCTTTTTTATTTCATAAAACTCTTCAGCATAGTCAAAAATACC
AGAAAATGCCAATCAAAGTATAATAGCTTGTACGGAAGTATTTTTTTAAAAATTGATAAAAATATATAAAAGCTGATTC
TTTCA
Nr of selected repeats 5 Similarity 1.000000
Consensus:
AAAATTCAAAAGTATGGGAAAATCATATGGAGTCATTCTTTTTTATTTCATAAAACTCTTCAGCATAGTCAAAAATACC
AGAAAATGCCAATCAAAGTATAATAGCTTGTACGGAAGTATTTTTTTAAAAATTGATAAAAATATATAAAAGCTGATTT
TTTTCA
>Cele-UNSB01_4:13759189-13759529 Satlength=341 Nr of Repeats=30 RepeatLength=11
seed=GCACATTTTT
GCACATTTTTA
GCACATTTTCT
GCACATTTTTA
GCACATTTTTG
GCACATTTTTA
GCACATTTTTGA
CACATTTTTG
GCACATTTTTT
GCACATTTTTG
GCACATTTTTATTGCGTTTTTA
GCACATTTTTG
GCACGTTTTTG

GCACATTTTCAA
CACATTTTTG
GCACATTTTTT
GCACATTTTTA
GCACATTTTTAA
CACATTTTTA
GCACATTTTTA
GCACAATTTTG
GCACATTTTTA
GCACATTTCTA
GCACATTTTTG
GCACATTTCTA
GCACTTTTTG
GCACATTTTTG
GCACATTATTA
GCACGTTTTTA
GCACATTTCTA
GCACATTTTTG
Nr of selected repeats 22 Similarity 0.772787
Consensus:
GCACATTTTTa
>Cele-UNSB01_4:13851511-13852113 Satlength=603 Nr of Repeats=12 RepeatLength=40
seed=AAAATTCTGG
AAAATTCTGGAAAGCTCTAGAACCTTTTGAAAAATTCGAG
AAAATTCTGGAATGTTCTAGAACCTTCTGGAAAGTTCGAG
AAAATTCTGGAATGTTCTAGAACCTTCTGGAAAAATTCGAA
AAAATTCTGGAATGTTCTAGAAGCCCCTGAAAAATTCGAG
AAAATTCTGGAATGTTCTAGAAGCTTCTAAAAAATTCGAG
AAAATTCTGGAATGTTCTAGAACCTTCTGGAATATTCGAG
AAAATTCTGGAATGTTCTAGAACCTTCTGGAATATTCGAG
AAAATTCTGGAATGTTCTAGAACCTTCTGGAATATTCGAG
AAAATTCTGGAATGTTCTAGAACCTTCTGGAAAAATTCGAAAA
AAAATTCTTGAATTTTCTAGAACCTTCTGAAAAATTCGTG
AAAATTCTGGAATTGTTGTGGTGAGACCTTGCTGAGACCCATTGTGGTGAGACCTGAGACCCATTGTGGTGAGACCCTT
A
AAAATTTGGGCGGGAAATTCAAAATTTATGAGAACATTGTTTTGAGGGAAATTCAAGTTTTCTGTGAAACTATTTTTGT
TTTAGTACCTTCTGGAAGTTTCAAGAACTGAAAAATTGGGG
Nr of selected repeats 9 Similarity 0.870370
Consensus:
AAAATTCTGGAATGTTCTAGAAcCTTCTGgAAaATTCGAG
>Cele-UNSB01_4:13917663-13921092 Satlength=3430 Nr of Repeats=37
RepeatLength=93 seed=AGGGCCGCAG
AGGGCCGCAGGCCCAATTAGGTGCAGAAATATTGGACATTCGTTAAGCCTATTCTAAGCCGAACAACCAAGTTTG
AGGGCCGCAGGCCCGAACACTCTCTGAAACTGTGGGCCAGCTTTAATCCTGTTCCAAGCAGATTGTTCTTCGCTCAAGC
CTACCAACCAGGTTTG
AGGGCCGCAGGCCCGAACACTCTTGGAAACTGGGCTGGCTTTAATCCTATTCCAAGCAGATTGTTATTCACTCAAGCCT
ATCAACCAAGTTTG
AGGGCCGCAGGCCCGAATAGTCTTGGAAACTGGGCTGGCTATAATCCTATTCCAAGCAGGTTGTTATTCACTCAAGCCT
ATCAACCAAGTTTG
AGGGCCGCAGGCCCAAATAGTCTTGGAAACTGGGCTGGCTATAATCCTATTCCAAGCAGGTTGTTATTCACTCAAGCCT
ATCAACCAAGTTTG
AGGGCCGCAGACCCGAATAGTCTTGGAAACTGGGCTGGCTATAATCCTATTCCAAGCAGGTTGTTTTTCACTCAAGCAT
ATCAACCAAGTTTG

AGGGCCGCAGGCCCAAATAGTCTTGGAAACTGGGCTGGCTATAATCCTATTCCAAGCAGGTTGTTATTCACTCAAGCCT
ATCAACCAAGTTTG
AGGGCCGCAGGCCCGAATAGTCTTGGAAACTGGGCTGGCTATAATCCTGTTCCAAGCAGATTGTTATTCGCTCAAGCCT
ACCAACCAAGTTTG
AGGGCCGCAGGCCCGAATAGTCTTGGAAACTGGGCTGGCTATAATCCTATTCCAAGCAGATTGTTATTCACTCAAGCCT
ATCAACCAAGTTTG
AGGGCCGCAGGCCCGAATAGTCTTGGAAACTGGGCTGGCTATAATCCTATTCCAAGCAGGTTGTTATTCACTCAAGCCT
ATCAACCAAGTTTG
AGGGCCGCAGGCCCGAATAGTCTTGGAAACTGGGCTGGCTATAATCCTATTCCAAGCAGGTTGTTATTCACTCAAGCCT
ATCAACCAAGTTTG
AGGGCCGCAGGCCCGAATAGTCTTGGAAACTGGGCTGGCTATAATCCTATTCCAAGCAGATTGTTATTCACTCAAGCCT
ATCAACCAAGTTTA
AGGGCCGCAGGCCCGAATAGTCTTGGAAACTGGGCTGGCTATAATCCTATTCCAAGCAGGTTGTTATTCACTCAAGCCT
ATCAACCAAGTTTG
AGGGCCGCAGGCCCGAATAGTCTTGGAAACTGGGCTAGCTATAATCCTATTCCAAGCAGGTTGTTATTCACTCAAGCCT
ATCAACCAAGTTTG
AGGGCCGCAGGCCCAAATAGTCTTGGAAACTGGGCTGGCTTTAATCCTATTCCAAGCAGATTGTTATTCGCTTAAGCCT
ACCAACCAGGTTTG
AGGGCCGCAGGCCCGAACACTCTTGGAAACTGTGAGCTGGCTATAACCCTATTCCAATCAGATTGCTATTCGCTCAAGC
CTACCAACCAGGTTTA
AGGGCCGCTGGCCCGAACACTCTTGGAAACTGGGCTGGCTATAATCCTATTCCAAGCAGGTTGTTATTCACTCAAGCCT
ATCAACCAGGTTTG
AGGGCCGCAGGCCCGAATAGTCTTGGAAACTGGGCTGGCTATAATCCTGTTCCAAGCAGATTGTTATTCGCTCAAGCCT
ACCAACCAAGTTTG
AGGGCCGCAGGCCCGAATAGTCTTGGAAACTGGGCTGGCTATAATCCTATTCCAAGCAGGTTGTTATTCACTCAAGCCT
ATCAACCAAGTTTG
AGGGCCGCAGGCCCGAATAGTCTTGGAAACTGGGCTGGCTATAATCCTATTCCAAGCAGGTTGTTATTCACTCAAGCCT
ATCAACCAAGTTTG
AGGGCCGCAGGCCCGAATAGTCTTGGAAACTGGGCTGGCTATAATCCTATTCCAAGCAGATTGTTATTCACTCAAGCCT
ATCAACCAAGTTTA
AGGGCCGCAGGCCCGAATAGTCTTGGAAACTGGGCTGGCTATAATCCTATTCCAAGCAGGTTGTTATTCACTCAAGCCT
ATCAACCAAGTTTG
AGGGCCGCAGGCCCGAATAGTCTTGGAAACTGGGCTAGCTATAATCCTATTCCAAGCAGGTTGTTATTCACTCAAGCCT
ATCAACCAAGTTTG
AGGGCCGCAGGCCCAAATAGTCTTGGAAACTGGGCTGGCTTTAATCCTATTCCAAGCAGATTGTTATTCGCTTAAGCCT
ACCAACCAGGTTTG
AGGGCCGCAGGCCCGAACACTCTTGGAAACTGTGAGCTGGCTATAACCCTATTCCAATCAGATTGCTATTCGCTCAAGC
CTACCAACCAGGTTTA
AGGGCCGCTGGCCCGAACACTCTTGGAAACTGGGCTGGCTATAATCCTATTCCAAGCAGGTTGTTATTCACTCAAGCCT
ATCAACCAGGTTTG
AGGGCCGCAGGCCCGAATAGTCTTGGAAACTGGGCTGGCTATAATCCTATTCCAAGCAGGTTGTTATTCACTCAAGCCT
ATCAACCAAGTTTG
AGGGCCGCAGACCCGAATAGTCTTGGAAACTGGGCTGGCTATAATCCTATTCCAAGCAGGTTGTTTTTCACTCAAGCAT
ATCAACCAAGTTTG
AGGGCCGCAGGCCCAAATAGTCTTGGAAACTGGGCTGGCTATAATCCTATTCCAAGCAGGTTGTTATTCACTCAAGCCT
ATCAACCAAGTTTG
AGGGCCGCAGGCCCGAATAGTCTTGGAAACTGGGCTGGCTATAATCCTGTTCCAAGCAGATTGTTATTCGCTCAAGCCT
ACCAACCAAGTTTG
AGGGCCGCAGGCCCGAATAGTCTTGGAAACTGGGCTGGCTATAATCCTATTCCAAGCAGATTGTTATTCACTCAAGCCT
ATCAACCAAGTTTG
AGGGCCGCAGGCCCGAATAGTCTTGGAAACTGGGCTGGCTATAATCCTATTCCAAGCAGGTTGTTATTCACTCAAGCCT
ATCAACCAGGTTTG
AGGGCCGCAGGCCCGAATAGTCTTGGAAACTGGGCTGGCTATAATCCTATTCCAAGCAGGTTGTTATTCACTCAAGCCT
ATCAACCAAGTTTG

AGGGCCGCAGGCCCAAATAGTCTTGGAAACTGGGCTGGCTATAATCCTATTCCAAGCAGGTTGTTATTCACTCAAGCCT
ATCAACCAAGTTTG
AGGGCCGCAGGCCCAAATAGTCTTGGAAACTGGGCTGGCTATAATCCTATTCCAAGCAGGTTGTTATTCACTCAAGCCT
ATCAACCAAGTTTG
AGGGCCGCAGGCCCGAATAGTCTTGGAAACTGGGCTGGCTATAATCCTATTCCAAGCAGGTTGTTATTCACTCAAGCCT
ATCAACCAGGTTTG
AGGGCCGCAGGCCCAAATAGTCTTGGAAACTGGGCTGGCTATAATCCTATTCCAAGCAGGTTGTTATTCACTCAAGCCT
ATCAACCAGGTTTG
Nr of selected repeats 33 Similarity 0.954220
Consensus:
AGGGCCGCAGGCCCgAATAGTCTTGGAAACTGGGCTGGCTATAATCCTATTCCAAGCAGgTTGTTATTCACTCAAGCCT
ATCAACCAaGTTTG
>Cele-UNSB01_4:14030114-14030248 Satlength=135 Nr of Repeats=6 RepeatLength=11
seed=AGTACTGTAG
AGTACTGTAGG
AGTACTGTAGG
AGTACGGTAGG
ATTACTGTAGTTTGGGAAAAATTGTGTTTTTGTCTTTTGAAGAGATATAGGTTTGGGGTTAGTAGTGGGATATGGTCGG
G
GTACTGTAGT
AGTACTGTAGG
Nr of selected repeats 4 Similarity 0.939394
Consensus:
AGTACtGTAGG
>Cele-UNSB01_4:14030460-14031210 Satlength=751 Nr of Repeats=45 RepeatLength=15
seed=CCCATCGTGG
CCCATCGTGGTGAGA
CCCATCGTGGCGAGA
CCCATCGTGGTGAGA
CCCATCGTGGCGAGA
CCCATCGTGGCGAGA
CCCATCGTTGCGAGACCAACCGTGGTGAGA
CCCGTCGTGGTGAGA
CCCATCGTGGTGAGG
CCCATCGTGGCGAGA
CCCATCGTGGCGAGA
CCCATCGTTGCGAGACCAACCGTGGTGAGA
CCCGTCGTGGTGAGA
CCCATCGTGGTGAGG
CCCATCGTGGCGAGA
CCCATCGTGGCGAGA
CCCATCGTTGCGAGACCAACCGTGGTGAGA
CCCGTCGTGGTGAGA
CCCATCGTGGTGAGG
CCCATCGTGGCGAGA
CCCATCGTGGCGAGA
CCCATCGTGGCGAGA
CCCATCGTGGTGAGG
CCCATCGTGGCGAGA
CCCATCGTGGCGAGA
CCCATCGTGGTGAGA
CCCATCGTGGTGAGG
CCCATCGTGGCGAGA
CCCATCGTGGCGAGA

CCCATCGTGGTGAGA
CCCATCGTGGCGAGA
CCCATCGTGGCGAGA
CCCATCGTTGCGAGACCAACCGTGGTGAGA
CCCGTCGTGGTGAGA
CCCATCGTGGTGAGG
CCCATCGTGGCGAGA
CCCATCGTGGCGAGA
CCCATCGTGGTGAGA
CCCATCGTGGCGAGG
CCCATCGTGGCGAGA
CCCATCGTGGCGAGA
CCCATCGTGGTGAGA
CCCATCGTGGAAAGA
CCCATCGTGGCGAGACCAACCGTGGTGAGA
CCCGTCGTGGTGAGA
CCCATCGTGGTGAGA
Nr of selected repeats 40 Similarity 0.901766
Consensus:
CCCATCGTGGcGAGA
>Cele-UNSB01_4:14031556-14031756 Satlength=201 Nr of Repeats=5 RepeatLength=40
seed=AATGTTCCAG
AATGTTCCAGAACTTTCTAGAAAAATCGAGAAAATTCTGG
AATGTTCCAGAACTTTCTAGAAAAATCGAGAAAATTCTGG
AATGTTCCAGAACTTTCTAGAAAAATCGAGAAAATTCTGG
AATGTTCCAGAACTTTCTAGAAAAATCGAGAAAATTCTGA
AATGTTCCAGAACTTTCTAGAAAAATCGAGAAAATTCTTG
Nr of selected repeats 5 Similarity 0.973333
Consensus:
AATGTTCCAGAACTTTCTAGAAAAATCGAGAAAATTCTgg
>Cele-UNSB01_4:14046078-14046558 Satlength=481 Nr of Repeats=12 RepeatLength=40
seed=CAGAACCTTC
CAGAACCTTCTAGAAAATCCGAGAAAATTCTGGAATGTTC
CAAAACCTTCTGGAAAATTTGAGAAAATGCTGGAATGTTC
CAGAACCTTCTGGAAAATTTGAGAAAATTCTGGAATGTTC
CAGAACCTTCTGGAAAATTTGAGAAAATTCTGGAATGTTC
CAGAACCTTCTGGAAAATTCGAGAAAATTCTGGAATGTTC
CAGAACCTTCTGGAAAATCCGAGAAAATTCTGGAATGTTC
CAGAACCTTCTGGAAACTTTGAAAAAATTCCGGAATGCTC
CAGAACCTTCTAGAAAATTCGAGAAAATTCTGGAATGTTC
CAGAACCTTCTGGAAAATCCGAGAAAATGCTGGAATGTTC
CAGAACCTTCTGGAAAATTTGAGAAAATTCTGGAATGTTC
CAGAACCTTCTGGAAAATTTGAGAAAATTCTGGAATGTTT
CAGAACCTTCTGGAAAATTCGAGAAAATTCTGGAATGTTC
Nr of selected repeats 12 Similarity 0.914646
Consensus:
CAGAACCTTCTGGAAAATtcGAGAAAATTCTGGAATGTTC
>Cele-UNSB01_4:14046107-14046777 Satlength=671 Nr of Repeats=11 RepeatLength=40
seed=CTGGAATGTT
CTGGAATGTTCCAAAACCTTCTGGAAAATTTGAGAAAATG
CTGGAATGTTCCAGAACCTTCTGGAAAATTTGAGAAAATT
CTGGAATGTTCCAGAACCTTCTGGAAAATTTGAGAAAATT
CTGGAATGTTCCAGAACCTTCTGGAAAATTCGAGAAAATT
CTGGAATGTTCCAGAACCTTCTGGAAAATCCGAGAAAATT

CTGGAATGTTCCAGAACCTTCTGGAAACTTTGAAAAAATTCCGGAATGCTCCAGAACCTTCTAGAAAATTCGAGAAAAT
T
CTGGAATGTTCCAGAACCTTCTGGAAAATCCGAGAAAATG
CTGGAATGTTCCAGAACCTTCTGGAAAATTTGAGAAAATT
CTGGAATGTTCCAGAACCTTCTGGAAAATTTGAGAAAATT
CTGGAATGTTTCAGAACCTTCTGGAAAATTCGAGAAAATT
CTGGAATGTTCCAGAACCTTCTGAAAAATGAAAAATTTTGGCGGGAATTCAAATTTTCTGTGAAAAATTGTGGCGGGAA
TTCAAAATTTCTGTGAAAAATTTTGGCGGGAATTCAAAATTTCTGTGAAAAATTTTTAGCGGGAATTCAAAATTTCTGT
GAAAAATTTTGGCGGGAATTCAAAATTTCTGTGAAAAATTTTGGCGGGAATTCAAAATTTCTGTGAAAAATT
Nr of selected repeats 9 Similarity 0.940741
Consensus:
CTGGAATGTTCCAGAACCTTCTGGAAAATttGAGAAAATt
>Cele-UNSB01_4:14068578-14068998 Satlength=421 Nr of Repeats=24 RepeatLength=15
seed=ACGATGGGTC
ACGATGGGTCTCACC
ACGATGGGTCTCGCC
ACGATGGGTCTCACC
ACGATGGGTCTCGCC
ACGACGGGTCTCACC
ACGATGGGTCTCGCC
ACGATGGGTCTCACC
ACGATGGGTCTCACC
ACGACGGGTCTCACC
ACGATGGGTCTTACC
ACGATGGGTCTCATC
ACGACGGGTCTCACC
ACGGTGGGTCTCGCAACGATGAGTATCACCACGACGAGTCTCACC
ACGATGGGTCTCACC
ACGATGGGTCTCGCCACGACGGGTATCGCC
ACGATGGGTCTCACTACGGTGGGTATCGCA
ACGATGGGTCTCACC
ACGATGGGTCTCGCC
ACGATGGGTCTCACC
ACGATGGGTCTCACC
ACGATGGGTCTCGCC
ACGACGGGTCTCACC
ACGATGGGTCTCGCC
ACGATGGGTCTCACC
Nr of selected repeats 21 Similarity 0.916191
Consensus:
ACGATGGGTCTCaCC
>Cele-UNSB01_4:14093954-14094851 Satlength=898 Nr of Repeats=32 RepeatLength=32
seed=CGACCTGCGA
CGACCTGCGACCTG
CGACCTAGCGACCTAGGTCGCTTTG
CGACCTGCGACCTAGCGACCTGGGTCGCTTTG
CGACCTGCGACCTAGCGACCTGGGTCGCTTTG
CGACCTGCGACCTAGCGACCTGGGTCGCTTTG
CGACCTGCGATCTAGCGACCTGGGTCGCTTTG
CGACCTGCGACCTAGCGACCTGGGTCGCTTTG
CGACCTGCGATCTAGCGACCTGGGTCGCTTTG
CGACCTGCGACCTAGCGACCTGGGTCGCTTTG
CGACCTGCGACCTAGCGACCTGGGTCGCTTTG
CGACCTGCGACCTAGCGACCTGGGTCGCTTTG

CGACCTGCGATCTAG
CGACCTGCGACCTAGCGACCTGGGTCGCTTTG
CGACCTGCGACCTAGCGACCTGGGTCGCTTTG
CGACCTGCGATCTAGCGACCTGGGTCGCTTTG
CGACCTGCGACCTAGCGACCTGGGTCGCTTTG
CGACCTGCGACCTAG
CGACCTGCGACCTAGCGACCTGGGTCGCTTTG
CGACCTGCGACCTAGCGACCTGGGTCGCTTTG
CGACCTGCGACCTAG
CGACCTGCGACCTAG
CGACCTGCGACCTAGCGACCTGGGTCGCTTTG
CGACCTGCGACCTAGCGACCTGGGTCGCTTTG
CGACCTGCGATCTAGCGACCTGGGTCGCTTTG
CGACCTGCGACCTAGCGACCTGGGTCGCTTTG
CGACCTGCGACCTAG
CGACCTGCGACCTAGCGACCTGGGTCGCTTTG
CGACCTGCGACCTAGCGACCTGGGTCGCTTTG
CGACCTGCGACCTAG
CGACCTGCGACCTAGCGACCTGGGTCGCTTTG
CGACCTGCGACCTAGCGACCTGGGTCGCTTTG
CGACCTGCGACCTAGCGACCTGGGTCGCTTTG
Nr of selected repeats 24 Similarity 0.987923
Consensus:
CGACCTGCGACCTAGCGACCTGGGTCGCTTTG
>Cele-UNSB01_4:14106561-14106695 Satlength=135 Nr of Repeats=6 RepeatLength=11
seed=AGTACTGTAG
AGTACTGTAGG
AGTACTGTAGG
AGTACGGTAGG
ATTACTGTAGTTTGGGAAAAAATTGTGTTTTTGTCTTTTGAAGAGATATAGGTTTGGGGTTAGTAGTGGAATATGGTCGG
G
GTACTGTAGT
AGTACTGTAGG
Nr of selected repeats 4 Similarity 0.939394
Consensus:
AGTACtGTAGG
>Cele-UNSB01_4:14106973-14107062 Satlength=90 Nr of Repeats=6 RepeatLength=15
seed=ACCCATCGTG
ACCCATCGTGGCGAG
ACCCATCGTGGTGAG
ACCCATCGTGGAAA
ACCCATCGTGGTGAG
ACCCATCGTGGCGAG
ACCCATCGTGGTGAG
Nr of selected repeats 5 Similarity 0.946667
Consensus:
ACCCATCGTGGtGAG
>Cele-UNSB01_4:14107092-14108860 Satlength=1769 Nr of Repeats=51
RepeatLength=35 seed=GGGAAATTTA
GGGAAATTTAAATTTTCTGAAAAAAATATTTTGGC
GGGAAATTTAAATTTTCTGAAAAAATATTTTGGC
GGGAAATTTAAATTTTCTGTGAAAAATATTTTGGA
GGGAAATTTAAATTTTCTGTGAAAAATATTTTGGA
GGGAAATTTAAATTTTCTGTGAAAATATTTTGGA

GGGAAATTTAAATTTTCTGAAAAAAAATATTTTGGC
GGGAAATTTAAATTTTCTGAAAAAAATATTTTGGC
GGGAAATTTAAATTTTCTGTGAAAAAATATTTTGGA
GGGAAATTTAAATTTTCTGTGAAAAAATATTTTGGA
GGGAAATTTAAATTTTCTGTGAAAAAATATTTTGGC
GGGAAATTTAAATTTTCTGAAAAAAAATATTTTGGC
GGGAAATTTAAATTTTCTGTGAAAAAATATTTTGGA
GGGAAATTTAAATTTTCTGTGAAAAAATATTTTGGC
GGGAAATTTAAATTTTCTGTGAAAAAATATTTTGGC
GGGAAATTTAAATTTTCTGAAAAAAAATATTTTGGC
GGGAAATTTAAATTTTCTGTGAAAAATATTTTGGA
GGGAAATTTAAATTTTCTGAAAAAAAATATTTTGGC
GGGAAATTTAAATTTTCTGAAAAAAAATATTTTGGC
GGGAAATTTAAATTTTCTGAAAAAAATATTTTGGC
GGGAAATTTAAATTTTCTGAAAAAAATATTTTGGC
GGGAAATTTAAATTTTCTGAAAAAAAATATTTTGGA
GGGAAATTTAAATTTTCTGTGAAAAATATTTTGGC
GGGAAATTTAAATTTTCTGTGAAAAAATATTTTGGC
GGGAAATTTAAATTTTCTGAAAAAAAATATTTTGGC
GGGAAATTTAAATTTTCTGAAAAAAATATTTTGGC
GGGAAATTTAAATTTTCTGTGAAAAAATATTTTGGC
GGGAAATTTAAATTTTCTGTGAAAAAATATTTTGGA
GGGAAATTTAAATTTTCTGTGAAAAAATATTTTGGC
GGGAAATTTAAATTTTCTGTGAAAAAATATTTTGGC
GGGAAATTTAAATTTTCTGAAAAAAAATATTTTGGC
GGGAAATTTAAATTTTCTGTGAAAAATATTTTGGA
GGGAAATTTAAATTTTCTGAAAAAAAATATTTTGGC
GGGAAATTTAAATTTTCTGAAAAAAAATATTTTGGC
GGGAAATTTAAATTTTCTGAAAAAAATATTTTGGC
GGGAAATTTAAATTTTCTGAAAAAAATATTTTGGC
GGGAAATTTAAATTTTCTGAAAAAAAATATTTTGGA
GGGAAATTTAAATTTTCTGTGAAAAATATTTTGGC
GGGAAATTTAAATTTTCTGTGAAAAAATATTTTGGC
GGGAAATTTAAATTTTCTGAAAAAAAATATTTTGGC
GGGAAATTTAAATTTTCTGAAAAAAATATTTTGGC
GGGAAATTTAAATTTTCTGAAAAAAAATATTTTGGC
GGGAAATTTAAATTTTCTGAAAAAAATATTTTGGC
GGGAAATTTAAATTTTCTGAAAAAAATATTTTGGC
GGGAAATTTAAATTTTCTGAAAAAAAATATTTTGGA
GGGAAATTTAAATTTTCTGTGAAAAATATTTTGGC
GGGAAATTTAAATTTTCTGTGAAAAAATATTTTGGC
GGGAAATTTAAATTTTCTGTGAAAAAATATTTTGGA
GGGAAATTTAAATTTTCTGTGAAAAAATATTTTGGA
GGGAAATTTAAATTTTCTGTGAAAAAATATTTTGGC
GGGAAATTTAAATTTTCTGTGAAAAATATTTTGGA
GGGAAATTTAAATTTTCTGAAAAAAAATATTTTGGC
Nr of selected repeats 34 Similarity 0.943706
Consensus:
GGGAAATTTAAATTTTCTGtgAAAAATATTTTGGc
>Cele-UNSB01_4:14107092-14111107 Satlength=4016 Nr of Repeats=106
RepeatLength=35 seed=GGGAAATTTA
GGGAAATTTAAATTTTCTGAAAAAAAATATTTTGGC
GGGAAATTTAAATTTTCTGAAAAAAATATTTTGGC
GGGAAATTTAAATTTTCTGTGAAAAAATATTTTGGA

GGGAAATTTAAATTTTCTGTGAAAAATATTTTGGA
GGGAAATTTAAATTTTCTGTGAAAATATTTTGGA
GGGAAATTTAAATTTTCTGAAAAAAATATTTTGGC
GGGAAATTTAAATTTTCTGAAAAAATATTTTGGC
GGGAAATTTAAATTTTCTGTGAAAAATATTTTGGA
GGGAAATTTAAATTTTCTGTGAAAAATATTTTGGA
GGGAAATTTAAATTTTCTGTGAAAAATATTTTGGC
GGGAAATTTAAATTTTCTGAAAAAAATATTTTGGC
GGGAAATTTAAATTTTCTGTGAAAAATATTTTGGA
GGGAAATTTAAATTTTCTGTGAAAAATATTTTGGC
GGGAAATTTAAATTTTCTGTGAAAAATATTTTGGC
GGGAAATTTAAATTTTCTGAAAAAAATATTTTGGC
GGGAAATTTAAATTTTCTGTGAAAATATTTTGGA
GGGAAATTTAAATTTTCTGAAAAAAATATTTTGGC
GGGAAATTTAAATTTTCTGAAAAAAATATTTTGGC
GGGAAATTTAAATTTTCTGAAAAAATATTTTGGC
GGGAAATTTAAATTTTCTGAAAAAATATTTTGGC
GGGAAATTTAAATTTTCTGAAAAAAATATTTTGGA
GGGAAATTTAAATTTTCTGTGAAAATATTTTGGC
GGGAAATTTAAATTTTCTGTGAAAAATATTTTGGC
GGGAAATTTAAATTTTCTGAAAAAAATATTTTGGC
GGGAAATTTAAATTTTCTGAAAAAATATTTTGGC
GGGAAATTTAAATTTTCTGTGAAAAATATTTTGGC
GGGAAATTTAAATTTTCTGTGAAAAATATTTTGGA
GGGAAATTTAAATTTTCTGTGAAAAATATTTTGGC
GGGAAATTTAAATTTTCTGTGAAAAATATTTTGGC
GGGAAATTTAAATTTTCTGAAAAAAATATTTTGGC
GGGAAATTTAAATTTTCTGTGAAAATATTTTGGA
GGGAAATTTAAATTTTCTGAAAAAAATATTTTGGC
GGGAAATTTAAATTTTCTGAAAAAAATATTTTGGC
GGGAAATTTAAATTTTCTGAAAAAATATTTTGGC
GGGAAATTTAAATTTTCTGAAAAAATATTTTGGC
GGGAAATTTAAATTTTCTGAAAAAAATATTTTGGA
GGGAAATTTAAATTTTCTGTGAAAATATTTTGGC
GGGAAATTTAAATTTTCTGTGAAAAATATTTTGGC
GGGAAATTTAAATTTTCTGAAAAAAATATTTTGGC
GGGAAATTTAAATTTTCTGAAAAAATATTTTGGC
GGGAAATTTAAATTTTCTGAAAAAAATATTTTGGC
GGGAAATTTAAATTTTCTGAAAAAATATTTTGGC
GGGAAATTTAAATTTTCTGAAAAAATATTTTGGC
GGGAAATTTAAATTTTCTGAAAAAAATATTTTGGA
GGGAAATTTAAATTTTCTGTGAAAATATTTTGGC
GGGAAATTTAAATTTTCTGTGAAAAATATTTTGGC
GGGAAATTTAAATTTTCTGTGAAAAATATTTTGGA
GGGAAATTTAAATTTTCTGTGAAAAATATTTTGGA
GGGAAATTTAAATTTTCTGTGAAAAATATTTTGGC
GGGAAATTTAAATTTTCTGTGAAAATATTTTGGA
GGGAAATTTAAATTTTCTGAAAAAAATATTTTGGC
GGGAAATTTAAATTTTCTGAAAAAAATATTTTTTTTAAAAAAAATGTTGCTGCGAGATCTTCGTGGCGAGACCCATTGTG
GCGAGACCCATCGTGGCGAGACCCATCGTGGTGAGACCCATCGTGGAAAACCCATCGTGGTGAGACCCATCGTGGCGAG
ACCCATCGTGGTGAGACCCATCGTGGTGGAAAACAAAATTTTGGC
GGGAAATTTAAATTTTCTGTGAAAAATATTTTGGA
GGGAAATTTAAATTTTCTGAAAAAATATTTTGGC
GGGAAATTTAAATTTTCTGAAAAAAATATTTTGGA

GGGAAATTTAAATTTTCTGTGAAAATATTTTGGC
GGGAAATTTAAATTTTCTGTGAAAAATATTTTGGC
GGGAAATTTAAATTTTCTGTGAAAAATATTTTGGA
GGGAAATTTAAATTTTCTGTGAAAAATATTTTGGA
GGGAAATTTAAATTTTCTGTGAAAAATATTTTGGC
GGGAAATTTAAATTTTCTGTGAAAATATTTTGGA
GGGAAATTTAAATTTTCTGAAAAAAATATTTTGGC
GGGAAATTTAAATTTTCTGAAAAAAATATTTTTTTTTAAAAAATGTTGCTGCGAGATCTTCGTGGCGAGACCCATTGTG
GCGAGACCCATCGTGGCGAGACCCATCGTGGTGAGACCCATCGTGGAAAACCCATCGTGGTGAGACCCATCGTGGCGAG
ACCCATCGTGGTGAGACCCATCGTGGTGGAAAACAAAATTTTGGC
GGGAAATTTAAATTTTCTGAAAAAAATATTTTGGC
GGGAAATTTAAATTTTCTGTGAAAATATTTTGGA
GGGAAATTTAAATTTTCTGAAAAAAATATTTTGGC
GGGAAATTTAAATTTTCTGAAAAAATATTTTGGC
GGGAAATTTAAATTTTCTGTGAAAAATATTTTGGA
GGGAAATTTAAATTTTCTGTGAAAAATATTTTGGC
GGGAAATTTAAATTTTCTGTGAAAAATATTTTGGA
GGGAAATTTAAATTTTCTGTGAAAAATATTTTGGA
GGGAAATTTAAATTTTCTGTGAAAAATATTTTGGC
GGGAAATTTAAATTTTCTGAAAAAAATATTTTGGC
GGGAAATTTAAATTTTCTGTGAAAATATTTTGGA
GGGAAATTTAAATTTTCTGAAAAAAATATTTTGGC
GGGAAATTTAAATTTTCTGAAAAAAATATTTTGGC
GGGAAATTTAAATTTTCTGAAAAAATATTTTGGC
GGGAAATTTAAATTTTCTGAAAAAATATTTTGGC
GGGAAATTTAAATTTTCTGAAAAAAATATTTTGGA
GGGAAATTTAAATTTTCTGTGAAAAATATTTTGAC
GGGAAATTTAAATTTTCTGTGAAAATATTTTGGA
GGGAAATTTAAATTTTCTGAAAAAAATATTTTGGC
GGGAAATTTAAATTTTCTGAAAAAAATATTTTGGC
GGGAAATTTAAATTTTCTGTGAAAAATATTTTGGA
GGGAAATTTAAATTTTCTGTGAAAAATATTTTGGC
GGGAAATTTAAATTTTCTGTGAAAAATATTTTGGA
GGGAAATTTAAATTTTCTGTGAAAAATATTTTGGA
GGGAAATTTAAATTTTCTGTGAAAAATATTTTGGC
GGGAAATTTAAATTTTCTGAAAAAAATATTTTGGC
GGGAAATTTAAATTTTCTGTGAAAATATTTTGGA
GGGAAATTTAAATTTTCTGAAAAAAATATTTTGGC
GGGAAATTTAAATTTTCTGAAAAAAATATTTTGGC
GGGAAATTTAAATTTTCTGAAAAAATATTTTGGC
GGGAAATTTAAATTTTCTGAAAAAATATTTTGGC
GGGAAATTTAAATTTTCTGAAAAAAATATTTTGGA
GGGAAATTTAAATTTTCTGTGAAAAATATTTTGAC
GGGAAATTTAAATTTTCTGTGAAAATATTTTGGA
GGGAAATTTAAATTTTCTGAAAAAAATATTTTGGC
GGGAAATTTAAATTTTCTGAAAAAAATATTTTGGC
GGGAAATTTAAATTTTCTGTGAAAAATATTTTGGA
GGGAAATTTAAATTTTCTGTGAAAAATATTTTGGC
GGGAAATTTAAATTTTCTGTGAAAAATATTTTGGA
GGGAAATTTAAATTTTCTGTGAAAAATATTTTGGA
GGGAAATTTAAATTTTCTGTGAAAAATATTTTGGC
GGGAAATTTAAATTTTCTGTGAAAATATTTTGGA
GGGAAATTTAAATTTTCTGAAAAAAATATTTTGGC
Nr of selected repeats 73 Similarity 0.941958

Consensus:
GGGAAATTTAAATTTTCTGtgAAAAATATTTTGGc
>Cele-UNSB01_4:14107093-14113320 Satlength=6228 Nr of Repeats=160
RepeatLength=35 seed=GGAAATTTAA
GGAAATTTAAATTTTCTGAAAAAAAATATTTTGGCG
GGAAATTTAAATTTTCTGAAAAAAATATTTTGGCG
GGAAATTTAAATTTTCTGTGAAAAAATATTTTGGAG
GGAAATTTAAATTTTCTGTGAAAAAATATTTTGGAG
GGAAATTTAAATTTTCTGTGAAAAATATTTTGGAG
GGAAATTTAAATTTTCTGAAAAAAAATATTTTGGCG
GGAAATTTAAATTTTCTGAAAAAAATATTTTGGCG
GGAAATTTAAATTTTCTGTGAAAAAATATTTTGGAG
GGAAATTTAAATTTTCTGTGAAAAAATATTTTGGAG
GGAAATTTAAATTTTCTGTGAAAAAATATTTTGGCG
GGAAATTTAAATTTTCTGAAAAAAAATATTTTGGCG
GGAAATTTAAATTTTCTGTGAAAAAATATTTTGGAG
GGAAATTTAAATTTTCTGTGAAAAAATATTTTGGCG
GGAAATTTAAATTTTCTGTGAAAAAATATTTTGGCG
GGAAATTTAAATTTTCTGAAAAAAAATATTTTGGCG
GGAAATTTAAATTTTCTGTGAAAAATATTTTGGAG
GGAAATTTAAATTTTCTGAAAAAAAATATTTTGGCG
GGAAATTTAAATTTTCTGAAAAAAAATATTTTGGCG
GGAAATTTAAATTTTCTGAAAAAAATATTTTGGCG
GGAAATTTAAATTTTCTGAAAAAAATATTTTGGCG
GGAAATTTAAATTTTCTGAAAAAAAATATTTTGGAG
GGAAATTTAAATTTTCTGTGAAAAATATTTTGGCG
GGAAATTTAAATTTTCTGTGAAAAAATATTTTGGCG
GGAAATTTAAATTTTCTGAAAAAAAATATTTTGGCG
GGAAATTTAAATTTTCTGAAAAAAATATTTTGGCG
GGAAATTTAAATTTTCTGTGAAAAAATATTTTGGCG
GGAAATTTAAATTTTCTGTGAAAAAATATTTTGGAG
GGAAATTTAAATTTTCTGTGAAAAAATATTTTGGCG
GGAAATTTAAATTTTCTGTGAAAAAATATTTTGGCG
GGAAATTTAAATTTTCTGAAAAAAAATATTTTGGCG
GGAAATTTAAATTTTCTGTGAAAAATATTTTGGAG
GGAAATTTAAATTTTCTGAAAAAAAATATTTTGGCG
GGAAATTTAAATTTTCTGAAAAAAAATATTTTGGCG
GGAAATTTAAATTTTCTGAAAAAAATATTTTGGCG
GGAAATTTAAATTTTCTGAAAAAAATATTTTGGCG
GGAAATTTAAATTTTCTGAAAAAAAATATTTTGGAG
GGAAATTTAAATTTTCTGTGAAAAATATTTTGGCG
GGAAATTTAAATTTTCTGTGAAAAAATATTTTGGCG
GGAAATTTAAATTTTCTGAAAAAAAATATTTTGGCG
GGAAATTTAAATTTTCTGAAAAAAATATTTTGGCG
GGAAATTTAAATTTTCTGAAAAAAAATATTTTGGCG
GGAAATTTAAATTTTCTGAAAAAAATATTTTGGCG
GGAAATTTAAATTTTCTGAAAAAAATATTTTGGCG
GGAAATTTAAATTTTCTGAAAAAAAATATTTTGGAG
GGAAATTTAAATTTTCTGTGAAAAATATTTTGGCG
GGAAATTTAAATTTTCTGTGAAAAAATATTTTGGCG
GGAAATTTAAATTTTCTGTGAAAAAATATTTTGGAG
GGAAATTTAAATTTTCTGTGAAAAAATATTTTGGAG
GGAAATTTAAATTTTCTGTGAAAAAATATTTTGGCG
GGAAATTTAAATTTTCTGTGAAAAATATTTTGGAG

GGAAATTTAAATTTTCTGAAAAAAAATATTTTGGCG
GGAAATTTAAATTTTCTGAAAAAAAATATTTTTTTTAAAAAAATGTTGCTGCGAGATCTTCGTGGCGAGACCCATTGTGG
CGAGACCCATCGTGGCGAGACCCATCGTGGTGAGACCCATCGTGGAAAACCCATCGTGGTGAGACCCATCGTGGCGAGA
CCCATCGTGGTGAGACCCATCGTGGTGGAAAACAAAATTTTGGCG
GGAAATTTAAATTTTCTGTGAAAAATATTTTGGAG
GGAAATTTAAATTTTCTGAAAAAAATATTTTGGCG
GGAAATTTAAATTTTCTGAAAAAAAATATTTTGGAG
GGAAATTTAAATTTTCTGTGAAAATATTTTGGCG
GGAAATTTAAATTTTCTGTGAAAAATATTTTGGCG
GGAAATTTAAATTTTCTGTGAAAAATATTTTGGAG
GGAAATTTAAATTTTCTGTGAAAAATATTTTGGAG
GGAAATTTAAATTTTCTGTGAAAAATATTTTGGCG
GGAAATTTAAATTTTCTGTGAAAATATTTTGGAG
GGAAATTTAAATTTTCTGAAAAAAAATATTTTGGCG
GGAAATTTAAATTTTCTGAAAAAAAATATTTTTTTTAAAAAAATGTTGCTGCGAGATCTTCGTGGCGAGACCCATTGTGG
CGAGACCCATCGTGGCGAGACCCATCGTGGTGAGACCCATCGTGGAAAACCCATCGTGGTGAGACCCATCGTGGCGAGA
CCCATCGTGGTGAGACCCATCGTGGTGGAAAACAAAATTTTGGCG
GGAAATTTAAATTTTCTGAAAAAAAATATTTTGGCG
GGAAATTTAAATTTTCTGTGAAAATATTTTGGAG
GGAAATTTAAATTTTCTGAAAAAAAATATTTTGGCG
GGAAATTTAAATTTTCTGAAAAAAATATTTTGGCG
GGAAATTTAAATTTTCTGTGAAAAATATTTTGGAG
GGAAATTTAAATTTTCTGTGAAAAATATTTTGGCG
GGAAATTTAAATTTTCTGTGAAAAATATTTTGGAG
GGAAATTTAAATTTTCTGTGAAAAATATTTTGGAG
GGAAATTTAAATTTTCTGTGAAAAATATTTTGGCG
GGAAATTTAAATTTTCTGAAAAAAAATATTTTGGCG
GGAAATTTAAATTTTCTGTGAAAATATTTTGGAG
GGAAATTTAAATTTTCTGAAAAAAAATATTTTGGCG
GGAAATTTAAATTTTCTGAAAAAAAATATTTTGGCG
GGAAATTTAAATTTTCTGAAAAAAATATTTTGGCG
GGAAATTTAAATTTTCTGAAAAAAATATTTTGGCG
GGAAATTTAAATTTTCTGAAAAAAAATATTTTGGAG
GGAAATTTAAATTTTCTGTGAAAAATATTTTGACG
GGAAATTTAAATTTTCTGTGAAAATATTTTGGAG
GGAAATTTAAATTTTCTGAAAAAAAATATTTTGGCG
GGAAATTTAAATTTTCTGAAAAAAAATATTTTGGCG
GGAAATTTAAATTTTCTGTGAAAAATATTTTGGAG
GGAAATTTAAATTTTCTGTGAAAAATATTTTGGCG
GGAAATTTAAATTTTCTGTGAAAAATATTTTGGAG
GGAAATTTAAATTTTCTGTGAAAAATATTTTGGAG
GGAAATTTAAATTTTCTGTGAAAAATATTTTGGCG
GGAAATTTAAATTTTCTGAAAAAAAATATTTTGGCG
GGAAATTTAAATTTTCTGTGAAAATATTTTGGAG
GGAAATTTAAATTTTCTGAAAAAAAATATTTTGGCG
GGAAATTTAAATTTTCTGAAAAAAAATATTTTGGCG
GGAAATTTAAATTTTCTGAAAAAAATATTTTGGCG
GGAAATTTAAATTTTCTGAAAAAAATATTTTGGCG
GGAAATTTAAATTTTCTGAAAAAAAATATTTTGGAG
GGAAATTTAAATTTTCTGTGAAAAATATTTTGACG
GGAAATTTAAATTTTCTGTGAAAATATTTTGGAG
GGAAATTTAAATTTTCTGAAAAAAAATATTTTGGCG
GGAAATTTAAATTTTCTGAAAAAAAATATTTTGGCG
GGAAATTTAAATTTTCTGTGAAAAATATTTTGGAG

GGAAATTTAAATTTTCTGTGAAAAATATTTTGGCG
GGAAATTTAAATTTTCTGTGAAAAATATTTTGGAG
GGAAATTTAAATTTTCTGTGAAAAATATTTTGGAG
GGAAATTTAAATTTTCTGTGAAAAATATTTTGGCG
GGAAATTTAAATTTTCTGTGAAAATATTTTGGAG
GGAAATTTAAATTTTCTGAAAAAAAATATTTTGGCG
GGAAATTTAAATTTTCTGAAAAAAAATATTTTTTTTTTTTAAATGTTGCTGCGAGATCTTCGTGGCGAGACCCATTGTGG
CGAGACCCATCGTGGCGAGACCCATCGTGGTGAGACCCATCGTGGAAAACCCATCGTGGTGAGACCCATCGTGGCGAGA
CCCATCGTGGTGAGACCCATCGTGGTGGAAAACAAAATTTTGGCG
GGAAATTTAAATTTTCTGAAAAAAAATATTTTGGCG
GGAAATTTAAATTTTCTGTGAAAATATTTTGGAG
GGAAATTTAAATTTTCTGTGAAAAATATTTTGGCG
GGAAATTTAAATTTTCTGAAAAAAAATATTTTGGCG
GGAAATTTAAATTTTCTGAAAAAAATATTTTGGCG
GGAAATTTAAATTTTCTGAAAAAAATATTTTGGCG
GGAAATTTAAATTTTCTGAAAAAAAATATTTTGGAG
GGAAATTTAAATTTTCTGTGAAAAATATTTTGACG
GGAAATTTAAATTTTCTGTGAAAATATTTTGGAG
GGAAATTTAAATTTTCTGAAAAAAAATATTTTGGCG
GGAAATTTAAATTTTCTGAAAAAAAATATTTTGGCG
GGAAATTTAAATTTTCTGTGAAAAATATTTTGGAG
GGAAATTTAAATTTTCTGTGAAAAATATTTTGGCG
GGAAATTTAAATTTTCTGTGAAAAATATTTTGGAG
GGAAATTTAAATTTTCTGTGAAAAATATTTTGGAG
GGAAATTTAAATTTTCTGTGAAAAATATTTTGGCG
GGAAATTTAAATTTTCTGTGAAAATATTTTGGAG
GGAAATTTAAATTTTCTGAAAAAAAATATTTTGGCG
GGAAATTTAAATTTTCTGAAAAAAAATATTTTTTTTTTAAAAATGTTGCTGCGAGATCTTCGTGGCGAGACCCATTGTGG
CGAGACCCATCGTGGCGAGACCCATCGTGGTGAGACCCATCGTGGAAAACCCATCGTGGTGAGACCCATCGTGGCGAGA
CCCATCGTGGTGAGACCCATCGTGGTGGAAAACAAAATTTTGGCG
GGAAATTTAAATTTTCTGAAAAAAAATATTTTGGCG
GGAAATTTAAATTTTCTGTGAAAATATTTTGGAG
GGAAATTTAAATTTTCTGAAAAAAAATATTTTGGCG
GGAAATTTAAATTTTCTGAAAAAAATATTTTGGCG
GGAAATTTAAATTTTCTGTGAAAAATATTTTGGAG
GGAAATTTAAATTTTCTGTGAAAAATATTTTGGAG
GGAAATTTAAATTTTCTGTGAAAAATATTTTGGCG
GGAAATTTAAATTTTCTGTGAAAAATATTTTGGAA
GGAAATTTAAATTTTCTGTGAAAAATATTTTGGAG
GGAAATTTAAATTTTCTGTGAAAAATATTTTGGCG
GGAAATTTAAATTTTCTGAAAAAAAATATTTTGGCG
GGAAATTTAAATTTTCTGTGAAAATATTTTGGAG
GGAAATTTAAATTTTCTGAAAAAAAATATTTTGGCG
GGAAATTTAAATTTTCTGAAAAAAAATATTTTGGCG
GGAAATTTAAATTTTCTGAAAAAAATATTTTGGCG
GGAAATTTAAATTTTCTGAAAAAAATATTTTGGCG
GGAAATTTAAATTTTCTGAAAAAAAATATTTTGGAG
GGAAATTTAAATTTTCTGTGAAAAATATTTTGGCG
GGAAATTTAAATTTTCTGTGAAAATATTTTGGAG
GGAAATTTAAATTTTCTGAAAAAAAATATTTTGGCG
GGAAATTTAAATTTTCTGAAAAAAAATATTTTGGCG
GGAAATTTAAATTTTCTGTGAAAAATATTTTGGAG
GGAAATTTAAATTTTCTGTGAAAAATATTTTGGCG
GGAAATTTAAATTTTCTGAAAAAAATATTTTGGCG

GGAAATTTAAATTTTCTGAAAAAAATATTTTGGCG
GGAAATTTAAATTTTCTGAAAAAAAATATTTTGGAG
GGAAATTTAAATTTTCTGTGAAAAAATATTTTGGCG
GGAAATTTAAATTTTCTGTGAAAAACATTTTGGCG
GGAAATTTAAATTTTCTGTGAAAAAATATTTTGGCG
GGAAATTTAAATTTTCTGTGAAAAAATATTTTGGAG
GGAAATTTAAATTTTCTGTGAAAAAATATTTTGGAG
GGAAATTTAAATTTTCTGTGAAAAAATATTTTGGCG
GGAAATTTAAATTTTCTGTGAAAATATTTTGGAG
GGAAATTTAAATTTTCTGAAAAAAAATATTTTGGCG
Nr of selected repeats 111 Similarity 0.941169
Consensus:
GGAAATTTAAATTTTCTGtgAAAAAATATTTTGGcG
>Cele-UNSB01_4:14154469-14154585 Satlength=117 Nr of Repeats=4 RepeatLength=29
seed=TACATTGCAA
TACATTGCAAGATTTTCTTACTCTGTAGG
TACATTGCAAGATTTTCTTACTATGTAGG
TACATTGCAAGATCTTCTTACTATGTAGG
TACATTGCAAGATCTTCTTACTATGTAGG
Nr of selected repeats 4 Similarity 0.946360
Consensus:
TACATTGCAAGATcTTCTTACTaTGTAGG
>Cele-UNSB01_4:14405343-14406331 Satlength=989 Nr of Repeats=13 RepeatLength=76
seed=AATGCTAGAG
AATGCTAGAGGACACTCATTTGCACGAAGGGAAGTGGGAGAGACTGTGGAGTTGTCGGTGTCTTTGTGCATGCTCG
AATGCTAGAGGACACTCCTTTGCACGAAAGGAAGTGGGAGAGACTGTGGAGTTGTCGGTGTCTTTGTGCATGCTCG
AATGCTAGAGGACACTCCTTTGCACGAAAGGAAGTGGGAGAGACTGTGGAGTTGTCGGTGTCTTTGTGCATGCTCG
AATGCTAGAGGACACTCCTTTGCACGAAAGGAAGTGGGAGAGACTGTGGAGTTGTCGGTGTCTTTGTGCATGCTCG
AATGCTAGAGGACACTCCTTTGCACGAAAGGAAGTGGGAGAGACTGTGGAGTTGTCGGTGTCTTTGTGCATGCTCG
AATGCTAGAGGACACTCCTTTGCACGAAAGGAAGTGGGAGAGACTGTGGAGTTGTCGGTGTCTTTGTGCATGCTCG
AATGCTAGAGGACACTCCTTTGCACGAAAGGAAGTGGGAGAGACTGTGGAGTTGTCGGTGTCTTTGTGCATGCTCA
AATGCTAGAGGACATTCCTTTGCACGAAAGGAAGTGGGAGAGACTGTGGAGTTGTCGGTGTCTTTGTGCATGCTCA
AATGCTAGAGGACATTCCTTTGCACGAAAGGAAGTGGGAGAGACTGTGGTGATGTCGGTGTCTTTGTGCATGCTCG
AATGCTAGAGGACACTCCTTTGCACGAAAGGAAGTGGGAGAGACTGTGGAGTTGTCGGTGTCTTTGTGCATGCTCA
AATGCTAGAGGACATTCCTTTGCACGAAAAGAAGTGGGAGAGACTGTGGAGTTGTCGGTGTCTTTGTGCATGCTCG
AATGCTAGAGGACACTCCTTTGCACGAAGGGAAGTGGGAGAGACTGTGGAGTTGTCGGTGTCTTTGTGCATGCTCG
AATGCTAGAGGACACTCCTTTGCACGAAGGGAAGTGGGAGAGACTGTGGAGTTGTCGGTGTCTTTGTGCATGCTCA
Nr of selected repeats 13 Similarity 0.967611
Consensus:
AATGCTAGAGGACAcTCCTTTGCACGAAaGGAAGTGGGAGAGACTGTGGAGTTGTCGGTGTCTTTGTGCATGCTCg
>Cele-UNSB01_4:14407623-14408822 Satlength=1200 Nr of Repeats=70
RepeatLength=14 seed=GCTATGCGCT
GCTATGCGCTGTTC
GCTATGCGCTGTTA
GCTATGCGCTGTAA
GCCATGCGCTGTAC
GCTATGCGCTGTAT
GCAATGCGCTATGCGCTGTTA
GCTATGCGCTGTAC
GCCATGCGCTGTAC
GCTATGCGCTGTAT
GCAATGCGCTATGCGCTGTTA
GCTGTGCGCTGTAC
GCCATGCGCTGTAC

GCTATGCGCTGTAT
GCAATGCGCTATGCGCTGTTA
GCTATGCGCTGTAC
GCCATGCGCTGTAC
GCTATGCGCTGTAT
GCAATGCGCTATGCGCTGTTA
GCTGTGCGCTGTAC
GCCATGCGCTGTAC
GCTATGCGCTGTAT
GCAATGCGCTATGCGCTGTTA
GCTGTGCGCTGTAC
GCCATGCGCTGTAC
GCTATGCGCTGTAT
GCAATGCGCTATGCGCTGTTA
GCTATGCGCTGTAC
GCCATGCGCTGTAC
GCTATGCGCTGTAT
GCAATGCGCTATGCGCTGTTAGCGGTGCGCTGTAC
GCCATGCGCTGTAC
GCTATGCGCTGTTA
GCTGTGCGCTGTAC
GCCATGCGCTGTAC
GCTATGCGCTGTTA
GCTGTGCGCTGTAC
GCCATGCGCTGTAC
GCTATGCGCTGTAT
GCAATGCGCTATGCGCTGTTA
GCTGTGCGCTGTAC
GCCATGCGCTGTAC
GCTATGCGCTGTTA
GCTATGCGCTGTAT
GCAATGCGCTATGCGCTGTTA
GCTGTGCGCTGTAC
GCCATGCGCTGTAC
GCTATGCGCTGTTA
GCTGTGCGCTGTAC
GCCATGCGCTGTAC
GCTATGCGCTGTAT
GCAATGCGCTATGCGCTGTTA
GCTATGCGCTGTAC
GCCATGCGCTGTAC
GCTATGCGCTGTAT
GCAATGCGCTATGCGCTGTTA
GCTGTGCGCTGTAC
GCCATGCGCTGTAC
GCTATGCGCTGTTA
GCTGTGCGCTGTAC
GCCATGCGCTGTAC
GCTATGCGCTGTAT
GCAATGCGCTATGCGCTGTAC
GCTATGAGCTATGCAACGATTGGACAATGGATGGATTCGCGATCGATCGGGAATGGACGACGACAATGAGGGCGAAGGA
TCCGATAG
GTATGCGCTATGCGCTGTTC
GCTATGCGCTGTACGCCATGCGCAGTAC

GCTATGCGCTGTAT
GCAATGCGCTATGCGCTGTTA
GCTATGCGCTGTAC
GCCATGCGCTGTACGATATGA
GCTATGCGCTATGCGCTGTACGCTGTAC
Nr of selected repeats 52 Similarity 0.786869
Consensus:
GCtATGCGCTGTAc
>Cele-UNSB01_4:14408867-14409192 Satlength=326 Nr of Repeats=13 RepeatLength=25 seed=TAATGCCAAT
TAATGCCAATTTATAGAATCTGCCA
TAATGCCAATTTATAGAATCTGCCG
TAATGCCAATTTATAGAATCTGCCA
TAATGCCAATTTATAGAATCTGCCG
TAATGCCAATTCATAGAATCTGCCA
TAATGCCAATTTATAGAATCTGCCG
TAATGCCAATTTATAGAATCTGCCG
TAATGCCAATTTTTAGAATCTGCCA
TAATGCCAATTTATAGAATCTGCCG
TAATGCCAATTTATAGAATCTGCCG
TAATGCCAATTTATAGAATCTGCCA
TAATGCCAATTTATAGAATCTGCCG
TAATGCCAATTTTTAGAATCTGCCA
Nr of selected repeats 13 Similarity 0.948034
Consensus:
TAATGCCAATTTATAGAATCTGCCg
>Cele-UNSB01_4:14409220-14409304 Satlength=85 Nr of Repeats=6 RepeatLength=14 seed=AGCGTATAGC
AGCGTATAGCGTAT
AGCGTATAGCGTAT
AGCGTATAGCGTAT
AGCGTATAGCGTAT
AGCGTATAGCGTAT
AGCGTATAGCGTAT
Nr of selected repeats 6 Similarity 1.000000
Consensus:
AGCGTATAGCGTAT
>Cele-UNSB01_4:14410457-14414735 Satlength=4279 Nr of Repeats=57 RepeatLength=76 seed=TCGTGCAAAG
TCGTGCAAAGGAGTGTCCTCTAGCATTCGAGCATGCACAAAGACACCGACAACTCCACAGTCTCTCCCACTTCCCT
TCGTGCAAAGGAGTGTCCTCTAGCATTCGAGCATGCACAAAGACACCGACAACTCCACAGTCTCTCCCACTTCTTT
TCGTGCAAAGGAATGTCCTCTAGCATTTGAGCATGCACAAAGACACCGACAACTCCACAGTCTCTCCCACTTCCTT
TCGTGCAAAGGAGTGTCCTCTAGCATTCGAGCATGCACAAAGACACCGACAACTCCACAGTCTCTCCCACTTCCCT
TCGTGCAAAGGAGTGTCCTCTAGCATTCGAGCATGCACAAAGACACCGACAACTCCACAGTCTCTCCCACTTCTTT
TCGTGCAAAGGAATGTCCTCTAGCATTTGAGCATGCACAAAGACACCGACAACTCCACAGTCTCTCCCACTTCCTT
TCGTGCAAAGGAATGTCCTCTAGCATTTGAGCATGCACAAAGACACCGACAACTCCACAGTCTCTCCCACTTCCTT
TCGTGCAAAGGAATGTCCTCTAGCATTCGAGCATGCACAAAGACACCGACATCACCACAGTCTCTCCCACTTCCTT
TCGTGCAAAGGAATGTCCTCTAGCATTTGAGCATGCACAAAGACACCGACAACTCCACAGTCTCTCCCACTTCCTT
TCGTGCAAAGGAATGTCCTCTAGCATTTGAGCATGCACAAAGACACCGACAACTCCACAGTCTCTCCCACTTCCTT
TCGTGCAAAGGAATGTCCTCTAGCATTTGAGCATGCACAAAGACACCGACAACTCCACAGTCTCTCCCACTTCCTT
TCGTGCAAAGGAATGTCCTCTAGCATTCGAGCATGCACAAAGACACCGACAACTCCACAGTCTCTCCCACTTCCTT
TCGTGCAAAGGAGTGTCCTCTAGCATTCGAGCATGCACAAAGACACCGACAACTCCACAGTCTCTCCCACTTCCTT
TCGTGCAAAGGAGTGTCCTCTAGCATGCGAGCATGCACAAAGACACCGACAACTCCACAGTCTCTCCCACTTCCTT
TCGTGCAAAGGAATGTCCTCTAGCATTTGAGCATGCACAAAGACACCGTCAACTCCACAGTCTCTCCCACTTCCTT

TCGTGCAAAGGAGTGTCCTCTAGCATTTGAGCATGCACAAAGACACCGTCAACTCCACAGTCTCTCCCACTTCCTT
TCGTGCAAAGGAATGTCCTCTAGCATTCGAGCATGCACAAAGACACCGACATCATCACAGTCTCTCCCACTTCCTT
TCGTGCAAAGGAATGTCCTCTAGCATTCGAGCATGCACAAAGACACCGACAACTCCACAGTCTCTCCCACTTCCTT
TCGTGCAAAGGAATGTCCTCTAGCATTCGAGCATGCACAAAGACACCGACATCATCACAGTCTCTCCCACTTCCTT
TCGTGCAAAGGAATGTCCTCTAGCATTCGAGCATGCACAAAGACACCGACATCATCACAGTCTCTCCCACTTCCTT
TCGTGCAAAGGAGTGTCCTCTAGCATTCGAGCATGCACAAAGACACCGACATCATCACAGTCTCTCCCACTTCCTT
TCGTGCAAAGGAGTGTCCTCTAGCATTCGAGCATGCACAAAGACACCGACATCATCACAGTCTCTCCCACTTCCTT
TCGTGCAAAGGAGTGTCCTCTAGCATTTGAGCATGCACAAAGACACCGTCAACTCCACAGTCTCTCCCACTTCCTT
TCGTGCAAAGGAGTGTCCTCTAGCATTCGAGCATGCACAAAGACACCGACATCATCACAGTCTCTCCCACTTCCTT
TCGTGCAAAGGAATGTCCTCTAGCATTCGAGCATGCACAAAGACACCGACATCATCACAGTCTCTCCCACTTCCTT
TCGTGCAAAGGAATGTCCTCTAGCATTCGAGCATGCACAAAGACACCGACATCATCACAGTCTCTCCCACTTCCTT
TCGTGCAAAGGAGTGTCCTCTAGCATTCGAGCATGCACAAAGACACCGACATCATCACAGTCTCTCCCACTTCCTT
TCGTGCAAAGGAGTGTCCTCTAGCATTCGAGCATGCACAAAGACACCGACATCATCACAGTCTCTCCCACTTCCTT
TCGTGCAAAGGAGTGTCCTCTAGCATTCGAGCATGCACAAAGACACCGACATCATCACAGTCTCTCCCACTTCCTT
TCGTGCAAAGGAGTGTCCTCTAGCATTCGAGCATGCACAAAGACACCGACATCATCACAGTCTCTCCCACTTCCTT
TCGTGCAAAGGAGTGTCCTCTAGCATTCGAGCATGCACAAAGACACCGACATCATCACAGTCTCTCCCACTTCCTT
TCGTGCAAAGGAGTGTCCTCTAGCATTCGAGCATGCACAAAGACACCGACATCATCACAGTCTCTCCCACTTCCTT
TCGTGCAAAGGAGTGTCCTCTAGCATTTGAGCATGCACAAAGACACCGTCAACTCCACAGTCTCTCCCACTTCCTT
TCGTGCAAAGGAGTGTCCTCTAGCATTCGAGCATGCACAAAGACACCGACATCATCACAGTCTCTCCCACTTCCTT
TCGTGCAAAGGAATGTCCTCTAGCATTCGAGCATGCACAAAGACACCGACAACTCCACAGTCTCTCCCACTTCCTT
TCGTGCAAAGGAATGTCCTCTAGCATTCGAGCATGCACAAAGACACCGACATCATCACAGTCTCTCCCACTTCCTT
TCGTGCAAAGGAGTGTCCTCTAGCATTCGAGCATGCACAAAGACACCGACATCATCACAGTCTCTCCCACTTCCTT
TCGTGCAAAGGAGTGTCCTCTAGCATTTGAGCATGCACAAAGACACCGTCAACTCCACAGTCTCTCCCACTTCCTT
TCGTGCAAAGGAGTGTCCTCTAGCATTCGAGCATGCACAAAGACACCGACATCATCACAGTCTCTCCCACTTCCTT
TCGTGCAAAGGAGTGTCCTCTAGCATTTGAGCATGCACAAAGACACCGTCAACTCCACAGTCTCTCCCACTTCCTT
TCGTGCAAAGGAATGTCCTCTAGCATTCGAGCATGCACAAAGACACCGACATCATCACAGTCTCTCCCACTTCCTT
TCGTGCAAAGGAATGTCCTCTAGCATTCGAGCATGCACAAAGACACCGACATCATCACAGTCTCTCCCACTTCCTT
TCGTGCAAAGGAGTGTCCTCTAGCATTCGAGCATGCACAAAGACACCGACATCATCACAGTCTCTCCCACTTCCTT
TCGTGCAAAGGAGTGTCCTCTAGCATTCGAGCATGCACAAAGACACCGACATCATCACAGTCTCTCCCACTTCCTT
TCGTGCAAAGGAGTGTCCTCTAGCATTTGAGCATGCACAAAGACACCGTCAACTCCACAGTCTCTCCCACTTCCTT
TCGTGCAAAGGAGTGTCCTCTAGCATTCGAGCATGCACAAAGACACCGACATCATCACAGTCTCTCCCACTTCCTT
TCGTGCAAAGGAATGTCCTCTAGCATTCGAGCATGCACAAAGACACCGACAACTCCACAGTCTCTCCCACTTCCTT
TCGTGCAAAGGAATGTCCTCTAGCATTTGAGCATGCACAAAGACACCGTCAACTCCACAGTCTCTCCCACTTCCTT
TCGTGCAAAGGAATGTCCTCTAGCATTCGAGCATGCACAAAGACACCGACATCATCACAGTCTCTCCCACTTCCTT
TCGTGCAAAGGAATGTCCTCTAGCATTCGAGCATGCACAAAGACACCGACAACTCCACAGTCTCTCCCACTTCCTT
TCGTGCAAAGGAATGTCCTCTAGCATTCGAGCATGCACAAAGACACCGACAACTCCACAGTCTCTCCCACTTCCTT
TCGTGCAAAGGAATGTCCTCTAGCATTCGAGCATGCACAAAGACACCGACATCATCACAGTCTCTCCCACTTCCTT
TCGTGCAAAGGAATGTCCTCTAGCATTCGAGCATGCACAAAGACACCGACATCATCACAGTCTCTCCCACTTCCTT
TCGTGCAAAGGAGTGTCCTCTAGCATTTGAGCATGCACAAAGACACCGTCAACTCCACAGTCTCTCCCACTTCCTT
TCGTGCAAAGGAATGTCCTCTAGCATTCGAGCATGCACAAAGACACCGACAACTCCACAGTCTCTCCCACTTCCTT
TCGTGCAAAGGAATGTCCTCTAGCATTCGAGCATGCACAAAGACACCGACAACTCCACAGTCTCTCCCACTTCCCT
TCGTGCAAAGGAATGTCTTCTG
Nr of selected repeats 56 Similarity 0.948849
Consensus:
TCGTGCAAAGGAaTGTCCTCTAGCATTcGAGCATGCACAAAGACACCGACAaCtcCACAGTCTCTCCCACTTCCTT
>Cele-UNSB01_4:14417295-14419575 Satlength=2281 Nr of Repeats=148
RepeatLength=15 seed=CTCACCACGA
CTCACCACGATGGGT
CTCACCACGAAGGGT
CTCACCACGATGGGC
CTCACTACGAAGGGT
CTCACCACGATGGGC
CTCACCACGAAGGGT
CTCACCACGAAGGGT

CTCACCACGAAGGGT
CTCACCACGATGGGT
CTCACCACGAAGGGT
CTCACCACGATGGGT
CTCACCACGAAGGGT
CTCACCACGAAGGGT
CTCACCACGATGGGT
CTCACCACGATGGTT
CTCACCACGAAGGGT
CTCACCACGATGGGT
CTCACCACGAAGGGTT
TCACCACGATGGGT
CTCACCACGATGGGC
CTCACCACGAAGGGT
CTCACCACGAAGGGT
CTCACCACGAAGGGT
CTCACCACGAAGGGT
CTCACCACGATGGGT
CTCACCACGAAGGGT
CTCACCACGATGGGC
CTCCCCACGATGGGT
CTCACCACGAAGGGC
CTCACCACGATGGGT
CTCCCCACGAAGGGT
CTCACCACGATGGGT
CTCACCACGAAGGGT
CTCACCACGATGGGT
CTCACCACGAAGGGT
CTCACCACGATGGGC
CTCACCACGAAGGGT
CTCACCACGATGGGTA
TCACCACGAAGGGC
CTCACCACGAAGGGC
CTCACCACGAAGGGT
CTCACCACGAAGGGT
CTCATCACGAAGGGT
CTCACCACGAAGGGT
CTCACCACGAAGGGT
CTCACCACGAAGGGT
CTCACCACGAAGGGT
CTCACCACGAAGGGT
CTCATCACGAAGGGT
CTCACCACGATGGGT
CTCACCACGAAGGGTT
TCACCACGATGGGT
CTCACCACGATGGGC
CTCACCACGAAGGGT
CTCACCACGAAGGGT
CTCACCACGAAGGGT
CTCACCACGAAGGGT
CTCACCACGATGGGT
CTCACCACGAAGGGT
CTCACCACGATGGGC
CTCCCCACGATGGGT

CTCACCACGAAGGGC
CTCACCACGATGGGT
CTCCCCACGAAGGGT
CTCACCACGATGGGT
CTCACCACGATGGGT
CTCACCACGATGGGT
CTCACCACGATGGGT
CTCACCACGATGGGAATCACCACGTAGGGT
CTCACCACGAAGGGT
CTCACCACAATGGGT
CTCACCACGAAGGGT
CTCACCACGATGGGT
CTCACCACGATGGGT
CTCACCACGAAGGGT
CTCACCACGATGGGT
CTCACCACGATGGGC
CTCCCCACGATGGGT
CTCACCACGAAGGGC
CTCACCACGATGGGT
CTCCCCACGAAGGGT
CTCACCACGATGGGT
CTCACCACGAAGGGT
CTCACCACGATGGGT
CTCACCACGAAGGGT
CTCACCACGATGGGC
CTCACCACGAAGGGT
CTCACCACGATGGGT
CTCACCACGAAGGGT
CTCACCACGATGGGT
CTCACCACGAAGGGT
CTCACCACGATGGGC
CTCACCACGAAGGGT
CTCACCACGAAGGGT
CTCATCACGAAGGGT
CTCACCACGAAGGGT
CTCACCACGAAGGGT
CTCACCACGAAGGGT
CTCACCACGATGGGT
CTCACCACGATGGGT
CTCACCACGATGGGT
CTCACCACGATGGGT
CTCACCACGATGGGAATCACCACGTAGGGT
CTCACCACGAAGGGT
CTCACCACAATGGGT
CTCACCACGAAGGGT
CTCACCACGATGGGT
CTCACCACGATGGGT
CTCACCACGAAGGGT
CTCACCACGATGGGT
CTCACCACGAAGGGT
CTCACCACGATGGGC
CTCACCACGATGGGT
CTCACCACGATGGGAATCACCACGTAGGGT
CTCACCACGAAGGGT

CTCACCACAATGGGT
CTCACCACGAAGGGT
CTCACCACGATGGGT
CTCACCACGATGGGT
CTCACCACGAAGGGT
CTCACCACGATGGGC
CTCACCACGAAGGGT
CTCACCACGAAGGGT
CTCATCACGAAGGGT
CTCACCACGAAGGGT
CTCACCACGAAGGGT
CTCACCACGATGGGT
CTCACCACGATGGGT
CTCACCACGATGGGAA
TCACCACGATGGGT
CTCACCACGATGGGT
CTCACCACGAAGGGT
CTCACCACGATGGGC
CTCACCACGAAGGGT
CTCACCACGAAGGGT
CTCATCACGAAGGGT
CTCACCACGAAGGGT
CTCACCACGAAGGGT
CTCACCACGATGGGT
CTCACCACGATGGGT
CTCACCACGATGGGTATCATCACGAAGGGT
CTCACCACGATGGGT
CTCACCACGAAGGGTA
TCACCACGAAGGGT
CTCACCACGATGGGT
CTCACCACGAAGGGT
CTCACCACGATGGGT
CTCACCACGAAGGGT
Nr of selected repeats 134 Similarity 0.914962
Consensus:
CTCACCACGAaGGGT
>Cele-UNSB01_4:14618655-14620445 Satlength=1791 Nr of Repeats=7
RepeatLength=200 seed=GAAAAAGTGA
GAAAAAGTGACATTTTCAAATGAAAATTAAAACAAAAAAATCAATAAATAGCAACACAAAACAAGGAAAAAAAAGAAAA
ATAAAGCGAAATAGGTGGAAATCCTAAAAATGCGACTATTTTTGAGAGCGCGCACCAGACTAATGTTCACCGTGAACTT
TTGTGTGCGGTACACTTTTGCCTACCGTACCCGCGGCAAATATGTGTTTCTCGGCAATTTTCATTGGGTTTCTGTAAAA
TTCAAGATTTTATGGCTAATTTCGGAATTTCACAAGGAAAAATGTTTTATTTGAAAAAAAAAATTTGCAATTTTCGAAA
ATTGTCGAATTAATTGAAAACATTGTTTAGAAAAAAAATTGCAGTTTCACTACTTCCATTTCTTACTTCTTATTTTACAT
TGCAAGTCTGCAAATTGAATGGTGTCGCTTTGACGTTTGCCAAAAAAAAAACGTACACTTCCAACAAAAATTTTAATTT
CTGGCTTCAAACTAACTTTTTTTTAAATTCCCGTAGCCATTTTGTAGCAAATTTTATGTTCTTTCAGAATTTAAAAACA
GAATTTCAAAAAATTTTGAAACCGCCAAGTTCAAGCA
GAAAAAGTGAATTTTCAGTCATACCTTGTAGACCACAATTTATATGCAAAATTAATAGATTTTCCAAACTTTGAAAATT
AATTTCTGGCTTCAAACTAACTTTTTTAAAAATTCCCGTAGCCATTTTGTAGCAAATTTTATGTTCTATTAGAATTTCA
AAAGAGAGTTTCAAAAAATTTTGAAACCATTAAGTTCAAGCA
GAAAAAGTGAATTTTCAGTCATACCTTGTAGACCACAATTTATATGCAAAATTAATAGGTTTTGCAAACTTTGAAAATT
AATTTCAGGCTTCAAACTAACTTTTTTTTAAATTCCCGTAGCCATTTTGTAGCAAATTTTATGTTCTTTCAGAATTTAA
AAACAGAATTTCAAAAAATTTTGAAACCGCCAAGTTCAAGCA

GAAAAAGTGAATTTTCAGTCATACCTTGTAGACCACAATTTTCATGCAAAATTAATAGATTTTCCAAACTTTGAAAATT
AATTTCTGGCTTCAACCTAACTTTTTTAAAAATTCCCGTAGCCATTTTGTAGCAAATTTTATGTTCTATTAGAATTTCA
AAAGAGAGTTTCAAAAAATTTTGAAACCATTAAGTTCAAGCA
GAAAAAGTGAATTTTCAGTCATACCTTGTAGACCACAATTTATATGCAAAATTAATAGGTTTTGCAAACTTTGAAAATT
AATTTCAGGCTTCAAACTAACTTTTTTTTAAATTCCCGTAGCCATTTTGTAGCAAATTTTATGTTCTTTCAGAATTTAA
AAACAGAATTTCAAAAAATTTTGAAACCGCCAAGTTCAAGCA
GAAAAAGTGAATTTTCAGTCATACCTTGTAGACCACAATTTTCATGCAAAATTAATAGATTTTCCAAACTTTGAAAATT
AATTTCTGGCTTCAACCTAACTTTTTTAAAAATTCCCGTAGCCATTTTGTAGCAAATTTTATGTTCTATTAGAATTTCA
AAAGAGAGTTTCAAAAAATTTTGAAACCATTAAGTTCAAGCA
GAAAAAGTGAATTTTCAGTCATACCTTGTAGACCACAATTTATATGCAAAATTAATAGGTTTTGCAAACTTTGAAAATT
AATTTCTGGCTTCAAACTAACTTTTTTTTAAATTCCCGTAGCCATTTTGTAGCAAATTTTATGTTCTTTCAGAATTTAA
AAACAGAATTTCAAAAAATTTTGAAACCGCCAAGTTCAAGCA
Nr of selected repeats 6 Similarity 0.937778
Consensus:
GAAAAAGTGAATTTTCAGTCATACCTTGTAGACCACAATTTatATGCAAAATTAATAGaTTTTcCAAACTTTGAAAATT
AATTTCtGGCTTCAAaCTAACTTTTTTaaAAATTCCCGTAGCCATTTTGTAGCAAATTTTATGTTCTaTcAGAATTTaA
AAAcAGAaTTTCAAAAAATTTTGAAACCaccAAGTTCAAGCA
>Cele-UNSB01_4:14628221-14628977 Satlength=757 Nr of Repeats=36 RepeatLength=21
seed=CAACTACCAC
CAACTACCACGACTACCGTAC
CAACTACCACAACTACCGTAC
CAACTACCACAACTACCGTAT
CAACTACCACAACTACCGTAC
CAACTACCACAACTACCGTAC
CAACTACCACAACTACCGTAT
CAACTACCACAACTACCGTAC
CAACTACCACAACTACCGTAC
CAACTACCACAACTACCGTAC
CAACTACCACAACTACCGTAC
CAACTACCACAACATCGGTAC
CAACTACCACGACTACCGTAC
CAACTACCACAACTACCGTAC
CAACTACCACAACTACCGTAT
CAACTACCACAACTACCGTAC
CAACTACCACAACTACCGTAT
CAACTACCACAACTACCGTAC
CAACTACCACAACTACCGTAC
CAACTACCACAACTACCGTAC
CAACTACCACAACTACCGTAC
CAACTACCACAACATCGGTAC
CAACTACCACGACTACCGTAC
CAACTACCACAACTACCGTAC
CAACTACCACAACTACCGTAT
CAACTACCACAACTACCGTAC
CAACTACCACAACTACCGTAC
CAACTACCACAACTACCGTAT
CAACTACCACAACTACCGTAC
CAACTACCACAACTACCGTAC
CAACTACCACAACTACCGTAT
CAACTACCACAACTACCGTAC
CAACTACCACAACTACCGCAC
CAACTACCACAACTACCGTAC
CAACTACCACAACTACCGTAC
CAACTACCACAACTACCGTAT

CAACTACCACAACTACCGTAC
Nr of selected repeats 36 Similarity 0.943361
Consensus:
CAACTACCACAACTACCGTAc
>Cele-UNSB01_4:14648431-14648725 Satlength=295 Nr of Repeats=13 RepeatLength=21 seed=ACTACCGTAC
ACTACCGTACCAACTACCACA
ACTACCGTACCAACTACCACA
ACTACCGCACCAACTACCACA
ACTACCGTACCAACTACCACA
ACTACCGTACCAACTACCACA
ACTACCGTACCAACTACTACA
ACTACCGTACCAACTACCACA
ACTACCGTACCAACTACCACAACCACCGTAACAACTACCACA
ACTACGGTACCAACAACCACG
ACTACCGTACCAACTACCACG
ACTACCGTACCTACTACCACG
ACTACCGTACCTACTACCACG
ACTACCGTACCAACTACAACG
Nr of selected repeats 12 Similarity 0.895142
Consensus:
ACTACCGTACCAACTACCACa
>Cele-UNSB01_4:14678240-14678405 Satlength=166 Nr of Repeats=11 RepeatLength=15 seed=GACCCATCGT
GACCCATCGTGGCGA
GACCTATCGTGGCGA
GACCCATCGTGGTGA
GACCCATCGTGGAAA
GACCCATCGTGGTGA
GACCCATCGTGGCGC
GACCCATCGTGGCGA
GACCCATCGTGGCGA
GACCCATCGTGTTGA
GACCCATCGTGGTGA
GACCCATCGTGGTGA
Nr of selected repeats 11 Similarity 0.878788
Consensus:
GACCCATCGTGGnGA
>Cele-UNSB01_4:14678232-14682258 Satlength=4027 Nr of Repeats=80 RepeatLength=15 seed=CGTGGTGAGA
CGTGGTGAGACCCAT
CGTGGCGAGACCTAT
CGTGGCGAGACCCAT
CGTGGTGAGACCCATCGTGGAAAGACCCAT
CGTGGTGAGACCCATCGTGGCGCGACCCAT
CGTGGCGAGACCCAT
CGTGGCGAGACCCAT
CGTGTTGAGACCCAT
CGTGGTGAGACCCAT
CGTGGTGAGACCCAT
CGTGGTGAGACCCGT
CGTGGTGAGACCCAC
CGTGGTAAGACCCAAAATTTTGGCGGGAAATTTAAATTTTCTGAGAAAAATATTTTGGCGGGAAATTTAAATTTTCTGT
GAAAAATGTTTTGGCGGGAAATTTAAATTTTCTGAGAAAAATATTTTGGCGGGAAATTTAAATTTTCTGTGAAAAATGT

TTTGGCGGGAAATTTAAATTTTCTGAGAAAAATATTTTGGGGGGAAATTTAAATTTTCTGTGAAAAATATTTTGGCGGG
AAATTTAAATTTTCTGTGAAAAATATTTTGGCGGGAAATAAGGTTCTGGAAAATTCTAGAAAATTCTGGAATGTTCCAG
AACTTTCTAGAAAAATCGGGAAATTTCTGGAATGTTCCAGAACCTTCTGGAAAATTCTAGAAAATTCTGGAATGTTCCA
GAACCTTCTGGAAAATTCGAAAAAATTCTGGATTGTTCTAGAACCTTCTGGAAAATTAAAAAAATGTTGCTGCGAGATC
TT
CGTGGTGAGACCCATCGTGGCGCGACCCATCGTGGCGCGACCCAT
CGTGGCGAGACCCAT
CGTGGTGAGACCCAT
CGTGGTGAGACCCAT
CGTGGTGAGACCCAT
CGTGGTGAGACCCGT
CGTGGTAAGACCCAC
CGTGGTAAGACCCAAAATTTTGGCGGGAAATTTAAATTTTCTGAGAAAAATATTTTGGCGGGAAATTTAAATTTTCTGT
GAAAAATATTTTGGCGGGAAATTTAAATATTCTGAGAAAAATATTTTGGAGGGAAATTTAAATTTTCTGTGAAAAATAT
TTTGGCGGGAAATTTAAATTTTCTGTGAAAAATGTTTTGGCGGGAAATTTAAATTTTCTGAGAAAAATATTTTGGGGGG
AAATTTAAATTTTCTGTGAAAAATATTTTGGCGGGAAATTTAAATTTTCTGTGAAAAATATTTTGGCGGGAAATAAGGT
TCTGGAAAATTCTAGAAAATTCTGGAATGTTCCAGAACTTTCTAGAAAAATCGGGAAATTTCTGGAATGTTCCAGAACC
TTCTGGAAAATTCTAGAAAATTCTGGAATGTTCCAGAACCTTCTGGAAAATTCGAAAAAATTCTGGATTGTTCTAGAAC
CTTCTGGAAAATTAAAAAAATGTTGCTGCGAGATCTT
CGTGGTGAGACCCATCGTGGCGCGACCCATCGTGGCGCGACCCAT
CGTGGCGAGACCCAT
CGTGGTGAGACCCAT
CGTGGTGAGACCCAT
CGTGGTGAGACCCAT
CGTGGTGAGACCCGT
CGTGGTAAGACCCAC
CGTGGTAAGACCCAAAATTTTGGCGGGAATTTTAAATTTTCTGAGAAAAATATTTTGGCGGGAAATTTAAATTTTCTGT
GAAAAATATTTTGGCGGGAAATTTAGATTTTCTGAGAAAAATATTTTGGAGGGAAATTTAAATTTTCTGTGAAAAATAT
TTTGGCGGGAAATTTAAATTTTCTGTGAAAAATATTTTGGCGGGAAATTTAAATTTTCTGTGAAAAATATTTTTGCGGG
AAATTGAAATTTTCTGAAAATTCTAAAATTCTGGAAATCTAGAATCTTCTGGAAATTTCGAAAAAATTCTCGAATGTTC
CAGAACTTTCTAGAAAAATCGAGAAAATTCTGGATTGTTCTAGAACCTTCTGGAAAATTAAAAAAATGTTGCTGCGAGA
GGTT
CGTGGCGAGACCCAC
CGTGGTGAGACCCAT
CGTGGTGAGACCAATCGTGGAAAGACCCAT
CGTGGTGAGACCCAT
CGTGGTGAGACCCGT
CGTGGTGAGACCCAC
CGTGGTGAGACCCGT
CGTGGTGAGACCCAT
CGTGGTGAGACCCAT
CGTGGTGAGACCCAT
CGTGGTGAGACCCGT
CGTGGTAAGACCCAC
CGTGGTAAGACCCAAAATTTTGGCGGGAATTTTAAATTTTCTGAGAAAAATATTTTGGCGGGAAATTTAAATTTTCTGT
GAAAAATATTTTGGCGGGAAATTTAGATTTTCTGAGAAAAATATTTTGGAGGGAAATTTAAATTTTCTGTGAAAAATAT
TTTGGCGGGAAATTTAAATTTTCTGTGAAAAATATTTTGGCGGGAAATTTAAATTTTCTGTGAAAAATATTTTTGCGGG
AAATTGAAATTTTCTGAAAATTCTAAAATTCTGGAAATCTAGAATCTTCTGGAAATTTCGAAAAAATTCTCGAATGTTC
CAGAACTTTCTAGAAAAATCGAGAAAATTCTGGATTGTTCTAGAACCTTCTGGAAAATTAAAAAAATGTTGCTGCGAGA
GGTT
CGTGGCGAGACCCAC
CGTGGTGAGACCCAT
CGTGGTGAGACCAATCGTGGAAAGACCCAT
CGTGGTGAGACCCAT

CGTGGTGAGACCCGT
CGTGGTGAGACCCAC
CGTGGTGAGACCCGT
CGTGGTGAGACCCAT
CGTGGTGAGACCCAT
CGTGGTGAGACCCAT
CGTGGTGAGACCCGT
CGTGGTAAGACCCAC
CGTGGTAAGACCCAAAATTTTGGCGGGAATTTTAAATTTTCTGAGAAAAATATTTTGGCGGGAAATTTAAATTTTCTGT
GAAAAATATTTTGGCGGGAAATTTAGATTTTCTGAGAAAAATATTTTGGAGGGAAATTTAAATTTTCTGTGAAAAATAT
TTTGGCGGGAAATTTAAATTTTCTGTGAAAAATATTTTGGCGGGAAATTTAAATTTTCTGTGAAAAATATTTTTGCGGG
AAATTGAAATTTTCTGAAAATTCTAAAATTCTGGAAATCTAGAATCTTCTGGAAATTTCGAAAAAATTCTCGAATGTTC
CAGAACTTTCTAGAAAAATCGAGAAAATTCTGGATTGTTCTAGAACCTTCTGGAAAATTAAAAAAATGTTGCTGCGAGA
GGTT
CGTGGCGAGACCCAC
CGTGGTGAGACCCAT
CGTGGTGAGACCAATCGTGGAAAGACCCAT
CGTGGTGAGACCCAT
CGTGGTGAGACCCGT
CGTGGTGAGACCCAC
CGTGGTGAGACCCGT
CGTGGTGAGACCCAC
CGTGGTGAGACCCGT
CGTGGTGAGACCCAC
CGTGGTAAGACCCAAAATTTTGGCGGGAAATTTAAATTTTCTGAGAAAAATATTTTGGCGGGAAATTTAAATTTTCTGT
GAAAAATATTTTGGCGGGAAATTTAAATTTTCTGAGAAAAATATTTTGGAGGGAAATTTAAATTTTCTGTGAAAAATAC
TTTGGCGGGAAATAAGGTTCTGGAAAATTCTAGAAAATTCTGGAATGTTCCAGAACCTTCTGGAAAATTCGAAAAAATT
CTGGATTGTTCTAGAACCTTCTGGAAAATTAAAAAAATGTTGCTGCGAGATCTT
CGTGGTGAGACCCATCGTGGCGCGACCCAT
CGTGGCGAGACCCAT
CGTGGTGAGACCCAT
CGTGGTGAGACCCAT
CGTGGTGAGACCCGT
CGTGGTGAGACCCAT
CGTGGTGAGACCCGT
CGTGGTAAGACCCAC
CGTGGTAAGACCCAAAATTTTGGCGGGAAATTTAAATTTTCTGTGAAAAATATTTTGGCGGGAAATTTAAATTTTCTGA
GAAAAATATTTTGGAGGGAAATTTAAATTTTCTGTGAAAAATATTTTGGCGGGAAATTTAAATTTTCTGTGAAAAATAC
TTTGGCGGGAAATAAGGTTCTGGAAAATTCTAGAAAATTCTGGAATGTTCCAGAACCTTCTGGAAAATTCGAAAAAATT
CTGGATTGTTCTAGAACCTTCTGGAAAATTAAAAAAATGTTGCTGCGAGAGGTT
CGTGGCGAGACCCAC
CGTGGTGAGACCCAT
CGTGGTGAGACCCATCGTGGAAAGACCCAT
CGTGGTGAGACCCAT
CGTGGTGAGACCCGT
Nr of selected repeats 64 Similarity 0.890917
Consensus:
CGTGGTGAGACCCat
>Cele-UNSB01_4:14942266-14942426 Satlength=161 Nr of Repeats=5 RepeatLength=40
seed=TTTTCTAGAA
TTTTCTAGAAAATTCTGGACCATTCCAGAACTTTCTCGGA
TTTTCTAGAAAATTCTGGACCATTCCGGAA
TTTTCTCGAA
TTTTCTAGAAAGTTCTAGAACATTGCCGATTTTTCCCGAT

TTTTCTAGAAAGTTCTGGAATATTCCAGAATTTTCCCGAA
Nr of selected repeats 3 Similarity 0.755556
Consensus:
TTTTCTAGAAAgTTCTgGAacATTcCaGAatTTTCcCGaa
>Cele-UNSB01_4:15002663-15002793 Satlength=131 Nr of Repeats=5 RepeatLength=26
seed=TGGTTAGAGT
TGGTTAGAGTCATTATTTTAGGTGAA
TGGTTAGAGTCATATTTTTAGGTGAA
TGGTTAGAGTCATTATTTTAGGTGAA
TGGTTAGAGTCATTATTTTAGGTGAA
TGGTTAGAGTCATTATTTTAGGTGAA
Nr of selected repeats 5 Similarity 0.958974
Consensus:
TGGTTAGAGTCATtaTTTTAGGTGAA
>Cele-UNSB01_4:15255716-15255837 Satlength=122 Nr of Repeats=9 RepeatLength=11
seed=TGTGCAAAAA
TGTGCAAAAAT
TGTGCAAAAAA
TGTGTAAAAAA
TGTGCAAAAAAAGCTCTAAAAA
TGTGTAAAAAA
TGTGCAAATAT
TGTGCAAAAAT
TGTGCAAAAAAAGCTCTAAAAA
TGTGCAAAAAT
Nr of selected repeats 7 Similarity 0.838384
Consensus:
TGTGcAAAAAt
>Cele-UNSB01_4:15280499-15283896 Satlength=3398 Nr of Repeats=88
RepeatLength=40 seed=AGAAAGTTCT
AGAAAGTTCTGGAACATTCCAGAAATTTCCCGACTTTTCT
AGAAAGTTCTGGAACATTCCAGAATTTTCTCGATTTTTCT
AGAAAGTTCTGGAACATTCCAGAATTTTCTCGATTTTTCT
AGAAAGTTCTGGAACATTCCAGAAATTTCCCGATTTTTCT
AGAAAGTTCTGGAACATTACAGAAGTTTCCAGATTTTTCT
AGAAAGTTTTGGAACATTCCAGAATTTTCTCGATTTTTCT
AGAAAGTTCTGGAACATTCCAGAAATTTCCCGACTTTTCT
AGAAAGTTCTGGAACATTCCAGAATTTTCTCGATTTTTCT
AGAAAGTTCTGGAACATTCCAGAATTTTCTCGATTTTTCT
AGAAAGTTCTGGAACATTCCAGAAATTTCCCGATTTTTCT
AGAAAGTTCTGGAACATTACAGAAATTTCCAGATTTTTCT
AGAAAGTTTTGGAACATTCCAGAATTTTCTCGATTTTTCT
AGAAAGTTCTGGAACATTCCAAAATTTTCCCGATTTTTCA
AGAAAGTTCTGGAACATTCTAGACTTTTCCCAATTTTTCT
AGAAAGTTCTGGAACATTCCAGAATTTTCTCGATTTTTCT
AGAAAGTTCTGGAACATTACAGAAATTTCCAGATTTTTCT
AGAAAGTTTTGGAACATTCCAGAATTTTCTCGATTTTTCT
AGAAAGTTCTGGAACATTCCAAAATTTTCCCGATTTTTCA
AGAAAGTTCTGGTACATTCTAGACTTTCCCAATTTTTCT
AGAAAGTTCTGGAACATTCCAGAATTTTCTCGATTTTTCT
AGAAAGTTCTGGAACATTCCAGAATTTTCCCGATTTTTCT
AGAAAGTTCTGGAACATTCCAGAATTTTCTCGATTTTTCT
AGAAAGTTCTGGAACATTCCAGAAATTTCCCGATTTTTCT
AGAAAGTTCTGGAACATTACAGAAATTTCCAGATTTTTCT

AGAAAGTTTTGGAACATTCCAGAATTTTCTCGATTTTTCT
AGAAAGTTCTGGAACATTCCAAAATTTTCCCGATTTTTCA
AGAAAGTTCTGGAACATTCC
AGAAATTTCTCGATTTTTCT
AGAAAGTTCTGGAACATTCCAAAATTTTCCCGATTTTTCT
AGAAAGTTCTGGAACATTCCAGAAATTTCCCGATTTTTCT
AGAAAGTTCTGGAACATTACAGAAATTTCCCGATTTTTCT
AGAAAGTTCTGGAACATTACAGAAATTTCCAGATTTTTCT
AGAAAGTTTTGGAACATTCCAGAATTTTCTCGATTTTTCT
AGAAAGTTCTGGAACATTCCAAAATTTTCCCGATTTTTCA
AGAAAGTTCTGGAACATTCTAGACTTTTCCCAATTTTTCT
AGAAAGTTCTGGAACATTACAGAAATTTCCAGATTTTTCT
AGAAAGTTTTGGAACATTCCAGAATTTTCCCGATTTTTCA
AGAAAGTTCTGGAACATTCTAGACTTTTCCCAATTTTTCT
AGAAAGTTCTGGAACATTACAGAAATTTCCAGATTTTTCT
AGAAAGTTCTGGAACATTCCAAAATTTTCCCGATTTTTCA
AGAAAGTTCTGGAACATTCC
AGAAATTTCTCGATTTTTCT
AGAAAGTTCTGGAACATTCCAAAATTTTCCCGATTTTTCA
AGAAAGTTCTGGAACATTCTAGACTTTTCCCAATTTTTCT
AGAAAGTTCTGGAACATTACAGAAATTTCCAGATTTTTCT
AGAAAGTTCTGGAACATTCCAAAATTTTCCCGATTTTTCA
AGAAAGTTCTGGAACATTCTAGACTTTTCCCAATTTTTCT
AGAAAGTTCTGGAACATTACAGAAATTTCCAGATTTTTCT
AGAAAGTTCTGGAACATTCCAAAATTTTCCCGATTTTTCA
AGAAAGTTCTGGAACATTCC
AGAAATTTCTCGATTTTTCT
AGAAAGTTCTGGAACATTCCAAAATTTTCCCGATTTTTCT
AGAAAGTTCTGGAACATTCCAGAATTTTCTCGATTTTTCT
AGAAAGTTCTGGAACATTCCAAAATTTTCCCGATTTTTCA
AGAAAGTTCTGGAACATTCTAGACTTTTCCCAATTTTTCT
AGAAAGTTCTGGAACATTACAGAAATTTCCAGATTTTTCT
AGAAAGTTTTGGAACATTCCAGAATTTTCTCGATTTTTCT
AGAAAGTTCTGGAACATTCCAAAATTTTCCCGATTTTTCA
AGAAAGTTCTGGTACATTCTAGACTTTCCCAATTTTTCT
AGAAAGTTCTGGAACATTCCAGAATTTTCTCGATTTTTCT
AGAAAGTTCTGGAACATTCCAGAATTTTCCCGATTTTTCT
AGAAAGTTCTGGAACATTCCAGAATTTTCTCGATTTTTCT
AGAAAGTTCTGGAACATTCCAGAAATTTCCCGATTTTTCT
AGAAAGTTCTGGAACATTACAGAAATTTCCAGATTTTTCT
AGAAAGTTTTGGAACATTCCAGAATTTTCTCGATTTTTCT
AGAAAGTTCTGGAACATTCCAAAATTTTCCCGATTTTTCA
AGAAAGTTCTGGAACATTCCAGAATTTTCCCGATTTTTCT
AGAAAGTTCTGGAACATTCCAGAATTTTCTCGATTTTTCT
AGAAAGTTCTGGAACATTCCAGAAATTTCCCGATTTTTCT
AGAAAGTTCTGGAACATTACAGAAATTTCCAGATTTTTCT
AGAAAGTTTTGGAACATTCCAGAATTTTCTCGATTTTTCT
AGAAAGTTCTGGAACATTCCAAAATTTTCCCGATTTTTCA
AGAAAGTTCTGGAACATTCTAGACTTTTCCCAATTTTTCT
AGAAAGTTCTGGAACATTCCAGAATTTTCTCGATTTTTCT
AGAAAGTTCTGGAACATTACAGAAATTTCCAGATTTTTCT
AGAAAGTTTTGGAACATTCCAGAATTTTCTCGATTTTTCT
AGAAAGTTCTGGAACATTCCAAAATTTTCCCGATTTTTCA
AGAAAGTTCTGGTACATTCTAGACTTTCCCAATTTTTCT

AGAAAGTTCTGGAACATTCCAGAATTTTCTCGATTTTTCT
AGAAAGTTCTGGAACATTCCAGAAATTTCCCGATTTTTCT
AGAAAGTTCTGGAACATTACAGAAATTTCCCGATTTTTCT
AGAAAGTTCTGGAACATTCCAGAATTTTCCCAATTTTTCT
AGAAAGTTCTGGACCATTCCAGACTTTTCCCAATTTTTCT
AGAAAGTTCTGGAACATTCCAGAAATTTCCCAATTTTTCT
AGAAAGTTCTGGAACATTCTAGACTTTTCCCAATTTTTCT
AGAAAGTTCCGGAACATTACAGAAATTTCCCGATTTTTCT
AGAAAGTTCTGGAACATTCCAGAAATGTCCCGATTTTTCT
AGAAAGTTCTGGAACATTCCAGAATTTTCCCAATTTTTCT
Nr of selected repeats 79 Similarity 0.897220
Consensus:
AGAAAGTTCTGGAACATTcCAGAAtTTTCcCGATTTTTCT
>Cele-UNSB01_4:15284214-15284904 Satlength=691 Nr of Repeats=39 RepeatLength=15
seed=GGTCTCACCA
GGTCTCACCACGACGGGTCTCGTCACGATG
GGTCTCACCACGATG
GGTCTCGCCACGATG
GGTCTCACCACGATG
GGTCTCGCCACGATG
GGTCTCACCACGAAG
GGTCTCACCACGAAG
GGTCTCGCCACGAAGGGTCTCGTCACGATG
GGTCTCACCACGATG
GGTCTCGCCACGATG
GGTCTCACCACGATG
GGTCTCGCCACGATG
GGTCTCACCACGAAG
GGTCTCACCACGAAG
GGTCTCGCCACGAAG
GGTCTCACCACGAAA
GGTCTCACCACGAAG
GGTCTCACCACGAAG
GGTCTCACCACGAAGGATCTTACCACGAAG
GGTCTCACCACGAAG
GGTCTCACCACGAAG
GGTCTCACCACGAAG
GGTCTCACCACGAAG
GGTCTCACCACGAAG
GGTCTCACCACGAAG
GGTCTCGCCACGAAAGGTCTCGCAACGAAG
GGTCTCACCACGAAG
GGTCTCACCACGAAG
GGTCTCGCCACGAAG
GGTCTCACCACGAAG
GGTCTCACCACGAAG
GGTCTCACCACGAAGGATCTTACCACGAAG
GGTCTCACCACGAAA
GGTCTCGCCACGAAAGGTCTCTCAACGAAG
GGTCTCACCACGAAG
GGTCTCGCCACGAAG
GGTCTCACCACGAAG
GGTCTCATCACGAAG
GGTCTCACCACGAAGGATCTTACCACGAAG

Nr of selected repeats 32 Similarity 0.917921
Consensus:
GGTCTCaCCACGAaG
>Cele-UNSB01_4:15313385-15313490 Satlength=106 Nr of Repeats=7 RepeatLength=15
seed=GGGTCTCACC
GGGTCTCACCACGAA
GGGTCTCACCACGAT
GGGTCTCACCACGAA
GGGTCTCACCACGAT
GGGTCTCACCACGAA
GGGTCTCACCACGAA
GGGTCTCACCACGAT
Nr of selected repeats 7 Similarity 0.949206
Consensus:
GGGTCTCACCACGAa
>Cele-UNSB01_4:15313710-15313830 Satlength=121 Nr of Repeats=8 RepeatLength=15
seed=TCGTGGTGAG
TCGTGGTGAGACCCA
TCGTGGTGAGAACCT
TCGTGGTGAGACCCA
TCGTGGTGAGACCCT
TCGTGGTGAGACCCA
TCGTGGTGAGACCCT
TCGTGGTGAGACCCA
TCGTGGTGAGACCCA
Nr of selected repeats 8 Similarity 0.930159
Consensus:
TCGTGGTGAGACCCa
>Cele-UNSB01_4:15399620-15399800 Satlength=181 Nr of Repeats=11 RepeatLength=15
seed=GAGACCCATC
GAGACCCATCGTGGC
GAGACCCATCGTGGC
GAGACCCATCGTGGC
GAGACCCATCGTGGT
GAGACCCGTCGTGGC
GAGACCCATCGTGAT
GAGACCCATCGTGGT
GAGACCCATCGTGGC
GAGACCCATCGTGGT
GAGACCCATCGTGGC
GAGACCCACCGTGGTAAGACCCGTCGTGGT
Nr of selected repeats 10 Similarity 0.917037
Consensus:
GAGACCCATCGTGGc
>Cele-UNSB01_4:15399887-15400027 Satlength=141 Nr of Repeats=4 RepeatLength=35
seed=AAATTTTCTG
AAATTTTCTGTGAAAAATATTTTGGCGGGAAATTT
AAATTTTCTGAGAAAACTATTTTGGAGGGAAATTT
AAATTTTCTGTGAAAAATATTTTGGCGGGAAATTT
AAATTTTCTGTGAAAAATATTTTTGCGGGAAATTG
Nr of selected repeats 4 Similarity 0.904762
Consensus:
AAATTTTCTGtGAAAAaTATTTTgGcGGGAAATTt

>Cele-UNSB01_4:15400092-15400412 Satlength=321 Nr of Repeats=8 RepeatLength=40 seed=GAATGTTCCA
GAATGTTCCAGAACTTTCTAGAACAATCGAGAAAATTCTG
GAATGTTCCAGAACTTTCTAGAAAAATCGAGAAAATTCTG
GAATGTTCCAGAATTTTCTAGAAAAATCGAGAAAATTCTG
GAATGTTCCAGAACTTTCTAGAAAATTCTGGAAAATTCTG
GAATGTTCCAGAACTTTCTAAAAAAATCGAGAAAATTCTG
GAATGTTCCAGAACTTTCTAGAAAAATTGGGAAATTTCTG
GAATGTTCCAGAACTTTCTAAAAAAATCGAGAAAATTCTG
GAATGTTCCAGCACTTTCTAGAAAAATTGGGAAATTTCTG
Nr of selected repeats 8 Similarity 0.897619
Consensus:
GAATGTTCCAGAACTTTCTAgAAAAATcGaGAAAaTTCTG
>Cele-UNSB01_4:15421750-15421870 Satlength=121 Nr of Repeats=8 RepeatLength=15 seed=GGTGAGACCC
GGTGAGACCCTTCGT
GGTGAGACCCATCGT
GGTGAGACCCTTCGT
GGTGAGACCCATCGT
GGTGAGACCCTTCGT
GGTGAGACCCATCGT
GGTGAGACCCATCGT
GGTGAGACCCTTCGC
Nr of selected repeats 8 Similarity 0.926984
Consensus:
GGTGAGACCCaTCGT
>Cele-UNSB01_4:15421887-15425906 Satlength=4020 Nr of Repeats=116 RepeatLength=34 seed=TGGCGGGAAA
TGGCGGGAAATTCAAATTTTCAGTGAAAAAATTT
TGGCGGGAAATTCAAATTTTCAGTGAAAAAATTT
TGGCGGGAAATTCAAATTTTCTGTGAAAAAAATTT
TGGCGGGAAATTCAAGTTTTCAGTGAAAAAATTT
TGGCGGGAAATTCAAATTTTCAGTGAAAAAATTT
TGGCGGGAAATTAAAATTTTCTGTGAAAAAAATAT
TGGCGGGAAATTCAAATTTTCAGTGAAAAAAATT
TGGCGGGAAATTCAAATTTTCAGTGAAAAAATAT
TGGCGGGAAATTCAAGTTTTCAGTGAAAAAATTT
TGGCGGGAAATTCAAGTTTTCTATGAAAAAAATAT
TGGCGGGAAATTCAAATTTTCAGTGAAAAAATAT
TGGCGGGAAATTCAAATTTTCAGTGAAAAAATTT
TGGCGGGAAATTCAAGTTTTCAGTGAAAAAATTT
TGGCGGGAAATTCAAATTTTCAGGGAAAAAATTT
TGGCGGGAAATTCAAATTTTCAGTGAAAAAAATT
TGGCGGGAAGTTCAAATTTTCTGTGAAAAAAATAT
TGGCGGGAAATTCAAATTTTCAGTGAAAAAATTT
TGGCGGGAAATTCAAGTTTTCAGTGAAAAAATTT
TGGCGGGAAATTCAAATTTTCAGTGAAAAAAATATT
TGGCGGGAAATTCAAATTTTCAGTGAAAAAATTT
TGGCGGGAAATTCAAATTTCAGTGAAAAAATTGCAAATTCAAATTCAGTGAAAAAATTT
TGGCGGGAAATTCAAATTTTCAGGTGAAAAAATTT
TGGCGGGAAATTCAAATTTTCAGTGAAAAAATTT
TGGCGGGAAATTCAAATTTTCAGTGAAAAAAATTT
TGGCGGGAAATTCAAATTTTCAGTGAAAAAAATTT
TGGCGGGAAATTCAAATTTTCAGTGAAAAAATTT

TGGCGGGAAATTCAAATTTTCAGTGAAAAAAATTT
TGGCGGGAAATTCAAATTTTCAGTGAAAAAAATTT
TGGCGGGAAATTCAAATTTTCAGTGAAAAAAATTT
TGGCGGGAAATTCAAATTTTCAGTGAAAAAAATTT
TGGCGGGAAATTCAAATTTTCAGTGAAAAAAATTT
TGGCGGGAAATTCAAATTTTCAGTGAAAAAAATT
TGGCGGGAAATTCAAATTTTCAGTGAAAAAAATTT
TGGCGGGAAATTCAAATTTTCAGTGAAAAAAAAAAAAAAAAAAAAT
TGGCGGGAAATTCAAATTTTCAGTGAAAAAAATTT
TGGCGGGAAATTCAAATTTTCAGTGAAAAAAATTT
TGGCGGGAAATTCAAATTTTCAGTGAAAAAAATT
TGGCGGGAAATTCAAATTTTCAGTGAAAAAAATTT
TGGCGGGAAATTCAAATTTTCAGTGAAAAAAATTT
TGGCGGGAAATTCAAATTTTCAGTGAAAAAAATTT
TGGCGGGAAATTCAAATTTTCAGTGAAAAAAATTT
TGGCGGGAAATTCAAATTTTCAGTGAAAAAAATTT
TGGCGGGAAATTCAAATTTTCAGTGAAAAAAATT
TGGCGGGAAATTCAAATTTTCAGTGAAAAAAATTT
TGGCGGGAAATTCAAATTTTCAGTGAAAAAAATTT
TGGCGGGAAATTCAAATTTTCAGTGAAAAAAATTT
TGGCGGGAAATTCAAATTTTCAGTGAAAAAAATT
TGGCGGGAAATTCAAATTTTCAGTGAAAAAAATTT
TGGCGGGAAAAATTCATGAAAAAATT
TGGCGGGAAATTCAAATTTTCAGTGAAAAAAATTT
TGGCGGGAAATTCAAATTTTCAGTGAAAAAAATTT
TGGCGGGAAATTCAAATTTTCAGTGAAAAAAAATTT
TGGCGGGAAATTCAAATTTTCAGTGAAAAAAATTT
TGGCGGGAAATTCAAATTTTCAGTGAAAAAAATTT
TGGCGGGAAATTCAAATTTTCAGTGAAAAAAATTT
TGGCGGGAAATTCAAATTTTCAGTGAAAAAAATTT
TGGCGGGAAATTCAAATTTTCAGTGAAAAAAATTT
TGGCGGGAAATTCAAATTTTCAGTGAAAAAAATTT
TGGCGGGAAATTCAAATTTTCAGTGAAAAAAATTT
TGGCGGGAAATTCAAATTTTCAGTGAAAAAAATTT
TGGCGGGAAATTCAAATTTTCAGTGAAAAAAATTT
TGGCGGGAAATTCAAATTTTCAGTGAAAAAAATTT
TGGCGGGAAATTCAAATTTTCAGTGAAAAAAATTT
TGGCGGGAAATTCAAATTTTCAGTGAAAAAAATTT
TGGCGGGAAATTCAAATTTTCAGTGAAAAAAATTT
TGGCGGGAAATTCAAATTTTCAGTGAAAAAAATTT
TGGCGGGAAATTCAAATTTTCAGTGAAAAAAATTT
TGGCGGGAAATTCAAATTTTCAGTGAAAAAAATTT
TGGCGGGAAATTCAAATTTTCAGTGAAAAAAATTT
TGGCGGGAAATTCAAATTTTCAGTGAAAAAAATTT
TGGCGGGAAATTCAAATTTTCAGTGAAAAAAATTT
TGGCGGGAAATTCAAATTTTCAGTGAAAAAAATTT
TGGCGGGAAATTCAAATTTTCAGTGAAAAAAAAAAAATAAAAAATTT
TGGCGGGAAATTCAAATTTTCAGTGAAAAAAATTT
TGGCGGGAAATTCAAATTTTCAGTGAAAAAAATTT
TGGCGGGAAATTCAAATTTTCAGTGAAAAAAATTT
TGGCGGGAAATTCAAATTTTCAGTGAAAAAAATTT
TGGCGGGAAATTCAAATTTTCAGTGAAAAAAATTT
TGGCGGGAAATTCAAATTTTCAGTGAAAAAAATTT
TGGCGGGAAATTCAAATTTTCAGTGAAAAAAATTT
TGGCGGGAAATTCAAATTTTCTGTGAAAAAAAATTT

TGGCGGGAAATTCAAATTTTCTGTGAAAAAATTT
TGGCGGGAAATTCAAATTTTCTGTGAAAAAAATTT
TGGCGGGAAATTCAAATTTTCTGTGAAAAAAATTTGGCG
TGGCGGGAAATTCAAATTTTCAGTGAAAAAATTT
TGGCGGGAAATTCAAATTTTCAGTGAAAAAATTTTGGCG
TGGCGGGAAATTCAAATTTTCAGTGAAAAAATTT
TGGCGGGAAATTCAAATTTTCAGTGAAAAAATTT
TGGCGGGAAATTCAAATTTTCTGTGAAAAAAATTT
TGGCGGGAAATTCAAATTTTCAGTGAAAAAATTT
TGGCGGGAAATTCGAAATTTCAGTGAAAAAATTT
TGGCGGGAAATTCAAATTTTCAGTGAAAAAAATT
TGGCGGGAAATTCAAATTTTCAGTGAAAAAATTT
TGGCGGGAAATTCAAATTTTCAGTGAAAAACATTT
TGGCGGGAAATTCAAATTTTCAGTGAAAAAATT
TGGCGGGAAATTCAAAATTTCAGTGAAAAAATTT
TGGCGGGAAATTCAAATTTTCAGTGAAAAAATTT
TGGCGGGAAATTCAAATTTTCAGTGAAAAAATTT
TGGCGGGAAATTCAAATTTTCAGTGAAAAAATTT
TGGCGGGAAATTCAAATTTTCAGTGAAAAAATTT
TGGCGGGAAATTCAAATTTTCAGTGAAAAAATTT
TGGCGGGAAATTCAAATTTTCTAGTGAAAAAAATAT
TGGCGGGAAATTCAAATTTTTCAGTGAAAAAATTT
TGGCGGGAAATTCAAATTTTCAGTGAAAAAAATTTTGGCG
TGGCGGGAAATTCAAATTTTCAGTGAAAAAATTT
TGGCGGGAAATTCAAATTTTCTGTGAAAAAAATAT
TGGCGGGAAATTCAAATTTTCAGTGAAAAAATTT
TGGCGGGAAATTCAAATTTTCAGTGAAAAAATTT
TGGCGGGAAATTCAAATTTTTTGTGAAAAAAAATT
TGGCGGGAAATTCAAATTTTCAGTGAAAAAATTT
TGGCGGGAAATTCAAATTTTCAGTAAAAACAATTT
TGGCGGGAAATTCAAATTTTCAGTGAAAAAAAATT
TGGCGGGAAATTCAAAATTTCAGTGAAAAAATTT
TGGCGGGAAATTCAAATTTTCAGTGAAAAAATTT
TGGCGGGAAATTCAAATTTTCAGTGAAAAAATTT
TGGCGGGAAATTCAAATTTTCTATGAAAAAAATAT
TGGCGGGAAATTCAAAATTTTCAGTGAAAAAAATTT
Nr of selected repeats 83 Similarity 0.986125
Consensus:
TGGCGGGAAATTCAAATTTTCAGTGAAAAAATTT
>Cele-UNSB01_4:15434528-15434620 Satlength=93 Nr of Repeats=6 RepeatLength=15
seed=CGAAAAGGGG
CGAAAAGGGGAGCAGAA
CGAAAAGGGGATCTG
CAAAAAGGGGATCTG
CGAAAAGGGGAGATA
CGAAAAGGGGAGATA
CGAAAAGGGGAGCAA
Nr of selected repeats 5 Similarity 0.768889
Consensus:
CgAAAAGGGGAgcta
>Cele-UNSB01_4:15434795-15434872 Satlength=78 Nr of Repeats=5 RepeatLength=15
seed=TCCCCTTTTC
TCCCCTTTTCGTTGC
TCCCCTTTTCGTATC

TCCCCTTTTCGCAGA
TCCCCTTTTTGCAGA
TCCCCTTTTCGTTCTGC
Nr of selected repeats 4 Similarity 0.748148
Consensus:
TCCCCTTTTcGcaga
>Cele-UNSB01_4:15436315-15436698 Satlength=384 Nr of Repeats=11 RepeatLength=35
seed=GGGAAATTCA
GGGAAATTCAAATTTTTGTGAAAAATATTTTGGC
GGGAAATTCAAATTTTTAGTAAAAAATATTTTGGC
GGGAAATTCAGATTTTTAGTGAAAAATATTTTGGC
GGGAAATTCAAATTTTTGTGAAAACTATTTTGGA
GGGAAATTCAAATTTTTAGTAAAAAATATTTTGGGA
GGAAATTCAAATTTTTAGTGAAAAATATTTTGGC
GGGAAATTCAAATTTTTAGTAAAAAATATTTTGGC
GGGAAATTCAAATTTTTAGTGAAAAATATTTTGGT
GGGAAATTCAAACTTTTAGTAAAAAATATTTTGGC
GGGAAATTCAAATTTTTAGTAAAAAATATTTTGGC
GGGAAATTCAAATTTTTAGTAAAAAATATTTTGGT
Nr of selected repeats 7 Similarity 0.941950
Consensus:
GGGAAATTCAAATTTTTAGTaAAAAATATTTTGGc
>Cele-UNSB01_4:15436367-15437145 Satlength=779 Nr of Repeats=9 RepeatLength=35
seed=GTAAAAAATA
GTAAAAAATATTTTGGCGGGAAATTCAGATTTTTA
GTGAAAAATATTTTGGCGGGAAATTCAAATTTTTGTGAAAACTATTTTGGAGGGAAATTCAAATTTTTA
GTAAAAAATATTTTGGGAGGAAATTCAAATTTTTA
GTGAAAAATATTTTGGCGGGAAATTCAAATTTTTA
GTAAAAAATATTTTGGCGGGAAATTCAAATTTTTA
GTGAAAAATATTTTGGTGGGAAATTCAAACTTTTA
GTAAAAAATATTTTGGCGGGAAATTCAAATTTTTA
GTAAAAAATATTTTGGCGGGAAATTCAAATTTTTA
GTAAAAAATATTTTGGTGGGAAATTCAAACTTTCTGAAATTTTAAAATTCTGGAAATTTCGAAAAAATTCTGGAACGTT
CCAGAACTTTCTAGAAAAATCGGGAAAAGTCTTCAATGTTCCAGAATTTTCTAGAAAAATCGGGAAATTCTGGAATGTT
CCAGAACTTTCTAGAAAAATCGGGAAAAGTCTTCAATGTTCCAGAAAATTCTAAAAAAAATCGGGAAAACTCCGGAACG
TTCCAGAACTTTCTAGAAAAATCGGGAAAAATTCTGGAATGTTCTAAATCGCTATAGAAAATTCAAGAATTGTCTGAAAT
GTTCCAGAACTTTCTAAAAAAATTCAACAAAACTCTGGAATGATCTTCTGGAAAAGTCTAGAAGTTCGTGGAGCGTTCTA
AAATTTTCTGAAATTTCTAGAATCTTTTAAAAAGATCAAAAGTTGTATAAATGCCATGAACTTAAAAAT
Nr of selected repeats 7 Similarity 0.929252
Consensus:
GTaAAAAATATTTTGGcGGGAAATTCAAATTTTTA
>Cele-UNSB01_4:15460070-15462017 Satlength=1948 Nr of Repeats=10
RepeatLength=168 seed=AAAAACAAAA
AAAAACAAAATTCAACAAAAAATGTACGAAAAAGTGTTTTTTTTTTCGGAGTGTTCATAACATTTGTGAAAAAAGTCTA
ACTATGTTTTCTCTAGTTATAAAGAAATTCGTGCTTTTTTCGGATTTAAAAGAATAATTATTGACTAAATATTCGACTT
ATATCTATTATTTCGGAACTTTCATATAATTGTAAAATCACTTTTGAAAACAATATGAAACAGTAGGTTTTTTCGGAAA
TTATTGGAGTTTTTCATTCGTGAGTGTAGCATTTGATATATGGAAAAATCGTTCTATCTAGAGACACAATCGTGTTTCA
GTGTGTTATTTAAAAAAAAGGAGTTTACAAAACATTTTATTGTGCCGATTGAATATTTATTAAAGTTACTTCAATAAAC
AAGAAGAAATTTCTGAAATTAACATTGCTGACAAAACTAAATCAGTTGAAAACCGCTACACACACTCCTTGAAACGGTA
TATGAACGAAAATTGGCATTCCTGTAAAAAGT
AAAAACAAAATGAATTTTCATAAAATCAGACGACTGGAGAAGTCTCAGCGGAAATTGTTTT
AAAATCAAAACTGATTCGAACAGTTTGTATTTCAACTTCAATATCACAGTGACACCGAAAAGAATTGTTTTCATTGAAA
GTTAATATCAGATTGAAACAGTAATGTTTGTAAATATGTGCATTTGATATATGGAAAAATCGTTCTATCTAGAGACACA
ATCGTGTTGGGGTTTTTGTCTGGAATTCCAATTTCAACAGGTACAA

AAAAACAAAAATAAAAATTTTGGAATGTTGGGAGAATTTTTATAGATTTGGATTCTAGATTTTTAACAAATATTTTTGG
TAAGTTTCTGGACCTAGATATCAATTTTCAGATGCGCTAGAGGGGTTTTTGTCTTGAATTCCAATTTCAACAGATATAT
ACAGGCACAA
AAAAACAAAAATAAAAATTTTGGAATGTTGGGAGAATTTTTATAGATTTGGATTCTAGATTTTTAACAAATATTTTTGG
TAAGTTTCTGGACCTAGATATCAATTTTCAGATGCGCTAGAGGGGTTTTTGTGTTGAAATTTATTTTTCACAGGTATAT
ACAGGCACAA
AAAAACAAAAATAAAAATTTTGGAATGTTGGGAGAATTTTTATAGATTTGGATTCTAGATTTTTAACAAATATTTTTGG
TAAGTTTCTGGACCTAGATATCAATTTTCAGATGCGCTAGAGGGGTTTTTGTCTTGAATTCCAATTTCAACAGATATAT
ACAGGCACAA
AAAAACAAAAATAAAAATTTTGGAATGTTGGGAGAATTTTTATAGATTTGGATTCTAGATTTTTAACAAATATTTTTGG
TAAGTTTCTGGACCTAGATATCAATTTTCAGATGCGCTAGAGGGGTTTTTGTCTTGAATTCCAATTTCAACAGATATAT
ACAGGCACAA
AAAAACAAAAATAAAAATTTTGGAATTTTGGGAGAATTTTTATAGATTTGGATTCTAGATTTTTAACAAATATTTTTGG
TAAGTTTCTGGACCTAGATATCAATTTTCAGATGCGCTAGAGGGGTTTTTGTGTTGAAATTTATTTTTCACAGGTATAT
ACAGGCACAA
AAAAACAAAAATAAAAATTTTGGAATTTTGGGAGAGTTTTTATAGATTTGGATTCTAGATTTTTAACAAATATTTTTGG
TAAGTTTCTGGACCTAGATATCAATTTTCAGATGCGCTAGAGGGGTTTTTGTCTTGAATTCCAATTTCAACAGATATAT
ACAGGCACAA
AAAAACAAAAATAAAAATTTTGGAATTTTGGGAGAATTTTTATAGATTTGGATTCTAGATTTTTAAAAAATATTTTTGG
TAAGTTTCTGGACCTAGATATCAATTTTCAGACGCGCTAGAGGGGTTTTTGTCTGGAATTCCAATTTCAACAGATATAT
ACAGGCACAA
Nr of selected repeats 7 Similarity 0.956160
Consensus:
AAAAACAAAAATAAAAATTTTGGAATgTTGGGAGAATTTTTATAGATTTGGATTCTAGATTTTTAACAAATATTTTTGG
TAAGTTTCTGGACCTAGATATCAATTTTCAGATGCGCTAGAGGGGTTTTTGTcTTGAAtTccAaTTTcaACAGaTATAT
ACAGGCACAA
>Cele-UNSB01_4:15460844-15462434 Satlength=1591 Nr of Repeats=9
RepeatLength=168 seed=AACAAAAATA
AACAAAAATAAAAATTTTGGAATGTTGGGAGAATTTTTATAGATTTGGATTCTAGATTTTTAACAAATATTTTTGGTAA
GTTTCTGGACCTAGATATCAATTTTCAGATGCGCTAGAGGGGTTTTTGTCTTGAATTCCAATTTCAACAGATATATACA
GGCACAAAAA
AACAAAAATAAAAATTTTGGAATGTTGGGAGAATTTTTATAGATTTGGATTCTAGATTTTTAACAAATATTTTTGGTAA
GTTTCTGGACCTAGATATCAATTTTCAGATGCGCTAGAGGGGTTTTTGTGTTGAAATTTATTTTTCACAGGTATATACA
GGCACAAAAA
AACAAAAATAAAAATTTTGGAATGTTGGGAGAATTTTTATAGATTTGGATTCTAGATTTTTAACAAATATTTTTGGTAA
GTTTCTGGACCTAGATATCAATTTTCAGATGCGCTAGAGGGGTTTTTGTCTTGAATTCCAATTTCAACAGATATATACA
GGCACAAAAA
AACAAAAATAAAAATTTTGGAATGTTGGGAGAATTTTTATAGATTTGGATTCTAGATTTTTAACAAATATTTTTGGTAA
GTTTCTGGACCTAGATATCAATTTTCAGATGCGCTAGAGGGGTTTTTGTCTTGAATTCCAATTTCAACAGATATATACA
GGCACAAAAA
AACAAAAATAAAAATTTTGGAATTTTGGGAGAATTTTTATAGATTTGGATTCTAGATTTTTAACAAATATTTTTGGTAA
GTTTCTGGACCTAGATATCAATTTTCAGATGCGCTAGAGGGGTTTTTGTGTTGAAATTTATTTTTCACAGGTATATACA
GGCACAAAAA
AACAAAAATAAAAATTTTGGAATTTTGGGAGAGTTTTTATAGATTTGGATTCTAGATTTTTAACAAATATTTTTGGTAA
GTTTCTGGACCTAGATATCAATTTTCAGATGCGCTAGAGGGGTTTTTGTCTTGAATTCCAATTTCAACAGATATATACA
GGCACAAAAA
AACAAAAATAAAAATTTTGGAATTTTGGGAGAATTTTTATAGATTTGGATTCTAGATTTTTAAAAAATATTTTTGGTAA
GTTTCTGGACCTAGATATCAATTTTCAGACGCGCTAGAGGGGTTTTTGTCTGGAATTCCAATTTCAACAGATATATACA
GGCACAAAAA
AACAAAAATAAAAATTTTGGAATTTTGGCAGCATTTTTATAGATCACATAATATTTTACTCTCTGGCTTCACAGAATAT
TTTATCCTCTGGCTTCACCGCAATAAATGTGCAAAAAATGTGCAAAAAATGTGCAAAAAATGTGCAATAAATGTGCAAT
AAATGTGCAAAAAATGTG
AAAAAAATAAATTTGTGAAATGGTGCAGAAAAATTCATAGATTGTATTCATAGATCCCAAAAATCTGAAAATGAGTTTT
CTTTTAGTAAATGCACAAATTTGGTTTTACTTTATGTAATTTCAAAAGTTGTAGTGGATTCGAGTATATGTCATTAGAC

TAATTTTGACTGTTTCAACAAGACTACCTATGTTAAAACTTATGATTTTTAATATTTTTGATTTTCGTTTTACATCGCT
C
Nr of selected repeats 7 Similarity 0.956160
Consensus:
AACAAAAATAAAAATTTTGGAATgTTGGGAGAATTTTTATAGATTTGGATTCTAGATTTTTAACAAATATTTTTGGTAA
GTTTCTGGACCTAGATATCAATTTTCAGATGCGCTAGAGGGGTTTTTGTcTTGAAtTccAaTTTcaACAGaTATATACA
GGCACAAAAA
>Cele-UNSB01_4:15529257-15530020 Satlength=764 Nr of Repeats=4 RepeatLength=14
seed=TTGTAGTTTG
TTGTAGTTTGTAGT
TTGTAATTTGTAGT
TTGTAGTTTGTAGT
TTATAGTTTGACCTTTGGTTTATCCTGTGATGAATACGCAGCCGACATAAACCAAAGTCTGCGTTGAGCACGCCTAATT
TTTTTTGGTTGAGTACAAGACCGTGAGTACTGTAGGATTACTGTATAGATATTCATGGAAAACGGTAAATATCACTATT
TTCAGGTTTTCTCAACAAACTTCAAGTCTCAGAAGTATCAGACGACGGATGTTTGTACGAAATTCTTTGCGCCCAAAAT
CAGCCAAATAGTTATTTGGCGGCCGGTCCGCCATCAGTTTTTACACTGAATCCATACCTACTGACCAGTTTTGTAAGTT
TTTGATAGTTATCCGCTTCCATTTTTTCTTAGTTTTCAGAGATTCTATCCGGTTTTCAACGGGGCCAACAAAATCGATT
ATTTCTCCCAATTTGGTATGCAATGTGGAGATGATCAGGACTGGTATATAACCAAAATCCCTGGAATGGTAGATATTTG
GAATTTAAATAGTTTAAGGAAATTGATTAACACATTTTTTTGAATTGAAGGTTGACTTGAGTCCCGGAGCAAATGAGCA
GAAAGTCAATGGAACTATGTGGAATGGGATGGGTTATCGCTTCAGAATCACCGAGATGCAATGTTTTGTGCCACTGGGA
TGATGAGCATATTAAAACATTTTTTCAAATGCAAAATTCAATATCGATTGAAGCTAAAAATTCGAATAAACTAATTTTA
ACACACTACA
Nr of selected repeats 3 Similarity 0.936508
Consensus:
TTGTAgTTTGTAGT
>Cele-UNSB01_4:15554617-15554707 Satlength=91 Nr of Repeats=6 RepeatLength=15
seed=TCGTGGTGAG
TCGTGGTGAGAACCA
TCGTGGTGGGACCCT
TCGTGGTGAGACCCA
TCGTGGTGAGACCCT
TCGTGGTGAGACCCA
TCGTGGTGAGACCCT
Nr of selected repeats 6 Similarity 0.887407
Consensus:
TCGTGGTGAGACCCa
>Cele-UNSB01_4:15554734-15555678 Satlength=945 Nr of Repeats=27 RepeatLength=35
seed=GAAATTCAAA
GAAATTCAAATTTTAAGTGAAAAAAAATTTTGGCGG
GAAATTCAAATTTTCAGTGAAAAAAAATTTTGGCGG
GAAATTCAAATTTTCAGTGAAAAAAAATTTTAGCAG
GAAATTCAAATTTTCAGTGAAAAAAATTTGGGCGG
GAAATTCAAATTTTCAGTGAAAAAAAATTTTAGCGG
GAAATTCAAATTTTCAGTAAAAAAAAATTTTGGCGA
GAAATTCAAATTTTCAGTGAAAAAAAATTTTAGCGG
GAAATTCAAATTTTCAGTGAAAAAAAATTTTGGCGG
GAAATTCAAATTTTCAGTGAAAAAAAATTTTTGGCGG
GAAATTCAAATTTTCAGTGAAAAAAAATTTTGGCGG
GAAATTCAAATTTTCAGTGAAAAAAAATTTTAGCGG
GAAATTCAAATTTTCAGTAAAAAAAAATTTTGGCGG
GAAATTCAAACTTTCAGTGAAAAAAAATTTTAGCGG
GAAATTCAAATTTTCAGTAAAAAAATATTTTAGCGG
GAAATTCAAATTTTCAGTAAAAAAAAATTTTGGCGG
GAAATTCAAACTTTCAGTGAAAAAAAATTTTAGCGG

GAAATTCAAATTTTCAGTAAAAAAAAATTTTGGCGG
GAAATTCAAATTTTCAGTGAAAAAAAATTTTGGCGG
GAAATTCAAATTTTTAGCGAAAAAAAAATTTTGGCGG
GAAATTCAAATTTTCAGTGAAAAAAAATTTTGGCGG
GAAATTCAAATTTTCAGTGAAAAAAATTTGGGCGG
GAAATTCAAATTTTCAGTGGAAAAAAAATTTTGGCGG
GAAATTCAAATTTTCAGTGAAAAAAATTTGGGCGG
GAAATTCAAATTTTCAGTGAAAAAAATTTGGGCGG
GAAATTCAAATTTTCAGTGAAAAAAAAAATTTGGCGG
GAAATTCAAACTTTCAGTGAAAAAAAATTTTGGCGG
GAAATTCAAATTTTCAGTGAAAAAAATTTTGGCGG
Nr of selected repeats 18 Similarity 0.936508
Consensus:
GAAATTCAAATTTTCAGTgAAAAAAATTTTgGCGG
>Cele-UNSB01_4:15607346-15608026 Satlength=681 Nr of Repeats=17 RepeatLength=40
seed=GAACATTCCA
GAACATTCCAGAATTTTCTCGATTTTTCTAGAAAGTTCTG
GAACATTCCAGAATTTTCTCAATTTTTCTAGAAAGTTCTT
GAACATTCCAGACTTTTCCCAATTTTTCTAGAAAGTTCTG
GAACATTCCAGAATTTTCTCAATTTTTCTAGAAAGTTCTT
GAACATTCCAGACTTTTCCCAATTTTTCTAGAAAGTTCTG
GAACATTCCAGAAATTTCCCGATTTTTCTAGAAAGTTCTG
GAACATTCCAGAATTTTCTCAATTTTTCTAGAAAGTTCTT
GAACATTCCAGACTTTTCCCAATTTTTCTAGAAAGTTCTG
GAACATTCCAGAATTTTTTTCGATTTTTCTAGAAAGTTCTG
GAACATTCCAGAATTTTCTCGATTTTTCTAGAAAGTTCTT
GAACATTCCAGAAATTTCCCGATTTTTCTAGAAAGTTCTG
GAACATTCCAGACTTTTCCCAATTTTTCTAGAAAGTTCTG
GAACATTCCAGACTTTTCCCAATTTTTCTAGAAAGTTCTG
GAACATTCCAGACTTTTCCCAATTTTTCTAGAAAGTTCTG
GAACATTCCAGAATTTTCTCGATTTTTCTAGAAAGTTCTT
GAACATTCCAGAAATTTCCCGATTTTTCTAGAAAGTTCTG
GAACATTCCAGACTTTTCCCAATTTTTCTAGAAAGTTCTG
Nr of selected repeats 17 Similarity 0.919608
Consensus:
GAACATTCCAGAaTTTTCcCaATTTTTCTAGAAAGTTCTg
>Cele-UNSB01_4:15608407-15609476 Satlength=1070 Nr of Repeats=51
RepeatLength=15 seed=CCACGATGGG
CCACGATGGGTCTCA
CCACGATGGGTCTCA
CCACGATGGGTCTCA
CCACGATGGGTCTCA
CCACGATGGGTCTCG
CCACGATGGGTCTCA
CCACGATGGGTCTTT
CCACGATGGGTCTCA
CCACGGTGGGTCTCG
CCACGATGGGTCTCG
CCACGATTGGTCTCGCCACGAAGATCTCGCAGCAACATTTTTTTAATTTTCCAGAAGGTTCTAGAACAATCCAGAATTT
TTTCGAATTTTCCAGAAGGTTCTGGAACATTCCAGAATTTTCTAGAATTTTCCAGAACCTTATTTCCCGCCAAAATATT
TTTCACAGAAAATTTAAATTTCCCGCCAAAATATTTTTCTCAGAAAATTTAAATTTCCCGCCAAAATTTTGGGTCTTA
CCACGGTGGGTCTCA
CCACGACGGGTCTCA
CCACGATGGGTCTCT

CCACGATGGGTCTCA
CCATGATGGGTCTCA
CCACGATGGGTCTCA
CCACGATGGGTCTCG
CCACGAAGGGTCTCG
CCACGATGGGTCTCG
CCACGAAGGGTCTCG
CCACGATGGGTCGCG
CCACGATGGGTCTCACCACGAAGATCTCG
CCACGATGGGTCTCA
CCACGATGGGTCTCA
CCACGATGGGTCTCA
CCACGATGGGTCTCG
CCACGATGGGTCTCACCACGAAGATCTCT
CCACGATGGGTCTCA
CCACGATGGGTCTCA
CCACGATGGGTCTCA
CCACGATGGGTCTCACCACGAAGATCTCG
CCACGATGGGTCTCA
CCACGATGGGTCTCA
CCACGATGGGTCTCA
CCACGATGGGTCTCG
CCACGATGGGTCTCACCACGAAGATCTCTCCACGAAGATCTCG
CCACGATGGGTCTCA
CCACGATGGGTCTCA
CCACGATGGGTCTCA
CCACGATGGGTCGCG
CCACGATGGGTCTCACCACGAAGATCTCT
CCACGATGGGTTCA
CCACGATGGGTCTCA
CCACGATGGGTCTCA
CCACGATGGGTCTCG
CCACGATGGGTCTCA
CCACGGTGGGTCTCG
CCACGATGGGTCTCA
CCACGGTGGGTCTCG
CCACGATGGGTCTCG
Nr of selected repeats 44 Similarity 0.911299
Consensus:
CCACGATGGGTCTCa
>Cele-UNSB01_4:15642758-15643070 Satlength=313 Nr of Repeats=12 RepeatLength=26
seed=CAGAGTCACT
CAGAGTCACTAAATTTGGTGAACGGC
CAGAGTCACTATTTTTGGTGAACGGC
CAGAGTCACTATTTTTGGTGAACGGC
CAGAGTCACTAAATTTGGTGAACGGC
CAGAGTCACTATTTCTGGTGAACGGC
CAGAGTCACTATTTTTGGTGAACGGC
CAGAGTCACTATTTTTGGTGAACGGT
CAGAGTCACTATTTTTGGTGATCGGC
CAGAGTCACTATTTTTGGTGAACGGC
CAGAGTCACTATTTTTGGTGAACGGC
CAGAGTCACTATTTTTGGTGAACGGC
CAGAGTCACTATTTTTGGTGAACGGC

Nr of selected repeats 12 Similarity 0.943279
Consensus:
CAGAGTCACTATTTTTGGTGAACGGC
>Cele-UNSB01_4:15644313-15644403 Satlength=91 Nr of Repeats=8 RepeatLength=10 seed=TGTTATGATA
TGTTATGATAA
GTTATGATAG
TGTTATGTTATGATA
TGTTATGATA
TGTTATGTTA
TGTTATGTTA
TGTTATGTTATGAG
GTTATGATAG
Nr of selected repeats 5 Similarity 0.763636
Consensus:
tGTTATGaTA
>Cele-UNSB01_4:15744856-15746870 Satlength=2015 Nr of Repeats=12 RepeatLength=168 seed=GAATTCCGGA
GAATTCCGGAAATGTTGTGATAGATTTGGATTCTAGATTTTTTAAAAATATTATTGAAAGTTTGTGGACCAGGAAAGTA
AATTTGAGCCGCGCTAAAGAGGTTTTTGTGTCGAGTTCCAATTTTCACAGTTATATACTGGCACAAAAAAACAAAAATA
AAAATTATG
GAATTCCGGAAATGTTTTGATAGATTTGGATTCTGGTTTTTTTTTAAATATTATTGGAAAGTTTGTGGACCAGGAAAGT
AAATTTAAGCCGCGCTAAAGAGGTTTTTGTGTCGAGTTCCAATTTTTACAGGTACTAGTAGGCGCAAAAAAACAAAAAT
AAAAATTATG
GAATTCCGGAAATGTTTTGATAGATTTGGATTCTAGATTTTTTAAAAAATATTATAGGTGAGTTTTTGGGACAGGAAAGT
AACTTTAAGCCGCGCTAGAGAGGTTTTTGTGTCGAGTTCCAATTTTTATAGGTACTAGTAGGCGCAAAAAAACAAAAAT
AAAAATTATG
GAATTCCGGAAATGTTTTGATAGATTTGGATTCTAGATTTTTTTTTAAATATTATAGGTGAGTTTTTGGGACAGGAAAGT
AACTTAAAGCCGCGCTAGAAAGGTTTTTGTGTCGAGTTACAATTTTCACAGGTACTAGTAGGCGCAAAAAAAAAAAATA
AAAATTATG
GAATTCCGGAAATGTTTTGATAGATTTGGATTCTAGATTTTTTAAAAAATATTATAGGTGAGTTTTTGGGACAGGAAAGT
AACTTTAAGCCGCGCTAGAGAGGTTTTTGTGTCGAGTTCCAATTTTTACAGGTACTAGTAGGCGCAAAAAAACAAAAAT
AAAAATTATG
GAATTCCGGAAATGTTTTGATAGATTTGGATTCTAGATTTTTTAAAAAATATTATAGGTGAGTTTTTGGGACAGGAAAGT
AACTTTAAGCCGCGCTAGAGAGGTTTTTGTGTCGAGTTACAATTTTCACAGGTACTAGTAGGCACAAAGAAACAAAAAT
AAAAATTATG
GAATTCCGGAAATGTTTTGATAGATTTGGATTCTAGATTTTTTAAAAAATATTATAGGTGAGTTTTTGGGACAGGAAAGT
AACTTAAAGCCGCGCTAGAAAGGTTTTTGTGTCGAGTTACAATTTTCACAGGTACTAGTAGGCACAAAGAAACAAAAAT
AAAAATTATG
GAATTCCGGAAATGTTTTGATAGATTTGGATTCTAGATTTTTTAAAAAATATTATAGGTGAGTTTTTGGGACAGGAAAGT
AACTTAAAGCCGCGCTAGAAAGGTTTTTGTGTCGAGTTACAATTTTCACAGGTACTAGTAGGCACAAAGAAACAAAAAT
AAAAATTATG
GAATTCCGGAAATGTTTTGATAGATTTGGATTCTAGATTTTTTAAAAAATATTATAGGTGAGTTTTTGGGACAGGAAAGT
AACTTAAAGCCGCGCTAGAAAGGTTTTTGTGTCGAGTTACAATTTTCACAGGTACTAGTAGGCACAAAGAAACAAAAAT
AAAAATTATG
GAATTCCGGAAATGTTTTGATAGATTTGGATTCTAGATTTTTTAAAAAATATTATAGGTGAGTTTTTGGGACAGGAAAGT
AACTTAAAGCCGCGCTAGAAAGGTTTTTGTGTCGAGTTACAATTTTCACAGGTACTAGTAGGCGCAAAAAAACAAAAAT
AAAAATTATG
GAATTCCGGAAATGTTTTGATAGATTTGGATTCTAGATTTTTTAAAAAATATTATAGGTGAGTTTTTGGGACAGGAAAGT
AACTTAAAGCCGCGCTAGAAAGGTTTTTGTGTCGAGTTACAATTTTCACAGGTACTAGTAGGCGCAAAAAAACAAAAAT
AAAAATTATG
GAATTCCGGAAATGTTTTGATAGATTTGGATTCTAGATTTTTTAAAAAATATTATAGGTGAGTTTTTGGGACAGGAAAGT
AACTTAAAGCCGCGCTAGAAAGGTTTTTGTGTCGAGTTACAATTTTCACAGGTACTAGTAGGCGCAAAAAAACAAAAAT
AAAAATTATG

Nr of selected repeats 10 Similarity 0.953439
Consensus:
GAATTCCGGAAATGTTTTGATAGATTTGGATTCTAGATTTTTTAAAAATATTATAGGTGAGTTTTTGGGACAGGAAAGT
AACTTaAAGCCGCGCTAGAaAGGTTTTTGTGTCGAGTTaCAATTTTcACAGGTACTAGTAGGCgCAAAaAAACAAAAAT
AAAAATTATG
>Cele-UNSB01_4:15750800-15751080 Satlength=281 Nr of Repeats=8 RepeatLength=35
seed=TTGCCGGATT
TTGCCGGATTTGAAAATTTATGGTAATTTGTCGTT
TTGCCGGATTTGAAAATTTATGGTAATTTGCCGTT
TTGCCGGATTTGAAAATTTATGGTAATTTGCCGTT
TTGCCGGATTTGAAAATTTATGGTAATTTGCCGTT
TTGCCGGATTTGAAAATTTATGGTAATTTGTCGTT
TTGCCGGATTTGAAAATTTATGGTAATTTGCCGTT
TTGCCGGATTTGAAAATTTATGGTAATTTGCCGTT
TTGCCGGATTTGAAAATTTATGGTAATTTGCCGTT
Nr of selected repeats 8 Similarity 0.983673
Consensus:
TTGCCGGATTTGAAAATTTATGGTAATTTGcCGTT
>Cele-UNSB01_4:15869737-15871646 Satlength=1910 Nr of Repeats=128
RepeatLength=14 seed=GGACTAGGGA
GGACTAGGGACTAG
GGACTAAGGACTAG
GGACTAGGGACTAG
GGACTAAGGACTAG
GGACTAGGGACTAG
GGACTAAGGACTAG
GGACTAGGGACTAG
GGACTAGGGACTAG
GGACTAGGGACTAG
GGACTAGGGACTAG
GGACTAGGGACTAG
GGACTAGGGACTGG
GGACTAGGGACTAG
GGACTGGGGACTAG
GGACTAGGGACTAG
GGACTAGGGACTGA
GGACTAGGGACTAG
GGACTAGGGACTAG
GGACTAGGGACTAG
GGACTAGGGACTAG
GGACTAGGGACTAG
GGACTGGGGACTAG
GGACTAGGGACTAG
GGACTAAGGACTAG
GGACTAGGGACTAG
GGACTAAGGACTAG
GGACTAGGGACTAG
GGACTAGGGACTAG
GGACTAGGGACTAG
GGACTAGGGACTAG
GGACTAGGGACTAG
GGACTAGGGACTAG
GGACTAGGGACTAG
GGACTAGGGACTAG

GGACTAGGGACTAG
GGACTAGGGACTAG
GGACTAGGGACTAG
GGACTAGGGACTAG
GGACTAGGGACTAG
GGACTAGGGACTGA
GGACTAGGGACTAG
GGACTAGGGACTA
GGACTAGGGACTAG
GGACTAGGACTAG
GGACTAGGGACTAG
GGACTAGGGACTAG
GGACTAGGGACTAG
GGACTAGGGACTAG
GGACTAGGGACTAG
GGACTAGGGACTAG
GGACTAGGGACTAG
GGACTAGGGACTAG
GGACTAGGGACTAG
GGACTAGGGACTAG
GGACTAGGGACTA
GGACTGGGACTAG
GGACTAGGGACTAG
GGACTAGGGACTAG
GGACTAGGGACTAG
GGACTAGGGACTAG
GGACTAGGGACTAG
GGACTAGGGACTAG
GGACTAGGGACTAG
GGACTGGGGACTAG
GGACTAGGGACTAGGGACTGA
GGACTAGGGACTAG
GGACTAGGGACTAG
GGACTAGGGACTAG
GGACTAGGGACTG
GGACTAGGGACTAG
GGACTGGGACTAG
GGACTAGGGACTG
GGACTAGGGACTAG
GGACTAGGGACTAG
GGACTAAGGACTAG
GGACTAGGGACTAG
GGACTAGGGACTAG
GGACTAGGGACTAG
GGACTAGGGACTAGGGACTGA
GGACTAGGGACTAG
GGACTAGGGACTGG
GGACTAGGGACTAG
GGACTAGGGACTGA
GGACTAGGGACTAG
GGACTAGGGACTAG
GGACTAGGGACTAG
GGACTAGGGACTGG
GGACTAGGGACTAG

GGACTAGGGACTGG
GGACTAGGGACTAG
GGACTAGGGACTAG
GGACTAGGGACTAG
GGACTAGGGACTAG
GGACTAGGGACTAG
GGACTGGGGACTAG
GGACTAGGGACTAGGGACTGA
GGACTAGGGACTAG
GGACTAGGGACTAG
GGACTAGGGACTGG
GGACTAGGGACTAG
GGACTAGGGACTGG
GGACTAGGGACTAG
GGACTAGGGACTAG
GGACTAGGGACTAG
GGACTGGGACTAG
GGACTAGGGACTAG
GGACTAGGGACTAG
GGACTAGGGACTAG
GGACTAGGGACTAG
GGACTAGGGACTG
GGACTAGGGACTAG
GGACTAGGGACTAG
GGACTAGGGACTAG
GGACTAGGGACTAG
GGACTAGGGACTAG
GGACTGGGGACTAG
GGACTAGGGACTAGGGACTGA
GGACTAGGGACTAG
GGACTAGGGACTAG
GGACTAGGGACTAG
GGACTAGGGACTGA
GGACTAGGGACTAG
GGACTAGGGACTAG
GGACTAGGGACTAGGGACTGAACTTGCAAAC
GGACTAGGGACTGCGCTTAAGATACCGAAAACCCCTGAATTTCAGGACGACTGTCTCTCCAAAATCAAAACGCTCTAG
GGACTAGGGACTGG
GGACTAGGGACTAG
GGACTAGGGACTAGGGACTGAACTTGCAAAC
Nr of selected repeats 112 Similarity 0.896439
Consensus:
GGACTAGGGACTAG
>Cele-UNSB01_4:15883454-15883531 Satlength=78 Nr of Repeats=6 RepeatLength=11
seed=TGCACATTTT
TGCACATTTTT
TGCACATTTTT
TGCACATTTTTCGCTCATTTTT
TGCTCATTTTT
TGCACATTTTG
TGCACATTTTT
Nr of selected repeats 5 Similarity 0.903030
Consensus:
TGCaCATTTTt

>Cele-UNSB01_4:15943206-15943656 Satlength=451 Nr of Repeats=14 RepeatLength=32
seed=TTGAATTCCC
TTGAATTCCCGCCAAAATTTTTCAAAGAAATT
TTGAATTCCCGCCAAAATTTTTCACAGAAAAT
TTGAATTCCCGCCAAAATTTTTCACAGAAAAC
TTGAATTCCCGTTATAATTTTTCACAGAAAAT
TTGAATTCCCGCCAAAATTTTTCACAGAAATT
TTGAATTCCCGCCAAAATTTTTCAAAGAAATT
TTGAATTCCCGCCAAAATTTTTCACAGAAAAT
TTGAATTCCCGCCAAAATTTTTCACAGAAAAC
TTGAATTCCCGTTATAATTTTTCACAGAAAAT
TTGAATTCCCGCCAAAATTTTTCACAGAAATT
TTGAATTCCCGCCAAAATTTTTCACAGAAAAT
TTGAATTCCCGCCAAAATTTTCCACAGAAATT
TTGAATTCCCGCCAAATTTTTTCACAGAAATT
TTGAATTCCCGCCAAAAATTTTTTCACAGAAAAT
Nr of selected repeats 13 Similarity 0.905983
Consensus:
TTGAATTCCCGCCAAAATTTTTCACAGAAAaT
>Cele-UNSB01_4:15944034-15944234 Satlength=201 Nr of Repeats=5 RepeatLength=40
seed=ATTTTCCAGA
ATTTTCCAGAAGGTTCTGGAACATTCCAGAATTTTCTCAA
ATTTTCCAGAAGGTTCTGGAACATTCCAGCATTTTCTCAA
ATTTTCCAGAAGGTTCTGGAACATTCCAGAATTTTCTCGG
ATTTTCCAGAAGGTTCTGGAACATTACAGACTTTTCTCGG
ATTTTCCAGAAGGTTCTGGAACATTCCAGAATTTTCTCAA
Nr of selected repeats 5 Similarity 0.920000
Consensus:
ATTTTCCAGAAGGTTCTGGAACATTcCAGaaTTTTCTCaa
>Cele-UNSB01_4:16000310-16000589 Satlength=280 Nr of Repeats=6 RepeatLength=40
seed=GATTTTTCTA
GATTTTTCTAGAAAAAAATCTGGAACATTCCAGAATTTTTTC
GATTTTTCTAGAAAGTTCTGGAACATTCCAGAATTTTCTC
GATTTTTCTAGAAAGTTCTGGAACATTCCAGAATTTTCTT
GATTTTTCTAGAAAGTTCTGGAACATTTCAGAATTTTCCC
GATTTTTCTACAAAGTTCTGGAACGTTCCAGAATTTTCCC
GATTTTTCTAGAAAGTTCTTGAACATTCGAGAATTTTTTCGAAATTTCCAGAAGATTCTAGATTTCCAGAATTTTCCC
Nr of selected repeats 4 Similarity 0.911111
Consensus:
GATTTTTCTAgAAAGTTCTGGAACaTTcCAGAATTTTCcc
>Cele-UNSB01_4:15999976-16000920 Satlength=945 Nr of Repeats=8 RepeatLength=35
seed=AAAATTTAAA
AAAATTTAAAAAAAAACCGTTTCAAAACCTCTACATTCAAAGTTGAAAGGCAAACAACTTATTGACTTCATAGTGATTTT
TTTTTAATTATTAAAAAAAATTATGTTTGAAATTATTTAAAAAGGCATAAATTCAAAACTGCCAAAAAATCTGATGAAC
TGAATTTAATAACTTCGAGAGAAAAAAAAAACAGTTTTTTTTTCGAGAATAAGCAAATGGAAAGTTTCTGAAAGTTATCG
AATTATTTAAATACCAGCCGCTGACCGCGCCTACGGCGCGGGCAACGACTGGCACCATTAAAAGTATTTGACACACATA
CACTTCCAGAATTTTCTCGATTTTTCTAGAAAAAAATCTGGAACATTCCAGAATTTTTTCGATTTTTCTAGAAAGTTCTG
GAACATTCCAGAATTTTCTCGATTTTTCTAGAAAGTTCTGGAACATTCCAGAATTTTCTTGATTTTTCTAGAAAGTTCT
GGAACATTTCAGAATTTTCCCGATTTTTCTACAAAGTTCTGGAACGTTCCAGAATTTTCCCGATTTTTCTAGAAAGTTC
TTGAACATTCGAGAATTTTTTCGAAATTTCCAGAAGATTCTAGATTTCCAGAATTTTCCCGATTTTTCTACAAAGTTCT
GGAACATTCGAGAATTTTTTCGAAATTTCCAGAAGATTCTAGATTTCCAGAATTTTAGAATTTTCAG
AAAATTTAAATTTCCCGCCAAAATATTTTTCTCAG
AAAATTTAAATTTCCCGGCAAAATATTTTTTTCAG
AAAATTTAAATTTCCCTCCAAAATTTTTTTCTCCG

AAAATTTAAATTACCCGCCAAAATATTTTTCTCAG
AAAATTAAAATTTCCCACCAAAATATTTTTTTCAG
AAAATTTAAATTTCCCTCCAAAATATTTTTTTCAG
AAAATTTAAATTTCCCACCAAAATATTTTTCACAG
Nr of selected repeats 7 Similarity 0.883900
Consensus:
AAAATTTAAATTTCCCnCCAAAATATTTTTcTCAG
>Cele-UNSB01_4:16000962-16001082 Satlength=121 Nr of Repeats=8 RepeatLength=15
seed=GGGTCTCACC
GGGTCTCACCACGAC
GGGTCTCACCACGAT
GGGTCTCACCACGAT
GGGTCTCACCACGAT
GGGTCTCACCTCGAT
GGGTCTCACCACGAT
GGGTCTCACCACGAT
GGGTCTCACCACGAC
Nr of selected repeats 8 Similarity 0.939683
Consensus:
GGGTCTCACCACGAt
>Cele-UNSB01_4:16000985-16001240 Satlength=256 Nr of Repeats=17 RepeatLength=15
seed=CCACGATGGG
CCACGATGGGTCTCA
CCACGATGGGTCTCA
CCACGATGGGTCTCA
CCTCGATGGGTCTCA
CCACGATGGGTCTCA
CCACGATGGGTCTCA
CCACGACGGGTCTCA
CCACGGTGGGTCTCG
CCACGATGGGCCTCG
CCACGATGGGTCTCG
CCACGATGGGTCTCG
CCACGATGGGTCTCG
CCACGATGGGTTTCA
CCACGATAGGTCTTT
CCACGATGGGTCTCA
CCACGATGGGTCTCG
CCACGATGGGTCTCG
Nr of selected repeats 17 Similarity 0.875163
Consensus:
CCACGATGGGTCTCa
>Cele-UNSB01_4:16001468-16002088 Satlength=621 Nr of Repeats=19 RepeatLength=11
seed=GTACTCCTAC
GTACTCCTACA
GTACTCCTACA
GTACTACTACAGTACCCCGACCATATTGCACTACTAATCGCAAACCTATATCTCTTCAAAAGACAAAAACACAATTTTT
CATAAACTACA
GTAATCCTACCGTACA
GTACTCCTACA
GTACTACTACAGTACCCCGACCATATCCCACTAATAACTCCAAACCTATATCTCTTCAAAAGACAAAAACTCAATTTTT
CCTAAACTACA
GTAATCCTACC
GTACTCCTACA

GTACTCCTACA
GTACTACTACAGTACCCCGACCATATCCCACTAATAACTCCAAACCTATATCTCTTCAAAAGACTAAAACACAATTTTT
CCTAAACTACA
GTAATCCTACC
GTACTCCTACA
GTACTCCTACAGTATTACTACGGTACCCCGACCATATCCCACTAATAACTCCAAACCTATATCTCTTCAAAAGACTAAA
ACACAATTTTTCCTAAACTACA
GTAATCCTACC
GTACTCCTACA
GTACTCCTACA
GTACTACTACAGTACCCCGACCATATCCCACTAATAACTCCAAACCTATATCTCTTCGAAAGACTAAAACACAATTTTT
CCTAAACTACA
GTAATCCTACC
GTACTCCTACA
Nr of selected repeats 13 Similarity 0.888112
Consensus:
GTAcTCCTACa
>Cele-UNSB01_4:16010822-16011211 Satlength=390 Nr of Repeats=5 RepeatLength=42
seed=GCTCGGCAAA
GCTCGGCAAATTTCGAAATTTGCCGCACACGTCAAAAATTTTACAGTACAATTTTGACTAAAATTATTGATTTTTTCGA
CAAAAATTTAGTAAAATGACACAAAATTGAGTTAGTTTTATGCTTAAGCAGACATACTACACGGAACTAATTCAGAAAC
CAGATGTGTGTTAAGAATTCGGTAGTTTTGGTGTTCCAAAAAACATCAAAAATTATCAAAATTTTCCGAGTTTGCTGA
GCTCGGCAAATTTGCCGAATTTGCCGT
GCTCGGCAAATATTGAAAAAAGAGATTTGCCGAATTTGCCGT
GCTCGGCAAATATTGAAAAAAGAGATTTGCCGAATTTGCCGT
GCTCGGCAAATATTGAAAAAAGAGATTTGCCGCATTTGCCGA
Nr of selected repeats 3 Similarity 0.957672
Consensus:
GCTCGGCAAATATTGAAAAAAGAGATTTGCCGaATTTGCCGt
>Cele-UNSB01_4:16019565-16019814 Satlength=250 Nr of Repeats=6 RepeatLength=31
seed=TCGACAGAAG
TCGACAGAAGCTGCCGGTCTTCACACTTTCCTTGACACACGCTGTCAGATCTCACAAATTTT
TCGACAGAAGCTGTCGATCATCAGACTTTCC
TCGACAGAAGCTGTCGGATATCACATTTTTCTCGGCAAAAGCTGCCGGAAACTCACACTTTCG
TCGACCGAAGCTGTCGGATATCACATTTCCC
TCGGCAGAAGCTGCCGGAACTCACACTTTCT
TCGACAGAAGCTGTCGGAACTTACAATTTCT
Nr of selected repeats 4 Similarity 0.665825
Consensus:
TCGaCaGAAGCTGtCgGAaaTcAcAcTTTCc
>Cele-UNSB01_4:16061332-16061647 Satlength=316 Nr of Repeats=21 RepeatLength=15
seed=CCCATCGTGG
CCCATCGTGGCGAGA
CCCATCGTGGCGAGA
CCCATCGTGGCGAGA
CCCATCGTGGCGAGG
CCCATCGTGGTGAGA
CCCATCGTGGCGAGA
CCCACCGTGGTGAGA
CCCGTCGTGGTGAGA
CCCATCGTGGTGAGA
CCCATCGTGGTGAGA
CCCGTCGTGGTGAGA
CCCATCGTGGTGAGA

CCCATCGTGGTGAGA
CCCATCGTGGTGAGA
CCCATCGTGGTGAGA
CCCATCGTGGTGAGA
CCCATCGTGGTGAGA
CCCATCGTGGTGAGA
CCCATCGTGGTGAGA
CCCATCGTGGTGAAA
CCCATCGTTGTGAGA
Nr of selected repeats 21 Similarity 0.916191
Consensus:
CCCATCGTGGtGAGA
>Cele-UNSB01_4:16061680-16061959 Satlength=280 Nr of Repeats=8 RepeatLength=35 seed=AATTTAAATT
AATTTAAATTTTCTGTGAAAAATATTTTGGCGGGA
AATTTAAATTTTCTGTGAAAAATATTTTGGCGGGA
AATTTAAATTTTCTGAAAAAAATATTTTGGAGGGA
AATTTAAATTTTCTGAAAAAAAAAATTTTGGAGGGA
AATTTAAATTTTCTGGAAAAAAAAATTTTGGAGGGA
AATTTAAATTTTCTGAAAAAAATATTTTGGCGGGA
AATTTAAATTTTCTGTGAAAAATATTTTGGCGGGT
AATTTAAATTTTCTGAGAAAAAAAATTTTGGAGGGA
Nr of selected repeats 7 Similarity 0.896599
Consensus:
AATTTAAATTTTCTGngAAAAAtATTTTGGcGGGA
>Cele-UNSB01_4:16061215-16062273 Satlength=1059 Nr of Repeats=8 RepeatLength=40 seed=GTTCCAGAAC
GTTCCAGAACCTTCTGGGAATTTCTGGAAAATTCTAGAAT
GTTCCAGAACCTTCTGGAAAATTCTGAAAAGCTTTAAAACCTTCTGGAAAATTCTAGAAAATCCAAAAAAAGAAAGACC
CATCGTGGCGAGACCCATCGTGGCGAGACCCATCGTGGCGAGACCCATCGTGGCGAGGCCCATCGTGGTGAGACCCATC
GTGGCGAGACCCACCGTGGTGAGACCCGTCGTGGTGAGACCCATCGTGGTGAGACCCATCGTGGTGAGACCCGTCGTGG
TGAGACCCATCGTGGTGAGACCCATCGTGGTGAGACCCATCGTGGTGAGACCCATCGTGGTGAGACCCATCGTGGTGAG
ACCCATCGTGGTGAGACCCATCGTGGTGAGACCCATCGTGGTGAGACCCATCGTGGTGAAACCCATCGTTGTGAGACCC
ATCGTGGTGAGACCCAAAATTTTGGCGGGAAATTTAAATTTTCTGTGAAAAATATTTTGGCGGGAAATTTAAATTTTCT
GTGAAAAATATTTTGGCGGGAAATTTAAATTTTCTGAAAAAAATATTTTGGAGGGAAATTTAAATTTTCTGAAAAAAAAA
TTTTGGAGGGAAATTTAAATTTTCTGGAAAAAAAAATTTTGGAGGGAAATTTAAATTTTCTGAAAAAAATATTTTGGCGG
GAAATTTAAATTTTCTGTGAAAAATATTTTGGCGGGTAATTTAAATTTTCTGAGAAAAAAAATTTTGGAGGGAAATTTAA
ATTTTCTGAAAATTCTAAAATACTGGAAATCTAGAATCTTCTGGAAATTTCGAAAAAATTCTCGAAT
GTTCCAGAACCTTCTAGAAAAATCGAGAAAATTCTGGAGT
GTTCCAGAACTTTCTAGAAAAATCGGTAAAATTCTGGAAT
GTTCCAGAACTTTCTATAAAAATTGGGAAAAGTATGGAGT
GTTCCAGAACTTTCTAGAAAAATCGAGAAAATTGTGGAAC
GTTCCAGAACTTTCTAGAAAAATCGAGAAAATTCTGGAAC
GTTCCAGAACTTTCTAGAAAAATCGGTAAAATTCTGGAAT
Nr of selected repeats 7 Similarity 0.814286
Consensus:
GTTCCAGAACtTTCTAGAAAAATCGggAAAATTcTGGAat
>Cele-UNSB01_4:16077133-16078206 Satlength=1074 Nr of Repeats=16 RepeatLength=37 seed=CGGGAATTCA
CGGGAATTCAAATTTTAATTTTTCTAAAACATTTTGG
CGGGAATTCAATTGTTAATTTTTCGAAATCATTTCGG
CGGGAATTCAAATTTTAATTTTCCGAAAAGATTTTGG
CGGGAATTCAAATTTTAATTTTTCTAAAACATTTTG
CGGGAATTCAAATTTTAATTTTCCGAAAAGATTTTGG

CGGGAATTCAAATTTTAATTTTTCTAAAACATTTTGG
CGGGAATTCAAATTTTAATTTTTTGAAATCATTTCGG
CGGGAATTCAAATTTGAATTTTTTGATCTCATTTTGG
CGGGAATTCAAATTTTAATTTTTTGAACTCATTTCGG
CGGGAATTCAAATTTGAATTTTTTGAACTCATTTCGG
CGGGAATTCAAATTCGAATTTTTTGAACTCATTTCGG
CGGGAATTCAAATTTTAATTTTTTGAACTCATTTCGG
CGGGAATTCAAATTTGAATTTTTTTAAATCATTTTGGCGCTTATTCCGTGTGTTCATAGTTTGTAGTGTATGTAGTCTT
TGTAGTCTGTGACGTCACAGCTTAAAGAGGCAATGCGTTTTGGGATGTTGGAGAGATATTGGTTTGGGGTTAGAAGGGG
GATATGGTCGGGGTACTGTAGTAGTACTGTAGTTGTAGAAAAATAAACAATTTTTCGAAAACATTTTGG
CGGGAATTCAAATTTAAATTTTTCGAAAACATTTTGA
CGGGAATTCAAATTTAAATGCTTTAATAATATTATTGTGGAATACTTTAGAACACTCTGGAAAATTCGAGAAAATTCTG
GAATGTTCCAGAACCTTCTTGAAAATTTGGGAAAGTTCTGGAATCTTCTTGAATCATCAGGAAAATTGGGGAAAATTCT
GGATTGTTCCAGAATCTTTTGAAAAATTCGAGAAAATTCTGAATGTTCGTGGCGAGACCTATCGTGGTTAGACCCATCA
TGGTGAGATCCTTAAAACTTTCGGTGGGAAATTGCTGGAAATAAATATTTTGCCGCGAAGTTCGAATTTTAATTTTTTT
GATATCATTTTGG
CGGGAATTCAAAATTTAATTTATTGAAATCACTTTGG
Nr of selected repeats 13 Similarity 0.831370
Consensus:
CGGGAATTCAAATTTtAATTTTTtGAAatCATTTtGG
>Cele-UNSB01_4:16093909-16094066 Satlength=158 Nr of Repeats=8 RepeatLength=20
seed=TGTTACACGA
TGTTACACGAAAAAATAGAT
TGTTACACGAAAAAAATAGAT
TGTTACACGAAAAAATAGAA
TGTTACACGAAAAAAATAGAT
TGTTACACGAAAAAATAGAA
TGTTACACGAAAAAAATAGAT
TGTTACACGAAAAAAATAGAT
TGTTACACGAAAAAAATAGAT
Nr of selected repeats 5 Similarity 1.000000
Consensus:
TGTTACACGAAAAAAATAGAT
>Cele-UNSB01_4:16130574-16144444 Satlength=13871 Nr of Repeats=346
RepeatLength=40 seed=TTTTTCTAGA
TTTTTCTAGAAAGTTCTGAACATTCCAGACTTTTCCCAA
TTTTTCTAGAAAGTTCTGGAACATTCCAGAATTTTCCCGA
TTTTTCTAGAAGTTCTGACATTTTTTTTTTTTTTTTTTTTTTTTTTTTTTTTTT
TTTTTCTAGAAAGTTCTGGAACATTCCAGAATTTTCTCA
TTTTTCTAGAAAGTTCTGGAACATTCCAGAATTTTCTCGA
TTTTTCTAGAAAGTTCTGGAACTTCCAGAATTTTCTCGA
TTTTTCTAGAAAGTTCTGGAACATTCCAGAATTTTCTCGA
TTTTTCTAGAAAGTTCTGGAACATTCCAGAATTTTCTCGA
TTTTTCTAGAAAGTTCTGGAACGTTCCAGAATTTTCTCGA
TTTTTCTAGAAAGTTCTGGAACATTCCAGAATTTTCTCGA
TTTTTCTAGAAAGTTCTGGAACATTCCAGAATTTTCTCGA
TTTTTCTAGAAAGTTCTGGAACTTCCAGAATTTTCTCGA
TTTTTCTAGAAAGTTCTGGAACATTCCAGAATTTTCTCGA
TTTTTCTAGAAAGTTCTGGAACATTCCAGAATTTTCTCGA
TTTTTCTAGAAAGTTCTGGAACATTCCAGAATTTTCTCGA
TTTTTCTAGAAAGTTCTGGAACATTCCAGAATTTTCTCGA
TTTTTCTAGAAAGTTCTGGAACATTCCAGAATTTTCTCGA
TTTTTCTAGAAAGTTCTGGAACATTCCAGAATTTTCTCGA
TTTTTCTAGAAAGTTCTGAACATTCCAGAATTTTCTTTTTTTTTTCTTAGTTTTCTCGA

TTTTTCTAGAAAGTTCTGGAACATTCCAGAATTTTCTCGA
TTTTTCTAGAAAGTTCTGGAACATTCCAGAATTTTCTCGA
TTTTTCTAGAAAGTTCTGGAACATTCCAGAATTTTCTCGA
TTTTTCTAGAAAGTTCTGGAACATTCCAGAATTTTCTCGA
TTTTTCTAGAAAGTTCTGGAACATTCCAGAATTTTCTCGA
TTTTTCTAGAAAGTTCTGAACATTCCAGAATTTTCTCGA
TTTTTCTAGAAAGTTCTGGAACATTCCAGAATTTTCTCGA
TTTTTCTAGAAAGTTCTGGAACATTCCAGAATTTTCTCGA
TTTTTCTAGAAAGTTCTGGAACATTCCAGAATTTTCTCGA
TTTTTCTAGAAAGTTCTGGAACATTCCAGAATTTTCTCGA
TTTTTCTAGAAAGTTCTGGAACATTCCAGAATTTTCTCGA
TTTTTCTAGAAAGTTCTGGAACATTCCAGAATTTTCTCGA
TTTTTCTAGAAAGTTCTGGAACATTCCAGAATTTTCCGA
TTTTTCTAGAAAGTTCTGGAACATTCCAGAATTTTCTCGA
TTTTTCTAGAAAGTTCTGGAACATTCCAGAATTTTCTCGA
TTTTTCTAGAAAGTTCTGGAACATTCCAGAATTTTCTCGA
TTTTTCTAGAAAGTTCTGGAACATTCCAGAATTTTCTCGA
TTTTTCTAGAAAGTTCTGGAACATTCCAGAATTTTCTCGA
TTTTTCTAGAAAGTTCTGGAACATTCCAGAATTTTCTCGA
TTTTTCTAGAAAGTTCTGGAACATTCCAGAATTTTCTCGA
TTTTTCTAGAAAGTTCTGGAACATTCCAGAATTTTCTCGA
TTTTTCTAGAAAGTTCTGGAACATTCCAGAATTTTCTCGA
TTTTTCTAGAAAGTTCTGGAACATTCCAGAATTTTCTCGA
TTTTTCTAGAAAGTTCTGGAACATTCCAGAATTTTCTCGA
TTTTTCTAGAAAGTTCTGGAACATTCCAGAATTTTCTCGA
TTTTTCTAGAAAGTTCTGGAACATTCCAGAATTTTCTCGA
TTTTTCTAGAAAGTTCTGGAACATTCCAGAATTTTCTCGA
TTTTTCTAGAAAGTTCTGGAACATTCCAGAATTTTCTCGA
TTTTTCTAGAAAGTTCTGGAACATTCCAGAATTTTCTCGA
TTTTTCTAGAAAGTTCTGGAACATTCCAGAATTTTCTCGA
TTTTTCTAGAAAGTTCTGGAACATTCCAGAATTTTCTCGA
TTTTTCTAGAAAGTTCTGGAACATTCCAGAATTTTCTCGA
TTTTTCTAGAAAGTTCTGGAACATTCCAGAATTTTCTCGA
TTTTTCTAGAAAGTTCTGGAACATTCCAGAATTTTCTCGA
TTTTTCTAGAAAGTTCTGGAACATTCCAGAATTTTCTCGA
TTTTTCTAGAAAGTTCTGGAACATTCCAGAATTTTCTCGA
TTTTTCTAGAAAGTTCTGGAACATTCCAGAATTTTCTCGA
TTTTTCTAGAAAGTTCTGGAACATTCCAGAATTTTCTCGA
TTTTTCTAGAAAGTTCTGGAACGTTCCAGAATTTTCTCGA
TTTTTCTAGAAAGTTCTGGAACATTCCAGAATTTTCTCGA
TTTTTCTAGAAAGTTCTGGAACATTCCAGAATTTTCTCGA
TTTTTCTAGAAAGTTCTGGAACATTCCAGAATTTTCTCGA
TTTTTCTAGAAAGTTCTGGAACATTCCAGAATTTTCTCGA
TTTTTCTAGAAAGTTCTGGAACATTCCAGAATTTTCTCGA
TTTTTCTAGAAAGTTCTGGAACATTCCAGAATTTTCTCGA
TTTTTCTAGAAAGTTCTGGAACATTCCAGAATTTTCTCGA
TTTTTCTAGAAAGTTCTGGAACATTCCAGAATTTTCTCGA
TTTTTCTAGAAAGTTCTGGAACATTCCAGAATTTTCTCGA
TTTTTCTAGAAAGTTCTGGAACATTCCAGAATTTTCTCGA
TTTTTCTAGAAAGTTCTGGAACATTCCAGAATTTTCTCGA
TTTTTCTAGAAAGTTCTGGAACATTCCAGAATTTTCTCGA
TTTTTCTAGAAAGTTCTGGAACATTCCAGAATTTTCTCGA
TTTTTCTAGAAAGTTCTGGAACATTCCAGAATTTTCTCGA
TTTTTCTAGAAAGTTCTGGAACATTCCAGAATTTTCTCGA

TTTTTCTAGAAAGTTCTGGAACATTCCAGAATTTTCTCGA
TTTTTCTAGAAAGTTCTGGAACATTCCAGAATTTTCTCGA
TTTTTCTAGAAAGTTCTGGAACATTCCAGAATTTTCTCGA
TTTTTCTAGAAAGTTCTGGAACATTCCAGAATTTTCTCGA
TTTTTCTAGAAAGTTCTGGAACATTCCAGAATTTTCTCGA
TTTTTCTAGAAAGTTCTGGAACATTCCAGAATTTTCTCGA
TTTTTCTAGAAAGTTCTGGAACATTCCAGAATTTTCTCGA
TTTTTCTAGAAAGTTCTGGAACATTCCAGAATTTTCTCGA
TTTTTCTAGAAAGTTCTGGAACATTCCAGAATTTTCTCGA
TTTTTCTAGAAAGTTCTGGAACATTCCAGAATTTTCTCGA
TTTTTCTAGAAAGTTCTGGAACATTCCAGAATTTTCTCGA
TTTTTCTAGAAAGTTCTGGAACATTCCAGAATTTTCTCGA
TTTTTCTAGAAAGTTCTGGAACATTCCAGAATTTTCTCGA
TTTTTCTAGAAAGTTCTGGAACATTCCAGAATTTTCTCGA
TTTTTCTAGAAAGTTCTGGAACATTCCAGAATTTTCTCGA
TTTTTCTAGAAAGTTCTGGAACATTCCAGAATTTTCTCGA
TTTTTCTAGAAAGTTCTGGAACATTCCAGAATTTTCTCGA
TTTTTCTAGAAAGTTCTGGAACATTCCAGAATTTTCTCGA
TTTTTCTAGAAAGTTCTGGAACATTCCAGAATTTTCTCGA
TTTTTCTAGAAAGTTCTGGAACGTTCCAGAATTTTCTCGA
TTTTTCTAGAAAGTTCTGGAACATTCCAGAATTTTCTCGA
TTTTTCTAGAAAGTTCTGGAACATTCCAGAATTTTCTCGA
TTTTTCTAGAAAGTTCTGGAACATTCCAGAATTTTCTCGA
TTTTTCTAGAAAGTTCTGGAACATTCCAGAATTTTCTCGA
TTTTTCTAGAAAGTTCTGGAACATTCCAGAATTTTCTCGA
TTTTTCTAGAAAGTTCTGGAACATTCCAGAATTTTCTCGA
TTTTTCTAGAAAGTTCTGGAACATTCCAGAATTTTCTCGA
TTTTTCTAGAAAGTTCTGGAACATTCCAGAATTTTCTCGA
TTTTTCTAGAAAGTTCTGGAACATTCCAGAATTTTCTCGA
TTTTTCTAGAAAGTTCTGGAACATTCCAGAATTTTCTCGA
TTTTTCTAGAAAGTTCTGGAACATTCCAGAATTTTCTCGA
TTTTTCTAGAAAGTTCTGGAACATTCCAGAATTTTCTCGA
TTTTTCTAGAAAGTTCTGGAACATTCCAGAATTTTCTCGA
TTTTTCTAGAAAGTTCTGGAACATTCCAGAATTTTCTCGA
TTTTTCTAGAAAGTTCTGGAACGTTCCAGAATTTTCTCGA
TTTTTCTAGAAAGTTCTGGAACATTCCAGAATTTTCTCGA
TTTTTCTAGAAAGTTCTGGAACATTCCAGAATTTTCTCGA
TTTTTCTAGAAAGTTCTGGAACATTCCAGAATTTTCTCGA
TTTTTCTAGAAAGTTCTGGAACATTCCAGAATTTTCTCGA
TTTTTCTAGAAAGTTCTGGAACATTCCAGAATTTTCTCGA
TTTTTCTAGAAAGTTTCTTCCAGAATTTTCTCGA
TTTTTCTAGAAAGTTCTGGAACATTCCAGAATTTTCTCGA
TTTTTCTAGAAAGTTCTGGAACATTCCAGAATTTTCTCGA
TTTTTCTAGAAAGTTCTGGAACATTCCAGAATTTTCTCGA
TTTTTCTAGAAAGTTCTGGAACATTCCAGAATTTTCTCGA
TTTTTCTAGAAAGTTCTGGAACATTCCAGAATTTTCTCGA
TTTTTCTAGAAAGTTCTGGAACATTCCAGAATTTTCTCGA
TTTTTCTAGAAAGTTCTGGAACATTCCAGAATTTTCTCGA
TTTTTCTAGAAAGTTCTGGAACATTCCAGAATTTTCTCGA
TTTTTCTAGAAAGTTCTGGAACATTCCAGAATTTTCTCGA
TTTTTCTAGAAAGTTCTGGAACATTCCAGAATTTTCTCGA
TTTTTCTAGAAAGTTCTGGAACATTCCAGAATTTTCTCGA
TTTTTCTAGAAAGTTCTGGAACATTCCAGAATTTTCTCGA
TTTTTCTAGAAAGTTCTGGAACATTCCAGAATTTTCTCGA

TTTTTCTAGAAAGTTCTGGAACATTCCAGAATTTTCTCGA
TTTTTCTAGAAAGTTCTGGAACATTCCAGAATTTTCTCGA
TTTTTCTAGAAAGTTCTGGAACATTCCAGAATTTTCTCGA
TTTTTCTAGAAAGTTCTGGAACATTCCAGAATTTTCTCGA
TTTTTCTAGAAAGTTCTGGAACATTCCAGAATTTTCTCGA
TTTTTCTAGAAAGTTCTGGAACATTCCAGAATTTTCTCGA
TTTTTCTAGAAAGTTCTGGAACATTCCAGAATTTTCTCGA
TTTTTCTAGAAAGTTCTGGAACATTCCAGAATTTTCTCGA
TTTTTCTAGAAAGTTCTGGAACATTCCAGAATTTTCTCGA
TTTTTCTAGAAAGTTCTGGAACATTCCAGAATTTTCTCGA
TTTTTCTAGAAAGTTCTGGAACATTCCAGAATTTTCTCGA
TTTTTCTAGAAAGTTCTGGAACATTCCAGAATTTTCTCGA
TTTTTCTAGAAAGTTCTGGAACATTCCAGAATTTTCTCGA
TTTTTCTAGAAAGTTCTGGAACATTCCAGAATTTTCTCGA
TTTTTCTAGAAAGTTCTGGAACATTCCAGAATTTTCTCGA
TTTTTCTAGAAAGTTCTGGAACATTCCAGAATTTTCTCGA
TTTTTCTAGAAAGTTCTGGAACATTCCAGAATTTTCTCGA
TTTTTCTAGAAAGTTCTGGAACATTCCAGAATTTTCTCGA
TTTTTCTAGAAAGTTCTGGAACATTCCAGAATTTTCTCGA
TTTTTCTAGAAAGTTCTGGAACATTCCAGAATTTTCTCGA
TTTTTCTAGAAAGTTCTGGAACATTCCAGAATTTTCTCGA
TTTTTCTAGAAAGTTCTGGAACATTCCAGAATTTTCTCGA
TTTTTCTAGAAAGTTCTGGAACATTCCAGAATTTTCTCGA
TTTTTCTAGAAAGTTCTGGAACATTCCAGAATTTTCTCGA
TTTTTCTAGAAAGTTCTGGAACATTCCAGAATTTTCTCGA
TTTTTCTAGAAAGTTCTGGAACATTCCAGAATTTTCTCGA
TTTTTCTAGAAAGTTCTGGAACATTCCAGAATTTTCTCGA
TTTTTCTAGAAAGTTCTGGAACATTCCAGAATTTTCTCGA
TTTTTCTAGAAAGTTCTGGAACATTCCAGAATTTTCTCGA
TTTTTCTAGAAAGTTCTGGAACATTCCAGAATTTTCTCGA
TTTTTCTAGAAAGTTCTGGAACATTCCAGAATTTTCTCGA
TTTTTCTAGAAAGTTCTGGAACATTCCAGAATTTTCTCGA
TTTTTCTAGAAAGTTCTGGAACATTCCAGAATTTTCTCGA
TTTTTCTAGAAAGTTCTGGAACATTCCAGAATTTTCTCGA
TTTTTCTAGAAAGTTCTGGAACATTCCAGAATTTTCTCGA
TTTTTCTAGAAAGTTCTGGAACATTCCAGAATTTTCTCGA
TTTTTCTAGAAAGTTCTGGAACATTCCAGAATTTTCTCGA
TTTTTCTAGAAAGTTCTGGAACATTCCAGAATTTTCTCGA
TTTTTCTAGAAAGTTCTGGAACATTCCAGAATTTTCTCGA
TTTTTCTAGAAAGTTCTGGAACATTCCAGAATTTTCTCGA
TTTTTCTAGAAAGTTCTGGAACATTCCAGAATTTTCTCGA
TTTTTCTAGAAAGTTCTGGAACATTCCAGAATTTTCTCGA
TTTTTCTAGAAAGTTCTGGAACATTCCAGAATTTTCTCGA
TTTTTCTAGAAAGTTCTGGAACATTCCAGAATTTTCTCGA
TTTTTCTAGAAAGTTCTGGAACATTCCAGAATTTTCTCGA
TTTTTCTAGAAAGTTCTGGAACATTCCAGAATTTTCTCGA
TTTTTCTAGAAAGTTCTGGAACATTCCAGAATTTTCTCGA
TTTTTCTAGAAAGTTCTGGAACATTCCAGAATTTTCTCGA
TTTTTCTAGAAAGTTCTGGAACATTCCAGAATTTTCTCGA
TTTTTCTAGAAAGTTCTGGAACATTCCAGAATTTTCTCGA
TTTTTCTAGAAAGTTCTGGAACATTCCAGAATTTTCTCGA
TTTTTCTAGAAAGTTCTGGAACATTCCAGAATTTTCTCGA
TTTTTCTAGAAAGTTCTGGAACATTCCAGAATTTTCTCGA
TTTTTCTAGAAAGTTCTGGAACATTCCAGAATTTTCTCGA

TTTTTCTAGAAAGTTCTGGAACATTCCAGAATTTTCTCGA
TTTTTCTAGAAAGTTCTGGAACATTCCAGAATTTTCTCGA
TTTTTCTAGAAAGTTCTGGAACATTCCAGAATTTTCTCGA
TTTTTCTAGAAAGTTCTGGAACATTCCAGAATTTTCTCGA
TTTTTCTAGAAAGTTCTGGAACATTCCAGAATTTTCTCGA
TTTTTCTAGAAAGTTCTGGAACATTCCAGAATTTTCTCGA
TTTTTCTAGAAAGTTCTGGAACATTCCAGAATTTTCTCGA
TTTTTCTAGAAAGTTCTGGAACATTCCAGAATTTTCTCGA
TTTTTCTAGAAAGTTCTGGAACATTCCAGAATTTTCTCGA
TTTTTCTAGAAAGTTCTGGAACATTCCAGAATTTTCTCGA
TTTTTCTAGAAAGTTCTGGAACATTCCAGAATTTTCTCGA
TTTTTCTAGAAAGTTCTGGAACATTCCAGAATTTTCTCGA
TTTTTCTAGAAAGTTCTGGAACATTCCAGAATTTTCTCGA
TTTTTCTAGAAAGTTCTGGAACATTCCAGAATTTTCTCGA
TTTTTCTAGAAAGTTCTGGAACATTCCAGAATTTTCTCGA
TTTTTCTAGAAAGTTCTGGAACATTCCAGAATTTTCTCGA
TTTTTCTAGAAAGTTCTGGAACATTCCAGAATTTTCTCGA
TTTTTCTAGAAAGTTCTGGAACATTCCAGAATTTTCTCGA
TTTTTCTAGAAAGTTCTGGAACATTCCAGAATTTTCTCGA
TTTTTCTAGAAAGTTCTGGAACATTCCAGAATTTTCTCGA
TTTTTCTAGAAAGTTCTGGAACATTCCAGAATTTTCTCGA
TTTTTCTAGAAAGTTCTGGAACATTCCAGAATTTTCTCGA
TTTTTCTAGAAAGTTCTGGAACATTCCAGAATTTTCTCGA
TTTTTCTAGAAAGTTCTGGAACATTCCAGAATTTTCTCGA
TTTTTCTAGAAAGTTCTGGAACATTCCAGAATTTTCTCGA
TTTTTCTAGAAAGTTCTGGAACATTCCAGAATTTTCTCGA
TTTTTCTAGAAAGTTCTGGAACATTCCAGAATTTTCTCGA
TTTTTCTAGAAAGTTCTGGAACATTCCAGAATTTTCTCGA
TTTTTCTAGAAAGTTCTGGAACATTCCAGAATTTTCTCGA
TTTTTCTAGAAAGTTCTGGAACATTCCAGAATTTTCTCGA
TTTTTCTAGAAAGTTCTGGAACATTCCAGAATTTTCTCGA
TTTTTCTAGAAAGTTCTGGAACATTCCAGAATTTTCTCGATTCTGAAATTCCAGAATTTTCTCGA
TTTTTCTAGAAAGTTCTGGAACATTCCAGAATTTTCTCGA
TTTTTCTAGAAAGTTCTGGAACATTCCAGAATTTTCTCGA
TTTTTCTAGAAAGTTCTGGAACATTCCAGAATTTTCTCGA
TTTTTCTAGAAAGTTCTGGAACATTCCAGAATTTTCTCGA
TTTTTCTAGAAAGTTCTGGAACATTCCAGAATTTTCTCGA
TTTTTCTAGAAAGTTCTGGAACATTCCAGAATTTTCTCGA
TTTTTCTAGAAAGTTCTGGAACATTCCAGAATTTTCTCGA
TTTTTCTAGAAAGTTCTGGAACATTCCAGAATTTTCTCGA
TTTTTCTAGAAAGTTCTGGAACATTCCAGAATTTTCTCGA
TTTTTCTAGAAAGTTCTGGAACATTCCAGAATTTTCTCGA
TTTTTCTAGAAAGTTCTGGAACATTCCAGAATTTTCTCGA
TTTTTCTAGAAAGTTCTGGAACATTCCAGAATTTTCTCGA
TTTTTCTAGAAAGTTCTGGAACATTCCAGAATTTTCTCGA
TTTTTCTAGAAAGTTCTGGAACATTCCAGAATTTTCTCGA
TTTTTCTAGAAAGTTCTGTTTCTCGA
TTTTTCTAGAAAGTTCTGGAACATTCCAGAATTTTCTCGA
TTTTTCTAGAAAGTTCTGGAACATTCCAGAATTTTCTCGA
TTTTTCTAGAAAGTTCTGGAACATTCCAGAATTTTCTCGA
TTTTTCTAGAAAGTTCTGGAACATTCCAGAATTTTCTCGA
TTTTTCTAGAAAGTTCTGGAACATTCCAGAATTTTCTCGA
TTTTTCTAGAAAGTTCTGGAACATTCCAGAATTTTCTCGA
TTTTTCTAGAAAGTTCTGGAACATTCCAGAATTTTCTCGA

TTTTTCTAGAAAGTTCTGGAACATTCCAGAATTTTCTCGA
TTTTTCTAGAAAGTTCTGGAACATTCCAGAATTTTCTCGA
TTTTTCTAGAAAGTTCTGGAACATTCCAGAATTTTCTCGA
TTTTTCTAGAAAGTTCTGGAACATTCCAGAATTTTCTCGA
TTTTTCTAGAAAGTTCTGGAACATTCCAGAATTTTCTCGA
TTTTTCTAGAAAGTTCTGGAACATTCCAGAATTTTCTCGA
TTTTTCTAGAAAGTTCTGGAACATTCCAGAATTTTCTCGA
TTTTTCTAGAAAGTTCTGGAACATTCCAGAATTTTCTCGA
TTTTTCTAGAAAGTTCTGGAACATTCCAGAATTTTCTCGA
TTTTTCTAGAAAGTTCTGGAACATTCCAGAATTTTCTCGA
TTTTTCTAGAAAGTTCTGGAACATTCCAGAATTTTCTCGA
TTTTTCTAGAAAGTTCTGGAACATTCCAGAATTTTCTCGA
TTTTTCTAGAAAGTTCTGGAACATTCCAGAATTTTCTCGA
TTTTTCTAGAAAGTTCTGGAACATTCCAGAATTTTCTCGA
TTTTTCTAGAAAGTTCTGGAACATTCCAGAATTTTCTCGA
TTTTTCTAGAAAGTTCTGGAACATTCCAGAATTTTCTCGA
TTTTTCTAGAAAGTTCTGGAACATTCCAGAATTTTCTCGA
TTTTTCTAGAAAGTTCTGGAACATTCCAGAATTTTCTCGA
TTTTTCTAGAAAGTTCTGGAACATTCCAGAATTTTCTCGA
TTTTTCTAGAAAGTTCTGGAACATTCCAGAATTTTCTCGA
TTTTTCTAGAAAGTTCTGGAACATTCCAGAATTTTCTCGA
TTTTTCTAGAAAGTTCTGGAACATTCCAGAATTTTCTCGA
TTTTTCTAGAAAGTTCTGGAACATTCCAGAATTTTCTCGA
TTTTTCTAGAAAGTTCTGGAACATTCCAGAATTTTCTCGA
TTTTTCTAGAAAGTTCTGGAACATTCCAGAATTTTCTCGA
TTTTTCTAGAAAGTTCTGGAACATTCCAGAATTTTCTCGA
TTTTTCTAGAAAGTTCTGGAACATTCCAGAATTTTCTCGA
TTTTTCTAGAAAGTTCTGGAACATTCCAGAATTTTCTCGA
TTTTTCTAGAAAGTTCTGGAACATTCCAGAATTTTCTCGA
TTTTTCTAGAAAGTTCTGGAACATTCCAGAATTTTCTCGA
TTTTTCTAGAAAGTTCTGGAACATTCCAGAATTTTCTCGA
TTTTTCTAGAAAGTTCTGGAACATTCCAGAATTTTCTCGA
TTTTTCTAGAAAGTTCTGGAACATTCCAGAATTTTCTCGA
TTTTTCTAGAAAGTTCTGGAACATTCCAGAATTTTCTCGA
TTTTTCTAGAAAGTTCTGGAACATTCCAGAATTTTCTCGA
TTTTTCTAGAAAGTTCTGGAACATTCCAGAATTTTCCCGA
TTTTTCTAGAAAGTTCTGAACATTCCAGAATTTTCTCGA
TTTTTCTAGAAAGTTCTGGAACATTCCAGAATTTTCTCGA
TTTTTCTAGAAAGTTCTGGAACATTCCAGAATTTTCTCGA
TTTTTCTAGAAAGTTCTGGAACATTCCAGAATTTTCTCGA
TTTTTCTAGAAAGTTCTGGAACATTCCAGAATTTTCTCGA
TTTTTCTAGAAAGTTCTGGAACATTCCAGAATTTTCTCGA
TTTTTCTAGAAAGTTCTGGAACATTCCAGAATTTTCTCGA
TTTTTCTAGAAAGTTCTGGAACATTCCAGAATTTTCCCGA
TTTTTCTAGAAAGTTCTGGAACATTCCAGAATTTTCTCGA
TTTTTCTAGAAAGTTCTGGAACATTCCAGAATTTTCTCGA
TTTTTCTAGAAAGTTCTGGAACATTCCAGAATTTTCTCGA
TTTTTCTAGAAAGTTCTGGAACATTCCAAAATTTTCTCGA
TTTTTCTAGAAAGTTCTGGAACATTTCAGAATTTTCCCGA
TTTTTCTAGAAAGTTCTGGAACATTCGAGAATTTTCTCGA
TTTTTCTAGAAAGTTCTGGAACGTTCCAGAATTTTCTCGA
TTTTTCTAGAAAGTGCTGGAACGTTCCAGAATTTTCTCGA
TTTTTCTAAAAAGTTCTGGAACATTCCAAAATTTTCTCCA
TTTTTCTAGAAAGTTCTGGAACATTCCAGAATTTTCCCGA

TTTTTCTAGAAAGTTCTGAACATTCCAGAATTTTCTCGA
TTTTTCTAGAAAGTTCTGGAACATTCCAGAATTTTCCCGA
TTTTTCTAGAAAGTTCTGGAACATTCGAGAATTTTCTCGA
TTTTTCTAGAAAGTTCTGGAACATTCCAGAATTTTCTCAA
TTTTTCTAGAAAGTTCTGGAACATTCCAGAATTTTCCCAA
TTTTTCTAGAAAGTTCTGGAACATTCCAGAATTTTCTCGA
TTTTTCTAGAAAGTTCTGGAACATTCCAGAATTTTCTCGA
TTTTTCTAAAAAGTTCTGGAACATTCCAAAATTTTCTCCA
TTTTTCTAGAAAGTTCTGGAACATTCCAGAATTTTCCCGA
TTTTTCTAGAAAGTTCTGAACATTCCAGAATTTTCTCGA
TTTTTCTAGAAAGTTCTGGAACATTCCAGAATTTTCCCAA
TTTTTCTAGAAAGTTCTGGAACGTTCCAGAATTTTCTCGA
TTTTTCTAGAAAGTTCTGGAACATTCCAGAATTTTCTCGA
TTTTTCTAGAAAGTTCTGGAACATTCCAAAATTTTCTCGA
TTTTTCTAGAAAGTTCTGGAACATTCCAGAATTTTCTCGA
TTTTTCTAGAAAGTTCTGGAACATTCCAAAATTTTCTCGA
TTTTTCTAGAAAATTCTGGAACATTTCAGAATTTTCTCGA
TTTTTCTAGAAAGTTCTGGAACATTCCAGAATTTTCCCGA
TTTTTCTAGAAAGTTCTGGAACATTCCAGAATTTTCTCGA
TTTTTCTAGAAAGTTCTGGAACATTCCAGAATTTTCTCGA
TTTTTCTAGAAAGTTCTGGAACATTCCAGAATTTTCTCGA
TTTTTCTAGAAAGTTCTGGAACATTCCAAAATTTTCTCGA
TTTTTCTAGAAAGTTCTGGAACATTCCAGAATTTTCTCGA
TTTTTCTAGAAAGTTCTGGAACATTCCAGAATTTTCTCGA
TTTTTCTAGAAAGTTCTGGAACATTCCAGAATTTTCTCGA
TTTTTCTAGAAAGTTCTGGAACATTCCAAAATTTTCTCGA
TTTTTCTAGAAAGTTCTGGAACATTCCAGAATTTTCTCGA
TTTTTCTAGAAAGTTCTGGAACATTCCAAAATTTTCTCGA
TTTTTCTAGAAAGTTCTGGAACATTCCAGAATTTTCTCGA
TTTTTCTAGAAAGTTCTGGAACATTCCAAAATTTTCTCGA
TTTTTCTAGAAAATTCTGGAACATTTCAGAATTTTCTCGA
TTTTTCTAGAAAGTTCTGGAACATTCCAGAATTTTCCCGA
TTTTTCTAGAAAGTTCTGGAACATTCCAGAATTTTCTCGA
TTTTTCTAGAAAGTTCTGGAACATTCCAGAATTTTCTCGA
TTTTTCTAGAAAGTTCTGGAACATTCCAGAATTTTCCCGA
TTTTTCTAGAAAGTTCTGGAACATTCCAGAATTTTCTCGA
TTTTTCTAGAAAGTTCTGGAACATTCCAGAATTTTCTCGA
TTTTTCTAGAAAGTTCTGGAACATTCCAGAATTTTCTCGA
TTTTTCTAGAAAGTTCTGGAACATTCCAGAATTTTCTCGA
TTTTTCTAGAAAGTTCTGGAACATTCCAGAATTTTCTCGA
TTTTTCTAGAAAGTTCTGGAACATTCCAGAATTTTCTCGA
TTTTTCTAGAAAGTTCTGGAACATTCCAGAATTTTCCCGA
TTTTTCTAGAAAGTTCTGGAGCATTCCAGAATTTTCTCGA
TTTTTCTAGAAAGTTCTGGAACATTCCAGAATTTTCCCGA
TTTTTCTAGAAAGTTCTGGAACATTCGAGAATTTTCTCGA
TTTTTCTAGAAAGTTCTGGAACATTCGAGAATTTTCTCGA
TTTTTCTAGAAAGTTCTGGAACATTCCAGAATTTTCCCGA
TTTTTCTAGAAAGTTCTGGAACGTTTCAGAATTTTCTCGA
TTATTCTAGAAAGTTCTGGAACATTCCAGAATTTTCCCGA
TTTTTCTAGAAAGTTCTGGAGCATTCCAGAATTTTCTCGA
TTTTTCTAGAAAGTTCTGGAACATTCCAGAATTTTCCCGA
TTTTTCTAGAAAGTTCTGGAACATTCGAGAATTTTCTCGA
TTTTTCTAGAAAGTTCTGGAACATTCCAGAATTTTCCCGA
TTTTTCTAGAAAGTTCTGGAACATTCGAGAATTTTCCCGA

TTTTTCTAGAAAGTTCTGAAACATTCGAGAATTTTCCCGA
TTTTTCAGAAAGTTCTGGAACGTTCCAGAATTTTCTCGA
TTTTTCTAGAAAGTTCTGGAACATTCCAGAATTTTCCCGA
Nr of selected repeats 331 Similarity 0.987691
Consensus:
TTTTTCTAGAAAGTTCTGGAACATTCCAGAATTTTCTCGA
>Cele-UNSB01_4:16144580-16144720 Satlength=141 Nr of Repeats=4 RepeatLength=35
seed=CCAAAATATT
CCAAAATATTTTTCACATAAAATTTAAATTTCCCG
CCAAAATATTTTTCTCAGAAAATTAAAATTTCCCT
CCAAAATATTTTTTTCAGAAAATTTAAATTTCCCT
CCAAAATATTTTTTTCAGAAAATTTAAATTTCCCG
Nr of selected repeats 4 Similarity 0.892063
Consensus:
CCAAAATATTTTTctCAgAAAATTtAAATTTCCCg
>Cele-UNSB01_4:16130581-16145036 Satlength=14456 Nr of Repeats=348
RepeatLength=40 seed=AGAAAGTTCT
AGAAAGTTCTGAACATTCCAGACTTTTCCCAATTTTTCT
AGAAAGTTCTGGAACATTCCAGAATTTTCCCGATTTTTCT
AGAAGTTCTGACATTTTTTTTTTTTTTTTTTTTTTTTTTTTTTTTTTTTTTTTTTTTTCT
AGAAAGTTCTGGAACATTCCAGAATTTTCTCATTTTTCT
AGAAAGTTCTGGAACATTCCAGAATTTTCTCGATTTTTCT
AGAAAGTTCTGGAACTTCCAGAATTTTCTCGATTTTTCT
AGAAAGTTCTGGAACATTCCAGAATTTTCTCGATTTTTCT
AGAAAGTTCTGGAACATTCCAGAATTTTCTCGATTTTTCT
AGAAAGTTCTGGAACGTTCCAGAATTTTCTCGATTTTTCT
AGAAAGTTCTGGAACATTCCAGAATTTTCTCGATTTTTCT
AGAAAGTTCTGGAACATTCCAGAATTTTCTCGATTTTTCT
AGAAAGTTCTGGAACTTCCAGAATTTTCTCGATTTTTCT
AGAAAGTTCTGGAACATTCCAGAATTTTCTCGATTTTTCT
AGAAAGTTCTGGAACATTCCAGAATTTTCTCGATTTTTCT
AGAAAGTTCTGGAACATTCCAGAATTTTCTCGATTTTTCT
AGAAAGTTCTGGAACATTCCAGAATTTTCTCGATTTTTCT
AGAAAGTTCTGGAACATTCCAGAATTTTCTCGATTTTTCT
AGAAAGTTCTGGAACATTCCAGAATTTTCTCGATTTTTCT
AGAAAGTTCTGAACATTCCAGAATTTTCTTTTTTTTTTCTTAGTTTTCTCGATTTTTCT
AGAAAGTTCTGGAACATTCCAGAATTTTCTCGATTTTTCT
AGAAAGTTCTGGAACATTCCAGAATTTTCTCGATTTTTCT
AGAAAGTTCTGGAACATTCCAGAATTTTCTCGATTTTTCT
AGAAAGTTCTGGAACATTCCAGAATTTTCTCGATTTTTCT
AGAAAGTTCTGGAACATTCCAGAATTTTCTCGATTTTTCT
AGAAAGTTCTGAACATTCCAGAATTTTCTCGATTTTTCT
AGAAAGTTCTGGAACATTCCAGAATTTTCTCGATTTTTCT
AGAAAGTTCTGGAACATTCCAGAATTTTCTCGATTTTTCT
AGAAAGTTCTGGAACATTCCAGAATTTTCTCGATTTTTCT
AGAAAGTTCTGGAACATTCCAGAATTTTCTCGATTTTTCT
AGAAAGTTCTGGAACATTCCAGAATTTTCTCGATTTTTCT
AGAAAGTTCTGGAACATTCCAGAATTTTCTCGATTTTTCT
AGAAAGTTCTGGAACATTCCAGAATTTTCCGATTTTTCT
AGAAAGTTCTGGAACATTCCAGAATTTTCTCGATTTTTCT
AGAAAGTTCTGGAACATTCCAGAATTTTCTCGATTTTTCT
AGAAAGTTCTGGAACATTCCAGAATTTTCTCGATTTTTCT
AGAAAGTTCTGGAACATTCCAGAATTTTCTCGATTTTTCT
AGAAAGTTCTGGAACATTCCAGAATTTTCTCGATTTTTCT

AGAAAGTTCTGGAACATTCCAGAATTTTCTCGATTTTTCT
AGAAAGTTCTGGAACATTCCAGAATTTTCTCGATTTTTCT
AGAAAGTTCTGGAACATTCCAGAATTTTCTCGATTTTTCT
AGAAAGTTCTGGAACATTCCAGAATTTTCTCGATTTTTCT
AGAAAGTTCTGGAACATTCCAGAATTTTCTCGATTTTTCT
AGAAAGTTCTGGAACATTCCAGAATTTTCTCGATTTTTCT
AGAAAGTTCTGGAACATTCCAGAATTTTCTCGATTTTTCT
AGAAAGTTCTGGAACATTCCAGAATTTTCTCGATTTTTCT
AGAAAGTTCTGGAACATTCCAGAATTTTCTCGATTTTTCT
AGAAAGTTCTGGAACATTCCAGAATTTTCTCGATTTTTCT
AGAAAGTTCTGGAACATTCCAGAATTTTCTCGATTTTTCT
AGAAAGTTCTGGAACATTCCAGAATTTTCTCGATTTTTCT
AGAAAGTTCTGGAACATTCCAGAATTTTCTCGATTTTTCT
AGAAAGTTCTGGAACATTCCAGAATTTTCTCGATTTTTCT
AGAAAGTTCTGGAACATTCCAGAATTTTCTCGATTTTTCT
AGAAAGTTCTGGAACATTCCAGAATTTTCTCGATTTTTCT
AGAAAGTTCTGGAACATTCCAGAATTTTCTCGATTTTTCT
AGAAAGTTCTGGAACATTCCAGAATTTTCTCGATTTTTCT
AGAAAGTTCTGGAACATTCCAGAATTTTCTCGATTTTTCT
AGAAAGTTCTGGAACATTCCAGAATTTTCTCGATTTTTCT
AGAAAGTTCTGGAACGTTCCAGAATTTTCTCGATTTTTCT
AGAAAGTTCTGGAACATTCCAGAATTTTCTCGATTTTTCT
AGAAAGTTCTGGAACATTCCAGAATTTTCTCGATTTTTCT
AGAAAGTTCTGGAACATTCCAGAATTTTCTCGATTTTTCT
AGAAAGTTCTGGAACATTCCAGAATTTTCTCGATTTTTCT
AGAAAGTTCTGGAACATTCCAGAATTTTCTCGATTTTTCT
AGAAAGTTCTGGAACATTCCAGAATTTTCTCGATTTTTCT
AGAAAGTTCTGGAACATTCCAGAATTTTCTCGATTTTTCT
AGAAAGTTCTGGAACATTCCAGAATTTTCTCGATTTTTCT
AGAAAGTTCTGGAACATTCCAGAATTTTCTCGATTTTTCT
AGAAAGTTCTGGAACATTCCAGAATTTTCTCGATTTTTCT
AGAAAGTTCTGGAACATTCCAGAATTTTCTCGATTTTTCT
AGAAAGTTCTGGAACATTCCAGAATTTTCTCGATTTTTCT
AGAAAGTTCTGGAACATTCCAGAATTTTCTCGATTTTTCT
AGAAAGTTCTGGAACATTCCAGAATTTTCTCGATTTTTCT
AGAAAGTTCTGGAACATTCCAGAATTTTCTCGATTTTTCT
AGAAAGTTCTGGAACATTCCAGAATTTTCTCGATTTTTCT
AGAAAGTTCTGGAACATTCCAGAATTTTCTCGATTTTTCT
AGAAAGTTCTGGAACATTCCAGAATTTTCTCGATTTTTCT
AGAAAGTTCTGGAACATTCCAGAATTTTCTCGATTTTTCT
AGAAAGTTCTGGAACATTCCAGAATTTTCTCGATTTTTCT
AGAAAGTTCTGGAACATTCCAGAATTTTCTCGATTTTTCT
AGAAAGTTCTGGAACATTCCAGAATTTTCTCGATTTTTCT
AGAAAGTTCTGGAACATTCCAGAATTTTCTCGATTTTTCT
AGAAAGTTCTGGAACATTCCAGAATTTTCTCGATTTTTCT
AGAAAGTTCTGGAACATTCCAGAATTTTCTCGATTTTTCT
AGAAAGTTCTGGAACATTCCAGAATTTTCTCGATTTTTCT
AGAAAGTTCTGGAACATTCCAGAATTTTCTCGATTTTTCT
AGAAAGTTCTGGAACATTCCAGAATTTTCTCGATTTTTCT
AGAAAGTTCTGGAACATTCCAGAATTTTCTCGATTTTTCT
AGAAAGTTCTGGAACATTCCAGAATTTTCTCGATTTTTCT
AGAAAGTTCTGGAACATTCCAGAATTTTCTCGATTTTTCT
AGAAAGTTCTGGAACATTCCAGAATTTTCTCGATTTTTCT
AGAAAGTTCTGGAACATTCCAGAATTTTCTCGATTTTTCT

AGAAAGTTCTGGAACATTCCAGAATTTTCTCGATTTTTCT
AGAAAGTTCTGGAACGTTCCAGAATTTTCTCGATTTTTCT
AGAAAGTTCTGGAACATTCCAGAATTTTCTCGATTTTTCT
AGAAAGTTCTGGAACATTCCAGAATTTTCTCGATTTTTCT
AGAAAGTTCTGGAACATTCCAGAATTTTCTCGATTTTTCT
AGAAAGTTCTGGAACATTCCAGAATTTTCTCGATTTTTCT
AGAAAGTTCTGGAACATTCCAGAATTTTCTCGATTTTTCT
AGAAAGTTCTGGAACATTCCAGAATTTTCTCGATTTTTCT
AGAAAGTTCTGGAACATTCCAGAATTTTCTCGATTTTTCT
AGAAAGTTCTGGAACATTCCAGAATTTTCTCGATTTTTCT
AGAAAGTTCTGGAACATTCCAGAATTTTCTCGATTTTTCT
AGAAAGTTCTGGAACATTCCAGAATTTTCTCGATTTTTCT
AGAAAGTTCTGGAACATTCCAGAATTTTCTCGATTTTTCT
AGAAAGTTCTGGAACATTCCAGAATTTTCTCGATTTTTCT
AGAAAGTTCTGGAACATTCCAGAATTTTCTCGATTTTTCT
AGAAAGTTCTGGAACATTCCAGAATTTTCTCGATTTTTCT
AGAAAGTTCTGGAACGTTCCAGAATTTTCTCGATTTTTCT
AGAAAGTTCTGGAACATTCCAGAATTTTCTCGATTTTTCT
AGAAAGTTCTGGAACATTCCAGAATTTTCTCGATTTTTCT
AGAAAGTTCTGGAACATTCCAGAATTTTCTCGATTTTTCT
AGAAAGTTCTGGAACATTCCAGAATTTTCTCGATTTTTCT
AGAAAGTTCTGGAACATTCCAGAATTTTCTCGATTTTTCT
AGAAAGTTTCTTCCAGAATTTTCTCGATTTTTCT
AGAAAGTTCTGGAACATTCCAGAATTTTCTCGATTTTTCT
AGAAAGTTCTGGAACATTCCAGAATTTTCTCGATTTTTCT
AGAAAGTTCTGGAACATTCCAGAATTTTCTCGATTTTTCT
AGAAAGTTCTGGAACATTCCAGAATTTTCTCGATTTTTCT
AGAAAGTTCTGGAACATTCCAGAATTTTCTCGATTTTTCT
AGAAAGTTCTGGAACATTCCAGAATTTTCTCGATTTTTCT
AGAAAGTTCTGGAACATTCCAGAATTTTCTCGATTTTTCT
AGAAAGTTCTGGAACATTCCAGAATTTTCTCGATTTTTCT
AGAAAGTTCTGGAACATTCCAGAATTTTCTCGATTTTTCT
AGAAAGTTCTGGAACATTCCAGAATTTTCTCGATTTTTCT
AGAAAGTTCTGGAACATTCCAGAATTTTCTCGATTTTTCT
AGAAAGTTCTGGAACATTCCAGAATTTTCTCGATTTTTCT
AGAAAGTTCTGGAACATTCCAGAATTTTCTCGATTTTTCT
AGAAAGTTCTGGAACATTCCAGAATTTTCTCGATTTTTCT
AGAAAGTTCTGGAACATTCCAGAATTTTCTCGATTTTTCT
AGAAAGTTCTGGAACATTCCAGAATTTTCTCGATTTTTCT
AGAAAGTTCTGGAACATTCCAGAATTTTCTCGATTTTTCT
AGAAAGTTCTGGAACATTCCAGAATTTTCTCGATTTTTCT
AGAAAGTTCTGGAACATTCCAGAATTTTCTCGATTTTTCT
AGAAAGTTCTGGAACATTCCAGAATTTTCTCGATTTTTCT
AGAAAGTTCTGGAACATTCCAGAATTTTCTCGATTTTTCT
AGAAAGTTCTGGAACATTCCAGAATTTTCTCGATTTTTCT
AGAAAGTTCTGGAACATTCCAGAATTTTCTCGATTTTTCT
AGAAAGTTCTGGAACATTCCAGAATTTTCTCGATTTTTCT
AGAAAGTTCTGGAACATTCCAGAATTTTCTCGATTTTTCT
AGAAAGTTCTGGAACATTCCAGAATTTTCTCGATTTTTCT
AGAAAGTTCTGGAACATTCCAGAATTTTCTCGATTTTTCT
AGAAAGTTCTGGAACATTCCAGAATTTTCTCGATTTTTCT
AGAAAGTTCTGGAACATTCCAGAATTTTCTCGATTTTTCT
AGAAAGTTCTGGAACATTCCAGAATTTTCTCGATTTTTCT
AGAAAGTTCTGGAACATTCCAGAATTTTCTCGATTTTTCT

AGAAAGTTCTGGAACATTCCAGAATTTTCTCGATTTTTCT
AGAAAGTTCTGGAACATTCCAGAATTTTCTCGATTTTTCT
AGAAAGTTCTGGAACATTCCAGAATTTTCTCGATTTTTCT
AGAAAGTTCTGGAACATTCCAGAATTTTCTCGATTTTTCT
AGAAAGTTCTGGAACATTCCAGAATTTTCTCGATTTTTCT
AGAAAGTTCTGGAACATTCCAGAATTTTCTCGATTTTTCT
AGAAAGTTCTGGAACATTCCAGAATTTTCTCGATTTTTCT
AGAAAGTTCTGGAACATTCCAGAATTTTCTCGATTTTTCT
AGAAAGTTCTGGAACATTCCAGAATTTTCTCGATTTTTCT
AGAAAGTTCTGGAACATTCCAGAATTTTCTCGATTTTTCT
AGAAAGTTCTGGAACATTCCAGAATTTTCTCGATTTTTCT
AGAAAGTTCTGGAACATTCCAGAATTTTCTCGATTTTTCT
AGAAAGTTCTGGAACATTCCAGAATTTTCTCGATTTTTCT
AGAAAGTTCTGGAACATTCCAGAATTTTCTCGATTTTTCT
AGAAAGTTCTGGAACATTCCAGAATTTTCTCGATTTTTCT
AGAAAGTTCTGGAACATTCCAGAATTTTCTCGATTTTTCT
AGAAAGTTCTGGAACATTCCAGAATTTTCTCGATTTTTCT
AGAAAGTTCTGGAACATTCCAGAATTTTCTCGATTTTTCT
AGAAAGTTCTGGAACATTCCAGAATTTTCTCGATTTTTCT
AGAAAGTTCTGGAACATTCCAGAATTTTCTCGATTTTTCT
AGAAAGTTCTGGAACATTCCAGAATTTTCTCGATTTTTCT
AGAAAGTTCTGGAACATTCCAGAATTTTCTCGATTTTTCT
AGAAAGTTCTGGAACATTCCAGAATTTTCTCGATTTTTCT
AGAAAGTTCTGGAACATTCCAGAATTTTCTCGATTTTTCT
AGAAAGTTCTGGAACATTCCAGAATTTTCTCGATTTTTCT
AGAAAGTTCTGGAACATTCCAGAATTTTCTCGATTTTTCT
AGAAAGTTCTGGAACATTCCAGAATTTTCTCGATTTTTCT
AGAAAGTTCTGGAACATTCCAGAATTTTCTCGATTTTTCT
AGAAAGTTCTGGAACATTCCAGAATTTTCTCGATTTTTCT
AGAAAGTTCTGGAACATTCCAGAATTTTCTCGATTTTTCT
AGAAAGTTCTGGAACATTCCAGAATTTTCTCGATTTTTCT
AGAAAGTTCTGGAACATTCCAGAATTTTCTCGATTTTTCT
AGAAAGTTCTGGAACATTCCAGAATTTTCTCGATTTTTCT
AGAAAGTTCTGGAACATTCCAGAATTTTCTCGATTTTTCT
AGAAAGTTCTGGAACATTCCAGAATTTTCTCGATTTTTCT
AGAAAGTTCTGGAACATTCCAGAATTTTCTCGATTTTTCT
AGAAAGTTCTGGAACATTCCAGAATTTTCTCGATTTTTCT
AGAAAGTTCTGGAACATTCCAGAATTTTCTCGATTTTTCT
AGAAAGTTCTGGAACATTCCAGAATTTTCTCGATTTTTCT
AGAAAGTTCTGGAACATTCCAGAATTTTCTCGATTTTTCT
AGAAAGTTCTGGAACATTCCAGAATTTTCTCGATTTTTCT
AGAAAGTTCTGGAACATTCCAGAATTTTCTCGATTTTTCT
AGAAAGTTCTGGAACATTCCAGAATTTTCTCGATTTTTCT
AGAAAGTTCTGGAACATTCCAGAATTTTCTCGATTTTTCT
AGAAAGTTCTGGAACATTCCAGAATTTTCTCGATTTTTCT
AGAAAGTTCTGGAACATTCCAGAATTTTCTCGATTTTTCT
AGAAAGTTCTGGAACATTCCAGAATTTTCTCGATTTTTCT
AGAAAGTTCTGGAACATTCCAGAATTTTCTCGATTTTTCT
AGAAAGTTCTGGAACATTCCAGAATTTTCTCGATTTTTCT
AGAAAGTTCTGGAACATTCCAGAATTTTCTCGATTTTTCT
AGAAAGTTCTGGAACATTCCAGAATTTTCTCGATTTTTCT
AGAAAGTTCTGGAACATTCCAGAATTTTCTCGATTTTTCT
AGAAAGTTCTGGAACATTCCAGAATTTTCTCGATTTTTCT
AGAAAGTTCTGGAACATTCCAGAATTTTCTCGATTTTTCT

AGAAAGTTCTGGAACATTCCAGAATTTTCTCGATTTTTCT
AGAAAGTTCTGGAACATTCCAGAATTTTCTCGATTTTTCT
AGAAAGTTCTGGAACATTCCAGAATTTTCTCGATTTTTCT
AGAAAGTTCTGGAACATTCCAGAATTTTCTCGATTTTTCT
AGAAAGTTCTGGAACATTCCAGAATTTTCTCGATTTTTCT
AGAAAGTTCTGGAACATTCCAGAATTTTCTCGATTTTTCT
AGAAAGTTCTGGAACATTCCAGAATTTTCTCGATTTTTCT
AGAAAGTTCTGGAACATTCCAGAATTTTCTCGATTTTTCT
AGAAAGTTCTGGAACATTCCAGAATTTTCTCGATTTTTCT
AGAAAGTTCTGGAACATTCCAGAATTTTCTCGATTTTTCT
AGAAAGTTCTGGAACATTCCAGAATTTTCTCGATTTTTCT
AGAAAGTTCTGGAACATTCCAGAATTTTCTCGATTTTTCT
AGAAAGTTCTGGAACATTCCAGAATTTTCTCGATTTTTCT
AGAAAGTTCTGGAACATTCCAGAATTTTCTCGATTCTGAAATTCCAGAATTTTCTCGATTTTTCT
AGAAAGTTCTGGAACATTCCAGAATTTTCTCGATTTTTCT
AGAAAGTTCTGGAACATTCCAGAATTTTCTCGATTTTTCT
AGAAAGTTCTGGAACATTCCAGAATTTTCTCGATTTTTCT
AGAAAGTTCTGGAACATTCCAGAATTTTCTCGATTTTTCT
AGAAAGTTCTGGAACATTCCAGAATTTTCTCGATTTTTCT
AGAAAGTTCTGGAACATTCCAGAATTTTCTCGATTTTTCT
AGAAAGTTCTGGAACATTCCAGAATTTTCTCGATTTTTCT
AGAAAGTTCTGGAACATTCCAGAATTTTCTCGATTTTTCT
AGAAAGTTCTGGAACATTCCAGAATTTTCTCGATTTTTCT
AGAAAGTTCTGGAACATTCCAGAATTTTCTCGATTTTTCT
AGAAAGTTCTGGAACATTCCAGAATTTTCTCGATTTTTCT
AGAAAGTTCTGGAACATTCCAGAATTTTCTCGATTTTTCT
AGAAAGTTCTGGAACATTCCAGAATTTTCTCGATTTTTCT
AGAAAGTTCTGGAACATTCCAGAATTTTCTCGATTTTTCT
AGAAAGTTCTGTTTCTCGATTTTTCT
AGAAAGTTCTGGAACATTCCAGAATTTTCTCGATTTTTCT
AGAAAGTTCTGGAACATTCCAGAATTTTCTCGATTTTTCT
AGAAAGTTCTGGAACATTCCAGAATTTTCTCGATTTTTCT
AGAAAGTTCTGGAACATTCCAGAATTTTCTCGATTTTTCT
AGAAAGTTCTGGAACATTCCAGAATTTTCTCGATTTTTCT
AGAAAGTTCTGGAACATTCCAGAATTTTCTCGATTTTTCT
AGAAAGTTCTGGAACATTCCAGAATTTTCTCGATTTTTCT
AGAAAGTTCTGGAACATTCCAGAATTTTCTCGATTTTTCT
AGAAAGTTCTGGAACATTCCAGAATTTTCTCGATTTTTCT
AGAAAGTTCTGGAACATTCCAGAATTTTCTCGATTTTTCT
AGAAAGTTCTGGAACATTCCAGAATTTTCTCGATTTTTCT
AGAAAGTTCTGGAACATTCCAGAATTTTCTCGATTTTTCT
AGAAAGTTCTGGAACATTCCAGAATTTTCTCGATTTTTCT
AGAAAGTTCTGGAACATTCCAGAATTTTCTCGATTTTTCT
AGAAAGTTCTGGAACATTCCAGAATTTTCTCGATTTTTCT
AGAAAGTTCTGGAACATTCCAGAATTTTCTCGATTTTTCT
AGAAAGTTCTGGAACATTCCAGAATTTTCTCGATTTTTCT
AGAAAGTTCTGGAACATTCCAGAATTTTCTCGATTTTTCT
AGAAAGTTCTGGAACATTCCAGAATTTTCTCGATTTTTCT
AGAAAGTTCTGGAACATTCCAGAATTTTCTCGATTTTTCT
AGAAAGTTCTGGAACATTCCAGAATTTTCTCGATTTTTCT
AGAAAGTTCTGGAACATTCCAGAATTTTCTCGATTTTTCT
AGAAAGTTCTGGAACATTCCAGAATTTTCTCGATTTTTCT
AGAAAGTTCTGGAACATTCCAGAATTTTCTCGATTTTTCT
AGAAAGTTCTGGAACATTCCAGAATTTTCTCGATTTTTCT

AGAAAGTTCTGGAACATTCCAGAATTTTCTCGATTTTTCT
AGAAAGTTCTGGAACATTCCAGAATTTTCTCGATTTTTCT
AGAAAGTTCTGGAACATTCCAGAATTTTCTCGATTTTTCT
AGAAAGTTCTGGAACATTCCAGAATTTTCTCGATTTTTCT
AGAAAGTTCTGGAACATTCCAGAATTTTCTCGATTTTTCT
AGAAAGTTCTGGAACATTCCAGAATTTTCTCGATTTTTCT
AGAAAGTTCTGGAACATTCCAGAATTTTCTCGATTTTTCT
AGAAAGTTCTGGAACATTCCAGAATTTTCTCGATTTTTCT
AGAAAGTTCTGGAACATTCCAGAATTTTCTCGATTTTTCT
AGAAAGTTCTGGAACATTCCAGAATTTTCTCGATTTTTCT
AGAAAGTTCTGGAACATTCCAGAATTTTCTCGATTTTTCT
AGAAAGTTCTGGAACATTCCAGAATTTTCTCGATTTTTCT
AGAAAGTTCTGGAACATTCCAGAATTTTCTCGATTTTTCT
AGAAAGTTCTGGAACATTCCAGAATTTTCTCGATTTTTCT
AGAAAGTTCTGGAACATTCCAGAATTTTCTCGATTTTTCT
AGAAAGTTCTGGAACATTCCAGAATTTTCTCGATTTTTCT
AGAAAGTTCTGGAACATTCCAGAATTTTCTCGATTTTTCT
AGAAAGTTCTGGAACATTCCAGAATTTTCCCGATTTTTCT
AGAAAGTTCTGAACATTCCAGAATTTTCTCGATTTTTCT
AGAAAGTTCTGGAACATTCCAGAATTTTCTCGATTTTTCT
AGAAAGTTCTGGAACATTCCAGAATTTTCTCGATTTTTCT
AGAAAGTTCTGGAACATTCCAGAATTTTCTCGATTTTTCT
AGAAAGTTCTGGAACATTCCAGAATTTTCTCGATTTTTCT
AGAAAGTTCTGGAACATTCCAGAATTTTCTCGATTTTTCT
AGAAAGTTCTGGAACATTCCAGAATTTTCTCGATTTTTCT
AGAAAGTTCTGGAACATTCCAGAATTTTCCCGATTTTTCT
AGAAAGTTCTGGAACATTCCAGAATTTTCTCGATTTTTCT
AGAAAGTTCTGGAACATTCCAGAATTTTCTCGATTTTTCT
AGAAAGTTCTGGAACATTCCAGAATTTTCTCGATTTTTCT
AGAAAGTTCTGGAACATTCCAAAATTTTCTCGATTTTTCT
AGAAAGTTCTGGAACATTTCAGAATTTTCCCGATTTTTCT
AGAAAGTTCTGGAACATTCGAGAATTTTCTCGATTTTTCT
AGAAAGTTCTGGAACGTTCCAGAATTTTCTCGATTTTTCT
AGAAAGTGCTGGAACGTTCCAGAATTTTCTCGATTTTTCTA
AAAAGTTCTGGAACATTCCAAAATTTTCTCCATTTTTCT
AGAAAGTTCTGGAACATTCCAGAATTTTCCCGATTTTTCT
AGAAAGTTCTGAACATTCCAGAATTTTCTCGATTTTTCT
AGAAAGTTCTGGAACATTCCAGAATTTTCCCGATTTTTCT
AGAAAGTTCTGGAACATTCGAGAATTTTCTCGATTTTTCT
AGAAAGTTCTGGAACATTCCAGAATTTTCTCAATTTTTCT
AGAAAGTTCTGGAACATTCCAGAATTTTCCCAATTTTTCT
AGAAAGTTCTGGAACATTCCAGAATTTTCTCGATTTTTCT
AGAAAGTTCTGGAACATTCCAGAATTTTCTCGATTTTTCTA
AAAAGTTCTGGAACATTCCAAAATTTTCTCCATTTTTCT
AGAAAGTTCTGGAACATTCCAGAATTTTCCCGATTTTTCT
AGAAAGTTCTGAACATTCCAGAATTTTCTCGATTTTTCT
AGAAAGTTCTGGAACATTCCAGAATTTTCCCAATTTTTCT
AGAAAGTTCTGGAACGTTCCAGAATTTTCTCGATTTTTCT
AGAAAGTTCTGGAACATTCCAGAATTTTCTCGATTTTTCT
AGAAAGTTCTGGAACATTCCAAAATTTTCTCGATTTTTCT
AGAAAGTTCTGGAACATTCCAGAATTTTCTCGATTTTTCT
AGAAAGTTCTGGAACATTCCAAAATTTTCTCGATTTTTCT
AGAAAATTCTGGAACATTTCAGAATTTTCTCGATTTTTCT
AGAAAGTTCTGGAACATTCCAGAATTTTCCCGATTTTTCT

AGAAAGTTCTGGAACATTCCAGAATTTTCTCGATTTTTCT
AGAAAGTTCTGGAACATTCCAGAATTTTCTCGATTTTTCT
AGAAAGTTCTGGAACATTCCAGAATTTTCTCGATTTTTCT
AGAAAGTTCTGGAACATTCCAAAATTTTCTCGATTTTTCT
AGAAAGTTCTGGAACATTCCAGAATTTTCTCGATTTTTCT
AGAAAGTTCTGGAACATTCCAGAATTTTCTCGATTTTTCT
AGAAAGTTCTGGAACATTCCAGAATTTTCTCGATTTTTCT
AGAAAGTTCTGGAACATTCCAAAATTTTCTCGATTTTTCT
AGAAAGTTCTGGAACATTCCAGAATTTTCTCGATTTTTCT
AGAAAGTTCTGGAACATTCCAAAATTTTCTCGATTTTTCT
AGAAAGTTCTGGAACATTCCAGAATTTTCTCGATTTTTCT
AGAAAGTTCTGGAACATTCCAAAATTTTCTCGATTTTTCT
AGAAAATTCTGGAACATTTCAGAATTTTCTCGATTTTTCT
AGAAAGTTCTGGAACATTCCAGAATTTTCCCGATTTTTCT
AGAAAGTTCTGGAACATTCCAGAATTTTCTCGATTTTTCT
AGAAAGTTCTGGAACATTCCAGAATTTTCTCGATTTTTCT
AGAAAGTTCTGGAACATTCCAGAATTTTCCCGATTTTTCT
AGAAAGTTCTGGAACATTCCAGAATTTTCTCGATTTTTCT
AGAAAGTTCTGGAACATTCCAGAATTTTCTCGATTTTTCT
AGAAAGTTCTGGAACATTCCAGAATTTTCTCGATTTTTCT
AGAAAGTTCTGGAACATTCCAGAATTTTCTCGATTTTTCT
AGAAAGTTCTGGAACATTCCAGAATTTTCTCGATTTTTCT
AGAAAGTTCTGGAACATTCCAGAATTTTCTCGATTTTTCT
AGAAAGTTCTGGAACATTCCAGAATTTTCCCGATTTTTCT
AGAAAGTTCTGGAGCATTCCAGAATTTTCTCGATTTTTCT
AGAAAGTTCTGGAACATTCCAGAATTTTCCCGATTTTTCT
AGAAAGTTCTGGAACATTCGAGAATTTTCTCGATTTTTCT
AGAAAGTTCTGGAACATTCGAGAATTTTCTCGATTTTTCT
AGAAAGTTCTGGAACATTCCAGAATTTTCCCGATTTTTCT
AGAAAGTTCTGGAACGTTTCAGAATTTTCTCGATTATTCT
AGAAAGTTCTGGAACATTCCAGAATTTTCCCGATTTTTCT
AGAAAGTTCTGGAGCATTCCAGAATTTTCTCGATTTTTCT
AGAAAGTTCTGGAACATTCCAGAATTTTCCCGATTTTTCT
AGAAAGTTCTGGAACATTCGAGAATTTTCTCGATTTTTCT
AGAAAGTTCTGGAACATTCCAGAATTTTCCCGATTTTTCT
AGAAAGTTCTGGAACATTCGAGAATTTTCCCGATTTTTCT
AGAAAGTTCTGAAACATTCGAGAATTTTCCCGATTTTTC
AGAAAGTTCTGGAACGTTCCAGAATTTTCTCGATTTTTCT
AGAAAGTTCTGGAACATTCCAGAATTTTCCCGATTTTTCT
AGAAAGTTCTGGAACATTCGAGAATTTTTTTCGAAATTTCCTGAAGATTCTAGATTTCCAGACTTTTAGAATTTTCAGAA
AATTTAAATTTCCCTCCAAAATTTTTTTCTCAGAATATTTAAATTACCCGCCAAAATATTTTTCACATAAAATTTAAAT
TTCCCGCCAAAATATTTTTCTCAGAAAATTAAAATTTCCCTCCAAAATATTTTTTTCAGAAAATTTAAATTTCCCTCCA
AAATATTTTTTTCAGAAAATTTAAATTTCCCGCCAAAATATTTTTCACAGAAAATTTAAATTTCCCGCCAAAATTTTGG
GTCTTACCACGGTGGGTCTCACCACGACGGGTCTCACCACGATGGGTCTCACCACGATGGGTCTCACCACGGTGGGTCT
CGCCACGATGGGTCTCACCACGATGGGCCTCGCCACGATGGGTCTCGCCACGATGGGTCTCGCCACGATGGGTCTCGCC
ACGATGGGTTTCACCACGAAGGGTCTCTCCACGCCACGAAGATCTCGCAGCAACATTTTTTTAAATTTTCC
AGAAGGTTCTAGAGCAATCCAGAATTTTTTCGATTTTTCC
Nr of selected repeats 328 Similarity 0.988340
Consensus:
AGAAAGTTCTGGAACATTCCAGAATTTTCTCGATTTTTCT
>Cele-UNSB01_4:16219937-16221099 Satlength=1163 Nr of Repeats=29
RepeatLength=35 seed=TTCCCGCCAA
TTCCCGCCAAAATTTTTCAAAGAAAATTTGAA
TTCCCGCCAAAATTTATTTTCTTATAAATTTTGAAG

TTCCCGCCCATATTTTTAAGGGTCTCACCACGATGGGTCTCGCCAGTTCCAGGTGGTACTTAAGCTAACAAAAAGTTTC
TCAGAAAATTTGAAT
TTCCCGCCAAAAATTTTTTCACTGAAAATTTGAAT
TTCCCGCCAAAATGTTTTTCACTGAAAATTTGAAA
TTCCCGCCAAAATGTTTTTCACTGAAAATTTGAAT
TTCCCGCCAAAATTTTTTTCACTGAAAATTTGAAT
TTCCCGCCAAAAATTTTTTCTCAGAAAATTTGAAT
TTCCCGCCGAAAATTTTTTCTCAGAAAATTTGAAT
TTCCCGCCAAAATTTTTCTCACAGAAAATTTGAAT
TTCCCGCCAAAATTTTTTTCACTGAAATTTGAAT
TTCCCGCCAAAATGTTTTTCACTGAAAATTTGAAT
TTCCCGCCAAAATTTTTTCACTGAAAATTTGAAT
TTCCCGCCAAAATTTATTTTCTTATAAATTTTGAAGTTACGGCCCATATTTTTAAGGGTCTCACCACGATGGGTCTCGC
CAGTTCCAGGTGGTACTTAAGCTAACAAAAAGTTTCTCAGAAAATTTGAAT
TTCCCGCCAAAAATTTTTTCTCAGTTAATTTGAAT
TTCCCGCCAAAATGTTTTTCACTGAAAATTTGAAA
TTCCCGCCAAAATGTTTTTCACTGAAAATTTGAAA
TTCCCGCCAAAATGTTTTTCACTGAAAATTTGAAT
TTCCCGCCAAAATTTTTTTCACTGAAAATTTGAAT
TTCCCGCCAAAAATTTTTTCTCAGAAAATTTGAAT
TTCCCGCCGAAAATTTTTTCTCAGAAAATTTGAAT
TTCCCGCCAAAATTTTTCTCACAGAAAATTTGAAT
TTCCCGCCAAAATTTTTTTCACTGAAATTTGAAT
TTCCCGCCAAAATGTTTTTCACTGAAAATTTGAAT
TTCCCGCCAAAATTTTTTTCACTGAAAATTTGAAT
TTCCCGCCAAAATTGTTTCACTGAAAATTGGAAT
TTCCCGCCAAAATTTTTCTCACTGAAAATTTGAAT
TTCCCGCCAAAATTTTTTTCACTGAAAATTTGAAT
TTCCCGCCAAAATTGTTTCACTGAAAATTGGAAT
Nr of selected repeats 20 Similarity 0.896341
Consensus:
TTCCCGCCAAAAttTTTTTCaCtGAAAATTTGAAT
>Cele-UNSB01_4:16220033-16221319 Satlength=1287 Nr of Repeats=15
RepeatLength=15 seed=CACGATGGGT
CACGATGGGTCTCGCCAGTTCCAGGTGGTACTTAAGCTAACAAAAAGTTTCTCAGAAAATTTGAATTTCCCGCCAAAAA
TTTTTTCACTGAAAATTTGAATTTCCCGCCAAAATGTTTTTCACTGAAAATTTGAAATTCCCGCCAAAATGTTTTTCAC
TGAAAATTTGAATTTCCCGCCAAAATTTTTTTCACTGAAAATTTGAATTTCCCGCCAAAAATTTTTTCTCAGAAAATTT
GAATTTCCCGCCGAAAATTTTTTCTCAGAAAATTTGAATTTCCCGCCAAAATTTTTCTCACAGAAAATTTGAATTTCCC
GCCAAAATTTTTTTCACTGAAATTTGAATTTCCCGCCAAAATGTTTTTCACTGAAAATTTGAATTTCCCGCCAAAATTT
TTTCACTGAAAATTTGAATTTCCCGCCAAAATTTATTTTCTTATAAATTTTGAAGTTACGGCCCATATTTTTAAGGGTC
TCAC
CACGATGGGTCTCGCCAGTTCCAGGTGGTACTTAAGCTAACAAAAAGTTTCTCAGAAAATTTGAATTTCCCGCCAAAAA
TTTTTTCTCAGTTAATTTGAATTTCCCGCCAAAATGTTTTTCACTGAAAATTTGAAATTCCCGCCAAAATGTTTTTCAC
TGAAAATTTGAAATTCCCGCCAAAATGTTTTTCACTGAAAATTTGAATTTCCCGCCAAAATTTTTTTCACTGAAAATTT
GAATTTCCCGCCAAAAATTTTTTCTCAGAAAATTTGAATTTCCCGCCGAAAATTTTTTCTCAGAAAATTTGAATTTCCC
GCCAAAATTTTTCTCACAGAAAATTTGAATTTCCCGCCAAAATTTTTTTCACTGAAATTTGAATTTCCCGCCAAAATGT
TTTTCACTGAAAATTTGAATTTCCCGCCAAAATTTTTTTCACTGAAAATTTGAATTTCCCGCCAAAATTGTTTCACTGA
AAATTGGAATTTCCCGCCAAAATTTTTCTCACTGAAAATTTGAATTTCCCGCCAAAATTTTTTTCACTGAAAATTTGAA
TTTCCCGCCAAAATTGTTTCACTGAAAATTGGAATTTCCCGCCAAAAATTTGGGTCTCAC
CACGATGGGTCTCGT
CACGATGGGTCTCAC
CACGATGGGTCTCAC
CACGAAGGGTCTCAC
CACGATGGGTCTCAC

CACGATGGGTCTCGT
CACAATGGGTCTCAC
CACGATGGGTCTCAC
CACGATGGGTCTCCC
CACGATGGGTCTCAC
CACGATGGGTCTCAC
CACGACGGGTCTTTC
CACGATGGGTCTTAC
Nr of selected repeats 13 Similarity 0.863248
Consensus:
CACGATGGGTCTCaC
>Cele-UNSB01_4:16343642-16344390 Satlength=749 Nr of Repeats=18 RepeatLength=11 seed=GTACTGTAGG
GTACTGTAGGA
GTACTGTAGGTATACGGTAGGGTTACTTTAGTTTGGGAAAAATTGAGTTTTTGTACACAGAAGAGTTATTGGGTTGGAA
GTTGCTGGGGGATAATGTCAAG
GTACTGTAGTA
GTACTGTAGGA
GTACTGTAGGTATACGGTAGGGTTACTGTAGTTTAGGAAAATTGAGTTTTTGTCTACAGAAGAGGTATTGGGTTGGGAG
TTGCTGGGGGATAATGTCAAG
GTACTGTAGTA
GTACTGTAGGA
GTACTGTAGGTATACGGTAGGGTTACTGTAGTTTGGGAAAAATTGAGTTTTTGTCTACAGAAGTGGTATTGGGTTGGGA
GTTGGTGAGGGATAATGTCAAGGTACAGTAGTG
GTACTGTAGGA
GTACTGCAGGTATACGGTAGGGTTACTGTAGTTTAGGAAAAATTGAGTTTTTGTCTACAGAAGAGGTATTGGGTTGGGA
GTTGCTGGGGGATAATGTCAAG
GTACTGTAGTG
GTACTGTAGGA
GTACTGTAGGTATACGGTAGGGTTACTGTAGTTCAGGAAAAATTGAGTTTTTGTCTACAGAAGAGGTATTGGGTTGGGA
GTTGTTGGGGGATAATGTCAAG
GTACTGTAGTA
GCACTGTAGGA
GTACTGTAGGTATACGGTAGGGTTACTGTAGTTTGGGAAAAATTGAGTTTTTGTCTACAGAAGAGGTATTGGGTTGGGA
GTTGCTGGGGGATAATGCCAAG
GTACTGTAGTG
GTACTGTAGGA
Nr of selected repeats 12 Similarity 0.878788
Consensus:
GTACTGTAGgA
>Cele-UNSB01_4:16376935-16378753 Satlength=1819 Nr of Repeats=17 RepeatLength=15 seed=GGGTCTCACC
GGGTCTCACCACGAATATTCCAGAATTTTTTTCGAATTTTCCAGAAGGTTCTGGAACTTTCCAGAAGGTTCTAGAACAT
TATAGAATTTTCTGGAATTTTCCAGATGATTCCAAAACCAAAATTGAAATTCCCGCCAAAACCTTTTTCTCAGAAAATT
TGAATTTCCCTTCAAAAATTTTTTTCTCAGAAAATTTGAATTTCCCTCCAAAAATTTTTTTCACTGAAAATTTGAATTT
CCCGCCAAATTTTTTTTCACTGAAAATTTGAATTTCCCGCCAAAAATTTTTTCTCAGAAATGTTGAGTTTTCGGCCAAA
ATTTAT
GGGTCTCACCACGAATATTCCAGAATTTTTTTCGAATTTTCCAGAAGGTTCTGGAACTTTCCAGAAGGTTCTAGAACAT
TATAGAATTTTCTGGAATTTTCCAGATGATTCCAAAACCAAAATTGAAATTCCCGCCAAAACCTTTTTCTCAGAAAATT
TGAATTTCCCTCCAAAAATTTTTTTCACTGAAAATTTGAATTTCCCGCCAAATTTTTTTCACTGAAAATTTGAATTTCC
CGCCAAAAATTTTTTCTCAGAAATGTTGAGTTTTCGGCCAAAATTTAT
GGGTCTCACCACGAATATTCCAGAAATTTTTTCGAATTTTCCAGCAGGTTCTGGAACTTTCCAGAAGGTTCTAGAACAT
TATAGAATTTTCTGGAATTTTCCAGATGATTCCAAAACCAAAATTGAAATTCCCGCCAAAACCTTTTTCTCAGAAAATT
TGAATTTCCCTTCAAAAATTTTTTTCTCAGAAAATTTGAATTTCCCTCCAAAAATTTTTTTCACTGAAAATTTGAATTT

CCCGCCAAATTTTTTTTCACTGAAAATTTGAATTTCCCGCCAAAAATTTTTTCTCAGAAATGTTGAGTTTTCGGCCAAA
ATTTAT
GGGTCTCACCACGAATATTCCAGAATTTTTTTCGAATTTTCCAGAAGGTTCTGGAACTTTCCAGAAGGTTCTAGAACAT
TATAGAATTTTCTGGAATTTTCCAGATGATTCCAAAACCAAAATTGAAATTCCCGCCAAAACCTTTTTCTCAGAAAATT
TGAATTTCCCTCCAAAAATTTTTTTCACTGAAAATTTGAATTTCCCGCCAAATTTTTTTCACTGAAAATTTGAATTTCC
CGCCAAAAATTTTTTCTCAGAAATGTTGAGTTTTCGGCCAAAATTTAT
GGGTCTCACCACGAATATTCCAGAAATTTTTTCGAATTTTCCAGCAGGTTCTGGAACTTTCCAGAAGGTTCTAGAACAT
TATAGAATTTTCTGGAATTTTCCAGATGATTCCAAAACCAAAATTGAAATTCCCGCCAAAACCTTTTTCTCAGAAAATT
TGAATTTCCCTTCAAAAATTTTTTTCTCAGAAAATTTGAATTTCCCTCCAAAAATTTTTTTCACTGAAAATTTGAATTT
CCCGCCAAATTTTTTTTCACTGAAAATTTGAATTTCCCGCCAAAAATTTTTTCTCAGAAATGTTGAGTTTTCGGCCAAA
ATTTAT
GGGTCTCACCACGAATATTCCAGAATTTTTTTCGAATTTTCCAGAAGGTTCTGGAACTTTCCAGAAGGTTCTAGAACAT
TATAGAATTTTCTGGAATTTTCCAGATGATTCCAATGT
GGGTCTCACCGCGAA
GGGTCTCACCACGAT
GGGTCTCACCACGAA
GGGTCTCACCACGAT
GGGTCTCACCACGAA
GGGTCTCACCACGAT
GGGTCTCACCACGAA
GGGTCTCACCACGAA
GGGTCTCACCACGAT
GGGTCTCACCACGAA
GGGTCTCACCACGAT
Nr of selected repeats 11 Similarity 0.935354
Consensus:
GGGTCTCACCACGAa
>Cele-UNSB01_4:16402456-16402561 Satlength=106 Nr of Repeats=7 RepeatLength=15
seed=GTGGTGAGAC
GTGGTGAGACCCATC
GTGGTGAGACCCTTC
GTGGTGAGACCCTTC
GTGGTGAGACCCATC
GTGGTGAGACCCTTA
GTGGTGAGACACATC
GTGGTGAGACCCTTC
Nr of selected repeats 7 Similarity 0.898413
Consensus:
GTGGTGAGACCCtTC
>Cele-UNSB01_4:16472875-16473271 Satlength=397 Nr of Repeats=25 RepeatLength=15
seed=TCTCACCACG
TCTCACCACGAAGTG
TCACACCACGAAAGG
TCTCACCACGATGGGTC
TCTCACCACGATGGA
TCTCACCACGAAGTG
TCTCACCACGAAAGG
TCTCACCACGATGCG
TCTCACCACGAAAGG
TCTCACCACGCTGGG
TCTCACCACGAAAGG
TCTCACCACGATGGGTC
TCTCACCACGATGGA
TCTCACCACGAAGTG

TCACACCACGAAAGG
TCTCACCACGATGGGTC
TCTCACCACGATGGA
TCTCACCACGAAAGG
TCTCACTACGATGGG
TCTCACCACGATGGG
TCTCACCACGAAGGG
TCTCACCACGATGGG
TCTCACCACGAAAGG
TCTCACCACGATGGGTCTTACCCCGATGGG
TATCACCACGAAGGG
TCTCACCACGAAGGG
Nr of selected repeats 21 Similarity 0.820106
Consensus:
TCTCACCACGAagGG
>Cele-UNSB01_4:16493225-16495520 Satlength=2296 Nr of Repeats=119
RepeatLength=19 seed=AAATAGATTG
AAATAGATTGTTACACGGA
AAATAGATTGTTACACGGA
AAATAGATTGTTACACGGA
AAATAGATTGTTACACGGA
AAATAGATTGTTACACGGA
AAATAGATTGTTACACGAA
AAATAGATTGTTACACGGAA
AAATAGATTGTTACACGAA
AAATAGATTGTTACACGAA
AAATAGATTGTTACACGGAT
AAATAGATTGTTACACGAAA
AAATAGATTGTTACACGGA
AAATAGATTGTTACACGGAA
AAATAGATTGTTACACGAA
AAATAGATTGCTACACGGA
AAATAGATTGTTACACGAA
AAATAGATTGTTACACGGA
AAATAGATTGTTACACAGA
AAATAGATTGTTACACGAA
AAATAGATTGTTACACGGAA
AAATAGATTGTTACACGAA
AAATAGATTGCTACACGGA
AAATAGATTGTTACACGAA
AAATAGATTGTTACACGGAA
AAATAGATTGTTACACGAA
AAATAGATTGTTACACGGA
AAATAGATTGTTACACAGA
AAATAGATTGTTACACGAA
AAATAGATTGTTACACGGAA
AAATAGATTGTTACACGAA
AAATAGATTGTTACACGAA
AAATAAATTGCTACACGGA
AAATAGATTGTTACACGAA
AAATAGATTGTTACACGGA
AAATAGATTGTTACACAGA
AAATAGATTGTTACACGAA
AAATAGATTGTTACACGGAA

AAATAGATTGTTACACGAA
AAATAGATTGCTACACGGA
AAATAGATTGTTACACGAA
AAATAGATTGTTACACGGAA
AAATAGATTGTTACACGAA
AAATAGATTGTTACACGGA
AAATAGATTGTTACACAGA
AAATAGATTGTTACACGAA
AAATAGATTGTTACACGGAA
AAATAGATTGTTACACGAA
AAATAGATTGCTACACGGA
AAATAGATTGTTACACGAA
AAATAGATTGTTACACGGA
AAATAGATTGTTACACAGA
AAATAGATTGTTACACGAA
AAATAGATTGTTACACGGAA
AAATAGATTGTTACACGAA
AAATAGATTGTTACACGAA
AAATAGATTGCTACACGGA
AAATAGATTGTTACACGAA
AAATAGATTGTTACACGGAA
AAATAGATTGTTACACGAAA
AAATAGATTGTTACACGGA
AAATAGATTGTTACACGAA
AAATAGATTGTTACACGAA
AAATAGATTGTTACACGGAT
AAATAGATTGTTACACGAAA
AAATAGATTGTTACACGGA
AAATAGATTGTTACACGGA
AAATAGATTGTTACACGGAT
AAATAGATTGTTACACGAAA
AAATAGATTGTTACACGGA
AAATAGATTGTTACACGAA
AAATAGATTGCTACACGGA
AAATAGATTGTTACACGAA
AAATAGATTGTTACACGGAT
AAATAGATTGTTACACGAAA
AAATAGATTGTTACACGGA
AAATAGATTGTTACACGAA
AAATAGATTGTTACACGGAA
AAATAGATTGTTACACGAA
AAATAGATTGCTACACGGA
AAATAGATTGTTACACGAA
AAATAGATTGTTACACGGA
AAATAGATTGTTACACAGA
AAATAGATTGTTACACGAA
AAATAGATTGTTACACGGAA
AAATAGATTGTTACACGGA
AAATAGATTGTTACACGAA
AAATAGATTGTTACACGGAT
AAATAGATTGTTACACGAAA
AAATAGATTGTTACACGGA
AAATAGATTGTTACACGGA
AAATAGATTGTTACACGGAT

AAATAGATTGTTACACGGAA
AAATAGATTGTTACACGAAA
AAATAGATTGTTACACGGA
AAATAGATTGTTACACGAA
AAATAGATTGTTACACGGAA
AAATAGATTGTTACACGAA
AAATAGATTGTTACACGGA
AAATAGATTGTTACACGAAA
AAATAGATTGTTACACGGA
AAATAGATTGTTACACGAA
AAATAGATTGTTACACGGAT
AAATAGATTGTTACACGAAA
AAATAGATTGTTACACGGA
AAATAGATTGTTACACGGA
AAATAGATTGTTACACGGA
AAATAGATTGTTACACGGAA
AAATAGATTGTTACACGAA
AAATAGATTGTTACACGGA
AAATAGATTGTTACACGAAA
AAATAGATTGTTACACGGA
AAATAGATTGTTACACGAA
AAATAGATTGTTACACGAA
AAATAGATTGTTACACGGAT
AAATAGATTGTTACACGAAA
AAATAGATTGTTACACGGA
AAATAGATTGTTACACGGA
AAATAGATTGTTACACGGA
AAATAGATTGTTACACGAA
Nr of selected repeats 85 Similarity 0.941540
Consensus:
AAATAGATTGTTACACGgA
>Cele-UNSB01_4:16653889-16654239 Satlength=351 Nr of Repeats=10 RepeatLength=35
seed=CAAATCGGCA
CAAATCGGCAAATTGCCGAAATTGTAAAGTTCCGG
CAAATCGGCAATATGCCAAAATTGAAAATTTCCGG
CAAATCGGCAATATGCCAAAACTGAAAATATCTGG
CAAATCGGCAATATGTCAAAATTGAAAATTTCTGG
CAAATCGGCAATATGCCAAAATTGAAAATTTTCGGC
CAATCGGCAAATTGCAAAAATGGAAAATTTTCCG
CAAATCGGTAATCTGTCAGAATTGAAAATTTCCGG
CAAATCGGCAATATGCCGAAATTGAAAATTTCCGA
CAAATCGGCAATATGTCAAAATTGAAAATTGCCTG
CAAATCGGCAATATGCCAAAATTGAAAATTTTCGG
Nr of selected repeats 8 Similarity 0.824490
Consensus:
CAAATCGGCAATaTGcCaAAATTGAAAATTTCcGG
>Cele-UNSB01_4:16658516-16659248 Satlength=733 Nr of Repeats=21 RepeatLength=35
seed=TTTGGCATAT
TTTGGCATATTGCTGATTTGCCGGAAAATTTCAAC
TTTGGTATATTGCCGGTTTGCCAGATATTTTCAAT
TTTGGCATATTGCCGGATTGCTGGAAAATTTTCAAT
TTTGGCATATTGCCGATTTGCCGGAAAATTTCAAC
TTTGGTATATTGCCGGTTTGCCGGAAAATTTCAAC
TTTGGTATATTGCCGGTTTGCCGGAAAATTTCAAT

TTTGGCATATTGCCGATTTGCCGGAAAATTTCAAC
TTTGGTATATTGCCGGTTTGCCGGAAAATTTCAAT
TTTGGCATATTGCCGATTTGCCGGAAAATTTCAAC
TTTGGCATATTGCTGATCTGCCGGAAGTTTTCAAT
TTTGGCATATTGCCGATTTGCCGGAAAATTCAAT
TTTGGCATATTGCCGATTTACCGGAAAATTTCAAT
TTTGGCATATTTCCGATTTGCCGGAAAATTCAAT
TTTGGCATATTGCCCATTTGCCGGAAAATTTCAAT
TTTGGCATATTGCTGATTTTCCGGAAATTTTCAAC
TTTGGCATATTGCCGATTTGCCGGAAAATTTCAAT
TTTGGCATATTGCCGGATTGCTGGAAATTTTCAAT
TTTGGCATATTGCTGATTTGCCGGAAACTTCAAT
TTTGGCATGTTGCCGATTTGCCGAAAATTTTCAAT
TTTGGCATATTGCCGATTTTCCGGAAATTTTCAAC
TTTGGCATATTGCTGATTTGCCGGAAATTTCAAT
Nr of selected repeats 16 Similarity 0.860000
Consensus:
TTTGGcATATTGCCGaTTTGCCGGAAAaTTTCAAt
>Cele-UNSB01_4:16659843-16660088 Satlength=246 Nr of Repeats=7 RepeatLength=35
seed=GCAAATCGGC
GCAAATCGGCAAAATGTCCGAATTGAAATTTTCCG
GCAAATCGGCAAAATGGCCGAATTGAAAATTTCCA
GCAAATCGGCAATATGCCAAAATTAAACATTTCCG
GCAAATCGGCAATATGTCAAAATTGAAACTTTCCG
GCAAATCGGCAATATGCCAAAGTTGAAAATTTCCG
GCAAATCGGCAATATGCCAAAGTTGAAAATTTCCG
GCAAATCGGCAATATGCCAAAATTAAAAATTTCCG
Nr of selected repeats 7 Similarity 0.842177
Consensus:
GCAAATCGGCAAtATGcCaaAaTTgAAAaTTTCCG
>Cele-UNSB01_4:16675153-16675373 Satlength=221 Nr of Repeats=8 RepeatLength=20
seed=TTTTAGTGAA
TTTTAGTGAAAATTTGAGAT
TTTTAGTGAAAATTTGAGAT
TTTTAGTGAAAATTTGAGAT
TTTTAGTGAAAATTTAAGATTTTTATGGAAAATTTGAGAT
TTTTAGTGAAAATTTGAGAT
TTTTAGTGAAAATTTGAGAT
TTTTAGTGAAAATTTTAGTTT
TTTAAGTGAAAATCTGGGATTTTTACTTGAAAAATCGTGAAAAAACCTGAAAATTGAGC
Nr of selected repeats 5 Similarity 1.000000
Consensus:
TTTTAGTGAAAATTTGAGAT
>Cele-UNSB01_4:16678025-16678676 Satlength=652 Nr of Repeats=13 RepeatLength=50
seed=GGGCGTGTTT
GGGCGTGTTTTACATGCCCCGCCCATGAAATGGAAGTTTCCACCGAAAT
GGGCGTGTTTTCACATGCCCCGCCCACAAAATTGATGTTTCCACCAAAAT
GGGCGTGTTTTTACATGTCCCGCCCACAAAATTGATGTTTCCACCAAAAT
GGGCGTGTTTTTACATGCCCCGCCCATAAAATTGATGTCACCACCAAAAG
GGGCGTGTTTTTACATGCCCCGCCCATAAAATTGATGTCACCACCAAAAT
GGGCGTGTTTTTACATGCCCCGCCCATAAAATGGATGTTTCCACCAAAAT
GGGCGTGTTTTTCACAAGCCCCGTCCATAAAATTGATGTCACCACCAAAAG
GGGCGTGTTTTCACATGCCCCGCCCATAAAATGGATGTTTCCACCAAAAT
GGGCGTGTTTTTACATGCCCCGCCCACAAAATTGACGTTTCCACAAAAAT

GGGCGTGTTTTTTACATGCCCCGCCCATAAAATTGATGTTTCCACTAAAAT
GGGCGTGTTTTCACAAGCCCCGCCCACAAAATTGATGTTTCCACTAAAAT
GGGCGTGTTTTCACAAGCCCCGCCCATAAAATTGATGTTTCCACTAAAAT
GGGCGTGTTTTCACAAGCCCCGCCCACAAAATTGATGTTTCCACTAAAAT
Nr of selected repeats 10 Similarity 0.897482
Consensus:
GGGCGTGTTTTcACAtGCCCCGCCCAcAAAATtGATGTttCCACcAAAAT
>Cele-UNSB01_4:16708641-16708839 Satlength=199 Nr of Repeats=5 RepeatLength=40
seed=TTCCAGAATT
TTCCAGAATTTTCTCGATTTTTCTAAAAAGTTCTGGAACA
TTCCAGAATTTTCTCGATTTTTCTAGAAAGTTCTGGAACG
TTCCAGAATTTTCTCGATTTTTCTAGAAAGTTCTGGAACA
TTCCAGAATTTTCTCGATTTTTCTAGAAAGTTCTGGAACA
TTCGAGAATTTTTTCGAAATTTCCAGAAGATTCTAGAT
Nr of selected repeats 4 Similarity 0.966667
Consensus:
TTCCAGAATTTTCTCGATTTTTCTAgAAAGTTCTGGAACa
>Cele-UNSB01_4:16709008-16709188 Satlength=181 Nr of Repeats=10 RepeatLength=15
seed=TGGGTCTCAC
TGGGTCTCACCACGA
TGGGTCTCACCACGA
TGGGTCTCACCACGA
TGGGTCTCACCACGA
TGGGTCTCACCACGG
TGGGTCTCGCCACGA
TGGGTCTCACCACGATGGGCCTCGCCACAA
TGGGTCTCGCCACGA
TGGGTCTCACGACGATTGGTCTCGCCACGA
TGGGTCTCACTTCGA
Nr of selected repeats 8 Similarity 0.895238
Consensus:
TGGGTCTCaCCACGA
>Cele-UNSB01_4:16721546-16722348 Satlength=803 Nr of Repeats=4 RepeatLength=37
seed=ATTCAAATTT
ATTCAAATTTCCGTATGTCAGAATAGCGGTAAATTCAAAGAAATTACGCAAGTTTTTGGGGTATGTGTACTTGAACATA
CGCACACACAGATTATTGCGTTACCACATTAGGACCACGGGGGTATAATGAGTTGGGGGGTGTTGGCCTTGTGGCCGTG
GAGGTGAAAAACCCTTTTGATCATCATTACTTCTGATGTGGAAAAGTAAATTTTTATACCACACTATTTATATAACATA
ACAACGTATTCTGAAATATCGAAAATTAAAAAAAAAGTAAAAGCAGTAAATTGCACAATAAATTGAAATTGCACGAAAA
ATGGAAATAAAATAATATGCAAGCAACTTATACTGTTTCAGTTAAAATTTAAATATGAAAATTTATTGGGAATACAGTA
TTCTAAGTGTTTACAGATCAAAACAGTTTTATGTGAGAAAAATAGACAAATAGAGTGTTTTTTGTAAACATGTTGTTTT
TGTCTGCAGGAAATAAAAGTTACTTTTTTGATTCCACTTTTCTAAAAAGAATAGAAAATATGCAAAAAAAAAAATACAAA
AGAAAAGTGTCATTCCTTATCATCATATCATATCGTACACTATATATGAAAGAAAATCGTCTGTTTGTCCCGTCCTTAA
ACTCGGCAGCGGACAGCGATCGAGGTCGGCGATGGACATCGCATATAATTTTGGCGGGA
ATTCAAATTTTAATTTTTTGAAAATATTTTGACGGGA
ATTCAAATTTTAATTTTTTGAAAATACTTTGGCGGGA
ATTCAAATTTTAATTTTTTGAAAATACTTTGGCGGGA
Nr of selected repeats 3 Similarity 0.951952
Consensus:
ATTCAAATTTTAATTTTTTGAAAATAcTTTGgCGGGA
>Cele-UNSB01_4:16741502-16741626 Satlength=125 Nr of Repeats=5 RepeatLength=21
seed=TTCAAGCCTT
TTCAAGCCTTTTAAGACCAAGCTAAAGCCTATTTAGACCAAT
TTCAAGCCTTTTCAGACCAAT
TTCAAGCCTTTTCAGACCAAT

TTCAAGCCTTTAGACCAAC
TTCAAGTCTTTTCAGGCCAAT
Nr of selected repeats 3 Similarity 0.915344
Consensus:
TTCAAGcCTTTTCAGaCCAAT
>Cele-UNSB01_4:16762223-16763229 Satlength=1007 Nr of Repeats=39
RepeatLength=11 seed=CTACAGTACT
CTACAGTACTC
CTACAGTACCT
CTACAGTACTA
CTACAGTACTCCGACCATATCCCACCACTAACCCCAAACCCATATCTCTCCAAAAGACAAAAACTCATTTTTTCCTAAA
CTACAGTAATC
CTACAGTACTC
CTACAGTACCT
CTACAGTACTA
CTACAGTATCCCGACCATATCCCACCACTAACCCCAAACCTATATCTCTTCAAAAGACAAAAACTCATTTTTTCCTAAA
CTACAGTAATC
CTACAGTACTC
CTACAGTACCT
CTACAGTACTA
CTACAGTACCCCGGCCATATTCCACTACTAACCCCAAACCTATATCTCTCCAAAAGACAAAAACTCATTTTTTCCTAAA
CTACAGTAATC
CTACAGTACTC
CTACAGTACCT
CTACAGTACTA
CTACAGTACTCCGACCATATCCCACCACTAACCCCAAACCTATATCTCTCCAAAAGACAAAAACTCATTTTTTCCTAAA
CTACAGTAATC
CTACAGTACTC
CTACAGTACCT
CTACAGTACTA
CTACAGTATCCCGACCATATCCCACCACTAACCCCAAACCTATATCTCTTCAAAAGACAAAAACTCATTTTTTCCTAAA
ATACAGTAATC
CTACAGTACTC
CTACAGTACCT
CTACAGTACTA
CTACAGTACTCCGACCATATCCCACCACTAACCCCAAACCTATATCTCTCCAAAAGACAAAAACTCATTTTTTCCTAAA
CTACAGTAATC
CTACAGTACTC
CTACAGTACCT
CTACAGTACTA
CTACAGTATCCCGACCATATCCCACCACTAACCCCAAACCTATATCTCTTCAAGAGACAAAAACTCATTTTTTCCTAAA
ATACAGTAATC
CTACAGTACTC
CTACAGTACCT
CTACAGTACTA
CTACAGTACTCCGACCATATCCCACCACTAACCCCAAACCTATATCTCTTCAAAAGACAAAAACTCATTTTTTCCTAAA
CTACAGTAATC
CTACAGTACTCCTACAGTACCT
Nr of selected repeats 30 Similarity 0.778225
Consensus:
CTACAGTAcTn
>Cele-UNSB01_4:16773369-16773770 Satlength=402 Nr of Repeats=28 RepeatLength=11
seed=CCCCTACAGT
CCCCTACAGTAT

CCCCACAGTA
CCCCTACAGAAAACCTAGAGTACGGTTACAGTACCTCTGTAGTACCTCTAATGTA
CCCCTACAGTA
CCCCTACAGTAC
CTCCTACAGTA
CCCCTACAGTAC
CCCCTACAGTA
CCCCTGCAGTA
CCCCTACAGTG
CCCCTACAGTA
CCCTTACAGTAAATCTAAAGTATCTCTACAGTA
CCCCTACAGTG
CCCCTACAGAA
CCCCTACAGTA
CCCCTACAGTGCCCATACAATA
CCACTACAGTA
CCCCTACAGTAC
CCCCTACAGTA
CCCCTACAGTA
CCCCTACAGTAC
CCCCTACAGTA
CCCCTACAGTA
CCCCTACAGTAC
CCCCTACAGTA
CCCCTAGAGTA
CCCCTACTGTA
CCCCTACAGTATGGCTACAGTA
Nr of selected repeats 17 Similarity 0.887701
Consensus:
CCCCTACAGTA
>Cele-UNSB01_4:16899632-16900079 Satlength=448 Nr of Repeats=15 RepeatLength=30
seed=AAAATCAAAA
AAAATCAAAAATTAGATGAATTTTCGAATA
AAAATCAAAAATTAGATGAATTTTCGATTA
AAAATCAAAAATGGGTGAATTTTCGATTA
AAAATCAAAAATTAGATGAATTTTCGATTA
AAAATCAAAAATGGGTGAATTTTCGATTA
AAAATCAAAAATTAGATGAATTTTCGATTA
AAAATCAAAAATGGGTGAATTTTCGATTA
AAAATCAAAAATTAGATGAATTTTCGATTA
AAAATCAAAAATTAGATGAATTTTCGATTA
AAAATCAAAAATTAGATGAATTTTCGATTA
AAAATCAAAAATTAGATGAATTTTCGATTA
AAAATCAAAAATTAGATGAATTTTCCATTC
AAAATCAAAAATTAGCTGAATTTTCGATTA
AAAATCAAAAATTAGATGAATTTTCCAATC
AAAATCAAAAATTAGCTAAATTTTCGATTA
Nr of selected repeats 12 Similarity 0.938721
Consensus:
AAAATCAAAAATTAGATGAATTTTCGATTA
>Cele-UNSB01_4:16926190-16926350 Satlength=161 Nr of Repeats=4 RepeatLength=40
seed=TTCCAGAATT
TTCCAGAATTTTCCCGATTTTTCTAGAAAGTTCTGGAACG
TTCCAGAATTTTCCCGATTTTTCTAGAAAGTTCTGGAACG

TTCCAGAATTTTCCCGACTTTTCTAGAAAGTTCTGGAACG
TTCCAGAATTTTCCCGATTTTTCTAGAAAGTTCTGGAGCA
Nr of selected repeats 4 Similarity 0.950000
Consensus:
TTCCAGAATTTTCCCGAtTTTTCTAGAAAGTTCTGGAaCg
>Cele-UNSB01_4:16926416-16926623 Satlength=208 Nr of Repeats=6 RepeatLength=35
seed=TTGAATTTCC
TTGAATTTCCCGCCAAAATATTTTTCTCAGAAAAT
TTGAATTTCCCGCCTAAATGTTTTCACTAAAAAT
TTGAATTTCCCGCCAAAATATTTTTCACTGAAAAT
TTGAATTTCCCGCCAAAATATTTCTCACAAAAAAT
TTGAATTTCCTGCCACGATTTTTTCACAAAAAA
TTGAATTTCCCGCCAAAATATTTTTCTCAGAAAAT
Nr of selected repeats 4 Similarity 0.917460
Consensus:
TTGAATTTCCCGCCAAAATATTTtTCaCagAAAAT
>Cele-UNSB01_4:16931676-16932199 Satlength=524 Nr of Repeats=4 RepeatLength=150
seed=TTGAGGGCCG
TTGAGGGCCGGAGGCCCGAATACACAAAAGTATTGGACAGCCGTTAAGCCTATACTGAGCCCAAAAACTCGAG
TTGAGGGCCGCAGTCCCGAATACACGAAGAAGTGTTGGAGATCCGTCAAGCCTATTCTAAGCATAATAATTCAGTTCGA
GTGTCGCAAGCCCAAATAGACGCAGAAGCGTTGGACAACTTTTAGGCCTATTCTGAGCCCAATAATTCATG
TTGAGGGCCGCAGTCCCGAATACACGAAGAAGTGTTGGAGATCCGTCAAGCCTATTCTAAGCATAATAATTCAGTTCGA
GTGTCGCAAGCCCAAATAGACGCAGAAGCGTTGGACAACTTTTAGGCCTATTCTGAGCCCAATAATTCATG
TTGAGGGCCGCAGGCCCGAATACACATAGAAGCGTTGGACAACTGTTAAGCCTATTCTTAGCATAATAATTCAGTTCGA
GTGTCGCAAGCCCAAATAGACGCAGAAGCGTTGGACAACTTTTAGGCCTATTCTAAGCCCAATAATTCATG
Nr of selected repeats 3 Similarity 0.940741
Consensus:
TTGAGGGCCGCAGtCCCGAATACACgaAGAAGtGTTGGAgAtCcGTcAAGCCTATTCTaAGCATAATAATTCAGTTCGA
GTGTCGCAAGCCCAAATAGACGCAGAAGCGTTGGACAACTTTTAGGCCTATTCTgAGCCCAATAATTCATG
>Cele-UNSB01_4:17138861-17138981 Satlength=121 Nr of Repeats=8 RepeatLength=15
seed=TCGTGGTGAG
TCGTGGTGAGAACCA
TCGTGGTGGGACCCT
TCGTGGTGAGACCCA
TCGTGGTGGGACCCT
TCGCGGTGAGACCCA
TCGTGGTGAGACCCT
TCGTGGTGAGACCCA
TCGTGGTGAGACCCT
Nr of selected repeats 8 Similarity 0.866667
Consensus:
TCGTGGTGaGACCCa
>Cele-UNSB01_4:17168442-17168576 Satlength=135 Nr of Repeats=6 RepeatLength=11
seed=AGTACTGTAG
AGTACTGTAGG
AGTACTGTAGG
AGTACGGTAGG
ATTACTGTAGTTTAGGAAAAATTGAGTTTTTGTCTTTTGAAGAGATATAGGTTTGGGGTTATTAGTGGGATATGGTCGG
G
GTACTGTAGT
AGTACTGTAGG
Nr of selected repeats 4 Similarity 0.939394
Consensus:
AGTACtGTAGG

>Cele-UNSB01_4:17168764-17168899 Satlength=136 Nr of Repeats=9 RepeatLength=15
seed=TCGTGGTGAG
TCGTGGTGAGACCCG
TCGTGGTGAGACCCA
TCGTGGTGAGACCCA
TCGTGGTGAGACCCA
TCGTGGTGAGACCCA
TCGTGGTGAGACCCA
TCGTGGTGAGACCCA
TCGTGGTGAGACCCA
TCGTGGTGAGACCCA
Nr of selected repeats 9 Similarity 0.980247
Consensus:
TCGTGGTGAGACCCA
>Cele-UNSB01_4:17168941-17169150 Satlength=210 Nr of Repeats=6 RepeatLength=35
seed=GAAATTTAAA
GAAATTTAAATTTTCTGTGAAAAATATTTTGGCGG
GAAATTTAAATTTTCTGTGAAAAATGTTTTGGCGG
GAAATTTAAATTTTCTGTAAAAAATATTTTGGAGG
GAAATTTAAATTTTCTGAAAAAATATTTTGGAGG
GAAATTTAAATTTTCTGAAAAAAATATTTTGGAGG
GAAATTTTAATTTTCTGAGAAAAATATTTTGGCGG
Nr of selected repeats 5 Similarity 0.900952
Consensus:
GAAATTTaAATTTTCTGtgAAAAATaTTTTGGcGG
>Cele-UNSB01_4:17169175-17169333 Satlength=159 Nr of Repeats=4 RepeatLength=40
seed=AAAATTCTGG
AAAATTCTGGAAATCTAGAATCTTCTGGAAATTTCGAA
AACATTCTGGAATGTTCCAGAACTTTCTAGAAAAATCGAG
AAAATTCTGGAATGTTCCAGAACTTTCTAGAAAAATCGAG
AAAATTCTGGAATGTTCCAGAACTTTCTAGAAAAATCGAG
Nr of selected repeats 3 Similarity 0.977778
Consensus:
AAaATTCTGGAATGTTCCAGAACTTTCTAGAAAAATCGAG
>Cele-UNSB01_4:17223540-17224619 Satlength=1080 Nr of Repeats=56
RepeatLength=19 seed=AAAAATAGAT
AAAAATAGATTGCTACACG
AAAAATAGATTGTTACACG
AAAAATAGATTGTTCCACG
AAAAATAGATTGTTACACG
AAAAATAGATTGTTACACG
AAAAATAGATTGTTACACG
AAAAATAGATTGTTACACG
AAAAATAGATTGTTCCACG
AAAAATAGATTGTTACACG
AAAAATAGATTGTTACACG
AAAAATAGATTGTTACACG
AAAAATAGATTGTTACACG
AAAAATAGATTGTTCCACG
AAAAATAGATTGTTACACGA
AAAAATAGATTGCTACACGA
AAAAATAGATTGCTACACGA
AAAAATAGATTGTTACACG
AAAAATAGATTGCTACACG

AAAAATAGATTGTTACACG
AAAAATAGATTGTTCCACG
AAAAATAGATTGTTACACG
AAAAATAGATTGTTACACG
AAAAATAGATTGTTACACG
AAAAATAGATTGTTACACG
AAAAATAGATTGTTACACGA
AAAAATAGATTGCTACACGA
AAAAATAGATTGCTACACGA
AAAAATAGATTGTTACACG
AAAAATAGATTGCTACACG
AAAAATAGATTGTTACACG
AAAAATAGATTGTTACACG
AAAAATAGATTGTTCCACG
AAAAATAGATTGTTACACGA
AAAAATAGATTGCTACACGA
AAAAATAGATTGTTACACG
AAAAATAGATTGCTACACG
AAAAATAGATTGTTACACG
AAAAATAGATTGCTACACG
AAAAATAGATTGTTACACG
AAAAATAGATTGCTACACG
AAAAATAGATTGTTACACG
AAAAATAGATTGTTACACG
AAAAATAGATTGTTACACG
AAAAATAGATTGTTCCACG
AAAAATAGATTGTTACACGA
AAAAATAGATTGCTACACG
AAAAATAGATTGTTCCACG
AAAAATAGATTGTTACACGA
AAAAATAGATTGCTACACGA
AAAAATAGATTGCTACACGA
AAAAATAGATTGTTACACGA
AAAAATAGATTGCTACACGA
AAAAATAGATTGCTACACGA
AAAAATAGATTGTTACACG
AAAAATAGATTGCTACACG
AAAAATAGATTGTTACACG
Nr of selected repeats 41 Similarity 0.957039
Consensus:
AAAAATAGATTGTTACACG
>Cele-UNSB01_4:17319515-17323871 Satlength=4357 Nr of Repeats=66
RepeatLength=66 seed=CAAATCAACC
CAAATCAACCTATTCAGTTGACAATGGTTTTCTTTTATCAAATTTTTAAAAATTTTAAAGAAAACA
CAAATCAACCTATTCAGTTGACAATGGTTTTCTTTTATCAAATTTTTTGAAATTTTAAAGAAAACA
CAAATCAACCTATTCAGTTGACAATGGTTTTCTTTTATCAAATTTTTTGAAATTTTAAAGAAAACA
CAAATCAACCTATTCAGTTGACAATGGTTTTCTTTTATCAAATTTTTTGAAATTTTAAAGAAAACA
CAAATCAACCTATTCAGTTGACAATGGTTTTCTTTTATCAAATTTTTTGAAATTTTAAAGAAAACA
CAAATCAACCTATTTAGTTGACAATTGTTTTCTTTTATCAAATTTTTTGAAATTTTAAAGAAAACA
CAAATCAACCTATTCAGTTGACAATGGTTTTCTTTTATCAAATTTTTTGAAATTTTAAAGAAAACA
CAAATCAACCTATTTAGTTGACAATTGTTTTCTTTTATCAAATTTTTTGAAATTTTAAAGAAAACA
CAAATCAACCTATTCAGTTGACAATGGTTTTCTTTTATCAAATTTTTTTAAATTTTAAAGAAAACA
CAAATCAACCTATTTAGTTGACAATGGTTTTCTTTTATCAAATTTTTTGAAATTTTAAAGAAAACA
CAAATCAACCTATTCAGTTGACAATGGTTTTCTTTTACCAAATTTTTTAAAATTTTAAAGAAAACA

CAAATCAACCTATTCAGTTGACAATGGTTTTCTTTTATCAAATTTTTTAAAATTTTAAAGAAAACA
CAAATCAACCTATTCAGTTGACAATGGTTTTCTTTTATCAAATTTTTTGAAATTTTAAAGAAAACA
CAAATCAACCTATTCAGTTGACAATGGTTTTCTTTTATCAAATTTTTTGAAATTTTAAAGAAAACA
CAAATCAACCTATTTAGTTGACAATTGTTTTCTTTTATCAAATTTTTTGAAATTTTAAAGAAAACA
CAAATCAACCTATTCAGTTGACAATGGTTTTCTTTTATCAAATTTTTTAAAATTTTAAAGAAAACA
CAAATCAACCTATTCAGTTGACAATGGTTTTCTTTTATCAAATTTTTTGAAATTTTAAAGAAAACA
CAAATCAACCTATTCAGTTGACAATGGTTTTCTTTTATCAAATTTTTTGAAATTTTAAAGAAAACA
CAAATCAACCTATTCAGTTGACAATGGTTTTCTTTTATCAAATTTTTTGAAATTTTAAAGAAAACA
CAAATCAACCTATTTAGTTGACAATTGTTTTCTTTTATCAAATTTTTTGAAATTTTAAAGAAAACA
CAAATCAACCTATTCAGTTGACAATGGTTTTCTTTTATCAAATTTTTTGAAATTTTAAAGAAAACA
CAAATCAACCTATTTAGTTGACAATTGTTTTCTTTTATCAAATTTTTTGAAATTTTAAAGAAAACA
CAAATCAACCTATTCAGTTGACAATGGTTTTCTTTTATCAAATTTTTTTAAATTTTAAAGAAAACA
CAAATCAACCTATTTAGTTGACAATGGTTTTCTTTTATCAAATTTTTTGAAATTTTAAAGAAAACA
CAAATCAACCTATTCAGTTGACAATGGTTTTCTTTTACCAAATTTTTTAAAATTTTAAAGAAAACA
CAAATCAACCTATTCAGTTGACAATGGTTTTCTTTTATCAAATTTTTTAAAATTTTAAAGAAAACA
CAAATCAACCTATTCAGTTGACAATGGTTTTCTTTTATCAAATTTTTTGAAATTTTAAAGAAAACA
CAAATCAACCTATTCAGTTGACAATGGTTTTCTTTTATCAAATTTTTTGAAATTTTAAAGAAAACA
CAAATCAACCTATTTAGTTGACAATTGTTTTCTTTTATCAAATTTTTTGAAATTTTAAAGAAAACA
CAAATCAACCTATTCAGTTGACAATGGTTTTCTTTTATCAAATTTTTTGAAATTTTAAAGAAAACA
CAAATCAACCTATTTAGTTGACAATTGTTTTCTTTTATCAAATTTTTTGAAATTTTAAAGAAAACA
CAAATCAACCTATTCAGTTGACAATGGTTTTCTTTTATCAAATTTTTTTAAATTTTAAAGAAAACA
CAAATCAACCTATTTAGTTGACAATGGTTTTCTTTTATCAAATTTTTTGAAATTTTAAAGAAAACA
CAAATCAACCTATTCAGTTGACAATGGTTTTCTTTTACCAAATTTTTTAAAATTTTAAAGAAAACA
CAAATCAACCTATTCAGTTGACAATGGTTTTCTTTTATCAAATTTTTTAAAATTTTAAAGAAAACA
CAAATCAACCTATTCAGTTGACAATGGTTTTCTTTTATCAAATTTTTTAAAATTTTAAAGAAAACA
CAAATCAACCTATTCAGTTGACAATGGTTTTCTTTTACCAAATTTTTTAAAATTTTAAAGAAAACA
CAAATCAACCTATTCAGTTGACAATGGTTTTCTTTTATCAAATTTTTTAAAATTTTAAAGAAAACA
CAAATCAACCTATTCAGTTGACAATGGTTTTCTTTTACCAAATTTTTTAAAATTTTAAAGAAAACA
CAAATCAACCTATTCAGTTGACAATGGTTTTCTTTTATCAAATTTTTTAAAATTTTAAAGAAAACA
CAAATCAACCTATTCAGTTGACAATGGTTTTCTTTTATCAAATTTTTTAAAATTTTAAAGAAAACA
CAAATCAACCTATTCAGTTGACAATGGTTTTCTTTTACCAAATTTTTTAAAATTTTAAAGAAAACA
CAAATCAACCTATTCAGTTGACAATGGTTTTCTTTTACCAAATTTTTTAAAAATTTAAAGAAAACA
CAAATCAACCTATTCAGTTGACAATGGTTTTCTTTTACCAAATTTTTTTAAATTTTAAAGAAAACA
CAAATCAACCTATTCAGTTGACAATGGTTTTCTTTTATCAAATTTTTTAAAATTTTAAAGAAAACA
CAAATCAACCTGTTTAGTTGACAATGGTTTTCTTTTACCAAATTTTTTAAAATTTAAAAGAAAACA
CAAATCAACCTATTCAGTTGACAATGGTTTTCTTTTATCAATTTTTTTAAAATTTTAAAGAAAACA
CAAATCAACCTATTCAGTTGACAATGGTTTTCTTTTATCAAATTTTTTAAAATTTTAAAGAAAACA
CAAATCAACCTATTTAGTTGACAATGGTTTTCTTTTACCAAATTTTTTAAAATTTTAAAGAAAACA
CAAATCAACCTATTTAGTTGACAATGGTTTTCTTTTACCAAATTTTTTTAAATTTTAAAGAAAACA
CAAATCAACCTATTCAGTTGACAATGGTTTTCTTTTACCAAATTTTTTAAAAATTTAAAGAAAACA
CAAATCAACCTATTCAGTTGACAATGGTTTTCTTTTACCAAATTTTTTTAAATTTTAAAGAAAACA
CAAATCAACCTATTCAGTTGACAATGGTTTTCTTTTATCAAATTTTTTAAAATTTTAAAGAAAACA
CAAATCAACCTGTTTAGTTGACAATGGTTTTCTTATACCAAATTTTTTAAAATTTAAAAGAAAACA
CAAATCAACCTATTCAGTTGACAATGGTTTTCTTTTATCAATTTTTTTAAAATTTAAAAGAAAACA
CAAATCAACCTATTCAGTTGACAATGGTTTTCTTTTACCAAATTTTTTAAAATTTAAAAGAAAACA
CAAATCAACCTATTCAGTTGACAATGGTTTTCTTTTATCAATTTTTTTAAAATTTTAAAGAAAACA
CAAATCAACCTATTCAGTTGACAATGGATTTCTTTTATCAAATTTTTTAAAATTTTAAAGAAAACA
CAAATCAACCTATTCAGTTGACAATGGTTTTCTTTTATCAAATTTTTTGAAATTTTAAAGAAAACA
CAAATCAACCTATTCAGTTGACAATGGTTTTCTTTTATCAAATTTTTTTAAATTTTAAAGAAAACA
CAAATCAACCTATTTAGTTGACAATGGTTTTCTTTTATCAAATTTTTTGAAATTTTAAAGAAAACA
CAAATCAACCTATTCAGTTGACAATGGTTTTCTTTTATCAAATTTTTTTAAATTTTAAAGAAAACA
CAAATCAACCTATTTAGTTGACAATGGTTTTCTTTTATCAAATTTTTTGAAATTTTAAAGAAAACA
CAAATCAACCTATTCAGTTGACAATGGTTTTCTTTTATCAAATTTTTTGAAATTTTAAAGAAAACA
CAAATCAACCTATTCAGTTGACAATTGTTTTCTTTTATCAAACTTTTTAAAATTTTAAAGAAAACG

CAAATCAACCTATTCAGTTGACAATGGTTTTCTTTTATCAAACTTTTTAAAATTTTAAAGAAAACA
Nr of selected repeats 66 Similarity 0.958428
Consensus:
CAAATCAACCTATTcAGTTGACAATGGTTTTCTTTTAtCAAATTTTTTnAAATTTTAAAGAAAACA
>Cele-UNSB01_4:17337235-17337502 Satlength=268 Nr of Repeats=10 RepeatLength=11
seed=CCTACAGTAC
CCTACAGTACT
CCTACAGTACCT
CTACAGTACTA
CTACAGTACCCCAACCATATCCCACTACTAACCGCAAACCTATATTCCCTCAAAAGACGAAGACACAGTTTTTCCTAAA
CTACAGTAAT
CCTACAGTACT
CCTAAAGTACC
CCTACAGTACTA
CTACAGTACCCCGACCATATCCCACTACTAACCCCAAACCTTTATCTCTTCAAAAGACAAAACTCAATTTTTCCTGAAT
TACAGAAAT
CCTACAGCACT
CCTAAAGTACA
Nr of selected repeats 6 Similarity 0.716667
Consensus:
CCTAcAGTACt
>Cele-UNSB01_4:17389165-17390089 Satlength=925 Nr of Repeats=21 RepeatLength=44
seed=GATTGATGGC
GATTGATGGCCGAAAGGCTGTTTTCTGAGCATTGTTGTATTTCG
GATTGATGGCCGAAAGGCTGTTTTCTGAGCATTGTAGATTTTTG
GATTGATGGCCGAAAGGCTGTTTTCTGAGCATTGTTGTATTTCG
GATTGATGGCCGAAAGGCTGTTTTCTGAGCATTTTAGATTTTCG
GATTGATGGCCGAAAGGCTGTTTTCTGAGCATTGTAGATTTTCG
GATTGATGGCCGAAAGGCTGTTTTCTGAGCATTGTAGATTTTCG
GATTGATGGCCGAAAGGCTGTTTTCTGAGCATTGTAGATTTTCG
GATTGATGGCCGAAAGGCTGTTTTCTGAGCATTGTAGATTTTCG
GATTGATGGCCGAAAGGCTGTTTTCTGAGCATTGTAGATTTTCG
GATTGATGGCCGAAAGGCTGTTTTCTGAGCATTTTAGATTTTCG
GATTGATGGCCGAAAGGCTGTTTTCTGAGCATTGTAGATTTTTG
GATTGATGGCCGAAAGGCTGTTTTCTGAGCATTGTTGTATTTCG
GATTGATGGCCGAAAGGCTGTTTTCTGAGCATTTTAGATTTTCG
GATTGATGGCCGAAAGGCTGTTTTCTGAGCATTGTAGATTTTCG
GATTGATGGCCGAAAGGCTGTTTTCTGAGCATTGTAGATTTTCG
GATTGATGGCCGAAAGGCTGTTTTCTGAGCATTGTAGATTTTCG
GATTGATGGCCGAAAGGCTGTTTTCTGAGCATTGTAGATTTTCG
GATTGATGGCCGAAAGGCTGTTTTCTGAGCATTGTAGATTTTCG
GATTGATGGCCGAAAGGCTGTTTTCTGAGCATTGTAGATTTTCG
GATTGATGGCCGAAAGGCTGTTTTCTGAGCATTGTAGATTTTCG
GATTGATGGCCGAAAGGCTGTTTTCTGAGCATTTTAGATTTTCG
Nr of selected repeats 21 Similarity 0.961328
Consensus:
GATTGATGGCCGAAAGGCTGTTTTCTGAGCATTGTAGATTTTCG
>Cele-UNSB01_4:17395956-17396303 Satlength=348 Nr of Repeats=5 RepeatLength=18
seed=GCACTCCCTA
GCACTCCCTATTAGTATTGCCCCTCGGAGACCTTCCGAAAATTAGTATG
GCACTCCCTATTAGTCTT
GCACTCTCTAATAGCCTTGCGTTTACTCTTTTTTATTGCAGGCCCGTCTTGTTAACTTCAGGATTTCAACGAAAAAAAT
TGCCTATTTTGCAACAGTTTTTCACAACATTTCTACGAGATTGAAGTTTTTGAACTTATATGTTACAATTAAAATCAAA

TTTTCTGAATTTTTACATAAGATTCGAGCTTTTTCAGAAAAAACTAGTAAGCTCAAAGTCAGAAAAATGTTCTGAAAAT
TAGTATT
GCACTCCCTAATAGTCTT
GCACTCCCTAATAGTCTT
Nr of selected repeats 3 Similarity 0.950617
Consensus:
GCACTCCCTAaTAGTCTT
>Cele-UNSB01_4:17401416-17402316 Satlength=901 Nr of Repeats=60 RepeatLength=15
seed=ACCCATCGTG
ACCCATCGTGGTGAG
ACCCATCGTGGTGAG
ACCCATCGTGGCGAG
ACCCACCGTGGTGAG
ACCCATCGCGGTGAG
ACCCACCGTGGTGAG
ACCCGTCGTGGTGAG
ACCCATCGTGGCGAG
ACCCATCGTGGCGAG
ACTCATCGTGGTGAG
ACCCATCGTGGTGAG
ACCCATCGTGGCGAG
ACCCACCGTGGTGAG
ACCCGTCGTGGTGAG
ACCCATCGTGGTAAG
ACCCACCGTGGTGAT
ACCCATCGTGGTGAG
ACCCATCGTGGTGAG
ACCCATCGTGATGAG
ACCCATCGTGGTGAG
ACCCGTCGTGGCGAG
ACCCACCGTGGTGAG
ACCCGTCGTGGTGAG
ACCCATCGTGGTGAG
ACCCATCGTGATGAG
ACCCATCGTGGTGAG
ACCCATCGTGGCGAG
ACCCACCGTGGTGAG
ACCCGTCGTGGTGAG
ACCCATCGTGGTAAG
ACCCACCGTGGTGAT
ACCCATCGTGGTGAG
ACCCATCGTGGTGAG
ACCCATCGTGATGAG
ACCCATCGTGGTGAG
ACCCATCGTGGTGAG
ACCCGTCGTGGCGAG
ACCCATCGTGGTGAG
ACCCGTCGTGGCGAG
ACCCATCGTGGTGAG
ACCCATCGTGGTGAG
ACCCACCGTGGTGAG
ACCCATCGTGGTGAG
ACCCATCGTGGCGAG
ACCCACCGTGGTGAG

ACCCATCGTGGTGAG
ACCCATCGTGGCGAG
ACCCATCGTGGTGAG
ACCCATCGTGGCGAG
ACCCACCGTGGTGAG
ACCCGTCGTGGTGAG
ACCCATCGTGGTAAG
ACCCATCGTGGTGAG
ACCCATCGTGGTAAG
ACCCATCGTGGTGAG
ACCCATCGTGGCGAG
ACCCACCGTGGTGAG
ACCCATCGTGGTGAG
ACCCATCGTGGCGAG
ACCCATCGTGGTGAG
Nr of selected repeats 60 Similarity 0.889768
Consensus:
ACCCATCGTGGtGAG
>Cele-UNSB01_4:17402400-17402610 Satlength=211 Nr of Repeats=6 RepeatLength=35
seed=AAATTTTCTG
AAATTTTCTGAGAAAAATATTTTGGCGGGAAATTT
AAATTTTCTGTGAAAAATATTTTGGCGGGAAATTT
AAATTTTCTGAGAAAAATCTTTTGGCAGGAAATTT
AAATTTTCTGTGAAAAATATTTTGGCGGGAAATTT
AAATTTTCTGTGAAAAATATTTTGGCGGGAAATTT
AAATTTTCTGTGAAAAATATTTTTGCGGGAAATTG
Nr of selected repeats 6 Similarity 0.928889
Consensus:
AAATTTTCTGtGAAAAATATTTTGGCGGGAAATTT
>Cele-UNSB01_4:17408961-17409411 Satlength=451 Nr of Repeats=28 RepeatLength=15
seed=TCTCACCACG
TCTCACCACGAAGTG
TCTCACCACGAAAGG
TCTCACCACGATGCG
TCTCACCACGATGCG
TCTCACCACGATAAG
TCTCACCACGATGGG
TCTCTCCACGAAAGG
TCTCACCACGCTGGG
TCTCACCACGATGGG
TCTCACCACGATGGG
TCTCACCACGATGGGC
CTCACCACGATGGGCCTCTCCACGAAAGG
TCTCACCACGAAAGG
TCTCACCACGATGGG
TCTCACCACGATGGG
TCTCACTACGAAAGG
TCTCACCACGATGGGTCTCTCCACAAAAGG
TCTCACCACGATGGG
TCTCACCACGAAAGG
TCTCACCACGATGGG
TCTCACCCCGATGGG
TCTCACCACGAAAGG
TCTCACCACGATGGG

TCTCACCCCGATGGG
TCTCACCACGAAGGG
TCTCACCACGGTGGG
TCTCACCACGATGGG
TCTCACCACGATGGT
Nr of selected repeats 25 Similarity 0.847111
Consensus:
TCTCACCACGAtgGG
>Cele-UNSB01_4:17408894-17409724 Satlength=831 Nr of Repeats=9 RepeatLength=37
seed=ATTCCCGCCA
ATTCCCGCCAAAATATTTCCAAAAAATTAAAATTTTA
ATTCCCGCCAATATGTTTCACTACGATGGGTCTCACCACGAAGTGTCTCACCACGAAAGGTCTCACCACGATGCGTCTC
ACCACGATGCGTCTCACCACGATAAGTCTCACCACGATGGGTCTCTCCACGAAAGGTCTCACCACGCTGGGTCTCACCA
CGATGGGTCTCACCACGATGGGTCTCACCACGATGGGCCTCACCACGATGGGCCTCTCCACGAAAGGTCTCACCACGAA
AGGTCTCACCACGATGGGTCTCACCACGATGGGTCTCACTACGAAAGGTCTCACCACGATGGGTCTCTCCACAAAAGGT
CTCACCACGATGGGTCTCACCACGAAAGGTCTCACCACGATGGGTCTCACCCCGATGGGTCTCACCACGAAAGGTCTCA
CCACGATGGGTCTCACCCCGATGGGTCTCACCACGAAGGGTCTCACCACGGTGGGTCTCACCACGATGGGTCTCACCAC
GATGGTTCTCACCACGATGGGTCTCGACACGAAGGTTCTTTTTCTCAGAAATTTTAA
ATTCCAGCCAAAATGTTTTCGAAAAATTAAATTTTGA
ATTCCCGCCAAAATATTTTCAAAAAATTAAACTTTTAA
ATTCCCGCCAAAATATTTTCAAAAAATTAAAATTTGA
ATTCCCGCCAAAATATTTTCAAAAAATTAAAATTTGA
ATTCCCGCCAAAATATTTTCAAAAAATTAAAATTTAA
ATTCCCGCCAAAATGTTTTCGAAAAATTAAATTTTGA
ATTCACGCCAAAATGATTTCTAAGAAAATTCAAATTTGA
Nr of selected repeats 6 Similarity 0.896697
Consensus:
ATTCCCGCCAAAATaTTTTCaAAAAATTAAAaTTTgA
>Cele-UNSB01_4:17523613-17523740 Satlength=128 Nr of Repeats=4 RepeatLength=32
seed=GAAGAAATTT
GAAGAAATTTGAATTTGCACCAAAATTTTTCT
GAACAAATTTGAAATCCCACCAAAATTTTCA
GAAGAAATTTGAATTCCCGCTAAAATTTTTCT
GAAGAAATTTGAGTTTGCACCAAAATTTTTCT
Nr of selected repeats 3 Similarity 0.861111
Consensus:
GAAGAAATTTGAaTTtgCaCcAAAATTTTTCT
>Cele-UNSB01_4:17545983-17546043 Satlength=61 Nr of Repeats=4 RepeatLength=15
seed=GTAGATCTTG
GTAGATCTTGTTTTG
GTAGATCTTGAAAAT
GTAGATCTTGTTTTG
GTGGATCTTGTTTTC
Nr of selected repeats 4 Similarity 0.675347
Consensus:
GTaGATCTTGtttTg
>Cele-UNSB01_4:17584126-17584294 Satlength=169 Nr of Repeats=7 RepeatLength=24
seed=TCAACAACAA
TCAACAACAACAGGAGAAGCTTCT
TCAACAACAACAGGAGAAGCTTCT
TCAACAACAACAGGAGAAGCTTCT
TCAACAACAACAGGAGAAGCTTCT
TCAACAACAACAGGAGAAGCTTCT
TCAACAACAACAGGAGAAGCTTCT

TCAACAACAACAGGAGAAGCTTCT
Nr of selected repeats 7 Similarity 1.000000
Consensus:
TCAACAACAACAGGAGAAGCTTCT
>Cele-UNSB01_4:17606143-17606220 Satlength=78 Nr of Repeats=5 RepeatLength=15
seed=TTCCCCTTTT
TTCCCCTTTTCGTAT
TTCCCCTTTTTGTAA
TTCCCCTTTTCGTAT
TTCCCCTTTTCGCAT
TTCCCCTTTTCGTTCAG
Nr of selected repeats 4 Similarity 0.866667
Consensus:
TTCCCCTTTTcGtAt
>Cele-UNSB01_4:17753355-17753780 Satlength=426 Nr of Repeats=17 RepeatLength=25
seed=AGCCAGAGAG
AGCCAGAGAGTAAAAAATACGGTGA
AGCCAGAGAGTAAAATAAACTGTGA
AGCCAGAGAGTAAAATAAACTGTGT
AGCCAGAGAGTAAAAAATACGGTGA
AGCCAGAGAGTAAAATAAACTGTGA
AGCCAGAGAGTAAAATATACGGTGA
AGCCAGAGAGTAAAATATACGGTGA
AGCCAGAGAGTAAAATATATGGTGA
AGCCAGAGAGTAAAATATACGGTGA
AGCCAGAGAGTAAAATATATGGTGA
AGCCAGAGAGTAAAATAAATTGTGA
AGCCAGAGAGTAAAAAATACGGTGA
AGCCAGAGAGTAAAATAAACTGTGT
AGCCAGAGAGTAAAAAATACGGTGA
AGCCAGAGAGTAAAATAAACTGTGA
AGCCAGAGAGTAAAATATACGGTGA
AGCCAGAGAGTAAAATAAACTGTGG
Nr of selected repeats 17 Similarity 0.890980
Consensus:
AGCCAGAGAGTAAAAtAtACgGTGA
>Cele-UNSB01_4:17753798-17754998 Satlength=1201 Nr of Repeats=48
RepeatLength=25 seed=ACTCTCTGGC
ACTCTCTGGCTTCACCATATATTTT
ACTCTCTGGCTTCACCGTATATTTT
ACTCTCTGGCTTCACCATATATTTT
ACTCTCTGGCTTCACAGTTTATTTT
ACTCTCTGGCTTCACCGTATTTTTT
ACTCTCTGGCTTCACAGTTTATTTT
ACTATCTGGCTTCACCATATATTTT
ACTCTCTGGCTTCACCGTATTTTTT
ACTCTCTGGCTTCACCATATATTTT
ACTCTCTGGCTTCACCGTATTTTTT
ACTCTCTGGCTTCACAGTTTATTTT
ACTCTCTGGCTTCGCCGTATTTTTT
ACTCTCTGGCTTCACCGTATTTTTA
ACTCTCTGGCTTCACCGTATATTTT
ACTCTCTGGCTTCACCGTATATTTT
ACTCTCTGGCTTCACCATATATTTT

ACTCTCTGGCTTCACCGTATATTTT
ACTCTCTGGCTTCACCGTATATTTT
ACTCTCTGGCTTCACCATATATTTT
ACTCTCTGGCTTCACCGTATATTTT
ACTCTCTGGCTTCACCGTATATTTT
ACTCTCTGGCTTCACAGTTTATTTT
ACTCTCTGGCTTCACCGTATTTTTT
ACTCTCTGGCTACACAGTTTATTTT
ACTCTCTGGCTTCACAGTTTATTTT
ACTCTCTGGCTTCACCGTATTTTTT
ACTCTCTGGCTTCACAGTTTATTTT
ACTCTCTGGCTTCGCCGTATTTTTT
ACTCTCTGGCTTCACCGTATTTTTA
ACTCTCTGGCTTCACCGTATATTTT
ACTCTCTGGCTTCACCGTATTTTTT
ACTCTCTGGCTTCACCGTATTTTTT
ACTCTCTGGCTACACAGTTTATTTT
ACTCTCTGGCTTCACCGTATTTTTT
ACTCTCTGGCTTCACAGTTTATTTT
ACTCTCTGGCTTCACCGTATTTTTT
ACTCTCTGGCTTCACAGTTTATTTT
ACTATCTGGCTTCACCGTATTTTTT
ACTCTCTGGCTTCACAATTTATTTT
ACTCTCTGGCTTCACCGTATTTTTT
ACTCTCTGGCATCACAGTTTATTTT
ACTCTCTGGCTTCACCGTATTTTTT
ACTCTCTGGCTTCACAGTTTATTTT
ACTATCTGGCTTCACCGTATTTTTT
ACTCTCTGGCTTCACAATTTATTTT
ACTCTCTGGCTTCACCGTATTTTTT
ACTCTCTGGCATCACAGTTTATTTT
ACTCTCTGGCTTCACCGTATTTTTT
Nr of selected repeats 48 Similarity 0.888227
Consensus:
ACTCTCTGGCTTCACcGTaTaTTTT
>Cele-UNSB01_4:17755008-17759186 Satlength=4179 Nr of Repeats=342
RepeatLength=12 seed=TTAGGCTTAG
TTAGGCTTAGGC
TTAGGCTTAGGC
TTAGGCTTAGGC
TTAGGCTTAGGC
TTAGGCTTAGGC
TTAGCTTAGGC
TTAGGCTTAGGC
TTAGGCTTAGGC
TTAGGCTTAGGC
TTAGGCTTAGGC
TTAGGCTTAGGC
TTAGGCTTAGGC
TTAGGCTTAGGC
TTAGGCTTAGGC
TTAGGCTTAGGC
TTAGGCTTAGGC
TTAGGCTTAGGC

TTAGGCTTAGGC
TTAGGCTTAGGC
TTAGGCTTAGGC
TTAGGCTTAGGC
TTAGGCTTAGGC
TTAGGCTTAGGC
TTAGGCTTAGGC
TTAGGCTTAGGC
TTAGGCTTAGGC
TTAGGCTTAGGC
TTAGGCTTAGGC
TTAGGCTTAGGC
TTAGGCTTAGGC
TTAGGCTTAGGC
TTAGGCTTAGGC
TTAGGCTTAGGC
TTAGGCTTAGGC
TTAGGCTTAGGC
TTAGGCTTAGGC
TTAGGCTTAGGGC
TTAGGCTTAGGC
TTAGGCTTAGGC
TTAGGCTTAGGC
TTAGGCTTAGGC
TTAGGCTTAGGC
TTAGGCTTAGGC
TTAGGCTTAGGGC
TTAGGCTTAGGC
TTAGGCTTAGGC
TTAGGCTTAGGC
TTAGGCTTAGGC
TTAGGCTTAGGC
TTAGGCTTAGGC
TTAGGCTTAGGC
TTAGGCTTAGGC
TTAGGCTTAGGC
TTAGGCTTAGGCTTGC
TTAGGCTTAGGC
TTAGGCTTAGGC
TTAGGCTTAGGC
TTAGGCTTAGGC
TTAGGCTTAGGAGC
TTAGGCTTAGGC
TTAGGCTTAGGC
TTAGGCTTAGGC
TTAGGCTTAGGC
TTAGGCCTTAGGC
TTAGGCTTAGGC
TTAGGCTTAGGC
TTAGGCTTAGGC
TTAGGCTTAGGC
TTAGGCTTAGGC
TTAGGCTTAGGC
TTAGGCTTAGGC

TTAGGCTTAGGC
TTAGGCTTAGGC
TTAGGCTTAGGC
TTAGGCTTAGGC
TTAGGCTTAGGC
TTAGGCTTAGGC
TTAGGCTTAGGC
TTAGGCTTAGGC
TTAGGCTTAGGC
TTAGGCTTAGGC
TTAGGCTTAGGC
TTAGGCTTAGGC
TTAGGCTTAGGC
TTAGGCTTAGGC
TTAGGCTTAGGC
TTAGGCTTAGGC
TTAGGCTTAGGC
TTAGGCTTAGGC
TTAGGCTTAGGC
TTAGGCTTAGGC
TTAGGCTTAGGC
TTAGGCTTAGGC
TTAGGCTTAGGC
TTAGGCTTAGGC
TTAGGCTTAGGC
TTAGGCTTAGGC
TTAGGCTTAGGCTTAGC
TAGGCTTAGGCTTAGC
TAGGCTTAGGCTTAGGCAGC
TTAGGCTTAGCTAGC
TTAGGCTTAGGC
TTAGGCTTAGGC
TTAGGCTTAGGC
TTAGGCTTAGGC
TTAGGCTTAGGC
TTAGGCTTAGGC
TTAGGCTTAGGC
TTAGGCTTAGGC
TTAGGCTTAGGC
TTAGGCTTAGGC
TTAGGCTTAGGC
TTAGGCTTAGGC
TTAGGCTTAGGC
TTAGGCTTAGGC
TTAGGCTTAGGC
TTAGGCTTAGGC
TTAGGCTTAGGC
TTAGGCTTAGGC
TTAGGCTTAGGC
TTAGGCTTAGGC
TTAGCTTAGGC
TTAGGCTTAGGC
TTAGGCTTAGGC
TTAGGCTTAGGC

TTAGGCTTAGGC
TTAGGCTTAGGC
TTAGGCTTAGGC
TTAGGCTTAGGC
TTAGGCTTAGGC
TTAGGCTTAGGC
TTAGGCTTAGGC
TTAGGCTTAGGC
TTAGGCTTAGGC
TTAGGCTTAGGC
TTAGGCTTAGGC
TTAGGCTTAGGC
TTAGGCTTAGGC
TTAGGCTTAGGC
TTAGGCTTAGGC
TTAGGCTTAGGC
TTAGGCTTAGC
TTAGGCTTAGGC
TTAGGCTAGC
TTAGGCTTAGGCTTAG
TTAGCTTAGGCTTAGGCGC
TTAGGCTTAGGC
TTAGGGCTTAGGC
TTAGGCTTAGGC
TTAGGCTTAGGC
TTAGGCTTAGGC
TTAGGCTTAGGC
TTAGGCTTAGGC
TTAGGCTTAGGC
TTAGGCTTAGGC
TTAGGCTTAGGC
TTAGGCTTTAGGC
TTAGGCTTAGGC
TTAGGCTTAGGC
TTAGGCTTAGGC
TTAGGCTTAGGC
TTAGGCTTAGGC
TTAGGCTTAGGC
TTAGGCTTAGGC
TTAGGCTTAGGC
TTAGGCTTAGGC
TTAGGCTTAGGC
TTAGGCTTAGGC
TTAGGCTTAGGC
TTAGGCTTAGGC
TTAGGCTTAGGC
TTAGGCTTAGGC
TTAGGCTTAGGC
TTAGGCTTAGGC
TTAGGCTTAGGC
TTAGGCTTAGGC
TTAGGCTTAGGC
TTAGGCTTAGGCT
TTAGGCTTAGGC

TTAGGCTTAGGC
TTAGGCTTAGGC
TTAGCTTAGGC
TTAGGCTTAGG
TTAGGCTTAGGC
TTAGGCTTAGGC
TTAGGCTTAGGCTTAGC
TAGGCTTAGGC
TTAGGCTTAGGCT
TTAGGCTTAGCGGCTTAGGCTTC
TTAGCTTAGGC
TTAGGCTTTAGGC
TTAGGCTTAGGC
TTAGGCTTAGGC
TTAGGCTTAGGC
TTAGGCTTAGGCC
TTAGGCTTAGGCTTGC
TTAGGCTTAGGCC
TTAGGCTTAGGCC
TTAGGCTTAGGC
TTAGGCTTAGGC
TTAGGCTTAGGC
TTAGGCTTAGGC
TTAGGCTTAGGC
TTAGGCTTAGGC
TTAGGCTTAGGC
TTAGGCTTAGGC
TTAGGCTTAGGC
TTAGGCTTAGGC
TTAGGCTTAGGC
TTAGGCTTAGGC
TTAGGCTTAGGC
TTAGGCTTAGGC
TTAGGCTTAGGC
TTAGGCTTAGGC
TTAGGCTTAGGC
TTAGGCTTAGGC
TTAGGCTTAGGC
TTAGGCTTAGGC
TTAGGCTTAGGC
TTAGGCTTAGGC
TTAGGCTTAGGC
TTAGGCTTAGGC
TTAGGCTTAGGC
TTAGGCTTAGGC
TTAGGCTTAGGC
TTAGGCTTAGGC
TTAGGCTTAGGC
TTAGGCTTAGGC
TTAGGCTTAGGC
TTAGGCTTAGGC
TTAGGCTTAGGC
TTAGGCTTAGGC

TTAGGCTTAGGC
TTAGGCTTAGGC
TTAGGCTTAGGC
TTAGGCTTAGGC
TTAGGCTTAGGC
TTAGGCTTAGGC
TTAGGCTTAGGC
TTAGGCTTAGGC
TTAGGCTTAGGC
TTAGGCTTAGGC
TTAGGCTTAGGC
TTAGGCTTAGGC
TTAGGCTTAGGC
TTAGGCTTAGGC
TTAGGCTTAGGC
TTAGGCTTAGGC
TTAGGCTTAGGC
TTAGGCTTAGGC
TTAGGCTTAGGC
TTAGGCTTAGGC
TTAGGCTTAGGC
TTAGGCTTAGGC
TTAGGCTTAGGC
TTAGGCTTAGGC
TTAGGCTTAGGC
TTAGGCTTAGGC
TTAGGCTTAGGC
TTAGGCTTAGGC
TTAGGCTTAGGC
TTAGGCTTAGGC
TTAGGCTTAGGC
TTAGGCTTAGGC
TTAGGCTTAGGC
TTAGGCTTAGGC
TTAGGCTTAGGC
TTAGGCTTAGGC
TTAGGCTTAGGC
TTAGGCTTAGGC
TTAGGCTTAGGC
TTAGGCTTAGGC
TTAGGCTTAGGC
TTAGGCTTAGGC
TTAGGCTTAGGC
TTAGGCTTAGGC
TTAGGCTTAGGC
TTAGGCTTAGGC
TTAGGCTTAGGC
TTAGGCTTAGGC
TTAGGCTTAGGC
TTAGGCTTAGGC
TTAGGCTTAGGC
TTAGGCTTAGGC
TTAGGCTTAGGC
TTAGGCTTAGGC

TTAGGCTTAGGC
TTAGGCTTAGGC
TTAGGCTTAGGC
TTAGGCTTAGGC
TTAGGCTTAGGC
TTAGGCTTAGGC
TTAGGCTTAGGC
TTAGGCTTAGGC
TTAGGCTTAGGC
TTAGGCTTAGGC
TTAGGCTTAGGC
TTAGGCTTAGGC
TTAGGCTTAGGC
TTAGGCTTAGGC
TTAGGCTTAGGC
TTAGGCTTAGGC
TTAGGCTTAGGC
TTAGGCTTAGC
TTAGGCTTAGGC
TTAGGCTTAGGC
TTAGGCTTAGGC
TTAGGCTTAGGC
TTAGGCTTAGGC
TTAGGCTTAGGC
TTAGGCTTAGGCTTAGCTAGC
TTAGGCTTAGGC
TTAGGCTTAGGC
TTAGGCTTAGGC
TTAGGCTTAGGC
TTAGGCTTAGGC
TTAGGCTAGC
TTAGGCTTAGGC
TTAGGCTTAGGC
TTAGGCTTAGGC
TTAGGCTTAGGC
TTAGGCTTAGGC
TTAGGCTTAGGC
TTAGGCTTAGGC
TTAGGCTTAGGC
TTAGGCTTAGGC
TTAGGCTTAGGC
TTAGGCTTAGGC
TTAGGCTTAGGC
TTAGGCTTAGGC
TTAGGCTTAGTAGGC
TTAGGCTTAGGC
TTAGGCTTAGGC
TTAGGCTTAGGC
TTAGGCTTAGC
TTAGGCTTAGGC
TTAGGCTTAGGC
TTAGGCTTAGGGC
TTAGGCTTAGGC
TTAGGCTTAGGC

TTAGGCTTAGGCTTAGGC
Nr of selected repeats 305 Similarity 1.000000
Consensus:
TTAGGCTTAGGC
>Cele-UNSB01_5:4-5075 Satlength=5072 Nr of Repeats=419 RepeatLength=12
seed=AAGCCTAAGC
AAGCCTAAGCCT
AAGCCTAAGCCT
AAGCCTAAGCCT
AAGCCTAAGCCT
AAGCCTAAGCCT
AAGCCTAAGCCT
AAGCCTAAGCCT
AAGCCTAAGCCT
AAGCCTAAGCCT
AAGCCTAAGCCT
AAGCCTAAGCCT
AAGCCTAAGCCT
AAGCCTAAGCCT
AAGCCTAAGCCT
AAGCCTAAGCCT
AAGCCTAAGCCT
AAGCCTAAGCCT
AAGCCTAAGCCT
AAGCCTAAGCCT
AAGCCTAAGCCT
AAGCCTAAGCCT
AAGCCTAAGCCT
AAGCCTAAGCCT
AAGCCTAAGCCT
AAGCCTAAGCCT
AAGCCTAAGCCT
AAGCCTAAGCCT
AAGCCTAAGCCT
AAGCCTAAGCCT
AAGCCTAAGCCT
AAGCCTAAGCCT
AAGCCTAAGCCT
AAGCCTAAGCCT
AAGCCTAAGCCT
AAGCCTAAGCCT
AAGCCTAAGCCT
AAGCCTAAGCCT
AAGCCTAAGCCT
AAGCCTAAGCCT
AAGCCTAAGCCT
AAGCCTAAGCCT
AAGCCTAAGCCT
AAGCCTAAGCCT
AAGCCTAAGCCT
AAGCCTAAGCCT
AAGCCTAAGCCT
AAGCCTAAGCCT
AAGCCTAAGCCT

AAGCTAAGCCT
AAGCCTAAGCCT
AAGCCTAAGCCT
AAGCCTAAGCCT
AAGCCTAAGCCT
AAGCCTAAGCCT
AAGCCTAAGCCT
AAGCCTAAGCCT
AAGCCTAAGCCT
AAGCCTAAGCCT
AAGCCTAAGCCT
AAGCCTAAGCCT
AAGCCTAAGCCT
AAGCCTAAGCCT
AAGCCTAAGCCT
AAGCCTAAGCCT
AAGCCTAAGCCT
AAGCCTAAGCCT
AAGCCTAAGCCT
AAGCCTAAGCCT
AAGCCTAAGCCT
AAGCCTAAGCCT
AAGCCTAAGCCT
AAGCCTAAGCCT
AAGCCTAAGCCT
AAGCCTAAGCCT
AAGCCTAAGCCT
AAGCCTAAGCCT
AAGCCTAAGCCT
AAGCCTAAGCCT
AAGCCTAAGCCT
AAGCCTAAGCCT
AAGCCTAAGCCT
AAGCCTAAGCCT
AAGCCTAAGCCT
AAGCCTAAGCCT
AAGCCTAAGCCT
AAGCCTAAGCCT
AAGCCTAAGCCT
AAGCCTAAGCCT
AAGCCTAAGCCT
AAGCCTAAGCCT
AAGCCTAAGCCT
AAGCCTAAGCCT
AAGCCTAAGCCT
AAGCCTAAGCCT
AAGCCTAAGCCT
AAGCCTAAGCCT
AAGCCTAAGCCT
AAGCCTAAGCCT
AAGCCTAAGCCT
AAGCCTAAGCCT
AAGCCTAAGCCT
AAGCCTAAGCCT

AAGCCTAAGCCT
AAGCCTAAGCCT
AAGCCTAAGCCT
AAGCCTAAGCCT
AAGCCTAAGCCT
AAGCCTAAGCCT
AAGCCTAAGCCT
AAGCCTAAGCCT
AAGCCTAAGCCT
AAGCCTAAGCCT
AAGCCTAAGCCT
AAGCCTAAGCCT
AAGCCTAAGCCT
AAGCCTAAGCCT
AAGCCTAAGCCT
AAGCCTAAGCCT
AAGCCTAAGCCT
AAGCCTAAGCCT
AAGCCTAAGCCT
AAGCCTAAGCCT
AAGCCTAAGCCT
AAGCCTAAGCCT
AAGCCTAAGCCT
AAGCCTAAGCCT
AAGCCTAAGCCT
AAGCCTAAGCCT
AAGCCTAAGCCT
AAGCCTAAGCCT
AAGCCTAAGCCT
AAGCCTAAGCCT
AAGCCTAAGCCT
AAGCCTAAGCCT
AAGCCTAAGCCT
AAGCCTAAGCCT
AAGCCTAAGCCT
AAGCCTAAGCCT
AAGCCTAAGCCT
AAGCCTAAGCCT
AAGCCTAAGCCT
AAGCCTAAGCCT
AAGCCTAAGCCT
AAGCCTAAGCCT
AAGCCTAAGCCT
AAGCCTAAGCCT
AAGCCTAAGCCT
AAGCCTAAGCCT
AAGCCTAAGCCT
AAGCCTAAGCCT
AAGCCTAAGCCT
AAGCCTAAGCCT
AAGCCTAAGCCT
AAGCCTAAGCCT
AAGCCTAAGCCT
AAGCCTAAGCCT

AAGCCTAAGCCT
AAGCCTAAGCCT
AAGCCTAAGCCT
AAGCCTAAGCCT
AAGCCTAAGCCT
AAGCCTAAGCCT
AAGCCTAAGCCT
AAGCCTAAGCCT
AAGCCTAAGCCT
AAGCCTAAGCCT
AAGCCTAAGCCT
AAGCCTAAGCCT
AAGCCTAAGCCT
AAGCCTAAGCCT
AAGCCTAAGCCT
AAGCCTAAGCCT
AAGCCTAAGCCT
AAGCCTAAGCCT
AAGCCTAAGCCT
AAGCCTAAGCCT
AAGCCTAAGCCT
AAGCCTAAGCCT
AAGCCTAAGCCT
AAGCCTAAGCCT
AAGCCTAAGCCT
AAGCCTAAGCCT
AAGCCTAAGCCT
AAGCCTAAGCCT
AAGCCTAAGCCT
AAGCCTAAGCGCT
AAGCCTAAGCCT
AAGCCTAAGCCTAAGCCAACT
AAGCCTAAGCCT
AAGCCTAAGCCT
AAGCCTAAGCT
AAGCCTAAGCCT
AAGCCTAAGCCT
AAGCCTAAGCCT
AAGCCCTAAGCCT
AAGCCTAAGCCT
AAGCCTAAGCCT
AAGCCTAAGCCT
AAGCCTAAGCCT
AAGCCTTAAGCCT
AAGCCTAAGCTCT
AAGCCTAAGCCT
AAGCCTAAGGCCT
AAGCCTAAGCCT
AAGCCTAAGCCT
AAGCCTAAGCCT
AAGCCTAAGCCT
AAGCCTAAGCCT
AAGCCTAAGCCT
AAGCCTAAGCCT

AAGCCTAAGCCT
AAGCCTAAGCCT
AAGCCTAAAGCCT
AAGCCTAAGCCT
AAGCCTAAGCCT
AAGCCTAAGCCT
AAGCCTAAGCCT
AAGCCTAAGCCT
AAGCCTAACT
AAGCCTAAAGCCT
AAGCTAAGCCT
AAGCCTAAGCCT
AAGCCTAAGCCT
AAGCTAAGCCT
AAGCCTAAGCCT
AAGCCTAAGCCT
AAGCCTAAGCCT
AAGCCTAAGGCCT
AAGCCTAAGCCT
AAGCCTAAGCCTAAGCCTACT
AAGCCTAAGCCT
AAGCCTAGCT
AAGCCTAAGCCT
AAGCTAAGCCT
AAGCCTAAGCT
AAGCCTAAGCCT
AAGCCTAAGCCT
AAGCCTAAGCCT
AAGCCTAAGCCT
AAGCCTAAGCCT
AAGCCTAAGCCT
AAGCCTAAGCCT
AAGCCTAAGCCT
AAGCCTAAGCCT
AAGCCTAAGCCT
AAGCCTAAGCCT
AAGCCTAAGCCT
AAGCCTAAGCCT
AAGCCTAAGCCT
AAGCCTAAGCCT
AAGCCCTAAGCCT
AAGCCTAAGCCT
AAGCCTAAGCCT
AAGCCTAAGCCT
AAGCCTAAGCCT
AAGCCTAAGCCT
AAGCCTAAGCCT
AAGCCTAAGCCT
AAGCCTAAGCCT
AAGCCTAAGCCT
AAGCCTAAGCCT
AAGCCTAAGCCT
AAGCCTAAGCCT
AAGCCTAAGCCT

AAGCCTAAGCCT
AAGCCTAAGCCT
AAGCCTAAGCCCT
AAGCCTAAGCCT
AAGCCTAAGCCT
AAGCCTAAGCCT
AAGCCTAAGGCCT
AAGCCTAAGTGCCT
AAGCCTAAGCGCCT
AAGCCCTAAGCT
AAGCCTAAGCCT
AAGCTAAGCCG
AAGCCTAAGCCT
AAGCCTAAGCCT
AAGCCTAAGCCT
AAGCCTAAGCCT
AAGCCTAAGCCT
AAGCCTAAGCCT
AAGCCTAAGCCT
AAGCCTAAGCCT
AAGCCTAAGCCT
AAGCCTAAGCCT
AAGCCTAAGCCT
AAGCCTAAGCCT
AAGCCTAAGCCT
AAGCCTAAGCCT
AAGCCTAAGCCT
AAGCCTAAGCCT
AAGCCTAAGCCT
AAGCCTAAGCCCT
AAGCCTAAGCCT
AAGCCTAAGCCT
AAGCCTAAGCCT
AAGCCTAAGCCT
AAGCCTAAGCCT
AAGCCTAAGCCT
AAGCCTAAGCCT
AAGCCTAAGCCT
AAGCCTAAGCCT
AAGCCTAAGCCT
AAGCCTAAGCCT
AAGCCCTAAGCCT
AAGCCTAAGCCT
AAGCCTAAGCCT
AAGCCTAAGCCT
AAGCCTAAGCCT
AAGCCTAAGCCT
AAGCCTAAGCCT
AAGCCTAAGCCT
AAGCCTAAGCCTAAGCGCCT
AAGCCTAAGCCT
AAGCCTAAGCCT
AAGCCTAAGCCT
AAGCCTTAAGCCT

AAGCCTAAGCCT
AAGCCTAAGCCT
AAGCCTAAGCCT
AAGCCTAAGCCT
AAGCCTAAGCCT
AAGCCTAAGCCT
AAGCCTAAGCCT
AAGCCTAAGCCT
AAGCCTAAGCCT
AAGCCTAAGCCT
AAGCCTAAGCCT
AAGCCTAAGCCT
AAGCCTAAGCCT
AAGCCTAAGCCT
AAGCCTAAGCCT
AAGCCTAAAGCCT
AAGCCTAAGCCT
AAGCCTAAGCCT
AAGCCTAAGCCT
AAGCCTAAGCCT
AAGCCTAAGCCT
AAGCCTAAGCCT
AAGCCTAAGCCT
AAGCCTAAGCCT
AAGCCTAAGCCT
AAGCCTAAGCCT
AAGCCTAAGCCT
AAGCCTAAGCCTA
AAGCCTAAGCCT
AAGCCTAAGCCT
AAGCCTAAGCCT
AAGCCTAAGCCCT
AAGCCTAAGCCT
AAGCCTAAGCCT
AAGCCTAAGCCT
AAGCCTAAGCCT
AAGCCTAAGCCT
AAGCCTAAGCCT
AAGCCTAAGCCT
AAGCCTAAGCCT
AAGCCTAAGCCT
AAGCCTAAGCCT
AAGCCTAAGCCT
AAGCCTAAGCCT
AAGCCTAAGCCT
AAGCCTAAGCCT
AAGCCTAAGCCT
AAGCCTAAGCCT
AAGCCTAAGCCT
AAGCCTAAGCCT
AAGCCTAAGCCCT
AAGCCTAAGCCT
AAGCCTAAGCCT
AAGCCTAAGCCT

AAGCCTAAGCCT
AAGCCTAAGCCT
AAGCCTAAGCCT
AAGCCTAAGCCT
AAGCCTAAGCCT
AAGCCTAAGCCT
AAGCCTAAGCCT
AAGCCTAAGCCT
AAGCCTAAGCCT
AAGCCTAAGCCT
AAGCCTAAGCCT
AAGCCTAAGCCT
AAGCCTAAGCCT
AAGCATAAGCCT
AAGCCTAAGCCT
AAGCCTAAGCCT
AAGCCTAAGCCT
AAGCCTAAGCCT
AAGCCTAAGCCT
AAGCCTAAGCCT
AAGCCTAAGCCT
AAGCATAAGCCT
AAGCCTAAGCCT
AAGCCTAAGCCT
AAGCCTAAGCCT
AAGCCTAAGCAT
AAGCCTAAGCCT
AAGCCTAAGCCT
AAGCCTAAGCCT
AAGCCTAAGCCT
AAGCCTAAGCCT
AAGCCTAAGCCT
AAGCCTAAGCCT
AAGCATAAGCCT
AAGCCTAAGCCT
AAGCCTAAGCCT
AAGCCTAAGCAT
AAGCCTAAGCCT
AAGCCTAAGCCT
AAGCCTAAGCCT
AAGCATAAGCCT
AAGCCTAAGCCT
AAGCCTAAGCAT
AAGCCTAAGCCT
AAGCCTAAGCCT
AAGCCTAAGCCT
AAGCATAAGCCTAAGCCT
Nr of selected repeats 386 Similarity 0.918190
Consensus:
AAGCCTAAGCCT
>Cele-UNSB01_5:105361-105451 Satlength=91 Nr of Repeats=6 RepeatLength=15
seed=AAAAGGAGAA
AAAAGGAGAATTTCG
AAAAGGAGAATTACG

AAAAGGAGAACGACG
AAAAGGAGAACAACG
AAAAGGAGAACAACC
AAAAGGAGAACGACG
Nr of selected repeats 6 Similarity 0.822222
Consensus:
AAAAGGAGAAcnACG
>Cele-UNSB01_5:105684-105774 Satlength=91 Nr of Repeats=6 RepeatLength=15
seed=TTCTCCTTTT
TTCTCCTTTTCGTCG
TTCTCCTTTTGGTTG
TTCTCCTTTTCGTTG
TTCTCCTTTTCGTCG
TTCTCGTTTTCGTAA
TTCTCCTTTTCGAAA
Nr of selected repeats 6 Similarity 0.792593
Consensus:
TTCTCCTTTTCGTng
>Cele-UNSB01_5:328030-329092 Satlength=1063 Nr of Repeats=6 RepeatLength=175
seed=AAATTTTGGG
AAATTTTGGGTTCACTCTTTTTACTAGACAGTGATTGTTGCATTGTAGGATTCGGTGGACTTGTCCAACAACTTACTTT
GGCATGAGTTTAATAAAAAGGTTTTACCATGAACTGAATCAATTTAAAATAAAAACCTCGAGAACGAAAACATCCTCAG
TAATTTCAATCCTGTTC
AAATTTTGGGTTCACTCTTTTCACTAGACAGTGATTGTTGCATTGTAGGATTCGGTGGACTTGTCCAACAACTTATTTT
GGCATGAGTTTAATGAAAAGTGTTTATAATGAACTGGATCAATTTAAAATAAAAACCTCGAGAATGAAAACATCCTCAG
TAATTTCAATCCTGTTC
AAATTTTGGGTTCACTCTTTTTACTAGACAGTGATTGTTGCATTGTAGGTTTCGGTGGACTTGTCCAACAACTTACTTT
GGCATGAGTTTAATAAAAAGGTTTTACCATGAACTGAATCAATTCAAAATAAAAACTTCGAGAATGAAAACATCTCCAG
TTGCCTCAATCATGTTC
AAATTTTGGGTTCACTCTTTTTACTAGACAGTGATTGTTGCATTGTAGGATTCGGTGGACTTGTCCAACAACTTAATTT
GGCATGCGTTTAATAAAAAGGTTTTACCATGAACTGATTCAATTCAAAATAAAAACTTCGAGAATGAAACCATCCTCAG
TAATTTCAATCCTGTTC
AAATTTTGGGTTCACTCTTTTTACTAGACAGTGATTGTTGCATTGTAGGATTGGGTGGACTTATCCAACAACTTACTTT
GGCATGAGTTTAATAAAAAGTGTTTACCATGAACTGAAACAAATTGGGAAATTGGGAAAAACTGCGAGAACCAAAATAT
CTCCAGTTAAAAAAAAACAGTTA
AAATTTTGGGTTCACTCTTATTACTTTACAGTGATTGTTACATTGTAGATTCTGTGGACTTGTCCAACTGTCTACGACG
GCATGAGTTTAGTAAAAAGTATTTACCATGAACTGAAACAAATTGGAAAATTGTGAAAAACTGCGAGAATCAAAATATC
TCCAGTTTTAAATAAAACAGTTC
Nr of selected repeats 4 Similarity 0.911111
Consensus:
AAATTTTGGGTTCACTCTTTTtACTAGACAGTGATTGTTGCATTGTAGGaTTCGGTGGACTTGTCCAACAACTTAcTTT
GGCATGaGTTTAATaAAAAGgtTTTAccATGAACTGaaTCAATTcAAAATAAAAACcTCGAGAAtGAAAaCATCctCAG
TaattTCAATCcTGTTC
>Cele-UNSB01_5:345871-345991 Satlength=121 Nr of Repeats=7 RepeatLength=16
seed=CATGCCTACA
CATGCCTACATGCCTA
CATACCTACATGCCTA
CATGCCTACATGCCTA
CATGCCTACATGCCTA
CATGCCTACATGCCTA
CATGCCTACAGGTTTACATACATA
CATGCCTACATGCCTA
Nr of selected repeats 6 Similarity 0.972222
Consensus:

CATGCCTACATGCCTA
>Cele-UNSB01_5:353635-353859 Satlength=225 Nr of Repeats=14 RepeatLength=16
seed=GGCATGTAGG
GGCATGTAGGCATGTA
GGCATGTAGGCATGTA
GGCATGTAGGCATGTA
GGCATGTAGGCATGTA
GGCGTGTAGGCATGTA
GGCATATAGGTATGTA
GGCATGTAGGCATGCA
GGCATGTAGGCATGTA
GGCATGTAGGCATGTA
GGCATGTTGGCATGTA
GGTATGTAGGCATGTA
GGCATGTAGGCATGTA
GGCATGTAGGCATGTA
GGCATGTAGGCATGTA
Nr of selected repeats 14 Similarity 0.928571
Consensus:
GGCATGTAGGCATGTA
>Cele-UNSB01_5:376477-376531 Satlength=55 Nr of Repeats=4 RepeatLength=12
seed=AGGCTTAGGC
AGGCTTAGGCTA
AGGCTTAGGCTA
AGGCTTAGGCTA
AGGCTTAGACCTAGGGTT
Nr of selected repeats 3 Similarity 1.000000
Consensus:
AGGCTTAGGCTA
>Cele-UNSB01_5:786342-786547 Satlength=206 Nr of Repeats=6 RepeatLength=34
seed=CAATTTGCCG
CAATTTGCCGGTTTGTCGGAAATTTCGAATCCGG
CAATTTGCCGATTTACCCGAAATTTCGATTCCGG
CAATTTGCCGGTTTGCCGGAAAATTCAATTTTAG
CAATCTGCCGATTTGCCGGAAATTTTCAATTCCGG
CAATTTGCCGGTTTGCCGGAAATTTAAATTTCGC
CAATTTGCCGATTTGCCGGAAATTTCAATTTCGG
Nr of selected repeats 5 Similarity 0.788235
Consensus:
CAATTTGCCGgTTTgcCgGAAAtTTcaAtTtcgg
>Cele-UNSB01_5:786696-786877 Satlength=182 Nr of Repeats=5 RepeatLength=35
seed=TTTCCGGCAA
TTTCCGGCAAACCGGCGAATTGCCGGAATTGAAAA
TTTCCGGTAAATCGGCCAATTGCTGGAATCGAAT
TTTCCGGTAAATCGGCCAATTGCCGGAATTGAAGA
TTTCCGGCAAACCGGCAAATTTCCGAAATTGAAGA
TTTCCGGCAAATCGGCAAACCGGCAAACTGCCGGAGTTGAAA
Nr of selected repeats 3 Similarity 0.834921
Consensus:
TTTCCGGcAAAcCGGCnAATTgCCGgAATTGAAgA
>Cele-UNSB01_5:788141-788221 Satlength=81 Nr of Repeats=5 RepeatLength=16
seed=CTACATGCCT
CTACATGCCTACATGC
CTACATGCCTACATGC

CTACATTCCTACATGC
CTACATGCCTACATGC
CTACATGTCTACATGC
Nr of selected repeats 5 Similarity 0.933333
Consensus:
CTACATgcCTACATGC
>Cele-UNSB01_5:788660-789185 Satlength=526 Nr of Repeats=12 RepeatLength=16
seed=AGGCATGTAG
AGGCATGTAGGCATGT
AGGTATGTAGGCATGT
AGGCATGTAGGCATGT
AGGCATGTAGGCATAT
AGGCATGTAGGCATGTAGGTACCAGGGAATTTTTCATCAAATTTTCAATTTTCAGCCATCACCGCCCATCTTGCCAAGT
TCGCCGAAGGAACCGGAAAATCAGTTGCCACAATTTTGAACGCCAAGTACGAGGGAGCCGGTGACTACCTCATCATCAC
TGCAGACTTCTCTGCTGAAACCGAAAAAGCTGGAACTGTGAAGGGAAAATTTTTGCAGATCTGGAAGAAGGAAGGCGAC
AGACACCTGGTTTTCCACGATGAATTTGAAATTAATCCATAGAAATTTGGGAACTTTTTTGTCTGATAACAATTATTGT
AATAAATTTTTTATTCTGAATTTTCCTCTCGTCTCTCGTA
AGCATGTAGGCATGT
AGGCATGTAGGCATGT
AGGCATGTAGGCATGT
AGACATGTAGGTATGT
AGGCATGTGT
AGGCATGTAGGCATGT
AGGCACGTAGGCATGT
Nr of selected repeats 9 Similarity 0.907407
Consensus:
AGGCATGTAGGCATGT
>Cele-UNSB01_5:790113-790281 Satlength=169 Nr of Repeats=10 RepeatLength=16
seed=AGGCATGTAG
AGGCATGTAGGCATGT
AGGCATGTAGGCATGT
AGGCATGTAGGCATGT
AGGCATGTAGGCATGT
AGGCATGTAGGCATGT
AGACATGTAGGCATGT
AGGCATGTAGGCATGT
AGGCATGTAGGCATGT
AGGCATGTAGGCAATT
AGGCATTTAGGCAATTAGGCATTT
Nr of selected repeats 9 Similarity 0.944444
Consensus:
AGGCATGTAGGCATGT
>Cele-UNSB01_5:853469-853759 Satlength=291 Nr of Repeats=23 RepeatLength=10
seed=GGCGTCAGCG
GGCGTCAGCGAGCTTCAGCGGGCATCACCAA
GCGTCAGCGGGCGTCAGCA
GGCGTCAGCGGGATTCAGTG
GGCGTCAGCG
GGCGGCAGCG
GGCGGCAGCG
GGCGTCAGCGGAAGTCAGCGGGAGTTAGCA
GGCGTCAGCG
GGCGTCGGCG
GGCGTCAGCG

GGCGTCGGCG
GGCGTCGGCG
GGCGTCAGCG
GGCGTCAGTG
GGCGTCAGCG
GGCGTCAGCG
GGCGTTAGCG
GGCGTCGGCG
GGCGTCAGCG
GGCGTCAGCG
GGCGTCAGCG
GGCGTCAGCGGGCGTCAGCG
Nr of selected repeats 17 Similarity 0.888235
Consensus:
GGCGTCaGCG
>Cele-UNSB01_5:896085-896285 Satlength=201 Nr of Repeats=12 RepeatLength=16
seed=ATGCCTACAT
ATGCCTACATGCCTAA
ATGCCTAGATGCCTAG
ATGCCTACATGCCTAC
ATGCCTACATGCCTAC
ATGCCTACATGCCTAC
ATGCCTACATGCCTAC
ATGCCTACATGCCTAC
ATGCCTACATGCCTAC
ATGCCTACATGCCTAC
ATGCCTACATGCCTAC
ATGCCTACATGCCTAC
ATGCCTCCATGCCTACATGCCTAC
Nr of selected repeats 11 Similarity 0.956061
Consensus:
ATGCCTACATGCCTAC
>Cele-UNSB01_5:915117-915271 Satlength=155 Nr of Repeats=7 RepeatLength=20
seed=CCTACCCCAG
CCTACCCCAGGCTGACCTAG
CCTACCCCAGGCTGACCTAG
CCTACCCCAGGCTGACCTAG
CCTACCCCAGGCAGGCCTAT
CCTACCCCAGGCAGGCCTAA
CCTACCCCAGGCAGACCTAGTCTACCCTAGGCAA
CCTACCCCAGGCAGGCCTAA
Nr of selected repeats 6 Similarity 0.871111
Consensus:
CCTACCCCAGGCaGaCCTAg
>Cele-UNSB01_5:915226-915380 Satlength=155 Nr of Repeats=8 RepeatLength=20
seed=GGCAGACCTA
GGCAGACCTAGTCTACCCTA
GGCAACCTACCCCA
GGCAGGCCTAACCTACCCCA
GGCAGGCCTAACCTACCATA
GGCAGACCTAGTCTACCATA
GGCAGACCTAGTCTACCATA
GGCAGACCTAGTCCACCCTG
GGCAGACCTAGTCTACCCTA

Nr of selected repeats 7 Similarity 0.809524
Consensus:
GGCAGaCCTAgtCTACCcTA
>Cele-UNSB01_5:978655-978943 Satlength=289 Nr of Repeats=7 RepeatLength=35
seed=TGCCGATTTG
TGCCGATTTGCCAGAAATTTTCAATTCCGGCAATTTGCAGGCTTCCCGGAAATTTTCAATTTCTCAAAAT
TGCCGATTTGCCGGAAATGTTCATTTTCGAGAATT
TGCCGATTTGCCGGAAATGTTCATTTTCGAGAATT
TGCCGGTTTGCCGGAAGTTTTTAATTTCGGCAATT
TGCCGATTTGCCGGAAATGTTCATTGTCGAGAAAT
TGCCGACTTGCCGGAAATTGTCATTTTCGGCAATT
TGCCGATTTGCCGAAAAATTTCAATTTCACCAATTTGCTGATC
Nr of selected repeats 5 Similarity 0.809524
Consensus:
TGCCGatTTGCCGGAAaTgtTcAtTtTCGagAAtT
>Cele-UNSB01_5:1026357-1026873 Satlength=517 Nr of Repeats=9 RepeatLength=35
seed=GCATTTGCCG
GCATTTGCCGATTTTCAAAATTTCCGACAGTTCCGGCATTTTCGGGGTTTTGGAATTTTCGGCAATTCCA
GCATTTGCCGGTTTCGGAAATTTTCGGCAATTTCG
GAATTTGCCGGTTTTTGGAATTTCCGGCAATTACGGTATTTACTGGTTTTGGGCACTTTCAGCAATTTCG
GCATTTGCCGATTTTTAAAATTTCTGGCAATTCCA
GCATTTGCCGATTTTTAAAATTTCTGGCAATTCCA
GCATTTGCCGATTTTTAAAATTTCTGGCAATTCCA
GCATTTGCCGATTTTTAAAATTTCTGGCAATTCCA
GCATTTGCCGGTTTCGGAAATTTTTGGCATTTATG
GAATTTGCCGGTTTTTGGAATTTTCGGCAATTCCTGTAATTACTGGTTTTGGGCACTGTCAGAAATTCTGACAATTGCC
GGATTCGGAAATTTTCGGTAATTAACGGTTTCGGGAAGTTCCGGCACATCCGGAATATGCCGGTTTTGGGAATTTTCGG
CGATTTCG
Nr of selected repeats 6 Similarity 0.817143
Consensus:
GCATTTGCCGaTTTttaAAATTTcTGGCAATTcCa
>Cele-UNSB01_5:1035824-1036308 Satlength=485 Nr of Repeats=20 RepeatLength=22
seed=ACAGTACTCC
ACAGTACTCCCACAGTACCACT
ACAGTACTCCTACAGTACCACT
ACAGTACTCCTACAGTACCAGT
ACAGTACTCCTACAGCACTATA
ACAGTACTCCTACAGTACCACT
ACAGTACTCCTACAGTAGCACT
ACAGTACTCCTACAGTACCAGT
ACAGTACTCCTACAGTACCAGT
ACAGTACTCCTACAGCACTATG
ACAGTACTCCTACATTACCGCT
ACAGTACTCCAACAGTGTCACTGCAATACTCCTACAGTACCACT
ACAGTACTCCTACAGTACCAGT
ACAGTACTCCTACAGCACTATA
ACAGTACTCCTACAGTACCACTT
CAGTACTCCTACAGTACCAGT
ACAGTACTCCTACAGCACTATG
ACAGTACTCCTACATGACCGCT
ACAGTACTCCAACAGTGTCACT
ACAATACTCCTACAGTACCACTTCACTACTCCTACCGTACCAGT
ACAGTACTCCTACAGCACTATA
Nr of selected repeats 16 Similarity 0.776630

Consensus:
ACAGTACTCCTACAGtACcAnT
>Cele-UNSB01_5:1038307-1038528 Satlength=222 Nr of Repeats=11 RepeatLength=13
seed=GCCTATGGCC
GCCTATGGCCTACGCCTATGACTTAC
GCCTATGGCAAAC
GCCTATGGCCTCC
GCCTATGGCCTACGCCTACGGTCTAC
GCCTATGGCCTACGCCTATTACCTACGCCTATTACCTAA
GCCTATGGCCTACGCCTATAGCAACCGTCTTTGACCAAT
GCCTATGGCCTAC
GCCTATGGCCTAC
GCCTATGGCCTAC
GCCTATGGCCTAC
GCCTATGGCCTAC
Nr of selected repeats 7 Similarity 0.912088
Consensus:
GCCTATGGCCTAC
>Cele-UNSB01_5:1039029-1039263 Satlength=235 Nr of Repeats=18 RepeatLength=13
seed=CGTAGGCCAT
CGTAGGCCATATA
CGTAGGCCATATA
CGTAGGCCATAGG
CGTAGGCCATAGG
CGTAGGCCATAGG
CGTAGGCCACAGG
CGTAGGCCACAGG
CGTAGGCCATATA
CGTAGGCCATATA
CGTAGGCCATAGG
CGTAGGCCATAGG
CGTAGGCCATAGG
CGTAGGCCACAGG
CGTAGGCCATAGG
CGTAGGCCATAGG
CGTAGGCCATAGG
CGTAGGCCATAGG
CGTAGGCCATAGG
Nr of selected repeats 18 Similarity 0.894754
Consensus:
CGTAGGCCATAgg
>Cele-UNSB01_5:1057921-1058017 Satlength=97 Nr of Repeats=6 RepeatLength=18
seed=AAGCCTAAGC
AAGCCTAAGCCTATGCCA
AAGCCTAAGCCT
AAGCCTAAGTCTAAATCT
AAGCCTAAGCCCAAACCT
AGGCCTAAGCCC
AAGCCTAAGCATGAGCCT
Nr of selected repeats 4 Similarity 0.691358
Consensus:
AAGCCTAAGcctaaacCt
>Cele-UNSB01_5:1115366-1136806 Satlength=21441 Nr of Repeats=722
RepeatLength=27 seed=TCTGTGGCTT

TCTGTGGCTTCCCACTATAATTTACTC
TCTGTGGCTTCACAGATTAAGTTACTC
TCTGTGGCTTCACAGTATATTTTACTC
TCTGTGGCTTCCCACTATATTTTACTC
TCTGTGGCTTCCCACTATATTTTACTC
TCTGTGGCTTCCCACTATATTTTACTC
TCTGTGGCTTCCCACTATATTTTACTC
TCTGTGGCTTCACAGTATATTTTACTC
TCTGTGGCTTCACAGTATATTTTACTC
TCTGTGGCTTCACAGTATATTTTACTC
TCTGTGGCTTCCCACTATATTTTACTC
TCTGTGGCTTCCCACTGTATTTTACTC
TCTGTGGCTTCACAGTATATTTTACTC
TCTGTGGCTTCACAGTATATTTTACTC
TCTGTGGCTTCACAGTATATTTTACTC
TCTGTGGCTTCCCACTATATTTTACTC
TCTGTGGCTTCCCACTATATTTTACTC
TCTGTGGCTTCCCACTATATTTTACTC
TCTGTGGCTTCCCACTATATTTTACTC
TCTGTGGCTTCCCACTATATTTTACTC
TCTGTGGCTTCACAGTATATTTTACTC
TCTGTGGCTTCCCACTATATTTTACTC
TCTGTGGCTTCCCACTGTATTTTACTC
TCTGTGGCTTCACAGTATATTTTACTC
TCTGTGGCTTCACAGTATATTTTACTC
TCTGTGGCTTCACAGTATATTTTACTC
TCTGTGGCTTCCCACTATATTTTACTC
TCTGTGGCTTCCCACTATATTTTACTC
TCTGTGGCTTCCCACTATATTTTACTC
TCTGTGGCTTCCCACTATATTTTACTC
TCTGTGGCTTCCCACTATATTTTACTC
TCTGTGGCTTCACAGTATATTTTACTC
TCTGTGGCTTCACAGTATATTTTACTC
TCTGTGGCTTCACAGTATATTTTACTC
TCTGTGGCTTCCCACTGTATTTTACTC
TCTGTGGCTTCACAGTATATTTTACTC
TCTGTGGCTTCCCACTGTATTTTACTC
TCTGTGGCTTCACAGTATATTTTACTC
TCTGTGGCTTCCCACTATATTTTACTC
TCTGTGGCTTCACAGTATATTTTACTC
TCTGTGGCTTCCCACTATATTTTACTC
TCTGTGGCTTCCCACTATATTTTACTC
TCTGTGGCTTCCCACTGTATTTTACTC
TCTGTGGCTTCACAGTATATTTTACTC
TCTGTGGCTTCACAGTATATTTTACTC
TCTGTGGCTTCACAGTATATTTTACTC
TCTGTGGCTTCCCACTATATTTTACTC
TCTGTGGCTTCCCACTATATTTTACTC
TCTGTGGCTTCCCACTATATTTTACTC
TCTGTGGCTTCCCACTATATTTTACTC
TCTGTGGCTTCCCACTATATTTTACTC
TCTGTGGCTTCACAGTATATTTTACTC
TCTGTGGCTTCACAGTATATTTTACTC
TCTGTGGCTTCACAGTATATTTTACTC

TCTGTGGCTTCCCACTGTATTTTACTC
TCTGTGGCTTCACAGTATATTTTACTC
TCTGTGGCTTCCCACTATATTTTACTC
TCTGTGGCTTCCCACTATATTTTACTC
TCTGTGGCTTCACAGTATATTTTACTC
TCTGTGGCTTTCCACTATATTTTACTC
TCTGTGGCTTCCCACTATATTTTACTC
TCTGTGGCTTCCCACTATATTTTACTC
TCTGTGGCTTCCCACTATATTTTACTC
TCTGTGGCTTCCCACTATATTTTACTC
TCTGTGGCTTCACAGTATATTTTACTC
TCTGTGGCTTCCCACTATATTTTACTC
TCTGTGGCTTCACAGTATATTTTACTC
TCTGTGGCTTCCCACTGTATTTTACTC
TCTGTGGCTTCACAGTATATTTTACTC
TCTGTGGCTTCACAGTATATTTTACTC
TCTGTGGCTTCACAGTATATTTTACTC
TCTGTGGCTTCCCACTATATTTTACTC
TCTGTGGCTTCACAGTATATTTTACTC
TCTGTGGCTTCCCACTGTATTTTACTC
TCTGTGGCTTCACAGTATATTTTACTC
TCTGTGGCTTCCCACTGTATTTTACTC
TCTGTGGCTTCACAGTATATTTTACTC
TCTGTGGCTTCCCACTATATTTTACTC
TCTGTGGCTTCACAGTATATTTTACTC
TCTGTGGCTTCCCACTATATTTTACTC
TCTGTGGCTTTCCACTATATTTTACTC
TCTGTGGCTTCCCACTGTATTTTACTC
TCTGTGGCTTCACAGTATATTTTACTC
TCTGTGGCTTCCCACTGTATTTTACTC
TCTGTGGCTTCACAGTATATTTTACTC
TCTGTGGCTTCCCACTATATTTTACTC
TCTGTGGCTTCACAGTATATTTTACTC
TCTGTGGCTTCCCACTATATTTTACTC
TCTGTGGCTTCCCACTGTATTTTACTC
TCTGTGGCTTCACAGTATATTTTACTC
TCTGTGGCTTCCCACTATATTTTACTC
TCTGTGGCTTCCCACTGTATTTTACTC
TCTGTGGCTTCACAGTATATTTTACTC
TCTGTGGCTTCACAGTATATTTTACTC
TCTGTGGCTTCACAGTATATTTTACTC
TCTGTGGCTTCCCACTATATTTTACTC
TCTGTGGCTTCCCACTATATTTTACTC
TCTGTGGCTTCCCACTATATTTTACTC
TCTGTGGCTTCCCACTATATTTTACTC
TCTGTGGCTTTCCACTATATTTTACTC
TCTGTGGCTTCCCACTATATTTTACTC
TCTGTGGCTTCCCACTATATTTTACTC
TCTGTGGCTTCCCACTATATTTTACTC
TCTGTGGCTTCCCACTATATTTTACTC
TCTGTGGCTTCCCACTGTATTTTACTC
TCTGTGGCTTCCCACTATATTTTACTC
TCTGTGGCTTCCCACTGTATTTTACTC
TCTGTGGCTTCACAGTATATTTTACTC

TCTGTGGCTTCCCACTATATTTTACTC
TCTGTGGCTTCCCACTGTATTTTACTC
TCTGTGGCTTCACAGTATATTTTACTC
TCTGTGGCTTCACAGTATATTTTACTC
TCTGTGGCTTCCCACTATATTTTACTC
TCTGTGGCTTCACAGTATATTTTACTC
TCTGTGGCTTCCCACTATATTTTACTC
TCTGTGGCTTCCCACTGTATTTTACTC
TCTGTGGCTTCACAGTATATTTTACTC
TCTGTGGCTTCACAGTATATTTTACTC
TCTGTGGCTTCACAGTATATTTTACTC
TCTGTGGCTTCCCACTGTATTTTACTC
TCTGTGGCTTCACAGTATATTTTACTC
TCTGTGGCTTCCCACTATATTTTACTC
TCTGTGGCTTTCCACTATATTTTACTC
TCTGTGGCTTCACAGTATATTTTACTC
TCTGTGGCTTCCCACTATATTTTACTC
TCTGTGGCTTCCCACTATATTTTACTC
TCTGTGGCTTCCCACTATATTTTACTC
TCTGTGGCTTCCCACTATATTTTACTC
TCTGTGGCTTTCCACTATATTTTACTC
TCTGTGGCTTCCCACTATATTTTACTC
TCTGTGGCTTCCCACTATATTTTACTC
TCTGTGGCTTCCCACTATATTTTACTC
TCTGTGGCTTCCCACTATATTTTACTC
TCTGTGGCTTCCCACTGTATTTTACTC
TCTGTGGCTTCCCACTATATTTTACTC
TCTGTGGCTTCCCACTGTATTTTACTC
TCTGTGGCTTCACAGTATATTTTACTC
TCTGTGGCTTCCCACTATATTTTACTC
TCTGTGGCTTCCCACTGTATTTTACTC
TCTGTGGCTTCACAGTATATTTTACTC
TCTGTGGCTTCACAGTATATTTTACTC
TCTGTGGCTTCCCACTATATTTTACTC
TCTGTGGCTTCACAGTATATTTTACTC
TCTGTGGCTTCCCACTATATTTTACTC
TCTGTGGCTTCCCACTGTATTTTACTC
TCTGTGGCTTCACAGTATATTTTACTC
TCTGTGGCTTCACAGTATATTTTACTC
TCTGTGGCTTCACAGTATATTTTACTC
TCTGTGGCTTCCCACTGTATTTTACTC
TCTGTGGCTTCACAGTATATTTTACTC
TCTGTGGCTTCCCACTATATTTTACTC
TCTGTGGCTTTCCACTATATTTTACTC
TCTGTGGCTTCACAGTATATTTTACTC
TCTGTGGCTTCACAGTATTTTTTACTC
TCTGTGGCTTCCCACTATATTTTACTC
TCTGTGGCTTCCCACTATATTTTACTC
TCTGTGGCTTCACAGTATATTTTACTC
TCTGTGGCTTCCCACTATATTTTACTC
TCTGTGGCTTCACAGTATATTTTACTC
TCTGTGGCTTCACAGTATTTTTTACTC
TCTGTGGCTTCCCACTATATTTTACTCTCCAAAAATAATTTCCAAGTTTTTAATGATTTGTTGCATATTGAAAAAAACA
TTTTTCGGGTTTTTTGAAATGAATATCGTAGCTACAGAAACGGTAATACACTCTTCTGAAAATACAAAAAATTTGCAAT

TTTTATAGCTAGGGCACTTTTTGTCTGCCCAAATATAGGCAACCAAAAATAATTTCCAAGTTTTTAATGATTTGTTGCA
TATTGAAAAAAACATTTTTTGGGTTTTTTGAAATGAATATCATAGCTATAGAAACGGTAGTATTTTACTC
TCTGTGGCTTCACAGTATATTTTACTC
TCTGTGGCTTCACAGTATATTTTACTC
TCTGTGGCTTCACAGTATATTTTACTC
TCTGTGGCTTCCCACTATATTTTACTC
TCTGTGGCTTCCCACTGTATTTTACTC
TCTGTGGCTTCACAGTATATTTTACTC
TCTGTGGCTTCACAGTATATTTTACTC
TCTGTGGCTTCACAGTATATTTTACTC
TCTGTGGCTTCCCACTGTATTTTACTC
TCTGTGGCTTCACAGTATATTTTACTC
TCTGTGGCTTCCCACTATATTTTACTC
TCTGTGGCTTTCCACTATATTTTACTC
TCTGTGGCTTCACAGTATATTTTACTC
TCTGTGGCTTCACAGTATTTTTTACTC
TCTGTGGCTTCCCACTATATTTTACTC
TCTGTGGCTTCCCACTATATTTTACTC
TCTGTGGCTTCACAGTATATTTTACTC
TCTGTGGCTTCCCACTATATTTTACTC
TCTGTGGCTTCCCACTATATTTTACTC
TCTGTGGCTTCACAGTATATTTTACTC
TCTGTGGCTTCACAGTATTTTTTACTC
TCTGTGGCTTCCCACTATATTTTACTCTCCAAAAATAATTTCCAAGTTTTTAATGATTTGTTGCATATTGAAAAAAACA
TTTTTCGGGTTTTTTGAAATGAATATCGTAGCTACAGAAACGGTAATACACTCTTCTGAAAATACAAAAAATTTGCAAT
TTTTATAGCTAGGGCACTTTTTGTCTGCCCAAATATAGGCAACCAAAAATAATTTCCAAGTTTTTAATGATTTGTTGCA
TATTGAAAAAAACATTTTTTGGGTTTTTTGAAATGAATATCATAGCTATAGAAACGGTAGTATTTTACTC
TCTGTGGCTTCACAGTATATTTTACAC
TCTGTGGCTTCACAGTATATTTTACTC
TCTGTGGCTTCACAGTATATTTTACTC
TCTGTGGCTTCCCACTATATTTTACTC
TCTGTGGCTTCACAGTATATTTTACAC
TCTGTGGCTTTCCACTATATTTTACTC
TCTGTGGCTTCACTGTATATTTTACTC
TCTGTGGCTTCACAGTATATTTTACTC
TCTGTGGCTTCCCACTATATTTTACTC
TCTGTGGCTTCCCACTATATTTTACTC
TCTGTGGCTTCACTGTATATTTTACTC
TCTGTGGCTTCACAGTATATTTTACTC
TCTGTGGCTTCACAGTATATTTTACTC
TCTGTGGCTTCCCACTATATTTTACTC
TCTGTGGCTTCACAGTATATTTTACTC
TCTGTGGCTTCACAGTATATTTTACTC
TCTGTGGCTTCCCACTATATTTTACTC
TCTGTGGCTTCCCACTATATTTTACTC
TCTGTGGCTTCGCACTATATTTTACTC
TCTGTGGCTTCACAGTATATTTTACTT
TCTGTGGCTTCACAGTATATTTTACTT
TCTGTGGCTTCACAGTATATTTTACTC
TCTGTGGCTTCCCACTATATTTTACTC
TCTGTGGCTTCACAGTATATTTTACTC
TCTGTGGCTTCCCACTATATTTTACTC
TCTGTGGCTTCCCACTATATTTTACTC

TCTGTGGCTTCACAGTATATTTTACTCTCCAAAAATAATTTCCAAGTTTTTAATGATTTGTTGCATATTGAAAAAAACA
TTTTTCGGGTTTTTTGAAATGAATATCGTAGCTACAGAAACGGTAATACACTCTTCTGAAAATACAAAAAATTTGCAAT
TTTTATAGCTAGGGCACTTTTTGTCTGCCCAAATATAGGCAACCAAAAATAATTTCCAAGTTTTTAATGATTTGTTGCA
TATTGAAAAAACATTTTTTGGGTTTTTTGAAATGAATATCATAGCTATAGAAACGGTAGTATTTTACTC
TCTGTGGCTTCACAGTATATTTTACTC
TCTGTGGCTTCACAGTATTTTTTACTC
TCTGTGGCTTCACAGTATATTTTACTC
TCTGTGGCTTCACAGTATATTTTACTT
TCTGTGGCTTCACAGTATATTTTACTT
TCTGTGGCTTCACAGTATATTTTACTC
TCTGTGGCTTCACAGTATATTTTACTC
TCTGTGGCTTCACAGTATATTTTACTC
TCTGTGGCTTCACAGTATATTTTACTA
TCTGTAGCTTCACAGTATATTTTACTC
TCTGTGGCTTCACAGTATATTTTACTC
TCTGTGGCTTCACAGTATATTTTACTA
TCTGTAGCTTCACAGTATATTTTACTC
TCTGTGGCTTCACAGTATATTTTACTC
TCTGTGGCTTCACAGTATATTTTACTC
TCTGTGGCTTCACAGTATATTTTACTC
TCTGTGGCTTCACAGTATATTTTACTC
TCTGTGGCTTCACAGTATATTTTACTC
TCTGTGGCTTCACAGTATATTTTACTA
TCTGTGGCTTCACAGTATATTTTACTC
TCTGTGGCTTCACAGTATATTTTACTC
TCTGTGGCTTCACAGTATATTTTACTC
TCTGTGGCTTCACAGTATATTTTACTC
TCTGTGGCTTCACAGTATATTTTACTC
TCTGTGGCTTCACAGTATATTTTACTC
TCTGTGGCTTCACAGTATATTTTACTC
TCTGTGGCTTCACAGTATATTTTACTC
TCTGTGGCTTCACAGTATATTTTACTA
TCTGTAGCTTCACAGTATATTTTACTC
TCTGTGGCTTCACAGTATATTTTACTC
TCTGTGGCTTCACAGTATATTTTACTC
TCTGTGGCTTCACAGTATATTTTACTC
TCTGTGGCTTCACAGTATATTTTACTC
TCTGTGGCTTCACAGTATATTTTACTC
TCTGTGGCTTCACAGTATATTTTACTC
TCTGTGGCTTCACAGTATATTTTACTC
TCTGTGGCTTCACAGTATATTTTACTC
TCTGTGGCTTCACAGTATATTTTACTA
TCTGTAGCTTCACAGTATATTTTACTC
TCTGTGGCTTCACAGTATATTTTACTC
TCTGTGGCTTCACAGTATATTTTACTC
TCTGTGGCTTCACAGTATATTTTACTC
TCTGTGGCTTCACAGTATATTTTACTC
TCTGTGGCTTCACAGTATATTTTACTC
TCTGTGGCTTCCCACTATATTTTACTC
TCTGTGGCTTCACAGTATATTTTACTC
TCTGTGGCTTCACAGTATTTTTACTC
TCTGTGGCTTCACAGTATTTTTTACTC
TCTGTGGCTTCACAGTATATTTTACTC
TCTGTGGCTTCACAGTATATTTTACAC

TCTGTGGCTTCCCACTATATTTTACTC
TCTGTGGCTTCACAGTATTTTTTACTC
TCTGTGGCTTCCCACTATATTTTACTC
TCTGTGGCTTCCCACTATATTTTACTC
TCTGTGGCTTCCCACTATATTTTACTC
TCTGTGGCTTCACAGTATATTTTACTC
TCTGTGGCTTCACAGTATATTTTACAC
TCTGTGGCTTCCCACTATATTTTACTC
TCTGTGGCTTCACAGTATATTTTACTC
TCTGTGGCTTCCCACTATATTTTACTC
TCTGTGGCTTCACAGTATATTTTACTC
TCTGTGGCTTCACAGTATATTTTACTC
TCTGTGGCTTCACAGTATATTTTACTC
TCTGTGGCTTCACAGTATATTTTACTC
TCTGTGGCTTCACAGTATATTTTACTA
TCTGTAGCTTCACAGTATATTTTACTC
TCTGTGGCTTCACAGTATATTTTACTC
TCTGTGGCTTCACAGTATATTTTACTC
TCTGTGGCTTCACAGTATATTTTACTC
TCTGTGGCTTCACAGTATATTTTACTC
TCTGTGGCTTCACAGTATATTTTACTC
TCTGTGGCTTCCCACTATATTTTACTC
TCTGTGGCTTCACAGTATATTTTACTC
TCTGTGGCTTCACAGTATTTTTACTC
TCTGTGGCTTCACAGTATTTTTTACTC
TCTGTGGCTTCACAGTATATTTTACTC
TCTGTGGCTTCACAGTATATTTTACAC
TCTGTGGCTTCCCACTATATTTTACTC
TCTGTGGCTTCACAGTATTTTTTACTC
TCTGTGGCTTCCCACTATATTTTACTC
TCTGTGGCTTCCCACTATATTTTACTC
TCTGTGGCTTCCCACTATATTTTACTC
TCTGTGGCTTCCCACTATATTTTACTC
TCTGTGGCTTCACAGTATATTTTACTC
TCTGTGGCTTCACAGTATATTTTACAC
TCTGTGGCTTCCCACTATATTTTACTC
TCTGTGGCTTCACAGTATTTTTTACTC
TCTGTGGCTTCCCACTATATTTTACTC
TCTGTGGCTTCCCACTATATTTTACTC
TCTGTGGCTTCCCACTATATTTTACTC
TCTGTGGCTTCCCACTATATTTTACTC
TCTGTGGCTTCACAGTATATTTTACTC
TCTGTGGCTTCCCACTATATTTTACTC
TCTGTGGCTTCCCACTATATTTTACTC
TCTGTGGCTTCACAGTATTTTTTACTC
TCTGTGGCTTCCCACTATATTTTACTCTCCAAAAATAATTTCCAAGTTTTTAATGATTTGTTGCATATTGAAAAAAACA
TTTTTCGGGTTTTTTGAAATGAATATCGTAGCTACAGAAACGGTAATACACTCTTCTGAAAATACAAAAAATTTGCAAT
TTTTATAGCTAGGGCACTTTTTGTCTGCCCAAATATAGGCAACCAAAAATAATTTCCAAGTTTTTAATGATTTGTTGCA
TATTGAAAAAACATTTTTTGGGTTTTTTGAAATGAATATCATAGCTATAGAAACGGTAGTATTTTACTC
TCTGTGGCTTCACAGTATATTTTACAC
TCTGTGGCTTCACAGTATATTTTACTC
TCTGTGGCTTCACAGTATATTTTACTC
TCTGTGGCTTCCCACTATATTTTACTC
TCTGTGGCTTCACAGTATATTTTACAC

TCTGTGGCTTTCCACTATATTTTACTC
TCTGTGGCTTCACTGTATATTTTACTC
TCTGTGGCTTCACAGTATATTTTACTC
TCTGTGGCTTCCCACTATATTTTACTC
TCTGTGGCTTCCCACTATATTTTACTC
TCTGTGGCTTCACTGTATATTTTACTC
TCTGTGGCTTCACAGTATATTTTACTC
TCTGTGGCTTCACAGTATATTTTACTC
TCTGTGGCTTCCCACTATATTTTACTC
TCTGTGGCTTCACAGTATATTTTACTC
TCTGTGGCTTCACAGTATTTTTTACTC
TCTGTGGCTTCGCACTATATTTTACTC
TCTGTGGCTTCACAGTATATTTTACTT
TCTGTGGCTTCACAGTATATTTTACTT
TCTGTGGCTTCACAGTATATTTTACTC
TCTGTGGCTTCCCACTATATTTTACTC
TCTGTGGCTTCACAGTATATTTTACTC
TCTGTGGCTTCCCACTATATTTTACTC
TCTGTGGCTTCCCACTATATTTTACTC
TCTGTGGCTTCACAGTATATTTTACTCTCCAAAAATAATTTCCAAGTTTTTAATGATTTGTTGCATATTGAAAAAAACA
TTTTTCGGGTTTTTTGAAATGAATATCGTAGCTACAGAAACGGTAATACACTCTTCTGAAAATACAAAAAATTTGCAAT
TTTTATAGCTAGGGCACTTTTTGTCTGCCCAAATATAGGCAACCAAAAATAATTTCCAAGTTTTTAATGATTTGTTGCA
TATTGAAAAAACATTTTTTGGGTTTTTTGAAATGAATATCATAGCTATAGAAACGGTAGTATTTTACTC
TCTGTGGCTTCACAGTATATTTTACTC
TCTGTGGCTTCACAGTATTTTTTACTC
TCTGTGGCTTCACAGTATATTTTACTC
TCTGTGGCTTCACAGTATATTTTACTT
TCTGTGGCTTCACAGTATATTTTACTT
TCTGTGGCTTCACAGTATATTTTACTC
[truncated: 572,881 more chars]
