# Supplementary material for: DNA Satellites Are Transcribed as Part of the Non-Coding Genome in Eukaryotes and Bacteria
Source: Genes (Basel). 2021 Oct 20;12(11):1651. doi: 10.3390/genes12111651 (PMC8625621; doi:10.3390/genes12111651)
Supplement: Supplementary file 1 [file genes-12-01651-s001.zip › Table S1. A list of all satellites in C.elegans.pdf]

Table S1. A list of all satellites in *C.elegans*

Genome	Satellite name	Contig	Start	End	Sat. length	Number repeats	Repeat length	Seed motif	repeat
UNSB01.fasta	Cele-UNSB01_1:1	1	1	2668	2668	204	12	CTAAGCCTAA	CTAAGCCTAAGC
UNSB01.fasta	Cele-UNSB01_1:224582	1	224582	224687	106	5	15	AGACCCATCG	AGACCCATCGTGGTG
UNSB01.fasta	Cele-UNSB01_1:282371	1	282371	283239	869	23	38	AAATTTTAAT	AAATTTTAATTTTTTGAAAATATTTTGGCGGGAATTTA
UNSB01.fasta	Cele-UNSB01_1:283314	1	283314	284124	811	52	15	TCGTGGTGAG	TCGTGGTGAGACCCA
UNSB01.fasta	Cele-UNSB01_1:309874	1	309874	309994	121	7	15	GAGACCCATC	GAGACCCATCGTGGC
UNSB01.fasta	Cele-UNSB01_1:336527	1	336527	337247	721	18	40	AATTTTCTCG	AATTTTCTCGAAATTTCCAAAAGGTTCTAGAACATTTCAG
UNSB01.fasta	Cele-UNSB01_1:340649	1	340649	342857	2209	12	184	GAATACAGAA	GAATACAGAACACCAATTATGCCCGAGAAAAAGATCCTACTCAGAATATAAACATAGTCAAATTTATCGGATGTATAAAGATTCCCGAAGACACTTTCCAATTACCCAAATTGTTCATATTCTAAATGAAATTCTTTTACTAGAACACTCTTGGCCAATGTACGCAGCCGAACGTATCATAAGT
UNSB01.fasta	Cele-UNSB01_1:356425	1	356425	357011	587	29	19	TATCGATTTT	TATCGATTTTCCTGAAATT
UNSB01.fasta	Cele-UNSB01_1:360798	1	360798	360950	153	8	19	GTACTACCTT	GTACTACCTTTTAATGTAT
UNSB01.fasta	Cele-UNSB01_1:479600	1	479600	479769	170	13	12	TAGGCTTAGG	TAGGCCTAGGCT
UNSB01.fasta	Cele-UNSB01_1:558852	1	558852	559000	149	8	12	CTAAGCCTAA	CTAAGCCTAAGC
UNSB01.fasta	Cele-UNSB01_1:564264	1	564264	564319	56	5	11	GTACTCCTAC	GTACTCCTAAA
UNSB01.fasta	Cele-UNSB01_1:567454	1	567454	567526	73	6	12	GCGTTGCGTG	GCGTTGCGTGCC
UNSB01.fasta	Cele-UNSB01_1:568805	1	568805	568943	139	8	12	AGGCTTAGGC	AGGCCTAGGCTT
UNSB01.fasta	Cele-UNSB01_1:625093	1	625093	626033	941	22	23	AAAAAAAATT	AAAAAAAATTAAATTTTTCAACG
UNSB01.fasta	Cele-UNSB01_1:642767	1	642767	642875	109	6	12	TAAGCCTAAG	TGAGCCTAAGCG
UNSB01.fasta	Cele-UNSB01_1:671513	1	671513	680928	9416	294	32	GAATTCAAAT	GAATTCAAATTTTCTGAGAAAAATTTAGGCGA
UNSB01.fasta	Cele-UNSB01_1:685710	1	685710	685978	269	6	45	TCTGATCCTT	TCTGATCCTTCAGATAAGAAGGATCGTAAGGGTACCTCCTGATGG
UNSB01.fasta	Cele-UNSB01_1:696260	1	696260	697073	814	14	44	TTTGCCGGTT	TTTGCCGGTTTGGCGATTTGCCCGAAATTTTTCAATTTCGGCAA
UNSB01.fasta	Cele-UNSB01_1:698928	1	698928	699108	181	12	15	GGTGAGACCC	GGTGAGACACATCGT
UNSB01.fasta	Cele-UNSB01_1:733992	1	733992	734160	169	4	42	TTTGATCTAC	TTTGATCTACAAAAAATGCGGGAATTTTCCCAGCATTGCTCA
UNSB01.fasta	Cele-UNSB01_1:736250	1	736250	736486	237	5	12	GCTTAGGCTT	GCTTAGGCCTGG
UNSB01.fasta	Cele-UNSB01_1:742856	1	742856	743285	430	11	12	GCAGGTAGGT	GTAGGTAGGTAG
UNSB01.fasta	Cele-UNSB01_1:758474	1	758474	759042	569	35	16	TTTCAGGCTT	TTTCAGGCTTTCAGGC
UNSB01.fasta	Cele-UNSB01_1:768290	1	768290	768741	452	13	34	TTTCTCAGAA	TTTATCAGAAAATTTGAATTTCCCACCAAAATTT

UNSB01.fasta	Cele-UNSB01_1:774871	1	774871	775189	319	26	12	TCTAGGTCTA
TCTAGGTTTAGG								
UNSB01.fasta	Cele-UNSB01_1:787252	1	787252	787531	280	6	40	GAACCTTCTG
GAACCTTTTGGAAATTTTGAGAAAATTCTGGAATGTTTTG								
UNSB01.fasta	Cele-UNSB01_1:788353	1	788353	788527	175	12	12	GCCTAAGCCT
GCCTAAGCCTAA								
UNSB01.fasta	Cele-UNSB01_1:789928	1	789928	790208	281	7	40	TCTAGAACAT
TCTAGAACATTCCAAACTTTTCTCAAAGTTTCCAAAAGAC								
UNSB01.fasta	Cele-UNSB01_1:815618	1	815618	815840	223	16	12	GCCTAAGCCT
GCCAAAGCCTGA								
UNSB01.fasta	Cele-UNSB01_1:817628	1	817628	817952	325	8	22	TTAAAATTTT
TTAAAATTTTGTCAGTTTGACA								
UNSB01.fasta	Cele-UNSB01_1:823660	1	823660	823840	181	13	12	GCTTAGGCTT
GCTTAGGCTTAG								
UNSB01.fasta	Cele-UNSB01_1:833000	1	833000	833653	654	29	21	TGGCCTAGAT
TGGCCTCGATTCTGTACAGGG								
UNSB01.fasta	Cele-UNSB01_1:842713	1	842713	843650	938	48	12	CTTAGGCTTA
CTCAGGCTTAGG								
UNSB01.fasta	Cele-UNSB01_1:855372	1	855372	855570	199	12	12	GCTTAGGCTT
GCTTAGACTTAG								
UNSB01.fasta	Cele-UNSB01_1:907957	1	907957	908415	459	21	22	TTTTAGCCAA
TTTTAACCAAAAAATCCGTTGA								
UNSB01.fasta	Cele-UNSB01_1:911764	1	911764	911915	152	8	15	ATCTTCTTTT
ATCTTCTTTTGGTAA								
UNSB01.fasta	Cele-UNSB01_1:927465	1	927465	927693	229	6	32	GAAAATTTGA
GAAAATTTGAATTCCCGCCAAGATTTTTTACA								
UNSB01.fasta	Cele-UNSB01_1:932742	1	932742	934750	2009	89	21	AATTTCTAGG
AATTTCTAGGCCACGATCTGA								
UNSB01.fasta	Cele-UNSB01_1:943054	1	943054	953346	10293	369	27	TTTTACTCTC
TTTTACTCTCTGTGGCTTCACCAATTA								
UNSB01.fasta	Cele-UNSB01_1:961004	1	961004	961082	79	6	12	AGCCTAAGCC
AGCCTAAGCCTA								
UNSB01.fasta	Cele-UNSB01_1:961195	1	961195	961471	277	23	12	CTAAGTCTAA
CTAAGTCTAAGT								
UNSB01.fasta	Cele-UNSB01_1:961002	1	961002	961808	807	44	12	TAAGCCTAAG
TAAGCCTAAGCC								
UNSB01.fasta	Cele-UNSB01_1:980318	1	980318	980990	673	16	42	CCGGAAATTT
CCGGAAATTTCGAAAACCGGCAATTGCCGAAATTGCCCATTG								
UNSB01.fasta	Cele-UNSB01_1:995161	1	995161	995964	804	5	162	ATCATATGAA
ATCATATGAAATGCTTTTTTTTTCTTTTTTAAACCTATTTAGAATTGTCAAAACTACCAGAAAATACTAAACAAAATAGCTTAGCTTGACCGGAAATATTTTTAAAAAATTGATAAAAATAATGAAAAGCTGATATTTTCAAAATTCCA								
GAATGACGGAAAA								
UNSB01.fasta	Cele-UNSB01_1:1009660	1	1009660	1009762	103	8	12	GGTCTAGGTC
GGTCTAGGTCTA								
UNSB01.fasta	Cele-UNSB01_1:1012680	1	1012680	1013098	419	4	32	CAAAATTTTT
CAAAATTTTTCTAAGAAAATTTGAATTCCCGC								
UNSB01.fasta	Cele-UNSB01_1:1018800	1	1018800	1018890	91	5	12	AGCCTAAGCC
AGCATAAGCCTA								
UNSB01.fasta	Cele-UNSB01_1:1018801	1	1018801	1019027	227	14	12	GCCTAAGCCT
GCATAAGCCTAA								
UNSB01.fasta	Cele-UNSB01_1:1031726	1	1031726	1032601	876	25	35	ATTTTCAATT
ATTTTCAATTCCGGCAATTTGCCGATTTGCCGGAA								
UNSB01.fasta	Cele-UNSB01_1:1103105	1	1103105	1103421	317	8	35	CGGCAAATCG
CGGCAAATCGGTATTTTGCCGGAATTGAAAATTTT								
UNSB01.fasta	Cele-UNSB01_1:1117625	1	1117625	1118154	530	23	23	GGCCTAGAAA
GGCCTAGAAATACTGAAAGTGGT								
UNSB01.fasta	Cele-UNSB01_1:1127917	1	1127917	1128577	661	21	20	ATTTCTAGGC
ATTTCTAGGCTATCGATGAG								
UNSB01.fasta	Cele-UNSB01_1:1149569	1	1149569	1149724	156	10	12	CCTAAGCCTA
CCTAAGCCTAAG								

UNSB01.fasta	Cele-UNSB01_1:1158888	1	1158888	1159076	189	5	31	ATTCCCGCCA	ATTCCCGCCAATTTTTTCTCAGAAATTTTGA
UNSB01.fasta	Cele-UNSB01_1:1232170	1	1232170	1233535	1366	32	43	CCAGAAATTA	CCAGAAATTAGAATTTTCGGCAAATCAACAAACTGACAAATTG
UNSB01.fasta	Cele-UNSB01_1:1234705	1	1234705	1235262	558	15	35	AAATCGGCAA	AAATCGGCAATTTTCCTAAAATGAAAGGTTCCGGC
UNSB01.fasta	Cele-UNSB01_1:1236946	1	1236946	1237103	158	7	11	GTACTGTAGG	GTACTGTAGGA
UNSB01.fasta	Cele-UNSB01_1:1302534	1	1302534	1303556	1023	33	19	TTCTAGGCCA	TTCTAGGCCATCTGATAAT
UNSB01.fasta	Cele-UNSB01_1:1341786	1	1341786	1343571	1786	47	38	GGGAATTCAA	GGGAATTCAAACTTTAATTTTTTGAAAACATTTTGGC
UNSB01.fasta	Cele-UNSB01_1:1346901	1	1346901	1347222	322	5	42	TTTGCCGGAA	TTTCCCGGAAGTTTTCATTTTCGACAAATTACCGATTTGTCG
UNSB01.fasta	Cele-UNSB01_1:1386048	1	1386048	1386208	161	5	32	AAAATTTTGG	AAAATTTTGGCGGGAATTCAAATTTTCTGAGA
UNSB01.fasta	Cele-UNSB01_1:1435639	1	1435639	1436319	681	8	71	ATTTATGTGA	ATTTATGTGAAAATTCAGGTTTTTAGGGCATTTTTTGAGGGAAATCTGATTTTTTTTCGATTTTCTGGCGA
UNSB01.fasta	Cele-UNSB01_1:1456315	1	1456315	1457241	927	24	35	AAATTTGAAT	AAATCTGAATTTCCCGCAAAATTTTTTTTCTCAGA
UNSB01.fasta	Cele-UNSB01_1:1457508	1	1457508	1457876	369	14	11	CTACAGTACT	CTACAGTAATT
UNSB01.fasta	Cele-UNSB01_1:1530606	1	1530606	1531290	685	18	31	GGTTACTGTA	GGTTACTGTAACTCAATTATAGGATTCTTCG
UNSB01.fasta	Cele-UNSB01_1:1591838	1	1591838	1592129	292	7	35	CGGCAAATCG	CGGCAAATCGGCAAATTGCCGGAATTGAAAATTTC
UNSB01.fasta	Cele-UNSB01_1:1620106	1	1620106	1621181	1076	5	189	TTTTTTTTGA	TTTTTTTTGACTATGGAATTATAAAGAAAATCGAATTTCAAAAAAGCATGTATTAGCCAATGTAAACGCGCTCCAATGATGATGTAGCCCACGTGGTATCAGAGTGTCTCTTTACGGCTTGATTTAAATACGTAGATCTACAAATTCACTTATCTGTTTTCTCACGGGTTTAGGATATTTTTTCTGTTA
UNSB01.fasta	Cele-UNSB01_1:1632570	1	1632570	1633118	549	6	35	AAATTTTCAA	AAATTTTCAATTCCGGCATTTTGCCGATTTGCCGG
UNSB01.fasta	Cele-UNSB01_1:1647038	1	1647038	1647577	540	9	49	CAATAATTTT	CAATAATTTTAAATGTAATTATCGATTTTTCTAAATTTTGCCGAAAAAT
UNSB01.fasta	Cele-UNSB01_1:1679297	1	1679297	1679633	337	16	21	TCTCCTGGTA	TCTCCTGGTAAATTTAAAAAT
UNSB01.fasta	Cele-UNSB01_1:1680231	1	1680231	1680351	121	7	12	AGCCTAAACC	AGCCTAAACCTG
UNSB01.fasta	Cele-UNSB01_1:1685357	1	1685357	1686631	1275	49	26	CAAAACCGGC	CAAAACCGGCAATTGCCGAAAATTCC
UNSB01.fasta	Cele-UNSB01_1:1691009	1	1691009	1691188	180	4	45	TCTGATCCTT	TCTGATCCTTCAGATAAGAAGGATCGTAAGGGTGCCACTTGATGG
UNSB01.fasta	Cele-UNSB01_1:1730783	1	1730783	1731662	880	24	30	AATTTGAATT	AATTTGATTATTTCCGAATTTTTTAGCGAA
UNSB01.fasta	Cele-UNSB01_1:1781850	1	1781850	1784996	3147	154	20	CCACCAAAAA	CCACCAAAAAGATTTCTAGG
UNSB01.fasta	Cele-UNSB01_1:1787086	1	1787086	1788479	1394	64	20	TTCTAGGCCA	TTCTAGGCCACCAAGAAAGT
UNSB01.fasta	Cele-UNSB01_1:1822719	1	1822719	1823169	451	9	43	AAATCGGCAA	AAATCGGCAACCCCGCAATTTGCCAGAATTGAAAATTTCCGGA
UNSB01.fasta	Cele-UNSB01_1:1824052	1	1824052	1824465	414	9	43	GCCGGAATTG	GCCGGAATTGAAAATTTCTGGCAAATCGGCAAACCGACACATA
UNSB01.fasta	Cele-UNSB01_1:1833668	1	1833668	1834030	363	11	26	TTGCCGGTTT	TTGCTGGTTTTGGGAATTATCGGCAC
UNSB01.fasta	Cele-UNSB01_1:1840655	1	1840655	1841028	374	9	35	TTTGCCGGAA	TTTGCCGGAAATTTTCAAATCCGGCAATTTGCCGA
UNSB01.fasta	Cele-UNSB01_1:1841619	1	1841619	1842039	421	18	19	AAAATCAATA	AAAATCAATAAATCCTAAG
UNSB01.fasta	Cele-UNSB01_1:1841619	1	1841619	1843394	1776	44	19	AAAATCAATA	AAAATCAATAAATCCTAAG

UNSB01.fasta	Cele-UNSB01_1:1878726	1	1878726	1878810	85	4	21	CCTACTCTAG	CCTACTCTAGGCAGCCCCTAG
UNSB01.fasta	Cele-UNSB01_1:1878816	1	1878816	1878916	101	5	20	CTAGGCAGAC	CTAGGCAGACTTAGCCTACC
UNSB01.fasta	Cele-UNSB01_1:1910732	1	1910732	1911187	456	11	35	AAATCGGCAA	AAATCGGCAAATTGCCGAAACTAAAAATTTCTGGT
UNSB01.fasta	Cele-UNSB01_1:1946077	1	1946077	1948348	2272	60	35	TTGCCGATTT	TTGCCGATTTGCCGGAAATTTTCATTTTTCGCAAA
UNSB01.fasta	Cele-UNSB01_1:1961569	1	1961569	1963172	1604	40	35	GAAATTTTCA	GAAATTTTCAGTTTCGGCAAATTGCCGATTTGCCC
UNSB01.fasta	Cele-UNSB01_1:1961569	1	1961569	1964216	2648	64	35	GAAATTTTCA	GAAATTTTCAGTTTCGGCAAATTGCCGATTTGCCC
UNSB01.fasta	Cele-UNSB01_1:1961569	1	1961569	1966139	4571	108	35	GAAATTTTCA	GAAATTTTCAGTTTCGGCAAATTGCCGATTTGCCC
UNSB01.fasta	Cele-UNSB01_1:1961569	1	1961569	1968269	6701	154	35	GAAATTTTCA	GAAATTTTCAGTTTCGGCAAATTGCCGATTTGCCC
UNSB01.fasta	Cele-UNSB01_1:1976543	1	1976543	1977056	514	5	35	TTTGCCGGAA	TTTGCCGGAAATTTTCATTTTTGGCAAAGTGCCGA
UNSB01.fasta	Cele-UNSB01_1:1980049	1	1980049	1980327	279	8	35	TTGCCGGAAA	TTGCCGGAAAGTATCAATTTCGGCTGATTGCCGAT
UNSB01.fasta	Cele-UNSB01_1:2017348	1	2017348	2017684	337	5	38	AAAATCGATA	AAAATCGATAATTTTCAGTATGCAGTGAAATTCTTGGA
UNSB01.fasta	Cele-UNSB01_1:2044939	1	2044939	2045157	219	13	11	TTTTGCCGAA	TTTCGCCGAAC
UNSB01.fasta	Cele-UNSB01_1:2141918	1	2141918	2142329	412	7	35	CAAACCGGCA	CAAACCGGCATTTTGCCGGAAATTAACATTTAGAG
UNSB01.fasta	Cele-UNSB01_1:2169410	1	2169410	2169690	281	8	35	CGGCAAATTG	CGGCAAATTGCCGATTTGCCGGAAACTTTCAATTC
UNSB01.fasta	Cele-UNSB01_1:2174082	1	2174082	2174248	167	10	15	AGATCTTGTT	AGATCATGTTTTCGG
UNSB01.fasta	Cele-UNSB01_1:2195476	1	2195476	2195914	439	10	35	TTTGCCGGAA	TTTGCCGGAATTTCGGAATTCTGACAGTTTGCCGG
UNSB01.fasta	Cele-UNSB01_1:2263475	1	2263475	2263726	252	6	43	CAAATCGGCA	CAAATCGGCAACTCGGCAAATTTCCGAAATTAAAAAATTTCAG
UNSB01.fasta	Cele-UNSB01_1:2281114	1	2281114	2281247	134	4	33	TAAGAAAAGT	TAAGAAAAGTTAGGCCACCAAACATTGGTGGAC
UNSB01.fasta	Cele-UNSB01_1:2284293	1	2284293	2285881	1589	82	12	CACGCAACGC	CACGCAACGCTG
UNSB01.fasta	Cele-UNSB01_1:2293586	1	2293586	2294094	509	12	43	AATTTCCGGC	AATTTCCGGCAAACCGGCAAACCGACATGTTGCCGAAAAAGAA
UNSB01.fasta	Cele-UNSB01_1:2301010	1	2301010	2301088	79	6	12	TAGGCTTAGG	TAGGCTTAGGCT
UNSB01.fasta	Cele-UNSB01_1:2308046	1	2308046	2308235	190	5	12	AGGCTTAGGC	AGGCTCAGGCTC
UNSB01.fasta	Cele-UNSB01_1:2365340	1	2365340	2366051	712	8	20	GGACTAGTTT	GGACTAGTTTTTGAGTAGAG
UNSB01.fasta	Cele-UNSB01_1:2365340	1	2365340	2366212	873	11	20	GGACTAGTTT	GGACTAGTTTTTGAGTAGAG
UNSB01.fasta	Cele-UNSB01_1:2396315	1	2396315	2396849	535	20	20	TTATTGATTT	TTATTAATTTTTCTAGGACA
UNSB01.fasta	Cele-UNSB01_1:2402282	1	2402282	2402378	97	6	12	AAGCCTAAGC	AAGCCTAAGCCT
UNSB01.fasta	Cele-UNSB01_1:2461739	1	2461739	2462720	982	5	175	GATGCACCAT	GATGCACCATGTCTTTGAAAGCCTATACTTCGGTTCATTTCGTAGCTGTCAAAAACTTTTTAACAACACATCTATAGAAATCAACATGCTTAATATTTCGTCAGTTGACAGTTTTTTGATAACTGCAATGAAATCCGAGATATGAGCTTTCAAAGTTGAGAGACCTCACTCGGTT
UNSB01.fasta	Cele-UNSB01_1:2556234	1	2556234	2556523	290	10	18	GCCTAGGAAT	GACTAGGAATCCAATATG
UNSB01.fasta	Cele-UNSB01_1:2574462	1	2574462	2585290	10829	565	19	TACACGAAAA	TACACGAAAAATAGATTGT

UNSB01.fasta	Cele-UNSB01_1:2599082	1	2599082	2599291	210	6	35	TTGCCGGAAT	TTGCCGGAATGGAAATTTTTTGGTAAATCGGCAAT
UNSB01.fasta	Cele-UNSB01_1:2691842	1	2691842	2691886	45	4	11	AAACTTGGGC	AAACTTGGGTA
UNSB01.fasta	Cele-UNSB01_1:2695367	1	2695367	2695539	173	4	43	TTGCCGATTT	TTGCCGATTTTCCTGAATTGTTCATTTCCGACAATATGACGAT
UNSB01.fasta	Cele-UNSB01_1:2780250	1	2780250	2780460	211	6	35	AATTTCAATT	AATTTCAATTTCGGCAATTTGCCGATTTGCCGGAA
UNSB01.fasta	Cele-UNSB01_1:2850958	1	2850958	2851167	210	5	35	GGAATTGAAA	GGAATTGAAAATTCCCGGCAAATCGGCAAATTGCC
UNSB01.fasta	Cele-UNSB01_1:2859395	1	2859395	2859671	277	5	43	TTGCCGATTT	TTGCCGGTTTCTCGATTTGCCGGATATTTACAATTTCACTATT
UNSB01.fasta	Cele-UNSB01_1:2881736	1	2881736	2881825	90	5	12	TAGGCTTAGG	TAGGCTGAGGCG
UNSB01.fasta	Cele-UNSB01_1:2886442	1	2886442	2886617	176	4	35	TTTGCCGATT	TTTGCCGACTTGTCAAAAATTGTTAATTTTAGCAC
UNSB01.fasta	Cele-UNSB01_1:2899706	1	2899706	2899878	173	4	43	AAATCGGCAA	AAATCGGCAATCCGGCCAATTGCAAAAAATGAAAATTCCCGGC
UNSB01.fasta	Cele-UNSB01_1:3070958	1	3070958	3071149	192	5	35	TTGCCGGAAA	TTGCCGGAAATTTGTAATTCCGACATATTGCCGAT
UNSB01.fasta	Cele-UNSB01_1:3156606	1	3156606	3157000	395	8	35	CGATTTGCCG	CGATTTGCCGGAAATATTCATTTTCTGCAAATTGC
UNSB01.fasta	Cele-UNSB01_1:3158079	1	3158079	3158367	289	21	12	CTAAGCCTAA	CTATGCCTAAAC
UNSB01.fasta	Cele-UNSB01_1:3159205	1	3159205	3159335	131	4	26	GGCAGGCGTC	GGCAGGCGTCAAGCAGGCAGGTTTTA
UNSB01.fasta	Cele-UNSB01_1:3213789	1	3213789	3217715	3927	151	26	CAGAGTCACT	CAGAGTCACTAAATTTGGTGAACGGC
UNSB01.fasta	Cele-UNSB01_1:3303901	1	3303901	3304753	853	71	12	CTAAGTCTAA	CTAAGTCTAAGC
UNSB01.fasta	Cele-UNSB01_1:3325616	1	3325616	3325694	79	6	12	AGGCTTAGGC	AGGCTTAGGCTT
UNSB01.fasta	Cele-UNSB01_1:3430435	1	3430435	3430513	79	6	12	GGCTTAGGCT	GGCTTAGGCTTC
UNSB01.fasta	Cele-UNSB01_1:3435421	1	3435421	3440265	4845	323	15	CTCACCACGA	CTCGCCACGATGGGT
UNSB01.fasta	Cele-UNSB01_1:3453387	1	3453387	3453600	214	5	32	AATTCAAATT	AATTCAAATTGTCAAGAATAATTTAGGTGGAA
UNSB01.fasta	Cele-UNSB01_1:3471234	1	3471234	3471484	251	6	43	TTGAAAATTT	TTGAAAATTTTCTAAAAATCGGCAAACCGGCAACTTGCCGGAA
UNSB01.fasta	Cele-UNSB01_1:3474380	1	3474380	3475480	1101	54	20	CTAGGCAGAC	CTAGGCAGACCTAGCCTACT
UNSB01.fasta	Cele-UNSB01_1:3474383	1	3474383	3476103	1721	83	20	GGCAGACCTA	GGCAGACCTAGCCTACTCTA
UNSB01.fasta	Cele-UNSB01_1:3489580	1	3489580	3490945	1366	56	35	TTTCGGCAAT	TTTCGGCAATTTCGGCAACTGCCGGTATCGTGAAT
UNSB01.fasta	Cele-UNSB01_1:3506179	1	3506179	3506592	414	8	35	TCCGGCAAAT	TCCGGCAAATCGGAAATCTGCCGGAAGTCAAAAAT
UNSB01.fasta	Cele-UNSB01_1:3532132	1	3532132	3534432	2301	115	20	AAAAAATAGA	AAAAAATAGATTGATATACC
UNSB01.fasta	Cele-UNSB01_1:3600639	1	3600639	3600909	271	6	45	CTGAATCTTC	CTGAATCTTCAGCTCCAAAGGATCACAAGGATCCTCCTTTATGAT
UNSB01.fasta	Cele-UNSB01_1:3650776	1	3650776	3650949	174	5	35	TTTGCCGGAA	TTTGCCGGAAAATTTTAATTCCAGCAATTTGCCGA
UNSB01.fasta	Cele-UNSB01_1:3711978	1	3711978	3718275	6298	242	26	ATGGTTAGAG	ATGGTTAGAGTCATATTTTTAGGTGA
UNSB01.fasta	Cele-UNSB01_1:3764869	1	3764869	3765030	162	5	14	TTTTTGTAGA	TTTTGTAGAACGTC

UNSB01.fasta	Cele-UNSB01_1:3798271	1	3798271	3798674	404	9	45	TCTGATCCTT	TCTGATCCTTCAGATAAGAAGGATCGTAAGGGTACCTCCTGATGG
UNSB01.fasta	Cele-UNSB01_1:3799094	1	3799094	3799407	314	7	45	CTGAAGGATC	CTGAAGTATCAGACCATCAGGTGGTACCCTTACGATCCTTCTTAT
UNSB01.fasta	Cele-UNSB01_1:3815334	1	3815334	3818659	3326	133	25	TTTACTCTCT	TTTACTCTCTGGCTTCACCAAATAT
UNSB01.fasta	Cele-UNSB01_1:3888641	1	3888641	3889727	1087	26	34	TGCCGGAATT	TGTCGGAATTGAAATTTCCAGCAAATCGGCAAAT
UNSB01.fasta	Cele-UNSB01_1:4033028	1	4033028	4033133	106	7	15	TCGTGGTGAG	TCGTGGTGAGACCCT
UNSB01.fasta	Cele-UNSB01_1:4033525	1	4033525	4033725	201	6	40	TTTCTAGAAA	TTTCTAGAAAATTCGGGAAAACTCTGGAATATTCCAGAAC
UNSB01.fasta	Cele-UNSB01_1:4042754	1	4042754	4055380	12627	214	59	AAAACCAGTG	AAAACCAGTGCGCATTTGAAATTCCAAATTCTCCATTAATTTCAGTTTAAAAAATTTTG
UNSB01.fasta	Cele-UNSB01_1:4183555	1	4183555	4184714	1160	5	223	TTTCAGAATT	TTTCAGAATTTAAAAACGGCATTCCAAAAACTTTCACAACCGCCAAGCTCAAGCAAAAAAAGTAAACTTCAGTCATACCTTTCAGAGCAAACTTTGAAATTGAATGAAATAGACCATTTATTTGCAAAATTAGTAGATTTTCCAATTTTTAAAAATTAATATCTGGCTTCTAAATAACTTTTTTGAAAATCCCCGTAGCCATTTTGTTGCAAATTTTGTGCTC
UNSB01.fasta	Cele-UNSB01_1:4232491	1	4232491	4233003	513	13	39	TATTTTTAAT	TATTTTTAATTTGGCCTACTTAAAAAGTAGGTCATGACT
UNSB01.fasta	Cele-UNSB01_1:4250268	1	4250268	4250328	61	4	15	TTTCCGGATC	TTTCCGGATCTACGT
UNSB01.fasta	Cele-UNSB01_1:4250510	1	4250510	4250645	136	9	15	AGATCCGGAA	AGATCCGGAAAACGT
UNSB01.fasta	Cele-UNSB01_1:4344690	1	4344690	4345849	1160	6	226	ACAAGAAGCT	ACAAGAAGCTTCACGTAGTTACAGAAATAGTAGATTTTTAGCCCTACCTTTTAGAGCGTATTTTATTATTTAATGAAAACTACCACTTATAGGCAAAATAGAGGGATTTTCGTAATTTTGAAAATTCATAAATCTCTTCAAAGTAATTTTTTTGCGAAATGTCTTTCAGAAACTTTGTAGTAAATTTTAAGCTCTTTTAGAATATATTAACAATATTCCAGTAGGT
UNSB01.fasta	Cele-UNSB01_1:4346155	1	4346155	4359862	13708	146	94	TCAAAATCTT	TCAAAATCTTCTGTTCTCTAAATATTGGGTGCTTTTCGATGCTCTTTGTAGACAAATCAATGGGAAAATTGTCAATTTCTGAAGGCAGTAATTC
UNSB01.fasta	Cele-UNSB01_1:4404167	1	4404167	4404863	697	4	174	CTTCAAGAGC	CTTCAAGAGCTCAGTGTTATTTCTGACATGCCAATCTCGACTCTAGACCTGAACATGTTGCAAGGAGTTGAAGACAACTTGAACAGTCTTCCAACTGAAGAAGCTGATAAGATCCGCGAGAAGATTAACGATCTGAGAAGAAGGAAACAAGAAAGCGATCTGGCTGAAGCTCTT
UNSB01.fasta	Cele-UNSB01_1:4530676	1	4530676	4530829	154	8	19	TTTTTCGTGT	TTTTTCGTGTAACAATCTA
UNSB01.fasta	Cele-UNSB01_1:4605678	1	4605678	4635032	29355	1129	26	TTTTCTAAAA	TTTTCTAAAATCCGCCGCAATGCCAT
UNSB01.fasta	Cele-UNSB01_1:4635066	1	4635066	4668893	33828	1253	27	TGGCTTCCCA	TGGCTTCCCACTATATTTACTCTCTG
UNSB01.fasta	Cele-UNSB01_1:4669638	1	4669638	4670054	417	33	12	TTTTTATTTT	TTTTTATTTTTA
UNSB01.fasta	Cele-UNSB01_1:4669638	1	4669638	4670196	559	41	12	TTTTTATTTT	TTTTTATTTTTA
UNSB01.fasta	Cele-UNSB01_1:4669638	1	4669638	4670946	1309	43	12	TTTTTATTTT	TTTTTATTTTTA
UNSB01.fasta	Cele-UNSB01_1:4694849	1	4694849	4695647	799	20	40	CATTCCAGAA	CATTCCAGAATTTTCTCGATTTTTCTAGAAAGTTCTGGAG
UNSB01.fasta	Cele-UNSB01_1:4695896	1	4695896	4696451	556	34	15	GGGTCTCACC	GGGTCTCACCACGGT
UNSB01.fasta	Cele-UNSB01_1:5080040	1	5080040	5080117	78	5	15	CGAAAAGGGG	CGAAAAGGGGATCTG
UNSB01.fasta	Cele-UNSB01_1:5080289	1	5080289	5080381	93	5	15	CTCCCCTTTT	CTCCCCTTTTCGTAT
UNSB01.fasta	Cele-UNSB01_1:5082880	1	5082880	5082987	108	6	15	CTCCCCTTTT	CTCCCCTTTTCGTAT
UNSB01.fasta	Cele-UNSB01_1:5266039	1	5266039	5266863	825	51	15	CGTGGTGAGA	CGTGGTGAGACCTTT
UNSB01.fasta	Cele-UNSB01_1:5273525	1	5273525	5290948	17424	599	29	TTGTAAGGAT	TTGTAAGGATTTCCCATTATGTCAATACA
UNSB01.fasta	Cele-UNSB01_1:5313372	1	5313372	5313447	76	5	15	TCCCCTTTTC	TCCCCTTTTCGTATC

UNSB01.fasta	Cele-UNSB01_1:5700313	1	5700313	5700505	193	16	12	GAAATTTTTT	GAAATTTTTTCT
UNSB01.fasta	Cele-UNSB01_1:5758991	1	5758991	5759083	93	6	15	TCCCCTTTTC	TCCCCTTTTCGTATC
UNSB01.fasta	Cele-UNSB01_1:5777645	1	5777645	5778359	715	6	119	GCCACCTTTA	GCCACCTTTAACCCTAATCCAAGCAGAGCAGGCCGTAGGCCTGCTAGGCGGTAGCGACACGCTCAAGCCCAACAGTCCGGGATGAGGGTCGCAGGCCCTCTTACTCTTAGAAACTTTAG
UNSB01.fasta	Cele-UNSB01_1:6112428	1	6112428	6113232	805	18	45	TCTGATCCTT	TCTGATCCTTCAGATAAGAAGGATCGTAAGGGTACCTTCTGATGG
UNSB01.fasta	Cele-UNSB01_1:6114632	1	6114632	6114855	224	5	45	AAGGATCAGA	AAGGATCAGATCATCAGAAGGTACCCATGTGATCCTTCGGAGCTG
UNSB01.fasta	Cele-UNSB01_1:6226496	1	6226496	6226583	88	5	17	AGGTAGTCAA	AGGTAGTCAATGAGCAA
UNSB01.fasta	Cele-UNSB01_1:6651710	1	6651710	6652545	836	13	30	TGGTGGAACT	TGGTGGAACTGGATTCGGCAGTGGAAATGC
UNSB01.fasta	Cele-UNSB01_1:6785229	1	6785229	6785394	166	11	15	GAGACCCATC	GAGACCCACCGTGGT
UNSB01.fasta	Cele-UNSB01_1:6785543	1	6785543	6785983	441	11	40	TTCTGGAATG	TTCTGGAATGTTCCAGAACTTTCTAGAAAAATCGAGAAAA
UNSB01.fasta	Cele-UNSB01_1:6909627	1	6909627	6919602	9976	399	25	GAAGCCAGAG	GAAGCCAGAGAGTAAATTATTATGT
UNSB01.fasta	Cele-UNSB01_1:7000180	1	7000180	7000255	76	5	15	AGATCCGGAA	AGATCCGGAAAACGT
UNSB01.fasta	Cele-UNSB01_1:7149236	1	7149236	7149296	61	4	15	CGAAAAGGGG	CAAAAAGGGGATCTG
UNSB01.fasta	Cele-UNSB01_1:7886332	1	7886332	7886407	76	5	15	TCGTGGTGAG	TCGTGGTGAGACCCT
UNSB01.fasta	Cele-UNSB01_1:7886430	1	7886430	7887346	917	26	35	GCGGGAAATT	GCGGGAAATTCAAATTTTAAGGGAAAAAATTTTTG
UNSB01.fasta	Cele-UNSB01_1:7887436	1	7887436	7887916	481	13	40	TTTCTAGAAA	TTTCTAGAAAAATCGAGAAATGTCTGCAATGTTCCAGAAG
UNSB01.fasta	Cele-UNSB01_1:7925712	1	7925712	7926382	671	39	16	AATTTTTCCG	AATTTTGCCGCGTCGA
UNSB01.fasta	Cele-UNSB01_1:8014213	1	8014213	8014468	256	17	15	ACCCATCGTG	ACCCATCGTGGCGAA
UNSB01.fasta	Cele-UNSB01_1:8014573	1	8014573	8014958	386	11	35	GGGAAATTTA	GGGAAATTTAAATTTTCTGTGAAAAATATTTTGGC
UNSB01.fasta	Cele-UNSB01_1:8015067	1	8015067	8016984	1918	48	40	GAATGTTCCA	GAATGTTCCAGAACTTTCTAGAAAAATCGAGAAAATTCTG
UNSB01.fasta	Cele-UNSB01_1:8640900	1	8640900	8641029	130	4	32	AAAATCGAAA	AAAATCGAAAAATTCTCGTTTTTCAATTTTTA
UNSB01.fasta	Cele-UNSB01_1:9793063	1	9793063	9794825	1763	41	44	CTGTTTTCTG	CTGTTTTCTGAGCATTGTAGATTTTCGGATTGATGGCCGAAAGG
UNSB01.fasta	Cele-UNSB01_1:9974009	1	9974009	9976809	2801	15	200	AACAGAATTT	AACAGAATTTTGAAAAATTTCGAAACCGCCAAGTTCAAGCAGAAAAAGTGAATTTTCAGTCATACCTTGTAGACCACAATTTATATACAAAATTAATAGATTTTCCAAACTTTGAAAATTAATTTCTGGCTTCAAACTAACTTTTTTAGAAATTCCCGTTGCCATTTTGTAGCACATTTTATGTTCTTTCAGAATTTGAA
UNSB01.fasta	Cele-UNSB01_1:9977112	1	9977112	9977772	661	41	15	CACGATGGGT	CACGAAGGGTATCAC
UNSB01.fasta	Cele-UNSB01_1:10089590	1	10089590	10089728	139	5	37	ATAATTTTTC	ATAATTTTTCTTGAGTAATGAGAGACTTTTTCATAAT
UNSB01.fasta	Cele-UNSB01_1:10295164	1	10295164	10303189	8026	319	25	TTACTCTAAT	TTACTCTAATTTTCTGCCAATTTAT
UNSB01.fasta	Cele-UNSB01_1:10374816	1	10374816	10387323	12508	186	68	AAAACCATTG	AAAACCATTGTCAACTGAATAGGTTGATTTGTGTTTTCTTTCTGAAATTCTAAGAATTTTGTTAAAAG
UNSB01.fasta	Cele-UNSB01_1:10434433	1	10434433	10461186	26754	116	231	GCTGAATTCA	GCTGAATTCAGTTGGGTAAGATGTGACTTCATGGCTGAGTGGAACATCTTGAACATCATCAGCACGATGTGTAGTGTCGAGTGGTCCATCGTATTGATCAGAAATCGGGTAATCAGAATGAGCAGAAGACTTCTTGAACAATCCAGTAATCTTCAATTTCAATGATGGCTCGTCCTTCTTCTCCGATTCTTCAATCACAACTGGGATGACATACTCTGGCAGCTTCTCATA
UNSB01.fasta	Cele-UNSB01_1:10475895	1	10475895	10475986	92	6	14	GCTATACGCT	GCTATACGCTATAC

UNSB01.fasta	Cele-UNSB01_1:10588945	1	10588945	10589028	84	6	12	TAGGCTTAGG	TAGGCTTAGTCT
UNSB01.fasta	Cele-UNSB01_1:10648848	1	10648848	10654019	5172	115	45	AAGGATCACA	AAGGATCACAAGGGTAGCTCCTGATGATCTGATCCTTCAGCTTCG
UNSB01.fasta	Cele-UNSB01_1:10660173	1	10660173	10660265	93	6	15	CGAAAAGGGG	CGAAAAGGGGATCTG
UNSB01.fasta	Cele-UNSB01_1:10660440	1	10660440	10660532	93	6	15	TCCCCTTTTC	TCCCCTTTTCGTATC
UNSB01.fasta	Cele-UNSB01_1:10842122	1	10842122	10842194	73	4	18	AAAAGTTACA	AAAAGTTACAGTGTCCTT
UNSB01.fasta	Cele-UNSB01_1:11042845	1	11042845	11046757	3913	24	163	GCATAGTCAA	GCATAGTCAAAAATACAAGAATATGCCTATCAAATTATAATAGTTTGTACGGAAGTATTTTTTAAAAACTTGATAAAAATATATAAAAGCTGATTTTTTAAAAAATTCAAAAGTATGGAAAATCATATGGAATCATTCTTTTTTATCTCAAAAAACTGTTCA
UNSB01.fasta	Cele-UNSB01_1:11104747	1	11104747	11105884	1138	5	94	TGTAGACAAA	TGTAGACAAATCAATGGGAGAATCATCAATTTCTGAAGGCAGTAATTCTCAAAATCTTCTGTTCTCTAAATATTGGGTGCTTTTCGATGTCCTT
UNSB01.fasta	Cele-UNSB01_1:11137320	1	11137320	11159590	22271	783	32	GCACCAAATA	GCACCAAATATCACCAATTTAGCACCGATAGA
UNSB01.fasta	Cele-UNSB01_1:11159617	1	11159617	11160082	466	4	26	TTTTTGAAAA	TTTTTGAAAAATGGCATTGCGGCGGA
UNSB01.fasta	Cele-UNSB01_1:11183622	1	11183622	11184094	473	10	35	TTGCCGATTT	TTACCGATTTGCCGGAATTTTTCAATTCCGGCAAT
UNSB01.fasta	Cele-UNSB01_1:11199905	1	11199905	11200227	323	8	35	GCCGGAATTG	GCCGGAATCGAAATTTTCCGGCAAATCTACAAACT
UNSB01.fasta	Cele-UNSB01_1:11239237	1	11239237	11239369	133	12	11	TATGCCTAGC	TATGCCTAGCC
UNSB01.fasta	Cele-UNSB01_1:11267568	1	11267568	11267793	226	15	15	CGATGCTCCA	CGATGCTCCACCCGG
UNSB01.fasta	Cele-UNSB01_1:11268535	1	11268535	11268966	432	11	34	TTCCGGCAAA	TTCCGGCAAATCGGAAAATTGCTGGAAACTAAAA
UNSB01.fasta	Cele-UNSB01_1:11276130	1	11276130	11276202	73	6	12	CGATCTCCAC	CGATCTCCACCA
UNSB01.fasta	Cele-UNSB01_1:11291527	1	11291527	11291639	113	7	14	AGCTTGAAGC	AGCTTGAAGCTGGA
UNSB01.fasta	Cele-UNSB01_1:11320046	1	11320046	11320214	169	4	42	TGCCGGAAAT	TGCCGGAAATTTCAAACCCGTCAATTTGTTGATTTGCCGATT
UNSB01.fasta	Cele-UNSB01_1:11351419	1	11351419	11351614	196	13	15	TCGTGGTGAG	TCGTAGTGAGACCCA
UNSB01.fasta	Cele-UNSB01_1:11352081	1	11352081	11352141	61	5	12	TATACATATA	TATACATATACA
UNSB01.fasta	Cele-UNSB01_1:11361560	1	11361560	11361658	99	7	14	AACTACTAAC	AACTACAAACTACA
UNSB01.fasta	Cele-UNSB01_1:11393763	1	11393763	11394894	1132	15	35	TTCCGGCAAA	TTCCGGCAAATCGGCAAATTGCCGGGACTGAAAAT
UNSB01.fasta	Cele-UNSB01_1:11398081	1	11398081	11398840	760	4	35	TCGGCAAATT	TCGGCAAATTACGGAAAATGAAAATTTCCGGCAAA
UNSB01.fasta	Cele-UNSB01_1:11408393	1	11408393	11409029	637	9	35	AATTGCCGAA	AATTGCCAAATTTCTCGTTTTTGGCAATTTTCAAA
UNSB01.fasta	Cele-UNSB01_1:11460977	1	11460977	11461721	745	60	12	CGTTGCGTGT	CGTTGCATGTCG
UNSB01.fasta	Cele-UNSB01_1:11481110	1	11481110	11481222	113	7	16	CTACATGCCT	CTACATGCCTATATGC
UNSB01.fasta	Cele-UNSB01_1:11481110	1	11481110	11482230	1121	68	16	CTACATGCCT	CTACATGCCTATATGC
UNSB01.fasta	Cele-UNSB01_1:11519985	1	11519985	11520529	545	18	26	ACCTACAGTA	ATCTACAGTACCCCTATTCTAGAGTG
UNSB01.fasta	Cele-UNSB01_1:11521003	1	11521003	11522550	1548	48	32	GCAATTTTTA	GCAATTTTTAGGCTAAAAATCACTAAAAAACT
UNSB01.fasta	Cele-UNSB01_1:11550058	1	11550058	11550130	73	6	12	TATATGTATA	TATATGTATATG

UNSB01.fasta	Cele-UNSB01_1:11556393	1	11556393	11558176	1784	47	35	GGCAAATCAG	GGCAAATCAGCAAGTTACCGGAATTGAAAATTTCC
UNSB01.fasta	Cele-UNSB01_1:11564527	1	11564527	11564628	102	5	20	TGTAACAATC	TGTAACAATCTATTTTTTCG
UNSB01.fasta	Cele-UNSB01_1:11591612	1	11591612	11591946	335	14	20	AAAAATCAAT	AAAAATCAATAATTTCATCG
UNSB01.fasta	Cele-UNSB01_1:11592271	1	11592271	11597890	5620	277	20	ATTGATTTTT	ATTGATTTTTTGAGAAAATT
UNSB01.fasta	Cele-UNSB01_1:11615647	1	11615647	11616001	355	10	30	CCTTGGGCTC	CCTTGGGCTCCCTGAGCTCCAAATTGTGTT
UNSB01.fasta	Cele-UNSB01_1:11631541	1	11631541	11632264	724	15	11	GTACTGTAGG	GTACTGTAGTG
UNSB01.fasta	Cele-UNSB01_1:11636321	1	11636321	11636670	350	9	35	ATTCCGGCAA	ATTCCGGCAATTTGCCGATTTTCCAGAAATTTTCA
UNSB01.fasta	Cele-UNSB01_1:11643779	1	11643779	11644036	258	6	34	ATGCACCATG	ATGCACCATGCCAAAAAATATATCCAAAACCCCG
UNSB01.fasta	Cele-UNSB01_1:11661653	1	11661653	11661731	79	6	12	AAGCCTAAGC	AAGCCTAAGCCT
UNSB01.fasta	Cele-UNSB01_1:11662428	1	11662428	11662542	115	9	12	GCTTAGGCTT	GCTTGGGCTTAG
UNSB01.fasta	Cele-UNSB01_1:11667166	1	11667166	11667423	258	6	34	ATGCACCATG	ATGCACCATGCCAAAAAATATATCCAAAACCCCG
UNSB01.fasta	Cele-UNSB01_1:11682141	1	11682141	11682225	85	7	12	GGCTTAGGCT	GGCTTAGGCTTA
UNSB01.fasta	Cele-UNSB01_1:11685636	1	11685636	11685851	216	5	43	CGGCAAATCG	CGGCAAATCGGCAAACCAGCAAGTTGCCAAAAATCAAAATTTC
UNSB01.fasta	Cele-UNSB01_1:11691383	1	11691383	11691503	121	8	15	TCGTGGTGAG	TCGTGGTGAGACCCT
UNSB01.fasta	Cele-UNSB01_1:11691539	1	11691539	11692754	1216	24	35	TGGCGGGAAA	TGGCGGGAAATTCAAATTTTCAGTGAAAAAAATTT
UNSB01.fasta	Cele-UNSB01_1:11696177	1	11696177	11696255	79	6	12	TAGGTCTAGG	TAGGTCTAGGTC
UNSB01.fasta	Cele-UNSB01_1:11696998	1	11696998	11697105	108	5	19	TCAAAAATCA	TCAAAAATCAATAATTTCA
UNSB01.fasta	Cele-UNSB01_1:11697315	1	11697315	11697448	134	7	19	TTATCGATTT	TTATCGATTTTTCCGGAAA
UNSB01.fasta	Cele-UNSB01_1:11696961	1	11696961	11698223	1263	20	19	AAAAATCGAT	AAAAATCAATAATTTCATC
UNSB01.fasta	Cele-UNSB01_1:11744458	1	11744458	11744933	476	17	19	TTTCACAAAA	TTTCACAAAAATCGATAAT
UNSB01.fasta	Cele-UNSB01_1:11743735	1	11743735	11745857	2123	45	19	AAAATCGATA	AAAATCGATAATTTCTCAT
UNSB01.fasta	Cele-UNSB01_1:11752079	1	11752079	11752337	259	16	12	AAGCCAAAGC	AAGCCAAAGAAT
UNSB01.fasta	Cele-UNSB01_1:11868010	1	11868010	11868741	732	60	12	AGGCTCAGGC	AGGGTCAGGCTC
UNSB01.fasta	Cele-UNSB01_1:11869240	1	11869240	11870103	864	53	16	GCATGTAGGC	GCATGTAGGCATGTAG
UNSB01.fasta	Cele-UNSB01_1:11907174	1	11907174	11907313	140	13	11	TTTTGCACAT	TTTTCCACATT
UNSB01.fasta	Cele-UNSB01_1:11925845	1	11925845	11930260	4416	27	164	ATACCAGAAA	ATACCAGAAAATGCCAATCAAAGTATATTAGCTTGTACGGAAGTAATTTTTTTTTTAATTGATAAAAATATATAAAAGCTGATTTTTTCAAAAATTCAAAAGTATGGGAAAGTCAATGGAGTCATTCTTTTTTATTCTATAAAACTGTTTACCATAGTCAAAA
UNSB01.fasta	Cele-UNSB01_1:11979429	1	11979429	11979575	147	6	11	AGTACTGTAG	AGTACTGTAGG
UNSB01.fasta	Cele-UNSB01_1:12045808	1	12045808	12046235	428	5	42	TCGGCAAATC	TCGGCAAATTCGGCAAATCTACTTTTTTGAAAATTGCCGAGC
UNSB01.fasta	Cele-UNSB01_1:12056400	1	12056400	12056477	78	7	11	CAAAAAATGT	CAAAAAATGTC

UNSB01.fasta	Cele-UNSB01_1:12149612	1	12149612	12149804	193	15	12	TTAGGCTTAG TTACGCTTAGGC
UNSB01.fasta	Cele-UNSB01_1:12200484	1	12200484	12200598	115	7	12	CTAAGCCTAA CGAAGCCTAAGC
UNSB01.fasta	Cele-UNSB01_1:12310642	1	12310642	12315079	4438	373	11	CACATTTTTT CACATTTTTTG
UNSB01.fasta	Cele-UNSB01_1:12330775	1	12330775	12330985	211	7	30	GTCATTATCA GTCATTATCAGCTTCTGGTGTCTTAGAAGA
UNSB01.fasta	Cele-UNSB01_1:12340678	1	12340678	12340816	139	10	12	TAAGCCTAAG TAAGCCTAAGCT
UNSB01.fasta	Cele-UNSB01_1:12350087	1	12350087	12350549	463	41	11	TTGCACATTT TTGCACATTTC
UNSB01.fasta	Cele-UNSB01_1:12396670	1	12396670	12396802	133	9	12	GCCTAAGCCT GCCTAAGCCTAT
UNSB01.fasta	Cele-UNSB01_1:12413207	1	12413207	12413533	327	4	73	AATAAAATAT AATAAAATATAGAAAATCCTTATGACACACTTAAGCCTAAAGGCCCGAAAAACATACTAGGATGCCCAACTGG
UNSB01.fasta	Cele-UNSB01_1:12444291	1	12444291	12444339	49	4	12	GCCTAAGCCT GCCTAAGCCTAG
UNSB01.fasta	Cele-UNSB01_1:12478910	1	12478910	12479068	159	5	34	ATGCACCATG ATGCACCGTGTCCAAAAGTATATATAAAATTGCG
UNSB01.fasta	Cele-UNSB01_1:12487096	1	12487096	12487168	73	5	12	GGCTTAGGCT GGCTTCGGCTTA
UNSB01.fasta	Cele-UNSB01_1:12700590	1	12700590	12700802	213	16	12	GAAATTTTTT GAAATTTTTTCC
UNSB01.fasta	Cele-UNSB01_1:12700867	1	12700867	12701490	624	50	12	AAAAAATTTC AAAAAATTTCGG
UNSB01.fasta	Cele-UNSB01_1:12702448	1	12702448	12702698	251	5	50	ACACATGCTA ACACATGCTATAACATTGTCTCCTTCCTCTAGTGCACGAGAGCGGCCGCC
UNSB01.fasta	Cele-UNSB01_1:12783664	1	12783664	12784000	337	20	16	TGATCTGATG TGATCTGATGATCTCA
UNSB01.fasta	Cele-UNSB01_1:12807224	1	12807224	12807329	106	5	21	ACCACCCCTA ACCACTCCTACAACAACCACT
UNSB01.fasta	Cele-UNSB01_1:12834373	1	12834373	12834766	394	5	32	GCTGATTGGT GCTGATTGGTTCAAAAGTGGGCTTGGCTTATG
UNSB01.fasta	Cele-UNSB01_1:12842011	1	12842011	12842077	67	5	12	TACCTATACC TACCTATACCTT
UNSB01.fasta	Cele-UNSB01_1:12905958	1	12905958	12906024	67	5	11	TTTGGAGCGG TTCGGAGCGGT
UNSB01.fasta	Cele-UNSB01_1:13114799	1	13114799	13114994	196	5	39	TATTTTTAAT TATTTTTAATTTAACCTACTTAAAAAGTAGGTCATGACC
UNSB01.fasta	Cele-UNSB01_1:13115312	1	13115312	13116088	777	11	23	ACTACAAACT ACTACAAACTACAAACTATGAAA
UNSB01.fasta	Cele-UNSB01_1:13121380	1	13121380	13121635	256	17	15	AAAAGGGGAT AAAAGGGGATCTGCG
UNSB01.fasta	Cele-UNSB01_1:13132164	1	13132164	13132288	125	6	21	AATCCAATAT AATCAAATATGGTTGAAATCT
UNSB01.fasta	Cele-UNSB01_1:13177981	1	13177981	13178228	248	13	19	CAAGGCACTA CAAGGTACTAGAAATGCGC
UNSB01.fasta	Cele-UNSB01_1:13199796	1	13199796	13200258	463	10	35	GCCGGAAATT GCCGGAAATTTTCAATTCTGGCAGTTTACCAATTT
UNSB01.fasta	Cele-UNSB01_1:13236201	1	13236201	13236854	654	16	35	ATTGCCGGAA ATTGCCGGAATTGAAAAATGCAGGCAAATCGGCAT
UNSB01.fasta	Cele-UNSB01_1:13241763	1	13241763	13242154	392	8	14	AGTTTGTAGT AGTCTGTAGTTTGT
UNSB01.fasta	Cele-UNSB01_1:13307625	1	13307625	13307715	91	4	22	TCGAGAAAAA TCGAGAAAAAATCTGAAAAAAT
UNSB01.fasta	Cele-UNSB01_1:13315136	1	13315136	13315216	81	5	16	AATACGGGAA AATACGGGAATACGGG

UNSB01.fasta	Cele-UNSB01_1:13324034	1	13324034	13324147	114	5	22	TTTTTTCTCG	TTTTTTCTCGAATTTTTTCAGA
UNSB01.fasta	Cele-UNSB01_1:13379937	1	13379937	13380822	886	18	39	TTAAAAAGTA	TTAAAAAGTAGGTCATGACCTAATTTTAATATGACCTAC
UNSB01.fasta	Cele-UNSB01_1:13456302	1	13456302	13456675	374	12	11	GTACTGTAGG	GTACTGTAGGA
UNSB01.fasta	Cele-UNSB01_1:13456767	1	13456767	13461871	5105	124	40	AATGTTCCAG	AATGTTCCAGAACTTTCTAGAAAAATCGAGAAAATTCTGA
UNSB01.fasta	Cele-UNSB01_1:13507191	1	13507191	13507550	360	18	20	AAAATAGATT	AAAATAGATTGTTACACGAA
UNSB01.fasta	Cele-UNSB01_1:13507576	1	13507576	13507758	183	7	26	CTCTGGCAGT	CTCTGGCAGTTCACTAAAATAAGTGA
UNSB01.fasta	Cele-UNSB01_1:13634561	1	13634561	13638668	4108	158	26	TTTTAGGTGA	TTTTAGGTGAATGGTTAGAGTCATAA
UNSB01.fasta	Cele-UNSB01_1:13683275	1	13683275	13683450	176	5	35	TGAAAATTTC	TGAAAATTTCCGGCAAAACGGCAAATTTTCGAAAA
UNSB01.fasta	Cele-UNSB01_1:13729567	1	13729567	13730578	1012	11	14	TAGTTTGTAG	TAGTTTGTAGTTTG
UNSB01.fasta	Cele-UNSB01_1:13730896	1	13730896	13731271	376	11	14	TACAAACTAC	TATAAACTACAAAC
UNSB01.fasta	Cele-UNSB01_1:13831060	1	13831060	13832042	983	52	14	TGTAGTATGT	TGTAGTATGTAGTT
UNSB01.fasta	Cele-UNSB01_1:13840435	1	13840435	13852243	11809	369	32	TGAATTCCCG	TGAATTCCCGCCAAAACTTTTCACAGAAAATT
UNSB01.fasta	Cele-UNSB01_1:13852339	1	13852339	13853459	1121	28	40	GAATTTTCTC	GAATTTTCTCGAATTTTCCAGAAGGTTCTGGAGCATTCCA
UNSB01.fasta	Cele-UNSB01_1:13927551	1	13927551	13927713	163	13	12	TATACCTATA	TATACCTATACC
UNSB01.fasta	Cele-UNSB01_1:13927986	1	13927986	13928124	139	11	12	ATAGGTATAG	ATAGGTATAGGC
UNSB01.fasta	Cele-UNSB01_1:14006347	1	14006347	14006515	169	7	21	CAAAATTCGT	CAAAATTCGCCCAAAAATGCT
UNSB01.fasta	Cele-UNSB01_1:14038349	1	14038349	14038662	314	9	35	AATTCCGGCA	AATTCCGCCAATTTGCCGATTTGCCGGAAAATTTA
UNSB01.fasta	Cele-UNSB01_1:14122632	1	14122632	14123925	1294	19	68	TTGGTTGAGG	TTGGTTGAGGCTAGAAAATGTTGTAGTTTGTAGTGTGTTAGCCTCAACCAAAATTAATTTTTTTTTAA
UNSB01.fasta	Cele-UNSB01_1:14164675	1	14164675	14164835	161	8	20	AAAAAATAGA	AAAAAATAGATTAGCATACC
UNSB01.fasta	Cele-UNSB01_1:14166580	1	14166580	14166640	61	5	12	GCCTAAGCCT	GCCTAAGCCTAC
UNSB01.fasta	Cele-UNSB01_1:14168111	1	14168111	14168331	221	5	63	TTTTTGACTA	TTTTTGACTACGAGTGTCTTTTTCGCTACTGAGCGAAAAAATAGCTACATTCGCACTTTTGTC
UNSB01.fasta	Cele-UNSB01_1:14174364	1	14174364	14175224	861	15	64	GTAGTCAAAA	GTAGTCAAAAAGTCAAAAGTGCGAATGTAGCTATTTTTTCAGTCAGTAGCGAAAAAGACATTCA
UNSB01.fasta	Cele-UNSB01_1:14199204	1	14199204	14199288	85	4	21	ATATTTTGTG	ATATTTTGTGACATTTTTATT
UNSB01.fasta	Cele-UNSB01_1:14207225	1	14207225	14207295	71	6	10	GATTTTCATA	GATTTTCATA
UNSB01.fasta	Cele-UNSB01_1:14240945	1	14240945	14242222	1278	80	16	ATCTAAGGAT	ATCTAAGGATCTAAGG
UNSB01.fasta	Cele-UNSB01_1:14240945	1	14240945	14243302	2358	147	16	ATCTAAGGAT	ATCTAAGGATCTAAGG
UNSB01.fasta	Cele-UNSB01_1:14245984	1	14245984	14247232	1249	59	16	AGATCCTTAG	AGATCCTTAGATCCTT
UNSB01.fasta	Cele-UNSB01_1:14248002	1	14248002	14248098	97	6	16	AGGATCTAAG	AGGATCTAAGGATCTA
UNSB01.fasta	Cele-UNSB01_1:14266018	1	14266018	14266935	918	5	179	CTACCCAAAT	CTACCCAAATTTTTGAAATTTGGAAATTTTTCAAAAATTCTCAAATTTTAAAAATCCCATAATTCAGCCAATTTTTGAGATTTTCAGATATTTTTTAATTGAAAATTCTGTTTGTCTTATCCATTAAACTATATCGCTGCATCTACATTTTTCACAGTACCCCATGACTTTTCCCGATT

UNSB01.fasta	Cele-UNSB01_1:14275073	1	14275073	14275913	841	24	40	TTCTGGAAAA	TTCTGGAAAATTTTAGAAAATTCGGGAATGTTCTGGAACC
UNSB01.fasta	Cele-UNSB01_1:14343732	1	14343732	14344621	890	4	62	GTAGTTTGTA	GTAGTTTGTAGTGTGAGCCTCAACCAAACTAGCCAAAGCTTGAGTGAGGCTATGGTGATTTT
UNSB01.fasta	Cele-UNSB01_1:14381938	1	14381938	14382071	134	7	19	CCCTGATACC	CCCTGATACCCCCGGTCCC
UNSB01.fasta	Cele-UNSB01_1:14392151	1	14392151	14393037	887	5	177	CTAACAAAAT	CTAACAAAATAATTAGCGTAAAATTTCCTACATTTTACTAGTTAACAATATTTTGATAGCATCAGTGGTTTTTGAGATACAGAAGTTATAAGATGTACGTCCAAAAATGATTGTGAGCTGCTCTTATCAAAAATTACCATAACTTTTTACTCACTAACGGTATCAAAAATGTTTTAA
UNSB01.fasta	Cele-UNSB01_1:14511380	1	14511380	14511819	440	17	22	GCACGGCCTG	GCATGGCCTGACGGTCCTGAGG
UNSB01.fasta	Cele-UNSB01_1:14535288	1	14535288	14535435	148	8	15	GGGGGCAACA	GGGGGCAACAAATTA
UNSB01.fasta	Cele-UNSB01_1:14537614	1	14537614	14537761	148	8	15	TGTTGCCCCC	TGTTGACCCCTATTA
UNSB01.fasta	Cele-UNSB01_1:14566637	1	14566637	14566717	81	4	20	ATATCAAAAA	ATACCAAAAAATAGATTGGC
UNSB01.fasta	Cele-UNSB01_1:14568940	1	14568940	14569168	229	4	14	TGTAGTTTGT	TGTAGTTTGTAGTG
UNSB01.fasta	Cele-UNSB01_1:14574460	1	14574460	14574865	406	4	101	GCATTTGCAT	GCATTTGCATATAGAGATCAGATAAAAGTTAGAATTTTCCAGCCGTTGCAAGTGCGCTCTATTGAGAACATTTTGAAAATTGAATGTTTTCAAATTAGAGC
UNSB01.fasta	Cele-UNSB01_1:14577219	1	14577219	14577388	170	5	23	GCACCATAAG	GCATCATAAGGACTCTAGTTGAT
UNSB01.fasta	Cele-UNSB01_1:14582954	1	14582954	14583490	537	26	18	TTGTAGATCA	TTGTAGATCAACGGGGTC
UNSB01.fasta	Cele-UNSB01_1:14647518	1	14647518	14647641	124	4	11	GTACTGTAGG	GTACTGTAGGA
UNSB01.fasta	Cele-UNSB01_1:14648049	1	14648049	14648258	210	6	35	AATTTAAATT	AATTTAAATTTTCTGAAAAAAATATTTTGGAGGGA
UNSB01.fasta	Cele-UNSB01_1:14674978	1	14674978	14675094	117	10	12	ATATGTATAT	ATATGTATTTGT
UNSB01.fasta	Cele-UNSB01_1:14687699	1	14687699	14688279	581	8	73	TGTCGGCCGC	TGTCGGCCGCTCTAAACTATTTTGCATACACACCAAGCTACTTGTTTCACGCCAAGCTGCGGAACCTCGAAAG
UNSB01.fasta	Cele-UNSB01_1:14694045	1	14694045	14694325	281	8	35	AAAATTTCCG	AAAATTTCCGGCAAATCGGCAATTTACCGAAAGTG
UNSB01.fasta	Cele-UNSB01_1:14697056	1	14697056	14704541	7486	214	35	GAAATTTTCA	GAAATTTTCACTTTCGGTAAACTGCCGATTTACCG
UNSB01.fasta	Cele-UNSB01_1:14726982	1	14726982	14727102	121	6	20	GTGAAATTTT	GTGAAATTTTAAATTTTTGA
UNSB01.fasta	Cele-UNSB01_1:14731269	1	14731269	14731934	666	19	35	GAAAATTTCC	GAAAATTTCCGGCAACCCGGCAAATTGCCGGAATC
UNSB01.fasta	Cele-UNSB01_1:14804070	1	14804070	14804449	380	9	43	TTTGCCGGTT	TTTGCCGGTTTGCCGATTTGCCGGAAATTTTTATTTTCGGCAC
UNSB01.fasta	Cele-UNSB01_1:14869199	1	14869199	14869444	246	6	35	CAAATCGGCA	CAAATCGGCAAATTTTCGGAATTGAAAATTTTCGG
UNSB01.fasta	Cele-UNSB01_1:14918515	1	14918515	14918575	61	4	15	CCACGATGGG	CCACGATGGGTCTCT
UNSB01.fasta	Cele-UNSB01_1:14918627	1	14918627	14918817	191	5	40	CCAGAAGGTT	CCAGAAGGTTCTAGAACAATCCAGAATTTTTTCGAATTTT
UNSB01.fasta	Cele-UNSB01_1:14936456	1	14936456	14937856	1401	35	40	TTTCTAGAAA	TTTCTAGAAAGTTCTGGAGCATTCCAGAATTTTCTCGATT
UNSB01.fasta	Cele-UNSB01_1:15014974	1	15014974	15015203	230	5	34	GAAATTTCAA	GAAACTTCAACTCCTGCAAATTGCCGATTTGCCG
UNSB01.fasta	Cele-UNSB01_1:15023397	1	15023397	15024044	648	19	34	CGGAAATTTC	CGGTAATTTCAATTCCGGCAAGTTGCCGATTTCC
UNSB01.fasta	Cele-UNSB01_1:15062491	1	15062491	15062841	351	10	35	AATTCCGGCA	AATTACGGCACTTTGTCGATTTTCCTGAAATTTTC
UNSB01.fasta	Cele-UNSB01_1:15073460	1	15073460	15073709	250	7	35	CGGTTTTGGA	CGGTTTGGGAAATTTTCGGTAATTTTGACAATTGC

UNSB01.fasta	Cele-UNSB01_1:15094323	1	15094323	15094532	210	6	35	TTTCAATTCC	TTTCAATTCCGGCAATTCGCCGATTTACCAAAAAC
UNSB01.fasta	Cele-UNSB01_1:15278589	1	15278589	15278861	273	10	21	AACTTTTTGG	ACCTTTTTGGCAGATTTTCAA
UNSB01.fasta	Cele-UNSB01_2:2	2	2	3054	3053	243	12	AAGCCTAAGC	AAGCCTAAGCCT
UNSB01.fasta	Cele-UNSB01_2:3110	2	3110	3266	157	6	26	GTTCACCAAA	GTTCACCAAAAATAGTGACTCTGACC
UNSB01.fasta	Cele-UNSB01_2:146899	2	146899	146982	84	6	12	ATTTCTTGCA	ATTTCTTGCAAC
UNSB01.fasta	Cele-UNSB01_2:236549	2	236549	238078	1530	40	35	TTTCCGGCAA	TTTCCGGCAAATCGGCAGCCTGTCGGCATTGAAAA
UNSB01.fasta	Cele-UNSB01_2:371286	2	371286	371494	209	10	18	TAAGCCTAAG	TAAGCCTATGTCTAAGAA
UNSB01.fasta	Cele-UNSB01_2:454595	2	454595	455440	846	32	15	TCTCACCACG	TCTCGCCACGAAGGG
UNSB01.fasta	Cele-UNSB01_2:466682	2	466682	467257	576	9	20	CAATTTTTGA	CAATTTTTGAGTTAAAAAAT
UNSB01.fasta	Cele-UNSB01_2:577469	2	577469	577719	251	20	12	TAGGCTTAGG	TAGGCTTAGGCT
UNSB01.fasta	Cele-UNSB01_2:590141	2	590141	590909	769	12	64	AAATTTTGTA	AAATTTTGTAGTTTGTAGTCTAGCAGACCCATTTAGAATACCAACAAGTTGGGTCTGCCAGATC
UNSB01.fasta	Cele-UNSB01_2:636344	2	636344	636664	321	8	40	TTTCTAGAAA	TTTCTAGAAAATTCTGGAACATTCCAGAATTTTCCCGATT
UNSB01.fasta	Cele-UNSB01_2:635957	2	635957	636850	894	4	35	GAAAATTTAA	GAAAATTTAAATTTCCCTCCAAAATTTTTTTCTCA
UNSB01.fasta	Cele-UNSB01_2:636908	2	636908	637328	421	27	15	GGGTCTCACC	GGGTCTCACCACGAT
UNSB01.fasta	Cele-UNSB01_2:637418	2	637418	637568	151	4	40	CAGAAGGTTC	CAGAAGGTTCTGCAACATTCCAGAATTTTCTAGAATTTTC
UNSB01.fasta	Cele-UNSB01_2:637664	2	637664	637798	135	4	11	GTACTCCTAC	GTAATCCTACC
UNSB01.fasta	Cele-UNSB01_2:646191	2	646191	646835	645	25	23	GGCCACCAAT	GGCCACCGATCTATAAAAATCTA
UNSB01.fasta	Cele-UNSB01_2:649028	2	649028	649082	55	4	12	GGCTCAGGCT	GGCTCAGGCTCA
UNSB01.fasta	Cele-UNSB01_2:677942	2	677942	678110	169	9	16	CCTACGTGCC	CCTAGGTGCCTTCGTG
UNSB01.fasta	Cele-UNSB01_2:694600	2	694600	694660	61	4	15	GTAGATCTTG	GTAGATCTTGAAAAT
UNSB01.fasta	Cele-UNSB01_2:698255	2	698255	706115	7861	432	15	GGTGAGACCC	GGTGAGACCCATCGT
UNSB01.fasta	Cele-UNSB01_2:698255	2	698255	706481	8227	433	15	GGTGAGACCC	GGTGAGACCCATCGT
UNSB01.fasta	Cele-UNSB01_2:858289	2	858289	858649	361	5	31	TGTCTAGCGA	TGTCGAGCGAGACCTCAGAAGAGAGCTGAGT
UNSB01.fasta	Cele-UNSB01_2:863874	2	863874	864986	1113	34	32	AATTCCCGCC	AATTCCCGCCAAAATTTTTCACAGAAAATTTG
UNSB01.fasta	Cele-UNSB01_2:865003	2	865003	865078	76	5	15	ATGGGTCTCG	ATGGGTCTCGCCACG
UNSB01.fasta	Cele-UNSB01_2:870904	2	870904	873513	2610	35	72	TCCATCCAGC	TCCATCCAGCTGTGGTTGGTCTCTGGGTGAATCCTCGAGAAGTATCACGAGTAGTACCATTGGCTCCGTTTG
UNSB01.fasta	Cele-UNSB01_2:882806	2	882806	882988	183	12	14	TAGGATCTAG	TAGGATCTACGATC
UNSB01.fasta	Cele-UNSB01_2:885470	2	885470	885668	199	15	12	ACCTAGACCT	ACCTAGACCTAG
UNSB01.fasta	Cele-UNSB01_2:889690	2	889690	889842	153	5	11	TGTAGGGGTA	TGTAGGGGTAC

UNSB01.fasta	Cele-UNSB01_2:891851	2	891851	892689	839	4	184	GTTCAAAAGA	GTTCAAAAGATACAAAATTTATCTACACATCTACAAGTTAAACTTGCAACTTAGTTAACCAAATTCAAAAGTTATGAGCCGGGGAACTATAATAACCAACGTTTCACACTAAAAAAGCCCAAAAAAATTCGAAAATCATTTTTATCTCATGACGCGGACAAGCTACAAATGTCTAGTTAGGCTT
UNSB01.fasta	Cele-UNSB01_2:894739	2	894739	894896	158	8	20	CAATCTATTT	CAATCTATTTTCTGGTATGC
UNSB01.fasta	Cele-UNSB01_2:898652	2	898652	902061	3410	58	59	TATGGAAAAT	TATGGAAAATATGGATTTTTAAATGTGCACTGGTTTTCAAATTTTTTTAAACTGAGAAT
UNSB01.fasta	Cele-UNSB01_2:1043954	2	1043954	1044457	504	17	11	GTACTGTAGG	GTACTGTAGTA
UNSB01.fasta	Cele-UNSB01_2:1044642	2	1044642	1047492	2851	181	15	TCGTGGTGAG	TCGTGGTGAGATCCA
UNSB01.fasta	Cele-UNSB01_2:1097712	2	1097712	1098091	380	5	26	GTCGGCTGCT	GTCGGCTGCTTTTTTTTTGCAAAAGT
UNSB01.fasta	Cele-UNSB01_2:1142725	2	1142725	1142802	78	5	15	CGAAAAGGGG	CGAAAAGGGGATCTG
UNSB01.fasta	Cele-UNSB01_2:1142992	2	1142992	1143084	93	6	15	TCCCCTTTTC	TCCCCTTTTCGTATC
UNSB01.fasta	Cele-UNSB01_2:1186731	2	1186731	1187015	285	8	26	TACTGTAGTT	TACTGTAGTTTGACTAACTTAGGACT
UNSB01.fasta	Cele-UNSB01_2:1212949	2	1212949	1213169	221	4	55	GGGATTATTG	GGGATTATTGGAGTTTCTTTTTCTAGAATTTTTTGAAAATTTTGGACAGTTTTTT
UNSB01.fasta	Cele-UNSB01_2:1233155	2	1233155	1233235	81	5	12	TTAGGCTTAG	TTAGGCTTAGGT
UNSB01.fasta	Cele-UNSB01_2:1258661	2	1258661	1259567	907	73	12	CTATGCCTAT	CTATGCCTATGC
UNSB01.fasta	Cele-UNSB01_2:1261170	2	1261170	1261684	515	42	12	CTATGCCTAT	CTATGCCTATGC
UNSB01.fasta	Cele-UNSB01_2:1316328	2	1316328	1316758	431	7	63	GGTCTGCTAA	GGTCTGCTAAATCGAAACTTGTAGTTTGTAGTCTAGCAGACCAAATTTCTTCAAAACTACTTG
UNSB01.fasta	Cele-UNSB01_2:1327419	2	1327419	1327790	372	5	73	AGTTTGTAGT	ACTTTGTAGTTTGTAGTCTAGCAGACCAAAATTTATTAAAAACTGCAGAAAATTGATGGGTCTGCTAATTAAG
UNSB01.fasta	Cele-UNSB01_2:1412228	2	1412228	1413706	1479	12	63	TAGTTTGTAG	TAGTTTGTAGTCTAGCAGACCATATTTCTTCAAAACCCCATAGGTCTGCTAAATCGAAACTTT
UNSB01.fasta	Cele-UNSB01_2:1416594	2	1416594	1416934	341	5	59	CTACAAACTA	CTACAAACTACAAAAGCCTATTTAGCAGACCCACGCCAACTTCTGATGGGTCTGCTAGA
UNSB01.fasta	Cele-UNSB01_2:1422798	2	1422798	1423926	1129	9	63	TTGGTCTGCT	TTGGTCTGCTAGACTACAAACTACAACTTTTGATTTAGCAGACCCATGGGGTTTTGAAGAAAA
UNSB01.fasta	Cele-UNSB01_2:1429641	2	1429641	1430207	567	41	12	CCTATGCCTA	CCTATGCCTATG
UNSB01.fasta	Cele-UNSB01_2:1488365	2	1488365	1488455	91	5	18	GGAGGTCCAG	GGAGGTCCAGGAACCCCT
UNSB01.fasta	Cele-UNSB01_2:1507976	2	1507976	1508324	349	19	15	GGGGGCAACA	GGGGACAACAAAAAA
UNSB01.fasta	Cele-UNSB01_2:1517382	2	1517382	1518287	906	6	150	TTGCAACATG	TTGCAACATGCGGCCGGCGGGAAATTTGAATTTTTGGGTCCAAAACACGTTTTTCGACTAATTTTTCATATTTTCAAGCTAAATTATGATAGAAAATTCCAATTTTCACTAAAAATCGGCAATTTTCAGGATTTTCTCACTGGGGCGCAT
UNSB01.fasta	Cele-UNSB01_2:1585165	2	1585165	1585860	696	7	96	GGCCTGCGGC	GGCCTGCGGCCCTCAAACCTGGTTATTAGGCTTGAGTGAATAACATATCTGCTTAGAATAGGATCAAAGCCAGCCCATAGTTTCCAAGAGTGTTCG
UNSB01.fasta	Cele-UNSB01_2:1599041	2	1599041	1599824	784	24	23	CGCTGTAAGA	CGCTGTAAGACCATTTGCTGTAT
UNSB01.fasta	Cele-UNSB01_2:1617453	2	1617453	1617521	69	4	17	TCATTCCGTG	TCATTCCGTGATTATTT
UNSB01.fasta	Cele-UNSB01_2:1625642	2	1625642	1627656	2015	131	14	GATCCTAGAT	GATCCTAGATCCTA
UNSB01.fasta	Cele-UNSB01_2:1652802	2	1652802	1653206	405	5	42	TTGCCGGTTG	TTGCCGGTTGCCGAAGTTTTCAGCGTTCGACAACTTCGGCAA
UNSB01.fasta	Cele-UNSB01_2:1657514	2	1657514	1657909	396	5	42	CAACCGGCAA	CAACCGGCAATTGCCGAAGTTGTCGAACGCTGAAAACTTCGG

UNSB01.fasta	Cele-UNSB01_2:1681965	2	1681965	1682083	119	7	17	GATGCCCCCT	GATGCCCCCTCTGAACG
UNSB01.fasta	Cele-UNSB01_2:1815608	2	1815608	1815814	207	6	34	ATTTGAATTT	ATTTGAATTTCCCGCCAAAATTTTTTCACTGAAA
UNSB01.fasta	Cele-UNSB01_2:1818966	2	1818966	1819342	377	11	34	CGGGAAATTC	CGGGAAATTCAAATTTTCAGTGAAAAAATTTTGC
UNSB01.fasta	Cele-UNSB01_2:1881035	2	1881035	1881350	316	5	65	TATCGAGAGA	TATCGAGAGAATGCTCTACATTCACTTTGAATTTCTTTTCCAATTTCAATAAGACTCTCAGTAAG
UNSB01.fasta	Cele-UNSB01_2:1915179	2	1915179	1915855	677	26	26	ATTTACGGAG	ATTTACGGAGCTTCAAGTTAATGTTG
UNSB01.fasta	Cele-UNSB01_2:1984417	2	1984417	1985378	962	31	31	AGAGCTGAGT	AGAGATGAGTTGTCGAGCGAGACCTCAGAAG
UNSB01.fasta	Cele-UNSB01_2:1991206	2	1991206	1991967	762	23	32	AATTCCCGCC	AATTCCCGCCAAAAAATTGTAAAGAAAATTTA
UNSB01.fasta	Cele-UNSB01_2:1992839	2	1992839	1994265	1427	46	31	AGAGCTGAGT	AGAGATGAGTTGTCGAGCGAGACCTCAGAAG
UNSB01.fasta	Cele-UNSB01_2:2000093	2	2000093	2000918	826	25	32	AATTCCCGCC	AATTCCCGCCAAAAAATTTTAAAGAAAATTTA
UNSB01.fasta	Cele-UNSB01_2:2015956	2	2015956	2016256	301	25	12	ATGTATATGT	ATGTATATGTAT
UNSB01.fasta	Cele-UNSB01_2:2017071	2	2017071	2017146	76	5	15	TCGTGGCGAG	TCGTTGCGAGACCCT
UNSB01.fasta	Cele-UNSB01_2:2017169	2	2017169	2017519	351	10	32	GGCGGGAATT	GGCGGGAATTCAAATTTTCTTTGAAAATTTTT
UNSB01.fasta	Cele-UNSB01_2:2023342	2	2023342	2024644	1303	42	31	GACAACTCAG	GACAACTCATCTCTTCTCTGCTATCTCGCTA
UNSB01.fasta	Cele-UNSB01_2:2065528	2	2065528	2066342	815	5	162	AGTTTTATGA	AGTTTTATGAAATAAAAAAGAATGACTCTATATGATTTTCCCATACTTTTGAATTTTTGAAAAAATCAGCTTTTATATATTTTTATCCATTTTTAAAAAATACTTCCGTACAAACTATTATACTTTGTTTAGCATTTTCTGGTATTTTTGACTATGCTGAAG
UNSB01.fasta	Cele-UNSB01_2:2125481	2	2125481	2125594	114	5	22	TGGTCAGTGC	TGGTCAGTGCAAACAATTTTAT
UNSB01.fasta	Cele-UNSB01_2:2168167	2	2168167	2168227	61	4	11	ACCGCAACGC	ACCGCAACGCA
UNSB01.fasta	Cele-UNSB01_2:2174137	2	2174137	2174267	131	5	26	TCAAGGCGAA	TCAAGGCGAAACGGGAGGTGAATCGC
UNSB01.fasta	Cele-UNSB01_2:2187430	2	2187430	2189107	1678	38	43	CGAGTTTGGC	CGAGTTTGGCCTACAGCAAATTTGGCAAAATCGGCAAATTTGC
UNSB01.fasta	Cele-UNSB01_2:2291158	2	2291158	2291371	214	8	21	TGTGCCCTGG	TGTGCCCTGGAAAGCTGGTGG
UNSB01.fasta	Cele-UNSB01_2:2296694	2	2296694	2296754	61	4	10	CAGCAGAGTG	CAGCAGAGTG
UNSB01.fasta	Cele-UNSB01_2:2366031	2	2366031	2366422	392	13	11	CTACAGTACC	CTACAGTACTC
UNSB01.fasta	Cele-UNSB01_2:2379059	2	2379059	2380853	1795	9	163	TGAATTTTTG	TGAATTTTTGAATATATCAGCTTTTGGTTAAATTCTGCATTTTTTAAAAATAAACTTCCGTACGAGCTAGCACCTTTTTTTAAGTATTTTCTGGTACATTTGACTATGCTGAACAGTTTTATAAAATAAAAAAGAATGACTTCATATAATTTTGAGGTACTTT
UNSB01.fasta	Cele-UNSB01_2:2403604	2	2403604	2403679	76	5	15	TCGTGGCGAG	TCGTTGCGAGACCCT
UNSB01.fasta	Cele-UNSB01_2:2547654	2	2547654	2548528	875	24	35	CCGGAAATTT	CCGGAAATTTTCATTTTCAACGATTTGCCGATTTG
UNSB01.fasta	Cele-UNSB01_2:2568294	2	2568294	2568366	73	6	12	TTAGGCTTAG	TTAGGCTTAGGC
UNSB01.fasta	Cele-UNSB01_2:2602717	2	2602717	2603767	1051	24	31	CTGAAATTCC	CTGAAATTCCAATTTTCCACAGTAAAAATGC
UNSB01.fasta	Cele-UNSB01_2:2619434	2	2619434	2619682	249	8	31	AGAGCTGAGT	AGAGATGAGTTGTCGAGCGAGACCTCAGAAG
UNSB01.fasta	Cele-UNSB01_2:2625510	2	2625510	2625733	224	7	32	AATTCCCGCC	AATTCCCGCCAAAAAATTTTAAAGAAAATTTA

UNSB01.fasta	Cele-UNSB01_2:2625547	2	2625547	2626102	556	7	32	CCGCCAAAAA	CCGCCAAAAATTTTCAGAGAAAATTTGAATTC
UNSB01.fasta	Cele-UNSB01_2:2648748	2	2648748	2648862	115	6	19	AAAATCGATA	AAAATCAATAGTTTCATCG
UNSB01.fasta	Cele-UNSB01_2:2655599	2	2655599	2656120	522	10	14	CTACAAACTA	CTACAACCTATAAA
UNSB01.fasta	Cele-UNSB01_2:2657840	2	2657840	2658242	403	31	13	TAAGCCTAAG	TAAGCCTAAGCTC
UNSB01.fasta	Cele-UNSB01_2:2659185	2	2659185	2659251	67	5	12	CCTAAGCCTA	CTTAAGCCTATG
UNSB01.fasta	Cele-UNSB01_2:2738496	2	2738496	2739194	699	7	95	TTCGGGCCTG	TTCGGGCCTGCGGCCCTCAAACCTGGTTTGTAGGCTTGAGCGAATAACAATCTGCTTGGAATAGGATTAAAGTTGGCCCACAGTTTCCAAGAGTG
UNSB01.fasta	Cele-UNSB01_2:2756689	2	2756689	2756749	61	4	12	GCCTAAGCCT	GCCTAAGCCTAA
UNSB01.fasta	Cele-UNSB01_2:2758878	2	2758878	2759540	663	24	22	ACTACAGTAA	ACTACAGTAATTCTACGTCGCT
UNSB01.fasta	Cele-UNSB01_2:2787652	2	2787652	2787836	185	11	16	CACGTAGGCA	CACGTAGGCAAGTAGG
UNSB01.fasta	Cele-UNSB01_2:2836927	2	2836927	2837967	1041	6	144	TGGCCTAGAA	TGGCCTAGAAATCCAAGTTTGCAAAAGTTAGGCCATCAACTTCAAATGCCTATAACTTTGCGGAAAATCTATATTTCAAATTAATTTTTGAACTTCTGTGATGCCATTAACATTGCGCATTTTTTTGGCCTAGTTAACGTTTGA
UNSB01.fasta	Cele-UNSB01_2:2842834	2	2842834	2842918	85	6	12	CCTAAGCCTA	CCTAAGTCTAAG
UNSB01.fasta	Cele-UNSB01_2:2923591	2	2923591	2929183	5593	231	21	CCAAAAATTG	CCAAAAATTGAGATTTTTAGT
UNSB01.fasta	Cele-UNSB01_2:2979318	2	2979318	2980713	1396	9	155	TCCCGCTGTG	TCCCGCTGTGGTTAAAAGTTTTTTGCGTGCTCAGTTCTGTACGGTAAATGTGTTAAAAACTTCTGGAAACTTGAAATTCCAAGTTATTCAGAAATTTGTCCAAATGGCGCAGTGGGAAATTCGATTTCTCTGACCCAAGAGGCCACGGG<br>TTCAAT
UNSB01.fasta	Cele-UNSB01_2:2986853	2	2986853	2986937	85	7	12	ATAGGCATAG	ATAGGCATAGGC
UNSB01.fasta	Cele-UNSB01_2:2995760	2	2995760	2997536	1777	53	14	CTACCTGCCT	CTACCTGCTTGTAC
UNSB01.fasta	Cele-UNSB01_2:3009682	2	3009682	3009822	141	5	28	AATGTCGGCT	AATGTCGGCTGCTTCTAGAAACTAAGCA
UNSB01.fasta	Cele-UNSB01_2:3063296	2	3063296	3063566	271	18	14	TTAGGGCCTT	TTAGGGCCTTGGGC
UNSB01.fasta	Cele-UNSB01_2:3065991	2	3065991	3066701	711	18	19	GCCAAGGCAC	GCCAAGGCACTGGAAATGC
UNSB01.fasta	Cele-UNSB01_2:3077809	2	3077809	3078033	225	15	14	CTTATACCTT	CTTATACCTTATAC
UNSB01.fasta	Cele-UNSB01_2:3099938	2	3099938	3100072	135	4	31	ATCGATTTTT	ATCGATTTTTTGGTTAAAAAATCAATAAAAT
UNSB01.fasta	Cele-UNSB01_2:3132033	2	3132033	3132195	163	6	27	GCTGCTGGAG	GCTGCTGGAGGATCAACTTCAACAACT
UNSB01.fasta	Cele-UNSB01_2:3135702	2	3135702	3137322	1621	60	27	TGCTGGAGGA	TGCTGGAGGATCAACTTCAACAACTGC
UNSB01.fasta	Cele-UNSB01_2:3141695	2	3141695	3142468	774	16	43	TTGCCGGAAT	TTGCCGGAGTTGAAAATTTCCTGTAAATCGGCAAACCGGCAAC
UNSB01.fasta	Cele-UNSB01_2:3154398	2	3154398	3154558	161	9	16	TAGGCACGTA	TAGGCACGTAAGCATG
UNSB01.fasta	Cele-UNSB01_2:3155808	2	3155808	3156160	353	18	16	TACGTGCCTA	TACGTGCCTACGTGCC
UNSB01.fasta	Cele-UNSB01_2:3166478	2	3166478	3166668	191	8	12	GGCTTAGGCT	GGCTTAGGCTTA
UNSB01.fasta	Cele-UNSB01_2:3175289	2	3175289	3175339	51	4	10	AACTTTCGAA	AACTTTCGAA
UNSB01.fasta	Cele-UNSB01_2:3182927	2	3182927	3183063	137	8	16	AGGCACGTAG	AGGCACGTAGGCATAT
UNSB01.fasta	Cele-UNSB01_2:3184073	2	3184073	3184138	66	4	14	CCAAGGCCCA	CCAAAGCCCAAGGC

UNSB01.fasta	Cele-UNSB01_2:3220556	2	3220556	3221123	568	21	16	GTAGGCACGT	GTAGGCACGTAGGCAC
UNSB01.fasta	Cele-UNSB01_2:3287465	2	3287465	3288008	544	19	20	GTAAATCTAC	GTAAATCTACACTTGCTTCC
UNSB01.fasta	Cele-UNSB01_2:3312389	2	3312389	3313069	681	42	16	CGTAGGCACG	CGTAGGCACGTGGGCG
UNSB01.fasta	Cele-UNSB01_2:3331473	2	3331473	3331608	136	5	27	TTTACTCTCT	TTTACTCTCTGTGGCTTCCCACTATAT
UNSB01.fasta	Cele-UNSB01_2:3341963	2	3341963	3342088	126	4	22	ATTCTGCGCA	ATTCTGCGCACCCCCTGAGCGG
UNSB01.fasta	Cele-UNSB01_2:3406743	2	3406743	3406803	61	4	15	GGTGAGACCC	GGTGAGACCCATCGC
UNSB01.fasta	Cele-UNSB01_2:3456136	2	3456136	3456276	141	4	28	TGCCTCGCCT	TGCCTCGCCTACGTGCCTATTTCAGGCA
UNSB01.fasta	Cele-UNSB01_2:3460910	2	3460910	3461075	166	5	16	GCACGTAGGC	GCACGTAAGCACGTAG
UNSB01.fasta	Cele-UNSB01_2:3500187	2	3500187	3500295	109	5	18	GGCTTAGGCT	GGCTTAGGCTTAGGCTCA
UNSB01.fasta	Cele-UNSB01_2:3515774	2	3515774	3515844	71	4	12	ATACATATAC	ATACATATACAT
UNSB01.fasta	Cele-UNSB01_2:3535061	2	3535061	3535729	669	54	12	TAGGTATAGG	TAGGTAAAGGCA
UNSB01.fasta	Cele-UNSB01_2:3539245	2	3539245	3539476	232	5	39	AATTTCCCGC	AATTTCCCGCCAAAATTGAATTTTTAAAAGAAAATTTA
UNSB01.fasta	Cele-UNSB01_2:3540761	2	3540761	3541492	732	18	31	GGTTACTGTA	GGTTACTGTAACTCAAGCCTGAGATTCTTCG
UNSB01.fasta	Cele-UNSB01_2:3557247	2	3557247	3557399	153	9	16	GTAGGCACGT	GTAGGTACGTAGGCAC
UNSB01.fasta	Cele-UNSB01_2:3617397	2	3617397	3617684	288	6	42	TTTGAAAACC	TTTGAAAACCGGCAATTGCCGAAATTGTCGAGTGCCGGAAAT
UNSB01.fasta	Cele-UNSB01_2:3687013	2	3687013	3687103	91	5	20	CTATCGCCTG	CTATCGCCTGCTATTGCCTA
UNSB01.fasta	Cele-UNSB01_2:3736361	2	3736361	3736609	249	8	31	GCTGATTGGT	GCTGATTGGTCGGAATAACTCTCCCAGAATC
UNSB01.fasta	Cele-UNSB01_2:3799242	2	3799242	3799338	97	4	24	TGCATCTGCC	TGCATCTGCCGCTGGAGTTGTTGC
UNSB01.fasta	Cele-UNSB01_2:3989433	2	3989433	3989722	290	6	45	AAGGATCAGA	AAGGATCAGATCATTAGGAGGTACCCTTCTGATCCTTCGGAGCTG
UNSB01.fasta	Cele-UNSB01_2:3993365	2	3993365	3994945	1581	20	79	GTGAATCTCC	GTGAATCTCCTCTCCACCTAATGATTCCTGACAGAATACCGGCAGTATTCTGGGGTCTCATTAGCTTTTAGTTTCTTCT
UNSB01.fasta	Cele-UNSB01_2:4043072	2	4043072	4059727	16656	833	20	ATTGTTACAC	ATTGTTACACGAAAAAATAG
UNSB01.fasta	Cele-UNSB01_2:4095823	2	4095823	4096643	821	4	16	TGCCTACGTG	TGCCTACGTGCCTACG
UNSB01.fasta	Cele-UNSB01_2:4119390	2	4119390	4119751	362	4	21	CTACGTAAGA	CTACGTAAGAGTAGCGCAGGA
UNSB01.fasta	Cele-UNSB01_2:4146856	2	4146856	4147072	217	14	12	CTTAGGCTTA	CTTAGGTTTAGA
UNSB01.fasta	Cele-UNSB01_2:4159337	2	4159337	4159593	257	13	11	GTACTGTAGG	GTACTGTAGGA
UNSB01.fasta	Cele-UNSB01_2:4203805	2	4203805	4204480	676	4	11	TGCCTGCCTA	TGCCTGCCTAC
UNSB01.fasta	Cele-UNSB01_2:4205915	2	4205915	4205959	45	4	11	GGCAGGCAGT	GGCAGGCAGTA
UNSB01.fasta	Cele-UNSB01_2:4270872	2	4270872	4271040	169	5	24	GTGAGGTACG	GTGAGGTACGAGATGAGGTACAAG
UNSB01.fasta	Cele-UNSB01_2:4272676	2	4272676	4272936	261	25	10	GCGTTAGCAG	GCGTTAGCAG

UNSB01.fasta	Cele-UNSB01_2:4275898	2	4275898	4276001	104	5	18	TGCAAGACTA	TGCAAGACTAATAGAGAC
UNSB01.fasta	Cele-UNSB01_2:4325046	2	4325046	4325721	676	27	25	GAGAGTAAAA	GAGAGTAAAAATATACGGTGAAGCCA
UNSB01.fasta	Cele-UNSB01_2:4325748	2	4325748	4326453	706	45	15	CTCACCACGA	CTCACCACGAAGGGT
UNSB01.fasta	Cele-UNSB01_2:4361834	2	4361834	4362159	326	8	35	TTCCGGCAAA	TTCCGGCAAAACGGCAAATTGTCAAAATTGAAAAT
UNSB01.fasta	Cele-UNSB01_2:4377229	2	4377229	4377482	254	23	11	CAGTTGTCAC	CAGTTATCACC
UNSB01.fasta	Cele-UNSB01_2:4380400	2	4380400	4380472	73	4	12	AGCCTAAGCC	AGCCTTAGCCTA
UNSB01.fasta	Cele-UNSB01_2:4433247	2	4433247	4433329	83	4	12	TAAGCCTAAG	TAAGCCTAAGAC
UNSB01.fasta	Cele-UNSB01_2:4510577	2	4510577	4510967	391	36	11	TGCACATTTT	TGCACATTTTT
UNSB01.fasta	Cele-UNSB01_2:4622617	2	4622617	4623337	721	18	40	TCCAGAACCT	TCCAGAACCTTCTGGAAAATCCGAGAAAATTCTGGAATGT
UNSB01.fasta	Cele-UNSB01_2:4623470	2	4623470	4624110	641	20	32	CGGGAATTCA	CGGGAATTCAAAATTTCTGTGAAAAATTTTGG
UNSB01.fasta	Cele-UNSB01_2:4637434	2	4637434	4637644	211	5	35	TTTGCCGGAA	TTTGCCAGAAAATTTCATTTTCGGCAAATTACCGA
UNSB01.fasta	Cele-UNSB01_2:4751417	2	4751417	4751507	91	6	15	GAGACCCATC	GAGACCCAGCGTGGT
UNSB01.fasta	Cele-UNSB01_2:4751413	2	4751413	4751956	544	21	15	TGGTGAGACC	TGGCGAGACCCAGCG
UNSB01.fasta	Cele-UNSB01_2:5140446	2	5140446	5146375	5930	77	76	CAATAGAAAA	CAATAGAAAAGATGGAAGACTACAGAAAAACTATTGAGAGACCCCGAAATACTGGCTGTATTTTGAAAGGTCATCT
UNSB01.fasta	Cele-UNSB01_2:5166221	2	5166221	5171016	4796	184	26	TAGACCATTA	TAGACCATTAGATGAATTCCCAAAGT
UNSB01.fasta	Cele-UNSB01_2:5217429	2	5217429	5217681	253	9	25	AGAGTAAAAT	AGAGTAAAATAATTGGTGAAGCCAG
UNSB01.fasta	Cele-UNSB01_2:5217526	2	5217526	5241162	23637	927	25	AGCCAGAGAG	AGCCACAGAGTAAAATAATTGGGAA
UNSB01.fasta	Cele-UNSB01_2:5722242	2	5722242	5722548	307	9	34	GGAATTGAAA	GGAATTGAAATTTCCAGCAAATCGGCAAATTGCC
UNSB01.fasta	Cele-UNSB01_2:5970897	2	5970897	5971730	834	4	203	GTTCAAGCAG	GTTCAAGCAGAACAAGTAGATCACATTTTGAGATTGAATAGAATAAACGAGTTATATGCAAAATTAGTAGACTTTCCAAACTTTGAAAATTAATATCTGGCTTCAAACTAACTTTTCTGAAAATCCCCGTAGCCATTTTGTTGCAAATTTTGTGCTCTTTCAGAATTTAGAAACGGCATTCCAAACATTTCCATATTCGCCAG
UNSB01.fasta	Cele-UNSB01_2:6168224	2	6168224	6172025	3802	21	181	TTTTTAACTT	TTTTTAACTTTTAAATCCACTAAAAATTGGAATGGTACAGAACATGAAAATTGTAATTTTAAAAACTGAATGTGGTTTCGAATATTTATGGATTCCTTTATTCTAAAACTGACTCTGAAATATTTTGCATCCATTTGACTATTAATTGCCATATTATACCGAACACTACTAACTTTACTGG
UNSB01.fasta	Cele-UNSB01_2:6176621	2	6176621	6177425	805	18	45	CTGAAGGATC	CTGAAGGATCAGACCATCAGGAGGTACCCTTACGATCCTTCTTAT
UNSB01.fasta	Cele-UNSB01_2:6280573	2	6280573	6282049	1477	96	14	CTATACGCTA	CTATACGCTATACG
UNSB01.fasta	Cele-UNSB01_2:6282823	2	6282823	6283646	824	34	10	TCCCGATCCA	TCCCGATCCA
UNSB01.fasta	Cele-UNSB01_2:6392491	2	6392491	6392539	49	4	12	CCACTGACCA	CCACTGACCATC
UNSB01.fasta	Cele-UNSB01_2:6392670	2	6392670	6395650	2981	44	68	AATAAAAATT	AATAAAAATTTAAAATTTTTATTAGTGTCTCTATTTTATCAAGAAAATACATTTTCAAAATAGAGACG
UNSB01.fasta	Cele-UNSB01_2:6532865	2	6532865	6533025	161	5	32	AAAAATTTTC	AAAAATTTTCACAGAAAATTTGAATTCCCGCC
UNSB01.fasta	Cele-UNSB01_2:6532865	2	6532865	6533618	754	6	32	AAAAATTTTC	AAAAATTTTCACAGAAAATTTGAATTCCCGCC
UNSB01.fasta	Cele-UNSB01_2:7497139	2	7497139	7497311	173	4	41	AAAACGGGAA	AAAACGGGAAAACGGGAATTCCCGTTCCCGTGAAAATTTTA

UNSB01.fasta	Cele-UNSB01_2:7497138	2	7497138	7497671	534	10	41	AAAAACGGGA	AAAAACGGGAAAACGGGAATTCCCGTTCCCGTGAAAATTTT
UNSB01.fasta	Cele-UNSB01_2:7564245	2	7564245	7565607	1363	34	14	GAAGGATCGG	GAAGGATCGGGCCG
UNSB01.fasta	Cele-UNSB01_2:7566750	2	7566750	7567567	818	42	16	CGGAAAAATT	CGGAAAACTTTCGCTG
UNSB01.fasta	Cele-UNSB01_2:7568262	2	7568262	7569624	1363	34	14	CCGATCCTTC	CCGATCCTTCCGAC
UNSB01.fasta	Cele-UNSB01_2:7569779	2	7569779	7570282	504	27	10	TCCATCCCGA	TCCATCCCAC
UNSB01.fasta	Cele-UNSB01_2:7594307	2	7594307	7594367	61	4	15	GTAGATCTTG	GTAGATCTTGAAAAT
UNSB01.fasta	Cele-UNSB01_2:8013550	2	8013550	8013642	93	6	15	CGAAAAGGGG	CGAAAAGGGGATCTG
UNSB01.fasta	Cele-UNSB01_2:8013818	2	8013818	8013910	93	6	15	CCCCTTTTCG	CCCCTTTTCGTATCT
UNSB01.fasta	Cele-UNSB01_2:8406477	2	8406477	8418390	11914	144	79	TGACAGAATA	TGACAGAATACCGACAGTATTTTGGGGTCTCATTAGGTTTCCTTTTCTTTTCTGAATCTCCACTCTTCCTAATGAGTCC
UNSB01.fasta	Cele-UNSB01_2:8585644	2	8585644	8586044	401	10	40	AAAGTTCTGG	AAAGTTCTGGAACATTCCAAAAATTTCCCAATTTTTCTAG
UNSB01.fasta	Cele-UNSB01_2:8586336	2	8586336	8586411	76	4	15	GGGTCTCACC	GGGTCTCACCACGAC
UNSB01.fasta	Cele-UNSB01_2:9101090	2	9101090	9103530	2441	29	86	AGCCAGTATT	AGCCAGTATTTCGGGGTCTGGCAATAGCTTCTCTTTAACTTCGAACTCTTCCATCTTTTCCCTATTGCCACGACCTTTCAAAATAC
UNSB01.fasta	Cele-UNSB01_2:9328143	2	9328143	9328548	406	27	15	TGGTGAGACC	TGGTGAGACCCTTCG
UNSB01.fasta	Cele-UNSB01_2:9328575	2	9328575	9329101	527	15	35	ATTCAAATTT	ATTCAAATTTTCAGTGAAAAAAATTTTGGCGGGAA
UNSB01.fasta	Cele-UNSB01_2:9328566	2	9328566	9329542	977	16	35	TGGCGGGAAA	TGGAGGGAAATTCAAATTTTCAGTGAAAAAAATTT
UNSB01.fasta	Cele-UNSB01_2:9530992	2	9530992	9537371	6380	247	25	CTCTCTGGCT	CTCTCCGGCTTCACAGTATATTTTA
UNSB01.fasta	Cele-UNSB01_2:9763711	2	9763711	9764616	906	23	40	TTCCAGAATT	TTCCAGAATTTTCTCGATTTTTCTAGAAAGTTCTGGAACA
UNSB01.fasta	Cele-UNSB01_2:9764656	2	9764656	9765286	631	18	35	CCAAAATATT	CCAAAATATTTTTTTCAGAAAATTTAAATTTCCCT
UNSB01.fasta	Cele-UNSB01_2:9765369	2	9765369	9765639	271	17	15	CCACGATGGG	CCACGAAGGGTATCA
UNSB01.fasta	Cele-UNSB01_2:9765885	2	9765885	9766019	135	5	11	GTACTCCTAC	GTACTCCTACA
UNSB01.fasta	Cele-UNSB01_2:9825153	2	9825153	9825520	368	4	42	CAGCCGACAG	CAGCCGACAGCCGAAAAACTTGGAATTTTCGGCTGGTAGCAC
UNSB01.fasta	Cele-UNSB01_2:10367690	2	10367690	10367782	93	6	15	TCCCCTTTTC	TCCCCTTTTCGTATC
UNSB01.fasta	Cele-UNSB01_2:10468271	2	10468271	10470517	2247	10	225	TTTTCGAAAT	TTTTCGAAATGTCTTTCAGAAACTTTGTAGTAAATTTTAAGCTCTTTCAGATTATATTAACAATATTCCAGTAGGTACAAGAAGCTTCAAGTAGTTACAGAAATAGTACATTTTCAGCCCTACCTTTTAGTGCGTATTTTATTATTAATGAAAACTACTATGTATAGGCAAAAATAGATGGATTTTACAAACTTTGAAAATTCATAAACCTCTTCAAAGTAACTG
UNSB01.fasta	Cele-UNSB01_2:10597740	2	10597740	10597833	94	5	18	TAAATAAGTG	TAAATAAGTGGCTATAAG
UNSB01.fasta	Cele-UNSB01_2:10844890	2	10844890	10845587	698	8	35	TTTCTGGCAA	TTTCTGGCAAACCGGCAATTTGCCGAAAATTAAAA
UNSB01.fasta	Cele-UNSB01_2:10964073	2	10964073	10965096	1024	24	11	GTACTGTAGG	GTACTGTAGTA
UNSB01.fasta	Cele-UNSB01_2:11255667	2	11255667	11256407	741	61	12	AAAAATAAAA	AAAAATAAAAAT
UNSB01.fasta	Cele-UNSB01_2:11255666	2	11255666	11256503	838	64	12	TAAAAATAAA	TAAAAATAAAAA
UNSB01.fasta	Cele-UNSB01_2:11256637	2	11256637	11258512	1876	15	124	AATTTTCAAA	AATTTTCAAAGTTTGGAAAATCTATTAATTTTACATATAAATTGTGGTCTACAAGGTATGACTGAAAATTCACTTTTTCTGCTTGAACTTGGCGGTTTCAAAATTTTTTGAAATTCTGTTTTTA

UNSB01.fasta	Cele-UNSB01_2:11366885	2	11366885	11367108	224	5	44	AAGGATCAGA	AAGGATCAGATCATCAGGCAGAGCCTCATGCTCTTTTGGAGCTG
UNSB01.fasta	Cele-UNSB01_2:11443617	2	11443617	11443668	52	4	12	ACTATAACTA	ACTACAACTACA
UNSB01.fasta	Cele-UNSB01_2:11502184	2	11502184	11502280	97	5	24	GCTTAGGCTT	GCTTAGGCTTAGGCTTGGGCTCAA
UNSB01.fasta	Cele-UNSB01_2:11560093	2	11560093	11560292	200	9	20	AAAAAATAGA	AAAAAATAGATTGGCATACG
UNSB01.fasta	Cele-UNSB01_2:11664501	2	11664501	11665796	1296	29	45	TCTGATCCTT	TCTGATCCTTCAGATAAGAAGAATCGTAAGGGTACCTCCTGATGG
UNSB01.fasta	Cele-UNSB01_2:11667411	2	11667411	11669597	2187	49	45	GAAGGATCAG	GAAGTATCAGACCATCAGGAGGTACCCTTACGATCCTTCTTATCT
UNSB01.fasta	Cele-UNSB01_2:11673801	2	11673801	11673893	93	6	15	CGAAAAGGGG	CGAAAAGGGGATCTG
UNSB01.fasta	Cele-UNSB01_2:11674068	2	11674068	11674143	76	5	15	TCCCCTTTTC	TCCCCTTTTCGTATC
UNSB01.fasta	Cele-UNSB01_2:11799205	2	11799205	11799469	265	8	33	AATTGAAAAT	AATTGAAAATCCGGCAAATGGGCAAATTGCCGT
UNSB01.fasta	Cele-UNSB01_2:11872286	2	11872286	11872364	79	6	12	CTTAGGCTTA	CTTAGGCTTAGG
UNSB01.fasta	Cele-UNSB01_2:12045935	2	12045935	12046042	108	8	12	AGGCTTAGGC	AGGCTTAAGCTT
UNSB01.fasta	Cele-UNSB01_2:12047204	2	12047204	12047947	744	32	20	TAAATCTACA	TAAATCTACACAGGGTGGTG
UNSB01.fasta	Cele-UNSB01_2:12070558	2	12070558	12070690	133	10	12	AAGCCTAAGC	AAGCCTAACCAT
UNSB01.fasta	Cele-UNSB01_2:12072605	2	12072605	12072779	175	10	12	AGGCTTAGGC	AGGCTTAGACTT
UNSB01.fasta	Cele-UNSB01_2:12142720	2	12142720	12142795	76	4	15	AAAACAAGAT	AAAAGAAGATCTACA
UNSB01.fasta	Cele-UNSB01_2:12311843	2	12311843	12313532	1690	56	30	CAAACTACAG	CAAACTACAGTAGCACTGTAGTACCATAAC
UNSB01.fasta	Cele-UNSB01_2:12381269	2	12381269	12381383	115	7	12	TTAGTCTTAG	TTAGTCTTAGGC
UNSB01.fasta	Cele-UNSB01_2:12388949	2	12388949	12389045	97	7	12	CCTAAGCCTA	CCTAACCCTAAG
UNSB01.fasta	Cele-UNSB01_2:12463695	2	12463695	12463773	79	5	12	TTAGGCTTAG	TTAGGCTCAGGC
UNSB01.fasta	Cele-UNSB01_2:12477614	2	12477614	12477893	280	4	69	AGGTAGGCCT	AGGTAGGCCTGTAGGCATTGACTGGCAGAAAAGTGTAGGCAGGCAGGCAGGTAAAAAGATACATGCGGC
UNSB01.fasta	Cele-UNSB01_2:12492091	2	12492091	12492147	57	5	11	CTACAGTACC	CTACAGTACCC
UNSB01.fasta	Cele-UNSB01_2:12494810	2	12494810	12494978	169	14	12	CCTATACCTA	CCTATACCTATA
UNSB01.fasta	Cele-UNSB01_2:12522288	2	12522288	12522472	185	13	12	AGCCTAAGCC	AGCCTAAGCCTA
UNSB01.fasta	Cele-UNSB01_2:12544046	2	12544046	12545336	1291	30	43	TTTCCGGCAA	TTTCCGGCAAATCGGCAAACCGGCATATTGCCAAAAATAAAAA
UNSB01.fasta	Cele-UNSB01_2:12597085	2	12597085	12597273	189	6	31	GACATCTCGC	GACATCTCGCAGGACAATATAAAAAGCGACC
UNSB01.fasta	Cele-UNSB01_2:12608492	2	12608492	12608559	68	5	12	GGCTTAGGCT	GGCTTAGGCTTA
UNSB01.fasta	Cele-UNSB01_2:12716681	2	12716681	12716766	86	5	17	ACTTGGTACA	ACTTGGTACATAGACCA
UNSB01.fasta	Cele-UNSB01_2:12718309	2	12718309	12718687	379	8	42	TTGCCGAACC	TTGCCGAACCGTAAAAATTTTGGCAACCTGGAACTGCCAAAG
UNSB01.fasta	Cele-UNSB01_2:12791702	2	12791702	12792113	412	11	37	ATTTTGGCGG	ATTTTGGCGGGAATTCAAATTTTAATTTTTTGAAAAT

UNSB01.fasta	Cele-UNSB01_2:12792220	2	12792220	12794215	1996	131	15	TCGTGGTGAG	TCGTGGTGAGACCCA
UNSB01.fasta	Cele-UNSB01_2:12843731	2	12843731	12843956	226	7	11	CTACAGTACC	CTACAGTACTC
UNSB01.fasta	Cele-UNSB01_2:12862834	2	12862834	12863019	186	5	43	TATTTTCATT	TATTTTCATTTTCGGCAAATTGCCGGTTTGCCGATTTGCCGGG
UNSB01.fasta	Cele-UNSB01_2:12892893	2	12892893	12893074	182	4	45	ATCTGATCCT	ATCTGATCCTTCAGCTCCGAAGGATCAGAAGGGTACCTCCTGGTG
UNSB01.fasta	Cele-UNSB01_2:12900948	2	12900948	12901228	281	8	35	ATTTCAGGCA	ATTTCAGGCAATTTACCGATTTGCCAGAAATGTTC
UNSB01.fasta	Cele-UNSB01_2:12905171	2	12905171	12905894	724	9	14	AACTACAAAC	AACTATAAACTACA
UNSB01.fasta	Cele-UNSB01_2:12906532	2	12906532	12906652	121	7	15	ATTTCCGGAT	ATTTCCGGATCTATG
UNSB01.fasta	Cele-UNSB01_2:12914019	2	12914019	12914481	463	11	35	TCAATTCCGG	TCAATTCCGGCAATTTTCCGGTTTGCCGGAAAATT
UNSB01.fasta	Cele-UNSB01_2:12938825	2	12938825	12939101	277	8	35	TGTGTAATAA	TGTGTAATAAGAAAATTTCAAATGTTTTTCACGAG
UNSB01.fasta	Cele-UNSB01_2:12968710	2	12968710	12969129	420	28	12	GGCTTAGGCT	GGCTTAGGCTTA
UNSB01.fasta	Cele-UNSB01_2:12988123	2	12988123	12990065	1943	72	27	GTGGGAAGCC	GTGGGAAGCCACAGAGAGTAAAATATA
UNSB01.fasta	Cele-UNSB01_2:12990090	2	12990090	13000798	10709	416	25	TGGCTTCCCA	TGGCTTCCCACTATATTTTACTCTC
UNSB01.fasta	Cele-UNSB01_2:13043920	2	13043920	13044316	397	21	18	ACTACTGTAT	ACTACTGTATCGCAAGAT
UNSB01.fasta	Cele-UNSB01_2:13060029	2	13060029	13060418	390	5	43	GATTTGCCGG	GATTTGCCGGAAATTTTCAATCCCGGCAAATTGCAGGTTTGCC
UNSB01.fasta	Cele-UNSB01_2:13097657	2	13097657	13097799	143	5	18	CCTAAGCCTA	CCTGAGCCTAAGCTCAAC
UNSB01.fasta	Cele-UNSB01_2:13106081	2	13106081	13106253	173	4	43	CGGCAAATTA	CGGCAAATTACCAATTTGCCGATTTGCCGGAAATTTTCATTTT
UNSB01.fasta	Cele-UNSB01_2:13133064	2	13133064	13133208	145	9	16	GCCTACATGC	GCCTACATGCCTACAT
UNSB01.fasta	Cele-UNSB01_2:13134300	2	13134300	13134450	151	4	30	TTCAGGCAGG	TTCAGGCAGGTATAGGTTTCTTTGAAAATT
UNSB01.fasta	Cele-UNSB01_2:13161799	2	13161799	13161943	145	6	19	TATGGTGCAT	TATGGTGCATTTAACTAGT
UNSB01.fasta	Cele-UNSB01_2:13192718	2	13192718	13192854	137	4	34	TTCAATTCCG	TTCAATTCCGGTAACTCGTCGATTTTTCGGAAAT
UNSB01.fasta	Cele-UNSB01_2:13216705	2	13216705	13216849	145	8	16	TAGGCACGTA	TAGGTACGTAGGCACA
UNSB01.fasta	Cele-UNSB01_2:13339084	2	13339084	13339231	148	7	21	AATCTAGCCA	AATCTAGCCAGACGCACACCT
UNSB01.fasta	Cele-UNSB01_2:13398823	2	13398823	13400207	1385	8	165	AAAATTCAAT	AAAATTCGATTTTGGACTTATCTATTCTCTCTTTTTTATAACATGTATTTCAGTTGATAGAGGACTCAAAATTATAATGCTCGTAATTATTTATAAGTGTGTGTTGTCTCAGAATCAATTCTAAAAAACAGATTTTTTTTGAAAGTTTA AGTAATATCCGCGGAG
UNSB01.fasta	Cele-UNSB01_2:13437444	2	13437444	13437847	404	10	31	CACGCCTATT	CACGCCTATTTTGTGGGAGGAGTCTACAAGC
UNSB01.fasta	Cele-UNSB01_2:13440285	2	13440285	13440859	575	12	48	TGCCTACGTG	TGCCTACGTGCCTGCAAAACAGGCGTGGGCAGCTTTAAAGGTGATGTA
UNSB01.fasta	Cele-UNSB01_2:13459229	2	13459229	13459607	379	13	14	TAGTTTGTAG	TAGTTTGTAGTTTA
UNSB01.fasta	Cele-UNSB01_2:13460078	2	13460078	13460334	257	15	16	GGCATGTAGG	GGAATGTAGGCATGTA
UNSB01.fasta	Cele-UNSB01_2:13465788	2	13465788	13466109	322	20	16	GGCATGTAGG	GGCATATAGGCATGTA
UNSB01.fasta	Cele-UNSB01_2:13474627	2	13474627	13476516	1890	78	20	AAAATTTGAA	AAAATTTGAATTTTTAAGCC

UNSB01.fasta	Cele-UNSB01_2:13490523	2	13490523	13491161	639	5	122	GATCTACAAA	GATCTACAAAAACGCGGGAAAAGTGACGCAGAGTTTTGAACTGATTTTGCATAAAAATTCCCGCATTTTTTGTAGATCAAACCGTTATGGGACAGCCTGGTGCCACGTGCTTGATCTACGCA
UNSB01.fasta	Cele-UNSB01_2:13499104	2	13499104	13499209	106	5	21	CTAGAAAAAC	CTAGAAAAACTCGGCCATCAA
UNSB01.fasta	Cele-UNSB01_2:13500094	2	13500094	13501231	1138	46	21	TTTTCTAGTT	TTTCCTAGTTGATAGCCGAGT
UNSB01.fasta	Cele-UNSB01_2:13509974	2	13509974	13510060	87	6	12	CTTAGGCTTA	CTTAGGCTTAGT
UNSB01.fasta	Cele-UNSB01_2:13528433	2	13528433	13528477	45	4	11	TGGAAAATTG	TGGAAAATTGA
UNSB01.fasta	Cele-UNSB01_2:13541965	2	13541965	13542724	760	8	95	GGCCTGCGGC	GGCCTGCGGCCCTCAAACCTGGTTGGTAGGCTTGAGCGAATAACAATCTGCTTGGAATAGGATTAAAGCTGGCCCTTATTTTTCATGAGTATTCA
UNSB01.fasta	Cele-UNSB01_2:13551182	2	13551182	13551985	804	20	35	GATTTGCCGG	GATTTGCCGGAAATGTTCAATTCCGGCAATTTACT
UNSB01.fasta	Cele-UNSB01_2:13571681	2	13571681	13571955	275	20	11	CCTACAGTAC	CCTATAGTACC
UNSB01.fasta	Cele-UNSB01_2:13579229	2	13579229	13580664	1436	41	35	GAATTTTTGA	GAGTTTTTGATTTTCGGCAATTTGCCAATTTGCCA
UNSB01.fasta	Cele-UNSB01_2:13658533	2	13658533	13659809	1277	26	27	AGCAGCCGAC	AGCAGCCGACGCCTTGCGGGTCGCTCA
UNSB01.fasta	Cele-UNSB01_2:13663688	2	13663688	13663784	97	6	16	ACATGCCTAC	ACATGCCTACATGCCT
UNSB01.fasta	Cele-UNSB01_2:13678541	2	13678541	13678601	61	5	12	AATACCAACC	AATACCAACCAC
UNSB01.fasta	Cele-UNSB01_2:13684820	2	13684820	13685366	547	24	20	GTAAATCGAC	GTAAATCGACAAAAGACATC
UNSB01.fasta	Cele-UNSB01_2:13754985	2	13754985	13755264	280	7	35	TTGCCGGAAT	TTGCCGGAATTGAATATTTGCGGCAAATCTGTAAT
UNSB01.fasta	Cele-UNSB01_2:13755448	2	13755448	13762935	7488	166	45	ACCAGGAGGT	ACCAGGAGGTACTCTTCTTATCCTTCGGAGCTGAATGATCAGATC
UNSB01.fasta	Cele-UNSB01_2:13794379	2	13794379	13794568	190	10	16	CCTACATGCC	CCTACATGCCTACATG
UNSB01.fasta	Cele-UNSB01_2:13805325	2	13805325	13805605	281	7	35	CTTGCCGATT	CTTGCCGATTTGCCGGAAATTTTCAATTCCGGCAA
UNSB01.fasta	Cele-UNSB01_2:13813351	2	13813351	13813423	73	6	12	TAGGCTTAGG	TAGGCTTAGGCT
UNSB01.fasta	Cele-UNSB01_2:13831609	2	13831609	13831953	345	12	27	TTCCGGCAAA	TTCCGGCAAATCGGCAATTTTGAAAAT
UNSB01.fasta	Cele-UNSB01_2:13844810	2	13844810	13844989	180	5	36	TTGCCTAAAA	TTGCCTAAAATTAAAATTTCCGGCAAATCAGCAAAT
UNSB01.fasta	Cele-UNSB01_2:13878619	2	13878619	13878763	145	10	12	GCCTAAGCCT	GCCGAAGCCTAA
UNSB01.fasta	Cele-UNSB01_2:13900624	2	13900624	13901003	380	15	21	CTCGTAAATC	CTCGTAGATCGACAAGAACTC
UNSB01.fasta	Cele-UNSB01_2:13903082	2	13903082	13903160	79	5	13	GGCTTCTAGT	GGCTTCTAGTCTA
UNSB01.fasta	Cele-UNSB01_2:13911573	2	13911573	13912594	1022	28	35	TTTGCCGGAA	TTTGCTGGAAATTTTCAAATCCGGCAAATTGCTGC
UNSB01.fasta	Cele-UNSB01_2:13932545	2	13932545	13934837	2293	68	35	CCGGAATTGA	CCGGAATTGAAAATTTCTGGAAAATCGGCAAATTG
UNSB01.fasta	Cele-UNSB01_2:13962674	2	13962674	13962948	275	23	10	CCGCTACCGC	CCGCTACCGC
UNSB01.fasta	Cele-UNSB01_2:13967191	2	13967191	13967257	67	5	11	CCGCTACCGC	CCGCTACCGCA
UNSB01.fasta	Cele-UNSB01_2:13968253	2	13968253	13968583	331	23	12	GCCTAAGCCT	GCCTAAGCCTAA
UNSB01.fasta	Cele-UNSB01_2:13971711	2	13971711	13972389	679	37	18	AATTTACCAA	AATTTACCAATTTACCAA

UNSB01.fasta	Cele-UNSB01_2:13977826	2	13977826	13977988	163	10	12	GCCTAAGCCT
GCCTAAGCCTTA								
UNSB01.fasta	Cele-UNSB01_2:13981365	2	13981365	13981461	97	7	12	TTAGGCTTAG
TTCGGCTTAGGA								
UNSB01.fasta	Cele-UNSB01_2:14003653	2	14003653	14004477	825	18	34	AATTCCGGCA
AATTCCGGCAACTTGCCTATTTTCCGGAAATTTC								
UNSB01.fasta	Cele-UNSB01_2:14078695	2	14078695	14078860	166	5	33	AAAATTTGAA
AAAATTCGAATTTGGCGCCAAAATTTTTCACAA								
UNSB01.fasta	Cele-UNSB01_2:14106829	2	14106829	14106943	115	7	12	AGCCTAAGCC
ACCTAAGCCTAC								
UNSB01.fasta	Cele-UNSB01_2:14136989	2	14136989	14138428	1440	41	14	TACAAACTAC
TACAAACTACAAAC								
UNSB01.fasta	Cele-UNSB01_2:14141898	2	14141898	14142455	558	7	62	TGCTAACTAG
TGCTAACTAGCGCGGGCCTGCTTAGGTGCCGGCTGCGAAAGCAGTCCTGGTTTAATGTCGGC								
UNSB01.fasta	Cele-UNSB01_2:14148263	2	14148263	14148341	79	6	12	GGCTTTGGCT
GGCTTTGGCTTT								
UNSB01.fasta	Cele-UNSB01_2:14148276	2	14148276	14148504	229	14	12	GCTTAGGCTT
GCTTCGGCTTTG								
UNSB01.fasta	Cele-UNSB01_2:14178132	2	14178132	14178499	368	11	11	GTACTGTAGG
GTACTGTAGGA								
UNSB01.fasta	Cele-UNSB01_2:14178691	2	14178691	14179588	898	30	30	TTTCTAGAAA
TTTCTAGAAACATTTGGAAACTTCTAGAAC								
UNSB01.fasta	Cele-UNSB01_2:14194765	2	14194765	14194942	178	5	14	GTAGTTTGTA
GTAGTTTGTAGCTT								
UNSB01.fasta	Cele-UNSB01_2:14195501	2	14195501	14195591	91	5	12	TAGGCTTAGG
TAGCCTTAGGCC								
UNSB01.fasta	Cele-UNSB01_2:14213145	2	14213145	14213210	66	4	12	TAGGCTTAGG
TAGGCTTAGGTT								
UNSB01.fasta	Cele-UNSB01_2:14289556	2	14289556	14289862	307	8	34	CCGTGCCTAC
CCGTGCCTACTAACCTACCGACCTTGCCTGCCTC								
UNSB01.fasta	Cele-UNSB01_2:14294397	2	14294397	14294650	254	11	18	CTAGGTGCCT
CTAGGTACCTATCCATTC								
UNSB01.fasta	Cele-UNSB01_2:14297875	2	14297875	14298051	177	10	16	TAGGCATATA
TAGGCATATAGGCTTA								
UNSB01.fasta	Cele-UNSB01_2:14303812	2	14303812	14304194	383	12	22	CTACAGTAAC
CTACAGTAACCCTAGTTTTGAA								
UNSB01.fasta	Cele-UNSB01_2:14320079	2	14320079	14320251	173	4	43	GAAAACTAGG
GAAAACTAGGCCACGGAGTTCAAATTGATGGCCGAGAAGTCTA								
UNSB01.fasta	Cele-UNSB01_2:14326178	2	14326178	14326688	511	14	34	CGGAATTGAA
CGGAATTGAAATTTCTGGCAAATTGGTAAAATGC								
UNSB01.fasta	Cele-UNSB01_2:14355758	2	14355758	14355994	237	18	12	ATAGGCATAG
ATAGGCATAGGC								
UNSB01.fasta	Cele-UNSB01_2:14379544	2	14379544	14380198	655	13	13	TATACAAAAA
TATACAAAATGAA								
UNSB01.fasta	Cele-UNSB01_2:14381587	2	14381587	14382260	674	16	42	AAAACCGGCA
AAAACTGGCAATTGCCAAAATTGCCGATTGCCGGGAAGATTG								
UNSB01.fasta	Cele-UNSB01_2:14401364	2	14401364	14401594	231	10	21	CAATTTTCTG
CAATTTTCTGTTTAACTTTGG								
UNSB01.fasta	Cele-UNSB01_2:14428274	2	14428274	14428974	701	18	35	GCAAATCGGC
GCAAATCGGCAAATTGTCGGCATGGAAATTTTCCG								
UNSB01.fasta	Cele-UNSB01_2:14453377	2	14453377	14453692	316	8	35	CGGCAAATCG
CGGCAAATCGGCAAATTGCCGGAACTGTTAATTTC								
UNSB01.fasta	Cele-UNSB01_2:14458676	2	14458676	14459058	383	11	35	CCGGCAAATC
CCGGCAAATGGGCAAATTGCCGAAAATGAAAGCAT								
UNSB01.fasta	Cele-UNSB01_2:14462473	2	14462473	14462688	216	5	35	CGGCAAATTG
CGGCAAATCGGCTAATTGCCGAAATTGAAAATTTC								
UNSB01.fasta	Cele-UNSB01_2:14464146	2	14464146	14465077	932	26	32	AACCAATCAG
AACCAATCAGCGATGGGCTCCACCCTTTTTGA								

UNSB01.fasta	Cele-UNSB01_2:14513028	2	14513028	14524678	11651	63	185	CTCGGACATA	CTCGGACATAATTGGTGTTCTGTATTCACTTATGATACGTTCGGCTGCGTACGTTGGCCAAGAGTGTTCTAGTAAGAGAATTTCATTTAGAATATGAACAATTTGGGTAATTGGAAAGTGTCTTCGGGAATCTTTATACATCCGACAAA<br>TTCGAGTATGTTAATATTCTGAATTGGATTCTTTTT
UNSB01.fasta	Cele-UNSB01_2:14526196	2	14526196	14534994	8799	53	166	TCGTAATTAT	TCGTAATTATTCATAAGTTTGTGTTGTCTCAGAGCCATGTCTGAAAAAACAGATTTTTTTCGAAAATTTTAAAAAGTTCGTAAAAATTGGATTTTTGACAAATCTATGCTCTCTTTTTTTGTTTGAAATTTAGTTCAGTGACATACAA<br>TTTCAAAAATATAATGT
UNSB01.fasta	Cele-UNSB01_2:14535014	2	14535014	14566604	31591	1170	27	TGTGGCTTCC	TGTGGCTTCCCACTATATTTTACTCTC
UNSB01.fasta	Cele-UNSB01_2:14573634	2	14573634	14573711	78	5	15	CGAAAAGGGG	CGAAAAGGGGATCTG
UNSB01.fasta	Cele-UNSB01_2:14573886	2	14573886	14573978	93	6	15	TCCCCTTTTC	TCCCCTTTTCGTATC
UNSB01.fasta	Cele-UNSB01_2:14582719	2	14582719	14582837	119	5	18	TAGTCTTGCA	TAGTCTTGCAGCCTCTAT
UNSB01.fasta	Cele-UNSB01_2:14966230	2	14966230	14966326	97	6	16	GCAGTTTGCC	GCCGTTTGCCGAATTG
UNSB01.fasta	Cele-UNSB01_2:15007973	2	15007973	15008319	347	4	35	TCCGGCAAAT	TCCGGCAATTTTCCGATTTGCCGGAAACTTTCAAT
UNSB01.fasta	Cele-UNSB01_2:15030456	2	15030456	15030601	146	7	21	ATTGGATTAG	ATTCGATTAGATTTCGTCCAT
UNSB01.fasta	Cele-UNSB01_2:15064033	2	15064033	15064173	141	5	30	TTCTGGAAAA	TTCTGGAAAATTCTAAAAAGCTTTAGAACC
UNSB01.fasta	Cele-UNSB01_2:15064232	2	15064232	15064367	136	9	15	AGACCCATCG	AGGCCCATCGTTGTG
UNSB01.fasta	Cele-UNSB01_2:15064421	2	15064421	15065299	879	25	35	TTAAATTTTC	TTAAATTTTCTGAAAAAAATATTTTGGAGGGAAAT
UNSB01.fasta	Cele-UNSB01_2:15097668	2	15097668	15098195	528	13	35	TTTCCGGCAA	TTTCCGGCAAATCGGCGAATTGCATTAATTTAAAA
UNSB01.fasta	Cele-UNSB01_2:15105694	2	15105694	15106081	388	11	35	CAGAAAATTT	CAGAAAATTTAAATTTCCCGCCAAAATATTTTTCA
UNSB01.fasta	Cele-UNSB01_2:15106134	2	15106134	15106254	121	7	15	CCACGATGGG	CCACGATGGGTCTCA
UNSB01.fasta	Cele-UNSB01_2:15106314	2	15106314	15106454	141	5	30	AATTTTCCAG	AATTTTCCAGAAAGTTCTGGAACATTCCAG
UNSB01.fasta	Cele-UNSB01_2:15121230	2	15121230	15121314	85	6	12	TTAGGCTTAG	TTAGGCTAAGGT
UNSB01.fasta	Cele-UNSB01_2:15182818	2	15182818	15182878	61	5	12	AAGCCTAAGC	AAGCCCAAGCCT
UNSB01.fasta	Cele-UNSB01_2:15212191	2	15212191	15212515	325	14	20	TTCAGTCAAA	TTCAGTCAAAATTTAGATTT
UNSB01.fasta	Cele-UNSB01_2:15219171	2	15219171	15219261	91	6	15	TCGTGGCGAG	TCGTTGCGAGACCCT
UNSB01.fasta	Cele-UNSB01_2:15219280	2	15219280	15219502	223	7	32	TTTTGGCGGG	TTTCGGCGGGAATTCAAATTTTCTGTGAAAAT
UNSB01.fasta	Cele-UNSB01_2:15221118	2	15221118	15221238	121	8	15	GGTGAGACCC	GGTGAGACCCATCGT
UNSB01.fasta	Cele-UNSB01_2:15221118	2	15221118	15221591	474	14	15	GGTGAGACCC	GGTGAGACCCATCGT
UNSB01.fasta	Cele-UNSB01_2:15221267	2	15221267	15222837	1571	42	32	TTTTGGCGGG	TTTTGGCGGGAATTCAAATTTTCTCTGAAAAT
UNSB01.fasta	Cele-UNSB01_2:15224452	2	15224452	15224572	121	8	15	GGTGAGACCC	GGTGAGACCCATCGT
UNSB01.fasta	Cele-UNSB01_2:15224452	2	15224452	15225368	917	26	15	GGTGAGACCC	GGTGAGACCCATCGT
UNSB01.fasta	Cele-UNSB01_2:15224601	2	15224601	15228185	3585	96	32	TTTTGGCGGG	TTTTGGCGGGAATTTAAATTTTCTTTGAAAAT
UNSB01.fasta	Cele-UNSB01_2:15229801	2	15229801	15229921	121	8	15	GGTGAGACCC	GGTGAGACCCATCGT

UNSB01.fasta	Cele-UNSB01_2:15229801	2	15229801	15230289	489	15	15	GGTGAGACCC	GGTGAGACCCATCGT
UNSB01.fasta	Cele-UNSB01_2:15229950	2	15229950	15234144	4195	125	32	TTTTGGCGGG	TTTTGGCGGGAATTCAAATTTTCTTTGAAAAT
UNSB01.fasta	Cele-UNSB01_2:15235758	2	15235758	15235848	91	6	15	GGTGAGACCC	GGTGAGACCCATCGT
UNSB01.fasta	Cele-UNSB01_2:15235864	2	15235864	15236243	380	11	34	TTGGCGGGAA	TTGGCGGGAAATTCAAATTTTCAGTGAAAAAATT
UNSB01.fasta	Cele-UNSB01_2:15242777	2	15242777	15242951	175	9	15	AGACCCATCG	AGACCCATCGTGGCG
UNSB01.fasta	Cele-UNSB01_2:15242891	2	15242891	15243160	270	5	35	ATTTTGGCGG	ATTTTGGCGGGTAATTTAAATATTCGGAGAAAAAT
UNSB01.fasta	Cele-UNSB01_2:15286316	2	15286316	15286491	176	5	35	TTTGCCGGAA	TTTGCCGGAAATTTTAAATTCTGGCAATTTGCCGA
UNSB01.fasta	Cele-UNSB01_2:15290141	2	15290141	15290702	562	16	35	TTTGCCGGAA	TTTGCCGGAAAGTTTCAATTCAGGCAACTTGTCGT
UNSB01.fasta	Cele-UNSB01_2:15354583	2	15354583	15354863	281	8	35	TTTGCCGGAA	TTTGCCGGAAATTTTCAATTCCGGCAATATACTGA
UNSB01.fasta	Cele-UNSB01_2:15381973	2	15381973	15383739	1767	38	15	GGGTCTCACC	GGGTCTCACCACAAC
UNSB01.fasta	Cele-UNSB01_2:15429658	2	15429658	15431122	1465	9	163	AAATAAAAAA	AAATAAAAAAGAATGACTCCATATGATTTTCCCATACTTTTGAATTTTTGAAAAAATCAGCTTTTATATATTTTTATCAATTTTTAAAAAAATACTTCCGTACAAGCTAATATACTTTGATTGGCATATTCTGGTAATTTTAACTATGCTGAACAGTTTTATG
UNSB01.fasta	Cele-UNSB01_2:15432049	2	15432049	15432695	647	18	34	TTTCAATTCC	TTTCAATTCCGGCAATTTGTCGATTTGCCGGAAA
UNSB01.fasta	Cele-UNSB01_2:15437952	2	15437952	15438032	81	4	20	TGTAGATTTA	TGTAGATTTACGCGGGATGG
UNSB01.fasta	Cele-UNSB01_2:15454038	2	15454038	15454614	577	12	35	CCGATTTGCC	CCGATTTTCCGGAAATTTCGAATTCCGGCAACTTC
UNSB01.fasta	Cele-UNSB01_2:15454990	2	15454990	15455468	479	9	35	TTGCCGGAAT	TTGCCAGAATTGAAAAATTCTGGCAAACCGCCAAA
UNSB01.fasta	Cele-UNSB01_2:15522229	2	15522229	15522469	241	12	23	GCTACAGTAC	GCTACAGTACCCCAGCTTGACTT
UNSB01.fasta	Cele-UNSB01_2:15522855	2	15522855	15525136	2282	188	12	TTAGGCTTAG	TTAGGCTTAGGC
UNSB01.fasta	Cele-UNSB01_3:11	3	11	3196	3186	261	12	TAAGCCTAAG	TAAGCCTAAGCC
UNSB01.fasta	Cele-UNSB01_3:81693	3	81693	82933	1241	39	31	TAAATAATTA	TAAATAATTAGCAGACAAACGTTGGGTCTGC
UNSB01.fasta	Cele-UNSB01_3:83964	3	83964	84341	378	14	21	AAATTTGGCT	AAATTTGGCTTAAAATGTTGA
UNSB01.fasta	Cele-UNSB01_3:86662	3	86662	86935	274	13	21	CCATTTTAAG	CCATTTTAAGCCAATTTTGGA
UNSB01.fasta	Cele-UNSB01_3:86662	3	86662	87180	519	25	21	CCATTTTAAG	CCATTTTAAGCCAATTTTGGA
UNSB01.fasta	Cele-UNSB01_3:88388	3	88388	88698	311	9	31	TGCTAAATAA	TGCTAAATAATTAGCAGACCATTTTTGGGTA
UNSB01.fasta	Cele-UNSB01_3:119691	3	119691	119763	73	5	12	GCCTAAGCCT	GCCTAAGCCTAA
UNSB01.fasta	Cele-UNSB01_3:134796	3	134796	135400	605	7	34	AATTCCGGCA	AATTCCGGCAATTTACCAATTTGCCAGAAATTTC
UNSB01.fasta	Cele-UNSB01_3:192738	3	192738	193322	585	4	31	TTAGCAGACC	TTAGCAGACCCAAGTATGGTCTGCTAATTAT
UNSB01.fasta	Cele-UNSB01_3:194647	3	194647	195253	607	5	31	TTAGCAGACC	TTAGCAGACCCAAGTATGGTCTGCTAAATAA
UNSB01.fasta	Cele-UNSB01_3:196367	3	196367	196735	369	7	31	GGTCTGCTAA	GGTCTGCTAAATATTTAGCATACCCAACTTT
UNSB01.fasta	Cele-UNSB01_3:198288	3	198288	198567	280	9	31	ACCCAAGTAT	ACCCAAGTATGGTCTGCTAATTATTTAGCAA

UNSB01.fasta	Cele-UNSB01_3:211856	3	211856	211976	121	7	16	TAGGCATCTA	TAGGCATCTAGGCAGG
UNSB01.fasta	Cele-UNSB01_3:214401	3	214401	214633	233	8	12	AAGCCTAAGC	AAGACTAAGCCT
UNSB01.fasta	Cele-UNSB01_3:249570	3	249570	249725	156	4	31	AGCTCAAAAT	AGCTCAAAATCTGTAAAAATGCCCATTTTTG
UNSB01.fasta	Cele-UNSB01_3:260141	3	260141	260567	427	12	30	TTTTTTGAGC	TGTTTTGAGCCAAAAATTCAAAAAATCTCG
UNSB01.fasta	Cele-UNSB01_3:265034	3	265034	265468	435	11	11	CCTACAGTAC	CCTACAGTACA
UNSB01.fasta	Cele-UNSB01_3:272176	3	272176	273482	1307	13	101	GGTTACTGTA	GGTTACTGTAGTTCCAAAAGTACGCAAACACTGACTACGGTTCCCTAATACATCAGATATTACGACAAAGTGCAGAAAATCGCTAAAAATGACTTTACTGG
UNSB01.fasta	Cele-UNSB01_3:344303	3	344303	344830	528	17	31	TGGTCTGCTA	TGGTCTGCTAAATTTTTAGCAGACCCAACAT
UNSB01.fasta	Cele-UNSB01_3:347263	3	347263	347821	559	17	31	TATGCTAAAT	TATGCTAAATATTTAGCAGACCAAAAATAGG
UNSB01.fasta	Cele-UNSB01_3:350896	3	350896	352295	1400	23	31	TTAGCAGACC	TTAGCAGACCCAAAGTAGGTATGCTAAATAT
UNSB01.fasta	Cele-UNSB01_3:355335	3	355335	355552	218	7	31	CTGCTAAATA	CTGCTAAATATTTAGCAGACCAACTTTGGGT
UNSB01.fasta	Cele-UNSB01_3:356762	3	356762	357146	385	13	31	TTAGCAGACC	TTAGCAGACCAAAGTTGGGTATGCTAAATAT
UNSB01.fasta	Cele-UNSB01_3:415402	3	415402	416126	725	7	40	TCCACGTGGA	TCTACGTGGAATTTTCAGAATTTTCTCAGAAATTTTGATT
UNSB01.fasta	Cele-UNSB01_3:421701	3	421701	421887	187	6	31	TTGGGTCTGC	TTGGGTCTGCTAAATAATTAGCAGACCATAA
UNSB01.fasta	Cele-UNSB01_3:437143	3	437143	437747	605	6	31	TATTTAGCAG	TATTTAGCAGACCCAATTATGGTCTGCTAAT
UNSB01.fasta	Cele-UNSB01_3:438816	3	438816	440336	1521	38	40	TAGAAAGTTC	TAGAAAGTTCTGGAACATTCCAGAAATTTCCCGATTTTTC
UNSB01.fasta	Cele-UNSB01_3:438790	3	438790	441355	2566	43	40	ACATTCCAGA	ACATTCCAGAATTTTCTCGATTTTTCTAGAAAGTTCTGGA
UNSB01.fasta	Cele-UNSB01_3:441483	3	441483	441987	505	18	11	CTACAGTACT	CTACAGTACTA
UNSB01.fasta	Cele-UNSB01_3:464201	3	464201	477164	12964	79	164	ATATAATGCT	ATATAATGCTCGTAATTATTAATAAATGTGTGTTGTCTCAGAGCCACGTCTGAAAATACAGATTTTTTCTCGAAAATTTCAAAAACTTTGTAGAAATTCGATTTTTGACATAAATGATCGCTCTTTTTTTCTGACATGTATTTCAGTGA CAACTAAACTAGAAA
UNSB01.fasta	Cele-UNSB01_3:495426	3	495426	496459	1034	16	31	TTTAGCAGAC	TTTAGCAGACCAAAGTTGGGTATGCTAAATA
UNSB01.fasta	Cele-UNSB01_3:503013	3	503013	503261	249	8	31	TTGGGTATGC	TTGGGTATGCTAAATATTTAGCAGACCAAAG
UNSB01.fasta	Cele-UNSB01_3:506857	3	506857	507105	249	8	31	GTATGCTAAA	GTATGCTAAATATTTAGCAGACCAAAGTTGG
UNSB01.fasta	Cele-UNSB01_3:516006	3	516006	516440	435	14	31	GGTCTGCTAA	GGTCTGCTAAATATTTAGCATACCCAACTTT
UNSB01.fasta	Cele-UNSB01_3:517798	3	517798	519152	1355	34	31	TTAGCAGACC	TTAGCAGACCAAAGATAGGTATGCTAAATAC
UNSB01.fasta	Cele-UNSB01_3:523916	3	523916	525062	1147	37	31	GGTCTGCTAA	GGTCTGCTAATTATTCAGCATACCCAAATTT
UNSB01.fasta	Cele-UNSB01_3:546017	3	546017	546152	136	6	11	CTACAGTACC	CTACAGTAACC
UNSB01.fasta	Cele-UNSB01_3:611245	3	611245	611586	342	11	31	GGTCTGCTAA	GGTCTGCTAATTATTTAGCATACCGAACTTC
UNSB01.fasta	Cele-UNSB01_3:614340	3	614340	614495	156	5	31	TGGGTATGCT	TGGGTATGCTAAATAATTAGCAGACCAAAGT
UNSB01.fasta	Cele-UNSB01_3:660202	3	660202	660394	193	6	31	TTAGCAGACC	TTAGCAGACTCATGGTAGGTATGCTAAATAT
UNSB01.fasta	Cele-UNSB01_3:662174	3	662174	662284	111	6	18	TAGGCTTAGG	TAGTCTTAGGCTAACCTT

UNSB01.fasta	Cele-UNSB01_3:665955	3	665955	666358	404	13	31	TGGGTCTGCT	TGGGTCTGCTAAATATTTAGCAGACCAACTA
UNSB01.fasta	Cele-UNSB01_3:727287	3	727287	727380	94	5	15	GGGGGCAACA	GGGGGCAACAAATTA
UNSB01.fasta	Cele-UNSB01_3:731921	3	731921	732069	149	8	15	TGTTGCCCCC	TGTTGACCCCCTATTA
UNSB01.fasta	Cele-UNSB01_3:882434	3	882434	882682	249	7	31	TAAATATTTA	TAAATATTTAGTAGACCAAAGTTGGGTATGC
UNSB01.fasta	Cele-UNSB01_3:949283	3	949283	949630	348	8	18	TGCAAGACTA	TGCAAGACTATTAGAAGC
UNSB01.fasta	Cele-UNSB01_3:949715	3	949715	950024	310	5	18	TAGTGCTGCA	TAGTGCTGCAGTCTCTAT
UNSB01.fasta	Cele-UNSB01_3:958005	3	958005	958109	105	4	26	CGGCCAGAGT	CGGCCAGAGTCACTATATTTGGTGAA
UNSB01.fasta	Cele-UNSB01_3:958146	3	958146	965823	7678	284	27	AGAGAGTAAA	AGAGAGTAAAATATAGTGGGAAGCCAC
UNSB01.fasta	Cele-UNSB01_3:958162	3	958162	973687	15526	569	27	TGGGAAGCCA	TGGGAAGCCACAGAGAGTAAAATATAG
UNSB01.fasta	Cele-UNSB01_3:1041359	3	1041359	1041618	260	12	22	TAAATCTACA	TAAATCTACACACTTGGCCTCG
UNSB01.fasta	Cele-UNSB01_3:1053032	3	1053032	1053110	79	5	16	TTATAGTGTT	TTATAGTGTTATAGTG
UNSB01.fasta	Cele-UNSB01_3:1059346	3	1059346	1075750	16405	279	59	AACTGAGAAT	AACTGAGAATTATGGAGAATATGGAAATTCATGTGTGCACTGGTTTTCAAAATTTTTTA
UNSB01.fasta	Cele-UNSB01_3:1107553	3	1107553	1107733	181	10	12	AAGCCTAAGC	AAGCCTAAGCCT
UNSB01.fasta	Cele-UNSB01_3:1112086	3	1112086	1112814	729	14	40	AAAATCCACG	AAAATCCACGTAGATTATCGATTTTTCATAGAATTTTGCA
UNSB01.fasta	Cele-UNSB01_3:1112079	3	1112079	1113479	1401	16	40	TTTTGCAAAA	TTTTGCAAAAATCCACGTAGATTATCGATTTTTCATAGAA
UNSB01.fasta	Cele-UNSB01_3:1175770	3	1175770	1176177	408	4	96	GAGGGCCGCA	GAGGGCCGCAGGCCCGAACACTCTTGGAAACTATGGGCTGGCTTTGATCCTATTCTAAGCAGATATGTTATTCACTCAAGCCTAATAACCAGGTTT
UNSB01.fasta	Cele-UNSB01_3:1204553	3	1204553	1205580	1028	13	35	TTTAAAATTT	TTGAAAATTTCGGGCAAATTGGCAACTTGCCGGAT
UNSB01.fasta	Cele-UNSB01_3:1210986	3	1210986	1211328	343	4	21	ACTACAGTAC	ACTACAGTACCCCCCACTACC
UNSB01.fasta	Cele-UNSB01_3:1301641	3	1301641	1302992	1352	116	11	TTTTTGCACA	TTTTTGCACAA
UNSB01.fasta	Cele-UNSB01_3:1307999	3	1307999	1309276	1278	65	20	AAAAATAGAT	AAAAATAAATTGGCAGACGA
UNSB01.fasta	Cele-UNSB01_3:1312329	3	1312329	1312425	97	5	12	AGGCTTAGGC	AGGCTTAGGCTT
UNSB01.fasta	Cele-UNSB01_3:1320709	3	1320709	1320805	97	5	12	AGGCTTAGGC	AGGCTTAGGCTT
UNSB01.fasta	Cele-UNSB01_3:1329089	3	1329089	1329185	97	5	12	AGGCTTAGGC	AGGCTTAGGCTT
UNSB01.fasta	Cele-UNSB01_3:1364177	3	1364177	1382615	18439	734	25	CAGAGAGTAA	CAGAGAGTAAAATAATTGGTGAAGC
UNSB01.fasta	Cele-UNSB01_3:1391886	3	1391886	1392275	390	16	21	TTTTTGGTAA	TTTTTGGTAAAATTTGATAAT
UNSB01.fasta	Cele-UNSB01_3:1394199	3	1394199	1394513	315	12	21	CAAAAAGTTA	CAAAAAGTTATCAAATTTTAC
UNSB01.fasta	Cele-UNSB01_3:1395088	3	1395088	1395322	235	5	35	CAAATCGGCA	CAAATCGACAAATTGCCGGAATTGAAAATTTCCGG
UNSB01.fasta	Cele-UNSB01_3:1399964	3	1399964	1400110	147	5	11	GTACTGTAGG	GTACTGTAGGA
UNSB01.fasta	Cele-UNSB01_3:1400275	3	1400275	1400335	61	4	15	TCGTGGTGAG	TCGTGGTGAGACCCA

UNSB01.fasta	Cele-UNSB01_3:1400394	3	1400394	1400521	128	4	31	AATTCAAATT
AATTCAAATTTCTTCAAAAAATTTTGGCGGG								
UNSB01.fasta	Cele-UNSB01_3:1406406	3	1406406	1406896	491	33	11	GGTACTGTAG
GGTGCTGTAGG								
UNSB01.fasta	Cele-UNSB01_3:1413910	3	1413910	1414798	889	40	14	ATACCTAATA
ATACCTTATACCTT								
UNSB01.fasta	Cele-UNSB01_3:1466668	3	1466668	1467327	660	5	43	GGCTCTCGGC
GGCTCTCGGCTCGCGCCGAGAGCCGAGCTCTATTTTCCGGTTC								
UNSB01.fasta	Cele-UNSB01_3:1616576	3	1616576	1616906	331	16	20	CATAAAAATT
CATAAAAATTCAGAAAAATG								
UNSB01.fasta	Cele-UNSB01_3:1617648	3	1617648	1618407	760	37	20	TTTCACTAAA
TTTCACTAAAAATTTCAAAT								
UNSB01.fasta	Cele-UNSB01_3:1642030	3	1642030	1642586	557	7	21	CCTAAAAATT
CCTAAAAATTCCAAATTTTAA								
UNSB01.fasta	Cele-UNSB01_3:1690415	3	1690415	1691093	679	5	72	ATGAATTTTA
ATGAATTTTAATACAATTTTCCTGACGATAAAGTCATAAAAACTCATTTTTTGACCTTTCAACTTTAGTTTT								
UNSB01.fasta	Cele-UNSB01_3:1715361	3	1715361	1716220	860	41	11	TACTGTAGGG
TACTGTAGAGG								
UNSB01.fasta	Cele-UNSB01_3:1724995	3	1724995	1725361	367	4	92	CTGCGGAACC
CTGCGGAACCCCGAACGTGTCGGCCGCTTCCAAAAACAACCACCTCTTCACACTTCATTGCGCACACACCAAACTACTAGTTTCACGTCAAA								
UNSB01.fasta	Cele-UNSB01_3:1738456	3	1738456	1739086	631	52	12	TGAGCCTGAG
TGAGCCTGAGCC								
UNSB01.fasta	Cele-UNSB01_3:1753459	3	1753459	1753580	122	11	11	GCAAAAAATG
GCAAAAAATGT								
UNSB01.fasta	Cele-UNSB01_3:1796051	3	1796051	1796519	469	35	12	CTAAGCCTAA
CTAAGCCTGAGC								
UNSB01.fasta	Cele-UNSB01_3:1833347	3	1833347	1833868	522	14	34	AATTGAAATT
AATTGAAATTTCCGGTAAATCGGAAAATTGCCGG								
UNSB01.fasta	Cele-UNSB01_3:1843958	3	1843958	1844048	91	6	12	AGGCTTAGGC
AGGCTGAGGCTT								
UNSB01.fasta	Cele-UNSB01_3:1887376	3	1887376	1889731	2356	56	19	AAAATCGATA
AAAATCAATATTTTATCGG								
UNSB01.fasta	Cele-UNSB01_3:1891974	3	1891974	1892414	441	36	11	ATTTTTTGCA
ATTTTTTGTAC								
UNSB01.fasta	Cele-UNSB01_3:1974698	3	1974698	1975558	861	44	19	AAAATCGATA
AAAATCGATAATTCCTCAA								
UNSB01.fasta	Cele-UNSB01_3:2017089	3	2017089	2018496	1408	57	21	TGGAAATTTT
TGGAAATTTTACCCCAAAAAT								
UNSB01.fasta	Cele-UNSB01_3:2046603	3	2046603	2047209	607	30	12	TAGGCTTAGG
TAGGCTTAGGCT								
UNSB01.fasta	Cele-UNSB01_3:2058475	3	2058475	2058618	144	11	11	GTCGCGTTGC
GTCGCATTGCG								
UNSB01.fasta	Cele-UNSB01_3:2068206	3	2068206	2068691	486	44	11	TGTGCAAAAA
TGTGCAAAAAA								
UNSB01.fasta	Cele-UNSB01_3:2092267	3	2092267	2093197	931	27	31	TGCGTCGGAA
TGCGTCGGAAATGCATTCTTATCATCAGCGG								
UNSB01.fasta	Cele-UNSB01_3:2094174	3	2094174	2094918	745	17	31	TCCGATGCAT
TCCGATGCATTGGAAATGATCGGAAAATGGT								
UNSB01.fasta	Cele-UNSB01_3:2095462	3	2095462	2096950	1489	36	19	TATCGATTTT
TATTGATTTTTTGCTAAAA								
UNSB01.fasta	Cele-UNSB01_3:2118334	3	2118334	2118593	260	20	11	CTACAGTACC
CTACAGTACTT								
UNSB01.fasta	Cele-UNSB01_3:2118949	3	2118949	2119137	189	9	20	TGTAGATTTA
TGTGGATTTACAGGAATCTA								
UNSB01.fasta	Cele-UNSB01_3:2149341	3	2149341	2150059	719	12	35	TTTGCCGGAA
TTTGCCGGAAATTTTCAATTCCGACAATTTGCCGA								
UNSB01.fasta	Cele-UNSB01_3:2160246	3	2160246	2184061	23816	142	168	TTTATTTTTG
TTTATTTTTGTTTTTTTGTGCCTACATATACCTGTTGAAATTGGAATTCAAGACAAAAACCCCTGTAGCGCGTCTGAAAATGGATATCTAGGTACAAAAACTTACCAATAATATTTTAAAACAATCTAGAATCCAAATCTATCAAAACA TTTCCGGAATTCCATAATG								

UNSB01.fasta	Cele-UNSB01_3:2204608	3	2204608	2205196	589	25	21	GCCTAAAAAC	GCATAAAAACGTAAATTTTAG
UNSB01.fasta	Cele-UNSB01_3:2212231	3	2212231	2213000	770	22	31	ATTTCCCGCC	ATTTCCCGCCAAAAAATATTTGAAAACTTGA
UNSB01.fasta	Cele-UNSB01_3:2214780	3	2214780	2216279	1500	39	39	TTTTTGGCGG	TTTTTGGCGGGAAATTTCAAAATTCAAATTTTTGAATAT
UNSB01.fasta	Cele-UNSB01_3:2219970	3	2219970	2221469	1500	39	39	TTTTTGGCGG	TTTTTGGCGGGAAATTTCAAAATTCAAATTTTTGAATAT
UNSB01.fasta	Cele-UNSB01_3:2223808	3	2223808	2223967	160	4	40	TCCCGCCAAA	TCCCGCCAAAAAATATTCAAAAAATTTGAATTTTGAAATT
UNSB01.fasta	Cele-UNSB01_3:2325106	3	2325106	2325938	833	23	32	CTCAGCCAAT	CTCAGCCAATCAGCAATTAGCTCTGCCTATTT
UNSB01.fasta	Cele-UNSB01_3:2342333	3	2342333	2342437	105	5	21	CTGACCAAAT	CTGACCAAATTTGTTTTAGCA
UNSB01.fasta	Cele-UNSB01_3:2351961	3	2351961	2352192	232	9	21	GTCAGTGCTG	GTCAGTGCTGTAACACATTTT
UNSB01.fasta	Cele-UNSB01_3:2562874	3	2562874	2563954	1081	40	10	AGGCAGGCTT	AGGCAGGCAT
UNSB01.fasta	Cele-UNSB01_3:2564180	3	2564180	2564252	73	5	12	CCAAGCCCAA	CCAAGCCCAATC
UNSB01.fasta	Cele-UNSB01_3:2578277	3	2578277	2578816	540	27	20	GACCCAGCCT	GACCCAGCCTACCCCAGGCA
UNSB01.fasta	Cele-UNSB01_3:2578282	3	2578282	2579101	820	41	20	AGCCTACCCC	AGCCTACCCCAGGCAGACCC
UNSB01.fasta	Cele-UNSB01_3:2604301	3	2604301	2604569	269	6	45	TCTGATCCTT	TCTGATCCTTCCGATCCGAAGGATCACATGGGTACCTCCTGATGG
UNSB01.fasta	Cele-UNSB01_3:2604631	3	2604631	2620875	16245	650	25	AGAGAGTAAA	AGAGAGTAAAATATATGGTGAAGCC
UNSB01.fasta	Cele-UNSB01_3:2620888	3	2620888	2621068	181	5	40	TTCCAGAATT	TTCCAGAATTTTCTCGATTTTTCTAGAAAGTTCTGGAACA
UNSB01.fasta	Cele-UNSB01_3:2621291	3	2621291	2621771	481	29	15	GGTCTCACCA	GGTCTCACCACGATG
UNSB01.fasta	Cele-UNSB01_3:2665588	3	2665588	2665980	393	11	35	TTTGCCGGAA	TTTGCTGGAAATGTTCAATTGCGGCAAATTGCCGA
UNSB01.fasta	Cele-UNSB01_3:2911307	3	2911307	2911655	349	10	35	TTTGCCGGAA	TTTGCCGGAAATTTTTAATTCTGGCAATTTGCCGA
UNSB01.fasta	Cele-UNSB01_3:2955858	3	2955858	2957390	1533	44	19	AAAATCGATA	AAAATTGATAATTTCCGGA
UNSB01.fasta	Cele-UNSB01_3:3099001	3	3099001	3099720	720	4	180	TGTCGGCCGC	TGTCGGCCGCTGTATCTTTTGCCTCAATTTTTTTTAAATTTTCAAACATACATATTCCATTAGGTAACATTATGAGCTACAGTTTGTTAGTTGGAATTTTATTTCTAAAATTACTAGTTTCTGAGTTATACTCATTTCAATATCCCCCAAGTGAAATCGCCTAAAAATGAACTCGCGAGG
UNSB01.fasta	Cele-UNSB01_3:3140206	3	3140206	3140936	731	5	16	TGTATATATA	TGTATATATATATATA
UNSB01.fasta	Cele-UNSB01_3:3145893	3	3145893	3146730	838	18	37	ATTTTGGCGG	ATTTTGGCGGGATTTCAAATTTGAATTTTTTGAAAAT
UNSB01.fasta	Cele-UNSB01_3:3152376	3	3152376	3152920	545	14	35	AATTGCCGGA	AATTGCCGGAATTGAAACTTTCCGGCGAATTGGAA
UNSB01.fasta	Cele-UNSB01_3:3159908	3	3159908	3160529	622	13	35	AAATCGGCAA	AAATCGGAAAATTGCCGGAATTGAAAATTTCCAGA
UNSB01.fasta	Cele-UNSB01_3:3164980	3	3164980	3165718	739	38	18	GTCGATTTAC	GTCGATTTACGAGATTTT
UNSB01.fasta	Cele-UNSB01_3:3200630	3	3200630	3201398	769	4	151	TTTAAAGGCG	TTTAAAGGAGCATAACCCAATTTCCCTCTTCATTTTCGAGGAAATCACTTAAAAATTTGAATTCTTTGAAAAATTCCAGTCAAAACACACTTTTATTAAGCCTCTAACGTCGATTTTTAACTATTTCGGGTCTCGCCACGAAAAATTGTAG
UNSB01.fasta	Cele-UNSB01_3:3207516	3	3207516	3208428	913	5	151	TTTAAAGGCG	TTTAAAGGCACATGACCCAATTTCCCCCTTCATTTTCGAGGAAATCACTTAAAAATTTGAATTCTTTGAAAAATTCCAGTCAAAACACACTTTTATTAAGCCTCTAACGTCGATTTTTAACTATTTCGGGTCTCGCCACGAAAAATTGTAG
UNSB01.fasta	Cele-UNSB01_3:3351031	3	3351031	3351269	239	7	30	TAGAACTTTC	TAGAACTTTTCAGTTTTTTCTAGAAAACTC

UNSB01.fasta	Cele-UNSB01_3:3352849	3	3352849	3353088	240	8	30	TAGAACTTTC	TAGAACTTTCCAAATTTCTCTAGAAAATTC
UNSB01.fasta	Cele-UNSB01_3:3356021	3	3356021	3356788	768	13	19	CTTGGCGCAA	CTTGGCGCAATTCTCGTGC
UNSB01.fasta	Cele-UNSB01_3:3394846	3	3394846	3394930	85	4	21	TCCAGCACTG	TCCAGCACTGACAAATTTTGT
UNSB01.fasta	Cele-UNSB01_3:3456121	3	3456121	3456336	216	18	12	AAAATAAAAA	AAAATAAAAATA
UNSB01.fasta	Cele-UNSB01_3:3456392	3	3456392	3470370	13979	556	25	ACTCTCTGGC	ACTCTCTGGCTTCCCACTATATTTT
UNSB01.fasta	Cele-UNSB01_3:3480039	3	3480039	3480224	186	12	12	TAGGCTTAGG	TAGGCTCAGGCT
UNSB01.fasta	Cele-UNSB01_3:3518362	3	3518362	3520167	1806	19	95	TAATCCTATT	TAATCCTATTCCAATCAGATTGTTATTCGCTCAAGCCTACAAATCAGGTTTGAGGGCCGCAGGCCCGAACACTCTTGGAAACTGTGGGCCAGCTT
UNSB01.fasta	Cele-UNSB01_3:3579672	3	3579672	3580411	740	47	15	GTGGTGAGAC	GTGGTGAGACCCGTC
UNSB01.fasta	Cele-UNSB01_3:3676441	3	3676441	3676612	172	5	34	AATTGAAATT	AATTGAAATTTCCTGCAAAGCGGCAAATTGCCGA
UNSB01.fasta	Cele-UNSB01_3:3780853	3	3780853	3780925	73	4	16	CCTACATACC	CCTACATACCTACATA
UNSB01.fasta	Cele-UNSB01_3:3795055	3	3795055	3795458	404	8	35	TTCCGGCAAA	TTCCGGCAAATCGACAATTTTCCGAAAATGAAAAT
UNSB01.fasta	Cele-UNSB01_3:3799869	3	3799869	3800537	669	5	65	AAGACACTAA	AAGACACTAATAAAAATATTATTTTTATTCGTCTTTTAATTTTACAAAATTTTCAATAAAAATTT
UNSB01.fasta	Cele-UNSB01_3:3818464	3	3818464	3818544	81	5	12	TAAGCCTAAG	TAAGCCTAAGCC
UNSB01.fasta	Cele-UNSB01_3:3873879	3	3873879	3874448	570	9	30	CCTTGTAGGC	CCTTGTAGGTAAGGAGGTAGGTGTAGGCCG
UNSB01.fasta	Cele-UNSB01_3:3880760	3	3880760	3880928	169	10	16	TAGGCATGTA	TAGACATGTAGGCATG
UNSB01.fasta	Cele-UNSB01_3:3928597	3	3928597	3928907	311	5	18	TGCAGCACTA	TGCAGCACTATTAGAGAC
UNSB01.fasta	Cele-UNSB01_3:4029920	3	4029920	4030220	301	8	40	AGAACCTTCT	AGAACCTTCTGAAAAATTCTAGAAAATTCTGGAATGGTCC
UNSB01.fasta	Cele-UNSB01_3:4030293	3	4030293	4031043	751	50	15	CGTGGTGAGA	CGTGGCGAGACCCAC
UNSB01.fasta	Cele-UNSB01_3:4031086	3	4031086	4031330	245	7	35	AATTTAAATT	AATTTAAATTTTCTGTGAGAAATATTTTGGCGGGA
UNSB01.fasta	Cele-UNSB01_3:4031404	3	4031404	4032964	1561	39	40	GTTCCAGAAC	GTTCCAGAACTTTCTAGAAAAATCGAGAAAATTCTGGAAT
UNSB01.fasta	Cele-UNSB01_3:4122367	3	4122367	4122965	599	31	19	AAAAATAGAT	AAAAATAGATTGTTACACG
UNSB01.fasta	Cele-UNSB01_3:4344679	3	4344679	4344767	89	4	22	CAAAACCCAA	CAAAACCCAAGTAGATCCCACT
UNSB01.fasta	Cele-UNSB01_3:4389614	3	4389614	4389732	119	7	17	AGGGGGCATC	AGGGGGCATTAGGTCAA
UNSB01.fasta	Cele-UNSB01_3:4392502	3	4392502	4392620	119	7	17	GATGCCCCCT	GATGCCCCCTCTGAATG
UNSB01.fasta	Cele-UNSB01_3:4778835	3	4778835	4779093	259	5	17	ATTATTTTCA	ATTATTTTCATTCCGTG
UNSB01.fasta	Cele-UNSB01_3:5426163	3	5426163	5426753	591	28	20	CTAGGTCTGC	CTAGGTCTGCCTAGGGTAGG
UNSB01.fasta	Cele-UNSB01_3:5426187	3	5426187	5427065	879	40	20	GGTAGGCTAG	GGTAGGCTAGGTCTGCCCAG
UNSB01.fasta	Cele-UNSB01_3:5494321	3	5494321	5534864	40544	1542	26	GTTAGAGTCA	GTTAGAGTCATTATTTTAGGTGAATG
UNSB01.fasta	Cele-UNSB01_3:5534873	3	5534873	5535003	131	5	26	ACCATTCACC	ACCATTCACCCAAAATAATGACTCTA

UNSB01.fasta	Cele-UNSB01_3:6120416	3	6120416	6122897	2482	10	228	CCACCAGATC	CCACCAGATCAGCAGCGTCTCATCTTCGCCGGAAAGCAGCTCGAGGACGGCCGCACCCTGTCTGACTACAACATCCAGAAGGAATCTACTCTTCACTTGGTTCTTCGTCTTAGAGGAGGAATGCAGATCTTTGTCAAGACTTTGACTGGAAAGACCATCACACTTGAAGTTGAAGCTTCCGACACGATCGAGAACGTCAAGGCCAAGATTCAAGACAAGGAGGGAATC
UNSB01.fasta	Cele-UNSB01_3:6192343	3	6192343	6192405	63	4	15	CGAAAAGGGG	CGAAAAGGGGATCTG
UNSB01.fasta	Cele-UNSB01_3:6192580	3	6192580	6192657	78	5	15	TCCCCTTTTC	TCCCCTTTTCGTATC
UNSB01.fasta	Cele-UNSB01_3:6364525	3	6364525	6365839	1315	65	19	AAAAATAGAT	AAAAATAGATTGTTACACG
UNSB01.fasta	Cele-UNSB01_3:6731291	3	6731291	6731428	138	9	15	CGAAAAGGGG	CGAAAAGGGGATCTG
UNSB01.fasta	Cele-UNSB01_3:6731603	3	6731603	6731679	77	5	15	TCCCCTTTTC	TCCCCTTTTCGTATC
UNSB01.fasta	Cele-UNSB01_3:6746945	3	6746945	6756350	9406	204	11	GTACTGTAGG	GTACTGTAGTA
UNSB01.fasta	Cele-UNSB01_3:6807960	3	6807960	6808176	217	6	21	AAATCTAATC	AAATCTAATCCAATATGGCCG
UNSB01.fasta	Cele-UNSB01_3:7587437	3	7587437	7632311	44875	1482	27	GTGGGAAGCC	GTGGGAAGCCACAGAGAGTAAAAAATA
UNSB01.fasta	Cele-UNSB01_3:7632425	3	7632425	7683091	50667	426	94	AAAGCACCCA	AAAGCACCCAATATTTAGAGAACAGAAGATTTTGAGAATTACTGCCTCCAGAAATTGATGATTTTCCCATTGATTTGTCTACATAGGGCATCGA
UNSB01.fasta	Cele-UNSB01_3:7683133	3	7683133	7684661	1529	131	11	TGGAGCGGTT	TGGAGCGGTTT
UNSB01.fasta	Cele-UNSB01_3:7825014	3	7825014	7825135	122	11	11	CGGATAATTA	CGGATAATTAA
UNSB01.fasta	Cele-UNSB01_3:7826007	3	7826007	7841897	15891	610	26	CATTAGATGA	CATTAGATGAATTTCCAAAGTTAGAC
UNSB01.fasta	Cele-UNSB01_3:7846966	3	7846966	7847368	403	9	45	TCTGATCCTT	TCTGATCCTTCAGATAAGAAGGATCGTAAGGGTACCTCCTGATGG
UNSB01.fasta	Cele-UNSB01_3:7847664	3	7847664	7848155	492	11	45	GAAGGATCAG	GAAGGATCAGACCATCAGGAGGTACCCTTACGATCCTTCTTATCT
UNSB01.fasta	Cele-UNSB01_3:7913456	3	7913456	7913988	533	28	19	AATCTATTTT	AATCTATTTTTCGTGTAAC
UNSB01.fasta	Cele-UNSB01_3:7922958	3	7922958	7923224	267	14	19	CAATCTATTT	CAATCTATTTTTCGTGGAA
UNSB01.fasta	Cele-UNSB01_3:7953449	3	7953449	7953511	63	4	15	CGAAAAGGGG	CGAAAAGGGGATCTG
UNSB01.fasta	Cele-UNSB01_3:7953695	3	7953695	7953787	93	6	15	TCCCCTTTTC	TCCCCTTTTCGTATC
UNSB01.fasta	Cele-UNSB01_3:8499608	3	8499608	8499713	106	5	21	AAGTTTTACT	AAGTTTTACTTTGATTTTTTT
UNSB01.fasta	Cele-UNSB01_3:8579889	3	8579889	8580089	201	12	16	AGGCACGTAG	AGGAACGTAGGCACGT
UNSB01.fasta	Cele-UNSB01_3:8580701	3	8580701	8581151	451	29	15	ACCCATCGTG	ACCCATCGTGGCGAG
UNSB01.fasta	Cele-UNSB01_3:8581179	3	8581179	8581353	175	5	35	GGGAAATTTA	GGGAAATTTAAATTTTCTGTGAAAAATATTTTGGC
UNSB01.fasta	Cele-UNSB01_3:8581474	3	8581474	8581754	281	7	40	TCTGGAATGT	TCTGGAATGTTCCAGAACTTTCTAGAAAAATTGGGAAAAG
UNSB01.fasta	Cele-UNSB01_3:8589669	3	8589669	8589949	281	7	40	TCTGGAATGT	TCTGGAATGTTCCAGAACTTTCTAGAAAAATTGGGAAAAG
UNSB01.fasta	Cele-UNSB01_3:8597864	3	8597864	8598144	281	7	40	TCTGGAATGT	TCTGGAATGTTCCAGAACTTTCTAGAAAAATTGGGAAAAG
UNSB01.fasta	Cele-UNSB01_3:8606059	3	8606059	8606339	281	7	40	TCTGGAATGT	TCTGGAATGTTCCAGAACTTTCTAGAAAAATTGGGAAAAG
UNSB01.fasta	Cele-UNSB01_3:9173467	3	9173467	9173588	122	11	11	ATTTGGAATA	ATTTGGAATAT
UNSB01.fasta	Cele-UNSB01_3:9234667	3	9234667	9236218	1552	21	59	TTCAAAACTT	TTCAAAACTTTTTAAACTTAGATTTATGGAGAATATGGAATTTCAAATGTGCACTGGTT

UNSB01.fasta	Cele-UNSB01_3:9809905	3	9809905	9810297	393	13	11	GTACTGTAGG	GTACTGTAGGG
UNSB01.fasta	Cele-UNSB01_3:9810383	3	9810383	9810703	321	8	40	TCCAGAACCT	TCCAGAACCTTCTGGAAAATCCGAGAAAATTCTGGAATGT
UNSB01.fasta	Cele-UNSB01_3:9810418	3	9810418	9811039	622	9	40	AATGTTCCAG	AATGTTCCAGAACCTTCTGGAAAATTTGAGAAAATGCTGG
UNSB01.fasta	Cele-UNSB01_3:10130069	3	10130069	10130193	125	4	31	TCTGCTAAAT	TCTGCTAAATAATTAGCAGACCATACTTGAT
UNSB01.fasta	Cele-UNSB01_3:10140093	3	10140093	10140163	71	4	19	TGGTTGAGGC	TGGTTGAGGCTCTAGATTT
UNSB01.fasta	Cele-UNSB01_3:10146078	3	10146078	10146173	96	5	19	ATTTTGGTTG	ATTTTGGTTGAGGTTGAGG
UNSB01.fasta	Cele-UNSB01_3:10214948	3	10214948	10215134	187	6	31	TCTGCTAAAT	TCTGCTAAATATTTAGCATACCCACATTGGG
UNSB01.fasta	Cele-UNSB01_3:10216874	3	10216874	10217114	241	12	20	AAAAATAGAT	AAAAATAGATTGACATACCA
UNSB01.fasta	Cele-UNSB01_3:10234987	3	10234987	10235185	199	4	31	TATGCTAAAT	TATGCTAAATATTTAGCAGACCCAACGTTGG
UNSB01.fasta	Cele-UNSB01_3:10399868	3	10399868	10399945	78	5	15	CGAAAAGGGG	CGAAAAGGGGATCTG
UNSB01.fasta	Cele-UNSB01_3:10400120	3	10400120	10400212	93	5	15	TCCCCTTTTC	TCCCCTTTTCGCAGA
UNSB01.fasta	Cele-UNSB01_3:10519446	3	10519446	10519548	103	8	12	AAAACTAAAA	AAAACTAAAAAT
UNSB01.fasta	Cele-UNSB01_3:10519570	3	10519570	10525291	5722	35	164	ATGCCAATCA	ATGCCAATCAAAGTATAATAGCTTATACGGAAGTATTTTTTTAAAAACTGATAAAAAATATATAAAAGCTGATTTTTTCAAAAATTCAAAAGTATGGGAAAATCATATGGAGTCATTCTTTTTTATTGCATAAAACTGTTCAGCGTAGTCAAAAATACCAGAAT
UNSB01.fasta	Cele-UNSB01_3:10614332	3	10614332	10615157	826	20	40	AAAAGTAGGT	AAAAGTAGGTCATGGCCTAGTTTTTAACGTGACCTCCTTA
UNSB01.fasta	Cele-UNSB01_3:10733639	3	10733639	10733701	63	4	15	CGAAAAGGGG	CGAAAAGGGGATCTG
UNSB01.fasta	Cele-UNSB01_3:10733861	3	10733861	10733953	93	6	15	TCCCCTTTTC	TCCCCTTTTCGTATC
UNSB01.fasta	Cele-UNSB01_3:10778310	3	10778310	10778713	404	14	16	GTAGGCACGT	GTAGGCAGGTAGGCAC
UNSB01.fasta	Cele-UNSB01_3:10787273	3	10787273	10787444	172	5	34	TTTCCGGCAA	TTTCCGGCAAATCGGCGAATTGCCGGAATTGAAT
UNSB01.fasta	Cele-UNSB01_3:10934450	3	10934450	10934819	370	11	29	GGCCTAGAAA	GGCCTAGAAAACCCATTTTTAAAGTTGGT
UNSB01.fasta	Cele-UNSB01_3:10975904	3	10975904	10976322	419	11	35	CCGCCAAAAT	CCGCCAAAATATTTTTCACTAAAAATTTGAATTTC
UNSB01.fasta	Cele-UNSB01_3:10980699	3	10980699	10982201	1503	43	35	AAATTCAAAT	AAATTCAAATTTTTAGTGAAAAATATTTTGGCGGA
UNSB01.fasta	Cele-UNSB01_3:11090269	3	11090269	11090626	358	8	45	TCTGATCCTT	TCTGATCCTTCAGATAAGAAAGATCGTAAGGGTACCTCCTGATGG
UNSB01.fasta	Cele-UNSB01_3:11094399	3	11094399	11094498	100	5	20	GTTACACGAA	GTTACACGAAAAAAATAGATT
UNSB01.fasta	Cele-UNSB01_3:11109268	3	11109268	11109343	76	5	15	TCCCCTTTTC	TCCCCTTTTCATATC
UNSB01.fasta	Cele-UNSB01_3:11222702	3	11222702	11222792	91	6	12	GGCTTAGGCT	GGCTTAGGCTTA
UNSB01.fasta	Cele-UNSB01_3:11241850	3	11241850	11242636	787	15	37	TTGGCGGGAA	TTGGCAGGAATTCAAATTTAATTTTTTTAAAATATT
UNSB01.fasta	Cele-UNSB01_3:11273157	3	11273157	11273241	85	5	12	GGCTTAGGCT	GGCTTAAGCTCA
UNSB01.fasta	Cele-UNSB01_3:11300832	3	11300832	11300986	155	5	31	GCAGCCGACG	GCAGCCGACGCTCTGCGAGGTTTGTAGAATG
UNSB01.fasta	Cele-UNSB01_3:11302620	3	11302620	11306800	4181	209	20	TCTATTTTTT	TCTATTTTTTGGTATGTCAA

UNSB01.fasta	Cele-UNSB01_3:11327560	3	11327560	11327604	45	4	11	AAACTTGGGC	AAACTTGGGTA
UNSB01.fasta	Cele-UNSB01_3:11452503	3	11452503	11452739	237	6	35	AATTGCCGGA	AATTGCCGGAATTAAAGATTTCCGGCAAATTGGCC
UNSB01.fasta	Cele-UNSB01_3:11461018	3	11461018	11461218	201	5	40	TCCAGAACCT	TCCAGAACCTTCTGGAAAATTTGAGAAAATGCTGGAATGT
UNSB01.fasta	Cele-UNSB01_3:11461401	3	11461401	11461721	321	10	32	TTTCTGTGAA	TTTCTGTGAAAAATTATAACGGGAATTCAAAA
UNSB01.fasta	Cele-UNSB01_3:11489719	3	11489719	11490125	407	12	31	TCTGAGCTAC	TCTGAGCTACAGTACTCCTGTCAAGAACAGT
UNSB01.fasta	Cele-UNSB01_3:11508351	3	11508351	11508507	157	10	12	GCTTAGGCTT	GCCTAGGCTTAG
UNSB01.fasta	Cele-UNSB01_3:11522131	3	11522131	11522299	169	11	14	GATAATTGAA	GATAATTGAAAATC
UNSB01.fasta	Cele-UNSB01_3:11543247	3	11543247	11543687	441	21	21	GGCAATTTTC	GGCAATTTTCCAGTGCAATTC
UNSB01.fasta	Cele-UNSB01_3:11612201	3	11612201	11612410	210	6	35	GATTTGCCGG	GATTTGCCGGAAATATTAAAATCCGGCAATTTATC
UNSB01.fasta	Cele-UNSB01_3:11614727	3	11614727	11615007	281	6	35	TTGCCGGATT	TTGCCGGATTTGAATATTTTCGGCAAATCTGCAGT
UNSB01.fasta	Cele-UNSB01_3:11631810	3	11631810	11631935	126	4	11	CTACAGTACT	CTACAGTACTC
UNSB01.fasta	Cele-UNSB01_3:11700261	3	11700261	11701084	824	59	14	AATTATAAAT	AATTATAAATTAGA
UNSB01.fasta	Cele-UNSB01_3:11723112	3	11723112	11723392	281	6	35	TTTGCCGGAA	TTTGCCGGAAATTTTTATTTCTGGCAATTTGACGA
UNSB01.fasta	Cele-UNSB01_3:11725694	3	11725694	11725970	277	12	12	TTAGGCTTAG	TTAGGCTTAGGC
UNSB01.fasta	Cele-UNSB01_3:11779688	3	11779688	11779808	121	10	12	CTTAGGCTTA	CTTAGACTTATG
UNSB01.fasta	Cele-UNSB01_3:11779996	3	11779996	11780140	145	7	16	TACAAGCCTA	TACACGCCTACAAGCC
UNSB01.fasta	Cele-UNSB01_3:11800186	3	11800186	11800737	552	15	34	AAATTTCAAT	AAATTTCAATTCCGCTAATTTGCCGATTTGCTGG
UNSB01.fasta	Cele-UNSB01_3:11834269	3	11834269	11834616	348	10	34	TTTTCGATTT	TTTTCGATTTTTCGATTTTTTGAAAAATCGGGAA
UNSB01.fasta	Cele-UNSB01_3:11851795	3	11851795	11852785	991	24	35	CGGCAAATCG	CGGCAAATCGGCAAGTTGCCAAAACTGAAAATTCC
UNSB01.fasta	Cele-UNSB01_3:11898352	3	11898352	11906615	8264	45	184	TACGCAGCCG	TACGCAGCCGAACGTATCATAAGTGAATACAGAACACCAATTATGTCCGAGAAAAAGATCCTACTCAGAATATGAACATATTCAAAAATATCGGAGGTATATAGATTCCCGAAGAGCCTTTCCAATTAACCAAACGGTTCAAATTCTAGCAAACATCTCCTTACTTGAACACTCTTGGCCAATG
UNSB01.fasta	Cele-UNSB01_3:11990778	3	11990778	11991299	522	14	35	CCGATTTGCC	CCGATTTGCCGGAAATTTTCAAATCCGGCAATTTT
UNSB01.fasta	Cele-UNSB01_3:12003957	3	12003957	12004292	336	7	11	GTACTGTAGA	GTACTGTAGAG
UNSB01.fasta	Cele-UNSB01_3:12010851	3	12010851	12011440	590	30	12	GGCTTAGGCT	GGCTTAGGTATA
UNSB01.fasta	Cele-UNSB01_3:12025839	3	12025839	12025976	138	8	12	TAAGCCTAAG	TAAGCCTAAACC
UNSB01.fasta	Cele-UNSB01_3:12040777	3	12040777	12040867	91	6	12	CCTAAGCCTA	CCTAACCCTAAC
UNSB01.fasta	Cele-UNSB01_3:12087067	3	12087067	12087157	91	4	12	AGCCTGAGCC	AGCCTGAGCCTC
UNSB01.fasta	Cele-UNSB01_3:12088649	3	12088649	12088899	251	9	12	TAAGCCTAAG	TAAGCCTAAGCC
UNSB01.fasta	Cele-UNSB01_3:12102276	3	12102276	12102493	218	6	34	TTGCCGAATT	TTGCCGAATTTGCTATTTGCCGAGCTTGTCAAAT
UNSB01.fasta	Cele-UNSB01_3:12120993	3	12120993	12121420	428	12	35	AATTGCCGGA	AATTGCTGGAATTGAAATTTTCCGGAAAATCGGCA

UNSB01.fasta	Cele-UNSB01_3:12166501	3	12166501	12166666	166	15	11	ACATTTTTTG	ACATTTTTTGA
UNSB01.fasta	Cele-UNSB01_3:12168292	3	12168292	12168722	431	13	34	AAGTATTTTT	AAGTATTTTTGCCGGTTTTTTTGCCTAAATTTCC
UNSB01.fasta	Cele-UNSB01_3:12227970	3	12227970	12228196	227	6	35	TTCCGGCAAA	TTCTGGCAAATTCACAAATTGCCGGAACTAAAAAA
UNSB01.fasta	Cele-UNSB01_3:12229422	3	12229422	12230200	779	21	35	TTCAATTCCG	TTCAATTCCGCAATTTGGCCAATTTGCCGGAAATT
UNSB01.fasta	Cele-UNSB01_3:12305276	3	12305276	12305678	403	10	40	ATTCCAGAAT	ATTCCAGAATTTCTTGGAATTTTCCAAAAGGTTCTAGAAC
UNSB01.fasta	Cele-UNSB01_3:12308090	3	12308090	12308555	466	12	35	AAAATTTCCG	AAAATTTCCGGTAAATCGGCAAATAGCCGGAATTG
UNSB01.fasta	Cele-UNSB01_3:12309471	3	12309471	12309813	343	8	35	GGAATTGAAA	GGAATTGAAAACTTCCGGGAAATCGGCAAATTGGC
UNSB01.fasta	Cele-UNSB01_3:12312970	3	12312970	12313186	217	11	20	AAATAGATTG	AAATAGATTGACAGACGGAA
UNSB01.fasta	Cele-UNSB01_3:12321003	3	12321003	12321555	553	10	43	AATTGCCGGT	AATTGCCGGTTTGCCGATTTGCCAGAAATTTCGGTTTTCGGCA
UNSB01.fasta	Cele-UNSB01_3:12376126	3	12376126	12377123	998	30	32	AAATTTGTGA	AAATTTGTGAAAATTTAGGCCCATTTTGCTCA
UNSB01.fasta	Cele-UNSB01_3:12433825	3	12433825	12433951	127	9	12	TAAGCCTAAG	TAAGTCTAAGCC
UNSB01.fasta	Cele-UNSB01_3:12445241	3	12445241	12445717	477	10	32	ATTCAAATTT	ATTCAATTTTTTACAGATACATTTTGGCGAAA
UNSB01.fasta	Cele-UNSB01_3:12447381	3	12447381	12447465	85	7	12	GGCTTAGGCT	GGCTTAGGCTTA
UNSB01.fasta	Cele-UNSB01_3:12475925	3	12475925	12476790	866	62	11	TTTTTGCACA	TTTTTGCACAT
UNSB01.fasta	Cele-UNSB01_3:12492090	3	12492090	12492261	172	7	19	CAATTTACCA	CAATTTACCAAAAATTTGT
UNSB01.fasta	Cele-UNSB01_3:12493882	3	12493882	12494280	399	13	16	ACGTAGGCAC	ACGCAGGCACGTAGGC
UNSB01.fasta	Cele-UNSB01_3:12499748	3	12499748	12499928	181	14	12	CTTAGACTTA	CTTAGGCTTAGG
UNSB01.fasta	Cele-UNSB01_3:12542724	3	12542724	12542833	110	5	15	CGACCTGCGA	CGACCTGCGACCTAG
UNSB01.fasta	Cele-UNSB01_3:12547936	3	12547936	12548181	246	7	35	AATTGCCGGA	AATTGCCGGAATTCAAAATTTCCGGCTAATCGGCA
UNSB01.fasta	Cele-UNSB01_3:12593157	3	12593157	12594697	1541	44	35	TTGCCAGAAT	TTGCCAGAATTAAAAATTTCCGGAAAATCGGCAAG
UNSB01.fasta	Cele-UNSB01_3:12605711	3	12605711	12605849	139	9	12	TAGGCATAGG	TAGGCATAGACT
UNSB01.fasta	Cele-UNSB01_3:12608882	3	12608882	12609486	605	14	43	AGCAGCCGAC	AGCAGCTGACAGTCACCGGTTGCCGAAAAATTGCAATAATTTC
UNSB01.fasta	Cele-UNSB01_3:12626974	3	12626974	12627042	69	4	17	TGAAAATAAT	TGAAAATAATCACGGAA
UNSB01.fasta	Cele-UNSB01_3:12644803	3	12644803	12645326	524	7	35	TTGCCGGAAT	TTGGCGGAATTGGAAATTTCCGCCAAACCGGCAAA
UNSB01.fasta	Cele-UNSB01_3:12692847	3	12692847	12693292	446	15	17	ATTTTTTCCG	ATTTTTCCGCCGGTTCG
UNSB01.fasta	Cele-UNSB01_3:12700775	3	12700775	12700871	97	4	24	AAGCACCACC	AAGCACCACCTCCTCCAACTACAA
UNSB01.fasta	Cele-UNSB01_3:12712606	3	12712606	12713078	473	10	35	GATTTGCCGG	GATTTGCCGGAAATTTTCGATTCCGGCAAATTGCC
UNSB01.fasta	Cele-UNSB01_3:12784240	3	12784240	12784411	172	5	35	AATTTGAATT	AATTTGAATTTCCCGCCAAAATCATTTTCTGAGAA
UNSB01.fasta	Cele-UNSB01_3:12788650	3	12788650	12788963	314	8	35	TTCAAATTTT	TTCAAATTTTCTGTGAAAGACTTTTTGGCGGTAAA

UNSB01.fasta	Cele-UNSB01_3:12817254	3	12817254	12818910	1657	46	36	GATATTTTCA	GATATTTTCATGTTCGGCAAAAGTACCGATTTGCCA
UNSB01.fasta	Cele-UNSB01_3:12834918	3	12834918	12835593	676	18	35	TTTCCGGCAA	TTTCCGGCAAAGCGGTAAATTGCCGGAATTGAAAA
UNSB01.fasta	Cele-UNSB01_3:12836433	3	12836433	12837192	760	21	34	TTTCAATTCC	TTTCAATTCCGCCAATATACCAATTTGCCGGAAG
UNSB01.fasta	Cele-UNSB01_3:12842209	3	12842209	12842347	139	11	12	TATACATATA	TATACATATACA
UNSB01.fasta	Cele-UNSB01_3:12885748	3	12885748	12886017	270	5	43	GGCAAATCGG	GGCAAATCGGCAAACCGGGGAATTATTGGAATTGAAAATTTCC
UNSB01.fasta	Cele-UNSB01_3:12889217	3	12889217	12889593	377	11	34	TTTCAATTCC	TTTCAATTCCGGCAATTTGCCGACTTGCCGGAAA
UNSB01.fasta	Cele-UNSB01_3:12934390	3	12934390	12934631	242	8	35	TTTTAATTCC	TTTTAATTCCGGCAATTTGCCGGATTGTCGGAAAT
UNSB01.fasta	Cele-UNSB01_3:12996870	3	12996870	12997291	422	8	35	GGAAATTTTA	GGAAATTTTAAATTCCGGCAATTTGCCGGTTAGCC
UNSB01.fasta	Cele-UNSB01_3:13000875	3	13000875	13002076	1202	5	32	GACCAATCAG	GACCAATCAGCGATTCGCCCCGCCCACTCTCT
UNSB01.fasta	Cele-UNSB01_3:13002319	3	13002319	13002507	189	5	38	TTTTGGCGGG	TTTTGGCGGGAATTCAAATTTTAATTTTTTTGAAAATA
UNSB01.fasta	Cele-UNSB01_3:13026111	3	13026111	13026534	424	13	35	AAATTTTGAT	AAATTTTGATTTTTGGCAATTATCCGATTTGCCGG
UNSB01.fasta	Cele-UNSB01_3:13035804	3	13035804	13036305	502	16	35	ATTTGCCGGA	ATTTGCCGGAACATTTGATTTTCTGGAATATACCG
UNSB01.fasta	Cele-UNSB01_3:13044209	3	13044209	13044762	554	5	32	ACCAATCAGC	ACCAATCAGCGATTTGCTCCGCCCACTTTTGA
UNSB01.fasta	Cele-UNSB01_3:13065987	3	13065987	13066581	595	17	35	AAATTCAAAT	AAATTCAAATTTTCTGATAAATATTTTATGGCGGT
UNSB01.fasta	Cele-UNSB01_3:13121321	3	13121321	13121505	185	5	31	GATTTTTTCA	GATTTTTTTCAGGGAAAAACCAAAAAAATTC
UNSB01.fasta	Cele-UNSB01_3:13127780	3	13127780	13127920	141	4	35	CGGCAATTTG	CGGCAATTTGCCAAAAATGAAAATTTCCGGCAAAT
UNSB01.fasta	Cele-UNSB01_3:13139705	3	13139705	13140905	1201	30	20	TGTAGATTTA	TGTAGATTTACGAAATTCTA
UNSB01.fasta	Cele-UNSB01_3:13143562	3	13143562	13144065	504	16	19	TTGGCAATTT	TTGGCATTTGACCAAAAAT
UNSB01.fasta	Cele-UNSB01_3:13181955	3	13181955	13182047	93	5	15	CGAAAAGGGG	CGAAAAGGGGAGATA
UNSB01.fasta	Cele-UNSB01_3:13182222	3	13182222	13182299	78	5	15	TCCCCTTTTC	TCCCCTTTTCGTATC
UNSB01.fasta	Cele-UNSB01_3:13188476	3	13188476	13189044	569	13	35	TGCCGGAATT	TGCCGGAATTTAGAATTTTCGACAAAGTGGCAAAT
UNSB01.fasta	Cele-UNSB01_3:13248196	3	13248196	13248346	151	9	12	TAAGCCTAAG	TGAGCCTAAGCA
UNSB01.fasta	Cele-UNSB01_3:13289132	3	13289132	13289912	781	10	78	CGAAGTTGCT	CGCAGTTGCTGGTCCAGAGATCACAACAGGCGGATTTGGCGGCATAACCTGAGCAACGGTAGTGTCATTTGGTTCTTC
UNSB01.fasta	Cele-UNSB01_3:13290888	3	13290888	13291020	133	7	12	CGCGACACGC	CGCGACACGCAA
UNSB01.fasta	Cele-UNSB01_3:13335994	3	13335994	13337798	1805	147	10	GCTACCGCCC	GCTACCGCCC
UNSB01.fasta	Cele-UNSB01_3:13409998	3	13409998	13411238	1241	25	43	CGGAAATTTT	CGGAAATTTTCAATTCCGGCAACTTGCCAGTTTGCCGGTTTGC
UNSB01.fasta	Cele-UNSB01_3:13415830	3	13415830	13416475	646	18	34	CGGAATTGAA	CGGAATTGAAATTTCCGACAAATCGGCAAATTAT
UNSB01.fasta	Cele-UNSB01_3:13432209	3	13432209	13432275	67	5	11	AAAAATGTGC	AAAAATGTGCA
UNSB01.fasta	Cele-UNSB01_3:13445252	3	13445252	13445836	585	45	12	AGTTATATAG	AGTTATATAGTA

UNSB01.fasta	Cele-UNSB01_3:13483516	3	13483516	13483661	146	5	31	CCAAAAATGA	CCAAAAATGACTGAAATTAGTCAAAAATTGG
UNSB01.fasta	Cele-UNSB01_3:13559329	3	13559329	13561136	1808	95	19	AAAAATAGAT	AAAAATAGATTGGCATACG
UNSB01.fasta	Cele-UNSB01_3:13583922	3	13583922	13584205	284	8	30	TTTCAGGCCA	TTTCAGGCCAATTTCGGTCAATTTTGGACA
UNSB01.fasta	Cele-UNSB01_3:13762939	3	13762939	13765339	2401	60	40	ATTTTTCTAG	ATTTTTCTAGAAAGTTCTGGAACATTCCAGAATTTTCCCA
UNSB01.fasta	Cele-UNSB01_3:13765752	3	13765752	13765977	226	15	15	CCACGATGGG	CCACGATGGGTCTCA
UNSB01.fasta	Cele-UNSB01_3:13900788	3	13900788	13900959	172	7	24	AAGAAGGATG	AAGAAAGATGCCGAAGAGAAGAAG
UNSB01.fasta	Cele-UNSB01_3:14059760	3	14059760	14060650	891	57	15	TCGTGGTGAG	TCGTGGTGAGACCCA
UNSB01.fasta	Cele-UNSB01_3:14063373	3	14063373	14064273	901	60	15	TCGTGGTGAG	TCGTGGTGAGACCCA
UNSB01.fasta	Cele-UNSB01_4:1	4	1	5637	5637	464	12	AAGCCTAAGC	AAGCCTAAGCCT
UNSB01.fasta	Cele-UNSB01_4:125359	4	125359	125607	249	13	19	TGGTTGAGGC	TGGTTGAGGCTCTCTATTT
UNSB01.fasta	Cele-UNSB01_4:128195	4	128195	129066	872	28	19	TTGGTTGAGG	TTGGTTGAGGCTCAAAATT
UNSB01.fasta	Cele-UNSB01_4:129890	4	129890	130903	1014	21	22	TTGGTTGAGG	TTGGTTGAGGCTCTCTGGATTT
UNSB01.fasta	Cele-UNSB01_4:160082	4	160082	160208	127	8	14	CCTAAGCCTA	CCTAAGCCTAAGGT
UNSB01.fasta	Cele-UNSB01_4:186112	4	186112	186245	134	7	19	CCTCAACCAA	CCTCAACCAAAATTCTGAG
UNSB01.fasta	Cele-UNSB01_4:201016	4	201016	202120	1105	53	20	CTCAACCAAA	CTCAACCAAAAATCCTTAGC
UNSB01.fasta	Cele-UNSB01_4:221202	4	221202	221862	661	32	20	CTCAGCCTCA	CTCAGCCTCAATCAAAAATT
UNSB01.fasta	Cele-UNSB01_4:236013	4	236013	237239	1227	63	18	CTCAACCAAA	CTCAACCAAACTCTTAGC
UNSB01.fasta	Cele-UNSB01_4:242271	4	242271	242555	285	12	22	TGAGGCTCTC	TGAGGCTCTCTAGATTTTTGGT
UNSB01.fasta	Cele-UNSB01_4:244645	4	244645	245490	846	8	18	AGCCTCAACC	AGCCTCAACCAAATTCTT
UNSB01.fasta	Cele-UNSB01_4:286805	4	286805	287284	480	13	37	TTGGCGGGAA	TTGGCGGGAATTCAAAATTTAATTTTTTGAACATATT
UNSB01.fasta	Cele-UNSB01_4:287372	4	287372	287852	481	32	15	TCGTGGTGAG	TCGTGGAGAGACCCT
UNSB01.fasta	Cele-UNSB01_4:355820	4	355820	356311	492	11	45	GAAGGATCAG	GAAGGATCAGACCATCAGGAGGTACCCTTACGATCCTTCTTATCT
UNSB01.fasta	Cele-UNSB01_4:388221	4	388221	388493	273	8	34	TTGCAGGAAA	TTGCAGGAAATTTCAATTCAGCCGATTTGCCGGT
UNSB01.fasta	Cele-UNSB01_4:391568	4	391568	391630	63	4	15	TCCCCTTTTC	TCCCCTTTTCGCAGA
UNSB01.fasta	Cele-UNSB01_4:552425	4	552425	552551	127	9	12	TAGGCTTAGG	TAGGCTAAGGCT
UNSB01.fasta	Cele-UNSB01_4:600866	4	600866	601084	219	10	16	AGGCATGTAG	AGGCATGTAGGCATGT
UNSB01.fasta	Cele-UNSB01_4:634851	4	634851	634943	93	6	15	TCCCCTTTTC	TCCCCTTTTCGTATC
UNSB01.fasta	Cele-UNSB01_4:646705	4	646705	646786	82	4	15	TAAGCCTAAG	TAAGCCTAAGCCCAC
UNSB01.fasta	Cele-UNSB01_4:695215	4	695215	705007	9793	96	102	TGATATGTCG	TGATATGTCGAACGATTGATAGTTTGGGAATCAAGAGAATCAGTTGGACGATCAATTCCATCTTTGGGTCTTGCTGCAGCATATCTTTCTCCAGTACCACCT

UNSB01.fasta	Cele-UNSB01_4:782171	4	782171	782295	125	4	31	CAAAAATCGG	CAAAAATCGGGAATTTTTCACAAAAAAATGT
UNSB01.fasta	Cele-UNSB01_4:984710	4	984710	985413	704	35	19	CAATAATTTC	CAATAATTTCATAAAAAAT
UNSB01.fasta	Cele-UNSB01_4:1076311	4	1076311	1076365	55	4	12	AGCCTAAGCC	AGCCTAAGCCTA
UNSB01.fasta	Cele-UNSB01_4:1078257	4	1078257	1078305	49	4	12	AGGCTTAGGC	AGGCTTAGGCTT
UNSB01.fasta	Cele-UNSB01_4:1160688	4	1160688	1160967	280	7	40	ATTTTTCTAG	ATTTTTCTAGAAAGTTCTGGAACATTTCAGACTTTTCCCA
UNSB01.fasta	Cele-UNSB01_4:1160589	4	1160589	1161121	533	4	35	AAAATATTTT	AAAATATTTTTCACAGAAAATTTAAATTTCCCTCC
UNSB01.fasta	Cele-UNSB01_4:1161180	4	1161180	1161420	241	16	15	GGTCTCACCA	GGTCTCACCACGATG
UNSB01.fasta	Cele-UNSB01_4:1160701	4	1160701	1161595	895	10	40	GTTCTGGAAC	GTTCTGGAACATTTCAGACTTTTCCCAATTTTTCTAGAAA
UNSB01.fasta	Cele-UNSB01_4:1161685	4	1161685	1161942	258	8	11	GTACTCCTAC	GTAATCCTACC
UNSB01.fasta	Cele-UNSB01_4:1309063	4	1309063	1309423	361	9	36	TTATTTTCTG	TTATTTTCTGACAAATTGCCGGTTTGCCGGAAATTT
UNSB01.fasta	Cele-UNSB01_4:1313914	4	1313914	1314237	324	8	36	TTTGCCGGAA	TTTGTCGGAAATTTTTATTTTCTGACAAATTGCCGG
UNSB01.fasta	Cele-UNSB01_4:1358775	4	1358775	1359527	753	22	34	ATTTCCCGCC	ATTTCCCGCCAAAATTTTTTCACTGAAAATTTGA
UNSB01.fasta	Cele-UNSB01_4:1359612	4	1359612	1359777	166	11	15	CCACGATGGG	CCACGATGGGTCTCG
UNSB01.fasta	Cele-UNSB01_4:1362949	4	1362949	1363282	334	9	34	ATTGCCGGAA	ATTGCCGGAATTCAAATTCCTAGCAAATCGGCAA
UNSB01.fasta	Cele-UNSB01_4:1370044	4	1370044	1370460	417	8	12	TAGGCTTAGG	TAGGCTTAGGCG
UNSB01.fasta	Cele-UNSB01_4:1429332	4	1429332	1429629	298	15	21	AAAATTGACT	AAAATCGACTAAAAATCGTCA
UNSB01.fasta	Cele-UNSB01_4:1444044	4	1444044	1444098	55	4	12	CATAGGCATA	CATAGGCATAGA
UNSB01.fasta	Cele-UNSB01_4:1452742	4	1452742	1452796	55	4	12	TATATGTATA	TATATGTATATG
UNSB01.fasta	Cele-UNSB01_4:1454445	4	1454445	1454610	166	11	15	AGACCCATCG	AGACCCATCGTGGTG
UNSB01.fasta	Cele-UNSB01_4:1461281	4	1461281	1461373	93	6	15	TCCCCTTTTC	TCCCCTTTTCGTATC
UNSB01.fasta	Cele-UNSB01_4:1490085	4	1490085	1491772	1688	46	35	AAAAAATTTT	AAAAAATTTTGGCGGGAAATTCAAATTTTCAGTGA
UNSB01.fasta	Cele-UNSB01_4:1491894	4	1491894	1492067	174	5	35	GAAAATTTGA	GAAAATTTGAATTTCCCGCCAAAATTTTTTTCACT
UNSB01.fasta	Cele-UNSB01_4:1492099	4	1492099	1492204	106	7	15	CTCACCACGA	CTCACCACGATGGGT
UNSB01.fasta	Cele-UNSB01_4:1508767	4	1508767	1509123	357	5	12	AGGCTTAGGC	AGGCTTAGGCCT
UNSB01.fasta	Cele-UNSB01_4:1530750	4	1530750	1530982	233	8	29	CCACCCGGGA	CCACCCGGGAGGCGTCGGCTGCTCTAAGC
UNSB01.fasta	Cele-UNSB01_4:1558589	4	1558589	1558964	376	4	94	TCCCATTGAT	TCCCATTGATTTGTCTACATAGGGCATTGAAAAGCACCCAATATTTAGAGAACAGAAGATTTTGAGAATTACTGCCTTCAGAAATTGATGATTT
UNSB01.fasta	Cele-UNSB01_4:1580035	4	1580035	1580182	148	7	21	CCATAGGTGG	CCATAGGTGGATGATCAGATA
UNSB01.fasta	Cele-UNSB01_4:1584655	4	1584655	1585480	826	7	101	TTGATTTTTC	TTGATTTTTCGTCTTTTGAAGGGATATTGGTTTCGGGGTAGTGGGCGGATATGGTCGGGGTACTGTAGGTATACGGTAGGGTTACTGTAGTTTTGGAAATT
UNSB01.fasta	Cele-UNSB01_4:1639262	4	1639262	1639353	92	6	14	ACTACACACT	ACTACACACTACAC

UNSB01.fasta	Cele-UNSB01_4:1643155	4	1643155	1643244	90	6	12	TTAGGCTTAG	TTAGGCTTCGGC
UNSB01.fasta	Cele-UNSB01_4:1647487	4	1647487	1647595	109	9	12	CTTAAGCTTA	CTTAAGCTTAGG
UNSB01.fasta	Cele-UNSB01_4:1654144	4	1654144	1654247	104	5	23	ATTTAATTAA	ATTTATTTAATTAATTAATATTT
UNSB01.fasta	Cele-UNSB01_4:1669669	4	1669669	1669879	211	6	35	ATTTCCGGCA	ATTTCCGGCAAATCGGCAATATGCCAAAAATGGAT
UNSB01.fasta	Cele-UNSB01_4:1695920	4	1695920	1696142	223	6	37	CGCCAAAATG	CGCCAAAATGATTCCAAAAAATTAAAATTTGAATTCC
UNSB01.fasta	Cele-UNSB01_4:1703835	4	1703835	1704045	211	11	12	CTAAGCCTAA	CTAAGCCTAATA
UNSB01.fasta	Cele-UNSB01_4:1729821	4	1729821	1729883	63	4	15	CGAAAAGGGG	CGAAAAGGGGATCTG
UNSB01.fasta	Cele-UNSB01_4:1755267	4	1755267	1755667	401	20	20	CAGAAAAATC	CAGAAAAATCAATAAATTCT
UNSB01.fasta	Cele-UNSB01_4:1766949	4	1766949	1767039	91	5	12	TAAGCCTAAG	TAAGCCTAAGAC
UNSB01.fasta	Cele-UNSB01_4:1777357	4	1777357	1777453	97	5	16	CACGTAGGCA	CACGTAAGCACGTAGG
UNSB01.fasta	Cele-UNSB01_4:1800043	4	1800043	1800133	91	7	12	TTAGGATTAG	TTAGGATTAGGA
UNSB01.fasta	Cele-UNSB01_4:1802551	4	1802551	1803063	513	30	16	TAGGCACGTA	TAGGTACGTAGGCACG
UNSB01.fasta	Cele-UNSB01_4:1866819	4	1866819	1867315	497	18	21	TGTCGATTTA	TGTCGATTTACGGGGGTTCAG
UNSB01.fasta	Cele-UNSB01_4:1908625	4	1908625	1908709	85	5	12	TAAGCCTAAG	TAAGTCTAAGCT
UNSB01.fasta	Cele-UNSB01_4:1946817	4	1946817	1947132	316	8	35	GAAATTTTCA	GAAATTTTCAATTCCGGCAGTTTGCCGATTTGCCG
UNSB01.fasta	Cele-UNSB01_4:1949645	4	1949645	1950620	976	28	35	CATTTTCGCC	CATTTTCGCCGAATTGCCGATTTGCCGGAAATTTT
UNSB01.fasta	Cele-UNSB01_4:1966969	4	1966969	1971964	4996	213	21	GTAAATCTAC	GTAAATCTACAAGAAACCCCC
UNSB01.fasta	Cele-UNSB01_4:1985632	4	1985632	1987358	1727	15	80	AATTTCTCAA	AATTTCTCAATAGCGCGCGCTTGCAACATCCGAACGGCGCGAAATTTGAGTTTTTGGCCATAAAATAACGAAAATCGGCA
UNSB01.fasta	Cele-UNSB01_4:2119001	4	2119001	2119076	76	5	15	GGGTCTCACC	GGGTCTCGCCACGAT
UNSB01.fasta	Cele-UNSB01_4:2158460	4	2158460	2158710	251	6	40	TTCCAGAATT	TTCCAGAATTTTCTCGAATATTCCAGAAGCTTCTAGAACA
UNSB01.fasta	Cele-UNSB01_4:2158825	4	2158825	2158968	144	5	11	CTACAGTACC	CTACAGTACTA
UNSB01.fasta	Cele-UNSB01_4:2163715	4	2163715	2164250	536	8	43	TTGCCGGAAA	TTGCCGGAAATTTTCAATCGCGGCATTTTGCCGGTTTGCTCAT
UNSB01.fasta	Cele-UNSB01_4:2179058	4	2179058	2179920	863	38	21	GATTTACGGG	GATTTACGGGATGAAAATGTG
UNSB01.fasta	Cele-UNSB01_4:2205280	4	2205280	2205605	326	13	21	AAAATAGCTT	AAAATAGCTGAAAATTCACTA
UNSB01.fasta	Cele-UNSB01_4:2267683	4	2267683	2267908	226	15	15	CGATGGGTCT	CGATGGGTCTCACCA
UNSB01.fasta	Cele-UNSB01_4:2268162	4	2268162	2268293	132	4	11	GTACTCCTAC	GTAATCCTACC
UNSB01.fasta	Cele-UNSB01_4:2419217	4	2419217	2421562	2346	23	70	AAAAATCGAT	AAAAATCGATGTTTTGAAGTTTTTCTCAATAGAGCGCACTTTCAACAGCGAGAAATTTGAATTTTCCACC
UNSB01.fasta	Cele-UNSB01_4:2422782	4	2422782	2423615	834	8	104	CCTGCGAAAG	CCTGCGAAAGCAAATCTCTGGCCTGACGGAGGGCCAGCTAACTCTCTCCCTTAAGCCTTCGGGTGCTTCCTGGGTTATCGGTGAATTTTTGGGTCTGTCAGGGT
UNSB01.fasta	Cele-UNSB01_4:2456640	4	2456640	2457356	717	5	144	TTCTAGGCCA	TTCTAGGCCACCAATTGGAAATCATCATAACTTGGCTTAAAATCCAGATTTTTGGAGTCTGGTAACTGCAACATGTTGCCCTGTAGTTTTTCTACAAGTTAGGATATATTGATTTTTGGTGGCCTAAAAGTCCAAATTATGGAT

UNSB01.fasta Cele-UNSB01_4:2599504 4 2599504 2602273 2770 17 164 CTTTTTTCTT
CTTTTTTCTTTTATAAAACTGTTTAGCATAGTCGAATATACCAGAAAATACCAAACAAAGTATGCTATCTTGTACGAAAGATTATTTTAAAAAATTCAGAAAATGCATCTAAAGCGGTTTTTTTTCAAAAATTCAAAAGTACCGGAAAA
TCATAAAAAGTGCTT
UNSB01.fasta Cele-UNSB01_4:2676914 4 2676914 2677180 267 11 19 CGCCAAGTAT
CGCCAAGTGTCCGAAAATA
UNSB01.fasta Cele-UNSB01_4:2701520 4 2701520 2702341 822 4 175 AACTTGTGAC
AACTTGTGACGGTGGGTATGGATTTTATGCACATATTTTGTTTGAAATAGTCTACCAAGTAAACTGGTGAAGTTTCAAAAACTTTGTGGATCTAGTTTTTGAAAAAAGTTCAAAATTGCGCCAATTTTGACCAATTTTGGTGGTTTTCT
GAGCTAAAATTGAAAAGTTGGGAATG
UNSB01.fasta Cele-UNSB01_4:2721172 4 2721172 2721362 191 5 40 GAATTTTCCA
GAATTTTTCAGAAAGTTCTAGAACATTCCAGAATTTTTTC
UNSB01.fasta Cele-UNSB01_4:2728247 4 2728247 2729274 1028 42 21 TTTGGATTTT
TTCGGATTTTTAGGCTTAAAA
UNSB01.fasta Cele-UNSB01_4:2780831 4 2780831 2781210 380 12 11 TACTGTAGGA
TACTGTAGAGA
UNSB01.fasta Cele-UNSB01_4:2843789 4 2843789 2844131 343 4 87 TGAAGTCGTA
TGAAGTCGTAGGATTCTGTGTAGCGACTGGTGTCGTTGTACTCTGTGAAGGGGACGTGGCTTCTTCTGTTGAGTCAGGCTTCTGAGG
UNSB01.fasta Cele-UNSB01_4:2889237 4 2889237 2889839 603 7 35 TTTTCAATTC
TTTTCAATTCAAGCAATTTGCAGTTTTGCCTGAAT
UNSB01.fasta Cele-UNSB01_4:2892401 4 2892401 2892617 217 5 24 GCTTCTGGAG
GCTTCTGGAGCCACAGGTTATCCA
UNSB01.fasta Cele-UNSB01_4:2898426 4 2898426 2898868 443 12 19 ACCTAGGCAT
ACCTATGCATAAGGCAGGC
UNSB01.fasta Cele-UNSB01_4:2909681 4 2909681 2910188 508 14 35 ATTCCGGCAA
ATTCCGGCAAATTACCGATTTGCCGAAAATTTTCA
UNSB01.fasta Cele-UNSB01_4:2933784 4 2933784 2933839 56 5 11 TTGTTTACAA
TTGTTTACAAG
UNSB01.fasta Cele-UNSB01_4:2990271 4 2990271 2990613 343 19 16 TAGGCATGTA
TAGGCATGTAGGAATG
UNSB01.fasta Cele-UNSB01_4:3002005 4 3002005 3002220 216 4 43 TTTGCCGGAA
TTTGCCGGAATTGAAAACTACCGGCAAATCTGCAAACTGGAAA
UNSB01.fasta Cele-UNSB01_4:3004201 4 3004201 3004345 145 6 24 GCGGCCGACA
GCGGCCGACACCTTACGGGTTGTA
UNSB01.fasta Cele-UNSB01_4:3018891 4 3018891 3018971 81 4 16 AGGTAGGAAG
AGATAGGAAGGTAGGA
UNSB01.fasta Cele-UNSB01_4:3087838 4 3087838 3087930 93 6 15 CGAAAAGGGG
CGAAAAGGGGATCTG
UNSB01.fasta Cele-UNSB01_4:3088105 4 3088105 3088197 93 6 15 TCCCCTTTTC
TCCCCTTTTCGTATC
UNSB01.fasta Cele-UNSB01_4:3090317 4 3090317 3090467 151 8 15 CACGATGGGT
CACGATGGGTCTCAT
UNSB01.fasta Cele-UNSB01_4:3135501 4 3135501 3135649 149 4 37 TGGCGGGAAT
TGGCGGGAATTCAAATTTCAATTTTTAGAACTCATTT
UNSB01.fasta Cele-UNSB01_4:3176916 4 3176916 3177039 124 7 12 TAGGCTTAGG
TAGGCGTAGGCA
UNSB01.fasta Cele-UNSB01_4:3192518 4 3192518 3194426 1909 53 35 CGGCAATTTG
CAGCAATTTGCCGATTTGCCGGAAATTTTCAGTTC
UNSB01.fasta Cele-UNSB01_4:3222029 4 3222029 3222419 391 5 16 TACATGCCTA
TACATGCCTACATGCC
UNSB01.fasta Cele-UNSB01_4:3226485 4 3226485 3226669 185 11 16 CATGCCTACA
CATGCCTACATACATA
UNSB01.fasta Cele-UNSB01_4:3230422 4 3230422 3232144 1723 67 16 ATGCCTACAT
ATGCCTACATGGCTAG
UNSB01.fasta Cele-UNSB01_4:3232737 4 3232737 3232899 163 6 18 CCTTCGCCTA
CCTTCGCCTACCTGCATG
UNSB01.fasta Cele-UNSB01_4:3243719 4 3243719 3271867 28149 1487 19 CCTCACTCAA
CCTCACTCAAAATCCTCAG
UNSB01.fasta Cele-UNSB01_4:3274030 4 3274030 3274126 97 6 16 AGGCATGTAG
AGGCATGTAGGCATGC

UNSB01.fasta	Cele-UNSB01_4:3353341	4	3353341	3353481	141	5	33	CGGCATATGC	CGGCATATGCCGTGAATTTTAAAATCGGCATAA
UNSB01.fasta	Cele-UNSB01_4:3371888	4	3371888	3372867	980	27	35	AATCGGCAAG	AATCGGCAAGTTACCGAAATTGAAAATTTCCGGCA
UNSB01.fasta	Cele-UNSB01_4:3428944	4	3428944	3430273	1330	24	45	TCTGATCCTT	TCTGATCCTTCAGCTCCAAAGGATCAGAAGGGTACCTCCTGGTGA
UNSB01.fasta	Cele-UNSB01_4:3517707	4	3517707	3518097	391	27	12	TAAGCCTAAG	TAAGCCTAAGCT
UNSB01.fasta	Cele-UNSB01_4:3630301	4	3630301	3630781	481	28	16	TGCCTACATG	TGCCTACATGCCCACA
UNSB01.fasta	Cele-UNSB01_4:3670951	4	3670951	3671065	115	7	12	TTAGGCTTAG	TTAGGATTAGGA
UNSB01.fasta	Cele-UNSB01_4:3714325	4	3714325	3715225	901	15	21	TGTAGATTTA	TGTAGATATACGGGAATCTTG
UNSB01.fasta	Cele-UNSB01_4:3733029	4	3733029	3733089	61	4	15	TCGTGGTGAG	TCGTGGTGAGACCCT
UNSB01.fasta	Cele-UNSB01_4:3733495	4	3733495	3734015	521	15	40	TTTCTAGAAA	TTTCTAGAAAATTCGGGAAAACTCTGGAATATTCCAGAAC
UNSB01.fasta	Cele-UNSB01_4:3758727	4	3758727	3758973	247	6	41	AAATTTTTAA	AAATTTTTAACTAAAAGTGGGCGAAAATCATTCAAAAATTC
UNSB01.fasta	Cele-UNSB01_4:3817232	4	3817232	3817391	160	5	21	TAGTTTGTAG	TAGTTTATAGTTCACAGTTTA
UNSB01.fasta	Cele-UNSB01_4:3888107	4	3888107	3888300	194	12	16	TAGGCATGTA	TAGGCATGTAGGCATG
UNSB01.fasta	Cele-UNSB01_4:3896416	4	3896416	3896737	322	5	18	ATTAGTATTG	ATTAGTATTGCCCTACCT
UNSB01.fasta	Cele-UNSB01_4:4053946	4	4053946	4054647	702	6	12	TAAGCCTAAG	TAAGCCTATGCC
UNSB01.fasta	Cele-UNSB01_4:4229494	4	4229494	4229583	90	7	12	GTTGCGTGTC	GTTGCGTGTCGC
UNSB01.fasta	Cele-UNSB01_4:4229497	4	4229497	4230359	863	71	12	GCGTGTCGCG	GCGTGTCGCGTT
UNSB01.fasta	Cele-UNSB01_4:4261575	4	4261575	4261676	102	6	17	GATGCCCCCT	GATGCCCCCTCTGAACG
UNSB01.fasta	Cele-UNSB01_4:4368763	4	4368763	4369020	258	5	11	GTACTGTAGG	GTACTGTAGGA
UNSB01.fasta	Cele-UNSB01_4:4388126	4	4388126	4388633	508	13	11	CTACAGTACT	CTACAGTACTA
UNSB01.fasta	Cele-UNSB01_4:4398109	4	4398109	4398181	73	4	18	TTTTTTGATA	TTTTTTGATAATTCTTGA
UNSB01.fasta	Cele-UNSB01_4:4418485	4	4418485	4418703	219	5	35	TTCCGGCAAA	TTCAGGCAAATCGGCAAATTGCCGGAATTGAATAC
UNSB01.fasta	Cele-UNSB01_4:4464716	4	4464716	4464793	78	5	15	CGAAAAGGGG	CGAAAAGGGGATCTG
UNSB01.fasta	Cele-UNSB01_4:4479166	4	4479166	4488089	8924	327	26	GTCTAACTTT	GTCTAACTTTGGAAATTCATCTAATG
UNSB01.fasta	Cele-UNSB01_4:4525521	4	4525521	4525683	163	12	12	GCCTAAGCCT	GCCTAAGACTAC
UNSB01.fasta	Cele-UNSB01_4:4598852	4	4598852	4599551	700	6	11	GCAGTAGGCA	GCAGTAGGAAG
UNSB01.fasta	Cele-UNSB01_4:4620647	4	4620647	4621153	507	13	11	CTACAGTACT	CTACAGTACTA
UNSB01.fasta	Cele-UNSB01_4:4665165	4	4665165	4665209	45	4	11	TGACAACTTT	TGACAACTTTG
UNSB01.fasta	Cele-UNSB01_4:4768182	4	4768182	4768259	78	5	15	CGAAAAGGGG	CGAAAAGGGGATCTG
UNSB01.fasta	Cele-UNSB01_4:4768434	4	4768434	4768526	93	6	15	TCCCCTTTTC	TCCCCTTTTCGTATC

UNSB01.fasta	Cele-UNSB01_4:4858228	4	4858228	4858874	647	17	35	TTGCCGGAAA
TTGCCGGAAATTTTGGTTTCCGGCAAGTTTCCGAT								
UNSB01.fasta	Cele-UNSB01_4:4963981	4	4963981	4964546	566	18	15	GAGACCCATC
GAGACCCGTCGTGGT								
UNSB01.fasta	Cele-UNSB01_4:4986658	4	4986658	4986858	201	5	40	TTCTAGAAAG
TTCTAGAAAGTTCTGGAACATTCTAGAATTTTCTCGATTT								
UNSB01.fasta	Cele-UNSB01_4:4986914	4	4986914	4987437	524	15	35	TTTTCAGAAA
TTTTCAGAAAATTAAAATTTCCCTCCAAAATATTT								
UNSB01.fasta	Cele-UNSB01_4:4987504	4	4987504	4988044	541	32	15	GGGTCTCACC
GGGTCTCACCACGAT								
UNSB01.fasta	Cele-UNSB01_4:4988071	4	4988071	4988281	211	14	15	ACCCATCGTG
ACCCATCGTGGCGAG								
UNSB01.fasta	Cele-UNSB01_4:4987504	4	4987504	4989222	1719	80	15	GGGTCTCACC
GGGTCTCACCACGAT								
UNSB01.fasta	Cele-UNSB01_4:4990413	4	4990413	4990623	211	6	35	AATATTTTTT
AATATTTTTTTCAGAAAATTAAAATTTCCCTCCAA								
UNSB01.fasta	Cele-UNSB01_4:4990696	4	4990696	4991348	653	40	15	GGGTCTCACC
GGGTCTCACCACGAT								
UNSB01.fasta	Cele-UNSB01_4:5061085	4	5061085	5061475	391	8	39	CCAACTGGAG
CCAACTGGAGCCAGCCCTATGACGGCCTTTGGAGGAGCA								
UNSB01.fasta	Cele-UNSB01_4:5124505	4	5124505	5124705	201	12	16	CTACGTGCCT
CTACGTGTCTACGTGC								
UNSB01.fasta	Cele-UNSB01_4:5337721	4	5337721	5338093	373	18	12	AGAAGGAGGA
AGAAAGAGGAGA								
UNSB01.fasta	Cele-UNSB01_4:5363528	4	5363528	5363903	376	15	25	TTTACTCTCT
TTTACTCTCTTGCTTCACAGAATAA								
UNSB01.fasta	Cele-UNSB01_4:5366596	4	5366596	5366788	193	10	12	TCCTCTTTCT
TCCTCTTTCTTC								
UNSB01.fasta	Cele-UNSB01_4:5521070	4	5521070	5521205	136	9	15	CCACGATGGG
CCACGATGGGTCTCG								
UNSB01.fasta	Cele-UNSB01_4:5529418	4	5529418	5534698	5281	110	48	TTGCCCGTAA
TTGCCTGTAATTTATATTTTTCTGAAGATGAGCAATTCAATTTTTGAT								
UNSB01.fasta	Cele-UNSB01_4:5758139	4	5758139	5758194	56	5	11	TCCACTGACC
TCCACTGACCA								
UNSB01.fasta	Cele-UNSB01_4:5945791	4	5945791	5945941	151	6	25	CCAGAGAGTA
CCAGAGAGTAAAATATACTGGGAAG								
UNSB01.fasta	Cele-UNSB01_4:6076861	4	6076861	6077186	326	6	55	AAGAGTCCCC
AAGAGTCCCCACACACTGAAAGGTTTAAGGAATTTTTTGAGGATTCCTAGGGTT								
UNSB01.fasta	Cele-UNSB01_4:6158202	4	6158202	6158352	151	6	25	TTTACTCTCT
TTTACTCTCTGGCTTCACAGTATAT								
UNSB01.fasta	Cele-UNSB01_4:6277867	4	6277867	6278002	136	7	19	ATTGACAGAC
ATTGACAGACGAAAAATAG								
UNSB01.fasta	Cele-UNSB01_4:6359332	4	6359332	6359386	55	4	13	TTTCATTTGT
TTTCATTTGTAAT								
UNSB01.fasta	Cele-UNSB01_4:6425603	4	6425603	6430447	4845	192	25	TTAGAGTAAA
TTAGAGTAAATAAATTAGCAGAACA								
UNSB01.fasta	Cele-UNSB01_4:6539759	4	6539759	6539908	150	10	12	CAGAATATAT
CAGAATATATTC								
UNSB01.fasta	Cele-UNSB01_4:6669007	4	6669007	6669090	84	4	22	AAGCGGTAAC
AAGCGGTAACGTCGTCTATATA								
UNSB01.fasta	Cele-UNSB01_4:6734929	4	6734929	6735237	309	9	31	CCCGCCAAAA
CCCGCCAAAATTTTTTGAAGAAATTCGGATT								
UNSB01.fasta	Cele-UNSB01_4:6945378	4	6945378	6946401	1024	11	93	TGCTTCTCGT
TGCTTCTCGTCGTCCACCACCACCACCGAAGGGAACTGGAACCCCACCACCACCACCAACTGGAGAACCACAGGATCTTTCTGCAGAAGGTAA								
UNSB01.fasta	Cele-UNSB01_4:7151737	4	7151737	7153750	2014	178	11	TGACAACTTC
TGACAACTTCA								
UNSB01.fasta	Cele-UNSB01_4:7464716	4	7464716	7464914	199	5	40	TCCAGAATTT
TCCAGAATTTTCCCGATTTTTCTAGAAAGTTCTGGAACAC								

UNSB01.fasta	Cele-UNSB01_4:7464933	4	7464933	7465143	211	6	35	CAGAAAATTT	CAGAAAATTTAAATTTCCCGCCAAAATATTTTTCA
UNSB01.fasta	Cele-UNSB01_4:7464716	4	7464716	7465331	616	6	40	TCCAGAATTT	TCCAGAATTTTCCCGATTTTTCTAGAAAGTTCTGGAACAC
UNSB01.fasta	Cele-UNSB01_4:7715733	4	7715733	7715854	122	6	17	CTCATTGCCT	CTCATTGACTACCCTTG
UNSB01.fasta	Cele-UNSB01_4:7767635	4	7767635	7768760	1126	25	45	AGCACCACCA	AGCACCACCATCAGGCGGACCACCAGGACTATTTGATCCATCAGG
UNSB01.fasta	Cele-UNSB01_4:8048486	4	8048486	8048578	93	6	15	CGAAAAGGGG	CGAAAAGGGGATCTG
UNSB01.fasta	Cele-UNSB01_4:8048753	4	8048753	8048845	93	6	15	TCCCCTTTTC	TCCCCTTTTCGTATC
UNSB01.fasta	Cele-UNSB01_4:8107145	4	8107145	8107415	271	18	15	CTCACCACGA	CTCACCACGATGGGT
UNSB01.fasta	Cele-UNSB01_4:8289359	4	8289359	8289627	269	6	45	TCTGATCCTT	TCTGATCCTTCAGCTCCGAAGGATCACATGGGCACCTTCTGATGA
UNSB01.fasta	Cele-UNSB01_4:8500583	4	8500583	8501255	673	21	32	TTTTCACAGA	TTTTCACAGAAAATTTGAATTCCCGCCAAAAT
UNSB01.fasta	Cele-UNSB01_4:8501471	4	8501471	8501871	401	10	40	AGAAGGTTCT	AGAAGGTTCTGGAACATTCCAGAATTTTCTCAAATTTTCC
UNSB01.fasta	Cele-UNSB01_4:8501476	4	8501476	8504168	2693	54	40	GTTCTGGAAC	GTTCTGGAACATTCCAGAATTTTCTCAAATTTTCCAGAAG
UNSB01.fasta	Cele-UNSB01_4:8504239	4	8504239	8504449	211	6	35	GAAAATTTAA	GAAAATTTAAATTTCCCACCAAAATATTTTTTTCA
UNSB01.fasta	Cele-UNSB01_4:8504495	4	8504495	8505035	541	35	15	TCTCACCACG	TCTCACCACGATGGG
UNSB01.fasta	Cele-UNSB01_4:8600392	4	8600392	8600896	505	13	11	CTACAGTACT	CTACAGTACTA
UNSB01.fasta	Cele-UNSB01_4:8607400	4	8607400	8623699	16300	831	20	ATACGATATA	ATACGATATAGCTATAAACA
UNSB01.fasta	Cele-UNSB01_4:8831351	4	8831351	8831453	103	6	17	TTCATTCCGT	TTCATTCCGTGATTATT
UNSB01.fasta	Cele-UNSB01_4:9041393	4	9041393	9041548	156	8	17	AGGCAATGAG	AGGCAATGAGAAAGAGT
UNSB01.fasta	Cele-UNSB01_4:9095021	4	9095021	9107795	12775	451	27	ATAGTGGGAA	ATAGTGGGAAGCCACAGAGAGTAAATA
UNSB01.fasta	Cele-UNSB01_4:9125039	4	9125039	9126314	1276	76	15	CTCACCACGA	CTCACCACGAAGGGT
UNSB01.fasta	Cele-UNSB01_4:9469814	4	9469814	9470654	841	56	15	ACGATGGGTC	ACGATGGGTCTCACC
UNSB01.fasta	Cele-UNSB01_4:9470711	4	9470711	9470851	141	5	30	AATTTTCCAG	AATTTTCCAGAAGGTTCTGGAACATTCCAG
UNSB01.fasta	Cele-UNSB01_4:9470964	4	9470964	9471243	280	10	11	GTACTCCTAC	GTACTCCTACA
UNSB01.fasta	Cele-UNSB01_4:9493015	4	9493015	9493149	135	4	11	CCCTACAGTA	CCCTACAGTAA
UNSB01.fasta	Cele-UNSB01_4:9825630	4	9825630	9825857	228	5	45	GGGTACCTCC	GGGTACCTCCTGGTGATCTGATCCTTCAGCTCCGAAAGATCAGAA
UNSB01.fasta	Cele-UNSB01_4:10086561	4	10086561	10087053	493	10	11	TACTGTAGGG	TACTGTAGGGG
UNSB01.fasta	Cele-UNSB01_4:10323949	4	10323949	10324337	389	26	15	GACCCATCGT	GACCCATCGTGGTGA
UNSB01.fasta	Cele-UNSB01_4:10324370	4	10324370	10326780	2411	67	35	GAAATTCAAA	GAAATTCAAATTTCAGTGAAAAAAATTTTTAGCGG
UNSB01.fasta	Cele-UNSB01_4:10610325	4	10610325	10610863	539	7	77	GGAAGCTTCA	GGAAGCTTCATCTTTTGAATTATAAAAAAATAAATTTCATTTCAAAATCAGTGTGTGCACTATCAAAAGTGTCCACT
UNSB01.fasta	Cele-UNSB01_4:10751931	4	10751931	10752311	381	20	19	ATTGTTACAC	ATTGTTACACGAAAAATAG

UNSB01.fasta	Cele-UNSB01_4:11013752	4	11013752	11031328	17577	392	45	TCCTGGTGAT

TCCTGGTGATGTGATCCTTAAGCTCCGAAGGATCACAGGGGTACC

UNSB01.fasta	Cele-UNSB01_4:11032975	4	11032975	11034460	1486	33	45	TCACCAGGAG

TCACCAGGAGGTACCCTTGTGATCCTTCGGAGCTGAAGGATCAGA

UNSB01.fasta	Cele-UNSB01_4:11073363	4	11073363	11083545	10183	395	26	GTTCACCAAA

GTTCACCAAATTTAGTGACTCTGGCC

UNSB01.fasta	Cele-UNSB01_4:11083607	4	11083607	11084360	754	4	11	TTTGAAATAT

TTTGAAATATA

UNSB01.fasta	Cele-UNSB01_4:11084695	4	11084695	11084799	105	4	26	CCAGAGTCAC

CCAGAGTCACAATTTTTGGTGAACAA

UNSB01.fasta	Cele-UNSB01_4:11101964	4	11101964	11102048	85	4	21	AAATCTAATC

AAATCTAATCCAATATGGCCG

UNSB01.fasta	Cele-UNSB01_4:11132963	4	11132963	11135728	2766	35	79	GAAACTAAAA

GAAACTAAAACCTAATGAGACCCCAGAATACTGCCGGTATTCTGTCAGGACTCATTAGGTAGAGAGGAGATTCACAGAA

UNSB01.fasta	Cele-UNSB01_4:11180885	4	11180885	11199179	18295	470	39	TTTATGGAAA

TTTATGGAAAAGTGGCTCTAGAGCCACTAGAAATACCAG

UNSB01.fasta	Cele-UNSB01_4:11302758	4	11302758	11302850	93	6	15	CGAAAAGGGG

CGAAAAGGGGATCTG

UNSB01.fasta	Cele-UNSB01_4:11303025	4	11303025	11303087	63	4	15	TCCCCTTTTC

TCCCCTTTTCGCAGA

UNSB01.fasta	Cele-UNSB01_4:11734646	4	11734646	11742696	8051	322	25	ATTACGGCAG

ATTACGGCAGATTCTATAAATTTGC

UNSB01.fasta	Cele-UNSB01_4:11742877	4	11742877	11743140	264	13	17	AAAATCAAAC

AAAATCAAACCGGAGAC

UNSB01.fasta	Cele-UNSB01_4:12149712	4	12149712	12149981	270	10	11	GTACTGTAGG

GTACTGTAGTA

UNSB01.fasta	Cele-UNSB01_4:12150067	4	12150067	12150386	320	8	40	TCCAGAACCT

TCCAGAACCTTCTGGAAAATTCGAGAAAATTCTGGAATGT

UNSB01.fasta	Cele-UNSB01_4:12150586	4	12150586	12159522	8937	274	32	CGGGAATTCA

CGGGAATTCAAAATTTCTGTGAAAAATTTTGG

UNSB01.fasta	Cele-UNSB01_4:12289740	4	12289740	12290350	611	8	35	AAATTTGAAT

AAATTTGAATTTCCCTCCAAAAATTTTTTACTGA

UNSB01.fasta	Cele-UNSB01_4:12303349	4	12303349	12310067	6719	128	59	TCCATAATTC

TCCATATTCTCCATAATTCTCGGTTTTAAAAATTTTGAAAACCAGTGAAGTCTTGATAT

UNSB01.fasta	Cele-UNSB01_4:12453898	4	12453898	12454471	574	6	96	GGTTGATTTG

GGTTGATTTGCGGCACTCTACAGATAGTGGATCTGCTTCTTACCAATCTTCCCGTTACCGCCACGCACTAAAGCTCCAAACTATTTTATTTTTTTG

UNSB01.fasta	Cele-UNSB01_4:12476016	4	12476016	12476336	321	18	16	GTGCCTACGT

GTGCCTACGTGCCTAC

UNSB01.fasta	Cele-UNSB01_4:12649750	4	12649750	12649885	136	5	27	ACAATAAAGT

ACAATAAAGTACTATGTATTACCGTAT

UNSB01.fasta	Cele-UNSB01_4:12734217	4	12734217	12734312	96	5	19	AAAAATAGAT

AAAAATAGATTGACAGACG

UNSB01.fasta	Cele-UNSB01_4:12770724	4	12770724	12770816	93	6	15	CGAAAAGGGG

CGAAAAGGGGATCTG

UNSB01.fasta	Cele-UNSB01_4:12770991	4	12770991	12771083	93	5	15	TCCCCTTTTC

TCCCCTTTTCGTATC

UNSB01.fasta	Cele-UNSB01_4:12787287	4	12787287	12803219	15933	166	96	GTTCCCATAA

GTTCCCATAATTGGGTTAAATACCACTCATAAGTAACTAGTATGGGACTGAAAAGATACTAAATGAGCTTATTCTAAGGGTGAAGCTTCTGGTAAT

UNSB01.fasta	Cele-UNSB01_4:12842983	4	12842983	12843155	173	5	35	AAACCGGCAA

AAACCGGCAATTGCCGAAATTGCCGAAAATTCTCG

UNSB01.fasta	Cele-UNSB01_4:12895275	4	12895275	12902336	7062	272	26	CATTGCGGCG

CATTGCGGCGGATTTTTGAAAAATGA

UNSB01.fasta	Cele-UNSB01_4:13061036	4	13061036	13070540	9505	198	48	ACGGGCAAAT

ACGGGCAAATCAAAAATTAAATTGCTCATCTTCAGAAAAATATAAATT

UNSB01.fasta	Cele-UNSB01_4:13110533	4	13110533	13113073	2541	14	168	TTTTTAAAAA

TTTTTAAAAAATCTAGAATCCAAATCTATCAAAACATTTCCGGAATTCGATAATTTTTATTTTTGTTTTTTTGAGCCTACTAGTACCTGTGAAAATTGGAACTCGACACAAAAACCTCTCTAGCGCGGCTTAAATTTACTTTCCTGGCC
CACAAACTTACCAATAATA

UNSB01.fasta	Cele-UNSB01_4:13187743	4	13187743	13188346	604	12	12	GCTTGGGTTT

GCTTGGGGTTAG

UNSB01.fasta	Cele-UNSB01_4:13187743	4	13187743	13188478	736	17	12	GCTTGGGTTT	GCTTGGGGTTAG
UNSB01.fasta	Cele-UNSB01_4:13295662	4	13295662	13296286	625	24	26	ATTAGATGAA	ATTAGATGAATTTCCAAAGTTAGACC
UNSB01.fasta	Cele-UNSB01_4:13402475	4	13402475	13402619	145	12	12	TATACCTATA	TATACCCATACC
UNSB01.fasta	Cele-UNSB01_4:13540735	4	13540735	13544977	4243	166	25	TTTACTCTCT	TTTACTCCTCTGCTTCACAGAATAA
UNSB01.fasta	Cele-UNSB01_4:13614335	4	13614335	13614410	76	5	15	CACAGGAGGC	CACAGGAGGCTGGGA
UNSB01.fasta	Cele-UNSB01_4:13645449	4	13645449	13645792	344	22	15	ACCCATCGTG	ACCCACCGTGGTGAG
UNSB01.fasta	Cele-UNSB01_4:13645884	4	13645884	13646555	672	18	35	GAAATTTAAA	GAAATTTAAATTTTCTGTGAAAAATATTTTGGCGG
UNSB01.fasta	Cele-UNSB01_4:13646609	4	13646609	13646697	89	4	23	AATATTTATT	AATATTTATTTATTTATTTAATA
UNSB01.fasta	Cele-UNSB01_4:13645885	4	13645885	13647427	1543	25	35	AAATTTAAAT	AAATTTAAATTTTCTGTGAAAAATATTTTGGCGGG
UNSB01.fasta	Cele-UNSB01_4:13647491	4	13647491	13647701	211	14	15	CCACGATGGG	CCACGATGGGTCTCG
UNSB01.fasta	Cele-UNSB01_4:13646820	4	13646820	13647869	1050	12	40	GTTCTGGAAC	GTTCTGGAACATTCCAGTATTTTCTCGATTTTTCTAGAAA
UNSB01.fasta	Cele-UNSB01_4:13647739	4	13647739	13648797	1059	13	27	TTTTTAAAAT	TTTTTAAAATTCATATACCGTTCTAAC
UNSB01.fasta	Cele-UNSB01_4:13661650	4	13661650	13661868	219	6	31	AATTCAAATT	AATTCAAATTTTCTCAAAAAATTTTGGCGGG
UNSB01.fasta	Cele-UNSB01_4:13730774	4	13730774	13730818	45	4	11	CACATTTTTT	CACATTTTTTC
UNSB01.fasta	Cele-UNSB01_4:13731432	4	13731432	13732897	1466	8	164	AAAATTCAAA	AAAATTCAAAAGTATGGAAAATCATATGGAGTCATTCTTTTTTATTTCATAAAACTCTTCAGCATAGTCAAAAATACCAGAAAATGCCAATCAAAGTATAATAGCTTGTACGGAAGTATTTTTTTAAAAATTGATAAAAATATATAAA AGCTGATTTTTTTCA
UNSB01.fasta	Cele-UNSB01_4:13759189	4	13759189	13759529	341	30	11	GCACATTTTT	GCACATTTTCT
UNSB01.fasta	Cele-UNSB01_4:13851511	4	13851511	13852113	603	12	40	AAAATTCTGG	AAAATTCTGGAATGTTCTAGAACCTTCTGGAAAGTTCGAG
UNSB01.fasta	Cele-UNSB01_4:13917663	4	13917663	13921092	3430	37	93	AGGGCCGCAG	AGGGCCGCAGGCCCGAACACTCTTGGAAACTGGGCTGGCTTTAATCCTATTCCAAGCAGATTGTTATTCACTCAAGCCTATCAACCAAGTTTG
UNSB01.fasta	Cele-UNSB01_4:14030114	4	14030114	14030248	135	6	11	AGTACTGTAG	AGTACTGTAGG
UNSB01.fasta	Cele-UNSB01_4:14030460	4	14030460	14031210	751	45	15	CCCATCGTGG	CCCATCGTGGCGAGA
UNSB01.fasta	Cele-UNSB01_4:14031556	4	14031556	14031756	201	5	40	AATGTTCCAG	AATGTTCCAGAACTTTCTAGAAAAATCGAGAAAATTCTGG
UNSB01.fasta	Cele-UNSB01_4:14046078	4	14046078	14046558	481	12	40	CAGAACCTTC	CAAAACCTTCTGGAAAATTTGAGAAAATGCTGGAATGTTC
UNSB01.fasta	Cele-UNSB01_4:14046107	4	14046107	14046777	671	11	40	CTGGAATGTT	CTGGAATGTTCCAGAACCTTCTGGAAAATTTGAGAAAATT
UNSB01.fasta	Cele-UNSB01_4:14068578	4	14068578	14068998	421	24	15	ACGATGGGTC	ACGATGGGTCTCGCC
UNSB01.fasta	Cele-UNSB01_4:14093954	4	14093954	14094851	898	32	32	CGACCTGCGA	CGACCTGCGACCTAGCGACCTGGGTCGCTTTG
UNSB01.fasta	Cele-UNSB01_4:14106561	4	14106561	14106695	135	6	11	AGTACTGTAG	AGTACTGTAGG
UNSB01.fasta	Cele-UNSB01_4:14106973	4	14106973	14107062	90	6	15	ACCCATCGTG	ACCCATCGTGGTGAG
UNSB01.fasta	Cele-UNSB01_4:14107092	4	14107092	14108860	1769	51	35	GGGAAATTTA	GGGAAATTTAAATTTTCTGTGAAAAATATTTTGGA
UNSB01.fasta	Cele-UNSB01_4:14107092	4	14107092	14111107	4016	106	35	GGGAAATTTA	GGGAAATTTAAATTTTCTGTGAAAAATATTTTGGA

UNSB01.fasta	Cele-UNSB01_4:14107093	4	14107093	14113320	6228	160	35	GGAAATTTAA	GGAAATTTAAATTTCTGTGAAAAATATTTTGGAG
UNSB01.fasta	Cele-UNSB01_4:14154469	4	14154469	14154585	117	4	29	TACATTGCAA	TACATTGCAAGATTTTCTTACTATGTAGG
UNSB01.fasta	Cele-UNSB01_4:14405343	4	14405343	14406331	989	13	76	AATGCTAGAG	AATGCTAGAGGACACTCCTTTGCACGAAAGGAAGTGGGAGAGACTGTGGAGTTGTCGGTGTCTTTGTGCATGCTCG
UNSB01.fasta	Cele-UNSB01_4:14407623	4	14407623	14408822	1200	70	14	GCTATGCGCT	GCTATGCGCTGTTA
UNSB01.fasta	Cele-UNSB01_4:14408867	4	14408867	14409192	326	13	25	TAATGCCAAT	TAATGCCAATTTATAGAATCTGCCG
UNSB01.fasta	Cele-UNSB01_4:14409220	4	14409220	14409304	85	6	14	AGCGTATAGC	AGCGTATAGCGTAT
UNSB01.fasta	Cele-UNSB01_4:14410457	4	14410457	14414735	4279	57	76	TCGTGCAAAG	TCGTGCAAAGGAGTGTCCTCTAGCATTCGAGCATGCACAAAGACACCGACAACTCCACAGTCTCTCCCACTTCTTT
UNSB01.fasta	Cele-UNSB01_4:14417295	4	14417295	14419575	2281	148	15	CTCACCACGA	CTCACCACGAAGGGT
UNSB01.fasta	Cele-UNSB01_4:14618655	4	14618655	14620445	1791	7	200	GAAAAAGTGA	GAAAAAGTGAATTTTCAGTCATACCTTGTAGACCACAATTTATATGCAAAATTAATAGATTTTCCAAACTTTGAAAATTAATTTCTGGCTTCAAACTAACTTTTTTAAAAATTCCCGTAGCCATTTTGTAGCAAATTTTATGTTCTATTAGAATTTCAAAAGAGAGTTTCAAAAAATTTTGAAACCATTAAGTTCAAGCA
UNSB01.fasta	Cele-UNSB01_4:14628221	4	14628221	14628977	757	36	21	CAACTACCAC	CAACTACCACAACTACCGTAC
UNSB01.fasta	Cele-UNSB01_4:14648431	4	14648431	14648725	295	13	21	ACTACCGTAC	ACTACCGTACCAACTACCACA
UNSB01.fasta	Cele-UNSB01_4:14678240	4	14678240	14678405	166	11	15	GACCCATCGT	GACCTATCGTGGCGA
UNSB01.fasta	Cele-UNSB01_4:14678232	4	14678232	14682258	4027	80	15	CGTGGTGAGA	CGTGGCGAGACCTAT
UNSB01.fasta	Cele-UNSB01_4:14942266	4	14942266	14942426	161	5	40	TTTTCTAGAA	TTTTCTAGAAAGTTCTAGAACATTGCCGATTTTTCCCGAT
UNSB01.fasta	Cele-UNSB01_4:15002663	4	15002663	15002793	131	5	26	TGGTTAGAGT	TGGTTAGAGTCATATTTTTAGGTGAA
UNSB01.fasta	Cele-UNSB01_4:15255716	4	15255716	15255837	122	9	11	TGTGCAAAAA	TGTGCAAAAAA
UNSB01.fasta	Cele-UNSB01_4:15280499	4	15280499	15283896	3398	88	40	AGAAAGTTCT	AGAAAGTTCTGGAACATTCCAGAATTTTCTCGATTTTTCT
UNSB01.fasta	Cele-UNSB01_4:15284214	4	15284214	15284904	691	39	15	GGTCTCACCA	GGTCTCACCACGATG
UNSB01.fasta	Cele-UNSB01_4:15313385	4	15313385	15313490	106	7	15	GGGTCTCACC	GGGTCTCACCACGAT
UNSB01.fasta	Cele-UNSB01_4:15313710	4	15313710	15313830	121	8	15	TCGTGGTGAG	TCGTGGTGAGAACCT
UNSB01.fasta	Cele-UNSB01_4:15399620	4	15399620	15399800	181	11	15	GAGACCCATC	GAGACCCATCGTGGC
UNSB01.fasta	Cele-UNSB01_4:15399887	4	15399887	15400027	141	4	35	AAATTTTCTG	AAATTTTCTGAGAAAACTATTTTGGAGGGAAATTT
UNSB01.fasta	Cele-UNSB01_4:15400092	4	15400092	15400412	321	8	40	GAATGTTCCA	GAATGTTCCAGAACTTTCTAGAAAAATCGAGAAAATTCTG
UNSB01.fasta	Cele-UNSB01_4:15421750	4	15421750	15421870	121	8	15	GGTGAGACCC	GGTGAGACCCATCGT
UNSB01.fasta	Cele-UNSB01_4:15421887	4	15421887	15425906	4020	116	34	TGGCGGGAAA	TGGCGGGAAATTCAAATTTTCAGTGAAAAAATTT
UNSB01.fasta	Cele-UNSB01_4:15434528	4	15434528	15434620	93	6	15	CGAAAAGGGG	CGAAAAGGGGATCTG
UNSB01.fasta	Cele-UNSB01_4:15434795	4	15434795	15434872	78	5	15	TCCCCTTTTC	TCCCCTTTTCGTATC
UNSB01.fasta	Cele-UNSB01_4:15436315	4	15436315	15436698	384	11	35	GGGAAATTCA	GGGAAATTCAAATTTTTAGTAAAAAATATTTTGGC
UNSB01.fasta	Cele-UNSB01_4:15436367	4	15436367	15437145	779	9	35	GTAAAAAATA	GTAAAAAATATTTTGGGAGGAAATTCAAATTTTTA

UNSB01.fasta	Cele-UNSB01_4:15460070	4	15460070	15462017	1948	10	168	AAAAACAAAA	AAAAACAAAAATAAAAATTTTGGAATGTTGGGAGAATTTTTATAGATTTGGATTCTAGATTTTTAACAAATATTTTTGGTAAGTTTCTGGACCTAGATATCAATTTTCAGATGCGCTAGAGGGGTTTTTGTCTTGAATTCCAATTTCAACAGATATATATACAGGCACAA
UNSB01.fasta	Cele-UNSB01_4:15460844	4	15460844	15462434	1591	9	168	AACAAAAATA	AACAAAAATAAAAATTTTGGAATGTTGGGAGAATTTTTATAGATTTGGATTCTAGATTTTTAACAAATATTTTTGGTAAGTTTCTGGACCTAGATATCAATTTTCAGATGCGCTAGAGGGGTTTTTGTGTTGAAATTTATTTTTCACAGGTATATACAGGCACAAAAA
UNSB01.fasta	Cele-UNSB01_4:15529257	4	15529257	15530020	764	4	14	TTGTAGTTTG	TTGTAATTTGTAGT
UNSB01.fasta	Cele-UNSB01_4:15554617	4	15554617	15554707	91	6	15	TCGTGGTGAG	TCGTGGTGGGACCCT
UNSB01.fasta	Cele-UNSB01_4:15554734	4	15554734	15555678	945	27	35	GAAATTCAAA	GAAATTCAAATTTTCAGTGAAAAAAATTTTGGCGG
UNSB01.fasta	Cele-UNSB01_4:15607346	4	15607346	15608026	681	17	40	GAACATTCCA	GAACATTCCAGAATTTTCTCAATTTTTCTAGAAAGTTCTT
UNSB01.fasta	Cele-UNSB01_4:15608407	4	15608407	15609476	1070	51	15	CCACGATGGG	CCACGATGGGTCTCA
UNSB01.fasta	Cele-UNSB01_4:15642758	4	15642758	15643070	313	12	26	CAGAGTCACT	CAGAGTCACTATTTTTGGTGAACGGC
UNSB01.fasta	Cele-UNSB01_4:15644313	4	15644313	15644403	91	8	10	TGTTATGATA	GTTATGATAG
UNSB01.fasta	Cele-UNSB01_4:15744856	4	15744856	15746870	2015	12	168	GAATTCCGGA	GAATTCCGGAAATGTTTTGATAGATTTGGATTCTGGTTTTTTTTTAAATATTATTGGAAAGTTTGTGGACCAGGAAAGTAAATTTAAGCCGCGCTAAAGAGGTTTTTGTGTCGAGTTCCAATTTTTACAGGTACTAGTAGGCGCAAAAAAACAAAAATAAAAATTATG
UNSB01.fasta	Cele-UNSB01_4:15750800	4	15750800	15751080	281	8	35	TTGCCGGATT	TTGCCGGATTTGAAAATTTATGGTAATTTGCCGTT
UNSB01.fasta	Cele-UNSB01_4:15869737	4	15869737	15871646	1910	128	14	GGACTAGGGA	GGACTAAGGACTAG
UNSB01.fasta	Cele-UNSB01_4:15883454	4	15883454	15883531	78	6	11	TGCACATTTT	TGCACATTTTT
UNSB01.fasta	Cele-UNSB01_4:15943206	4	15943206	15943656	451	14	32	TTGAATTCCC	TTGAATTCCCGCCAAAATTTTTCACAGAAAAT
UNSB01.fasta	Cele-UNSB01_4:15944034	4	15944034	15944234	201	5	40	ATTTTCCAGA	ATTTTCCAGAAGGTTCTGGAACATTCCAGCATTTTCTCAA
UNSB01.fasta	Cele-UNSB01_4:16000310	4	16000310	16000589	280	6	40	GATTTTTCTA	GATTTTTCTAGAAAGTTCTGGAACATTCCAGAATTTTCTC
UNSB01.fasta	Cele-UNSB01_4:15999976	4	15999976	16000920	945	8	35	AAAATTTAAA	AAAATTTAAATTTCCCGCCAAAATATTTTTCTCAG
UNSB01.fasta	Cele-UNSB01_4:16000962	4	16000962	16001082	121	8	15	GGGTCTCACC	GGGTCTCACCACGAT
UNSB01.fasta	Cele-UNSB01_4:16000985	4	16000985	16001240	256	17	15	CCACGATGGG	CCACGATGGGTCTCA
UNSB01.fasta	Cele-UNSB01_4:16001468	4	16001468	16002088	621	19	11	GTACTCCTAC	GTACTCCTACA
UNSB01.fasta	Cele-UNSB01_4:16010822	4	16010822	16011211	390	5	42	GCTCGGCAAA	GCTCGGCAAATATTGAAAAAAGAGATTTGCCGAATTTGCCGT
UNSB01.fasta	Cele-UNSB01_4:16019565	4	16019565	16019814	250	6	31	TCGACAGAAG	TCGACAGAAGCTGTCGATCATCAGACTTTCC
UNSB01.fasta	Cele-UNSB01_4:16061332	4	16061332	16061647	316	21	15	CCCATCGTGG	CCCATCGTGGCGAGA
UNSB01.fasta	Cele-UNSB01_4:16061680	4	16061680	16061959	280	8	35	AATTTAAATT	AATTTAAATTTTCTGTGAAAAATATTTTGGCGGGA
UNSB01.fasta	Cele-UNSB01_4:16061215	4	16061215	16062273	1059	8	40	GTTCCAGAAC	GTTCCAGAACCTTCTAGAAAAATCGAGAAAATTCTGGAGT
UNSB01.fasta	Cele-UNSB01_4:16077133	4	16077133	16078206	1074	16	37	CGGGAATTCA	CGGGAATTCAATTGTTAATTTTTCGAAATCATTTCGG
UNSB01.fasta	Cele-UNSB01_4:16093909	4	16093909	16094066	158	8	20	TGTTACACGA	TGTTACACGAAAAAAATAGAT
UNSB01.fasta	Cele-UNSB01_4:16130574	4	16130574	16144444	13871	346	40	TTTTTCTAGA	TTTTTCTAGAAAGTTCTGGAACATTCCAGAATTTTCCCGA

UNSB01.fasta	Cele-UNSB01_4:16144580	4	16144580	16144720	141	4	35	CCAAAATATT	CCAAAATATTTTTCTCAGAAAATTAAAATTTCCCT
UNSB01.fasta	Cele-UNSB01_4:16130581	4	16130581	16145036	14456	348	40	AGAAAGTTCT	AGAAAGTTCTGGAACATTCCAGAATTTTCCCGATTTTTCT
UNSB01.fasta	Cele-UNSB01_4:16219937	4	16219937	16221099	1163	29	35	TTCCCGCCAA	TTCCCGCCAAAAAATTTTTTCACTGAAAATTTGAAT
UNSB01.fasta	Cele-UNSB01_4:16220033	4	16220033	16221319	1287	15	15	CACGATGGGT	CACGATGGGTCTCGT
UNSB01.fasta	Cele-UNSB01_4:16343642	4	16343642	16344390	749	18	11	GTACTGTAGG	GTACTGTAGTA
UNSB01.fasta	Cele-UNSB01_4:16376935	4	16376935	16378753	1819	17	15	GGGTCTCACC	GGGTCTCACCGCGAA
UNSB01.fasta	Cele-UNSB01_4:16402456	4	16402456	16402561	106	7	15	GTGGTGAGAC	GTGGTGAGACCCTTC
UNSB01.fasta	Cele-UNSB01_4:16472875	4	16472875	16473271	397	25	15	TCTCACCACG	TCACACCACGAAAGG
UNSB01.fasta	Cele-UNSB01_4:16493225	4	16493225	16495520	2296	119	19	AAATAGATTG	AAATAGATTGTTACACGGA
UNSB01.fasta	Cele-UNSB01_4:16653889	4	16653889	16654239	351	10	35	CAAATCGGCA	CAAATCGGCAATATGCCAAAATTGAAAATTTCCGG
UNSB01.fasta	Cele-UNSB01_4:16658516	4	16658516	16659248	733	21	35	TTTGGCATAT	TTTGGTATATTGCCGGTTTGCCAGATATTTTCAAT
UNSB01.fasta	Cele-UNSB01_4:16659843	4	16659843	16660088	246	7	35	GCAAATCGGC	GCAAATCGGCAAAATGGCCGAATTGAAAATTTCCA
UNSB01.fasta	Cele-UNSB01_4:16675153	4	16675153	16675373	221	8	20	TTTTAGTGAA	TTTTAGTGAAAATTTGAGAT
UNSB01.fasta	Cele-UNSB01_4:16678025	4	16678025	16678676	652	13	50	GGGCGTGTTT	GGGCGTGTTTTCACATGCCCCGCCCACAAAATTGATGTTTCCACCAAAAT
UNSB01.fasta	Cele-UNSB01_4:16708641	4	16708641	16708839	199	5	40	TTCCAGAATT	TTCCAGAATTTTCTCGATTTTTCTAGAAAGTTCTGGAACG
UNSB01.fasta	Cele-UNSB01_4:16709008	4	16709008	16709188	181	10	15	TGGGTCTCAC	TGGGTCTCACCACGA
UNSB01.fasta	Cele-UNSB01_4:16721546	4	16721546	16722348	803	4	37	ATTCAAATTT	ATTCAAATTTTAATTTTTTGAAAATATTTTGACGGGA
UNSB01.fasta	Cele-UNSB01_4:16741502	4	16741502	16741626	125	5	21	TTCAAGCCTT	TTCAAGCCTTTTCAGACCAAT
UNSB01.fasta	Cele-UNSB01_4:16762223	4	16762223	16763229	1007	39	11	CTACAGTACT	CTACAGTACCT
UNSB01.fasta	Cele-UNSB01_4:16773369	4	16773369	16773770	402	28	11	CCCCTACAGT	CCCCTACAGTA
UNSB01.fasta	Cele-UNSB01_4:16899632	4	16899632	16900079	448	15	30	AAAATCAAAA	AAAATCAAAAATTAGATGAATTTTCGATTA
UNSB01.fasta	Cele-UNSB01_4:16926190	4	16926190	16926350	161	4	40	TTCCAGAATT	TTCCAGAATTTTCCCGATTTTTCTAGAAAGTTCTGGAACG
UNSB01.fasta	Cele-UNSB01_4:16926416	4	16926416	16926623	208	6	35	TTGAATTTCC	TTGAATTTCCCGCCAAAATATTTTTCACTGAAAAT
UNSB01.fasta	Cele-UNSB01_4:16931676	4	16931676	16932199	524	4	150	TTGAGGGCCG	TTGAGGGCCGCAGTCCCGAATACACGAAGAAGTGTTGGAGATCCGTCAAGCCTATTCTAAGCATAATAATTCAGTTCGAGTGTCGCAAGCCCAAATAGACGCAGAAGCGTTGGACAACTTTTAGGCCTATTCTGAGCCCAATAATTCATG
UNSB01.fasta	Cele-UNSB01_4:17138861	4	17138861	17138981	121	8	15	TCGTGGTGAG	TCGTGGTGGGACCCT
UNSB01.fasta	Cele-UNSB01_4:17168442	4	17168442	17168576	135	6	11	AGTACTGTAG	AGTACTGTAGG
UNSB01.fasta	Cele-UNSB01_4:17168764	4	17168764	17168899	136	9	15	TCGTGGTGAG	TCGTGGTGAGACCCA
UNSB01.fasta	Cele-UNSB01_4:17168941	4	17168941	17169150	210	6	35	GAAATTTAAA	GAAATTTAAATTTTCTGTGAAAAATGTTTTGGCGG
UNSB01.fasta	Cele-UNSB01_4:17169175	4	17169175	17169333	159	4	40	AAAATTCTGG	AACATTCTGGAATGTTCCAGAACTTTCTAGAAAAATCGAG

UNSB01.fasta	Cele-UNSB01_4:17223540	4	17223540	17224619	1080	56	19	AAAAATAGAT	AAAAATAGATTGTTACACG
UNSB01.fasta	Cele-UNSB01_4:17319515	4	17319515	17323871	4357	66	66	CAAATCAACC	CAAATCAACCTATTCAGTTGACAATGGTTTTCTTTTATCAAATTTTTTGAAATTTTAAAGAAAACA
UNSB01.fasta	Cele-UNSB01_4:17337235	4	17337235	17337502	268	10	11	CCTACAGTAC	CTACAGTACTA
UNSB01.fasta	Cele-UNSB01_4:17389165	4	17389165	17390089	925	21	44	GATTGATGGC	GATTGATGGCCGAAAGGCTGTTTTCTGAGCATTGTAGATTTTTG
UNSB01.fasta	Cele-UNSB01_4:17395956	4	17395956	17396303	348	5	18	GCACTCCCTA	GCACTCCCTATTAGTCTT
UNSB01.fasta	Cele-UNSB01_4:17401416	4	17401416	17402316	901	60	15	ACCCATCGTG	ACCCATCGTGGTGAG
UNSB01.fasta	Cele-UNSB01_4:17402400	4	17402400	17402610	211	6	35	AAATTTTCTG	AAATTTTCTGTGAAAAATATTTTGGCGGGAAATTT
UNSB01.fasta	Cele-UNSB01_4:17408961	4	17408961	17409411	451	28	15	TCTCACCACG	TCTCACCACGAAAGG
UNSB01.fasta	Cele-UNSB01_4:17408894	4	17408894	17409724	831	9	37	ATTCCCGCCA	ATTCCAGCCAAAATGTTTCGAAAAATTAAATTTTGA
UNSB01.fasta	Cele-UNSB01_4:17523613	4	17523613	17523740	128	4	32	GAAGAAATTT	GAAGAAATTTGAATTCCCGCTAAAATTTTTCT
UNSB01.fasta	Cele-UNSB01_4:17545983	4	17545983	17546043	61	4	15	GTAGATCTTG	GTAGATCTTGAAAAT
UNSB01.fasta	Cele-UNSB01_4:17584126	4	17584126	17584294	169	7	24	TCAACAACAA	TCAACAACAACAGGAGAAGCTTCT
UNSB01.fasta	Cele-UNSB01_4:17606143	4	17606143	17606220	78	5	15	TTCCCCTTTT	TTCCCCTTTTTGTAA
UNSB01.fasta	Cele-UNSB01_4:17753355	4	17753355	17753780	426	17	25	AGCCAGAGAG	AGCCAGAGAGTAAAATAAACTGTGA
UNSB01.fasta	Cele-UNSB01_4:17753798	4	17753798	17754998	1201	48	25	ACTCTCTGGC	ACTCTCTGGCTTCACCGTATATTTT
UNSB01.fasta	Cele-UNSB01_4:17755008	4	17755008	17759186	4179	342	12	TTAGGCTTAG	TTAGGCTTAGGC
UNSB01.fasta	Cele-UNSB01_5:4	5	4	5075	5072	419	12	AAGCCTAAGC	AAGCCTAAGCCT
UNSB01.fasta	Cele-UNSB01_5:105361	5	105361	105451	91	6	15	AAAAGGAGAA	AAAAGGAGAATTACG
UNSB01.fasta	Cele-UNSB01_5:105684	5	105684	105774	91	6	15	TTCTCCTTTT	TTCTCCTTTTGGTTG
UNSB01.fasta	Cele-UNSB01_5:328030	5	328030	329092	1063	6	175	AAATTTTGGG	AAATTTTGGGTTCACTCTTTTTACTAGACAGTGATTGTTGCATTGTAGGATTCGGTGGACTTGTCCAACAACTTACTTTGGCATGAGTTTAATAAAAAGGTTTTACCATGAACTGAATCAATTTAAAATAAAAACCTCGAGAACGAAAACATCCTCAGTAATTTCAATCCTGTTC
UNSB01.fasta	Cele-UNSB01_5:345871	5	345871	345991	121	7	16	CATGCCTACA	CATACCTACATGCCTA
UNSB01.fasta	Cele-UNSB01_5:353635	5	353635	353859	225	14	16	GGCATGTAGG	GGCATGTAGGCATGTA
UNSB01.fasta	Cele-UNSB01_5:376477	5	376477	376531	55	4	12	AGGCTTAGGC	AGGCTTAGGCTA
UNSB01.fasta	Cele-UNSB01_5:786342	5	786342	786547	206	6	34	CAATTTGCCG	CAATTTGCCGATTTACCCGAAATTTCGATTCCGG
UNSB01.fasta	Cele-UNSB01_5:786696	5	786696	786877	182	5	35	TTTCCGGCAA	TTTCCGGTAAATCGGCCAATTGCCGGAATTGAAGA
UNSB01.fasta	Cele-UNSB01_5:788141	5	788141	788221	81	5	16	CTACATGCCT	CTACATGCCTACATGC
UNSB01.fasta	Cele-UNSB01_5:788660	5	788660	789185	526	12	16	AGGCATGTAG	AGGTATGTAGGCATGT
UNSB01.fasta	Cele-UNSB01_5:790113	5	790113	790281	169	10	16	AGGCATGTAG	AGGCATGTAGGCATGT
UNSB01.fasta	Cele-UNSB01_5:853469	5	853469	853759	291	23	10	GGCGTCAGCG	GGCGTCAGCG

UNSB01.fasta	Cele-UNSB01_5:896085	5	896085	896285	201	12	16	ATGCCTACAT	ATGCCTAGATGCCTAG
UNSB01.fasta	Cele-UNSB01_5:915117	5	915117	915271	155	7	20	CCTACCCCAG	CCTACCCCAGGCTGACCTAG
UNSB01.fasta	Cele-UNSB01_5:915226	5	915226	915380	155	8	20	GGCAGACCTA	GGCAGGCCTAACCTACCCCA
UNSB01.fasta	Cele-UNSB01_5:978655	5	978655	978943	289	7	35	TGCCGATTTG	TGCCGATTTGCCGGAAATGTTCATTTTCGAGAATT
UNSB01.fasta	Cele-UNSB01_5:1026357	5	1026357	1026873	517	9	35	GCATTTGCCG	GCATTTGCCGGTTTCGGAAATTTTCGGCAATTTCG
UNSB01.fasta	Cele-UNSB01_5:1035824	5	1035824	1036308	485	20	22	ACAGTACTCC	ACAGTACTCCTACAGTACCACT
UNSB01.fasta	Cele-UNSB01_5:1038307	5	1038307	1038528	222	11	13	GCCTATGGCC	GCCTATGGCAAAC
UNSB01.fasta	Cele-UNSB01_5:1039029	5	1039029	1039263	235	18	13	CGTAGGCCAT	CGTAGGCCATATA
UNSB01.fasta	Cele-UNSB01_5:1057921	5	1057921	1058017	97	6	18	AAGCCTAAGC	AAGCCTAAGTCTAAATCT
UNSB01.fasta	Cele-UNSB01_5:1115366	5	1115366	1136806	21441	722	27	TCTGTGGCTT	TCTGTGGCTTCACAGATTAAGTTACTC
UNSB01.fasta	Cele-UNSB01_5:1161256	5	1161256	1161400	145	10	12	GGCTTAGGCT	GGCTTCGGCTTA
UNSB01.fasta	Cele-UNSB01_5:1174135	5	1174135	1174237	103	5	12	CTAAGCCTAA	CTGAGCCTAAGC
UNSB01.fasta	Cele-UNSB01_5:1229040	5	1229040	1229117	78	5	15	CGAAAAGGGG	CGAAAAGGGGATCTG
UNSB01.fasta	Cele-UNSB01_5:1229292	5	1229292	1229367	76	5	15	TCCCCTTTTC	TCCCCTTTTCGTATC
UNSB01.fasta	Cele-UNSB01_5:1233778	5	1233778	1237846	4069	22	185	AATATAAACA	AATATAAACATACACGAATTTGTCTGATGTATAAAGATTCCCGAAGACACTTTCCAATTACCCAAATTGTTCATATTCTAAATGAGATTCTCTTACTAGAACACTCTTGGCCAATGTACGCAGCCGAACGTATCATAAGTGAATACAGA ACACCAGTTATGTCCGAGCAAAAAGATCCGATTCAG
UNSB01.fasta	Cele-UNSB01_5:1262246	5	1262246	1262294	49	4	12	AAGCCTAAGC	AAGCCTAAGCCT
UNSB01.fasta	Cele-UNSB01_5:1265623	5	1265623	1265875	253	19	12	AAGCCTAAGC	AAGCTTAAGCCT
UNSB01.fasta	Cele-UNSB01_5:1271695	5	1271695	1271973	279	15	12	CTAAGCCTAA	CTAGGCCTAAAC
UNSB01.fasta	Cele-UNSB01_5:1274939	5	1274939	1275298	360	10	35	GAAATTTTCA	GAAATTTTCAATTTCGGCAAATTGCCAAATTGCCA
UNSB01.fasta	Cele-UNSB01_5:1278621	5	1278621	1278717	97	7	12	CTTAGGCTTA	CTTAGGCTTAGT
UNSB01.fasta	Cele-UNSB01_5:1284626	5	1284626	1284947	322	10	11	TACCTACTAG	TACCTACTGGA
UNSB01.fasta	Cele-UNSB01_5:1289399	5	1289399	1289474	76	5	15	TCGTGGTGAG	TCGTGGTGAGACCCT
UNSB01.fasta	Cele-UNSB01_5:1289402	5	1289402	1291686	2285	44	15	TGGTGAGACC	TGGTGAGACCCTTCG
UNSB01.fasta	Cele-UNSB01_5:1289520	5	1289520	1292435	2916	32	35	GCGGGAAATT	GCGGGAAATTCAAATTTTCAGTGAAAAAAATTTTG
UNSB01.fasta	Cele-UNSB01_5:1308044	5	1308044	1308391	348	8	35	AATTCTGGCA	AATTCTGGCAAATTACCGATTTGCCGGAAATTTTC
UNSB01.fasta	Cele-UNSB01_5:1308547	5	1308547	1312321	3775	192	20	AAAATAGATT	AAAATAGATTGTTACACGAA
UNSB01.fasta	Cele-UNSB01_5:1343973	5	1343973	1344069	97	7	12	AGGCTTAGGC	AGGCTTAGGCTC
UNSB01.fasta	Cele-UNSB01_5:1379498	5	1379498	1379672	175	13	12	CTAAGCCTAA	CTAAGCTTAAGC
UNSB01.fasta	Cele-UNSB01_5:1390382	5	1390382	1390822	441	9	40	AATGTTCTAG	AATGTTCTAGACCCTTCTGGACAACTCGTGAAAATTCTGA

UNSB01.fasta	Cele-UNSB01_5:1393460	5	1393460	1393720	261	6	40	CTAGAACATT	CTAGAACTTTCCAGAATTTTCTCAAACTTTCTAGAAGGTT
UNSB01.fasta	Cele-UNSB01_5:1406535	5	1406535	1406737	203	6	34	AAATTCAAAT	AAATTCAAATTTTCTGAGAAATAAATTTGACGGA
UNSB01.fasta	Cele-UNSB01_5:1415140	5	1415140	1415840	701	21	35	ATTTTCAATT	ATTTTCAATTCCAGCAATTTGCCGATTTTCTTGAA
UNSB01.fasta	Cele-UNSB01_5:1423918	5	1423918	1424416	499	9	35	TTTGCCGGAA	TTTGCCGGAAAATTTCAATTCCGGCAAAATGTGGA
UNSB01.fasta	Cele-UNSB01_5:1427061	5	1427061	1427411	351	8	35	TGCCGGAAAT	TGCCGGAAATTTTGATTTTTGGTAATTTGCCGATT
UNSB01.fasta	Cele-UNSB01_5:1441594	5	1441594	1441873	280	7	40	ATTTTTCTAG	ATTTTTCTAGAAAGTTCTGGAACATTCCAGACTTTTCCCA
UNSB01.fasta	Cele-UNSB01_5:1441948	5	1441948	1442263	316	21	15	CGATGGGTCT	CGATGGGTCTCACCA
UNSB01.fasta	Cele-UNSB01_5:1442495	5	1442495	1442763	269	8	11	CTACAGTACC	CTACAGTACTA
UNSB01.fasta	Cele-UNSB01_5:1461442	5	1461442	1461802	361	24	12	CCTAAGCCTA	CCTAAGCCTAAG
UNSB01.fasta	Cele-UNSB01_5:1519749	5	1519749	1519965	217	10	12	AGCCTAAGCC	AGCCTAAGCCTA
UNSB01.fasta	Cele-UNSB01_5:1568984	5	1568984	1569144	161	4	40	GTTCTAGAAC	GTTCTAGAACGTTCCAGAATTTTCTCGAATTTACCAGGAA
UNSB01.fasta	Cele-UNSB01_5:1577782	5	1577782	1578190	409	5	89	GCACTACGTT	GCACAACGTTGTGCACACACTAAGCTACTCGTTTCACGCCAAGCTACGGAACCCTAAACGTGTCGGCCGCTCCAAAAAGCTACCTTCTT
UNSB01.fasta	Cele-UNSB01_5:1676007	5	1676007	1687585	11579	242	48	AATTTCTGAA	AATTTCTGAAGATGAGCAATTTAAATTTTGAGTTGCCCGTAATTTATA
UNSB01.fasta	Cele-UNSB01_5:1707873	5	1707873	1707951	79	5	18	GCCTAAGCCT	GCCTAGGCCTACAACCTA
UNSB01.fasta	Cele-UNSB01_5:1765405	5	1765405	1765535	131	9	13	GCTCAAGCTA	GCTCAAGCTAAGA
UNSB01.fasta	Cele-UNSB01_5:1860337	5	1860337	1860547	211	7	35	TCCGGCAATT	TCCGGCAATTTTCCGATTTGCCGGTAATTTTAAAT
UNSB01.fasta	Cele-UNSB01_5:1872631	5	1872631	1872733	103	7	12	AGCCTGAGCC	AGCCTGAGCCTG
UNSB01.fasta	Cele-UNSB01_5:1895975	5	1895975	1896260	286	14	17	TAAGCCTAAG	TGAGCCTAAGCTTAGCT
UNSB01.fasta	Cele-UNSB01_5:1897354	5	1897354	1897674	321	23	10	GGGCGTCAGC	GGGCGTCAGC
UNSB01.fasta	Cele-UNSB01_5:1955153	5	1955153	1955723	571	7	93	CAGTACCCTC	CAGTACCCTCAAGGATCATCTGCTCCACGGTATCCTGGTAACTACAATGCCTTTGCCAGCACTTCCGATGCTCCACAGTATCCAGGATCCTCC
UNSB01.fasta	Cele-UNSB01_5:1958361	5	1958361	1958506	146	7	11	CTACAGTACT	CTACAGTACTA
UNSB01.fasta	Cele-UNSB01_5:1987432	5	1987432	1987712	281	8	35	GGCAAATCGG	GGCAAATCGGCAATTTTCCGAAAATGAAAGTTTCC
UNSB01.fasta	Cele-UNSB01_5:2151047	5	2151047	2151352	306	21	13	GCCTAAGCCT	GCTTAAGCCTCGG
UNSB01.fasta	Cele-UNSB01_5:2189835	5	2189835	2190349	515	18	11	CTACAGTACC	CTACAGTACTA
UNSB01.fasta	Cele-UNSB01_5:2213030	5	2213030	2213142	113	6	16	ATGCCTACAT	ATGCCTACATGCCTAC
UNSB01.fasta	Cele-UNSB01_5:2439353	5	2439353	2440062	710	4	177	GAGTCTCACG	GAGTCTCACGATGTGTCAACGTGTCTCAGTCGGCTTGATCTACGTAGATCTACAAAAAATGCGGGAGAAGAGACGCAGACTTATTCTCTGATTTTGCATGAGTAAGAGCGTGCTGGCGTCACATTTTTGTCGGCAAAAAATTCCCGCATTTTTTGTAGATCAAACCGTAATGGGACG
UNSB01.fasta	Cele-UNSB01_5:2441369	5	2441369	2441565	197	4	35	AAATTTTCAA	AAATTTTCAATTCCTCCAGTTTGCCAATTTGCCGG
UNSB01.fasta	Cele-UNSB01_5:2444425	5	2444425	2444473	49	4	12	AGGCTTAGGC	AGGCTTAAGCTC
UNSB01.fasta	Cele-UNSB01_5:2527844	5	2527844	2527991	148	6	12	CCTAAGCCTA	CCTAGGCCTAAA

UNSB01.fasta	Cele-UNSB01_5:2582229	5	2582229	2582433	205	7	20	CCCCGACGCT	CCCCGACGCTCGAGCTCAAA
UNSB01.fasta	Cele-UNSB01_5:2582360	5	2582360	2582560	201	10	20	GCTCGAGCTC	GCTCGAGCTCAAACCCCGAC
UNSB01.fasta	Cele-UNSB01_5:2584213	5	2584213	2584387	175	6	21	GATTAGATTT	GATTAGATTTCGACCATATTG
UNSB01.fasta	Cele-UNSB01_5:2587851	5	2587851	2588904	1054	15	35	TCAATTCCGG	TCAATTCCGGCAATTTTCCGATTTGCCGGAAATTG
UNSB01.fasta	Cele-UNSB01_5:2602720	5	2602720	2603297	578	16	35	TTTGCCGGAA	TTTGCCGGAAATTTTCAATTCCGACAATTTGCCGG
UNSB01.fasta	Cele-UNSB01_5:2608578	5	2608578	2609028	451	10	35	TTTCCGGCAA	TTTCCGGCAAATCGGAAAAGTGTCCAAGTTTAAAA
UNSB01.fasta	Cele-UNSB01_5:2619946	5	2619946	2620165	220	6	35	GCCGGAAATT	GCCGGAAATTTTCAATTCCGGCAACTCCCCGATTT
UNSB01.fasta	Cele-UNSB01_5:2625951	5	2625951	2626161	211	14	15	CTCACCACGA	CTCACCACGAAGGGT
UNSB01.fasta	Cele-UNSB01_5:2661964	5	2661964	2662120	157	13	12	CTAAGCCTAA	CTAAGCCTAAGC
UNSB01.fasta	Cele-UNSB01_5:2665615	5	2665615	2665738	124	4	35	TTTCTCAGAA	TTTCTCAGAAAATTTGAATTTCCCGCCAAAATGTT
UNSB01.fasta	Cele-UNSB01_5:2726947	5	2726947	2727107	161	12	10	GCTACCGCCC	GCTACCGCCC
UNSB01.fasta	Cele-UNSB01_5:2726947	5	2726947	2728543	1597	110	10	GCTACCGCCC	GCTACCGCCC
UNSB01.fasta	Cele-UNSB01_5:2836474	5	2836474	2836692	219	20	11	GCACATTTTT	GCACATTTTTT
UNSB01.fasta	Cele-UNSB01_5:3006278	5	3006278	3006868	591	10	59	AATTTCAAAT	AATTTCAAATGTGCACTGGTTTTCAAAAATTTTTTAACTGAGAATTATGGAAAATTGGG
UNSB01.fasta	Cele-UNSB01_5:3035465	5	3035465	3035958	494	4	16	GCATGTAGGC	GCATGTAGGCATGTAG
UNSB01.fasta	Cele-UNSB01_5:3042873	5	3042873	3042939	67	5	11	TTTGGCAAGA	TTTGGCAAGAA
UNSB01.fasta	Cele-UNSB01_5:3102518	5	3102518	3102978	461	41	10	GGTAGCGGGC	GATAGCGGGC
UNSB01.fasta	Cele-UNSB01_5:3289995	5	3289995	3290260	266	4	18	TGCAAGACTA	TGCAAGACTATTAGAGGC
UNSB01.fasta	Cele-UNSB01_5:3330728	5	3330728	3331463	736	20	21	CTAACATTTT	CTAACATTTTTCAATTTTCGT
UNSB01.fasta	Cele-UNSB01_5:3492580	5	3492580	3492838	259	7	11	TACTGTAGGA	TACTGTAGAGG
UNSB01.fasta	Cele-UNSB01_5:3492923	5	3492923	3493163	241	6	40	TCCAGAACCT	TCCAGAACCTTCTGGAAAATCCGAGAAAATTCTGGAATGT
UNSB01.fasta	Cele-UNSB01_5:3493344	5	3493344	3494338	995	31	32	CGGGAATTCA	CGGGAATTCAACATTTCTGTGAAAAATTTTGG
UNSB01.fasta	Cele-UNSB01_5:3593686	5	3593686	3593761	76	5	15	CGTGGTGAGA	CGTGGCGAGACCCAC
UNSB01.fasta	Cele-UNSB01_5:3602166	5	3602166	3602210	45	4	11	CAAAAAATGT	CAAAAAATGTG
UNSB01.fasta	Cele-UNSB01_5:3604514	5	3604514	3604619	106	5	21	TTTCAGTTTT	TTTCAGTTTTCGTCTAACATT
UNSB01.fasta	Cele-UNSB01_5:3623960	5	3623960	3624037	78	4	21	AGTTTGTAGT	AGTCTGTAGTTTGTCGTTTGT
UNSB01.fasta	Cele-UNSB01_5:3651691	5	3651691	3651860	170	4	42	TTTCCAGCAA	TTTCCAGAATATCGGCAAGTCAGTGATTTGCCGGAATTGAGA
UNSB01.fasta	Cele-UNSB01_5:3666468	5	3666468	3666780	313	8	39	GATTTACGGG	GATTTACGGGCTTTAATCAGTTTCGAAAAAGCTTGCGTC
UNSB01.fasta	Cele-UNSB01_5:3674779	5	3674779	3675094	316	9	35	AAAATTTCCG	AAAATTTCCGGCAAACAGGCAACTTGCCGGAATTG

UNSB01.fasta	Cele-UNSB01_5:3703054	5	3703054	3703534	481	23	11	AGGATTACTG	AGGATTACTGT
UNSB01.fasta	Cele-UNSB01_5:3735730	5	3735730	3736160	431	4	35	GCCGATTTGC	GCCGATTTGCCGGAAATGTTCAGTTCTGGCAAATT
UNSB01.fasta	Cele-UNSB01_5:3737155	5	3737155	3737311	157	4	35	TTGCCGATTT	TTGCAGATTTGCCGGAAAAATTCAATTCCGGCAAT
UNSB01.fasta	Cele-UNSB01_5:3739928	5	3739928	3740028	101	6	10	TAGCCATGCC	TAGCCATGCC
UNSB01.fasta	Cele-UNSB01_5:3789009	5	3789009	3789261	253	6	42	ACCGGCAATT	ACCGGAAATTGCCGGAATTGTCGATTGCTGAAAATTTCCAAA
UNSB01.fasta	Cele-UNSB01_5:4001570	5	4001570	4002151	582	5	42	CCAGCCGACA	CCAGCCGACAGCCGAAATTTTCACTATTTTCGGCTGGTGCCA
UNSB01.fasta	Cele-UNSB01_5:4022711	5	4022711	4023271	561	14	40	GAACCTTCTG	GAACCTTCTGGAAAATTCGAAAAAATTCTGAAATGTTCTA
UNSB01.fasta	Cele-UNSB01_5:4024546	5	4024546	4025389	844	21	40	CTAGAAAGTT	CTAGAATGTTCTGGAACATTCTAGAATTTTCTTGAATTTC
UNSB01.fasta	Cele-UNSB01_5:4025406	5	4025406	4025890	485	15	32	TTTCACAGAA	TTTCACAGAAAATTTGAATTCCCGCCAAAATT
UNSB01.fasta	Cele-UNSB01_5:4118450	5	4118450	4118598	149	4	35	TTTGCCGGAA	TTTGCCGGAAATTTTTTTTTTTGGTAAATTGCTGT
UNSB01.fasta	Cele-UNSB01_5:4162356	5	4162356	4162767	412	11	35	TTCCGGCAAA	TTCCGGCAAACCGGCAATTTCCCGAAAACGAAAAA
UNSB01.fasta	Cele-UNSB01_5:4165711	5	4165711	4166017	307	5	35	AATTTTCAAT	AATTTTAAATTCCTGCAATTTGCCAGTTTGCCAGA
UNSB01.fasta	Cele-UNSB01_5:4169850	5	4169850	4170172	323	8	35	TTTCAATTCC	TTTCAATTCCGGAAGTTTGCCGATTTGCCGGAAAT
UNSB01.fasta	Cele-UNSB01_5:4216093	5	4216093	4219558	3466	18	200	TGTGGTCCAG	TGTGGTCCAGAAGGTATGACTAAAAATTCACTTTTTCTGCTTGAACTTGGCAGATTTTAAATTTTTTGAAATGCCGTTTTCAAATTCTGAAAGAACTTAAAATTTGCTACAAAATGGCTACTGGAATTTCTGAAAAAGTTAGTTTCAAG CCAGAAATTAATATTCAAAGTTTGGAAAATCTATTAATTTTGCATAAATAT
UNSB01.fasta	Cele-UNSB01_5:4240718	5	4240718	4240800	83	4	18	AAATATTCTT	AAATATTCTTATTTTCAG
UNSB01.fasta	Cele-UNSB01_5:4305444	5	4305444	4315394	9951	398	25	AGTAAAATAT	AGTAAAATATTCTGTGAAGCCAGAG
UNSB01.fasta	Cele-UNSB01_5:4422681	5	4422681	4422875	195	5	35	ATTCCGGCAA	ATTCCGGCAATTTGCCGATCTGCCGGACCTATTAA
UNSB01.fasta	Cele-UNSB01_5:4531686	5	4531686	4531763	78	5	15	CCCCTTTTCG	CCCCTTTTCGTATCT
UNSB01.fasta	Cele-UNSB01_5:4631559	5	4631559	4631760	202	8	18	CCTGGCTGTC	CCTGGTTGTCCTGGGCCT
UNSB01.fasta	Cele-UNSB01_5:4664068	5	4664068	4664530	463	10	35	GCAAATCGGC	GCAAATCGGCAAATTGCCGTATTTGAAAATGCCCG
UNSB01.fasta	Cele-UNSB01_5:4696924	5	4696924	4697115	192	5	35	CGGCAAATCG	CGGCAAATTGTCGGAATTGAATATTTCCGACAAAC
UNSB01.fasta	Cele-UNSB01_5:4698132	5	4698132	4698369	238	5	35	CAATTCCGGC	CAATTCCGGCAATCTGCCGATTTGCCAGAAATTTA
UNSB01.fasta	Cele-UNSB01_5:4751883	5	4751883	4752158	276	22	11	CACGAAGTTG	CACAAAGTTGT
UNSB01.fasta	Cele-UNSB01_5:4763134	5	4763134	4763326	193	11	16	TAGGCATGTA	TAGGCATGTAGGAAAG
UNSB01.fasta	Cele-UNSB01_5:4844742	5	4844742	4844913	172	5	17	AAATGCCAAA	AAATGCCAAAACTGCCA
UNSB01.fasta	Cele-UNSB01_5:4982997	5	4982997	4984483	1487	35	11	GTACTGTAGG	GTACTGTAGGA
UNSB01.fasta	Cele-UNSB01_5:4984650	5	4984650	4985356	707	46	15	TCGTGGTGAG	TCGTGGTGAGATCCT
UNSB01.fasta	Cele-UNSB01_5:4989154	5	4989154	4989259	106	7	15	GGGTCTCACC	GGGTCTCACCACGAT
UNSB01.fasta	Cele-UNSB01_5:5106861	5	5106861	5107246	386	10	35	TGCCGGAAAT	TGCCGAAAATTTCCTTTTCCGGCAACTTGTCGATT

UNSB01.fasta	Cele-UNSB01_5:5114820	5	5114820	5115220	401	8	35	TTGCCGATTT	TTGCCGATTTGCCGGAACTTTCCAATTTCGACAAG
UNSB01.fasta	Cele-UNSB01_5:5204356	5	5204356	5204779	424	9	35	CGATTTGCCG	CGATTTGCCGGAAATTTTTATTTTTGGCAAATTGC
UNSB01.fasta	Cele-UNSB01_5:5272176	5	5272176	5275211	3036	112	26	CAGAGTCACT	CAGAGTCACTAATTTTGGTGAACGGT
UNSB01.fasta	Cele-UNSB01_5:5413359	5	5413359	5414150	792	8	96	TCTGCTTAGA	TCTGCTTAGAATAGGATTAAAGCCAGCCCACAGTTTTCAAGAGTATTCGGGCCTGCGGCCCTCAAACCTGGTTATTAGGCTTGAGTGAATAACATA
UNSB01.fasta	Cele-UNSB01_5:5638035	5	5638035	5638095	61	4	15	CGTGGTGAGA	CGTGGTGAGACCCTT
UNSB01.fasta	Cele-UNSB01_5:5649265	5	5649265	5649420	156	8	19	AAAAATAGAT	AAAAATAGATTGGCAGACG
UNSB01.fasta	Cele-UNSB01_5:5757268	5	5757268	5757343	76	5	15	CGAAAAGGGG	CAAAAAGGGGATCTG
UNSB01.fasta	Cele-UNSB01_5:5757528	5	5757528	5757620	93	6	15	TCCCCTTTTC	TCCCCTTTTCGTATC
UNSB01.fasta	Cele-UNSB01_5:5757785	5	5757785	5758298	514	19	27	TGGAGGCGGC	TGGAGGCGGCTTCTGGCCTGGTGGTGA
UNSB01.fasta	Cele-UNSB01_5:6073958	5	6073958	6074258	301	20	15	ACCCATCGTG	ACCCATCGTGGTGAG
UNSB01.fasta	Cele-UNSB01_5:6074295	5	6074295	6074435	141	4	35	ATTTTGGCGG	ATTTTGGCGGGAAATTTAAATTTTCTGTGAAAAAT
UNSB01.fasta	Cele-UNSB01_5:6074472	5	6074472	6074951	480	12	40	TTCCAGAACT	TTCCAGAACTTTCTAGAAAAATCGGGAAAATTCTGGAATG
UNSB01.fasta	Cele-UNSB01_5:6179564	5	6179564	6194498	14935	110	117	TACTTCAGAT	TACTTCAGATGTTTCCTTTGATTCCACAGGTGCAAGTTTCTCCTTTGTTGGTTCGACAGTAGTTGCACTAGTCTCTGGAACTGGAACATCCTTTTGCTCGACGACTGCAGCTTGTTG
UNSB01.fasta	Cele-UNSB01_5:6199698	5	6199698	6200391	694	15	42	AGGTGCTGCT	AGGTGCTGCTTCCTTCGGAACATTTTCTTGAGTTGGCTCCGA
UNSB01.fasta	Cele-UNSB01_5:6230981	5	6230981	6231799	819	13	59	ATGTGCACTG	ATGTGCACTGGTTTTCAAAAATTTTTAAACTGAGAATAATGGAGAATATGGAATTTCAA
UNSB01.fasta	Cele-UNSB01_5:6307822	5	6307822	6308103	282	8	35	AATTTCCCGC	AATTTCCCGCCAAAATTTTTTACACTGCAAATTTG
UNSB01.fasta	Cele-UNSB01_5:6309373	5	6309373	6309654	282	8	35	AATTTCCCGC	AATTTCCCGCCAAAATTTTTTACACTGCAAATTTG
UNSB01.fasta	Cele-UNSB01_5:6310933	5	6310933	6311214	282	8	35	AATTTCCCGC	AATTTCCCGCCAAAATTTTTTACACTGCAAATTTG
UNSB01.fasta	Cele-UNSB01_5:6314778	5	6314778	6314883	106	7	15	GGGTCTCACC	GGGTCTCACCACGAT
UNSB01.fasta	Cele-UNSB01_5:6410601	5	6410601	6410727	127	6	21	TTGGTGACAA	TTGGTGACAATGTGGAAACAA
UNSB01.fasta	Cele-UNSB01_5:6605586	5	6605586	6605676	91	6	15	GGTGAGACCC	GGTGAGACCCTTCGT
UNSB01.fasta	Cele-UNSB01_5:6928533	5	6928533	6928671	139	7	17	CTCATTGCCT	CTCATTGACTACCCTTG
UNSB01.fasta	Cele-UNSB01_5:6955453	5	6955453	6955644	192	10	19	AACAATCTAT	AACAATCTATTTTTCGTGT
UNSB01.fasta	Cele-UNSB01_5:6963271	5	6963271	6963507	237	4	59	TGGAAATTCA	TGGAAATTCAAGACTTCACTGGTTTTCAAAATTTTTAAAACCGAGAATTATGGGGAATA
UNSB01.fasta	Cele-UNSB01_5:6963686	5	6963686	6964031	346	23	15	AGACCCATCG	AGACCCATCGTGGTG
UNSB01.fasta	Cele-UNSB01_5:6964092	5	6964092	6964484	393	9	35	CGGGAAATTT	CGGGAAATTTGAATTTTCTGTGAAAAATATTTTGG
UNSB01.fasta	Cele-UNSB01_5:6964823	5	6964823	6965938	1116	19	59	ACCAGTGAAG	ACCAGTGAAGTCTTGAATTTCCATATTCCCCAATATTCTCGGTTTTAAATTTTTTGAAT
UNSB01.fasta	Cele-UNSB01_5:6966002	5	6966002	6966297	296	5	59	TGGAAATTCA	TGGAAATTCAAGACTTCACTGGTTTTCAAGATTTTTAAAACCGAGAATTATGGGGAATA
UNSB01.fasta	Cele-UNSB01_5:6966476	5	6966476	6966776	301	20	15	AGACCCATCG	AGACCCATCGTGGTG

UNSB01.fasta	Cele-UNSB01_5:6967498	5	6967498	6969084	1587	27	59	ACCAGTGAAG	ACCAGTGAAGTCTTGAATTGCCATATTCCCCATAATTCTCGGTTTTAAATTTTTTGAAA
UNSB01.fasta	Cele-UNSB01_5:6969386	5	6969386	6969716	331	22	15	AGACCCATCG	AGACCCATCGTGGTG
UNSB01.fasta	Cele-UNSB01_5:6971634	5	6971634	6982576	10943	186	59	ATGGAAATTC	ATGGAAATTCAAGACTTCACTGGTTTTCAAAAAATTTAAAACCGAGAATATTGGGGAAT
UNSB01.fasta	Cele-UNSB01_5:6983222	5	6983222	6984063	842	56	12	TTTTAATTTT	TTTTAATTTTAA
UNSB01.fasta	Cele-UNSB01_5:7024350	5	7024350	7024712	363	11	32	AATTCAAATT	AATTCAAATTTTTTGTGAAAAATTTTGGCGGG
UNSB01.fasta	Cele-UNSB01_5:7301688	5	7301688	7302048	361	6	60	CTAGCTAGAT	CTAGCTAGATTGAACGCTCTCAAGAGAAAGCATCTTGTTGCTGCCCACAAGAAGAAGGCT
UNSB01.fasta	Cele-UNSB01_5:7379892	5	7379892	7380037	146	8	18	TTTCATAAGA	TTTCATAAGAAGTAAATT
UNSB01.fasta	Cele-UNSB01_5:7485041	5	7485041	7485201	161	4	40	GAAGGTTCTA	GAAGGTTCTAGAACATTTCAGCATTTTCTGAGAATTTCTA
UNSB01.fasta	Cele-UNSB01_5:7485330	5	7485330	7487566	2237	74	11	CTACAGTACC	CTACAGTACTA
UNSB01.fasta	Cele-UNSB01_5:7745857	5	7745857	7745978	122	6	17	CTCATTGCCT	CTCATTGACTACCTGTG
UNSB01.fasta	Cele-UNSB01_5:7764570	5	7764570	7764720	151	10	15	GGGTCTCACC	GGGTCTCACCACGAT
UNSB01.fasta	Cele-UNSB01_5:7956964	5	7956964	7957604	641	16	40	TCTGGAACAT	TCTGGAACATTCCAGAATTTTCTCGATTTTTCTAAAAAGT
UNSB01.fasta	Cele-UNSB01_5:7957689	5	7957689	7957969	281	8	35	CAGAAAATTT	CAGAAAATTTAAATTTCCCGCAAAAATATTTTTCA
UNSB01.fasta	Cele-UNSB01_5:7958037	5	7958037	7958202	166	11	15	CCACGATGGG	CCACGACGGGTCTCA
UNSB01.fasta	Cele-UNSB01_5:7959905	5	7959905	7960085	181	11	15	ACCCATCGTG	ACCCATCGTGGTGAG
UNSB01.fasta	Cele-UNSB01_5:8047889	5	8047889	8048164	276	11	25	CCAGAGAGTA	CCAGAGAGTAAATTATATTGTGAAG
UNSB01.fasta	Cele-UNSB01_5:8372367	5	8372367	8372880	514	17	15	GGTGAGACCC	GGTGAGACCCTTCGT
UNSB01.fasta	Cele-UNSB01_5:8373901	5	8373901	8376688	2788	161	17	TCATTGACTA	TCATTGACTACCTTTGC
UNSB01.fasta	Cele-UNSB01_5:8388026	5	8388026	8392649	4624	67	69	GTCTGGACTG	GTCTGGACTGGTGTAGTAGAGCTGCTTGGAGGTTCTGTACTGGTTGTCTCTGGAGCAGTGGTAGTAGTA
UNSB01.fasta	Cele-UNSB01_5:8761530	5	8761530	8782970	21441	357	60	ACACAATTGG	ACACAATTGGAGTGGTGCTTGAGGAGCTGGATTCAGTTGAGCTCGTTATGTCGGTTGAAG
UNSB01.fasta	Cele-UNSB01_5:8785418	5	8785418	8789720	4303	37	114	GTGTTTCAGA	GTGTTTCAGAATTACATGGAGTCTCACTACTTGCAGAATTTGTGGCTGTAGACGATTGACCTGCTGTAGTTGAATCGCTTGAAACTGTCATAGTGGATGTTCCATCAGACGTTT
UNSB01.fasta	Cele-UNSB01_5:8793961	5	8793961	8799031	5071	65	78	TGCAAGGCTC	TGCAAGGCTCCGATGATGATACCACTTCCAAAGTCGAGGTGTTACAATTTTTGGATGTAATAGAACTTGAATCAGTGA
UNSB01.fasta	Cele-UNSB01_5:8921030	5	8921030	8921237	208	8	26	TTCGCCCTGA	TTCGCCCTGAGCGATTTACCTCCCGT
UNSB01.fasta	Cele-UNSB01_5:9471886	5	9471886	9475930	4045	19	214	GTAACTGCTC	GTAACTGCTCAAAGCTTCTGGCACCTATTGGGATGTTGTTGGTAAATTCTGAAAAAACTTGGAATTTGCAACAAAGTTTCTGAAAGAATTTTCGAAAAAAGTTGATTTGAAGTGATTTGGGAATTTTCAAAGTTTCATAAATTTACCTATTTTGCCTATAAATGGTCGTTTTCATTTAATAATAAAATACGCACAAAGGTACTGTAGGTTTTCT
UNSB01.fasta	Cele-UNSB01_5:9479420	5	9479420	9479630	211	6	35	AGAAAATTTA	AGAAAATTTAAATTACCCGCCAAAATATTTTTCAC
UNSB01.fasta	Cele-UNSB01_5:9479685	5	9479685	9479850	166	11	15	CGATGGGTCT	CGATGGGTCTCACCA
UNSB01.fasta	Cele-UNSB01_5:9480075	5	9480075	9480332	258	8	11	GTACTCCTAC	GTACTCCTACA
UNSB01.fasta	Cele-UNSB01_5:9642570	5	9642570	9642900	331	22	15	GGTGAGACCC	GGTGAGACCCATCGT
UNSB01.fasta	Cele-UNSB01_5:9642989	5	9642989	9644244	1256	36	35	GCGGGAAATT	GCGGGAAATTCAAATTTTCAGTGAAAAAAATTTTG

UNSB01.fasta	Cele-UNSB01_5:9644299	5	9644299	9645659	1361	40	40	TTTCTAGAAA	TTTCTAGAAAATTCGGGAAAACTCTGGAATATTCCAGAAC
UNSB01.fasta	Cele-UNSB01_5:9975370	5	9975370	9975475	106	5	21	TTCTAATTCG	TTCTAATTCGACGAGCATCCC
UNSB01.fasta	Cele-UNSB01_5:9977175	5	9977175	9978242	1068	56	19	TGTAACAATC	TGTAACAATCTATTTTTCG
UNSB01.fasta	Cele-UNSB01_5:10311486	5	10311486	10312622	1137	33	34	TTGGCGGGAA	TTGGCGGGAAATTCAAATTTTCAGTGAAAAAATT
UNSB01.fasta	Cele-UNSB01_5:10490005	5	10490005	10492889	2885	111	26	CCAGAGTCAC	CCAGAGTCACTATAATTGGTGAACGG
UNSB01.fasta	Cele-UNSB01_5:10495318	5	10495318	10500018	4701	187	25	TTTACTCTCT	TTTACTCTCTGGCTTCACAGAATAA
UNSB01.fasta	Cele-UNSB01_5:10500053	5	10500053	10500266	214	19	11	AGATACACTT	AGATACACTTC
UNSB01.fasta	Cele-UNSB01_5:10655040	5	10655040	10675549	20510	527	39	CCACCAGAAA	CCACCAGAAATACCAGTTTATGAAAAAGTGGCTCTAGGG
UNSB01.fasta	Cele-UNSB01_5:11125880	5	11125880	11126094	215	10	11	CACATTTTTT	CACATTTTTTG
UNSB01.fasta	Cele-UNSB01_5:11176286	5	11176286	11176509	224	5	45	TCTGATCCTT	TCTGATCCTTCAGATAAGAAGAATCGTAAGGGTACCTCCTGATGG
UNSB01.fasta	Cele-UNSB01_5:11176622	5	11176622	11177248	627	14	45	CTGAAGGATC	CTGAAGGATCAGAACATCAGAAGGTACCCATGTGATCCTTCGGAG
UNSB01.fasta	Cele-UNSB01_5:11342057	5	11342057	11345882	3826	85	45	CCAAGCACAT	CCAAGCACATCTACAGAGACACCTACAACTGAATCAACTGCAGCA
UNSB01.fasta	Cele-UNSB01_5:12149471	5	12149471	12149551	81	5	16	AAAAATTATT	AAAAATTATTGAATAC
UNSB01.fasta	Cele-UNSB01_5:12442637	5	12442637	12442985	349	5	18	GCACTCCCTA	GCACTCCCTATTAGTCTT
UNSB01.fasta	Cele-UNSB01_5:12601066	5	12601066	12601185	120	8	14	CTATACGCTA	CTATACGCTATACG
UNSB01.fasta	Cele-UNSB01_5:12723614	5	12723614	12724001	388	10	39	TGGTACGGTA	TGGTACGGTAGTTGTAGTGGCAGTAGAGGTTGTTGTTGC
UNSB01.fasta	Cele-UNSB01_5:13457267	5	13457267	13457761	495	26	19	GTTACACGAA	GTTACACGAAAAAATAGATT
UNSB01.fasta	Cele-UNSB01_5:13461673	5	13461673	13462167	495	26	19	GTTACACGAA	GTTACACGAAAAAATAGATT
UNSB01.fasta	Cele-UNSB01_5:13535913	5	13535913	13536380	468	12	40	TCTGGAATGT	TCTGGAATGTTCCAGAACTTTCTAGAAAAATCGAGAAAAT
UNSB01.fasta	Cele-UNSB01_5:13735981	5	13735981	13739186	3206	193	16	GAAAAATTTT	GAAAAATTTTGCTGCT
UNSB01.fasta	Cele-UNSB01_5:13735981	5	13735981	13740432	4452	256	16	GAAAAATTTT	GAAAAATTTTGCTGCT
UNSB01.fasta	Cele-UNSB01_5:13735981	5	13735981	13741243	5263	280	16	GAAAAATTTT	GAAAAATTTTGCTGCT
UNSB01.fasta	Cele-UNSB01_5:13735981	5	13735981	13743674	7694	334	16	GAAAAATTTT	GAAAAATTTTGCTGCT
UNSB01.fasta	Cele-UNSB01_5:13979410	5	13979410	13979500	91	6	15	TCGTGGTGAG	TCGTGGTGAGACCCT
UNSB01.fasta	Cele-UNSB01_5:13979523	5	13979523	13981831	2309	66	35	GCGGGAAATT	GCGGGAAATTCAAATTTTCAGTGAAAAAAATTTTG
UNSB01.fasta	Cele-UNSB01_5:13979521	5	13979521	13982440	2920	68	35	TGGCGGGAAA	TGGCGGGAAATTCAAATTTTCAGTGAAAAAAATTT
UNSB01.fasta	Cele-UNSB01_5:14055771	5	14055771	14055849	79	6	12	TCTTTCTCCT	TCTTTCTCCTTT
UNSB01.fasta	Cele-UNSB01_5:14155473	5	14155473	14156076	604	4	151	CATTTCAGAT	CATTTCAGATATTCCATCACCACCAACACCTTCATCCACTTCTTCGCCTGGAACGACGGGATTTTCTACAAAAATCGGATTTTTTCTCTATGTCAGTTTAATTGATGTATAATTCCGAAAAGTCAGTCTTTCCTAGGTTTTTAAATTGAAA
UNSB01.fasta	Cele-UNSB01_5:14159253	5	14159253	14159397	145	6	11	CTACAGTACC	CTACAGTACTG

UNSB01.fasta	Cele-UNSB01_5:14206302	5	14206302	14207970	1669	22	57	GAACTCAACA	GAACTCAACAAGCTGATCAAGAGCAACACCGTGGATCCCGATCTGCCGGACAGAAGG
UNSB01.fasta	Cele-UNSB01_5:14209723	5	14209723	14211643	1921	32	60	CTGCTGATTC	CTGCTGATTCCGAGAAGAAGGCCCCAACCAAGAGAGAATCTGATAAGCCAAAGACTGAAT
UNSB01.fasta	Cele-UNSB01_5:14226001	5	14226001	14226091	91	6	15	CGTGGTGAGA	CGTGTTGAGACCCTA
UNSB01.fasta	Cele-UNSB01_5:14226151	5	14226151	14226968	818	21	35	GGAAATTCAA	GGAAATTCAAATTTTCAGTAAAAAAAATTTTGGCG
UNSB01.fasta	Cele-UNSB01_5:14260478	5	14260478	14260568	91	6	15	GGGTCTCACC	GGGTCTCACCACGAT
UNSB01.fasta	Cele-UNSB01_5:14309212	5	14309212	14310008	797	14	64	GTAGTCAAAA	GTAGTCAAAAAGACAAAAGTGCGAATGTAGCTATTTTTTCAGTCAGTAGCGAAAAAGACATTCA
UNSB01.fasta	Cele-UNSB01_5:14425209	5	14425209	14425464	256	5	24	GCCGCTCCAG	GCCGCTCCAGCACCAGCACCAGAA
UNSB01.fasta	Cele-UNSB01_5:14452450	5	14452450	14453298	849	19	45	TCTGATCCTT	TCTGATCCTTCAGATAAGAAGGATCGTAAGGGTACCTCCTGATGG
UNSB01.fasta	Cele-UNSB01_5:14454544	5	14454544	14454902	359	8	45	AAGGATCAGA	AAGGATCAGAACATCAGAAGGTACCCATGTGATCCTTCGGAGCTG
UNSB01.fasta	Cele-UNSB01_5:14550286	5	14550286	14550378	93	6	15	TCCCCTTTTC	TCCCCTTTTCGTATC
UNSB01.fasta	Cele-UNSB01_5:14856483	5	14856483	14857645	1163	45	25	TGTGAAGCCA	TGTGAAGCCAGAGAGTAAATAATAC
UNSB01.fasta	Cele-UNSB01_5:14878525	5	14878525	14883699	5175	182	27	GGAAGCCACA	GGAAGCCACAGAGAGTAGAATATGGTG
UNSB01.fasta	Cele-UNSB01_5:15130293	5	15130293	15130341	49	4	12	TCTAAGTCTA	TCTAAGTCTAAA
UNSB01.fasta	Cele-UNSB01_5:15289065	5	15289065	15289259	195	6	35	GGAATTGAAA	GGAATTGAAAATTTCCGACAAATCGGCAAATTGGC
UNSB01.fasta	Cele-UNSB01_5:15290454	5	15290454	15290610	157	6	26	TTCACCTAAA	TTCACTTAAAATAATGACTCTAACAA
UNSB01.fasta	Cele-UNSB01_5:15415879	5	15415879	15416665	787	8	15	TCTCACCACG	TCTCACCACGAAGGG
UNSB01.fasta	Cele-UNSB01_5:15441112	5	15441112	15445831	4720	118	40	ATTTTTCTAG	ATTTTTCTAGAAAATTCTGGAACACTCCAGAATTTTCCCG
UNSB01.fasta	Cele-UNSB01_5:15445975	5	15445975	15447055	1081	72	15	CCACGATGGG	CCACGATGGGTCTCA
UNSB01.fasta	Cele-UNSB01_5:15447313	5	15447313	15447447	135	5	11	GTACTCCTAC	GTACTCCTACA
UNSB01.fasta	Cele-UNSB01_5:15668468	5	15668468	15668848	381	5	73	TTAGGCTTAA	TTAGGCTTAAGTGTGTCATAAGGATTTCCAATATTTTCTTCCAGTTGGGCATCCTAGTATGTTTTTCGGGCCT
UNSB01.fasta	Cele-UNSB01_5:15725489	5	15725489	15725564	76	5	15	AGACCCATCG	AGACCCATCGTAGTG
UNSB01.fasta	Cele-UNSB01_5:15725600	5	15725600	15726976	1377	36	35	TTCAAATTTT	TTCAAATTTTCTGTGAGAAAAATTTTGGGGGGAAA
UNSB01.fasta	Cele-UNSB01_5:15757927	5	15757927	15757998	72	5	12	CTTAGGCTTA	CTTAGACTTAGG
UNSB01.fasta	Cele-UNSB01_5:15913508	5	15913508	15913600	93	6	15	CGAAAAGGGG	CGAAAAGGGGATCTG
UNSB01.fasta	Cele-UNSB01_5:15966117	5	15966117	15966182	66	4	11	GTACTGTAGG	GTACTGTAGGG
UNSB01.fasta	Cele-UNSB01_5:16082522	5	16082522	16083430	909	7	37	GCGGGAATTC	GCGGGAATTCAAATTTTAATTATTTGAAATCATTTTG
UNSB01.fasta	Cele-UNSB01_5:16332671	5	16332671	16332882	212	6	42	TTTCGGCAAC	TTTCGGCAACCGGCAATTGCCGATGTTGCCGAACCCGAAAAA
UNSB01.fasta	Cele-UNSB01_5:16414638	5	16414638	16414819	182	5	36	GGGAATTCAA	GGGAATTCAAATTTCAATTTTTGAAAACATTTTGGC
UNSB01.fasta	Cele-UNSB01_5:16414904	5	16414904	16415369	466	31	15	TGGTGAGACC	TGGTGAGACCTTTCG

UNSB01.fasta	Cele-UNSB01_5:16471613	5	16471613	16475389	3777	23	164	CGTACAAGCT	CGTACAAGCTATTATACTTTGATTGGCATATTCTGGTGTTTTTGACTATGCTGAACATTTTTATGAAATAAAAAAGAATGACTCCATATGATTTTCTAGGACTTTTGAATTTTTGAAAAAAATCAGATTTTATATATTTTTATCAATTTTTAAAAAAAATACTTC
UNSB01.fasta	Cele-UNSB01_5:16591935	5	16591935	16597985	6051	50	121	GTTGCAAACC	GTTGCAAACCGCAAGAGCAAACTTGCCAAATGACAAGCCACCCAAGTACTCGTTGCCCCTACCTATCATATAATCGTAGACTATAATTTCTCTAAAAAGATAGTTGCCCTAATAGCAAAAG
UNSB01.fasta	Cele-UNSB01_5:16677048	5	16677048	16677311	264	16	12	CATTGACTTG	CATTGACTTGAG
UNSB01.fasta	Cele-UNSB01_5:16682435	5	16682435	16683237	803	14	22	GGGTACTGTA	GGGTACTGTAGGAATACTGTGG
UNSB01.fasta	Cele-UNSB01_5:16693408	5	16693408	16693990	583	10	11	GTACTGTAGG	GTACTGTAGGG
UNSB01.fasta	Cele-UNSB01_5:16694107	5	16694107	16694865	759	17	40	CTGGAATGTT	CTGGAATGTTCCAAAACCTTCTGGAAAATTTGAGAAAATG
UNSB01.fasta	Cele-UNSB01_5:16694107	5	16694107	16695183	1077	18	40	CTGGAATGTT	CTGGAATGTTCCAAAACCTTCTGGAAAATTTGAGAAAATG
UNSB01.fasta	Cele-UNSB01_5:16699381	5	16699381	16699441	61	4	15	CGAAAAGGGG	CAAAAAGGGGATCTG
UNSB01.fasta	Cele-UNSB01_5:16699651	5	16699651	16699786	136	7	15	GCAGATCCCC	GCAGATCCCCCTTTTT
UNSB01.fasta	Cele-UNSB01_5:16730521	5	16730521	16730649	129	4	32	GCTGATTGGT	GCTGATTGGTAAGATAAAACTGGTGGTGAAAC
UNSB01.fasta	Cele-UNSB01_5:16772051	5	16772051	16772198	148	7	21	ATTTTTCAAT	ATTTTTCAATTTTCGTCTAAC
UNSB01.fasta	Cele-UNSB01_5:16819160	5	16819160	16819347	188	11	17	TAACCTGACT	TAACCTGACTGCTATAT
UNSB01.fasta	Cele-UNSB01_5:16845717	5	16845717	16846129	413	25	16	ACATACCTAC	ACATACCTACATACCT
UNSB01.fasta	Cele-UNSB01_5:17041242	5	17041242	17041488	247	7	11	CTACAGTACC	CTACAGTACCT
UNSB01.fasta	Cele-UNSB01_5:17104481	5	17104481	17104658	178	5	21	CTAATTAGCG	CTAAGTAGCGTCGCTTCCTTT
UNSB01.fasta	Cele-UNSB01_5:17108364	5	17108364	17108469	106	5	21	TGCTAATTAG	TGCTAATTAGCGCGGCTGCGT
UNSB01.fasta	Cele-UNSB01_5:17207041	5	17207041	17207511	471	12	40	AAAATTCTGG	AATATTCTGGGATGTTCTAGAACCTTCTGGAAAAATTGAG
UNSB01.fasta	Cele-UNSB01_5:17249920	5	17249920	17250872	953	8	119	AACTGTGGGC	AACTGTGGGCTGGCTTTAATCCTAACCCAAGCAGAGCAGGATGTAGGCCTGAAACGTGGTTGTTATTCAATCAAGCCTAACAACCAGGTTTGAGGGCCGCAGGCCCGAAAACTCTTGGA
UNSB01.fasta	Cele-UNSB01_5:17487338	5	17487338	17487635	298	8	37	AAAACATTTT	AAAACATTTTGGCGGGAATTCAAAATTTAATTTTTTG
UNSB01.fasta	Cele-UNSB01_5:17569173	5	17569173	17569281	109	9	12	CGCAACGCGA	CGCAACGCGATA
UNSB01.fasta	Cele-UNSB01_5:17588702	5	17588702	17608641	19940	337	59	TTATGGTGAA	TTATGGTGAATATGGAAATTCAAATGTGCACTGGTTTTCAAAATTTTTAAAACCGAGAA
UNSB01.fasta	Cele-UNSB01_5:17626210	5	17626210	17626494	285	15	19	TTTTCGTGTA	TTTTCGTGTAACCATCTAT
UNSB01.fasta	Cele-UNSB01_5:17709602	5	17709602	17709736	135	5	11	CCTACAGTAC	CCTACAGTACC
UNSB01.fasta	Cele-UNSB01_5:17737089	5	17737089	17738916	1828	7	255	AACAAGTCGG	AACAAGTCGGGAACGTTCGGAAAGCTCTGAGGAAAACTCCGACAGTATTATGCTTAAGTACGCAATTGAGCTATCTTTATTAGATTCGAAAGAGGACGCTTCAAAAATTGAAGCAATAACGAAGAAGCTTAAGTTGGAACTGGCGAAGCTTAAATTGAGAATTACGCAGTCTTTATCAGATTCAAAAGACGACTCTTTGTCCAATGAAGCAAATCTGCAGAACGATCTACCTTCGACTTCTGATAGCAAAACTCC
UNSB01.fasta	Cele-UNSB01_5:17801402	5	17801402	17801541	140	10	12	ACGCGACACG	ACGCGGCACGCA
UNSB01.fasta	Cele-UNSB01_5:17834079	5	17834079	17834336	258	8	11	CCTACAGTAC	CCTACAGTACC
UNSB01.fasta	Cele-UNSB01_5:17844176	5	17844176	17844688	513	32	16	CCAGGGTTCC	CCAGGGTTCCAGGGTT
UNSB01.fasta	Cele-UNSB01_5:17844176	5	17844176	17845043	868	35	16	CCAGGGTTCC	CCAGGGTTCCAGGGTT

UNSB01.fasta	Cele-UNSB01_5:17868232	5	17868232	17868328	97	6	16	GAATAATATT	GAATAATATTGGAGAT
UNSB01.fasta	Cele-UNSB01_5:17962175	5	17962175	17963525	1351	6	143	CAGTGGGATT	CAGTGGGATTCTGAATTTATTCCAATCAAACGTCCGGGGTTCGAGTCCGCACAGGGGTATTTATTTTTGCAAGCTGTGATTTACAAGTTTATAGCTGCAAAAGTGATTTTTATCGAATTTTTAGGAAAATGTCCGAATGGCA
UNSB01.fasta	Cele-UNSB01_5:17995705	5	17995705	17996016	312	5	20	ATTTGACCTA	ATTTGACCTACTTTTTAAAA
UNSB01.fasta	Cele-UNSB01_5:18032604	5	18032604	18033134	531	13	34	TCAAATTTTC	TCAGATTTTCTGAAAAAAAACTTTATCGGGAAAT
UNSB01.fasta	Cele-UNSB01_5:18100660	5	18100660	18102309	1650	32	44	TTGCCGGAAA	TTGCCGGAAATAGTAATTTTTGGCAAATTGCCGGTTTGCTGAAT
UNSB01.fasta	Cele-UNSB01_5:18206252	5	18206252	18206609	358	5	35	AATTCCGGCA	ACTTCCGGCAAGTTGCCGATTTGCCGGAAATCTTC
UNSB01.fasta	Cele-UNSB01_5:18261348	5	18261348	18261785	438	10	35	TTTCCGGCAA	TTTCCGGCAAATCGGCGATTTGCCTGAATTGAAAG
UNSB01.fasta	Cele-UNSB01_5:18345564	5	18345564	18345809	246	7	35	AAATTTTCGG	AAATTTTCGGCAAATTCGATAATTGCCGGTTTTGT
UNSB01.fasta	Cele-UNSB01_5:18406789	5	18406789	18410349	3561	114	31	GTCAATTTTT	GTCAATTTTTGGCGGGAAATTCAAATTTTCA
UNSB01.fasta	Cele-UNSB01_5:18412081	5	18412081	18412792	712	10	78	GAGCTTTGGG	GAGCTTTGGGGCTTTTGGCTTGATCTCAGTCGACTTGATGGATTCTGGTTTGGGTTCTGCAGGCTTTTCGGGCTCCTT
UNSB01.fasta	Cele-UNSB01_5:18413578	5	18413578	18417093	3516	121	19	TTGACGCAAT	TTGACGCAGTTCTCGTGCC
UNSB01.fasta	Cele-UNSB01_5:18442188	5	18442188	18442272	85	4	21	GCACTGACCA	GCACTGACCAAATGCAATGTT
UNSB01.fasta	Cele-UNSB01_5:18556604	5	18556604	18557205	602	6	45	GGGGGACCCT	GGGGGACCCTTATGATCCTTTGGAGCTGAAGATTCAAGTCATAAG
UNSB01.fasta	Cele-UNSB01_5:18596071	5	18596071	18596370	300	14	14	ACAAACTACA	ACAAACTACAAACT
UNSB01.fasta	Cele-UNSB01_5:18630095	5	18630095	18630532	438	22	19	CAAGGCACTA	CAAGGCACGAGAATTGCGC
UNSB01.fasta	Cele-UNSB01_5:18631640	5	18631640	18632148	509	18	19	TGACGCAATT	TGACGCATTTCTAGTTCAT
UNSB01.fasta	Cele-UNSB01_5:18642200	5	18642200	18655941	13742	679	20	ACACGAAAAA	ACACGAAAAAAATAGATTGTT
UNSB01.fasta	Cele-UNSB01_5:18664756	5	18664756	18664882	127	9	14	GTCTGGGGTC	GTCTGGGGTCTGGG
UNSB01.fasta	Cele-UNSB01_5:18669912	5	18669912	18670449	538	16	31	TTGGCCCAGA	TTGACCCAGAGCTTTCTTGACCCAAGCCCCC
UNSB01.fasta	Cele-UNSB01_5:18701783	5	18701783	18702035	253	9	28	CCAAGAGAAT	CCAAGAGAATTTCAACCACCTGGAAATT
UNSB01.fasta	Cele-UNSB01_5:18728400	5	18728400	18728559	160	4	32	GAATTCAAAT	GAATTCAAATTTTCTGAAAAATTTTTTGGCGT
UNSB01.fasta	Cele-UNSB01_5:18730021	5	18730021	18731423	1403	114	12	GTATATGTAT	GTATATGTATAT
UNSB01.fasta	Cele-UNSB01_5:18793643	5	18793643	18794338	696	5	31	AAAATTTTGA	AAAATTTTGACCCAGAAATCGAAAAATTCGC
UNSB01.fasta	Cele-UNSB01_5:18824768	5	18824768	18825020	253	16	12	AGGCTTAGGC	AGGCCTAGGCTT
UNSB01.fasta	Cele-UNSB01_5:18831834	5	18831834	18832119	286	11	19	TTGACGCAAT	TTGACACAATTTTAGTACC
UNSB01.fasta	Cele-UNSB01_5:18833669	5	18833669	18833973	305	15	19	TAGTGCCTTG	TAGTGCCTTGATGCAATTT
UNSB01.fasta	Cele-UNSB01_5:18836263	5	18836263	18836629	367	11	33	TGTCGGCTGC	TGTCGGCTGCTAAAACTATAGCCTCCCGGTAGG
UNSB01.fasta	Cele-UNSB01_5:18839756	5	18839756	18840116	361	22	12	AGCCTAAGCC	AGCCTAAGCCTA
UNSB01.fasta	Cele-UNSB01_5:18842565	5	18842565	18843912	1348	50	20	GTAAATCTAC	GTAAATTTACATTGGCCTCC

UNSB01.fasta	Cele-UNSB01_5:18846370	5	18846370	18846628	259	18	12	TAAGCCTAAG	TAAACCTAAGCG
UNSB01.fasta	Cele-UNSB01_5:18856264	5	18856264	18856396	133	9	12	AGCCTAAGCC	AGCCTAAGCCTA
UNSB01.fasta	Cele-UNSB01_5:18874657	5	18874657	18874753	97	6	12	CTAAGCCTAA	CTAATCCTAAGC
UNSB01.fasta	Cele-UNSB01_5:18877478	5	18877478	18878086	609	29	19	GTGCCTTGAC	GTGCTTTGACGCAATTCTA
UNSB01.fasta	Cele-UNSB01_5:18890478	5	18890478	18890638	161	8	20	GATTTTTCTG	GATTTTTTTGAAAATTTATT
UNSB01.fasta	Cele-UNSB01_5:18891023	5	18891023	18891077	55	4	12	TAAGCCTAAG	TAAGCCTAGGCC
UNSB01.fasta	Cele-UNSB01_5:18895119	5	18895119	18895676	558	15	39	GTAATTTATA	GTAATTTATAATTTTTTAAATATGAGTTATGTATTGCCC
UNSB01.fasta	Cele-UNSB01_5:18898055	5	18898055	18898199	145	9	12	GCTTAGGCTT	GCTTCGGCTTAG
UNSB01.fasta	Cele-UNSB01_5:18899719	5	18899719	18900018	300	5	11	TTTCAGGCCT	TTTAGGCCTAC
UNSB01.fasta	Cele-UNSB01_5:18902702	5	18902702	18903276	575	24	21	AAAAATCGAT	AAAAATCGATAAAATATGGAG
UNSB01.fasta	Cele-UNSB01_5:18924789	5	18924789	18925018	230	6	35	GGAAATTCAA	GAAAATTCAAATTTTCTGAGAAAATTTTTTTGGC
UNSB01.fasta	Cele-UNSB01_5:18933758	5	18933758	18934184	427	12	20	AGAAAAATCA	AGAAAAATCAATAAATTTTT
UNSB01.fasta	Cele-UNSB01_5:18934684	5	18934684	18935273	590	23	19	TGACGCAATT	TGACGCATTTTTAGTGCCT
UNSB01.fasta	Cele-UNSB01_5:18935616	5	18935616	18936186	571	26	19	ACTAGAATTG	ACTAGAAGTGCGCCAATAC
UNSB01.fasta	Cele-UNSB01_5:18937116	5	18937116	18937194	79	6	12	GGCTCAGGCT	GGCTCCGGCTCA
UNSB01.fasta	Cele-UNSB01_5:18944459	5	18944459	18944687	229	8	19	TTCTAGTGCC	TTCTAGTGCCTTGGCGCTT
UNSB01.fasta	Cele-UNSB01_5:18982935	5	18982935	18983047	113	7	12	AGCCTGAGCC	AGCCTGAGCCTG
UNSB01.fasta	Cele-UNSB01_5:19004973	5	19004973	19005085	113	7	12	AGCCTGAGCC	AGCCTGAGCCTG
UNSB01.fasta	Cele-UNSB01_5:19009672	5	19009672	19010877	1206	61	20	AAAAATAGAT	AAAAATAGATTGTTACACGA
UNSB01.fasta	Cele-UNSB01_5:19020255	5	19020255	19020473	219	6	31	TGTAAAAATG	TGAAAAAATGTCTGAAACTTCCAATTTACAA
UNSB01.fasta	Cele-UNSB01_5:19059372	5	19059372	19059511	140	4	35	TCAGAAAATT	TCAGAAAATTTAAATTTCCCTCCAAAATTTTTTTC
UNSB01.fasta	Cele-UNSB01_5:19079815	5	19079815	19080004	190	5	24	GCCTAAGCCT	GCCTAAACCTAACCCCGAGTCTAG
UNSB01.fasta	Cele-UNSB01_5:19138115	5	19138115	19138319	205	6	31	TCGTTTTTTC	TCGTTTTTTCATGAAAAAACTCAATTTTCCG
UNSB01.fasta	Cele-UNSB01_5:19147729	5	19147729	19148084	356	7	40	GAATTTTTTG	GAATTTTTTGCAATTTTATTGAATTTTCCAAATTTTCAGC
UNSB01.fasta	Cele-UNSB01_5:19162213	5	19162213	19162392	180	5	40	TTTTGCCGAA	TTTTACCGAAAATTGCAAATTTTGTGGAATGTATCACATT
UNSB01.fasta	Cele-UNSB01_5:19164473	5	19164473	19167625	3153	148	17	ATTAGCAGCG	TTAGCAGCGACCAAAAA
UNSB01.fasta	Cele-UNSB01_5:19170107	5	19170107	19171106	1000	12	18	AATTAGCAGC	AATTAGCAGCGACCAAAA
UNSB01.fasta	Cele-UNSB01_5:19172684	5	19172684	19173158	475	14	32	TTGTAGTTTG	TTGTAGTTTGTAGTCTAAGCAGCGCCCAAAAC
UNSB01.fasta	Cele-UNSB01_5:19225003	5	19225003	19225171	169	8	21	ATTATTGATT	ATTATCGATTTTTGAAGGGAA

UNSB01.fasta	Cele-UNSB01_5:19249579	5	19249579	19249688	110	6	18	AGCAGCGCCC
AGCAGCGCCCAAAAAAAT								
UNSB01.fasta	Cele-UNSB01_5:19251983	5	19251983	19252110	128	5	18	GCAGCGCCCA
GCAGCGCCCAAAAGATTA								
UNSB01.fasta	Cele-UNSB01_5:19255197	5	19255197	19255501	305	17	18	TGCTAATTTT
TGCTAATTTTTTTGGCGC								
UNSB01.fasta	Cele-UNSB01_5:19270843	5	19270843	19271693	851	31	20	ATTTATTGAT
ATTTATCGATTTTTCTGTAA								
UNSB01.fasta	Cele-UNSB01_5:19272660	5	19272660	19272781	122	6	20	ATAAATTTTC
ATAAATTTTCAGAAAAATCG								
UNSB01.fasta	Cele-UNSB01_5:19273514	5	19273514	19273585	72	4	18	TAGCAGCGCC
TAGCAGCGCCCAAGAAAT								
UNSB01.fasta	Cele-UNSB01_5:19275863	5	19275863	19276354	492	14	32	TGTAGTTTGT
TGTAGTTTGTAGTTTAGCAGCGCCCAAAATTG								
UNSB01.fasta	Cele-UNSB01_5:19279992	5	19279992	19280235	244	8	20	TATCGATTTT
TATCGATTTTTCTGCAAATT								
UNSB01.fasta	Cele-UNSB01_5:19309007	5	19309007	19310074	1068	10	19	TGGCGCATTT
TGGCGCGTTTTCGGTGTGT								
UNSB01.fasta	Cele-UNSB01_5:19313130	5	19313130	19313974	845	33	20	AAAATCGATA
ATAATCGATAAATTCCCAGA								
UNSB01.fasta	Cele-UNSB01_5:19325271	5	19325271	19325379	109	9	12	ATACATATAC
ATACATATACAT								
UNSB01.fasta	Cele-UNSB01_5:19329973	5	19329973	19330169	197	8	12	ATATGTATAT
ATATGTATATGT								
UNSB01.fasta	Cele-UNSB01_5:19334298	5	19334298	19334625	328	23	14	TATATGTTAT
TATATGTTATATGT								
UNSB01.fasta	Cele-UNSB01_5:19338537	5	19338537	19338764	228	7	33	GGAATTCAAA
GGAATTCAAATTTCTTCAAAAAAAATTTTGGCG								
UNSB01.fasta	Cele-UNSB01_5:19352170	5	19352170	19352944	775	29	26	TGAAATTTCA
TGAAATTTCACAAAATATCGATTTTC								
UNSB01.fasta	Cele-UNSB01_5:19385136	5	19385136	19386466	1331	44	20	AAAAATCAAT
AAAAATCAATCAATTTCCGA								
UNSB01.fasta	Cele-UNSB01_5:19386514	5	19386514	19386960	447	10	20	ATTGATTTTT
ATTGATTTTTCTGAAAATTT								
UNSB01.fasta	Cele-UNSB01_5:19387732	5	19387732	19388133	402	17	20	CAGAAAAATC
CAGAAAAATCGATAAATTTT								
UNSB01.fasta	Cele-UNSB01_5:19392335	5	19392335	19392460	126	6	18	ATTAGCAGCG
ATTAGCAGCGACCAAAAA								
UNSB01.fasta	Cele-UNSB01_5:19398669	5	19398669	19400674	2006	80	20	TATCGATTTT
TATTGATTTTTCTGAAAATT								
UNSB01.fasta	Cele-UNSB01_5:19404337	5	19404337	19404490	154	5	33	TTAGCAGCGC
TTAGCAGCGCCCAAAATCTTGTAGTTTGTAGTC								
UNSB01.fasta	Cele-UNSB01_5:19407563	5	19407563	19408121	559	25	20	AATTTATTGA
AATTTATTGATTTTTCCGGA								
UNSB01.fasta	Cele-UNSB01_5:19408877	5	19408877	19410842	1966	65	33	AGCAGCGCCC
AGCAGCGCCCAAAATCTTGTAGTTTGTAGTCTT								
UNSB01.fasta	Cele-UNSB01_5:19416239	5	19416239	19417040	802	34	20	GATTTTTCTG
GATTTTTCTGAAAATATATC								
UNSB01.fasta	Cele-UNSB01_5:19434732	5	19434732	19435093	362	16	19	TGGCGCATTT
TGGCGCATTTCTTGAGCCT								
UNSB01.fasta	Cele-UNSB01_5:19480245	5	19480245	19483641	3397	189	18	GCTGCTAATT
GCTGCTAATTTTTTGGGC								
UNSB01.fasta	Cele-UNSB01_5:19517858	5	19517858	19518362	505	37	12	AGGCTCAGGC
AGGCTCAGGCTC								
UNSB01.fasta	Cele-UNSB01_5:19521326	5	19521326	19521536	211	15	12	GCCTAAGCCT
GCCTGAGCCTGA								
UNSB01.fasta	Cele-UNSB01_5:19549755	5	19549755	19549902	148	7	21	CACACGAGCT
CACACGATCTTCAGATTCCAC								

UNSB01.fasta	Cele-UNSB01_5:19567297	5	19567297	19567464	168	5	29	CTCGTCAGTG	CTCGTCAGTGGGTGGACGAGGAATGAGCG
UNSB01.fasta	Cele-UNSB01_5:19604230	5	19604230	19604355	126	4	22	GTGCGCAGAA	GTGCGCAGAATCTGCTCAGGGG
UNSB01.fasta	Cele-UNSB01_5:19643678	5	19643678	19643940	263	11	21	TCTAATTAGA	TCAAATTAGATTACCTTTTTT
UNSB01.fasta	Cele-UNSB01_5:19645516	5	19645516	19645794	279	13	21	TTCTAATTAG	TTCTAATTAGATGCGCATTCC
UNSB01.fasta	Cele-UNSB01_5:19666114	5	19666114	19666380	267	11	19	GATCCTTGGC	GATCCTTAGCGCTTTTTTT
UNSB01.fasta	Cele-UNSB01_5:19667001	5	19667001	19667210	210	9	19	TTGCGCCAAG	TTGCGTCAAGACACTAAAA
UNSB01.fasta	Cele-UNSB01_5:19670399	5	19670399	19670678	280	9	31	CGGGAAATTC	CGGGAAATTTGAATTTTCGAATAATTTTTGG
UNSB01.fasta	Cele-UNSB01_5:19673680	5	19673680	19673832	153	5	19	AGAATTGCGC	AGAATTGCGCCAAGGCACG
UNSB01.fasta	Cele-UNSB01_5:19673879	5	19673879	19674316	438	16	19	GCCTTGACGC	GCCTTGACGCAATTCTAGC
UNSB01.fasta	Cele-UNSB01_5:19696042	5	19696042	19696279	238	14	17	GAATTAGAAA	GAATTAGAAAACTTTGA
UNSB01.fasta	Cele-UNSB01_5:19714208	5	19714208	19714335	128	5	25	AAAAATCTGT	AAAAATCTGTTCTCAGAGGTCCCTG
UNSB01.fasta	Cele-UNSB01_5:19721189	5	19721189	19721423	235	7	22	ACGTATACAG	ACGTATACAGTCATATACACAT
UNSB01.fasta	Cele-UNSB01_5:19721079	5	19721079	19721595	517	5	21	TATTTTCAAA	TATTTTCAAAAAAATTTAAAA
UNSB01.fasta	Cele-UNSB01_5:19764010	5	19764010	19764338	329	4	82	CCCCGAACGT	CCCCGAACGTGTCGGCCGCTTCCAAATAACCACCTCCTTGCACTACGTTGCGCACACACTCATTTCACGCCAAGCTGCGGAA
UNSB01.fasta	Cele-UNSB01_5:19784949	5	19784949	19785086	138	6	23	TTTTCAGAAA	TTTTCAGAAATAGACATTTCAGA
UNSB01.fasta	Cele-UNSB01_5:19785568	5	19785568	19787058	1491	85	17	TTGCAAGAAA	TTGCAAGAAAATCTGCA
UNSB01.fasta	Cele-UNSB01_5:19792773	5	19792773	19792843	71	5	10	TGGCGAAAGC	TGGCGAAAGC
UNSB01.fasta	Cele-UNSB01_5:19792863	5	19792863	19792928	66	5	13	TTGGGGTGGG	TTGGGGTGGGCCT
UNSB01.fasta	Cele-UNSB01_5:19795988	5	19795988	19796719	732	4	183	TTTCTCTGCG	TTTCTCTGCGTCTCGCCTTGGCTCACAGTATTTTTCACACCAAACTTCGAGGCTATGTATCTTTGCAACGGGTTGAGATATTGAAAAATTTCCAACTAACAAAATGCTCTAAAATTTATTCTGTATCTTGTGAAATAAACTTTAAAACC CTAGGCAAATCGCAAACAGCGAAATACACCCAAG
UNSB01.fasta	Cele-UNSB01_5:19815492	5	19815492	19815850	359	22	12	CTTAGGCTTA	CTTGGGCTTAGG
UNSB01.fasta	Cele-UNSB01_5:19826165	5	19826165	19826303	139	11	12	CTTAGGCTTA	CTTAGGCTTAGG
UNSB01.fasta	Cele-UNSB01_5:19832755	5	19832755	19833004	250	6	31	AATTTCCCGC	AATTTCCCGCTAAAAATTAACTGAAAATTTG
UNSB01.fasta	Cele-UNSB01_5:19848468	5	19848468	19849404	937	7	14	TTTGTAGTTT	TTTGTACTTTGTAG
UNSB01.fasta	Cele-UNSB01_5:19849444	5	19849444	19849810	367	11	14	ACAAACTACA	ACAAACTACATACT
UNSB01.fasta	Cele-UNSB01_5:19852888	5	19852888	19853192	305	8	27	GCAGCCGACA	GCAGCCGACACCTCACAGGTCCCTGTA
UNSB01.fasta	Cele-UNSB01_5:19913255	5	19913255	19913615	361	8	31	TTCAAATTTT	TTCAAATTTTCAGTCAATTTTTGGCGGGAGT
UNSB01.fasta	Cele-UNSB01_5:19928437	5	19928437	19928836	400	12	31	GGCGGGAAAT	GGCGGGAAATTCAAATTTTCAGTCAATTTTT
UNSB01.fasta	Cele-UNSB01_5:19938977	5	19938977	19939376	400	12	31	GGCGGGAAAT	GGCGGGAAATTCAAATTTTCAGTCAATTTTT
UNSB01.fasta	Cele-UNSB01_5:19953202	5	19953202	19953370	169	6	21	ACCCCGATTT	ACTCCGATTTTCATCCAAAAA

UNSB01.fasta	Cele-UNSB01_5:19987417	5	19987417	19987506	90	4	22	ATTGGATTTT	ATTGGATTTTTCACCCCAAAAA
UNSB01.fasta	Cele-UNSB01_5:19998052	5	19998052	19998541	490	10	31	AAAATTTGAA	AAAATTTGAATTTCCCGCCAAAAATTGACTG
UNSB01.fasta	Cele-UNSB01_5:20061429	5	20061429	20063397	1969	117	16	CTTAGATCTC	CTTAGATCTCAATTCA
UNSB01.fasta	Cele-UNSB01_5:20065513	5	20065513	20066023	511	14	34	TTTCCCGCCA	TTTCCCGCCAAAATAATTTCTCCGAAAATTTGTA
UNSB01.fasta	Cele-UNSB01_5:20072393	5	20072393	20073779	1387	40	35	CCGGCAAATC	CCGGCAAATCGACAAATTGCCGGATTTGTAAATTT
UNSB01.fasta	Cele-UNSB01_5:20105608	5	20105608	20107135	1528	40	35	GAAATTTTCA	GAAATTTTCATTTCCGGCAAATTACTGATCTGCCG
UNSB01.fasta	Cele-UNSB01_5:20109112	5	20109112	20109540	429	12	35	AATTTCCGGC	AATTTCCGGCAATACGGCAAATTACCAGAATTAAA
UNSB01.fasta	Cele-UNSB01_5:20111577	5	20111577	20111694	118	5	12	AAGCCTAAGC	AAGCCTAATCTT
UNSB01.fasta	Cele-UNSB01_5:20131328	5	20131328	20132164	837	37	19	TAGTGCCTTG	TAGTGCATTGGCGCAATTC
UNSB01.fasta	Cele-UNSB01_5:20173699	5	20173699	20174897	1199	28	31	TTCCCGCCTA	TTCCCGCCTAAAACTACTCGAAAATTCAAAT
UNSB01.fasta	Cele-UNSB01_5:20176707	5	20176707	20177444	738	21	33	TTTGAATTTC	TTTGAATTTCCCACCAAATATTTTCTCAAAAAT
UNSB01.fasta	Cele-UNSB01_5:20212391	5	20212391	20212631	241	10	20	TTATTGATTT	TTATTGATTTTTCTGGGAAT
UNSB01.fasta	Cele-UNSB01_5:20217981	5	20217981	20218181	201	9	20	CAGAAAAATC	CAGAAAAATCAATAAATTTT
UNSB01.fasta	Cele-UNSB01_5:20220047	5	20220047	20220618	572	12	20	TTATTGATTT	TTATCGATTTTTATGGAAAT
UNSB01.fasta	Cele-UNSB01_5:20243571	5	20243571	20245480	1910	58	32	ACTACAAACT	ACTACAAACTACAAGTTTTTGGCGCTGCTAA
UNSB01.fasta	Cele-UNSB01_5:20247169	5	20247169	20247404	236	11	20	TTTGAATTTT	TTTGAATTTTTGAGTGAAAA
UNSB01.fasta	Cele-UNSB01_5:20269010	5	20269010	20269323	314	8	12	TATGTATATG	TATGTATATGTA
UNSB01.fasta	Cele-UNSB01_5:20275164	5	20275164	20275248	85	4	21	CTCAATTCAA	CTCAATTCAATCAAGGAAATT
UNSB01.fasta	Cele-UNSB01_5:20277753	5	20277753	20277891	139	11	12	ATATACATAT	ATATACATATAC
UNSB01.fasta	Cele-UNSB01_5:20304611	5	20304611	20305126	516	14	32	AAATTTGAAT	AAATTTGAATTCCCGCCAAAATTTTTCACCGA
UNSB01.fasta	Cele-UNSB01_5:20312857	5	20312857	20314295	1439	50	12	GCTTGGGCTT	GCTTGGGCTTAG
UNSB01.fasta	Cele-UNSB01_5:20413138	5	20413138	20414402	1265	44	32	TTTTGGGCGC	TTTTGGGCGCTGCTTAGACTATAAACTACAAT
UNSB01.fasta	Cele-UNSB01_5:20465532	5	20465532	20465791	260	7	37	AATTTGAATT	AATTTGAATTCCCGCCAAAATATTTTTGAAAAATTAA
UNSB01.fasta	Cele-UNSB01_5:20522553	5	20522553	20523111	559	29	18	CATTGTCACT	CATTGTCACGTTCTTCTC
UNSB01.fasta	Cele-UNSB01_5:20548445	5	20548445	20548748	304	5	41	GCATTTTTCG	GCATTTTTCGCCGATTTTTCACTATTTTTTCGTGAAAATTC
UNSB01.fasta	Cele-UNSB01_5:20558603	5	20558603	20558932	330	11	30	CTCAAATTTG	CTCAAATTTGCACTAAAAATTCCAATTTTT
UNSB01.fasta	Cele-UNSB01_5:20572226	5	20572226	20572853	628	16	11	CTACAGTACT	CTACAGTACTA
UNSB01.fasta	Cele-UNSB01_5:20575835	5	20575835	20576061	227	7	32	TTAGCAGCGC	TTAGCAGCGCCCAAAAACTTGTAGTTTGTAGT
UNSB01.fasta	Cele-UNSB01_5:20579964	5	20579964	20580501	538	15	31	CTACAAACTA	CTACAAACTACTAGTTTTGGGCGCTGCTAAG

UNSB01.fasta	Cele-UNSB01_5:20584979	5	20584979	20587201	2223	68	31	GGCGCTGCTA	GGCGCTGCTAAACTACAAACTACAAGAATTG
UNSB01.fasta	Cele-UNSB01_5:20627832	5	20627832	20628586	755	12	42	ATTGCCGGTT	ATTGCCGGTTTTGAACATTTTCGGCAATCGGCAATTTCGGCA
UNSB01.fasta	Cele-UNSB01_5:20719213	5	20719213	20719394	182	9	20	AAAATTCAAA	AAAATTCAAATTTTTGACTG
UNSB01.fasta	Cele-UNSB01_5:20720350	5	20720350	20720568	219	4	31	GAAAAAGTGC	GAAAAAGTGCTGAAAAGCACATTTTCAGAAG
UNSB01.fasta	Cele-UNSB01_5:20786599	5	20786599	20786882	284	9	32	CCGCCAAAAT	CCGCCAAAATGTTTCTTTGAAAATTTGAATTC
UNSB01.fasta	Cele-UNSB01_5:20839531	5	20839531	20839647	117	5	16	GTACTAGGGT	GTACTGGGGTACTGGG
UNSB01.fasta	Cele-UNSB01_5:20903761	5	20903761	20903966	206	5	11	CCTACAGTAC	CCTACAGTACT
UNSB01.fasta	Cele-UNSB01_5:20920761	5	20920761	20921207	447	40	11	GTTGCGGTTG	GTTGCGATTGC
UNSB01.fasta	Cele-UNSB01_5:21020210	5	21020210	21020336	127	4	32	GTGCGTCCGG	GTGCGTCCGGCGTCCGAAAAAAGCGGACGCGT
UNSB01.fasta	Cele-UNSB01_5:21154449	5	21154449	21154526	78	5	15	CGAAAAGGGG	CGAAAAGGGGATCTG
UNSB01.fasta	Cele-UNSB01_5:21154702	5	21154702	21154779	78	5	15	CCCCTTTTCG	CCCCTTTTCGTATCT
UNSB01.fasta	Cele-UNSB01_5:21238953	5	21238953	21243221	4269	353	12	TTAGGCTTAG	TTAGGCTTAGGC
UNSB01.fasta	Cele-UNSB01_6:1	6	1	2653	2653	219	12	AAGCCTAAGC	AAGCCTAAGCCT
UNSB01.fasta	Cele-UNSB01_6:40792	6	40792	40926	135	5	11	GTACTGTAGT	GTACTGTAGTA
UNSB01.fasta	Cele-UNSB01_6:122121	6	122121	133095	10975	405	27	ACAGAGAGTA	ACAGAGAGTAAAATAATCGATGAAGCC
UNSB01.fasta	Cele-UNSB01_6:161019	6	161019	161648	630	17	35	GGGAAATTCA	GGGAAATTCAAATTTTTTATGAAAATATTTTTGGC
UNSB01.fasta	Cele-UNSB01_6:161741	6	161741	162501	761	19	40	GAAAATTCTG	GAAAATTCTGGAATGTTCCACAACTTTCTAGAAAAATCGG
UNSB01.fasta	Cele-UNSB01_6:179889	6	179889	180069	181	5	32	TTAGCAGCGC	TTAGCAGCGCCCAAATCTTGTAGTTTGTAGTG
UNSB01.fasta	Cele-UNSB01_6:180452	6	180452	180557	106	7	15	GTCTCACCAC	GTCTCACCACGATGG
UNSB01.fasta	Cele-UNSB01_6:187518	6	187518	187756	239	6	40	TTCCAGAATT	TTCCAGAATTTTCTCGATTTTTCTAGAAAGTTCTGGAACA
UNSB01.fasta	Cele-UNSB01_6:188088	6	188088	188493	406	27	15	ACGATGGGTC	ACGACGGGTCTCACT
UNSB01.fasta	Cele-UNSB01_6:207322	6	207322	209932	2611	80	32	TTTGGGCGCT	TTTGGGCGCTGCTAAGACTACAAACTACAATG
UNSB01.fasta	Cele-UNSB01_6:210105	6	210105	210613	509	10	20	AAAAATCAAT	AAAAATCAATAAATTTCCAA
UNSB01.fasta	Cele-UNSB01_6:229151	6	229151	229455	305	8	36	ATAAAAATTT	ATAAAAATTTCCGGCAAACCGGCAATTTGTCAGAAA
UNSB01.fasta	Cele-UNSB01_6:276570	6	276570	276632	63	4	15	CGAAAAGGGG	CGAAAAGGGGATCTG
UNSB01.fasta	Cele-UNSB01_6:276806	6	276806	276883	78	5	15	TCCCCTTTTC	TCCCCTTTTCGTAGA
UNSB01.fasta	Cele-UNSB01_6:309569	6	309569	317496	7928	66	119	GGCCACCGTT	GGCCACCGTTCCATATTTCGCAATAATTTTTACGGGAACGTAATCTCCACATCACCAGCTTTCATTTAAGTACCCACACGGCTTGGTTTAATTTTTAAAATTTGGTCGCGGATTTTCTC
UNSB01.fasta	Cele-UNSB01_6:350789	6	350789	351447	659	7	94	GCAGTAATTC	GCAGTAATTCTCAAAATCTTCTGTTCTCTAAATATTGGGTGCTTTTCGATGCCCTATGTAGACCATCCAATGGGAAAATTGTCAATTTCTGAAG
UNSB01.fasta	Cele-UNSB01_6:356210	6	356210	356329	120	8	14	ATAGCGTATA	ATAGCGTATAGCGT

UNSB01.fasta	Cele-UNSB01_6:444285	6	444285	444373	89	7	11	CATAACTGAG	CATAACTGAGA
UNSB01.fasta	Cele-UNSB01_6:483296	6	483296	484174	879	19	40	AAAATTCTGG	AAAATTCTGGAATGTTCTAGAACCTTCTGGAAAGTTCGAG
UNSB01.fasta	Cele-UNSB01_6:556288	6	556288	556934	647	15	15	CCACGATGGG	CCACGGTGGGTCTCA
UNSB01.fasta	Cele-UNSB01_6:556993	6	556993	557132	140	5	30	AATTTTCCAG	AATTTTCCAGAAGGTTCTGGAACATTCCAG
UNSB01.fasta	Cele-UNSB01_6:743518	6	743518	743644	127	5	21	GGTCAGTGCA	GGTCAGTGCATTACCAATGTT
UNSB01.fasta	Cele-UNSB01_6:763886	6	763886	764130	245	5	22	TGGTCAGTGC	TGGTCAGAGCTGGCGGCCAATC
UNSB01.fasta	Cele-UNSB01_6:903956	6	903956	904040	85	4	21	ACTGACCAAA	ACTGACCAAAAATAGACATGC
UNSB01.fasta	Cele-UNSB01_6:960884	6	960884	960987	104	5	20	ATGCACTGAC	ATGCACTGACCGAAAATATG
UNSB01.fasta	Cele-UNSB01_6:983566	6	983566	988244	4679	290	15	CACGATGGGT	CACGATGGGTCTCGT
UNSB01.fasta	Cele-UNSB01_6:988331	6	988331	988610	280	13	11	CTACAGTACT	CTACAGTACCT
UNSB01.fasta	Cele-UNSB01_6:998781	6	998781	998865	85	4	21	CAGTGCATTC	CAGTGCATTCCGAACTTTCGT
UNSB01.fasta	Cele-UNSB01_6:998801	6	998801	998992	192	6	21	TCAGTGCATT	TCAGTTCATTCAGAAGTTTGG
UNSB01.fasta	Cele-UNSB01_6:1033071	6	1033071	1033155	85	4	21	TGGTCAGTGC	TGGTCAGTGCAAGAAACAATT
UNSB01.fasta	Cele-UNSB01_6:1052300	6	1052300	1052972	673	4	21	TGACAAAACA	TGACAAAACATAACTTTGCAC
UNSB01.fasta	Cele-UNSB01_6:1060978	6	1060978	1061104	127	6	21	GTCAGTGCAT	GTCAGTGCATTGATAGTTTCT
UNSB01.fasta	Cele-UNSB01_6:1066955	6	1066955	1067057	103	5	20	TTGGTCAGTG	TTGGTCAGTGCATTTTAGTC
UNSB01.fasta	Cele-UNSB01_6:1102792	6	1102792	1102897	106	4	21	GTCAGTGCAA	GTCAGTGTAATTAGAATAATG
UNSB01.fasta	Cele-UNSB01_6:1106813	6	1106813	1106897	85	4	21	GTCAGTGCAA	GTCAGTGCAAATTCAAATATT
UNSB01.fasta	Cele-UNSB01_6:1138488	6	1138488	1138593	106	5	21	TTGGTCAGTG	TTGGTCAGTGCGATTTTTTAA
UNSB01.fasta	Cele-UNSB01_6:1180467	6	1180467	1185764	5298	52	102	TGTACACTTC	TGTACACTTCTCCCAAAATGACTGTAAGAATGGACTCCTTATATGATTTAAACCCGACTCTGGACATACAAACTTCTCTCAAAAGAAAATAGACTTCACA
UNSB01.fasta	Cele-UNSB01_6:1259650	6	1259650	1260300	651	6	21	GTCAGTGCAA	GTCAGTGCAAATGCATTGTTG
UNSB01.fasta	Cele-UNSB01_6:1324054	6	1324054	1324372	319	7	22	GTCAGTGCAA	GTCAGTGCAAATTGTGCACATG
UNSB01.fasta	Cele-UNSB01_6:1338339	6	1338339	1338633	295	14	21	TTTGCACTGA	TTTGCACTGACCAACACGAGT
UNSB01.fasta	Cele-UNSB01_6:1342228	6	1342228	1342338	111	5	22	TCGCACTGAC	TCGCACTGACCAAACTTAGAAA
UNSB01.fasta	Cele-UNSB01_6:1365926	6	1365926	1366333	408	5	21	TTGCACTGAC	TTGCACTGACAAAATTTCGTT
UNSB01.fasta	Cele-UNSB01_6:1371051	6	1371051	1371242	192	7	21	TTGCACTGAC	TTGAACTGACAAAGTGCAAAT
UNSB01.fasta	Cele-UNSB01_6:1379546	6	1379546	1379614	69	4	17	TGGTCAGTGC	TGGTCAGTGCAAAAAGT
UNSB01.fasta	Cele-UNSB01_6:1420400	6	1420400	1420757	358	9	40	ATTTTTCTAG	ATTTTTCTAGAAAGTTCTGGAACATTCCAGAGTTTTCTCG
UNSB01.fasta	Cele-UNSB01_6:1420856	6	1420856	1421521	666	19	35	CCCGCCAAAA	CCCGCCAAAATTTTTTTTCAGAAAATTTAAATTT

UNSB01.fasta	Cele-UNSB01_6:1421556	6	1421556	1421900	345	23	15	CGATGGGTCT	CGATGGGTCTCACCA
UNSB01.fasta	Cele-UNSB01_6:1421957	6	1421957	1422097	141	5	30	AATTTTCCAG	AATTTTCCAGAAAGTTCTGGAACATTCCAG
UNSB01.fasta	Cele-UNSB01_6:1422210	6	1422210	1422590	381	13	11	GTACTCCTAC	GTACTCCTACA
UNSB01.fasta	Cele-UNSB01_6:1553970	6	1553970	1554390	421	18	20	AGGCAGACCT	AGGCAGACCTAGCCTACCCT
UNSB01.fasta	Cele-UNSB01_6:1555230	6	1555230	1555302	73	5	12	CTAAGCCTAA	CTAAGCCAAAGA
UNSB01.fasta	Cele-UNSB01_6:1569817	6	1569817	1569931	115	5	23	AAAAAATTTG	AAAAAATTTGTTTCGCACTGACC
UNSB01.fasta	Cele-UNSB01_6:1590254	6	1590254	1590338	85	4	21	TCTGAGCACT	TCTGAGCACTGACAAAAATTA
UNSB01.fasta	Cele-UNSB01_6:1594210	6	1594210	1594900	691	19	30	ACTACCCAAC	ACTACTCAACCGGCTCCGGCAACTAGTCCG
UNSB01.fasta	Cele-UNSB01_6:1595379	6	1595379	1595859	481	5	108	ATCCTCAACA	ATCCTCAACACCATCAGCGTCATCTAGTTCCTCCGGAACTATGTCCACAAATTCGGGATCAACTGGTTCTACAGTTACTGTTGCACCAGTATCGTCTAGTACATTTGG
UNSB01.fasta	Cele-UNSB01_6:1598138	6	1598138	1600934	2797	10	249	CTGGAAGCAC	CTGGAAGCACTGTAACCATGGGATCATCAAGCACTTCCGGTGTTTCCACATCTTCAGCATCCAGTACTCAGCCTCAGATGAGCACTTCACAAGGAAGTTCAGCAGGATCCACAGTTGCTTCTTCAACAGCTAGCCCTGCTGCGAGTTCCACAGCTCCATCTTCAACTGGAACTATGAGCTCCACTTCTAGTGGAACAGTTGGATCTACAATTTCTGAATCATCGACAACAGCATCTGCAAGTTCTCAGA
UNSB01.fasta	Cele-UNSB01_6:1657466	6	1657466	1657754	289	10	11	GTACTGTAGG	GTACTGTAGTA
UNSB01.fasta	Cele-UNSB01_6:1682621	6	1682621	1693139	10519	404	26	AACGGTCAGA	AACGGTCAGAGTCACTATTTTTGGTG
UNSB01.fasta	Cele-UNSB01_6:1697161	6	1697161	1700255	3095	119	26	GACTCTAACC	GACTCTAACCATTCACCTAAAAATAT
UNSB01.fasta	Cele-UNSB01_6:1700308	6	1700308	1706287	5980	323	11	GGAGCGGTTT	GGAGCGGTTTG
UNSB01.fasta	Cele-UNSB01_6:1706298	6	1706298	1706662	365	14	26	GCCGCAATGC	GCCGCAATGCCATTTTTCTGAAATCC
UNSB01.fasta	Cele-UNSB01_6:1780465	6	1780465	1781641	1177	4	165	CTTTTCCAAA	CTTTTCCAAAATGGCAATTTTTGATGAAAAAAATCCGAATTTTCACAAATTTCAAAAAAATTTTTAAACTTTATAATTTTAGCTTTGAATATTCCAAATGGAACTGTCATAACCTATCAATTTGATATATAAACTCGTGCCTACCTTCAGGCATTCAGTTTTCAC
UNSB01.fasta	Cele-UNSB01_6:1814276	6	1814276	1830201	15926	595	27	TTACTCTCTG	TTACTCTCTGTGGCTTCCCACCATATT
UNSB01.fasta	Cele-UNSB01_6:1911366	6	1911366	1912554	1189	59	18	AAGCCTATGC	AAGCCTATGCCGAAGCCG
UNSB01.fasta	Cele-UNSB01_6:1926117	6	1926117	1926284	168	11	14	TAGGCCCTCA	TAGGCCCTCAAGCG
UNSB01.fasta	Cele-UNSB01_6:1991346	6	1991346	1991764	419	14	22	CCGGCGTGGT	CCGGCGTGGTTTGAAGCCGACA
UNSB01.fasta	Cele-UNSB01_6:2020674	6	2020674	2021510	837	42	20	GTTACACGAA	GTTACACGAAAAAAATAGATT
UNSB01.fasta	Cele-UNSB01_6:2029404	6	2029404	2030161	758	38	20	ACACGAAAAA	ACACGAAAAAAATAGATTGTT
UNSB01.fasta	Cele-UNSB01_6:2038051	6	2038051	2039066	1016	51	20	GTTACACGAA	GTTACACGAAAAAAATAGATT
UNSB01.fasta	Cele-UNSB01_6:2065387	6	2065387	2065569	183	4	46	TGGTAAATTG	TGGTAAATTGCTTCCCGGATCACGCACTTTTCTTATTTCCTAACTA
UNSB01.fasta	Cele-UNSB01_6:2087511	6	2087511	2088871	1361	34	40	GAATTTTCTC	GAATTTTCTCGATTTTTCTAGAAAGTTCTGGAACATTCCA
UNSB01.fasta	Cele-UNSB01_6:2088902	6	2088902	2090459	1558	79	15	CCACGATGGG	CCACGATGGGTCTCA
UNSB01.fasta	Cele-UNSB01_6:2088902	6	2088902	2090835	1934	85	15	CCACGATGGG	CCACGATGGGTCTCA
UNSB01.fasta	Cele-UNSB01_6:2091376	6	2091376	2093692	2317	86	25	TTAGAGTAAA	TTAGAGTAAATTATTTGGCAGAACG

UNSB01.fasta	Cele-UNSB01_6:2093711	6	2093711	2093945	235	9	26	ATAGTGACTC	ATAGTGACTCTGGCCGTTCTCCAAAA
UNSB01.fasta	Cele-UNSB01_6:2093985	6	2093985	2094613	629	30	19	TAATATTTAT	TAATATTTATTTATTTAAC
UNSB01.fasta	Cele-UNSB01_6:2094704	6	2094704	2095024	321	8	40	TTCCAGAATT	TTCCAGAATTTTCTCGATTTTTCTAGAAAGTTCTGGAACA
UNSB01.fasta	Cele-UNSB01_6:2095079	6	2095079	2095359	281	7	35	CAGAAAATTT	CAGAAAATTTAAATTTCCCGCCAAAATATTTTTCT
UNSB01.fasta	Cele-UNSB01_6:2094705	6	2094705	2095572	868	10	40	TCCAGAATTT	TCCAGAATTTTCTCGATTTTTCTAGAAAGTTCTGGAACAT
UNSB01.fasta	Cele-UNSB01_6:2095085	6	2095085	2096434	1350	17	35	ATTTAAATTT	ATTTAAATTTCCCGCCAAAATATTTTTCTCAGAAA
UNSB01.fasta	Cele-UNSB01_6:2097795	6	2097795	2102366	4572	207	19	AATTAATATT	AATTAATATTTATTTATTT
UNSB01.fasta	Cele-UNSB01_6:2097795	6	2097795	2102461	4667	212	19	AATTAATATT	AATTAATATTTATTTATTT
UNSB01.fasta	Cele-UNSB01_6:2109492	6	2109492	2110453	962	78	12	AATAAATAAT	AATAAATAAATA
UNSB01.fasta	Cele-UNSB01_6:2154760	6	2154760	2154935	176	7	25	TATATTTTAC	TATATTTTACTCTCTGGCATCACAC
UNSB01.fasta	Cele-UNSB01_6:2156238	6	2156238	2156563	326	12	25	TCTGGCTTCA	TCTGGCTTCACAGTAAAATTTACTA
UNSB01.fasta	Cele-UNSB01_6:2157154	6	2157154	2162472	5319	133	40	AAGTTCTGGA	AAGTTCTGGAACATTCCAGAATTTTCTCGATTTTTCTAGA
UNSB01.fasta	Cele-UNSB01_6:2157131	6	2157131	2163386	6256	140	40	AGAATTTTCT	AGAATTTTCTCGATATTTCTAGAAAGTTCTGGAACATTCC
UNSB01.fasta	Cele-UNSB01_6:2369427	6	2369427	2369729	303	24	11	CCATACCTAT	CCATACCTATA
UNSB01.fasta	Cele-UNSB01_6:2413161	6	2413161	2413272	112	4	32	CGACCTGCGA	CGACCTGCGACCTAGCGACCTAGGTCGCTTTG
UNSB01.fasta	Cele-UNSB01_6:2485227	6	2485227	2486657	1431	55	26	TTTTAGTGAA	TTTTAGTGAACTGCCAGAGTCACTAA
UNSB01.fasta	Cele-UNSB01_6:2669709	6	2669709	2669769	61	5	12	AGGCTTAGGC	AGGCTTAGGCTT
UNSB01.fasta	Cele-UNSB01_6:3096507	6	3096507	3096571	65	4	16	AGAGAGTATA	AGAGAGTATAGATTAG
UNSB01.fasta	Cele-UNSB01_6:3098438	6	3098438	3098774	337	7	48	AAGATGAGGA	AAGATGAGGAATTTATTTTTTGATTTGCCCGTAATTTATATTTTTCTG
UNSB01.fasta	Cele-UNSB01_6:3115311	6	3115311	3123060	7750	398	19	AATCTATTTT	AATCTATTTTTCGTGTAAC
UNSB01.fasta	Cele-UNSB01_6:3190243	6	3190243	3190348	106	7	15	TCACCACGAT	TCACCACGATTGGTC
UNSB01.fasta	Cele-UNSB01_6:3377659	6	3377659	3377780	122	11	11	AAAATGTGCT	AAAATGTGCTA
UNSB01.fasta	Cele-UNSB01_6:3455223	6	3455223	3455778	556	27	18	TTTTGTCAGT	TTTTGTCAGTTCAATAGT
UNSB01.fasta	Cele-UNSB01_6:3463610	6	3463610	3463814	205	10	17	TTTTAACGTG	TTTTAACGTGACCTAGG
UNSB01.fasta	Cele-UNSB01_6:3482169	6	3482169	3482345	177	12	11	TAGCTCAGCT	TAGCTCAGCTT
UNSB01.fasta	Cele-UNSB01_6:3508288	6	3508288	3508508	221	10	18	TTGTCAGTGC	TTGTCAGTGCTATATTTT
UNSB01.fasta	Cele-UNSB01_6:3747622	6	3747622	3752392	4771	406	11	CAGTTGTCAC	CAGTTGTCACT
UNSB01.fasta	Cele-UNSB01_6:3814487	6	3814487	3814588	102	5	20	TTTGGTCAGT	TTTGGTCAGTGCTTTTCGAC
UNSB01.fasta	Cele-UNSB01_6:3834410	6	3834410	3834530	121	5	20	CCATGTAGCT	CAATGTAGCTACATAGTTTC

UNSB01.fasta	Cele-UNSB01_6:4047987	6	4047987	4048126	140	7	20	AAAATAGATT	AAAATAGATTGACATACCAG
UNSB01.fasta	Cele-UNSB01_6:4149433	6	4149433	4175058	25626	1025	25	AATGTTCTGC	AATGTTCTGCCAATTTATTTACTCC
UNSB01.fasta	Cele-UNSB01_6:4175079	6	4175079	4227144	52066	2000	20	CTATATCGTA	CTATATCGTATCGTAAATAG
UNSB01.fasta	Cele-UNSB01_6:4456017	6	4456017	4456156	140	7	20	ACAGACGAAA	ACAGACGAAAAAATAGATTA
UNSB01.fasta	Cele-UNSB01_6:5238384	6	5238384	5284176	45793	1482	27	TGTGGCTTCC	TGTGGCTTCCCACTATATTTTACTCTC
UNSB01.fasta	Cele-UNSB01_6:5352204	6	5352204	5352282	79	6	13	GCCATAGGCG	GCCATAGGCGTAA
UNSB01.fasta	Cele-UNSB01_6:5454734	6	5454734	5454854	121	8	15	TGGTGAGACC	TGGTGAGACCCATCC
UNSB01.fasta	Cele-UNSB01_6:5456252	6	5456252	5456359	108	5	23	AATAAATATT	AATAAATATTAATTAATTAAATG
UNSB01.fasta	Cele-UNSB01_6:5456777	6	5456777	5457242	466	31	15	CCACGATGGG	CCACGATGGGTCTCA
UNSB01.fasta	Cele-UNSB01_6:5457466	6	5457466	5457723	258	10	11	CTACAGTACT	CTACAGTACTA
UNSB01.fasta	Cele-UNSB01_6:5769379	6	5769379	5769561	183	7	26	TAGTGACTCT	TAGTGACTCTGGCCGTTCACCATAAA
UNSB01.fasta	Cele-UNSB01_6:6133294	6	6133294	6134199	906	4	226	CAAGAAGCTT	CAAGAAGCTTCACGTAGTTACAGAAATAGTACATTTTCAGAGCTACCTTTTAGCGCGTATTTTATTATTTAATGAAAACTACCATTTATAGGCAAAAATAGATAAATTTTCGAAACTTTGAAAATTCATAAATCTCTTCAATGTAACTTTTTTTGAAATGTCTTTCAGAAACTTTGTAGTAAATTTTAAGCTCTTTTAGAATATACCAACAATACTCCAGTAGGTG
UNSB01.fasta	Cele-UNSB01_6:6135861	6	6135861	6144415	8555	329	26	TTCTAAATTT	TTCTAAATTTGAAAATTCATCTACCG
UNSB01.fasta	Cele-UNSB01_6:6343035	6	6343035	6343121	87	5	17	TAATTACTGG	TAATTACTGGAATAAAA
UNSB01.fasta	Cele-UNSB01_6:6441827	6	6441827	6441956	130	8	16	GCACTGACAA	GCACTGACAATTTTTA
UNSB01.fasta	Cele-UNSB01_6:6516089	6	6516089	6517558	1470	50	19	GTCAGTGCAA	GTCAGTGCAACTGCAACTG
UNSB01.fasta	Cele-UNSB01_6:7055357	6	7055357	7058647	3291	35	94	TGCCTTCAGA	TGCCTTCAGAAATTGACAATTTTCCCATTGATTTGTCTACAAAGGGCATCGAAAAGCACCCAATATTTAGAGAACAGAAGATTTTGAGAATTAC
UNSB01.fasta	Cele-UNSB01_6:7306204	6	7306204	7313772	7569	373	20	ATAGCTATGT	ATAGCTATGTCGTATCATAA
UNSB01.fasta	Cele-UNSB01_6:7315610	6	7315610	7315788	179	9	20	TTTTTCGTCT	TTTTTCGTCTGTCAATCTAT
UNSB01.fasta	Cele-UNSB01_6:7586233	6	7586233	7592821	6589	165	40	TTACCCTTAA	TTACCCTTAAATGGGCGCCGGTTTCGCTTTGAGCGATTCC
UNSB01.fasta	Cele-UNSB01_6:7806159	6	7806159	7806450	292	5	18	TAGTCTTGCA	TAGTCTTGCAGTCTCTAA
UNSB01.fasta	Cele-UNSB01_6:7918402	6	7918402	7918617	216	6	12	TCTTCCTCCT	TCTTCTTCCTCC
UNSB01.fasta	Cele-UNSB01_6:8111999	6	8111999	8112167	169	7	24	GGTGGCTGGT	GGTGGCTGGTCACAAAAGTAAGTT
UNSB01.fasta	Cele-UNSB01_6:8127661	6	8127661	8127776	116	6	19	CAGACGAAAA	CAGACGAAAAAATATATTGG
UNSB01.fasta	Cele-UNSB01_6:8150139	6	8150139	8150195	57	4	14	TTACCAAAAT	TTACCAAAATCTAG
UNSB01.fasta	Cele-UNSB01_6:8414337	6	8414337	8415392	1056	85	11	GAGCGGTTTT	GAGCGGTCTTG
UNSB01.fasta	Cele-UNSB01_6:8836090	6	8836090	8836155	66	5	11	GCAAAAAATG	GCAAAAAATGT
UNSB01.fasta	Cele-UNSB01_6:8885720	6	8885720	8885804	85	5	17	AGGGGGCATC	AGGGGGCATTAGGTCAA
UNSB01.fasta	Cele-UNSB01_6:8886112	6	8886112	8886213	102	6	17	GATGCCCCCT	GATGCCCCCTCTGAACG

UNSB01.fasta	Cele-UNSB01_6:9243247	6	9243247	9243516	270	10	11	GTACTGTAGG	GTACTGTAGTA
UNSB01.fasta	Cele-UNSB01_6:9243610	6	9243610	9244090	481	12	40	CTTCTGGAAA	CTTCTGGAAAATCCGAGAAAATTCTGGAATGTTCCAGAAC
UNSB01.fasta	Cele-UNSB01_6:9244298	6	9244298	9244938	641	20	32	CGGGAATTCA	CGGGAATTCAAAATTTCTGTGAAAATTTTTAG
UNSB01.fasta	Cele-UNSB01_6:9421607	6	9421607	9443178	21572	857	25	AGAGAGTAAA	AGAGAGTAAACTATACTGTGAAGCC
UNSB01.fasta	Cele-UNSB01_6:9443244	6	9443244	9443487	244	7	35	AAAATTTGAA	AAAATTTGAATTTCCCGCCAAAATTTTTTTCACTG
UNSB01.fasta	Cele-UNSB01_6:9866116	6	9866116	9867908	1793	48	32	TCCCGCCAAA	TCACGCCAAAAATTTTCAAAGAAAATTTGAAT
UNSB01.fasta	Cele-UNSB01_6:9867922	6	9867922	9867997	76	5	15	ATGGGTCTCG	ATGGGTCTCGCCACG
UNSB01.fasta	Cele-UNSB01_6:10621309	6	10621309	10621525	217	4	25	ATTGGCAGAA	ATTGGCAGAACATTAGAGTAAATAT
UNSB01.fasta	Cele-UNSB01_6:10621572	6	10621572	10641051	19480	772	25	CAGAGAGTAA	CAGAGAGTAAAATATATGGTGAAGC
UNSB01.fasta	Cele-UNSB01_6:10641130	6	10641130	10642329	1200	107	11	TGGAGCGGTT	TGGAGCGGTAT
UNSB01.fasta	Cele-UNSB01_6:11021354	6	11021354	11026794	5441	27	214	AAAACCTACA	AAAACCTACAGTAACTTTGTGCGTATTTTATTATTAAATGAAAACGACCATTTATAGGCAAAATAGGTAAATTTATGAAACTTTGAAAACTCCCAAATCACTTCAAACCAACTTTTTTTGAAAATTCTTTCAGAAACTTTGTTGCAAATTCCAAGCTTTTTCAGAAGTTACCAACAACATCCCAGTAGGTGCCAGAAGCTCCGAGCAGTTACAG
UNSB01.fasta	Cele-UNSB01_6:11249167	6	11249167	11249852	686	21	27	CACTGACCAA	CACTGGCCAAAGAAAAAATTACACGTG
UNSB01.fasta	Cele-UNSB01_6:11535780	6	11535780	11535884	105	4	26	AAATTAATCT	AAATTAATCTGTCGTTCTAACTTGTT
UNSB01.fasta	Cele-UNSB01_6:11722389	6	11722389	11722452	64	4	14	CTATACGCTA	CTATACGCTACACG
UNSB01.fasta	Cele-UNSB01_6:11722486	6	11722486	11725761	3276	130	25	CTGCCGTAAT	CTGCCGTAATGCCAATTTATAGAAT
UNSB01.fasta	Cele-UNSB01_6:11725790	6	11725790	11730325	4536	175	26	CGGCGGATTT	CGGCGGATTTTAGAAAAATGGCATTG
UNSB01.fasta	Cele-UNSB01_6:11775904	6	11775904	11776150	247	7	11	TCCTACAGTA	TCCTACCGTAA
UNSB01.fasta	Cele-UNSB01_6:11975065	6	11975065	11975494	430	4	108	TTTCACGTTT	TTTCACGTTTTCAGGTGATTATATAGGTCACGAACTAATTTATTTTGAAAATTGACCTGTGGACCTATAACTTTGGAGATATGGTAAATTGAGTATGGGCTTTTAGAT
UNSB01.fasta	Cele-UNSB01_6:12031687	6	12031687	12031907	221	10	22	TCAGTGCAAA	TCAGTGCAAAGTGACAATTATG
UNSB01.fasta	Cele-UNSB01_6:12072301	6	12072301	12080487	8187	312	26	ATGACTCTAA	ATGACTCTAACCATTCACCTAAAATA
UNSB01.fasta	Cele-UNSB01_6:12080509	6	12080509	12089472	8964	344	26	AAGTTAGACC	AAGTTAGACCATTAGATGAATTTCTA
UNSB01.fasta	Cele-UNSB01_6:12089493	6	12089493	12089669	177	16	11	CGAAGTTGTC	CAAAGTTGTCA
UNSB01.fasta	Cele-UNSB01_6:12159416	6	12159416	12159737	322	5	18	TGCAATACTA	TGCAATACTAATAGGGAG
UNSB01.fasta	Cele-UNSB01_6:12186349	6	12186349	12201499	15151	591	25	CTCTCTGGCT	CTCTCTGGCTTCCCACTATATTTTA
UNSB01.fasta	Cele-UNSB01_6:12201539	6	12201539	12202895	1357	6	226	TGTACTATTT	TGTACTATTTCTGTAACTACGTGAAGCTTCTTGTACCTACTGAAATATTGTTGGTATATTCTAAAAGAGCTTGAAATTTACTACAAAGTTTCTGAAAGACATTTCAAAAAAAGTTACTTTGAAGAGATTTATGAATTTTCAAACTTACGAAAATTCGTCTATTTTTGCCTATAAATGGTAGTTTTCATTAAATAATAAAATACGCATTATAAGGTAGGGCTGAAAA
UNSB01.fasta	Cele-UNSB01_6:12589371	6	12589371	12589487	117	6	19	ATAGATTGTT	ATAGATTGTTACACGAAAA
UNSB01.fasta	Cele-UNSB01_6:12589530	6	12589530	12590986	1457	71	19	TTAATTAATA	TTAATTAATATTTATTTAT
UNSB01.fasta	Cele-UNSB01_6:12591006	6	12591006	12601690	10685	320	32	GCTATTTGGT	GCTATTTGGTGCTCCATCGGTGTTAAAGTGGT

UNSB01.fasta	Cele-UNSB01_6:12591003	6	12591003	12604866	13864	415	32	GGTGCTATTT	GGTGCTATTTGGTGCTCCATCGGTGTTAAAGT
UNSB01.fasta	Cele-UNSB01_6:12827464	6	12827464	12828819	1356	70	19	AAAAATAGAT	AAAAATAGATTGTTACACG
UNSB01.fasta	Cele-UNSB01_6:12847168	6	12847168	12848031	864	10	15	GGGTCTCACC	GGGTCTCACCGCGAA
UNSB01.fasta	Cele-UNSB01_6:12904034	6	12904034	12904836	803	12	32	CGACCTAGGT	CGACCTAGGTCGCATTGCGACCTGCGACCTAG
UNSB01.fasta	Cele-UNSB01_6:12932636	6	12932636	12932914	279	9	31	TCGACAGAAG	TCGACAGAAGCTGTCGGATCTATTATCTTCC
UNSB01.fasta	Cele-UNSB01_6:12933662	6	12933662	12933969	308	5	26	AAAAATGGCA	AAAAATGGCATTGCGGCGGATTTCAG
UNSB01.fasta	Cele-UNSB01_6:13568461	6	13568461	13570473	2013	18	108	TTTCAGATTT	TTTCAGATTTTCAGATTTTCACTTGATTATATAGGTCACGACCCAAGTTATTTTGAAAATTGAGCTACACGGCTGTAACTTTGGAGCTATGGGTAATCAAGTATGAGC
UNSB01.fasta	Cele-UNSB01_6:13750177	6	13750177	13750509	333	8	18	TGCAAGACTA	TGCAAGACTATTAAAGGC
UNSB01.fasta	Cele-UNSB01_6:13887397	6	13887397	13887577	181	11	15	TCGTGGTGAG	TCGTGGTGAGACCCT
UNSB01.fasta	Cele-UNSB01_6:14010915	6	14010915	14011665	751	7	108	TGGGTAATCA	TGGGTAATCAGGTGCGAGCTTTCAGATTTTTACGTTTTCACGTGAATATCTAGGTCACGAACCAATTTCTTTTCGGAATGGGACTAGGGACATGAAACTTTTGGTTTA
UNSB01.fasta	Cele-UNSB01_6:14101701	6	14101701	14101756	56	5	11	GTGCTTGGTC	GTGCTTGGTCA
UNSB01.fasta	Cele-UNSB01_6:14121790	6	14121790	14122309	520	13	40	GAACATTCCA	GAACATTCCAGAAATTTCCCGATTTTTCTAGAAAGTTCTG
UNSB01.fasta	Cele-UNSB01_6:14122412	6	14122412	14122622	211	6	35	CAGAAAATTT	CAGAAAATTTAAATTTCCCGCCAAAATATTTTTCA
UNSB01.fasta	Cele-UNSB01_6:14122652	6	14122652	14123413	762	19	15	GGGTCTCACC	GGGTCTCACCACGAC
UNSB01.fasta	Cele-UNSB01_6:14123669	6	14123669	14124172	504	19	11	CTACAGTACT	CTACAGTACTA
UNSB01.fasta	Cele-UNSB01_6:14250466	6	14250466	14251066	601	4	153	TGAGGTGCAG	TGAGGTGCAGGGCGTGATGAGCTCTGTCCCATTCGGAACTGTTGATTCCCGGAGCCGTGATTCATAGATGAGTTGGAGGGACCTGCTGTGGAACTTGTTTGAATTTGAGAGAGTCCATGAGCCATCAAGAAGCTTTCCGGCAGATTGGG CAAC
UNSB01.fasta	Cele-UNSB01_6:14307881	6	14307881	14308131	251	10	25	TCTCTGGCTT	TCTCTGGCTTCACCGTATATTTTAC
UNSB01.fasta	Cele-UNSB01_6:14447847	6	14447847	14448957	1111	73	15	TCGTGGTGAG	TCGTGGTGAGAACTT
UNSB01.fasta	Cele-UNSB01_6:14452976	6	14452976	14453126	151	12	12	ATAGGCATAG	ATAGGCATAGGC
UNSB01.fasta	Cele-UNSB01_6:14480301	6	14480301	14480556	256	13	20	TAACAATCTA	TAACAATCTATTTTTTCGTG
UNSB01.fasta	Cele-UNSB01_6:14589132	6	14589132	14589372	241	5	48	GGAGGTGGAG	GGAGGTGGAGAAAGTGTAGTTCCCGGTGGTCGCGGTTCTTTAGAGGGT
UNSB01.fasta	Cele-UNSB01_6:14650166	6	14650166	14651272	1107	10	18	TGCAAGACTA	TGCAAGACTATTAGAGGC
UNSB01.fasta	Cele-UNSB01_6:14688507	6	14688507	14688582	76	5	15	TCGTGGCGAG	TCGTTGCGAGACCCT
UNSB01.fasta	Cele-UNSB01_6:14688602	6	14688602	14689240	639	20	32	TTTGGCGGGA	TTCGGCGGGAATTCAAATTTTCTGTGAAAATT
UNSB01.fasta	Cele-UNSB01_6:14711854	6	14711854	14734555	22702	477	48	GGCAAATCAA	GGCAAATCAACAATTGAATTCCTCATCTTCAAAAAAATATAAATTACG
UNSB01.fasta	Cele-UNSB01_6:14983794	6	14983794	14983948	155	5	22	TTTGTGGGAG	TTTGTGGGAGTGAACTTACACT
UNSB01.fasta	Cele-UNSB01_6:15068529	6	15068529	15068945	417	13	32	TTGAATTCCC	TTGAATTCCCGCCAAAATTTTTCACAGAAAAT
UNSB01.fasta	Cele-UNSB01_6:15068534	6	15068534	15069086	553	16	32	TTCCCGCCAA	TTCCCGCCAAAATTTTTCACAGAAAATTTGAA
UNSB01.fasta	Cele-UNSB01_6:15068455	6	15068455	15069305	851	5	40	TTCTGGAACA	TTCTGGAACATTCCAGCATTTTCTCAAGTTTTCCAGAAGG

UNSB01.fasta	Cele-UNSB01_6:15069428	6	15069428	15069697	270	10	11	CCTACAGTAC	CTACAGTACAA
UNSB01.fasta	Cele-UNSB01_6:15253053	6	15253053	15270737	17685	696	25	TGGCTTCACA	TGGCTTCACACTATATTTTACTCTG
UNSB01.fasta	Cele-UNSB01_6:15298701	6	15298701	15298945	245	7	35	CAGAAAATTT	CAGAAAATTTAAATTTCCCGCCAAAAGGTTTTTTT
UNSB01.fasta	Cele-UNSB01_6:15412985	6	15412985	15413295	311	5	72	TGGTTGTGTT	TGGTTGTGTTCCCAAAAGTGAGAAAAGAGGGTACAGACCTCTGTACGAATATGTCTTATCAGCTTCAAAAGA
UNSB01.fasta	Cele-UNSB01_6:15588433	6	15588433	15598390	9958	92	108	AAGTCTTCTT	AAGTCTTCTTGGTTCTGTGTCTTTGCTTCGAAATGTTGACCATCTCCTTGCAGGATCTTGTTGTCAGATGGAACCTTCTTGGCATACCGTTCTCCACTGATGTGAGTG
UNSB01.fasta	Cele-UNSB01_6:15665052	6	15665052	15665283	232	11	21	TGAAAGATTG	TGAAAGATTGGTCAGTGCCTA
UNSB01.fasta	Cele-UNSB01_6:15839450	6	15839450	15840189	740	22	10	TATCTAGATC	TATCTATATC
UNSB01.fasta	Cele-UNSB01_6:15851631	6	15851631	15851806	176	5	35	ATTTAAATTT	ATTTAAATTTCCCGCCAAAATATTTTTCACAGAAA
UNSB01.fasta	Cele-UNSB01_6:15851847	6	15851847	15853377	1531	97	15	GTCTCACCAC	GTCTCACCGCGAAGG
UNSB01.fasta	Cele-UNSB01_6:15876707	6	15876707	15876856	150	4	21	TGTCCGTGAT	TGTCCGTGATGATCGTGAGCA
UNSB01.fasta	Cele-UNSB01_6:15966442	6	15966442	15966650	209	8	26	GGTCTAACTT	GGTCTAACTTTATAAATGGATCTGAT
UNSB01.fasta	Cele-UNSB01_6:16004509	6	16004509	16004749	241	6	40	AAAGTTCTGG	AAAGTTCTGGAACATTCCAGAATTTTCCCGATTTTTCTAA
UNSB01.fasta	Cele-UNSB01_6:16004822	6	16004822	16005172	351	10	35	CAGAAAATTT	CAGAAAATTTAAATTACCCGCCAAAATATCTTTCA
UNSB01.fasta	Cele-UNSB01_6:16005217	6	16005217	16005457	241	15	15	GGGTCTCACC	GGGTCTCACCACGAT
UNSB01.fasta	Cele-UNSB01_6:16057246	6	16057246	16058243	998	38	26	AATTCATCTA	AATTCATCTAGTGGTCTAACTCTGGA
UNSB01.fasta	Cele-UNSB01_6:16108332	6	16108332	16108500	169	8	21	GGCTGCCGAG	GGCTGCCGAGGGTAGGCTAGG
UNSB01.fasta	Cele-UNSB01_6:16177777	6	16177777	16179557	1781	89	20	CATACCAAAA	CATACCAAAAAAATAGATTGG
UNSB01.fasta	Cele-UNSB01_6:16179578	6	16179578	16182464	2887	111	26	GTGAACGGCC	GTGAAGGGCCAGAGTCACTATTTTTG
UNSB01.fasta	Cele-UNSB01_6:16182513	6	16182513	16182592	80	4	20	TAACAATCTA	TAACAATCTATTTTTTTCGTG
UNSB01.fasta	Cele-UNSB01_6:16300389	6	16300389	16301032	644	4	158	TATCAATTTT	TATCAATTTTCAGACGCGCTACGGGGGTTTTTGTCTTGAATTCCAATTTCAACAGGCGCAAAAAAACAAAAATAAAATTTTTGGATTTTTGGAAAAGTTTTTATAGATTTGTATTCTAGATTGTTTAAAAATATTATTGGTACGTTTTTGGACCTATA
UNSB01.fasta	Cele-UNSB01_6:16300466	6	16300466	16301134	669	4	158	CAAAAATAAA	CAAAAATAAAATTTTTGGATTTTTGGAAAAGTTTTTATAGATTTGTATTCTAGATTGTTTAAAAATATTATTGGTACGTTTTTGGACCTATATATCAATTTTCAGACGCGCTACGGGGGTTTTTGTCTTGAATTCCAATTTCAACAGGCGCAAAAAAA
UNSB01.fasta	Cele-UNSB01_6:16421783	6	16421783	16421902	120	6	20	CCACCCTGTA	CCACCCTGTAATATCATGGA
UNSB01.fasta	Cele-UNSB01_6:16471702	6	16471702	16471949	248	8	11	GTACTGTAGG	GTACTGTAGTA
UNSB01.fasta	Cele-UNSB01_6:16472035	6	16472035	16472475	441	11	40	TCCAGAACCT	TCCAGAACCTTCTGGAAAATCCGAGAAAATTCTGGAATGT
UNSB01.fasta	Cele-UNSB01_6:16472071	6	16472071	16473145	1075	13	40	ATGTTCCAGA	ATGTTCCAGAACCTTCTGGAAAATTTGAGAAAATGCTGGA
UNSB01.fasta	Cele-UNSB01_6:16506358	6	16506358	16506686	329	5	18	ATTAGTTTTG	ATTAGTTTTGCACTCTCT
UNSB01.fasta	Cele-UNSB01_6:16529882	6	16529882	16530026	145	9	14	TACCATATAC	TACCAAATACCATA
UNSB01.fasta	Cele-UNSB01_6:16570815	6	16570815	16570983	169	12	14	CTTAGGGCTT	CTTAGGGCTTCAGG

UNSB01.fasta	Cele-UNSB01_6:16657827	6	16657827	16657967	141	7	20	TACAGGGTGG	TACAGGGTGGCCCATAATTA
UNSB01.fasta	Cele-UNSB01_6:16744464	6	16744464	16744698	235	10	16	TTCCGGCAAT	TTCCGGCAATCGGCTA
UNSB01.fasta	Cele-UNSB01_6:16876782	6	16876782	16877097	316	18	15	TCACCACGAA	TCACCACGAAGGGTC
UNSB01.fasta	Cele-UNSB01_6:17130007	6	17130007	17135013	5007	66	76	AGCATTAGAG	AGCATTAGAGCATGTACACAGACACCGACAACACCACAGTCTCTCCCACTTTCCTTCGTGCAAAGGAGTGTCTTCT
UNSB01.fasta	Cele-UNSB01_6:17211035	6	17211035	17211529	495	4	52	GCATGTCTTC	GCATGTCTTCCAAGCGTTTTGAGCCACGCATGGGATTTCCTATACACGGCTG
UNSB01.fasta	Cele-UNSB01_6:17224721	6	17224721	17225161	441	13	40	TTTCTAGAAA	TTTCTAGAAAATTCTGGACCATTCCAGAACTTTCTCGGAT
UNSB01.fasta	Cele-UNSB01_6:17225477	6	17225477	17225567	91	6	15	GGGTCTCACC	GGGTCTCACCACGAT
UNSB01.fasta	Cele-UNSB01_6:17257914	6	17257914	17259280	1367	37	10	TGGATCGGGA	TGGATCGGAA
UNSB01.fasta	Cele-UNSB01_6:17259878	6	17259878	17260337	460	20	14	ATAGCGCATA	ATAGCGTATAGCGT
UNSB01.fasta	Cele-UNSB01_6:17463543	6	17463543	17463648	106	5	21	GGTGGACCCA	GGTGGATCCAGGGGTTCCTAG
UNSB01.fasta	Cele-UNSB01_6:17501177	6	17501177	17501366	190	5	38	CCGTTTCGCC	CCGTTTCGCCTTGAGCGATGCACCCTTAAATGGGCCTC
UNSB01.fasta	Cele-UNSB01_6:17555071	6	17555071	17555695	625	16	39	ATTGATACTT	ATTGATACTTTGTCAGGTTTAGACCATCGGCCGAGCTTT
UNSB01.fasta	Cele-UNSB01_6:17663802	6	17663802	17664403	602	6	40	TTTCTCGAAT	TTTCTCGAATTTTCCAGAAGGTTCTAGAACATTCCAGAAA
UNSB01.fasta	Cele-UNSB01_6:17686729	6	17686729	17686834	106	7	15	GAGACCCATC	GAGACCCATCATGGC
UNSB01.fasta	Cele-UNSB01_6:17686891	6	17686891	17687206	316	9	35	AAATTTTCTG	AAATTTTCTGTGAAAAATATTTTGGCGGGAAATTT
UNSB01.fasta	Cele-UNSB01_6:17686552	6	17686552	17689936	3385	70	40	AATGTTCCAG	AATGTTCCAGAACCTTCTGGAAAATTCTAGAAAAATCTGG
UNSB01.fasta	Cele-UNSB01_6:17722232	6	17722232	17722379	148	8	15	GGGGGCAACA	GGGGGCAACAAATTA
UNSB01.fasta	Cele-UNSB01_6:17820421	6	17820421	17836619	16199	357	45	CTCCATCCAC	CTCCATCCACCAGCACTGCTTCGAGTACGACAGAAAGCACTTCGA
UNSB01.fasta	Cele-UNSB01_6:18063681	6	18063681	18064057	377	7	36	CCACCAACTC	CCACCAACACCAAAATCAGCTGCTCCATCAGAGGTT
UNSB01.fasta	Cele-UNSB01_6:18106897	6	18106897	18110840	3944	258	12	TTAGGCTTAG	TCAGGCTTAGGC
