# Supplementary material for: DNA Satellites Are Transcribed as Part of the Non-Coding Genome in Eukaryotes and Bacteria
Source: Genes (Basel). 2021 Oct 20;12(11):1651. doi: 10.3390/genes12111651 (PMC8625621; doi:10.3390/genes12111651)
Supplement: Supplementary file 1 [file genes-12-01651-s001.zip › Table S3. Alignment of satellites in families.pdf]

Table S3. Alignment of satellites in families

Number of sequences=4066
There are 408 alignments:

>Cele-UNSB01_Fam_1_12_169 Nr. of seq. 169 Alignment length(with gaps) = 14 Alignment score = 0.769097
Cele-UNSB01_3:2562874-2563954 Satlength=1081 Nr of Repeats=40 RepeatLength=10 seed=AGGCAGGCTT Num.seqs=27 Similarity=0.883001
0   AG-GC---AGGCTT
Rev.of_Cele-UNSB01_1:1-2668 Satlength=2668 Nr of Repeats=204 RepeatLength=12 seed=CTAAGCCTAA Num.seqs=173 Similarity=0.987409
2   AG-GCTT-AGGCTT
Rev.of_Cele-UNSB01_1:558852-559000 Satlength=149 Nr of Repeats=8 RepeatLength=12 seed=CTAAGCCTAA Num.seqs=5 Similarity=0.811111
2   AG-GCTT-AGGCTT
Cele-UNSB01_1:568805-568943 Satlength=139 Nr of Repeats=8 RepeatLength=12 seed=AGGCTTAGGC Num.seqs=5 Similarity=0.844444
12   AG-GCTT-AGGCTT
Rev.of_Cele-UNSB01_1:642767-642875 Satlength=109 Nr of Repeats=6 RepeatLength=12 seed=TAAGCCTAAG Num.seqs=4 Similarity=0.759259
13   AG-GCTT-AGGCTT
Cele-UNSB01_1:736250-736486 Satlength=237 Nr of Repeats=5 RepeatLength=12 seed=GCTTAGGCTT Num.seqs=3 Similarity=0.777778
2   AG-GCTT-AGGCTT
Rev.of_Cele-UNSB01_1:788353-788527 Satlength=175 Nr of Repeats=12 RepeatLength=12 seed=GCCTAAGCCT Num.seqs=10 Similarity=0.933333
10   AG-GCTT-AGGCTT
Rev.of_Cele-UNSB01_1:815618-815840 Satlength=223 Nr of Repeats=16 RepeatLength=12 seed=GCCTAAGCCT Num.seqs=10 Similarity=0.782716
10   AG-GCTT-AGGCTT
Cele-UNSB01_1:823660-823840 Satlength=181 Nr of Repeats=13 RepeatLength=12 seed=GCTTAGGCTT Num.seqs=9 Similarity=0.895062
2   AG-GCTT-AGGCTT
Cele-UNSB01_1:842713-843650 Satlength=938 Nr of Repeats=48 RepeatLength=12 seed=CTTAGGCTTA Num.seqs=32 Similarity=0.819668
9   AG-GCTT-AGGCTT
Cele-UNSB01_1:855372-855570 Satlength=199 Nr of Repeats=12 RepeatLength=12 seed=GCTTAGGCTT Num.seqs=8 Similarity=0.888889
2   AG-GCTT-AGGCTT
Rev.of_Cele-UNSB01_1:961004-961082 Satlength=79 Nr of Repeats=6 RepeatLength=12 seed=AGCCTAAGCC Num.seqs=5 Similarity=0.866667
11   AG-GCTT-AGGCTT
Rev.of_Cele-UNSB01_1:1018801-1019027 Satlength=227 Nr of Repeats=14 RepeatLength=12 seed=GCCTAAGCCT Num.seqs=9 Similarity=0.854938
10   AG-GCTT-AGGCTT
Rev.of_Cele-UNSB01_1:1149569-1149724 Satlength=156 Nr of Repeats=10 RepeatLength=12 seed=CCTAAGCCTA Num.seqs=6 Similarity=0.866667
9   AG-GCTT-AGGCTT
Cele-UNSB01_1:2301010-2301088 Satlength=79 Nr of Repeats=6 RepeatLength=12 seed=TAGGCTTAGG Num.seqs=5 Similarity=0.844444
11   AG-GCTT-AGGCTT

```
Cele-UNSB01_1:2308046-2308235 Satlength=190 Nr of Repeats=5
RepeatLength=12 seed=AGGCTTAGGC  Num.seqs=3 Similarity=0.703704
12    AG-GCTT-AGGCTT
Rev.of_Cele-UNSB01_1:2402282-2402378 Satlength=97 Nr of Repeats=6
RepeatLength=12 seed=AAGCCTAAGC  Num.seqs=4 Similarity=0.839744
12    AG-GCTT-AGGCTT
Cele-UNSB01_1:2881736-2881825 Satlength=90 Nr of Repeats=5
RepeatLength=12 seed=TAGGCTTAGG  Num.seqs=4 Similarity=0.833333
11    AG-GCTT-AGGCTT
Rev.of_Cele-UNSB01_1:3158079-3158367 Satlength=289 Nr of Repeats=21
RepeatLength=12 seed=CTAAGCCTAA  Num.seqs=15 Similarity=0.766138
2    AG-GCTT-AGGCTT
Cele-UNSB01_1:3325616-3325694 Satlength=79 Nr of Repeats=6
RepeatLength=12 seed=AGGCTTAGGC  Num.seqs=5 Similarity=0.888889
12    AG-GCTT-AGGCTT
Cele-UNSB01_1:3430435-3430513 Satlength=79 Nr of Repeats=6
RepeatLength=12 seed=GGCTTAGGCT  Num.seqs=5 Similarity=0.788889
13    AG-GCTT-AGGCTT
Cele-UNSB01_1:10588945-10589028 Satlength=84 Nr of Repeats=6
RepeatLength=12 seed=TAGGCTTAGG  Num.seqs=4 Similarity=0.777778
11    AG-GCTT-AGGCTT
Rev.of_Cele-UNSB01_1:11661653-11661731 Satlength=79 Nr of Repeats=6
RepeatLength=12 seed=AAGCCTAAGC  Num.seqs=5 Similarity=1.000000
12    AG-GCTT-AGGCTT
Cele-UNSB01_1:11662428-11662542 Satlength=115 Nr of Repeats=9
RepeatLength=12 seed=GCTTAGGCTT  Num.seqs=8 Similarity=0.813492
2    AG-GCTT-AGGCTT
Cele-UNSB01_1:11682141-11682225 Satlength=85 Nr of Repeats=7
RepeatLength=12 seed=GGCTTAGGCT  Num.seqs=7 Similarity=0.968254
13    AG-GCTT-AGGCTT
Cele-UNSB01_1:12149612-12149804 Satlength=193 Nr of Repeats=15
RepeatLength=12 seed=TTAGGCTTAG  Num.seqs=11 Similarity=0.838695
10    AG-GCTT-AGGCTT
Rev.of_Cele-UNSB01_1:12340678-12340816 Satlength=139 Nr of Repeats=10
RepeatLength=12 seed=TAAGCCTAAG  Num.seqs=6 Similarity=0.859259
13    AG-GCTT-AGGCTT
Rev.of_Cele-UNSB01_1:12444291-12444339 Satlength=49 Nr of Repeats=4
RepeatLength=12 seed=GCCTAAGCCT  Num.seqs=4 Similarity=0.796296
10    AG-GCTT-AGGCTT
Cele-UNSB01_1:12487096-12487168 Satlength=73 Nr of Repeats=5
RepeatLength=12 seed=GGCTTAGGCT  Num.seqs=4 Similarity=0.814815
13    AG-GCTT-AGGCTT
Rev.of_Cele-UNSB01_1:14166580-14166640 Satlength=61 Nr of Repeats=5
RepeatLength=12 seed=GCCTAAGCCT  Num.seqs=5 Similarity=0.866667
10    AG-GCTT-AGGCTT
Rev.of_Cele-UNSB01_2:2-3054 Satlength=3053 Nr of Repeats=243
RepeatLength=12 seed=AAGCCTAAGC  Num.seqs=197 Similarity=0.998872
12    AG-GCTT-AGGCTT
Cele-UNSB01_2:577469-577719 Satlength=251 Nr of Repeats=20
RepeatLength=12 seed=TAGGCTTAGG  Num.seqs=18 Similarity=0.816993
11    AG-GCTT-AGGCTT
Cele-UNSB01_2:2568294-2568366 Satlength=73 Nr of Repeats=6
RepeatLength=12 seed=TTAGGCTTAG  Num.seqs=6 Similarity=0.785185
10    AG-GCTT-AGGCTT
```

Rev.of_Cele-UNSB01_2:2659185-2659251 Satlength=67 Nr of Repeats=5
RepeatLength=12 seed=CCTAAGCCTA Num.seqs=4 Similarity=0.833333
9   AG-GCTT-AGGCTT
Rev.of_Cele-UNSB01_2:2756689-2756749 Satlength=61 Nr of Repeats=4
RepeatLength=12 seed=GCCTAAGCCT Num.seqs=3 Similarity=0.777778
10   AG-GCTT-AGGCTT
Cele-UNSB01_2:3166478-3166668 Satlength=191 Nr of Repeats=8
RepeatLength=12 seed=GGCTTAGGCT Num.seqs=6 Similarity=0.785185
13   AG-GCTT-AGGCTT
Cele-UNSB01_2:4146856-4147072 Satlength=217 Nr of Repeats=14
RepeatLength=12 seed=CTTAGGCTTA Num.seqs=10 Similarity=0.748148
9   AG-GCTT-AGGCTT
Rev.of_Cele-UNSB01_2:4380400-4380472 Satlength=73 Nr of Repeats=4
RepeatLength=12 seed=AGCCTAAGCC Num.seqs=3 Similarity=0.851852
11   AG-GCTT-AGGCTT
Cele-UNSB01_2:11872286-11872364 Satlength=79 Nr of Repeats=6
RepeatLength=12 seed=CTTAGGCTTA Num.seqs=5 Similarity=0.844444
9   AG-GCTT-AGGCTT
Cele-UNSB01_2:12045935-12046042 Satlength=108 Nr of Repeats=8
RepeatLength=12 seed=AGGCTTAGGC Num.seqs=6 Similarity=0.748148
12   AG-GCTT-AGGCTT
Rev.of_Cele-UNSB01_2:12070558-12070690 Satlength=133 Nr of Repeats=10
RepeatLength=12 seed=AAGCCTAAGC Num.seqs=6 Similarity=0.792593
12   AG-GCTT-AGGCTT
Cele-UNSB01_2:12072605-12072779 Satlength=175 Nr of Repeats=10
RepeatLength=12 seed=AGGCTTAGGC Num.seqs=6 Similarity=0.822222
12   AG-GCTT-AGGCTT
Rev.of_Cele-UNSB01_2:12388949-12389045 Satlength=97 Nr of Repeats=7
RepeatLength=12 seed=CCTAAGCCTA Num.seqs=5 Similarity=0.741026
9   AG-GCTT-AGGCTT
Cele-UNSB01_2:12463695-12463773 Satlength=79 Nr of Repeats=5
RepeatLength=12 seed=TTAGGCTTAG Num.seqs=4 Similarity=0.777778
10   AG-GCTT-AGGCTT
Rev.of_Cele-UNSB01_2:12522288-12522472 Satlength=185 Nr of Repeats=13
RepeatLength=12 seed=AGCCTAAGCC Num.seqs=8 Similarity=0.884921
11   AG-GCTT-AGGCTT
Cele-UNSB01_2:12608492-12608559 Satlength=68 Nr of Repeats=5
RepeatLength=12 seed=GGCTTAGGCT Num.seqs=4 Similarity=0.944444
13   AG-GCTT-AGGCTT
Cele-UNSB01_2:12968710-12969129 Satlength=420 Nr of Repeats=28
RepeatLength=12 seed=GGCTTAGGCT Num.seqs=17 Similarity=0.799020
13   AG-GCTT-AGGCTT
Cele-UNSB01_2:13813351-13813423 Satlength=73 Nr of Repeats=6
RepeatLength=12 seed=TAGGCTTAGG Num.seqs=6 Similarity=0.962963
11   AG-GCTT-AGGCTT
Rev.of_Cele-UNSB01_2:13878619-13878763 Satlength=145 Nr of Repeats=10
RepeatLength=12 seed=GCCTAAGCCT Num.seqs=6 Similarity=0.725926
10   AG-GCTT-AGGCTT
Rev.of_Cele-UNSB01_2:13968253-13968583 Satlength=331 Nr of Repeats=23
RepeatLength=12 seed=GCCTAAGCCT Num.seqs=15 Similarity=0.803175
10   AG-GCTT-AGGCTT
Rev.of_Cele-UNSB01_2:13977826-13977988 Satlength=163 Nr of Repeats=10
RepeatLength=12 seed=GCCTAAGCCT Num.seqs=7 Similarity=0.798942
10   AG-GCTT-AGGCTT

Cele-UNSB01_2:13981365-13981461 Satlength=97 Nr of Repeats=7
RepeatLength=12 seed=TTAGGCTTAG Num.seqs=6 Similarity=0.755556
10   AG-GCTT-AGGCTT
Rev.of_Cele-UNSB01_2:14106829-14106943 Satlength=115 Nr of Repeats=7
RepeatLength=12 seed=AGCCTAAGCC Num.seqs=5 Similarity=0.733333
11   AG-GCTT-AGGCTT
Cele-UNSB01_2:14148276-14148504 Satlength=229 Nr of Repeats=14
RepeatLength=12 seed=GCTTAGGCTT Num.seqs=11 Similarity=0.711888
2   AG-GCTT-AGGCTT
Cele-UNSB01_2:14195501-14195591 Satlength=91 Nr of Repeats=5
RepeatLength=12 seed=TAGGCTTAGG Num.seqs=3 Similarity=0.851852
11   AG-GCTT-AGGCTT
Cele-UNSB01_2:14213145-14213210 Satlength=66 Nr of Repeats=4
RepeatLength=12 seed=TAGGCTTAGG Num.seqs=3 Similarity=0.925926
11   AG-GCTT-AGGCTT
Cele-UNSB01_2:15121230-15121314 Satlength=85 Nr of Repeats=6
RepeatLength=12 seed=TTAGGCTTAG Num.seqs=4 Similarity=0.814815
10   AG-GCTT-AGGCTT
Rev.of_Cele-UNSB01_2:15182818-15182878 Satlength=61 Nr of Repeats=5
RepeatLength=12 seed=AAGCCTAAGC Num.seqs=5 Similarity=0.888889
12   AG-GCTT-AGGCTT
Cele-UNSB01_2:15522855-15525136 Satlength=2282 Nr of Repeats=188
RepeatLength=12 seed=TTAGGCTTAG Num.seqs=173 Similarity=1.000000
10   AG-GCTT-AGGCTT
Rev.of_Cele-UNSB01_3:11-3196 Satlength=3186 Nr of Repeats=261
RepeatLength=12 seed=TAAGCCTAAG Num.seqs=239 Similarity=1.000000
13   AG-GCTT-AGGCTT
Rev.of_Cele-UNSB01_3:119691-119763 Satlength=73 Nr of Repeats=5
RepeatLength=12 seed=GCCTAAGCCT Num.seqs=3 Similarity=0.851852
10   AG-GCTT-AGGCTT
Rev.of_Cele-UNSB01_3:214401-214633 Satlength=233 Nr of Repeats=8
RepeatLength=12 seed=AAGCCTAAGC Num.seqs=5 Similarity=0.822222
12   AG-GCTT-AGGCTT
Rev.of_Cele-UNSB01_3:1107553-1107733 Satlength=181 Nr of Repeats=10
RepeatLength=12 seed=AAGCCTAAGC Num.seqs=7 Similarity=0.841270
12   AG-GCTT-AGGCTT
Rev.of_Cele-UNSB01_3:1796051-1796519 Satlength=469 Nr of Repeats=35
RepeatLength=12 seed=CTAAGCCTAA Num.seqs=27 Similarity=0.758492
2   AG-GCTT-AGGCTT
Cele-UNSB01_3:2046603-2047209 Satlength=607 Nr of Repeats=30
RepeatLength=12 seed=TAGGCTTAGG Num.seqs=18 Similarity=0.824256
11   AG-GCTT-AGGCTT
Cele-UNSB01_3:3480039-3480224 Satlength=186 Nr of Repeats=12
RepeatLength=12 seed=TAGGCTTAGG Num.seqs=9 Similarity=0.901235
11   AG-GCTT-AGGCTT
Rev.of_Cele-UNSB01_3:3818464-3818544 Satlength=81 Nr of Repeats=5
RepeatLength=12 seed=TAAGCCTAAG Num.seqs=3 Similarity=0.777778
13   AG-GCTT-AGGCTT
Cele-UNSB01_3:11222702-11222792 Satlength=91 Nr of Repeats=6
RepeatLength=12 seed=GGCTTAGGCT Num.seqs=5 Similarity=0.822222
13   AG-GCTT-AGGCTT
Cele-UNSB01_3:11273157-11273241 Satlength=85 Nr of Repeats=5
RepeatLength=12 seed=GGCTTAGGCT Num.seqs=3 Similarity=0.777778
13   AG-GCTT-AGGCTT

Cele-UNSB01_3:11508351-11508507 Satlength=157 Nr of Repeats=10 RepeatLength=12 seed=GCTTAGGCTT Num.seqs=6 Similarity=0.755556
2   AG-GCTT-AGGCTT
Cele-UNSB01_3:11725694-11725970 Satlength=277 Nr of Repeats=12 RepeatLength=12 seed=TTAGGCTTAG Num.seqs=8 Similarity=0.916667
10   AG-GCTT-AGGCTT
Cele-UNSB01_3:11779688-11779808 Satlength=121 Nr of Repeats=10 RepeatLength=12 seed=CTTAGGCTTA Num.seqs=8 Similarity=0.797619
9   AG-GCTT-AGGCTT
Cele-UNSB01_3:12010851-12011440 Satlength=590 Nr of Repeats=30 RepeatLength=12 seed=GGCTTAGGCT Num.seqs=20 Similarity=0.774854
13   AG-GCTT-AGGCTT
Rev.of_Cele-UNSB01_3:12025839-12025976 Satlength=138 Nr of Repeats=8 RepeatLength=12 seed=TAAGCCTAAG Num.seqs=5 Similarity=0.822222
13   AG-GCTT-AGGCTT
Rev.of_Cele-UNSB01_3:12040777-12040867 Satlength=91 Nr of Repeats=6 RepeatLength=12 seed=CCTAAGCCTA Num.seqs=5 Similarity=0.833333
9   AG-GCTT-AGGCTT
Rev.of_Cele-UNSB01_3:12088649-12088899 Satlength=251 Nr of Repeats=9 RepeatLength=12 seed=TAAGCCTAAG Num.seqs=7 Similarity=0.936508
13   AG-GCTT-AGGCTT
Rev.of_Cele-UNSB01_3:12433825-12433951 Satlength=127 Nr of Repeats=9 RepeatLength=12 seed=TAAGCCTAAG Num.seqs=6 Similarity=0.814815
13   AG-GCTT-AGGCTT
Cele-UNSB01_3:12447381-12447465 Satlength=85 Nr of Repeats=7 RepeatLength=12 seed=GGCTTAGGCT Num.seqs=7 Similarity=0.968254
13   AG-GCTT-AGGCTT
Rev.of_Cele-UNSB01_3:13248196-13248346 Satlength=151 Nr of Repeats=9 RepeatLength=12 seed=TAAGCCTAAG Num.seqs=7 Similarity=0.830688
13   AG-GCTT-AGGCTT
Rev.of_Cele-UNSB01_4:1-5637 Satlength=5637 Nr of Repeats=464 RepeatLength=12 seed=AAGCCTAAGC Num.seqs=403 Similarity=0.920412
12   AG-GCTT-AGGCTT
Cele-UNSB01_4:552425-552551 Satlength=127 Nr of Repeats=9 RepeatLength=12 seed=TAGGCTTAGG Num.seqs=7 Similarity=0.788360
11   AG-GCTT-AGGCTT
Rev.of_Cele-UNSB01_4:1076311-1076365 Satlength=55 Nr of Repeats=4 RepeatLength=12 seed=AGCCTAAGCC Num.seqs=3 Similarity=0.851852
11   AG-GCTT-AGGCTT
Cele-UNSB01_4:1078257-1078305 Satlength=49 Nr of Repeats=4 RepeatLength=12 seed=AGGCTTAGGC Num.seqs=4 Similarity=0.888889
12   AG-GCTT-AGGCTT
Cele-UNSB01_4:1370044-1370460 Satlength=417 Nr of Repeats=8 RepeatLength=12 seed=TAGGCTTAGG Num.seqs=5 Similarity=0.822222
11   AG-GCTT-AGGCTT
Cele-UNSB01_4:1508767-1509123 Satlength=357 Nr of Repeats=5 RepeatLength=12 seed=AGGCTTAGGC Num.seqs=4 Similarity=0.740741
12   AG-GCTT-AGGCTT
Cele-UNSB01_4:1643155-1643244 Satlength=90 Nr of Repeats=6 RepeatLength=12 seed=TTAGGCTTAG Num.seqs=4 Similarity=0.888889
10   AG-GCTT-AGGCTT
Rev.of_Cele-UNSB01_4:1703835-1704045 Satlength=211 Nr of Repeats=11 RepeatLength=12 seed=CTAAGCCTAA Num.seqs=7 Similarity=0.777778
2   AG-GCTT-AGGCTT

Rev.of_Cele-UNSB01_4:1766949-1767039 Satlength=91 Nr of Repeats=5
RepeatLength=12 seed=TAAGCCTAAG Num.seqs=4 Similarity=0.796296
13   AG-GCTT-AGGCTT
Cele-UNSB01_4:3176916-3177039 Satlength=124 Nr of Repeats=7
RepeatLength=12 seed=TAGGCTTAGG Num.seqs=5 Similarity=0.755556
11   AG-GCTT-AGGCTT
Rev.of_Cele-UNSB01_4:4053946-4054647 Satlength=702 Nr of Repeats=6
RepeatLength=12 seed=TAAGCCTAAG Num.seqs=4 Similarity=0.759259
13   AG-GCTT-AGGCTT
Cele-UNSB01_4:17755008-17759186 Satlength=4179 Nr of Repeats=342
RepeatLength=12 seed=TTAGGCTTAG Num.seqs=305 Similarity=1.000000
10   AG-GCTT-AGGCTT
Rev.of_Cele-UNSB01_5:4-5075 Satlength=5072 Nr of Repeats=419
RepeatLength=12 seed=AAGCCTAAGC Num.seqs=386 Similarity=0.918190
12   AG-GCTT-AGGCTT
Cele-UNSB01_5:1161256-1161400 Satlength=145 Nr of Repeats=10
RepeatLength=12 seed=GGCTTAGGCT Num.seqs=7 Similarity=0.814815
13   AG-GCTT-AGGCTT
Rev.of_Cele-UNSB01_5:1174135-1174237 Satlength=103 Nr of Repeats=5
RepeatLength=12 seed=CTAAGCCTAA Num.seqs=3 Similarity=0.851852
2   AG-GCTT-AGGCTT
Rev.of_Cele-UNSB01_5:1262246-1262294 Satlength=49 Nr of Repeats=4
RepeatLength=12 seed=AAGCCTAAGC Num.seqs=4 Similarity=0.944444
12   AG-GCTT-AGGCTT
Rev.of_Cele-UNSB01_5:1265623-1265875 Satlength=253 Nr of Repeats=19
RepeatLength=12 seed=AAGCCTAAGC Num.seqs=14 Similarity=0.804640
12   AG-GCTT-AGGCTT
Rev.of_Cele-UNSB01_5:1271695-1271973 Satlength=279 Nr of Repeats=15
RepeatLength=12 seed=CTAAGCCTAA Num.seqs=9 Similarity=0.836420
2   AG-GCTT-AGGCTT
Cele-UNSB01_5:1278621-1278717 Satlength=97 Nr of Repeats=7
RepeatLength=12 seed=CTTAGGCTTA Num.seqs=6 Similarity=0.822222
9   AG-GCTT-AGGCTT
Rev.of_Cele-UNSB01_5:1379498-1379672 Satlength=175 Nr of Repeats=13
RepeatLength=12 seed=CTAAGCCTAA Num.seqs=11 Similarity=0.826263
2   AG-GCTT-AGGCTT
Rev.of_Cele-UNSB01_5:1461442-1461802 Satlength=361 Nr of Repeats=24
RepeatLength=12 seed=CCTAAGCCTA Num.seqs=17 Similarity=0.861928
9   AG-GCTT-AGGCTT
Rev.of_Cele-UNSB01_5:1519749-1519965 Satlength=217 Nr of Repeats=10
RepeatLength=12 seed=AGCCTAAGCC Num.seqs=7 Similarity=0.788360
11   AG-GCTT-AGGCTT
Rev.of_Cele-UNSB01_5:2527844-2527991 Satlength=148 Nr of Repeats=6
RepeatLength=12 seed=CCTAAGCCTA Num.seqs=4 Similarity=0.833333
9   AG-GCTT-AGGCTT
Rev.of_Cele-UNSB01_5:2661964-2662120 Satlength=157 Nr of Repeats=13
RepeatLength=12 seed=CTAAGCCTAA Num.seqs=13 Similarity=0.839031
2   AG-GCTT-AGGCTT
Cele-UNSB01_5:15757927-15757998 Satlength=72 Nr of Repeats=5
RepeatLength=12 seed=CTTAGGCTTA Num.seqs=3 Similarity=0.925926
9   AG-GCTT-AGGCTT
Cele-UNSB01_5:18824768-18825020 Satlength=253 Nr of Repeats=16
RepeatLength=12 seed=AGGCTTAGGC Num.seqs=10 Similarity=0.935802
12   AG-GCTT-AGGCTT

Rev.of_Cele-UNSB01_5:18839756-18840116 Satlength=361 Nr of Repeats=22
RepeatLength=12 seed=AGCCTAAGCC Num.seqs=15 Similarity=0.860317
11   AG-GCTT-AGGCTT
Rev.of_Cele-UNSB01_5:18846370-18846628 Satlength=259 Nr of Repeats=18
RepeatLength=12 seed=TAAGCCTAAG Num.seqs=13 Similarity=0.874644
13   AG-GCTT-AGGCTT
Rev.of_Cele-UNSB01_5:18874657-18874753 Satlength=97 Nr of Repeats=6
RepeatLength=12 seed=CTAAGCCTAA Num.seqs=4 Similarity=0.888889
2   AG-GCTT-AGGCTT
Rev.of_Cele-UNSB01_5:18891023-18891077 Satlength=55 Nr of Repeats=4
RepeatLength=12 seed=TAAGCCTAAG Num.seqs=3 Similarity=0.851852
13   AG-GCTT-AGGCTT
Cele-UNSB01_5:18898055-18898199 Satlength=145 Nr of Repeats=9
RepeatLength=12 seed=GCTTAGGCTT Num.seqs=6 Similarity=0.925926
2   AG-GCTT-AGGCTT
Rev.of_Cele-UNSB01_5:19521326-19521536 Satlength=211 Nr of Repeats=15
RepeatLength=12 seed=GCCTAAGCCT Num.seqs=11 Similarity=0.818182
10   AG-GCTT-AGGCTT
Cele-UNSB01_5:19815492-19815850 Satlength=359 Nr of Repeats=22
RepeatLength=12 seed=CTTAGGCTTA Num.seqs=15 Similarity=0.813757
9   AG-GCTT-AGGCTT
Cele-UNSB01_5:19826165-19826303 Satlength=139 Nr of Repeats=11
RepeatLength=12 seed=CTTAGGCTTA Num.seqs=10 Similarity=0.938272
9   AG-GCTT-AGGCTT
Rev.of_Cele-UNSB01_5:20111577-20111694 Satlength=118 Nr of Repeats=5
RepeatLength=12 seed=AAGCCTAAGC Num.seqs=3 Similarity=0.851852
12   AG-GCTT-AGGCTT
Cele-UNSB01_5:21238953-21243221 Satlength=4269 Nr of Repeats=353
RepeatLength=12 seed=TTAGGCTTAG Num.seqs=315 Similarity=0.999295
10   AG-GCTT-AGGCTT
Rev.of_Cele-UNSB01_6:1-2653 Satlength=2653 Nr of Repeats=219
RepeatLength=12 seed=AAGCCTAAGC Num.seqs=188 Similarity=0.920213
12   AG-GCTT-AGGCTT
Cele-UNSB01_6:2669709-2669769 Satlength=61 Nr of Repeats=5
RepeatLength=12 seed=AGGCTTAGGC Num.seqs=5 Similarity=0.888889
12   AG-GCTT-AGGCTT
Cele-UNSB01_6:18106897-18110840 Satlength=3944 Nr of Repeats=258
RepeatLength=12 seed=TTAGGCTTAG Num.seqs=218 Similarity=0.919666
10   AG-GCTT-AGGCTT
Cele-UNSB01_1:479600-479769 Satlength=170 Nr of Repeats=13
RepeatLength=12 seed=TAGGCTTAGG Num.seqs=8 Similarity=0.813492
11   AG-GCNT-AGGCTT
Rev.of_Cele-UNSB01_1:1018800-1018890 Satlength=91 Nr of Repeats=5
RepeatLength=12 seed=AGCCTAAGCC Num.seqs=3 Similarity=0.740741
11   AG-GCNT-AGGCTT
Rev.of_Cele-UNSB01_1:12396670-12396802 Satlength=133 Nr of Repeats=9
RepeatLength=12 seed=GCCTAAGCCT Num.seqs=7 Similarity=0.767196
10   AG-GCTT-AGGCNT
Rev.of_Cele-UNSB01_2:2842834-2842918 Satlength=85 Nr of Repeats=6
RepeatLength=12 seed=CCTAAGCCTA Num.seqs=4 Similarity=0.754274
21   AG-GCTT-AGGCGT
Rev.of_Cele-UNSB01_4:4525521-4525683 Satlength=163 Nr of Repeats=12
RepeatLength=12 seed=GCCTAAGCCT Num.seqs=11 Similarity=0.751515
22   AG-GCTT-AGGCGT

Cele-UNSB01_2:1233155-1233235 Satlength=81 Nr of Repeats=5 RepeatLength=12 seed=TTAGGCTTAG Num.seqs=3 Similarity=0.925926
10   AG-GCTT-AGGTTT
Cele-UNSB01_4:3670951-3671065 Satlength=115 Nr of Repeats=7 RepeatLength=12 seed=TTAGGCTTAG Num.seqs=5 Similarity=0.866667
10   AG-GCTT-AGGATT
Cele-UNSB01_4:1647487-1647595 Satlength=109 Nr of Repeats=9 RepeatLength=12 seed=CTTAAGCTTA Num.seqs=9 Similarity=0.975309
9   AA-GCTT-AGGCTT
Rev.of_Cele-UNSB01_4:1908625-1908709 Satlength=85 Nr of Repeats=5 RepeatLength=12 seed=TAAGCCTAAG Num.seqs=3 Similarity=0.851852
13   AA-GCTT-AGGCTT
Rev.of_Cele-UNSB01_4:3517707-3518097 Satlength=391 Nr of Repeats=27 RepeatLength=12 seed=TAAGCCTAAG Num.seqs=21 Similarity=0.808059
13   AA-GCTT-AGGCTT
Cele-UNSB01_5:376477-376531 Satlength=55 Nr of Repeats=4 RepeatLength=12 seed=AGGCTTAGGC Num.seqs=3 Similarity=1.000000
12   AG-GCTT-AGGCTA
Rev.of_Cele-UNSB01_2:4433247-4433329 Satlength=83 Nr of Repeats=4 RepeatLength=12 seed=TAAGCCTAAG Num.seqs=3 Similarity=0.925926
13   AG-TCTT-AGGCTT
Cele-UNSB01_2:12381269-12381383 Satlength=115 Nr of Repeats=7 RepeatLength=12 seed=TTAGTCTTAG Num.seqs=5 Similarity=0.833333
22   AG-TCTT-AGGCTT
Rev.of_Cele-UNSB01_6:1555230-1555302 Satlength=73 Nr of Repeats=5 RepeatLength=12 seed=CTAAGCCTAA Num.seqs=4 Similarity=0.851852
14   AG-TCTT-TGGCTT
Rev.of_Cele-UNSB01_2:2657840-2658242 Satlength=403 Nr of Repeats=31 RepeatLength=13 seed=TAAGCCTAAG Num.seqs=28 Similarity=0.872449
14   AGAGCTT-AGGCTT
Rev.of_Cele-UNSB01_1:12200484-12200598 Satlength=115 Nr of Repeats=7 RepeatLength=12 seed=CTAAGCCTAA Num.seqs=5 Similarity=0.766667
2   AG-GTTT-AGGCTT
Cele-UNSB01_4:13187743-13188346 Satlength=604 Nr of Repeats=12 RepeatLength=12 seed=GCTTGGGTTT Num.seqs=9 Similarity=0.907407
8   GG-GTTT-AGGCTT
Cele-UNSB01_4:13187743-13188478 Satlength=736 Nr of Repeats=17 RepeatLength=12 seed=GCTTGGGTTT Num.seqs=11 Similarity=0.903030
8   GG-GTTT-AGGCTT
Rev.of_Cele-UNSB01_1:1680231-1680351 Satlength=121 Nr of Repeats=7 RepeatLength=12 seed=AGCCTAAACC Num.seqs=5 Similarity=0.702564
11   AG-GTTT-AGGCTC
Cele-UNSB01_3:1312329-1312425 Satlength=97 Nr of Repeats=5 RepeatLength=12 seed=AGGCTTAGGC Num.seqs=3 Similarity=0.925926
12   AG-GCTT-AGGCTC
Cele-UNSB01_3:1320709-1320805 Satlength=97 Nr of Repeats=5 RepeatLength=12 seed=AGGCTTAGGC Num.seqs=3 Similarity=0.925926
12   AG-GCTT-AGGCTC
Cele-UNSB01_3:1329089-1329185 Satlength=97 Nr of Repeats=5 RepeatLength=12 seed=AGGCTTAGGC Num.seqs=3 Similarity=0.925926
12   AG-GCTT-AGGCTC
Cele-UNSB01_3:1843958-1844048 Satlength=91 Nr of Repeats=6 RepeatLength=12 seed=AGGCTTAGGC Num.seqs=4 Similarity=0.668803
12   AG-GCTT-AGGCTC

Cele-UNSB01_5:1343973-1344069 Satlength=97 Nr of Repeats=7 RepeatLength=12 seed=AGGCTTAGGC Num.seqs=5 Similarity=1.000000
12 AG-GCTT-AGGCTC
Rev.of_Cele-UNSB01_5:1872631-1872733 Satlength=103 Nr of Repeats=7 RepeatLength=12 seed=AGCCTGAGCC Num.seqs=5 Similarity=0.955556
17 AG-GCTT-AGGCTC
Cele-UNSB01_5:2444425-2444473 Satlength=49 Nr of Repeats=4 RepeatLength=12 seed=AGGCTTAGGC Num.seqs=4 Similarity=0.870370
12 AG-GCTT-AGGCTC
Rev.of_Cele-UNSB01_5:18856264-18856396 Satlength=133 Nr of Repeats=9 RepeatLength=12 seed=AGCCTAAGCC Num.seqs=7 Similarity=0.841270
23 AG-GCTT-AGGCTC
Cele-UNSB01_1:11868010-11868741 Satlength=732 Nr of Repeats=60 RepeatLength=12 seed=AGGCTCAGGC Num.seqs=57 Similarity=0.962545
18 AG-GCTC-AGGCTC
Cele-UNSB01_2:649028-649082 Satlength=55 Nr of Repeats=4 RepeatLength=12 seed=GGCTCAGGCT Num.seqs=3 Similarity=1.000000
19 AG-GCTC-AGGCTC
Rev.of_Cele-UNSB01_3:1738456-1739086 Satlength=631 Nr of Repeats=52 RepeatLength=12 seed=TGAGCCTGAG Num.seqs=51 Similarity=0.969848
19 AG-GCTC-AGGCTC
Rev.of_Cele-UNSB01_3:12087067-12087157 Satlength=91 Nr of Repeats=4 RepeatLength=12 seed=AGCCTGAGCC Num.seqs=3 Similarity=0.851852
23 AG-GCTC-AGGCTC
Cele-UNSB01_5:18937116-18937194 Satlength=79 Nr of Repeats=6 RepeatLength=12 seed=GGCTCAGGCT Num.seqs=5 Similarity=0.955556
19 AG-GCTC-AGGCTC
Rev.of_Cele-UNSB01_5:18982935-18983047 Satlength=113 Nr of Repeats=7 RepeatLength=12 seed=AGCCTGAGCC Num.seqs=6 Similarity=1.000000
23 AG-GCTC-AGGCTC
Rev.of_Cele-UNSB01_5:19004973-19005085 Satlength=113 Nr of Repeats=7 RepeatLength=12 seed=AGCCTGAGCC Num.seqs=6 Similarity=1.000000
23 AG-GCTC-AGGCTC
Cele-UNSB01_5:19517858-19518362 Satlength=505 Nr of Repeats=37 RepeatLength=12 seed=AGGCTCAGGC Num.seqs=32 Similarity=0.903226
18 AG-GCTC-AGGCTC
Cele-UNSB01_2:13509974-13510060 Satlength=87 Nr of Repeats=6 RepeatLength=12 seed=CTTAGGCTTA Num.seqs=4 Similarity=0.743590
10 AG-GCTTAAGNCTT
Cele-UNSB01_4:1800043-1800133 Satlength=91 Nr of Repeats=7 RepeatLength=12 seed=TTAGGATTAG Num.seqs=6 Similarity=0.962963
10 AG-GATT-AGGATT
Rev.of_Cele-UNSB01_1:11752079-11752337 Satlength=259 Nr of Repeats=16 RepeatLength=12 seed=AAGCCAAAGC Num.seqs=13 Similarity=0.854701
12 TG-GCTT-TGGCTT
Cele-UNSB01_2:14148263-14148341 Satlength=79 Nr of Repeats=6 RepeatLength=12 seed=GGCTTTGGCT Num.seqs=5 Similarity=1.000000
19 TG-GCTT-TGGCTT
Rev.of_Cele-UNSB01_3:2564180-2564252 Satlength=73 Nr of Repeats=5 RepeatLength=12 seed=CCAAGCCCAA Num.seqs=3 Similarity=0.851852
20 GG-GCTT-GGGCTT
Cele-UNSB01_5:20312857-20314295 Satlength=1439 Nr of Repeats=50 RepeatLength=12 seed=GCTTGGGCTT Num.seqs=36 Similarity=0.903704
20 GG-GCTT-GGGCTT

Rev.of_Cele-UNSB01_5:3739928-3740028 Satlength=101 Nr of Repeats=6
RepeatLength=10 seed=TAGCCATGCC Num.seqs=4 Similarity=0.933333
1   AG-GCAT--GGC-T
Rev.of_Cele-UNSB01_1:11239237-11239369 Satlength=133 Nr of Repeats=12
RepeatLength=11 seed=TATGCCTAGC Num.seqs=12 Similarity=1.000000
7   AG-GCAT-AGGC-T
Rev.of_Cele-UNSB01_2:1258661-1259567 Satlength=907 Nr of Repeats=73
RepeatLength=12 seed=CTATGCCTAT Num.seqs=70 Similarity=0.936416
14   AG-GCAT-AGGCAT
Rev.of_Cele-UNSB01_2:1261170-1261684 Satlength=515 Nr of Repeats=42
RepeatLength=12 seed=CTATGCCTAT Num.seqs=38 Similarity=0.936621
14   AG-GCAT-AGGCAT
Rev.of_Cele-UNSB01_2:1429641-1430207 Satlength=567 Nr of Repeats=41
RepeatLength=12 seed=CCTATGCCTA Num.seqs=30 Similarity=0.874585
21   AG-GCAT-AGGCAT
Cele-UNSB01_2:2986853-2986937 Satlength=85 Nr of Repeats=7
RepeatLength=12 seed=ATAGGCATAG Num.seqs=7 Similarity=1.000000
22   AG-GCAT-AGGCAT
Cele-UNSB01_2:14355758-14355994 Satlength=237 Nr of Repeats=18
RepeatLength=12 seed=ATAGGCATAG Num.seqs=11 Similarity=0.834343
22   AG-GCAT-AGGCAT
Cele-UNSB01_3:12605711-12605849 Satlength=139 Nr of Repeats=9
RepeatLength=12 seed=TAGGCATAGG Num.seqs=6 Similarity=0.829630
23   AG-GCAT-AGGCAT
Cele-UNSB01_4:1444044-1444098 Satlength=55 Nr of Repeats=4
RepeatLength=12 seed=CATAGGCATA Num.seqs=3 Similarity=0.925926
21   AG-GCAT-AGGCAT
Cele-UNSB01_6:14452976-14453126 Satlength=151 Nr of Repeats=12
RepeatLength=12 seed=ATAGGCATAG Num.seqs=11 Similarity=0.979798
22   AG-GCAT-AGGCAT

*
Consensus:

AGGCTTAGGCTT

>Cele-UNSB01_Fam_2_35_166 Nr. of seq. 166 Alignment length(with gaps) = 46 Alignment score = 0.624350
Cele-UNSB01_2:11799205-11799469 Satlength=265 Nr of Repeats=8
RepeatLength=33 seed=AATTGAAAAT Num.seqs=8 Similarity=0.834055
0   AATT--G-AAA--A---TCCGGCAAATG--GGC-AAATTGCCG-G-
Cele-UNSB01_1:3888641-3889727 Satlength=1087 Nr of Repeats=26
RepeatLength=34 seed=TGCCGGAATT Num.seqs=16 Similarity=0.878105
28   AATT--G--AA--A-TTTCCAGCAAATC--GGC-AAATTGCCG-G-
Cele-UNSB01_2:5722242-5722548 Satlength=307 Nr of Repeats=9
RepeatLength=34 seed=GGAATTGAAA Num.seqs=9 Similarity=0.831481
32   AATT--G--AA--A-TTTCCGGCAAATC--GGC-AAATTGCCG-G-
Rev.of_Cele-UNSB01_2:15432049-15432695 Satlength=647 Nr of Repeats=18
RepeatLength=34 seed=TTTCAATTCC Num.seqs=13 Similarity=0.912016
42   AATT--G--AA--A-TTTCCGGCAAATC--GGC-AAATTGCCG-G-
Cele-UNSB01_3:1833347-1833868 Satlength=522 Nr of Repeats=14
RepeatLength=34 seed=AATTGAAATT Num.seqs=9 Similarity=0.808279
34   AATT--G--AA--A-TTTCCGGCAAATC--GGC-AAATTGCCG-G-

Rev.of_Cele-UNSB01_3:11800186-11800737 Satlength=552 Nr of Repeats=15
RepeatLength=34 seed=AAATTTCAAT Num.seqs=10 Similarity=0.841394
45   AATT--G--AA--A-TTTCCGGCAAATC--GGC-AAATTGCCG-G-
Cele-UNSB01_3:13415830-13416475 Satlength=646 Nr of Repeats=18
RepeatLength=34 seed=CGGAATTGAA Num.seqs=15 Similarity=0.854715
65   AATT--G--AA--A-TTTCCGGCAAATC--GGC-AAATTGCCG-G-
Rev.of_Cele-UNSB01_3:12889217-12889593 Satlength=377 Nr of Repeats=11
RepeatLength=34 seed=TTTCAATTCC Num.seqs=7 Similarity=0.788982
42   AATT--G--AA--A-TTTCCGGTAAATC--GGC-AAATTGCCG-G-
Rev.of_Cele-UNSB01_1:15023397-15024044 Satlength=648 Nr of Repeats=19
RepeatLength=34 seed=CGGAAATTTC Num.seqs=18 Similarity=0.965142
48   AATT--G--AA--A-TTTCCGGCAAATC--GGC-AAATTGCTG-G-
Cele-UNSB01_2:15097668-15098195 Satlength=528 Nr of Repeats=13
RepeatLength=35 seed=TTTCCGGCAA Num.seqs=8 Similarity=0.791837
77   AATT--GA-AA--A-TTTCCGGCAAATC--GGC-AAATTGCTG-G-
Rev.of_Cele-UNSB01_4:388221-388493 Satlength=273 Nr of Repeats=8
RepeatLength=34 seed=TTGCAGGAAA Num.seqs=8 Similarity=0.823529
52   AATT--G--AA--A-TTTCCTGCAAACC--GGC-AAATTGCCG-G-
Rev.of_Cele-UNSB01_5:786342-786547 Satlength=206 Nr of Repeats=6
RepeatLength=34 seed=CAATTTGCCG Num.seqs=5 Similarity=0.788235
64   AATT--G--AA--A-TTTCCGGCAAACC--GGC-AAATTGCCG-A-
Rev.of_Cele-UNSB01_2:13192718-13192854 Satlength=137 Nr of Repeats=4
RepeatLength=34 seed=TTCAATTCCG Num.seqs=4 Similarity=0.803922
41   AATT--G--AA--A-TTTCCGGCAAATC--GAC-GAATTGCCG-G-
Cele-UNSB01_1:11268535-11268966 Satlength=432 Nr of Repeats=11
RepeatLength=34 seed=TTCCGGCAAA Num.seqs=9 Similarity=0.844227
43   AATT--T--AA--A-TTTCCGGCAAATC--GGA-AAATTGCCG-G-
Rev.of_Cele-UNSB01_1:1031726-1032601 Satlength=876 Nr of Repeats=25
RepeatLength=35 seed=ATTTTCAATT Num.seqs=25 Similarity=0.921397
44   AATT--G-AAA--A-TTTCCGGCAAATC--GGC-AAACTGCCG-G-
Cele-UNSB01_1:1591838-1592129 Satlength=292 Nr of Repeats=7
RepeatLength=35 seed=CGGCAAATCG Num.seqs=6 Similarity=0.895873
47   AATT--G-AAA--A-TTTCCGGCAAATC--GGC-AAATTGCCG-G-
Rev.of_Cele-UNSB01_1:1632570-1633118 Satlength=549 Nr of Repeats=6
RepeatLength=35 seed=AAATTTTCAA Num.seqs=5 Similarity=0.878095
81   AATT--G-AAA--A-TTTCCGGCAAATC--GGC-AAATTGCCG-G-
Rev.of_Cele-UNSB01_1:1976543-1977056 Satlength=514 Nr of Repeats=5
RepeatLength=35 seed=TTTGCCGGAA Num.seqs=3 Similarity=0.759259
54   AATT--G-AAA--A-TTTCCGGCAAATC--GGC-AAATTGCCG-G-
Cele-UNSB01_1:2850958-2851167 Satlength=210 Nr of Repeats=5
RepeatLength=35 seed=GGAATTGAAA Num.seqs=3 Similarity=0.898413
67   AATT--G-AAA--A-TTTCCGGCAAATC--GGC-AAATTGCCG-G-
Rev.of_Cele-UNSB01_1:11183622-11184094 Satlength=473 Nr of Repeats=10
RepeatLength=35 seed=TTGCCGATTT Num.seqs=6 Similarity=0.702469
61   AATT--G-AAA--A-TTTCCGGCAAATC--GGC-AAATTGCCG-G-
Rev.of_Cele-UNSB01_2:12914019-12914481 Satlength=463 Nr of Repeats=11
RepeatLength=35 seed=TCAATTCCGG Num.seqs=10 Similarity=0.869630
75   AATT--G-AAA--A-TTTCCGGCAAATC--GGC-AAATTGCCG-G-
Rev.of_Cele-UNSB01_2:13551182-13551985 Satlength=804 Nr of Repeats=20
RepeatLength=35 seed=GATTTGCCGG Num.seqs=15 Similarity=0.816490
56   AATT--G-AAA--A-TTTCCGGCAAATC--GGC-AAATTGCCG-G-
Cele-UNSB01_2:14428274-14428974 Satlength=701 Nr of Repeats=18
RepeatLength=35 seed=GCAAATCGGC Num.seqs=14 Similarity=0.835060
49   AATT--G-AAA--A-TTTCCGGCAAATC--GGC-AAATTGCCG-G-

Rev.of_Cele-UNSB01_2:15354583-15354863 Satlength=281 Nr of Repeats=8
RepeatLength=35 seed=TTTGCCGGAA Num.seqs=8 Similarity=0.858503
54   AATT--G-AAA--A-TTTCCGGCAAATC--GGC-AAATTGCCG-G-
Cele-UNSB01_3:1204553-1205580 Satlength=1028 Nr of Repeats=13
RepeatLength=35 seed=TTTAAAATTT Num.seqs=8 Similarity=0.863946
71   AATT--G-AAA--A-TTTCCGGCAAATC--GGC-AAATTGCCG-G-
Cele-UNSB01_3:1395088-1395322 Satlength=235 Nr of Repeats=5
RepeatLength=35 seed=CAAATCGGCA Num.seqs=3 Similarity=0.695238
50   AATT--G-AAA--A-TTTCCGGCAAATC--GGC-AAATTGCCG-G-
Rev.of_Cele-UNSB01_3:2665588-2665980 Satlength=393 Nr of Repeats=11
RepeatLength=35 seed=TTTGCCGGAA Num.seqs=7 Similarity=0.807710
54   AATT--G-AAA--A-TTTCCGGCAAATC--GGC-AAATTGCCG-G-
Cele-UNSB01_3:3795055-3795458 Satlength=404 Nr of Repeats=8
RepeatLength=35 seed=TTCCGGCAAA Num.seqs=5 Similarity=0.760000
79   AATT--G-AAA--A-TTTCCGGCAAATC--GGC-AAATTGCCG-G-
Cele-UNSB01_3:12120993-12121420 Satlength=428 Nr of Repeats=12
RepeatLength=35 seed=AATTGCCGGA Num.seqs=10 Similarity=0.840847
60   AATT--G-AAA--A-TTTCCGGCAAATC--GGC-AAATTGCCG-G-
Rev.of_Cele-UNSB01_3:12712606-12713078 Satlength=473 Nr of Repeats=10
RepeatLength=35 seed=GATTTGCCGG Num.seqs=7 Similarity=0.791446
56   AATT--G-AAA--A-TTTCCGGCAAATC--GGC-AAATTGCCG-G-
Rev.of_Cele-UNSB01_5:4169850-4170172 Satlength=323 Nr of Repeats=8
RepeatLength=35 seed=TTTCAATTCC Num.seqs=7 Similarity=0.885714
77   AATT--G-AAA--A-TTTCCGGCAAATC--GGC-AAATTGCCG-G-
Cele-UNSB01_5:4696924-4697115 Satlength=192 Nr of Repeats=5
RepeatLength=35 seed=CGGCAAATCG Num.seqs=3 Similarity=0.479798
47   AATT--G-AAA--A-TTTCCGGCAAATC--GGC-AAATTGCCG-G-
Cele-UNSB01_1:13236201-13236854 Satlength=654 Nr of Repeats=16
RepeatLength=35 seed=ATTGCCGGAA Num.seqs=10 Similarity=0.780741
61   AATT--G-AAA--A-TTTCCGGCAAATC--GGN-AAATTGCCG-G-
Cele-UNSB01_2:236549-238078 Satlength=1530 Nr of Repeats=40
RepeatLength=35 seed=TTTCCGGCAA Num.seqs=27 Similarity=0.708272
78   AATT--G-AAA--A-TTTCCGGCAAATC--GGC-ANATTGCCG-G-
Rev.of_Cele-UNSB01_3:11990778-11991299 Satlength=522 Nr of Repeats=14
RepeatLength=35 seed=CCGATTTGCC Num.seqs=10 Similarity=0.809053
58   AATT--G-AAA--A-TTTCNGGCAAATC--GGC-AAATTGCCG-G-
Rev.of_Cele-UNSB01_5:1415140-1415840 Satlength=701 Nr of Repeats=21
RepeatLength=35 seed=ATTTTCAATT Num.seqs=15 Similarity=0.700680
79   AATT--G-AAA--A-TTTCNGGCAAATC--GGC-AAATTGCCG-G-
Rev.of_Cele-UNSB01_5:2441369-2441565 Satlength=197 Nr of Repeats=4
RepeatLength=35 seed=AAATTTTCAA Num.seqs=3 Similarity=0.780864
81   AATT--G-AAA--A-TTTCCGGCNAATC--GGC-AAATTGCCG-G-
Cele-UNSB01_2:14453377-14453692 Satlength=316 Nr of Repeats=8
RepeatLength=35 seed=CGGCAAATCG Num.seqs=7 Similarity=0.780864
47   AATT--G-NAA--A-TTTCCGGCAAATC--GGC-AAATTGCCG-G-
Rev.of_Cele-UNSB01_5:4698132-4698369 Satlength=238 Nr of Repeats=5
RepeatLength=35 seed=CAATTCCGGC Num.seqs=3 Similarity=0.873016
74   AATT--G-TAA--A-TTTCCGGCAAATC--GGC-AAATTGCCG-G-
Rev.of_Cele-UNSB01_1:1840655-1841028 Satlength=374 Nr of Repeats=9
RepeatLength=35 seed=TTTGCCGGAA Num.seqs=8 Similarity=0.766700
54   AATT--G-AAA--A-TTTCCGGCAAATC--GGC-AACTTGCCG-G-
Rev.of_Cele-UNSB01_2:15290141-15290702 Satlength=562 Nr of Repeats=16
RepeatLength=35 seed=TTTGCCGGAA Num.seqs=11 Similarity=0.731650
54   AATT--G-AAA--A-TTTCCGGCAAATC--GGC-AANTTGCCG-G-

Rev.of_Cele-UNSB01_1:2169410-2169690 Satlength=281 Nr of Repeats=8
RepeatLength=35 seed=CGGCAAATTG Num.seqs=5 Similarity=0.878095
68   AATT--G-AAA--A-TTTCCGGCAAATC--GGC-AATTTGCCG-G-
Rev.of_Cele-UNSB01_5:2619946-2620165 Satlength=220 Nr of Repeats=6
RepeatLength=35 seed=GCCGGAAATT Num.seqs=4 Similarity=0.752381
51   AATT--G-AAA--A-TTTCCGGCAAATC--GGG-AAATTGCCG-G-
Cele-UNSB01_1:11556393-11558176 Satlength=1784 Nr of Repeats=47
RepeatLength=35 seed=GGCAAATCAG Num.seqs=42 Similarity=0.911023
48   AATT--G-AAA--A-TTTCCGGCAAATC--AGC-AAATTGCCG-G-
Cele-UNSB01_2:13754985-13755264 Satlength=280 Nr of Repeats=7
RepeatLength=35 seed=TTGCCGGAAT Num.seqs=6 Similarity=0.784127
62   AATT--G-AAT--A-TTTCCGGCAAATC--GGC-AAATTGCCG-G-
Rev.of_Cele-UNSB01_5:3737155-3737311 Satlength=157 Nr of Repeats=4
RepeatLength=35 seed=TTGCCGATTT Num.seqs=3 Similarity=0.784127
96   AATT--G-AAT--N-TTTCCGGCAAATC--GGC-AAATTGCCG-G-
Rev.of_Cele-UNSB01_2:15286316-15286491 Satlength=176 Nr of Repeats=5
RepeatLength=35 seed=TTTGCCGGAA Num.seqs=3 Similarity=0.771429
54   AATT--N-AAA--A-TTTCCGGCAAATC--GGC-AAATTGCCN-G-
Rev.of_Cele-UNSB01_2:15454038-15454614 Satlength=577 Nr of Repeats=12
RepeatLength=35 seed=CCGATTTGCC Num.seqs=11 Similarity=0.807446
58   AATT--C-AAA--A-TTTCCGGCAAATC--GGC-AAATTGCCG-G-
Cele-UNSB01_3:12547936-12548181 Satlength=246 Nr of Repeats=7
RepeatLength=35 seed=AATTGCCGGA Num.seqs=7 Similarity=0.912925
60   AATT--C-AAA--A-TTTCCGGCAAATC--GGC-AAATTGCCG-G-
Rev.of_Cele-UNSB01_3:2911307-2911655 Satlength=349 Nr of Repeats=10
RepeatLength=35 seed=TTTGCCGGAA Num.seqs=8 Similarity=0.859864
89   AATT--A-AAA--A-TTTCCGGCAAATC--GGC-AAATTGCCG-G-
Cele-UNSB01_3:13188476-13189044 Satlength=569 Nr of Repeats=13
RepeatLength=35 seed=TGCCGGAATT Num.seqs=8 Similarity=0.817687
63   AATT--T-AAA--A-TTTCCGGCAAATC--GGC-AAATTGCCG-G-
Cele-UNSB01_5:2608578-2609028 Satlength=451 Nr of Repeats=10
RepeatLength=35 seed=TTTCCGGCAA Num.seqs=7 Similarity=0.807710
78   AATT--T-AAA--A-TTTCCGGCAAATC--GGC-AAATTGCCG-G-
Rev.of_Cele-UNSB01_1:13199796-13200258 Satlength=463 Nr of Repeats=10
RepeatLength=35 seed=GCCGGAAATT Num.seqs=7 Similarity=0.776871
51   AATT--N-ANA--A-TTTCCGGCAAATC--GGC-AAATTGCCG-G-
Rev.of_Cele-UNSB01_5:2587851-2588904 Satlength=1054 Nr of Repeats=15
RepeatLength=35 seed=TCAATTCCGG Num.seqs=10 Similarity=0.832381
75   AATT--G-AGA--A-TTTCCGGCAAATC--GGC-AAATTGCCG-G-
Cele-UNSB01_1:1910732-1911187 Satlength=456 Nr of Repeats=11
RepeatLength=35 seed=AAATCGGCAA Num.seqs=8 Similarity=0.753742
51   AATT--G-AAA--A-TTTCCGGCAAATC--GGC-AAATTGCCG-A-
Rev.of_Cele-UNSB01_2:13911573-13912594 Satlength=1022 Nr of Repeats=28
RepeatLength=35 seed=TTTGCCGGAA Num.seqs=18 Similarity=0.750016
54   AATT--C-AAA--A-TTTCCGGCAAATC--GGC-AAATTGCCG-A-
Cele-UNSB01_2:14462473-14462688 Satlength=216 Nr of Repeats=5
RepeatLength=35 seed=CGGCAAATTG Num.seqs=3 Similarity=0.586563
55   AATT--G-AAA--A-TTTCCGNNAAATC--GGC-AAATTGCCG-G-
Rev.of_Cele-UNSB01_4:1946817-1947132 Satlength=316 Nr of Repeats=8
RepeatLength=35 seed=GAAATTTTCA Num.seqs=7 Similarity=0.927438
82   AATT--G-AAA--A-TTTCCGTCAAATC--GGC-AAATTGCCG-G-
Rev.of_Cele-UNSB01_1:2195476-2195914 Satlength=439 Nr of Repeats=10
RepeatLength=35 seed=TTTGCCGGAA Num.seqs=6 Similarity=0.735873
54   AATT--C-AAA--A-TTTCCGGCAAATC--GGC-AAATTGTCG-G-

Cele-UNSB01_1:13683275-13683450 Satlength=176 Nr of Repeats=5
RepeatLength=35 seed=TGAAAATTTC Num.seqs=5 Similarity=0.878095
72 AATT--G-AAA--A-TTTCCGGCAAATC--GGC-AAATTGTCG-G-
Cele-UNSB01_1:14869199-14869444 Satlength=246 Nr of Repeats=6
RepeatLength=35 seed=CAAATCGGCA Num.seqs=4 Similarity=0.733333
85 AATT--G-AAA--A-TTTCCGGCAAATC--GGC-AAATTGTCG-G-
Rev.of_Cele-UNSB01_3:2149341-2150059 Satlength=719 Nr of Repeats=12
RepeatLength=35 seed=TTTGCCGGAA Num.seqs=10 Similarity=0.926349
89 AATT--G-AAA--A-TTTCCGGCAAATC--GGC-AAATTGTCG-G-
Rev.of_Cele-UNSB01_5:5114820-5115220 Satlength=401 Nr of Repeats=8
RepeatLength=35 seed=TTGCCGATTT Num.seqs=5 Similarity=0.767619
61 AATT--G-AAA--A-TTTCCGGCAAATC--GGC-AANTTGNCG-G-
Cele-UNSB01_2:14458676-14459058 Satlength=383 Nr of Repeats=11
RepeatLength=35 seed=CCGGCAAATC Num.seqs=8 Similarity=0.757275
81 AATT--G-AAA--C-TTTCCGGCAAATC--GGC-AAATTGGCG-G-
Cele-UNSB01_3:12309471-12309813 Satlength=343 Nr of Repeats=8
RepeatLength=35 seed=GGAATTGAAA Num.seqs=5 Similarity=0.843810
102 AATT--G-AAA--A-TTTCCGGCAAATC--GGC-AAATTGGCG-G-
Cele-UNSB01_5:15289065-15289259 Satlength=195 Nr of Repeats=6
RepeatLength=35 seed=GGAATTGAAA Num.seqs=4 Similarity=0.904762
102 AATT--G-AAA--A-TTTCCGGCAAATC--GGC-AAATTGGCG-G-
Rev.of_Cele-UNSB01_5:1860337-1860547 Satlength=211 Nr of Repeats=7
RepeatLength=35 seed=TCCGGCAATT Num.seqs=5 Similarity=0.833333
70 ATTT--N-AAA--A-TTTCCGGCAAATC--GGC-AAATTGCCG-G-
Rev.of_Cele-UNSB01_1:15094323-15094532 Satlength=210 Nr of Repeats=6
RepeatLength=35 seed=TTTCAATTCC Num.seqs=5 Similarity=0.878095
77 AATT--G-AAA--G-TTTTCGGCAAATC--GGC-AAATTGCCG-G-
Rev.of_Cele-UNSB01_4:2909681-2910188 Satlength=508 Nr of Repeats=14
RepeatLength=35 seed=ATTCCGGCAA Num.seqs=11 Similarity=0.820606
107 AATT--G-AAA--A-TTTTCGGCAAATC--GGC-AAATTGCCG-G-
Cele-UNSB01_1:14731269-14731934 Satlength=666 Nr of Repeats=19
RepeatLength=35 seed=GAAAATTTCC Num.seqs=19 Similarity=0.939850
73 AATT--G-AAA--A-TTTCCGGCAAACC--GGC-AAATTGCCG-G-
Rev.of_Cele-UNSB01_3:12934390-12934631 Satlength=242 Nr of Repeats=8
RepeatLength=35 seed=TTTTAATTCC Num.seqs=6 Similarity=0.888254
77 AATT--A-AAA--A-TTTCCGGCAAACC--GGC-AAATTGCCG-G-
Rev.of_Cele-UNSB01_3:12996870-12997291 Satlength=422 Nr of Repeats=8
RepeatLength=35 seed=GGAAATTTTA Num.seqs=5 Similarity=0.820952
83 AATT--T-AAA--A-TTTCCGGCAAACC--GGC-AAATTGCCG-G-
Rev.of_Cele-UNSB01_5:4165711-4166017 Satlength=307 Nr of Repeats=5
RepeatLength=35 seed=AATTTTCAAT Num.seqs=4 Similarity=0.847619
80 AATT--G-AAA--A-TTTCCGGCAAACC--GGC-AAATTGCAG-G-
Cele-UNSB01_5:786696-786877 Satlength=182 Nr of Repeats=5 RepeatLength=35
seed=TTTCCGGCAA Num.seqs=3 Similarity=0.834921
78 AATT--G-AAG--A-TTTCCGGCAAACC--GGC-NAATTGCCG-G-
Rev.of_Cele-UNSB01_1:2780250-2780460 Satlength=211 Nr of Repeats=6
RepeatLength=35 seed=AATTTCAATT Num.seqs=6 Similarity=0.860317
44 AATT--G-AAA--T-TTTCCGGCAAATC--GGC-AAGTTGCCG-G-
Cele-UNSB01_1:11199905-11200227 Satlength=323 Nr of Repeats=8
RepeatLength=35 seed=GCCGGAATTG Num.seqs=7 Similarity=0.829478
64 AATT--G-AAA--T-TTTCCGGCAAATC--GGC-AAATTGCCG-G-
Rev.of_Cele-UNSB01_2:13805325-13805605 Satlength=281 Nr of Repeats=7
RepeatLength=35 seed=CTTGCCGATT Num.seqs=6 Similarity=0.862857
62 AATT--G-AAA--A-TTTCTGGCAAATC--GGC-AAGTTGCCG-G-

Cele-UNSB01_4:3371888-3372867 Satlength=980 Nr of Repeats=27
RepeatLength=35 seed=AATCGGCAAG Num.seqs=25 Similarity=0.852698
87 AATT--G-AAA--A-TTTCCGGCAAATC--GGC-AAGTTACCG-G-
Cele-UNSB01_3:12834918-12835593 Satlength=676 Nr of Repeats=18
RepeatLength=35 seed=TTTCCGGCAA Num.seqs=13 Similarity=0.878877
78 AATT--G-AAC--A-TTTCCGGCAAATC--GGT-AAATTGCCG-G-
Rev.of_Cele-UNSB01_4:3192518-3194426 Satlength=1909 Nr of Repeats=53
RepeatLength=35 seed=CGGCAATTTG Num.seqs=43 Similarity=0.870738
103 AATT--G-AAC--A-TTTCCGGCAAATN--GGC-AAATTGCCG-G-
Cele-UNSB01_1:1103105-1103421 Satlength=317 Nr of Repeats=8
RepeatLength=35 seed=CGGCAAATCG Num.seqs=7 Similarity=0.873016
47 AATT--G-AAA--A-TTTCCGGCAAATC--GGC-ATTTTGCCG-G-
Rev.of_Cele-UNSB01_5:1423918-1424416 Satlength=499 Nr of Repeats=9
RepeatLength=35 seed=TTTGCCGGAA Num.seqs=6 Similarity=0.779048
54 AATT--G-AAA--A-TTTCCGGCAAATC--CGC-AATTTGCCG-G-
Rev.of_Cele-UNSB01_5:18206252-18206609 Satlength=358 Nr of Repeats=5
RepeatLength=35 seed=AATTCCGGCA Num.seqs=3 Similarity=0.822222
73 AATT--G-AAA--A-TTTCCGGCAAAAC--GGC-ATATTGCCG-G-
Rev.of_Cele-UNSB01_1:14038349-14038662 Satlength=314 Nr of Repeats=9
RepeatLength=35 seed=AATTCCGGCA Num.seqs=7 Similarity=0.756173
73 AATT--T-AAA--T-TTTCCGGCAAATN--GGC-AAATTGCCG-G-
Cele-UNSB01_3:3152376-3152920 Satlength=545 Nr of Repeats=14
RepeatLength=35 seed=AATTGCCGGA Num.seqs=11 Similarity=0.843463
95 AATT--T-AAA--A-TTTCCGGCAAATT--GGC-AAATTGCCG-G-
Cele-UNSB01_3:11452503-11452739 Satlength=237 Nr of Repeats=6
RepeatLength=35 seed=AATTGCCGGA Num.seqs=4 Similarity=0.720635
95 AATT--A-AAA--A-TTTCCGGCAAATT--GGC-AAATTGCCG-G-
Cele-UNSB01_3:12227970-12228196 Satlength=227 Nr of Repeats=6
RepeatLength=35 seed=TTCCGGCAAA Num.seqs=4 Similarity=0.676190
114 AATT--G-AAA--A-TTTCCGGCAAATT--GGC-AAATTGCCG-G-
Rev.of_Cele-UNSB01_5:2602720-2603297 Satlength=578 Nr of Repeats=16
RepeatLength=35 seed=TTTGCCGGAA Num.seqs=10 Similarity=0.790053
124 AATT--G-AAA--A-TTTCCGGCAAATT--GGC-AAATTGNCG-G-
Rev.of_Cele-UNSB01_1:11636321-11636670 Satlength=350 Nr of Repeats=9
RepeatLength=35 seed=ATTCCGGCAA Num.seqs=6 Similarity=0.888254
72 AATT--G-AAA--A-TTTCTGGAAAATC--GGC-AAATTGCCG-G-
Rev.of_Cele-UNSB01_1:15062491-15062841 Satlength=351 Nr of Repeats=10
RepeatLength=35 seed=AATTCCGGCA Num.seqs=10 Similarity=0.804115
73 AATT--G-AAA--A-TTTCAGGAAAATC--GGC-AAATTGCCG-G-
Cele-UNSB01_2:13932545-13934837 Satlength=2293 Nr of Repeats=68
RepeatLength=35 seed=CCGGAATTGA Num.seqs=48 Similarity=0.862310
100 AATT--G-AAA--A-TTTCTGGAAAATC--GAC-AAATTGCCG-G-
Rev.of_Cele-UNSB01_3:12229422-12230200 Satlength=779 Nr of Repeats=21
RepeatLength=35 seed=TTCAATTCCG Num.seqs=15 Similarity=0.834492
111 AATT--G-AAA--A-TTTCTGGCAAATC--GAC-AAATTGCCG-G-
Cele-UNSB01_3:3159908-3160529 Satlength=622 Nr of Repeats=13
RepeatLength=35 seed=AAATCGGCAA Num.seqs=8 Similarity=0.885714
86 AATT--G-AAA--A-TTTCCAGAAAATC--GGC-AAATTGCCG-G-
Cele-UNSB01_3:3676441-3676612 Satlength=172 Nr of Repeats=5
RepeatLength=34 seed=AATTGAAATT Num.seqs=4 Similarity=0.800794
34 AATT--G--AA--A-TTTCCGGCAAAGC--GGC-AAATTGTCG-G-
Rev.of_Cele-UNSB01_1:3650776-3650949 Satlength=174 Nr of Repeats=5
RepeatLength=35 seed=TTTGCCGGAA Num.seqs=3 Similarity=0.822222
54 AATT--G--AAC-A-TTTCCGGCAAACC--GGC-AAATTGTCG-G-

Cele-UNSB01_1:11393763-11394894 Satlength=1132 Nr of Repeats=15
RepeatLength=35 seed=TTCCGGCAAA Num.seqs=9 Similarity=0.743915
44   AACT--G-AAA--A-TTTCCGGCAAATC--GGC-AAATTGCCN-G-
Cele-UNSB01_3:11851795-11852785 Satlength=991 Nr of Repeats=24
RepeatLength=35 seed=CGGCAAATCG Num.seqs=16 Similarity=0.809524
47   AACT--G-AAA--A-TTTCCGGCAAATC--GGC-AAATTGCCA-A-
Cele-UNSB01_2:4361834-4362159 Satlength=326 Nr of Repeats=8
RepeatLength=35 seed=TTCCGGCAAA Num.seqs=5 Similarity=0.790476
44   AATT--G-AAA--A-TTTCCGGCAAAAC--GGC-AAATTGCCA-A-
Cele-UNSB01_3:12593157-12594697 Satlength=1541 Nr of Repeats=44
RepeatLength=35 seed=TTGCCAGAAT Num.seqs=44 Similarity=0.791362
62   AATT--G-AAA--A-ATTCCGGNAAATC--GGC-AAATTGCCA-G-
Rev.of_Cele-UNSB01_2:12900948-12901228 Satlength=281 Nr of Repeats=8
RepeatLength=35 seed=ATTTCAGGCA Num.seqs=8 Similarity=0.791832
73   AAAT--G-AAA--A-TTTCTGGCAAATC--GGC-AAATTGCCT-G-
Cele-UNSB01_3:12308090-12308555 Satlength=466 Nr of Repeats=12
RepeatLength=35 seed=AAAATTTCCG Num.seqs=9 Similarity=0.868783
74   AATT--G-AAA--A-TTTCCGGCAAATCA-GCT-AA-TTGCCA-G-
Rev.of_Cele-UNSB01_5:1308044-1308391 Satlength=348 Nr of Repeats=8
RepeatLength=35 seed=AATTCTGGCA Num.seqs=7 Similarity=0.902041
108   AATT--G-AAA--A-TTTCCGGCAAATC--GGT-AATTTGCCA-G-
Rev.of_Cele-UNSB01_2:14003653-14004477 Satlength=825 Nr of Repeats=18
RepeatLength=34 seed=AATTCCGGCA Num.seqs=12 Similarity=0.808081
38   AATT--G--AA--A-TTTCCGATAAATC--AGC-AAATTGCCG-G-
Cele-UNSB01_2:15007973-15008319 Satlength=347 Nr of Repeats=4
RepeatLength=35 seed=TCCGGCAAAT Num.seqs=3 Similarity=0.586420
45   AATT--T-ANA--A-TNTCCGGCAAATC--GGC-AAATTGCCG-A-
Cele-UNSB01_5:20109112-20109540 Satlength=429 Nr of Repeats=12
RepeatLength=35 seed=AATTTCCGGC Num.seqs=11 Similarity=0.926580
76   AATT--T-AAA--A-TTTCCGGCAAATC--GGC-AAATAACCG-G-
Cele-UNSB01_5:4664068-4664530 Satlength=463 Nr of Repeats=10
RepeatLength=35 seed=GCAAATCGGC Num.seqs=6 Similarity=0.837460
15   -ATTC-G-AAA--A-TGTCCGGCAAATC--GGC-AAATTGCCG-G-
Rev.of_Cele-UNSB01_1:15014974-15015203 Satlength=230 Nr of Repeats=5
RepeatLength=34 seed=GAAATTTCAA Num.seqs=4 Similarity=0.901961
12   AGTT--G--AA--A-TTTCCGGCAAATT--GGC-TAATTGCCG-G-
Cele-UNSB01_2:14326178-14326688 Satlength=511 Nr of Repeats=14
RepeatLength=34 seed=CGGAATTGAA Num.seqs=13 Similarity=0.833082
31   AATT--G--AA--A-TTTCCGGCAAATT--GGT-AAAATGCCG-G-
Rev.of_Cele-UNSB01_3:12836433-12837192 Satlength=760 Nr of Repeats=21
RepeatLength=34 seed=TTTCAATTCC Num.seqs=15 Similarity=0.747664
42   AATT--G--AA--A-NTTCCGGCAAATT--GGT-AAATTGCCG-G-
Rev.of_Cele-UNSB01_3:134796-135400 Satlength=605 Nr of Repeats=7
RepeatLength=34 seed=AATTCCGGCA Num.seqs=5 Similarity=0.976471
38   AATT--G--AA--A-TTTTTGGCAAATT--GGT-AAATTGCCG-G-
Cele-UNSB01_4:1362949-1363282 Satlength=334 Nr of Repeats=9
RepeatLength=34 seed=ATTGCCGGAA Num.seqs=7 Similarity=0.886088
60   AATT--G--AA--A-TTTCTGGCAAATT--GGC-AAATTGCCG-G-
Cele-UNSB01_3:10787273-10787444 Satlength=172 Nr of Repeats=5
RepeatLength=34 seed=TTTCCGGCAA Num.seqs=4 Similarity=0.819841
42   AATC--A--AA--A-TTTCCGGCAAATT--GAC-AAATTGCCG-G-
Cele-UNSB01_5:3674779-3675094 Satlength=316 Nr of Repeats=9
RepeatLength=35 seed=AAAATTTCCG Num.seqs=9 Similarity=0.834921
5   AATT--G-AAA--A-TTTCCGGCAAA-C-AGGC-AAATTTCTG-G-

```
Cele-UNSB01_1:2599082-2599291 Satlength=210 Nr of Repeats=6
RepeatLength=35 seed=TTGCCGGAAT  Num.seqs=5 Similarity=0.794286
28    AATT--G-AAA--A-TTTTTGGNAAATC--GGC-AATTTGCCG-G-
Rev.of_Cele-UNSB01_1:2886442-2886617 Satlength=176 Nr of Repeats=4
RepeatLength=35 seed=TTTGCCGATT  Num.seqs=3 Similarity=0.733333
28    AATT--A-AAA--A-TTTTTGNCAAATC--GGC-AAATTGCCG-G-
Rev.of_Cele-UNSB01_5:1274939-1275298 Satlength=360 Nr of Repeats=10
RepeatLength=35 seed=GAAATTTTCA  Num.seqs=8 Similarity=0.842177
48    AATT--G-AAA--A-TTTCTGGCAATTT--GGC-AATTTGCCG-G-
Cele-UNSB01_2:10844890-10845587 Satlength=698 Nr of Repeats=8
RepeatLength=35 seed=TTTCTGGCAA  Num.seqs=5 Similarity=0.900952
44    AAAT--T-AAA--A-TTTCTGGCAAACC--GGC-AATTTGCCG-A-
Cele-UNSB01_3:12644803-12645326 Satlength=524 Nr of Repeats=7
RepeatLength=35 seed=TTGCCGGAAT  Num.seqs=5 Similarity=0.729630
63    AATT--G-NAA--A-TTTCTGGCAAACC--GGC-AATTTGCCG-G-
Cele-UNSB01_2:15454990-15455468 Satlength=479 Nr of Repeats=9
RepeatLength=35 seed=TTGCCGGAAT  Num.seqs=6 Similarity=0.740952
63    AATT--T-AAA--A-TTTCTGGCAAACC--GCC-AAATTGCCG-G-
Rev.of_Cele-UNSB01_1:3070958-3071149 Satlength=192 Nr of Repeats=5
RepeatLength=35 seed=TTGCCGGAAA  Num.seqs=3 Similarity=0.808642
54    AATT-AC-AA---A-TTTCCGGCAAATC--GGC-AATTTGTCG-G-
Cele-UNSB01_5:18261348-18261785 Satlength=438 Nr of Repeats=10
RepeatLength=35 seed=TTTCCGGCAA  Num.seqs=6 Similarity=0.790432
80    AATT-TG-AA---AGTTTCCGGCAAATC--GGC-ATTTTGCCG-G-
Rev.of_Cele-UNSB01_1:3156606-3157000 Satlength=395 Nr of Repeats=8
RepeatLength=35 seed=CGATTTGCCG  Num.seqs=6 Similarity=0.862857
58    AAAT--G-AAT--A-TTTCCGGCAAATC--GGC-AGTTTGCCG-G-
Cele-UNSB01_3:11614727-11615007 Satlength=281 Nr of Repeats=6
RepeatLength=35 seed=TTGCCGGATT  Num.seqs=4 Similarity=0.815873
63    ATTG--G-AAT--A-TTTTCGGCAAATC--GGC-AATTTGCCG-G-
Rev.of_Cele-UNSB01_3:11612201-11612410 Satlength=210 Nr of Repeats=6
RepeatLength=35 seed=GATTTGCCGG  Num.seqs=5 Similarity=0.850926
22    ATTT----TAA-TA-TTTCCGGCAAATC--GAT-AAATTGCCG-G-
Rev.of_Cele-UNSB01_5:4422681-4422875 Satlength=195 Nr of Repeats=5
RepeatLength=35 seed=ATTCCGGCAA  Num.seqs=3 Similarity=0.822222
38    AATT----TAA-TA-GTTCCGGCAAATC--GGC-AAATTGCCG-G-
Cele-UNSB01_5:20072393-20073779 Satlength=1387 Nr of Repeats=40
RepeatLength=35 seed=CCGGCAAATC  Num.seqs=26 Similarity=0.887707
47    ATTT--G-TAA--A-TTTCCGGCAAATC--GAN-AAATTACCG-G-
Rev.of_Cele-UNSB01_4:2889237-2889839 Satlength=603 Nr of Repeats=7
RepeatLength=35 seed=TTTTCAATTC  Num.seqs=5 Similarity=0.801905
9    AATT--G-AAA--A-ATTCAGGCAAATC--TGC-AAATTGCTT-G-
Rev.of_Cele-UNSB01_5:3735730-3736160 Satlength=431 Nr of Repeats=4
RepeatLength=35 seed=GCCGATTTGC  Num.seqs=3 Similarity=0.743827
25    ANTT--G-AAC--A-TTTCAGGCAAATC--GGC-AAATTGNCA-G-
Cele-UNSB01_1:1234705-1235262 Satlength=558 Nr of Repeats=15
RepeatLength=35 seed=AAATCGGCAA  Num.seqs=12 Similarity=0.802020
17    AAAT--G-AAA--G-ATTCCGGCAAATC--GGC-AATTTGCCN-A-
Rev.of_Cele-UNSB01_1:1946077-1948348 Satlength=2272 Nr of Repeats=60
RepeatLength=35 seed=TTGCCGATTT  Num.seqs=44 Similarity=0.891070
27    AAAT--G-AAA--A-TTTCCGGCAAATC--GGC-AATTTGCNA-A-
Cele-UNSB01_5:4162356-4162767 Satlength=412 Nr of Repeats=11
RepeatLength=35 seed=TTCCGGCAAA  Num.seqs=7 Similarity=0.865760
45    AAAT--G-AAA--A-TTTCCGGCAAACC--GGC-AATTTGCCA-A-
```

Rev.of_Cele-UNSB01_3:11723112-11723392 Satlength=281 Nr of Repeats=6 RepeatLength=35 seed=TTTGCCGGAA Num.seqs=4 Similarity=0.790476
55   AAAT--C-AAA--A-TTTCCGGCAAATC--GGC-AATTTGCCA-G-
Cele-UNSB01_3:13127780-13127920 Satlength=141 Nr of Repeats=4 RepeatLength=35 seed=CGGCAATTTG Num.seqs=4 Similarity=0.936508
56   AAAT--C-AAA--A-TTTCCGGCAAATC--GGC-AATTTGCCA-A-
Rev.of_Cele-UNSB01_5:1427061-1427411 Satlength=351 Nr of Repeats=8 RepeatLength=35 seed=TGCCGGAAAT Num.seqs=5 Similarity=0.790476
88   AAAT--C-AAA--A-TTTCCGGCAAATC--GGC-AATTTGCCA-A-
Rev.of_Cele-UNSB01_3:13026111-13026534 Satlength=424 Nr of Repeats=13 RepeatLength=35 seed=AAATTTTGAT Num.seqs=9 Similarity=0.900529
82   AAAT--C-AAA--A-TTTCCGGCAAATC--GGC-AAATTGCCA-A-
Cele-UNSB01_4:1669669-1669879 Satlength=211 Nr of Repeats=6 RepeatLength=35 seed=ATTTCCGGCA Num.seqs=6 Similarity=0.819683
43   AAAT--G-AAA--A-TTTCCGGCAAATC--GGC-AATATGCCG-A-
Cele-UNSB01_4:16653889-16654239 Satlength=351 Nr of Repeats=10 RepeatLength=35 seed=CAAATCGGCA Num.seqs=8 Similarity=0.824490
51   AATT--G-AAA--A-TTTCCGGCAAATC--GGC-AATATGCCA-A-
Cele-UNSB01_4:16659843-16660088 Satlength=246 Nr of Repeats=7 RepeatLength=35 seed=GCAAATCGGC Num.seqs=7 Similarity=0.842177
85   AATT--G-AAA--A-TTTCCGGCAAATC--GGC-AATATGCCA-A-
Rev.of_Cele-UNSB01_4:16658516-16659248 Satlength=733 Nr of Repeats=21 RepeatLength=35 seed=TTTGGCATAT Num.seqs=16 Similarity=0.860000
71   AATT--G-AAA--T-TTTCCGGCAAATC--GGC-AATATGCCA-A-
Rev.of_Cele-UNSB01_5:5204356-5204779 Satlength=424 Nr of Repeats=9 RepeatLength=35 seed=CGATTTGCCG Num.seqs=6 Similarity=0.576768
58   AAAT--A-AAA--A-TTTCCGGCAAATC--GGC-AATTTGCC-GA-
Cele-UNSB01_6:229151-229455 Satlength=305 Nr of Repeats=8 RepeatLength=36 seed=ATAAAAATTT Num.seqs=7 Similarity=0.865961
73   AAAT--A-AAA--A-TTTCCGGCAAATC--GGC-AATTTGCCAGA-
Rev.of_Cele-UNSB01_4:1309063-1309423 Satlength=361 Nr of Repeats=9 RepeatLength=36 seed=TTATTTTCTG Num.seqs=8 Similarity=0.838624
77   AAAT--A-AAA--A-TTTCCGGCAAATG--GGC-AATTTGTCAGA-
Rev.of_Cele-UNSB01_4:1313914-1314237 Satlength=324 Nr of Repeats=8 RepeatLength=36 seed=TTTGCCGGAA Num.seqs=5 Similarity=0.859259
91   AAAT--A-AAA--A-TTTCCGGCAAATC--GGC-AATTTGTCAGA-
Rev.of_Cele-UNSB01_1:1961569-1963172 Satlength=1604 Nr of Repeats=40 RepeatLength=35 seed=GAAATTTTCA Num.seqs=33 Similarity=0.890620
48   AACT--G-AAA--A-TTTCGGGCAAATC--GGC-AATTTGCCG-A-
Rev.of_Cele-UNSB01_1:1961569-1964216 Satlength=2648 Nr of Repeats=64 RepeatLength=35 seed=GAAATTTTCA Num.seqs=47 Similarity=0.883388
48   AACT--G-AAA--A-TTTCGGGCAAATC--GGC-AATTTGCCG-A-
Rev.of_Cele-UNSB01_1:1961569-1966139 Satlength=4571 Nr of Repeats=108 RepeatLength=35 seed=GAAATTTTCA Num.seqs=73 Similarity=0.839524
48   AACT--G-AAA--A-TTTCNGGCAAATC--GGC-AATTTGCCG-A-
Rev.of_Cele-UNSB01_1:1961569-1968269 Satlength=6701 Nr of Repeats=154 RepeatLength=35 seed=GAAATTTTCA Num.seqs=94 Similarity=0.828463
48   AACT--G-AAA--A-TTTCCGGCAAATC--GGC-AATTTGCCG-A-
Rev.of_Cele-UNSB01_1:1980049-1980327 Satlength=279 Nr of Repeats=8 RepeatLength=35 seed=TTGCCGGAAA Num.seqs=6 Similarity=0.654655
54   AATT--G-AAC--A-TTTCCGGCAAATC--GGC-AATTTGCCG-A-
Cele-UNSB01_1:14694045-14694325 Satlength=281 Nr of Repeats=8 RepeatLength=35 seed=AAAATTTCCG Num.seqs=8 Similarity=0.952381
75   AAGT--G-AAA--A-TTTCCGGCAAATC--GGC-AATTTACCG-A-

```
Rev.of_Cele-UNSB01_1:14697056-14704541 Satlength=7486 Nr of Repeats=214
RepeatLength=35 seed=GAAATTTTCA  Num.seqs=213 Similarity=0.929447
83    AAGT--G-AAA--A-TTTCCGGCAAATC--GGC-AATTTACCG-A-
Cele-UNSB01_5:1987432-1987712 Satlength=281 Nr of Repeats=8
RepeatLength=35 seed=GGCAAATCGG  Num.seqs=8 Similarity=0.914286
84    AAAT--G-AAA--A-TTTCCGGCAAATC--GGC-AATTTTCCG-A-
Rev.of_Cele-UNSB01_2:4637434-4637644 Satlength=211 Nr of Repeats=5
RepeatLength=35 seed=TTTGCCGGAA  Num.seqs=4 Similarity=0.757716
55    AAAT--G-AAA--T-TTTCCGGCAAATC--GGT-AATTTGCCG-A-
Rev.of_Cele-UNSB01_5:20105608-20107135 Satlength=1528 Nr of Repeats=40
RepeatLength=35 seed=GAAATTTTCA  Num.seqs=29 Similarity=0.756979
83    AAAT--G-AAA--A-TTTCCGGCAAATC--NGT-AATTTGCCG-A-
Rev.of_Cele-UNSB01_5:4118450-4118598 Satlength=149 Nr of Repeats=4
RepeatLength=35 seed=TTTGCCGGAA  Num.seqs=3 Similarity=0.682540
55    AAAT--N-AAA--A-TTTCCGGCAAAAC--GGC-AATTTACCA-A-
Cele-UNSB01_1:11398081-11398840 Satlength=760 Nr of Repeats=4
RepeatLength=35 seed=TCGGCAAATT  Num.seqs=3 Similarity=1.000000
20    AAAT--G-AAA--A-TTTCCGGCAAATC--GGC-AAATTACGG-A-
Rev.of_Cele-UNSB01_2:2547654-2548528 Satlength=875 Nr of Repeats=24
RepeatLength=35 seed=CCGGAAATTT  Num.seqs=16 Similarity=0.798413
51    AAAT--G-AAA--A-TTTCCGGCAAATC--GGC-AAATCGCCG-A-
Rev.of_Cele-UNSB01_5:978655-978943 Satlength=289 Nr of Repeats=7
RepeatLength=35 seed=TGCCGATTTG  Num.seqs=5 Similarity=0.809524
26    AAAT--G-AAC--A-TTTCCGGCAAATC--GGC-AAATTCTCG-A-
Rev.of_Cele-UNSB01_2:13579229-13580664 Satlength=1436 Nr of Repeats=41
RepeatLength=35 seed=GAATTTTTGA  Num.seqs=41 Similarity=0.852172
48    AAAT--C-AAA--A-ATTCCGGCAAATC--GGC-AAATTGCCG-A-
Rev.of_Cele-UNSB01_3:13035804-13036305 Satlength=502 Nr of Repeats=16
RepeatLength=35 seed=ATTTGCCGGA  Num.seqs=10 Similarity=0.867936
56    AAAT--C-AAA--A-TTTCCGGCAAATC--GGC-ATATTGCCG-G-
Rev.of_Cele-UNSB01_5:5106861-5107246 Satlength=386 Nr of Repeats=10
RepeatLength=35 seed=TGCCGGAAAT  Num.seqs=6 Similarity=0.730794
88    AAAT--C-AAA--A-TTTCCGGCAAATC--GGC-ATTTTGCCG-G-
Cele-UNSB01_4:4418485-4418703 Satlength=219 Nr of Repeats=5
RepeatLength=35 seed=TTCCGGCAAA  Num.seqs=3 Similarity=0.675676
45    AAAT--C-AGA--G-TTTCCGGCAAATC--GAC-AATTTGCCG-A-
Cele-UNSB01_2:13844810-13844989 Satlength=180 Nr of Repeats=5
RepeatLength=36 seed=TTGCCTAAAA  Num.seqs=4 Similarity=0.938272
29    AAAT--T-AAA--A-TTTCCGGCAAATC--AGCAAATTTGCCT-A-
Rev.of_Cele-UNSB01_4:1949645-1950620 Satlength=976 Nr of Repeats=28
RepeatLength=35 seed=CATTTTCGCC  Num.seqs=22 Similarity=0.945083
40    AAAT--G-GAA--A-TTTCCGGCAAACC--GGC-AATTCGGCG-A-
Cele-UNSB01_1:3506179-3506592 Satlength=414 Nr of Repeats=8
RepeatLength=35 seed=TCCGGCAAAT  Num.seqs=5 Similarity=0.939048
46    AAGT--C-AAA--A-ATTCCGGCAAATC--GGA-AATCTGCCG-G-
Rev.of_Cele-UNSB01_4:4858228-4858874 Satlength=647 Nr of Repeats=17
RepeatLength=35 seed=TTGCCGGAAA  Num.seqs=13 Similarity=0.909646
54    AAAC--C-AAA--A-TTTCCGGCAAATC--GGA-AACTTGCCG-G-
Cele-UNSB01_1:2141918-2142329 Satlength=412 Nr of Repeats=7
RepeatLength=35 seed=CAAACCGGCA  Num.seqs=5 Similarity=0.675926
15    AATT----AAC--A-TTTNGAGCAAACC--GGC-AANTTGCCG-AA

*      *
Consensus:
```

AAtTgAAAATTTCCGGCAAATCGGCAAaTTGCCGg

>Cele-UNSB01_Fam_3_15_146 Nr. of seq. 146 Alignment length(with gaps) = 15 Alignment score = 0.943142
Cele-UNSB01_1:224582-224687 Satlength=106 Nr of Repeats=5 RepeatLength=15 seed=AGACCCATCG Num.seqs=4 Similarity=0.911111
0   AGACCCATCGTGGTG
Cele-UNSB01_1:283314-284124 Satlength=811 Nr of Repeats=52 RepeatLength=15 seed=TCGTGGTGAG Num.seqs=48 Similarity=0.862569
7   AGACCCATCGTGGTG
Cele-UNSB01_1:698928-699108 Satlength=181 Nr of Repeats=12 RepeatLength=15 seed=GGTGAGACCC Num.seqs=12 Similarity=0.900337
11   AGACCCATCGTGGTG
Rev.of_Cele-UNSB01_1:4695896-4696451 Satlength=556 Nr of Repeats=34 RepeatLength=15 seed=GGGTCTCACC Num.seqs=31 Similarity=0.896583
6   AGACCCATCGTGGTG
Cele-UNSB01_1:8014213-8014468 Satlength=256 Nr of Repeats=17 RepeatLength=15 seed=ACCCATCGTG Num.seqs=17 Similarity=0.847712
2   AGACCCATCGTGGTG
Rev.of_Cele-UNSB01_1:9977112-9977772 Satlength=661 Nr of Repeats=41 RepeatLength=15 seed=CACGATGGGT Num.seqs=38 Similarity=0.852821
12   AGACCCATCGTGGTG
Cele-UNSB01_1:11351419-11351614 Satlength=196 Nr of Repeats=13 RepeatLength=15 seed=TCGTGGTGAG Num.seqs=13 Similarity=0.842735
7   AGACCCATCGTGGTG
Cele-UNSB01_1:11691383-11691503 Satlength=121 Nr of Repeats=8 RepeatLength=15 seed=TCGTGGTGAG Num.seqs=8 Similarity=0.930159
7   AGACCCATCGTGGTG
Rev.of_Cele-UNSB01_2:454595-455440 Satlength=846 Nr of Repeats=32 RepeatLength=15 seed=TCTCACCACG Num.seqs=30 Similarity=0.884138
3   AGACCCATCGTGGTG
Rev.of_Cele-UNSB01_2:636908-637328 Satlength=421 Nr of Repeats=27 RepeatLength=15 seed=GGGTCTCACC Num.seqs=26 Similarity=0.898256
6   AGACCCATCGTGGTG
Cele-UNSB01_2:698255-706115 Satlength=7861 Nr of Repeats=432 RepeatLength=15 seed=GGTGAGACCC Num.seqs=346 Similarity=0.920561
11   AGACCCATCGTGGTG
Cele-UNSB01_2:698255-706481 Satlength=8227 Nr of Repeats=433 RepeatLength=15 seed=GGTGAGACCC Num.seqs=346 Similarity=0.920561
11   AGACCCATCGTGGTG
Cele-UNSB01_2:1044642-1047492 Satlength=2851 Nr of Repeats=181 RepeatLength=15 seed=TCGTGGTGAG Num.seqs=173 Similarity=0.911565
7   AGACCCATCGTGGTG
Cele-UNSB01_2:3406743-3406803 Satlength=61 Nr of Repeats=4 RepeatLength=15 seed=GGTGAGACCC Num.seqs=4 Similarity=0.911111
11   AGACCCATCGTGGTG
Cele-UNSB01_2:4751413-4751956 Satlength=544 Nr of Repeats=21 RepeatLength=15 seed=TGGTGAGACC Num.seqs=13 Similarity=0.879202
10   AGACCCATCGTGGTG
Rev.of_Cele-UNSB01_2:8586336-8586411 Satlength=76 Nr of Repeats=4 RepeatLength=15 seed=GGGTCTCACC Num.seqs=3 Similarity=0.822222
6   AGACCCATCGTGGTG

Rev.of_Cele-UNSB01_2:9765369-9765639 Satlength=271 Nr of Repeats=17
RepeatLength=15 seed=CCACGATGGG Num.seqs=16 Similarity=0.891852
13   AGACCCATCGTGGTG
Cele-UNSB01_2:12792220-12794215 Satlength=1996 Nr of Repeats=131
RepeatLength=15 seed=TCGTGGTGAG Num.seqs=129 Similarity=0.876421
7   AGACCCATCGTGGTG
Cele-UNSB01_2:15064232-15064367 Satlength=136 Nr of Repeats=9
RepeatLength=15 seed=AGACCCATCG Num.seqs=9 Similarity=0.881481
0   AGACCCATCGTGGTG
Rev.of_Cele-UNSB01_2:15106134-15106254 Satlength=121 Nr of Repeats=7
RepeatLength=15 seed=CCACGATGGG Num.seqs=6 Similarity=0.881481
13   AGACCCATCGTGGTG
Cele-UNSB01_2:15221118-15221238 Satlength=121 Nr of Repeats=8
RepeatLength=15 seed=GGTGAGACCC Num.seqs=8 Similarity=0.926984
11   AGACCCATCGTGGTG
Cele-UNSB01_2:15224452-15224572 Satlength=121 Nr of Repeats=8
RepeatLength=15 seed=GGTGAGACCC Num.seqs=8 Similarity=0.926984
11   AGACCCATCGTGGTG
Cele-UNSB01_2:15229801-15229921 Satlength=121 Nr of Repeats=8
RepeatLength=15 seed=GGTGAGACCC Num.seqs=8 Similarity=0.926984
11   AGACCCATCGTGGTG
Rev.of_Cele-UNSB01_3:2621291-2621771 Satlength=481 Nr of Repeats=29
RepeatLength=15 seed=GGTCTCACCA Num.seqs=26 Similarity=0.920957
5   AGACCCATCGTGGTG
Cele-UNSB01_3:4030293-4031043 Satlength=751 Nr of Repeats=50
RepeatLength=15 seed=CGTGGTGAGA Num.seqs=50 Similarity=0.939483
8   AGACCCATCGTGGTG
Cele-UNSB01_3:8580701-8581151 Satlength=451 Nr of Repeats=29
RepeatLength=15 seed=ACCCATCGTG Num.seqs=28 Similarity=0.904762
2   AGACCCATCGTGGTG
Rev.of_Cele-UNSB01_3:13765752-13765977 Satlength=226 Nr of Repeats=15
RepeatLength=15 seed=CCACGATGGG Num.seqs=15 Similarity=0.930582
13   AGACCCATCGTGGTG
Cele-UNSB01_3:14063373-14064273 Satlength=901 Nr of Repeats=60
RepeatLength=15 seed=TCGTGGTGAG Num.seqs=60 Similarity=0.880778
7   AGACCCATCGTGGTG
Cele-UNSB01_4:287372-287852 Satlength=481 Nr of Repeats=32
RepeatLength=15 seed=TCGTGGTGAG Num.seqs=32 Similarity=0.882258
7   AGACCCATCGTGGTG
Rev.of_Cele-UNSB01_4:1161180-1161420 Satlength=241 Nr of Repeats=16
RepeatLength=15 seed=GGTCTCACCA Num.seqs=16 Similarity=0.880741
5   AGACCCATCGTGGTG
Cele-UNSB01_4:1454445-1454610 Satlength=166 Nr of Repeats=11
RepeatLength=15 seed=AGACCCATCG Num.seqs=11 Similarity=0.880404
0   AGACCCATCGTGGTG
Rev.of_Cele-UNSB01_4:2119001-2119076 Satlength=76 Nr of Repeats=5
RepeatLength=15 seed=GGGTCTCACC Num.seqs=5 Similarity=0.840000
6   AGACCCATCGTGGTG
Rev.of_Cele-UNSB01_4:2267683-2267908 Satlength=226 Nr of Repeats=15
RepeatLength=15 seed=CGATGGGTCT Num.seqs=15 Similarity=0.914497
10   AGACCCATCGTGGTG
Rev.of_Cele-UNSB01_4:3090317-3090467 Satlength=151 Nr of Repeats=8
RepeatLength=15 seed=CACGATGGGT Num.seqs=7 Similarity=0.843386
12   AGACCCATCGTGGTG

Cele-UNSB01_4:3733029-3733089 Satlength=61 Nr of Repeats=4
RepeatLength=15 seed=TCGTGGTGAG Num.seqs=4 Similarity=0.940741
7   AGACCCATCGTGGTG
Cele-UNSB01_4:4963981-4964546 Satlength=566 Nr of Repeats=18
RepeatLength=15 seed=GAGACCCATC Num.seqs=15 Similarity=0.903492
14   AGACCCATCGTGGTG
Rev.of_Cele-UNSB01_4:4987504-4988044 Satlength=541 Nr of Repeats=32
RepeatLength=15 seed=GGGTCTCACC Num.seqs=28 Similarity=0.940506
6   AGACCCATCGTGGTG
Cele-UNSB01_4:4988071-4988281 Satlength=211 Nr of Repeats=14
RepeatLength=15 seed=ACCCATCGTG Num.seqs=14 Similarity=0.916972
2   AGACCCATCGTGGTG
Rev.of_Cele-UNSB01_4:4987504-4989222 Satlength=1719 Nr of Repeats=80
RepeatLength=15 seed=GGGTCTCACC Num.seqs=69 Similarity=0.943090
6   AGACCCATCGTGGTG
Rev.of_Cele-UNSB01_4:4990696-4991348 Satlength=653 Nr of Repeats=40
RepeatLength=15 seed=GGGTCTCACC Num.seqs=33 Similarity=0.951515
6   AGACCCATCGTGGTG
Rev.of_Cele-UNSB01_4:5521070-5521205 Satlength=136 Nr of Repeats=9
RepeatLength=15 seed=CCACGATGGG Num.seqs=7 Similarity=0.898413
13   AGACCCATCGTGGTG
Rev.of_Cele-UNSB01_4:8107145-8107415 Satlength=271 Nr of Repeats=18
RepeatLength=15 seed=CTCACCACGA Num.seqs=18 Similarity=0.902977
2   AGACCCATCGTGGTG
Rev.of_Cele-UNSB01_4:8504495-8505035 Satlength=541 Nr of Repeats=35
RepeatLength=15 seed=TCTCACCACG Num.seqs=34 Similarity=0.956744
3   AGACCCATCGTGGTG
Rev.of_Cele-UNSB01_4:9125039-9126314 Satlength=1276 Nr of Repeats=76
RepeatLength=15 seed=CTCACCACGA Num.seqs=55 Similarity=0.889240
2   AGACCCATCGTGGTG
Cele-UNSB01_4:10323949-10324337 Satlength=389 Nr of Repeats=26
RepeatLength=15 seed=GACCCATCGT Num.seqs=25 Similarity=0.898074
1   AGACCCATCGTGGTG
Cele-UNSB01_4:13645449-13645792 Satlength=344 Nr of Repeats=22
RepeatLength=15 seed=ACCCATCGTG Num.seqs=19 Similarity=0.894997
2   AGACCCATCGTGGTG
Rev.of_Cele-UNSB01_4:14068578-14068998 Satlength=421 Nr of Repeats=24
RepeatLength=15 seed=ACGATGGGTC Num.seqs=21 Similarity=0.916191
11   AGACCCATCGTGGTG
Cele-UNSB01_4:14106973-14107062 Satlength=90 Nr of Repeats=6
RepeatLength=15 seed=ACCCATCGTG Num.seqs=5 Similarity=0.946667
2   AGACCCATCGTGGTG
Cele-UNSB01_4:14678232-14682258 Satlength=4027 Nr of Repeats=80
RepeatLength=15 seed=CGTGGTGAGA Num.seqs=64 Similarity=0.890917
8   AGACCCATCGTGGTG
Cele-UNSB01_4:15313710-15313830 Satlength=121 Nr of Repeats=8
RepeatLength=15 seed=TCGTGGTGAG Num.seqs=8 Similarity=0.930159
7   AGACCCATCGTGGTG
Cele-UNSB01_4:15421750-15421870 Satlength=121 Nr of Repeats=8
RepeatLength=15 seed=GGTGAGACCC Num.seqs=8 Similarity=0.926984
11   AGACCCATCGTGGTG
Cele-UNSB01_4:15554617-15554707 Satlength=91 Nr of Repeats=6
RepeatLength=15 seed=TCGTGGTGAG Num.seqs=6 Similarity=0.887407
7   AGACCCATCGTGGTG

Rev.of_Cele-UNSB01_4:15608407-15609476 Satlength=1070 Nr of Repeats=51
RepeatLength=15 seed=CCACGATGGG Num.seqs=44 Similarity=0.911299
13   AGACCCATCGTGGTG
Rev.of_Cele-UNSB01_4:16000962-16001082 Satlength=121 Nr of Repeats=8
RepeatLength=15 seed=GGGTCTCACC Num.seqs=8 Similarity=0.939683
6   AGACCCATCGTGGTG
Rev.of_Cele-UNSB01_4:16000985-16001240 Satlength=256 Nr of Repeats=17
RepeatLength=15 seed=CCACGATGGG Num.seqs=17 Similarity=0.875163
13   AGACCCATCGTGGTG
Cele-UNSB01_4:16061332-16061647 Satlength=316 Nr of Repeats=21
RepeatLength=15 seed=CCCATCGTGG Num.seqs=21 Similarity=0.916191
3   AGACCCATCGTGGTG
Rev.of_Cele-UNSB01_4:16220033-16221319 Satlength=1287 Nr of Repeats=15
RepeatLength=15 seed=CACGATGGGT Num.seqs=13 Similarity=0.863248
12   AGACCCATCGTGGTG
Rev.of_Cele-UNSB01_4:16709008-16709188 Satlength=181 Nr of Repeats=10
RepeatLength=15 seed=TGGGTCTCAC Num.seqs=8 Similarity=0.895238
7   AGACCCATCGTGGTG
Cele-UNSB01_4:17138861-17138981 Satlength=121 Nr of Repeats=8
RepeatLength=15 seed=TCGTGGTGAG Num.seqs=8 Similarity=0.866667
7   AGACCCATCGTGGTG
Cele-UNSB01_4:17168764-17168899 Satlength=136 Nr of Repeats=9
RepeatLength=15 seed=TCGTGGTGAG Num.seqs=9 Similarity=0.980247
7   AGACCCATCGTGGTG
Cele-UNSB01_4:17401416-17402316 Satlength=901 Nr of Repeats=60
RepeatLength=15 seed=ACCCATCGTG Num.seqs=60 Similarity=0.889768
2   AGACCCATCGTGGTG
Rev.of_Cele-UNSB01_4:17408961-17409411 Satlength=451 Nr of Repeats=28
RepeatLength=15 seed=TCTCACCACG Num.seqs=25 Similarity=0.847111
3   AGACCCATCGTGGTG
Rev.of_Cele-UNSB01_5:1441948-1442263 Satlength=316 Nr of Repeats=21
RepeatLength=15 seed=CGATGGGTCT Num.seqs=21 Similarity=0.927196
10   AGACCCATCGTGGTG
Rev.of_Cele-UNSB01_5:2625951-2626161 Satlength=211 Nr of Repeats=14
RepeatLength=15 seed=CTCACCACGA Num.seqs=14 Similarity=0.840316
2   AGACCCATCGTGGTG
Cele-UNSB01_5:3593686-3593761 Satlength=76 Nr of Repeats=5
RepeatLength=15 seed=CGTGGTGAGA Num.seqs=5 Similarity=0.840000
8   AGACCCATCGTGGTG
Rev.of_Cele-UNSB01_5:4989154-4989259 Satlength=106 Nr of Repeats=7
RepeatLength=15 seed=GGGTCTCACC Num.seqs=7 Similarity=0.898413
6   AGACCCATCGTGGTG
Cele-UNSB01_5:6073958-6074258 Satlength=301 Nr of Repeats=20
RepeatLength=15 seed=ACCCATCGTG Num.seqs=20 Similarity=0.901287
2   AGACCCATCGTGGTG
Rev.of_Cele-UNSB01_5:6314778-6314883 Satlength=106 Nr of Repeats=7
RepeatLength=15 seed=GGGTCTCACC Num.seqs=7 Similarity=0.881481
6   AGACCCATCGTGGTG
Cele-UNSB01_5:6605586-6605676 Satlength=91 Nr of Repeats=6
RepeatLength=15 seed=GGTGAGACCC Num.seqs=6 Similarity=0.917037
11   AGACCCATCGTGGTG
Cele-UNSB01_5:6963686-6964031 Satlength=346 Nr of Repeats=23
RepeatLength=15 seed=AGACCCATCG Num.seqs=23 Similarity=0.901625
0   AGACCCATCGTGGTG

Cele-UNSB01_5:6966476-6966776 Satlength=301 Nr of Repeats=20
RepeatLength=15 seed=AGACCCATCG Num.seqs=20 Similarity=0.894269
0   AGACCCATCGTGGTG
Rev.of_Cele-UNSB01_5:7958037-7958202 Satlength=166 Nr of Repeats=11
RepeatLength=15 seed=CCACGATGGG Num.seqs=11 Similarity=0.877172
13   AGACCCATCGTGGTG
Cele-UNSB01_5:7959905-7960085 Satlength=181 Nr of Repeats=11
RepeatLength=15 seed=ACCCATCGTG Num.seqs=10 Similarity=0.907161
2   AGACCCATCGTGGTG
Cele-UNSB01_5:8372367-8372880 Satlength=514 Nr of Repeats=17
RepeatLength=15 seed=GGTGAGACCC Num.seqs=16 Similarity=0.932593
11   AGACCCATCGTGGTG
Rev.of_Cele-UNSB01_5:9479685-9479850 Satlength=166 Nr of Repeats=11
RepeatLength=15 seed=CGATGGGTCT Num.seqs=11 Similarity=0.899798
10   AGACCCATCGTGGTG
Rev.of_Cele-UNSB01_5:15445975-15447055 Satlength=1081 Nr of Repeats=72
RepeatLength=15 seed=CCACGATGGG Num.seqs=70 Similarity=0.859308
13   AGACCCATCGTGGTG
Cele-UNSB01_5:15725489-15725564 Satlength=76 Nr of Repeats=5
RepeatLength=15 seed=AGACCCATCG Num.seqs=5 Similarity=0.928889
0   AGACCCATCGTGGTG
Rev.of_Cele-UNSB01_6:180452-180557 Satlength=106 Nr of Repeats=7
RepeatLength=15 seed=GTCTCACCAC Num.seqs=7 Similarity=0.932275
4   AGACCCATCGTGGTG
Rev.of_Cele-UNSB01_6:188088-188493 Satlength=406 Nr of Repeats=27
RepeatLength=15 seed=ACGATGGGTC Num.seqs=27 Similarity=0.913897
11   AGACCCATCGTGGTG
Rev.of_Cele-UNSB01_6:983566-988244 Satlength=4679 Nr of Repeats=290
RepeatLength=15 seed=CACGATGGGT Num.seqs=284 Similarity=0.839014
12   AGACCCATCGTGGTG
Rev.of_Cele-UNSB01_6:1421556-1421900 Satlength=345 Nr of Repeats=23
RepeatLength=15 seed=CGATGGGTCT Num.seqs=22 Similarity=0.845695
10   AGACCCATCGTGGTG
Rev.of_Cele-UNSB01_6:2088902-2090459 Satlength=1558 Nr of Repeats=79
RepeatLength=15 seed=CCACGATGGG Num.seqs=73 Similarity=0.860748
13   AGACCCATCGTGGTG
Rev.of_Cele-UNSB01_6:2088902-2090835 Satlength=1934 Nr of Repeats=85
RepeatLength=15 seed=CCACGATGGG Num.seqs=78 Similarity=0.862715
13   AGACCCATCGTGGTG
Rev.of_Cele-UNSB01_6:3190243-3190348 Satlength=106 Nr of Repeats=7
RepeatLength=15 seed=TCACCACGAT Num.seqs=7 Similarity=0.923810
1   AGACCCATCGTGGTG
Cele-UNSB01_6:5454734-5454854 Satlength=121 Nr of Repeats=8
RepeatLength=15 seed=TGGTGAGACC Num.seqs=8 Similarity=0.860317
10   AGACCCATCGTGGTG
Rev.of_Cele-UNSB01_6:14122652-14123413 Satlength=762 Nr of Repeats=19
RepeatLength=15 seed=GGGTCTCACC Num.seqs=15 Similarity=0.911958
6   AGACCCATCGTGGTG
Rev.of_Cele-UNSB01_6:16005217-16005457 Satlength=241 Nr of Repeats=15
RepeatLength=15 seed=GGGTCTCACC Num.seqs=14 Similarity=0.906227
6   AGACCCATCGTGGTG
Rev.of_Cele-UNSB01_6:17225477-17225567 Satlength=91 Nr of Repeats=6
RepeatLength=15 seed=GGGTCTCACC Num.seqs=6 Similarity=0.922963
6   AGACCCATCGTGGTG

Rev.of_Cele-UNSB01_1:3435421-3440265 Satlength=4845 Nr of Repeats=323
RepeatLength=15 seed=CTCACCACGA Num.seqs=294 Similarity=0.881418
2   AGACCCNTCGTGGTG
Rev.of_Cele-UNSB01_2:15381973-15383739 Satlength=1767 Nr of Repeats=38
RepeatLength=15 seed=GGGTCTCACC Num.seqs=35 Similarity=0.915593
6   AGACCCNTCGTGGTG
Cele-UNSB01_5:16414904-16415369 Satlength=466 Nr of Repeats=31
RepeatLength=15 seed=TGGTGAGACC Num.seqs=31 Similarity=0.865806
10   AGACCCNTCGTGGTG
Rev.of_Cele-UNSB01_6:12847168-12848031 Satlength=864 Nr of Repeats=10
RepeatLength=15 seed=GGGTCTCACC Num.seqs=9 Similarity=0.920988
6   AGACCCNTCGTGGTG
Cele-UNSB01_1:6785229-6785394 Satlength=166 Nr of Repeats=11
RepeatLength=15 seed=GAGACCCATC Num.seqs=11 Similarity=0.883636
14   AGACCCATCGTGGCG
Rev.of_Cele-UNSB01_2:865003-865078 Satlength=76 Nr of Repeats=5
RepeatLength=15 seed=ATGGGTCTCG Num.seqs=3 Similarity=1.000000
23   AGACCCATCGTGGCG
Cele-UNSB01_2:2017071-2017146 Satlength=76 Nr of Repeats=5
RepeatLength=15 seed=TCGTGGCGAG Num.seqs=5 Similarity=0.928889
22   AGACCCATCGTGGCG
Cele-UNSB01_2:2403604-2403679 Satlength=76 Nr of Repeats=5
RepeatLength=15 seed=TCGTGGCGAG Num.seqs=5 Similarity=0.928889
22   AGACCCATCGTGGCG
Cele-UNSB01_2:4751417-4751507 Satlength=91 Nr of Repeats=6
RepeatLength=15 seed=GAGACCCATC Num.seqs=6 Similarity=0.899259
14   AGACCCATCGTGGCG
Cele-UNSB01_2:15219171-15219261 Satlength=91 Nr of Repeats=6
RepeatLength=15 seed=TCGTGGCGAG Num.seqs=6 Similarity=0.940741
22   AGACCCATCGTGGCG
Cele-UNSB01_2:15242777-15242951 Satlength=175 Nr of Repeats=9
RepeatLength=15 seed=AGACCCATCG Num.seqs=8 Similarity=0.895238
15   AGACCCATCGTGGCG
Cele-UNSB01_4:14030460-14031210 Satlength=751 Nr of Repeats=45
RepeatLength=15 seed=CCCATCGTGG Num.seqs=40 Similarity=0.901766
18   AGACCCATCGTGGCG
Cele-UNSB01_4:15399620-15399800 Satlength=181 Nr of Repeats=11
RepeatLength=15 seed=GAGACCCATC Num.seqs=10 Similarity=0.917037
14   AGACCCATCGTGGCG
Cele-UNSB01_5:6969386-6969716 Satlength=331 Nr of Repeats=22
RepeatLength=15 seed=AGACCCATCG Num.seqs=22 Similarity=0.899182
15   AGACCCATCGTGGCG
Rev.of_Cele-UNSB01_6:9867922-9867997 Satlength=76 Nr of Repeats=5
RepeatLength=15 seed=ATGGGTCTCG Num.seqs=3 Similarity=1.000000
23   AGACCCATCGTGGCG
Cele-UNSB01_6:14688507-14688582 Satlength=76 Nr of Repeats=5
RepeatLength=15 seed=TCGTGGCGAG Num.seqs=5 Similarity=0.928889
22   AGACCCATCGTGGCG
Cele-UNSB01_6:17686729-17686834 Satlength=106 Nr of Repeats=7
RepeatLength=15 seed=GAGACCCATC Num.seqs=7 Similarity=0.847619
14   AGACCCATCGTGGCG
Rev.of_Cele-UNSB01_4:1359612-1359777 Satlength=166 Nr of Repeats=11
RepeatLength=15 seed=CCACGATGGG Num.seqs=11 Similarity=0.880404
28   AGACCCATCGTGGNG

```
Rev.of_Cele-UNSB01_4:9469814-9470654 Satlength=841 Nr of Repeats=56
RepeatLength=15 seed=ACGATGGGTC  Num.seqs=56 Similarity=0.902684
26    AGACCCATCGTGGNG
Rev.of_Cele-UNSB01_4:13647491-13647701 Satlength=211 Nr of Repeats=14
RepeatLength=15 seed=CCACGATGGG  Num.seqs=14 Similarity=0.902320
28    AGACCCATCGTGGNG
Cele-UNSB01_4:14678240-14678405 Satlength=166 Nr of Repeats=11
RepeatLength=15 seed=GACCCATCGT  Num.seqs=11 Similarity=0.878788
16    AGACCCATCGTGGNG
Rev.of_Cele-UNSB01_6:556288-556934 Satlength=647 Nr of Repeats=15
RepeatLength=15 seed=CCACGATGGG  Num.seqs=14 Similarity=0.873016
28    AGACCCATCGTGGNG
Rev.of_Cele-UNSB01_6:5456777-5457242 Satlength=466 Nr of Repeats=31
RepeatLength=15 seed=CCACGATGGG  Num.seqs=27 Similarity=0.802469
28    AGACCCATCGTGGNG
Rev.of_Cele-UNSB01_1:14918515-14918575 Satlength=61 Nr of Repeats=4
RepeatLength=15 seed=CCACGATGGG  Num.seqs=4 Similarity=0.881481
28    AGACCCATCGTGGAG
Cele-UNSB01_1:4033028-4033133 Satlength=106 Nr of Repeats=7
RepeatLength=15 seed=TCGTGGTGAG  Num.seqs=7 Similarity=0.949206
7    AGACCCTTCGTGGTG
Cele-UNSB01_1:5266039-5266863 Satlength=825 Nr of Repeats=51
RepeatLength=15 seed=CGTGGTGAGA  Num.seqs=47 Similarity=0.827485
8    AGACCCTTCGTGGTG
Cele-UNSB01_1:7886332-7886407 Satlength=76 Nr of Repeats=5
RepeatLength=15 seed=TCGTGGTGAG  Num.seqs=5 Similarity=0.911111
7    AGACCCTTCGTGGTG
Rev.of_Cele-UNSB01_2:4325748-4326453 Satlength=706 Nr of Repeats=45
RepeatLength=15 seed=CTCACCACGA  Num.seqs=40 Similarity=0.798251
17    AGACCCTTCGTGGTG
Cele-UNSB01_2:9328143-9328548 Satlength=406 Nr of Repeats=27
RepeatLength=15 seed=TGGTGAGACC  Num.seqs=25 Similarity=0.902519
10    AGACCCTTCGTGGTG
Cele-UNSB01_2:15221118-15221591 Satlength=474 Nr of Repeats=14
RepeatLength=15 seed=GGTGAGACCC  Num.seqs=12 Similarity=0.916498
11    AGACCCTTCGTGGTG
Cele-UNSB01_2:15224452-15225368 Satlength=917 Nr of Repeats=26
RepeatLength=15 seed=GGTGAGACCC  Num.seqs=22 Similarity=0.913805
11    AGACCCTTCGTGGTG
Cele-UNSB01_2:15229801-15230289 Satlength=489 Nr of Repeats=15
RepeatLength=15 seed=GGTGAGACCC  Num.seqs=13 Similarity=0.913390
11    AGACCCTTCGTGGTG
Cele-UNSB01_2:15235758-15235848 Satlength=91 Nr of Repeats=6
RepeatLength=15 seed=GGTGAGACCC  Num.seqs=6 Similarity=0.905185
11    AGACCCTTCGTGGTG
Cele-UNSB01_3:1400275-1400335 Satlength=61 Nr of Repeats=4
RepeatLength=15 seed=TCGTGGTGAG  Num.seqs=4 Similarity=0.955556
7    AGACCCTTCGTGGTG
Cele-UNSB01_3:3579672-3580411 Satlength=740 Nr of Repeats=47
RepeatLength=15 seed=GTGGTGAGAC  Num.seqs=44 Similarity=0.837350
9    AGACCCTTCGTGGTG
Cele-UNSB01_3:14059760-14060650 Satlength=891 Nr of Repeats=57
RepeatLength=15 seed=TCGTGGTGAG  Num.seqs=54 Similarity=0.868872
7    AGACCCTTCGTGGTG
```

Rev.of_Cele-UNSB01_4:1492099-1492204 Satlength=106 Nr of Repeats=7
RepeatLength=15 seed=CTCACCACGA Num.seqs=7 Similarity=0.873016
17   AGACCCTTCGTGGTG
Rev.of_Cele-UNSB01_4:14417295-14419575 Satlength=2281 Nr of Repeats=148
RepeatLength=15 seed=CTCACCACGA Num.seqs=134 Similarity=0.914962
17   AGACCCTTCGTGGTG
Rev.of_Cele-UNSB01_4:15284214-15284904 Satlength=691 Nr of Repeats=39
RepeatLength=15 seed=GGTCTCACCA Num.seqs=32 Similarity=0.917921
20   AGACCCTTCGTGGTG
Rev.of_Cele-UNSB01_4:15313385-15313490 Satlength=106 Nr of Repeats=7
RepeatLength=15 seed=GGGTCTCACC Num.seqs=7 Similarity=0.949206
21   AGACCCTTCGTGGTG
Rev.of_Cele-UNSB01_4:16376935-16378753 Satlength=1819 Nr of Repeats=17
RepeatLength=15 seed=GGGTCTCACC Num.seqs=11 Similarity=0.935354
21   AGACCCTTCGTGGTG
Cele-UNSB01_4:16402456-16402561 Satlength=106 Nr of Repeats=7
RepeatLength=15 seed=GTGGTGAGAC Num.seqs=7 Similarity=0.898413
9   AGACCCTTCGTGGTG
Rev.of_Cele-UNSB01_4:16472875-16473271 Satlength=397 Nr of Repeats=25
RepeatLength=15 seed=TCTCACCACG Num.seqs=21 Similarity=0.820106
18   AGACCCTTCGTGGTG
Cele-UNSB01_5:1289399-1289474 Satlength=76 Nr of Repeats=5
RepeatLength=15 seed=TCGTGGTGAG Num.seqs=5 Similarity=0.911111
7   AGACCCTTCGTGGTG
Cele-UNSB01_5:1289402-1291686 Satlength=2285 Nr of Repeats=44
RepeatLength=15 seed=TGGTGAGACC Num.seqs=41 Similarity=0.901138
10   AGACCCTTCGTGGTG
Cele-UNSB01_5:4984650-4985356 Satlength=707 Nr of Repeats=46
RepeatLength=15 seed=TCGTGGTGAG Num.seqs=38 Similarity=0.852948
7   AGACCCTTCGTGGTG
Cele-UNSB01_5:5638035-5638095 Satlength=61 Nr of Repeats=4
RepeatLength=15 seed=CGTGGTGAGA Num.seqs=4 Similarity=0.911111
8   AGACCCTTCGTGGTG
Rev.of_Cele-UNSB01_5:7764570-7764720 Satlength=151 Nr of Repeats=10
RepeatLength=15 seed=GGGTCTCACC Num.seqs=10 Similarity=0.934815
21   AGACCCTTCGTGGTG
Cele-UNSB01_5:9642570-9642900 Satlength=331 Nr of Repeats=22
RepeatLength=15 seed=GGTGAGACCC Num.seqs=22 Similarity=0.929582
11   AGACCCTTCGTGGTG
Cele-UNSB01_5:13979410-13979500 Satlength=91 Nr of Repeats=6
RepeatLength=15 seed=TCGTGGTGAG Num.seqs=6 Similarity=0.857778
7   AGACCCTTCGTGGTG
Cele-UNSB01_5:14226001-14226091 Satlength=91 Nr of Repeats=6
RepeatLength=15 seed=CGTGGTGAGA Num.seqs=6 Similarity=0.893333
8   AGACCCTTCGTGGTG
Rev.of_Cele-UNSB01_5:14260478-14260568 Satlength=91 Nr of Repeats=6
RepeatLength=15 seed=GGGTCTCACC Num.seqs=6 Similarity=0.917037
21   AGACCCTTCGTGGTG
Rev.of_Cele-UNSB01_5:15415879-15416665 Satlength=787 Nr of Repeats=8
RepeatLength=15 seed=TCTCACCACG Num.seqs=7 Similarity=0.923810
18   AGACCCTTCGTGGTG
Cele-UNSB01_6:13887397-13887577 Satlength=181 Nr of Repeats=11
RepeatLength=15 seed=TCGTGGTGAG Num.seqs=10 Similarity=0.857778
7   AGACCCTTCGTGGTG

Rev.of_Cele-UNSB01_6:15851847-15853377 Satlength=1531 Nr of Repeats=97 RepeatLength=15 seed=GTCTCACCAC Num.seqs=92 Similarity=0.888135
19   AGACCCTTCGTGGTG
Rev.of_Cele-UNSB01_6:16876782-16877097 Satlength=316 Nr of Repeats=18 RepeatLength=15 seed=TCACCACGAA Num.seqs=15 Similarity=0.947513
16   AGACCCTTCGTGGTG
Cele-UNSB01_6:14447847-14448957 Satlength=1111 Nr of Repeats=73 RepeatLength=15 seed=TCGTGGTGAG Num.seqs=70 Similarity=0.770484
7   AGACCTATCGTGGTG
Cele-UNSB01_1:309874-309994 Satlength=121 Nr of Repeats=7 RepeatLength=15 seed=GAGACCCATC Num.seqs=6 Similarity=0.764583
14   AGACCCATCGTGACG

***** ***** *
Consensus:

AGACCCaTCGTGGTG

>Cele-UNSB01_Fam_4_35_122 Nr. of seq. 122 Alignment length(with gaps) = 43 Alignment score = 0.628454
Cele-UNSB01_1:1158888-1159076 Satlength=189 Nr of Repeats=5 RepeatLength=31 seed=ATTCCCGCCA Num.seqs=3 Similarity=0.628472
0   ATT--CCCGCC--AATT------TTTT-CTCAGGAAATTTGA-
Rev.of_Cele-UNSB01_3:1400394-1400521 Satlength=128 Nr of Repeats=4 RepeatLength=31 seed=AATTCAAATT Num.seqs=3 Similarity=0.885305
3   ATT--CCCGCC--AAAA------TTTT-TTGAAGAAATTTGA-
Cele-UNSB01_4:6734929-6735237 Satlength=309 Nr of Repeats=9 RepeatLength=31 seed=CCCGCCAAAA Num.seqs=7 Similarity=0.897593
3   ATT--CCCGCC--AAAA------TTTT-TTGAAAAAATTTGA-
Rev.of_Cele-UNSB01_4:13661650-13661868 Satlength=219 Nr of Repeats=6 RepeatLength=31 seed=AATTCAAATT Num.seqs=4 Similarity=0.942652
3   ATT--CCCGCC--AAAA------TTTT-TTGAGGAAATTTGA-
Rev.of_Cele-UNSB01_5:19338537-19338764 Satlength=228 Nr of Repeats=7 RepeatLength=33 seed=GGAATTCAAA Num.seqs=5 Similarity=0.967677
6   AT-T-CCCGCC--AAAATT----TTTT-TTGAATAAATTTGA-
Rev.of_Cele-UNSB01_3:12445241-12445717 Satlength=477 Nr of Repeats=10 RepeatLength=32 seed=ATTCAAATTT Num.seqs=6 Similarity=0.602694
34   ATT--TTCGCC--AAAA-N----TTTT-CTGAAGAAATTTGA-
Cele-UNSB01_4:17523613-17523740 Satlength=128 Nr of Repeats=4 RepeatLength=32 seed=GAAGAAATTT Num.seqs=3 Similarity=0.861111
52   ATT--TGCACC--AAAA-T----TTTT-CTGAAGAAATTTGA-
Rev.of_Cele-UNSB01_1:671513-680928 Satlength=9416 Nr of Repeats=294 RepeatLength=32 seed=GAATTCAAAT Num.seqs=192 Similarity=0.889807
4   ATT-C-CCGCC--AAAA-T----TTTT-CTCAGAAAATTTGA-
Rev.of_Cele-UNSB01_1:1386048-1386208 Satlength=161 Nr of Repeats=5 RepeatLength=32 seed=AAAATTTTGG Num.seqs=5 Similarity=0.983333
17   ATT-C-CCGCC--AAAA-T----TTTT-CTCAGAAAATTTGA-
Cele-UNSB01_4:15943206-15943656 Satlength=451 Nr of Repeats=14 RepeatLength=32 seed=TTGAATTCCC Num.seqs=13 Similarity=0.905983
28   ATT-C-CCGCC--AAAA-T----TTTT-CACAGAAAATTTGA-
Cele-UNSB01_5:4025406-4025890 Satlength=485 Nr of Repeats=15 RepeatLength=32 seed=TTTCACAGAA Num.seqs=11 Similarity=0.921212
47   ATT-C-CCGCC--AAAA-T----TTTT-CACAGAAAATTTGA-

```
Cele-UNSB01_6:15068529-15068945 Satlength=417 Nr of Repeats=13
RepeatLength=32 seed=TTGAATTCCC  Num.seqs=13 Similarity=0.927885
28    ATT-C-CCGCC--AAAA-T----TTTT-CACAGAAAATTTGA-
Cele-UNSB01_5:20304611-20305126 Satlength=516 Nr of Repeats=14
RepeatLength=32 seed=AAATTTGAAT  Num.seqs=9 Similarity=0.806958
24    ATT-T-CCGCC--AAAA-T----TTTT-CTCAGAAAATTTGA-
Cele-UNSB01_1:13840435-13852243 Satlength=11809 Nr of Repeats=369
RepeatLength=32 seed=TGAATTCCCG  Num.seqs=365 Similarity=0.924622
29    ATT-C-CCGCC--AAAA-T----TTTT-CACAGAAATTTTGA-
Rev.of_Cele-UNSB01_2:4623470-4624110 Satlength=641 Nr of Repeats=20
RepeatLength=32 seed=CGGGAATTCA  Num.seqs=20 Similarity=0.921491
39    ATT-C-CCGCC--AAAA-T----TTTT-CACAGAAATTTTGA-
Rev.of_Cele-UNSB01_3:11461401-11461721 Satlength=321 Nr of Repeats=10
RepeatLength=32 seed=TTTCTGTGAA  Num.seqs=10 Similarity=0.896296
58    ATT-C-CCGCC--AAAA-T----TTTT-CACAGAAATTTTGA-
Cele-UNSB01_4:8500583-8501255 Satlength=673 Nr of Repeats=21
RepeatLength=32 seed=TTTTCACAGA  Num.seqs=21 Similarity=0.903571
46    ATT-C-CCGCC--AAAA-T----TTTT-CACAGAAATTTTGA-
Rev.of_Cele-UNSB01_4:12150586-12159522 Satlength=8937 Nr of Repeats=274
RepeatLength=32 seed=CGGGAATTCA  Num.seqs=262 Similarity=0.923743
39    ATT-C-CCGCC--AAAA-T----TTTT-CACAGAAATTTTGA-
Rev.of_Cele-UNSB01_5:3493344-3494338 Satlength=995 Nr of Repeats=31
RepeatLength=32 seed=CGGGAATTCA  Num.seqs=29 Similarity=0.920874
39    ATT-C-CCGCC--AAAA-T----TTTT-CACAGAAATTTTGA-
Rev.of_Cele-UNSB01_6:9244298-9244938 Satlength=641 Nr of Repeats=20
RepeatLength=32 seed=CGGGAATTCA  Num.seqs=20 Similarity=0.894298
39    ATT-C-CCGCC--AAAA-T----TTTT-CACAGAAATTTTGA-
Cele-UNSB01_6:15068534-15069086 Satlength=553 Nr of Repeats=16
RepeatLength=32 seed=TTCCCGCCAA  Num.seqs=11 Similarity=0.947727
33    ATT-C-CCGCC--AAAA-T----TTTT-CACAGAAATTTTGA-
Rev.of_Cele-UNSB01_5:7024350-7024712 Satlength=363 Nr of Repeats=11
RepeatLength=32 seed=AATTCAAATT  Num.seqs=7 Similarity=0.908730
35    ATT-C-CCGCC--AAAA-T----TTTT-CACAAAAAATTTGA-
Cele-UNSB01_2:863874-864986 Satlength=1113 Nr of Repeats=34
RepeatLength=32 seed=AATTCCCGCC  Num.seqs=26 Similarity=0.854615
31    ATT-C-CCGCC--AAAA-A----TTTT-CAAAGAAAATTTGA-
Rev.of_Cele-UNSB01_2:2017169-2017519 Satlength=351 Nr of Repeats=10
RepeatLength=32 seed=GGCGGGAATT  Num.seqs=7 Similarity=0.843675
41    ATT-C-CCGCC--AAAA-A----TTTT-CAAAGAAAATTTGA-
Cele-UNSB01_6:9866116-9867908 Satlength=1793 Nr of Repeats=48
RepeatLength=32 seed=TCCCGCCAAA  Num.seqs=33 Similarity=0.845249
34    ATT-C-CCGCC--AAAA-A----TTTT-CAAAGAAAATTTGA-
Cele-UNSB01_2:1991206-1991967 Satlength=762 Nr of Repeats=23
RepeatLength=32 seed=AATTCCCGCC  Num.seqs=16 Similarity=0.855208
31    ATT-C-CCGCC--AAAA-A----TTTT-CANAGAAAATTTGA-
Cele-UNSB01_2:2000093-2000918 Satlength=826 Nr of Repeats=25
RepeatLength=32 seed=AATTCCCGCC  Num.seqs=18 Similarity=0.865196
31    ATT-C-CCGCC--AAAA-A----TTTT-CANAGAAAATTTGA-
Rev.of_Cele-UNSB01_2:15219280-15219502 Satlength=223 Nr of Repeats=7
RepeatLength=32 seed=TTTTGGCGGG  Num.seqs=5 Similarity=0.841667
45    ATT-C-CCGCC--AAAA-A----TTTT-CANAGAAAATTTGA-
Rev.of_Cele-UNSB01_2:15221267-15222837 Satlength=1571 Nr of Repeats=42
RepeatLength=32 seed=TTTTGGCGGG  Num.seqs=27 Similarity=0.855679
45    ATT-C-CCGCC--AAAA-A----TTTT-CANAGAAAATTTGA-
```

Rev.of_Cele-UNSB01_2:15224601-15228185 Satlength=3585 Nr of Repeats=96 RepeatLength=32 seed=TTTTGGCGGG Num.seqs=67 Similarity=0.850591
45   ATT-C-CCGCC--AAAA-A----TTTT-CANAGAAAATTTGA-
Rev.of_Cele-UNSB01_2:15229950-15234144 Satlength=4195 Nr of Repeats=125 RepeatLength=32 seed=TTTTGGCGGG Num.seqs=93 Similarity=0.859221
45   ATT-C-CCGCC--AAAA-A----TTTT-CANAGAAAATTTGA-
Rev.of_Cele-UNSB01_6:14688602-14689240 Satlength=639 Nr of Repeats=20 RepeatLength=32 seed=TTTGGCGGGA Num.seqs=15 Similarity=0.817027
44   ATT-C-CCGCC--AAAA-A----TTTT-CANAGAAAATTTGA-
Cele-UNSB01_2:6532865-6533025 Satlength=161 Nr of Repeats=5 RepeatLength=32 seed=AAAAATTTTC Num.seqs=5 Similarity=0.916667
41   ATT-C-CCGCC--AAAA-A----TTTT-CACAGAAAATTTGA-
Cele-UNSB01_2:6532865-6533618 Satlength=754 Nr of Repeats=6 RepeatLength=32 seed=AAAAATTTTC Num.seqs=5 Similarity=0.916667
41   ATT-C-CCGCC--AAAA-A----TTTT-CACAGAAAATTTGA-
Cele-UNSB01_2:2625510-2625733 Satlength=224 Nr of Repeats=7 RepeatLength=32 seed=AATTCCCGCC Num.seqs=6 Similarity=0.855556
31   ATT-C-CCGCC--AAAA-A----ATTT-CAAAGAAAATTTAA-
Cele-UNSB01_2:2625547-2626102 Satlength=556 Nr of Repeats=7 RepeatLength=32 seed=CCGCCAAAAA Num.seqs=5 Similarity=0.866667
36   ATT-C-CCGCC--AAAA-A----ATTT-CAAAGAAAATTTAA-
Cele-UNSB01_1:927465-927693 Satlength=229 Nr of Repeats=6 RepeatLength=32 seed=GAAAATTTGA Num.seqs=4 Similarity=0.727941
22   ATT-C-CCGCC--AAAA-T----TTTT-CTTTGAAAATTTGA-
Cele-UNSB01_5:20786599-20786882 Satlength=284 Nr of Repeats=9 RepeatLength=32 seed=CCGCCAAAAT Num.seqs=7 Similarity=0.853175
36   ATT-C-CCGCC--AAAA-T----TTTT-CTTTGAAAATTTGA-
Cele-UNSB01_1:1012680-1013098 Satlength=419 Nr of Repeats=4 RepeatLength=32 seed=CAAAATTTTT Num.seqs=3 Similarity=0.888889
9   A-T-CGCCGCC--AAAA-T----TTTT-CTCAGAAAATTTGA-
Rev.of_Cele-UNSB01_5:18728400-18728559 Satlength=160 Nr of Repeats=4 RepeatLength=32 seed=GAATTCAAAT Num.seqs=3 Similarity=0.888889
4   ATT-C-ACGCC--AAAA-A----TTTT-TTCAGAAAATTTGA-
Cele-UNSB01_2:14078695-14078860 Satlength=166 Nr of Repeats=5 RepeatLength=33 seed=AAAATTTGAA Num.seqs=5 Similarity=0.749495
24   ATT-TCCCGCC--AAAA-T---T-TTT-CTCAGAAAATTTGA-
Rev.of_Cele-UNSB01_5:1406535-1406737 Satlength=203 Nr of Repeats=6 RepeatLength=34 seed=AAATTCAAAT Num.seqs=5 Similarity=0.823529
37   ATT-TCCCGCC--AAAA-T---TATTT-CTCAGAAAATTTGA-
Cele-UNSB01_1:768290-768741 Satlength=452 Nr of Repeats=13 RepeatLength=34 seed=TTTCTCAGAA Num.seqs=8 Similarity=0.787245
50   ATT-TCCCACC--AAAA-T---TTTTT-CTCAGAAAATTTGA-
Cele-UNSB01_5:2665615-2665738 Satlength=124 Nr of Repeats=4 RepeatLength=35 seed=TTTCTCAGAA Num.seqs=3 Similarity=0.923810
51   ATT-TCCCGCC--AAAA-TG--TTTTT-CTCAGAAAATTTGA-
Cele-UNSB01_3:12784240-12784411 Satlength=172 Nr of Repeats=5 RepeatLength=35 seed=AATTTGAATT Num.seqs=3 Similarity=0.923810
26   ATT-TCCCGCC--AAAA-T-CAT-TTT-CTGAGAAAATTTGA-
Cele-UNSB01_2:1815608-1815814 Satlength=207 Nr of Repeats=6 RepeatLength=34 seed=ATTTGAATTT Num.seqs=4 Similarity=0.980392
27   ATT-TCCCGCC--AAAA----TTTTTT-CACTGAAAATTTGA-
Rev.of_Cele-UNSB01_2:1818966-1819342 Satlength=377 Nr of Repeats=11 RepeatLength=34 seed=CGGGAAATTC Num.seqs=9 Similarity=0.956427
41   ATT-TCCCGCC--AAAA----TTTTTT-CACTGAAAATTTGA-

Rev.of_Cele-UNSB01_2:15235864-15236243 Satlength=380 Nr of Repeats=11
RepeatLength=34 seed=TTGGCGGGAA Num.seqs=7 Similarity=0.880511
45   ATT-TCCCGCC--AAAA----TTTTTT-CACTGAAAATTTGA-
Cele-UNSB01_4:1358775-1359527 Satlength=753 Nr of Repeats=22
RepeatLength=34 seed=ATTTCCCGCC Num.seqs=18 Similarity=0.995643
33   ATT-TCCCGCC--AAAA----TTTTTT-CACTGAAAATTTGA-
Rev.of_Cele-UNSB01_4:15421887-15425906 Satlength=4020 Nr of Repeats=116
RepeatLength=34 seed=TGGCGGGAAA Num.seqs=83 Similarity=0.986125
44   ATT-TCCCGCC--AAAA----TTTTTT-CACTGAAAATTTGA-
Rev.of_Cele-UNSB01_5:10311486-10312622 Satlength=1137 Nr of Repeats=33
RepeatLength=34 seed=TTGGCGGGAA Num.seqs=20 Similarity=0.915765
45   ATT-TCCCGCC--AAAA----TTTTTT-CACTGAAAATTTGA-
Cele-UNSB01_1:1456315-1457241 Satlength=927 Nr of Repeats=24
RepeatLength=35 seed=AAATTTGAAT Num.seqs=15 Similarity=0.827628
60   ATT-TCCCGCC--AAAA-T--TTTTTT-CACTGAAAATTTGA-
Rev.of_Cele-UNSB01_1:7886430-7887346 Satlength=917 Nr of Repeats=26
RepeatLength=35 seed=GCGGGAAATT Num.seqs=17 Similarity=0.870370
77   ATT-TCCCGCC--AAAA-T--TTTTTT-CACTGAAAATTTGA-
Rev.of_Cele-UNSB01_1:11691539-11692754 Satlength=1216 Nr of Repeats=24
RepeatLength=35 seed=TGGCGGGAAA Num.seqs=17 Similarity=0.860771
79   ATT-TCCCGCC--AAAA-T--TTTTTT-CACTGAAAATTTGA-
Rev.of_Cele-UNSB01_2:9328575-9329101 Satlength=527 Nr of Repeats=15
RepeatLength=35 seed=ATTCAAATTT Num.seqs=10 Similarity=0.900952
70   ATT-TCCCGCC--AAAA-T--TTTTTT-CACTGAAAATTTGA-
Rev.of_Cele-UNSB01_2:9328566-9329542 Satlength=977 Nr of Repeats=16
RepeatLength=35 seed=TGGCGGGAAA Num.seqs=10 Similarity=0.909418
79   ATT-TCCCGCC--AAAA-T--TTTTTT-CACTGAAAATTTGA-
Rev.of_Cele-UNSB01_4:1490085-1491772 Satlength=1688 Nr of Repeats=46
RepeatLength=35 seed=AAAAAATTTT Num.seqs=30 Similarity=0.963442
88   ATT-TCCCGCC--AAAA-T--TTTTTT-CACTGAAAATTTGA-
Cele-UNSB01_4:1491894-1492067 Satlength=174 Nr of Repeats=5
RepeatLength=35 seed=GAAAATTTGA Num.seqs=3 Similarity=0.949206
93   ATT-TCCCGCC--AAAA-T--TTTTTT-CACTGAAAATTTGA-
Rev.of_Cele-UNSB01_4:10324370-10326780 Satlength=2411 Nr of Repeats=67
RepeatLength=35 seed=GAAATTCAAA Num.seqs=43 Similarity=0.847276
73   ATT-TCCCGCC--AAAA-T--TTTTTT-CACTGAAAATTTGA-
Cele-UNSB01_4:12289740-12290350 Satlength=611 Nr of Repeats=8
RepeatLength=35 seed=AAATTTGAAT Num.seqs=5 Similarity=0.847619
60   ATT-TCCCGCC--AAAA-T--TTTTTT-CACTGAAAATTTGA-
Rev.of_Cele-UNSB01_4:15554734-15555678 Satlength=945 Nr of Repeats=27
RepeatLength=35 seed=GAAATTCAAA Num.seqs=18 Similarity=0.936508
73   ATT-TCCCGCC--AAAA-T--TTTTTT-CACTGAAAATTTGA-
Cele-UNSB01_4:16219937-16221099 Satlength=1163 Nr of Repeats=29
RepeatLength=35 seed=TTCCCGCCAA Num.seqs=20 Similarity=0.896341
70   ATT-TCCCGCC--AAAA-T--TTTTTT-CACTGAAAATTTGA-
Rev.of_Cele-UNSB01_5:1289520-1292435 Satlength=2916 Nr of Repeats=32
RepeatLength=35 seed=GCGGGAAATT Num.seqs=20 Similarity=0.939850
77   ATT-TCCCGCC--AAAA-T--TTTTTT-CACTGAAAATTTGA-
Cele-UNSB01_5:6307822-6308103 Satlength=282 Nr of Repeats=8
RepeatLength=35 seed=AATTTCCCGC Num.seqs=7 Similarity=0.909297
67   ATT-TCCCGCC--AAAA-T--TTTTTT-CACTGAAAATTTGA-
Cele-UNSB01_5:6309373-6309654 Satlength=282 Nr of Repeats=8
RepeatLength=35 seed=AATTTCCCGC Num.seqs=7 Similarity=0.909297
67   ATT-TCCCGCC--AAAA-T--TTTTTT-CACTGAAAATTTGA-

Cele-UNSB01_5:6310933-6311214 Satlength=282 Nr of Repeats=8
RepeatLength=35 seed=AATTTCCCGC Num.seqs=7 Similarity=0.909297
67   ATT-TCCCGCC--AAAA-T--TTTTTT-CACTGAAAATTTGA-
Rev.of_Cele-UNSB01_5:9642989-9644244 Satlength=1256 Nr of Repeats=36
RepeatLength=35 seed=GCGGGAAATT Num.seqs=25 Similarity=0.936127
77   ATT-TCCCGCC--AAAA-T--TTTTTT-CACTGAAAATTTGA-
Rev.of_Cele-UNSB01_5:13979523-13981831 Satlength=2309 Nr of Repeats=66
RepeatLength=35 seed=GCGGGAAATT Num.seqs=50 Similarity=0.968964
77   ATT-TCCCGCC--AAAA-T--TTTTTT-CACTGAAAATTTGA-
Rev.of_Cele-UNSB01_5:13979521-13982440 Satlength=2920 Nr of Repeats=68
RepeatLength=35 seed=TGGCGGGAAA Num.seqs=50 Similarity=0.947124
79   ATT-TCCCGCC--AAAA-T--TTTTTT-CACTGAAAATTTGA-
Rev.of_Cele-UNSB01_5:14226151-14226968 Satlength=818 Nr of Repeats=21
RepeatLength=35 seed=GGAAATTCAA Num.seqs=14 Similarity=0.922554
74   ATT-TCCCGCC--AAAA-T--TTTTTT-CACTGAAAATTTGA-
Rev.of_Cele-UNSB01_5:15725600-15726976 Satlength=1377 Nr of Repeats=36
RepeatLength=35 seed=TTCAAATTTT Num.seqs=30 Similarity=0.828501
69   ATT-TCCCGCC--AAAA-T--TTTTTT-CACTGAAAATTTGA-
Cele-UNSB01_6:9443244-9443487 Satlength=244 Nr of Repeats=7
RepeatLength=35 seed=AAAATTTGAA Num.seqs=5 Similarity=0.908571
94   ATT-TCCCGCC--AAAA-T--TTTTTT-CACTGAAAATTTGA-
Cele-UNSB01_3:10975904-10976322 Satlength=419 Nr of Repeats=11
RepeatLength=35 seed=CCGCCAAAAT Num.seqs=10 Similarity=0.943280
38   ATT-TCCCGCC--AAAA-T--ATTTTT-CACTAAAAATTTGA-
Rev.of_Cele-UNSB01_3:10980699-10982201 Satlength=1503 Nr of Repeats=43
RepeatLength=35 seed=AAATTCAAAT Num.seqs=40 Similarity=0.893520
72   ATT-TCCCGCC--AAAA-T--ATTTTT-CACTAAAAATTTGA-
Rev.of_Cele-UNSB01_4:15436315-15436698 Satlength=384 Nr of Repeats=11
RepeatLength=35 seed=GGGAAATTCA Num.seqs=7 Similarity=0.941950
40   ATT-TCCCGCC--AAAA-T--ATTTTT-TACTAAAAATTTGA-
Rev.of_Cele-UNSB01_4:15436367-15437145 Satlength=779 Nr of Repeats=9
RepeatLength=35 seed=GTAAAAAATA Num.seqs=7 Similarity=0.929252
57   ATT-TCCCGCC--AAAA-T--ATTTTT-TACTAAAAATTTGA-
Rev.of_Cele-UNSB01_1:8014573-8014958 Satlength=386 Nr of Repeats=11
RepeatLength=35 seed=GGGAAATTTA Num.seqs=11 Similarity=0.944589
40   ATT-TCCCGCC--AAAA-T--ATTTTT-CACAGAAAATTTAA-
Cele-UNSB01_2:15105694-15106081 Satlength=388 Nr of Repeats=11
RepeatLength=35 seed=CAGAAAATTT Num.seqs=9 Similarity=0.949206
56   ATT-TCCCGCC--AAAA-T--ATTTTT-CACAGAAAATTTAA-
Rev.of_Cele-UNSB01_4:13645884-13646555 Satlength=672 Nr of Repeats=18
RepeatLength=35 seed=GAAATTTAAA Num.seqs=15 Similarity=0.948481
73   ATT-TCCCGCC--AAAA-T--ATTTTT-CACAGAAAATTTAA-
Rev.of_Cele-UNSB01_4:13645885-13647427 Satlength=1543 Nr of Repeats=25
RepeatLength=35 seed=AAATTTAAAT Num.seqs=19 Similarity=0.791694
72   ATT-TCCCGCC--AAAA-T--ATTTTT-CACAGAAAATTTAA-
Rev.of_Cele-UNSB01_4:14107092-14108860 Satlength=1769 Nr of Repeats=51
RepeatLength=35 seed=GGGAAATTTA Num.seqs=34 Similarity=0.943706
40   ATT-TCCCGCC--AAAA-T--ATTTTT-CACAGAAAATTTAA-
Rev.of_Cele-UNSB01_4:14107092-14111107 Satlength=4016 Nr of Repeats=106
RepeatLength=35 seed=GGGAAATTTA Num.seqs=73 Similarity=0.941958
40   ATT-TCCCGCC--AAAA-T--ATTTTT-CACAGAAAATTTAA-
Rev.of_Cele-UNSB01_4:14107093-14113320 Satlength=6228 Nr of Repeats=160
RepeatLength=35 seed=GGAAATTTAA Num.seqs=111 Similarity=0.941169
74   ATT-TCCCGCC--AAAA-T--ATTTTT-CACAGAAAATTTAA-

Rev.of_Cele-UNSB01_4:15399887-15400027 Satlength=141 Nr of Repeats=4 RepeatLength=35 seed=AAATTTTCTG Num.seqs=4 Similarity=0.904762
66   ATT-TCCCGCC--AAAA-T--ATTTTT-CACAGAAAATTTAA-
Rev.of_Cele-UNSB01_4:17168941-17169150 Satlength=210 Nr of Repeats=6 RepeatLength=35 seed=GAAATTTAAA Num.seqs=5 Similarity=0.900952
73   ATT-TCCCGCC--AAAA-T--ATTTTT-CACAGAAAATTTAA-
Rev.of_Cele-UNSB01_4:17402400-17402610 Satlength=211 Nr of Repeats=6 RepeatLength=35 seed=AAATTTTCTG Num.seqs=6 Similarity=0.928889
66   ATT-TCCCGCC--AAAA-T--ATTTTT-CACAGAAAATTTAA-
Rev.of_Cele-UNSB01_5:6074295-6074435 Satlength=141 Nr of Repeats=4 RepeatLength=35 seed=ATTTTGGCGG Num.seqs=4 Similarity=0.961905
48   ATT-TCCCGCC--AAAA-T--ATTTTT-CACAGAAAATTTAA-
Cele-UNSB01_5:7957689-7957969 Satlength=281 Nr of Repeats=8 RepeatLength=35 seed=CAGAAAATTT Num.seqs=8 Similarity=0.964626
56   ATT-TCCCGCC--AAAA-T--ATTTTT-CACAGAAAATTTAA-
Cele-UNSB01_6:1420856-1421521 Satlength=666 Nr of Repeats=19 RepeatLength=35 seed=CCCGCCAAAA Num.seqs=19 Similarity=0.932275
72   ATT-TCCCGCC--AAAA-T--ATTTTT-CACAGAAAATTTAA-
Cele-UNSB01_6:14122412-14122622 Satlength=211 Nr of Repeats=6 RepeatLength=35 seed=CAGAAAATTT Num.seqs=6 Similarity=0.949206
56   ATT-TCCCGCC--AAAA-T--ATTTTT-CACAGAAAATTTAA-
Cele-UNSB01_6:15851631-15851806 Satlength=176 Nr of Repeats=5 RepeatLength=35 seed=ATTTAAATTT Num.seqs=5 Similarity=0.939048
62   ATT-TCCCGCC--AAAA-T--ATTTTT-CACAGAAAATTTAA-
Rev.of_Cele-UNSB01_6:17686891-17687206 Satlength=316 Nr of Repeats=9 RepeatLength=35 seed=AAATTTTCTG Num.seqs=7 Similarity=0.909297
66   ATT-TCCCGCC--AAAA-T--ATTTTT-CACAGAAAATTTAA-
Rev.of_Cele-UNSB01_4:16061680-16061959 Satlength=280 Nr of Repeats=8 RepeatLength=35 seed=AATTTAAATT Num.seqs=7 Similarity=0.896599
71   ATT-TCCCGCC--AAAA-T--ATTTTT-CNCAGAAAATTTAA-
Cele-UNSB01_2:635957-636850 Satlength=894 Nr of Repeats=4 RepeatLength=35 seed=GAAAATTTAA Num.seqs=3 Similarity=0.923810
58   ATT-TCCCTCC--AAAA-T--ATTTTT-CACAGAAAATTTAA-
Rev.of_Cele-UNSB01_2:15064421-15065299 Satlength=879 Nr of Repeats=25 RepeatLength=35 seed=TTAAATTTTC Num.seqs=22 Similarity=0.939312
68   ATT-TCCCTCC--AAAA-T--ATTTTT-CACAGAAAATTTAA-
Rev.of_Cele-UNSB01_3:8581179-8581353 Satlength=175 Nr of Repeats=5 RepeatLength=35 seed=GGGAAATTTA Num.seqs=4 Similarity=0.974603
75   ATT-TCCCTCC--AAAA-T--ATTTTT-CACAGAAAATTTAA-
Rev.of_Cele-UNSB01_5:6964092-6964484 Satlength=393 Nr of Repeats=9 RepeatLength=35 seed=CGGGAAATTT Num.seqs=6 Similarity=0.921270
41   ATT-TCCCGCC--AAAA-T--ATTTTT-CACAGAAAATTCAA-
Rev.of_Cele-UNSB01_1:14648049-14648258 Satlength=210 Nr of Repeats=6 RepeatLength=35 seed=AATTTAAATT Num.seqs=5 Similarity=0.859048
71   ATT-TCCCGCC--AAAA-T--ATTTTT-CTCAGAAAATTTAA-
Cele-UNSB01_4:7464933-7465143 Satlength=211 Nr of Repeats=6 RepeatLength=35 seed=CAGAAAATTT Num.seqs=6 Similarity=0.926349
91   ATT-TCCCGCC--AAAA-T--ATTTTT-CTCAGAAAATTTAA-
Cele-UNSB01_4:16144580-16144720 Satlength=141 Nr of Repeats=4 RepeatLength=35 seed=CCAAAATATT Num.seqs=4 Similarity=0.892063
76   ATT-TCCCGCC--AAAA-T--ATTTTT-CTCAGAAAATTTAA-
Cele-UNSB01_5:9479420-9479630 Satlength=211 Nr of Repeats=6 RepeatLength=35 seed=AGAAAATTTA Num.seqs=6 Similarity=0.888254
92   ATT-TCCCGCC--AAAA-T--ATTTTT-CTCAGAAAATTTAA-

Cele-UNSB01_5:19059372-19059511 Satlength=140 Nr of Repeats=4
RepeatLength=35 seed=TCAGAAAATT Num.seqs=3 Similarity=0.923810
90   ATT-TCCCGCC--AAAA-T--ATTTTT-CTCAGAAAATTTAA-
Cele-UNSB01_6:15298701-15298945 Satlength=245 Nr of Repeats=7
RepeatLength=35 seed=CAGAAAATTT Num.seqs=6 Similarity=0.893333
91   ATT-TCCCGCC--AAAA-T--ATTTTT-CTCAGAAAATTTAA-
Cele-UNSB01_4:8504239-8504449 Satlength=211 Nr of Repeats=6
RepeatLength=35 seed=GAAAATTTAA Num.seqs=6 Similarity=0.898413
93   ATT-TCCCTCC--AAAA-T--ATTTTT-CTCAGAAAATTTAA-
Cele-UNSB01_6:16004822-16005172 Satlength=351 Nr of Repeats=10
RepeatLength=35 seed=CAGAAAATTT Num.seqs=10 Similarity=0.875556
126   ATT-TCCCTCC--AAAA-T--ATTTTT-CTCAGAAAATTTAA-
Cele-UNSB01_4:15999976-16000920 Satlength=945 Nr of Repeats=8
RepeatLength=35 seed=AAAATTTAAA Num.seqs=7 Similarity=0.883900
94   ATT-TCCCNCC--AAAA-T--ATTTTT-CTCAGAAAATTTAA-
Rev.of_Cele-UNSB01_2:15242891-15243160 Satlength=270 Nr of Repeats=5
RepeatLength=35 seed=ATTTTGGCGG Num.seqs=4 Similarity=0.904762
83   ATT-TCCCGCC--AAAA-T--ATTTTT-CTCCGAAAATTTAA-
Cele-UNSB01_4:16926416-16926623 Satlength=208 Nr of Repeats=6
RepeatLength=35 seed=TTGAATTTCC Num.seqs=4 Similarity=0.917460
64   ATT-TCCCGCC--AAAA-T--ATTTTT-CACAGAAAATTTGA-
Rev.of_Cele-UNSB01_6:161019-161648 Satlength=630 Nr of Repeats=17
RepeatLength=35 seed=GGGAAATTCA Num.seqs=14 Similarity=0.796654
75   ATT-TCCCGCC--AAAA-T--ATTTTT-CACAAAAAATTTGA-
Cele-UNSB01_2:9764656-9765286 Satlength=631 Nr of Repeats=18
RepeatLength=35 seed=CCAAAATATT Num.seqs=18 Similarity=0.925801
41   ATT-TCCCTCC--AAAA-T--ATTTTT-TTCAGAAAATTTAA-
Cele-UNSB01_4:1160589-1161121 Satlength=533 Nr of Repeats=4
RepeatLength=35 seed=AAAATATTTT Num.seqs=3 Similarity=0.898413
43   ATT-TCCCTCC--AAAA-T--ATTTTT-TTCAGAAAATTTAA-
Rev.of_Cele-UNSB01_3:4031086-4031330 Satlength=245 Nr of Repeats=7
RepeatLength=35 seed=AATTTAAATT Num.seqs=6 Similarity=0.890794
71   ATT-TCCCGCC--AAAA-T--ATTTTT-TTCAGAAAATTTAA-
Cele-UNSB01_6:2095079-2095359 Satlength=281 Nr of Repeats=7
RepeatLength=35 seed=CAGAAAATTT Num.seqs=6 Similarity=0.903492
91   ATT-TCCCGCC--AAAA-T--ATTTTT-TTCAGAAAATTTAA-
Cele-UNSB01_6:2095085-2096434 Satlength=1350 Nr of Repeats=17
RepeatLength=35 seed=ATTTAAATTT Num.seqs=12 Similarity=0.912266
97   ATT-TCCCGCC--AAAA-T--ATTTTT-TTCAGAAAATTTAA-
Cele-UNSB01_4:4986914-4987437 Satlength=524 Nr of Repeats=15
RepeatLength=35 seed=TTTTCAGAAA Num.seqs=13 Similarity=0.973626
52   ATT-TCCCTCC--AAAA-T--ATTTTT-TTCAGAAAATTAAA-
Cele-UNSB01_4:4990413-4990623 Satlength=211 Nr of Repeats=6
RepeatLength=35 seed=AATATTTTTT Num.seqs=6 Similarity=0.964444
80   ATT-TCCCTCC--AAAA-T--ATTTTT-TTCAGAAAATTAAA-
Rev.of_Cele-UNSB01_3:12788650-12788963 Satlength=314 Nr of Repeats=8
RepeatLength=35 seed=TTCAAATTTT Num.seqs=5 Similarity=0.658559
34   ATT-TNCCGCC--AAAA-T--GTCTTT-CACAGAAAATTTGA-
Cele-UNSB01_5:20176707-20177444 Satlength=738 Nr of Repeats=21
RepeatLength=33 seed=TTTGAATTTC Num.seqs=17 Similarity=0.722086
26   -TT-TCCCACC--AAAA-T----TTTC--TCAAAAAAATTTGAA
Cele-UNSB01_5:20065513-20066023 Satlength=511 Nr of Repeats=14
RepeatLength=34 seed=TTTCCCGCCA Num.seqs=12 Similarity=0.859774
32   -TT-TCCCGCC--AAAA-T----ATTCACTCNAAAAATTTGAA

```
Rev.of_Cele-UNSB01_3:13065987-13066581 Satlength=595 Nr of Repeats=17
RepeatLength=35 seed=AAATTCAAAT  Num.seqs=16 Similarity=0.851746
35    -TT-TCCCGCCATAAAA-T----ATTT-CTCAAATAATTTGAA
Rev.of_Cele-UNSB01_5:18032604-18033134 Satlength=531 Nr of Repeats=13
RepeatLength=34 seed=TCAAATTTTC  Num.seqs=8 Similarity=0.860204
0    ATT-T-CCGCC--AAAG-T--TTTTTT-AAAAGAAAATTTGA-
Rev.of_Cele-UNSB01_1:3453387-3453600 Satlength=214 Nr of Repeats=5
RepeatLength=32 seed=AATTCAAATT  Num.seqs=3 Similarity=0.602694
2    -TT-TTCCACC--TAAA-T----TATT-CT-TGAAAATTTGAA

* **   *          *         * **  *
Consensus:

ATTtcCCGCCAAAAtntTTTTCaCaGAAAATTTgA

>Cele-UNSB01_Fam_5_40_94  Nr. of seq. 94 Alignment length(with gaps) = 41
Alignment score = 0.668259
Cele-UNSB01_1:336527-337247 Satlength=721 Nr of Repeats=18
RepeatLength=40 seed=AATTTTCTCG  Num.seqs=18 Similarity=0.920044
0    AATTTTCTCGAAATTTCCAAAAGGTTCTAGAACATTTCA-G
Rev.of_Cele-UNSB01_5:1390382-1390822 Satlength=441 Nr of Repeats=9
RepeatLength=40 seed=AATGTTCTAG  Num.seqs=6 Similarity=0.738753
36    AATTTTCTCGAATTTTCCAGAAGGTTCTAGAACATTTCA-G
Cele-UNSB01_1:13852339-13853459 Satlength=1121 Nr of Repeats=28
RepeatLength=40 seed=GAATTTTCTC  Num.seqs=28 Similarity=0.956702
39    AATTTTCTCGAATTTTCCAGAAGGTTCTGGAGCATTCCA-G
Rev.of_Cele-UNSB01_6:16472071-16473145 Satlength=1075 Nr of Repeats=13
RepeatLength=40 seed=ATGTTCCAGA  Num.seqs=12 Similarity=0.873676
75    AATTTTCTCGAATTTTCCAGAAGGTTCTGGAACATTCCA-G
Cele-UNSB01_4:2158460-2158710 Satlength=251 Nr of Repeats=6
RepeatLength=40 seed=TTCCAGAATT  Num.seqs=5 Similarity=0.866667
74    AATTTTCTCGAATTTTCCAGAAGGTTCTAGAACATTCCA-G
Rev.of_Cele-UNSB01_4:13851511-13852113 Satlength=603 Nr of Repeats=12
RepeatLength=40 seed=AAAATTCTGG  Num.seqs=9 Similarity=0.870370
86    AATTTTCTCGAATTTTCCAGAAGGTTCTAGAACATTCCA-G
Rev.of_Cele-UNSB01_6:483296-484174 Satlength=879 Nr of Repeats=19
RepeatLength=40 seed=AAAATTCTGG  Num.seqs=12 Similarity=0.943434
86    AATTTTCTCGAATTTTCCAGAAGGTTCTAGAACATTCCA-G
Rev.of_Cele-UNSB01_4:14046107-14046777 Satlength=671 Nr of Repeats=11
RepeatLength=40 seed=CTGGAATGTT  Num.seqs=9 Similarity=0.940741
40    AATTTTCTCAAATTTTCCAGAAGGTTCTGGAACATTCCA-G
Rev.of_Cele-UNSB01_5:17207041-17207511 Satlength=471 Nr of Repeats=12
RepeatLength=40 seed=AAAATTCTGG  Num.seqs=11 Similarity=0.899394
6    AATTTTCTCAAATTTTCCAAAAGGCTCTAGAACATTCCA-G
Cele-UNSB01_6:17663802-17664403 Satlength=602 Nr of Repeats=6
RepeatLength=40 seed=TTTCTCGAAT  Num.seqs=4 Similarity=0.866667
43    AAATTTCTCGAATCTTCCAGAAGGTTCTAGAACATTCCA-G
Cele-UNSB01_4:2721172-2721362 Satlength=191 Nr of Repeats=5
RepeatLength=40 seed=GAATTTTCCA  Num.seqs=4 Similarity=0.822222
9    AATTTTTTCGAATTTTCCAGAAAGTTCTAGAAAATTCCA-G
Cele-UNSB01_1:4694849-4695647 Satlength=799 Nr of Repeats=20
RepeatLength=40 seed=CATTCCAGAA  Num.seqs=18 Similarity=0.964052
22    ATTTTTCTAGAAAGTTCTGGAACATTCCAGAATTTTCCC-G
```

Cele-UNSB01_2:9763711-9764616 Satlength=906 Nr of Repeats=23
RepeatLength=40 seed=TTCCAGAATT Num.seqs=22 Similarity=0.947763
24   ATTTTTCTAGAAAGTTCTGGAACATTCCAGAATTTTCCC-G
Cele-UNSB01_3:2620888-2621068 Satlength=181 Nr of Repeats=5
RepeatLength=40 seed=TTCCAGAATT Num.seqs=4 Similarity=0.966667
24   ATTTTTCTAGAAAGTTCTGGAACATTCCAGAATTTTCCC-G
Cele-UNSB01_4:16000310-16000589 Satlength=280 Nr of Repeats=6
RepeatLength=40 seed=GATTTTTCTA Num.seqs=4 Similarity=0.911111
39   ATTTTTCTAGAAAGTTCTGGAACATTCCAGAATTTTCCC-G
Rev.of_Cele-UNSB01_5:13535913-13536380 Satlength=468 Nr of Repeats=12
RepeatLength=40 seed=TCTGGAATGT Num.seqs=11 Similarity=0.922424
31   ATTTTTCTAGAAAGTTCTGGAACATTCCAGAATTTTCCC-G
Rev.of_Cele-UNSB01_6:161741-162501 Satlength=761 Nr of Repeats=19
RepeatLength=40 seed=GAAAATTCTG Num.seqs=19 Similarity=0.974269
37   ATTTTTCTAGAAAGTTCTGGAACATTCCAGAATTTTCCC-G
Cele-UNSB01_6:14121790-14122309 Satlength=520 Nr of Repeats=13
RepeatLength=40 seed=GAACATTCCA Num.seqs=12 Similarity=0.904545
59   ATTTTTCTAGAAAGTTCTGGAACATTCCAGAATTTTCCC-G
Cele-UNSB01_4:16130574-16144444 Satlength=13871 Nr of Repeats=346
RepeatLength=40 seed=TTTTTCTAGA Num.seqs=331 Similarity=0.987691
41   ATTTTTCTAGAAAGTTCTGGAACATTCCAGAATTTTCTC-G
Rev.of_Cele-UNSB01_4:17169175-17169333 Satlength=159 Nr of Repeats=4
RepeatLength=40 seed=AAAATTCTGG Num.seqs=3 Similarity=0.977778
76   ATTTTTCTAGAAAGTTCTGGAACATTCCAGAATTTTCTC-G
Cele-UNSB01_5:15441112-15445831 Satlength=4720 Nr of Repeats=118
RepeatLength=40 seed=ATTTTTCTAG Num.seqs=117 Similarity=0.948354
80   ATTTTTCTAGAAAGTTCTGGAACATTCCAGAATTTTCTC-G
Cele-UNSB01_6:1420400-1420757 Satlength=358 Nr of Repeats=9
RepeatLength=40 seed=ATTTTTCTAG Num.seqs=6 Similarity=0.937778
80   ATTTTTCTAGAAAGTTCTGGAACATTCCAGAATTTTCTC-G
Rev.of_Cele-UNSB01_3:8581474-8581754 Satlength=281 Nr of Repeats=7
RepeatLength=40 seed=TCTGGAATGT Num.seqs=7 Similarity=0.971429
31   ATTTTTCTAGAAAGTTCTGGAACATTCCAGACTTTTCCC-A
Rev.of_Cele-UNSB01_3:8589669-8589949 Satlength=281 Nr of Repeats=7
RepeatLength=40 seed=TCTGGAATGT Num.seqs=7 Similarity=0.971429
31   ATTTTTCTAGAAAGTTCTGGAACATTCCAGACTTTTCCC-A
Rev.of_Cele-UNSB01_3:8597864-8598144 Satlength=281 Nr of Repeats=7
RepeatLength=40 seed=TCTGGAATGT Num.seqs=7 Similarity=0.971429
31   ATTTTTCTAGAAAGTTCTGGAACATTCCAGACTTTTCCC-A
Rev.of_Cele-UNSB01_3:8606059-8606339 Satlength=281 Nr of Repeats=7
RepeatLength=40 seed=TCTGGAATGT Num.seqs=7 Similarity=0.971429
31   ATTTTTCTAGAAAGTTCTGGAACATTCCAGACTTTTCCC-A
Cele-UNSB01_4:1160688-1160967 Satlength=280 Nr of Repeats=7
RepeatLength=40 seed=ATTTTTCTAG Num.seqs=6 Similarity=0.911111
40   ATTTTTCTAGAAAGTTCTGGAACATTCCAGACTTTTCCC-A
Cele-UNSB01_4:1160701-1161595 Satlength=895 Nr of Repeats=10
RepeatLength=40 seed=GTTCTGGAAC Num.seqs=7 Similarity=0.844444
53   ATTTTTCTAGAAAGTTCTGGAACATTCCAGACTTTTCCC-A
Cele-UNSB01_5:1441594-1441873 Satlength=280 Nr of Repeats=7
RepeatLength=40 seed=ATTTTTCTAG Num.seqs=6 Similarity=0.880000
40   ATTTTTCTAGAAAGTTCTGGAACATTCCAGACTTTTCCC-A
Rev.of_Cele-UNSB01_1:6785543-6785983 Satlength=441 Nr of Repeats=11
RepeatLength=40 seed=TTCTGGAATG Num.seqs=11 Similarity=0.960000
32   ATTTTTCTAGAAAGTTCTGGAACATTCCAGAATTTTCTC-G

Rev.of_Cele-UNSB01_1:8015067-8016984 Satlength=1918 Nr of Repeats=48
RepeatLength=40 seed=GAATGTTCCA Num.seqs=45 Similarity=0.944512
67   ATTTTTCTAGAAAGTTCTGGAACATTCCAGAATTTTCTC-G
Cele-UNSB01_3:438790-441355 Satlength=2566 Nr of Repeats=43
RepeatLength=40 seed=ACATTCCAGA Num.seqs=41 Similarity=0.892358
61   ATTTTTCTAGAAAGTTCTGGAACATTCCAGAATTTTCTC-G
Rev.of_Cele-UNSB01_3:4031404-4032964 Satlength=1561 Nr of Repeats=39
RepeatLength=40 seed=GTTCCAGAAC Num.seqs=39 Similarity=0.942330
63   ATTTTTCTAGAAAGTTCTGGAACATTCCAGAATTTTCTC-G
Cele-UNSB01_4:7464716-7464914 Satlength=199 Nr of Repeats=5
RepeatLength=40 seed=TCCAGAATTT Num.seqs=4 Similarity=0.966667
65   ATTTTTCTAGAAAGTTCTGGAACATTCCAGAATTTTCTC-G
Cele-UNSB01_4:7464716-7465331 Satlength=616 Nr of Repeats=6
RepeatLength=40 seed=TCCAGAATTT Num.seqs=4 Similarity=0.966667
65   ATTTTTCTAGAAAGTTCTGGAACATTCCAGAATTTTCTC-G
Rev.of_Cele-UNSB01_4:14031556-14031756 Satlength=201 Nr of Repeats=5
RepeatLength=40 seed=AATGTTCCAG Num.seqs=5 Similarity=0.973333
66   ATTTTTCTAGAAAGTTCTGGAACATTCCAGAATTTTCTC-G
Rev.of_Cele-UNSB01_4:15400092-15400412 Satlength=321 Nr of Repeats=8
RepeatLength=40 seed=GAATGTTCCA Num.seqs=8 Similarity=0.897619
67   ATTTTTCTAGAAAGTTCTGGAACATTCCAGAATTTTCTC-G
Cele-UNSB01_4:16708641-16708839 Satlength=199 Nr of Repeats=5
RepeatLength=40 seed=TTCCAGAATT Num.seqs=4 Similarity=0.966667
64   ATTTTTCTAGAAAGTTCTGGAACATTCCAGAATTTTCTC-G
Cele-UNSB01_6:187518-187756 Satlength=239 Nr of Repeats=6 RepeatLength=40
seed=TTCCAGAATT Num.seqs=5 Similarity=0.986667
64   ATTTTTCTAGAAAGTTCTGGAACATTCCAGAATTTTCTC-G
Cele-UNSB01_6:2087511-2088871 Satlength=1361 Nr of Repeats=34
RepeatLength=40 seed=GAATTTTCTC Num.seqs=34 Similarity=0.961200
69   ATTTTTCTAGAAAGTTCTGGAACATTCCAGAATTTTCTC-G
Cele-UNSB01_6:2094704-2095024 Satlength=321 Nr of Repeats=8
RepeatLength=40 seed=TTCCAGAATT Num.seqs=6 Similarity=0.911111
64   ATTTTTCTAGAAAGTTCTGGAACATTCCAGAATTTTCTC-G
Cele-UNSB01_6:2094705-2095572 Satlength=868 Nr of Repeats=10
RepeatLength=40 seed=TCCAGAATTT Num.seqs=6 Similarity=0.911111
65   ATTTTTCTAGAAAGTTCTGGAACATTCCAGAATTTTCTC-G
Cele-UNSB01_6:2157131-2163386 Satlength=6256 Nr of Repeats=140
RepeatLength=40 seed=AGAATTTTCT Num.seqs=131 Similarity=0.954680
68   ATTTTTCTAGAAAGTTCTGGAACATTCCAGAATTTTCTC-G
Rev.of_Cele-UNSB01_1:13456767-13461871 Satlength=5105 Nr of Repeats=124
RepeatLength=40 seed=AATGTTCCAG Num.seqs=118 Similarity=0.974311
66   ATTTTTCTAGAAAGTTCTGGAACATTTCAGAATTTTCTC-G
Rev.of_Cele-UNSB01_6:17686552-17689936 Satlength=3385 Nr of Repeats=70
RepeatLength=40 seed=AATGTTCCAG Num.seqs=69 Similarity=0.965771
66   ATTTTTCTAGAAAGTTCTGGAACATTTCAGAATTTTCTC-G
Cele-UNSB01_4:16926190-16926350 Satlength=161 Nr of Repeats=4
RepeatLength=40 seed=TTCCAGAATT Num.seqs=4 Similarity=0.950000
64   ATTTTTCTAGAAAGTTCTGGAACGTTCCAGAATTTTCCC-G
Cele-UNSB01_5:4024546-4025389 Satlength=844 Nr of Repeats=21
RepeatLength=40 seed=CTAGAAAGTT Num.seqs=18 Similarity=0.796514
46   AATTTCCTAGAAAGTTCTGGAACATTCCAGAATTTTCTT-G
Rev.of_Cele-UNSB01_1:787252-787531 Satlength=280 Nr of Repeats=6
RepeatLength=40 seed=GAACCTTCTG Num.seqs=4 Similarity=0.850000
7   AAGGTTCTAGAACATTCCAGAATTTTCTCAAATTTTCCA-G

Rev.of_Cele-UNSB01_2:4622617-4623337 Satlength=721 Nr of Repeats=18 RepeatLength=40 seed=TCCAGAACCT Num.seqs=18 Similarity=0.957734
11 AAGGTTCTGGAACATTCCAGAATTTTCTCAAATTTTCCA-G
Cele-UNSB01_4:8501471-8501871 Satlength=401 Nr of Repeats=10 RepeatLength=40 seed=AGAAGGTTCT Num.seqs=10 Similarity=0.953333
38 AAGGTTCTGGAACATTCCAGAATTTTCTCAAATTTTCCA-G
Cele-UNSB01_4:15944034-15944234 Satlength=201 Nr of Repeats=5 RepeatLength=40 seed=ATTTTCCAGA Num.seqs=5 Similarity=0.920000
31 AAGGTTCTGGAACATTCCAGAATTTTCTCAAATTTTCCA-G
Cele-UNSB01_6:15068455-15069305 Satlength=851 Nr of Repeats=5 RepeatLength=40 seed=TTCTGGAACA Num.seqs=4 Similarity=0.866667
44 AAGGTTCTGGAACATTCCAGAATTTTCTCAAATTTTCCA-G
Rev.of_Cele-UNSB01_6:16472035-16472475 Satlength=441 Nr of Repeats=11 RepeatLength=40 seed=TCCAGAACCT Num.seqs=11 Similarity=0.950303
11 AAGGTTCTGGAACATTCCAGAATTTTCTCAAATTTTCCA-G
Cele-UNSB01_5:7485041-7485201 Satlength=161 Nr of Repeats=4 RepeatLength=40 seed=GAAGGTTCTA Num.seqs=4 Similarity=0.794444
39 AAGGTTCTAGAACATTCCAGAATTTTCTNAAAATTTCCA-G
Cele-UNSB01_1:14918627-14918817 Satlength=191 Nr of Repeats=5 RepeatLength=40 seed=CCAGAAGGTT Num.seqs=4 Similarity=0.800000
36 AAGGTTCTAGAACAATCCAGAATTTTCTCGAATTTTCCA-G
Cele-UNSB01_2:637418-637568 Satlength=151 Nr of Repeats=4 RepeatLength=40 seed=CAGAAGGTTC Num.seqs=3 Similarity=0.800000
37 AAGGTTCTGGAACATTCCAGAATTTTCTAGAATTTTCCA-G
Rev.of_Cele-UNSB01_3:4029920-4030220 Satlength=301 Nr of Repeats=8 RepeatLength=40 seed=AGAACCTTCT Num.seqs=6 Similarity=0.824444
48 AAGGTTCTGGAACATTCCAGAATTTTCTAGAATTTTCCA-G
Rev.of_Cele-UNSB01_3:9810383-9810703 Satlength=321 Nr of Repeats=8 RepeatLength=40 seed=TCCAGAACCT Num.seqs=8 Similarity=0.945238
51 AAGGTTCTGGAACATTCCAGAATTTTCTCGAATTTTCCA-G
Rev.of_Cele-UNSB01_3:9810418-9811039 Satlength=622 Nr of Repeats=9 RepeatLength=40 seed=AATGTTCCAG Num.seqs=8 Similarity=0.903571
56 AAGGTTCTGGAACATTCCAGAATTTTCTCGAATTTTCCA-G
Rev.of_Cele-UNSB01_4:12150067-12150386 Satlength=320 Nr of Repeats=8 RepeatLength=40 seed=TCCAGAACCT Num.seqs=7 Similarity=0.946032
51 AAGGTTCTGGAACATTCCAGAATTTTCTCGAATTTTCCA-G
Rev.of_Cele-UNSB01_4:14046078-14046558 Satlength=481 Nr of Repeats=12 RepeatLength=40 seed=CAGAACCTTC Num.seqs=12 Similarity=0.914646
89 AAGGTTCTGGAACATTCCAGAATTTTCTCGAATTTTCCA-G
Rev.of_Cele-UNSB01_5:3492923-3493163 Satlength=241 Nr of Repeats=6 RepeatLength=40 seed=TCCAGAACCT Num.seqs=6 Similarity=0.931111
51 AAGGTTCTGGAACATTCCAGAATTTTCTCGAATTTTCCA-G
Rev.of_Cele-UNSB01_6:9243610-9244090 Satlength=481 Nr of Repeats=12 RepeatLength=40 seed=CTTCTGGAAA Num.seqs=12 Similarity=0.953030
83 AAGGTTCTGGAACATTCCAGAATTTTCTCGAATTTTCCA-G
Cele-UNSB01_1:789928-790208 Satlength=281 Nr of Repeats=7 RepeatLength=40 seed=TCTAGAACAT Num.seqs=7 Similarity=0.838095
45 AAGGTTCTAGAACATTCCAGAATTTTCTCGAATTTTCCA-A
Cele-UNSB01_5:1393460-1393720 Satlength=261 Nr of Repeats=6 RepeatLength=40 seed=CTAGAACATT Num.seqs=5 Similarity=0.712195
46 AANGTTCTAGAACATTCCAGAATTTTCTCGAATTTTCCA-G
Rev.of_Cele-UNSB01_1:14275073-14275913 Satlength=841 Nr of Repeats=24 RepeatLength=40 seed=TTCTGGAAAA Num.seqs=18 Similarity=0.755338
82 AAGGTTCTAGAACATTCCAGAATTTTATCGAATTTTCCA-G

```
Rev.of_Cele-UNSB01_5:4022711-4023271 Satlength=561 Nr of Repeats=14
RepeatLength=40 seed=GAACCTTCTG  Num.seqs=12 Similarity=0.857071
47    AAGGTTCAAAAACATTCCAGAATTTTCTCGAATTTTCCA-G
Cele-UNSB01_5:1568984-1569144 Satlength=161 Nr of Repeats=4
RepeatLength=40 seed=GTTCTAGAAC  Num.seqs=4 Similarity=0.875339
84    AAGGTTCTAGAACATTCCAGAATTTTCTCGAATTTACCAGG
Rev.of_Cele-UNSB01_3:11461018-11461218 Satlength=201 Nr of Repeats=5
RepeatLength=40 seed=TCCAGAACCT  Num.seqs=5 Similarity=0.960000
11    AAGGTTCTGGAACATTCCAGCATTTTCTCAAATTTTCCA-G
Rev.of_Cele-UNSB01_5:16694107-16694865 Satlength=759 Nr of Repeats=17
RepeatLength=40 seed=CTGGAATGTT  Num.seqs=13 Similarity=0.950427
20    AAGGTTCTGGAACATTCCAGCATTTTCTCAAATTTTCCA-G
Rev.of_Cele-UNSB01_5:16694107-16695183 Satlength=1077 Nr of Repeats=18
RepeatLength=40 seed=CTGGAATGTT  Num.seqs=13 Similarity=0.950427
20    AAGGTTCTGGAACATTCCAGCATTTTCTCAAATTTTCCA-G
Cele-UNSB01_3:12305276-12305678 Satlength=403 Nr of Repeats=10
RepeatLength=40 seed=ATTCCAGAAT  Num.seqs=8 Similarity=0.860714
13    AAGGTTCTAGAACATTCCAGAATTTTTTAAAATTTTCCA-G
Rev.of_Cele-UNSB01_1:4033525-4033725 Satlength=201 Nr of Repeats=6
RepeatLength=40 seed=TTTCTAGAAA  Num.seqs=4 Similarity=0.816667
43    AAAATTCTGGACCATTCCGGAATTTTCTCGAATTTTCTA-G
Rev.of_Cele-UNSB01_4:3733495-3734015 Satlength=521 Nr of Repeats=15
RepeatLength=40 seed=TTTCTAGAAA  Num.seqs=11 Similarity=0.820606
43    AAAATTCTGGACCATTCCNGAATTTTCTCGAATTTTCTA-G
Rev.of_Cele-UNSB01_1:7887436-7887916 Satlength=481 Nr of Repeats=13
RepeatLength=40 seed=TTTCTAGAAA  Num.seqs=11 Similarity=0.818182
43    AAANTTCTGGAACATTCCAGANTTTTCCCGAATTTTCTA-G
Cele-UNSB01_4:14942266-14942426 Satlength=161 Nr of Repeats=5
RepeatLength=40 seed=TTTTCTAGAA  Num.seqs=3 Similarity=0.755556
72    AAAGTTCTGGAACATTCCAGAATTTTCCCGAATTTTCTA-G
Cele-UNSB01_6:17224721-17225161 Satlength=441 Nr of Repeats=13
RepeatLength=40 seed=TTTCTAGAAA  Num.seqs=9 Similarity=0.814815
73    AAANTTCTGGAACATTCCAGAATTTTCTCGAATTTTCTA-G
Cele-UNSB01_1:14936456-14937856 Satlength=1401 Nr of Repeats=35
RepeatLength=40 seed=TTTCTAGAAA  Num.seqs=35 Similarity=0.907675
73    AAAGTTCTGGAACATTCCAGAATTTTCTCGATTTTTCTA-G
Cele-UNSB01_4:8501476-8504168 Satlength=2693 Nr of Repeats=54
RepeatLength=40 seed=GTTCTGGAAC  Num.seqs=51 Similarity=0.884078
83    AAAGTTCTGGAACATTCCAGAATTTTCTCGATTTTTCTA-G
Cele-UNSB01_4:13646820-13647869 Satlength=1050 Nr of Repeats=12
RepeatLength=40 seed=GTTCTGGAAC  Num.seqs=10 Similarity=0.891111
83    AAAGTTCTGGAACATTCCAGAATTTTCTCGATTTTTCTA-G
Cele-UNSB01_4:16130581-16145036 Satlength=14456 Nr of Repeats=348
RepeatLength=40 seed=AGAAAGTTCT  Num.seqs=328 Similarity=0.988340
78    AAAGTTCTGGAACATTCCAGAATTTTCTCGATTTTTCTA-G
Cele-UNSB01_6:2157154-2162472 Satlength=5319 Nr of Repeats=133
RepeatLength=40 seed=AAGTTCTGGA  Num.seqs=127 Similarity=0.964104
81    AAAGTTCTGGAACATTCCAGAATTTTCTCGATTTTTCTA-G
Cele-UNSB01_3:438816-440336 Satlength=1521 Nr of Repeats=38
RepeatLength=40 seed=TAGAAAGTTC  Num.seqs=38 Similarity=0.903556
77    AAAGTTCTGGAACATTCCAGAATTTTCCCGATTTTTCTA-G
Cele-UNSB01_4:15280499-15283896 Satlength=3398 Nr of Repeats=88
RepeatLength=40 seed=AGAAAGTTCT  Num.seqs=79 Similarity=0.897220
78    AAAGTTCTGGAACATTCCAGAATTTTCCCGATTTTTCTA-G
```

Rev.of_Cele-UNSB01_4:16061215-16062273 Satlength=1059 Nr of Repeats=8 RepeatLength=40 seed=GTTCCAGAAC Num.seqs=7 Similarity=0.814286
93   AAAGTTCTGGAACATTCCAGAATTTTCCCGATTTTTCTA-G
Rev.of_Cele-UNSB01_5:6074472-6074951 Satlength=480 Nr of Repeats=12 RepeatLength=40 seed=TTCCAGAACT Num.seqs=11 Similarity=0.960000
92   AAAGTTCTGGAACATTCCAGAATTTTCCCGATTTTTCTA-G
Cele-UNSB01_4:4986658-4986858 Satlength=201 Nr of Repeats=5 RepeatLength=40 seed=TTCTAGAAAG Num.seqs=5 Similarity=0.913333
114   AAAGTTCTGGAACATTCNAGAATTTTCCCGATTTTTCTA-G
Cele-UNSB01_2:636344-636664 Satlength=321 Nr of Repeats=8 RepeatLength=40 seed=TTTCTAGAAA Num.seqs=8 Similarity=0.917857
73   AAAATTCTGGAACATTCCAGAATTTTCCCGATTTTTCTA-G
Cele-UNSB01_2:8585644-8586044 Satlength=401 Nr of Repeats=10 RepeatLength=40 seed=AAAGTTCTGG Num.seqs=10 Similarity=0.901482
80   AAAGTTCTGGAACATTCCAGAATTTTCCCAATTTTTCTA-G
Cele-UNSB01_3:13762939-13765339 Satlength=2401 Nr of Repeats=60 RepeatLength=40 seed=ATTTTTCTAG Num.seqs=60 Similarity=0.908418
110   AAAGTTCTGGAACATTCCAGAATTTTCCCAATTTTTCTA-G
Cele-UNSB01_4:15607346-15608026 Satlength=681 Nr of Repeats=17 RepeatLength=40 seed=GAACATTCCA Num.seqs=17 Similarity=0.919608
89   AAAGTTCTGGAACATTCCAGAATTTTCCCAATTTTTCTA-G
Cele-UNSB01_5:7956964-7957604 Satlength=641 Nr of Repeats=16 RepeatLength=40 seed=TCTGGAACAT Num.seqs=16 Similarity=0.859790
85   AAAGTTCTGGAACATTCCAGAATTTTCCCAATTTTTCTA-G
Cele-UNSB01_6:16004509-16004749 Satlength=241 Nr of Repeats=6 RepeatLength=40 seed=AAAGTTCTGG Num.seqs=6 Similarity=0.904444
80   AAAGTTCTGGAACATTCCAGAATTTTCCCAATTTTTCTA-G
Rev.of_Cele-UNSB01_5:9644299-9645659 Satlength=1361 Nr of Repeats=40 RepeatLength=40 seed=TTTCTAGAAA Num.seqs=28 Similarity=0.851676
83   AAANTTCTGGAATATTCCAGAATTTTCCCGAATTTTCTA-G

*   *   *   **   *   *   *
Consensus:

AantTTCTnGAAnaTTCcaGAAttTTCcaGAaTTTTCcaG

>Cele-UNSB01_Fam_6_11_76 Nr. of seq. 76 Alignment length(with gaps) = 12 Alignment score = 0.808392
Cele-UNSB01_1:564264-564319 Satlength=56 Nr of Repeats=5 RepeatLength=11 seed=GTACTCCTAC Num.seqs=5 Similarity=0.830303
0   GTACTC-CTACC
Rev.of_Cele-UNSB01_1:1236946-1237103 Satlength=158 Nr of Repeats=7 RepeatLength=11 seed=GTACTGTAGG Num.seqs=6 Similarity=0.870707
4   GTACTC-CTACA
Rev.of_Cele-UNSB01_1:13456302-13456675 Satlength=374 Nr of Repeats=12 RepeatLength=11 seed=GTACTGTAGG Num.seqs=9 Similarity=0.952862
4   GTACTC-CTACA
Rev.of_Cele-UNSB01_1:14647518-14647641 Satlength=124 Nr of Repeats=4 RepeatLength=11 seed=GTACTGTAGG Num.seqs=3 Similarity=0.919192
4   GTACTC-CTACA
Cele-UNSB01_2:637664-637798 Satlength=135 Nr of Repeats=4 RepeatLength=11 seed=GTACTCCTAC Num.seqs=3 Similarity=0.838384
11   GTACTC-CTACA

Cele-UNSB01_2:9765885-9766019 Satlength=135 Nr of Repeats=5
RepeatLength=11 seed=GTACTCCTAC Num.seqs=4 Similarity=0.878788
11   GTACTC-CTACA
Rev.of_Cele-UNSB01_2:14178132-14178499 Satlength=368 Nr of Repeats=11
RepeatLength=11 seed=GTACTGTAGG Num.seqs=8 Similarity=0.878788
4   GTACTC-CTACA
Cele-UNSB01_3:441483-441987 Satlength=505 Nr of Repeats=18
RepeatLength=11 seed=CTACAGTACT Num.seqs=12 Similarity=0.773779
6   GTACTC-CTACA
Rev.of_Cele-UNSB01_3:1399964-1400110 Satlength=147 Nr of Repeats=5
RepeatLength=11 seed=GTACTGTAGG Num.seqs=3 Similarity=0.919192
4   GTACTC-CTACA
Rev.of_Cele-UNSB01_3:9809905-9810297 Satlength=393 Nr of Repeats=13
RepeatLength=11 seed=GTACTGTAGG Num.seqs=8 Similarity=0.796537
4   GTACTC-CTACA
Cele-UNSB01_4:1161685-1161942 Satlength=258 Nr of Repeats=8
RepeatLength=11 seed=GTACTCCTAC Num.seqs=5 Similarity=0.854545
11   GTACTC-CTACA
Cele-UNSB01_4:2268162-2268293 Satlength=132 Nr of Repeats=4
RepeatLength=11 seed=GTACTCCTAC Num.seqs=3 Similarity=0.838384
11   GTACTC-CTACA
Rev.of_Cele-UNSB01_4:2780831-2781210 Satlength=380 Nr of Repeats=12
RepeatLength=11 seed=TACTGTAGGA Num.seqs=8 Similarity=0.787698
14   GTACTC-CTACA
Rev.of_Cele-UNSB01_4:4368763-4369020 Satlength=258 Nr of Repeats=5
RepeatLength=11 seed=GTACTGTAGG Num.seqs=3 Similarity=1.000000
4   GTACTC-CTACA
Cele-UNSB01_4:4388126-4388633 Satlength=508 Nr of Repeats=13
RepeatLength=11 seed=CTACAGTACT Num.seqs=9 Similarity=0.878788
6   GTACTC-CTACA
Cele-UNSB01_4:4620647-4621153 Satlength=507 Nr of Repeats=13
RepeatLength=11 seed=CTACAGTACT Num.seqs=9 Similarity=0.878788
6   GTACTC-CTACA
Cele-UNSB01_4:9470964-9471243 Satlength=280 Nr of Repeats=10
RepeatLength=11 seed=GTACTCCTAC Num.seqs=8 Similarity=0.883117
11   GTACTC-CTACA
Rev.of_Cele-UNSB01_4:14030114-14030248 Satlength=135 Nr of Repeats=6
RepeatLength=11 seed=AGTACTGTAG Num.seqs=4 Similarity=0.939394
5   GTACTC-CTACA
Rev.of_Cele-UNSB01_4:14106561-14106695 Satlength=135 Nr of Repeats=6
RepeatLength=11 seed=AGTACTGTAG Num.seqs=4 Similarity=0.939394
5   GTACTC-CTACA
Cele-UNSB01_4:16001468-16002088 Satlength=621 Nr of Repeats=19
RepeatLength=11 seed=GTACTCCTAC Num.seqs=13 Similarity=0.888112
11   GTACTC-CTACA
Rev.of_Cele-UNSB01_4:16343642-16344390 Satlength=749 Nr of Repeats=18
RepeatLength=11 seed=GTACTGTAGG Num.seqs=12 Similarity=0.878788
4   GTACTC-CTACA
Rev.of_Cele-UNSB01_4:17168442-17168576 Satlength=135 Nr of Repeats=6
RepeatLength=11 seed=AGTACTGTAG Num.seqs=4 Similarity=0.939394
5   GTACTC-CTACA
Cele-UNSB01_4:17337235-17337502 Satlength=268 Nr of Repeats=10
RepeatLength=11 seed=CCTACAGTAC Num.seqs=6 Similarity=0.716667
5   GTACTC-CTACA

Rev.of_Cele-UNSB01_5:3492580-3492838 Satlength=259 Nr of Repeats=7
RepeatLength=11 seed=TACTGTAGGA Num.seqs=5 Similarity=0.763889
14   GTACTC-CTACA
Rev.of_Cele-UNSB01_5:4982997-4984483 Satlength=1487 Nr of Repeats=35
RepeatLength=11 seed=GTACTGTAGG Num.seqs=22 Similarity=0.888233
4   GTACTC-CTACA
Cele-UNSB01_5:9480075-9480332 Satlength=258 Nr of Repeats=8
RepeatLength=11 seed=GTACTCCTAC Num.seqs=6 Similarity=0.870707
11   GTACTC-CTACA
Cele-UNSB01_5:15447313-15447447 Satlength=135 Nr of Repeats=5
RepeatLength=11 seed=GTACTCCTAC Num.seqs=4 Similarity=0.878788
11   GTACTC-CTACA
Rev.of_Cele-UNSB01_5:16693408-16693990 Satlength=583 Nr of Repeats=10
RepeatLength=11 seed=GTACTGTAGG Num.seqs=6 Similarity=0.846465
4   GTACTC-CTACA
Cele-UNSB01_5:17709602-17709736 Satlength=135 Nr of Repeats=5
RepeatLength=11 seed=CCTACAGTAC Num.seqs=3 Similarity=0.919192
5   GTACTC-CTACA
Cele-UNSB01_5:20903761-20903966 Satlength=206 Nr of Repeats=5
RepeatLength=11 seed=CCTACAGTAC Num.seqs=3 Similarity=1.000000
5   GTACTC-CTACA
Cele-UNSB01_6:1422210-1422590 Satlength=381 Nr of Repeats=13
RepeatLength=11 seed=GTACTCCTAC Num.seqs=10 Similarity=0.886869
11   GTACTC-CTACA
Cele-UNSB01_6:5457466-5457723 Satlength=258 Nr of Repeats=10
RepeatLength=11 seed=CTACAGTACT Num.seqs=7 Similarity=0.849928
6   GTACTC-CTACA
Cele-UNSB01_6:11775904-11776150 Satlength=247 Nr of Repeats=7
RepeatLength=11 seed=TCCTACAGTA Num.seqs=5 Similarity=0.854545
4   GTACTC-CTACA
Cele-UNSB01_6:14123669-14124172 Satlength=504 Nr of Repeats=19
RepeatLength=11 seed=CTACAGTACT Num.seqs=14 Similarity=0.771368
6   GTACTC-CTACA
Cele-UNSB01_6:15069428-15069697 Satlength=270 Nr of Repeats=10
RepeatLength=11 seed=CCTACAGTAC Num.seqs=6 Similarity=0.738889
5   GTACTC-CTACA
Cele-UNSB01_1:1457508-1457876 Satlength=369 Nr of Repeats=14
RepeatLength=11 seed=CTACAGTACT Num.seqs=10 Similarity=0.753704
6   GTACTN-CTACA
Rev.of_Cele-UNSB01_2:1043954-1044457 Satlength=504 Nr of Repeats=17
RepeatLength=11 seed=GTACTGTAGG Num.seqs=11 Similarity=0.819284
4   GTACTN-CTACA
Cele-UNSB01_2:2366031-2366422 Satlength=392 Nr of Repeats=13
RepeatLength=11 seed=CTACAGTACC Num.seqs=9 Similarity=0.763889
6   GTACTN-CTACA
Cele-UNSB01_2:12843731-12843956 Satlength=226 Nr of Repeats=7
RepeatLength=11 seed=CTACAGTACC Num.seqs=5 Similarity=0.775000
6   GTACTN-CTACA
Cele-UNSB01_4:8600392-8600896 Satlength=505 Nr of Repeats=13
RepeatLength=11 seed=CTACAGTACT Num.seqs=9 Similarity=0.865320
6   GTACTN-CTACA
Rev.of_Cele-UNSB01_4:12149712-12149981 Satlength=270 Nr of Repeats=10
RepeatLength=11 seed=GTACTGTAGG Num.seqs=7 Similarity=0.832612
4   GTACTN-CTACA

Cele-UNSB01_4:16762223-16763229 Satlength=1007 Nr of Repeats=39
RepeatLength=11 seed=CTACAGTACT Num.seqs=30 Similarity=0.778225
6   GTACTN-CTACA
Cele-UNSB01_5:1958361-1958506 Satlength=146 Nr of Repeats=7
RepeatLength=11 seed=CTACAGTACT Num.seqs=6 Similarity=0.723148
6   GTACTN-CTACA
Cele-UNSB01_5:17041242-17041488 Satlength=247 Nr of Repeats=7
RepeatLength=11 seed=CTACAGTACC Num.seqs=5 Similarity=0.786111
6   GTACTN-CTACA
Cele-UNSB01_5:20572226-20572853 Satlength=628 Nr of Repeats=16
RepeatLength=11 seed=CTACAGTACT Num.seqs=11 Similarity=0.854545
6   GTACTN-CTACA
Cele-UNSB01_6:988331-988610 Satlength=280 Nr of Repeats=13
RepeatLength=11 seed=CTACAGTACT Num.seqs=11 Similarity=0.823691
6   GTACTN-CTACA
Rev.of_Cele-UNSB01_6:1657466-1657754 Satlength=289 Nr of Repeats=10
RepeatLength=11 seed=GTACTGTAGG Num.seqs=6 Similarity=0.790741
4   GTACTN-CTACA
Rev.of_Cele-UNSB01_6:9243247-9243516 Satlength=270 Nr of Repeats=10
RepeatLength=11 seed=GTACTGTAGG Num.seqs=7 Similarity=0.832612
4   GTACTN-CTACA
Rev.of_Cele-UNSB01_6:16471702-16471949 Satlength=248 Nr of Repeats=8
RepeatLength=11 seed=GTACTGTAGG Num.seqs=5 Similarity=0.755556
4   GTACTN-CTACA
Rev.of_Cele-UNSB01_2:10964073-10965096 Satlength=1024 Nr of Repeats=24
RepeatLength=11 seed=GTACTGTAGG Num.seqs=16 Similarity=0.896970
4   GTACTA-CTACA
Rev.of_Cele-UNSB01_3:6746945-6756350 Satlength=9406 Nr of Repeats=204
RepeatLength=11 seed=GTACTGTAGG Num.seqs=123 Similarity=0.909143
4   GTACTA-CTACA
Cele-UNSB01_5:1442495-1442763 Satlength=269 Nr of Repeats=8
RepeatLength=11 seed=CTACAGTACC Num.seqs=6 Similarity=0.798148
6   GTACTA-CTACA
Rev.of_Cele-UNSB01_6:40792-40926 Satlength=135 Nr of Repeats=5
RepeatLength=11 seed=GTACTGTAGT Num.seqs=3 Similarity=1.000000
4   GTACTA-CTACA
Rev.of_Cele-UNSB01_1:11631541-11632264 Satlength=724 Nr of Repeats=15
RepeatLength=11 seed=GTACTGTAGG Num.seqs=9 Similarity=0.841751
4   GTACCC-CTACA
Rev.of_Cele-UNSB01_2:889690-889842 Satlength=153 Nr of Repeats=5
RepeatLength=11 seed=TGTAGGGGTA Num.seqs=3 Similarity=1.000000
11   GTACCC-CTACA
Cele-UNSB01_2:12492091-12492147 Satlength=57 Nr of Repeats=5
RepeatLength=11 seed=CTACAGTACC Num.seqs=4 Similarity=0.919192
6   GTACCC-CTACA
Cele-UNSB01_2:13571681-13571955 Satlength=275 Nr of Repeats=20
RepeatLength=11 seed=CCTACAGTAC Num.seqs=12 Similarity=0.853076
5   GTACCC-CTACA
Cele-UNSB01_3:265034-265468 Satlength=435 Nr of Repeats=11
RepeatLength=11 seed=CCTACAGTAC Num.seqs=7 Similarity=0.965368
5   GTACCC-CTACA
Rev.of_Cele-UNSB01_3:1406406-1406896 Satlength=491 Nr of Repeats=33
RepeatLength=11 seed=GGTACTGTAG Num.seqs=21 Similarity=0.844156
5   GTACCC-CTACA

```
Rev.of_Cele-UNSB01_3:1715361-1716220 Satlength=860 Nr of Repeats=41
RepeatLength=11 seed=TACTGTAGGG  Num.seqs=30 Similarity=0.811076
14    GTACCC-CTACA
Cele-UNSB01_4:9493015-9493149 Satlength=135 Nr of Repeats=4
RepeatLength=11 seed=CCCTACAGTA  Num.seqs=3 Similarity=0.919192
4    GTACCC-CTACA
Cele-UNSB01_4:16773369-16773770 Satlength=402 Nr of Repeats=28
RepeatLength=11 seed=CCCCTACAGT  Num.seqs=17 Similarity=0.887701
14    GTACCC-CTACA
Cele-UNSB01_5:14159253-14159397 Satlength=145 Nr of Repeats=6
RepeatLength=11 seed=CTACAGTACC  Num.seqs=4 Similarity=0.818182
6    GTACCC-CTACA
Rev.of_Cele-UNSB01_5:15966117-15966182 Satlength=66 Nr of Repeats=4
RepeatLength=11 seed=GTACTGTAGG  Num.seqs=3 Similarity=0.919192
4    GTACCC-CTACA
Cele-UNSB01_5:17834079-17834336 Satlength=258 Nr of Repeats=8
RepeatLength=11 seed=CCTACAGTAC  Num.seqs=6 Similarity=0.886869
5    GTACCC-CTACA
Rev.of_Cele-UNSB01_2:4159337-4159593 Satlength=257 Nr of Repeats=13
RepeatLength=11 seed=GTACTGTAGG  Num.seqs=9 Similarity=0.735043
4    GTACNC-CTACA
Cele-UNSB01_3:546017-546152 Satlength=136 Nr of Repeats=6 RepeatLength=11
seed=CTACAGTACC  Num.seqs=4 Similarity=0.858586
6    GTACCA-CTACA
Cele-UNSB01_3:2118334-2118593 Satlength=260 Nr of Repeats=20
RepeatLength=11 seed=CTACAGTACC  Num.seqs=14 Similarity=0.795788
6    GTACCN-CTACA
Cele-UNSB01_5:2189835-2190349 Satlength=515 Nr of Repeats=18
RepeatLength=11 seed=CTACAGTACC  Num.seqs=14 Similarity=0.742063
6    GTACCN-CTACA
Rev.of_Cele-UNSB01_4:10086561-10087053 Satlength=493 Nr of Repeats=10
RepeatLength=11 seed=TACTGTAGGG  Num.seqs=6 Similarity=0.935354
14    GTAACC-CTACA
Rev.of_Cele-UNSB01_5:3703054-3703534 Satlength=481 Nr of Repeats=23
RepeatLength=11 seed=AGGATTACTG  Num.seqs=15 Similarity=0.843001
19    GTAATC-CTACA
Cele-UNSB01_3:11631810-11631935 Satlength=126 Nr of Repeats=4
RepeatLength=11 seed=CTACAGTACT  Num.seqs=3 Similarity=0.838384
6    GTACTT-CTACA
Rev.of_Cele-UNSB01_3:12003957-12004292 Satlength=336 Nr of Repeats=7
RepeatLength=11 seed=GTACTGTAGA  Num.seqs=5 Similarity=0.854545
15    GTACNT-CTACA
Cele-UNSB01_5:7485330-7487566 Satlength=2237 Nr of Repeats=74
RepeatLength=11 seed=CTACAGTACC  Num.seqs=56 Similarity=0.675400
6    GTACCT-CTACA
Cele-UNSB01_4:2158825-2158968 Satlength=144 Nr of Repeats=5
RepeatLength=11 seed=CTACAGTACC  Num.seqs=4 Similarity=0.796296
6    GTACCTACTACA
Rev.of_Cele-UNSB01_1:11979429-11979575 Satlength=147 Nr of Repeats=6
RepeatLength=11 seed=AGTACTGTAG  Num.seqs=4 Similarity=0.731481
16    GTACTNACTACA

***    ****
Consensus:
```

GTACtcCTACA

>Cele-UNSB01_Fam_7_15_70 Nr. of seq. 70 Alignment length(with gaps) = 15 Alignment score = 0.830265
Cele-UNSB01_1:911764-911915 Satlength=152 Nr of Repeats=8 RepeatLength=15 seed=ATCTTCTTTT Num.seqs=5 Similarity=0.911111
0 ATCTTCTTTTCGTAG
Cele-UNSB01_1:2174082-2174248 Satlength=167 Nr of Repeats=10 RepeatLength=15 seed=AGATCTTGTT Num.seqs=8 Similarity=0.831746
13 ATCTTGTTTTCGTAG
Rev.of_Cele-UNSB01_2:12142720-12142795 Satlength=76 Nr of Repeats=4 RepeatLength=15 seed=AAAACAAGAT Num.seqs=3 Similarity=0.792593
25 ATCTTGTTTTNGTAG
Cele-UNSB01_2:694600-694660 Satlength=61 Nr of Repeats=4 RepeatLength=15 seed=GTAGATCTTG Num.seqs=4 Similarity=0.675347
26 ATCTTGTTTTGGTAG
Cele-UNSB01_2:7594307-7594367 Satlength=61 Nr of Repeats=4 RepeatLength=15 seed=GTAGATCTTG Num.seqs=4 Similarity=0.675347
26 ATCTTGTTTTGGTAG
Cele-UNSB01_4:17545983-17546043 Satlength=61 Nr of Repeats=4 RepeatLength=15 seed=GTAGATCTTG Num.seqs=4 Similarity=0.675347
26 ATCTTGTTTTGGTAG
Rev.of_Cele-UNSB01_5:105361-105451 Satlength=91 Nr of Repeats=6 RepeatLength=15 seed=AAAAGGAGAA Num.seqs=6 Similarity=0.822222
10 TTCTCCTTTTCGTNG
Cele-UNSB01_5:105684-105774 Satlength=91 Nr of Repeats=6 RepeatLength=15 seed=TTCTCCTTTT Num.seqs=6 Similarity=0.792593
15 TTCTCCTTTTCGTNG
Rev.of_Cele-UNSB01_1:5080040-5080117 Satlength=78 Nr of Repeats=5 RepeatLength=15 seed=CGAAAAGGGG Num.seqs=4 Similarity=0.807407
12 CTCCCCTTTTCGTAT
Cele-UNSB01_1:5080289-5080381 Satlength=93 Nr of Repeats=5 RepeatLength=15 seed=CTCCCCTTTT Num.seqs=3 Similarity=0.847222
15 CTCCCCTTTTCGTAT
Cele-UNSB01_1:5082880-5082987 Satlength=108 Nr of Repeats=6 RepeatLength=15 seed=CTCCCCTTTT Num.seqs=4 Similarity=0.869792
15 CTCCCCTTTTCGTAT
Rev.of_Cele-UNSB01_2:1142725-1142802 Satlength=78 Nr of Repeats=5 RepeatLength=15 seed=CGAAAAGGGG Num.seqs=4 Similarity=0.777778
12 CTCCCCTTTTCGTAT
Rev.of_Cele-UNSB01_3:6731291-6731428 Satlength=138 Nr of Repeats=9 RepeatLength=15 seed=CGAAAAGGGG Num.seqs=8 Similarity=0.847619
12 CTCCCCTTTTCGTAT
Rev.of_Cele-UNSB01_3:13181955-13182047 Satlength=93 Nr of Repeats=5 RepeatLength=15 seed=CGAAAAGGGG Num.seqs=3 Similarity=0.881481
12 CTCCCCTTTTCGTAT
Cele-UNSB01_4:1461281-1461373 Satlength=93 Nr of Repeats=6 RepeatLength=15 seed=TCCCCTTTTC Num.seqs=5 Similarity=0.840000
16 CTCCCCTTTTCGTAT
Rev.of_Cele-UNSB01_4:4768182-4768259 Satlength=78 Nr of Repeats=5 RepeatLength=15 seed=CGAAAAGGGG Num.seqs=4 Similarity=0.777778
12 CTCCCCTTTTCGTAT

Cele-UNSB01_4:12770991-12771083 Satlength=93 Nr of Repeats=5
RepeatLength=15 seed=TCCCCTTTTC Num.seqs=3 Similarity=0.881481
16   CTCCCCTTTTCGTAT
Cele-UNSB01_1:5313372-5313447 Satlength=76 Nr of Repeats=5
RepeatLength=15 seed=TCCCCTTTTC Num.seqs=5 Similarity=0.768889
16   CTCCCCTTTTCGTAG
Rev.of_Cele-UNSB01_1:10660173-10660265 Satlength=93 Nr of Repeats=6
RepeatLength=15 seed=CGAAAAGGGG Num.seqs=5 Similarity=0.768889
27   CTCCCCTTTTCGTAG
Cele-UNSB01_1:10660440-10660532 Satlength=93 Nr of Repeats=6
RepeatLength=15 seed=TCCCCTTTTC Num.seqs=5 Similarity=0.768889
16   CTCCCCTTTTCGTAG
Cele-UNSB01_2:1142992-1143084 Satlength=93 Nr of Repeats=6
RepeatLength=15 seed=TCCCCTTTTC Num.seqs=5 Similarity=0.768889
16   CTCCCCTTTTCGTAG
Rev.of_Cele-UNSB01_2:8013550-8013642 Satlength=93 Nr of Repeats=6
RepeatLength=15 seed=CGAAAAGGGG Num.seqs=5 Similarity=0.768889
27   CTCCCCTTTTCGTAG
Cele-UNSB01_2:8013818-8013910 Satlength=93 Nr of Repeats=6
RepeatLength=15 seed=CCCCTTTTCG Num.seqs=5 Similarity=0.733333
17   CTCCCCTTTTCGTAG
Cele-UNSB01_2:10367690-10367782 Satlength=93 Nr of Repeats=6
RepeatLength=15 seed=TCCCCTTTTC Num.seqs=5 Similarity=0.733333
16   CTCCCCTTTTCGTAG
Rev.of_Cele-UNSB01_2:11673801-11673893 Satlength=93 Nr of Repeats=6
RepeatLength=15 seed=CGAAAAGGGG Num.seqs=5 Similarity=0.768889
27   CTCCCCTTTTCGTAG
Cele-UNSB01_2:11674068-11674143 Satlength=76 Nr of Repeats=5
RepeatLength=15 seed=TCCCCTTTTC Num.seqs=5 Similarity=0.768889
16   CTCCCCTTTTCGTAG
Rev.of_Cele-UNSB01_2:14573634-14573711 Satlength=78 Nr of Repeats=5
RepeatLength=15 seed=CGAAAAGGGG Num.seqs=4 Similarity=0.748148
27   CTCCCCTTTTCGTAG
Cele-UNSB01_3:7953695-7953787 Satlength=93 Nr of Repeats=6
RepeatLength=15 seed=TCCCCTTTTC Num.seqs=5 Similarity=0.768889
16   CTCCCCTTTTCGTAG
Rev.of_Cele-UNSB01_3:10399868-10399945 Satlength=78 Nr of Repeats=5
RepeatLength=15 seed=CGAAAAGGGG Num.seqs=4 Similarity=0.748148
27   CTCCCCTTTTCGTAG
Cele-UNSB01_3:10733861-10733953 Satlength=93 Nr of Repeats=6
RepeatLength=15 seed=TCCCCTTTTC Num.seqs=5 Similarity=0.768889
16   CTCCCCTTTTCGTAG
Cele-UNSB01_4:634851-634943 Satlength=93 Nr of Repeats=6 RepeatLength=15
seed=TCCCCTTTTC Num.seqs=5 Similarity=0.768889
16   CTCCCCTTTTCGTAG
Rev.of_Cele-UNSB01_4:3087838-3087930 Satlength=93 Nr of Repeats=6
RepeatLength=15 seed=CGAAAAGGGG Num.seqs=5 Similarity=0.760000
27   CTCCCCTTTTCGTAG
Cele-UNSB01_4:3088105-3088197 Satlength=93 Nr of Repeats=6
RepeatLength=15 seed=TCCCCTTTTC Num.seqs=5 Similarity=0.768889
16   CTCCCCTTTTCGTAG
Rev.of_Cele-UNSB01_4:4464716-4464793 Satlength=78 Nr of Repeats=5
RepeatLength=15 seed=CGAAAAGGGG Num.seqs=4 Similarity=0.748148
27   CTCCCCTTTTCGTAG

Cele-UNSB01_4:4768434-4768526 Satlength=93 Nr of Repeats=6
RepeatLength=15 seed=TCCCCTTTTC Num.seqs=5 Similarity=0.768889
16   CTCCCCTTTTCGTAG
Rev.of_Cele-UNSB01_4:8048486-8048578 Satlength=93 Nr of Repeats=6
RepeatLength=15 seed=CGAAAAGGGG Num.seqs=5 Similarity=0.768889
27   CTCCCCTTTTCGTAG
Cele-UNSB01_4:8048753-8048845 Satlength=93 Nr of Repeats=6
RepeatLength=15 seed=TCCCCTTTTC Num.seqs=5 Similarity=0.768889
16   CTCCCCTTTTCGTAG
Rev.of_Cele-UNSB01_4:11302758-11302850 Satlength=93 Nr of Repeats=6
RepeatLength=15 seed=CGAAAAGGGG Num.seqs=5 Similarity=0.768889
27   CTCCCCTTTTCGTAG
Rev.of_Cele-UNSB01_4:12770724-12770816 Satlength=93 Nr of Repeats=6
RepeatLength=15 seed=CGAAAAGGGG Num.seqs=5 Similarity=0.768889
27   CTCCCCTTTTCGTAG
Rev.of_Cele-UNSB01_4:15434528-15434620 Satlength=93 Nr of Repeats=6
RepeatLength=15 seed=CGAAAAGGGG Num.seqs=5 Similarity=0.768889
27   CTCCCCTTTTCGTAG
Rev.of_Cele-UNSB01_5:1229040-1229117 Satlength=78 Nr of Repeats=5
RepeatLength=15 seed=CGAAAAGGGG Num.seqs=4 Similarity=0.748148
27   CTCCCCTTTTCGTAG
Cele-UNSB01_5:1229292-1229367 Satlength=76 Nr of Repeats=5
RepeatLength=15 seed=TCCCCTTTTC Num.seqs=5 Similarity=0.768889
16   CTCCCCTTTTCGTAG
Rev.of_Cele-UNSB01_5:5757268-5757343 Satlength=76 Nr of Repeats=5
RepeatLength=15 seed=CGAAAAGGGG Num.seqs=5 Similarity=0.768889
27   CTCCCCTTTTCGTAG
Cele-UNSB01_5:5757528-5757620 Satlength=93 Nr of Repeats=6
RepeatLength=15 seed=TCCCCTTTTC Num.seqs=5 Similarity=0.733333
16   CTCCCCTTTTCGTAG
Cele-UNSB01_5:14550286-14550378 Satlength=93 Nr of Repeats=6
RepeatLength=15 seed=TCCCCTTTTC Num.seqs=5 Similarity=0.768889
16   CTCCCCTTTTCGTAG
Rev.of_Cele-UNSB01_5:15913508-15913600 Satlength=93 Nr of Repeats=6
RepeatLength=15 seed=CGAAAAGGGG Num.seqs=5 Similarity=0.768889
27   CTCCCCTTTTCGTAG
Rev.of_Cele-UNSB01_5:16699381-16699441 Satlength=61 Nr of Repeats=4
RepeatLength=15 seed=CGAAAAGGGG Num.seqs=4 Similarity=0.748148
27   CTCCCCTTTTCGTAG
Cele-UNSB01_4:17606143-17606220 Satlength=78 Nr of Repeats=5
RepeatLength=15 seed=TTCCCCTTTT Num.seqs=4 Similarity=0.866667
15   TTCCCCTTTTCGTAT
Cele-UNSB01_1:5758991-5759083 Satlength=93 Nr of Repeats=6
RepeatLength=15 seed=TCCCCTTTTC Num.seqs=5 Similarity=0.768889
16   ATCCCCTTTTCGCAG
Cele-UNSB01_2:14573886-14573978 Satlength=93 Nr of Repeats=6
RepeatLength=15 seed=TCCCCTTTTC Num.seqs=5 Similarity=0.768889
16   ATCCCCTTTTCGCAG
Rev.of_Cele-UNSB01_3:6192343-6192405 Satlength=63 Nr of Repeats=4
RepeatLength=15 seed=CGAAAAGGGG Num.seqs=3 Similarity=0.762963
27   ATCCCCTTTTCGCAG
Cele-UNSB01_3:6192580-6192657 Satlength=78 Nr of Repeats=5
RepeatLength=15 seed=TCCCCTTTTC Num.seqs=4 Similarity=0.748148
16   ATCCCCTTTTCGCAG

Cele-UNSB01_3:6731603-6731679 Satlength=77 Nr of Repeats=5 RepeatLength=15 seed=TCCCCTTTTC Num.seqs=4 Similarity=0.748148
16 ATCCCCTTTTCGCAG
Rev.of_Cele-UNSB01_3:7953449-7953511 Satlength=63 Nr of Repeats=4 RepeatLength=15 seed=CGAAAAGGGG Num.seqs=3 Similarity=0.762963
27 ATCCCCTTTTCGCAG
Cele-UNSB01_3:10400120-10400212 Satlength=93 Nr of Repeats=5 RepeatLength=15 seed=TCCCCTTTTC Num.seqs=3 Similarity=0.762963
16 ATCCCCTTTTCGCAG
Rev.of_Cele-UNSB01_3:10733639-10733701 Satlength=63 Nr of Repeats=4 RepeatLength=15 seed=CGAAAAGGGG Num.seqs=3 Similarity=0.762963
27 ATCCCCTTTTCGCAG
Cele-UNSB01_3:11109268-11109343 Satlength=76 Nr of Repeats=5 RepeatLength=15 seed=TCCCCTTTTC Num.seqs=5 Similarity=0.751111
16 ATCCCCTTTTCGCAG
Cele-UNSB01_3:13182222-13182299 Satlength=78 Nr of Repeats=5 RepeatLength=15 seed=TCCCCTTTTC Num.seqs=4 Similarity=0.777778
16 ATCCCCTTTTCGCAG
Cele-UNSB01_4:391568-391630 Satlength=63 Nr of Repeats=4 RepeatLength=15 seed=TCCCCTTTTC Num.seqs=3 Similarity=0.762963
16 ATCCCCTTTTCGCAG
Rev.of_Cele-UNSB01_4:1729821-1729883 Satlength=63 Nr of Repeats=4 RepeatLength=15 seed=CGAAAAGGGG Num.seqs=3 Similarity=0.703704
27 ATCCCCTTTTCGCAG
Cele-UNSB01_4:11303025-11303087 Satlength=63 Nr of Repeats=4 RepeatLength=15 seed=TCCCCTTTTC Num.seqs=3 Similarity=0.762963
16 ATCCCCTTTTCGCAG
Cele-UNSB01_4:15434795-15434872 Satlength=78 Nr of Repeats=5 RepeatLength=15 seed=TCCCCTTTTC Num.seqs=4 Similarity=0.748148
16 ATCCCCTTTTCGCAG
Cele-UNSB01_5:4531686-4531763 Satlength=78 Nr of Repeats=5 RepeatLength=15 seed=CCCCTTTTCG Num.seqs=4 Similarity=0.748148
17 ATCCCCTTTTCGCAG
Cele-UNSB01_5:16699651-16699786 Satlength=136 Nr of Repeats=7 RepeatLength=15 seed=GCAGATCCCC Num.seqs=6 Similarity=0.946667
26 ATCCCCTTTTCGCAG
Rev.of_Cele-UNSB01_5:21154449-21154526 Satlength=78 Nr of Repeats=5 RepeatLength=15 seed=CGAAAAGGGG Num.seqs=4 Similarity=0.822222
27 ATCCCCTTTTCGCAG
Cele-UNSB01_5:21154702-21154779 Satlength=78 Nr of Repeats=5 RepeatLength=15 seed=CCCCTTTTCG Num.seqs=4 Similarity=0.748148
17 ATCCCCTTTTCGCAG
Rev.of_Cele-UNSB01_6:276570-276632 Satlength=63 Nr of Repeats=4 RepeatLength=15 seed=CGAAAAGGGG Num.seqs=3 Similarity=0.762963
27 ATCCCCTTTTCGCAG
Rev.of_Cele-UNSB01_1:7149236-7149296 Satlength=61 Nr of Repeats=4 RepeatLength=15 seed=CGAAAAGGGG Num.seqs=4 Similarity=0.807407
27 ATCCCCTTTTCGCTG
Rev.of_Cele-UNSB01_1:13121380-13121635 Satlength=256 Nr of Repeats=17 RepeatLength=15 seed=AAAAGGGGAT Num.seqs=15 Similarity=0.935661
25 ATCCCCTTTTTGCAG
Cele-UNSB01_6:276806-276883 Satlength=78 Nr of Repeats=5 RepeatLength=15 seed=TCCCCTTTTC Num.seqs=4 Similarity=0.762963
16 ATCCCCTTTTCGTAG

```
**   **** *
Consensus:

cTCCCCTTTTCGtAG

>Cele-UNSB01_Fam_8_16_50  Nr. of seq. 50 Alignment length(with gaps) = 16
Alignment score = 0.867364
Cele-UNSB01_1:11481110-11481222 Satlength=113 Nr of Repeats=7
RepeatLength=16 seed=CTACATGCCT  Num.seqs=7 Similarity=0.805556
0    CTACATGCCTACATGC
Cele-UNSB01_1:11481110-11482230 Satlength=1121 Nr of Repeats=68
RepeatLength=16 seed=CTACATGCCT  Num.seqs=64 Similarity=0.868304
0    CTACATGCCTACATGC
Rev.of_Cele-UNSB01_1:11869240-11870103 Satlength=864 Nr of Repeats=53
RepeatLength=16 seed=GCATGTAGGC  Num.seqs=50 Similarity=0.957891
0    CTACATGCCTACATGC
Cele-UNSB01_2:13133064-13133208 Satlength=145 Nr of Repeats=9
RepeatLength=16 seed=GCCTACATGC  Num.seqs=7 Similarity=0.861111
6    CTACATGCCTACATGC
Rev.of_Cele-UNSB01_2:13460078-13460334 Satlength=257 Nr of Repeats=15
RepeatLength=16 seed=GGCATGTAGG  Num.seqs=13 Similarity=0.840812
1    CTACATGCCTACATGC
Rev.of_Cele-UNSB01_2:13465788-13466109 Satlength=322 Nr of Repeats=20
RepeatLength=16 seed=GGCATGTAGG  Num.seqs=19 Similarity=0.773879
1    CTACATGCCTACATGC
Cele-UNSB01_2:13663688-13663784 Satlength=97 Nr of Repeats=6
RepeatLength=16 seed=ACATGCCTAC  Num.seqs=6 Similarity=0.888889
2    CTACATGCCTACATGC
Cele-UNSB01_2:13794379-13794568 Satlength=190 Nr of Repeats=10
RepeatLength=16 seed=CCTACATGCC  Num.seqs=7 Similarity=0.976190
7    CTACATGCCTACATGC
Rev.of_Cele-UNSB01_3:3880760-3880928 Satlength=169 Nr of Repeats=10
RepeatLength=16 seed=TAGGCATGTA  Num.seqs=9 Similarity=0.912037
3    CTACATGCCTACATGC
Rev.of_Cele-UNSB01_4:600866-601084 Satlength=219 Nr of Repeats=10
RepeatLength=16 seed=AGGCATGTAG  Num.seqs=9 Similarity=0.796296
2    CTACATGCCTACATGC
Rev.of_Cele-UNSB01_4:2990271-2990613 Satlength=343 Nr of Repeats=19
RepeatLength=16 seed=TAGGCATGTA  Num.seqs=16 Similarity=0.859028
3    CTACATGCCTACATGC
Cele-UNSB01_4:3222029-3222419 Satlength=391 Nr of Repeats=5
RepeatLength=16 seed=TACATGCCTA  Num.seqs=4 Similarity=0.958333
1    CTACATGCCTACATGC
Cele-UNSB01_4:3226485-3226669 Satlength=185 Nr of Repeats=11
RepeatLength=16 seed=CATGCCTACA  Num.seqs=10 Similarity=0.816667
3    CTACATGCCTACATGC
Cele-UNSB01_4:3230422-3232144 Satlength=1723 Nr of Repeats=67
RepeatLength=16 seed=ATGCCTACAT  Num.seqs=46 Similarity=0.819565
4    CTACATGCCTACATGC
Rev.of_Cele-UNSB01_4:3274030-3274126 Satlength=97 Nr of Repeats=6
RepeatLength=16 seed=AGGCATGTAG  Num.seqs=6 Similarity=0.872222
2    CTACATGCCTACATGC
```

Cele-UNSB01_4:3630301-3630781 Satlength=481 Nr of Repeats=28 RepeatLength=16 seed=TGCCTACATG Num.seqs=23 Similarity=0.914032
5   CTACATGCCTACATGC
Rev.of_Cele-UNSB01_4:3888107-3888300 Satlength=194 Nr of Repeats=12 RepeatLength=16 seed=TAGGCATGTA Num.seqs=9 Similarity=0.981481
3   CTACATGCCTACATGC
Cele-UNSB01_5:345871-345991 Satlength=121 Nr of Repeats=7 RepeatLength=16 seed=CATGCCTACA Num.seqs=6 Similarity=0.972222
3   CTACATGCCTACATGC
Rev.of_Cele-UNSB01_5:353635-353859 Satlength=225 Nr of Repeats=14 RepeatLength=16 seed=GGCATGTAGG Num.seqs=14 Similarity=0.928571
1   CTACATGCCTACATGC
Cele-UNSB01_5:788141-788221 Satlength=81 Nr of Repeats=5 RepeatLength=16 seed=CTACATGCCT Num.seqs=5 Similarity=0.933333
0   CTACATGCCTACATGC
Rev.of_Cele-UNSB01_5:788660-789185 Satlength=526 Nr of Repeats=12 RepeatLength=16 seed=AGGCATGTAG Num.seqs=9 Similarity=0.907407
2   CTACATGCCTACATGC
Rev.of_Cele-UNSB01_5:790113-790281 Satlength=169 Nr of Repeats=10 RepeatLength=16 seed=AGGCATGTAG Num.seqs=9 Similarity=0.944444
2   CTACATGCCTACATGC
Cele-UNSB01_5:896085-896285 Satlength=201 Nr of Repeats=12 RepeatLength=16 seed=ATGCCTACAT Num.seqs=11 Similarity=0.956061
4   CTACATGCCTACATGC
Cele-UNSB01_5:2213030-2213142 Satlength=113 Nr of Repeats=6 RepeatLength=16 seed=ATGCCTACAT Num.seqs=4 Similarity=0.875000
4   CTACATGCCTACATGC
Rev.of_Cele-UNSB01_5:4763134-4763326 Satlength=193 Nr of Repeats=11 RepeatLength=16 seed=TAGGCATGTA Num.seqs=9 Similarity=0.944444
3   CTACATGCCTACATGC
Rev.of_Cele-UNSB01_5:3035465-3035958 Satlength=494 Nr of Repeats=4 RepeatLength=16 seed=GCATGTAGGC Num.seqs=3 Similarity=0.944444
0   CTACAAGCCTACATGC
Cele-UNSB01_3:11779996-11780140 Satlength=145 Nr of Repeats=7 RepeatLength=16 seed=TACAAGCCTA Num.seqs=5 Similarity=0.866667
1   CTACAAGCCTACAAGC
Cele-UNSB01_2:677942-678110 Satlength=169 Nr of Repeats=9 RepeatLength=16 seed=CCTACGTGCC Num.seqs=6 Similarity=0.844444
7   CTACGTGCCTACGTGC
Rev.of_Cele-UNSB01_2:2787652-2787836 Satlength=185 Nr of Repeats=11 RepeatLength=16 seed=CACGTAGGCA Num.seqs=10 Similarity=0.966667
7   CTACGTGCCTACGTGC
Rev.of_Cele-UNSB01_2:3154398-3154558 Satlength=161 Nr of Repeats=9 RepeatLength=16 seed=TAGGCACGTA Num.seqs=7 Similarity=0.849206
11   CTACGTGCCTACGTGC
Cele-UNSB01_2:3155808-3156160 Satlength=353 Nr of Repeats=18 RepeatLength=16 seed=TACGTGCCTA Num.seqs=12 Similarity=0.845960
9   CTACGTGCCTACGTGC
Rev.of_Cele-UNSB01_2:3220556-3221123 Satlength=568 Nr of Repeats=21 RepeatLength=16 seed=GTAGGCACGT Num.seqs=18 Similarity=0.936819
12   CTACGTGCCTACGTGC
Rev.of_Cele-UNSB01_2:3312389-3313069 Satlength=681 Nr of Repeats=42 RepeatLength=16 seed=CGTAGGCACG Num.seqs=39 Similarity=0.967611
13   CTACGTGCCTACGTGC

Rev.of_Cele-UNSB01_2:3460910-3461075 Satlength=166 Nr of Repeats=5
RepeatLength=16 seed=GCACGTAGGC Num.seqs=3 Similarity=0.944444
8   CTACGTGCCTACGTGC
Rev.of_Cele-UNSB01_2:3557247-3557399 Satlength=153 Nr of Repeats=9
RepeatLength=16 seed=GTAGGCACGT Num.seqs=8 Similarity=0.958333
12   CTACGTGCCTACGTGC
Cele-UNSB01_2:4095823-4096643 Satlength=821 Nr of Repeats=4
RepeatLength=16 seed=TGCCTACGTG Num.seqs=3 Similarity=1.000000
13   CTACGTGCCTACGTGC
Rev.of_Cele-UNSB01_2:13216705-13216849 Satlength=145 Nr of Repeats=8
RepeatLength=16 seed=TAGGCACGTA Num.seqs=6 Similarity=0.916667
11   CTACGTGCCTACGTGC
Rev.of_Cele-UNSB01_3:8579889-8580089 Satlength=201 Nr of Repeats=12
RepeatLength=16 seed=AGGCACGTAG Num.seqs=11 Similarity=0.887879
10   CTACGTGCCTACGTGC
Rev.of_Cele-UNSB01_3:10778310-10778713 Satlength=404 Nr of Repeats=14
RepeatLength=16 seed=GTAGGCACGT Num.seqs=9 Similarity=0.981481
12   CTACGTGCCTACGTGC
Rev.of_Cele-UNSB01_3:12493882-12494280 Satlength=399 Nr of Repeats=13
RepeatLength=16 seed=ACGTAGGCAC Num.seqs=10 Similarity=0.916667
14   CTACGTGCCTACGTGC
Rev.of_Cele-UNSB01_4:1777357-1777453 Satlength=97 Nr of Repeats=5
RepeatLength=16 seed=CACGTAGGCA Num.seqs=4 Similarity=0.916667
7   CTACGTGCCTACGTGC
Rev.of_Cele-UNSB01_4:1802551-1803063 Satlength=513 Nr of Repeats=30
RepeatLength=16 seed=TAGGCACGTA Num.seqs=26 Similarity=0.962564
11   CTACGTGCCTACGTGC
Cele-UNSB01_4:5124505-5124705 Satlength=201 Nr of Repeats=12
RepeatLength=16 seed=CTACGTGCCT Num.seqs=11 Similarity=0.969697
8   CTACGTGCCTACGTGC
Cele-UNSB01_4:12476016-12476336 Satlength=321 Nr of Repeats=18
RepeatLength=16 seed=GTGCCTACGT Num.seqs=14 Similarity=0.887363
12   CTACGTGCCTACGTGC
Rev.of_Cele-UNSB01_2:3182927-3183063 Satlength=137 Nr of Repeats=8
RepeatLength=16 seed=AGGCACGTAG Num.seqs=7 Similarity=0.853175
10   CTACGTGCCTACNTGC
Rev.of_Cele-UNSB01_2:14297875-14298051 Satlength=177 Nr of Repeats=10
RepeatLength=16 seed=TAGGCATATA Num.seqs=9 Similarity=0.842593
3   CTATATGCCTATATGC
Cele-UNSB01_3:3780853-3780925 Satlength=73 Nr of Repeats=4
RepeatLength=16 seed=CCTACATACC Num.seqs=3 Similarity=0.888889
7   CTACATACCTACATAC
Cele-UNSB01_5:16845717-16846129 Satlength=413 Nr of Repeats=25
RepeatLength=16 seed=ACATACCTAC Num.seqs=22 Similarity=0.969697
10   CTACATACCTACATAC
Rev.of_Cele-UNSB01_3:211856-211976 Satlength=121 Nr of Repeats=7
RepeatLength=16 seed=TAGGCATCTA Num.seqs=6 Similarity=0.827778
3   CTAGGTGCATAGATGC
Rev.of_Cele-UNSB01_4:3018891-3018971 Satlength=81 Nr of Repeats=4
RepeatLength=16 seed=AGGTAGGAAG Num.seqs=3 Similarity=0.722222
2   CTACCTACCTTCCTAC

***   * *   *
Consensus:

```
CTACaTGCCTACaTGC

>Cele-UNSB01_Fam_9_20_48  Nr. of seq. 48 Alignment length(with gaps) = 20
Alignment score = 0.805585
Cele-UNSB01_1:2574462-2585290 Satlength=10829 Nr of Repeats=565
RepeatLength=19 seed=TACACGAAAA  Num.seqs=539 Similarity=0.979754
0    TACACG-AAAAATAGATTGT
Rev.of_Cele-UNSB01_1:4530676-4530829 Satlength=154 Nr of Repeats=8
RepeatLength=19 seed=TTTTTCGTGT  Num.seqs=7 Similarity=0.900000
11    TACACG-AAAAATAGATTGT
Cele-UNSB01_3:4122367-4122965 Satlength=599 Nr of Repeats=31
RepeatLength=19 seed=AAAAATAGAT  Num.seqs=22 Similarity=1.000000
6    TACACG-AAAAATAGATTGT
Cele-UNSB01_3:6364525-6365839 Satlength=1315 Nr of Repeats=65
RepeatLength=19 seed=AAAAATAGAT  Num.seqs=61 Similarity=0.968479
6    TACACG-AAAAATAGATTGT
Rev.of_Cele-UNSB01_3:7913456-7913988 Satlength=533 Nr of Repeats=28
RepeatLength=19 seed=AATCTATTTT  Num.seqs=28 Similarity=0.920040
17    TACACG-AAAAATAGATTGT
Rev.of_Cele-UNSB01_3:7922958-7923224 Satlength=267 Nr of Repeats=14
RepeatLength=19 seed=CAATCTATTT  Num.seqs=14 Similarity=0.974552
18    TACACG-AAAAATAGATTGT
Cele-UNSB01_3:13559329-13561136 Satlength=1808 Nr of Repeats=95
RepeatLength=19 seed=AAAAATAGAT  Num.seqs=93 Similarity=0.940307
6    TACACG-AAAAATAGATTGT
Cele-UNSB01_4:10751931-10752311 Satlength=381 Nr of Repeats=20
RepeatLength=19 seed=ATTGTTACAC  Num.seqs=20 Similarity=0.967867
14    TACACG-AAAAATAGATTGT
Cele-UNSB01_4:17223540-17224619 Satlength=1080 Nr of Repeats=56
RepeatLength=19 seed=AAAAATAGAT  Num.seqs=41 Similarity=0.957039
6    TACACG-AAAAATAGATTGT
Rev.of_Cele-UNSB01_5:6955453-6955644 Satlength=192 Nr of Repeats=10
RepeatLength=19 seed=AACAATCTAT  Num.seqs=9 Similarity=1.000000
1    TACACG-AAAAATAGATTGT
Rev.of_Cele-UNSB01_5:9977175-9978242 Satlength=1068 Nr of Repeats=56
RepeatLength=19 seed=TGTAACAATC  Num.seqs=53 Similarity=0.979833
4    TACACG-AAAAATAGATTGT
Cele-UNSB01_5:13457267-13457761 Satlength=495 Nr of Repeats=26
RepeatLength=19 seed=GTTACACGAA  Num.seqs=26 Similarity=0.979703
17    TACACG-AAAAATAGATTGT
Cele-UNSB01_5:13461673-13462167 Satlength=495 Nr of Repeats=26
RepeatLength=19 seed=GTTACACGAA  Num.seqs=26 Similarity=0.979703
17    TACACG-AAAAATAGATTGT
Rev.of_Cele-UNSB01_5:17626210-17626494 Satlength=285 Nr of Repeats=15
RepeatLength=19 seed=TTTTCGTGTA  Num.seqs=14 Similarity=0.934452
10    TACACG-AAAAATAGATTGT
Rev.of_Cele-UNSB01_6:3115311-3123060 Satlength=7750 Nr of Repeats=398
RepeatLength=19 seed=AATCTATTTT  Num.seqs=272 Similarity=0.892683
17    TACACG-AAAAATAGATTGT
Cele-UNSB01_6:12589371-12589487 Satlength=117 Nr of Repeats=6
RepeatLength=19 seed=ATAGATTGTT  Num.seqs=4 Similarity=1.000000
10    TACACG-AAAAATAGATTGT
```

Cele-UNSB01_6:12827464-12828819 Satlength=1356 Nr of Repeats=70
RepeatLength=19 seed=AAAAATAGAT Num.seqs=45 Similarity=0.943434
6   TACACG-AAAAATAGATTGT
Cele-UNSB01_4:16493225-16495520 Satlength=2296 Nr of Repeats=119
RepeatLength=19 seed=AAATAGATTG Num.seqs=85 Similarity=0.941540
8   TACACG-GAAAATAGATTGT
Rev.of_Cele-UNSB01_1:11564527-11564628 Satlength=102 Nr of Repeats=5
RepeatLength=20 seed=TGTAACAATC Num.seqs=4 Similarity=0.933333
4   TACACGAAAAAAATAGATTGT
Cele-UNSB01_1:13507191-13507550 Satlength=360 Nr of Repeats=18
RepeatLength=20 seed=AAAATAGATT Num.seqs=17 Similarity=0.992157
8   TACACGAAAAAAATAGATTGT
Cele-UNSB01_3:11094399-11094498 Satlength=100 Nr of Repeats=5
RepeatLength=20 seed=GTTACACGAA Num.seqs=4 Similarity=1.000000
18   TACACGAAAAAAATAGATTGT
Cele-UNSB01_4:16093909-16094066 Satlength=158 Nr of Repeats=8
RepeatLength=20 seed=TGTTACACGA Num.seqs=5 Similarity=1.000000
17   TACACGAAAAAAATAGATTGT
Cele-UNSB01_5:1308547-1312321 Satlength=3775 Nr of Repeats=192
RepeatLength=20 seed=AAAATAGATT Num.seqs=127 Similarity=0.995817
8   TACACGAAAAAAATAGATTGT
Cele-UNSB01_5:18642200-18655941 Satlength=13742 Nr of Repeats=679
RepeatLength=20 seed=ACACGAAAAA Num.seqs=525 Similarity=0.915785
21   TACACGAAAAAAATAGATTGT
Cele-UNSB01_5:19009672-19010877 Satlength=1206 Nr of Repeats=61
RepeatLength=20 seed=AAAAATAGAT Num.seqs=52 Similarity=1.000000
7   TACACGAAAAAAATAGATTGT
Cele-UNSB01_6:2020674-2021510 Satlength=837 Nr of Repeats=42
RepeatLength=20 seed=GTTACACGAA Num.seqs=38 Similarity=1.000000
18   TACACGAAAAAAATAGATTGT
Cele-UNSB01_6:2029404-2030161 Satlength=758 Nr of Repeats=38
RepeatLength=20 seed=ACACGAAAAA Num.seqs=33 Similarity=1.000000
21   TACACGAAAAAAATAGATTGT
Cele-UNSB01_6:2038051-2039066 Satlength=1016 Nr of Repeats=51
RepeatLength=20 seed=GTTACACGAA Num.seqs=46 Similarity=1.000000
18   TACACGAAAAAAATAGATTGT
Rev.of_Cele-UNSB01_6:14480301-14480556 Satlength=256 Nr of Repeats=13
RepeatLength=20 seed=TAACAATCTA Num.seqs=8 Similarity=0.966667
22   TACACGAAAAAAATAGATTGT
Rev.of_Cele-UNSB01_6:16182513-16182592 Satlength=80 Nr of Repeats=4
RepeatLength=20 seed=TAACAATCTA Num.seqs=3 Similarity=0.955556
22   TACACGAAAAAAATAGATTGT
Cele-UNSB01_2:4043072-4059727 Satlength=16656 Nr of Repeats=833
RepeatLength=20 seed=ATTGTTACAC Num.seqs=812 Similarity=0.960325
15   TACACTAAAAAAATAGATTGT
Cele-UNSB01_4:6277867-6278002 Satlength=136 Nr of Repeats=7
RepeatLength=19 seed=ATTGACAGAC Num.seqs=5 Similarity=0.971930
14   CAGACG-AAAAATAGATTGA
Cele-UNSB01_4:12734217-12734312 Satlength=96 Nr of Repeats=5
RepeatLength=19 seed=AAAAATAGAT Num.seqs=5 Similarity=0.971930
25   CAGACG-AAAAATAGATTGA
Cele-UNSB01_5:5649265-5649420 Satlength=156 Nr of Repeats=8
RepeatLength=19 seed=AAAAATAGAT Num.seqs=5 Similarity=1.000000
25   CAGACG-AAAAATAGATTGG

Cele-UNSB01_3:1307999-1309276 Satlength=1278 Nr of Repeats=65
RepeatLength=20 seed=AAAAATAGAT Num.seqs=40 Similarity=0.893203
26   CAGACGAAAAAATAGATTGA
Cele-UNSB01_3:12312970-12313186 Satlength=217 Nr of Repeats=11
RepeatLength=20 seed=AAATAGATTG Num.seqs=7 Similarity=0.904762
28   CAGACGAAAAAATAGATTGA
Cele-UNSB01_6:4456017-4456156 Satlength=140 Nr of Repeats=7
RepeatLength=20 seed=ACAGACGAAA Num.seqs=6 Similarity=0.937778
38   CAGACGAAAAAATAGATTGA
Rev.of_Cele-UNSB01_6:7315610-7315788 Satlength=179 Nr of Repeats=9
RepeatLength=20 seed=TTTTTCGTCT Num.seqs=7 Similarity=0.961905
30   CAGACGAAAAAATAGATTGA
Cele-UNSB01_6:8127661-8127776 Satlength=116 Nr of Repeats=6
RepeatLength=19 seed=CAGACGAAAA Num.seqs=5 Similarity=0.887719
19   CAGACG-AAAAATATATTGG
Cele-UNSB01_1:3532132-3534432 Satlength=2301 Nr of Repeats=115
RepeatLength=20 seed=AAAAAATAGA Num.seqs=115 Similarity=0.971320
25   CATACCAAAAAATAGATTGA
Cele-UNSB01_1:14164675-14164835 Satlength=161 Nr of Repeats=8
RepeatLength=20 seed=AAAAAATAGA Num.seqs=8 Similarity=0.900000
25   CATACCAAAAAATAGATTGA
Cele-UNSB01_3:10216874-10217114 Satlength=241 Nr of Repeats=12
RepeatLength=20 seed=AAAAATAGAT Num.seqs=8 Similarity=0.950000
26   CATACCAAAAAATAGATTGA
Rev.of_Cele-UNSB01_3:11302620-11306800 Satlength=4181 Nr of Repeats=209
RepeatLength=20 seed=TCTATTTTTT Num.seqs=209 Similarity=0.952647
35   CATACCAAAAAATAGATTGA
Cele-UNSB01_6:4047987-4048126 Satlength=140 Nr of Repeats=7
RepeatLength=20 seed=AAAATAGATT Num.seqs=6 Similarity=0.955556
27   CATACCAAAAAATAGATTGA
Rev.of_Cele-UNSB01_2:894739-894896 Satlength=158 Nr of Repeats=8
RepeatLength=20 seed=CAATCTATTT Num.seqs=6 Similarity=0.902222
38   CATACCAAAAAATAGATTGN
Cele-UNSB01_6:16177777-16179557 Satlength=1781 Nr of Repeats=89
RepeatLength=20 seed=CATACCAAAA Num.seqs=85 Similarity=0.936956
39   CATACCAAAAAATAGATTGG
Cele-UNSB01_2:11560093-11560292 Satlength=200 Nr of Repeats=9
RepeatLength=20 seed=AAAAAATAGA Num.seqs=8 Similarity=0.892857
25   CATACGAAAAAATAGATTGG
Cele-UNSB01_1:14566637-14566717 Satlength=81 Nr of Repeats=4
RepeatLength=20 seed=ATATCAAAAA Num.seqs=4 Similarity=0.855556
40   CATATCAAAAAATAAATTGG

* *   ****** ****
Consensus:

tAcACGaAAAAATAGATTGt

>Cele-UNSB01_Fam_10_25_41 Nr. of seq. 41 Alignment length(with gaps) = 27 Alignment score = 0.791177
Cele-UNSB01_1:3815334-3818659 Satlength=3326 Nr of Repeats=133
RepeatLength=25 seed=TTTACTCTCT Num.seqs=133 Similarity=0.898680
0   TTTACTCTC--TGGCTTCACCAAATAT

Rev.of_Cele-UNSB01_6:10621572-10641051 Satlength=19480 Nr of Repeats=772
RepeatLength=25 seed=CAGAGAGTAA Num.seqs=526 Similarity=0.847899
11   TTTACTCTC--TGGCTTCACCAAATAT
Rev.of_Cele-UNSB01_2:5217526-5241162 Satlength=23637 Nr of Repeats=927
RepeatLength=25 seed=AGCCAGAGAG Num.seqs=627 Similarity=0.736670
14   TTTACTCTC--TGGCTTCACCAATTAT
Rev.of_Cele-UNSB01_3:1364177-1382615 Satlength=18439 Nr of Repeats=734
RepeatLength=25 seed=CAGAGAGTAA Num.seqs=587 Similarity=0.896061
36   TTTACTCTC--TGGCTTCACCAATTAT
Rev.of_Cele-UNSB01_2:5217429-5217681 Satlength=253 Nr of Repeats=9
RepeatLength=25 seed=AGAGTAAAAT Num.seqs=6 Similarity=0.823932
8   TTTACTCTC--TGGCTTTCCCAATTAT
Rev.of_Cele-UNSB01_1:6909627-6919602 Satlength=9976 Nr of Repeats=399
RepeatLength=25 seed=GAAGCCAGAG Num.seqs=399 Similarity=0.903264
16   TTTACTCTC--TGGCTTCACAGTATAT
Cele-UNSB01_2:9530992-9537371 Satlength=6380 Nr of Repeats=247
RepeatLength=25 seed=CTCTCTGGCT Num.seqs=194 Similarity=0.906588
29   TTTACTCTC--TGGCTTCACAGTATAT
Cele-UNSB01_4:5363528-5363903 Satlength=376 Nr of Repeats=15
RepeatLength=25 seed=TTTACTCTCT Num.seqs=15 Similarity=0.890286
25   TTTACTCTC--TGGCTTCACAGTATAT
Cele-UNSB01_5:10495318-10500018 Satlength=4701 Nr of Repeats=187
RepeatLength=25 seed=TTTACTCTCT Num.seqs=186 Similarity=0.897636
25   TTTACTCTC--TGGCTTCACAGTATAT
Rev.of_Cele-UNSB01_6:9421607-9443178 Satlength=21572 Nr of Repeats=857
RepeatLength=25 seed=AGAGAGTAAA Num.seqs=649 Similarity=0.731868
35   TTTACTCTC--TGGCTTCACAGTATAT
Cele-UNSB01_6:2154760-2154935 Satlength=176 Nr of Repeats=7
RepeatLength=25 seed=TATATTTTAC Num.seqs=7 Similarity=0.852698
20   TTTACTCTC--TGGCTTCACANTATAT
Cele-UNSB01_6:2156238-2156563 Satlength=326 Nr of Repeats=12
RepeatLength=25 seed=TCTGGCTTCA Num.seqs=11 Similarity=0.885576
32   TTTACTCTC--TGGCTTCACACTATAT
Cele-UNSB01_6:15253053-15270737 Satlength=17685 Nr of Repeats=696
RepeatLength=25 seed=TGGCTTCACA Num.seqs=628 Similarity=0.913347
34   TTTACTCTC--TGGCTTCACAATATAT
Rev.of_Cele-UNSB01_5:8047889-8048164 Satlength=276 Nr of Repeats=11
RepeatLength=25 seed=CCAGAGAGTA Num.seqs=11 Similarity=0.870061
37   TTTACTCTC--TGGCTTCACAGTATAA
Rev.of_Cele-UNSB01_2:4325046-4325721 Satlength=676 Nr of Repeats=27
RepeatLength=25 seed=GAGAGTAAAA Num.seqs=27 Similarity=0.936486
34   TTTACTCTC--TGGCTTCACCGTATAT
Rev.of_Cele-UNSB01_4:17753355-17753780 Satlength=426 Nr of Repeats=17
RepeatLength=25 seed=AGCCAGAGAG Num.seqs=17 Similarity=0.890980
39   TTTACTCTC--TGGCTTCACCGTATAT
Cele-UNSB01_4:17753798-17754998 Satlength=1201 Nr of Repeats=48
RepeatLength=25 seed=ACTCTCTGGC Num.seqs=48 Similarity=0.888227
53   TTTACTCTC--TGGCTTCACCGTATAT
Rev.of_Cele-UNSB01_3:2604631-2620875 Satlength=16245 Nr of Repeats=650
RepeatLength=25 seed=AGAGAGTAAA Num.seqs=546 Similarity=0.862999
35   TTTACTCTC--TGGCTTCACCATATAT
Cele-UNSB01_6:12186349-12201499 Satlength=15151 Nr of Repeats=591
RepeatLength=25 seed=CTCTCTGGCT Num.seqs=490 Similarity=0.859225
54   TTTACTCTC--TGGCTTCACCATATAT

Cele-UNSB01_4:6158202-6158352 Satlength=151 Nr of Repeats=6
RepeatLength=25 seed=TTTACTCTCT Num.seqs=6 Similarity=0.882667
25   TTTACTCTC--TGGCTTCACAGAATAA
Rev.of_Cele-UNSB01_5:14856483-14857645 Satlength=1163 Nr of Repeats=45
RepeatLength=25 seed=TGTGAAGCCA Num.seqs=41 Similarity=0.923265
44   TTTACTCTC--TGGCTTCACAGAATAA
Cele-UNSB01_4:13540735-13544977 Satlength=4243 Nr of Repeats=166
RepeatLength=25 seed=TTTACTCTCT Num.seqs=131 Similarity=0.786650
25   TTTACTCTC--TGGCTTCACAGAATAT
Rev.of_Cele-UNSB01_5:4305444-4315394 Satlength=9951 Nr of Repeats=398
RepeatLength=25 seed=AGTAAAATAT Num.seqs=346 Similarity=0.935129
31   TTTACTCTC--TGGCTTCACAGAATAT
Cele-UNSB01_6:14307881-14308131 Satlength=251 Nr of Repeats=10
RepeatLength=25 seed=TCTCTGGCTT Num.seqs=10 Similarity=0.913481
30   TTTACTCTC--TGGCTTCACAGAATAT
Rev.of_Cele-UNSB01_4:5945791-5945941 Satlength=151 Nr of Repeats=6
RepeatLength=25 seed=CCAGAGAGTA Num.seqs=6 Similarity=0.875556
37   TTTACTCTC--TGGTTTCCCAGTATAT
Cele-UNSB01_3:3456392-3470370 Satlength=13979 Nr of Repeats=556
RepeatLength=25 seed=ACTCTCTGGC Num.seqs=537 Similarity=0.870368
28   TTTACTCTC--TGGCTTCCCATCATAT
Cele-UNSB01_2:12990090-13000798 Satlength=10709 Nr of Repeats=416
RepeatLength=25 seed=TGGCTTCCCA Num.seqs=292 Similarity=0.913730
9   TTTACTCTC--TGGCTTCCCACTATAT
Cele-UNSB01_1:943054-953346 Satlength=10293 Nr of Repeats=369
RepeatLength=27 seed=TTTTACTCTC Num.seqs=309 Similarity=0.884164
24   TTTACTCTCTGTGGCTTCCCACTATAT
Cele-UNSB01_1:4635066-4668893 Satlength=33828 Nr of Repeats=1253
RepeatLength=27 seed=TGGCTTCCCA Num.seqs=1251 Similarity=1.000000
36   TTTACTCTCTGTGGCTTCCCACTATAT
Cele-UNSB01_2:3331473-3331608 Satlength=136 Nr of Repeats=5
RepeatLength=27 seed=TTTACTCTCT Num.seqs=5 Similarity=1.000000
25   TTTACTCTCTGTGGCTTCCCACTATAT
Cele-UNSB01_2:14535014-14566604 Satlength=31591 Nr of Repeats=1170
RepeatLength=27 seed=TGTGGCTTCC Num.seqs=1170 Similarity=0.999916
34   TTTACTCTCTGTGGCTTCCCACTATAT
Rev.of_Cele-UNSB01_3:958162-973687 Satlength=15526 Nr of Repeats=569
RepeatLength=27 seed=TGGGAAGCCA Num.seqs=519 Similarity=0.929610
46   TTTACTCTCTGTGGCTTCCCACTATAT
Rev.of_Cele-UNSB01_3:7587437-7632311 Satlength=44875 Nr of Repeats=1482
RepeatLength=27 seed=GTGGGAAGCC Num.seqs=1481 Similarity=0.999867
47   TTTACTCTCTGTGGCTTCCCACTATAT
Rev.of_Cele-UNSB01_4:9095021-9107795 Satlength=12775 Nr of Repeats=451
RepeatLength=27 seed=ATAGTGGGAA Num.seqs=334 Similarity=0.885216
50   TTTACTCTCTGTGGCTTCCCACTATAT
Rev.of_Cele-UNSB01_6:122121-133095 Satlength=10975 Nr of Repeats=405
RepeatLength=27 seed=ACAGAGAGTA Num.seqs=396 Similarity=0.948514
37   TTTACTCTCTGTGGCTTCCCACTATAT
Cele-UNSB01_6:5238384-5284176 Satlength=45793 Nr of Repeats=1482
RepeatLength=27 seed=TGTGGCTTCC Num.seqs=1481 Similarity=1.000000
34   TTTACTCTCTGTGGCTTCCCACTATAT
Rev.of_Cele-UNSB01_5:14878525-14883699 Satlength=5175 Nr of Repeats=182
RepeatLength=27 seed=GGAAGCCACA Num.seqs=139 Similarity=0.948635
44   TTTACTCTCTGTGGCTTCCCACCATAT

Cele-UNSB01_6:1814276-1830201 Satlength=15926 Nr of Repeats=595
RepeatLength=27 seed=TTACTCTCTG Num.seqs=528 Similarity=0.899607
53   TTTACTCTCTGTGGCTTCCCACCATAT
Rev.of_Cele-UNSB01_2:12988123-12990065 Satlength=1943 Nr of Repeats=72
RepeatLength=27 seed=GTGGGAAGCC Num.seqs=71 Similarity=0.975358
47   TTTACTCTCTATGGCTTCCCACTATAT
Rev.of_Cele-UNSB01_3:958146-965823 Satlength=7678 Nr of Repeats=284
RepeatLength=27 seed=AGAGAGTAAA Num.seqs=246 Similarity=0.923613
35   TTTACTCTCTGTGGCTTCCCAATATAT
Cele-UNSB01_5:1115366-1136806 Satlength=21441 Nr of Repeats=722
RepeatLength=27 seed=TCTGTGGCTT Num.seqs=708 Similarity=0.928835
32   TTTACTCTCTGTGGCTTCACAGTATAT

********* *** ** *   **
Consensus:

TTTACTCTCTGGCTTCaCantATAT

>Cele-UNSB01_Fam_11_45_30 Nr. of seq. 30 Alignment length(with gaps) =
46 Alignment score = 0.806030
Cele-UNSB01_2:11366885-11367108 Satlength=224 Nr of Repeats=5
RepeatLength=44 seed=AAGGATCAGA Num.seqs=3 Similarity=0.696296
0   AAGGATCAGAACATCA-GGAAG-AGCCTCGTGATCCTTTGGAGCTG
Rev.of_Cele-UNSB01_1:3600639-3600909 Satlength=271 Nr of Repeats=6
RepeatLength=45 seed=CTGAATCTTC Num.seqs=6 Similarity=0.990123
9   AAGATTCAGATCATAAAGGAGG-ATCCTTGTGATCCTTTGGAGCTG
Cele-UNSB01_5:18556604-18557205 Satlength=602 Nr of Repeats=6
RepeatLength=45 seed=GGGGGACCCT Num.seqs=5 Similarity=0.940741
17   AAGATTCAGTTCATAAGGGGGG-ACCCTTATGATCCTTTGGAGCTG
Rev.of_Cele-UNSB01_1:685710-685978 Satlength=269 Nr of Repeats=6
RepeatLength=45 seed=TCTGATCCTT Num.seqs=4 Similarity=0.792593
10   AAGGATCAGACCATCA-GGAGGTACCCTTGTGATCCTTCTTATCTG
Rev.of_Cele-UNSB01_3:7846966-7847368 Satlength=403 Nr of Repeats=9
RepeatLength=45 seed=TCTGATCCTT Num.seqs=6 Similarity=0.857778
10   AAGGATCAGACCATCA-GGAGGTACCCTTGTGATCCTTCTTATCTG
Cele-UNSB01_1:6114632-6114855 Satlength=224 Nr of Repeats=5
RepeatLength=45 seed=AAGGATCAGA Num.seqs=3 Similarity=0.841975
45   AAGGATCAGACCATCA-GAAGGTACCCTTACGATCCTTCTTATCTG
Rev.of_Cele-UNSB01_5:11176286-11176509 Satlength=224 Nr of Repeats=5
RepeatLength=45 seed=TCTGATCCTT Num.seqs=3 Similarity=0.822222
55   AAGGATCAGACCATCA-GGAGGTACCCTTACGATTCTTCTTATCTG
Rev.of_Cele-UNSB01_1:1691009-1691188 Satlength=180 Nr of Repeats=4
RepeatLength=45 seed=TCTGATCCTT Num.seqs=3 Similarity=0.743210
10   AAGGATCAGAACATCA-GAAGGTACCCATGTGATCCTTCGGAGCTG
Rev.of_Cele-UNSB01_5:14452450-14453298 Satlength=849 Nr of Repeats=19
RepeatLength=45 seed=TCTGATCCTT Num.seqs=12 Similarity=0.868013
10   AAGGATCAGAACATCA-GAAGGTACCCATGTGATCCTTCGGAGCTG
Rev.of_Cele-UNSB01_1:3798271-3798674 Satlength=404 Nr of Repeats=9
RepeatLength=45 seed=TCTGATCCTT Num.seqs=7 Similarity=0.825044
10   AAGGATCAGANCATCA-GAAGGTACCCATGTGATCCTTCGGAGCTG
Cele-UNSB01_1:3799094-3799407 Satlength=314 Nr of Repeats=7
RepeatLength=45 seed=CTGAAGGATC Num.seqs=5 Similarity=0.792593
42   AAGGATCAGANCATCA-GAAGGTACCCATGTGATCCTTCGGAGCTG

Cele-UNSB01_2:11667411-11669597 Satlength=2187 Nr of Repeats=49
RepeatLength=45 seed=GAAGGATCAG Num.seqs=30 Similarity=0.857233
44   AAGGATCAGANCATCA-GAAGGTACCCATGTGATCCTTCGGAGCTG
Rev.of_Cele-UNSB01_1:6112428-6113232 Satlength=805 Nr of Repeats=18
RepeatLength=45 seed=TCTGATCCTT Num.seqs=12 Similarity=0.835690
10   AAGGATCAGAACATCA-GAAGGTACCCTTGTGATCCTTCGGATCTG
Rev.of_Cele-UNSB01_2:11664501-11665796 Satlength=1296 Nr of Repeats=29
RepeatLength=45 seed=TCTGATCCTT Num.seqs=19 Similarity=0.857050
10   AAGGATCAGANCATCA-GAAGGTACCCATGTGATCCTTCGGATCTG
Cele-UNSB01_2:6176621-6177425 Satlength=805 Nr of Repeats=18
RepeatLength=45 seed=CTGAAGGATC Num.seqs=12 Similarity=0.850954
42   AAGGATCAGAACATCA-GAAGGTACCCATACGATCCTTCGGAGCTG
Cele-UNSB01_5:11176622-11177248 Satlength=627 Nr of Repeats=14
RepeatLength=45 seed=CTGAAGGATC Num.seqs=10 Similarity=0.868313
42   AAGGATCAGAACATCA-GAAGGTACCCATACGATCCTTCGGAGCTG
Cele-UNSB01_5:14454544-14454902 Satlength=359 Nr of Repeats=8
RepeatLength=45 seed=AAGGATCAGA Num.seqs=6 Similarity=0.857778
45   AAGGATCAGAACATCA-GAAGGTACCCATACGATCCTTCGGAGCTG
Rev.of_Cele-UNSB01_3:2604301-2604569 Satlength=269 Nr of Repeats=6
RepeatLength=45 seed=TCTGATCCTT Num.seqs=4 Similarity=0.802469
10   AAGGATCAGACCATCA-GGAGGTACCCATGTGATCCTTCGGATCTG
Cele-UNSB01_3:7847664-7848155 Satlength=492 Nr of Repeats=11
RepeatLength=45 seed=GAAGGATCAG Num.seqs=7 Similarity=0.820811
44   AAGGATCAGANCATCA-GGAGGTACCCATGTGATCCTTCGGATCTG
Rev.of_Cele-UNSB01_3:11090269-11090626 Satlength=358 Nr of Repeats=8
RepeatLength=45 seed=TCTGATCCTT Num.seqs=5 Similarity=0.801481
10   AAGGATCAGANCATCA-GGAGGTACCCATGTGATCCTTCGGATCTG
Cele-UNSB01_4:355820-356311 Satlength=492 Nr of Repeats=11
RepeatLength=45 seed=GAAGGATCAG Num.seqs=7 Similarity=0.826455
44   AAGGATCAGANCATCA-GGAGGTACCCATGTGATCCTTCGGATCTG
Rev.of_Cele-UNSB01_4:8289359-8289627 Satlength=269 Nr of Repeats=6
RepeatLength=45 seed=TCTGATCCTT Num.seqs=4 Similarity=0.851852
10   AAGGATCAGACCATCA-GGAGGTACCCTTACGATCCTTCGGAGCTG
Cele-UNSB01_2:3989433-3989722 Satlength=290 Nr of Repeats=6
RepeatLength=45 seed=AAGGATCAGA Num.seqs=5 Similarity=0.643210
45   AAGGATCAGATCANCA-GGAGGTACCCTTCTGATCCTTCGGAGCTG
Rev.of_Cele-UNSB01_2:12892893-12893074 Satlength=182 Nr of Repeats=4
RepeatLength=45 seed=ATCTGATCCT Num.seqs=3 Similarity=0.950617
56   AAGGATCAGATCACCA-GGAGGTACCCTTNTGATCCTTCGGAGCTG
Cele-UNSB01_4:3428944-3430273 Satlength=1330 Nr of Repeats=24
RepeatLength=45 seed=TCTGATCCTT Num.seqs=17 Similarity=0.598811
72   AAGGATCAGATCANCA-GGAGGTACCCTTCTGATCCTTCGNAGCTG
Rev.of_Cele-UNSB01_4:9825630-9825857 Satlength=228 Nr of Repeats=5
RepeatLength=45 seed=GGGTACCTCC Num.seqs=3 Similarity=0.920988
71   AAGGATCAGATCACCA-GGAGGTACCCTTCTGATCTTTCGGAGCTG
Rev.of_Cele-UNSB01_4:11013752-11031328 Satlength=17577 Nr of Repeats=392
RepeatLength=45 seed=TCCTGGTGAT Num.seqs=347 Similarity=0.928761
64   AAGGATCAGATCACCA-GGAGGTACCCTTCTGATCCTTCGGAGCTT
Cele-UNSB01_4:11032975-11034460 Satlength=1486 Nr of Repeats=33
RepeatLength=45 seed=TCACCAGGAG Num.seqs=33 Similarity=0.911616
100   AAGGATCAGATCACCA-GGAGGTACCCTTCTGATCCTTCGGAGCTT
Cele-UNSB01_2:13755448-13762935 Satlength=7488 Nr of Repeats=166
RepeatLength=45 seed=ACCAGGAGGT Num.seqs=165 Similarity=0.932682
57   AATGATCAGATCACCA-GGAGGTACCCTTCTTATCCTTCGGAGCTG

Rev.of_Cele-UNSB01_1:10648848-10654019 Satlength=5172 Nr of Repeats=115
RepeatLength=45 seed=AAGGATCACA Num.seqs=114 Similarity=0.941734
37 AAGGATCAGATCATCA-GAAACTACCCTTGTGATCCTTCGAAGCTG

**   **** ** * *   * **   ** **   * **
Consensus:

AAGGATCAGAnCAtCAGgAGGTACCCtTgtGATCCTTCGgAgCTG

>Cele-UNSB01_Fam_12_20_29 Nr. of seq. 29 Alignment length(with gaps) = 25 Alignment score = 0.605057
Cele-UNSB01_1:356425-357011 Satlength=587 Nr of Repeats=29
RepeatLength=19 seed=TATCGATTTT Num.seqs=25 Similarity=0.914854
0 TATCGATTTT-C---TG-G-AATT
Rev.of_Cele-UNSB01_5:19313130-19313974 Satlength=845 Nr of Repeats=33
RepeatLength=20 seed=AAAATCGATA Num.seqs=22 Similarity=0.814643
10 TATCGATTTT-C---TG-GAAATT
Cele-UNSB01_5:19270843-19271693 Satlength=851 Nr of Repeats=31
RepeatLength=20 seed=ATTTATTGAT Num.seqs=20 Similarity=0.886667
17 TATCGATTTTT-C---TG-GAAATT
Cele-UNSB01_5:19398669-19400674 Satlength=2006 Nr of Repeats=80
RepeatLength=20 seed=TATCGATTTT Num.seqs=72 Similarity=0.876291
20 TATCGATTTTT-C---TG-GAAATT
Cele-UNSB01_5:19279992-19280235 Satlength=244 Nr of Repeats=8
RepeatLength=20 seed=TATCGATTTT Num.seqs=5 Similarity=0.900000
20 TATCGATTTTT-C---TG-CAAATT
Cele-UNSB01_5:20220047-20220618 Satlength=572 Nr of Repeats=12
RepeatLength=20 seed=TTATTGATTT Num.seqs=8 Similarity=0.876190
19 TATTGATTTTT-A---TG-GAAATT
Rev.of_Cele-UNSB01_1:1841619-1842039 Satlength=421 Nr of Repeats=18
RepeatLength=19 seed=AAAATCAATA Num.seqs=14 Similarity=0.796414
10 TATTGATTTT--C---TT-AGAATT
Rev.of_Cele-UNSB01_1:1841619-1843394 Satlength=1776 Nr of Repeats=44
RepeatLength=19 seed=AAAATCAATA Num.seqs=32 Similarity=0.769949
10 TATTGATTTT--C---TN-NGAATT
Rev.of_Cele-UNSB01_4:1755267-1755667 Satlength=401 Nr of Repeats=20
RepeatLength=20 seed=CAGAAAAATC Num.seqs=20 Similarity=0.835088
14 TATTGATTTTT-C---TG-AGAATT
Cele-UNSB01_5:18890478-18890638 Satlength=161 Nr of Repeats=8
RepeatLength=20 seed=GATTTTTCTG Num.seqs=8 Similarity=0.871429
24 TATTGATTTTT-C---TG-AAAATT
Rev.of_Cele-UNSB01_5:18933758-18934184 Satlength=427 Nr of Repeats=12
RepeatLength=20 seed=AGAAAAATCA Num.seqs=8 Similarity=0.902381
33 TATTGATTTTT-C---TG-AAAATT
Rev.of_Cele-UNSB01_5:19272660-19272781 Satlength=122 Nr of Repeats=6
RepeatLength=20 seed=ATAAATTTTC Num.seqs=5 Similarity=0.960000
43 TATTGATTTTT-C---TG-AAAATT
Rev.of_Cele-UNSB01_5:19385136-19386466 Satlength=1331 Nr of Repeats=44
RepeatLength=20 seed=AAAAATCAAT Num.seqs=31 Similarity=0.770186
31 TATTGATTTTT-C---TG-AAAATT
Cele-UNSB01_5:19386514-19386960 Satlength=447 Nr of Repeats=10
RepeatLength=20 seed=ATTGATTTTT Num.seqs=8 Similarity=0.861905
41 TATTGATTTTT-C---TG-AAAATT

Cele-UNSB01_5:19416239-19417040 Satlength=802 Nr of Repeats=34
RepeatLength=20 seed=GATTTTTCTG Num.seqs=28 Similarity=0.875661
24   TATTGATTTTT-C---TG-AAAATT
Rev.of_Cele-UNSB01_5:19387732-19388133 Satlength=402 Nr of Repeats=17
RepeatLength=20 seed=CAGAAAAATC Num.seqs=12 Similarity=0.905051
14   TATTGATTTTT-C---TG-GAAATT
Cele-UNSB01_5:20212391-20212631 Satlength=241 Nr of Repeats=10
RepeatLength=20 seed=TTATTGATTT Num.seqs=8 Similarity=0.866667
19   TATTGATTTTT-C---TG-GAAATT
Rev.of_Cele-UNSB01_5:20217981-20218181 Satlength=201 Nr of Repeats=9
RepeatLength=20 seed=CAGAAAAATC Num.seqs=8 Similarity=0.873810
14   TATTGATTTTT-C---TG-GAAATT
Cele-UNSB01_5:19407563-19408121 Satlength=559 Nr of Repeats=25
RepeatLength=20 seed=AATTTATTGA Num.seqs=16 Similarity=0.823333
16   TATTGATTTTT-T---TG-GAAATT
Rev.of_Cele-UNSB01_6:210105-210613 Satlength=509 Nr of Repeats=10
RepeatLength=20 seed=AAAAATCAAT Num.seqs=9 Similarity=0.907407
31   TATTGATTTTT-N---TG-GAAATT
Rev.of_Cele-UNSB01_1:11696998-11697105 Satlength=108 Nr of Repeats=5
RepeatLength=19 seed=TCAAAAATCA Num.seqs=4 Similarity=0.873611
12   -ATTGATTTTT-GA--TG--AAATT
Rev.of_Cele-UNSB01_1:11591612-11591946 Satlength=335 Nr of Repeats=14
RepeatLength=20 seed=AAAAATCAAT Num.seqs=11 Similarity=0.883636
29   -ATTGATTTTTCGA--TG--AAATT
Rev.of_Cele-UNSB01_1:11696961-11698223 Satlength=1263 Nr of Repeats=20
RepeatLength=19 seed=AAAAATCGAT Num.seqs=12 Similarity=0.725830
29   -ATCGATTTTT-GA--NG--AAATT
Rev.of_Cele-UNSB01_2:2648748-2648862 Satlength=115 Nr of Repeats=6
RepeatLength=19 seed=AAAATCGATA Num.seqs=6 Similarity=0.601058
47   -ATCGATTTTC-GA--TG--AAATT
Rev.of_Cele-UNSB01_1:11744458-11744933 Satlength=476 Nr of Repeats=17
RepeatLength=19 seed=TTTCACAAAA Num.seqs=12 Similarity=0.943647
17   -ATTGATTTTT-G---TGA-AAATT
Rev.of_Cele-UNSB01_1:11743735-11745857 Satlength=2123 Nr of Repeats=45
RepeatLength=19 seed=AAAATCGATA Num.seqs=29 Similarity=0.730706
29   -ATCGATTTTT-N---TGA-AAATT
Rev.of_Cele-UNSB01_4:984710-985413 Satlength=704 Nr of Repeats=35
RepeatLength=19 seed=CAATAATTTC Num.seqs=27 Similarity=0.905233
24   -ATTGATTTTT-T-G-GG--AAATT
Cele-UNSB01_1:11592271-11597890 Satlength=5620 Nr of Repeats=277
RepeatLength=20 seed=ATTGATTTTT Num.seqs=273 Similarity=0.884518
40   -ATTGATTTTT-T-GAGG--AAATT
Cele-UNSB01_1:2396315-2396849 Satlength=535 Nr of Repeats=20
RepeatLength=20 seed=TTATTGATTT Num.seqs=12 Similarity=0.816162
38   -ATTGATTTTT-C-TAGG--ACATT

** ******   ***
Consensus:

tATtGATTTTTcTGnAAATT

>Cele-UNSB01_Fam_13_31_27 Nr. of seq. 27 Alignment length(with gaps) =
32 Alignment score = 0.817159

Cele-UNSB01_3:81693-82933 Satlength=1241 Nr of Repeats=39 RepeatLength=31 seed=TAAATAATTA Num.seqs=38 Similarity=0.865584
0 TAAATAATTAGCAGA-CCAAAGTTGGGTCTGC
Cele-UNSB01_3:88388-88698 Satlength=311 Nr of Repeats=9 RepeatLength=31 seed=TGCTAAATAA Num.seqs=8 Similarity=0.907834
28 TAAATAATTAGCAGA-CCAAAGTTGGGTATGC
Rev.of_Cele-UNSB01_3:523916-525062 Satlength=1147 Nr of Repeats=37 RepeatLength=31 seed=GGTCTGCTAA Num.seqs=31 Similarity=0.865048
48 TAAATAATTAGCAGA-CCAAAGTTGGGTATGC
Cele-UNSB01_3:614340-614495 Satlength=156 Nr of Repeats=5 RepeatLength=31 seed=TGGGTATGCT Num.seqs=5 Similarity=1.000000
53 TAAATAATTAGCAGA-CCAAAGTTGGGTATGC
Rev.of_Cele-UNSB01_3:611245-611586 Satlength=342 Nr of Repeats=11 RepeatLength=31 seed=GGTCTGCTAA Num.seqs=11 Similarity=0.968719
17 TAAATAATTAGCAGA-CCGAAGTTGGGTATGC
Rev.of_Cele-UNSB01_3:344303-344830 Satlength=528 Nr of Repeats=17 RepeatLength=31 seed=TGGTCTGCTA Num.seqs=15 Similarity=0.868920
18 TAAATATTTAGCAGA-CCAAAGTTGGGTATGC
Cele-UNSB01_3:356762-357146 Satlength=385 Nr of Repeats=13 RepeatLength=31 seed=TTAGCAGACC Num.seqs=11 Similarity=0.960899
38 TAAATATTTAGCAGA-CCAAAGTTGGGTATGC
Cele-UNSB01_3:495426-496459 Satlength=1034 Nr of Repeats=16 RepeatLength=31 seed=TTTAGCAGAC Num.seqs=10 Similarity=0.931183
37 TAAATATTTAGCAGA-CCAAAGTTGGGTATGC
Cele-UNSB01_3:503013-503261 Satlength=249 Nr of Repeats=8 RepeatLength=31 seed=TTGGGTATGC Num.seqs=8 Similarity=0.970814
21 TAAATATTTAGCAGA-CCAAAGTTGGGTATGC
Cele-UNSB01_3:506857-507105 Satlength=249 Nr of Repeats=8 RepeatLength=31 seed=GTATGCTAAA Num.seqs=8 Similarity=0.967742
25 TAAATATTTAGCAGA-CCAAAGTTGGGTATGC
Rev.of_Cele-UNSB01_3:516006-516440 Satlength=435 Nr of Repeats=14 RepeatLength=31 seed=GGTCTGCTAA Num.seqs=14 Similarity=0.954626
48 TAAATATTTAGCAGA-CCAAAGTTGGGTATGC
Cele-UNSB01_3:517798-519152 Satlength=1355 Nr of Repeats=34 RepeatLength=31 seed=TTAGCAGACC Num.seqs=25 Similarity=0.924301
38 TAAATATTTAGCAGA-CCAAAGATGGGTATGC
Cele-UNSB01_3:882434-882682 Satlength=249 Nr of Repeats=7 RepeatLength=31 seed=TAAATATTTA Num.seqs=6 Similarity=0.862366
31 TAAATATTTAGCAGA-CCAAAGTTAGGTATGC
Rev.of_Cele-UNSB01_3:192738-193322 Satlength=585 Nr of Repeats=4 RepeatLength=31 seed=TTAGCAGACC Num.seqs=3 Similarity=0.942652
3 TAAATAATTAGCAGA-CCATAGTTGGGTCTGC
Cele-UNSB01_3:421701-421887 Satlength=187 Nr of Repeats=6 RepeatLength=31 seed=TTGGGTCTGC Num.seqs=6 Similarity=0.868100
21 TAAATAATTAGCAGA-CCATAATTGGGTCTGC
Rev.of_Cele-UNSB01_3:437143-437747 Satlength=605 Nr of Repeats=6 RepeatLength=31 seed=TATTTAGCAG Num.seqs=5 Similarity=0.901075
37 TAAATAATTAGCAGA-CCATAATTGGGTCTGC
Rev.of_Cele-UNSB01_3:194647-195253 Satlength=607 Nr of Repeats=5 RepeatLength=31 seed=TTAGCAGACC Num.seqs=3 Similarity=0.971326
3 TAATTATTTAGCAGA-CCATACTTGGGTCTGC
Rev.of_Cele-UNSB01_3:196367-196735 Satlength=369 Nr of Repeats=7 RepeatLength=31 seed=GGTCTGCTAA Num.seqs=6 Similarity=0.868100
17 TAAATATTTAGCAGA-CCATACTTGGGTCTGC

Rev.of_Cele-UNSB01_3:198288-198567 Satlength=280 Nr of Repeats=9
RepeatLength=31 seed=ACCCAAGTAT Num.seqs=9 Similarity=0.906810
27   TAAACATTTAGCAGA-CCATACTTGGGTCTGC
Cele-UNSB01_3:10130069-10130193 Satlength=125 Nr of Repeats=4
RepeatLength=31 seed=TCTGCTAAAT Num.seqs=4 Similarity=0.878136
26   TAAATAATTAGCAGA-CCATACTTGATTCTGC
Cele-UNSB01_3:347263-347821 Satlength=559 Nr of Repeats=17
RepeatLength=31 seed=TATGCTAAAT Num.seqs=16 Similarity=0.883154
26   TAAATATTTAGCAGA-CCAAAAGTAGGTATGC
Cele-UNSB01_3:350896-352295 Satlength=1400 Nr of Repeats=23
RepeatLength=31 seed=TTAGCAGACC Num.seqs=22 Similarity=0.867430
38   TAAATATTTAGCAGA-CCAAAANTAGGTATGC
Rev.of_Cele-UNSB01_3:355335-355552 Satlength=218 Nr of Repeats=7
RepeatLength=31 seed=CTGCTAAATA Num.seqs=7 Similarity=0.938556
45   TAAATATTTAGCAGA-CCCAAAGTTGGTATGC
Rev.of_Cele-UNSB01_3:665955-666358 Satlength=404 Nr of Repeats=13
RepeatLength=31 seed=TGGGTCTGCT Num.seqs=13 Similarity=0.912876
50   TAAATATTTAGCAGA-CCCACAGTTGGTCTGC
Rev.of_Cele-UNSB01_3:10214948-10215134 Satlength=187 Nr of Repeats=6
RepeatLength=31 seed=TCTGCTAAAT Num.seqs=6 Similarity=0.962724
46   TAAATATTTAGCAGA-CCCAATGTGGGTATGC
Cele-UNSB01_3:10234987-10235185 Satlength=199 Nr of Repeats=4
RepeatLength=31 seed=TATGCTAAAT Num.seqs=3 Similarity=0.812500
57   TAAATATTTAGCAGA-CCCAANGTGGNTATGC
Cele-UNSB01_3:660202-660394 Satlength=193 Nr of Repeats=6 RepeatLength=31
seed=TTAGCAGACC Num.seqs=4 Similarity=0.781250
7   TAAATAATTAGCAGACCCATAGGTAGGTATGC

*** * ******** **   *   * ***
Consensus:

TAAATAtTTAGCAGACCAaAgtTgGGTaTGC

>Cele-UNSB01_Fam_14_43_26 Nr. of seq. 26 Alignment length(with gaps) =
46 Alignment score = 0.721271
Cele-UNSB01_1:1346901-1347222 Satlength=322 Nr of Repeats=5
RepeatLength=42 seed=TTTGCCGGAA Num.seqs=3 Similarity=0.705426
0   TTTGCCGGAAATT-TTCATTTCCGGCAA-ATTGCCGATTTGNCG--
Cele-UNSB01_2:13106081-13106253 Satlength=173 Nr of Repeats=4
RepeatLength=43 seed=CGGCAAATTA Num.seqs=4 Similarity=0.803618
21   TTTGCCGGAAATT-TTCATTTCCGGCAA-ATTACCAATTTGCCGA-
Rev.of_Cele-UNSB01_1:1822719-1823169 Satlength=451 Nr of Repeats=9
RepeatLength=43 seed=AAATCGGCAA Num.seqs=7 Similarity=0.793282
3   TTTGCCGGAAATT-TTCAATTCCGGCAA-ATTGCCGGTTTGCCGA-
Cele-UNSB01_2:13060029-13060418 Satlength=390 Nr of Repeats=5
RepeatLength=43 seed=GATTTGCCGG Num.seqs=3 Similarity=0.875969
41   TTTGCCGGAAATT-TTCAATCCCGGCAA-ATTGCAGGTTTGCCGA-
Cele-UNSB01_4:2163715-2164250 Satlength=536 Nr of Repeats=8
RepeatLength=43 seed=TTGCCGGAAA Num.seqs=5 Similarity=0.868148
44   TTTGCCGGAAATT-TTCAATCCCGGCAT-TTTGCCGGTTTGCCGA-
Rev.of_Cele-UNSB01_1:3471234-3471484 Satlength=251 Nr of Repeats=6
RepeatLength=43 seed=TTGAAAATTT Num.seqs=5 Similarity=0.810853
18   TTTGCCGAAAATT-TTCAATTCCGCCAA-TTTGCCGGTTTGCCGA-

Rev.of_Cele-UNSB01_2:3141695-3142468 Satlength=774 Nr of Repeats=16
RepeatLength=43 seed=TTGCCGGAAT Num.seqs=13 Similarity=0.825084
27   TTTACAGGAAATT-TTCAATTCCGGCAA-GTTGCCGGTTTGCCGA-
Rev.of_Cele-UNSB01_4:3002005-3002220 Satlength=216 Nr of Repeats=4
RepeatLength=43 seed=TTTGCCGGAA Num.seqs=3 Similarity=0.739899
28   TTTNCAGGAAATT-TTCAATTCCGGCAA-ACTGCCGGTTTGCAGA-
Rev.of_Cele-UNSB01_1:2293586-2294094 Satlength=509 Nr of Repeats=12
RepeatLength=43 seed=AATTTCCGGC Num.seqs=11 Similarity=0.790839
13   TTTGCCGGAAATT-TTTATTTTCGGCAA-NTTGCCGGTTTGCCGA-
Rev.of_Cele-UNSB01_1:11685636-11685851 Satlength=216 Nr of Repeats=5
RepeatLength=43 seed=CGGCAAATCG Num.seqs=5 Similarity=0.801550
50   TTTGCCGGAAATT-TTCATTTTCGGCAA-NTTGCCGGTTTGCCGA-
Rev.of_Cele-UNSB01_2:12544046-12545336 Satlength=1291 Nr of Repeats=30
RepeatLength=43 seed=TTTCCGGCAA Num.seqs=30 Similarity=0.867914
54   TTTGCCGGAAATT-TTCATTTTCGGCAA-ATTGCCGGTTTGCCGA-
Cele-UNSB01_3:12321003-12321555 Satlength=553 Nr of Repeats=10
RepeatLength=43 seed=AATTGCCGGT Num.seqs=7 Similarity=0.748854
69   TTTGCCGGAAAAT-TTCATTTTCGGCAA-ATTGCCGGTTTGCCGA-
Cele-UNSB01_3:13409998-13411238 Satlength=1241 Nr of Repeats=25
RepeatLength=43 seed=CGGAAATTTT Num.seqs=17 Similarity=0.776106
48   TTTGCCGGAAATT-TTNATTTTCGGCAA-TTTGCCNGTTTGCCGA-
Cele-UNSB01_2:12862834-12863019 Satlength=186 Nr of Repeats=5
RepeatLength=43 seed=TATTTTCATT Num.seqs=3 Similarity=0.979328
52   TTTGCCGGGTATT-TTCATTTTCGGCAA-ATTGCCGGTTTGCCGA-
Cele-UNSB01_1:696260-697073 Satlength=814 Nr of Repeats=14
RepeatLength=44 seed=TTTGCCGGTT Num.seqs=9 Similarity=0.920875
28   TTTGCCCGAAATTTTTCAATTTCGGCAA-TTTGCCGGTTTGCCGA-
Rev.of_Cele-UNSB01_1:1824052-1824465 Satlength=414 Nr of Repeats=9
RepeatLength=43 seed=GCCGGAATTG Num.seqs=7 Similarity=0.864157
25   TTTGCCAGAAATT-TTCAATTCCGGCTA-TGTGCCGGTTTGCCGA-
Cele-UNSB01_1:2859395-2859671 Satlength=277 Nr of Repeats=5
RepeatLength=43 seed=TTGCCGATTT Num.seqs=3 Similarity=0.834625
29   TTTGCCGGAAATT-TACAATTCC-ACTATTTTGCCGGTTTGCCGA-
Rev.of_Cele-UNSB01_1:1232170-1233535 Satlength=1366 Nr of Repeats=32
RepeatLength=43 seed=CCAGAAATTA Num.seqs=22 Similarity=0.892211
24   TTTGCCGAAAATT-CTAATTTCTGGCAA-TTTGCCGGTTTGCTGA-
Rev.of_Cele-UNSB01_1:2899706-2899878 Satlength=173 Nr of Repeats=4
RepeatLength=43 seed=AAATCGGCAA Num.seqs=4 Similarity=0.906977
46   TTTGCCGGGAATT-TTCATTTTTTGCAA-TTTGCCGGATTGCCGA-
Rev.of_Cele-UNSB01_3:12885748-12886017 Satlength=270 Nr of Repeats=5
RepeatLength=43 seed=GGCAAATCGG Num.seqs=3 Similarity=0.689394
49   TTTGCCGGAAATT-TTNATATTTNACAA-ATTGCCGGTTTGCCGA-
Cele-UNSB01_1:2695367-2695539 Satlength=173 Nr of Repeats=4
RepeatLength=43 seed=TTGCCGATTT Num.seqs=4 Similarity=0.798450
36   TTTGCCGGAAATG-TTTATTTCCGACAA-TTGGCAGATTTGCCGA-
Cele-UNSB01_1:14804070-14804449 Satlength=380 Nr of Repeats=9
RepeatLength=43 seed=TTTGCCGGTT Num.seqs=8 Similarity=0.931340
27   TTCGACGGAAATT-TTTATTTTCGGCAC-TTTGCCGGTTTGCCGG-
Rev.of_Cele-UNSB01_5:3651691-3651860 Satlength=170 Nr of Repeats=4
RepeatLength=42 seed=TTTCCAGCAA Num.seqs=3 Similarity=0.767196
11   TTTGCTGGAAA-T-CTCAATTCCGGCAA-TTCGCTGATTTGCCGA-
Rev.of_Cele-UNSB01_1:2263475-2263726 Satlength=252 Nr of Repeats=6
RepeatLength=43 seed=CAAATCGGCA Num.seqs=5 Similarity=0.782946
47   TTTGCTGGAAATT-TTTAATTTCGGAAA-TTTGCCGAGTTGCCGA-

Cele-UNSB01_5:18100660-18102309 Satlength=1650 Nr of Repeats=32
RepeatLength=44 seed=TTGCCGGAAA Num.seqs=27 Similarity=0.807908
1   TTTGCCGGAAATA-TTCAATTTTGGCAA-ATTGNCGGTTTGCTGAA
Cele-UNSB01_1:11320046-11320214 Satlength=169 Nr of Repeats=4
RepeatLength=42 seed=TGCCGGAAAT Num.seqs=4 Similarity=0.788360
2   GCTGCCGGAAATT--TCAAACCCGGCAA-TTTGCCCATTTGCCGA-

*   *   ***   *
Consensus:

TTTGCCGGAAATTTTcAaTTcCGGCAAtTTGCCGgTTTGCCGA

>Cele-UNSB01_Fam_15_26_22 Nr. of seq. 22 Alignment length(with gaps) = 26 Alignment score = 0.774226
Cele-UNSB01_1:3213789-3217715 Satlength=3927 Nr of Repeats=151
RepeatLength=26 seed=CAGAGTCACT Num.seqs=151 Similarity=0.930474
0   CAGAGTCACTATTTTTGGGGAACGGC
Cele-UNSB01_4:15642758-15643070 Satlength=313 Nr of Repeats=12
RepeatLength=26 seed=CAGAGTCACT Num.seqs=12 Similarity=0.943279
0   CAGAGTCACTATTTTTGGTGAACGGC
Cele-UNSB01_6:16179578-16182464 Satlength=2887 Nr of Repeats=111
RepeatLength=26 seed=GTGAACGGCC Num.seqs=111 Similarity=0.930666
17   CAGAGTCACTATTTTTGGTGAACGGC
Rev.of_Cele-UNSB01_2:3110-3266 Satlength=157 Nr of Repeats=6
RepeatLength=26 seed=GTTCACCAAA Num.seqs=6 Similarity=0.921368
23   CAGAGTCACTATTTTTGGTGAACGGT
Cele-UNSB01_6:1682621-1693139 Satlength=10519 Nr of Repeats=404
RepeatLength=26 seed=AACGGTCAGA Num.seqs=393 Similarity=0.943434
46   CAGAGTCACTATTTTTGGTGAACGGT
Rev.of_Cele-UNSB01_4:11073363-11083545 Satlength=10183 Nr of Repeats=395
RepeatLength=26 seed=GTTCACCAAA Num.seqs=331 Similarity=0.919481
23   CAGAGTCACTAAATTTGGTGAACGGC
Cele-UNSB01_5:5272176-5275211 Satlength=3036 Nr of Repeats=112
RepeatLength=26 seed=CAGAGTCACT Num.seqs=99 Similarity=0.819649
26   CAGAGTCACTAATTTTGGTGAACGGC
Rev.of_Cele-UNSB01_6:5769379-5769561 Satlength=183 Nr of Repeats=7
RepeatLength=26 seed=TAGTGACTCT Num.seqs=7 Similarity=0.836567
37   CAGAGTCACTAATTTTGGTGAACGGC
Cele-UNSB01_5:10490005-10492889 Satlength=2885 Nr of Repeats=111
RepeatLength=26 seed=CCAGAGTCAC Num.seqs=109 Similarity=0.974141
25   CAGAGTCACTATATTTGGTGAACGGC
Rev.of_Cele-UNSB01_6:2093711-2093945 Satlength=235 Nr of Repeats=9
RepeatLength=26 seed=ATAGTGACTC Num.seqs=9 Similarity=0.897436
38   CAGAGTCACTATATTTGGTGAACGGC
Cele-UNSB01_3:958005-958109 Satlength=105 Nr of Repeats=4 RepeatLength=26
seed=CGGCCAGAGT Num.seqs=4 Similarity=0.863248
22   CAGAGTCACTACTTATGGTGAACGGC
Rev.of_Cele-UNSB01_1:13507576-13507758 Satlength=183 Nr of Repeats=7
RepeatLength=26 seed=CTCTGGCAGT Num.seqs=7 Similarity=0.941392
5   CAGAGCCACTTATTTTAGTGAACTGC
Cele-UNSB01_4:11084695-11084799 Satlength=105 Nr of Repeats=4
RepeatLength=26 seed=CCAGAGTCAC Num.seqs=4 Similarity=0.863248
25   CAGAGTCACTATTTTTAGTGAACTGC

Cele-UNSB01_6:2485227-2486657 Satlength=1431 Nr of Repeats=55
RepeatLength=26 seed=TTTTAGTGAA Num.seqs=55 Similarity=0.934801
38   CAGAGTCACTAATTTTAGTGAACTGC
Cele-UNSB01_1:3711978-3718275 Satlength=6298 Nr of Repeats=242
RepeatLength=26 seed=ATGGTTAGAG Num.seqs=238 Similarity=0.970067
21   TAGAGTCATATTTTAGGTGAATGGT
Rev.of_Cele-UNSB01_6:1697161-1700255 Satlength=3095 Nr of Repeats=119
RepeatLength=26 seed=GACTCTAACC Num.seqs=119 Similarity=0.954495
33   TAGAGTCATATTTTAGGTGAATGGT
Cele-UNSB01_1:13634561-13638668 Satlength=4108 Nr of Repeats=158
RepeatLength=26 seed=TTTTAGGTGA Num.seqs=157 Similarity=0.972948
37   TAGAGTCATAATTTTAGGTGAATGGT
Cele-UNSB01_3:5494321-5534864 Satlength=40544 Nr of Repeats=1542
RepeatLength=26 seed=GTTAGAGTCA Num.seqs=1416 Similarity=0.990443
24   TAGAGTCATTATTTTAGGTGAATGGT
Rev.of_Cele-UNSB01_3:5534873-5535003 Satlength=131 Nr of Repeats=5
RepeatLength=26 seed=ACCATTCACC Num.seqs=5 Similarity=0.917949
26   TAGAGTCATTATTTTAGGTGAATGGT
Cele-UNSB01_4:15002663-15002793 Satlength=131 Nr of Repeats=5
RepeatLength=26 seed=TGGTTAGAGT Num.seqs=5 Similarity=0.958974
48   TAGAGTCATTATTTTAGGTGAATGGT
Rev.of_Cele-UNSB01_5:15290454-15290610 Satlength=157 Nr of Repeats=6
RepeatLength=26 seed=TTCACCTAAA Num.seqs=6 Similarity=0.846154
48   TAGAGTCATTATTTTAGGTGAATGGT
Rev.of_Cele-UNSB01_6:12072301-12080487 Satlength=8187 Nr of Repeats=312
RepeatLength=26 seed=ATGACTCTAA Num.seqs=264 Similarity=0.927423
35   TAGAGTCATTATTTTAGGTGAATGGT

**** **   *   * *** *
Consensus:

cAGAGTCAcTAtTTTtGGTGAAcGGc

>Cele-UNSB01_Fam_16_11_21 Nr. of seq. 21 Alignment length(with gaps) = 11 Alignment score = 0.924964
Cele-UNSB01_1:11907174-11907313 Satlength=140 Nr of Repeats=13
RepeatLength=11 seed=TTTTGCACAT Num.seqs=9 Similarity=0.838384
0   TTTTGCACATT
Cele-UNSB01_1:12310642-12315079 Satlength=4438 Nr of Repeats=373
RepeatLength=11 seed=CACATTTTTT Num.seqs=292 Similarity=0.843776
5   TTTTGCACATT
Cele-UNSB01_1:12350087-12350549 Satlength=463 Nr of Repeats=41
RepeatLength=11 seed=TTGCACATTT Num.seqs=31 Similarity=0.961681
2   TTTTGCACATT
Cele-UNSB01_2:4510577-4510967 Satlength=391 Nr of Repeats=36
RepeatLength=11 seed=TGCACATTTT Num.seqs=30 Similarity=0.991919
3   TTTTGCACATT
Cele-UNSB01_3:1301641-1302992 Satlength=1352 Nr of Repeats=116
RepeatLength=11 seed=TTTTTGCACA Num.seqs=73 Similarity=0.910060
10   TTTTGCACATT
Rev.of_Cele-UNSB01_3:1753459-1753580 Satlength=122 Nr of Repeats=11
RepeatLength=11 seed=GCAAAAAATG Num.seqs=11 Similarity=0.960331
6   TTTTGCACATT

Cele-UNSB01_3:1891974-1892414 Satlength=441 Nr of Repeats=36
RepeatLength=11 seed=ATTTTTTGCA Num.seqs=32 Similarity=0.920088
8   TTTTGCACATT
Rev.of_Cele-UNSB01_3:2068206-2068691 Satlength=486 Nr of Repeats=44
RepeatLength=11 seed=TGTGCAAAAA Num.seqs=31 Similarity=0.939785
9   TTTTGCACATT
Cele-UNSB01_3:12475925-12476790 Satlength=866 Nr of Repeats=62
RepeatLength=11 seed=TTTTTGCACA Num.seqs=46 Similarity=1.000000
10   TTTTGCACATT
Rev.of_Cele-UNSB01_3:13432209-13432275 Satlength=67 Nr of Repeats=5
RepeatLength=11 seed=AAAAATGTGC Num.seqs=4 Similarity=1.000000
3   TTTTGCACATT
Cele-UNSB01_4:15883454-15883531 Satlength=78 Nr of Repeats=6
RepeatLength=11 seed=TGCACATTTT Num.seqs=5 Similarity=0.903030
3   TTTTGCACATT
Rev.of_Cele-UNSB01_5:3602166-3602210 Satlength=45 Nr of Repeats=4
RepeatLength=11 seed=CAAAAAATGT Num.seqs=4 Similarity=0.878788
5   TTTTGCACATT
Cele-UNSB01_5:11125880-11126094 Satlength=215 Nr of Repeats=10
RepeatLength=11 seed=CACATTTTTT Num.seqs=9 Similarity=0.946128
5   TTTTGCACATT
Rev.of_Cele-UNSB01_6:8836090-8836155 Satlength=66 Nr of Repeats=5
RepeatLength=11 seed=GCAAAAAATG Num.seqs=4 Similarity=0.878788
6   TTTTGCACATT
Cele-UNSB01_3:12166501-12166666 Satlength=166 Nr of Repeats=15
RepeatLength=11 seed=ACATTTTTTG Num.seqs=13 Similarity=0.919192
6   TTTTGNACATT
Rev.of_Cele-UNSB01_1:12056400-12056477 Satlength=78 Nr of Repeats=7
RepeatLength=11 seed=CAAAAAATGT Num.seqs=7 Similarity=0.942280
5   TTTTGGACATT
Cele-UNSB01_4:13730774-13730818 Satlength=45 Nr of Repeats=4
RepeatLength=11 seed=CACATTTTTT Num.seqs=4 Similarity=0.919192
5   TTTTCCACATT
Rev.of_Cele-UNSB01_4:15255716-15255837 Satlength=122 Nr of Repeats=9
RepeatLength=11 seed=TGTGCAAAAA Num.seqs=7 Similarity=0.838384
9   TTTTGCACAAT
Cele-UNSB01_4:13759189-13759529 Satlength=341 Nr of Repeats=30
RepeatLength=11 seed=GCACATTTTT Num.seqs=22 Similarity=0.772787
4   TTTAGCACATT
Rev.of_Cele-UNSB01_6:3377659-3377780 Satlength=122 Nr of Repeats=11
RepeatLength=11 seed=AAAATGTGCT Num.seqs=7 Similarity=0.826720
13   TTTAGCACATT
Cele-UNSB01_5:2836474-2836692 Satlength=219 Nr of Repeats=20
RepeatLength=11 seed=GCACATTTTT Num.seqs=18 Similarity=0.909685
4   TTTCGCACATT

***   *** *
Consensus:

TTTTGCACATT

>Cele-UNSB01_Fam_17_37_19 Nr. of seq. 19 Alignment length(with gaps) =
44 Alignment score = 0.712431

```
Cele-UNSB01_5:16414638-16414819 Satlength=182 Nr of Repeats=5
RepeatLength=36 seed=GGGAATTCAA  Num.seqs=4 Similarity=0.962963
0    GGGAA-TTCAAATTTT---AA--TTTTTGAAAAC-ATTTTGGC-
Cele-UNSB01_1:1341786-1343571 Satlength=1786 Nr of Repeats=47
RepeatLength=38 seed=GGGAATTCAA  Num.seqs=46 Similarity=0.937825
0    GGGAA-TTCAAATTTT---AATTTTTTTGAAAAC-ATTTTGGC-
Cele-UNSB01_3:3145893-3146730 Satlength=838 Nr of Repeats=18
RepeatLength=37 seed=ATTTTGGCGG  Num.seqs=16 Similarity=0.920120
29    GGGAA-TTCAAAATTT---AA-TTTTTCGAAAAC-ATTTTGGC-
Cele-UNSB01_5:17487338-17487635 Satlength=298 Nr of Repeats=8
RepeatLength=37 seed=AAAACATTTT  Num.seqs=5 Similarity=0.894737
61    GGGAA-TTCAAAATTT---AA-TTTTTTGAAAAC-ATTTTGGC-
Cele-UNSB01_4:3135501-3135649 Satlength=149 Nr of Repeats=4
RepeatLength=37 seed=TGGCGGGAAT  Num.seqs=4 Similarity=0.867868
33    GGGAA-TTCAAATTTT---AA-TTTTCCGAAAAC-ATTTTGGC-
Cele-UNSB01_2:12791702-12792113 Satlength=412 Nr of Repeats=11
RepeatLength=37 seed=ATTTTGGCGG  Num.seqs=7 Similarity=0.951952
29    GGGAA-TTCAAATTTT---AA-TTTTTTGAAAAT-ATTTTGGC-
Cele-UNSB01_3:11241850-11242636 Satlength=787 Nr of Repeats=15
RepeatLength=37 seed=TTGGCGGGAA  Num.seqs=10 Similarity=0.935135
32    GGGAA-TTCAAATTTT---AA-TTTTTTGAAAAT-ATTTTGGC-
Cele-UNSB01_4:286805-287284 Satlength=480 Nr of Repeats=13
RepeatLength=37 seed=TTGGCGGGAA  Num.seqs=9 Similarity=0.933934
32    GGGAA-TTCAAATTTT---AA-TTTTTTGAAAAT-ATTTTGGC-
Rev.of_Cele-UNSB01_4:17408894-17409724 Satlength=831 Nr of Repeats=9
RepeatLength=37 seed=ATTCCCGCCA  Num.seqs=6 Similarity=0.896697
43    GGGAA-TTCAAATTTT---AA-TTTTTTGAAAAT-ATTTTGGC-
Rev.of_Cele-UNSB01_4:1695920-1696142 Satlength=223 Nr of Repeats=6
RepeatLength=37 seed=CGCCAAAATG  Num.seqs=6 Similarity=0.894294
38    GGGAA-TTCAAATTTT---AA-TTTTTTGAAAAC-ATTTTGGC-
Cele-UNSB01_4:16721546-16722348 Satlength=803 Nr of Repeats=4
RepeatLength=37 seed=ATTCAAATTT  Num.seqs=3 Similarity=0.951952
41    GGGAA-TTCAAATTTT---AA-TTTTTTGAAAAT-ACTTTGGC-
Cele-UNSB01_3:13002319-13002507 Satlength=189 Nr of Repeats=5
RepeatLength=38 seed=TTTTGGCGGG  Num.seqs=3 Similarity=0.811966
30    GGGAA-TTCAAATTTTN--AA-TTTTTTGAAAAT-ATTTTGGC-
Rev.of_Cele-UNSB01_5:20465532-20465791 Satlength=260 Nr of Repeats=7
RepeatLength=37 seed=AATTTGAATT  Num.seqs=7 Similarity=0.941656
50    GGGAA-TTCAAATTTT---AA-TTTTTCAAAAAT-ATTTTGGC-
Cele-UNSB01_4:16077133-16078206 Satlength=1074 Nr of Repeats=16
RepeatLength=37 seed=CGGGAATTCA  Num.seqs=13 Similarity=0.831370
36    GGGAA-TTCAAATTTT---AA-TTTTTTG-AAATCATTTTGGC-
Cele-UNSB01_5:16082522-16083430 Satlength=909 Nr of Repeats=7
RepeatLength=37 seed=GCGGGAATTC  Num.seqs=5 Similarity=0.877477
72    GGGAA-TTCAAATTTT---AA-TTATTTG-AAATCATTTTGGC-
Cele-UNSB01_1:282371-283239 Satlength=869 Nr of Repeats=23
RepeatLength=38 seed=AAATTTTAAT  Num.seqs=17 Similarity=0.995872
9    GGGAATTTAAAATTTT---AA-TTTTTTGAAAAT-ATTTTGGC-
Cele-UNSB01_3:2214780-2216279 Satlength=1500 Nr of Repeats=39
RepeatLength=39 seed=TTTTTGGCGG  Num.seqs=29 Similarity=0.914530
30    GGAAA-TTCAAAAATT-CAAA--TTTTCAAATAT-TTTTTGGCG
Cele-UNSB01_3:2219970-2221469 Satlength=1500 Nr of Repeats=39
RepeatLength=39 seed=TTTTTGGCGG  Num.seqs=29 Similarity=0.914530
30    GGAAA-TTCAAAAATT-CAAA--TTTTCAAATAT-TTTTTGGCG
```

Rev.of_Cele-UNSB01_3:2223808-2223967 Satlength=160 Nr of Repeats=4
RepeatLength=40 seed=TCCCGCCAAA Num.seqs=3 Similarity=0.866667
42   GGAAA-TTCAAAAATT-TAAA-TTTTTTGAATAT-TTTTTGGCG

** ** ** *** **   ** * *   * *   ******
Consensus:

GGGAATTCAAAtTTTAATTTTTtGAAAAtATTTTGGC

>Cele-UNSB01_Fam_18_19_18 Nr. of seq. 18 Alignment length(with gaps) = 19 Alignment score = 0.859420
Cele-UNSB01_1:13177981-13178228 Satlength=248 Nr of Repeats=13
RepeatLength=19 seed=CAAGGCACTA Num.seqs=13 Similarity=0.816464
0   CAAGGCACTAGAAATGCGC
Rev.of_Cele-UNSB01_5:18833669-18833973 Satlength=305 Nr of Repeats=15
RepeatLength=19 seed=TAGTGCCTTG Num.seqs=12 Similarity=0.808612
10   CAAGGCACTAGAATTGCGC
Rev.of_Cele-UNSB01_5:18944459-18944687 Satlength=229 Nr of Repeats=8
RepeatLength=19 seed=TTCTAGTGCC Num.seqs=5 Similarity=0.817544
13   CAAGGCACTAGAATTGCGC
Cele-UNSB01_5:19667001-19667210 Satlength=210 Nr of Repeats=9
RepeatLength=19 seed=TTGCGCCAAG Num.seqs=7 Similarity=0.744444
13   CAAGGCACTAGAATTGCGC
Rev.of_Cele-UNSB01_3:3356021-3356788 Satlength=768 Nr of Repeats=13
RepeatLength=19 seed=CTTGGCGCAA Num.seqs=8 Similarity=0.764411
4   CAAGGCACGAGAATTGCGC
Cele-UNSB01_5:19673680-19673832 Satlength=153 Nr of Repeats=5
RepeatLength=19 seed=AGAATTGCGC Num.seqs=3 Similarity=0.859649
9   CAAGGCACGAGAATTGCGC
Rev.of_Cele-UNSB01_5:19434732-19435093 Satlength=362 Nr of Repeats=16
RepeatLength=19 seed=TGGCGCATTT Num.seqs=14 Similarity=0.787160
21   CAAGGCACGAGAAATGCGC
Rev.of_Cele-UNSB01_5:18413578-18417093 Satlength=3516 Nr of Repeats=121
RepeatLength=19 seed=TTGACGCAAT Num.seqs=77 Similarity=0.809381
3   CAAGNCACTAGAATTGCGT
Rev.of_Cele-UNSB01_5:18631640-18632148 Satlength=509 Nr of Repeats=18
RepeatLength=19 seed=TGACGCAATT Num.seqs=14 Similarity=0.787931
21   CAAGGCACTAGAATTGCGT
Rev.of_Cele-UNSB01_5:18934684-18935273 Satlength=590 Nr of Repeats=23
RepeatLength=19 seed=TGACGCAATT Num.seqs=15 Similarity=0.830476
21   CAAGGCACTAGAATTGCGT
Cele-UNSB01_5:18935616-18936186 Satlength=571 Nr of Repeats=26
RepeatLength=19 seed=ACTAGAATTG Num.seqs=23 Similarity=0.820539
25   CAAGGCACTAGAATTGCGT
Rev.of_Cele-UNSB01_5:19673879-19674316 Satlength=438 Nr of Repeats=16
RepeatLength=19 seed=GCCTTGACGC Num.seqs=11 Similarity=0.888995
25   CAAGGCACTAGAATTGCGT
Rev.of_Cele-UNSB01_5:20131328-20132164 Satlength=837 Nr of Repeats=37
RepeatLength=19 seed=TAGTGCCTTG Num.seqs=30 Similarity=0.844807
29   CAAGGCACTAGAATTGCGT
Cele-UNSB01_5:18630095-18630532 Satlength=438 Nr of Repeats=22
RepeatLength=19 seed=CAAGGCACTA Num.seqs=21 Similarity=0.750042
19   CAAGGCACTANAATTGCGT

Rev.of_Cele-UNSB01_5:18831834-18832119 Satlength=286 Nr of Repeats=11
RepeatLength=19 seed=TTGACGCAAT Num.seqs=9 Similarity=0.666667
3   CAANGCACTAGAATTGCGT
Rev.of_Cele-UNSB01_5:18877478-18878086 Satlength=609 Nr of Repeats=29
RepeatLength=19 seed=GTGCCTTGAC Num.seqs=26 Similarity=0.876923
8   CAAGGCACTAGAANTGCGT
Cele-UNSB01_2:3065991-3066701 Satlength=711 Nr of Repeats=18
RepeatLength=19 seed=GCCAAGGCAC Num.seqs=11 Similarity=0.680606
17   CAAGGCACTNGAAANGCGC
Rev.of_Cele-UNSB01_5:19309007-19310074 Satlength=1068 Nr of Repeats=10
RepeatLength=19 seed=TGGCGCATTT Num.seqs=6 Similarity=0.714620
2   CAACTCACCAAAATTGCGC

*** ***   ** ***
Consensus:

CAAGGCACtAGAAtTGCGc

>Cele-UNSB01_Fam_19_14_17 Nr. of seq. 17 Alignment length(with gaps) = 14 Alignment score = 0.892682
Cele-UNSB01_2:11443617-11443668 Satlength=52 Nr of Repeats=4
RepeatLength=12 seed=ACTATAACTA Num.seqs=3 Similarity=0.851852
0   ACTATA-ACTACA-
Cele-UNSB01_1:11361560-11361658 Satlength=99 Nr of Repeats=7
RepeatLength=14 seed=AACTACTAAC Num.seqs=7 Similarity=0.803175
6   ACTACTAACTACTA
Rev.of_Cele-UNSB01_1:13241763-13242154 Satlength=392 Nr of Repeats=8
RepeatLength=14 seed=AGTTTGTAGT Num.seqs=5 Similarity=0.885714
10   ACTACAAACTACAA
Rev.of_Cele-UNSB01_1:13729567-13730578 Satlength=1012 Nr of Repeats=11
RepeatLength=14 seed=TAGTTTGTAG Num.seqs=7 Similarity=0.845805
18   ACTACAAACTACAA
Cele-UNSB01_1:13730896-13731271 Satlength=376 Nr of Repeats=11
RepeatLength=14 seed=TACAAACTAC Num.seqs=7 Similarity=0.918367
23   ACTACAAACTACAA
Rev.of_Cele-UNSB01_1:14568940-14569168 Satlength=229 Nr of Repeats=4
RepeatLength=14 seed=TGTAGTTTGT Num.seqs=3 Similarity=0.936508
20   ACTACAAACTACAA
Cele-UNSB01_2:2655599-2656120 Satlength=522 Nr of Repeats=10
RepeatLength=14 seed=CTACAAACTA Num.seqs=6 Similarity=0.796825
22   ACTACAAACTACAA
Cele-UNSB01_2:12905171-12905894 Satlength=724 Nr of Repeats=9
RepeatLength=14 seed=AACTACAAAC Num.seqs=7 Similarity=0.845805
20   ACTACAAACTACAA
Rev.of_Cele-UNSB01_2:13459229-13459607 Satlength=379 Nr of Repeats=13
RepeatLength=14 seed=TAGTTTGTAG Num.seqs=8 Similarity=0.829932
18   ACTACAAACTACAA
Cele-UNSB01_2:14136989-14138428 Satlength=1440 Nr of Repeats=41
RepeatLength=14 seed=TACAAACTAC Num.seqs=27 Similarity=0.871659
23   ACTACAAACTACAA
Rev.of_Cele-UNSB01_2:14194765-14194942 Satlength=178 Nr of Repeats=5
RepeatLength=14 seed=GTAGTTTGTA Num.seqs=3 Similarity=0.809524
19   ACTACAAACTACAA

Rev.of_Cele-UNSB01_4:15529257-15530020 Satlength=764 Nr of Repeats=4
RepeatLength=14 seed=TTGTAGTTTG Num.seqs=3 Similarity=0.936508
21   ACTACAAACTACAA
Cele-UNSB01_5:18596071-18596370 Satlength=300 Nr of Repeats=14
RepeatLength=14 seed=ACAAACTACA Num.seqs=10 Similarity=0.942857
10   ACTACAAACTACAA
Rev.of_Cele-UNSB01_5:19848468-19849404 Satlength=937 Nr of Repeats=7
RepeatLength=14 seed=TTTGTAGTTT Num.seqs=5 Similarity=0.804444
22   ACTACAAACTACAA
Cele-UNSB01_5:19849444-19849810 Satlength=367 Nr of Repeats=11
RepeatLength=14 seed=ACAAACTACA Num.seqs=7 Similarity=0.931973
10   ACTACANACTACAA
Rev.of_Cele-UNSB01_1:13831060-13832042 Satlength=983 Nr of Repeats=52
RepeatLength=14 seed=TGTAGTATGT Num.seqs=44 Similarity=0.966375
20   ACTACATACTACAT
Cele-UNSB01_4:1639262-1639353 Satlength=92 Nr of Repeats=6
RepeatLength=14 seed=ACTACACACT Num.seqs=5 Similarity=1.000000
21   ACTACACACTACAC

****   *****
Consensus:

ACTACAaACTACAA

>Cele-UNSB01_Fam_20_12_15 Nr. of seq. 15 Alignment length(with gaps) = 14 Alignment score = 0.745805
Cele-UNSB01_4:15644313-15644403 Satlength=91 Nr of Repeats=8
RepeatLength=10 seed=TGTTATGATA Num.seqs=5 Similarity=0.763636
0   TG-T-TATG--ATA
Rev.of_Cele-UNSB01_1:11352081-11352141 Satlength=61 Nr of Repeats=5
RepeatLength=12 seed=TATACATATA Num.seqs=5 Similarity=1.000000
0   TG-TATATG-TATA
Cele-UNSB01_1:11550058-11550130 Satlength=73 Nr of Repeats=6
RepeatLength=12 seed=TATATGTATA Num.seqs=6 Similarity=1.000000
8   TG-TATATG-TATA
Cele-UNSB01_1:14674978-14675094 Satlength=117 Nr of Repeats=10
RepeatLength=12 seed=ATATGTATAT Num.seqs=6 Similarity=0.888889
9   TG-TATATG-TATA
Cele-UNSB01_2:2015956-2016256 Satlength=301 Nr of Repeats=25
RepeatLength=12 seed=ATGTATATGT Num.seqs=25 Similarity=0.948889
11   TG-TATATG-TATA
Rev.of_Cele-UNSB01_2:3515774-3515844 Satlength=71 Nr of Repeats=4
RepeatLength=12 seed=ATACATATAC Num.seqs=3 Similarity=1.000000
11   TG-TATATG-TATA
Rev.of_Cele-UNSB01_3:12842209-12842347 Satlength=139 Nr of Repeats=11
RepeatLength=12 seed=TATACATATA Num.seqs=10 Similarity=1.000000
0   TG-TATATG-TATA
Cele-UNSB01_4:1452742-1452796 Satlength=55 Nr of Repeats=4
RepeatLength=12 seed=TATATGTATA Num.seqs=3 Similarity=1.000000
8   TG-TATATG-TATA
Cele-UNSB01_5:18730021-18731423 Satlength=1403 Nr of Repeats=114
RepeatLength=12 seed=GTATATGTAT Num.seqs=110 Similarity=0.972866
7   TG-TATATG-TATA

Rev.of_Cele-UNSB01_5:19325271-19325379 Satlength=109 Nr of Repeats=9 RepeatLength=12 seed=ATACATATAC Num.seqs=9 Similarity=0.975309
11   TG-TATATG-TATA
Cele-UNSB01_5:20269010-20269323 Satlength=314 Nr of Repeats=8 RepeatLength=12 seed=TATGTATATG Num.seqs=6 Similarity=1.000000
10   TG-TATATG-TATA
Rev.of_Cele-UNSB01_5:20277753-20277891 Satlength=139 Nr of Repeats=11 RepeatLength=12 seed=ATATACATAT Num.seqs=10 Similarity=1.000000
7   TG-TATATG-TATA
Cele-UNSB01_5:19329973-19330169 Satlength=197 Nr of Repeats=8 RepeatLength=12 seed=ATATGTATAT Num.seqs=5 Similarity=0.777778
11   TA-TNTATA-TNTA
Cele-UNSB01_5:19334298-19334625 Satlength=328 Nr of Repeats=23 RepeatLength=14 seed=TATATGTTAT Num.seqs=20 Similarity=0.980952
10   TGTTATATGTTATA
Rev.of_Cele-UNSB01_6:16529882-16530026 Satlength=145 Nr of Repeats=9 RepeatLength=14 seed=TACCATATAC Num.seqs=7 Similarity=0.841270
12   TGGTATATGGTATA

* * ***   **
Consensus:

TGTATATGTATA

>Cele-UNSB01_Fam_21_18_14 Nr. of seq. 14 Alignment length(with gaps) = 18 Alignment score = 0.786121
Cele-UNSB01_2:4275898-4276001 Satlength=104 Nr of Repeats=5 RepeatLength=18 seed=TGCAAGACTA Num.seqs=3 Similarity=0.950617
0   TGCAAGACTAATAGAGAC
Cele-UNSB01_3:949283-949630 Satlength=348 Nr of Repeats=8 RepeatLength=18 seed=TGCAAGACTA Num.seqs=5 Similarity=0.822222
0   TGCAAGACTAATAGAGAC
Rev.of_Cele-UNSB01_2:14582719-14582837 Satlength=119 Nr of Repeats=5 RepeatLength=18 seed=TAGTCTTGCA Num.seqs=3 Similarity=0.901235
10   TGCAAGACTAATAGAGGC
Cele-UNSB01_6:13750177-13750509 Satlength=333 Nr of Repeats=8 RepeatLength=18 seed=TGCAAGACTA Num.seqs=5 Similarity=0.851852
18   TGCAAGACTAATAGAGGC
Cele-UNSB01_5:3289995-3290260 Satlength=266 Nr of Repeats=4 RepeatLength=18 seed=TGCAAGACTA Num.seqs=3 Similarity=0.876543
18   TGCAAGACTANTAGAGGC
Rev.of_Cele-UNSB01_6:7806159-7806450 Satlength=292 Nr of Repeats=5 RepeatLength=18 seed=TAGTCTTGCA Num.seqs=3 Similarity=0.901235
10   TGCAAGACTATTAGAGGC
Cele-UNSB01_6:14650166-14651272 Satlength=1107 Nr of Repeats=10 RepeatLength=18 seed=TGCAAGACTA Num.seqs=6 Similarity=0.841975
0   TGCAAGACTAATAAAGGC
Rev.of_Cele-UNSB01_4:3896416-3896737 Satlength=322 Nr of Repeats=5 RepeatLength=18 seed=ATTAGTATTG Num.seqs=3 Similarity=0.802469
12   TGCAATACTAATAGGGAG
Cele-UNSB01_6:12159416-12159737 Satlength=322 Nr of Repeats=5 RepeatLength=18 seed=TGCAATACTA Num.seqs=3 Similarity=0.901235
18   TGCAAGACTAATAGGGAG

Rev.of_Cele-UNSB01_4:17395956-17396303 Satlength=348 Nr of Repeats=5
RepeatLength=18 seed=GCACTCCCTA Num.seqs=3 Similarity=0.950617
21   TGCAAGACTATTAGGGAG
Rev.of_Cele-UNSB01_5:12442637-12442985 Satlength=349 Nr of Repeats=5
RepeatLength=18 seed=GCACTCCCTA Num.seqs=3 Similarity=0.950617
21   TGCAAGACTATTAGGGAG
Rev.of_Cele-UNSB01_6:16506358-16506686 Satlength=329 Nr of Repeats=5
RepeatLength=18 seed=ATTAGTTTTG Num.seqs=3 Similarity=0.950617
12   TGCAAAACTAATAGAGAG
Rev.of_Cele-UNSB01_3:949715-950024 Satlength=310 Nr of Repeats=5
RepeatLength=18 seed=TAGTGCTGCA Num.seqs=3 Similarity=0.777778
10   TGCAGCACTANTAGAGAC
Cele-UNSB01_3:3928597-3928907 Satlength=311 Nr of Repeats=5
RepeatLength=18 seed=TGCAGCACTA Num.seqs=3 Similarity=0.851852
18   TGCAGCACTATTAGAGAC

**** **** ** *
Consensus:

TGCAAgACTAaTAGaGac

>Cele-UNSB01_Fam_22_59_13 Nr. of seq. 13 Alignment length(with gaps) = 60 Alignment score = 0.818661
Cele-UNSB01_1:4042754-4055380 Satlength=12627 Nr of Repeats=214
RepeatLength=59 seed=AAAACCAGTG Num.seqs=214 Similarity=0.852508
0   AAAACCAGTGCACATTTGAAATT-TTATATTCTCCATAATTCTCGGTTTTAAATATTTTG
Rev.of_Cele-UNSB01_2:898652-902061 Satlength=3410 Nr of Repeats=58
RepeatLength=59 seed=TATGGAAAAT Num.seqs=56 Similarity=0.981848
37   AAAACCAGTGCACATTTAAAAAT-CCATATTTTCCATAATTCTCAGTTTAAAAAAATTTG
Rev.of_Cele-UNSB01_3:1059346-1075750 Satlength=16405 Nr of Repeats=279
RepeatLength=59 seed=AACTGAGAAT Num.seqs=241 Similarity=0.923220
47   AAAACCAGTGCACATTTAAATTT-CCATATTCTCCATAATTCTCAGTTTAAAAAATTTTG
Rev.of_Cele-UNSB01_5:17588702-17608641 Satlength=19940 Nr of Repeats=337
RepeatLength=59 seed=TTATGGTGAA Num.seqs=283 Similarity=0.948621
97   AAAACCAGTGCACATTTGAATTT-CCATATTCACCATAATTCTCGGTTTAAAAAATTTTG
Rev.of_Cele-UNSB01_3:9234667-9236218 Satlength=1552 Nr of Repeats=21
RepeatLength=59 seed=TTCAAAACTT Num.seqs=20 Similarity=0.972643
61   AAAACCAGTGCACATTTGAAATT-CCATATTCTCCATAAATCTCAGTTTAAAAAGTTTTG
Rev.of_Cele-UNSB01_5:6230981-6231799 Satlength=819 Nr of Repeats=13
RepeatLength=59 seed=ATGTGCACTG Num.seqs=10 Similarity=0.954300
74   AAAACCAGTGCACATTTGAAATT-CCATATTCTCCATTATTCTCAGTTTAAAAATTTTTG
Rev.of_Cele-UNSB01_5:3006278-3006868 Satlength=591 Nr of Repeats=10
RepeatLength=59 seed=AATTTCAAAT Num.seqs=10 Similarity=0.895123
82   AAAACCAGTGCACATTTGAAATTNCCATATTTTCCATAATTCTCAGTTTAAAAATTTTTG
Cele-UNSB01_4:12303349-12310067 Satlength=6719 Nr of Repeats=128
RepeatLength=59 seed=TCCATAATTC Num.seqs=91 Similarity=0.837481
31   AAAACCAGTGAAGTCTTGATATT-CCATATTTTCCATAATTCTCAGTTTTAAAAATTTTG
Rev.of_Cele-UNSB01_5:6963271-6963507 Satlength=237 Nr of Repeats=4
RepeatLength=59 seed=TGGAAATTCA Num.seqs=4 Similarity=0.951036
85   AAAACCAGTGAAGTCTTGAATTT-CCATTTTCCCCATAATTCTCGGTTTTAAAAATTTTG
Rev.of_Cele-UNSB01_5:6966002-6966297 Satlength=296 Nr of Repeats=5
RepeatLength=59 seed=TGGAAATTCA Num.seqs=5 Similarity=0.941243
85   AAAACCAGTGAAGTCTTGAATTT-CCATATTCCCCATAATTCTCGGTTTTAAAAATTTTG

Rev.of_Cele-UNSB01_5:6971634-6982576 Satlength=10943 Nr of Repeats=186 RepeatLength=59 seed=ATGGAAATTC Num.seqs=182 Similarity=0.914233
86   AAAACCAGTGAAGTCTTGAATTT-CCATATTCCCCATAATTCTCGGTTTTAAANATTTTG
Cele-UNSB01_5:6964823-6965938 Satlength=1116 Nr of Repeats=19 RepeatLength=59 seed=ACCAGTGAAG Num.seqs=13 Similarity=0.950167
121   AAAACCAGTGAAGTCTTGAATTT-CCATATTCCCCATAATTCTCGGTTTTAAATTTTTTG
Cele-UNSB01_5:6967498-6969084 Satlength=1587 Nr of Repeats=27 RepeatLength=59 seed=ACCAGTGAAG Num.seqs=20 Similarity=0.961939
121   AAAACCAGTGAAGTCTTGAATTT-CCATATTCCCCATAATTCTCGGTTTTAAATTTTTTG

```
********** *   ** *   *   ** **  **** * **** **** ***   ****
```

Consensus:

AAAACCAGTGcAcatTTGAAtTTCCATATTctCCATAATTCTCgGTTTtAAAaaTTTTG

>Cele-UNSB01_Fam_23_20_12 Nr. of seq. 12 Alignment length(with gaps) = 21 Alignment score = 0.831890
Cele-UNSB01_1:1878816-1878916 Satlength=101 Nr of Repeats=5 RepeatLength=20 seed=CTAGGCAGAC Num.seqs=5 Similarity=1.000000
0   CTAGGCAG-ACTTAGCCTACC
Cele-UNSB01_1:3474380-3475480 Satlength=1101 Nr of Repeats=54 RepeatLength=20 seed=CTAGGCAGAC Num.seqs=53 Similarity=0.846734
0   CTAGGCAG-ACCTAGCCTACC
Cele-UNSB01_1:3474383-3476103 Satlength=1721 Nr of Repeats=83 RepeatLength=20 seed=GGCAGACCTA Num.seqs=80 Similarity=0.844789
3   CTAGGCAG-ACCTAGCCTACC
Rev.of_Cele-UNSB01_3:5426163-5426753 Satlength=591 Nr of Repeats=28 RepeatLength=20 seed=CTAGGTCTGC Num.seqs=25 Similarity=0.872444
14   CTAGGCAG-ACCTAGCCTACC
Rev.of_Cele-UNSB01_3:5426187-5427065 Satlength=879 Nr of Repeats=40 RepeatLength=20 seed=GGTAGGCTAG Num.seqs=28 Similarity=0.844293
0   CTAGGCAG-ACCTAGCCTACC
Cele-UNSB01_6:1553970-1554390 Satlength=421 Nr of Repeats=18 RepeatLength=20 seed=AGGCAGACCT Num.seqs=16 Similarity=0.885556
2   CTAGGCAG-ACCTAGCCTACC
Cele-UNSB01_5:915226-915380 Satlength=155 Nr of Repeats=8 RepeatLength=20 seed=GGCAGACCTA Num.seqs=7 Similarity=0.809524
3   CTAGGCAG-ACCTAGTCTACC
Cele-UNSB01_3:2578277-2578816 Satlength=540 Nr of Repeats=27 RepeatLength=20 seed=GACCCAGCCT Num.seqs=26 Similarity=0.985026
7   CCAGGCAG-ACCCAGCCTACC
Cele-UNSB01_3:2578282-2579101 Satlength=820 Nr of Repeats=41 RepeatLength=20 seed=AGCCTACCCC Num.seqs=40 Similarity=0.907436
12   CCAGGCAG-ACCCAGCCTACC
Cele-UNSB01_5:915117-915271 Satlength=155 Nr of Repeats=7 RepeatLength=20 seed=CCTACCCCAG Num.seqs=6 Similarity=0.871111
14   CCAGGCAG-ACCTAGCCTACC
Cele-UNSB01_1:1878726-1878810 Satlength=85 Nr of Repeats=4 RepeatLength=21 seed=CCTACTCTAG Num.seqs=4 Similarity=1.000000
15   CTAGGCAGCCCCTAGCCTACT
Rev.of_Cele-UNSB01_6:16108332-16108500 Satlength=169 Nr of Repeats=8 RepeatLength=21 seed=GGCTGCCGAG Num.seqs=8 Similarity=0.916100
31   CTCGGCAGCCCCTAGCCTACC

```
*  *****  *  ** ****
Consensus:

CtAGGCAGACCTAGCCTACC

>Cele-UNSB01_Fam_24_32_11  Nr. of seq. 11 Alignment length(with gaps) =
34 Alignment score = 0.834759
Cele-UNSB01_5:20579964-20580501 Satlength=538 Nr of Repeats=15
RepeatLength=31 seed=CTACAAACTA  Num.seqs=9 Similarity=0.899642
0    CTACAAACTACG-AG-TTTTGGGCGCTGCTAAG-
Cele-UNSB01_5:20584979-20587201 Satlength=2223 Nr of Repeats=68
RepeatLength=31 seed=GGCGCTGCTA  Num.seqs=43 Similarity=0.966992
19    CTACAAACTACA-AG-ATTTGGGCGCTGCTAA-A
Rev.of_Cele-UNSB01_5:20575835-20576061 Satlength=227 Nr of Repeats=7
RepeatLength=32 seed=TTAGCAGCGC  Num.seqs=5 Similarity=0.931313
30    CTACAAACTACA-AGTTTTTGGGCGCTGCTAA-A
Rev.of_Cele-UNSB01_5:19404337-19404490 Satlength=154 Nr of Repeats=5
RepeatLength=33 seed=TTAGCAGCGC  Num.seqs=4 Similarity=0.979798
30    CTACAAACTACA-AGATTTTGGGCGCTGCTAAGA
Rev.of_Cele-UNSB01_5:19408877-19410842 Satlength=1966 Nr of Repeats=65
RepeatLength=33 seed=AGCAGCGCCC  Num.seqs=49 Similarity=0.900143
61    CTACAAACTACA-AGATTTTGGGCGCTGCTAAGA
Rev.of_Cele-UNSB01_6:179889-180069 Satlength=181 Nr of Repeats=5
RepeatLength=32 seed=TTAGCAGCGC  Num.seqs=3 Similarity=0.944444
30    CTACAAACTACA-AG-TTTTGGGCGCTGCTAACA
Cele-UNSB01_6:207322-209932 Satlength=2611 Nr of Repeats=80
RepeatLength=32 seed=TTTGGGCGCT  Num.seqs=62 Similarity=0.938886
47    CTACAAACTACA-AG-TTTTGGGCGCTGCTAAGA
Rev.of_Cele-UNSB01_5:19172684-19173158 Satlength=475 Nr of Repeats=14
RepeatLength=32 seed=TTGTAGTTTG  Num.seqs=9 Similarity=0.939815
13    CTACAAACTACA-AA-TTTTGGGCGCTGCTTAGA
Cele-UNSB01_5:20243571-20245480 Satlength=1910 Nr of Repeats=58
RepeatLength=32 seed=ACTACAAACT  Num.seqs=46 Similarity=0.942073
31    CTACAAACTACA-AG-TTTTGGGCGCTGCTTAGA
Cele-UNSB01_5:20413138-20414402 Satlength=1265 Nr of Repeats=44
RepeatLength=32 seed=TTTTGGGCGC  Num.seqs=27 Similarity=0.907170
14    CTACAAACTACA-AT-TTTTGGGCGCTGCTTAGA
Rev.of_Cele-UNSB01_5:19275863-19276354 Satlength=492 Nr of Repeats=14
RepeatLength=32 seed=TGTAGTTTGT  Num.seqs=9 Similarity=0.905443
12    CTACAAACTACACAA-TTTTGGGCGCTGCTAA-A

***********  *   ************* *
Consensus:

CTACAAACTACAAgTTTTGGGCGCTGCTaAgA

>Cele-UNSB01_Fam_25_12_11  Nr. of seq. 11 Alignment length(with gaps) =
12 Alignment score = 0.746970
Cele-UNSB01_2:2168167-2168227 Satlength=61 Nr of Repeats=4
RepeatLength=11 seed=ACCGCAACGC  Num.seqs=3 Similarity=1.000000
0    AC-CGCAACGCA
```

Rev.of_Cele-UNSB01_5:20920761-20921207 Satlength=447 Nr of Repeats=40
RepeatLength=11 seed=GTTGCGGTTG Num.seqs=39 Similarity=0.965976
8   AC-CGCAACGCA
Rev.of_Cele-UNSB01_3:2058475-2058618 Satlength=144 Nr of Repeats=11
RepeatLength=11 seed=GTCGCGTTGC Num.seqs=9 Similarity=0.885522
2   AC-CGCAACGCG
Rev.of_Cele-UNSB01_1:567454-567526 Satlength=73 Nr of Repeats=6
RepeatLength=12 seed=GCGTTGCGTG Num.seqs=6 Similarity=0.896296
4   ACGCGGCACGCA
Cele-UNSB01_1:2284293-2285881 Satlength=1589 Nr of Repeats=82
RepeatLength=12 seed=CACGCAACGC Num.seqs=74 Similarity=0.933811
6   ACGCAGCACGCA
Rev.of_Cele-UNSB01_1:11460977-11461721 Satlength=745 Nr of Repeats=60
RepeatLength=12 seed=CGTTGCGTGT Num.seqs=58 Similarity=0.901727
15   ACGCGACACGCA
Cele-UNSB01_3:13290888-13291020 Satlength=133 Nr of Repeats=7
RepeatLength=12 seed=CGCGACACGC Num.seqs=5 Similarity=0.822222
25   ACGCGACACGCA
Rev.of_Cele-UNSB01_4:4229494-4229583 Satlength=90 Nr of Repeats=7
RepeatLength=12 seed=GTTGCGTGTC Num.seqs=6 Similarity=0.962963
26   ACGCGACACGCA
Rev.of_Cele-UNSB01_4:4229497-4230359 Satlength=863 Nr of Repeats=71
RepeatLength=12 seed=GCGTGTCGCG Num.seqs=69 Similarity=0.901298
23   ACGCGACACGCA
Cele-UNSB01_5:17801402-17801541 Satlength=140 Nr of Repeats=10
RepeatLength=12 seed=ACGCGACACG Num.seqs=8 Similarity=0.972222
24   ACGCGACACGCA
Cele-UNSB01_5:17569173-17569281 Satlength=109 Nr of Repeats=9
RepeatLength=12 seed=CGCAACGCGA Num.seqs=9 Similarity=0.907407
8   ACGCGATACGCA

** *   ****
Consensus:

ACgCGacACGCA

>Cele-UNSB01_Fam_26_163_10 Nr. of seq. 10 Alignment length(with gaps) = 172 Alignment score = 0.762446
Cele-UNSB01_1:995161-995964 Satlength=804 Nr of Repeats=5
RepeatLength=162 seed=ATCATATGAA Num.seqs=4 Similarity=0.781893
0   ATCATATGAAGCGATTCTTTTTTCTTTTATAAACCTGTTCAGAATTGGCAAAACTACCAGAAAATAC-
TAAACAAATTAGGCTAGCATGAACGGAAGT-A-T--TTTTTAAAAATTGAT-AAAAATA-ATG-
AAAAGCTGA--TATTTTCAAAATTCCAGAATGACGGAAAA
Rev.of_Cele-UNSB01_2:2065528-2066342 Satlength=815 Nr of Repeats=5
RepeatLength=162 seed=AGTTTTATGA Num.seqs=3 Similarity=0.983539
36
ATCATATAGAGTCATTCTTTTTTATTTCATAAAACTCTTCAGCATAGTCAAAAATACCAGAAAATGC-
TAAACAAAGTATAATAGTTTGTACGGAAGT-A-T--TTTTTAAAAATGGAT-AAAAATATAT--
AAAAGCTGA--TTTTTTCAAAAATTCAAAAGTATGGGAAA
Rev.of_Cele-UNSB01_2:15429658-15431122 Satlength=1465 Nr of Repeats=9
RepeatLength=163 seed=AAATAAAAAA Num.seqs=8 Similarity=0.966696
190
ATCATATGGAGTCATTCTTTTTTATTTCATAAAACTGTTCAGCATAGTTAAAATTACCAGAATATGC-

```
CAATCAAAGTATATTAGCTTGTACGGAAGT-ATT--TTTTTAAAAATTGAT-AAAAATATAT--
AAAAGCTGA--TTTTTTCAAAAATTCAAAAGTATGGGAAA
Cele-UNSB01_3:10519570-10525291 Satlength=5722 Nr of Repeats=35
RepeatLength=164 seed=ATGCCAATCA  Num.seqs=23 Similarity=0.972621
226
ATCATATGGAGTCATTCTTTTTTATTGCATAAAACTGTTCAGTATAGTCAAAAATACCAGAATATGC-
CAATCAAAGTATAATAGCTTGTACGGAAGT-ATT--TTTTTAAAAATTGATAAAAAATATAT--
AAAAGCTGA--TTTTTTCAAAAATTCAAAAGTATGGGAAA
Cele-UNSB01_4:13731432-13732897 Satlength=1466 Nr of Repeats=8
RepeatLength=164 seed=AAAATTCAAA  Num.seqs=5 Similarity=1.000000
306
ATCATATGGAGTCATTCTTTTTTATTTCATAAAACTCTTCAGCATAGTCAAAAATACCAGAAAATGC-
CAATCAAAGTATAATAGCTTGTACGGAAGT-ATT--TTTTTAAAAATTGAT-AAAAATATAT--
AAAAGCTGAT-TTTTTTCAAAAATTCAAAAGTATGGGAAA
Cele-UNSB01_1:11925845-11930260 Satlength=4416 Nr of Repeats=27
RepeatLength=164 seed=ATACCAGAAA  Num.seqs=20 Similarity=0.942191
216
ATCAAATGGAGTCATTCTTTTTTATTCCATAAAACTGTTCAGCATAGTCAAAAATACCAGAAAATGC-
CAATCAAAGTATATTAGCTTGTACGGAAGTAATT--TTTTTTTAAATTGAT-AAAAATATAT--
AAAAGCTGA--TTTTTTCAAAAATTCAAAAGTATGGGAAA
Cele-UNSB01_1:11042845-11046757 Satlength=3913 Nr of Repeats=24
RepeatLength=163 seed=GCATAGTCAA  Num.seqs=24 Similarity=0.936516
41
ATCATATGGAGTCATTCTTTTTTATCTCAAAAAACTGTTCAGCATAGTCAAAAATACCAGCAAATAC-
CAAACAGAGTATATTAGCTTGTACGGAAGT-ATT--TTTTAGAAAATTGAT-AAAAATATAT--
AAAAGCTGA--TTTTTTCAAAAATTCAAAAGTATGGGAAA
Rev.of_Cele-UNSB01_5:16471613-16475389 Satlength=3777 Nr of Repeats=23
RepeatLength=164 seed=CGTACAAGCT  Num.seqs=17 Similarity=0.961741
92
ATCATATGGAGTCATTCTTTTTTATTTCATAAAAATGTTCAGCATAGTCAAAAACACCAGAATATGC-
CAATCAAAGTATAATAGCTTGTACGGAAGT-ATT--CTTTAAAAAATTGAT-AAAAATATAT--
AAAATCTGA-TTTTTTTCAAAAATTCAAAAGTCCTAGAAA
Rev.of_Cele-UNSB01_2:2379059-2380853 Satlength=1795 Nr of Repeats=9
RepeatLength=163 seed=TGAATTTTTG  Num.seqs=7 Similarity=0.906387
150
ATCATATGAAGTCATTCTTTTTTATTTTATAAAACTGTTCAGCATAGTCAAATGTACCAGAAAATACTTAAAA
AAAG-GTGCTAGCTCGTACGGAAGT--TTATTTTTAAAAAATGCAG---AATTTAAC-CAAAAGCTG--
TTATTTTCAAAAATTCAAAAGTACCTCAAA
Cele-UNSB01_4:2599504-2602273 Satlength=2770 Nr of Repeats=17
RepeatLength=164 seed=CTTTTTTCTT  Num.seqs=16 Similarity=0.982520
179
ATCATAAAAAGTGCTTCTTTTTTCTTTTATAACACTGTTTAGCATAGTCAAATATACCAGAAAATAC-
CAAACAAAGTATGCTATCTTGTACGAAAGA--TTA-TTTTAAAAAATTCAG--AAAATGCAT-
CTAAAGCGGT-TTTTTTTCAAAAATTCAAAAGTACCGGAAA

**** *   **   ********* *   * **   * ** ** ** *  ***   ***** * ** *  **
* *      **    * *** ***    *   ***   ****  *    ** *  *    *** * *   *
********* * ** **       ***
Consensus:

ATCATATggAGTcATTCTTTTTTaTTtcATAAAACTgTTCAGcATAGTCAAAaaTACCAGAAaATgCcAAaCA
AAGTATanTAGCTTGTACGGAAGTatTTTTTtaAAAATtgAtaAAAATatATAAAAGCTGaTtTTTTCAAAAA
TTCAAAAGTAtgggAAA
```

>Cele-UNSB01_Fam_27_31_10 Nr. of seq. 10 Alignment length(with gaps) = 37 Alignment score = 0.720120
Cele-UNSB01_3:2212231-2213000 Satlength=770 Nr of Repeats=22
RepeatLength=31 seed=ATTTCCCGCC Num.seqs=14 Similarity=0.922486
0   ATTTCCCGCCAAAAAATA---TTT---GAAAATTTGA
Rev.of_Cele-UNSB01_5:18406789-18410349 Satlength=3561 Nr of Repeats=114
RepeatLength=31 seed=GTCAATTTTT Num.seqs=88 Similarity=0.885902
20   ATTTCCCGCCAAAAATTG---ACT---GAAAATTTGA
Cele-UNSB01_5:19832755-19833004 Satlength=250 Nr of Repeats=6
RepeatLength=31 seed=AATTTCCCGC Num.seqs=5 Similarity=0.756250
30   ATTTCCCGCCAAAAATTG---ACT---GAAAATTTGA
Rev.of_Cele-UNSB01_5:19928437-19928836 Satlength=400 Nr of Repeats=12
RepeatLength=31 seed=GGCGGGAAAT Num.seqs=8 Similarity=0.930876
41   ATTTCCCGCCAAAAATTG---ACT---GAAAATTTGA
Rev.of_Cele-UNSB01_5:19938977-19939376 Satlength=400 Nr of Repeats=12
RepeatLength=31 seed=GGCGGGAAAT Num.seqs=8 Similarity=0.930876
41   ATTTCCCGCCAAAAATTG---ACT---GAAAATTTGA
Cele-UNSB01_5:19998052-19998541 Satlength=490 Nr of Repeats=10
RepeatLength=31 seed=AAAATTTGAA Num.seqs=9 Similarity=0.942652
22   ATTTCCCGCCAAAAATTG---ACT---GAAAATTTGA
Rev.of_Cele-UNSB01_5:19670399-19670678 Satlength=280 Nr of Repeats=9
RepeatLength=31 seed=CGGGAAATTC Num.seqs=7 Similarity=0.774706
39   ATTTCCCGCCAAAAATTA---ACT---GAAAATTTGA
Rev.of_Cele-UNSB01_5:19913255-19913615 Satlength=361 Nr of Repeats=8
RepeatLength=31 seed=TTCAAATTTT Num.seqs=7 Similarity=0.819764
32   AATTCCCGCCAAAAATTG---ACT---GAAAATTTGA
Cele-UNSB01_5:20173699-20174897 Satlength=1199 Nr of Repeats=28
RepeatLength=31 seed=TTCCCGCCTA Num.seqs=17 Similarity=0.854243
2   ATTTCCCGCCTAAAACT----ACTC--GAAAATTCAA
Rev.of_Cele-UNSB01_5:18924789-18925018 Satlength=230 Nr of Repeats=6
RepeatLength=35 seed=GGAAATTCAA Num.seqs=4 Similarity=0.776235
6   ATTTTCCGCCAAAAAATATTTTCT-CCGAAAATTTGA

* ** ***** **** *   *   ******* *
Consensus:

ATTTCCCGCCAAAAAtTgaCTGAAAATTTGA

>Cele-UNSB01_Fam_28_26_10 Nr. of seq. 10 Alignment length(with gaps) = 27 Alignment score = 0.716324
Cele-UNSB01_2:5166221-5171016 Satlength=4796 Nr of Repeats=184
RepeatLength=26 seed=TAGACCATTA Num.seqs=155 Similarity=0.871050
0   TAGACCATTAGATGAATTTC-CAAAGT
Rev.of_Cele-UNSB01_4:4479166-4488089 Satlength=8924 Nr of Repeats=327
RepeatLength=26 seed=GTCTAACTTT Num.seqs=305 Similarity=0.965297
5   TAGACCATTAGATGAATTTC-CAAAGT
Cele-UNSB01_4:13295662-13296286 Satlength=625 Nr of Repeats=24
RepeatLength=26 seed=ATTAGATGAA Num.seqs=24 Similarity=0.974173
6   TAGACCATTAGATGAATTTC-CAAAGT
Cele-UNSB01_3:7826007-7841897 Satlength=15891 Nr of Repeats=610
RepeatLength=26 seed=CATTAGATGA Num.seqs=417 Similarity=0.923292
5   TAGACCATTAGATGAATTCC-CAAAGT

Rev.of_Cele-UNSB01_6:16057246-16058243 Satlength=998 Nr of Repeats=38
RepeatLength=26 seed=AATTCATCTA Num.seqs=36 Similarity=0.954009
18   TAGACCAGTAGATGAATTCC-CAAAGT
Cele-UNSB01_6:12080509-12089472 Satlength=8964 Nr of Repeats=344
RepeatLength=26 seed=AAGTTAGACC Num.seqs=291 Similarity=0.954261
22   TAGACCATTAGATGAATTTA-TAAAGT
Rev.of_Cele-UNSB01_6:6135861-6144415 Satlength=8555 Nr of Repeats=329
RepeatLength=26 seed=TTCTAAATTT Num.seqs=329 Similarity=0.936466
5   TAGAACGGTAGATGAATTTT-CAAATT
Rev.of_Cele-UNSB01_4:13647739-13648797 Satlength=1059 Nr of Repeats=13
RepeatLength=27 seed=TTTTTAAAAT Num.seqs=11 Similarity=0.892256
25   TAGAACGGTATATGAATTTTAAAAAGT
Rev.of_Cele-UNSB01_6:11535780-11535884 Satlength=105 Nr of Repeats=4
RepeatLength=26 seed=AAATTAATCT Num.seqs=4 Similarity=0.803419
19   TAGAGTGACAGATTAATTTA-AAAAGT
Rev.of_Cele-UNSB01_6:15966442-15966650 Satlength=209 Nr of Repeats=8
RepeatLength=26 seed=GGTCTAACTT Num.seqs=8 Similarity=0.899267
32   TAGACCGTCAGATCCATTTA-TAAAGT

****   * ** ***   *** *
Consensus:

TAGAcCattAGATgAATTtccAAAGT

>Cele-UNSB01_Fam_29_21_10 Nr. of seq. 10 Alignment length(with gaps) = 23 Alignment score = 0.611433
Cele-UNSB01_6:960884-960987 Satlength=104 Nr of Repeats=5 RepeatLength=20
seed=ATGCACTGAC Num.seqs=3 Similarity=0.866667
0   AT-GCACTGACCAAAAATATG--
Rev.of_Cele-UNSB01_6:998781-998865 Satlength=85 Nr of Repeats=4
RepeatLength=21 seed=CAGTGCATTC Num.seqs=4 Similarity=0.830688
8   AT-GCACTGACCAAAGTTCTGA-
Rev.of_Cele-UNSB01_6:998801-998992 Satlength=192 Nr of Repeats=6
RepeatLength=21 seed=TCAGTGCATT Num.seqs=4 Similarity=0.735450
9   AT-GCACTGACCAAACTTCTGA-
Rev.of_Cele-UNSB01_6:1060978-1061104 Satlength=127 Nr of Repeats=6
RepeatLength=21 seed=GTCAGTGCAT Num.seqs=6 Similarity=0.944974
10   AT-GCACTGACCGAAACTATCA-
Rev.of_Cele-UNSB01_6:1066955-1067057 Satlength=103 Nr of Repeats=5
RepeatLength=20 seed=TTGGTCAGTG Num.seqs=3 Similarity=0.955556
13   AT-GCACTGACCAAGACTA-AA-
Rev.of_Cele-UNSB01_6:3814487-3814588 Satlength=102 Nr of Repeats=5
RepeatLength=20 seed=TTTGGTCAGT Num.seqs=4 Similarity=0.933333
14   AA-GCACTGACCAAA-ATA-GAA
Cele-UNSB01_6:903956-904040 Satlength=85 Nr of Repeats=4 RepeatLength=21
seed=ACTGACCAAA Num.seqs=4 Similarity=0.936508
25   AT-GCACTGACCAAAAATA-GAT
Cele-UNSB01_6:1342228-1342338 Satlength=111 Nr of Repeats=5
RepeatLength=22 seed=TCGCACTGAC Num.seqs=5 Similarity=0.915152
22   ATCGCACTGACCAAACTTA-GAA
Rev.of_Cele-UNSB01_6:743518-743644 Satlength=127 Nr of Repeats=5
RepeatLength=21 seed=GGTCAGTGCA Num.seqs=4 Similarity=0.904762
32   AT-GCACTGACCAAAATTG-GTA

Rev.of_Cele-UNSB01_6:1102792-1102897 Satlength=106 Nr of Repeats=4
RepeatLength=21 seed=GTCAGTGCAA Num.seqs=3 Similarity=0.661376
10   TT-GCACTGACCANNACTCTAA-

*********   *
Consensus:

ATGCACTGACCAAaanTatga

>Cele-UNSB01_Fam_30_12_10 Nr. of seq. 10 Alignment length(with gaps) = 14 Alignment score = 0.713757
Cele-UNSB01_1:4669638-4670054 Satlength=417 Nr of Repeats=33
RepeatLength=12 seed=TTTTTATTTT Num.seqs=26 Similarity=0.967179
0   TTTTTATTT-TTA-
Cele-UNSB01_1:4669638-4670196 Satlength=559 Nr of Repeats=41
RepeatLength=12 seed=TTTTTATTTT Num.seqs=27 Similarity=0.968344
0   TTTTTATTT-TTA-
Cele-UNSB01_1:4669638-4670946 Satlength=1309 Nr of Repeats=43
RepeatLength=12 seed=TTTTTATTTT Num.seqs=27 Similarity=0.968344
0   TTTTTATTT-TTA-
Rev.of_Cele-UNSB01_2:11255667-11256407 Satlength=741 Nr of Repeats=61
RepeatLength=12 seed=AAAAATAAAA Num.seqs=52 Similarity=1.000000
5   TTTTTATTT-TTA-
Rev.of_Cele-UNSB01_2:11255666-11256503 Satlength=838 Nr of Repeats=64
RepeatLength=12 seed=TAAAAATAAA Num.seqs=57 Similarity=0.988443
0   TTTTTATTT-TTA-
Rev.of_Cele-UNSB01_3:3456121-3456336 Satlength=216 Nr of Repeats=18
RepeatLength=12 seed=AAAATAAAAA Num.seqs=17 Similarity=0.986928
4   TTTTTATTT-TTA-
Rev.of_Cele-UNSB01_3:10519446-10519548 Satlength=103 Nr of Repeats=8
RepeatLength=12 seed=AAAACTAAAA Num.seqs=5 Similarity=0.815385
5   GTTTTATTT-TTA-
Cele-UNSB01_5:6983222-6984063 Satlength=842 Nr of Repeats=56
RepeatLength=12 seed=TTTTAATTTT Num.seqs=46 Similarity=1.000000
0   -TTTTAATT-TTAA
Cele-UNSB01_4:6359332-6359386 Satlength=55 Nr of Repeats=4
RepeatLength=13 seed=TTTCATTTGT Num.seqs=3 Similarity=0.931624
1   TTTTCATTT-GTAA
Rev.of_Cele-UNSB01_6:2109492-2110453 Satlength=962 Nr of Repeats=78
RepeatLength=12 seed=AATAAATAAT Num.seqs=54 Similarity=1.000000
0   TATTTATTTATT--

** * ** *
Consensus:

tTTTTATTTTTA

>Cele-UNSB01_Fam_31_42_9 Nr. of seq. 9 Alignment length(with gaps) = 42 Alignment score = 0.733686
Cele-UNSB01_1:980318-980990 Satlength=673 Nr of Repeats=16
RepeatLength=42 seed=CCGGAAATTT Num.seqs=16 Similarity=0.888889
0   CCGGAAATTTCGAAAACCGGCAATTGCCGAAATTGCCGATTG

Cele-UNSB01_2:3617397-3617684 Satlength=288 Nr of Repeats=6
RepeatLength=42 seed=TTTGAAAACC Num.seqs=4 Similarity=0.851852
8   CCGGAAATTTTGAAAACCGGCAATAGCCGAAATTGCCGAGTG
Rev.of_Cele-UNSB01_5:20627832-20628586 Satlength=755 Nr of Repeats=12
RepeatLength=42 seed=ATTGCCGGTT Num.seqs=9 Similarity=0.803351
24   CCGAAAATTTTAAAAACCGGCAATTGCCGAAATTGCCGATTG
Cele-UNSB01_5:3789009-3789261 Satlength=253 Nr of Repeats=6
RepeatLength=42 seed=ACCGGCAATT Num.seqs=6 Similarity=0.881481
15   CTGAAAATTTCCAAAACCGGCAATTGCCAAAATTGCCGATTG
Cele-UNSB01_2:14381587-14382260 Satlength=674 Nr of Repeats=16
RepeatLength=42 seed=AAAACCGGCA Num.seqs=10 Similarity=0.749832
12   CCGGNAATATTAAAAACCGGCAATTGCCGGAATTGCCGATTG
Rev.of_Cele-UNSB01_2:1652802-1653206 Satlength=405 Nr of Repeats=5
RepeatLength=42 seed=TTGCCGGTTG Num.seqs=3 Similarity=0.809524
23   CAAAAAATTTCGGCAACCGGCAATTGCCGAAGTTGCCGAACC
Cele-UNSB01_2:1657514-1657909 Satlength=396 Nr of Repeats=5
RepeatLength=42 seed=CAACCGGCAA Num.seqs=3 Similarity=0.809524
55   CAAAAAATTTCGGCAACCGGCAATTGCCGAAGTTGCCGAACC
Cele-UNSB01_5:16332671-16332882 Satlength=212 Nr of Repeats=6
RepeatLength=42 seed=TTTCGGCAAC Num.seqs=4 Similarity=0.899471
49   CGAAAAAATTTCGGCAACCGGCAATTGCCGATGTTGCCGAACC
Cele-UNSB01_2:12718309-12718687 Satlength=379 Nr of Repeats=8
RepeatLength=42 seed=TTGCCGAACC Num.seqs=7 Similarity=0.797619
32   GTAAAAATTTCGGCAACCNGGAATTGCCAAAGTTGCCGAACC

*** *   **** * *** ***   *******
Consensus:

CngaAAATTTcgaaAACCGGCAATTGCCgAAaTTGCCGAntg

>Cele-UNSB01_Fam_32_26_9 Nr. of seq. 9 Alignment length(with gaps) = 26
Alignment score = 0.800214
Cele-UNSB01_4:11734646-11742696 Satlength=8051 Nr of Repeats=322
RepeatLength=25 seed=ATTACGGCAG Num.seqs=322 Similarity=0.947627
0   ATTACGGCAGATTCTA-TAAATTGGC
Rev.of_Cele-UNSB01_4:14408867-14409192 Satlength=326 Nr of Repeats=13
RepeatLength=25 seed=TAATGCCAAT Num.seqs=13 Similarity=0.948034
4   ATTACGGCAGATTCTA-TAAATTGGC
Rev.of_Cele-UNSB01_6:11722486-11725761 Satlength=3276 Nr of Repeats=130
RepeatLength=25 seed=CTGCCGTAAT Num.seqs=129 Similarity=0.942132
10   ATTACGGCAGATTCTA-TAAATTGGC
Rev.of_Cele-UNSB01_1:4605678-4635032 Satlength=29355 Nr of Repeats=1129
RepeatLength=26 seed=TTTTCTAAAA Num.seqs=1129 Similarity=0.999909
21   ATTGCGGCGGATTTTAGAAAAATGGC
Rev.of_Cele-UNSB01_6:1706298-1706662 Satlength=365 Nr of Repeats=14
RepeatLength=26 seed=GCCGCAATGC Num.seqs=14 Similarity=0.970132
34   ATTGCGGCGGATTTTAGAAAAATGGC
Cele-UNSB01_6:11725790-11730325 Satlength=4536 Nr of Repeats=175
RepeatLength=26 seed=CGGCGGATTT Num.seqs=160 Similarity=0.928249
30   ATTGCGGCGGATTTTAGAAAAATGGC
Cele-UNSB01_4:12895275-12902336 Satlength=7062 Nr of Repeats=272
RepeatLength=26 seed=CATTGCGGCG Num.seqs=261 Similarity=0.919236
25   ATTGCGGCGGATTTTNGAAAAATGGC

Cele-UNSB01_1:11159617-11160082 Satlength=466 Nr of Repeats=4
RepeatLength=26 seed=TTTTTGAAAA Num.seqs=3 Similarity=0.931624
37   ATTGCGGCGGATTTTTGAAAAATGGC
Cele-UNSB01_6:12933662-12933969 Satlength=308 Nr of Repeats=5
RepeatLength=26 seed=AAAAATGGCA Num.seqs=4 Similarity=0.897436
43   ATTGCGGCGGATTTCAGAAAAATGGC

*** **** ****   *** ****
Consensus:

ATTgCGGCgGATTtTagaAAAaTGGC

>Cele-UNSB01_Fam_33_18_9 Nr. of seq. 9 Alignment length(with gaps) = 23
Alignment score = 0.628019
Cele-UNSB01_1:11696998-11697105 Satlength=108 Nr of Repeats=5
RepeatLength=19 seed=TCAAAAATCA Num.seqs=4 Similarity=0.873611
21   CA--TC-AAAAATCAATAA-TTT
Cele-UNSB01_1:11591612-11591946 Satlength=335 Nr of Repeats=14
RepeatLength=20 seed=AAAAATCAAT Num.seqs=11 Similarity=0.883636
24   CA--TCGAAAAATCAATAA-TTT
Cele-UNSB01_1:11696961-11698223 Satlength=1263 Nr of Repeats=20
RepeatLength=19 seed=AAAAATCGAT Num.seqs=12 Similarity=0.725830
23   CN--TC-AAAAATCGATAA-TTT
Cele-UNSB01_2:2648748-2648862 Satlength=115 Nr of Repeats=6
RepeatLength=19 seed=AAAATCGATA Num.seqs=6 Similarity=0.601058
24   CA--TC-GAAAATCGATAA-TTT
Cele-UNSB01_1:11744458-11744933 Satlength=476 Nr of Repeats=17
RepeatLength=19 seed=TTTCACAAAA Num.seqs=12 Similarity=0.943647
35   CA---C-AAAAATCAATAATTTT
Cele-UNSB01_1:11743735-11745857 Satlength=2123 Nr of Repeats=45
RepeatLength=19 seed=AAAATCGATA Num.seqs=29 Similarity=0.730706
42   CA---N-AAAAATCGATAATTTT
Cele-UNSB01_4:984710-985413 Satlength=704 Nr of Repeats=35
RepeatLength=19 seed=CAATAATTTC Num.seqs=27 Similarity=0.905233
28   CC-C-A-AAAAATCAATAA-TTT
Rev.of_Cele-UNSB01_1:11592271-11597890 Satlength=5620 Nr of Repeats=277
RepeatLength=20 seed=ATTGATTTTT Num.seqs=273 Similarity=0.884518
32   CCTC-A-AAAAATCAATAA-TTT
Rev.of_Cele-UNSB01_1:2396315-2396849 Satlength=535 Nr of Repeats=20
RepeatLength=20 seed=TTATTGATTT Num.seqs=12 Similarity=0.816162
34   CCTA-G-AAAAATCAATAA-TGT

****** **** * **
Consensus:

cAAAAATCaATAATTTCa

>Cele-UNSB01_Fam_34_12_9 Nr. of seq. 9 Alignment length(with gaps) = 15
Alignment score = 0.680247
Cele-UNSB01_6:2369427-2369729 Satlength=303 Nr of Repeats=24
RepeatLength=11 seed=CCATACCTAT Num.seqs=20 Similarity=1.000000
0   -CC-ATACCT-ATA-

Cele-UNSB01_1:12842011-12842077 Satlength=67 Nr of Repeats=5
RepeatLength=12 seed=TACCTATACC Num.seqs=4 Similarity=0.944444
3   -CT-ATACCT-ATAC
Cele-UNSB01_1:13927551-13927713 Satlength=163 Nr of Repeats=13
RepeatLength=12 seed=TATACCTATA Num.seqs=10 Similarity=0.955556
7   -CT-ATACCT-ATAC
Rev.of_Cele-UNSB01_1:13927986-13928124 Satlength=139 Nr of Repeats=11
RepeatLength=12 seed=ATAGGTATAG Num.seqs=10 Similarity=0.955556
4   -CT-ATACCT-ATAC
Rev.of_Cele-UNSB01_2:3535061-3535729 Satlength=669 Nr of Repeats=54
RepeatLength=12 seed=TAGGTATAGG Num.seqs=46 Similarity=0.876973
3   -CT-ATACCT-ATAC
Cele-UNSB01_2:12494810-12494978 Satlength=169 Nr of Repeats=14
RepeatLength=12 seed=CCTATACCTA Num.seqs=14 Similarity=0.968254
5   -CT-ATACCT-ATAC
Cele-UNSB01_4:13402475-13402619 Satlength=145 Nr of Repeats=12
RepeatLength=12 seed=TATACCTATA Num.seqs=12 Similarity=0.944444
7   -CT-ATACCT-ATAC
Cele-UNSB01_2:3077809-3078033 Satlength=225 Nr of Repeats=15
RepeatLength=14 seed=CTTATACCTT Num.seqs=14 Similarity=0.947671
1   CCTTATACCTTATA-
Cele-UNSB01_3:1413910-1414798 Satlength=889 Nr of Repeats=40
RepeatLength=14 seed=ATACCTAATA Num.seqs=28 Similarity=0.877047
4   CCTAATACCTAATA-

* ****** ***
Consensus:

CTATACCTATAc

>Cele-UNSB01_Fam_35_12_9 Nr. of seq. 9 Alignment length(with gaps) = 12
Alignment score = 0.876543
Cele-UNSB01_1:774871-775189 Satlength=319 Nr of Repeats=26
RepeatLength=12 seed=TCTAGGTCTA Num.seqs=25 Similarity=0.948148
0   TCTAGGTCTAGG
Cele-UNSB01_1:1009660-1009762 Satlength=103 Nr of Repeats=8
RepeatLength=12 seed=GGTCTAGGTC Num.seqs=7 Similarity=0.936508
4   TCTAGGTCTAGG
Cele-UNSB01_1:11696177-11696255 Satlength=79 Nr of Repeats=6
RepeatLength=12 seed=TAGGTCTAGG Num.seqs=5 Similarity=1.000000
2   TCTAGGTCTAGG
Rev.of_Cele-UNSB01_2:885470-885668 Satlength=199 Nr of Repeats=15
RepeatLength=12 seed=ACCTAGACCT Num.seqs=12 Similarity=0.858586
1   TCTAGGTCTAGG
Cele-UNSB01_1:961195-961471 Satlength=277 Nr of Repeats=23
RepeatLength=12 seed=CTAAGTCTAA Num.seqs=23 Similarity=1.000000
1   TCTAAGTCTAAG
Cele-UNSB01_1:961002-961808 Satlength=807 Nr of Repeats=44
RepeatLength=12 seed=TAAGCCTAAG Num.seqs=39 Similarity=0.765770
2   TCTAAGTCTAAG
Cele-UNSB01_1:3303901-3304753 Satlength=853 Nr of Repeats=71
RepeatLength=12 seed=CTAAGTCTAA Num.seqs=69 Similarity=0.908497
1   TCTAAGTCTAAG

Rev.of_Cele-UNSB01_3:12499748-12499928 Satlength=181 Nr of Repeats=14 RepeatLength=12 seed=CTTAGACTTA Num.seqs=12 Similarity=0.929293
6   TCTAAGTCTAAG
Cele-UNSB01_5:15130293-15130341 Satlength=49 Nr of Repeats=4 RepeatLength=12 seed=TCTAAGTCTA Num.seqs=4 Similarity=0.944444
6   TCTAAGTCTAAG

**** ***** *
Consensus:

TCTAaGTCTAaG

>Cele-UNSB01_Fam_36_10_9 Nr. of seq. 9 Alignment length(with gaps) = 11 Alignment score = 0.771465
Cele-UNSB01_2:4272676-4272936 Satlength=261 Nr of Repeats=25 RepeatLength=10 seed=GCGTTAGCAG Num.seqs=24 Similarity=0.977778
0   GCGTTAGCAG-
Cele-UNSB01_5:853469-853759 Satlength=291 Nr of Repeats=23 RepeatLength=10 seed=GGCGTCAGCG Num.seqs=17 Similarity=0.888235
9   GCGTCAGCGG-
Cele-UNSB01_5:1897354-1897674 Satlength=321 Nr of Repeats=23 RepeatLength=10 seed=GGGCGTCAGC Num.seqs=15 Similarity=0.949206
18   GCGTCAGCGG-
Rev.of_Cele-UNSB01_2:13962674-13962948 Satlength=275 Nr of Repeats=23 RepeatLength=10 seed=CCGCTACCGC Num.seqs=19 Similarity=1.000000
0   GCGGTAGCGG-
Rev.of_Cele-UNSB01_3:13335994-13337798 Satlength=1805 Nr of Repeats=147 RepeatLength=10 seed=GCTACCGCCC Num.seqs=101 Similarity=0.923248
8   GCGGTAGCGG-
Rev.of_Cele-UNSB01_5:2726947-2727107 Satlength=161 Nr of Repeats=12 RepeatLength=10 seed=GCTACCGCCC Num.seqs=9 Similarity=1.000000
8   GCGGTAGCGG-
Rev.of_Cele-UNSB01_5:2726947-2728543 Satlength=1597 Nr of Repeats=110 RepeatLength=10 seed=GCTACCGCCC Num.seqs=99 Similarity=0.951240
8   GCGGTAGCGG-
Cele-UNSB01_5:3102518-3102978 Satlength=461 Nr of Repeats=41 RepeatLength=10 seed=GGTAGCGGGC Num.seqs=37 Similarity=0.925125
2   GCGGTAGCGG-
Rev.of_Cele-UNSB01_2:13967191-13967257 Satlength=67 Nr of Repeats=5 RepeatLength=11 seed=CCGCTACCGC Num.seqs=4 Similarity=0.919192
0   GCGGTAGCGGT

*** *** *
Consensus:

GCGgtAGCGG

>Cele-UNSB01_Fam_37_18_8 Nr. of seq. 8 Alignment length(with gaps) = 19 Alignment score = 0.681391
Cele-UNSB01_4:646705-646786 Satlength=82 Nr of Repeats=4 RepeatLength=15 seed=TAAGCCTAAG Num.seqs=3 Similarity=1.000000
0   TAAGCCTA-AGCC-CA-C-

```
Cele-UNSB01_5:1895975-1896260 Satlength=286 Nr of Repeats=14
RepeatLength=17 seed=TAAGCCTAAG  Num.seqs=9 Similarity=0.930283
0    TAAGCCTA-AG-CTTAGCT
Rev.of_Cele-UNSB01_3:662174-662284 Satlength=111 Nr of Repeats=6
RepeatLength=18 seed=TAGGCTTAGG  Num.seqs=4 Similarity=0.527778
8    TAAGCCTACAG-CTAAGCC
Cele-UNSB01_2:371286-371494 Satlength=209 Nr of Repeats=10
RepeatLength=18 seed=TAAGCCTAAG  Num.seqs=6 Similarity=0.619753
12    TAAGCCTA-AGTCTAAGCC
Cele-UNSB01_2:13097657-13097799 Satlength=143 Nr of Repeats=5
RepeatLength=18 seed=CCTAAGCCTA  Num.seqs=3 Similarity=0.660819
16    TAAGCCTA-AGNCTAAACC
Cele-UNSB01_5:1057921-1058017 Satlength=97 Nr of Repeats=6
RepeatLength=18 seed=AAGCCTAAGC  Num.seqs=4 Similarity=0.691358
19    TAAGCCTA-AGCCTAAACC
Cele-UNSB01_5:1707873-1707951 Satlength=79 Nr of Repeats=5
RepeatLength=18 seed=GCCTAAGCCT  Num.seqs=3 Similarity=0.614035
21    TANGCCTA-AGCCTAAGCA
Rev.of_Cele-UNSB01_2:3500187-3500295 Satlength=109 Nr of Repeats=5
RepeatLength=18 seed=GGCTTAGGCT  Num.seqs=3 Similarity=0.901235
12    TAAGCCTA-AGCCTGAGCT

** ***** ** *  * *
Consensus:

TAAGCCTAAGcCTaAgCc

>Cele-UNSB01_Fam_38_18_8  Nr. of seq. 8 Alignment length(with gaps) = 19
Alignment score = 0.829261
Cele-UNSB01_5:19164473-19167625 Satlength=3153 Nr of Repeats=148
RepeatLength=17 seed=ATTAGCAGCG  Num.seqs=97 Similarity=0.880902
0    -ATTAGCAGCGCCCAAAA-
Cele-UNSB01_5:19251983-19252110 Satlength=128 Nr of Repeats=5
RepeatLength=18 seed=GCAGCGCCCA  Num.seqs=3 Similarity=0.950617
4    -ATTAGCAGCGCCCAAAAA
Rev.of_Cele-UNSB01_5:19255197-19255501 Satlength=305 Nr of Repeats=17
RepeatLength=18 seed=TGCTAATTTT  Num.seqs=15 Similarity=0.936508
7    -ATTAGCAGCGCCCAAAAA
Cele-UNSB01_5:19273514-19273585 Satlength=72 Nr of Repeats=4
RepeatLength=18 seed=TAGCAGCGCC  Num.seqs=3 Similarity=0.901235
2    -ATTAGCAGCGCCCAAGAA
Cele-UNSB01_5:19170107-19171106 Satlength=1000 Nr of Repeats=12
RepeatLength=18 seed=AATTAGCAGC  Num.seqs=10 Similarity=0.960494
0    AATTAGCAGCGACCAAAA-
Cele-UNSB01_5:19392335-19392460 Satlength=126 Nr of Repeats=6
RepeatLength=18 seed=ATTAGCAGCG  Num.seqs=4 Similarity=1.000000
1    AATTAGCAGCGACCAAAA-
Rev.of_Cele-UNSB01_5:19480245-19483641 Satlength=3397 Nr of Repeats=189
RepeatLength=18 seed=GCTGCTAATT  Num.seqs=161 Similarity=0.900696
10    AATTAGCAGCGACCAAAA-
Cele-UNSB01_5:19249579-19249688 Satlength=110 Nr of Repeats=6
RepeatLength=18 seed=AGCAGCGCCC  Num.seqs=5 Similarity=0.896296
3    -AATAGCAGCGCCCAAAAA
```

* ******** **** *
Consensus:

ATTAGCAGCGcCCAAAAa

>Cele-UNSB01_Fam_39_14_8 Nr. of seq. 8 Alignment length(with gaps) = 14
Alignment score = 0.894558
Cele-UNSB01_1:10475895-10475986 Satlength=92 Nr of Repeats=6
RepeatLength=14 seed=GCTATACGCT Num.seqs=5 Similarity=1.000000
0   GCTATACGCTATAC
Cele-UNSB01_2:6280573-6282049 Satlength=1477 Nr of Repeats=96
RepeatLength=14 seed=CTATACGCTA Num.seqs=79 Similarity=1.000000
1   GCTATACGCTATAC
Rev.of_Cele-UNSB01_4:14409220-14409304 Satlength=85 Nr of Repeats=6
RepeatLength=14 seed=AGCGTATAGC Num.seqs=6 Similarity=1.000000
3   GCTATACGCTATAC
Rev.of_Cele-UNSB01_6:356210-356329 Satlength=120 Nr of Repeats=8
RepeatLength=14 seed=ATAGCGTATA Num.seqs=7 Similarity=0.891156
5   GCTATACGCTATAC
Cele-UNSB01_6:11722389-11722452 Satlength=64 Nr of Repeats=4
RepeatLength=14 seed=CTATACGCTA Num.seqs=3 Similarity=0.936508
1   GCTATACGCTATAC
Cele-UNSB01_5:12601066-12601185 Satlength=120 Nr of Repeats=8
RepeatLength=14 seed=CTATACGCTA Num.seqs=7 Similarity=0.891156
1   GCTATACGCTATGC
Rev.of_Cele-UNSB01_6:17259878-17260337 Satlength=460 Nr of Repeats=20
RepeatLength=14 seed=ATAGCGCATA Num.seqs=18 Similarity=0.808279
5   GCTATGCGCTATGC
Cele-UNSB01_4:14407623-14408822 Satlength=1200 Nr of Repeats=70
RepeatLength=14 seed=GCTATGCGCT Num.seqs=52 Similarity=0.786869
0   GCTATGCGCTGTAC

***** **** * *
Consensus:

GCTATaCGCTATaC

>Cele-UNSB01_Fam_40_166_7 Nr. of seq. 7 Alignment length(with gaps) =
173 Alignment score = 0.740343
Cele-UNSB01_6:16300389-16301032 Satlength=644 Nr of Repeats=4
RepeatLength=158 seed=TATCAATTTT Num.seqs=3 Similarity=0.924051
0   TATCAATTTTCAGACGCGCTACGGGGGTTTTTGTCTTGAATTCCAATTTCAAC-----------
AGGCGCAAAAAAACAAAAATAAAATTTTTGGATT-TTTGGAAAAGT-TTTT-
ATAGATTTCTATTCTAGATTGTTTAA-AAATATTATTGGTACGTTTTTGTACCAANA
Cele-UNSB01_6:16300466-16301134 Satlength=669 Nr of Repeats=4
RepeatLength=158 seed=CAAAAATAAA Num.seqs=3 Similarity=0.935302
66   TATCAATTTTCAGACGCGCTACGGGGGTTTTTGTCTTGAATTCCAATTTCAAC-----------
AGGCGCAAAAAAACAAAAATAAAATTTTTGGATT-TTTGGAAAAGT-TTTT-
ATAGATTTGTATTCTAGATTGTTTAA-AAATATTATTGGTACGTTTTTGTACCAANA
Rev.of_Cele-UNSB01_3:2160246-2184061 Satlength=23816 Nr of Repeats=142
RepeatLength=168 seed=TTTATTTTTG Num.seqs=110 Similarity=0.984798
86   TATCCATTTTCAGACGCGCTACAGGGGTTTTTGTCTTGAATTCCAATTTCAACAGGTA-

TATGTAGGCACAAAAAAACAAAAATAAACATTATGGAAT-TCCGGA-AA-
TGTTTTGATAGATTTGGATTCTAGATTGTTTTA-AAATATTATTGGTAAGTTTTTGTACCTAGA
Cele-UNSB01_4:15460070-15462017 Satlength=1948 Nr of Repeats=10
RepeatLength=168 seed=AAAAACAAAA Num.seqs=7 Similarity=0.956160
239   TATCAATTTTCAGATGCGCTAGAGGGGTTTTTGTCTTGAATTCCAATTTCAACAGATA-
TATACAGGCACAAAAAAACAAAAATAAAAATTTTGGAATGTTGGGAGAA-T-TTTT-
ATAGATTTGGATTCTAGATT-TTTAACAAATATTTTTGGTAAGTTTCTGGACCTAGA
Cele-UNSB01_4:15460844-15462434 Satlength=1591 Nr of Repeats=9
RepeatLength=168 seed=AACAAAAATA Num.seqs=7 Similarity=0.956160
242   TATCAATTTTCAGATGCGCTAGAGGGGTTTTTGTCTTGAATTCCAATTTCAACAGATA-
TATACAGGCACAAAAAAACAAAAATAAAAATTTTGGAATGTTGGGAGAA-T-TTTT-
ATAGATTTGGATTCTAGATT-TTTAACAAATATTTTTGGTAAGTTTCTGGACCTAGA
Rev.of_Cele-UNSB01_4:13110533-13113073 Satlength=2541 Nr of Repeats=14
RepeatLength=168 seed=TTTTTAAAAA Num.seqs=12 Similarity=0.993146
141   AAGTAAATTTAAGCCGCGCTAGAGAGGTTTTTGTGTCGAGTTCCAATTTTCACAGGTACTA-
GTAGGCTCAAAAAAACAAAAATAAAAATTATCGAAT-TCCGGA-AA-
TGTTTTGATAGATTTGGATTCTAGATTTTTTAA-AAATATTATTGGTAAGTTTGTGGGCCAGGA
Cele-UNSB01_4:15744856-15746870 Satlength=2015 Nr of Repeats=12
RepeatLength=168 seed=GAATTCCGGA Num.seqs=10 Similarity=0.953439
261   AAGTAACTTAAAGCCGCGCTAGAAAGGTTTTTGTGTCGAGTTACAATTTTCACAGGTACTA-
GTAGGCGCAAAAAAACAAAAATAAAAATTATGGAAT-TCCGGA-AA-
TGTTTTGATAGATTTGGATTCTAGATTTTTTAA-AAATATTATAGGTGAGTTTTTGGGACAGGA

*   * ** ** ******   ********* * ** ** ****** **   ****
****************** ** * ** * * *** ** * **** ******** ********** *** *
******* * *** **** **   *   *
Consensus:

tAtcAAtTTTcAGacGCGCTAgaGgGGTTTTTGTcTtGAaTTCCAATTTcaACagntatannAGGCnCAAAAA
AACAAAAATAAAAaTTtTGGAaTTtnGGAnAATTTTTATAGATTTGgATTCTAGATTnTTTAAAAATATTaTT
GGTAaGTTTtTGgaCCaagA

>Cele-UNSB01_Fam_41_95_7 Nr. of seq. 7 Alignment length(with gaps) = 96
Alignment score = 0.868386
Cele-UNSB01_4:13917663-13921092 Satlength=3430 Nr of Repeats=37
RepeatLength=93 seed=AGGGCCGCAG Num.seqs=33 Similarity=0.954220
0   AGGGCCGCAGGCCCGAATAGTCTTGGAAACT--GGGCTGGCTATAATCCTATTCCAAGCAGGT-
TGTTATTCACTCAAGCCTATCAACCAAGTTTG
Rev.of_Cele-UNSB01_2:2738496-2739194 Satlength=699 Nr of Repeats=7
RepeatLength=95 seed=TTCGGGCCTG Num.seqs=6 Similarity=0.981287
17   AGGGCCGCAGGCCCGAATACTCATGGAAACTGTGGGCCAGCTTTAATCCTATTCCAAGCAGAT-
TGTTATTCGCTCAAGCCTACAAACCAGGTTTG
Cele-UNSB01_3:3518362-3520167 Satlength=1806 Nr of Repeats=19
RepeatLength=95 seed=TAATCCTATT Num.seqs=19 Similarity=0.948948
43   AGGGCCGCAGGCCCGAATACTCATGGAAACTGTGGGCCAGCTTTAATCCTATTCCAAGCAGAT-
TGTTATTCGCTCAAGCCTACAAACCAGGTTTG
Rev.of_Cele-UNSB01_2:13541965-13542724 Satlength=760 Nr of Repeats=8
RepeatLength=95 seed=GGCCTGCGGC Num.seqs=5 Similarity=0.859649
108   AGGGCCGCAGGCCCGAATACTCATGGAAACTAAGGGCCAGCTTTAATCCTATTCCAAGCAGAT-
TGTTATTCGCTCAAGCCTACCAACCAGGTTTG
Rev.of_Cele-UNSB01_2:1585165-1585860 Satlength=696 Nr of Repeats=7
RepeatLength=96 seed=GGCCTGCGGC Num.seqs=6 Similarity=0.968518
109

AGGGCCGCAGGCCCGAACACTCTTGGAAACTATGGGCTGGCTTTGATCCTATTCTAAGCAGATATGTTATTCA
CTCAAGCCTAACAACCAGGTTCG
Cele-UNSB01_3:1175770-1176177 Satlength=408 Nr of Repeats=4
RepeatLength=96 seed=GAGGGCCGCA Num.seqs=3 Similarity=0.949074
191
AGGGCCGCAGGCCCGAACACTCTTGGAAACTATGGGCTGGCTTTGATCCTATTCTAAGCAGATATGTTATTCA
CTCAAGCCTAATAACCAGGTTNG
Rev.of_Cele-UNSB01_5:5413359-5414150 Satlength=792 Nr of Repeats=8
RepeatLength=96 seed=TCTGCTTAGA Num.seqs=7 Similarity=0.949735
254
AGGGCCGCAGGCCCGAACACTCTTGGAAACTATGGGCTGGCTTTGATCCTATTCTAAGCAGATATGTTATTCA
CTCAAGCCTAATAACCAGGTTTG

**************** * ** ******** **** *** * ********* ****** * ********
********** ***** *** *
Consensus:

AGGGCCGCAGGCCCGAAtACTCtTGGAAACTatGGGCtgGCTTTaATCCTATTCcAAGCAGATTGTTATTCaC
TCAAGCCTAnnAACCAGGTTtG

>Cele-UNSB01_Fam_42_19_7 Nr. of seq. 7 Alignment length(with gaps) = 23
Alignment score = 0.619048
Cele-UNSB01_6:2093985-2094613 Satlength=629 Nr of Repeats=30
RepeatLength=19 seed=TAATATTTAT Num.seqs=22 Similarity=0.883952
0   ----TAATATTTATTTATTTAAT
Cele-UNSB01_6:12589530-12590986 Satlength=1457 Nr of Repeats=71
RepeatLength=19 seed=TTAATTAATA Num.seqs=50 Similarity=0.869728
14   ----TAATATTTATTTATTTAAT
Cele-UNSB01_6:2097795-2102366 Satlength=4572 Nr of Repeats=207
RepeatLength=19 seed=AATTAATATT Num.seqs=127 Similarity=0.940253
1   ----TAATTAATATTTATTTATT
Cele-UNSB01_6:2097795-2102461 Satlength=4667 Nr of Repeats=212
RepeatLength=19 seed=AATTAATATT Num.seqs=132 Similarity=0.937235
1   ----TAATTAATATTTATTTATT
Cele-UNSB01_4:1654144-1654247 Satlength=104 Nr of Repeats=5
RepeatLength=23 seed=ATTTAATTAA Num.seqs=3 Similarity=0.729469
1   TATTTATTTAATTAATATTTATT
Rev.of_Cele-UNSB01_6:5456252-5456359 Satlength=108 Nr of Repeats=5
RepeatLength=23 seed=AATAAATATT Num.seqs=3 Similarity=0.922705
23   TATTTAATTAATTAATATTTATT
Cele-UNSB01_4:13646609-13646697 Satlength=89 Nr of Repeats=4
RepeatLength=23 seed=AATATTTATT Num.seqs=3 Similarity=0.961353
9   TATTTAATAAATATTTATTTATT

** *   *   ****** *
Consensus:

TAATtaaTattTATTTAtT

>Cele-UNSB01_Fam_43_19_7 Nr. of seq. 7 Alignment length(with gaps) = 21
Alignment score = 0.666667
Cele-UNSB01_4:236013-237239 Satlength=1227 Nr of Repeats=63
RepeatLength=18 seed=CTCAACCAAA Num.seqs=43 Similarity=0.898938
0   CTCAACCAA--AC-TCTTAGC

Cele-UNSB01_4:244645-245490 Satlength=846 Nr of Repeats=8 RepeatLength=18
seed=AGCCTCAACC Num.seqs=5 Similarity=0.925926
15   CTCAACCAA--AC-TCTTAGC
Rev.of_Cele-UNSB01_4:128195-129066 Satlength=872 Nr of Repeats=28
RepeatLength=19 seed=TTGGTTGAGG Num.seqs=19 Similarity=0.809582
9   CTCAACCAA-AAT-TGTTAGC
Cele-UNSB01_4:221202-221862 Satlength=661 Nr of Repeats=32
RepeatLength=20 seed=CTCAGCCTCA Num.seqs=30 Similarity=0.934713
14   CTCAACCAAAAAT-TCTCAGC
Rev.of_Cele-UNSB01_3:10146078-10146173 Satlength=96 Nr of Repeats=5
RepeatLength=19 seed=ATTTTGGTTG Num.seqs=5 Similarity=0.818333
12   CTCAACCAA-AAT-CCTGAGC
Cele-UNSB01_4:201016-202120 Satlength=1105 Nr of Repeats=53
RepeatLength=20 seed=CTCAACCAAA Num.seqs=32 Similarity=0.882930
20   CTCAACCAA-AAATCCTGAGC
Cele-UNSB01_4:3243719-3271867 Satlength=28149 Nr of Repeats=1487
RepeatLength=19 seed=CCTCACTCAA Num.seqs=1365 Similarity=0.827742
18   CTCACTCAA-AAT-CCTGAGC

**** *** *   * ***
Consensus:

CTCAACCAAaAttCTnAGC

>Cele-UNSB01_Fam_44_21_7 Nr. of seq. 7 Alignment length(with gaps) = 21
Alignment score = 0.628118
Cele-UNSB01_2:466682-467257 Satlength=576 Nr of Repeats=9 RepeatLength=20
seed=CAATTTTTGA Num.seqs=7 Similarity=0.942857
0   CAATTTTTGAGTTAAAAATT-
Rev.of_Cele-UNSB01_2:13474627-13476516 Satlength=1890 Nr of Repeats=78
RepeatLength=20 seed=AAAATTTGAA Num.seqs=57 Similarity=0.826829
8   CAAATTTTGACTTAAAAATT-
Rev.of_Cele-UNSB01_3:2017089-2018496 Satlength=1408 Nr of Repeats=57
RepeatLength=21 seed=TGGAAATTTT Num.seqs=45 Similarity=0.855059
2   CAATTTTTGGGTTAAAATTTC
Cele-UNSB01_3:1642030-1642586 Satlength=557 Nr of Repeats=7
RepeatLength=21 seed=CCTAAAAATT Num.seqs=5 Similarity=0.695238
10   CAATTTTTNACCTAAAAATTN
Rev.of_Cele-UNSB01_2:2923591-2929183 Satlength=5593 Nr of Repeats=231
RepeatLength=21 seed=CCAAAAATTG Num.seqs=201 Similarity=0.731595
10   CAATTTTTGGACTAAAAACCT
Cele-UNSB01_3:2204608-2205196 Satlength=589 Nr of Repeats=25
RepeatLength=21 seed=GCCTAAAAAC Num.seqs=16 Similarity=0.805291
30   AAATTTTAAGCCTAAAAACGT
Rev.of_Cele-UNSB01_4:2728247-2729274 Satlength=1028 Nr of Repeats=42
RepeatLength=21 seed=TTTGGATTTT Num.seqs=35 Similarity=0.859037
45   AAATTTTAAGCCTAAAAATCC

** ***   *****
Consensus:

cAATTTTtggccTAAAAAttn

>Cele-UNSB01_Fam_45_16_7 Nr. of seq. 7 Alignment length(with gaps) = 16
Alignment score = 0.732143
Cele-UNSB01_2:882806-882988 Satlength=183 Nr of Repeats=12
RepeatLength=14 seed=TAGGATCTAG Num.seqs=10 Similarity=0.807407
2   TC-TAGGATCT-AGGA
Rev.of_Cele-UNSB01_2:1625642-1627656 Satlength=2015 Nr of Repeats=131
RepeatLength=14 seed=GATCCTAGAT Num.seqs=112 Similarity=0.963106
2   TC-TAGGATCT-AGGA
Cele-UNSB01_1:14240945-14242222 Satlength=1278 Nr of Repeats=80
RepeatLength=16 seed=ATCTAAGGAT Num.seqs=79 Similarity=0.956183
6   TCTAAGGATCTAAGGA
Cele-UNSB01_1:14240945-14243302 Satlength=2358 Nr of Repeats=147
RepeatLength=16 seed=ATCTAAGGAT Num.seqs=145 Similarity=0.924178
6   TCTAAGGATCTAAGGA
Rev.of_Cele-UNSB01_1:14245984-14247232 Satlength=1249 Nr of Repeats=59
RepeatLength=16 seed=AGATCCTTAG Num.seqs=48 Similarity=0.862780
10   TCTAAGGATCTAAGGA
Cele-UNSB01_1:14248002-14248098 Satlength=97 Nr of Repeats=6
RepeatLength=16 seed=AGGATCTAAG Num.seqs=6 Similarity=0.972222
11   TCTAAGGATCTAAGGA
Cele-UNSB01_4:15869737-15871646 Satlength=1910 Nr of Repeats=128
RepeatLength=14 seed=GGACTAGGGA Num.seqs=112 Similarity=0.896439
3   TAGGGACTAGGGA-C-

*   *
Consensus:

TCtaAGGATCTaAGGA

>Cele-UNSB01_Fam_46_226_6 Nr. of seq. 6 Alignment length(with gaps) =
228 Alignment score = 0.781092
Cele-UNSB01_5:9471886-9475930 Satlength=4045 Nr of Repeats=19
RepeatLength=214 seed=GTAACTGCTC Num.seqs=12 Similarity=0.965543
0
GTAACTGCTCAAAGCTTCTGGCACCTATTGGGATGTTGTTGGTAAATTCTGAAAAAGCTTGGAATTTGCAACA
AAGTTTCTGAAAGAATTTT--
CGAAAAAAGTTGATTTGAAGTGATTTGGGAATTTTCAAAGTTTCATAAATTTACCTA-
TTTTGCCTATAAATGGTCGTTTTCATTTAATAATAAAATACGCAC--AAAGGTA---CTG--TAGGT----
TTTCT
Rev.of_Cele-UNSB01_6:11021354-11026794 Satlength=5441 Nr of Repeats=27
RepeatLength=214 seed=AAAACCTACA Num.seqs=21 Similarity=0.959472
212
GTAACTGCTCGAAGCTTCTGGCACCTACTGGGATGTTGTTGGTAAATTCTGAAAAAGCTTGGAATTTGCAACA
AAGTTTCTGAAAGAAATTT--
CAAAAAAAGTTGGTTTGAAGTGATTTGGGAATTTTCAAAGTTTCATAAATTTACCTA-
TTTTGCCTATAAATGGTCGTTTTCATTTAATAATAAAATACGCAC--AAAGGTA---CTG--TAGGT----
TTTCT
Rev.of_Cele-UNSB01_2:10468271-10470517 Satlength=2247 Nr of Repeats=10
RepeatLength=225 seed=TTTTCGAAAT Num.seqs=6 Similarity=1.000000
98
GTAACTACTTGAAGCTTCTTGTACCTACTGGAATATTGTTAATATAATCTGAAAGAGCTTAAAATTTACTACA
AAGTTTCTGAAAGACATTTCGAAAA--
CAGTTACTTTGAAGAGGTTTATGAATTTTCAAAGTTTGTAAAATCCATCTATTTTTGCCTATACATAGTAGTT
TTCATT-AATAATAAAATACGCACTAAAAGGTAGGGCTGAAAATGTACTATTTCT

Rev.of_Cele-UNSB01_1:4344690-4345849 Satlength=1160 Nr of Repeats=6
RepeatLength=226 seed=ACAAGAAGCT Num.seqs=5 Similarity=0.915789
248
GTAACTACGTGAAGCTTCTTGTACCTACTGGAATATTGTTAATATATTCTAAAAGAGCTTAAAATTTACTACA
AAGTTTCTGAAAGACATTTCGCAAAAAAA-
TTACTTTGAAGAGATTTATGAATTTTCAAAATTACGAAAATCCCTCTA-
TTTTGCCTATAAGTGGTAGTTTTCATTAAATAATAAAATACGCACTAAAAGGTAGGGCTGAAAATGTACTATT
TCT
Rev.of_Cele-UNSB01_6:6133294-6134199 Satlength=906 Nr of Repeats=4
RepeatLength=226 seed=CAAGAAGCTT Num.seqs=3 Similarity=0.929204
473
GTAACTACGTGAAGCTTCTTGCACCTACTGGAGTATTGTTGGTATATTCTAAAAGAGCTTAAAATTTACTACA
AAGTTTCTGAAAGACATTT--
CAAAAAAAGTTACATTGAAGAGATTTATGAATTTTCAAAGTTTCGAAAATTTATCTATTTTTGCCTATAAATG
GTAGTTTTCATTAAATAATAAAATACGCGCTAAAAGGTAGCTCTGAAAATGTACTATTTCT
Cele-UNSB01_6:12201539-12202895 Satlength=1357 Nr of Repeats=6
RepeatLength=226 seed=TGTACTATTT Num.seqs=6 Similarity=0.964602
666
GTAACTACGTGAAGCTTCTTGTACCTACTGAAATATTGTTGGTATATTCTAAAAGAGCTTAAAATTTACTACA
AAGTTTCTAAAAGACATTT--
CAAAAAAAGTTACTTTGAAGAGATTTATGAATTTTCAAAGTTACGAAAATTCGTCTATTTTTGCCTATAAATG
GTAGTTTTCATTAAATAATAAAATACGCATTATAAGGTAGGGCTGAAAATGTACTATTTCT

****** *   ******** * ***** **   * *****   ** * *** *** ***** ***** *
*********** ***** ***   **   * **   ****** * *** *********** **
****   *** *********** * ** ********* ***************   ******   ***
* **   *****
Consensus:

GTAACTaCgtGAAGCTTCTtGcACCTACTGGaATaTTGTTggTAtATTCTaAAAgAGCTTaaAATTTaCtACA
AAGTTTCTGAAAGAcATTTCAAAAAAAGTTacTTTGAAGaGATTTatGAATTTTCAAAGTTtCgaAAATtcat
CTAtTTTTGCCTATAAATGGTaGTTTTCATTaAATAATAAAATACGCACtaAAAGGTAgggCTGaaaAtGTac
taTTTCT

>Cele-UNSB01_Fam_47_94_6 Nr. of seq. 6 Alignment length(with gaps) = 94
Alignment score = 0.946099
Cele-UNSB01_1:4346155-4359862 Satlength=13708 Nr of Repeats=146
RepeatLength=94 seed=TCAAAATCTT Num.seqs=130 Similarity=0.962485
0
TCAAAATCTTCTGTTCTCTAAATATTGGGTGCTTTTCGATGCCCTATGTAGACAAATCAATGGGAAAATCATC
AATTTCTGAAGGCAGTAATTC
Rev.of_Cele-UNSB01_3:7632425-7683091 Satlength=50667 Nr of Repeats=426
RepeatLength=94 seed=AAAGCACCCA Num.seqs=425 Similarity=1.000000
35
TCAAAATCTTCTGTTCTCTAAATATTGGGTGCTTTTCGATGCCCTATGTAGACAAATCAATGGGAAAATCATC
AATTTCTGGAGGCAGTAATTC
Cele-UNSB01_1:11104747-11105884 Satlength=1138 Nr of Repeats=5
RepeatLength=94 seed=TGTAGACAAA Num.seqs=4 Similarity=0.971631
46
TCAAAATCTTCTGTTCTCTAAATATTGGGTGCTTTTCGATGTCCTTTGTAGACAAATCAATGGGAGAATCATC
AATTTCTGAAGGCAGTAATTC
Rev.of_Cele-UNSB01_4:1558589-1558964 Satlength=376 Nr of Repeats=4
RepeatLength=94 seed=TCCCATTGAT Num.seqs=3 Similarity=0.924350
65

TCAAAATCTTCTGTTCTCTAAATATTGGGTGCTTTTCGATGCCCTTTGTAGACAAATCAATGGGAAAATTGTC
CATTTCTGAAGGCAGTAATTC
Cele-UNSB01_6:350789-351447 Satlength=659 Nr of Repeats=7 RepeatLength=94
seed=GCAGTAATTC Num.seqs=7 Similarity=0.944613
84
TCAAAATCTTCTGTTCTCTAAATATTGGGTGCTTTTCGATGCCCTTTGTAGACAAATCAATGAGAAAATTGTC
AATTTCTGAAGGCAGTAATTC
Rev.of_Cele-UNSB01_6:7055357-7058647 Satlength=3291 Nr of Repeats=35
RepeatLength=94 seed=TGCCTTCAGA Num.seqs=35 Similarity=0.978211
87
TCAAAATCTTCTGTTCTCTAAATATTGAGTGCTTTTCGATGCCCTATGTAGACAAATCAATGGGAAAATTGTC
AATTTCTGAAGGCAGTAATTC

************************** ************* *** **************** ** ***   **
******* ************
Consensus:

TCAAAATCTTCTGTTCTCTAAATATTGGGTGCTTTTCGATGCCCTaTGTAGACAAATCAATGGGAAAATcaTC
AATTTCTGAAGGCAGTAATTC

>Cele-UNSB01_Fam_48_33_6 Nr. of seq. 6 Alignment length(with gaps) = 33
Alignment score = 0.649832
Cele-UNSB01_1:12834373-12834766 Satlength=394 Nr of Repeats=5
RepeatLength=32 seed=GCTGATTGGT Num.seqs=3 Similarity=0.861111
0   GCTGATTGGTTCA-AAAGTGGGCGTGGCTAATC
Rev.of_Cele-UNSB01_3:13044209-13044762 Satlength=554 Nr of Repeats=5
RepeatLength=32 seed=ACCAATCAGC Num.seqs=3 Similarity=0.916667
10   GCTGATTGGTTCA-AAAGTGGGCGGAGCAAATC
Rev.of_Cele-UNSB01_3:13000875-13002076 Satlength=1202 Nr of Repeats=5
RepeatLength=32 seed=GACCAATCAG Num.seqs=3 Similarity=0.861111
11   GCTGATTGG-TCAGAGAGTGGGCGGGGCGAATC
Rev.of_Cele-UNSB01_2:14464146-14465077 Satlength=932 Nr of Repeats=26
RepeatLength=32 seed=AACCAATCAG Num.seqs=19 Similarity=0.803296
11   GCTGATTGGTTCC-AAAATGGGTGGAGCTGAGC
Cele-UNSB01_5:16730521-16730649 Satlength=129 Nr of Repeats=4
RepeatLength=32 seed=GCTGATTGGT Num.seqs=4 Similarity=0.508488
0   GCTGATTGG-TAAGAAACTGGGCGTGGCTGAAA
Rev.of_Cele-UNSB01_3:2325106-2325938 Satlength=833 Nr of Repeats=23
RepeatLength=32 seed=CTCAGCCAAT Num.seqs=17 Similarity=0.871936
14   GTTGATTGGCTGAGAAA-TAGGCGGAGCTAAAC

* ******* *   * * * ** * ** *
Consensus:

GCTGATTGGtTcAgAAAgTGGGCGgaGCtaAtC

>Cele-UNSB01_Fam_49_21_6 Nr. of seq. 6 Alignment length(with gaps) = 25
Alignment score = 0.624889
Cele-UNSB01_3:2342333-2342437 Satlength=105 Nr of Repeats=5
RepeatLength=21 seed=CTGACCAAAT Num.seqs=4 Similarity=0.841270
0   CTGACC---AAATTTGT-TTCAGCA
Rev.of_Cele-UNSB01_3:2351961-2352192 Satlength=232 Nr of Repeats=9
RepeatLength=21 seed=GTCAGTGCTG Num.seqs=6 Similarity=0.856085
5   CTGACA---AAATTTGT-TCCAGCA

```
Cele-UNSB01_3:3394846-3394930 Satlength=85 Nr of Repeats=4
RepeatLength=21 seed=TCCAGCACTG  Num.seqs=4 Similarity=0.936508
14    CTGACA---AAATTTGT-TCCAGCA
Rev.of_Cele-UNSB01_6:763886-764130 Satlength=245 Nr of Repeats=5
RepeatLength=22 seed=TGGTCAGTGC  Num.seqs=4 Similarity=0.777778
7    CTGACC---AAATTGGTCGCCAGCA
Rev.of_Cele-UNSB01_6:1033071-1033155 Satlength=85 Nr of Repeats=4
RepeatLength=21 seed=TGGTCAGTGC  Num.seqs=4 Similarity=0.968254
7    CTGACC---AAATTGTT-TCTTGCA
Cele-UNSB01_6:1569817-1569931 Satlength=115 Nr of Repeats=5
RepeatLength=23 seed=AAAAAATTTG  Num.seqs=4 Similarity=0.971014
6    CTGACCAAAAAATTTGT-TTC-GCA

*****    *****  *     ***
Consensus:

CTGACcAAATTtGTTcCaGCA

>Cele-UNSB01_Fam_50_21_6  Nr. of seq. 6 Alignment length(with gaps) = 22
Alignment score = 0.621212
Cele-UNSB01_6:1052300-1052972 Satlength=673 Nr of Repeats=4
RepeatLength=21 seed=TGACAAAACA  Num.seqs=3 Similarity=1.000000
0    TGACAAAA-CATAACTTTGCAC
Rev.of_Cele-UNSB01_6:12031687-12031907 Satlength=221 Nr of Repeats=10
RepeatLength=22 seed=TCAGTGCAAA  Num.seqs=8 Similarity=0.818182
3    TGACAAAAGTGTCACTTTGCAC
Rev.of_Cele-UNSB01_6:1106813-1106897 Satlength=85 Nr of Repeats=4
RepeatLength=21 seed=GTCAGTGCAA  Num.seqs=4 Similarity=0.841270
4    TGACAATA-TTTGAATTTGCAC
Cele-UNSB01_6:1365926-1366333 Satlength=408 Nr of Repeats=5
RepeatLength=21 seed=TTGCACTGAC  Num.seqs=3 Similarity=0.747475
15    TGACAAAA-TTTCGTNTTGCAC
Rev.of_Cele-UNSB01_6:1259650-1260300 Satlength=651 Nr of Repeats=6
RepeatLength=21 seed=GTCAGTGCAA  Num.seqs=4 Similarity=0.809524
4    TGACCAAA-AATGCATTTGCAC
Cele-UNSB01_6:1371051-1371242 Satlength=192 Nr of Repeats=7
RepeatLength=21 seed=TTGCACTGAC  Num.seqs=6 Similarity=0.630303
15    TGACCAAC-TCTTCATTTGCAC

**** *     *    ******
Consensus:

TGACaAAAtnTnaaTTTGCAC

>Cele-UNSB01_Fam_51_19_6  Nr. of seq. 6 Alignment length(with gaps) = 21
Alignment score = 0.706878
Cele-UNSB01_1:11697315-11697448 Satlength=134 Nr of Repeats=7
RepeatLength=19 seed=TTATCGATTT  Num.seqs=5 Similarity=0.873684
46    TCNA--GAAATTATCGATTTT
Rev.of_Cele-UNSB01_3:2955858-2957390 Satlength=1533 Nr of Repeats=44
RepeatLength=19 seed=AAAATCGATA  Num.seqs=31 Similarity=0.912620
57    TCCG--GAAATTATCGATTTT
```

Rev.of_Cele-UNSB01_3:1887376-1889731 Satlength=2356 Nr of Repeats=56
RepeatLength=19 seed=AAAATCGATA Num.seqs=36 Similarity=0.647665
57   CCGA--TAAATTATCGATTTT
Rev.of_Cele-UNSB01_3:1974698-1975558 Satlength=861 Nr of Repeats=44
RepeatLength=19 seed=AAAATCGATA Num.seqs=39 Similarity=0.805668
57   CCGA--GNAATTATCGATTTT
Cele-UNSB01_3:2095462-2096950 Satlength=1489 Nr of Repeats=36
RepeatLength=19 seed=TATCGATTTT Num.seqs=22 Similarity=0.729931
47   TCGT--AAAATTATCGATTTT
Cele-UNSB01_5:19225003-19225171 Satlength=169 Nr of Repeats=8
RepeatLength=21 seed=ATTATTGATT Num.seqs=8 Similarity=0.778409
23   GAAAAATTATTGATTTTTCAA

* * **   *
Consensus:

gaAATTATCGATTTTtCga

>Cele-UNSB01_Fam_52_18_6 Nr. of seq. 6 Alignment length(with gaps) = 19
Alignment score = 0.691228
Cele-UNSB01_1:6226496-6226583 Satlength=88 Nr of Repeats=5
RepeatLength=17 seed=AGGTAGTCAA Num.seqs=3 Similarity=1.000000
0   AGGTAGTCAATGAGCA-A-
Rev.of_Cele-UNSB01_5:8373901-8376688 Satlength=2788 Nr of Repeats=161
RepeatLength=17 seed=TCATTGACTA Num.seqs=112 Similarity=0.914313
13   AGGTAGTCAATGAGCA-A-
Rev.of_Cele-UNSB01_4:7715733-7715854 Satlength=122 Nr of Repeats=6
RepeatLength=17 seed=CTCATTGCCT Num.seqs=4 Similarity=0.764706
14   GGGTAGTCAATGAGCA-C-
Cele-UNSB01_4:9041393-9041548 Satlength=156 Nr of Repeats=8
RepeatLength=17 seed=AGGCAATGAG Num.seqs=5 Similarity=0.781482
3   -AGTAGGCAATGAGAA-AG
Rev.of_Cele-UNSB01_5:6928533-6928671 Satlength=139 Nr of Repeats=7
RepeatLength=17 seed=CTCATTGCCT Num.seqs=5 Similarity=0.703704
13   -NGTAGGCAATGAGCA-AG
Rev.of_Cele-UNSB01_5:7745857-7745978 Satlength=122 Nr of Repeats=6
RepeatLength=17 seed=CTCATTGCCT Num.seqs=4 Similarity=0.697531
13   -NGTAGGCAATGAGCACAG

**** ******* *
Consensus:

ngGTAGgCAATGAGCAAg

>Cele-UNSB01_Fam_53_17_6 Nr. of seq. 6 Alignment length(with gaps) = 18
Alignment score = 0.858025
Cele-UNSB01_2:1681965-1682083 Satlength=119 Nr of Repeats=7
RepeatLength=17 seed=GATGCCCCCT Num.seqs=5 Similarity=0.729630
0   GATGCCCCCTTTGAACG-
Rev.of_Cele-UNSB01_3:4389614-4389732 Satlength=119 Nr of Repeats=7
RepeatLength=17 seed=AGGGGGCATC Num.seqs=5 Similarity=0.749020
10   GATGCCCCCTCTGAACG-

Cele-UNSB01_3:4392502-4392620 Satlength=119 Nr of Repeats=7
RepeatLength=17 seed=GATGCCCCCT Num.seqs=5 Similarity=0.729630
17   GATGCCCCCTCTGAACG-
Cele-UNSB01_4:4261575-4261676 Satlength=102 Nr of Repeats=6
RepeatLength=17 seed=GATGCCCCCT Num.seqs=4 Similarity=0.722222
0   GATGCCCCCTCTGAACGN
Cele-UNSB01_6:8886112-8886213 Satlength=102 Nr of Repeats=6
RepeatLength=17 seed=GATGCCCCCT Num.seqs=4 Similarity=0.722222
0   GATGCCCCCTCTGAACGN
Rev.of_Cele-UNSB01_6:8885720-8885804 Satlength=85 Nr of Repeats=5
RepeatLength=17 seed=AGGGGGCATC Num.seqs=3 Similarity=0.686275
10   AATGCCCCCTNTGAACN-

********* *****
Consensus:

GATGCCCCCTcTGAACG

>Cele-UNSB01_Fam_54_16_6 Nr. of seq. 6 Alignment length(with gaps) = 16
Alignment score = 0.865278
Cele-UNSB01_1:7925712-7926382 Satlength=671 Nr of Repeats=39
RepeatLength=16 seed=AATTTTTCCG Num.seqs=31 Similarity=0.813978
0   AATTTTTCCGCNGCGA
Rev.of_Cele-UNSB01_2:7566750-7567567 Satlength=818 Nr of Repeats=42
RepeatLength=16 seed=CGGAAAAATT Num.seqs=27 Similarity=0.719401
10   AATTTTTCCGCNGCNA
Rev.of_Cele-UNSB01_5:13735981-13739186 Satlength=3206 Nr of Repeats=193
RepeatLength=16 seed=GAAAAATTTT Num.seqs=167 Similarity=0.786698
8   AATTTTTCAGCAGCAA
Rev.of_Cele-UNSB01_5:13735981-13740432 Satlength=4452 Nr of Repeats=256
RepeatLength=16 seed=GAAAAATTTT Num.seqs=212 Similarity=0.783548
8   AATTTTTCAGCAGCAA
Rev.of_Cele-UNSB01_5:13735981-13741243 Satlength=5263 Nr of Repeats=280
RepeatLength=16 seed=GAAAAATTTT Num.seqs=227 Similarity=0.784063
8   AATTTTTCNGCAGCAA
Rev.of_Cele-UNSB01_5:13735981-13743674 Satlength=7694 Nr of Repeats=334
RepeatLength=16 seed=GAAAAATTTT Num.seqs=260 Similarity=0.786925
8   AATTTTTCCGCAGCAA

******** ** ** *
Consensus:

AATTTTTCcGCaGCaA

>Cele-UNSB01_Fam_55_15_6 Nr. of seq. 6 Alignment length(with gaps) = 15
Alignment score = 0.952593
Cele-UNSB01_1:14535288-14535435 Satlength=148 Nr of Repeats=8
RepeatLength=15 seed=GGGGGCAACA Num.seqs=5 Similarity=0.822222
0   GGGGGCAACAAAATA
Rev.of_Cele-UNSB01_1:14537614-14537761 Satlength=148 Nr of Repeats=8
RepeatLength=15 seed=TGTTGCCCCC Num.seqs=5 Similarity=0.822222
10   GGGGGCAACAAAATA

Rev.of_Cele-UNSB01_3:731921-732069 Satlength=149 Nr of Repeats=8 RepeatLength=15 seed=TGTTGCCCCC Num.seqs=5 Similarity=0.795556
10   GGGGGCAACAAAATA
Cele-UNSB01_6:17722232-17722379 Satlength=148 Nr of Repeats=8 RepeatLength=15 seed=GGGGGCAACA Num.seqs=5 Similarity=0.822222
0   GGGGGCAACAAAATA
Cele-UNSB01_2:1507976-1508324 Satlength=349 Nr of Repeats=19 RepeatLength=15 seed=GGGGGCAACA Num.seqs=12 Similarity=0.807407
0   GGGGGCAACAAAAAA
Cele-UNSB01_3:727287-727380 Satlength=94 Nr of Repeats=5 RepeatLength=15 seed=GGGGGCAACA Num.seqs=3 Similarity=0.822222
0   GGGGGCAACAAAAAA

************* *
Consensus:

GGGGGCAACAAAAtA

>Cele-UNSB01_Fam_56_11_6 Nr. of seq. 6 Alignment length(with gaps) = 11 Alignment score = 0.838384
Cele-UNSB01_2:4377229-4377482 Satlength=254 Nr of Repeats=23 RepeatLength=11 seed=CAGTTGTCAC Num.seqs=19 Similarity=0.897927
0   CAGTTGTCACT
Cele-UNSB01_6:3747622-3752392 Satlength=4771 Nr of Repeats=406 RepeatLength=11 seed=CAGTTGTCAC Num.seqs=340 Similarity=0.892175
0   CAGTTGTCACC
Rev.of_Cele-UNSB01_4:4665165-4665209 Satlength=45 Nr of Repeats=4 RepeatLength=11 seed=TGACAACTTT Num.seqs=4 Similarity=1.000000
9   AAGTTGTCACA
Rev.of_Cele-UNSB01_4:7151737-7153750 Satlength=2014 Nr of Repeats=178 RepeatLength=11 seed=TGACAACTTC Num.seqs=131 Similarity=0.801993
9   AAGTTGTCACG
Cele-UNSB01_5:4751883-4752158 Satlength=276 Nr of Repeats=22 RepeatLength=11 seed=CACGAAGTTG Num.seqs=19 Similarity=0.914939
18   AAGTTGTCACG
Cele-UNSB01_6:12089493-12089669 Satlength=177 Nr of Repeats=16 RepeatLength=11 seed=CGAAGTTGTC Num.seqs=16 Similarity=0.870707
9   AAGTTGTCACG

****
Consensus:

aAGTTGTCACg

>Cele-UNSB01_Fam_57_200_5 Nr. of seq. 5 Alignment length(with gaps) = 228 Alignment score = 0.666228
Cele-UNSB01_1:9974009-9976809 Satlength=2801 Nr of Repeats=15 RepeatLength=200 seed=AACAGAATTT Num.seqs=13 Similarity=0.975385
0   AACAGAATTTTAAAAAATTTCGAAACCGCCAA--------------------
GTTCAAGCAGAAAAAGT-GA----ATTTTCAGTCATACCTTG---
TAGACCACAATTTATATACAAAATTAATAGATTTTCCAAACTTTGAAAATTAATTTCTGGCTTCAAACTAACT
TTTTTAGAAATTCCCGTTGCCATTTTGTAGCAAATTTTATGTTCTTTCAGAATTTGAA
Cele-UNSB01_4:14618655-14620445 Satlength=1791 Nr of Repeats=7 RepeatLength=200 seed=GAAAAAGTGA Num.seqs=6 Similarity=0.937778

```
41    AACAGAATTTCAAAAAATTTTGAAACCACCAA--------------------
GTTCAAGCAGAAAAAGT-GA----ATTTTCAGTCATACCTTG---
TAGACCACAATTTATATGCAAAATTAATAGATTTTCCAAACTTTGAAAATTAATTTCTGGCTTCAAACTAACT
TTTTTAAAAATTCCCGTAGCCATTTTGTAGCAAATTTTATGTTCTATCAGAATTTAAA
Rev.of_Cele-UNSB01_5:4216093-4219558 Satlength=3466 Nr of Repeats=18
RepeatLength=200 seed=TGTGGTCCAG  Num.seqs=13 Similarity=0.916410
78    AACGGCATTTCAAAAAATTTAAAATCTGCCAA--------------------
GTTCAAGCAGAAAAAGT-GA----ATTTTCAGTCATACCTTC---
TGGACCACAATTTATATGCAAAATTAATAGATTTTCCAAACTTTGAAAATTAATTTCTGGCTTGAAACTAACT
TTTTTAGAAATTCCCGTAGCCATTTTGTAGCAAATTTTAAGTTCTTTCAGAATTTGAA
Cele-UNSB01_2:5970897-5971730 Satlength=834 Nr of Repeats=4
RepeatLength=203 seed=GTTCAAGCAG  Num.seqs=3 Similarity=0.935730
32    AACGGCATTCCAAACATTTCCATATTCGCCGA--------------------
GTTCAAGCAGAACAAGTAGATCACATTTTGAG--ATTGAATGGAATAAACG---
AGTTATATGCAAAATTAGTAGACTTTCCAAACTTTGAAAATTAATATCTGGCTTCAAACTAACTTTTCTGAAA
ATCCCCGTAGCCATTTTGTTGCAAATTTTGTGCTCTTTCAGAATTTAAA
Cele-UNSB01_1:4183555-4184714 Satlength=1160 Nr of Repeats=5
RepeatLength=223 seed=TTTCAGAATT  Num.seqs=4 Similarity=0.971101
189
AACGGCATTCCAAAAACTTTCACAACCGCCAAGTTCAAGCAAAAAAAGTAAACTTCAGTCATACCTTTCAGAG
CAAACTTTGAA--ATTGAATGAAATAGACC---
ATTTATTTGCAAAATTAGTAGATTTTCCAATTTTTAAAAATTAATATCTGGCTTCAAAATAACTTTTTTGAAA
ATCCCCGTAGCCATTTTGTTGCAAATTTTGTGCTCTTTCAGAATTTAAA

*** * ***  *** * **    *    ** *                     ****  ** *       **
* *** *   **    *    *  **    * **** * ******** **** *******  ***
********* ******** *** ******** *  **** ***** ********** *********  * ***
********* **
Consensus:

AACgGcATTtcAAAaAaTTtcaaAaccgCCaAgTTCAagCAgAaaaagtGAAtTTTcAgtcATacctTgTagA
CcacaAtTTATaTgCAAAATTAaTAGAtTTTCCAAacTTTgAAAATTAATtTCTGGCTTcAAAcTAACTTTTt
TaaAAATtCCCGTaGCCATTTTGTaGCAAATTTTatGtTCTtTCAGAATTTaAA

>Cele-UNSB01_Fam_58_48_5  Nr. of seq. 5 Alignment length(with gaps) = 48
Alignment score = 0.900000
Cele-UNSB01_4:5529418-5534698 Satlength=5281 Nr of Repeats=110
RepeatLength=48 seed=TTGCCCGTAA  Num.seqs=110 Similarity=0.963752
0    TTGCCCGTAATTTATATTTTTCTGAAGATGAGCAATTCAATTTTTGAT
Rev.of_Cele-UNSB01_4:13061036-13070540 Satlength=9505 Nr of Repeats=198
RepeatLength=48 seed=ACGGGCAAAT  Num.seqs=198 Similarity=0.966024
8    TTGCCCGTAATTTATAATTTTCTGAAGATGAGCAATTTAATTTTTGAT
Cele-UNSB01_6:3098438-3098774 Satlength=337 Nr of Repeats=7
RepeatLength=48 seed=AAGATGAGGA  Num.seqs=7 Similarity=0.933862
24    TTGCCCGTAATTTATATTTTTCTGAAGATGAGGAATTTATTTTTTGAT
Rev.of_Cele-UNSB01_6:14711854-14734555 Satlength=22702 Nr of Repeats=477
RepeatLength=48 seed=GGCAAATCAA  Num.seqs=293 Similarity=0.961435
53    TTGCCCGTAATTTATATTTTTCTGAAGATGAGGAATTTAATTGTTGAT
Cele-UNSB01_5:1676007-1687585 Satlength=11579 Nr of Repeats=242
RepeatLength=48 seed=AATTTCTGAA  Num.seqs=199 Similarity=0.901202
16    TTGCCCGTAATTTATAAATTTCTGAAGATGAGCAATTTAAATTTTGAG

***************  ************* **** *  * ****
Consensus:
```

TTGCCCGTAATTTATAttTTTCTGAAGATGAGcAATTtAatTtTTGAt

>Cele-UNSB01_Fam_59_31_5 Nr. of seq. 5 Alignment length(with gaps) = 32
Alignment score = 0.826042
Cele-UNSB01_2:858289-858649 Satlength=361 Nr of Repeats=5 RepeatLength=31
seed=TGTCTAGCGA Num.seqs=3 Similarity=0.799283
0   TGTCGAGCGAGACCTCAGAAG-AGAGCTGAGT
Cele-UNSB01_2:2619434-2619682 Satlength=249 Nr of Repeats=8
RepeatLength=31 seed=AGAGCTGAGT Num.seqs=8 Similarity=0.806548
22   TGTCGAGCGAGACAGCAGAAGAAGAGCTGAGT
Cele-UNSB01_2:1984417-1985378 Satlength=962 Nr of Repeats=31
RepeatLength=31 seed=AGAGCTGAGT Num.seqs=31 Similarity=0.809341
21   TGTCTAGCGAGATAGCAGANA-AGAGCTGAGT
Cele-UNSB01_2:1992839-1994265 Satlength=1427 Nr of Repeats=46
RepeatLength=31 seed=AGAGCTGAGT Num.seqs=46 Similarity=0.811262
21   TGTCTAGCGAGATAGCAGANA-AGAGCTGAGT
Rev.of_Cele-UNSB01_2:2023342-2024644 Satlength=1303 Nr of Repeats=42
RepeatLength=31 seed=GACAACTCAG Num.seqs=42 Similarity=0.826558
35   TGTCTAGCGAGATAGCAGAAA-AGAGCTGAGT

**** *******   ****   **********
Consensus:

TGTCtAGCGAGAtagCAGAaaAGAGCTGAGT

>Cele-UNSB01_Fam_60_25_5 Nr. of seq. 5 Alignment length(with gaps) = 25
Alignment score = 0.904000
Cele-UNSB01_1:10295164-10303189 Satlength=8026 Nr of Repeats=319
RepeatLength=25 seed=TTACTCTAAT Num.seqs=293 Similarity=0.953127
0   TTACTCTAATGTTCTGCCAATATAT
Rev.of_Cele-UNSB01_6:10621309-10621525 Satlength=217 Nr of Repeats=4
RepeatLength=25 seed=ATTGGCAGAA Num.seqs=3 Similarity=0.837607
21   TTACTCTAATGTTCTGCCAATATAT
Cele-UNSB01_6:4149433-4175058 Satlength=25626 Nr of Repeats=1025
RepeatLength=25 seed=AATGTTCTGC Num.seqs=1025 Similarity=0.999688
7   TTACTCTAATGTTCTGCCAATTTAT
Rev.of_Cele-UNSB01_4:6425603-6430447 Satlength=4845 Nr of Repeats=192
RepeatLength=25 seed=TTAGAGTAAA Num.seqs=177 Similarity=0.908867
9   TTACTCTAATGTTCTGCTAATTTAT
Rev.of_Cele-UNSB01_6:2091376-2093692 Satlength=2317 Nr of Repeats=86
RepeatLength=25 seed=TTAGAGTAAA Num.seqs=71 Similarity=0.941451
9   TTACTCTAATGTTCTGCCAAATAAT

***************** **   **
Consensus:

TTACTCTAATGTTCTGCcAAtttAT

>Cele-UNSB01_Fam_61_19_5 Nr. of seq. 5 Alignment length(with gaps) = 22
Alignment score = 0.775758
Cele-UNSB01_3:10140093-10140163 Satlength=71 Nr of Repeats=4
RepeatLength=19 seed=TGGTTGAGGC Num.seqs=3 Similarity=0.906433
0   -TGGTTGAGGCTCTA--GATTT

```
Rev.of_Cele-UNSB01_4:186112-186245 Satlength=134 Nr of Repeats=7
RepeatLength=19 seed=CCTCAACCAA  Num.seqs=7 Similarity=0.826232
9    -TGGTTGAGGCTCTN--GATTT
Cele-UNSB01_4:125359-125607 Satlength=249 Nr of Repeats=13
RepeatLength=19 seed=TGGTTGAGGC  Num.seqs=12 Similarity=0.880914
0    -TGGTTGAGGCTCTC--TATTT
Cele-UNSB01_4:129890-130903 Satlength=1014 Nr of Repeats=21
RepeatLength=22 seed=TTGGTTGAGG  Num.seqs=16 Similarity=0.915152
0    TTGGTTGAGGCTCTCTGGATTT
Cele-UNSB01_4:242271-242555 Satlength=285 Nr of Repeats=12
RepeatLength=22 seed=TGAGGCTCTC  Num.seqs=11 Similarity=0.886501
5    TTGGTTGAGGCTCTCTGGATTT

*************    ****
Consensus:

TGGTTGAGGCTCTcgATTT

>Cele-UNSB01_Fam_62_21_5  Nr. of seq. 5 Alignment length(with gaps) = 22
Alignment score = 0.863636
Cele-UNSB01_1:13132164-13132288 Satlength=125 Nr of Repeats=6
RepeatLength=21 seed=AATCCAATAT  Num.seqs=5 Similarity=0.790476
0    AATCCAATATGG-ANGAAATCT
Rev.of_Cele-UNSB01_2:15030456-15030601 Satlength=146 Nr of Repeats=7
RepeatLength=21 seed=ATTGGATTAG  Num.seqs=6 Similarity=0.792593
8    AATCCAATATGG-AAGAAATCT
Cele-UNSB01_3:6807960-6808176 Satlength=217 Nr of Repeats=6
RepeatLength=21 seed=AAATCTAATC  Num.seqs=5 Similarity=0.822727
15    AATCCAATATGG-NCGAAATCT
Rev.of_Cele-UNSB01_5:2584213-2584387 Satlength=175 Nr of Repeats=6
RepeatLength=21 seed=GATTAGATTT  Num.seqs=5 Similarity=0.841270
25    AATCCAATATGG-ACNAAATCT
Cele-UNSB01_4:11101964-11102048 Satlength=85 Nr of Repeats=4
RepeatLength=21 seed=AAATCTAATC  Num.seqs=4 Similarity=0.777778
16    AATCCAATATGGAACGAAATCT

************    ******
Consensus:

AATCCAATATGGacgAAATCT

>Cele-UNSB01_Fam_63_19_5  Nr. of seq. 5 Alignment length(with gaps) = 21
Alignment score = 0.695238
Cele-UNSB01_2:13971711-13972389 Satlength=679 Nr of Repeats=37
RepeatLength=18 seed=AATTTACCAA  Num.seqs=29 Similarity=0.745813
0    AATTTACC-AA-TTTACCAA-
Cele-UNSB01_3:12492090-12492261 Satlength=172 Nr of Repeats=7
RepeatLength=19 seed=CAATTTACCA  Num.seqs=5 Similarity=0.791667
7    AATTTGNC-AA-TTTACCAAA
Cele-UNSB01_3:13143562-13144065 Satlength=504 Nr of Repeats=16
RepeatLength=19 seed=TTGGCAATTT  Num.seqs=10 Similarity=0.809747
22    AATTTGGC-AA-TTTACCAAA
```

```
Rev.of_Cele-UNSB01_3:1391886-1392275 Satlength=390 Nr of Repeats=16
RepeatLength=21 seed=TTTTTGGTAA  Num.seqs=13 Similarity=0.873016
2    AAGTTACCAAATTTTACCAAA
Cele-UNSB01_3:1394199-1394513 Satlength=315 Nr of Repeats=12
RepeatLength=21 seed=CAAAAAGTTA  Num.seqs=10 Similarity=0.923810
17    AAGTTACCAAATTTTAACAAA

** **  * ** **** ***
Consensus:

AAtTTacCAATTTAcCAAa

>Cele-UNSB01_Fam_64_17_5  Nr. of seq. 5 Alignment length(with gaps) = 18
Alignment score = 0.848148
Cele-UNSB01_2:1617453-1617521 Satlength=69 Nr of Repeats=4
RepeatLength=17 seed=TCATTCCGTG  Num.seqs=4 Similarity=1.000000
0    TCATTCC-GTGATTATTT
Cele-UNSB01_3:4778835-4779093 Satlength=259 Nr of Repeats=5
RepeatLength=17 seed=ATTATTTTCA  Num.seqs=4 Similarity=1.000000
10    TCATTCC-GTGATTATTT
Rev.of_Cele-UNSB01_3:12626974-12627042 Satlength=69 Nr of Repeats=4
RepeatLength=17 seed=TGAAAATAAT  Num.seqs=4 Similarity=1.000000
3    TCATTCC-GTGATTATTT
Cele-UNSB01_4:8831351-8831453 Satlength=103 Nr of Repeats=6
RepeatLength=17 seed=TTCATTCCGT  Num.seqs=6 Similarity=1.000000
16    TCATTCC-GTGATTATTT
Rev.of_Cele-UNSB01_6:6343035-6343121 Satlength=87 Nr of Repeats=5
RepeatLength=17 seed=TAATTACTGG  Num.seqs=4 Similarity=0.921569
14    T-ATTCCAGTAATTATTT

* ***** ** *******
Consensus:

TcATTCCGTgATTATTT

>Cele-UNSB01_Fam_65_11_5  Nr. of seq. 5 Alignment length(with gaps) = 14
Alignment score = 0.697619
Cele-UNSB01_2:4203805-4204480 Satlength=676 Nr of Repeats=4
RepeatLength=11 seed=TGCCTGCCTA  Num.seqs=3 Similarity=0.919192
0    TGCCT--GCCTAC-
Rev.of_Cele-UNSB01_2:4205915-4205959 Satlength=45 Nr of Repeats=4
RepeatLength=11 seed=GGCAGGCAGT  Num.seqs=4 Similarity=0.898990
8    TGCCT--GCCTAC-
Rev.of_Cele-UNSB01_4:4598852-4599551 Satlength=700 Nr of Repeats=6
RepeatLength=11 seed=GCAGTAGGCA  Num.seqs=5 Similarity=0.878788
3    TGCCT--GCCTAC-
Rev.of_Cele-UNSB01_1:742856-743285 Satlength=430 Nr of Repeats=11
RepeatLength=12 seed=GCAGGTAGGT  Num.seqs=7 Similarity=0.841270
3    TGCCTA--CCTACC
Cele-UNSB01_2:2995760-2997536 Satlength=1777 Nr of Repeats=53
RepeatLength=14 seed=CTACCTGCCT  Num.seqs=34 Similarity=0.798659
9    TGCCTATGCCTACC

*****   *****
```

Consensus:

TGCCTgCCTAC

>Cele-UNSB01_Fam_66_11_5 Nr. of seq. 5 Alignment length(with gaps) = 11 Alignment score = 0.951515
Cele-UNSB01_1:12905958-12906024 Satlength=67 Nr of Repeats=5 RepeatLength=11 seed=TTTGGAGCGG Num.seqs=4 Similarity=0.777778
0   TTTGGAGCGGA
Cele-UNSB01_3:7683133-7684661 Satlength=1529 Nr of Repeats=131 RepeatLength=11 seed=TGGAGCGGTT Num.seqs=122 Similarity=0.884864
2   TTTGGAGCGGT
Cele-UNSB01_6:1700308-1706287 Satlength=5980 Nr of Repeats=323 RepeatLength=11 seed=GGAGCGGTTT Num.seqs=251 Similarity=0.936599
3   TTTGGAGCGGT
Cele-UNSB01_6:8414337-8415392 Satlength=1056 Nr of Repeats=85 RepeatLength=11 seed=GAGCGGTTTT Num.seqs=64 Similarity=0.790068
4   TTTGGAGCGGT
Cele-UNSB01_6:10641130-10642329 Satlength=1200 Nr of Repeats=107 RepeatLength=11 seed=TGGAGCGGTT Num.seqs=105 Similarity=0.866467
2   TTTGGAGCGGT

****
Consensus:

TTTGGAGCGGt

>Cele-UNSB01_Fam_67_184_4 Nr. of seq. 4 Alignment length(with gaps) = 189 Alignment score = 0.862140
Cele-UNSB01_1:340649-342857 Satlength=2209 Nr of Repeats=12 RepeatLength=184 seed=GAATACAGAA Num.seqs=12 Similarity=0.974089
0   GAATACAGAACACCAATTATGCCCGAG-AAAAAG-ATCCTACTCAGAATATAAACATAGTCGAATTTATCGGATGTATAAAGATTCCCGAAGACACTTTCCAATTACCCAAATTGTTCATATTCTA--AATGAAAT-TCTCTTACTAGAACACTCTTGGCCAATGTACGCAGCCGAACGTATCATAAGT
Rev.of_Cele-UNSB01_2:14513028-14524678 Satlength=11651 Nr of Repeats=63 RepeatLength=185 seed=CTCGGACATA Num.seqs=58 Similarity=0.981369
27   GAATACAGAACACCAATTATGTCCGAG-AAAAAGAATCCAATTCAGAATATTAACATACTCGAATTTGTCGGATGTATAAAGATTCCCGAAGACACTTTCCAATTACCCAAATTGTTCATATTCTA--AATGAAAT-TCTCTTACTAGAACACTCTTGGCCAATGTACGCAGCCGAACGTATCATAAGT
Cele-UNSB01_5:1233778-1237846 Satlength=4069 Nr of Repeats=22 RepeatLength=185 seed=AATATAAACA Num.seqs=15 Similarity=0.974054
45   GAATACAGAACACCAGTTATGTCCGAGCAAAAAG-ATCCGATTCAGAATATAAACATATTTGAATTTGACGGATGTATAAAGATTCCCGAAGACACTTTCCAATTACCCAAATTGTTCATATTCTA--AATGAGAT-TCTCTTACTAGAACACTCTTGGCCAATGTACGCAGCCGAACGTATCATAAGT
Cele-UNSB01_3:11898352-11906615 Satlength=8264 Nr of Repeats=45 RepeatLength=184 seed=TACGCAGCCG Num.seqs=41 Similarity=0.970891
160   GAATACAGAACACCAATTATGTCCGAG-AAAAAG-ATCCTACTCAGAATATGAACATATTCGAATACATCGGAGGTATATAGATTCCCGAAGAGCCTTTCCAATTAACCAAACGGTTCAAATTCTAGCAA--ACATCTC-CTTACTTGAACACTCTTGGCCAATGTACGCAGCCGAACGTATCATAAGT

```
*************** ***** ***** ****** **** * ********* ****** * ****   ****
***** *************  *********** *****  ***** ******  **  * ** ** ******
******************************************
Consensus:

GAATACAGAACACCAaTTATGtCCGAGAAAAAGATCCtAcTCAGAATATaAACATAtTcGAATttatCGGAtG
TATAaAGATTCCCGAAGAcaCTTTCCAATTAcCCAAAttGTTCAtATTCTAAAtgAaATTCtCTTACTaGAAC
ACTCTTGGCCAATGTACGCAGCCGAACGTATCATAAGT

>Cele-UNSB01_Fam_68_64_4  Nr. of seq. 4 Alignment length(with gaps) = 67
Alignment score = 0.721393
Cele-UNSB01_2:1316328-1316758 Satlength=431 Nr of Repeats=7
RepeatLength=63 seed=GGTCTGCTAA  Num.seqs=6 Similarity=0.855035
0    GGTCTGCTAAATCGAAACTTGTAGTTTGTAGCCTAGCAGACCAAATTTCTTCAAAACT-AC---TTG
Cele-UNSB01_2:1412228-1413706 Satlength=1479 Nr of Repeats=12
RepeatLength=63 seed=TAGTTTGTAG  Num.seqs=8 Similarity=0.873016
20    -GTCTGCTAAATCGAAACTTGTAGTTTGTAGTCTAGCAGACCAAATTTCTTCAAAACC-CC--ATTG
Rev.of_Cele-UNSB01_2:1422798-1423926 Satlength=1129 Nr of Repeats=9
RepeatLength=63 seed=TTGGTCTGCT  Num.seqs=6 Similarity=0.976014
43    -GTCTGCTAAATCAAAAGTTGTAGTTTGTAGTCTAGCAGACCAATTTTCTTCAAAACC-CC--ATGG
Cele-UNSB01_2:590141-590909 Satlength=769 Nr of Repeats=12
RepeatLength=64 seed=AAATTTTGTA  Num.seqs=12 Similarity=0.921402
13    GGTCTGCCAGATCAAATTTTGTAGTTTGTAGTCTAGCAGACCCA--TT-TAGAATACCAACAAATTG

****** * *** **  ************* ********** *  ** *  ** **   *   * *
Consensus:

gGTCTGCtAaATCaAAacTTGTAGTTTGTAGtCTAGCAGACCaAatTTcTtcAAaACcaCaTtG

>Cele-UNSB01_Fam_69_39_4  Nr. of seq. 4 Alignment length(with gaps) = 40
Alignment score = 0.856944
Cele-UNSB01_1:4232491-4233003 Satlength=513 Nr of Repeats=13
RepeatLength=39 seed=TATTTTTAAT  Num.seqs=8 Similarity=0.954823
0    TA-TTTTTAATTTGGCCTACTTAAAAAGTAGGTCATGACC
Cele-UNSB01_1:13379937-13380822 Satlength=886 Nr of Repeats=18
RepeatLength=39 seed=TTAAAAAGTA  Num.seqs=13 Similarity=0.822863
19    TA-ATTTTAATTTGGCCTACTTAAAAAGTAGGTCATGACC
Cele-UNSB01_1:13114799-13114994 Satlength=196 Nr of Repeats=5
RepeatLength=39 seed=TATTTTTAAT  Num.seqs=5 Similarity=0.965812
0    TA-TTTTTAATTTAACCTACTTAAAAAGTAGGTCATGACC
Cele-UNSB01_3:10614332-10615157 Satlength=826 Nr of Repeats=20
RepeatLength=40 seed=AAAAGTAGGT  Num.seqs=16 Similarity=0.951944
23    TAGTTTTTAACGTGACCTCCTTAAAAAGTAGGTCATGACC

**  ******  *  *** *********************
Consensus:

TAtTTTTAAttTgaCCTaCTTAAAAAGTAGGTCATGACC

>Cele-UNSB01_Fam_70_36_4  Nr. of seq. 4 Alignment length(with gaps) = 36
Alignment score = 0.754630
```

Cele-UNSB01_1:3489580-3490945 Satlength=1366 Nr of Repeats=56
RepeatLength=35 seed=TTTCGGCAAT Num.seqs=35 Similarity=0.808371
0   TTTCGGCAATTTCGGCAATTGCCGGTTTCNTGAAT-
Rev.of_Cele-UNSB01_4:12842983-12843155 Satlength=173 Nr of Repeats=5
RepeatLength=35 seed=AAACCGGCAA Num.seqs=3 Similarity=0.759259
28   TTTCGGCAATTTCGGCAATTGCCGGTTTTGAGAAT-
Cele-UNSB01_1:15073460-15073709 Satlength=250 Nr of Repeats=7
RepeatLength=35 seed=CGGTTTTGGA Num.seqs=5 Similarity=0.885714
21   -TTCGGCAATTTCGATAATTGCCGGTTTTGGAAAAT
Cele-UNSB01_5:18345564-18345809 Satlength=246 Nr of Repeats=7
RepeatLength=35 seed=AAATTTTCGG Num.seqs=7 Similarity=0.945578
30   -TTCGGCAAATTTGATAATTGCCGGTTTTGGAAATT

******** ** * ************   **
Consensus:

tTTCGGCAAtTTcGacAATTGCCGGTTTtggaAAtt

>Cele-UNSB01_Fam_71_30_4 Nr. of seq. 4 Alignment length(with gaps) = 30
Alignment score = 0.777778
Cele-UNSB01_2:14178691-14179588 Satlength=898 Nr of Repeats=30
RepeatLength=30 seed=TTTCTAGAAA Num.seqs=24 Similarity=0.857649
0   TTTCTAGAAAAAATCTGGAAACTTCTAGAAC
Rev.of_Cele-UNSB01_3:3351031-3351269 Satlength=239 Nr of Repeats=7
RepeatLength=30 seed=TAGAACTTTC Num.seqs=5 Similarity=0.786667
26   TTTCTAGAAAAATTTGGAAAGTTCTAGAAT
Rev.of_Cele-UNSB01_3:3352849-3353088 Satlength=240 Nr of Repeats=8
RepeatLength=30 seed=TAGAACTTTC Num.seqs=6 Similarity=0.925926
26   TTTCTAGAGAAATTTGGAAAGTTCTAGAAT
Rev.of_Cele-UNSB01_2:15064033-15064173 Satlength=141 Nr of Repeats=5
RepeatLength=30 seed=TTCTGGAAAA Num.seqs=3 Similarity=0.762963
9   TTTCCAGAAGGTTCTGGAACATTCTAGAAT

**** ***   * ***** ********
Consensus:

TTTCtAGAaaaaTcTGGAAagTTCTAGAAt

>Cele-UNSB01_Fam_72_30_4 Nr. of seq. 4 Alignment length(with gaps) = 30
Alignment score = 0.983333
Cele-UNSB01_2:15106314-15106454 Satlength=141 Nr of Repeats=5
RepeatLength=30 seed=AATTTTCCAG Num.seqs=3 Similarity=0.792593
0   AATTTTCCAGAAGGTTCTGGAACATTCCAG
Cele-UNSB01_4:9470711-9470851 Satlength=141 Nr of Repeats=5
RepeatLength=30 seed=AATTTTCCAG Num.seqs=3 Similarity=0.822222
0   AATTTTCCAGAAGGTTCTGGAACATTCCAG
Cele-UNSB01_6:1421957-1422097 Satlength=141 Nr of Repeats=5
RepeatLength=30 seed=AATTTTCCAG Num.seqs=3 Similarity=0.792593
0   AATTTTCCAGAAGGTTCTGGAACATTCCAG
Cele-UNSB01_6:556993-557132 Satlength=140 Nr of Repeats=5 RepeatLength=30
seed=AATTTTCCAG Num.seqs=3 Similarity=0.807407
0   AATTTTCCAGAAGGTTCTGGAACNTTCCAG

*********************** ******

Consensus:

AATTTTCCAGAAGGTTCTGGAACaTTCCAG

>Cele-UNSB01_Fam_73_20_4 Nr. of seq. 4 Alignment length(with gaps) = 23
Alignment score = 0.677536
Cele-UNSB01_3:1617648-1618407 Satlength=760 Nr of Repeats=37
RepeatLength=20 seed=TTTCACTAAA Num.seqs=36 Similarity=0.904019
0   TTTCACTAAAAAT--C-TCAAAA
Rev.of_Cele-UNSB01_4:16675153-16675373 Satlength=221 Nr of Repeats=8
RepeatLength=20 seed=TTTTAGTGAA Num.seqs=5 Similarity=1.000000
11   TTTCACTAAAAAT--C-TCAAAT
Cele-UNSB01_4:1429332-1429629 Satlength=298 Nr of Repeats=15
RepeatLength=21 seed=AAAATTGACT Num.seqs=10 Similarity=0.908289
18   ATTGACTAAAAAT--CGTCAAAA
Cele-UNSB01_4:2205280-2205605 Satlength=326 Nr of Repeats=13
RepeatLength=21 seed=AAAATAGCTT Num.seqs=9 Similarity=0.765993
7   -TTGACTAAAAATAGC-TTAAAA

** ********* * * ***
Consensus:

tTTcACTAAAAAATCTcAAAa

>Cele-UNSB01_Fam_74_21_4 Nr. of seq. 4 Alignment length(with gaps) = 22
Alignment score = 0.651515
Cele-UNSB01_6:6516089-6517558 Satlength=1470 Nr of Repeats=50
RepeatLength=19 seed=GTCAGTGCAA Num.seqs=37 Similarity=0.811390
0   GTCAGTGCAAC---TGCAANTG
Rev.of_Cele-UNSB01_5:18442188-18442272 Satlength=85 Nr of Repeats=4
RepeatLength=21 seed=GCACTGACCA Num.seqs=4 Similarity=1.000000
8   GTCAGTGCAACAT-TGCATTTG
Cele-UNSB01_6:1138488-1138593 Satlength=106 Nr of Repeats=5
RepeatLength=21 seed=TTGGTCAGTG Num.seqs=5 Similarity=0.746032
18   GTCAGTGCAATTT-TNNAATTG
Cele-UNSB01_6:1324054-1324372 Satlength=319 Nr of Repeats=7
RepeatLength=22 seed=GTCAGTGCAA Num.seqs=5 Similarity=0.692754
0   GTCAGTGCAAATTGTGCACATG

**********   * * **
Consensus:

GTCAGTGCAActtTgcAatTG

>Cele-UNSB01_Fam_75_20_4 Nr. of seq. 4 Alignment length(with gaps) = 20
Alignment score = 0.744444
Cele-UNSB01_1:14726982-14727102 Satlength=121 Nr of Repeats=6
RepeatLength=20 seed=GTGAAATTTT Num.seqs=6 Similarity=0.705820
0   GTGAAATTTTAAATTTTTAA
Cele-UNSB01_2:15212191-15212515 Satlength=325 Nr of Repeats=14
RepeatLength=20 seed=TTCAGTCAAA Num.seqs=10 Similarity=0.802963
16   GTCAAAATTTAAATTTTTCA

Cele-UNSB01_5:20247169-20247404 Satlength=236 Nr of Repeats=11
RepeatLength=20 seed=TTTGAATTTT Num.seqs=7 Similarity=0.936508
7   GTGAAAATTTGAATTTTTGA
Cele-UNSB01_5:20719213-20719394 Satlength=182 Nr of Repeats=9
RepeatLength=20 seed=AAAATTCAAA Num.seqs=6 Similarity=0.778836
3   CTGAAAATTCAAATTTCTGA

* *** ** ***** * *
Consensus:

gTgAAAaTTtaAATTTtTgA

>Cele-UNSB01_Fam_76_14_4 Nr. of seq. 4 Alignment length(with gaps) = 15
Alignment score = 0.727778
Cele-UNSB01_2:3063296-3063566 Satlength=271 Nr of Repeats=18
RepeatLength=14 seed=TTAGGGCCTT Num.seqs=17 Similarity=0.949580
0   TTAGGGCCTTGGGC-
Rev.of_Cele-UNSB01_4:160082-160208 Satlength=127 Nr of Repeats=8
RepeatLength=14 seed=CCTAAGCCTA Num.seqs=7 Similarity=0.918367
5   TTAGGGCCTTAGGC-
Cele-UNSB01_6:16570815-16570983 Satlength=169 Nr of Repeats=12
RepeatLength=14 seed=CTTAGGGCTT Num.seqs=12 Similarity=0.894661
6   TTAGGGCTTAGGGC-
Rev.of_Cele-UNSB01_2:3184073-3184138 Satlength=66 Nr of Repeats=4
RepeatLength=14 seed=CCAAGGCCCA Num.seqs=3 Similarity=0.746032
4   TT-GGGCCTTGGGCC

** **** * ***
Consensus:

TTaGGGCcTtgGGC

>Cele-UNSB01_Fam_77_15_4 Nr. of seq. 4 Alignment length(with gaps) = 15
Alignment score = 0.955556
Cele-UNSB01_1:4250268-4250328 Satlength=61 Nr of Repeats=4
RepeatLength=15 seed=TTTCCGGATC Num.seqs=4 Similarity=0.911111
0   TTTCCGGATCTACGT
Rev.of_Cele-UNSB01_1:4250510-4250645 Satlength=136 Nr of Repeats=9
RepeatLength=15 seed=AGATCCGGAA Num.seqs=9 Similarity=1.000000
11   TTTCCGGATCTACGT
Rev.of_Cele-UNSB01_1:7000180-7000255 Satlength=76 Nr of Repeats=5
RepeatLength=15 seed=AGATCCGGAA Num.seqs=5 Similarity=0.928889
11   TTTCCGGATCTACGT
Cele-UNSB01_2:12906532-12906652 Satlength=121 Nr of Repeats=7
RepeatLength=15 seed=ATTTCCGGAT Num.seqs=6 Similarity=0.922963
14   TTTCCGGATCTACGA

****
Consensus:

TTTCCGGATCTACGt

>Cele-UNSB01_Fam_78_13_4 Nr. of seq. 4 Alignment length(with gaps) = 13
Alignment score = 0.897436

Cele-UNSB01_5:1038307-1038528 Satlength=222 Nr of Repeats=11
RepeatLength=13 seed=GCCTATGGCC Num.seqs=7 Similarity=0.912088
0   GCCTATGGCCTAC
Rev.of_Cele-UNSB01_5:1039029-1039263 Satlength=235 Nr of Repeats=18
RepeatLength=13 seed=CGTAGGCCAT Num.seqs=18 Similarity=0.894754
1   GCCTATGGCCTAC
Cele-UNSB01_5:2151047-2151352 Satlength=306 Nr of Repeats=21
RepeatLength=13 seed=GCCTAAGCCT Num.seqs=17 Similarity=0.930618
7   GCCTATGGCCTAA
Rev.of_Cele-UNSB01_6:5352204-5352282 Satlength=79 Nr of Repeats=6
RepeatLength=13 seed=GCCATAGGCG Num.seqs=6 Similarity=0.931624
9   GCCTATGGCTTAC

********* **
Consensus:

GCCTATGGCcTAc

>Cele-UNSB01_Fam_79_12_4 Nr. of seq. 4 Alignment length(with gaps) = 12
Alignment score = 0.840278
Cele-UNSB01_1:2044939-2045157 Satlength=219 Nr of Repeats=13
RepeatLength=11 seed=TTTTGCCGAA Num.seqs=10 Similarity=0.811448
0   TTTTGCCGAAC-
Cele-UNSB01_1:5700313-5700505 Satlength=193 Nr of Repeats=16
RepeatLength=12 seed=GAAATTTTTT Num.seqs=16 Similarity=0.949074
7   TTTTTCCGAAAT
Cele-UNSB01_1:12700590-12700802 Satlength=213 Nr of Repeats=16
RepeatLength=12 seed=GAAATTTTTT Num.seqs=12 Similarity=0.915825
7   TTTTTCCGAAAT
Rev.of_Cele-UNSB01_1:12700867-12701490 Satlength=624 Nr of Repeats=50
RepeatLength=12 seed=AAAAAATTTC Num.seqs=37 Similarity=0.905572
17   TTTTTCCGAAAT

**** *****
Consensus:

TTTTtCCGAAat

>Cele-UNSB01_Fam_80_12_4 Nr. of seq. 4 Alignment length(with gaps) = 12
Alignment score = 0.662037
Cele-UNSB01_4:5337721-5338093 Satlength=373 Nr of Repeats=18
RepeatLength=12 seed=AGAAGGAGGA Num.seqs=11 Similarity=0.864646
0   AGAAGGAGGAAA
Rev.of_Cele-UNSB01_4:5366596-5366788 Satlength=193 Nr of Repeats=10
RepeatLength=12 seed=TCCTCTTTCT Num.seqs=8 Similarity=0.896825
10   AGAAAGAGGAGA
Rev.of_Cele-UNSB01_6:7918402-7918617 Satlength=216 Nr of Repeats=6
RepeatLength=12 seed=TCTTCCTCCT Num.seqs=4 Similarity=0.870370
1   AGGAGGAGGAAG
Rev.of_Cele-UNSB01_5:14055771-14055849 Satlength=79 Nr of Repeats=6
RepeatLength=12 seed=TCTTTCTCCT Num.seqs=5 Similarity=0.866667
1   ANAAGGAGAAAG

* * *** *

Consensus:

AgaAgGAGgAaa

>Cele-UNSB01_Fam_81_10_4 Nr. of seq. 4 Alignment length(with gaps) = 10 Alignment score = 0.800000
Cele-UNSB01_2:6282823-6283646 Satlength=824 Nr of Repeats=34 RepeatLength=10 seed=TCCCGATCCA Num.seqs=22 Similarity=0.952670
0 TCCCGATCCA
Cele-UNSB01_2:7569779-7570282 Satlength=504 Nr of Repeats=27 RepeatLength=10 seed=TCCATCCCGA Num.seqs=19 Similarity=0.831650
6 TCCCGATCCA
Rev.of_Cele-UNSB01_6:17257914-17259280 Satlength=1367 Nr of Repeats=37 RepeatLength=10 seed=TGGATCGGGA Num.seqs=23 Similarity=0.908300
0 TCCCGATCCA
Cele-UNSB01_6:15839450-15840189 Satlength=740 Nr of Repeats=22 RepeatLength=10 seed=TATCTAGATC Num.seqs=15 Similarity=0.964444
8 TCTAGATCTA

** **** *
Consensus:

TCccGATCcA

>Cele-UNSB01_Fam_82_167_3 Nr. of seq. 3 Alignment length(with gaps) = 172 Alignment score = 0.651809
Cele-UNSB01_3:464201-477164 Satlength=12964 Nr of Repeats=79 RepeatLength=164 seed=ATATAATGCT Num.seqs=68 Similarity=0.967912
0
ATATAATGCTCGTAATTATTTATAAGTGTGTGTTGTCTCAGAGCCACGTCTGAAAATACAGATTTTTTCTCGA
AAATTT---CAAAAACTTTGTA-GAAATTCGATTTTTGAC--
ATAAATGATCGCTCTTTTTTTCTGACATGTATTTCAGTGACAAA-TGGANT-AGAAA
Cele-UNSB01_2:14526196-14534994 Satlength=8799 Nr of Repeats=53 RepeatLength=166 seed=TCGTAATTAT Num.seqs=53 Similarity=0.999091
8 -
TATAATGTTCGTAATTATTCATAAGTTTGTGTTGTCTCAGAGCCATGTCTGAAAAAACAGATTTTTTTTCGAA
AATTT---TAAAAAGTTCGTA-
AAAATTGGATTTTTGACAAATCTATGCTCTCTTTTTTTGTTTGAAATTTAGTTCAGTGACATA-
CAATTTCAAAAA
Cele-UNSB01_2:13398823-13400207 Satlength=1385 Nr of Repeats=8 RepeatLength=165 seed=AAAATTCAAT Num.seqs=7 Similarity=0.943050
94 TTATAATGCTCGTAATTATTTATAAGTGTGTGTTGTCTCAGAATCAATTCT-
AAAAAACAGATTTTTTTT-GAAAGTTTAAGTAATATCCGCGGAGAAAATTCAATTTTGGACATTTCTAT--
TATCT-TTTTTTTAAAACATGTATTTCAGTGA-ATAGAAGACTCAAAA-

******* *********** ****** ************** ** *** **** *********** *
**** *** ** * * * ***** ***** *** * ** * ** ***** * * **
** ******** * * * * **
Consensus:

nTATAATGcTCGTAATTATTtATAAGTgTGTGTTGTCTCAGAgcCAngTCTgAAAAaACAGATTTTTTtTcGA
AAaTTTtAAaAacttcGtAaAAATTcgATTTTtGACanaTctATgnTctCTnTTTTTtTntgAcATgTAtTTC
AGTGAcAtAnaganTcAaAAa

>Cele-UNSB01_Fam_83_90_3 Nr. of seq. 3 Alignment length(with gaps) = 95
Alignment score = 0.673684
Cele-UNSB01_5:19764010-19764338 Satlength=329 Nr of Repeats=4
RepeatLength=82 seed=CCCCGAACGT Num.seqs=4 Similarity=0.948510
0   CCCCGAACGTGTCGGCCGCTTCC--AAATAACCACCTCCTTGCACTACGTTGCGCACACA--------
CTCATTTCACGCCAAGCTGCGGAA---
Cele-UNSB01_5:1577782-1578190 Satlength=409 Nr of Repeats=5
RepeatLength=89 seed=GCACTACGTT Num.seqs=3 Similarity=0.740741
38   CCCTGAACGTGTCGGCCGC-TCC--
AAATAACTACCTTCTNGCACTACGTTGCGCACACACTAAGCTACTCGTTTCACGCCAAGCTACGGAA---
Cele-UNSB01_3:1724995-1725361 Satlength=367 Nr of Repeats=4
RepeatLength=92 seed=CTGCGGAACC Num.seqs=3 Similarity=1.000000
81   ---
CGAACGTGTCGGCCGCTTCCAAAAACAACCACCTCTTCACACTTCATTGCGCACACACCAAACTACTAGTTTC
ACGTCAAACTGCGGAACCC

************** *** *** *** **** * **** * ***********   **
******* *** ** *****
Consensus:

ccccGAACGTGTCGGCCGCtTCCAAAtAACcACCTccTngCACTaCgTTGCGCACACAcnaanctaCTcgTTT
CACGcCAAgCTgCGGAA

>Cele-UNSB01_Fam_84_79_3 Nr. of seq. 3 Alignment length(with gaps) = 79
Alignment score = 0.909986
Cele-UNSB01_2:3993365-3994945 Satlength=1581 Nr of Repeats=20
RepeatLength=79 seed=GTGAATCTCC Num.seqs=20 Similarity=0.984544
0
GTGAATCTCCTCTCCACCTAATGATTCCTGACAGAATACCGGCAGTATTCTGGGGTCTCATTAGGTTTTAGTT
TCTTCT
Rev.of_Cele-UNSB01_4:11132963-11135728 Satlength=2766 Nr of Repeats=35
RepeatLength=79 seed=GAAACTAAAA Num.seqs=35 Similarity=0.918347
75
GTGAATCTCCTCTCTACCTAATGAGTCCTGACAGAATACCGGCAGTATTCTGGGGTCTCATTAGGTTTTAGTT
TCTTCT
Cele-UNSB01_2:8406477-8418390 Satlength=11914 Nr of Repeats=144
RepeatLength=79 seed=TGACAGAATA Num.seqs=143 Similarity=0.910204
28
CTGAATCTCCACTCTTCCTAATGAGTCCTGACAGAATACCGGCAGTATTTTGGGGTCTCATTAGGTTTTCGTT
TCTTTT

********* *** ******** ********************** ******************
******* *
Consensus:

gTGAATCTCCtCTCtaCCTAATGAgTCCTGACAGAATACCGGCAGTATTcTGGGGTCTCATTAGGTTTTaGTT
TCTTcT

>Cele-UNSB01_Fam_85_76_3 Nr. of seq. 3 Alignment length(with gaps) = 76
Alignment score = 0.906433
Cele-UNSB01_4:14405343-14406331 Satlength=989 Nr of Repeats=13
RepeatLength=76 seed=AATGCTAGAG Num.seqs=13 Similarity=0.967611
0

AATGCTAGAGGACACTCCTTTGCACGAAAGGAAGTGGGAGAGACTGTGGAGTTGTCGGTGTCTTTGTGCATGC
TCG
Rev.of_Cele-UNSB01_4:14410457-14414735 Satlength=4279 Nr of Repeats=57
RepeatLength=76 seed=TCGTGCAAAG Num.seqs=56 Similarity=0.948849
27
AATGCTAGAGGACATTCCTTTGCACGAAAGGAAGTGGGAGAGACTGTGGAGTTGTCGGTGTCTTTGTGCATGC
TCG
Rev.of_Cele-UNSB01_6:17130007-17135013 Satlength=5007 Nr of Repeats=66
RepeatLength=76 seed=AGCATTAGAG Num.seqs=56 Similarity=0.950911
6
AATGCTAGAAGACACTCCTTTGCACGAAGGAAAGTGGGAGAGACTGTGGTGTTGTCGGTGTCTGTGTACATGC
TCT

********* **** ************* * ***************** ************ ***
****
Consensus:

AATGCTAGAgGACAcTCCTTTGCACGAAaGgAAGTGGGAGAGACTGTGGaGTTGTCGGTGTCTtTGTgCATGC
TCg

>Cele-UNSB01_Fam_86_64_3 Nr. of seq. 3 Alignment length(with gaps) = 64
Alignment score = 0.954861
Cele-UNSB01_1:14168111-14168331 Satlength=221 Nr of Repeats=5
RepeatLength=63 seed=TTTTTGACTA Num.seqs=3 Similarity=0.876736
0   TTTTTGACTACGGAATGTCTTTTTCGCTACTGACTG-AAAAATAGCTACATTCGCACTTTTGNC
Rev.of_Cele-UNSB01_1:14174364-14175224 Satlength=861 Nr of Repeats=15
RepeatLength=64 seed=GTAGTCAAAA Num.seqs=10 Similarity=0.982870
11   TTTTTGACTACTGAATGTCTTTTTCGCTACTGACTGAAAAAATAGCTACATTCGCACTTTTGTC
Rev.of_Cele-UNSB01_5:14309212-14310008 Satlength=797 Nr of Repeats=14
RepeatLength=64 seed=GTAGTCAAAA Num.seqs=9 Similarity=0.983796
11   TTTTTGACTACTGAATGTCTTTTTCGCTACTGACTGAAAAAATAGCTACATTCGCACTTTTGTC

*********** *********************** ************************* *
Consensus:

TTTTTGACTACtGAATGTCTTTTTCGCTACTGACTGaAAAAATAGCTACATTCGCACTTTTGtC

>Cele-UNSB01_Fam_87_34_3 Nr. of seq. 3 Alignment length(with gaps) = 34
Alignment score = 0.980392
Cele-UNSB01_1:11643779-11644036 Satlength=258 Nr of Repeats=6
RepeatLength=34 seed=ATGCACCATG Num.seqs=4 Similarity=0.679739
0   ATGCACCATGTCAAAAAATATATCCAAAAGTACG
Cele-UNSB01_1:11667166-11667423 Satlength=258 Nr of Repeats=6
RepeatLength=34 seed=ATGCACCATG Num.seqs=4 Similarity=0.679739
0   ATGCACCATGTCAAAAAATATATCCAAAAGTACG
Cele-UNSB01_1:12478910-12479068 Satlength=159 Nr of Repeats=5
RepeatLength=34 seed=ATGCACCATG Num.seqs=4 Similarity=0.712418
0   ATGCACCATGTCAAAAAATATATCCAAAANTACG

***************************** ****
Consensus:

ATGCACCATGTCAAAAAATATATCCAAAAgTACG

```
>Cele-UNSB01_Fam_88_32_3  Nr. of seq. 3 Alignment length(with gaps) = 32
Alignment score = 0.972222
Cele-UNSB01_1:11137320-11159590 Satlength=22271 Nr of Repeats=783
RepeatLength=32 seed=GCACCAAATA  Num.seqs=516 Similarity=0.905773
0    GCACCAAATAGCACCAATTTAGCACCGATGGA
Rev.of_Cele-UNSB01_6:12591006-12601690 Satlength=10685 Nr of Repeats=320
RepeatLength=32 seed=GCTATTTGGT  Num.seqs=268 Similarity=0.880720
12    GCACCAAATAGCACCAATGTAGCACCGATGGA
Rev.of_Cele-UNSB01_6:12591003-12604866 Satlength=13864 Nr of Repeats=415
RepeatLength=32 seed=GGTGCTATTT  Num.seqs=356 Similarity=0.868768
15    GCACCAAATAGCACCAATGTAGCACCGATGGA

****************** *************
Consensus:

GCACCAAATAGCACCAATgTAGCACCGATGGA

>Cele-UNSB01_Fam_89_32_3  Nr. of seq. 3 Alignment length(with gaps) = 32
Alignment score = 0.972222
Cele-UNSB01_4:14093954-14094851 Satlength=898 Nr of Repeats=32
RepeatLength=32 seed=CGACCTGCGA  Num.seqs=24 Similarity=0.987923
0    CGACCTGCGACCTAGCGACCTGGGTCGCTTTG
Cele-UNSB01_6:2413161-2413272 Satlength=112 Nr of Repeats=4
RepeatLength=32 seed=CGACCTGCGA  Num.seqs=3 Similarity=0.805556
0    CGACCTGCGACCTAGCGACCTAGGTCGCTTTG
Cele-UNSB01_6:12904034-12904836 Satlength=803 Nr of Repeats=12
RepeatLength=32 seed=CGACCTAGGT  Num.seqs=8 Similarity=0.956845
15    CGACCTGCGACCTAGCGACCTAGGTCGCTTTG

********************* **********
Consensus:

CGACCTGCGACCTAGCGACCTaGGTCGCTTTG

>Cele-UNSB01_Fam_90_27_3  Nr. of seq. 3 Alignment length(with gaps) = 27
Alignment score = 0.658436
Cele-UNSB01_4:3004201-3004345 Satlength=145 Nr of Repeats=6
RepeatLength=24 seed=GCGGCCGACA  Num.seqs=6 Similarity=0.855556
0    -GCGGCCGACACCTTACGGGTTG--TA
Cele-UNSB01_2:13658533-13659809 Satlength=1277 Nr of Repeats=26
RepeatLength=27 seed=AGCAGCCGAC  Num.seqs=20 Similarity=0.791813
0    AGCAGCCGACACCTTGCNGGTTGCTTA
Cele-UNSB01_5:19852888-19853192 Satlength=305 Nr of Repeats=8
RepeatLength=27 seed=GCAGCCGACA  Num.seqs=6 Similarity=0.584291
1    AGCAGCCGACACCTCACTGGTCCCTNA

** **********  * ***     *
Consensus:

aGCaGCCGACACCTtaCnGGTtgcttA

>Cele-UNSB01_Fam_91_21_3  Nr. of seq. 3 Alignment length(with gaps) = 23
Alignment score = 0.801932
```

Cele-UNSB01_2:13499104-13499209 Satlength=106 Nr of Repeats=5
RepeatLength=21 seed=CTAGAAAAAC Num.seqs=5 Similarity=0.898413
0   CTA-GAAAAACTCGGCCATCAA-
Rev.of_Cele-UNSB01_2:13500094-13501231 Satlength=1138 Nr of Repeats=46
RepeatLength=21 seed=TTTTCTAGTT Num.seqs=34 Similarity=0.794245
8   CTA-GAAAAACTCGGCCATCAA-
Cele-UNSB01_2:646191-646835 Satlength=645 Nr of Repeats=25
RepeatLength=23 seed=GGCCACCAAT Num.seqs=20 Similarity=0.753776
13   CTATAAAAAACTCGGCCACCAAT

*** ************* ***
Consensus:

CTAgAAAAACTCGGCCAtCAA

>Cele-UNSB01_Fam_92_21_3 Nr. of seq. 3 Alignment length(with gaps) = 23
Alignment score = 0.748792
Cele-UNSB01_4:3817232-3817391 Satlength=160 Nr of Repeats=5
RepeatLength=21 seed=TAGTTTGTAG Num.seqs=3 Similarity=0.661376
0   TAG-TTT-ATAGTTTGCAGTTTG
Cele-UNSB01_5:3623960-3624037 Satlength=78 Nr of Repeats=4
RepeatLength=21 seed=AGTTTGTAGT Num.seqs=3 Similarity=0.640212
1   TAG-TTT-GTAGTTTGTNGTTTG
Rev.of_Cele-UNSB01_1:13115312-13116088 Satlength=777 Nr of Repeats=11
RepeatLength=23 seed=ACTACAAACT Num.seqs=10 Similarity=1.000000
4   TAGTTTTCATAGTTTGTAGTTTG

*** *** ******* *****
Consensus:

TAGTTTaTAGTTTGtaGTTTG

>Cele-UNSB01_Fam_93_20_3 Nr. of seq. 3 Alignment length(with gaps) = 22
Alignment score = 0.656566
Cele-UNSB01_3:3164980-3165718 Satlength=739 Nr of Repeats=38
RepeatLength=18 seed=GTCGATTTAC Num.seqs=33 Similarity=0.778900
0   GTCGATTTACG-AGA-TTN--T
Rev.of_Cele-UNSB01_2:13900624-13901003 Satlength=380 Nr of Repeats=15
RepeatLength=21 seed=CTCGTAAATC Num.seqs=9 Similarity=0.754630
13   GTCGATTTACGAGGAGTTC-TT
Cele-UNSB01_4:1866819-1867315 Satlength=497 Nr of Repeats=18
RepeatLength=21 seed=TGTCGATTTA Num.seqs=15 Similarity=0.738095
21   GTCGATTTACG-GGAGTTCAGT

*********** ** **   *
Consensus:

GTCGATTTACGgGAgTTcnT

>Cele-UNSB01_Fam_94_21_3 Nr. of seq. 3 Alignment length(with gaps) = 22
Alignment score = 0.843434
Cele-UNSB01_3:86662-86935 Satlength=274 Nr of Repeats=13 RepeatLength=21
seed=CCATTTTAAG Num.seqs=13 Similarity=0.947904
0   CCATTTTAAGCC-AATTTTGGA

Cele-UNSB01_3:86662-87180 Satlength=519 Nr of Repeats=25 RepeatLength=21 seed=CCATTTTAAG Num.seqs=16 Similarity=0.877588
0   CCATTTTAAGCC-AATTTTGGA
Rev.of_Cele-UNSB01_3:83964-84341 Satlength=378 Nr of Repeats=14 RepeatLength=21 seed=AAATTTGGCT Num.seqs=11 Similarity=0.774105
18   CCATTTTAAGCCAAATTTTCAA

************ ****** *
Consensus:

CCATTTTAAGCCAATTTTggA

>Cele-UNSB01_Fam_95_22_3 Nr. of seq. 3 Alignment length(with gaps) = 22 Alignment score = 0.777778
Cele-UNSB01_2:2758878-2759540 Satlength=663 Nr of Repeats=24 RepeatLength=22 seed=ACTACAGTAA Num.seqs=18 Similarity=0.729847
0   ACTACAGTAACCCTACANTACC
Cele-UNSB01_5:1035824-1036308 Satlength=485 Nr of Repeats=20 RepeatLength=22 seed=ACAGTACTCC Num.seqs=16 Similarity=0.776630
3   ANTACAGTACTCCTACAGTACC
Rev.of_Cele-UNSB01_5:16682435-16683237 Satlength=803 Nr of Repeats=14 RepeatLength=22 seed=GGGTACTGTA Num.seqs=9 Similarity=0.840067
12   ACTACAGTACCCATACAGTACT

* ******* * **** ***
Consensus:

AcTACAGTAccCcTACAgTACc

>Cele-UNSB01_Fam_96_19_3 Nr. of seq. 3 Alignment length(with gaps) = 21 Alignment score = 0.608466
Cele-UNSB01_1:2556234-2556523 Satlength=290 Nr of Repeats=10 RepeatLength=18 seed=GCCTAGGAAT Num.seqs=8 Similarity=0.772487
0   GCCTAGGAAT-CCAAT--ATG
Rev.of_Cele-UNSB01_1:1302534-1303556 Satlength=1023 Nr of Repeats=33 RepeatLength=19 seed=TTCTAGGCCA Num.seqs=21 Similarity=0.753383
8   GCCTAGAAATCCTCA--GATG
Rev.of_Cele-UNSB01_1:1127917-1128577 Satlength=661 Nr of Repeats=21 RepeatLength=20 seed=ATTTCTAGGC Num.seqs=13 Similarity=0.625153
10   GCCTAGAAAT-CTNATNGATG

****** *** * *   ***
Consensus:

GCCTAGaAATCtnAtgATG

>Cele-UNSB01_Fam_97_18_3 Nr. of seq. 3 Alignment length(with gaps) = 21 Alignment score = 0.761905
Cele-UNSB01_6:3455223-3455778 Satlength=556 Nr of Repeats=27 RepeatLength=18 seed=TTTTGTCAGT Num.seqs=20 Similarity=0.766579
0   TTTTGTCAGTGCT---ATAGT
Cele-UNSB01_6:3508288-3508508 Satlength=221 Nr of Repeats=10 RepeatLength=18 seed=TTGTCAGTGC Num.seqs=7 Similarity=0.848325
2   TTTTGTCAGTGCT---ATAGT

```
Rev.of_Cele-UNSB01_6:1590254-1590338 Satlength=85 Nr of Repeats=4
RepeatLength=21 seed=TCTGAGCACT  Num.seqs=4 Similarity=0.925926
17    TTTTGTCAGTGCTCAGATAAT

*************   *** *
Consensus:

TTTTGTCAGTGCTATAgT

>Cele-UNSB01_Fam_98_20_3  Nr. of seq. 3 Alignment length(with gaps) = 21
Alignment score = 0.719577
Cele-UNSB01_2:3287465-3288008 Satlength=544 Nr of Repeats=19
RepeatLength=20 seed=GTAAATCTAC  Num.seqs=12 Similarity=0.565197
0    GTAAATCTACACTA-GCTTCC
Rev.of_Cele-UNSB01_4:3714325-3715225 Satlength=901 Nr of Repeats=15
RepeatLength=21 seed=TGTAGATTTA  Num.seqs=10 Similarity=0.670875
11    GTAAATCTACANTAGGCTCCC
Cele-UNSB01_2:12047204-12047947 Satlength=744 Nr of Repeats=32
RepeatLength=20 seed=TAAATCTACA  Num.seqs=25 Similarity=0.700222
1    GTAAATCTACACNG-GCTNGC

***********    ***  *
Consensus:

GTAAATCTACActaGCTncC

>Cele-UNSB01_Fam_99_21_3  Nr. of seq. 3 Alignment length(with gaps) = 21
Alignment score = 0.767196
Cele-UNSB01_3:1210986-1211328 Satlength=343 Nr of Repeats=4
RepeatLength=21 seed=ACTACAGTAC  Num.seqs=3 Similarity=0.788360
0    ACTACAGTACCCCCNACNACT
Cele-UNSB01_4:14628221-14628977 Satlength=757 Nr of Repeats=36
RepeatLength=21 seed=CAACTACCAC  Num.seqs=36 Similarity=0.943361
10    ACTACCGTACCAACTACCACA
Cele-UNSB01_4:14648431-14648725 Satlength=295 Nr of Repeats=13
RepeatLength=21 seed=ACTACCGTAC  Num.seqs=12 Similarity=0.895142
21    ACTACCGTACCAACTACCACA

***** *****  * ** **
Consensus:

ACTACcGTACCaaCtACcACa

>Cele-UNSB01_Fam_100_21_3  Nr. of seq. 3 Alignment length(with gaps) = 21
Alignment score = 0.957672
Cele-UNSB01_5:3330728-3331463 Satlength=736 Nr of Repeats=20
RepeatLength=21 seed=CTAACATTTT  Num.seqs=12 Similarity=0.927850
0    CTAACATTTTTCAATTTTCGT
Cele-UNSB01_5:16772051-16772198 Satlength=148 Nr of Repeats=7
RepeatLength=21 seed=ATTTTTCAAT  Num.seqs=7 Similarity=0.848828
5    CTAACATTTTTCAATTTTCGT
Cele-UNSB01_5:3604514-3604619 Satlength=106 Nr of Repeats=5
RepeatLength=21 seed=TTTCAGTTTT  Num.seqs=5 Similarity=0.911111
8    CTAACATTTTTCAGTTTTCGT
```

```
************* *******
Consensus:

CTAACATTTTTCAaTTTTCGT

>Cele-UNSB01_Fam_101_21_3  Nr. of seq. 3 Alignment length(with gaps) = 21
Alignment score = 0.682540
Cele-UNSB01_6:1259650-1260300 Satlength=651 Nr of Repeats=6
RepeatLength=21 seed=GTCAGTGCAA  Num.seqs=4 Similarity=0.809524
34    AAATGCATTTTTGGTCAGTGC
Rev.of_Cele-UNSB01_6:1371051-1371242 Satlength=192 Nr of Repeats=7
RepeatLength=21 seed=TTGCACTGAC  Num.seqs=6 Similarity=0.630303
44    AAATGAAGAGTTGGTCAGTGC
Cele-UNSB01_6:15665052-15665283 Satlength=232 Nr of Repeats=11
RepeatLength=21 seed=TGAAAGATTG  Num.seqs=9 Similarity=0.957672
28    GTGCCTATGAAAGATTGGTCA

**        *     * *
Consensus:

GTgcaaatgnAtnnttGgTca

>Cele-UNSB01_Fam_102_20_3  Nr. of seq. 3 Alignment length(with gaps) = 20
Alignment score = 0.688889
Cele-UNSB01_2:13684820-13685366 Satlength=547 Nr of Repeats=24
RepeatLength=20 seed=GTAAATCGAC  Num.seqs=16 Similarity=0.655886
10    ACACNACGTCGTAAATCGAC
Rev.of_Cele-UNSB01_3:2118949-2119137 Satlength=189 Nr of Repeats=9
RepeatLength=20 seed=TGTAGATTTA  Num.seqs=6 Similarity=0.697778
21    ATAGAACCCCGTAAATCTAC
Rev.of_Cele-UNSB01_3:13139705-13140905 Satlength=1201 Nr of Repeats=30
RepeatLength=20 seed=TGTAGATTTA  Num.seqs=22 Similarity=0.674981
21    ATAGANCTTCGTAAATCTAC

******* *** *   *  *
Consensus:

GTAAATCtACAtAgaaCntC

>Cele-UNSB01_Fam_103_20_3  Nr. of seq. 3 Alignment length(with gaps) = 20
Alignment score = 0.822222
Cele-UNSB01_4:8607400-8623699 Satlength=16300 Nr of Repeats=831
RepeatLength=20 seed=ATACGATATA  Num.seqs=721 Similarity=0.881205
0    ATACGATATAGCTATTTACG
Rev.of_Cele-UNSB01_6:4175079-4227144 Satlength=52066 Nr of Repeats=2000
RepeatLength=20 seed=CTATATCGTA  Num.seqs=1999 Similarity=1.000000
11    ATACGATATAGCTATTTACG
Rev.of_Cele-UNSB01_6:7306204-7313772 Satlength=7569 Nr of Repeats=373
RepeatLength=20 seed=ATAGCTATGT  Num.seqs=332 Similarity=0.894033
15    ACACGACATAGCTATAAACG

* **** ********  ***
Consensus:
```

AtACGAtATAGCTATttACG

>Cele-UNSB01_Fam_104_16_3 Nr. of seq. 3 Alignment length(with gaps) = 16
Alignment score = 0.777778
Cele-UNSB01_1:758474-759042 Satlength=569 Nr of Repeats=35
RepeatLength=16 seed=TTTCAGGCTT Num.seqs=34 Similarity=0.947564
0   TTTCAGGCTTTCAGGC
Cele-UNSB01_5:17844176-17844688 Satlength=513 Nr of Repeats=32
RepeatLength=16 seed=CCAGGGTTCC Num.seqs=32 Similarity=1.000000
2   TTCCAGGGTTCCAGGG
Cele-UNSB01_5:17844176-17845043 Satlength=868 Nr of Repeats=35
RepeatLength=16 seed=CCAGGGTTCC Num.seqs=33 Similarity=0.989899
2   TTCCAGGGTTCCAGGG

** **** ** ****
Consensus:

TTcCAGGgTTcCAGGg

>Cele-UNSB01_Fam_105_11_3 Nr. of seq. 3 Alignment length(with gaps) = 12
Alignment score = 0.787037
Cele-UNSB01_3:9173467-9173588 Satlength=122 Nr of Repeats=11
RepeatLength=11 seed=ATTTGGAATA Num.seqs=11 Similarity=0.903030
0   ATT-TGGAATAT
Cele-UNSB01_4:11083607-11084360 Satlength=754 Nr of Repeats=4
RepeatLength=11 seed=TTTGAAATAT Num.seqs=3 Similarity=1.000000
1   ATT-TGAAATAT
Rev.of_Cele-UNSB01_4:6539759-6539908 Satlength=150 Nr of Repeats=10
RepeatLength=12 seed=CAGAATATAT Num.seqs=7 Similarity=0.915344
6   ATTCTGGAATAT

*** ** *****
Consensus:

ATTTGgAATAT

>Cele-UNSB01_Fam_106_11_3 Nr. of seq. 3 Alignment length(with gaps) = 12
Alignment score = 0.722222
Cele-UNSB01_4:5758139-5758194 Satlength=56 Nr of Repeats=5
RepeatLength=11 seed=TCCACTGACC Num.seqs=5 Similarity=0.951515
0   TCCACTGACCA-
Cele-UNSB01_2:6392491-6392539 Satlength=49 Nr of Repeats=4
RepeatLength=12 seed=CCACTGACCA Num.seqs=4 Similarity=0.944444
1   CCCACTGACCAT
Rev.of_Cele-UNSB01_6:14101701-14101756 Satlength=56 Nr of Repeats=5
RepeatLength=11 seed=GTGCTTGGTC Num.seqs=5 Similarity=0.951515
5   AGCACTGACCA-

****
Consensus:

ncCACTGACCA

>Cele-UNSB01_Fam_107_151_2 Nr. of seq. 2 Alignment length(with gaps) = 151 Alignment score = 0.924945
Cele-UNSB01_3:3200630-3201398 Satlength=769 Nr of Repeats=4 RepeatLength=151 seed=TTTAAAGGCG Num.seqs=3 Similarity=0.500000
0
TTTAAAGGCGCATAGGTAGNTCAAANTTCAAANTTGACGGATTCTAGTCAATATGGGGCTTATTTTAAAGCTC
TCAGTAAGCTNAATNTANTTNAGCTACTNAAAATTGAANATTTTNAAATTTTTTGGGTCTCGCAGCGAAAATT
TCGAA
Cele-UNSB01_3:3207516-3208428 Satlength=913 Nr of Repeats=5 RepeatLength=151 seed=TTTAAAGGCG Num.seqs=3 Similarity=0.500000
0
TTTAAAGGCGCATAGGTAGTTCAAANTTCAAANTTGACGGATTCTAGTCAATATGGGGCTTATTTTAAAGCTC
TCAGTAAGCTNAATNTANTTNAGCTACTAACAATTGAANATTTTNAAATTTTTTGGGTCTCGCAGCGAAAATT
TCGAA

```
*******************
*************************************************************************
******** * ***********************************************
```

Consensus:

TTTAAAGGCGCATAGGTAGtTCAAANTTCAAANTTGACGGATTCTAGTCAATATGGGGCTTATTTTAAAGCTC
TCAGTAAGCTNAATNTANTTNAGCTACTaAaAATTGAANATTTTNAAATTTTTTGGGTCTCGCAGCGAAAATT
TCGAA

>Cele-UNSB01_Fam_108_119_2 Nr. of seq. 2 Alignment length(with gaps) = 119 Alignment score = 0.607843
Cele-UNSB01_1:5777645-5778359 Satlength=715 Nr of Repeats=6 RepeatLength=119 seed=GCCACCTTTA Num.seqs=6 Similarity=0.967880
0
GCCACCTTTAACCCTAATCCAAGCAGAGCAGGCCGTAGGCCTGCTATGCGGTAGCGACACGCTCAGGCCCAAT
AGTCCGGGATGAGGGCCGCAGGCCCTCTTACTCTTAGAAACTTTAG
Cele-UNSB01_5:17249920-17250872 Satlength=953 Nr of Repeats=8 RepeatLength=119 seed=AACTGTGGGC Num.seqs=8 Similarity=0.947979
111
GCTGGCCTTAATCCTAACCCACGCAGAGCAGGATGTAGGCCTGAAACGTGGTTGTTATTCAATCAAGCCTAAC
AACCAGGTTTGAGGGCCGCAGGCCCGAAAACTCTTGGAAACTGTGG

```
**   * **** ***** *** **********  *********  * * *** *  *  *  *** *** **
*  * **  ****************    ****** ****** * *
```

Consensus:

GCcacCcTTAAcCCTAAcCCAaGCAGAGCAGGacGTAGGCCTGaaAcGcGGTaGcgAcaCaaTCAaGCCcAAc
AacCaGGgaTGAGGGCCGCAGGCCCgaaaACTCTTaGAAACTgTaG

>Cele-UNSB01_Fam_109_110_2 Nr. of seq. 2 Alignment length(with gaps) = 110 Alignment score = 0.745455
Cele-UNSB01_6:11975065-11975494 Satlength=430 Nr of Repeats=4 RepeatLength=108 seed=TTTCACGTTT Num.seqs=3 Similarity=0.860082
0   TTTCACGTTTTCAGGTGATTATCTAGGTCACGAACTAATTTATTTTGAAAATTGACCT--
GTGGACCTATAACTTTGGAGATATGGGAAATTGAGTATGGGCTTTCAGAT
Cele-UNSB01_6:13568461-13570473 Satlength=2013 Nr of Repeats=18 RepeatLength=108 seed=TTTCAGATTT Num.seqs=17 Similarity=0.875000
100   TTTCACATTTTCACTTGATTATATAGGTCACGACCCAAATTATTTTGAAAATTGAGCTACACGG--
CTGTAACTTTGGAGCTATGGGTAATCAAGTATGAGCTTTCAGAT

```
****** ******  ******* ********** * ** **************** **     **   **
*********** ****** ***  ****** **********
Consensus:

TTTCACaTTTTCAcgTGATTATaTAGGTCACGAaCcAAaTTATTTTGAAAATTGAcCTacacGGacCTaTAAC
TTTGGAGaTATGGGaAATcaAGTATGaGCTTTCAGAT

>Cele-UNSB01_Fam_110_86_2  Nr. of seq. 2 Alignment length(with gaps) = 86
Alignment score = 0.689922
Cele-UNSB01_2:5140446-5146375 Satlength=5930 Nr of Repeats=77
RepeatLength=76 seed=CAATAGAAAA  Num.seqs=56 Similarity=0.988380
0    CAATA--GAAAAGATGGAAGACTAC--AG--AA--A-AA-
CTATTGAGAGACCCCGAAATACTGGCTGTATTTTGAAAGGTCATCT
Rev.of_Cele-UNSB01_2:9101090-9103530 Satlength=2441 Nr of Repeats=29
RepeatLength=86 seed=AGCCAGTATT  Num.seqs=28 Similarity=0.980066
67
CAATAGGGAAAAGATGGAAGAGTTCGAAGTTAACGAGAAGCTATTGCCAGACCCCGAAATACTGGCTGTATTT
TGAAAGGTCGTGG

*****  ************** * *  **  **  * ** ******
********************************* *
Consensus:

CAATAggGAAAAGATGGAAGAcTaCgaAGttAAcgAgAAgCTATTGacAGACCCCGAAATACTGGCTGTATTT
TGAAAGGTCaTcg

>Cele-UNSB01_Fam_111_73_2  Nr. of seq. 2 Alignment length(with gaps) = 73
Alignment score = 0.945205
Cele-UNSB01_1:12413207-12413533 Satlength=327 Nr of Repeats=4
RepeatLength=73 seed=AATAAAATAT  Num.seqs=3 Similarity=0.963470
0
AATAAAATATTGGAAATCCCTATGACACACTTAAGCCTAAAGGCCCGAAAAACATACTAGGATGCCCAACTGG
Rev.of_Cele-UNSB01_5:15668468-15668848 Satlength=381 Nr of Repeats=5
RepeatLength=73 seed=TTAGGCTTAA  Num.seqs=3 Similarity=0.939117
40
AATAAAATATTGGAAATCCTTATGACACACTTAAGCCTAAAGGCCCGAAAAAAAAACTAGGATGCCCAACTGG

******************* ******************************** * ******************
Consensus:

AATAAAATATTGGAAATCCcTATGACACACTTAAGCCTAAAGGCCCGAAAAAaAaACTAGGATGCCCAACTGG

>Cele-UNSB01_Fam_112_71_2  Nr. of seq. 2 Alignment length(with gaps) = 71
Alignment score = 0.626761
Cele-UNSB01_3:3799869-3800537 Satlength=669 Nr of Repeats=5
RepeatLength=65 seed=AAGACACTAA  Num.seqs=3 Similarity=0.945299
0    AAGACACTAATAAAAATATT--ATTTTTATTCGTCTTTTAATTTTAGAA-A-ATTTTC--
AATAAAAATTT
Rev.of_Cele-UNSB01_2:6392670-6395650 Satlength=2981 Nr of Repeats=44
RepeatLength=68 seed=AATAAAAATT  Num.seqs=38 Similarity=0.936325
30    -AGACACTAATAAAAATTTTAAATTTTTATTCGTC-TCTATTTTGAAAATATATTTTCTTGAT-
AAAATAG
```

**************** ** ************* * ** *** * ** * ******   ** *****
Consensus:

aAGACACTAATAAAAATaTTaaATTTTTATTCGTCtTcTAaTTTgAaAAtAtATTTTCttaATaAAAATag

>Cele-UNSB01_Fam_113_68_2 Nr. of seq. 2 Alignment length(with gaps) = 68
Alignment score = 0.838235
Cele-UNSB01_4:17319515-17323871 Satlength=4357 Nr of Repeats=66
RepeatLength=66 seed=CAAATCAACC Num.seqs=66 Similarity=0.958428
0   CAAATCAACCTATTCAGTTGACAATGGTTTTCTTTTATCAAATTTTTTNAAATTT--TAAAGAAAACA
Rev.of_Cele-UNSB01_1:10374816-10387323 Satlength=12508 Nr of Repeats=186
RepeatLength=68 seed=AAAACCATTG Num.seqs=123 Similarity=0.979909
31
CAAATCAACCTATTCAGTTGACAATGGTTTTCTTTTACCAAAATTCTTAGAATTTCAGAAAGAAAACA

************************************* **** ** ** *****   **********
Consensus:

CAAATCAACCTATTCAGTTGACAATGGTTTTCTTTTAcCAAAaTTcTTaaAATTTcagAAAGAAAACA

>Cele-UNSB01_Fam_114_44_2 Nr. of seq. 2 Alignment length(with gaps) = 44
Alignment score = 0.681818
Cele-UNSB01_2:9825153-9825520 Satlength=368 Nr of Repeats=4
RepeatLength=42 seed=CAGCCGACAG Num.seqs=3 Similarity=0.533769
0   -CAGCCGACAGCCGAAAAATT--GGAATTTTCGGCTGGTAGCAC
Cele-UNSB01_5:4001570-4002151 Satlength=582 Nr of Repeats=5
RepeatLength=42 seed=CCAGCCGACA Num.seqs=3 Similarity=0.684755
0   CCAGCCGACAGCCGAAATTTTCACG-ATTTTCGGCTGGTNGCG-

**************** **   * ************* **
Consensus:

cCAGCCGACAGCCGAAAaaTTcacGaATTTTCGGCTGGTaGCac

>Cele-UNSB01_Fam_115_44_2 Nr. of seq. 2 Alignment length(with gaps) = 44
Alignment score = 0.704545
Cele-UNSB01_1:12045808-12046235 Satlength=428 Nr of Repeats=5
RepeatLength=42 seed=TCGGCAAATC Num.seqs=3 Similarity=0.723514
0   TCGGCAAA-TCGGCAATTNCTGGTTTTTTNAA-ATTTGCCGAGC
Rev.of_Cele-UNSB01_4:16010822-16011211 Satlength=390 Nr of Repeats=5
RepeatLength=42 seed=GCTCGGCAAA Num.seqs=3 Similarity=0.957672
0   ACGGCAAATTCGGCAAAT-CT-CTTTTTTCAATATTTGCCGAGC

******* ******* * ** ****** ** ***********
Consensus:

aCGGCAAAtTCGGCAAaTnCTgcTTTTTTcAAtATTTGCCGAGC

>Cele-UNSB01_Fam_116_44_2 Nr. of seq. 2 Alignment length(with gaps) = 44
Alignment score = 0.969697
Cele-UNSB01_1:9793063-9794825 Satlength=1763 Nr of Repeats=41
RepeatLength=44 seed=CTGTTTTCTG Num.seqs=35 Similarity=0.988999
0   CTGTTTTCTGAGCATTTTAGATTTTCGGATTGATGGCCGAAAGG

Cele-UNSB01_4:17389165-17390089 Satlength=925 Nr of Repeats=21
RepeatLength=44 seed=GATTGATGGC Num.seqs=21 Similarity=0.961328
27   CTGTTTTCTGAGCATTGTAGATTTTCGGATTGATGGCCGAAAGG

**************** ***************************
Consensus:

CTGTTTTCTGAGCATTgTAGATTTTCGGATTGATGGCCGAAAGG

>Cele-UNSB01_Fam_117_41_2 Nr. of seq. 2 Alignment length(with gaps) = 41
Alignment score = 0.711382
Cele-UNSB01_6:17501177-17501366 Satlength=190 Nr of Repeats=5
RepeatLength=38 seed=CCGTTTCGCC Num.seqs=4 Similarity=0.982456
0   CCGTTTCGCCTTGAGCGATT-C--ACCCTTAAATGGGCCTC
Cele-UNSB01_6:7586233-7592821 Satlength=6589 Nr of Repeats=165
RepeatLength=40 seed=TTACCCTTAA Num.seqs=156 Similarity=0.968630
21   -GGTTTCGCTTTGAGCGATTCCTTACCCTTAAATGGGCGCC

******* ********** * ************** *
Consensus:

ccGTTTCGCcTTGAGCGATTcCttACCCTTAAATGGGCccC

>Cele-UNSB01_Fam_118_41_2 Nr. of seq. 2 Alignment length(with gaps) = 41
Alignment score = 1.000000
Cele-UNSB01_2:7497139-7497311 Satlength=173 Nr of Repeats=4
RepeatLength=41 seed=AAAACGGGAA Num.seqs=3 Similarity=0.978320
0   AAAACGGGAAAACGGGAATTCCCGTTCCCGTGAAAATTTTA
Cele-UNSB01_2:7497138-7497671 Satlength=534 Nr of Repeats=10
RepeatLength=41 seed=AAAAACGGGA Num.seqs=6 Similarity=0.956640
40   AAAACGGGAAAACGGGAATTCCCGTTCCCGTGAAAATTTTA

****
Consensus:

AAAACGGGAAAACGGGAATTCCCGTTCCCGTGAAAATTTTA

>Cele-UNSB01_Fam_119_40_2 Nr. of seq. 2 Alignment length(with gaps) = 40
Alignment score = 0.975000
Cele-UNSB01_3:1112086-1112814 Satlength=729 Nr of Repeats=14
RepeatLength=40 seed=AAAATCCACG Num.seqs=12 Similarity=0.883838
0   AAAATCCACGTAGATTATTGATTTTTCATNGAATTTTGCA
Cele-UNSB01_3:1112079-1113479 Satlength=1401 Nr of Repeats=16
RepeatLength=40 seed=TTTTGCAAAA Num.seqs=11 Similarity=0.759596
33   AAAATCCACGTAGATTATTGATTTTTCATGGAATTTTGCA

***************************** **********
Consensus:

AAAATCCACGTAGATTATTGATTTTTCATgGAATTTTGCA

>Cele-UNSB01_Fam_120_39_2 Nr. of seq. 2 Alignment length(with gaps) = 39
Alignment score = 0.897436

```
Cele-UNSB01_4:11180885-11199179 Satlength=18295 Nr of Repeats=470
RepeatLength=39 seed=TTTATGGAAA  Num.seqs=408 Similarity=0.953106
0    TTTATGGAAAAGTGGCTCTAGAGCCACAAGAAATACCAG
Cele-UNSB01_5:10655040-10675549 Satlength=20510 Nr of Repeats=527
RepeatLength=39 seed=CCACCAGAAA  Num.seqs=467 Similarity=0.955996
23    TTTATGAAAAAGTGGCTCTAGGGCCACCAGAAATACCAG

****** ************** ***** ***********
Consensus:

TTTATGaAAAAGTGGCTCTAGaGCCACaAGAAATACCAG

>Cele-UNSB01_Fam_121_36_2  Nr. of seq. 2 Alignment length(with gaps) = 36
Alignment score = 0.601852
Cele-UNSB01_4:3353341-3353481 Satlength=141 Nr of Repeats=5
RepeatLength=33 seed=CGGCATATGC  Num.seqs=3 Similarity=0.946128
0    CGGCATA-TGCCGTGAATTTTAAAAT-CGGCATAA-
Rev.of_Cele-UNSB01_4:15750800-15751080 Satlength=281 Nr of Repeats=8
RepeatLength=35 seed=TTGCCGGATT  Num.seqs=8 Similarity=0.983673
32    -GGCAAATTACCATAAATTTTCAAATCCGGCAAAAC

**** * * ** * ****** **** ***** **
Consensus:

cGGCAaAtTaCCaTaAATTTTaAAATcCGGCAaAAc

>Cele-UNSB01_Fam_122_35_2  Nr. of seq. 2 Alignment length(with gaps) = 35
Alignment score = 0.666667
Cele-UNSB01_1:8640900-8641029 Satlength=130 Nr of Repeats=4
RepeatLength=32 seed=AAAATCGAAA  Num.seqs=3 Similarity=1.000000
0    AAAATCGAAAAATTCTCG-TTTTTCAATTTTTA--
Cele-UNSB01_3:11834269-11834616 Satlength=348 Nr of Repeats=10
RepeatLength=34 seed=TTTTCGATTT  Num.seqs=8 Similarity=0.935574
11    AAAATCG-GGAATTTTCGATTTTTCAATTTTTTGA

*******   **** *** *************
Consensus:

AAAATCGaaaAATTcTCGaTTTTTCAATTTTTaga

>Cele-UNSB01_Fam_123_34_2  Nr. of seq. 2 Alignment length(with gaps) = 34
Alignment score = 0.612745
Cele-UNSB01_4:1530750-1530982 Satlength=233 Nr of Repeats=8
RepeatLength=29 seed=CCACCCGGGA  Num.seqs=8 Similarity=0.850575
0    CCACCCGGGAGGTGTCGGCTGCT----CTA-AAC
Cele-UNSB01_5:18836263-18836629 Satlength=367 Nr of Repeats=11
RepeatLength=33 seed=TGTCGGCTGC  Num.seqs=10 Similarity=0.876094
11    -CTCCCGGCAGGTGTCGGCTGCTAAAACTATAGC

* ***** **************    *** * *
Consensus:

cCaCCCGGcAGGTGTCGGCTGCTaaaaCTAtAaC
```

>Cele-UNSB01_Fam_124_32_2 Nr. of seq. 2 Alignment length(with gaps) = 32
Alignment score = 0.671875
Cele-UNSB01_3:260141-260567 Satlength=427 Nr of Repeats=12
RepeatLength=30 seed=TTTTTTGAGC Num.seqs=9 Similarity=0.761574
0   TTTTTTGAGCCAAAAATTC-AAAAAAT-CTCG
Cele-UNSB01_3:249570-249725 Satlength=156 Nr of Repeats=4 RepeatLength=31
seed=AGCTCAAAAT Num.seqs=3 Similarity=0.711806
6   -TTTTTGAGCTCAAAATTCTGAAAAATGCTCA

********* ******* ****** ***
Consensus:

tTTTTTGAGCcaAAAATTCtaAAAAATgCTCa

>Cele-UNSB01_Fam_125_31_2 Nr. of seq. 2 Alignment length(with gaps) = 31
Alignment score = 0.752688
Cele-UNSB01_1:1530606-1531290 Satlength=685 Nr of Repeats=18
RepeatLength=31 seed=GGTTACTGTA Num.seqs=11 Similarity=0.738025
0   GGTTACTGTAACTCAATTATAGGATACTTNG
Cele-UNSB01_2:3540761-3541492 Satlength=732 Nr of Repeats=18
RepeatLength=31 seed=GGTTACTGTA Num.seqs=11 Similarity=0.669318
0   GGTTACTGTAGATCGATCCTAGGATACTTTG

********** ** ** ********** *
Consensus:

GGTTACTGTAaaTCaATcaTAGGATACTTtG

>Cele-UNSB01_Fam_126_31_2 Nr. of seq. 2 Alignment length(with gaps) = 31
Alignment score = 0.784946
Cele-UNSB01_2:2602717-2603767 Satlength=1051 Nr of Repeats=24
RepeatLength=31 seed=CTGAAATTCC Num.seqs=16 Similarity=0.855556
0   CTGAAATTCCAATTTTCCAGTGTAAAAATGT
Cele-UNSB01_5:19020255-19020473 Satlength=219 Nr of Repeats=6
RepeatLength=31 seed=TGTAAAAATG Num.seqs=5 Similarity=0.651613
20   CTGAAATTATGAATTTCCAATGTAAAAATGT

********   * ****** ***********
Consensus:

CTGAAATTacaAaTTTCCAaTGTAAAAATGT

>Cele-UNSB01_Fam_127_31_2 Nr. of seq. 2 Alignment length(with gaps) = 31
Alignment score = 0.956989
Cele-UNSB01_4:16019565-16019814 Satlength=250 Nr of Repeats=6
RepeatLength=31 seed=TCGACAGAAG Num.seqs=4 Similarity=0.665825
0   TCGACAGAAGCTGTCGGAAATCACACTTTCC
Cele-UNSB01_6:12932636-12932914 Satlength=279 Nr of Repeats=9
RepeatLength=31 seed=TCGACAGAAG Num.seqs=8 Similarity=0.729437
0   TCGACAGAAGCTGTCGGACATCACACTTTCC

****************** ************
Consensus:

TCGACAGAAGCTGTCGGAaATCACACTTTCC

>Cele-UNSB01_Fam_128_27_2 Nr. of seq. 2 Alignment length(with gaps) = 27
Alignment score = 0.901235
Cele-UNSB01_2:3132033-3132195 Satlength=163 Nr of Repeats=6
RepeatLength=27 seed=GCTGCTGGAG Num.seqs=6 Similarity=0.881481
0   GCTGCTGGAGGATCCACTGCATCAACT
Cele-UNSB01_2:3135702-3137322 Satlength=1621 Nr of Repeats=60
RepeatLength=27 seed=TGCTGGAGGA Num.seqs=60 Similarity=0.923806
2   GCTGCTGGAGGATCAACTGCATCAACC

************** ***********
Consensus:

GCTGCTGGAGGATCaACTGCATCAACc

>Cele-UNSB01_Fam_129_26_2 Nr. of seq. 2 Alignment length(with gaps) = 26
Alignment score = 1.000000
Cele-UNSB01_1:1685357-1686631 Satlength=1275 Nr of Repeats=49
RepeatLength=26 seed=CAAAACCGGC Num.seqs=49 Similarity=0.924036
0   CAAAACCGGCAATTGCCGAAAATTCC
Rev.of_Cele-UNSB01_1:1833668-1834030 Satlength=363 Nr of Repeats=11
RepeatLength=26 seed=TTGCCGGTTT Num.seqs=8 Similarity=0.798535
12   CAAAACCGGCAATTGCCGAAAATTCC

****
Consensus:

CAAAACCGGCAATTGCCGAAAATTCC

>Cele-UNSB01_Fam_130_26_2 Nr. of seq. 2 Alignment length(with gaps) = 26
Alignment score = 0.948718
Cele-UNSB01_2:2174137-2174267 Satlength=131 Nr of Repeats=5
RepeatLength=26 seed=TCAAGGCGAA Num.seqs=5 Similarity=0.979487
0   TCAAGGCGAAACGGGAGGTGAATCGC
Rev.of_Cele-UNSB01_5:8921030-8921237 Satlength=208 Nr of Repeats=8
RepeatLength=26 seed=TTCGCCCTGA Num.seqs=7 Similarity=0.907204
10   TCAGGGCGAAACGGGAGGTGAATCGC

*** **********************
Consensus:

TCAaGGCGAAACGGGAGGTGAATCGC

>Cele-UNSB01_Fam_131_24_2 Nr. of seq. 2 Alignment length(with gaps) = 24
Alignment score = 0.722222
Cele-UNSB01_2:3799242-3799338 Satlength=97 Nr of Repeats=4
RepeatLength=24 seed=TGCATCTGCC Num.seqs=4 Similarity=0.750000
0   TGCATCTGCCGCTGGAGCTGGCGC
Rev.of_Cele-UNSB01_5:14425209-14425464 Satlength=256 Nr of Repeats=5
RepeatLength=24 seed=GCCGCTCCAG Num.seqs=3 Similarity=0.814815
3   GGCATCTGGTGCCGGAGCTGGAGC

```
*******  ** ******** **
Consensus:

gGCATCTGccGCcGGAGCTGGaGC

>Cele-UNSB01_Fam_132_24_2  Nr. of seq. 2 Alignment length(with gaps) = 24
Alignment score = 0.680556
Cele-UNSB01_2:11502184-11502280 Satlength=97 Nr of Repeats=5
RepeatLength=24 seed=GCTTAGGCTT  Num.seqs=3 Similarity=0.648148
0    GCTTAGGCTTGGGCTTAGGCTNAA
Rev.of_Cele-UNSB01_5:19079815-19080004 Satlength=190 Nr of Repeats=5
RepeatLength=24 seed=GCCTAAGCCT  Num.seqs=3 Similarity=0.666667
2    GCCTAGACTCAGGCTTAGGCTTAG

** *** **  ********** *
Consensus:

GCcTAGaCTcaGGCTTAGGCTtAa

>Cele-UNSB01_Fam_133_23_2  Nr. of seq. 2 Alignment length(with gaps) = 23
Alignment score = 0.630435
Cele-UNSB01_1:932742-934750 Satlength=2009 Nr of Repeats=89
RepeatLength=21 seed=AATTTCTAGG  Num.seqs=60 Similarity=0.911757
0    AATTTCTAGGCCACGA-TTTGA-
Rev.of_Cele-UNSB01_1:1117625-1118154 Satlength=530 Nr of Repeats=23
RepeatLength=23 seed=GGCCTAGAAA  Num.seqs=23 Similarity=0.818525
12    TATTTCTAGGCCACCACGTTCAG

************* *  ** *
Consensus:

aATTTCTAGGCCACcAcgTTcAg

>Cele-UNSB01_Fam_134_23_2  Nr. of seq. 2 Alignment length(with gaps) = 23
Alignment score = 0.673913
Cele-UNSB01_5:19643678-19643940 Satlength=263 Nr of Repeats=11
RepeatLength=21 seed=TCTAATTAGA  Num.seqs=10 Similarity=0.939330
0    TCTAATTAGAT-CACCTT-TTTT
Cele-UNSB01_5:19645516-19645794 Satlength=279 Nr of Repeats=13
RepeatLength=21 seed=TTCTAATTAG  Num.seqs=10 Similarity=0.650000
20    -CTAATTAGATNCGCCNTCTTTT

********** * ** * ****
Consensus:

tCTAATTAGATnCaCCtTcTTTT

>Cele-UNSB01_Fam_135_23_2  Nr. of seq. 2 Alignment length(with gaps) = 23
Alignment score = 0.659420
Cele-UNSB01_5:19987417-19987506 Satlength=90 Nr of Repeats=4
RepeatLength=22 seed=ATTGGATTTT  Num.seqs=3 Similarity=0.959596
0    ATTGGATTTTTCACCCCAAAAA-
```

```
Cele-UNSB01_1:625093-626033 Satlength=941 Nr of Repeats=22
RepeatLength=23 seed=AAAAAAAATT  Num.seqs=14 Similarity=0.950948
16    ATTAAATTTTTCAACAAAAAAAA

***  ******** *  *****
Consensus:

ATTaaATTTTTCAaCaaAAAAAa

>Cele-UNSB01_Fam_136_22_2  Nr. of seq. 2 Alignment length(with gaps) = 22
Alignment score = 0.643939
Cele-UNSB01_3:1616576-1616906 Satlength=331 Nr of Repeats=16
RepeatLength=20 seed=CATAAAAATT  Num.seqs=13 Similarity=0.947009
0    CATAAAAAT-TCAGAAAA-ATG
Rev.of_Cele-UNSB01_1:14199204-14199288 Satlength=85 Nr of Repeats=4
RepeatLength=21 seed=ATATTTTGTG  Num.seqs=4 Similarity=0.936508
0    AATAAAAATGTCACAAAATAT-

******** *** **** **
Consensus:

aATAAAAATgTCAcAAAAtATg

>Cele-UNSB01_Fam_137_22_2  Nr. of seq. 2 Alignment length(with gaps) = 22
Alignment score = 0.681818
Cele-UNSB01_4:1966969-1971964 Satlength=4996 Nr of Repeats=213
RepeatLength=21 seed=GTAAATCTAC  Num.seqs=158 Similarity=0.856783
0    GTAAATCTACAGTAAT-NCCCC
Cele-UNSB01_3:1041359-1041618 Satlength=260 Nr of Repeats=12
RepeatLength=22 seed=TAAATCTACA  Num.seqs=10 Similarity=0.692929
1    GTAAATCTACACACATAGCCCC

***********   **  ****
Consensus:

GTAAATCTACAcaaATagCCCC

>Cele-UNSB01_Fam_138_22_2  Nr. of seq. 2 Alignment length(with gaps) = 22
Alignment score = 0.621212
Cele-UNSB01_4:16741502-16741626 Satlength=125 Nr of Repeats=5
RepeatLength=21 seed=TTCAAGCCTT  Num.seqs=3 Similarity=0.915344
0    TTCAAGCC-TTTTCAGACCAAT
Rev.of_Cele-UNSB01_1:14006347-14006515 Satlength=169 Nr of Repeats=7
RepeatLength=21 seed=CAAAATTCGT  Num.seqs=6 Similarity=0.814646
4    TTTGAGCNATTTTCGGACGAAT

**  ***  ***** *** ***
Consensus:

TTcaAGCcaTTTTCaGACcAAT

>Cele-UNSB01_Fam_139_22_2  Nr. of seq. 2 Alignment length(with gaps) = 22
Alignment score = 0.765152
```

Cele-UNSB01_6:1338339-1338633 Satlength=295 Nr of Repeats=14
RepeatLength=21 seed=TTTGCACTGA Num.seqs=14 Similarity=0.853480
0   TTTGCACTGACCAACACGAAT-
Rev.of_Cele-UNSB01_2:2125481-2125594 Satlength=114 Nr of Repeats=5
RepeatLength=22 seed=TGGTCAGTGC Num.seqs=3 Similarity=0.959596
13   TTTGCACTGACCAATAAAAATG

************** * ***
Consensus:

TTTGCACTGACCAAcAaaAATg

>Cele-UNSB01_Fam_140_22_2 Nr. of seq. 2 Alignment length(with gaps) = 22
Alignment score = 1.000000
Cele-UNSB01_1:13307625-13307715 Satlength=91 Nr of Repeats=4
RepeatLength=22 seed=TCGAGAAAAA Num.seqs=3 Similarity=1.000000
0   TCGAGAAAAAATCTGAAAAAAT
Rev.of_Cele-UNSB01_1:13324034-13324147 Satlength=114 Nr of Repeats=5
RepeatLength=22 seed=TTTTTTCTCG Num.seqs=4 Similarity=1.000000
11   TCGAGAAAAAATCTGAAAAAAT

****
Consensus:

TCGAGAAAAAATCTGAAAAAAT

>Cele-UNSB01_Fam_141_22_2 Nr. of seq. 2 Alignment length(with gaps) = 22
Alignment score = 0.954545
Cele-UNSB01_2:3341963-3342088 Satlength=126 Nr of Repeats=4
RepeatLength=22 seed=ATTCTGCGCA Num.seqs=3 Similarity=0.656566
0   ATTCTGCGCACCCCCTGCGCNG
Rev.of_Cele-UNSB01_5:19604230-19604355 Satlength=126 Nr of Repeats=4
RepeatLength=22 seed=GTGCGCAGAA Num.seqs=3 Similarity=0.676768
11   ATTCTGCGCACCCCCTGCGCAG

******************** *
Consensus:

ATTCTGCGCACCCCCTGCGCaG

>Cele-UNSB01_Fam_142_20_2 Nr. of seq. 2 Alignment length(with gaps) = 20
Alignment score = 0.933333
Cele-UNSB01_1:1781850-1784996 Satlength=3147 Nr of Repeats=154
RepeatLength=20 seed=CCACCAAAAA Num.seqs=145 Similarity=0.874815
0   CCACCAAAAAGGTTTCTAGG
Cele-UNSB01_1:1787086-1788479 Satlength=1394 Nr of Repeats=64
RepeatLength=20 seed=TTCTAGGCCA Num.seqs=51 Similarity=0.812235
13   CCACCAAAAATGTTTCTAGG

********** *********
Consensus:

CCACCAAAAAgGTTTCTAGG

>Cele-UNSB01_Fam_143_20_2 Nr. of seq. 2 Alignment length(with gaps) = 20
Alignment score = 0.950000
Cele-UNSB01_1:2365340-2366051 Satlength=712 Nr of Repeats=8
RepeatLength=20 seed=GGACTAGTTT Num.seqs=6 Similarity=0.880000
0   GGACTAGTTTTCGAGTAGAG
Cele-UNSB01_1:2365340-2366212 Satlength=873 Nr of Repeats=11
RepeatLength=20 seed=GGACTAGTTT Num.seqs=7 Similarity=0.745276
0   GGACTAGTTTTNGAGTAGAG

*********** ********
Consensus:

GGACTAGTTTTcGAGTAGAG

>Cele-UNSB01_Fam_144_20_2 Nr. of seq. 2 Alignment length(with gaps) = 20
Alignment score = 0.750000
Cele-UNSB01_2:15437952-15438032 Satlength=81 Nr of Repeats=4
RepeatLength=20 seed=TGTAGATTTA Num.seqs=4 Similarity=0.688889
0   TGTAGATTTACAAAGGACAA
Rev.of_Cele-UNSB01_5:18842565-18843912 Satlength=1348 Nr of Repeats=50
RepeatLength=20 seed=GTAAATCTAC Num.seqs=35 Similarity=0.635294
11   TGTAGATTTACGGAGGTCNA

*********** *** * *
Consensus:

TGTAGATTTACaaAGGaCaA

>Cele-UNSB01_Fam_145_20_2 Nr. of seq. 2 Alignment length(with gaps) = 20
Alignment score = 0.866667
Cele-UNSB01_5:2582229-2582433 Satlength=205 Nr of Repeats=7
RepeatLength=20 seed=CCCCGACGCT Num.seqs=5 Similarity=0.920000
0   CCCCGACGCTCGAGCTCAAA
Cele-UNSB01_5:2582360-2582560 Satlength=201 Nr of Repeats=10
RepeatLength=20 seed=GCTCGAGCTC Num.seqs=10 Similarity=0.928889
7   CCCCGAAGCTCGAGCTCAAG

****** ************
Consensus:

CCCCGAaGCTCGAGCTCAAa

>Cele-UNSB01_Fam_146_20_2 Nr. of seq. 2 Alignment length(with gaps) = 20
Alignment score = 0.800000
Cele-UNSB01_6:16421783-16421902 Satlength=120 Nr of Repeats=6
RepeatLength=20 seed=CCACCCTGTA Num.seqs=5 Similarity=0.706349
0   CCACCCTGTAATATCATGGA
Rev.of_Cele-UNSB01_6:16657827-16657967 Satlength=141 Nr of Repeats=7
RepeatLength=20 seed=TACAGGGTGG Num.seqs=5 Similarity=0.920000
10   CCACCCTGTATAATTATGGA

********** ** *****
Consensus:

CCACCCTGTAaaATcATGGA

>Cele-UNSB01_Fam_147_19_2 Nr. of seq. 2 Alignment length(with gaps) = 19
Alignment score = 0.684211
Cele-UNSB01_2:14294397-14294650 Satlength=254 Nr of Repeats=11
RepeatLength=18 seed=CTAGGTGCCT Num.seqs=8 Similarity=0.804233
0   CTAGGTGCCTA-TCCATTC
Rev.of_Cele-UNSB01_4:2898426-2898868 Satlength=443 Nr of Repeats=12
RepeatLength=19 seed=ACCTAGGCAT Num.seqs=8 Similarity=0.751880
6   CTAGGTGCCTACTTTATGC

*********** * ** *
Consensus:

CTAGGTGCCTAcTccATgC

>Cele-UNSB01_Fam_148_19_2 Nr. of seq. 2 Alignment length(with gaps) = 19
Alignment score = 0.929825
Cele-UNSB01_4:2676914-2677180 Satlength=267 Nr of Repeats=11
RepeatLength=19 seed=CGCCAAGTAT Num.seqs=8 Similarity=0.694345
0   CGCCAAGTATCGAGAAAAG
Rev.of_Cele-UNSB01_5:19666114-19666380 Satlength=267 Nr of Repeats=11
RepeatLength=19 seed=GATCCTTGGC Num.seqs=9 Similarity=0.898636
11   CGCCAAGGATCGAGAAAAG

******* ***********
Consensus:

CGCCAAGgATCGAGAAAAG

>Cele-UNSB01_Fam_149_17_2 Nr. of seq. 2 Alignment length(with gaps) = 17
Alignment score = 0.656863
Cele-UNSB01_2:14966230-14966326 Satlength=97 Nr of Repeats=6
RepeatLength=16 seed=GCAGTTTGCC Num.seqs=6 Similarity=0.794444
0   GCAGTTTGCC-GAATTG
Rev.of_Cele-UNSB01_6:16744464-16744698 Satlength=235 Nr of Repeats=10
RepeatLength=16 seed=TTCCGGCAAT Num.seqs=6 Similarity=0.972222
13   -CCGATTGCCGGAATTG

* * ***** ******
Consensus:

gCaGaTTGCCgGAATTG

>Cele-UNSB01_Fam_150_17_2 Nr. of seq. 2 Alignment length(with gaps) = 17
Alignment score = 0.803922
Cele-UNSB01_6:6441827-6441956 Satlength=130 Nr of Repeats=8
RepeatLength=16 seed=GCACTGACAA Num.seqs=7 Similarity=0.876984
0   GCACTGA-CAATTTTTT
Rev.of_Cele-UNSB01_6:1379546-1379614 Satlength=69 Nr of Repeats=4
RepeatLength=17 seed=TGGTCAGTGC Num.seqs=4 Similarity=0.882353
10   GCACTGACCAACTTTTT

```
******* *** *****
Consensus:

GCACTGAcCAAcTTTTT

>Cele-UNSB01_Fam_151_16_2  Nr. of seq. 2 Alignment length(with gaps) = 16
Alignment score = 0.750000
Cele-UNSB01_5:18664756-18664882 Satlength=127 Nr of Repeats=9
RepeatLength=14 seed=GTCTGGGGTC  Num.seqs=9 Similarity=0.978836
1    GGT-CTGGGGT-CTGG
Cele-UNSB01_5:20839531-20839647 Satlength=117 Nr of Repeats=5
RepeatLength=16 seed=GTACTAGGGT  Num.seqs=3 Similarity=0.888889
1    GGTACTGGGGTACTGG

** ******* *****
Consensus:

GTaCTGGGGTaCTGGG

>Cele-UNSB01_Fam_152_15_2  Nr. of seq. 2 Alignment length(with gaps) = 15
Alignment score = 0.622222
Cele-UNSB01_1:11276130-11276202 Satlength=73 Nr of Repeats=6
RepeatLength=12 seed=CGATCTCCAC  Num.seqs=6 Similarity=0.933333
0    CGAT-CTCCACCA--
Cele-UNSB01_1:11267568-11267793 Satlength=226 Nr of Repeats=15
RepeatLength=15 seed=CGATGCTCCA  Num.seqs=15 Similarity=0.900106
0    CGATGCTCCACCCGA

**** *******
Consensus:

CGATgCTCCACCaga

>Cele-UNSB01_Fam_153_14_2  Nr. of seq. 2 Alignment length(with gaps) = 14
Alignment score = 0.761905
Cele-UNSB01_2:14379544-14380198 Satlength=655 Nr of Repeats=13
RepeatLength=13 seed=TATACAAAAA  Num.seqs=9 Similarity=0.837607
0    TATACAAAAA-GTA
Rev.of_Cele-UNSB01_1:3764869-3765030 Satlength=162 Nr of Repeats=5
RepeatLength=14 seed=TTTTTGTAGA  Num.seqs=3 Similarity=0.588235
10    TCTACAAAAANGTA

* ******** ***
Consensus:

TaTACAAAAAnGTA

>Cele-UNSB01_Fam_154_14_2  Nr. of seq. 2 Alignment length(with gaps) = 14
Alignment score = 0.666667
Cele-UNSB01_5:1765405-1765535 Satlength=131 Nr of Repeats=9
RepeatLength=13 seed=GCTCAAGCTA  Num.seqs=6 Similarity=0.815385
0    GCTCAAGC-TAGGA
```

Cele-UNSB01_6:1926117-1926284 Satlength=168 Nr of Repeats=11
RepeatLength=14 seed=TAGGCCCTCA Num.seqs=8 Similarity=0.823129
9   CCTCAAGCGTAGGC

******* ****
Consensus:

cCTCAAGCgTAGGa

>Cele-UNSB01_Fam_155_14_2 Nr. of seq. 2 Alignment length(with gaps) = 14
Alignment score = 1.000000
Cele-UNSB01_2:7564245-7565607 Satlength=1363 Nr of Repeats=34
RepeatLength=14 seed=GAAGGATCGG Num.seqs=26 Similarity=0.910916
0   GAAGGATCGGGCCG
Rev.of_Cele-UNSB01_2:7568262-7569624 Satlength=1363 Nr of Repeats=34
RepeatLength=14 seed=CCGATCCTTC Num.seqs=26 Similarity=0.898608
10   GAAGGATCGGGCCG

****
Consensus:

GAAGGATCGGGCCG

>Cele-UNSB01_Fam_156_14_2 Nr. of seq. 2 Alignment length(with gaps) = 14
Alignment score = 0.619048
Cele-UNSB01_3:11522131-11522299 Satlength=169 Nr of Repeats=11
RepeatLength=14 seed=GATAATTGAA Num.seqs=10 Similarity=0.980952
0   GATAATTGAAAATC
Rev.of_Cele-UNSB01_3:11700261-11701084 Satlength=824 Nr of Repeats=59
RepeatLength=14 seed=AATTATAAAT Num.seqs=56 Similarity=0.980334
0   TATAATTTATAATT

****** * ***
Consensus:

gATAATTgAaAATc

>Cele-UNSB01_Fam_157_12_2 Nr. of seq. 2 Alignment length(with gaps) = 12
Alignment score = 0.680556
Cele-UNSB01_5:3042873-3042939 Satlength=67 Nr of Repeats=5
RepeatLength=11 seed=TTTGGCAAGA Num.seqs=4 Similarity=0.818182
0   TTGGGCAAGAA-
Rev.of_Cele-UNSB01_2:146899-146982 Satlength=84 Nr of Repeats=6
RepeatLength=12 seed=ATTTCTTGCA Num.seqs=5 Similarity=0.844444
1   TTTTGCAAGAAA

** *******
Consensus:

TTggGCAAGAAa

>Cele-UNSB01_Fam_158_11_2 Nr. of seq. 2 Alignment length(with gaps) = 11
Alignment score = 0.878788

Cele-UNSB01_1:2691842-2691886 Satlength=45 Nr of Repeats=4 RepeatLength=11 seed=AAACTTGGGC Num.seqs=4 Similarity=0.770833
0   AAACTTGGGCT
Cele-UNSB01_3:11327560-11327604 Satlength=45 Nr of Repeats=4 RepeatLength=11 seed=AAACTTGGGC Num.seqs=4 Similarity=0.858586
0   AAACTTGGGCA

****
Consensus:

AAACTTGGGCa

>Cele-UNSB01_Fam_159_11_2 Nr. of seq. 2 Alignment length(with gaps) = 11 Alignment score = 0.636364
Cele-UNSB01_2:13528433-13528477 Satlength=45 Nr of Repeats=4 RepeatLength=11 seed=TGGAAAATTG Num.seqs=4 Similarity=1.000000
0   TGGAAAATTGA
Cele-UNSB01_3:7825014-7825135 Satlength=122 Nr of Repeats=11 RepeatLength=11 seed=CGGATAATTA Num.seqs=11 Similarity=1.000000
0   CGGATAATTAA

*** **** *
Consensus:

cGGAaAATTaA

>Cele-UNSB01_Fam_160_11_2 Nr. of seq. 2 Alignment length(with gaps) = 11 Alignment score = 0.636364
Cele-UNSB01_6:444285-444373 Satlength=89 Nr of Repeats=7 RepeatLength=11 seed=CATAACTGAG Num.seqs=6 Similarity=1.000000
0   CATAACTGAGA
Rev.of_Cele-UNSB01_6:3482169-3482345 Satlength=177 Nr of Repeats=12 RepeatLength=11 seed=TAGCTCAGCT Num.seqs=8 Similarity=0.883117
2   TATAGCTGAGC

*** *****
Consensus:

cATAaCTGAGa

>Cele-UNSB01_Fam_161_255_1 Nr. of seq. 1 Alignment length(with gaps) = 255 Alignment score = 0.000000
Cele-UNSB01_5:17737089-17738916 Satlength=1828 Nr of Repeats=7 RepeatLength=255 seed=AACAAGTCGG Num.seqs=5 Similarity=0.720930
0
AACAAGTCGGGAACGTTCGGAAAGCTCTGAGGAAAACTCCGACAATATCATGCTTAAGTACGCAATTGAGCTA
TCTTTATTAGATTCGAAAAAGGACTCTTCAAAAATTGAAGCAATAACGAAGAAGCTTAAGTTGGAACTGGCGA
AGCTTAAATTGGGANTTATGCAGTCTTTATCAGATTCAAAAGACGATTCTTCGACCAATGAAGCAAGTCCGGA
GNACGATCTACCTTCGACTTCTGATGGCAGAACTCC

****
****
****
****

Consensus:

AACAAGTCGGGAACGTTCGGAAAGCTCTGAGGAAAACTCCGACAATATCATGCTTAAGTACGCAATTGAGCTA
TCTTTATTAGATTCGAAAAAGGACTCTTCAAAAATTGAAGCAATAACGAAGAAGCTTAAGTTGGAACTGGCGA
AGCTTAAATTGGGANTTATGCAGTCTTTATCAGATTCAAAAGACGATTCTTCGACCAATGAAGCAAGTCCGGA
GNACGATCTACCTTCGACTTCTGATGGCAGAACTCC

>Cele-UNSB01_Fam_162_249_1 Nr. of seq. 1 Alignment length(with gaps) =
249 Alignment score = 0.000000
Cele-UNSB01_6:1598138-1600934 Satlength=2797 Nr of Repeats=10
RepeatLength=249 seed=CTGGAAGCAC Num.seqs=7 Similarity=0.929623
0
CTGGAAGCACTGTAACCATGGGATCATCAAGCACTTCCGGTGTTTCCACATCTTCAGCATCCAGTACTCAGCC
TCAGATGAGCACTTCACAAGGAAGTTCAGCAGGATCCACAGTTGCTTCATCAACCGCTGGCCTTGTTTCAACT
TCCACAGTTCCATCCTCGACAGGAACAATGGGCTCCACTTCTAGTGGAACAGTTGGATCTACAATTTCTGAAT
CATCGACAACAGCATCTGCAAGTTCTCAGA

****
****
****
****
Consensus:

CTGGAAGCACTGTAACCATGGGATCATCAAGCACTTCCGGTGTTTCCACATCTTCAGCATCCAGTACTCAGCC
TCAGATGAGCACTTCACAAGGAAGTTCAGCAGGATCCACAGTTGCTTCATCAACCGCTGGCCTTGTTTCAACT
TCCACAGTTCCATCCTCGACAGGAACAATGGGCTCCACTTCTAGTGGAACAGTTGGATCTACAATTTCTGAAT
CATCGACAACAGCATCTGCAAGTTCTCAGA

>Cele-UNSB01_Fam_163_231_1 Nr. of seq. 1 Alignment length(with gaps) =
231 Alignment score = 0.000000
Cele-UNSB01_1:10434433-10461186 Satlength=26754 Nr of Repeats=116
RepeatLength=231 seed=GCTGAATTCA Num.seqs=92 Similarity=0.829532
0
GCTGAATTCAGTTGGATATGAATGCACTTCCGTTGTGAGTGGAATATCATGAATGTTNGATGCTCGTGAAGTA
GANTCNAGTGGTCCATCATAAGATTCAGACACTGGATAATCAAGATGAGATGGTGATTTCTTGAACAATCCAG
TGATCTTAGATTTCAGTGATGGCTCGTCCTTCTTCTCTGTCTCCTCGATCACAACTGGAATGACATGCTCTGG
CAGCTTCTCATA

****
****
****
****
Consensus:

GCTGAATTCAGTTGGATATGAATGCACTTCCGTTGTGAGTGGAATATCATGAATGTTNGATGCTCGTGAAGTA
GANTCNAGTGGTCCATCATAAGATTCAGACACTGGATAATCAAGATGAGATGGTGATTTCTTGAACAATCCAG
TGATCTTAGATTTCAGTGATGGCTCGTCCTTCTTCTCTGTCTCCTCGATCACAACTGGAATGACATGCTCTGG
CAGCTTCTCATA

>Cele-UNSB01_Fam_164_228_1 Nr. of seq. 1 Alignment length(with gaps) =
228 Alignment score = 0.000000
Cele-UNSB01_3:6120416-6122897 Satlength=2482 Nr of Repeats=10
RepeatLength=228 seed=CCACCAGATC Num.seqs=6 Similarity=0.843765
0

CCACCAGATCAGCAACGTCTCATCTTCGCCGGAAAGCAACTCGAGGACGGCCGCACCCTTTCGGACTACAACA
TCCAGAAGGAATCAACTCTTCATTTGGTTCTCCGTCTGAGAGGAGGTATGCAGATCTTCGTCAAGACATTGAC
CGGAAAGACCATCACCCTCGAAGTCGAAGCCTCCGACACCATCGAAAATGTCAAGGCCAAGATCCAAGACAAG
GAAGGAATC

****
****
****
****
Consensus:

CCACCAGATCAGCAACGTCTCATCTTCGCCGGAAAGCAACTCGAGGACGGCCGCACCCTTTCGGACTACAACA
TCCAGAAGGAATCAACTCTTCATTTGGTTCTCCGTCTGAGAGGAGGTATGCAGATCTTCGTCAAGACATTGAC
CGGAAAGACCATCACCCTCGAAGTCGAAGCCTCCGACACCATCGAAAATGTCAAGGCCAAGATCCAAGACAAG
GAAGGAATC

>Cele-UNSB01_Fam_165_189_1 Nr. of seq. 1 Alignment length(with gaps) =
189 Alignment score = 0.000000
Cele-UNSB01_1:1620106-1621181 Satlength=1076 Nr of Repeats=5
RepeatLength=189 seed=TTTTTTTTGA Num.seqs=3 Similarity=1.000000
0
TTTTTTTTGACTATGGAATTATAAAGAAAATCGAATTTCAAAAAAGCATGTATTAGCCAATGTAAACGCGCTC
CAATGATGATGTAGCCCACGTGGTATCAGAGTGTCTCTTTACGGCTTGATTTAAATACGTAGATCTACAAATT
CACTTATCTGTTTTCTCACGGGTTTAGGATATTTTTTCTGTTA

****
****
****
Consensus:

TTTTTTTTGACTATGGAATTATAAAGAAAATCGAATTTCAAAAAAGCATGTATTAGCCAATGTAAACGCGCTC
CAATGATGATGTAGCCCACGTGGTATCAGAGTGTCTCTTTACGGCTTGATTTAAATACGTAGATCTACAAATT
CACTTATCTGTTTTCTCACGGGTTTAGGATATTTTTTCTGTTA

>Cele-UNSB01_Fam_166_184_1 Nr. of seq. 1 Alignment length(with gaps) =
184 Alignment score = 0.000000
Cele-UNSB01_2:891851-892689 Satlength=839 Nr of Repeats=4
RepeatLength=184 seed=GTTCAAAAGA Num.seqs=3 Similarity=1.000000
0
GTTCAAAAGATACAAAATTTATCTACACATCTACAAGTTAAACTTGCAACTTAGTTAACCAAATTCAAAAGTT
ATGAGCCGGGGAACTATAATAACCAACGTTTCACACTAAAAAAGCCCAAAAAAATTCGAAAATCATTTTTATC
TCATGACGCGGACAAGCTACAAATGTCTAGTTAGGCTT

****
****
****
Consensus:

GTTCAAAAGATACAAAATTTATCTACACATCTACAAGTTAAACTTGCAACTTAGTTAACCAAATTCAAAAGTT
ATGAGCCGGGGAACTATAATAACCAACGTTTCACACTAAAAAAGCCCAAAAAAATTCGAAAATCATTTTTATC
TCATGACGCGGACAAGCTACAAATGTCTAGTTAGGCTT

>Cele-UNSB01_Fam_167_183_1 Nr. of seq. 1 Alignment length(with gaps) =
183 Alignment score = 0.000000

Cele-UNSB01_5:19795988-19796719 Satlength=732 Nr of Repeats=4 RepeatLength=183 seed=TTTCTCTGCG Num.seqs=3 Similarity=0.615789
0
TTTCTCTGCGTCTCGCCTCGCCTCACACTATTTTNCACACCAAACTTTGAGNCTATGTATCTCTGTAACGGGT
TGCGCTTTCTGAAAAATTCCAACTGACGGGATATATAAAATTTNGAATTCTTTCTCACCGTTAAAAAACTAAA
CACCTAGGCAAGACGCAGACAGCGAGANACCCCCAAT

****
****
****
Consensus:

TTTCTCTGCGTCTCGCCTCGCCTCACACTATTTTNCACACCAAACTTTGAGNCTATGTATCTCTGTAACGGGT
TGCGCTTTCTGAAAAATTCCAACTGACGGGATATATAAAATTTNGAATTCTTTCTCACCGTTAAAAAACTAAA
CACCTAGGCAAGACGCAGACAGCGAGANACCCCCAAT

>Cele-UNSB01_Fam_168_181_1 Nr. of seq. 1 Alignment length(with gaps) = 181 Alignment score = 0.000000
Cele-UNSB01_2:6168224-6172025 Satlength=3802 Nr of Repeats=21 RepeatLength=181 seed=TTTTTAACTT Num.seqs=21 Similarity=0.964746
0
TTTTTAACTTTTAAATCCACTAAAACTCGGAATGGTACAGAACATGCAAATTGTAATTTCAAGAACTGAATGT
GGTTTCGAATATTTATGGATTGCTTTATTCTAAAACTGACTCTGAAATATTTTGCATCCATTTGACTACTAAT
TGTCATATTATACCGAACACTACTAACTTTAGTGG

****
****
****
Consensus:

TTTTTAACTTTTAAATCCACTAAAACTCGGAATGGTACAGAACATGCAAATTGTAATTTCAAGAACTGAATGT
GGTTTCGAATATTTATGGATTGCTTTATTCTAAAACTGACTCTGAAATATTTTGCATCCATTTGACTACTAAT
TGTCATATTATACCGAACACTACTAACTTTAGTGG

>Cele-UNSB01_Fam_169_180_1 Nr. of seq. 1 Alignment length(with gaps) = 180 Alignment score = 0.000000
Cele-UNSB01_3:3099001-3099720 Satlength=720 Nr of Repeats=4 RepeatLength=180 seed=TGTCGGCCGC Num.seqs=3 Similarity=0.750153
0
TGTCGGCCGCTGTATCTTTTGCCTCAATTTTTTTTNAATTTTNAAACATNCATATTCCAGTAGATAACATTAT
GAGCTACAGTTTGTTAGTTAAAATTTTATTTCTAAAATTACTAGTTTCTGAGTTATACTCATTCCAACCCACC
CCAAGAGAAATTTCCTAAAAATGAACTCGCGAGG

****
****
****
Consensus:

TGTCGGCCGCTGTATCTTTTGCCTCAATTTTTTTTNAATTTTNAAACATNCATATTCCAGTAGATAACATTAT
GAGCTACAGTTTGTTAGTTAAAATTTTATTTCTAAAATTACTAGTTTCTGAGTTATACTCATTCCAACCCACC
CCAAGAGAAATTTCCTAAAAATGAACTCGCGAGG

>Cele-UNSB01_Fam_170_179_1 Nr. of seq. 1 Alignment length(with gaps) = 179 Alignment score = 0.000000

Cele-UNSB01_1:14266018-14266935 Satlength=918 Nr of Repeats=5
RepeatLength=179 seed=CTACCCAAAT Num.seqs=3 Similarity=0.995034
0
CTACCCAAATTTTTGAAATTTGGAAATTTTTCAAAAATTCTCAAATTTTCAAAATCCCATAATTCAGCCAATT
TTTGAGATTTTCAGATATTTTTTAATTGAAAATTCTGTTTGTCTTATCCATTAAACTATATCGCTGCATCTAC
ATTTTTCACAGTACCCCATGACTTTTCCCGATT

****
****
****
Consensus:

CTACCCAAATTTTTGAAATTTGGAAATTTTTCAAAAATTCTCAAATTTTCAAAATCCCATAATTCAGCCAATT
TTTGAGATTTTCAGATATTTTTTAATTGAAAATTCTGTTTGTCTTATCCATTAAACTATATCGCTGCATCTAC
ATTTTTCACAGTACCCCATGACTTTTCCCGATT

>Cele-UNSB01_Fam_171_177_1 Nr. of seq. 1 Alignment length(with gaps) =
177 Alignment score = 0.000000
Cele-UNSB01_1:14392151-14393037 Satlength=887 Nr of Repeats=5
RepeatLength=177 seed=CTAACAAAAT Num.seqs=4 Similarity=0.721286
0
CTAACAAAATAATCAGCGTAAAATTTCCTACATTTTACTAGTTAACAATATTTTGATAACATCAGTGGTTTTT
GAGATACGCAAGTTTTAAGGTGGACGCCCAAAAATAGTTGTGAGCAGCTTTAATCAAAAACCGACATAACTTT
TTACTCACTAACGGTATCAAAAATGTTTTAA

****
****
****
Consensus:

CTAACAAAATAATCAGCGTAAAATTTCCTACATTTTACTAGTTAACAATATTTTGATAACATCAGTGGTTTTT
GAGATACGCAAGTTTTAAGGTGGACGCCCAAAAATAGTTGTGAGCAGCTTTAATCAAAAACCGACATAACTTT
TTACTCACTAACGGTATCAAAAATGTTTTAA

>Cele-UNSB01_Fam_172_177_1 Nr. of seq. 1 Alignment length(with gaps) =
177 Alignment score = 0.000000
Cele-UNSB01_5:2439353-2440062 Satlength=710 Nr of Repeats=4
RepeatLength=177 seed=GAGTCTCACG Num.seqs=3 Similarity=0.943196
0
GAGTCTCACGATGTGTCAACGTGTCTCAGTCGGCTTGATCTACGTAGATCTACAAAAAATGCGGGAGAAGAGA
CGCAGACTTATTCTCTGATTTTGCATGAGTAAGAGCGTGCTGGCGTCACATTTTTGTCGGCAAAAAATTCCCG
CATTTTTTGTAGATCAAACCGTAATGGGACG

****
****
****
Consensus:

GAGTCTCACGATGTGTCAACGTGTCTCAGTCGGCTTGATCTACGTAGATCTACAAAAAATGCGGGAGAAGAGA
CGCAGACTTATTCTCTGATTTTGCATGAGTAAGAGCGTGCTGGCGTCACATTTTTGTCGGCAAAAAATTCCCG
CATTTTTTGTAGATCAAACCGTAATGGGACG

>Cele-UNSB01_Fam_173_175_1 Nr. of seq. 1 Alignment length(with gaps) =
175 Alignment score = 0.000000

Cele-UNSB01_1:2461739-2462720 Satlength=982 Nr of Repeats=5
RepeatLength=175 seed=GATGCACCAT Num.seqs=3 Similarity=0.840552
0
GATGCACCATGTCTTTGACAGTCAATATCTCGGTTTATTTTGTAGCTATCAAAAAATTTTCAACTACAAATCT
ATAGAAAATTAAATGTTCAACATTTTCTCAGTTGACAGTTTTTTGATAACTGCAATGACATCCGAGATATGAG
CTTTCAAAGTTGAGAGACCTCACTCGGTT

****
****
****
Consensus:

GATGCACCATGTCTTTGACAGTCAATATCTCGGTTTATTTTGTAGCTATCAAAAAATTTTCAACTACAAATCT
ATAGAAAATTAAATGTTCAACATTTTCTCAGTTGACAGTTTTTTGATAACTGCAATGACATCCGAGATATGAG
CTTTCAAAGTTGAGAGACCTCACTCGGTT

>Cele-UNSB01_Fam_174_175_1 Nr. of seq. 1 Alignment length(with gaps) =
175 Alignment score = 0.000000
Cele-UNSB01_4:2701520-2702341 Satlength=822 Nr of Repeats=4
RepeatLength=175 seed=AACTTGTGAC Num.seqs=3 Similarity=0.715980
0
AACTTGTGACGGTGGGTATGGATTTTATGCACATAATTTGTTCGAAATAGTCTACCNAGTAAACTGGTGAAGT
TTCAAAAACTTTGTGGATCTAGTTTTTGAAAAAAGTTCAAAATTGTGCCAATTTTGACCAATTTTGGTGGTTT
TCTGAGCTAAAATTGAAAAGTTGGGAATG

****
****
****
Consensus:

AACTTGTGACGGTGGGTATGGATTTTATGCACATAATTTGTTCGAAATAGTCTACCNAGTAAACTGGTGAAGT
TTCAAAAACTTTGTGGATCTAGTTTTTGAAAAAAGTTCAAAATTGTGCCAATTTTGACCAATTTTGGTGGTTT
TCTGAGCTAAAATTGAAAAGTTGGGAATG

>Cele-UNSB01_Fam_175_175_1 Nr. of seq. 1 Alignment length(with gaps) =
175 Alignment score = 0.000000
Cele-UNSB01_5:328030-329092 Satlength=1063 Nr of Repeats=6
RepeatLength=175 seed=AAATTTTGGG Num.seqs=4 Similarity=0.911111
0
AAATTTTGGGTTCACTCTTTTTACTAGACAGTGATTGTTGCATTGTAGGATTCGGTGGACTTGTCCAACAACT
TACTTTGGCATGAGTTTAATAAAAAGGTTTTACCATGAACTGAATCAATTCAAAATAAAAACCTCGAGAATGA
AAACATCCTCAGTAATTTCAATCCTGTTC

****
****
****
Consensus:

AAATTTTGGGTTCACTCTTTTTACTAGACAGTGATTGTTGCATTGTAGGATTCGGTGGACTTGTCCAACAACT
TACTTTGGCATGAGTTTAATAAAAAGGTTTTACCATGAACTGAATCAATTCAAAATAAAAACCTCGAGAATGA
AAACATCCTCAGTAATTTCAATCCTGTTC

>Cele-UNSB01_Fam_176_174_1 Nr. of seq. 1 Alignment length(with gaps) =
174 Alignment score = 0.000000

Cele-UNSB01_1:4404167-4404863 Satlength=697 Nr of Repeats=4
RepeatLength=174 seed=CTTCAAGAGC Num.seqs=4 Similarity=0.868455
0
CTTCAAGAGCTCAGTGTTATTTATGACATGCCAACCTCGACAATTGACCTGAACATGCTCCAAGGAATCGAAG
ACAACTTGAACAGCCTTCCAGCTGAAGAATCTGATAAGATCCGAGAGAAGATTAACGATCTGAGAAGAAGGAA
ACAAGAAAGCGATCAAGCTGAAGCTCTT

****
****
****
Consensus:

CTTCAAGAGCTCAGTGTTATTTATGACATGCCAACCTCGACAATTGACCTGAACATGCTCCAAGGAATCGAAG
ACAACTTGAACAGCCTTCCAGCTGAAGAATCTGATAAGATCCGAGAGAAGATTAACGATCTGAGAAGAAGGAA
ACAAGAAAGCGATCAAGCTGAAGCTCTT

>Cele-UNSB01_Fam_177_165_1 Nr. of seq. 1 Alignment length(with gaps) =
165 Alignment score = 0.000000
Cele-UNSB01_6:1780465-1781641 Satlength=1177 Nr of Repeats=4
RepeatLength=165 seed=CTTTTCCAAA Num.seqs=3 Similarity=1.000000
0
CTTTTCCAAAATGGCAATTTTTGATGAAAAAAAATCCGAATTTTCACAAATTTCAAAAAAATTTTTAAACTTTA
TAATTTTAGCTTTGAATATTCCAAATGGAACTGTCATAACCTATCAATTTGATATATAAACTCGTGCCTACCT
TCAGGCATTCAGTTTTCAC

****
****
****
Consensus:

CTTTTCCAAAATGGCAATTTTTGATGAAAAAAAATCCGAATTTTCACAAATTTCAAAAAAATTTTTAAACTTTA
TAATTTTAGCTTTGAATATTCCAAATGGAACTGTCATAACCTATCAATTTGATATATAAACTCGTGCCTACCT
TCAGGCATTCAGTTTTCAC

>Cele-UNSB01_Fam_178_155_1 Nr. of seq. 1 Alignment length(with gaps) =
155 Alignment score = 0.000000
Cele-UNSB01_2:2979318-2980713 Satlength=1396 Nr of Repeats=9
RepeatLength=155 seed=TCCCGCTGTG Num.seqs=6 Similarity=0.781691
0
TCCCGCTGTGGTTAAAAGTTTTTTGCGTGCTCAGTTCTGTACGGTAAATGTGTTAAAAACTTCTGGAAACTTG
AAATTCCAAGTTATTCAGAAAATTGTTCAAATGGCGCAGTGGGAAATTCGATTTCTCAGACCCAAGAGGCCAC
GGGTTCAAT

****
****
****
Consensus:

TCCCGCTGTGGTTAAAAGTTTTTTGCGTGCTCAGTTCTGTACGGTAAATGTGTTAAAAACTTCTGGAAACTTG
AAATTCCAAGTTATTCAGAAAATTGTTCAAATGGCGCAGTGGGAAATTCGATTTCTCAGACCCAAGAGGCCAC
GGGTTCAAT

>Cele-UNSB01_Fam_179_153_1 Nr. of seq. 1 Alignment length(with gaps) =
153 Alignment score = 0.000000

Cele-UNSB01_6:14250466-14251066 Satlength=601 Nr of Repeats=4 RepeatLength=153 seed=TGAGGTGCAG Num.seqs=3 Similarity=0.872186
0
TGAGGTGCAGGGCGTGATGAGCTTTGTTGAACTGGGAACTGTTGATTCCCGGAGCCGTGATTCATAGATGAGT
TGGAGGGACCTGCTGTGGAACTTGTTTCAACTTGAGCAATTCCATGAGCCATCAAGAAGCTTTCCGGCAGATT
GGGCAAC

****
****
****
Consensus:

TGAGGTGCAGGGCGTGATGAGCTTTGTTGAACTGGGAACTGTTGATTCCCGGAGCCGTGATTCATAGATGAGT
TGGAGGGACCTGCTGTGGAACTTGTTTCAACTTGAGCAATTCCATGAGCCATCAAGAAGCTTTCCGGCAGATT
GGGCAAC

>Cele-UNSB01_Fam_180_151_1 Nr. of seq. 1 Alignment length(with gaps) = 151 Alignment score = 0.000000
Cele-UNSB01_5:14155473-14156076 Satlength=604 Nr of Repeats=4 RepeatLength=151 seed=CATTTCAGAT Num.seqs=3 Similarity=0.988227
0
CATTTCAGATATTCCATCACCACCAACACCTTCATCCACTTCTTCGCCTGGAACGACGGGATTTTCTACAAAA
ATCGGATTTTTTCTCTATGTCAGTTTAATTGATGTATAATTCCGAAAAGTCAGTCTTTCCTAGGTTTTTAAAT
TGAAA

****
****
*****
Consensus:

CATTTCAGATATTCCATCACCACCAACACCTTCATCCACTTCTTCGCCTGGAACGACGGGATTTTCTACAAAA
ATCGGATTTTTTCTCTATGTCAGTTTAATTGATGTATAATTCCGAAAAGTCAGTCTTTCCTAGGTTTTTAAAT
TGAAA

>Cele-UNSB01_Fam_181_150_1 Nr. of seq. 1 Alignment length(with gaps) = 150 Alignment score = 0.000000
Cele-UNSB01_2:1517382-1518287 Satlength=906 Nr of Repeats=6 RepeatLength=150 seed=TTGCAACATG Num.seqs=4 Similarity=0.817145
0
TTGCAACATGCGGTCGGCGGAAAATTCGAATTTTTCAGTCCAAAACAGGTTTTTCGACCAATTTTTCATATTT
TCAAGCGAAATTATGATAGAAAATTCAAATTTTCACTAAAAATCGCCAATTTTCCACATTTTCTCATTGGAGC
GCAC

****
****
****
Consensus:

TTGCAACATGCGGTCGGCGGAAAATTCGAATTTTTCAGTCCAAAACAGGTTTTTCGACCAATTTTTCATATTT
TCAAGCGAAATTATGATAGAAAATTCAAATTTTCACTAAAAATCGCCAATTTTCCACATTTTCTCATTGGAGC
GCAC

>Cele-UNSB01_Fam_182_150_1 Nr. of seq. 1 Alignment length(with gaps) = 150 Alignment score = 0.000000

Cele-UNSB01_4:16931676-16932199 Satlength=524 Nr of Repeats=4
RepeatLength=150 seed=TTGAGGGCCG Num.seqs=3 Similarity=0.940741
0
TTGAGGGCCGCAGTCCCGAATACACGAAGAAGTGTTGGAGATCCGTCAAGCCTATTCTAAGCATAATAATTCA
GTTCGAGTGTCGCAAGCCCAAATAGACGCAGAAGCGTTGGACAACTTTTAGGCCTATTCTGAGCCCAATAATT
CATG

****
****
****
Consensus:

TTGAGGGCCGCAGTCCCGAATACACGAAGAAGTGTTGGAGATCCGTCAAGCCTATTCTAAGCATAATAATTCA
GTTCGAGTGTCGCAAGCCCAAATAGACGCAGAAGCGTTGGACAACTTTTAGGCCTATTCTGAGCCCAATAATT
CATG

>Cele-UNSB01_Fam_183_146_1 Nr. of seq. 1 Alignment length(with gaps) =
146 Alignment score = 0.000000
Cele-UNSB01_2:2836927-2837967 Satlength=1041 Nr of Repeats=6
RepeatLength=144 seed=TGGCCTAGAA Num.seqs=4 Similarity=0.634815
0
TGGCCTAGAAATCNCAAGTTTGCAAAAGTTAGGCCACCAACTTCAAATGCCTATAACTCAGCGGAAAATCAAT
ATTTTCCATTGATTTTAACTGCTAAGATGTAGCCAATTACGTAGCGCAACTTTTTGGCCTACTTAACAATTGG

****
****
Consensus:

TGGCCTAGAAATCNCAAGTTTGCAAAAGTTAGGCCACCAACTTCAAATGCCTATAACTCAGCGGAAAATCAAT
ATTTTCCATTGATTTTAACTGCTAAGATGTAGCCAATTACGTAGCGCAACTTTTTGGCCTACTTAACAATTGG

>Cele-UNSB01_Fam_184_144_1 Nr. of seq. 1 Alignment length(with gaps) =
144 Alignment score = 0.000000
Cele-UNSB01_4:2456640-2457356 Satlength=717 Nr of Repeats=5
RepeatLength=144 seed=TTCTAGGCCA Num.seqs=4 Similarity=0.915326
0
TTCTAGGCCACCAATTGGAAATCATCATAACTTGGCTTAAAATCCAGATTTTTGGAGTCTGGTAACTGCAACA
TGTTGCCCTGTAGTTTTTCTACAAGTTAGGATATATTAATTTTTGGTGGCCTAAAAGTCCAAATTATGGAT

****
****
Consensus:

TTCTAGGCCACCAATTGGAAATCATCATAACTTGGCTTAAAATCCAGATTTTTGGAGTCTGGTAACTGCAACA
TGTTGCCCTGTAGTTTTTCTACAAGTTAGGATATATTAATTTTTGGTGGCCTAAAAGTCCAAATTATGGAT

>Cele-UNSB01_Fam_185_143_1 Nr. of seq. 1 Alignment length(with gaps) =
143 Alignment score = 0.000000
Cele-UNSB01_5:17962175-17963525 Satlength=1351 Nr of Repeats=6
RepeatLength=143 seed=CAGTGGGATT Num.seqs=4 Similarity=0.759132
0
CAGTGGGATTTTCAATGTCTTCCAATCAAACGTCCGGGGTTCGAGTCCGCACAGGGGTATTTATTTTTTGCAA
GCTGTGATTAACAAGTTTATAGCTGCAAAAGTCATTTTAATCGAACTTTCAGGAAAATGTCCGAATGGCG

****
****
Consensus:

CAGTGGGATTTTCAATGTCTTCCAATCAAACGTCCGGGGTTCGAGTCCGCACAGGGGTATTTATTTTTTGCAA
GCTGTGATTAACAAGTTTATAGCTGCAAAAGTCATTTTAATCGAACTTTCAGGAAAATGTCCGAATGGCG

>Cele-UNSB01_Fam_186_124_1 Nr. of seq. 1 Alignment length(with gaps) =
124 Alignment score = 0.000000
Cele-UNSB01_2:11256637-11258512 Satlength=1876 Nr of Repeats=15
RepeatLength=124 seed=AATTTTCAAA Num.seqs=9 Similarity=0.708025
0
AATTTTCAAAGTTTGGAAAATCTATTAATTTTGCATATAATTTTTATAAATTCTGAAAGAACATAAAATTTGC
TACAAAATGGCTACGGGAATTTCTAAAAAATTTAGTTTGAAGNCAGAAATT

****
****
Consensus:

AATTTTCAAAGTTTGGAAAATCTATTAATTTTGCATATAATTTTTATAAATTCTGAAAGAACATAAAATTTGC
TACAAAATGGCTACGGGAATTTCTAAAAAATTTAGTTTGAAGNCAGAAATT

>Cele-UNSB01_Fam_187_122_1 Nr. of seq. 1 Alignment length(with gaps) =
122 Alignment score = 0.000000
Cele-UNSB01_2:13490523-13491161 Satlength=639 Nr of Repeats=5
RepeatLength=122 seed=GATCTACAAA Num.seqs=3 Similarity=0.927140
0
GATCTACAAAAATGCGGGAAAAGTGACACAGAGTTCTCAACTGATTTTGCATAAAAATTCCCGCATTTTTTGG
AGATCAAACCGTGATGGGACAGCCTGGCACCACTTGCTTGATCTACGTA

****
****
Consensus:

GATCTACAAAAATGCGGGAAAAGTGACACAGAGTTCTCAACTGATTTTGCATAAAAATTCCCGCATTTTTTGG
AGATCAAACCGTGATGGGACAGCCTGGCACCACTTGCTTGATCTACGTA

>Cele-UNSB01_Fam_188_121_1 Nr. of seq. 1 Alignment length(with gaps) =
121 Alignment score = 0.000000
Cele-UNSB01_5:16591935-16597985 Satlength=6051 Nr of Repeats=50
RepeatLength=121 seed=GTTGCAAACC Num.seqs=50 Similarity=0.958478
0
GTTGCAAACCGCAAGAGCAAACTTGCCAAATGAAAAGCCACCCAAGTACTCGTTGCACCTACCTATCATAAGC
TCGTAGACTATAATTTCTCTGAAAAGATAGTTGCCCTAATAGCAGAAG

****
****
Consensus:

GTTGCAAACCGCAAGAGCAAACTTGCCAAATGAAAAGCCACCCAAGTACTCGTTGCACCTACCTATCATAAGC
TCGTAGACTATAATTTCTCTGAAAAGATAGTTGCCCTAATAGCAGAAG

>Cele-UNSB01_Fam_189_119_1 Nr. of seq. 1 Alignment length(with gaps) = 119 Alignment score = 0.000000
Cele-UNSB01_6:309569-317496 Satlength=7928 Nr of Repeats=66 RepeatLength=119 seed=GGCCACCGTT Num.seqs=63 Similarity=0.981664
0
GGCCACCGTTGCATATTTCGCAATAATTTTTACGGGAACGTAATCTCCACGTCACCAGCTTTCATTTAAGTAC
CCACACGGCAAGGTTTAATTTTTGAAATTTGGTCGCGGATTTTCTC

****
****
Consensus:

GGCCACCGTTGCATATTTCGCAATAATTTTTACGGGAACGTAATCTCCACGTCACCAGCTTTCATTTAAGTAC
CCACACGGCAAGGTTTAATTTTTGAAATTTGGTCGCGGATTTTCTC

>Cele-UNSB01_Fam_190_117_1 Nr. of seq. 1 Alignment length(with gaps) = 117 Alignment score = 0.000000
Cele-UNSB01_5:6179564-6194498 Satlength=14935 Nr of Repeats=110 RepeatLength=117 seed=TACTTCAGAT Num.seqs=94 Similarity=0.905974
0
TACTTCAGATGTTTCCTTTGATTCCACAGGTGCAAGTTTCTCAACTGTTGGTTCGACAGTAGGTGCACTAGTC
TCTGGCACTGGAACATCCTTTTGCTCAACAATTTCAGCTGGTTG

****
****
Consensus:

TACTTCAGATGTTTCCTTTGATTCCACAGGTGCAAGTTTCTCAACTGTTGGTTCGACAGTAGGTGCACTAGTC
TCTGGCACTGGAACATCCTTTTGCTCAACAATTTCAGCTGGTTG

>Cele-UNSB01_Fam_191_114_1 Nr. of seq. 1 Alignment length(with gaps) = 114 Alignment score = 0.000000
Cele-UNSB01_5:8785418-8789720 Satlength=4303 Nr of Repeats=37 RepeatLength=114 seed=GTGTTTCAGA Num.seqs=35 Similarity=0.970986
0
GTGTTTCAGAATTACATGGAGTCTCACTACTTGCAGAATTTGTGGCTGTAGACGATGGGCCTGCTGTAGTTGA
ATCGTTTGGAACTGTCATAGTGGATGTTCCATCAGACGTTT

****
****
Consensus:

GTGTTTCAGAATTACATGGAGTCTCACTACTTGCAGAATTTGTGGCTGTAGACGATGGGCCTGCTGTAGTTGA
ATCGTTTGGAACTGTCATAGTGGATGTTCCATCAGACGTTT

>Cele-UNSB01_Fam_192_108_1 Nr. of seq. 1 Alignment length(with gaps) = 108 Alignment score = 0.000000
Cele-UNSB01_6:1595379-1595859 Satlength=481 Nr of Repeats=5 RepeatLength=108 seed=ATCCTCAACA Num.seqs=3 Similarity=0.732510
0
ATCCTCAACACCATCNGCGTCATCTAGTTCCTCCGGAACTATGTCGACAATTTCAGGATCAACTGGTTCNACA
GTTACAGTTGTACCAGGATCNTCTAGCACATTTGG

****
****
Consensus:

ATCCTCAACACCATCNGCGTCATCTAGTTCCTCCGGAACTATGTCGACAATTTCAGGATCAACTGGTTCNACA
GTTACAGTTGTACCAGGATCNTCTAGCACATTTGG

>Cele-UNSB01_Fam_193_108_1 Nr. of seq. 1 Alignment length(with gaps) =
108 Alignment score = 0.000000
Cele-UNSB01_6:14010915-14011665 Satlength=751 Nr of Repeats=7
RepeatLength=108 seed=TGGGTAATCA Num.seqs=6 Similarity=0.845267
0
TGGGTAATCAGGTACGGGCTTTCAGATTTTCAAGTTTTCACGTGAATATCTAGGTCACGAGCCAATTTCAGCC
AGGAGTGGGACTAGAGATATGAAACTCTTGGGTTA

****
****
Consensus:

TGGGTAATCAGGTACGGGCTTTCAGATTTTCAAGTTTTCACGTGAATATCTAGGTCACGAGCCAATTTCAGCC
AGGAGTGGGACTAGAGATATGAAACTCTTGGGTTA

>Cele-UNSB01_Fam_194_108_1 Nr. of seq. 1 Alignment length(with gaps) =
108 Alignment score = 0.000000
Cele-UNSB01_6:15588433-15598390 Satlength=9958 Nr of Repeats=92
RepeatLength=108 seed=AAGTCTTCTT Num.seqs=90 Similarity=0.968330
0
AAGTCTTCTTGATTCTGAGTTCTTGCTTCGAACTGTTGACCATCTCCTTGCAGGATCTTATTGTCAGATGGAA
CCTTCTTGGCATACCGTTCTCCACTGATGTGAGTG

****
****
Consensus:

AAGTCTTCTTGATTCTGAGTTCTTGCTTCGAACTGTTGACCATCTCCTTGCAGGATCTTATTGTCAGATGGAA
CCTTCTTGGCATACCGTTCTCCACTGATGTGAGTG

>Cele-UNSB01_Fam_195_104_1 Nr. of seq. 1 Alignment length(with gaps) =
104 Alignment score = 0.000000
Cele-UNSB01_4:2422782-2423615 Satlength=834 Nr of Repeats=8
RepeatLength=104 seed=CCTGCGAAAG Num.seqs=7 Similarity=0.985348
0
CCTGCGAAAGCGAATCTCTGGCCTGGCGGAGGGCCAACTAACTCTCTCCCTCAAGCCTTCGGGTGCTTCCTGG
GTTATCGGTGAATTTTTGGGTCTGTCAGGGT

****
****
Consensus:

CCTGCGAAAGCGAATCTCTGGCCTGGCGGAGGGCCAACTAACTCTCTCCCTCAAGCCTTCGGGTGCTTCCTGG
GTTATCGGTGAATTTTTGGGTCTGTCAGGGT

>Cele-UNSB01_Fam_196_102_1 Nr. of seq. 1 Alignment length(with gaps) = 102 Alignment score = 0.000000
Cele-UNSB01_4:695215-705007 Satlength=9793 Nr of Repeats=96 RepeatLength=102 seed=TGATATGTCG Num.seqs=96 Similarity=0.972641
0
TGATATGTCGAACGATTGATAGTTTGAGAATCAAGAGAATCTGTTGGACGATCAATTCCATCTTTGGGTCTTG
CTGCAGCATATCTTTCTCCAGTACCACCT

****
Consensus:

TGATATGTCGAACGATTGATAGTTTGAGAATCAAGAGAATCTGTTGGACGATCAATTCCATCTTTGGGTCTTG
CTGCAGCATATCTTTCTCCAGTACCACCT

>Cele-UNSB01_Fam_197_102_1 Nr. of seq. 1 Alignment length(with gaps) = 102 Alignment score = 0.000000
Cele-UNSB01_6:1180467-1185764 Satlength=5298 Nr of Repeats=52 RepeatLength=102 seed=TGTACACTTC Num.seqs=37 Similarity=0.984062
0
TGTACACTTCTCCCAAATTGACTGTAAGAATGGACTCCTTATATGATTTTAAACCCGACTCTGGACATACAAA
CTTCTCTCAAAAAGAAAATAGACTTCACA

****
Consensus:

TGTACACTTCTCCCAAATTGACTGTAAGAATGGACTCCTTATATGATTTTAAACCCGACTCTGGACATACAAA
CTTCTCTCAAAAAGAAAATAGACTTCACA

>Cele-UNSB01_Fam_198_101_1 Nr. of seq. 1 Alignment length(with gaps) = 101 Alignment score = 0.000000
Cele-UNSB01_1:14574460-14574865 Satlength=406 Nr of Repeats=4 RepeatLength=101 seed=GCATTTGCAT Num.seqs=3 Similarity=0.826797
0
GCATTTGCATATNGAGATCAGATAAAAGTTAGAATTTTCCAGCCGTTGCAAGTGCGCTCTATTGAGAACATTT
TGAAAATTGAATGTTTTCACATTAGAGC

****
Consensus:

GCATTTGCATATNGAGATCAGATAAAAGTTAGAATTTTCCAGCCGTTGCAAGTGCGCTCTATTGAGAACATTT
TGAAAATTGAATGTTTTCACATTAGAGC

>Cele-UNSB01_Fam_199_101_1 Nr. of seq. 1 Alignment length(with gaps) = 101 Alignment score = 0.000000
Cele-UNSB01_3:272176-273482 Satlength=1307 Nr of Repeats=13 RepeatLength=101 seed=GGTTACTGTA Num.seqs=11 Similarity=0.942394
0
GGTTACTGTAGCTCCAGAAATACGCAAACACCGACTACGGTTCCCTAATACATCAGATATTACGACAAAGTGC
AGAAAATCGCTAAAAATGACTTTACTGG

****
****
Consensus:

GGTTACTGTAGCTCCAGAAATACGCAAACACCGACTACGGTTCCCTAATACATCAGATATTACGACAAAGTGC
AGAAAATCGCTAAAAATGACTTTACTGG

>Cele-UNSB01_Fam_200_101_1 Nr. of seq. 1 Alignment length(with gaps) = 101 Alignment score = 0.000000
Cele-UNSB01_4:1584655-1585480 Satlength=826 Nr of Repeats=7 RepeatLength=101 seed=TTGATTTTTC Num.seqs=5 Similarity=0.952475
0
TTGATTTTTCGTCTTATGAAGGAATATTGGTTTCGGGGTAGTGGGCGGATATGGTCGGGGTACTGTAGGTATA
CGGTAGGGTTACTGTAGTTTTGGAAATT

****
****
Consensus:

TTGATTTTTCGTCTTATGAAGGAATATTGGTTTCGGGGTAGTGGGCGGATATGGTCGGGGTACTGTAGGTATA
CGGTAGGGTTACTGTAGTTTTGGAAATT

>Cele-UNSB01_Fam_201_96_1 Nr. of seq. 1 Alignment length(with gaps) = 96 Alignment score = 0.000000
Cele-UNSB01_4:12453898-12454471 Satlength=574 Nr of Repeats=6 RepeatLength=96 seed=GGTTGATTTG Num.seqs=5 Similarity=0.991667
0
GGTTGATTTGCGGCACTCTACAGATAGTGGATCTGCTTCTTACCAATCTTCCCGTTACCGCCACGCACTTAAG
CTCCAAACTATTTTATTTTTTTG

****
****
Consensus:

GGTTGATTTGCGGCACTCTACAGATAGTGGATCTGCTTCTTACCAATCTTCCCGTTACCGCCACGCACTTAAG
CTCCAAACTATTTTATTTTTTTG

>Cele-UNSB01_Fam_202_96_1 Nr. of seq. 1 Alignment length(with gaps) = 96 Alignment score = 0.000000
Cele-UNSB01_4:12787287-12803219 Satlength=15933 Nr of Repeats=166 RepeatLength=96 seed=GTTCCCATAA Num.seqs=162 Similarity=0.969395
0
GTTCCCATAATTGGGTTAAATACCACTCATAAGTAACTAGTATGGGACTGAAAAGATACTAAATGAGCTTATT
CTAAGGGTGAAGCTTCTGGTAAT

****
****
Consensus:

GTTCCCATAATTGGGTTAAATACCACTCATAAGTAACTAGTATGGGACTGAAAAGATACTAAATGAGCTTATT
CTAAGGGTGAAGCTTCTGGTAAT

>Cele-UNSB01_Fam_203_93_1 Nr. of seq. 1 Alignment length(with gaps) = 93
Alignment score = 0.000000
Cele-UNSB01_4:6945378-6946401 Satlength=1024 Nr of Repeats=11
RepeatLength=93 seed=TGCTTCTCGT Num.seqs=11 Similarity=0.961942
0
TGCTTCTCGTCGTCCACCACCACCACCGAAGGGAACTGGAAGCCCACCACCACCACCAACTGGAGAACCACAG
GATCTTTCTGGAGAAGGTAA

****
****
Consensus:

TGCTTCTCGTCGTCCACCACCACCACCGAAGGGAACTGGAAGCCCACCACCACCACCAACTGGAGAACCACAG
GATCTTTCTGGAGAAGGTAA

>Cele-UNSB01_Fam_204_93_1 Nr. of seq. 1 Alignment length(with gaps) = 93
Alignment score = 0.000000
Cele-UNSB01_5:1955153-1955723 Satlength=571 Nr of Repeats=7
RepeatLength=93 seed=CAGTACCCTC Num.seqs=5 Similarity=0.954122
0
CAGTACCCTCAAGGATCATCTGCTCCACGGTATCCCGGTAACTACAATGCCTTTGCCAGCACTTCCGATGCTC
CACAGTATCCAGGATCCTCC

****
****
Consensus:

CAGTACCCTCAAGGATCATCTGCTCCACGGTATCCCGGTAACTACAATGCCTTTGCCAGCACTTCCGATGCTC
CACAGTATCCAGGATCCTCC

>Cele-UNSB01_Fam_205_87_1 Nr. of seq. 1 Alignment length(with gaps) = 87
Alignment score = 0.000000
Cele-UNSB01_4:2843789-2844131 Satlength=343 Nr of Repeats=4
RepeatLength=87 seed=TGAAGTCGTA Num.seqs=3 Similarity=0.685393
0
TGAAGTCGTAGGATTCTGTGTGGTGACTGGTGTCGTTGTANTCTGTGAAGGGGACGTGGCTTCTTCCGTTGTG
TCATGCTTCTNNGG

****
****
Consensus:

TGAAGTCGTAGGATTCTGTGTGGTGACTGGTGTCGTTGTANTCTGTGAAGGGGACGTGGCTTCTTCCGTTGTG
TCATGCTTCTNNGG

>Cele-UNSB01_Fam_206_81_1 Nr. of seq. 1 Alignment length(with gaps) = 81
Alignment score = 0.000000
Cele-UNSB01_1:14687699-14688279 Satlength=581 Nr of Repeats=8
RepeatLength=73 seed=TGTCGGCCGC Num.seqs=6 Similarity=0.624829
0
TGTCGGCCGCTACCAAACTATTTAGCANACACACCAAGCACTACGTTGCGCACACACACCAAACTGCGGAACC
CCGAACAG

****
Consensus:

TGTCGGCCGCTACCAAACTATTTAGCANACACACCAAGCACTACGTTGCGCACACACACCAAACTGCGGAACCCCGAACAG

>Cele-UNSB01_Fam_207_80_1 Nr. of seq. 1 Alignment length(with gaps) = 80
Alignment score = 0.000000
Cele-UNSB01_4:1985632-1987358 Satlength=1727 Nr of Repeats=15
RepeatLength=80 seed=AATTTCTCAA Num.seqs=14 Similarity=0.854212
0
AATTTCTCAATAGGGCGCGCTTGCAACATCTGAACGGCGCGAAATTTGAATTTTTGGCCATAAAATAACGAAAACTAACT

****
Consensus:

AATTTCTCAATAGGGCGCGCTTGCAACATCTGAACGGCGCGAAATTTGAATTTTTGGCCATAAAATAACGAAAACTAACT

>Cele-UNSB01_Fam_208_78_1 Nr. of seq. 1 Alignment length(with gaps) = 78
Alignment score = 0.000000
Cele-UNSB01_3:13289132-13289912 Satlength=781 Nr of Repeats=10
RepeatLength=78 seed=CGAAGTTGCT Num.seqs=10 Similarity=0.831974
0
CGAAGTTGCTGGACCAAAGATCACAACAGGCGGATTTGGCGGCATAACCGGAGCAACAGTAGTGTCATTTGGTTCTTC

****
Consensus:

CGAAGTTGCTGGACCAAAGATCACAACAGGCGGATTTGGCGGCATAACCGGAGCAACAGTAGTGTCATTTGGTTCTTC

>Cele-UNSB01_Fam_209_78_1 Nr. of seq. 1 Alignment length(with gaps) = 78
Alignment score = 0.000000
Cele-UNSB01_5:8793961-8799031 Satlength=5071 Nr of Repeats=65
RepeatLength=78 seed=TGCAAGGCTC Num.seqs=65 Similarity=0.932885
0
TGCAAGGCTCCGATGATGATACCACTTCTAAAGTCGAGGTGTCACAATTTTCGGGTGTCGTAGAACTTGAATCAGTGA

****
Consensus:

TGCAAGGCTCCGATGATGATACCACTTCTAAAGTCGAGGTGTCACAATTTTCGGGTGTCGTAGAACTTGAATCAGTGA

>Cele-UNSB01_Fam_210_78_1 Nr. of seq. 1 Alignment length(with gaps) = 78
Alignment score = 0.000000
Cele-UNSB01_5:18412081-18412792 Satlength=712 Nr of Repeats=10
RepeatLength=78 seed=GAGCTTTGGG Num.seqs=7 Similarity=0.947904
0
GAGCTTTGGGGCTTTTGGCTTGATCTCAGTCGACTTGATGGATTCTGGCTTGGGTTCTGCAGGCTTTTCGGGC
TCCTT

****
*****
Consensus:

GAGCTTTGGGGCTTTTGGCTTGATCTCAGTCGACTTGATGGATTCTGGCTTGGGTTCTGCAGGCTTTTCGGGC
TCCTT

>Cele-UNSB01_Fam_211_77_1 Nr. of seq. 1 Alignment length(with gaps) = 77
Alignment score = 0.000000
Cele-UNSB01_4:10610325-10610863 Satlength=539 Nr of Repeats=7
RepeatLength=77 seed=GGAAGCTTCA Num.seqs=6 Similarity=0.984993
0
GGAAGCTTCATCTTTTGAATTATAAAAAAATAAATTTCATTTCAAAATCAGTGTGTGCACTATCAAAAGTGTC
CACT

****
****
Consensus:

GGAAGCTTCATCTTTTGAATTATAAAAAAATAAATTTCATTTCAAAATCAGTGTGTGCACTATCAAAAGTGTC
CACT

>Cele-UNSB01_Fam_212_73_1 Nr. of seq. 1 Alignment length(with gaps) = 73
Alignment score = 0.000000
Cele-UNSB01_2:1327419-1327790 Satlength=372 Nr of Repeats=5
RepeatLength=73 seed=AGTTTGTAGT Num.seqs=3 Similarity=0.920852
0
ANTTTGTAGTTTGTAGTCTAGCAGACCAAAATTTATTAAAAACTGCAGAAAATTGATGGGTCTGCTAATTAAG

****
Consensus:

ANTTTGTAGTTTGTAGTCTAGCAGACCAAAATTTATTAAAAACTGCAGAAAATTGATGGGTCTGCTAATTAAG

>Cele-UNSB01_Fam_213_72_1 Nr. of seq. 1 Alignment length(with gaps) = 72
Alignment score = 0.000000
Cele-UNSB01_2:870904-873513 Satlength=2610 Nr of Repeats=35
RepeatLength=72 seed=TCCATCCAGC Num.seqs=29 Similarity=0.928845
0
TCCATCCAGCTGTGGTTGGTCTCTGGGTGAATCCTCGAGAAGTATCACTAGTAGTACCATTGGCTCCGTTCG

****
Consensus:

TCCATCCAGCTGTGGTTGGTCTCTGGGTGAATCCTCGAGAAGTATCACTAGTAGTACCATTGGCTCCGTTCG

>Cele-UNSB01_Fam_214_72_1 Nr. of seq. 1 Alignment length(with gaps) = 72
Alignment score = 0.000000
Cele-UNSB01_3:1690415-1691093 Satlength=679 Nr of Repeats=5
RepeatLength=72 seed=ATGAATTTTA Num.seqs=3 Similarity=0.975309
0
ATGAATTTTAATACAATTTTCCTGACGATGAAGTCATAAAAATTCATTTTTTGACCTTTCAACTTTAGTTTT

****
Consensus:

ATGAATTTTAATACAATTTTCCTGACGATGAAGTCATAAAAATTCATTTTTTGACCTTTCAACTTTAGTTTT

>Cele-UNSB01_Fam_215_72_1 Nr. of seq. 1 Alignment length(with gaps) = 72
Alignment score = 0.000000
Cele-UNSB01_6:15412985-15413295 Satlength=311 Nr of Repeats=5
RepeatLength=72 seed=TGGTTGTGTT Num.seqs=4 Similarity=0.990741
0
TGGTTGTGTTCCCAAAAGTGAGAAAAGAGGGTACAGACCTCTGTACGAATATGTCTTATCAGCTTCAAAAGA

****
Consensus:

TGGTTGTGTTCCCAAAAGTGAGAAAAGAGGGTACAGACCTCTGTACGAATATGTCTTATCAGCTTCAAAAGA

>Cele-UNSB01_Fam_216_71_1 Nr. of seq. 1 Alignment length(with gaps) = 71
Alignment score = 0.000000
Cele-UNSB01_1:1435639-1436319 Satlength=681 Nr of Repeats=8
RepeatLength=71 seed=ATTTATGTGA Num.seqs=6 Similarity=0.847840
0
ATTTATGTGAAAATTCAGGTTTTTAGGGCATTTTTTGAGGGAAATCTGAATTTTTCCGATTTTTCTGACGA

****
Consensus:

ATTTATGTGAAAATTCAGGTTTTTAGGGCATTTTTTGAGGGAAATCTGAATTTTTCCGATTTTTCTGACGA

>Cele-UNSB01_Fam_217_70_1 Nr. of seq. 1 Alignment length(with gaps) = 70
Alignment score = 0.000000
Cele-UNSB01_4:2419217-2421562 Satlength=2346 Nr of Repeats=23
RepeatLength=70 seed=AAAAATCGAT Num.seqs=14 Similarity=0.884668
0
AAAAATCGATTTTTTTGAAGTTTTTCTCAATAGAGCGCATTTTCAACAGCGAAAAAATTGAATTTTCCGCC

****
Consensus:

AAAAATCGATTTTTTTGAAGTTTTTCTCAATAGAGCGCATTTTCAACAGCGAAAAAATTGAATTTTCCGCC

>Cele-UNSB01_Fam_218_69_1 Nr. of seq. 1 Alignment length(with gaps) = 69
Alignment score = 0.000000
Cele-UNSB01_2:12477614-12477893 Satlength=280 Nr of Repeats=4
RepeatLength=69 seed=AGGTAGGCCT Num.seqs=3 Similarity=0.747619
0
AGGTAGGCCTGTAGGCATTGACTGNCAGAAAAGCGTAGACAGGCAAGCAGGTAAAAAGATACATGAGGC

****
Consensus:

AGGTAGGCCTGTAGGCATTGACTGNCAGAAAAGCGTAGACAGGCAAGCAGGTAAAAAGATACATGAGGC

>Cele-UNSB01_Fam_219_69_1 Nr. of seq. 1 Alignment length(with gaps) = 69
Alignment score = 0.000000
Cele-UNSB01_5:8388026-8392649 Satlength=4624 Nr of Repeats=67
RepeatLength=69 seed=GTCTGGACTG Num.seqs=67 Similarity=0.948872
0
GTCTGGACTGGTGTAGTAGAACTGCTTGGAGGTTCTGTACTGGTTGTCTCTGGAGCCGTGGTAGTAGTA

****
Consensus:

GTCTGGACTGGTGTAGTAGAACTGCTTGGAGGTTCTGTACTGGTTGTCTCTGGAGCCGTGGTAGTAGTA

>Cele-UNSB01_Fam_220_68_1 Nr. of seq. 1 Alignment length(with gaps) = 68
Alignment score = 0.000000
Cele-UNSB01_1:14122632-14123925 Satlength=1294 Nr of Repeats=19
RepeatLength=68 seed=TTGGTTGAGG Num.seqs=14 Similarity=0.987503
0   TTGGTTGAGGCTAGAAAATGTTGTAGTTTGTAGTGTGTTAGCCTCAACCAAAATTAATTTTTTTTTAA

****
Consensus:

TTGGTTGAGGCTAGAAAATGTTGTAGTTTGTAGTGTGTTAGCCTCAACCAAAATTAATTTTTTTTTAA

>Cele-UNSB01_Fam_221_65_1 Nr. of seq. 1 Alignment length(with gaps) = 65
Alignment score = 0.000000
Cele-UNSB01_2:1881035-1881350 Satlength=316 Nr of Repeats=5
RepeatLength=65 seed=TATCGAGAGA Num.seqs=3 Similarity=0.658497
0   TATCGAGAGAATNTTCCACATTCACTTTGAATTCCNTTTCCAATTTCAATAAGACTCTCAGTAAG

****
Consensus:

TATCGAGAGAATNTTCCACATTCACTTTGAATTCCNTTTCCAATTTCAATAAGACTCTCAGTAAG

>Cele-UNSB01_Fam_222_62_1 Nr. of seq. 1 Alignment length(with gaps) = 62
Alignment score = 0.000000
Cele-UNSB01_1:14343732-14344621 Satlength=890 Nr of Repeats=4
RepeatLength=62 seed=GTAGTTTGTA Num.seqs=3 Similarity=0.928315
0   GTAGTTTGTAGCTTTAGCCTCAACCAAATTAGCTAAAGCTTGAGTGAGGCTATGGTGATTTT

****
Consensus:

GTAGTTTGTAGCTTTAGCCTCAACCAAATTAGCTAAAGCTTGAGTGAGGCTATGGTGATTTT

>Cele-UNSB01_Fam_223_62_1 Nr. of seq. 1 Alignment length(with gaps) = 62
Alignment score = 0.000000

Cele-UNSB01_2:14141898-14142455 Satlength=558 Nr of Repeats=7
RepeatLength=62 seed=TGCTAACTAG Num.seqs=6 Similarity=0.822222
0   TGCTAACTAGCTACGGCCAGCTTAGGTGCCGGCTGCACGAGCAGTCCTGGTTTAATGTCGGC

****
Consensus:

TGCTAACTAGCTACGGCCAGCTTAGGTGCCGGCTGCACGAGCAGTCCTGGTTTAATGTCGGC

>Cele-UNSB01_Fam_224_60_1 Nr. of seq. 1 Alignment length(with gaps) = 60
Alignment score = 0.000000
Cele-UNSB01_5:7301688-7302048 Satlength=361 Nr of Repeats=6
RepeatLength=60 seed=CTAGCTAGAT Num.seqs=6 Similarity=0.943704
0   CTAGCTAGATTAAACGCTCTCAAGAGACAGCATGTTGTTGTTGCCCACAAGAAGAAGGCT

****
Consensus:

CTAGCTAGATTAAACGCTCTCAAGAGACAGCATGTTGTTGTTGCCCACAAGAAGAAGGCT

>Cele-UNSB01_Fam_225_60_1 Nr. of seq. 1 Alignment length(with gaps) = 60
Alignment score = 0.000000
Cele-UNSB01_5:8761530-8782970 Satlength=21441 Nr of Repeats=357
RepeatLength=60 seed=ACACAATTGG Num.seqs=349 Similarity=0.921038
0   ACACAATTGGAGTGGTGCTTGAGGAGCTGGATTCAGTTGAGCTCGTTATGTCGGTTGAAG

****
Consensus:

ACACAATTGGAGTGGTGCTTGAGGAGCTGGATTCAGTTGAGCTCGTTATGTCGGTTGAAG

>Cele-UNSB01_Fam_226_60_1 Nr. of seq. 1 Alignment length(with gaps) = 60
Alignment score = 0.000000
Cele-UNSB01_5:14209723-14211643 Satlength=1921 Nr of Repeats=32
RepeatLength=60 seed=CTGCTGATTC Num.seqs=32 Similarity=0.925493
0   CTGCTGATTCCGAGAAGAAGGCCCCAGCCAAGAGAGAAGCTGATAAGCCAAAGACTGAAT

****
Consensus:

CTGCTGATTCCGAGAAGAAGGCCCCAGCCAAGAGAGAAGCTGATAAGCCAAAGACTGAAT

>Cele-UNSB01_Fam_227_59_1 Nr. of seq. 1 Alignment length(with gaps) = 59
Alignment score = 0.000000
Cele-UNSB01_2:1416594-1416934 Satlength=341 Nr of Repeats=5
RepeatLength=59 seed=CTACAAACTA Num.seqs=3 Similarity=0.969868
0   CTACAAACTACAAAAGCCTATTTAGCAGACCCACGCCAACTTCTGATGGGTCTGCTAGA

****
Consensus:

CTACAAACTACAAAAGCCTATTTAGCAGACCCACGCCAACTTCTGATGGGTCTGCTAGA

```
>Cele-UNSB01_Fam_228_57_1  Nr. of seq. 1 Alignment length(with gaps) = 57
Alignment score = 0.000000
Cele-UNSB01_5:14206302-14207970 Satlength=1669 Nr of Repeats=22
RepeatLength=57 seed=GAACTCAACA  Num.seqs=15 Similarity=0.952771
0    GAACTCAACAAGCTGATCAAGAGCAACACCGTGGATCCCGAGCTGCCGGACAGAAGG

*********************************************************
Consensus:

GAACTCAACAAGCTGATCAAGAGCAACACCGTGGATCCCGAGCTGCCGGACAGAAGG

>Cele-UNSB01_Fam_229_55_1  Nr. of seq. 1 Alignment length(with gaps) = 55
Alignment score = 0.000000
Cele-UNSB01_2:1212949-1213169 Satlength=221 Nr of Repeats=4
RepeatLength=55 seed=GGGATTATTG  Num.seqs=4 Similarity=0.903030
0    GGGATTATTGGAGTTTCTTTTTCTAGAATTTTTTGAAAATTTTGGACAGTTTTTT

*******************************************************
Consensus:

GGGATTATTGGAGTTTCTTTTTCTAGAATTTTTTGAAAATTTTGGACAGTTTTTT

>Cele-UNSB01_Fam_230_55_1  Nr. of seq. 1 Alignment length(with gaps) = 55
Alignment score = 0.000000
Cele-UNSB01_4:6076861-6077186 Satlength=326 Nr of Repeats=6
RepeatLength=55 seed=AAGAGTCCCC  Num.seqs=4 Similarity=1.000000
0    AAGAGTCCCCACACACTGAAAGGTTTTAAGGAATTTTTTGAGGATTCCTAGGGTT

*******************************************************
Consensus:

AAGAGTCCCCACACACTGAAAGGTTTTAAGGAATTTTTTGAGGATTCCTAGGGTT

>Cele-UNSB01_Fam_231_52_1  Nr. of seq. 1 Alignment length(with gaps) = 52
Alignment score = 0.000000
Cele-UNSB01_6:17211035-17211529 Satlength=495 Nr of Repeats=4
RepeatLength=52 seed=GCATGTCTTC  Num.seqs=3 Similarity=0.626263
0    GCATGTCTTCCAAGCGTTTTGAGCCACGCNTGGGATTTCCTATACACGGCTG

****************************************************
Consensus:

GCATGTCTTCCAAGCGTTTTGAGCCACGCNTGGGATTTCCTATACACGGCTG

>Cele-UNSB01_Fam_232_50_1  Nr. of seq. 1 Alignment length(with gaps) = 50
Alignment score = 0.000000
Cele-UNSB01_1:12702448-12702698 Satlength=251 Nr of Repeats=5
RepeatLength=50 seed=ACACATGCTA  Num.seqs=5 Similarity=0.952000
0    ACACATGCTATAACATTGTCTCCTTCCCCTAGTGCACGAGAGCGGCCGTT

**************************************************
Consensus:

ACACATGCTATAACATTGTCTCCTTCCCCTAGTGCACGAGAGCGGCCGTT
```

```
>Cele-UNSB01_Fam_233_50_1  Nr. of seq. 1 Alignment length(with gaps) = 50
Alignment score = 0.000000
Cele-UNSB01_4:16678025-16678676 Satlength=652 Nr of Repeats=13
RepeatLength=50 seed=GGGCGTGTTT  Num.seqs=10 Similarity=0.897482
0    GGGCGTGTTTTCACATGCCCCGCCCACAAAATTGATGTTTCCACCAAAAT

**************************************************
Consensus:

GGGCGTGTTTTCACATGCCCCGCCCACAAAATTGATGTTTCCACCAAAAT

>Cele-UNSB01_Fam_234_49_1  Nr. of seq. 1 Alignment length(with gaps) = 49
Alignment score = 0.000000
Cele-UNSB01_1:1647038-1647577 Satlength=540 Nr of Repeats=9
RepeatLength=49 seed=CAATAATTTT  Num.seqs=7 Similarity=0.909297
0    CAATAATTTTAAATTAAATTATCGATTTTTCTAAATTTTTCCGAAAAAT

*************************************************
Consensus:

CAATAATTTTAAATTAAATTATCGATTTTTCTAAATTTTTCCGAAAAAT

>Cele-UNSB01_Fam_235_48_1  Nr. of seq. 1 Alignment length(with gaps) = 48
Alignment score = 0.000000
Cele-UNSB01_2:13440285-13440859 Satlength=575 Nr of Repeats=12
RepeatLength=48 seed=TGCCTACGTG  Num.seqs=10 Similarity=0.812346
0    TGCCTACGTGCCTACAAAACAGGCGTAGGCAGCTTTAAAGGTGATGCA

************************************************
Consensus:

TGCCTACGTGCCTACAAAACAGGCGTAGGCAGCTTTAAAGGTGATGCA

>Cele-UNSB01_Fam_236_48_1  Nr. of seq. 1 Alignment length(with gaps) = 48
Alignment score = 0.000000
Cele-UNSB01_6:14589132-14589372 Satlength=241 Nr of Repeats=5
RepeatLength=48 seed=GGAGGTGGAG  Num.seqs=5 Similarity=0.841667
0    GGAGGTGGAGAAAGTGTAGTTCCCGGTGGTCGNGGCTCTTCNGAGGGT

************************************************
Consensus:

GGAGGTGGAGAAAGTGTAGTTCCCGGTGGTCGNGGCTCTTCNGAGGGT

>Cele-UNSB01_Fam_237_46_1  Nr. of seq. 1 Alignment length(with gaps) = 46
Alignment score = 0.000000
Cele-UNSB01_6:2065387-2065569 Satlength=183 Nr of Repeats=4
RepeatLength=46 seed=TGGTAAATTG  Num.seqs=3 Similarity=0.835749
0    TGGTAAATTGCCTCCCGGANCACCCACTTTTCTTATTTCCCAACTA

**********************************************
Consensus:
```

TGGTAAATTGCCTCCCGGANCACCCACTTTTCTTATTTCCCAACTA

>Cele-UNSB01_Fam_238_45_1 Nr. of seq. 1 Alignment length(with gaps) = 45
Alignment score = 0.000000
Cele-UNSB01_4:7767635-7768760 Satlength=1126 Nr of Repeats=25
RepeatLength=45 seed=AGCACCACCA Num.seqs=25 Similarity=0.965432
0   AGCACCACCATCAGGCGGACCACCAGGACCATTTGATCCATCAGG

****
Consensus:

AGCACCACCATCAGGCGGACCACCAGGACCATTTGATCCATCAGG

>Cele-UNSB01_Fam_239_45_1 Nr. of seq. 1 Alignment length(with gaps) = 45
Alignment score = 0.000000
Cele-UNSB01_5:11342057-11345882 Satlength=3826 Nr of Repeats=85
RepeatLength=45 seed=CCAAGCACAT Num.seqs=85 Similarity=0.985127
0   CCAAGCACATCTACAGAGACACCTACAACTGAATCAACTGCAGCA

****
Consensus:

CCAAGCACATCTACAGAGACACCTACAACTGAATCAACTGCAGCA

>Cele-UNSB01_Fam_240_45_1 Nr. of seq. 1 Alignment length(with gaps) = 45
Alignment score = 0.000000
Cele-UNSB01_6:17820421-17836619 Satlength=16199 Nr of Repeats=357
RepeatLength=45 seed=CTCCATCCAC Num.seqs=275 Similarity=0.944464
0   CTCCATCCACCAGCACTGCTTCGAGTACGACAGAAAGCACTTCGA

****
Consensus:

CTCCATCCACCAGCACTGCTTCGAGTACGACAGAAAGCACTTCGA

>Cele-UNSB01_Fam_241_43_1 Nr. of seq. 1 Alignment length(with gaps) = 43
Alignment score = 0.000000
Cele-UNSB01_2:2187430-2189107 Satlength=1678 Nr of Repeats=38
RepeatLength=43 seed=CGAGTTTGGC Num.seqs=37 Similarity=0.823312
0   CGAGTTTGGCCTACANCAAATTTGGCAAATTCGGCAAATTTGC

****
Consensus:

CGAGTTTGGCCTACANCAAATTTGGCAAATTCGGCAAATTTGC

>Cele-UNSB01_Fam_242_43_1 Nr. of seq. 1 Alignment length(with gaps) = 43
Alignment score = 0.000000
Cele-UNSB01_2:14320079-14320251 Satlength=173 Nr of Repeats=4
RepeatLength=43 seed=GAAAACTAGG Num.seqs=4 Similarity=0.715762
0   GAAAACTAGGCCACAGAGTTCAAATTGATGGCCTAGAAATCTA

****

Consensus:

GAAAACTAGGCCACAGAGTTCAAATTGATGGCCTAGAAATCTA

>Cele-UNSB01_Fam_243_43_1 Nr. of seq. 1 Alignment length(with gaps) = 43
Alignment score = 0.000000
Cele-UNSB01_3:1466668-1467327 Satlength=660 Nr of Repeats=5
RepeatLength=43 seed=GGCTCTCGGC Num.seqs=3 Similarity=0.627907
0   GGCTCTCGGCTCGCGCCGAGAGCCGAGNTCTACTNAAAGTTTC

****
Consensus:

GGCTCTCGGCTCGCGCCGAGAGCCGAGNTCTACTNAAAGTTTC

>Cele-UNSB01_Fam_244_43_1 Nr. of seq. 1 Alignment length(with gaps) = 43
Alignment score = 0.000000
Cele-UNSB01_3:12608882-12609486 Satlength=605 Nr of Repeats=14
RepeatLength=43 seed=AGCAGCCGAC Num.seqs=11 Similarity=0.800423
0   AGCAGCCGACAGTTGCCGGTTGCCGAAAAATTGCAGTAATTTC

****
Consensus:

AGCAGCCGACAGTTGCCGGTTGCCGAAAAATTGCAGTAATTTC

>Cele-UNSB01_Fam_245_42_1 Nr. of seq. 1 Alignment length(with gaps) = 42
Alignment score = 0.000000
Cele-UNSB01_1:733992-734160 Satlength=169 Nr of Repeats=4 RepeatLength=42
seed=TTTGATCTAC Num.seqs=4 Similarity=0.968254
0   TTTGATCTACAAAAAATGCGGGAATTTTCCCAGCATTGCTCA

****
Consensus:

TTTGATCTACAAAAAATGCGGGAATTTTCCCAGCATTGCTCA

>Cele-UNSB01_Fam_246_42_1 Nr. of seq. 1 Alignment length(with gaps) = 42
Alignment score = 0.000000
Cele-UNSB01_5:6199698-6200391 Satlength=694 Nr of Repeats=15
RepeatLength=42 seed=AGGTGCTGCT Num.seqs=9 Similarity=0.859788
0   AGGTGCTGCTTCCTTTGGAACATCTGCTTGGGTTGGCTCCGA

****
Consensus:

AGGTGCTGCTTCCTTTGGAACATCTGCTTGGGTTGGCTCCGA

>Cele-UNSB01_Fam_247_41_1 Nr. of seq. 1 Alignment length(with gaps) = 41
Alignment score = 0.000000
Cele-UNSB01_4:3758727-3758973 Satlength=247 Nr of Repeats=6
RepeatLength=41 seed=AAATTTTTAA Num.seqs=6 Similarity=0.945799
0   AAATTTTTAACGAGAAGTGGGCGAAAATCATTCAAAAATTC

****
Consensus:

AAATTTTTAACGAGAAGTGGGCGAAAATCATTCAAAAATTC

>Cele-UNSB01_Fam_248_41_1 Nr. of seq. 1 Alignment length(with gaps) = 41
Alignment score = 0.000000
Cele-UNSB01_5:20548445-20548748 Satlength=304 Nr of Repeats=5
RepeatLength=41 seed=GCATTTTTCG Num.seqs=3 Similarity=0.772358
0   GCATTTTTCGCCGATTTTTNATTAATTTTCAGTGAAAATTC

****
Consensus:

GCATTTTTCGCCGATTTTTNATTAATTTTCAGTGAAAATTC

>Cele-UNSB01_Fam_249_40_1 Nr. of seq. 1 Alignment length(with gaps) = 40
Alignment score = 0.000000
Cele-UNSB01_3:415402-416126 Satlength=725 Nr of Repeats=7 RepeatLength=40
seed=TCCACGTGGA Num.seqs=5 Similarity=0.650794
0   TCCACGTGGAATTTTCAGAATTTTCNCAGAAAATTTGAAA

****
Consensus:

TCCACGTGGAATTTTCAGAATTTTCNCAGAAAATTTGAAA

>Cele-UNSB01_Fam_250_40_1 Nr. of seq. 1 Alignment length(with gaps) = 40
Alignment score = 0.000000
Cele-UNSB01_5:19147729-19148084 Satlength=356 Nr of Repeats=7
RepeatLength=40 seed=GAATTTTTTG Num.seqs=6 Similarity=0.926667
0   GAATTTTTTGCAATTCTACTGAATTTTCCAAATTTTCAGC

****
Consensus:

GAATTTTTTGCAATTCTACTGAATTTTCCAAATTTTCAGC

>Cele-UNSB01_Fam_251_40_1 Nr. of seq. 1 Alignment length(with gaps) = 40
Alignment score = 0.000000
Cele-UNSB01_5:19162213-19162392 Satlength=180 Nr of Repeats=5
RepeatLength=40 seed=TTTTGCCGAA Num.seqs=3 Similarity=0.629630
0   TTTTGCCAAAAATNTGTCAATTTTGTGGAATTTCTNTCAT

****
Consensus:

TTTTGCCAAAAATNTGTCAATTTTGTGGAATTTCTNTCAT

>Cele-UNSB01_Fam_252_39_1 Nr. of seq. 1 Alignment length(with gaps) = 39
Alignment score = 0.000000

Cele-UNSB01_2:3539245-3539476 Satlength=232 Nr of Repeats=5
RepeatLength=39 seed=AATTTCCCGC Num.seqs=3 Similarity=0.769444
0   AATTTCCCGCCAAAATTGAATTTTTTTAAAGAAAATTTA

****
Consensus:

AATTTCCCGCCAAAATTGAATTTTTTTAAAGAAAATTTA

>Cele-UNSB01_Fam_253_39_1 Nr. of seq. 1 Alignment length(with gaps) = 39
Alignment score = 0.000000
Cele-UNSB01_4:5061085-5061475 Satlength=391 Nr of Repeats=8
RepeatLength=39 seed=CCAACTGGAG Num.seqs=7 Similarity=0.784921
0   CCAACTGGAGCCAGCACNATGACCGCTGTTGGAGGAGCA

****
Consensus:

CCAACTGGAGCCAGCACNATGACCGCTGTTGGAGGAGCA

>Cele-UNSB01_Fam_254_39_1 Nr. of seq. 1 Alignment length(with gaps) = 39
Alignment score = 0.000000
Cele-UNSB01_5:3666468-3666780 Satlength=313 Nr of Repeats=8
RepeatLength=39 seed=GATTTACGGG Num.seqs=6 Similarity=0.849573
0   GATTTACGGGCTTTAATCAGTTTCGAAAAAGCTTGTGTC

****
Consensus:

GATTTACGGGCTTTAATCAGTTTCGAAAAAGCTTGTGTC

>Cele-UNSB01_Fam_255_39_1 Nr. of seq. 1 Alignment length(with gaps) = 39
Alignment score = 0.000000
Cele-UNSB01_5:12723614-12724001 Satlength=388 Nr of Repeats=10
RepeatLength=39 seed=TGGTACGGTA Num.seqs=9 Similarity=0.880342
0   TGGTACGGTAGTTGTAGTGGTAGTGGAGGTTGTTGTTGG

****
Consensus:

TGGTACGGTAGTTGTAGTGGTAGTGGAGGTTGTTGTTGG

>Cele-UNSB01_Fam_256_39_1 Nr. of seq. 1 Alignment length(with gaps) = 39
Alignment score = 0.000000
Cele-UNSB01_5:18895119-18895676 Satlength=558 Nr of Repeats=15
RepeatLength=39 seed=GTAATTTATA Num.seqs=13 Similarity=0.917598
0   GTAATTTATAATTTTTTAAAGATGAGTAATATATTGCCC

****
Consensus:

GTAATTTATAATTTTTTAAAGATGAGTAATATATTGCCC

>Cele-UNSB01_Fam_257_39_1 Nr. of seq. 1 Alignment length(with gaps) = 39
Alignment score = 0.000000
Cele-UNSB01_6:17555071-17555695 Satlength=625 Nr of Repeats=16
RepeatLength=39 seed=ATTGATACTT Num.seqs=16 Similarity=0.929345
0   ATTGATACTTTGTCGGGTTTAGACCATCGGCCGAGCTTC

****
Consensus:

ATTGATACTTTGTCGGGTTTAGACCATCGGCCGAGCTTC

>Cele-UNSB01_Fam_258_38_1 Nr. of seq. 1 Alignment length(with gaps) = 38
Alignment score = 0.000000
Cele-UNSB01_1:2017348-2017684 Satlength=337 Nr of Repeats=5
RepeatLength=38 seed=AAAATCGATA Num.seqs=3 Similarity=1.000000
0   AAAATCGATAATTTTCAGTATGCAGTGAAATTCTTGGA

****
Consensus:

AAAATCGATAATTTTCAGTATGCAGTGAAATTCTTGGA

>Cele-UNSB01_Fam_259_37_1 Nr. of seq. 1 Alignment length(with gaps) = 37
Alignment score = 0.000000
Cele-UNSB01_1:10089590-10089728 Satlength=139 Nr of Repeats=5
RepeatLength=37 seed=ATAATTTTTC Num.seqs=3 Similarity=1.000000
0   ATAATTTTTCTTGAGTAATGAGAGACTTTTTCATAAT

****
Consensus:

ATAATTTTTCTTGAGTAATGAGAGACTTTTTCATAAT

>Cele-UNSB01_Fam_260_36_1 Nr. of seq. 1 Alignment length(with gaps) = 36
Alignment score = 0.000000
Cele-UNSB01_3:12817254-12818910 Satlength=1657 Nr of Repeats=46
RepeatLength=36 seed=GATATTTTCA Num.seqs=46 Similarity=0.952800
0   GATATTTTCATGTTCGGCAAAAGTACCGATTTGCCA

****
Consensus:

GATATTTTCATGTTCGGCAAAAGTACCGATTTGCCA

>Cele-UNSB01_Fam_261_36_1 Nr. of seq. 1 Alignment length(with gaps) = 36
Alignment score = 0.000000
Cele-UNSB01_6:18063681-18064057 Satlength=377 Nr of Repeats=7
RepeatLength=36 seed=CCACCAACTC Num.seqs=6 Similarity=0.728395
0   CCACCAACTCCAAAATCAGCTGCTCCATCAGAAGTC

****
Consensus:

CCACCAACTCCAAAATCAGCTGCTCCATCAGAAGTC

```
>Cele-UNSB01_Fam_262_35_1  Nr. of seq. 1 Alignment length(with gaps) = 35
Alignment score = 0.000000
Cele-UNSB01_1:11408393-11409029 Satlength=637 Nr of Repeats=9
RepeatLength=35 seed=AATTGCCGAA  Num.seqs=7 Similarity=0.793210
0    AATTGCCGAATTTCTCGTTTTTGGCAATTTACCAA

***********************************
Consensus:

AATTGCCGAATTTCTCGTTTTTGGCAATTTACCAA

>Cele-UNSB01_Fam_263_35_1  Nr. of seq. 1 Alignment length(with gaps) = 35
Alignment score = 0.000000
Cele-UNSB01_2:12938825-12939101 Satlength=277 Nr of Repeats=8
RepeatLength=35 seed=TGTGTAATAA  Num.seqs=5 Similarity=0.794286
0    TGTGTAATAAGAAAATTTCAAATTTTTTTCACGAA

***********************************
Consensus:

TGTGTAATAAGAAAATTTCAAATTTTTTTCACGAA

>Cele-UNSB01_Fam_264_35_1  Nr. of seq. 1 Alignment length(with gaps) = 35
Alignment score = 0.000000
Cele-UNSB01_5:1026357-1026873 Satlength=517 Nr of Repeats=9
RepeatLength=35 seed=GCATTTGCCG  Num.seqs=6 Similarity=0.817143
0    GCATTTGCCGATTTTTAAAATTTCTGGCAATTCCA

***********************************
Consensus:

GCATTTGCCGATTTTTAAAATTTCTGGCAATTCCA

>Cele-UNSB01_Fam_265_34_1  Nr. of seq. 1 Alignment length(with gaps) = 34
Alignment score = 0.000000
Cele-UNSB01_2:14289556-14289862 Satlength=307 Nr of Repeats=8
RepeatLength=34 seed=CCGTGCCTAC  Num.seqs=7 Similarity=0.828198
0    CCGTGCCTACTAACCTACCGACCTTGCCTGCCTT

**********************************
Consensus:

CCGTGCCTACTAACCTACCGACCTTGCCTGCCTT

>Cele-UNSB01_Fam_266_34_1  Nr. of seq. 1 Alignment length(with gaps) = 34
Alignment score = 0.000000
Cele-UNSB01_3:12102276-12102493 Satlength=218 Nr of Repeats=6
RepeatLength=34 seed=TTGCCGAATT  Num.seqs=5 Similarity=0.811765
0    TTGCCGAATTTGCCGTTTGCCGAGCTCGTCAAAT

**********************************
Consensus:
```

TTGCCGAATTTGCCGTTTGCCGAGCTCGTCAAAT

>Cele-UNSB01_Fam_267_34_1 Nr. of seq. 1 Alignment length(with gaps) = 34
Alignment score = 0.000000
Cele-UNSB01_3:12168292-12168722 Satlength=431 Nr of Repeats=13
RepeatLength=34 seed=AAGTATTTTT Num.seqs=12 Similarity=0.954843
0   AAGTATTTTTGCCGGTTTTTTTGCCGAAATTTCC

****
Consensus:

AAGTATTTTTGCCGGTTTTTTTGCCGAAATTTCC

>Cele-UNSB01_Fam_268_33_1 Nr. of seq. 1 Alignment length(with gaps) = 33
Alignment score = 0.000000
Cele-UNSB01_1:2281114-2281247 Satlength=134 Nr of Repeats=4
RepeatLength=33 seed=TAAGAAAAGT Num.seqs=3 Similarity=0.892256
0   TAAGAAAAGTTAGGCCACCAAACTTTGGTGAAC

****
Consensus:

TAAGAAAAGTTAGGCCACCAAACTTTGGTGAAC

>Cele-UNSB01_Fam_269_32_1 Nr. of seq. 1 Alignment length(with gaps) = 32
Alignment score = 0.000000
Cele-UNSB01_1:11521003-11522550 Satlength=1548 Nr of Repeats=48
RepeatLength=32 seed=GCAATTTTTA Num.seqs=37 Similarity=0.890140
0   GCAATTTTTAGGCTAAAAATCACTAAAAAACT

****
Consensus:

GCAATTTTTAGGCTAAAAATCACTAAAAAACT

>Cele-UNSB01_Fam_270_32_1 Nr. of seq. 1 Alignment length(with gaps) = 32
Alignment score = 0.000000
Cele-UNSB01_3:12376126-12377123 Satlength=998 Nr of Repeats=30
RepeatLength=32 seed=AAATTTGTGA Num.seqs=18 Similarity=0.874992
0   AAATTTGTGAAAATTTAGGCCCATTTTGCTCA

****
Consensus:

AAATTTGTGAAAATTTAGGCCCATTTTGCTCA

>Cele-UNSB01_Fam_271_32_1 Nr. of seq. 1 Alignment length(with gaps) = 32
Alignment score = 0.000000
Cele-UNSB01_5:21020210-21020336 Satlength=127 Nr of Repeats=4
RepeatLength=32 seed=GTGCGTCCGG Num.seqs=3 Similarity=0.888889
0   GTGCGTCCGGCGTCCGGAAAAAGCGGACGCGT

****

Consensus:

GTGCGTCCGGCGTCCGGAAAAAGCGGACGCGT

>Cele-UNSB01_Fam_272_31_1 Nr. of seq. 1 Alignment length(with gaps) = 31
Alignment score = 0.000000
Cele-UNSB01_2:3099938-3100072 Satlength=135 Nr of Repeats=4
RepeatLength=31 seed=ATCGATTTTT Num.seqs=3 Similarity=0.700337
0   ATCGATTTTTTCGTTAAAAAATCAATAAAAT

****
Consensus:

ATCGATTTTTTCGTTAAAAAATCAATAAAAT

>Cele-UNSB01_Fam_273_31_1 Nr. of seq. 1 Alignment length(with gaps) = 31
Alignment score = 0.000000
Cele-UNSB01_2:3736361-3736609 Satlength=249 Nr of Repeats=8
RepeatLength=31 seed=GCTGATTGGT Num.seqs=8 Similarity=0.553571
0   GCTGATTGGTCGGCGNGGTTCGCCCATCNTT

****
Consensus:

GCTGATTGGTCGGCGNGGTTCGCCCATCNTT

>Cele-UNSB01_Fam_274_31_1 Nr. of seq. 1 Alignment length(with gaps) = 31
Alignment score = 0.000000
Cele-UNSB01_2:12597085-12597273 Satlength=189 Nr of Repeats=6
RepeatLength=31 seed=GACATCTCGC Num.seqs=4 Similarity=0.584229
0   GACATCTCGCAGGACATTATAAGAAGCGACC

****
Consensus:

GACATCTCGCAGGACATTATAAGAAGCGACC

>Cele-UNSB01_Fam_275_31_1 Nr. of seq. 1 Alignment length(with gaps) = 31
Alignment score = 0.000000
Cele-UNSB01_2:13437444-13437847 Satlength=404 Nr of Repeats=10
RepeatLength=31 seed=CACGCCTATT Num.seqs=7 Similarity=0.811572
0   CACGCCTATTTTATGGGCGGAGTCTACAAGC

****
Consensus:

CACGCCTATTTTATGGGCGGAGTCTACAAGC

>Cele-UNSB01_Fam_276_31_1 Nr. of seq. 1 Alignment length(with gaps) = 31
Alignment score = 0.000000
Cele-UNSB01_3:2092267-2093197 Satlength=931 Nr of Repeats=27
RepeatLength=31 seed=TGCGTCGGAA Num.seqs=23 Similarity=0.733775
0   TGCGTCGGAAATTNATTCCGATCATCGCCGG

****
Consensus:

TGCGTCGGAAATTNATTCCGATCATCGCCGG

>Cele-UNSB01_Fam_277_31_1 Nr. of seq. 1 Alignment length(with gaps) = 31
Alignment score = 0.000000
Cele-UNSB01_3:2094174-2094918 Satlength=745 Nr of Repeats=17
RepeatLength=31 seed=TCCGATGCAT Num.seqs=13 Similarity=0.625486
0   TCCGATGCATTGCCGATAATCGGAACCTGGT

****
Consensus:

TCCGATGCATTGCCGATAATCGGAACCTGGT

>Cele-UNSB01_Fam_278_31_1 Nr. of seq. 1 Alignment length(with gaps) = 31
Alignment score = 0.000000
Cele-UNSB01_3:11300832-11300986 Satlength=155 Nr of Repeats=5
RepeatLength=31 seed=GCAGCCGACG Num.seqs=4 Similarity=0.524411
0   GCAGCCGACGCTATACGGGTCTGATGGAGTA

****
Consensus:

GCAGCCGACGCTATACGGGTCTGATGGAGTA

>Cele-UNSB01_Fam_279_31_1 Nr. of seq. 1 Alignment length(with gaps) = 31
Alignment score = 0.000000
Cele-UNSB01_3:11489719-11490125 Satlength=407 Nr of Repeats=12
RepeatLength=31 seed=TCTGAGCTAC Num.seqs=11 Similarity=0.755258
0   TCTGAGCTACAGTACTCCTGTCAAGAACAAT

****
Consensus:

TCTGAGCTACAGTACTCCTGTCAAGAACAAT

>Cele-UNSB01_Fam_280_31_1 Nr. of seq. 1 Alignment length(with gaps) = 31
Alignment score = 0.000000
Cele-UNSB01_3:13121321-13121505 Satlength=185 Nr of Repeats=5
RepeatLength=31 seed=GATTTTTTCA Num.seqs=3 Similarity=0.659722
0   GATTTTTTCAGNGAAAANCNAAAAATTTTCG

****
Consensus:

GATTTTTTCAGNGAAAANCNAAAAATTTTCG

>Cele-UNSB01_Fam_281_31_1 Nr. of seq. 1 Alignment length(with gaps) = 31
Alignment score = 0.000000

Cele-UNSB01_3:13483516-13483661 Satlength=146 Nr of Repeats=5 RepeatLength=31 seed=CCAAAAATGA Num.seqs=4 Similarity=0.863799
0   CCAAAAATGACTGAAAATAGTCAAAAATTAG

****
Consensus:

CCAAAAATGACTGAAAATAGTCAAAAATTAG

>Cele-UNSB01_Fam_282_31_1 Nr. of seq. 1 Alignment length(with gaps) = 31 Alignment score = 0.000000
Cele-UNSB01_4:782171-782295 Satlength=125 Nr of Repeats=4 RepeatLength=31 seed=CAAAAATCGG Num.seqs=4 Similarity=0.949821
0   CAAAAATCGGGAAATTTTCACAAAAAAAATGC

****
Consensus:

CAAAAATCGGGAAATTTTCACAAAAAAAATGC

>Cele-UNSB01_Fam_283_31_1 Nr. of seq. 1 Alignment length(with gaps) = 31 Alignment score = 0.000000
Cele-UNSB01_5:18669912-18670449 Satlength=538 Nr of Repeats=16 RepeatLength=31 seed=TTGGCCCAGA Num.seqs=11 Similarity=0.885826
0   TTGGCCCAGAGCTCTCTTGACCCAAGCCCCC

****
Consensus:

TTGGCCCAGAGCTCTCTTGACCCAAGCCCCC

>Cele-UNSB01_Fam_284_31_1 Nr. of seq. 1 Alignment length(with gaps) = 31 Alignment score = 0.000000
Cele-UNSB01_5:18793643-18794338 Satlength=696 Nr of Repeats=5 RepeatLength=31 seed=AAAATTTTGA Num.seqs=3 Similarity=0.971326
0   AAAATTTTGACCCAGAAATCGAAAAATTCGC

****
Consensus:

AAAATTTTGACCCAGAAATCGAAAAATTCGC

>Cele-UNSB01_Fam_285_31_1 Nr. of seq. 1 Alignment length(with gaps) = 31 Alignment score = 0.000000
Cele-UNSB01_5:19138115-19138319 Satlength=205 Nr of Repeats=6 RepeatLength=31 seed=TCGTTTTTTC Num.seqs=5 Similarity=0.819355
0   TCGTTTTTTCATGAAAAAACTCAATTTTCGG

****
Consensus:

TCGTTTTTTCATGAAAAAACTCAATTTTCGG

>Cele-UNSB01_Fam_286_31_1 Nr. of seq. 1 Alignment length(with gaps) = 31
Alignment score = 0.000000
Cele-UNSB01_5:20720350-20720568 Satlength=219 Nr of Repeats=4
RepeatLength=31 seed=GAAAAAGTGC Num.seqs=3 Similarity=0.784722
0   GAAAAAGTGCTGAAAATCACATTTTTGAACT

****
Consensus:

GAAAAAGTGCTGAAAATCACATTTTTGAACT

>Cele-UNSB01_Fam_287_30_1 Nr. of seq. 1 Alignment length(with gaps) = 30
Alignment score = 0.000000
Cele-UNSB01_1:1730783-1731662 Satlength=880 Nr of Repeats=24
RepeatLength=30 seed=AATTTGAATT Num.seqs=15 Similarity=0.732487
0   AATTTGAATTTTCCCGAATTTTTNAACGAA

****
Consensus:

AATTTGAATTTTCCCGAATTTTTNAACGAA

>Cele-UNSB01_Fam_288_30_1 Nr. of seq. 1 Alignment length(with gaps) = 30
Alignment score = 0.000000
Cele-UNSB01_1:6651710-6652545 Satlength=836 Nr of Repeats=13
RepeatLength=30 seed=TGGTGGAACT Num.seqs=11 Similarity=0.953131
0   TGGTGGAACTGGATTCGGCAGTGGAAATGC

****
Consensus:

TGGTGGAACTGGATTCGGCAGTGGAAATGC

>Cele-UNSB01_Fam_289_30_1 Nr. of seq. 1 Alignment length(with gaps) = 30
Alignment score = 0.000000
Cele-UNSB01_1:11615647-11616001 Satlength=355 Nr of Repeats=10
RepeatLength=30 seed=CCTTGGGCTC Num.seqs=7 Similarity=0.875132
0   CCTTGGGCTCCCTGAGCTCCAAATTGTGCT

****
Consensus:

CCTTGGGCTCCCTGAGCTCCAAATTGTGCT

>Cele-UNSB01_Fam_290_30_1 Nr. of seq. 1 Alignment length(with gaps) = 30
Alignment score = 0.000000
Cele-UNSB01_1:12330775-12330985 Satlength=211 Nr of Repeats=7
RepeatLength=30 seed=GTCATTATCA Num.seqs=7 Similarity=0.974603
0   GTCATTATCAGCTTCTGGTGTCTTAGAAGA

****
Consensus:

GTCATTATCAGCTTCTGGTGTCTTAGAAGA

```
>Cele-UNSB01_Fam_291_30_1  Nr. of seq. 1 Alignment length(with gaps) = 30
Alignment score = 0.000000
Cele-UNSB01_2:12311843-12313532 Satlength=1690 Nr of Repeats=56
RepeatLength=30 seed=CAAACTACAG  Num.seqs=47 Similarity=0.880029
0    CAAACTACAGTAGTACTGTAGTACCATAAC

******************************
Consensus:

CAAACTACAGTAGTACTGTAGTACCATAAC

>Cele-UNSB01_Fam_292_30_1  Nr. of seq. 1 Alignment length(with gaps) = 30
Alignment score = 0.000000
Cele-UNSB01_2:13134300-13134450 Satlength=151 Nr of Repeats=4
RepeatLength=30 seed=TTCAGGCAGG  Num.seqs=3 Similarity=0.644444
0    TTCAGGCAGGCATAGGTTACTTTGAAAATT

******************************
Consensus:

TTCAGGCAGGCATAGGTTACTTTGAAAATT

>Cele-UNSB01_Fam_293_30_1  Nr. of seq. 1 Alignment length(with gaps) = 30
Alignment score = 0.000000
Cele-UNSB01_3:3873879-3874448 Satlength=570 Nr of Repeats=9
RepeatLength=30 seed=CCTTGTAGGC  Num.seqs=6 Similarity=0.842963
0    CCTTGTAGGCAAGGAGGTAGGCGTAGGTCA

******************************
Consensus:

CCTTGTAGGCAAGGAGGTAGGCGTAGGTCA

>Cele-UNSB01_Fam_294_30_1  Nr. of seq. 1 Alignment length(with gaps) = 30
Alignment score = 0.000000
Cele-UNSB01_3:13583922-13584205 Satlength=284 Nr of Repeats=8
RepeatLength=30 seed=TTTCAGGCCA  Num.seqs=5 Similarity=0.822222
0    TTTCAGGCCAATTTCNGTCATTTTTGGACA

******************************
Consensus:

TTTCAGGCCAATTTCNGTCATTTTTGGACA

>Cele-UNSB01_Fam_295_30_1  Nr. of seq. 1 Alignment length(with gaps) = 30
Alignment score = 0.000000
Cele-UNSB01_4:16899632-16900079 Satlength=448 Nr of Repeats=15
RepeatLength=30 seed=AAAATCAAAA  Num.seqs=12 Similarity=0.938721
0    AAAATCAAAAATTAGATGAATTTTCGATTA

******************************
Consensus:
```

AAAATCAAAAATTAGATGAATTTTCGATTA

>Cele-UNSB01_Fam_296_30_1 Nr. of seq. 1 Alignment length(with gaps) = 30
Alignment score = 0.000000
Cele-UNSB01_5:20558603-20558932 Satlength=330 Nr of Repeats=11
RepeatLength=30 seed=CTCAAATTTG Num.seqs=10 Similarity=0.911111
0   CTCAAATTTGCACTAAAAATTCCAATTTTT

****
Consensus:

CTCAAATTTGCACTAAAAATTCCAATTTTT

>Cele-UNSB01_Fam_297_30_1 Nr. of seq. 1 Alignment length(with gaps) = 30
Alignment score = 0.000000
Cele-UNSB01_6:1594210-1594900 Satlength=691 Nr of Repeats=19
RepeatLength=30 seed=ACTACCCAAC Num.seqs=18 Similarity=0.920407
0   ACTACCCAACCGGCTCCGGCAACCAGTCCA

****
Consensus:

ACTACCCAACCGGCTCCGGCAACCAGTCCA

>Cele-UNSB01_Fam_298_29_1 Nr. of seq. 1 Alignment length(with gaps) = 29
Alignment score = 0.000000
Cele-UNSB01_1:5273525-5290948 Satlength=17424 Nr of Repeats=599
RepeatLength=29 seed=TTGTAAGGAT Num.seqs=565 Similarity=0.955515
0   TTGTAAGGATTTCCCACTATGTCAATACA

****
Consensus:

TTGTAAGGATTTCCCACTATGTCAATACA

>Cele-UNSB01_Fam_299_29_1 Nr. of seq. 1 Alignment length(with gaps) = 29
Alignment score = 0.000000
Cele-UNSB01_3:10934450-10934819 Satlength=370 Nr of Repeats=11
RepeatLength=29 seed=GGCCTAGAAA Num.seqs=7 Similarity=0.700055
0   GGCCTAGAAANTTCTATTTGAAAGTTGAT

****
Consensus:

GGCCTAGAAANTTCTATTTGAAAGTTGAT

>Cele-UNSB01_Fam_300_29_1 Nr. of seq. 1 Alignment length(with gaps) = 29
Alignment score = 0.000000
Cele-UNSB01_4:14154469-14154585 Satlength=117 Nr of Repeats=4
RepeatLength=29 seed=TACATTGCAA Num.seqs=4 Similarity=0.946360
25   TTGCAAGATCTTCTTACTATGTAGGTACA

****

Consensus:

TACATTGCAAGATCTTCTTACTATGTAGG

>Cele-UNSB01_Fam_301_29_1 Nr. of seq. 1 Alignment length(with gaps) = 29
Alignment score = 0.000000
Cele-UNSB01_5:19567297-19567464 Satlength=168 Nr of Repeats=5
RepeatLength=29 seed=CTCGTCAGTG Num.seqs=4 Similarity=0.954023
0   CTCGTCAGTGGGTGGACGAGGAATGAGCG

****
Consensus:

CTCGTCAGTGGGTGGACGAGGAATGAGCG

>Cele-UNSB01_Fam_302_28_1 Nr. of seq. 1 Alignment length(with gaps) = 28
Alignment score = 0.000000
Cele-UNSB01_2:3009682-3009822 Satlength=141 Nr of Repeats=5
RepeatLength=28 seed=AATGTCGGCT Num.seqs=5 Similarity=0.847619
0   AATGTCGGCTGCTTCTAGAAACCAGGCA

****
Consensus:

AATGTCGGCTGCTTCTAGAAACCAGGCA

>Cele-UNSB01_Fam_303_28_1 Nr. of seq. 1 Alignment length(with gaps) = 28
Alignment score = 0.000000
Cele-UNSB01_2:3456136-3456276 Satlength=141 Nr of Repeats=4
RepeatLength=28 seed=TGCCTCGCCT Num.seqs=3 Similarity=0.968254
0   TGCCTCGCCTACGTGCTTATTTCAGGCA

****
Consensus:

TGCCTCGCCTACGTGCTTATTTCAGGCA

>Cele-UNSB01_Fam_304_28_1 Nr. of seq. 1 Alignment length(with gaps) = 28
Alignment score = 0.000000
Cele-UNSB01_5:18701783-18702035 Satlength=253 Nr of Repeats=9
RepeatLength=28 seed=CCAAGAGAAT Num.seqs=9 Similarity=0.915344
0   CCAAGAGAATTTCAACCACCTGGAAATT

****
Consensus:

CCAAGAGAATTTCAACCACCTGGAAATT

>Cele-UNSB01_Fam_305_27_1 Nr. of seq. 1 Alignment length(with gaps) = 27
Alignment score = 0.000000
Cele-UNSB01_2:13831609-13831953 Satlength=345 Nr of Repeats=12
RepeatLength=27 seed=TTCCGGCAAA Num.seqs=8 Similarity=0.904762
0   TTCCGGCAAATCGGCAAATTGCAAAAT

****
Consensus:

TTCCGGCAAATCGGCAAATTGCAAAAT

>Cele-UNSB01_Fam_306_27_1 Nr. of seq. 1 Alignment length(with gaps) = 27
Alignment score = 0.000000
Cele-UNSB01_4:12649750-12649885 Satlength=136 Nr of Repeats=5
RepeatLength=27 seed=ACAATAAAGT Num.seqs=5 Similarity=1.000000
0   ACAATAAAGTACTATGTATTACCGTAT

****
Consensus:

ACAATAAAGTACTATGTATTACCGTAT

>Cele-UNSB01_Fam_307_27_1 Nr. of seq. 1 Alignment length(with gaps) = 27
Alignment score = 0.000000
Cele-UNSB01_5:5757785-5758298 Satlength=514 Nr of Repeats=19
RepeatLength=27 seed=TGGAGGCGGC Num.seqs=19 Similarity=0.948018
0   TGGAGGCGGCTTCTGGCCTGGTGGTGG

****
Consensus:

TGGAGGCGGCTTCTGGCCTGGTGGTGG

>Cele-UNSB01_Fam_308_27_1 Nr. of seq. 1 Alignment length(with gaps) = 27
Alignment score = 0.000000
Cele-UNSB01_6:11249167-11249852 Satlength=686 Nr of Repeats=21
RepeatLength=27 seed=CACTGACCAA Num.seqs=15 Similarity=0.793063
0   CACTGACCAAAGAAAATATTANACGTG

****
Consensus:

CACTGACCAAAGAAAATATTANACGTG

>Cele-UNSB01_Fam_309_26_1 Nr. of seq. 1 Alignment length(with gaps) = 26
Alignment score = 0.000000
Cele-UNSB01_1:3159205-3159335 Satlength=131 Nr of Repeats=4
RepeatLength=26 seed=GGCAGGCGTC Num.seqs=3 Similarity=0.931624
0   GGCAGGCGTCAAGCAGGCAGGTTTCA

****
Consensus:

GGCAGGCGTCAAGCAGGCAGGTTTCA

>Cele-UNSB01_Fam_310_26_1 Nr. of seq. 1 Alignment length(with gaps) = 26
Alignment score = 0.000000

Cele-UNSB01_1:11519985-11520529 Satlength=545 Nr of Repeats=18
RepeatLength=26 seed=ACCTACAGTA Num.seqs=12 Similarity=0.881896
0   ACCTACAGTACCCCTGTCCTAGAATG

****
Consensus:

ACCTACAGTACCCCTGTCCTAGAATG

>Cele-UNSB01_Fam_311_26_1 Nr. of seq. 1 Alignment length(with gaps) = 26
Alignment score = 0.000000
Cele-UNSB01_2:1186731-1187015 Satlength=285 Nr of Repeats=8
RepeatLength=26 seed=TACTGTAGTT Num.seqs=5 Similarity=0.621429
0   TACTGTAGTTTGACCTACTTANGGAT

****
Consensus:

TACTGTAGTTTGACCTACTTANGGAT

>Cele-UNSB01_Fam_312_26_1 Nr. of seq. 1 Alignment length(with gaps) = 26
Alignment score = 0.000000
Cele-UNSB01_2:1915179-1915855 Satlength=677 Nr of Repeats=26
RepeatLength=26 seed=ATTTACGGAG Num.seqs=22 Similarity=0.829282
0   ATTTACGGAGCTTCAAGCTTGTGTCG

****
Consensus:

ATTTACGGAGCTTCAAGCTTGTGTCG

>Cele-UNSB01_Fam_313_26_1 Nr. of seq. 1 Alignment length(with gaps) = 26
Alignment score = 0.000000
Cele-UNSB01_5:19352170-19352944 Satlength=775 Nr of Repeats=29
RepeatLength=26 seed=TGAAATTTCA Num.seqs=20 Similarity=0.904453
0   TGAAATTTCACAAAATATCGATTTTC

****
Consensus:

TGAAATTTCACAAAATATCGATTTTC

>Cele-UNSB01_Fam_314_25_1 Nr. of seq. 1 Alignment length(with gaps) = 25
Alignment score = 0.000000
Cele-UNSB01_2:1097712-1098091 Satlength=380 Nr of Repeats=5
RepeatLength=26 seed=GTCGGCTGCT Num.seqs=3 Similarity=0.339869
0   GTCGGCTGCTTNCAAACCCTGAGTT

****
Consensus:

GTCGGCTGCTTNCAAACCCTGAGTT

>Cele-UNSB01_Fam_315_25_1 Nr. of seq. 1 Alignment length(with gaps) = 25
Alignment score = 0.000000
Cele-UNSB01_5:19714208-19714335 Satlength=128 Nr of Repeats=5
RepeatLength=25 seed=AAAAATCTGT Num.seqs=3 Similarity=0.822222
0   AAAAATCTGTTCTNNGAGGTCCCTG

****
Consensus:

AAAAATCTGTTCTNNGAGGTCCCTG

>Cele-UNSB01_Fam_316_24_1 Nr. of seq. 1 Alignment length(with gaps) = 24
Alignment score = 0.000000
Cele-UNSB01_2:4270872-4271040 Satlength=169 Nr of Repeats=5
RepeatLength=24 seed=GTGAGGTACG Num.seqs=3 Similarity=0.611111
0   GTGAGGTACGAGGTGAGGTANGAG

****
Consensus:

GTGAGGTACGAGGTGAGGTANGAG

>Cele-UNSB01_Fam_317_24_1 Nr. of seq. 1 Alignment length(with gaps) = 24
Alignment score = 0.000000
Cele-UNSB01_3:12700775-12700871 Satlength=97 Nr of Repeats=4
RepeatLength=24 seed=AAGCACCACC Num.seqs=4 Similarity=0.972222
0   AAGCACCACCTCCTCCAACTACAA

****
Consensus:

AAGCACCACCTCCTCCAACTACAA

>Cele-UNSB01_Fam_318_24_1 Nr. of seq. 1 Alignment length(with gaps) = 24
Alignment score = 0.000000
Cele-UNSB01_3:13900788-13900959 Satlength=172 Nr of Repeats=7
RepeatLength=24 seed=AAGAAGGATG Num.seqs=6 Similarity=0.825926
0   AAGAAGGATGCTGAAGAGAAGAAG

****
Consensus:

AAGAAGGATGCTGAAGAGAAGAAG

>Cele-UNSB01_Fam_319_24_1 Nr. of seq. 1 Alignment length(with gaps) = 24
Alignment score = 0.000000
Cele-UNSB01_4:2892401-2892617 Satlength=217 Nr of Repeats=5
RepeatLength=24 seed=GCTTCTGGAG Num.seqs=3 Similarity=0.777778
0   GCTTCTGGAGCTACAGGTTATCCA

****
Consensus:

GCTTCTGGAGCTACAGGTTATCCA

>Cele-UNSB01_Fam_320_24_1 Nr. of seq. 1 Alignment length(with gaps) = 24
Alignment score = 0.000000
Cele-UNSB01_4:17584126-17584294 Satlength=169 Nr of Repeats=7
RepeatLength=24 seed=TCAACAACAA Num.seqs=7 Similarity=1.000000
0 TCAACAACAACAGGAGAAGCTTCT

****
Consensus:

TCAACAACAACAGGAGAAGCTTCT

>Cele-UNSB01_Fam_321_24_1 Nr. of seq. 1 Alignment length(with gaps) = 24
Alignment score = 0.000000
Cele-UNSB01_6:8111999-8112167 Satlength=169 Nr of Repeats=7
RepeatLength=24 seed=GGTGGCTGGT Num.seqs=7 Similarity=1.000000
0 GGTGGCTGGTCACAAAAGTAAGTT

****
Consensus:

GGTGGCTGGTCACAAAAGTAAGTT

>Cele-UNSB01_Fam_322_23_1 Nr. of seq. 1 Alignment length(with gaps) = 23
Alignment score = 0.000000
Cele-UNSB01_1:14577219-14577388 Satlength=170 Nr of Repeats=5
RepeatLength=23 seed=GCACCATAAG Num.seqs=3 Similarity=0.726852
0 GCATCATAAGGATCATAGTTGAT

****
Consensus:

GCATCATAAGGATCATAGTTGAT

>Cele-UNSB01_Fam_323_23_1 Nr. of seq. 1 Alignment length(with gaps) = 23
Alignment score = 0.000000
Cele-UNSB01_2:1599041-1599824 Satlength=784 Nr of Repeats=24
RepeatLength=23 seed=CGCTGTAAGA Num.seqs=16 Similarity=0.806763
0 CGCTGTAAGACCCTTCGCTGTAC

****
Consensus:

CGCTGTAAGACCCTTCGCTGTAC

>Cele-UNSB01_Fam_324_23_1 Nr. of seq. 1 Alignment length(with gaps) = 23
Alignment score = 0.000000
Cele-UNSB01_2:15522229-15522469 Satlength=241 Nr of Repeats=12
RepeatLength=23 seed=GCTACAGTAC Num.seqs=8 Similarity=0.821946
0 GCTACAGTACCCCAGCTTGACTT

****
Consensus:

GCTACAGTACCCCAGCTTGACTT

>Cele-UNSB01_Fam_325_23_1 Nr. of seq. 1 Alignment length(with gaps) = 23
Alignment score = 0.000000
Cele-UNSB01_5:19784949-19785086 Satlength=138 Nr of Repeats=6
RepeatLength=23 seed=TTTTCAGAAA Num.seqs=5 Similarity=0.631944
0 TTTTCAGAAATAGGTATTTCAGA

****
Consensus:

TTTTCAGAAATAGGTATTTCAGA

>Cele-UNSB01_Fam_326_22_1 Nr. of seq. 1 Alignment length(with gaps) = 22
Alignment score = 0.000000
Cele-UNSB01_1:817628-817952 Satlength=325 Nr of Repeats=8 RepeatLength=22
seed=TTAAAATTTT Num.seqs=6 Similarity=0.886869
0 TTAAAATTTTGTCAGTTTGACC

****
Consensus:

TTAAAATTTTGTCAGTTTGACC

>Cele-UNSB01_Fam_327_22_1 Nr. of seq. 1 Alignment length(with gaps) = 22
Alignment score = 0.000000
Cele-UNSB01_1:907957-908415 Satlength=459 Nr of Repeats=21
RepeatLength=22 seed=TTTTAGCCAA Num.seqs=17 Similarity=0.862299
0 TTTTAGCCAAAAAAATCCGTTAA

****
Consensus:

TTTTAGCCAAAAAAATCCGTTAA

>Cele-UNSB01_Fam_328_22_1 Nr. of seq. 1 Alignment length(with gaps) = 22
Alignment score = 0.000000
Cele-UNSB01_1:14511380-14511819 Satlength=440 Nr of Repeats=17
RepeatLength=22 seed=GCACGGCCTG Num.seqs=13 Similarity=0.701226
0 GCACGGCCTGGCGGTCCTGAGG

****
Consensus:

GCACGGCCTGGCGGTCCTGAGG

>Cele-UNSB01_Fam_329_22_1 Nr. of seq. 1 Alignment length(with gaps) = 22
Alignment score = 0.000000
Cele-UNSB01_2:14303812-14304194 Satlength=383 Nr of Repeats=12
RepeatLength=22 seed=CTACAGTAAC Num.seqs=8 Similarity=0.798395
0 CTACAGTAACCCTAGTATTGAA

****

Consensus:

CTACAGTAACCCTAGTATTGAA

>Cele-UNSB01_Fam_330_22_1 Nr. of seq. 1 Alignment length(with gaps) = 22
Alignment score = 0.000000
Cele-UNSB01_3:4344679-4344767 Satlength=89 Nr of Repeats=4
RepeatLength=22 seed=CAAAACCCAA Num.seqs=4 Similarity=0.939394
0   CAAAACCCAAGTAGATCCCACT

****
Consensus:

CAAAACCCAAGTAGATCCCACT

>Cele-UNSB01_Fam_331_22_1 Nr. of seq. 1 Alignment length(with gaps) = 22
Alignment score = 0.000000
Cele-UNSB01_4:6669007-6669090 Satlength=84 Nr of Repeats=4
RepeatLength=22 seed=AAGCGGTAAC Num.seqs=3 Similarity=1.000000
0   AAGCGGTAACGTCGTCTATATA

****
Consensus:

AAGCGGTAACGTCGTCTATATA

>Cele-UNSB01_Fam_332_22_1 Nr. of seq. 1 Alignment length(with gaps) = 22
Alignment score = 0.000000
Cele-UNSB01_5:17108364-17108469 Satlength=106 Nr of Repeats=5
RepeatLength=21 seed=TGCTAATTAG Num.seqs=3 Similarity=0.545455
0   TGCTAATTAGCGCGGNTGCGTN

****
Consensus:

TGCTAATTAGCGCGGNTGCGTN

>Cele-UNSB01_Fam_333_22_1 Nr. of seq. 1 Alignment length(with gaps) = 22
Alignment score = 0.000000
Cele-UNSB01_5:19721189-19721423 Satlength=235 Nr of Repeats=7
RepeatLength=22 seed=ACGTATACAG Num.seqs=5 Similarity=0.915152
0   ACGTATACAGGCATATACACAT

****
Consensus:

ACGTATACAGGCATATACACAT

>Cele-UNSB01_Fam_334_22_1 Nr. of seq. 1 Alignment length(with gaps) = 22
Alignment score = 0.000000
Cele-UNSB01_6:1991346-1991764 Satlength=419 Nr of Repeats=14
RepeatLength=22 seed=CCGGCGTGGT Num.seqs=9 Similarity=0.677134
0   CCGGCGTGGTNTTGAAGCGGTC

****
Consensus:

CCGGCGTGGTNTTGAAGCGGTC

>Cele-UNSB01_Fam_335_22_1 Nr. of seq. 1 Alignment length(with gaps) = 22
Alignment score = 0.000000
Cele-UNSB01_6:14983794-14983948 Satlength=155 Nr of Repeats=5
RepeatLength=22 seed=TTTGTGGGAG Num.seqs=3 Similarity=0.757576
0   TTTGTGGGAGGCGACATATACC

****
Consensus:

TTTGTGGGAGGCGACATATACC

>Cele-UNSB01_Fam_336_21_1 Nr. of seq. 1 Alignment length(with gaps) = 21
Alignment score = 0.000000
Cele-UNSB01_1:833000-833653 Satlength=654 Nr of Repeats=29
RepeatLength=21 seed=TGGCCTAGAT Num.seqs=21 Similarity=0.658586
0   TGGCCTAGATTNTTTACAGGG

****
Consensus:

TGGCCTAGATTNTTTACAGGG

>Cele-UNSB01_Fam_337_21_1 Nr. of seq. 1 Alignment length(with gaps) = 21
Alignment score = 0.000000
Cele-UNSB01_1:1679297-1679633 Satlength=337 Nr of Repeats=16
RepeatLength=21 seed=TCTCCTGGTA Num.seqs=16 Similarity=0.963492
0   TCTCCTGGTAAATTTAAAAAT

****
Consensus:

TCTCCTGGTAAATTTAAAAAT

>Cele-UNSB01_Fam_338_21_1 Nr. of seq. 1 Alignment length(with gaps) = 21
Alignment score = 0.000000
Cele-UNSB01_1:12807224-12807329 Satlength=106 Nr of Repeats=5
RepeatLength=21 seed=ACCACCCCTA Num.seqs=5 Similarity=0.815873
0   ACCACCCCTACAACNACCACC

****
Consensus:

ACCACCCCTACAACNACCACC

>Cele-UNSB01_Fam_339_21_1 Nr. of seq. 1 Alignment length(with gaps) = 21
Alignment score = 0.000000

Cele-UNSB01_1:15278589-15278861 Satlength=273 Nr of Repeats=10
RepeatLength=21 seed=AACTTTTTGG Num.seqs=6 Similarity=0.791919
0   AACTTTTTGGCAATTTTCCAA

****
Consensus:

AACTTTTTGGCAATTTTCCAA

>Cele-UNSB01_Fam_340_21_1 Nr. of seq. 1 Alignment length(with gaps) = 21
Alignment score = 0.000000
Cele-UNSB01_2:2291158-2291371 Satlength=214 Nr of Repeats=8
RepeatLength=21 seed=TGTGCCCTGG Num.seqs=6 Similarity=0.936508
0   TGTGCCCTGGAAAGCTGGCAG

****
Consensus:

TGTGCCCTGGAAAGCTGGCAG

>Cele-UNSB01_Fam_341_21_1 Nr. of seq. 1 Alignment length(with gaps) = 21
Alignment score = 0.000000
Cele-UNSB01_2:4119390-4119751 Satlength=362 Nr of Repeats=4
RepeatLength=21 seed=CTACGTAAGA Num.seqs=3 Similarity=0.830688
0   CTACGTAAGAGTAGCGCAGGA

****
Consensus:

CTACGTAAGAGTAGCGCAGGA

>Cele-UNSB01_Fam_342_21_1 Nr. of seq. 1 Alignment length(with gaps) = 21
Alignment score = 0.000000
Cele-UNSB01_2:13339084-13339231 Satlength=148 Nr of Repeats=7
RepeatLength=21 seed=AATCTAGCCA Num.seqs=7 Similarity=0.930461
0   AATCTAGCCAGACGCACACCT

****
Consensus:

AATCTAGCCAGACGCACACCT

>Cele-UNSB01_Fam_343_21_1 Nr. of seq. 1 Alignment length(with gaps) = 21
Alignment score = 0.000000
Cele-UNSB01_2:14401364-14401594 Satlength=231 Nr of Repeats=10
RepeatLength=21 seed=CAATTTTCTG Num.seqs=8 Similarity=0.841270
0   CAATTTTCTGTTNAACTTTGA

****
Consensus:

CAATTTTCTGTTNAACTTTGA

```
>Cele-UNSB01_Fam_344_21_1  Nr. of seq. 1 Alignment length(with gaps) = 21
Alignment score = 0.000000
Cele-UNSB01_3:8499608-8499713 Satlength=106 Nr of Repeats=5
RepeatLength=21 seed=AAGTTTTACT  Num.seqs=5 Similarity=0.974603
0    AAGTTTTACTTTGATTTTTTT

*********************
Consensus:

AAGTTTTACTTTGATTTTTTT

>Cele-UNSB01_Fam_345_21_1  Nr. of seq. 1 Alignment length(with gaps) = 21
Alignment score = 0.000000
Cele-UNSB01_3:11543247-11543687 Satlength=441 Nr of Repeats=21
RepeatLength=21 seed=GGCAATTTTC  Num.seqs=20 Similarity=0.903425
0    GGCAATTTTCCAGTGCAATTC

*********************
Consensus:

GGCAATTTTCCAGTGCAATTC

>Cele-UNSB01_Fam_346_21_1  Nr. of seq. 1 Alignment length(with gaps) = 21
Alignment score = 0.000000
Cele-UNSB01_4:1580035-1580182 Satlength=148 Nr of Repeats=7
RepeatLength=21 seed=CCATAGGTGG  Num.seqs=7 Similarity=0.794407
0    CCATAGGTGGAAGATCTNATA

*********************
Consensus:

CCATAGGTGGAAGATCTNATA

>Cele-UNSB01_Fam_347_21_1  Nr. of seq. 1 Alignment length(with gaps) = 21
Alignment score = 0.000000
Cele-UNSB01_4:2179058-2179920 Satlength=863 Nr of Repeats=38
RepeatLength=21 seed=GATTTACGGG  Num.seqs=31 Similarity=0.686520
0    GATTTACGGGGTGAACATGTG

*********************
Consensus:

GATTTACGGGGTGAACATGTG

>Cele-UNSB01_Fam_348_21_1  Nr. of seq. 1 Alignment length(with gaps) = 21
Alignment score = 0.000000
Cele-UNSB01_5:6410601-6410727 Satlength=127 Nr of Repeats=6
RepeatLength=21 seed=TTGGTGACAA  Num.seqs=6 Similarity=1.000000
0    TTGGTGACAATGTGGAAACAA

*********************
Consensus:

TTGGTGACAATGTGGAAACAA
```

```
>Cele-UNSB01_Fam_349_21_1  Nr. of seq. 1 Alignment length(with gaps) = 21
Alignment score = 0.000000
Cele-UNSB01_5:9975370-9975475 Satlength=106 Nr of Repeats=5
RepeatLength=21 seed=TTCTAATTCG  Num.seqs=5 Similarity=0.726984
0    TTCTAATTCGACGCGCATNGT

*********************
Consensus:

TTCTAATTCGACGCGCATNGT

>Cele-UNSB01_Fam_350_21_1  Nr. of seq. 1 Alignment length(with gaps) = 21
Alignment score = 0.000000
Cele-UNSB01_5:17104481-17104658 Satlength=178 Nr of Repeats=5
RepeatLength=21 seed=CTAATTAGCG  Num.seqs=3 Similarity=0.640212
0    CTAATTAGCGTCGCTTCCNTT

*********************
Consensus:

CTAATTAGCGTCGCTTCCNTT

>Cele-UNSB01_Fam_351_21_1  Nr. of seq. 1 Alignment length(with gaps) = 21
Alignment score = 0.000000
Cele-UNSB01_5:18902702-18903276 Satlength=575 Nr of Repeats=24
RepeatLength=21 seed=AAAAATCGAT  Num.seqs=18 Similarity=0.739602
0    AAAAATCGATAAAATTTGGAN

*********************
Consensus:

AAAAATCGATAAAATTTGGAN

>Cele-UNSB01_Fam_352_21_1  Nr. of seq. 1 Alignment length(with gaps) = 21
Alignment score = 0.000000
Cele-UNSB01_5:19549755-19549902 Satlength=148 Nr of Repeats=7
RepeatLength=21 seed=CACACGAGCT  Num.seqs=7 Similarity=0.727891
0    CACACGAGCTTGAGAGCCCGG

*********************
Consensus:

CACACGAGCTTGAGAGCCCGG

>Cele-UNSB01_Fam_353_21_1  Nr. of seq. 1 Alignment length(with gaps) = 21
Alignment score = 0.000000
Cele-UNSB01_5:19721079-19721595 Satlength=517 Nr of Repeats=5
RepeatLength=21 seed=TATTTTCAAA  Num.seqs=3 Similarity=0.957672
0    TATTTTCAAAAAAATTTAAAA

*********************
Consensus:
```

TATTTTCAAAAAAATTTAAAA

>Cele-UNSB01_Fam_354_21_1 Nr. of seq. 1 Alignment length(with gaps) = 21
Alignment score = 0.000000
Cele-UNSB01_5:19953202-19953370 Satlength=169 Nr of Repeats=6
RepeatLength=21 seed=ACCCCGATTT Num.seqs=5 Similarity=0.911111
0   ACCCCGATTTTCACCCAAAAA

****
Consensus:

ACCCCGATTTTCACCCAAAAA

>Cele-UNSB01_Fam_355_21_1 Nr. of seq. 1 Alignment length(with gaps) = 21
Alignment score = 0.000000
Cele-UNSB01_5:20275164-20275248 Satlength=85 Nr of Repeats=4
RepeatLength=21 seed=CTCAATTCAA Num.seqs=4 Similarity=0.883598
0   CTCAATTCAATCAAGGAAATC

****
Consensus:

CTCAATTCAATCAAGGAAATC

>Cele-UNSB01_Fam_356_21_1 Nr. of seq. 1 Alignment length(with gaps) = 21
Alignment score = 0.000000
Cele-UNSB01_6:3834410-3834530 Satlength=121 Nr of Repeats=5
RepeatLength=20 seed=CCATGTAGCT Num.seqs=4 Similarity=0.561869
0   CCATGTAGCTTCAAAATTNCT

****
Consensus:

CCATGTAGCTTCAAAATTNCT

>Cele-UNSB01_Fam_357_21_1 Nr. of seq. 1 Alignment length(with gaps) = 21
Alignment score = 0.000000
Cele-UNSB01_6:15876707-15876856 Satlength=150 Nr of Repeats=4
RepeatLength=21 seed=TGTCCGTGAT Num.seqs=3 Similarity=0.666667
0   TGTCCGTGATGATCATGNGCA

****
Consensus:

TGTCCGTGATGATCATGNGCA

>Cele-UNSB01_Fam_358_21_1 Nr. of seq. 1 Alignment length(with gaps) = 21
Alignment score = 0.000000
Cele-UNSB01_6:17463543-17463648 Satlength=106 Nr of Repeats=5
RepeatLength=21 seed=GGTGGACCCA Num.seqs=5 Similarity=0.822222
0   GGTGGACCCANGGGTTCCTAG

****

Consensus:

GGTGGACCCANGGGTTCCTAG

>Cele-UNSB01_Fam_359_20_1 Nr. of seq. 1 Alignment length(with gaps) = 20
Alignment score = 0.000000
Cele-UNSB01_2:3687013-3687103 Satlength=91 Nr of Repeats=5
RepeatLength=20 seed=CTATCGCCTG Num.seqs=4 Similarity=0.833333
0   CTATCGCCTGCTATCGCCTA

****
Consensus:

CTATCGCCTGCTATCGCCTA

>Cele-UNSB01_Fam_360_20_1 Nr. of seq. 1 Alignment length(with gaps) = 20
Alignment score = 0.000000
Cele-UNSB01_5:17995705-17996016 Satlength=312 Nr of Repeats=5
RepeatLength=20 seed=ATTTGACCTA Num.seqs=4 Similarity=0.900000
0   ATTTGACCTACTTTTTAAAA

****
Consensus:

ATTTGACCTACTTTTTAAAA

>Cele-UNSB01_Fam_361_19_1 Nr. of seq. 1 Alignment length(with gaps) = 19
Alignment score = 0.000000
Cele-UNSB01_1:360798-360950 Satlength=153 Nr of Repeats=8 RepeatLength=19
seed=GTACTACCTT Num.seqs=8 Similarity=0.982456
0   GTACTACCTTTTAATGTAT

****
Consensus:

GTACTACCTTTTAATGTAT

>Cele-UNSB01_Fam_362_19_1 Nr. of seq. 1 Alignment length(with gaps) = 19
Alignment score = 0.000000
Cele-UNSB01_1:14381938-14382071 Satlength=134 Nr of Repeats=7
RepeatLength=19 seed=CCCTGATACC Num.seqs=7 Similarity=0.788889
0   CCCTGATACCCCCTAGTCC

****
Consensus:

CCCTGATACCCCCTAGTCC

>Cele-UNSB01_Fam_363_19_1 Nr. of seq. 1 Alignment length(with gaps) = 19
Alignment score = 0.000000
Cele-UNSB01_2:13161799-13161943 Satlength=145 Nr of Repeats=6
RepeatLength=19 seed=TATGGTGCAT Num.seqs=4 Similarity=0.707602
0   TATGGTGCATCTAACTAGT

****
Consensus:

TATGGTGCATCTAACTAGT

>Cele-UNSB01_Fam_364_18_1 Nr. of seq. 1 Alignment length(with gaps) = 18
Alignment score = 0.000000
Cele-UNSB01_1:10842122-10842194 Satlength=73 Nr of Repeats=4
RepeatLength=18 seed=AAAAGTTACA Num.seqs=4 Similarity=1.000000
0   AAAAGTTACAGTGTCCTT

****
Consensus:

AAAAGTTACAGTGTCCTT

>Cele-UNSB01_Fam_365_18_1 Nr. of seq. 1 Alignment length(with gaps) = 18
Alignment score = 0.000000
Cele-UNSB01_1:14582954-14583490 Satlength=537 Nr of Repeats=26
RepeatLength=18 seed=TTGTAGATCA Num.seqs=22 Similarity=0.869489
0   TTGTAGATCAACGGGGTC

****
Consensus:

TTGTAGATCAACGGGGTC

>Cele-UNSB01_Fam_366_18_1 Nr. of seq. 1 Alignment length(with gaps) = 18
Alignment score = 0.000000
Cele-UNSB01_2:1488365-1488455 Satlength=91 Nr of Repeats=5
RepeatLength=18 seed=GGAGGTCCAG Num.seqs=5 Similarity=0.859259
0   GGAGGTCCAGGTACCCCG

****
Consensus:

GGAGGTCCAGGTACCCCG

>Cele-UNSB01_Fam_367_18_1 Nr. of seq. 1 Alignment length(with gaps) = 18
Alignment score = 0.000000
Cele-UNSB01_2:10597740-10597833 Satlength=94 Nr of Repeats=5
RepeatLength=18 seed=TAAATAAGTG Num.seqs=3 Similarity=0.950617
0   TAAATAAGTGGCTATAAT

****
Consensus:

TAAATAAGTGGCTATAAT

>Cele-UNSB01_Fam_368_18_1 Nr. of seq. 1 Alignment length(with gaps) = 18
Alignment score = 0.000000

Cele-UNSB01_2:13043920-13044316 Satlength=397 Nr of Repeats=21
RepeatLength=18 seed=ACTACTGTAT Num.seqs=18 Similarity=0.797628
0   ACTACTGTATCNCAGGAG

****
Consensus:

ACTACTGTATCNCAGGAG

>Cele-UNSB01_Fam_369_18_1 Nr. of seq. 1 Alignment length(with gaps) = 18
Alignment score = 0.000000
Cele-UNSB01_4:3232737-3232899 Satlength=163 Nr of Repeats=6
RepeatLength=18 seed=CCTTCGCCTA Num.seqs=4 Similarity=0.765432
0   CCTTCGCCTACCTACGTG

****
Consensus:

CCTTCGCCTACCTACGTG

>Cele-UNSB01_Fam_370_18_1 Nr. of seq. 1 Alignment length(with gaps) = 18
Alignment score = 0.000000
Cele-UNSB01_4:4398109-4398181 Satlength=73 Nr of Repeats=4
RepeatLength=18 seed=TTTTTTGATA Num.seqs=4 Similarity=0.962963
0   TTTTTTGATAATTCTTGA

****
Consensus:

TTTTTTGATAATTCTTGA

>Cele-UNSB01_Fam_371_18_1 Nr. of seq. 1 Alignment length(with gaps) = 18
Alignment score = 0.000000
Cele-UNSB01_5:4240718-4240800 Satlength=83 Nr of Repeats=4
RepeatLength=18 seed=AAATATTCTT Num.seqs=3 Similarity=0.851852
0   AAATATTCTTATTTTCAG

****
Consensus:

AAATATTCTTATTTTCAG

>Cele-UNSB01_Fam_372_18_1 Nr. of seq. 1 Alignment length(with gaps) = 18
Alignment score = 0.000000
Cele-UNSB01_5:4631559-4631760 Satlength=202 Nr of Repeats=8
RepeatLength=18 seed=CCTGGCTGTC Num.seqs=5 Similarity=0.607407
0   CCTGGTTGTCCNTCTNNT

****
Consensus:

CCTGGTTGTCCNTCTNNT

```
>Cele-UNSB01_Fam_373_18_1  Nr. of seq. 1 Alignment length(with gaps) = 18
Alignment score = 0.000000
Cele-UNSB01_5:7379892-7380037 Satlength=146 Nr of Repeats=8
RepeatLength=18 seed=TTTCATAAGA  Num.seqs=7 Similarity=0.943563
0    TTTCATAAGAAGTAAATN

******************
Consensus:

TTTCATAAGAAGTAAATN

>Cele-UNSB01_Fam_374_18_1  Nr. of seq. 1 Alignment length(with gaps) = 18
Alignment score = 0.000000
Cele-UNSB01_5:20522553-20523111 Satlength=559 Nr of Repeats=29
RepeatLength=18 seed=CATTGTCACT  Num.seqs=27 Similarity=0.884774
0    CATTGTCACTTTCTTCTC

******************
Consensus:

CATTGTCACTTTCTTCTC

>Cele-UNSB01_Fam_375_18_1  Nr. of seq. 1 Alignment length(with gaps) = 18
Alignment score = 0.000000
Cele-UNSB01_6:1911366-1912554 Satlength=1189 Nr of Repeats=59
RepeatLength=18 seed=AAGCCTATGC  Num.seqs=44 Similarity=0.776212
0    AAGCCTATGCCTTTCCCA

******************
Consensus:

AAGCCTATGCCTTTCCCA

>Cele-UNSB01_Fam_376_17_1  Nr. of seq. 1 Alignment length(with gaps) = 17
Alignment score = 0.000000
Cele-UNSB01_2:12716681-12716766 Satlength=86 Nr of Repeats=5
RepeatLength=17 seed=ACTTGGTACA  Num.seqs=5 Similarity=0.890196
0    ACTTGGTACATAGGCCA

*****************
Consensus:

ACTTGGTACATAGGCCA

>Cele-UNSB01_Fam_377_17_1  Nr. of seq. 1 Alignment length(with gaps) = 17
Alignment score = 0.000000
Cele-UNSB01_3:12692847-12693292 Satlength=446 Nr of Repeats=15
RepeatLength=17 seed=ATTTTTTCCG  Num.seqs=10 Similarity=0.687449
0    ATTTTTTCCGCAAGTAG

*****************
Consensus:

ATTTTTTCCGCAAGTAG
```

>Cele-UNSB01_Fam_378_17_1 Nr. of seq. 1 Alignment length(with gaps) = 17
Alignment score = 0.000000
Cele-UNSB01_4:11742877-11743140 Satlength=264 Nr of Repeats=13
RepeatLength=17 seed=AAAATCAAAC Num.seqs=10 Similarity=0.809259
0   AAAATCAAACCGGCGGA

****
Consensus:

AAAATCAAACCGGCGGA

>Cele-UNSB01_Fam_379_17_1 Nr. of seq. 1 Alignment length(with gaps) = 17
Alignment score = 0.000000
Cele-UNSB01_5:4844742-4844913 Satlength=172 Nr of Repeats=5
RepeatLength=17 seed=AAATGCCAAA Num.seqs=3 Similarity=0.641975
0   AAATGCCAAAANTGCCA

****
Consensus:

AAATGCCAAAANTGCCA

>Cele-UNSB01_Fam_380_17_1 Nr. of seq. 1 Alignment length(with gaps) = 17
Alignment score = 0.000000
Cele-UNSB01_5:16819160-16819347 Satlength=188 Nr of Repeats=11
RepeatLength=17 seed=TAACCTGACT Num.seqs=11 Similarity=0.957219
0   TAACCTGACTGCTACAG

****
Consensus:

TAACCTGACTGCTACAG

>Cele-UNSB01_Fam_381_17_1 Nr. of seq. 1 Alignment length(with gaps) = 17
Alignment score = 0.000000
Cele-UNSB01_5:19696042-19696279 Satlength=238 Nr of Repeats=14
RepeatLength=17 seed=GAATTAGAAA Num.seqs=13 Similarity=0.554584
0   GAATTAGAAAAATTTGA

****
Consensus:

GAATTAGAAAAATTTGA

>Cele-UNSB01_Fam_382_17_1 Nr. of seq. 1 Alignment length(with gaps) = 17
Alignment score = 0.000000
Cele-UNSB01_5:19785568-19787058 Satlength=1491 Nr of Repeats=85
RepeatLength=17 seed=TTGCAAGAAA Num.seqs=83 Similarity=0.953951
0   TTGCAAGAAAATCTGCA

****
Consensus:

TTGCAAGAAAATCTGCA

>Cele-UNSB01_Fam_383_17_1 Nr. of seq. 1 Alignment length(with gaps) = 17
Alignment score = 0.000000
Cele-UNSB01_6:3463610-3463814 Satlength=205 Nr of Repeats=10
RepeatLength=17 seed=TTTTAACGTG Num.seqs=8 Similarity=0.901961
0   TTTTAACGTGACCTAGT

****
Consensus:

TTTTAACGTGACCTAGT

>Cele-UNSB01_Fam_384_16_1 Nr. of seq. 1 Alignment length(with gaps) = 16
Alignment score = 0.000000
Cele-UNSB01_1:12783664-12784000 Satlength=337 Nr of Repeats=20
RepeatLength=16 seed=TGATCTGATG Num.seqs=18 Similarity=0.831699
0   TGATCTGATGATCTGA

****
Consensus:

TGATCTGATGATCTGA

>Cele-UNSB01_Fam_385_16_1 Nr. of seq. 1 Alignment length(with gaps) = 16
Alignment score = 0.000000
Cele-UNSB01_1:13315136-13315216 Satlength=81 Nr of Repeats=5
RepeatLength=16 seed=AATACGGGAA Num.seqs=5 Similarity=1.000000
0   AATACGGGAATACGGG

****
Consensus:

AATACGGGAATACGGG

>Cele-UNSB01_Fam_386_16_1 Nr. of seq. 1 Alignment length(with gaps) = 16
Alignment score = 0.000000
Cele-UNSB01_3:1053032-1053110 Satlength=79 Nr of Repeats=5
RepeatLength=16 seed=TTATAGTGTT Num.seqs=3 Similarity=0.888889
0   TTATAGTGTTATAGTG

****
Consensus:

TTATAGTGTTATAGTG

>Cele-UNSB01_Fam_387_16_1 Nr. of seq. 1 Alignment length(with gaps) = 16
Alignment score = 0.000000
Cele-UNSB01_3:3140206-3140936 Satlength=731 Nr of Repeats=5
RepeatLength=16 seed=TGTATATATA Num.seqs=3 Similarity=1.000000
0   TGTATATATATATATA

****

Consensus:

TGTATATATATATATA

>Cele-UNSB01_Fam_388_16_1 Nr. of seq. 1 Alignment length(with gaps) = 16
Alignment score = 0.000000
Cele-UNSB01_5:12149471-12149551 Satlength=81 Nr of Repeats=5
RepeatLength=16 seed=AAAAATTATT Num.seqs=5 Similarity=1.000000
0   AAAAATTATTGAATAC

****
Consensus:

AAAAATTATTGAATAC

>Cele-UNSB01_Fam_389_16_1 Nr. of seq. 1 Alignment length(with gaps) = 16
Alignment score = 0.000000
Cele-UNSB01_5:17868232-17868328 Satlength=97 Nr of Repeats=6
RepeatLength=16 seed=GAATAATATT Num.seqs=6 Similarity=0.972222
0   GAATAATATTGGAGAA

****
Consensus:

GAATAATATTGGAGAA

>Cele-UNSB01_Fam_390_16_1 Nr. of seq. 1 Alignment length(with gaps) = 16
Alignment score = 0.000000
Cele-UNSB01_5:20061429-20063397 Satlength=1969 Nr of Repeats=117
RepeatLength=16 seed=CTTAGATCTC Num.seqs=111 Similarity=0.886787
0   CTTAGATCTCAATTCC

****
Consensus:

CTTAGATCTCAATTCC

>Cele-UNSB01_Fam_391_16_1 Nr. of seq. 1 Alignment length(with gaps) = 16
Alignment score = 0.000000
Cele-UNSB01_6:3096507-3096571 Satlength=65 Nr of Repeats=4
RepeatLength=16 seed=AGAGAGTATA Num.seqs=4 Similarity=1.000000
0   AGAGAGTATAGATTAG

****
Consensus:

AGAGAGTATAGATTAG

>Cele-UNSB01_Fam_392_15_1 Nr. of seq. 1 Alignment length(with gaps) = 15
Alignment score = 0.000000
Cele-UNSB01_3:12542724-12542833 Satlength=110 Nr of Repeats=5
RepeatLength=15 seed=CGACCTGCGA Num.seqs=3 Similarity=0.940741
0   CGACCTGCGACCTAG

****
Consensus:

CGACCTGCGACCTAG

>Cele-UNSB01_Fam_393_15_1 Nr. of seq. 1 Alignment length(with gaps) = 15
Alignment score = 0.000000
Cele-UNSB01_4:13614335-13614410 Satlength=76 Nr of Repeats=5
RepeatLength=15 seed=CACAGGAGGC Num.seqs=5 Similarity=1.000000
0   CACAGGAGGCTGGGA

****
Consensus:

CACAGGAGGCTGGGA

>Cele-UNSB01_Fam_394_14_1 Nr. of seq. 1 Alignment length(with gaps) = 14
Alignment score = 0.000000
Cele-UNSB01_1:11291527-11291639 Satlength=113 Nr of Repeats=7
RepeatLength=14 seed=AGCTTGAAGC Num.seqs=5 Similarity=0.800000
0   AGCTTGAAGCTTGG

****
Consensus:

AGCTTGAAGCTTGG

>Cele-UNSB01_Fam_395_14_1 Nr. of seq. 1 Alignment length(with gaps) = 14
Alignment score = 0.000000
Cele-UNSB01_6:8150139-8150195 Satlength=57 Nr of Repeats=4
RepeatLength=14 seed=TTACCAAAAT Num.seqs=4 Similarity=0.888889
0   TTACCAAAATCTAA

****
Consensus:

TTACCAAAATCTAA

>Cele-UNSB01_Fam_396_13_1 Nr. of seq. 1 Alignment length(with gaps) = 13
Alignment score = 0.000000
Cele-UNSB01_2:13903082-13903160 Satlength=79 Nr of Repeats=5
RepeatLength=13 seed=GGCTTCTAGT Num.seqs=4 Similarity=0.880342
0   GGCTTCTAGTCCA

****
Consensus:

GGCTTCTAGTCCA

>Cele-UNSB01_Fam_397_13_1 Nr. of seq. 1 Alignment length(with gaps) = 13
Alignment score = 0.000000

Cele-UNSB01_5:19792863-19792928 Satlength=66 Nr of Repeats=5
RepeatLength=13 seed=TTGGGGTGGG Num.seqs=5 Similarity=1.000000
0   TTGGGGTGGGCCT

****
Consensus:

TTGGGGTGGGCCT

>Cele-UNSB01_Fam_398_12_1 Nr. of seq. 1 Alignment length(with gaps) = 12
Alignment score = 0.000000
Cele-UNSB01_2:13678541-13678601 Satlength=61 Nr of Repeats=5
RepeatLength=12 seed=AATACCAACC Num.seqs=5 Similarity=0.933333
0   AATACCAACCAC

****
Consensus:

AATACCAACCAC

>Cele-UNSB01_Fam_399_12_1 Nr. of seq. 1 Alignment length(with gaps) = 12
Alignment score = 0.000000
Cele-UNSB01_3:13445252-13445836 Satlength=585 Nr of Repeats=45
RepeatLength=12 seed=AGTTATATAG Num.seqs=41 Similarity=0.931707
0   AGTTATATAGTA

****
Consensus:

AGTTATATAGTA

>Cele-UNSB01_Fam_400_12_1 Nr. of seq. 1 Alignment length(with gaps) = 12
Alignment score = 0.000000
Cele-UNSB01_5:16677048-16677311 Satlength=264 Nr of Repeats=16
RepeatLength=12 seed=CATTGACTTG Num.seqs=13 Similarity=0.842209
0   CATTGACTTGAG

****
Consensus:

CATTGACTTGAG

>Cele-UNSB01_Fam_401_11_1 Nr. of seq. 1 Alignment length(with gaps) = 11
Alignment score = 0.000000
Cele-UNSB01_4:2933784-2933839 Satlength=56 Nr of Repeats=5
RepeatLength=11 seed=TTGTTTACAA Num.seqs=5 Similarity=1.000000
0   TTGTTTACAAG

****
Consensus:

TTGTTTACAAG

>Cele-UNSB01_Fam_402_11_1 Nr. of seq. 1 Alignment length(with gaps) = 11
Alignment score = 0.000000
Cele-UNSB01_5:1284626-1284947 Satlength=322 Nr of Repeats=10
RepeatLength=11 seed=TACCTACTAG Num.seqs=6 Similarity=0.789899
0   TACCTACTAGA

****
Consensus:

TACCTACTAGA

>Cele-UNSB01_Fam_403_11_1 Nr. of seq. 1 Alignment length(with gaps) = 11
Alignment score = 0.000000
Cele-UNSB01_5:10500053-10500266 Satlength=214 Nr of Repeats=19
RepeatLength=11 seed=AGATACACTT Num.seqs=15 Similarity=0.983838
0   AGATACACTTC

****
Consensus:

AGATACACTTC

>Cele-UNSB01_Fam_404_11_1 Nr. of seq. 1 Alignment length(with gaps) = 11
Alignment score = 0.000000
Cele-UNSB01_5:18899719-18900018 Satlength=300 Nr of Repeats=5
RepeatLength=11 seed=TTTCAGGCCT Num.seqs=3 Similarity=0.796296
0   TTTCAGGCCTA

****
Consensus:

TTTCAGGCCTA

>Cele-UNSB01_Fam_405_10_1 Nr. of seq. 1 Alignment length(with gaps) = 10
Alignment score = 0.000000
Cele-UNSB01_1:14207225-14207295 Satlength=71 Nr of Repeats=6
RepeatLength=10 seed=GATTTTCATA Num.seqs=5 Similarity=1.000000
0   GATTTTCATA

****
Consensus:

GATTTTCATA

>Cele-UNSB01_Fam_406_10_1 Nr. of seq. 1 Alignment length(with gaps) = 10
Alignment score = 0.000000
Cele-UNSB01_2:2296694-2296754 Satlength=61 Nr of Repeats=4
RepeatLength=10 seed=CAGCAGAGTG Num.seqs=3 Similarity=1.000000
0   CAGCAGAGTG

****
Consensus:

CAGCAGAGTG

>Cele-UNSB01_Fam_407_10_1 Nr. of seq. 1 Alignment length(with gaps) = 10
Alignment score = 0.000000
Cele-UNSB01_2:3175289-3175339 Satlength=51 Nr of Repeats=4
RepeatLength=10 seed=AACTTTCGAA Num.seqs=3 Similarity=1.000000
0   AACTTTCGAA

****
Consensus:

AACTTTCGAA

>Cele-UNSB01_Fam_408_10_1 Nr. of seq. 1 Alignment length(with gaps) = 10
Alignment score = 0.000000
Cele-UNSB01_5:19792773-19792843 Satlength=71 Nr of Repeats=5
RepeatLength=10 seed=TGGCGAAAGC Num.seqs=3 Similarity=1.000000
12   GCTGGCGAAA

****
Consensus:

TGGCGAAAGC
