# Supplementary material for: DNA Satellites Are Transcribed as Part of the Non-Coding Genome in Eukaryotes and Bacteria
Source: Genes (Basel). 2021 Oct 20;12(11):1651. doi: 10.3390/genes12111651 (PMC8625621; doi:10.3390/genes12111651)
Supplement: Supplementary file 1 [file genes-12-01651-s001.zip › Table S4. Satellite families in C.elegans.pdf]

## Table S4. Satellite families in *C.elegans*

| Genome | Family name | Number of sequences | Alignment length | Score | Consensus without gaps |
|---|---|---|---|---|---|
| Cele-UNSB01 | Cele-UNSB01_Fam_1_12_169 | 169 | 14 | 0.769097 | AGGCTTAGGCTT |
| Cele-UNSB01 | Cele-UNSB01_Fam_2_35_166 | 166 | 46 | 0.624350 | AAtTgAAAATTTCCGGCAAATCGGCAAaTTGCCGg |
| Cele-UNSB01 | Cele-UNSB01_Fam_3_15_146 | 146 | 15 | 0.943142 | AGACCCaTCGTGGTG |
| Cele-UNSB01 | Cele-UNSB01_Fam_4_35_122 | 122 | 43 | 0.628454 | ATTtcCCGCCAAAAtntTTTTCaCaGAAAATTTgA |
| Cele-UNSB01 | Cele-UNSB01_Fam_5_40_94 | 94 | 41 | 0.668259 | AantTTCTnGAAnaTTCcaGAAttTTCcaGAaTTTTCcaG |
| Cele-UNSB01 | Cele-UNSB01_Fam_6_11_76 | 76 | 12 | 0.808392 | GTACtcCTACA |
| Cele-UNSB01 | Cele-UNSB01_Fam_7_15_70 | 70 | 15 | 0.830265 | cTCCCCTTTTCGtAG |
| Cele-UNSB01 | Cele-UNSB01_Fam_8_16_50 | 50 | 16 | 0.867364 | CTACaTGCCTACaTGC |
| Cele-UNSB01 | Cele-UNSB01_Fam_9_20_48 | 48 | 20 | 0.805585 | tAcACGaAAAAATAGATTGt |
| Cele-UNSB01 | Cele-UNSB01_Fam_10_25_41 | 41 | 27 | 0.791177 | TTTACTCTCTGGCTTCaCantATAT |
| Cele-UNSB01 | Cele-UNSB01_Fam_11_45_30 | 30 | 46 | 0.806030 | AAGGATCAGAnCAtCAGgAGGTACCCtTgtGATCCTTCGgAgCTG |
| Cele-UNSB01 | Cele-UNSB01_Fam_12_20_29 | 29 | 25 | 0.605057 | tATtGATTTTTcTGnAAATT |
| Cele-UNSB01 | Cele-UNSB01_Fam_13_31_27 | 27 | 32 | 0.817159 | TAAATAtTTAGCAGACCAaAgtTgGGTaTGC |
| Cele-UNSB01 | Cele-UNSB01_Fam_14_43_26 | 26 | 46 | 0.721271 | TTTGCCGGAAATTTTcAaTTcCGGCAAtTTGCCGgTTTGCCGA |
| Cele-UNSB01 | Cele-UNSB01_Fam_15_26_22 | 22 | 26 | 0.774226 | cAGAGTCAcTAtTTTtGGTGAAcGGc |
| Cele-UNSB01 | Cele-UNSB01_Fam_16_11_21 | 21 | 11 | 0.924964 | TTTTGCACATT |
| Cele-UNSB01 | Cele-UNSB01_Fam_17_37_19 | 19 | 44 | 0.712431 | GGGAATTCAAAtTTTAATTTTTtGAAAAtATTTTGGC |
| Cele-UNSB01 | Cele-UNSB01_Fam_18_19_18 | 18 | 19 | 0.859420 | CAAGGCACtAGAAtTGCGc |
| Cele-UNSB01 | Cele-UNSB01_Fam_19_14_17 | 17 | 14 | 0.892682 | ACTACAaACTACAA |
| Cele-UNSB01 | Cele-UNSB01_Fam_20_12_15 | 15 | 14 | 0.745805 | TGTATATGTATA |
| Cele-UNSB01 | Cele-UNSB01_Fam_21_18_14 | 14 | 18 | 0.786121 | TGCAAgACTAaTAGaGac |
| Cele-UNSB01 | Cele-UNSB01_Fam_22_59_13 | 13 | 60 | 0.818661 | AAAACCAGTGcAcatTTGAAtTTCCATATTctCCATAATTCTCgGTTTtAAAaaTTTTG |
| Cele-UNSB01 | Cele-UNSB01_Fam_23_20_12 | 12 | 21 | 0.831890 | CtAGGCAGACCTAGCCTACC |
| Cele-UNSB01 | Cele-UNSB01_Fam_24_32_11 | 11 | 34 | 0.834759 | CTACAAACTACAAgTTTTGGGCGCTGCTaAgA |
| Cele-UNSB01 | Cele-UNSB01_Fam_25_12_11 | 11 | 12 | 0.746970 | ACgCGacACGCA |
| Cele-UNSB01 | Cele-UNSB01_Fam_26_163_10 | 10 | 172 | 0.762446 | ATCATATggAGTcATTCTTTTTTaTTtcATAAAACTgTTCAGcATAGTCAAAaaTACCAGAAaATgCcAAaCAAAGTATanTAGCTTGTACGGAAGTatTTTTTtaAAAATtgAtaAAAATatATAAAAGCTGaTtTTTTCAAAAATTCAAAAGTAtgggAAA |
| Cele-UNSB01 | Cele-UNSB01_Fam_27_31_10 | 10 | 37 | 0.720120 | ATTTCCCGCCAAAAAtTgaCTGAAAATTTGA |
| Cele-UNSB01 | Cele-UNSB01_Fam_28_26_10 | 10 | 27 | 0.716324 | TAGAcCattAGATgAATTtccAAAGT |

| | | | | | |
|---|---|---|---|---|---|
| Cele-UNSB01 | Cele-UNSB01_Fam_29_21_10 | 10 | 23 | 0.611433 | ATGCACTGACCAAaanTatga |
| Cele-UNSB01 | Cele-UNSB01_Fam_30_12_10 | 10 | 14 | 0.713757 | tTTTTATTTTTA |
| Cele-UNSB01 | Cele-UNSB01_Fam_31_42_9 | 9 | 42 | 0.733686 | CngaAAATTTcgaaAACCGGCAATTGCCgAAaTTGCCGAntg |
| Cele-UNSB01 | Cele-UNSB01_Fam_32_26_9 | 9 | 26 | 0.800214 | ATTgCGGCgGATTtTagaAAAaTGGC |
| Cele-UNSB01 | Cele-UNSB01_Fam_33_18_9 | 9 | 23 | 0.628019 | cAAAAATCaATAATTTCa |
| Cele-UNSB01 | Cele-UNSB01_Fam_34_12_9 | 9 | 15 | 0.680247 | CTATACCTATAc |
| Cele-UNSB01 | Cele-UNSB01_Fam_35_12_9 | 9 | 12 | 0.876543 | TCTAaGTCTAaG |
| Cele-UNSB01 | Cele-UNSB01_Fam_36_10_9 | 9 | 11 | 0.771465 | GCGgtAGCGG |
| Cele-UNSB01 | Cele-UNSB01_Fam_37_18_8 | 8 | 19 | 0.681391 | TAAGCCTAAGcCTaAgCc |
| Cele-UNSB01 | Cele-UNSB01_Fam_38_18_8 | 8 | 19 | 0.829261 | ATTAGCAGCGcCCAAAAa |
| Cele-UNSB01 | Cele-UNSB01_Fam_39_14_8 | 8 | 14 | 0.894558 | GCTATaCGCTATaC |
| Cele-UNSB01 | Cele-UNSB01_Fam_40_166_7 | 7 | 173 | 0.740343 | tAtcAAtTTTcAGacGCGCTAgaGgGGTTTTTGTcTtGAaTTCCAATTTcaACagntatannAGGCnCAAAAAAACAAAAATAAAAaTTtTGGAaTTtnGGAnAATTTTTATAGATTTGgATTCTAGATTnTTTAAAAATATTaTTGGTAaGTTTtTGgaCCaagA |
| Cele-UNSB01 | Cele-UNSB01_Fam_41_95_7 | 7 | 96 | 0.868386 | AGGGCCGCAGGCCCGAAtACTCtTGGAAACTatGGGCtgGCTTTaATCCTATTCcAAGCAGATTGTTATTCaCTCAAGCCTAnnAACCAGGTTtG |
| Cele-UNSB01 | Cele-UNSB01_Fam_42_19_7 | 7 | 23 | 0.619048 | TAATtaaTattTATTTAtT |
| Cele-UNSB01 | Cele-UNSB01_Fam_43_19_7 | 7 | 21 | 0.666667 | CTCAACCAAaAttCTnAGC |
| Cele-UNSB01 | Cele-UNSB01_Fam_44_21_7 | 7 | 21 | 0.628118 | cAATTTTtggccTAAAAAttn |
| Cele-UNSB01 | Cele-UNSB01_Fam_45_16_7 | 7 | 16 | 0.732143 | TCtaAGGATCTaAGGA |
| Cele-UNSB01 | Cele-UNSB01_Fam_46_226_6 | 6 | 228 | 0.781092 | GTAACTaCgtGAAGCTTCTtGcACCTACTGGaATaTTGTTggTAtATTCTaAAAgAGCTTaaAATTTaCtACAAAGTTTCTGAAAGAcATTTCAAAAAAAGTTacTTTGAAGaGATTTatGAATTTTCAAAGTTtCgaAAATtcatCTAtTTTTGCCTATAAATGGTaGTTTTCATTaAATAATAAAATACGCACtaAAAGGTAgggCTGaaaAtGTactaTTTCT |
| Cele-UNSB01 | Cele-UNSB01_Fam_47_94_6 | 6 | 94 | 0.946099 | TCAAAATCTTCTGTTCTCTAAATATTGGGTGCTTTTCGATGCCCTaTGTAGACAAATCAATGGGAAAATcaTCAATTTCTGAAGGCAGTAATTC |
| Cele-UNSB01 | Cele-UNSB01_Fam_48_33_6 | 6 | 33 | 0.649832 | GCTGATTGGtTcAgAAAgTGGGCGgaGCtaAtC |
| Cele-UNSB01 | Cele-UNSB01_Fam_49_21_6 | 6 | 25 | 0.624889 | CTGACcAAATTtGTTcCaGCA |
| Cele-UNSB01 | Cele-UNSB01_Fam_50_21_6 | 6 | 22 | 0.621212 | TGACaAAAtnTnaaTTTGCAC |
| Cele-UNSB01 | Cele-UNSB01_Fam_51_19_6 | 6 | 21 | 0.706878 | gaAATTATCGATTTTTtCga |
| Cele-UNSB01 | Cele-UNSB01_Fam_52_18_6 | 6 | 19 | 0.691228 | ngGTAGgCAATGAGCAAg |
| Cele-UNSB01 | Cele-UNSB01_Fam_53_17_6 | 6 | 18 | 0.858025 | GATGCCCCCTcTGAACG |
| Cele-UNSB01 | Cele-UNSB01_Fam_54_16_6 | 6 | 16 | 0.865278 | AATTTTTCcGCaGCaA |
| Cele-UNSB01 | Cele-UNSB01_Fam_55_15_6 | 6 | 15 | 0.952593 | GGGGGCAACAAAAtA |
| Cele-UNSB01 | Cele-UNSB01_Fam_56_11_6 | 6 | 11 | 0.838384 | aAGTTGTCACg |
| Cele-UNSB01 | Cele-UNSB01_Fam_57_200_5 | 5 | 228 | 0.666228 | AACgGcATTtcAAAaAaTTtcaaAaccgCCaAgTTCAagCAgAaaaagtGAAtTTTcAgtcATacctTgTagACcacaAtTTATaTgCAAAATTAaTAGAtTTTCCAAacTTTgAAAATTAATtTCTGGCTTcAAAcTAACTTTTtTaaAAATtCCCGTaGCCATTTTGTaGCAAATTTTatGtTCTtTCAGAATTTaAA |
| Cele-UNSB01 | Cele-UNSB01_Fam_58_48_5 | 5 | 48 | 0.900000 | TTGCCCGTAATTTATAttTTTCTGAAGATGAGcAATTtAatTtTTGAt |
| Cele-UNSB01 | Cele-UNSB01_Fam_59_31_5 | 5 | 32 | 0.826042 | TGTCtAGCGAGAtagCAGAaaAGAGCTGAGT |
| Cele-UNSB01 | Cele-UNSB01_Fam_60_25_5 | 5 | 25 | 0.904000 | TTACTCTAATGTTCTGCcAAtttAT |
| Cele-UNSB01 | Cele-UNSB01_Fam_61_19_5 | 5 | 22 | 0.775758 | TGGTTGAGGCTCTcgATTT |

Cele-UNSB01 Cele-UNSB01_Fam_62_21_5 5 22 0.863636 AATCCAATATGGacgAAATCT
Cele-UNSB01 Cele-UNSB01_Fam_63_19_5 5 21 0.695238 AAtTTacCAATTTAcCAAa
Cele-UNSB01 Cele-UNSB01_Fam_64_17_5 5 18 0.848148 TcATTCCGTgATTATTT
Cele-UNSB01 Cele-UNSB01_Fam_65_11_5 5 14 0.697619 TGCCTgCCTAC
Cele-UNSB01 Cele-UNSB01_Fam_66_11_5 5 11 0.951515 TTTGGAGCGGt
Cele-UNSB01 Cele-UNSB01_Fam_67_184_4 4 189 0.862140
GAATACAGAACACCAaTTATGtCCGAGAAAAAGATCCtAcTCAGAATATaAACATAtTcGAATttatCGGAtGTATAaAGATTCCCGAAGAcaCTTTCCAATTAcCCAAAttGT
TCAtATTCTAAAtgAaATTCtCTTACTaGAACACTCTTGGCCAATGTACGCAGCCGAACGTATCATAAGT
Cele-UNSB01 Cele-UNSB01_Fam_68_64_4 4 67 0.721393
gGTCTGCtAaATCaAAacTTGTAGTTTGTAGtCTAGCAGACCaAatTTcTtcAAaACcaCaTtG
Cele-UNSB01 Cele-UNSB01_Fam_69_39_4 4 40 0.856944
TAtTTTTAAttTgaCCTaCTTAAAAAGTAGGTCATGACC
Cele-UNSB01 Cele-UNSB01_Fam_70_36_4 4 36 0.754630
tTTCGGCAAtTTcGacAATTGCCGGTTTtggaAAtt
Cele-UNSB01 Cele-UNSB01_Fam_71_30_4 4 30 0.777778
TTTCtAGAaaaaTcTGGAAagTTCTAGAAt
Cele-UNSB01 Cele-UNSB01_Fam_72_30_4 4 30 0.983333
AATTTTCCAGAAGGTTCTGGAACaTTCCAG
Cele-UNSB01 Cele-UNSB01_Fam_73_20_4 4 23 0.677536 tTTcACTAAAAATCTcAAAa
Cele-UNSB01 Cele-UNSB01_Fam_74_21_4 4 22 0.651515 GTCAGTGCAActtTgcAatTG
Cele-UNSB01 Cele-UNSB01_Fam_75_20_4 4 20 0.744444 gTgAAAaTTtaAATTTtTgA
Cele-UNSB01 Cele-UNSB01_Fam_76_14_4 4 15 0.727778 TTaGGGCcTtgGGC
Cele-UNSB01 Cele-UNSB01_Fam_77_15_4 4 15 0.955556 TTTCCGGATCTACGt
Cele-UNSB01 Cele-UNSB01_Fam_78_13_4 4 13 0.897436 GCCTATGGCcTAc
Cele-UNSB01 Cele-UNSB01_Fam_79_12_4 4 12 0.840278 TTTTtCCGAAat
Cele-UNSB01 Cele-UNSB01_Fam_80_12_4 4 12 0.662037 AgaAgGAGgAaa
Cele-UNSB01 Cele-UNSB01_Fam_81_10_4 4 10 0.800000 TCccGATCcA
Cele-UNSB01 Cele-UNSB01_Fam_82_167_3 3 172 0.651809
nTATAATGcTCGTAATTATTtATAAGTgTGTGTTGTCTCAGAgcCAngTCTgAAAAaACAGATTTTTTtTcGAAAaTTTtAAaAacttcGtAaAAATTcgATTTTtGACanaTc
tATgnTctCTnTTTTTtTntgAcATgTAtTTCAGTGAcAtAnaganTcAaAAa
Cele-UNSB01 Cele-UNSB01_Fam_83_90_3 3 95 0.673684
ccccGAACGTGTCGGCCGCtTCCAAAtAACcACCTccTngCACTaCgTTGCGCACACAcnaanctaCTcgTTTCACGcCAAgCTgCGGAA
Cele-UNSB01 Cele-UNSB01_Fam_84_79_3 3 79 0.909986
gTGAATCTCCtCTCtaCCTAATGAgTCCTGACAGAATACCGGCAGTATTcTGGGGTCTCATTAGGTTTTaGTTTCTTcT
Cele-UNSB01 Cele-UNSB01_Fam_85_76_3 3 76 0.906433
AATGCTAGAgGACAcTCCTTTGCACGAAaGgAAGTGGGAGAGACTGTGGaGTTGTCGGTGTCTtTGTgCATGCTCg
Cele-UNSB01 Cele-UNSB01_Fam_86_64_3 3 64 0.954861
TTTTTGACTACtGAATGTCTTTTTCGCTACTGACTGaAAAAATAGCTACATTCGCACTTTTGtC
Cele-UNSB01 Cele-UNSB01_Fam_87_34_3 3 34 0.980392
ATGCACCATGTCAAAAAATATATCCAAAAgTACG
Cele-UNSB01 Cele-UNSB01_Fam_88_32_3 3 32 0.972222
GCACCAAATAGCACCAATgTAGCACCGATGGA
Cele-UNSB01 Cele-UNSB01_Fam_89_32_3 3 32 0.972222
CGACCTGCGACCTAGCGACCTaGGTCGCTTTG
Cele-UNSB01 Cele-UNSB01_Fam_90_27_3 3 27 0.658436 aGCaGCCGACACCTTaCnGGTtgcttA

| | | | | | |
|---|---|---|---|---|---|
| Cele-UNSB01 | Cele-UNSB01_Fam_91_21_3 | 3 | 23 | 0.801932 | CTAgAAAAACTCGGCCAtCAA |
| Cele-UNSB01 | Cele-UNSB01_Fam_92_21_3 | 3 | 23 | 0.748792 | TAGTTTaTAGTTTGtaGTTTG |
| Cele-UNSB01 | Cele-UNSB01_Fam_93_20_3 | 3 | 22 | 0.656566 | GTCGATTTACGgGAgTTcnT |
| Cele-UNSB01 | Cele-UNSB01_Fam_94_21_3 | 3 | 22 | 0.843434 | CCATTTTAAGCCAATTTTggA |
| Cele-UNSB01 | Cele-UNSB01_Fam_95_22_3 | 3 | 22 | 0.777778 | AcTACAGTAccCcTACAgTACc |
| Cele-UNSB01 | Cele-UNSB01_Fam_96_19_3 | 3 | 21 | 0.608466 | GCCTAGaAATCtnAtgATG |
| Cele-UNSB01 | Cele-UNSB01_Fam_97_18_3 | 3 | 21 | 0.761905 | TTTTGTCAGTGCTATAgT |
| Cele-UNSB01 | Cele-UNSB01_Fam_98_20_3 | 3 | 21 | 0.719577 | GTAAATCTACActaGCTncC |
| Cele-UNSB01 | Cele-UNSB01_Fam_99_21_3 | 3 | 21 | 0.767196 | ACTACcGTACCaaCtACcACa |
| Cele-UNSB01 | Cele-UNSB01_Fam_100_21_3 | 3 | 21 | 0.957672 | CTAACATTTTTCAaTTTTCGT |
| Cele-UNSB01 | Cele-UNSB01_Fam_101_21_3 | 3 | 21 | 0.682540 | GTgcaaatgnAtnnttGgTca |
| Cele-UNSB01 | Cele-UNSB01_Fam_102_20_3 | 3 | 20 | 0.688889 | GTAAATCtACAtAgaaCntC |
| Cele-UNSB01 | Cele-UNSB01_Fam_103_20_3 | 3 | 20 | 0.822222 | AtACGAtATAGCTATttACG |
| Cele-UNSB01 | Cele-UNSB01_Fam_104_16_3 | 3 | 16 | 0.777778 | TTcCAGGgTTcCAGGg |
| Cele-UNSB01 | Cele-UNSB01_Fam_105_11_3 | 3 | 12 | 0.787037 | ATTTGgAATAT |
| Cele-UNSB01 | Cele-UNSB01_Fam_106_11_3 | 3 | 12 | 0.722222 | ncCACTGACCA |
| Cele-UNSB01 | Cele-UNSB01_Fam_107_151_2 | 2 | 151 | 0.924945 | TTTAAAGGCGCATAGGTAGtTCAAANTTCAAANTTGACGGATTCTAGTCAATATGGGGCTTATTTTAAAGCTCTCAGTAAGCTNAATNTANTTNAGCTACTaAaAATTGAANATTTTNAAATTTTTTGGGTCTCGCAGCGAAAATTTCGAA |
| Cele-UNSB01 | Cele-UNSB01_Fam_108_119_2 | 2 | 119 | 0.607843 | GCcacCcTTAAcCCTAAcCCAaGCAGAGCAGGacGTAGGCCTGaaAcGcGGTaGcgAcaCaaTCAaGCCcAAcAacCaGGgaTGAGGGCCGCAGGCCCgaaaACTCTTaGAAACTgTaG |
| Cele-UNSB01 | Cele-UNSB01_Fam_109_110_2 | 2 | 110 | 0.745455 | TTTCACaTTTTCAcgTGATTATaTAGGTCACGAaCcAAaTTATTTTGAAAATTGAcCTacacGGacCTaTAACTTTGGAGaTATGGGaAATcaAGTATGaGCTTTCAGAT |
| Cele-UNSB01 | Cele-UNSB01_Fam_110_86_2 | 2 | 86 | 0.689922 | CAATAggGAAAAGATGGAAGAcTaCgaAGttAAcgAgAAgCTATTGacAGACCCCGAAATACTGGCTGTATTTTGAAAGGTCaTcg |
| Cele-UNSB01 | Cele-UNSB01_Fam_111_73_2 | 2 | 73 | 0.945205 | AATAAAATATTGGAAATCCcTATGACACACTTAAGCCTAAAGGCCCGAAAAAaAaACTAGGATGCCCAACTGG |
| Cele-UNSB01 | Cele-UNSB01_Fam_112_71_2 | 2 | 71 | 0.626761 | aAGACACTAATAAAAATaTTaaATTTTTATTCGTCtTcTAaTTTgAaAAtAtATTTTCttaATaAAAATag |
| Cele-UNSB01 | Cele-UNSB01_Fam_113_68_2 | 2 | 68 | 0.838235 | CAAATCAACCTATTCAGTTGACAATGGTTTTCTTTTAcCAAAaTTcTTaaAATTTcagAAAGAAAACA |
| Cele-UNSB01 | Cele-UNSB01_Fam_114_44_2 | 2 | 44 | 0.681818 | cCAGCCGACAGCCGAAAaaTTcacGaATTTTCGGCTGGTaGCac |
| Cele-UNSB01 | Cele-UNSB01_Fam_115_44_2 | 2 | 44 | 0.704545 | aCGGCAAAtTCGGCAAaTnCTgcTTTTTTcAAtATTTGCCGAGC |
| Cele-UNSB01 | Cele-UNSB01_Fam_116_44_2 | 2 | 44 | 0.969697 | CTGTTTTCTGAGCATTgTAGATTTTCGGATTGATGGCCGAAAGG |
| Cele-UNSB01 | Cele-UNSB01_Fam_117_41_2 | 2 | 41 | 0.711382 | ccGTTTCGCcTTGAGCGATTcCttACCCTTAAATGGGCccC |
| Cele-UNSB01 | Cele-UNSB01_Fam_118_41_2 | 2 | 41 | 1.000000 | AAAACGGGAAAACGGGAATTCCCGTTCCCGTGAAAATTTTA |
| Cele-UNSB01 | Cele-UNSB01_Fam_119_40_2 | 2 | 40 | 0.975000 | AAAATCCACGTAGATTATTGATTTTTCATgGAATTTTGCA |

| | | | | | |
|---|---|---|---|---|---|
| Cele-UNSB01 | Cele-UNSB01_Fam_120_39_2 | 2 | 39 | 0.897436 | TTTATGaAAAAGTGGCTCTAGaGCCACaAGAAATACCAG |
| Cele-UNSB01 | Cele-UNSB01_Fam_121_36_2 | 2 | 36 | 0.601852 | cGGCAaAtTaCCaTaAATTTTaAAATcCGGCAaAAc |
| Cele-UNSB01 | Cele-UNSB01_Fam_122_35_2 | 2 | 35 | 0.666667 | AAAATCGaaaAATTcTCGaTTTTTCAATTTTTaga |
| Cele-UNSB01 | Cele-UNSB01_Fam_123_34_2 | 2 | 34 | 0.612745 | cCaCCCGGcAGGTGTCGGCTGCTaaaaCTAtAaC |
| Cele-UNSB01 | Cele-UNSB01_Fam_124_32_2 | 2 | 32 | 0.671875 | tTTTTTGAGCcaAAAATTCtaAAAAATgCTCa |
| Cele-UNSB01 | Cele-UNSB01_Fam_125_31_2 | 2 | 31 | 0.752688 | GGTTACTGTAaaTCaATcaTAGGATACTTtG |
| Cele-UNSB01 | Cele-UNSB01_Fam_126_31_2 | 2 | 31 | 0.784946 | CTGAAATTacaAaTTTCCAaTGTAAAAATGT |
| Cele-UNSB01 | Cele-UNSB01_Fam_127_31_2 | 2 | 31 | 0.956989 | TCGACAGAAGCTGTCGGAaATCACACTTTCC |
| Cele-UNSB01 | Cele-UNSB01_Fam_128_27_2 | 2 | 27 | 0.901235 | GCTGCTGGAGGATCaACTGCATCAACc |
| Cele-UNSB01 | Cele-UNSB01_Fam_129_26_2 | 2 | 26 | 1.000000 | CAAAACCGGCAATTGCCGAAAATTCC |
| Cele-UNSB01 | Cele-UNSB01_Fam_130_26_2 | 2 | 26 | 0.948718 | TCAaGGCGAAACGGGAGGTGAATCGC |
| Cele-UNSB01 | Cele-UNSB01_Fam_131_24_2 | 2 | 24 | 0.722222 | gGCATCTGccGCcGGAGCTGGaGC |
| Cele-UNSB01 | Cele-UNSB01_Fam_132_24_2 | 2 | 24 | 0.680556 | GCcTAGaCTcaGGCTTAGGCTtAa |
| Cele-UNSB01 | Cele-UNSB01_Fam_133_23_2 | 2 | 23 | 0.630435 | aATTTCTAGGCCACcAcgTTcAg |
| Cele-UNSB01 | Cele-UNSB01_Fam_134_23_2 | 2 | 23 | 0.673913 | tCTAATTAGATnCaCCtTcTTTT |
| Cele-UNSB01 | Cele-UNSB01_Fam_135_23_2 | 2 | 23 | 0.659420 | ATTaaATTTTTCAaCaaAAAAAa |
| Cele-UNSB01 | Cele-UNSB01_Fam_136_22_2 | 2 | 22 | 0.643939 | aATAAAAATgTCAcAAAAtATg |
| Cele-UNSB01 | Cele-UNSB01_Fam_137_22_2 | 2 | 22 | 0.681818 | GTAAATCTACAcaaATagCCCC |
| Cele-UNSB01 | Cele-UNSB01_Fam_138_22_2 | 2 | 22 | 0.621212 | TTcaAGCcaTTTTCaGACcAAT |
| Cele-UNSB01 | Cele-UNSB01_Fam_139_22_2 | 2 | 22 | 0.765152 | TTTGCACTGACCAAcAaaAATg |
| Cele-UNSB01 | Cele-UNSB01_Fam_140_22_2 | 2 | 22 | 1.000000 | TCGAGAAAAAATCTGAAAAAAT |
| Cele-UNSB01 | Cele-UNSB01_Fam_141_22_2 | 2 | 22 | 0.954545 | ATTCTGCGCACCCCCTGCGCaG |
| Cele-UNSB01 | Cele-UNSB01_Fam_142_20_2 | 2 | 20 | 0.933333 | CCACCAAAAAgGTTTCTAGG |
| Cele-UNSB01 | Cele-UNSB01_Fam_143_20_2 | 2 | 20 | 0.950000 | GGACTAGTTTTcGAGTAGAG |
| Cele-UNSB01 | Cele-UNSB01_Fam_144_20_2 | 2 | 20 | 0.750000 | TGTAGATTTACaaAGGaCaA |
| Cele-UNSB01 | Cele-UNSB01_Fam_145_20_2 | 2 | 20 | 0.866667 | CCCCGAaGCTCGAGCTCAAa |
| Cele-UNSB01 | Cele-UNSB01_Fam_146_20_2 | 2 | 20 | 0.800000 | CCACCCTGTAaaATcATGGA |
| Cele-UNSB01 | Cele-UNSB01_Fam_147_19_2 | 2 | 19 | 0.684211 | CTAGGTGCCTAcTccATgC |
| Cele-UNSB01 | Cele-UNSB01_Fam_148_19_2 | 2 | 19 | 0.929825 | CGCCAAGgATCGAGAAAAG |
| Cele-UNSB01 | Cele-UNSB01_Fam_149_17_2 | 2 | 17 | 0.656863 | gCaGaTTGCCgGAATTG |
| Cele-UNSB01 | Cele-UNSB01_Fam_150_17_2 | 2 | 17 | 0.803922 | GCACTGAcCAAcTTTTT |
| Cele-UNSB01 | Cele-UNSB01_Fam_151_16_2 | 2 | 16 | 0.750000 | GTaCTGGGGTaCTGGG |
| Cele-UNSB01 | Cele-UNSB01_Fam_152_15_2 | 2 | 15 | 0.622222 | CGATgCTCCACCaga |
| Cele-UNSB01 | Cele-UNSB01_Fam_153_14_2 | 2 | 14 | 0.761905 | TaTACAAAAAnGTA |
| Cele-UNSB01 | Cele-UNSB01_Fam_154_14_2 | 2 | 14 | 0.666667 | cCTCAAGCgTAGGa |
| Cele-UNSB01 | Cele-UNSB01_Fam_155_14_2 | 2 | 14 | 1.000000 | GAAGGATCGGGCCG |
| Cele-UNSB01 | Cele-UNSB01_Fam_156_14_2 | 2 | 14 | 0.619048 | gATAATTgAaAATc |

Cele-UNSB01 Cele-UNSB01_Fam_157_12_2 2 12 0.680556 TTggGCAAGAAa
Cele-UNSB01 Cele-UNSB01_Fam_158_11_2 2 11 0.878788 AAACTTGGGCa
Cele-UNSB01 Cele-UNSB01_Fam_159_11_2 2 11 0.636364 cGGAaAATTaA
Cele-UNSB01 Cele-UNSB01_Fam_160_11_2 2 11 0.636364 cATAaCTGAGa
Cele-UNSB01 Cele-UNSB01_Fam_161_255_1 1 255 0.000000
AACAAGTCGGGAACGTTCGGAAAGCTCTGAGGAAAACTCCGACAATATCATGCTTAAGTACGCAATTGAGCTATCTTTATTAGATTCGAAAAGGACTCTTCAAAAATTGAAGC
AATAACGAAGAAGCTTAAGTTGGAACTGGCGAAGCTTAAATTGGGANTTATGCAGTCTTTATCAGATTCAAAAGACGATTCTTCGACCAATGAAGCAAGTCCGGAGNACGATCT
ACCTTCGACTTCTGATGGCAGAACTCC
Cele-UNSB01 Cele-UNSB01_Fam_162_249_1 1 249 0.000000
CTGGAAGCACTGTAACCATGGGATCATCAAGCACTTCCGGTGTTTCCACATCTTCAGCATCCAGTACTCAGCCTCAGATGAGCACTTCACAAGGAAGTTCAGCAGGATCCACAG
TTGCTTCATCAACCGCTGGCCTTGTTTCAACTTCCACAGTTCCATCCTCGACAGGAACAATGGGCTCCACTTCTAGTGGAACAGTTGGATCTACAATTTCTGAATCATCGACAA
CAGCATCTGCAAGTTCTCAGA
Cele-UNSB01 Cele-UNSB01_Fam_163_231_1 1 231 0.000000
GCTGAATTCAGTTGGATATGAATGCACTTCCGTTGTGAGTGGAATATCATGAATGTTNGATGCTCGTGAAGTAGANTCNAGTGGTCCATCATAAGATTCAGACACTGGATAATC
AAGATGAGATGGTGATTTCTTGAACAATCCAGTGATCTTAGATTTCAGTGATGGCTCGTCCTTCTTCTCTGTCTCCTCGATCACAACTGGAATGACATGCTCTGGCAGCTTCTC
ATA
Cele-UNSB01 Cele-UNSB01_Fam_164_228_1 1 228 0.000000
CCACCAGATCAGCAACGTCTCATCTTCGCCGGAAAGCAACTCGAGGACGGCCGCACCCTTTCGGACTACAACATCCAGAAGGAATCAACTCTTCATTTGGTTCTCCGTCTGAGA
GGAGGTATGCAGATCTTCGTCAAGACATTGACCGGAAAGACCATCACCCTCGAAGTCGAAGCCTCCGACACCATCGAAAATGTCAAGGCCAAGATCCAAGACAAGGAAGGAATC
Cele-UNSB01 Cele-UNSB01_Fam_165_189_1 1 189 0.000000
TTTTTTTTGACTATGGAATTATAAAGAAAATCGAATTTCAAAAAAGCATGTATTAGCCAATGTAAACGCGCTCCAATGATGATGTAGCCCACGTGGTATCAGAGTGTCTCTTTA
CGGCTTGATTTAAATACGTAGATCTACAAATTCACTTATCTGTTTTCTCACGGGTTTAGGATATTTTTTCTGTTA
Cele-UNSB01 Cele-UNSB01_Fam_166_184_1 1 184 0.000000
GTTCAAAAGATACAAAATTTATCTACACATCTACAAGTTAAACTTGCAACTTAGTTAACCAAATTCAAAAGTTATGAGCCGGGGAACTATAATAACCAACGTTTCACACTAAAA
AAGCCCAAAAAAAATTCGAAAATCATTTTTATCTCATGACGCGGACAAGCTACAAATGTCTAGTTAGGCTT
Cele-UNSB01 Cele-UNSB01_Fam_167_183_1 1 183 0.000000
TTTCTCTGCGTCTCGCCTCGCCTCACACTATTTTNCACACCAAACTTTGAGNCTATGTATCTCTGTAACGGGTTGCGCTTTCTGAAAAATTCCAACTGACGGGATATATAAAAT
TTNGAATTCTTTCTCACCGTTAAAAAACTAAACACCTAGGCAAGACGCAGACAGCGAGANACCCCCAAT
Cele-UNSB01 Cele-UNSB01_Fam_168_181_1 1 181 0.000000
TTTTTAACTTTTAAATCCACTAAAACTCGGAATGGTACAGAACATGCAAATTGTAATTTCAAGAACTGAATGTGGTTTCGAATATTTATGGATTGCTTTATTCTAAAACTGACT
CTGAAATATTTTGCATCCATTTGACTACTAATTGTCATATTATACCGAACACTACTAACTTTAGTGG
Cele-UNSB01 Cele-UNSB01_Fam_169_180_1 1 180 0.000000
TGTCGGCCGCTGTATCTTTTGCCTCAATTTTTTTTNAATTTTNAAACATNCATATTCCAGTAGATAACATTATGAGCTACAGTTTGTTAGTTAAAATTTTATTTCTAAAATTAC
TAGTTTCTGAGTTATACTCATTCCAACCCACCCCAAGAGAAATTTCCTAAAAATGAACTCGCGAGG
Cele-UNSB01 Cele-UNSB01_Fam_170_179_1 1 179 0.000000
CTACCCAAATTTTTGAAATTTGGAAATTTTTCAAAAATTCTCAAATTTTCAAAATCCCATAATTCAGCCAATTTTTGAGATTTTCAGATATTTTTTAATTGAAAATTCTGTTTG
TCTTATCCATTAAACTATATCGCTGCATCTACATTTTTCACAGTACCCCATGACTTTTCCCGATT
Cele-UNSB01 Cele-UNSB01_Fam_171_177_1 1 177 0.000000
CTAACAAAATAATCAGCGTAAAATTTCCTACATTTTACTAGTTAACAATATTTTGATAACATCAGTGGTTTTTGAGATACGCAAGTTTTAAGGTGGACGCCCAAAAATAGTTGT
GAGCAGCTTTAATCAAAAACCGACATAACTTTTTACTCACTAACGGTATCAAAAATGTTTTAA
Cele-UNSB01 Cele-UNSB01_Fam_172_177_1 1 177 0.000000
GAGTCTCACGATGTGTCAACGTGTCTCAGTCGGCTTGATCTACGTAGATCTACAAAAAATGCGGGAGAAGAGACGCAGACTTATTCTCTGATTTTGCATGAGTAAGAGCGTGCT
GGCGTCACATTTTTGTCGGCAAAAAATTCCCGCATTTTTTGTAGATCAAACCGTAATGGGACG

Cele-UNSB01 Cele-UNSB01_Fam_173_175_1 1 175 0.000000
GATGCACCATGTCTTTGACAGTCAATATCTCGGTTTATTTTGTAGCTATCAAAAAATTTTCAACTACAAATCTATAGAAAATTAAATGTTCAACATTTTCTCAGTTGACAGTTT
TTTGATAACTGCAATGACATCCGAGATATGAGCTTTCAAAGTTGAGAGACCTCACTCGGTT
Cele-UNSB01 Cele-UNSB01_Fam_174_175_1 1 175 0.000000
AACTTGTGACGGTGGGTATGGATTTTATGCACATAATTTGTTCGAAATAGTCTACCNAGTAAACTGGTGAAGTTTCAAAAACTTTGTGGATCTAGTTTTTGAAAAAAGTTCAAA
ATTGTGCCAATTTTGACCAATTTTGGTGGTTTTCTGAGCTAAAATTGAAAAGTTGGGAATG
Cele-UNSB01 Cele-UNSB01_Fam_175_175_1 1 175 0.000000
AAATTTTGGGTTCACTCTTTTTACTAGACAGTGATTGTTGCATTGTAGGATTCGGTGGACTTGTCCAACAACTTACTTTGGCATGAGTTTAATAAAAAGGTTTTACCATGAACT
GAATCAATTCAAAATAAAAACCTCGAGAATGAAAACATCCTCAGTAATTTCAATCCTGTTC
Cele-UNSB01 Cele-UNSB01_Fam_176_174_1 1 174 0.000000
CTTCAAGAGCTCAGTGTTATTTATGACATGCCAACCTCGACAATTGACCTGAACATGCTCCAAGGAATCGAAGACAACTTGAACAGCCTTCCAGCTGAAGAATCTGATAAGATC
CGAGAGAAGATTAACGATCTGAGAAGAAGGAAACAAGAAAGCGATCAAGCTGAAGCTCTT
Cele-UNSB01 Cele-UNSB01_Fam_177_165_1 1 165 0.000000
CTTTTCCAAAATGGCAATTTTTGATGAAAAAAATCCGAATTTTCACAAATTTCAAAAAAATTTTTAAACTTTATAATTTTAGCTTTGAATATTCCAAATGGAACTGTCATAACC
TATCAATTTGATATATAAACTCGTGCCTACCTTCAGGCATTCAGTTTTCAC
Cele-UNSB01 Cele-UNSB01_Fam_178_155_1 1 155 0.000000
TCCCGCTGTGGTTAAAAGTTTTTTGCGTGCTCAGTTCTGTACGGTAAATGTGTTAAAAACTTCTGGAAACTTGAAATTCCAAGTTATTCAGAAAATTGTTCAAATGGCGCAGTG
GGAAATTCGATTTCTCAGACCCAAGAGGCCACGGGTTCAAT
Cele-UNSB01 Cele-UNSB01_Fam_179_153_1 1 153 0.000000
TGAGGTGCAGGGCGTGATGAGCTTTGTTGAACTGGGAACTGTTGATTCCCGGAGCCGTGATTCATAGATGAGTTGGAGGGACCTGCTGTGGAACTTGTTTCAACTTGAGCAATT
CCATGAGCCATCAAGAAGCTTTCCGGCAGATTGGGCAAC
Cele-UNSB01 Cele-UNSB01_Fam_180_151_1 1 151 0.000000
CATTTCAGATATTCCATCACCACCAACACCTTCATCCACTTCTTCGCCTGGAACGACGGGATTTTCTACAAAAATCGGATTTTTTCTCTATGTCAGTTTAATTGATGTATAATT
CCGAAAAGTCAGTCTTTCCTAGGTTTTTAAATTGAAA
Cele-UNSB01 Cele-UNSB01_Fam_181_150_1 1 150 0.000000
TTGCAACATGCGGTCGGCGGAAAATTCGAATTTTTCAGTCCAAAACAGGTTTTTCGACCAATTTTTCATATTTTCAAGCGAAATTATGATAGAAAATTCAAATTTTCACTAAAA
ATCGCCAATTTTCCACATTTTCTCATTGGAGCGCAC
Cele-UNSB01 Cele-UNSB01_Fam_182_150_1 1 150 0.000000
TTGAGGGCCGCAGTCCCGAATACACGAAGAAGTGTTGGAGATCCGTCAAGCCTATTCTAAGCATAATAATTCAGTTCGAGTGTCGCAAGCCCAAATAGACGCAGAAGCGTTGGA
CAACTTTTAGGCCTATTCTGAGCCCAATAATTCATG
Cele-UNSB01 Cele-UNSB01_Fam_183_146_1 1 146 0.000000
TGGCCTAGAAATCNCAAGTTTGCAAAAGTTAGGCCACCAACTTCAAATGCCTATAACTCAGCGGAAAATCAATATTTTCCATTGATTTTAACTGCTAAGATGTAGCCAATTACG
TAGCGCAACTTTTTGGCCTACTTAACAATTGG
Cele-UNSB01 Cele-UNSB01_Fam_184_144_1 1 144 0.000000
TTCTAGGCCACCAATTGGAAATCATCATAACTTGGCTTAAAATCCAGATTTTTGGAGTCTGGTAACTGCAACATGTTGCCCTGTAGTTTTTCTACAAGTTAGGATATATTAATT
TTTGGTGGCCTAAAAGTCCAAATTATGGAT
Cele-UNSB01 Cele-UNSB01_Fam_185_143_1 1 143 0.000000
CAGTGGGATTTTCAATGTCTTCCAATCAAACGTCCGGGGTTCGAGTCCGCACAGGGGTATTTATTTTTTGCAAGCTGTGATTAACAAGTTTATAGCTGCAAAAGTCATTTTAAT
CGAACTTTCAGGAAAATGTCCGAATGGCG
Cele-UNSB01 Cele-UNSB01_Fam_186_124_1 1 124 0.000000
AATTTTCAAAGTTTGGAAAATCTATTAATTTTGCATATAATTTTTATAAATTCTGAAAGAACATAAAATTTGCTACAAAATGGCTACGGGAATTTCTAAAAAATTTAGTTTGAA
GNCAGAAATT
Cele-UNSB01 Cele-UNSB01_Fam_187_122_1 1 122 0.000000
GATCTACAAAAATGCGGGAAAAGTGACACAGAGTTCTCAACTGATTTTGCATAAAAATTCCCGCATTTTTTGGAGATCAAACCGTGATGGGACAGCCTGGCACCACTTGCTTGA
TCTACGTA

```
Cele-UNSB01          Cele-UNSB01_Fam_188_121_1         1         121    0.000000
GTTGCAAACCGCAAGAGCAAACTTGCCAAATGAAAAGCCACCCAAGTACTCGTTGCACCTACCTATCATAAGCTCGTAGACTATAATTTCTCTGAAAAGATAGTTGCCCTAATA
GCAGAAG
Cele-UNSB01          Cele-UNSB01_Fam_189_119_1         1         119    0.000000
GGCCACCGTTGCATATTTCGCAATAATTTTTACGGGAACGTAATCTCCACGTCACCAGCTTTCATTTAAGTACCCACACGGCAAGGTTTAATTTTTGAAATTTGGTCGCGGATT
TTCTC
Cele-UNSB01          Cele-UNSB01_Fam_190_117_1         1         117    0.000000
TACTTCAGATGTTTCCTTTGATTCCACAGGTGCAAGTTTCTCAACTGTTGGTTCGACAGTAGGTGCACTAGTCTCTGGCACTGGAACATCCTTTTGCTCAACAATTTCAGCTGG
TTG
Cele-UNSB01          Cele-UNSB01_Fam_191_114_1         1         114    0.000000
GTGTTTCAGAATTACATGGAGTCTCACTACTTGCAGAATTTGTGGCTGTAGACGATGGGCCTGCTGTAGTTGAATCGTTTGGAACTGTCATAGTGGATGTTCCATCAGACGTTT
Cele-UNSB01          Cele-UNSB01_Fam_192_108_1         1         108    0.000000
ATCCTCAACACCATCNGCGTCATCTAGTTCCTCCGGAACTATGTCGACAATTTCAGGATCAACTGGTTCNACAGTTACAGTTGTACCAGGATCNTCTAGCACATTTGG
Cele-UNSB01          Cele-UNSB01_Fam_193_108_1         1         108    0.000000
TGGGTAATCAGGTACGGGCTTTCAGATTTTCAAGTTTCACGTGAATATCTAGGTCACGAGCCAATTTCAGCCAGGAGTGGGACTAGAGATATGAAACTCTTGGGTTA
Cele-UNSB01          Cele-UNSB01_Fam_194_108_1         1         108    0.000000
AAGTCTTCTTGATTCTGAGTTCTTGCTTCGAACTGTTGACCATCTCCTTGCAGGATCTTATTGTCAGATGGAACCTTCTTGGCATACCGTTCTCCACTGATGTGAGTG
Cele-UNSB01          Cele-UNSB01_Fam_195_104_1         1         104    0.000000
CCTGCGAAAGCGAATCTCTGGCCTGGCGGAGGGCCAACTAACTCTCTCCCTCAAGCCTTCGGGTGCTTCCTGGGTTATCGGTGAATTTTTGGGTCTGTCAGGGT
Cele-UNSB01          Cele-UNSB01_Fam_196_102_1         1         102    0.000000
TGATATGTCGAACGATTGATAGTTTGAGAATCAAGAGAATCTGTTGGACGATCAATTCCATCTTTGGGTCTTGCTGCAGCATATCTTTCTCCAGTACCACCT
Cele-UNSB01          Cele-UNSB01_Fam_197_102_1         1         102    0.000000
TGTACACTTCTCCCAAATTGACTGTAAGAATGGACTCCTTATATGATTTTAAACCCGACTCTGGACATACAAACTTCTCTCAAAAAGAAAATAGACTTCACA
Cele-UNSB01          Cele-UNSB01_Fam_198_101_1         1         101    0.000000
GCATTTGCATATNGAGATCAGATAAAAGTTAGAATTTTCCAGCCGTTGCAAGTGCGCTCTATTGAGAACATTTTGAAAATTGAATGTTTTCACATTAGAGC
Cele-UNSB01          Cele-UNSB01_Fam_199_101_1         1         101    0.000000
GGTTACTGTAGCTCCAGAAATACGCAAACACCGACTACGGTTCCCTAATACATCAGATATTACGACAAAGTGCAGAAAATCGCTAAAAATGACTTTACTGG
Cele-UNSB01          Cele-UNSB01_Fam_200_101_1         1         101    0.000000
TTGATTTTTCGTCTTATGAAGGAATATTGGTTTCGGGGTAGTGGGCGGATATGGTCGGGGTACTGTAGGTATACGGTAGGGTTACTGTAGTTTTGGAAATT
Cele-UNSB01          Cele-UNSB01_Fam_201_96_1         1         96    0.000000
GGTTGATTTGCGGCACTCTACAGATAGTGGATCTGCTTCTTACCAATCTTCCCGTTACCGCCACGCACTTAAGCTCCAAACTATTTTATTTTTTTG
Cele-UNSB01          Cele-UNSB01_Fam_202_96_1         1         96    0.000000
GTTCCCATAATTGGGTTAAATACCACTCATAAGTAACTAGTATGGGACTGAAAAGATACTAAATGAGCTTATTCTAAGGGTGAAGCTTCTGGTAAT
Cele-UNSB01          Cele-UNSB01_Fam_203_93_1         1         93    0.000000
TGCTTCTCGTCGTCCACCACCACCACCGAAGGGAACTGGAAGCCCACCACCACCACCAACTGGAGAACCACAGGATCTTTCTGGAGAAGGTAA
Cele-UNSB01          Cele-UNSB01_Fam_204_93_1         1         93    0.000000
CAGTACCCTCAAGGATCATCTGCTCCACGGTATCCCGGTAACTACAATGCCTTTGCCAGCACTTCCGATGCTCCACAGTATCCAGGATCCTCC
Cele-UNSB01          Cele-UNSB01_Fam_205_87_1         1         87    0.000000
TGAAGTCGTAGGATTCTGTGTGGTGACTGGTGTCGTTGTANTCTGTGAAGGGGACGTGGCTTCTTCCGTTGTGTCATGCTTCTNNGG
Cele-UNSB01          Cele-UNSB01_Fam_206_81_1         1         81    0.000000
TGTCGGCCGCTACCAAACTATTTAGCANACACACCAAGCACTACGTTGCGCACACACACCAAACTGCGGAACCCCGAACAG
Cele-UNSB01          Cele-UNSB01_Fam_207_80_1         1         80    0.000000
AATTTCTCAATAGGGCGCGCTTGCAACATCTGAACGGCGCGAAATTTGAATTTTTGGCCATAAAATAACGAAAACTAACT
Cele-UNSB01          Cele-UNSB01_Fam_208_78_1         1         78    0.000000
CGAAGTTGCTGGACCAAAGATCACAACAGGCGGATTTGGCGGCATAACCGGAGCAACAGTAGTGTCATTTGGTTCTTC
```

```
Cele-UNSB01          Cele-UNSB01_Fam_209_78_1          1          78    0.000000
TGCAAGGCTCCGATGATGATACCACTTCTAAAGTCGAGGTGTCACAATTTTCGGGTGTCGTAGAACTTGAATCAGTGA
Cele-UNSB01          Cele-UNSB01_Fam_210_78_1          1          78    0.000000
GAGCTTTGGGGCTTTTGGCTTGATCTCAGTCGACTTGATGGATTCTGGCTTGGGTTCTGCAGGCTTTTCGGGCTCCTT
Cele-UNSB01          Cele-UNSB01_Fam_211_77_1          1          77    0.000000
GGAAGCTTCATCTTTTGAATTATAAAAAAATAAATTTCATTTCAAAATCAGTGTGTGCACTATCAAAAGTGTCCACT
Cele-UNSB01          Cele-UNSB01_Fam_212_73_1          1          73    0.000000
ANTTTGTAGTTTGTAGTCTAGCAGACCAAAATTTATTAAAAACTGCAGAAAATTGATGGGTCTGCTAATTAAG
Cele-UNSB01          Cele-UNSB01_Fam_213_72_1          1          72    0.000000
TCCATCCAGCTGTGGTTGGTCTCTGGGTGAATCCTCGAGAAGTATCACTAGTAGTACCATTGGCTCCGTTCG
Cele-UNSB01          Cele-UNSB01_Fam_214_72_1          1          72    0.000000
ATGAATTTTAATACAATTTTCCTGACGATGAAGTCATAAAAATTCATTTTTTGACCTTTCAACTTTAGTTTT
Cele-UNSB01          Cele-UNSB01_Fam_215_72_1          1          72    0.000000
TGGTTGTGTTCCCAAAAGTGAGAAAAGAGGGTACAGACCTCTGTACGAATATGTCTTATCAGCTTCAAAAGA
Cele-UNSB01          Cele-UNSB01_Fam_216_71_1          1          71    0.000000
ATTTATGTGAAAATTCAGGTTTTTAGGGCATTTTTTGAGGGAAATCTGAATTTTTCCGATTTTTCTGACGA
Cele-UNSB01          Cele-UNSB01_Fam_217_70_1          1          70    0.000000
AAAAATCGATTTTTTGAAGTTTTTCTCAATAGAGCGCATTTTCAACAGCGAAAAAATTGAATTTTCCGCC
Cele-UNSB01          Cele-UNSB01_Fam_218_69_1          1          69    0.000000
AGGTAGGCCTGTAGGCATTGACTGNCAGAAAAGCGTAGACAGGCAAGCAGGTAAAAAGATACATGAGGC
Cele-UNSB01          Cele-UNSB01_Fam_219_69_1          1          69    0.000000
GTCTGGACTGGTGTAGTAGAACTGCTTGGAGGTTCTGTACTGGTTGTCTCTGGAGCCGTGGTAGTAGTA
Cele-UNSB01          Cele-UNSB01_Fam_220_68_1          1          68    0.000000
TTGGTTGAGGCTAGAAAATGTTGTAGTTTGTAGTGTGTTAGCCTCAACCAAAATTAATTTTTTTTTAA
Cele-UNSB01          Cele-UNSB01_Fam_221_65_1          1          65    0.000000
TATCGAGAGAATNTTCCACATTCACTTTGAATTCCNTTTCCAATTTCAATAAGACTCTCAGTAAG
Cele-UNSB01          Cele-UNSB01_Fam_222_62_1          1          62    0.000000
GTAGTTTGTAGCTTTAGCCTCAACCAAATTAGCTAAAGCTTGAGTGAGGCTATGGTGATTTT
Cele-UNSB01          Cele-UNSB01_Fam_223_62_1          1          62    0.000000
TGCTAACTAGCTACGGCCAGCTTAGGTGCCGGCTGCACGAGCAGTCCTGGTTTAATGTCGGC
Cele-UNSB01          Cele-UNSB01_Fam_224_60_1          1          60    0.000000
CTAGCTAGATTAAACGCTCTCAAGAGACAGCATGTTGTTGTTGCCCACAAGAAGAAGGCT
Cele-UNSB01          Cele-UNSB01_Fam_225_60_1          1          60    0.000000
ACACAATTGGAGTGGTGCTTGAGGAGCTGGATTCAGTTGAGCTCGTTATGTCGGTTGAAG
Cele-UNSB01          Cele-UNSB01_Fam_226_60_1          1          60    0.000000
CTGCTGATTCCGAGAAGAAGGCCCCAGCCAAGAGAGAAGCTGATAAGCCAAAGACTGAAT
Cele-UNSB01          Cele-UNSB01_Fam_227_59_1          1          59    0.000000
CTACAAACTACAAAAGCCTATTTAGCAGACCCACGCCAACTTCTGATGGGTCTGCTAGA
Cele-UNSB01          Cele-UNSB01_Fam_228_57_1          1          57    0.000000
GAACTCAACAAGCTGATCAAGAGCAACACCGTGGATCCCGAGCTGCCGGACAGAAGG
Cele-UNSB01          Cele-UNSB01_Fam_229_55_1          1          55    0.000000
GGGATTATTGGAGTTTCTTTTTCTAGAATTTTTTGAAAATTTTGGACAGTTTTTT
Cele-UNSB01          Cele-UNSB01_Fam_230_55_1          1          55    0.000000
AAGAGTCCCCACACACTGAAAGGTTTTAAGGAATTTTTTGAGGATTCCTAGGGTT
```

Cele-UNSB01 Cele-UNSB01_Fam_231_52_1 1 52 0.000000
GCATGTCTTCCAAGCGTTTTGAGCCACGCNTGGGATTTCCTATACACGGCTG
Cele-UNSB01 Cele-UNSB01_Fam_232_50_1 1 50 0.000000
ACACATGCTATAACATTGTCTCCTTCCCCTAGTGCACGAGAGCGGCCGTT
Cele-UNSB01 Cele-UNSB01_Fam_233_50_1 1 50 0.000000
GGGCGTGTTTTCACATGCCCCGCCCACAAAATTGATGTTTCCACCAAAAT
Cele-UNSB01 Cele-UNSB01_Fam_234_49_1 1 49 0.000000
CAATAATTTTAAATTAAATTATCGATTTTTCTAAATTTTTCCGAAAAAT
Cele-UNSB01 Cele-UNSB01_Fam_235_48_1 1 48 0.000000
TGCCTACGTGCCTACAAAACAGGCGTAGGCAGCTTTAAAGGTGATGCA
Cele-UNSB01 Cele-UNSB01_Fam_236_48_1 1 48 0.000000
GGAGGTGGAGAAAGTGTAGTTCCCGGTGGTCGNGGCTCTTCNGAGGGT
Cele-UNSB01 Cele-UNSB01_Fam_237_46_1 1 46 0.000000
TGGTAAATTGCCTCCCGGANCACCCACTTTTCTTATTTCCCAACTA
Cele-UNSB01 Cele-UNSB01_Fam_238_45_1 1 45 0.000000
AGCACCACCATCAGGCGGACCACCAGGACCATTTGATCCATCAGG
Cele-UNSB01 Cele-UNSB01_Fam_239_45_1 1 45 0.000000
CCAAGCACATCTACAGAGACACCTACAACTGAATCAACTGCAGCA
Cele-UNSB01 Cele-UNSB01_Fam_240_45_1 1 45 0.000000
CTCCATCCACCAGCACTGCTTCGAGTACGACAGAAAGCACTTCGA
Cele-UNSB01 Cele-UNSB01_Fam_241_43_1 1 43 0.000000
CGAGTTTGGCCTACANCAAATTTGGCAAATTCGGCAAATTTGC
Cele-UNSB01 Cele-UNSB01_Fam_242_43_1 1 43 0.000000
GAAAACTAGGCCACAGAGTTCAAATTGATGGCCTAGAAATCTA
Cele-UNSB01 Cele-UNSB01_Fam_243_43_1 1 43 0.000000
GGCTCTCGGCTCGCGCCGAGAGCCGAGNTCTACTNAAAGTTTC
Cele-UNSB01 Cele-UNSB01_Fam_244_43_1 1 43 0.000000
AGCAGCCGACAGTTGCCGGTTGCCGAAAAATTGCAGTAATTTC
Cele-UNSB01 Cele-UNSB01_Fam_245_42_1 1 42 0.000000
TTTGATCTACAAAAAATGCGGGAATTTTCCCAGCATTGCTCA
Cele-UNSB01 Cele-UNSB01_Fam_246_42_1 1 42 0.000000
AGGTGCTGCTTCCTTTGGAACATCTGCTTGGGTTGGCTCCGA
Cele-UNSB01 Cele-UNSB01_Fam_247_41_1 1 41 0.000000
AAATTTTTAACGAGAAGTGGGCGAAAATCATTCAAAAATTC
Cele-UNSB01 Cele-UNSB01_Fam_248_41_1 1 41 0.000000
GCATTTTTCGCCGATTTTTNATTAATTTTCAGTGAAAATTC
Cele-UNSB01 Cele-UNSB01_Fam_249_40_1 1 40 0.000000
TCCACGTGGAATTTTCAGAATTTTCNCAGAAAATTTGAAA
Cele-UNSB01 Cele-UNSB01_Fam_250_40_1 1 40 0.000000
GAATTTTTTGCAATTCTACTGAATTTTCCAAATTTTCAGC
Cele-UNSB01 Cele-UNSB01_Fam_251_40_1 1 40 0.000000
TTTTGCCAAAAATNTGTCAATTTTGTGGAATTTCTNTCAT
Cele-UNSB01 Cele-UNSB01_Fam_252_39_1 1 39 0.000000
AATTTCCCGCCAAAATTGAATTTTTTTAAAGAAAATTTA

Cele-UNSB01 Cele-UNSB01_Fam_253_39_1 1 39 0.000000
CCAACTGGAGCCAGCACNATGACCGCTGTTGGAGGAGCA
Cele-UNSB01 Cele-UNSB01_Fam_254_39_1 1 39 0.000000
GATTTACGGGCTTTAATCAGTTTCGAAAAAGCTTGTGTC
Cele-UNSB01 Cele-UNSB01_Fam_255_39_1 1 39 0.000000
TGGTACGGTAGTTGTAGTGGTAGTGGAGGTTGTTGTTGG
Cele-UNSB01 Cele-UNSB01_Fam_256_39_1 1 39 0.000000
GTAATTTATAATTTTTTAAAGATGAGTAATATATTGCCC
Cele-UNSB01 Cele-UNSB01_Fam_257_39_1 1 39 0.000000
ATTGATACTTTGTCGGGTTTAGACCATCGGCCGAGCTTC
Cele-UNSB01 Cele-UNSB01_Fam_258_38_1 1 38 0.000000
AAAATCGATAATTTTCAGTATGCAGTGAAATTCTTGGA
Cele-UNSB01 Cele-UNSB01_Fam_259_37_1 1 37 0.000000
ATAATTTTTCTTGAGTAATGAGAGACTTTTTCATAAT
Cele-UNSB01 Cele-UNSB01_Fam_260_36_1 1 36 0.000000
GATATTTTCATGTTCGGCAAAAGTACCGATTTGCCA
Cele-UNSB01 Cele-UNSB01_Fam_261_36_1 1 36 0.000000
CCACCAACTCCAAAATCAGCTGCTCCATCAGAAGTC
Cele-UNSB01 Cele-UNSB01_Fam_262_35_1 1 35 0.000000
AATTGCCGAATTTCTCGTTTTTGGCAATTTACCAA
Cele-UNSB01 Cele-UNSB01_Fam_263_35_1 1 35 0.000000
TGTGTAATAAGAAAATTTCAAATTTTTTTCACGAA
Cele-UNSB01 Cele-UNSB01_Fam_264_35_1 1 35 0.000000
GCATTTGCCGATTTTTAAAATTTCTGGCAATTCCA
Cele-UNSB01 Cele-UNSB01_Fam_265_34_1 1 34 0.000000
CCGTGCCTACTAACCTACCGACCTTGCCTGCCTT
Cele-UNSB01 Cele-UNSB01_Fam_266_34_1 1 34 0.000000
TTGCCGAATTTGCCGTTTGCCGAGCTCGTCAAAT
Cele-UNSB01 Cele-UNSB01_Fam_267_34_1 1 34 0.000000
AAGTATTTTTGCCGGTTTTTTTGCCGAAATTTCC
Cele-UNSB01 Cele-UNSB01_Fam_268_33_1 1 33 0.000000
TAAGAAAAGTTAGGCCACCAAACTTTGGTGAAC
Cele-UNSB01 Cele-UNSB01_Fam_269_32_1 1 32 0.000000
GCAATTTTTAGGCTAAAAATCACTAAAAAACT
Cele-UNSB01 Cele-UNSB01_Fam_270_32_1 1 32 0.000000
AAATTTGTGAAAATTTAGGCCCATTTTGCTCA
Cele-UNSB01 Cele-UNSB01_Fam_271_32_1 1 32 0.000000
GTGCGTCCGGCGTCCGGAAAAAGCGGACGCGT
Cele-UNSB01 Cele-UNSB01_Fam_272_31_1 1 31 0.000000
ATCGATTTTTTCGTTAAAAAATCAATAAAAT
Cele-UNSB01 Cele-UNSB01_Fam_273_31_1 1 31 0.000000
GCTGATTGGTCGGCGNGGTTCGCCCATCNTT
Cele-UNSB01 Cele-UNSB01_Fam_274_31_1 1 31 0.000000
GACATCTCGCAGGACATTATAAGAAGCGACC

Cele-UNSB01 Cele-UNSB01_Fam_275_31_1 1 31 0.000000
CACGCCTATTTTATGGGCGGAGTCTACAAGC
Cele-UNSB01 Cele-UNSB01_Fam_276_31_1 1 31 0.000000
TGCGTCGGAAATTNATTCCGATCATCGCCGG
Cele-UNSB01 Cele-UNSB01_Fam_277_31_1 1 31 0.000000
TCCGATGCATTGCCGATAATCGGAACCTGGT
Cele-UNSB01 Cele-UNSB01_Fam_278_31_1 1 31 0.000000
GCAGCCGACGCTATACGGGTCTGATGGAGTA
Cele-UNSB01 Cele-UNSB01_Fam_279_31_1 1 31 0.000000
TCTGAGCTACAGTACTCCTGTCAAGAACAAT
Cele-UNSB01 Cele-UNSB01_Fam_280_31_1 1 31 0.000000
GATTTTTTCAGNGAAAANCNAAAAATTTTCG
Cele-UNSB01 Cele-UNSB01_Fam_281_31_1 1 31 0.000000
CCAAAAATGACTGAAAATAGTCAAAAATTAG
Cele-UNSB01 Cele-UNSB01_Fam_282_31_1 1 31 0.000000
CAAAAATCGGGAAATTTTCACAAAAAAATGC
Cele-UNSB01 Cele-UNSB01_Fam_283_31_1 1 31 0.000000
TTGGCCCAGAGCTCTCTTGACCCAAGCCCCC
Cele-UNSB01 Cele-UNSB01_Fam_284_31_1 1 31 0.000000
AAAATTTTGACCCAGAAATCGAAAAATTCGC
Cele-UNSB01 Cele-UNSB01_Fam_285_31_1 1 31 0.000000
TCGTTTTTTCATGAAAAAACTCAATTTTCGG
Cele-UNSB01 Cele-UNSB01_Fam_286_31_1 1 31 0.000000
GAAAAAGTGCTGAAAATCACATTTTTGAACT
Cele-UNSB01 Cele-UNSB01_Fam_287_30_1 1 30 0.000000
AATTTGAATTTTCCCGAATTTTTNAACGAA
Cele-UNSB01 Cele-UNSB01_Fam_288_30_1 1 30 0.000000
TGGTGGAACTGGATTCGGCAGTGGAAATGC
Cele-UNSB01 Cele-UNSB01_Fam_289_30_1 1 30 0.000000
CCTTGGGCTCCCTGAGCTCCAAATTGTGCT
Cele-UNSB01 Cele-UNSB01_Fam_290_30_1 1 30 0.000000
GTCATTATCAGCTTCTGGTGTCTTAGAAGA
Cele-UNSB01 Cele-UNSB01_Fam_291_30_1 1 30 0.000000
CAAACTACAGTAGTACTGTAGTACCATAAC
Cele-UNSB01 Cele-UNSB01_Fam_292_30_1 1 30 0.000000
TTCAGGCAGGCATAGGTTACTTTGAAAATT
Cele-UNSB01 Cele-UNSB01_Fam_293_30_1 1 30 0.000000
CCTTGTAGGCAAGGAGGTAGGCGTAGGTCA
Cele-UNSB01 Cele-UNSB01_Fam_294_30_1 1 30 0.000000
TTTCAGGCCAATTTCNGTCATTTTTGGACA
Cele-UNSB01 Cele-UNSB01_Fam_295_30_1 1 30 0.000000
AAAATCAAAAATTAGATGAATTTTCGATTA
Cele-UNSB01 Cele-UNSB01_Fam_296_30_1 1 30 0.000000
CTCAAATTTGCACTAAAAATTCCAATTTTT

| | | | | | |
|---|---|---|---|---|---|
| Cele-UNSB01 | Cele-UNSB01_Fam_297_30_1 | 1 | 30 | 0.000000 | |
| ACTACCCAACCGGCTCCGGCAACCAGTCCA | | | | | |
| Cele-UNSB01 | Cele-UNSB01_Fam_298_29_1 | 1 | 29 | 0.000000 | |
| TTGTAAGGATTTCCCACTATGTCAATACA | | | | | |
| Cele-UNSB01 | Cele-UNSB01_Fam_299_29_1 | 1 | 29 | 0.000000 | |
| GGCCTAGAAANTTCTATTTGAAAGTTGAT | | | | | |
| Cele-UNSB01 | Cele-UNSB01_Fam_300_29_1 | 1 | 29 | 0.000000 | |
| TACATTGCAAGATCTTCTTACTATGTAGG | | | | | |
| Cele-UNSB01 | Cele-UNSB01_Fam_301_29_1 | 1 | 29 | 0.000000 | |
| CTCGTCAGTGGGTGGACGAGGAATGAGCG | | | | | |
| Cele-UNSB01 | Cele-UNSB01_Fam_302_28_1 | 1 | 28 | 0.000000 | |
| AATGTCGGCTGCTTCTAGAAACCAGGCA | | | | | |
| Cele-UNSB01 | Cele-UNSB01_Fam_303_28_1 | 1 | 28 | 0.000000 | |
| TGCCTCGCCTACGTGCTTATTTCAGGCA | | | | | |
| Cele-UNSB01 | Cele-UNSB01_Fam_304_28_1 | 1 | 28 | 0.000000 | |
| CCAAGAGAATTTCAACCACCTGGAAATT | | | | | |
| Cele-UNSB01 | Cele-UNSB01_Fam_305_27_1 | 1 | 27 | 0.000000 | TTCCGGCAAATCGGCAAATTGCAAAAT |
| Cele-UNSB01 | Cele-UNSB01_Fam_306_27_1 | 1 | 27 | 0.000000 | ACAATAAAGTACTATGTATTACCGTAT |
| Cele-UNSB01 | Cele-UNSB01_Fam_307_27_1 | 1 | 27 | 0.000000 | TGGAGGCGGCTTCTGGCCTGGTGGTGG |
| Cele-UNSB01 | Cele-UNSB01_Fam_308_27_1 | 1 | 27 | 0.000000 | CACTGACCAAAGAAAATATTANACGTG |
| Cele-UNSB01 | Cele-UNSB01_Fam_309_26_1 | 1 | 26 | 0.000000 | GGCAGGCGTCAAGCAGGCAGGTTTCA |
| Cele-UNSB01 | Cele-UNSB01_Fam_310_26_1 | 1 | 26 | 0.000000 | ACCTACAGTACCCCTGTCCTAGAATG |
| Cele-UNSB01 | Cele-UNSB01_Fam_311_26_1 | 1 | 26 | 0.000000 | TACTGTAGTTTGACCTACTTANGGAT |
| Cele-UNSB01 | Cele-UNSB01_Fam_312_26_1 | 1 | 26 | 0.000000 | ATTTACGGAGCTTCAAGCTTGTGTCG |
| Cele-UNSB01 | Cele-UNSB01_Fam_313_26_1 | 1 | 26 | 0.000000 | TGAAATTTCACAAAATATCGATTTTC |
| Cele-UNSB01 | Cele-UNSB01_Fam_314_25_1 | 1 | 25 | 0.000000 | GTCGGCTGCTTNCAAACCCTGAGTT |
| Cele-UNSB01 | Cele-UNSB01_Fam_315_25_1 | 1 | 25 | 0.000000 | AAAAATCTGTTCTNNGAGGTCCCTG |
| Cele-UNSB01 | Cele-UNSB01_Fam_316_24_1 | 1 | 24 | 0.000000 | GTGAGGTACGAGGTGAGGTANGAG |
| Cele-UNSB01 | Cele-UNSB01_Fam_317_24_1 | 1 | 24 | 0.000000 | AAGCACCACCTCCTCCAACTACAA |
| Cele-UNSB01 | Cele-UNSB01_Fam_318_24_1 | 1 | 24 | 0.000000 | AAGAAGGATGCTGAAGAGAAGAAG |
| Cele-UNSB01 | Cele-UNSB01_Fam_319_24_1 | 1 | 24 | 0.000000 | GCTTCTGGAGCTACAGGTTATCCA |
| Cele-UNSB01 | Cele-UNSB01_Fam_320_24_1 | 1 | 24 | 0.000000 | TCAACAACAACAGGAGAAGCTTCT |
| Cele-UNSB01 | Cele-UNSB01_Fam_321_24_1 | 1 | 24 | 0.000000 | GGTGGCTGGTCACAAAAGTAAGTT |
| Cele-UNSB01 | Cele-UNSB01_Fam_322_23_1 | 1 | 23 | 0.000000 | GCATCATAAGGATCATAGTTGAT |
| Cele-UNSB01 | Cele-UNSB01_Fam_323_23_1 | 1 | 23 | 0.000000 | CGCTGTAAGACCCTTCGCTGTAC |
| Cele-UNSB01 | Cele-UNSB01_Fam_324_23_1 | 1 | 23 | 0.000000 | GCTACAGTACCCCAGCTTGACTT |
| Cele-UNSB01 | Cele-UNSB01_Fam_325_23_1 | 1 | 23 | 0.000000 | TTTTCAGAAATAGGTATTTCAGA |
| Cele-UNSB01 | Cele-UNSB01_Fam_326_22_1 | 1 | 22 | 0.000000 | TTAAAATTTTGTCAGTTTGACC |
| Cele-UNSB01 | Cele-UNSB01_Fam_327_22_1 | 1 | 22 | 0.000000 | TTTTAGCCAAAAAATCCGTTAA |
| Cele-UNSB01 | Cele-UNSB01_Fam_328_22_1 | 1 | 22 | 0.000000 | GCACGGCCTGGCGGTCCTGAGG |
| Cele-UNSB01 | Cele-UNSB01_Fam_329_22_1 | 1 | 22 | 0.000000 | CTACAGTAACCCTAGTATTGAA |
| Cele-UNSB01 | Cele-UNSB01_Fam_330_22_1 | 1 | 22 | 0.000000 | CAAAACCCAAGTAGATCCCACT |
| Cele-UNSB01 | Cele-UNSB01_Fam_331_22_1 | 1 | 22 | 0.000000 | AAGCGGTAACGTCGTCTATATA |
| Cele-UNSB01 | Cele-UNSB01_Fam_332_22_1 | 1 | 22 | 0.000000 | TGCTAATTAGCGCGGNTGCGTN |
| Cele-UNSB01 | Cele-UNSB01_Fam_333_22_1 | 1 | 22 | 0.000000 | ACGTATACAGGCATATACACAT |

| | | | | | |
|---|---|---|---|---|---|
| Cele-UNSB01 | Cele-UNSB01_Fam_334_22_1 | 1 | 22 | 0.000000 | CCGGCGTGGTNTTGAAGCGGTC |
| Cele-UNSB01 | Cele-UNSB01_Fam_335_22_1 | 1 | 22 | 0.000000 | TTTGTGGGAGGCGACATATACC |
| Cele-UNSB01 | Cele-UNSB01_Fam_336_21_1 | 1 | 21 | 0.000000 | TGGCCTAGATTNTTTACAGGG |
| Cele-UNSB01 | Cele-UNSB01_Fam_337_21_1 | 1 | 21 | 0.000000 | TCTCCTGGTAAATTTAAAAAT |
| Cele-UNSB01 | Cele-UNSB01_Fam_338_21_1 | 1 | 21 | 0.000000 | ACCACCCCTACAACNACCACC |
| Cele-UNSB01 | Cele-UNSB01_Fam_339_21_1 | 1 | 21 | 0.000000 | AACTTTTTGGCAATTTTCCAA |
| Cele-UNSB01 | Cele-UNSB01_Fam_340_21_1 | 1 | 21 | 0.000000 | TGTGCCCTGGAAAGCTGGCAG |
| Cele-UNSB01 | Cele-UNSB01_Fam_341_21_1 | 1 | 21 | 0.000000 | CTACGTAAGAGTAGCGCAGGA |
| Cele-UNSB01 | Cele-UNSB01_Fam_342_21_1 | 1 | 21 | 0.000000 | AATCTAGCCAGACGCACACCT |
| Cele-UNSB01 | Cele-UNSB01_Fam_343_21_1 | 1 | 21 | 0.000000 | CAATTTTCTGTTNAACTTTGA |
| Cele-UNSB01 | Cele-UNSB01_Fam_344_21_1 | 1 | 21 | 0.000000 | AAGTTTTACTTTGATTTTTTT |
| Cele-UNSB01 | Cele-UNSB01_Fam_345_21_1 | 1 | 21 | 0.000000 | GGCAATTTTCCAGTGCAATTC |
| Cele-UNSB01 | Cele-UNSB01_Fam_346_21_1 | 1 | 21 | 0.000000 | CCATAGGTGGAAGATCTNATA |
| Cele-UNSB01 | Cele-UNSB01_Fam_347_21_1 | 1 | 21 | 0.000000 | GATTTACGGGGTGAACATGTG |
| Cele-UNSB01 | Cele-UNSB01_Fam_348_21_1 | 1 | 21 | 0.000000 | TTGGTGACAATGTGGAAACAA |
| Cele-UNSB01 | Cele-UNSB01_Fam_349_21_1 | 1 | 21 | 0.000000 | TTCTAATTCGACGCGCATNGT |
| Cele-UNSB01 | Cele-UNSB01_Fam_350_21_1 | 1 | 21 | 0.000000 | CTAATTAGCGTCGCTTCCNTT |
| Cele-UNSB01 | Cele-UNSB01_Fam_351_21_1 | 1 | 21 | 0.000000 | AAAAATCGATAAAATTTGGAN |
| Cele-UNSB01 | Cele-UNSB01_Fam_352_21_1 | 1 | 21 | 0.000000 | CACACGAGCTTGAGAGCCCGG |
| Cele-UNSB01 | Cele-UNSB01_Fam_353_21_1 | 1 | 21 | 0.000000 | TATTTTCAAAAAAATTTAAAA |
| Cele-UNSB01 | Cele-UNSB01_Fam_354_21_1 | 1 | 21 | 0.000000 | ACCCCGATTTTCACCCAAAAA |
| Cele-UNSB01 | Cele-UNSB01_Fam_355_21_1 | 1 | 21 | 0.000000 | CTCAATTCAATCAAGGAAATC |
| Cele-UNSB01 | Cele-UNSB01_Fam_356_21_1 | 1 | 21 | 0.000000 | CCATGTAGCTTCAAAATTNCT |
| Cele-UNSB01 | Cele-UNSB01_Fam_357_21_1 | 1 | 21 | 0.000000 | TGTCCGTGATGATCATGNGCA |
| Cele-UNSB01 | Cele-UNSB01_Fam_358_21_1 | 1 | 21 | 0.000000 | GGTGGACCCANGGGTTCCTAG |
| Cele-UNSB01 | Cele-UNSB01_Fam_359_20_1 | 1 | 20 | 0.000000 | CTATCGCCTGCTATCGCCTA |
| Cele-UNSB01 | Cele-UNSB01_Fam_360_20_1 | 1 | 20 | 0.000000 | ATTTGACCTACTTTTTAAAA |
| Cele-UNSB01 | Cele-UNSB01_Fam_361_19_1 | 1 | 19 | 0.000000 | GTACTACCTTTTAATGTAT |
| Cele-UNSB01 | Cele-UNSB01_Fam_362_19_1 | 1 | 19 | 0.000000 | CCCTGATACCCCCTAGTCC |
| Cele-UNSB01 | Cele-UNSB01_Fam_363_19_1 | 1 | 19 | 0.000000 | TATGGTGCATCTAACTAGT |
| Cele-UNSB01 | Cele-UNSB01_Fam_364_18_1 | 1 | 18 | 0.000000 | AAAAGTTACAGTGTCCTT |
| Cele-UNSB01 | Cele-UNSB01_Fam_365_18_1 | 1 | 18 | 0.000000 | TTGTAGATCAACGGGGTC |
| Cele-UNSB01 | Cele-UNSB01_Fam_366_18_1 | 1 | 18 | 0.000000 | GGAGGTCCAGGTACCCCG |
| Cele-UNSB01 | Cele-UNSB01_Fam_367_18_1 | 1 | 18 | 0.000000 | TAAATAAGTGGCTATAAT |
| Cele-UNSB01 | Cele-UNSB01_Fam_368_18_1 | 1 | 18 | 0.000000 | ACTACTGTATCNCAGGAG |
| Cele-UNSB01 | Cele-UNSB01_Fam_369_18_1 | 1 | 18 | 0.000000 | CCTTCGCCTACCTACGTG |
| Cele-UNSB01 | Cele-UNSB01_Fam_370_18_1 | 1 | 18 | 0.000000 | TTTTTTGATAATTCTTGA |
| Cele-UNSB01 | Cele-UNSB01_Fam_371_18_1 | 1 | 18 | 0.000000 | AAATATTCTTATTTTCAG |
| Cele-UNSB01 | Cele-UNSB01_Fam_372_18_1 | 1 | 18 | 0.000000 | CCTGGTTGTCCNTCTNNT |
| Cele-UNSB01 | Cele-UNSB01_Fam_373_18_1 | 1 | 18 | 0.000000 | TTTCATAAGAAGTAAATN |
| Cele-UNSB01 | Cele-UNSB01_Fam_374_18_1 | 1 | 18 | 0.000000 | CATTGTCACTTTCTTCTC |
| Cele-UNSB01 | Cele-UNSB01_Fam_375_18_1 | 1 | 18 | 0.000000 | AAGCCTATGCCTTTCCCA |
| Cele-UNSB01 | Cele-UNSB01_Fam_376_17_1 | 1 | 17 | 0.000000 | ACTTGGTACATAGGCCA |
| Cele-UNSB01 | Cele-UNSB01_Fam_377_17_1 | 1 | 17 | 0.000000 | ATTTTTTCCGCAAGTAG |
| Cele-UNSB01 | Cele-UNSB01_Fam_378_17_1 | 1 | 17 | 0.000000 | AAAATCAAACCGGCGGA |

| | | | | | |
|---|---|---|---|---|---|
| Cele-UNSB01 | Cele-UNSB01_Fam_379_17_1 | 1 | 17 | 0.000000 | AAATGCCAAAANTGCCA |
| Cele-UNSB01 | Cele-UNSB01_Fam_380_17_1 | 1 | 17 | 0.000000 | TAACCTGACTGCTACAG |
| Cele-UNSB01 | Cele-UNSB01_Fam_381_17_1 | 1 | 17 | 0.000000 | GAATTAGAAAAATTTGA |
| Cele-UNSB01 | Cele-UNSB01_Fam_382_17_1 | 1 | 17 | 0.000000 | TTGCAAGAAAATCTGCA |
| Cele-UNSB01 | Cele-UNSB01_Fam_383_17_1 | 1 | 17 | 0.000000 | TTTTAACGTGACCTAGT |
| Cele-UNSB01 | Cele-UNSB01_Fam_384_16_1 | 1 | 16 | 0.000000 | TGATCTGATGATCTGA |
| Cele-UNSB01 | Cele-UNSB01_Fam_385_16_1 | 1 | 16 | 0.000000 | AATACGGGAATACGGG |
| Cele-UNSB01 | Cele-UNSB01_Fam_386_16_1 | 1 | 16 | 0.000000 | TTATAGTGTTATAGTG |
| Cele-UNSB01 | Cele-UNSB01_Fam_387_16_1 | 1 | 16 | 0.000000 | TGTATATATATATATA |
| Cele-UNSB01 | Cele-UNSB01_Fam_388_16_1 | 1 | 16 | 0.000000 | AAAAATTATTGAATAC |
| Cele-UNSB01 | Cele-UNSB01_Fam_389_16_1 | 1 | 16 | 0.000000 | GAATAATATTGGAGAA |
| Cele-UNSB01 | Cele-UNSB01_Fam_390_16_1 | 1 | 16 | 0.000000 | CTTAGATCTCAATTCC |
| Cele-UNSB01 | Cele-UNSB01_Fam_391_16_1 | 1 | 16 | 0.000000 | AGAGAGTATAGATTAG |
| Cele-UNSB01 | Cele-UNSB01_Fam_392_15_1 | 1 | 15 | 0.000000 | CGACCTGCGACCTAG |
| Cele-UNSB01 | Cele-UNSB01_Fam_393_15_1 | 1 | 15 | 0.000000 | CACAGGAGGCTGGGA |
| Cele-UNSB01 | Cele-UNSB01_Fam_394_14_1 | 1 | 14 | 0.000000 | AGCTTGAAGCTTGG |
| Cele-UNSB01 | Cele-UNSB01_Fam_395_14_1 | 1 | 14 | 0.000000 | TTACCAAAATCTAA |
| Cele-UNSB01 | Cele-UNSB01_Fam_396_13_1 | 1 | 13 | 0.000000 | GGCTTCTAGTCCA |
| Cele-UNSB01 | Cele-UNSB01_Fam_397_13_1 | 1 | 13 | 0.000000 | TTGGGGTGGGCCT |
| Cele-UNSB01 | Cele-UNSB01_Fam_398_12_1 | 1 | 12 | 0.000000 | AATACCAACCAC |
| Cele-UNSB01 | Cele-UNSB01_Fam_399_12_1 | 1 | 12 | 0.000000 | AGTTATATAGTA |
| Cele-UNSB01 | Cele-UNSB01_Fam_400_12_1 | 1 | 12 | 0.000000 | CATTGACTTGAG |
| Cele-UNSB01 | Cele-UNSB01_Fam_401_11_1 | 1 | 11 | 0.000000 | TTGTTTACAAG |
| Cele-UNSB01 | Cele-UNSB01_Fam_402_11_1 | 1 | 11 | 0.000000 | TACCTACTAGA |
| Cele-UNSB01 | Cele-UNSB01_Fam_403_11_1 | 1 | 11 | 0.000000 | AGATACACTTC |
| Cele-UNSB01 | Cele-UNSB01_Fam_404_11_1 | 1 | 11 | 0.000000 | TTTCAGGCCTA |
| Cele-UNSB01 | Cele-UNSB01_Fam_405_10_1 | 1 | 10 | 0.000000 | GATTTTCATA |
| Cele-UNSB01 | Cele-UNSB01_Fam_406_10_1 | 1 | 10 | 0.000000 | CAGCAGAGTG |
| Cele-UNSB01 | Cele-UNSB01_Fam_407_10_1 | 1 | 10 | 0.000000 | AACTTTCGAA |
| Cele-UNSB01 | Cele-UNSB01_Fam_408_10_1 | 1 | 10 | 0.000000 | TGGCGAAAGC |
