# Supplementary material for: DNA Satellites Are Transcribed as Part of the Non-Coding Genome in Eukaryotes and Bacteria
Source: Genes (Basel). 2021 Oct 20;12(11):1651. doi: 10.3390/genes12111651 (PMC8625621; doi:10.3390/genes12111651)
Supplement: Supplementary file 1 [file genes-12-01651-s001.zip › Table S5. Sequence of main families of B.coagulans satellites.pdf]

Table S5.
Sequence of main families of *B.coagulans* satellites

Family name	Consensus Sequence
1_52_139	GTgAAGgAAGgcCnTCnTTTTTcCncGCTTcCTTAACGTAGACGcgCTCTAT
2_52_35	TTTTGTCCTTTTGaCaGcTTCAAAAnGACATTTCGgGCCCgGATgCAgCntG
8_52_18	TGTCCTTCATaagggtGATGAAaGACAAAACaCnGGcCgggaAAcGgCgAAt
360_52_1	TTTTGTCTTTTTGAACCGTTGTAAAAGACAGTTCGGTGGAAAGCTGGAACGC

We have used the consensus sequence of the main satellite families previously determined [15] from the genome sequence of seven strains of *B.coagulans*. We have also used a unique satellite (360_52_1), which is only present in the 2-6 strain studied by Qin et al. [11]. Note that part of the consensus sequence of this satellite (shown in red) is identical with the consensus sequence of the 2_52_35 family.
