# Supplementary material for: DNA Satellites Are Transcribed as Part of the Non-Coding Genome in Eukaryotes and Bacteria
Source: Genes (Basel). 2021 Oct 20;12(11):1651. doi: 10.3390/genes12111651 (PMC8625621; doi:10.3390/genes12111651)
Supplement: Supplementary file 1 [file genes-12-01651-s001.zip › Table S6. Example of perfect hits.pdf]

Table S6. Examples of perfect hits

SRR7443983. Spot 236610013 Score 127 Length 140 nt Identity 100 %

GGCGGCAAATCGGCAAATTGTCGG-AATTGAAAATTTCCGGCAAATCGGCAAATTGCCGG-AATTGAAAATTTCCGGCAAATCGGCAAATTGCCGG-AATTGAAAATTTCCGGCAAATCGACAAATTGCCGA-AATTGGAAATT
TCCGGCAAATCGGCAAaTTGCCGg-AAtTgAAAATTTCCGGCAAATCGGCAAaTTGCCGg-AAtTgAAAATTTCCGGCAAATCGGCAAaTTGCCGg-AAtTgAAAATTTCCGGCAAATCGGCAAaTTGCCGg-AAtTgAAAATT

SRR7443983. Spot 213225154 Score 118 Length 137 nt Identity 97.14%

TCTTAGGTATGGTATATT-TCTTGAAAATTTCCGGCAAATCGGTAAATCGCCGG-AATTGAAAATTTCCGGCAAATCGGTAAATTGCCGG-AATTGAAAATTTCCGGCAAATCGGTAAATTGCCGG-AATTGAAAATTTTC
AAATCGGCAAaTTGCCGg-AAtTgAAAATTTCCGGCAAATCGGCAAaTTGCCGg-AAtTgAAAATTTCCGGCAAATCGGCAAaTTGCCGg-AAtTgAAAATTTCCGGCAAATCGGCAAaTTGCCGg-AAtTgAAAATTTCC

SRR7443983. Spot 150997260 Score 106 Length 140 nt Identity 95.45%

GCAAATCGACAAATTGCCGG-AATTCAAA--TTCCGGCAAATCAACAAATCGTCGG-AATTGAAAATTTCCGGCAAATCGGCAAGTTCCCGA-AATTGAAAATTTCCGGCAAATCGGCAAATTGTCAG-AATTGAAAATTCCCGGC
GCAAATCGGCAAaTTGCCGg-AAtTgAAAATTTCCGGCAAATCGGCAAaTTGCCGg-AAtTgAAAATTTCCGGCAAATCGGCAAaTTGCCGg-AAtTgAAAATTTCCGGCAAATCGGCAAaTTGCCGg-AAtTgAAAATTTCCGGC

Above we compare the sequence of different RNA-seq spots (upper lines) with a theoretical satellite (lower lines) in *C.elegans.* Dashes separate individual repeats. The RNA-seq spots were obtained from a run of SRX4314512 with a query sequence of two repeats of the consensus sequence of Fam_2_35_166 (AAtTgAAAATTTCCGGCAAATCGGCAAaTTGCCGg). The theoretical satellite is formed by a string of repeats with this consensus sequence. Differences between both are highlighted in red. All spots in this run present a string of several repeats of this family, which differ slightly from the consensus sequence. In this way different regions of satellites in this family are captured in the RNA-seq experiment. Occasionally part of the sequence corresponds to the terminal region of one satellite with its neighboring region in the genome, as shown above in the second example. Below we show similar results in *B.coagulans*, with the reverse sequence of Fam_360_52_1 (Table S5).

>gnl|SRA|SRR1574894.4395888.1 HWI-ST531R:246:C348YACXX:1:2112:12873:55937 *(Biological)*

TTTCCAGCGAACTGTCCTTTACAACGGTTCAAAAAGACAAAA-GCGTTCCATCTTTCCGCCGAACTGTCTTTTACAACGATCCAAAAAGACAAAA-GCATTCC
TTTCCACCGAACTGTCTTTTACAACGGTTCAAAAAGACAAAA-GCGTTCCAGCTTTCCACCGAACTGTCTTTTACAACGGTTCAAAAAGACAAAA-GCGTTCC....

>gnl|SRA|SRR1574894.6119208.1 HWI-ST531R:246:C348YACXX:1:2303:10326:69539 *(Biological)*

GTTCAAAAAGACAAAA--TCGTTCCATCTTTCCGCCGAACTGTCTTTTACAACGATCCAAAAAGACAAAA--GCATTCCAGCTTTCCACCAAAATGTTCTTTACA
GTTCAAAA-GACAAAAA-GCGTTCCAGCTTTCCACCGAACTGTCTTTTACAACGGTTCAAAA-GACAAAAA-GCGTTCCAGCTTTCCACCGAACTGTCTTTTACA....
